# Current status of proton pump inhibitor use in Japanese elderly patients with non-valvular atrial fibrillation: A subanalysis of the ANAFIE Registry

**DOI:** 10.1371/journal.pone.0240859

**Published:** 2020-11-05

**Authors:** Yuji Mizokami, Takatsugu Yamamoto, Hirotsugu Atarashi, Takeshi Yamashita, Masaharu Akao, Takanori Ikeda, Yukihiro Koretsune, Ken Okumura, Wataru Shimizu, Hiroyuki Tsutsui, Kazunori Toyoda, Atsushi Hirayama, Masahiro Yasaka, Takenori Yamaguchi, Satoshi Teramukai, Tetsuya Kimura, Jumpei Kaburagi, Atsushi Takita, Hiroshi Inoue

**Affiliations:** 1 Department of Gastroenterology, University of Tsukuba Hospital, Tsukuba, Ibaraki, Japan; 2 Department of Medicine, Teikyo University, Itabashi City, Tokyo, Japan; 3 Minami Hachioji Hospital, Hachioji, Tokyo, Japan; 4 The Cardiovascular Research Institute, Minato City, Tokyo, Japan; 5 National Hospital Organization Kyoto Medical Center, Fushimi Ward, Kyoto, Japan; 6 Toho University Omori Medical Center, Ota City, Tokyo, Japan; 7 National Hospital Organization Osaka National Hospital, Chuo Ward, Osaka, Japan; 8 Saiseikai Kumamoto Hospital, Minami Ward, Kumamoto, Japan; 9 Nippon Medical School, Bunkyo City, Tokyo, Japan; 10 Kyushu University, Higashi Ward, Fukuoka, Japan; 11 National Cerebral and Cardiovascular Center, Suita, Osaka, Japan; 12 Osaka Police Hospital, Tennoji Ward, Osaka, Japan; 13 National Hospital Organization Kyushu Medical Center, Chuo Ward, Fukuoka, Japan; 14 Kyoto Prefectural University of Medicine, Kamigyo Ward, Kyoto, Japan; 15 Medical Science Department, Daiichi Sankyo Co., Ltd., Chuo City, Tokyo, Japan; 16 Biostatistics and Data Management Department, Daiichi Sankyo Co., Ltd., Shinagawa-ku, Tokyo, Japan; 17 Saiseikai Toyama Hospital, Toyama, Toyama, Japan; Ohio State University, UNITED STATES

## Abstract

The real-world status of proton pump inhibitor (PPI) use in patients with atrial fibrillation (AF) receiving antithrombotic treatment is largely unknown. The All Nippon AF In the Elderly (ANAFIE) Registry, a prospective, multicenter, observational study, aimed to determine treatment patterns, risk factors, and outcomes among elderly (aged ≥75 years) Japanese non-valvular AF (NVAF) patients in the real-world clinical setting. The present subanalysis of the ANAFIE Registry determined the PPI prescription status of 32,490 elderly Japanese NVAF patients. Patients were stratified by PPI use (PPI+) or no PPI use (PPI−). Risk scores for stroke (CHADS_2_, CHA_2_DS_2_-VASc) and bleeding (HAS-BLED), anticoagulant use, time in therapeutic range (TTR) for warfarin, and anticoagulant/antiplatelet combination use were evaluated. PPIs were used in 11,981 (36.9%) patients. Compared with the PPI− group, the PPI+ group included a greater proportion of female patients (45.2% vs 41.3%; *P* <0.0001) and had significantly higher CHADS_2_, CHA_2_DS_2_-VASc, and HAS-BLED scores (*P* <0.0001 for each) as well as higher prevalences of several comorbidities. In the PPI+ group, 54.6% of patients did not have gastrointestinal (GI) disorders and were likely prescribed a PPI to prevent GI bleeding events. Most of the patients with a GI disorder in the PPI+ group had reflux esophagitis. Compared with patients not receiving anticoagulants, a significantly higher proportion of patients receiving anticoagulants received PPIs. For patients receiving anticoagulants, antiplatelet drugs, and both drugs, rates of PPI use were 34.1%, 44.1%, and 53.5%, respectively (P <0.01). Although the rate of PPI use was the highest for NVAF patients receiving both antiplatelet and anticoagulants, no clear differences were observed in the anticoagulants used. These data suggest that PPIs were actively prescribed in high-risk cases and may have been used to prevent GI bleeding among elderly NVAF patients receiving antithrombotic drugs.

**Trial registration:** UMIN000024006

## Introduction

Globally, atrial fibrillation (AF) is a leading cause of morbidity and mortality [1, 2] and is an important risk factor for stroke [3, 4]. Because patients with AF have a high risk of embolism, long-term oral anticoagulation is recommended for most AF patients [5]. Both direct oral anticoagulants (DOACs) and vitamin K antagonists (such as warfarin) have shown effectiveness in preventing strokes due to AF [6–8].

As the prevalence of non-valvular AF (NVAF) increases with age [9], appropriate anticoagulation is particularly important among the elderly, as this group is not only at the greatest risk of stroke attributable to AF [3, 10] but also of bleeding [11, 12]. Clinicians must consider both the benefits and risks before prescribing an anticoagulant, as such therapy may increase the risk of bleeding [13, 14]. Older patients receiving anticoagulation may be at a particularly high risk of gastrointestinal (GI) bleeding events and other major bleeding events [15, 16]. GI bleeding often leads to treatment discontinuation, which may, in turn, result in increased risks of thromboembolism and mortality [17, 18].

Elderly patients are commonly prescribed proton pump inhibitors (PPIs) for several acid-related conditions, such as gastroesophageal reflux disease [19] and nonsteroidal anti-inflammatory drug-induced GI adverse events [20]. Thus, it is expected that many elderly NVAF patients are receiving PPIs. A recent retrospective analysis study that included >1.6 million patients treated with anticoagulants suggested that concomitant use of PPIs would reduce the risk of GI bleeding [21]. Conversely, associations between PPIs and serious adverse events, as well as increased risk of mortality, have been previously reported [22, 23]. A recent retrospective analysis of a large primary cohort (from the Department of Veterans Affairs databases) followed for over 5 years reported that PPI users, particularly those without GI conditions, had an excess risk of death, which increased with prolonged PPI use [22].

The real-world status of PPI administration in AF patients receiving antithrombotic treatment is largely unknown. Furthermore, there is a lack of information and considerable controversy surrounding the use of PPIs and the related risk-benefit balance, particularly for elderly NVAF patients who are at high risk of bleeding. The All Nippon AF In the Elderly (ANAFIE) Registry aims to determine treatment patterns, risk factors, and outcomes among elderly Japanese NVAF patients in the real-world clinical setting [24, 25]. As this database provides an ideal opportunity to examine concomitant PPI and anticoagulant/antiplatelet use, the present subanalysis aimed to determine the actual status of PPI prescription in elderly Japanese NVAF patients.

## Materials and methods

### Study design

Details of the ANAFIE Registry study design have been published [24]. In brief, this was a prospective, multicenter, observational cohort study that included 32,726 patients who were enrolled between October 2016 and January 2018. The ANAFIE Registry was conducted in accordance with the Declaration of Helsinki, and all applicable local and national requirements for clinical studies. The study was approved by the Ethics Committees of The Cardiovascular Research Institute (Tokyo, Japan) and is registered with the University hospital Medical Information Network with the identifier UMIN000024006. Participating patients provided written informed consent and could withdraw from the registry at any time.

### Patients

Detailed inclusion/exclusion criteria for the ANAFIE Registry are available elsewhere [24] and baseline patient data were recently published [25]. Key enrollment criteria included age ≥75 years, ambulatory, NVAF diagnosis (by electrocardiogram), and the ability to visit the study site for specified visits. Exclusion criteria included a definitive diagnosis of mitral stenosis, mechanical or bioprosthetic valve replacement, a recent history of stroke, myocardial infarction, cardiac intervention, or heart failure, or <1 year of life expectancy.

### Measures

For the present analyses, patients were stratified according to PPI use (PPI+) or no PPI use (PPI−). Evaluations included presence/absence of GI disease, risk scores for stroke (CHADS_2_, CHA_2_DS_2_-VASc) and bleeding (HAS-BLED), anticoagulant use, time in therapeutic range (TTR) for warfarin, and anticoagulant/antiplatelet combination use, all according to PPI use.

### Statistical methods

Data are presented as mean ± standard deviation or percentage (%). For categorical variables, frequency tables were created, and *P* values were calculated using the chi-squared test. For continuous variables, summary statistics were produced, and *P* values were calculated using a two-sample t-test. No imputations were made for missing data, which were not included in the analyses. A two-sided *P* value <0.05 was considered to indicate statistical significance. All statistical analyses were performed using SAS version 9.4 (SAS Institute, Tokyo, Japan).

## Results

### Patients

Of the total ANAFIE population of 32,726 patients, 32,490 were included in the present analyses; 236 (0.7%) patients were excluded as their PPI status was unknown. Table 1 describes the patient background characteristics. Of the 32,490 patients evaluated, 11,981 (36.9%) were prescribed PPIs. The proportion of female patients was statistically significantly higher in the PPI+ group (45.2%) compared with that in the PPI− group (41.3%). Statistically significant differences were noted in the mean age and weight between the PPI+ group and PPI− group. The PPI+ group had statistically significantly higher CHADS_2_, CHA_2_DS_2_-VASc, and HAS-BLED scores than the PPI− group. Additionally, the prevalences of comorbidities were statistically significantly higher in the PPI+ group compared with the PPI− group (Table 1).

**Table 1:** Patient background and clinical characteristics according to PPI use.

	Total population^a^	PPI+ patients	PPI− patients	*P* value^b^
	N = 32,490	n = 11,981	n = 20,509	
Age, years	81.5 ± 4.8	81.7 ± 4.9	81.3 ± 4.8	<0.0001
Female	13,889 (42.7)	5420 (45.2)	8469 (41.3)	<0.0001
Height, cm	157.2 ± 9.5	156.6 ± 9.6	157.6 ± 9.4	<0.0001
Weight, kg	57.8 ± 11.2	57.3 ± 11.2	58.1 ± 11.1	<0.0001
BMI, kg/m^2^	23.3 ± 3.6	23.3 ± 3.6	23.3 ± 3.5	0.7499
SBP, mmHg	127.3 ± 17.0	126.9 ± 17.4	127.6 ± 16.8	0.0003
DBP, mmHg	70.6 ± 11.6	70.2 ± 11.8	70.9 ± 11.5	<0.0001
CCr, mL/min^c^	48.4 ± 21.8	46.3 ± 25.3	49.8 ± 19.1	<0.0001
CHADS_2_ score	2.9 ± 1.2	3.0 ± 1.2	2.8 ± 1.2	<0.0001
CHA_2_DS_2_-VASc score	4.5 ± 1.4	4.7 ± 1.4	4.3 ± 1.4	<0.0001
HAS-BLED score	1.9 ± 0.9	2.0 ± 0.9	1.8 ± 0.8	<0.0001
Comorbidities				
Hypertension	24,475 (75.3)	9340 (78.0)	15,135 (73.8)	<0.0001
Dyslipidemia	13,815 (42.5)	5827 (48.6)	7988 (38.9)	<0.0001
Heart failure	12,188 (37.5)	4946 (41.3)	7242 (35.3)	<0.0001
Coronary artery disease (myocardial infarction + angina)	6751 (20.8)	3288 (27.4)	3463 (16.9)	<0.0001
GI disorders	9524 (29.3)	5440 (45.4)	4084 (19.9)	<0.0001
Diabetes	8750 (26.9)	3484 (29.1)	5266 (25.7)	<0.0001
Cerebrovascular disease	7357 (22.6)	3125 (26.1)	4232 (20.6)	<0.0001
Hyperuricemia	7378 (22.7)	3009 (25.1)	4369 (21.3)	<0.0001
Chronic kidney disease	6758 (20.8)	2980 (24.9)	3778 (18.4)	<0.0001
Respiratory disease	4164 (12.8)	1798 (15.0)	2366 (11.5)	<0.0001
Cancer	3559 (11.0)	1363 (11.4)	2196 (10.7)	0.0625
Thromboembolic disease	2781 (8.6)	1225 (10.2)	1556 (7.6)	<0.0001
Dementia	2553 (7.9)	986 (8.2)	1567 (7.6)	0.0569
Fall within the past year	2369 (7.3)	999 (8.3)	1370 (6.7)	<0.0001

Data are shown as mean ± standard deviation or n (%).

BMI, body mass index; CCr, creatinine clearance; DBP, diastolic blood pressure; GI, gastrointestinal; PPI, proton pump inhibitor; SBP, systolic blood pressure

^a^Excludes patients with unknown PPI use (n = 236, 0.7%).

^b^Comparison of PPI+ vs. PPI−.

^c^Creatinine clearance was calculated using the Cockcroft-Gault formula: Ccr (mL/min) = (140 − age) × body weight (kg) / (72 × serum creatinine [mg/dL]) for males, and Ccr (mL/min) = [male Ccr] × 0.85 for females.

In the PPI+ group, the proportion of patients with GI disorders was 45.4% (n = 6541) (Table 2). The most prevalent GI disorder was reflux esophagitis in the PPI+ group.

**Table 2:** PPI use by presence or absence of GI disorder.

	Total population	PPI+ patients	PPI− patients	*P*-value^a^
	N = 32,490	n = 11,981	n = 20,509	
Presence of GI disorders				
Yes	9524 (29.3)	5440 (45.4)	4084 (19.9)	<0.0001
Type of disorder				
Reflux esophagitis	5119 (15.8)	3841 (32.1)	1278 (6.2)	<0.0001
Others	4421 (13.6)	1938 (16.2)	2483 (12.1)	<0.0001

Data are shown as n (%).

GI, gastrointestinal; PPI, proton pump inhibitor.

^a^PPI+ vs PPI−.

### PPI use and risk scores for stroke and bleeding

**Fig 1:**
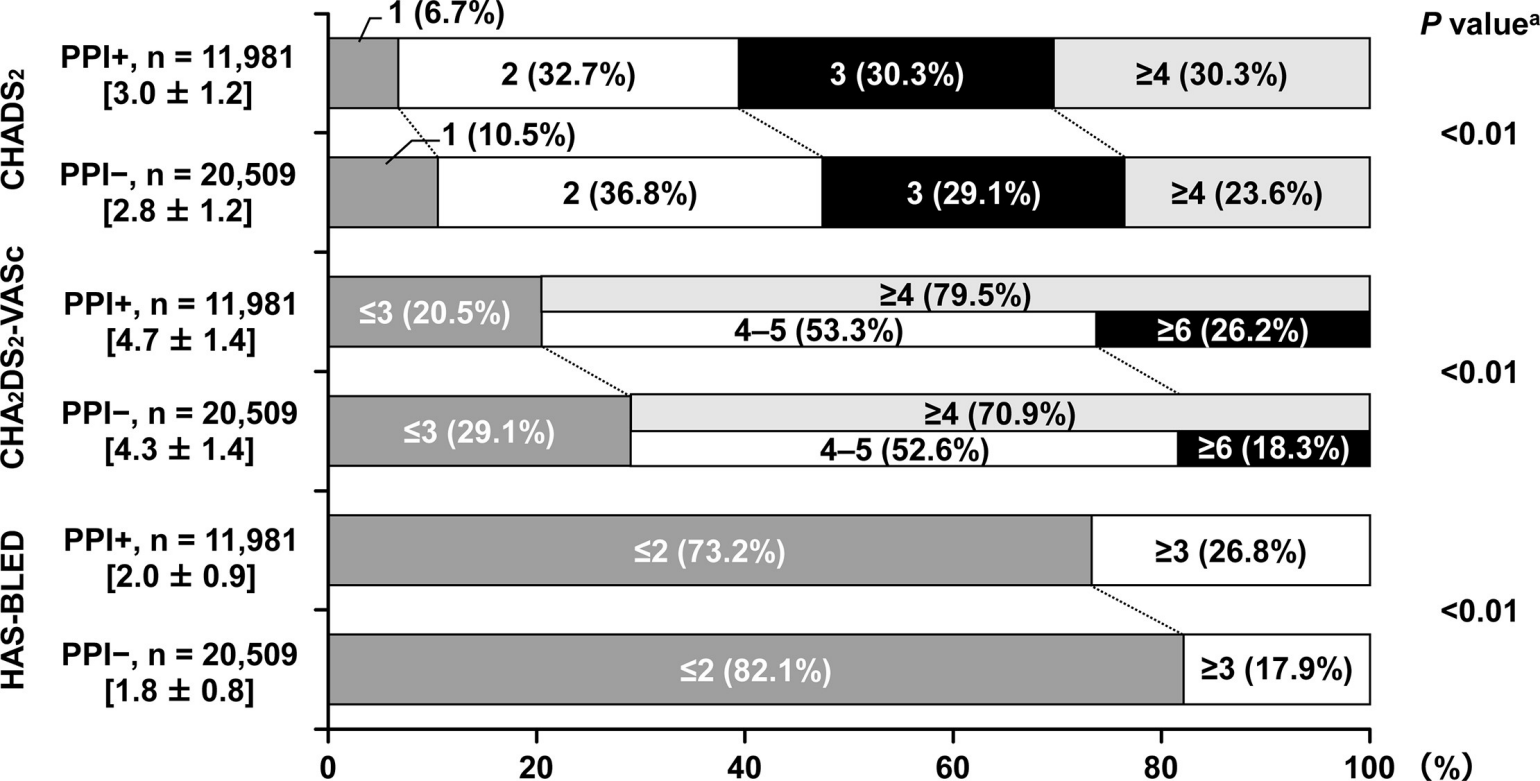
PPI use and risk scores. Data are shown as n (%) or mean ± standard deviation. ^a^ PPI+ vs PPI−. PPI, proton pump inhibitor.

For each risk scale (CHADS_2_, CHA_2_DS_2_-VASc, and HAS-BLED), scores were statistically significantly higher in the PPI+ group than in the PPI− group (*P* <0.01 for each; Fig 1).

### PPI use and anticoagulants

**Fig 2:**
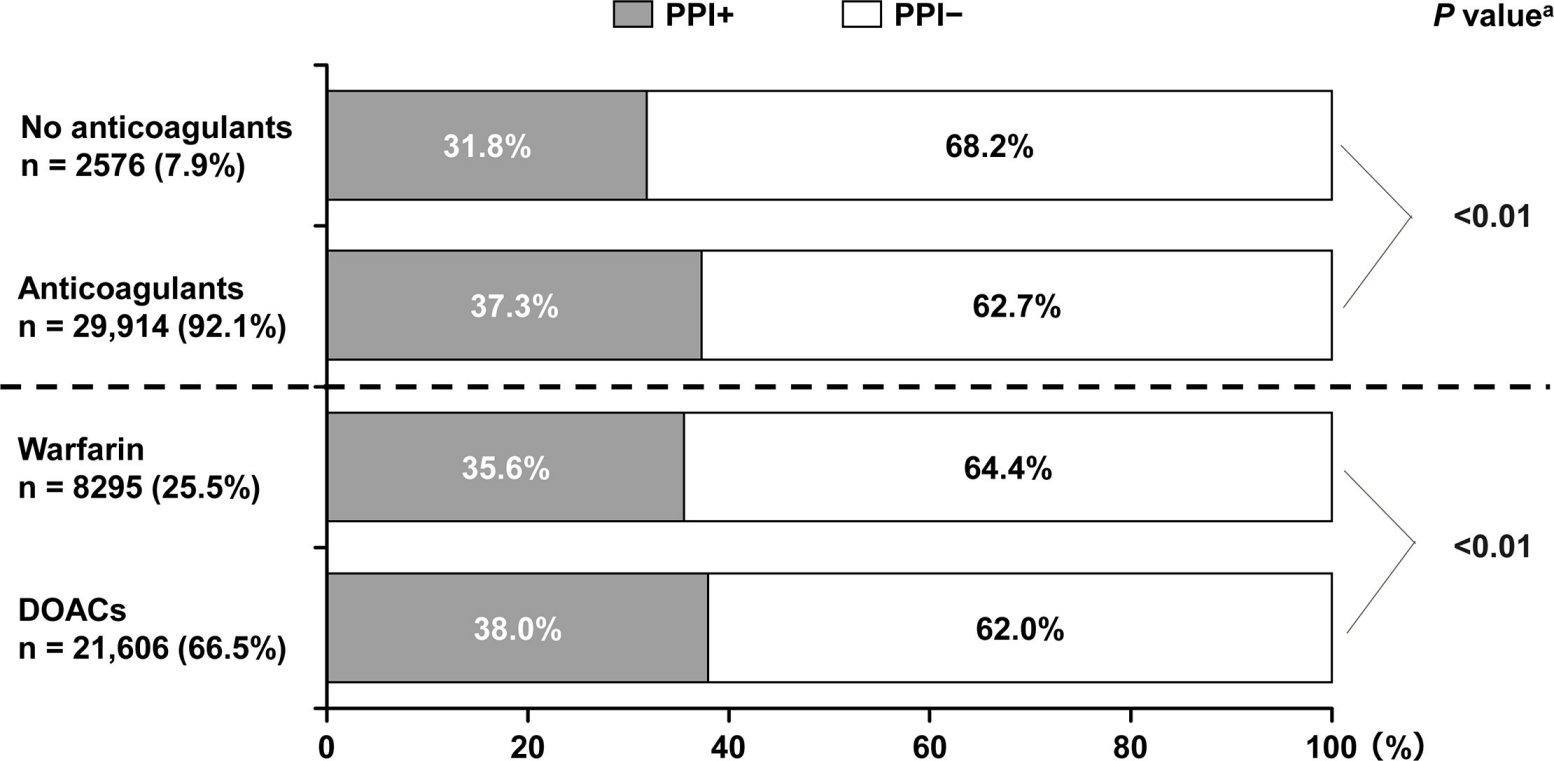
PPI use by type of anticoagulant. ^a^Difference in the proportion of PPI+ between the no anticoagulant group and anticoagulant group, and between warfarin and DOACs. DOAC, direct oral anticoagulant; PPI, proton pump inhibitor.

Of patients treated with anticoagulants, a statistically significantly higher proportion of patients received PPIs compared with patients not receiving anticoagulants (Fig 2). This remained the case for both DOACs and warfarin. Patients receiving DOACs had a significantly higher prescription rate of PPIs than those receiving warfarin.

Among the 8206 patients who were receiving DOACs and PPIs, 906 (11.0%) were receiving a factor IIa inhibitor (i.e., dabigatran) and 7300 (89.0%) were receiving a factor Xa inhibitor (i.e., apixaban, rivaroxaban, or edoxaban). Among the 13,400 patients who were receiving DOACs but not receiving PPIs, 1433 (10.7%) were receiving factor IIa inhibitor (i.e., dabigatran) and 11,967 (89.3%) were receiving a factor Xa inhibitor (i.e., apixaban, rivaroxaban, or edoxaban).

**Fig 3:**
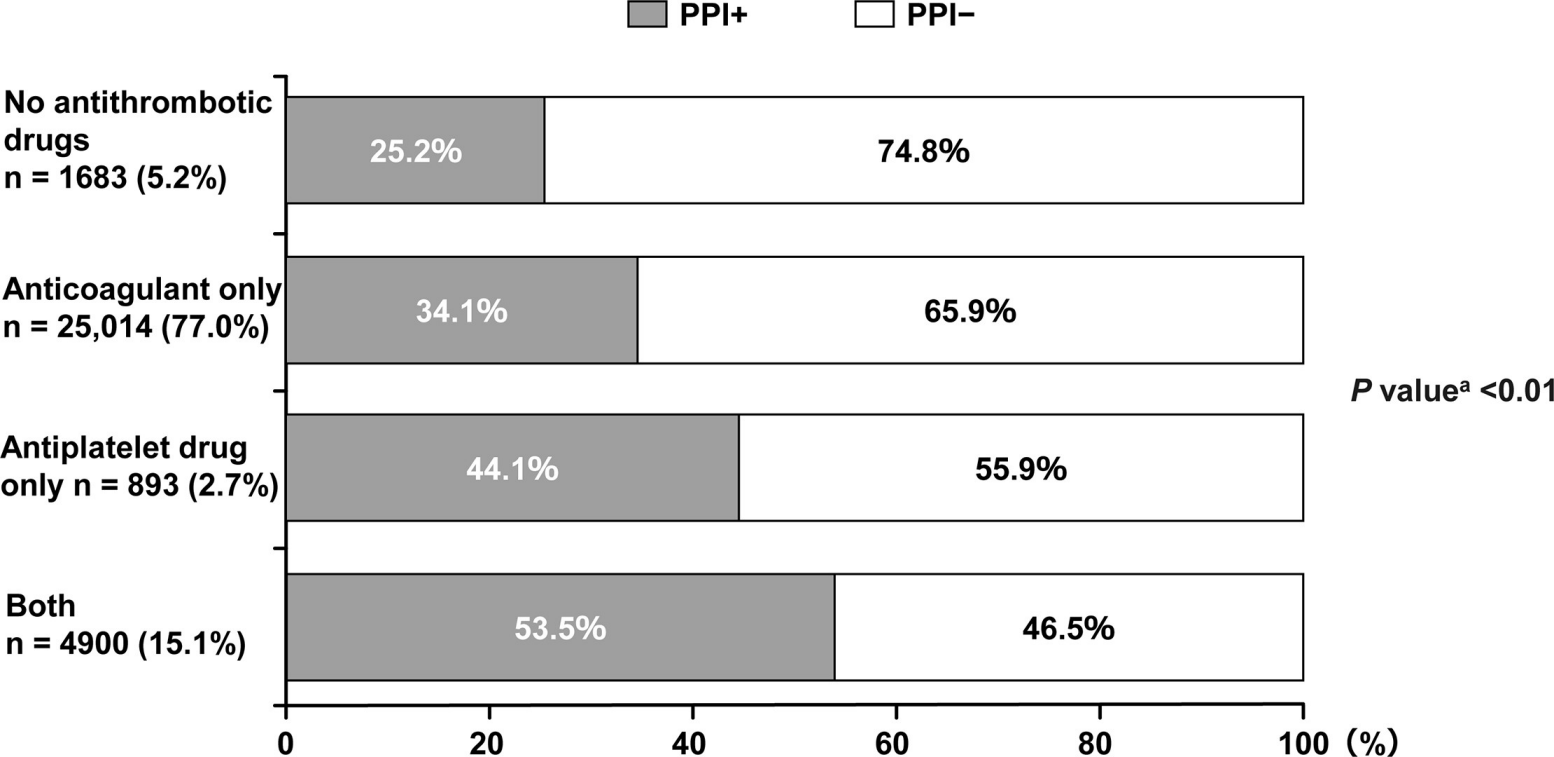
PPI use and presence/absence of anticoagulant/antiplatelet combination. ^a^Difference in the proportion of PPI+ use among the four groups. PPI, proton pump inhibitor.

Regarding the use of antithrombotic drugs, 77.0% of patients were using anticoagulants only, 15.1% were using both anticoagulants and antiplatelet drugs, 2.7% were using antiplatelet drugs only, and 5.2% were not using antithrombotic drugs. The main reasons for not using antithrombotic drugs in 5.2% of patients were older age, renal functional decline, and high HAS-BLED score. Notably, the rate of PPI use was significantly different by pattern of antithrombotic agent use. PPIs were more frequently used among patients receiving antiplatelet drugs only (44.1%) compared with those receiving anticoagulants only (34.1%). Additionally, more than half of the patients who used both anticoagulants and antiplatelet drugs received PPIs (53.5%). (Fig 3).

## Discussion

Elderly NVAF patients are not only at an increased risk of stroke but also at an increased risk of bleeding [3, 10, 11]; thus, the benefits of anticoagulation must be weighed against the potential for bleeding events, including GI bleeding [15, 16]. PPIs have been shown to reduce GI bleeding in patients receiving antiplatelet therapy [26], and a recent study suggested that PPIs may also reduce the GI bleeding risk in patients receiving anticoagulants [19]. However, other studies have found that long-term use of PPIs may be associated with increased mortality [22, 23]. Therefore, great uncertainty remains regarding the benefit–risk balance of the use of PPIs, particularly for elderly patients with NVAF receiving anticoagulant therapy. This subanalysis of the ANAFIE Registry determined the real-world clinical use of PPIs in patients with NVAF.

The key findings of the present subanalysis were as follows. There was a higher proportion of female NVAF patients in the PPI+ group compared with the PPI− group. The PPI+ group included more patients with coronary artery disease (myocardial infarction + angina), cerebrovascular disease, and chronic kidney disease, and significantly higher CHADS_2_, CHA_2_DS_2_-VASc, and HAS-BLED scores compared with the PPI− group. Over 54% of patients receiving PPIs did not have GI disorders. No clear differences were observed in the rate of PPI use by type of anticoagulant used. PPIs tended to be prescribed more frequently among those treated with DOACs than those treated with warfarin. The rate of PPI use varied according to the use of antithrombotic drugs, with the highest rates among patients using both anticoagulants and antiplatelet drugs (53.5%).

As stated above, it is noteworthy that fewer than half of those receiving PPIs had evidence of GI disorders. A possible explanation for this finding is that the PPI+ group had a high prevalence of coronary artery disease (myocardial infarction + angina pectoris) and cerebrovascular disease, and thus, a high proportion of patients were being treated with antiplatelet drugs. Another interesting finding is that the rate of PPI use increased even with antiplatelet drugs alone and was the highest among patients receiving both antiplatelet drugs and anticoagulants. From these findings, it can be inferred that cardiovascular physicians tend to use PPIs as a preventative measure to reduce the risk of GI bleeding, even in cases where there is no GI disorder.

It has long been known that PPIs can reduce the risk of GI bleeding [27]; as such, PPIs are now considered for prescription to a wide range of patients for various conditions requiring bleeding prevention [28–31]. Nevertheless, it is important to note that the long-term use of PPIs may be associated with an excess risk of mortality from cardiovascular disease, chronic kidney diseases, and upper GI cancer among users [22, 23], and this excess burden is reportedly greater among patients exposed to PPIs without an appropriate indication [23]. Recent reports emphasize the need for increased awareness of these potential adverse events, and physicians are encouraged to limit PPI exposure only to patients in which the benefits of this treatment clearly outweigh its risks. Additionally, it is advocated that PPIs should be prescribed for a specific treatment duration and such treatment reassessed accordingly to avoid inadequately indicated PPI prescriptions.

In patients receiving antiplatelet therapy, coadministration of PPIs can improve clinical outcomes [32, 33] and reduce health care costs [34]; similar benefits might be expected for AF patients receiving anticoagulants. However, it is recommended that patients should be properly assessed in terms of risk of PPI-related adverse events before initiating PPIs [23], and those receiving PPIs as a preventive measure should be carefully monitored.

We consider that not all patients receiving anticoagulant therapy should receive PPIs. In the future, careful assessment of NVAF patients will be necessary to identify patients who can really benefit from PPI administration. Because PPIs, particularly omeprazole, lansoprazole, and pantoprazole, are metabolized by CYP2C-19 [35], care must be taken when using PPIs concomitantly with other drugs that are metabolized by the same enzyme, such as the antiplatelet drug clopidogrel [36, 37]. When combined with warfarin [38], PPIs can affect the quality of the anticoagulation with warfarin, as indicated by a reduced TTR. In the present subanalysis, PPIs tended to be more frequently prescribed among DOAC users than those receiving warfarin, suggesting that physicians are aware of these relevant drug interactions. In addition, it is necessary to monitor changes in the gut microbiota of patients receiving long-term administration of PPIs as long-term PPI use may predispose them to *Clostridium difficile* infection [39–41]. This is relevant when considering elderly patients are already at risk of *C*. *difficile* infection because of age-related changes in their gut flora and immunological defenses [42].

Limitations associated with the overall ANAFIE Registry have already been reported; these are mainly related to the observational, registry-based design and the fact that the registry was restricted to the enrollment of Japanese patients only [24, 25]. In the present subanalysis, only baseline characteristics of patients were analyzed; detailed analyses of the follow-up data will be reported separately. Other limitations specific to the present subanalysis are that data on relevant details of PPI use, such as dose, administration period, and adherence were not collected. Thus, further analyses of PPI use in NVAF patients are warranted.

## Conclusions

The results of the present subanalysis suggest that PPIs were actively prescribed in high-risk cases and were likely to be prescribed to prevent GI bleeding events. The use of antiplatelet plus anticoagulant combinations was associated with an increase in PPI use, but no clear differences were observed among the various anticoagulant agents used.

## Supporting information

S1 Checklist TREND statement checklist. (PDF)

S1 File (DOCX)

S2 File (PDF)

S3 File (PDF)

S4 File (PDF)

S5 File (PDF)
