# Supplementary material for: Current status of proton pump inhibitor use in Japanese elderly patients with non-valvular atrial fibrillation: A subanalysis of the ANAFIE Registry
Source: PLoS One. 2020 Nov 5;15(11):e0240859. doi: 10.1371/journal.pone.0240859 (PMC7644054; doi:10.1371/journal.pone.0240859)
Supplement: S1 File — (DOCX) [file pone.0240859.s002.docx]

　CLINICAL STUDY PROTOCOL NUMBER: LIX-DS-15022

In late-stage elderly patients with non-valvular atrial fibrillation

Prospective observational study

All Nippon AF In Elderly Registry

- ANAFIE Registry-

(Company-initiated clinical research)

Clinical Study Protocol

Representative Caregivers: Hiroshi Inoue
 Toyama Hospital, Saiseikai, Toyama Prefecture

Principal Investigator: Shigeshi Yamashita
 Cardiovascular Inst.

Research Sponsor: Ryowa Fukuchi
 Daiichi Sankyo Company, Limited

Prepared 10 April 2017 (Ver 1. 21)

Table of contents

[Table of contents 1](#_Toc452725640)

[Tables 4](#_Toc452725641)

[Figures 4](#_Toc452725642)

[Appendix 4](#_Toc452725643)

Summary of studies [5](#_Toc452725644)

[List of abbreviations and definitions 9](#_Toc452725645)

[1 Background 11](#_Toc452725646)

[2 Purpose 13](#_Toc452725647)

[3 Study design 15](#_Toc452725648)

[4 Patients 15](#_Toc452725649)

[5 Inclusion criteria 15](#_Toc452725650)

[6 Exclusion criteria 16](#_Toc452725651)

[7 Informed consent 16](#_Toc452725652)

[8 Procedures for study participation and registration of subjects 17](#_Toc452725653)

[8.1 Procedures for study participation 17](#_Toc452725654)

[8.2 Contract procedure 18](#_Toc452725655)

[8.3 registration of study subjects 18](#_Toc452725656)

[9 Method 18](#_Toc452725657)

[10 Adverse event 20](#_Toc452725658)

[10.1 Definition of adverse event 20](#_Toc452725659)

[10.1.1 Adverse event 20](#_Toc452725660)

[10.1.2 Serious adverse event 20](#_Toc452725661)

[10.2 Investigation Items and Collection Procedures for Adverse Events 20](#_Toc452725662)

[10.2.1 Check items of adverse events 20](#_Toc452725663)

[10.2.2 Procedures for Response in the Event of an Adverse Event 22](#_Toc452725664)

[10.3 Responding to Serious Adverse Event 22](#_Toc452725665)

[10.3.1 Response of study collaborators, etc. 22](#_Toc452725666)

[10.3.2 Response by investigators, etc. 22](#_Toc452725667)

[10.3.3 Response of the head of the study site 23](#_Toc452725668)

[11 Observations 23](#_Toc452725669)

[11.1 Enrollment survey 23](#_Toc452725670)

[11.2 Baseline survey 24](#_Toc452725671)

[11.3 12-month and 24-month surveys 26](#_Toc452725672)

[12 Schedule of research 30](#_Toc452725673)

[13 Number of target registrations of study subjects 32](#_Toc452725674)

[14 Study Period 32](#_Toc452725675)

[15 Endpoint 33](#_Toc452725676)

[15.1 Primary endpoint 33](#_Toc452725677)

[15.2 Secondary endpoint 33](#_Toc452725678)

[15.3 Secondary endpoint 33](#_Toc452725679)

[16 Implementation of Sub-Cohort Study (Sub-Study 1) 33](#_Toc452725680)

[16.1 Sub-cohort study A: assessment markers 34](#_Toc452725681)

[16.2 Sub-cohort study B: Echocardiogram 34](#_Toc452725682)

[16.3 Sub-cohort study C: Heart Rate 35](#_Toc452725683)

[16.4 Sub-cohort study D: Hypertension 35](#_Toc452725684)

[16.5 Sub-cohort study E: cognitive function 35](#_Toc452725685)

[16.6 Sub-cohort study F: Frail 36](#_Toc452725686)

[16.7 Sub-cohort study G: adherence 36](#_Toc452725687)

[17 Statistical Analysis 37](#_Toc452725688)

[17.1 Analysis Set 37](#_Toc452725689)

[17.2 Analysis of Baseline Survey Items 37](#_Toc452725690)

[17.3 Analysis of follow-up data 37](#_Toc452725691)

[17.4 Subgroup analysis (Sub-study 2) 38](#_Toc452725692)

[17.5 Analysis of a sub-cohort study 38](#_Toc452725693)

[17.5.1 Sub-cohort study A: assessment markers 38](#_Toc452725694)

[17.5.2 Sub-cohort study B: Echocardiogram 39](#_Toc452725695)

[17.5.3 Sub-cohort study C: Heart Rate 39](#_Toc452725696)

[17.5.4 Sub-cohort study D: Hypertension 39](#_Toc452725697)

[17.5.5 Sub-cohort study E: cognitive function 39](#_Toc452725698)

[17.5.6 Sub-cohort study F: Frail 39](#_Toc452725699)

[17.5.7 Sub-cohort study G: adherence 40](#_Toc452725700)

[18 Preparation and Submission of Case Report Form 40](#_Toc452725701)

[18.1 Format and Submission of Case Report Form 40](#_Toc452725702)

[18.2 Method of creating the Case Report Form 41](#_Toc452725703)

[19 Monitoring and Auditing 41](#_Toc452725704)

[19.1 Monitoring and Auditing 41](#_Toc452725705)

[19.2 Monitoring 41](#_Toc452725706)

[19.3 Auditing 41](#_Toc452725707)

[19.4 Direct access to source documents 42](#_Toc452725708)

[20 Ethic 42](#_Toc452725709)

[20.1 Ethical Review Board 42](#_Toc452725710)

[20.2 Expected benefits to the study audience 42](#_Toc452725711)

[20.3 Potential risks and disadvantages to the study population 43](#_Toc452725712)

[20.4 Compensation for health damage 43](#_Toc452725713)

[20.5 Protection of personal information 43](#_Toc452725714)

[21 Matters concerning the cost of the study 44](#_Toc452725715)

[21.1 Research Funding and Conflicts of Interest 44](#_Toc452725716)

[21.2 Cost burden for the study subjects 44](#_Toc452725717)

[22 Amendment or amendment of the study protocol 44](#_Toc452725718)

[22.1 Amendment of the study protocol 44](#_Toc452725719)

[22.2 Amendment of the study protocol 45](#_Toc452725720)

[23 Completion, Interruption, and Termination of the Study 45](#_Toc452725721)

[23.1 End of the study 45](#_Toc452725722)

[23.2 Discontinuation/discontinuation of the study 45](#_Toc452725723)

[24 Storage of documents related to study, etc. 46](#_Toc452725724)

[25 Response to Consultations, etc. from Research Subjects, etc. and Their Relevant Persons 46](#_Toc452725725)

[26 Publication of Study and Attribution of Results 47](#_Toc452725726)

[26.1 Clinical Research Registry 47](#_Toc452725727)

[26.2 Publication of the study 47](#_Toc452725728)

[26.3 Ownership of results 47](#_Toc452725729)

[27 Research organization 47](#_Toc452725730)

[27.1 Representative caregiver 47](#_Toc452725731)

[27.2 Research Advisor 47](#_Toc452725732)

[27.3 Principal investigator 48](#_Toc452725733)

[27.4 Central Research Committee 48](#_Toc452725734)

[27.5 Research Promotion and Publication Committee 48](#_Toc452725735)

[27.6 Event Evaluation Committee Members 48](#_Toc452725736)

[27.7 Sub-investigators 48](#_Toc452725737)

[27.8 District promotion physician 49](#_Toc452725738)

[27.9 Responsible statistician 49](#_Toc452725739)

[27.10 Institute responsible for research 49](#_Toc452725740)

[27.11 Research Secretariat 49](#_Toc452725741)

[27.12 Research Sponsor 50](#_Toc452725742)

[27.13 Data center 50](#_Toc452725743)

[27.14 Monitoring agency 50](#_Toc452725744)

[27.15 Auditing organization 50](#_Toc452725745)

[28 Reference 52](#_Toc452725746)

[Appendix 54](#_Toc452725747)

Table of Contents

[表 1 Observation and examination schedule 8](#_Toc452553583)

[表 2 AF Type 24](#_Toc452553584)

[表 3 Office blood pressure measurement 26](#_Toc452553585)

[表 4 Definition of Bleeding Grouping 29](#_Toc452553586)

[表 5 Observation and examination schedule 30](#_Toc452553587)

List of figures

[図 1 Flow of the study 15](#_Toc451259731)

Appendix Table of Contents

[付録 1 CHADS_2_ Score 54](#_Toc468213245)

[付録 2 CHA_2_DS_2_-VASc Score 54](#_Toc468213246)

[付録 3 HAS-BLED score 55](#_Toc468213247)

[付録 4 P-Glycoprotein Inhibitors with Interactions with Individual DOAC 56](#_Toc468213248)

Summary of studies

| Purpose of the study | | The aim of this study was to clarify the current status and prognosis of anticoagulation in late-stage elderly patients (75 years of age or older) with non-valvular atrial fibrillation (NVAF), identify risk factors for stroke/systemic embolism and intracranial hemorrhage, and define the optimal treatment population for direct oral anticoagulation (DOAC) and its use. We will also examine a variety of secondary clinical questions related to NVAF. |
| --- | --- | --- |
| Study Design | | It is a multicenter, prospective registry, observational study, and there is no intervention in the treatment by this study.   |
| Study patients | | Late-stage elderly patients (75 years or older) with NVAF  (Regardless of whether anticoagulants are administered or not, and whether anticoagulants are used.) |
| Eligibility criteria | Inclusion criteria | 1. Patients with a definite diagnosis of NVAF who are able to attend the hospital 2. Patients aged 75 years or older at the time of informed consent (regardless of gender) 3. Written informed consent patients |
|  | Exclusion criteria | 1. Patients who are currently participating in or are scheduled to participate in an intervention trial. 2. Patients with a definite diagnosis of mitral stenosis 3. Patients with prosthetic (mechanical, bioprosthetic) valves 4. Patients who developed cardiovascular events (stroke, myocardial infarction, cardiac intervention other than myocardial infarction, heart failure requiring hospitalization) or hemorrhage with hospitalization within 1 month before enrollment. 5. Patients who are diagnosed with any disease within one year of life expectancy 6. Other patients determined by the investigator to be ineligible for participation in the study |
| Methodologies | | Prospective observational study |
| Enrollment method | | Continuous registration system |
| Follow-up data collection method | | Data from the main study will be collected in Electric data capturing system (EDCs). Data from subcohort studies will be collected from electronic medical records and other output data, EDC or paper questionnaires. |
| Examination/observation and examination items | | Patient characteristics, drug status, health status, status of concomitant medications other than anticoagulants and concomitant administration, status of non-pharmacotherapy for NVAF, implementation of invasive procedures other than NVAF therapy (including minor surgical ^※^), blood coagulation tests, clinical course and laboratory values, occurrence of cardiovascular events, occurrence of other haemorrhagic adverse events, occurrence of falls and fractures, and other adverse events  ※ Minor surgery includes procedures involving biopsy and puncture |
| Follow-up period by study subject | | For 2 years |
| Endpoint | Primary endpoint | Incidence of Stroke/Systemic Embolism during the Observation Period |
|  | Secondary endpoint | Occurrence of the following events during the observation period   1. Hemorrhagic Adverse Events (Major Bleeding) 2. Stroke 3. Systemic embolism 4. Ischemic stroke 5. Hemorrhagic stroke 6. Intracranial hemorrhage 7. Cardiovascular events (stroke, myocardial infarction, non-myocardial infarction cardiac intervention, heart failure requiring hospitalization) 8. Death due to cardiovascular disease (death due to cardiovascular causes undeniable) 9. All deaths |
|  | Other Endpoints | Falls and fractures |
| Main Analytical Methods | | Survival time analysis (COX proportional hazards regression analysis, etc.) |
| Planned Study Period (Observation Period and Patient Enrollment Period) | | Date of approval by the ethics review committee of the study site-September 2020  (Registration period: Date of approval by the ethics review committee of the study site-September 2018, follow-up period of 2 years)  However, the registration period and the scheduled period may be shortened or extended in accordance with the agreement of the principal investigator and the sponsor. |
| Planned participating medical institutions | | Approximately 1,000 sites (add as appropriate, depending on the registration status of the study subject) |

Table 11 Observation and examination schedule

The investigation items are as follows and will be conducted within the scope of daily medical care.

| Time of investigation  Survey item | Eligibility Confirm | Baseline Study  (-180-0 days) | 12 months Study  (±60 days) | 24 months Study  (±60 days) |
| --- | --- | --- | --- | --- |
| Confirmation of consent, inclusion and exclusion criteria | ○ |  |  |  |
| Withdrawal of Informed Consent, Visit Status, and Confirmation of Health |  |  | ○ | ○ |
| Patient characteristics, history of previous treatment for AF, surgical history, history of bleeding, past medical history (other than bleeding), complications, etc. |  | ○ |  |  |
| Status of anticoagulation administration |  | ○ | ○ | ○ |
| Status of administration and compliance with drugs other than anticoagulants |  | ○ | ○ | ○ |
| Status of non-pharmacologic therapy for AF |  | ○ | ○ | ○ |
| Implementation status of invasive procedures (including minor surgical ^1^) other than AF treatment |  |  | ○ | ○ |
| Clotting studies (PT-INR) |  | ○ | ○ | ○ |
| Clinical course and laboratory values |  | ○ | ○ | ○ |
| Presence or absence of cardiovascular events, gastrointestinal bleeding, bleeding adverse events, falls, fractures, deaths, or other adverse events |  |  | ○ | ○ |
| Sub-cohort study |  |  |  |  |
| A. Assessment marker |  | ○  (central measurement) |  |  |
| B. Echocardiogram |  | ○ | ○ | ○ |
| C. Heart rate |  | ○ | ○ | ○ |
| D. Hypertension |  | ○ |  |  |
| E. Cognitive functioning |  | ○ |  | ○ |
| F. Frail |  | ○ |  |  |
| G. Adherence |  | ○ |  | ○ |

AF, atrial fibrillation; PT-INR, prothrombin-international normalized ratio

1 Minor surgery includes procedures involving biopsy and puncture

List of Abbreviations and Definitions

List of abbreviations

| Abbreviation | Terms not abbreviated | |
| --- | --- | --- |
|  | English | Japanese |
| A |  | Left ventricular inflow atrial systolic peak velocity |
| AF | Atrial fibrillation | Atrial fibrillation |
| AHA | American Heart Association | American Heart Association |
| APTT | Activated partial thromboplastin time | Activated partial thromboplastin time |
| Ccr | Creatinine clearance | Creatinine clearance |
| CHADS_2_ | - | CHADS_2_ Score |
| CHA_2_DS_2_-VASc | - | CHA_2_DS_2_-VASc Score |
| COPD | Chronic obstructive pulmonary disease | Chronic obstructive pulmonary disease |
| CYP2C9 | Cytochrome P450 2C9 | Cytochrome P4502C9 |
| DOAC | Direct oral anticoagulant | Direct oral anticoagulants |
| E |  | Left ventricular inflow early peak diastolic velocity |
| eCRF | Electronic case report form | Electronic case report forms |
| EDC | Electronic data capturing | Electronic data capturing |
| F1+2 | Prothrombin fragment 1+2 | Prothrombin fragment 1+2 |
| HbA1c | Hemoglobin A1c | Hemoglobin A 1 c |
| IVCD | Inferior vena cava diameter | Inferior vena cava diameter |
| IVSTD | Interventricular septum thickness | Interventricular septal wall thickness |
| LA | Left atrium | Left atrial |
| LAD | Left atrial dimension | Left atrial dimension |
| LV | Left ventricle | Left ventricle |
| LVDd | Left ventricular end-diastolic dimension | Left ventricular end-diastolic dimension |
| LVDs | Left ventricular end-systolic dimension | Left ventricular end-systolic dimension |
| LVEDV | Left ventricular end-diastolic volume | Left ventricular end-diastolic volume |
| LVEF | Left ventricular ejection fraction | Left ventricular ejection fraction |
| LVESV | Left ventricular end-systolic volume | Left ventricular end-systolic volume |
| MMSE | Mini-Mental State Examination | Mini-mental state examination |
| MMAS-8 | Morisky Medication Adherence Scale | Morrisky Medication Adherence Scale |
| NT-proBNP | N-terminal pro B-type natriuretic peptide | N-terminal precursor fragment of brain natriuretic peptide |
| NVAF | Non-valvular atrial fibrillation | Non-valvular atrial fibrillation |
| PASP | Pulmonary artery systolic pressure | Pulmonary arterial systolic pressure |
| PDC | Proportion of days covered | Proportion of days prescribed |
| P-gp | P-glycoprotein | P- glycoprotein |
| PT | Prothrombin time | Prothrombin time |
| PT-INR | Prothrombin time-international normalized ratio | Prothrombin time-international normalized ratio |
| RV | Right ventricle | Right ventricle |
| SFMC | Soluble fibrinmonomer complex | Soluble fibrin-monomer complex |
| SRI | Severe renal impairment | Severe renal impairment |
| SV | Stroke volume | Stroke volume |
| TAPSE | Tricuspid annual plane systolic excursion | Tricuspid annular plane systolic travel distance |
| TAT | Thrombin-antithrombin complex | Thrombin-antithrombin iii complex |
| TDI | Tissue doppler imaging | Tissue Doppler imaging |
| TDIa' | - | Mitral annular atrial systolic velocity |
| TDIe' | - | Early mitral annular dilatation velocity |
| TIA | Transient ischemic attacks | Transient ischemic attack |
| TRPG | Tricuspid regurgitation pressure gradient | Tricuspid systolic pressure gradient |
| TTR | Time in therapeutic range | International Normalized Ratio Time Within Optimal Range |
| TVS | Tricuspid valve systolic speed | Tricuspid annular systolic velocity |
| t_1/2_ | - | Elimination half-life |
| UMIN | University hospital medical information network | University hospital Medical Information Network |
| VKORC1 | Vitamin K epoxide reductase complex, subunit 1 | Vitamin K epoxide reductase |

List of definitions

| Term | Definition |
| --- | --- |
| Scheduled Period of Implementation | Time to study subject enrollment and completion of the 2-year observation by all study subjects |
| Case registration period | After study subject enrollment until the baseline survey is completed |
| Principal investigator | A person who is a research representative who oversees research at joint research institutes in the Guidelines for Ethical Guidelines on Medical Research for Humans (partially revised on March 31, 2015) |
| Investigator | Principal investigator at the participating institution |
| Investigators, etc. | Principal investigators and physicians in charge at the participating institutions |
| Volunteers in a study | Clinical research coordinators (nurses, pharmacists, etc.) appointed by the principal investigator |
| Patient Registration Identification Number | A number that identifies the study-specific subject replaced by the site patient chart number. |
| Contract research organization | Companies entrusting all or part of the work related to the preparation or management of such research |

※ Terms not specified in this study plan shall conform to the Ethical Guidelines for Medical Research Involving Human Subjects.

# Background of the study

The prevalence of atrial fibrillation (AF) is known to increase with aging. The prevalence of age-specific AF in ^1^ in the United States in ATRIA study was 5.0% or less in men and 3.4% or less in women younger than 75 years, compared with 7.3% in men and 5.0% in women aged 75 to 80 years, 10.6% in men and 8.0% in women older than 80 years, particularly in those older than 75 years. In Japan, the ^2^ is estimated to be 3.4% for men and 1.1% for women in their 70s, 4.4% for men and 2.2% for women in their 80s or older. As the population ages, the number of NVAF patients is increasing, and the incidence of stroke in patients with NVAF is reported to be approximately 6% per year, about five times higher than in patients with sinus rhythm, ^3^.^123^

Cardiogenic cerebral embolism, in which NVAF is a major risk factor, is a disease type with easy seriousness and poor prognosis even in the cerebral apoplexy. Therefore, it is important to prevent embolism by anticoagulation in the daily medical care of AF patients. On the other hand, it has been reported that the older age of patients with AF is the risk of embolism, and that the risk of developing cardiogenic cerebral embolism is higher in patients 75 years of age or older. However, there are concerns that the risk of developing hemorrhagic adverse events is also higher ^4^, and the risk of developing anticoagulant therapy is somewhat ^5^.^4^

Warfarin has been used as an anticoagulant therapy for many years since its introduction in Japan in 1962. In J-RHYTHM Registry, a large Japanese study of AF patients, warfarin was used by many of the low-risk subjects, and the incidence of cerebral infarction was ^7^ low. On the other hand, bleeding complications such as intracranial hemorrhage and digestive tract hemorrhage are the most feared under the warfarin dose. In addition, there are many ^9^ factors that lead to a decrease in patients' QOL, such as the fact that warfarin is often treated with concomitant medication with caution due to drug interactions, ^8^, and the fact that ingestion of vitamin K-containing foods by an action mechanism that inhibits biosynthesis of vitamin K-dependent coagulation factors reduces the anticoagulant effect of warfarin, which requires dietary restrictions on vitamin K-containing foods.^6789^

The target molecules of warfarin are vitamin K epoxide reductase (VKORC1), which is involved in the generation of vitamin K-dependent coagulation factors, and the main metabolizing enzyme of S-warfarin, which exhibits pharmacological action, is cytochrome P4502C9(CYP2C9, but there are ^10^ genetic polymorphisms in these enzymes. That is to say, the individual difference is big for drug efficacy expression and metabolism potency of the warfarin, and it also fluctuates by the interaction with drug and food in the individual. Therefore, in order to obtain adequate safety-conscious efficacy, the ^8,11^ requires that the prothrombin time-to-international standard ratio (PT-INR) be monitored regularly to determine and adjust the dosage.^10811^

To resolve these problems with warfarin administration, the direct oral anticoagulant (DOAC) dabigatran ^12^, the factor Xa inhibitor rivaroxaban ^13^, apixaban ^14^, and edoxaban ^15^ were available in clinical practice in the 2010s. The benefits of these DOAC compared with warfarin include the ^16^ that it does not require periodic testing for response assessment or dose adjustments for each patient, that it has a certain anticoagulant effect, that it acts directly on coagulation factors, that it has a low incidence of intracranial hemorrhage, that drugs are not directly related to vitamin K metabolism, that diet has little effect, that it interacts with other drugs, and that it has a short half-life. In particular, the short half-life of a single dose of these agents, ^8^ 55-133 hours of warfarin and ^16,17^ 10-14 hours of factor Xa inhibitors, facilitated pharmacokinetic control. However, these DOAC have the disadvantages that they are inexperienced and cannot be administered to patients with severe renal impairment (creatinine clearance (Ccr) less than 15 mL/min), have a short half-life and are quickly reduced by forgetting to take them, have not sufficiently established measures to deal with serious bleeding adverse events, and have the possibility of increasing the cost of patients. ^18^, more data based on the experience needs to be accumulated for more appropriate use. The most recent "Guideline for the Treatment of Atrial Fibrillation (Drugs)" (Revised 2013) ^18^ is also difficult to determine the degree of recommendation based on the results of large clinical trials conducted as part of a clinical development trial and on information obtained up to December 2013. It is therefore desirable to collect information on the use of each drug as soon as possible.^1213^14^1516816171818^

In NVAF, because the risk of cerebral infarction increases when the risk of cerebral infarction is accumulated, CHADS score and CHA _2_ DS _2_-VASc score are advocated as risk assessment, and both are used in decision of the anticoagulation therapy for NVAF. The CHADS score is a score named by the acronym Congestive heart failure (heart failure), Hypertension (hypertension), Age >=75 (age 75 years or older), Diabetics (diabetes mellitus), Stroke/TIA (history of cerebral infarction or transient ischemic attack (TIA)), and the previous four items are scored as 1, and the previous cerebral infarction or TIA is scored as 2, and the risk is evaluated by the sum score of each item (0-6). The CHA_2_ DS_2_-VASc score is a score named by the acronym Congestive heart failure (heart failure), Hypertension (hypertension), Age >=75 (age 75 years or older), Diabetics (diabetes mellitus), Stroke/TIA (previous cerebral infarction, TIA, thromboembolism), Vascular Disease (vascular disease), Age 65-74 (age 65-74 years), and Sex category (sex (women)). The score is defined as age 75 years or older, 2 points each for previous cerebral infarction, TIA, and thromboembolism, and 1 point each for other items, and the risk is evaluated by the sum score (0-9 points) of each item. In ^18^ the DOAC is recommended or considered for other risks (cardiomyopathy, age 65 to 74 years, vascular disease) even if these scores are at least 1 or 0.^18^

However, in advancing anticoagulation, it is important to evaluate the hemorrhagic risk in conjunction with the prevention of bleeding events. The ^19^ in which HAS-BLED scores that allow relatively simple and clinically significant bleeding events to be evaluated were proposed as scores for predicting bleeding from various bleeding risk factors. HAS-BLED scores were adopted in the guidelines by the European Society of Cardiology in 2010 and also in Japan. HAS-BLED risk factors are as follows: hypertension, renal dysfunction, hepatic dysfunction, stroke, history of bleeding/bleeding tendency, unstable INR, elderly patients (65 years or older), drugs (antiplatelet drugs or NSAIDs), or alcoholism, if any, each score is 1. The total score is the ^18^ to evaluate the risk of bleeding as low risk (0 points), moderate risk (1-2 points), and high risk (3 points or more). However, the HAS-BLED scores assume warfarin therapy such as poor control of PT-INR, and it is currently not sufficiently verified when applied to DOAC. On the other hand, it has been pointed out that the major bleeding-related factors during anticoagulant therapy are elderly aged 75 years or older, low body weight of 50 kg or less, renal dysfunction, and concomitant use of antiplatelet drugs, and intracranial bleeding factors are age, history of cerebral infarction and TIAs, aspirin use, warfarin administration, and non-white ^20^. These risk factors must also be closely monitored during NVAF anticoagulation.^191820^

Especially in the elderly, the manifestation of illness and response to treatment differ from that in the younger ^21^, and there are many points to consider, such as the complications of multiple diseases due to aging, concomitant use of multiple drugs, changes in living functions, and diversity of living environments. The ^22^ of the Guideline for Clinical Evaluation of Drugs Used in the Elderly (New Drug Notification No. 104 dated December 2, 1993) recommends that more appropriate information should be provided for pharmacotherapy in the elderly, because the elderly often have reduced physiological functions such as renal function due to senescence, accompanying multiple organ diseases, and the possibility of interaction due to drug coadministration is increased. In addition, the Japanese Geriatrics Association's "Guidelines for Safe Pharmacotherapy for the Elderly 2015" published in 2015 was also prepared for the purpose of enhancing the safety of pharmacotherapy for the elderly due to the high incidence of adverse drug events and the high number of severe cases in the elderly. From these guideline enactment, it is clear that the demand of safety and effective medical treatment for the elderly is increasing in the old people, especially in the modern Japanese society in which the old people over 75-year-old are increasing.^212223^

Against this background, this study was designed to target late-stage elderly patients with NVAF and to inform the definition of therapeutic issues and optimal anticoagulation. And, the clinical question from the clinician who actually treats the NVAF was noticed, and assessment markers, heart rate control, cardiac insufficiency, recognition function, blood pressure control, frail, adherence, etc. were multilaterally evaluated. These investigations and evaluations are expected to lead to the creation of information that contributes to the total care of patients with NVAF.

# Purpose of the study

The aim of this study was to clarify the current status and prognosis of anticoagulation in late-stage elderly patients (75 years of age or older) with non-valvular atrial fibrillation (NVAF), identify risk factors for stroke/systemic embolism and intracranial hemorrhage, and define the optimal treatment population for DOAC and its use. We will also examine a variety of secondary clinical questions related to NVAF.

# Study Design

It is a multicenter, prospective registry, observational study, and there is no intervention in the treatment by this study.

7 Sub-cohort studies (sites participating in sub-cohort studies)

Elderly people 75 year old and over

Patients with NVAF

Overall target: 30,000 cases

**Observations periods**

Obtaining consent

Qualification

Case Registration

Bases

Line

12 months

24 months

Follow-up 2 years: Investigation of anticoagulation under daily medical care, etc.

[Each study site].

Figure 11 Flow of the study

# Study patients

Late-stage elderly patients (75 years or older) with NVAF
(Regardless of whether anticoagulants are administered or not, and whether anticoagulants are used.)

# Inclusion criteria

1. Patients with a definite diagnosis of NVAF who are able to attend the hospital. In addition, when newly diagnosed as NVAF, electrocardiograms, Holter electrocardiograms, portable electrocardiograms, echocardiography, etc. are performed to confirm the diagnosis.
2. Patients aged 75 years or older at the time of informed consent (regardless of gender)
3. Written informed consent patients

Rationale

1. Considering the observation period of 2 years, patients who were able to attend the hospital at the time of enrollment were included.
2. As described in section 2 "Background of the Study", patients aged 75 years or older at the time of enrollment were included in the study because this study was designed to consider appropriate treatment for late-stage elderly patients with NVAF.2
3. It was established in compliance with the Ethical Guidelines for Medical Research Involving Human Subjects ^24^.^24^

# Exclusion criteria

1. Patients who are currently or are scheduled to participate in an intervention trial
2. Patients with a definite diagnosis of mitral stenosis
3. Patients with prosthetic (mechanical, bioprosthetic) valves
4. Patients who developed cardiovascular events (stroke, myocardial infarction, cardiac intervention other than myocardial infarction, heart failure requiring hospitalization) or bleeding with hospitalization within 1 month before enrollment.
5. Patients who are diagnosed with any disease within one year of life expectancy
6. Other patients determined by the investigator to be ineligible for participation in the study

Rationale
(1) This was set for an observational study of therapeutic in general practice.
(2) ,(3) It was set for the NVAF of observational diseases.
(4) ,(5) Considering the impact on the primary endpoint, the study population was set to ensure that the observation period could be completed.

# Informed Consent

Prior to the start of the study, the investigator will obtain the approval of the ethics review committee and the permission of the director of the research institution for the informed consent form and consent form for participation in the study. When revising the informed consent form and the consent form, the revised informed consent form and the consent form shall also be approved by the ethical review committee and approved by the head of the research institution. In addition, the subjects of this study are the late elderly, and if it is difficult to obtain consent from the research subject, due to cognitive decline, etc., consent from the proxy consenter may be obtained. However, the proxy consenter shall be limited to the person who exercises the parental rights of the test subject, the spouse, the guardian, or other persons similar to the same.

<<Explanatory>>

The matters to be explained to the study subjects or proxy consenters, etc. when obtaining informed consent shall be as follows:

1. The name of the clinical study and the fact that the head of the research institution has obtained permission for the conduct of the study;
2. Name of the research institution and the name of the principal investigator (including the name of the research institution participating in the clinical study and the name of the principal investigator)
3. Objectives and Significance of the Clinical Study
4. Method and duration of this clinical study (including the purpose of using the specimens and information obtained from the research subjects)
5. Reasons for selection as study subjects
6. The burden and anticipated risks and benefits incurred by the study subjects.
7. The fact that, even if the clinical trial has agreed to be conducted or continued, it may be withdrawn from the clinical trial at any time (when it becomes difficult to take actions according to the details of withdrawal from the research subject, etc., the fact and the reason therefor)
8. That the research subject, etc. does not receive disadvantageous treatment by not agreeing to conduct or continue the clinical research or withdrawing the consent;
9. Method of disclosure of information on this clinical study
10. The fact that, upon the request of the research subject, the research protocol and research methods can be obtained or accessed within the scope that does not interfere with the protection of personal information, etc. of other research subjects, etc. and the assurance of the originality of the research, and the method of obtaining or accessing the protocol and research methods.
11. Handling of personal information, etc. (including methods when anonymized)
12. Method of storage and disposal of samples and information
13. Conflicts of interest related to research at research institutions, such as sources of funding for this clinical research, personal profits, and conflicts of interest related to research by researchers, etc.
14. Response to Consultations, etc. from Research Subjects, etc. and Their Relevant Persons
15. Where there is financial burden or honoraria to study subjects, etc., the fact and the details thereof.
16. The possibility of using the information obtained from a study subject for future research or, if it is possible to provide the information to another research institution that is not identified at the time of obtaining consent from the research subject, the details assumed at the time of obtaining consent to that effect.

# Procedures for Study Participation and Registration of Subjects

## Procedures for Research Participation

When participating in this study, the principal investigator at each study site shall affiliate with the "Agreement on the Clinical Study Protocol" and enter the necessary information such as the name of the study site and submit it to the Research Secretariat. The participation in each subcohort study (16. Conduct of subcohort study) should also be specified (participation in subcohort study is optional).16

After deciding to participate in this study, the ethics review committee will be requested to review the clinical study protocol, the clinical study protocol (Appendix version) of the participating subcohort study, and the informed consent form including the content of the subcohort study, and obtain approval.

## Contract Procedure

After approval by the Ethics Review Committee at each study site, a contract will be concluded between the CRO, which entrusts research activities, and each study site.

## Registration of study subjects

Registration of the study subjects will be performed on the EDC system of this study. The investigator or sub-investigator will obtain written informed consent from NVAF patient who may be eligible for the study, promptly access the registration system, and enter all of the following information:
　　・ Sex
　　・ Date of birth or age at the time of obtaining informed consent
　　・ Date of consent

The registered information will be promptly issued from the system, only if the eligibility criteria for this study are met (details will be in accordance with the EDC System Procedures).

# Methodologies

1. Application for Account to EDC System

The investigator will appoint a Research Doctor and Research Coordinator (e.g., Clinical Research Coordinator) and submit an application for an EDC system account for the study after the contract is concluded. After issuing accounts and passwords, the Study Doctor and Research Collaborator will log in to the EDC system and set the user name. Investigators and research collaborators should, as far as possible, be trained in operating procedures prior to the start of the study.

1. Written informed consent obtained from patients, etc.

During the conduct of this study, investigators and others will inform patients of patients who meet eligibility criteria using informed consent forms according to the present study and the sub-cohort study of the study in which they participate, and obtain written informed consent. If the patient is incapacitated, the consent of the legally acceptable representative is acceptable (see "7. Informed Consent" for details).7

1. Registration of study subjects

After obtaining written informed consent from the patients eligible for the study, the case registration system will be used to promptly enroll the patients. At the same time, investigators and others will create a corresponding table "Patient Identification Number Management Table" for patient chart number and patient registration number for the purpose of anonymisation of patient data in a linkable fashion so that individuals cannot be identified. In addition, strictly store it in a locked place within the study site.

1. Investigation of Observations

This study creates a form on the EDC system that enters survey information on the following observations:

- Baseline Survey: Data on the subject's background at entry (hereinafter, at baseline) (most recent data within 180 days of the date of informed consent)
- 12-Month Survey: The most recent data at 12 months after the date of informed consent (the most recent data at 12 months after baseline is preferred (tolerance: 1 year after the date of informed consent ± 60 days))
- 24-Month Survey: Most recent data at 24 months after the date of informed consent (most recent data at 24 months after baseline is preferred (tolerance: 2 years after the date of informed consent ± 60 days))

Investigators and research collaborators shall enter the data into the EDC system at each of the above time points for all registered research subjects and submit (transmit) the eCRF by electronic signature of the investigators etc. For data obtained in writing, such as output data from electronic medical records, patient questionnaires, etc. in subcohort studies, the Study Secretariat will register the data in the database.

1. Confirmation of health (including confirmation of occurrence of events and adverse events)

The investigator or sub-investigator will confirm the patient's well-being and the occurrence of events or adverse events as far as possible at 12 and 24 months after enrollment. Study subjects who do not have visits will be contacted by telephone, letter, or other means to search for event and adverse event information whenever possible. In addition, when transferring the patient to another department or another medical institution, the physician in charge of therapeutic at the transferring patient will listen to events, adverse events, and other necessary information as much as possible and enter this information into the EDC system.

1. Query correspondence

If the data center issues a query requiring checks, additions, or amendments to the EDC system input, the investigator or study collaborator shall confirm the EDC system data, then add and amend it accordingly, and submit the eCRF (transmit) after electronic signatures are signed by the investigator or sub-investigator. Data from sub-cohort studies will be handled as described in the respective sub-cohort study Appendix.

# Adverse event

## Definition of adverse events

### Adverse event

Adverse events are any unfavorable or unintended sign (including an abnormal laboratory finding), symptom, or disease that occurs during the observation period from the date of acquisition of consent, regardless of causal relationship with the drug, etc. Adverse events include those related to surgical stress.

### Serious Adverse Events

A serious adverse event is any of the following:

1. Death
2. May result in death
3. Requires hospitalization in a hospital or clinic or prolongation of hospitalization for therapeutic
4. Disorder
5. May lead to disability
6. Other serious cases according to 1 to 5
7. Any congenital disease or anomaly in the offspring of a treated patient.

## Investigation Items and Collection Procedures for Adverse Events

### Check items of adverse events

When the investigator or sub-investigator confirms the occurrence of an adverse event, the following items will be investigated.

1. Name of adverse event
2. Day of onset
3. Seriousness

- Serious or non-serious
- Severity level

Serious adverse events were defined as "10.1.2.Serious Adverse Events".10.1.2

1. Outcome, date of confirmation of outcome

Outcome and confirmation date of each adverse event will be collected.

- Outcome

Outcomes will be either "recovered," "improved," "recovered but with sequelae (and sequelae)," "not recovered," "died of the event," or "unknown" (and reasons)"

- Date of confirming outcome

When the outcome is "recovered" the day of resolution, when the event is "improved," "recovered but with sequelae," "not recovered" or "unknown" the date of confirmation of outcome, and when the event is "died" the date of death.

1. Presence or absence of causally related drugs

- Name of drug related

The judgment of causality with the drug shall be based on the following criteria.

[Causality]

When there is a reasonable temporal correlation between the occurrence of an adverse event and the administration of the drug, and it is not appropriate to judge it to be due to the condition of the subject or a factor other than the drug concerned (primary disease, complication, concomitant drug, etc.), and the relationship to the drug concerned cannot be denied.

Not related

When there is no reasonable temporal correlation between the occurrence of an adverse event and the administration of the drug, or when it is reasonable to judge that the occurrence of an adverse event is due to the status of the subject or to factors other than the relevant drug (primary disease, complications, concomitant drugs, etc.), and the relevance of the drug can be denied.

If Ricciana is administered within one week before the onset of the adverse event, or if the drug concerned is a product approved for marketing by Daiichi Sankyo Company, Limited or a product marketed by another company, the following items should be investigated.

1. Procedure

- Administration of anticoagulants (continued (no dose reduction), "decreased dose," "withdrawal/discontinue of administration," or "developed after completion of administration")
- Presence or absence of other treatment

If any, describe details such as drug treatment.

1. Other Possible Factors

- Presence or absence of other possible factors

　　If any, describe the details.

1. Details of the status of administration of the drug related to the drug (if the drug related to the drug is "Yes")

- Drug name
- Daily dose
- Treatment period

In addition, in the case of a product of Daiichi Sankyo Company, Limited that has been treated with Ricciana within one week before the onset of the adverse event, or a drug related to the event, and in the case of a serious adverse event, the following items will be examined in addition to the above.

- Details of concomitant medications used in the event of an adverse event: name, daily dose, and duration of treatment
- Related laboratory values at the time of onset of adverse events: name, date, and laboratory values
- Clinical course

### Procedures for Response in the Event of an Adverse Event

If an adverse event occurs, the investigator or sub-investigator shall explain the event to the study population and take necessary measures. In addition, research collaborators, including the principal investigators, will investigate the items required for adverse events (see 10.2.1 "Investigation items of Adverse Events") and promptly enter the investigation results and interpretation results into the EDC. The principal investigator, etc. will determine the causal relationship with the drug.10.2.1

## Responding to Serious Adverse Event

Within this study, only subcohort study A will perform the examination with minor invasion, so the correspondence to serious adverse events described in this section will only apply to subcohort study A.

### Response of study collaborators, etc.

When the research collaborators, etc. are aware of the occurrence of serious adverse events arising from blood sampling conducted in Sub-Cohort Study A during the course of this clinical study, they shall explain the information to the research subjects, etc. and take necessary measures in accordance with the procedure manual prepared by the head of the research institution. Research collaborators should promptly report the event to the principal investigator.

### Response by investigators, etc.

If the investigator or sub-cohort study A becomes aware of the occurrence of serious adverse events arising from blood sampling during the course of this clinical study, the investigator or sub-investigator will promptly report this to the head of the study site and take appropriate measures in accordance with the procedures prepared by the head of the study site.

The investigator or sub-investigator should promptly report the adverse event to the Study Secretariat (or enter it into the EDC system). Subsequently, the Research Secretariat, which has received reports from the principal investigators, etc., shall promptly share information on the occurrence of such adverse events with research collaborators, etc. involved in this subcohort study and investigators of other participating medical institutions.

(Cooperation with the Study Sponsor)

If adverse reactions related to a drug are observed during the course of this clinical study, the investigator or sub-investigator shall report the event to the pharmaceutical company that manufactures and markets the drug and cooperate with the post-marketing safety measures. Adverse reactions for which reporting is required by the Law Concerning the Assurance of Quality, Efficacy, and Safety of Drugs, Medical Devices, etc., of products sold by Daiichi Sankyo Company Limited shall be reported to the Pharmaceuticals and Medical Devices Agency by Daiichi Sankyo Company, Limited. The study subjects reported are posted as "Information on Case Reports Suspected for Adverse Drug Reactions" on the website of the Pharmaceuticals and Medical Devices Agency (http://www.pmda.go.jp/ [(Link)](http://www.pmda.go.jp/)). In such cases, the source of the report and the privacy of the study subject are regarded as unpublished.

### Response of the head of the study site

The head of the study site participating in Sub-Cohort Study A prepares a procedure manual on the matters to be implemented by the investigators etc. in the event of a serious adverse event arising from blood sampling conducted in Sub-Cohort Study A in advance, and takes necessary measures to ensure that appropriate and smooth responses are taken in accordance with the procedure manual. If the investigator or sub-cohort study A reports the occurrence of serious adverse events resulting from blood sampling, the investigator or sub-cohort study A will promptly take necessary actions in accordance with the documented procedure and seek the ethics review committee's opinion on the relevant adverse events and take necessary measures.

# Observations

The principal investigators and research collaborators will investigate the following items in accordance with "12. Survey Schedule" for all enrolled research subjects, enter them in the EDC system Case Study Form, and submit (transmit) the eCRF by electronic signatures of the investigators etc.12

## Enrollment survey

1. Patient identifying information

- Patient Registration Identification Number (excl Patient Chart Number)
- Sex
- Date of birth or age at the time of informed consent
- Date of consent

## Baseline survey (most recent data within 180 days after the date of obtaining informed consent)

(1) Patient Characteristics

- Physical finding

Height, weight

- - AF Treatment history

AF subtype (Table 2), prior non-pharmacological treatment for AF

Table 22 AF Type

| Disease type ^1^ | Criteria |
| --- | --- |
| Paroxysmal AF | Restoration to sinus rhythm within 7 days of onset |
| Persistent AF | Persistent AF >7 days after onset |
| Long-standing persistent AF | Persistent AF > 1 yr after onset and persistent AF |
| Permanent AF | Electrically or pharmacologically cardiovertable |

1 This classification is adapted with or without pharmacotherapy, non-pharmacotherapy, and when duration is extensive, it is substituted with the representative AF duration that the patient exhibits.

Referenced Origin: Guideline for the Treatment of Atrial Fibrillation (Drugs), p. 14 ^1616^

- - Medical history other than AF treatment

History of major surgery other than atrial fibrillation, major bleeding, or drug allergy

- - Complications/history

Hypertension, diabetes mellitus (including diabetic complications), dyslipidemia, hyperuricemia, kidney disease, severe hepatic dysfunction, respiratory disease, heart disease, cerebrovascular disease, other vascular disease, thromboembolism-related disease, hyperthyroidism, gastrointestinal disease, malignancy (cancer-bearing condition), dementia, presence or absence of cerebral imaging, and presence or absence of falls within one year

- - Lifestyle habits

Smoking habit

1. Administration of anticoagulants at the time of enrollment
   - With or without anticoagulants
   - Type of anticoagulant
   - Time of the start of administration
   - Daily Dose and Number of Daily Doses (DOAC Only) at Enrollment
2. Concomitant medications other than anticoagulants at the time of enrollment
   - The use of the following concomitant medications will be investigated:

Arrhythmias, antiplatelet drugs, antihypertensives, lipids, diabetes, dementia drugs, anticancer drugs, chronic obstructive pulmonary disease (COPD) drugs, psychotropic drugs, proton pump inhibitors (PPIs), P-glycoprotein (p-gp) inhibitors (Appendix 4)Appendix 44

- - Number of oral medications at enrollment (all medications, including anticoagulants and antiplatelet agents, except topical and over-the-counter medications)

1. Blood clotting tests (only in patients taking warfarin)

If the following test items are measured in the daily medical care, they are entered in the eCRF.

- - PT-INR level and date of measurement
    (Enter up to 6 values measured in the first half year of enrollment [1-month interval as a guide].)

1. Clinical course and laboratory values

If the following test items are measured in the daily medical care, they are entered in the eCRF.

- - Office blood pressure

Systolic and diastolic blood pressures will be measured (see Table 3 for measurement methods)Table 33

- - Hemoglobin
  - Serum creatinine
  - HbA1c

Table 33 Office blood pressure measurement

| 1. Device | a. Auscultation by mercury sphygmomanometer and aneroid sphygmomanometer with precision test is used. Accuracy-calibrated digital blood pressure monitors are also ^1^ to be used  b. Cuffs 13 cm in width and 22-24 cm in length of the internal cuff rubber sac are used  [Pediatric cuff for children < 27 cm and large cuff for adults for large arms (> 34 cm arm circumference)]. |
| --- | --- |
| 2. Measurement conditions | a. Quiet, suitable room-temperature environment  b. After a few minutes of rest in a backrest chair with the feet set first  c. Don't know the conversation  d. Do not smoke, drink alcohol, or consume caffeine before measurement |
| 3. Assay | a. Cuff position is maintained at heart height  b. Rapidly pressurize the cuff  c. Cuff exhaust rate is 2 to 3 mmHg per beat or sec  d. In auscultation method, the start of Korotkoff phase I is made to be a systolic blood pressure, and phase V is made to be a diastolic blood pressure. |
| 4. Number of the measurements | At least two measurements at 1-to 2-minute intervals. If the two measurements differ greatly, perform additional measurements. |
| 5. Determination | a. The mean of two stable values of ^2^ is taken as the blood pressure value.  b. Diagnosis of hypertension is based on BP levels at least on 2 or more different occasions |
| 6. Other precautions | a. Blood pressure difference between the right and left brachials was confirmed at the initial examination.  b. Do not wrap cuffs on thick shirts or overwear. Do not put thick shirts on the upper arm.  c. In cases of orthostatic hypotension, such as diabetes mellitus and the elderly, blood pressure should be measured 1 minute and 3 minutes after standing to check for orthostatic hypotension.  d. The auscultator should be a person with sufficient hearing and sufficient guidance for measurement.  e. Pulse rate must be measured and recorded |

1. Recently, the use of the electronic sphygmomanometer is advised from environmental effects of mercury, quality control of the mercury column, issues of the accuracy of the aneroid sphygmomanometer, etc.. Hybrid sphygmomanometers using electronic analog pillars instead of mercury meters are also available. Large errors arise when automatically wrapped blood pressure monitors are used in waiting rooms, etc. if they are not measured under sufficient guidance and management.

2. A stable value is an approximate value where the difference in the measured value is less than about the 5 mmHg as a guide.

Reference Source: Hypertension Treatment Guidelines 2014, p.16

## 12-month and 24-month surveys (permissible range: within 1 year after the date of obtaining informed consent, and within ±60 days 2 years after the date of obtaining informed consent)

1. Patient identifying information

- Patient Registration Identification Number (excl Patient Chart Number)

1. Confirmation of the status of continuation of consent
   - Whether consent was withdrawn or not, date on which consent was withdrawn
2. Checking visit status and health status
   - Confirmation of health (date of confirmation, method of confirmation, results of confirmation)
3. Administration of anticoagulants
   - Presence or absence of anticoagulant treatment at the start of the observation period
   - Type of anticoagulant at the start of the observation period
   - Daily anticoagulant dose and number of daily doses (DOAC only) at the beginning of the run-in period
   - Starting day of anticoagulant therapy
   - Date of completion of anticoagulation
   - Reason for change or discontinue of administration
4. Investigation of concomitant medications and therapies other than anticoagulants
   - At the end of each observation period, the use of the following drugs will be investigated

Antiplatelet drugs, p-gp inhibitors

- - The maximum number of oral medications used during the observation period (all medications including anticoagulants and antiplatelet agents, except topical and over-the-counter medications)

1. Status of non-pharmacologic therapy for AF

- Presence or absence of non-pharmacological treatment during the study period
  - If yes, the date of implementation, status of administration of antiplatelet drugs before and after surgery, and presence or absence of heparin bridges.

1. Implementation status of invasive procedures other than AF treatment (including minor surgery*)
   - ^[[1]](#footnote-1)^Presence/absence of invasive procedures (including minor operations*) other than NVAF therapeutic during the study period**^エラー! ブックマークが定義されていません。^**

If yes, date of implementation, type of invasive surgery (major surgery, minor surgery*), status of administration of antiplatelet drugs before and after surgery, and presence or absence of heparin bridges.**^エラー! ブックマークが定義されていません。^**

1. Blood clotting tests (only in patients taking warfarin)

If the following test items are measured in the daily medical care, they are entered in the eCRF.

- - PT-INR values, dates of measurements
    (Maximum six measured values during the observation period (based on two-month intervals).)

1. Clinical course and laboratory values

If the following test items are measured in the daily medical care, they are entered in the eCRF.

- - Body weight
  - Office blood pressure (systolic and diastolic; see Table 3 for measurement methods)
  - Pulse rate
  - Hemoglobin
  - Serum creatinine
  - HbA1c

1. Cardiovascular events

For events (1) to (8) below, the name of the event, details of the event, PT-INR level immediately before the event, presence or absence of administration of antiplatelet drug immediately before the event, type of oral drug immediately before the event, number of types of oral drug immediately before the event, seriousness, treatment, other measures, outcome, and date of outcome (if the outcome is "death" the date of death), causally related drugs, factors other than the drug, and comments on the adverse event will be investigated.

1. Stroke (including transient ischemic attack)

Details of events: type of stroke (atherogenic, cardiogenic, lacunar, haemorrhagic, unclassifiable, other stroke, TIA), presence of neurological symptoms, presence of imaging, Japanese version of modified Rankin Scale (mRS), National Institutes of Health Stroke Scale(NIHSS), acute-phase treatment processes, and acute-phase outcomes

1. Systemic embolism

Details of events: presence or absence of imaging

1. Intracranial haemorrhages other than haemorrhagic stroke (including subarachnoid haemorrhage, subdural/epidural haemorrhage)

Details of events: type of intracranial hemorrhage (subarachnoid hemorrhage, subdural/epidural hemorrhage, other), presence of neurological symptoms, presence of imaging, acute treatment process, and acute-phase outcome

1. Cardiac sudden death
2. Myocardial infarction

Details of events: clinical presentation, rationale for diagnosis, presence of cardiac intervention, and type

1. Cardiac intervention other than myocardial infarction

Details of events: primary disease name, type of cardiac intervention

1. Heart failure requiring hospitalization

Details of Events: Clinical Presentation

1. ① Deaths other than (7)

Details of events: Cause of death type (cardiovascular death, non-cardiovascular death, unknown), cause of death

1. Gastrointestinal bleeding

Site (name of adverse event), presence or absence of ulcer/reflux esophagitis, bleeding classification (Table 4), shock index, treatment, presence or absence of pylori, treatment, hemoglobin level before and after bleeding, PT-INR level immediately before the event, presence or absence of antiplatelet therapy immediately before the event, type of antiplatelet therapy immediately before the event, presence or absence of PPI therapy immediately before the event, number of oral medication immediately before the event, seriousness, procedure, other procedures, outcome, date of outcome, drugs with causal relationship, comments on adverse events, possible factors other than the drug, and adverse events.Table 44

1. Other Haemorrhagic Adverse Events (Other than Hemorrhagic Stroke, Intracranial Hemorrhage, and Gastrointestinal Bleeding)

Adverse event name, bleeding class (Table 4), date of onset, PT-INR level immediately before the event, type, type of oral drug immediately before the event, type of oral drug immediately before the event, seriousness, procedure, other procedure, outcome, date of outcome, drug causally related, drug other possible factors, comments on adverse events

Table 44 Definition of Bleeding Grouping

| Haemorrhage Grouping ^1^ | Definition |
| --- | --- |
| Major bleeding (Major bleeding) | Clinically evident bleeding (bleeding visually evident by examination or radiologic imaging) that meets at least one of the following  ・ Fatal loss of blood  ・ Symptomatic intramuscular hemorrhage with retroperitoneal, intracranial, intraocular, intrathecal, intraarticular, pericardial, or compartment syndrome  ・ Clinically evident haemorrhage ^※^ with a decrease in haemoglobin of 2.0 g/dL or more requiring transfusions  When 2 units (400 cc) of ^※^ concentrated red blood cells or whole blood are transfused, it is converted to hemoglobin reduction of 1.0 g/dL. Haemorrhage associated with a surgical procedure is defined as bleeding that is greater than the amount of bleeding observed during routine operation or procedure. If no hemoglobin data are available, the hematocrit level is reduced by at least 6.0% and bleeding requiring blood transfusion is considered. |
| Clinically relevant bleeding (Clinically relevant non-major bleeding) | Clinically evident bleeding requiring treatment. Examples include, but are not limited to, diagnostic tests and treatments listed below. Outpatient visits without any of the following or similar medical procedures (diagnostic tests and treatments) do not fall under "require treatment."  ・ Inpatient hospitalization or prolongation of existing hospitalization  ・ Laboratory tests  ・ Imaging test  ・ Endoscopy; colonoscopy; cystoscopy; bronchoscopy  ・ Nasal packing  ・ Compression tourniquet  ・ Ultrasound-guided aneurysm closure  ・ Coil embolization  ・ Inotropic support  ・ Surgery  ・ discontinue or discontinuation of anticoagulant therapy as directed by the physician  ・ Change of concomitant treatment other than anticoagulants (reduction or discontinue of aspirin, etc.) as directed by the physician |
| Minor bleeding [Minor (not clinically relevant) bleeding] | Other obvious bleeding that does not meet the criteria for major bleeding or clinically important bleeding (e.g., epistaxis without treatment). |

1 Modified definitions of phase III, MNCT ^2525^

1. Other events

Presence or absence of falls and fractures (extrinsic or intrinsic)

If yes,

Name of the adverse event, date of onset, PT-INR immediately before the event, presence or absence of antiplatelet therapy immediately before the event, type, seriousness, procedure, other procedure, outcome, date of outcome, drug(s) related to the event, drug(s) possibly related to the event, comments about the adverse event(s)

1. Other adverse events

If a serious adverse event occurs or if Licciana is administered within one week before the onset of the adverse event, the following items will be investigated.

Name of adverse event, date of onset, seriousness, procedure, other procedure, outcome, date of outcome, drug(s) of causal relationship, drug(s) other possible cause(s), adverse event(s)

(10) For (14), the study subject will be contacted by telephone or letter even if there is no visit, and the event status will be checked at 12 and 24 months.

# Schedule of research

Table 55 Observation and examination schedule

The investigation items are as follows and will be conducted within the scope of daily medical care.

| Time of investigation  Survey item | Eligibility Confirm | Baseline Study  (-180-0 days) | 12 months Study  (±60 days) | 24 months Study  (±60 days) |
| --- | --- | --- | --- | --- |
| Confirmation of consent, inclusion and exclusion criteria | ○ |  |  |  |
| Withdrawal of Informed Consent, Visit Status, and Confirmation of Health |  |  | ○ | ○ |
| Patient characteristics, history of previous treatment for AF, surgical history, history of bleeding, past medical history (other than bleeding), complications, etc. |  | ○ |  |  |
| Status of anticoagulation administration |  | ○ | ○ | ○ |
| Status of administration and compliance with drugs other than anticoagulants |  | ○ | ○ | ○ |
| Status of non-pharmacologic therapy for AF |  | ○ | ○ | ○ |
| Implementation status of invasive procedures (including minor surgical ^1^) other than AF treatment |  |  | ○ | ○ |
| Clotting studies (PT-INR) |  | ○ | ○ | ○ |
| Clinical course and laboratory values |  | ○ | ○ | ○ |
| Presence or absence of cardiovascular events, gastrointestinal bleeding, bleeding adverse events, falls, fractures, deaths, or other adverse events |  |  | ○ | ○ |
| Sub-cohort study |  |  |  |  |
| A. Assessment marker |  | ○  (central measurement) |  |  |
| B. Echocardiogram |  | ○ | ○ | ○ |
| C. Heart rate |  | ○ | ○ | ○ |
| D. Hypertension |  | ○ |  |  |
| E. Cognitive functioning |  | ○ |  | ○ |
| F. Frail |  | ○ |  |  |
| G. Adherence |  | ○ |  | ○ |

AF, atrial fibrillation; PT-INR, prothrombin-international normalized ratio

1 Minor surgery includes procedures involving biopsy and puncture

# Number of target registrations of study subjects

30,000 patients (consecutive enrollment) in the study as a whole

Rationale for target enrollment

The incidence of stroke/systemic embolism in Fushimi AF Registry's ^5^ has been reported to be 5.1/100 patients aged 85 years or older, 2.0/100 patients aged 84 years or younger, and a hazard ratio of 1.77 for patients aged 85 years or older to patients aged 75 years or older and 84 years or younger.

Based on this assumption, the incidence rates in patients aged 75 to 84 years and those aged 85 years and older are 2.9 per 100 person-years and 5.1 per 100 person-years, respectively, and the overall incidence rate is estimated to be about 3.5 per 100 person-years, assuming a patient composition ratio of 3:1 in this study.

We estimate the incidence of stroke/systemic embolism in patients without risk factors for this study to be 3.4/100 person-years. Assuming an exponential distribution (constant hazard) in time to onset, if the proportion of patients with risk factors is 5%, enrolling 30,000 patients will detect a hazard ratio of 1.3 for the development of stroke/systemic embolism with risk factors to no risk factors over a 2-year follow-up period of at least 5% with a two-sided significance level of at least 80% power.

For 30,000 patients, the power of 80% is to detect risk factors with a hazard ratio of about 1.5, even for intracranial hemorrhage, which is a low incidence but of particular concern, among the events planned to be studied. An estimated incidence of 1.2% per year of intracranial bleeding and a risk factor of 5% were assumed [e.g., a potential intracranial bleeding risk of 1.5 times greater than that of patients without SRI in patients with severe renal dysfunction (SRI)] would detect a statistically significant risk increase of 80% even with an SRI of about 5% of patients overall.

# Study Period

Date of approval by the ethics review committee of the responsible medical institution-September 2020

(Registration period: Date of approval by the ethics review committee of the research institution-September 2018, follow-up period of 2 years)

However, the registration period may be shortened and the planned implementation period may be shortened or extended by the agreement of the Central Committee for Research, the principal investigator and the sponsor.

# Endpoint

## Primary endpoint

Incidence of Stroke/Systemic Embolism during the Observation Period

## Secondary endpoint

Occurrence of the following events during the observation period

1. Hemorrhagic Adverse Events (Major Bleeding)
2. Stroke
3. Systemic embolism
4. Ischemic stroke
5. Hemorrhagic stroke
6. Intracranial hemorrhage
7. Cardiovascular events (stroke, myocardial infarction, non-myocardial infarction cardiac intervention, heart failure requiring hospitalization)
8. Death due to cardiovascular disease (death due to cardiovascular causes undeniable)
9. All deaths

## Other evaluation items

Incidence of falls and fractures

# Implementation of Sub-Cohort Study (Sub-Study 1)

Along with the conduct of this study, we conduct the following subcohort studies:As far as possible, investigators at participating medical institutions that have declared their cooperation in each sub-cohort study at the time of their participation in this clinical study will conduct a survey of all research subjects registered in the relevant study institution, which is set out for each of the following sub-cohort studies.

Each subcohort study will be conducted at the time of the survey in Table 1 Observations and Tests Schedule, and the details will be in accordance with the separately developed Sub-Cohort Study Protocol (Appendix).Table 11Observation and examination schedule

Acceptable

Baseline (most recent information within 180 days of the date of obtaining informed consent, unless otherwise specified)
12-month and 24-month surveys: both ±60 days

## Sub-cohort study A: assessment markers

To compare the association of coagulation and fibrinolytic molecular markers, antithrombotic activity with stroke/systemic embolism, and hemorrhagic adverse events by non-anticoagulant, warfarin, and DOAC.

Endpoint

1. Prothrombin time (PT), PT-INR, activated partial thromboplastin time (APTT), TTR, D-dimer, soluble fibrin monomer complex (SFMC), prothrombin fragment 1+2 (F1+2), thrombin-antithrombin complex (TAT), sensitive troponin I, brain natriuretic polypeptide (BNP) precursor N-terminal fragment (NT-pro BNP).
2. Stroke/systemic embolism
3. Hemorrhagic adverse events

## Subcohort study B: Echocardiogram

To investigate underlying heart disease by echocardiography, left ventricular (LV) and left atrial (LA) structure and function, and their association with stroke/systemic embolism.

Endpoint

1. Structure and function of LV:

- Left ventricular end-diastolic dimension (LVDd)/left ventricular end-systolic dimension (LVDs)/interventricular septal wall thickness (IVSTD)/left ventricular posterior wall thickness (PWTD)
- Left ventricular end-diastolic volume (LVEDV)/left ventricular end-systolic volume (LVESV)/left ventricular ejection fraction (LVEF)
- Stroke volume (SV)
- LV mass index (LV mass index)
- LV sphericity index (1) Left ventricular end-diastolic volume (LVEDV) /π*LV length: ^3^ /6, 2) LVDd/LV length)
- LV wall abnormal motion

1. LA structure and function:

- Left atrial diameter (LAD)
- LA expansion index by left atrial volume* (LA volume) (peak LA volume [LA _max_], minimum LA volume [LA _min_] and atrial pre-contraction LA volume [LA _pre-A_ ]), calculated values of total/passive/active LA emptying fraction

1. Structure and function of the right ventricle (RV):

- Tricuspid systolic pressure gradient (TRPG), inferior vena cava diameter (IVCD), estimated right atrial pressure (RAP) from respiratory variability, and estimated pulmonary artery systolic pressure (PASP)
- RV function [Tricuspid Annular Systolic Migration Distance (TAPSE) and Tricuspid Annular Systolic Velocity (TVS)].

1. Doppler imaging

- LV inflow early diastolic wave (E), LV inflow atrial systolic wave (A), mitral annular early diastolic velocity (TDI e'), and mitral annular atrial systolic velocity (TDI a').

1. Valvular heart lesions (mitral, aortic, tricuspid, pulmonic, prosthetic, and valve repair)
2. Stroke/systemic embolism

## Sub-Cohort Study C: Heart Rate

To evaluate the association between heart rate during the development of AF recorded on an electrocardiogram and the risk of developing stroke/systemic embolism.

Endpoint

1. Heart rate
2. Stroke/systemic embolism

## Sub-cohort study D: Hypertension

To investigate the relationship between blood pressure (office and home blood pressure) and stroke/systemic embolism and bleeding adverse events.

Endpoint

1. Office blood pressure and home blood pressure
2. Stroke/systemic embolism
3. Hemorrhagic adverse events

## Sub-cohort study E: cognitive function

To clarify the actual status of cognitive function and to investigate the factors affecting cognitive function in late-stage elderly patients with NVAF. Information on cognitive function and social activity will be collected using the Mini-Mental State Examination (Mini Mental State Examination,MMSE), which measures cognitive function (see Sub-Cohort Study E Protocol, Appendix 1) and the Social Activity Questionnaire (see Sub-Cohort Study E Protocol, Appendix 2).

Endpoint

1. Mini-Mental State Examination(MMSE) scores.
2. Social activity level

## Sub-cohort study F: Frail

To ascertain the percentage of frail patients in late-stage elderly patients with NVAF and to check the status and status of use of anticoagulation therapy in frail patients. We will also explore the relationship between frail and stroke/systemic embolism and bleeding adverse events. Information on the frail will be collected using questionnaires related to the frail [Abbreviated Flail Scale (National Center for Longevity Medical Research) (Sub-Cohort Study F Implementation plan, Appendix 1), Basic Checklist for the Elderly (Ministry of Health, Labour and Welfare) (see Sub-Cohort Study F Protocol, Appendix 2), and questionnaires on living conditions (presence and grade of certification of need for nursing care, presence or absence of a cohabiting person, composition of a cohabiting family)].

Endpoint

1. Frailty score
2. Basic checklist evaluation value for the elderly
3. Questionnaires on living conditions
4. Stroke/systemic embolism
5. Hemorrhagic adverse events

## Sub-cohort study G: adherence

To investigate the comprehensibility of medication adherence and NVAF in older patients with NVAF, and to investigate the association between medication adherence and NVAF comprehension, stroke/systemic embolism, and hemorrhagic adverse events. Adherence information will be collected by the Adherence Questionnaire [Morisky Medication Adherence Scale(MMAS] ^26^ Score and NVAF Comprehensibility Questionnaire. There are two types of MMAS: a MMAS-4 consisting of four-item questions and a MMAS-8 consisting of eight-item questions (Sub-Cohort Study G Protocol, see Appendix 1). This subcohort research employs a MMAS-8 that provides a more accurate picture of patients' medication adherence. We will also investigate the percentage of days prescribed (Proportion of days covered, PDCs) to the time of enrollment.^26^

Endpoint

1. MMAS-8 Score
2. NVAF comprehensibility
3. Percentage of days prescribed (Proportion of days covered, PDCs)
4. Stroke/systemic embolism
5. Hemorrhagic adverse events

# Statistical Analysis

Statistical analysis will be performed by tabulating survey information based on the following provisions. Detailed analysis methods and items not described below will be subject to a separate Statistical Analysis Plan.

## Analysis Sets

Of all research subjects enrolled in this study, the population will include the study subjects, excluding significant deviations from the following study protocol:

- Incompatibility of inclusion criteria
- To meet the exclusion criterion

## Analysis of Baseline Survey Items

Frequency tables will be generated for categorical variables and summary statistics (number of subjects, mean, standard deviation, minimum, median, and maximum) will be obtained for continuous variables.

1. Distribution of background factors
2. Type, administration, and number of daily doses of anticoagulation used at enrollment
3. Type of antiplatelet drug used at the time of enrollment
4. Types of other concomitant medications used at the time of enrollment
5. CHADS scores (Appendix 1) or the CHA _2_ DS _2_-VASc scores (Appendix 2) calculated from patient data at enrollmentAppendix 11Appendix 22

## Analysis of follow-up data

Frequencies and event rates and their 95% confidence intervals will be calculated for the following items:Cox proportional hazards models and logistic regression analysis are used to search for risk factors.

1. Type, dose, number of daily doses, and frequency of changes of anticoagulants and antiplatelet agents [at 12 and 24 months of follow-up]
2. Incidence of stroke/systemic embolism at 24-month follow-up

- Stroke (atherosclerotic, cardiogenic, lacunar, haemorrhagic, unclassifiable, other stroke, TIA)
- Systemic embolism

1. Incidence rate of bleeding adverse events [at 24-month investigation] (see Table 4 for classification of bleeding and definitions)Table 44

- Major bleeding
- Clinically relevant bleeding
- Minor bleeding
- Intracranial hemorrhage
- Gastrointestinal bleeding (upper and lower)

1. Number of falls
2. Incidence of bone fractures
3. Mortality (all deaths, cardiovascular deaths, non-cardiovascular deaths)
4. Incidence of myocardial infarction, cardiac intervention, and heart failure requiring hospitalization

(1) For ~(7), the CHADS score, the CHA _2_ DS_2_-VASc score, the HAS-BLED score and its constituent factors, the factors identified as risk factors in the guideline, the factors identified as risk factors in the present study, the presence or absence of anticoagulant therapy and type (DOAC, warfarin), dosage, PT-INR, etc. will be examined by subgroup.

## Subgroup analysis (Sub-study 2)

Items to be examined by subgroup analysis are shown below.

1. By renal function (Ccr value)
2. By HbA1c
3. By the maximum number of drugs administered
4. Cases of ischemic heart disease
5. Cases of gastrointestinal bleeding
6. Cases of stroke

## Analysis of a subcohort study

Depending on the tabulated items, analyses will be conducted in accordance with Sections 17.2 and 17.3. A separate Statistical Analysis Plan should be followed for further analyses and for analyses not described below.17.217.3

### Sub-cohort study A: assessment markers

1. Distributions of PT; PT-INR; APTT; TTR; D-dimer; SFMC; F1+2; TAT; High-sensitivity troponin I; NT-pro BNP at baseline
2. Incidence of major bleeding, clinically important bleeding, and stroke/systemic embolism by TTR (warfarin administration patients) (at 24-month follow-up)
3. Incidence of major haemorrhage, clinically significant bleeding, and stroke/systemic embolism summarized by PT and APTT (at 24-month follow-up)
4. Incidence of major haemorrhage, clinically significant bleeding, and stroke/systemic embolism summarized by D-dimer, SFMC, F1+2, TAT, high-sensitivity troponin I, and NT-pro BNP levels at 24-month follow-up.

### Subcohort study B: Echocardiogram

1. Breakdown of underlying heart disease
2. Echocardiographic parameter values (at baseline) by type of NVAF (paroxysmal, persistent, permanent)
3. Echocardiographic parameter values (at baseline) by CHADS score and _2_ score.
4. Changes in Echocardiographic Parameters Over Time (Baseline, 12-Month, and 24-Month Surveys)
5. Echocardiogram defined factors (at baseline) in high _2_ scores of CHADS
6. Echocardiographic predictors of events, including stroke (at 24-month follow-up)

### Sub-Cohort Study C: Heart Rate

1. Distribution of heart rate (at baseline) during AF expression recorded on a 12-induced resting ELECTROCARDIOGRAM.
2. Changes in the frequency of AF recorded on 12-induced resting ELECTROCARDIOGRAM at baseline, 12-month follow-up, and 24-month follow-up
3. Changes in Heart Rate During AF Recorded on 12-Induced Resting ELECTROCARDIOGRAM (Baseline, 12-Month, and 24-Month Investigations)
4. Occurrence of Stroke and Systemic Embolism during AF Recorded by 12-Lead Electrocardiogram at Rest by Heart Rate (at 24-Month Investigation)

### Sub-cohort study D: Hypertension

1. Distribution of office blood pressure and home blood pressure values at enrollment (at baseline)
2. Incidence of stroke and systemic embolism based on distribution of office blood pressure and home blood pressure at enrollment (at 24-month survey)
3. Incidence of bleeding adverse events based on distribution of office blood pressure and home blood pressure at enrollment (at 24-month survey)

### Sub-cohort study E: cognitive function

1. Distributions of MMSE scores at different time points (baseline, 24-month survey)
2. Distribution of Social Activity at Enrollment (Baseline)
3. Change from Baseline in MMSE Score (Month 24)

### Sub-cohort study F: Frail

1. Percentage of frail and non-frail patients at enrollment (at baseline)
2. Patient Characteristics of Filled and Nonfrailed Patients at Enrollment (Baseline)
3. Frail and non-frail patients at enrollment, type of anticoagulant, dosage (DOAC only), and duration of treatment (at baseline)
4. Distribution of basic checklist evaluation values for the elderly (at baseline)
5. Distribution of questionnaires on living conditions (at baseline)
6. Incidence of stroke/systemic embolism by frail and non-frail patients at enrollment (at 24-month survey)
7. Incidence rate of bleeding adverse events by frail and non-frail patients at enrollment (at 24-month investigation)

### Sub-cohort study G: adherence

(1) Distributions of MMAS-8 scores at different time points (baseline, 24-month survey)

(2) Distribution of PDC (at baseline)

(3) Background Factors of Study Subjects with Improved MMAS-8 Scores (at Baseline and Month 24 Survey)

(4) MMAS-8 scores, comprehensibility of PDCs and NVAF, and their association (at baseline, at 24-month survey)

(5) Association of MMAS-8 scores with stroke/systemic embolism and hemorrhagic adverse events at 24-month follow-up

(6) Association between NVAF comprehensibility and stroke/systemic embolism and hemorrhagic adverse events at 24-month follow-up

(7) Association of PDC with stroke/systemic embolism and bleeding adverse events at 24-month follow-up

# Preparation and Submission of Case Report Form

## Format and Submission of Case Report Form

Investigations specified in this study protocol will be recorded on the Study Specific Case Report Form (eCRF). The format of the eCRF is as follows. The form will be entered at baseline, 12-month, and 24-month surveys and submitted (transmitted) promptly.

- Registration information for the study population
- Time of the baseline survey
- Months 12 and 24
- At withdrawal of consent and at the time of onset of adverse events, unless an emergent serious adverse event occurs.

## Method of creating the Case Report Form

Investigators and research collaborators shall access the EDC system dedicated to this study using the issued ID and password and enter the search information (the entry procedure for EDC shall follow the EDC entry manual, which is created separately).

# Monitoring and Auditing

## Monitoring and Auditing

The sponsor consults with the principal investigator to ensure the conduct of the study and the reliability of the records, and to ensure that the monitoring and audits are outsourced to the contract research organization, etc. Investigators and research institutions cooperate in monitoring and auditing. The principal investigator or others shall explain to the study subject that the source documents will be accessed by the monitors and the auditors and obtain consent from the subject in the Informed Consent Form.

## Monitoring

The monitors shall confirm that the study is conducted in compliance with the Ethical Guidelines for Medical Research Involving Human Subjects and the study protocol in accordance with the Monitoring Procedures developed separately. For direct access, the procedures specified in this study will be checked to check the source documents against those entered in the EDC to verify the integrity, accuracy and consistency of the collected data. After completion of the monitoring activities, a monitoring report will be prepared and reported to the principal investigator. In addition, the monitors shall divulge information obtained in the course of their duties without any justifiable reason, even after they have ceased to be engaged in the duties.

The research sponsor (Daiichi Sankyo Company, Limited) shall not be directly involved in the monitoring work in order to maintain the neutrality to this study.

## Auditing

The auditor maintains a standpoint independent of the monitoring duties and conducts written and on-site inspections in accordance with the Audit Operating Procedures prepared separately. The monitoring report shall be verified to ensure the conduct of the study and the reliability of the records thereof, and to guarantee this. Each investigator or sub-investigator shall, upon request of an auditor, provide all study-related records of the study subject for direct access. The auditor shall submit an audit report and an audit certificate to the principal investigator and the head of the study site. In addition, the auditor shall divulge information obtained in the course of his/her duties without justifiable reason, even after he/she has stopped engaging in the duties.

## Direct access to source documents

The director of a medical institution and the principal investigator provide direct access to the records related to clinical research, such as source documents, during monitoring, audits, and investigations by regulatory authorities and ethics review committees. The study sponsor (Daiichi Sankyo Company, Limited) does not conduct direct access.

# Ethic

This study will be conducted in accordance with the spirit of the latest Declaration on Helsinki by the World Medical Association, while protecting the human rights and welfare of the trainees, and in compliance with the Ethical Guidelines for Medical Research for Humans (MEXT, MHLW Notification No. 415 of 2014) ^24^ and related notices.^24^

## Ethical Review Board

After consultation with the principal investigator and the Study Central Committee, the sponsor will prepare a research protocol and informed consent form (sample) and obtain approval from an external ethics review committee designated by the research sponsor and from the Ethics Review Committee of the Cardiovascular Research Institute, which has jurisdiction over the Research Representative Facility. When the research protocol is revised, the research sponsor and the principal investigator shall prepare an revised version of the research protocol, etc. (informed consent form, if necessary) after requesting opinions from the Research Central Committee if necessary. After requesting and obtaining approval from the respective ethics review committees for reexamination, the revised version shall be implemented. At least once a year after the start of the study, the ethics review committee shall be informed of the continuation of the study.

The principal investigator at each participating center will initiate the study after obtaining the approval of the ethics review committee of each participating center. In the case of a facility that does not have an ethics review committee, an external ethics review committee designated by the facility concerned or an external ethics review committee designated by the study sponsor or an ethics review committee at the cardiovascular research institute shall be subject to executive deliberations.

## Expected benefits to the study audience

Since this study is a registry study that uses approved drugs within the scope of indications, there is no change in treatment policy by study subjects to participate in this study, and there is no specific benefit from participating in this study. Findings from this study are expected to benefit future patients with AF, including those studied.

## Potential risks and disadvantages to the study population

This study prospectively observes the current treatment status of late-stage elderly patients with NVAF, and no treatment-related adverse events are other than those that can occur with conventional treatment in general practice. In addition, blood sampling, examinations, and survey questionnaires administered to the study subjects do not deviate from the range of therapeutic normally performed in general practice.

## Compensation for health damage

This is a prospective observational study in general practice. If any health damage occurs after participation in this study, in principle, the health insurance provided by the research subject will be used to treat the health damage, and the cost of treatment will not be paid to the research subject from the research institution.

In the event of a health hazard, the principal investigator, etc., of the study institution shall promptly take treatment and other necessary measures. Health hazards caused by adverse reactions that occur after administration and dosage according to the instructions of the principal investigator, etc. are subject to the application for relief benefits under the Relief System for Sufferers from Adverse Drug Reactions.

The sponsor will also purchase clinical research insurance for health damages caused by the study as compensation or liability for health disorder during the study period, and as compensation for health damage caused by the study plan (death, sequelae class 1 or 2).

## Protection of personal information

The persons involved in this study shall comply with the Law Concerning the Protection of Personal Information (Law No. 57 of 2003) and related notifications applicable to the protection of personal information, etc. of research subjects.

Personnel involved should be fully aware of the personal information and the protection of privacy of the study subjects, and the patient record number identifying the patient information will be anonymized with the patient registration identification number given at the time of enrollment in the study (concatenated anonymisation). The EDC does not include any personal identifiable information, such as patient chart number, name, and contact information. Correspondence tables between patient chart numbers and patient registration identification numbers will be kept strictly in locked locations within the study site.

The data of the research subjects obtained in this study are not used for purposes other than this study, and when publishing the results do not include information that identifies the research subjects.

Personal information obtained in conducting this study must be disclosed without justifiable reason. The same shall apply even after the persons concerned have retired from the job.

# Matters concerning the cost of the study

## Research Funding and Conflicts of Interest

This is a company-initiated clinical study funded by Daiichi Sankyo Company, Limited.

The funding source will be involved in the development of the study protocol, selection of contract research organizations and advice to the institutions concerned, planning of the statistical analysis plan, and interpretation of the research results, but will not be directly involved in data management, direct access, or statistical analysis.

The funding source shall appropriately manage conflicts of interest and conduct research appropriately, maintaining neutrality and publicity, in conducting the study and in publishing the results.

Principal investigators and investigators will be reviewed by the Institutional Review Board or Conflict of Interest Committee in accordance with institutional regulations regarding the plan of the study, the status of conflicts of interest that may affect the interpretation of the results. The details will be described in the explanatory documents and the research subject will be informed of the study and consent to participate in the study will be obtained. In addition, when publishing research results, they shall comply with the guidelines of academic organizations and organizations that publish the results, and shall disclose the exact situation in a self-report format.

## Cost burden for the study subjects

Expenses incurred within the scope of routine insurance treatment, including drug, haematology test, and other treatments and tests, shall be paid for by the study subjects.
Traffic expenses will not be paid by this study, but may be offered honoraria for research cooperation at the end of the study in accordance with local regulations. Institutions providing research cooperation honoraria should be described in the informed consent form and approved by the Institutional Review Board.

# Amendment or amendment of the study protocol

## Amendment of the study protocol

If the investigator decides that a change in the study protocol is necessary, the investigator or sub-investigator shall obtain approval from the ethical review board after approval from the principal investigator. If it is deemed necessary to change the informed consent form due to the content of the amendment of the study protocol, the ethics review committee shall approve the changed informed consent form together with the research protocol, and obtain the research subject's reconsent in writing using the relevant informed consent form.

## Amendment of the study protocol

When revising the study protocol, the principal investigator must submit the revised research protocol to the head of the research institution and obtain approval from the ethical review committee.

After obtaining approval for revision, the principal investigator shall promptly communicate the details of the revision through the Study Secretariat to the principal investigators, data centers, etc. of each research institution.

# Completion, Interruption, and Termination of the Study

## End of the study

After completion of the study, the principal investigator at each study site shall promptly submit the research completion report to the head of the study site.

When the director of a research institution receives a report as described above from the principal investigator of each research institution, he/she submits a study completion report to the ethics review committee who reviewed the study.

## Discontinuation/discontinuation of the study

The principal investigator shall consider whether to continue the study after obtaining the opinion of the Central Committee if any of the following items apply:If the principal investigator decides to suspend or discontinue the study, he/she will prepare a document for each reason and how to respond to the subject of the study and promptly notify the study site.

If a research institution (including the principal investigator) is found to have committed a serious violation of the "Ethical Guidelines for Medical Research on Humans," the study protocol or contract, or if it is no longer possible to conduct appropriate research, the research institution may request the principal investigator to discontinue the research.

If the research is suspended or prematurely terminated due to the following reasons, etc., at the study site, the principal investigator shall promptly report the information suspended or prematurely to the principal investigator.

1. When significant information on the quality, safety and efficacy of a medicinal product related to this study is available
2. When it is determined that recruitment of study subjects is difficult and that it is difficult to achieve the enrollment of the planned number of study subjects.
3. When the Ethics Review Board recommends or instructs discontinuation
4. When it is determined by the ethical review committee that it is difficult to accept an instruction for a change in the study protocol, etc.

# Storage of documents related to study, etc.

The researcher, etc. shall ensure that the documents related to the conduct of the study, etc. (such as the documents required to retain the application form, notification documents from the head of the research institution, various applications and reports, a list of patient registration numbers of the research subjects who have been anonymized in a linkable manner, the consent form, and other documents or records required to ensure the reliability of the data) are accurate.

When retaining specimens obtained from research subjects and documents related to the conduct of research, the principal investigator shall instruct and manage the researchers, etc. to ensure that the documents related to the conduct of research are accurate in accordance with the documented procedures prepared by the head of the research institution, and shall control such documents so that they do not leak, mix-up, stolen, or lose. In addition, the management status shall be reported to the head of the study site according to the documented procedure.

The director of the study institution shall prepare written operating procedures for the retention of specimens obtained from research subjects and documents related to the conduct of the research, and appropriately store specimens and documents in accordance with these procedures. The head of the study site shall appropriately store the documents related to the conduct of the study for at least five years after the date reported on the completion of the study or three years after the date reported on the final publication of the results of the study, whichever is later. Corresponding tables for linkable anonymised information correspond similarly. After the storage period has passed, the personal information of the subject to be studied is disposed of promptly by an appropriate method that is not identified.

Each study institution establishes a personal information manager to manage personal information.

Data and specimens from the study subjects obtained in this study will not be used for purposes other than those specified in this protocol.

# Response to Consultations, etc. from Research Subjects, etc. and Their Relevant Persons

The principal investigator shall establish a consultation center for the study from the research subjects and their relevant parties and describe the contact method in the explanatory documents. Researchers should promptly answer and explain the details to all questions received from the study subjects. Provided, however, that this provision shall not apply to the contents which must be disclosed to protect the rights and interests of study subjects and their interests of the persons concerned, researchers, etc. and their interests, and which are approved by the head of the research institution based on the opinions of the ethics review committee.

# Publication of Study and Attribution of Results

## Clinical Research Registry

Prior to the start of the study (prior to the start of enrollment of the first study subject), the principal investigator will register and publish the study plan content in the Public Registration System [University Hospital Medical Information Network (UMIN)].

## Publication of the study

The principal investigator will publish the results without delay after completion of the study. The person who publishes the research result obtains the approval of the research promotion and publication committee in advance. Authors of articles and conference presentations will be appropriately decided by the Research Promotion and Publication Committee in accordance with the International Committee of Medical Journal Editors Author Requirements (authorship).

## Ownership of results

The results obtained in the study have the right to be used exclusively by Daiichi Sankyo Company, Limited. When the principal investigator, etc. publishes the research results externally, he/she requests the principal investigator, etc. before publication to ensure that the statement of conflicts of interest is appropriate for the paper, etc. in accordance with the provisions of the contract.

In addition, the name of the study subject and personal information such as the name of the injury and illness should be minded when reporting as a conference presentation or paper.

# Research organization

## Representative caregiver

Coordinate between multiple centers as a representative of the research organization.

Hiroshi Inoue Director, Saiseikai Toyama Hospital, Toyama Prefecture

〒931-8533 33-1 Kusuki, Toyama-shi, Toyama Japan

Phone: 076-437-1111(s)

## Research Advisor

Multicenter coordination is done as a study advisor.

Mt. Yamaguchi President Emeritus, National Cardiovascular Research Center

〒565-8565 5-7-1 Fujishiradai, Suita-shi, Osaka Japan

Phone: 06-6833-5012(s)

## Principal investigator

In the "Guidance on Ethical Guidelines for Medical Sciences Research for Persons (partially revised on March 31, 2015)", the Research Representative who supervises research at joint research institutions is responsible for the implementation of this research.

Shigeshi Yamashita Director of the Cardiovascular Institute

〒106-0031 3-2-19 Nishiamabu, Minato-ku, Tokyo

Phone: 03-3408-2151 (Ph.D.) Fax:03-3408-2159

## Central Research Committee

Medical judgment will be given to the study program, and a policy for the management of the research organization will be established to facilitate the smooth implementation of this research.

※ See the separate volume for a list of committee members.

## Research Promotion and Publication Committee

Promote research such as patient enrollment and follow-up. In addition, the policy for publication of research results and the content of publication will be decided.

※ See the separate volume for a list of committee members.

## Event Evaluation Committee Members

To determine whether stroke events, cardiac events, and bleeding adverse events are considered evaluable events in this study.

※ See the separate volume for a list of committee members.

## Sub-investigators

It is responsible for planning each of the 16 sub-cohort studies (sub-study 1) and the 17,4 subgroup analyses (sub-study 2) and for promoting the implementation of sub-studies. It also offers suggestions for interpretation of sub-study results and publication of results.

※ See separate volume for a list of sub-investigators.

## District promotion physician

As a coordinator of the related facilities in the region, it is in charge of promoting patient registration and patient follow-up.

※ See separate volume for a list of district promotion physicians.

## Responsible statistician

It is responsible for the planning, implementation of the statistical analysis plan and the results of the statistical analysis. They will also advise on the interpretation of the study results.

Satoshi Hando Professor of Biostatistics, Graduate School of Medicine, Kyoto Prefectural University of Medicine

〒602-8566 465 Kajii-cho, Hirojiro-Kojikami, Kawahara-cho, Kamikyo-ku, Kyoto Japan

Phone :075-251-5966、075-251-5944 Fax:075-251-6588

## Institute responsible for research

As a responsible institute for conducting this study, it plays the role of "research institutes" in the Ethical Guidelines for Medical Research Involving Human Subjects. The ethical review committee of the research institute will be the central ethical committee of this study.

Cardiovascular Institute Hospital

〒106-0031 Res. Inst. of Cardiovascular Research, Nishi-amabu 3-2-19 Minato-ku, Tokyo

Phone: 03-3408-2151 (Ph.D.) Fax:03-3408-2159

## Research Secretariat

To assist research principal investigators in their work and to take the necessary actions to ensure that the study is carried out smoothly.

Cardiovascular Inst. CVI-ARO

〒106-0031 Res. Inst. of Cardiovascular Research, Nishi-amabu 3-2-19 Minato-ku, Tokyo

Phone: 03-3408-2151 (Ph.D.) Fax:03-3408-2159

In addition, the following organizations perform administrative tasks related to research management.

Quintiles Transnational Japan Co., Ltd.

〒532-0003 Nissei Shin Osaka Building, 3-4-30 Miyahara, Yodogawa-ku, Osaka-shi, Osaka Japan

Phone :06-7668-9053 Fax:06-4807-9801

E-mail: ANAFIE_office@quintiles.com

## Research Sponsor

As a research sponsor, it has the overall responsibility for this study.

Medical Science Department, Fukuchi Ryowa Daiichi Sankyo Company, Limited

〒103-8426 3-5-1 Nihonbashi Honmachi, Chuo-ku, Tokyo

Phone :03-6225-1053 Fax:03-6225-1959

## Data center

Appropriately manage data and materials collected from each participating institution.

Quintiles Transnational Japan Co., Ltd.

〒532-0003 Nissei Shin Osaka Building, 3-4-30 Miyahara, Yodogawa-ku, Osaka-shi, Osaka Japan

Phone :06-7668-9053 Fax:06-4807-9801

## Monitoring agency

An organization that conducts monitoring services on consignment from a study contractor.

① Quintiles Transnational Japan Co., Ltd.

〒532-0003 Nissei Shin Osaka Building, 3-4-30 Miyahara, Yodogawa-ku, Osaka-shi, Osaka Japan

Phone :06-7668-9053 Fax:06-4807-9801

② EP Cruz K.K.

Acropolis Tokyo, 6-29 Shinkokawa-cho, Shinjuku-ku, Tokyo 162-0821 Japan

Phone:03-5804-5045 Fax:03-5684-8052

## Auditing organization

An organization that conducts audit duties outsourced by a study contractor.

LINICAL CORPORATION

10th floor of the Shin Osaka Brick Bldg Bldg. Bldg. Bldg., 6-1 Miyahara 1-chome, Yodogawa-ku, Osaka 532-0003 Japan

Phone:06-6150-2478 Fax:06-6150-2675

# Reference

1. Go AS, Hylek EM, Phillips KA, et al.: Prevalence of diagnosed atrial fibrillation in adults. National implications for rhythm management and stroke prevention: the AnTicoagulation and Risk Factors in Atrial Fibrillation (ATRIA) study. JAMA 2001; 285(18):2370-5.
2. Inoue H, Fujiki A, Origasa H, et al. Prevalence of atrial fibrillation in the general population of Japan: an analysis based on periodic health examination. Int J Cardiol 2009;137:102-7.
3. Wolf PA, Abbott RD, Kannel WB: Atrial fibrillation as an independent risk factor for stroke: the Framingham Study. Stroke. 1991;22(8):983-8.
4. Hart RG, Pearce LA, Rothbart RM, et al.: Stroke with intermittent atrial fibrillation: incidence and predictors during aspirin therapy. Stroke Prevention in Atrial Fibrillation Investigators. J Am Coll Cardiol. 2000;35(1):183-7.
5. Yamashita Y, Hamatani Y, Esato M, Chun YH, Tsuji H, Wada H, Hasegawa K, Abe M, Lip GY, Akao M. Clinical Characteristics and Outcomes in Extreme Elderly (Age >= 85 Years) Japanese Patients With Atrial Fibrillation: The Fushimi AF Registry. Chest. 2016 ;149:401-12.
6. Warfarin Tablets, 23rd Edition, Package Insert
7. Atarashi H, Inoue H, Okumura K, et al. Present status of anticoagulation treatment in Japanese patients with atrial fibrillation. Circ J. 2011;75:1328-33.
8. Ogawa S, Yamashita T, Yamazaki T, et al. Optimal treatment strategy for patients with paroxysmal atrial fibrillation: J-RHYTHM Study. Circ J. 2009 Feb;73(2):242-8.
9. Yoko Sato, Yuki Murata, Tsuyoshi Chiba, and Keizo Umegaki. A systematic review of the tolerance of vitamin K intake in warfarin users. Journal of Food Hygiene. 2015;56(4):157-65.
10. Ministry of HealthPharmaceuticals and Medical Devices Safety Information No. 235, Ref. 2 "Perspectives on Pharmacogenomics (Genetic Polymorphisms Related to the Treatment of Warfarin). 2007;235:22-3.
11. Ministry of HealthManuals for Measures for Individual Serious Adverse Drug Reactions-Bleeding Trends (June 2007). 2007:11.
12. Plazaxa ^®^, 8th edition of the package insert
13. Exalert ^®^ Tablets, 6th Edition, Package Insert
14. Package insert, Ericus ^®^ Tablets, 6th Edition
15. Ricciana ^®^ Tablets 5th Edition of the Package Insert
16. Mendell J, Zahir H, Matsushima N, et al. Drug-drug interaction studies of cardiovascular drugs involving P-glycoprotein, an efflux transporter, on the pharmacokinetics of edoxaban, an oral factor Xa inhibitor.Am J Cardiovasc Drugs. 2013 Oct;13(5):331-2.
17. Abstracts of the 60th Annual Meeting of the Scientific and Standardization Committee of the International Society on Thrombosis and Haemostasis, June 23-26, 2014, Milwaukee, WI. J Thromb Haemost. 2014;12(Suppl 1):17.
18. Guidelines for the diagnosis and treatment of cardiovascular diseases (2012 Joint Research Team Report). Non-valvular atrial fibrillation treatment (drug) guidelines (2013 updated). 2013
19. Apostolakis S, Lane DA, Buller H, et al. Comparison of the CHADS_2_, CHA_2_DS_2_-VASc and HAS-BLED scores for the prediction of clinically relevant bleeding in anticoagulated patients with atrial fibrillation: the AMADEUS trial. Thromb Haemost. 2013 Nov;110(5):1074-9.
20. Connolly SJ, Ezekowitz MD, Yusuf S, et al. RE-LY Steering Committee and Investigators. Dabigatran versus warfarin in patients with atrial fibrillation. N Engl J Med. 2009;361(12):1139-51.
21. Japan Geriatrics Soc. Guide to Appropriate Provision of Health Care for the Elderly 2011.
22. Ministry of HealthGuidelines for Clinical Evaluation of Drugs Used in the Elderly (Notification No. 104 of the New Drug Division, PAB dated December 2, 1993) 1997.
23. Japan Geriatrics Soc. Safety Pharmacotherapy Guidelines for the Elderly 2015.2015
24. Ministry of Education, Culture, Sports, Science and Technology and Ministry of Health, Labour and Welfare. Ethical Guidelines for Medical Research for Humans (December 22, 2014). 2014
25. Ruff CT, Giugliano RP, Antman EM, et al. Evaluation of the novel factor Xa inhibitor edoxaban compared with warfarin in patients with atrial fibrillation: design and rationale for the Effective aNticoaGulation with factor xA next GEneration in Atrial Fibrillation-Thrombolysis In Myocardial Infarction study 48 (ENGAGE AF-TIMI 48). Am Heart J. 2010 Oct;160(4):635-41.
26. Moriski DE, Green LW, Levine DM. Concurrent and predictive validity of a self-reported measure of medicatio adherence. Med Care. 1986;24:67-74

Appendix:

Appendix 11 CHADS_2_ Score

|  | Risk Factors | | Score |
| --- | --- | --- | --- |
| C | Congestive heart failure/LV dysfunction | Heart failure, left ventricular dysfunction | 1 |
| H | Hypertension | Hypertension | 1 |
| A | Age >= 75 years | 75 years of age or older | 1 |
| D | Diabetes mellitus | Diabetes mellitus | 1 |
| S_2_ | Stroke/TIA | Previous cerebral infarction, TIA | 2 |
|  | Total | | 0~6 |

TIA: transient ischemic attack

Reference Source: Guidelines for the Treatment of Atrial Fibrillation (Drugs) 2013 Revised Edition, p.22

Appendix 22 CHA_2_DS_2_-VASc Score

|  | Risk Factors | | Score |
| --- | --- | --- | --- |
| C | Congestive heart failure/LV dysfunction | Heart failure, left ventricular dysfunction | 1 |
| H | Hypertension | Hypertension | 1 |
| A_2_ | Age >= 75 years | 75 years of age or older | 2 |
| D | Diabetes mellitus | Diabetes mellitus | 1 |
| S_2_ | Stroke/TIA/TE | History of cerebral infarction, TIA, or thromboembolism | 2 |
| V | Vascular disease (prior myocardial infarction, peripheral artery desease, or aortic plaque) | Vascular disease (previous MYOCARDIAL INFARCTION, peripheral arterial disease, aortic plaque) | 1 |
| A | Age 65 – 74 years |  | 1 |
| Sc | Sex category (i.e., female gender) | Female gender | 1 |
|  | Total | | 0~9* |

TIA: transient ischemic attack

* Because 0, 1, and 2 points are assigned according to age, the total is at most 9 points.

Reference Source: Guidelines for the Treatment of Atrial Fibrillation (Drugs) 2013 Revised Edition, p.24

Appendix 33 HAS-BLED scores

|  | CLINICAL PRESENTATION | Key Notes |
| --- | --- | --- |
| H | Hypertensive ^1^ | 1 |
| A | ^2^ of renal or hepatic dysfunction (1 point each) | 2 |
| S | Stroke | 1 |
| B | Hemorrhage^3^ | 1 |
| L | Unstable international normalized ratio (INR) ^4^ | 1 |
| E | Elderly (>65 years) | 1 |
| D | Drug/alcohol ^5^ (1 point each) | 2 |
|  | Total | 9 |

1. Systolic blood pressure> 160 mmHg
2. Renal impairment: chronic dialysis, kidney transplantation, serum creatinine 200 μmol/L
    (2.26 mg/dL) or higher.
   Liver function disorder: Chronic liver disorder (e.g., cirrhosis) or abnormal laboratory test values
    (Bilirubin > 2 times upper limit of normal, AST, ALT, ALP
    > 3 times the upper limit of normal)
3. Bleeding history, bleeding tendency (bleeding diathesis, anaemia, etc.)
4. INR-unstable, high or TTR (time in therapeutic range) <60%
5. Antiplatelet drugs, concomitant NSAIDs use, alcoholism

Reference Source: Guidelines for the Treatment of Atrial Fibrillation (Drugs) 2013 Revised Edition, p.25

Appendix 44 P-Glycoprotein Inhibitors with Interactions with Individual DOAC

| DOAC names | p-gp inhibitor | |
| --- | --- | --- |
| Exalert | Contraindication of concomitant use | Ritonavir  Lopinavir/ritonavir  Atazanavir  Indinavir  Saquinavir  Darunavir  Fosamprenavir  Nelfinavir  Itraconazole  Voriconazole  Miconazole  Ketoconazole (not marketed in Japan) |
|  | Precautions for Coadministration | Clarithromycin  Erythromycin |
| Elique | Precautions for Coadministration | Itraconazole  Voriconazole  HIV protease inhibitors (e.g., ritonavir)  Macrolides (clarithromycin, erythromycin, etc.)  Fluconazole  Naproxen  Diltiazem |
| Prazaxa | Contraindication of concomitant use | Itraconazole |
|  | Precautions for Coadministration | Verapamil hydrochloric  Amiodarone hydrochloride  Quinidine Sulfate Hydrate  Tacrolimus  Cyclosporine  Clarithromycin  Ritonavir  Nelfinavir  Saquinavir, etc. |
| Lixiana | Precautions for Coadministration | Quinidine Sulfate Hydrate  Verapamil hydrochloric  Erythromycin  Cyclosporine  Azithromycin  Clarithromyline  Itraconazole  Diltiazem  Amiodarone hydrochloride  HIV protease inhibitors (ritonavir, etc.) |

Reference: 8th edition ^12^ of "Plazaxa ^®^" in the package insert, 6th edition ^13^ of "Exalert ^®^ Tablets" in the package insert, 6th edition ^14^ of "Elicus ^®^ Tablets" in the package insert, and 5th edition ^15^ of "Ricciana ^®^ Tablets" in the package insert.^12131415^

1. ※ Minor surgery includes procedures involving biopsy and puncture [↑](#footnote-ref-1)
