# Supplementary material for: Current status of proton pump inhibitor use in Japanese elderly patients with non-valvular atrial fibrillation: A subanalysis of the ANAFIE Registry
Source: PLoS One. 2020 Nov 5;15(11):e0240859. doi: 10.1371/journal.pone.0240859 (PMC7644054; doi:10.1371/journal.pone.0240859)
Supplement: S4 File — (PDF) [file pone.0240859.s005.pdf]

Laboratory tests at the time of obtaining Table 2-6 consent, according to the presence or absence of proton-pump inhibitors

Unique Subject Identifier=6298000000-0169

MEANS Procedure

Assay variable : PTINR_VAL1 PT-INR Value

N	Mean	Standard Deviation	Minimum	Maximum
6	1.9033333	0.2122891	1.6400000	2.1500000

Unique Subject Identifier=6298000000-0170

Assay variable : PTINR_VAL1 PT-INR Value

N	Mean	Standard Deviation	Minimum	Maximum
4	1.8300000	0.7481978	1.3300000	2.9400000

Unique Subject Identifier=6304000000-0005

Assay variable : PTINR_VAL1 PT-INR Value

N	Mean	Standard Deviation	Minimum	Maximum
4	2.2475000	0.4780080	1.6400000	2.7200000

Unique Subject Identifier=6304000000-0011

Assay variable : PTINR_VAL1 PT-INR Value

N	Mean	Standard Deviation	Minimum	Maximum
1	1.9600000		1.9600000	1.9600000

Laboratory tests at the time of obtaining Table 2-6 consent, according to the presence or absence of proton-pump inhibitors

Unique Subject Identifier=6304000000-0012

MEANS Procedure

Assay variable : PTINR_VAL1 PT-INR Value

N	Mean	Standard Deviation	Minimum	Maximum
1	1.7000000		1.7000000	1.7000000

Unique Subject Identifier=6304000000-0021

Assay variable : PTINR_VAL1 PT-INR Value

N	Mean	Standard Deviation	Minimum	Maximum
3	1.7233333	0.4135618	1.4600000	2.2000000

Unique Subject Identifier=6304000000-0023

Assay variable : PTINR_VAL1 PT-INR Value

N	Mean	Standard Deviation	Minimum	Maximum
2	2.1250000	0.3464823	1.8800000	2.3700000

Unique Subject Identifier=6304000000-0024

Assay variable : PTINR_VAL1 PT-INR Value

N	Mean	Standard Deviation	Minimum	Maximum
3	1.7866667	0.1101514	1.6600000	1.8600000

Laboratory tests at the time of obtaining Table 2-6 consent, according to the presence or absence of proton-pump inhibitors

Unique Subject Identifier=6304000000-0025

MEANS Procedure

Assay variable : PTINR_VAL1 PT-INR Value

N	Mean	Standard Deviation	Minimum	Maximum
1	2.5100000		2.5100000	2.5100000

Unique Subject Identifier=6309000000-0005

Assay variable : PTINR_VAL1 PT-INR Value

N	Mean	Standard Deviation	Minimum	Maximum
3	2.7133333	0.0757188	2.6600000	2.8000000

Unique Subject Identifier=6309000000-0007

Assay variable : PTINR_VAL1 PT-INR Value

N	Mean	Standard Deviation	Minimum	Maximum
2	2.8000000	0.0141421	2.7900000	2.8100000

Unique Subject Identifier=6309000000-0013

Assay variable : PTINR_VAL1 PT-INR Value

N	Mean	Standard Deviation	Minimum	Maximum
5	2.2200000	0.3342155	1.6900000	2.5700000

Laboratory tests at the time of obtaining Table 2-6 consent, according to the presence or absence of proton-pump inhibitors

Unique Subject Identifier=6309000000-0014

MEANS Procedure

Assay variable : PTINR_VAL1 PT-INR Value

N	Mean	Standard Deviation	Minimum	Maximum
4	1.9350000	0.1100000	1.7900000	2.0300000

Unique Subject Identifier=6309000000-0017

Assay variable : PTINR_VAL1 PT-INR Value

N	Mean	Standard Deviation	Minimum	Maximum
3	1.8566667	0.2532456	1.5700000	2.0500000

Unique Subject Identifier=6309000000-0018

Assay variable : PTINR_VAL1 PT-INR Value

N	Mean	Standard Deviation	Minimum	Maximum
3	2.5700000	0.2426932	2.3700000	2.8400000

Unique Subject Identifier=6309000000-0030

Assay variable : PTINR_VAL1 PT-INR Value

N	Mean	Standard Deviation	Minimum	Maximum
2	2.5000000	0.3959798	2.2200000	2.7800000

Laboratory tests at the time of obtaining Table 2-6 consent, according to the presence or absence of proton-pump inhibitors

Unique Subject Identifier=6310000100-0001

MEANS Procedure

Assay variable : PTINR_VAL1 PT-INR Value

N	Mean	Standard Deviation	Minimum	Maximum
5	1.8760000	0.1974335	1.6300000	2.1000000

Unique Subject Identifier=6310000100-0003

Assay variable : PTINR_VAL1 PT-INR Value

N	Mean	Standard Deviation	Minimum	Maximum
6	2.0233333	0.2492923	1.6700000	2.2600000

Unique Subject Identifier=6310000100-0005

Assay variable : PTINR_VAL1 PT-INR Value

N	Mean	Standard Deviation	Minimum	Maximum
6	2.0816667	0.4285518	1.6600000	2.7200000

Unique Subject Identifier=6310000100-0007

Assay variable : PTINR_VAL1 PT-INR Value

N	Mean	Standard Deviation	Minimum	Maximum
5	2.3380000	0.5558507	1.6600000	2.8900000

Laboratory tests at the time of obtaining Table 2-6 consent, according to the presence or absence of proton-pump inhibitors

Unique Subject Identifier=6310000100-0008

MEANS Procedure

Assay variable : PTINR_VAL1 PT-INR Value

N	Mean	Standard Deviation	Minimum	Maximum
5	2.4180000	0.5912867	1.7600000	3.3200000

Unique Subject Identifier=6310000100-0009

Assay variable : PTINR_VAL1 PT-INR Value

N	Mean	Standard Deviation	Minimum	Maximum
5	1.9400000	0.4992494	1.4000000	2.7400000

Unique Subject Identifier=6310000100-0012

Assay variable : PTINR_VAL1 PT-INR Value

N	Mean	Standard Deviation	Minimum	Maximum
6	2.3900000	0.6134493	1.5300000	3.2800000

Unique Subject Identifier=6310000100-0014

Assay variable : PTINR_VAL1 PT-INR Value

N	Mean	Standard Deviation	Minimum	Maximum
5	2.3340000	0.8346436	1.5700000	3.7600000

Laboratory tests at the time of obtaining Table 2-6 consent, according to the presence or absence of proton-pump inhibitors

Unique Subject Identifier=6310000100-0016

MEANS Procedure

Assay variable : PTINR_VAL1 PT-INR Value

N	Mean	Standard Deviation	Minimum	Maximum
5	1.9120000	0.4343040	1.1900000	2.3500000

Unique Subject Identifier=6310000100-0019

Assay variable : PTINR_VAL1 PT-INR Value

N	Mean	Standard Deviation	Minimum	Maximum
5	2.3420000	0.4076395	1.6800000	2.6700000

Unique Subject Identifier=6310000100-0021

Assay variable : PTINR_VAL1 PT-INR Value

N	Mean	Standard Deviation	Minimum	Maximum
6	2.2133333	0.6199892	1.5300000	3.1600000

Unique Subject Identifier=6310000100-0024

Assay variable : PTINR_VAL1 PT-INR Value

N	Mean	Standard Deviation	Minimum	Maximum
5	2.0120000	0.5511533	1.3400000	2.8200000

Laboratory tests at the time of obtaining Table 2-6 consent, according to the presence or absence of proton-pump inhibitors

Unique Subject Identifier=6310000100-0026

MEANS Procedure

Assay variable : PTINR_VAL1 PT-INR Value

N	Mean	Standard Deviation	Minimum	Maximum
4	1.9675000	0.4823812	1.4300000	2.5900000

Unique Subject Identifier=6310000100-0030

Assay variable : PTINR_VAL1 PT-INR Value

N	Mean	Standard Deviation	Minimum	Maximum
6	1.8666667	0.1127239	1.7100000	1.9700000

Unique Subject Identifier=6310000100-0031

Assay variable : PTINR_VAL1 PT-INR Value

N	Mean	Standard Deviation	Minimum	Maximum
5	2.4940000	0.2847455	2.0800000	2.8600000

Unique Subject Identifier=6310000100-0033

Assay variable : PTINR_VAL1 PT-INR Value

N	Mean	Standard Deviation	Minimum	Maximum
5	1.9360000	0.5511170	1.3400000	2.6400000

Laboratory tests at the time of obtaining Table 2-6 consent, according to the presence or absence of proton-pump inhibitors

Unique Subject Identifier=6310000100-0035

MEANS Procedure

Assay variable : PTINR_VAL1 PT-INR Value

N	Mean	Standard Deviation	Minimum	Maximum
5	1.8880000	0.3094673	1.5500000	2.2200000

Unique Subject Identifier=6310000100-0036

Assay variable : PTINR_VAL1 PT-INR Value

N	Mean	Standard Deviation	Minimum	Maximum
6	2.1116667	0.3047239	1.7000000	2.5100000

Unique Subject Identifier=6333000000-0001

Assay variable : PTINR_VAL1 PT-INR Value

N	Mean	Standard Deviation	Minimum	Maximum
4	2.4175000	0.7544258	1.4700000	3.2300000

Unique Subject Identifier=6340011000-0002

Assay variable : PTINR_VAL1 PT-INR Value

N	Mean	Standard Deviation	Minimum	Maximum
6	1.7666667	0.1966384	1.6000000	2.1000000

Laboratory tests at the time of obtaining Table 2-6 consent, according to the presence or absence of proton-pump inhibitors

Unique Subject Identifier=6340011000-0008

MEANS Procedure

Assay variable : PTINR_VAL1 PT-INR Value

N	Mean	Standard Deviation	Minimum	Maximum
3	1.5666667	0.0577350	1.5000000	1.6000000

Unique Subject Identifier=6340011000-0009

Assay variable : PTINR_VAL1 PT-INR Value

N	Mean	Standard Deviation	Minimum	Maximum
5	1.8200000	0.1303840	1.7000000	2.0000000

Unique Subject Identifier=6340011000-0010

Assay variable : PTINR_VAL1 PT-INR Value

N	Mean	Standard Deviation	Minimum	Maximum
4	1.9750000	0.3593976	1.5000000	2.3000000

Unique Subject Identifier=6366000000-0015

Assay variable : PTINR_VAL1 PT-INR Value

N	Mean	Standard Deviation	Minimum	Maximum
6	2.2650000	0.2011716	1.9300000	2.5400000

Laboratory tests at the time of obtaining Table 2-6 consent, according to the presence or absence of proton-pump inhibitors

Unique Subject Identifier=6426000000-0001

MEANS Procedure

Assay variable : PTINR_VAL1 PT-INR Value

N	Mean	Standard Deviation	Minimum	Maximum
5	2.3820000	0.2289541	2.2200000	2.7800000

Unique Subject Identifier=6426000000-0002

Assay variable : PTINR_VAL1 PT-INR Value

N	Mean	Standard Deviation	Minimum	Maximum
6	1.8916667	0.5451024	1.2100000	2.4500000

Unique Subject Identifier=6426000000-0006

Assay variable : PTINR_VAL1 PT-INR Value

N	Mean	Standard Deviation	Minimum	Maximum
3	2.4766667	0.3066486	2.1400000	2.7400000

Unique Subject Identifier=6426000000-0008

Assay variable : PTINR_VAL1 PT-INR Value

N	Mean	Standard Deviation	Minimum	Maximum
6	1.7933333	0.3691973	1.4400000	2.4900000

Laboratory tests at the time of obtaining Table 2-6 consent, according to the presence or absence of proton-pump inhibitors

Unique Subject Identifier=6426000000-0009

MEANS Procedure

Assay variable : PTINR_VAL1 PT-INR Value

N	Mean	Standard Deviation	Minimum	Maximum
5	1.7180000	0.2357329	1.4800000	2.0600000

Unique Subject Identifier=6426000000-0010

Assay variable : PTINR_VAL1 PT-INR Value

N	Mean	Standard Deviation	Minimum	Maximum
5	1.5780000	0.0995992	1.4300000	1.6800000

Unique Subject Identifier=6426000000-0014

Assay variable : PTINR_VAL1 PT-INR Value

N	Mean	Standard Deviation	Minimum	Maximum
3	1.7433333	0.0808290	1.6700000	1.8300000

Unique Subject Identifier=6426000000-0015

Assay variable : PTINR_VAL1 PT-INR Value

N	Mean	Standard Deviation	Minimum	Maximum
5	1.9220000	0.0967988	1.8000000	2.0300000

Laboratory tests at the time of obtaining Table 2-6 consent, according to the presence or absence of proton-pump inhibitors

Unique Subject Identifier=6426000000-0016

MEANS Procedure

Assay variable : PTINR_VAL1 PT-INR Value

N	Mean	Standard Deviation	Minimum	Maximum
3	2.1966667	0.7054313	1.6400000	2.9900000

Unique Subject Identifier=6426000000-0017

Assay variable : PTINR_VAL1 PT-INR Value

N	Mean	Standard Deviation	Minimum	Maximum
1	1.0400000		1.0400000	1.0400000

Unique Subject Identifier=6426000000-0019

Assay variable : PTINR_VAL1 PT-INR Value

N	Mean	Standard Deviation	Minimum	Maximum
5	1.6720000	0.4934268	1.1700000	2.4900000

Unique Subject Identifier=6426000000-0021

Assay variable : PTINR_VAL1 PT-INR Value

N	Mean	Standard Deviation	Minimum	Maximum
4	2.3925000	0.3836991	1.8900000	2.8100000

Laboratory tests at the time of obtaining Table 2-6 consent, according to the presence or absence of proton-pump inhibitors

Unique Subject Identifier=6426000000-0023

MEANS Procedure

Assay variable : PTINR_VAL1 PT-INR Value

N	Mean	Standard Deviation	Minimum	Maximum
6	2.2016667	0.3270117	1.7600000	2.6600000

Unique Subject Identifier=6542010000-0005

Assay variable : PTINR_VAL1 PT-INR Value

N	Mean	Standard Deviation	Minimum	Maximum
5	1.5580000	0.1697645	1.3000000	1.7500000

Unique Subject Identifier=6542010000-0009

Assay variable : PTINR_VAL1 PT-INR Value

N	Mean	Standard Deviation	Minimum	Maximum
2	2.4000000	0.2828427	2.2000000	2.6000000

Unique Subject Identifier=6542010000-0010

Assay variable : PTINR_VAL1 PT-INR Value

N	Mean	Standard Deviation	Minimum	Maximum
5	1.8400000	0.1516575	1.7000000	2.1000000

Laboratory tests at the time of obtaining Table 2-6 consent, according to the presence or absence of proton-pump inhibitors

Unique Subject Identifier=6542010000-0011

MEANS Procedure

Assay variable : PTINR_VAL1 PT-INR Value

N	Mean	Standard Deviation	Minimum	Maximum
5	1.8000000	0.3082207	1.5000000	2.2000000

Unique Subject Identifier=6542010000-0012

Assay variable : PTINR_VAL1 PT-INR Value

N	Mean	Standard Deviation	Minimum	Maximum
6	1.4866667	0.1143095	1.4000000	1.7000000

Unique Subject Identifier=6542010000-0017

Assay variable : PTINR_VAL1 PT-INR Value

N	Mean	Standard Deviation	Minimum	Maximum
6	2.2666667	0.5921711	1.6000000	3.0000000

Unique Subject Identifier=6542010000-0019

Assay variable : PTINR_VAL1 PT-INR Value

N	Mean	Standard Deviation	Minimum	Maximum
5	1.8560000	0.3077012	1.6000000	2.2000000

Laboratory tests at the time of obtaining Table 2-6 consent, according to the presence or absence of proton-pump inhibitors

Unique Subject Identifier=6614001000-0004

MEANS Procedure

Assay variable : PTINR_VAL1 PT-INR Value

N	Mean	Standard Deviation	Minimum	Maximum
1	2.7900000		2.7900000	2.7900000

Unique Subject Identifier=6614001000-0006

Assay variable : PTINR_VAL1 PT-INR Value

N	Mean	Standard Deviation	Minimum	Maximum
3	2.1066667	0.4781562	1.7500000	2.6500000

Unique Subject Identifier=6614001000-0007

Assay variable : PTINR_VAL1 PT-INR Value

N	Mean	Standard Deviation	Minimum	Maximum
5	2.0940000	0.1668233	1.8100000	2.2200000

Unique Subject Identifier=6614001000-0009

Assay variable : PTINR_VAL1 PT-INR Value

N	Mean	Standard Deviation	Minimum	Maximum
2	1.6600000	0.4242641	1.3600000	1.9600000

Laboratory tests at the time of obtaining Table 2-6 consent, according to the presence or absence of proton-pump inhibitors

Unique Subject Identifier=6623000000-0003

MEANS Procedure

Assay variable : PTINR_VAL1 PT-INR Value

N	Mean	Standard Deviation	Minimum	Maximum
6	1.8516667	0.1552310	1.6500000	2.1000000

Unique Subject Identifier=6623000000-0004

Assay variable : PTINR_VAL1 PT-INR Value

N	Mean	Standard Deviation	Minimum	Maximum
4	2.1275000	0.1711481	2.0000000	2.3800000

Unique Subject Identifier=6623000000-0012

Assay variable : PTINR_VAL1 PT-INR Value

N	Mean	Standard Deviation	Minimum	Maximum
3	1.2633333	0.0776745	1.2000000	1.3500000

Unique Subject Identifier=6623000000-0016

Assay variable : PTINR_VAL1 PT-INR Value

N	Mean	Standard Deviation	Minimum	Maximum
5	1.7220000	0.3189357	1.2500000	2.0300000

Laboratory tests at the time of obtaining Table 2-6 consent, according to the presence or absence of proton-pump inhibitors

Unique Subject Identifier=6642010010-0003

MEANS Procedure

Assay variable : PTINR_VAL1 PT-INR Value

N	Mean	Standard Deviation	Minimum	Maximum
6	1.8100000	0.1882551	1.5800000	2.1000000

Unique Subject Identifier=6642010010-0004

Assay variable : PTINR_VAL1 PT-INR Value

N	Mean	Standard Deviation	Minimum	Maximum
6	1.7483333	0.1020621	1.6000000	1.8400000

Unique Subject Identifier=6642010010-0010

Assay variable : PTINR_VAL1 PT-INR Value

N	Mean	Standard Deviation	Minimum	Maximum
5	2.2960000	0.5373360	1.8000000	3.0600000

Unique Subject Identifier=6642010010-0015

Assay variable : PTINR_VAL1 PT-INR Value

N	Mean	Standard Deviation	Minimum	Maximum
6	1.6866667	0.2398889	1.4700000	2.0100000

Laboratory tests at the time of obtaining Table 2-6 consent, according to the presence or absence of proton-pump inhibitors

Unique Subject Identifier=6748000000-0001

MEANS Procedure

Assay variable : PTINR_VAL1 PT-INR Value

N	Mean	Standard Deviation	Minimum	Maximum
6	1.8666667	0.2338090	1.6000000	2.2000000

Unique Subject Identifier=6748000000-0002

Assay variable : PTINR_VAL1 PT-INR Value

N	Mean	Standard Deviation	Minimum	Maximum
5	2.1400000	0.2880972	1.7000000	2.5000000

Unique Subject Identifier=6748000000-0004

Assay variable : PTINR_VAL1 PT-INR Value

N	Mean	Standard Deviation	Minimum	Maximum
6	1.8833333	0.4308906	1.5000000	2.7000000

Unique Subject Identifier=7001010000-0004

Assay variable : PTINR_VAL1 PT-INR Value

N	Mean	Standard Deviation	Minimum	Maximum
6	1.3983333	0.1105290	1.2700000	1.5600000

Laboratory tests at the time of obtaining Table 2-6 consent, according to the presence or absence of proton-pump inhibitors

Unique Subject Identifier=7001010000-0005

MEANS Procedure

Assay variable : PTINR_VAL1 PT-INR Value

N	Mean	Standard Deviation	Minimum	Maximum
4	1.4525000	0.1034005	1.3300000	1.5800000

Unique Subject Identifier=7002000000-0004

Assay variable : PTINR_VAL1 PT-INR Value

N	Mean	Standard Deviation	Minimum	Maximum
3	2.1266667	0.3200521	1.7600000	2.3500000

Unique Subject Identifier=7002000000-0011

Assay variable : PTINR_VAL1 PT-INR Value

N	Mean	Standard Deviation	Minimum	Maximum
6	1.9516667	0.2183957	1.6700000	2.2000000

Unique Subject Identifier=7002000000-0013

Assay variable : PTINR_VAL1 PT-INR Value

N	Mean	Standard Deviation	Minimum	Maximum
3	2.0033333	0.2441994	1.7300000	2.2000000

Laboratory tests at the time of obtaining Table 2-6 consent, according to the presence or absence of proton-pump inhibitors

Unique Subject Identifier=7002000000-0016

MEANS Procedure

Assay variable : PTINR_VAL1 PT-INR Value

N	Mean	Standard Deviation	Minimum	Maximum
6	1.9100000	0.1604992	1.7600000	2.2000000

Unique Subject Identifier=7002000000-0017

Assay variable : PTINR_VAL1 PT-INR Value

N	Mean	Standard Deviation	Minimum	Maximum
6	2.0300000	0.5932285	1.3200000	3.1000000

Unique Subject Identifier=7002000000-0018

Assay variable : PTINR_VAL1 PT-INR Value

N	Mean	Standard Deviation	Minimum	Maximum
2	2.6450000	0.6858936	2.1600000	3.1300000

Unique Subject Identifier=7002000000-0020

Assay variable : PTINR_VAL1 PT-INR Value

N	Mean	Standard Deviation	Minimum	Maximum
3	1.7266667	0.2631223	1.4900000	2.0100000

Laboratory tests at the time of obtaining Table 2-6 consent, according to the presence or absence of proton-pump inhibitors

Unique Subject Identifier=7002000000-0026

MEANS Procedure

Assay variable : PTINR_VAL1 PT-INR Value

N	Mean	Standard Deviation	Minimum	Maximum
3	2.2900000	0.0754983	2.2200000	2.3700000

Unique Subject Identifier=7002000000-0032

Assay variable : PTINR_VAL1 PT-INR Value

N	Mean	Standard Deviation	Minimum	Maximum
4	2.1050000	0.4320880	1.7400000	2.7300000

Unique Subject Identifier=7002000000-0038

Assay variable : PTINR_VAL1 PT-INR Value

N	Mean	Standard Deviation	Minimum	Maximum
4	2.1075000	0.4912145	1.5400000	2.5800000

Unique Subject Identifier=7002000000-0039

Assay variable : PTINR_VAL1 PT-INR Value

N	Mean	Standard Deviation	Minimum	Maximum
4	2.4050000	0.7684834	1.6700000	3.3800000

Laboratory tests at the time of obtaining Table 2-6 consent, according to the presence or absence of proton-pump inhibitors

Unique Subject Identifier=7002000000-0041

MEANS Procedure

Assay variable : PTINR_VAL1 PT-INR Value

N	Mean	Standard Deviation	Minimum	Maximum
4	2.3250000	0.4141256	1.8700000	2.7100000

Unique Subject Identifier=7002000000-0042

Assay variable : PTINR_VAL1 PT-INR Value

N	Mean	Standard Deviation	Minimum	Maximum
4	2.2700000	0.0734847	2.1700000	2.3300000

Unique Subject Identifier=7004000000-0002

Assay variable : PTINR_VAL1 PT-INR Value

N	Mean	Standard Deviation	Minimum	Maximum
6	1.6400000	0.1906830	1.3200000	1.8100000

Unique Subject Identifier=7004000000-0008

Assay variable : PTINR_VAL1 PT-INR Value

N	Mean	Standard Deviation	Minimum	Maximum
3	2.1933333	0.1305118	2.0900000	2.3400000

Laboratory tests at the time of obtaining Table 2-6 consent, according to the presence or absence of proton-pump inhibitors

Unique Subject Identifier=7004000000-0011

MEANS Procedure

Assay variable : PTINR_VAL1 PT-INR Value

N	Mean	Standard Deviation	Minimum	Maximum
3	2.2066667	0.0971253	2.1000000	2.2900000

Unique Subject Identifier=7004000000-0018

Assay variable : PTINR_VAL1 PT-INR Value

N	Mean	Standard Deviation	Minimum	Maximum
3	2.0600000	0.1400000	1.9000000	2.1600000

Unique Subject Identifier=7005000000-0001

Assay variable : PTINR_VAL1 PT-INR Value

N	Mean	Standard Deviation	Minimum	Maximum
3	1.9966667	0.3552933	1.7300000	2.4000000

Unique Subject Identifier=7005000000-0002

Assay variable : PTINR_VAL1 PT-INR Value

N	Mean	Standard Deviation	Minimum	Maximum
3	1.8633333	0.3758102	1.5500000	2.2800000

Laboratory tests at the time of obtaining Table 2-6 consent, according to the presence or absence of proton-pump inhibitors

Unique Subject Identifier=7005000000-0003

MEANS Procedure

Assay variable : PTINR_VAL1 PT-INR Value

N	Mean	Standard Deviation	Minimum	Maximum
3	1.4366667	0.2177919	1.2600000	1.6800000

Unique Subject Identifier=7005000000-0006

Assay variable : PTINR_VAL1 PT-INR Value

N	Mean	Standard Deviation	Minimum	Maximum
5	2.3880000	0.1505656	2.1400000	2.5200000

Unique Subject Identifier=7005000000-0007

Assay variable : PTINR_VAL1 PT-INR Value

N	Mean	Standard Deviation	Minimum	Maximum
4	2.3500000	0.7673330	1.6300000	3.3900000

Unique Subject Identifier=7005000000-0008

Assay variable : PTINR_VAL1 PT-INR Value

N	Mean	Standard Deviation	Minimum	Maximum
4	1.5600000	0.5383308	1.1400000	2.3100000

Laboratory tests at the time of obtaining Table 2-6 consent, according to the presence or absence of proton-pump inhibitors

Unique Subject Identifier=7005000000-0009

MEANS Procedure

Assay variable : PTINR_VAL1 PT-INR Value

N	Mean	Standard Deviation	Minimum	Maximum
6	2.0566667	0.1980572	1.8200000	2.2800000

Unique Subject Identifier=7005000000-0010

Assay variable : PTINR_VAL1 PT-INR Value

N	Mean	Standard Deviation	Minimum	Maximum
3	1.8400000	0.2022375	1.6900000	2.0700000

Unique Subject Identifier=7005000000-0011

Assay variable : PTINR_VAL1 PT-INR Value

N	Mean	Standard Deviation	Minimum	Maximum
3	1.7033333	0.8807005	1.0300000	2.7000000

Unique Subject Identifier=7005000000-0012

Assay variable : PTINR_VAL1 PT-INR Value

N	Mean	Standard Deviation	Minimum	Maximum
3	2.1700000	0.6636264	1.4700000	2.7900000

Laboratory tests at the time of obtaining Table 2-6 consent, according to the presence or absence of proton-pump inhibitors

Unique Subject Identifier=7005000000-0013

MEANS Procedure

Assay variable : PTINR_VAL1 PT-INR Value

N	Mean	Standard Deviation	Minimum	Maximum
4	1.5550000	0.1569501	1.4000000	1.7000000

Unique Subject Identifier=7005000000-0014

Assay variable : PTINR_VAL1 PT-INR Value

N	Mean	Standard Deviation	Minimum	Maximum
3	1.8066667	0.1800926	1.6000000	1.9300000

Unique Subject Identifier=7005000000-0015

Assay variable : PTINR_VAL1 PT-INR Value

N	Mean	Standard Deviation	Minimum	Maximum
5	2.4800000	0.4851288	1.8800000	3.1100000

Unique Subject Identifier=7005000000-0016

Assay variable : PTINR_VAL1 PT-INR Value

N	Mean	Standard Deviation	Minimum	Maximum
3	2.0133333	0.1464013	1.8800000	2.1700000

Laboratory tests at the time of obtaining Table 2-6 consent, according to the presence or absence of proton-pump inhibitors

Unique Subject Identifier=7008000000-0005

MEANS Procedure

Assay variable : PTINR_VAL1 PT-INR Value

N	Mean	Standard Deviation	Minimum	Maximum
6	2.2400000	0.3352014	1.8000000	2.8000000

Unique Subject Identifier=7008000000-0007

Assay variable : PTINR_VAL1 PT-INR Value

N	Mean	Standard Deviation	Minimum	Maximum
6	1.6850000	0.2126735	1.4700000	2.0400000

Unique Subject Identifier=7008000000-0011

Assay variable : PTINR_VAL1 PT-INR Value

N	Mean	Standard Deviation	Minimum	Maximum
4	2.2325000	0.3472151	1.9200000	2.5900000

Unique Subject Identifier=7008000000-0013

Assay variable : PTINR_VAL1 PT-INR Value

N	Mean	Standard Deviation	Minimum	Maximum
3	1.7566667	0.0907377	1.6900000	1.8600000

Laboratory tests at the time of obtaining Table 2-6 consent, according to the presence or absence of proton-pump inhibitors

Unique Subject Identifier=7008000000-0022

MEANS Procedure

Assay variable : PTINR_VAL1 PT-INR Value

N	Mean	Standard Deviation	Minimum	Maximum
5	1.9140000	0.4508104	1.2900000	2.5100000

Unique Subject Identifier=7008000000-0023

Assay variable : PTINR_VAL1 PT-INR Value

N	Mean	Standard Deviation	Minimum	Maximum
3	2.2800000	0.3551056	2.0700000	2.6900000

Unique Subject Identifier=7008000000-0027

Assay variable : PTINR_VAL1 PT-INR Value

N	Mean	Standard Deviation	Minimum	Maximum
6	1.8600000	0.4904692	1.1700000	2.4500000

Unique Subject Identifier=7009010000-0003

Assay variable : PTINR_VAL1 PT-INR Value

N	Mean	Standard Deviation	Minimum	Maximum
3	2.1500000	0.2457641	1.9700000	2.4300000

Laboratory tests at the time of obtaining Table 2-6 consent, according to the presence or absence of proton-pump inhibitors

Unique Subject Identifier=7009010000-0004

MEANS Procedure

Assay variable : PTINR_VAL1 PT-INR Value

N	Mean	Standard Deviation	Minimum	Maximum
6	2.4316667	0.5638587	1.5500000	3.2100000

Unique Subject Identifier=7009010000-0007

Assay variable : PTINR_VAL1 PT-INR Value

N	Mean	Standard Deviation	Minimum	Maximum
4	1.7175000	0.2490482	1.5400000	2.0800000

Unique Subject Identifier=7009010000-0016

Assay variable : PTINR_VAL1 PT-INR Value

N	Mean	Standard Deviation	Minimum	Maximum
3	2.6766667	0.1955335	2.4900000	2.8800000

Unique Subject Identifier=7014000000-0003

Assay variable : PTINR_VAL1 PT-INR Value

N	Mean	Standard Deviation	Minimum	Maximum
6	2.2066667	0.1984607	1.9800000	2.5500000

Laboratory tests at the time of obtaining Table 2-6 consent, according to the presence or absence of proton-pump inhibitors

Unique Subject Identifier=7014000000-0005

MEANS Procedure

Assay variable : PTINR_VAL1 PT-INR Value

N	Mean	Standard Deviation	Minimum	Maximum
6	2.4600000	0.5049752	1.7300000	3.0800000

Unique Subject Identifier=7014000000-0007

Assay variable : PTINR_VAL1 PT-INR Value

N	Mean	Standard Deviation	Minimum	Maximum
5	2.2220000	0.4557631	1.7900000	2.9300000

Unique Subject Identifier=7014000000-0008

Assay variable : PTINR_VAL1 PT-INR Value

N	Mean	Standard Deviation	Minimum	Maximum
1	1.6500000		1.6500000	1.6500000

Unique Subject Identifier=7014000000-0010

Assay variable : PTINR_VAL1 PT-INR Value

N	Mean	Standard Deviation	Minimum	Maximum
2	2.3950000	0.4879037	2.0500000	2.7400000

Laboratory tests at the time of obtaining Table 2-6 consent, according to the presence or absence of proton-pump inhibitors

Unique Subject Identifier=7026000000-0012

MEANS Procedure

Assay variable : PTINR_VAL1 PT-INR Value

N	Mean	Standard Deviation	Minimum	Maximum
6	2.6666667	0.9492242	1.5000000	4.2800000

Unique Subject Identifier=7026000000-0013

Assay variable : PTINR_VAL1 PT-INR Value

N	Mean	Standard Deviation	Minimum	Maximum
5	2.5980000	1.4820830	1.1000000	5.0000000

Unique Subject Identifier=7026000000-0020

Assay variable : PTINR_VAL1 PT-INR Value

N	Mean	Standard Deviation	Minimum	Maximum
6	1.9583333	0.5064550	1.2000000	2.7400000

Unique Subject Identifier=7026000000-0021

Assay variable : PTINR_VAL1 PT-INR Value

N	Mean	Standard Deviation	Minimum	Maximum
6	1.6650000	0.1202913	1.5200000	1.8000000

Laboratory tests at the time of obtaining Table 2-6 consent, according to the presence or absence of proton-pump inhibitors

Unique Subject Identifier=7026000000-0023

MEANS Procedure

Assay variable : PTINR_VAL1 PT-INR Value

N	Mean	Standard Deviation	Minimum	Maximum
4	1.5375000	0.2137561	1.2600000	1.7500000

Unique Subject Identifier=7026000000-0025

Assay variable : PTINR_VAL1 PT-INR Value

N	Mean	Standard Deviation	Minimum	Maximum
3	2.0766667	0.8183724	1.4100000	2.9900000

Unique Subject Identifier=7026000000-0026

Assay variable : PTINR_VAL1 PT-INR Value

N	Mean	Standard Deviation	Minimum	Maximum
2	1.7650000	0.3181981	1.5400000	1.9900000

Unique Subject Identifier=7026000000-0029

Assay variable : PTINR_VAL1 PT-INR Value

N	Mean	Standard Deviation	Minimum	Maximum
4	2.2500000	0.3271085	1.9800000	2.7200000

Laboratory tests at the time of obtaining Table 2-6 consent, according to the presence or absence of proton-pump inhibitors

Unique Subject Identifier=7026000000-0034

MEANS Procedure

Assay variable : PTINR_VAL1 PT-INR Value

N	Mean	Standard Deviation	Minimum	Maximum
4	1.7175000	0.1732772	1.5400000	1.8900000

Unique Subject Identifier=7026000000-0035

Assay variable : PTINR_VAL1 PT-INR Value

N	Mean	Standard Deviation	Minimum	Maximum
5	1.4540000	0.1748714	1.2800000	1.7200000

Unique Subject Identifier=7026000000-0036

Assay variable : PTINR_VAL1 PT-INR Value

N	Mean	Standard Deviation	Minimum	Maximum
2	1.8600000	0.5656854	1.4600000	2.2600000

Unique Subject Identifier=7026000000-0037

Assay variable : PTINR_VAL1 PT-INR Value

N	Mean	Standard Deviation	Minimum	Maximum
2	1.7150000	0.0070711	1.7100000	1.7200000

Laboratory tests at the time of obtaining Table 2-6 consent, according to the presence or absence of proton-pump inhibitors

Unique Subject Identifier=7026000000-0040

MEANS Procedure

Assay variable : PTINR_VAL1 PT-INR Value

N	Mean	Standard Deviation	Minimum	Maximum
3	1.8066667	0.2173323	1.5600000	1.9700000

Unique Subject Identifier=7030010000-0006

Assay variable : PTINR_VAL1 PT-INR Value

N	Mean	Standard Deviation	Minimum	Maximum
5	2.5320000	0.6437934	1.9800000	3.6000000

Unique Subject Identifier=7030010000-0008

Assay variable : PTINR_VAL1 PT-INR Value

N	Mean	Standard Deviation	Minimum	Maximum
4	1.8550000	0.3162805	1.4400000	2.1600000

Unique Subject Identifier=7030010000-0009

Assay variable : PTINR_VAL1 PT-INR Value

N	Mean	Standard Deviation	Minimum	Maximum
5	2.1940000	0.4893669	1.6100000	2.6300000

Laboratory tests at the time of obtaining Table 2-6 consent, according to the presence or absence of proton-pump inhibitors

Unique Subject Identifier=7030010000-0013

MEANS Procedure

Assay variable : PTINR_VAL1 PT-INR Value

N	Mean	Standard Deviation	Minimum	Maximum
5	2.1060000	0.1677200	1.9200000	2.3100000

Unique Subject Identifier=7030010000-0018

Assay variable : PTINR_VAL1 PT-INR Value

N	Mean	Standard Deviation	Minimum	Maximum
4	2.1800000	0.1272792	2.0400000	2.3400000

Unique Subject Identifier=7030010000-0020

Assay variable : PTINR_VAL1 PT-INR Value

N	Mean	Standard Deviation	Minimum	Maximum
5	1.5300000	0.1873499	1.2800000	1.8000000

Unique Subject Identifier=7030010000-0023

Assay variable : PTINR_VAL1 PT-INR Value

N	Mean	Standard Deviation	Minimum	Maximum
5	1.8140000	0.1689083	1.5500000	2.0000000

Laboratory tests at the time of obtaining Table 2-6 consent, according to the presence or absence of proton-pump inhibitors

Unique Subject Identifier=7030010000-0027

MEANS Procedure

Assay variable : PTINR_VAL1 PT-INR Value

N	Mean	Standard Deviation	Minimum	Maximum
4	1.9275000	0.4454492	1.5900000	2.5700000

Unique Subject Identifier=7030010000-0028

Assay variable : PTINR_VAL1 PT-INR Value

N	Mean	Standard Deviation	Minimum	Maximum
4	2.0875000	0.6229700	1.6400000	2.9800000

Unique Subject Identifier=7030010000-0031

Assay variable : PTINR_VAL1 PT-INR Value

N	Mean	Standard Deviation	Minimum	Maximum
4	2.3525000	0.3063087	2.0600000	2.6600000

Unique Subject Identifier=7030010000-0032

Assay variable : PTINR_VAL1 PT-INR Value

N	Mean	Standard Deviation	Minimum	Maximum
5	2.4060000	0.4752683	1.6900000	2.8700000

Laboratory tests at the time of obtaining Table 2-6 consent, according to the presence or absence of proton-pump inhibitors

Unique Subject Identifier=7030010000-0033

MEANS Procedure

Assay variable : PTINR_VAL1 PT-INR Value

N	Mean	Standard Deviation	Minimum	Maximum
4	2.3750000	0.9238146	1.7700000	3.7500000

Unique Subject Identifier=7030010000-0034

Assay variable : PTINR_VAL1 PT-INR Value

N	Mean	Standard Deviation	Minimum	Maximum
4	1.9250000	0.3516153	1.4800000	2.3400000

Unique Subject Identifier=7030010000-0039

Assay variable : PTINR_VAL1 PT-INR Value

N	Mean	Standard Deviation	Minimum	Maximum
4	1.9325000	0.0917878	1.8400000	2.0300000

Unique Subject Identifier=7030010000-0040

Assay variable : PTINR_VAL1 PT-INR Value

N	Mean	Standard Deviation	Minimum	Maximum
4	1.8700000	0.2855404	1.6500000	2.2700000

Laboratory tests at the time of obtaining Table 2-6 consent, according to the presence or absence of proton-pump inhibitors

Unique Subject Identifier=7030010000-0041

MEANS Procedure

Assay variable : PTINR_VAL1 PT-INR Value

N	Mean	Standard Deviation	Minimum	Maximum
4	2.6000000	1.2533422	1.6500000	4.3400000

Unique Subject Identifier=7030010000-0042

Assay variable : PTINR_VAL1 PT-INR Value

N	Mean	Standard Deviation	Minimum	Maximum
4	2.2000000	0.2679552	1.8700000	2.4600000

Unique Subject Identifier=7031011111-0006

Assay variable : PTINR_VAL1 PT-INR Value

N	Mean	Standard Deviation	Minimum	Maximum
6	1.7516667	0.4393821	1.1700000	2.4100000

Unique Subject Identifier=7031011111-0008

Assay variable : PTINR_VAL1 PT-INR Value

N	Mean	Standard Deviation	Minimum	Maximum
4	1.8225000	0.3039051	1.4300000	2.1700000

Laboratory tests at the time of obtaining Table 2-6 consent, according to the presence or absence of proton-pump inhibitors

Unique Subject Identifier=7031011111-0012

MEANS Procedure

Assay variable : PTINR_VAL1 PT-INR Value

N	Mean	Standard Deviation	Minimum	Maximum
3	1.9900000	0.3862642	1.6500000	2.4100000

Unique Subject Identifier=7031011111-0013

Assay variable : PTINR_VAL1 PT-INR Value

N	Mean	Standard Deviation	Minimum	Maximum
3	2.1766667	0.2983845	1.9800000	2.5200000

Unique Subject Identifier=7031011111-0015

Assay variable : PTINR_VAL1 PT-INR Value

N	Mean	Standard Deviation	Minimum	Maximum
4	1.9800000	0.2738613	1.7000000	2.2200000

Unique Subject Identifier=7031011111-0019

Assay variable : PTINR_VAL1 PT-INR Value

N	Mean	Standard Deviation	Minimum	Maximum
5	2.1200000	0.2667396	1.8500000	2.5300000

Laboratory tests at the time of obtaining Table 2-6 consent, according to the presence or absence of proton-pump inhibitors

Unique Subject Identifier=7031011111-0022

MEANS Procedure

Assay variable : PTINR_VAL1 PT-INR Value

N	Mean	Standard Deviation	Minimum	Maximum
4	1.7775000	0.2590206	1.4800000	2.1000000

Unique Subject Identifier=7031011111-0026

Assay variable : PTINR_VAL1 PT-INR Value

N	Mean	Standard Deviation	Minimum	Maximum
6	2.6250000	0.1683746	2.4700000	2.9300000

Unique Subject Identifier=7031011111-0027

Assay variable : PTINR_VAL1 PT-INR Value

N	Mean	Standard Deviation	Minimum	Maximum
3	2.2533333	0.4850086	1.7700000	2.7400000

Unique Subject Identifier=7031011111-0029

Assay variable : PTINR_VAL1 PT-INR Value

N	Mean	Standard Deviation	Minimum	Maximum
4	2.3375000	0.2215664	2.0400000	2.5300000

Laboratory tests at the time of obtaining Table 2-6 consent, according to the presence or absence of proton-pump inhibitors

Unique Subject Identifier=7031011111-0030

MEANS Procedure

Assay variable : PTINR_VAL1 PT-INR Value

N	Mean	Standard Deviation	Minimum	Maximum
5	1.8260000	0.2589981	1.5000000	2.1900000

Unique Subject Identifier=7031011111-0034

Assay variable : PTINR_VAL1 PT-INR Value

N	Mean	Standard Deviation	Minimum	Maximum
4	2.1175000	0.6155418	1.4500000	2.9000000

Unique Subject Identifier=7031011111-0037

Assay variable : PTINR_VAL1 PT-INR Value

N	Mean	Standard Deviation	Minimum	Maximum
4	1.7200000	0.1718527	1.5500000	1.9500000

Unique Subject Identifier=7031011111-0043

Assay variable : PTINR_VAL1 PT-INR Value

N	Mean	Standard Deviation	Minimum	Maximum
4	2.7425000	0.2351418	2.4500000	3.0100000

Laboratory tests at the time of obtaining Table 2-6 consent, according to the presence or absence of proton-pump inhibitors

Unique Subject Identifier=7035000000-0009

MEANS Procedure

Assay variable : PTINR_VAL1 PT-INR Value

N	Mean	Standard Deviation	Minimum	Maximum
3	2.8666667	0.2811287	2.6800000	3.1900000

Unique Subject Identifier=7035000000-0010

Assay variable : PTINR_VAL1 PT-INR Value

N	Mean	Standard Deviation	Minimum	Maximum
3	1.9400000	0.2007486	1.7100000	2.0800000

Unique Subject Identifier=7035000000-0011

Assay variable : PTINR_VAL1 PT-INR Value

N	Mean	Standard Deviation	Minimum	Maximum
6	1.7483333	0.3171382	1.3600000	2.1500000

Unique Subject Identifier=7035000000-0012

Assay variable : PTINR_VAL1 PT-INR Value

N	Mean	Standard Deviation	Minimum	Maximum
5	1.5500000	0.0790569	1.4700000	1.6600000

Laboratory tests at the time of obtaining Table 2-6 consent, according to the presence or absence of proton-pump inhibitors

Unique Subject Identifier=7035000000-0022

MEANS Procedure

Assay variable : PTINR_VAL1 PT-INR Value

N	Mean	Standard Deviation	Minimum	Maximum
3	1.8900000	0.2688866	1.7200000	2.2000000

Unique Subject Identifier=7035000000-0026

Assay variable : PTINR_VAL1 PT-INR Value

N	Mean	Standard Deviation	Minimum	Maximum
4	1.5450000	0.1337909	1.3900000	1.6800000

Unique Subject Identifier=7035000000-0027

Assay variable : PTINR_VAL1 PT-INR Value

N	Mean	Standard Deviation	Minimum	Maximum
6	2.2966667	0.6932147	1.9700000	3.7100000

Unique Subject Identifier=7035000000-0028

Assay variable : PTINR_VAL1 PT-INR Value

N	Mean	Standard Deviation	Minimum	Maximum
4	1.7625000	0.1931105	1.5900000	2.0300000

Laboratory tests at the time of obtaining Table 2-6 consent, according to the presence or absence of proton-pump inhibitors

Unique Subject Identifier=7035000000-0032

MEANS Procedure

Assay variable : PTINR_VAL1 PT-INR Value

N	Mean	Standard Deviation	Minimum	Maximum
4	2.2075000	0.1721191	1.9900000	2.3600000

Unique Subject Identifier=7035000000-0038

Assay variable : PTINR_VAL1 PT-INR Value

N	Mean	Standard Deviation	Minimum	Maximum
4	2.5200000	0.8057709	1.8400000	3.5400000

Unique Subject Identifier=7035000000-0039

Assay variable : PTINR_VAL1 PT-INR Value

N	Mean	Standard Deviation	Minimum	Maximum
4	2.3600000	0.3371449	1.8800000	2.6000000

Unique Subject Identifier=7035000000-0041

Assay variable : PTINR_VAL1 PT-INR Value

N	Mean	Standard Deviation	Minimum	Maximum
3	1.9266667	0.3431229	1.5600000	2.2400000

Laboratory tests at the time of obtaining Table 2-6 consent, according to the presence or absence of proton-pump inhibitors

Unique Subject Identifier=7035000000-0043

MEANS Procedure

Assay variable : PTINR_VAL1 PT-INR Value

N	Mean	Standard Deviation	Minimum	Maximum
4	1.8850000	0.3070831	1.6300000	2.3300000

Unique Subject Identifier=7035000000-0050

Assay variable : PTINR_VAL1 PT-INR Value

N	Mean	Standard Deviation	Minimum	Maximum
4	1.4325000	0.0518813	1.3600000	1.4700000

Unique Subject Identifier=7035000000-0052

Assay variable : PTINR_VAL1 PT-INR Value

N	Mean	Standard Deviation	Minimum	Maximum
3	2.0600000	0.2351595	1.9000000	2.3300000

Unique Subject Identifier=7035000000-0054

Assay variable : PTINR_VAL1 PT-INR Value

N	Mean	Standard Deviation	Minimum	Maximum
3	1.7533333	0.0503322	1.7000000	1.8000000

Laboratory tests at the time of obtaining Table 2-6 consent, according to the presence or absence of proton-pump inhibitors

Unique Subject Identifier=7035000000-0055

MEANS Procedure

Assay variable : PTINR_VAL1 PT-INR Value

N	Mean	Standard Deviation	Minimum	Maximum
3	2.2566667	0.1877054	2.0400000	2.3700000

Unique Subject Identifier=7035000000-0057

Assay variable : PTINR_VAL1 PT-INR Value

N	Mean	Standard Deviation	Minimum	Maximum
1	2.3200000		2.3200000	2.3200000

Unique Subject Identifier=7035000000-0061

Assay variable : PTINR_VAL1 PT-INR Value

N	Mean	Standard Deviation	Minimum	Maximum
3	2.4400000	0.1609348	2.2700000	2.5900000

Unique Subject Identifier=7035000000-0062

Assay variable : PTINR_VAL1 PT-INR Value

N	Mean	Standard Deviation	Minimum	Maximum
2	2.0050000	0.2474874	1.8300000	2.1800000

Laboratory tests at the time of obtaining Table 2-6 consent, according to the presence or absence of proton-pump inhibitors

Unique Subject Identifier=7035000000-0064

MEANS Procedure

Assay variable : PTINR_VAL1 PT-INR Value

N	Mean	Standard Deviation	Minimum	Maximum
3	1.8733333	0.3552933	1.4700000	2.1400000

Unique Subject Identifier=7035000000-0066

Assay variable : PTINR_VAL1 PT-INR Value

N	Mean	Standard Deviation	Minimum	Maximum
2	1.5500000	0.2121320	1.4000000	1.7000000

Unique Subject Identifier=7035000000-0068

Assay variable : PTINR_VAL1 PT-INR Value

N	Mean	Standard Deviation	Minimum	Maximum
2	2.0100000	0.4101219	1.7200000	2.3000000

Unique Subject Identifier=7035000000-0071

Assay variable : PTINR_VAL1 PT-INR Value

N	Mean	Standard Deviation	Minimum	Maximum
3	2.0800000	0.1228821	1.9400000	2.1700000

Laboratory tests at the time of obtaining Table 2-6 consent, according to the presence or absence of proton-pump inhibitors

Unique Subject Identifier=7035000000-0072

MEANS Procedure

Assay variable : PTINR_VAL1 PT-INR Value

N	Mean	Standard Deviation	Minimum	Maximum
4	1.9850000	0.4655105	1.5200000	2.4700000

Unique Subject Identifier=7035000000-0074

Assay variable : PTINR_VAL1 PT-INR Value

N	Mean	Standard Deviation	Minimum	Maximum
3	1.6933333	0.0577350	1.6600000	1.7600000

Unique Subject Identifier=7035000000-0075

Assay variable : PTINR_VAL1 PT-INR Value

N	Mean	Standard Deviation	Minimum	Maximum
2	2.4450000	0.5444722	2.0600000	2.8300000

Unique Subject Identifier=7035000000-0076

Assay variable : PTINR_VAL1 PT-INR Value

N	Mean	Standard Deviation	Minimum	Maximum
3	1.8600000	0.4214262	1.4200000	2.2600000

Laboratory tests at the time of obtaining Table 2-6 consent, according to the presence or absence of proton-pump inhibitors

Unique Subject Identifier=7035000000-0079

MEANS Procedure

Assay variable : PTINR_VAL1 PT-INR Value

N	Mean	Standard Deviation	Minimum	Maximum
3	2.3800000	0.3961060	2.0300000	2.8100000

Unique Subject Identifier=7035000000-0084

Assay variable : PTINR_VAL1 PT-INR Value

N	Mean	Standard Deviation	Minimum	Maximum
3	2.7966667	0.2750152	2.5200000	3.0700000

Unique Subject Identifier=7035000000-0086

Assay variable : PTINR_VAL1 PT-INR Value

N	Mean	Standard Deviation	Minimum	Maximum
2	1.6950000	0.2050610	1.5500000	1.8400000

Unique Subject Identifier=7035000000-0088

Assay variable : PTINR_VAL1 PT-INR Value

N	Mean	Standard Deviation	Minimum	Maximum
4	1.9900000	0.2070427	1.7500000	2.2200000

Laboratory tests at the time of obtaining Table 2-6 consent, according to the presence or absence of proton-pump inhibitors

Unique Subject Identifier=7035000000-0090

MEANS Procedure

Assay variable : PTINR_VAL1 PT-INR Value

N	Mean	Standard Deviation	Minimum	Maximum
3	2.2766667	0.1858315	2.1500000	2.4900000

Unique Subject Identifier=7035000000-0095

Assay variable : PTINR_VAL1 PT-INR Value

N	Mean	Standard Deviation	Minimum	Maximum
4	2.1625000	0.7162576	1.5900000	3.2100000

Unique Subject Identifier=7035000000-0097

Assay variable : PTINR_VAL1 PT-INR Value

N	Mean	Standard Deviation	Minimum	Maximum
1	2.4600000		2.4600000	2.4600000

Unique Subject Identifier=7036000000-0003

Assay variable : PTINR_VAL1 PT-INR Value

N	Mean	Standard Deviation	Minimum	Maximum
5	2.2800000	0.5263079	1.7000000	3.1000000

Laboratory tests at the time of obtaining Table 2-6 consent, according to the presence or absence of proton-pump inhibitors

Unique Subject Identifier=7037000000-0012

MEANS Procedure

Assay variable : PTINR_VAL1 PT-INR Value

N	Mean	Standard Deviation	Minimum	Maximum
6	2.1016667	0.5417164	1.7000000	3.1000000

Unique Subject Identifier=7040011000-0001

Assay variable : PTINR_VAL1 PT-INR Value

N	Mean	Standard Deviation	Minimum	Maximum
6	2.3516667	0.5745752	1.3200000	2.9400000

Unique Subject Identifier=7040011000-0002

Assay variable : PTINR_VAL1 PT-INR Value

N	Mean	Standard Deviation	Minimum	Maximum
5	2.5740000	0.3576031	2.0600000	2.9600000

Unique Subject Identifier=7042010110-0003

Assay variable : PTINR_VAL1 PT-INR Value

N	Mean	Standard Deviation	Minimum	Maximum
6	1.8033333	0.6052988	1.3700000	2.9500000

Laboratory tests at the time of obtaining Table 2-6 consent, according to the presence or absence of proton-pump inhibitors

Unique Subject Identifier=7042010110-0005

MEANS Procedure

Assay variable : PTINR_VAL1 PT-INR Value

N	Mean	Standard Deviation	Minimum	Maximum
4	1.6250000	0.5434765	1.2300000	2.4200000

Unique Subject Identifier=7042010110-0006

Assay variable : PTINR_VAL1 PT-INR Value

N	Mean	Standard Deviation	Minimum	Maximum
3	2.2100000	0.1100000	2.1000000	2.3200000

Unique Subject Identifier=7042010110-0010

Assay variable : PTINR_VAL1 PT-INR Value

N	Mean	Standard Deviation	Minimum	Maximum
6	2.0633333	0.1961292	1.8600000	2.3900000

Unique Subject Identifier=7042010110-0011

Assay variable : PTINR_VAL1 PT-INR Value

N	Mean	Standard Deviation	Minimum	Maximum
3	1.9200000	0.1252996	1.8000000	2.0500000

Laboratory tests at the time of obtaining Table 2-6 consent, according to the presence or absence of proton-pump inhibitors

Unique Subject Identifier=7042010110-0013

MEANS Procedure

Assay variable : PTINR_VAL1 PT-INR Value

N	Mean	Standard Deviation	Minimum	Maximum
6	2.2466667	0.2058802	1.8900000	2.4400000

Unique Subject Identifier=7042010110-0014

Assay variable : PTINR_VAL1 PT-INR Value

N	Mean	Standard Deviation	Minimum	Maximum
6	1.3150000	0.2433721	1.0300000	1.7600000

Unique Subject Identifier=7042010110-0017

Assay variable : PTINR_VAL1 PT-INR Value

N	Mean	Standard Deviation	Minimum	Maximum
6	2.1166667	1.2655697	1.4100000	4.6700000

Unique Subject Identifier=7042010110-0018

Assay variable : PTINR_VAL1 PT-INR Value

N	Mean	Standard Deviation	Minimum	Maximum
6	1.4266667	0.0799166	1.3100000	1.5200000

Laboratory tests at the time of obtaining Table 2-6 consent, according to the presence or absence of proton-pump inhibitors

Unique Subject Identifier=7042010110-0021

MEANS Procedure

Assay variable : PTINR_VAL1 PT-INR Value

N	Mean	Standard Deviation	Minimum	Maximum
3	2.2633333	0.0208167	2.2400000	2.2800000

Unique Subject Identifier=7042010110-0022

Assay variable : PTINR_VAL1 PT-INR Value

N	Mean	Standard Deviation	Minimum	Maximum
5	1.5120000	0.1613072	1.2300000	1.6300000

Unique Subject Identifier=7042010110-0030

Assay variable : PTINR_VAL1 PT-INR Value

N	Mean	Standard Deviation	Minimum	Maximum
3	1.3633333	0.1266228	1.2500000	1.5000000

Unique Subject Identifier=7042010110-0033

Assay variable : PTINR_VAL1 PT-INR Value

N	Mean	Standard Deviation	Minimum	Maximum
3	2.2200000	0.3934463	1.8000000	2.5800000

Laboratory tests at the time of obtaining Table 2-6 consent, according to the presence or absence of proton-pump inhibitors

Unique Subject Identifier=7042010110-0036

MEANS Procedure

Assay variable : PTINR_VAL1 PT-INR Value

N	Mean	Standard Deviation	Minimum	Maximum
4	1.9200000	0.3194787	1.6200000	2.2700000

Unique Subject Identifier=7045000000-0001

Assay variable : PTINR_VAL1 PT-INR Value

N	Mean	Standard Deviation	Minimum	Maximum
5	1.9640000	0.2596729	1.7400000	2.4000000

Unique Subject Identifier=7045000000-0002

Assay variable : PTINR_VAL1 PT-INR Value

N	Mean	Standard Deviation	Minimum	Maximum
6	2.0250000	0.2338162	1.8300000	2.4900000

Unique Subject Identifier=7045000000-0009

Assay variable : PTINR_VAL1 PT-INR Value

N	Mean	Standard Deviation	Minimum	Maximum
4	2.0150000	0.2143984	1.7300000	2.1900000

Laboratory tests at the time of obtaining Table 2-6 consent, according to the presence or absence of proton-pump inhibitors

Unique Subject Identifier=7045000000-0010

MEANS Procedure

Assay variable : PTINR_VAL1 PT-INR Value

N	Mean	Standard Deviation	Minimum	Maximum
6	2.3050000	0.2608256	1.8900000	2.6200000

Unique Subject Identifier=7045000000-0011

Assay variable : PTINR_VAL1 PT-INR Value

N	Mean	Standard Deviation	Minimum	Maximum
6	1.9750000	0.2833196	1.5400000	2.2500000

Unique Subject Identifier=7045000000-0012

Assay variable : PTINR_VAL1 PT-INR Value

N	Mean	Standard Deviation	Minimum	Maximum
6	2.5050000	0.2514558	2.0500000	2.6800000

Unique Subject Identifier=7045000000-0013

Assay variable : PTINR_VAL1 PT-INR Value

N	Mean	Standard Deviation	Minimum	Maximum
6	2.1566667	0.6226288	1.3300000	3.0500000

Laboratory tests at the time of obtaining Table 2-6 consent, according to the presence or absence of proton-pump inhibitors

Unique Subject Identifier=7045000000-0020

MEANS Procedure

Assay variable : PTINR_VAL1 PT-INR Value

N	Mean	Standard Deviation	Minimum	Maximum
5	2.0620000	0.0849706	1.9600000	2.1400000

Unique Subject Identifier=7045000000-0021

Assay variable : PTINR_VAL1 PT-INR Value

N	Mean	Standard Deviation	Minimum	Maximum
6	2.1316667	0.5024109	1.6700000	2.8300000

Unique Subject Identifier=7045000000-0023

Assay variable : PTINR_VAL1 PT-INR Value

N	Mean	Standard Deviation	Minimum	Maximum
4	2.2825000	0.4265657	1.8300000	2.6900000

Unique Subject Identifier=7045000000-0024

Assay variable : PTINR_VAL1 PT-INR Value

N	Mean	Standard Deviation	Minimum	Maximum
5	2.1500000	0.3277194	1.6700000	2.5900000

Laboratory tests at the time of obtaining Table 2-6 consent, according to the presence or absence of proton-pump inhibitors

Unique Subject Identifier=7045000000-0026

MEANS Procedure

Assay variable : PTINR_VAL1 PT-INR Value

N	Mean	Standard Deviation	Minimum	Maximum
4	1.7650000	0.1415392	1.5600000	1.8800000

Unique Subject Identifier=7045000000-0028

Assay variable : PTINR_VAL1 PT-INR Value

N	Mean	Standard Deviation	Minimum	Maximum
6	2.1716667	0.4511060	1.4500000	2.6600000

Unique Subject Identifier=7045000000-0029

Assay variable : PTINR_VAL1 PT-INR Value

N	Mean	Standard Deviation	Minimum	Maximum
4	2.0725000	0.1486327	1.9300000	2.2800000

Unique Subject Identifier=7045000000-0030

Assay variable : PTINR_VAL1 PT-INR Value

N	Mean	Standard Deviation	Minimum	Maximum
4	2.0825000	0.3003748	1.6700000	2.3900000

Laboratory tests at the time of obtaining Table 2-6 consent, according to the presence or absence of proton-pump inhibitors

Unique Subject Identifier=7047000000-0001

MEANS Procedure

Assay variable : PTINR_VAL1 PT-INR Value

N	Mean	Standard Deviation	Minimum	Maximum
6	1.6450000	0.5950714	1.2000000	2.6400000

Unique Subject Identifier=7047000000-0004

Assay variable : PTINR_VAL1 PT-INR Value

N	Mean	Standard Deviation	Minimum	Maximum
6	1.6333333	0.2356834	1.4200000	2.0300000

Unique Subject Identifier=7047000000-0005

Assay variable : PTINR_VAL1 PT-INR Value

N	Mean	Standard Deviation	Minimum	Maximum
5	1.4900000	0.1770593	1.2500000	1.7000000

Unique Subject Identifier=7047000000-0007

Assay variable : PTINR_VAL1 PT-INR Value

N	Mean	Standard Deviation	Minimum	Maximum
2	1.4050000	0.0636396	1.3600000	1.4500000

Laboratory tests at the time of obtaining Table 2-6 consent, according to the presence or absence of proton-pump inhibitors

Unique Subject Identifier=7047000000-0008

MEANS Procedure

Assay variable : PTINR_VAL1 PT-INR Value

N	Mean	Standard Deviation	Minimum	Maximum
2	1.5200000	0.3252691	1.2900000	1.7500000

Unique Subject Identifier=7047000000-0009

Assay variable : PTINR_VAL1 PT-INR Value

N	Mean	Standard Deviation	Minimum	Maximum
3	1.7633333	0.3592121	1.4600000	2.1600000

Unique Subject Identifier=7055000000-0007

Assay variable : PTINR_VAL1 PT-INR Value

N	Mean	Standard Deviation	Minimum	Maximum
6	2.0216667	0.1614208	1.8200000	2.2600000

Unique Subject Identifier=7061010010-0002

Assay variable : PTINR_VAL1 PT-INR Value

N	Mean	Standard Deviation	Minimum	Maximum
6	1.9500000	0.2588436	1.7000000	2.4000000

Laboratory tests at the time of obtaining Table 2-6 consent, according to the presence or absence of proton-pump inhibitors

Unique Subject Identifier=7061010010-0004

MEANS Procedure

Assay variable : PTINR_VAL1 PT-INR Value

N	Mean	Standard Deviation	Minimum	Maximum
6	1.5500000	0.3563706	1.1000000	2.1000000

Unique Subject Identifier=7061010010-0007

Assay variable : PTINR_VAL1 PT-INR Value

N	Mean	Standard Deviation	Minimum	Maximum
3	1.9666667	0.5033223	1.5000000	2.5000000

Unique Subject Identifier=7061010010-0009

Assay variable : PTINR_VAL1 PT-INR Value

N	Mean	Standard Deviation	Minimum	Maximum
4	2.3250000	0.5737305	1.8000000	3.1000000

Unique Subject Identifier=7066000000-0002

Assay variable : PTINR_VAL1 PT-INR Value

N	Mean	Standard Deviation	Minimum	Maximum
6	2.6683333	1.5619913	1.1900000	5.4500000

Laboratory tests at the time of obtaining Table 2-6 consent, according to the presence or absence of proton-pump inhibitors

Unique Subject Identifier=7066000000-0007

MEANS Procedure

Assay variable : PTINR_VAL1 PT-INR Value

N	Mean	Standard Deviation	Minimum	Maximum
6	1.9766667	0.2334666	1.6000000	2.2900000

Unique Subject Identifier=7066000000-0008

Assay variable : PTINR_VAL1 PT-INR Value

N	Mean	Standard Deviation	Minimum	Maximum
5	2.1600000	0.5041825	1.4600000	2.7900000

Unique Subject Identifier=7066000000-0013

Assay variable : PTINR_VAL1 PT-INR Value

N	Mean	Standard Deviation	Minimum	Maximum
6	1.9066667	0.1186030	1.7200000	2.0300000

Unique Subject Identifier=7066000000-0021

Assay variable : PTINR_VAL1 PT-INR Value

N	Mean	Standard Deviation	Minimum	Maximum
6	1.7133333	0.3478889	1.3200000	2.2000000

Laboratory tests at the time of obtaining Table 2-6 consent, according to the presence or absence of proton-pump inhibitors

Unique Subject Identifier=7066000000-0028

MEANS Procedure

Assay variable : PTINR_VAL1 PT-INR Value

N	Mean	Standard Deviation	Minimum	Maximum
6	2.3533333	0.7609380	1.6100000	3.5400000

Unique Subject Identifier=7066000000-0029

Assay variable : PTINR_VAL1 PT-INR Value

N	Mean	Standard Deviation	Minimum	Maximum
5	2.1020000	0.1020784	1.9600000	2.2200000

Unique Subject Identifier=7066000000-0030

Assay variable : PTINR_VAL1 PT-INR Value

N	Mean	Standard Deviation	Minimum	Maximum
4	1.9500000	0.6216108	1.3900000	2.6100000

Unique Subject Identifier=7104010000-0010

Assay variable : PTINR_VAL1 PT-INR Value

N	Mean	Standard Deviation	Minimum	Maximum
6	2.2700000	0.3199375	1.7500000	2.6700000

Laboratory tests at the time of obtaining Table 2-6 consent, according to the presence or absence of proton-pump inhibitors

Unique Subject Identifier=7104010000-0020

MEANS Procedure

Assay variable : PTINR_VAL1 PT-INR Value

N	Mean	Standard Deviation	Minimum	Maximum
6	2.4650000	0.1415274	2.2900000	2.6500000

Unique Subject Identifier=7132010000-0011

Assay variable : PTINR_VAL1 PT-INR Value

N	Mean	Standard Deviation	Minimum	Maximum
4	2.2025000	0.5558402	1.5000000	2.7100000

Unique Subject Identifier=7132010000-0017

Assay variable : PTINR_VAL1 PT-INR Value

N	Mean	Standard Deviation	Minimum	Maximum
4	2.4975000	0.8896582	1.5000000	3.6100000

Unique Subject Identifier=7132010000-0018

Assay variable : PTINR_VAL1 PT-INR Value

N	Mean	Standard Deviation	Minimum	Maximum
3	1.9233333	0.0650641	1.8600000	1.9900000

Laboratory tests at the time of obtaining Table 2-6 consent, according to the presence or absence of proton-pump inhibitors

Unique Subject Identifier=7132010000-0020

MEANS Procedure

Assay variable : PTINR_VAL1 PT-INR Value

N	Mean	Standard Deviation	Minimum	Maximum
3	1.8400000	0.2260531	1.6900000	2.1000000

Unique Subject Identifier=7133000000-0001

Assay variable : PTINR_VAL1 PT-INR Value

N	Mean	Standard Deviation	Minimum	Maximum
6	2.3316667	1.1864471	1.0700000	4.5200000

Unique Subject Identifier=7136000000-0002

Assay variable : PTINR_VAL1 PT-INR Value

N	Mean	Standard Deviation	Minimum	Maximum
3	2.3066667	0.4752192	1.8400000	2.7900000

Unique Subject Identifier=7136000000-0003

Assay variable : PTINR_VAL1 PT-INR Value

N	Mean	Standard Deviation	Minimum	Maximum
4	2.3200000	0.2786874	2.0500000	2.7100000

Laboratory tests at the time of obtaining Table 2-6 consent, according to the presence or absence of proton-pump inhibitors

Unique Subject Identifier=7136000000-0005

MEANS Procedure

Assay variable : PTINR_VAL1 PT-INR Value

N	Mean	Standard Deviation	Minimum	Maximum
3	1.7033333	0.0814453	1.6100000	1.7600000

Unique Subject Identifier=7136000000-0006

Assay variable : PTINR_VAL1 PT-INR Value

N	Mean	Standard Deviation	Minimum	Maximum
4	1.5450000	0.2235322	1.2900000	1.7700000

Unique Subject Identifier=7136000000-0007

Assay variable : PTINR_VAL1 PT-INR Value

N	Mean	Standard Deviation	Minimum	Maximum
5	2.3600000	0.6914478	1.3700000	3.0000000

Unique Subject Identifier=7136000000-0008

Assay variable : PTINR_VAL1 PT-INR Value

N	Mean	Standard Deviation	Minimum	Maximum
6	1.6066667	0.7731666	1.1100000	3.1000000

Laboratory tests at the time of obtaining Table 2-6 consent, according to the presence or absence of proton-pump inhibitors

Unique Subject Identifier=7136000000-0011

MEANS Procedure

Assay variable : PTINR_VAL1 PT-INR Value

N	Mean	Standard Deviation	Minimum	Maximum
5	1.4300000	0.1609348	1.2200000	1.6200000

Unique Subject Identifier=7136000000-0013

Assay variable : PTINR_VAL1 PT-INR Value

N	Mean	Standard Deviation	Minimum	Maximum
4	2.0000000	0.1647220	1.7700000	2.1600000

Unique Subject Identifier=7136000000-0014

Assay variable : PTINR_VAL1 PT-INR Value

N	Mean	Standard Deviation	Minimum	Maximum
3	2.3633333	0.4532475	1.8400000	2.6300000

Unique Subject Identifier=7136000000-0015

Assay variable : PTINR_VAL1 PT-INR Value

N	Mean	Standard Deviation	Minimum	Maximum
4	2.2800000	0.1390444	2.1500000	2.4100000

Laboratory tests at the time of obtaining Table 2-6 consent, according to the presence or absence of proton-pump inhibitors

Unique Subject Identifier=7136000000-0016

MEANS Procedure

Assay variable : PTINR_VAL1 PT-INR Value

N	Mean	Standard Deviation	Minimum	Maximum
3	1.9100000	0.3477068	1.5900000	2.2800000

Unique Subject Identifier=7136000000-0017

Assay variable : PTINR_VAL1 PT-INR Value

N	Mean	Standard Deviation	Minimum	Maximum
6	2.2816667	0.7896940	1.4800000	3.5500000

Unique Subject Identifier=7136000000-0020

Assay variable : PTINR_VAL1 PT-INR Value

N	Mean	Standard Deviation	Minimum	Maximum
3	2.4700000	0.3996248	2.0400000	2.8300000

Unique Subject Identifier=7136000000-0021

Assay variable : PTINR_VAL1 PT-INR Value

N	Mean	Standard Deviation	Minimum	Maximum
6	2.1450000	0.4427528	1.6400000	2.7900000

Laboratory tests at the time of obtaining Table 2-6 consent, according to the presence or absence of proton-pump inhibitors

Unique Subject Identifier=7136000000-0025

MEANS Procedure

Assay variable : PTINR_VAL1 PT-INR Value

N	Mean	Standard Deviation	Minimum	Maximum
6	1.7150000	0.3321897	1.3700000	2.1200000

Unique Subject Identifier=7136000000-0028

Assay variable : PTINR_VAL1 PT-INR Value

N	Mean	Standard Deviation	Minimum	Maximum
4	1.9450000	0.4199603	1.5500000	2.4900000

Unique Subject Identifier=7136000000-0029

Assay variable : PTINR_VAL1 PT-INR Value

N	Mean	Standard Deviation	Minimum	Maximum
3	2.4300000	0.0871780	2.3700000	2.5300000

Unique Subject Identifier=7136000000-0035

Assay variable : PTINR_VAL1 PT-INR Value

N	Mean	Standard Deviation	Minimum	Maximum
4	2.4650000	0.2752575	2.1200000	2.7700000

Laboratory tests at the time of obtaining Table 2-6 consent, according to the presence or absence of proton-pump inhibitors

Unique Subject Identifier=7136000000-0037

MEANS Procedure

Assay variable : PTINR_VAL1 PT-INR Value

N	Mean	Standard Deviation	Minimum	Maximum
3	2.1100000	0.1300000	2.0300000	2.2600000

Unique Subject Identifier=7136000000-0039

Assay variable : PTINR_VAL1 PT-INR Value

N	Mean	Standard Deviation	Minimum	Maximum
3	2.2033333	0.1616581	2.1100000	2.3900000

Unique Subject Identifier=7136000000-0040

Assay variable : PTINR_VAL1 PT-INR Value

N	Mean	Standard Deviation	Minimum	Maximum
3	1.9866667	0.1569501	1.8100000	2.1100000

Unique Subject Identifier=7136000000-0042

Assay variable : PTINR_VAL1 PT-INR Value

N	Mean	Standard Deviation	Minimum	Maximum
4	1.7850000	0.3411256	1.3800000	2.2000000

Laboratory tests at the time of obtaining Table 2-6 consent, according to the presence or absence of proton-pump inhibitors

Unique Subject Identifier=7136000000-0044

MEANS Procedure

Assay variable : PTINR_VAL1 PT-INR Value

N	Mean	Standard Deviation	Minimum	Maximum
3	2.1866667	0.3338163	1.9600000	2.5700000

Unique Subject Identifier=7136000000-0049

Assay variable : PTINR_VAL1 PT-INR Value

N	Mean	Standard Deviation	Minimum	Maximum
3	2.3166667	0.3942503	1.9600000	2.7400000

Unique Subject Identifier=7137010000-0001

Assay variable : PTINR_VAL1 PT-INR Value

N	Mean	Standard Deviation	Minimum	Maximum
5	1.5740000	0.2757354	1.1700000	1.8700000

Unique Subject Identifier=7154000000-0002

Assay variable : PTINR_VAL1 PT-INR Value

N	Mean	Standard Deviation	Minimum	Maximum
5	2.1620000	0.6197338	1.5500000	3.1000000

Laboratory tests at the time of obtaining Table 2-6 consent, according to the presence or absence of proton-pump inhibitors

Unique Subject Identifier=7154000000-0009

MEANS Procedure

Assay variable : PTINR_VAL1 PT-INR Value

N	Mean	Standard Deviation	Minimum	Maximum
5	2.2640000	0.3001333	1.9800000	2.7700000

Unique Subject Identifier=7154000000-0012

Assay variable : PTINR_VAL1 PT-INR Value

N	Mean	Standard Deviation	Minimum	Maximum
3	2.3266667	0.3888873	1.8800000	2.5900000

Unique Subject Identifier=7154000000-0018

Assay variable : PTINR_VAL1 PT-INR Value

N	Mean	Standard Deviation	Minimum	Maximum
5	1.9240000	0.2447039	1.6800000	2.3100000

Unique Subject Identifier=7154000000-0024

Assay variable : PTINR_VAL1 PT-INR Value

N	Mean	Standard Deviation	Minimum	Maximum
4	2.0525000	0.3732180	1.7000000	2.5300000

Laboratory tests at the time of obtaining Table 2-6 consent, according to the presence or absence of proton-pump inhibitors

Unique Subject Identifier=7154000000-0025

MEANS Procedure

Assay variable : PTINR_VAL1 PT-INR Value

N	Mean	Standard Deviation	Minimum	Maximum
4	2.2275000	0.0974252	2.1500000	2.3700000

Unique Subject Identifier=7154000000-0028

Assay variable : PTINR_VAL1 PT-INR Value

N	Mean	Standard Deviation	Minimum	Maximum
3	1.4433333	0.1001665	1.3400000	1.5400000

Unique Subject Identifier=7154000000-0030

Assay variable : PTINR_VAL1 PT-INR Value

N	Mean	Standard Deviation	Minimum	Maximum
3	2.0633333	0.5601190	1.5100000	2.6300000

Unique Subject Identifier=7154000000-0031

Assay variable : PTINR_VAL1 PT-INR Value

N	Mean	Standard Deviation	Minimum	Maximum
6	2.3266667	0.2047112	2.1100000	2.6100000

Laboratory tests at the time of obtaining Table 2-6 consent, according to the presence or absence of proton-pump inhibitors

Unique Subject Identifier=7155000000-0004

MEANS Procedure

Assay variable : PTINR_VAL1 PT-INR Value

N	Mean	Standard Deviation	Minimum	Maximum
5	1.2500000	0.0574456	1.1600000	1.3000000

Unique Subject Identifier=7155000000-0007

Assay variable : PTINR_VAL1 PT-INR Value

N	Mean	Standard Deviation	Minimum	Maximum
2	1.8200000	0.4666905	1.4900000	2.1500000

Unique Subject Identifier=7155000000-0014

Assay variable : PTINR_VAL1 PT-INR Value

N	Mean	Standard Deviation	Minimum	Maximum
6	1.4666667	0.3201041	1.1100000	1.9000000

Unique Subject Identifier=7155000000-0016

Assay variable : PTINR_VAL1 PT-INR Value

N	Mean	Standard Deviation	Minimum	Maximum
1	1.0500000		1.0500000	1.0500000

Laboratory tests at the time of obtaining Table 2-6 consent, according to the presence or absence of proton-pump inhibitors

Unique Subject Identifier=7155000000-0020

MEANS Procedure

Assay variable : PTINR_VAL1 PT-INR Value

N	Mean	Standard Deviation	Minimum	Maximum
4	2.0050000	0.1793507	1.8100000	2.2400000

Unique Subject Identifier=7155000000-0031

Assay variable : PTINR_VAL1 PT-INR Value

N	Mean	Standard Deviation	Minimum	Maximum
3	1.6966667	0.1569501	1.5200000	1.8200000

Unique Subject Identifier=7155000000-0039

Assay variable : PTINR_VAL1 PT-INR Value

N	Mean	Standard Deviation	Minimum	Maximum
4	2.3650000	0.4262628	1.8300000	2.7900000

Unique Subject Identifier=7167000000-0001

Assay variable : PTINR_VAL1 PT-INR Value

N	Mean	Standard Deviation	Minimum	Maximum
6	1.1450000	0.1162325	0.9800000	1.2700000

Laboratory tests at the time of obtaining Table 2-6 consent, according to the presence or absence of proton-pump inhibitors

Unique Subject Identifier=7167000000-0003

MEANS Procedure

Assay variable : PTINR_VAL1 PT-INR Value

N	Mean	Standard Deviation	Minimum	Maximum
3	2.1766667	0.2000833	1.9800000	2.3800000

Unique Subject Identifier=7171010000-0001

Assay variable : PTINR_VAL1 PT-INR Value

N	Mean	Standard Deviation	Minimum	Maximum
6	1.9683333	0.3200260	1.6800000	2.5600000

Unique Subject Identifier=7171010000-0002

Assay variable : PTINR_VAL1 PT-INR Value

N	Mean	Standard Deviation	Minimum	Maximum
6	1.7250000	0.1789693	1.4700000	1.9700000

Unique Subject Identifier=7171010000-0004

Assay variable : PTINR_VAL1 PT-INR Value

N	Mean	Standard Deviation	Minimum	Maximum
5	1.7980000	0.1628496	1.6200000	2.0200000

Laboratory tests at the time of obtaining Table 2-6 consent, according to the presence or absence of proton-pump inhibitors

Unique Subject Identifier=7198010000-0001

MEANS Procedure

Assay variable : PTINR_VAL1 PT-INR Value

N	Mean	Standard Deviation	Minimum	Maximum
3	1.5100000	0.1670329	1.3300000	1.6600000

Unique Subject Identifier=7198010000-0011

Assay variable : PTINR_VAL1 PT-INR Value

N	Mean	Standard Deviation	Minimum	Maximum
6	1.8333333	0.1242041	1.6800000	2.0100000

Unique Subject Identifier=7198010000-0017

Assay variable : PTINR_VAL1 PT-INR Value

N	Mean	Standard Deviation	Minimum	Maximum
6	2.1916667	0.4857743	1.5900000	3.0000000

Unique Subject Identifier=7198010000-0019

Assay variable : PTINR_VAL1 PT-INR Value

N	Mean	Standard Deviation	Minimum	Maximum
4	1.6625000	0.1850000	1.4500000	1.9000000

Laboratory tests at the time of obtaining Table 2-6 consent, according to the presence or absence of proton-pump inhibitors

Unique Subject Identifier=7198010000-0031

MEANS Procedure

Assay variable : PTINR_VAL1 PT-INR Value

N	Mean	Standard Deviation	Minimum	Maximum
5	1.8700000	0.3157531	1.6400000	2.4200000

Unique Subject Identifier=7198010000-0038

Assay variable : PTINR_VAL1 PT-INR Value

N	Mean	Standard Deviation	Minimum	Maximum
6	1.9550000	0.4408968	1.3800000	2.5300000

Unique Subject Identifier=7198010000-0039

Assay variable : PTINR_VAL1 PT-INR Value

N	Mean	Standard Deviation	Minimum	Maximum
4	2.3550000	0.0838650	2.2700000	2.4700000

Unique Subject Identifier=7198010000-0040

Assay variable : PTINR_VAL1 PT-INR Value

N	Mean	Standard Deviation	Minimum	Maximum
4	1.9075000	0.1607016	1.7000000	2.0900000

Laboratory tests at the time of obtaining Table 2-6 consent, according to the presence or absence of proton-pump inhibitors

Unique Subject Identifier=7198010000-0044

MEANS Procedure

Assay variable : PTINR_VAL1 PT-INR Value

N	Mean	Standard Deviation	Minimum	Maximum
2	1.9250000	0.1767767	1.8000000	2.0500000

Unique Subject Identifier=7198010000-0048

Assay variable : PTINR_VAL1 PT-INR Value

N	Mean	Standard Deviation	Minimum	Maximum
1	1.5200000		1.5200000	1.5200000

Unique Subject Identifier=7198010000-0051

Assay variable : PTINR_VAL1 PT-INR Value

N	Mean	Standard Deviation	Minimum	Maximum
4	2.1575000	0.5864228	1.6400000	3.0000000

Unique Subject Identifier=7198010000-0053

Assay variable : PTINR_VAL1 PT-INR Value

N	Mean	Standard Deviation	Minimum	Maximum
6	1.9600000	0.1584929	1.8100000	2.2200000

Laboratory tests at the time of obtaining Table 2-6 consent, according to the presence or absence of proton-pump inhibitors

Unique Subject Identifier=7198010000-0057

MEANS Procedure

Assay variable : PTINR_VAL1 PT-INR Value

N	Mean	Standard Deviation	Minimum	Maximum
5	2.1120000	0.2903791	1.7700000	2.4200000

Unique Subject Identifier=7198010000-0067

Assay variable : PTINR_VAL1 PT-INR Value

N	Mean	Standard Deviation	Minimum	Maximum
3	2.3033333	0.5783886	1.6500000	2.7500000

Unique Subject Identifier=7198010000-0074

Assay variable : PTINR_VAL1 PT-INR Value

N	Mean	Standard Deviation	Minimum	Maximum
6	1.7716667	0.4282250	1.4100000	2.3100000

Unique Subject Identifier=7198010000-0085

Assay variable : PTINR_VAL1 PT-INR Value

N	Mean	Standard Deviation	Minimum	Maximum
3	1.8200000	0.4014972	1.4400000	2.2400000

Laboratory tests at the time of obtaining Table 2-6 consent, according to the presence or absence of proton-pump inhibitors

Unique Subject Identifier=7198010000-0092

MEANS Procedure

Assay variable : PTINR_VAL1 PT-INR Value

N	Mean	Standard Deviation	Minimum	Maximum
3	1.7466667	0.3002221	1.4400000	2.0400000

Unique Subject Identifier=7204000000-0002

Assay variable : PTINR_VAL1 PT-INR Value

N	Mean	Standard Deviation	Minimum	Maximum
5	1.8680000	0.2940578	1.6000000	2.3500000

Unique Subject Identifier=7204000000-0010

Assay variable : PTINR_VAL1 PT-INR Value

N	Mean	Standard Deviation	Minimum	Maximum
4	1.6300000	0.3236253	1.2800000	1.9200000

Unique Subject Identifier=7204000000-0017

Assay variable : PTINR_VAL1 PT-INR Value

N	Mean	Standard Deviation	Minimum	Maximum
6	1.1933333	0.0588784	1.1100000	1.2800000

Laboratory tests at the time of obtaining Table 2-6 consent, according to the presence or absence of proton-pump inhibitors

Unique Subject Identifier=7204000000-0024

MEANS Procedure

Assay variable : PTINR_VAL1 PT-INR Value

N	Mean	Standard Deviation	Minimum	Maximum
5	1.3140000	0.1689083	1.0900000	1.5600000

Unique Subject Identifier=7204000000-0026

Assay variable : PTINR_VAL1 PT-INR Value

N	Mean	Standard Deviation	Minimum	Maximum
6	1.8383333	0.5723431	1.1400000	2.7900000

Unique Subject Identifier=7204000000-0028

Assay variable : PTINR_VAL1 PT-INR Value

N	Mean	Standard Deviation	Minimum	Maximum
6	1.7533333	0.1148332	1.6400000	1.9100000

Unique Subject Identifier=7204000000-0030

Assay variable : PTINR_VAL1 PT-INR Value

N	Mean	Standard Deviation	Minimum	Maximum
5	1.5080000	0.2537124	1.2800000	1.8900000

Laboratory tests at the time of obtaining Table 2-6 consent, according to the presence or absence of proton-pump inhibitors

Unique Subject Identifier=7204000000-0033

MEANS Procedure

Assay variable : PTINR_VAL1 PT-INR Value

N	Mean	Standard Deviation	Minimum	Maximum
6	2.4266667	1.2527197	1.4600000	4.8300000

Unique Subject Identifier=7205000000-0001

Assay variable : PTINR_VAL1 PT-INR Value

N	Mean	Standard Deviation	Minimum	Maximum
3	1.9266667	0.5118919	1.4700000	2.4800000

Unique Subject Identifier=7205000000-0027

Assay variable : PTINR_VAL1 PT-INR Value

N	Mean	Standard Deviation	Minimum	Maximum
3	2.0700000	0.3593049	1.8100000	2.4800000

Unique Subject Identifier=7205000000-0061

Assay variable : PTINR_VAL1 PT-INR Value

N	Mean	Standard Deviation	Minimum	Maximum
4	2.7075000	1.1173891	1.5500000	3.9700000

Laboratory tests at the time of obtaining Table 2-6 consent, according to the presence or absence of proton-pump inhibitors

Unique Subject Identifier=7205000000-0075

MEANS Procedure

Assay variable : PTINR_VAL1 PT-INR Value

N	Mean	Standard Deviation	Minimum	Maximum
4	1.9125000	0.4852748	1.5700000	2.6300000

Unique Subject Identifier=7205000000-0080

Assay variable : PTINR_VAL1 PT-INR Value

N	Mean	Standard Deviation	Minimum	Maximum
3	2.4900000	1.1468653	1.5300000	3.7600000

Unique Subject Identifier=7208011010-0005

Assay variable : PTINR_VAL1 PT-INR Value

N	Mean	Standard Deviation	Minimum	Maximum
4	2.1100000	0.1925271	1.8700000	2.3100000

Unique Subject Identifier=7208011010-0007

Assay variable : PTINR_VAL1 PT-INR Value

N	Mean	Standard Deviation	Minimum	Maximum
4	1.8925000	0.2736634	1.6600000	2.2600000

Laboratory tests at the time of obtaining Table 2-6 consent, according to the presence or absence of proton-pump inhibitors

Unique Subject Identifier=7208011010-0009

MEANS Procedure

Assay variable : PTINR_VAL1 PT-INR Value

N	Mean	Standard Deviation	Minimum	Maximum
6	2.2666667	1.2165141	1.2300000	4.6400000

Unique Subject Identifier=7208011010-0011

Assay variable : PTINR_VAL1 PT-INR Value

N	Mean	Standard Deviation	Minimum	Maximum
6	1.7300000	0.2324651	1.4900000	1.9900000

Unique Subject Identifier=7208011010-0012

Assay variable : PTINR_VAL1 PT-INR Value

N	Mean	Standard Deviation	Minimum	Maximum
3	1.4966667	0.0503322	1.4500000	1.5500000

Unique Subject Identifier=7208011010-0013

Assay variable : PTINR_VAL1 PT-INR Value

N	Mean	Standard Deviation	Minimum	Maximum
1	2.3100000		2.3100000	2.3100000

Laboratory tests at the time of obtaining Table 2-6 consent, according to the presence or absence of proton-pump inhibitors

Unique Subject Identifier=7208011010-0015

MEANS Procedure

Assay variable : PTINR_VAL1 PT-INR Value

N	Mean	Standard Deviation	Minimum	Maximum
3	1.5433333	0.4148895	1.1700000	1.9900000

Unique Subject Identifier=7209000000-0002

Assay variable : PTINR_VAL1 PT-INR Value

N	Mean	Standard Deviation	Minimum	Maximum
3	2.1400000	0.2651415	1.8500000	2.3700000

Unique Subject Identifier=7209000000-0003

Assay variable : PTINR_VAL1 PT-INR Value

N	Mean	Standard Deviation	Minimum	Maximum
3	2.3333333	0.1594783	2.1500000	2.4400000

Unique Subject Identifier=7209000000-0006

Assay variable : PTINR_VAL1 PT-INR Value

N	Mean	Standard Deviation	Minimum	Maximum
1	1.5700000		1.5700000	1.5700000

Laboratory tests at the time of obtaining Table 2-6 consent, according to the presence or absence of proton-pump inhibitors

Unique Subject Identifier=7209000000-0007

MEANS Procedure

Assay variable : PTINR_VAL1 PT-INR Value

N	Mean	Standard Deviation	Minimum	Maximum
4	1.1625000	0.0994569	1.0800000	1.2800000

Unique Subject Identifier=7209000000-0008

Assay variable : PTINR_VAL1 PT-INR Value

N	Mean	Standard Deviation	Minimum	Maximum
4	1.9075000	0.2458150	1.7500000	2.2700000

Unique Subject Identifier=7209000000-0010

Assay variable : PTINR_VAL1 PT-INR Value

N	Mean	Standard Deviation	Minimum	Maximum
4	1.8575000	0.3522665	1.5500000	2.3500000

Unique Subject Identifier=7209000000-0011

Assay variable : PTINR_VAL1 PT-INR Value

N	Mean	Standard Deviation	Minimum	Maximum
4	1.9475000	0.1793274	1.6800000	2.0500000

Laboratory tests at the time of obtaining Table 2-6 consent, according to the presence or absence of proton-pump inhibitors

Unique Subject Identifier=7209000000-0014

MEANS Procedure

Assay variable : PTINR_VAL1 PT-INR Value

N	Mean	Standard Deviation	Minimum	Maximum
4	2.0500000	0.3359563	1.5700000	2.3400000

Unique Subject Identifier=7209000000-0019

Assay variable : PTINR_VAL1 PT-INR Value

N	Mean	Standard Deviation	Minimum	Maximum
4	2.2150000	0.1398809	2.0500000	2.3500000

Unique Subject Identifier=7209000000-0021

Assay variable : PTINR_VAL1 PT-INR Value

N	Mean	Standard Deviation	Minimum	Maximum
3	1.9133333	0.3455913	1.5800000	2.2700000

Unique Subject Identifier=7209000000-0025

Assay variable : PTINR_VAL1 PT-INR Value

N	Mean	Standard Deviation	Minimum	Maximum
3	1.8266667	0.2761038	1.5900000	2.1300000

Laboratory tests at the time of obtaining Table 2-6 consent, according to the presence or absence of proton-pump inhibitors

Unique Subject Identifier=7209000000-0026

MEANS Procedure

Assay variable : PTINR_VAL1 PT-INR Value

N	Mean	Standard Deviation	Minimum	Maximum
2	1.5100000	0.0707107	1.4600000	1.5600000

Unique Subject Identifier=7209000000-0027

Assay variable : PTINR_VAL1 PT-INR Value

N	Mean	Standard Deviation	Minimum	Maximum
4	1.8250000	0.4704962	1.2300000	2.3800000

Unique Subject Identifier=7209000000-0028

Assay variable : PTINR_VAL1 PT-INR Value

N	Mean	Standard Deviation	Minimum	Maximum
4	2.2975000	0.0877021	2.2000000	2.3900000

Unique Subject Identifier=7209000000-0034

Assay variable : PTINR_VAL1 PT-INR Value

N	Mean	Standard Deviation	Minimum	Maximum
4	1.8975000	0.0758837	1.8200000	2.0000000

Laboratory tests at the time of obtaining Table 2-6 consent, according to the presence or absence of proton-pump inhibitors

Unique Subject Identifier=7209000000-0036

MEANS Procedure

Assay variable : PTINR_VAL1 PT-INR Value

N	Mean	Standard Deviation	Minimum	Maximum
4	1.8700000	0.4377975	1.4200000	2.2700000

Unique Subject Identifier=7209000000-0037

Assay variable : PTINR_VAL1 PT-INR Value

N	Mean	Standard Deviation	Minimum	Maximum
4	2.0650000	0.1690168	1.8900000	2.2900000

Unique Subject Identifier=7209000000-0048

Assay variable : PTINR_VAL1 PT-INR Value

N	Mean	Standard Deviation	Minimum	Maximum
3	1.6966667	0.1721434	1.5600000	1.8900000

Unique Subject Identifier=7209000000-0049

Assay variable : PTINR_VAL1 PT-INR Value

N	Mean	Standard Deviation	Minimum	Maximum
6	2.9650000	0.6056979	2.1300000	3.9400000

Laboratory tests at the time of obtaining Table 2-6 consent, according to the presence or absence of proton-pump inhibitors

Unique Subject Identifier=7209000000-0050

MEANS Procedure

Assay variable : PTINR_VAL1 PT-INR Value

N	Mean	Standard Deviation	Minimum	Maximum
3	1.2866667	0.3592121	0.9000000	1.6100000

Unique Subject Identifier=7209000000-0056

Assay variable : PTINR_VAL1 PT-INR Value

N	Mean	Standard Deviation	Minimum	Maximum
6	1.5700000	0.1340149	1.3900000	1.7700000

Unique Subject Identifier=7209000000-0057

Assay variable : PTINR_VAL1 PT-INR Value

N	Mean	Standard Deviation	Minimum	Maximum
6	1.8100000	0.3024566	1.4600000	2.2100000

Unique Subject Identifier=7209000000-0060

Assay variable : PTINR_VAL1 PT-INR Value

N	Mean	Standard Deviation	Minimum	Maximum
4	2.0250000	0.1470827	1.8100000	2.1200000

Laboratory tests at the time of obtaining Table 2-6 consent, according to the presence or absence of proton-pump inhibitors

Unique Subject Identifier=7209000000-0061

MEANS Procedure

Assay variable : PTINR_VAL1 PT-INR Value

N	Mean	Standard Deviation	Minimum	Maximum
4	1.8175000	0.4486554	1.2500000	2.3100000

Unique Subject Identifier=7209000000-0062

Assay variable : PTINR_VAL1 PT-INR Value

N	Mean	Standard Deviation	Minimum	Maximum
4	2.8925000	0.0906918	2.8000000	2.9800000

Unique Subject Identifier=7209000000-0066

Assay variable : PTINR_VAL1 PT-INR Value

N	Mean	Standard Deviation	Minimum	Maximum
4	1.6500000	0.3350622	1.3500000	2.1300000

Unique Subject Identifier=7209000000-0068

Assay variable : PTINR_VAL1 PT-INR Value

N	Mean	Standard Deviation	Minimum	Maximum
4	2.3250000	0.2700000	2.1000000	2.7000000

Laboratory tests at the time of obtaining Table 2-6 consent, according to the presence or absence of proton-pump inhibitors

Unique Subject Identifier=7209000000-0072

MEANS Procedure

Assay variable : PTINR_VAL1 PT-INR Value

N	Mean	Standard Deviation	Minimum	Maximum
4	2.4225000	0.2519755	2.1200000	2.6400000

Unique Subject Identifier=7209000000-0073

Assay variable : PTINR_VAL1 PT-INR Value

N	Mean	Standard Deviation	Minimum	Maximum
2	1.8300000	0.8202439	1.2500000	2.4100000

Unique Subject Identifier=7209000000-0074

Assay variable : PTINR_VAL1 PT-INR Value

N	Mean	Standard Deviation	Minimum	Maximum
4	1.9750000	0.8438602	1.2800000	3.0200000

Unique Subject Identifier=7209000000-0085

Assay variable : PTINR_VAL1 PT-INR Value

N	Mean	Standard Deviation	Minimum	Maximum
3	2.8000000	0.2971532	2.4600000	3.0100000

Laboratory tests at the time of obtaining Table 2-6 consent, according to the presence or absence of proton-pump inhibitors

Unique Subject Identifier=7209000000-0089

MEANS Procedure

Assay variable : PTINR_VAL1 PT-INR Value

N	Mean	Standard Deviation	Minimum	Maximum
3	1.7333333	0.5928181	1.0500000	2.1100000

Unique Subject Identifier=7209000000-0090

Assay variable : PTINR_VAL1 PT-INR Value

N	Mean	Standard Deviation	Minimum	Maximum
3	2.5233333	0.5186842	2.1800000	3.1200000

Unique Subject Identifier=7209000000-0093

Assay variable : PTINR_VAL1 PT-INR Value

N	Mean	Standard Deviation	Minimum	Maximum
4	1.8825000	0.4947979	1.2900000	2.3200000

Unique Subject Identifier=7209000000-0094

Assay variable : PTINR_VAL1 PT-INR Value

N	Mean	Standard Deviation	Minimum	Maximum
3	2.0833333	0.2569695	1.8100000	2.3200000

Laboratory tests at the time of obtaining Table 2-6 consent, according to the presence or absence of proton-pump inhibitors

Unique Subject Identifier=7209000000-0095

MEANS Procedure

Assay variable : PTINR_VAL1 PT-INR Value

N	Mean	Standard Deviation	Minimum	Maximum
1	1.8800000		1.8800000	1.8800000

Unique Subject Identifier=7209000000-0097

Assay variable : PTINR_VAL1 PT-INR Value

N	Mean	Standard Deviation	Minimum	Maximum
2	2.3950000	1.3364318	1.4500000	3.3400000

Unique Subject Identifier=7209000000-0098

Assay variable : PTINR_VAL1 PT-INR Value

N	Mean	Standard Deviation	Minimum	Maximum
3	2.5200000	0.1345362	2.3700000	2.6300000

Unique Subject Identifier=7209000000-0099

Assay variable : PTINR_VAL1 PT-INR Value

N	Mean	Standard Deviation	Minimum	Maximum
4	1.9275000	0.1412740	1.7200000	2.0300000

Laboratory tests at the time of obtaining Table 2-6 consent, according to the presence or absence of proton-pump inhibitors

Unique Subject Identifier=7209000000-0110

MEANS Procedure

Assay variable : PTINR_VAL1 PT-INR Value

N	Mean	Standard Deviation	Minimum	Maximum
1	2.8100000		2.8100000	2.8100000

Unique Subject Identifier=7209000000-0111

Assay variable : PTINR_VAL1 PT-INR Value

N	Mean	Standard Deviation	Minimum	Maximum
1	1.5700000		1.5700000	1.5700000

Unique Subject Identifier=7209000000-0112

Assay variable : PTINR_VAL1 PT-INR Value

N	Mean	Standard Deviation	Minimum	Maximum
1	1.6600000		1.6600000	1.6600000

Unique Subject Identifier=7209000000-0114

Assay variable : PTINR_VAL1 PT-INR Value

N	Mean	Standard Deviation	Minimum	Maximum
4	2.2475000	0.5292999	1.6200000	2.8900000

Laboratory tests at the time of obtaining Table 2-6 consent, according to the presence or absence of proton-pump inhibitors

Unique Subject Identifier=7209000000-0116

MEANS Procedure

Assay variable : PTINR_VAL1 PT-INR Value

N	Mean	Standard Deviation	Minimum	Maximum
3	1.6533333	0.1715615	1.4700000	1.8100000

Unique Subject Identifier=7209000000-0136

Assay variable : PTINR_VAL1 PT-INR Value

N	Mean	Standard Deviation	Minimum	Maximum
3	1.9733333	0.1436431	1.8100000	2.0800000

Unique Subject Identifier=7209000000-0148

Assay variable : PTINR_VAL1 PT-INR Value

N	Mean	Standard Deviation	Minimum	Maximum
4	1.7325000	0.0623832	1.6500000	1.8000000

Unique Subject Identifier=7209000000-0149

Assay variable : PTINR_VAL1 PT-INR Value

N	Mean	Standard Deviation	Minimum	Maximum
3	1.9566667	0.4895236	1.5800000	2.5100000

Laboratory tests at the time of obtaining Table 2-6 consent, according to the presence or absence of proton-pump inhibitors

Unique Subject Identifier=7221000000-0003

MEANS Procedure

Assay variable : PTINR_VAL1 PT-INR Value

N	Mean	Standard Deviation	Minimum	Maximum
5	1.9000000	0.3807887	1.4000000	2.4000000

Unique Subject Identifier=7221000000-0008

Assay variable : PTINR_VAL1 PT-INR Value

N	Mean	Standard Deviation	Minimum	Maximum
5	1.7600000	0.2073644	1.4000000	1.9000000

Unique Subject Identifier=7221000000-0016

Assay variable : PTINR_VAL1 PT-INR Value

N	Mean	Standard Deviation	Minimum	Maximum
5	2.3400000	0.3435113	2.0000000	2.8000000

Unique Subject Identifier=7221000000-0020

Assay variable : PTINR_VAL1 PT-INR Value

N	Mean	Standard Deviation	Minimum	Maximum
5	2.1800000	0.1923538	1.9000000	2.4000000

Laboratory tests at the time of obtaining Table 2-6 consent, according to the presence or absence of proton-pump inhibitors

Unique Subject Identifier=7224011010-0002

MEANS Procedure

Assay variable : PTINR_VAL1 PT-INR Value

N	Mean	Standard Deviation	Minimum	Maximum
1	2.3800000		2.3800000	2.3800000

Unique Subject Identifier=7224011010-0004

Assay variable : PTINR_VAL1 PT-INR Value

N	Mean	Standard Deviation	Minimum	Maximum
1	2.0300000		2.0300000	2.0300000

Unique Subject Identifier=7224011010-0006

Assay variable : PTINR_VAL1 PT-INR Value

N	Mean	Standard Deviation	Minimum	Maximum
1	1.9000000		1.9000000	1.9000000

Unique Subject Identifier=7224011010-0009

Assay variable : PTINR_VAL1 PT-INR Value

N	Mean	Standard Deviation	Minimum	Maximum
5	1.7500000	0.2431049	1.5200000	2.1400000

Laboratory tests at the time of obtaining Table 2-6 consent, according to the presence or absence of proton-pump inhibitors

Unique Subject Identifier=7224011010-0011

MEANS Procedure

Assay variable : PTINR_VAL1 PT-INR Value

N	Mean	Standard Deviation	Minimum	Maximum
3	1.4700000	0.2389561	1.2600000	1.7300000

Unique Subject Identifier=7226000000-0001

Assay variable : PTINR_VAL1 PT-INR Value

N	Mean	Standard Deviation	Minimum	Maximum
3	1.4700000	0.1587451	1.2900000	1.5900000

Unique Subject Identifier=7226000000-0005

Assay variable : PTINR_VAL1 PT-INR Value

N	Mean	Standard Deviation	Minimum	Maximum
4	1.7500000	0.2714160	1.4200000	2.0100000

Unique Subject Identifier=7226000000-0006

Assay variable : PTINR_VAL1 PT-INR Value

N	Mean	Standard Deviation	Minimum	Maximum
4	2.2900000	1.0512532	1.4400000	3.7100000

Laboratory tests at the time of obtaining Table 2-6 consent, according to the presence or absence of proton-pump inhibitors

Unique Subject Identifier=7226000000-0012

MEANS Procedure

Assay variable : PTINR_VAL1 PT-INR Value

N	Mean	Standard Deviation	Minimum	Maximum
3	2.3866667	0.3066486	2.1900000	2.7400000

Unique Subject Identifier=7226000000-0013

Assay variable : PTINR_VAL1 PT-INR Value

N	Mean	Standard Deviation	Minimum	Maximum
4	2.2375000	0.3244868	1.9300000	2.6900000

Unique Subject Identifier=7226000000-0017

Assay variable : PTINR_VAL1 PT-INR Value

N	Mean	Standard Deviation	Minimum	Maximum
5	1.8540000	0.3089984	1.4200000	2.2900000

Unique Subject Identifier=7226000000-0019

Assay variable : PTINR_VAL1 PT-INR Value

N	Mean	Standard Deviation	Minimum	Maximum
3	1.9366667	0.4315476	1.5900000	2.4200000

Laboratory tests at the time of obtaining Table 2-6 consent, according to the presence or absence of proton-pump inhibitors

Unique Subject Identifier=7226000000-0020

MEANS Procedure

Assay variable : PTINR_VAL1 PT-INR Value

N	Mean	Standard Deviation	Minimum	Maximum
3	1.8766667	0.5612783	1.3700000	2.4800000

Unique Subject Identifier=7226000000-0022

Assay variable : PTINR_VAL1 PT-INR Value

N	Mean	Standard Deviation	Minimum	Maximum
3	2.0766667	0.6251666	1.4600000	2.7100000

Unique Subject Identifier=7226000000-0024

Assay variable : PTINR_VAL1 PT-INR Value

N	Mean	Standard Deviation	Minimum	Maximum
5	1.6380000	0.4292086	1.2000000	2.3500000

Unique Subject Identifier=7226000000-0026

Assay variable : PTINR_VAL1 PT-INR Value

N	Mean	Standard Deviation	Minimum	Maximum
3	1.9100000	0.6080296	1.3400000	2.5500000

Laboratory tests at the time of obtaining Table 2-6 consent, according to the presence or absence of proton-pump inhibitors

Unique Subject Identifier=7226000000-0029

MEANS Procedure

Assay variable : PTINR_VAL1 PT-INR Value

N	Mean	Standard Deviation	Minimum	Maximum
3	1.6866667	0.3027100	1.4100000	2.0100000

Unique Subject Identifier=7233000000-0007

Assay variable : PTINR_VAL1 PT-INR Value

N	Mean	Standard Deviation	Minimum	Maximum
4	2.2800000	0.1663330	2.0900000	2.4600000

Unique Subject Identifier=7235000000-0002

Assay variable : PTINR_VAL1 PT-INR Value

N	Mean	Standard Deviation	Minimum	Maximum
1	2.9300000		2.9300000	2.9300000

Unique Subject Identifier=7235000000-0003

Assay variable : PTINR_VAL1 PT-INR Value

N	Mean	Standard Deviation	Minimum	Maximum
1	2.2700000		2.2700000	2.2700000

Laboratory tests at the time of obtaining Table 2-6 consent, according to the presence or absence of proton-pump inhibitors

Unique Subject Identifier=7235000000-0011

MEANS Procedure

Assay variable : PTINR_VAL1 PT-INR Value

N	Mean	Standard Deviation	Minimum	Maximum
2	2.1850000	0.2616295	2.0000000	2.3700000

Unique Subject Identifier=7235000000-0012

Assay variable : PTINR_VAL1 PT-INR Value

N	Mean	Standard Deviation	Minimum	Maximum
1	3.0300000		3.0300000	3.0300000

Unique Subject Identifier=7235000000-0013

Assay variable : PTINR_VAL1 PT-INR Value

N	Mean	Standard Deviation	Minimum	Maximum
2	1.7250000	0.5161880	1.3600000	2.0900000

Unique Subject Identifier=7235000000-0015

Assay variable : PTINR_VAL1 PT-INR Value

N	Mean	Standard Deviation	Minimum	Maximum
1	2.2800000		2.2800000	2.2800000

Laboratory tests at the time of obtaining Table 2-6 consent, according to the presence or absence of proton-pump inhibitors

Unique Subject Identifier=7235000000-0018

MEANS Procedure

Assay variable : PTINR_VAL1 PT-INR Value

N	Mean	Standard Deviation	Minimum	Maximum
1	1.4900000		1.4900000	1.4900000

Unique Subject Identifier=7237010000-0001

Assay variable : PTINR_VAL1 PT-INR Value

N	Mean	Standard Deviation	Minimum	Maximum
3	1.5800000	0.0346410	1.5400000	1.6000000

Unique Subject Identifier=7237010000-0002

Assay variable : PTINR_VAL1 PT-INR Value

N	Mean	Standard Deviation	Minimum	Maximum
3	1.8833333	0.4650090	1.4200000	2.3500000

Unique Subject Identifier=7237010000-0003

Assay variable : PTINR_VAL1 PT-INR Value

N	Mean	Standard Deviation	Minimum	Maximum
3	1.4000000	0.3143247	1.0400000	1.6200000

Laboratory tests at the time of obtaining Table 2-6 consent, according to the presence or absence of proton-pump inhibitors

Unique Subject Identifier=7242010000-0001

MEANS Procedure

Assay variable : PTINR_VAL1 PT-INR Value

N	Mean	Standard Deviation	Minimum	Maximum
2	1.3250000	0.2050610	1.1800000	1.4700000

Unique Subject Identifier=7242010000-0003

Assay variable : PTINR_VAL1 PT-INR Value

N	Mean	Standard Deviation	Minimum	Maximum
2	2.2050000	0.1060660	2.1300000	2.2800000

Unique Subject Identifier=7242010000-0008

Assay variable : PTINR_VAL1 PT-INR Value

N	Mean	Standard Deviation	Minimum	Maximum
2	1.6850000	0.0070711	1.6800000	1.6900000

Unique Subject Identifier=7242010000-0009

Assay variable : PTINR_VAL1 PT-INR Value

N	Mean	Standard Deviation	Minimum	Maximum
2	2.3100000	0.4101219	2.0200000	2.6000000

Laboratory tests at the time of obtaining Table 2-6 consent, according to the presence or absence of proton-pump inhibitors

Unique Subject Identifier=7242010000-0012

MEANS Procedure

Assay variable : PTINR_VAL1 PT-INR Value

N	Mean	Standard Deviation	Minimum	Maximum
2	1.9250000	0.4737615	1.5900000	2.2600000

Unique Subject Identifier=7314000000-0009

Assay variable : PTINR_VAL1 PT-INR Value

N	Mean	Standard Deviation	Minimum	Maximum
4	1.7750000	0.2362908	1.6000000	2.1000000

Unique Subject Identifier=7314000000-0016

Assay variable : PTINR_VAL1 PT-INR Value

N	Mean	Standard Deviation	Minimum	Maximum
5	1.5800000	0.0836660	1.5000000	1.7000000

Unique Subject Identifier=7314000000-0020

Assay variable : PTINR_VAL1 PT-INR Value

N	Mean	Standard Deviation	Minimum	Maximum
6	1.7666667	0.1632993	1.6000000	2.0000000

Laboratory tests at the time of obtaining Table 2-6 consent, according to the presence or absence of proton-pump inhibitors

Unique Subject Identifier=7326000000-0001

MEANS Procedure

Assay variable : PTINR_VAL1 PT-INR Value

N	Mean	Standard Deviation	Minimum	Maximum
5	1.6100000	0.3037269	1.2800000	2.0500000

Unique Subject Identifier=7326000000-0004

Assay variable : PTINR_VAL1 PT-INR Value

N	Mean	Standard Deviation	Minimum	Maximum
3	2.1566667	0.2074448	1.9700000	2.3800000

Unique Subject Identifier=7326000000-0005

Assay variable : PTINR_VAL1 PT-INR Value

N	Mean	Standard Deviation	Minimum	Maximum
5	2.4660000	0.3561320	1.9400000	2.9100000

Unique Subject Identifier=7326000000-0006

Assay variable : PTINR_VAL1 PT-INR Value

N	Mean	Standard Deviation	Minimum	Maximum
5	1.9400000	0.2518928	1.6100000	2.2600000

Laboratory tests at the time of obtaining Table 2-6 consent, according to the presence or absence of proton-pump inhibitors

Unique Subject Identifier=7326000000-0007

MEANS Procedure

Assay variable : PTINR_VAL1 PT-INR Value

N	Mean	Standard Deviation	Minimum	Maximum
4	1.8825000	0.2154646	1.5800000	2.0600000

Unique Subject Identifier=7332010000-0002

Assay variable : PTINR_VAL1 PT-INR Value

N	Mean	Standard Deviation	Minimum	Maximum
5	2.1080000	1.0816977	1.3600000	3.9600000

Unique Subject Identifier=7332010000-0004

Assay variable : PTINR_VAL1 PT-INR Value

N	Mean	Standard Deviation	Minimum	Maximum
4	1.8125000	0.2139120	1.5300000	2.0500000

Unique Subject Identifier=7332010000-0005

Assay variable : PTINR_VAL1 PT-INR Value

N	Mean	Standard Deviation	Minimum	Maximum
4	2.2150000	0.1815673	2.0400000	2.4500000

Laboratory tests at the time of obtaining Table 2-6 consent, according to the presence or absence of proton-pump inhibitors

Unique Subject Identifier=7332010000-0006

MEANS Procedure

Assay variable : PTINR_VAL1 PT-INR Value

N	Mean	Standard Deviation	Minimum	Maximum
6	1.8366667	0.2407211	1.5800000	2.2400000

Unique Subject Identifier=7332010000-0007

Assay variable : PTINR_VAL1 PT-INR Value

N	Mean	Standard Deviation	Minimum	Maximum
6	2.3350000	0.3577569	1.9900000	2.9900000

Unique Subject Identifier=7332010000-0008

Assay variable : PTINR_VAL1 PT-INR Value

N	Mean	Standard Deviation	Minimum	Maximum
5	1.6520000	0.0990959	1.5300000	1.7900000

Unique Subject Identifier=7332010000-0014

Assay variable : PTINR_VAL1 PT-INR Value

N	Mean	Standard Deviation	Minimum	Maximum
3	1.8333333	0.1021437	1.7600000	1.9500000

Laboratory tests at the time of obtaining Table 2-6 consent, according to the presence or absence of proton-pump inhibitors

Unique Subject Identifier=7332010000-0016

MEANS Procedure

Assay variable : PTINR_VAL1 PT-INR Value

N	Mean	Standard Deviation	Minimum	Maximum
3	1.8733333	0.1674316	1.6800000	1.9700000

Unique Subject Identifier=7332010000-0017

Assay variable : PTINR_VAL1 PT-INR Value

N	Mean	Standard Deviation	Minimum	Maximum
3	2.2433333	0.5253887	1.9400000	2.8500000

Unique Subject Identifier=7332010000-0018

Assay variable : PTINR_VAL1 PT-INR Value

N	Mean	Standard Deviation	Minimum	Maximum
3	2.0533333	0.3925982	1.6000000	2.2800000

Unique Subject Identifier=7332010000-0023

Assay variable : PTINR_VAL1 PT-INR Value

N	Mean	Standard Deviation	Minimum	Maximum
4	2.0575000	0.3232517	1.7500000	2.4500000

Laboratory tests at the time of obtaining Table 2-6 consent, according to the presence or absence of proton-pump inhibitors

Unique Subject Identifier=7332010000-0026

MEANS Procedure

Assay variable : PTINR_VAL1 PT-INR Value

N	Mean	Standard Deviation	Minimum	Maximum
4	2.0350000	0.4239890	1.6600000	2.5400000

Unique Subject Identifier=7332010000-0029

Assay variable : PTINR_VAL1 PT-INR Value

N	Mean	Standard Deviation	Minimum	Maximum
3	1.9233333	0.2400694	1.6600000	2.1300000

Unique Subject Identifier=7332010000-0032

Assay variable : PTINR_VAL1 PT-INR Value

N	Mean	Standard Deviation	Minimum	Maximum
3	1.7233333	0.1446836	1.6300000	1.8900000

Unique Subject Identifier=7340011000-0001

Assay variable : PTINR_VAL1 PT-INR Value

N	Mean	Standard Deviation	Minimum	Maximum
6	2.0333333	0.0816497	1.9000000	2.1000000

Laboratory tests at the time of obtaining Table 2-6 consent, according to the presence or absence of proton-pump inhibitors

Unique Subject Identifier=7345000010-0004

MEANS Procedure

Assay variable : PTINR_VAL1 PT-INR Value

N	Mean	Standard Deviation	Minimum	Maximum
6	2.0566667	0.4411651	1.6700000	2.9000000

Unique Subject Identifier=7345000010-0005

Assay variable : PTINR_VAL1 PT-INR Value

N	Mean	Standard Deviation	Minimum	Maximum
4	1.8300000	0.3754997	1.3200000	2.2000000

Unique Subject Identifier=7345000010-0008

Assay variable : PTINR_VAL1 PT-INR Value

N	Mean	Standard Deviation	Minimum	Maximum
6	2.2266667	0.4322345	1.5300000	2.6900000

Unique Subject Identifier=7345000010-0009

Assay variable : PTINR_VAL1 PT-INR Value

N	Mean	Standard Deviation	Minimum	Maximum
6	2.1466667	0.4553973	1.6800000	2.8100000

Laboratory tests at the time of obtaining Table 2-6 consent, according to the presence or absence of proton-pump inhibitors

Unique Subject Identifier=7345000010-0011

MEANS Procedure

Assay variable : PTINR_VAL1 PT-INR Value

N	Mean	Standard Deviation	Minimum	Maximum
6	2.0033333	0.2483277	1.7300000	2.4300000

Unique Subject Identifier=7345000010-0012

Assay variable : PTINR_VAL1 PT-INR Value

N	Mean	Standard Deviation	Minimum	Maximum
6	1.7850000	0.4234973	1.2100000	2.1900000

Unique Subject Identifier=7345000010-0014

Assay variable : PTINR_VAL1 PT-INR Value

N	Mean	Standard Deviation	Minimum	Maximum
4	2.2275000	0.6051102	1.4200000	2.8700000

Unique Subject Identifier=7345000010-0015

Assay variable : PTINR_VAL1 PT-INR Value

N	Mean	Standard Deviation	Minimum	Maximum
5	1.8660000	0.1965452	1.5900000	2.1400000

Laboratory tests at the time of obtaining Table 2-6 consent, according to the presence or absence of proton-pump inhibitors

Unique Subject Identifier=7345000010-0017

MEANS Procedure

Assay variable : PTINR_VAL1 PT-INR Value

N	Mean	Standard Deviation	Minimum	Maximum
6	1.5000000	0.5067544	1.1100000	2.4100000

Unique Subject Identifier=7345000010-0020

Assay variable : PTINR_VAL1 PT-INR Value

N	Mean	Standard Deviation	Minimum	Maximum
6	2.1600000	0.3951202	1.7000000	2.7200000

Unique Subject Identifier=7345000010-0023

Assay variable : PTINR_VAL1 PT-INR Value

N	Mean	Standard Deviation	Minimum	Maximum
6	1.9916667	0.3581852	1.5400000	2.4100000

Unique Subject Identifier=7345000010-0024

Assay variable : PTINR_VAL1 PT-INR Value

N	Mean	Standard Deviation	Minimum	Maximum
5	2.0580000	0.2605187	1.8100000	2.4600000

Laboratory tests at the time of obtaining Table 2-6 consent, according to the presence or absence of proton-pump inhibitors

Unique Subject Identifier=7345000010-0025

MEANS Procedure

Assay variable : PTINR_VAL1 PT-INR Value

N	Mean	Standard Deviation	Minimum	Maximum
6	1.7316667	0.3022196	1.3400000	2.0900000

Unique Subject Identifier=7345000010-0026

Assay variable : PTINR_VAL1 PT-INR Value

N	Mean	Standard Deviation	Minimum	Maximum
3	2.2166667	0.2746513	1.9100000	2.4400000

Unique Subject Identifier=7345000010-0027

Assay variable : PTINR_VAL1 PT-INR Value

N	Mean	Standard Deviation	Minimum	Maximum
6	2.2650000	0.3276431	1.9300000	2.7700000

Unique Subject Identifier=7345000010-0029

Assay variable : PTINR_VAL1 PT-INR Value

N	Mean	Standard Deviation	Minimum	Maximum
4	1.6425000	0.2725650	1.3000000	1.9200000

Laboratory tests at the time of obtaining Table 2-6 consent, according to the presence or absence of proton-pump inhibitors

Unique Subject Identifier=7345000010-0039

MEANS Procedure

Assay variable : PTINR_VAL1 PT-INR Value

N	Mean	Standard Deviation	Minimum	Maximum
3	1.4266667	0.0723418	1.3800000	1.5100000

Unique Subject Identifier=7345000010-0040

Assay variable : PTINR_VAL1 PT-INR Value

N	Mean	Standard Deviation	Minimum	Maximum
6	1.7333333	0.2383834	1.3900000	1.9200000

Unique Subject Identifier=7345000010-0044

Assay variable : PTINR_VAL1 PT-INR Value

N	Mean	Standard Deviation	Minimum	Maximum
3	1.9400000	0.0888819	1.8400000	2.0100000

Unique Subject Identifier=7366011100-0002

Assay variable : PTINR_VAL1 PT-INR Value

N	Mean	Standard Deviation	Minimum	Maximum
6	1.7066667	0.3946982	1.2400000	2.3800000

Laboratory tests at the time of obtaining Table 2-6 consent, according to the presence or absence of proton-pump inhibitors

Unique Subject Identifier=7366011100-0006

MEANS Procedure

Assay variable : PTINR_VAL1 PT-INR Value

N	Mean	Standard Deviation	Minimum	Maximum
6	1.9866667	0.1116542	1.8400000	2.1100000

Unique Subject Identifier=7366011100-0009

Assay variable : PTINR_VAL1 PT-INR Value

N	Mean	Standard Deviation	Minimum	Maximum
6	1.8883333	0.3343900	1.4900000	2.4700000

Unique Subject Identifier=7366011100-0010

Assay variable : PTINR_VAL1 PT-INR Value

N	Mean	Standard Deviation	Minimum	Maximum
4	2.4100000	0.3554340	1.8800000	2.6400000

Unique Subject Identifier=7366011100-0013

Assay variable : PTINR_VAL1 PT-INR Value

N	Mean	Standard Deviation	Minimum	Maximum
5	2.0740000	0.2734593	1.7400000	2.3200000

Laboratory tests at the time of obtaining Table 2-6 consent, according to the presence or absence of proton-pump inhibitors

Unique Subject Identifier=7366011100-0016

MEANS Procedure

Assay variable : PTINR_VAL1 PT-INR Value

N	Mean	Standard Deviation	Minimum	Maximum
6	2.1350000	0.4511652	1.7400000	3.0100000

Unique Subject Identifier=7414010000-0001

Assay variable : PTINR_VAL1 PT-INR Value

N	Mean	Standard Deviation	Minimum	Maximum
6	1.7383333	0.2650597	1.3300000	2.1000000

Unique Subject Identifier=7414010000-0004

Assay variable : PTINR_VAL1 PT-INR Value

N	Mean	Standard Deviation	Minimum	Maximum
6	1.8016667	0.4156160	1.3900000	2.5300000

Unique Subject Identifier=7414010000-0014

Assay variable : PTINR_VAL1 PT-INR Value

N	Mean	Standard Deviation	Minimum	Maximum
4	2.3650000	0.1204159	2.2100000	2.4700000

Laboratory tests at the time of obtaining Table 2-6 consent, according to the presence or absence of proton-pump inhibitors

Unique Subject Identifier=7414010000-0016

MEANS Procedure

Assay variable : PTINR_VAL1 PT-INR Value

N	Mean	Standard Deviation	Minimum	Maximum
5	2.1760000	0.1050238	2.0800000	2.3500000

Unique Subject Identifier=7414010000-0019

Assay variable : PTINR_VAL1 PT-INR Value

N	Mean	Standard Deviation	Minimum	Maximum
5	2.1620000	0.6068113	1.3700000	2.6800000

Unique Subject Identifier=7414010000-0020

Assay variable : PTINR_VAL1 PT-INR Value

N	Mean	Standard Deviation	Minimum	Maximum
5	1.9640000	0.6578982	1.4600000	2.9700000

Unique Subject Identifier=7414010000-0021

Assay variable : PTINR_VAL1 PT-INR Value

N	Mean	Standard Deviation	Minimum	Maximum
4	1.6950000	0.4900680	1.2800000	2.3400000

Laboratory tests at the time of obtaining Table 2-6 consent, according to the presence or absence of proton-pump inhibitors

Unique Subject Identifier=7414010000-0025

MEANS Procedure

Assay variable : PTINR_VAL1 PT-INR Value

N	Mean	Standard Deviation	Minimum	Maximum
6	1.7083333	0.4653780	1.3200000	2.5600000

Unique Subject Identifier=7414010000-0028

Assay variable : PTINR_VAL1 PT-INR Value

N	Mean	Standard Deviation	Minimum	Maximum
4	1.3650000	0.0519615	1.3100000	1.4300000

Unique Subject Identifier=7414010000-0033

Assay variable : PTINR_VAL1 PT-INR Value

N	Mean	Standard Deviation	Minimum	Maximum
6	1.9616667	0.1038107	1.8300000	2.1000000

Unique Subject Identifier=7414010000-0034

Assay variable : PTINR_VAL1 PT-INR Value

N	Mean	Standard Deviation	Minimum	Maximum
6	1.6100000	0.1550484	1.4200000	1.8700000

Laboratory tests at the time of obtaining Table 2-6 consent, according to the presence or absence of proton-pump inhibitors

Unique Subject Identifier=7414010000-0035

MEANS Procedure

Assay variable : PTINR_VAL1 PT-INR Value

N	Mean	Standard Deviation	Minimum	Maximum
3	2.1000000	0.2451530	1.8600000	2.3500000

Unique Subject Identifier=7414010000-0038

Assay variable : PTINR_VAL1 PT-INR Value

N	Mean	Standard Deviation	Minimum	Maximum
6	1.7816667	0.1220519	1.6200000	1.9600000

Unique Subject Identifier=7414010000-0041

Assay variable : PTINR_VAL1 PT-INR Value

N	Mean	Standard Deviation	Minimum	Maximum
4	2.1425000	0.2148449	1.9200000	2.4000000

Unique Subject Identifier=7414010000-0044

Assay variable : PTINR_VAL1 PT-INR Value

N	Mean	Standard Deviation	Minimum	Maximum
6	2.0483333	0.1449713	1.8800000	2.2900000

Laboratory tests at the time of obtaining Table 2-6 consent, according to the presence or absence of proton-pump inhibitors

Unique Subject Identifier=7414010000-0045

MEANS Procedure

Assay variable : PTINR_VAL1 PT-INR Value

N	Mean	Standard Deviation	Minimum	Maximum
6	1.3216667	0.1364429	1.2200000	1.5700000

Unique Subject Identifier=7414010000-0054

Assay variable : PTINR_VAL1 PT-INR Value

N	Mean	Standard Deviation	Minimum	Maximum
5	1.2300000	0.0479583	1.1900000	1.3000000

Unique Subject Identifier=7423000000-0003

Assay variable : PTINR_VAL1 PT-INR Value

N	Mean	Standard Deviation	Minimum	Maximum
4	1.7500000	0.1264911	1.5900000	1.8700000

Unique Subject Identifier=7423000000-0005

Assay variable : PTINR_VAL1 PT-INR Value

N	Mean	Standard Deviation	Minimum	Maximum
6	1.8283333	0.4879105	1.3300000	2.4600000

Laboratory tests at the time of obtaining Table 2-6 consent, according to the presence or absence of proton-pump inhibitors

Unique Subject Identifier=7423000000-0006

MEANS Procedure

Assay variable : PTINR_VAL1 PT-INR Value

N	Mean	Standard Deviation	Minimum	Maximum
4	1.6550000	0.1668333	1.5400000	1.9000000

Unique Subject Identifier=7423000000-0007

Assay variable : PTINR_VAL1 PT-INR Value

N	Mean	Standard Deviation	Minimum	Maximum
1	2.0500000		2.0500000	2.0500000

Unique Subject Identifier=7423000000-0012

Assay variable : PTINR_VAL1 PT-INR Value

N	Mean	Standard Deviation	Minimum	Maximum
3	1.9333333	0.3265476	1.7300000	2.3100000

Unique Subject Identifier=7423000000-0014

Assay variable : PTINR_VAL1 PT-INR Value

N	Mean	Standard Deviation	Minimum	Maximum
3	2.2466667	0.3239341	2.0400000	2.6200000

Laboratory tests at the time of obtaining Table 2-6 consent, according to the presence or absence of proton-pump inhibitors

Unique Subject Identifier=7423000000-0017

MEANS Procedure

Assay variable : PTINR_VAL1 PT-INR Value

N	Mean	Standard Deviation	Minimum	Maximum
4	1.4725000	0.1138347	1.3500000	1.6200000

Unique Subject Identifier=7423000000-0020

Assay variable : PTINR_VAL1 PT-INR Value

N	Mean	Standard Deviation	Minimum	Maximum
2	1.2050000	0.0636396	1.1600000	1.2500000

Unique Subject Identifier=7423000000-0025

Assay variable : PTINR_VAL1 PT-INR Value

N	Mean	Standard Deviation	Minimum	Maximum
5	1.5060000	0.0770065	1.4400000	1.6200000

Unique Subject Identifier=7423000000-0026

Assay variable : PTINR_VAL1 PT-INR Value

N	Mean	Standard Deviation	Minimum	Maximum
4	2.5350000	0.8121781	1.5200000	3.4300000

Laboratory tests at the time of obtaining Table 2-6 consent, according to the presence or absence of proton-pump inhibitors

Unique Subject Identifier=7423000000-0028

MEANS Procedure

Assay variable : PTINR_VAL1 PT-INR Value

N	Mean	Standard Deviation	Minimum	Maximum
2	1.8000000	0.4101219	1.5100000	2.0900000

Unique Subject Identifier=7423000000-0029

Assay variable : PTINR_VAL1 PT-INR Value

N	Mean	Standard Deviation	Minimum	Maximum
5	1.6200000	0.1680774	1.4900000	1.8800000

Unique Subject Identifier=7423000000-0030

Assay variable : PTINR_VAL1 PT-INR Value

N	Mean	Standard Deviation	Minimum	Maximum
5	1.8160000	0.3728002	1.3200000	2.1100000

Unique Subject Identifier=7423000000-0038

Assay variable : PTINR_VAL1 PT-INR Value

N	Mean	Standard Deviation	Minimum	Maximum
2	1.7950000	0.0353553	1.7700000	1.8200000

Laboratory tests at the time of obtaining Table 2-6 consent, according to the presence or absence of proton-pump inhibitors

Unique Subject Identifier=7423000000-0039

MEANS Procedure

Assay variable : PTINR_VAL1 PT-INR Value

N	Mean	Standard Deviation	Minimum	Maximum
6	1.7416667	0.5770760	1.1300000	2.4800000

Unique Subject Identifier=7423000000-0040

Assay variable : PTINR_VAL1 PT-INR Value

N	Mean	Standard Deviation	Minimum	Maximum
2	2.4000000	0.0989949	2.3300000	2.4700000

Unique Subject Identifier=7423000000-0043

Assay variable : PTINR_VAL1 PT-INR Value

N	Mean	Standard Deviation	Minimum	Maximum
3	2.8633333	0.7456764	2.3600000	3.7200000

Unique Subject Identifier=7423000000-0044

Assay variable : PTINR_VAL1 PT-INR Value

N	Mean	Standard Deviation	Minimum	Maximum
5	1.6500000	0.2155226	1.4200000	1.9200000

Laboratory tests at the time of obtaining Table 2-6 consent, according to the presence or absence of proton-pump inhibitors

Unique Subject Identifier=7423000000-0052

MEANS Procedure

Assay variable : PTINR_VAL1 PT-INR Value

N	Mean	Standard Deviation	Minimum	Maximum
4	2.3200000	0.2422120	2.0800000	2.6400000

Unique Subject Identifier=7423000000-0053

Assay variable : PTINR_VAL1 PT-INR Value

N	Mean	Standard Deviation	Minimum	Maximum
6	2.0233333	0.2628815	1.7500000	2.4100000

Unique Subject Identifier=7423000000-0058

Assay variable : PTINR_VAL1 PT-INR Value

N	Mean	Standard Deviation	Minimum	Maximum
2	1.8750000	0.3606245	1.6200000	2.1300000

Unique Subject Identifier=7423000000-0060

Assay variable : PTINR_VAL1 PT-INR Value

N	Mean	Standard Deviation	Minimum	Maximum
2	1.7700000	0.4242641	1.4700000	2.0700000

Laboratory tests at the time of obtaining Table 2-6 consent, according to the presence or absence of proton-pump inhibitors

Unique Subject Identifier=7423000000-0061

MEANS Procedure

Assay variable : PTINR_VAL1 PT-INR Value

N	Mean	Standard Deviation	Minimum	Maximum
5	1.7380000	0.1310343	1.5800000	1.8900000

Unique Subject Identifier=7423000000-0062

Assay variable : PTINR_VAL1 PT-INR Value

N	Mean	Standard Deviation	Minimum	Maximum
5	1.7300000	0.1811077	1.4800000	1.9400000

Unique Subject Identifier=7423000000-0063

Assay variable : PTINR_VAL1 PT-INR Value

N	Mean	Standard Deviation	Minimum	Maximum
6	1.6400000	0.2395830	1.3500000	2.0000000

Unique Subject Identifier=7423000000-0064

Assay variable : PTINR_VAL1 PT-INR Value

N	Mean	Standard Deviation	Minimum	Maximum
4	1.8525000	0.1408013	1.6600000	1.9800000

Laboratory tests at the time of obtaining Table 2-6 consent, according to the presence or absence of proton-pump inhibitors

Unique Subject Identifier=7423000000-0065

MEANS Procedure

Assay variable : PTINR_VAL1 PT-INR Value

N	Mean	Standard Deviation	Minimum	Maximum
2	1.9850000	0.2474874	1.8100000	2.1600000

Unique Subject Identifier=7423000000-0066

Assay variable : PTINR_VAL1 PT-INR Value

N	Mean	Standard Deviation	Minimum	Maximum
2	2.2600000	0.0707107	2.2100000	2.3100000

Unique Subject Identifier=7423000000-0070

Assay variable : PTINR_VAL1 PT-INR Value

N	Mean	Standard Deviation	Minimum	Maximum
3	1.7100000	0.0556776	1.6500000	1.7600000

Unique Subject Identifier=7423000000-0071

Assay variable : PTINR_VAL1 PT-INR Value

N	Mean	Standard Deviation	Minimum	Maximum
1	1.5600000		1.5600000	1.5600000

Laboratory tests at the time of obtaining Table 2-6 consent, according to the presence or absence of proton-pump inhibitors

Unique Subject Identifier=7423000000-0072

MEANS Procedure

Assay variable : PTINR_VAL1 PT-INR Value

N	Mean	Standard Deviation	Minimum	Maximum
4	1.6525000	0.2732978	1.3500000	1.9900000

Unique Subject Identifier=7423000000-0074

Assay variable : PTINR_VAL1 PT-INR Value

N	Mean	Standard Deviation	Minimum	Maximum
2	1.9250000	0.0070711	1.9200000	1.9300000

Unique Subject Identifier=7423000000-0075

Assay variable : PTINR_VAL1 PT-INR Value

N	Mean	Standard Deviation	Minimum	Maximum
2	1.5850000	0.0494975	1.5500000	1.6200000

Unique Subject Identifier=7423000000-0076

Assay variable : PTINR_VAL1 PT-INR Value

N	Mean	Standard Deviation	Minimum	Maximum
3	2.2800000	0.3604164	1.9100000	2.6300000

Laboratory tests at the time of obtaining Table 2-6 consent, according to the presence or absence of proton-pump inhibitors

Unique Subject Identifier=7423000000-0080

MEANS Procedure

Assay variable : PTINR_VAL1 PT-INR Value

N	Mean	Standard Deviation	Minimum	Maximum
3	1.7333333	0.0378594	1.6900000	1.7600000

Unique Subject Identifier=7423000000-0081

Assay variable : PTINR_VAL1 PT-INR Value

N	Mean	Standard Deviation	Minimum	Maximum
1	1.5700000		1.5700000	1.5700000

Unique Subject Identifier=7423000000-0086

Assay variable : PTINR_VAL1 PT-INR Value

N	Mean	Standard Deviation	Minimum	Maximum
3	2.2666667	0.0404145	2.2200000	2.2900000

Unique Subject Identifier=7423000000-0087

Assay variable : PTINR_VAL1 PT-INR Value

N	Mean	Standard Deviation	Minimum	Maximum
2	1.9600000	0.0989949	1.8900000	2.0300000

Laboratory tests at the time of obtaining Table 2-6 consent, according to the presence or absence of proton-pump inhibitors

Unique Subject Identifier=7423000000-0088

MEANS Procedure

Assay variable : PTINR_VAL1 PT-INR Value

N	Mean	Standard Deviation	Minimum	Maximum
3	1.8633333	0.3108590	1.6500000	2.2200000

Unique Subject Identifier=7423000000-0090

Assay variable : PTINR_VAL1 PT-INR Value

N	Mean	Standard Deviation	Minimum	Maximum
5	1.6720000	0.6956795	1.0500000	2.6600000

Unique Subject Identifier=7423000000-0095

Assay variable : PTINR_VAL1 PT-INR Value

N	Mean	Standard Deviation	Minimum	Maximum
3	1.7266667	0.0680686	1.6500000	1.7800000

Unique Subject Identifier=7423000000-0097

Assay variable : PTINR_VAL1 PT-INR Value

N	Mean	Standard Deviation	Minimum	Maximum
4	1.7925000	0.1867931	1.6200000	2.0000000

Laboratory tests at the time of obtaining Table 2-6 consent, according to the presence or absence of proton-pump inhibitors

Unique Subject Identifier=7423000000-0099

MEANS Procedure

Assay variable : PTINR_VAL1 PT-INR Value

N	Mean	Standard Deviation	Minimum	Maximum
3	1.6133333	0.0152753	1.6000000	1.6300000

Unique Subject Identifier=7542000000-0003

Assay variable : PTINR_VAL1 PT-INR Value

N	Mean	Standard Deviation	Minimum	Maximum
6	2.0333333	0.6377042	1.6000000	3.3000000

Unique Subject Identifier=7542000000-0005

Assay variable : PTINR_VAL1 PT-INR Value

N	Mean	Standard Deviation	Minimum	Maximum
6	2.5833333	1.2416387	1.5000000	4.5000000

Unique Subject Identifier=7542000000-0006

Assay variable : PTINR_VAL1 PT-INR Value

N	Mean	Standard Deviation	Minimum	Maximum
5	1.4200000	0.2167948	1.3000000	1.8000000

Laboratory tests at the time of obtaining Table 2-6 consent, according to the presence or absence of proton-pump inhibitors

Unique Subject Identifier=7542000000-0007

MEANS Procedure

Assay variable : PTINR_VAL1 PT-INR Value

N	Mean	Standard Deviation	Minimum	Maximum
6	2.5066667	1.1654641	1.4600000	4.3000000

Unique Subject Identifier=7542000000-0008

Assay variable : PTINR_VAL1 PT-INR Value

N	Mean	Standard Deviation	Minimum	Maximum
6	2.2666667	0.5391351	1.4000000	2.9000000

Unique Subject Identifier=7542000000-0009

Assay variable : PTINR_VAL1 PT-INR Value

N	Mean	Standard Deviation	Minimum	Maximum
6	2.2166667	0.1602082	1.9000000	2.3000000

Unique Subject Identifier=7542000000-0015

Assay variable : PTINR_VAL1 PT-INR Value

N	Mean	Standard Deviation	Minimum	Maximum
6	2.1900000	0.2202726	1.9900000	2.5800000

Laboratory tests at the time of obtaining Table 2-6 consent, according to the presence or absence of proton-pump inhibitors

Unique Subject Identifier=7542000000-0017

MEANS Procedure

Assay variable : PTINR_VAL1 PT-INR Value

N	Mean	Standard Deviation	Minimum	Maximum
6	2.3833333	0.9368387	1.5000000	3.9000000

Unique Subject Identifier=7542000000-0021

Assay variable : PTINR_VAL1 PT-INR Value

N	Mean	Standard Deviation	Minimum	Maximum
6	1.9666667	0.2804758	1.7000000	2.5000000

Unique Subject Identifier=7542000000-0023

Assay variable : PTINR_VAL1 PT-INR Value

N	Mean	Standard Deviation	Minimum	Maximum
6	2.1333333	0.1632993	1.9000000	2.3000000

Unique Subject Identifier=7542000000-0025

Assay variable : PTINR_VAL1 PT-INR Value

N	Mean	Standard Deviation	Minimum	Maximum
5	2.1400000	0.2073644	1.9000000	2.4000000

Laboratory tests at the time of obtaining Table 2-6 consent, according to the presence or absence of proton-pump inhibitors

Unique Subject Identifier=7542000000-0026

MEANS Procedure

Assay variable : PTINR_VAL1 PT-INR Value

N	Mean	Standard Deviation	Minimum	Maximum
6	1.7833333	0.1329160	1.6000000	2.0000000

Unique Subject Identifier=7542000000-0029

Assay variable : PTINR_VAL1 PT-INR Value

N	Mean	Standard Deviation	Minimum	Maximum
6	2.3000000	0.7014271	1.4000000	3.3000000

Unique Subject Identifier=7542000000-0030

Assay variable : PTINR_VAL1 PT-INR Value

N	Mean	Standard Deviation	Minimum	Maximum
2	2.1500000	0.3535534	1.9000000	2.4000000

Unique Subject Identifier=7542000000-0031

Assay variable : PTINR_VAL1 PT-INR Value

N	Mean	Standard Deviation	Minimum	Maximum
3	1.6666667	0.4041452	1.3000000	2.1000000

Laboratory tests at the time of obtaining Table 2-6 consent, according to the presence or absence of proton-pump inhibitors

Unique Subject Identifier=7542000000-0032

MEANS Procedure

Assay variable : PTINR_VAL1 PT-INR Value

N	Mean	Standard Deviation	Minimum	Maximum
6	1.5666667	0.2804758	1.3000000	2.0000000

Unique Subject Identifier=7542000000-0037

Assay variable : PTINR_VAL1 PT-INR Value

N	Mean	Standard Deviation	Minimum	Maximum
5	1.9200000	0.2167948	1.7000000	2.2000000

Unique Subject Identifier=7642010000-0001

Assay variable : PTINR_VAL1 PT-INR Value

N	Mean	Standard Deviation	Minimum	Maximum
3	2.2033333	0.4838733	1.8300000	2.7500000

Unique Subject Identifier=7642010000-0002

Assay variable : PTINR_VAL1 PT-INR Value

N	Mean	Standard Deviation	Minimum	Maximum
3	2.3966667	0.0737111	2.3400000	2.4800000

Laboratory tests at the time of obtaining Table 2-6 consent, according to the presence or absence of proton-pump inhibitors

Unique Subject Identifier=7642010000-0003

MEANS Procedure

Assay variable : PTINR_VAL1 PT-INR Value

N	Mean	Standard Deviation	Minimum	Maximum
4	2.1725000	0.4658594	1.7000000	2.8000000

Unique Subject Identifier=8005000000-0001

Assay variable : PTINR_VAL1 PT-INR Value

N	Mean	Standard Deviation	Minimum	Maximum
6	1.6983333	0.2394508	1.3400000	1.9300000

Unique Subject Identifier=8005000000-0002

Assay variable : PTINR_VAL1 PT-INR Value

N	Mean	Standard Deviation	Minimum	Maximum
6	2.0000000	0.1436663	1.8400000	2.2500000

Unique Subject Identifier=8005000000-0006

Assay variable : PTINR_VAL1 PT-INR Value

N	Mean	Standard Deviation	Minimum	Maximum
5	2.2320000	0.2921815	1.9600000	2.6400000

Laboratory tests at the time of obtaining Table 2-6 consent, according to the presence or absence of proton-pump inhibitors

Unique Subject Identifier=8005000000-0007

MEANS Procedure

Assay variable : PTINR_VAL1 PT-INR Value

N	Mean	Standard Deviation	Minimum	Maximum
4	1.8350000	0.5570458	1.2300000	2.4600000

Unique Subject Identifier=8005000000-0010

Assay variable : PTINR_VAL1 PT-INR Value

N	Mean	Standard Deviation	Minimum	Maximum
5	1.9360000	0.4195593	1.4700000	2.5200000

Unique Subject Identifier=8005000000-0011

Assay variable : PTINR_VAL1 PT-INR Value

N	Mean	Standard Deviation	Minimum	Maximum
5	1.7220000	0.2052316	1.5300000	2.0500000

Unique Subject Identifier=8009000000-0001

Assay variable : PTINR_VAL1 PT-INR Value

N	Mean	Standard Deviation	Minimum	Maximum
6	1.7416667	0.4793085	1.1800000	2.5100000

Laboratory tests at the time of obtaining Table 2-6 consent, according to the presence or absence of proton-pump inhibitors

Unique Subject Identifier=8009000000-0002

MEANS Procedure

Assay variable : PTINR_VAL1 PT-INR Value

N	Mean	Standard Deviation	Minimum	Maximum
5	2.2660000	0.5860717	1.8800000	3.3000000

Unique Subject Identifier=8009000000-0003

Assay variable : PTINR_VAL1 PT-INR Value

N	Mean	Standard Deviation	Minimum	Maximum
4	2.1725000	0.4624842	1.5800000	2.5500000

Unique Subject Identifier=8009000000-0005

Assay variable : PTINR_VAL1 PT-INR Value

N	Mean	Standard Deviation	Minimum	Maximum
4	1.6425000	0.0939415	1.5200000	1.7300000

Unique Subject Identifier=8009000000-0014

Assay variable : PTINR_VAL1 PT-INR Value

N	Mean	Standard Deviation	Minimum	Maximum
4	2.3925000	0.2822971	2.0400000	2.7300000

Laboratory tests at the time of obtaining Table 2-6 consent, according to the presence or absence of proton-pump inhibitors

Unique Subject Identifier=8009000000-0016

MEANS Procedure

Assay variable : PTINR_VAL1 PT-INR Value

N	Mean	Standard Deviation	Minimum	Maximum
4	2.2550000	1.0195914	1.3800000	3.6200000

Unique Subject Identifier=8009000000-0023

Assay variable : PTINR_VAL1 PT-INR Value

N	Mean	Standard Deviation	Minimum	Maximum
5	2.1700000	1.0790042	0.9600000	3.6500000

Unique Subject Identifier=8009000000-0029

Assay variable : PTINR_VAL1 PT-INR Value

N	Mean	Standard Deviation	Minimum	Maximum
5	1.9920000	0.2108791	1.7200000	2.2500000

Unique Subject Identifier=8009000000-0030

Assay variable : PTINR_VAL1 PT-INR Value

N	Mean	Standard Deviation	Minimum	Maximum
4	1.5125000	0.1631717	1.3600000	1.7300000

Laboratory tests at the time of obtaining Table 2-6 consent, according to the presence or absence of proton-pump inhibitors

Unique Subject Identifier=8010000100-0007

MEANS Procedure

Assay variable : PTINR_VAL1 PT-INR Value

N	Mean	Standard Deviation	Minimum	Maximum
3	1.4333333	0.1305118	1.3300000	1.5800000

Unique Subject Identifier=8010000100-0012

Assay variable : PTINR_VAL1 PT-INR Value

N	Mean	Standard Deviation	Minimum	Maximum
6	2.0383333	0.2545912	1.5800000	2.3100000

Unique Subject Identifier=8010000100-0016

Assay variable : PTINR_VAL1 PT-INR Value

N	Mean	Standard Deviation	Minimum	Maximum
5	2.3700000	0.5013482	1.7600000	3.1300000

Unique Subject Identifier=8010000100-0022

Assay variable : PTINR_VAL1 PT-INR Value

N	Mean	Standard Deviation	Minimum	Maximum
6	1.5650000	0.3614831	1.1400000	1.9600000

Laboratory tests at the time of obtaining Table 2-6 consent, according to the presence or absence of proton-pump inhibitors

Unique Subject Identifier=8010000100-0029

MEANS Procedure

Assay variable : PTINR_VAL1 PT-INR Value

N	Mean	Standard Deviation	Minimum	Maximum
5	2.0400000	0.7243273	1.2900000	3.1100000

Unique Subject Identifier=8010000100-0030

Assay variable : PTINR_VAL1 PT-INR Value

N	Mean	Standard Deviation	Minimum	Maximum
3	2.5700000	0.1212436	2.4300000	2.6400000

Unique Subject Identifier=8010000100-0060

Assay variable : PTINR_VAL1 PT-INR Value

N	Mean	Standard Deviation	Minimum	Maximum
3	2.1866667	0.2967041	1.9100000	2.5000000

Unique Subject Identifier=8014000000-0001

Assay variable : PTINR_VAL1 PT-INR Value

N	Mean	Standard Deviation	Minimum	Maximum
2	2.7150000	0.0636396	2.6700000	2.7600000

Laboratory tests at the time of obtaining Table 2-6 consent, according to the presence or absence of proton-pump inhibitors

Unique Subject Identifier=8017000000-0001

MEANS Procedure

Assay variable : PTINR_VAL1 PT-INR Value

N	Mean	Standard Deviation	Minimum	Maximum
3	1.9800000	0.3386739	1.6000000	2.2500000

Unique Subject Identifier=8020011000-0002

Assay variable : PTINR_VAL1 PT-INR Value

N	Mean	Standard Deviation	Minimum	Maximum
1	2.3500000		2.3500000	2.3500000

Unique Subject Identifier=8020011000-0007

Assay variable : PTINR_VAL1 PT-INR Value

N	Mean	Standard Deviation	Minimum	Maximum
1	1.8400000		1.8400000	1.8400000

Unique Subject Identifier=8020011000-0029

Assay variable : PTINR_VAL1 PT-INR Value

N	Mean	Standard Deviation	Minimum	Maximum
1	2.3600000		2.3600000	2.3600000

Laboratory tests at the time of obtaining Table 2-6 consent, according to the presence or absence of proton-pump inhibitors

Unique Subject Identifier=8020011000-0032

MEANS Procedure

Assay variable : PTINR_VAL1 PT-INR Value

N	Mean	Standard Deviation	Minimum	Maximum
1	1.3700000		1.3700000	1.3700000

Unique Subject Identifier=8020011000-0040

Assay variable : PTINR_VAL1 PT-INR Value

N	Mean	Standard Deviation	Minimum	Maximum
2	1.8000000	0.5798276	1.3900000	2.2100000

Unique Subject Identifier=8020011000-0042

Assay variable : PTINR_VAL1 PT-INR Value

N	Mean	Standard Deviation	Minimum	Maximum
1	2.9400000		2.9400000	2.9400000

Unique Subject Identifier=8020011000-0043

Assay variable : PTINR_VAL1 PT-INR Value

N	Mean	Standard Deviation	Minimum	Maximum
2	1.5300000	0.1838478	1.4000000	1.6600000

Laboratory tests at the time of obtaining Table 2-6 consent, according to the presence or absence of proton-pump inhibitors

Unique Subject Identifier=8020011000-0046

MEANS Procedure

Assay variable : PTINR_VAL1 PT-INR Value

N	Mean	Standard Deviation	Minimum	Maximum
1	1.3200000		1.3200000	1.3200000

Unique Subject Identifier=8023000000-0003

Assay variable : PTINR_VAL1 PT-INR Value

N	Mean	Standard Deviation	Minimum	Maximum
2	2.1800000	0.2404163	2.0100000	2.3500000

Unique Subject Identifier=8023000000-0008

Assay variable : PTINR_VAL1 PT-INR Value

N	Mean	Standard Deviation	Minimum	Maximum
1	2.0100000		2.0100000	2.0100000

Unique Subject Identifier=8023000000-0010

Assay variable : PTINR_VAL1 PT-INR Value

N	Mean	Standard Deviation	Minimum	Maximum
4	1.9550000	0.6821779	1.5200000	2.9700000

Laboratory tests at the time of obtaining Table 2-6 consent, according to the presence or absence of proton-pump inhibitors

Unique Subject Identifier=8023000000-0011

MEANS Procedure

Assay variable : PTINR_VAL1 PT-INR Value

N	Mean	Standard Deviation	Minimum	Maximum
6	1.4300000	0.1912067	1.2400000	1.8000000

Unique Subject Identifier=8023000000-0012

Assay variable : PTINR_VAL1 PT-INR Value

N	Mean	Standard Deviation	Minimum	Maximum
3	2.0833333	0.2829016	1.9200000	2.4100000

Unique Subject Identifier=8023000000-0013

Assay variable : PTINR_VAL1 PT-INR Value

N	Mean	Standard Deviation	Minimum	Maximum
3	2.0533333	0.1258306	1.9200000	2.1700000

Unique Subject Identifier=8023000000-0016

Assay variable : PTINR_VAL1 PT-INR Value

N	Mean	Standard Deviation	Minimum	Maximum
3	2.0233333	0.2557994	1.7800000	2.2900000

Laboratory tests at the time of obtaining Table 2-6 consent, according to the presence or absence of proton-pump inhibitors

Unique Subject Identifier=8023000000-0018

MEANS Procedure

Assay variable : PTINR_VAL1 PT-INR Value

N	Mean	Standard Deviation	Minimum	Maximum
3	2.0666667	0.2950141	1.7700000	2.3600000

Unique Subject Identifier=8023000000-0019

Assay variable : PTINR_VAL1 PT-INR Value

N	Mean	Standard Deviation	Minimum	Maximum
3	1.4900000	0.0556776	1.4300000	1.5400000

Unique Subject Identifier=8023000000-0020

Assay variable : PTINR_VAL1 PT-INR Value

N	Mean	Standard Deviation	Minimum	Maximum
3	1.9100000	0.1802776	1.7600000	2.1100000

Unique Subject Identifier=8023000000-0021

Assay variable : PTINR_VAL1 PT-INR Value

N	Mean	Standard Deviation	Minimum	Maximum
3	2.0333333	0.1550269	1.9200000	2.2100000

Laboratory tests at the time of obtaining Table 2-6 consent, according to the presence or absence of proton-pump inhibitors

Unique Subject Identifier=8023000000-0025

MEANS Procedure

Assay variable : PTINR_VAL1 PT-INR Value

N	Mean	Standard Deviation	Minimum	Maximum
3	2.1266667	0.1115049	2.0000000	2.2100000

Unique Subject Identifier=8023000000-0033

Assay variable : PTINR_VAL1 PT-INR Value

N	Mean	Standard Deviation	Minimum	Maximum
3	1.4900000	0.3051229	1.1400000	1.7000000

Unique Subject Identifier=8023000000-0034

Assay variable : PTINR_VAL1 PT-INR Value

N	Mean	Standard Deviation	Minimum	Maximum
3	2.5033333	0.1266228	2.3600000	2.6000000

Unique Subject Identifier=8023000000-0036

Assay variable : PTINR_VAL1 PT-INR Value

N	Mean	Standard Deviation	Minimum	Maximum
3	1.8033333	0.1250333	1.6600000	1.8900000

Laboratory tests at the time of obtaining Table 2-6 consent, according to the presence or absence of proton-pump inhibitors

Unique Subject Identifier=8023000000-0037

MEANS Procedure

Assay variable : PTINR_VAL1 PT-INR Value

N	Mean	Standard Deviation	Minimum	Maximum
4	1.6125000	0.2650000	1.2800000	1.8800000

Unique Subject Identifier=8023000000-0041

Assay variable : PTINR_VAL1 PT-INR Value

N	Mean	Standard Deviation	Minimum	Maximum
2	1.9000000	0.2828427	1.7000000	2.1000000

Unique Subject Identifier=8023000000-0042

Assay variable : PTINR_VAL1 PT-INR Value

N	Mean	Standard Deviation	Minimum	Maximum
2	2.3350000	0.4454773	2.0200000	2.6500000

Unique Subject Identifier=8023000000-0043

Assay variable : PTINR_VAL1 PT-INR Value

N	Mean	Standard Deviation	Minimum	Maximum
1	1.7700000		1.7700000	1.7700000

Laboratory tests at the time of obtaining Table 2-6 consent, according to the presence or absence of proton-pump inhibitors

Unique Subject Identifier=8023000000-0045

MEANS Procedure

Assay variable : PTINR_VAL1 PT-INR Value

N	Mean	Standard Deviation	Minimum	Maximum
3	1.6966667	0.4239497	1.3100000	2.1500000

Unique Subject Identifier=8023000000-0048

Assay variable : PTINR_VAL1 PT-INR Value

N	Mean	Standard Deviation	Minimum	Maximum
1	2.3200000		2.3200000	2.3200000

Unique Subject Identifier=8023000000-0049

Assay variable : PTINR_VAL1 PT-INR Value

N	Mean	Standard Deviation	Minimum	Maximum
2	1.7100000	0	1.7100000	1.7100000

Unique Subject Identifier=8024000000-0003

Assay variable : PTINR_VAL1 PT-INR Value

N	Mean	Standard Deviation	Minimum	Maximum
5	1.6740000	0.0901665	1.5800000	1.7700000

Laboratory tests at the time of obtaining Table 2-6 consent, according to the presence or absence of proton-pump inhibitors

Unique Subject Identifier=8026000000-0002

MEANS Procedure

Assay variable : PTINR_VAL1 PT-INR Value

N	Mean	Standard Deviation	Minimum	Maximum
4	2.1300000	0.5430776	1.4300000	2.7500000

Unique Subject Identifier=8026000000-0003

Assay variable : PTINR_VAL1 PT-INR Value

N	Mean	Standard Deviation	Minimum	Maximum
3	2.2133333	0.2136196	1.9700000	2.3700000

Unique Subject Identifier=8026000000-0004

Assay variable : PTINR_VAL1 PT-INR Value

N	Mean	Standard Deviation	Minimum	Maximum
3	1.9600000	1.0142485	1.3300000	3.1300000

Unique Subject Identifier=8026000000-0007

Assay variable : PTINR_VAL1 PT-INR Value

N	Mean	Standard Deviation	Minimum	Maximum
2	1.8300000	0.4384062	1.5200000	2.1400000

Laboratory tests at the time of obtaining Table 2-6 consent, according to the presence or absence of proton-pump inhibitors

Unique Subject Identifier=8026000000-0008

MEANS Procedure

Assay variable : PTINR_VAL1 PT-INR Value

N	Mean	Standard Deviation	Minimum	Maximum
2	1.1350000	0.0636396	1.0900000	1.1800000

Unique Subject Identifier=8026000000-0012

Assay variable : PTINR_VAL1 PT-INR Value

N	Mean	Standard Deviation	Minimum	Maximum
3	1.9900000	0.4513314	1.5200000	2.4200000

Unique Subject Identifier=8026000000-0018

Assay variable : PTINR_VAL1 PT-INR Value

N	Mean	Standard Deviation	Minimum	Maximum
3	1.6266667	0.0945163	1.5200000	1.7000000

Unique Subject Identifier=8026000000-0026

Assay variable : PTINR_VAL1 PT-INR Value

N	Mean	Standard Deviation	Minimum	Maximum
3	2.3400000	0.8098765	1.6700000	3.2400000

Laboratory tests at the time of obtaining Table 2-6 consent, according to the presence or absence of proton-pump inhibitors

Unique Subject Identifier=8026000000-0027

MEANS Procedure

Assay variable : PTINR_VAL1 PT-INR Value

N	Mean	Standard Deviation	Minimum	Maximum
2	2.2950000	0.3606245	2.0400000	2.5500000

Unique Subject Identifier=8026000000-0028

Assay variable : PTINR_VAL1 PT-INR Value

N	Mean	Standard Deviation	Minimum	Maximum
2	1.8650000	0.2474874	1.6900000	2.0400000

Unique Subject Identifier=8026000000-0029

Assay variable : PTINR_VAL1 PT-INR Value

N	Mean	Standard Deviation	Minimum	Maximum
3	2.2333333	0.1209683	2.1400000	2.3700000

Unique Subject Identifier=8026000000-0030

Assay variable : PTINR_VAL1 PT-INR Value

N	Mean	Standard Deviation	Minimum	Maximum
3	1.9200000	0.2402082	1.7300000	2.1900000

Laboratory tests at the time of obtaining Table 2-6 consent, according to the presence or absence of proton-pump inhibitors

Unique Subject Identifier=8030011000-0001

MEANS Procedure

Assay variable : PTINR_VAL1 PT-INR Value

N	Mean	Standard Deviation	Minimum	Maximum
4	1.8025000	0.3365883	1.4100000	2.2300000

Unique Subject Identifier=8030011000-0002

Assay variable : PTINR_VAL1 PT-INR Value

N	Mean	Standard Deviation	Minimum	Maximum
6	1.9350000	0.2510578	1.7200000	2.4200000

Unique Subject Identifier=8030011000-0007

Assay variable : PTINR_VAL1 PT-INR Value

N	Mean	Standard Deviation	Minimum	Maximum
6	2.5100000	0.6117189	1.9100000	3.6600000

Unique Subject Identifier=8030011000-0008

Assay variable : PTINR_VAL1 PT-INR Value

N	Mean	Standard Deviation	Minimum	Maximum
4	2.3600000	0.4857297	1.7500000	2.8800000

Laboratory tests at the time of obtaining Table 2-6 consent, according to the presence or absence of proton-pump inhibitors

Unique Subject Identifier=8030011000-0018

MEANS Procedure

Assay variable : PTINR_VAL1 PT-INR Value

N	Mean	Standard Deviation	Minimum	Maximum
4	1.5875000	0.2430878	1.2700000	1.8500000

Unique Subject Identifier=8030011000-0021

Assay variable : PTINR_VAL1 PT-INR Value

N	Mean	Standard Deviation	Minimum	Maximum
6	2.3433333	0.1875811	2.1000000	2.5500000

Unique Subject Identifier=8030011000-0026

Assay variable : PTINR_VAL1 PT-INR Value

N	Mean	Standard Deviation	Minimum	Maximum
4	2.3300000	0.2210581	2.1400000	2.6300000

Unique Subject Identifier=8030011000-0027

Assay variable : PTINR_VAL1 PT-INR Value

N	Mean	Standard Deviation	Minimum	Maximum
6	2.1783333	0.2555321	1.8500000	2.5100000

Laboratory tests at the time of obtaining Table 2-6 consent, according to the presence or absence of proton-pump inhibitors

Unique Subject Identifier=8030011000-0029

MEANS Procedure

Assay variable : PTINR_VAL1 PT-INR Value

N	Mean	Standard Deviation	Minimum	Maximum
5	1.9740000	0.1647119	1.7500000	2.1900000

Unique Subject Identifier=8031000000-0005

Assay variable : PTINR_VAL1 PT-INR Value

N	Mean	Standard Deviation	Minimum	Maximum
4	1.9000000	0.1016530	1.7700000	2.0100000

Unique Subject Identifier=8031000000-0009

Assay variable : PTINR_VAL1 PT-INR Value

N	Mean	Standard Deviation	Minimum	Maximum
4	1.9700000	0.8517824	1.2300000	2.9800000

Unique Subject Identifier=8031000000-0014

Assay variable : PTINR_VAL1 PT-INR Value

N	Mean	Standard Deviation	Minimum	Maximum
5	1.8780000	0.1722498	1.6200000	2.0500000

Laboratory tests at the time of obtaining Table 2-6 consent, according to the presence or absence of proton-pump inhibitors

Unique Subject Identifier=8031000000-0023

MEANS Procedure

Assay variable : PTINR_VAL1 PT-INR Value

N	Mean	Standard Deviation	Minimum	Maximum
5	1.9620000	0.4747315	1.3400000	2.4400000

Unique Subject Identifier=8031000000-0028

Assay variable : PTINR_VAL1 PT-INR Value

N	Mean	Standard Deviation	Minimum	Maximum
2	1.9250000	0.0636396	1.8800000	1.9700000

Unique Subject Identifier=8031000000-0040

Assay variable : PTINR_VAL1 PT-INR Value

N	Mean	Standard Deviation	Minimum	Maximum
6	2.5016667	1.3092962	1.1200000	4.3600000

Unique Subject Identifier=8031000000-0046

Assay variable : PTINR_VAL1 PT-INR Value

N	Mean	Standard Deviation	Minimum	Maximum
4	2.0850000	0.4438093	1.7500000	2.7000000

Laboratory tests at the time of obtaining Table 2-6 consent, according to the presence or absence of proton-pump inhibitors

Unique Subject Identifier=8031000000-0048

MEANS Procedure

Assay variable : PTINR_VAL1 PT-INR Value

N	Mean	Standard Deviation	Minimum	Maximum
6	1.6966667	0.1569289	1.4000000	1.8600000

Unique Subject Identifier=8031000000-0049

Assay variable : PTINR_VAL1 PT-INR Value

N	Mean	Standard Deviation	Minimum	Maximum
5	2.3820000	0.7620171	1.7900000	3.4100000

Unique Subject Identifier=8031000000-0050

Assay variable : PTINR_VAL1 PT-INR Value

N	Mean	Standard Deviation	Minimum	Maximum
6	1.9216667	0.2844234	1.6600000	2.4000000

Unique Subject Identifier=8033000000-0003

Assay variable : PTINR_VAL1 PT-INR Value

N	Mean	Standard Deviation	Minimum	Maximum
6	1.6350000	1.0143323	0.9200000	3.6300000

Laboratory tests at the time of obtaining Table 2-6 consent, according to the presence or absence of proton-pump inhibitors

Unique Subject Identifier=8035000000-0005

MEANS Procedure

Assay variable : PTINR_VAL1 PT-INR Value

N	Mean	Standard Deviation	Minimum	Maximum
2	1.9000000	0.1414214	1.8000000	2.0000000

Unique Subject Identifier=8035000000-0010

Assay variable : PTINR_VAL1 PT-INR Value

N	Mean	Standard Deviation	Minimum	Maximum
3	2.1333333	0.2386071	1.9400000	2.4000000

Unique Subject Identifier=8035000000-0014

Assay variable : PTINR_VAL1 PT-INR Value

N	Mean	Standard Deviation	Minimum	Maximum
3	1.8766667	0.1436431	1.7700000	2.0400000

Unique Subject Identifier=8035000000-0015

Assay variable : PTINR_VAL1 PT-INR Value

N	Mean	Standard Deviation	Minimum	Maximum
3	2.0900000	0.2475884	1.9000000	2.3700000

Laboratory tests at the time of obtaining Table 2-6 consent, according to the presence or absence of proton-pump inhibitors

Unique Subject Identifier=8035000000-0016

MEANS Procedure

Assay variable : PTINR_VAL1 PT-INR Value

N	Mean	Standard Deviation	Minimum	Maximum
1	2.5900000		2.5900000	2.5900000

Unique Subject Identifier=8036000000-0001

Assay variable : PTINR_VAL1 PT-INR Value

N	Mean	Standard Deviation	Minimum	Maximum
4	2.1950000	0.8135724	1.4600000	3.3100000

Unique Subject Identifier=8036000000-0007

Assay variable : PTINR_VAL1 PT-INR Value

N	Mean	Standard Deviation	Minimum	Maximum
6	1.7033333	0.1907529	1.4600000	1.9500000

Unique Subject Identifier=8040011000-0007

Assay variable : PTINR_VAL1 PT-INR Value

N	Mean	Standard Deviation	Minimum	Maximum
6	2.1116667	0.2406173	1.8700000	2.4400000

Laboratory tests at the time of obtaining Table 2-6 consent, according to the presence or absence of proton-pump inhibitors

Unique Subject Identifier=8047000000-0015

MEANS Procedure

Assay variable : PTINR_VAL1 PT-INR Value

N	Mean	Standard Deviation	Minimum	Maximum
3	1.8966667	0.7503555	1.3400000	2.7500000

Unique Subject Identifier=8047000000-0016

Assay variable : PTINR_VAL1 PT-INR Value

N	Mean	Standard Deviation	Minimum	Maximum
5	1.3080000	0.1616168	1.1300000	1.5300000

Unique Subject Identifier=8047000000-0025

Assay variable : PTINR_VAL1 PT-INR Value

N	Mean	Standard Deviation	Minimum	Maximum
3	1.9100000	0.1113553	1.7900000	2.0100000

Unique Subject Identifier=8047000000-0028

Assay variable : PTINR_VAL1 PT-INR Value

N	Mean	Standard Deviation	Minimum	Maximum
3	1.5933333	0.0472582	1.5400000	1.6300000

Laboratory tests at the time of obtaining Table 2-6 consent, according to the presence or absence of proton-pump inhibitors

Unique Subject Identifier=8047000000-0029

MEANS Procedure

Assay variable : PTINR_VAL1 PT-INR Value

N	Mean	Standard Deviation	Minimum	Maximum
3	1.6866667	0.2311565	1.4700000	1.9300000

Unique Subject Identifier=8048010011-0002

Assay variable : PTINR_VAL1 PT-INR Value

N	Mean	Standard Deviation	Minimum	Maximum
2	1.3900000	0.0565685	1.3500000	1.4300000

Unique Subject Identifier=8048010011-0007

Assay variable : PTINR_VAL1 PT-INR Value

N	Mean	Standard Deviation	Minimum	Maximum
3	1.9533333	0.4188476	1.6100000	2.4200000

Unique Subject Identifier=8048010011-0028

Assay variable : PTINR_VAL1 PT-INR Value

N	Mean	Standard Deviation	Minimum	Maximum
6	2.0916667	0.1070358	1.9700000	2.2800000

Laboratory tests at the time of obtaining Table 2-6 consent, according to the presence or absence of proton-pump inhibitors

Unique Subject Identifier=8048010011-0034

MEANS Procedure

Assay variable : PTINR_VAL1 PT-INR Value

N	Mean	Standard Deviation	Minimum	Maximum
2	1.6400000	0.6363961	1.1900000	2.0900000

Unique Subject Identifier=8054010000-0001

Assay variable : PTINR_VAL1 PT-INR Value

N	Mean	Standard Deviation	Minimum	Maximum
4	2.5475000	0.5144819	1.9100000	3.1200000

Unique Subject Identifier=8055000000-0004

Assay variable : PTINR_VAL1 PT-INR Value

N	Mean	Standard Deviation	Minimum	Maximum
4	1.8375000	0.0997914	1.7300000	1.9600000

Unique Subject Identifier=8055000000-0006

Assay variable : PTINR_VAL1 PT-INR Value

N	Mean	Standard Deviation	Minimum	Maximum
4	2.1900000	0.6639779	1.7200000	3.1700000

Laboratory tests at the time of obtaining Table 2-6 consent, according to the presence or absence of proton-pump inhibitors

Unique Subject Identifier=8057011000-0001

MEANS Procedure

Assay variable : PTINR_VAL1 PT-INR Value

N	Mean	Standard Deviation	Minimum	Maximum
1	1.3800000		1.3800000	1.3800000

Unique Subject Identifier=8057011000-0012

Assay variable : PTINR_VAL1 PT-INR Value

N	Mean	Standard Deviation	Minimum	Maximum
6	1.7183333	0.3896879	1.3100000	2.2200000

Unique Subject Identifier=8057011000-0014

Assay variable : PTINR_VAL1 PT-INR Value

N	Mean	Standard Deviation	Minimum	Maximum
3	2.2566667	0.3689625	1.8500000	2.5700000

Unique Subject Identifier=8057011000-0015

Assay variable : PTINR_VAL1 PT-INR Value

N	Mean	Standard Deviation	Minimum	Maximum
6	2.1483333	0.6102923	1.4900000	3.1000000

Laboratory tests at the time of obtaining Table 2-6 consent, according to the presence or absence of proton-pump inhibitors

Unique Subject Identifier=8060011000-0001

MEANS Procedure

Assay variable : PTINR_VAL1 PT-INR Value

N	Mean	Standard Deviation	Minimum	Maximum
6	2.3666667	0.4273952	1.9000000	3.1000000

Unique Subject Identifier=8060011000-0002

Assay variable : PTINR_VAL1 PT-INR Value

N	Mean	Standard Deviation	Minimum	Maximum
3	2.7333333	1.0066446	1.8000000	3.8000000

Unique Subject Identifier=8060011000-0003

Assay variable : PTINR_VAL1 PT-INR Value

N	Mean	Standard Deviation	Minimum	Maximum
3	2.6000000	0.4582576	2.1000000	3.0000000

Unique Subject Identifier=8060011000-0004

Assay variable : PTINR_VAL1 PT-INR Value

N	Mean	Standard Deviation	Minimum	Maximum
1	1.8000000		1.8000000	1.8000000

Laboratory tests at the time of obtaining Table 2-6 consent, according to the presence or absence of proton-pump inhibitors

Unique Subject Identifier=8060011000-0008

MEANS Procedure

Assay variable : PTINR_VAL1 PT-INR Value

N	Mean	Standard Deviation	Minimum	Maximum
6	2.1166667	0.7305249	1.4000000	3.0000000

Unique Subject Identifier=8060011000-0009

Assay variable : PTINR_VAL1 PT-INR Value

N	Mean	Standard Deviation	Minimum	Maximum
6	2.4000000	0.0894427	2.3000000	2.5000000

Unique Subject Identifier=8060011000-0011

Assay variable : PTINR_VAL1 PT-INR Value

N	Mean	Standard Deviation	Minimum	Maximum
2	1.9500000	0.4949747	1.6000000	2.3000000

Unique Subject Identifier=8060011000-0012

Assay variable : PTINR_VAL1 PT-INR Value

N	Mean	Standard Deviation	Minimum	Maximum
5	1.9200000	0.4438468	1.5000000	2.4000000

Laboratory tests at the time of obtaining Table 2-6 consent, according to the presence or absence of proton-pump inhibitors

Unique Subject Identifier=8060011000-0013

MEANS Procedure

Assay variable : PTINR_VAL1 PT-INR Value

N	Mean	Standard Deviation	Minimum	Maximum
6	1.9333333	0.3932768	1.5000000	2.5000000

Unique Subject Identifier=8060011000-0017

Assay variable : PTINR_VAL1 PT-INR Value

N	Mean	Standard Deviation	Minimum	Maximum
3	2.2333333	0.1527525	2.1000000	2.4000000

Unique Subject Identifier=8060011000-0020

Assay variable : PTINR_VAL1 PT-INR Value

N	Mean	Standard Deviation	Minimum	Maximum
6	2.3166667	0.3125167	1.9000000	2.8000000

Unique Subject Identifier=8060011000-0021

Assay variable : PTINR_VAL1 PT-INR Value

N	Mean	Standard Deviation	Minimum	Maximum
6	1.9666667	0.1632993	1.7000000	2.2000000

Laboratory tests at the time of obtaining Table 2-6 consent, according to the presence or absence of proton-pump inhibitors

Unique Subject Identifier=8060011000-0022

MEANS Procedure

Assay variable : PTINR_VAL1 PT-INR Value

N	Mean	Standard Deviation	Minimum	Maximum
3	2.0666667	0.1527525	1.9000000	2.2000000

Unique Subject Identifier=8060011000-0023

Assay variable : PTINR_VAL1 PT-INR Value

N	Mean	Standard Deviation	Minimum	Maximum
6	1.9833333	0.3311596	1.7000000	2.6000000

Unique Subject Identifier=8060011000-0026

Assay variable : PTINR_VAL1 PT-INR Value

N	Mean	Standard Deviation	Minimum	Maximum
4	2.3000000	0.2449490	2.0000000	2.6000000

Unique Subject Identifier=8060011000-0029

Assay variable : PTINR_VAL1 PT-INR Value

N	Mean	Standard Deviation	Minimum	Maximum
2	2.4500000	0.0707107	2.4000000	2.5000000

Laboratory tests at the time of obtaining Table 2-6 consent, according to the presence or absence of proton-pump inhibitors

Unique Subject Identifier=8060011000-0030

MEANS Procedure

Assay variable : PTINR_VAL1 PT-INR Value

N	Mean	Standard Deviation	Minimum	Maximum
3	1.9000000	0.2645751	1.6000000	2.1000000

Unique Subject Identifier=8061010010-0002

Assay variable : PTINR_VAL1 PT-INR Value

N	Mean	Standard Deviation	Minimum	Maximum
4	1.7425000	0.4847938	1.0900000	2.2500000

Unique Subject Identifier=8067000000-0001

Assay variable : PTINR_VAL1 PT-INR Value

N	Mean	Standard Deviation	Minimum	Maximum
6	2.0366667	0.2960856	1.7900000	2.4900000

Unique Subject Identifier=8100000000-0002

Assay variable : PTINR_VAL1 PT-INR Value

N	Mean	Standard Deviation	Minimum	Maximum
5	2.1960000	0.2891021	1.8900000	2.5300000

Laboratory tests at the time of obtaining Table 2-6 consent, according to the presence or absence of proton-pump inhibitors

Unique Subject Identifier=8100000000-0005

MEANS Procedure

Assay variable : PTINR_VAL1 PT-INR Value

N	Mean	Standard Deviation	Minimum	Maximum
4	1.8875000	0.2543456	1.5400000	2.1500000

Unique Subject Identifier=8104000000-0001

Assay variable : PTINR_VAL1 PT-INR Value

N	Mean	Standard Deviation	Minimum	Maximum
2	1.8600000	0.0141421	1.8500000	1.8700000

Unique Subject Identifier=8104000000-0002

Assay variable : PTINR_VAL1 PT-INR Value

N	Mean	Standard Deviation	Minimum	Maximum
2	2.8700000	0.6081118	2.4400000	3.3000000

Unique Subject Identifier=8104000000-0003

Assay variable : PTINR_VAL1 PT-INR Value

N	Mean	Standard Deviation	Minimum	Maximum
2	1.3500000	0.0707107	1.3000000	1.4000000

Laboratory tests at the time of obtaining Table 2-6 consent, according to the presence or absence of proton-pump inhibitors

Unique Subject Identifier=8104000000-0005

MEANS Procedure

Assay variable : PTINR_VAL1 PT-INR Value

N	Mean	Standard Deviation	Minimum	Maximum
4	2.3900000	0.1764464	2.1600000	2.5900000

Unique Subject Identifier=8104000000-0007

Assay variable : PTINR_VAL1 PT-INR Value

N	Mean	Standard Deviation	Minimum	Maximum
4	1.5350000	0.1473092	1.3900000	1.6900000

Unique Subject Identifier=8104000000-0008

Assay variable : PTINR_VAL1 PT-INR Value

N	Mean	Standard Deviation	Minimum	Maximum
4	1.8350000	0.2037155	1.6000000	2.0200000

Unique Subject Identifier=8104000000-0018

Assay variable : PTINR_VAL1 PT-INR Value

N	Mean	Standard Deviation	Minimum	Maximum
6	2.0150000	0.2504196	1.6900000	2.4400000

Laboratory tests at the time of obtaining Table 2-6 consent, according to the presence or absence of proton-pump inhibitors

Unique Subject Identifier=8104000000-0021

MEANS Procedure

Assay variable : PTINR_VAL1 PT-INR Value

N	Mean	Standard Deviation	Minimum	Maximum
3	1.6700000	0.4063250	1.3300000	2.1200000

Unique Subject Identifier=8104000000-0027

Assay variable : PTINR_VAL1 PT-INR Value

N	Mean	Standard Deviation	Minimum	Maximum
3	1.9133333	0.5225259	1.3100000	2.2200000

Unique Subject Identifier=8108111100-0026

Assay variable : PTINR_VAL1 PT-INR Value

N	Mean	Standard Deviation	Minimum	Maximum
1	2.2600000		2.2600000	2.2600000

Unique Subject Identifier=8110000100-0001

Assay variable : PTINR_VAL1 PT-INR Value

N	Mean	Standard Deviation	Minimum	Maximum
3	1.6433333	0.1755942	1.4600000	1.8100000

Laboratory tests at the time of obtaining Table 2-6 consent, according to the presence or absence of proton-pump inhibitors

Unique Subject Identifier=8110000100-0002

MEANS Procedure

Assay variable : PTINR_VAL1 PT-INR Value

N	Mean	Standard Deviation	Minimum	Maximum
3	2.1833333	0.1167619	2.0800000	2.3100000

Unique Subject Identifier=8110000100-0003

Assay variable : PTINR_VAL1 PT-INR Value

N	Mean	Standard Deviation	Minimum	Maximum
5	2.2760000	0.3549366	1.9500000	2.7100000

Unique Subject Identifier=8110000100-0004

Assay variable : PTINR_VAL1 PT-INR Value

N	Mean	Standard Deviation	Minimum	Maximum
3	2.2466667	0.4954123	1.8200000	2.7900000

Unique Subject Identifier=8110000100-0006

Assay variable : PTINR_VAL1 PT-INR Value

N	Mean	Standard Deviation	Minimum	Maximum
5	2.0980000	0.1888650	1.8400000	2.2900000

Laboratory tests at the time of obtaining Table 2-6 consent, according to the presence or absence of proton-pump inhibitors

Unique Subject Identifier=8114000000-0002

MEANS Procedure

Assay variable : PTINR_VAL1 PT-INR Value

N	Mean	Standard Deviation	Minimum	Maximum
4	1.7950000	0.3923859	1.5600000	2.3800000

Unique Subject Identifier=8114000000-0006

Assay variable : PTINR_VAL1 PT-INR Value

N	Mean	Standard Deviation	Minimum	Maximum
3	1.4600000	0.1374773	1.3400000	1.6100000

Unique Subject Identifier=8114000000-0008

Assay variable : PTINR_VAL1 PT-INR Value

N	Mean	Standard Deviation	Minimum	Maximum
6	1.7950000	0.2055967	1.4900000	2.0900000

Unique Subject Identifier=8114000000-0009

Assay variable : PTINR_VAL1 PT-INR Value

N	Mean	Standard Deviation	Minimum	Maximum
4	1.4150000	0.3961902	1.0400000	1.9500000

Laboratory tests at the time of obtaining Table 2-6 consent, according to the presence or absence of proton-pump inhibitors

Unique Subject Identifier=8117011010-0002

MEANS Procedure

Assay variable : PTINR_VAL1 PT-INR Value

N	Mean	Standard Deviation	Minimum	Maximum
3	1.6833333	0.4539089	1.1600000	1.9700000

Unique Subject Identifier=8117011010-0004

Assay variable : PTINR_VAL1 PT-INR Value

N	Mean	Standard Deviation	Minimum	Maximum
1	2.0900000		2.0900000	2.0900000

Unique Subject Identifier=8117011010-0005

Assay variable : PTINR_VAL1 PT-INR Value

N	Mean	Standard Deviation	Minimum	Maximum
2	1.7700000	0.2121320	1.6200000	1.9200000

Unique Subject Identifier=8117011010-0009

Assay variable : PTINR_VAL1 PT-INR Value

N	Mean	Standard Deviation	Minimum	Maximum
5	1.9260000	0.1119375	1.7700000	2.0400000

Laboratory tests at the time of obtaining Table 2-6 consent, according to the presence or absence of proton-pump inhibitors

Unique Subject Identifier=8117011010-0010

MEANS Procedure

Assay variable : PTINR_VAL1 PT-INR Value

N	Mean	Standard Deviation	Minimum	Maximum
6	1.7433333	0.2410532	1.4200000	2.1300000

Unique Subject Identifier=8117011010-0016

Assay variable : PTINR_VAL1 PT-INR Value

N	Mean	Standard Deviation	Minimum	Maximum
3	1.7100000	0.1708801	1.5500000	1.8900000

Unique Subject Identifier=8117011010-0024

Assay variable : PTINR_VAL1 PT-INR Value

N	Mean	Standard Deviation	Minimum	Maximum
2	2.3050000	0.0777817	2.2500000	2.3600000

Unique Subject Identifier=8117011010-0025

Assay variable : PTINR_VAL1 PT-INR Value

N	Mean	Standard Deviation	Minimum	Maximum
5	2.0960000	0.2261194	1.8200000	2.3200000

Laboratory tests at the time of obtaining Table 2-6 consent, according to the presence or absence of proton-pump inhibitors

Unique Subject Identifier=8117011010-0028

MEANS Procedure

Assay variable : PTINR_VAL1 PT-INR Value

N	Mean	Standard Deviation	Minimum	Maximum
6	1.9283333	0.3702117	1.3900000	2.3500000

Unique Subject Identifier=8123000000-0002

Assay variable : PTINR_VAL1 PT-INR Value

N	Mean	Standard Deviation	Minimum	Maximum
6	1.9000000	0.2909983	1.5900000	2.3800000

Unique Subject Identifier=8123000000-0004

Assay variable : PTINR_VAL1 PT-INR Value

N	Mean	Standard Deviation	Minimum	Maximum
6	1.8983333	0.2920559	1.4500000	2.2300000

Unique Subject Identifier=8123000000-0005

Assay variable : PTINR_VAL1 PT-INR Value

N	Mean	Standard Deviation	Minimum	Maximum
3	2.0866667	0.2542309	1.8100000	2.3100000

Laboratory tests at the time of obtaining Table 2-6 consent, according to the presence or absence of proton-pump inhibitors

Unique Subject Identifier=8123000000-0010

MEANS Procedure

Assay variable : PTINR_VAL1 PT-INR Value

N	Mean	Standard Deviation	Minimum	Maximum
5	1.9240000	0.3630840	1.4900000	2.4600000

Unique Subject Identifier=8123000000-0011

Assay variable : PTINR_VAL1 PT-INR Value

N	Mean	Standard Deviation	Minimum	Maximum
4	1.8825000	0.2151550	1.6800000	2.1500000

Unique Subject Identifier=8123000000-0012

Assay variable : PTINR_VAL1 PT-INR Value

N	Mean	Standard Deviation	Minimum	Maximum
4	1.9625000	0.1668083	1.7300000	2.1100000

Unique Subject Identifier=8123000000-0013

Assay variable : PTINR_VAL1 PT-INR Value

N	Mean	Standard Deviation	Minimum	Maximum
5	2.1160000	0.1350185	1.9500000	2.3000000

Laboratory tests at the time of obtaining Table 2-6 consent, according to the presence or absence of proton-pump inhibitors

Unique Subject Identifier=8123000000-0018

MEANS Procedure

Assay variable : PTINR_VAL1 PT-INR Value

N	Mean	Standard Deviation	Minimum	Maximum
6	1.8600000	0.3850714	1.5200000	2.5700000

Unique Subject Identifier=8124000000-0001

Assay variable : PTINR_VAL1 PT-INR Value

N	Mean	Standard Deviation	Minimum	Maximum
1	3.6500000		3.6500000	3.6500000

Unique Subject Identifier=8133000000-0001

Assay variable : PTINR_VAL1 PT-INR Value

N	Mean	Standard Deviation	Minimum	Maximum
1	2.3400000		2.3400000	2.3400000

Unique Subject Identifier=8135000000-0002

Assay variable : PTINR_VAL1 PT-INR Value

N	Mean	Standard Deviation	Minimum	Maximum
1	1.8500000		1.8500000	1.8500000

Laboratory tests at the time of obtaining Table 2-6 consent, according to the presence or absence of proton-pump inhibitors

Unique Subject Identifier=8135000000-0004

MEANS Procedure

Assay variable : PTINR_VAL1 PT-INR Value

N	Mean	Standard Deviation	Minimum	Maximum
2	1.4900000	0.1555635	1.3800000	1.6000000

Unique Subject Identifier=8135000000-0006

Assay variable : PTINR_VAL1 PT-INR Value

N	Mean	Standard Deviation	Minimum	Maximum
2	1.5700000	0.0282843	1.5500000	1.5900000

Unique Subject Identifier=8135000000-0007

Assay variable : PTINR_VAL1 PT-INR Value

N	Mean	Standard Deviation	Minimum	Maximum
3	2.0833333	0.2635021	1.7900000	2.3000000

Unique Subject Identifier=8135000000-0009

Assay variable : PTINR_VAL1 PT-INR Value

N	Mean	Standard Deviation	Minimum	Maximum
3	2.1200000	0.3815757	1.6800000	2.3600000

Laboratory tests at the time of obtaining Table 2-6 consent, according to the presence or absence of proton-pump inhibitors

Unique Subject Identifier=8135000000-0013

MEANS Procedure

Assay variable : PTINR_VAL1 PT-INR Value

N	Mean	Standard Deviation	Minimum	Maximum
3	2.1000000	0.5543465	1.7700000	2.7400000

Unique Subject Identifier=8135000000-0014

Assay variable : PTINR_VAL1 PT-INR Value

N	Mean	Standard Deviation	Minimum	Maximum
3	1.7500000	0.5369358	1.4400000	2.3700000

Unique Subject Identifier=8136000000-0002

Assay variable : PTINR_VAL1 PT-INR Value

N	Mean	Standard Deviation	Minimum	Maximum
6	1.8233333	0.2530349	1.4000000	2.1300000

Unique Subject Identifier=8136000000-0010

Assay variable : PTINR_VAL1 PT-INR Value

N	Mean	Standard Deviation	Minimum	Maximum
4	1.8400000	0.0294392	1.8100000	1.8800000

Laboratory tests at the time of obtaining Table 2-6 consent, according to the presence or absence of proton-pump inhibitors

Unique Subject Identifier=8136000000-0015

MEANS Procedure

Assay variable : PTINR_VAL1 PT-INR Value

N	Mean	Standard Deviation	Minimum	Maximum
4	2.1425000	0.8701485	1.5400000	3.4300000

Unique Subject Identifier=8136000000-0022

Assay variable : PTINR_VAL1 PT-INR Value

N	Mean	Standard Deviation	Minimum	Maximum
4	2.0050000	0.6165225	1.5300000	2.9100000

Unique Subject Identifier=8137010000-0002

Assay variable : PTINR_VAL1 PT-INR Value

N	Mean	Standard Deviation	Minimum	Maximum
3	2.3833333	0.7011657	1.8600000	3.1800000

Unique Subject Identifier=8137010000-0004

Assay variable : PTINR_VAL1 PT-INR Value

N	Mean	Standard Deviation	Minimum	Maximum
3	1.8800000	0.1705872	1.6900000	2.0200000

Laboratory tests at the time of obtaining Table 2-6 consent, according to the presence or absence of proton-pump inhibitors

Unique Subject Identifier=8137010000-0007

MEANS Procedure

Assay variable : PTINR_VAL1 PT-INR Value

N	Mean	Standard Deviation	Minimum	Maximum
5	1.9260000	0.2608256	1.5500000	2.2100000

Unique Subject Identifier=8137010000-0013

Assay variable : PTINR_VAL1 PT-INR Value

N	Mean	Standard Deviation	Minimum	Maximum
2	1.8400000	0.4242641	1.5400000	2.1400000

Unique Subject Identifier=8137010000-0015

Assay variable : PTINR_VAL1 PT-INR Value

N	Mean	Standard Deviation	Minimum	Maximum
2	1.9550000	0.2757716	1.7600000	2.1500000

Unique Subject Identifier=8137010000-0019

Assay variable : PTINR_VAL1 PT-INR Value

N	Mean	Standard Deviation	Minimum	Maximum
3	1.6933333	0.0709460	1.6300000	1.7700000

Laboratory tests at the time of obtaining Table 2-6 consent, according to the presence or absence of proton-pump inhibitors

Unique Subject Identifier=8148010111-0001

MEANS Procedure

Assay variable : PTINR_VAL1 PT-INR Value

N	Mean	Standard Deviation	Minimum	Maximum
6	2.5416667	0.6447144	1.7300000	3.3800000

Unique Subject Identifier=8148010111-0002

Assay variable : PTINR_VAL1 PT-INR Value

N	Mean	Standard Deviation	Minimum	Maximum
3	1.7133333	0.1650253	1.5500000	1.8800000

Unique Subject Identifier=8148010111-0015

Assay variable : PTINR_VAL1 PT-INR Value

N	Mean	Standard Deviation	Minimum	Maximum
5	1.9880000	0.3409839	1.6400000	2.4500000

Unique Subject Identifier=8154011000-0003

Assay variable : PTINR_VAL1 PT-INR Value

N	Mean	Standard Deviation	Minimum	Maximum
3	1.4433333	0.1715615	1.2600000	1.6000000

Laboratory tests at the time of obtaining Table 2-6 consent, according to the presence or absence of proton-pump inhibitors

Unique Subject Identifier=8154011000-0020

MEANS Procedure

Assay variable : PTINR_VAL1 PT-INR Value

N	Mean	Standard Deviation	Minimum	Maximum
6	2.1566667	0.4464378	1.8000000	3.0400000

Unique Subject Identifier=8154011000-0021

Assay variable : PTINR_VAL1 PT-INR Value

N	Mean	Standard Deviation	Minimum	Maximum
5	2.1940000	0.2634008	1.9800000	2.6500000

Unique Subject Identifier=8154011000-0027

Assay variable : PTINR_VAL1 PT-INR Value

N	Mean	Standard Deviation	Minimum	Maximum
4	1.8125000	0.2051625	1.6000000	2.0300000

Unique Subject Identifier=8160000000-0003

Assay variable : PTINR_VAL1 PT-INR Value

N	Mean	Standard Deviation	Minimum	Maximum
6	1.8100000	0.2372341	1.6000000	2.2000000

Laboratory tests at the time of obtaining Table 2-6 consent, according to the presence or absence of proton-pump inhibitors

Unique Subject Identifier=8160000000-0004

MEANS Procedure

Assay variable : PTINR_VAL1 PT-INR Value

N	Mean	Standard Deviation	Minimum	Maximum
6	1.9666667	0.1751190	1.7000000	2.2000000

Unique Subject Identifier=8160000000-0006

Assay variable : PTINR_VAL1 PT-INR Value

N	Mean	Standard Deviation	Minimum	Maximum
6	1.7850000	0.3574213	1.4000000	2.3000000

Unique Subject Identifier=8160000000-0007

Assay variable : PTINR_VAL1 PT-INR Value

N	Mean	Standard Deviation	Minimum	Maximum
5	1.9880000	0.1475466	1.7700000	2.1000000

Unique Subject Identifier=8160000000-0008

Assay variable : PTINR_VAL1 PT-INR Value

N	Mean	Standard Deviation	Minimum	Maximum
6	2.1683333	0.4108731	1.7100000	2.8000000

Laboratory tests at the time of obtaining Table 2-6 consent, according to the presence or absence of proton-pump inhibitors

Unique Subject Identifier=8160000000-0009

MEANS Procedure

Assay variable : PTINR_VAL1 PT-INR Value

N	Mean	Standard Deviation	Minimum	Maximum
6	1.5600000	0.1019804	1.4000000	1.7000000

Unique Subject Identifier=8160000000-0010

Assay variable : PTINR_VAL1 PT-INR Value

N	Mean	Standard Deviation	Minimum	Maximum
6	1.8516667	0.2371005	1.6300000	2.3000000

Unique Subject Identifier=8160000000-0011

Assay variable : PTINR_VAL1 PT-INR Value

N	Mean	Standard Deviation	Minimum	Maximum
5	1.5800000	0.2774887	1.3000000	2.0000000

Unique Subject Identifier=8160000000-0013

Assay variable : PTINR_VAL1 PT-INR Value

N	Mean	Standard Deviation	Minimum	Maximum
6	2.0950000	0.4871858	1.5000000	2.9700000

Laboratory tests at the time of obtaining Table 2-6 consent, according to the presence or absence of proton-pump inhibitors

Unique Subject Identifier=8160000000-0017

MEANS Procedure

Assay variable : PTINR_VAL1 PT-INR Value

N	Mean	Standard Deviation	Minimum	Maximum
6	1.8283333	0.1000833	1.7000000	2.0000000

Unique Subject Identifier=8160000000-0018

Assay variable : PTINR_VAL1 PT-INR Value

N	Mean	Standard Deviation	Minimum	Maximum
6	1.6333333	0.2111556	1.4000000	2.0000000

Unique Subject Identifier=8160000000-0022

Assay variable : PTINR_VAL1 PT-INR Value

N	Mean	Standard Deviation	Minimum	Maximum
6	1.7250000	0.2403123	1.5000000	2.0000000

Unique Subject Identifier=8160000000-0025

Assay variable : PTINR_VAL1 PT-INR Value

N	Mean	Standard Deviation	Minimum	Maximum
6	1.7800000	0.1707630	1.5000000	2.0000000

Laboratory tests at the time of obtaining Table 2-6 consent, according to the presence or absence of proton-pump inhibitors

Unique Subject Identifier=8160000000-0026

MEANS Procedure

Assay variable : PTINR_VAL1 PT-INR Value

N	Mean	Standard Deviation	Minimum	Maximum
6	1.6266667	0.1751190	1.3600000	1.9000000

Unique Subject Identifier=8161010010-0002

Assay variable : PTINR_VAL1 PT-INR Value

N	Mean	Standard Deviation	Minimum	Maximum
4	1.9625000	0.3406244	1.5400000	2.3600000

Unique Subject Identifier=8161010010-0003

Assay variable : PTINR_VAL1 PT-INR Value

N	Mean	Standard Deviation	Minimum	Maximum
3	2.6700000	0.3439477	2.3000000	2.9800000

Unique Subject Identifier=8161010010-0006

Assay variable : PTINR_VAL1 PT-INR Value

N	Mean	Standard Deviation	Minimum	Maximum
2	1.8250000	0.2333452	1.6600000	1.9900000

Laboratory tests at the time of obtaining Table 2-6 consent, according to the presence or absence of proton-pump inhibitors

Unique Subject Identifier=8161010010-0008

MEANS Procedure

Assay variable : PTINR_VAL1 PT-INR Value

N	Mean	Standard Deviation	Minimum	Maximum
2	1.6500000	0.1555635	1.5400000	1.7600000

Unique Subject Identifier=8161010010-0010

Assay variable : PTINR_VAL1 PT-INR Value

N	Mean	Standard Deviation	Minimum	Maximum
3	1.3700000	0.2260531	1.1200000	1.5600000

Unique Subject Identifier=8161010010-0011

Assay variable : PTINR_VAL1 PT-INR Value

N	Mean	Standard Deviation	Minimum	Maximum
5	1.7800000	0.1607794	1.6000000	1.9800000

Unique Subject Identifier=8161010010-0016

Assay variable : PTINR_VAL1 PT-INR Value

N	Mean	Standard Deviation	Minimum	Maximum
4	2.3000000	0.5686827	1.7900000	2.9900000

Laboratory tests at the time of obtaining Table 2-6 consent, according to the presence or absence of proton-pump inhibitors

Unique Subject Identifier=8161010010-0018

MEANS Procedure

Assay variable : PTINR_VAL1 PT-INR Value

N	Mean	Standard Deviation	Minimum	Maximum
5	1.5080000	0.2834078	1.2900000	1.9900000

Unique Subject Identifier=8161010010-0022

Assay variable : PTINR_VAL1 PT-INR Value

N	Mean	Standard Deviation	Minimum	Maximum
3	2.8366667	0.3043572	2.6000000	3.1800000

Unique Subject Identifier=8161010010-0023

Assay variable : PTINR_VAL1 PT-INR Value

N	Mean	Standard Deviation	Minimum	Maximum
3	2.5333333	0.4272392	2.2200000	3.0200000

Unique Subject Identifier=8161010010-0024

Assay variable : PTINR_VAL1 PT-INR Value

N	Mean	Standard Deviation	Minimum	Maximum
5	2.1140000	0.6029760	1.6600000	3.1600000

Laboratory tests at the time of obtaining Table 2-6 consent, according to the presence or absence of proton-pump inhibitors

Unique Subject Identifier=8161010010-0025

MEANS Procedure

Assay variable : PTINR_VAL1 PT-INR Value

N	Mean	Standard Deviation	Minimum	Maximum
4	2.4100000	0.2201515	2.2300000	2.7300000

Unique Subject Identifier=8164000000-0001

Assay variable : PTINR_VAL1 PT-INR Value

N	Mean	Standard Deviation	Minimum	Maximum
6	2.1366667	0.1568014	1.9800000	2.3300000

Unique Subject Identifier=8164000000-0003

Assay variable : PTINR_VAL1 PT-INR Value

N	Mean	Standard Deviation	Minimum	Maximum
5	2.1320000	0.2025339	1.8700000	2.3400000

Unique Subject Identifier=8164000000-0004

Assay variable : PTINR_VAL1 PT-INR Value

N	Mean	Standard Deviation	Minimum	Maximum
3	1.5166667	0.2302897	1.3200000	1.7700000

Laboratory tests at the time of obtaining Table 2-6 consent, according to the presence or absence of proton-pump inhibitors

Unique Subject Identifier=8171010100-0002

MEANS Procedure

Assay variable : PTINR_VAL1 PT-INR Value

N	Mean	Standard Deviation	Minimum	Maximum
4	1.7700000	0.5373391	1.1600000	2.4700000

Unique Subject Identifier=8171010100-0003

Assay variable : PTINR_VAL1 PT-INR Value

N	Mean	Standard Deviation	Minimum	Maximum
2	1.7250000	0.1626346	1.6100000	1.8400000

Unique Subject Identifier=8171010100-0004

Assay variable : PTINR_VAL1 PT-INR Value

N	Mean	Standard Deviation	Minimum	Maximum
2	2.0500000	0.1838478	1.9200000	2.1800000

Unique Subject Identifier=8171010100-0006

Assay variable : PTINR_VAL1 PT-INR Value

N	Mean	Standard Deviation	Minimum	Maximum
4	2.0450000	0.1247664	1.8800000	2.1600000

Laboratory tests at the time of obtaining Table 2-6 consent, according to the presence or absence of proton-pump inhibitors

Unique Subject Identifier=8171010100-0007

MEANS Procedure

Assay variable : PTINR_VAL1 PT-INR Value

N	Mean	Standard Deviation	Minimum	Maximum
3	1.8766667	0.3955165	1.4700000	2.2600000

Unique Subject Identifier=8171010100-0008

Assay variable : PTINR_VAL1 PT-INR Value

N	Mean	Standard Deviation	Minimum	Maximum
3	1.9600000	0.1835756	1.8300000	2.1700000

Unique Subject Identifier=8198010000-0036

Assay variable : PTINR_VAL1 PT-INR Value

N	Mean	Standard Deviation	Minimum	Maximum
6	2.2000000	0.4560702	1.8000000	3.1000000

Unique Subject Identifier=8198010000-0039

Assay variable : PTINR_VAL1 PT-INR Value

N	Mean	Standard Deviation	Minimum	Maximum
4	2.1500000	0.3696846	1.7000000	2.6000000

Laboratory tests at the time of obtaining Table 2-6 consent, according to the presence or absence of proton-pump inhibitors

Unique Subject Identifier=8198010000-0045

MEANS Procedure

Assay variable : PTINR_VAL1 PT-INR Value

N	Mean	Standard Deviation	Minimum	Maximum
5	1.9200000	0.1095445	1.8000000	2.1000000

Unique Subject Identifier=8198010000-0047

Assay variable : PTINR_VAL1 PT-INR Value

N	Mean	Standard Deviation	Minimum	Maximum
6	1.6833333	0.3060501	1.4000000	2.2000000

Unique Subject Identifier=8204000000-0002

Assay variable : PTINR_VAL1 PT-INR Value

N	Mean	Standard Deviation	Minimum	Maximum
3	1.9800000	0.2095233	1.7500000	2.1600000

Unique Subject Identifier=8204000000-0010

Assay variable : PTINR_VAL1 PT-INR Value

N	Mean	Standard Deviation	Minimum	Maximum
5	1.8460000	0.6179239	1.1200000	2.7900000

Laboratory tests at the time of obtaining Table 2-6 consent, according to the presence or absence of proton-pump inhibitors

Unique Subject Identifier=8204000000-0014

MEANS Procedure

Assay variable : PTINR_VAL1 PT-INR Value

N	Mean	Standard Deviation	Minimum	Maximum
3	1.6766667	0.1123981	1.5800000	1.8000000

Unique Subject Identifier=8204000000-0015

Assay variable : PTINR_VAL1 PT-INR Value

N	Mean	Standard Deviation	Minimum	Maximum
3	2.0000000	0.6939741	1.4000000	2.7600000

Unique Subject Identifier=8204000000-0019

Assay variable : PTINR_VAL1 PT-INR Value

N	Mean	Standard Deviation	Minimum	Maximum
5	2.0960000	0.4102804	1.7200000	2.6600000

Unique Subject Identifier=8205000000-0001

Assay variable : PTINR_VAL1 PT-INR Value

N	Mean	Standard Deviation	Minimum	Maximum
4	1.6725000	0.1225765	1.5000000	1.7900000

Laboratory tests at the time of obtaining Table 2-6 consent, according to the presence or absence of proton-pump inhibitors

Unique Subject Identifier=8205000000-0008

MEANS Procedure

Assay variable : PTINR_VAL1 PT-INR Value

N	Mean	Standard Deviation	Minimum	Maximum
3	2.4466667	0.2569695	2.2100000	2.7200000

Unique Subject Identifier=8205000000-0020

Assay variable : PTINR_VAL1 PT-INR Value

N	Mean	Standard Deviation	Minimum	Maximum
3	2.6433333	0.1877054	2.5300000	2.8600000

Unique Subject Identifier=8205000000-0027

Assay variable : PTINR_VAL1 PT-INR Value

N	Mean	Standard Deviation	Minimum	Maximum
4	2.0325000	0.2025463	1.8800000	2.3300000

Unique Subject Identifier=8205000000-0028

Assay variable : PTINR_VAL1 PT-INR Value

N	Mean	Standard Deviation	Minimum	Maximum
3	1.8200000	0.2463737	1.5900000	2.0800000

Laboratory tests at the time of obtaining Table 2-6 consent, according to the presence or absence of proton-pump inhibitors

Unique Subject Identifier=8205000000-0036

MEANS Procedure

Assay variable : PTINR_VAL1 PT-INR Value

N	Mean	Standard Deviation	Minimum	Maximum
4	2.2500000	0.2450850	2.0400000	2.5800000

Unique Subject Identifier=8205000000-0042

Assay variable : PTINR_VAL1 PT-INR Value

N	Mean	Standard Deviation	Minimum	Maximum
4	1.8975000	0.3986122	1.6300000	2.4800000

Unique Subject Identifier=8209000000-0003

Assay variable : PTINR_VAL1 PT-INR Value

N	Mean	Standard Deviation	Minimum	Maximum
6	2.2900000	0.3209984	1.9000000	2.6400000

Unique Subject Identifier=8209000000-0004

Assay variable : PTINR_VAL1 PT-INR Value

N	Mean	Standard Deviation	Minimum	Maximum
6	2.2233333	0.7048593	1.6500000	3.2500000

Laboratory tests at the time of obtaining Table 2-6 consent, according to the presence or absence of proton-pump inhibitors

Unique Subject Identifier=8209000000-0005

MEANS Procedure

Assay variable : PTINR_VAL1 PT-INR Value

N	Mean	Standard Deviation	Minimum	Maximum
6	2.3283333	0.4630515	1.7800000	2.9800000

Unique Subject Identifier=8209000000-0007

Assay variable : PTINR_VAL1 PT-INR Value

N	Mean	Standard Deviation	Minimum	Maximum
5	1.6820000	0.2108791	1.3700000	1.9200000

Unique Subject Identifier=8209000000-0008

Assay variable : PTINR_VAL1 PT-INR Value

N	Mean	Standard Deviation	Minimum	Maximum
6	2.0366667	0.4864018	1.5400000	2.8300000

Unique Subject Identifier=8209000000-0010

Assay variable : PTINR_VAL1 PT-INR Value

N	Mean	Standard Deviation	Minimum	Maximum
4	2.2750000	0.3152248	1.9200000	2.6500000

Laboratory tests at the time of obtaining Table 2-6 consent, according to the presence or absence of proton-pump inhibitors

Unique Subject Identifier=8209000000-0014

MEANS Procedure

Assay variable : PTINR_VAL1 PT-INR Value

N	Mean	Standard Deviation	Minimum	Maximum
6	2.3583333	0.3791262	1.8000000	2.8700000

Unique Subject Identifier=8209000000-0017

Assay variable : PTINR_VAL1 PT-INR Value

N	Mean	Standard Deviation	Minimum	Maximum
4	2.0750000	0.2619796	1.7800000	2.4000000

Unique Subject Identifier=8209000000-0019

Assay variable : PTINR_VAL1 PT-INR Value

N	Mean	Standard Deviation	Minimum	Maximum
3	2.2633333	0.1154701	2.1300000	2.3300000

Unique Subject Identifier=8209000000-0023

Assay variable : PTINR_VAL1 PT-INR Value

N	Mean	Standard Deviation	Minimum	Maximum
6	1.8800000	0.3602222	1.5000000	2.5700000

Laboratory tests at the time of obtaining Table 2-6 consent, according to the presence or absence of proton-pump inhibitors

Unique Subject Identifier=8209000000-0024

MEANS Procedure

Assay variable : PTINR_VAL1 PT-INR Value

N	Mean	Standard Deviation	Minimum	Maximum
4	1.7000000	0.3987480	1.2600000	2.2200000

Unique Subject Identifier=8209000000-0026

Assay variable : PTINR_VAL1 PT-INR Value

N	Mean	Standard Deviation	Minimum	Maximum
4	1.9675000	0.5758689	1.3100000	2.5400000

Unique Subject Identifier=8209000000-0027

Assay variable : PTINR_VAL1 PT-INR Value

N	Mean	Standard Deviation	Minimum	Maximum
6	2.4133333	0.2027478	2.1700000	2.7200000

Unique Subject Identifier=8209000000-0028

Assay variable : PTINR_VAL1 PT-INR Value

N	Mean	Standard Deviation	Minimum	Maximum
6	2.2266667	0.2068494	1.9600000	2.5000000

Laboratory tests at the time of obtaining Table 2-6 consent, according to the presence or absence of proton-pump inhibitors

Unique Subject Identifier=8210000100-0002

MEANS Procedure

Assay variable : PTINR_VAL1 PT-INR Value

N	Mean	Standard Deviation	Minimum	Maximum
1	1.0800000		1.0800000	1.0800000

Unique Subject Identifier=8223000000-0004

Assay variable : PTINR_VAL1 PT-INR Value

N	Mean	Standard Deviation	Minimum	Maximum
3	1.8933333	0.3043572	1.6700000	2.2400000

Unique Subject Identifier=8223000000-0016

Assay variable : PTINR_VAL1 PT-INR Value

N	Mean	Standard Deviation	Minimum	Maximum
6	1.8766667	0.8429630	1.3900000	3.5600000

Unique Subject Identifier=8223000000-0022

Assay variable : PTINR_VAL1 PT-INR Value

N	Mean	Standard Deviation	Minimum	Maximum
3	2.0200000	0.3835362	1.6700000	2.4300000

Laboratory tests at the time of obtaining Table 2-6 consent, according to the presence or absence of proton-pump inhibitors

Unique Subject Identifier=8223000000-0023

MEANS Procedure

Assay variable : PTINR_VAL1 PT-INR Value

N	Mean	Standard Deviation	Minimum	Maximum
3	1.8933333	0.4461315	1.3900000	2.2400000

Unique Subject Identifier=8223000000-0024

Assay variable : PTINR_VAL1 PT-INR Value

N	Mean	Standard Deviation	Minimum	Maximum
5	1.8820000	0.5756040	1.2900000	2.5300000

Unique Subject Identifier=8223000000-0025

Assay variable : PTINR_VAL1 PT-INR Value

N	Mean	Standard Deviation	Minimum	Maximum
1	2.1500000		2.1500000	2.1500000

Unique Subject Identifier=8233000000-0006

Assay variable : PTINR_VAL1 PT-INR Value

N	Mean	Standard Deviation	Minimum	Maximum
5	1.6120000	0.1308434	1.4000000	1.7600000

Laboratory tests at the time of obtaining Table 2-6 consent, according to the presence or absence of proton-pump inhibitors

Unique Subject Identifier=8233000000-0018

MEANS Procedure

Assay variable : PTINR_VAL1 PT-INR Value

N	Mean	Standard Deviation	Minimum	Maximum
2	1.3900000	0.2687006	1.2000000	1.5800000

Unique Subject Identifier=8233000000-0019

Assay variable : PTINR_VAL1 PT-INR Value

N	Mean	Standard Deviation	Minimum	Maximum
2	2.6950000	0.1909188	2.5600000	2.8300000

Unique Subject Identifier=8237011000-0002

Assay variable : PTINR_VAL1 PT-INR Value

N	Mean	Standard Deviation	Minimum	Maximum
2	1.8500000	0.0707107	1.8000000	1.9000000

Unique Subject Identifier=8237011000-0003

Assay variable : PTINR_VAL1 PT-INR Value

N	Mean	Standard Deviation	Minimum	Maximum
3	2.2333333	0.2886751	1.9000000	2.4000000

Laboratory tests at the time of obtaining Table 2-6 consent, according to the presence or absence of proton-pump inhibitors

Unique Subject Identifier=8237011000-0005

MEANS Procedure

Assay variable : PTINR_VAL1 PT-INR Value

N	Mean	Standard Deviation	Minimum	Maximum
2	2.3000000	0.1414214	2.2000000	2.4000000

Unique Subject Identifier=8237011000-0006

Assay variable : PTINR_VAL1 PT-INR Value

N	Mean	Standard Deviation	Minimum	Maximum
2	1.8000000	0.2828427	1.6000000	2.0000000

Unique Subject Identifier=8237011000-0008

Assay variable : PTINR_VAL1 PT-INR Value

N	Mean	Standard Deviation	Minimum	Maximum
3	1.9666667	0.2081666	1.8000000	2.2000000

Unique Subject Identifier=8237011000-0011

Assay variable : PTINR_VAL1 PT-INR Value

N	Mean	Standard Deviation	Minimum	Maximum
3	2.1000000	0.1732051	1.9000000	2.2000000

Laboratory tests at the time of obtaining Table 2-6 consent, according to the presence or absence of proton-pump inhibitors

Unique Subject Identifier=8237011000-0014

MEANS Procedure

Assay variable : PTINR_VAL1 PT-INR Value

N	Mean	Standard Deviation	Minimum	Maximum
6	2.1166667	0.6675827	1.4000000	3.1000000

Unique Subject Identifier=8237011000-0015

Assay variable : PTINR_VAL1 PT-INR Value

N	Mean	Standard Deviation	Minimum	Maximum
2	2.5000000	0.9899495	1.8000000	3.2000000

Unique Subject Identifier=8237011000-0017

Assay variable : PTINR_VAL1 PT-INR Value

N	Mean	Standard Deviation	Minimum	Maximum
2	1.4000000	0.1414214	1.3000000	1.5000000

Unique Subject Identifier=8237011000-0018

Assay variable : PTINR_VAL1 PT-INR Value

N	Mean	Standard Deviation	Minimum	Maximum
4	2.0500000	0.1732051	1.9000000	2.3000000

Laboratory tests at the time of obtaining Table 2-6 consent, according to the presence or absence of proton-pump inhibitors

Unique Subject Identifier=8237011000-0023

MEANS Procedure

Assay variable : PTINR_VAL1 PT-INR Value

N	Mean	Standard Deviation	Minimum	Maximum
3	2.5666667	0.3214550	2.2000000	2.8000000

Unique Subject Identifier=8237011000-0025

Assay variable : PTINR_VAL1 PT-INR Value

N	Mean	Standard Deviation	Minimum	Maximum
3	2.4000000	0.2000000	2.2000000	2.6000000

Unique Subject Identifier=8237011000-0028

Assay variable : PTINR_VAL1 PT-INR Value

N	Mean	Standard Deviation	Minimum	Maximum
1	2.4000000		2.4000000	2.4000000

Unique Subject Identifier=8237011000-0029

Assay variable : PTINR_VAL1 PT-INR Value

N	Mean	Standard Deviation	Minimum	Maximum
6	1.7166667	0.4400758	1.3000000	2.5000000

Laboratory tests at the time of obtaining Table 2-6 consent, according to the presence or absence of proton-pump inhibitors

Unique Subject Identifier=8240011000-0004

MEANS Procedure

Assay variable : PTINR_VAL1 PT-INR Value

N	Mean	Standard Deviation	Minimum	Maximum
4	2.3950000	0.3850974	1.8500000	2.7200000

Unique Subject Identifier=8240011000-0006

Assay variable : PTINR_VAL1 PT-INR Value

N	Mean	Standard Deviation	Minimum	Maximum
6	2.1900000	0.3000667	1.7700000	2.5500000

Unique Subject Identifier=8240011000-0007

Assay variable : PTINR_VAL1 PT-INR Value

N	Mean	Standard Deviation	Minimum	Maximum
6	2.3183333	0.5312030	1.8300000	3.2400000

Unique Subject Identifier=8240011000-0008

Assay variable : PTINR_VAL1 PT-INR Value

N	Mean	Standard Deviation	Minimum	Maximum
6	2.2466667	0.2787592	1.9700000	2.6700000

Laboratory tests at the time of obtaining Table 2-6 consent, according to the presence or absence of proton-pump inhibitors

Unique Subject Identifier=8240011000-0009

MEANS Procedure

Assay variable : PTINR_VAL1 PT-INR Value

N	Mean	Standard Deviation	Minimum	Maximum
6	2.0766667	0.1127239	1.9200000	2.2100000

Unique Subject Identifier=8240011000-0020

Assay variable : PTINR_VAL1 PT-INR Value

N	Mean	Standard Deviation	Minimum	Maximum
6	1.8100000	0.3050246	1.5200000	2.3700000

Unique Subject Identifier=8240011000-0021

Assay variable : PTINR_VAL1 PT-INR Value

N	Mean	Standard Deviation	Minimum	Maximum
5	1.8580000	0.2609023	1.5700000	2.2700000

Unique Subject Identifier=8240011000-0025

Assay variable : PTINR_VAL1 PT-INR Value

N	Mean	Standard Deviation	Minimum	Maximum
6	1.6516667	0.1531557	1.4100000	1.7700000

Laboratory tests at the time of obtaining Table 2-6 consent, according to the presence or absence of proton-pump inhibitors

Unique Subject Identifier=8240011000-0035

MEANS Procedure

Assay variable : PTINR_VAL1 PT-INR Value

N	Mean	Standard Deviation	Minimum	Maximum
6	1.8183333	0.3077282	1.4000000	2.1500000

Unique Subject Identifier=8240011000-0042

Assay variable : PTINR_VAL1 PT-INR Value

N	Mean	Standard Deviation	Minimum	Maximum
5	2.2280000	0.4447696	1.5800000	2.6500000

Unique Subject Identifier=8240011000-0043

Assay variable : PTINR_VAL1 PT-INR Value

N	Mean	Standard Deviation	Minimum	Maximum
6	1.8783333	0.2213971	1.6400000	2.2500000

Unique Subject Identifier=8240011000-0044

Assay variable : PTINR_VAL1 PT-INR Value

N	Mean	Standard Deviation	Minimum	Maximum
6	2.5533333	0.4300543	2.1700000	3.3200000

Laboratory tests at the time of obtaining Table 2-6 consent, according to the presence or absence of proton-pump inhibitors

Unique Subject Identifier=8241000000-0001

MEANS Procedure

Assay variable : PTINR_VAL1 PT-INR Value

N	Mean	Standard Deviation	Minimum	Maximum
2	2.3550000	0.1767767	2.2300000	2.4800000

Unique Subject Identifier=8241000000-0010

Assay variable : PTINR_VAL1 PT-INR Value

N	Mean	Standard Deviation	Minimum	Maximum
4	2.0475000	0.3894761	1.7500000	2.6000000

Unique Subject Identifier=8241000000-0014

Assay variable : PTINR_VAL1 PT-INR Value

N	Mean	Standard Deviation	Minimum	Maximum
1	1.8500000		1.8500000	1.8500000

Unique Subject Identifier=8241000000-0018

Assay variable : PTINR_VAL1 PT-INR Value

N	Mean	Standard Deviation	Minimum	Maximum
5	1.5240000	0.1637987	1.3200000	1.7500000

Laboratory tests at the time of obtaining Table 2-6 consent, according to the presence or absence of proton-pump inhibitors

Unique Subject Identifier=8241000000-0021

MEANS Procedure

Assay variable : PTINR_VAL1 PT-INR Value

N	Mean	Standard Deviation	Minimum	Maximum
6	1.8233333	0.1009290	1.6800000	1.9600000

Unique Subject Identifier=8241000000-0023

Assay variable : PTINR_VAL1 PT-INR Value

N	Mean	Standard Deviation	Minimum	Maximum
4	1.7300000	0.3918333	1.2400000	2.1900000

Unique Subject Identifier=8241000000-0024

Assay variable : PTINR_VAL1 PT-INR Value

N	Mean	Standard Deviation	Minimum	Maximum
4	1.9350000	0.1491085	1.7500000	2.1100000

Unique Subject Identifier=8241000000-0027

Assay variable : PTINR_VAL1 PT-INR Value

N	Mean	Standard Deviation	Minimum	Maximum
4	1.8400000	0.4162531	1.3100000	2.3100000

Laboratory tests at the time of obtaining Table 2-6 consent, according to the presence or absence of proton-pump inhibitors

Unique Subject Identifier=8241000000-0028

MEANS Procedure

Assay variable : PTINR_VAL1 PT-INR Value

N	Mean	Standard Deviation	Minimum	Maximum
4	2.4300000	0.2320919	2.2500000	2.7700000

Unique Subject Identifier=8241000000-0029

Assay variable : PTINR_VAL1 PT-INR Value

N	Mean	Standard Deviation	Minimum	Maximum
3	2.0566667	0.2107922	1.9300000	2.3000000

Unique Subject Identifier=8241000000-0030

Assay variable : PTINR_VAL1 PT-INR Value

N	Mean	Standard Deviation	Minimum	Maximum
4	2.2750000	0.5987487	1.8400000	3.1200000

Unique Subject Identifier=8245000000-0003

Assay variable : PTINR_VAL1 PT-INR Value

N	Mean	Standard Deviation	Minimum	Maximum
3	2.8333333	0.1365040	2.7100000	2.9800000

Laboratory tests at the time of obtaining Table 2-6 consent, according to the presence or absence of proton-pump inhibitors

Unique Subject Identifier=8245000000-0004

MEANS Procedure

Assay variable : PTINR_VAL1 PT-INR Value

N	Mean	Standard Deviation	Minimum	Maximum
4	1.7650000	0.2053452	1.5600000	2.0000000

Unique Subject Identifier=8245000000-0005

Assay variable : PTINR_VAL1 PT-INR Value

N	Mean	Standard Deviation	Minimum	Maximum
2	2.0600000	0.1838478	1.9300000	2.1900000

Unique Subject Identifier=8245000000-0006

Assay variable : PTINR_VAL1 PT-INR Value

N	Mean	Standard Deviation	Minimum	Maximum
2	1.9400000	0.0565685	1.9000000	1.9800000

Unique Subject Identifier=8245000000-0007

Assay variable : PTINR_VAL1 PT-INR Value

N	Mean	Standard Deviation	Minimum	Maximum
2	1.7900000	0.0989949	1.7200000	1.8600000

Laboratory tests at the time of obtaining Table 2-6 consent, according to the presence or absence of proton-pump inhibitors

Unique Subject Identifier=8245000000-0008

MEANS Procedure

Assay variable : PTINR_VAL1 PT-INR Value

N	Mean	Standard Deviation	Minimum	Maximum
2	2.5000000	1.0889444	1.7300000	3.2700000

Unique Subject Identifier=8245000000-0009

Assay variable : PTINR_VAL1 PT-INR Value

N	Mean	Standard Deviation	Minimum	Maximum
1	2.5000000		2.5000000	2.5000000

Unique Subject Identifier=8245000000-0010

Assay variable : PTINR_VAL1 PT-INR Value

N	Mean	Standard Deviation	Minimum	Maximum
2	2.0550000	0.2192031	1.9000000	2.2100000

Unique Subject Identifier=8245000000-0012

Assay variable : PTINR_VAL1 PT-INR Value

N	Mean	Standard Deviation	Minimum	Maximum
2	2.5850000	0.8131728	2.0100000	3.1600000

Laboratory tests at the time of obtaining Table 2-6 consent, according to the presence or absence of proton-pump inhibitors

Unique Subject Identifier=8245000000-0013

MEANS Procedure

Assay variable : PTINR_VAL1 PT-INR Value

N	Mean	Standard Deviation	Minimum	Maximum
3	2.0166667	0.1761628	1.8200000	2.1600000

Unique Subject Identifier=8245000000-0016

Assay variable : PTINR_VAL1 PT-INR Value

N	Mean	Standard Deviation	Minimum	Maximum
3	1.8500000	0.2029778	1.6300000	2.0300000

Unique Subject Identifier=8245000000-0019

Assay variable : PTINR_VAL1 PT-INR Value

N	Mean	Standard Deviation	Minimum	Maximum
2	1.9850000	0.2899138	1.7800000	2.1900000

Unique Subject Identifier=8245000000-0025

Assay variable : PTINR_VAL1 PT-INR Value

N	Mean	Standard Deviation	Minimum	Maximum
2	2.6200000	0.3818377	2.3500000	2.8900000

Laboratory tests at the time of obtaining Table 2-6 consent, according to the presence or absence of proton-pump inhibitors

Unique Subject Identifier=8245000000-0027

MEANS Procedure

Assay variable : PTINR_VAL1 PT-INR Value

N	Mean	Standard Deviation	Minimum	Maximum
3	2.3433333	0.2650157	2.0800000	2.6100000

Unique Subject Identifier=8245000000-0028

Assay variable : PTINR_VAL1 PT-INR Value

N	Mean	Standard Deviation	Minimum	Maximum
4	1.9225000	0.2891799	1.5900000	2.2200000

Unique Subject Identifier=8257010001-0002

Assay variable : PTINR_VAL1 PT-INR Value

N	Mean	Standard Deviation	Minimum	Maximum
6	1.5250000	0.4353734	1.1300000	2.3600000

Unique Subject Identifier=8257010001-0003

Assay variable : PTINR_VAL1 PT-INR Value

N	Mean	Standard Deviation	Minimum	Maximum
4	2.2125000	0.7436117	1.3100000	3.0400000

Laboratory tests at the time of obtaining Table 2-6 consent, according to the presence or absence of proton-pump inhibitors

Unique Subject Identifier=8257010001-0004

MEANS Procedure

Assay variable : PTINR_VAL1 PT-INR Value

N	Mean	Standard Deviation	Minimum	Maximum
6	1.9466667	0.0480278	1.9100000	2.0400000

Unique Subject Identifier=8257010001-0006

Assay variable : PTINR_VAL1 PT-INR Value

N	Mean	Standard Deviation	Minimum	Maximum
3	2.4566667	0.8939985	1.4400000	3.1200000

Unique Subject Identifier=8257010001-0010

Assay variable : PTINR_VAL1 PT-INR Value

N	Mean	Standard Deviation	Minimum	Maximum
2	1.2300000	0.1838478	1.1000000	1.3600000

Unique Subject Identifier=8257010001-0011

Assay variable : PTINR_VAL1 PT-INR Value

N	Mean	Standard Deviation	Minimum	Maximum
6	2.1350000	0.2946693	1.7500000	2.6500000

Laboratory tests at the time of obtaining Table 2-6 consent, according to the presence or absence of proton-pump inhibitors

Unique Subject Identifier=8257010001-0012

MEANS Procedure

Assay variable : PTINR_VAL1 PT-INR Value

N	Mean	Standard Deviation	Minimum	Maximum
6	1.5666667	0.4684300	1.1700000	2.4900000

Unique Subject Identifier=8257010001-0015

Assay variable : PTINR_VAL1 PT-INR Value

N	Mean	Standard Deviation	Minimum	Maximum
3	1.4633333	0.2557994	1.2200000	1.7300000

Unique Subject Identifier=8257010001-0016

Assay variable : PTINR_VAL1 PT-INR Value

N	Mean	Standard Deviation	Minimum	Maximum
3	2.2433333	0.3477547	1.8500000	2.5100000

Unique Subject Identifier=8257010001-0024

Assay variable : PTINR_VAL1 PT-INR Value

N	Mean	Standard Deviation	Minimum	Maximum
2	1.7650000	0.4171930	1.4700000	2.0600000

Laboratory tests at the time of obtaining Table 2-6 consent, according to the presence or absence of proton-pump inhibitors

Unique Subject Identifier=8257010001-0029

MEANS Procedure

Assay variable : PTINR_VAL1 PT-INR Value

N	Mean	Standard Deviation	Minimum	Maximum
3	1.7733333	0.3181719	1.5700000	2.1400000

Unique Subject Identifier=8257010001-0030

Assay variable : PTINR_VAL1 PT-INR Value

N	Mean	Standard Deviation	Minimum	Maximum
4	2.1250000	0.7816436	1.1700000	3.0100000

Unique Subject Identifier=8266000000-0017

Assay variable : PTINR_VAL1 PT-INR Value

N	Mean	Standard Deviation	Minimum	Maximum
3	2.4100000	0.2389561	2.2000000	2.6700000

Unique Subject Identifier=8266000000-0020

Assay variable : PTINR_VAL1 PT-INR Value

N	Mean	Standard Deviation	Minimum	Maximum
6	2.5250000	0.3613170	2.0700000	3.1400000

Laboratory tests at the time of obtaining Table 2-6 consent, according to the presence or absence of proton-pump inhibitors

Unique Subject Identifier=8266000000-0026

MEANS Procedure

Assay variable : PTINR_VAL1 PT-INR Value

N	Mean	Standard Deviation	Minimum	Maximum
4	2.7025000	0.4548535	2.2100000	3.2400000

Unique Subject Identifier=8266000000-0027

Assay variable : PTINR_VAL1 PT-INR Value

N	Mean	Standard Deviation	Minimum	Maximum
6	2.6733333	0.5607733	2.1000000	3.4200000

Unique Subject Identifier=8301010000-0004

Assay variable : PTINR_VAL1 PT-INR Value

N	Mean	Standard Deviation	Minimum	Maximum
6	1.7666667	0.3141125	1.2000000	2.1000000

Unique Subject Identifier=8326000000-0003

Assay variable : PTINR_VAL1 PT-INR Value

N	Mean	Standard Deviation	Minimum	Maximum
3	1.6633333	0.2281082	1.4000000	1.8000000

Laboratory tests at the time of obtaining Table 2-6 consent, according to the presence or absence of proton-pump inhibitors

Unique Subject Identifier=8326000000-0006

MEANS Procedure

Assay variable : PTINR_VAL1 PT-INR Value

N	Mean	Standard Deviation	Minimum	Maximum
4	2.2975000	1.4179886	1.3000000	4.4000000

Unique Subject Identifier=8326000000-0009

Assay variable : PTINR_VAL1 PT-INR Value

N	Mean	Standard Deviation	Minimum	Maximum
4	2.4150000	0.3139533	2.1000000	2.8500000

Unique Subject Identifier=8326000000-0012

Assay variable : PTINR_VAL1 PT-INR Value

N	Mean	Standard Deviation	Minimum	Maximum
4	2.3250000	0.2564501	1.9500000	2.5200000

Unique Subject Identifier=8326000000-0013

Assay variable : PTINR_VAL1 PT-INR Value

N	Mean	Standard Deviation	Minimum	Maximum
6	2.0000000	1.3607057	1.1100000	4.7100000

Laboratory tests at the time of obtaining Table 2-6 consent, according to the presence or absence of proton-pump inhibitors

Unique Subject Identifier=8326000000-0014

MEANS Procedure

Assay variable : PTINR_VAL1 PT-INR Value

N	Mean	Standard Deviation	Minimum	Maximum
6	2.1600000	0.2707767	1.8200000	2.5000000

Unique Subject Identifier=8326000000-0020

Assay variable : PTINR_VAL1 PT-INR Value

N	Mean	Standard Deviation	Minimum	Maximum
5	2.0620000	0.4699149	1.5900000	2.7800000

Unique Subject Identifier=8326000000-0021

Assay variable : PTINR_VAL1 PT-INR Value

N	Mean	Standard Deviation	Minimum	Maximum
2	2.3350000	0.2474874	2.1600000	2.5100000

Unique Subject Identifier=8326000000-0023

Assay variable : PTINR_VAL1 PT-INR Value

N	Mean	Standard Deviation	Minimum	Maximum
4	2.1175000	0.1120640	2.0000000	2.2700000

Laboratory tests at the time of obtaining Table 2-6 consent, according to the presence or absence of proton-pump inhibitors

Unique Subject Identifier=8326000000-0024

MEANS Procedure

Assay variable : PTINR_VAL1 PT-INR Value

N	Mean	Standard Deviation	Minimum	Maximum
3	1.7500000	0.3148015	1.4900000	2.1000000

Unique Subject Identifier=8326000000-0026

Assay variable : PTINR_VAL1 PT-INR Value

N	Mean	Standard Deviation	Minimum	Maximum
5	2.1620000	0.2448877	1.9000000	2.5300000

Unique Subject Identifier=8326000000-0028

Assay variable : PTINR_VAL1 PT-INR Value

N	Mean	Standard Deviation	Minimum	Maximum
3	2.3600000	0.2286919	2.1000000	2.5300000

Unique Subject Identifier=8326000000-0029

Assay variable : PTINR_VAL1 PT-INR Value

N	Mean	Standard Deviation	Minimum	Maximum
3	2.4400000	0.6109828	1.8500000	3.0700000

Laboratory tests at the time of obtaining Table 2-6 consent, according to the presence or absence of proton-pump inhibitors

Unique Subject Identifier=8326000000-0031

MEANS Procedure

Assay variable : PTINR_VAL1 PT-INR Value

N	Mean	Standard Deviation	Minimum	Maximum
5	1.8400000	0.2701851	1.4000000	2.1000000

Unique Subject Identifier=8326000000-0033

Assay variable : PTINR_VAL1 PT-INR Value

N	Mean	Standard Deviation	Minimum	Maximum
6	1.8983333	0.4345304	1.5000000	2.5000000

Unique Subject Identifier=8326000000-0035

Assay variable : PTINR_VAL1 PT-INR Value

N	Mean	Standard Deviation	Minimum	Maximum
5	1.7980000	0.1549839	1.6000000	1.9900000

Unique Subject Identifier=8326000000-0036

Assay variable : PTINR_VAL1 PT-INR Value

N	Mean	Standard Deviation	Minimum	Maximum
6	1.6400000	0.1035374	1.4900000	1.7600000

Laboratory tests at the time of obtaining Table 2-6 consent, according to the presence or absence of proton-pump inhibitors

Unique Subject Identifier=8326000000-0038

MEANS Procedure

Assay variable : PTINR_VAL1 PT-INR Value

N	Mean	Standard Deviation	Minimum	Maximum
6	1.4966667	0.0941630	1.3800000	1.6000000

Unique Subject Identifier=8326000000-0040

Assay variable : PTINR_VAL1 PT-INR Value

N	Mean	Standard Deviation	Minimum	Maximum
3	2.0766667	0.2916048	1.7400000	2.2500000

Unique Subject Identifier=8332010000-0005

Assay variable : PTINR_VAL1 PT-INR Value

N	Mean	Standard Deviation	Minimum	Maximum
5	1.7620000	0.3076849	1.5100000	2.2200000

Unique Subject Identifier=8332010000-0008

Assay variable : PTINR_VAL1 PT-INR Value

N	Mean	Standard Deviation	Minimum	Maximum
4	2.1475000	0.1114675	2.0000000	2.2700000

Laboratory tests at the time of obtaining Table 2-6 consent, according to the presence or absence of proton-pump inhibitors

Unique Subject Identifier=8332010000-0009

MEANS Procedure

Assay variable : PTINR_VAL1 PT-INR Value

N	Mean	Standard Deviation	Minimum	Maximum
5	2.6960000	0.3958282	2.1100000	3.1800000

Unique Subject Identifier=8332010000-0015

Assay variable : PTINR_VAL1 PT-INR Value

N	Mean	Standard Deviation	Minimum	Maximum
6	2.4466667	0.6553981	1.7400000	3.3300000

Unique Subject Identifier=8332010000-0017

Assay variable : PTINR_VAL1 PT-INR Value

N	Mean	Standard Deviation	Minimum	Maximum
4	2.1225000	0.2233644	1.8100000	2.3400000

Unique Subject Identifier=8332010000-0020

Assay variable : PTINR_VAL1 PT-INR Value

N	Mean	Standard Deviation	Minimum	Maximum
6	2.6250000	0.7922563	1.9700000	4.1100000

Laboratory tests at the time of obtaining Table 2-6 consent, according to the presence or absence of proton-pump inhibitors

Unique Subject Identifier=8332010000-0028

MEANS Procedure

Assay variable : PTINR_VAL1 PT-INR Value

N	Mean	Standard Deviation	Minimum	Maximum
6	2.7833333	0.3792976	2.1700000	3.2100000

Unique Subject Identifier=8332010000-0043

Assay variable : PTINR_VAL1 PT-INR Value

N	Mean	Standard Deviation	Minimum	Maximum
4	1.5925000	0.0689807	1.4900000	1.6400000

Unique Subject Identifier=8332010000-0046

Assay variable : PTINR_VAL1 PT-INR Value

N	Mean	Standard Deviation	Minimum	Maximum
3	2.1833333	0.3803069	1.8800000	2.6100000

Unique Subject Identifier=8332010000-0052

Assay variable : PTINR_VAL1 PT-INR Value

N	Mean	Standard Deviation	Minimum	Maximum
6	2.0950000	0.3677907	1.7000000	2.6000000

Laboratory tests at the time of obtaining Table 2-6 consent, according to the presence or absence of proton-pump inhibitors

Unique Subject Identifier=8332010000-0055

MEANS Procedure

Assay variable : PTINR_VAL1 PT-INR Value

N	Mean	Standard Deviation	Minimum	Maximum
6	1.9950000	0.5670185	1.5500000	3.0300000

Unique Subject Identifier=8332010000-0056

Assay variable : PTINR_VAL1 PT-INR Value

N	Mean	Standard Deviation	Minimum	Maximum
6	2.2566667	0.4317715	1.6200000	2.7300000

Unique Subject Identifier=8332010000-0068

Assay variable : PTINR_VAL1 PT-INR Value

N	Mean	Standard Deviation	Minimum	Maximum
5	1.9500000	0.3262668	1.5800000	2.2300000

Unique Subject Identifier=8332010000-0072

Assay variable : PTINR_VAL1 PT-INR Value

N	Mean	Standard Deviation	Minimum	Maximum
5	2.8200000	0.2271563	2.6100000	3.2000000

Laboratory tests at the time of obtaining Table 2-6 consent, according to the presence or absence of proton-pump inhibitors

Unique Subject Identifier=8332010000-0093

MEANS Procedure

Assay variable : PTINR_VAL1 PT-INR Value

N	Mean	Standard Deviation	Minimum	Maximum
1	2.0300000		2.0300000	2.0300000

Unique Subject Identifier=8332010000-0095

Assay variable : PTINR_VAL1 PT-INR Value

N	Mean	Standard Deviation	Minimum	Maximum
1	1.6800000		1.6800000	1.6800000

Unique Subject Identifier=8332010000-0101

Assay variable : PTINR_VAL1 PT-INR Value

N	Mean	Standard Deviation	Minimum	Maximum
2	1.9700000	0.0424264	1.9400000	2.0000000

Unique Subject Identifier=8332010000-0121

Assay variable : PTINR_VAL1 PT-INR Value

N	Mean	Standard Deviation	Minimum	Maximum
2	1.7250000	0.2333452	1.5600000	1.8900000

Laboratory tests at the time of obtaining Table 2-6 consent, according to the presence or absence of proton-pump inhibitors

Unique Subject Identifier=8332010000-0141

MEANS Procedure

Assay variable : PTINR_VAL1 PT-INR Value

N	Mean	Standard Deviation	Minimum	Maximum
3	2.0033333	0.0416333	1.9700000	2.0500000

Unique Subject Identifier=8332010000-0142

Assay variable : PTINR_VAL1 PT-INR Value

N	Mean	Standard Deviation	Minimum	Maximum
4	2.3750000	0.1707825	2.2000000	2.6000000

Unique Subject Identifier=8332010000-0145

Assay variable : PTINR_VAL1 PT-INR Value

N	Mean	Standard Deviation	Minimum	Maximum
1	1.8200000		1.8200000	1.8200000

Unique Subject Identifier=8332010000-0151

Assay variable : PTINR_VAL1 PT-INR Value

N	Mean	Standard Deviation	Minimum	Maximum
6	2.1100000	0.2538503	1.7200000	2.3300000

Laboratory tests at the time of obtaining Table 2-6 consent, according to the presence or absence of proton-pump inhibitors

Unique Subject Identifier=8332010000-0180

MEANS Procedure

Assay variable : PTINR_VAL1 PT-INR Value

N	Mean	Standard Deviation	Minimum	Maximum
4	1.8625000	0.1791415	1.7000000	2.1100000

Unique Subject Identifier=8332010000-0192

Assay variable : PTINR_VAL1 PT-INR Value

N	Mean	Standard Deviation	Minimum	Maximum
1	1.9600000		1.9600000	1.9600000

Unique Subject Identifier=8332010000-0194

Assay variable : PTINR_VAL1 PT-INR Value

N	Mean	Standard Deviation	Minimum	Maximum
4	1.3850000	0.1550269	1.2400000	1.5600000

Unique Subject Identifier=8340011000-0013

Assay variable : PTINR_VAL1 PT-INR Value

N	Mean	Standard Deviation	Minimum	Maximum
4	1.7675000	0.2636127	1.5300000	2.0200000

Laboratory tests at the time of obtaining Table 2-6 consent, according to the presence or absence of proton-pump inhibitors

Unique Subject Identifier=8340011000-0022

MEANS Procedure

Assay variable : PTINR_VAL1 PT-INR Value

N	Mean	Standard Deviation	Minimum	Maximum
4	2.1000000	0.3261901	1.7600000	2.5400000

Unique Subject Identifier=8340011000-0025

Assay variable : PTINR_VAL1 PT-INR Value

N	Mean	Standard Deviation	Minimum	Maximum
5	2.1240000	0.4209869	1.5300000	2.6200000

Unique Subject Identifier=8345000000-0006

Assay variable : PTINR_VAL1 PT-INR Value

N	Mean	Standard Deviation	Minimum	Maximum
6	1.4600000	0.0876356	1.3400000	1.5700000

Unique Subject Identifier=8345000000-0007

Assay variable : PTINR_VAL1 PT-INR Value

N	Mean	Standard Deviation	Minimum	Maximum
3	2.5100000	0.4132796	2.0700000	2.8900000

Laboratory tests at the time of obtaining Table 2-6 consent, according to the presence or absence of proton-pump inhibitors

Unique Subject Identifier=8345000000-0009

MEANS Procedure

Assay variable : PTINR_VAL1 PT-INR Value

N	Mean	Standard Deviation	Minimum	Maximum
6	2.0716667	0.4374205	1.5300000	2.8300000

Unique Subject Identifier=8345000000-0020

Assay variable : PTINR_VAL1 PT-INR Value

N	Mean	Standard Deviation	Minimum	Maximum
3	2.1166667	0.1985783	1.8900000	2.2600000

Unique Subject Identifier=8345000000-0023

Assay variable : PTINR_VAL1 PT-INR Value

N	Mean	Standard Deviation	Minimum	Maximum
3	2.2200000	0.3380828	2.0100000	2.6100000

Unique Subject Identifier=8345000000-0025

Assay variable : PTINR_VAL1 PT-INR Value

N	Mean	Standard Deviation	Minimum	Maximum
1	1.6600000		1.6600000	1.6600000

Laboratory tests at the time of obtaining Table 2-6 consent, according to the presence or absence of proton-pump inhibitors

Unique Subject Identifier=8417010011-0004

MEANS Procedure

Assay variable : PTINR_VAL1 PT-INR Value

N	Mean	Standard Deviation	Minimum	Maximum
6	2.2533333	0.2838779	1.9400000	2.6300000

Unique Subject Identifier=8442010000-0002

Assay variable : PTINR_VAL1 PT-INR Value

N	Mean	Standard Deviation	Minimum	Maximum
3	1.7133333	0.2074448	1.4900000	1.9000000

Unique Subject Identifier=8442010000-0005

Assay variable : PTINR_VAL1 PT-INR Value

N	Mean	Standard Deviation	Minimum	Maximum
4	2.2075000	0.5354982	1.4600000	2.6100000

Unique Subject Identifier=8442010000-0008

Assay variable : PTINR_VAL1 PT-INR Value

N	Mean	Standard Deviation	Minimum	Maximum
3	1.6866667	0.2514624	1.4900000	1.9700000

Laboratory tests at the time of obtaining Table 2-6 consent, according to the presence or absence of proton-pump inhibitors

Unique Subject Identifier=8442010000-0010

MEANS Procedure

Assay variable : PTINR_VAL1 PT-INR Value

N	Mean	Standard Deviation	Minimum	Maximum
3	2.1933333	0.2502665	1.9500000	2.4500000

Unique Subject Identifier=8442010000-0017

Assay variable : PTINR_VAL1 PT-INR Value

N	Mean	Standard Deviation	Minimum	Maximum
2	1.7450000	0.0919239	1.6800000	1.8100000

Unique Subject Identifier=8442010000-0021

Assay variable : PTINR_VAL1 PT-INR Value

N	Mean	Standard Deviation	Minimum	Maximum
3	1.6566667	0.1877054	1.4900000	1.8600000

Unique Subject Identifier=8514000000-0001

Assay variable : PTINR_VAL1 PT-INR Value

N	Mean	Standard Deviation	Minimum	Maximum
5	1.8760000	0.5155386	1.0700000	2.4900000

Laboratory tests at the time of obtaining Table 2-6 consent, according to the presence or absence of proton-pump inhibitors

Unique Subject Identifier=8514000000-0004

MEANS Procedure

Assay variable : PTINR_VAL1 PT-INR Value

N	Mean	Standard Deviation	Minimum	Maximum
6	2.0583333	0.1905168	1.7900000	2.3600000

Unique Subject Identifier=8623000000-0001

Assay variable : PTINR_VAL1 PT-INR Value

N	Mean	Standard Deviation	Minimum	Maximum
4	2.0100000	0.0787401	1.9200000	2.0900000

Unique Subject Identifier=8623000000-0004

Assay variable : PTINR_VAL1 PT-INR Value

N	Mean	Standard Deviation	Minimum	Maximum
4	2.1550000	0.5980803	1.4900000	2.9300000

Unique Subject Identifier=8623000000-0005

Assay variable : PTINR_VAL1 PT-INR Value

N	Mean	Standard Deviation	Minimum	Maximum
2	1.8250000	0.1060660	1.7500000	1.9000000

Laboratory tests at the time of obtaining Table 2-6 consent, according to the presence or absence of proton-pump inhibitors

Unique Subject Identifier=8623000000-0009

MEANS Procedure

Assay variable : PTINR_VAL1 PT-INR Value

N	Mean	Standard Deviation	Minimum	Maximum
2	1.9350000	0.0494975	1.9000000	1.9700000

Unique Subject Identifier=8623000000-0010

Assay variable : PTINR_VAL1 PT-INR Value

N	Mean	Standard Deviation	Minimum	Maximum
1	1.7200000		1.7200000	1.7200000

Unique Subject Identifier=8623000000-0011

Assay variable : PTINR_VAL1 PT-INR Value

N	Mean	Standard Deviation	Minimum	Maximum
3	2.4300000	0.2662705	2.1800000	2.7100000

Unique Subject Identifier=8623000000-0016

Assay variable : PTINR_VAL1 PT-INR Value

N	Mean	Standard Deviation	Minimum	Maximum
6	2.1816667	0.2010390	1.9700000	2.4500000

Laboratory tests at the time of obtaining Table 2-6 consent, according to the presence or absence of proton-pump inhibitors

Unique Subject Identifier=8623000000-0025

MEANS Procedure

Assay variable : PTINR_VAL1 PT-INR Value

N	Mean	Standard Deviation	Minimum	Maximum
3	2.6033333	1.0813109	1.9200000	3.8500000

Unique Subject Identifier=8623000000-0026

Assay variable : PTINR_VAL1 PT-INR Value

N	Mean	Standard Deviation	Minimum	Maximum
4	1.9875000	0.5948319	1.4900000	2.8400000

Unique Subject Identifier=8623000000-0027

Assay variable : PTINR_VAL1 PT-INR Value

N	Mean	Standard Deviation	Minimum	Maximum
5	1.5660000	0.0808084	1.4900000	1.6800000

Unique Subject Identifier=8623000000-0029

Assay variable : PTINR_VAL1 PT-INR Value

N	Mean	Standard Deviation	Minimum	Maximum
4	1.4850000	0.1933046	1.2500000	1.6800000

Laboratory tests at the time of obtaining Table 2-6 consent, according to the presence or absence of proton-pump inhibitors

Unique Subject Identifier=8623000000-0030

MEANS Procedure

Assay variable : PTINR_VAL1 PT-INR Value

N	Mean	Standard Deviation	Minimum	Maximum
2	2.0000000	1.2727922	1.1000000	2.9000000

Unique Subject Identifier=8748000000-0003

Assay variable : PTINR_VAL1 PT-INR Value

N	Mean	Standard Deviation	Minimum	Maximum
1	1.4300000		1.4300000	1.4300000

Unique Subject Identifier=9001010000-0009

Assay variable : PTINR_VAL1 PT-INR Value

N	Mean	Standard Deviation	Minimum	Maximum
6	1.8500000	0.3271085	1.4000000	2.2000000

Unique Subject Identifier=9001010000-0010

Assay variable : PTINR_VAL1 PT-INR Value

N	Mean	Standard Deviation	Minimum	Maximum
6	2.1833333	0.3311596	1.8000000	2.7000000

Laboratory tests at the time of obtaining Table 2-6 consent, according to the presence or absence of proton-pump inhibitors

Unique Subject Identifier=9002010000-0051

MEANS Procedure

Assay variable : PTINR_VAL1 PT-INR Value

N	Mean	Standard Deviation	Minimum	Maximum
5	2.0380000	0.3810118	1.5400000	2.5500000

Unique Subject Identifier=9002010000-0057

Assay variable : PTINR_VAL1 PT-INR Value

N	Mean	Standard Deviation	Minimum	Maximum
4	1.6625000	0.1725060	1.4400000	1.8600000

Unique Subject Identifier=9002010000-0067

Assay variable : PTINR_VAL1 PT-INR Value

N	Mean	Standard Deviation	Minimum	Maximum
5	1.8720000	0.1495660	1.6300000	2.0200000

Unique Subject Identifier=9002010000-0069

Assay variable : PTINR_VAL1 PT-INR Value

N	Mean	Standard Deviation	Minimum	Maximum
6	1.9333333	0.3015073	1.5700000	2.3700000

Laboratory tests at the time of obtaining Table 2-6 consent, according to the presence or absence of proton-pump inhibitors

Unique Subject Identifier=9002010000-0079

MEANS Procedure

Assay variable : PTINR_VAL1 PT-INR Value

N	Mean	Standard Deviation	Minimum	Maximum
4	2.0350000	1.1125197	1.0500000	3.6300000

Unique Subject Identifier=9002010000-0088

Assay variable : PTINR_VAL1 PT-INR Value

N	Mean	Standard Deviation	Minimum	Maximum
4	1.5800000	0.2423496	1.3800000	1.9300000

Unique Subject Identifier=9002010000-0139

Assay variable : PTINR_VAL1 PT-INR Value

N	Mean	Standard Deviation	Minimum	Maximum
4	2.1200000	0.4318179	1.6100000	2.6000000

Unique Subject Identifier=9002010000-0164

Assay variable : PTINR_VAL1 PT-INR Value

N	Mean	Standard Deviation	Minimum	Maximum
6	2.1116667	0.3227021	1.8600000	2.7100000

Laboratory tests at the time of obtaining Table 2-6 consent, according to the presence or absence of proton-pump inhibitors

Unique Subject Identifier=9002010000-0223

MEANS Procedure

Assay variable : PTINR_VAL1 PT-INR Value

N	Mean	Standard Deviation	Minimum	Maximum
6	1.7866667	0.2677810	1.5100000	2.2500000

Unique Subject Identifier=9005000000-0004

Assay variable : PTINR_VAL1 PT-INR Value

N	Mean	Standard Deviation	Minimum	Maximum
3	1.7166667	0.2285461	1.5700000	1.9800000

Unique Subject Identifier=9005000000-0005

Assay variable : PTINR_VAL1 PT-INR Value

N	Mean	Standard Deviation	Minimum	Maximum
3	1.7833333	0.2150194	1.6000000	2.0200000

Unique Subject Identifier=9005000000-0007

Assay variable : PTINR_VAL1 PT-INR Value

N	Mean	Standard Deviation	Minimum	Maximum
3	1.5000000	0.1442221	1.3800000	1.6600000

Laboratory tests at the time of obtaining Table 2-6 consent, according to the presence or absence of proton-pump inhibitors

Unique Subject Identifier=9005000000-0009

MEANS Procedure

Assay variable : PTINR_VAL1 PT-INR Value

N	Mean	Standard Deviation	Minimum	Maximum
6	2.4033333	0.3593698	1.9600000	3.0200000

Unique Subject Identifier=9005000000-0015

Assay variable : PTINR_VAL1 PT-INR Value

N	Mean	Standard Deviation	Minimum	Maximum
3	1.9733333	0.1357694	1.8300000	2.1000000

Unique Subject Identifier=9005000000-0023

Assay variable : PTINR_VAL1 PT-INR Value

N	Mean	Standard Deviation	Minimum	Maximum
3	1.4833333	0.0923760	1.4300000	1.5900000

Unique Subject Identifier=9005000000-0033

Assay variable : PTINR_VAL1 PT-INR Value

N	Mean	Standard Deviation	Minimum	Maximum
3	1.8233333	0.2218859	1.6200000	2.0600000

Laboratory tests at the time of obtaining Table 2-6 consent, according to the presence or absence of proton-pump inhibitors

Unique Subject Identifier=9008010000-0006

MEANS Procedure

Assay variable : PTINR_VAL1 PT-INR Value

N	Mean	Standard Deviation	Minimum	Maximum
3	1.9466667	0.1858315	1.7400000	2.1000000

Unique Subject Identifier=9008010000-0012

Assay variable : PTINR_VAL1 PT-INR Value

N	Mean	Standard Deviation	Minimum	Maximum
3	2.3766667	0.2554082	2.0900000	2.5800000

Unique Subject Identifier=9008010000-0017

Assay variable : PTINR_VAL1 PT-INR Value

N	Mean	Standard Deviation	Minimum	Maximum
2	1.8100000	0.1414214	1.7100000	1.9100000

Unique Subject Identifier=9008010000-0021

Assay variable : PTINR_VAL1 PT-INR Value

N	Mean	Standard Deviation	Minimum	Maximum
4	1.8275000	0.2637391	1.5600000	2.1300000

Laboratory tests at the time of obtaining Table 2-6 consent, according to the presence or absence of proton-pump inhibitors

Unique Subject Identifier=9008010000-0027

MEANS Procedure

Assay variable : PTINR_VAL1 PT-INR Value

N	Mean	Standard Deviation	Minimum	Maximum
4	1.3375000	0.2895255	1.0500000	1.7100000

Unique Subject Identifier=9009010000-0001

Assay variable : PTINR_VAL1 PT-INR Value

N	Mean	Standard Deviation	Minimum	Maximum
6	1.7666667	0.1512173	1.6000000	2.0100000

Unique Subject Identifier=9009010000-0003

Assay variable : PTINR_VAL1 PT-INR Value

N	Mean	Standard Deviation	Minimum	Maximum
4	1.7750000	0.1660321	1.5900000	1.9900000

Unique Subject Identifier=9009010000-0013

Assay variable : PTINR_VAL1 PT-INR Value

N	Mean	Standard Deviation	Minimum	Maximum
4	1.3275000	0.0419325	1.2700000	1.3700000

Laboratory tests at the time of obtaining Table 2-6 consent, according to the presence or absence of proton-pump inhibitors

Unique Subject Identifier=9009010000-0015

MEANS Procedure

Assay variable : PTINR_VAL1 PT-INR Value

N	Mean	Standard Deviation	Minimum	Maximum
6	2.2666667	0.3688722	1.8900000	2.9200000

Unique Subject Identifier=9009010000-0017

Assay variable : PTINR_VAL1 PT-INR Value

N	Mean	Standard Deviation	Minimum	Maximum
4	1.6525000	0.2736634	1.4200000	2.0400000

Unique Subject Identifier=9009010000-0022

Assay variable : PTINR_VAL1 PT-INR Value

N	Mean	Standard Deviation	Minimum	Maximum
3	1.4166667	0.0665833	1.3600000	1.4900000

Unique Subject Identifier=9009010000-0030

Assay variable : PTINR_VAL1 PT-INR Value

N	Mean	Standard Deviation	Minimum	Maximum
4	1.6875000	0.1787689	1.5200000	1.9400000

Laboratory tests at the time of obtaining Table 2-6 consent, according to the presence or absence of proton-pump inhibitors

Unique Subject Identifier=9014000000-0002

MEANS Procedure

Assay variable : PTINR_VAL1 PT-INR Value

N	Mean	Standard Deviation	Minimum	Maximum
5	1.8480000	0.1758408	1.7000000	2.1400000

Unique Subject Identifier=9014000000-0003

Assay variable : PTINR_VAL1 PT-INR Value

N	Mean	Standard Deviation	Minimum	Maximum
4	2.1700000	0.1529706	2.0300000	2.3800000

Unique Subject Identifier=9014000000-0004

Assay variable : PTINR_VAL1 PT-INR Value

N	Mean	Standard Deviation	Minimum	Maximum
5	2.2540000	0.2352233	2.0200000	2.6400000

Unique Subject Identifier=9014000000-0007

Assay variable : PTINR_VAL1 PT-INR Value

N	Mean	Standard Deviation	Minimum	Maximum
3	2.3133333	0.1569501	2.1900000	2.4900000

Laboratory tests at the time of obtaining Table 2-6 consent, according to the presence or absence of proton-pump inhibitors

Unique Subject Identifier=9014000000-0008

MEANS Procedure

Assay variable : PTINR_VAL1 PT-INR Value

N	Mean	Standard Deviation	Minimum	Maximum
4	1.9625000	0.1302242	1.8600000	2.1400000

Unique Subject Identifier=9014000000-0010

Assay variable : PTINR_VAL1 PT-INR Value

N	Mean	Standard Deviation	Minimum	Maximum
3	2.0033333	0.3700450	1.7700000	2.4300000

Unique Subject Identifier=9014000000-0013

Assay variable : PTINR_VAL1 PT-INR Value

N	Mean	Standard Deviation	Minimum	Maximum
6	2.0133333	0.2587405	1.7200000	2.4800000

Unique Subject Identifier=9014000000-0019

Assay variable : PTINR_VAL1 PT-INR Value

N	Mean	Standard Deviation	Minimum	Maximum
5	2.3220000	0.7481110	1.5400000	3.1100000

Laboratory tests at the time of obtaining Table 2-6 consent, according to the presence or absence of proton-pump inhibitors

Unique Subject Identifier=9014000000-0021

MEANS Procedure

Assay variable : PTINR_VAL1 PT-INR Value

N	Mean	Standard Deviation	Minimum	Maximum
5	2.3020000	0.6430941	1.5200000	3.0900000

Unique Subject Identifier=9014000000-0022

Assay variable : PTINR_VAL1 PT-INR Value

N	Mean	Standard Deviation	Minimum	Maximum
6	1.7750000	0.3111752	1.3500000	2.2400000

Unique Subject Identifier=9014000000-0023

Assay variable : PTINR_VAL1 PT-INR Value

N	Mean	Standard Deviation	Minimum	Maximum
4	2.0475000	0.0981071	1.9500000	2.1700000

Unique Subject Identifier=9014000000-0025

Assay variable : PTINR_VAL1 PT-INR Value

N	Mean	Standard Deviation	Minimum	Maximum
4	1.9575000	0.2604323	1.6400000	2.2700000

Laboratory tests at the time of obtaining Table 2-6 consent, according to the presence or absence of proton-pump inhibitors

Unique Subject Identifier=9014000000-0027

MEANS Procedure

Assay variable : PTINR_VAL1 PT-INR Value

N	Mean	Standard Deviation	Minimum	Maximum
3	1.8500000	0.0692820	1.8100000	1.9300000

Unique Subject Identifier=9014000000-0028

Assay variable : PTINR_VAL1 PT-INR Value

N	Mean	Standard Deviation	Minimum	Maximum
5	2.5640000	0.8219976	1.8500000	3.8300000

Unique Subject Identifier=9014000000-0029

Assay variable : PTINR_VAL1 PT-INR Value

N	Mean	Standard Deviation	Minimum	Maximum
5	2.6740000	0.6759290	1.9800000	3.4800000

Unique Subject Identifier=9014000000-0030

Assay variable : PTINR_VAL1 PT-INR Value

N	Mean	Standard Deviation	Minimum	Maximum
5	1.8000000	0.0751665	1.7200000	1.8800000

Laboratory tests at the time of obtaining Table 2-6 consent, according to the presence or absence of proton-pump inhibitors

Unique Subject Identifier=9020011000-0001

MEANS Procedure

Assay variable : PTINR_VAL1 PT-INR Value

N	Mean	Standard Deviation	Minimum	Maximum
5	2.2440000	0.3773990	1.6800000	2.7000000

Unique Subject Identifier=9020011000-0005

Assay variable : PTINR_VAL1 PT-INR Value

N	Mean	Standard Deviation	Minimum	Maximum
3	1.5033333	0.1850225	1.2900000	1.6200000

Unique Subject Identifier=9020011000-0010

Assay variable : PTINR_VAL1 PT-INR Value

N	Mean	Standard Deviation	Minimum	Maximum
3	1.4700000	0.1126943	1.4000000	1.6000000

Unique Subject Identifier=9020011000-0028

Assay variable : PTINR_VAL1 PT-INR Value

N	Mean	Standard Deviation	Minimum	Maximum
3	1.8933333	0.2294196	1.6300000	2.0500000

Laboratory tests at the time of obtaining Table 2-6 consent, according to the presence or absence of proton-pump inhibitors

Unique Subject Identifier=9024000000-0002

MEANS Procedure

Assay variable : PTINR_VAL1 PT-INR Value

N	Mean	Standard Deviation	Minimum	Maximum
6	2.2916667	0.2705858	1.9500000	2.5700000

Unique Subject Identifier=9024000000-0005

Assay variable : PTINR_VAL1 PT-INR Value

N	Mean	Standard Deviation	Minimum	Maximum
6	1.6800000	0.4543567	1.1000000	2.2200000

Unique Subject Identifier=9024000000-0007

Assay variable : PTINR_VAL1 PT-INR Value

N	Mean	Standard Deviation	Minimum	Maximum
3	2.5966667	0.2138535	2.4100000	2.8300000

Unique Subject Identifier=9031010000-0016

Assay variable : PTINR_VAL1 PT-INR Value

N	Mean	Standard Deviation	Minimum	Maximum
3	2.0600000	0.2981610	1.8100000	2.3900000

Laboratory tests at the time of obtaining Table 2-6 consent, according to the presence or absence of proton-pump inhibitors

Unique Subject Identifier=9032011000-0005

MEANS Procedure

Assay variable : PTINR_VAL1 PT-INR Value

N	Mean	Standard Deviation	Minimum	Maximum
4	2.1000000	0.2160247	1.8000000	2.3000000

Unique Subject Identifier=9032011000-0008

Assay variable : PTINR_VAL1 PT-INR Value

N	Mean	Standard Deviation	Minimum	Maximum
4	1.8500000	0.6608076	1.4000000	2.8000000

Unique Subject Identifier=9032011000-0009

Assay variable : PTINR_VAL1 PT-INR Value

N	Mean	Standard Deviation	Minimum	Maximum
4	2.0000000	0.3162278	1.6000000	2.3000000

Unique Subject Identifier=9035000000-0005

Assay variable : PTINR_VAL1 PT-INR Value

N	Mean	Standard Deviation	Minimum	Maximum
3	2.3500000	0.1300000	2.2000000	2.4300000

Laboratory tests at the time of obtaining Table 2-6 consent, according to the presence or absence of proton-pump inhibitors

Unique Subject Identifier=9035000000-0045

MEANS Procedure

Assay variable : PTINR_VAL1 PT-INR Value

N	Mean	Standard Deviation	Minimum	Maximum
2	2.0350000	0.0212132	2.0200000	2.0500000

Unique Subject Identifier=9035000000-0058

Assay variable : PTINR_VAL1 PT-INR Value

N	Mean	Standard Deviation	Minimum	Maximum
4	2.0100000	0.5874805	1.2400000	2.6600000

Unique Subject Identifier=9035000000-0061

Assay variable : PTINR_VAL1 PT-INR Value

N	Mean	Standard Deviation	Minimum	Maximum
5	1.7380000	0.2872629	1.5100000	2.1700000

Unique Subject Identifier=9035000000-0062

Assay variable : PTINR_VAL1 PT-INR Value

N	Mean	Standard Deviation	Minimum	Maximum
3	1.8933333	0.3875994	1.5900000	2.3300000

Laboratory tests at the time of obtaining Table 2-6 consent, according to the presence or absence of proton-pump inhibitors

Unique Subject Identifier=9035000000-0069

MEANS Procedure

Assay variable : PTINR_VAL1 PT-INR Value

N	Mean	Standard Deviation	Minimum	Maximum
3	1.8800000	0.1915724	1.7500000	2.1000000

Unique Subject Identifier=9035000000-0078

Assay variable : PTINR_VAL1 PT-INR Value

N	Mean	Standard Deviation	Minimum	Maximum
3	1.7866667	0.3807011	1.4200000	2.1800000

Unique Subject Identifier=9035000000-0081

Assay variable : PTINR_VAL1 PT-INR Value

N	Mean	Standard Deviation	Minimum	Maximum
6	1.6900000	0.2090933	1.3500000	1.9500000

Unique Subject Identifier=9035000000-0084

Assay variable : PTINR_VAL1 PT-INR Value

N	Mean	Standard Deviation	Minimum	Maximum
3	1.4033333	0.1871719	1.1900000	1.5400000

Laboratory tests at the time of obtaining Table 2-6 consent, according to the presence or absence of proton-pump inhibitors

Unique Subject Identifier=9035000000-0094

MEANS Procedure

Assay variable : PTINR_VAL1 PT-INR Value

N	Mean	Standard Deviation	Minimum	Maximum
3	1.6166667	0.2956913	1.3600000	1.9400000

Unique Subject Identifier=9035000000-0095

Assay variable : PTINR_VAL1 PT-INR Value

N	Mean	Standard Deviation	Minimum	Maximum
3	1.9266667	0.0650641	1.8600000	1.9900000

Unique Subject Identifier=9035000000-0100

Assay variable : PTINR_VAL1 PT-INR Value

N	Mean	Standard Deviation	Minimum	Maximum
4	1.7525000	0.6388205	0.9800000	2.4400000

Unique Subject Identifier=9036000000-0015

Assay variable : PTINR_VAL1 PT-INR Value

N	Mean	Standard Deviation	Minimum	Maximum
3	1.9733333	0.0503322	1.9200000	2.0200000

Laboratory tests at the time of obtaining Table 2-6 consent, according to the presence or absence of proton-pump inhibitors

Unique Subject Identifier=9037000010-0003

MEANS Procedure

Assay variable : PTINR_VAL1 PT-INR Value

N	Mean	Standard Deviation	Minimum	Maximum
5	2.2560000	1.0411676	1.5800000	4.0700000

Unique Subject Identifier=9037000010-0006

Assay variable : PTINR_VAL1 PT-INR Value

N	Mean	Standard Deviation	Minimum	Maximum
2	1.8450000	0.3606245	1.5900000	2.1000000

Unique Subject Identifier=9037000010-0007

Assay variable : PTINR_VAL1 PT-INR Value

N	Mean	Standard Deviation	Minimum	Maximum
2	2.0700000	0.0424264	2.0400000	2.1000000

Unique Subject Identifier=9037000010-0009

Assay variable : PTINR_VAL1 PT-INR Value

N	Mean	Standard Deviation	Minimum	Maximum
3	2.1466667	0.8800189	1.1400000	2.7700000

Laboratory tests at the time of obtaining Table 2-6 consent, according to the presence or absence of proton-pump inhibitors

Unique Subject Identifier=9037000010-0010

MEANS Procedure

Assay variable : PTINR_VAL1 PT-INR Value

N	Mean	Standard Deviation	Minimum	Maximum
5	1.7520000	0.4846855	1.3200000	2.5800000

Unique Subject Identifier=9040011000-0003

Assay variable : PTINR_VAL1 PT-INR Value

N	Mean	Standard Deviation	Minimum	Maximum
4	1.8200000	0.0336650	1.7800000	1.8600000

Unique Subject Identifier=9047000000-0011

Assay variable : PTINR_VAL1 PT-INR Value

N	Mean	Standard Deviation	Minimum	Maximum
3	1.1000000	0.1153256	1.0100000	1.2300000

Unique Subject Identifier=9047000000-0012

Assay variable : PTINR_VAL1 PT-INR Value

N	Mean	Standard Deviation	Minimum	Maximum
4	1.5700000	0.2437212	1.4200000	1.9300000

Laboratory tests at the time of obtaining Table 2-6 consent, according to the presence or absence of proton-pump inhibitors

Unique Subject Identifier=9047000000-0013

MEANS Procedure

Assay variable : PTINR_VAL1 PT-INR Value

N	Mean	Standard Deviation	Minimum	Maximum
3	1.7666667	0.1361372	1.6600000	1.9200000

Unique Subject Identifier=9047000000-0014

Assay variable : PTINR_VAL1 PT-INR Value

N	Mean	Standard Deviation	Minimum	Maximum
4	2.3525000	0.5212405	1.6800000	2.8900000

Unique Subject Identifier=9047000000-0015

Assay variable : PTINR_VAL1 PT-INR Value

N	Mean	Standard Deviation	Minimum	Maximum
2	1.7500000	0.2404163	1.5800000	1.9200000

Unique Subject Identifier=9047000000-0017

Assay variable : PTINR_VAL1 PT-INR Value

N	Mean	Standard Deviation	Minimum	Maximum
5	1.8880000	0.1759830	1.6800000	2.0700000

Laboratory tests at the time of obtaining Table 2-6 consent, according to the presence or absence of proton-pump inhibitors

Unique Subject Identifier=9047000000-0019

MEANS Procedure

Assay variable : PTINR_VAL1 PT-INR Value

N	Mean	Standard Deviation	Minimum	Maximum
4	1.6550000	0.4173328	1.1100000	2.0200000

Unique Subject Identifier=9055000000-0001

Assay variable : PTINR_VAL1 PT-INR Value

N	Mean	Standard Deviation	Minimum	Maximum
4	1.6950000	0.2852484	1.4300000	2.1000000

Unique Subject Identifier=9055000000-0002

Assay variable : PTINR_VAL1 PT-INR Value

N	Mean	Standard Deviation	Minimum	Maximum
4	1.6700000	0.1842100	1.5000000	1.9300000

Unique Subject Identifier=9057000000-0006

Assay variable : PTINR_VAL1 PT-INR Value

N	Mean	Standard Deviation	Minimum	Maximum
3	2.0800000	0.0600000	2.0200000	2.1400000

Laboratory tests at the time of obtaining Table 2-6 consent, according to the presence or absence of proton-pump inhibitors

Unique Subject Identifier=9057000000-0007

MEANS Procedure

Assay variable : PTINR_VAL1 PT-INR Value

N	Mean	Standard Deviation	Minimum	Maximum
5	1.7260000	0.1522498	1.5600000	1.9500000

Unique Subject Identifier=9057000000-0010

Assay variable : PTINR_VAL1 PT-INR Value

N	Mean	Standard Deviation	Minimum	Maximum
3	1.8133333	0.2470493	1.5500000	2.0400000

Unique Subject Identifier=9057000000-0011

Assay variable : PTINR_VAL1 PT-INR Value

N	Mean	Standard Deviation	Minimum	Maximum
5	1.8460000	0.3114964	1.5000000	2.3300000

Unique Subject Identifier=9057000000-0014

Assay variable : PTINR_VAL1 PT-INR Value

N	Mean	Standard Deviation	Minimum	Maximum
5	2.0500000	0.2982449	1.7800000	2.5100000

Laboratory tests at the time of obtaining Table 2-6 consent, according to the presence or absence of proton-pump inhibitors

Unique Subject Identifier=9057000000-0015

MEANS Procedure

Assay variable : PTINR_VAL1 PT-INR Value

N	Mean	Standard Deviation	Minimum	Maximum
4	2.1025000	0.4014453	1.8500000	2.7000000

Unique Subject Identifier=9071010000-0003

Assay variable : PTINR_VAL1 PT-INR Value

N	Mean	Standard Deviation	Minimum	Maximum
5	1.8520000	0.3769217	1.5400000	2.4300000

Unique Subject Identifier=9071010000-0004

Assay variable : PTINR_VAL1 PT-INR Value

N	Mean	Standard Deviation	Minimum	Maximum
1	2.4800000		2.4800000	2.4800000

Unique Subject Identifier=9100001000-0004

Assay variable : PTINR_VAL1 PT-INR Value

N	Mean	Standard Deviation	Minimum	Maximum
3	2.2366667	0.1761628	2.0400000	2.3800000

Laboratory tests at the time of obtaining Table 2-6 consent, according to the presence or absence of proton-pump inhibitors

Unique Subject Identifier=9100001000-0005

MEANS Procedure

Assay variable : PTINR_VAL1 PT-INR Value

N	Mean	Standard Deviation	Minimum	Maximum
5	1.8000000	0.3383046	1.4400000	2.2900000

Unique Subject Identifier=9100001000-0006

Assay variable : PTINR_VAL1 PT-INR Value

N	Mean	Standard Deviation	Minimum	Maximum
3	1.9733333	0.0986577	1.8600000	2.0400000

Unique Subject Identifier=9100001000-0010

Assay variable : PTINR_VAL1 PT-INR Value

N	Mean	Standard Deviation	Minimum	Maximum
4	1.8100000	0.8195934	1.0700000	2.7300000

Unique Subject Identifier=9100001000-0011

Assay variable : PTINR_VAL1 PT-INR Value

N	Mean	Standard Deviation	Minimum	Maximum
4	1.8975000	0.0754431	1.7900000	1.9500000

Laboratory tests at the time of obtaining Table 2-6 consent, according to the presence or absence of proton-pump inhibitors

Unique Subject Identifier=9100001000-0012

MEANS Procedure

Assay variable : PTINR_VAL1 PT-INR Value

N	Mean	Standard Deviation	Minimum	Maximum
4	1.4100000	0.1902630	1.2100000	1.6300000

Unique Subject Identifier=9100001000-0013

Assay variable : PTINR_VAL1 PT-INR Value

N	Mean	Standard Deviation	Minimum	Maximum
3	1.9266667	0.3707200	1.5100000	2.2200000

Unique Subject Identifier=9100001000-0014

Assay variable : PTINR_VAL1 PT-INR Value

N	Mean	Standard Deviation	Minimum	Maximum
5	2.4900000	0.4428882	1.7600000	2.8500000

Unique Subject Identifier=9100001000-0015

Assay variable : PTINR_VAL1 PT-INR Value

N	Mean	Standard Deviation	Minimum	Maximum
3	1.8333333	0.7793801	1.1900000	2.7000000

Laboratory tests at the time of obtaining Table 2-6 consent, according to the presence or absence of proton-pump inhibitors

Unique Subject Identifier=9100001000-0016

MEANS Procedure

Assay variable : PTINR_VAL1 PT-INR Value

N	Mean	Standard Deviation	Minimum	Maximum
4	1.8700000	0.4128761	1.4600000	2.3500000

Unique Subject Identifier=9100001000-0019

Assay variable : PTINR_VAL1 PT-INR Value

N	Mean	Standard Deviation	Minimum	Maximum
3	1.5066667	0.2318045	1.2600000	1.7200000

Unique Subject Identifier=9100001000-0021

Assay variable : PTINR_VAL1 PT-INR Value

N	Mean	Standard Deviation	Minimum	Maximum
2	1.7300000	0.0848528	1.6700000	1.7900000

Unique Subject Identifier=9100001000-0024

Assay variable : PTINR_VAL1 PT-INR Value

N	Mean	Standard Deviation	Minimum	Maximum
3	2.0100000	0.1873499	1.8600000	2.2200000

Laboratory tests at the time of obtaining Table 2-6 consent, according to the presence or absence of proton-pump inhibitors

Unique Subject Identifier=9100001000-0026

MEANS Procedure

Assay variable : PTINR_VAL1 PT-INR Value

N	Mean	Standard Deviation	Minimum	Maximum
4	2.3650000	0.7005474	1.6800000	3.2600000

Unique Subject Identifier=9100001000-0027

Assay variable : PTINR_VAL1 PT-INR Value

N	Mean	Standard Deviation	Minimum	Maximum
4	2.6375000	2.0399244	1.4300000	5.6900000

Unique Subject Identifier=9100001000-0030

Assay variable : PTINR_VAL1 PT-INR Value

N	Mean	Standard Deviation	Minimum	Maximum
4	1.8500000	0.2499333	1.6100000	2.0900000

Unique Subject Identifier=9101010000-0001

Assay variable : PTINR_VAL1 PT-INR Value

N	Mean	Standard Deviation	Minimum	Maximum
3	2.0000000	0.3605551	1.7000000	2.4000000

Laboratory tests at the time of obtaining Table 2-6 consent, according to the presence or absence of proton-pump inhibitors

Unique Subject Identifier=9101010000-0002

MEANS Procedure

Assay variable : PTINR_VAL1 PT-INR Value

N	Mean	Standard Deviation	Minimum	Maximum
4	2.8000000	0.8602325	1.9000000	3.9000000

Unique Subject Identifier=9101010000-0003

Assay variable : PTINR_VAL1 PT-INR Value

N	Mean	Standard Deviation	Minimum	Maximum
2	1.2500000	0.0707107	1.2000000	1.3000000

Unique Subject Identifier=9101010000-0004

Assay variable : PTINR_VAL1 PT-INR Value

N	Mean	Standard Deviation	Minimum	Maximum
5	2.1400000	0.2190890	1.8000000	2.4000000

Unique Subject Identifier=9101010000-0007

Assay variable : PTINR_VAL1 PT-INR Value

N	Mean	Standard Deviation	Minimum	Maximum
2	1.6000000	0.1414214	1.5000000	1.7000000

Laboratory tests at the time of obtaining Table 2-6 consent, according to the presence or absence of proton-pump inhibitors

Unique Subject Identifier=9101010000-0008

MEANS Procedure

Assay variable : PTINR_VAL1 PT-INR Value

N	Mean	Standard Deviation	Minimum	Maximum
2	1.7000000	0.1414214	1.6000000	1.8000000

Unique Subject Identifier=9101010000-0010

Assay variable : PTINR_VAL1 PT-INR Value

N	Mean	Standard Deviation	Minimum	Maximum
3	1.5666667	0.1154701	1.5000000	1.7000000

Unique Subject Identifier=9101010000-0011

Assay variable : PTINR_VAL1 PT-INR Value

N	Mean	Standard Deviation	Minimum	Maximum
3	1.5333333	0.1154701	1.4000000	1.6000000

Unique Subject Identifier=9101010000-0013

Assay variable : PTINR_VAL1 PT-INR Value

N	Mean	Standard Deviation	Minimum	Maximum
4	2.1250000	1.3400871	1.2000000	4.1000000

Laboratory tests at the time of obtaining Table 2-6 consent, according to the presence or absence of proton-pump inhibitors

Unique Subject Identifier=9101010000-0018

MEANS Procedure

Assay variable : PTINR_VAL1 PT-INR Value

N	Mean	Standard Deviation	Minimum	Maximum
3	2.0000000	0.2000000	1.8000000	2.2000000

Unique Subject Identifier=9101010000-0021

Assay variable : PTINR_VAL1 PT-INR Value

N	Mean	Standard Deviation	Minimum	Maximum
3	1.3666667	0.1154701	1.3000000	1.5000000

Unique Subject Identifier=9101010000-0022

Assay variable : PTINR_VAL1 PT-INR Value

N	Mean	Standard Deviation	Minimum	Maximum
3	2.8000000	1.5000000	1.3000000	4.3000000

Unique Subject Identifier=9101010000-0025

Assay variable : PTINR_VAL1 PT-INR Value

N	Mean	Standard Deviation	Minimum	Maximum
3	2.1333333	0.8020806	1.3000000	2.9000000

Laboratory tests at the time of obtaining Table 2-6 consent, according to the presence or absence of proton-pump inhibitors

Unique Subject Identifier=9101010000-0028

MEANS Procedure

Assay variable : PTINR_VAL1 PT-INR Value

N	Mean	Standard Deviation	Minimum	Maximum
2	1.3000000	0.1414214	1.2000000	1.4000000

Unique Subject Identifier=9101010000-0029

Assay variable : PTINR_VAL1 PT-INR Value

N	Mean	Standard Deviation	Minimum	Maximum
3	1.6666667	0.1154701	1.6000000	1.8000000

Unique Subject Identifier=9101010000-0031

Assay variable : PTINR_VAL1 PT-INR Value

N	Mean	Standard Deviation	Minimum	Maximum
3	2.0666667	0.1527525	1.9000000	2.2000000

Unique Subject Identifier=9101010000-0032

Assay variable : PTINR_VAL1 PT-INR Value

N	Mean	Standard Deviation	Minimum	Maximum
3	2.1000000	0.2645751	1.8000000	2.3000000

Laboratory tests at the time of obtaining Table 2-6 consent, according to the presence or absence of proton-pump inhibitors

Unique Subject Identifier=9101010000-0033

MEANS Procedure

Assay variable : PTINR_VAL1 PT-INR Value

N	Mean	Standard Deviation	Minimum	Maximum
4	1.4250000	0.0957427	1.3000000	1.5000000

Unique Subject Identifier=9101010000-0037

Assay variable : PTINR_VAL1 PT-INR Value

N	Mean	Standard Deviation	Minimum	Maximum
3	1.2666667	0.2886751	1.1000000	1.6000000

Unique Subject Identifier=9101010000-0038

Assay variable : PTINR_VAL1 PT-INR Value

N	Mean	Standard Deviation	Minimum	Maximum
6	2.4000000	0.3687818	2.1000000	3.0000000

Unique Subject Identifier=9101010000-0039

Assay variable : PTINR_VAL1 PT-INR Value

N	Mean	Standard Deviation	Minimum	Maximum
2	1.9000000	0.1414214	1.8000000	2.0000000

Laboratory tests at the time of obtaining Table 2-6 consent, according to the presence or absence of proton-pump inhibitors

Unique Subject Identifier=9101010000-0040

MEANS Procedure

Assay variable : PTINR_VAL1 PT-INR Value

N	Mean	Standard Deviation	Minimum	Maximum
4	1.4750000	0.2629956	1.2000000	1.7000000

Unique Subject Identifier=9101010000-0045

Assay variable : PTINR_VAL1 PT-INR Value

N	Mean	Standard Deviation	Minimum	Maximum
3	1.0666667	0.0577350	1.0000000	1.1000000

Unique Subject Identifier=9101010000-0048

Assay variable : PTINR_VAL1 PT-INR Value

N	Mean	Standard Deviation	Minimum	Maximum
2	1.2500000	0.0707107	1.2000000	1.3000000

Unique Subject Identifier=9104000000-0002

Assay variable : PTINR_VAL1 PT-INR Value

N	Mean	Standard Deviation	Minimum	Maximum
4	2.2725000	0.3339037	2.0700000	2.7700000

Laboratory tests at the time of obtaining Table 2-6 consent, according to the presence or absence of proton-pump inhibitors

Unique Subject Identifier=9105000000-0011

MEANS Procedure

Assay variable : PTINR_VAL1 PT-INR Value

N	Mean	Standard Deviation	Minimum	Maximum
4	2.0950000	0.2757414	1.7000000	2.3300000

Unique Subject Identifier=9105000000-0016

Assay variable : PTINR_VAL1 PT-INR Value

N	Mean	Standard Deviation	Minimum	Maximum
2	2.4500000	0.2545584	2.2700000	2.6300000

Unique Subject Identifier=9105000000-0017

Assay variable : PTINR_VAL1 PT-INR Value

N	Mean	Standard Deviation	Minimum	Maximum
5	1.9320000	0.5714630	1.2000000	2.7800000

Unique Subject Identifier=9105000000-0023

Assay variable : PTINR_VAL1 PT-INR Value

N	Mean	Standard Deviation	Minimum	Maximum
3	1.8933333	0.2514624	1.7100000	2.1800000

Laboratory tests at the time of obtaining Table 2-6 consent, according to the presence or absence of proton-pump inhibitors

Unique Subject Identifier=9108011111-0005

MEANS Procedure

Assay variable : PTINR_VAL1 PT-INR Value

N	Mean	Standard Deviation	Minimum	Maximum
6	2.2700000	0.2148488	1.9700000	2.5000000

Unique Subject Identifier=9108011111-0007

Assay variable : PTINR_VAL1 PT-INR Value

N	Mean	Standard Deviation	Minimum	Maximum
3	1.9500000	0.4334743	1.6800000	2.4500000

Unique Subject Identifier=9124000000-0001

Assay variable : PTINR_VAL1 PT-INR Value

N	Mean	Standard Deviation	Minimum	Maximum
3	2.2400000	0.2357965	2.0400000	2.5000000

Unique Subject Identifier=9127000000-0003

Assay variable : PTINR_VAL1 PT-INR Value

N	Mean	Standard Deviation	Minimum	Maximum
1	1.9300000		1.9300000	1.9300000

Laboratory tests at the time of obtaining Table 2-6 consent, according to the presence or absence of proton-pump inhibitors

Unique Subject Identifier=9127000000-0010

MEANS Procedure

Assay variable : PTINR_VAL1 PT-INR Value

N	Mean	Standard Deviation	Minimum	Maximum
5	2.1300000	0.1070047	1.9700000	2.2200000

Unique Subject Identifier=9127000000-0011

Assay variable : PTINR_VAL1 PT-INR Value

N	Mean	Standard Deviation	Minimum	Maximum
5	2.0780000	0.3126020	1.7800000	2.5200000

Unique Subject Identifier=9127000000-0012

Assay variable : PTINR_VAL1 PT-INR Value

N	Mean	Standard Deviation	Minimum	Maximum
3	2.4033333	0.1001665	2.2900000	2.4800000

Unique Subject Identifier=9135000000-0008

Assay variable : PTINR_VAL1 PT-INR Value

N	Mean	Standard Deviation	Minimum	Maximum
1	2.3900000		2.3900000	2.3900000

Laboratory tests at the time of obtaining Table 2-6 consent, according to the presence or absence of proton-pump inhibitors

Unique Subject Identifier=9136010000-0003

MEANS Procedure

Assay variable : PTINR_VAL1 PT-INR Value

N	Mean	Standard Deviation	Minimum	Maximum
3	2.0733333	0.2050203	1.9500000	2.3100000

Unique Subject Identifier=9136010000-0005

Assay variable : PTINR_VAL1 PT-INR Value

N	Mean	Standard Deviation	Minimum	Maximum
4	1.8575000	0.3407223	1.5800000	2.3100000

Unique Subject Identifier=9136010000-0006

Assay variable : PTINR_VAL1 PT-INR Value

N	Mean	Standard Deviation	Minimum	Maximum
3	2.1933333	0.3382800	1.8100000	2.4500000

Unique Subject Identifier=9136010000-0008

Assay variable : PTINR_VAL1 PT-INR Value

N	Mean	Standard Deviation	Minimum	Maximum
3	1.4733333	0.2055075	1.3400000	1.7100000

Laboratory tests at the time of obtaining Table 2-6 consent, according to the presence or absence of proton-pump inhibitors

Unique Subject Identifier=9136010000-0009

MEANS Procedure

Assay variable : PTINR_VAL1 PT-INR Value

N	Mean	Standard Deviation	Minimum	Maximum
3	2.0366667	0.3555747	1.6700000	2.3800000

Unique Subject Identifier=9136010000-0010

Assay variable : PTINR_VAL1 PT-INR Value

N	Mean	Standard Deviation	Minimum	Maximum
3	1.8500000	0.2858321	1.5300000	2.0800000

Unique Subject Identifier=9136010000-0012

Assay variable : PTINR_VAL1 PT-INR Value

N	Mean	Standard Deviation	Minimum	Maximum
4	2.1550000	0.1791647	1.9600000	2.3600000

Unique Subject Identifier=9136010000-0014

Assay variable : PTINR_VAL1 PT-INR Value

N	Mean	Standard Deviation	Minimum	Maximum
5	1.7640000	0.1040673	1.6700000	1.9200000

Laboratory tests at the time of obtaining Table 2-6 consent, according to the presence or absence of proton-pump inhibitors

Unique Subject Identifier=9136010000-0015

MEANS Procedure

Assay variable : PTINR_VAL1 PT-INR Value

N	Mean	Standard Deviation	Minimum	Maximum
3	2.5133333	0.4801389	2.1000000	3.0400000

Unique Subject Identifier=9136010000-0018

Assay variable : PTINR_VAL1 PT-INR Value

N	Mean	Standard Deviation	Minimum	Maximum
3	2.4366667	0.4005413	2.0800000	2.8700000

Unique Subject Identifier=9136010000-0019

Assay variable : PTINR_VAL1 PT-INR Value

N	Mean	Standard Deviation	Minimum	Maximum
3	1.8533333	0.1662328	1.7000000	2.0300000

Unique Subject Identifier=9136010000-0025

Assay variable : PTINR_VAL1 PT-INR Value

N	Mean	Standard Deviation	Minimum	Maximum
3	2.5166667	0.1913984	2.3600000	2.7300000

Laboratory tests at the time of obtaining Table 2-6 consent, according to the presence or absence of proton-pump inhibitors

Unique Subject Identifier=9136010000-0035

MEANS Procedure

Assay variable : PTINR_VAL1 PT-INR Value

N	Mean	Standard Deviation	Minimum	Maximum
3	1.7700000	0.2264950	1.5600000	2.0100000

Unique Subject Identifier=9136010000-0036

Assay variable : PTINR_VAL1 PT-INR Value

N	Mean	Standard Deviation	Minimum	Maximum
4	2.2800000	0.2290560	2.0400000	2.5900000

Unique Subject Identifier=9136010000-0040

Assay variable : PTINR_VAL1 PT-INR Value

N	Mean	Standard Deviation	Minimum	Maximum
3	1.4800000	0.3364521	1.2200000	1.8600000

Unique Subject Identifier=9137010000-0001

Assay variable : PTINR_VAL1 PT-INR Value

N	Mean	Standard Deviation	Minimum	Maximum
4	1.8950000	0.5202884	1.1200000	2.2300000

Laboratory tests at the time of obtaining Table 2-6 consent, according to the presence or absence of proton-pump inhibitors

Unique Subject Identifier=9141000000-0001

MEANS Procedure

Assay variable : PTINR_VAL1 PT-INR Value

N	Mean	Standard Deviation	Minimum	Maximum
4	2.0550000	0.1330413	1.8600000	2.1600000

Unique Subject Identifier=9141000000-0002

Assay variable : PTINR_VAL1 PT-INR Value

N	Mean	Standard Deviation	Minimum	Maximum
5	1.9540000	0.2758260	1.5100000	2.1600000

Unique Subject Identifier=9141000000-0003

Assay variable : PTINR_VAL1 PT-INR Value

N	Mean	Standard Deviation	Minimum	Maximum
5	2.1600000	0.3426368	1.5700000	2.4600000

Unique Subject Identifier=9141000000-0004

Assay variable : PTINR_VAL1 PT-INR Value

N	Mean	Standard Deviation	Minimum	Maximum
3	1.9000000	0.1833030	1.7000000	2.0600000

Laboratory tests at the time of obtaining Table 2-6 consent, according to the presence or absence of proton-pump inhibitors

Unique Subject Identifier=9141000000-0005

MEANS Procedure

Assay variable : PTINR_VAL1 PT-INR Value

N	Mean	Standard Deviation	Minimum	Maximum
5	2.0400000	0.2370654	1.7200000	2.3800000

Unique Subject Identifier=9141000000-0006

Assay variable : PTINR_VAL1 PT-INR Value

N	Mean	Standard Deviation	Minimum	Maximum
4	1.6450000	0.1408309	1.4600000	1.8000000

Unique Subject Identifier=9141000000-0007

Assay variable : PTINR_VAL1 PT-INR Value

N	Mean	Standard Deviation	Minimum	Maximum
6	1.9016667	0.2760012	1.5000000	2.1600000

Unique Subject Identifier=9142010000-0005

Assay variable : PTINR_VAL1 PT-INR Value

N	Mean	Standard Deviation	Minimum	Maximum
3	1.7000000	0.2879236	1.5000000	2.0300000

Laboratory tests at the time of obtaining Table 2-6 consent, according to the presence or absence of proton-pump inhibitors

Unique Subject Identifier=9142010000-0009

MEANS Procedure

Assay variable : PTINR_VAL1 PT-INR Value

N	Mean	Standard Deviation	Minimum	Maximum
3	1.3900000	0.0818535	1.3200000	1.4800000

Unique Subject Identifier=9142010000-0013

Assay variable : PTINR_VAL1 PT-INR Value

N	Mean	Standard Deviation	Minimum	Maximum
2	1.7500000	0.3252691	1.5200000	1.9800000

Unique Subject Identifier=9142010000-0014

Assay variable : PTINR_VAL1 PT-INR Value

N	Mean	Standard Deviation	Minimum	Maximum
1	1.6200000		1.6200000	1.6200000

Unique Subject Identifier=9142010000-0017

Assay variable : PTINR_VAL1 PT-INR Value

N	Mean	Standard Deviation	Minimum	Maximum
1	2.0800000		2.0800000	2.0800000

Laboratory tests at the time of obtaining Table 2-6 consent, according to the presence or absence of proton-pump inhibitors

Unique Subject Identifier=9142010000-0026

MEANS Procedure

Assay variable : PTINR_VAL1 PT-INR Value

N	Mean	Standard Deviation	Minimum	Maximum
3	2.2733333	0.0351188	2.2400000	2.3100000

Unique Subject Identifier=9142010000-0027

Assay variable : PTINR_VAL1 PT-INR Value

N	Mean	Standard Deviation	Minimum	Maximum
2	2.4250000	0.4313351	2.1200000	2.7300000

Unique Subject Identifier=9142010000-0029

Assay variable : PTINR_VAL1 PT-INR Value

N	Mean	Standard Deviation	Minimum	Maximum
3	1.8100000	0.0556776	1.7600000	1.8700000

Unique Subject Identifier=9142010000-0034

Assay variable : PTINR_VAL1 PT-INR Value

N	Mean	Standard Deviation	Minimum	Maximum
1	1.3500000		1.3500000	1.3500000

Laboratory tests at the time of obtaining Table 2-6 consent, according to the presence or absence of proton-pump inhibitors

Unique Subject Identifier=9142010000-0036

MEANS Procedure

Assay variable : PTINR_VAL1 PT-INR Value

N	Mean	Standard Deviation	Minimum	Maximum
3	1.5100000	0.1646208	1.3200000	1.6100000

Unique Subject Identifier=9142010000-0037

Assay variable : PTINR_VAL1 PT-INR Value

N	Mean	Standard Deviation	Minimum	Maximum
3	2.0333333	0.4867580	1.6000000	2.5600000

Unique Subject Identifier=9148000000-0006

Assay variable : PTINR_VAL1 PT-INR Value

N	Mean	Standard Deviation	Minimum	Maximum
6	2.4450000	0.6390853	1.5200000	3.4200000

Unique Subject Identifier=9148000000-0007

Assay variable : PTINR_VAL1 PT-INR Value

N	Mean	Standard Deviation	Minimum	Maximum
5	1.9100000	0.3306055	1.4400000	2.3000000

Laboratory tests at the time of obtaining Table 2-6 consent, according to the presence or absence of proton-pump inhibitors

Unique Subject Identifier=9148000000-0010

MEANS Procedure

Assay variable : PTINR_VAL1 PT-INR Value

N	Mean	Standard Deviation	Minimum	Maximum
2	2.1450000	0.3323402	1.9100000	2.3800000

Unique Subject Identifier=9148000000-0013

Assay variable : PTINR_VAL1 PT-INR Value

N	Mean	Standard Deviation	Minimum	Maximum
4	1.6500000	0.0860233	1.5500000	1.7600000

Unique Subject Identifier=9154010000-0001

Assay variable : PTINR_VAL1 PT-INR Value

N	Mean	Standard Deviation	Minimum	Maximum
4	1.7475000	0.1175798	1.5800000	1.8500000

Unique Subject Identifier=9154010000-0005

Assay variable : PTINR_VAL1 PT-INR Value

N	Mean	Standard Deviation	Minimum	Maximum
4	2.4000000	0.1449138	2.2300000	2.5600000

Laboratory tests at the time of obtaining Table 2-6 consent, according to the presence or absence of proton-pump inhibitors

Unique Subject Identifier=9154010000-0009

MEANS Procedure

Assay variable : PTINR_VAL1 PT-INR Value

N	Mean	Standard Deviation	Minimum	Maximum
5	1.9180000	0.6155648	1.4200000	2.9800000

Unique Subject Identifier=9154010000-0011

Assay variable : PTINR_VAL1 PT-INR Value

N	Mean	Standard Deviation	Minimum	Maximum
4	2.0875000	0.3872445	1.5300000	2.3600000

Unique Subject Identifier=9154010000-0012

Assay variable : PTINR_VAL1 PT-INR Value

N	Mean	Standard Deviation	Minimum	Maximum
4	2.4750000	0.9984488	1.6500000	3.7900000

Unique Subject Identifier=9154010000-0014

Assay variable : PTINR_VAL1 PT-INR Value

N	Mean	Standard Deviation	Minimum	Maximum
4	2.0575000	0.1883923	1.8400000	2.3000000

Laboratory tests at the time of obtaining Table 2-6 consent, according to the presence or absence of proton-pump inhibitors

Unique Subject Identifier=9154010000-0015

MEANS Procedure

Assay variable : PTINR_VAL1 PT-INR Value

N	Mean	Standard Deviation	Minimum	Maximum
4	2.3175000	0.1291962	2.1800000	2.4900000

Unique Subject Identifier=9154010000-0019

Assay variable : PTINR_VAL1 PT-INR Value

N	Mean	Standard Deviation	Minimum	Maximum
6	1.9866667	0.4823553	1.2700000	2.6300000

Unique Subject Identifier=9154010000-0027

Assay variable : PTINR_VAL1 PT-INR Value

N	Mean	Standard Deviation	Minimum	Maximum
4	2.6075000	0.2267708	2.3500000	2.8600000

Unique Subject Identifier=9154010000-0028

Assay variable : PTINR_VAL1 PT-INR Value

N	Mean	Standard Deviation	Minimum	Maximum
6	1.2216667	0.0861201	1.1400000	1.3900000

Laboratory tests at the time of obtaining Table 2-6 consent, according to the presence or absence of proton-pump inhibitors

Unique Subject Identifier=9154010000-0029

MEANS Procedure

Assay variable : PTINR_VAL1 PT-INR Value

N	Mean	Standard Deviation	Minimum	Maximum
5	1.5140000	0.3269251	1.1900000	2.0000000

Unique Subject Identifier=9154010000-0030

Assay variable : PTINR_VAL1 PT-INR Value

N	Mean	Standard Deviation	Minimum	Maximum
4	2.2300000	0.0559762	2.1500000	2.2800000

Unique Subject Identifier=9164000000-0002

Assay variable : PTINR_VAL1 PT-INR Value

N	Mean	Standard Deviation	Minimum	Maximum
6	2.3750000	0.1931580	2.0500000	2.6100000

Unique Subject Identifier=9164000000-0003

Assay variable : PTINR_VAL1 PT-INR Value

N	Mean	Standard Deviation	Minimum	Maximum
6	2.1133333	0.5858214	1.2700000	2.7400000

Laboratory tests at the time of obtaining Table 2-6 consent, according to the presence or absence of proton-pump inhibitors

Unique Subject Identifier=9164000000-0005

MEANS Procedure

Assay variable : PTINR_VAL1 PT-INR Value

N	Mean	Standard Deviation	Minimum	Maximum
6	1.9916667	0.2276327	1.6500000	2.3600000

Unique Subject Identifier=9164000000-0006

Assay variable : PTINR_VAL1 PT-INR Value

N	Mean	Standard Deviation	Minimum	Maximum
3	2.4333333	0.6278800	1.9800000	3.1500000

Unique Subject Identifier=9164000000-0012

Assay variable : PTINR_VAL1 PT-INR Value

N	Mean	Standard Deviation	Minimum	Maximum
3	2.0300000	0.1212436	1.9000000	2.1400000

Unique Subject Identifier=9164000000-0013

Assay variable : PTINR_VAL1 PT-INR Value

N	Mean	Standard Deviation	Minimum	Maximum
1	1.8300000		1.8300000	1.8300000

Laboratory tests at the time of obtaining Table 2-6 consent, according to the presence or absence of proton-pump inhibitors

Unique Subject Identifier=9166000000-0005

MEANS Procedure

Assay variable : PTINR_VAL1 PT-INR Value

N	Mean	Standard Deviation	Minimum	Maximum
3	1.7233333	0.1890326	1.5100000	1.8700000

Unique Subject Identifier=9166000000-0008

Assay variable : PTINR_VAL1 PT-INR Value

N	Mean	Standard Deviation	Minimum	Maximum
3	1.8900000	0.4396590	1.5300000	2.3800000

Unique Subject Identifier=9166000000-0009

Assay variable : PTINR_VAL1 PT-INR Value

N	Mean	Standard Deviation	Minimum	Maximum
5	2.1800000	0.1788854	2.0000000	2.4100000

Unique Subject Identifier=9210000100-0007

Assay variable : PTINR_VAL1 PT-INR Value

N	Mean	Standard Deviation	Minimum	Maximum
5	2.2660000	0.4350057	1.7700000	2.7200000

Laboratory tests at the time of obtaining Table 2-6 consent, according to the presence or absence of proton-pump inhibitors

Unique Subject Identifier=9210000100-0017

MEANS Procedure

Assay variable : PTINR_VAL1 PT-INR Value

N	Mean	Standard Deviation	Minimum	Maximum
4	2.0425000	0.5600818	1.3900000	2.7300000

Unique Subject Identifier=9210000100-0027

Assay variable : PTINR_VAL1 PT-INR Value

N	Mean	Standard Deviation	Minimum	Maximum
5	1.7020000	0.1355360	1.6100000	1.9400000

Unique Subject Identifier=9210000100-0030

Assay variable : PTINR_VAL1 PT-INR Value

N	Mean	Standard Deviation	Minimum	Maximum
6	1.5966667	0.2411362	1.3400000	1.9800000

Unique Subject Identifier=9214000000-0002

Assay variable : PTINR_VAL1 PT-INR Value

N	Mean	Standard Deviation	Minimum	Maximum
1	1.7300000		1.7300000	1.7300000

Laboratory tests at the time of obtaining Table 2-6 consent, according to the presence or absence of proton-pump inhibitors

Unique Subject Identifier=9214000000-0022

MEANS Procedure

Assay variable : PTINR_VAL1 PT-INR Value

N	Mean	Standard Deviation	Minimum	Maximum
4	1.5850000	0.3219213	1.2000000	1.9700000

Unique Subject Identifier=9235000000-0002

Assay variable : PTINR_VAL1 PT-INR Value

N	Mean	Standard Deviation	Minimum	Maximum
3	1.7000000	0.1000000	1.6000000	1.8000000

Unique Subject Identifier=9235000000-0008

Assay variable : PTINR_VAL1 PT-INR Value

N	Mean	Standard Deviation	Minimum	Maximum
2	1.7250000	0.3889087	1.4500000	2.0000000

Unique Subject Identifier=9235000000-0009

Assay variable : PTINR_VAL1 PT-INR Value

N	Mean	Standard Deviation	Minimum	Maximum
3	1.5000000	0.1000000	1.4000000	1.6000000

Laboratory tests at the time of obtaining Table 2-6 consent, according to the presence or absence of proton-pump inhibitors

Unique Subject Identifier=9235000000-0012

MEANS Procedure

Assay variable : PTINR_VAL1 PT-INR Value

N	Mean	Standard Deviation	Minimum	Maximum
3	1.9000000	0.2000000	1.7000000	2.1000000

Unique Subject Identifier=9235000000-0019

Assay variable : PTINR_VAL1 PT-INR Value

N	Mean	Standard Deviation	Minimum	Maximum
2	2.1500000	0.0707107	2.1000000	2.2000000

Unique Subject Identifier=9241000000-0003

Assay variable : PTINR_VAL1 PT-INR Value

N	Mean	Standard Deviation	Minimum	Maximum
3	2.2033333	0.3728717	1.8600000	2.6000000

Unique Subject Identifier=9241000000-0005

Assay variable : PTINR_VAL1 PT-INR Value

N	Mean	Standard Deviation	Minimum	Maximum
3	1.5566667	0.0709460	1.4800000	1.6200000

Laboratory tests at the time of obtaining Table 2-6 consent, according to the presence or absence of proton-pump inhibitors

Unique Subject Identifier=9241000000-0007

MEANS Procedure

Assay variable : PTINR_VAL1 PT-INR Value

N	Mean	Standard Deviation	Minimum	Maximum
3	2.1000000	0.1587451	1.9200000	2.2200000

Unique Subject Identifier=9241000000-0011

Assay variable : PTINR_VAL1 PT-INR Value

N	Mean	Standard Deviation	Minimum	Maximum
3	1.4533333	0.2223361	1.3200000	1.7100000

Unique Subject Identifier=9241000000-0012

Assay variable : PTINR_VAL1 PT-INR Value

N	Mean	Standard Deviation	Minimum	Maximum
3	1.8133333	0.2557994	1.5200000	1.9900000

Unique Subject Identifier=9241000000-0015

Assay variable : PTINR_VAL1 PT-INR Value

N	Mean	Standard Deviation	Minimum	Maximum
3	2.1733333	0.3837100	1.8900000	2.6100000

Laboratory tests at the time of obtaining Table 2-6 consent, according to the presence or absence of proton-pump inhibitors

Unique Subject Identifier=9241000000-0017

MEANS Procedure

Assay variable : PTINR_VAL1 PT-INR Value

N	Mean	Standard Deviation	Minimum	Maximum
3	1.1666667	0.2478575	0.9900000	1.4500000

Unique Subject Identifier=9242011011-0003

Assay variable : PTINR_VAL1 PT-INR Value

N	Mean	Standard Deviation	Minimum	Maximum
6	1.1233333	0.0571548	1.0500000	1.2000000

Unique Subject Identifier=9257010000-0001

Assay variable : PTINR_VAL1 PT-INR Value

N	Mean	Standard Deviation	Minimum	Maximum
3	1.3333333	0.0305505	1.3000000	1.3600000

Unique Subject Identifier=9257010000-0002

Assay variable : PTINR_VAL1 PT-INR Value

N	Mean	Standard Deviation	Minimum	Maximum
3	1.1666667	0.1050397	1.0600000	1.2700000

Laboratory tests at the time of obtaining Table 2-6 consent, according to the presence or absence of proton-pump inhibitors

Unique Subject Identifier=9257010000-0003

MEANS Procedure

Assay variable : PTINR_VAL1 PT-INR Value

N	Mean	Standard Deviation	Minimum	Maximum
5	1.9600000	0.7876865	1.0700000	3.0600000

Unique Subject Identifier=9257010000-0004

Assay variable : PTINR_VAL1 PT-INR Value

N	Mean	Standard Deviation	Minimum	Maximum
3	1.9866667	0.1096966	1.8600000	2.0500000

Unique Subject Identifier=9257010000-0005

Assay variable : PTINR_VAL1 PT-INR Value

N	Mean	Standard Deviation	Minimum	Maximum
5	1.5140000	0.0971082	1.4000000	1.6300000

Unique Subject Identifier=9257010000-0006

Assay variable : PTINR_VAL1 PT-INR Value

N	Mean	Standard Deviation	Minimum	Maximum
3	1.5133333	0.2610236	1.2200000	1.7200000

Laboratory tests at the time of obtaining Table 2-6 consent, according to the presence or absence of proton-pump inhibitors

Unique Subject Identifier=9257010000-0007

MEANS Procedure

Assay variable : PTINR_VAL1 PT-INR Value

N	Mean	Standard Deviation	Minimum	Maximum
4	1.4750000	0.1464013	1.2700000	1.5900000

Unique Subject Identifier=9257010000-0008

Assay variable : PTINR_VAL1 PT-INR Value

N	Mean	Standard Deviation	Minimum	Maximum
4	1.4725000	0.1961929	1.2300000	1.6600000

Unique Subject Identifier=9257010000-0009

Assay variable : PTINR_VAL1 PT-INR Value

N	Mean	Standard Deviation	Minimum	Maximum
6	2.0683333	1.0299013	1.4400000	4.1400000

Unique Subject Identifier=9257010000-0010

Assay variable : PTINR_VAL1 PT-INR Value

N	Mean	Standard Deviation	Minimum	Maximum
2	2.2350000	0.3889087	1.9600000	2.5100000

Laboratory tests at the time of obtaining Table 2-6 consent, according to the presence or absence of proton-pump inhibitors

Unique Subject Identifier=9257010000-0012

MEANS Procedure

Assay variable : PTINR_VAL1 PT-INR Value

N	Mean	Standard Deviation	Minimum	Maximum
3	1.4000000	0.0435890	1.3700000	1.4500000

Unique Subject Identifier=9257010000-0013

Assay variable : PTINR_VAL1 PT-INR Value

N	Mean	Standard Deviation	Minimum	Maximum
3	1.5366667	0.1703917	1.3400000	1.6400000

Unique Subject Identifier=9257010000-0014

Assay variable : PTINR_VAL1 PT-INR Value

N	Mean	Standard Deviation	Minimum	Maximum
3	1.2100000	0.1000000	1.1100000	1.3100000

Unique Subject Identifier=9257010000-0019

Assay variable : PTINR_VAL1 PT-INR Value

N	Mean	Standard Deviation	Minimum	Maximum
5	1.4320000	0.2583989	1.1800000	1.8200000

Laboratory tests at the time of obtaining Table 2-6 consent, according to the presence or absence of proton-pump inhibitors

Unique Subject Identifier=9257010000-0020

MEANS Procedure

Assay variable : PTINR_VAL1 PT-INR Value

N	Mean	Standard Deviation	Minimum	Maximum
3	1.2266667	0.0057735	1.2200000	1.2300000

Unique Subject Identifier=9257010000-0021

Assay variable : PTINR_VAL1 PT-INR Value

N	Mean	Standard Deviation	Minimum	Maximum
4	1.5275000	0.1359841	1.4000000	1.7000000

Unique Subject Identifier=9257010000-0023

Assay variable : PTINR_VAL1 PT-INR Value

N	Mean	Standard Deviation	Minimum	Maximum
3	2.3233333	0.6915442	1.6600000	3.0400000

Unique Subject Identifier=9257010000-0025

Assay variable : PTINR_VAL1 PT-INR Value

N	Mean	Standard Deviation	Minimum	Maximum
6	1.4900000	0.5108424	0.9700000	2.3000000

Laboratory tests at the time of obtaining Table 2-6 consent, according to the presence or absence of proton-pump inhibitors

Unique Subject Identifier=9326000000-0004

MEANS Procedure

Assay variable : PTINR_VAL1 PT-INR Value

N	Mean	Standard Deviation	Minimum	Maximum
6	1.9966667	0.1367723	1.8500000	2.1600000

Unique Subject Identifier=9342010000-0002

Assay variable : PTINR_VAL1 PT-INR Value

N	Mean	Standard Deviation	Minimum	Maximum
6	1.6016667	0.1135635	1.5100000	1.7700000

Unique Subject Identifier=9345000010-0006

Assay variable : PTINR_VAL1 PT-INR Value

N	Mean	Standard Deviation	Minimum	Maximum
3	2.4233333	0.2084067	2.2400000	2.6500000

Unique Subject Identifier=9345000010-0007

Assay variable : PTINR_VAL1 PT-INR Value

N	Mean	Standard Deviation	Minimum	Maximum
1	2.1300000		2.1300000	2.1300000

Laboratory tests at the time of obtaining Table 2-6 consent, according to the presence or absence of proton-pump inhibitors

Unique Subject Identifier=9348010000-0001

MEANS Procedure

Assay variable : PTINR_VAL1 PT-INR Value

N	Mean	Standard Deviation	Minimum	Maximum
4	2.4325000	0.5248730	1.9600000	3.1700000

Unique Subject Identifier=9348010000-0002

Assay variable : PTINR_VAL1 PT-INR Value

N	Mean	Standard Deviation	Minimum	Maximum
2	1.8700000	0.1272792	1.7800000	1.9600000

Unique Subject Identifier=9348010000-0004

Assay variable : PTINR_VAL1 PT-INR Value

N	Mean	Standard Deviation	Minimum	Maximum
6	1.8600000	0.2059126	1.6200000	2.2300000

Unique Subject Identifier=9348010000-0005

Assay variable : PTINR_VAL1 PT-INR Value

N	Mean	Standard Deviation	Minimum	Maximum
3	1.7166667	0.1101514	1.6100000	1.8300000

Laboratory tests at the time of obtaining Table 2-6 consent, according to the presence or absence of proton-pump inhibitors

Unique Subject Identifier=9348010000-0007

MEANS Procedure

Assay variable : PTINR_VAL1 PT-INR Value

N	Mean	Standard Deviation	Minimum	Maximum
6	2.1533333	0.6930993	1.5300000	3.3200000

Unique Subject Identifier=9348010000-0010

Assay variable : PTINR_VAL1 PT-INR Value

N	Mean	Standard Deviation	Minimum	Maximum
3	2.4533333	0.2354428	2.2100000	2.6800000

Unique Subject Identifier=9348010000-0020

Assay variable : PTINR_VAL1 PT-INR Value

N	Mean	Standard Deviation	Minimum	Maximum
3	1.8866667	0.2367136	1.7500000	2.1600000

Unique Subject Identifier=9348010000-0028

Assay variable : PTINR_VAL1 PT-INR Value

N	Mean	Standard Deviation	Minimum	Maximum
2	1.7800000	0.2545584	1.6000000	1.9600000

Laboratory tests at the time of obtaining Table 2-6 consent, according to the presence or absence of proton-pump inhibitors

Unique Subject Identifier=9348010000-0029

MEANS Procedure

Assay variable : PTINR_VAL1 PT-INR Value

N	Mean	Standard Deviation	Minimum	Maximum
6	1.4150000	0.2147324	1.2000000	1.7300000

Unique Subject Identifier=9348010000-0033

Assay variable : PTINR_VAL1 PT-INR Value

N	Mean	Standard Deviation	Minimum	Maximum
3	1.9400000	0.2193171	1.7800000	2.1900000

Unique Subject Identifier=9348010000-0039

Assay variable : PTINR_VAL1 PT-INR Value

N	Mean	Standard Deviation	Minimum	Maximum
5	1.6740000	0.2322283	1.2700000	1.8500000

Unique Subject Identifier=9348010000-0040

Assay variable : PTINR_VAL1 PT-INR Value

N	Mean	Standard Deviation	Minimum	Maximum
4	2.0700000	0.5057008	1.4300000	2.5900000

Laboratory tests at the time of obtaining Table 2-6 consent, according to the presence or absence of proton-pump inhibitors

Unique Subject Identifier=9348010000-0046

MEANS Procedure

Assay variable : PTINR_VAL1 PT-INR Value

N	Mean	Standard Deviation	Minimum	Maximum
6	2.0550000	0.8034613	1.1700000	3.4400000

Unique Subject Identifier=9348010000-0055

Assay variable : PTINR_VAL1 PT-INR Value

N	Mean	Standard Deviation	Minimum	Maximum
3	2.1500000	0.1216553	2.0100000	2.2300000

Unique Subject Identifier=9348010000-0058

Assay variable : PTINR_VAL1 PT-INR Value

N	Mean	Standard Deviation	Minimum	Maximum
6	2.3483333	0.5566836	1.5900000	3.2400000

Unique Subject Identifier=9348010000-0063

Assay variable : PTINR_VAL1 PT-INR Value

N	Mean	Standard Deviation	Minimum	Maximum
2	1.9800000	0.2545584	1.8000000	2.1600000

Laboratory tests at the time of obtaining Table 2-6 consent, according to the presence or absence of proton-pump inhibitors

Unique Subject Identifier=9348010000-0065

MEANS Procedure

Assay variable : PTINR_VAL1 PT-INR Value

N	Mean	Standard Deviation	Minimum	Maximum
3	1.9866667	0.2112660	1.7900000	2.2100000

Unique Subject Identifier=9348010000-0072

Assay variable : PTINR_VAL1 PT-INR Value

N	Mean	Standard Deviation	Minimum	Maximum
3	2.6566667	0.1650253	2.4900000	2.8200000

Unique Subject Identifier=9348010000-0083

Assay variable : PTINR_VAL1 PT-INR Value

N	Mean	Standard Deviation	Minimum	Maximum
3	2.6333333	0.3501904	2.2900000	2.9900000

Unique Subject Identifier=9348010000-0088

Assay variable : PTINR_VAL1 PT-INR Value

N	Mean	Standard Deviation	Minimum	Maximum
3	2.2800000	0.2051828	2.0700000	2.4800000

Laboratory tests at the time of obtaining Table 2-6 consent, according to the presence or absence of proton-pump inhibitors

Unique Subject Identifier=9348010000-0093

MEANS Procedure

Assay variable : PTINR_VAL1 PT-INR Value

N	Mean	Standard Deviation	Minimum	Maximum
4	1.7250000	0.2547548	1.3900000	1.9900000

Unique Subject Identifier=9348010000-0094

Assay variable : PTINR_VAL1 PT-INR Value

N	Mean	Standard Deviation	Minimum	Maximum
3	2.0833333	0.8114391	1.5000000	3.0100000

Unique Subject Identifier=9348010000-0095

Assay variable : PTINR_VAL1 PT-INR Value

N	Mean	Standard Deviation	Minimum	Maximum
1	1.9300000		1.9300000	1.9300000

Unique Subject Identifier=9348010000-0096

Assay variable : PTINR_VAL1 PT-INR Value

N	Mean	Standard Deviation	Minimum	Maximum
4	2.4150000	0.6133243	1.8600000	3.1300000

Laboratory tests at the time of obtaining Table 2-6 consent, according to the presence or absence of proton-pump inhibitors

Unique Subject Identifier=9348010000-0097

MEANS Procedure

Assay variable : PTINR_VAL1 PT-INR Value

N	Mean	Standard Deviation	Minimum	Maximum
3	2.6066667	0.3995414	2.1600000	2.9300000

Unique Subject Identifier=9348010000-0098

Assay variable : PTINR_VAL1 PT-INR Value

N	Mean	Standard Deviation	Minimum	Maximum
4	2.4000000	0.9819369	1.6200000	3.6900000

Unique Subject Identifier=9348010000-0105

Assay variable : PTINR_VAL1 PT-INR Value

N	Mean	Standard Deviation	Minimum	Maximum
4	2.0250000	0.2706166	1.7900000	2.3800000

Unique Subject Identifier=9348010000-0114

Assay variable : PTINR_VAL1 PT-INR Value

N	Mean	Standard Deviation	Minimum	Maximum
5	1.7580000	0.2359449	1.4200000	2.0100000

Laboratory tests at the time of obtaining Table 2-6 consent, according to the presence or absence of proton-pump inhibitors

Unique Subject Identifier=9348010000-0115

MEANS Procedure

Assay variable : PTINR_VAL1 PT-INR Value

N	Mean	Standard Deviation	Minimum	Maximum
4	2.3700000	0.3962323	2.1300000	2.9600000

Unique Subject Identifier=9348010000-0117

Assay variable : PTINR_VAL1 PT-INR Value

N	Mean	Standard Deviation	Minimum	Maximum
3	1.5300000	0.3803945	1.1400000	1.9000000

Unique Subject Identifier=9348010000-0127

Assay variable : PTINR_VAL1 PT-INR Value

N	Mean	Standard Deviation	Minimum	Maximum
2	2.1100000	0.7495332	1.5800000	2.6400000

Unique Subject Identifier=9348010000-0131

Assay variable : PTINR_VAL1 PT-INR Value

N	Mean	Standard Deviation	Minimum	Maximum
3	1.1200000	0.0458258	1.0700000	1.1600000

Laboratory tests at the time of obtaining Table 2-6 consent, according to the presence or absence of proton-pump inhibitors

Unique Subject Identifier=9348010000-0132

MEANS Procedure

Assay variable : PTINR_VAL1 PT-INR Value

N	Mean	Standard Deviation	Minimum	Maximum
3	1.5533333	0.4306197	1.1100000	1.9700000

Unique Subject Identifier=9366000000-0001

Assay variable : PTINR_VAL1 PT-INR Value

N	Mean	Standard Deviation	Minimum	Maximum
6	2.3066667	0.4933829	1.6900000	2.9500000

Unique Subject Identifier=9366000000-0002

Assay variable : PTINR_VAL1 PT-INR Value

N	Mean	Standard Deviation	Minimum	Maximum
6	2.7283333	0.6265275	1.6400000	3.4200000

Unique Subject Identifier=9366000000-0003

Assay variable : PTINR_VAL1 PT-INR Value

N	Mean	Standard Deviation	Minimum	Maximum
6	2.2566667	0.7819378	1.5100000	3.7200000

Laboratory tests at the time of obtaining Table 2-6 consent, according to the presence or absence of proton-pump inhibitors

Unique Subject Identifier=9366000000-0004

MEANS Procedure

Assay variable : PTINR_VAL1 PT-INR Value

N	Mean	Standard Deviation	Minimum	Maximum
6	2.6233333	0.1745470	2.3500000	2.8300000

Unique Subject Identifier=9366000000-0006

Assay variable : PTINR_VAL1 PT-INR Value

N	Mean	Standard Deviation	Minimum	Maximum
6	1.8183333	0.8000854	1.1300000	3.2400000

Unique Subject Identifier=9514000000-0002

Assay variable : PTINR_VAL1 PT-INR Value

N	Mean	Standard Deviation	Minimum	Maximum
3	2.3766667	1.0442860	1.6300000	3.5700000

Unique Subject Identifier=9514000000-0006

Assay variable : PTINR_VAL1 PT-INR Value

N	Mean	Standard Deviation	Minimum	Maximum
3	1.7633333	0.3178574	1.5100000	2.1200000

Laboratory tests at the time of obtaining Table 2-6 consent, according to the presence or absence of proton-pump inhibitors

Unique Subject Identifier=9514000000-0007

MEANS Procedure

Assay variable : PTINR_VAL1 PT-INR Value

N	Mean	Standard Deviation	Minimum	Maximum
5	1.6980000	0.1586506	1.5200000	1.8900000

Unique Subject Identifier=9514000000-0009

Assay variable : PTINR_VAL1 PT-INR Value

N	Mean	Standard Deviation	Minimum	Maximum
4	1.5000000	0.0883176	1.4400000	1.6300000

Unique Subject Identifier=9514000000-0011

Assay variable : PTINR_VAL1 PT-INR Value

N	Mean	Standard Deviation	Minimum	Maximum
4	1.7575000	0.2359908	1.5100000	2.0600000

Unique Subject Identifier=9514000000-0016

Assay variable : PTINR_VAL1 PT-INR Value

N	Mean	Standard Deviation	Minimum	Maximum
4	1.6700000	0.3421501	1.3900000	2.1300000

Laboratory tests at the time of obtaining Table 2-6 consent, according to the presence or absence of proton-pump inhibitors

Unique Subject Identifier=9514000000-0018

MEANS Procedure

Assay variable : PTINR_VAL1 PT-INR Value

N	Mean	Standard Deviation	Minimum	Maximum
4	2.1200000	0.3594440	1.6700000	2.4100000

Unique Subject Identifier=9514000000-0021

Assay variable : PTINR_VAL1 PT-INR Value

N	Mean	Standard Deviation	Minimum	Maximum
4	2.0400000	0.1831211	1.7900000	2.2300000

Unique Subject Identifier=9514000000-0022

Assay variable : PTINR_VAL1 PT-INR Value

N	Mean	Standard Deviation	Minimum	Maximum
4	1.5775000	0.0590903	1.5300000	1.6600000

Unique Subject Identifier=9514000000-0023

Assay variable : PTINR_VAL1 PT-INR Value

N	Mean	Standard Deviation	Minimum	Maximum
4	1.5075000	0.1209339	1.4000000	1.6800000

Laboratory tests at the time of obtaining Table 2-6 consent, according to the presence or absence of proton-pump inhibitors

Unique Subject Identifier=9514000000-0026

MEANS Procedure

Assay variable : PTINR_VAL1 PT-INR Value

N	Mean	Standard Deviation	Minimum	Maximum
4	2.3200000	0.0979796	2.2000000	2.4400000

Unique Subject Identifier=9514000000-0034

Assay variable : PTINR_VAL1 PT-INR Value

N	Mean	Standard Deviation	Minimum	Maximum
4	2.1250000	0.1417745	2.0000000	2.3000000

Unique Subject Identifier=9548000000-0002

Assay variable : PTINR_VAL1 PT-INR Value

N	Mean	Standard Deviation	Minimum	Maximum
2	1.2150000	0.0212132	1.2000000	1.2300000

Unique Subject Identifier=9623100000-0003

Assay variable : PTINR_VAL1 PT-INR Value

N	Mean	Standard Deviation	Minimum	Maximum
2	1.3450000	0.1060660	1.2700000	1.4200000

Laboratory tests at the time of obtaining Table 2-6 consent, according to the presence or absence of proton-pump inhibitors

Unique Subject Identifier=9623100000-0004

MEANS Procedure

Assay variable : PTINR_VAL1 PT-INR Value

N	Mean	Standard Deviation	Minimum	Maximum
2	1.7900000	0.2687006	1.6000000	1.9800000

Unique Subject Identifier=9623100000-0005

Assay variable : PTINR_VAL1 PT-INR Value

N	Mean	Standard Deviation	Minimum	Maximum
3	1.4600000	0.1732051	1.2600000	1.5600000

Unique Subject Identifier=9623100000-0007

Assay variable : PTINR_VAL1 PT-INR Value

N	Mean	Standard Deviation	Minimum	Maximum
5	1.6000000	0.1528071	1.4400000	1.8200000

Unique Subject Identifier=9648000000-0006

Assay variable : PTINR_VAL1 PT-INR Value

N	Mean	Standard Deviation	Minimum	Maximum
3	2.6066667	1.1305898	1.8900000	3.9100000

Laboratory tests at the time of obtaining Table 2-6 consent, according to the presence or absence of proton-pump inhibitors

Unique Subject Identifier=9648000000-0012

MEANS Procedure

Assay variable : PTINR_VAL1 PT-INR Value

N	Mean	Standard Deviation	Minimum	Maximum
2	2.6800000	0.0141421	2.6700000	2.6900000

Unique Subject Identifier=9648000000-0014

Assay variable : PTINR_VAL1 PT-INR Value

N	Mean	Standard Deviation	Minimum	Maximum
2	1.7100000	0.0989949	1.6400000	1.7800000

Unique Subject Identifier=9648000000-0021

Assay variable : PTINR_VAL1 PT-INR Value

N	Mean	Standard Deviation	Minimum	Maximum
3	2.4466667	0.3758102	2.2100000	2.8800000

Unique Subject Identifier=9648000000-0023

Assay variable : PTINR_VAL1 PT-INR Value

N	Mean	Standard Deviation	Minimum	Maximum
1	1.7800000		1.7800000	1.7800000

Laboratory tests at the time of obtaining Table 2-6 consent, according to the presence or absence of proton-pump inhibitors

FREQ Procedure

Table : ALL * _PTINRCT

ALL _PTINRCT

Power |
Percent |
Percent of rows
Percentage of columns | 1| 2| 3| 4| 9| Total

	1	2	3	4	9	Total
1	1074	3266	3316	324	315	8295
	12.95	39.37	39.98	3.91	3.80	100.00
	12.95	39.37	39.98	3.91	3.80	
	100.00	100.00	100.00	100.00	100.00	
Total	1074	3266	3316	324	315	8295
	12.95	39.37	39.98	3.91	3.80	100.00

Laboratory tests at the time of obtaining Table 2-6 consent, according to the presence or absence of proton-pump inhibitors

FREQ Procedure

Table : ALL * _PTINRCT

ALL _PTINRCT

Power |
Percent |
Percent of rows
Percentage of columns | 1| 2| 3| 4| 9| Total

1	1074	3266	3316	324	315	8295
	12.95	39.37	39.98	3.91	3.80	100.00
	12.95	39.37	39.98	3.91	3.80	
	100.00	100.00	100.00	100.00	100.00	
Total	1074	3266	3316	324	315	8295
	12.95	39.37	39.98	3.91	3.80	100.00

Laboratory tests at the time of obtaining Table 2-6 consent, according to the presence or absence of proton-pump inhibitors

FREQ Procedure

Table : ALL * _PTINRCT

ALL _PTINRCT

Power |
Percent |
Percent of rows
Percentage of columns | 1| 2| 3| 4| 9| Total

	1	2	3	4	9	Total
1	1074	3266	3316	324	315	8295
	12.95	39.37	39.98	3.91	3.80	100.00
	12.95	39.37	39.98	3.91	3.80	
	100.00	100.00	100.00	100.00	100.00	
Total	1074	3266	3316	324	315	8295
	12.95	39.37	39.98	3.91	3.80	100.00

Laboratory tests at the time of obtaining Table 2-6 consent, according to the presence or absence of proton-pump inhibitors

FREQ Procedure

Table : ALL * _PTINRCT

ALL _PTINRCT

Power |
Percent |
Percent of rows
Percentage of columns | 1| 2| 3| 4| 9| Total

	1	2	3	4	9	Total
1	1074	3266	3316	324	315	8295
	12.95	39.37	39.98	3.91	3.80	100.00
	12.95	39.37	39.98	3.91	3.80	
	100.00	100.00	100.00	100.00	100.00	
Total	1074	3266	3316	324	315	8295
	12.95	39.37	39.98	3.91	3.80	100.00

Laboratory tests at the time of obtaining Table 2-6 consent, according to the presence or absence of proton-pump inhibitors

FREQ Procedure

Table : ALL * _PTINRCT

ALL _PTINRCT

Power |
Percent |
Percent of rows
Percentage of columns | 1| 2| 3| 4| 9| Total

	1	2	3	4	9	Total
1	1074	3266	3316	324	315	8295
	12.95	39.37	39.98	3.91	3.80	100.00
	12.95	39.37	39.98	3.91	3.80	
	100.00	100.00	100.00	100.00	100.00	
Total	1074	3266	3316	324	315	8295
	12.95	39.37	39.98	3.91	3.80	100.00

Laboratory tests at the time of obtaining Table 2-6 consent, according to the presence or absence of proton-pump inhibitors

FREQ Procedure

Table : ALL * _TTRCT

ALL _TTRCT

Power |
Percent |
Percent of rows
Percentage of columns | 1| 2| 3| 4| 9| Total

	1	2	3	4	9	Total
1	1043	736	1166	4003	1347	8295
	12.57	8.87	14.06	48.26	16.24	100.00
	12.57	8.87	14.06	48.26	16.24	
	100.00	100.00	100.00	100.00	100.00	
Total	1043	736	1166	4003	1347	8295
	12.57	8.87	14.06	48.26	16.24	100.00

Laboratory tests at the time of obtaining Table 2-6 consent, according to the presence or absence of proton-pump inhibitors

FREQ Procedure

Table : ALL * _TTRCT

ALL _TTRCT

Power |
Percent |
Percent of rows
Percentage of columns | 1| 2| 3| 4| 9| Total
______________+________+________+________+________+________+
1 | 1043 | 736 | 1166 | 4003 | 1347 | 8295
| 12.57 | 8.87 | 14.06 | 48.26 | 16.24 | 100.00
| 12.57 | 8.87 | 14.06 | 48.26 | 16.24 |
| 100.00 | 100.00 | 100.00 | 100.00 | 100.00 |
______________+________+________+________+________+________+
Total 1043 736 1166 4003 1347 8295
12.57 8.87 14.06 48.26 16.24 100.00

Laboratory tests at the time of obtaining Table 2-6 consent, according to the presence or absence of proton-pump inhibitors

FREQ Procedure

Table : ALL * _TTRCT

ALL _TTRCT

Power |
Percent |
Percent of rows
Percentage of columns | 1| 2| 3| 4| 9| Total

1	1043	736	1166	4003	1347	8295
	12.57	8.87	14.06	48.26	16.24	100.00
	12.57	8.87	14.06	48.26	16.24	
	100.00	100.00	100.00	100.00	100.00	
Total	1043	736	1166	4003	1347	8295
	12.57	8.87	14.06	48.26	16.24	100.00

Laboratory tests at the time of obtaining Table 2-6 consent, according to the presence or absence of proton-pump inhibitors

FREQ Procedure

Table : ALL * _TTRCT

ALL _TTRCT

Power |
Percent |
Percent of rows
Percentage of columns | 1| 2| 3| 4| 9| Total

1	1043	736	1166	4003	1347	8295
	12.57	8.87	14.06	48.26	16.24	100.00
	12.57	8.87	14.06	48.26	16.24	
	100.00	100.00	100.00	100.00	100.00	
Total	1043	736	1166	4003	1347	8295
	12.57	8.87	14.06	48.26	16.24	100.00

Laboratory tests at the time of obtaining Table 2-6 consent, according to the presence or absence of proton-pump inhibitors

FREQ Procedure

Table : ALL * _TTRCT

ALL _TTRCT

Power<br>Percent<br>Percent of rows<br>Percentage of columns	1	2	3	4	9	Total
1	1043<br>12.57<br>12.57<br>100.00	736<br>8.87<br>8.87<br>100.00	1166<br>14.06<br>14.06<br>100.00	4003<br>48.26<br>48.26<br>100.00	1347<br>16.24<br>16.24<br>100.00	8295<br>100.00
Total	1043<br>12.57	736<br>8.87	1166<br>14.06	4003<br>48.26	1347<br>16.24	8295<br>100.00

Laboratory tests at the time of obtaining Table 2-6 consent, according to the presence or absence of proton-pump inhibitors

FREQ Procedure

Table : ALL * _FIRCT

ALL _FIRCT

Power |
Percent |
Percent of rows
Percentage of columns | 1| 2| 3| 4| 9| Total

1	1033	823	1621	3517	1301	8295
	12.45	9.92	19.54	42.40	15.68	100.00
	12.45	9.92	19.54	42.40	15.68	
	100.00	100.00	100.00	100.00	100.00	
Total	1033	823	1621	3517	1301	8295
	12.45	9.92	19.54	42.40	15.68	100.00

Laboratory tests at the time of obtaining Table 2-6 consent, according to the presence or absence of proton-pump inhibitors

FREQ Procedure

Table : ALL * _FIRCT

ALL _FIRCT

Power |
Percent |
Percent of rows
Percentage of columns | 1| 2| 3| 4| 9| Total

	1	2	3	4	9	Total
1	1033	823	1621	3517	1301	8295
	12.45	9.92	19.54	42.40	15.68	100.00
	12.45	9.92	19.54	42.40	15.68	
	100.00	100.00	100.00	100.00	100.00	
Total	1033	823	1621	3517	1301	8295
	12.45	9.92	19.54	42.40	15.68	100.00

Laboratory tests at the time of obtaining Table 2-6 consent, according to the presence or absence of proton-pump inhibitors

FREQ Procedure

Table : ALL * _FIRCT

ALL _FIRCT

Power |
Percent |
Percent of rows
Percentage of columns | 1| 2| 3| 4| 9| Total

	1	2	3	4	9	Total
1	1033	823	1621	3517	1301	8295
	12.45	9.92	19.54	42.40	15.68	100.00
	12.45	9.92	19.54	42.40	15.68	
	100.00	100.00	100.00	100.00	100.00	
Total	1033	823	1621	3517	1301	8295
	12.45	9.92	19.54	42.40	15.68	100.00

Laboratory tests at the time of obtaining Table 2-6 consent, according to the presence or absence of proton-pump inhibitors

FREQ Procedure

Table : ALL * _FIRCT

ALL _FIRCT

Power |
Percent |
Percent of rows
Percentage of columns | 1| 2| 3| 4| 9| Total

1	1033	823	1621	3517	1301	8295
	12.45	9.92	19.54	42.40	15.68	100.00
	12.45	9.92	19.54	42.40	15.68	
	100.00	100.00	100.00	100.00	100.00	
Total	1033	823	1621	3517	1301	8295
	12.45	9.92	19.54	42.40	15.68	100.00

Laboratory tests at the time of obtaining Table 2-6 consent, according to the presence or absence of proton-pump inhibitors

FREQ Procedure

Table : ALL * _FIRCT

ALL _FIRCT

Power |
Percent |
Percent of rows
Percentage of columns | 1| 2| 3| 4| 9| Total

1	1033	823	1621	3517	1301	8295
	12.45	9.92	19.54	42.40	15.68	100.00
	12.45	9.92	19.54	42.40	15.68	
	100.00	100.00	100.00	100.00	100.00	
Total	1033	823	1621	3517	1301	8295
	12.45	9.92	19.54	42.40	15.68	100.00

Laboratory tests at the time of obtaining Table 2-6 consent, according to the presence or absence of proton-pump inhibitors

FREQ Procedure

Table : ALL * t_CRCC1

ALL t_CRCC1

Power |
Percent |
Percent of rows
Percentage of columns | 1| 2| 3| 4| 5| Total

	1	2	3	4	5	Total
1	423	3506	10833	10525	1117	26404
	1.60	13.28	41.03	39.86	4.23	100.00
	1.60	13.28	41.03	39.86	4.23	
	100.00	100.00	100.00	100.00	100.00	
Total	423	3506	10833	10525	1117	26404
	1.60	13.28	41.03	39.86	4.23	100.00

Degree of missing values = 6086

Laboratory tests at the time of obtaining Table 2-6 consent, according to the presence or absence of proton-pump inhibitors

FREQ Procedure

Table : ALL * t_CRCC1

ALL t_CRCC1

Power |
Percent |
Percent of rows
Percentage of columns | 1| 2| 3| 4| 5| Total

1	423	3506	10833	10525	1117	26404
	1.60	13.28	41.03	39.86	4.23	100.00
	1.60	13.28	41.03	39.86	4.23	
	100.00	100.00	100.00	100.00	100.00	
Total	423	3506	10833	10525	1117	26404
	1.60	13.28	41.03	39.86	4.23	100.00

Degree of missing values = 6086

Laboratory tests at the time of obtaining Table 2-6 consent, according to the presence or absence of proton-pump inhibitors

FREQ Procedure

Table : ALL * t_CRCC1

ALL t_CRCC1

Power / Percent / Percent of rows / Percentage of columns	1	2	3	4	5	Total
1	423	3506	10833	10525	1117	26404
	1.60	13.28	41.03	39.86	4.23	100.00
	1.60	13.28	41.03	39.86	4.23	
	100.00	100.00	100.00	100.00	100.00	
Total	423	3506	10833	10525	1117	26404
	1.60	13.28	41.03	39.86	4.23	100.00

Degree of missing values = 6086

Laboratory tests at the time of obtaining Table 2-6 consent, according to the presence or absence of proton-pump inhibitors

FREQ Procedure

Table : ALL * t_CRCC1

ALL t_CRCC1

Power |
Percent |
Percent of rows
Percentage of columns | 1| 2| 3| 4| 5| Total

	1	2	3	4	5	Total
1	423	3506	10833	10525	1117	26404
	1.60	13.28	41.03	39.86	4.23	100.00
	1.60	13.28	41.03	39.86	4.23	
	100.00	100.00	100.00	100.00	100.00	
Total	423	3506	10833	10525	1117	26404
	1.60	13.28	41.03	39.86	4.23	100.00

Degree of missing values = 6086

Laboratory tests at the time of obtaining Table 2-6 consent, according to the presence or absence of proton-pump inhibitors

FREQ Procedure

Table : ALL * t_CRCC1

ALL t_CRCC1

Power |
Percent |
Percent of rows
Percentage of columns | 1| 2| 3| 4| 5| Total

	1	2	3	4	5	Total
1	423	3506	10833	10525	1117	26404
	1.60	13.28	41.03	39.86	4.23	100.00
	1.60	13.28	41.03	39.86	4.23	
	100.00	100.00	100.00	100.00	100.00	
Total	423	3506	10833	10525	1117	26404
	1.60	13.28	41.03	39.86	4.23	100.00

Degree of missing values = 6086

Laboratory tests at the time of obtaining Table 2-6 consent, according to the presence or absence of proton-pump inhibitors

FREQ Procedure

Table : ALL * non_CRCC1

ALL non_CRCC1

Power |
Percent |
Percent of rows
Percent of columns | 1| Total
______________+________+
1 | 6086 | 6086
| 100.00 | 100.00
| 100.00 |
| 100.00 |
______________+________+
Total 6086 6086
100.00 100.00

Degree of missing values = 26404

Laboratory tests at the time of obtaining Table 2-6 consent, according to the presence or absence of proton-pump inhibitors

FREQ Procedure

Table : ALL * t_EGFR1

ALL t_EGFR1

Power |
Percent |
Percent of rows
Percentage of columns | 1| 2| 3| 4| 5| 6| Total

	1	2	3	4	5	6	Total
1	276	1923	6383	10204	9237	575	28598
	0.97	6.72	22.32	35.68	32.30	2.01	100.00
	0.97	6.72	22.32	35.68	32.30	2.01	
	100.00	100.00	100.00	100.00	100.00	100.00	
Total	276	1923	6383	10204	9237	575	28598
	0.97	6.72	22.32	35.68	32.30	2.01	100.00

Degree of missing values = 3892

Laboratory tests at the time of obtaining Table 2-6 consent, according to the presence or absence of proton-pump inhibitors

FREQ Procedure

Table : ALL * t_EGFR1

ALL t_EGFR1

Power |
Percent |
Percent of rows
Percentage of columns | 1| 2| 3| 4| 5| 6| Total

	1	2	3	4	5	6	Total
1	276	1923	6383	10204	9237	575	28598
	0.97	6.72	22.32	35.68	32.30	2.01	100.00
	0.97	6.72	22.32	35.68	32.30	2.01	
	100.00	100.00	100.00	100.00	100.00	100.00	
Total	276	1923	6383	10204	9237	575	28598
	0.97	6.72	22.32	35.68	32.30	2.01	100.00

Degree of missing values = 3892

Laboratory tests at the time of obtaining Table 2-6 consent, according to the presence or absence of proton-pump inhibitors

FREQ Procedure

Table : ALL * t_EGFR1

ALL t_EGFR1

Power |
Percent |
Percent of rows
Percentage of columns | 1| 2| 3| 4| 5| 6| Total

	1	2	3	4	5	6	Total
1	276	1923	6383	10204	9237	575	28598
	0.97	6.72	22.32	35.68	32.30	2.01	100.00
	0.97	6.72	22.32	35.68	32.30	2.01	
	100.00	100.00	100.00	100.00	100.00	100.00	
Total	276	1923	6383	10204	9237	575	28598
	0.97	6.72	22.32	35.68	32.30	2.01	100.00

Degree of missing values = 3892

Laboratory tests at the time of obtaining Table 2-6 consent, according to the presence or absence of proton-pump inhibitors

FREQ Procedure

Table : ALL * t_EGFR1

ALL t_EGFR1

Power |
Percent |
Percent of rows
Percentage of columns | 1| 2| 3| 4| 5| 6| Total

1	276	1923	6383	10204	9237	575	28598
	0.97	6.72	22.32	35.68	32.30	2.01	100.00
	0.97	6.72	22.32	35.68	32.30	2.01	
	100.00	100.00	100.00	100.00	100.00	100.00	
Total	276	1923	6383	10204	9237	575	28598
	0.97	6.72	22.32	35.68	32.30	2.01	100.00

Degree of missing values = 3892

Laboratory tests at the time of obtaining Table 2-6 consent, according to the presence or absence of proton-pump inhibitors

FREQ Procedure

Table : ALL * t_EGFR1

ALL t_EGFR1

Power |
Percent |
Percent of rows
Percentage of columns | 1| 2| 3| 4| 5| 6| Total

	1	2	3	4	5	6	Total
1	276	1923	6383	10204	9237	575	28598
	0.97	6.72	22.32	35.68	32.30	2.01	100.00
	0.97	6.72	22.32	35.68	32.30	2.01	
	100.00	100.00	100.00	100.00	100.00	100.00	
Total	276	1923	6383	10204	9237	575	28598
	0.97	6.72	22.32	35.68	32.30	2.01	100.00

Degree of missing values = 3892

Laboratory tests at the time of obtaining Table 2-6 consent, according to the presence or absence of proton-pump inhibitors

FREQ Procedure

Table : ALL * t_EGFR1

ALL t_EGFR1

Power |
Percent |
Percent of rows
Percentage of columns | 1| 2| 3| 4| 5| 6| Total

	1	2	3	4	5	6	Total
1	276	1923	6383	10204	9237	575	28598
	0.97	6.72	22.32	35.68	32.30	2.01	100.00
	0.97	6.72	22.32	35.68	32.30	2.01	
	100.00	100.00	100.00	100.00	100.00	100.00	
Total	276	1923	6383	10204	9237	575	28598
	0.97	6.72	22.32	35.68	32.30	2.01	100.00

Degree of missing values = 3892

Laboratory tests at the time of obtaining Table 2-6 consent, according to the presence or absence of proton-pump inhibitors

FREQ Procedure

Table : ALL * non_EGFR1

ALL non_EGFR1

Power |
Percent |
Percent of rows
Percent of columns | 1| Total
______________+________+
1 | 3892 | 3892
| 100.00 | 100.00
| 100.00 |
| 100.00 |
______________+________+
Total 3892 3892
100.00 100.00

Frequency of missing values = 28598

Laboratory tests at the time of obtaining Table 2-6 consent, according to the presence or absence of proton-pump inhibitors

FREQ Procedure

Table : ALL * HBAC_BL

ALL HBAC_BL(Category of Hemoglobin A1c at Baseline)

Power / Percent / Percent of rows / Column Percent	<6.0%	6.0% More than < 7.0%	7.0% More than < 8.0%	8.0%	Missing	Total
1	8796	6754	1565	556	14819	32490
	27.07	20.79	4.82	1.71	45.61	100.00
	27.07	20.79	4.82	1.71	45.61	
	100.00	100.00	100.00	100.00	100.00	
Total	8796	6754	1565	556	14819	32490
	27.07	20.79	4.82	1.71	45.61	100.00

Laboratory tests at the time of obtaining Table 2-6 consent, according to the presence or absence of proton-pump inhibitors

FREQ Procedure

Table : ALL * HBAC_BL

ALL HBAC_BL(Category of Hemoglobin A1c at Baseline)

Power / Percent / Percent of rows / Column Percent	<6.0%	6.0% < 7.0%	More than 7.0% < 8.0%	More than 8.0%	Missing	Total
1	8796	6754	1565	556	14819	32490
	27.07	20.79	4.82	1.71	45.61	100.00
	27.07	20.79	4.82	1.71	45.61	
	100.00	100.00	100.00	100.00	100.00	
Total	8796	6754	1565	556	14819	32490
	27.07	20.79	4.82	1.71	45.61	100.00

Laboratory tests at the time of obtaining Table 2-6 consent, according to the presence or absence of proton-pump inhibitors

FREQ Procedure

Table : ALL * HBAC_BL

ALL HBAC_BL(Category of Hemoglobin A1c at Baseline)

Power / Percent / Percent of rows / Column Percent	<6.0%	6.0% < 7.0%	More than 7.0% < 8.0%	More than 8.0%	Missing	Total
1	8796	6754	1565	556	14819	32490
	27.07	20.79	4.82	1.71	45.61	100.00
	27.07	20.79	4.82	1.71	45.61	
	100.00	100.00	100.00	100.00	100.00	
Total	8796	6754	1565	556	14819	32490
	27.07	20.79	4.82	1.71	45.61	100.00

Laboratory tests at the time of obtaining Table 2-6 consent, according to the presence or absence of proton-pump inhibitors

FREQ Procedure

Table : ALL * HBAC_BL

ALL HBAC_BL(Category of Hemoglobin A1c at Baseline)

Power |
Percent |
Percent of rows
Column Percent|<6.0%|6.0%|More than |7.0%|More than |8.0%|Missing|Total
| | < 7.0%| < 8.0%| | |

	<6.0%	6.0% < 7.0%	7.0% < 8.0%	More than 8.0%	Missing	Total
1	8796	6754	1565	556	14819	32490
	27.07	20.79	4.82	1.71	45.61	100.00
	27.07	20.79	4.82	1.71	45.61	
	100.00	100.00	100.00	100.00	100.00	
Total	8796	6754	1565	556	14819	32490
	27.07	20.79	4.82	1.71	45.61	100.00

Laboratory tests at the time of obtaining Table 2-6 consent, according to the presence or absence of proton-pump inhibitors

FREQ Procedure

Table : ALL * HBAC_BL

ALL HBAC_BL(Category of Hemoglobin A1c at Baseline)

Power<br>Percent<br>Percent of rows<br>Column Percent	<6.0%	6.0%<br>< 7.0%	More than 7.0%<br>< 8.0%	More than 8.0%	Missing	Total
1	8796<br>27.07<br>27.07<br>100.00	6754<br>20.79<br>20.79<br>100.00	1565<br>4.82<br>4.82<br>100.00	556<br>1.71<br>1.71<br>100.00	14819<br>45.61<br>45.61<br>100.00	32490<br>100.00
Total	8796<br>27.07	6754<br>20.79	1565<br>4.82	556<br>1.71	14819<br>45.61	32490<br>100.00

Laboratory tests at the time of obtaining Table 2-6 consent, according to the presence or absence of proton-pump inhibitors

FREQ Procedure

Cumulative

Percent of ALL power Percent of power

1	11981	100.00	11981	100.00

Laboratory tests at the time of obtaining Table 2-6 consent, according to the presence or absence of proton-pump inhibitors

FREQ Procedure

Tables: ALL* ACTYP

ALL
ACTYP(Type of AF)

Power |
Percent |
Percent of rows
Percent of column|Warfa| Total
|Phosphate|
| |
______________+________+
1 | 2955 | 2955
| 100.00 | 100.00
| 100.00 |
| 100.00 |
______________+________+
Total 2955 2955
100.00 100.00

Laboratory tests at the time of obtaining Table 2-6 consent, according to the presence or absence of proton-pump inhibitors

Unique Subject Identifier=0001110010-0003

MEANS Procedure

Assay variable : PTINR_VAL1 PT-INR Value

N	Mean	Standard Deviation	Minimum	Maximum
3	2.0366667	0.2542309	1.7600000	2.2600000

Unique Subject Identifier=0001110010-0004

Assay variable : PTINR_VAL1 PT-INR Value

N	Mean	Standard Deviation	Minimum	Maximum
4	2.7500000	0.3389199	2.3000000	3.0700000

Unique Subject Identifier=0001110010-0015

Assay variable : PTINR_VAL1 PT-INR Value

N	Mean	Standard Deviation	Minimum	Maximum
3	2.0866667	0.1006645	1.9800000	2.1800000

Unique Subject Identifier=0001110010-0032

Assay variable : PTINR_VAL1 PT-INR Value

N	Mean	Standard Deviation	Minimum	Maximum
4	2.3300000	0.4302712	1.8600000	2.7700000

Laboratory tests at the time of obtaining Table 2-6 consent, according to the presence or absence of proton-pump inhibitors

Unique Subject Identifier=0004110010-0010

MEANS Procedure

Assay variable : PTINR_VAL1 PT-INR Value

N	Mean	Standard Deviation	Minimum	Maximum
6	1.8166667	0.2108712	1.5500000	2.0100000

Unique Subject Identifier=0004110010-0026

Assay variable : PTINR_VAL1 PT-INR Value

N	Mean	Standard Deviation	Minimum	Maximum
6	1.8650000	0.2438647	1.4900000	2.1800000

Unique Subject Identifier=0004110010-0031

Assay variable : PTINR_VAL1 PT-INR Value

N	Mean	Standard Deviation	Minimum	Maximum
4	2.1100000	0.1602082	1.9300000	2.3000000

Unique Subject Identifier=0004110010-0042

Assay variable : PTINR_VAL1 PT-INR Value

N	Mean	Standard Deviation	Minimum	Maximum
4	1.7100000	0.1927001	1.4800000	1.9100000

Laboratory tests at the time of obtaining Table 2-6 consent, according to the presence or absence of proton-pump inhibitors

Unique Subject Identifier=0004110010-0047

MEANS Procedure

Assay variable : PTINR_VAL1 PT-INR Value

N	Mean	Standard Deviation	Minimum	Maximum
3	1.8500000	0.3874274	1.4900000	2.2600000

Unique Subject Identifier=0005010000-0003

Assay variable : PTINR_VAL1 PT-INR Value

N	Mean	Standard Deviation	Minimum	Maximum
6	1.9066667	0.4583739	1.3500000	2.6800000

Unique Subject Identifier=0005010000-0007

Assay variable : PTINR_VAL1 PT-INR Value

N	Mean	Standard Deviation	Minimum	Maximum
3	1.9800000	0.4900000	1.4900000	2.4700000

Unique Subject Identifier=0005010000-0020

Assay variable : PTINR_VAL1 PT-INR Value

N	Mean	Standard Deviation	Minimum	Maximum
3	1.8400000	0.1216553	1.7000000	1.9200000

Laboratory tests at the time of obtaining Table 2-6 consent, according to the presence or absence of proton-pump inhibitors

Unique Subject Identifier=0005010000-0022

MEANS Procedure

Assay variable : PTINR_VAL1 PT-INR Value

N	Mean	Standard Deviation	Minimum	Maximum
6	2.2233333	0.7559012	1.6600000	3.6100000

Unique Subject Identifier=0005010000-0034

Assay variable : PTINR_VAL1 PT-INR Value

N	Mean	Standard Deviation	Minimum	Maximum
6	1.2433333	0.0997330	1.1400000	1.4000000

Unique Subject Identifier=0005010000-0050

Assay variable : PTINR_VAL1 PT-INR Value

N	Mean	Standard Deviation	Minimum	Maximum
6	1.7866667	0.2182353	1.5600000	2.1200000

Unique Subject Identifier=0005010000-0052

Assay variable : PTINR_VAL1 PT-INR Value

N	Mean	Standard Deviation	Minimum	Maximum
2	1.0900000	0.0707107	1.0400000	1.1400000

Laboratory tests at the time of obtaining Table 2-6 consent, according to the presence or absence of proton-pump inhibitors

Unique Subject Identifier=0005010000-0053

MEANS Procedure

Assay variable : PTINR_VAL1 PT-INR Value

N	Mean	Standard Deviation	Minimum	Maximum
6	1.9983333	0.3748822	1.4700000	2.5400000

Unique Subject Identifier=0005010000-0058

Assay variable : PTINR_VAL1 PT-INR Value

N	Mean	Standard Deviation	Minimum	Maximum
2	1.6950000	0.1484924	1.5900000	1.8000000

Unique Subject Identifier=0006100000-0006

Assay variable : PTINR_VAL1 PT-INR Value

N	Mean	Standard Deviation	Minimum	Maximum
3	2.3633333	0.2098412	2.2000000	2.6000000

Unique Subject Identifier=0006100000-0016

Assay variable : PTINR_VAL1 PT-INR Value

N	Mean	Standard Deviation	Minimum	Maximum
3	1.9400000	1.0544667	1.0900000	3.1200000

Laboratory tests at the time of obtaining Table 2-6 consent, according to the presence or absence of proton-pump inhibitors

Unique Subject Identifier=0006100000-0054

MEANS Procedure

Assay variable : PTINR_VAL1 PT-INR Value

N	Mean	Standard Deviation	Minimum	Maximum
5	1.7740000	0.1986957	1.5800000	2.0200000

Unique Subject Identifier=0006100000-0056

Assay variable : PTINR_VAL1 PT-INR Value

N	Mean	Standard Deviation	Minimum	Maximum
3	1.7166667	0.0986577	1.6500000	1.8300000

Unique Subject Identifier=0006100000-0062

Assay variable : PTINR_VAL1 PT-INR Value

N	Mean	Standard Deviation	Minimum	Maximum
6	1.9066667	0.3507516	1.7100000	2.6200000

Unique Subject Identifier=0006100000-0064

Assay variable : PTINR_VAL1 PT-INR Value

N	Mean	Standard Deviation	Minimum	Maximum
4	1.7800000	0.1888562	1.5600000	1.9700000

Laboratory tests at the time of obtaining Table 2-6 consent, according to the presence or absence of proton-pump inhibitors

Unique Subject Identifier=0006100000-0065

MEANS Procedure

Assay variable : PTINR_VAL1 PT-INR Value

N	Mean	Standard Deviation	Minimum	Maximum
6	1.8983333	1.0817470	1.2700000	4.0900000

Unique Subject Identifier=0006100000-0068

Assay variable : PTINR_VAL1 PT-INR Value

N	Mean	Standard Deviation	Minimum	Maximum
1	1.7700000		1.7700000	1.7700000

Unique Subject Identifier=0006100000-0069

Assay variable : PTINR_VAL1 PT-INR Value

N	Mean	Standard Deviation	Minimum	Maximum
4	2.0075000	0.4908751	1.7100000	2.7400000

Unique Subject Identifier=0007011000-0001

Assay variable : PTINR_VAL1 PT-INR Value

N	Mean	Standard Deviation	Minimum	Maximum
3	1.8966667	0.2950141	1.6000000	2.1900000

Laboratory tests at the time of obtaining Table 2-6 consent, according to the presence or absence of proton-pump inhibitors

Unique Subject Identifier=0007011000-0002

MEANS Procedure

Assay variable : PTINR_VAL1 PT-INR Value

N	Mean	Standard Deviation	Minimum	Maximum
3	1.9133333	0.8891194	1.4000000	2.9400000

Unique Subject Identifier=0007011000-0005

Assay variable : PTINR_VAL1 PT-INR Value

N	Mean	Standard Deviation	Minimum	Maximum
5	2.0900000	0.3537655	1.7300000	2.4700000

Unique Subject Identifier=0007011000-0022

Assay variable : PTINR_VAL1 PT-INR Value

N	Mean	Standard Deviation	Minimum	Maximum
6	2.0483333	0.5314665	1.3000000	2.8300000

Unique Subject Identifier=0007011000-0043

Assay variable : PTINR_VAL1 PT-INR Value

N	Mean	Standard Deviation	Minimum	Maximum
3	1.6100000	0.1014889	1.5000000	1.7000000

Laboratory tests at the time of obtaining Table 2-6 consent, according to the presence or absence of proton-pump inhibitors

Unique Subject Identifier=0007011000-0078

MEANS Procedure

Assay variable : PTINR_VAL1 PT-INR Value

N	Mean	Standard Deviation	Minimum	Maximum
4	1.7925000	0.1004573	1.7000000	1.9300000

Unique Subject Identifier=0007011000-0095

Assay variable : PTINR_VAL1 PT-INR Value

N	Mean	Standard Deviation	Minimum	Maximum
5	1.1160000	0.0545894	1.0500000	1.1800000

Unique Subject Identifier=0007011000-0097

Assay variable : PTINR_VAL1 PT-INR Value

N	Mean	Standard Deviation	Minimum	Maximum
6	1.6150000	0.1943965	1.3700000	1.9600000

Unique Subject Identifier=0007011000-0102

Assay variable : PTINR_VAL1 PT-INR Value

N	Mean	Standard Deviation	Minimum	Maximum
1	1.6600000		1.6600000	1.6600000

Laboratory tests at the time of obtaining Table 2-6 consent, according to the presence or absence of proton-pump inhibitors

Unique Subject Identifier=0007011000-0112

MEANS Procedure

Assay variable : PTINR_VAL1 PT-INR Value

N	Mean	Standard Deviation	Minimum	Maximum
3	2.1133333	0.5084617	1.8000000	2.7000000

Unique Subject Identifier=0007011000-0118

Assay variable : PTINR_VAL1 PT-INR Value

N	Mean	Standard Deviation	Minimum	Maximum
3	1.5900000	0.3000000	1.2900000	1.8900000

Unique Subject Identifier=0007011000-0120

Assay variable : PTINR_VAL1 PT-INR Value

N	Mean	Standard Deviation	Minimum	Maximum
3	1.9666667	0.1527525	1.8000000	2.1000000

Unique Subject Identifier=0007011000-0122

Assay variable : PTINR_VAL1 PT-INR Value

N	Mean	Standard Deviation	Minimum	Maximum
6	1.7816667	0.3644402	1.2000000	2.2000000

Laboratory tests at the time of obtaining Table 2-6 consent, according to the presence or absence of proton-pump inhibitors

Unique Subject Identifier=0007011000-0135

MEANS Procedure

Assay variable : PTINR_VAL1 PT-INR Value

N	Mean	Standard Deviation	Minimum	Maximum
5	2.1000000	0.4484417	1.5700000	2.8100000

Unique Subject Identifier=0007011000-0137

Assay variable : PTINR_VAL1 PT-INR Value

N	Mean	Standard Deviation	Minimum	Maximum
3	1.3900000	0.0781025	1.3400000	1.4800000

Unique Subject Identifier=0008000110-0001

Assay variable : PTINR_VAL1 PT-INR Value

N	Mean	Standard Deviation	Minimum	Maximum
1	2.2800000		2.2800000	2.2800000

Unique Subject Identifier=0009010000-0013

Assay variable : PTINR_VAL1 PT-INR Value

N	Mean	Standard Deviation	Minimum	Maximum
3	2.4933333	0.3326159	2.2400000	2.8700000

Laboratory tests at the time of obtaining Table 2-6 consent, according to the presence or absence of proton-pump inhibitors

Unique Subject Identifier=0009010000-0041

MEANS Procedure

Assay variable : PTINR_VAL1 PT-INR Value

N	Mean	Standard Deviation	Minimum	Maximum
3	1.5933333	0.0723418	1.5100000	1.6400000

Unique Subject Identifier=0009010000-0043

Assay variable : PTINR_VAL1 PT-INR Value

N	Mean	Standard Deviation	Minimum	Maximum
2	2.5200000	0.4525483	2.2000000	2.8400000

Unique Subject Identifier=0009010000-0044

Assay variable : PTINR_VAL1 PT-INR Value

N	Mean	Standard Deviation	Minimum	Maximum
2	2.7550000	0.4737615	2.4200000	3.0900000

Unique Subject Identifier=0009010000-0072

Assay variable : PTINR_VAL1 PT-INR Value

N	Mean	Standard Deviation	Minimum	Maximum
6	2.0783333	0.4169612	1.6500000	2.8700000

Laboratory tests at the time of obtaining Table 2-6 consent, according to the presence or absence of proton-pump inhibitors

Unique Subject Identifier=0009010000-0085

MEANS Procedure

Assay variable : PTINR_VAL1 PT-INR Value

N	Mean	Standard Deviation	Minimum	Maximum
3	2.0700000	0.2946184	1.8100000	2.3900000

Unique Subject Identifier=0009010000-0087

Assay variable : PTINR_VAL1 PT-INR Value

N	Mean	Standard Deviation	Minimum	Maximum
5	1.4800000	0.0930054	1.3800000	1.6100000

Unique Subject Identifier=0009010000-0090

Assay variable : PTINR_VAL1 PT-INR Value

N	Mean	Standard Deviation	Minimum	Maximum
5	1.8860000	0.4982269	1.2900000	2.5700000

Unique Subject Identifier=0009010000-0112

Assay variable : PTINR_VAL1 PT-INR Value

N	Mean	Standard Deviation	Minimum	Maximum
3	1.8366667	0.5005330	1.3500000	2.3500000

Laboratory tests at the time of obtaining Table 2-6 consent, according to the presence or absence of proton-pump inhibitors

Unique Subject Identifier=0009010000-0114

MEANS Procedure

Assay variable : PTINR_VAL1 PT-INR Value

N	Mean	Standard Deviation	Minimum	Maximum
3	2.2966667	0.6668083	1.6600000	2.9900000

Unique Subject Identifier=0009010000-0130

Assay variable : PTINR_VAL1 PT-INR Value

N	Mean	Standard Deviation	Minimum	Maximum
4	1.2725000	0.2150000	1.0400000	1.5600000

Unique Subject Identifier=0009010000-0137

Assay variable : PTINR_VAL1 PT-INR Value

N	Mean	Standard Deviation	Minimum	Maximum
5	1.6860000	0.3005495	1.2200000	1.9700000

Unique Subject Identifier=0009010000-0149

Assay variable : PTINR_VAL1 PT-INR Value

N	Mean	Standard Deviation	Minimum	Maximum
4	2.3825000	0.2696139	2.0200000	2.6600000

Laboratory tests at the time of obtaining Table 2-6 consent, according to the presence or absence of proton-pump inhibitors

Unique Subject Identifier=0009010000-0178

MEANS Procedure

Assay variable : PTINR_VAL1 PT-INR Value

N	Mean	Standard Deviation	Minimum	Maximum
2	1.9800000	0.3959798	1.7000000	2.2600000

Unique Subject Identifier=0009010000-0223

Assay variable : PTINR_VAL1 PT-INR Value

N	Mean	Standard Deviation	Minimum	Maximum
6	2.1433333	0.2331237	1.8100000	2.4600000

Unique Subject Identifier=0009010000-0239

Assay variable : PTINR_VAL1 PT-INR Value

N	Mean	Standard Deviation	Minimum	Maximum
3	1.6366667	0.0493288	1.5800000	1.6700000

Unique Subject Identifier=0009010000-0246

Assay variable : PTINR_VAL1 PT-INR Value

N	Mean	Standard Deviation	Minimum	Maximum
4	2.5500000	0.3987480	1.9700000	2.8800000

Laboratory tests at the time of obtaining Table 2-6 consent, according to the presence or absence of proton-pump inhibitors

Unique Subject Identifier=0009010000-0254

MEANS Procedure

Assay variable : PTINR_VAL1 PT-INR Value

N	Mean	Standard Deviation	Minimum	Maximum
2	1.3400000	0.1131371	1.2600000	1.4200000

Unique Subject Identifier=0016011100-0001

Assay variable : PTINR_VAL1 PT-INR Value

N	Mean	Standard Deviation	Minimum	Maximum
2	2.2550000	0.0070711	2.2500000	2.2600000

Unique Subject Identifier=0017010000-0003

Assay variable : PTINR_VAL1 PT-INR Value

N	Mean	Standard Deviation	Minimum	Maximum
4	2.1775000	0.1350000	1.9900000	2.2900000

Unique Subject Identifier=0017010000-0015

Assay variable : PTINR_VAL1 PT-INR Value

N	Mean	Standard Deviation	Minimum	Maximum
4	2.0900000	0.2738613	1.8500000	2.4600000

Laboratory tests at the time of obtaining Table 2-6 consent, according to the presence or absence of proton-pump inhibitors

Unique Subject Identifier=0017010000-0028

MEANS Procedure

Assay variable : PTINR_VAL1 PT-INR Value

N	Mean	Standard Deviation	Minimum	Maximum
4	2.5500000	0.5575542	1.7700000	3.0400000

Unique Subject Identifier=0017010000-0038

Assay variable : PTINR_VAL1 PT-INR Value

N	Mean	Standard Deviation	Minimum	Maximum
3	2.1200000	0.1228821	1.9800000	2.2100000

Unique Subject Identifier=0017010000-0071

Assay variable : PTINR_VAL1 PT-INR Value

N	Mean	Standard Deviation	Minimum	Maximum
3	1.9066667	0.0472582	1.8700000	1.9600000

Unique Subject Identifier=0017010000-0082

Assay variable : PTINR_VAL1 PT-INR Value

N	Mean	Standard Deviation	Minimum	Maximum
3	2.3800000	0.7171471	1.7700000	3.1700000

Laboratory tests at the time of obtaining Table 2-6 consent, according to the presence or absence of proton-pump inhibitors

Unique Subject Identifier=0019010000-0026

MEANS Procedure

Assay variable : PTINR_VAL1 PT-INR Value

N	Mean	Standard Deviation	Minimum	Maximum
4	1.8475000	0.2385197	1.5600000	2.1400000

Unique Subject Identifier=0019010000-0031

Assay variable : PTINR_VAL1 PT-INR Value

N	Mean	Standard Deviation	Minimum	Maximum
2	1.6300000	0.0989949	1.5600000	1.7000000

Unique Subject Identifier=0019010000-0041

Assay variable : PTINR_VAL1 PT-INR Value

N	Mean	Standard Deviation	Minimum	Maximum
3	2.2533333	0.2742870	1.9400000	2.4500000

Unique Subject Identifier=0019010000-0043

Assay variable : PTINR_VAL1 PT-INR Value

N	Mean	Standard Deviation	Minimum	Maximum
2	2.2850000	0.0636396	2.2400000	2.3300000

Laboratory tests at the time of obtaining Table 2-6 consent, according to the presence or absence of proton-pump inhibitors

Unique Subject Identifier=0019010000-0057

MEANS Procedure

Assay variable : PTINR_VAL1 PT-INR Value

N	Mean	Standard Deviation	Minimum	Maximum
3	1.8700000	0.0173205	1.8600000	1.8900000

Unique Subject Identifier=0019010000-0086

Assay variable : PTINR_VAL1 PT-INR Value

N	Mean	Standard Deviation	Minimum	Maximum
3	2.5833333	0.4772141	2.0500000	2.9700000

Unique Subject Identifier=0019010000-0097

Assay variable : PTINR_VAL1 PT-INR Value

N	Mean	Standard Deviation	Minimum	Maximum
3	2.3900000	0.0888819	2.3200000	2.4900000

Unique Subject Identifier=0019010000-0112

Assay variable : PTINR_VAL1 PT-INR Value

N	Mean	Standard Deviation	Minimum	Maximum
3	2.9333333	1.3891124	1.4100000	4.1300000

Laboratory tests at the time of obtaining Table 2-6 consent, according to the presence or absence of proton-pump inhibitors

Unique Subject Identifier=0021000000-0009

MEANS Procedure

Assay variable : PTINR_VAL1 PT-INR Value

N	Mean	Standard Deviation	Minimum	Maximum
6	2.1216667	0.1859480	1.9100000	2.3800000

Unique Subject Identifier=0021000000-0012

Assay variable : PTINR_VAL1 PT-INR Value

N	Mean	Standard Deviation	Minimum	Maximum
3	1.7833333	0.1365040	1.6900000	1.9400000

Unique Subject Identifier=0021000000-0017

Assay variable : PTINR_VAL1 PT-INR Value

N	Mean	Standard Deviation	Minimum	Maximum
6	1.6866667	0.1303329	1.6000000	1.9500000

Unique Subject Identifier=0021000000-0027

Assay variable : PTINR_VAL1 PT-INR Value

N	Mean	Standard Deviation	Minimum	Maximum
5	2.2300000	0.2109502	1.9200000	2.4600000

Laboratory tests at the time of obtaining Table 2-6 consent, according to the presence or absence of proton-pump inhibitors

Unique Subject Identifier=0027000000-0022

MEANS Procedure

Assay variable : PTINR_VAL1 PT-INR Value

N	Mean	Standard Deviation	Minimum	Maximum
3	1.9033333	0.1656301	1.7300000	2.0600000

Unique Subject Identifier=0030010010-0012

Assay variable : PTINR_VAL1 PT-INR Value

N	Mean	Standard Deviation	Minimum	Maximum
6	1.7750000	0.1925357	1.4900000	2.0500000

Unique Subject Identifier=0031110001-0007

Assay variable : PTINR_VAL1 PT-INR Value

N	Mean	Standard Deviation	Minimum	Maximum
3	2.6366667	0.3496188	2.4200000	3.0400000

Unique Subject Identifier=0031110001-0023

Assay variable : PTINR_VAL1 PT-INR Value

N	Mean	Standard Deviation	Minimum	Maximum
3	2.1600000	0.0781025	2.0700000	2.2100000

Laboratory tests at the time of obtaining Table 2-6 consent, according to the presence or absence of proton-pump inhibitors

Unique Subject Identifier=0031110001-0025

MEANS Procedure

Assay variable : PTINR_VAL1 PT-INR Value

N	Mean	Standard Deviation	Minimum	Maximum
3	2.2066667	0.2615977	1.9300000	2.4500000

Unique Subject Identifier=0032110000-0008

Assay variable : PTINR_VAL1 PT-INR Value

N	Mean	Standard Deviation	Minimum	Maximum
6	1.3133333	0.1148332	1.1300000	1.4200000

Unique Subject Identifier=0038111111-0011

Assay variable : PTINR_VAL1 PT-INR Value

N	Mean	Standard Deviation	Minimum	Maximum
6	1.5683333	0.1190658	1.4300000	1.7500000

Unique Subject Identifier=0038111111-0019

Assay variable : PTINR_VAL1 PT-INR Value

N	Mean	Standard Deviation	Minimum	Maximum
5	2.0840000	0.3230015	1.7300000	2.4800000

Laboratory tests at the time of obtaining Table 2-6 consent, according to the presence or absence of proton-pump inhibitors

Unique Subject Identifier=0038111111-0022

MEANS Procedure

Assay variable : PTINR_VAL1 PT-INR Value

N	Mean	Standard Deviation	Minimum	Maximum
6	2.3133333	0.6361027	1.2200000	3.1000000

Unique Subject Identifier=0041110000-0011

Assay variable : PTINR_VAL1 PT-INR Value

N	Mean	Standard Deviation	Minimum	Maximum
2	1.9750000	0.3889087	1.7000000	2.2500000

Unique Subject Identifier=0042100010-0003

Assay variable : PTINR_VAL1 PT-INR Value

N	Mean	Standard Deviation	Minimum	Maximum
5	1.8320000	0.2375289	1.5900000	2.1300000

Unique Subject Identifier=0042100010-0006

Assay variable : PTINR_VAL1 PT-INR Value

N	Mean	Standard Deviation	Minimum	Maximum
6	2.2916667	0.3726884	1.9300000	2.8300000

Laboratory tests at the time of obtaining Table 2-6 consent, according to the presence or absence of proton-pump inhibitors

Unique Subject Identifier=0043010010-0001

MEANS Procedure

Assay variable : PTINR_VAL1 PT-INR Value

N	Mean	Standard Deviation	Minimum	Maximum
4	1.8025000	0.2833578	1.4100000	2.0200000

Unique Subject Identifier=0043010010-0003

Assay variable : PTINR_VAL1 PT-INR Value

N	Mean	Standard Deviation	Minimum	Maximum
2	1.4700000	0.0565685	1.4300000	1.5100000

Unique Subject Identifier=0043010010-0007

Assay variable : PTINR_VAL1 PT-INR Value

N	Mean	Standard Deviation	Minimum	Maximum
5	2.0780000	0.7081102	1.4600000	3.2300000

Unique Subject Identifier=0046100001-0002

Assay variable : PTINR_VAL1 PT-INR Value

N	Mean	Standard Deviation	Minimum	Maximum
6	2.2733333	0.2268627	2.0200000	2.6100000

Laboratory tests at the time of obtaining Table 2-6 consent, according to the presence or absence of proton-pump inhibitors

Unique Subject Identifier=0046100001-0003

MEANS Procedure

Assay variable : PTINR_VAL1 PT-INR Value

N	Mean	Standard Deviation	Minimum	Maximum
4	2.0625000	0.3096638	1.7700000	2.3400000

Unique Subject Identifier=0046100001-0011

Assay variable : PTINR_VAL1 PT-INR Value

N	Mean	Standard Deviation	Minimum	Maximum
6	1.7933333	0.5809877	1.1400000	2.7400000

Unique Subject Identifier=0046100001-0022

Assay variable : PTINR_VAL1 PT-INR Value

N	Mean	Standard Deviation	Minimum	Maximum
6	3.1633333	1.9392335	1.1800000	6.4300000

Unique Subject Identifier=0046100001-0027

Assay variable : PTINR_VAL1 PT-INR Value

N	Mean	Standard Deviation	Minimum	Maximum
6	1.8350000	0.4404884	1.3100000	2.4700000

Laboratory tests at the time of obtaining Table 2-6 consent, according to the presence or absence of proton-pump inhibitors

Unique Subject Identifier=0046100001-0047

MEANS Procedure

Assay variable : PTINR_VAL1 PT-INR Value

N	Mean	Standard Deviation	Minimum	Maximum
3	1.6033333	0.1365040	1.4800000	1.7500000

Unique Subject Identifier=0046100001-0049

Assay variable : PTINR_VAL1 PT-INR Value

N	Mean	Standard Deviation	Minimum	Maximum
3	1.7633333	0.3695042	1.5500000	2.1900000

Unique Subject Identifier=0046100001-0066

Assay variable : PTINR_VAL1 PT-INR Value

N	Mean	Standard Deviation	Minimum	Maximum
2	2.2950000	0.4879037	1.9500000	2.6400000

Unique Subject Identifier=0046100001-0073

Assay variable : PTINR_VAL1 PT-INR Value

N	Mean	Standard Deviation	Minimum	Maximum
4	1.2925000	0.0750000	1.2300000	1.3800000

Laboratory tests at the time of obtaining Table 2-6 consent, according to the presence or absence of proton-pump inhibitors

Unique Subject Identifier=0046100001-0082

MEANS Procedure

Assay variable : PTINR_VAL1 PT-INR Value

N	Mean	Standard Deviation	Minimum	Maximum
1	2.3100000		2.3100000	2.3100000

Unique Subject Identifier=0048000010-0003

Assay variable : PTINR_VAL1 PT-INR Value

N	Mean	Standard Deviation	Minimum	Maximum
3	1.6566667	0.2050203	1.4500000	1.8600000

Unique Subject Identifier=0049000010-0005

Assay variable : PTINR_VAL1 PT-INR Value

N	Mean	Standard Deviation	Minimum	Maximum
6	2.2850000	0.2167718	2.0400000	2.5900000

Unique Subject Identifier=0049000010-0006

Assay variable : PTINR_VAL1 PT-INR Value

N	Mean	Standard Deviation	Minimum	Maximum
5	1.9750000	0.6737025	1.3400000	3.1000000

Laboratory tests at the time of obtaining Table 2-6 consent, according to the presence or absence of proton-pump inhibitors

Unique Subject Identifier=0049000010-0008

MEANS Procedure

Assay variable : PTINR_VAL1 PT-INR Value

N	Mean	Standard Deviation	Minimum	Maximum
5	1.9020000	0.0957601	1.7700000	2.0200000

Unique Subject Identifier=0049000010-0011

Assay variable : PTINR_VAL1 PT-INR Value

N	Mean	Standard Deviation	Minimum	Maximum
4	1.8350000	0.2335951	1.6200000	2.1200000

Unique Subject Identifier=0049000010-0015

Assay variable : PTINR_VAL1 PT-INR Value

N	Mean	Standard Deviation	Minimum	Maximum
6	1.8416667	0.4710166	1.4100000	2.7300000

Unique Subject Identifier=0054010000-0020

Assay variable : PTINR_VAL1 PT-INR Value

N	Mean	Standard Deviation	Minimum	Maximum
1	2.2300000		2.2300000	2.2300000

Laboratory tests at the time of obtaining Table 2-6 consent, according to the presence or absence of proton-pump inhibitors

Unique Subject Identifier=0054010000-0027

MEANS Procedure

Assay variable : PTINR_VAL1 PT-INR Value

N	Mean	Standard Deviation	Minimum	Maximum
3	1.7700000	0.1646208	1.6700000	1.9600000

Unique Subject Identifier=0055100000-0001

Assay variable : PTINR_VAL1 PT-INR Value

N	Mean	Standard Deviation	Minimum	Maximum
4	1.8175000	0.0754431	1.7700000	1.9300000

Unique Subject Identifier=0059100000-0003

Assay variable : PTINR_VAL1 PT-INR Value

N	Mean	Standard Deviation	Minimum	Maximum
6	1.7716667	0.1251266	1.6400000	1.9700000

Unique Subject Identifier=0059100000-0005

Assay variable : PTINR_VAL1 PT-INR Value

N	Mean	Standard Deviation	Minimum	Maximum
5	1.5380000	0.2161481	1.3400000	1.8400000

Laboratory tests at the time of obtaining Table 2-6 consent, according to the presence or absence of proton-pump inhibitors

Unique Subject Identifier=0061000000-0005

MEANS Procedure

Assay variable : PTINR_VAL1 PT-INR Value

N	Mean	Standard Deviation	Minimum	Maximum
3	2.1266667	0.4099187	1.8900000	2.6000000

Unique Subject Identifier=0061000000-0018

Assay variable : PTINR_VAL1 PT-INR Value

N	Mean	Standard Deviation	Minimum	Maximum
1	1.0800000		1.0800000	1.0800000

Unique Subject Identifier=0061000000-0019

Assay variable : PTINR_VAL1 PT-INR Value

N	Mean	Standard Deviation	Minimum	Maximum
2	2.4500000	0.9616652	1.7700000	3.1300000

Unique Subject Identifier=0063010000-0029

Assay variable : PTINR_VAL1 PT-INR Value

N	Mean	Standard Deviation	Minimum	Maximum
3	1.9566667	0.2571640	1.7700000	2.2500000

Laboratory tests at the time of obtaining Table 2-6 consent, according to the presence or absence of proton-pump inhibitors

Unique Subject Identifier=0063010000-0041

MEANS Procedure

Assay variable : PTINR_VAL1 PT-INR Value

N	Mean	Standard Deviation	Minimum	Maximum
2	1.7050000	0.5586144	1.3100000	2.1000000

Unique Subject Identifier=0063010000-0044

Assay variable : PTINR_VAL1 PT-INR Value

N	Mean	Standard Deviation	Minimum	Maximum
3	2.4366667	0.3066486	2.1900000	2.7800000

Unique Subject Identifier=0063110000-0004

Assay variable : PTINR_VAL1 PT-INR Value

N	Mean	Standard Deviation	Minimum	Maximum
6	1.8750000	0.0531977	1.8300000	1.9800000

Unique Subject Identifier=0063110000-0005

Assay variable : PTINR_VAL1 PT-INR Value

N	Mean	Standard Deviation	Minimum	Maximum
5	2.7820000	0.5848248	1.9100000	3.3800000

Laboratory tests at the time of obtaining Table 2-6 consent, according to the presence or absence of proton-pump inhibitors

Unique Subject Identifier=0063110000-0007

MEANS Procedure

Assay variable : PTINR_VAL1 PT-INR Value

N	Mean	Standard Deviation	Minimum	Maximum
2	1.8050000	0.0919239	1.7400000	1.8700000

Unique Subject Identifier=0063110000-0012

Assay variable : PTINR_VAL1 PT-INR Value

N	Mean	Standard Deviation	Minimum	Maximum
5	1.9900000	0.9109336	1.2200000	3.5600000

Unique Subject Identifier=0063110000-0015

Assay variable : PTINR_VAL1 PT-INR Value

N	Mean	Standard Deviation	Minimum	Maximum
4	2.0775000	1.0203717	1.3600000	3.5900000

Unique Subject Identifier=0064010110-0011

Assay variable : PTINR_VAL1 PT-INR Value

N	Mean	Standard Deviation	Minimum	Maximum
6	1.7750000	0.1013410	1.6100000	1.9100000

Laboratory tests at the time of obtaining Table 2-6 consent, according to the presence or absence of proton-pump inhibitors

Unique Subject Identifier=0069000000-0003

MEANS Procedure

Assay variable : PTINR_VAL1 PT-INR Value

N	Mean	Standard Deviation	Minimum	Maximum
6	1.8816667	0.5136698	1.5700000	2.9000000

Unique Subject Identifier=0069000000-0014

Assay variable : PTINR_VAL1 PT-INR Value

N	Mean	Standard Deviation	Minimum	Maximum
6	3.1800000	2.1056116	1.4200000	6.8400000

Unique Subject Identifier=0070000000-0013

Assay variable : PTINR_VAL1 PT-INR Value

N	Mean	Standard Deviation	Minimum	Maximum
3	2.1933333	0.2554082	1.9900000	2.4800000

Unique Subject Identifier=0070000000-0026

Assay variable : PTINR_VAL1 PT-INR Value

N	Mean	Standard Deviation	Minimum	Maximum
6	2.6550000	0.1619568	2.4700000	2.8500000

Laboratory tests at the time of obtaining Table 2-6 consent, according to the presence or absence of proton-pump inhibitors

Unique Subject Identifier=0070000000-0028

MEANS Procedure

Assay variable : PTINR_VAL1 PT-INR Value

N	Mean	Standard Deviation	Minimum	Maximum
3	1.8166667	0.2800595	1.6500000	2.1400000

Unique Subject Identifier=0070000000-0029

Assay variable : PTINR_VAL1 PT-INR Value

N	Mean	Standard Deviation	Minimum	Maximum
4	2.5775000	0.5146763	2.1100000	3.2100000

Unique Subject Identifier=0070000000-0031

Assay variable : PTINR_VAL1 PT-INR Value

N	Mean	Standard Deviation	Minimum	Maximum
3	1.7400000	0.1967232	1.5300000	1.9200000

Unique Subject Identifier=0070000000-0033

Assay variable : PTINR_VAL1 PT-INR Value

N	Mean	Standard Deviation	Minimum	Maximum
3	1.8100000	0.0655744	1.7500000	1.8800000

Laboratory tests at the time of obtaining Table 2-6 consent, according to the presence or absence of proton-pump inhibitors

Unique Subject Identifier=0070000000-0043

MEANS Procedure

Assay variable : PTINR_VAL1 PT-INR Value

N	Mean	Standard Deviation	Minimum	Maximum
4	1.7825000	0.1664081	1.5700000	1.9700000

Unique Subject Identifier=0070000000-0050

Assay variable : PTINR_VAL1 PT-INR Value

N	Mean	Standard Deviation	Minimum	Maximum
6	2.2550000	1.2352935	1.4300000	4.7300000

Unique Subject Identifier=0070000000-0054

Assay variable : PTINR_VAL1 PT-INR Value

N	Mean	Standard Deviation	Minimum	Maximum
5	2.2600000	0.3611094	1.8900000	2.6900000

Unique Subject Identifier=0070000000-0058

Assay variable : PTINR_VAL1 PT-INR Value

N	Mean	Standard Deviation	Minimum	Maximum
3	2.2500000	0.3811824	2.0200000	2.6900000

Laboratory tests at the time of obtaining Table 2-6 consent, according to the presence or absence of proton-pump inhibitors

Unique Subject Identifier=0070000000-0067

MEANS Procedure

Assay variable : PTINR_VAL1 PT-INR Value

N	Mean	Standard Deviation	Minimum	Maximum
4	2.3400000	0.0496655	2.2900000	2.4000000

Unique Subject Identifier=0071000000-0002

Assay variable : PTINR_VAL1 PT-INR Value

N	Mean	Standard Deviation	Minimum	Maximum
1	2.1300000		2.1300000	2.1300000

Unique Subject Identifier=0071000000-0005

Assay variable : PTINR_VAL1 PT-INR Value

N	Mean	Standard Deviation	Minimum	Maximum
6	1.9083333	0.1915637	1.7200000	2.2000000

Unique Subject Identifier=0071000000-0008

Assay variable : PTINR_VAL1 PT-INR Value

N	Mean	Standard Deviation	Minimum	Maximum
3	1.7433333	0.1556706	1.5800000	1.8900000

Laboratory tests at the time of obtaining Table 2-6 consent, according to the presence or absence of proton-pump inhibitors

Unique Subject Identifier=0071000000-0010

MEANS Procedure

Assay variable : PTINR_VAL1 PT-INR Value

N	Mean	Standard Deviation	Minimum	Maximum
4	1.8125000	0.2233644	1.5500000	2.0700000

Unique Subject Identifier=0071000000-0016

Assay variable : PTINR_VAL1 PT-INR Value

N	Mean	Standard Deviation	Minimum	Maximum
1	2.0500000		2.0500000	2.0500000

Unique Subject Identifier=0071000000-0019

Assay variable : PTINR_VAL1 PT-INR Value

N	Mean	Standard Deviation	Minimum	Maximum
5	1.9220000	0.0683374	1.8700000	2.0400000

Unique Subject Identifier=0071000000-0020

Assay variable : PTINR_VAL1 PT-INR Value

N	Mean	Standard Deviation	Minimum	Maximum
4	2.4150000	0.4956813	2.0700000	3.1500000

Laboratory tests at the time of obtaining Table 2-6 consent, according to the presence or absence of proton-pump inhibitors

Unique Subject Identifier=0071000000-0027

MEANS Procedure

Assay variable : PTINR_VAL1 PT-INR Value

N	Mean	Standard Deviation	Minimum	Maximum
4	1.5950000	0.3139533	1.2600000	1.9100000

Unique Subject Identifier=0072000000-0004

Assay variable : PTINR_VAL1 PT-INR Value

N	Mean	Standard Deviation	Minimum	Maximum
6	1.8583333	0.3816499	1.4300000	2.2900000

Unique Subject Identifier=0072000000-0011

Assay variable : PTINR_VAL1 PT-INR Value

N	Mean	Standard Deviation	Minimum	Maximum
6	1.6583333	0.2003414	1.4000000	2.0200000

Unique Subject Identifier=0076011000-0005

Assay variable : PTINR_VAL1 PT-INR Value

N	Mean	Standard Deviation	Minimum	Maximum
5	1.8240000	1.0294319	1.0100000	3.5100000

Laboratory tests at the time of obtaining Table 2-6 consent, according to the presence or absence of proton-pump inhibitors

Unique Subject Identifier=0076011000-0015

MEANS Procedure

Assay variable : PTINR_VAL1 PT-INR Value

N	Mean	Standard Deviation	Minimum	Maximum
2	1.8200000	0.1131371	1.7400000	1.9000000

Unique Subject Identifier=0076011000-0017

Assay variable : PTINR_VAL1 PT-INR Value

N	Mean	Standard Deviation	Minimum	Maximum
4	1.2225000	0.0330404	1.2000000	1.2700000

Unique Subject Identifier=0077000000-0004

Assay variable : PTINR_VAL1 PT-INR Value

N	Mean	Standard Deviation	Minimum	Maximum
4	2.1100000	1.0274564	1.0300000	3.4900000

Unique Subject Identifier=0078001000-0005

Assay variable : PTINR_VAL1 PT-INR Value

N	Mean	Standard Deviation	Minimum	Maximum
3	2.1133333	0.2804164	1.8700000	2.4200000

Laboratory tests at the time of obtaining Table 2-6 consent, according to the presence or absence of proton-pump inhibitors

Unique Subject Identifier=0078001000-0016

MEANS Procedure

Assay variable : PTINR_VAL1 PT-INR Value

N	Mean	Standard Deviation	Minimum	Maximum
4	2.2425000	1.1359101	1.5000000	3.9200000

Unique Subject Identifier=0079000000-0002

Assay variable : PTINR_VAL1 PT-INR Value

N	Mean	Standard Deviation	Minimum	Maximum
3	1.9466667	0.2103172	1.7300000	2.1500000

Unique Subject Identifier=0079000000-0009

Assay variable : PTINR_VAL1 PT-INR Value

N	Mean	Standard Deviation	Minimum	Maximum
3	1.7866667	0.0850490	1.6900000	1.8500000

Unique Subject Identifier=0079000000-0020

Assay variable : PTINR_VAL1 PT-INR Value

N	Mean	Standard Deviation	Minimum	Maximum
4	1.8125000	0.2628529	1.4700000	2.0600000

Laboratory tests at the time of obtaining Table 2-6 consent, according to the presence or absence of proton-pump inhibitors

Unique Subject Identifier=0079000000-0029

MEANS Procedure

Assay variable : PTINR_VAL1 PT-INR Value

N	Mean	Standard Deviation	Minimum	Maximum
3	1.9366667	0.5179125	1.3400000	2.2700000

Unique Subject Identifier=0081000000-0005

Assay variable : PTINR_VAL1 PT-INR Value

N	Mean	Standard Deviation	Minimum	Maximum
4	1.6250000	0.2651415	1.3800000	2.0000000

Unique Subject Identifier=0081000000-0006

Assay variable : PTINR_VAL1 PT-INR Value

N	Mean	Standard Deviation	Minimum	Maximum
3	1.6066667	0.2400694	1.4300000	1.8800000

Unique Subject Identifier=0081000000-0010

Assay variable : PTINR_VAL1 PT-INR Value

N	Mean	Standard Deviation	Minimum	Maximum
4	2.0850000	0.1552417	1.8900000	2.2700000

Laboratory tests at the time of obtaining Table 2-6 consent, according to the presence or absence of proton-pump inhibitors

Unique Subject Identifier=0081000000-0011

MEANS Procedure

Assay variable : PTINR_VAL1 PT-INR Value

N	Mean	Standard Deviation	Minimum	Maximum
6	2.3900000	0.7361522	1.3800000	3.6200000

Unique Subject Identifier=0085000000-0007

Assay variable : PTINR_VAL1 PT-INR Value

N	Mean	Standard Deviation	Minimum	Maximum
6	1.8800000	0.5933633	1.2700000	2.9200000

Unique Subject Identifier=0086100000-0010

Assay variable : PTINR_VAL1 PT-INR Value

N	Mean	Standard Deviation	Minimum	Maximum
1	2.5800000		2.5800000	2.5800000

Unique Subject Identifier=0086100000-0019

Assay variable : PTINR_VAL1 PT-INR Value

N	Mean	Standard Deviation	Minimum	Maximum
4	1.6750000	0.2030599	1.4400000	1.8900000

Laboratory tests at the time of obtaining Table 2-6 consent, according to the presence or absence of proton-pump inhibitors

Unique Subject Identifier=0094001000-0016

MEANS Procedure

Assay variable : PTINR_VAL1 PT-INR Value

N	Mean	Standard Deviation	Minimum	Maximum
4	1.5175000	0.2064582	1.2500000	1.6900000

Unique Subject Identifier=0094001000-0018

Assay variable : PTINR_VAL1 PT-INR Value

N	Mean	Standard Deviation	Minimum	Maximum
3	2.0966667	0.1850225	1.9800000	2.3100000

Unique Subject Identifier=0094001000-0023

Assay variable : PTINR_VAL1 PT-INR Value

N	Mean	Standard Deviation	Minimum	Maximum
3	1.9300000	0.0818535	1.8600000	2.0200000

Unique Subject Identifier=0094001000-0049

Assay variable : PTINR_VAL1 PT-INR Value

N	Mean	Standard Deviation	Minimum	Maximum
4	1.9250000	0.4018706	1.4100000	2.3400000

Laboratory tests at the time of obtaining Table 2-6 consent, according to the presence or absence of proton-pump inhibitors

Unique Subject Identifier=0098000000-0002

MEANS Procedure

Assay variable : PTINR_VAL1 PT-INR Value

N	Mean	Standard Deviation	Minimum	Maximum
6	1.9750000	0.3007158	1.5600000	2.4900000

Unique Subject Identifier=0098000000-0007

Assay variable : PTINR_VAL1 PT-INR Value

N	Mean	Standard Deviation	Minimum	Maximum
5	1.9360000	0.3231563	1.5900000	2.4600000

Unique Subject Identifier=0098000000-0012

Assay variable : PTINR_VAL1 PT-INR Value

N	Mean	Standard Deviation	Minimum	Maximum
6	2.0800000	0.2728369	1.8000000	2.4900000

Unique Subject Identifier=0098000000-0013

Assay variable : PTINR_VAL1 PT-INR Value

N	Mean	Standard Deviation	Minimum	Maximum
6	1.4966667	0.3375599	1.2000000	1.9700000

Laboratory tests at the time of obtaining Table 2-6 consent, according to the presence or absence of proton-pump inhibitors

Unique Subject Identifier=0098000000-0016

MEANS Procedure

Assay variable : PTINR_VAL1 PT-INR Value

N	Mean	Standard Deviation	Minimum	Maximum
6	2.1116667	0.4949108	1.5200000	2.9500000

Unique Subject Identifier=0098000000-0017

Assay variable : PTINR_VAL1 PT-INR Value

N	Mean	Standard Deviation	Minimum	Maximum
6	1.5916667	0.2320704	1.1500000	1.8100000

Unique Subject Identifier=0102000000-0002

Assay variable : PTINR_VAL1 PT-INR Value

N	Mean	Standard Deviation	Minimum	Maximum
6	1.5333333	0.1966384	1.2000000	1.7000000

Unique Subject Identifier=0102000000-0008

Assay variable : PTINR_VAL1 PT-INR Value

N	Mean	Standard Deviation	Minimum	Maximum
6	1.8833333	0.8035339	1.2000000	3.2000000

Laboratory tests at the time of obtaining Table 2-6 consent, according to the presence or absence of proton-pump inhibitors

Unique Subject Identifier=0102000000-0009

MEANS Procedure

Assay variable : PTINR_VAL1 PT-INR Value

N	Mean	Standard Deviation	Minimum	Maximum
6	1.7166667	0.3188521	1.4000000	2.3000000

Unique Subject Identifier=0103000000-0009

Assay variable : PTINR_VAL1 PT-INR Value

N	Mean	Standard Deviation	Minimum	Maximum
6	1.8450000	0.2712748	1.5500000	2.3300000

Unique Subject Identifier=0103000000-0015

Assay variable : PTINR_VAL1 PT-INR Value

N	Mean	Standard Deviation	Minimum	Maximum
6	1.8083333	0.1024532	1.6900000	1.9800000

Unique Subject Identifier=0103000000-0017

Assay variable : PTINR_VAL1 PT-INR Value

N	Mean	Standard Deviation	Minimum	Maximum
4	2.3025000	0.2242580	1.9800000	2.5000000

Laboratory tests at the time of obtaining Table 2-6 consent, according to the presence or absence of proton-pump inhibitors

Unique Subject Identifier=0120000000-0003

MEANS Procedure

Assay variable : PTINR_VAL1 PT-INR Value

N	Mean	Standard Deviation	Minimum	Maximum
2	2.0250000	0.3323402	1.7900000	2.2600000

Unique Subject Identifier=0120000000-0004

Assay variable : PTINR_VAL1 PT-INR Value

N	Mean	Standard Deviation	Minimum	Maximum
3	1.7433333	0.2494661	1.4600000	1.9300000

Unique Subject Identifier=0121010000-0005

Assay variable : PTINR_VAL1 PT-INR Value

N	Mean	Standard Deviation	Minimum	Maximum
6	1.9666667	0.2250926	1.6000000	2.3000000

Unique Subject Identifier=0121010000-0012

Assay variable : PTINR_VAL1 PT-INR Value

N	Mean	Standard Deviation	Minimum	Maximum
6	2.2166667	0.2857738	1.8000000	2.6000000

Laboratory tests at the time of obtaining Table 2-6 consent, according to the presence or absence of proton-pump inhibitors

Unique Subject Identifier=0121010000-0014

MEANS Procedure

Assay variable : PTINR_VAL1 PT-INR Value

N	Mean	Standard Deviation	Minimum	Maximum
6	2.1500000	0.1048809	2.0000000	2.3000000

Unique Subject Identifier=0121010000-0023

Assay variable : PTINR_VAL1 PT-INR Value

N	Mean	Standard Deviation	Minimum	Maximum
6	2.1500000	0.6379655	1.6000000	3.4000000

Unique Subject Identifier=0132000000-0003

Assay variable : PTINR_VAL1 PT-INR Value

N	Mean	Standard Deviation	Minimum	Maximum
2	2.4950000	0.1909188	2.3600000	2.6300000

Unique Subject Identifier=0132000000-0009

Assay variable : PTINR_VAL1 PT-INR Value

N	Mean	Standard Deviation	Minimum	Maximum
4	1.6600000	0.3465064	1.1700000	1.9100000

Laboratory tests at the time of obtaining Table 2-6 consent, according to the presence or absence of proton-pump inhibitors

Unique Subject Identifier=0132000000-0012

MEANS Procedure

Assay variable : PTINR_VAL1 PT-INR Value

N	Mean	Standard Deviation	Minimum	Maximum
3	1.5266667	0.3661056	1.1200000	1.8300000

Unique Subject Identifier=0133000000-0001

Assay variable : PTINR_VAL1 PT-INR Value

N	Mean	Standard Deviation	Minimum	Maximum
4	1.3675000	0.2090255	1.0800000	1.5800000

Unique Subject Identifier=0133000000-0005

Assay variable : PTINR_VAL1 PT-INR Value

N	Mean	Standard Deviation	Minimum	Maximum
3	1.8500000	0.0556776	1.7900000	1.9000000

Unique Subject Identifier=0133000000-0010

Assay variable : PTINR_VAL1 PT-INR Value

N	Mean	Standard Deviation	Minimum	Maximum
3	2.2966667	0.6230837	1.7600000	2.9800000

Laboratory tests at the time of obtaining Table 2-6 consent, according to the presence or absence of proton-pump inhibitors

Unique Subject Identifier=0133000000-0011

MEANS Procedure

Assay variable : PTINR_VAL1 PT-INR Value

N	Mean	Standard Deviation	Minimum	Maximum
5	1.7120000	0.1485598	1.5300000	1.9300000

Unique Subject Identifier=0133000000-0012

Assay variable : PTINR_VAL1 PT-INR Value

N	Mean	Standard Deviation	Minimum	Maximum
3	1.5366667	0.0288675	1.5200000	1.5700000

Unique Subject Identifier=0133000000-0013

Assay variable : PTINR_VAL1 PT-INR Value

N	Mean	Standard Deviation	Minimum	Maximum
2	1.4750000	0.0494975	1.4400000	1.5100000

Unique Subject Identifier=0133000000-0015

Assay variable : PTINR_VAL1 PT-INR Value

N	Mean	Standard Deviation	Minimum	Maximum
1	2.0400000		2.0400000	2.0400000

Laboratory tests at the time of obtaining Table 2-6 consent, according to the presence or absence of proton-pump inhibitors

Unique Subject Identifier=0133000000-0045

MEANS Procedure

Assay variable : PTINR_VAL1 PT-INR Value

N	Mean	Standard Deviation	Minimum	Maximum
3	2.2766667	0.7092484	1.5000000	2.8900000

Unique Subject Identifier=0133000000-0052

Assay variable : PTINR_VAL1 PT-INR Value

N	Mean	Standard Deviation	Minimum	Maximum
4	1.6150000	0.0974679	1.5200000	1.7500000

Unique Subject Identifier=0133000000-0061

Assay variable : PTINR_VAL1 PT-INR Value

N	Mean	Standard Deviation	Minimum	Maximum
3	1.8633333	0.3716629	1.4500000	2.1700000

Unique Subject Identifier=0133000000-0068

Assay variable : PTINR_VAL1 PT-INR Value

N	Mean	Standard Deviation	Minimum	Maximum
3	1.7433333	0.3837100	1.4600000	2.1800000

Laboratory tests at the time of obtaining Table 2-6 consent, according to the presence or absence of proton-pump inhibitors

Unique Subject Identifier=0135000000-0002

MEANS Procedure

Assay variable : PTINR_VAL1 PT-INR Value

N	Mean	Standard Deviation	Minimum	Maximum
3	1.7400000	0.1571623	1.6000000	1.9100000

Unique Subject Identifier=0135000000-0010

Assay variable : PTINR_VAL1 PT-INR Value

N	Mean	Standard Deviation	Minimum	Maximum
3	1.6866667	0.0378594	1.6600000	1.7300000

Unique Subject Identifier=0135000000-0020

Assay variable : PTINR_VAL1 PT-INR Value

N	Mean	Standard Deviation	Minimum	Maximum
3	1.7466667	0.1101514	1.6400000	1.8600000

Unique Subject Identifier=0135000000-0026

Assay variable : PTINR_VAL1 PT-INR Value

N	Mean	Standard Deviation	Minimum	Maximum
3	1.6366667	0.1436431	1.5300000	1.8000000

Laboratory tests at the time of obtaining Table 2-6 consent, according to the presence or absence of proton-pump inhibitors

Unique Subject Identifier=0135000000-0028

MEANS Procedure

Assay variable : PTINR_VAL1 PT-INR Value

N	Mean	Standard Deviation	Minimum	Maximum
4	2.0575000	0.2653771	1.7000000	2.3000000

Unique Subject Identifier=0136000010-0003

Assay variable : PTINR_VAL1 PT-INR Value

N	Mean	Standard Deviation	Minimum	Maximum
6	2.1200000	0.4215211	1.3000000	2.4500000

Unique Subject Identifier=0138000010-0001

Assay variable : PTINR_VAL1 PT-INR Value

N	Mean	Standard Deviation	Minimum	Maximum
6	1.6950000	0.2682350	1.4500000	2.0600000

Unique Subject Identifier=0138000010-0002

Assay variable : PTINR_VAL1 PT-INR Value

N	Mean	Standard Deviation	Minimum	Maximum
6	1.7716667	0.3066214	1.3300000	2.1600000

Laboratory tests at the time of obtaining Table 2-6 consent, according to the presence or absence of proton-pump inhibitors

Unique Subject Identifier=0138000010-0003

MEANS Procedure

Assay variable : PTINR_VAL1 PT-INR Value

N	Mean	Standard Deviation	Minimum	Maximum
3	1.9933333	0.4215843	1.6400000	2.4600000

Unique Subject Identifier=0138000010-0004

Assay variable : PTINR_VAL1 PT-INR Value

N	Mean	Standard Deviation	Minimum	Maximum
3	1.8566667	0.1096966	1.7300000	1.9200000

Unique Subject Identifier=0138000010-0014

Assay variable : PTINR_VAL1 PT-INR Value

N	Mean	Standard Deviation	Minimum	Maximum
4	1.8300000	0.4009988	1.3500000	2.1900000

Unique Subject Identifier=0138000010-0015

Assay variable : PTINR_VAL1 PT-INR Value

N	Mean	Standard Deviation	Minimum	Maximum
3	2.1300000	0.0953939	2.0400000	2.2300000

Laboratory tests at the time of obtaining Table 2-6 consent, according to the presence or absence of proton-pump inhibitors

Unique Subject Identifier=0138000010-0016

MEANS Procedure

Assay variable : PTINR_VAL1 PT-INR Value

N	Mean	Standard Deviation	Minimum	Maximum
3	1.2733333	0.0288675	1.2400000	1.2900000

Unique Subject Identifier=0138000010-0022

Assay variable : PTINR_VAL1 PT-INR Value

N	Mean	Standard Deviation	Minimum	Maximum
2	1.7550000	0.1202082	1.6700000	1.8400000

Unique Subject Identifier=0138000010-0031

Assay variable : PTINR_VAL1 PT-INR Value

N	Mean	Standard Deviation	Minimum	Maximum
3	1.5700000	0.1500000	1.4200000	1.7200000

Unique Subject Identifier=0138000010-0033

Assay variable : PTINR_VAL1 PT-INR Value

N	Mean	Standard Deviation	Minimum	Maximum
3	1.9066667	0.2657693	1.6300000	2.1600000

Laboratory tests at the time of obtaining Table 2-6 consent, according to the presence or absence of proton-pump inhibitors

Unique Subject Identifier=0138000010-0035

MEANS Procedure

Assay variable : PTINR_VAL1 PT-INR Value

N	Mean	Standard Deviation	Minimum	Maximum
2	1.5750000	0.2192031	1.4200000	1.7300000

Unique Subject Identifier=0138000010-0036

Assay variable : PTINR_VAL1 PT-INR Value

N	Mean	Standard Deviation	Minimum	Maximum
3	1.8566667	0.1501111	1.7700000	2.0300000

Unique Subject Identifier=0138000010-0037

Assay variable : PTINR_VAL1 PT-INR Value

N	Mean	Standard Deviation	Minimum	Maximum
3	1.8733333	0.1285820	1.7800000	2.0200000

Unique Subject Identifier=0139000010-0017

Assay variable : PTINR_VAL1 PT-INR Value

N	Mean	Standard Deviation	Minimum	Maximum
2	1.4250000	0.5020458	1.0700000	1.7800000

Laboratory tests at the time of obtaining Table 2-6 consent, according to the presence or absence of proton-pump inhibitors

Unique Subject Identifier=0140000010-0002

MEANS Procedure

Assay variable : PTINR_VAL1 PT-INR Value

N	Mean	Standard Deviation	Minimum	Maximum
6	2.2400000	0.5415903	1.3400000	2.8500000

Unique Subject Identifier=0140000010-0008

Assay variable : PTINR_VAL1 PT-INR Value

N	Mean	Standard Deviation	Minimum	Maximum
4	2.3675000	0.2835930	2.0100000	2.6100000

Unique Subject Identifier=0140000010-0016

Assay variable : PTINR_VAL1 PT-INR Value

N	Mean	Standard Deviation	Minimum	Maximum
4	1.7725000	0.6115758	1.3100000	2.6000000

Unique Subject Identifier=0140000010-0017

Assay variable : PTINR_VAL1 PT-INR Value

N	Mean	Standard Deviation	Minimum	Maximum
6	1.8600000	0.7626008	1.4200000	3.4000000

Laboratory tests at the time of obtaining Table 2-6 consent, according to the presence or absence of proton-pump inhibitors

Unique Subject Identifier=0140000010-0025

MEANS Procedure

Assay variable : PTINR_VAL1 PT-INR Value

N	Mean	Standard Deviation	Minimum	Maximum
3	1.4900000	0.0624500	1.4200000	1.5400000

Unique Subject Identifier=0143010000-0003

Assay variable : PTINR_VAL1 PT-INR Value

N	Mean	Standard Deviation	Minimum	Maximum
6	2.1150000	0.3043518	1.8500000	2.5100000

Unique Subject Identifier=0143010000-0011

Assay variable : PTINR_VAL1 PT-INR Value

N	Mean	Standard Deviation	Minimum	Maximum
5	2.3660000	0.6961537	1.5700000	3.4500000

Unique Subject Identifier=0143010000-0012

Assay variable : PTINR_VAL1 PT-INR Value

N	Mean	Standard Deviation	Minimum	Maximum
6	1.6550000	0.1595932	1.5100000	1.9200000

Laboratory tests at the time of obtaining Table 2-6 consent, according to the presence or absence of proton-pump inhibitors

Unique Subject Identifier=0143010000-0031

MEANS Procedure

Assay variable : PTINR_VAL1 PT-INR Value

N	Mean	Standard Deviation	Minimum	Maximum
6	2.4433333	0.3251256	1.8200000	2.7800000

Unique Subject Identifier=0143010000-0036

Assay variable : PTINR_VAL1 PT-INR Value

N	Mean	Standard Deviation	Minimum	Maximum
6	1.8383333	0.1904118	1.5600000	2.0500000

Unique Subject Identifier=0143010000-0043

Assay variable : PTINR_VAL1 PT-INR Value

N	Mean	Standard Deviation	Minimum	Maximum
6	1.7733333	0.3512074	1.4200000	2.2500000

Unique Subject Identifier=0143010000-0044

Assay variable : PTINR_VAL1 PT-INR Value

N	Mean	Standard Deviation	Minimum	Maximum
6	1.7583333	0.2982225	1.3700000	1.9800000

Laboratory tests at the time of obtaining Table 2-6 consent, according to the presence or absence of proton-pump inhibitors

Unique Subject Identifier=0143010000-0056

MEANS Procedure

Assay variable : PTINR_VAL1 PT-INR Value

N	Mean	Standard Deviation	Minimum	Maximum
4	2.0625000	0.3895617	1.7200000	2.6200000

Unique Subject Identifier=0143010000-0064

Assay variable : PTINR_VAL1 PT-INR Value

N	Mean	Standard Deviation	Minimum	Maximum
6	2.2883333	0.3831144	1.9200000	2.8400000

Unique Subject Identifier=0143010000-0072

Assay variable : PTINR_VAL1 PT-INR Value

N	Mean	Standard Deviation	Minimum	Maximum
6	2.1366667	0.6937915	1.4600000	3.3900000

Unique Subject Identifier=0143010000-0073

Assay variable : PTINR_VAL1 PT-INR Value

N	Mean	Standard Deviation	Minimum	Maximum
6	2.0133333	0.2307957	1.7300000	2.2800000

Laboratory tests at the time of obtaining Table 2-6 consent, according to the presence or absence of proton-pump inhibitors

Unique Subject Identifier=0143010000-0075

MEANS Procedure

Assay variable : PTINR_VAL1 PT-INR Value

N	Mean	Standard Deviation	Minimum	Maximum
6	2.1666667	0.2559427	1.8800000	2.6100000

Unique Subject Identifier=0143010000-0076

Assay variable : PTINR_VAL1 PT-INR Value

N	Mean	Standard Deviation	Minimum	Maximum
6	1.9283333	0.3119241	1.5600000	2.4000000

Unique Subject Identifier=0143010000-0079

Assay variable : PTINR_VAL1 PT-INR Value

N	Mean	Standard Deviation	Minimum	Maximum
3	2.4166667	0.2302897	2.1800000	2.6400000

Unique Subject Identifier=0143010000-0093

Assay variable : PTINR_VAL1 PT-INR Value

N	Mean	Standard Deviation	Minimum	Maximum
5	2.0500000	0.4650269	1.6700000	2.6900000

Laboratory tests at the time of obtaining Table 2-6 consent, according to the presence or absence of proton-pump inhibitors

Unique Subject Identifier=0143010000-0099

MEANS Procedure

Assay variable : PTINR_VAL1 PT-INR Value

N	Mean	Standard Deviation	Minimum	Maximum
6	2.2566667	0.2694934	1.8800000	2.6700000

Unique Subject Identifier=0143010000-0100

Assay variable : PTINR_VAL1 PT-INR Value

N	Mean	Standard Deviation	Minimum	Maximum
6	2.0233333	0.5109468	1.2100000	2.6900000

Unique Subject Identifier=0143010000-0108

Assay variable : PTINR_VAL1 PT-INR Value

N	Mean	Standard Deviation	Minimum	Maximum
6	2.1850000	0.6755664	1.8200000	3.5600000

Unique Subject Identifier=0143010000-0111

Assay variable : PTINR_VAL1 PT-INR Value

N	Mean	Standard Deviation	Minimum	Maximum
5	1.7480000	0.1564289	1.5800000	1.9800000

Laboratory tests at the time of obtaining Table 2-6 consent, according to the presence or absence of proton-pump inhibitors

Unique Subject Identifier=0143010000-0112

MEANS Procedure

Assay variable : PTINR_VAL1 PT-INR Value

N	Mean	Standard Deviation	Minimum	Maximum
6	1.8850000	0.1078425	1.7700000	2.0600000

Unique Subject Identifier=0143010000-0118

Assay variable : PTINR_VAL1 PT-INR Value

N	Mean	Standard Deviation	Minimum	Maximum
6	1.7116667	0.3252332	1.4400000	2.3400000

Unique Subject Identifier=0143010000-0129

Assay variable : PTINR_VAL1 PT-INR Value

N	Mean	Standard Deviation	Minimum	Maximum
4	2.1300000	0.1134313	1.9800000	2.2500000

Unique Subject Identifier=0143010000-0132

Assay variable : PTINR_VAL1 PT-INR Value

N	Mean	Standard Deviation	Minimum	Maximum
4	1.3600000	0.5643285	1.0000000	2.2000000

Laboratory tests at the time of obtaining Table 2-6 consent, according to the presence or absence of proton-pump inhibitors

Unique Subject Identifier=0143010000-0141

MEANS Procedure

Assay variable : PTINR_VAL1 PT-INR Value

N	Mean	Standard Deviation	Minimum	Maximum
4	2.1150000	0.1844813	2.0000000	2.3900000

Unique Subject Identifier=0143010000-0153

Assay variable : PTINR_VAL1 PT-INR Value

N	Mean	Standard Deviation	Minimum	Maximum
6	1.9733333	0.4101544	1.4300000	2.5900000

Unique Subject Identifier=0143010000-0154

Assay variable : PTINR_VAL1 PT-INR Value

N	Mean	Standard Deviation	Minimum	Maximum
6	2.0316667	0.6977798	1.1400000	2.8200000

Unique Subject Identifier=0143010000-0155

Assay variable : PTINR_VAL1 PT-INR Value

N	Mean	Standard Deviation	Minimum	Maximum
6	2.3250000	0.2429609	1.9500000	2.6100000

Laboratory tests at the time of obtaining Table 2-6 consent, according to the presence or absence of proton-pump inhibitors

Unique Subject Identifier=0143010000-0163

MEANS Procedure

Assay variable : PTINR_VAL1 PT-INR Value

N	Mean	Standard Deviation	Minimum	Maximum
5	1.9640000	0.1141490	1.7900000	2.0800000

Unique Subject Identifier=0143010000-0164

Assay variable : PTINR_VAL1 PT-INR Value

N	Mean	Standard Deviation	Minimum	Maximum
6	2.4100000	0.2503597	2.0900000	2.7800000

Unique Subject Identifier=0143010000-0173

Assay variable : PTINR_VAL1 PT-INR Value

N	Mean	Standard Deviation	Minimum	Maximum
6	2.3616667	0.3055105	1.8700000	2.8200000

Unique Subject Identifier=0143010000-0175

Assay variable : PTINR_VAL1 PT-INR Value

N	Mean	Standard Deviation	Minimum	Maximum
2	1.8800000	0.2828427	1.6800000	2.0800000

Laboratory tests at the time of obtaining Table 2-6 consent, according to the presence or absence of proton-pump inhibitors

Unique Subject Identifier=0143010000-0182

MEANS Procedure

Assay variable : PTINR_VAL1 PT-INR Value

N	Mean	Standard Deviation	Minimum	Maximum
6	2.0133333	0.7044336	1.6300000	3.4200000

Unique Subject Identifier=0143010000-0188

Assay variable : PTINR_VAL1 PT-INR Value

N	Mean	Standard Deviation	Minimum	Maximum
1	1.6400000		1.6400000	1.6400000

Unique Subject Identifier=0143010000-0196

Assay variable : PTINR_VAL1 PT-INR Value

N	Mean	Standard Deviation	Minimum	Maximum
2	1.8900000	0.0141421	1.8800000	1.9000000

Unique Subject Identifier=0144000000-0006

Assay variable : PTINR_VAL1 PT-INR Value

N	Mean	Standard Deviation	Minimum	Maximum
6	1.9683333	0.2527779	1.6300000	2.4100000

Laboratory tests at the time of obtaining Table 2-6 consent, according to the presence or absence of proton-pump inhibitors

Unique Subject Identifier=0144000000-0014

MEANS Procedure

Assay variable : PTINR_VAL1 PT-INR Value

N	Mean	Standard Deviation	Minimum	Maximum
2	1.2000000	0.1555635	1.0900000	1.3100000

Unique Subject Identifier=0145000000-0005

Assay variable : PTINR_VAL1 PT-INR Value

N	Mean	Standard Deviation	Minimum	Maximum
4	2.6175000	0.3463500	2.2700000	3.0100000

Unique Subject Identifier=0145000000-0008

Assay variable : PTINR_VAL1 PT-INR Value

N	Mean	Standard Deviation	Minimum	Maximum
2	2.4200000	0.5232590	2.0500000	2.7900000

Unique Subject Identifier=0145000000-0014

Assay variable : PTINR_VAL1 PT-INR Value

N	Mean	Standard Deviation	Minimum	Maximum
3	2.2100000	0.2861818	2.0300000	2.5400000

Laboratory tests at the time of obtaining Table 2-6 consent, according to the presence or absence of proton-pump inhibitors

Unique Subject Identifier=0145000000-0015

MEANS Procedure

Assay variable : PTINR_VAL1 PT-INR Value

N	Mean	Standard Deviation	Minimum	Maximum
4	1.7450000	0.0974679	1.6100000	1.8300000

Unique Subject Identifier=0146000000-0007

Assay variable : PTINR_VAL1 PT-INR Value

N	Mean	Standard Deviation	Minimum	Maximum
3	2.2566667	0.2608320	1.9600000	2.4500000

Unique Subject Identifier=0146000000-0009

Assay variable : PTINR_VAL1 PT-INR Value

N	Mean	Standard Deviation	Minimum	Maximum
3	1.8233333	0.1700980	1.6300000	1.9500000

Unique Subject Identifier=0146000000-0015

Assay variable : PTINR_VAL1 PT-INR Value

N	Mean	Standard Deviation	Minimum	Maximum
4	2.3250000	0.3706301	1.8300000	2.7200000

Laboratory tests at the time of obtaining Table 2-6 consent, according to the presence or absence of proton-pump inhibitors

Unique Subject Identifier=0146000000-0021

MEANS Procedure

Assay variable : PTINR_VAL1 PT-INR Value

N	Mean	Standard Deviation	Minimum	Maximum
2	2.1050000	0.0212132	2.0900000	2.1200000

Unique Subject Identifier=0147000000-0009

Assay variable : PTINR_VAL1 PT-INR Value

N	Mean	Standard Deviation	Minimum	Maximum
3	1.8133333	0.0152753	1.8000000	1.8300000

Unique Subject Identifier=0147000000-0013

Assay variable : PTINR_VAL1 PT-INR Value

N	Mean	Standard Deviation	Minimum	Maximum
4	1.9850000	0.5911853	1.6500000	2.8700000

Unique Subject Identifier=0155010000-0004

Assay variable : PTINR_VAL1 PT-INR Value

N	Mean	Standard Deviation	Minimum	Maximum
3	2.1666667	0.1154701	2.1000000	2.3000000

Laboratory tests at the time of obtaining Table 2-6 consent, according to the presence or absence of proton-pump inhibitors

Unique Subject Identifier=0155010000-0006

MEANS Procedure

Assay variable : PTINR_VAL1 PT-INR Value

N	Mean	Standard Deviation	Minimum	Maximum
4	2.1250000	0.2872281	1.7000000	2.3000000

Unique Subject Identifier=0155010000-0007

Assay variable : PTINR_VAL1 PT-INR Value

N	Mean	Standard Deviation	Minimum	Maximum
3	1.9333333	0.1154701	1.8000000	2.0000000

Unique Subject Identifier=0155010000-0008

Assay variable : PTINR_VAL1 PT-INR Value

N	Mean	Standard Deviation	Minimum	Maximum
4	2.7000000	0.4760952	2.2000000	3.2000000

Unique Subject Identifier=0155010000-0009

Assay variable : PTINR_VAL1 PT-INR Value

N	Mean	Standard Deviation	Minimum	Maximum
4	1.7750000	0.1500000	1.6000000	1.9000000

Laboratory tests at the time of obtaining Table 2-6 consent, according to the presence or absence of proton-pump inhibitors

Unique Subject Identifier=0155010000-0010

MEANS Procedure

Assay variable : PTINR_VAL1 PT-INR Value

N	Mean	Standard Deviation	Minimum	Maximum
4	2.2000000	0.2943920	1.9000000	2.5000000

Unique Subject Identifier=0161000000-0011

Assay variable : PTINR_VAL1 PT-INR Value

N	Mean	Standard Deviation	Minimum	Maximum
6	2.0733333	0.3310992	1.5700000	2.5900000

Unique Subject Identifier=0161000000-0017

Assay variable : PTINR_VAL1 PT-INR Value

N	Mean	Standard Deviation	Minimum	Maximum
6	1.8400000	0.3245921	1.3300000	2.2500000

Unique Subject Identifier=0165000000-0013

Assay variable : PTINR_VAL1 PT-INR Value

N	Mean	Standard Deviation	Minimum	Maximum
5	2.2620000	0.5316672	1.6800000	3.1300000

Laboratory tests at the time of obtaining Table 2-6 consent, according to the presence or absence of proton-pump inhibitors

Unique Subject Identifier=0165000000-0017

MEANS Procedure

Assay variable : PTINR_VAL1 PT-INR Value

N	Mean	Standard Deviation	Minimum	Maximum
6	1.7750000	0.1590912	1.5600000	2.0400000

Unique Subject Identifier=0166000000-0009

Assay variable : PTINR_VAL1 PT-INR Value

N	Mean	Standard Deviation	Minimum	Maximum
2	2.1050000	0.1060660	2.0300000	2.1800000

Unique Subject Identifier=0166000000-0017

Assay variable : PTINR_VAL1 PT-INR Value

N	Mean	Standard Deviation	Minimum	Maximum
6	1.1783333	0.1292156	1.0700000	1.4300000

Unique Subject Identifier=0166000000-0021

Assay variable : PTINR_VAL1 PT-INR Value

N	Mean	Standard Deviation	Minimum	Maximum
2	2.6400000	0.3394113	2.4000000	2.8800000

Laboratory tests at the time of obtaining Table 2-6 consent, according to the presence or absence of proton-pump inhibitors

Unique Subject Identifier=0166000000-0023

MEANS Procedure

Assay variable : PTINR_VAL1 PT-INR Value

N	Mean	Standard Deviation	Minimum	Maximum
1	1.6900000		1.6900000	1.6900000

Unique Subject Identifier=0166000000-0039

Assay variable : PTINR_VAL1 PT-INR Value

N	Mean	Standard Deviation	Minimum	Maximum
3	2.1466667	0.1357694	1.9900000	2.2300000

Unique Subject Identifier=0166000000-0040

Assay variable : PTINR_VAL1 PT-INR Value

N	Mean	Standard Deviation	Minimum	Maximum
3	2.2566667	0.2030599	2.1200000	2.4900000

Unique Subject Identifier=0166000000-0047

Assay variable : PTINR_VAL1 PT-INR Value

N	Mean	Standard Deviation	Minimum	Maximum
2	2.1550000	0.0212132	2.1400000	2.1700000

Laboratory tests at the time of obtaining Table 2-6 consent, according to the presence or absence of proton-pump inhibitors

Unique Subject Identifier=0166000000-0054

MEANS Procedure

Assay variable : PTINR_VAL1 PT-INR Value

N	Mean	Standard Deviation	Minimum	Maximum
2	1.8250000	0.0353553	1.8000000	1.8500000

Unique Subject Identifier=0166000000-0062

Assay variable : PTINR_VAL1 PT-INR Value

N	Mean	Standard Deviation	Minimum	Maximum
2	2.0200000	0.2969848	1.8100000	2.2300000

Unique Subject Identifier=0170000000-0008

Assay variable : PTINR_VAL1 PT-INR Value

N	Mean	Standard Deviation	Minimum	Maximum
4	1.6825000	0.2304163	1.4600000	1.9600000

Unique Subject Identifier=0170000000-0013

Assay variable : PTINR_VAL1 PT-INR Value

N	Mean	Standard Deviation	Minimum	Maximum
3	1.7366667	0.0986577	1.6700000	1.8500000

Laboratory tests at the time of obtaining Table 2-6 consent, according to the presence or absence of proton-pump inhibitors

Unique Subject Identifier=0173000000-0001

MEANS Procedure

Assay variable : PTINR_VAL1 PT-INR Value

N	Mean	Standard Deviation	Minimum	Maximum
4	1.6525000	0.1883923	1.4300000	1.8900000

Unique Subject Identifier=0177100000-0007

Assay variable : PTINR_VAL1 PT-INR Value

N	Mean	Standard Deviation	Minimum	Maximum
5	1.8680000	0.4621904	1.5500000	2.6700000

Unique Subject Identifier=0177100000-0065

Assay variable : PTINR_VAL1 PT-INR Value

N	Mean	Standard Deviation	Minimum	Maximum
4	2.3825000	0.4011130	1.9700000	2.8500000

Unique Subject Identifier=0178100000-0004

Assay variable : PTINR_VAL1 PT-INR Value

N	Mean	Standard Deviation	Minimum	Maximum
4	2.2875000	0.1978846	2.1200000	2.5400000

Laboratory tests at the time of obtaining Table 2-6 consent, according to the presence or absence of proton-pump inhibitors

Unique Subject Identifier=0178100000-0007

MEANS Procedure

Assay variable : PTINR_VAL1 PT-INR Value

N	Mean	Standard Deviation	Minimum	Maximum
5	2.1760000	0.5141790	1.7100000	2.9900000

Unique Subject Identifier=0178100000-0011

Assay variable : PTINR_VAL1 PT-INR Value

N	Mean	Standard Deviation	Minimum	Maximum
5	1.6000000	0.1048809	1.4400000	1.7200000

Unique Subject Identifier=0178100000-0013

Assay variable : PTINR_VAL1 PT-INR Value

N	Mean	Standard Deviation	Minimum	Maximum
4	2.2000000	0.0898146	2.1100000	2.3200000

Unique Subject Identifier=0178100000-0017

Assay variable : PTINR_VAL1 PT-INR Value

N	Mean	Standard Deviation	Minimum	Maximum
6	2.0333333	0.2592811	1.5800000	2.2600000

Laboratory tests at the time of obtaining Table 2-6 consent, according to the presence or absence of proton-pump inhibitors

Unique Subject Identifier=0178100000-0041

MEANS Procedure

Assay variable : PTINR_VAL1 PT-INR Value

N	Mean	Standard Deviation	Minimum	Maximum
3	1.9866667	0.2354428	1.7600000	2.2300000

Unique Subject Identifier=0178100000-0042

Assay variable : PTINR_VAL1 PT-INR Value

N	Mean	Standard Deviation	Minimum	Maximum
4	1.9625000	0.1602862	1.8500000	2.1900000

Unique Subject Identifier=0178100000-0053

Assay variable : PTINR_VAL1 PT-INR Value

N	Mean	Standard Deviation	Minimum	Maximum
6	1.8166667	0.3120684	1.5200000	2.4000000

Unique Subject Identifier=0178100000-0058

Assay variable : PTINR_VAL1 PT-INR Value

N	Mean	Standard Deviation	Minimum	Maximum
4	2.0675000	0.3244868	1.7600000	2.5200000

Laboratory tests at the time of obtaining Table 2-6 consent, according to the presence or absence of proton-pump inhibitors

Unique Subject Identifier=0178100000-0065

MEANS Procedure

Assay variable : PTINR_VAL1 PT-INR Value

N	Mean	Standard Deviation	Minimum	Maximum
4	2.3025000	0.7097124	1.5900000	3.2800000

Unique Subject Identifier=0180000000-0001

Assay variable : PTINR_VAL1 PT-INR Value

N	Mean	Standard Deviation	Minimum	Maximum
5	1.7280000	0.2413918	1.4800000	1.9900000

Unique Subject Identifier=0180000000-0004

Assay variable : PTINR_VAL1 PT-INR Value

N	Mean	Standard Deviation	Minimum	Maximum
6	2.2966667	0.1778389	2.1000000	2.5200000

Unique Subject Identifier=0180000000-0024

Assay variable : PTINR_VAL1 PT-INR Value

N	Mean	Standard Deviation	Minimum	Maximum
2	1.8900000	0.0282843	1.8700000	1.9100000

Laboratory tests at the time of obtaining Table 2-6 consent, according to the presence or absence of proton-pump inhibitors

Unique Subject Identifier=0180000000-0026

MEANS Procedure

Assay variable : PTINR_VAL1 PT-INR Value

N	Mean	Standard Deviation	Minimum	Maximum
6	2.2150000	0.4043637	1.7900000	2.9400000

Unique Subject Identifier=0189000000-0020

Assay variable : PTINR_VAL1 PT-INR Value

N	Mean	Standard Deviation	Minimum	Maximum
3	2.4733333	0.6214767	1.9100000	3.1400000

Unique Subject Identifier=0189000000-0030

Assay variable : PTINR_VAL1 PT-INR Value

N	Mean	Standard Deviation	Minimum	Maximum
4	1.8200000	0.0743864	1.7100000	1.8700000

Unique Subject Identifier=0189000000-0033

Assay variable : PTINR_VAL1 PT-INR Value

N	Mean	Standard Deviation	Minimum	Maximum
3	1.9033333	0.2003331	1.7100000	2.1100000

Laboratory tests at the time of obtaining Table 2-6 consent, according to the presence or absence of proton-pump inhibitors

Unique Subject Identifier=0195011000-0016

MEANS Procedure

Assay variable : PTINR_VAL1 PT-INR Value

N	Mean	Standard Deviation	Minimum	Maximum
3	1.7366667	0.1305118	1.6000000	1.8600000

Unique Subject Identifier=0201000000-0006

Assay variable : PTINR_VAL1 PT-INR Value

N	Mean	Standard Deviation	Minimum	Maximum
3	2.2900000	0.5406478	1.8700000	2.9000000

Unique Subject Identifier=0207100000-0002

Assay variable : PTINR_VAL1 PT-INR Value

N	Mean	Standard Deviation	Minimum	Maximum
6	1.9150000	0.3571414	1.6400000	2.6100000

Unique Subject Identifier=0207100000-0008

Assay variable : PTINR_VAL1 PT-INR Value

N	Mean	Standard Deviation	Minimum	Maximum
1	1.6500000		1.6500000	1.6500000

Laboratory tests at the time of obtaining Table 2-6 consent, according to the presence or absence of proton-pump inhibitors

Unique Subject Identifier=0207100000-0013

MEANS Procedure

Assay variable : PTINR_VAL1 PT-INR Value

N	Mean	Standard Deviation	Minimum	Maximum
3	1.7533333	0.1193035	1.6700000	1.8900000

Unique Subject Identifier=0207100000-0015

Assay variable : PTINR_VAL1 PT-INR Value

N	Mean	Standard Deviation	Minimum	Maximum
2	3.0850000	0.1060660	3.0100000	3.1600000

Unique Subject Identifier=0207100000-0017

Assay variable : PTINR_VAL1 PT-INR Value

N	Mean	Standard Deviation	Minimum	Maximum
3	2.2733333	0.4908496	1.9800000	2.8400000

Unique Subject Identifier=0210000000-0003

Assay variable : PTINR_VAL1 PT-INR Value

N	Mean	Standard Deviation	Minimum	Maximum
6	2.4666667	0.5715476	1.7000000	3.3000000

Laboratory tests at the time of obtaining Table 2-6 consent, according to the presence or absence of proton-pump inhibitors

Unique Subject Identifier=0210000000-0017

MEANS Procedure

Assay variable : PTINR_VAL1 PT-INR Value

N	Mean	Standard Deviation	Minimum	Maximum
5	1.7600000	0.1140175	1.6000000	1.9000000

Unique Subject Identifier=0213000000-0001

Assay variable : PTINR_VAL1 PT-INR Value

N	Mean	Standard Deviation	Minimum	Maximum
6	2.1350000	0.1533297	1.9100000	2.3700000

Unique Subject Identifier=0217000000-0004

Assay variable : PTINR_VAL1 PT-INR Value

N	Mean	Standard Deviation	Minimum	Maximum
6	1.7750000	0.2757354	1.5000000	2.2200000

Unique Subject Identifier=0217000000-0006

Assay variable : PTINR_VAL1 PT-INR Value

N	Mean	Standard Deviation	Minimum	Maximum
1	1.6500000		1.6500000	1.6500000

Laboratory tests at the time of obtaining Table 2-6 consent, according to the presence or absence of proton-pump inhibitors

Unique Subject Identifier=0220000000-0004

MEANS Procedure

Assay variable : PTINR_VAL1 PT-INR Value

N	Mean	Standard Deviation	Minimum	Maximum
5	2.3160000	0.4197976	1.9700000	2.9400000

Unique Subject Identifier=0220000000-0005

Assay variable : PTINR_VAL1 PT-INR Value

N	Mean	Standard Deviation	Minimum	Maximum
5	2.5600000	0.7336212	1.8100000	3.7900000

Unique Subject Identifier=0220000000-0007

Assay variable : PTINR_VAL1 PT-INR Value

N	Mean	Standard Deviation	Minimum	Maximum
6	1.9116667	0.0919601	1.8000000	2.0800000

Unique Subject Identifier=0220000000-0008

Assay variable : PTINR_VAL1 PT-INR Value

N	Mean	Standard Deviation	Minimum	Maximum
4	2.2100000	0.4927474	1.7800000	2.9200000

Laboratory tests at the time of obtaining Table 2-6 consent, according to the presence or absence of proton-pump inhibitors

Unique Subject Identifier=0220000000-0010

MEANS Procedure

Assay variable : PTINR_VAL1 PT-INR Value

N	Mean	Standard Deviation	Minimum	Maximum
4	2.0350000	0.1047219	1.9300000	2.1700000

Unique Subject Identifier=0220000000-0013

Assay variable : PTINR_VAL1 PT-INR Value

N	Mean	Standard Deviation	Minimum	Maximum
6	2.1783333	0.2587212	1.9100000	2.4800000

Unique Subject Identifier=0220000000-0015

Assay variable : PTINR_VAL1 PT-INR Value

N	Mean	Standard Deviation	Minimum	Maximum
4	1.6975000	0.4239792	1.1300000	2.0600000

Unique Subject Identifier=0220000000-0016

Assay variable : PTINR_VAL1 PT-INR Value

N	Mean	Standard Deviation	Minimum	Maximum
5	2.1600000	0.4509989	1.6400000	2.6600000

Laboratory tests at the time of obtaining Table 2-6 consent, according to the presence or absence of proton-pump inhibitors

Unique Subject Identifier=0220000000-0020

MEANS Procedure

Assay variable : PTINR_VAL1 PT-INR Value

N	Mean	Standard Deviation	Minimum	Maximum
4	1.9100000	0.2201515	1.7300000	2.2300000

Unique Subject Identifier=0220000000-0025

Assay variable : PTINR_VAL1 PT-INR Value

N	Mean	Standard Deviation	Minimum	Maximum
4	2.1150000	0.4969574	1.6500000	2.6600000

Unique Subject Identifier=0220000000-0032

Assay variable : PTINR_VAL1 PT-INR Value

N	Mean	Standard Deviation	Minimum	Maximum
3	2.6033333	0.2802380	2.3300000	2.8900000

Unique Subject Identifier=0222011000-0001

Assay variable : PTINR_VAL1 PT-INR Value

N	Mean	Standard Deviation	Minimum	Maximum
6	1.9800000	0.3700811	1.5700000	2.3900000

Laboratory tests at the time of obtaining Table 2-6 consent, according to the presence or absence of proton-pump inhibitors

Unique Subject Identifier=0222011000-0005

MEANS Procedure

Assay variable : PTINR_VAL1 PT-INR Value

N	Mean	Standard Deviation	Minimum	Maximum
5	2.0880000	0.2135884	1.8900000	2.4000000

Unique Subject Identifier=0222011000-0009

Assay variable : PTINR_VAL1 PT-INR Value

N	Mean	Standard Deviation	Minimum	Maximum
4	2.0425000	0.2069420	1.8600000	2.3400000

Unique Subject Identifier=0222011000-0010

Assay variable : PTINR_VAL1 PT-INR Value

N	Mean	Standard Deviation	Minimum	Maximum
5	2.4220000	0.7858562	1.8800000	3.8100000

Unique Subject Identifier=0222011000-0013

Assay variable : PTINR_VAL1 PT-INR Value

N	Mean	Standard Deviation	Minimum	Maximum
5	2.3140000	0.1659217	2.0500000	2.4600000

Laboratory tests at the time of obtaining Table 2-6 consent, according to the presence or absence of proton-pump inhibitors

Unique Subject Identifier=0228100000-0012

MEANS Procedure

Assay variable : PTINR_VAL1 PT-INR Value

N	Mean	Standard Deviation	Minimum	Maximum
6	1.3550000	0.1380942	1.1200000	1.5100000

Unique Subject Identifier=0228100000-0014

Assay variable : PTINR_VAL1 PT-INR Value

N	Mean	Standard Deviation	Minimum	Maximum
6	2.0833333	0.2852134	1.8300000	2.6200000

Unique Subject Identifier=0228100000-0052

Assay variable : PTINR_VAL1 PT-INR Value

N	Mean	Standard Deviation	Minimum	Maximum
1	1.8000000		1.8000000	1.8000000

Unique Subject Identifier=0229100000-0001

Assay variable : PTINR_VAL1 PT-INR Value

N	Mean	Standard Deviation	Minimum	Maximum
4	1.7550000	0.1178983	1.6400000	1.8800000

Laboratory tests at the time of obtaining Table 2-6 consent, according to the presence or absence of proton-pump inhibitors

Unique Subject Identifier=0229100000-0004

MEANS Procedure

Assay variable : PTINR_VAL1 PT-INR Value

N	Mean	Standard Deviation	Minimum	Maximum
6	1.5983333	0.4860213	1.1700000	2.4600000

Unique Subject Identifier=0229100000-0018

Assay variable : PTINR_VAL1 PT-INR Value

N	Mean	Standard Deviation	Minimum	Maximum
6	1.9466667	0.1880071	1.7800000	2.2800000

Unique Subject Identifier=0229100000-0029

Assay variable : PTINR_VAL1 PT-INR Value

N	Mean	Standard Deviation	Minimum	Maximum
6	2.1550000	0.2954827	1.7900000	2.5900000

Unique Subject Identifier=0229100000-0030

Assay variable : PTINR_VAL1 PT-INR Value

N	Mean	Standard Deviation	Minimum	Maximum
4	2.0775000	0.1252664	1.9500000	2.1900000

Laboratory tests at the time of obtaining Table 2-6 consent, according to the presence or absence of proton-pump inhibitors

Unique Subject Identifier=0230100000-0004

MEANS Procedure

Assay variable : PTINR_VAL1 PT-INR Value

N	Mean	Standard Deviation	Minimum	Maximum
5	2.4640000	0.3543021	2.0000000	2.8100000

Unique Subject Identifier=0231100000-0008

Assay variable : PTINR_VAL1 PT-INR Value

N	Mean	Standard Deviation	Minimum	Maximum
5	1.7260000	0.2921986	1.2700000	2.0600000

Unique Subject Identifier=0231100000-0013

Assay variable : PTINR_VAL1 PT-INR Value

N	Mean	Standard Deviation	Minimum	Maximum
4	1.8125000	0.2224672	1.4900000	1.9700000

Unique Subject Identifier=0231100000-0017

Assay variable : PTINR_VAL1 PT-INR Value

N	Mean	Standard Deviation	Minimum	Maximum
2	1.3900000	0.0282843	1.3700000	1.4100000

Laboratory tests at the time of obtaining Table 2-6 consent, according to the presence or absence of proton-pump inhibitors

Unique Subject Identifier=0231100000-0019

MEANS Procedure

Assay variable : PTINR_VAL1 PT-INR Value

N	Mean	Standard Deviation	Minimum	Maximum
5	1.7680000	0.3922627	1.4200000	2.3500000

Unique Subject Identifier=0231100000-0038

Assay variable : PTINR_VAL1 PT-INR Value

N	Mean	Standard Deviation	Minimum	Maximum
4	2.0575000	0.2807579	1.7800000	2.3800000

Unique Subject Identifier=0231100000-0046

Assay variable : PTINR_VAL1 PT-INR Value

N	Mean	Standard Deviation	Minimum	Maximum
6	2.1883333	0.1718623	1.8800000	2.4000000

Unique Subject Identifier=1003000000-0010

Assay variable : PTINR_VAL1 PT-INR Value

N	Mean	Standard Deviation	Minimum	Maximum
5	2.2020000	0.2882187	1.8400000	2.5900000

Laboratory tests at the time of obtaining Table 2-6 consent, according to the presence or absence of proton-pump inhibitors

Unique Subject Identifier=1005000000-0010

MEANS Procedure

Assay variable : PTINR_VAL1 PT-INR Value

N	Mean	Standard Deviation	Minimum	Maximum
6	1.5200000	0.0850882	1.4200000	1.6700000

Unique Subject Identifier=1009010010-0007

Assay variable : PTINR_VAL1 PT-INR Value

N	Mean	Standard Deviation	Minimum	Maximum
3	2.3333333	0.2309401	2.2000000	2.6000000

Unique Subject Identifier=1009010010-0013

Assay variable : PTINR_VAL1 PT-INR Value

N	Mean	Standard Deviation	Minimum	Maximum
6	2.5333333	0.1966384	2.3000000	2.8000000

Unique Subject Identifier=1009010010-0014

Assay variable : PTINR_VAL1 PT-INR Value

N	Mean	Standard Deviation	Minimum	Maximum
6	2.3833333	0.6080022	1.9000000	3.5000000

Laboratory tests at the time of obtaining Table 2-6 consent, according to the presence or absence of proton-pump inhibitors

Unique Subject Identifier=1009010010-0027

MEANS Procedure

Assay variable : PTINR_VAL1 PT-INR Value

N	Mean	Standard Deviation	Minimum	Maximum
6	2.1166667	0.7250287	1.6000000	3.5000000

Unique Subject Identifier=1010010000-0003

Assay variable : PTINR_VAL1 PT-INR Value

N	Mean	Standard Deviation	Minimum	Maximum
6	1.7833333	0.1329160	1.6000000	2.0000000

Unique Subject Identifier=1010010000-0005

Assay variable : PTINR_VAL1 PT-INR Value

N	Mean	Standard Deviation	Minimum	Maximum
1	2.0000000		2.0000000	2.0000000

Unique Subject Identifier=1013010110-0006

Assay variable : PTINR_VAL1 PT-INR Value

N	Mean	Standard Deviation	Minimum	Maximum
6	1.9666667	0.2160247	1.6000000	2.2000000

Laboratory tests at the time of obtaining Table 2-6 consent, according to the presence or absence of proton-pump inhibitors

Unique Subject Identifier=1019010100-0009

MEANS Procedure

Assay variable : PTINR_VAL1 PT-INR Value

N	Mean	Standard Deviation	Minimum	Maximum
6	3.2066667	1.1550180	1.7200000	4.9100000

Unique Subject Identifier=1019010100-0013

Assay variable : PTINR_VAL1 PT-INR Value

N	Mean	Standard Deviation	Minimum	Maximum
5	1.6940000	0.3864324	1.2700000	2.1700000

Unique Subject Identifier=1028000000-0019

Assay variable : PTINR_VAL1 PT-INR Value

N	Mean	Standard Deviation	Minimum	Maximum
2	1.9950000	0.0212132	1.9800000	2.0100000

Unique Subject Identifier=1031010000-0111

Assay variable : PTINR_VAL1 PT-INR Value

N	Mean	Standard Deviation	Minimum	Maximum
6	1.7900000	0.2429815	1.5900000	2.2300000

Laboratory tests at the time of obtaining Table 2-6 consent, according to the presence or absence of proton-pump inhibitors

Unique Subject Identifier=1031010000-0120

MEANS Procedure

Assay variable : PTINR_VAL1 PT-INR Value

N	Mean	Standard Deviation	Minimum	Maximum
2	1.7400000	0.3535534	1.4900000	1.9900000

Unique Subject Identifier=1032000000-0001

Assay variable : PTINR_VAL1 PT-INR Value

N	Mean	Standard Deviation	Minimum	Maximum
3	1.6900000	0.0300000	1.6600000	1.7200000

Unique Subject Identifier=1032000000-0004

Assay variable : PTINR_VAL1 PT-INR Value

N	Mean	Standard Deviation	Minimum	Maximum
3	2.0666667	0.5291818	1.4900000	2.5300000

Unique Subject Identifier=1032000000-0006

Assay variable : PTINR_VAL1 PT-INR Value

N	Mean	Standard Deviation	Minimum	Maximum
6	1.7933333	0.4071200	1.4600000	2.5000000

Laboratory tests at the time of obtaining Table 2-6 consent, according to the presence or absence of proton-pump inhibitors

Unique Subject Identifier=1032000000-0007

MEANS Procedure

Assay variable : PTINR_VAL1 PT-INR Value

N	Mean	Standard Deviation	Minimum	Maximum
6	2.4366667	0.6166252	1.6900000	3.1000000

Unique Subject Identifier=1032000000-0008

Assay variable : PTINR_VAL1 PT-INR Value

N	Mean	Standard Deviation	Minimum	Maximum
6	1.6883333	0.5840348	1.2900000	2.5600000

Unique Subject Identifier=1032000000-0010

Assay variable : PTINR_VAL1 PT-INR Value

N	Mean	Standard Deviation	Minimum	Maximum
6	2.2250000	0.2714959	1.8800000	2.6200000

Unique Subject Identifier=1032000000-0011

Assay variable : PTINR_VAL1 PT-INR Value

N	Mean	Standard Deviation	Minimum	Maximum
6	2.1600000	0.1288410	1.9200000	2.2900000

Laboratory tests at the time of obtaining Table 2-6 consent, according to the presence or absence of proton-pump inhibitors

Unique Subject Identifier=1032000000-0013

MEANS Procedure

Assay variable : PTINR_VAL1 PT-INR Value

N	Mean	Standard Deviation	Minimum	Maximum
6	1.9833333	0.4921653	1.6500000	2.9300000

Unique Subject Identifier=1032000000-0017

Assay variable : PTINR_VAL1 PT-INR Value

N	Mean	Standard Deviation	Minimum	Maximum
6	2.1833333	0.3101398	1.7600000	2.7000000

Unique Subject Identifier=1032000000-0018

Assay variable : PTINR_VAL1 PT-INR Value

N	Mean	Standard Deviation	Minimum	Maximum
6	1.9116667	0.3806529	1.4900000	2.5500000

Unique Subject Identifier=1032000000-0021

Assay variable : PTINR_VAL1 PT-INR Value

N	Mean	Standard Deviation	Minimum	Maximum
6	1.8950000	0.1712016	1.6500000	2.0500000

Laboratory tests at the time of obtaining Table 2-6 consent, according to the presence or absence of proton-pump inhibitors

Unique Subject Identifier=1032000000-0025

MEANS Procedure

Assay variable : PTINR_VAL1 PT-INR Value

N	Mean	Standard Deviation	Minimum	Maximum
6	1.5883333	0.3950907	1.2800000	2.3500000

Unique Subject Identifier=1032000000-0028

Assay variable : PTINR_VAL1 PT-INR Value

N	Mean	Standard Deviation	Minimum	Maximum
6	2.3250000	0.2562616	1.9800000	2.7200000

Unique Subject Identifier=1032000000-0030

Assay variable : PTINR_VAL1 PT-INR Value

N	Mean	Standard Deviation	Minimum	Maximum
6	1.7050000	0.0939681	1.5900000	1.8300000

Unique Subject Identifier=1034000000-0009

Assay variable : PTINR_VAL1 PT-INR Value

N	Mean	Standard Deviation	Minimum	Maximum
1	1.2000000		1.2000000	1.2000000

Laboratory tests at the time of obtaining Table 2-6 consent, according to the presence or absence of proton-pump inhibitors

Unique Subject Identifier=1035000000-0013

MEANS Procedure

Assay variable : PTINR_VAL1 PT-INR Value

N	Mean	Standard Deviation	Minimum	Maximum
5	2.0740000	0.1984439	1.8000000	2.3300000

Unique Subject Identifier=1038000000-0004

Assay variable : PTINR_VAL1 PT-INR Value

N	Mean	Standard Deviation	Minimum	Maximum
6	2.0833333	0.1722401	1.9000000	2.3000000

Unique Subject Identifier=1041011110-0033

Assay variable : PTINR_VAL1 PT-INR Value

N	Mean	Standard Deviation	Minimum	Maximum
6	1.9833333	0.1471960	1.8000000	2.2000000

Unique Subject Identifier=1041011110-0036

Assay variable : PTINR_VAL1 PT-INR Value

N	Mean	Standard Deviation	Minimum	Maximum
6	2.2666667	0.6186006	1.2000000	2.9000000

Laboratory tests at the time of obtaining Table 2-6 consent, according to the presence or absence of proton-pump inhibitors

Unique Subject Identifier=1041011110-0043

MEANS Procedure

Assay variable : PTINR_VAL1 PT-INR Value

N	Mean	Standard Deviation	Minimum	Maximum
5	1.7200000	0.1303840	1.6000000	1.9000000

Unique Subject Identifier=1052000000-0003

Assay variable : PTINR_VAL1 PT-INR Value

N	Mean	Standard Deviation	Minimum	Maximum
5	1.7960000	0.1668233	1.6300000	2.0000000

Unique Subject Identifier=1060000000-0010

Assay variable : PTINR_VAL1 PT-INR Value

N	Mean	Standard Deviation	Minimum	Maximum
6	1.9666667	0.2771041	1.6800000	2.3600000

Unique Subject Identifier=1060000000-0022

Assay variable : PTINR_VAL1 PT-INR Value

N	Mean	Standard Deviation	Minimum	Maximum
4	1.8100000	0.1570563	1.6200000	2.0000000

Laboratory tests at the time of obtaining Table 2-6 consent, according to the presence or absence of proton-pump inhibitors

Unique Subject Identifier=1060000000-0043

MEANS Procedure

Assay variable : PTINR_VAL1 PT-INR Value

N	Mean	Standard Deviation	Minimum	Maximum
2	1.8950000	0.2899138	1.6900000	2.1000000

Unique Subject Identifier=1065010011-0008

Assay variable : PTINR_VAL1 PT-INR Value

N	Mean	Standard Deviation	Minimum	Maximum
6	2.4666667	0.5163978	1.6000000	3.0000000

Unique Subject Identifier=1065010011-0012

Assay variable : PTINR_VAL1 PT-INR Value

N	Mean	Standard Deviation	Minimum	Maximum
6	2.2333333	0.7580677	1.5000000	3.4000000

Unique Subject Identifier=1068010111-0006

Assay variable : PTINR_VAL1 PT-INR Value

N	Mean	Standard Deviation	Minimum	Maximum
3	1.9266667	0.1331666	1.7800000	2.0400000

Laboratory tests at the time of obtaining Table 2-6 consent, according to the presence or absence of proton-pump inhibitors

Unique Subject Identifier=1068010111-0026

MEANS Procedure

Assay variable : PTINR_VAL1 PT-INR Value

N	Mean	Standard Deviation	Minimum	Maximum
5	2.1220000	0.7623451	1.4100000	3.2600000

Unique Subject Identifier=1072000000-0022

Assay variable : PTINR_VAL1 PT-INR Value

N	Mean	Standard Deviation	Minimum	Maximum
6	1.7950000	0.1415274	1.6500000	2.0600000

Unique Subject Identifier=1072000000-0034

Assay variable : PTINR_VAL1 PT-INR Value

N	Mean	Standard Deviation	Minimum	Maximum
6	1.9483333	0.1212298	1.7300000	2.0400000

Unique Subject Identifier=1074011010-0002

Assay variable : PTINR_VAL1 PT-INR Value

N	Mean	Standard Deviation	Minimum	Maximum
5	2.3260000	0.6975529	1.7600000	3.5000000

Laboratory tests at the time of obtaining Table 2-6 consent, according to the presence or absence of proton-pump inhibitors

Unique Subject Identifier=1074011010-0010

MEANS Procedure

Assay variable : PTINR_VAL1 PT-INR Value

N	Mean	Standard Deviation	Minimum	Maximum
5	1.7600000	0.2302173	1.5100000	2.1000000

Unique Subject Identifier=1080000000-0001

Assay variable : PTINR_VAL1 PT-INR Value

N	Mean	Standard Deviation	Minimum	Maximum
6	2.1783333	0.2145150	1.9600000	2.4400000

Unique Subject Identifier=1080000000-0007

Assay variable : PTINR_VAL1 PT-INR Value

N	Mean	Standard Deviation	Minimum	Maximum
6	2.5350000	0.1775106	2.2700000	2.7300000

Unique Subject Identifier=1087000000-0004

Assay variable : PTINR_VAL1 PT-INR Value

N	Mean	Standard Deviation	Minimum	Maximum
6	1.9983333	0.4471875	1.5400000	2.5400000

Laboratory tests at the time of obtaining Table 2-6 consent, according to the presence or absence of proton-pump inhibitors

Unique Subject Identifier=1090001110-0011

MEANS Procedure

Assay variable : PTINR_VAL1 PT-INR Value

N	Mean	Standard Deviation	Minimum	Maximum
6	2.4000000	0.3098387	1.9000000	2.8000000

Unique Subject Identifier=1090001110-0012

Assay variable : PTINR_VAL1 PT-INR Value

N	Mean	Standard Deviation	Minimum	Maximum
6	1.8666667	0.2732520	1.5000000	2.3000000

Unique Subject Identifier=1091010000-0007

Assay variable : PTINR_VAL1 PT-INR Value

N	Mean	Standard Deviation	Minimum	Maximum
6	2.3666667	0.5046451	1.6000000	3.1000000

Unique Subject Identifier=1091010000-0010

Assay variable : PTINR_VAL1 PT-INR Value

N	Mean	Standard Deviation	Minimum	Maximum
6	2.0500000	0.2073644	1.8000000	2.4000000

Laboratory tests at the time of obtaining Table 2-6 consent, according to the presence or absence of proton-pump inhibitors

Unique Subject Identifier=1091010000-0028

MEANS Procedure

Assay variable : PTINR_VAL1 PT-INR Value

N	Mean	Standard Deviation	Minimum	Maximum
5	2.1800000	0.8043631	1.4000000	3.3000000

Unique Subject Identifier=1091010000-0030

Assay variable : PTINR_VAL1 PT-INR Value

N	Mean	Standard Deviation	Minimum	Maximum
6	1.7000000	0.2000000	1.5000000	2.0000000

Unique Subject Identifier=1091010000-0038

Assay variable : PTINR_VAL1 PT-INR Value

N	Mean	Standard Deviation	Minimum	Maximum
6	1.8666667	0.2065591	1.7000000	2.2000000

Unique Subject Identifier=1091010000-0042

Assay variable : PTINR_VAL1 PT-INR Value

N	Mean	Standard Deviation	Minimum	Maximum
6	1.8833333	0.2401388	1.6000000	2.3000000

Laboratory tests at the time of obtaining Table 2-6 consent, according to the presence or absence of proton-pump inhibitors

Unique Subject Identifier=1094000000-0003

MEANS Procedure

Assay variable : PTINR_VAL1 PT-INR Value

N	Mean	Standard Deviation	Minimum	Maximum
6	1.5350000	0.0736885	1.4400000	1.6300000

Unique Subject Identifier=1094000000-0005

Assay variable : PTINR_VAL1 PT-INR Value

N	Mean	Standard Deviation	Minimum	Maximum
5	2.5000000	0.4803644	2.1200000	3.3000000

Unique Subject Identifier=1095000000-0025

Assay variable : PTINR_VAL1 PT-INR Value

N	Mean	Standard Deviation	Minimum	Maximum
6	2.0666667	0.3204164	1.8000000	2.6000000

Unique Subject Identifier=1096000000-0001

Assay variable : PTINR_VAL1 PT-INR Value

N	Mean	Standard Deviation	Minimum	Maximum
1	2.0600000		2.0600000	2.0600000

Laboratory tests at the time of obtaining Table 2-6 consent, according to the presence or absence of proton-pump inhibitors

Unique Subject Identifier=1096000000-0018

MEANS Procedure

Assay variable : PTINR_VAL1 PT-INR Value

N	Mean	Standard Deviation	Minimum	Maximum
3	1.5666667	0.2516611	1.3000000	1.8000000

Unique Subject Identifier=1099000000-0002

Assay variable : PTINR_VAL1 PT-INR Value

N	Mean	Standard Deviation	Minimum	Maximum
3	1.4300000	0.1300000	1.3500000	1.5800000

Unique Subject Identifier=1099000000-0004

Assay variable : PTINR_VAL1 PT-INR Value

N	Mean	Standard Deviation	Minimum	Maximum
1	1.6400000		1.6400000	1.6400000

Unique Subject Identifier=1102000000-0008

Assay variable : PTINR_VAL1 PT-INR Value

N	Mean	Standard Deviation	Minimum	Maximum
2	1.5700000	0.1131371	1.4900000	1.6500000

Laboratory tests at the time of obtaining Table 2-6 consent, according to the presence or absence of proton-pump inhibitors

Unique Subject Identifier=1102000000-0021

MEANS Procedure

Assay variable : PTINR_VAL1 PT-INR Value

N	Mean	Standard Deviation	Minimum	Maximum
2	1.7950000	0.0353553	1.7700000	1.8200000

Unique Subject Identifier=1102000000-0022

Assay variable : PTINR_VAL1 PT-INR Value

N	Mean	Standard Deviation	Minimum	Maximum
3	2.0666667	0.4441096	1.5800000	2.4500000

Unique Subject Identifier=1110000000-0002

Assay variable : PTINR_VAL1 PT-INR Value

N	Mean	Standard Deviation	Minimum	Maximum
5	2.1000000	0.1581139	1.9000000	2.3000000

Unique Subject Identifier=1111000000-0005

Assay variable : PTINR_VAL1 PT-INR Value

N	Mean	Standard Deviation	Minimum	Maximum
6	1.5600000	0.1181524	1.4800000	1.7800000

Laboratory tests at the time of obtaining Table 2-6 consent, according to the presence or absence of proton-pump inhibitors

Unique Subject Identifier=1111000000-0012

MEANS Procedure

Assay variable : PTINR_VAL1 PT-INR Value

N	Mean	Standard Deviation	Minimum	Maximum
2	2.1050000	0.1484924	2.0000000	2.2100000

Unique Subject Identifier=1112000000-0002

Assay variable : PTINR_VAL1 PT-INR Value

N	Mean	Standard Deviation	Minimum	Maximum
6	1.8966667	0.5702865	1.3100000	2.7600000

Unique Subject Identifier=1112000000-0015

Assay variable : PTINR_VAL1 PT-INR Value

N	Mean	Standard Deviation	Minimum	Maximum
6	2.1833333	0.6064212	1.5500000	3.1900000

Unique Subject Identifier=1118011000-0002

Assay variable : PTINR_VAL1 PT-INR Value

N	Mean	Standard Deviation	Minimum	Maximum
5	2.1000000	0.2549510	1.8000000	2.4000000

Laboratory tests at the time of obtaining Table 2-6 consent, according to the presence or absence of proton-pump inhibitors

Unique Subject Identifier=1118011000-0006

MEANS Procedure

Assay variable : PTINR_VAL1 PT-INR Value

N	Mean	Standard Deviation	Minimum	Maximum
6	2.1833333	0.5307228	1.6000000	3.0000000

Unique Subject Identifier=1118011000-0009

Assay variable : PTINR_VAL1 PT-INR Value

N	Mean	Standard Deviation	Minimum	Maximum
6	1.9666667	0.2503331	1.7000000	2.3000000

Unique Subject Identifier=1118011000-0021

Assay variable : PTINR_VAL1 PT-INR Value

N	Mean	Standard Deviation	Minimum	Maximum
5	1.5600000	0.1140175	1.4000000	1.7000000

Unique Subject Identifier=1118011000-0023

Assay variable : PTINR_VAL1 PT-INR Value

N	Mean	Standard Deviation	Minimum	Maximum
6	2.0000000	0.5513620	1.5000000	2.8000000

Laboratory tests at the time of obtaining Table 2-6 consent, according to the presence or absence of proton-pump inhibitors

Unique Subject Identifier=1118011000-0027

MEANS Procedure

Assay variable : PTINR_VAL1 PT-INR Value

N	Mean	Standard Deviation	Minimum	Maximum
5	2.0800000	0.4549725	1.6000000	2.8000000

Unique Subject Identifier=1118011000-0045

Assay variable : PTINR_VAL1 PT-INR Value

N	Mean	Standard Deviation	Minimum	Maximum
6	1.8833333	0.1169045	1.7000000	2.0000000

Unique Subject Identifier=1118011000-0083

Assay variable : PTINR_VAL1 PT-INR Value

N	Mean	Standard Deviation	Minimum	Maximum
6	2.2833333	0.4792355	1.9000000	2.9000000

Unique Subject Identifier=1118011000-0087

Assay variable : PTINR_VAL1 PT-INR Value

N	Mean	Standard Deviation	Minimum	Maximum
5	1.8000000	0.3000000	1.5000000	2.1000000

Laboratory tests at the time of obtaining Table 2-6 consent, according to the presence or absence of proton-pump inhibitors

Unique Subject Identifier=1118011000-0090

MEANS Procedure

Assay variable : PTINR_VAL1 PT-INR Value

N	Mean	Standard Deviation	Minimum	Maximum
6	2.5833333	0.2483277	2.3000000	2.9000000

Unique Subject Identifier=1118011000-0102

Assay variable : PTINR_VAL1 PT-INR Value

N	Mean	Standard Deviation	Minimum	Maximum
6	1.9000000	0.3521363	1.7000000	2.6000000

Unique Subject Identifier=1118011000-0109

Assay variable : PTINR_VAL1 PT-INR Value

N	Mean	Standard Deviation	Minimum	Maximum
5	2.2200000	0.2774887	1.8000000	2.5000000

Unique Subject Identifier=1118011000-0120

Assay variable : PTINR_VAL1 PT-INR Value

N	Mean	Standard Deviation	Minimum	Maximum
6	1.8166667	0.2136976	1.6000000	2.2000000

Laboratory tests at the time of obtaining Table 2-6 consent, according to the presence or absence of proton-pump inhibitors

Unique Subject Identifier=1119000000-0005

MEANS Procedure

Assay variable : PTINR_VAL1 PT-INR Value

N	Mean	Standard Deviation	Minimum	Maximum
1	1.6300000		1.6300000	1.6300000

Unique Subject Identifier=1119000000-0016

Assay variable : PTINR_VAL1 PT-INR Value

N	Mean	Standard Deviation	Minimum	Maximum
2	1.9800000	0.0282843	1.9600000	2.0000000

Unique Subject Identifier=1119000000-0019

Assay variable : PTINR_VAL1 PT-INR Value

N	Mean	Standard Deviation	Minimum	Maximum
6	2.1466667	0.3225937	1.9000000	2.7400000

Unique Subject Identifier=1119000000-0044

Assay variable : PTINR_VAL1 PT-INR Value

N	Mean	Standard Deviation	Minimum	Maximum
5	1.8400000	0.2966479	1.5000000	2.3000000

Laboratory tests at the time of obtaining Table 2-6 consent, according to the presence or absence of proton-pump inhibitors

Unique Subject Identifier=1122001010-0018

MEANS Procedure

Assay variable : PTINR_VAL1 PT-INR Value

N	Mean	Standard Deviation	Minimum	Maximum
1	1.6400000		1.6400000	1.6400000

Unique Subject Identifier=1122001010-0031

Assay variable : PTINR_VAL1 PT-INR Value

N	Mean	Standard Deviation	Minimum	Maximum
6	2.4833333	0.4684727	1.7500000	2.9500000

Unique Subject Identifier=1122001010-0038

Assay variable : PTINR_VAL1 PT-INR Value

N	Mean	Standard Deviation	Minimum	Maximum
6	2.0683333	0.1979310	1.8600000	2.3300000

Unique Subject Identifier=1122001010-0052

Assay variable : PTINR_VAL1 PT-INR Value

N	Mean	Standard Deviation	Minimum	Maximum
6	1.9266667	0.1954141	1.6200000	2.1300000

Laboratory tests at the time of obtaining Table 2-6 consent, according to the presence or absence of proton-pump inhibitors

Unique Subject Identifier=1122001010-0055

MEANS Procedure

Assay variable : PTINR_VAL1 PT-INR Value

N	Mean	Standard Deviation	Minimum	Maximum
6	1.7900000	0.1943193	1.6000000	2.1300000

Unique Subject Identifier=1122001010-0056

Assay variable : PTINR_VAL1 PT-INR Value

N	Mean	Standard Deviation	Minimum	Maximum
5	2.4940000	0.3922754	2.1100000	2.9300000

Unique Subject Identifier=1122001010-0060

Assay variable : PTINR_VAL1 PT-INR Value

N	Mean	Standard Deviation	Minimum	Maximum
6	1.7200000	0.7457882	1.1800000	3.1800000

Unique Subject Identifier=1122001010-0061

Assay variable : PTINR_VAL1 PT-INR Value

N	Mean	Standard Deviation	Minimum	Maximum
6	2.4133333	0.2000667	2.1300000	2.7100000

Laboratory tests at the time of obtaining Table 2-6 consent, according to the presence or absence of proton-pump inhibitors

Unique Subject Identifier=1122001010-0063

MEANS Procedure

Assay variable : PTINR_VAL1 PT-INR Value

N	Mean	Standard Deviation	Minimum	Maximum
6	2.2633333	0.2497732	1.8100000	2.5600000

Unique Subject Identifier=1122001010-0066

Assay variable : PTINR_VAL1 PT-INR Value

N	Mean	Standard Deviation	Minimum	Maximum
6	2.0233333	0.2823945	1.6200000	2.3800000

Unique Subject Identifier=1122001010-0072

Assay variable : PTINR_VAL1 PT-INR Value

N	Mean	Standard Deviation	Minimum	Maximum
3	1.6566667	0.4454586	1.2000000	2.0900000

Unique Subject Identifier=1122001010-0073

Assay variable : PTINR_VAL1 PT-INR Value

N	Mean	Standard Deviation	Minimum	Maximum
6	1.7566667	0.2808321	1.4700000	2.2500000

Laboratory tests at the time of obtaining Table 2-6 consent, according to the presence or absence of proton-pump inhibitors

Unique Subject Identifier=1127010000-0001

MEANS Procedure

Assay variable : PTINR_VAL1 PT-INR Value

N	Mean	Standard Deviation	Minimum	Maximum
6	1.6833333	0.1834848	1.4000000	1.9000000

Unique Subject Identifier=1154000000-0006

Assay variable : PTINR_VAL1 PT-INR Value

N	Mean	Standard Deviation	Minimum	Maximum
5	1.5900000	0.1168332	1.4500000	1.7200000

Unique Subject Identifier=1156000000-0003

Assay variable : PTINR_VAL1 PT-INR Value

N	Mean	Standard Deviation	Minimum	Maximum
6	1.1816667	0.2081746	0.9600000	1.4500000

Unique Subject Identifier=1156000000-0006

Assay variable : PTINR_VAL1 PT-INR Value

N	Mean	Standard Deviation	Minimum	Maximum
5	2.6020000	0.5514708	2.0500000	3.4700000

Laboratory tests at the time of obtaining Table 2-6 consent, according to the presence or absence of proton-pump inhibitors

Unique Subject Identifier=1156000000-0007

MEANS Procedure

Assay variable : PTINR_VAL1 PT-INR Value

N	Mean	Standard Deviation	Minimum	Maximum
5	3.6280000	2.4042400	1.6800000	7.8100000

Unique Subject Identifier=1156000000-0008

Assay variable : PTINR_VAL1 PT-INR Value

N	Mean	Standard Deviation	Minimum	Maximum
6	2.1600000	0.5060040	1.7400000	3.1300000

Unique Subject Identifier=1156000000-0010

Assay variable : PTINR_VAL1 PT-INR Value

N	Mean	Standard Deviation	Minimum	Maximum
6	1.6116667	0.2249815	1.3200000	1.8600000

Unique Subject Identifier=1156000000-0011

Assay variable : PTINR_VAL1 PT-INR Value

N	Mean	Standard Deviation	Minimum	Maximum
4	2.6725000	0.2568235	2.4200000	3.0300000

Laboratory tests at the time of obtaining Table 2-6 consent, according to the presence or absence of proton-pump inhibitors

Unique Subject Identifier=1156000000-0012

MEANS Procedure

Assay variable : PTINR_VAL1 PT-INR Value

N	Mean	Standard Deviation	Minimum	Maximum
5	1.4780000	0.2141728	1.3100000	1.8400000

Unique Subject Identifier=1156000000-0021

Assay variable : PTINR_VAL1 PT-INR Value

N	Mean	Standard Deviation	Minimum	Maximum
2	1.2700000	0.3394113	1.0300000	1.5100000

Unique Subject Identifier=1156000000-0059

Assay variable : PTINR_VAL1 PT-INR Value

N	Mean	Standard Deviation	Minimum	Maximum
6	1.6850000	0.2930358	1.2700000	1.9800000

Unique Subject Identifier=1157000000-0014

Assay variable : PTINR_VAL1 PT-INR Value

N	Mean	Standard Deviation	Minimum	Maximum
6	2.2783333	0.4660651	1.8000000	3.0000000

Laboratory tests at the time of obtaining Table 2-6 consent, according to the presence or absence of proton-pump inhibitors

Unique Subject Identifier=1157000000-0028

MEANS Procedure

Assay variable : PTINR_VAL1 PT-INR Value

N	Mean	Standard Deviation	Minimum	Maximum
6	1.6500000	0.2509980	1.5000000	2.1000000

Unique Subject Identifier=1157000000-0029

Assay variable : PTINR_VAL1 PT-INR Value

N	Mean	Standard Deviation	Minimum	Maximum
6	2.1000000	0.4560702	1.5000000	2.8000000

Unique Subject Identifier=1158010000-0010

Assay variable : PTINR_VAL1 PT-INR Value

N	Mean	Standard Deviation	Minimum	Maximum
6	2.1350000	0.4554887	1.6400000	2.9800000

Unique Subject Identifier=1158010000-0015

Assay variable : PTINR_VAL1 PT-INR Value

N	Mean	Standard Deviation	Minimum	Maximum
6	2.4800000	0.4456905	1.8800000	2.8700000

Laboratory tests at the time of obtaining Table 2-6 consent, according to the presence or absence of proton-pump inhibitors

Unique Subject Identifier=1158010000-0026

MEANS Procedure

Assay variable : PTINR_VAL1 PT-INR Value

N	Mean	Standard Deviation	Minimum	Maximum
6	2.0000000	0.3034469	1.4700000	2.3300000

Unique Subject Identifier=1158010000-0031

Assay variable : PTINR_VAL1 PT-INR Value

N	Mean	Standard Deviation	Minimum	Maximum
6	2.3666667	0.3695763	1.9000000	3.0100000

Unique Subject Identifier=1158010000-0043

Assay variable : PTINR_VAL1 PT-INR Value

N	Mean	Standard Deviation	Minimum	Maximum
6	2.3216667	0.2634704	1.8300000	2.6300000

Unique Subject Identifier=1158010000-0058

Assay variable : PTINR_VAL1 PT-INR Value

N	Mean	Standard Deviation	Minimum	Maximum
6	1.9850000	0.1488288	1.8800000	2.2800000

Laboratory tests at the time of obtaining Table 2-6 consent, according to the presence or absence of proton-pump inhibitors

Unique Subject Identifier=1158010000-0067

MEANS Procedure

Assay variable : PTINR_VAL1 PT-INR Value

N	Mean	Standard Deviation	Minimum	Maximum
6	2.2433333	0.6172412	1.4900000	3.2600000

Unique Subject Identifier=1158010000-0071

Assay variable : PTINR_VAL1 PT-INR Value

N	Mean	Standard Deviation	Minimum	Maximum
6	2.4450000	0.6404295	1.4800000	3.2000000

Unique Subject Identifier=1158010000-0076

Assay variable : PTINR_VAL1 PT-INR Value

N	Mean	Standard Deviation	Minimum	Maximum
6	2.0983333	0.4266810	1.6000000	2.6300000

Unique Subject Identifier=1158010000-0093

Assay variable : PTINR_VAL1 PT-INR Value

N	Mean	Standard Deviation	Minimum	Maximum
6	2.0983333	0.4264935	1.5700000	2.8400000

Laboratory tests at the time of obtaining Table 2-6 consent, according to the presence or absence of proton-pump inhibitors

Unique Subject Identifier=1158010000-0095

MEANS Procedure

Assay variable : PTINR_VAL1 PT-INR Value

N	Mean	Standard Deviation	Minimum	Maximum
6	2.2450000	0.2022622	1.9700000	2.4800000

Unique Subject Identifier=1158010000-0105

Assay variable : PTINR_VAL1 PT-INR Value

N	Mean	Standard Deviation	Minimum	Maximum
6	2.0800000	0.3533836	1.5500000	2.5300000

Unique Subject Identifier=1158010000-0114

Assay variable : PTINR_VAL1 PT-INR Value

N	Mean	Standard Deviation	Minimum	Maximum
6	2.3900000	0.3985975	1.7700000	2.9800000

Unique Subject Identifier=1158010000-0124

Assay variable : PTINR_VAL1 PT-INR Value

N	Mean	Standard Deviation	Minimum	Maximum
6	1.9900000	0.7699610	1.1200000	3.2900000

Laboratory tests at the time of obtaining Table 2-6 consent, according to the presence or absence of proton-pump inhibitors

Unique Subject Identifier=1158010000-0125

MEANS Procedure

Assay variable : PTINR_VAL1 PT-INR Value

N	Mean	Standard Deviation	Minimum	Maximum
6	2.2600000	0.9979780	1.6400000	4.2600000

Unique Subject Identifier=1159000000-0001

Assay variable : PTINR_VAL1 PT-INR Value

N	Mean	Standard Deviation	Minimum	Maximum
6	2.1000000	0.1897367	1.8000000	2.3000000

Unique Subject Identifier=1159000000-0007

Assay variable : PTINR_VAL1 PT-INR Value

N	Mean	Standard Deviation	Minimum	Maximum
6	2.3000000	0.5549775	1.8000000	3.1000000

Unique Subject Identifier=1161010100-0025

Assay variable : PTINR_VAL1 PT-INR Value

N	Mean	Standard Deviation	Minimum	Maximum
2	2.3350000	0.8980256	1.7000000	2.9700000

Laboratory tests at the time of obtaining Table 2-6 consent, according to the presence or absence of proton-pump inhibitors

Unique Subject Identifier=1168011000-0006

MEANS Procedure

Assay variable : PTINR_VAL1 PT-INR Value

N	Mean	Standard Deviation	Minimum	Maximum
3	2.3966667	0.1674316	2.3000000	2.5900000

Unique Subject Identifier=1168011000-0011

Assay variable : PTINR_VAL1 PT-INR Value

N	Mean	Standard Deviation	Minimum	Maximum
3	2.1066667	0.1934770	1.9900000	2.3300000

Unique Subject Identifier=1168011000-0021

Assay variable : PTINR_VAL1 PT-INR Value

N	Mean	Standard Deviation	Minimum	Maximum
2	2.2450000	0.0777817	2.1900000	2.3000000

Unique Subject Identifier=1171000000-0004

Assay variable : PTINR_VAL1 PT-INR Value

N	Mean	Standard Deviation	Minimum	Maximum
6	1.3383333	0.0617792	1.2900000	1.4400000

Laboratory tests at the time of obtaining Table 2-6 consent, according to the presence or absence of proton-pump inhibitors

Unique Subject Identifier=1172011000-0007

MEANS Procedure

Assay variable : PTINR_VAL1 PT-INR Value

N	Mean	Standard Deviation	Minimum	Maximum
6	1.6833333	0.1329160	1.5000000	1.9000000

Unique Subject Identifier=1173011100-0002

Assay variable : PTINR_VAL1 PT-INR Value

N	Mean	Standard Deviation	Minimum	Maximum
6	1.7833333	0.1329160	1.6000000	2.0000000

Unique Subject Identifier=1173011100-0027

Assay variable : PTINR_VAL1 PT-INR Value

N	Mean	Standard Deviation	Minimum	Maximum
6	2.1666667	0.7174027	1.6000000	3.6000000

Unique Subject Identifier=1173011100-0041

Assay variable : PTINR_VAL1 PT-INR Value

N	Mean	Standard Deviation	Minimum	Maximum
5	1.6800000	0.3346640	1.4000000	2.2000000

Laboratory tests at the time of obtaining Table 2-6 consent, according to the presence or absence of proton-pump inhibitors

Unique Subject Identifier=1175011000-0002

MEANS Procedure

Assay variable : PTINR_VAL1 PT-INR Value

N	Mean	Standard Deviation	Minimum	Maximum
6	1.7333333	0.0516398	1.7000000	1.8000000

Unique Subject Identifier=1175011000-0008

Assay variable : PTINR_VAL1 PT-INR Value

N	Mean	Standard Deviation	Minimum	Maximum
6	2.0166667	0.2041241	1.7000000	2.3000000

Unique Subject Identifier=1175011000-0011

Assay variable : PTINR_VAL1 PT-INR Value

N	Mean	Standard Deviation	Minimum	Maximum
6	2.0666667	0.4885352	1.6000000	3.0000000

Unique Subject Identifier=1175011000-0018

Assay variable : PTINR_VAL1 PT-INR Value

N	Mean	Standard Deviation	Minimum	Maximum
6	2.3500000	0.6090977	1.5000000	3.3000000

Laboratory tests at the time of obtaining Table 2-6 consent, according to the presence or absence of proton-pump inhibitors

Unique Subject Identifier=1179000000-0001

MEANS Procedure

Assay variable : PTINR_VAL1 PT-INR Value

N	Mean	Standard Deviation	Minimum	Maximum
6	1.7000000	0.2280351	1.3000000	1.9000000

Unique Subject Identifier=1181000000-0011

Assay variable : PTINR_VAL1 PT-INR Value

N	Mean	Standard Deviation	Minimum	Maximum
2	1.3500000	0.0282843	1.3300000	1.3700000

Unique Subject Identifier=1181000000-0014

Assay variable : PTINR_VAL1 PT-INR Value

N	Mean	Standard Deviation	Minimum	Maximum
3	2.1600000	0.4396590	1.8000000	2.6500000

Unique Subject Identifier=1194000000-0008

Assay variable : PTINR_VAL1 PT-INR Value

N	Mean	Standard Deviation	Minimum	Maximum
3	2.3300000	0.4942671	1.9000000	2.8700000

Laboratory tests at the time of obtaining Table 2-6 consent, according to the presence or absence of proton-pump inhibitors

Unique Subject Identifier=1203010000-0001

MEANS Procedure

Assay variable : PTINR_VAL1 PT-INR Value

N	Mean	Standard Deviation	Minimum	Maximum
5	2.3280000	0.3598194	2.0300000	2.9400000

Unique Subject Identifier=1203010000-0007

Assay variable : PTINR_VAL1 PT-INR Value

N	Mean	Standard Deviation	Minimum	Maximum
6	2.6633333	0.5982530	2.0900000	3.7300000

Unique Subject Identifier=1203010000-0012

Assay variable : PTINR_VAL1 PT-INR Value

N	Mean	Standard Deviation	Minimum	Maximum
6	2.4400000	1.1452161	1.0100000	4.1600000

Unique Subject Identifier=1203010000-0016

Assay variable : PTINR_VAL1 PT-INR Value

N	Mean	Standard Deviation	Minimum	Maximum
6	2.3400000	0.4595215	1.6400000	3.0600000

Laboratory tests at the time of obtaining Table 2-6 consent, according to the presence or absence of proton-pump inhibitors

Unique Subject Identifier=1203010000-0024

MEANS Procedure

Assay variable : PTINR_VAL1 PT-INR Value

N	Mean	Standard Deviation	Minimum	Maximum
6	2.2000000	0.0822192	2.0600000	2.3000000

Unique Subject Identifier=1211000000-0028

Assay variable : PTINR_VAL1 PT-INR Value

N	Mean	Standard Deviation	Minimum	Maximum
5	2.1600000	0.3847077	1.7000000	2.5000000

Unique Subject Identifier=1211000000-0036

Assay variable : PTINR_VAL1 PT-INR Value

N	Mean	Standard Deviation	Minimum	Maximum
5	1.5200000	0.1303840	1.4000000	1.7000000

Unique Subject Identifier=1212000000-0003

Assay variable : PTINR_VAL1 PT-INR Value

N	Mean	Standard Deviation	Minimum	Maximum
6	2.2216667	0.3413747	1.9000000	2.6500000

Laboratory tests at the time of obtaining Table 2-6 consent, according to the presence or absence of proton-pump inhibitors

Unique Subject Identifier=1212000000-0005

MEANS Procedure

Assay variable : PTINR_VAL1 PT-INR Value

N	Mean	Standard Deviation	Minimum	Maximum
6	1.9483333	0.2242692	1.7200000	2.3500000

Unique Subject Identifier=1212000000-0011

Assay variable : PTINR_VAL1 PT-INR Value

N	Mean	Standard Deviation	Minimum	Maximum
6	1.7366667	0.2789743	1.3100000	2.1300000

Unique Subject Identifier=1212000000-0013

Assay variable : PTINR_VAL1 PT-INR Value

N	Mean	Standard Deviation	Minimum	Maximum
6	1.8283333	0.2626341	1.4700000	2.0900000

Unique Subject Identifier=1212000000-0017

Assay variable : PTINR_VAL1 PT-INR Value

N	Mean	Standard Deviation	Minimum	Maximum
5	2.4480000	0.3210452	2.1600000	2.9800000

Laboratory tests at the time of obtaining Table 2-6 consent, according to the presence or absence of proton-pump inhibitors

Unique Subject Identifier=1216000000-0003

MEANS Procedure

Assay variable : PTINR_VAL1 PT-INR Value

N	Mean	Standard Deviation	Minimum	Maximum
5	1.7820000	0.6529701	1.2300000	2.6000000

Unique Subject Identifier=1219000000-0002

Assay variable : PTINR_VAL1 PT-INR Value

N	Mean	Standard Deviation	Minimum	Maximum
4	2.3425000	0.6785954	1.7700000	3.2500000

Unique Subject Identifier=1219000000-0004

Assay variable : PTINR_VAL1 PT-INR Value

N	Mean	Standard Deviation	Minimum	Maximum
3	2.2533333	0.1289703	2.1100000	2.3600000

Unique Subject Identifier=1219000000-0007

Assay variable : PTINR_VAL1 PT-INR Value

N	Mean	Standard Deviation	Minimum	Maximum
2	2.1700000	0	2.1700000	2.1700000

Laboratory tests at the time of obtaining Table 2-6 consent, according to the presence or absence of proton-pump inhibitors

Unique Subject Identifier=1219000000-0010

MEANS Procedure

Assay variable : PTINR_VAL1 PT-INR Value

N	Mean	Standard Deviation	Minimum	Maximum
3	1.2733333	0.1700980	1.0800000	1.4000000

Unique Subject Identifier=1219000000-0028

Assay variable : PTINR_VAL1 PT-INR Value

N	Mean	Standard Deviation	Minimum	Maximum
4	2.2550000	0.4258717	1.6700000	2.6300000

Unique Subject Identifier=1219000000-0037

Assay variable : PTINR_VAL1 PT-INR Value

N	Mean	Standard Deviation	Minimum	Maximum
4	2.3400000	0.2230097	2.0900000	2.6300000

Unique Subject Identifier=1221000000-0003

Assay variable : PTINR_VAL1 PT-INR Value

N	Mean	Standard Deviation	Minimum	Maximum
5	1.9200000	0.3271085	1.7000000	2.5000000

Laboratory tests at the time of obtaining Table 2-6 consent, according to the presence or absence of proton-pump inhibitors

Unique Subject Identifier=1222000000-0003

MEANS Procedure

Assay variable : PTINR_VAL1 PT-INR Value

N	Mean	Standard Deviation	Minimum	Maximum
5	2.3000000	0.1224745	2.1000000	2.4000000

Unique Subject Identifier=1222000000-0007

Assay variable : PTINR_VAL1 PT-INR Value

N	Mean	Standard Deviation	Minimum	Maximum
4	2.0750000	0.0957427	2.0000000	2.2000000

Unique Subject Identifier=1222000000-0008

Assay variable : PTINR_VAL1 PT-INR Value

N	Mean	Standard Deviation	Minimum	Maximum
5	1.8200000	0.0836660	1.7000000	1.9000000

Unique Subject Identifier=1222000000-0011

Assay variable : PTINR_VAL1 PT-INR Value

N	Mean	Standard Deviation	Minimum	Maximum
5	1.6800000	0.1788854	1.5000000	1.9000000

Laboratory tests at the time of obtaining Table 2-6 consent, according to the presence or absence of proton-pump inhibitors

Unique Subject Identifier=1223010000-0007

MEANS Procedure

Assay variable : PTINR_VAL1 PT-INR Value

N	Mean	Standard Deviation	Minimum	Maximum
6	1.8666667	0.3932768	1.1000000	2.2000000

Unique Subject Identifier=1223010000-0013

Assay variable : PTINR_VAL1 PT-INR Value

N	Mean	Standard Deviation	Minimum	Maximum
5	1.7800000	0.1303840	1.6000000	1.9000000

Unique Subject Identifier=1223010000-0018

Assay variable : PTINR_VAL1 PT-INR Value

N	Mean	Standard Deviation	Minimum	Maximum
5	2.0200000	0.5761944	1.5000000	2.9000000

Unique Subject Identifier=1223010000-0021

Assay variable : PTINR_VAL1 PT-INR Value

N	Mean	Standard Deviation	Minimum	Maximum
6	1.8666667	0.1632993	1.7000000	2.1000000

Laboratory tests at the time of obtaining Table 2-6 consent, according to the presence or absence of proton-pump inhibitors

Unique Subject Identifier=1223010000-0038

MEANS Procedure

Assay variable : PTINR_VAL1 PT-INR Value

N	Mean	Standard Deviation	Minimum	Maximum
4	2.1500000	0.7937254	1.0000000	2.8000000

Unique Subject Identifier=1226000000-0007

Assay variable : PTINR_VAL1 PT-INR Value

N	Mean	Standard Deviation	Minimum	Maximum
3	1.7700000	0.2137756	1.6000000	2.0100000

Unique Subject Identifier=1226000000-0013

Assay variable : PTINR_VAL1 PT-INR Value

N	Mean	Standard Deviation	Minimum	Maximum
4	1.7850000	0.1103026	1.6400000	1.9000000

Unique Subject Identifier=1226000000-0030

Assay variable : PTINR_VAL1 PT-INR Value

N	Mean	Standard Deviation	Minimum	Maximum
4	2.1225000	0.4256270	1.6100000	2.6500000

Laboratory tests at the time of obtaining Table 2-6 consent, according to the presence or absence of proton-pump inhibitors

Unique Subject Identifier=1227000000-0028

MEANS Procedure

Assay variable : PTINR_VAL1 PT-INR Value

N	Mean	Standard Deviation	Minimum	Maximum
6	2.3833333	0.2228602	2.1000000	2.7000000

Unique Subject Identifier=1227000000-0034

Assay variable : PTINR_VAL1 PT-INR Value

N	Mean	Standard Deviation	Minimum	Maximum
5	2.2200000	0.5263079	1.4000000	2.8000000

Unique Subject Identifier=1227000000-0078

Assay variable : PTINR_VAL1 PT-INR Value

N	Mean	Standard Deviation	Minimum	Maximum
5	1.8600000	0.0894427	1.8000000	2.0000000

Unique Subject Identifier=1229000000-0016

Assay variable : PTINR_VAL1 PT-INR Value

N	Mean	Standard Deviation	Minimum	Maximum
6	2.1666667	0.2732520	1.8000000	2.5000000

Laboratory tests at the time of obtaining Table 2-6 consent, according to the presence or absence of proton-pump inhibitors

Unique Subject Identifier=1232000000-0005

MEANS Procedure

Assay variable : PTINR_VAL1 PT-INR Value

N	Mean	Standard Deviation	Minimum	Maximum
6	2.0833333	0.3920034	1.6000000	2.6000000

Unique Subject Identifier=1232000000-0008

Assay variable : PTINR_VAL1 PT-INR Value

N	Mean	Standard Deviation	Minimum	Maximum
5	2.0400000	0.2073644	1.7000000	2.2000000

Unique Subject Identifier=1234001000-0006

Assay variable : PTINR_VAL1 PT-INR Value

N	Mean	Standard Deviation	Minimum	Maximum
1	1.0300000		1.0300000	1.0300000

Unique Subject Identifier=1235000000-0006

Assay variable : PTINR_VAL1 PT-INR Value

N	Mean	Standard Deviation	Minimum	Maximum
3	1.4233333	0.1234234	1.3200000	1.5600000

Laboratory tests at the time of obtaining Table 2-6 consent, according to the presence or absence of proton-pump inhibitors

Unique Subject Identifier=1235000000-0009

MEANS Procedure

Assay variable : PTINR_VAL1 PT-INR Value

N	Mean	Standard Deviation	Minimum	Maximum
3	1.6466667	0.1616581	1.5000000	1.8200000

Unique Subject Identifier=1235000000-0010

Assay variable : PTINR_VAL1 PT-INR Value

N	Mean	Standard Deviation	Minimum	Maximum
6	1.6300000	0.2368122	1.2000000	1.8800000

Unique Subject Identifier=1235000000-0013

Assay variable : PTINR_VAL1 PT-INR Value

N	Mean	Standard Deviation	Minimum	Maximum
6	1.4433333	0.0871015	1.3600000	1.5500000

Unique Subject Identifier=1235000000-0017

Assay variable : PTINR_VAL1 PT-INR Value

N	Mean	Standard Deviation	Minimum	Maximum
5	1.5700000	0.1234909	1.4200000	1.7300000

Laboratory tests at the time of obtaining Table 2-6 consent, according to the presence or absence of proton-pump inhibitors

Unique Subject Identifier=1235000000-0021

MEANS Procedure

Assay variable : PTINR_VAL1 PT-INR Value

N	Mean	Standard Deviation	Minimum	Maximum
6	1.2966667	0.0920145	1.2100000	1.4100000

Unique Subject Identifier=1235000000-0024

Assay variable : PTINR_VAL1 PT-INR Value

N	Mean	Standard Deviation	Minimum	Maximum
6	1.3716667	0.0793515	1.2700000	1.5000000

Unique Subject Identifier=1235000000-0027

Assay variable : PTINR_VAL1 PT-INR Value

N	Mean	Standard Deviation	Minimum	Maximum
2	2.0100000	0.1131371	1.9300000	2.0900000

Unique Subject Identifier=1235000000-0034

Assay variable : PTINR_VAL1 PT-INR Value

N	Mean	Standard Deviation	Minimum	Maximum
6	1.6650000	0.3907301	1.3300000	2.3500000

Laboratory tests at the time of obtaining Table 2-6 consent, according to the presence or absence of proton-pump inhibitors

Unique Subject Identifier=1235000000-0042

MEANS Procedure

Assay variable : PTINR_VAL1 PT-INR Value

N	Mean	Standard Deviation	Minimum	Maximum
6	1.7666667	0.6168198	1.2600000	2.9900000

Unique Subject Identifier=1237000000-0011

Assay variable : PTINR_VAL1 PT-INR Value

N	Mean	Standard Deviation	Minimum	Maximum
3	2.0000000	0.1732051	1.9000000	2.2000000

Unique Subject Identifier=1237000000-0012

Assay variable : PTINR_VAL1 PT-INR Value

N	Mean	Standard Deviation	Minimum	Maximum
3	1.5333333	0.1527525	1.4000000	1.7000000

Unique Subject Identifier=1241000000-0005

Assay variable : PTINR_VAL1 PT-INR Value

N	Mean	Standard Deviation	Minimum	Maximum
1	2.7900000		2.7900000	2.7900000

Laboratory tests at the time of obtaining Table 2-6 consent, according to the presence or absence of proton-pump inhibitors

Unique Subject Identifier=1241000000-0006

MEANS Procedure

Assay variable : PTINR_VAL1 PT-INR Value

N	Mean	Standard Deviation	Minimum	Maximum
5	2.8220000	0.7436868	1.5500000	3.4700000

Unique Subject Identifier=1248000000-0001

Assay variable : PTINR_VAL1 PT-INR Value

N	Mean	Standard Deviation	Minimum	Maximum
2	2.4450000	0.3323402	2.2100000	2.6800000

Unique Subject Identifier=1255011110-0002

Assay variable : PTINR_VAL1 PT-INR Value

N	Mean	Standard Deviation	Minimum	Maximum
2	2.4200000	0.3535534	2.1700000	2.6700000

Unique Subject Identifier=1255011110-0004

Assay variable : PTINR_VAL1 PT-INR Value

N	Mean	Standard Deviation	Minimum	Maximum
1	2.3000000		2.3000000	2.3000000

Laboratory tests at the time of obtaining Table 2-6 consent, according to the presence or absence of proton-pump inhibitors

Unique Subject Identifier=1258000000-0005

MEANS Procedure

Assay variable : PTINR_VAL1 PT-INR Value

N	Mean	Standard Deviation	Minimum	Maximum
6	2.0050000	0.3219161	1.8500000	2.6600000

Unique Subject Identifier=1262000000-0016

Assay variable : PTINR_VAL1 PT-INR Value

N	Mean	Standard Deviation	Minimum	Maximum
2	1.5000000	0	1.5000000	1.5000000

Unique Subject Identifier=1262000000-0020

Assay variable : PTINR_VAL1 PT-INR Value

N	Mean	Standard Deviation	Minimum	Maximum
2	1.6000000	0.1414214	1.5000000	1.7000000

Unique Subject Identifier=1262000000-0023

Assay variable : PTINR_VAL1 PT-INR Value

N	Mean	Standard Deviation	Minimum	Maximum
1	1.7000000		1.7000000	1.7000000

Laboratory tests at the time of obtaining Table 2-6 consent, according to the presence or absence of proton-pump inhibitors

Unique Subject Identifier=1262000000-0027

MEANS Procedure

Assay variable : PTINR_VAL1 PT-INR Value

N	Mean	Standard Deviation	Minimum	Maximum
2	1.9500000	0.6363961	1.5000000	2.4000000

Unique Subject Identifier=1262000000-0058

Assay variable : PTINR_VAL1 PT-INR Value

N	Mean	Standard Deviation	Minimum	Maximum
1	1.3000000		1.3000000	1.3000000

Unique Subject Identifier=1262000000-0064

Assay variable : PTINR_VAL1 PT-INR Value

N	Mean	Standard Deviation	Minimum	Maximum
2	1.9000000	0.2828427	1.7000000	2.1000000

Unique Subject Identifier=1262000000-0075

Assay variable : PTINR_VAL1 PT-INR Value

N	Mean	Standard Deviation	Minimum	Maximum
2	1.4500000	0.2121320	1.3000000	1.6000000

Laboratory tests at the time of obtaining Table 2-6 consent, according to the presence or absence of proton-pump inhibitors

Unique Subject Identifier=1262000000-0079

MEANS Procedure

Assay variable : PTINR_VAL1 PT-INR Value

N	Mean	Standard Deviation	Minimum	Maximum
2	2.2500000	0.3535534	2.0000000	2.5000000

Unique Subject Identifier=1263011000-0004

Assay variable : PTINR_VAL1 PT-INR Value

N	Mean	Standard Deviation	Minimum	Maximum
6	2.3133333	0.2189673	2.0900000	2.5800000

Unique Subject Identifier=1263011000-0007

Assay variable : PTINR_VAL1 PT-INR Value

N	Mean	Standard Deviation	Minimum	Maximum
4	2.5700000	0.9076343	1.6000000	3.6700000

Unique Subject Identifier=1263011000-0018

Assay variable : PTINR_VAL1 PT-INR Value

N	Mean	Standard Deviation	Minimum	Maximum
6	2.3416667	0.2632426	1.9700000	2.6100000

Laboratory tests at the time of obtaining Table 2-6 consent, according to the presence or absence of proton-pump inhibitors

Unique Subject Identifier=1264001000-0007

MEANS Procedure

Assay variable : PTINR_VAL1 PT-INR Value

N	Mean	Standard Deviation	Minimum	Maximum
6	2.1166667	0.6080022	1.4000000	2.8000000

Unique Subject Identifier=1266000000-0001

Assay variable : PTINR_VAL1 PT-INR Value

N	Mean	Standard Deviation	Minimum	Maximum
6	2.0283333	0.3773283	1.4000000	2.4500000

Unique Subject Identifier=1266000000-0020

Assay variable : PTINR_VAL1 PT-INR Value

N	Mean	Standard Deviation	Minimum	Maximum
6	1.4316667	0.1837843	1.2000000	1.7000000

Unique Subject Identifier=1266000000-0021

Assay variable : PTINR_VAL1 PT-INR Value

N	Mean	Standard Deviation	Minimum	Maximum
6	2.1716667	0.3237540	1.8000000	2.7000000

Laboratory tests at the time of obtaining Table 2-6 consent, according to the presence or absence of proton-pump inhibitors

Unique Subject Identifier=1266000000-0029

MEANS Procedure

Assay variable : PTINR_VAL1 PT-INR Value

N	Mean	Standard Deviation	Minimum	Maximum
6	1.9283333	0.2204011	1.6400000	2.2800000

Unique Subject Identifier=1266000000-0038

Assay variable : PTINR_VAL1 PT-INR Value

N	Mean	Standard Deviation	Minimum	Maximum
6	2.1250000	0.1740977	1.9000000	2.4000000

Unique Subject Identifier=1266000000-0047

Assay variable : PTINR_VAL1 PT-INR Value

N	Mean	Standard Deviation	Minimum	Maximum
6	1.4850000	0.2843062	1.1900000	1.8700000

Unique Subject Identifier=1266000000-0049

Assay variable : PTINR_VAL1 PT-INR Value

N	Mean	Standard Deviation	Minimum	Maximum
6	2.5066667	1.1082719	1.5000000	4.6000000

Laboratory tests at the time of obtaining Table 2-6 consent, according to the presence or absence of proton-pump inhibitors

Unique Subject Identifier=1266000000-0051

MEANS Procedure

Assay variable : PTINR_VAL1 PT-INR Value

N	Mean	Standard Deviation	Minimum	Maximum
5	2.0340000	0.3240062	1.6100000	2.5000000

Unique Subject Identifier=1266000000-0053

Assay variable : PTINR_VAL1 PT-INR Value

N	Mean	Standard Deviation	Minimum	Maximum
6	2.4983333	0.1980320	2.2000000	2.8000000

Unique Subject Identifier=1266000000-0055

Assay variable : PTINR_VAL1 PT-INR Value

N	Mean	Standard Deviation	Minimum	Maximum
6	2.1800000	0.2497999	1.8000000	2.5800000

Unique Subject Identifier=1266000000-0059

Assay variable : PTINR_VAL1 PT-INR Value

N	Mean	Standard Deviation	Minimum	Maximum
6	2.2900000	0.4923007	1.9000000	3.2000000

Laboratory tests at the time of obtaining Table 2-6 consent, according to the presence or absence of proton-pump inhibitors

Unique Subject Identifier=1266000000-0063

MEANS Procedure

Assay variable : PTINR_VAL1 PT-INR Value

N	Mean	Standard Deviation	Minimum	Maximum
6	2.3433333	0.5746883	1.6000000	2.9000000

Unique Subject Identifier=1266000000-0068

Assay variable : PTINR_VAL1 PT-INR Value

N	Mean	Standard Deviation	Minimum	Maximum
6	1.8616667	0.1397736	1.7000000	2.1000000

Unique Subject Identifier=1269000000-0002

Assay variable : PTINR_VAL1 PT-INR Value

N	Mean	Standard Deviation	Minimum	Maximum
6	1.6083333	0.4269153	1.0500000	2.2500000

Unique Subject Identifier=1269000000-0007

Assay variable : PTINR_VAL1 PT-INR Value

N	Mean	Standard Deviation	Minimum	Maximum
4	1.8625000	0.1990603	1.6400000	2.1200000

Laboratory tests at the time of obtaining Table 2-6 consent, according to the presence or absence of proton-pump inhibitors

Unique Subject Identifier=1269000000-0009

MEANS Procedure

Assay variable : PTINR_VAL1 PT-INR Value

N	Mean	Standard Deviation	Minimum	Maximum
6	1.8533333	0.3991324	1.1900000	2.1600000

Unique Subject Identifier=1269000000-0010

Assay variable : PTINR_VAL1 PT-INR Value

N	Mean	Standard Deviation	Minimum	Maximum
5	1.8400000	0.2164486	1.5600000	2.0500000

Unique Subject Identifier=1269000000-0012

Assay variable : PTINR_VAL1 PT-INR Value

N	Mean	Standard Deviation	Minimum	Maximum
6	1.8933333	0.1742029	1.7200000	2.1600000

Unique Subject Identifier=1269000000-0020

Assay variable : PTINR_VAL1 PT-INR Value

N	Mean	Standard Deviation	Minimum	Maximum
6	1.5016667	0.1826928	1.2100000	1.7000000

Laboratory tests at the time of obtaining Table 2-6 consent, according to the presence or absence of proton-pump inhibitors

Unique Subject Identifier=1270000000-0002

MEANS Procedure

Assay variable : PTINR_VAL1 PT-INR Value

N	Mean	Standard Deviation	Minimum	Maximum
3	2.0933333	0.0404145	2.0500000	2.1300000

Unique Subject Identifier=1270000000-0004

Assay variable : PTINR_VAL1 PT-INR Value

N	Mean	Standard Deviation	Minimum	Maximum
2	1.9000000	0.1414214	1.8000000	2.0000000

Unique Subject Identifier=1276000000-0004

Assay variable : PTINR_VAL1 PT-INR Value

N	Mean	Standard Deviation	Minimum	Maximum
4	1.7250000	0.2747726	1.4300000	2.0600000

Unique Subject Identifier=1276000000-0005

Assay variable : PTINR_VAL1 PT-INR Value

N	Mean	Standard Deviation	Minimum	Maximum
3	2.1000000	0.2951271	1.8000000	2.3900000

Laboratory tests at the time of obtaining Table 2-6 consent, according to the presence or absence of proton-pump inhibitors

Unique Subject Identifier=1276000000-0008

MEANS Procedure

Assay variable : PTINR_VAL1 PT-INR Value

N	Mean	Standard Deviation	Minimum	Maximum
3	1.8633333	0.0665833	1.8200000	1.9400000

Unique Subject Identifier=1278011100-0013

Assay variable : PTINR_VAL1 PT-INR Value

N	Mean	Standard Deviation	Minimum	Maximum
2	1.6900000	0.0141421	1.6800000	1.7000000

Unique Subject Identifier=1286000000-0026

Assay variable : PTINR_VAL1 PT-INR Value

N	Mean	Standard Deviation	Minimum	Maximum
6	2.2833333	0.9453394	1.4000000	4.0000000

Unique Subject Identifier=1286000000-0027

Assay variable : PTINR_VAL1 PT-INR Value

N	Mean	Standard Deviation	Minimum	Maximum
6	2.1166667	0.1602082	2.0000000	2.4000000

Laboratory tests at the time of obtaining Table 2-6 consent, according to the presence or absence of proton-pump inhibitors

Unique Subject Identifier=1286000000-0028

MEANS Procedure

Assay variable : PTINR_VAL1 PT-INR Value

N	Mean	Standard Deviation	Minimum	Maximum
6	1.9333333	0.7501111	1.3000000	3.4000000

Unique Subject Identifier=1289000000-0006

Assay variable : PTINR_VAL1 PT-INR Value

N	Mean	Standard Deviation	Minimum	Maximum
3	1.6366667	0.1833939	1.4300000	1.7800000

Unique Subject Identifier=1289000000-0009

Assay variable : PTINR_VAL1 PT-INR Value

N	Mean	Standard Deviation	Minimum	Maximum
3	2.1033333	0.4000417	1.7000000	2.5000000

Unique Subject Identifier=1290011000-0004

Assay variable : PTINR_VAL1 PT-INR Value

N	Mean	Standard Deviation	Minimum	Maximum
3	1.5233333	0.0680686	1.4700000	1.6000000

Laboratory tests at the time of obtaining Table 2-6 consent, according to the presence or absence of proton-pump inhibitors

Unique Subject Identifier=1290011000-0005

MEANS Procedure

Assay variable : PTINR_VAL1 PT-INR Value

N	Mean	Standard Deviation	Minimum	Maximum
2	1.7150000	0.2333452	1.5500000	1.8800000

Unique Subject Identifier=1290011000-0011

Assay variable : PTINR_VAL1 PT-INR Value

N	Mean	Standard Deviation	Minimum	Maximum
2	1.7600000	0.6929646	1.2700000	2.2500000

Unique Subject Identifier=1291000000-0001

Assay variable : PTINR_VAL1 PT-INR Value

N	Mean	Standard Deviation	Minimum	Maximum
4	2.0500000	0.4509250	1.7000000	2.7000000

Unique Subject Identifier=1291000000-0006

Assay variable : PTINR_VAL1 PT-INR Value

N	Mean	Standard Deviation	Minimum	Maximum
5	2.1400000	0.5727128	1.4000000	2.8000000

Laboratory tests at the time of obtaining Table 2-6 consent, according to the presence or absence of proton-pump inhibitors

Unique Subject Identifier=1291000000-0007

MEANS Procedure

Assay variable : PTINR_VAL1 PT-INR Value

N	Mean	Standard Deviation	Minimum	Maximum
4	2.5500000	0.6557439	2.0000000	3.5000000

Unique Subject Identifier=1291000000-0008

Assay variable : PTINR_VAL1 PT-INR Value

N	Mean	Standard Deviation	Minimum	Maximum
3	2.1333333	0.3511885	1.8000000	2.5000000

Unique Subject Identifier=1291000000-0010

Assay variable : PTINR_VAL1 PT-INR Value

N	Mean	Standard Deviation	Minimum	Maximum
4	1.8500000	0.1732051	1.6000000	2.0000000

Unique Subject Identifier=1291000000-0013

Assay variable : PTINR_VAL1 PT-INR Value

N	Mean	Standard Deviation	Minimum	Maximum
6	1.9166667	0.6013873	1.2000000	3.0000000

Laboratory tests at the time of obtaining Table 2-6 consent, according to the presence or absence of proton-pump inhibitors

Unique Subject Identifier=1291000000-0017

MEANS Procedure

Assay variable : PTINR_VAL1 PT-INR Value

N	Mean	Standard Deviation	Minimum	Maximum
6	1.5333333	0.2732520	1.1000000	1.9000000

Unique Subject Identifier=1293000000-0006

Assay variable : PTINR_VAL1 PT-INR Value

N	Mean	Standard Deviation	Minimum	Maximum
3	1.3333333	0.4163332	1.0000000	1.8000000

Unique Subject Identifier=1293000000-0022

Assay variable : PTINR_VAL1 PT-INR Value

N	Mean	Standard Deviation	Minimum	Maximum
5	1.3000000	0.1000000	1.2000000	1.4000000

Unique Subject Identifier=1293000000-0030

Assay variable : PTINR_VAL1 PT-INR Value

N	Mean	Standard Deviation	Minimum	Maximum
6	1.3333333	0.1751190	1.0000000	1.5000000

Laboratory tests at the time of obtaining Table 2-6 consent, according to the presence or absence of proton-pump inhibitors

Unique Subject Identifier=1294011001-0009

MEANS Procedure

Assay variable : PTINR_VAL1 PT-INR Value

N	Mean	Standard Deviation	Minimum	Maximum
6	2.4700000	0.3459480	1.9100000	2.9000000

Unique Subject Identifier=1298011000-0006

Assay variable : PTINR_VAL1 PT-INR Value

N	Mean	Standard Deviation	Minimum	Maximum
6	2.2666667	0.3723797	1.9000000	2.9000000

Unique Subject Identifier=1298011000-0025

Assay variable : PTINR_VAL1 PT-INR Value

N	Mean	Standard Deviation	Minimum	Maximum
5	1.5800000	0.2774887	1.2000000	1.9000000

Unique Subject Identifier=1298011000-0027

Assay variable : PTINR_VAL1 PT-INR Value

N	Mean	Standard Deviation	Minimum	Maximum
6	2.4666667	0.2943920	2.1000000	2.8000000

Laboratory tests at the time of obtaining Table 2-6 consent, according to the presence or absence of proton-pump inhibitors

Unique Subject Identifier=1303000000-0001

MEANS Procedure

Assay variable : PTINR_VAL1 PT-INR Value

N	Mean	Standard Deviation	Minimum	Maximum
5	1.8600000	0.4159327	1.4000000	2.5000000

Unique Subject Identifier=1303000000-0004

Assay variable : PTINR_VAL1 PT-INR Value

N	Mean	Standard Deviation	Minimum	Maximum
5	2.1800000	0.2774887	1.8000000	2.5000000

Unique Subject Identifier=1303000000-0012

Assay variable : PTINR_VAL1 PT-INR Value

N	Mean	Standard Deviation	Minimum	Maximum
5	1.9800000	0.5848077	1.6000000	3.0000000

Unique Subject Identifier=1303000000-0013

Assay variable : PTINR_VAL1 PT-INR Value

N	Mean	Standard Deviation	Minimum	Maximum
6	1.8500000	0.2073644	1.5000000	2.1000000

Laboratory tests at the time of obtaining Table 2-6 consent, according to the presence or absence of proton-pump inhibitors

Unique Subject Identifier=1303000000-0026

MEANS Procedure

Assay variable : PTINR_VAL1 PT-INR Value

N	Mean	Standard Deviation	Minimum	Maximum
5	1.9200000	0.3271085	1.5000000	2.3000000

Unique Subject Identifier=1303000000-0027

Assay variable : PTINR_VAL1 PT-INR Value

N	Mean	Standard Deviation	Minimum	Maximum
5	1.7400000	0.3577709	1.4000000	2.2000000

Unique Subject Identifier=1308000000-0003

Assay variable : PTINR_VAL1 PT-INR Value

N	Mean	Standard Deviation	Minimum	Maximum
6	1.9550000	0.3385705	1.4200000	2.4200000

Unique Subject Identifier=1313000000-0014

Assay variable : PTINR_VAL1 PT-INR Value

N	Mean	Standard Deviation	Minimum	Maximum
1	1.5100000		1.5100000	1.5100000

Laboratory tests at the time of obtaining Table 2-6 consent, according to the presence or absence of proton-pump inhibitors

Unique Subject Identifier=1314010000-0007

MEANS Procedure

Assay variable : PTINR_VAL1 PT-INR Value

N	Mean	Standard Deviation	Minimum	Maximum
1	1.5000000		1.5000000	1.5000000

Unique Subject Identifier=1319000000-0009

Assay variable : PTINR_VAL1 PT-INR Value

N	Mean	Standard Deviation	Minimum	Maximum
5	1.9000000	0.3464102	1.4000000	2.2000000

Unique Subject Identifier=1319000000-0019

Assay variable : PTINR_VAL1 PT-INR Value

N	Mean	Standard Deviation	Minimum	Maximum
6	2.5166667	1.0068101	1.7000000	4.4000000

Unique Subject Identifier=1320000000-0006

Assay variable : PTINR_VAL1 PT-INR Value

N	Mean	Standard Deviation	Minimum	Maximum
4	1.9150000	0.3267517	1.4400000	2.1400000

Laboratory tests at the time of obtaining Table 2-6 consent, according to the presence or absence of proton-pump inhibitors

Unique Subject Identifier=1320000000-0008

MEANS Procedure

Assay variable : PTINR_VAL1 PT-INR Value

N	Mean	Standard Deviation	Minimum	Maximum
5	2.0800000	0.2589401	1.7800000	2.3200000

Unique Subject Identifier=1320000000-0012

Assay variable : PTINR_VAL1 PT-INR Value

N	Mean	Standard Deviation	Minimum	Maximum
6	1.7816667	0.0879583	1.6700000	1.9000000

Unique Subject Identifier=1320000000-0016

Assay variable : PTINR_VAL1 PT-INR Value

N	Mean	Standard Deviation	Minimum	Maximum
6	2.5616667	0.6723218	1.6800000	3.2600000

Unique Subject Identifier=1320000000-0018

Assay variable : PTINR_VAL1 PT-INR Value

N	Mean	Standard Deviation	Minimum	Maximum
5	1.9200000	0.3896794	1.3300000	2.2700000

Laboratory tests at the time of obtaining Table 2-6 consent, according to the presence or absence of proton-pump inhibitors

Unique Subject Identifier=1320000000-0028

MEANS Procedure

Assay variable : PTINR_VAL1 PT-INR Value

N	Mean	Standard Deviation	Minimum	Maximum
6	1.9716667	0.1146153	1.8000000	2.1000000

Unique Subject Identifier=1320000000-0030

Assay variable : PTINR_VAL1 PT-INR Value

N	Mean	Standard Deviation	Minimum	Maximum
6	1.9950000	0.1315675	1.7700000	2.1500000

Unique Subject Identifier=1321011110-0025

Assay variable : PTINR_VAL1 PT-INR Value

N	Mean	Standard Deviation	Minimum	Maximum
6	2.9550000	0.5216416	2.4000000	3.7400000

Unique Subject Identifier=1324111000-0004

Assay variable : PTINR_VAL1 PT-INR Value

N	Mean	Standard Deviation	Minimum	Maximum
6	2.0333333	0.6501282	1.2000000	3.1000000

Laboratory tests at the time of obtaining Table 2-6 consent, according to the presence or absence of proton-pump inhibitors

Unique Subject Identifier=1324111000-0013

MEANS Procedure

Assay variable : PTINR_VAL1 PT-INR Value

N	Mean	Standard Deviation	Minimum	Maximum
6	1.6500000	0.2428992	1.4000000	2.0000000

Unique Subject Identifier=1324111000-0015

Assay variable : PTINR_VAL1 PT-INR Value

N	Mean	Standard Deviation	Minimum	Maximum
5	2.0800000	0.3962323	1.6000000	2.6000000

Unique Subject Identifier=1324111000-0019

Assay variable : PTINR_VAL1 PT-INR Value

N	Mean	Standard Deviation	Minimum	Maximum
6	1.2333333	0.0516398	1.2000000	1.3000000

Unique Subject Identifier=1326000000-0005

Assay variable : PTINR_VAL1 PT-INR Value

N	Mean	Standard Deviation	Minimum	Maximum
5	0.9000000	0	0.9000000	0.9000000

Laboratory tests at the time of obtaining Table 2-6 consent, according to the presence or absence of proton-pump inhibitors

Unique Subject Identifier=1326000000-0008

MEANS Procedure

Assay variable : PTINR_VAL1 PT-INR Value

N	Mean	Standard Deviation	Minimum	Maximum
6	1.3500000	0.0836660	1.3000000	1.5000000

Unique Subject Identifier=1326000000-0009

Assay variable : PTINR_VAL1 PT-INR Value

N	Mean	Standard Deviation	Minimum	Maximum
6	1.0000000	0	1.0000000	1.0000000

Unique Subject Identifier=1326000000-0010

Assay variable : PTINR_VAL1 PT-INR Value

N	Mean	Standard Deviation	Minimum	Maximum
6	1.1000000	0.0632456	1.0000000	1.2000000

Unique Subject Identifier=1328011111-0043

Assay variable : PTINR_VAL1 PT-INR Value

N	Mean	Standard Deviation	Minimum	Maximum
6	1.8166667	0.2701604	1.4700000	2.2800000

Laboratory tests at the time of obtaining Table 2-6 consent, according to the presence or absence of proton-pump inhibitors

Unique Subject Identifier=1328011111-0050

MEANS Procedure

Assay variable : PTINR_VAL1 PT-INR Value

N	Mean	Standard Deviation	Minimum	Maximum
6	1.7950000	0.0926823	1.6300000	1.9000000

Unique Subject Identifier=1328011111-0060

Assay variable : PTINR_VAL1 PT-INR Value

N	Mean	Standard Deviation	Minimum	Maximum
2	1.2050000	0.2333452	1.0400000	1.3700000

Unique Subject Identifier=1328011111-0075

Assay variable : PTINR_VAL1 PT-INR Value

N	Mean	Standard Deviation	Minimum	Maximum
1	1.9500000		1.9500000	1.9500000

Unique Subject Identifier=1328011111-0086

Assay variable : PTINR_VAL1 PT-INR Value

N	Mean	Standard Deviation	Minimum	Maximum
6	1.6766667	0.2522433	1.3700000	2.0900000

Laboratory tests at the time of obtaining Table 2-6 consent, according to the presence or absence of proton-pump inhibitors

Unique Subject Identifier=1341011111-0008

MEANS Procedure

Assay variable : PTINR_VAL1 PT-INR Value

N	Mean	Standard Deviation	Minimum	Maximum
5	1.9160000	0.1283745	1.7000000	2.0400000

Unique Subject Identifier=1351010111-0003

Assay variable : PTINR_VAL1 PT-INR Value

N	Mean	Standard Deviation	Minimum	Maximum
6	2.1666667	0.3386247	1.9000000	2.8000000

Unique Subject Identifier=1351010111-0018

Assay variable : PTINR_VAL1 PT-INR Value

N	Mean	Standard Deviation	Minimum	Maximum
6	2.1166667	0.2994439	1.7000000	2.5000000

Unique Subject Identifier=1353000000-0050

Assay variable : PTINR_VAL1 PT-INR Value

N	Mean	Standard Deviation	Minimum	Maximum
1	1.2000000		1.2000000	1.2000000

Laboratory tests at the time of obtaining Table 2-6 consent, according to the presence or absence of proton-pump inhibitors

Unique Subject Identifier=1354010000-0012

MEANS Procedure

Assay variable : PTINR_VAL1 PT-INR Value

N	Mean	Standard Deviation	Minimum	Maximum
6	1.8100000	0.1186592	1.6400000	1.9700000

Unique Subject Identifier=1354010000-0013

Assay variable : PTINR_VAL1 PT-INR Value

N	Mean	Standard Deviation	Minimum	Maximum
5	1.9380000	0.4510211	1.6000000	2.7000000

Unique Subject Identifier=1354010000-0016

Assay variable : PTINR_VAL1 PT-INR Value

N	Mean	Standard Deviation	Minimum	Maximum
3	1.9233333	0.2315887	1.7300000	2.1800000

Unique Subject Identifier=1354010000-0034

Assay variable : PTINR_VAL1 PT-INR Value

N	Mean	Standard Deviation	Minimum	Maximum
6	1.8083333	0.1801573	1.5800000	2.0800000

Laboratory tests at the time of obtaining Table 2-6 consent, according to the presence or absence of proton-pump inhibitors

Unique Subject Identifier=1354010000-0037

MEANS Procedure

Assay variable : PTINR_VAL1 PT-INR Value

N	Mean	Standard Deviation	Minimum	Maximum
6	1.4383333	0.1360025	1.3000000	1.6500000

Unique Subject Identifier=1354010000-0045

Assay variable : PTINR_VAL1 PT-INR Value

N	Mean	Standard Deviation	Minimum	Maximum
6	1.8366667	0.3957609	1.3400000	2.5100000

Unique Subject Identifier=1354010000-0052

Assay variable : PTINR_VAL1 PT-INR Value

N	Mean	Standard Deviation	Minimum	Maximum
6	2.2083333	1.4976838	1.1600000	5.1000000

Unique Subject Identifier=1354010000-0056

Assay variable : PTINR_VAL1 PT-INR Value

N	Mean	Standard Deviation	Minimum	Maximum
6	1.7100000	0.1572260	1.5300000	1.9800000

Laboratory tests at the time of obtaining Table 2-6 consent, according to the presence or absence of proton-pump inhibitors

Unique Subject Identifier=1365010000-0002

MEANS Procedure

Assay variable : PTINR_VAL1 PT-INR Value

N	Mean	Standard Deviation	Minimum	Maximum
4	2.0400000	0.2174090	1.7600000	2.2900000

Unique Subject Identifier=1365010000-0007

Assay variable : PTINR_VAL1 PT-INR Value

N	Mean	Standard Deviation	Minimum	Maximum
6	2.0833333	0.4950421	1.5000000	2.7000000

Unique Subject Identifier=1365010000-0008

Assay variable : PTINR_VAL1 PT-INR Value

N	Mean	Standard Deviation	Minimum	Maximum
5	1.8940000	0.3383489	1.6100000	2.3200000

Unique Subject Identifier=1365010000-0019

Assay variable : PTINR_VAL1 PT-INR Value

N	Mean	Standard Deviation	Minimum	Maximum
3	1.7700000	0.2000000	1.5700000	1.9700000

Laboratory tests at the time of obtaining Table 2-6 consent, according to the presence or absence of proton-pump inhibitors

Unique Subject Identifier=1370010000-0012

MEANS Procedure

Assay variable : PTINR_VAL1 PT-INR Value

N	Mean	Standard Deviation	Minimum	Maximum
1	2.1800000		2.1800000	2.1800000

Unique Subject Identifier=1371000000-0011

Assay variable : PTINR_VAL1 PT-INR Value

N	Mean	Standard Deviation	Minimum	Maximum
5	1.8440000	0.2234502	1.4800000	2.0200000

Unique Subject Identifier=1373000000-0002

Assay variable : PTINR_VAL1 PT-INR Value

N	Mean	Standard Deviation	Minimum	Maximum
5	1.4800000	0.2774887	1.2000000	1.9000000

Unique Subject Identifier=1377011000-0003

Assay variable : PTINR_VAL1 PT-INR Value

N	Mean	Standard Deviation	Minimum	Maximum
4	1.5925000	0.0780491	1.4900000	1.6800000

Laboratory tests at the time of obtaining Table 2-6 consent, according to the presence or absence of proton-pump inhibitors

Unique Subject Identifier=1377011000-0011

MEANS Procedure

Assay variable : PTINR_VAL1 PT-INR Value

N	Mean	Standard Deviation	Minimum	Maximum
6	2.2766667	0.4296821	1.7900000	2.8700000

Unique Subject Identifier=1380000110-0008

Assay variable : PTINR_VAL1 PT-INR Value

N	Mean	Standard Deviation	Minimum	Maximum
6	1.8500000	0.5576737	1.4000000	2.8000000

Unique Subject Identifier=1380000110-0011

Assay variable : PTINR_VAL1 PT-INR Value

N	Mean	Standard Deviation	Minimum	Maximum
6	2.0000000	0.2366432	1.6000000	2.3000000

Unique Subject Identifier=1380000110-0022

Assay variable : PTINR_VAL1 PT-INR Value

N	Mean	Standard Deviation	Minimum	Maximum
6	2.0666667	0.3444803	1.6000000	2.5000000

Laboratory tests at the time of obtaining Table 2-6 consent, according to the presence or absence of proton-pump inhibitors

Unique Subject Identifier=1380000110-0024

MEANS Procedure

Assay variable : PTINR_VAL1 PT-INR Value

N	Mean	Standard Deviation	Minimum	Maximum
6	1.8666667	0.2875181	1.4000000	2.2000000

Unique Subject Identifier=1385011000-0011

Assay variable : PTINR_VAL1 PT-INR Value

N	Mean	Standard Deviation	Minimum	Maximum
5	2.0820000	0.2811050	1.7200000	2.4100000

Unique Subject Identifier=1385011000-0014

Assay variable : PTINR_VAL1 PT-INR Value

N	Mean	Standard Deviation	Minimum	Maximum
3	2.4733333	0.7379928	1.7000000	3.1700000

Unique Subject Identifier=1385011000-0016

Assay variable : PTINR_VAL1 PT-INR Value

N	Mean	Standard Deviation	Minimum	Maximum
3	2.1500000	0.0888819	2.0800000	2.2500000

Laboratory tests at the time of obtaining Table 2-6 consent, according to the presence or absence of proton-pump inhibitors

Unique Subject Identifier=1385011000-0027

MEANS Procedure

Assay variable : PTINR_VAL1 PT-INR Value

N	Mean	Standard Deviation	Minimum	Maximum
2	2.1200000	0.0282843	2.1000000	2.1400000

Unique Subject Identifier=1385011000-0028

Assay variable : PTINR_VAL1 PT-INR Value

N	Mean	Standard Deviation	Minimum	Maximum
3	2.2933333	0.0450925	2.2500000	2.3400000

Unique Subject Identifier=1385011000-0044

Assay variable : PTINR_VAL1 PT-INR Value

N	Mean	Standard Deviation	Minimum	Maximum
3	2.1200000	0.6100000	1.5100000	2.7300000

Unique Subject Identifier=1385011000-0059

Assay variable : PTINR_VAL1 PT-INR Value

N	Mean	Standard Deviation	Minimum	Maximum
4	1.6200000	0.1346601	1.4200000	1.7000000

Laboratory tests at the time of obtaining Table 2-6 consent, according to the presence or absence of proton-pump inhibitors

Unique Subject Identifier=1385011000-0061

MEANS Procedure

Assay variable : PTINR_VAL1 PT-INR Value

N	Mean	Standard Deviation	Minimum	Maximum
3	2.2866667	0.0152753	2.2700000	2.3000000

Unique Subject Identifier=1385011000-0065

Assay variable : PTINR_VAL1 PT-INR Value

N	Mean	Standard Deviation	Minimum	Maximum
3	2.2500000	0.0600000	2.1900000	2.3100000

Unique Subject Identifier=1386000000-0017

Assay variable : PTINR_VAL1 PT-INR Value

N	Mean	Standard Deviation	Minimum	Maximum
2	1.8150000	0.5444722	1.4300000	2.2000000

Unique Subject Identifier=1386000000-0018

Assay variable : PTINR_VAL1 PT-INR Value

N	Mean	Standard Deviation	Minimum	Maximum
4	1.9650000	0.3481858	1.6000000	2.3900000

Laboratory tests at the time of obtaining Table 2-6 consent, according to the presence or absence of proton-pump inhibitors

Unique Subject Identifier=1392011110-0003

MEANS Procedure

Assay variable : PTINR_VAL1 PT-INR Value

N	Mean	Standard Deviation	Minimum	Maximum
6	1.6166667	0.2483277	1.3000000	2.0000000

Unique Subject Identifier=1392011110-0006

Assay variable : PTINR_VAL1 PT-INR Value

N	Mean	Standard Deviation	Minimum	Maximum
6	2.0833333	0.4020779	1.6000000	2.6000000

Unique Subject Identifier=1392011110-0011

Assay variable : PTINR_VAL1 PT-INR Value

N	Mean	Standard Deviation	Minimum	Maximum
6	1.8166667	0.1602082	1.6000000	2.0000000

Unique Subject Identifier=1394011010-0004

Assay variable : PTINR_VAL1 PT-INR Value

N	Mean	Standard Deviation	Minimum	Maximum
3	1.9666667	1.0016653	1.2000000	3.1000000

Laboratory tests at the time of obtaining Table 2-6 consent, according to the presence or absence of proton-pump inhibitors

Unique Subject Identifier=1395000000-0007

MEANS Procedure

Assay variable : PTINR_VAL1 PT-INR Value

N	Mean	Standard Deviation	Minimum	Maximum
5	2.3880000	0.4996699	1.9000000	3.2300000

Unique Subject Identifier=1396000000-0006

Assay variable : PTINR_VAL1 PT-INR Value

N	Mean	Standard Deviation	Minimum	Maximum
1	1.5000000		1.5000000	1.5000000

Unique Subject Identifier=1396000000-0007

Assay variable : PTINR_VAL1 PT-INR Value

N	Mean	Standard Deviation	Minimum	Maximum
1	2.0000000		2.0000000	2.0000000

Unique Subject Identifier=1398010000-0022

Assay variable : PTINR_VAL1 PT-INR Value

N	Mean	Standard Deviation	Minimum	Maximum
5	2.2000000	0.3082207	1.9000000	2.6000000

Laboratory tests at the time of obtaining Table 2-6 consent, according to the presence or absence of proton-pump inhibitors

Unique Subject Identifier=1400000000-0007

MEANS Procedure

Assay variable : PTINR_VAL1 PT-INR Value

N	Mean	Standard Deviation	Minimum	Maximum
3	1.7200000	0.0781025	1.6300000	1.7700000

Unique Subject Identifier=1405000000-0003

Assay variable : PTINR_VAL1 PT-INR Value

N	Mean	Standard Deviation	Minimum	Maximum
6	2.2500000	0.3674235	1.9000000	2.8000000

Unique Subject Identifier=1405000000-0009

Assay variable : PTINR_VAL1 PT-INR Value

N	Mean	Standard Deviation	Minimum	Maximum
6	2.2166667	0.6306082	1.7000000	3.4000000

Unique Subject Identifier=1405000000-0013

Assay variable : PTINR_VAL1 PT-INR Value

N	Mean	Standard Deviation	Minimum	Maximum
6	2.0333333	0.1366260	1.9000000	2.3000000

Laboratory tests at the time of obtaining Table 2-6 consent, according to the presence or absence of proton-pump inhibitors

Unique Subject Identifier=1405000000-0016

MEANS Procedure

Assay variable : PTINR_VAL1 PT-INR Value

N	Mean	Standard Deviation	Minimum	Maximum
6	2.4233333	0.4233989	1.8000000	2.8000000

Unique Subject Identifier=1405000000-0019

Assay variable : PTINR_VAL1 PT-INR Value

N	Mean	Standard Deviation	Minimum	Maximum
6	2.4666667	0.6742897	1.7000000	3.6000000

Unique Subject Identifier=1405000000-0020

Assay variable : PTINR_VAL1 PT-INR Value

N	Mean	Standard Deviation	Minimum	Maximum
6	2.0500000	0.3271085	1.7000000	2.5000000

Unique Subject Identifier=1405000000-0022

Assay variable : PTINR_VAL1 PT-INR Value

N	Mean	Standard Deviation	Minimum	Maximum
6	2.0166667	0.4996666	1.6000000	3.0000000

Laboratory tests at the time of obtaining Table 2-6 consent, according to the presence or absence of proton-pump inhibitors

Unique Subject Identifier=1405000000-0027

MEANS Procedure

Assay variable : PTINR_VAL1 PT-INR Value

N	Mean	Standard Deviation	Minimum	Maximum
6	2.6833333	1.0534072	1.3000000	4.4000000

Unique Subject Identifier=1405000000-0033

Assay variable : PTINR_VAL1 PT-INR Value

N	Mean	Standard Deviation	Minimum	Maximum
6	1.9833333	0.4622409	1.4000000	2.6000000

Unique Subject Identifier=1406000000-0009

Assay variable : PTINR_VAL1 PT-INR Value

N	Mean	Standard Deviation	Minimum	Maximum
1	1.2900000		1.2900000	1.2900000

Unique Subject Identifier=1406000000-0021

Assay variable : PTINR_VAL1 PT-INR Value

N	Mean	Standard Deviation	Minimum	Maximum
1	1.0500000		1.0500000	1.0500000

Laboratory tests at the time of obtaining Table 2-6 consent, according to the presence or absence of proton-pump inhibitors

Unique Subject Identifier=1407000000-0004

MEANS Procedure

Assay variable : PTINR_VAL1 PT-INR Value

N	Mean	Standard Deviation	Minimum	Maximum
6	1.9650000	0.1980656	1.7300000	2.3300000

Unique Subject Identifier=1407000000-0016

Assay variable : PTINR_VAL1 PT-INR Value

N	Mean	Standard Deviation	Minimum	Maximum
6	2.3616667	0.2377744	2.0800000	2.7100000

Unique Subject Identifier=1407000000-0027

Assay variable : PTINR_VAL1 PT-INR Value

N	Mean	Standard Deviation	Minimum	Maximum
5	2.1520000	0.2169562	1.8900000	2.4900000

Unique Subject Identifier=1407000000-0030

Assay variable : PTINR_VAL1 PT-INR Value

N	Mean	Standard Deviation	Minimum	Maximum
6	1.8800000	0.3470447	1.3400000	2.2600000

Laboratory tests at the time of obtaining Table 2-6 consent, according to the presence or absence of proton-pump inhibitors

Unique Subject Identifier=1408010000-0029

MEANS Procedure

Assay variable : PTINR_VAL1 PT-INR Value

N	Mean	Standard Deviation	Minimum	Maximum
4	2.5700000	0.1476482	2.4800000	2.7900000

Unique Subject Identifier=1412000000-0002

Assay variable : PTINR_VAL1 PT-INR Value

N	Mean	Standard Deviation	Minimum	Maximum
6	2.0500000	0.0836660	1.9000000	2.1000000

Unique Subject Identifier=1412000000-0023

Assay variable : PTINR_VAL1 PT-INR Value

N	Mean	Standard Deviation	Minimum	Maximum
6	1.7833333	0.1329160	1.7000000	2.0000000

Unique Subject Identifier=1412000000-0029

Assay variable : PTINR_VAL1 PT-INR Value

N	Mean	Standard Deviation	Minimum	Maximum
5	1.5400000	0.1140175	1.4000000	1.7000000

Laboratory tests at the time of obtaining Table 2-6 consent, according to the presence or absence of proton-pump inhibitors

Unique Subject Identifier=1420010000-0004

MEANS Procedure

Assay variable : PTINR_VAL1 PT-INR Value

N	Mean	Standard Deviation	Minimum	Maximum
6	1.8333333	0.3881580	1.4000000	2.4000000

Unique Subject Identifier=1420010000-0010

Assay variable : PTINR_VAL1 PT-INR Value

N	Mean	Standard Deviation	Minimum	Maximum
6	1.8333333	0.1751190	1.5000000	2.0000000

Unique Subject Identifier=1420010000-0017

Assay variable : PTINR_VAL1 PT-INR Value

N	Mean	Standard Deviation	Minimum	Maximum
3	1.6333333	0.4041452	1.2000000	2.0000000

Unique Subject Identifier=1423000000-0021

Assay variable : PTINR_VAL1 PT-INR Value

N	Mean	Standard Deviation	Minimum	Maximum
1	2.0200000		2.0200000	2.0200000

Laboratory tests at the time of obtaining Table 2-6 consent, according to the presence or absence of proton-pump inhibitors

Unique Subject Identifier=1423000000-0022

MEANS Procedure

Assay variable : PTINR_VAL1 PT-INR Value

N	Mean	Standard Deviation	Minimum	Maximum
2	1.2700000	0.0282843	1.2500000	1.2900000

Unique Subject Identifier=1427000000-0003

Assay variable : PTINR_VAL1 PT-INR Value

N	Mean	Standard Deviation	Minimum	Maximum
6	1.8000000	0.3521363	1.4000000	2.3000000

Unique Subject Identifier=1430011010-0003

Assay variable : PTINR_VAL1 PT-INR Value

N	Mean	Standard Deviation	Minimum	Maximum
6	1.8166667	0.2786874	1.5000000	2.1000000

Unique Subject Identifier=1432000000-0011

Assay variable : PTINR_VAL1 PT-INR Value

N	Mean	Standard Deviation	Minimum	Maximum
6	1.1833333	0.0752773	1.1000000	1.3000000

Laboratory tests at the time of obtaining Table 2-6 consent, according to the presence or absence of proton-pump inhibitors

Unique Subject Identifier=1432000000-0015

MEANS Procedure

Assay variable : PTINR_VAL1 PT-INR Value

N	Mean	Standard Deviation	Minimum	Maximum
6	1.8333333	0.7865537	1.1000000	3.3000000

Unique Subject Identifier=1432000000-0017

Assay variable : PTINR_VAL1 PT-INR Value

N	Mean	Standard Deviation	Minimum	Maximum
6	2.3500000	0.4549725	1.8000000	3.0000000

Unique Subject Identifier=1432000000-0020

Assay variable : PTINR_VAL1 PT-INR Value

N	Mean	Standard Deviation	Minimum	Maximum
5	1.9400000	0.4449719	1.6000000	2.6000000

Unique Subject Identifier=1432000000-0023

Assay variable : PTINR_VAL1 PT-INR Value

N	Mean	Standard Deviation	Minimum	Maximum
6	1.8500000	0.5648008	1.2000000	2.7000000

Laboratory tests at the time of obtaining Table 2-6 consent, according to the presence or absence of proton-pump inhibitors

Unique Subject Identifier=1432000000-0028

MEANS Procedure

Assay variable : PTINR_VAL1 PT-INR Value

N	Mean	Standard Deviation	Minimum	Maximum
6	2.3333333	0.5465040	1.7000000	3.2000000

Unique Subject Identifier=1432000000-0034

Assay variable : PTINR_VAL1 PT-INR Value

N	Mean	Standard Deviation	Minimum	Maximum
6	2.1000000	0.3162278	1.7000000	2.6000000

Unique Subject Identifier=1432000000-0037

Assay variable : PTINR_VAL1 PT-INR Value

N	Mean	Standard Deviation	Minimum	Maximum
6	1.4333333	0.0516398	1.4000000	1.5000000

Unique Subject Identifier=1432000000-0042

Assay variable : PTINR_VAL1 PT-INR Value

N	Mean	Standard Deviation	Minimum	Maximum
5	1.7600000	0.5176872	1.3000000	2.6000000

Laboratory tests at the time of obtaining Table 2-6 consent, according to the presence or absence of proton-pump inhibitors

Unique Subject Identifier=1432000000-0072

MEANS Procedure

Assay variable : PTINR_VAL1 PT-INR Value

N	Mean	Standard Deviation	Minimum	Maximum
6	1.7000000	0.3033150	1.3000000	2.2000000

Unique Subject Identifier=1435000000-0021

Assay variable : PTINR_VAL1 PT-INR Value

N	Mean	Standard Deviation	Minimum	Maximum
1	1.1600000		1.1600000	1.1600000

Unique Subject Identifier=1435000000-0072

Assay variable : PTINR_VAL1 PT-INR Value

N	Mean	Standard Deviation	Minimum	Maximum
4	1.8275000	0.2848830	1.5900000	2.2000000

Unique Subject Identifier=1435000000-0145

Assay variable : PTINR_VAL1 PT-INR Value

N	Mean	Standard Deviation	Minimum	Maximum
6	1.5983333	0.3660829	1.1900000	2.1900000

Laboratory tests at the time of obtaining Table 2-6 consent, according to the presence or absence of proton-pump inhibitors

Unique Subject Identifier=1435000000-0177

MEANS Procedure

Assay variable : PTINR_VAL1 PT-INR Value

N	Mean	Standard Deviation	Minimum	Maximum
5	1.9480000	0.3900897	1.5500000	2.4600000

Unique Subject Identifier=1436000000-0003

Assay variable : PTINR_VAL1 PT-INR Value

N	Mean	Standard Deviation	Minimum	Maximum
3	1.7666667	0.1709776	1.5700000	1.8800000

Unique Subject Identifier=1436000000-0013

Assay variable : PTINR_VAL1 PT-INR Value

N	Mean	Standard Deviation	Minimum	Maximum
1	2.2400000		2.2400000	2.2400000

Unique Subject Identifier=1436000000-0018

Assay variable : PTINR_VAL1 PT-INR Value

N	Mean	Standard Deviation	Minimum	Maximum
1	1.7200000		1.7200000	1.7200000

Laboratory tests at the time of obtaining Table 2-6 consent, according to the presence or absence of proton-pump inhibitors

Unique Subject Identifier=1436000000-0020

MEANS Procedure

Assay variable : PTINR_VAL1 PT-INR Value

N	Mean	Standard Deviation	Minimum	Maximum
2	2.4400000	0.3252691	2.2100000	2.6700000

Unique Subject Identifier=1436000000-0021

Assay variable : PTINR_VAL1 PT-INR Value

N	Mean	Standard Deviation	Minimum	Maximum
1	1.3700000		1.3700000	1.3700000

Unique Subject Identifier=1436000000-0022

Assay variable : PTINR_VAL1 PT-INR Value

N	Mean	Standard Deviation	Minimum	Maximum
1	2.2200000		2.2200000	2.2200000

Unique Subject Identifier=1436000000-0034

Assay variable : PTINR_VAL1 PT-INR Value

N	Mean	Standard Deviation	Minimum	Maximum
1	1.3800000		1.3800000	1.3800000

Laboratory tests at the time of obtaining Table 2-6 consent, according to the presence or absence of proton-pump inhibitors

Unique Subject Identifier=1436000000-0047

MEANS Procedure

Assay variable : PTINR_VAL1 PT-INR Value

N	Mean	Standard Deviation	Minimum	Maximum
1	2.0700000		2.0700000	2.0700000

Unique Subject Identifier=1436000000-0063

Assay variable : PTINR_VAL1 PT-INR Value

N	Mean	Standard Deviation	Minimum	Maximum
1	1.8600000		1.8600000	1.8600000

Unique Subject Identifier=1438000000-0001

Assay variable : PTINR_VAL1 PT-INR Value

N	Mean	Standard Deviation	Minimum	Maximum
2	1.6550000	0.1343503	1.5600000	1.7500000

Unique Subject Identifier=1440000000-0001

Assay variable : PTINR_VAL1 PT-INR Value

N	Mean	Standard Deviation	Minimum	Maximum
4	1.3000000	0.1920069	1.1200000	1.4900000

Laboratory tests at the time of obtaining Table 2-6 consent, according to the presence or absence of proton-pump inhibitors

Unique Subject Identifier=1440000000-0011

MEANS Procedure

Assay variable : PTINR_VAL1 PT-INR Value

N	Mean	Standard Deviation	Minimum	Maximum
3	1.9133333	0.5744853	1.2500000	2.2500000

Unique Subject Identifier=1440000000-0014

Assay variable : PTINR_VAL1 PT-INR Value

N	Mean	Standard Deviation	Minimum	Maximum
5	2.0280000	0.2251000	1.7000000	2.3000000

Unique Subject Identifier=1446000000-0004

Assay variable : PTINR_VAL1 PT-INR Value

N	Mean	Standard Deviation	Minimum	Maximum
5	1.6200000	0.1095445	1.5000000	1.8000000

Unique Subject Identifier=1446000000-0010

Assay variable : PTINR_VAL1 PT-INR Value

N	Mean	Standard Deviation	Minimum	Maximum
3	1.7666667	0.2081666	1.6000000	2.0000000

Laboratory tests at the time of obtaining Table 2-6 consent, according to the presence or absence of proton-pump inhibitors

Unique Subject Identifier=1446000000-0012

MEANS Procedure

Assay variable : PTINR_VAL1 PT-INR Value

N	Mean	Standard Deviation	Minimum	Maximum
3	1.8666667	0.0577350	1.8000000	1.9000000

Unique Subject Identifier=1446000000-0014

Assay variable : PTINR_VAL1 PT-INR Value

N	Mean	Standard Deviation	Minimum	Maximum
6	1.9666667	0.2160247	1.6000000	2.2000000

Unique Subject Identifier=1448000000-0002

Assay variable : PTINR_VAL1 PT-INR Value

N	Mean	Standard Deviation	Minimum	Maximum
1	1.4300000		1.4300000	1.4300000

Unique Subject Identifier=1448000000-0004

Assay variable : PTINR_VAL1 PT-INR Value

N	Mean	Standard Deviation	Minimum	Maximum
2	1.6800000	0.1697056	1.5600000	1.8000000

Laboratory tests at the time of obtaining Table 2-6 consent, according to the presence or absence of proton-pump inhibitors

Unique Subject Identifier=1448000000-0009

MEANS Procedure

Assay variable : PTINR_VAL1 PT-INR Value

N	Mean	Standard Deviation	Minimum	Maximum
2	1.6750000	0.0070711	1.6700000	1.6800000

Unique Subject Identifier=1448000000-0016

Assay variable : PTINR_VAL1 PT-INR Value

N	Mean	Standard Deviation	Minimum	Maximum
2	2.1150000	0.6434672	1.6600000	2.5700000

Unique Subject Identifier=1449000000-0003

Assay variable : PTINR_VAL1 PT-INR Value

N	Mean	Standard Deviation	Minimum	Maximum
5	2.2800000	0.2863564	1.9000000	2.7000000

Unique Subject Identifier=1451000000-0013

Assay variable : PTINR_VAL1 PT-INR Value

N	Mean	Standard Deviation	Minimum	Maximum
6	1.8166667	0.2714160	1.5000000	2.3000000

Laboratory tests at the time of obtaining Table 2-6 consent, according to the presence or absence of proton-pump inhibitors

Unique Subject Identifier=1451000000-0023

MEANS Procedure

Assay variable : PTINR_VAL1 PT-INR Value

N	Mean	Standard Deviation	Minimum	Maximum
6	1.7833333	0.1329160	1.7000000	2.0000000

Unique Subject Identifier=1451000000-0025

Assay variable : PTINR_VAL1 PT-INR Value

N	Mean	Standard Deviation	Minimum	Maximum
1	2.9000000		2.9000000	2.9000000

Unique Subject Identifier=1451000000-0026

Assay variable : PTINR_VAL1 PT-INR Value

N	Mean	Standard Deviation	Minimum	Maximum
6	1.9000000	0.4098780	1.4000000	2.5000000

Unique Subject Identifier=1451000000-0027

Assay variable : PTINR_VAL1 PT-INR Value

N	Mean	Standard Deviation	Minimum	Maximum
5	2.0800000	0.1788854	1.9000000	2.3000000

Laboratory tests at the time of obtaining Table 2-6 consent, according to the presence or absence of proton-pump inhibitors

Unique Subject Identifier=1451000000-0028

MEANS Procedure

Assay variable : PTINR_VAL1 PT-INR Value

N	Mean	Standard Deviation	Minimum	Maximum
6	2.2500000	0.4183300	1.6000000	2.8000000

Unique Subject Identifier=1451000000-0029

Assay variable : PTINR_VAL1 PT-INR Value

N	Mean	Standard Deviation	Minimum	Maximum
6	1.8333333	0.2250926	1.6000000	2.2000000

Unique Subject Identifier=1451000000-0032

Assay variable : PTINR_VAL1 PT-INR Value

N	Mean	Standard Deviation	Minimum	Maximum
6	1.8500000	0.3728270	1.4000000	2.3000000

Unique Subject Identifier=1451000000-0043

Assay variable : PTINR_VAL1 PT-INR Value

N	Mean	Standard Deviation	Minimum	Maximum
6	1.8833333	0.2786874	1.6000000	2.3000000

Laboratory tests at the time of obtaining Table 2-6 consent, according to the presence or absence of proton-pump inhibitors

Unique Subject Identifier=1451000000-0048

MEANS Procedure

Assay variable : PTINR_VAL1 PT-INR Value

N	Mean	Standard Deviation	Minimum	Maximum
6	1.8000000	0.6066300	1.1000000	2.9000000

Unique Subject Identifier=1451000000-0049

Assay variable : PTINR_VAL1 PT-INR Value

N	Mean	Standard Deviation	Minimum	Maximum
6	2.0833333	0.3544949	1.7000000	2.6000000

Unique Subject Identifier=1451000000-0052

Assay variable : PTINR_VAL1 PT-INR Value

N	Mean	Standard Deviation	Minimum	Maximum
6	2.0666667	0.6314006	1.1000000	2.8000000

Unique Subject Identifier=1451000000-0053

Assay variable : PTINR_VAL1 PT-INR Value

N	Mean	Standard Deviation	Minimum	Maximum
6	1.8666667	0.2338090	1.5000000	2.2000000

Laboratory tests at the time of obtaining Table 2-6 consent, according to the presence or absence of proton-pump inhibitors

Unique Subject Identifier=1451000000-0054

MEANS Procedure

Assay variable : PTINR_VAL1 PT-INR Value

N	Mean	Standard Deviation	Minimum	Maximum
6	2.2000000	0.4472136	1.4000000	2.7000000

Unique Subject Identifier=1452000000-0006

Assay variable : PTINR_VAL1 PT-INR Value

N	Mean	Standard Deviation	Minimum	Maximum
6	2.5516667	0.2155381	2.3600000	2.9500000

Unique Subject Identifier=1452000000-0018

Assay variable : PTINR_VAL1 PT-INR Value

N	Mean	Standard Deviation	Minimum	Maximum
5	2.4340000	0.4462398	1.6900000	2.8000000

Unique Subject Identifier=1452000000-0025

Assay variable : PTINR_VAL1 PT-INR Value

N	Mean	Standard Deviation	Minimum	Maximum
6	1.7983333	0.2129241	1.5100000	2.1600000

Laboratory tests at the time of obtaining Table 2-6 consent, according to the presence or absence of proton-pump inhibitors

Unique Subject Identifier=1452000000-0028

MEANS Procedure

Assay variable : PTINR_VAL1 PT-INR Value

N	Mean	Standard Deviation	Minimum	Maximum
6	2.2400000	0.5422914	1.6100000	3.2200000

Unique Subject Identifier=1452000000-0034

Assay variable : PTINR_VAL1 PT-INR Value

N	Mean	Standard Deviation	Minimum	Maximum
6	2.0716667	0.2058559	1.8700000	2.3000000

Unique Subject Identifier=1452000000-0040

Assay variable : PTINR_VAL1 PT-INR Value

N	Mean	Standard Deviation	Minimum	Maximum
3	1.8100000	0.3704052	1.5300000	2.2300000

Unique Subject Identifier=1452000000-0042

Assay variable : PTINR_VAL1 PT-INR Value

N	Mean	Standard Deviation	Minimum	Maximum
6	1.8716667	0.3365363	1.5500000	2.3800000

Laboratory tests at the time of obtaining Table 2-6 consent, according to the presence or absence of proton-pump inhibitors

Unique Subject Identifier=1452000000-0048

MEANS Procedure

Assay variable : PTINR_VAL1 PT-INR Value

N	Mean	Standard Deviation	Minimum	Maximum
5	2.0620000	0.6736245	1.3600000	3.0500000

Unique Subject Identifier=1452000000-0049

Assay variable : PTINR_VAL1 PT-INR Value

N	Mean	Standard Deviation	Minimum	Maximum
5	2.5280000	0.4078235	2.0800000	3.1000000

Unique Subject Identifier=1452000000-0067

Assay variable : PTINR_VAL1 PT-INR Value

N	Mean	Standard Deviation	Minimum	Maximum
6	2.0500000	0.1951410	1.8000000	2.3000000

Unique Subject Identifier=1452000000-0090

Assay variable : PTINR_VAL1 PT-INR Value

N	Mean	Standard Deviation	Minimum	Maximum
6	1.9233333	0.3040833	1.6300000	2.3100000

Laboratory tests at the time of obtaining Table 2-6 consent, according to the presence or absence of proton-pump inhibitors

Unique Subject Identifier=1452000000-0095

MEANS Procedure

Assay variable : PTINR_VAL1 PT-INR Value

N	Mean	Standard Deviation	Minimum	Maximum
3	1.9766667	0.5643876	1.3300000	2.3700000

Unique Subject Identifier=1454000000-0022

Assay variable : PTINR_VAL1 PT-INR Value

N	Mean	Standard Deviation	Minimum	Maximum
6	1.9100000	0.6382789	1.1400000	2.5600000

Unique Subject Identifier=1454000000-0061

Assay variable : PTINR_VAL1 PT-INR Value

N	Mean	Standard Deviation	Minimum	Maximum
5	2.0200000	0.2285826	1.7200000	2.3300000

Unique Subject Identifier=1454000000-0066

Assay variable : PTINR_VAL1 PT-INR Value

N	Mean	Standard Deviation	Minimum	Maximum
1	2.1100000		2.1100000	2.1100000

Laboratory tests at the time of obtaining Table 2-6 consent, according to the presence or absence of proton-pump inhibitors

Unique Subject Identifier=1463010000-0017

MEANS Procedure

Assay variable : PTINR_VAL1 PT-INR Value

N	Mean	Standard Deviation	Minimum	Maximum
6	1.7950000	0.1617096	1.6400000	2.0700000

Unique Subject Identifier=1463010000-0025

Assay variable : PTINR_VAL1 PT-INR Value

N	Mean	Standard Deviation	Minimum	Maximum
6	2.2816667	0.2688804	1.8900000	2.6000000

Unique Subject Identifier=1463010000-0035

Assay variable : PTINR_VAL1 PT-INR Value

N	Mean	Standard Deviation	Minimum	Maximum
5	2.4900000	0.4252646	1.8200000	2.8700000

Unique Subject Identifier=1469010110-0011

Assay variable : PTINR_VAL1 PT-INR Value

N	Mean	Standard Deviation	Minimum	Maximum
3	1.5800000	0.1252996	1.4500000	1.7000000

Laboratory tests at the time of obtaining Table 2-6 consent, according to the presence or absence of proton-pump inhibitors

Unique Subject Identifier=1470000000-0003

MEANS Procedure

Assay variable : PTINR_VAL1 PT-INR Value

N	Mean	Standard Deviation	Minimum	Maximum
4	1.6775000	0.1960230	1.4700000	1.9400000

Unique Subject Identifier=1475000000-0005

Assay variable : PTINR_VAL1 PT-INR Value

N	Mean	Standard Deviation	Minimum	Maximum
3	1.9000000	0.1000000	1.8000000	2.0000000

Unique Subject Identifier=1475000000-0006

Assay variable : PTINR_VAL1 PT-INR Value

N	Mean	Standard Deviation	Minimum	Maximum
3	2.6333333	0.8020806	1.8000000	3.4000000

Unique Subject Identifier=1475000000-0012

Assay variable : PTINR_VAL1 PT-INR Value

N	Mean	Standard Deviation	Minimum	Maximum
2	1.7000000	0	1.7000000	1.7000000

Laboratory tests at the time of obtaining Table 2-6 consent, according to the presence or absence of proton-pump inhibitors

Unique Subject Identifier=1477000000-0005

MEANS Procedure

Assay variable : PTINR_VAL1 PT-INR Value

N	Mean	Standard Deviation	Minimum	Maximum
6	2.0833333	0.1717750	1.9900000	2.4300000

Unique Subject Identifier=1478011111-0034

Assay variable : PTINR_VAL1 PT-INR Value

N	Mean	Standard Deviation	Minimum	Maximum
3	1.6966667	0.2200757	1.4800000	1.9200000

Unique Subject Identifier=1478011111-0037

Assay variable : PTINR_VAL1 PT-INR Value

N	Mean	Standard Deviation	Minimum	Maximum
3	2.6400000	0.3451087	2.3000000	2.9900000

Unique Subject Identifier=1478011111-0041

Assay variable : PTINR_VAL1 PT-INR Value

N	Mean	Standard Deviation	Minimum	Maximum
3	2.3966667	0.0602771	2.3400000	2.4600000

Laboratory tests at the time of obtaining Table 2-6 consent, according to the presence or absence of proton-pump inhibitors

Unique Subject Identifier=1478011111-0051

MEANS Procedure

Assay variable : PTINR_VAL1 PT-INR Value

N	Mean	Standard Deviation	Minimum	Maximum
2	1.1900000	0.0565685	1.1500000	1.2300000

Unique Subject Identifier=1478011111-0070

Assay variable : PTINR_VAL1 PT-INR Value

N	Mean	Standard Deviation	Minimum	Maximum
3	1.9900000	0.4450843	1.4800000	2.3000000

Unique Subject Identifier=1478011111-0079

Assay variable : PTINR_VAL1 PT-INR Value

N	Mean	Standard Deviation	Minimum	Maximum
4	2.7100000	0.9296236	1.9100000	3.9700000

Unique Subject Identifier=1478011111-0096

Assay variable : PTINR_VAL1 PT-INR Value

N	Mean	Standard Deviation	Minimum	Maximum
2	2.5350000	0.1626346	2.4200000	2.6500000

Laboratory tests at the time of obtaining Table 2-6 consent, according to the presence or absence of proton-pump inhibitors

Unique Subject Identifier=1478011111-0120

MEANS Procedure

Assay variable : PTINR_VAL1 PT-INR Value

N	Mean	Standard Deviation	Minimum	Maximum
4	2.0225000	0.5209846	1.4000000	2.5100000

Unique Subject Identifier=1478011111-0144

Assay variable : PTINR_VAL1 PT-INR Value

N	Mean	Standard Deviation	Minimum	Maximum
2	2.2250000	0.1909188	2.0900000	2.3600000

Unique Subject Identifier=1480011111-0002

Assay variable : PTINR_VAL1 PT-INR Value

N	Mean	Standard Deviation	Minimum	Maximum
1	1.0800000		1.0800000	1.0800000

Unique Subject Identifier=1487000000-0002

Assay variable : PTINR_VAL1 PT-INR Value

N	Mean	Standard Deviation	Minimum	Maximum
6	2.1166667	0.3710346	1.7000000	2.7000000

Laboratory tests at the time of obtaining Table 2-6 consent, according to the presence or absence of proton-pump inhibitors

Unique Subject Identifier=1487000000-0007

MEANS Procedure

Assay variable : PTINR_VAL1 PT-INR Value

N	Mean	Standard Deviation	Minimum	Maximum
6	1.9166667	0.1834848	1.6000000	2.1000000

Unique Subject Identifier=1487000000-0031

Assay variable : PTINR_VAL1 PT-INR Value

N	Mean	Standard Deviation	Minimum	Maximum
6	2.0833333	0.4622409	1.5000000	2.8000000

Unique Subject Identifier=1491000000-0002

Assay variable : PTINR_VAL1 PT-INR Value

N	Mean	Standard Deviation	Minimum	Maximum
4	2.3225000	0.9042262	1.7200000	3.6500000

Unique Subject Identifier=1491000000-0006

Assay variable : PTINR_VAL1 PT-INR Value

N	Mean	Standard Deviation	Minimum	Maximum
4	2.1075000	0.2436357	1.7600000	2.3300000

Laboratory tests at the time of obtaining Table 2-6 consent, according to the presence or absence of proton-pump inhibitors

Unique Subject Identifier=1491000000-0007

MEANS Procedure

Assay variable : PTINR_VAL1 PT-INR Value

N	Mean	Standard Deviation	Minimum	Maximum
6	1.6833333	0.2338090	1.4200000	2.0600000

Unique Subject Identifier=1491000000-0020

Assay variable : PTINR_VAL1 PT-INR Value

N	Mean	Standard Deviation	Minimum	Maximum
3	1.9133333	0.1601041	1.7500000	2.0700000

Unique Subject Identifier=1491000000-0041

Assay variable : PTINR_VAL1 PT-INR Value

N	Mean	Standard Deviation	Minimum	Maximum
4	2.0400000	0.5090514	1.6000000	2.6300000

Unique Subject Identifier=1491000000-0045

Assay variable : PTINR_VAL1 PT-INR Value

N	Mean	Standard Deviation	Minimum	Maximum
4	1.9100000	0.2947315	1.6100000	2.3000000

Laboratory tests at the time of obtaining Table 2-6 consent, according to the presence or absence of proton-pump inhibitors

Unique Subject Identifier=1491000000-0047

MEANS Procedure

Assay variable : PTINR_VAL1 PT-INR Value

N	Mean	Standard Deviation	Minimum	Maximum
6	1.8016667	0.1619156	1.6200000	2.0900000

Unique Subject Identifier=1491000000-0050

Assay variable : PTINR_VAL1 PT-INR Value

N	Mean	Standard Deviation	Minimum	Maximum
3	1.8200000	0.0818535	1.7300000	1.8900000

Unique Subject Identifier=1491000000-0057

Assay variable : PTINR_VAL1 PT-INR Value

N	Mean	Standard Deviation	Minimum	Maximum
3	2.0800000	0.2718455	1.7800000	2.3100000

Unique Subject Identifier=1491000000-0074

Assay variable : PTINR_VAL1 PT-INR Value

N	Mean	Standard Deviation	Minimum	Maximum
4	2.0675000	0.1901534	1.8100000	2.2300000

Laboratory tests at the time of obtaining Table 2-6 consent, according to the presence or absence of proton-pump inhibitors

Unique Subject Identifier=1491000000-0082

MEANS Procedure

Assay variable : PTINR_VAL1 PT-INR Value

N	Mean	Standard Deviation	Minimum	Maximum
4	2.3575000	0.3834384	2.1200000	2.9300000

Unique Subject Identifier=1491000000-0084

Assay variable : PTINR_VAL1 PT-INR Value

N	Mean	Standard Deviation	Minimum	Maximum
3	2.0066667	0.2441994	1.8100000	2.2800000

Unique Subject Identifier=1491000000-0088

Assay variable : PTINR_VAL1 PT-INR Value

N	Mean	Standard Deviation	Minimum	Maximum
4	2.7900000	0.8003749	2.3600000	3.9900000

Unique Subject Identifier=1491000000-0089

Assay variable : PTINR_VAL1 PT-INR Value

N	Mean	Standard Deviation	Minimum	Maximum
4	1.5925000	0.1152895	1.4200000	1.6600000

Laboratory tests at the time of obtaining Table 2-6 consent, according to the presence or absence of proton-pump inhibitors

Unique Subject Identifier=1491000000-0101

MEANS Procedure

Assay variable : PTINR_VAL1 PT-INR Value

N	Mean	Standard Deviation	Minimum	Maximum
4	1.7700000	0.2044505	1.4700000	1.9300000

Unique Subject Identifier=1491000000-0103

Assay variable : PTINR_VAL1 PT-INR Value

N	Mean	Standard Deviation	Minimum	Maximum
4	2.5075000	0.2325762	2.2900000	2.8200000

Unique Subject Identifier=1492011100-0027

Assay variable : PTINR_VAL1 PT-INR Value

N	Mean	Standard Deviation	Minimum	Maximum
6	2.1500000	0.2509980	1.8000000	2.4000000

Unique Subject Identifier=1494000000-0005

Assay variable : PTINR_VAL1 PT-INR Value

N	Mean	Standard Deviation	Minimum	Maximum
2	0.9500000	0.0424264	0.9200000	0.9800000

Laboratory tests at the time of obtaining Table 2-6 consent, according to the presence or absence of proton-pump inhibitors

Unique Subject Identifier=1494000000-0007

MEANS Procedure

Assay variable : PTINR_VAL1 PT-INR Value

N	Mean	Standard Deviation	Minimum	Maximum
4	1.5500000	0.2585859	1.2000000	1.7900000

Unique Subject Identifier=1494000000-0008

Assay variable : PTINR_VAL1 PT-INR Value

N	Mean	Standard Deviation	Minimum	Maximum
3	1.9500000	0.1058301	1.8300000	2.0300000

Unique Subject Identifier=1494000000-0017

Assay variable : PTINR_VAL1 PT-INR Value

N	Mean	Standard Deviation	Minimum	Maximum
4	1.6825000	0.2443188	1.3900000	1.9300000

Unique Subject Identifier=1494000000-0033

Assay variable : PTINR_VAL1 PT-INR Value

N	Mean	Standard Deviation	Minimum	Maximum
2	1.8150000	0.1484924	1.7100000	1.9200000

Laboratory tests at the time of obtaining Table 2-6 consent, according to the presence or absence of proton-pump inhibitors

Unique Subject Identifier=1495011010-0003

MEANS Procedure

Assay variable : PTINR_VAL1 PT-INR Value

N	Mean	Standard Deviation	Minimum	Maximum
3	2.3600000	0.2193171	2.1700000	2.6000000

Unique Subject Identifier=1499000000-0001

Assay variable : PTINR_VAL1 PT-INR Value

N	Mean	Standard Deviation	Minimum	Maximum
2	2.0850000	0.1343503	1.9900000	2.1800000

Unique Subject Identifier=1501000000-0009

Assay variable : PTINR_VAL1 PT-INR Value

N	Mean	Standard Deviation	Minimum	Maximum
5	1.5920000	0.0729383	1.5000000	1.7000000

Unique Subject Identifier=1501000000-0010

Assay variable : PTINR_VAL1 PT-INR Value

N	Mean	Standard Deviation	Minimum	Maximum
4	1.9700000	0.5339788	1.5100000	2.6700000

Laboratory tests at the time of obtaining Table 2-6 consent, according to the presence or absence of proton-pump inhibitors

Unique Subject Identifier=1501000000-0035

MEANS Procedure

Assay variable : PTINR_VAL1 PT-INR Value

N	Mean	Standard Deviation	Minimum	Maximum
2	1.6400000	0.2262742	1.4800000	1.8000000

Unique Subject Identifier=1502000000-0001

Assay variable : PTINR_VAL1 PT-INR Value

N	Mean	Standard Deviation	Minimum	Maximum
4	1.6900000	0.2336664	1.4600000	1.9700000

Unique Subject Identifier=1502000000-0003

Assay variable : PTINR_VAL1 PT-INR Value

N	Mean	Standard Deviation	Minimum	Maximum
1	1.5600000		1.5600000	1.5600000

Unique Subject Identifier=1502000000-0004

Assay variable : PTINR_VAL1 PT-INR Value

N	Mean	Standard Deviation	Minimum	Maximum
4	2.1600000	0.4730750	1.8000000	2.8500000

Laboratory tests at the time of obtaining Table 2-6 consent, according to the presence or absence of proton-pump inhibitors

Unique Subject Identifier=1502000000-0008

MEANS Procedure

Assay variable : PTINR_VAL1 PT-INR Value

N	Mean	Standard Deviation	Minimum	Maximum
2	1.9900000	0.0141421	1.9800000	2.0000000

Unique Subject Identifier=1502000000-0010

Assay variable : PTINR_VAL1 PT-INR Value

N	Mean	Standard Deviation	Minimum	Maximum
1	1.3700000		1.3700000	1.3700000

Unique Subject Identifier=1503000000-0010

Assay variable : PTINR_VAL1 PT-INR Value

N	Mean	Standard Deviation	Minimum	Maximum
2	1.4200000	0.1838478	1.2900000	1.5500000

Unique Subject Identifier=1504000000-0009

Assay variable : PTINR_VAL1 PT-INR Value

N	Mean	Standard Deviation	Minimum	Maximum
1	2.3800000		2.3800000	2.3800000

Laboratory tests at the time of obtaining Table 2-6 consent, according to the presence or absence of proton-pump inhibitors

Unique Subject Identifier=1513000000-0011

MEANS Procedure

Assay variable : PTINR_VAL1 PT-INR Value

N	Mean	Standard Deviation	Minimum	Maximum
3	1.5200000	0.2306513	1.2600000	1.7000000

Unique Subject Identifier=1516011000-0013

Assay variable : PTINR_VAL1 PT-INR Value

N	Mean	Standard Deviation	Minimum	Maximum
6	2.0500000	0.1516575	1.9000000	2.3000000

Unique Subject Identifier=1516011000-0020

Assay variable : PTINR_VAL1 PT-INR Value

N	Mean	Standard Deviation	Minimum	Maximum
5	2.1400000	0.5549775	1.3000000	2.7000000

Unique Subject Identifier=1516011000-0041

Assay variable : PTINR_VAL1 PT-INR Value

N	Mean	Standard Deviation	Minimum	Maximum
6	2.1233333	0.4757590	1.4500000	2.5600000

Laboratory tests at the time of obtaining Table 2-6 consent, according to the presence or absence of proton-pump inhibitors

Unique Subject Identifier=1516011000-0051

MEANS Procedure

Assay variable : PTINR_VAL1 PT-INR Value

N	Mean	Standard Deviation	Minimum	Maximum
2	2.0000000	0.9899495	1.3000000	2.7000000

Unique Subject Identifier=1516011000-0079

Assay variable : PTINR_VAL1 PT-INR Value

N	Mean	Standard Deviation	Minimum	Maximum
6	1.8500000	0.4086563	1.5000000	2.6000000

Unique Subject Identifier=1517010100-0005

Assay variable : PTINR_VAL1 PT-INR Value

N	Mean	Standard Deviation	Minimum	Maximum
3	1.8766667	0.2650157	1.6100000	2.1400000

Unique Subject Identifier=1517010100-0008

Assay variable : PTINR_VAL1 PT-INR Value

N	Mean	Standard Deviation	Minimum	Maximum
4	1.2925000	0.0330404	1.2600000	1.3300000

Laboratory tests at the time of obtaining Table 2-6 consent, according to the presence or absence of proton-pump inhibitors

Unique Subject Identifier=1522000000-0009

MEANS Procedure

Assay variable : PTINR_VAL1 PT-INR Value

N	Mean	Standard Deviation	Minimum	Maximum
6	2.0883333	0.5870747	1.5500000	3.1100000

Unique Subject Identifier=1522000000-0022

Assay variable : PTINR_VAL1 PT-INR Value

N	Mean	Standard Deviation	Minimum	Maximum
4	2.5100000	0.3154890	2.1600000	2.8900000

Unique Subject Identifier=1522000000-0023

Assay variable : PTINR_VAL1 PT-INR Value

N	Mean	Standard Deviation	Minimum	Maximum
5	2.0600000	0.1140175	1.8900000	2.2100000

Unique Subject Identifier=1522000000-0033

Assay variable : PTINR_VAL1 PT-INR Value

N	Mean	Standard Deviation	Minimum	Maximum
3	1.2466667	0.3384277	0.9600000	1.6200000

Laboratory tests at the time of obtaining Table 2-6 consent, according to the presence or absence of proton-pump inhibitors

Unique Subject Identifier=1523010111-0011

MEANS Procedure

Assay variable : PTINR_VAL1 PT-INR Value

N	Mean	Standard Deviation	Minimum	Maximum
6	1.8616667	0.3711828	1.5900000	2.5800000

Unique Subject Identifier=1524010000-0003

Assay variable : PTINR_VAL1 PT-INR Value

N	Mean	Standard Deviation	Minimum	Maximum
1	2.3200000		2.3200000	2.3200000

Unique Subject Identifier=1524010000-0006

Assay variable : PTINR_VAL1 PT-INR Value

N	Mean	Standard Deviation	Minimum	Maximum
1	2.4200000		2.4200000	2.4200000

Unique Subject Identifier=1524010000-0007

Assay variable : PTINR_VAL1 PT-INR Value

N	Mean	Standard Deviation	Minimum	Maximum
3	2.2233333	0.1154701	2.0900000	2.2900000

Laboratory tests at the time of obtaining Table 2-6 consent, according to the presence or absence of proton-pump inhibitors

Unique Subject Identifier=1524010000-0008

MEANS Procedure

Assay variable : PTINR_VAL1 PT-INR Value

N	Mean	Standard Deviation	Minimum	Maximum
4	1.8575000	0.3262284	1.5700000	2.3000000

Unique Subject Identifier=1524010000-0009

Assay variable : PTINR_VAL1 PT-INR Value

N	Mean	Standard Deviation	Minimum	Maximum
4	1.8200000	0.4307358	1.2300000	2.2000000

Unique Subject Identifier=1527010000-0005

Assay variable : PTINR_VAL1 PT-INR Value

N	Mean	Standard Deviation	Minimum	Maximum
5	2.4140000	0.5662861	2.0300000	3.3900000

Unique Subject Identifier=1528000000-0001

Assay variable : PTINR_VAL1 PT-INR Value

N	Mean	Standard Deviation	Minimum	Maximum
6	1.7533333	0.1539697	1.5100000	1.9700000

Laboratory tests at the time of obtaining Table 2-6 consent, according to the presence or absence of proton-pump inhibitors

Unique Subject Identifier=1528000000-0004

MEANS Procedure

Assay variable : PTINR_VAL1 PT-INR Value

N	Mean	Standard Deviation	Minimum	Maximum
4	1.8200000	0.2580698	1.4900000	2.1000000

Unique Subject Identifier=1530000000-0002

Assay variable : PTINR_VAL1 PT-INR Value

N	Mean	Standard Deviation	Minimum	Maximum
5	1.5800000	0.1923538	1.4000000	1.9000000

Unique Subject Identifier=1600011000-0016

Assay variable : PTINR_VAL1 PT-INR Value

N	Mean	Standard Deviation	Minimum	Maximum
3	1.3533333	0.0680686	1.3000000	1.4300000

Unique Subject Identifier=1600011000-0018

Assay variable : PTINR_VAL1 PT-INR Value

N	Mean	Standard Deviation	Minimum	Maximum
6	2.0333333	0.5442671	1.3900000	2.7200000

Laboratory tests at the time of obtaining Table 2-6 consent, according to the presence or absence of proton-pump inhibitors

Unique Subject Identifier=1600011000-0023

MEANS Procedure

Assay variable : PTINR_VAL1 PT-INR Value

N	Mean	Standard Deviation	Minimum	Maximum
3	2.0266667	0.2706166	1.8100000	2.3300000

Unique Subject Identifier=1600011000-0024

Assay variable : PTINR_VAL1 PT-INR Value

N	Mean	Standard Deviation	Minimum	Maximum
1	1.8300000		1.8300000	1.8300000

Unique Subject Identifier=1600011000-0027

Assay variable : PTINR_VAL1 PT-INR Value

N	Mean	Standard Deviation	Minimum	Maximum
6	1.7750000	0.3326710	1.3100000	2.1700000

Unique Subject Identifier=1603000000-0015

Assay variable : PTINR_VAL1 PT-INR Value

N	Mean	Standard Deviation	Minimum	Maximum
5	1.7600000	0.4669047	1.3000000	2.4000000

Laboratory tests at the time of obtaining Table 2-6 consent, according to the presence or absence of proton-pump inhibitors

Unique Subject Identifier=1701010000-0011

MEANS Procedure

Assay variable : PTINR_VAL1 PT-INR Value

N	Mean	Standard Deviation	Minimum	Maximum
4	1.4125000	0.2327194	1.1000000	1.5900000

Unique Subject Identifier=1702010000-0009

Assay variable : PTINR_VAL1 PT-INR Value

N	Mean	Standard Deviation	Minimum	Maximum
2	2.1850000	0.1343503	2.0900000	2.2800000

Unique Subject Identifier=1705010000-0002

Assay variable : PTINR_VAL1 PT-INR Value

N	Mean	Standard Deviation	Minimum	Maximum
6	1.8116667	0.1802683	1.5000000	1.9700000

Unique Subject Identifier=1710000000-0001

Assay variable : PTINR_VAL1 PT-INR Value

N	Mean	Standard Deviation	Minimum	Maximum
6	2.2450000	0.2115420	1.9800000	2.4700000

Laboratory tests at the time of obtaining Table 2-6 consent, according to the presence or absence of proton-pump inhibitors

Unique Subject Identifier=1710000000-0009

MEANS Procedure

Assay variable : PTINR_VAL1 PT-INR Value

N	Mean	Standard Deviation	Minimum	Maximum
5	1.9600000	0.4259108	1.5300000	2.5900000

Unique Subject Identifier=1716010000-0006

Assay variable : PTINR_VAL1 PT-INR Value

N	Mean	Standard Deviation	Minimum	Maximum
2	2.0500000	0.4808326	1.7100000	2.3900000

Unique Subject Identifier=1718000000-0008

Assay variable : PTINR_VAL1 PT-INR Value

N	Mean	Standard Deviation	Minimum	Maximum
6	1.7333333	0.3559026	1.2000000	2.1000000

Unique Subject Identifier=1750100000-0005

Assay variable : PTINR_VAL1 PT-INR Value

N	Mean	Standard Deviation	Minimum	Maximum
3	1.8033333	0.1550269	1.6500000	1.9600000

Laboratory tests at the time of obtaining Table 2-6 consent, according to the presence or absence of proton-pump inhibitors

Unique Subject Identifier=1750100000-0006

MEANS Procedure

Assay variable : PTINR_VAL1 PT-INR Value

N	Mean	Standard Deviation	Minimum	Maximum
6	1.7966667	0.1076414	1.6300000	1.9000000

Unique Subject Identifier=1750100000-0018

Assay variable : PTINR_VAL1 PT-INR Value

N	Mean	Standard Deviation	Minimum	Maximum
3	2.0766667	0.2402776	1.8300000	2.3100000

Unique Subject Identifier=1750100000-0040

Assay variable : PTINR_VAL1 PT-INR Value

N	Mean	Standard Deviation	Minimum	Maximum
6	1.8750000	0.1408190	1.6800000	2.0400000

Unique Subject Identifier=1750100000-0043

Assay variable : PTINR_VAL1 PT-INR Value

N	Mean	Standard Deviation	Minimum	Maximum
5	1.8100000	0.2511971	1.6400000	2.2500000

Laboratory tests at the time of obtaining Table 2-6 consent, according to the presence or absence of proton-pump inhibitors

Unique Subject Identifier=1750100000-0047

MEANS Procedure

Assay variable : PTINR_VAL1 PT-INR Value

N	Mean	Standard Deviation	Minimum	Maximum
4	1.8800000	0.7425631	1.4300000	2.9900000

Unique Subject Identifier=1810000000-0014

Assay variable : PTINR_VAL1 PT-INR Value

N	Mean	Standard Deviation	Minimum	Maximum
6	1.9666667	0.2732520	1.5900000	2.2300000

Unique Subject Identifier=1811000000-0006

Assay variable : PTINR_VAL1 PT-INR Value

N	Mean	Standard Deviation	Minimum	Maximum
6	2.0950000	0.7683684	1.2700000	3.2500000

Unique Subject Identifier=1811000000-0015

Assay variable : PTINR_VAL1 PT-INR Value

N	Mean	Standard Deviation	Minimum	Maximum
4	2.5000000	0.3900427	2.1300000	3.0500000

Laboratory tests at the time of obtaining Table 2-6 consent, according to the presence or absence of proton-pump inhibitors

Unique Subject Identifier=1811000000-0021

MEANS Procedure

Assay variable : PTINR_VAL1 PT-INR Value

N	Mean	Standard Deviation	Minimum	Maximum
5	2.3140000	0.4056230	1.8800000	2.9500000

Unique Subject Identifier=1811000000-0028

Assay variable : PTINR_VAL1 PT-INR Value

N	Mean	Standard Deviation	Minimum	Maximum
3	2.5866667	0.4980295	2.0300000	2.9900000

Unique Subject Identifier=1812000000-0018

Assay variable : PTINR_VAL1 PT-INR Value

N	Mean	Standard Deviation	Minimum	Maximum
6	1.5166667	0.0983192	1.4000000	1.6000000

Unique Subject Identifier=1813100000-0009

Assay variable : PTINR_VAL1 PT-INR Value

N	Mean	Standard Deviation	Minimum	Maximum
6	2.0850000	0.3976305	1.7300000	2.6200000

Laboratory tests at the time of obtaining Table 2-6 consent, according to the presence or absence of proton-pump inhibitors

Unique Subject Identifier=1813100000-0015

MEANS Procedure

Assay variable : PTINR_VAL1 PT-INR Value

N	Mean	Standard Deviation	Minimum	Maximum
4	1.9550000	0.1250333	1.8100000	2.1100000

Unique Subject Identifier=1813100000-0024

Assay variable : PTINR_VAL1 PT-INR Value

N	Mean	Standard Deviation	Minimum	Maximum
3	2.1166667	0.1201388	2.0000000	2.2400000

Unique Subject Identifier=1813100000-0025

Assay variable : PTINR_VAL1 PT-INR Value

N	Mean	Standard Deviation	Minimum	Maximum
1	1.6400000		1.6400000	1.6400000

Unique Subject Identifier=1813100000-0046

Assay variable : PTINR_VAL1 PT-INR Value

N	Mean	Standard Deviation	Minimum	Maximum
2	2.1900000	0.1697056	2.0700000	2.3100000

Laboratory tests at the time of obtaining Table 2-6 consent, according to the presence or absence of proton-pump inhibitors

Unique Subject Identifier=1813100000-0048

MEANS Procedure

Assay variable : PTINR_VAL1 PT-INR Value

N	Mean	Standard Deviation	Minimum	Maximum
4	1.7750000	0.2340228	1.4300000	1.9300000

Unique Subject Identifier=1814100000-0017

Assay variable : PTINR_VAL1 PT-INR Value

N	Mean	Standard Deviation	Minimum	Maximum
6	2.0050000	0.6320680	1.2700000	2.9600000

Unique Subject Identifier=1814100000-0022

Assay variable : PTINR_VAL1 PT-INR Value

N	Mean	Standard Deviation	Minimum	Maximum
6	1.8650000	0.2738430	1.4500000	2.1600000

Unique Subject Identifier=1814100000-0026

Assay variable : PTINR_VAL1 PT-INR Value

N	Mean	Standard Deviation	Minimum	Maximum
6	2.6150000	0.3059902	2.2300000	2.9600000

Laboratory tests at the time of obtaining Table 2-6 consent, according to the presence or absence of proton-pump inhibitors

Unique Subject Identifier=1814100000-0050

MEANS Procedure

Assay variable : PTINR_VAL1 PT-INR Value

N	Mean	Standard Deviation	Minimum	Maximum
4	1.7250000	0.9980815	1.1600000	3.2200000

Unique Subject Identifier=1814100000-0053

Assay variable : PTINR_VAL1 PT-INR Value

N	Mean	Standard Deviation	Minimum	Maximum
5	1.7200000	0.2049390	1.5000000	1.9000000

Unique Subject Identifier=1814100000-0055

Assay variable : PTINR_VAL1 PT-INR Value

N	Mean	Standard Deviation	Minimum	Maximum
3	2.1200000	0.4233202	1.6400000	2.4400000

Unique Subject Identifier=1815100000-0004

Assay variable : PTINR_VAL1 PT-INR Value

N	Mean	Standard Deviation	Minimum	Maximum
1	1.0500000		1.0500000	1.0500000

Laboratory tests at the time of obtaining Table 2-6 consent, according to the presence or absence of proton-pump inhibitors

Unique Subject Identifier=1973000000-0002

MEANS Procedure

Assay variable : PTINR_VAL1 PT-INR Value

N	Mean	Standard Deviation	Minimum	Maximum
5	1.8800000	0.1923538	1.6000000	2.1000000

Unique Subject Identifier=1973000000-0004

Assay variable : PTINR_VAL1 PT-INR Value

N	Mean	Standard Deviation	Minimum	Maximum
6	2.3000000	0.8602325	1.7000000	4.0000000

Unique Subject Identifier=1973000000-0016

Assay variable : PTINR_VAL1 PT-INR Value

N	Mean	Standard Deviation	Minimum	Maximum
6	1.7166667	0.2926887	1.5000000	2.3000000

Unique Subject Identifier=1973000000-0017

Assay variable : PTINR_VAL1 PT-INR Value

N	Mean	Standard Deviation	Minimum	Maximum
5	1.6000000	0.5787918	1.2000000	2.6000000

Laboratory tests at the time of obtaining Table 2-6 consent, according to the presence or absence of proton-pump inhibitors

Unique Subject Identifier=2008010000-0031

MEANS Procedure

Assay variable : PTINR_VAL1 PT-INR Value

N	Mean	Standard Deviation	Minimum	Maximum
6	1.8166667	0.3656045	1.4000000	2.3000000

Unique Subject Identifier=2008010000-0038

Assay variable : PTINR_VAL1 PT-INR Value

N	Mean	Standard Deviation	Minimum	Maximum
6	1.9500000	0.1643168	1.8000000	2.2000000

Unique Subject Identifier=2008010000-0041

Assay variable : PTINR_VAL1 PT-INR Value

N	Mean	Standard Deviation	Minimum	Maximum
6	1.8166667	0.5913262	1.5000000	3.0000000

Unique Subject Identifier=2016011111-0018

Assay variable : PTINR_VAL1 PT-INR Value

N	Mean	Standard Deviation	Minimum	Maximum
4	1.7375000	0.3456757	1.5200000	2.2500000

Laboratory tests at the time of obtaining Table 2-6 consent, according to the presence or absence of proton-pump inhibitors

Unique Subject Identifier=2016011111-0023

MEANS Procedure

Assay variable : PTINR_VAL1 PT-INR Value

N	Mean	Standard Deviation	Minimum	Maximum
6	2.3000000	0.6515213	1.6300000	3.1600000

Unique Subject Identifier=2017011100-0006

Assay variable : PTINR_VAL1 PT-INR Value

N	Mean	Standard Deviation	Minimum	Maximum
5	1.9760000	0.1540454	1.8400000	2.2100000

Unique Subject Identifier=2017011100-0012

Assay variable : PTINR_VAL1 PT-INR Value

N	Mean	Standard Deviation	Minimum	Maximum
6	2.0150000	0.3148174	1.6300000	2.4300000

Unique Subject Identifier=2017011100-0025

Assay variable : PTINR_VAL1 PT-INR Value

N	Mean	Standard Deviation	Minimum	Maximum
4	1.7850000	0.1317826	1.6400000	1.9600000

Laboratory tests at the time of obtaining Table 2-6 consent, according to the presence or absence of proton-pump inhibitors

Unique Subject Identifier=2017011100-0035

MEANS Procedure

Assay variable : PTINR_VAL1 PT-INR Value

N	Mean	Standard Deviation	Minimum	Maximum
1	2.2700000		2.2700000	2.2700000

Unique Subject Identifier=2017011100-0059

Assay variable : PTINR_VAL1 PT-INR Value

N	Mean	Standard Deviation	Minimum	Maximum
5	2.0640000	0.5264314	1.4000000	2.8500000

Unique Subject Identifier=2017011100-0088

Assay variable : PTINR_VAL1 PT-INR Value

N	Mean	Standard Deviation	Minimum	Maximum
6	2.1233333	0.2399722	1.8400000	2.5100000

Unique Subject Identifier=2017011100-0093

Assay variable : PTINR_VAL1 PT-INR Value

N	Mean	Standard Deviation	Minimum	Maximum
6	1.8216667	0.1090718	1.6400000	1.9600000

Laboratory tests at the time of obtaining Table 2-6 consent, according to the presence or absence of proton-pump inhibitors

Unique Subject Identifier=2017011100-0113

MEANS Procedure

Assay variable : PTINR_VAL1 PT-INR Value

N	Mean	Standard Deviation	Minimum	Maximum
4	1.3725000	0.1711481	1.2000000	1.5900000

Unique Subject Identifier=2017011100-0116

Assay variable : PTINR_VAL1 PT-INR Value

N	Mean	Standard Deviation	Minimum	Maximum
6	1.9816667	0.2195829	1.7300000	2.3700000

Unique Subject Identifier=2018000000-0005

Assay variable : PTINR_VAL1 PT-INR Value

N	Mean	Standard Deviation	Minimum	Maximum
5	1.8820000	0.1771158	1.6800000	2.0500000

Unique Subject Identifier=2018000000-0006

Assay variable : PTINR_VAL1 PT-INR Value

N	Mean	Standard Deviation	Minimum	Maximum
2	2.5900000	0.2828427	2.3900000	2.7900000

Laboratory tests at the time of obtaining Table 2-6 consent, according to the presence or absence of proton-pump inhibitors

Unique Subject Identifier=2018000000-0010

MEANS Procedure

Assay variable : PTINR_VAL1 PT-INR Value

N	Mean	Standard Deviation	Minimum	Maximum
5	1.8100000	0.3000000	1.3900000	2.1500000

Unique Subject Identifier=2026011111-0004

Assay variable : PTINR_VAL1 PT-INR Value

N	Mean	Standard Deviation	Minimum	Maximum
6	1.6000000	0.2000000	1.3000000	1.8000000

Unique Subject Identifier=2026011111-0006

Assay variable : PTINR_VAL1 PT-INR Value

N	Mean	Standard Deviation	Minimum	Maximum
6	1.9500000	0.1870829	1.7000000	2.2000000

Unique Subject Identifier=2027010010-0004

Assay variable : PTINR_VAL1 PT-INR Value

N	Mean	Standard Deviation	Minimum	Maximum
6	2.0166667	0.1966384	1.6600000	2.1700000

Laboratory tests at the time of obtaining Table 2-6 consent, according to the presence or absence of proton-pump inhibitors

Unique Subject Identifier=2027010010-0007

MEANS Procedure

Assay variable : PTINR_VAL1 PT-INR Value

N	Mean	Standard Deviation	Minimum	Maximum
3	1.6766667	0.0665833	1.6000000	1.7200000

Unique Subject Identifier=2047011111-0012

Assay variable : PTINR_VAL1 PT-INR Value

N	Mean	Standard Deviation	Minimum	Maximum
2	2.4900000	0.2687006	2.3000000	2.6800000

Unique Subject Identifier=2048011011-0035

Assay variable : PTINR_VAL1 PT-INR Value

N	Mean	Standard Deviation	Minimum	Maximum
3	1.9000000	0.1000000	1.8000000	2.0000000

Unique Subject Identifier=2048011011-0067

Assay variable : PTINR_VAL1 PT-INR Value

N	Mean	Standard Deviation	Minimum	Maximum
4	2.2000000	0.1825742	2.0000000	2.4000000

Laboratory tests at the time of obtaining Table 2-6 consent, according to the presence or absence of proton-pump inhibitors

Unique Subject Identifier=2064011111-0008

MEANS Procedure

Assay variable : PTINR_VAL1 PT-INR Value

N	Mean	Standard Deviation	Minimum	Maximum
1	1.9700000		1.9700000	1.9700000

Unique Subject Identifier=2064011111-0011

Assay variable : PTINR_VAL1 PT-INR Value

N	Mean	Standard Deviation	Minimum	Maximum
3	2.6533333	0.8775154	1.7400000	3.4900000

Unique Subject Identifier=2069000000-0002

Assay variable : PTINR_VAL1 PT-INR Value

N	Mean	Standard Deviation	Minimum	Maximum
4	2.2425000	0.2984823	1.8600000	2.5600000

Unique Subject Identifier=2077011111-0003

Assay variable : PTINR_VAL1 PT-INR Value

N	Mean	Standard Deviation	Minimum	Maximum
5	2.2580000	0.3998375	1.8400000	2.8100000

Laboratory tests at the time of obtaining Table 2-6 consent, according to the presence or absence of proton-pump inhibitors

Unique Subject Identifier=2077011111-0004

MEANS Procedure

Assay variable : PTINR_VAL1 PT-INR Value

N	Mean	Standard Deviation	Minimum	Maximum
6	1.9366667	0.6416749	1.3200000	2.6800000

Unique Subject Identifier=2077011111-0022

Assay variable : PTINR_VAL1 PT-INR Value

N	Mean	Standard Deviation	Minimum	Maximum
5	1.9780000	0.1329286	1.8300000	2.1600000

Unique Subject Identifier=2107011000-0024

Assay variable : PTINR_VAL1 PT-INR Value

N	Mean	Standard Deviation	Minimum	Maximum
6	2.1416667	0.4149418	1.5100000	2.6100000

Unique Subject Identifier=2107011000-0037

Assay variable : PTINR_VAL1 PT-INR Value

N	Mean	Standard Deviation	Minimum	Maximum
3	2.4533333	0.6792888	1.9700000	3.2300000

Laboratory tests at the time of obtaining Table 2-6 consent, according to the presence or absence of proton-pump inhibitors

Unique Subject Identifier=2117011111-0001

MEANS Procedure

Assay variable : PTINR_VAL1 PT-INR Value

N	Mean	Standard Deviation	Minimum	Maximum
6	1.7750000	0.4429334	1.1200000	2.3800000

Unique Subject Identifier=2125011111-0014

Assay variable : PTINR_VAL1 PT-INR Value

N	Mean	Standard Deviation	Minimum	Maximum
5	1.8920000	0.3536524	1.4200000	2.2900000

Unique Subject Identifier=2127011011-0003

Assay variable : PTINR_VAL1 PT-INR Value

N	Mean	Standard Deviation	Minimum	Maximum
2	1.4000000	0	1.4000000	1.4000000

Unique Subject Identifier=2127011011-0009

Assay variable : PTINR_VAL1 PT-INR Value

N	Mean	Standard Deviation	Minimum	Maximum
3	1.5000000	0.4358899	1.2000000	2.0000000

Laboratory tests at the time of obtaining Table 2-6 consent, according to the presence or absence of proton-pump inhibitors

Unique Subject Identifier=2127011011-0011

MEANS Procedure

Assay variable : PTINR_VAL1 PT-INR Value

N	Mean	Standard Deviation	Minimum	Maximum
6	1.5333333	0.2943920	1.1000000	1.9000000

Unique Subject Identifier=2127011011-0023

Assay variable : PTINR_VAL1 PT-INR Value

N	Mean	Standard Deviation	Minimum	Maximum
5	1.6400000	0.1341641	1.5000000	1.8000000

Unique Subject Identifier=2127011011-0039

Assay variable : PTINR_VAL1 PT-INR Value

N	Mean	Standard Deviation	Minimum	Maximum
5	1.2400000	0.1140175	1.1000000	1.4000000

Unique Subject Identifier=2127011011-0041

Assay variable : PTINR_VAL1 PT-INR Value

N	Mean	Standard Deviation	Minimum	Maximum
6	2.0166667	0.4578937	1.6000000	2.6000000

Laboratory tests at the time of obtaining Table 2-6 consent, according to the presence or absence of proton-pump inhibitors

Unique Subject Identifier=2138011111-0002

MEANS Procedure

Assay variable : PTINR_VAL1 PT-INR Value

N	Mean	Standard Deviation	Minimum	Maximum
4	1.4375000	0.1021029	1.3100000	1.5600000

Unique Subject Identifier=2138011111-0009

Assay variable : PTINR_VAL1 PT-INR Value

N	Mean	Standard Deviation	Minimum	Maximum
5	2.0520000	0.1943451	1.8100000	2.3400000

Unique Subject Identifier=2138011111-0028

Assay variable : PTINR_VAL1 PT-INR Value

N	Mean	Standard Deviation	Minimum	Maximum
5	1.5300000	0.2331309	1.1900000	1.7800000

Unique Subject Identifier=2138011111-0031

Assay variable : PTINR_VAL1 PT-INR Value

N	Mean	Standard Deviation	Minimum	Maximum
4	1.7425000	0.1851801	1.6000000	2.0100000

Laboratory tests at the time of obtaining Table 2-6 consent, according to the presence or absence of proton-pump inhibitors

Unique Subject Identifier=2138011111-0046

MEANS Procedure

Assay variable : PTINR_VAL1 PT-INR Value

N	Mean	Standard Deviation	Minimum	Maximum
3	1.6033333	0.3744774	1.1900000	1.9200000

Unique Subject Identifier=2138011111-0059

Assay variable : PTINR_VAL1 PT-INR Value

N	Mean	Standard Deviation	Minimum	Maximum
6	1.8566667	0.4131182	1.2200000	2.3000000

Unique Subject Identifier=2140011100-0001

Assay variable : PTINR_VAL1 PT-INR Value

N	Mean	Standard Deviation	Minimum	Maximum
3	2.2000000	0.9165151	1.4000000	3.2000000

Unique Subject Identifier=2163000000-0035

Assay variable : PTINR_VAL1 PT-INR Value

N	Mean	Standard Deviation	Minimum	Maximum
1	1.1000000		1.1000000	1.1000000

Laboratory tests at the time of obtaining Table 2-6 consent, according to the presence or absence of proton-pump inhibitors

Unique Subject Identifier=2163000000-0047

MEANS Procedure

Assay variable : PTINR_VAL1 PT-INR Value

N	Mean	Standard Deviation	Minimum	Maximum
2	3.4000000	1.2727922	2.5000000	4.3000000

Unique Subject Identifier=2163000000-0060

Assay variable : PTINR_VAL1 PT-INR Value

N	Mean	Standard Deviation	Minimum	Maximum
2	1.6500000	0.0707107	1.6000000	1.7000000

Unique Subject Identifier=2174011011-0021

Assay variable : PTINR_VAL1 PT-INR Value

N	Mean	Standard Deviation	Minimum	Maximum
6	2.6333333	0.2732520	2.2000000	3.0000000

Unique Subject Identifier=2176011011-0013

Assay variable : PTINR_VAL1 PT-INR Value

N	Mean	Standard Deviation	Minimum	Maximum
2	2.2250000	0.8697413	1.6100000	2.8400000

Laboratory tests at the time of obtaining Table 2-6 consent, according to the presence or absence of proton-pump inhibitors

Unique Subject Identifier=2176011011-0015

MEANS Procedure

Assay variable : PTINR_VAL1 PT-INR Value

N	Mean	Standard Deviation	Minimum	Maximum
4	1.2325000	0.0419325	1.1900000	1.2900000

Unique Subject Identifier=2176011011-0022

Assay variable : PTINR_VAL1 PT-INR Value

N	Mean	Standard Deviation	Minimum	Maximum
4	1.8175000	0.2969147	1.4000000	2.1000000

Unique Subject Identifier=2176011011-0026

Assay variable : PTINR_VAL1 PT-INR Value

N	Mean	Standard Deviation	Minimum	Maximum
2	2.1450000	0.0636396	2.1000000	2.1900000

Unique Subject Identifier=2176011011-0034

Assay variable : PTINR_VAL1 PT-INR Value

N	Mean	Standard Deviation	Minimum	Maximum
1	2.5200000		2.5200000	2.5200000

Laboratory tests at the time of obtaining Table 2-6 consent, according to the presence or absence of proton-pump inhibitors

Unique Subject Identifier=2176011011-0044

MEANS Procedure

Assay variable : PTINR_VAL1 PT-INR Value

N	Mean	Standard Deviation	Minimum	Maximum
1	2.2900000		2.2900000	2.2900000

Unique Subject Identifier=2183011011-0009

Assay variable : PTINR_VAL1 PT-INR Value

N	Mean	Standard Deviation	Minimum	Maximum
3	1.5333333	0.2081666	1.3000000	1.7000000

Unique Subject Identifier=2183011011-0014

Assay variable : PTINR_VAL1 PT-INR Value

N	Mean	Standard Deviation	Minimum	Maximum
2	1.6000000	0.5656854	1.2000000	2.0000000

Unique Subject Identifier=2185010010-0005

Assay variable : PTINR_VAL1 PT-INR Value

N	Mean	Standard Deviation	Minimum	Maximum
6	1.9500000	0.3391165	1.6000000	2.5000000

Laboratory tests at the time of obtaining Table 2-6 consent, according to the presence or absence of proton-pump inhibitors

Unique Subject Identifier=2185010010-0008

MEANS Procedure

Assay variable : PTINR_VAL1 PT-INR Value

N	Mean	Standard Deviation	Minimum	Maximum
6	1.9666667	0.5645057	1.4000000	2.9000000

Unique Subject Identifier=2205001111-0027

Assay variable : PTINR_VAL1 PT-INR Value

N	Mean	Standard Deviation	Minimum	Maximum
6	1.7333333	0.1632993	1.6000000	2.0000000

Unique Subject Identifier=2205001111-0049

Assay variable : PTINR_VAL1 PT-INR Value

N	Mean	Standard Deviation	Minimum	Maximum
6	1.9450000	0.7634854	1.1700000	3.2300000

Unique Subject Identifier=2211011111-0001

Assay variable : PTINR_VAL1 PT-INR Value

N	Mean	Standard Deviation	Minimum	Maximum
1	2.6000000		2.6000000	2.6000000

Laboratory tests at the time of obtaining Table 2-6 consent, according to the presence or absence of proton-pump inhibitors

Unique Subject Identifier=2211011111-0037

MEANS Procedure

Assay variable : PTINR_VAL1 PT-INR Value

N	Mean	Standard Deviation	Minimum	Maximum
6	1.7000000	0.6131884	1.0000000	2.6000000

Unique Subject Identifier=2211011111-0039

Assay variable : PTINR_VAL1 PT-INR Value

N	Mean	Standard Deviation	Minimum	Maximum
5	1.4000000	0.1870829	1.2000000	1.7000000

Unique Subject Identifier=2211011111-0041

Assay variable : PTINR_VAL1 PT-INR Value

N	Mean	Standard Deviation	Minimum	Maximum
1	2.5000000		2.5000000	2.5000000

Unique Subject Identifier=2211011111-0044

Assay variable : PTINR_VAL1 PT-INR Value

N	Mean	Standard Deviation	Minimum	Maximum
5	1.0940000	0.0531977	1.0500000	1.1800000

Laboratory tests at the time of obtaining Table 2-6 consent, according to the presence or absence of proton-pump inhibitors

Unique Subject Identifier=2211011111-0058

MEANS Procedure

Assay variable : PTINR_VAL1 PT-INR Value

N	Mean	Standard Deviation	Minimum	Maximum
1	1.1900000		1.1900000	1.1900000

Unique Subject Identifier=2211011111-0060

Assay variable : PTINR_VAL1 PT-INR Value

N	Mean	Standard Deviation	Minimum	Maximum
1	1.9100000		1.9100000	1.9100000

Unique Subject Identifier=2233011111-0014

Assay variable : PTINR_VAL1 PT-INR Value

N	Mean	Standard Deviation	Minimum	Maximum
4	1.4800000	0.2044505	1.1800000	1.6100000

Unique Subject Identifier=2233011111-0023

Assay variable : PTINR_VAL1 PT-INR Value

N	Mean	Standard Deviation	Minimum	Maximum
5	1.7000000	0.2354782	1.3500000	1.9700000

Laboratory tests at the time of obtaining Table 2-6 consent, according to the presence or absence of proton-pump inhibitors

Unique Subject Identifier=2233011111-0024

MEANS Procedure

Assay variable : PTINR_VAL1 PT-INR Value

N	Mean	Standard Deviation	Minimum	Maximum
6	1.5616667	0.2039036	1.3400000	1.8800000

Unique Subject Identifier=2233011111-0025

Assay variable : PTINR_VAL1 PT-INR Value

N	Mean	Standard Deviation	Minimum	Maximum
3	1.5566667	0.1556706	1.4100000	1.7200000

Unique Subject Identifier=2233011111-0034

Assay variable : PTINR_VAL1 PT-INR Value

N	Mean	Standard Deviation	Minimum	Maximum
4	1.3150000	0.2056696	1.1200000	1.5800000

Unique Subject Identifier=2241011111-0011

Assay variable : PTINR_VAL1 PT-INR Value

N	Mean	Standard Deviation	Minimum	Maximum
5	1.9700000	0.1996246	1.8000000	2.2600000

Laboratory tests at the time of obtaining Table 2-6 consent, according to the presence or absence of proton-pump inhibitors

Unique Subject Identifier=2247010011-0006

MEANS Procedure

Assay variable : PTINR_VAL1 PT-INR Value

N	Mean	Standard Deviation	Minimum	Maximum
4	1.8550000	0.1123981	1.7100000	1.9700000

Unique Subject Identifier=2247010011-0010

Assay variable : PTINR_VAL1 PT-INR Value

N	Mean	Standard Deviation	Minimum	Maximum
4	2.5575000	0.3375772	2.1200000	2.8700000

Unique Subject Identifier=2247010011-0012

Assay variable : PTINR_VAL1 PT-INR Value

N	Mean	Standard Deviation	Minimum	Maximum
4	1.5250000	0.2338803	1.1900000	1.7200000

Unique Subject Identifier=2247010011-0013

Assay variable : PTINR_VAL1 PT-INR Value

N	Mean	Standard Deviation	Minimum	Maximum
4	2.3450000	0.2517274	2.0700000	2.6600000

Laboratory tests at the time of obtaining Table 2-6 consent, according to the presence or absence of proton-pump inhibitors

Unique Subject Identifier=2247010011-0015

MEANS Procedure

Assay variable : PTINR_VAL1 PT-INR Value

N	Mean	Standard Deviation	Minimum	Maximum
5	1.8440000	0.1425833	1.6400000	2.0300000

Unique Subject Identifier=2247010011-0016

Assay variable : PTINR_VAL1 PT-INR Value

N	Mean	Standard Deviation	Minimum	Maximum
4	1.5300000	0.1219289	1.4300000	1.6900000

Unique Subject Identifier=2247010011-0018

Assay variable : PTINR_VAL1 PT-INR Value

N	Mean	Standard Deviation	Minimum	Maximum
4	1.7825000	0.3397425	1.2800000	2.0300000

Unique Subject Identifier=2247010011-0020

Assay variable : PTINR_VAL1 PT-INR Value

N	Mean	Standard Deviation	Minimum	Maximum
3	1.9966667	0.1965536	1.7700000	2.1200000

Laboratory tests at the time of obtaining Table 2-6 consent, according to the presence or absence of proton-pump inhibitors

Unique Subject Identifier=2247010011-0021

MEANS Procedure

Assay variable : PTINR_VAL1 PT-INR Value

N	Mean	Standard Deviation	Minimum	Maximum
6	1.4100000	0.3959798	1.0800000	2.0700000

Unique Subject Identifier=2247010011-0022

Assay variable : PTINR_VAL1 PT-INR Value

N	Mean	Standard Deviation	Minimum	Maximum
4	1.5900000	0.1227464	1.4400000	1.7400000

Unique Subject Identifier=2247010011-0023

Assay variable : PTINR_VAL1 PT-INR Value

N	Mean	Standard Deviation	Minimum	Maximum
4	2.3800000	0.3067029	2.1700000	2.8200000

Unique Subject Identifier=2247010011-0024

Assay variable : PTINR_VAL1 PT-INR Value

N	Mean	Standard Deviation	Minimum	Maximum
4	2.0825000	0.1840969	1.8500000	2.3000000

Laboratory tests at the time of obtaining Table 2-6 consent, according to the presence or absence of proton-pump inhibitors

Unique Subject Identifier=2247010011-0028

MEANS Procedure

Assay variable : PTINR_VAL1 PT-INR Value

N	Mean	Standard Deviation	Minimum	Maximum
4	2.2100000	0.1311488	2.0200000	2.3200000

Unique Subject Identifier=2247010011-0029

Assay variable : PTINR_VAL1 PT-INR Value

N	Mean	Standard Deviation	Minimum	Maximum
4	2.2475000	0.3852597	1.8100000	2.7400000

Unique Subject Identifier=2247010011-0033

Assay variable : PTINR_VAL1 PT-INR Value

N	Mean	Standard Deviation	Minimum	Maximum
4	1.6950000	0.3319136	1.2900000	2.0000000

Unique Subject Identifier=2247010011-0035

Assay variable : PTINR_VAL1 PT-INR Value

N	Mean	Standard Deviation	Minimum	Maximum
5	2.4340000	0.4240637	2.0900000	3.1500000

Laboratory tests at the time of obtaining Table 2-6 consent, according to the presence or absence of proton-pump inhibitors

Unique Subject Identifier=2247010011-0036

MEANS Procedure

Assay variable : PTINR_VAL1 PT-INR Value

N	Mean	Standard Deviation	Minimum	Maximum
6	1.6916667	0.1110705	1.5600000	1.8800000

Unique Subject Identifier=2247010011-0039

Assay variable : PTINR_VAL1 PT-INR Value

N	Mean	Standard Deviation	Minimum	Maximum
4	1.9850000	0.2522565	1.7200000	2.2600000

Unique Subject Identifier=2247010011-0040

Assay variable : PTINR_VAL1 PT-INR Value

N	Mean	Standard Deviation	Minimum	Maximum
4	1.8800000	0.1446836	1.7100000	2.0100000

Unique Subject Identifier=2247010011-0041

Assay variable : PTINR_VAL1 PT-INR Value

N	Mean	Standard Deviation	Minimum	Maximum
4	2.4325000	0.6373578	1.5500000	3.0200000

Laboratory tests at the time of obtaining Table 2-6 consent, according to the presence or absence of proton-pump inhibitors

Unique Subject Identifier=2247010011-0042

MEANS Procedure

Assay variable : PTINR_VAL1 PT-INR Value

N	Mean	Standard Deviation	Minimum	Maximum
5	1.7160000	0.4074678	1.3000000	2.2000000

Unique Subject Identifier=2247010011-0043

Assay variable : PTINR_VAL1 PT-INR Value

N	Mean	Standard Deviation	Minimum	Maximum
4	1.8125000	0.2784930	1.6000000	2.2000000

Unique Subject Identifier=2247010011-0044

Assay variable : PTINR_VAL1 PT-INR Value

N	Mean	Standard Deviation	Minimum	Maximum
4	2.3600000	0.2565151	1.9800000	2.5200000

Unique Subject Identifier=2247010011-0045

Assay variable : PTINR_VAL1 PT-INR Value

N	Mean	Standard Deviation	Minimum	Maximum
4	1.9025000	0.1508587	1.7500000	2.1000000

Laboratory tests at the time of obtaining Table 2-6 consent, according to the presence or absence of proton-pump inhibitors

Unique Subject Identifier=2247010011-0046

MEANS Procedure

Assay variable : PTINR_VAL1 PT-INR Value

N	Mean	Standard Deviation	Minimum	Maximum
4	2.0975000	0.2850000	1.7900000	2.4600000

Unique Subject Identifier=2247010011-0047

Assay variable : PTINR_VAL1 PT-INR Value

N	Mean	Standard Deviation	Minimum	Maximum
4	2.1025000	0.0942956	1.9900000	2.1900000

Unique Subject Identifier=2247010011-0048

Assay variable : PTINR_VAL1 PT-INR Value

N	Mean	Standard Deviation	Minimum	Maximum
4	3.2375000	0.8573360	2.4900000	4.4400000

Unique Subject Identifier=2247010011-0052

Assay variable : PTINR_VAL1 PT-INR Value

N	Mean	Standard Deviation	Minimum	Maximum
1	1.4300000		1.4300000	1.4300000

Laboratory tests at the time of obtaining Table 2-6 consent, according to the presence or absence of proton-pump inhibitors

Unique Subject Identifier=2247010011-0053

MEANS Procedure

Assay variable : PTINR_VAL1 PT-INR Value

N	Mean	Standard Deviation	Minimum	Maximum
6	2.3283333	0.4026620	1.5600000	2.6900000

Unique Subject Identifier=2247010011-0056

Assay variable : PTINR_VAL1 PT-INR Value

N	Mean	Standard Deviation	Minimum	Maximum
6	2.1866667	0.2207865	1.8800000	2.5200000

Unique Subject Identifier=2247010011-0057

Assay variable : PTINR_VAL1 PT-INR Value

N	Mean	Standard Deviation	Minimum	Maximum
4	2.1075000	0.6416840	1.6100000	3.0500000

Unique Subject Identifier=2247010011-0058

Assay variable : PTINR_VAL1 PT-INR Value

N	Mean	Standard Deviation	Minimum	Maximum
3	1.5500000	0.0700000	1.4700000	1.6000000

Laboratory tests at the time of obtaining Table 2-6 consent, according to the presence or absence of proton-pump inhibitors

Unique Subject Identifier=2247010011-0060

MEANS Procedure

Assay variable : PTINR_VAL1 PT-INR Value

N	Mean	Standard Deviation	Minimum	Maximum
6	2.1866667	0.6503743	1.4200000	2.9700000

Unique Subject Identifier=2247010011-0061

Assay variable : PTINR_VAL1 PT-INR Value

N	Mean	Standard Deviation	Minimum	Maximum
6	2.1000000	0.1716974	1.8400000	2.2900000

Unique Subject Identifier=2247010011-0064

Assay variable : PTINR_VAL1 PT-INR Value

N	Mean	Standard Deviation	Minimum	Maximum
4	1.6325000	0.2839454	1.2800000	1.9400000

Unique Subject Identifier=2247010011-0066

Assay variable : PTINR_VAL1 PT-INR Value

N	Mean	Standard Deviation	Minimum	Maximum
5	1.9060000	0.2160555	1.7300000	2.1700000

Laboratory tests at the time of obtaining Table 2-6 consent, according to the presence or absence of proton-pump inhibitors

Unique Subject Identifier=2247010011-0067

MEANS Procedure

Assay variable : PTINR_VAL1 PT-INR Value

N	Mean	Standard Deviation	Minimum	Maximum
4	1.4775000	0.1908533	1.2100000	1.6600000

Unique Subject Identifier=2247010011-0068

Assay variable : PTINR_VAL1 PT-INR Value

N	Mean	Standard Deviation	Minimum	Maximum
5	2.0100000	0.3930649	1.6100000	2.5200000

Unique Subject Identifier=2247010011-0069

Assay variable : PTINR_VAL1 PT-INR Value

N	Mean	Standard Deviation	Minimum	Maximum
5	1.5540000	0.2886694	1.2900000	1.9200000

Unique Subject Identifier=2247010011-0070

Assay variable : PTINR_VAL1 PT-INR Value

N	Mean	Standard Deviation	Minimum	Maximum
4	2.2375000	0.3467348	2.0200000	2.7500000

Laboratory tests at the time of obtaining Table 2-6 consent, according to the presence or absence of proton-pump inhibitors

Unique Subject Identifier=2247010011-0071

MEANS Procedure

Assay variable : PTINR_VAL1 PT-INR Value

N	Mean	Standard Deviation	Minimum	Maximum
4	2.2725000	0.6687987	1.7000000	3.0300000

Unique Subject Identifier=2247010011-0073

Assay variable : PTINR_VAL1 PT-INR Value

N	Mean	Standard Deviation	Minimum	Maximum
3	1.2500000	0.2981610	1.0000000	1.5800000

Unique Subject Identifier=2247010011-0074

Assay variable : PTINR_VAL1 PT-INR Value

N	Mean	Standard Deviation	Minimum	Maximum
3	1.6533333	0.2177919	1.4100000	1.8300000

Unique Subject Identifier=2247010011-0078

Assay variable : PTINR_VAL1 PT-INR Value

N	Mean	Standard Deviation	Minimum	Maximum
3	1.7633333	0.0750555	1.6900000	1.8400000

Laboratory tests at the time of obtaining Table 2-6 consent, according to the presence or absence of proton-pump inhibitors

Unique Subject Identifier=2258010111-0014

MEANS Procedure

Assay variable : PTINR_VAL1 PT-INR Value

N	Mean	Standard Deviation	Minimum	Maximum
1	1.1000000		1.1000000	1.1000000

Unique Subject Identifier=2268011111-0013

Assay variable : PTINR_VAL1 PT-INR Value

N	Mean	Standard Deviation	Minimum	Maximum
4	1.8500000	0.1290994	1.7000000	2.0000000

Unique Subject Identifier=2268011111-0029

Assay variable : PTINR_VAL1 PT-INR Value

N	Mean	Standard Deviation	Minimum	Maximum
6	1.8833333	0.5776389	1.5000000	3.0000000

Unique Subject Identifier=2292000000-0014

Assay variable : PTINR_VAL1 PT-INR Value

N	Mean	Standard Deviation	Minimum	Maximum
6	2.2500000	0.3619392	1.8000000	2.9000000

Laboratory tests at the time of obtaining Table 2-6 consent, according to the presence or absence of proton-pump inhibitors

Unique Subject Identifier=2296010111-0002

MEANS Procedure

Assay variable : PTINR_VAL1 PT-INR Value

N	Mean	Standard Deviation	Minimum	Maximum
5	1.7000000	0.0707107	1.6000000	1.8000000

Unique Subject Identifier=2301011111-0022

Assay variable : PTINR_VAL1 PT-INR Value

N	Mean	Standard Deviation	Minimum	Maximum
2	1.4950000	0.0919239	1.4300000	1.5600000

Unique Subject Identifier=2301011111-0027

Assay variable : PTINR_VAL1 PT-INR Value

N	Mean	Standard Deviation	Minimum	Maximum
4	1.7500000	0.2745906	1.3600000	2.0000000

Unique Subject Identifier=2301011111-0031

Assay variable : PTINR_VAL1 PT-INR Value

N	Mean	Standard Deviation	Minimum	Maximum
1	1.9500000		1.9500000	1.9500000

Laboratory tests at the time of obtaining Table 2-6 consent, according to the presence or absence of proton-pump inhibitors

Unique Subject Identifier=2310011111-0011

MEANS Procedure

Assay variable : PTINR_VAL1 PT-INR Value

N	Mean	Standard Deviation	Minimum	Maximum
3	1.7933333	0.1800926	1.6100000	1.9700000

Unique Subject Identifier=2312011111-0010

Assay variable : PTINR_VAL1 PT-INR Value

N	Mean	Standard Deviation	Minimum	Maximum
4	1.6750000	0.0957427	1.6000000	1.8000000

Unique Subject Identifier=2327011011-0005

Assay variable : PTINR_VAL1 PT-INR Value

N	Mean	Standard Deviation	Minimum	Maximum
4	1.7100000	0.2096028	1.4600000	1.9400000

Unique Subject Identifier=2327011011-0007

Assay variable : PTINR_VAL1 PT-INR Value

N	Mean	Standard Deviation	Minimum	Maximum
3	2.0733333	0.9042308	1.3300000	3.0800000

Laboratory tests at the time of obtaining Table 2-6 consent, according to the presence or absence of proton-pump inhibitors

Unique Subject Identifier=2327011011-0020

MEANS Procedure

Assay variable : PTINR_VAL1 PT-INR Value

N	Mean	Standard Deviation	Minimum	Maximum
4	2.2000000	0.1512173	2.0000000	2.3400000

Unique Subject Identifier=2327011011-0022

Assay variable : PTINR_VAL1 PT-INR Value

N	Mean	Standard Deviation	Minimum	Maximum
4	1.9525000	0.1477329	1.7700000	2.1000000

Unique Subject Identifier=2327011011-0028

Assay variable : PTINR_VAL1 PT-INR Value

N	Mean	Standard Deviation	Minimum	Maximum
3	1.9733333	0.1137248	1.8800000	2.1000000

Unique Subject Identifier=2327011011-0031

Assay variable : PTINR_VAL1 PT-INR Value

N	Mean	Standard Deviation	Minimum	Maximum
4	1.8800000	0.2592296	1.5600000	2.1600000

Laboratory tests at the time of obtaining Table 2-6 consent, according to the presence or absence of proton-pump inhibitors

Unique Subject Identifier=2327011011-0034

MEANS Procedure

Assay variable : PTINR_VAL1 PT-INR Value

N	Mean	Standard Deviation	Minimum	Maximum
4	1.8725000	0.0531507	1.8300000	1.9500000

Unique Subject Identifier=2327011011-0039

Assay variable : PTINR_VAL1 PT-INR Value

N	Mean	Standard Deviation	Minimum	Maximum
4	2.1100000	0.2201515	1.9000000	2.4200000

Unique Subject Identifier=2327011011-0041

Assay variable : PTINR_VAL1 PT-INR Value

N	Mean	Standard Deviation	Minimum	Maximum
4	1.9600000	0.5112729	1.5600000	2.7100000

Unique Subject Identifier=2327011011-0047

Assay variable : PTINR_VAL1 PT-INR Value

N	Mean	Standard Deviation	Minimum	Maximum
6	2.3916667	0.1847611	2.0900000	2.6100000

Laboratory tests at the time of obtaining Table 2-6 consent, according to the presence or absence of proton-pump inhibitors

Unique Subject Identifier=2341001000-0003

MEANS Procedure

Assay variable : PTINR_VAL1 PT-INR Value

N	Mean	Standard Deviation	Minimum	Maximum
1	1.4200000		1.4200000	1.4200000

Unique Subject Identifier=2341001000-0013

Assay variable : PTINR_VAL1 PT-INR Value

N	Mean	Standard Deviation	Minimum	Maximum
1	1.6000000		1.6000000	1.6000000

Unique Subject Identifier=2341001000-0015

Assay variable : PTINR_VAL1 PT-INR Value

N	Mean	Standard Deviation	Minimum	Maximum
1	1.4600000		1.4600000	1.4600000

Unique Subject Identifier=2347011111-0002

Assay variable : PTINR_VAL1 PT-INR Value

N	Mean	Standard Deviation	Minimum	Maximum
6	2.6183333	0.4615373	2.1500000	3.4700000

Laboratory tests at the time of obtaining Table 2-6 consent, according to the presence or absence of proton-pump inhibitors

Unique Subject Identifier=2347011111-0005

MEANS Procedure

Assay variable : PTINR_VAL1 PT-INR Value

N	Mean	Standard Deviation	Minimum	Maximum
5	1.4620000	0.3902179	0.9700000	2.0500000

Unique Subject Identifier=2347011111-0009

Assay variable : PTINR_VAL1 PT-INR Value

N	Mean	Standard Deviation	Minimum	Maximum
6	2.5550000	0.4134126	2.0100000	3.1600000

Unique Subject Identifier=2347011111-0011

Assay variable : PTINR_VAL1 PT-INR Value

N	Mean	Standard Deviation	Minimum	Maximum
6	2.3883333	0.4908937	1.8900000	3.0600000

Unique Subject Identifier=2347011111-0012

Assay variable : PTINR_VAL1 PT-INR Value

N	Mean	Standard Deviation	Minimum	Maximum
5	2.3960000	0.3449348	1.9700000	2.7800000

Laboratory tests at the time of obtaining Table 2-6 consent, according to the presence or absence of proton-pump inhibitors

Unique Subject Identifier=2347011111-0016

MEANS Procedure

Assay variable : PTINR_VAL1 PT-INR Value

N	Mean	Standard Deviation	Minimum	Maximum
6	1.9300000	0.1825377	1.6200000	2.1500000

Unique Subject Identifier=2347011111-0018

Assay variable : PTINR_VAL1 PT-INR Value

N	Mean	Standard Deviation	Minimum	Maximum
6	1.4783333	0.1919809	1.2800000	1.8100000

Unique Subject Identifier=2347011111-0019

Assay variable : PTINR_VAL1 PT-INR Value

N	Mean	Standard Deviation	Minimum	Maximum
6	1.7483333	0.2143284	1.4900000	1.9500000

Unique Subject Identifier=2347011111-0021

Assay variable : PTINR_VAL1 PT-INR Value

N	Mean	Standard Deviation	Minimum	Maximum
6	1.8633333	0.1841376	1.5400000	2.0700000

Laboratory tests at the time of obtaining Table 2-6 consent, according to the presence or absence of proton-pump inhibitors

Unique Subject Identifier=2347011111-0026

MEANS Procedure

Assay variable : PTINR_VAL1 PT-INR Value

N	Mean	Standard Deviation	Minimum	Maximum
6	1.8900000	0.3367492	1.3300000	2.2000000

Unique Subject Identifier=2347011111-0030

Assay variable : PTINR_VAL1 PT-INR Value

N	Mean	Standard Deviation	Minimum	Maximum
6	1.3700000	0.1685230	1.1600000	1.6000000

Unique Subject Identifier=2347011111-0032

Assay variable : PTINR_VAL1 PT-INR Value

N	Mean	Standard Deviation	Minimum	Maximum
6	1.9483333	0.3411402	1.3800000	2.3000000

Unique Subject Identifier=2347011111-0034

Assay variable : PTINR_VAL1 PT-INR Value

N	Mean	Standard Deviation	Minimum	Maximum
6	2.0850000	0.3237746	1.6800000	2.4400000

Laboratory tests at the time of obtaining Table 2-6 consent, according to the presence or absence of proton-pump inhibitors

Unique Subject Identifier=2347011111-0043

MEANS Procedure

Assay variable : PTINR_VAL1 PT-INR Value

N	Mean	Standard Deviation	Minimum	Maximum
6	1.9233333	0.2250926	1.6900000	2.3300000

Unique Subject Identifier=2348000000-0006

Assay variable : PTINR_VAL1 PT-INR Value

N	Mean	Standard Deviation	Minimum	Maximum
3	1.7866667	0.1680278	1.6400000	1.9700000

Unique Subject Identifier=2354011111-0001

Assay variable : PTINR_VAL1 PT-INR Value

N	Mean	Standard Deviation	Minimum	Maximum
5	1.7280000	0.2139392	1.4100000	1.9500000

Unique Subject Identifier=2354011111-0012

Assay variable : PTINR_VAL1 PT-INR Value

N	Mean	Standard Deviation	Minimum	Maximum
4	2.3900000	0.0824621	2.2800000	2.4800000

Laboratory tests at the time of obtaining Table 2-6 consent, according to the presence or absence of proton-pump inhibitors

Unique Subject Identifier=2354011111-0014

MEANS Procedure

Assay variable : PTINR_VAL1 PT-INR Value

N	Mean	Standard Deviation	Minimum	Maximum
6	1.5150000	0.2960912	1.1900000	1.9300000

Unique Subject Identifier=2356011111-0007

Assay variable : PTINR_VAL1 PT-INR Value

N	Mean	Standard Deviation	Minimum	Maximum
2	2.0700000	0.5232590	1.7000000	2.4400000

Unique Subject Identifier=2356011111-0019

Assay variable : PTINR_VAL1 PT-INR Value

N	Mean	Standard Deviation	Minimum	Maximum
2	1.9900000	0.1555635	1.8800000	2.1000000

Unique Subject Identifier=2357011010-0018

Assay variable : PTINR_VAL1 PT-INR Value

N	Mean	Standard Deviation	Minimum	Maximum
3	2.3866667	1.3743847	1.0800000	3.8200000

Laboratory tests at the time of obtaining Table 2-6 consent, according to the presence or absence of proton-pump inhibitors

Unique Subject Identifier=2357011010-0021

MEANS Procedure

Assay variable : PTINR_VAL1 PT-INR Value

N	Mean	Standard Deviation	Minimum	Maximum
4	1.8350000	0.8066598	1.2700000	3.0200000

Unique Subject Identifier=2357011010-0027

Assay variable : PTINR_VAL1 PT-INR Value

N	Mean	Standard Deviation	Minimum	Maximum
3	2.5733333	0.2154840	2.3300000	2.7400000

Unique Subject Identifier=2358000000-0001

Assay variable : PTINR_VAL1 PT-INR Value

N	Mean	Standard Deviation	Minimum	Maximum
6	1.6133333	0.1127239	1.4400000	1.7500000

Unique Subject Identifier=2361011111-0012

Assay variable : PTINR_VAL1 PT-INR Value

N	Mean	Standard Deviation	Minimum	Maximum
1	1.4900000		1.4900000	1.4900000

Laboratory tests at the time of obtaining Table 2-6 consent, according to the presence or absence of proton-pump inhibitors

Unique Subject Identifier=2366011111-0017

MEANS Procedure

Assay variable : PTINR_VAL1 PT-INR Value

N	Mean	Standard Deviation	Minimum	Maximum
1	2.1000000		2.1000000	2.1000000

Unique Subject Identifier=2370011111-0006

Assay variable : PTINR_VAL1 PT-INR Value

N	Mean	Standard Deviation	Minimum	Maximum
6	2.0500000	0.2866357	1.6200000	2.3800000

Unique Subject Identifier=2378000010-0013

Assay variable : PTINR_VAL1 PT-INR Value

N	Mean	Standard Deviation	Minimum	Maximum
6	1.5733333	0.1177568	1.4000000	1.7000000

Unique Subject Identifier=2378000010-0034

Assay variable : PTINR_VAL1 PT-INR Value

N	Mean	Standard Deviation	Minimum	Maximum
6	1.3833333	0.1169045	1.2000000	1.5000000

Laboratory tests at the time of obtaining Table 2-6 consent, according to the presence or absence of proton-pump inhibitors

Unique Subject Identifier=2378000010-0043

MEANS Procedure

Assay variable : PTINR_VAL1 PT-INR Value

N	Mean	Standard Deviation	Minimum	Maximum
5	1.3000000	0.0707107	1.2000000	1.4000000

Unique Subject Identifier=2383011110-0013

Assay variable : PTINR_VAL1 PT-INR Value

N	Mean	Standard Deviation	Minimum	Maximum
4	2.0225000	0.2067809	1.8300000	2.2300000

Unique Subject Identifier=2383011110-0030

Assay variable : PTINR_VAL1 PT-INR Value

N	Mean	Standard Deviation	Minimum	Maximum
3	1.7966667	0.0513160	1.7400000	1.8400000

Unique Subject Identifier=2386011111-0003

Assay variable : PTINR_VAL1 PT-INR Value

N	Mean	Standard Deviation	Minimum	Maximum
6	1.8833333	0.3060501	1.6000000	2.4000000

Laboratory tests at the time of obtaining Table 2-6 consent, according to the presence or absence of proton-pump inhibitors

Unique Subject Identifier=2386011111-0010

MEANS Procedure

Assay variable : PTINR_VAL1 PT-INR Value

N	Mean	Standard Deviation	Minimum	Maximum
5	2.3000000	0.3807887	1.7000000	2.7000000

Unique Subject Identifier=2393011111-0008

Assay variable : PTINR_VAL1 PT-INR Value

N	Mean	Standard Deviation	Minimum	Maximum
6	1.4066667	0.1474675	1.1900000	1.5600000

Unique Subject Identifier=2393011111-0009

Assay variable : PTINR_VAL1 PT-INR Value

N	Mean	Standard Deviation	Minimum	Maximum
6	1.6000000	0.2359661	1.2900000	2.0100000

Unique Subject Identifier=2393011111-0021

Assay variable : PTINR_VAL1 PT-INR Value

N	Mean	Standard Deviation	Minimum	Maximum
1	1.4200000		1.4200000	1.4200000

Laboratory tests at the time of obtaining Table 2-6 consent, according to the presence or absence of proton-pump inhibitors

Unique Subject Identifier=2393011111-0023

MEANS Procedure

Assay variable : PTINR_VAL1 PT-INR Value

N	Mean	Standard Deviation	Minimum	Maximum
1	1.0400000		1.0400000	1.0400000

Unique Subject Identifier=2396000000-0005

Assay variable : PTINR_VAL1 PT-INR Value

N	Mean	Standard Deviation	Minimum	Maximum
6	2.3800000	1.3431902	1.0100000	4.6600000

Unique Subject Identifier=2399011111-0014

Assay variable : PTINR_VAL1 PT-INR Value

N	Mean	Standard Deviation	Minimum	Maximum
5	2.0540000	0.2409979	1.7000000	2.3700000

Unique Subject Identifier=2403010000-0002

Assay variable : PTINR_VAL1 PT-INR Value

N	Mean	Standard Deviation	Minimum	Maximum
6	1.9816667	0.5278036	1.3400000	2.8600000

Laboratory tests at the time of obtaining Table 2-6 consent, according to the presence or absence of proton-pump inhibitors

Unique Subject Identifier=2403010000-0003

MEANS Procedure

Assay variable : PTINR_VAL1 PT-INR Value

N	Mean	Standard Deviation	Minimum	Maximum
6	2.2700000	0.4328048	1.6200000	2.7500000

Unique Subject Identifier=2403010000-0010

Assay variable : PTINR_VAL1 PT-INR Value

N	Mean	Standard Deviation	Minimum	Maximum
6	2.1766667	0.3940389	1.6100000	2.6700000

Unique Subject Identifier=2403010000-0015

Assay variable : PTINR_VAL1 PT-INR Value

N	Mean	Standard Deviation	Minimum	Maximum
1	2.1800000		2.1800000	2.1800000

Unique Subject Identifier=2403010000-0016

Assay variable : PTINR_VAL1 PT-INR Value

N	Mean	Standard Deviation	Minimum	Maximum
5	1.7780000	0.5202596	1.1900000	2.6100000

Laboratory tests at the time of obtaining Table 2-6 consent, according to the presence or absence of proton-pump inhibitors

Unique Subject Identifier=2403010000-0027

MEANS Procedure

Assay variable : PTINR_VAL1 PT-INR Value

N	Mean	Standard Deviation	Minimum	Maximum
6	1.7783333	0.1731377	1.5200000	1.9800000

Unique Subject Identifier=2403010000-0048

Assay variable : PTINR_VAL1 PT-INR Value

N	Mean	Standard Deviation	Minimum	Maximum
6	2.1666667	0.1751190	1.9000000	2.4000000

Unique Subject Identifier=2403010000-0077

Assay variable : PTINR_VAL1 PT-INR Value

N	Mean	Standard Deviation	Minimum	Maximum
1	2.0800000		2.0800000	2.0800000

Unique Subject Identifier=2403110000-0004

Assay variable : PTINR_VAL1 PT-INR Value

N	Mean	Standard Deviation	Minimum	Maximum
4	2.2075000	0.8871443	1.3300000	3.4200000

Laboratory tests at the time of obtaining Table 2-6 consent, according to the presence or absence of proton-pump inhibitors

Unique Subject Identifier=2403110000-0005

MEANS Procedure

Assay variable : PTINR_VAL1 PT-INR Value

N	Mean	Standard Deviation	Minimum	Maximum
3	1.5200000	0.3235738	1.1500000	1.7500000

Unique Subject Identifier=2403110000-0013

Assay variable : PTINR_VAL1 PT-INR Value

N	Mean	Standard Deviation	Minimum	Maximum
5	1.8800000	0.1172604	1.7400000	2.0200000

Unique Subject Identifier=2403110000-0018

Assay variable : PTINR_VAL1 PT-INR Value

N	Mean	Standard Deviation	Minimum	Maximum
6	2.2583333	0.5069287	1.8100000	2.8900000

Unique Subject Identifier=2403110000-0021

Assay variable : PTINR_VAL1 PT-INR Value

N	Mean	Standard Deviation	Minimum	Maximum
4	2.1075000	0.1560716	1.8800000	2.2300000

Laboratory tests at the time of obtaining Table 2-6 consent, according to the presence or absence of proton-pump inhibitors

Unique Subject Identifier=2403110000-0022

MEANS Procedure

Assay variable : PTINR_VAL1 PT-INR Value

N	Mean	Standard Deviation	Minimum	Maximum
6	1.9016667	0.5437800	1.1300000	2.7500000

Unique Subject Identifier=2404011111-0003

Assay variable : PTINR_VAL1 PT-INR Value

N	Mean	Standard Deviation	Minimum	Maximum
6	1.9833333	0.3430258	1.3000000	2.2000000

Unique Subject Identifier=2404011111-0006

Assay variable : PTINR_VAL1 PT-INR Value

N	Mean	Standard Deviation	Minimum	Maximum
6	1.8833333	0.2857738	1.5000000	2.3000000

Unique Subject Identifier=2405011111-0008

Assay variable : PTINR_VAL1 PT-INR Value

N	Mean	Standard Deviation	Minimum	Maximum
6	2.1950000	0.1857687	2.0300000	2.5000000

Laboratory tests at the time of obtaining Table 2-6 consent, according to the presence or absence of proton-pump inhibitors

Unique Subject Identifier=2410011111-0013

MEANS Procedure

Assay variable : PTINR_VAL1 PT-INR Value

N	Mean	Standard Deviation	Minimum	Maximum
2	2.3000000	0.4242641	2.0000000	2.6000000

Unique Subject Identifier=2410011111-0017

Assay variable : PTINR_VAL1 PT-INR Value

N	Mean	Standard Deviation	Minimum	Maximum
1	2.2800000		2.2800000	2.2800000

Unique Subject Identifier=2410011111-0019

Assay variable : PTINR_VAL1 PT-INR Value

N	Mean	Standard Deviation	Minimum	Maximum
1	1.7700000		1.7700000	1.7700000

Unique Subject Identifier=2410011111-0020

Assay variable : PTINR_VAL1 PT-INR Value

N	Mean	Standard Deviation	Minimum	Maximum
2	1.7100000	0.8343860	1.1200000	2.3000000

Laboratory tests at the time of obtaining Table 2-6 consent, according to the presence or absence of proton-pump inhibitors

Unique Subject Identifier=2410011111-0032

MEANS Procedure

Assay variable : PTINR_VAL1 PT-INR Value

N	Mean	Standard Deviation	Minimum	Maximum
2	1.8400000	0.7636753	1.3000000	2.3800000

Unique Subject Identifier=2412011111-0001

Assay variable : PTINR_VAL1 PT-INR Value

N	Mean	Standard Deviation	Minimum	Maximum
2	1.4300000	0.0282843	1.4100000	1.4500000

Unique Subject Identifier=2421010000-0003

Assay variable : PTINR_VAL1 PT-INR Value

N	Mean	Standard Deviation	Minimum	Maximum
2	1.5850000	0.0212132	1.5700000	1.6000000

Unique Subject Identifier=2421010000-0041

Assay variable : PTINR_VAL1 PT-INR Value

N	Mean	Standard Deviation	Minimum	Maximum
1	1.8100000		1.8100000	1.8100000

Laboratory tests at the time of obtaining Table 2-6 consent, according to the presence or absence of proton-pump inhibitors

Unique Subject Identifier=2421010000-0064

MEANS Procedure

Assay variable : PTINR_VAL1 PT-INR Value

N	Mean	Standard Deviation	Minimum	Maximum
4	1.3700000	0.1749286	1.1100000	1.4900000

Unique Subject Identifier=2421010000-0079

Assay variable : PTINR_VAL1 PT-INR Value

N	Mean	Standard Deviation	Minimum	Maximum
1	1.4200000		1.4200000	1.4200000

Unique Subject Identifier=2421010000-0095

Assay variable : PTINR_VAL1 PT-INR Value

N	Mean	Standard Deviation	Minimum	Maximum
4	1.7950000	0.3303029	1.3900000	2.1200000

Unique Subject Identifier=2421010000-0097

Assay variable : PTINR_VAL1 PT-INR Value

N	Mean	Standard Deviation	Minimum	Maximum
6	2.2066667	0.5789185	1.5500000	2.9500000

Laboratory tests at the time of obtaining Table 2-6 consent, according to the presence or absence of proton-pump inhibitors

Unique Subject Identifier=2421010000-0119

MEANS Procedure

Assay variable : PTINR_VAL1 PT-INR Value

N	Mean	Standard Deviation	Minimum	Maximum
1	2.4900000		2.4900000	2.4900000

Unique Subject Identifier=2421010000-0144

Assay variable : PTINR_VAL1 PT-INR Value

N	Mean	Standard Deviation	Minimum	Maximum
2	2.1350000	0.1909188	2.0000000	2.2700000

Unique Subject Identifier=2421010000-0150

Assay variable : PTINR_VAL1 PT-INR Value

N	Mean	Standard Deviation	Minimum	Maximum
4	1.9025000	0.2670050	1.7000000	2.2700000

Unique Subject Identifier=2421010000-0157

Assay variable : PTINR_VAL1 PT-INR Value

N	Mean	Standard Deviation	Minimum	Maximum
4	2.3325000	1.0938426	1.2200000	3.8400000

Laboratory tests at the time of obtaining Table 2-6 consent, according to the presence or absence of proton-pump inhibitors

Unique Subject Identifier=2421010000-0232

MEANS Procedure

Assay variable : PTINR_VAL1 PT-INR Value

N	Mean	Standard Deviation	Minimum	Maximum
1	1.1400000		1.1400000	1.1400000

Unique Subject Identifier=2421010000-0238

Assay variable : PTINR_VAL1 PT-INR Value

N	Mean	Standard Deviation	Minimum	Maximum
6	2.1800000	0.1436663	1.9500000	2.3400000

Unique Subject Identifier=2423010010-0001

Assay variable : PTINR_VAL1 PT-INR Value

N	Mean	Standard Deviation	Minimum	Maximum
1	1.7100000		1.7100000	1.7100000

Unique Subject Identifier=2423010010-0006

Assay variable : PTINR_VAL1 PT-INR Value

N	Mean	Standard Deviation	Minimum	Maximum
1	3.8400000		3.8400000	3.8400000

Laboratory tests at the time of obtaining Table 2-6 consent, according to the presence or absence of proton-pump inhibitors

Unique Subject Identifier=2425010000-0003

MEANS Procedure

Assay variable : PTINR_VAL1 PT-INR Value

N	Mean	Standard Deviation	Minimum	Maximum
1	1.0600000		1.0600000	1.0600000

Unique Subject Identifier=2425010000-0008

Assay variable : PTINR_VAL1 PT-INR Value

N	Mean	Standard Deviation	Minimum	Maximum
1	1.1700000		1.1700000	1.1700000

Unique Subject Identifier=2425010000-0009

Assay variable : PTINR_VAL1 PT-INR Value

N	Mean	Standard Deviation	Minimum	Maximum
1	2.0800000		2.0800000	2.0800000

Unique Subject Identifier=2425010000-0017

Assay variable : PTINR_VAL1 PT-INR Value

N	Mean	Standard Deviation	Minimum	Maximum
1	1.7900000		1.7900000	1.7900000

Laboratory tests at the time of obtaining Table 2-6 consent, according to the presence or absence of proton-pump inhibitors

Unique Subject Identifier=2425010000-0029

MEANS Procedure

Assay variable : PTINR_VAL1 PT-INR Value

N	Mean	Standard Deviation	Minimum	Maximum
1	1.4200000		1.4200000	1.4200000

Unique Subject Identifier=2425010000-0035

Assay variable : PTINR_VAL1 PT-INR Value

N	Mean	Standard Deviation	Minimum	Maximum
1	1.4300000		1.4300000	1.4300000

Unique Subject Identifier=2425010000-0041

Assay variable : PTINR_VAL1 PT-INR Value

N	Mean	Standard Deviation	Minimum	Maximum
1	2.3700000		2.3700000	2.3700000

Unique Subject Identifier=2425010000-0050

Assay variable : PTINR_VAL1 PT-INR Value

N	Mean	Standard Deviation	Minimum	Maximum
1	1.8600000		1.8600000	1.8600000

Laboratory tests at the time of obtaining Table 2-6 consent, according to the presence or absence of proton-pump inhibitors

Unique Subject Identifier=2425010000-0051

MEANS Procedure

Assay variable : PTINR_VAL1 PT-INR Value

N	Mean	Standard Deviation	Minimum	Maximum
1	2.1200000		2.1200000	2.1200000

Unique Subject Identifier=2425010000-0061

Assay variable : PTINR_VAL1 PT-INR Value

N	Mean	Standard Deviation	Minimum	Maximum
3	2.4133333	0.1069268	2.3200000	2.5300000

Unique Subject Identifier=2426011100-0001

Assay variable : PTINR_VAL1 PT-INR Value

N	Mean	Standard Deviation	Minimum	Maximum
6	2.1000000	0.4049691	1.7000000	2.8000000

Unique Subject Identifier=2426011100-0003

Assay variable : PTINR_VAL1 PT-INR Value

N	Mean	Standard Deviation	Minimum	Maximum
6	1.6833333	0.3710346	1.1000000	2.1000000

Laboratory tests at the time of obtaining Table 2-6 consent, according to the presence or absence of proton-pump inhibitors

Unique Subject Identifier=2427000000-0006

MEANS Procedure

Assay variable : PTINR_VAL1 PT-INR Value

N	Mean	Standard Deviation	Minimum	Maximum
6	1.7833333	0.8420135	0.9900000	2.9600000

Unique Subject Identifier=2434011000-0007

Assay variable : PTINR_VAL1 PT-INR Value

N	Mean	Standard Deviation	Minimum	Maximum
3	2.8000000	0.7937254	2.2000000	3.7000000

Unique Subject Identifier=2434011000-0015

Assay variable : PTINR_VAL1 PT-INR Value

N	Mean	Standard Deviation	Minimum	Maximum
4	2.5000000	0.2581989	2.2000000	2.8000000

Unique Subject Identifier=2437000000-0006

Assay variable : PTINR_VAL1 PT-INR Value

N	Mean	Standard Deviation	Minimum	Maximum
3	1.6300000	0.1216553	1.4900000	1.7100000

Laboratory tests at the time of obtaining Table 2-6 consent, according to the presence or absence of proton-pump inhibitors

Unique Subject Identifier=2438010011-0006

MEANS Procedure

Assay variable : PTINR_VAL1 PT-INR Value

N	Mean	Standard Deviation	Minimum	Maximum
6	1.9350000	0.1947049	1.7500000	2.2800000

Unique Subject Identifier=2438010011-0017

Assay variable : PTINR_VAL1 PT-INR Value

N	Mean	Standard Deviation	Minimum	Maximum
4	1.8500000	0.2362202	1.5900000	2.0600000

Unique Subject Identifier=2438010011-0036

Assay variable : PTINR_VAL1 PT-INR Value

N	Mean	Standard Deviation	Minimum	Maximum
5	1.7040000	0.1722788	1.4600000	1.8700000

Unique Subject Identifier=2438010011-0048

Assay variable : PTINR_VAL1 PT-INR Value

N	Mean	Standard Deviation	Minimum	Maximum
5	2.4760000	0.6522500	2.0400000	3.6100000

Laboratory tests at the time of obtaining Table 2-6 consent, according to the presence or absence of proton-pump inhibitors

Unique Subject Identifier=2448011111-0001

MEANS Procedure

Assay variable : PTINR_VAL1 PT-INR Value

N	Mean	Standard Deviation	Minimum	Maximum
1	1.1800000		1.1800000	1.1800000

Unique Subject Identifier=2449011111-0042

Assay variable : PTINR_VAL1 PT-INR Value

N	Mean	Standard Deviation	Minimum	Maximum
6	1.7833333	0.1329160	1.7000000	2.0000000

Unique Subject Identifier=2449011111-0043

Assay variable : PTINR_VAL1 PT-INR Value

N	Mean	Standard Deviation	Minimum	Maximum
6	1.7666667	0.5354126	1.3000000	2.7000000

Unique Subject Identifier=2453011111-0001

Assay variable : PTINR_VAL1 PT-INR Value

N	Mean	Standard Deviation	Minimum	Maximum
5	2.0720000	0.1876699	1.8500000	2.3600000

Laboratory tests at the time of obtaining Table 2-6 consent, according to the presence or absence of proton-pump inhibitors

Unique Subject Identifier=2480011111-0013

MEANS Procedure

Assay variable : PTINR_VAL1 PT-INR Value

N	Mean	Standard Deviation	Minimum	Maximum
3	2.3933333	0.0450925	2.3500000	2.4400000

Unique Subject Identifier=2485000000-0002

Assay variable : PTINR_VAL1 PT-INR Value

N	Mean	Standard Deviation	Minimum	Maximum
4	1.7050000	0.1144552	1.5700000	1.8000000

Unique Subject Identifier=2487000000-0004

Assay variable : PTINR_VAL1 PT-INR Value

N	Mean	Standard Deviation	Minimum	Maximum
3	1.5000000	0.2645751	1.3000000	1.8000000

Unique Subject Identifier=2498010111-0010

Assay variable : PTINR_VAL1 PT-INR Value

N	Mean	Standard Deviation	Minimum	Maximum
5	1.8400000	0.1516575	1.6000000	2.0000000

Laboratory tests at the time of obtaining Table 2-6 consent, according to the presence or absence of proton-pump inhibitors

Unique Subject Identifier=2498010111-0013

MEANS Procedure

Assay variable : PTINR_VAL1 PT-INR Value

N	Mean	Standard Deviation	Minimum	Maximum
5	1.7600000	0.1140175	1.6000000	1.9000000

Unique Subject Identifier=2511011000-0001

Assay variable : PTINR_VAL1 PT-INR Value

N	Mean	Standard Deviation	Minimum	Maximum
6	2.1833333	0.7521081	1.5000000	3.4000000

Unique Subject Identifier=2511011000-0065

Assay variable : PTINR_VAL1 PT-INR Value

N	Mean	Standard Deviation	Minimum	Maximum
6	1.7333333	0.1632993	1.5000000	1.9000000

Unique Subject Identifier=2522011111-0002

Assay variable : PTINR_VAL1 PT-INR Value

N	Mean	Standard Deviation	Minimum	Maximum
2	2.2150000	1.3364318	1.2700000	3.1600000

Laboratory tests at the time of obtaining Table 2-6 consent, according to the presence or absence of proton-pump inhibitors

Unique Subject Identifier=2527000000-0009

MEANS Procedure

Assay variable : PTINR_VAL1 PT-INR Value

N	Mean	Standard Deviation	Minimum	Maximum
6	1.9833333	0.2483277	1.5000000	2.2000000

Unique Subject Identifier=2527000000-0022

Assay variable : PTINR_VAL1 PT-INR Value

N	Mean	Standard Deviation	Minimum	Maximum
5	1.8600000	0.6465292	1.4000000	3.0000000

Unique Subject Identifier=2527000000-0024

Assay variable : PTINR_VAL1 PT-INR Value

N	Mean	Standard Deviation	Minimum	Maximum
6	2.1166667	0.5154286	1.5000000	2.9000000

Unique Subject Identifier=2527000000-0037

Assay variable : PTINR_VAL1 PT-INR Value

N	Mean	Standard Deviation	Minimum	Maximum
6	2.0333333	0.2422120	1.8000000	2.4000000

Laboratory tests at the time of obtaining Table 2-6 consent, according to the presence or absence of proton-pump inhibitors

Unique Subject Identifier=2527000000-0042

MEANS Procedure

Assay variable : PTINR_VAL1 PT-INR Value

N	Mean	Standard Deviation	Minimum	Maximum
6	2.0666667	0.4273952	1.6000000	2.8000000

Unique Subject Identifier=2527000000-0048

Assay variable : PTINR_VAL1 PT-INR Value

N	Mean	Standard Deviation	Minimum	Maximum
6	1.6833333	0.2401388	1.3000000	1.9000000

Unique Subject Identifier=2527000000-0050

Assay variable : PTINR_VAL1 PT-INR Value

N	Mean	Standard Deviation	Minimum	Maximum
6	1.9500000	0.1224745	1.8000000	2.1000000

Unique Subject Identifier=2527000000-0060

Assay variable : PTINR_VAL1 PT-INR Value

N	Mean	Standard Deviation	Minimum	Maximum
5	1.5800000	0.1303840	1.4000000	1.7000000

Laboratory tests at the time of obtaining Table 2-6 consent, according to the presence or absence of proton-pump inhibitors

Unique Subject Identifier=2527000000-0074

MEANS Procedure

Assay variable : PTINR_VAL1 PT-INR Value

N	Mean	Standard Deviation	Minimum	Maximum
6	2.2500000	0.4929503	1.6000000	2.9000000

Unique Subject Identifier=2527000000-0076

Assay variable : PTINR_VAL1 PT-INR Value

N	Mean	Standard Deviation	Minimum	Maximum
5	1.5800000	0.3420526	1.2000000	2.1000000

Unique Subject Identifier=2527000000-0077

Assay variable : PTINR_VAL1 PT-INR Value

N	Mean	Standard Deviation	Minimum	Maximum
6	1.8333333	0.2065591	1.6000000	2.2000000

Unique Subject Identifier=2582010000-0002

Assay variable : PTINR_VAL1 PT-INR Value

N	Mean	Standard Deviation	Minimum	Maximum
4	2.4450000	0.3622614	2.1800000	2.9800000

Laboratory tests at the time of obtaining Table 2-6 consent, according to the presence or absence of proton-pump inhibitors

Unique Subject Identifier=2582010000-0007

MEANS Procedure

Assay variable : PTINR_VAL1 PT-INR Value

N	Mean	Standard Deviation	Minimum	Maximum
6	2.0100000	0.1256981	1.8000000	2.1500000

Unique Subject Identifier=2582010000-0015

Assay variable : PTINR_VAL1 PT-INR Value

N	Mean	Standard Deviation	Minimum	Maximum
6	1.7083333	0.2526196	1.3400000	2.0500000

Unique Subject Identifier=2582010000-0016

Assay variable : PTINR_VAL1 PT-INR Value

N	Mean	Standard Deviation	Minimum	Maximum
6	1.4516667	0.0798540	1.3800000	1.5800000

Unique Subject Identifier=2582010000-0018

Assay variable : PTINR_VAL1 PT-INR Value

N	Mean	Standard Deviation	Minimum	Maximum
6	1.5866667	0.0688961	1.4700000	1.6500000

Laboratory tests at the time of obtaining Table 2-6 consent, according to the presence or absence of proton-pump inhibitors

Unique Subject Identifier=2582010000-0019

MEANS Procedure

Assay variable : PTINR_VAL1 PT-INR Value

N	Mean	Standard Deviation	Minimum	Maximum
6	1.4000000	0.4267552	1.0100000	2.1000000

Unique Subject Identifier=2582010000-0025

Assay variable : PTINR_VAL1 PT-INR Value

N	Mean	Standard Deviation	Minimum	Maximum
6	1.7133333	0.1846799	1.6300000	2.0900000

Unique Subject Identifier=2600000000-0002

Assay variable : PTINR_VAL1 PT-INR Value

N	Mean	Standard Deviation	Minimum	Maximum
6	1.7333333	0.1505545	1.6000000	1.9000000

Unique Subject Identifier=2603011011-0001

Assay variable : PTINR_VAL1 PT-INR Value

N	Mean	Standard Deviation	Minimum	Maximum
6	2.1666667	0.3669696	1.5000000	2.6000000

Laboratory tests at the time of obtaining Table 2-6 consent, according to the presence or absence of proton-pump inhibitors

Unique Subject Identifier=2603011011-0002

MEANS Procedure

Assay variable : PTINR_VAL1 PT-INR Value

N	Mean	Standard Deviation	Minimum	Maximum
6	2.0333333	0.3141125	1.8000000	2.6000000

Unique Subject Identifier=2603011011-0012

Assay variable : PTINR_VAL1 PT-INR Value

N	Mean	Standard Deviation	Minimum	Maximum
6	2.1833333	0.4445972	1.7000000	2.9000000

Unique Subject Identifier=2603011011-0015

Assay variable : PTINR_VAL1 PT-INR Value

N	Mean	Standard Deviation	Minimum	Maximum
6	2.2000000	0.4289522	1.7000000	2.9000000

Unique Subject Identifier=2603011011-0025

Assay variable : PTINR_VAL1 PT-INR Value

N	Mean	Standard Deviation	Minimum	Maximum
6	2.2833333	0.1940790	2.0000000	2.5000000

Laboratory tests at the time of obtaining Table 2-6 consent, according to the presence or absence of proton-pump inhibitors

Unique Subject Identifier=2603011011-0032

MEANS Procedure

Assay variable : PTINR_VAL1 PT-INR Value

N	Mean	Standard Deviation	Minimum	Maximum
6	2.0666667	0.2065591	1.7000000	2.3000000

Unique Subject Identifier=2603011011-0041

Assay variable : PTINR_VAL1 PT-INR Value

N	Mean	Standard Deviation	Minimum	Maximum
6	2.2166667	0.3600926	1.6000000	2.7000000

Unique Subject Identifier=2605010000-0003

Assay variable : PTINR_VAL1 PT-INR Value

N	Mean	Standard Deviation	Minimum	Maximum
3	1.4800000	0.3666061	1.1600000	1.8800000

Unique Subject Identifier=2605010000-0014

Assay variable : PTINR_VAL1 PT-INR Value

N	Mean	Standard Deviation	Minimum	Maximum
5	2.0000000	0.3970516	1.4700000	2.5400000

Laboratory tests at the time of obtaining Table 2-6 consent, according to the presence or absence of proton-pump inhibitors

Unique Subject Identifier=2605010000-0015

MEANS Procedure

Assay variable : PTINR_VAL1 PT-INR Value

N	Mean	Standard Deviation	Minimum	Maximum
5	2.0100000	0.2220360	1.6900000	2.2400000

Unique Subject Identifier=2606011111-0091

Assay variable : PTINR_VAL1 PT-INR Value

N	Mean	Standard Deviation	Minimum	Maximum
4	1.7650000	0.1866369	1.5800000	2.0100000

Unique Subject Identifier=2606011111-0098

Assay variable : PTINR_VAL1 PT-INR Value

N	Mean	Standard Deviation	Minimum	Maximum
5	1.3240000	0.0832466	1.2600000	1.4200000

Unique Subject Identifier=2606011111-0104

Assay variable : PTINR_VAL1 PT-INR Value

N	Mean	Standard Deviation	Minimum	Maximum
3	1.8133333	0.4743768	1.5100000	2.3600000

Laboratory tests at the time of obtaining Table 2-6 consent, according to the presence or absence of proton-pump inhibitors

Unique Subject Identifier=2606011111-0124

MEANS Procedure

Assay variable : PTINR_VAL1 PT-INR Value

N	Mean	Standard Deviation	Minimum	Maximum
1	3.5100000		3.5100000	3.5100000

Unique Subject Identifier=2606011111-0130

Assay variable : PTINR_VAL1 PT-INR Value

N	Mean	Standard Deviation	Minimum	Maximum
6	1.8350000	0.1123833	1.6300000	1.9700000

Unique Subject Identifier=2607010010-0004

Assay variable : PTINR_VAL1 PT-INR Value

N	Mean	Standard Deviation	Minimum	Maximum
6	2.4316667	0.3943307	1.7500000	2.7600000

Unique Subject Identifier=2607010010-0007

Assay variable : PTINR_VAL1 PT-INR Value

N	Mean	Standard Deviation	Minimum	Maximum
4	2.1750000	0.1454877	1.9800000	2.3200000

Laboratory tests at the time of obtaining Table 2-6 consent, according to the presence or absence of proton-pump inhibitors

Unique Subject Identifier=2607010010-0023

MEANS Procedure

Assay variable : PTINR_VAL1 PT-INR Value

N	Mean	Standard Deviation	Minimum	Maximum
3	1.6366667	0.2059935	1.4200000	1.8300000

Unique Subject Identifier=2607010010-0058

Assay variable : PTINR_VAL1 PT-INR Value

N	Mean	Standard Deviation	Minimum	Maximum
6	2.1583333	0.5357394	1.5700000	3.1000000

Unique Subject Identifier=2607010010-0072

Assay variable : PTINR_VAL1 PT-INR Value

N	Mean	Standard Deviation	Minimum	Maximum
6	2.0650000	0.3690935	1.4600000	2.5800000

Unique Subject Identifier=2607010010-0076

Assay variable : PTINR_VAL1 PT-INR Value

N	Mean	Standard Deviation	Minimum	Maximum
6	1.9866667	0.4856199	1.4700000	2.8200000

Laboratory tests at the time of obtaining Table 2-6 consent, according to the presence or absence of proton-pump inhibitors

Unique Subject Identifier=2608010010-0027

MEANS Procedure

Assay variable : PTINR_VAL1 PT-INR Value

N	Mean	Standard Deviation	Minimum	Maximum
3	1.2100000	0.1044031	1.0900000	1.2800000

Unique Subject Identifier=2608010010-0035

Assay variable : PTINR_VAL1 PT-INR Value

N	Mean	Standard Deviation	Minimum	Maximum
5	2.1140000	0.3235429	1.7700000	2.6300000

Unique Subject Identifier=2608010010-0056

Assay variable : PTINR_VAL1 PT-INR Value

N	Mean	Standard Deviation	Minimum	Maximum
5	1.5960000	0.2307163	1.4200000	1.9000000

Unique Subject Identifier=2608010010-0061

Assay variable : PTINR_VAL1 PT-INR Value

N	Mean	Standard Deviation	Minimum	Maximum
5	1.7180000	0.1935717	1.4900000	2.0100000

Laboratory tests at the time of obtaining Table 2-6 consent, according to the presence or absence of proton-pump inhibitors

Unique Subject Identifier=2608010010-0067

MEANS Procedure

Assay variable : PTINR_VAL1 PT-INR Value

N	Mean	Standard Deviation	Minimum	Maximum
5	1.7880000	0.2141728	1.5100000	2.1100000

Unique Subject Identifier=2608010010-0077

Assay variable : PTINR_VAL1 PT-INR Value

N	Mean	Standard Deviation	Minimum	Maximum
1	1.4700000		1.4700000	1.4700000

Unique Subject Identifier=2608010010-0084

Assay variable : PTINR_VAL1 PT-INR Value

N	Mean	Standard Deviation	Minimum	Maximum
4	1.5075000	0.0788987	1.4200000	1.6000000

Unique Subject Identifier=2608010010-0097

Assay variable : PTINR_VAL1 PT-INR Value

N	Mean	Standard Deviation	Minimum	Maximum
2	2.5050000	0.6717514	2.0300000	2.9800000

Laboratory tests at the time of obtaining Table 2-6 consent, according to the presence or absence of proton-pump inhibitors

Unique Subject Identifier=2608010010-0102

MEANS Procedure

Assay variable : PTINR_VAL1 PT-INR Value

N	Mean	Standard Deviation	Minimum	Maximum
1	2.3300000		2.3300000	2.3300000

Unique Subject Identifier=2614000000-0001

Assay variable : PTINR_VAL1 PT-INR Value

N	Mean	Standard Deviation	Minimum	Maximum
3	1.9033333	0.7136059	1.4000000	2.7200000

Unique Subject Identifier=2614000000-0008

Assay variable : PTINR_VAL1 PT-INR Value

N	Mean	Standard Deviation	Minimum	Maximum
3	2.0466667	0.1386843	1.9300000	2.2000000

Unique Subject Identifier=2614000000-0010

Assay variable : PTINR_VAL1 PT-INR Value

N	Mean	Standard Deviation	Minimum	Maximum
3	1.7500000	0.1637071	1.6100000	1.9300000

Laboratory tests at the time of obtaining Table 2-6 consent, according to the presence or absence of proton-pump inhibitors

Unique Subject Identifier=2614000000-0047

MEANS Procedure

Assay variable : PTINR_VAL1 PT-INR Value

N	Mean	Standard Deviation	Minimum	Maximum
3	2.0400000	0.4243819	1.7900000	2.5300000

Unique Subject Identifier=2614000000-0061

Assay variable : PTINR_VAL1 PT-INR Value

N	Mean	Standard Deviation	Minimum	Maximum
3	2.5233333	0.0680686	2.4700000	2.6000000

Unique Subject Identifier=2614000000-0073

Assay variable : PTINR_VAL1 PT-INR Value

N	Mean	Standard Deviation	Minimum	Maximum
4	1.3475000	0.3710683	1.0700000	1.8800000

Unique Subject Identifier=2614000000-0081

Assay variable : PTINR_VAL1 PT-INR Value

N	Mean	Standard Deviation	Minimum	Maximum
2	1.5550000	0.0353553	1.5300000	1.5800000

Laboratory tests at the time of obtaining Table 2-6 consent, according to the presence or absence of proton-pump inhibitors

Unique Subject Identifier=2614000000-0083

MEANS Procedure

Assay variable : PTINR_VAL1 PT-INR Value

N	Mean	Standard Deviation	Minimum	Maximum
3	2.2300000	0.0953939	2.1200000	2.2900000

Unique Subject Identifier=2614000000-0085

Assay variable : PTINR_VAL1 PT-INR Value

N	Mean	Standard Deviation	Minimum	Maximum
3	1.7400000	0.0655744	1.6800000	1.8100000

Unique Subject Identifier=2614000000-0091

Assay variable : PTINR_VAL1 PT-INR Value

N	Mean	Standard Deviation	Minimum	Maximum
2	1.6500000	0.1555635	1.5400000	1.7600000

Unique Subject Identifier=2614000000-0095

Assay variable : PTINR_VAL1 PT-INR Value

N	Mean	Standard Deviation	Minimum	Maximum
3	2.6200000	0.8020598	1.9100000	3.4900000

Laboratory tests at the time of obtaining Table 2-6 consent, according to the presence or absence of proton-pump inhibitors

Unique Subject Identifier=2616000000-0005

MEANS Procedure

Assay variable : PTINR_VAL1 PT-INR Value

N	Mean	Standard Deviation	Minimum	Maximum
3	1.5200000	0.1216553	1.3800000	1.6000000

Unique Subject Identifier=2616000000-0006

Assay variable : PTINR_VAL1 PT-INR Value

N	Mean	Standard Deviation	Minimum	Maximum
4	2.7000000	0.4152911	2.2300000	3.2400000

Unique Subject Identifier=2616000000-0009

Assay variable : PTINR_VAL1 PT-INR Value

N	Mean	Standard Deviation	Minimum	Maximum
4	2.6675000	0.9233048	1.7600000	3.9400000

Unique Subject Identifier=2616000000-0011

Assay variable : PTINR_VAL1 PT-INR Value

N	Mean	Standard Deviation	Minimum	Maximum
5	1.9420000	0.4100854	1.5800000	2.6500000

Laboratory tests at the time of obtaining Table 2-6 consent, according to the presence or absence of proton-pump inhibitors

Unique Subject Identifier=2616000000-0013

MEANS Procedure

Assay variable : PTINR_VAL1 PT-INR Value

N	Mean	Standard Deviation	Minimum	Maximum
3	2.1300000	0.4744470	1.7800000	2.6700000

Unique Subject Identifier=2616000000-0017

Assay variable : PTINR_VAL1 PT-INR Value

N	Mean	Standard Deviation	Minimum	Maximum
4	1.6800000	0.1319091	1.5200000	1.8300000

Unique Subject Identifier=2616000000-0021

Assay variable : PTINR_VAL1 PT-INR Value

N	Mean	Standard Deviation	Minimum	Maximum
3	2.3666667	0.0802081	2.2900000	2.4500000

Unique Subject Identifier=2616000000-0022

Assay variable : PTINR_VAL1 PT-INR Value

N	Mean	Standard Deviation	Minimum	Maximum
4	1.8850000	0.2046949	1.5800000	2.0200000

Laboratory tests at the time of obtaining Table 2-6 consent, according to the presence or absence of proton-pump inhibitors

Unique Subject Identifier=2616000000-0045

MEANS Procedure

Assay variable : PTINR_VAL1 PT-INR Value

N	Mean	Standard Deviation	Minimum	Maximum
6	2.1783333	0.1834575	1.9200000	2.3900000

Unique Subject Identifier=2616000000-0046

Assay variable : PTINR_VAL1 PT-INR Value

N	Mean	Standard Deviation	Minimum	Maximum
3	1.9466667	0.1738774	1.7500000	2.0800000

Unique Subject Identifier=2616000000-0052

Assay variable : PTINR_VAL1 PT-INR Value

N	Mean	Standard Deviation	Minimum	Maximum
2	1.8850000	0.5444722	1.5000000	2.2700000

Unique Subject Identifier=2616000000-0053

Assay variable : PTINR_VAL1 PT-INR Value

N	Mean	Standard Deviation	Minimum	Maximum
4	1.4200000	0.1180395	1.3400000	1.5900000

Laboratory tests at the time of obtaining Table 2-6 consent, according to the presence or absence of proton-pump inhibitors

Unique Subject Identifier=2616000000-0055

MEANS Procedure

Assay variable : PTINR_VAL1 PT-INR Value

N	Mean	Standard Deviation	Minimum	Maximum
2	2.5750000	0.5020458	2.2200000	2.9300000

Unique Subject Identifier=2616000000-0056

Assay variable : PTINR_VAL1 PT-INR Value

N	Mean	Standard Deviation	Minimum	Maximum
2	2.1350000	0.3606245	1.8800000	2.3900000

Unique Subject Identifier=2616000000-0058

Assay variable : PTINR_VAL1 PT-INR Value

N	Mean	Standard Deviation	Minimum	Maximum
2	1.7150000	0.4171930	1.4200000	2.0100000

Unique Subject Identifier=2616000000-0059

Assay variable : PTINR_VAL1 PT-INR Value

N	Mean	Standard Deviation	Minimum	Maximum
3	2.0733333	0.0950438	1.9800000	2.1700000

Laboratory tests at the time of obtaining Table 2-6 consent, according to the presence or absence of proton-pump inhibitors

Unique Subject Identifier=2616000000-0061

MEANS Procedure

Assay variable : PTINR_VAL1 PT-INR Value

N	Mean	Standard Deviation	Minimum	Maximum
2	3.1000000	0.2545584	2.9200000	3.2800000

Unique Subject Identifier=2616000000-0069

Assay variable : PTINR_VAL1 PT-INR Value

N	Mean	Standard Deviation	Minimum	Maximum
2	2.2350000	0.0494975	2.2000000	2.2700000

Unique Subject Identifier=2616000000-0074

Assay variable : PTINR_VAL1 PT-INR Value

N	Mean	Standard Deviation	Minimum	Maximum
3	2.0833333	0.3987898	1.7200000	2.5100000

Unique Subject Identifier=2616000000-0078

Assay variable : PTINR_VAL1 PT-INR Value

N	Mean	Standard Deviation	Minimum	Maximum
2	1.3950000	0.3040559	1.1800000	1.6100000

Laboratory tests at the time of obtaining Table 2-6 consent, according to the presence or absence of proton-pump inhibitors

Unique Subject Identifier=2617000000-0001

MEANS Procedure

Assay variable : PTINR_VAL1 PT-INR Value

N	Mean	Standard Deviation	Minimum	Maximum
5	2.3840000	0.1942421	2.0900000	2.5800000

Unique Subject Identifier=2617000000-0004

Assay variable : PTINR_VAL1 PT-INR Value

N	Mean	Standard Deviation	Minimum	Maximum
3	2.0966667	0.4186088	1.8500000	2.5800000

Unique Subject Identifier=2617000000-0013

Assay variable : PTINR_VAL1 PT-INR Value

N	Mean	Standard Deviation	Minimum	Maximum
2	2.5400000	1.3010765	1.6200000	3.4600000

Unique Subject Identifier=2617000000-0017

Assay variable : PTINR_VAL1 PT-INR Value

N	Mean	Standard Deviation	Minimum	Maximum
6	2.3483333	0.1633911	2.1900000	2.5400000

Laboratory tests at the time of obtaining Table 2-6 consent, according to the presence or absence of proton-pump inhibitors

Unique Subject Identifier=2617000000-0020

MEANS Procedure

Assay variable : PTINR_VAL1 PT-INR Value

N	Mean	Standard Deviation	Minimum	Maximum
4	1.8500000	0.4354308	1.2500000	2.2700000

Unique Subject Identifier=2617000000-0031

Assay variable : PTINR_VAL1 PT-INR Value

N	Mean	Standard Deviation	Minimum	Maximum
4	2.1925000	0.5868773	1.3400000	2.5900000

Unique Subject Identifier=2617000000-0041

Assay variable : PTINR_VAL1 PT-INR Value

N	Mean	Standard Deviation	Minimum	Maximum
4	1.7750000	0.1234234	1.6500000	1.9000000

Unique Subject Identifier=2617000000-0044

Assay variable : PTINR_VAL1 PT-INR Value

N	Mean	Standard Deviation	Minimum	Maximum
2	1.7150000	0.0212132	1.7000000	1.7300000

Laboratory tests at the time of obtaining Table 2-6 consent, according to the presence or absence of proton-pump inhibitors

Unique Subject Identifier=2617000000-0047

MEANS Procedure

Assay variable : PTINR_VAL1 PT-INR Value

N	Mean	Standard Deviation	Minimum	Maximum
4	2.1525000	0.1925920	1.9500000	2.3500000

Unique Subject Identifier=2617000000-0056

Assay variable : PTINR_VAL1 PT-INR Value

N	Mean	Standard Deviation	Minimum	Maximum
6	1.4783333	0.2339587	1.2400000	1.7100000

Unique Subject Identifier=2617000000-0058

Assay variable : PTINR_VAL1 PT-INR Value

N	Mean	Standard Deviation	Minimum	Maximum
6	1.6966667	0.3600926	1.3300000	2.2100000

Unique Subject Identifier=2617000000-0061

Assay variable : PTINR_VAL1 PT-INR Value

N	Mean	Standard Deviation	Minimum	Maximum
3	1.7833333	0.3910669	1.3500000	2.1100000

Laboratory tests at the time of obtaining Table 2-6 consent, according to the presence or absence of proton-pump inhibitors

Unique Subject Identifier=2617000000-0068

MEANS Procedure

Assay variable : PTINR_VAL1 PT-INR Value

N	Mean	Standard Deviation	Minimum	Maximum
3	1.7166667	0.3875994	1.2800000	2.0200000

Unique Subject Identifier=2617000000-0076

Assay variable : PTINR_VAL1 PT-INR Value

N	Mean	Standard Deviation	Minimum	Maximum
4	2.2675000	0.4007805	1.8800000	2.7800000

Unique Subject Identifier=2617000000-0082

Assay variable : PTINR_VAL1 PT-INR Value

N	Mean	Standard Deviation	Minimum	Maximum
4	2.2950000	0.2372762	2.0500000	2.6200000

Unique Subject Identifier=2617000000-0083

Assay variable : PTINR_VAL1 PT-INR Value

N	Mean	Standard Deviation	Minimum	Maximum
1	1.9100000		1.9100000	1.9100000

Laboratory tests at the time of obtaining Table 2-6 consent, according to the presence or absence of proton-pump inhibitors

Unique Subject Identifier=2617000000-0087

MEANS Procedure

Assay variable : PTINR_VAL1 PT-INR Value

N	Mean	Standard Deviation	Minimum	Maximum
3	1.9233333	0.3312602	1.6100000	2.2700000

Unique Subject Identifier=2617000000-0109

Assay variable : PTINR_VAL1 PT-INR Value

N	Mean	Standard Deviation	Minimum	Maximum
4	2.3750000	0.5471441	1.9300000	3.1500000

Unique Subject Identifier=2617000000-0115

Assay variable : PTINR_VAL1 PT-INR Value

N	Mean	Standard Deviation	Minimum	Maximum
3	1.7333333	0.0737111	1.6500000	1.7900000

Unique Subject Identifier=2617000000-0119

Assay variable : PTINR_VAL1 PT-INR Value

N	Mean	Standard Deviation	Minimum	Maximum
3	1.9000000	0.0519615	1.8400000	1.9300000

Laboratory tests at the time of obtaining Table 2-6 consent, according to the presence or absence of proton-pump inhibitors

Unique Subject Identifier=2617000000-0128

MEANS Procedure

Assay variable : PTINR_VAL1 PT-INR Value

N	Mean	Standard Deviation	Minimum	Maximum
2	1.7500000	0.2262742	1.5900000	1.9100000

Unique Subject Identifier=2617000000-0136

Assay variable : PTINR_VAL1 PT-INR Value

N	Mean	Standard Deviation	Minimum	Maximum
1	1.4600000		1.4600000	1.4600000

Unique Subject Identifier=2617000000-0140

Assay variable : PTINR_VAL1 PT-INR Value

N	Mean	Standard Deviation	Minimum	Maximum
6	1.2533333	0.0691857	1.1500000	1.3400000

Unique Subject Identifier=2617000000-0148

Assay variable : PTINR_VAL1 PT-INR Value

N	Mean	Standard Deviation	Minimum	Maximum
3	2.0666667	0.2600641	1.8100000	2.3300000

Laboratory tests at the time of obtaining Table 2-6 consent, according to the presence or absence of proton-pump inhibitors

Unique Subject Identifier=2617000000-0149

MEANS Procedure

Assay variable : PTINR_VAL1 PT-INR Value

N	Mean	Standard Deviation	Minimum	Maximum
3	1.6800000	0.1705872	1.5400000	1.8700000

Unique Subject Identifier=2617000000-0152

Assay variable : PTINR_VAL1 PT-INR Value

N	Mean	Standard Deviation	Minimum	Maximum
4	2.2000000	0.2255364	1.9400000	2.4900000

Unique Subject Identifier=2617000000-0153

Assay variable : PTINR_VAL1 PT-INR Value

N	Mean	Standard Deviation	Minimum	Maximum
2	2.0400000	0.1414214	1.9400000	2.1400000

Unique Subject Identifier=2617000000-0162

Assay variable : PTINR_VAL1 PT-INR Value

N	Mean	Standard Deviation	Minimum	Maximum
6	2.2416667	0.2527779	1.9100000	2.6000000

Laboratory tests at the time of obtaining Table 2-6 consent, according to the presence or absence of proton-pump inhibitors

Unique Subject Identifier=2617000000-0169

MEANS Procedure

Assay variable : PTINR_VAL1 PT-INR Value

N	Mean	Standard Deviation	Minimum	Maximum
4	2.1750000	0.2441994	1.9600000	2.4200000

Unique Subject Identifier=2617000000-0177

Assay variable : PTINR_VAL1 PT-INR Value

N	Mean	Standard Deviation	Minimum	Maximum
3	2.2433333	0.0321455	2.2200000	2.2800000

Unique Subject Identifier=2617000000-0178

Assay variable : PTINR_VAL1 PT-INR Value

N	Mean	Standard Deviation	Minimum	Maximum
5	1.8060000	0.5015775	1.0800000	2.4800000

Unique Subject Identifier=2617000000-0179

Assay variable : PTINR_VAL1 PT-INR Value

N	Mean	Standard Deviation	Minimum	Maximum
3	2.2233333	0.5672154	1.8100000	2.8700000

Laboratory tests at the time of obtaining Table 2-6 consent, according to the presence or absence of proton-pump inhibitors

Unique Subject Identifier=2617000000-0188

MEANS Procedure

Assay variable : PTINR_VAL1 PT-INR Value

N	Mean	Standard Deviation	Minimum	Maximum
3	1.9066667	0.3707200	1.6400000	2.3300000

Unique Subject Identifier=2617000000-0190

Assay variable : PTINR_VAL1 PT-INR Value

N	Mean	Standard Deviation	Minimum	Maximum
4	1.8875000	0.3312980	1.5300000	2.2300000

Unique Subject Identifier=2617000000-0200

Assay variable : PTINR_VAL1 PT-INR Value

N	Mean	Standard Deviation	Minimum	Maximum
3	1.9633333	0.6379133	1.4300000	2.6700000

Unique Subject Identifier=2617000000-0204

Assay variable : PTINR_VAL1 PT-INR Value

N	Mean	Standard Deviation	Minimum	Maximum
5	1.2140000	0.0901665	1.0600000	1.2800000

Laboratory tests at the time of obtaining Table 2-6 consent, according to the presence or absence of proton-pump inhibitors

Unique Subject Identifier=2617000000-0210

MEANS Procedure

Assay variable : PTINR_VAL1 PT-INR Value

N	Mean	Standard Deviation	Minimum	Maximum
3	1.7466667	0.1250333	1.6200000	1.8700000

Unique Subject Identifier=2617000000-0232

Assay variable : PTINR_VAL1 PT-INR Value

N	Mean	Standard Deviation	Minimum	Maximum
3	2.0600000	0.2981610	1.7300000	2.3100000

Unique Subject Identifier=2617000000-0233

Assay variable : PTINR_VAL1 PT-INR Value

N	Mean	Standard Deviation	Minimum	Maximum
3	1.7766667	0.0850490	1.6900000	1.8600000

Unique Subject Identifier=2617000000-0239

Assay variable : PTINR_VAL1 PT-INR Value

N	Mean	Standard Deviation	Minimum	Maximum
3	2.1333333	0.1106044	2.0300000	2.2500000

Laboratory tests at the time of obtaining Table 2-6 consent, according to the presence or absence of proton-pump inhibitors

Unique Subject Identifier=2617000000-0251

MEANS Procedure

Assay variable : PTINR_VAL1 PT-INR Value

N	Mean	Standard Deviation	Minimum	Maximum
4	1.7475000	0.1723127	1.5000000	1.8700000

Unique Subject Identifier=2617000000-0256

Assay variable : PTINR_VAL1 PT-INR Value

N	Mean	Standard Deviation	Minimum	Maximum
3	1.5733333	0.1497776	1.4500000	1.7400000

Unique Subject Identifier=2617000000-0261

Assay variable : PTINR_VAL1 PT-INR Value

N	Mean	Standard Deviation	Minimum	Maximum
3	2.6266667	0.2400694	2.4200000	2.8900000

Unique Subject Identifier=2617000000-0270

Assay variable : PTINR_VAL1 PT-INR Value

N	Mean	Standard Deviation	Minimum	Maximum
5	1.8940000	0.5493906	1.0500000	2.5500000

Laboratory tests at the time of obtaining Table 2-6 consent, according to the presence or absence of proton-pump inhibitors

Unique Subject Identifier=2617000000-0272

MEANS Procedure

Assay variable : PTINR_VAL1 PT-INR Value

N	Mean	Standard Deviation	Minimum	Maximum
2	1.9200000	0.0282843	1.9000000	1.9400000

Unique Subject Identifier=2617000000-0278

Assay variable : PTINR_VAL1 PT-INR Value

N	Mean	Standard Deviation	Minimum	Maximum
2	1.2200000	0.2828427	1.0200000	1.4200000

Unique Subject Identifier=2617000000-0279

Assay variable : PTINR_VAL1 PT-INR Value

N	Mean	Standard Deviation	Minimum	Maximum
4	2.0075000	0.2286737	1.6800000	2.2100000

Unique Subject Identifier=2617000000-0282

Assay variable : PTINR_VAL1 PT-INR Value

N	Mean	Standard Deviation	Minimum	Maximum
4	2.2625000	0.8172464	1.5000000	3.3800000

Laboratory tests at the time of obtaining Table 2-6 consent, according to the presence or absence of proton-pump inhibitors

Unique Subject Identifier=2617000000-0290

MEANS Procedure

Assay variable : PTINR_VAL1 PT-INR Value

N	Mean	Standard Deviation	Minimum	Maximum
3	1.7466667	0.0896289	1.6900000	1.8500000

Unique Subject Identifier=2617000000-0295

Assay variable : PTINR_VAL1 PT-INR Value

N	Mean	Standard Deviation	Minimum	Maximum
2	1.9150000	0.2899138	1.7100000	2.1200000

Unique Subject Identifier=2617000000-0296

Assay variable : PTINR_VAL1 PT-INR Value

N	Mean	Standard Deviation	Minimum	Maximum
4	1.9500000	0.1373560	1.7500000	2.0400000

Unique Subject Identifier=2617000000-0301

Assay variable : PTINR_VAL1 PT-INR Value

N	Mean	Standard Deviation	Minimum	Maximum
1	1.5500000		1.5500000	1.5500000

Laboratory tests at the time of obtaining Table 2-6 consent, according to the presence or absence of proton-pump inhibitors

Unique Subject Identifier=2617000000-0305

MEANS Procedure

Assay variable : PTINR_VAL1 PT-INR Value

N	Mean	Standard Deviation	Minimum	Maximum
3	2.5733333	0.5810622	1.9300000	3.0600000

Unique Subject Identifier=2617000000-0312

Assay variable : PTINR_VAL1 PT-INR Value

N	Mean	Standard Deviation	Minimum	Maximum
3	1.5666667	0.5598512	1.1900000	2.2100000

Unique Subject Identifier=2617000000-0315

Assay variable : PTINR_VAL1 PT-INR Value

N	Mean	Standard Deviation	Minimum	Maximum
2	2.0850000	0.3606245	1.8300000	2.3400000

Unique Subject Identifier=2617000000-0325

Assay variable : PTINR_VAL1 PT-INR Value

N	Mean	Standard Deviation	Minimum	Maximum
3	2.2000000	0.1014889	2.0900000	2.2900000

Laboratory tests at the time of obtaining Table 2-6 consent, according to the presence or absence of proton-pump inhibitors

Unique Subject Identifier=2617000000-0328

MEANS Procedure

Assay variable : PTINR_VAL1 PT-INR Value

N	Mean	Standard Deviation	Minimum	Maximum
6	1.8083333	0.5617977	1.2100000	2.5100000

Unique Subject Identifier=2617000000-0344

Assay variable : PTINR_VAL1 PT-INR Value

N	Mean	Standard Deviation	Minimum	Maximum
1	0.9800000		0.9800000	0.9800000

Unique Subject Identifier=2617000000-0356

Assay variable : PTINR_VAL1 PT-INR Value

N	Mean	Standard Deviation	Minimum	Maximum
4	1.7250000	0.1470827	1.5500000	1.9100000

Unique Subject Identifier=2617000000-0359

Assay variable : PTINR_VAL1 PT-INR Value

N	Mean	Standard Deviation	Minimum	Maximum
4	1.6275000	0.1982213	1.4800000	1.9100000

Laboratory tests at the time of obtaining Table 2-6 consent, according to the presence or absence of proton-pump inhibitors

Unique Subject Identifier=2617000000-0366

MEANS Procedure

Assay variable : PTINR_VAL1 PT-INR Value

N	Mean	Standard Deviation	Minimum	Maximum
3	1.8433333	0.1850225	1.6300000	1.9600000

Unique Subject Identifier=2621011111-0002

Assay variable : PTINR_VAL1 PT-INR Value

N	Mean	Standard Deviation	Minimum	Maximum
1	1.5600000		1.5600000	1.5600000

Unique Subject Identifier=2621011111-0004

Assay variable : PTINR_VAL1 PT-INR Value

N	Mean	Standard Deviation	Minimum	Maximum
5	1.7460000	0.1094532	1.6100000	1.8800000

Unique Subject Identifier=2623000000-0009

Assay variable : PTINR_VAL1 PT-INR Value

N	Mean	Standard Deviation	Minimum	Maximum
6	1.8883333	0.7314757	1.0700000	2.9900000

Laboratory tests at the time of obtaining Table 2-6 consent, according to the presence or absence of proton-pump inhibitors

Unique Subject Identifier=2623000000-0016

MEANS Procedure

Assay variable : PTINR_VAL1 PT-INR Value

N	Mean	Standard Deviation	Minimum	Maximum
3	2.0500000	0.1637071	1.8700000	2.1900000

Unique Subject Identifier=2623000000-0018

Assay variable : PTINR_VAL1 PT-INR Value

N	Mean	Standard Deviation	Minimum	Maximum
6	1.6016667	0.1927088	1.3200000	1.8800000

Unique Subject Identifier=2623000000-0034

Assay variable : PTINR_VAL1 PT-INR Value

N	Mean	Standard Deviation	Minimum	Maximum
3	2.5966667	0.2315887	2.3400000	2.7900000

Unique Subject Identifier=2623000000-0035

Assay variable : PTINR_VAL1 PT-INR Value

N	Mean	Standard Deviation	Minimum	Maximum
5	2.4620000	0.2728919	2.2700000	2.9400000

Laboratory tests at the time of obtaining Table 2-6 consent, according to the presence or absence of proton-pump inhibitors

Unique Subject Identifier=2623000000-0051

MEANS Procedure

Assay variable : PTINR_VAL1 PT-INR Value

N	Mean	Standard Deviation	Minimum	Maximum
1	2.5000000		2.5000000	2.5000000

Unique Subject Identifier=2623000000-0059

Assay variable : PTINR_VAL1 PT-INR Value

N	Mean	Standard Deviation	Minimum	Maximum
4	2.1325000	0.2364142	1.7900000	2.3200000

Unique Subject Identifier=2623000000-0063

Assay variable : PTINR_VAL1 PT-INR Value

N	Mean	Standard Deviation	Minimum	Maximum
3	2.0000000	0.0346410	1.9800000	2.0400000

Unique Subject Identifier=2623000000-0069

Assay variable : PTINR_VAL1 PT-INR Value

N	Mean	Standard Deviation	Minimum	Maximum
4	1.7950000	0.1027943	1.6600000	1.9100000

Laboratory tests at the time of obtaining Table 2-6 consent, according to the presence or absence of proton-pump inhibitors

Unique Subject Identifier=2623000000-0075

MEANS Procedure

Assay variable : PTINR_VAL1 PT-INR Value

N	Mean	Standard Deviation	Minimum	Maximum
2	2.1300000	0.3535534	1.8800000	2.3800000

Unique Subject Identifier=2623000000-0086

Assay variable : PTINR_VAL1 PT-INR Value

N	Mean	Standard Deviation	Minimum	Maximum
2	1.5900000	0.0424264	1.5600000	1.6200000

Unique Subject Identifier=2626011111-0005

Assay variable : PTINR_VAL1 PT-INR Value

N	Mean	Standard Deviation	Minimum	Maximum
3	1.7066667	0.4697162	1.2800000	2.2100000

Unique Subject Identifier=2626011111-0006

Assay variable : PTINR_VAL1 PT-INR Value

N	Mean	Standard Deviation	Minimum	Maximum
3	1.6000000	0.1389244	1.4400000	1.6900000

Laboratory tests at the time of obtaining Table 2-6 consent, according to the presence or absence of proton-pump inhibitors

Unique Subject Identifier=2626011111-0012

MEANS Procedure

Assay variable : PTINR_VAL1 PT-INR Value

N	Mean	Standard Deviation	Minimum	Maximum
4	1.8325000	0.4608959	1.2600000	2.3700000

Unique Subject Identifier=2626011111-0021

Assay variable : PTINR_VAL1 PT-INR Value

N	Mean	Standard Deviation	Minimum	Maximum
5	1.8620000	0.4428544	1.2700000	2.4900000

Unique Subject Identifier=2626011111-0035

Assay variable : PTINR_VAL1 PT-INR Value

N	Mean	Standard Deviation	Minimum	Maximum
3	1.6600000	0.0600000	1.6000000	1.7200000

Unique Subject Identifier=2626011111-0043

Assay variable : PTINR_VAL1 PT-INR Value

N	Mean	Standard Deviation	Minimum	Maximum
4	1.7700000	0.2409703	1.5100000	1.9800000

Laboratory tests at the time of obtaining Table 2-6 consent, according to the presence or absence of proton-pump inhibitors

Unique Subject Identifier=2627011111-0002

MEANS Procedure

Assay variable : PTINR_VAL1 PT-INR Value

N	Mean	Standard Deviation	Minimum	Maximum
6	2.1716667	0.1154845	1.9700000	2.2900000

Unique Subject Identifier=2627011111-0033

Assay variable : PTINR_VAL1 PT-INR Value

N	Mean	Standard Deviation	Minimum	Maximum
4	1.9225000	0.1543535	1.7200000	2.0900000

Unique Subject Identifier=2628011111-0004

Assay variable : PTINR_VAL1 PT-INR Value

N	Mean	Standard Deviation	Minimum	Maximum
4	2.1850000	0.3445287	1.7000000	2.4500000

Unique Subject Identifier=2628011111-0006

Assay variable : PTINR_VAL1 PT-INR Value

N	Mean	Standard Deviation	Minimum	Maximum
4	2.2875000	0.5296776	1.8500000	2.9500000

Laboratory tests at the time of obtaining Table 2-6 consent, according to the presence or absence of proton-pump inhibitors

Unique Subject Identifier=2628011111-0007

MEANS Procedure

Assay variable : PTINR_VAL1 PT-INR Value

N	Mean	Standard Deviation	Minimum	Maximum
3	1.9333333	0.2064784	1.7900000	2.1700000

Unique Subject Identifier=2628011111-0011

Assay variable : PTINR_VAL1 PT-INR Value

N	Mean	Standard Deviation	Minimum	Maximum
3	1.8900000	0.2389561	1.6300000	2.1000000

Unique Subject Identifier=2628011111-0025

Assay variable : PTINR_VAL1 PT-INR Value

N	Mean	Standard Deviation	Minimum	Maximum
5	1.8240000	0.1524139	1.6400000	2.0500000

Unique Subject Identifier=2628011111-0042

Assay variable : PTINR_VAL1 PT-INR Value

N	Mean	Standard Deviation	Minimum	Maximum
4	1.4575000	0.2721978	1.2100000	1.8000000

Laboratory tests at the time of obtaining Table 2-6 consent, according to the presence or absence of proton-pump inhibitors

Unique Subject Identifier=2628011111-0043

MEANS Procedure

Assay variable : PTINR_VAL1 PT-INR Value

N	Mean	Standard Deviation	Minimum	Maximum
5	1.8300000	0.3910882	1.4100000	2.2400000

Unique Subject Identifier=2628011111-0061

Assay variable : PTINR_VAL1 PT-INR Value

N	Mean	Standard Deviation	Minimum	Maximum
6	1.4683333	0.0453505	1.4200000	1.5300000

Unique Subject Identifier=2628011111-0066

Assay variable : PTINR_VAL1 PT-INR Value

N	Mean	Standard Deviation	Minimum	Maximum
4	2.3500000	0.2585859	2.0600000	2.6500000

Unique Subject Identifier=2634000000-0002

Assay variable : PTINR_VAL1 PT-INR Value

N	Mean	Standard Deviation	Minimum	Maximum
3	2.2466667	0.2888483	2.0700000	2.5800000

Laboratory tests at the time of obtaining Table 2-6 consent, according to the presence or absence of proton-pump inhibitors

Unique Subject Identifier=2636011000-0004

MEANS Procedure

Assay variable : PTINR_VAL1 PT-INR Value

N	Mean	Standard Deviation	Minimum	Maximum
5	2.0400000	0.2709243	1.7300000	2.3400000

Unique Subject Identifier=2636011000-0007

Assay variable : PTINR_VAL1 PT-INR Value

N	Mean	Standard Deviation	Minimum	Maximum
3	1.8400000	0.1249000	1.7000000	1.9400000

Unique Subject Identifier=2636011000-0010

Assay variable : PTINR_VAL1 PT-INR Value

N	Mean	Standard Deviation	Minimum	Maximum
4	2.0000000	0.0571548	1.9300000	2.0700000

Unique Subject Identifier=2636011000-0019

Assay variable : PTINR_VAL1 PT-INR Value

N	Mean	Standard Deviation	Minimum	Maximum
1	2.2100000		2.2100000	2.2100000

Laboratory tests at the time of obtaining Table 2-6 consent, according to the presence or absence of proton-pump inhibitors

Unique Subject Identifier=2636011000-0032

MEANS Procedure

Assay variable : PTINR_VAL1 PT-INR Value

N	Mean	Standard Deviation	Minimum	Maximum
6	2.1766667	0.5461380	1.5700000	2.8800000

Unique Subject Identifier=2636011000-0043

Assay variable : PTINR_VAL1 PT-INR Value

N	Mean	Standard Deviation	Minimum	Maximum
3	2.1000000	0.8351647	1.3500000	3.0000000

Unique Subject Identifier=2638000000-0010

Assay variable : PTINR_VAL1 PT-INR Value

N	Mean	Standard Deviation	Minimum	Maximum
4	1.2550000	0.0412311	1.2100000	1.2900000

Unique Subject Identifier=2638000000-0020

Assay variable : PTINR_VAL1 PT-INR Value

N	Mean	Standard Deviation	Minimum	Maximum
3	2.1800000	0.0655744	2.1200000	2.2500000

Laboratory tests at the time of obtaining Table 2-6 consent, according to the presence or absence of proton-pump inhibitors

Unique Subject Identifier=2640011000-0004

MEANS Procedure

Assay variable : PTINR_VAL1 PT-INR Value

N	Mean	Standard Deviation	Minimum	Maximum
6	2.4166667	0.4273952	1.9800000	3.0700000

Unique Subject Identifier=2640011000-0006

Assay variable : PTINR_VAL1 PT-INR Value

N	Mean	Standard Deviation	Minimum	Maximum
6	1.9783333	0.6804533	1.2000000	3.0600000

Unique Subject Identifier=2641010000-0009

Assay variable : PTINR_VAL1 PT-INR Value

N	Mean	Standard Deviation	Minimum	Maximum
3	2.3300000	0.3726929	1.9000000	2.5600000

Unique Subject Identifier=2641010000-0011

Assay variable : PTINR_VAL1 PT-INR Value

N	Mean	Standard Deviation	Minimum	Maximum
5	2.3120000	0.2875239	1.9200000	2.6200000

Laboratory tests at the time of obtaining Table 2-6 consent, according to the presence or absence of proton-pump inhibitors

Unique Subject Identifier=2656011000-0005

MEANS Procedure

Assay variable : PTINR_VAL1 PT-INR Value

N	Mean	Standard Deviation	Minimum	Maximum
3	2.1766667	0.1078579	2.1000000	2.3000000

Unique Subject Identifier=2656011000-0013

Assay variable : PTINR_VAL1 PT-INR Value

N	Mean	Standard Deviation	Minimum	Maximum
2	1.6850000	0.4171930	1.3900000	1.9800000

Unique Subject Identifier=2656011000-0016

Assay variable : PTINR_VAL1 PT-INR Value

N	Mean	Standard Deviation	Minimum	Maximum
2	1.6550000	0.0919239	1.5900000	1.7200000

Unique Subject Identifier=2662011111-0004

Assay variable : PTINR_VAL1 PT-INR Value

N	Mean	Standard Deviation	Minimum	Maximum
6	1.9166667	0.3656045	1.5000000	2.4000000

Laboratory tests at the time of obtaining Table 2-6 consent, according to the presence or absence of proton-pump inhibitors

Unique Subject Identifier=2662011111-0005

MEANS Procedure

Assay variable : PTINR_VAL1 PT-INR Value

N	Mean	Standard Deviation	Minimum	Maximum
6	1.9000000	0.2966479	1.4000000	2.2000000

Unique Subject Identifier=2662011111-0036

Assay variable : PTINR_VAL1 PT-INR Value

N	Mean	Standard Deviation	Minimum	Maximum
3	1.5666667	0.3511885	1.2000000	1.9000000

Unique Subject Identifier=2662011111-0039

Assay variable : PTINR_VAL1 PT-INR Value

N	Mean	Standard Deviation	Minimum	Maximum
6	1.9666667	0.2250926	1.7000000	2.2000000

Unique Subject Identifier=2662011111-0046

Assay variable : PTINR_VAL1 PT-INR Value

N	Mean	Standard Deviation	Minimum	Maximum
4	1.8250000	0.1258306	1.7000000	2.0000000

Laboratory tests at the time of obtaining Table 2-6 consent, according to the presence or absence of proton-pump inhibitors

Unique Subject Identifier=2662011111-0047

MEANS Procedure

Assay variable : PTINR_VAL1 PT-INR Value

N	Mean	Standard Deviation	Minimum	Maximum
3	1.6433333	0.2040425	1.4200000	1.8200000

Unique Subject Identifier=2662011111-0049

Assay variable : PTINR_VAL1 PT-INR Value

N	Mean	Standard Deviation	Minimum	Maximum
4	1.8750000	0.5188127	1.4000000	2.5000000

Unique Subject Identifier=2662011111-0063

Assay variable : PTINR_VAL1 PT-INR Value

N	Mean	Standard Deviation	Minimum	Maximum
4	1.4750000	0.4573474	1.0000000	2.1000000

Unique Subject Identifier=2668011000-0030

Assay variable : PTINR_VAL1 PT-INR Value

N	Mean	Standard Deviation	Minimum	Maximum
4	1.9975000	0.1977161	1.7700000	2.2000000

Laboratory tests at the time of obtaining Table 2-6 consent, according to the presence or absence of proton-pump inhibitors

Unique Subject Identifier=2669000000-0001

MEANS Procedure

Assay variable : PTINR_VAL1 PT-INR Value

N	Mean	Standard Deviation	Minimum	Maximum
6	1.8700000	0.4251588	1.3500000	2.5500000

Unique Subject Identifier=2669000000-0015

Assay variable : PTINR_VAL1 PT-INR Value

N	Mean	Standard Deviation	Minimum	Maximum
3	2.2933333	0.1850225	2.1100000	2.4800000

Unique Subject Identifier=2671000000-0023

Assay variable : PTINR_VAL1 PT-INR Value

N	Mean	Standard Deviation	Minimum	Maximum
3	2.4033333	0.1379613	2.3000000	2.5600000

Unique Subject Identifier=2671000000-0055

Assay variable : PTINR_VAL1 PT-INR Value

N	Mean	Standard Deviation	Minimum	Maximum
5	1.9900000	0.2740438	1.7500000	2.4000000

Laboratory tests at the time of obtaining Table 2-6 consent, according to the presence or absence of proton-pump inhibitors

Unique Subject Identifier=2671000000-0058

MEANS Procedure

Assay variable : PTINR_VAL1 PT-INR Value

N	Mean	Standard Deviation	Minimum	Maximum
6	2.4900000	0.9104504	1.5700000	3.6100000

Unique Subject Identifier=2671000000-0064

Assay variable : PTINR_VAL1 PT-INR Value

N	Mean	Standard Deviation	Minimum	Maximum
5	1.8620000	0.5265169	1.2400000	2.6400000

Unique Subject Identifier=2672000000-0020

Assay variable : PTINR_VAL1 PT-INR Value

N	Mean	Standard Deviation	Minimum	Maximum
3	2.0666667	0.2804164	1.7600000	2.3100000

Unique Subject Identifier=2672000000-0027

Assay variable : PTINR_VAL1 PT-INR Value

N	Mean	Standard Deviation	Minimum	Maximum
2	2.3000000	0.2687006	2.1100000	2.4900000

Laboratory tests at the time of obtaining Table 2-6 consent, according to the presence or absence of proton-pump inhibitors

Unique Subject Identifier=2672000000-0028

MEANS Procedure

Assay variable : PTINR_VAL1 PT-INR Value

N	Mean	Standard Deviation	Minimum	Maximum
2	1.6800000	0.2687006	1.4900000	1.8700000

Unique Subject Identifier=2675000000-0005

Assay variable : PTINR_VAL1 PT-INR Value

N	Mean	Standard Deviation	Minimum	Maximum
4	2.3200000	0.3329665	1.9900000	2.7500000

Unique Subject Identifier=2675000000-0013

Assay variable : PTINR_VAL1 PT-INR Value

N	Mean	Standard Deviation	Minimum	Maximum
5	1.9460000	0.3591379	1.3600000	2.2100000

Unique Subject Identifier=2678010000-0002

Assay variable : PTINR_VAL1 PT-INR Value

N	Mean	Standard Deviation	Minimum	Maximum
3	2.2166667	0.2020726	2.1000000	2.4500000

Laboratory tests at the time of obtaining Table 2-6 consent, according to the presence or absence of proton-pump inhibitors

Unique Subject Identifier=2678010000-0005

MEANS Procedure

Assay variable : PTINR_VAL1 PT-INR Value

N	Mean	Standard Deviation	Minimum	Maximum
5	1.9020000	0.2669644	1.6500000	2.2800000

Unique Subject Identifier=2678010000-0014

Assay variable : PTINR_VAL1 PT-INR Value

N	Mean	Standard Deviation	Minimum	Maximum
4	2.0950000	0.3875134	1.6900000	2.6000000

Unique Subject Identifier=2678010000-0034

Assay variable : PTINR_VAL1 PT-INR Value

N	Mean	Standard Deviation	Minimum	Maximum
3	1.5233333	0.0251661	1.5000000	1.5500000

Unique Subject Identifier=2687001000-0001

Assay variable : PTINR_VAL1 PT-INR Value

N	Mean	Standard Deviation	Minimum	Maximum
5	1.8400000	0.1673320	1.6000000	2.0000000

Laboratory tests at the time of obtaining Table 2-6 consent, according to the presence or absence of proton-pump inhibitors

Unique Subject Identifier=2687001000-0016

MEANS Procedure

Assay variable : PTINR_VAL1 PT-INR Value

N	Mean	Standard Deviation	Minimum	Maximum
5	2.2000000	0.2236068	1.9000000	2.5000000

Unique Subject Identifier=2687001000-0036

Assay variable : PTINR_VAL1 PT-INR Value

N	Mean	Standard Deviation	Minimum	Maximum
5	2.1200000	0.2863564	1.9000000	2.6000000

Unique Subject Identifier=2687001000-0042

Assay variable : PTINR_VAL1 PT-INR Value

N	Mean	Standard Deviation	Minimum	Maximum
5	1.6000000	0.0707107	1.5000000	1.7000000

Unique Subject Identifier=2688011111-0001

Assay variable : PTINR_VAL1 PT-INR Value

N	Mean	Standard Deviation	Minimum	Maximum
6	2.5000000	0.4604346	2.0000000	3.3000000

Laboratory tests at the time of obtaining Table 2-6 consent, according to the presence or absence of proton-pump inhibitors

Unique Subject Identifier=2688011111-0003

MEANS Procedure

Assay variable : PTINR_VAL1 PT-INR Value

N	Mean	Standard Deviation	Minimum	Maximum
5	2.0400000	0.2302173	1.8000000	2.4000000

Unique Subject Identifier=2688011111-0006

Assay variable : PTINR_VAL1 PT-INR Value

N	Mean	Standard Deviation	Minimum	Maximum
6	1.5500000	0.0836660	1.5000000	1.7000000

Unique Subject Identifier=2688011111-0007

Assay variable : PTINR_VAL1 PT-INR Value

N	Mean	Standard Deviation	Minimum	Maximum
6	2.3500000	0.5958188	1.3000000	3.0000000

Unique Subject Identifier=2688011111-0008

Assay variable : PTINR_VAL1 PT-INR Value

N	Mean	Standard Deviation	Minimum	Maximum
6	1.6666667	0.1505545	1.5000000	1.8000000

Laboratory tests at the time of obtaining Table 2-6 consent, according to the presence or absence of proton-pump inhibitors

Unique Subject Identifier=2688011111-0010

MEANS Procedure

Assay variable : PTINR_VAL1 PT-INR Value

N	Mean	Standard Deviation	Minimum	Maximum
6	2.0166667	0.3970726	1.7000000	2.7000000

Unique Subject Identifier=2688011111-0012

Assay variable : PTINR_VAL1 PT-INR Value

N	Mean	Standard Deviation	Minimum	Maximum
5	1.9600000	0.1140175	1.8000000	2.1000000

Unique Subject Identifier=2688011111-0013

Assay variable : PTINR_VAL1 PT-INR Value

N	Mean	Standard Deviation	Minimum	Maximum
6	1.6833333	0.2136976	1.4000000	2.0000000

Unique Subject Identifier=2688011111-0015

Assay variable : PTINR_VAL1 PT-INR Value

N	Mean	Standard Deviation	Minimum	Maximum
5	1.8000000	0.1581139	1.6000000	2.0000000

Laboratory tests at the time of obtaining Table 2-6 consent, according to the presence or absence of proton-pump inhibitors

Unique Subject Identifier=2688011111-0016

MEANS Procedure

Assay variable : PTINR_VAL1 PT-INR Value

N	Mean	Standard Deviation	Minimum	Maximum
5	2.3800000	0.3898718	2.0000000	3.0000000

Unique Subject Identifier=2688011111-0018

Assay variable : PTINR_VAL1 PT-INR Value

N	Mean	Standard Deviation	Minimum	Maximum
6	1.5666667	0.2160247	1.3000000	1.9000000

Unique Subject Identifier=2688011111-0020

Assay variable : PTINR_VAL1 PT-INR Value

N	Mean	Standard Deviation	Minimum	Maximum
5	1.8600000	0.2408319	1.6000000	2.1000000

Unique Subject Identifier=2688011111-0023

Assay variable : PTINR_VAL1 PT-INR Value

N	Mean	Standard Deviation	Minimum	Maximum
5	2.0800000	0.3271085	1.7000000	2.6000000

Laboratory tests at the time of obtaining Table 2-6 consent, according to the presence or absence of proton-pump inhibitors

Unique Subject Identifier=2688011111-0026

MEANS Procedure

Assay variable : PTINR_VAL1 PT-INR Value

N	Mean	Standard Deviation	Minimum	Maximum
1	1.5000000		1.5000000	1.5000000

Unique Subject Identifier=2688011111-0027

Assay variable : PTINR_VAL1 PT-INR Value

N	Mean	Standard Deviation	Minimum	Maximum
5	2.2600000	0.5224940	1.7000000	3.1000000

Unique Subject Identifier=2688011111-0028

Assay variable : PTINR_VAL1 PT-INR Value

N	Mean	Standard Deviation	Minimum	Maximum
5	1.6000000	0.2915476	1.2000000	1.9000000

Unique Subject Identifier=2688011111-0034

Assay variable : PTINR_VAL1 PT-INR Value

N	Mean	Standard Deviation	Minimum	Maximum
5	1.7600000	0.1516575	1.6000000	2.0000000

Laboratory tests at the time of obtaining Table 2-6 consent, according to the presence or absence of proton-pump inhibitors

Unique Subject Identifier=2688011111-0036

MEANS Procedure

Assay variable : PTINR_VAL1 PT-INR Value

N	Mean	Standard Deviation	Minimum	Maximum
5	1.6600000	0.1816590	1.4000000	1.9000000

Unique Subject Identifier=2688011111-0037

Assay variable : PTINR_VAL1 PT-INR Value

N	Mean	Standard Deviation	Minimum	Maximum
5	1.6800000	0.5718391	1.2000000	2.6000000

Unique Subject Identifier=2688011111-0040

Assay variable : PTINR_VAL1 PT-INR Value

N	Mean	Standard Deviation	Minimum	Maximum
3	2.1666667	0.3511885	1.8000000	2.5000000

Unique Subject Identifier=2688011111-0041

Assay variable : PTINR_VAL1 PT-INR Value

N	Mean	Standard Deviation	Minimum	Maximum
6	1.4333333	0.2943920	1.2000000	2.0000000

Laboratory tests at the time of obtaining Table 2-6 consent, according to the presence or absence of proton-pump inhibitors

Unique Subject Identifier=2688011111-0044

MEANS Procedure

Assay variable : PTINR_VAL1 PT-INR Value

N	Mean	Standard Deviation	Minimum	Maximum
5	1.7800000	0.1095445	1.6000000	1.9000000

Unique Subject Identifier=2688011111-0047

Assay variable : PTINR_VAL1 PT-INR Value

N	Mean	Standard Deviation	Minimum	Maximum
6	1.5500000	0.1378405	1.4000000	1.8000000

Unique Subject Identifier=2688011111-0048

Assay variable : PTINR_VAL1 PT-INR Value

N	Mean	Standard Deviation	Minimum	Maximum
6	2.1000000	0.4774935	1.4000000	2.7000000

Unique Subject Identifier=2688011111-0049

Assay variable : PTINR_VAL1 PT-INR Value

N	Mean	Standard Deviation	Minimum	Maximum
5	2.0000000	0.2000000	1.7000000	2.2000000

Laboratory tests at the time of obtaining Table 2-6 consent, according to the presence or absence of proton-pump inhibitors

Unique Subject Identifier=2688011111-0050

MEANS Procedure

Assay variable : PTINR_VAL1 PT-INR Value

N	Mean	Standard Deviation	Minimum	Maximum
4	2.3250000	0.6994045	1.4000000	3.0000000

Unique Subject Identifier=2688011111-0052

Assay variable : PTINR_VAL1 PT-INR Value

N	Mean	Standard Deviation	Minimum	Maximum
4	2.0000000	0.1414214	1.9000000	2.2000000

Unique Subject Identifier=2688011111-0054

Assay variable : PTINR_VAL1 PT-INR Value

N	Mean	Standard Deviation	Minimum	Maximum
5	2.0600000	0.3286335	1.7000000	2.4000000

Unique Subject Identifier=2688011111-0057

Assay variable : PTINR_VAL1 PT-INR Value

N	Mean	Standard Deviation	Minimum	Maximum
5	2.3200000	0.1923538	2.1000000	2.6000000

Laboratory tests at the time of obtaining Table 2-6 consent, according to the presence or absence of proton-pump inhibitors

Unique Subject Identifier=2688011111-0058

MEANS Procedure

Assay variable : PTINR_VAL1 PT-INR Value

N	Mean	Standard Deviation	Minimum	Maximum
5	1.9600000	0.2302173	1.6000000	2.2000000

Unique Subject Identifier=2688011111-0059

Assay variable : PTINR_VAL1 PT-INR Value

N	Mean	Standard Deviation	Minimum	Maximum
6	1.8000000	0.3898718	1.3000000	2.4000000

Unique Subject Identifier=2688011111-0061

Assay variable : PTINR_VAL1 PT-INR Value

N	Mean	Standard Deviation	Minimum	Maximum
5	1.6200000	0.2167948	1.3000000	1.8000000

Unique Subject Identifier=2688011111-0062

Assay variable : PTINR_VAL1 PT-INR Value

N	Mean	Standard Deviation	Minimum	Maximum
6	2.5833333	1.0008330	1.3000000	4.1000000

Laboratory tests at the time of obtaining Table 2-6 consent, according to the presence or absence of proton-pump inhibitors

Unique Subject Identifier=2688011111-0064

MEANS Procedure

Assay variable : PTINR_VAL1 PT-INR Value

N	Mean	Standard Deviation	Minimum	Maximum
5	1.8400000	0.8443933	1.2000000	3.3000000

Unique Subject Identifier=2688011111-0065

Assay variable : PTINR_VAL1 PT-INR Value

N	Mean	Standard Deviation	Minimum	Maximum
5	1.9400000	0.2966479	1.5000000	2.2000000

Unique Subject Identifier=2688011111-0074

Assay variable : PTINR_VAL1 PT-INR Value

N	Mean	Standard Deviation	Minimum	Maximum
5	2.1400000	0.3130495	1.8000000	2.6000000

Unique Subject Identifier=2688011111-0078

Assay variable : PTINR_VAL1 PT-INR Value

N	Mean	Standard Deviation	Minimum	Maximum
5	2.3400000	0.2190890	2.0000000	2.6000000

Laboratory tests at the time of obtaining Table 2-6 consent, according to the presence or absence of proton-pump inhibitors

Unique Subject Identifier=2688011111-0082

MEANS Procedure

Assay variable : PTINR_VAL1 PT-INR Value

N	Mean	Standard Deviation	Minimum	Maximum
6	2.2333333	0.1032796	2.1000000	2.4000000

Unique Subject Identifier=2692000000-0005

Assay variable : PTINR_VAL1 PT-INR Value

N	Mean	Standard Deviation	Minimum	Maximum
6	1.8283333	0.3561694	1.4500000	2.4900000

Unique Subject Identifier=2692000000-0009

Assay variable : PTINR_VAL1 PT-INR Value

N	Mean	Standard Deviation	Minimum	Maximum
6	1.9716667	0.4512834	1.5800000	2.6200000

Unique Subject Identifier=2703000000-0010

Assay variable : PTINR_VAL1 PT-INR Value

N	Mean	Standard Deviation	Minimum	Maximum
1	2.1000000		2.1000000	2.1000000

Laboratory tests at the time of obtaining Table 2-6 consent, according to the presence or absence of proton-pump inhibitors

Unique Subject Identifier=2703000000-0019

MEANS Procedure

Assay variable : PTINR_VAL1 PT-INR Value

N	Mean	Standard Deviation	Minimum	Maximum
3	1.5533333	0.3233162	1.2600000	1.9000000

Unique Subject Identifier=2737011111-0002

Assay variable : PTINR_VAL1 PT-INR Value

N	Mean	Standard Deviation	Minimum	Maximum
6	1.5500000	0.1048809	1.4000000	1.7000000

Unique Subject Identifier=2737011111-0007

Assay variable : PTINR_VAL1 PT-INR Value

N	Mean	Standard Deviation	Minimum	Maximum
4	1.9500000	0.4123106	1.4000000	2.4000000

Unique Subject Identifier=2737011111-0008

Assay variable : PTINR_VAL1 PT-INR Value

N	Mean	Standard Deviation	Minimum	Maximum
6	1.4666667	0.1505545	1.3000000	1.7000000

Laboratory tests at the time of obtaining Table 2-6 consent, according to the presence or absence of proton-pump inhibitors

Unique Subject Identifier=2748011000-0036

MEANS Procedure

Assay variable : PTINR_VAL1 PT-INR Value

N	Mean	Standard Deviation	Minimum	Maximum
3	2.6733333	0.1761628	2.5100000	2.8600000

Unique Subject Identifier=2748011000-0042

Assay variable : PTINR_VAL1 PT-INR Value

N	Mean	Standard Deviation	Minimum	Maximum
3	2.0900000	0.2722132	1.8400000	2.3800000

Unique Subject Identifier=2748011000-0059

Assay variable : PTINR_VAL1 PT-INR Value

N	Mean	Standard Deviation	Minimum	Maximum
4	2.7425000	0.2808766	2.4100000	3.0500000

Unique Subject Identifier=2749000000-0003

Assay variable : PTINR_VAL1 PT-INR Value

N	Mean	Standard Deviation	Minimum	Maximum
2	2.2600000	0.2545584	2.0800000	2.4400000

Laboratory tests at the time of obtaining Table 2-6 consent, according to the presence or absence of proton-pump inhibitors

Unique Subject Identifier=2749000000-0007

MEANS Procedure

Assay variable : PTINR_VAL1 PT-INR Value

N	Mean	Standard Deviation	Minimum	Maximum
6	2.1766667	0.2716370	1.8900000	2.4800000

Unique Subject Identifier=2765000000-0002

Assay variable : PTINR_VAL1 PT-INR Value

N	Mean	Standard Deviation	Minimum	Maximum
5	1.7420000	0.4029516	1.2400000	2.3500000

Unique Subject Identifier=2765000000-0009

Assay variable : PTINR_VAL1 PT-INR Value

N	Mean	Standard Deviation	Minimum	Maximum
4	1.9475000	0.1848197	1.8100000	2.2200000

Unique Subject Identifier=2777011010-0013

Assay variable : PTINR_VAL1 PT-INR Value

N	Mean	Standard Deviation	Minimum	Maximum
3	1.7033333	0.2413158	1.5000000	1.9700000

Laboratory tests at the time of obtaining Table 2-6 consent, according to the presence or absence of proton-pump inhibitors

Unique Subject Identifier=2777011010-0021

MEANS Procedure

Assay variable : PTINR_VAL1 PT-INR Value

N	Mean	Standard Deviation	Minimum	Maximum
4	1.6625000	0.4193149	1.1500000	2.0200000

Unique Subject Identifier=2777011010-0023

Assay variable : PTINR_VAL1 PT-INR Value

N	Mean	Standard Deviation	Minimum	Maximum
5	1.9680000	0.2752635	1.5800000	2.2700000

Unique Subject Identifier=2777011010-0025

Assay variable : PTINR_VAL1 PT-INR Value

N	Mean	Standard Deviation	Minimum	Maximum
3	2.0800000	0.0264575	2.0600000	2.1100000

Unique Subject Identifier=2777011010-0031

Assay variable : PTINR_VAL1 PT-INR Value

N	Mean	Standard Deviation	Minimum	Maximum
2	1.7950000	0.6010408	1.3700000	2.2200000

Laboratory tests at the time of obtaining Table 2-6 consent, according to the presence or absence of proton-pump inhibitors

Unique Subject Identifier=2777011010-0033

MEANS Procedure

Assay variable : PTINR_VAL1 PT-INR Value

N	Mean	Standard Deviation	Minimum	Maximum
3	1.8100000	0.1907878	1.5900000	1.9300000

Unique Subject Identifier=2777011010-0043

Assay variable : PTINR_VAL1 PT-INR Value

N	Mean	Standard Deviation	Minimum	Maximum
3	2.0100000	0.2605763	1.7400000	2.2600000

Unique Subject Identifier=2777011010-0046

Assay variable : PTINR_VAL1 PT-INR Value

N	Mean	Standard Deviation	Minimum	Maximum
3	2.0400000	0.3996248	1.6800000	2.4700000

Unique Subject Identifier=2782000000-0007

Assay variable : PTINR_VAL1 PT-INR Value

N	Mean	Standard Deviation	Minimum	Maximum
3	2.0633333	0.1537314	1.9600000	2.2400000

Laboratory tests at the time of obtaining Table 2-6 consent, according to the presence or absence of proton-pump inhibitors

Unique Subject Identifier=2784011111-0009

MEANS Procedure

Assay variable : PTINR_VAL1 PT-INR Value

N	Mean	Standard Deviation	Minimum	Maximum
5	1.7400000	0.2073644	1.6000000	2.1000000

Unique Subject Identifier=2784011111-0013

Assay variable : PTINR_VAL1 PT-INR Value

N	Mean	Standard Deviation	Minimum	Maximum
5	1.4800000	0.2683282	1.2000000	1.8000000

Unique Subject Identifier=2791010010-0004

Assay variable : PTINR_VAL1 PT-INR Value

N	Mean	Standard Deviation	Minimum	Maximum
6	1.8000000	0.1095445	1.6000000	1.9000000

Unique Subject Identifier=2809000000-0009

Assay variable : PTINR_VAL1 PT-INR Value

N	Mean	Standard Deviation	Minimum	Maximum
5	2.0760000	0.1779888	1.8600000	2.2800000

Laboratory tests at the time of obtaining Table 2-6 consent, according to the presence or absence of proton-pump inhibitors

Unique Subject Identifier=2872000000-0003

MEANS Procedure

Assay variable : PTINR_VAL1 PT-INR Value

N	Mean	Standard Deviation	Minimum	Maximum
3	1.7366667	0.2746513	1.4200000	1.9100000

Unique Subject Identifier=2875000000-0004

Assay variable : PTINR_VAL1 PT-INR Value

N	Mean	Standard Deviation	Minimum	Maximum
1	1.0200000		1.0200000	1.0200000

Unique Subject Identifier=2875000000-0017

Assay variable : PTINR_VAL1 PT-INR Value

N	Mean	Standard Deviation	Minimum	Maximum
4	1.9275000	0.2639918	1.6800000	2.2800000

Unique Subject Identifier=2875000000-0026

Assay variable : PTINR_VAL1 PT-INR Value

N	Mean	Standard Deviation	Minimum	Maximum
5	1.5100000	0.1721918	1.3400000	1.7100000

Laboratory tests at the time of obtaining Table 2-6 consent, according to the presence or absence of proton-pump inhibitors

Unique Subject Identifier=2875000000-0027

MEANS Procedure

Assay variable : PTINR_VAL1 PT-INR Value

N	Mean	Standard Deviation	Minimum	Maximum
6	1.7183333	0.3345096	1.3500000	2.2000000

Unique Subject Identifier=2875000000-0030

Assay variable : PTINR_VAL1 PT-INR Value

N	Mean	Standard Deviation	Minimum	Maximum
5	2.1120000	0.2107605	1.8400000	2.3700000

Unique Subject Identifier=2875000000-0035

Assay variable : PTINR_VAL1 PT-INR Value

N	Mean	Standard Deviation	Minimum	Maximum
4	1.5375000	0.3564992	1.2000000	1.9200000

Unique Subject Identifier=2889010000-0003

Assay variable : PTINR_VAL1 PT-INR Value

N	Mean	Standard Deviation	Minimum	Maximum
5	1.7200000	1.1529744	0.9200000	3.7100000

Laboratory tests at the time of obtaining Table 2-6 consent, according to the presence or absence of proton-pump inhibitors

Unique Subject Identifier=2890001000-0003

MEANS Procedure

Assay variable : PTINR_VAL1 PT-INR Value

N	Mean	Standard Deviation	Minimum	Maximum
4	2.6925000	0.2248518	2.4500000	2.9300000

Unique Subject Identifier=2890001000-0006

Assay variable : PTINR_VAL1 PT-INR Value

N	Mean	Standard Deviation	Minimum	Maximum
3	1.2100000	0.0435890	1.1800000	1.2600000

Unique Subject Identifier=2890001000-0007

Assay variable : PTINR_VAL1 PT-INR Value

N	Mean	Standard Deviation	Minimum	Maximum
4	2.3225000	0.1635797	2.1900000	2.5600000

Unique Subject Identifier=2898010000-0012

Assay variable : PTINR_VAL1 PT-INR Value

N	Mean	Standard Deviation	Minimum	Maximum
5	1.9440000	0.2618778	1.7100000	2.2800000

Laboratory tests at the time of obtaining Table 2-6 consent, according to the presence or absence of proton-pump inhibitors

Unique Subject Identifier=2898010000-0018

MEANS Procedure

Assay variable : PTINR_VAL1 PT-INR Value

N	Mean	Standard Deviation	Minimum	Maximum
6	2.1266667	0.6186976	1.3800000	2.8400000

Unique Subject Identifier=2898010000-0020

Assay variable : PTINR_VAL1 PT-INR Value

N	Mean	Standard Deviation	Minimum	Maximum
3	1.6000000	0.0888819	1.5300000	1.7000000

Unique Subject Identifier=2898010000-0021

Assay variable : PTINR_VAL1 PT-INR Value

N	Mean	Standard Deviation	Minimum	Maximum
3	1.8000000	0.3605551	1.5000000	2.2000000

Unique Subject Identifier=2898010000-0025

Assay variable : PTINR_VAL1 PT-INR Value

N	Mean	Standard Deviation	Minimum	Maximum
5	1.6100000	0.1612452	1.4600000	1.8800000

Laboratory tests at the time of obtaining Table 2-6 consent, according to the presence or absence of proton-pump inhibitors

Unique Subject Identifier=2898010000-0030

MEANS Procedure

Assay variable : PTINR_VAL1 PT-INR Value

N	Mean	Standard Deviation	Minimum	Maximum
3	2.4900000	0.5565070	1.8800000	2.9700000

Unique Subject Identifier=2910000000-0010

Assay variable : PTINR_VAL1 PT-INR Value

N	Mean	Standard Deviation	Minimum	Maximum
2	2.2300000	0.1555635	2.1200000	2.3400000

Unique Subject Identifier=2910000000-0018

Assay variable : PTINR_VAL1 PT-INR Value

N	Mean	Standard Deviation	Minimum	Maximum
3	1.9100000	0.2193171	1.6600000	2.0700000

Unique Subject Identifier=2910000000-0020

Assay variable : PTINR_VAL1 PT-INR Value

N	Mean	Standard Deviation	Minimum	Maximum
6	2.3700000	0.4295579	1.8500000	2.8900000

Laboratory tests at the time of obtaining Table 2-6 consent, according to the presence or absence of proton-pump inhibitors

Unique Subject Identifier=2910000000-0027

MEANS Procedure

Assay variable : PTINR_VAL1 PT-INR Value

N	Mean	Standard Deviation	Minimum	Maximum
2	2.1300000	0.1131371	2.0500000	2.2100000

Unique Subject Identifier=2913000000-0004

Assay variable : PTINR_VAL1 PT-INR Value

N	Mean	Standard Deviation	Minimum	Maximum
3	1.6633333	0.0776745	1.6000000	1.7500000

Unique Subject Identifier=2913000000-0005

Assay variable : PTINR_VAL1 PT-INR Value

N	Mean	Standard Deviation	Minimum	Maximum
5	2.3100000	0.2511971	1.9200000	2.6000000

Unique Subject Identifier=2913000000-0016

Assay variable : PTINR_VAL1 PT-INR Value

N	Mean	Standard Deviation	Minimum	Maximum
5	1.7400000	0.5002499	1.0600000	2.4000000

Laboratory tests at the time of obtaining Table 2-6 consent, according to the presence or absence of proton-pump inhibitors

Unique Subject Identifier=2913000000-0023

MEANS Procedure

Assay variable : PTINR_VAL1 PT-INR Value

N	Mean	Standard Deviation	Minimum	Maximum
6	1.7716667	0.2058559	1.4800000	2.0900000

Unique Subject Identifier=2913000000-0027

Assay variable : PTINR_VAL1 PT-INR Value

N	Mean	Standard Deviation	Minimum	Maximum
4	2.3625000	0.9694113	1.5200000	3.7600000

Unique Subject Identifier=2913000000-0034

Assay variable : PTINR_VAL1 PT-INR Value

N	Mean	Standard Deviation	Minimum	Maximum
6	1.8983333	0.4070586	1.3500000	2.4300000

Unique Subject Identifier=2926011010-0001

Assay variable : PTINR_VAL1 PT-INR Value

N	Mean	Standard Deviation	Minimum	Maximum
4	1.8650000	0.2991655	1.6000000	2.2600000

Laboratory tests at the time of obtaining Table 2-6 consent, according to the presence or absence of proton-pump inhibitors

Unique Subject Identifier=2926011010-0016

MEANS Procedure

Assay variable : PTINR_VAL1 PT-INR Value

N	Mean	Standard Deviation	Minimum	Maximum
5	1.6280000	0.1584929	1.4000000	1.7700000

Unique Subject Identifier=2941010110-0016

Assay variable : PTINR_VAL1 PT-INR Value

N	Mean	Standard Deviation	Minimum	Maximum
5	1.8400000	0.3435113	1.5000000	2.3000000

Unique Subject Identifier=2949000000-0012

Assay variable : PTINR_VAL1 PT-INR Value

N	Mean	Standard Deviation	Minimum	Maximum
1	2.0300000		2.0300000	2.0300000

Unique Subject Identifier=2949000000-0016

Assay variable : PTINR_VAL1 PT-INR Value

N	Mean	Standard Deviation	Minimum	Maximum
2	1.8300000	0.2404163	1.6600000	2.0000000

Laboratory tests at the time of obtaining Table 2-6 consent, according to the presence or absence of proton-pump inhibitors

Unique Subject Identifier=2949000000-0018

MEANS Procedure

Assay variable : PTINR_VAL1 PT-INR Value

N	Mean	Standard Deviation	Minimum	Maximum
1	2.1100000		2.1100000	2.1100000

Unique Subject Identifier=2949000000-0021

Assay variable : PTINR_VAL1 PT-INR Value

N	Mean	Standard Deviation	Minimum	Maximum
2	2.2150000	0.4737615	1.8800000	2.5500000

Unique Subject Identifier=2949000000-0026

Assay variable : PTINR_VAL1 PT-INR Value

N	Mean	Standard Deviation	Minimum	Maximum
3	2.1533333	0.0709460	2.0900000	2.2300000

Unique Subject Identifier=2949000000-0039

Assay variable : PTINR_VAL1 PT-INR Value

N	Mean	Standard Deviation	Minimum	Maximum
3	1.8733333	0.6087966	1.3700000	2.5500000

Laboratory tests at the time of obtaining Table 2-6 consent, according to the presence or absence of proton-pump inhibitors

Unique Subject Identifier=2954000000-0003

MEANS Procedure

Assay variable : PTINR_VAL1 PT-INR Value

N	Mean	Standard Deviation	Minimum	Maximum
4	2.6250000	0.8148006	1.8000000	3.7500000

Unique Subject Identifier=2954000000-0015

Assay variable : PTINR_VAL1 PT-INR Value

N	Mean	Standard Deviation	Minimum	Maximum
3	2.1066667	0.4186088	1.8600000	2.5900000

Unique Subject Identifier=2957000000-0016

Assay variable : PTINR_VAL1 PT-INR Value

N	Mean	Standard Deviation	Minimum	Maximum
6	2.0816667	0.2756387	1.6300000	2.4100000

Unique Subject Identifier=2957000000-0018

Assay variable : PTINR_VAL1 PT-INR Value

N	Mean	Standard Deviation	Minimum	Maximum
6	1.8816667	0.2513497	1.5900000	2.3300000

Laboratory tests at the time of obtaining Table 2-6 consent, according to the presence or absence of proton-pump inhibitors

Unique Subject Identifier=3001010001-0002

MEANS Procedure

Assay variable : PTINR_VAL1 PT-INR Value

N	Mean	Standard Deviation	Minimum	Maximum
4	1.8975000	0.2953388	1.4900000	2.1600000

Unique Subject Identifier=3001010001-0010

Assay variable : PTINR_VAL1 PT-INR Value

N	Mean	Standard Deviation	Minimum	Maximum
4	2.0650000	0.2483948	1.7700000	2.3700000

Unique Subject Identifier=3001010001-0014

Assay variable : PTINR_VAL1 PT-INR Value

N	Mean	Standard Deviation	Minimum	Maximum
6	2.1550000	0.2165872	1.9100000	2.4800000

Unique Subject Identifier=3001010001-0015

Assay variable : PTINR_VAL1 PT-INR Value

N	Mean	Standard Deviation	Minimum	Maximum
6	2.1016667	0.3437101	1.6300000	2.5100000

Laboratory tests at the time of obtaining Table 2-6 consent, according to the presence or absence of proton-pump inhibitors

Unique Subject Identifier=3001010001-0016

MEANS Procedure

Assay variable : PTINR_VAL1 PT-INR Value

N	Mean	Standard Deviation	Minimum	Maximum
6	1.9816667	0.4285985	1.6800000	2.8300000

Unique Subject Identifier=3001010001-0017

Assay variable : PTINR_VAL1 PT-INR Value

N	Mean	Standard Deviation	Minimum	Maximum
6	1.7166667	0.3188521	1.4000000	2.3000000

Unique Subject Identifier=3001010001-0018

Assay variable : PTINR_VAL1 PT-INR Value

N	Mean	Standard Deviation	Minimum	Maximum
6	2.1366667	0.2878657	1.7900000	2.6000000

Unique Subject Identifier=3001010001-0020

Assay variable : PTINR_VAL1 PT-INR Value

N	Mean	Standard Deviation	Minimum	Maximum
6	1.8433333	0.1123684	1.6700000	1.9900000

Laboratory tests at the time of obtaining Table 2-6 consent, according to the presence or absence of proton-pump inhibitors

Unique Subject Identifier=3001010001-0026

MEANS Procedure

Assay variable : PTINR_VAL1 PT-INR Value

N	Mean	Standard Deviation	Minimum	Maximum
5	1.9760000	0.1064425	1.8900000	2.1200000

Unique Subject Identifier=3001010001-0037

Assay variable : PTINR_VAL1 PT-INR Value

N	Mean	Standard Deviation	Minimum	Maximum
4	1.5500000	0.1329160	1.3800000	1.6900000

Unique Subject Identifier=3001010001-0041

Assay variable : PTINR_VAL1 PT-INR Value

N	Mean	Standard Deviation	Minimum	Maximum
3	1.9433333	0.1457166	1.7900000	2.0800000

Unique Subject Identifier=3001010001-0051

Assay variable : PTINR_VAL1 PT-INR Value

N	Mean	Standard Deviation	Minimum	Maximum
6	1.8866667	0.3142398	1.4500000	2.2700000

Laboratory tests at the time of obtaining Table 2-6 consent, according to the presence or absence of proton-pump inhibitors

Unique Subject Identifier=3001010001-0055

MEANS Procedure

Assay variable : PTINR_VAL1 PT-INR Value

N	Mean	Standard Deviation	Minimum	Maximum
5	1.7960000	0.1291511	1.6200000	1.9200000

Unique Subject Identifier=3001010001-0062

Assay variable : PTINR_VAL1 PT-INR Value

N	Mean	Standard Deviation	Minimum	Maximum
5	1.9160000	0.3692289	1.5200000	2.4600000

Unique Subject Identifier=3001010001-0064

Assay variable : PTINR_VAL1 PT-INR Value

N	Mean	Standard Deviation	Minimum	Maximum
4	1.6625000	0.2707243	1.2700000	1.8800000

Unique Subject Identifier=3003011110-0007

Assay variable : PTINR_VAL1 PT-INR Value

N	Mean	Standard Deviation	Minimum	Maximum
3	1.3933333	0.3564174	1.0200000	1.7300000

Laboratory tests at the time of obtaining Table 2-6 consent, according to the presence or absence of proton-pump inhibitors

Unique Subject Identifier=3003011110-0015

MEANS Procedure

Assay variable : PTINR_VAL1 PT-INR Value

N	Mean	Standard Deviation	Minimum	Maximum
6	2.1916667	0.2978870	1.8400000	2.6300000

Unique Subject Identifier=3009011111-0012

Assay variable : PTINR_VAL1 PT-INR Value

N	Mean	Standard Deviation	Minimum	Maximum
3	1.6000000	0.2913760	1.2800000	1.8500000

Unique Subject Identifier=3009011111-0019

Assay variable : PTINR_VAL1 PT-INR Value

N	Mean	Standard Deviation	Minimum	Maximum
3	2.0800000	0.0700000	2.0100000	2.1500000

Unique Subject Identifier=3009011111-0020

Assay variable : PTINR_VAL1 PT-INR Value

N	Mean	Standard Deviation	Minimum	Maximum
2	2.0500000	0.1414214	1.9500000	2.1500000

Laboratory tests at the time of obtaining Table 2-6 consent, according to the presence or absence of proton-pump inhibitors

Unique Subject Identifier=3010011111-0002

MEANS Procedure

Assay variable : PTINR_VAL1 PT-INR Value

N	Mean	Standard Deviation	Minimum	Maximum
6	1.9800000	0.2705550	1.5500000	2.3400000

Unique Subject Identifier=3016111111-0026

Assay variable : PTINR_VAL1 PT-INR Value

N	Mean	Standard Deviation	Minimum	Maximum
6	1.6416667	0.3370114	1.2000000	2.0300000

Unique Subject Identifier=3016111111-0037

Assay variable : PTINR_VAL1 PT-INR Value

N	Mean	Standard Deviation	Minimum	Maximum
1	1.2000000		1.2000000	1.2000000

Unique Subject Identifier=3019011110-0021

Assay variable : PTINR_VAL1 PT-INR Value

N	Mean	Standard Deviation	Minimum	Maximum
3	1.7200000	0.1212436	1.6100000	1.8500000

Laboratory tests at the time of obtaining Table 2-6 consent, according to the presence or absence of proton-pump inhibitors

Unique Subject Identifier=3019011110-0026

MEANS Procedure

Assay variable : PTINR_VAL1 PT-INR Value

N	Mean	Standard Deviation	Minimum	Maximum
3	1.5400000	0.1646208	1.3500000	1.6400000

Unique Subject Identifier=3019011110-0035

Assay variable : PTINR_VAL1 PT-INR Value

N	Mean	Standard Deviation	Minimum	Maximum
5	1.6800000	0.1743560	1.5400000	1.9600000

Unique Subject Identifier=3019011110-0038

Assay variable : PTINR_VAL1 PT-INR Value

N	Mean	Standard Deviation	Minimum	Maximum
2	1.9100000	0.1272792	1.8200000	2.0000000

Unique Subject Identifier=3019011110-0039

Assay variable : PTINR_VAL1 PT-INR Value

N	Mean	Standard Deviation	Minimum	Maximum
3	1.9833333	0.7320064	1.2000000	2.6500000

Laboratory tests at the time of obtaining Table 2-6 consent, according to the presence or absence of proton-pump inhibitors

Unique Subject Identifier=3019011110-0044

MEANS Procedure

Assay variable : PTINR_VAL1 PT-INR Value

N	Mean	Standard Deviation	Minimum	Maximum
3	1.9200000	0.1178983	1.8200000	2.0500000

Unique Subject Identifier=3019011110-0046

Assay variable : PTINR_VAL1 PT-INR Value

N	Mean	Standard Deviation	Minimum	Maximum
4	1.8450000	0.2100000	1.6000000	2.1000000

Unique Subject Identifier=3019011110-0052

Assay variable : PTINR_VAL1 PT-INR Value

N	Mean	Standard Deviation	Minimum	Maximum
1	1.5800000		1.5800000	1.5800000

Unique Subject Identifier=3021011010-0005

Assay variable : PTINR_VAL1 PT-INR Value

N	Mean	Standard Deviation	Minimum	Maximum
3	2.3466667	1.0304045	1.3000000	3.3600000

Laboratory tests at the time of obtaining Table 2-6 consent, according to the presence or absence of proton-pump inhibitors

Unique Subject Identifier=3022000000-0002

MEANS Procedure

Assay variable : PTINR_VAL1 PT-INR Value

N	Mean	Standard Deviation	Minimum	Maximum
6	1.4950000	0.6555227	1.1000000	2.8000000

Unique Subject Identifier=3022000000-0005

Assay variable : PTINR_VAL1 PT-INR Value

N	Mean	Standard Deviation	Minimum	Maximum
3	1.6166667	0.4532475	1.3500000	2.1400000

Unique Subject Identifier=3026011100-0028

Assay variable : PTINR_VAL1 PT-INR Value

N	Mean	Standard Deviation	Minimum	Maximum
3	1.8900000	0.2882707	1.5800000	2.1500000

Unique Subject Identifier=3026011100-0031

Assay variable : PTINR_VAL1 PT-INR Value

N	Mean	Standard Deviation	Minimum	Maximum
3	2.0366667	0.4331666	1.5500000	2.3800000

Laboratory tests at the time of obtaining Table 2-6 consent, according to the presence or absence of proton-pump inhibitors

Unique Subject Identifier=3026011100-0039

MEANS Procedure

Assay variable : PTINR_VAL1 PT-INR Value

N	Mean	Standard Deviation	Minimum	Maximum
3	2.7100000	1.0318430	1.5700000	3.5800000

Unique Subject Identifier=3026011100-0040

Assay variable : PTINR_VAL1 PT-INR Value

N	Mean	Standard Deviation	Minimum	Maximum
6	1.5600000	0.1363818	1.3100000	1.6800000

Unique Subject Identifier=3026011100-0043

Assay variable : PTINR_VAL1 PT-INR Value

N	Mean	Standard Deviation	Minimum	Maximum
2	1.6750000	0.3606245	1.4200000	1.9300000

Unique Subject Identifier=3026011100-0055

Assay variable : PTINR_VAL1 PT-INR Value

N	Mean	Standard Deviation	Minimum	Maximum
3	2.6866667	0.2437895	2.4100000	2.8700000

Laboratory tests at the time of obtaining Table 2-6 consent, according to the presence or absence of proton-pump inhibitors

Unique Subject Identifier=3026011100-0064

MEANS Procedure

Assay variable : PTINR_VAL1 PT-INR Value

N	Mean	Standard Deviation	Minimum	Maximum
5	1.6760000	0.7995186	1.0800000	3.0400000

Unique Subject Identifier=3056000000-0007

Assay variable : PTINR_VAL1 PT-INR Value

N	Mean	Standard Deviation	Minimum	Maximum
5	1.6560000	0.1505988	1.5100000	1.9000000

Unique Subject Identifier=3060011100-0004

Assay variable : PTINR_VAL1 PT-INR Value

N	Mean	Standard Deviation	Minimum	Maximum
6	1.7333333	0.5202563	1.2000000	2.6000000

Unique Subject Identifier=3060011100-0016

Assay variable : PTINR_VAL1 PT-INR Value

N	Mean	Standard Deviation	Minimum	Maximum
6	1.5666667	0.1966384	1.4000000	1.9000000

Laboratory tests at the time of obtaining Table 2-6 consent, according to the presence or absence of proton-pump inhibitors

Unique Subject Identifier=3077010011-0014

MEANS Procedure

Assay variable : PTINR_VAL1 PT-INR Value

N	Mean	Standard Deviation	Minimum	Maximum
3	1.5033333	0.0461880	1.4500000	1.5300000

Unique Subject Identifier=3084010000-0002

Assay variable : PTINR_VAL1 PT-INR Value

N	Mean	Standard Deviation	Minimum	Maximum
2	1.2550000	0.2333452	1.0900000	1.4200000

Unique Subject Identifier=3084010000-0004

Assay variable : PTINR_VAL1 PT-INR Value

N	Mean	Standard Deviation	Minimum	Maximum
2	2.2200000	0.2121320	2.0700000	2.3700000

Unique Subject Identifier=3084010000-0018

Assay variable : PTINR_VAL1 PT-INR Value

N	Mean	Standard Deviation	Minimum	Maximum
2	1.9150000	0.1484924	1.8100000	2.0200000

Laboratory tests at the time of obtaining Table 2-6 consent, according to the presence or absence of proton-pump inhibitors

Unique Subject Identifier=3084010000-0019

MEANS Procedure

Assay variable : PTINR_VAL1 PT-INR Value

N	Mean	Standard Deviation	Minimum	Maximum
3	1.6133333	0.2565801	1.3300000	1.8300000

Unique Subject Identifier=3084010000-0032

Assay variable : PTINR_VAL1 PT-INR Value

N	Mean	Standard Deviation	Minimum	Maximum
1	1.5700000		1.5700000	1.5700000

Unique Subject Identifier=3085010000-0007

Assay variable : PTINR_VAL1 PT-INR Value

N	Mean	Standard Deviation	Minimum	Maximum
3	1.5333333	0.0585947	1.4900000	1.6000000

Unique Subject Identifier=3085010000-0010

Assay variable : PTINR_VAL1 PT-INR Value

N	Mean	Standard Deviation	Minimum	Maximum
3	1.9633333	0.4086971	1.5900000	2.4000000

Laboratory tests at the time of obtaining Table 2-6 consent, according to the presence or absence of proton-pump inhibitors

Unique Subject Identifier=3085010000-0025

MEANS Procedure

Assay variable : PTINR_VAL1 PT-INR Value

N	Mean	Standard Deviation	Minimum	Maximum
3	1.9366667	0.1285820	1.7900000	2.0300000

Unique Subject Identifier=3096000000-0003

Assay variable : PTINR_VAL1 PT-INR Value

N	Mean	Standard Deviation	Minimum	Maximum
5	1.8800000	0.2774887	1.6000000	2.3000000

Unique Subject Identifier=3097011000-0004

Assay variable : PTINR_VAL1 PT-INR Value

N	Mean	Standard Deviation	Minimum	Maximum
4	1.4775000	0.0708872	1.3900000	1.5400000

Unique Subject Identifier=3102111011-0013

Assay variable : PTINR_VAL1 PT-INR Value

N	Mean	Standard Deviation	Minimum	Maximum
2	1.8500000	0.0707107	1.8000000	1.9000000

Laboratory tests at the time of obtaining Table 2-6 consent, according to the presence or absence of proton-pump inhibitors

Unique Subject Identifier=3102111011-0045

MEANS Procedure

Assay variable : PTINR_VAL1 PT-INR Value

N	Mean	Standard Deviation	Minimum	Maximum
3	1.6300000	0.2151743	1.4200000	1.8500000

Unique Subject Identifier=3102111011-0057

Assay variable : PTINR_VAL1 PT-INR Value

N	Mean	Standard Deviation	Minimum	Maximum
4	1.5350000	0.1405940	1.3900000	1.6700000

Unique Subject Identifier=3102111011-0059

Assay variable : PTINR_VAL1 PT-INR Value

N	Mean	Standard Deviation	Minimum	Maximum
5	1.7100000	0.3766298	1.4000000	2.3500000

Unique Subject Identifier=3102111011-0067

Assay variable : PTINR_VAL1 PT-INR Value

N	Mean	Standard Deviation	Minimum	Maximum
3	1.7966667	0.1250333	1.6700000	1.9200000

Laboratory tests at the time of obtaining Table 2-6 consent, according to the presence or absence of proton-pump inhibitors

Unique Subject Identifier=3102111011-0071

MEANS Procedure

Assay variable : PTINR_VAL1 PT-INR Value

N	Mean	Standard Deviation	Minimum	Maximum
2	1.3500000	0.1131371	1.2700000	1.4300000

Unique Subject Identifier=3102111011-0073

Assay variable : PTINR_VAL1 PT-INR Value

N	Mean	Standard Deviation	Minimum	Maximum
3	2.0200000	0.3950949	1.6300000	2.4200000

Unique Subject Identifier=3102111011-0085

Assay variable : PTINR_VAL1 PT-INR Value

N	Mean	Standard Deviation	Minimum	Maximum
2	1.7150000	0.0919239	1.6500000	1.7800000

Unique Subject Identifier=3102111011-0088

Assay variable : PTINR_VAL1 PT-INR Value

N	Mean	Standard Deviation	Minimum	Maximum
5	2.0980000	0.5374663	1.5100000	2.6000000

Laboratory tests at the time of obtaining Table 2-6 consent, according to the presence or absence of proton-pump inhibitors

Unique Subject Identifier=3102111011-0112

MEANS Procedure

Assay variable : PTINR_VAL1 PT-INR Value

N	Mean	Standard Deviation	Minimum	Maximum
3	2.5833333	0.3231615	2.3400000	2.9500000

Unique Subject Identifier=3102111011-0115

Assay variable : PTINR_VAL1 PT-INR Value

N	Mean	Standard Deviation	Minimum	Maximum
1	1.3300000		1.3300000	1.3300000

Unique Subject Identifier=3102111011-0119

Assay variable : PTINR_VAL1 PT-INR Value

N	Mean	Standard Deviation	Minimum	Maximum
4	1.7300000	0.3841007	1.2900000	2.1300000

Unique Subject Identifier=3105011000-0007

Assay variable : PTINR_VAL1 PT-INR Value

N	Mean	Standard Deviation	Minimum	Maximum
4	2.0450000	0.5570458	1.4700000	2.7300000

Laboratory tests at the time of obtaining Table 2-6 consent, according to the presence or absence of proton-pump inhibitors

Unique Subject Identifier=3105011000-0009

MEANS Procedure

Assay variable : PTINR_VAL1 PT-INR Value

N	Mean	Standard Deviation	Minimum	Maximum
3	1.6966667	0.2811287	1.5100000	2.0200000

Unique Subject Identifier=3114011111-0014

Assay variable : PTINR_VAL1 PT-INR Value

N	Mean	Standard Deviation	Minimum	Maximum
3	1.2133333	0.1556706	1.0500000	1.3600000

Unique Subject Identifier=3122010111-0017

Assay variable : PTINR_VAL1 PT-INR Value

N	Mean	Standard Deviation	Minimum	Maximum
1	1.9500000		1.9500000	1.9500000

Unique Subject Identifier=3122010111-0024

Assay variable : PTINR_VAL1 PT-INR Value

N	Mean	Standard Deviation	Minimum	Maximum
4	2.1075000	0.2934138	1.8900000	2.5400000

Laboratory tests at the time of obtaining Table 2-6 consent, according to the presence or absence of proton-pump inhibitors

Unique Subject Identifier=3122010111-0030

MEANS Procedure

Assay variable : PTINR_VAL1 PT-INR Value

N	Mean	Standard Deviation	Minimum	Maximum
2	2.0750000	0.7283200	1.5600000	2.5900000

Unique Subject Identifier=3122010111-0050

Assay variable : PTINR_VAL1 PT-INR Value

N	Mean	Standard Deviation	Minimum	Maximum
6	2.3250000	0.6476033	1.4300000	3.2800000

Unique Subject Identifier=3122010111-0058

Assay variable : PTINR_VAL1 PT-INR Value

N	Mean	Standard Deviation	Minimum	Maximum
1	1.7600000		1.7600000	1.7600000

Unique Subject Identifier=3122010111-0061

Assay variable : PTINR_VAL1 PT-INR Value

N	Mean	Standard Deviation	Minimum	Maximum
4	2.6700000	0.5993886	2.0600000	3.4900000

Laboratory tests at the time of obtaining Table 2-6 consent, according to the presence or absence of proton-pump inhibitors

Unique Subject Identifier=3122010111-0071

MEANS Procedure

Assay variable : PTINR_VAL1 PT-INR Value

N	Mean	Standard Deviation	Minimum	Maximum
3	2.0633333	0.1942507	1.8500000	2.2300000

Unique Subject Identifier=3122010111-0079

Assay variable : PTINR_VAL1 PT-INR Value

N	Mean	Standard Deviation	Minimum	Maximum
4	1.7450000	0.1466288	1.5400000	1.8800000

Unique Subject Identifier=3122010111-0080

Assay variable : PTINR_VAL1 PT-INR Value

N	Mean	Standard Deviation	Minimum	Maximum
3	1.4966667	0.1101514	1.3700000	1.5700000

Unique Subject Identifier=3122010111-0084

Assay variable : PTINR_VAL1 PT-INR Value

N	Mean	Standard Deviation	Minimum	Maximum
3	1.8600000	0.1178983	1.7600000	1.9900000

Laboratory tests at the time of obtaining Table 2-6 consent, according to the presence or absence of proton-pump inhibitors

Unique Subject Identifier=3122010111-0106

MEANS Procedure

Assay variable : PTINR_VAL1 PT-INR Value

N	Mean	Standard Deviation	Minimum	Maximum
4	1.9350000	0.1126943	1.8300000	2.0600000

Unique Subject Identifier=3122010111-0126

Assay variable : PTINR_VAL1 PT-INR Value

N	Mean	Standard Deviation	Minimum	Maximum
1	1.8300000		1.8300000	1.8300000

Unique Subject Identifier=3122010111-0145

Assay variable : PTINR_VAL1 PT-INR Value

N	Mean	Standard Deviation	Minimum	Maximum
3	2.0733333	0.2757414	1.8100000	2.3600000

Unique Subject Identifier=3122010111-0148

Assay variable : PTINR_VAL1 PT-INR Value

N	Mean	Standard Deviation	Minimum	Maximum
4	2.5925000	0.6295700	2.0400000	3.3900000

Laboratory tests at the time of obtaining Table 2-6 consent, according to the presence or absence of proton-pump inhibitors

Unique Subject Identifier=3122010111-0150

MEANS Procedure

Assay variable : PTINR_VAL1 PT-INR Value

N	Mean	Standard Deviation	Minimum	Maximum
1	2.2200000		2.2200000	2.2200000

Unique Subject Identifier=3122010111-0170

Assay variable : PTINR_VAL1 PT-INR Value

N	Mean	Standard Deviation	Minimum	Maximum
1	2.0900000		2.0900000	2.0900000

Unique Subject Identifier=3122010111-0172

Assay variable : PTINR_VAL1 PT-INR Value

N	Mean	Standard Deviation	Minimum	Maximum
1	1.3200000		1.3200000	1.3200000

Unique Subject Identifier=3122010111-0173

Assay variable : PTINR_VAL1 PT-INR Value

N	Mean	Standard Deviation	Minimum	Maximum
1	2.5300000		2.5300000	2.5300000

Laboratory tests at the time of obtaining Table 2-6 consent, according to the presence or absence of proton-pump inhibitors

Unique Subject Identifier=3122010111-0176

MEANS Procedure

Assay variable : PTINR_VAL1 PT-INR Value

N	Mean	Standard Deviation	Minimum	Maximum
5	2.5260000	0.5103724	1.9300000	3.2100000

Unique Subject Identifier=3122010111-0187

Assay variable : PTINR_VAL1 PT-INR Value

N	Mean	Standard Deviation	Minimum	Maximum
1	1.6700000		1.6700000	1.6700000

Unique Subject Identifier=3122010111-0191

Assay variable : PTINR_VAL1 PT-INR Value

N	Mean	Standard Deviation	Minimum	Maximum
1	2.1400000		2.1400000	2.1400000

Unique Subject Identifier=3122010111-0201

Assay variable : PTINR_VAL1 PT-INR Value

N	Mean	Standard Deviation	Minimum	Maximum
3	2.3000000	0.0700000	2.2200000	2.3500000

Laboratory tests at the time of obtaining Table 2-6 consent, according to the presence or absence of proton-pump inhibitors

Unique Subject Identifier=3122010111-0212

MEANS Procedure

Assay variable : PTINR_VAL1 PT-INR Value

N	Mean	Standard Deviation	Minimum	Maximum
1	2.7100000		2.7100000	2.7100000

Unique Subject Identifier=3122010111-0213

Assay variable : PTINR_VAL1 PT-INR Value

N	Mean	Standard Deviation	Minimum	Maximum
4	1.5050000	0.1126943	1.3900000	1.6600000

Unique Subject Identifier=3122010111-0216

Assay variable : PTINR_VAL1 PT-INR Value

N	Mean	Standard Deviation	Minimum	Maximum
1	2.1900000		2.1900000	2.1900000

Unique Subject Identifier=3122010111-0254

Assay variable : PTINR_VAL1 PT-INR Value

N	Mean	Standard Deviation	Minimum	Maximum
1	2.5500000		2.5500000	2.5500000

Laboratory tests at the time of obtaining Table 2-6 consent, according to the presence or absence of proton-pump inhibitors

Unique Subject Identifier=3122010111-0263

MEANS Procedure

Assay variable : PTINR_VAL1 PT-INR Value

N	Mean	Standard Deviation	Minimum	Maximum
5	1.8240000	0.1383112	1.7100000	2.0600000

Unique Subject Identifier=3131010000-0012

Assay variable : PTINR_VAL1 PT-INR Value

N	Mean	Standard Deviation	Minimum	Maximum
4	2.1800000	1.7153037	1.1300000	4.7300000

Unique Subject Identifier=3131010000-0021

Assay variable : PTINR_VAL1 PT-INR Value

N	Mean	Standard Deviation	Minimum	Maximum
1	2.2300000		2.2300000	2.2300000

Unique Subject Identifier=3131010000-0044

Assay variable : PTINR_VAL1 PT-INR Value

N	Mean	Standard Deviation	Minimum	Maximum
4	1.7825000	0.4825194	1.0600000	2.0600000

Laboratory tests at the time of obtaining Table 2-6 consent, according to the presence or absence of proton-pump inhibitors

Unique Subject Identifier=3133000000-0009

MEANS Procedure

Assay variable : PTINR_VAL1 PT-INR Value

N	Mean	Standard Deviation	Minimum	Maximum
3	2.5533333	2.1909207	1.1800000	5.0800000

Unique Subject Identifier=3133000000-0010

Assay variable : PTINR_VAL1 PT-INR Value

N	Mean	Standard Deviation	Minimum	Maximum
5	1.9100000	0.3012474	1.5400000	2.3500000

Unique Subject Identifier=3133000000-0012

Assay variable : PTINR_VAL1 PT-INR Value

N	Mean	Standard Deviation	Minimum	Maximum
3	1.0800000	0.0360555	1.0500000	1.1200000

Unique Subject Identifier=3133000000-0015

Assay variable : PTINR_VAL1 PT-INR Value

N	Mean	Standard Deviation	Minimum	Maximum
4	1.9100000	0.0989949	1.7700000	1.9800000

Laboratory tests at the time of obtaining Table 2-6 consent, according to the presence or absence of proton-pump inhibitors

Unique Subject Identifier=3133000000-0022

MEANS Procedure

Assay variable : PTINR_VAL1 PT-INR Value

N	Mean	Standard Deviation	Minimum	Maximum
2	2.1000000	0.6222540	1.6600000	2.5400000

Unique Subject Identifier=3133000000-0026

Assay variable : PTINR_VAL1 PT-INR Value

N	Mean	Standard Deviation	Minimum	Maximum
3	0.9866667	0.0472582	0.9500000	1.0400000

Unique Subject Identifier=3133000000-0031

Assay variable : PTINR_VAL1 PT-INR Value

N	Mean	Standard Deviation	Minimum	Maximum
2	2.2500000	0.9899495	1.5500000	2.9500000

Unique Subject Identifier=3133000000-0032

Assay variable : PTINR_VAL1 PT-INR Value

N	Mean	Standard Deviation	Minimum	Maximum
5	2.5380000	0.7537042	1.8200000	3.6500000

Laboratory tests at the time of obtaining Table 2-6 consent, according to the presence or absence of proton-pump inhibitors

Unique Subject Identifier=3133000000-0054

MEANS Procedure

Assay variable : PTINR_VAL1 PT-INR Value

N	Mean	Standard Deviation	Minimum	Maximum
5	2.0560000	0.9046436	1.3100000	3.5900000

Unique Subject Identifier=3133000000-0061

Assay variable : PTINR_VAL1 PT-INR Value

N	Mean	Standard Deviation	Minimum	Maximum
6	1.6583333	0.2319842	1.2900000	1.8900000

Unique Subject Identifier=3133000000-0086

Assay variable : PTINR_VAL1 PT-INR Value

N	Mean	Standard Deviation	Minimum	Maximum
3	2.3833333	0.2950141	2.1700000	2.7200000

Unique Subject Identifier=3133000000-0090

Assay variable : PTINR_VAL1 PT-INR Value

N	Mean	Standard Deviation	Minimum	Maximum
4	1.2775000	0.0377492	1.2300000	1.3200000

Laboratory tests at the time of obtaining Table 2-6 consent, according to the presence or absence of proton-pump inhibitors

Unique Subject Identifier=3135000000-0009

MEANS Procedure

Assay variable : PTINR_VAL1 PT-INR Value

N	Mean	Standard Deviation	Minimum	Maximum
2	1.9400000	0.0707107	1.8900000	1.9900000

Unique Subject Identifier=3135000000-0034

Assay variable : PTINR_VAL1 PT-INR Value

N	Mean	Standard Deviation	Minimum	Maximum
2	2.0350000	0.2333452	1.8700000	2.2000000

Unique Subject Identifier=3139000000-0021

Assay variable : PTINR_VAL1 PT-INR Value

N	Mean	Standard Deviation	Minimum	Maximum
2	1.9200000	0.0282843	1.9000000	1.9400000

Unique Subject Identifier=3143000000-0001

Assay variable : PTINR_VAL1 PT-INR Value

N	Mean	Standard Deviation	Minimum	Maximum
4	1.3000000	0.1414214	1.1000000	1.4000000

Laboratory tests at the time of obtaining Table 2-6 consent, according to the presence or absence of proton-pump inhibitors

Unique Subject Identifier=3143000000-0010

MEANS Procedure

Assay variable : PTINR_VAL1 PT-INR Value

N	Mean	Standard Deviation	Minimum	Maximum
6	1.7066667	0.2299275	1.4900000	2.0900000

Unique Subject Identifier=3150000000-0002

Assay variable : PTINR_VAL1 PT-INR Value

N	Mean	Standard Deviation	Minimum	Maximum
6	2.3833333	0.6400521	1.9000000	3.5000000

Unique Subject Identifier=3150000000-0003

Assay variable : PTINR_VAL1 PT-INR Value

N	Mean	Standard Deviation	Minimum	Maximum
6	1.9833333	0.4792355	1.5000000	2.6000000

Unique Subject Identifier=3150000000-0006

Assay variable : PTINR_VAL1 PT-INR Value

N	Mean	Standard Deviation	Minimum	Maximum
6	1.8000000	0.3405877	1.2000000	2.2000000

Laboratory tests at the time of obtaining Table 2-6 consent, according to the presence or absence of proton-pump inhibitors

Unique Subject Identifier=3151000000-0001

MEANS Procedure

Assay variable : PTINR_VAL1 PT-INR Value

N	Mean	Standard Deviation	Minimum	Maximum
5	1.4560000	0.2850088	1.1100000	1.7800000

Unique Subject Identifier=3151000000-0003

Assay variable : PTINR_VAL1 PT-INR Value

N	Mean	Standard Deviation	Minimum	Maximum
6	2.2800000	0.0933809	2.1100000	2.3700000

Unique Subject Identifier=3151000000-0011

Assay variable : PTINR_VAL1 PT-INR Value

N	Mean	Standard Deviation	Minimum	Maximum
3	0.9800000	0.0100000	0.9700000	0.9900000

Unique Subject Identifier=3152011000-0019

Assay variable : PTINR_VAL1 PT-INR Value

N	Mean	Standard Deviation	Minimum	Maximum
4	1.6500000	0.1914854	1.4000000	1.8000000

Laboratory tests at the time of obtaining Table 2-6 consent, according to the presence or absence of proton-pump inhibitors

Unique Subject Identifier=3158111111-0003

MEANS Procedure

Assay variable : PTINR_VAL1 PT-INR Value

N	Mean	Standard Deviation	Minimum	Maximum
4	2.1125000	0.2077458	1.9200000	2.3800000

Unique Subject Identifier=3158111111-0006

Assay variable : PTINR_VAL1 PT-INR Value

N	Mean	Standard Deviation	Minimum	Maximum
6	2.1500000	0.4448820	1.6000000	2.7500000

Unique Subject Identifier=3158111111-0007

Assay variable : PTINR_VAL1 PT-INR Value

N	Mean	Standard Deviation	Minimum	Maximum
6	1.9750000	0.4844275	1.4500000	2.7500000

Unique Subject Identifier=3158111111-0015

Assay variable : PTINR_VAL1 PT-INR Value

N	Mean	Standard Deviation	Minimum	Maximum
4	2.0750000	0.2230844	1.9000000	2.4000000

Laboratory tests at the time of obtaining Table 2-6 consent, according to the presence or absence of proton-pump inhibitors

Unique Subject Identifier=3158111111-0019

MEANS Procedure

Assay variable : PTINR_VAL1 PT-INR Value

N	Mean	Standard Deviation	Minimum	Maximum
3	1.9700000	0.1915724	1.8400000	2.1900000

Unique Subject Identifier=3158111111-0020

Assay variable : PTINR_VAL1 PT-INR Value

N	Mean	Standard Deviation	Minimum	Maximum
6	1.3966667	0.1936664	1.2200000	1.7000000

Unique Subject Identifier=3158111111-0024

Assay variable : PTINR_VAL1 PT-INR Value

N	Mean	Standard Deviation	Minimum	Maximum
3	2.5300000	0.8201829	1.7000000	3.3400000

Unique Subject Identifier=3158111111-0027

Assay variable : PTINR_VAL1 PT-INR Value

N	Mean	Standard Deviation	Minimum	Maximum
4	1.9050000	0.3092464	1.4800000	2.2100000

Laboratory tests at the time of obtaining Table 2-6 consent, according to the presence or absence of proton-pump inhibitors

Unique Subject Identifier=3158111111-0036

MEANS Procedure

Assay variable : PTINR_VAL1 PT-INR Value

N	Mean	Standard Deviation	Minimum	Maximum
4	1.9800000	0.0752773	1.8900000	2.0600000

Unique Subject Identifier=3158111111-0042

Assay variable : PTINR_VAL1 PT-INR Value

N	Mean	Standard Deviation	Minimum	Maximum
4	2.2750000	0.1717556	2.1600000	2.5300000

Unique Subject Identifier=3158111111-0046

Assay variable : PTINR_VAL1 PT-INR Value

N	Mean	Standard Deviation	Minimum	Maximum
3	1.6600000	0.3041381	1.4600000	2.0100000

Unique Subject Identifier=3158111111-0048

Assay variable : PTINR_VAL1 PT-INR Value

N	Mean	Standard Deviation	Minimum	Maximum
4	2.0875000	0.3432565	1.7500000	2.4400000

Laboratory tests at the time of obtaining Table 2-6 consent, according to the presence or absence of proton-pump inhibitors

Unique Subject Identifier=3158111111-0050

MEANS Procedure

Assay variable : PTINR_VAL1 PT-INR Value

N	Mean	Standard Deviation	Minimum	Maximum
6	2.0600000	0.1970787	1.8400000	2.3100000

Unique Subject Identifier=3158111111-0056

Assay variable : PTINR_VAL1 PT-INR Value

N	Mean	Standard Deviation	Minimum	Maximum
2	1.1700000	0.0424264	1.1400000	1.2000000

Unique Subject Identifier=3158111111-0057

Assay variable : PTINR_VAL1 PT-INR Value

N	Mean	Standard Deviation	Minimum	Maximum
6	1.7616667	0.4397007	1.2800000	2.5100000

Unique Subject Identifier=3158111111-0060

Assay variable : PTINR_VAL1 PT-INR Value

N	Mean	Standard Deviation	Minimum	Maximum
6	2.5533333	0.3623074	2.1100000	3.0200000

Laboratory tests at the time of obtaining Table 2-6 consent, according to the presence or absence of proton-pump inhibitors

Unique Subject Identifier=3158111111-0062

MEANS Procedure

Assay variable : PTINR_VAL1 PT-INR Value

N	Mean	Standard Deviation	Minimum	Maximum
4	1.9500000	0.3721111	1.4400000	2.3000000

Unique Subject Identifier=3158111111-0064

Assay variable : PTINR_VAL1 PT-INR Value

N	Mean	Standard Deviation	Minimum	Maximum
3	2.2800000	0.0624500	2.2300000	2.3500000

Unique Subject Identifier=3158111111-0068

Assay variable : PTINR_VAL1 PT-INR Value

N	Mean	Standard Deviation	Minimum	Maximum
6	1.7716667	0.4928455	1.4300000	2.7500000

Unique Subject Identifier=3162011000-0001

Assay variable : PTINR_VAL1 PT-INR Value

N	Mean	Standard Deviation	Minimum	Maximum
4	2.1300000	0.3909817	1.6500000	2.5600000

Laboratory tests at the time of obtaining Table 2-6 consent, according to the presence or absence of proton-pump inhibitors

Unique Subject Identifier=3162011000-0003

MEANS Procedure

Assay variable : PTINR_VAL1 PT-INR Value

N	Mean	Standard Deviation	Minimum	Maximum
3	1.9200000	0.2051828	1.7200000	2.1300000

Unique Subject Identifier=3162011000-0007

Assay variable : PTINR_VAL1 PT-INR Value

N	Mean	Standard Deviation	Minimum	Maximum
1	2.0600000		2.0600000	2.0600000

Unique Subject Identifier=3162011000-0012

Assay variable : PTINR_VAL1 PT-INR Value

N	Mean	Standard Deviation	Minimum	Maximum
1	1.5000000		1.5000000	1.5000000

Unique Subject Identifier=3162011000-0018

Assay variable : PTINR_VAL1 PT-INR Value

N	Mean	Standard Deviation	Minimum	Maximum
4	1.8050000	0.3591193	1.3100000	2.1200000

Laboratory tests at the time of obtaining Table 2-6 consent, according to the presence or absence of proton-pump inhibitors

Unique Subject Identifier=3162011000-0024

MEANS Procedure

Assay variable : PTINR_VAL1 PT-INR Value

N	Mean	Standard Deviation	Minimum	Maximum
4	2.1125000	0.2831225	1.6900000	2.2900000

Unique Subject Identifier=3163011011-0002

Assay variable : PTINR_VAL1 PT-INR Value

N	Mean	Standard Deviation	Minimum	Maximum
5	1.9200000	0.7823043	1.2000000	3.2000000

Unique Subject Identifier=3164011100-0003

Assay variable : PTINR_VAL1 PT-INR Value

N	Mean	Standard Deviation	Minimum	Maximum
4	1.3925000	0.1678044	1.1500000	1.5100000

Unique Subject Identifier=3175011000-0002

Assay variable : PTINR_VAL1 PT-INR Value

N	Mean	Standard Deviation	Minimum	Maximum
4	1.7375000	0.1878608	1.5800000	2.0100000

Laboratory tests at the time of obtaining Table 2-6 consent, according to the presence or absence of proton-pump inhibitors

Unique Subject Identifier=3175011000-0005

MEANS Procedure

Assay variable : PTINR_VAL1 PT-INR Value

N	Mean	Standard Deviation	Minimum	Maximum
6	1.8583333	0.1468900	1.6500000	2.0500000

Unique Subject Identifier=3179010000-0007

Assay variable : PTINR_VAL1 PT-INR Value

N	Mean	Standard Deviation	Minimum	Maximum
3	1.5833333	0.5345403	0.9700000	1.9500000

Unique Subject Identifier=3180010000-0002

Assay variable : PTINR_VAL1 PT-INR Value

N	Mean	Standard Deviation	Minimum	Maximum
6	1.8366667	0.6839201	1.2700000	3.1600000

Unique Subject Identifier=3185010010-0018

Assay variable : PTINR_VAL1 PT-INR Value

N	Mean	Standard Deviation	Minimum	Maximum
3	1.8466667	0.2844878	1.5200000	2.0400000

Laboratory tests at the time of obtaining Table 2-6 consent, according to the presence or absence of proton-pump inhibitors

Unique Subject Identifier=3185010010-0019

MEANS Procedure

Assay variable : PTINR_VAL1 PT-INR Value

N	Mean	Standard Deviation	Minimum	Maximum
3	2.4433333	0.7543430	1.7400000	3.2400000

Unique Subject Identifier=3185010010-0020

Assay variable : PTINR_VAL1 PT-INR Value

N	Mean	Standard Deviation	Minimum	Maximum
6	1.2866667	0.0668331	1.2200000	1.4000000

Unique Subject Identifier=3185010010-0024

Assay variable : PTINR_VAL1 PT-INR Value

N	Mean	Standard Deviation	Minimum	Maximum
5	1.5640000	0.3812873	1.1900000	2.0900000

Unique Subject Identifier=3185010010-0029

Assay variable : PTINR_VAL1 PT-INR Value

N	Mean	Standard Deviation	Minimum	Maximum
6	1.5650000	0.8708329	1.0500000	3.2800000

Laboratory tests at the time of obtaining Table 2-6 consent, according to the presence or absence of proton-pump inhibitors

Unique Subject Identifier=3185010010-0030

MEANS Procedure

Assay variable : PTINR_VAL1 PT-INR Value

N	Mean	Standard Deviation	Minimum	Maximum
4	1.3100000	0.1505545	1.1300000	1.4900000

Unique Subject Identifier=3185010010-0032

Assay variable : PTINR_VAL1 PT-INR Value

N	Mean	Standard Deviation	Minimum	Maximum
4	2.5475000	0.7944547	1.6600000	3.4000000

Unique Subject Identifier=3185010010-0040

Assay variable : PTINR_VAL1 PT-INR Value

N	Mean	Standard Deviation	Minimum	Maximum
6	1.5733333	0.1688392	1.3800000	1.8200000

Unique Subject Identifier=3185010010-0042

Assay variable : PTINR_VAL1 PT-INR Value

N	Mean	Standard Deviation	Minimum	Maximum
3	2.3966667	0.7870409	1.7700000	3.2800000

Laboratory tests at the time of obtaining Table 2-6 consent, according to the presence or absence of proton-pump inhibitors

Unique Subject Identifier=3185010010-0046

MEANS Procedure

Assay variable : PTINR_VAL1 PT-INR Value

N	Mean	Standard Deviation	Minimum	Maximum
6	1.6483333	0.3326510	1.4000000	2.3000000

Unique Subject Identifier=3187000000-0008

Assay variable : PTINR_VAL1 PT-INR Value

N	Mean	Standard Deviation	Minimum	Maximum
6	1.9483333	0.3566745	1.4600000	2.2500000

Unique Subject Identifier=3190111111-0004

Assay variable : PTINR_VAL1 PT-INR Value

N	Mean	Standard Deviation	Minimum	Maximum
4	1.8375000	0.2088660	1.6800000	2.1400000

Unique Subject Identifier=3197000000-0005

Assay variable : PTINR_VAL1 PT-INR Value

N	Mean	Standard Deviation	Minimum	Maximum
6	2.3916667	0.4574458	1.8700000	3.1900000

Laboratory tests at the time of obtaining Table 2-6 consent, according to the presence or absence of proton-pump inhibitors

Unique Subject Identifier=3197000000-0017

MEANS Procedure

Assay variable : PTINR_VAL1 PT-INR Value

N	Mean	Standard Deviation	Minimum	Maximum
6	2.1116667	0.9602378	1.1900000	3.6400000

Unique Subject Identifier=3197000000-0058

Assay variable : PTINR_VAL1 PT-INR Value

N	Mean	Standard Deviation	Minimum	Maximum
6	2.2750000	0.5256900	1.6700000	3.1000000

Unique Subject Identifier=3205011100-0001

Assay variable : PTINR_VAL1 PT-INR Value

N	Mean	Standard Deviation	Minimum	Maximum
5	1.6800000	0.1095445	1.5000000	1.8000000

Unique Subject Identifier=3205011100-0019

Assay variable : PTINR_VAL1 PT-INR Value

N	Mean	Standard Deviation	Minimum	Maximum
6	1.7000000	0.1788854	1.5000000	2.0000000

Laboratory tests at the time of obtaining Table 2-6 consent, according to the presence or absence of proton-pump inhibitors

Unique Subject Identifier=3206011010-0004

MEANS Procedure

Assay variable : PTINR_VAL1 PT-INR Value

N	Mean	Standard Deviation	Minimum	Maximum
2	2.5150000	0.2192031	2.3600000	2.6700000

Unique Subject Identifier=3208000000-0006

Assay variable : PTINR_VAL1 PT-INR Value

N	Mean	Standard Deviation	Minimum	Maximum
6	1.7600000	0.5092347	0.9800000	2.3000000

Unique Subject Identifier=3214111000-0004

Assay variable : PTINR_VAL1 PT-INR Value

N	Mean	Standard Deviation	Minimum	Maximum
1	2.1100000		2.1100000	2.1100000

Unique Subject Identifier=3301010000-0013

Assay variable : PTINR_VAL1 PT-INR Value

N	Mean	Standard Deviation	Minimum	Maximum
6	1.6000000	0.1095445	1.4000000	1.7000000

Laboratory tests at the time of obtaining Table 2-6 consent, according to the presence or absence of proton-pump inhibitors

Unique Subject Identifier=3301010000-0027

MEANS Procedure

Assay variable : PTINR_VAL1 PT-INR Value

N	Mean	Standard Deviation	Minimum	Maximum
6	1.8166667	0.2228602	1.4000000	2.0000000

Unique Subject Identifier=3302010000-0023

Assay variable : PTINR_VAL1 PT-INR Value

N	Mean	Standard Deviation	Minimum	Maximum
6	1.9566667	0.3199167	1.6300000	2.5000000

Unique Subject Identifier=3304011000-0005

Assay variable : PTINR_VAL1 PT-INR Value

N	Mean	Standard Deviation	Minimum	Maximum
4	1.8000000	0.2160247	1.6000000	2.1000000

Unique Subject Identifier=3344010000-0006

Assay variable : PTINR_VAL1 PT-INR Value

N	Mean	Standard Deviation	Minimum	Maximum
1	2.1000000		2.1000000	2.1000000

Laboratory tests at the time of obtaining Table 2-6 consent, according to the presence or absence of proton-pump inhibitors

Unique Subject Identifier=3347010000-0008

MEANS Procedure

Assay variable : PTINR_VAL1 PT-INR Value

N	Mean	Standard Deviation	Minimum	Maximum
6	2.1000000	0.3577709	1.7000000	2.7000000

Unique Subject Identifier=3347010000-0017

Assay variable : PTINR_VAL1 PT-INR Value

N	Mean	Standard Deviation	Minimum	Maximum
6	1.7200000	0.2433105	1.4200000	2.1000000

Unique Subject Identifier=3347010000-0020

Assay variable : PTINR_VAL1 PT-INR Value

N	Mean	Standard Deviation	Minimum	Maximum
6	2.1083333	0.1855173	1.8000000	2.3000000

Unique Subject Identifier=3347010000-0027

Assay variable : PTINR_VAL1 PT-INR Value

N	Mean	Standard Deviation	Minimum	Maximum
6	1.5000000	0.0894427	1.4000000	1.6000000

Laboratory tests at the time of obtaining Table 2-6 consent, according to the presence or absence of proton-pump inhibitors

Unique Subject Identifier=3347010000-0039

MEANS Procedure

Assay variable : PTINR_VAL1 PT-INR Value

N	Mean	Standard Deviation	Minimum	Maximum
6	1.9050000	0.4900510	1.4000000	2.8000000

Unique Subject Identifier=3347010000-0041

Assay variable : PTINR_VAL1 PT-INR Value

N	Mean	Standard Deviation	Minimum	Maximum
6	1.8800000	0.2262742	1.6000000	2.1800000

Unique Subject Identifier=3347010000-0045

Assay variable : PTINR_VAL1 PT-INR Value

N	Mean	Standard Deviation	Minimum	Maximum
6	2.5000000	0.5796551	1.9000000	3.3000000

Unique Subject Identifier=3349010000-0003

Assay variable : PTINR_VAL1 PT-INR Value

N	Mean	Standard Deviation	Minimum	Maximum
6	1.8500000	0.1643168	1.6000000	2.0000000

Laboratory tests at the time of obtaining Table 2-6 consent, according to the presence or absence of proton-pump inhibitors

Unique Subject Identifier=3349010000-0010

MEANS Procedure

Assay variable : PTINR_VAL1 PT-INR Value

N	Mean	Standard Deviation	Minimum	Maximum
6	2.1000000	0.7694154	1.2000000	3.4000000

Unique Subject Identifier=3350010000-0002

Assay variable : PTINR_VAL1 PT-INR Value

N	Mean	Standard Deviation	Minimum	Maximum
4	2.1750000	0.4645787	1.6000000	2.6000000

Unique Subject Identifier=3350010000-0004

Assay variable : PTINR_VAL1 PT-INR Value

N	Mean	Standard Deviation	Minimum	Maximum
6	1.5833333	0.1329160	1.4000000	1.8000000

Unique Subject Identifier=3401000000-0016

Assay variable : PTINR_VAL1 PT-INR Value

N	Mean	Standard Deviation	Minimum	Maximum
5	1.8900000	0.6411708	1.3300000	2.9000000

Laboratory tests at the time of obtaining Table 2-6 consent, according to the presence or absence of proton-pump inhibitors

Unique Subject Identifier=3401000000-0017

MEANS Procedure

Assay variable : PTINR_VAL1 PT-INR Value

N	Mean	Standard Deviation	Minimum	Maximum
6	1.5116667	0.2470155	1.2700000	1.9700000

Unique Subject Identifier=3401000000-0026

Assay variable : PTINR_VAL1 PT-INR Value

N	Mean	Standard Deviation	Minimum	Maximum
4	1.9875000	0.2729316	1.7100000	2.3600000

Unique Subject Identifier=3402010011-0040

Assay variable : PTINR_VAL1 PT-INR Value

N	Mean	Standard Deviation	Minimum	Maximum
6	2.0000000	0.2097618	1.7000000	2.2000000

Unique Subject Identifier=3406010000-0008

Assay variable : PTINR_VAL1 PT-INR Value

N	Mean	Standard Deviation	Minimum	Maximum
3	1.9800000	0.9184770	1.2800000	3.0200000

Laboratory tests at the time of obtaining Table 2-6 consent, according to the presence or absence of proton-pump inhibitors

Unique Subject Identifier=3407010011-0006

MEANS Procedure

Assay variable : PTINR_VAL1 PT-INR Value

N	Mean	Standard Deviation	Minimum	Maximum
2	2.0600000	0.0282843	2.0400000	2.0800000

Unique Subject Identifier=3412000000-0005

Assay variable : PTINR_VAL1 PT-INR Value

N	Mean	Standard Deviation	Minimum	Maximum
6	2.1700000	1.4140438	1.1700000	5.0000000

Unique Subject Identifier=3415011000-0011

Assay variable : PTINR_VAL1 PT-INR Value

N	Mean	Standard Deviation	Minimum	Maximum
4	2.3175000	0.1071992	2.2300000	2.4600000

Unique Subject Identifier=3421000000-0002

Assay variable : PTINR_VAL1 PT-INR Value

N	Mean	Standard Deviation	Minimum	Maximum
6	2.1883333	0.3823829	1.7300000	2.8500000

Laboratory tests at the time of obtaining Table 2-6 consent, according to the presence or absence of proton-pump inhibitors

Unique Subject Identifier=3421000000-0019

MEANS Procedure

Assay variable : PTINR_VAL1 PT-INR Value

N	Mean	Standard Deviation	Minimum	Maximum
5	1.7980000	0.1801943	1.6100000	1.9800000

Unique Subject Identifier=3421000000-0023

Assay variable : PTINR_VAL1 PT-INR Value

N	Mean	Standard Deviation	Minimum	Maximum
3	1.7433333	0.0152753	1.7300000	1.7600000

Unique Subject Identifier=3421000000-0028

Assay variable : PTINR_VAL1 PT-INR Value

N	Mean	Standard Deviation	Minimum	Maximum
3	2.0400000	0.3576311	1.7100000	2.4200000

Unique Subject Identifier=3421000000-0030

Assay variable : PTINR_VAL1 PT-INR Value

N	Mean	Standard Deviation	Minimum	Maximum
4	2.3175000	0.2288194	2.0300000	2.5900000

Laboratory tests at the time of obtaining Table 2-6 consent, according to the presence or absence of proton-pump inhibitors

Unique Subject Identifier=3422000000-0026

MEANS Procedure

Assay variable : PTINR_VAL1 PT-INR Value

N	Mean	Standard Deviation	Minimum	Maximum
4	2.0750000	0.6184658	1.7000000	3.0000000

Unique Subject Identifier=3422000000-0046

Assay variable : PTINR_VAL1 PT-INR Value

N	Mean	Standard Deviation	Minimum	Maximum
2	2.0500000	0.2121320	1.9000000	2.2000000

Unique Subject Identifier=3422000000-0098

Assay variable : PTINR_VAL1 PT-INR Value

N	Mean	Standard Deviation	Minimum	Maximum
4	1.6750000	0.2872281	1.5000000	2.1000000

Unique Subject Identifier=3422000000-0115

Assay variable : PTINR_VAL1 PT-INR Value

N	Mean	Standard Deviation	Minimum	Maximum
4	1.7500000	0.2081666	1.5000000	2.0000000

Laboratory tests at the time of obtaining Table 2-6 consent, according to the presence or absence of proton-pump inhibitors

Unique Subject Identifier=3423000000-0021

MEANS Procedure

Assay variable : PTINR_VAL1 PT-INR Value

N	Mean	Standard Deviation	Minimum	Maximum
3	2.3600000	0.0888819	2.2600000	2.4300000

Unique Subject Identifier=3423000000-0036

Assay variable : PTINR_VAL1 PT-INR Value

N	Mean	Standard Deviation	Minimum	Maximum
2	2.0200000	0	2.0200000	2.0200000

Unique Subject Identifier=3423000000-0037

Assay variable : PTINR_VAL1 PT-INR Value

N	Mean	Standard Deviation	Minimum	Maximum
4	1.8475000	0.6827091	1.0700000	2.4600000

Unique Subject Identifier=3423000000-0041

Assay variable : PTINR_VAL1 PT-INR Value

N	Mean	Standard Deviation	Minimum	Maximum
2	1.7300000	0.3535534	1.4800000	1.9800000

Laboratory tests at the time of obtaining Table 2-6 consent, according to the presence or absence of proton-pump inhibitors

Unique Subject Identifier=3424000000-0035

MEANS Procedure

Assay variable : PTINR_VAL1 PT-INR Value

N	Mean	Standard Deviation	Minimum	Maximum
4	2.3150000	0.1968925	2.2100000	2.6100000

Unique Subject Identifier=3424000000-0036

Assay variable : PTINR_VAL1 PT-INR Value

N	Mean	Standard Deviation	Minimum	Maximum
6	1.9500000	0.4419502	1.4000000	2.7300000

Unique Subject Identifier=3424000000-0038

Assay variable : PTINR_VAL1 PT-INR Value

N	Mean	Standard Deviation	Minimum	Maximum
3	1.7366667	0.1222020	1.6300000	1.8700000

Unique Subject Identifier=3424000000-0040

Assay variable : PTINR_VAL1 PT-INR Value

N	Mean	Standard Deviation	Minimum	Maximum
3	2.3266667	0.3850108	1.9400000	2.7100000

Laboratory tests at the time of obtaining Table 2-6 consent, according to the presence or absence of proton-pump inhibitors

Unique Subject Identifier=3424000000-0042

MEANS Procedure

Assay variable : PTINR_VAL1 PT-INR Value

N	Mean	Standard Deviation	Minimum	Maximum
5	2.6560000	0.6932027	1.8700000	3.5200000

Unique Subject Identifier=3424000000-0046

Assay variable : PTINR_VAL1 PT-INR Value

N	Mean	Standard Deviation	Minimum	Maximum
3	2.4666667	0.8658137	1.5800000	3.3100000

Unique Subject Identifier=3424000000-0058

Assay variable : PTINR_VAL1 PT-INR Value

N	Mean	Standard Deviation	Minimum	Maximum
2	2.3650000	1.4919953	1.3100000	3.4200000

Unique Subject Identifier=3424000000-0060

Assay variable : PTINR_VAL1 PT-INR Value

N	Mean	Standard Deviation	Minimum	Maximum
4	2.2725000	0.6992079	1.5900000	3.1600000

Laboratory tests at the time of obtaining Table 2-6 consent, according to the presence or absence of proton-pump inhibitors

Unique Subject Identifier=3425001000-0006

MEANS Procedure

Assay variable : PTINR_VAL1 PT-INR Value

N	Mean	Standard Deviation	Minimum	Maximum
3	1.8233333	0.0115470	1.8100000	1.8300000

Unique Subject Identifier=3426100000-0008

Assay variable : PTINR_VAL1 PT-INR Value

N	Mean	Standard Deviation	Minimum	Maximum
3	1.8866667	0.7471501	1.3500000	2.7400000

Unique Subject Identifier=3426100000-0031

Assay variable : PTINR_VAL1 PT-INR Value

N	Mean	Standard Deviation	Minimum	Maximum
5	2.6220000	0.3401029	2.0700000	2.9900000

Unique Subject Identifier=3426100000-0033

Assay variable : PTINR_VAL1 PT-INR Value

N	Mean	Standard Deviation	Minimum	Maximum
1	0.9900000		0.9900000	0.9900000

Laboratory tests at the time of obtaining Table 2-6 consent, according to the presence or absence of proton-pump inhibitors

Unique Subject Identifier=3426100000-0036

MEANS Procedure

Assay variable : PTINR_VAL1 PT-INR Value

N	Mean	Standard Deviation	Minimum	Maximum
1	1.5800000		1.5800000	1.5800000

Unique Subject Identifier=3428000000-0002

Assay variable : PTINR_VAL1 PT-INR Value

N	Mean	Standard Deviation	Minimum	Maximum
6	2.0283333	0.3252947	1.6300000	2.5000000

Unique Subject Identifier=3443011001-0004

Assay variable : PTINR_VAL1 PT-INR Value

N	Mean	Standard Deviation	Minimum	Maximum
6	2.0316667	0.4054833	1.5800000	2.5500000

Unique Subject Identifier=3443011001-0011

Assay variable : PTINR_VAL1 PT-INR Value

N	Mean	Standard Deviation	Minimum	Maximum
6	2.2616667	0.2148876	1.8600000	2.4400000

Laboratory tests at the time of obtaining Table 2-6 consent, according to the presence or absence of proton-pump inhibitors

Unique Subject Identifier=3444000000-0006

MEANS Procedure

Assay variable : PTINR_VAL1 PT-INR Value

N	Mean	Standard Deviation	Minimum	Maximum
3	2.1733333	0.1755942	1.9900000	2.3400000

Unique Subject Identifier=3444000000-0009

Assay variable : PTINR_VAL1 PT-INR Value

N	Mean	Standard Deviation	Minimum	Maximum
4	2.2650000	0.3875994	1.7300000	2.5700000

Unique Subject Identifier=3444000000-0010

Assay variable : PTINR_VAL1 PT-INR Value

N	Mean	Standard Deviation	Minimum	Maximum
4	2.3450000	0.1586401	2.1800000	2.5400000

Unique Subject Identifier=3444000000-0025

Assay variable : PTINR_VAL1 PT-INR Value

N	Mean	Standard Deviation	Minimum	Maximum
4	2.2175000	0.2156193	1.9100000	2.4100000

Laboratory tests at the time of obtaining Table 2-6 consent, according to the presence or absence of proton-pump inhibitors

Unique Subject Identifier=3444000000-0030

MEANS Procedure

Assay variable : PTINR_VAL1 PT-INR Value

N	Mean	Standard Deviation	Minimum	Maximum
3	1.8300000	0.2251666	1.6100000	2.0600000

Unique Subject Identifier=3444000000-0032

Assay variable : PTINR_VAL1 PT-INR Value

N	Mean	Standard Deviation	Minimum	Maximum
6	2.6150000	0.5350421	1.6100000	3.1400000

Unique Subject Identifier=3444000000-0036

Assay variable : PTINR_VAL1 PT-INR Value

N	Mean	Standard Deviation	Minimum	Maximum
3	1.6233333	0.0288675	1.5900000	1.6400000

Unique Subject Identifier=3444000000-0038

Assay variable : PTINR_VAL1 PT-INR Value

N	Mean	Standard Deviation	Minimum	Maximum
3	1.7700000	0.0556776	1.7100000	1.8200000

Laboratory tests at the time of obtaining Table 2-6 consent, according to the presence or absence of proton-pump inhibitors

Unique Subject Identifier=3444000000-0041

MEANS Procedure

Assay variable : PTINR_VAL1 PT-INR Value

N	Mean	Standard Deviation	Minimum	Maximum
5	2.3560000	0.1558204	2.1300000	2.5100000

Unique Subject Identifier=3444000000-0043

Assay variable : PTINR_VAL1 PT-INR Value

N	Mean	Standard Deviation	Minimum	Maximum
6	2.4000000	0.2827720	1.9600000	2.6500000

Unique Subject Identifier=3444000000-0046

Assay variable : PTINR_VAL1 PT-INR Value

N	Mean	Standard Deviation	Minimum	Maximum
4	1.8350000	0.0810350	1.7200000	1.9100000

Unique Subject Identifier=3444000000-0050

Assay variable : PTINR_VAL1 PT-INR Value

N	Mean	Standard Deviation	Minimum	Maximum
4	1.9400000	0.4112582	1.3900000	2.2600000

Laboratory tests at the time of obtaining Table 2-6 consent, according to the presence or absence of proton-pump inhibitors

Unique Subject Identifier=3444000000-0051

MEANS Procedure

Assay variable : PTINR_VAL1 PT-INR Value

N	Mean	Standard Deviation	Minimum	Maximum
4	2.4825000	0.1286792	2.2900000	2.5600000

Unique Subject Identifier=3444000000-0052

Assay variable : PTINR_VAL1 PT-INR Value

N	Mean	Standard Deviation	Minimum	Maximum
4	1.7050000	0.1517674	1.5800000	1.9200000

Unique Subject Identifier=3444000000-0054

Assay variable : PTINR_VAL1 PT-INR Value

N	Mean	Standard Deviation	Minimum	Maximum
5	2.0200000	0.1488288	1.8800000	2.1900000

Unique Subject Identifier=3444000000-0063

Assay variable : PTINR_VAL1 PT-INR Value

N	Mean	Standard Deviation	Minimum	Maximum
3	1.9566667	0.1446836	1.7900000	2.0500000

Laboratory tests at the time of obtaining Table 2-6 consent, according to the presence or absence of proton-pump inhibitors

Unique Subject Identifier=3444000000-0064

MEANS Procedure

Assay variable : PTINR_VAL1 PT-INR Value

N	Mean	Standard Deviation	Minimum	Maximum
3	2.0033333	0.1803700	1.8300000	2.1900000

Unique Subject Identifier=3444000000-0066

Assay variable : PTINR_VAL1 PT-INR Value

N	Mean	Standard Deviation	Minimum	Maximum
3	1.6233333	0.0057735	1.6200000	1.6300000

Unique Subject Identifier=3444000000-0067

Assay variable : PTINR_VAL1 PT-INR Value

N	Mean	Standard Deviation	Minimum	Maximum
3	2.0766667	0.3900427	1.6900000	2.4700000

Unique Subject Identifier=3444000000-0071

Assay variable : PTINR_VAL1 PT-INR Value

N	Mean	Standard Deviation	Minimum	Maximum
3	1.8800000	0.2600000	1.5800000	2.0400000

Laboratory tests at the time of obtaining Table 2-6 consent, according to the presence or absence of proton-pump inhibitors

Unique Subject Identifier=3444000000-0072

MEANS Procedure

Assay variable : PTINR_VAL1 PT-INR Value

N	Mean	Standard Deviation	Minimum	Maximum
4	1.6925000	0.1664081	1.4800000	1.8800000

Unique Subject Identifier=3444000000-0073

Assay variable : PTINR_VAL1 PT-INR Value

N	Mean	Standard Deviation	Minimum	Maximum
3	1.8633333	0.1677299	1.6700000	1.9700000

Unique Subject Identifier=3444000000-0075

Assay variable : PTINR_VAL1 PT-INR Value

N	Mean	Standard Deviation	Minimum	Maximum
4	2.0800000	0.6319810	1.6200000	3.0100000

Unique Subject Identifier=3444000000-0083

Assay variable : PTINR_VAL1 PT-INR Value

N	Mean	Standard Deviation	Minimum	Maximum
3	2.2700000	0.3477068	1.9000000	2.5900000

Laboratory tests at the time of obtaining Table 2-6 consent, according to the presence or absence of proton-pump inhibitors

Unique Subject Identifier=3444000000-0086

MEANS Procedure

Assay variable : PTINR_VAL1 PT-INR Value

N	Mean	Standard Deviation	Minimum	Maximum
4	2.6000000	0.1230176	2.4600000	2.7600000

Unique Subject Identifier=3452000000-0004

Assay variable : PTINR_VAL1 PT-INR Value

N	Mean	Standard Deviation	Minimum	Maximum
4	2.1925000	0.2632331	1.9600000	2.5200000

Unique Subject Identifier=3452000000-0005

Assay variable : PTINR_VAL1 PT-INR Value

N	Mean	Standard Deviation	Minimum	Maximum
5	2.1920000	0.2230919	1.8700000	2.3800000

Unique Subject Identifier=3452000000-0006

Assay variable : PTINR_VAL1 PT-INR Value

N	Mean	Standard Deviation	Minimum	Maximum
4	2.2675000	0.4836235	1.7800000	2.9100000

Laboratory tests at the time of obtaining Table 2-6 consent, according to the presence or absence of proton-pump inhibitors

Unique Subject Identifier=3452000000-0008

MEANS Procedure

Assay variable : PTINR_VAL1 PT-INR Value

N	Mean	Standard Deviation	Minimum	Maximum
4	1.7400000	0.4661902	1.2800000	2.2600000

Unique Subject Identifier=3462011111-0002

Assay variable : PTINR_VAL1 PT-INR Value

N	Mean	Standard Deviation	Minimum	Maximum
4	2.0175000	0.0946485	1.9100000	2.1400000

Unique Subject Identifier=3462011111-0011

Assay variable : PTINR_VAL1 PT-INR Value

N	Mean	Standard Deviation	Minimum	Maximum
6	1.9033333	0.1689576	1.7200000	2.1100000

Unique Subject Identifier=3462011111-0023

Assay variable : PTINR_VAL1 PT-INR Value

N	Mean	Standard Deviation	Minimum	Maximum
6	2.1783333	0.3364174	1.9000000	2.7700000

Laboratory tests at the time of obtaining Table 2-6 consent, according to the presence or absence of proton-pump inhibitors

Unique Subject Identifier=3472000000-0013

MEANS Procedure

Assay variable : PTINR_VAL1 PT-INR Value

N	Mean	Standard Deviation	Minimum	Maximum
1	2.3600000		2.3600000	2.3600000

Unique Subject Identifier=3481010111-0004

Assay variable : PTINR_VAL1 PT-INR Value

N	Mean	Standard Deviation	Minimum	Maximum
4	1.8000000	0.0816497	1.7000000	1.9000000

Unique Subject Identifier=3481010111-0014

Assay variable : PTINR_VAL1 PT-INR Value

N	Mean	Standard Deviation	Minimum	Maximum
3	1.1666667	0.0577350	1.1000000	1.2000000

Unique Subject Identifier=3491001000-0032

Assay variable : PTINR_VAL1 PT-INR Value

N	Mean	Standard Deviation	Minimum	Maximum
1	2.2200000		2.2200000	2.2200000

Laboratory tests at the time of obtaining Table 2-6 consent, according to the presence or absence of proton-pump inhibitors

Unique Subject Identifier=3501111111-0012

MEANS Procedure

Assay variable : PTINR_VAL1 PT-INR Value

N	Mean	Standard Deviation	Minimum	Maximum
2	1.9600000	0.1131371	1.8800000	2.0400000

Unique Subject Identifier=5001010000-0033

Assay variable : PTINR_VAL1 PT-INR Value

N	Mean	Standard Deviation	Minimum	Maximum
4	1.8225000	0.1948290	1.5400000	1.9700000

Unique Subject Identifier=5002010010-0002

Assay variable : PTINR_VAL1 PT-INR Value

N	Mean	Standard Deviation	Minimum	Maximum
5	1.8800000	0.1519868	1.6300000	2.0300000

Unique Subject Identifier=5002010010-0008

Assay variable : PTINR_VAL1 PT-INR Value

N	Mean	Standard Deviation	Minimum	Maximum
4	2.6300000	0.4993996	2.0800000	3.2200000

Laboratory tests at the time of obtaining Table 2-6 consent, according to the presence or absence of proton-pump inhibitors

Unique Subject Identifier=5002010010-0015

MEANS Procedure

Assay variable : PTINR_VAL1 PT-INR Value

N	Mean	Standard Deviation	Minimum	Maximum
2	1.8750000	0.1909188	1.7400000	2.0100000

Unique Subject Identifier=5002010010-0016

Assay variable : PTINR_VAL1 PT-INR Value

N	Mean	Standard Deviation	Minimum	Maximum
3	1.7700000	0.0781025	1.7200000	1.8600000

Unique Subject Identifier=5002010010-0030

Assay variable : PTINR_VAL1 PT-INR Value

N	Mean	Standard Deviation	Minimum	Maximum
6	2.4650000	0.1486943	2.2800000	2.6900000

Unique Subject Identifier=5002010010-0037

Assay variable : PTINR_VAL1 PT-INR Value

N	Mean	Standard Deviation	Minimum	Maximum
3	2.3233333	0.3696395	2.1000000	2.7500000

Laboratory tests at the time of obtaining Table 2-6 consent, according to the presence or absence of proton-pump inhibitors

Unique Subject Identifier=5002010010-0045

MEANS Procedure

Assay variable : PTINR_VAL1 PT-INR Value

N	Mean	Standard Deviation	Minimum	Maximum
6	1.8216667	0.3260930	1.5300000	2.4200000

Unique Subject Identifier=5002010010-0050

Assay variable : PTINR_VAL1 PT-INR Value

N	Mean	Standard Deviation	Minimum	Maximum
5	2.2620000	0.4080686	1.8400000	2.9300000

Unique Subject Identifier=5002010010-0070

Assay variable : PTINR_VAL1 PT-INR Value

N	Mean	Standard Deviation	Minimum	Maximum
4	2.1350000	0.3702702	1.8600000	2.6600000

Unique Subject Identifier=5002010010-0074

Assay variable : PTINR_VAL1 PT-INR Value

N	Mean	Standard Deviation	Minimum	Maximum
3	1.8133333	0.1814754	1.6200000	1.9800000

Laboratory tests at the time of obtaining Table 2-6 consent, according to the presence or absence of proton-pump inhibitors

Unique Subject Identifier=5002010010-0079

MEANS Procedure

Assay variable : PTINR_VAL1 PT-INR Value

N	Mean	Standard Deviation	Minimum	Maximum
3	1.9600000	0.1081665	1.8400000	2.0500000

Unique Subject Identifier=5002010010-0080

Assay variable : PTINR_VAL1 PT-INR Value

N	Mean	Standard Deviation	Minimum	Maximum
2	1.9150000	0.0494975	1.8800000	1.9500000

Unique Subject Identifier=5002010010-0091

Assay variable : PTINR_VAL1 PT-INR Value

N	Mean	Standard Deviation	Minimum	Maximum
3	1.7033333	0.0230940	1.6900000	1.7300000

Unique Subject Identifier=5004000111-0024

Assay variable : PTINR_VAL1 PT-INR Value

N	Mean	Standard Deviation	Minimum	Maximum
2	1.9250000	0.0494975	1.8900000	1.9600000

Laboratory tests at the time of obtaining Table 2-6 consent, according to the presence or absence of proton-pump inhibitors

Unique Subject Identifier=5004000111-0033

MEANS Procedure

Assay variable : PTINR_VAL1 PT-INR Value

N	Mean	Standard Deviation	Minimum	Maximum
3	2.4300000	0.2165641	2.1800000	2.5600000

Unique Subject Identifier=5004000111-0037

Assay variable : PTINR_VAL1 PT-INR Value

N	Mean	Standard Deviation	Minimum	Maximum
4	2.4375000	0.1645955	2.2000000	2.5700000

Unique Subject Identifier=5004000111-0059

Assay variable : PTINR_VAL1 PT-INR Value

N	Mean	Standard Deviation	Minimum	Maximum
2	2.1600000	0.1979899	2.0200000	2.3000000

Unique Subject Identifier=5004000111-0062

Assay variable : PTINR_VAL1 PT-INR Value

N	Mean	Standard Deviation	Minimum	Maximum
2	2.1250000	0.4737615	1.7900000	2.4600000

Laboratory tests at the time of obtaining Table 2-6 consent, according to the presence or absence of proton-pump inhibitors

Unique Subject Identifier=5004000111-0063

MEANS Procedure

Assay variable : PTINR_VAL1 PT-INR Value

N	Mean	Standard Deviation	Minimum	Maximum
3	2.2166667	0.1882374	2.0000000	2.3400000

Unique Subject Identifier=5004000111-0073

Assay variable : PTINR_VAL1 PT-INR Value

N	Mean	Standard Deviation	Minimum	Maximum
4	2.0550000	0.4651523	1.6200000	2.6800000

Unique Subject Identifier=5004000111-0076

Assay variable : PTINR_VAL1 PT-INR Value

N	Mean	Standard Deviation	Minimum	Maximum
4	2.4300000	0.6195159	2.0000000	3.3500000

Unique Subject Identifier=5004000111-0079

Assay variable : PTINR_VAL1 PT-INR Value

N	Mean	Standard Deviation	Minimum	Maximum
5	1.4820000	0.1173456	1.2900000	1.6100000

Laboratory tests at the time of obtaining Table 2-6 consent, according to the presence or absence of proton-pump inhibitors

Unique Subject Identifier=5007111000-0024

MEANS Procedure

Assay variable : PTINR_VAL1 PT-INR Value

N	Mean	Standard Deviation	Minimum	Maximum
3	2.1866667	0.1331666	2.0400000	2.3000000

Unique Subject Identifier=5007111000-0025

Assay variable : PTINR_VAL1 PT-INR Value

N	Mean	Standard Deviation	Minimum	Maximum
3	2.2133333	0.3153305	1.8900000	2.5200000

Unique Subject Identifier=5007111000-0030

Assay variable : PTINR_VAL1 PT-INR Value

N	Mean	Standard Deviation	Minimum	Maximum
3	1.7200000	0.3966106	1.3900000	2.1600000

Unique Subject Identifier=5007111000-0036

Assay variable : PTINR_VAL1 PT-INR Value

N	Mean	Standard Deviation	Minimum	Maximum
6	1.9566667	0.3189148	1.5900000	2.4400000

Laboratory tests at the time of obtaining Table 2-6 consent, according to the presence or absence of proton-pump inhibitors

Unique Subject Identifier=5007111000-0060

MEANS Procedure

Assay variable : PTINR_VAL1 PT-INR Value

N	Mean	Standard Deviation	Minimum	Maximum
4	1.7900000	0.1512173	1.5700000	1.9100000

Unique Subject Identifier=5009000010-0010

Assay variable : PTINR_VAL1 PT-INR Value

N	Mean	Standard Deviation	Minimum	Maximum
6	1.9866667	0.2273910	1.7700000	2.3800000

Unique Subject Identifier=5010000100-0005

Assay variable : PTINR_VAL1 PT-INR Value

N	Mean	Standard Deviation	Minimum	Maximum
2	2.0550000	0.2899138	1.8500000	2.2600000

Unique Subject Identifier=5010000100-0011

Assay variable : PTINR_VAL1 PT-INR Value

N	Mean	Standard Deviation	Minimum	Maximum
3	1.7133333	0.4266536	1.2500000	2.0900000

Laboratory tests at the time of obtaining Table 2-6 consent, according to the presence or absence of proton-pump inhibitors

Unique Subject Identifier=5010000100-0026

MEANS Procedure

Assay variable : PTINR_VAL1 PT-INR Value

N	Mean	Standard Deviation	Minimum	Maximum
1	2.0400000		2.0400000	2.0400000

Unique Subject Identifier=5010000100-0038

Assay variable : PTINR_VAL1 PT-INR Value

N	Mean	Standard Deviation	Minimum	Maximum
6	2.1550000	0.7552682	1.4500000	3.2200000

Unique Subject Identifier=5010000100-0041

Assay variable : PTINR_VAL1 PT-INR Value

N	Mean	Standard Deviation	Minimum	Maximum
3	2.1866667	0.2182506	1.9500000	2.3800000

Unique Subject Identifier=5011010000-0022

Assay variable : PTINR_VAL1 PT-INR Value

N	Mean	Standard Deviation	Minimum	Maximum
3	2.2466667	0.0757188	2.1600000	2.3000000

Laboratory tests at the time of obtaining Table 2-6 consent, according to the presence or absence of proton-pump inhibitors

Unique Subject Identifier=5011010000-0035

MEANS Procedure

Assay variable : PTINR_VAL1 PT-INR Value

N	Mean	Standard Deviation	Minimum	Maximum
5	2.1540000	0.1908664	1.9900000	2.4800000

Unique Subject Identifier=5011010000-0040

Assay variable : PTINR_VAL1 PT-INR Value

N	Mean	Standard Deviation	Minimum	Maximum
3	2.0000000	0.0700000	1.9200000	2.0500000

Unique Subject Identifier=5011010000-0048

Assay variable : PTINR_VAL1 PT-INR Value

N	Mean	Standard Deviation	Minimum	Maximum
4	2.4225000	0.3162673	2.0000000	2.7600000

Unique Subject Identifier=5011010000-0049

Assay variable : PTINR_VAL1 PT-INR Value

N	Mean	Standard Deviation	Minimum	Maximum
4	1.6875000	0.2389386	1.4700000	1.9700000

Laboratory tests at the time of obtaining Table 2-6 consent, according to the presence or absence of proton-pump inhibitors

Unique Subject Identifier=5011010000-0052

MEANS Procedure

Assay variable : PTINR_VAL1 PT-INR Value

N	Mean	Standard Deviation	Minimum	Maximum
3	2.4233333	0.3372437	2.1900000	2.8100000

Unique Subject Identifier=5012000000-0004

Assay variable : PTINR_VAL1 PT-INR Value

N	Mean	Standard Deviation	Minimum	Maximum
5	1.6800000	0.0961769	1.5400000	1.7700000

Unique Subject Identifier=5012000000-0016

Assay variable : PTINR_VAL1 PT-INR Value

N	Mean	Standard Deviation	Minimum	Maximum
4	2.0800000	0.1505545	1.9000000	2.2400000

Unique Subject Identifier=5012000000-0020

Assay variable : PTINR_VAL1 PT-INR Value

N	Mean	Standard Deviation	Minimum	Maximum
4	1.5025000	0.0457347	1.4400000	1.5500000

Laboratory tests at the time of obtaining Table 2-6 consent, according to the presence or absence of proton-pump inhibitors

Unique Subject Identifier=5013000000-0016

MEANS Procedure

Assay variable : PTINR_VAL1 PT-INR Value

N	Mean	Standard Deviation	Minimum	Maximum
6	1.8733333	0.2174090	1.5400000	2.1000000

Unique Subject Identifier=5013000000-0019

Assay variable : PTINR_VAL1 PT-INR Value

N	Mean	Standard Deviation	Minimum	Maximum
3	1.7566667	0.0416333	1.7100000	1.7900000

Unique Subject Identifier=5013000000-0032

Assay variable : PTINR_VAL1 PT-INR Value

N	Mean	Standard Deviation	Minimum	Maximum
4	2.0975000	0.1286792	1.9800000	2.2800000

Unique Subject Identifier=5013000000-0040

Assay variable : PTINR_VAL1 PT-INR Value

N	Mean	Standard Deviation	Minimum	Maximum
6	1.6983333	0.3235686	1.1400000	2.0000000

Laboratory tests at the time of obtaining Table 2-6 consent, according to the presence or absence of proton-pump inhibitors

Unique Subject Identifier=5013000000-0045

MEANS Procedure

Assay variable : PTINR_VAL1 PT-INR Value

N	Mean	Standard Deviation	Minimum	Maximum
3	2.0633333	0.2891943	1.7500000	2.3200000

Unique Subject Identifier=5013000000-0051

Assay variable : PTINR_VAL1 PT-INR Value

N	Mean	Standard Deviation	Minimum	Maximum
3	2.2133333	0.2309401	2.0800000	2.4800000

Unique Subject Identifier=5013000000-0055

Assay variable : PTINR_VAL1 PT-INR Value

N	Mean	Standard Deviation	Minimum	Maximum
4	1.7875000	0.3181588	1.3600000	2.0300000

Unique Subject Identifier=5013000000-0071

Assay variable : PTINR_VAL1 PT-INR Value

N	Mean	Standard Deviation	Minimum	Maximum
1	2.5300000		2.5300000	2.5300000

Laboratory tests at the time of obtaining Table 2-6 consent, according to the presence or absence of proton-pump inhibitors

Unique Subject Identifier=5013000000-0099

MEANS Procedure

Assay variable : PTINR_VAL1 PT-INR Value

N	Mean	Standard Deviation	Minimum	Maximum
6	2.0050000	0.2776869	1.5000000	2.3100000

Unique Subject Identifier=5013000000-0102

Assay variable : PTINR_VAL1 PT-INR Value

N	Mean	Standard Deviation	Minimum	Maximum
2	2.2250000	0.1060660	2.1500000	2.3000000

Unique Subject Identifier=5013000000-0106

Assay variable : PTINR_VAL1 PT-INR Value

N	Mean	Standard Deviation	Minimum	Maximum
6	1.5150000	0.2665896	1.2700000	1.9600000

Unique Subject Identifier=5013000000-0120

Assay variable : PTINR_VAL1 PT-INR Value

N	Mean	Standard Deviation	Minimum	Maximum
3	1.6633333	0.1855622	1.4700000	1.8400000

Laboratory tests at the time of obtaining Table 2-6 consent, according to the presence or absence of proton-pump inhibitors

Unique Subject Identifier=5013000000-0126

MEANS Procedure

Assay variable : PTINR_VAL1 PT-INR Value

N	Mean	Standard Deviation	Minimum	Maximum
3	2.7233333	0.1942507	2.5100000	2.8900000

Unique Subject Identifier=5013000000-0142

Assay variable : PTINR_VAL1 PT-INR Value

N	Mean	Standard Deviation	Minimum	Maximum
3	1.8033333	0.2023199	1.5700000	1.9300000

Unique Subject Identifier=5013000000-0157

Assay variable : PTINR_VAL1 PT-INR Value

N	Mean	Standard Deviation	Minimum	Maximum
6	1.7683333	0.0968332	1.6900000	1.9100000

Unique Subject Identifier=5013000000-0174

Assay variable : PTINR_VAL1 PT-INR Value

N	Mean	Standard Deviation	Minimum	Maximum
2	1.6700000	0.5091169	1.3100000	2.0300000

Laboratory tests at the time of obtaining Table 2-6 consent, according to the presence or absence of proton-pump inhibitors

Unique Subject Identifier=5013000000-0176

MEANS Procedure

Assay variable : PTINR_VAL1 PT-INR Value

N	Mean	Standard Deviation	Minimum	Maximum
3	2.1200000	0.1135782	2.0400000	2.2500000

Unique Subject Identifier=5013000000-0203

Assay variable : PTINR_VAL1 PT-INR Value

N	Mean	Standard Deviation	Minimum	Maximum
4	2.0350000	0.4690771	1.7000000	2.7000000

Unique Subject Identifier=5013000000-0204

Assay variable : PTINR_VAL1 PT-INR Value

N	Mean	Standard Deviation	Minimum	Maximum
4	2.0300000	0.0778888	1.9300000	2.1200000

Unique Subject Identifier=5013000000-0205

Assay variable : PTINR_VAL1 PT-INR Value

N	Mean	Standard Deviation	Minimum	Maximum
1	1.9600000		1.9600000	1.9600000

Laboratory tests at the time of obtaining Table 2-6 consent, according to the presence or absence of proton-pump inhibitors

Unique Subject Identifier=5013000000-0209

MEANS Procedure

Assay variable : PTINR_VAL1 PT-INR Value

N	Mean	Standard Deviation	Minimum	Maximum
3	1.9533333	0.1890326	1.7400000	2.1000000

Unique Subject Identifier=5013000000-0227

Assay variable : PTINR_VAL1 PT-INR Value

N	Mean	Standard Deviation	Minimum	Maximum
4	2.3125000	0.7481254	1.6600000	3.2900000

Unique Subject Identifier=5013000000-0261

Assay variable : PTINR_VAL1 PT-INR Value

N	Mean	Standard Deviation	Minimum	Maximum
3	1.9133333	0.3685557	1.6300000	2.3300000

Unique Subject Identifier=5013000000-0263

Assay variable : PTINR_VAL1 PT-INR Value

N	Mean	Standard Deviation	Minimum	Maximum
6	2.5300000	0.5399630	1.7400000	3.3300000

Laboratory tests at the time of obtaining Table 2-6 consent, according to the presence or absence of proton-pump inhibitors

Unique Subject Identifier=5013000000-0282

MEANS Procedure

Assay variable : PTINR_VAL1 PT-INR Value

N	Mean	Standard Deviation	Minimum	Maximum
5	1.8660000	0.6208704	1.3200000	2.8600000

Unique Subject Identifier=5013000000-0301

Assay variable : PTINR_VAL1 PT-INR Value

N	Mean	Standard Deviation	Minimum	Maximum
4	1.7025000	0.3012612	1.3900000	2.1100000

Unique Subject Identifier=5013000000-0312

Assay variable : PTINR_VAL1 PT-INR Value

N	Mean	Standard Deviation	Minimum	Maximum
3	2.1366667	0.1497776	1.9700000	2.2600000

Unique Subject Identifier=5013000000-0338

Assay variable : PTINR_VAL1 PT-INR Value

N	Mean	Standard Deviation	Minimum	Maximum
3	2.3600000	0.3508561	1.9700000	2.6500000

Laboratory tests at the time of obtaining Table 2-6 consent, according to the presence or absence of proton-pump inhibitors

Unique Subject Identifier=5013000000-0345

MEANS Procedure

Assay variable : PTINR_VAL1 PT-INR Value

N	Mean	Standard Deviation	Minimum	Maximum
6	2.0850000	1.1294556	0.9500000	3.9600000

Unique Subject Identifier=5013000000-0356

Assay variable : PTINR_VAL1 PT-INR Value

N	Mean	Standard Deviation	Minimum	Maximum
5	2.0840000	1.1571646	1.1400000	3.8200000

Unique Subject Identifier=5013000000-0366

Assay variable : PTINR_VAL1 PT-INR Value

N	Mean	Standard Deviation	Minimum	Maximum
3	1.8333333	0.0950438	1.7400000	1.9300000

Unique Subject Identifier=5013000000-0372

Assay variable : PTINR_VAL1 PT-INR Value

N	Mean	Standard Deviation	Minimum	Maximum
6	1.4833333	0.1162182	1.3100000	1.6500000

Laboratory tests at the time of obtaining Table 2-6 consent, according to the presence or absence of proton-pump inhibitors

Unique Subject Identifier=5013000000-0374

MEANS Procedure

Assay variable : PTINR_VAL1 PT-INR Value

N	Mean	Standard Deviation	Minimum	Maximum
3	2.5533333	0.1692139	2.4100000	2.7400000

Unique Subject Identifier=5013000000-0377

Assay variable : PTINR_VAL1 PT-INR Value

N	Mean	Standard Deviation	Minimum	Maximum
2	1.7450000	0.0494975	1.7100000	1.7800000

Unique Subject Identifier=5013000000-0379

Assay variable : PTINR_VAL1 PT-INR Value

N	Mean	Standard Deviation	Minimum	Maximum
6	2.2350000	0.9610567	1.3700000	3.7700000

Unique Subject Identifier=5013000000-0394

Assay variable : PTINR_VAL1 PT-INR Value

N	Mean	Standard Deviation	Minimum	Maximum
3	1.6366667	0.0814453	1.5800000	1.7300000

Laboratory tests at the time of obtaining Table 2-6 consent, according to the presence or absence of proton-pump inhibitors

Unique Subject Identifier=5013000000-0419

MEANS Procedure

Assay variable : PTINR_VAL1 PT-INR Value

N	Mean	Standard Deviation	Minimum	Maximum
2	2.0100000	0.1131371	1.9300000	2.0900000

Unique Subject Identifier=5013000000-0476

Assay variable : PTINR_VAL1 PT-INR Value

N	Mean	Standard Deviation	Minimum	Maximum
1	1.8200000		1.8200000	1.8200000

Unique Subject Identifier=5013000000-0486

Assay variable : PTINR_VAL1 PT-INR Value

N	Mean	Standard Deviation	Minimum	Maximum
1	2.9500000		2.9500000	2.9500000

Unique Subject Identifier=5013000000-0493

Assay variable : PTINR_VAL1 PT-INR Value

N	Mean	Standard Deviation	Minimum	Maximum
2	1.8400000	0.1555635	1.7300000	1.9500000

Laboratory tests at the time of obtaining Table 2-6 consent, according to the presence or absence of proton-pump inhibitors

Unique Subject Identifier=5013000000-0506

MEANS Procedure

Assay variable : PTINR_VAL1 PT-INR Value

N	Mean	Standard Deviation	Minimum	Maximum
3	2.1766667	0.4821134	1.8000000	2.7200000

Unique Subject Identifier=5013000000-0543

Assay variable : PTINR_VAL1 PT-INR Value

N	Mean	Standard Deviation	Minimum	Maximum
1	1.4800000		1.4800000	1.4800000

Unique Subject Identifier=5013000000-0544

Assay variable : PTINR_VAL1 PT-INR Value

N	Mean	Standard Deviation	Minimum	Maximum
3	2.7800000	0.1969772	2.6200000	3.0000000

Unique Subject Identifier=5013000000-0546

Assay variable : PTINR_VAL1 PT-INR Value

N	Mean	Standard Deviation	Minimum	Maximum
3	2.5366667	0.3855299	2.1400000	2.9100000

Laboratory tests at the time of obtaining Table 2-6 consent, according to the presence or absence of proton-pump inhibitors

Unique Subject Identifier=5013000000-0557

MEANS Procedure

Assay variable : PTINR_VAL1 PT-INR Value

N	Mean	Standard Deviation	Minimum	Maximum
1	1.7800000		1.7800000	1.7800000

Unique Subject Identifier=5013000000-0560

Assay variable : PTINR_VAL1 PT-INR Value

N	Mean	Standard Deviation	Minimum	Maximum
4	2.5125000	0.7355893	1.8700000	3.5700000

Unique Subject Identifier=5013000000-0576

Assay variable : PTINR_VAL1 PT-INR Value

N	Mean	Standard Deviation	Minimum	Maximum
6	1.9100000	0.7090839	1.1900000	2.8200000

Unique Subject Identifier=5013000000-0580

Assay variable : PTINR_VAL1 PT-INR Value

N	Mean	Standard Deviation	Minimum	Maximum
4	1.6100000	0.2672078	1.3600000	1.9600000

Laboratory tests at the time of obtaining Table 2-6 consent, according to the presence or absence of proton-pump inhibitors

Unique Subject Identifier=5014010001-0024

MEANS Procedure

Assay variable : PTINR_VAL1 PT-INR Value

N	Mean	Standard Deviation	Minimum	Maximum
1	2.6400000		2.6400000	2.6400000

Unique Subject Identifier=5015000000-0012

Assay variable : PTINR_VAL1 PT-INR Value

N	Mean	Standard Deviation	Minimum	Maximum
3	1.7666667	0.0472582	1.7300000	1.8200000

Unique Subject Identifier=5015000000-0025

Assay variable : PTINR_VAL1 PT-INR Value

N	Mean	Standard Deviation	Minimum	Maximum
3	1.7700000	0.0984886	1.6900000	1.8800000

Unique Subject Identifier=5016000000-0016

Assay variable : PTINR_VAL1 PT-INR Value

N	Mean	Standard Deviation	Minimum	Maximum
3	1.9133333	0.2742870	1.7500000	2.2300000

Laboratory tests at the time of obtaining Table 2-6 consent, according to the presence or absence of proton-pump inhibitors

Unique Subject Identifier=5016000000-0018

MEANS Procedure

Assay variable : PTINR_VAL1 PT-INR Value

N	Mean	Standard Deviation	Minimum	Maximum
2	2.1750000	0.3747666	1.9100000	2.4400000

Unique Subject Identifier=5016000000-0021

Assay variable : PTINR_VAL1 PT-INR Value

N	Mean	Standard Deviation	Minimum	Maximum
6	1.6933333	0.0542832	1.6200000	1.7600000

Unique Subject Identifier=5016000000-0035

Assay variable : PTINR_VAL1 PT-INR Value

N	Mean	Standard Deviation	Minimum	Maximum
3	1.9333333	0.0503322	1.8800000	1.9800000

Unique Subject Identifier=5016000000-0038

Assay variable : PTINR_VAL1 PT-INR Value

N	Mean	Standard Deviation	Minimum	Maximum
3	1.2966667	0.1069268	1.1800000	1.3900000

Laboratory tests at the time of obtaining Table 2-6 consent, according to the presence or absence of proton-pump inhibitors

Unique Subject Identifier=5016000000-0047

MEANS Procedure

Assay variable : PTINR_VAL1 PT-INR Value

N	Mean	Standard Deviation	Minimum	Maximum
4	2.1975000	0.6012418	1.3800000	2.7400000

Unique Subject Identifier=5016000000-0052

Assay variable : PTINR_VAL1 PT-INR Value

N	Mean	Standard Deviation	Minimum	Maximum
3	1.8100000	0.4677606	1.2700000	2.0900000

Unique Subject Identifier=5016000000-0053

Assay variable : PTINR_VAL1 PT-INR Value

N	Mean	Standard Deviation	Minimum	Maximum
5	1.6480000	0.3802236	1.2400000	2.2600000

Unique Subject Identifier=5016000000-0058

Assay variable : PTINR_VAL1 PT-INR Value

N	Mean	Standard Deviation	Minimum	Maximum
4	2.4200000	0.4606517	1.7700000	2.8500000

Laboratory tests at the time of obtaining Table 2-6 consent, according to the presence or absence of proton-pump inhibitors

Unique Subject Identifier=5016000000-0090

MEANS Procedure

Assay variable : PTINR_VAL1 PT-INR Value

N	Mean	Standard Deviation	Minimum	Maximum
4	2.1400000	0.5934644	1.5800000	2.8900000

Unique Subject Identifier=5016000000-0094

Assay variable : PTINR_VAL1 PT-INR Value

N	Mean	Standard Deviation	Minimum	Maximum
2	1.7000000	0.0282843	1.6800000	1.7200000

Unique Subject Identifier=5016000000-0095

Assay variable : PTINR_VAL1 PT-INR Value

N	Mean	Standard Deviation	Minimum	Maximum
3	2.6333333	0.0513160	2.5900000	2.6900000

Unique Subject Identifier=5016000000-0109

Assay variable : PTINR_VAL1 PT-INR Value

N	Mean	Standard Deviation	Minimum	Maximum
2	1.9900000	0.5374012	1.6100000	2.3700000

Laboratory tests at the time of obtaining Table 2-6 consent, according to the presence or absence of proton-pump inhibitors

Unique Subject Identifier=5016000000-0112

MEANS Procedure

Assay variable : PTINR_VAL1 PT-INR Value

N	Mean	Standard Deviation	Minimum	Maximum
1	2.2600000		2.2600000	2.2600000

Unique Subject Identifier=5016000000-0137

Assay variable : PTINR_VAL1 PT-INR Value

N	Mean	Standard Deviation	Minimum	Maximum
3	1.9800000	0.1705872	1.8400000	2.1700000

Unique Subject Identifier=5016000000-0142

Assay variable : PTINR_VAL1 PT-INR Value

N	Mean	Standard Deviation	Minimum	Maximum
4	2.2250000	0.4744470	1.9000000	2.9300000

Unique Subject Identifier=5016000000-0146

Assay variable : PTINR_VAL1 PT-INR Value

N	Mean	Standard Deviation	Minimum	Maximum
3	2.2000000	0.8861151	1.1800000	2.7800000

Laboratory tests at the time of obtaining Table 2-6 consent, according to the presence or absence of proton-pump inhibitors

Unique Subject Identifier=5016000000-0147

MEANS Procedure

Assay variable : PTINR_VAL1 PT-INR Value

N	Mean	Standard Deviation	Minimum	Maximum
4	1.7075000	0.0713559	1.6200000	1.7900000

Unique Subject Identifier=5016000000-0153

Assay variable : PTINR_VAL1 PT-INR Value

N	Mean	Standard Deviation	Minimum	Maximum
4	2.1400000	0.1013246	2.0100000	2.2300000

Unique Subject Identifier=5016000000-0157

Assay variable : PTINR_VAL1 PT-INR Value

N	Mean	Standard Deviation	Minimum	Maximum
3	2.3133333	0.5451911	1.7300000	2.8100000

Unique Subject Identifier=5016000000-0164

Assay variable : PTINR_VAL1 PT-INR Value

N	Mean	Standard Deviation	Minimum	Maximum
5	2.1540000	0.4639289	1.6800000	2.8000000

Laboratory tests at the time of obtaining Table 2-6 consent, according to the presence or absence of proton-pump inhibitors

Unique Subject Identifier=5016000000-0168

MEANS Procedure

Assay variable : PTINR_VAL1 PT-INR Value

N	Mean	Standard Deviation	Minimum	Maximum
5	1.9020000	0.5697982	1.3700000	2.8700000

Unique Subject Identifier=5016000000-0174

Assay variable : PTINR_VAL1 PT-INR Value

N	Mean	Standard Deviation	Minimum	Maximum
2	1.8050000	1.1101576	1.0200000	2.5900000

Unique Subject Identifier=5016000000-0188

Assay variable : PTINR_VAL1 PT-INR Value

N	Mean	Standard Deviation	Minimum	Maximum
5	2.4440000	0.3880464	2.0600000	2.8500000

Unique Subject Identifier=5016000000-0213

Assay variable : PTINR_VAL1 PT-INR Value

N	Mean	Standard Deviation	Minimum	Maximum
6	2.0316667	0.5348800	1.4800000	2.8400000

Laboratory tests at the time of obtaining Table 2-6 consent, according to the presence or absence of proton-pump inhibitors

Unique Subject Identifier=5016000000-0242

MEANS Procedure

Assay variable : PTINR_VAL1 PT-INR Value

N	Mean	Standard Deviation	Minimum	Maximum
4	1.9025000	0.1637834	1.6900000	2.0800000

Unique Subject Identifier=5016000000-0251

Assay variable : PTINR_VAL1 PT-INR Value

N	Mean	Standard Deviation	Minimum	Maximum
6	1.9333333	0.8179405	1.0800000	3.2100000

Unique Subject Identifier=5016000000-0255

Assay variable : PTINR_VAL1 PT-INR Value

N	Mean	Standard Deviation	Minimum	Maximum
3	1.6133333	0.2722744	1.4000000	1.9200000

Unique Subject Identifier=5016000000-0265

Assay variable : PTINR_VAL1 PT-INR Value

N	Mean	Standard Deviation	Minimum	Maximum
4	2.2125000	0.1332604	2.0900000	2.3600000

Laboratory tests at the time of obtaining Table 2-6 consent, according to the presence or absence of proton-pump inhibitors

Unique Subject Identifier=5016000000-0272

MEANS Procedure

Assay variable : PTINR_VAL1 PT-INR Value

N	Mean	Standard Deviation	Minimum	Maximum
3	2.4600000	0.6478426	1.8500000	3.1400000

Unique Subject Identifier=5016000000-0284

Assay variable : PTINR_VAL1 PT-INR Value

N	Mean	Standard Deviation	Minimum	Maximum
6	2.1316667	0.3020210	1.8500000	2.6300000

Unique Subject Identifier=5016000000-0285

Assay variable : PTINR_VAL1 PT-INR Value

N	Mean	Standard Deviation	Minimum	Maximum
3	1.8233333	0.1792577	1.7100000	2.0300000

Unique Subject Identifier=5016000000-0317

Assay variable : PTINR_VAL1 PT-INR Value

N	Mean	Standard Deviation	Minimum	Maximum
3	1.8300000	0.4714870	1.5000000	2.3700000

Laboratory tests at the time of obtaining Table 2-6 consent, according to the presence or absence of proton-pump inhibitors

Unique Subject Identifier=5016000000-0341

MEANS Procedure

Assay variable : PTINR_VAL1 PT-INR Value

N	Mean	Standard Deviation	Minimum	Maximum
4	2.1125000	0.1337597	1.9700000	2.2800000

Unique Subject Identifier=5016000000-0348

Assay variable : PTINR_VAL1 PT-INR Value

N	Mean	Standard Deviation	Minimum	Maximum
6	1.9631667	0.3504628	1.3400000	2.3100000

Unique Subject Identifier=5017011110-0001

Assay variable : PTINR_VAL1 PT-INR Value

N	Mean	Standard Deviation	Minimum	Maximum
4	2.6750000	0.3767846	2.2800000	3.1400000

Unique Subject Identifier=5017011110-0008

Assay variable : PTINR_VAL1 PT-INR Value

N	Mean	Standard Deviation	Minimum	Maximum
4	2.1975000	0.3617895	1.7300000	2.5300000

Laboratory tests at the time of obtaining Table 2-6 consent, according to the presence or absence of proton-pump inhibitors

Unique Subject Identifier=5017011110-0015

MEANS Procedure

Assay variable : PTINR_VAL1 PT-INR Value

N	Mean	Standard Deviation	Minimum	Maximum
3	2.0833333	0.5263396	1.5800000	2.6300000

Unique Subject Identifier=5017011110-0016

Assay variable : PTINR_VAL1 PT-INR Value

N	Mean	Standard Deviation	Minimum	Maximum
5	1.2760000	0.1040673	1.1800000	1.4200000

Unique Subject Identifier=5017011110-0019

Assay variable : PTINR_VAL1 PT-INR Value

N	Mean	Standard Deviation	Minimum	Maximum
6	1.9083333	0.6652343	1.3500000	3.1200000

Unique Subject Identifier=5017011110-0032

Assay variable : PTINR_VAL1 PT-INR Value

N	Mean	Standard Deviation	Minimum	Maximum
6	2.0500000	0.6796764	1.4900000	3.0700000

Laboratory tests at the time of obtaining Table 2-6 consent, according to the presence or absence of proton-pump inhibitors

Unique Subject Identifier=5020011000-0004

MEANS Procedure

Assay variable : PTINR_VAL1 PT-INR Value

N	Mean	Standard Deviation	Minimum	Maximum
3	2.8800000	0.5499091	2.4000000	3.4800000

Unique Subject Identifier=5020011000-0014

Assay variable : PTINR_VAL1 PT-INR Value

N	Mean	Standard Deviation	Minimum	Maximum
3	2.0200000	0.4003748	1.6100000	2.4100000

Unique Subject Identifier=5020011000-0016

Assay variable : PTINR_VAL1 PT-INR Value

N	Mean	Standard Deviation	Minimum	Maximum
3	2.7800000	0.1997498	2.5600000	2.9500000

Unique Subject Identifier=5020011000-0020

Assay variable : PTINR_VAL1 PT-INR Value

N	Mean	Standard Deviation	Minimum	Maximum
4	2.3350000	0.5791085	1.5000000	2.8300000

Laboratory tests at the time of obtaining Table 2-6 consent, according to the presence or absence of proton-pump inhibitors

Unique Subject Identifier=5020011000-0030

MEANS Procedure

Assay variable : PTINR_VAL1 PT-INR Value

N	Mean	Standard Deviation	Minimum	Maximum
6	1.6700000	0.1637071	1.4600000	1.9000000

Unique Subject Identifier=5020011000-0036

Assay variable : PTINR_VAL1 PT-INR Value

N	Mean	Standard Deviation	Minimum	Maximum
4	1.8025000	0.1547848	1.5900000	1.9400000

Unique Subject Identifier=5020011000-0039

Assay variable : PTINR_VAL1 PT-INR Value

N	Mean	Standard Deviation	Minimum	Maximum
3	2.2300000	0.3061046	1.9400000	2.5500000

Unique Subject Identifier=5020011000-0042

Assay variable : PTINR_VAL1 PT-INR Value

N	Mean	Standard Deviation	Minimum	Maximum
3	2.2200000	0.3011644	1.8900000	2.4800000

Laboratory tests at the time of obtaining Table 2-6 consent, according to the presence or absence of proton-pump inhibitors

Unique Subject Identifier=5020011000-0043

MEANS Procedure

Assay variable : PTINR_VAL1 PT-INR Value

N	Mean	Standard Deviation	Minimum	Maximum
6	2.3666667	0.3654951	1.8200000	2.8600000

Unique Subject Identifier=5020011000-0048

Assay variable : PTINR_VAL1 PT-INR Value

N	Mean	Standard Deviation	Minimum	Maximum
4	1.8675000	0.2920474	1.4400000	2.1000000

Unique Subject Identifier=5020011000-0064

Assay variable : PTINR_VAL1 PT-INR Value

N	Mean	Standard Deviation	Minimum	Maximum
4	1.5050000	0.1420094	1.3700000	1.6600000

Unique Subject Identifier=5021011111-0039

Assay variable : PTINR_VAL1 PT-INR Value

N	Mean	Standard Deviation	Minimum	Maximum
6	1.9983333	0.1591750	1.7400000	2.1900000

Laboratory tests at the time of obtaining Table 2-6 consent, according to the presence or absence of proton-pump inhibitors

Unique Subject Identifier=5021011111-0048

MEANS Procedure

Assay variable : PTINR_VAL1 PT-INR Value

N	Mean	Standard Deviation	Minimum	Maximum
4	2.0650000	0.4353160	1.5800000	2.5600000

Unique Subject Identifier=5025000100-0001

Assay variable : PTINR_VAL1 PT-INR Value

N	Mean	Standard Deviation	Minimum	Maximum
4	2.5750000	0.9542012	1.8100000	3.9700000

Unique Subject Identifier=5025000100-0009

Assay variable : PTINR_VAL1 PT-INR Value

N	Mean	Standard Deviation	Minimum	Maximum
2	1.7400000	0.2969848	1.5300000	1.9500000

Unique Subject Identifier=5025000100-0013

Assay variable : PTINR_VAL1 PT-INR Value

N	Mean	Standard Deviation	Minimum	Maximum
3	2.5633333	0.2968726	2.3100000	2.8900000

Laboratory tests at the time of obtaining Table 2-6 consent, according to the presence or absence of proton-pump inhibitors

Unique Subject Identifier=5025000100-0015

MEANS Procedure

Assay variable : PTINR_VAL1 PT-INR Value

N	Mean	Standard Deviation	Minimum	Maximum
6	1.7016667	0.1265570	1.5500000	1.8900000

Unique Subject Identifier=5026101000-0010

Assay variable : PTINR_VAL1 PT-INR Value

N	Mean	Standard Deviation	Minimum	Maximum
3	1.6133333	0.3288363	1.2400000	1.8600000

Unique Subject Identifier=5026101000-0011

Assay variable : PTINR_VAL1 PT-INR Value

N	Mean	Standard Deviation	Minimum	Maximum
6	1.8550000	0.2261637	1.7100000	2.3000000

Unique Subject Identifier=5026101000-0016

Assay variable : PTINR_VAL1 PT-INR Value

N	Mean	Standard Deviation	Minimum	Maximum
5	1.9960000	0.0952890	1.8400000	2.0700000

Laboratory tests at the time of obtaining Table 2-6 consent, according to the presence or absence of proton-pump inhibitors

Unique Subject Identifier=5026101000-0018

MEANS Procedure

Assay variable : PTINR_VAL1 PT-INR Value

N	Mean	Standard Deviation	Minimum	Maximum
6	1.5350000	0.2157545	1.2500000	1.7700000

Unique Subject Identifier=5026101000-0020

Assay variable : PTINR_VAL1 PT-INR Value

N	Mean	Standard Deviation	Minimum	Maximum
6	1.9700000	0.3825180	1.5000000	2.5400000

Unique Subject Identifier=5026101000-0023

Assay variable : PTINR_VAL1 PT-INR Value

N	Mean	Standard Deviation	Minimum	Maximum
5	1.9020000	0.5618897	1.5600000	2.9000000

Unique Subject Identifier=5026101000-0026

Assay variable : PTINR_VAL1 PT-INR Value

N	Mean	Standard Deviation	Minimum	Maximum
6	1.9766667	0.2718578	1.5400000	2.3100000

Laboratory tests at the time of obtaining Table 2-6 consent, according to the presence or absence of proton-pump inhibitors

Unique Subject Identifier=5026101000-0031

MEANS Procedure

Assay variable : PTINR_VAL1 PT-INR Value

N	Mean	Standard Deviation	Minimum	Maximum
3	1.9700000	0.3044667	1.7000000	2.3000000

Unique Subject Identifier=5026101000-0037

Assay variable : PTINR_VAL1 PT-INR Value

N	Mean	Standard Deviation	Minimum	Maximum
3	1.5166667	0.2454248	1.2800000	1.7700000

Unique Subject Identifier=5026101000-0039

Assay variable : PTINR_VAL1 PT-INR Value

N	Mean	Standard Deviation	Minimum	Maximum
3	2.1100000	0.2773085	1.7900000	2.2800000

Unique Subject Identifier=5026101000-0040

Assay variable : PTINR_VAL1 PT-INR Value

N	Mean	Standard Deviation	Minimum	Maximum
6	2.1850000	0.1707337	1.9800000	2.3900000

Laboratory tests at the time of obtaining Table 2-6 consent, according to the presence or absence of proton-pump inhibitors

Unique Subject Identifier=5026101000-0041

MEANS Procedure

Assay variable : PTINR_VAL1 PT-INR Value

N	Mean	Standard Deviation	Minimum	Maximum
4	1.8425000	0.5897104	1.3000000	2.6800000

Unique Subject Identifier=5026101000-0044

Assay variable : PTINR_VAL1 PT-INR Value

N	Mean	Standard Deviation	Minimum	Maximum
3	1.5833333	0.1418920	1.4300000	1.7100000

Unique Subject Identifier=5026101000-0045

Assay variable : PTINR_VAL1 PT-INR Value

N	Mean	Standard Deviation	Minimum	Maximum
3	2.2400000	0.3551056	1.8900000	2.6000000

Unique Subject Identifier=5026101000-0049

Assay variable : PTINR_VAL1 PT-INR Value

N	Mean	Standard Deviation	Minimum	Maximum
6	1.9166667	0.3360754	1.3900000	2.2500000

Laboratory tests at the time of obtaining Table 2-6 consent, according to the presence or absence of proton-pump inhibitors

Unique Subject Identifier=5026101000-0051

MEANS Procedure

Assay variable : PTINR_VAL1 PT-INR Value

N	Mean	Standard Deviation	Minimum	Maximum
6	1.9233333	0.5225961	1.3500000	2.8100000

Unique Subject Identifier=5026101000-0058

Assay variable : PTINR_VAL1 PT-INR Value

N	Mean	Standard Deviation	Minimum	Maximum
3	2.9366667	2.0850020	1.4500000	5.3200000

Unique Subject Identifier=5026101000-0065

Assay variable : PTINR_VAL1 PT-INR Value

N	Mean	Standard Deviation	Minimum	Maximum
4	2.2250000	0.3145897	1.9200000	2.5700000

Unique Subject Identifier=5026101000-0066

Assay variable : PTINR_VAL1 PT-INR Value

N	Mean	Standard Deviation	Minimum	Maximum
6	2.3000000	0.3119615	1.9300000	2.8800000

Laboratory tests at the time of obtaining Table 2-6 consent, according to the presence or absence of proton-pump inhibitors

Unique Subject Identifier=5026101000-0068

MEANS Procedure

Assay variable : PTINR_VAL1 PT-INR Value

N	Mean	Standard Deviation	Minimum	Maximum
2	2.2050000	0.2899138	2.0000000	2.4100000

Unique Subject Identifier=5026101000-0071

Assay variable : PTINR_VAL1 PT-INR Value

N	Mean	Standard Deviation	Minimum	Maximum
3	1.6766667	0.4675824	1.3000000	2.2000000

Unique Subject Identifier=5026101000-0074

Assay variable : PTINR_VAL1 PT-INR Value

N	Mean	Standard Deviation	Minimum	Maximum
5	2.4080000	0.8267829	1.1200000	3.1600000

Unique Subject Identifier=5026101000-0100

Assay variable : PTINR_VAL1 PT-INR Value

N	Mean	Standard Deviation	Minimum	Maximum
6	2.0300000	0.2504396	1.6800000	2.4500000

Laboratory tests at the time of obtaining Table 2-6 consent, according to the presence or absence of proton-pump inhibitors

Unique Subject Identifier=5026101000-0111

MEANS Procedure

Assay variable : PTINR_VAL1 PT-INR Value

N	Mean	Standard Deviation	Minimum	Maximum
3	1.9033333	0.0665833	1.8600000	1.9800000

Unique Subject Identifier=5026101000-0116

Assay variable : PTINR_VAL1 PT-INR Value

N	Mean	Standard Deviation	Minimum	Maximum
4	1.8525000	0.0830161	1.7400000	1.9400000

Unique Subject Identifier=5026101000-0119

Assay variable : PTINR_VAL1 PT-INR Value

N	Mean	Standard Deviation	Minimum	Maximum
6	2.5766667	1.0478295	1.4800000	3.9100000

Unique Subject Identifier=5030010000-0008

Assay variable : PTINR_VAL1 PT-INR Value

N	Mean	Standard Deviation	Minimum	Maximum
6	1.5750000	0.2702406	1.2700000	2.0200000

Laboratory tests at the time of obtaining Table 2-6 consent, according to the presence or absence of proton-pump inhibitors

Unique Subject Identifier=5030010000-0031

MEANS Procedure

Assay variable : PTINR_VAL1 PT-INR Value

N	Mean	Standard Deviation	Minimum	Maximum
5	2.2100000	0.2586503	1.9400000	2.6400000

Unique Subject Identifier=5030010000-0035

Assay variable : PTINR_VAL1 PT-INR Value

N	Mean	Standard Deviation	Minimum	Maximum
6	1.7733333	0.1737431	1.5700000	2.0200000

Unique Subject Identifier=5030010000-0047

Assay variable : PTINR_VAL1 PT-INR Value

N	Mean	Standard Deviation	Minimum	Maximum
2	1.2850000	0.0212132	1.2700000	1.3000000

Unique Subject Identifier=5030010000-0057

Assay variable : PTINR_VAL1 PT-INR Value

N	Mean	Standard Deviation	Minimum	Maximum
1	1.2900000		1.2900000	1.2900000

Laboratory tests at the time of obtaining Table 2-6 consent, according to the presence or absence of proton-pump inhibitors

Unique Subject Identifier=5031111000-0018

MEANS Procedure

Assay variable : PTINR_VAL1 PT-INR Value

N	Mean	Standard Deviation	Minimum	Maximum
3	2.0000000	0.0793725	1.9100000	2.0600000

Unique Subject Identifier=5032010000-0001

Assay variable : PTINR_VAL1 PT-INR Value

N	Mean	Standard Deviation	Minimum	Maximum
6	2.3283333	0.0913053	2.2000000	2.4500000

Unique Subject Identifier=5032010000-0005

Assay variable : PTINR_VAL1 PT-INR Value

N	Mean	Standard Deviation	Minimum	Maximum
5	2.4120000	0.2832314	2.0700000	2.8300000

Unique Subject Identifier=5032010000-0006

Assay variable : PTINR_VAL1 PT-INR Value

N	Mean	Standard Deviation	Minimum	Maximum
5	1.7180000	0.3067083	1.1800000	1.9000000

Laboratory tests at the time of obtaining Table 2-6 consent, according to the presence or absence of proton-pump inhibitors

Unique Subject Identifier=5032010000-0009

MEANS Procedure

Assay variable : PTINR_VAL1 PT-INR Value

N	Mean	Standard Deviation	Minimum	Maximum
5	2.4320000	0.5597946	1.6800000	3.1200000

Unique Subject Identifier=5032010000-0010

Assay variable : PTINR_VAL1 PT-INR Value

N	Mean	Standard Deviation	Minimum	Maximum
6	1.2650000	0.3125860	1.0800000	1.8900000

Unique Subject Identifier=5032010000-0013

Assay variable : PTINR_VAL1 PT-INR Value

N	Mean	Standard Deviation	Minimum	Maximum
6	2.4083333	0.1874478	2.2400000	2.6800000

Unique Subject Identifier=5034000010-0002

Assay variable : PTINR_VAL1 PT-INR Value

N	Mean	Standard Deviation	Minimum	Maximum
3	2.0033333	0.2565801	1.7200000	2.2200000

Laboratory tests at the time of obtaining Table 2-6 consent, according to the presence or absence of proton-pump inhibitors

Unique Subject Identifier=5034000010-0004

MEANS Procedure

Assay variable : PTINR_VAL1 PT-INR Value

N	Mean	Standard Deviation	Minimum	Maximum
2	1.7950000	0.0070711	1.7900000	1.8000000

Unique Subject Identifier=5034000010-0024

Assay variable : PTINR_VAL1 PT-INR Value

N	Mean	Standard Deviation	Minimum	Maximum
4	1.8900000	0.1930458	1.7100000	2.0900000

Unique Subject Identifier=5035010010-0004

Assay variable : PTINR_VAL1 PT-INR Value

N	Mean	Standard Deviation	Minimum	Maximum
3	1.8266667	0.3231615	1.4700000	2.1000000

Unique Subject Identifier=5035010010-0012

Assay variable : PTINR_VAL1 PT-INR Value

N	Mean	Standard Deviation	Minimum	Maximum
3	1.7266667	0.4821134	1.3500000	2.2700000

Laboratory tests at the time of obtaining Table 2-6 consent, according to the presence or absence of proton-pump inhibitors

Unique Subject Identifier=5035010010-0019

MEANS Procedure

Assay variable : PTINR_VAL1 PT-INR Value

N	Mean	Standard Deviation	Minimum	Maximum
6	1.7366667	0.1521403	1.5500000	1.9800000

Unique Subject Identifier=5035010010-0036

Assay variable : PTINR_VAL1 PT-INR Value

N	Mean	Standard Deviation	Minimum	Maximum
4	2.1125000	0.4731719	1.6100000	2.7300000

Unique Subject Identifier=5035010010-0045

Assay variable : PTINR_VAL1 PT-INR Value

N	Mean	Standard Deviation	Minimum	Maximum
3	1.7666667	0.2914332	1.5600000	2.1000000

Unique Subject Identifier=5035010010-0053

Assay variable : PTINR_VAL1 PT-INR Value

N	Mean	Standard Deviation	Minimum	Maximum
6	2.0250000	0.1277106	1.8900000	2.2100000

Laboratory tests at the time of obtaining Table 2-6 consent, according to the presence or absence of proton-pump inhibitors

Unique Subject Identifier=5035010010-0055

MEANS Procedure

Assay variable : PTINR_VAL1 PT-INR Value

N	Mean	Standard Deviation	Minimum	Maximum
5	1.5440000	0.5030209	1.1400000	2.1700000

Unique Subject Identifier=5036010000-0003

Assay variable : PTINR_VAL1 PT-INR Value

N	Mean	Standard Deviation	Minimum	Maximum
4	1.7175000	0.2444552	1.5500000	2.0800000

Unique Subject Identifier=5036010000-0007

Assay variable : PTINR_VAL1 PT-INR Value

N	Mean	Standard Deviation	Minimum	Maximum
5	2.2340000	0.4251235	1.8000000	2.9400000

Unique Subject Identifier=5036010000-0012

Assay variable : PTINR_VAL1 PT-INR Value

N	Mean	Standard Deviation	Minimum	Maximum
5	1.8520000	0.3199531	1.4000000	2.1300000

Laboratory tests at the time of obtaining Table 2-6 consent, according to the presence or absence of proton-pump inhibitors

Unique Subject Identifier=5036010000-0021

MEANS Procedure

Assay variable : PTINR_VAL1 PT-INR Value

N	Mean	Standard Deviation	Minimum	Maximum
1	1.9600000		1.9600000	1.9600000

Unique Subject Identifier=5036010000-0034

Assay variable : PTINR_VAL1 PT-INR Value

N	Mean	Standard Deviation	Minimum	Maximum
6	1.6266667	0.2657568	1.2400000	1.9400000

Unique Subject Identifier=5036010000-0050

Assay variable : PTINR_VAL1 PT-INR Value

N	Mean	Standard Deviation	Minimum	Maximum
4	1.9450000	0.2318764	1.6400000	2.1400000

Unique Subject Identifier=5040011000-0003

Assay variable : PTINR_VAL1 PT-INR Value

N	Mean	Standard Deviation	Minimum	Maximum
3	2.3100000	0.3559494	2.0800000	2.7200000

Laboratory tests at the time of obtaining Table 2-6 consent, according to the presence or absence of proton-pump inhibitors

Unique Subject Identifier=5040011000-0006

MEANS Procedure

Assay variable : PTINR_VAL1 PT-INR Value

N	Mean	Standard Deviation	Minimum	Maximum
4	1.7900000	0.1023067	1.6600000	1.8900000

Unique Subject Identifier=5040011000-0008

Assay variable : PTINR_VAL1 PT-INR Value

N	Mean	Standard Deviation	Minimum	Maximum
5	2.0520000	0.7066612	1.5500000	3.2900000

Unique Subject Identifier=5040011000-0010

Assay variable : PTINR_VAL1 PT-INR Value

N	Mean	Standard Deviation	Minimum	Maximum
4	2.8625000	0.3236639	2.4100000	3.1200000

Unique Subject Identifier=5041011000-0007

Assay variable : PTINR_VAL1 PT-INR Value

N	Mean	Standard Deviation	Minimum	Maximum
3	1.9166667	0.2454248	1.6800000	2.1700000

Laboratory tests at the time of obtaining Table 2-6 consent, according to the presence or absence of proton-pump inhibitors

Unique Subject Identifier=5044000000-0013

MEANS Procedure

Assay variable : PTINR_VAL1 PT-INR Value

N	Mean	Standard Deviation	Minimum	Maximum
2	2.2100000	0.1979899	2.0700000	2.3500000

Unique Subject Identifier=5044000000-0043

Assay variable : PTINR_VAL1 PT-INR Value

N	Mean	Standard Deviation	Minimum	Maximum
6	1.9166667	0.3020375	1.5600000	2.3600000

Unique Subject Identifier=5046010000-0007

Assay variable : PTINR_VAL1 PT-INR Value

N	Mean	Standard Deviation	Minimum	Maximum
3	1.9066667	0.3302020	1.5700000	2.2300000

Unique Subject Identifier=5046010000-0010

Assay variable : PTINR_VAL1 PT-INR Value

N	Mean	Standard Deviation	Minimum	Maximum
4	1.9700000	0.4588391	1.5400000	2.4600000

Laboratory tests at the time of obtaining Table 2-6 consent, according to the presence or absence of proton-pump inhibitors

Unique Subject Identifier=5046010000-0023

MEANS Procedure

Assay variable : PTINR_VAL1 PT-INR Value

N	Mean	Standard Deviation	Minimum	Maximum
5	1.6520000	0.2872629	1.2400000	1.9800000

Unique Subject Identifier=5046010000-0028

Assay variable : PTINR_VAL1 PT-INR Value

N	Mean	Standard Deviation	Minimum	Maximum
6	2.2566667	0.2976351	1.7600000	2.5400000

Unique Subject Identifier=5047000010-0001

Assay variable : PTINR_VAL1 PT-INR Value

N	Mean	Standard Deviation	Minimum	Maximum
6	1.8633333	0.2439399	1.5700000	2.2700000

Unique Subject Identifier=5047000010-0002

Assay variable : PTINR_VAL1 PT-INR Value

N	Mean	Standard Deviation	Minimum	Maximum
6	1.9133333	0.2501733	1.5900000	2.3200000

Laboratory tests at the time of obtaining Table 2-6 consent, according to the presence or absence of proton-pump inhibitors

Unique Subject Identifier=5048011000-0015

MEANS Procedure

Assay variable : PTINR_VAL1 PT-INR Value

N	Mean	Standard Deviation	Minimum	Maximum
6	1.2033333	0.2780408	0.9700000	1.7300000

Unique Subject Identifier=5048011000-0017

Assay variable : PTINR_VAL1 PT-INR Value

N	Mean	Standard Deviation	Minimum	Maximum
6	1.7366667	0.2778249	1.3600000	2.1300000

Unique Subject Identifier=5048011000-0032

Assay variable : PTINR_VAL1 PT-INR Value

N	Mean	Standard Deviation	Minimum	Maximum
6	1.5533333	0.3556215	1.3600000	2.2700000

Unique Subject Identifier=5048011000-0063

Assay variable : PTINR_VAL1 PT-INR Value

N	Mean	Standard Deviation	Minimum	Maximum
4	1.9700000	0.1555635	1.8700000	2.2000000

Laboratory tests at the time of obtaining Table 2-6 consent, according to the presence or absence of proton-pump inhibitors

Unique Subject Identifier=5048011000-0068

MEANS Procedure

Assay variable : PTINR_VAL1 PT-INR Value

N	Mean	Standard Deviation	Minimum	Maximum
4	1.3250000	0.0772442	1.2200000	1.4000000

Unique Subject Identifier=5048011000-0075

Assay variable : PTINR_VAL1 PT-INR Value

N	Mean	Standard Deviation	Minimum	Maximum
6	1.6916667	0.2980884	1.3700000	2.0500000

Unique Subject Identifier=5048011000-0087

Assay variable : PTINR_VAL1 PT-INR Value

N	Mean	Standard Deviation	Minimum	Maximum
4	2.1875000	0.1873277	1.9700000	2.4000000

Unique Subject Identifier=5048011000-0105

Assay variable : PTINR_VAL1 PT-INR Value

N	Mean	Standard Deviation	Minimum	Maximum
3	2.1900000	0.1300000	2.1100000	2.3400000

Laboratory tests at the time of obtaining Table 2-6 consent, according to the presence or absence of proton-pump inhibitors

Unique Subject Identifier=5050011100-0002

MEANS Procedure

Assay variable : PTINR_VAL1 PT-INR Value

N	Mean	Standard Deviation	Minimum	Maximum
5	2.3140000	0.4131344	1.8600000	2.8300000

Unique Subject Identifier=5050011100-0005

Assay variable : PTINR_VAL1 PT-INR Value

N	Mean	Standard Deviation	Minimum	Maximum
5	2.3660000	0.4697127	2.0700000	3.1700000

Unique Subject Identifier=5050011100-0007

Assay variable : PTINR_VAL1 PT-INR Value

N	Mean	Standard Deviation	Minimum	Maximum
4	2.3125000	0.4699202	1.9100000	2.9600000

Unique Subject Identifier=5050011100-0008

Assay variable : PTINR_VAL1 PT-INR Value

N	Mean	Standard Deviation	Minimum	Maximum
6	2.0516667	0.5068892	1.5400000	2.9100000

Laboratory tests at the time of obtaining Table 2-6 consent, according to the presence or absence of proton-pump inhibitors

Unique Subject Identifier=5050011100-0009

MEANS Procedure

Assay variable : PTINR_VAL1 PT-INR Value

N	Mean	Standard Deviation	Minimum	Maximum
5	2.3420000	0.1531992	2.1800000	2.5300000

Unique Subject Identifier=5050011100-0010

Assay variable : PTINR_VAL1 PT-INR Value

N	Mean	Standard Deviation	Minimum	Maximum
4	2.0425000	0.5867637	1.5700000	2.9000000

Unique Subject Identifier=5050011100-0011

Assay variable : PTINR_VAL1 PT-INR Value

N	Mean	Standard Deviation	Minimum	Maximum
3	1.9266667	0.1059874	1.8300000	2.0400000

Unique Subject Identifier=5050011100-0012

Assay variable : PTINR_VAL1 PT-INR Value

N	Mean	Standard Deviation	Minimum	Maximum
4	2.1525000	0.1813606	1.9200000	2.3200000

Laboratory tests at the time of obtaining Table 2-6 consent, according to the presence or absence of proton-pump inhibitors

Unique Subject Identifier=5050011100-0026

MEANS Procedure

Assay variable : PTINR_VAL1 PT-INR Value

N	Mean	Standard Deviation	Minimum	Maximum
5	1.9080000	0.1536880	1.7700000	2.1100000

Unique Subject Identifier=5051100000-0021

Assay variable : PTINR_VAL1 PT-INR Value

N	Mean	Standard Deviation	Minimum	Maximum
4	1.8375000	0.4241364	1.2600000	2.1800000

Unique Subject Identifier=5052000000-0009

Assay variable : PTINR_VAL1 PT-INR Value

N	Mean	Standard Deviation	Minimum	Maximum
4	2.2775000	0.8479141	1.6200000	3.4300000

Unique Subject Identifier=5052000000-0022

Assay variable : PTINR_VAL1 PT-INR Value

N	Mean	Standard Deviation	Minimum	Maximum
3	2.2800000	0.1307670	2.1900000	2.4300000

Laboratory tests at the time of obtaining Table 2-6 consent, according to the presence or absence of proton-pump inhibitors

Unique Subject Identifier=5052000000-0023

MEANS Procedure

Assay variable : PTINR_VAL1 PT-INR Value

N	Mean	Standard Deviation	Minimum	Maximum
3	1.8233333	0.1270171	1.7500000	1.9700000

Unique Subject Identifier=5052000000-0024

Assay variable : PTINR_VAL1 PT-INR Value

N	Mean	Standard Deviation	Minimum	Maximum
3	2.1366667	0.5468394	1.7000000	2.7500000

Unique Subject Identifier=5052000000-0032

Assay variable : PTINR_VAL1 PT-INR Value

N	Mean	Standard Deviation	Minimum	Maximum
5	1.8520000	0.1704993	1.7000000	2.0900000

Unique Subject Identifier=5052000000-0035

Assay variable : PTINR_VAL1 PT-INR Value

N	Mean	Standard Deviation	Minimum	Maximum
4	1.9400000	0.0658281	1.8700000	2.0100000

Laboratory tests at the time of obtaining Table 2-6 consent, according to the presence or absence of proton-pump inhibitors

Unique Subject Identifier=5052000000-0038

MEANS Procedure

Assay variable : PTINR_VAL1 PT-INR Value

N	Mean	Standard Deviation	Minimum	Maximum
4	1.6425000	0.2814694	1.4200000	2.0500000

Unique Subject Identifier=5052000000-0041

Assay variable : PTINR_VAL1 PT-INR Value

N	Mean	Standard Deviation	Minimum	Maximum
6	1.7566667	0.1502886	1.6000000	1.9700000

Unique Subject Identifier=5052000000-0042

Assay variable : PTINR_VAL1 PT-INR Value

N	Mean	Standard Deviation	Minimum	Maximum
3	1.5200000	0.1513275	1.3500000	1.6400000

Unique Subject Identifier=5052000000-0045

Assay variable : PTINR_VAL1 PT-INR Value

N	Mean	Standard Deviation	Minimum	Maximum
4	1.9025000	0.0946485	1.7800000	2.0100000

Laboratory tests at the time of obtaining Table 2-6 consent, according to the presence or absence of proton-pump inhibitors

Unique Subject Identifier=5053000000-0006

MEANS Procedure

Assay variable : PTINR_VAL1 PT-INR Value

N	Mean	Standard Deviation	Minimum	Maximum
3	2.0400000	0.0200000	2.0200000	2.0600000

Unique Subject Identifier=5053000000-0010

Assay variable : PTINR_VAL1 PT-INR Value

N	Mean	Standard Deviation	Minimum	Maximum
5	1.8140000	0.3134167	1.4000000	2.1100000

Unique Subject Identifier=5053000000-0011

Assay variable : PTINR_VAL1 PT-INR Value

N	Mean	Standard Deviation	Minimum	Maximum
3	1.6400000	0.1249000	1.5000000	1.7400000

Unique Subject Identifier=5053000000-0027

Assay variable : PTINR_VAL1 PT-INR Value

N	Mean	Standard Deviation	Minimum	Maximum
3	1.8300000	0.1374773	1.6800000	1.9500000

Laboratory tests at the time of obtaining Table 2-6 consent, according to the presence or absence of proton-pump inhibitors

Unique Subject Identifier=5053000000-0028

MEANS Procedure

Assay variable : PTINR_VAL1 PT-INR Value

N	Mean	Standard Deviation	Minimum	Maximum
3	1.5800000	0.3345146	1.2000000	1.8300000

Unique Subject Identifier=5054010000-0006

Assay variable : PTINR_VAL1 PT-INR Value

N	Mean	Standard Deviation	Minimum	Maximum
6	1.4033333	0.2957476	1.0600000	1.8700000

Unique Subject Identifier=5054010000-0011

Assay variable : PTINR_VAL1 PT-INR Value

N	Mean	Standard Deviation	Minimum	Maximum
6	2.2150000	0.4840558	1.7800000	3.0700000

Unique Subject Identifier=5054010000-0018

Assay variable : PTINR_VAL1 PT-INR Value

N	Mean	Standard Deviation	Minimum	Maximum
4	1.8250000	0.3304038	1.5000000	2.2000000

Laboratory tests at the time of obtaining Table 2-6 consent, according to the presence or absence of proton-pump inhibitors

Unique Subject Identifier=5054010000-0020

MEANS Procedure

Assay variable : PTINR_VAL1 PT-INR Value

N	Mean	Standard Deviation	Minimum	Maximum
6	2.0433333	0.7488569	1.1300000	3.0200000

Unique Subject Identifier=5054010000-0025

Assay variable : PTINR_VAL1 PT-INR Value

N	Mean	Standard Deviation	Minimum	Maximum
3	1.1966667	0.1401190	1.0600000	1.3400000

Unique Subject Identifier=5054010000-0026

Assay variable : PTINR_VAL1 PT-INR Value

N	Mean	Standard Deviation	Minimum	Maximum
2	1.9000000	0.1697056	1.7800000	2.0200000

Unique Subject Identifier=5055000000-0003

Assay variable : PTINR_VAL1 PT-INR Value

N	Mean	Standard Deviation	Minimum	Maximum
6	1.7266667	0.3415065	1.4200000	2.2900000

Laboratory tests at the time of obtaining Table 2-6 consent, according to the presence or absence of proton-pump inhibitors

Unique Subject Identifier=5055000000-0013

MEANS Procedure

Assay variable : PTINR_VAL1 PT-INR Value

N	Mean	Standard Deviation	Minimum	Maximum
4	1.2450000	0.0288675	1.2100000	1.2800000

Unique Subject Identifier=5055000000-0018

Assay variable : PTINR_VAL1 PT-INR Value

N	Mean	Standard Deviation	Minimum	Maximum
6	1.7750000	0.2356905	1.5000000	2.1100000

Unique Subject Identifier=5055000000-0019

Assay variable : PTINR_VAL1 PT-INR Value

N	Mean	Standard Deviation	Minimum	Maximum
3	1.5633333	0.3262412	1.2800000	1.9200000

Unique Subject Identifier=5055000000-0022

Assay variable : PTINR_VAL1 PT-INR Value

N	Mean	Standard Deviation	Minimum	Maximum
6	2.2383333	0.3947869	1.8900000	2.9900000

Laboratory tests at the time of obtaining Table 2-6 consent, according to the presence or absence of proton-pump inhibitors

Unique Subject Identifier=5055000000-0025

MEANS Procedure

Assay variable : PTINR_VAL1 PT-INR Value

N	Mean	Standard Deviation	Minimum	Maximum
6	2.0750000	0.2662893	1.6000000	2.4000000

Unique Subject Identifier=5055000000-0026

Assay variable : PTINR_VAL1 PT-INR Value

N	Mean	Standard Deviation	Minimum	Maximum
5	1.8460000	0.1716974	1.6600000	2.0600000

Unique Subject Identifier=5055000000-0028

Assay variable : PTINR_VAL1 PT-INR Value

N	Mean	Standard Deviation	Minimum	Maximum
5	1.8840000	0.0907193	1.7300000	1.9600000

Unique Subject Identifier=5058011111-0028

Assay variable : PTINR_VAL1 PT-INR Value

N	Mean	Standard Deviation	Minimum	Maximum
5	2.0800000	0.3664014	1.5400000	2.4200000

Laboratory tests at the time of obtaining Table 2-6 consent, according to the presence or absence of proton-pump inhibitors

Unique Subject Identifier=5060111000-0004

MEANS Procedure

Assay variable : PTINR_VAL1 PT-INR Value

N	Mean	Standard Deviation	Minimum	Maximum
5	1.8600000	0.4219005	1.3000000	2.4000000

Unique Subject Identifier=5063000000-0008

Assay variable : PTINR_VAL1 PT-INR Value

N	Mean	Standard Deviation	Minimum	Maximum
3	1.9233333	0.1955335	1.7600000	2.1400000

Unique Subject Identifier=5064000000-0003

Assay variable : PTINR_VAL1 PT-INR Value

N	Mean	Standard Deviation	Minimum	Maximum
6	2.4566667	0.7464226	1.4700000	3.4000000

Unique Subject Identifier=5066000100-0015

Assay variable : PTINR_VAL1 PT-INR Value

N	Mean	Standard Deviation	Minimum	Maximum
6	2.0683333	0.1002829	1.9300000	2.2300000

Laboratory tests at the time of obtaining Table 2-6 consent, according to the presence or absence of proton-pump inhibitors

Unique Subject Identifier=5066000100-0030

MEANS Procedure

Assay variable : PTINR_VAL1 PT-INR Value

N	Mean	Standard Deviation	Minimum	Maximum
3	2.0833333	0.2542309	1.8600000	2.3600000

Unique Subject Identifier=5068011111-0023

Assay variable : PTINR_VAL1 PT-INR Value

N	Mean	Standard Deviation	Minimum	Maximum
3	1.7666667	0.0650641	1.7000000	1.8300000

Unique Subject Identifier=5071110000-0021

Assay variable : PTINR_VAL1 PT-INR Value

N	Mean	Standard Deviation	Minimum	Maximum
6	1.2183333	0.2004412	1.0300000	1.6000000

Unique Subject Identifier=5071110000-0022

Assay variable : PTINR_VAL1 PT-INR Value

N	Mean	Standard Deviation	Minimum	Maximum
5	2.0000000	0.4756049	1.5000000	2.7800000

Laboratory tests at the time of obtaining Table 2-6 consent, according to the presence or absence of proton-pump inhibitors

Unique Subject Identifier=5073000010-0003

MEANS Procedure

Assay variable : PTINR_VAL1 PT-INR Value

N	Mean	Standard Deviation	Minimum	Maximum
6	1.9633333	0.9886287	1.0700000	3.7200000

Unique Subject Identifier=5073000010-0005

Assay variable : PTINR_VAL1 PT-INR Value

N	Mean	Standard Deviation	Minimum	Maximum
6	1.8433333	0.5318521	1.1900000	2.3700000

Unique Subject Identifier=5073000010-0010

Assay variable : PTINR_VAL1 PT-INR Value

N	Mean	Standard Deviation	Minimum	Maximum
6	1.7983333	0.6251853	1.1700000	2.7100000

Unique Subject Identifier=5073000010-0013

Assay variable : PTINR_VAL1 PT-INR Value

N	Mean	Standard Deviation	Minimum	Maximum
4	1.8775000	0.1372042	1.7000000	2.0000000

Laboratory tests at the time of obtaining Table 2-6 consent, according to the presence or absence of proton-pump inhibitors

Unique Subject Identifier=5073000010-0015

MEANS Procedure

Assay variable : PTINR_VAL1 PT-INR Value

N	Mean	Standard Deviation	Minimum	Maximum
5	1.6880000	0.3448478	1.4300000	2.2900000

Unique Subject Identifier=5073000010-0017

Assay variable : PTINR_VAL1 PT-INR Value

N	Mean	Standard Deviation	Minimum	Maximum
3	2.1266667	0.1242310	2.0500000	2.2700000

Unique Subject Identifier=5073000010-0031

Assay variable : PTINR_VAL1 PT-INR Value

N	Mean	Standard Deviation	Minimum	Maximum
4	1.7225000	0.1839157	1.5000000	1.9100000

Unique Subject Identifier=5073000010-0048

Assay variable : PTINR_VAL1 PT-INR Value

N	Mean	Standard Deviation	Minimum	Maximum
4	2.0825000	0.2471673	1.8500000	2.4200000

Laboratory tests at the time of obtaining Table 2-6 consent, according to the presence or absence of proton-pump inhibitors

Unique Subject Identifier=5073000010-0051

MEANS Procedure

Assay variable : PTINR_VAL1 PT-INR Value

N	Mean	Standard Deviation	Minimum	Maximum
3	2.6566667	0.1201388	2.5400000	2.7800000

Unique Subject Identifier=5073000010-0055

Assay variable : PTINR_VAL1 PT-INR Value

N	Mean	Standard Deviation	Minimum	Maximum
2	2.4400000	0.2121320	2.2900000	2.5900000

Unique Subject Identifier=5073000010-0066

Assay variable : PTINR_VAL1 PT-INR Value

N	Mean	Standard Deviation	Minimum	Maximum
4	2.7025000	0.2994857	2.3900000	3.1000000

Unique Subject Identifier=5073000010-0083

Assay variable : PTINR_VAL1 PT-INR Value

N	Mean	Standard Deviation	Minimum	Maximum
3	2.0100000	0.6153861	1.6300000	2.7200000

Laboratory tests at the time of obtaining Table 2-6 consent, according to the presence or absence of proton-pump inhibitors

Unique Subject Identifier=5073000010-0086

MEANS Procedure

Assay variable : PTINR_VAL1 PT-INR Value

N	Mean	Standard Deviation	Minimum	Maximum
4	1.6675000	0.5208567	1.1400000	2.3500000

Unique Subject Identifier=5098010011-0007

Assay variable : PTINR_VAL1 PT-INR Value

N	Mean	Standard Deviation	Minimum	Maximum
5	2.1400000	0.3208582	1.7600000	2.6400000

Unique Subject Identifier=5098010011-0017

Assay variable : PTINR_VAL1 PT-INR Value

N	Mean	Standard Deviation	Minimum	Maximum
6	2.1183333	0.4181587	1.6800000	2.8900000

Unique Subject Identifier=5098010011-0021

Assay variable : PTINR_VAL1 PT-INR Value

N	Mean	Standard Deviation	Minimum	Maximum
4	1.9525000	0.2828869	1.6300000	2.2800000

Laboratory tests at the time of obtaining Table 2-6 consent, according to the presence or absence of proton-pump inhibitors

Unique Subject Identifier=5098010011-0027

MEANS Procedure

Assay variable : PTINR_VAL1 PT-INR Value

N	Mean	Standard Deviation	Minimum	Maximum
4	1.6925000	0.3346018	1.2700000	2.0400000

Unique Subject Identifier=5098010011-0028

Assay variable : PTINR_VAL1 PT-INR Value

N	Mean	Standard Deviation	Minimum	Maximum
6	1.6383333	0.0915241	1.5000000	1.7300000

Unique Subject Identifier=5098010011-0030

Assay variable : PTINR_VAL1 PT-INR Value

N	Mean	Standard Deviation	Minimum	Maximum
6	2.0366667	0.8263091	1.2600000	3.6100000

Unique Subject Identifier=5098010011-0038

Assay variable : PTINR_VAL1 PT-INR Value

N	Mean	Standard Deviation	Minimum	Maximum
4	2.9025000	0.7606740	2.1600000	3.9300000

Laboratory tests at the time of obtaining Table 2-6 consent, according to the presence or absence of proton-pump inhibitors

Unique Subject Identifier=5098010011-0061

MEANS Procedure

Assay variable : PTINR_VAL1 PT-INR Value

N	Mean	Standard Deviation	Minimum	Maximum
4	1.8800000	0.2367840	1.6300000	2.1800000

Unique Subject Identifier=5098010011-0063

Assay variable : PTINR_VAL1 PT-INR Value

N	Mean	Standard Deviation	Minimum	Maximum
5	2.3260000	0.3467420	1.9200000	2.6700000

Unique Subject Identifier=5098010011-0066

Assay variable : PTINR_VAL1 PT-INR Value

N	Mean	Standard Deviation	Minimum	Maximum
4	2.2750000	0.4327817	1.8500000	2.8700000

Unique Subject Identifier=5098010011-0069

Assay variable : PTINR_VAL1 PT-INR Value

N	Mean	Standard Deviation	Minimum	Maximum
4	2.3350000	0.6630485	1.5600000	3.1800000

Laboratory tests at the time of obtaining Table 2-6 consent, according to the presence or absence of proton-pump inhibitors

Unique Subject Identifier=5098010011-0076

MEANS Procedure

Assay variable : PTINR_VAL1 PT-INR Value

N	Mean	Standard Deviation	Minimum	Maximum
4	2.1050000	0.4198809	1.5400000	2.5000000

Unique Subject Identifier=5098010011-0087

Assay variable : PTINR_VAL1 PT-INR Value

N	Mean	Standard Deviation	Minimum	Maximum
4	2.1250000	0.1135782	2.0100000	2.2500000

Unique Subject Identifier=5098010011-0089

Assay variable : PTINR_VAL1 PT-INR Value

N	Mean	Standard Deviation	Minimum	Maximum
5	1.9860000	0.2320129	1.6700000	2.2600000

Unique Subject Identifier=5100001000-0001

Assay variable : PTINR_VAL1 PT-INR Value

N	Mean	Standard Deviation	Minimum	Maximum
4	1.4300000	0.1205543	1.3200000	1.6000000

Laboratory tests at the time of obtaining Table 2-6 consent, according to the presence or absence of proton-pump inhibitors

Unique Subject Identifier=5100001000-0006

MEANS Procedure

Assay variable : PTINR_VAL1 PT-INR Value

N	Mean	Standard Deviation	Minimum	Maximum
4	1.7250000	0.2724579	1.4600000	1.9600000

Unique Subject Identifier=5103000000-0050

Assay variable : PTINR_VAL1 PT-INR Value

N	Mean	Standard Deviation	Minimum	Maximum
6	2.1166667	0.2786874	1.7000000	2.4000000

Unique Subject Identifier=5103000000-0058

Assay variable : PTINR_VAL1 PT-INR Value

N	Mean	Standard Deviation	Minimum	Maximum
6	1.5833333	0.1329160	1.4000000	1.8000000

Unique Subject Identifier=5103000000-0067

Assay variable : PTINR_VAL1 PT-INR Value

N	Mean	Standard Deviation	Minimum	Maximum
6	2.0500000	0.6090977	1.2000000	3.0000000

Laboratory tests at the time of obtaining Table 2-6 consent, according to the presence or absence of proton-pump inhibitors

Unique Subject Identifier=5103000000-0075

MEANS Procedure

Assay variable : PTINR_VAL1 PT-INR Value

N	Mean	Standard Deviation	Minimum	Maximum
6	1.8333333	0.1632993	1.6000000	2.1000000

Unique Subject Identifier=5103000000-0079

Assay variable : PTINR_VAL1 PT-INR Value

N	Mean	Standard Deviation	Minimum	Maximum
6	2.1500000	0.2345208	1.7000000	2.3000000

Unique Subject Identifier=5103000000-0142

Assay variable : PTINR_VAL1 PT-INR Value

N	Mean	Standard Deviation	Minimum	Maximum
6	2.3666667	1.1395906	1.4000000	4.6000000

Unique Subject Identifier=5103000000-0150

Assay variable : PTINR_VAL1 PT-INR Value

N	Mean	Standard Deviation	Minimum	Maximum
6	2.2333333	1.1570076	1.1000000	4.1000000

Laboratory tests at the time of obtaining Table 2-6 consent, according to the presence or absence of proton-pump inhibitors

Unique Subject Identifier=5108001000-0017

MEANS Procedure

Assay variable : PTINR_VAL1 PT-INR Value

N	Mean	Standard Deviation	Minimum	Maximum
4	1.3500000	0.1290994	1.2000000	1.5000000

Unique Subject Identifier=5108001000-0023

Assay variable : PTINR_VAL1 PT-INR Value

N	Mean	Standard Deviation	Minimum	Maximum
6	1.2166667	0.0752773	1.1000000	1.3000000

Unique Subject Identifier=5114000000-0001

Assay variable : PTINR_VAL1 PT-INR Value

N	Mean	Standard Deviation	Minimum	Maximum
1	3.6800000		3.6800000	3.6800000

Unique Subject Identifier=5114000000-0007

Assay variable : PTINR_VAL1 PT-INR Value

N	Mean	Standard Deviation	Minimum	Maximum
2	1.8350000	0.1202082	1.7500000	1.9200000

Laboratory tests at the time of obtaining Table 2-6 consent, according to the presence or absence of proton-pump inhibitors

Unique Subject Identifier=5114000000-0009

MEANS Procedure

Assay variable : PTINR_VAL1 PT-INR Value

N	Mean	Standard Deviation	Minimum	Maximum
6	1.4816667	0.2443290	1.2500000	1.9600000

Unique Subject Identifier=5114000000-0010

Assay variable : PTINR_VAL1 PT-INR Value

N	Mean	Standard Deviation	Minimum	Maximum
1	1.0300000		1.0300000	1.0300000

Unique Subject Identifier=5114000000-0028

Assay variable : PTINR_VAL1 PT-INR Value

N	Mean	Standard Deviation	Minimum	Maximum
4	2.0700000	0.6102459	1.3400000	2.7800000

Unique Subject Identifier=5120011000-0008

Assay variable : PTINR_VAL1 PT-INR Value

N	Mean	Standard Deviation	Minimum	Maximum
4	1.7975000	0.4035984	1.4300000	2.2600000

Laboratory tests at the time of obtaining Table 2-6 consent, according to the presence or absence of proton-pump inhibitors

Unique Subject Identifier=5120011000-0014

MEANS Procedure

Assay variable : PTINR_VAL1 PT-INR Value

N	Mean	Standard Deviation	Minimum	Maximum
3	2.0666667	0.2967041	1.7300000	2.2900000

Unique Subject Identifier=5125000000-0001

Assay variable : PTINR_VAL1 PT-INR Value

N	Mean	Standard Deviation	Minimum	Maximum
6	1.9500000	0.1974842	1.7000000	2.3000000

Unique Subject Identifier=5131011111-0005

Assay variable : PTINR_VAL1 PT-INR Value

N	Mean	Standard Deviation	Minimum	Maximum
3	2.1333333	0.3098925	1.9300000	2.4900000

Unique Subject Identifier=5131011111-0019

Assay variable : PTINR_VAL1 PT-INR Value

N	Mean	Standard Deviation	Minimum	Maximum
5	2.2800000	0.8635103	1.2800000	3.1800000

Laboratory tests at the time of obtaining Table 2-6 consent, according to the presence or absence of proton-pump inhibitors

Unique Subject Identifier=5136000000-0003

MEANS Procedure

Assay variable : PTINR_VAL1 PT-INR Value

N	Mean	Standard Deviation	Minimum	Maximum
4	2.1800000	0.6470446	1.6000000	2.7600000

Unique Subject Identifier=5136000000-0005

Assay variable : PTINR_VAL1 PT-INR Value

N	Mean	Standard Deviation	Minimum	Maximum
6	1.3500000	0.3485972	1.0400000	1.9900000

Unique Subject Identifier=5136000000-0011

Assay variable : PTINR_VAL1 PT-INR Value

N	Mean	Standard Deviation	Minimum	Maximum
6	2.1800000	1.1414552	0.9600000	3.6700000

Unique Subject Identifier=5136000000-0016

Assay variable : PTINR_VAL1 PT-INR Value

N	Mean	Standard Deviation	Minimum	Maximum
5	2.3200000	0.1864135	2.0400000	2.5300000

Laboratory tests at the time of obtaining Table 2-6 consent, according to the presence or absence of proton-pump inhibitors

Unique Subject Identifier=5136000000-0022

MEANS Procedure

Assay variable : PTINR_VAL1 PT-INR Value

N	Mean	Standard Deviation	Minimum	Maximum
3	1.6100000	0.2505993	1.3500000	1.8500000

Unique Subject Identifier=5136000000-0026

Assay variable : PTINR_VAL1 PT-INR Value

N	Mean	Standard Deviation	Minimum	Maximum
5	1.7460000	0.4400341	1.1700000	2.2900000

Unique Subject Identifier=5137010111-0002

Assay variable : PTINR_VAL1 PT-INR Value

N	Mean	Standard Deviation	Minimum	Maximum
4	2.4050000	0.3504759	2.0400000	2.8600000

Unique Subject Identifier=5141111100-0001

Assay variable : PTINR_VAL1 PT-INR Value

N	Mean	Standard Deviation	Minimum	Maximum
6	1.7983333	0.2667146	1.4300000	2.1700000

Laboratory tests at the time of obtaining Table 2-6 consent, according to the presence or absence of proton-pump inhibitors

Unique Subject Identifier=5141111100-0012

MEANS Procedure

Assay variable : PTINR_VAL1 PT-INR Value

N	Mean	Standard Deviation	Minimum	Maximum
1	1.6800000		1.6800000	1.6800000

Unique Subject Identifier=5141111100-0018

Assay variable : PTINR_VAL1 PT-INR Value

N	Mean	Standard Deviation	Minimum	Maximum
3	1.7200000	0.2515949	1.5600000	2.0100000

Unique Subject Identifier=5141111100-0030

Assay variable : PTINR_VAL1 PT-INR Value

N	Mean	Standard Deviation	Minimum	Maximum
3	1.1266667	0.1266228	1.0300000	1.2700000

Unique Subject Identifier=5155000000-0012

Assay variable : PTINR_VAL1 PT-INR Value

N	Mean	Standard Deviation	Minimum	Maximum
4	1.6750000	0.0939858	1.5600000	1.7700000

Laboratory tests at the time of obtaining Table 2-6 consent, according to the presence or absence of proton-pump inhibitors

Unique Subject Identifier=5157010010-0014

MEANS Procedure

Assay variable : PTINR_VAL1 PT-INR Value

N	Mean	Standard Deviation	Minimum	Maximum
3	2.3300000	0.2463737	2.0700000	2.5600000

Unique Subject Identifier=5157010010-0015

Assay variable : PTINR_VAL1 PT-INR Value

N	Mean	Standard Deviation	Minimum	Maximum
3	2.3833333	0.1422439	2.2200000	2.4800000

Unique Subject Identifier=5161010010-0004

Assay variable : PTINR_VAL1 PT-INR Value

N	Mean	Standard Deviation	Minimum	Maximum
4	1.9275000	0.2324328	1.7300000	2.2500000

Unique Subject Identifier=5171010000-0025

Assay variable : PTINR_VAL1 PT-INR Value

N	Mean	Standard Deviation	Minimum	Maximum
3	1.9033333	0.2914332	1.6700000	2.2300000

Laboratory tests at the time of obtaining Table 2-6 consent, according to the presence or absence of proton-pump inhibitors

Unique Subject Identifier=5198000001-0019

MEANS Procedure

Assay variable : PTINR_VAL1 PT-INR Value

N	Mean	Standard Deviation	Minimum	Maximum
1	1.3000000		1.3000000	1.3000000

Unique Subject Identifier=5198000001-0025

Assay variable : PTINR_VAL1 PT-INR Value

N	Mean	Standard Deviation	Minimum	Maximum
2	1.9250000	0.2899138	1.7200000	2.1300000

Unique Subject Identifier=5198000001-0053

Assay variable : PTINR_VAL1 PT-INR Value

N	Mean	Standard Deviation	Minimum	Maximum
4	2.2575000	0.3925451	1.9900000	2.8400000

Unique Subject Identifier=5198000001-0057

Assay variable : PTINR_VAL1 PT-INR Value

N	Mean	Standard Deviation	Minimum	Maximum
1	2.4800000		2.4800000	2.4800000

Laboratory tests at the time of obtaining Table 2-6 consent, according to the presence or absence of proton-pump inhibitors

Unique Subject Identifier=5198000001-0071

MEANS Procedure

Assay variable : PTINR_VAL1 PT-INR Value

N	Mean	Standard Deviation	Minimum	Maximum
1	2.2000000		2.2000000	2.2000000

Unique Subject Identifier=5198000001-0081

Assay variable : PTINR_VAL1 PT-INR Value

N	Mean	Standard Deviation	Minimum	Maximum
4	2.1250000	0.7048641	1.5300000	3.1400000

Unique Subject Identifier=5198000001-0101

Assay variable : PTINR_VAL1 PT-INR Value

N	Mean	Standard Deviation	Minimum	Maximum
5	1.7960000	0.7152832	0.9600000	2.8300000

Unique Subject Identifier=5198000001-0116

Assay variable : PTINR_VAL1 PT-INR Value

N	Mean	Standard Deviation	Minimum	Maximum
2	1.4900000	0.5939697	1.0700000	1.9100000

Laboratory tests at the time of obtaining Table 2-6 consent, according to the presence or absence of proton-pump inhibitors

Unique Subject Identifier=5198000001-0117

MEANS Procedure

Assay variable : PTINR_VAL1 PT-INR Value

N	Mean	Standard Deviation	Minimum	Maximum
3	2.1300000	0.2851315	1.8500000	2.4200000

Unique Subject Identifier=5198000001-0137

Assay variable : PTINR_VAL1 PT-INR Value

N	Mean	Standard Deviation	Minimum	Maximum
1	1.4100000		1.4100000	1.4100000

Unique Subject Identifier=5198000001-0138

Assay variable : PTINR_VAL1 PT-INR Value

N	Mean	Standard Deviation	Minimum	Maximum
1	2.4300000		2.4300000	2.4300000

Unique Subject Identifier=5198000001-0152

Assay variable : PTINR_VAL1 PT-INR Value

N	Mean	Standard Deviation	Minimum	Maximum
5	1.9880000	0.6437546	1.2200000	2.9700000

Laboratory tests at the time of obtaining Table 2-6 consent, according to the presence or absence of proton-pump inhibitors

Unique Subject Identifier=5198000001-0165

MEANS Procedure

Assay variable : PTINR_VAL1 PT-INR Value

N	Mean	Standard Deviation	Minimum	Maximum
1	2.2000000		2.2000000	2.2000000

Unique Subject Identifier=5198000001-0186

Assay variable : PTINR_VAL1 PT-INR Value

N	Mean	Standard Deviation	Minimum	Maximum
3	1.1200000	0.1757840	0.9900000	1.3200000

Unique Subject Identifier=5198000001-0230

Assay variable : PTINR_VAL1 PT-INR Value

N	Mean	Standard Deviation	Minimum	Maximum
2	2.2850000	0.4313351	1.9800000	2.5900000

Unique Subject Identifier=5198000001-0234

Assay variable : PTINR_VAL1 PT-INR Value

N	Mean	Standard Deviation	Minimum	Maximum
1	1.9500000		1.9500000	1.9500000

Laboratory tests at the time of obtaining Table 2-6 consent, according to the presence or absence of proton-pump inhibitors

Unique Subject Identifier=5210000100-0026

MEANS Procedure

Assay variable : PTINR_VAL1 PT-INR Value

N	Mean	Standard Deviation	Minimum	Maximum
3	2.4066667	0.1193035	2.3100000	2.5400000

Unique Subject Identifier=5223000000-0001

Assay variable : PTINR_VAL1 PT-INR Value

N	Mean	Standard Deviation	Minimum	Maximum
6	1.1266667	0.0868716	1.0000000	1.2100000

Unique Subject Identifier=5223000000-0010

Assay variable : PTINR_VAL1 PT-INR Value

N	Mean	Standard Deviation	Minimum	Maximum
4	2.2075000	0.2393568	1.9200000	2.4700000

Unique Subject Identifier=5223000000-0012

Assay variable : PTINR_VAL1 PT-INR Value

N	Mean	Standard Deviation	Minimum	Maximum
3	3.0733333	0.4499259	2.5900000	3.4800000

Laboratory tests at the time of obtaining Table 2-6 consent, according to the presence or absence of proton-pump inhibitors

Unique Subject Identifier=5223000000-0014

MEANS Procedure

Assay variable : PTINR_VAL1 PT-INR Value

N	Mean	Standard Deviation	Minimum	Maximum
3	1.4166667	0.1550269	1.2400000	1.5300000

Unique Subject Identifier=5223000000-0019

Assay variable : PTINR_VAL1 PT-INR Value

N	Mean	Standard Deviation	Minimum	Maximum
3	1.9466667	0.1069268	1.8800000	2.0700000

Unique Subject Identifier=5223000000-0021

Assay variable : PTINR_VAL1 PT-INR Value

N	Mean	Standard Deviation	Minimum	Maximum
3	1.8433333	0.2345918	1.6400000	2.1000000

Unique Subject Identifier=5224000000-0001

Assay variable : PTINR_VAL1 PT-INR Value

N	Mean	Standard Deviation	Minimum	Maximum
4	1.6775000	0.1925920	1.4800000	1.9400000

Laboratory tests at the time of obtaining Table 2-6 consent, according to the presence or absence of proton-pump inhibitors

Unique Subject Identifier=5232010000-0007

MEANS Procedure

Assay variable : PTINR_VAL1 PT-INR Value

N	Mean	Standard Deviation	Minimum	Maximum
5	1.7300000	0.2442335	1.4900000	2.0500000

Unique Subject Identifier=5232010000-0012

Assay variable : PTINR_VAL1 PT-INR Value

N	Mean	Standard Deviation	Minimum	Maximum
4	1.7875000	0.1767060	1.5300000	1.9300000

Unique Subject Identifier=5233000000-0008

Assay variable : PTINR_VAL1 PT-INR Value

N	Mean	Standard Deviation	Minimum	Maximum
4	1.9125000	0.8410460	1.1000000	3.0500000

Unique Subject Identifier=5235000000-0015

Assay variable : PTINR_VAL1 PT-INR Value

N	Mean	Standard Deviation	Minimum	Maximum
6	2.1500000	0.2073644	1.9000000	2.5000000

Laboratory tests at the time of obtaining Table 2-6 consent, according to the presence or absence of proton-pump inhibitors

Unique Subject Identifier=5235000000-0030

MEANS Procedure

Assay variable : PTINR_VAL1 PT-INR Value

N	Mean	Standard Deviation	Minimum	Maximum
6	2.0616667	0.1830209	1.9500000	2.4200000

Unique Subject Identifier=5235000000-0050

Assay variable : PTINR_VAL1 PT-INR Value

N	Mean	Standard Deviation	Minimum	Maximum
6	1.6666667	0.1005319	1.5500000	1.8100000

Unique Subject Identifier=5236000000-0019

Assay variable : PTINR_VAL1 PT-INR Value

N	Mean	Standard Deviation	Minimum	Maximum
5	2.0580000	0.2839366	1.8600000	2.5600000

Unique Subject Identifier=5236000000-0021

Assay variable : PTINR_VAL1 PT-INR Value

N	Mean	Standard Deviation	Minimum	Maximum
6	2.2716667	0.7649161	1.4800000	3.2800000

Laboratory tests at the time of obtaining Table 2-6 consent, according to the presence or absence of proton-pump inhibitors

Unique Subject Identifier=5237010000-0010

MEANS Procedure

Assay variable : PTINR_VAL1 PT-INR Value

N	Mean	Standard Deviation	Minimum	Maximum
6	2.1066667	0.1736280	1.9400000	2.3300000

Unique Subject Identifier=5255000000-0006

Assay variable : PTINR_VAL1 PT-INR Value

N	Mean	Standard Deviation	Minimum	Maximum
4	2.3950000	0.3145897	2.1300000	2.7700000

Unique Subject Identifier=5260010000-0013

Assay variable : PTINR_VAL1 PT-INR Value

N	Mean	Standard Deviation	Minimum	Maximum
4	2.1075000	0.2550000	1.8500000	2.4600000

Unique Subject Identifier=5260010000-0019

Assay variable : PTINR_VAL1 PT-INR Value

N	Mean	Standard Deviation	Minimum	Maximum
5	1.6580000	0.2629068	1.2500000	1.9700000

Laboratory tests at the time of obtaining Table 2-6 consent, according to the presence or absence of proton-pump inhibitors

Unique Subject Identifier=5260010000-0025

MEANS Procedure

Assay variable : PTINR_VAL1 PT-INR Value

N	Mean	Standard Deviation	Minimum	Maximum
3	1.8800000	0.3897435	1.4600000	2.2300000

Unique Subject Identifier=5261010010-0014

Assay variable : PTINR_VAL1 PT-INR Value

N	Mean	Standard Deviation	Minimum	Maximum
3	1.8066667	0.1331666	1.6600000	1.9200000

Unique Subject Identifier=5261010010-0018

Assay variable : PTINR_VAL1 PT-INR Value

N	Mean	Standard Deviation	Minimum	Maximum
4	1.6675000	0.3820449	1.2000000	2.0400000

Unique Subject Identifier=5305000000-0009

Assay variable : PTINR_VAL1 PT-INR Value

N	Mean	Standard Deviation	Minimum	Maximum
6	2.0983333	0.5871769	1.3300000	2.8800000

Laboratory tests at the time of obtaining Table 2-6 consent, according to the presence or absence of proton-pump inhibitors

Unique Subject Identifier=5305000000-0016

MEANS Procedure

Assay variable : PTINR_VAL1 PT-INR Value

N	Mean	Standard Deviation	Minimum	Maximum
4	1.8925000	0.3764195	1.4200000	2.3200000

Unique Subject Identifier=5305000000-0039

Assay variable : PTINR_VAL1 PT-INR Value

N	Mean	Standard Deviation	Minimum	Maximum
3	2.2633333	0.5253887	1.8700000	2.8600000

Unique Subject Identifier=5342011001-0023

Assay variable : PTINR_VAL1 PT-INR Value

N	Mean	Standard Deviation	Minimum	Maximum
6	1.9283333	0.3761604	1.3700000	2.2700000

Unique Subject Identifier=5355000000-0019

Assay variable : PTINR_VAL1 PT-INR Value

N	Mean	Standard Deviation	Minimum	Maximum
6	2.0066667	0.8450010	1.6100000	3.7300000

Laboratory tests at the time of obtaining Table 2-6 consent, according to the presence or absence of proton-pump inhibitors

Unique Subject Identifier=5355000000-0027

MEANS Procedure

Assay variable : PTINR_VAL1 PT-INR Value

N	Mean	Standard Deviation	Minimum	Maximum
2	2.0350000	0.0212132	2.0200000	2.0500000

Unique Subject Identifier=5414010000-0006

Assay variable : PTINR_VAL1 PT-INR Value

N	Mean	Standard Deviation	Minimum	Maximum
5	1.4400000	0.4939636	1.0700000	2.2900000

Unique Subject Identifier=5414010000-0007

Assay variable : PTINR_VAL1 PT-INR Value

N	Mean	Standard Deviation	Minimum	Maximum
5	2.2880000	0.6480895	1.6300000	3.3500000

Unique Subject Identifier=5414010000-0009

Assay variable : PTINR_VAL1 PT-INR Value

N	Mean	Standard Deviation	Minimum	Maximum
5	1.2900000	0.5819364	0.9400000	2.2900000

Laboratory tests at the time of obtaining Table 2-6 consent, according to the presence or absence of proton-pump inhibitors

Unique Subject Identifier=5414010000-0012

MEANS Procedure

Assay variable : PTINR_VAL1 PT-INR Value

N	Mean	Standard Deviation	Minimum	Maximum
6	1.5450000	0.1681368	1.2600000	1.7700000

Unique Subject Identifier=5414010000-0014

Assay variable : PTINR_VAL1 PT-INR Value

N	Mean	Standard Deviation	Minimum	Maximum
6	1.7450000	0.2980436	1.2800000	2.1900000

Unique Subject Identifier=5414010000-0033

Assay variable : PTINR_VAL1 PT-INR Value

N	Mean	Standard Deviation	Minimum	Maximum
6	1.7183333	0.1582930	1.5600000	1.9900000

Unique Subject Identifier=5414010000-0034

Assay variable : PTINR_VAL1 PT-INR Value

N	Mean	Standard Deviation	Minimum	Maximum
4	1.4325000	0.1167262	1.2800000	1.5500000

Laboratory tests at the time of obtaining Table 2-6 consent, according to the presence or absence of proton-pump inhibitors

Unique Subject Identifier=5414010000-0046

MEANS Procedure

Assay variable : PTINR_VAL1 PT-INR Value

N	Mean	Standard Deviation	Minimum	Maximum
2	2.0000000	0.6081118	1.5700000	2.4300000

Unique Subject Identifier=5414010000-0057

Assay variable : PTINR_VAL1 PT-INR Value

N	Mean	Standard Deviation	Minimum	Maximum
1	2.3000000		2.3000000	2.3000000

Unique Subject Identifier=5414010000-0059

Assay variable : PTINR_VAL1 PT-INR Value

N	Mean	Standard Deviation	Minimum	Maximum
5	2.0720000	0.8327785	1.2200000	3.4200000

Unique Subject Identifier=5414010000-0064

Assay variable : PTINR_VAL1 PT-INR Value

N	Mean	Standard Deviation	Minimum	Maximum
4	1.9275000	0.5612709	1.2800000	2.4500000

Laboratory tests at the time of obtaining Table 2-6 consent, according to the presence or absence of proton-pump inhibitors

Unique Subject Identifier=5466000000-0003

MEANS Procedure

Assay variable : PTINR_VAL1 PT-INR Value

N	Mean	Standard Deviation	Minimum	Maximum
6	2.3516667	0.4270090	2.0200000	3.1400000

Unique Subject Identifier=5466000000-0004

Assay variable : PTINR_VAL1 PT-INR Value

N	Mean	Standard Deviation	Minimum	Maximum
5	2.5000000	0.1102270	2.4100000	2.6200000

Unique Subject Identifier=5466000000-0005

Assay variable : PTINR_VAL1 PT-INR Value

N	Mean	Standard Deviation	Minimum	Maximum
4	2.3175000	0.3972719	2.0600000	2.9100000

Unique Subject Identifier=5466000000-0010

Assay variable : PTINR_VAL1 PT-INR Value

N	Mean	Standard Deviation	Minimum	Maximum
6	2.4283333	0.6023094	1.6900000	3.1400000

Laboratory tests at the time of obtaining Table 2-6 consent, according to the presence or absence of proton-pump inhibitors

Unique Subject Identifier=5514000000-0004

MEANS Procedure

Assay variable : PTINR_VAL1 PT-INR Value

N	Mean	Standard Deviation	Minimum	Maximum
6	1.5866667	0.2106814	1.3000000	1.8400000

Unique Subject Identifier=5514000000-0006

Assay variable : PTINR_VAL1 PT-INR Value

N	Mean	Standard Deviation	Minimum	Maximum
4	1.5150000	0.0810350	1.4400000	1.6300000

Unique Subject Identifier=5514000000-0016

Assay variable : PTINR_VAL1 PT-INR Value

N	Mean	Standard Deviation	Minimum	Maximum
6	1.8500000	0.3519091	1.2700000	2.2700000

Unique Subject Identifier=5514000000-0026

Assay variable : PTINR_VAL1 PT-INR Value

N	Mean	Standard Deviation	Minimum	Maximum
6	2.2283333	0.2108475	1.8300000	2.4300000

Laboratory tests at the time of obtaining Table 2-6 consent, according to the presence or absence of proton-pump inhibitors

Unique Subject Identifier=5514000000-0038

MEANS Procedure

Assay variable : PTINR_VAL1 PT-INR Value

N	Mean	Standard Deviation	Minimum	Maximum
4	1.8175000	0.2489143	1.5000000	2.0900000

Unique Subject Identifier=5514000000-0041

Assay variable : PTINR_VAL1 PT-INR Value

N	Mean	Standard Deviation	Minimum	Maximum
4	2.6175000	0.3613286	2.1200000	2.9500000

Unique Subject Identifier=5542000000-0008

Assay variable : PTINR_VAL1 PT-INR Value

N	Mean	Standard Deviation	Minimum	Maximum
2	2.3800000	0.6222540	1.9400000	2.8200000

Unique Subject Identifier=5542000000-0013

Assay variable : PTINR_VAL1 PT-INR Value

N	Mean	Standard Deviation	Minimum	Maximum
5	1.1120000	0.0303315	1.0800000	1.1600000

Laboratory tests at the time of obtaining Table 2-6 consent, according to the presence or absence of proton-pump inhibitors

Unique Subject Identifier=5542000000-0016

MEANS Procedure

Assay variable : PTINR_VAL1 PT-INR Value

N	Mean	Standard Deviation	Minimum	Maximum
1	1.5300000		1.5300000	1.5300000

Unique Subject Identifier=5542000000-0017

Assay variable : PTINR_VAL1 PT-INR Value

N	Mean	Standard Deviation	Minimum	Maximum
6	2.1666667	0.4706449	1.6600000	2.8900000

Unique Subject Identifier=5542000000-0019

Assay variable : PTINR_VAL1 PT-INR Value

N	Mean	Standard Deviation	Minimum	Maximum
6	1.8383333	0.3072730	1.4400000	2.1600000

Unique Subject Identifier=5542000000-0024

Assay variable : PTINR_VAL1 PT-INR Value

N	Mean	Standard Deviation	Minimum	Maximum
2	1.9250000	0.1626346	1.8100000	2.0400000

Laboratory tests at the time of obtaining Table 2-6 consent, according to the presence or absence of proton-pump inhibitors

Unique Subject Identifier=5542000000-0027

MEANS Procedure

Assay variable : PTINR_VAL1 PT-INR Value

N	Mean	Standard Deviation	Minimum	Maximum
5	1.7600000	0.6942622	1.1400000	2.8700000

Unique Subject Identifier=5542000000-0031

Assay variable : PTINR_VAL1 PT-INR Value

N	Mean	Standard Deviation	Minimum	Maximum
1	2.0600000		2.0600000	2.0600000

Unique Subject Identifier=5542000000-0034

Assay variable : PTINR_VAL1 PT-INR Value

N	Mean	Standard Deviation	Minimum	Maximum
2	1.4250000	0.0212132	1.4100000	1.4400000

Unique Subject Identifier=5542000000-0041

Assay variable : PTINR_VAL1 PT-INR Value

N	Mean	Standard Deviation	Minimum	Maximum
6	1.7100000	0.1954482	1.5000000	2.0000000

Laboratory tests at the time of obtaining Table 2-6 consent, according to the presence or absence of proton-pump inhibitors

Unique Subject Identifier=5542000000-0043

MEANS Procedure

Assay variable : PTINR_VAL1 PT-INR Value

N	Mean	Standard Deviation	Minimum	Maximum
4	1.7350000	0.7977259	1.2900000	2.9300000

Unique Subject Identifier=5717000000-0005

Assay variable : PTINR_VAL1 PT-INR Value

N	Mean	Standard Deviation	Minimum	Maximum
6	2.0900000	0.1194990	1.9500000	2.3000000

Unique Subject Identifier=6000010011-0001

Assay variable : PTINR_VAL1 PT-INR Value

N	Mean	Standard Deviation	Minimum	Maximum
4	2.4150000	0.2066398	2.2200000	2.6600000

Unique Subject Identifier=6002010000-0030

Assay variable : PTINR_VAL1 PT-INR Value

N	Mean	Standard Deviation	Minimum	Maximum
5	2.4440000	0.9090820	1.4900000	3.5800000

Laboratory tests at the time of obtaining Table 2-6 consent, according to the presence or absence of proton-pump inhibitors

Unique Subject Identifier=6002010000-0102

MEANS Procedure

Assay variable : PTINR_VAL1 PT-INR Value

N	Mean	Standard Deviation	Minimum	Maximum
4	2.3100000	0.1235584	2.1300000	2.4000000

Unique Subject Identifier=6009000000-0016

Assay variable : PTINR_VAL1 PT-INR Value

N	Mean	Standard Deviation	Minimum	Maximum
5	1.9400000	0.4449719	1.2000000	2.4000000

Unique Subject Identifier=6009000000-0017

Assay variable : PTINR_VAL1 PT-INR Value

N	Mean	Standard Deviation	Minimum	Maximum
6	1.6666667	0.6772493	1.1000000	3.0000000

Unique Subject Identifier=6009000000-0026

Assay variable : PTINR_VAL1 PT-INR Value

N	Mean	Standard Deviation	Minimum	Maximum
6	1.5333333	0.1966384	1.3000000	1.8000000

Laboratory tests at the time of obtaining Table 2-6 consent, according to the presence or absence of proton-pump inhibitors

Unique Subject Identifier=6009000000-0027

MEANS Procedure

Assay variable : PTINR_VAL1 PT-INR Value

N	Mean	Standard Deviation	Minimum	Maximum
5	1.8600000	0.8763561	1.2000000	3.4000000

Unique Subject Identifier=6009000000-0032

Assay variable : PTINR_VAL1 PT-INR Value

N	Mean	Standard Deviation	Minimum	Maximum
4	1.6250000	0.2061553	1.4000000	1.8000000

Unique Subject Identifier=6014011010-0002

Assay variable : PTINR_VAL1 PT-INR Value

N	Mean	Standard Deviation	Minimum	Maximum
1	2.3200000		2.3200000	2.3200000

Unique Subject Identifier=6014011010-0009

Assay variable : PTINR_VAL1 PT-INR Value

N	Mean	Standard Deviation	Minimum	Maximum
5	2.6520000	0.2508386	2.2800000	2.8700000

Laboratory tests at the time of obtaining Table 2-6 consent, according to the presence or absence of proton-pump inhibitors

Unique Subject Identifier=6014011010-0021

MEANS Procedure

Assay variable : PTINR_VAL1 PT-INR Value

N	Mean	Standard Deviation	Minimum	Maximum
5	2.1020000	0.8499235	1.4500000	3.3500000

Unique Subject Identifier=6014011010-0032

Assay variable : PTINR_VAL1 PT-INR Value

N	Mean	Standard Deviation	Minimum	Maximum
4	2.0000000	0.4690416	1.4000000	2.5000000

Unique Subject Identifier=6022000000-0008

Assay variable : PTINR_VAL1 PT-INR Value

N	Mean	Standard Deviation	Minimum	Maximum
2	1.7150000	0.2757716	1.5200000	1.9100000

Unique Subject Identifier=6022000000-0024

Assay variable : PTINR_VAL1 PT-INR Value

N	Mean	Standard Deviation	Minimum	Maximum
4	2.2525000	0.4459727	1.7900000	2.8600000

Laboratory tests at the time of obtaining Table 2-6 consent, according to the presence or absence of proton-pump inhibitors

Unique Subject Identifier=6022000000-0030

MEANS Procedure

Assay variable : PTINR_VAL1 PT-INR Value

N	Mean	Standard Deviation	Minimum	Maximum
4	2.7850000	1.8992016	1.2400000	5.5100000

Unique Subject Identifier=6023000000-0003

Assay variable : PTINR_VAL1 PT-INR Value

N	Mean	Standard Deviation	Minimum	Maximum
3	1.7666667	0.3785939	1.5000000	2.2000000

Unique Subject Identifier=6023000000-0007

Assay variable : PTINR_VAL1 PT-INR Value

N	Mean	Standard Deviation	Minimum	Maximum
4	1.9250000	0.4573474	1.4000000	2.4000000

Unique Subject Identifier=6023000000-0009

Assay variable : PTINR_VAL1 PT-INR Value

N	Mean	Standard Deviation	Minimum	Maximum
3	1.6666667	0.0577350	1.6000000	1.7000000

Laboratory tests at the time of obtaining Table 2-6 consent, according to the presence or absence of proton-pump inhibitors

Unique Subject Identifier=6023000000-0010

MEANS Procedure

Assay variable : PTINR_VAL1 PT-INR Value

N	Mean	Standard Deviation	Minimum	Maximum
6	1.6000000	0.2966479	1.2000000	1.9000000

Unique Subject Identifier=6023000000-0013

Assay variable : PTINR_VAL1 PT-INR Value

N	Mean	Standard Deviation	Minimum	Maximum
6	2.1833333	0.1722401	2.0000000	2.4000000

Unique Subject Identifier=6023000000-0014

Assay variable : PTINR_VAL1 PT-INR Value

N	Mean	Standard Deviation	Minimum	Maximum
3	1.7666667	0.0577350	1.7000000	1.8000000

Unique Subject Identifier=6023000000-0022

Assay variable : PTINR_VAL1 PT-INR Value

N	Mean	Standard Deviation	Minimum	Maximum
6	2.5000000	0.9591663	1.6000000	3.8000000

Laboratory tests at the time of obtaining Table 2-6 consent, according to the presence or absence of proton-pump inhibitors

Unique Subject Identifier=6023000000-0025

MEANS Procedure

Assay variable : PTINR_VAL1 PT-INR Value

N	Mean	Standard Deviation	Minimum	Maximum
2	1.9500000	0.0707107	1.9000000	2.0000000

Unique Subject Identifier=6023000000-0043

Assay variable : PTINR_VAL1 PT-INR Value

N	Mean	Standard Deviation	Minimum	Maximum
6	2.3500000	1.1040833	1.0000000	3.7000000

Unique Subject Identifier=6023000000-0045

Assay variable : PTINR_VAL1 PT-INR Value

N	Mean	Standard Deviation	Minimum	Maximum
3	2.2666667	0.1527525	2.1000000	2.4000000

Unique Subject Identifier=6023000000-0048

Assay variable : PTINR_VAL1 PT-INR Value

N	Mean	Standard Deviation	Minimum	Maximum
5	1.6000000	0.2915476	1.2000000	2.0000000

Laboratory tests at the time of obtaining Table 2-6 consent, according to the presence or absence of proton-pump inhibitors

Unique Subject Identifier=6023000000-0049

MEANS Procedure

Assay variable : PTINR_VAL1 PT-INR Value

N	Mean	Standard Deviation	Minimum	Maximum
3	1.7333333	0.2516611	1.5000000	2.0000000

Unique Subject Identifier=6026000000-0021

Assay variable : PTINR_VAL1 PT-INR Value

N	Mean	Standard Deviation	Minimum	Maximum
4	1.8675000	0.2617091	1.5600000	2.2000000

Unique Subject Identifier=6027000000-0003

Assay variable : PTINR_VAL1 PT-INR Value

N	Mean	Standard Deviation	Minimum	Maximum
3	1.7766667	0.2802380	1.4900000	2.0500000

Unique Subject Identifier=6027000000-0015

Assay variable : PTINR_VAL1 PT-INR Value

N	Mean	Standard Deviation	Minimum	Maximum
3	2.3333333	0.3784618	2.1000000	2.7700000

Laboratory tests at the time of obtaining Table 2-6 consent, according to the presence or absence of proton-pump inhibitors

Unique Subject Identifier=6027000000-0020

MEANS Procedure

Assay variable : PTINR_VAL1 PT-INR Value

N	Mean	Standard Deviation	Minimum	Maximum
3	2.6366667	0.3239341	2.4300000	3.0100000

Unique Subject Identifier=6029000000-0001

Assay variable : PTINR_VAL1 PT-INR Value

N	Mean	Standard Deviation	Minimum	Maximum
6	2.1400000	0.1475127	1.9900000	2.4100000

Unique Subject Identifier=6029000000-0005

Assay variable : PTINR_VAL1 PT-INR Value

N	Mean	Standard Deviation	Minimum	Maximum
6	2.0750000	0.3825049	1.5500000	2.6800000

Unique Subject Identifier=6029000000-0011

Assay variable : PTINR_VAL1 PT-INR Value

N	Mean	Standard Deviation	Minimum	Maximum
4	1.4875000	0.3539656	1.1800000	1.9900000

Laboratory tests at the time of obtaining Table 2-6 consent, according to the presence or absence of proton-pump inhibitors

Unique Subject Identifier=6029000000-0012

MEANS Procedure

Assay variable : PTINR_VAL1 PT-INR Value

N	Mean	Standard Deviation	Minimum	Maximum
4	2.0125000	0.1699755	1.8200000	2.1700000

Unique Subject Identifier=6031011010-0003

Assay variable : PTINR_VAL1 PT-INR Value

N	Mean	Standard Deviation	Minimum	Maximum
3	1.6800000	0.0360555	1.6400000	1.7100000

Unique Subject Identifier=6031011010-0006

Assay variable : PTINR_VAL1 PT-INR Value

N	Mean	Standard Deviation	Minimum	Maximum
6	1.7933333	0.2618142	1.4000000	2.1600000

Unique Subject Identifier=6031011010-0009

Assay variable : PTINR_VAL1 PT-INR Value

N	Mean	Standard Deviation	Minimum	Maximum
4	2.1325000	0.3088014	1.7600000	2.4500000

Laboratory tests at the time of obtaining Table 2-6 consent, according to the presence or absence of proton-pump inhibitors

Unique Subject Identifier=6031011010-0017

MEANS Procedure

Assay variable : PTINR_VAL1 PT-INR Value

N	Mean	Standard Deviation	Minimum	Maximum
6	2.4200000	0.3189357	2.0400000	2.9200000

Unique Subject Identifier=6031011010-0023

Assay variable : PTINR_VAL1 PT-INR Value

N	Mean	Standard Deviation	Minimum	Maximum
6	1.6983333	0.1800463	1.5400000	2.0200000

Unique Subject Identifier=6031011010-0027

Assay variable : PTINR_VAL1 PT-INR Value

N	Mean	Standard Deviation	Minimum	Maximum
5	2.2720000	0.2816381	1.9100000	2.5900000

Unique Subject Identifier=6032010000-0018

Assay variable : PTINR_VAL1 PT-INR Value

N	Mean	Standard Deviation	Minimum	Maximum
3	1.9066667	0.0577350	1.8400000	1.9400000

Laboratory tests at the time of obtaining Table 2-6 consent, according to the presence or absence of proton-pump inhibitors

Unique Subject Identifier=6032010000-0074

MEANS Procedure

Assay variable : PTINR_VAL1 PT-INR Value

N	Mean	Standard Deviation	Minimum	Maximum
3	2.2933333	0.2138535	2.0600000	2.4800000

Unique Subject Identifier=6032010000-0080

Assay variable : PTINR_VAL1 PT-INR Value

N	Mean	Standard Deviation	Minimum	Maximum
2	2.4400000	0.4808326	2.1000000	2.7800000

Unique Subject Identifier=6032010000-0081

Assay variable : PTINR_VAL1 PT-INR Value

N	Mean	Standard Deviation	Minimum	Maximum
4	2.9875000	0.5244918	2.4000000	3.4300000

Unique Subject Identifier=6035000000-0018

Assay variable : PTINR_VAL1 PT-INR Value

N	Mean	Standard Deviation	Minimum	Maximum
6	2.0516667	0.5956985	1.4500000	3.0500000

Laboratory tests at the time of obtaining Table 2-6 consent, according to the presence or absence of proton-pump inhibitors

Unique Subject Identifier=6035000000-0019

MEANS Procedure

Assay variable : PTINR_VAL1 PT-INR Value

N	Mean	Standard Deviation	Minimum	Maximum
3	2.0300000	0.2306513	1.7900000	2.2500000

Unique Subject Identifier=6035000000-0022

Assay variable : PTINR_VAL1 PT-INR Value

N	Mean	Standard Deviation	Minimum	Maximum
3	1.5866667	0.1703917	1.3900000	1.6900000

Unique Subject Identifier=6035000000-0023

Assay variable : PTINR_VAL1 PT-INR Value

N	Mean	Standard Deviation	Minimum	Maximum
1	2.2000000		2.2000000	2.2000000

Unique Subject Identifier=6036000000-0006

Assay variable : PTINR_VAL1 PT-INR Value

N	Mean	Standard Deviation	Minimum	Maximum
6	1.7333333	0.4803193	1.3000000	2.6400000

Laboratory tests at the time of obtaining Table 2-6 consent, according to the presence or absence of proton-pump inhibitors

Unique Subject Identifier=6040011000-0001

MEANS Procedure

Assay variable : PTINR_VAL1 PT-INR Value

N	Mean	Standard Deviation	Minimum	Maximum
5	2.2780000	0.3104352	1.8600000	2.6700000

Unique Subject Identifier=6041000000-0001

Assay variable : PTINR_VAL1 PT-INR Value

N	Mean	Standard Deviation	Minimum	Maximum
3	1.6700000	0.1307670	1.5200000	1.7600000

Unique Subject Identifier=6041000000-0006

Assay variable : PTINR_VAL1 PT-INR Value

N	Mean	Standard Deviation	Minimum	Maximum
6	1.5233333	0.4314935	1.1200000	2.2500000

Unique Subject Identifier=6041000000-0007

Assay variable : PTINR_VAL1 PT-INR Value

N	Mean	Standard Deviation	Minimum	Maximum
3	1.9133333	0.1861003	1.7400000	2.1100000

Laboratory tests at the time of obtaining Table 2-6 consent, according to the presence or absence of proton-pump inhibitors

Unique Subject Identifier=6041000000-0013

MEANS Procedure

Assay variable : PTINR_VAL1 PT-INR Value

N	Mean	Standard Deviation	Minimum	Maximum
3	1.8533333	0.2458319	1.6200000	2.1100000

Unique Subject Identifier=6041000000-0020

Assay variable : PTINR_VAL1 PT-INR Value

N	Mean	Standard Deviation	Minimum	Maximum
6	2.0016667	0.3270117	1.3700000	2.3100000

Unique Subject Identifier=6041000000-0022

Assay variable : PTINR_VAL1 PT-INR Value

N	Mean	Standard Deviation	Minimum	Maximum
3	2.0866667	0.2484619	1.8000000	2.2400000

Unique Subject Identifier=6041000000-0024

Assay variable : PTINR_VAL1 PT-INR Value

N	Mean	Standard Deviation	Minimum	Maximum
6	1.9033333	0.2281812	1.6100000	2.1300000

Laboratory tests at the time of obtaining Table 2-6 consent, according to the presence or absence of proton-pump inhibitors

Unique Subject Identifier=6041000000-0029

MEANS Procedure

Assay variable : PTINR_VAL1 PT-INR Value

N	Mean	Standard Deviation	Minimum	Maximum
6	1.5833333	0.3390968	1.2400000	2.1600000

Unique Subject Identifier=6041000000-0032

Assay variable : PTINR_VAL1 PT-INR Value

N	Mean	Standard Deviation	Minimum	Maximum
3	1.4500000	0.0529150	1.3900000	1.4900000

Unique Subject Identifier=6041000000-0038

Assay variable : PTINR_VAL1 PT-INR Value

N	Mean	Standard Deviation	Minimum	Maximum
6	1.5633333	0.1440370	1.4400000	1.7800000

Unique Subject Identifier=6041000000-0042

Assay variable : PTINR_VAL1 PT-INR Value

N	Mean	Standard Deviation	Minimum	Maximum
3	1.5166667	0.1150362	1.4000000	1.6300000

Laboratory tests at the time of obtaining Table 2-6 consent, according to the presence or absence of proton-pump inhibitors

Unique Subject Identifier=6041000000-0059

MEANS Procedure

Assay variable : PTINR_VAL1 PT-INR Value

N	Mean	Standard Deviation	Minimum	Maximum
3	1.6933333	0.2157159	1.5400000	1.9400000

Unique Subject Identifier=6045010111-0002

Assay variable : PTINR_VAL1 PT-INR Value

N	Mean	Standard Deviation	Minimum	Maximum
3	2.5033333	1.0830205	1.5300000	3.6700000

Unique Subject Identifier=6048010000-0043

Assay variable : PTINR_VAL1 PT-INR Value

N	Mean	Standard Deviation	Minimum	Maximum
2	2.1000000	0.4242641	1.8000000	2.4000000

Unique Subject Identifier=6048010000-0046

Assay variable : PTINR_VAL1 PT-INR Value

N	Mean	Standard Deviation	Minimum	Maximum
2	3.4500000	0.0707107	3.4000000	3.5000000

Laboratory tests at the time of obtaining Table 2-6 consent, according to the presence or absence of proton-pump inhibitors

Unique Subject Identifier=6048010000-0047

MEANS Procedure

Assay variable : PTINR_VAL1 PT-INR Value

N	Mean	Standard Deviation	Minimum	Maximum
1	2.3000000		2.3000000	2.3000000

Unique Subject Identifier=6048010000-0049

Assay variable : PTINR_VAL1 PT-INR Value

N	Mean	Standard Deviation	Minimum	Maximum
2	1.9000000	0.4242641	1.6000000	2.2000000

Unique Subject Identifier=6048010000-0050

Assay variable : PTINR_VAL1 PT-INR Value

N	Mean	Standard Deviation	Minimum	Maximum
1	3.5000000		3.5000000	3.5000000

Unique Subject Identifier=6048010000-0054

Assay variable : PTINR_VAL1 PT-INR Value

N	Mean	Standard Deviation	Minimum	Maximum
2	2.8000000	0.7071068	2.3000000	3.3000000

Laboratory tests at the time of obtaining Table 2-6 consent, according to the presence or absence of proton-pump inhibitors

Unique Subject Identifier=6048010000-0058

MEANS Procedure

Assay variable : PTINR_VAL1 PT-INR Value

N	Mean	Standard Deviation	Minimum	Maximum
3	2.4666667	0.6027714	1.9000000	3.1000000

Unique Subject Identifier=6048010000-0066

Assay variable : PTINR_VAL1 PT-INR Value

N	Mean	Standard Deviation	Minimum	Maximum
1	2.2000000		2.2000000	2.2000000

Unique Subject Identifier=6048010000-0076

Assay variable : PTINR_VAL1 PT-INR Value

N	Mean	Standard Deviation	Minimum	Maximum
2	2.2500000	0.4949747	1.9000000	2.6000000

Unique Subject Identifier=6048010000-0080

Assay variable : PTINR_VAL1 PT-INR Value

N	Mean	Standard Deviation	Minimum	Maximum
6	1.6333333	0.4179314	1.2000000	2.3000000

Laboratory tests at the time of obtaining Table 2-6 consent, according to the presence or absence of proton-pump inhibitors

Unique Subject Identifier=6048010000-0082

MEANS Procedure

Assay variable : PTINR_VAL1 PT-INR Value

N	Mean	Standard Deviation	Minimum	Maximum
3	1.0666667	0.0577350	1.0000000	1.1000000

Unique Subject Identifier=6048010000-0086

Assay variable : PTINR_VAL1 PT-INR Value

N	Mean	Standard Deviation	Minimum	Maximum
6	2.1333333	0.9584710	1.5000000	3.8000000

Unique Subject Identifier=6048010000-0092

Assay variable : PTINR_VAL1 PT-INR Value

N	Mean	Standard Deviation	Minimum	Maximum
3	1.3666667	0.0577350	1.3000000	1.4000000

Unique Subject Identifier=6048010000-0093

Assay variable : PTINR_VAL1 PT-INR Value

N	Mean	Standard Deviation	Minimum	Maximum
1	1.8000000		1.8000000	1.8000000

Laboratory tests at the time of obtaining Table 2-6 consent, according to the presence or absence of proton-pump inhibitors

Unique Subject Identifier=6048010000-0100

MEANS Procedure

Assay variable : PTINR_VAL1 PT-INR Value

N	Mean	Standard Deviation	Minimum	Maximum
1	2.3000000		2.3000000	2.3000000

Unique Subject Identifier=6050011100-0001

Assay variable : PTINR_VAL1 PT-INR Value

N	Mean	Standard Deviation	Minimum	Maximum
3	2.3033333	0.4990324	1.7400000	2.6900000

Unique Subject Identifier=6050011100-0002

Assay variable : PTINR_VAL1 PT-INR Value

N	Mean	Standard Deviation	Minimum	Maximum
3	1.8866667	0.0838650	1.7900000	1.9400000

Unique Subject Identifier=6050011100-0004

Assay variable : PTINR_VAL1 PT-INR Value

N	Mean	Standard Deviation	Minimum	Maximum
4	2.3300000	0.3524202	2.0700000	2.8500000

Laboratory tests at the time of obtaining Table 2-6 consent, according to the presence or absence of proton-pump inhibitors

Unique Subject Identifier=6050011100-0009

MEANS Procedure

Assay variable : PTINR_VAL1 PT-INR Value

N	Mean	Standard Deviation	Minimum	Maximum
2	2.1150000	0.2616295	1.9300000	2.3000000

Unique Subject Identifier=6050011100-0012

Assay variable : PTINR_VAL1 PT-INR Value

N	Mean	Standard Deviation	Minimum	Maximum
3	2.0933333	0.6208328	1.5500000	2.7700000

Unique Subject Identifier=6050011100-0016

Assay variable : PTINR_VAL1 PT-INR Value

N	Mean	Standard Deviation	Minimum	Maximum
3	2.1500000	0.0529150	2.1100000	2.2100000

Unique Subject Identifier=6050011100-0022

Assay variable : PTINR_VAL1 PT-INR Value

N	Mean	Standard Deviation	Minimum	Maximum
2	2.0900000	0.1414214	1.9900000	2.1900000

Laboratory tests at the time of obtaining Table 2-6 consent, according to the presence or absence of proton-pump inhibitors

Unique Subject Identifier=6050011100-0026

MEANS Procedure

Assay variable : PTINR_VAL1 PT-INR Value

N	Mean	Standard Deviation	Minimum	Maximum
5	2.1080000	0.8909938	1.2500000	3.4200000

Unique Subject Identifier=6055000000-0021

Assay variable : PTINR_VAL1 PT-INR Value

N	Mean	Standard Deviation	Minimum	Maximum
4	2.0050000	0.8294777	1.4700000	3.2300000

Unique Subject Identifier=6058010011-0003

Assay variable : PTINR_VAL1 PT-INR Value

N	Mean	Standard Deviation	Minimum	Maximum
6	2.0733333	0.5177515	1.4000000	2.6400000

Unique Subject Identifier=6058010011-0004

Assay variable : PTINR_VAL1 PT-INR Value

N	Mean	Standard Deviation	Minimum	Maximum
6	2.4633333	0.2251814	2.1300000	2.8100000

Laboratory tests at the time of obtaining Table 2-6 consent, according to the presence or absence of proton-pump inhibitors

Unique Subject Identifier=6058010011-0005

MEANS Procedure

Assay variable : PTINR_VAL1 PT-INR Value

N	Mean	Standard Deviation	Minimum	Maximum
6	2.7083333	1.3459631	1.6700000	5.3000000

Unique Subject Identifier=6058010011-0013

Assay variable : PTINR_VAL1 PT-INR Value

N	Mean	Standard Deviation	Minimum	Maximum
6	2.1533333	0.1315548	1.9400000	2.2900000

Unique Subject Identifier=6058010011-0014

Assay variable : PTINR_VAL1 PT-INR Value

N	Mean	Standard Deviation	Minimum	Maximum
6	1.9000000	0.3553027	1.2800000	2.2200000

Unique Subject Identifier=6058010011-0015

Assay variable : PTINR_VAL1 PT-INR Value

N	Mean	Standard Deviation	Minimum	Maximum
6	1.8283333	0.1251266	1.6400000	2.0000000

Laboratory tests at the time of obtaining Table 2-6 consent, according to the presence or absence of proton-pump inhibitors

Unique Subject Identifier=6058010011-0019

MEANS Procedure

Assay variable : PTINR_VAL1 PT-INR Value

N	Mean	Standard Deviation	Minimum	Maximum
6	2.2000000	0.2450306	1.9600000	2.5700000

Unique Subject Identifier=6058010011-0021

Assay variable : PTINR_VAL1 PT-INR Value

N	Mean	Standard Deviation	Minimum	Maximum
6	2.4066667	0.4194123	2.1300000	3.2400000

Unique Subject Identifier=6058010011-0022

Assay variable : PTINR_VAL1 PT-INR Value

N	Mean	Standard Deviation	Minimum	Maximum
6	2.3033333	0.2956800	1.9300000	2.7600000

Unique Subject Identifier=6058010011-0027

Assay variable : PTINR_VAL1 PT-INR Value

N	Mean	Standard Deviation	Minimum	Maximum
6	2.1966667	0.1939759	2.0200000	2.5500000

Laboratory tests at the time of obtaining Table 2-6 consent, according to the presence or absence of proton-pump inhibitors

Unique Subject Identifier=6060011000-0002

MEANS Procedure

Assay variable : PTINR_VAL1 PT-INR Value

N	Mean	Standard Deviation	Minimum	Maximum
4	2.4750000	0.0675771	2.4000000	2.5500000

Unique Subject Identifier=6060011000-0014

Assay variable : PTINR_VAL1 PT-INR Value

N	Mean	Standard Deviation	Minimum	Maximum
3	1.8866667	0.2433790	1.6700000	2.1500000

Unique Subject Identifier=6061000000-0004

Assay variable : PTINR_VAL1 PT-INR Value

N	Mean	Standard Deviation	Minimum	Maximum
3	2.3900000	0.2858321	2.2200000	2.7200000

Unique Subject Identifier=6071010010-0002

Assay variable : PTINR_VAL1 PT-INR Value

N	Mean	Standard Deviation	Minimum	Maximum
4	2.0525000	0.2657536	1.8300000	2.4000000

Laboratory tests at the time of obtaining Table 2-6 consent, according to the presence or absence of proton-pump inhibitors

Unique Subject Identifier=6071010010-0006

MEANS Procedure

Assay variable : PTINR_VAL1 PT-INR Value

N	Mean	Standard Deviation	Minimum	Maximum
4	2.0900000	0.3558089	1.6500000	2.5200000

Unique Subject Identifier=6071010010-0008

Assay variable : PTINR_VAL1 PT-INR Value

N	Mean	Standard Deviation	Minimum	Maximum
6	2.3166667	0.0852447	2.2300000	2.4300000

Unique Subject Identifier=6071010010-0016

Assay variable : PTINR_VAL1 PT-INR Value

N	Mean	Standard Deviation	Minimum	Maximum
3	2.7133333	0.5978573	2.0300000	3.1400000

Unique Subject Identifier=6098000000-0021

Assay variable : PTINR_VAL1 PT-INR Value

N	Mean	Standard Deviation	Minimum	Maximum
3	1.9000000	0.1732051	1.8000000	2.1000000

Laboratory tests at the time of obtaining Table 2-6 consent, according to the presence or absence of proton-pump inhibitors

Unique Subject Identifier=6104000000-0002

MEANS Procedure

Assay variable : PTINR_VAL1 PT-INR Value

N	Mean	Standard Deviation	Minimum	Maximum
6	1.9000000	0.0843801	1.7900000	2.0000000

Unique Subject Identifier=6104000000-0004

Assay variable : PTINR_VAL1 PT-INR Value

N	Mean	Standard Deviation	Minimum	Maximum
6	1.9866667	0.6669533	1.2700000	3.2500000

Unique Subject Identifier=6104000000-0008

Assay variable : PTINR_VAL1 PT-INR Value

N	Mean	Standard Deviation	Minimum	Maximum
6	1.6550000	0.3602638	1.1500000	2.0600000

Unique Subject Identifier=6104000000-0012

Assay variable : PTINR_VAL1 PT-INR Value

N	Mean	Standard Deviation	Minimum	Maximum
5	2.0900000	0.2411431	1.7200000	2.3600000

Laboratory tests at the time of obtaining Table 2-6 consent, according to the presence or absence of proton-pump inhibitors

Unique Subject Identifier=6104000000-0013

MEANS Procedure

Assay variable : PTINR_VAL1 PT-INR Value

N	Mean	Standard Deviation	Minimum	Maximum
3	1.9400000	0.4103657	1.5200000	2.3400000

Unique Subject Identifier=6105000000-0004

Assay variable : PTINR_VAL1 PT-INR Value

N	Mean	Standard Deviation	Minimum	Maximum
5	1.6520000	0.1347961	1.5200000	1.8200000

Unique Subject Identifier=6105000000-0010

Assay variable : PTINR_VAL1 PT-INR Value

N	Mean	Standard Deviation	Minimum	Maximum
4	2.0025000	0.7981802	1.3400000	3.1200000

Unique Subject Identifier=6105000000-0014

Assay variable : PTINR_VAL1 PT-INR Value

N	Mean	Standard Deviation	Minimum	Maximum
5	1.8740000	0.2150116	1.5100000	2.0200000

Laboratory tests at the time of obtaining Table 2-6 consent, according to the presence or absence of proton-pump inhibitors

Unique Subject Identifier=6105000000-0015

MEANS Procedure

Assay variable : PTINR_VAL1 PT-INR Value

N	Mean	Standard Deviation	Minimum	Maximum
6	1.7683333	0.3649338	1.4800000	2.3900000

Unique Subject Identifier=6105000000-0018

Assay variable : PTINR_VAL1 PT-INR Value

N	Mean	Standard Deviation	Minimum	Maximum
3	1.9766667	0.4052571	1.5800000	2.3900000

Unique Subject Identifier=6105000000-0021

Assay variable : PTINR_VAL1 PT-INR Value

N	Mean	Standard Deviation	Minimum	Maximum
6	2.1183333	0.2009395	1.8000000	2.3500000

Unique Subject Identifier=6109000000-0007

Assay variable : PTINR_VAL1 PT-INR Value

N	Mean	Standard Deviation	Minimum	Maximum
5	1.9740000	0.3146903	1.6000000	2.3100000

Laboratory tests at the time of obtaining Table 2-6 consent, according to the presence or absence of proton-pump inhibitors

Unique Subject Identifier=6109000000-0034

MEANS Procedure

Assay variable : PTINR_VAL1 PT-INR Value

N	Mean	Standard Deviation	Minimum	Maximum
6	2.6500000	0.4605214	1.9200000	3.3000000

Unique Subject Identifier=6109000000-0036

Assay variable : PTINR_VAL1 PT-INR Value

N	Mean	Standard Deviation	Minimum	Maximum
3	1.5533333	0.0351188	1.5200000	1.5900000

Unique Subject Identifier=6117010000-0003

Assay variable : PTINR_VAL1 PT-INR Value

N	Mean	Standard Deviation	Minimum	Maximum
6	1.7133333	0.1693123	1.5000000	1.9900000

Unique Subject Identifier=6117010000-0004

Assay variable : PTINR_VAL1 PT-INR Value

N	Mean	Standard Deviation	Minimum	Maximum
5	1.9080000	0.7322022	1.2300000	3.0700000

Laboratory tests at the time of obtaining Table 2-6 consent, according to the presence or absence of proton-pump inhibitors

Unique Subject Identifier=6117010000-0006

MEANS Procedure

Assay variable : PTINR_VAL1 PT-INR Value

N	Mean	Standard Deviation	Minimum	Maximum
6	1.9516667	0.2314663	1.5900000	2.1600000

Unique Subject Identifier=6117010000-0008

Assay variable : PTINR_VAL1 PT-INR Value

N	Mean	Standard Deviation	Minimum	Maximum
4	1.9425000	0.2324328	1.6200000	2.1400000

Unique Subject Identifier=6117010000-0009

Assay variable : PTINR_VAL1 PT-INR Value

N	Mean	Standard Deviation	Minimum	Maximum
6	2.2366667	0.5143021	1.7700000	3.0500000

Unique Subject Identifier=6117010000-0010

Assay variable : PTINR_VAL1 PT-INR Value

N	Mean	Standard Deviation	Minimum	Maximum
4	1.8275000	0.2718302	1.6100000	2.2200000

Laboratory tests at the time of obtaining Table 2-6 consent, according to the presence or absence of proton-pump inhibitors

Unique Subject Identifier=6124000000-0006

MEANS Procedure

Assay variable : PTINR_VAL1 PT-INR Value

N	Mean	Standard Deviation	Minimum	Maximum
5	1.9800000	0.1923538	1.7000000	2.2000000

Unique Subject Identifier=6125000000-0006

Assay variable : PTINR_VAL1 PT-INR Value

N	Mean	Standard Deviation	Minimum	Maximum
6	1.7200000	0.5220728	1.1900000	2.5000000

Unique Subject Identifier=6125000000-0007

Assay variable : PTINR_VAL1 PT-INR Value

N	Mean	Standard Deviation	Minimum	Maximum
3	2.4566667	0.3957693	2.0000000	2.7000000

Unique Subject Identifier=6125000000-0009

Assay variable : PTINR_VAL1 PT-INR Value

N	Mean	Standard Deviation	Minimum	Maximum
5	1.8400000	0.3435113	1.5000000	2.3000000

Laboratory tests at the time of obtaining Table 2-6 consent, according to the presence or absence of proton-pump inhibitors

Unique Subject Identifier=6125000000-0010

MEANS Procedure

Assay variable : PTINR_VAL1 PT-INR Value

N	Mean	Standard Deviation	Minimum	Maximum
5	2.3800000	0.2167948	2.2000000	2.7000000

Unique Subject Identifier=6125000000-0013

Assay variable : PTINR_VAL1 PT-INR Value

N	Mean	Standard Deviation	Minimum	Maximum
6	2.6166667	0.5231316	2.0000000	3.3000000

Unique Subject Identifier=6125000000-0014

Assay variable : PTINR_VAL1 PT-INR Value

N	Mean	Standard Deviation	Minimum	Maximum
6	2.2616667	0.4834632	1.8000000	3.1600000

Unique Subject Identifier=6125000000-0019

Assay variable : PTINR_VAL1 PT-INR Value

N	Mean	Standard Deviation	Minimum	Maximum
4	1.7025000	0.3671852	1.4000000	2.2200000

Laboratory tests at the time of obtaining Table 2-6 consent, according to the presence or absence of proton-pump inhibitors

Unique Subject Identifier=6125000000-0020

MEANS Procedure

Assay variable : PTINR_VAL1 PT-INR Value

N	Mean	Standard Deviation	Minimum	Maximum
6	2.2766667	0.4222164	1.7000000	2.8600000

Unique Subject Identifier=6125000000-0023

Assay variable : PTINR_VAL1 PT-INR Value

N	Mean	Standard Deviation	Minimum	Maximum
3	1.1900000	0.0435890	1.1400000	1.2200000

Unique Subject Identifier=6125000000-0032

Assay variable : PTINR_VAL1 PT-INR Value

N	Mean	Standard Deviation	Minimum	Maximum
5	1.9120000	0.2340299	1.8000000	2.3300000

Unique Subject Identifier=6125000000-0035

Assay variable : PTINR_VAL1 PT-INR Value

N	Mean	Standard Deviation	Minimum	Maximum
3	2.4600000	0.3421988	2.0700000	2.7100000

Laboratory tests at the time of obtaining Table 2-6 consent, according to the presence or absence of proton-pump inhibitors

Unique Subject Identifier=6125000000-0040

MEANS Procedure

Assay variable : PTINR_VAL1 PT-INR Value

N	Mean	Standard Deviation	Minimum	Maximum
2	1.8200000	0.3818377	1.5500000	2.0900000

Unique Subject Identifier=6125000000-0041

Assay variable : PTINR_VAL1 PT-INR Value

N	Mean	Standard Deviation	Minimum	Maximum
3	1.8033333	0.3326159	1.5500000	2.1800000

Unique Subject Identifier=6125000000-0044

Assay variable : PTINR_VAL1 PT-INR Value

N	Mean	Standard Deviation	Minimum	Maximum
3	1.7333333	0.2081666	1.5000000	1.9000000

Unique Subject Identifier=6125000000-0045

Assay variable : PTINR_VAL1 PT-INR Value

N	Mean	Standard Deviation	Minimum	Maximum
5	2.0560000	0.7491195	1.5100000	3.3700000

Laboratory tests at the time of obtaining Table 2-6 consent, according to the presence or absence of proton-pump inhibitors

Unique Subject Identifier=6125000000-0050

MEANS Procedure

Assay variable : PTINR_VAL1 PT-INR Value

N	Mean	Standard Deviation	Minimum	Maximum
4	1.9400000	0.5424021	1.3800000	2.4900000

Unique Subject Identifier=6125000000-0061

Assay variable : PTINR_VAL1 PT-INR Value

N	Mean	Standard Deviation	Minimum	Maximum
4	2.1075000	0.2066196	1.9300000	2.3900000

Unique Subject Identifier=6125000000-0074

Assay variable : PTINR_VAL1 PT-INR Value

N	Mean	Standard Deviation	Minimum	Maximum
4	2.1900000	0.1518771	2.0600000	2.4000000

Unique Subject Identifier=6125000000-0086

Assay variable : PTINR_VAL1 PT-INR Value

N	Mean	Standard Deviation	Minimum	Maximum
5	1.5580000	0.1559487	1.3000000	1.7000000

Laboratory tests at the time of obtaining Table 2-6 consent, according to the presence or absence of proton-pump inhibitors

Unique Subject Identifier=6126000000-0003

MEANS Procedure

Assay variable : PTINR_VAL1 PT-INR Value

N	Mean	Standard Deviation	Minimum	Maximum
3	1.9700000	0.5204805	1.3700000	2.3000000

Unique Subject Identifier=6127000000-0011

Assay variable : PTINR_VAL1 PT-INR Value

N	Mean	Standard Deviation	Minimum	Maximum
5	1.8040000	0.1327780	1.6400000	1.9500000

Unique Subject Identifier=6127000000-0019

Assay variable : PTINR_VAL1 PT-INR Value

N	Mean	Standard Deviation	Minimum	Maximum
6	1.8316667	0.5717838	1.3100000	2.9100000

Unique Subject Identifier=6127000000-0021

Assay variable : PTINR_VAL1 PT-INR Value

N	Mean	Standard Deviation	Minimum	Maximum
6	1.6133333	0.3310388	1.1800000	2.0300000

Laboratory tests at the time of obtaining Table 2-6 consent, according to the presence or absence of proton-pump inhibitors

Unique Subject Identifier=6136110000-0004

MEANS Procedure

Assay variable : PTINR_VAL1 PT-INR Value

N	Mean	Standard Deviation	Minimum	Maximum
4	1.9325000	1.5902909	1.0200000	4.3100000

Unique Subject Identifier=6142000000-0014

Assay variable : PTINR_VAL1 PT-INR Value

N	Mean	Standard Deviation	Minimum	Maximum
6	1.8433333	0.2362767	1.5300000	2.2100000

Unique Subject Identifier=6142000000-0015

Assay variable : PTINR_VAL1 PT-INR Value

N	Mean	Standard Deviation	Minimum	Maximum
5	1.7940000	0.1411737	1.6600000	2.0300000

Unique Subject Identifier=6142000000-0026

Assay variable : PTINR_VAL1 PT-INR Value

N	Mean	Standard Deviation	Minimum	Maximum
5	2.1560000	0.4077744	1.6100000	2.5700000

Laboratory tests at the time of obtaining Table 2-6 consent, according to the presence or absence of proton-pump inhibitors

Unique Subject Identifier=6142000000-0027

MEANS Procedure

Assay variable : PTINR_VAL1 PT-INR Value

N	Mean	Standard Deviation	Minimum	Maximum
3	1.9166667	0.6803920	1.2500000	2.6100000

Unique Subject Identifier=6142000000-0028

Assay variable : PTINR_VAL1 PT-INR Value

N	Mean	Standard Deviation	Minimum	Maximum
6	1.8650000	0.4125409	1.5200000	2.6600000

Unique Subject Identifier=6147000000-0003

Assay variable : PTINR_VAL1 PT-INR Value

N	Mean	Standard Deviation	Minimum	Maximum
2	2.1300000	0.5091169	1.7700000	2.4900000

Unique Subject Identifier=6147000000-0005

Assay variable : PTINR_VAL1 PT-INR Value

N	Mean	Standard Deviation	Minimum	Maximum
1	1.8900000		1.8900000	1.8900000

Laboratory tests at the time of obtaining Table 2-6 consent, according to the presence or absence of proton-pump inhibitors

Unique Subject Identifier=6166000000-0016

MEANS Procedure

Assay variable : PTINR_VAL1 PT-INR Value

N	Mean	Standard Deviation	Minimum	Maximum
6	2.1366667	0.3290390	1.6800000	2.6600000

Unique Subject Identifier=6166000000-0026

Assay variable : PTINR_VAL1 PT-INR Value

N	Mean	Standard Deviation	Minimum	Maximum
6	1.1700000	0.0456070	1.1300000	1.2600000

Unique Subject Identifier=6171011010-0004

Assay variable : PTINR_VAL1 PT-INR Value

N	Mean	Standard Deviation	Minimum	Maximum
5	2.1380000	0.2712379	1.9300000	2.5900000

Unique Subject Identifier=6171011010-0020

Assay variable : PTINR_VAL1 PT-INR Value

N	Mean	Standard Deviation	Minimum	Maximum
6	2.0083333	0.3429529	1.6500000	2.4500000

Laboratory tests at the time of obtaining Table 2-6 consent, according to the presence or absence of proton-pump inhibitors

Unique Subject Identifier=6171011010-0021

MEANS Procedure

Assay variable : PTINR_VAL1 PT-INR Value

N	Mean	Standard Deviation	Minimum	Maximum
5	2.4160000	0.6340978	1.5800000	3.2600000

Unique Subject Identifier=6171011010-0022

Assay variable : PTINR_VAL1 PT-INR Value

N	Mean	Standard Deviation	Minimum	Maximum
6	1.9416667	0.3609109	1.5900000	2.6400000

Unique Subject Identifier=6198011000-0002

Assay variable : PTINR_VAL1 PT-INR Value

N	Mean	Standard Deviation	Minimum	Maximum
6	1.7216667	0.1783723	1.5700000	1.9800000

Unique Subject Identifier=6198011000-0043

Assay variable : PTINR_VAL1 PT-INR Value

N	Mean	Standard Deviation	Minimum	Maximum
6	1.8183333	0.7112923	1.2500000	3.2300000

Laboratory tests at the time of obtaining Table 2-6 consent, according to the presence or absence of proton-pump inhibitors

Unique Subject Identifier=6214000000-0010

MEANS Procedure

Assay variable : PTINR_VAL1 PT-INR Value

N	Mean	Standard Deviation	Minimum	Maximum
4	2.0100000	0.7942712	1.4100000	3.1800000

Unique Subject Identifier=6214000000-0016

Assay variable : PTINR_VAL1 PT-INR Value

N	Mean	Standard Deviation	Minimum	Maximum
6	1.7383333	0.5724130	1.2900000	2.8000000

Unique Subject Identifier=6214000000-0040

Assay variable : PTINR_VAL1 PT-INR Value

N	Mean	Standard Deviation	Minimum	Maximum
5	1.6740000	0.1550161	1.4900000	1.8000000

Unique Subject Identifier=6214000000-0045

Assay variable : PTINR_VAL1 PT-INR Value

N	Mean	Standard Deviation	Minimum	Maximum
4	2.4100000	0.4181706	1.9100000	2.7700000

Laboratory tests at the time of obtaining Table 2-6 consent, according to the presence or absence of proton-pump inhibitors

Unique Subject Identifier=6214000000-0061

MEANS Procedure

Assay variable : PTINR_VAL1 PT-INR Value

N	Mean	Standard Deviation	Minimum	Maximum
3	1.7900000	0.0754983	1.7200000	1.8700000

Unique Subject Identifier=6223000000-0002

Assay variable : PTINR_VAL1 PT-INR Value

N	Mean	Standard Deviation	Minimum	Maximum
3	2.2266667	0.2663331	2.0000000	2.5200000

Unique Subject Identifier=6235000000-0017

Assay variable : PTINR_VAL1 PT-INR Value

N	Mean	Standard Deviation	Minimum	Maximum
4	1.9500000	0.2380476	1.7000000	2.2000000

Unique Subject Identifier=6236010000-0001

Assay variable : PTINR_VAL1 PT-INR Value

N	Mean	Standard Deviation	Minimum	Maximum
6	1.5750000	0.3858886	1.1300000	2.1100000

Laboratory tests at the time of obtaining Table 2-6 consent, according to the presence or absence of proton-pump inhibitors

Unique Subject Identifier=6236010000-0010

MEANS Procedure

Assay variable : PTINR_VAL1 PT-INR Value

N	Mean	Standard Deviation	Minimum	Maximum
6	1.8783333	0.3113465	1.5300000	2.2000000

Unique Subject Identifier=6236010000-0013

Assay variable : PTINR_VAL1 PT-INR Value

N	Mean	Standard Deviation	Minimum	Maximum
3	1.9400000	0.7847930	1.2100000	2.7700000

Unique Subject Identifier=6236010000-0016

Assay variable : PTINR_VAL1 PT-INR Value

N	Mean	Standard Deviation	Minimum	Maximum
6	1.6433333	0.4206503	1.1700000	2.3700000

Unique Subject Identifier=6240011000-0011

Assay variable : PTINR_VAL1 PT-INR Value

N	Mean	Standard Deviation	Minimum	Maximum
6	1.7300000	0.2445404	1.3700000	2.1100000

Laboratory tests at the time of obtaining Table 2-6 consent, according to the presence or absence of proton-pump inhibitors

Unique Subject Identifier=6240011000-0014

MEANS Procedure

Assay variable : PTINR_VAL1 PT-INR Value

N	Mean	Standard Deviation	Minimum	Maximum
5	2.2900000	0.1367479	2.0600000	2.4000000

Unique Subject Identifier=6240011000-0018

Assay variable : PTINR_VAL1 PT-INR Value

N	Mean	Standard Deviation	Minimum	Maximum
6	2.3033333	0.4195553	1.6200000	2.9300000

Unique Subject Identifier=6240011000-0024

Assay variable : PTINR_VAL1 PT-INR Value

N	Mean	Standard Deviation	Minimum	Maximum
6	2.0950000	0.1867351	1.7900000	2.2800000

Unique Subject Identifier=6245000000-0023

Assay variable : PTINR_VAL1 PT-INR Value

N	Mean	Standard Deviation	Minimum	Maximum
4	1.8700000	0.1570563	1.7000000	2.0800000

Laboratory tests at the time of obtaining Table 2-6 consent, according to the presence or absence of proton-pump inhibitors

Unique Subject Identifier=6248110111-0006

MEANS Procedure

Assay variable : PTINR_VAL1 PT-INR Value

N	Mean	Standard Deviation	Minimum	Maximum
3	1.4266667	0.0850490	1.3300000	1.4900000

Unique Subject Identifier=6298000000-0001

Assay variable : PTINR_VAL1 PT-INR Value

N	Mean	Standard Deviation	Minimum	Maximum
4	1.8100000	0.2816617	1.5000000	2.1800000

Unique Subject Identifier=6298000000-0017

Assay variable : PTINR_VAL1 PT-INR Value

N	Mean	Standard Deviation	Minimum	Maximum
4	2.1225000	0.1532699	1.9800000	2.3300000

Unique Subject Identifier=6298000000-0026

Assay variable : PTINR_VAL1 PT-INR Value

N	Mean	Standard Deviation	Minimum	Maximum
4	1.6875000	0.2535580	1.3300000	1.9200000

Laboratory tests at the time of obtaining Table 2-6 consent, according to the presence or absence of proton-pump inhibitors

Unique Subject Identifier=6298000000-0028

MEANS Procedure

Assay variable : PTINR_VAL1 PT-INR Value

N	Mean	Standard Deviation	Minimum	Maximum
5	1.7580000	0.0605805	1.6900000	1.8300000

Unique Subject Identifier=6298000000-0029

Assay variable : PTINR_VAL1 PT-INR Value

N	Mean	Standard Deviation	Minimum	Maximum
6	1.5116667	0.3346291	1.1200000	1.8900000

Unique Subject Identifier=6298000000-0037

Assay variable : PTINR_VAL1 PT-INR Value

N	Mean	Standard Deviation	Minimum	Maximum
4	2.2650000	0.1738774	2.0300000	2.4500000

Unique Subject Identifier=6298000000-0046

Assay variable : PTINR_VAL1 PT-INR Value

N	Mean	Standard Deviation	Minimum	Maximum
4	1.9975000	0.0801561	1.9200000	2.1100000

Laboratory tests at the time of obtaining Table 2-6 consent, according to the presence or absence of proton-pump inhibitors

Unique Subject Identifier=6298000000-0064

MEANS Procedure

Assay variable : PTINR_VAL1 PT-INR Value

N	Mean	Standard Deviation	Minimum	Maximum
3	1.7366667	0.1908752	1.5200000	1.8800000

Unique Subject Identifier=6298000000-0068

Assay variable : PTINR_VAL1 PT-INR Value

N	Mean	Standard Deviation	Minimum	Maximum
3	2.4133333	0.9972629	1.5400000	3.5000000

Unique Subject Identifier=6298000000-0075

Assay variable : PTINR_VAL1 PT-INR Value

N	Mean	Standard Deviation	Minimum	Maximum
3	1.7733333	0.1123981	1.6500000	1.8700000

Unique Subject Identifier=6298000000-0082

Assay variable : PTINR_VAL1 PT-INR Value

N	Mean	Standard Deviation	Minimum	Maximum
1	1.2400000		1.2400000	1.2400000

Laboratory tests at the time of obtaining Table 2-6 consent, according to the presence or absence of proton-pump inhibitors

Unique Subject Identifier=6298000000-0096

MEANS Procedure

Assay variable : PTINR_VAL1 PT-INR Value

N	Mean	Standard Deviation	Minimum	Maximum
1	1.8400000		1.8400000	1.8400000

Unique Subject Identifier=6298000000-0120

Assay variable : PTINR_VAL1 PT-INR Value

N	Mean	Standard Deviation	Minimum	Maximum
3	1.9733333	0.5163655	1.5100000	2.5300000

Unique Subject Identifier=6298000000-0167

Assay variable : PTINR_VAL1 PT-INR Value

N	Mean	Standard Deviation	Minimum	Maximum
4	1.9150000	0.1927866	1.6600000	2.1200000

Unique Subject Identifier=6298000000-0169

Assay variable : PTINR_VAL1 PT-INR Value

N	Mean	Standard Deviation	Minimum	Maximum
6	1.9033333	0.2122891	1.6400000	2.1500000

Laboratory tests at the time of obtaining Table 2-6 consent, according to the presence or absence of proton-pump inhibitors

Unique Subject Identifier=6304000000-0011

MEANS Procedure

Assay variable : PTINR_VAL1 PT-INR Value

N	Mean	Standard Deviation	Minimum	Maximum
1	1.9600000		1.9600000	1.9600000

Unique Subject Identifier=6304000000-0025

Assay variable : PTINR_VAL1 PT-INR Value

N	Mean	Standard Deviation	Minimum	Maximum
1	2.5100000		2.5100000	2.5100000

Unique Subject Identifier=6309000000-0005

Assay variable : PTINR_VAL1 PT-INR Value

N	Mean	Standard Deviation	Minimum	Maximum
3	2.7133333	0.0757188	2.6600000	2.8000000

Unique Subject Identifier=6309000000-0013

Assay variable : PTINR_VAL1 PT-INR Value

N	Mean	Standard Deviation	Minimum	Maximum
5	2.2200000	0.3342155	1.6900000	2.5700000

Laboratory tests at the time of obtaining Table 2-6 consent, according to the presence or absence of proton-pump inhibitors

Unique Subject Identifier=6309000000-0017

MEANS Procedure

Assay variable : PTINR_VAL1 PT-INR Value

N	Mean	Standard Deviation	Minimum	Maximum
3	1.8566667	0.2532456	1.5700000	2.0500000

Unique Subject Identifier=6309000000-0030

Assay variable : PTINR_VAL1 PT-INR Value

N	Mean	Standard Deviation	Minimum	Maximum
2	2.5000000	0.3959798	2.2200000	2.7800000

Unique Subject Identifier=6310000100-0009

Assay variable : PTINR_VAL1 PT-INR Value

N	Mean	Standard Deviation	Minimum	Maximum
5	1.9400000	0.4992494	1.4000000	2.7400000

Unique Subject Identifier=6310000100-0012

Assay variable : PTINR_VAL1 PT-INR Value

N	Mean	Standard Deviation	Minimum	Maximum
6	2.3900000	0.6134493	1.5300000	3.2800000

Laboratory tests at the time of obtaining Table 2-6 consent, according to the presence or absence of proton-pump inhibitors

Unique Subject Identifier=6310000100-0016

MEANS Procedure

Assay variable : PTINR_VAL1 PT-INR Value

N	Mean	Standard Deviation	Minimum	Maximum
5	1.9120000	0.4343040	1.1900000	2.3500000

Unique Subject Identifier=6310000100-0019

Assay variable : PTINR_VAL1 PT-INR Value

N	Mean	Standard Deviation	Minimum	Maximum
5	2.3420000	0.4076395	1.6800000	2.6700000

Unique Subject Identifier=6366000000-0015

Assay variable : PTINR_VAL1 PT-INR Value

N	Mean	Standard Deviation	Minimum	Maximum
6	2.2650000	0.2011716	1.9300000	2.5400000

Unique Subject Identifier=6426000000-0001

Assay variable : PTINR_VAL1 PT-INR Value

N	Mean	Standard Deviation	Minimum	Maximum
5	2.3820000	0.2289541	2.2200000	2.7800000

Laboratory tests at the time of obtaining Table 2-6 consent, according to the presence or absence of proton-pump inhibitors

Unique Subject Identifier=6426000000-0009

MEANS Procedure

Assay variable : PTINR_VAL1 PT-INR Value

N	Mean	Standard Deviation	Minimum	Maximum
5	1.7180000	0.2357329	1.4800000	2.0600000

Unique Subject Identifier=6426000000-0015

Assay variable : PTINR_VAL1 PT-INR Value

N	Mean	Standard Deviation	Minimum	Maximum
5	1.9220000	0.0967988	1.8000000	2.0300000

Unique Subject Identifier=6426000000-0016

Assay variable : PTINR_VAL1 PT-INR Value

N	Mean	Standard Deviation	Minimum	Maximum
3	2.1966667	0.7054313	1.6400000	2.9900000

Unique Subject Identifier=6426000000-0017

Assay variable : PTINR_VAL1 PT-INR Value

N	Mean	Standard Deviation	Minimum	Maximum
1	1.0400000		1.0400000	1.0400000

Laboratory tests at the time of obtaining Table 2-6 consent, according to the presence or absence of proton-pump inhibitors

Unique Subject Identifier=6426000000-0019

MEANS Procedure

Assay variable : PTINR_VAL1 PT-INR Value

N	Mean	Standard Deviation	Minimum	Maximum
5	1.6720000	0.4934268	1.1700000	2.4900000

Unique Subject Identifier=6426000000-0021

Assay variable : PTINR_VAL1 PT-INR Value

N	Mean	Standard Deviation	Minimum	Maximum
4	2.3925000	0.3836991	1.8900000	2.8100000

Unique Subject Identifier=6642010010-0003

Assay variable : PTINR_VAL1 PT-INR Value

N	Mean	Standard Deviation	Minimum	Maximum
6	1.8100000	0.1882551	1.5800000	2.1000000

Unique Subject Identifier=7001010000-0004

Assay variable : PTINR_VAL1 PT-INR Value

N	Mean	Standard Deviation	Minimum	Maximum
6	1.3983333	0.1105290	1.2700000	1.5600000

Laboratory tests at the time of obtaining Table 2-6 consent, according to the presence or absence of proton-pump inhibitors

Unique Subject Identifier=7002000000-0017

MEANS Procedure

Assay variable : PTINR_VAL1 PT-INR Value

N	Mean	Standard Deviation	Minimum	Maximum
6	2.0300000	0.5932285	1.3200000	3.1000000

Unique Subject Identifier=7002000000-0018

Assay variable : PTINR_VAL1 PT-INR Value

N	Mean	Standard Deviation	Minimum	Maximum
2	2.6450000	0.6858936	2.1600000	3.1300000

Unique Subject Identifier=7002000000-0020

Assay variable : PTINR_VAL1 PT-INR Value

N	Mean	Standard Deviation	Minimum	Maximum
3	1.7266667	0.2631223	1.4900000	2.0100000

Unique Subject Identifier=7002000000-0039

Assay variable : PTINR_VAL1 PT-INR Value

N	Mean	Standard Deviation	Minimum	Maximum
4	2.4050000	0.7684834	1.6700000	3.3800000

Laboratory tests at the time of obtaining Table 2-6 consent, according to the presence or absence of proton-pump inhibitors

Unique Subject Identifier=7004000000-0002

MEANS Procedure

Assay variable : PTINR_VAL1 PT-INR Value

N	Mean	Standard Deviation	Minimum	Maximum
6	1.6400000	0.1906830	1.3200000	1.8100000

Unique Subject Identifier=7004000000-0008

Assay variable : PTINR_VAL1 PT-INR Value

N	Mean	Standard Deviation	Minimum	Maximum
3	2.1933333	0.1305118	2.0900000	2.3400000

Unique Subject Identifier=7004000000-0018

Assay variable : PTINR_VAL1 PT-INR Value

N	Mean	Standard Deviation	Minimum	Maximum
3	2.0600000	0.1400000	1.9000000	2.1600000

Unique Subject Identifier=7005000000-0008

Assay variable : PTINR_VAL1 PT-INR Value

N	Mean	Standard Deviation	Minimum	Maximum
4	1.5600000	0.5383308	1.1400000	2.3100000

Laboratory tests at the time of obtaining Table 2-6 consent, according to the presence or absence of proton-pump inhibitors

Unique Subject Identifier=7005000000-0015

MEANS Procedure

Assay variable : PTINR_VAL1 PT-INR Value

N	Mean	Standard Deviation	Minimum	Maximum
5	2.4800000	0.4851288	1.8800000	3.1100000

Unique Subject Identifier=7008000000-0007

Assay variable : PTINR_VAL1 PT-INR Value

N	Mean	Standard Deviation	Minimum	Maximum
6	1.6850000	0.2126735	1.4700000	2.0400000

Unique Subject Identifier=7008000000-0027

Assay variable : PTINR_VAL1 PT-INR Value

N	Mean	Standard Deviation	Minimum	Maximum
6	1.8600000	0.4904692	1.1700000	2.4500000

Unique Subject Identifier=7009010000-0007

Assay variable : PTINR_VAL1 PT-INR Value

N	Mean	Standard Deviation	Minimum	Maximum
4	1.7175000	0.2490482	1.5400000	2.0800000

Laboratory tests at the time of obtaining Table 2-6 consent, according to the presence or absence of proton-pump inhibitors

Unique Subject Identifier=7009010000-0016

MEANS Procedure

Assay variable : PTINR_VAL1 PT-INR Value

N	Mean	Standard Deviation	Minimum	Maximum
3	2.6766667	0.1955335	2.4900000	2.8800000

Unique Subject Identifier=7014000000-0005

Assay variable : PTINR_VAL1 PT-INR Value

N	Mean	Standard Deviation	Minimum	Maximum
6	2.4600000	0.5049752	1.7300000	3.0800000

Unique Subject Identifier=7014000000-0010

Assay variable : PTINR_VAL1 PT-INR Value

N	Mean	Standard Deviation	Minimum	Maximum
2	2.3950000	0.4879037	2.0500000	2.7400000

Unique Subject Identifier=7026000000-0020

Assay variable : PTINR_VAL1 PT-INR Value

N	Mean	Standard Deviation	Minimum	Maximum
6	1.9583333	0.5064550	1.2000000	2.7400000

Laboratory tests at the time of obtaining Table 2-6 consent, according to the presence or absence of proton-pump inhibitors

Unique Subject Identifier=7026000000-0025

MEANS Procedure

Assay variable : PTINR_VAL1 PT-INR Value

N	Mean	Standard Deviation	Minimum	Maximum
3	2.0766667	0.8183724	1.4100000	2.9900000

Unique Subject Identifier=7026000000-0026

Assay variable : PTINR_VAL1 PT-INR Value

N	Mean	Standard Deviation	Minimum	Maximum
2	1.7650000	0.3181981	1.5400000	1.9900000

Unique Subject Identifier=7026000000-0029

Assay variable : PTINR_VAL1 PT-INR Value

N	Mean	Standard Deviation	Minimum	Maximum
4	2.2500000	0.3271085	1.9800000	2.7200000

Unique Subject Identifier=7026000000-0034

Assay variable : PTINR_VAL1 PT-INR Value

N	Mean	Standard Deviation	Minimum	Maximum
4	1.7175000	0.1732772	1.5400000	1.8900000

Laboratory tests at the time of obtaining Table 2-6 consent, according to the presence or absence of proton-pump inhibitors

Unique Subject Identifier=7026000000-0037

MEANS Procedure

Assay variable : PTINR_VAL1 PT-INR Value

N	Mean	Standard Deviation	Minimum	Maximum
2	1.7150000	0.0070711	1.7100000	1.7200000

Unique Subject Identifier=7030010000-0013

Assay variable : PTINR_VAL1 PT-INR Value

N	Mean	Standard Deviation	Minimum	Maximum
5	2.1060000	0.1677200	1.9200000	2.3100000

Unique Subject Identifier=7030010000-0023

Assay variable : PTINR_VAL1 PT-INR Value

N	Mean	Standard Deviation	Minimum	Maximum
5	1.8140000	0.1689083	1.5500000	2.0000000

Unique Subject Identifier=7030010000-0031

Assay variable : PTINR_VAL1 PT-INR Value

N	Mean	Standard Deviation	Minimum	Maximum
4	2.3525000	0.3063087	2.0600000	2.6600000

Laboratory tests at the time of obtaining Table 2-6 consent, according to the presence or absence of proton-pump inhibitors

Unique Subject Identifier=7030010000-0034

MEANS Procedure

Assay variable : PTINR_VAL1 PT-INR Value

N	Mean	Standard Deviation	Minimum	Maximum
4	1.9250000	0.3516153	1.4800000	2.3400000

Unique Subject Identifier=7031011111-0013

Assay variable : PTINR_VAL1 PT-INR Value

N	Mean	Standard Deviation	Minimum	Maximum
3	2.1766667	0.2983845	1.9800000	2.5200000

Unique Subject Identifier=7031011111-0019

Assay variable : PTINR_VAL1 PT-INR Value

N	Mean	Standard Deviation	Minimum	Maximum
5	2.1200000	0.2667396	1.8500000	2.5300000

Unique Subject Identifier=7031011111-0026

Assay variable : PTINR_VAL1 PT-INR Value

N	Mean	Standard Deviation	Minimum	Maximum
6	2.6250000	0.1683746	2.4700000	2.9300000

Laboratory tests at the time of obtaining Table 2-6 consent, according to the presence or absence of proton-pump inhibitors

Unique Subject Identifier=7031011111-0034

MEANS Procedure

Assay variable : PTINR_VAL1 PT-INR Value

N	Mean	Standard Deviation	Minimum	Maximum
4	2.1175000	0.6155418	1.4500000	2.9000000

Unique Subject Identifier=7031011111-0037

Assay variable : PTINR_VAL1 PT-INR Value

N	Mean	Standard Deviation	Minimum	Maximum
4	1.7200000	0.1718527	1.5500000	1.9500000

Unique Subject Identifier=7035000000-0026

Assay variable : PTINR_VAL1 PT-INR Value

N	Mean	Standard Deviation	Minimum	Maximum
4	1.5450000	0.1337909	1.3900000	1.6800000

Unique Subject Identifier=7035000000-0028

Assay variable : PTINR_VAL1 PT-INR Value

N	Mean	Standard Deviation	Minimum	Maximum
4	1.7625000	0.1931105	1.5900000	2.0300000

Laboratory tests at the time of obtaining Table 2-6 consent, according to the presence or absence of proton-pump inhibitors

Unique Subject Identifier=7035000000-0050

MEANS Procedure

Assay variable : PTINR_VAL1 PT-INR Value

N	Mean	Standard Deviation	Minimum	Maximum
4	1.4325000	0.0518813	1.3600000	1.4700000

Unique Subject Identifier=7035000000-0054

Assay variable : PTINR_VAL1 PT-INR Value

N	Mean	Standard Deviation	Minimum	Maximum
3	1.7533333	0.0503322	1.7000000	1.8000000

Unique Subject Identifier=7035000000-0055

Assay variable : PTINR_VAL1 PT-INR Value

N	Mean	Standard Deviation	Minimum	Maximum
3	2.2566667	0.1877054	2.0400000	2.3700000

Unique Subject Identifier=7035000000-0057

Assay variable : PTINR_VAL1 PT-INR Value

N	Mean	Standard Deviation	Minimum	Maximum
1	2.3200000		2.3200000	2.3200000

Laboratory tests at the time of obtaining Table 2-6 consent, according to the presence or absence of proton-pump inhibitors

Unique Subject Identifier=7035000000-0068

MEANS Procedure

Assay variable : PTINR_VAL1 PT-INR Value

N	Mean	Standard Deviation	Minimum	Maximum
2	2.0100000	0.4101219	1.7200000	2.3000000

Unique Subject Identifier=7035000000-0071

Assay variable : PTINR_VAL1 PT-INR Value

N	Mean	Standard Deviation	Minimum	Maximum
3	2.0800000	0.1228821	1.9400000	2.1700000

Unique Subject Identifier=7035000000-0072

Assay variable : PTINR_VAL1 PT-INR Value

N	Mean	Standard Deviation	Minimum	Maximum
4	1.9850000	0.4655105	1.5200000	2.4700000

Unique Subject Identifier=7035000000-0084

Assay variable : PTINR_VAL1 PT-INR Value

N	Mean	Standard Deviation	Minimum	Maximum
3	2.7966667	0.2750152	2.5200000	3.0700000

Laboratory tests at the time of obtaining Table 2-6 consent, according to the presence or absence of proton-pump inhibitors

Unique Subject Identifier=7035000000-0086

MEANS Procedure

Assay variable : PTINR_VAL1 PT-INR Value

N	Mean	Standard Deviation	Minimum	Maximum
2	1.6950000	0.2050610	1.5500000	1.8400000

Unique Subject Identifier=7035000000-0090

Assay variable : PTINR_VAL1 PT-INR Value

N	Mean	Standard Deviation	Minimum	Maximum
3	2.2766667	0.1858315	2.1500000	2.4900000

Unique Subject Identifier=7035000000-0097

Assay variable : PTINR_VAL1 PT-INR Value

N	Mean	Standard Deviation	Minimum	Maximum
1	2.4600000		2.4600000	2.4600000

Unique Subject Identifier=7037000000-0012

Assay variable : PTINR_VAL1 PT-INR Value

N	Mean	Standard Deviation	Minimum	Maximum
6	2.1016667	0.5417164	1.7000000	3.1000000

Laboratory tests at the time of obtaining Table 2-6 consent, according to the presence or absence of proton-pump inhibitors

Unique Subject Identifier=7042010110-0006

MEANS Procedure

Assay variable : PTINR_VAL1 PT-INR Value

N	Mean	Standard Deviation	Minimum	Maximum
3	2.2100000	0.1100000	2.1000000	2.3200000

Unique Subject Identifier=7042010110-0011

Assay variable : PTINR_VAL1 PT-INR Value

N	Mean	Standard Deviation	Minimum	Maximum
3	1.9200000	0.1252996	1.8000000	2.0500000

Unique Subject Identifier=7042010110-0013

Assay variable : PTINR_VAL1 PT-INR Value

N	Mean	Standard Deviation	Minimum	Maximum
6	2.2466667	0.2058802	1.8900000	2.4400000

Unique Subject Identifier=7042010110-0014

Assay variable : PTINR_VAL1 PT-INR Value

N	Mean	Standard Deviation	Minimum	Maximum
6	1.3150000	0.2433721	1.0300000	1.7600000

Laboratory tests at the time of obtaining Table 2-6 consent, according to the presence or absence of proton-pump inhibitors

Unique Subject Identifier=7042010110-0017

MEANS Procedure

Assay variable : PTINR_VAL1 PT-INR Value

N	Mean	Standard Deviation	Minimum	Maximum
6	2.1166667	1.2655697	1.4100000	4.6700000

Unique Subject Identifier=7042010110-0018

Assay variable : PTINR_VAL1 PT-INR Value

N	Mean	Standard Deviation	Minimum	Maximum
6	1.4266667	0.0799166	1.3100000	1.5200000

Unique Subject Identifier=7042010110-0021

Assay variable : PTINR_VAL1 PT-INR Value

N	Mean	Standard Deviation	Minimum	Maximum
3	2.2633333	0.0208167	2.2400000	2.2800000

Unique Subject Identifier=7042010110-0022

Assay variable : PTINR_VAL1 PT-INR Value

N	Mean	Standard Deviation	Minimum	Maximum
5	1.5120000	0.1613072	1.2300000	1.6300000

Laboratory tests at the time of obtaining Table 2-6 consent, according to the presence or absence of proton-pump inhibitors

Unique Subject Identifier=7045000000-0001

MEANS Procedure

Assay variable : PTINR_VAL1 PT-INR Value

N	Mean	Standard Deviation	Minimum	Maximum
5	1.9640000	0.2596729	1.7400000	2.4000000

Unique Subject Identifier=7045000000-0010

Assay variable : PTINR_VAL1 PT-INR Value

N	Mean	Standard Deviation	Minimum	Maximum
6	2.3050000	0.2608256	1.8900000	2.6200000

Unique Subject Identifier=7045000000-0011

Assay variable : PTINR_VAL1 PT-INR Value

N	Mean	Standard Deviation	Minimum	Maximum
6	1.9750000	0.2833196	1.5400000	2.2500000

Unique Subject Identifier=7045000000-0024

Assay variable : PTINR_VAL1 PT-INR Value

N	Mean	Standard Deviation	Minimum	Maximum
5	2.1500000	0.3277194	1.6700000	2.5900000

Laboratory tests at the time of obtaining Table 2-6 consent, according to the presence or absence of proton-pump inhibitors

Unique Subject Identifier=7045000000-0026

MEANS Procedure

Assay variable : PTINR_VAL1 PT-INR Value

N	Mean	Standard Deviation	Minimum	Maximum
4	1.7650000	0.1415392	1.5600000	1.8800000

Unique Subject Identifier=7045000000-0028

Assay variable : PTINR_VAL1 PT-INR Value

N	Mean	Standard Deviation	Minimum	Maximum
6	2.1716667	0.4511060	1.4500000	2.6600000

Unique Subject Identifier=7045000000-0029

Assay variable : PTINR_VAL1 PT-INR Value

N	Mean	Standard Deviation	Minimum	Maximum
4	2.0725000	0.1486327	1.9300000	2.2800000

Unique Subject Identifier=7047000000-0001

Assay variable : PTINR_VAL1 PT-INR Value

N	Mean	Standard Deviation	Minimum	Maximum
6	1.6450000	0.5950714	1.2000000	2.6400000

Laboratory tests at the time of obtaining Table 2-6 consent, according to the presence or absence of proton-pump inhibitors

Unique Subject Identifier=7055000000-0007

MEANS Procedure

Assay variable : PTINR_VAL1 PT-INR Value

N	Mean	Standard Deviation	Minimum	Maximum
6	2.0216667	0.1614208	1.8200000	2.2600000

Unique Subject Identifier=7061010010-0002

Assay variable : PTINR_VAL1 PT-INR Value

N	Mean	Standard Deviation	Minimum	Maximum
6	1.9500000	0.2588436	1.7000000	2.4000000

Unique Subject Identifier=7061010010-0004

Assay variable : PTINR_VAL1 PT-INR Value

N	Mean	Standard Deviation	Minimum	Maximum
6	1.5500000	0.3563706	1.1000000	2.1000000

Unique Subject Identifier=7061010010-0007

Assay variable : PTINR_VAL1 PT-INR Value

N	Mean	Standard Deviation	Minimum	Maximum
3	1.9666667	0.5033223	1.5000000	2.5000000

Laboratory tests at the time of obtaining Table 2-6 consent, according to the presence or absence of proton-pump inhibitors

Unique Subject Identifier=7061010010-0009

MEANS Procedure

Assay variable : PTINR_VAL1 PT-INR Value

N	Mean	Standard Deviation	Minimum	Maximum
4	2.3250000	0.5737305	1.8000000	3.1000000

Unique Subject Identifier=7066000000-0002

Assay variable : PTINR_VAL1 PT-INR Value

N	Mean	Standard Deviation	Minimum	Maximum
6	2.6683333	1.5619913	1.1900000	5.4500000

Unique Subject Identifier=7066000000-0007

Assay variable : PTINR_VAL1 PT-INR Value

N	Mean	Standard Deviation	Minimum	Maximum
6	1.9766667	0.2334666	1.6000000	2.2900000

Unique Subject Identifier=7066000000-0013

Assay variable : PTINR_VAL1 PT-INR Value

N	Mean	Standard Deviation	Minimum	Maximum
6	1.9066667	0.1186030	1.7200000	2.0300000

Laboratory tests at the time of obtaining Table 2-6 consent, according to the presence or absence of proton-pump inhibitors

Unique Subject Identifier=7066000000-0021

MEANS Procedure

Assay variable : PTINR_VAL1 PT-INR Value

N	Mean	Standard Deviation	Minimum	Maximum
6	1.7133333	0.3478889	1.3200000	2.2000000

Unique Subject Identifier=7104010000-0020

Assay variable : PTINR_VAL1 PT-INR Value

N	Mean	Standard Deviation	Minimum	Maximum
6	2.4650000	0.1415274	2.2900000	2.6500000

Unique Subject Identifier=7132010000-0017

Assay variable : PTINR_VAL1 PT-INR Value

N	Mean	Standard Deviation	Minimum	Maximum
4	2.4975000	0.8896582	1.5000000	3.6100000

Unique Subject Identifier=7132010000-0020

Assay variable : PTINR_VAL1 PT-INR Value

N	Mean	Standard Deviation	Minimum	Maximum
3	1.8400000	0.2260531	1.6900000	2.1000000

Laboratory tests at the time of obtaining Table 2-6 consent, according to the presence or absence of proton-pump inhibitors

Unique Subject Identifier=7133000000-0001

MEANS Procedure

Assay variable : PTINR_VAL1 PT-INR Value

N	Mean	Standard Deviation	Minimum	Maximum
6	2.3316667	1.1864471	1.0700000	4.5200000

Unique Subject Identifier=7136000000-0003

Assay variable : PTINR_VAL1 PT-INR Value

N	Mean	Standard Deviation	Minimum	Maximum
4	2.3200000	0.2786874	2.0500000	2.7100000

Unique Subject Identifier=7136000000-0005

Assay variable : PTINR_VAL1 PT-INR Value

N	Mean	Standard Deviation	Minimum	Maximum
3	1.7033333	0.0814453	1.6100000	1.7600000

Unique Subject Identifier=7136000000-0006

Assay variable : PTINR_VAL1 PT-INR Value

N	Mean	Standard Deviation	Minimum	Maximum
4	1.5450000	0.2235322	1.2900000	1.7700000

Laboratory tests at the time of obtaining Table 2-6 consent, according to the presence or absence of proton-pump inhibitors

Unique Subject Identifier=7136000000-0007

MEANS Procedure

Assay variable : PTINR_VAL1 PT-INR Value

N	Mean	Standard Deviation	Minimum	Maximum
5	2.3600000	0.6914478	1.3700000	3.0000000

Unique Subject Identifier=7136000000-0011

Assay variable : PTINR_VAL1 PT-INR Value

N	Mean	Standard Deviation	Minimum	Maximum
5	1.4300000	0.1609348	1.2200000	1.6200000

Unique Subject Identifier=7136000000-0015

Assay variable : PTINR_VAL1 PT-INR Value

N	Mean	Standard Deviation	Minimum	Maximum
4	2.2800000	0.1390444	2.1500000	2.4100000

Unique Subject Identifier=7136000000-0016

Assay variable : PTINR_VAL1 PT-INR Value

N	Mean	Standard Deviation	Minimum	Maximum
3	1.9100000	0.3477068	1.5900000	2.2800000

Laboratory tests at the time of obtaining Table 2-6 consent, according to the presence or absence of proton-pump inhibitors

Unique Subject Identifier=7136000000-0017

MEANS Procedure

Assay variable : PTINR_VAL1 PT-INR Value

N	Mean	Standard Deviation	Minimum	Maximum
6	2.2816667	0.7896940	1.4800000	3.5500000

Unique Subject Identifier=7136000000-0021

Assay variable : PTINR_VAL1 PT-INR Value

N	Mean	Standard Deviation	Minimum	Maximum
6	2.1450000	0.4427528	1.6400000	2.7900000

Unique Subject Identifier=7136000000-0025

Assay variable : PTINR_VAL1 PT-INR Value

N	Mean	Standard Deviation	Minimum	Maximum
6	1.7150000	0.3321897	1.3700000	2.1200000

Unique Subject Identifier=7136000000-0028

Assay variable : PTINR_VAL1 PT-INR Value

N	Mean	Standard Deviation	Minimum	Maximum
4	1.9450000	0.4199603	1.5500000	2.4900000

Laboratory tests at the time of obtaining Table 2-6 consent, according to the presence or absence of proton-pump inhibitors

Unique Subject Identifier=7136000000-0029

MEANS Procedure

Assay variable : PTINR_VAL1 PT-INR Value

N	Mean	Standard Deviation	Minimum	Maximum
3	2.4300000	0.0871780	2.3700000	2.5300000

Unique Subject Identifier=7136000000-0037

Assay variable : PTINR_VAL1 PT-INR Value

N	Mean	Standard Deviation	Minimum	Maximum
3	2.1100000	0.1300000	2.0300000	2.2600000

Unique Subject Identifier=7136000000-0039

Assay variable : PTINR_VAL1 PT-INR Value

N	Mean	Standard Deviation	Minimum	Maximum
3	2.2033333	0.1616581	2.1100000	2.3900000

Unique Subject Identifier=7136000000-0040

Assay variable : PTINR_VAL1 PT-INR Value

N	Mean	Standard Deviation	Minimum	Maximum
3	1.9866667	0.1569501	1.8100000	2.1100000

Laboratory tests at the time of obtaining Table 2-6 consent, according to the presence or absence of proton-pump inhibitors

Unique Subject Identifier=7136000000-0042

MEANS Procedure

Assay variable : PTINR_VAL1 PT-INR Value

N	Mean	Standard Deviation	Minimum	Maximum
4	1.7850000	0.3411256	1.3800000	2.2000000

Unique Subject Identifier=7136000000-0049

Assay variable : PTINR_VAL1 PT-INR Value

N	Mean	Standard Deviation	Minimum	Maximum
3	2.3166667	0.3942503	1.9600000	2.7400000

Unique Subject Identifier=7137010000-0001

Assay variable : PTINR_VAL1 PT-INR Value

N	Mean	Standard Deviation	Minimum	Maximum
5	1.5740000	0.2757354	1.1700000	1.8700000

Unique Subject Identifier=7154000000-0002

Assay variable : PTINR_VAL1 PT-INR Value

N	Mean	Standard Deviation	Minimum	Maximum
5	2.1620000	0.6197338	1.5500000	3.1000000

Laboratory tests at the time of obtaining Table 2-6 consent, according to the presence or absence of proton-pump inhibitors

Unique Subject Identifier=7154000000-0009

MEANS Procedure

Assay variable : PTINR_VAL1 PT-INR Value

N	Mean	Standard Deviation	Minimum	Maximum
5	2.2640000	0.3001333	1.9800000	2.7700000

Unique Subject Identifier=7154000000-0018

Assay variable : PTINR_VAL1 PT-INR Value

N	Mean	Standard Deviation	Minimum	Maximum
5	1.9240000	0.2447039	1.6800000	2.3100000

Unique Subject Identifier=7154000000-0024

Assay variable : PTINR_VAL1 PT-INR Value

N	Mean	Standard Deviation	Minimum	Maximum
4	2.0525000	0.3732180	1.7000000	2.5300000

Unique Subject Identifier=7154000000-0025

Assay variable : PTINR_VAL1 PT-INR Value

N	Mean	Standard Deviation	Minimum	Maximum
4	2.2275000	0.0974252	2.1500000	2.3700000

Laboratory tests at the time of obtaining Table 2-6 consent, according to the presence or absence of proton-pump inhibitors

Unique Subject Identifier=7155000000-0014

MEANS Procedure

Assay variable : PTINR_VAL1 PT-INR Value

N	Mean	Standard Deviation	Minimum	Maximum
6	1.4666667	0.3201041	1.1100000	1.9000000

Unique Subject Identifier=7155000000-0016

Assay variable : PTINR_VAL1 PT-INR Value

N	Mean	Standard Deviation	Minimum	Maximum
1	1.0500000		1.0500000	1.0500000

Unique Subject Identifier=7155000000-0039

Assay variable : PTINR_VAL1 PT-INR Value

N	Mean	Standard Deviation	Minimum	Maximum
4	2.3650000	0.4262628	1.8300000	2.7900000

Unique Subject Identifier=7171010000-0001

Assay variable : PTINR_VAL1 PT-INR Value

N	Mean	Standard Deviation	Minimum	Maximum
6	1.9683333	0.3200260	1.6800000	2.5600000

Laboratory tests at the time of obtaining Table 2-6 consent, according to the presence or absence of proton-pump inhibitors

Unique Subject Identifier=7171010000-0004

MEANS Procedure

Assay variable : PTINR_VAL1 PT-INR Value

N	Mean	Standard Deviation	Minimum	Maximum
5	1.7980000	0.1628496	1.6200000	2.0200000

Unique Subject Identifier=7198010000-0019

Assay variable : PTINR_VAL1 PT-INR Value

N	Mean	Standard Deviation	Minimum	Maximum
4	1.6625000	0.1850000	1.4500000	1.9000000

Unique Subject Identifier=7198010000-0031

Assay variable : PTINR_VAL1 PT-INR Value

N	Mean	Standard Deviation	Minimum	Maximum
5	1.8700000	0.3157531	1.6400000	2.4200000

Unique Subject Identifier=7198010000-0044

Assay variable : PTINR_VAL1 PT-INR Value

N	Mean	Standard Deviation	Minimum	Maximum
2	1.9250000	0.1767767	1.8000000	2.0500000

Laboratory tests at the time of obtaining Table 2-6 consent, according to the presence or absence of proton-pump inhibitors

Unique Subject Identifier=7198010000-0048

MEANS Procedure

Assay variable : PTINR_VAL1 PT-INR Value

N	Mean	Standard Deviation	Minimum	Maximum
1	1.5200000		1.5200000	1.5200000

Unique Subject Identifier=7198010000-0051

Assay variable : PTINR_VAL1 PT-INR Value

N	Mean	Standard Deviation	Minimum	Maximum
4	2.1575000	0.5864228	1.6400000	3.0000000

Unique Subject Identifier=7198010000-0053

Assay variable : PTINR_VAL1 PT-INR Value

N	Mean	Standard Deviation	Minimum	Maximum
6	1.9600000	0.1584929	1.8100000	2.2200000

Unique Subject Identifier=7198010000-0067

Assay variable : PTINR_VAL1 PT-INR Value

N	Mean	Standard Deviation	Minimum	Maximum
3	2.3033333	0.5783886	1.6500000	2.7500000

Laboratory tests at the time of obtaining Table 2-6 consent, according to the presence or absence of proton-pump inhibitors

Unique Subject Identifier=7198010000-0092

MEANS Procedure

Assay variable : PTINR_VAL1 PT-INR Value

N	Mean	Standard Deviation	Minimum	Maximum
3	1.7466667	0.3002221	1.4400000	2.0400000

Unique Subject Identifier=7204000000-0002

Assay variable : PTINR_VAL1 PT-INR Value

N	Mean	Standard Deviation	Minimum	Maximum
5	1.8680000	0.2940578	1.6000000	2.3500000

Unique Subject Identifier=7204000000-0024

Assay variable : PTINR_VAL1 PT-INR Value

N	Mean	Standard Deviation	Minimum	Maximum
5	1.3140000	0.1689083	1.0900000	1.5600000

Unique Subject Identifier=7204000000-0026

Assay variable : PTINR_VAL1 PT-INR Value

N	Mean	Standard Deviation	Minimum	Maximum
6	1.8383333	0.5723431	1.1400000	2.7900000

Laboratory tests at the time of obtaining Table 2-6 consent, according to the presence or absence of proton-pump inhibitors

Unique Subject Identifier=7204000000-0030

MEANS Procedure

Assay variable : PTINR_VAL1 PT-INR Value

N	Mean	Standard Deviation	Minimum	Maximum
5	1.5080000	0.2537124	1.2800000	1.8900000

Unique Subject Identifier=7204000000-0033

Assay variable : PTINR_VAL1 PT-INR Value

N	Mean	Standard Deviation	Minimum	Maximum
6	2.4266667	1.2527197	1.4600000	4.8300000

Unique Subject Identifier=7205000000-0027

Assay variable : PTINR_VAL1 PT-INR Value

N	Mean	Standard Deviation	Minimum	Maximum
3	2.0700000	0.3593049	1.8100000	2.4800000

Unique Subject Identifier=7208011010-0011

Assay variable : PTINR_VAL1 PT-INR Value

N	Mean	Standard Deviation	Minimum	Maximum
6	1.7300000	0.2324651	1.4900000	1.9900000

Laboratory tests at the time of obtaining Table 2-6 consent, according to the presence or absence of proton-pump inhibitors

Unique Subject Identifier=7208011010-0012

MEANS Procedure

Assay variable : PTINR_VAL1 PT-INR Value

N	Mean	Standard Deviation	Minimum	Maximum
3	1.4966667	0.0503322	1.4500000	1.5500000

Unique Subject Identifier=7209000000-0002

Assay variable : PTINR_VAL1 PT-INR Value

N	Mean	Standard Deviation	Minimum	Maximum
3	2.1400000	0.2651415	1.8500000	2.3700000

Unique Subject Identifier=7209000000-0008

Assay variable : PTINR_VAL1 PT-INR Value

N	Mean	Standard Deviation	Minimum	Maximum
4	1.9075000	0.2458150	1.7500000	2.2700000

Unique Subject Identifier=7209000000-0014

Assay variable : PTINR_VAL1 PT-INR Value

N	Mean	Standard Deviation	Minimum	Maximum
4	2.0500000	0.3359563	1.5700000	2.3400000

Laboratory tests at the time of obtaining Table 2-6 consent, according to the presence or absence of proton-pump inhibitors

Unique Subject Identifier=7209000000-0034

MEANS Procedure

Assay variable : PTINR_VAL1 PT-INR Value

N	Mean	Standard Deviation	Minimum	Maximum
4	1.8975000	0.0758837	1.8200000	2.0000000

Unique Subject Identifier=7209000000-0037

Assay variable : PTINR_VAL1 PT-INR Value

N	Mean	Standard Deviation	Minimum	Maximum
4	2.0650000	0.1690168	1.8900000	2.2900000

Unique Subject Identifier=7209000000-0048

Assay variable : PTINR_VAL1 PT-INR Value

N	Mean	Standard Deviation	Minimum	Maximum
3	1.6966667	0.1721434	1.5600000	1.8900000

Unique Subject Identifier=7209000000-0049

Assay variable : PTINR_VAL1 PT-INR Value

N	Mean	Standard Deviation	Minimum	Maximum
6	2.9650000	0.6056979	2.1300000	3.9400000

Laboratory tests at the time of obtaining Table 2-6 consent, according to the presence or absence of proton-pump inhibitors

Unique Subject Identifier=7209000000-0061

MEANS Procedure

Assay variable : PTINR_VAL1 PT-INR Value

N	Mean	Standard Deviation	Minimum	Maximum
4	1.8175000	0.4486554	1.2500000	2.3100000

Unique Subject Identifier=7209000000-0073

Assay variable : PTINR_VAL1 PT-INR Value

N	Mean	Standard Deviation	Minimum	Maximum
2	1.8300000	0.8202439	1.2500000	2.4100000

Unique Subject Identifier=7209000000-0074

Assay variable : PTINR_VAL1 PT-INR Value

N	Mean	Standard Deviation	Minimum	Maximum
4	1.9750000	0.8438602	1.2800000	3.0200000

Unique Subject Identifier=7209000000-0089

Assay variable : PTINR_VAL1 PT-INR Value

N	Mean	Standard Deviation	Minimum	Maximum
3	1.7333333	0.5928181	1.0500000	2.1100000

Laboratory tests at the time of obtaining Table 2-6 consent, according to the presence or absence of proton-pump inhibitors

Unique Subject Identifier=7209000000-0090

MEANS Procedure

Assay variable : PTINR_VAL1 PT-INR Value

N	Mean	Standard Deviation	Minimum	Maximum
3	2.5233333	0.5186842	2.1800000	3.1200000

Unique Subject Identifier=7209000000-0095

Assay variable : PTINR_VAL1 PT-INR Value

N	Mean	Standard Deviation	Minimum	Maximum
1	1.8800000		1.8800000	1.8800000

Unique Subject Identifier=7209000000-0114

Assay variable : PTINR_VAL1 PT-INR Value

N	Mean	Standard Deviation	Minimum	Maximum
4	2.2475000	0.5292999	1.6200000	2.8900000

Unique Subject Identifier=7209000000-0116

Assay variable : PTINR_VAL1 PT-INR Value

N	Mean	Standard Deviation	Minimum	Maximum
3	1.6533333	0.1715615	1.4700000	1.8100000

Laboratory tests at the time of obtaining Table 2-6 consent, according to the presence or absence of proton-pump inhibitors

Unique Subject Identifier=7209000000-0148

MEANS Procedure

Assay variable : PTINR_VAL1 PT-INR Value

N	Mean	Standard Deviation	Minimum	Maximum
4	1.7325000	0.0623832	1.6500000	1.8000000

Unique Subject Identifier=7221000000-0016

Assay variable : PTINR_VAL1 PT-INR Value

N	Mean	Standard Deviation	Minimum	Maximum
5	2.3400000	0.3435113	2.0000000	2.8000000

Unique Subject Identifier=7224011010-0006

Assay variable : PTINR_VAL1 PT-INR Value

N	Mean	Standard Deviation	Minimum	Maximum
1	1.9000000		1.9000000	1.9000000

Unique Subject Identifier=7224011010-0011

Assay variable : PTINR_VAL1 PT-INR Value

N	Mean	Standard Deviation	Minimum	Maximum
3	1.4700000	0.2389561	1.2600000	1.7300000

Laboratory tests at the time of obtaining Table 2-6 consent, according to the presence or absence of proton-pump inhibitors

Unique Subject Identifier=7226000000-0005

MEANS Procedure

Assay variable : PTINR_VAL1 PT-INR Value

N	Mean	Standard Deviation	Minimum	Maximum
4	1.7500000	0.2714160	1.4200000	2.0100000

Unique Subject Identifier=7226000000-0013

Assay variable : PTINR_VAL1 PT-INR Value

N	Mean	Standard Deviation	Minimum	Maximum
4	2.2375000	0.3244868	1.9300000	2.6900000

Unique Subject Identifier=7235000000-0011

Assay variable : PTINR_VAL1 PT-INR Value

N	Mean	Standard Deviation	Minimum	Maximum
2	2.1850000	0.2616295	2.0000000	2.3700000

Unique Subject Identifier=7314000000-0009

Assay variable : PTINR_VAL1 PT-INR Value

N	Mean	Standard Deviation	Minimum	Maximum
4	1.7750000	0.2362908	1.6000000	2.1000000

Laboratory tests at the time of obtaining Table 2-6 consent, according to the presence or absence of proton-pump inhibitors

Unique Subject Identifier=7314000000-0016

MEANS Procedure

Assay variable : PTINR_VAL1 PT-INR Value

N	Mean	Standard Deviation	Minimum	Maximum
5	1.5800000	0.0836660	1.5000000	1.7000000

Unique Subject Identifier=7326000000-0001

Assay variable : PTINR_VAL1 PT-INR Value

N	Mean	Standard Deviation	Minimum	Maximum
5	1.6100000	0.3037269	1.2800000	2.0500000

Unique Subject Identifier=7326000000-0004

Assay variable : PTINR_VAL1 PT-INR Value

N	Mean	Standard Deviation	Minimum	Maximum
3	2.1566667	0.2074448	1.9700000	2.3800000

Unique Subject Identifier=7326000000-0006

Assay variable : PTINR_VAL1 PT-INR Value

N	Mean	Standard Deviation	Minimum	Maximum
5	1.9400000	0.2518928	1.6100000	2.2600000

Laboratory tests at the time of obtaining Table 2-6 consent, according to the presence or absence of proton-pump inhibitors

Unique Subject Identifier=7326000000-0007

MEANS Procedure

Assay variable : PTINR_VAL1 PT-INR Value

N	Mean	Standard Deviation	Minimum	Maximum
4	1.8825000	0.2154646	1.5800000	2.0600000

Unique Subject Identifier=7332010000-0002

Assay variable : PTINR_VAL1 PT-INR Value

N	Mean	Standard Deviation	Minimum	Maximum
5	2.1080000	1.0816977	1.3600000	3.9600000

Unique Subject Identifier=7332010000-0005

Assay variable : PTINR_VAL1 PT-INR Value

N	Mean	Standard Deviation	Minimum	Maximum
4	2.2150000	0.1815673	2.0400000	2.4500000

Unique Subject Identifier=7332010000-0029

Assay variable : PTINR_VAL1 PT-INR Value

N	Mean	Standard Deviation	Minimum	Maximum
3	1.9233333	0.2400694	1.6600000	2.1300000

Laboratory tests at the time of obtaining Table 2-6 consent, according to the presence or absence of proton-pump inhibitors

Unique Subject Identifier=7345000010-0008

MEANS Procedure

Assay variable : PTINR_VAL1 PT-INR Value

N	Mean	Standard Deviation	Minimum	Maximum
6	2.2266667	0.4322345	1.5300000	2.6900000

Unique Subject Identifier=7345000010-0011

Assay variable : PTINR_VAL1 PT-INR Value

N	Mean	Standard Deviation	Minimum	Maximum
6	2.0033333	0.2483277	1.7300000	2.4300000

Unique Subject Identifier=7345000010-0012

Assay variable : PTINR_VAL1 PT-INR Value

N	Mean	Standard Deviation	Minimum	Maximum
6	1.7850000	0.4234973	1.2100000	2.1900000

Unique Subject Identifier=7345000010-0014

Assay variable : PTINR_VAL1 PT-INR Value

N	Mean	Standard Deviation	Minimum	Maximum
4	2.2275000	0.6051102	1.4200000	2.8700000

Laboratory tests at the time of obtaining Table 2-6 consent, according to the presence or absence of proton-pump inhibitors

Unique Subject Identifier=7345000010-0017

MEANS Procedure

Assay variable : PTINR_VAL1 PT-INR Value

N	Mean	Standard Deviation	Minimum	Maximum
6	1.5000000	0.5067544	1.1100000	2.4100000

Unique Subject Identifier=7345000010-0023

Assay variable : PTINR_VAL1 PT-INR Value

N	Mean	Standard Deviation	Minimum	Maximum
6	1.9916667	0.3581852	1.5400000	2.4100000

Unique Subject Identifier=7345000010-0024

Assay variable : PTINR_VAL1 PT-INR Value

N	Mean	Standard Deviation	Minimum	Maximum
5	2.0580000	0.2605187	1.8100000	2.4600000

Unique Subject Identifier=7345000010-0025

Assay variable : PTINR_VAL1 PT-INR Value

N	Mean	Standard Deviation	Minimum	Maximum
6	1.7316667	0.3022196	1.3400000	2.0900000

Laboratory tests at the time of obtaining Table 2-6 consent, according to the presence or absence of proton-pump inhibitors

Unique Subject Identifier=7345000010-0040

MEANS Procedure

Assay variable : PTINR_VAL1 PT-INR Value

N	Mean	Standard Deviation	Minimum	Maximum
6	1.7333333	0.2383834	1.3900000	1.9200000

Unique Subject Identifier=7366011100-0002

Assay variable : PTINR_VAL1 PT-INR Value

N	Mean	Standard Deviation	Minimum	Maximum
6	1.7066667	0.3946982	1.2400000	2.3800000

Unique Subject Identifier=7366011100-0010

Assay variable : PTINR_VAL1 PT-INR Value

N	Mean	Standard Deviation	Minimum	Maximum
4	2.4100000	0.3554340	1.8800000	2.6400000

Unique Subject Identifier=7366011100-0016

Assay variable : PTINR_VAL1 PT-INR Value

N	Mean	Standard Deviation	Minimum	Maximum
6	2.1350000	0.4511652	1.7400000	3.0100000

Laboratory tests at the time of obtaining Table 2-6 consent, according to the presence or absence of proton-pump inhibitors

Unique Subject Identifier=7414010000-0001

MEANS Procedure

Assay variable : PTINR_VAL1 PT-INR Value

N	Mean	Standard Deviation	Minimum	Maximum
6	1.7383333	0.2650597	1.3300000	2.1000000

Unique Subject Identifier=7414010000-0019

Assay variable : PTINR_VAL1 PT-INR Value

N	Mean	Standard Deviation	Minimum	Maximum
5	2.1620000	0.6068113	1.3700000	2.6800000

Unique Subject Identifier=7414010000-0033

Assay variable : PTINR_VAL1 PT-INR Value

N	Mean	Standard Deviation	Minimum	Maximum
6	1.9616667	0.1038107	1.8300000	2.1000000

Unique Subject Identifier=7414010000-0034

Assay variable : PTINR_VAL1 PT-INR Value

N	Mean	Standard Deviation	Minimum	Maximum
6	1.6100000	0.1550484	1.4200000	1.8700000

Laboratory tests at the time of obtaining Table 2-6 consent, according to the presence or absence of proton-pump inhibitors

Unique Subject Identifier=7423000000-0003

MEANS Procedure

Assay variable : PTINR_VAL1 PT-INR Value

N	Mean	Standard Deviation	Minimum	Maximum
4	1.7500000	0.1264911	1.5900000	1.8700000

Unique Subject Identifier=7423000000-0005

Assay variable : PTINR_VAL1 PT-INR Value

N	Mean	Standard Deviation	Minimum	Maximum
6	1.8283333	0.4879105	1.3300000	2.4600000

Unique Subject Identifier=7423000000-0006

Assay variable : PTINR_VAL1 PT-INR Value

N	Mean	Standard Deviation	Minimum	Maximum
4	1.6550000	0.1668333	1.5400000	1.9000000

Unique Subject Identifier=7423000000-0014

Assay variable : PTINR_VAL1 PT-INR Value

N	Mean	Standard Deviation	Minimum	Maximum
3	2.2466667	0.3239341	2.0400000	2.6200000

Laboratory tests at the time of obtaining Table 2-6 consent, according to the presence or absence of proton-pump inhibitors

Unique Subject Identifier=7423000000-0017

MEANS Procedure

Assay variable : PTINR_VAL1 PT-INR Value

N	Mean	Standard Deviation	Minimum	Maximum
4	1.4725000	0.1138347	1.3500000	1.6200000

Unique Subject Identifier=7423000000-0020

Assay variable : PTINR_VAL1 PT-INR Value

N	Mean	Standard Deviation	Minimum	Maximum
2	1.2050000	0.0636396	1.1600000	1.2500000

Unique Subject Identifier=7423000000-0026

Assay variable : PTINR_VAL1 PT-INR Value

N	Mean	Standard Deviation	Minimum	Maximum
4	2.5350000	0.8121781	1.5200000	3.4300000

Unique Subject Identifier=7423000000-0029

Assay variable : PTINR_VAL1 PT-INR Value

N	Mean	Standard Deviation	Minimum	Maximum
5	1.6200000	0.1680774	1.4900000	1.8800000

Laboratory tests at the time of obtaining Table 2-6 consent, according to the presence or absence of proton-pump inhibitors

Unique Subject Identifier=7423000000-0030

MEANS Procedure

Assay variable : PTINR_VAL1 PT-INR Value

N	Mean	Standard Deviation	Minimum	Maximum
5	1.8160000	0.3728002	1.3200000	2.1100000

Unique Subject Identifier=7423000000-0040

Assay variable : PTINR_VAL1 PT-INR Value

N	Mean	Standard Deviation	Minimum	Maximum
2	2.4000000	0.0989949	2.3300000	2.4700000

Unique Subject Identifier=7423000000-0044

Assay variable : PTINR_VAL1 PT-INR Value

N	Mean	Standard Deviation	Minimum	Maximum
5	1.6500000	0.2155226	1.4200000	1.9200000

Unique Subject Identifier=7423000000-0052

Assay variable : PTINR_VAL1 PT-INR Value

N	Mean	Standard Deviation	Minimum	Maximum
4	2.3200000	0.2422120	2.0800000	2.6400000

Laboratory tests at the time of obtaining Table 2-6 consent, according to the presence or absence of proton-pump inhibitors

Unique Subject Identifier=7423000000-0053

MEANS Procedure

Assay variable : PTINR_VAL1 PT-INR Value

N	Mean	Standard Deviation	Minimum	Maximum
6	2.0233333	0.2628815	1.7500000	2.4100000

Unique Subject Identifier=7423000000-0061

Assay variable : PTINR_VAL1 PT-INR Value

N	Mean	Standard Deviation	Minimum	Maximum
5	1.7380000	0.1310343	1.5800000	1.8900000

Unique Subject Identifier=7423000000-0062

Assay variable : PTINR_VAL1 PT-INR Value

N	Mean	Standard Deviation	Minimum	Maximum
5	1.7300000	0.1811077	1.4800000	1.9400000

Unique Subject Identifier=7423000000-0064

Assay variable : PTINR_VAL1 PT-INR Value

N	Mean	Standard Deviation	Minimum	Maximum
4	1.8525000	0.1408013	1.6600000	1.9800000

Laboratory tests at the time of obtaining Table 2-6 consent, according to the presence or absence of proton-pump inhibitors

Unique Subject Identifier=7423000000-0071

MEANS Procedure

Assay variable : PTINR_VAL1 PT-INR Value

N	Mean	Standard Deviation	Minimum	Maximum
1	1.5600000		1.5600000	1.5600000

Unique Subject Identifier=7423000000-0072

Assay variable : PTINR_VAL1 PT-INR Value

N	Mean	Standard Deviation	Minimum	Maximum
4	1.6525000	0.2732978	1.3500000	1.9900000

Unique Subject Identifier=7423000000-0074

Assay variable : PTINR_VAL1 PT-INR Value

N	Mean	Standard Deviation	Minimum	Maximum
2	1.9250000	0.0070711	1.9200000	1.9300000

Unique Subject Identifier=7423000000-0075

Assay variable : PTINR_VAL1 PT-INR Value

N	Mean	Standard Deviation	Minimum	Maximum
2	1.5850000	0.0494975	1.5500000	1.6200000

Laboratory tests at the time of obtaining Table 2-6 consent, according to the presence or absence of proton-pump inhibitors

Unique Subject Identifier=7423000000-0076

MEANS Procedure

Assay variable : PTINR_VAL1 PT-INR Value

N	Mean	Standard Deviation	Minimum	Maximum
3	2.2800000	0.3604164	1.9100000	2.6300000

Unique Subject Identifier=7423000000-0081

Assay variable : PTINR_VAL1 PT-INR Value

N	Mean	Standard Deviation	Minimum	Maximum
1	1.5700000		1.5700000	1.5700000

Unique Subject Identifier=7423000000-0090

Assay variable : PTINR_VAL1 PT-INR Value

N	Mean	Standard Deviation	Minimum	Maximum
5	1.6720000	0.6956795	1.0500000	2.6600000

Unique Subject Identifier=7423000000-0097

Assay variable : PTINR_VAL1 PT-INR Value

N	Mean	Standard Deviation	Minimum	Maximum
4	1.7925000	0.1867931	1.6200000	2.0000000

Laboratory tests at the time of obtaining Table 2-6 consent, according to the presence or absence of proton-pump inhibitors

Unique Subject Identifier=7542000000-0003

MEANS Procedure

Assay variable : PTINR_VAL1 PT-INR Value

N	Mean	Standard Deviation	Minimum	Maximum
6	2.0333333	0.6377042	1.6000000	3.3000000

Unique Subject Identifier=7542000000-0005

Assay variable : PTINR_VAL1 PT-INR Value

N	Mean	Standard Deviation	Minimum	Maximum
6	2.5833333	1.2416387	1.5000000	4.5000000

Unique Subject Identifier=7542000000-0008

Assay variable : PTINR_VAL1 PT-INR Value

N	Mean	Standard Deviation	Minimum	Maximum
6	2.2666667	0.5391351	1.4000000	2.9000000

Unique Subject Identifier=7542000000-0009

Assay variable : PTINR_VAL1 PT-INR Value

N	Mean	Standard Deviation	Minimum	Maximum
6	2.2166667	0.1602082	1.9000000	2.3000000

Laboratory tests at the time of obtaining Table 2-6 consent, according to the presence or absence of proton-pump inhibitors

Unique Subject Identifier=7542000000-0015

MEANS Procedure

Assay variable : PTINR_VAL1 PT-INR Value

N	Mean	Standard Deviation	Minimum	Maximum
6	2.1900000	0.2202726	1.9900000	2.5800000

Unique Subject Identifier=7542000000-0017

Assay variable : PTINR_VAL1 PT-INR Value

N	Mean	Standard Deviation	Minimum	Maximum
6	2.3833333	0.9368387	1.5000000	3.9000000

Unique Subject Identifier=7542000000-0025

Assay variable : PTINR_VAL1 PT-INR Value

N	Mean	Standard Deviation	Minimum	Maximum
5	2.1400000	0.2073644	1.9000000	2.4000000

Unique Subject Identifier=7542000000-0026

Assay variable : PTINR_VAL1 PT-INR Value

N	Mean	Standard Deviation	Minimum	Maximum
6	1.7833333	0.1329160	1.6000000	2.0000000

Laboratory tests at the time of obtaining Table 2-6 consent, according to the presence or absence of proton-pump inhibitors

Unique Subject Identifier=7642010000-0001

MEANS Procedure

Assay variable : PTINR_VAL1 PT-INR Value

N	Mean	Standard Deviation	Minimum	Maximum
3	2.2033333	0.4838733	1.8300000	2.7500000

Unique Subject Identifier=8005000000-0002

Assay variable : PTINR_VAL1 PT-INR Value

N	Mean	Standard Deviation	Minimum	Maximum
6	2.0000000	0.1436663	1.8400000	2.2500000

Unique Subject Identifier=8005000000-0010

Assay variable : PTINR_VAL1 PT-INR Value

N	Mean	Standard Deviation	Minimum	Maximum
5	1.9360000	0.4195593	1.4700000	2.5200000

Unique Subject Identifier=8005000000-0011

Assay variable : PTINR_VAL1 PT-INR Value

N	Mean	Standard Deviation	Minimum	Maximum
5	1.7220000	0.2052316	1.5300000	2.0500000

Laboratory tests at the time of obtaining Table 2-6 consent, according to the presence or absence of proton-pump inhibitors

Unique Subject Identifier=8009000000-0001

MEANS Procedure

Assay variable : PTINR_VAL1 PT-INR Value

N	Mean	Standard Deviation	Minimum	Maximum
6	1.7416667	0.4793085	1.1800000	2.5100000

Unique Subject Identifier=8009000000-0002

Assay variable : PTINR_VAL1 PT-INR Value

N	Mean	Standard Deviation	Minimum	Maximum
5	2.2660000	0.5860717	1.8800000	3.3000000

Unique Subject Identifier=8009000000-0005

Assay variable : PTINR_VAL1 PT-INR Value

N	Mean	Standard Deviation	Minimum	Maximum
4	1.6425000	0.0939415	1.5200000	1.7300000

Unique Subject Identifier=8009000000-0030

Assay variable : PTINR_VAL1 PT-INR Value

N	Mean	Standard Deviation	Minimum	Maximum
4	1.5125000	0.1631717	1.3600000	1.7300000

Laboratory tests at the time of obtaining Table 2-6 consent, according to the presence or absence of proton-pump inhibitors

Unique Subject Identifier=8010000100-0012

MEANS Procedure

Assay variable : PTINR_VAL1 PT-INR Value

N	Mean	Standard Deviation	Minimum	Maximum
6	2.0383333	0.2545912	1.5800000	2.3100000

Unique Subject Identifier=8010000100-0060

Assay variable : PTINR_VAL1 PT-INR Value

N	Mean	Standard Deviation	Minimum	Maximum
3	2.1866667	0.2967041	1.9100000	2.5000000

Unique Subject Identifier=8020011000-0032

Assay variable : PTINR_VAL1 PT-INR Value

N	Mean	Standard Deviation	Minimum	Maximum
1	1.3700000		1.3700000	1.3700000

Unique Subject Identifier=8020011000-0040

Assay variable : PTINR_VAL1 PT-INR Value

N	Mean	Standard Deviation	Minimum	Maximum
2	1.8000000	0.5798276	1.3900000	2.2100000

Laboratory tests at the time of obtaining Table 2-6 consent, according to the presence or absence of proton-pump inhibitors

Unique Subject Identifier=8020011000-0042

MEANS Procedure

Assay variable : PTINR_VAL1 PT-INR Value

N	Mean	Standard Deviation	Minimum	Maximum
1	2.9400000		2.9400000	2.9400000

Unique Subject Identifier=8020011000-0046

Assay variable : PTINR_VAL1 PT-INR Value

N	Mean	Standard Deviation	Minimum	Maximum
1	1.3200000		1.3200000	1.3200000

Unique Subject Identifier=8023000000-0010

Assay variable : PTINR_VAL1 PT-INR Value

N	Mean	Standard Deviation	Minimum	Maximum
4	1.9550000	0.6821779	1.5200000	2.9700000

Unique Subject Identifier=8023000000-0011

Assay variable : PTINR_VAL1 PT-INR Value

N	Mean	Standard Deviation	Minimum	Maximum
6	1.4300000	0.1912067	1.2400000	1.8000000

Laboratory tests at the time of obtaining Table 2-6 consent, according to the presence or absence of proton-pump inhibitors

Unique Subject Identifier=8023000000-0016

MEANS Procedure

Assay variable : PTINR_VAL1 PT-INR Value

N	Mean	Standard Deviation	Minimum	Maximum
3	2.0233333	0.2557994	1.7800000	2.2900000

Unique Subject Identifier=8023000000-0020

Assay variable : PTINR_VAL1 PT-INR Value

N	Mean	Standard Deviation	Minimum	Maximum
3	1.9100000	0.1802776	1.7600000	2.1100000

Unique Subject Identifier=8023000000-0021

Assay variable : PTINR_VAL1 PT-INR Value

N	Mean	Standard Deviation	Minimum	Maximum
3	2.0333333	0.1550269	1.9200000	2.2100000

Unique Subject Identifier=8023000000-0033

Assay variable : PTINR_VAL1 PT-INR Value

N	Mean	Standard Deviation	Minimum	Maximum
3	1.4900000	0.3051229	1.1400000	1.7000000

Laboratory tests at the time of obtaining Table 2-6 consent, according to the presence or absence of proton-pump inhibitors

Unique Subject Identifier=8023000000-0037

MEANS Procedure

Assay variable : PTINR_VAL1 PT-INR Value

N	Mean	Standard Deviation	Minimum	Maximum
4	1.6125000	0.2650000	1.2800000	1.8800000

Unique Subject Identifier=8023000000-0042

Assay variable : PTINR_VAL1 PT-INR Value

N	Mean	Standard Deviation	Minimum	Maximum
2	2.3350000	0.4454773	2.0200000	2.6500000

Unique Subject Identifier=8023000000-0045

Assay variable : PTINR_VAL1 PT-INR Value

N	Mean	Standard Deviation	Minimum	Maximum
3	1.6966667	0.4239497	1.3100000	2.1500000

Unique Subject Identifier=8026000000-0003

Assay variable : PTINR_VAL1 PT-INR Value

N	Mean	Standard Deviation	Minimum	Maximum
3	2.2133333	0.2136196	1.9700000	2.3700000

Laboratory tests at the time of obtaining Table 2-6 consent, according to the presence or absence of proton-pump inhibitors

Unique Subject Identifier=8026000000-0004

MEANS Procedure

Assay variable : PTINR_VAL1 PT-INR Value

N	Mean	Standard Deviation	Minimum	Maximum
3	1.9600000	1.0142485	1.3300000	3.1300000

Unique Subject Identifier=8026000000-0007

Assay variable : PTINR_VAL1 PT-INR Value

N	Mean	Standard Deviation	Minimum	Maximum
2	1.8300000	0.4384062	1.5200000	2.1400000

Unique Subject Identifier=8026000000-0027

Assay variable : PTINR_VAL1 PT-INR Value

N	Mean	Standard Deviation	Minimum	Maximum
2	2.2950000	0.3606245	2.0400000	2.5500000

Unique Subject Identifier=8026000000-0028

Assay variable : PTINR_VAL1 PT-INR Value

N	Mean	Standard Deviation	Minimum	Maximum
2	1.8650000	0.2474874	1.6900000	2.0400000

Laboratory tests at the time of obtaining Table 2-6 consent, according to the presence or absence of proton-pump inhibitors

Unique Subject Identifier=8026000000-0030

MEANS Procedure

Assay variable : PTINR_VAL1 PT-INR Value

N	Mean	Standard Deviation	Minimum	Maximum
3	1.9200000	0.2402082	1.7300000	2.1900000

Unique Subject Identifier=8030011000-0021

Assay variable : PTINR_VAL1 PT-INR Value

N	Mean	Standard Deviation	Minimum	Maximum
6	2.3433333	0.1875811	2.1000000	2.5500000

Unique Subject Identifier=8030011000-0027

Assay variable : PTINR_VAL1 PT-INR Value

N	Mean	Standard Deviation	Minimum	Maximum
6	2.1783333	0.2555321	1.8500000	2.5100000

Unique Subject Identifier=8031000000-0005

Assay variable : PTINR_VAL1 PT-INR Value

N	Mean	Standard Deviation	Minimum	Maximum
4	1.9000000	0.1016530	1.7700000	2.0100000

Laboratory tests at the time of obtaining Table 2-6 consent, according to the presence or absence of proton-pump inhibitors

Unique Subject Identifier=8031000000-0009

MEANS Procedure

Assay variable : PTINR_VAL1 PT-INR Value

N	Mean	Standard Deviation	Minimum	Maximum
4	1.9700000	0.8517824	1.2300000	2.9800000

Unique Subject Identifier=8031000000-0014

Assay variable : PTINR_VAL1 PT-INR Value

N	Mean	Standard Deviation	Minimum	Maximum
5	1.8780000	0.1722498	1.6200000	2.0500000

Unique Subject Identifier=8031000000-0023

Assay variable : PTINR_VAL1 PT-INR Value

N	Mean	Standard Deviation	Minimum	Maximum
5	1.9620000	0.4747315	1.3400000	2.4400000

Unique Subject Identifier=8031000000-0048

Assay variable : PTINR_VAL1 PT-INR Value

N	Mean	Standard Deviation	Minimum	Maximum
6	1.6966667	0.1569289	1.4000000	1.8600000

Laboratory tests at the time of obtaining Table 2-6 consent, according to the presence or absence of proton-pump inhibitors

Unique Subject Identifier=8031000000-0049

MEANS Procedure

Assay variable : PTINR_VAL1 PT-INR Value

N	Mean	Standard Deviation	Minimum	Maximum
5	2.3820000	0.7620171	1.7900000	3.4100000

Unique Subject Identifier=8035000000-0005

Assay variable : PTINR_VAL1 PT-INR Value

N	Mean	Standard Deviation	Minimum	Maximum
2	1.9000000	0.1414214	1.8000000	2.0000000

Unique Subject Identifier=8035000000-0010

Assay variable : PTINR_VAL1 PT-INR Value

N	Mean	Standard Deviation	Minimum	Maximum
3	2.1333333	0.2386071	1.9400000	2.4000000

Unique Subject Identifier=8035000000-0016

Assay variable : PTINR_VAL1 PT-INR Value

N	Mean	Standard Deviation	Minimum	Maximum
1	2.5900000		2.5900000	2.5900000

Laboratory tests at the time of obtaining Table 2-6 consent, according to the presence or absence of proton-pump inhibitors

Unique Subject Identifier=8047000000-0015

MEANS Procedure

Assay variable : PTINR_VAL1 PT-INR Value

N	Mean	Standard Deviation	Minimum	Maximum
3	1.8966667	0.7503555	1.3400000	2.7500000

Unique Subject Identifier=8047000000-0029

Assay variable : PTINR_VAL1 PT-INR Value

N	Mean	Standard Deviation	Minimum	Maximum
3	1.6866667	0.2311565	1.4700000	1.9300000

Unique Subject Identifier=8048010011-0002

Assay variable : PTINR_VAL1 PT-INR Value

N	Mean	Standard Deviation	Minimum	Maximum
2	1.3900000	0.0565685	1.3500000	1.4300000

Unique Subject Identifier=8048010011-0028

Assay variable : PTINR_VAL1 PT-INR Value

N	Mean	Standard Deviation	Minimum	Maximum
6	2.0916667	0.1070358	1.9700000	2.2800000

Laboratory tests at the time of obtaining Table 2-6 consent, according to the presence or absence of proton-pump inhibitors

Unique Subject Identifier=8048010011-0034

MEANS Procedure

Assay variable : PTINR_VAL1 PT-INR Value

N	Mean	Standard Deviation	Minimum	Maximum
2	1.6400000	0.6363961	1.1900000	2.0900000

Unique Subject Identifier=8057011000-0001

Assay variable : PTINR_VAL1 PT-INR Value

N	Mean	Standard Deviation	Minimum	Maximum
1	1.3800000		1.3800000	1.3800000

Unique Subject Identifier=8057011000-0012

Assay variable : PTINR_VAL1 PT-INR Value

N	Mean	Standard Deviation	Minimum	Maximum
6	1.7183333	0.3896879	1.3100000	2.2200000

Unique Subject Identifier=8057011000-0014

Assay variable : PTINR_VAL1 PT-INR Value

N	Mean	Standard Deviation	Minimum	Maximum
3	2.2566667	0.3689625	1.8500000	2.5700000

Laboratory tests at the time of obtaining Table 2-6 consent, according to the presence or absence of proton-pump inhibitors

Unique Subject Identifier=8057011000-0015

MEANS Procedure

Assay variable : PTINR_VAL1 PT-INR Value

N	Mean	Standard Deviation	Minimum	Maximum
6	2.1483333	0.6102923	1.4900000	3.1000000

Unique Subject Identifier=8060011000-0001

Assay variable : PTINR_VAL1 PT-INR Value

N	Mean	Standard Deviation	Minimum	Maximum
6	2.3666667	0.4273952	1.9000000	3.1000000

Unique Subject Identifier=8060011000-0002

Assay variable : PTINR_VAL1 PT-INR Value

N	Mean	Standard Deviation	Minimum	Maximum
3	2.7333333	1.0066446	1.8000000	3.8000000

Unique Subject Identifier=8060011000-0003

Assay variable : PTINR_VAL1 PT-INR Value

N	Mean	Standard Deviation	Minimum	Maximum
3	2.6000000	0.4582576	2.1000000	3.0000000

Laboratory tests at the time of obtaining Table 2-6 consent, according to the presence or absence of proton-pump inhibitors

Unique Subject Identifier=8060011000-0012

MEANS Procedure

Assay variable : PTINR_VAL1 PT-INR Value

N	Mean	Standard Deviation	Minimum	Maximum
5	1.9200000	0.4438468	1.5000000	2.4000000

Unique Subject Identifier=8060011000-0013

Assay variable : PTINR_VAL1 PT-INR Value

N	Mean	Standard Deviation	Minimum	Maximum
6	1.9333333	0.3932768	1.5000000	2.5000000

Unique Subject Identifier=8060011000-0020

Assay variable : PTINR_VAL1 PT-INR Value

N	Mean	Standard Deviation	Minimum	Maximum
6	2.3166667	0.3125167	1.9000000	2.8000000

Unique Subject Identifier=8060011000-0023

Assay variable : PTINR_VAL1 PT-INR Value

N	Mean	Standard Deviation	Minimum	Maximum
6	1.9833333	0.3311596	1.7000000	2.6000000

Laboratory tests at the time of obtaining Table 2-6 consent, according to the presence or absence of proton-pump inhibitors

Unique Subject Identifier=8104000000-0002

MEANS Procedure

Assay variable : PTINR_VAL1 PT-INR Value

N	Mean	Standard Deviation	Minimum	Maximum
2	2.8700000	0.6081118	2.4400000	3.3000000

Unique Subject Identifier=8104000000-0003

Assay variable : PTINR_VAL1 PT-INR Value

N	Mean	Standard Deviation	Minimum	Maximum
2	1.3500000	0.0707107	1.3000000	1.4000000

Unique Subject Identifier=8104000000-0007

Assay variable : PTINR_VAL1 PT-INR Value

N	Mean	Standard Deviation	Minimum	Maximum
4	1.5350000	0.1473092	1.3900000	1.6900000

Unique Subject Identifier=8104000000-0018

Assay variable : PTINR_VAL1 PT-INR Value

N	Mean	Standard Deviation	Minimum	Maximum
6	2.0150000	0.2504196	1.6900000	2.4400000

Laboratory tests at the time of obtaining Table 2-6 consent, according to the presence or absence of proton-pump inhibitors

Unique Subject Identifier=8104000000-0021

MEANS Procedure

Assay variable : PTINR_VAL1 PT-INR Value

N	Mean	Standard Deviation	Minimum	Maximum
3	1.6700000	0.4063250	1.3300000	2.1200000

Unique Subject Identifier=8104000000-0027

Assay variable : PTINR_VAL1 PT-INR Value

N	Mean	Standard Deviation	Minimum	Maximum
3	1.9133333	0.5225259	1.3100000	2.2200000

Unique Subject Identifier=8110000100-0002

Assay variable : PTINR_VAL1 PT-INR Value

N	Mean	Standard Deviation	Minimum	Maximum
3	2.1833333	0.1167619	2.0800000	2.3100000

Unique Subject Identifier=8110000100-0004

Assay variable : PTINR_VAL1 PT-INR Value

N	Mean	Standard Deviation	Minimum	Maximum
3	2.2466667	0.4954123	1.8200000	2.7900000

Laboratory tests at the time of obtaining Table 2-6 consent, according to the presence or absence of proton-pump inhibitors

Unique Subject Identifier=8114000000-0008

MEANS Procedure

Assay variable : PTINR_VAL1 PT-INR Value

N	Mean	Standard Deviation	Minimum	Maximum
6	1.7950000	0.2055967	1.4900000	2.0900000

Unique Subject Identifier=8117011010-0002

Assay variable : PTINR_VAL1 PT-INR Value

N	Mean	Standard Deviation	Minimum	Maximum
3	1.6833333	0.4539089	1.1600000	1.9700000

Unique Subject Identifier=8117011010-0005

Assay variable : PTINR_VAL1 PT-INR Value

N	Mean	Standard Deviation	Minimum	Maximum
2	1.7700000	0.2121320	1.6200000	1.9200000

Unique Subject Identifier=8117011010-0009

Assay variable : PTINR_VAL1 PT-INR Value

N	Mean	Standard Deviation	Minimum	Maximum
5	1.9260000	0.1119375	1.7700000	2.0400000

Laboratory tests at the time of obtaining Table 2-6 consent, according to the presence or absence of proton-pump inhibitors

Unique Subject Identifier=8123000000-0004

MEANS Procedure

Assay variable : PTINR_VAL1 PT-INR Value

N	Mean	Standard Deviation	Minimum	Maximum
6	1.8983333	0.2920559	1.4500000	2.2300000

Unique Subject Identifier=8123000000-0010

Assay variable : PTINR_VAL1 PT-INR Value

N	Mean	Standard Deviation	Minimum	Maximum
5	1.9240000	0.3630840	1.4900000	2.4600000

Unique Subject Identifier=8123000000-0011

Assay variable : PTINR_VAL1 PT-INR Value

N	Mean	Standard Deviation	Minimum	Maximum
4	1.8825000	0.2151550	1.6800000	2.1500000

Unique Subject Identifier=8123000000-0012

Assay variable : PTINR_VAL1 PT-INR Value

N	Mean	Standard Deviation	Minimum	Maximum
4	1.9625000	0.1668083	1.7300000	2.1100000

Laboratory tests at the time of obtaining Table 2-6 consent, according to the presence or absence of proton-pump inhibitors

Unique Subject Identifier=8123000000-0013

MEANS Procedure

Assay variable : PTINR_VAL1 PT-INR Value

N	Mean	Standard Deviation	Minimum	Maximum
5	2.1160000	0.1350185	1.9500000	2.3000000

Unique Subject Identifier=8123000000-0018

Assay variable : PTINR_VAL1 PT-INR Value

N	Mean	Standard Deviation	Minimum	Maximum
6	1.8600000	0.3850714	1.5200000	2.5700000

Unique Subject Identifier=8136000000-0002

Assay variable : PTINR_VAL1 PT-INR Value

N	Mean	Standard Deviation	Minimum	Maximum
6	1.8233333	0.2530349	1.4000000	2.1300000

Unique Subject Identifier=8136000000-0010

Assay variable : PTINR_VAL1 PT-INR Value

N	Mean	Standard Deviation	Minimum	Maximum
4	1.8400000	0.0294392	1.8100000	1.8800000

Laboratory tests at the time of obtaining Table 2-6 consent, according to the presence or absence of proton-pump inhibitors

Unique Subject Identifier=8136000000-0015

MEANS Procedure

Assay variable : PTINR_VAL1 PT-INR Value

N	Mean	Standard Deviation	Minimum	Maximum
4	2.1425000	0.8701485	1.5400000	3.4300000

Unique Subject Identifier=8137010000-0002

Assay variable : PTINR_VAL1 PT-INR Value

N	Mean	Standard Deviation	Minimum	Maximum
3	2.3833333	0.7011657	1.8600000	3.1800000

Unique Subject Identifier=8137010000-0007

Assay variable : PTINR_VAL1 PT-INR Value

N	Mean	Standard Deviation	Minimum	Maximum
5	1.9260000	0.2608256	1.5500000	2.2100000

Unique Subject Identifier=8154011000-0020

Assay variable : PTINR_VAL1 PT-INR Value

N	Mean	Standard Deviation	Minimum	Maximum
6	2.1566667	0.4464378	1.8000000	3.0400000

Laboratory tests at the time of obtaining Table 2-6 consent, according to the presence or absence of proton-pump inhibitors

Unique Subject Identifier=8154011000-0021

MEANS Procedure

Assay variable : PTINR_VAL1 PT-INR Value

N	Mean	Standard Deviation	Minimum	Maximum
5	2.1940000	0.2634008	1.9800000	2.6500000

Unique Subject Identifier=8160000000-0003

Assay variable : PTINR_VAL1 PT-INR Value

N	Mean	Standard Deviation	Minimum	Maximum
6	1.8100000	0.2372341	1.6000000	2.2000000

Unique Subject Identifier=8160000000-0006

Assay variable : PTINR_VAL1 PT-INR Value

N	Mean	Standard Deviation	Minimum	Maximum
6	1.7850000	0.3574213	1.4000000	2.3000000

Unique Subject Identifier=8160000000-0007

Assay variable : PTINR_VAL1 PT-INR Value

N	Mean	Standard Deviation	Minimum	Maximum
5	1.9880000	0.1475466	1.7700000	2.1000000

Laboratory tests at the time of obtaining Table 2-6 consent, according to the presence or absence of proton-pump inhibitors

Unique Subject Identifier=8160000000-0008

MEANS Procedure

Assay variable : PTINR_VAL1 PT-INR Value

N	Mean	Standard Deviation	Minimum	Maximum
6	2.1683333	0.4108731	1.7100000	2.8000000

Unique Subject Identifier=8160000000-0010

Assay variable : PTINR_VAL1 PT-INR Value

N	Mean	Standard Deviation	Minimum	Maximum
6	1.8516667	0.2371005	1.6300000	2.3000000

Unique Subject Identifier=8160000000-0017

Assay variable : PTINR_VAL1 PT-INR Value

N	Mean	Standard Deviation	Minimum	Maximum
6	1.8283333	0.1000833	1.7000000	2.0000000

Unique Subject Identifier=8160000000-0018

Assay variable : PTINR_VAL1 PT-INR Value

N	Mean	Standard Deviation	Minimum	Maximum
6	1.6333333	0.2111556	1.4000000	2.0000000

Laboratory tests at the time of obtaining Table 2-6 consent, according to the presence or absence of proton-pump inhibitors

Unique Subject Identifier=8160000000-0022

MEANS Procedure

Assay variable : PTINR_VAL1 PT-INR Value

N	Mean	Standard Deviation	Minimum	Maximum
6	1.7250000	0.2403123	1.5000000	2.0000000

Unique Subject Identifier=8160000000-0026

Assay variable : PTINR_VAL1 PT-INR Value

N	Mean	Standard Deviation	Minimum	Maximum
6	1.6266667	0.1751190	1.3600000	1.9000000

Unique Subject Identifier=8161010010-0002

Assay variable : PTINR_VAL1 PT-INR Value

N	Mean	Standard Deviation	Minimum	Maximum
4	1.9625000	0.3406244	1.5400000	2.3600000

Unique Subject Identifier=8161010010-0003

Assay variable : PTINR_VAL1 PT-INR Value

N	Mean	Standard Deviation	Minimum	Maximum
3	2.6700000	0.3439477	2.3000000	2.9800000

Laboratory tests at the time of obtaining Table 2-6 consent, according to the presence or absence of proton-pump inhibitors

Unique Subject Identifier=8161010010-0011

MEANS Procedure

Assay variable : PTINR_VAL1 PT-INR Value

N	Mean	Standard Deviation	Minimum	Maximum
5	1.7800000	0.1607794	1.6000000	1.9800000

Unique Subject Identifier=8161010010-0018

Assay variable : PTINR_VAL1 PT-INR Value

N	Mean	Standard Deviation	Minimum	Maximum
5	1.5080000	0.2834078	1.2900000	1.9900000

Unique Subject Identifier=8161010010-0024

Assay variable : PTINR_VAL1 PT-INR Value

N	Mean	Standard Deviation	Minimum	Maximum
5	2.1140000	0.6029760	1.6600000	3.1600000

Unique Subject Identifier=8161010010-0025

Assay variable : PTINR_VAL1 PT-INR Value

N	Mean	Standard Deviation	Minimum	Maximum
4	2.4100000	0.2201515	2.2300000	2.7300000

Laboratory tests at the time of obtaining Table 2-6 consent, according to the presence or absence of proton-pump inhibitors

Unique Subject Identifier=8205000000-0001

MEANS Procedure

Assay variable : PTINR_VAL1 PT-INR Value

N	Mean	Standard Deviation	Minimum	Maximum
4	1.6725000	0.1225765	1.5000000	1.7900000

Unique Subject Identifier=8205000000-0020

Assay variable : PTINR_VAL1 PT-INR Value

N	Mean	Standard Deviation	Minimum	Maximum
3	2.6433333	0.1877054	2.5300000	2.8600000

Unique Subject Identifier=8205000000-0027

Assay variable : PTINR_VAL1 PT-INR Value

N	Mean	Standard Deviation	Minimum	Maximum
4	2.0325000	0.2025463	1.8800000	2.3300000

Unique Subject Identifier=8205000000-0028

Assay variable : PTINR_VAL1 PT-INR Value

N	Mean	Standard Deviation	Minimum	Maximum
3	1.8200000	0.2463737	1.5900000	2.0800000

Laboratory tests at the time of obtaining Table 2-6 consent, according to the presence or absence of proton-pump inhibitors

Unique Subject Identifier=8205000000-0042

MEANS Procedure

Assay variable : PTINR_VAL1 PT-INR Value

N	Mean	Standard Deviation	Minimum	Maximum
4	1.8975000	0.3986122	1.6300000	2.4800000

Unique Subject Identifier=8209000000-0003

Assay variable : PTINR_VAL1 PT-INR Value

N	Mean	Standard Deviation	Minimum	Maximum
6	2.2900000	0.3209984	1.9000000	2.6400000

Unique Subject Identifier=8209000000-0004

Assay variable : PTINR_VAL1 PT-INR Value

N	Mean	Standard Deviation	Minimum	Maximum
6	2.2233333	0.7048593	1.6500000	3.2500000

Unique Subject Identifier=8209000000-0007

Assay variable : PTINR_VAL1 PT-INR Value

N	Mean	Standard Deviation	Minimum	Maximum
5	1.6820000	0.2108791	1.3700000	1.9200000

Laboratory tests at the time of obtaining Table 2-6 consent, according to the presence or absence of proton-pump inhibitors

Unique Subject Identifier=8209000000-0008

MEANS Procedure

Assay variable : PTINR_VAL1 PT-INR Value

N	Mean	Standard Deviation	Minimum	Maximum
6	2.0366667	0.4864018	1.5400000	2.8300000

Unique Subject Identifier=8209000000-0024

Assay variable : PTINR_VAL1 PT-INR Value

N	Mean	Standard Deviation	Minimum	Maximum
4	1.7000000	0.3987480	1.2600000	2.2200000

Unique Subject Identifier=8209000000-0028

Assay variable : PTINR_VAL1 PT-INR Value

N	Mean	Standard Deviation	Minimum	Maximum
6	2.2266667	0.2068494	1.9600000	2.5000000

Unique Subject Identifier=8223000000-0023

Assay variable : PTINR_VAL1 PT-INR Value

N	Mean	Standard Deviation	Minimum	Maximum
3	1.8933333	0.4461315	1.3900000	2.2400000

Laboratory tests at the time of obtaining Table 2-6 consent, according to the presence or absence of proton-pump inhibitors

Unique Subject Identifier=8223000000-0024

MEANS Procedure

Assay variable : PTINR_VAL1 PT-INR Value

N	Mean	Standard Deviation	Minimum	Maximum
5	1.8820000	0.5756040	1.2900000	2.5300000

Unique Subject Identifier=8233000000-0018

Assay variable : PTINR_VAL1 PT-INR Value

N	Mean	Standard Deviation	Minimum	Maximum
2	1.3900000	0.2687006	1.2000000	1.5800000

Unique Subject Identifier=8233000000-0019

Assay variable : PTINR_VAL1 PT-INR Value

N	Mean	Standard Deviation	Minimum	Maximum
2	2.6950000	0.1909188	2.5600000	2.8300000

Unique Subject Identifier=8237011000-0003

Assay variable : PTINR_VAL1 PT-INR Value

N	Mean	Standard Deviation	Minimum	Maximum
3	2.2333333	0.2886751	1.9000000	2.4000000

Laboratory tests at the time of obtaining Table 2-6 consent, according to the presence or absence of proton-pump inhibitors

Unique Subject Identifier=8237011000-0005

MEANS Procedure

Assay variable : PTINR_VAL1 PT-INR Value

N	Mean	Standard Deviation	Minimum	Maximum
2	2.3000000	0.1414214	2.2000000	2.4000000

Unique Subject Identifier=8237011000-0017

Assay variable : PTINR_VAL1 PT-INR Value

N	Mean	Standard Deviation	Minimum	Maximum
2	1.4000000	0.1414214	1.3000000	1.5000000

Unique Subject Identifier=8237011000-0023

Assay variable : PTINR_VAL1 PT-INR Value

N	Mean	Standard Deviation	Minimum	Maximum
3	2.5666667	0.3214550	2.2000000	2.8000000

Unique Subject Identifier=8237011000-0029

Assay variable : PTINR_VAL1 PT-INR Value

N	Mean	Standard Deviation	Minimum	Maximum
6	1.7166667	0.4400758	1.3000000	2.5000000

Laboratory tests at the time of obtaining Table 2-6 consent, according to the presence or absence of proton-pump inhibitors

Unique Subject Identifier=8240011000-0008

MEANS Procedure

Assay variable : PTINR_VAL1 PT-INR Value

N	Mean	Standard Deviation	Minimum	Maximum
6	2.2466667	0.2787592	1.9700000	2.6700000

Unique Subject Identifier=8241000000-0014

Assay variable : PTINR_VAL1 PT-INR Value

N	Mean	Standard Deviation	Minimum	Maximum
1	1.8500000		1.8500000	1.8500000

Unique Subject Identifier=8241000000-0023

Assay variable : PTINR_VAL1 PT-INR Value

N	Mean	Standard Deviation	Minimum	Maximum
4	1.7300000	0.3918333	1.2400000	2.1900000

Unique Subject Identifier=8241000000-0024

Assay variable : PTINR_VAL1 PT-INR Value

N	Mean	Standard Deviation	Minimum	Maximum
4	1.9350000	0.1491085	1.7500000	2.1100000

Laboratory tests at the time of obtaining Table 2-6 consent, according to the presence or absence of proton-pump inhibitors

Unique Subject Identifier=8241000000-0027

MEANS Procedure

Assay variable : PTINR_VAL1 PT-INR Value

N	Mean	Standard Deviation	Minimum	Maximum
4	1.8400000	0.4162531	1.3100000	2.3100000

Unique Subject Identifier=8241000000-0029

Assay variable : PTINR_VAL1 PT-INR Value

N	Mean	Standard Deviation	Minimum	Maximum
3	2.0566667	0.2107922	1.9300000	2.3000000

Unique Subject Identifier=8245000000-0007

Assay variable : PTINR_VAL1 PT-INR Value

N	Mean	Standard Deviation	Minimum	Maximum
2	1.7900000	0.0989949	1.7200000	1.8600000

Unique Subject Identifier=8245000000-0008

Assay variable : PTINR_VAL1 PT-INR Value

N	Mean	Standard Deviation	Minimum	Maximum
2	2.5000000	1.0889444	1.7300000	3.2700000

Laboratory tests at the time of obtaining Table 2-6 consent, according to the presence or absence of proton-pump inhibitors

Unique Subject Identifier=8245000000-0013

MEANS Procedure

Assay variable : PTINR_VAL1 PT-INR Value

N	Mean	Standard Deviation	Minimum	Maximum
3	2.0166667	0.1761628	1.8200000	2.1600000

Unique Subject Identifier=8245000000-0016

Assay variable : PTINR_VAL1 PT-INR Value

N	Mean	Standard Deviation	Minimum	Maximum
3	1.8500000	0.2029778	1.6300000	2.0300000

Unique Subject Identifier=8257010001-0011

Assay variable : PTINR_VAL1 PT-INR Value

N	Mean	Standard Deviation	Minimum	Maximum
6	2.1350000	0.2946693	1.7500000	2.6500000

Unique Subject Identifier=8257010001-0015

Assay variable : PTINR_VAL1 PT-INR Value

N	Mean	Standard Deviation	Minimum	Maximum
3	1.4633333	0.2557994	1.2200000	1.7300000

Laboratory tests at the time of obtaining Table 2-6 consent, according to the presence or absence of proton-pump inhibitors

Unique Subject Identifier=8257010001-0016

MEANS Procedure

Assay variable : PTINR_VAL1 PT-INR Value

N	Mean	Standard Deviation	Minimum	Maximum
3	2.2433333	0.3477547	1.8500000	2.5100000

Unique Subject Identifier=8257010001-0030

Assay variable : PTINR_VAL1 PT-INR Value

N	Mean	Standard Deviation	Minimum	Maximum
4	2.1250000	0.7816436	1.1700000	3.0100000

Unique Subject Identifier=8266000000-0017

Assay variable : PTINR_VAL1 PT-INR Value

N	Mean	Standard Deviation	Minimum	Maximum
3	2.4100000	0.2389561	2.2000000	2.6700000

Unique Subject Identifier=8266000000-0020

Assay variable : PTINR_VAL1 PT-INR Value

N	Mean	Standard Deviation	Minimum	Maximum
6	2.5250000	0.3613170	2.0700000	3.1400000

Laboratory tests at the time of obtaining Table 2-6 consent, according to the presence or absence of proton-pump inhibitors

Unique Subject Identifier=8301010000-0004

MEANS Procedure

Assay variable : PTINR_VAL1 PT-INR Value

N	Mean	Standard Deviation	Minimum	Maximum
6	1.7666667	0.3141125	1.2000000	2.1000000

Unique Subject Identifier=8326000000-0014

Assay variable : PTINR_VAL1 PT-INR Value

N	Mean	Standard Deviation	Minimum	Maximum
6	2.1600000	0.2707767	1.8200000	2.5000000

Unique Subject Identifier=8326000000-0026

Assay variable : PTINR_VAL1 PT-INR Value

N	Mean	Standard Deviation	Minimum	Maximum
5	2.1620000	0.2448877	1.9000000	2.5300000

Unique Subject Identifier=8326000000-0033

Assay variable : PTINR_VAL1 PT-INR Value

N	Mean	Standard Deviation	Minimum	Maximum
6	1.8983333	0.4345304	1.5000000	2.5000000

Laboratory tests at the time of obtaining Table 2-6 consent, according to the presence or absence of proton-pump inhibitors

Unique Subject Identifier=8326000000-0035

MEANS Procedure

Assay variable : PTINR_VAL1 PT-INR Value

N	Mean	Standard Deviation	Minimum	Maximum
5	1.7980000	0.1549839	1.6000000	1.9900000

Unique Subject Identifier=8326000000-0038

Assay variable : PTINR_VAL1 PT-INR Value

N	Mean	Standard Deviation	Minimum	Maximum
6	1.4966667	0.0941630	1.3800000	1.6000000

Unique Subject Identifier=8332010000-0005

Assay variable : PTINR_VAL1 PT-INR Value

N	Mean	Standard Deviation	Minimum	Maximum
5	1.7620000	0.3076849	1.5100000	2.2200000

Unique Subject Identifier=8332010000-0017

Assay variable : PTINR_VAL1 PT-INR Value

N	Mean	Standard Deviation	Minimum	Maximum
4	2.1225000	0.2233644	1.8100000	2.3400000

Laboratory tests at the time of obtaining Table 2-6 consent, according to the presence or absence of proton-pump inhibitors

Unique Subject Identifier=8332010000-0028

MEANS Procedure

Assay variable : PTINR_VAL1 PT-INR Value

N	Mean	Standard Deviation	Minimum	Maximum
6	2.7833333	0.3792976	2.1700000	3.2100000

Unique Subject Identifier=8332010000-0043

Assay variable : PTINR_VAL1 PT-INR Value

N	Mean	Standard Deviation	Minimum	Maximum
4	1.5925000	0.0689807	1.4900000	1.6400000

Unique Subject Identifier=8332010000-0052

Assay variable : PTINR_VAL1 PT-INR Value

N	Mean	Standard Deviation	Minimum	Maximum
6	2.0950000	0.3677907	1.7000000	2.6000000

Unique Subject Identifier=8332010000-0055

Assay variable : PTINR_VAL1 PT-INR Value

N	Mean	Standard Deviation	Minimum	Maximum
6	1.9950000	0.5670185	1.5500000	3.0300000

Laboratory tests at the time of obtaining Table 2-6 consent, according to the presence or absence of proton-pump inhibitors

Unique Subject Identifier=8332010000-0056

MEANS Procedure

Assay variable : PTINR_VAL1 PT-INR Value

N	Mean	Standard Deviation	Minimum	Maximum
6	2.2566667	0.4317715	1.6200000	2.7300000

Unique Subject Identifier=8332010000-0093

Assay variable : PTINR_VAL1 PT-INR Value

N	Mean	Standard Deviation	Minimum	Maximum
1	2.0300000		2.0300000	2.0300000

Unique Subject Identifier=8332010000-0095

Assay variable : PTINR_VAL1 PT-INR Value

N	Mean	Standard Deviation	Minimum	Maximum
1	1.6800000		1.6800000	1.6800000

Unique Subject Identifier=8332010000-0121

Assay variable : PTINR_VAL1 PT-INR Value

N	Mean	Standard Deviation	Minimum	Maximum
2	1.7250000	0.2333452	1.5600000	1.8900000

Laboratory tests at the time of obtaining Table 2-6 consent, according to the presence or absence of proton-pump inhibitors

Unique Subject Identifier=8332010000-0141

MEANS Procedure

Assay variable : PTINR_VAL1 PT-INR Value

N	Mean	Standard Deviation	Minimum	Maximum
3	2.0033333	0.0416333	1.9700000	2.0500000

Unique Subject Identifier=8332010000-0145

Assay variable : PTINR_VAL1 PT-INR Value

N	Mean	Standard Deviation	Minimum	Maximum
1	1.8200000		1.8200000	1.8200000

Unique Subject Identifier=8332010000-0151

Assay variable : PTINR_VAL1 PT-INR Value

N	Mean	Standard Deviation	Minimum	Maximum
6	2.1100000	0.2538503	1.7200000	2.3300000

Unique Subject Identifier=8332010000-0180

Assay variable : PTINR_VAL1 PT-INR Value

N	Mean	Standard Deviation	Minimum	Maximum
4	1.8625000	0.1791415	1.7000000	2.1100000

Laboratory tests at the time of obtaining Table 2-6 consent, according to the presence or absence of proton-pump inhibitors

Unique Subject Identifier=8332010000-0192

MEANS Procedure

Assay variable : PTINR_VAL1 PT-INR Value

N	Mean	Standard Deviation	Minimum	Maximum
1	1.9600000		1.9600000	1.9600000

Unique Subject Identifier=8332010000-0194

Assay variable : PTINR_VAL1 PT-INR Value

N	Mean	Standard Deviation	Minimum	Maximum
4	1.3850000	0.1550269	1.2400000	1.5600000

Unique Subject Identifier=8340011000-0025

Assay variable : PTINR_VAL1 PT-INR Value

N	Mean	Standard Deviation	Minimum	Maximum
5	2.1240000	0.4209869	1.5300000	2.6200000

Unique Subject Identifier=8345000000-0006

Assay variable : PTINR_VAL1 PT-INR Value

N	Mean	Standard Deviation	Minimum	Maximum
6	1.4600000	0.0876356	1.3400000	1.5700000

Laboratory tests at the time of obtaining Table 2-6 consent, according to the presence or absence of proton-pump inhibitors

Unique Subject Identifier=8345000000-0009

MEANS Procedure

Assay variable : PTINR_VAL1 PT-INR Value

N	Mean	Standard Deviation	Minimum	Maximum
6	2.0716667	0.4374205	1.5300000	2.8300000

Unique Subject Identifier=8345000000-0023

Assay variable : PTINR_VAL1 PT-INR Value

N	Mean	Standard Deviation	Minimum	Maximum
3	2.2200000	0.3380828	2.0100000	2.6100000

Unique Subject Identifier=8442010000-0002

Assay variable : PTINR_VAL1 PT-INR Value

N	Mean	Standard Deviation	Minimum	Maximum
3	1.7133333	0.2074448	1.4900000	1.9000000

Unique Subject Identifier=8514000000-0001

Assay variable : PTINR_VAL1 PT-INR Value

N	Mean	Standard Deviation	Minimum	Maximum
5	1.8760000	0.5155386	1.0700000	2.4900000

Laboratory tests at the time of obtaining Table 2-6 consent, according to the presence or absence of proton-pump inhibitors

Unique Subject Identifier=8514000000-0004

MEANS Procedure

Assay variable : PTINR_VAL1 PT-INR Value

N	Mean	Standard Deviation	Minimum	Maximum
6	2.0583333	0.1905168	1.7900000	2.3600000

Unique Subject Identifier=8623000000-0001

Assay variable : PTINR_VAL1 PT-INR Value

N	Mean	Standard Deviation	Minimum	Maximum
4	2.0100000	0.0787401	1.9200000	2.0900000

Unique Subject Identifier=8623000000-0005

Assay variable : PTINR_VAL1 PT-INR Value

N	Mean	Standard Deviation	Minimum	Maximum
2	1.8250000	0.1060660	1.7500000	1.9000000

Unique Subject Identifier=8623000000-0016

Assay variable : PTINR_VAL1 PT-INR Value

N	Mean	Standard Deviation	Minimum	Maximum
6	2.1816667	0.2010390	1.9700000	2.4500000

Laboratory tests at the time of obtaining Table 2-6 consent, according to the presence or absence of proton-pump inhibitors

Unique Subject Identifier=8623000000-0026

MEANS Procedure

Assay variable : PTINR_VAL1 PT-INR Value

N	Mean	Standard Deviation	Minimum	Maximum
4	1.9875000	0.5948319	1.4900000	2.8400000

Unique Subject Identifier=8623000000-0027

Assay variable : PTINR_VAL1 PT-INR Value

N	Mean	Standard Deviation	Minimum	Maximum
5	1.5660000	0.0808084	1.4900000	1.6800000

Unique Subject Identifier=8623000000-0029

Assay variable : PTINR_VAL1 PT-INR Value

N	Mean	Standard Deviation	Minimum	Maximum
4	1.4850000	0.1933046	1.2500000	1.6800000

Unique Subject Identifier=8748000000-0003

Assay variable : PTINR_VAL1 PT-INR Value

N	Mean	Standard Deviation	Minimum	Maximum
1	1.4300000		1.4300000	1.4300000

Laboratory tests at the time of obtaining Table 2-6 consent, according to the presence or absence of proton-pump inhibitors

Unique Subject Identifier=9001010000-0009

MEANS Procedure

Assay variable : PTINR_VAL1 PT-INR Value

N	Mean	Standard Deviation	Minimum	Maximum
6	1.8500000	0.3271085	1.4000000	2.2000000

Unique Subject Identifier=9001010000-0010

Assay variable : PTINR_VAL1 PT-INR Value

N	Mean	Standard Deviation	Minimum	Maximum
6	2.1833333	0.3311596	1.8000000	2.7000000

Unique Subject Identifier=9002010000-0057

Assay variable : PTINR_VAL1 PT-INR Value

N	Mean	Standard Deviation	Minimum	Maximum
4	1.6625000	0.1725060	1.4400000	1.8600000

Unique Subject Identifier=9002010000-0069

Assay variable : PTINR_VAL1 PT-INR Value

N	Mean	Standard Deviation	Minimum	Maximum
6	1.9333333	0.3015073	1.5700000	2.3700000

Laboratory tests at the time of obtaining Table 2-6 consent, according to the presence or absence of proton-pump inhibitors

Unique Subject Identifier=9005000000-0004

MEANS Procedure

Assay variable : PTINR_VAL1 PT-INR Value

N	Mean	Standard Deviation	Minimum	Maximum
3	1.7166667	0.2285461	1.5700000	1.9800000

Unique Subject Identifier=9005000000-0005

Assay variable : PTINR_VAL1 PT-INR Value

N	Mean	Standard Deviation	Minimum	Maximum
3	1.7833333	0.2150194	1.6000000	2.0200000

Unique Subject Identifier=9005000000-0009

Assay variable : PTINR_VAL1 PT-INR Value

N	Mean	Standard Deviation	Minimum	Maximum
6	2.4033333	0.3593698	1.9600000	3.0200000

Unique Subject Identifier=9008010000-0012

Assay variable : PTINR_VAL1 PT-INR Value

N	Mean	Standard Deviation	Minimum	Maximum
3	2.3766667	0.2554082	2.0900000	2.5800000

Laboratory tests at the time of obtaining Table 2-6 consent, according to the presence or absence of proton-pump inhibitors

Unique Subject Identifier=9008010000-0017

MEANS Procedure

Assay variable : PTINR_VAL1 PT-INR Value

N	Mean	Standard Deviation	Minimum	Maximum
2	1.8100000	0.1414214	1.7100000	1.9100000

Unique Subject Identifier=9008010000-0021

Assay variable : PTINR_VAL1 PT-INR Value

N	Mean	Standard Deviation	Minimum	Maximum
4	1.8275000	0.2637391	1.5600000	2.1300000

Unique Subject Identifier=9008010000-0027

Assay variable : PTINR_VAL1 PT-INR Value

N	Mean	Standard Deviation	Minimum	Maximum
4	1.3375000	0.2895255	1.0500000	1.7100000

Unique Subject Identifier=9009010000-0003

Assay variable : PTINR_VAL1 PT-INR Value

N	Mean	Standard Deviation	Minimum	Maximum
4	1.7750000	0.1660321	1.5900000	1.9900000

Laboratory tests at the time of obtaining Table 2-6 consent, according to the presence or absence of proton-pump inhibitors

Unique Subject Identifier=9009010000-0013

MEANS Procedure

Assay variable : PTINR_VAL1 PT-INR Value

N	Mean	Standard Deviation	Minimum	Maximum
4	1.3275000	0.0419325	1.2700000	1.3700000

Unique Subject Identifier=9009010000-0015

Assay variable : PTINR_VAL1 PT-INR Value

N	Mean	Standard Deviation	Minimum	Maximum
6	2.2666667	0.3688722	1.8900000	2.9200000

Unique Subject Identifier=9009010000-0022

Assay variable : PTINR_VAL1 PT-INR Value

N	Mean	Standard Deviation	Minimum	Maximum
3	1.4166667	0.0665833	1.3600000	1.4900000

Unique Subject Identifier=9014000000-0008

Assay variable : PTINR_VAL1 PT-INR Value

N	Mean	Standard Deviation	Minimum	Maximum
4	1.9625000	0.1302242	1.8600000	2.1400000

Laboratory tests at the time of obtaining Table 2-6 consent, according to the presence or absence of proton-pump inhibitors

Unique Subject Identifier=9014000000-0013

MEANS Procedure

Assay variable : PTINR_VAL1 PT-INR Value

N	Mean	Standard Deviation	Minimum	Maximum
6	2.0133333	0.2587405	1.7200000	2.4800000

Unique Subject Identifier=9014000000-0019

Assay variable : PTINR_VAL1 PT-INR Value

N	Mean	Standard Deviation	Minimum	Maximum
5	2.3220000	0.7481110	1.5400000	3.1100000

Unique Subject Identifier=9014000000-0023

Assay variable : PTINR_VAL1 PT-INR Value

N	Mean	Standard Deviation	Minimum	Maximum
4	2.0475000	0.0981071	1.9500000	2.1700000

Unique Subject Identifier=9020011000-0001

Assay variable : PTINR_VAL1 PT-INR Value

N	Mean	Standard Deviation	Minimum	Maximum
5	2.2440000	0.3773990	1.6800000	2.7000000

Laboratory tests at the time of obtaining Table 2-6 consent, according to the presence or absence of proton-pump inhibitors

Unique Subject Identifier=9020011000-0010

MEANS Procedure

Assay variable : PTINR_VAL1 PT-INR Value

N	Mean	Standard Deviation	Minimum	Maximum
3	1.4700000	0.1126943	1.4000000	1.6000000

Unique Subject Identifier=9024000000-0005

Assay variable : PTINR_VAL1 PT-INR Value

N	Mean	Standard Deviation	Minimum	Maximum
6	1.6800000	0.4543567	1.1000000	2.2200000

Unique Subject Identifier=9032011000-0009

Assay variable : PTINR_VAL1 PT-INR Value

N	Mean	Standard Deviation	Minimum	Maximum
4	2.0000000	0.3162278	1.6000000	2.3000000

Unique Subject Identifier=9035000000-0061

Assay variable : PTINR_VAL1 PT-INR Value

N	Mean	Standard Deviation	Minimum	Maximum
5	1.7380000	0.2872629	1.5100000	2.1700000

Laboratory tests at the time of obtaining Table 2-6 consent, according to the presence or absence of proton-pump inhibitors

Unique Subject Identifier=9035000000-0062

MEANS Procedure

Assay variable : PTINR_VAL1 PT-INR Value

N	Mean	Standard Deviation	Minimum	Maximum
3	1.8933333	0.3875994	1.5900000	2.3300000

Unique Subject Identifier=9035000000-0078

Assay variable : PTINR_VAL1 PT-INR Value

N	Mean	Standard Deviation	Minimum	Maximum
3	1.7866667	0.3807011	1.4200000	2.1800000

Unique Subject Identifier=9035000000-0084

Assay variable : PTINR_VAL1 PT-INR Value

N	Mean	Standard Deviation	Minimum	Maximum
3	1.4033333	0.1871719	1.1900000	1.5400000

Unique Subject Identifier=9035000000-0094

Assay variable : PTINR_VAL1 PT-INR Value

N	Mean	Standard Deviation	Minimum	Maximum
3	1.6166667	0.2956913	1.3600000	1.9400000

Laboratory tests at the time of obtaining Table 2-6 consent, according to the presence or absence of proton-pump inhibitors

Unique Subject Identifier=9036000000-0015

MEANS Procedure

Assay variable : PTINR_VAL1 PT-INR Value

N	Mean	Standard Deviation	Minimum	Maximum
3	1.9733333	0.0503322	1.9200000	2.0200000

Unique Subject Identifier=9037000010-0003

Assay variable : PTINR_VAL1 PT-INR Value

N	Mean	Standard Deviation	Minimum	Maximum
5	2.2560000	1.0411676	1.5800000	4.0700000

Unique Subject Identifier=9037000010-0007

Assay variable : PTINR_VAL1 PT-INR Value

N	Mean	Standard Deviation	Minimum	Maximum
2	2.0700000	0.0424264	2.0400000	2.1000000

Unique Subject Identifier=9037000010-0009

Assay variable : PTINR_VAL1 PT-INR Value

N	Mean	Standard Deviation	Minimum	Maximum
3	2.1466667	0.8800189	1.1400000	2.7700000

Laboratory tests at the time of obtaining Table 2-6 consent, according to the presence or absence of proton-pump inhibitors

Unique Subject Identifier=9037000010-0010

MEANS Procedure

Assay variable : PTINR_VAL1 PT-INR Value

N	Mean	Standard Deviation	Minimum	Maximum
5	1.7520000	0.4846855	1.3200000	2.5800000

Unique Subject Identifier=9057000000-0014

Assay variable : PTINR_VAL1 PT-INR Value

N	Mean	Standard Deviation	Minimum	Maximum
5	2.0500000	0.2982449	1.7800000	2.5100000

Unique Subject Identifier=9071010000-0003

Assay variable : PTINR_VAL1 PT-INR Value

N	Mean	Standard Deviation	Minimum	Maximum
5	1.8520000	0.3769217	1.5400000	2.4300000

Unique Subject Identifier=9071010000-0004

Assay variable : PTINR_VAL1 PT-INR Value

N	Mean	Standard Deviation	Minimum	Maximum
1	2.4800000		2.4800000	2.4800000

Laboratory tests at the time of obtaining Table 2-6 consent, according to the presence or absence of proton-pump inhibitors

Unique Subject Identifier=9100001000-0010

MEANS Procedure

Assay variable : PTINR_VAL1 PT-INR Value

N	Mean	Standard Deviation	Minimum	Maximum
4	1.8100000	0.8195934	1.0700000	2.7300000

Unique Subject Identifier=9100001000-0012

Assay variable : PTINR_VAL1 PT-INR Value

N	Mean	Standard Deviation	Minimum	Maximum
4	1.4100000	0.1902630	1.2100000	1.6300000

Unique Subject Identifier=9100001000-0015

Assay variable : PTINR_VAL1 PT-INR Value

N	Mean	Standard Deviation	Minimum	Maximum
3	1.8333333	0.7793801	1.1900000	2.7000000

Unique Subject Identifier=9100001000-0019

Assay variable : PTINR_VAL1 PT-INR Value

N	Mean	Standard Deviation	Minimum	Maximum
3	1.5066667	0.2318045	1.2600000	1.7200000

Laboratory tests at the time of obtaining Table 2-6 consent, according to the presence or absence of proton-pump inhibitors

Unique Subject Identifier=9100001000-0024

MEANS Procedure

Assay variable : PTINR_VAL1 PT-INR Value

N	Mean	Standard Deviation	Minimum	Maximum
3	2.0100000	0.1873499	1.8600000	2.2200000

Unique Subject Identifier=9100001000-0030

Assay variable : PTINR_VAL1 PT-INR Value

N	Mean	Standard Deviation	Minimum	Maximum
4	1.8500000	0.2499333	1.6100000	2.0900000

Unique Subject Identifier=9101010000-0003

Assay variable : PTINR_VAL1 PT-INR Value

N	Mean	Standard Deviation	Minimum	Maximum
2	1.2500000	0.0707107	1.2000000	1.3000000

Unique Subject Identifier=9101010000-0007

Assay variable : PTINR_VAL1 PT-INR Value

N	Mean	Standard Deviation	Minimum	Maximum
2	1.6000000	0.1414214	1.5000000	1.7000000

Laboratory tests at the time of obtaining Table 2-6 consent, according to the presence or absence of proton-pump inhibitors

Unique Subject Identifier=9101010000-0013

MEANS Procedure

Assay variable : PTINR_VAL1 PT-INR Value

N	Mean	Standard Deviation	Minimum	Maximum
4	2.1250000	1.3400871	1.2000000	4.1000000

Unique Subject Identifier=9101010000-0029

Assay variable : PTINR_VAL1 PT-INR Value

N	Mean	Standard Deviation	Minimum	Maximum
3	1.6666667	0.1154701	1.6000000	1.8000000

Unique Subject Identifier=9101010000-0031

Assay variable : PTINR_VAL1 PT-INR Value

N	Mean	Standard Deviation	Minimum	Maximum
3	2.0666667	0.1527525	1.9000000	2.2000000

Unique Subject Identifier=9101010000-0032

Assay variable : PTINR_VAL1 PT-INR Value

N	Mean	Standard Deviation	Minimum	Maximum
3	2.1000000	0.2645751	1.8000000	2.3000000

Laboratory tests at the time of obtaining Table 2-6 consent, according to the presence or absence of proton-pump inhibitors

Unique Subject Identifier=9101010000-0033

MEANS Procedure

Assay variable : PTINR_VAL1 PT-INR Value

N	Mean	Standard Deviation	Minimum	Maximum
4	1.4250000	0.0957427	1.3000000	1.5000000

Unique Subject Identifier=9101010000-0039

Assay variable : PTINR_VAL1 PT-INR Value

N	Mean	Standard Deviation	Minimum	Maximum
2	1.9000000	0.1414214	1.8000000	2.0000000

Unique Subject Identifier=9101010000-0045

Assay variable : PTINR_VAL1 PT-INR Value

N	Mean	Standard Deviation	Minimum	Maximum
3	1.0666667	0.0577350	1.0000000	1.1000000

Unique Subject Identifier=9101010000-0048

Assay variable : PTINR_VAL1 PT-INR Value

N	Mean	Standard Deviation	Minimum	Maximum
2	1.2500000	0.0707107	1.2000000	1.3000000

Laboratory tests at the time of obtaining Table 2-6 consent, according to the presence or absence of proton-pump inhibitors

Unique Subject Identifier=9105000000-0017

MEANS Procedure

Assay variable : PTINR_VAL1 PT-INR Value

N	Mean	Standard Deviation	Minimum	Maximum
5	1.9320000	0.5714630	1.2000000	2.7800000

Unique Subject Identifier=9108011111-0007

Assay variable : PTINR_VAL1 PT-INR Value

N	Mean	Standard Deviation	Minimum	Maximum
3	1.9500000	0.4334743	1.6800000	2.4500000

Unique Subject Identifier=9127000000-0003

Assay variable : PTINR_VAL1 PT-INR Value

N	Mean	Standard Deviation	Minimum	Maximum
1	1.9300000		1.9300000	1.9300000

Unique Subject Identifier=9127000000-0010

Assay variable : PTINR_VAL1 PT-INR Value

N	Mean	Standard Deviation	Minimum	Maximum
5	2.1300000	0.1070047	1.9700000	2.2200000

Laboratory tests at the time of obtaining Table 2-6 consent, according to the presence or absence of proton-pump inhibitors

Unique Subject Identifier=9136010000-0006

MEANS Procedure

Assay variable : PTINR_VAL1 PT-INR Value

N	Mean	Standard Deviation	Minimum	Maximum
3	2.1933333	0.3382800	1.8100000	2.4500000

Unique Subject Identifier=9136010000-0014

Assay variable : PTINR_VAL1 PT-INR Value

N	Mean	Standard Deviation	Minimum	Maximum
5	1.7640000	0.1040673	1.6700000	1.9200000

Unique Subject Identifier=9136010000-0018

Assay variable : PTINR_VAL1 PT-INR Value

N	Mean	Standard Deviation	Minimum	Maximum
3	2.4366667	0.4005413	2.0800000	2.8700000

Unique Subject Identifier=9136010000-0025

Assay variable : PTINR_VAL1 PT-INR Value

N	Mean	Standard Deviation	Minimum	Maximum
3	2.5166667	0.1913984	2.3600000	2.7300000

Laboratory tests at the time of obtaining Table 2-6 consent, according to the presence or absence of proton-pump inhibitors

Unique Subject Identifier=9136010000-0036

MEANS Procedure

Assay variable : PTINR_VAL1 PT-INR Value

N	Mean	Standard Deviation	Minimum	Maximum
4	2.2800000	0.2290560	2.0400000	2.5900000

Unique Subject Identifier=9137010000-0001

Assay variable : PTINR_VAL1 PT-INR Value

N	Mean	Standard Deviation	Minimum	Maximum
4	1.8950000	0.5202884	1.1200000	2.2300000

Unique Subject Identifier=9141000000-0002

Assay variable : PTINR_VAL1 PT-INR Value

N	Mean	Standard Deviation	Minimum	Maximum
5	1.9540000	0.2758260	1.5100000	2.1600000

Unique Subject Identifier=9141000000-0005

Assay variable : PTINR_VAL1 PT-INR Value

N	Mean	Standard Deviation	Minimum	Maximum
5	2.0400000	0.2370654	1.7200000	2.3800000

Laboratory tests at the time of obtaining Table 2-6 consent, according to the presence or absence of proton-pump inhibitors

Unique Subject Identifier=9141000000-0006

MEANS Procedure

Assay variable : PTINR_VAL1 PT-INR Value

N	Mean	Standard Deviation	Minimum	Maximum
4	1.6450000	0.1408309	1.4600000	1.8000000

Unique Subject Identifier=9142010000-0005

Assay variable : PTINR_VAL1 PT-INR Value

N	Mean	Standard Deviation	Minimum	Maximum
3	1.7000000	0.2879236	1.5000000	2.0300000

Unique Subject Identifier=9142010000-0013

Assay variable : PTINR_VAL1 PT-INR Value

N	Mean	Standard Deviation	Minimum	Maximum
2	1.7500000	0.3252691	1.5200000	1.9800000

Unique Subject Identifier=9142010000-0014

Assay variable : PTINR_VAL1 PT-INR Value

N	Mean	Standard Deviation	Minimum	Maximum
1	1.6200000		1.6200000	1.6200000

Laboratory tests at the time of obtaining Table 2-6 consent, according to the presence or absence of proton-pump inhibitors

Unique Subject Identifier=9142010000-0027

MEANS Procedure

Assay variable : PTINR_VAL1 PT-INR Value

N	Mean	Standard Deviation	Minimum	Maximum
2	2.4250000	0.4313351	2.1200000	2.7300000

Unique Subject Identifier=9142010000-0036

Assay variable : PTINR_VAL1 PT-INR Value

N	Mean	Standard Deviation	Minimum	Maximum
3	1.5100000	0.1646208	1.3200000	1.6100000

Unique Subject Identifier=9142010000-0037

Assay variable : PTINR_VAL1 PT-INR Value

N	Mean	Standard Deviation	Minimum	Maximum
3	2.0333333	0.4867580	1.6000000	2.5600000

Unique Subject Identifier=9154010000-0001

Assay variable : PTINR_VAL1 PT-INR Value

N	Mean	Standard Deviation	Minimum	Maximum
4	1.7475000	0.1175798	1.5800000	1.8500000

Laboratory tests at the time of obtaining Table 2-6 consent, according to the presence or absence of proton-pump inhibitors

Unique Subject Identifier=9154010000-0009

MEANS Procedure

Assay variable : PTINR_VAL1 PT-INR Value

N	Mean	Standard Deviation	Minimum	Maximum
5	1.9180000	0.6155648	1.4200000	2.9800000

Unique Subject Identifier=9154010000-0015

Assay variable : PTINR_VAL1 PT-INR Value

N	Mean	Standard Deviation	Minimum	Maximum
4	2.3175000	0.1291962	2.1800000	2.4900000

Unique Subject Identifier=9154010000-0027

Assay variable : PTINR_VAL1 PT-INR Value

N	Mean	Standard Deviation	Minimum	Maximum
4	2.6075000	0.2267708	2.3500000	2.8600000

Unique Subject Identifier=9154010000-0029

Assay variable : PTINR_VAL1 PT-INR Value

N	Mean	Standard Deviation	Minimum	Maximum
5	1.5140000	0.3269251	1.1900000	2.0000000

Laboratory tests at the time of obtaining Table 2-6 consent, according to the presence or absence of proton-pump inhibitors

Unique Subject Identifier=9154010000-0030

MEANS Procedure

Assay variable : PTINR_VAL1 PT-INR Value

N	Mean	Standard Deviation	Minimum	Maximum
4	2.2300000	0.0559762	2.1500000	2.2800000

Unique Subject Identifier=9166000000-0005

Assay variable : PTINR_VAL1 PT-INR Value

N	Mean	Standard Deviation	Minimum	Maximum
3	1.7233333	0.1890326	1.5100000	1.8700000

Unique Subject Identifier=9166000000-0009

Assay variable : PTINR_VAL1 PT-INR Value

N	Mean	Standard Deviation	Minimum	Maximum
5	2.1800000	0.1788854	2.0000000	2.4100000

Unique Subject Identifier=9210000100-0007

Assay variable : PTINR_VAL1 PT-INR Value

N	Mean	Standard Deviation	Minimum	Maximum
5	2.2660000	0.4350057	1.7700000	2.7200000

Laboratory tests at the time of obtaining Table 2-6 consent, according to the presence or absence of proton-pump inhibitors

Unique Subject Identifier=9214000000-0002

MEANS Procedure

Assay variable : PTINR_VAL1 PT-INR Value

N	Mean	Standard Deviation	Minimum	Maximum
1	1.7300000		1.7300000	1.7300000

Unique Subject Identifier=9214000000-0022

Assay variable : PTINR_VAL1 PT-INR Value

N	Mean	Standard Deviation	Minimum	Maximum
4	1.5850000	0.3219213	1.2000000	1.9700000

Unique Subject Identifier=9235000000-0002

Assay variable : PTINR_VAL1 PT-INR Value

N	Mean	Standard Deviation	Minimum	Maximum
3	1.7000000	0.1000000	1.6000000	1.8000000

Unique Subject Identifier=9235000000-0009

Assay variable : PTINR_VAL1 PT-INR Value

N	Mean	Standard Deviation	Minimum	Maximum
3	1.5000000	0.1000000	1.4000000	1.6000000

Laboratory tests at the time of obtaining Table 2-6 consent, according to the presence or absence of proton-pump inhibitors

Unique Subject Identifier=9235000000-0012

MEANS Procedure

Assay variable : PTINR_VAL1 PT-INR Value

N	Mean	Standard Deviation	Minimum	Maximum
3	1.9000000	0.2000000	1.7000000	2.1000000

Unique Subject Identifier=9241000000-0003

Assay variable : PTINR_VAL1 PT-INR Value

N	Mean	Standard Deviation	Minimum	Maximum
3	2.2033333	0.3728717	1.8600000	2.6000000

Unique Subject Identifier=9241000000-0005

Assay variable : PTINR_VAL1 PT-INR Value

N	Mean	Standard Deviation	Minimum	Maximum
3	1.5566667	0.0709460	1.4800000	1.6200000

Unique Subject Identifier=9241000000-0007

Assay variable : PTINR_VAL1 PT-INR Value

N	Mean	Standard Deviation	Minimum	Maximum
3	2.1000000	0.1587451	1.9200000	2.2200000

Laboratory tests at the time of obtaining Table 2-6 consent, according to the presence or absence of proton-pump inhibitors

Unique Subject Identifier=9241000000-0012

MEANS Procedure

Assay variable : PTINR_VAL1 PT-INR Value

N	Mean	Standard Deviation	Minimum	Maximum
3	1.8133333	0.2557994	1.5200000	1.9900000

Unique Subject Identifier=9242011011-0003

Assay variable : PTINR_VAL1 PT-INR Value

N	Mean	Standard Deviation	Minimum	Maximum
6	1.1233333	0.0571548	1.0500000	1.2000000

Unique Subject Identifier=9257010000-0009

Assay variable : PTINR_VAL1 PT-INR Value

N	Mean	Standard Deviation	Minimum	Maximum
6	2.0683333	1.0299013	1.4400000	4.1400000

Unique Subject Identifier=9257010000-0012

Assay variable : PTINR_VAL1 PT-INR Value

N	Mean	Standard Deviation	Minimum	Maximum
3	1.4000000	0.0435890	1.3700000	1.4500000

Laboratory tests at the time of obtaining Table 2-6 consent, according to the presence or absence of proton-pump inhibitors

Unique Subject Identifier=9257010000-0025

MEANS Procedure

Assay variable : PTINR_VAL1 PT-INR Value

N	Mean	Standard Deviation	Minimum	Maximum
6	1.4900000	0.5108424	0.9700000	2.3000000

Unique Subject Identifier=9342010000-0002

Assay variable : PTINR_VAL1 PT-INR Value

N	Mean	Standard Deviation	Minimum	Maximum
6	1.6016667	0.1135635	1.5100000	1.7700000

Unique Subject Identifier=9345000010-0006

Assay variable : PTINR_VAL1 PT-INR Value

N	Mean	Standard Deviation	Minimum	Maximum
3	2.4233333	0.2084067	2.2400000	2.6500000

Unique Subject Identifier=9345000010-0007

Assay variable : PTINR_VAL1 PT-INR Value

N	Mean	Standard Deviation	Minimum	Maximum
1	2.1300000		2.1300000	2.1300000

Laboratory tests at the time of obtaining Table 2-6 consent, according to the presence or absence of proton-pump inhibitors

Unique Subject Identifier=9348010000-0005

MEANS Procedure

Assay variable : PTINR_VAL1 PT-INR Value

N	Mean	Standard Deviation	Minimum	Maximum
3	1.7166667	0.1101514	1.6100000	1.8300000

Unique Subject Identifier=9348010000-0028

Assay variable : PTINR_VAL1 PT-INR Value

N	Mean	Standard Deviation	Minimum	Maximum
2	1.7800000	0.2545584	1.6000000	1.9600000

Unique Subject Identifier=9348010000-0029

Assay variable : PTINR_VAL1 PT-INR Value

N	Mean	Standard Deviation	Minimum	Maximum
6	1.4150000	0.2147324	1.2000000	1.7300000

Unique Subject Identifier=9348010000-0040

Assay variable : PTINR_VAL1 PT-INR Value

N	Mean	Standard Deviation	Minimum	Maximum
4	2.0700000	0.5057008	1.4300000	2.5900000

Laboratory tests at the time of obtaining Table 2-6 consent, according to the presence or absence of proton-pump inhibitors

Unique Subject Identifier=9348010000-0072

MEANS Procedure

Assay variable : PTINR_VAL1 PT-INR Value

N	Mean	Standard Deviation	Minimum	Maximum
3	2.6566667	0.1650253	2.4900000	2.8200000

Unique Subject Identifier=9348010000-0094

Assay variable : PTINR_VAL1 PT-INR Value

N	Mean	Standard Deviation	Minimum	Maximum
3	2.0833333	0.8114391	1.5000000	3.0100000

Unique Subject Identifier=9348010000-0098

Assay variable : PTINR_VAL1 PT-INR Value

N	Mean	Standard Deviation	Minimum	Maximum
4	2.4000000	0.9819369	1.6200000	3.6900000

Unique Subject Identifier=9348010000-0114

Assay variable : PTINR_VAL1 PT-INR Value

N	Mean	Standard Deviation	Minimum	Maximum
5	1.7580000	0.2359449	1.4200000	2.0100000

Laboratory tests at the time of obtaining Table 2-6 consent, according to the presence or absence of proton-pump inhibitors

Unique Subject Identifier=9348010000-0117

MEANS Procedure

Assay variable : PTINR_VAL1 PT-INR Value

N	Mean	Standard Deviation	Minimum	Maximum
3	1.5300000	0.3803945	1.1400000	1.9000000

Unique Subject Identifier=9348010000-0131

Assay variable : PTINR_VAL1 PT-INR Value

N	Mean	Standard Deviation	Minimum	Maximum
3	1.1200000	0.0458258	1.0700000	1.1600000

Unique Subject Identifier=9348010000-0132

Assay variable : PTINR_VAL1 PT-INR Value

N	Mean	Standard Deviation	Minimum	Maximum
3	1.5533333	0.4306197	1.1100000	1.9700000

Unique Subject Identifier=9366000000-0001

Assay variable : PTINR_VAL1 PT-INR Value

N	Mean	Standard Deviation	Minimum	Maximum
6	2.3066667	0.4933829	1.6900000	2.9500000

Laboratory tests at the time of obtaining Table 2-6 consent, according to the presence or absence of proton-pump inhibitors

Unique Subject Identifier=9366000000-0002

MEANS Procedure

Assay variable : PTINR_VAL1 PT-INR Value

N	Mean	Standard Deviation	Minimum	Maximum
6	2.7283333	0.6265275	1.6400000	3.4200000

Unique Subject Identifier=9366000000-0003

Assay variable : PTINR_VAL1 PT-INR Value

N	Mean	Standard Deviation	Minimum	Maximum
6	2.2566667	0.7819378	1.5100000	3.7200000

Unique Subject Identifier=9548000000-0002

Assay variable : PTINR_VAL1 PT-INR Value

N	Mean	Standard Deviation	Minimum	Maximum
2	1.2150000	0.0212132	1.2000000	1.2300000

Unique Subject Identifier=9623100000-0003

Assay variable : PTINR_VAL1 PT-INR Value

N	Mean	Standard Deviation	Minimum	Maximum
2	1.3450000	0.1060660	1.2700000	1.4200000

Laboratory tests at the time of obtaining Table 2-6 consent, according to the presence or absence of proton-pump inhibitors

Unique Subject Identifier=9623100000-0007

MEANS Procedure

Assay variable : PTINR_VAL1 PT-INR Value

N	Mean	Standard Deviation	Minimum	Maximum
5	1.6000000	0.1528071	1.4400000	1.8200000

Laboratory tests at the time of obtaining Table 2-6 consent, according to the presence or absence of proton-pump inhibitors

FREQ Procedure

Table : ALL * _PTINRCT

ALL _PTINRCT

Power |
Percent |
Percent of rows
Percentage of columns | 1| 2| 3| 4| 9| Total

	1	2	3	4	9	Total
1	399	1204	1159	99	94	2955
	13.50	40.74	39.22	3.35	3.18	100.00
	13.50	40.74	39.22	3.35	3.18	
	100.00	100.00	100.00	100.00	100.00	
Total	399	1204	1159	99	94	2955
	13.50	40.74	39.22	3.35	3.18	100.00

Laboratory tests at the time of obtaining Table 2-6 consent, according to the presence or absence of proton-pump inhibitors

FREQ Procedure

Table : ALL * _PTINRCT

ALL _PTINRCT

Power |
Percent |
Percent of rows
Percentage of columns | 1| 2| 3| 4| 9| Total

	1	2	3	4	9	Total
1	399	1204	1159	99	94	2955
	13.50	40.74	39.22	3.35	3.18	100.00
	13.50	40.74	39.22	3.35	3.18	
	100.00	100.00	100.00	100.00	100.00	
Total	399	1204	1159	99	94	2955
	13.50	40.74	39.22	3.35	3.18	100.00

Laboratory tests at the time of obtaining Table 2-6 consent, according to the presence or absence of proton-pump inhibitors

FREQ Procedure

Table : ALL * _PTINRCT

ALL _PTINRCT

Power |
Percent |
Percent of rows
Percentage of columns | 1| 2| 3| 4| 9| Total

ALL	1	2	3	4	9	Total
1	399	1204	1159	99	94	2955
	13.50	40.74	39.22	3.35	3.18	100.00
	13.50	40.74	39.22	3.35	3.18	
	100.00	100.00	100.00	100.00	100.00	
Total	399	1204	1159	99	94	2955
	13.50	40.74	39.22	3.35	3.18	100.00

Laboratory tests at the time of obtaining Table 2-6 consent, according to the presence or absence of proton-pump inhibitors

FREQ Procedure

Table : ALL * _PTINRCT

ALL _PTINRCT

Power |
Percent |
Percent of rows
Percentage of columns | 1| 2| 3| 4| 9| Total

	1	2	3	4	9	Total
1	399	1204	1159	99	94	2955
	13.50	40.74	39.22	3.35	3.18	100.00
	13.50	40.74	39.22	3.35	3.18	
	100.00	100.00	100.00	100.00	100.00	
Total	399	1204	1159	99	94	2955
	13.50	40.74	39.22	3.35	3.18	100.00

Laboratory tests at the time of obtaining Table 2-6 consent, according to the presence or absence of proton-pump inhibitors

FREQ Procedure

Table : ALL * _PTINRCT

ALL _PTINRCT

Power |
Percent |
Percent of rows
Percentage of columns | 1| 2| 3| 4| 9| Total

	1	2	3	4	9	Total
1	399	1204	1159	99	94	2955
	13.50	40.74	39.22	3.35	3.18	100.00
	13.50	40.74	39.22	3.35	3.18	
	100.00	100.00	100.00	100.00	100.00	
Total	399	1204	1159	99	94	2955
	13.50	40.74	39.22	3.35	3.18	100.00

Laboratory tests at the time of obtaining Table 2-6 consent, according to the presence or absence of proton-pump inhibitors

FREQ Procedure

Table : ALL * _TTRCT

ALL _TTRCT

Power |
Percent |
Percent of rows
Percentage of columns | 1| 2| 3| 4| 9| Total

1	385	297	443	1405	425	2955
	13.03	10.05	14.99	47.55	14.38	100.00
	13.03	10.05	14.99	47.55	14.38	
	100.00	100.00	100.00	100.00	100.00	
Total	385	297	443	1405	425	2955
	13.03	10.05	14.99	47.55	14.38	100.00

Laboratory tests at the time of obtaining Table 2-6 consent, according to the presence or absence of proton-pump inhibitors

FREQ Procedure

Table : ALL * _TTRCT

ALL _TTRCT

Power |
Percent |
Percent of rows
Percentage of columns | 1| 2| 3| 4| 9| Total

	1	2	3	4	9	Total
1	385	297	443	1405	425	2955
	13.03	10.05	14.99	47.55	14.38	100.00
	13.03	10.05	14.99	47.55	14.38	
	100.00	100.00	100.00	100.00	100.00	
Total	385	297	443	1405	425	2955
	13.03	10.05	14.99	47.55	14.38	100.00

Laboratory tests at the time of obtaining Table 2-6 consent, according to the presence or absence of proton-pump inhibitors

FREQ Procedure

Table : ALL * _TTRCT

ALL _TTRCT

Power |
Percent |
Percent of rows
Percentage of columns | 1| 2| 3| 4| 9| Total

	1	2	3	4	9	Total
1	385	297	443	1405	425	2955
	13.03	10.05	14.99	47.55	14.38	100.00
	13.03	10.05	14.99	47.55	14.38	
	100.00	100.00	100.00	100.00	100.00	
Total	385	297	443	1405	425	2955
	13.03	10.05	14.99	47.55	14.38	100.00

Laboratory tests at the time of obtaining Table 2-6 consent, according to the presence or absence of proton-pump inhibitors

FREQ Procedure

Table : ALL * _TTRCT

ALL _TTRCT

Power |
Percent |
Percent of rows
Percentage of columns | 1| 2| 3| 4| 9| Total

1	385	297	443	1405	425	2955
	13.03	10.05	14.99	47.55	14.38	100.00
	13.03	10.05	14.99	47.55	14.38	
	100.00	100.00	100.00	100.00	100.00	
Total	385	297	443	1405	425	2955
	13.03	10.05	14.99	47.55	14.38	100.00

Laboratory tests at the time of obtaining Table 2-6 consent, according to the presence or absence of proton-pump inhibitors

FREQ Procedure

Table : ALL * _TTRCT

ALL _TTRCT

Power |
Percent |
Percent of rows
Percentage of columns | 1| 2| 3| 4| 9| Total

1	385	297	443	1405	425	2955
	13.03	10.05	14.99	47.55	14.38	100.00
	13.03	10.05	14.99	47.55	14.38	
	100.00	100.00	100.00	100.00	100.00	
Total	385	297	443	1405	425	2955
	13.03	10.05	14.99	47.55	14.38	100.00

Laboratory tests at the time of obtaining Table 2-6 consent, according to the presence or absence of proton-pump inhibitors

FREQ Procedure

Table : ALL * _FIRCT

ALL _FIRCT

Power |
Percent |
Percent of rows
Percentage of columns | 1| 2| 3| 4| 9| Total

	1	2	3	4	9	Total
1	396	313	588	1250	408	2955
	13.40	10.59	19.90	42.30	13.81	100.00
	13.40	10.59	19.90	42.30	13.81	
	100.00	100.00	100.00	100.00	100.00	
Total	396	313	588	1250	408	2955
	13.40	10.59	19.90	42.30	13.81	100.00

Laboratory tests at the time of obtaining Table 2-6 consent, according to the presence or absence of proton-pump inhibitors

FREQ Procedure

Table : ALL * _FIRCT

ALL _FIRCT

Power |
Percent |
Percent of rows
Percentage of columns | 1| 2| 3| 4| 9| Total

	1	2	3	4	9	Total
1	396	313	588	1250	408	2955
	13.40	10.59	19.90	42.30	13.81	100.00
	13.40	10.59	19.90	42.30	13.81	
	100.00	100.00	100.00	100.00	100.00	
Total	396	313	588	1250	408	2955
	13.40	10.59	19.90	42.30	13.81	100.00

Laboratory tests at the time of obtaining Table 2-6 consent, according to the presence or absence of proton-pump inhibitors

FREQ Procedure

Table : ALL * _FIRCT

ALL _FIRCT

Power |
Percent |
Percent of rows
Percentage of columns | 1| 2| 3| 4| 9| Total

1	396	313	588	1250	408	2955
	13.40	10.59	19.90	42.30	13.81	100.00
	13.40	10.59	19.90	42.30	13.81	
	100.00	100.00	100.00	100.00	100.00	
Total	396	313	588	1250	408	2955
	13.40	10.59	19.90	42.30	13.81	100.00

Laboratory tests at the time of obtaining Table 2-6 consent, according to the presence or absence of proton-pump inhibitors

FREQ Procedure

Table : ALL * _FIRCT

ALL _FIRCT

Power |
Percent |
Percent of rows
Percentage of columns | 1| 2| 3| 4| 9| Total

	1	2	3	4	9	Total
1	396	313	588	1250	408	2955
	13.40	10.59	19.90	42.30	13.81	100.00
	13.40	10.59	19.90	42.30	13.81	
	100.00	100.00	100.00	100.00	100.00	
Total	396	313	588	1250	408	2955
	13.40	10.59	19.90	42.30	13.81	100.00

Laboratory tests at the time of obtaining Table 2-6 consent, according to the presence or absence of proton-pump inhibitors

FREQ Procedure

Table : ALL * _FIRCT

ALL _FIRCT

Power |
Percent |
Percent of rows
Percentage of columns | 1| 2| 3| 4| 9| Total

	1	2	3	4	9	Total
1	396	313	588	1250	408	2955
	13.40	10.59	19.90	42.30	13.81	100.00
	13.40	10.59	19.90	42.30	13.81	
	100.00	100.00	100.00	100.00	100.00	
Total	396	313	588	1250	408	2955
	13.40	10.59	19.90	42.30	13.81	100.00

Laboratory tests at the time of obtaining Table 2-6 consent, according to the presence or absence of proton-pump inhibitors

FREQ Procedure

Table : ALL * t_CRCC1

ALL t_CRCC1

Power |
Percent |
Percent of rows
Percentage of columns | 1| 2| 3| 4| 5| Total

1	223	1626	4292	3539	355	10035
	2.22	16.20	42.77	35.27	3.54	100.00
	2.22	16.20	42.77	35.27	3.54	
	100.00	100.00	100.00	100.00	100.00	
Total	223	1626	4292	3539	355	10035
	2.22	16.20	42.77	35.27	3.54	100.00

Frequency of missing values = 1946

Laboratory tests at the time of obtaining Table 2-6 consent, according to the presence or absence of proton-pump inhibitors

FREQ Procedure

Table : ALL * t_CRCC1

ALL t_CRCC1

Power |
Percent |
Percent of rows
Percentage of columns | 1| 2| 3| 4| 5| Total

	1	2	3	4	5	Total
1	223	1626	4292	3539	355	10035
	2.22	16.20	42.77	35.27	3.54	100.00
	2.22	16.20	42.77	35.27	3.54	
	100.00	100.00	100.00	100.00	100.00	
Total	223	1626	4292	3539	355	10035
	2.22	16.20	42.77	35.27	3.54	100.00

Frequency of missing values = 1946

Laboratory tests at the time of obtaining Table 2-6 consent, according to the presence or absence of proton-pump inhibitors

FREQ Procedure

Table : ALL * t_CRCC1

ALL t_CRCC1

Power<br>Percent<br>Percent of rows<br>Percentage of columns	1	2	3	4	5	Total
1	223<br>2.22<br>2.22<br>100.00	1626<br>16.20<br>16.20<br>100.00	4292<br>42.77<br>42.77<br>100.00	3539<br>35.27<br>35.27<br>100.00	355<br>3.54<br>3.54<br>100.00	10035<br>100.00
Total	223<br>2.22	1626<br>16.20	4292<br>42.77	3539<br>35.27	355<br>3.54	10035<br>100.00

Frequency of missing values = 1946

Laboratory tests at the time of obtaining Table 2-6 consent, according to the presence or absence of proton-pump inhibitors

FREQ Procedure

Table : ALL * t_CRCC1

ALL t_CRCC1

Power |
Percent |
Percent of rows
Percentage of columns | 1| 2| 3| 4| 5| Total

1	223	1626	4292	3539	355	10035
	2.22	16.20	42.77	35.27	3.54	100.00
	2.22	16.20	42.77	35.27	3.54	
	100.00	100.00	100.00	100.00	100.00	
Total	223	1626	4292	3539	355	10035
	2.22	16.20	42.77	35.27	3.54	100.00

Frequency of missing values = 1946

Laboratory tests at the time of obtaining Table 2-6 consent, according to the presence or absence of proton-pump inhibitors

FREQ Procedure

Table : ALL * t_CRCC1

ALL t_CRCC1

Power |
Percent |
Percent of rows
Percentage of columns | 1| 2| 3| 4| 5| Total

ALL	1	2	3	4	5	Total
1	223	1626	4292	3539	355	10035
	2.22	16.20	42.77	35.27	3.54	100.00
	2.22	16.20	42.77	35.27	3.54	
	100.00	100.00	100.00	100.00	100.00	
Total	223	1626	4292	3539	355	10035
	2.22	16.20	42.77	35.27	3.54	100.00

Frequency of missing values = 1946

Laboratory tests at the time of obtaining Table 2-6 consent, according to the presence or absence of proton-pump inhibitors

FREQ Procedure

Table : ALL * non_CRCC1

ALL non_CRCC1

Power |
Percent |
Percent of rows
Percent of columns | 1| Total

1	1946	1946
	100.00	100.00
	100.00	
	100.00	
Total	1946	1946
	100.00	100.00

Degree of missing values = 10035

Laboratory tests at the time of obtaining Table 2-6 consent, according to the presence or absence of proton-pump inhibitors

FREQ Procedure

Table : ALL * t_EGFR1

ALL t_EGFR1

Power |
Percent |
Percent of rows
Percentage of columns | 1| 2| 3| 4| 5| 6| Total

1	134	919	2806	3672	3082	176	10789
	1.24	8.52	26.01	34.03	28.57	1.63	100.00
	1.24	8.52	26.01	34.03	28.57	1.63	
	100.00	100.00	100.00	100.00	100.00	100.00	
Total	134	919	2806	3672	3082	176	10789
	1.24	8.52	26.01	34.03	28.57	1.63	100.00

Frequency of missing values = 1192

Laboratory tests at the time of obtaining Table 2-6 consent, according to the presence or absence of proton-pump inhibitors

FREQ Procedure

Table : ALL * t_EGFR1

ALL t_EGFR1

Power |
Percent |
Percent of rows
Percentage of columns | 1| 2| 3| 4| 5| 6| Total

	1	2	3	4	5	6	Total
1	134	919	2806	3672	3082	176	10789
	1.24	8.52	26.01	34.03	28.57	1.63	100.00
	1.24	8.52	26.01	34.03	28.57	1.63	
	100.00	100.00	100.00	100.00	100.00	100.00	
Total	134	919	2806	3672	3082	176	10789
	1.24	8.52	26.01	34.03	28.57	1.63	100.00

Frequency of missing values = 1192

Laboratory tests at the time of obtaining Table 2-6 consent, according to the presence or absence of proton-pump inhibitors

FREQ Procedure

Table : ALL * t_EGFR1

ALL t_EGFR1

Power |
Percent |
Percent of rows
Percentage of columns | 1| 2| 3| 4| 5| 6| Total

	1	2	3	4	5	6	Total
1	134	919	2806	3672	3082	176	10789
	1.24	8.52	26.01	34.03	28.57	1.63	100.00
	1.24	8.52	26.01	34.03	28.57	1.63	
	100.00	100.00	100.00	100.00	100.00	100.00	
Total	134	919	2806	3672	3082	176	10789
	1.24	8.52	26.01	34.03	28.57	1.63	100.00

Frequency of missing values = 1192

Laboratory tests at the time of obtaining Table 2-6 consent, according to the presence or absence of proton-pump inhibitors

FREQ Procedure

Table : ALL * t_EGFR1

ALL t_EGFR1

Power |
Percent |
Percent of rows
Percentage of columns | 1| 2| 3| 4| 5| 6| Total

	1	2	3	4	5	6	Total
1	134	919	2806	3672	3082	176	10789
	1.24	8.52	26.01	34.03	28.57	1.63	100.00
	1.24	8.52	26.01	34.03	28.57	1.63	
	100.00	100.00	100.00	100.00	100.00	100.00	
Total	134	919	2806	3672	3082	176	10789
	1.24	8.52	26.01	34.03	28.57	1.63	100.00

Frequency of missing values = 1192

Laboratory tests at the time of obtaining Table 2-6 consent, according to the presence or absence of proton-pump inhibitors

FREQ Procedure

Table : ALL * t_EGFR1

ALL t_EGFR1

Power |
Percent |
Percent of rows
Percentage of columns | 1| 2| 3| 4| 5| 6| Total

1	134	919	2806	3672	3082	176	10789
	1.24	8.52	26.01	34.03	28.57	1.63	100.00
	1.24	8.52	26.01	34.03	28.57	1.63	
	100.00	100.00	100.00	100.00	100.00	100.00	
Total	134	919	2806	3672	3082	176	10789
	1.24	8.52	26.01	34.03	28.57	1.63	100.00

Frequency of missing values = 1192

Laboratory tests at the time of obtaining Table 2-6 consent, according to the presence or absence of proton-pump inhibitors

FREQ Procedure

Table : ALL * t_EGFR1

ALL t_EGFR1

Power |
Percent |
Percent of rows
Percentage of columns | 1| 2| 3| 4| 5| 6| Total

	1	2	3	4	5	6	Total
1	134	919	2806	3672	3082	176	10789
	1.24	8.52	26.01	34.03	28.57	1.63	100.00
	1.24	8.52	26.01	34.03	28.57	1.63	
	100.00	100.00	100.00	100.00	100.00	100.00	
Total	134	919	2806	3672	3082	176	10789
	1.24	8.52	26.01	34.03	28.57	1.63	100.00

Frequency of missing values = 1192

Laboratory tests at the time of obtaining Table 2-6 consent, according to the presence or absence of proton-pump inhibitors

FREQ Procedure

Table : ALL * non_EGFR1

ALL non_EGFR1

Power |
Percent |
Percent of rows
Percent of columns | 1| Total
______________+________+
1 | 1192 | 1192
| 100.00 | 100.00
| 100.00 |
| 100.00 |
______________+________+
Total 1192 1192
100.00 100.00

Degree of missing values = 10789

Laboratory tests at the time of obtaining Table 2-6 consent, according to the presence or absence of proton-pump inhibitors

FREQ Procedure

Table : ALL * HBAC_BL

ALL HBAC_BL(Category of Hemoglobin A1c at Baseline)

Power / Percent / Percent of rows / Column Percent	<6.0%	6.0% < 7.0%	More than 7.0% < 8.0%	More than 8.0%	Missing	Total
1	3184	2745	679	231	5142	11981
	26.58	22.91	5.67	1.93	42.92	100.00
	26.58	22.91	5.67	1.93	42.92	
	100.00	100.00	100.00	100.00	100.00	
Total	3184	2745	679	231	5142	11981
	26.58	22.91	5.67	1.93	42.92	100.00

Laboratory tests at the time of obtaining Table 2-6 consent, according to the presence or absence of proton-pump inhibitors

FREQ Procedure

Table : ALL * HBAC_BL

ALL HBAC_BL(Category of Hemoglobin A1c at Baseline)

Power |
Percent |
Percent of rows
Column Percent

Column Percent	<6.0%	6.0% < 7.0%	More than 7.0% < 8.0%	More than 8.0%	Missing	Total
1	3184	2745	679	231	5142	11981
	26.58	22.91	5.67	1.93	42.92	100.00
	26.58	22.91	5.67	1.93	42.92	
	100.00	100.00	100.00	100.00	100.00	
Total	3184	2745	679	231	5142	11981
	26.58	22.91	5.67	1.93	42.92	100.00

Laboratory tests at the time of obtaining Table 2-6 consent, according to the presence or absence of proton-pump inhibitors

FREQ Procedure

Table : ALL * HBAC_BL

ALL HBAC_BL(Category of Hemoglobin A1c at Baseline)

Power |
Percent |
Percent of rows
Column Percent|<6.0%|6.0%|More than |7.0%|More than |8.0%|Missing|Total
| | < 7.0%| < 8.0%| | |

Power / Percent / Percent of rows / Column Percent	<6.0%	6.0% < 7.0%	More than 7.0% < 8.0%	More than 8.0%	Missing	Total
1	3184	2745	679	231	5142	11981
	26.58	22.91	5.67	1.93	42.92	100.00
	26.58	22.91	5.67	1.93	42.92	
	100.00	100.00	100.00	100.00	100.00	
Total	3184	2745	679	231	5142	11981
	26.58	22.91	5.67	1.93	42.92	100.00

Laboratory tests at the time of obtaining Table 2-6 consent, according to the presence or absence of proton-pump inhibitors

FREQ Procedure

Table : ALL * HBAC_BL

ALL HBAC_BL(Category of Hemoglobin A1c at Baseline)

Power |
Percent |
Percent of rows
Column Percent|<6.0%|6.0%|More than |7.0%|More than |8.0%|Missing|Total
| | < 7.0%| < 8.0%| | |

1	3184	2745	679	231	5142	11981
	26.58	22.91	5.67	1.93	42.92	100.00
	26.58	22.91	5.67	1.93	42.92	
	100.00	100.00	100.00	100.00	100.00	
Total	3184	2745	679	231	5142	11981
	26.58	22.91	5.67	1.93	42.92	100.00

Laboratory tests at the time of obtaining Table 2-6 consent, according to the presence or absence of proton-pump inhibitors

FREQ Procedure

Table : ALL * HBAC_BL

ALL HBAC_BL(Category of Hemoglobin A1c at Baseline)

Power |
Percent |
Percent of rows

Column Percent	<6.0%	6.0% < 7.0%	More than 7.0% < 8.0%	More than 8.0%	Missing	Total
1	3184	2745	679	231	5142	11981
	26.58	22.91	5.67	1.93	42.92	100.00
	26.58	22.91	5.67	1.93	42.92	
	100.00	100.00	100.00	100.00	100.00	
Total	3184	2745	679	231	5142	11981
	26.58	22.91	5.67	1.93	42.92	100.00

Laboratory tests at the time of obtaining Table 2-6 consent, according to the presence or absence of proton-pump inhibitors

FREQ Procedure

			Cumulative	
Percent of ALL power	Percent of power			
1	20509	100.00	20509	100.00

Laboratory tests at the time of obtaining Table 2-6 consent, according to the presence or absence of proton-pump inhibitors

FREQ Procedure

Tables: ALL* ACTYP

ALL
ACTYP(Type of AF)

```
Power |
Percent |
Percent of rows
Percent of column|Warfa| Total
                 |Phosphate|
                 |         |
_______________+_________+
               1 |    5340 |    5340
                 | 100.00 | 100.00
                 | 100.00 |
                 | 100.00 |
_______________+_________+
Total 5340 5340
                   100.00    100.00
```

Laboratory tests at the time of obtaining Table 2-6 consent, according to the presence or absence of proton-pump inhibitors

Unique Subject Identifier=0001110010-0005

MEANS Procedure

Assay variable : PTINR_VAL1 PT-INR Value

N	Mean	Standard Deviation	Minimum	Maximum
5	2.1760000	0.2298478	2.0100000	2.5600000

Unique Subject Identifier=0001110010-0018

Assay variable : PTINR_VAL1 PT-INR Value

N	Mean	Standard Deviation	Minimum	Maximum
4	2.7475000	0.4163632	2.4500000	3.3500000

Unique Subject Identifier=0001110010-0027

Assay variable : PTINR_VAL1 PT-INR Value

N	Mean	Standard Deviation	Minimum	Maximum
4	2.2725000	0.0665207	2.1900000	2.3400000

Unique Subject Identifier=0004110010-0007

Assay variable : PTINR_VAL1 PT-INR Value

N	Mean	Standard Deviation	Minimum	Maximum
5	1.7880000	0.1658915	1.5600000	1.9300000

Laboratory tests at the time of obtaining Table 2-6 consent, according to the presence or absence of proton-pump inhibitors

Unique Subject Identifier=0004110010-0008

MEANS Procedure

Assay variable : PTINR_VAL1 PT-INR Value

N	Mean	Standard Deviation	Minimum	Maximum
2	1.6800000	0.3535534	1.4300000	1.9300000

Unique Subject Identifier=0004110010-0009

Assay variable : PTINR_VAL1 PT-INR Value

N	Mean	Standard Deviation	Minimum	Maximum
6	2.1000000	0.4030385	1.7400000	2.8000000

Unique Subject Identifier=0004110010-0011

Assay variable : PTINR_VAL1 PT-INR Value

N	Mean	Standard Deviation	Minimum	Maximum
4	2.0425000	0.1662077	1.8600000	2.2200000

Unique Subject Identifier=0004110010-0017

Assay variable : PTINR_VAL1 PT-INR Value

N	Mean	Standard Deviation	Minimum	Maximum
5	1.9240000	0.8984320	1.3900000	3.5100000

Laboratory tests at the time of obtaining Table 2-6 consent, according to the presence or absence of proton-pump inhibitors

Unique Subject Identifier=0004110010-0021

MEANS Procedure

Assay variable : PTINR_VAL1 PT-INR Value

N	Mean	Standard Deviation	Minimum	Maximum
3	1.8900000	0.2233831	1.7100000	2.1400000

Unique Subject Identifier=0004110010-0022

Assay variable : PTINR_VAL1 PT-INR Value

N	Mean	Standard Deviation	Minimum	Maximum
4	1.6350000	0.3158586	1.2900000	2.0100000

Unique Subject Identifier=0004110010-0029

Assay variable : PTINR_VAL1 PT-INR Value

N	Mean	Standard Deviation	Minimum	Maximum
4	2.0075000	0.2956772	1.5900000	2.2800000

Unique Subject Identifier=0004110010-0032

Assay variable : PTINR_VAL1 PT-INR Value

N	Mean	Standard Deviation	Minimum	Maximum
3	2.2100000	0.1900000	2.0200000	2.4000000

Laboratory tests at the time of obtaining Table 2-6 consent, according to the presence or absence of proton-pump inhibitors

Unique Subject Identifier=0004110010-0033

MEANS Procedure

Assay variable : PTINR_VAL1 PT-INR Value

N	Mean	Standard Deviation	Minimum	Maximum
4	2.1400000	0.1019804	2.0000000	2.2400000

Unique Subject Identifier=0004110010-0034

Assay variable : PTINR_VAL1 PT-INR Value

N	Mean	Standard Deviation	Minimum	Maximum
4	1.9650000	0.1391642	1.8000000	2.1400000

Unique Subject Identifier=0004110010-0037

Assay variable : PTINR_VAL1 PT-INR Value

N	Mean	Standard Deviation	Minimum	Maximum
3	2.4966667	0.5108163	2.0900000	3.0700000

Unique Subject Identifier=0004110010-0040

Assay variable : PTINR_VAL1 PT-INR Value

N	Mean	Standard Deviation	Minimum	Maximum
4	2.3200000	0.2684524	1.9700000	2.6200000

Laboratory tests at the time of obtaining Table 2-6 consent, according to the presence or absence of proton-pump inhibitors

Unique Subject Identifier=0004110010-0043

MEANS Procedure

Assay variable : PTINR_VAL1 PT-INR Value

N	Mean	Standard Deviation	Minimum	Maximum
3	2.5533333	0.0776745	2.4900000	2.6400000

Unique Subject Identifier=0004110010-0044

Assay variable : PTINR_VAL1 PT-INR Value

N	Mean	Standard Deviation	Minimum	Maximum
3	2.5333333	0.1386843	2.3800000	2.6500000

Unique Subject Identifier=0004110010-0046

Assay variable : PTINR_VAL1 PT-INR Value

N	Mean	Standard Deviation	Minimum	Maximum
3	2.4433333	0.5312564	1.8300000	2.7600000

Unique Subject Identifier=0004110010-0048

Assay variable : PTINR_VAL1 PT-INR Value

N	Mean	Standard Deviation	Minimum	Maximum
4	1.7050000	0.2266422	1.5400000	2.0400000

Laboratory tests at the time of obtaining Table 2-6 consent, according to the presence or absence of proton-pump inhibitors

Unique Subject Identifier=0004110010-0049

MEANS Procedure

Assay variable : PTINR_VAL1 PT-INR Value

N	Mean	Standard Deviation	Minimum	Maximum
4	2.3000000	0.2121320	2.0500000	2.5000000

Unique Subject Identifier=0004110010-0051

Assay variable : PTINR_VAL1 PT-INR Value

N	Mean	Standard Deviation	Minimum	Maximum
6	2.4366667	0.3922074	1.8300000	2.9100000

Unique Subject Identifier=0004110010-0052

Assay variable : PTINR_VAL1 PT-INR Value

N	Mean	Standard Deviation	Minimum	Maximum
6	2.1983333	0.2008399	1.9600000	2.4600000

Unique Subject Identifier=0004110010-0053

Assay variable : PTINR_VAL1 PT-INR Value

N	Mean	Standard Deviation	Minimum	Maximum
6	2.4883333	0.3756816	2.2400000	3.1700000

Laboratory tests at the time of obtaining Table 2-6 consent, according to the presence or absence of proton-pump inhibitors

Unique Subject Identifier=0005010000-0012

MEANS Procedure

Assay variable : PTINR_VAL1 PT-INR Value

N	Mean	Standard Deviation	Minimum	Maximum
6	2.6266667	0.3273327	2.1800000	3.1700000

Unique Subject Identifier=0005010000-0028

Assay variable : PTINR_VAL1 PT-INR Value

N	Mean	Standard Deviation	Minimum	Maximum
3	1.9700000	0.6065476	1.6000000	2.6700000

Unique Subject Identifier=0005010000-0036

Assay variable : PTINR_VAL1 PT-INR Value

N	Mean	Standard Deviation	Minimum	Maximum
3	2.1866667	0.1721434	1.9900000	2.3100000

Unique Subject Identifier=0005010000-0045

Assay variable : PTINR_VAL1 PT-INR Value

N	Mean	Standard Deviation	Minimum	Maximum
4	1.8525000	0.2305609	1.5800000	2.1400000

Laboratory tests at the time of obtaining Table 2-6 consent, according to the presence or absence of proton-pump inhibitors

Unique Subject Identifier=0006100000-0012

MEANS Procedure

Assay variable : PTINR_VAL1 PT-INR Value

N	Mean	Standard Deviation	Minimum	Maximum
2	1.9450000	0.3181981	1.7200000	2.1700000

Unique Subject Identifier=0006100000-0014

Assay variable : PTINR_VAL1 PT-INR Value

N	Mean	Standard Deviation	Minimum	Maximum
4	2.1925000	0.0713559	2.1300000	2.2900000

Unique Subject Identifier=0006100000-0021

Assay variable : PTINR_VAL1 PT-INR Value

N	Mean	Standard Deviation	Minimum	Maximum
1	1.5100000		1.5100000	1.5100000

Unique Subject Identifier=0006100000-0032

Assay variable : PTINR_VAL1 PT-INR Value

N	Mean	Standard Deviation	Minimum	Maximum
6	2.3083333	0.3635611	1.8600000	2.7700000

Laboratory tests at the time of obtaining Table 2-6 consent, according to the presence or absence of proton-pump inhibitors

Unique Subject Identifier=0006100000-0034

MEANS Procedure

Assay variable : PTINR_VAL1 PT-INR Value

N	Mean	Standard Deviation	Minimum	Maximum
1	2.8900000		2.8900000	2.8900000

Unique Subject Identifier=0006100000-0036

Assay variable : PTINR_VAL1 PT-INR Value

N	Mean	Standard Deviation	Minimum	Maximum
3	1.7233333	0.6992377	1.2900000	2.5300000

Unique Subject Identifier=0006100000-0050

Assay variable : PTINR_VAL1 PT-INR Value

N	Mean	Standard Deviation	Minimum	Maximum
4	2.6000000	0.2682039	2.3800000	2.9500000

Unique Subject Identifier=0006100000-0055

Assay variable : PTINR_VAL1 PT-INR Value

N	Mean	Standard Deviation	Minimum	Maximum
4	1.8725000	0.1350000	1.6800000	1.9800000

Laboratory tests at the time of obtaining Table 2-6 consent, according to the presence or absence of proton-pump inhibitors

Unique Subject Identifier=0006100000-0057

MEANS Procedure

Assay variable : PTINR_VAL1 PT-INR Value

N	Mean	Standard Deviation	Minimum	Maximum
3	2.4100000	0.2206808	2.2000000	2.6400000

Unique Subject Identifier=0007011000-0004

Assay variable : PTINR_VAL1 PT-INR Value

N	Mean	Standard Deviation	Minimum	Maximum
4	1.8250000	0.2629956	1.6000000	2.1000000

Unique Subject Identifier=0007011000-0011

Assay variable : PTINR_VAL1 PT-INR Value

N	Mean	Standard Deviation	Minimum	Maximum
3	2.5433333	0.1193035	2.4600000	2.6800000

Unique Subject Identifier=0007011000-0012

Assay variable : PTINR_VAL1 PT-INR Value

N	Mean	Standard Deviation	Minimum	Maximum
5	1.7060000	0.0861974	1.6100000	1.8200000

Laboratory tests at the time of obtaining Table 2-6 consent, according to the presence or absence of proton-pump inhibitors

Unique Subject Identifier=0007011000-0014

MEANS Procedure

Assay variable : PTINR_VAL1 PT-INR Value

N	Mean	Standard Deviation	Minimum	Maximum
3	1.8666667	0.0404145	1.8200000	1.8900000

Unique Subject Identifier=0007011000-0028

Assay variable : PTINR_VAL1 PT-INR Value

N	Mean	Standard Deviation	Minimum	Maximum
3	2.0833333	0.5838093	1.4500000	2.6000000

Unique Subject Identifier=0007011000-0031

Assay variable : PTINR_VAL1 PT-INR Value

N	Mean	Standard Deviation	Minimum	Maximum
3	1.9800000	0.4073082	1.5500000	2.3600000

Unique Subject Identifier=0007011000-0034

Assay variable : PTINR_VAL1 PT-INR Value

N	Mean	Standard Deviation	Minimum	Maximum
4	1.7350000	0.2042058	1.5700000	2.0100000

Laboratory tests at the time of obtaining Table 2-6 consent, according to the presence or absence of proton-pump inhibitors

Unique Subject Identifier=0007011000-0037

MEANS Procedure

Assay variable : PTINR_VAL1 PT-INR Value

N	Mean	Standard Deviation	Minimum	Maximum
3	2.2366667	0.6185736	1.5300000	2.6800000

Unique Subject Identifier=0007011000-0042

Assay variable : PTINR_VAL1 PT-INR Value

N	Mean	Standard Deviation	Minimum	Maximum
1	1.7900000		1.7900000	1.7900000

Unique Subject Identifier=0007011000-0048

Assay variable : PTINR_VAL1 PT-INR Value

N	Mean	Standard Deviation	Minimum	Maximum
3	1.7733333	0.0945163	1.7000000	1.8800000

Unique Subject Identifier=0007011000-0053

Assay variable : PTINR_VAL1 PT-INR Value

N	Mean	Standard Deviation	Minimum	Maximum
3	1.8866667	0.0230940	1.8600000	1.9000000

Laboratory tests at the time of obtaining Table 2-6 consent, according to the presence or absence of proton-pump inhibitors

Unique Subject Identifier=0007011000-0057

MEANS Procedure

Assay variable : PTINR_VAL1 PT-INR Value

N	Mean	Standard Deviation	Minimum	Maximum
4	2.0075000	0.1024288	1.9400000	2.1600000

Unique Subject Identifier=0007011000-0058

Assay variable : PTINR_VAL1 PT-INR Value

N	Mean	Standard Deviation	Minimum	Maximum
4	2.1775000	0.2439091	1.8400000	2.4000000

Unique Subject Identifier=0007011000-0067

Assay variable : PTINR_VAL1 PT-INR Value

N	Mean	Standard Deviation	Minimum	Maximum
1	1.2900000		1.2900000	1.2900000

Unique Subject Identifier=0007011000-0070

Assay variable : PTINR_VAL1 PT-INR Value

N	Mean	Standard Deviation	Minimum	Maximum
2	3.1950000	1.1101576	2.4100000	3.9800000

Laboratory tests at the time of obtaining Table 2-6 consent, according to the presence or absence of proton-pump inhibitors

Unique Subject Identifier=0007011000-0072

MEANS Procedure

Assay variable : PTINR_VAL1 PT-INR Value

N	Mean	Standard Deviation	Minimum	Maximum
2	2.2050000	0.4454773	1.8900000	2.5200000

Unique Subject Identifier=0007011000-0075

Assay variable : PTINR_VAL1 PT-INR Value

N	Mean	Standard Deviation	Minimum	Maximum
6	1.9633333	0.6006219	1.4100000	2.8300000

Unique Subject Identifier=0007011000-0079

Assay variable : PTINR_VAL1 PT-INR Value

N	Mean	Standard Deviation	Minimum	Maximum
2	2.2000000	0.4242641	1.9000000	2.5000000

Unique Subject Identifier=0007011000-0094

Assay variable : PTINR_VAL1 PT-INR Value

N	Mean	Standard Deviation	Minimum	Maximum
4	1.6375000	0.4458980	1.0400000	2.1200000

Laboratory tests at the time of obtaining Table 2-6 consent, according to the presence or absence of proton-pump inhibitors

Unique Subject Identifier=0007011000-0100

MEANS Procedure

Assay variable : PTINR_VAL1 PT-INR Value

N	Mean	Standard Deviation	Minimum	Maximum
4	2.0300000	0.6737457	1.3000000	2.7700000

Unique Subject Identifier=0007011000-0116

Assay variable : PTINR_VAL1 PT-INR Value

N	Mean	Standard Deviation	Minimum	Maximum
3	2.0733333	0.5900282	1.4000000	2.5000000

Unique Subject Identifier=0007011000-0121

Assay variable : PTINR_VAL1 PT-INR Value

N	Mean	Standard Deviation	Minimum	Maximum
3	2.1766667	0.5600298	1.5300000	2.5000000

Unique Subject Identifier=0007011000-0130

Assay variable : PTINR_VAL1 PT-INR Value

N	Mean	Standard Deviation	Minimum	Maximum
1	1.7600000		1.7600000	1.7600000

Laboratory tests at the time of obtaining Table 2-6 consent, according to the presence or absence of proton-pump inhibitors

Unique Subject Identifier=0007011000-0139

MEANS Procedure

Assay variable : PTINR_VAL1 PT-INR Value

N	Mean	Standard Deviation	Minimum	Maximum
3	2.9266667	0.2052641	2.7000000	3.1000000

Unique Subject Identifier=0008000110-0007

Assay variable : PTINR_VAL1 PT-INR Value

N	Mean	Standard Deviation	Minimum	Maximum
6	1.9166667	0.3415650	1.3700000	2.3000000

Unique Subject Identifier=0008000110-0016

Assay variable : PTINR_VAL1 PT-INR Value

N	Mean	Standard Deviation	Minimum	Maximum
4	1.8175000	0.2196019	1.5600000	2.0100000

Unique Subject Identifier=0009010000-0002

Assay variable : PTINR_VAL1 PT-INR Value

N	Mean	Standard Deviation	Minimum	Maximum
4	2.0575000	0.1802544	1.8400000	2.2700000

Laboratory tests at the time of obtaining Table 2-6 consent, according to the presence or absence of proton-pump inhibitors

Unique Subject Identifier=0009010000-0004

MEANS Procedure

Assay variable : PTINR_VAL1 PT-INR Value

N	Mean	Standard Deviation	Minimum	Maximum
3	2.0366667	0.1985783	1.8800000	2.2600000

Unique Subject Identifier=0009010000-0006

Assay variable : PTINR_VAL1 PT-INR Value

N	Mean	Standard Deviation	Minimum	Maximum
6	1.6366667	0.0915787	1.5500000	1.7500000

Unique Subject Identifier=0009010000-0008

Assay variable : PTINR_VAL1 PT-INR Value

N	Mean	Standard Deviation	Minimum	Maximum
2	2.4600000	0.1131371	2.3800000	2.5400000

Unique Subject Identifier=0009010000-0010

Assay variable : PTINR_VAL1 PT-INR Value

N	Mean	Standard Deviation	Minimum	Maximum
2	2.1800000	0.0282843	2.1600000	2.2000000

Laboratory tests at the time of obtaining Table 2-6 consent, according to the presence or absence of proton-pump inhibitors

Unique Subject Identifier=0009010000-0018

MEANS Procedure

Assay variable : PTINR_VAL1 PT-INR Value

N	Mean	Standard Deviation	Minimum	Maximum
6	2.0583333	0.2135806	1.7500000	2.3100000

Unique Subject Identifier=0009010000-0019

Assay variable : PTINR_VAL1 PT-INR Value

N	Mean	Standard Deviation	Minimum	Maximum
5	2.4300000	0.5772781	1.9300000	3.4200000

Unique Subject Identifier=0009010000-0021

Assay variable : PTINR_VAL1 PT-INR Value

N	Mean	Standard Deviation	Minimum	Maximum
6	1.8750000	0.5326443	1.1600000	2.7900000

Unique Subject Identifier=0009010000-0024

Assay variable : PTINR_VAL1 PT-INR Value

N	Mean	Standard Deviation	Minimum	Maximum
6	2.0600000	0.1930803	1.7100000	2.2700000

Laboratory tests at the time of obtaining Table 2-6 consent, according to the presence or absence of proton-pump inhibitors

Unique Subject Identifier=0009010000-0029

MEANS Procedure

Assay variable : PTINR_VAL1 PT-INR Value

N	Mean	Standard Deviation	Minimum	Maximum
5	2.8740000	0.5623433	2.3800000	3.5500000

Unique Subject Identifier=0009010000-0032

Assay variable : PTINR_VAL1 PT-INR Value

N	Mean	Standard Deviation	Minimum	Maximum
4	1.7675000	0.1362290	1.6300000	1.9500000

Unique Subject Identifier=0009010000-0033

Assay variable : PTINR_VAL1 PT-INR Value

N	Mean	Standard Deviation	Minimum	Maximum
3	2.3200000	0.0264575	2.2900000	2.3400000

Unique Subject Identifier=0009010000-0036

Assay variable : PTINR_VAL1 PT-INR Value

N	Mean	Standard Deviation	Minimum	Maximum
3	2.3766667	0.4796179	1.9400000	2.8900000

Laboratory tests at the time of obtaining Table 2-6 consent, according to the presence or absence of proton-pump inhibitors

Unique Subject Identifier=0009010000-0037

MEANS Procedure

Assay variable : PTINR_VAL1 PT-INR Value

N	Mean	Standard Deviation	Minimum	Maximum
3	1.6200000	0.1664332	1.4300000	1.7400000

Unique Subject Identifier=0009010000-0039

Assay variable : PTINR_VAL1 PT-INR Value

N	Mean	Standard Deviation	Minimum	Maximum
3	1.7366667	0.1357694	1.5800000	1.8200000

Unique Subject Identifier=0009010000-0046

Assay variable : PTINR_VAL1 PT-INR Value

N	Mean	Standard Deviation	Minimum	Maximum
3	2.1500000	0.4313931	1.7000000	2.5600000

Unique Subject Identifier=0009010000-0047

Assay variable : PTINR_VAL1 PT-INR Value

N	Mean	Standard Deviation	Minimum	Maximum
3	2.0633333	0.7159143	1.6500000	2.8900000

Laboratory tests at the time of obtaining Table 2-6 consent, according to the presence or absence of proton-pump inhibitors

Unique Subject Identifier=0009010000-0048

MEANS Procedure

Assay variable : PTINR_VAL1 PT-INR Value

N	Mean	Standard Deviation	Minimum	Maximum
3	1.9066667	0.3288363	1.6600000	2.2800000

Unique Subject Identifier=0009010000-0050

Assay variable : PTINR_VAL1 PT-INR Value

N	Mean	Standard Deviation	Minimum	Maximum
3	2.1800000	0.1300000	2.1000000	2.3300000

Unique Subject Identifier=0009010000-0061

Assay variable : PTINR_VAL1 PT-INR Value

N	Mean	Standard Deviation	Minimum	Maximum
2	2.2950000	0.5161880	1.9300000	2.6600000

Unique Subject Identifier=0009010000-0062

Assay variable : PTINR_VAL1 PT-INR Value

N	Mean	Standard Deviation	Minimum	Maximum
3	2.0800000	0.2690725	1.7800000	2.3000000

Laboratory tests at the time of obtaining Table 2-6 consent, according to the presence or absence of proton-pump inhibitors

Unique Subject Identifier=0009010000-0078

MEANS Procedure

Assay variable : PTINR_VAL1 PT-INR Value

N	Mean	Standard Deviation	Minimum	Maximum
3	1.9533333	0.1517674	1.7900000	2.0900000

Unique Subject Identifier=0009010000-0079

Assay variable : PTINR_VAL1 PT-INR Value

N	Mean	Standard Deviation	Minimum	Maximum
4	2.4000000	0.3892728	1.9700000	2.8400000

Unique Subject Identifier=0009010000-0084

Assay variable : PTINR_VAL1 PT-INR Value

N	Mean	Standard Deviation	Minimum	Maximum
5	1.9940000	0.1761533	1.8200000	2.2600000

Unique Subject Identifier=0009010000-0089

Assay variable : PTINR_VAL1 PT-INR Value

N	Mean	Standard Deviation	Minimum	Maximum
3	2.4633333	0.5451911	1.8800000	2.9600000

Laboratory tests at the time of obtaining Table 2-6 consent, according to the presence or absence of proton-pump inhibitors

Unique Subject Identifier=0009010000-0093

MEANS Procedure

Assay variable : PTINR_VAL1 PT-INR Value

N	Mean	Standard Deviation	Minimum	Maximum
6	2.1100000	0.2913417	1.7500000	2.4600000

Unique Subject Identifier=0009010000-0097

Assay variable : PTINR_VAL1 PT-INR Value

N	Mean	Standard Deviation	Minimum	Maximum
3	2.3400000	0.2594224	2.1300000	2.6300000

Unique Subject Identifier=0009010000-0099

Assay variable : PTINR_VAL1 PT-INR Value

N	Mean	Standard Deviation	Minimum	Maximum
3	2.7266667	0.6957250	2.3200000	3.5300000

Unique Subject Identifier=0009010000-0102

Assay variable : PTINR_VAL1 PT-INR Value

N	Mean	Standard Deviation	Minimum	Maximum
3	1.6266667	0.3937427	1.3700000	2.0800000

Laboratory tests at the time of obtaining Table 2-6 consent, according to the presence or absence of proton-pump inhibitors

Unique Subject Identifier=0009010000-0104

MEANS Procedure

Assay variable : PTINR_VAL1 PT-INR Value

N	Mean	Standard Deviation	Minimum	Maximum
6	2.1083333	0.6375395	1.3300000	3.0000000

Unique Subject Identifier=0009010000-0106

Assay variable : PTINR_VAL1 PT-INR Value

N	Mean	Standard Deviation	Minimum	Maximum
3	1.9833333	0.3879863	1.7300000	2.4300000

Unique Subject Identifier=0009010000-0107

Assay variable : PTINR_VAL1 PT-INR Value

N	Mean	Standard Deviation	Minimum	Maximum
3	2.2800000	0.3174902	2.0400000	2.6400000

Unique Subject Identifier=0009010000-0108

Assay variable : PTINR_VAL1 PT-INR Value

N	Mean	Standard Deviation	Minimum	Maximum
3	1.9233333	0.0568624	1.8600000	1.9700000

Laboratory tests at the time of obtaining Table 2-6 consent, according to the presence or absence of proton-pump inhibitors

Unique Subject Identifier=0009010000-0109

MEANS Procedure

Assay variable : PTINR_VAL1 PT-INR Value

N	Mean	Standard Deviation	Minimum	Maximum
4	2.4000000	0.2871701	2.0300000	2.7000000

Unique Subject Identifier=0009010000-0117

Assay variable : PTINR_VAL1 PT-INR Value

N	Mean	Standard Deviation	Minimum	Maximum
3	2.1800000	0.0435890	2.1500000	2.2300000

Unique Subject Identifier=0009010000-0139

Assay variable : PTINR_VAL1 PT-INR Value

N	Mean	Standard Deviation	Minimum	Maximum
3	2.1466667	0.6335877	1.6900000	2.8700000

Unique Subject Identifier=0009010000-0141

Assay variable : PTINR_VAL1 PT-INR Value

N	Mean	Standard Deviation	Minimum	Maximum
4	2.4450000	0.3221283	2.0300000	2.7000000

Laboratory tests at the time of obtaining Table 2-6 consent, according to the presence or absence of proton-pump inhibitors

Unique Subject Identifier=0009010000-0142

MEANS Procedure

Assay variable : PTINR_VAL1 PT-INR Value

N	Mean	Standard Deviation	Minimum	Maximum
4	2.0500000	0.2815434	1.7200000	2.3800000

Unique Subject Identifier=0009010000-0143

Assay variable : PTINR_VAL1 PT-INR Value

N	Mean	Standard Deviation	Minimum	Maximum
2	1.8850000	0.2474874	1.7100000	2.0600000

Unique Subject Identifier=0009010000-0147

Assay variable : PTINR_VAL1 PT-INR Value

N	Mean	Standard Deviation	Minimum	Maximum
4	1.7300000	0.1726268	1.5900000	1.9700000

Unique Subject Identifier=0009010000-0150

Assay variable : PTINR_VAL1 PT-INR Value

N	Mean	Standard Deviation	Minimum	Maximum
2	1.8450000	0.2050610	1.7000000	1.9900000

Laboratory tests at the time of obtaining Table 2-6 consent, according to the presence or absence of proton-pump inhibitors

Unique Subject Identifier=0009010000-0151

MEANS Procedure

Assay variable : PTINR_VAL1 PT-INR Value

N	Mean	Standard Deviation	Minimum	Maximum
3	1.5366667	0.1504438	1.4400000	1.7100000

Unique Subject Identifier=0009010000-0153

Assay variable : PTINR_VAL1 PT-INR Value

N	Mean	Standard Deviation	Minimum	Maximum
4	2.2850000	0.2056696	2.0300000	2.5300000

Unique Subject Identifier=0009010000-0154

Assay variable : PTINR_VAL1 PT-INR Value

N	Mean	Standard Deviation	Minimum	Maximum
5	2.0180000	0.3054832	1.7100000	2.3700000

Unique Subject Identifier=0009010000-0156

Assay variable : PTINR_VAL1 PT-INR Value

N	Mean	Standard Deviation	Minimum	Maximum
3	2.3700000	0.1345362	2.2200000	2.4800000

Laboratory tests at the time of obtaining Table 2-6 consent, according to the presence or absence of proton-pump inhibitors

Unique Subject Identifier=0009010000-0162

MEANS Procedure

Assay variable : PTINR_VAL1 PT-INR Value

N	Mean	Standard Deviation	Minimum	Maximum
3	1.6933333	0.1205543	1.5800000	1.8200000

Unique Subject Identifier=0009010000-0163

Assay variable : PTINR_VAL1 PT-INR Value

N	Mean	Standard Deviation	Minimum	Maximum
3	2.2966667	0.1677299	2.1900000	2.4900000

Unique Subject Identifier=0009010000-0166

Assay variable : PTINR_VAL1 PT-INR Value

N	Mean	Standard Deviation	Minimum	Maximum
3	1.8800000	0.1479865	1.7100000	1.9800000

Unique Subject Identifier=0009010000-0167

Assay variable : PTINR_VAL1 PT-INR Value

N	Mean	Standard Deviation	Minimum	Maximum
3	2.0033333	0.3251666	1.7500000	2.3700000

Laboratory tests at the time of obtaining Table 2-6 consent, according to the presence or absence of proton-pump inhibitors

Unique Subject Identifier=0009010000-0168

MEANS Procedure

Assay variable : PTINR_VAL1 PT-INR Value

N	Mean	Standard Deviation	Minimum	Maximum
3	2.2400000	0.0400000	2.2000000	2.2800000

Unique Subject Identifier=0009010000-0180

Assay variable : PTINR_VAL1 PT-INR Value

N	Mean	Standard Deviation	Minimum	Maximum
3	2.2466667	0.0585947	2.1800000	2.2900000

Unique Subject Identifier=0009010000-0197

Assay variable : PTINR_VAL1 PT-INR Value

N	Mean	Standard Deviation	Minimum	Maximum
3	1.8233333	0.0351188	1.7900000	1.8600000

Unique Subject Identifier=0009010000-0198

Assay variable : PTINR_VAL1 PT-INR Value

N	Mean	Standard Deviation	Minimum	Maximum
6	1.5600000	0.1628496	1.3500000	1.8200000

Laboratory tests at the time of obtaining Table 2-6 consent, according to the presence or absence of proton-pump inhibitors

Unique Subject Identifier=0009010000-0200

MEANS Procedure

Assay variable : PTINR_VAL1 PT-INR Value

N	Mean	Standard Deviation	Minimum	Maximum
2	2.3700000	0.1131371	2.2900000	2.4500000

Unique Subject Identifier=0009010000-0204

Assay variable : PTINR_VAL1 PT-INR Value

N	Mean	Standard Deviation	Minimum	Maximum
5	2.2680000	0.1293058	2.1400000	2.4400000

Unique Subject Identifier=0009010000-0217

Assay variable : PTINR_VAL1 PT-INR Value

N	Mean	Standard Deviation	Minimum	Maximum
6	1.3750000	0.0768765	1.2500000	1.4700000

Unique Subject Identifier=0009010000-0220

Assay variable : PTINR_VAL1 PT-INR Value

N	Mean	Standard Deviation	Minimum	Maximum
6	2.0316667	0.0970395	1.8700000	2.1300000

Laboratory tests at the time of obtaining Table 2-6 consent, according to the presence or absence of proton-pump inhibitors

Unique Subject Identifier=0009010000-0222

MEANS Procedure

Assay variable : PTINR_VAL1 PT-INR Value

N	Mean	Standard Deviation	Minimum	Maximum
6	1.7883333	0.1853016	1.5200000	2.0600000

Unique Subject Identifier=0009010000-0224

Assay variable : PTINR_VAL1 PT-INR Value

N	Mean	Standard Deviation	Minimum	Maximum
5	2.2180000	0.0772658	2.1000000	2.3100000

Unique Subject Identifier=0009010000-0229

Assay variable : PTINR_VAL1 PT-INR Value

N	Mean	Standard Deviation	Minimum	Maximum
3	1.8700000	0.1307670	1.7800000	2.0200000

Unique Subject Identifier=0009010000-0234

Assay variable : PTINR_VAL1 PT-INR Value

N	Mean	Standard Deviation	Minimum	Maximum
1	2.7500000		2.7500000	2.7500000

Laboratory tests at the time of obtaining Table 2-6 consent, according to the presence or absence of proton-pump inhibitors

Unique Subject Identifier=0009010000-0235

MEANS Procedure

Assay variable : PTINR_VAL1 PT-INR Value

N	Mean	Standard Deviation	Minimum	Maximum
6	1.5416667	0.1703428	1.3300000	1.7400000

Unique Subject Identifier=0009010000-0238

Assay variable : PTINR_VAL1 PT-INR Value

N	Mean	Standard Deviation	Minimum	Maximum
3	2.2100000	0.5456189	1.8900000	2.8400000

Unique Subject Identifier=0009010000-0244

Assay variable : PTINR_VAL1 PT-INR Value

N	Mean	Standard Deviation	Minimum	Maximum
4	2.1200000	0.6906519	1.5500000	3.0400000

Unique Subject Identifier=0009010000-0247

Assay variable : PTINR_VAL1 PT-INR Value

N	Mean	Standard Deviation	Minimum	Maximum
3	1.6566667	0.3817504	1.3700000	2.0900000

Laboratory tests at the time of obtaining Table 2-6 consent, according to the presence or absence of proton-pump inhibitors

Unique Subject Identifier=0013011100-0002

MEANS Procedure

Assay variable : PTINR_VAL1 PT-INR Value

N	Mean	Standard Deviation	Minimum	Maximum
5	2.1560000	0.0944987	2.0300000	2.2800000

Unique Subject Identifier=0013011100-0008

Assay variable : PTINR_VAL1 PT-INR Value

N	Mean	Standard Deviation	Minimum	Maximum
6	1.8916667	0.1126795	1.7700000	2.0700000

Unique Subject Identifier=0013011100-0016

Assay variable : PTINR_VAL1 PT-INR Value

N	Mean	Standard Deviation	Minimum	Maximum
4	1.9625000	0.1768945	1.7600000	2.1700000

Unique Subject Identifier=0013011100-0022

Assay variable : PTINR_VAL1 PT-INR Value

N	Mean	Standard Deviation	Minimum	Maximum
3	1.4700000	0.2351595	1.2000000	1.6300000

Laboratory tests at the time of obtaining Table 2-6 consent, according to the presence or absence of proton-pump inhibitors

Unique Subject Identifier=0013011100-0027

MEANS Procedure

Assay variable : PTINR_VAL1 PT-INR Value

N	Mean	Standard Deviation	Minimum	Maximum
3	2.4366667	0.3259346	2.1700000	2.8000000

Unique Subject Identifier=0014010000-0001

Assay variable : PTINR_VAL1 PT-INR Value

N	Mean	Standard Deviation	Minimum	Maximum
4	2.2700000	0.2674572	2.1100000	2.6700000

Unique Subject Identifier=0014010000-0004

Assay variable : PTINR_VAL1 PT-INR Value

N	Mean	Standard Deviation	Minimum	Maximum
6	1.5366667	0.3691432	1.2200000	2.2400000

Unique Subject Identifier=0017010000-0001

Assay variable : PTINR_VAL1 PT-INR Value

N	Mean	Standard Deviation	Minimum	Maximum
3	1.7100000	0.1969772	1.5500000	1.9300000

Laboratory tests at the time of obtaining Table 2-6 consent, according to the presence or absence of proton-pump inhibitors

Unique Subject Identifier=0017010000-0007

MEANS Procedure

Assay variable : PTINR_VAL1 PT-INR Value

N	Mean	Standard Deviation	Minimum	Maximum
2	2.1550000	0.2192031	2.0000000	2.3100000

Unique Subject Identifier=0017010000-0014

Assay variable : PTINR_VAL1 PT-INR Value

N	Mean	Standard Deviation	Minimum	Maximum
6	2.1916667	0.2579470	1.9200000	2.5500000

Unique Subject Identifier=0017010000-0017

Assay variable : PTINR_VAL1 PT-INR Value

N	Mean	Standard Deviation	Minimum	Maximum
3	2.1833333	0.2482606	2.0400000	2.4700000

Unique Subject Identifier=0017010000-0020

Assay variable : PTINR_VAL1 PT-INR Value

N	Mean	Standard Deviation	Minimum	Maximum
3	1.9500000	0.1228821	1.8100000	2.0400000

Laboratory tests at the time of obtaining Table 2-6 consent, according to the presence or absence of proton-pump inhibitors

Unique Subject Identifier=0017010000-0024

MEANS Procedure

Assay variable : PTINR_VAL1 PT-INR Value

N	Mean	Standard Deviation	Minimum	Maximum
3	2.0533333	0.0208167	2.0300000	2.0700000

Unique Subject Identifier=0017010000-0029

Assay variable : PTINR_VAL1 PT-INR Value

N	Mean	Standard Deviation	Minimum	Maximum
4	2.6250000	0.7736709	1.8500000	3.6000000

Unique Subject Identifier=0017010000-0031

Assay variable : PTINR_VAL1 PT-INR Value

N	Mean	Standard Deviation	Minimum	Maximum
2	2.3350000	0.0070711	2.3300000	2.3400000

Unique Subject Identifier=0017010000-0032

Assay variable : PTINR_VAL1 PT-INR Value

N	Mean	Standard Deviation	Minimum	Maximum
2	2.0100000	0.1131371	1.9300000	2.0900000

Laboratory tests at the time of obtaining Table 2-6 consent, according to the presence or absence of proton-pump inhibitors

Unique Subject Identifier=0017010000-0035

MEANS Procedure

Assay variable : PTINR_VAL1 PT-INR Value

N	Mean	Standard Deviation	Minimum	Maximum
2	1.8100000	0.5232590	1.4400000	2.1800000

Unique Subject Identifier=0017010000-0039

Assay variable : PTINR_VAL1 PT-INR Value

N	Mean	Standard Deviation	Minimum	Maximum
4	1.7600000	0.1032796	1.6400000	1.8800000

Unique Subject Identifier=0017010000-0040

Assay variable : PTINR_VAL1 PT-INR Value

N	Mean	Standard Deviation	Minimum	Maximum
3	1.6533333	0.2050203	1.4500000	1.8600000

Unique Subject Identifier=0017010000-0042

Assay variable : PTINR_VAL1 PT-INR Value

N	Mean	Standard Deviation	Minimum	Maximum
3	2.2000000	0.0888819	2.1300000	2.3000000

Laboratory tests at the time of obtaining Table 2-6 consent, according to the presence or absence of proton-pump inhibitors

Unique Subject Identifier=0017010000-0044

MEANS Procedure

Assay variable : PTINR_VAL1 PT-INR Value

N	Mean	Standard Deviation	Minimum	Maximum
5	1.9300000	0.1246996	1.7900000	2.1300000

Unique Subject Identifier=0017010000-0056

Assay variable : PTINR_VAL1 PT-INR Value

N	Mean	Standard Deviation	Minimum	Maximum
3	1.9366667	0.5896892	1.2700000	2.3900000

Unique Subject Identifier=0017010000-0060

Assay variable : PTINR_VAL1 PT-INR Value

N	Mean	Standard Deviation	Minimum	Maximum
3	2.2133333	0.6601767	1.6300000	2.9300000

Unique Subject Identifier=0017010000-0069

Assay variable : PTINR_VAL1 PT-INR Value

N	Mean	Standard Deviation	Minimum	Maximum
3	1.7033333	0.2052641	1.5300000	1.9300000

Laboratory tests at the time of obtaining Table 2-6 consent, according to the presence or absence of proton-pump inhibitors

Unique Subject Identifier=0017010000-0076

MEANS Procedure

Assay variable : PTINR_VAL1 PT-INR Value

N	Mean	Standard Deviation	Minimum	Maximum
3	2.5266667	0.2640707	2.2400000	2.7600000

Unique Subject Identifier=0017010000-0084

Assay variable : PTINR_VAL1 PT-INR Value

N	Mean	Standard Deviation	Minimum	Maximum
3	1.8366667	0.1965536	1.6100000	1.9600000

Unique Subject Identifier=0019010000-0003

Assay variable : PTINR_VAL1 PT-INR Value

N	Mean	Standard Deviation	Minimum	Maximum
3	1.8966667	0.4006661	1.5100000	2.3100000

Unique Subject Identifier=0019010000-0010

Assay variable : PTINR_VAL1 PT-INR Value

N	Mean	Standard Deviation	Minimum	Maximum
2	1.8950000	0.0777817	1.8400000	1.9500000

Laboratory tests at the time of obtaining Table 2-6 consent, according to the presence or absence of proton-pump inhibitors

Unique Subject Identifier=0019010000-0012

MEANS Procedure

Assay variable : PTINR_VAL1 PT-INR Value

N	Mean	Standard Deviation	Minimum	Maximum
6	1.8150000	0.3632492	1.3100000	2.3500000

Unique Subject Identifier=0019010000-0017

Assay variable : PTINR_VAL1 PT-INR Value

N	Mean	Standard Deviation	Minimum	Maximum
6	1.9300000	0.4606083	1.0600000	2.3900000

Unique Subject Identifier=0019010000-0035

Assay variable : PTINR_VAL1 PT-INR Value

N	Mean	Standard Deviation	Minimum	Maximum
3	1.9733333	0.2138535	1.7400000	2.1600000

Unique Subject Identifier=0019010000-0038

Assay variable : PTINR_VAL1 PT-INR Value

N	Mean	Standard Deviation	Minimum	Maximum
3	1.3966667	0.2657693	1.1200000	1.6500000

Laboratory tests at the time of obtaining Table 2-6 consent, according to the presence or absence of proton-pump inhibitors

Unique Subject Identifier=0019010000-0039

MEANS Procedure

Assay variable : PTINR_VAL1 PT-INR Value

N	Mean	Standard Deviation	Minimum	Maximum
2	2.0800000	0.2121320	1.9300000	2.2300000

Unique Subject Identifier=0019010000-0045

Assay variable : PTINR_VAL1 PT-INR Value

N	Mean	Standard Deviation	Minimum	Maximum
4	1.8050000	0.3639139	1.6000000	2.3500000

Unique Subject Identifier=0019010000-0056

Assay variable : PTINR_VAL1 PT-INR Value

N	Mean	Standard Deviation	Minimum	Maximum
4	2.4125000	0.4027716	1.8800000	2.7800000

Unique Subject Identifier=0019010000-0065

Assay variable : PTINR_VAL1 PT-INR Value

N	Mean	Standard Deviation	Minimum	Maximum
4	1.4900000	0.2083267	1.2400000	1.6900000

Laboratory tests at the time of obtaining Table 2-6 consent, according to the presence or absence of proton-pump inhibitors

Unique Subject Identifier=0019010000-0070

MEANS Procedure

Assay variable : PTINR_VAL1 PT-INR Value

N	Mean	Standard Deviation	Minimum	Maximum
2	2.1300000	0.2545584	1.9500000	2.3100000

Unique Subject Identifier=0019010000-0092

Assay variable : PTINR_VAL1 PT-INR Value

N	Mean	Standard Deviation	Minimum	Maximum
2	2.0850000	0.2192031	1.9300000	2.2400000

Unique Subject Identifier=0019010000-0094

Assay variable : PTINR_VAL1 PT-INR Value

N	Mean	Standard Deviation	Minimum	Maximum
3	1.4366667	0.1680278	1.2900000	1.6200000

Unique Subject Identifier=0019010000-0096

Assay variable : PTINR_VAL1 PT-INR Value

N	Mean	Standard Deviation	Minimum	Maximum
2	1.8300000	0.4101219	1.5400000	2.1200000

Laboratory tests at the time of obtaining Table 2-6 consent, according to the presence or absence of proton-pump inhibitors

Unique Subject Identifier=0019010000-0098

MEANS Procedure

Assay variable : PTINR_VAL1 PT-INR Value

N	Mean	Standard Deviation	Minimum	Maximum
2	2.0450000	0.0070711	2.0400000	2.0500000

Unique Subject Identifier=0019010000-0114

Assay variable : PTINR_VAL1 PT-INR Value

N	Mean	Standard Deviation	Minimum	Maximum
5	2.1180000	0.2438647	1.9400000	2.5400000

Unique Subject Identifier=0019010000-0118

Assay variable : PTINR_VAL1 PT-INR Value

N	Mean	Standard Deviation	Minimum	Maximum
3	1.8733333	0.3617089	1.6400000	2.2900000

Unique Subject Identifier=0021000000-0008

Assay variable : PTINR_VAL1 PT-INR Value

N	Mean	Standard Deviation	Minimum	Maximum
6	1.9783333	0.5227013	1.6500000	3.0300000

Laboratory tests at the time of obtaining Table 2-6 consent, according to the presence or absence of proton-pump inhibitors

Unique Subject Identifier=0024010000-0001

MEANS Procedure

Assay variable : PTINR_VAL1 PT-INR Value

N	Mean	Standard Deviation	Minimum	Maximum
6	2.1833333	0.1940790	1.9000000	2.4000000

Unique Subject Identifier=0027000000-0001

Assay variable : PTINR_VAL1 PT-INR Value

N	Mean	Standard Deviation	Minimum	Maximum
4	4.1550000	1.3986779	2.1300000	5.1600000

Unique Subject Identifier=0027000000-0012

Assay variable : PTINR_VAL1 PT-INR Value

N	Mean	Standard Deviation	Minimum	Maximum
6	1.9250000	0.4174087	1.2100000	2.4200000

Unique Subject Identifier=0027000000-0018

Assay variable : PTINR_VAL1 PT-INR Value

N	Mean	Standard Deviation	Minimum	Maximum
6	2.2250000	0.6775470	1.0600000	3.0800000

Laboratory tests at the time of obtaining Table 2-6 consent, according to the presence or absence of proton-pump inhibitors

Unique Subject Identifier=0027000000-0027

MEANS Procedure

Assay variable : PTINR_VAL1 PT-INR Value

N	Mean	Standard Deviation	Minimum	Maximum
2	2.9300000	0.2262742	2.7700000	3.0900000

Unique Subject Identifier=0027000000-0028

Assay variable : PTINR_VAL1 PT-INR Value

N	Mean	Standard Deviation	Minimum	Maximum
6	2.0700000	0.1446375	1.9100000	2.2700000

Unique Subject Identifier=0027000000-0029

Assay variable : PTINR_VAL1 PT-INR Value

N	Mean	Standard Deviation	Minimum	Maximum
2	2.5500000	0.2545584	2.3700000	2.7300000

Unique Subject Identifier=0030010010-0001

Assay variable : PTINR_VAL1 PT-INR Value

N	Mean	Standard Deviation	Minimum	Maximum
3	1.7333333	0.3326159	1.4800000	2.1100000

Laboratory tests at the time of obtaining Table 2-6 consent, according to the presence or absence of proton-pump inhibitors

Unique Subject Identifier=0030010010-0005

MEANS Procedure

Assay variable : PTINR_VAL1 PT-INR Value

N	Mean	Standard Deviation	Minimum	Maximum
3	2.1000000	0.1442221	1.9400000	2.2200000

Unique Subject Identifier=0030010010-0007

Assay variable : PTINR_VAL1 PT-INR Value

N	Mean	Standard Deviation	Minimum	Maximum
6	1.8966667	0.4332974	1.5100000	2.4700000

Unique Subject Identifier=0030010010-0011

Assay variable : PTINR_VAL1 PT-INR Value

N	Mean	Standard Deviation	Minimum	Maximum
3	2.4633333	0.5852635	1.7900000	2.8500000

Unique Subject Identifier=0031110001-0005

Assay variable : PTINR_VAL1 PT-INR Value

N	Mean	Standard Deviation	Minimum	Maximum
5	2.1180000	0.4717203	1.7100000	2.8900000

Laboratory tests at the time of obtaining Table 2-6 consent, according to the presence or absence of proton-pump inhibitors

Unique Subject Identifier=0031110001-0008

MEANS Procedure

Assay variable : PTINR_VAL1 PT-INR Value

N	Mean	Standard Deviation	Minimum	Maximum
2	2.3400000	0.3535534	2.0900000	2.5900000

Unique Subject Identifier=0031110001-0016

Assay variable : PTINR_VAL1 PT-INR Value

N	Mean	Standard Deviation	Minimum	Maximum
3	2.0766667	0.2354428	1.8500000	2.3200000

Unique Subject Identifier=0031110001-0018

Assay variable : PTINR_VAL1 PT-INR Value

N	Mean	Standard Deviation	Minimum	Maximum
5	2.0640000	0.4643598	1.3600000	2.5100000

Unique Subject Identifier=0031110001-0020

Assay variable : PTINR_VAL1 PT-INR Value

N	Mean	Standard Deviation	Minimum	Maximum
3	2.9833333	0.1939931	2.7600000	3.1100000

Laboratory tests at the time of obtaining Table 2-6 consent, according to the presence or absence of proton-pump inhibitors

Unique Subject Identifier=0031110001-0026

MEANS Procedure

Assay variable : PTINR_VAL1 PT-INR Value

N	Mean	Standard Deviation	Minimum	Maximum
4	1.6300000	0.3492850	1.1600000	2.0000000

Unique Subject Identifier=0032110000-0004

Assay variable : PTINR_VAL1 PT-INR Value

N	Mean	Standard Deviation	Minimum	Maximum
4	1.6025000	0.1512999	1.4900000	1.8200000

Unique Subject Identifier=0032110000-0007

Assay variable : PTINR_VAL1 PT-INR Value

N	Mean	Standard Deviation	Minimum	Maximum
4	2.0075000	0.2425387	1.7700000	2.3100000

Unique Subject Identifier=0032110000-0009

Assay variable : PTINR_VAL1 PT-INR Value

N	Mean	Standard Deviation	Minimum	Maximum
6	1.5466667	0.1057670	1.4200000	1.6900000

Laboratory tests at the time of obtaining Table 2-6 consent, according to the presence or absence of proton-pump inhibitors

Unique Subject Identifier=0038111111-0002

MEANS Procedure

Assay variable : PTINR_VAL1 PT-INR Value

N	Mean	Standard Deviation	Minimum	Maximum
5	1.7800000	0.1973575	1.5500000	1.9800000

Unique Subject Identifier=0038111111-0008

Assay variable : PTINR_VAL1 PT-INR Value

N	Mean	Standard Deviation	Minimum	Maximum
5	2.3140000	0.2132604	1.9600000	2.4800000

Unique Subject Identifier=0038111111-0023

Assay variable : PTINR_VAL1 PT-INR Value

N	Mean	Standard Deviation	Minimum	Maximum
5	2.2580000	0.6187649	1.3000000	2.8500000

Unique Subject Identifier=0041110000-0002

Assay variable : PTINR_VAL1 PT-INR Value

N	Mean	Standard Deviation	Minimum	Maximum
6	1.9350000	0.4455895	1.5400000	2.7500000

Laboratory tests at the time of obtaining Table 2-6 consent, according to the presence or absence of proton-pump inhibitors

Unique Subject Identifier=0041110000-0003

MEANS Procedure

Assay variable : PTINR_VAL1 PT-INR Value

N	Mean	Standard Deviation	Minimum	Maximum
3	2.1166667	0.0611010	2.0500000	2.1700000

Unique Subject Identifier=0041110000-0017

Assay variable : PTINR_VAL1 PT-INR Value

N	Mean	Standard Deviation	Minimum	Maximum
2	2.2000000	0.1838478	2.0700000	2.3300000

Unique Subject Identifier=0041110000-0018

Assay variable : PTINR_VAL1 PT-INR Value

N	Mean	Standard Deviation	Minimum	Maximum
4	1.6275000	0.5288588	1.1700000	2.1800000

Unique Subject Identifier=0041110000-0020

Assay variable : PTINR_VAL1 PT-INR Value

N	Mean	Standard Deviation	Minimum	Maximum
3	2.0300000	0.5715768	1.3700000	2.3600000

Laboratory tests at the time of obtaining Table 2-6 consent, according to the presence or absence of proton-pump inhibitors

Unique Subject Identifier=0041110000-0022

MEANS Procedure

Assay variable : PTINR_VAL1 PT-INR Value

N	Mean	Standard Deviation	Minimum	Maximum
4	1.6175000	0.2218671	1.3100000	1.7800000

Unique Subject Identifier=0042100010-0001

Assay variable : PTINR_VAL1 PT-INR Value

N	Mean	Standard Deviation	Minimum	Maximum
4	2.0150000	0.7527948	1.3900000	3.1000000

Unique Subject Identifier=0042100010-0002

Assay variable : PTINR_VAL1 PT-INR Value

N	Mean	Standard Deviation	Minimum	Maximum
3	1.7866667	0.3384277	1.5000000	2.1600000

Unique Subject Identifier=0042100010-0004

Assay variable : PTINR_VAL1 PT-INR Value

N	Mean	Standard Deviation	Minimum	Maximum
5	1.9240000	0.0756968	1.8200000	2.0200000

Laboratory tests at the time of obtaining Table 2-6 consent, according to the presence or absence of proton-pump inhibitors

Unique Subject Identifier=0043010010-0005

MEANS Procedure

Assay variable : PTINR_VAL1 PT-INR Value

N	Mean	Standard Deviation	Minimum	Maximum
3	1.7700000	0.1587451	1.6500000	1.9500000

Unique Subject Identifier=0043010010-0009

Assay variable : PTINR_VAL1 PT-INR Value

N	Mean	Standard Deviation	Minimum	Maximum
3	2.5600000	0.1824829	2.3500000	2.6800000

Unique Subject Identifier=0044110000-0014

Assay variable : PTINR_VAL1 PT-INR Value

N	Mean	Standard Deviation	Minimum	Maximum
3	1.6800000	0.2605763	1.3800000	1.8500000

Unique Subject Identifier=0046100001-0015

Assay variable : PTINR_VAL1 PT-INR Value

N	Mean	Standard Deviation	Minimum	Maximum
2	1.5500000	0.0707107	1.5000000	1.6000000

Laboratory tests at the time of obtaining Table 2-6 consent, according to the presence or absence of proton-pump inhibitors

Unique Subject Identifier=0046100001-0018

MEANS Procedure

Assay variable : PTINR_VAL1 PT-INR Value

N	Mean	Standard Deviation	Minimum	Maximum
3	2.1766667	0.5727419	1.5300000	2.6200000

Unique Subject Identifier=0046100001-0051

Assay variable : PTINR_VAL1 PT-INR Value

N	Mean	Standard Deviation	Minimum	Maximum
2	1.6000000	0.3111270	1.3800000	1.8200000

Unique Subject Identifier=0046100001-0065

Assay variable : PTINR_VAL1 PT-INR Value

N	Mean	Standard Deviation	Minimum	Maximum
1	1.9200000		1.9200000	1.9200000

Unique Subject Identifier=0046100001-0079

Assay variable : PTINR_VAL1 PT-INR Value

N	Mean	Standard Deviation	Minimum	Maximum
5	2.0460000	0.8770006	1.3100000	3.4600000

Laboratory tests at the time of obtaining Table 2-6 consent, according to the presence or absence of proton-pump inhibitors

Unique Subject Identifier=0048000010-0002

MEANS Procedure

Assay variable : PTINR_VAL1 PT-INR Value

N	Mean	Standard Deviation	Minimum	Maximum
3	2.1433333	0.7721615	1.2600000	2.6900000

Unique Subject Identifier=0048000010-0004

Assay variable : PTINR_VAL1 PT-INR Value

N	Mean	Standard Deviation	Minimum	Maximum
5	2.2000000	0.0984886	2.0300000	2.2700000

Unique Subject Identifier=0048000010-0010

Assay variable : PTINR_VAL1 PT-INR Value

N	Mean	Standard Deviation	Minimum	Maximum
1	1.7300000		1.7300000	1.7300000

Unique Subject Identifier=0049000010-0002

Assay variable : PTINR_VAL1 PT-INR Value

N	Mean	Standard Deviation	Minimum	Maximum
6	1.8866667	0.2731056	1.5600000	2.3300000

Laboratory tests at the time of obtaining Table 2-6 consent, according to the presence or absence of proton-pump inhibitors

Unique Subject Identifier=0049000010-0004

MEANS Procedure

Assay variable : PTINR_VAL1 PT-INR Value

N	Mean	Standard Deviation	Minimum	Maximum
6	1.9850000	0.1707337	1.7600000	2.1700000

Unique Subject Identifier=0049000010-0029

Assay variable : PTINR_VAL1 PT-INR Value

N	Mean	Standard Deviation	Minimum	Maximum
6	1.9883333	0.2657380	1.6800000	2.2900000

Unique Subject Identifier=0049000010-0031

Assay variable : PTINR_VAL1 PT-INR Value

N	Mean	Standard Deviation	Minimum	Maximum
6	1.9050000	0.2059854	1.5900000	2.1400000

Unique Subject Identifier=0049000010-0036

Assay variable : PTINR_VAL1 PT-INR Value

N	Mean	Standard Deviation	Minimum	Maximum
6	2.2200000	0.4799167	1.7700000	3.0900000

Laboratory tests at the time of obtaining Table 2-6 consent, according to the presence or absence of proton-pump inhibitors

Unique Subject Identifier=0049000010-0037

MEANS Procedure

Assay variable : PTINR_VAL1 PT-INR Value

N	Mean	Standard Deviation	Minimum	Maximum
6	2.5400000	0.3212476	2.1400000	2.9400000

Unique Subject Identifier=0052000000-0001

Assay variable : PTINR_VAL1 PT-INR Value

N	Mean	Standard Deviation	Minimum	Maximum
5	2.0080000	0.6680344	1.0900000	2.6800000

Unique Subject Identifier=0052000000-0003

Assay variable : PTINR_VAL1 PT-INR Value

N	Mean	Standard Deviation	Minimum	Maximum
6	1.7916667	0.1185608	1.6900000	2.0000000

Unique Subject Identifier=0052000000-0004

Assay variable : PTINR_VAL1 PT-INR Value

N	Mean	Standard Deviation	Minimum	Maximum
6	1.9250000	0.1330789	1.6900000	2.0600000

Laboratory tests at the time of obtaining Table 2-6 consent, according to the presence or absence of proton-pump inhibitors

Unique Subject Identifier=0054010000-0004

MEANS Procedure

Assay variable : PTINR_VAL1 PT-INR Value

N	Mean	Standard Deviation	Minimum	Maximum
3	2.4300000	0.6701492	1.8900000	3.1800000

Unique Subject Identifier=0054010000-0005

Assay variable : PTINR_VAL1 PT-INR Value

N	Mean	Standard Deviation	Minimum	Maximum
5	2.2800000	0.1093161	2.1800000	2.4300000

Unique Subject Identifier=0054010000-0007

Assay variable : PTINR_VAL1 PT-INR Value

N	Mean	Standard Deviation	Minimum	Maximum
3	2.1000000	0.1931321	1.9300000	2.3100000

Unique Subject Identifier=0054010000-0017

Assay variable : PTINR_VAL1 PT-INR Value

N	Mean	Standard Deviation	Minimum	Maximum
4	1.8950000	0.3164912	1.6100000	2.3400000

Laboratory tests at the time of obtaining Table 2-6 consent, according to the presence or absence of proton-pump inhibitors

Unique Subject Identifier=0054010000-0022

MEANS Procedure

Assay variable : PTINR_VAL1 PT-INR Value

N	Mean	Standard Deviation	Minimum	Maximum
6	1.3066667	0.1929421	1.1300000	1.6700000

Unique Subject Identifier=0054010000-0026

Assay variable : PTINR_VAL1 PT-INR Value

N	Mean	Standard Deviation	Minimum	Maximum
3	1.6966667	0.3189566	1.4300000	2.0500000

Unique Subject Identifier=0054010000-0028

Assay variable : PTINR_VAL1 PT-INR Value

N	Mean	Standard Deviation	Minimum	Maximum
5	2.3900000	0.2317326	2.0800000	2.7100000

Unique Subject Identifier=0054010000-0029

Assay variable : PTINR_VAL1 PT-INR Value

N	Mean	Standard Deviation	Minimum	Maximum
3	2.4200000	0.2351595	2.2600000	2.6900000

Laboratory tests at the time of obtaining Table 2-6 consent, according to the presence or absence of proton-pump inhibitors

Unique Subject Identifier=0054010000-0033

MEANS Procedure

Assay variable : PTINR_VAL1 PT-INR Value

N	Mean	Standard Deviation	Minimum	Maximum
3	2.5566667	0.1266228	2.4600000	2.7000000

Unique Subject Identifier=0058000100-0007

Assay variable : PTINR_VAL1 PT-INR Value

N	Mean	Standard Deviation	Minimum	Maximum
2	1.6900000	0.8343860	1.1000000	2.2800000

Unique Subject Identifier=0059100000-0001

Assay variable : PTINR_VAL1 PT-INR Value

N	Mean	Standard Deviation	Minimum	Maximum
3	1.6000000	0.0953939	1.5000000	1.6900000

Unique Subject Identifier=0059100000-0002

Assay variable : PTINR_VAL1 PT-INR Value

N	Mean	Standard Deviation	Minimum	Maximum
5	1.5640000	0.1342758	1.3600000	1.7300000

Laboratory tests at the time of obtaining Table 2-6 consent, according to the presence or absence of proton-pump inhibitors

Unique Subject Identifier=0061000000-0001

MEANS Procedure

Assay variable : PTINR_VAL1 PT-INR Value

N	Mean	Standard Deviation	Minimum	Maximum
3	1.9800000	0.2170253	1.8400000	2.2300000

Unique Subject Identifier=0061000000-0006

Assay variable : PTINR_VAL1 PT-INR Value

N	Mean	Standard Deviation	Minimum	Maximum
2	2.2100000	0.2828427	2.0100000	2.4100000

Unique Subject Identifier=0061000000-0010

Assay variable : PTINR_VAL1 PT-INR Value

N	Mean	Standard Deviation	Minimum	Maximum
4	2.0625000	0.4855495	1.6000000	2.7400000

Unique Subject Identifier=0061000000-0021

Assay variable : PTINR_VAL1 PT-INR Value

N	Mean	Standard Deviation	Minimum	Maximum
6	1.9633333	0.6763333	1.2700000	3.1700000

Laboratory tests at the time of obtaining Table 2-6 consent, according to the presence or absence of proton-pump inhibitors

Unique Subject Identifier=0063010000-0012

MEANS Procedure

Assay variable : PTINR_VAL1 PT-INR Value

N	Mean	Standard Deviation	Minimum	Maximum
3	1.6033333	0.0757188	1.5500000	1.6900000

Unique Subject Identifier=0063010000-0013

Assay variable : PTINR_VAL1 PT-INR Value

N	Mean	Standard Deviation	Minimum	Maximum
1	2.7000000		2.7000000	2.7000000

Unique Subject Identifier=0063010000-0015

Assay variable : PTINR_VAL1 PT-INR Value

N	Mean	Standard Deviation	Minimum	Maximum
2	1.5100000	0.0707107	1.4600000	1.5600000

Unique Subject Identifier=0063010000-0016

Assay variable : PTINR_VAL1 PT-INR Value

N	Mean	Standard Deviation	Minimum	Maximum
3	1.5333333	0.1761628	1.3700000	1.7200000

Laboratory tests at the time of obtaining Table 2-6 consent, according to the presence or absence of proton-pump inhibitors

Unique Subject Identifier=0063010000-0018

MEANS Procedure

Assay variable : PTINR_VAL1 PT-INR Value

N	Mean	Standard Deviation	Minimum	Maximum
4	1.9150000	0.4522905	1.5300000	2.5700000

Unique Subject Identifier=0063010000-0024

Assay variable : PTINR_VAL1 PT-INR Value

N	Mean	Standard Deviation	Minimum	Maximum
6	2.0466667	0.4078071	1.6100000	2.6000000

Unique Subject Identifier=0063010000-0030

Assay variable : PTINR_VAL1 PT-INR Value

N	Mean	Standard Deviation	Minimum	Maximum
3	1.9133333	0.2902298	1.6300000	2.2100000

Unique Subject Identifier=0063010000-0033

Assay variable : PTINR_VAL1 PT-INR Value

N	Mean	Standard Deviation	Minimum	Maximum
3	1.7900000	0.3328663	1.5500000	2.1700000

Laboratory tests at the time of obtaining Table 2-6 consent, according to the presence or absence of proton-pump inhibitors

Unique Subject Identifier=0063010000-0036

MEANS Procedure

Assay variable : PTINR_VAL1 PT-INR Value

N	Mean	Standard Deviation	Minimum	Maximum
3	2.0900000	0.1509967	1.9300000	2.2300000

Unique Subject Identifier=0064010110-0003

Assay variable : PTINR_VAL1 PT-INR Value

N	Mean	Standard Deviation	Minimum	Maximum
6	2.4583333	0.1922932	2.2600000	2.7800000

Unique Subject Identifier=0064010110-0012

Assay variable : PTINR_VAL1 PT-INR Value

N	Mean	Standard Deviation	Minimum	Maximum
3	1.5666667	0.3768731	1.1700000	1.9200000

Unique Subject Identifier=0064010110-0014

Assay variable : PTINR_VAL1 PT-INR Value

N	Mean	Standard Deviation	Minimum	Maximum
6	1.8866667	0.1789600	1.7200000	2.1100000

Laboratory tests at the time of obtaining Table 2-6 consent, according to the presence or absence of proton-pump inhibitors

Unique Subject Identifier=0064010110-0015

MEANS Procedure

Assay variable : PTINR_VAL1 PT-INR Value

N	Mean	Standard Deviation	Minimum	Maximum
5	1.6900000	0.3061862	1.4100000	2.1700000

Unique Subject Identifier=0070000000-0003

Assay variable : PTINR_VAL1 PT-INR Value

N	Mean	Standard Deviation	Minimum	Maximum
6	2.0000000	0.3207491	1.7300000	2.4700000

Unique Subject Identifier=0070000000-0005

Assay variable : PTINR_VAL1 PT-INR Value

N	Mean	Standard Deviation	Minimum	Maximum
3	1.8533333	0.4343194	1.4800000	2.3300000

Unique Subject Identifier=0070000000-0008

Assay variable : PTINR_VAL1 PT-INR Value

N	Mean	Standard Deviation	Minimum	Maximum
4	2.2175000	0.1260622	2.0600000	2.3600000

Laboratory tests at the time of obtaining Table 2-6 consent, according to the presence or absence of proton-pump inhibitors

Unique Subject Identifier=0070000000-0009

MEANS Procedure

Assay variable : PTINR_VAL1 PT-INR Value

N	Mean	Standard Deviation	Minimum	Maximum
5	2.0500000	0.6826419	1.4600000	3.0000000

Unique Subject Identifier=0070000000-0016

Assay variable : PTINR_VAL1 PT-INR Value

N	Mean	Standard Deviation	Minimum	Maximum
5	2.7660000	0.8777984	1.9700000	4.2300000

Unique Subject Identifier=0070000000-0018

Assay variable : PTINR_VAL1 PT-INR Value

N	Mean	Standard Deviation	Minimum	Maximum
5	1.5540000	0.3332867	1.0600000	1.8600000

Unique Subject Identifier=0070000000-0022

Assay variable : PTINR_VAL1 PT-INR Value

N	Mean	Standard Deviation	Minimum	Maximum
4	2.1075000	0.3629853	1.7900000	2.5300000

Laboratory tests at the time of obtaining Table 2-6 consent, according to the presence or absence of proton-pump inhibitors

Unique Subject Identifier=0070000000-0030

MEANS Procedure

Assay variable : PTINR_VAL1 PT-INR Value

N	Mean	Standard Deviation	Minimum	Maximum
3	1.9400000	0.3465545	1.7300000	2.3400000

Unique Subject Identifier=0070000000-0032

Assay variable : PTINR_VAL1 PT-INR Value

N	Mean	Standard Deviation	Minimum	Maximum
3	2.1766667	0.1250333	2.0900000	2.3200000

Unique Subject Identifier=0070000000-0035

Assay variable : PTINR_VAL1 PT-INR Value

N	Mean	Standard Deviation	Minimum	Maximum
3	1.6000000	0.1000000	1.5000000	1.7000000

Unique Subject Identifier=0070000000-0039

Assay variable : PTINR_VAL1 PT-INR Value

N	Mean	Standard Deviation	Minimum	Maximum
4	1.5500000	0.4002499	1.1300000	2.0700000

Laboratory tests at the time of obtaining Table 2-6 consent, according to the presence or absence of proton-pump inhibitors

Unique Subject Identifier=0070000000-0041

MEANS Procedure

Assay variable : PTINR_VAL1 PT-INR Value

N	Mean	Standard Deviation	Minimum	Maximum
4	2.1800000	0.2409703	1.8200000	2.3300000

Unique Subject Identifier=0070000000-0046

Assay variable : PTINR_VAL1 PT-INR Value

N	Mean	Standard Deviation	Minimum	Maximum
3	2.0866667	0.1504438	1.9300000	2.2300000

Unique Subject Identifier=0070000000-0048

Assay variable : PTINR_VAL1 PT-INR Value

N	Mean	Standard Deviation	Minimum	Maximum
4	1.8150000	0.1554563	1.6800000	2.0200000

Unique Subject Identifier=0070000000-0049

Assay variable : PTINR_VAL1 PT-INR Value

N	Mean	Standard Deviation	Minimum	Maximum
5	2.0280000	0.4447134	1.5700000	2.6600000

Laboratory tests at the time of obtaining Table 2-6 consent, according to the presence or absence of proton-pump inhibitors

Unique Subject Identifier=0070000000-0053

MEANS Procedure

Assay variable : PTINR_VAL1 PT-INR Value

N	Mean	Standard Deviation	Minimum	Maximum
3	1.7166667	0.1001665	1.6400000	1.8300000

Unique Subject Identifier=0070000000-0056

Assay variable : PTINR_VAL1 PT-INR Value

N	Mean	Standard Deviation	Minimum	Maximum
3	2.4266667	0.7861510	1.5900000	3.1500000

Unique Subject Identifier=0070000000-0061

Assay variable : PTINR_VAL1 PT-INR Value

N	Mean	Standard Deviation	Minimum	Maximum
5	1.8480000	0.3226763	1.3500000	2.2100000

Unique Subject Identifier=0070000000-0063

Assay variable : PTINR_VAL1 PT-INR Value

N	Mean	Standard Deviation	Minimum	Maximum
3	1.5533333	0.0838650	1.5000000	1.6500000

Laboratory tests at the time of obtaining Table 2-6 consent, according to the presence or absence of proton-pump inhibitors

Unique Subject Identifier=0070000000-0064

MEANS Procedure

Assay variable : PTINR_VAL1 PT-INR Value

N	Mean	Standard Deviation	Minimum	Maximum
3	2.1933333	0.3875994	1.8900000	2.6300000

Unique Subject Identifier=0070000000-0066

Assay variable : PTINR_VAL1 PT-INR Value

N	Mean	Standard Deviation	Minimum	Maximum
3	1.8166667	0.1484363	1.6900000	1.9800000

Unique Subject Identifier=0070000000-0070

Assay variable : PTINR_VAL1 PT-INR Value

N	Mean	Standard Deviation	Minimum	Maximum
4	2.2600000	0.6172520	1.6200000	3.0400000

Unique Subject Identifier=0070000000-0071

Assay variable : PTINR_VAL1 PT-INR Value

N	Mean	Standard Deviation	Minimum	Maximum
3	1.9133333	0.2454248	1.6600000	2.1500000

Laboratory tests at the time of obtaining Table 2-6 consent, according to the presence or absence of proton-pump inhibitors

Unique Subject Identifier=0070000000-0073

MEANS Procedure

Assay variable : PTINR_VAL1 PT-INR Value

N	Mean	Standard Deviation	Minimum	Maximum
5	1.8960000	0.4698191	1.4000000	2.4600000

Unique Subject Identifier=0070000000-0074

Assay variable : PTINR_VAL1 PT-INR Value

N	Mean	Standard Deviation	Minimum	Maximum
4	1.7525000	0.0991211	1.6100000	1.8200000

Unique Subject Identifier=0071000000-0001

Assay variable : PTINR_VAL1 PT-INR Value

N	Mean	Standard Deviation	Minimum	Maximum
4	1.6275000	0.1065755	1.4900000	1.7300000

Unique Subject Identifier=0071000000-0003

Assay variable : PTINR_VAL1 PT-INR Value

N	Mean	Standard Deviation	Minimum	Maximum
4	2.5250000	0.7388505	1.9500000	3.5600000

Laboratory tests at the time of obtaining Table 2-6 consent, according to the presence or absence of proton-pump inhibitors

Unique Subject Identifier=0071000000-0004

MEANS Procedure

Assay variable : PTINR_VAL1 PT-INR Value

N	Mean	Standard Deviation	Minimum	Maximum
4	2.1550000	0.2315887	1.9500000	2.4800000

Unique Subject Identifier=0071000000-0006

Assay variable : PTINR_VAL1 PT-INR Value

N	Mean	Standard Deviation	Minimum	Maximum
3	1.8133333	0.4452340	1.3600000	2.2500000

Unique Subject Identifier=0071000000-0007

Assay variable : PTINR_VAL1 PT-INR Value

N	Mean	Standard Deviation	Minimum	Maximum
6	1.8166667	0.3808762	1.4000000	2.3400000

Unique Subject Identifier=0071000000-0009

Assay variable : PTINR_VAL1 PT-INR Value

N	Mean	Standard Deviation	Minimum	Maximum
4	1.7650000	0.1239624	1.6100000	1.8700000

Laboratory tests at the time of obtaining Table 2-6 consent, according to the presence or absence of proton-pump inhibitors

Unique Subject Identifier=0071000000-0013

MEANS Procedure

Assay variable : PTINR_VAL1 PT-INR Value

N	Mean	Standard Deviation	Minimum	Maximum
4	2.0150000	0.1584298	1.9200000	2.2500000

Unique Subject Identifier=0071000000-0015

Assay variable : PTINR_VAL1 PT-INR Value

N	Mean	Standard Deviation	Minimum	Maximum
4	1.8825000	0.1547848	1.7500000	2.0900000

Unique Subject Identifier=0071000000-0018

Assay variable : PTINR_VAL1 PT-INR Value

N	Mean	Standard Deviation	Minimum	Maximum
3	1.8100000	0.2343075	1.6600000	2.0800000

Unique Subject Identifier=0071000000-0021

Assay variable : PTINR_VAL1 PT-INR Value

N	Mean	Standard Deviation	Minimum	Maximum
5	1.9080000	0.2037646	1.7200000	2.1900000

Laboratory tests at the time of obtaining Table 2-6 consent, according to the presence or absence of proton-pump inhibitors

Unique Subject Identifier=0071000000-0022

MEANS Procedure

Assay variable : PTINR_VAL1 PT-INR Value

N	Mean	Standard Deviation	Minimum	Maximum
5	1.9380000	0.1781011	1.7000000	2.2000000

Unique Subject Identifier=0071000000-0025

Assay variable : PTINR_VAL1 PT-INR Value

N	Mean	Standard Deviation	Minimum	Maximum
3	1.4066667	0.2466441	1.2400000	1.6900000

Unique Subject Identifier=0071000000-0028

Assay variable : PTINR_VAL1 PT-INR Value

N	Mean	Standard Deviation	Minimum	Maximum
3	2.1533333	0.6351640	1.4200000	2.5300000

Unique Subject Identifier=0071000000-0030

Assay variable : PTINR_VAL1 PT-INR Value

N	Mean	Standard Deviation	Minimum	Maximum
3	2.5500000	0.3465545	2.3400000	2.9500000

Laboratory tests at the time of obtaining Table 2-6 consent, according to the presence or absence of proton-pump inhibitors

Unique Subject Identifier=0072000000-0006

MEANS Procedure

Assay variable : PTINR_VAL1 PT-INR Value

N	Mean	Standard Deviation	Minimum	Maximum
5	2.2060000	0.1829754	2.0400000	2.5200000

Unique Subject Identifier=0072000000-0010

Assay variable : PTINR_VAL1 PT-INR Value

N	Mean	Standard Deviation	Minimum	Maximum
6	2.3000000	0.4325737	1.6100000	2.7300000

Unique Subject Identifier=0073000000-0003

Assay variable : PTINR_VAL1 PT-INR Value

N	Mean	Standard Deviation	Minimum	Maximum
5	1.8840000	0.3686190	1.3200000	2.3000000

Unique Subject Identifier=0073000000-0022

Assay variable : PTINR_VAL1 PT-INR Value

N	Mean	Standard Deviation	Minimum	Maximum
6	1.4116667	0.5947577	1.0300000	2.5800000

Laboratory tests at the time of obtaining Table 2-6 consent, according to the presence or absence of proton-pump inhibitors

Unique Subject Identifier=0073000000-0035

MEANS Procedure

Assay variable : PTINR_VAL1 PT-INR Value

N	Mean	Standard Deviation	Minimum	Maximum
3	2.1766667	0.1150362	2.0600000	2.2900000

Unique Subject Identifier=0076011000-0003

Assay variable : PTINR_VAL1 PT-INR Value

N	Mean	Standard Deviation	Minimum	Maximum
1	1.5300000		1.5300000	1.5300000

Unique Subject Identifier=0076011000-0004

Assay variable : PTINR_VAL1 PT-INR Value

N	Mean	Standard Deviation	Minimum	Maximum
1	1.6900000		1.6900000	1.6900000

Unique Subject Identifier=0076011000-0008

Assay variable : PTINR_VAL1 PT-INR Value

N	Mean	Standard Deviation	Minimum	Maximum
6	1.4433333	0.3104620	1.2000000	1.9800000

Laboratory tests at the time of obtaining Table 2-6 consent, according to the presence or absence of proton-pump inhibitors

Unique Subject Identifier=0076011000-0009

MEANS Procedure

Assay variable : PTINR_VAL1 PT-INR Value

N	Mean	Standard Deviation	Minimum	Maximum
1	1.2100000		1.2100000	1.2100000

Unique Subject Identifier=0076011000-0020

Assay variable : PTINR_VAL1 PT-INR Value

N	Mean	Standard Deviation	Minimum	Maximum
2	2.1250000	0.2757716	1.9300000	2.3200000

Unique Subject Identifier=0076011000-0024

Assay variable : PTINR_VAL1 PT-INR Value

N	Mean	Standard Deviation	Minimum	Maximum
1	2.0300000		2.0300000	2.0300000

Unique Subject Identifier=0076011000-0025

Assay variable : PTINR_VAL1 PT-INR Value

N	Mean	Standard Deviation	Minimum	Maximum
2	2.0250000	0.6858936	1.5400000	2.5100000

Laboratory tests at the time of obtaining Table 2-6 consent, according to the presence or absence of proton-pump inhibitors

Unique Subject Identifier=0077000000-0005

MEANS Procedure

Assay variable : PTINR_VAL1 PT-INR Value

N	Mean	Standard Deviation	Minimum	Maximum
1	1.6400000		1.6400000	1.6400000

Unique Subject Identifier=0077000000-0010

Assay variable : PTINR_VAL1 PT-INR Value

N	Mean	Standard Deviation	Minimum	Maximum
3	2.1966667	0.7328256	1.4100000	2.8600000

Unique Subject Identifier=0078001000-0001

Assay variable : PTINR_VAL1 PT-INR Value

N	Mean	Standard Deviation	Minimum	Maximum
6	2.1783333	0.1894642	1.8300000	2.3400000

Unique Subject Identifier=0078001000-0003

Assay variable : PTINR_VAL1 PT-INR Value

N	Mean	Standard Deviation	Minimum	Maximum
3	2.0466667	0.5746593	1.7000000	2.7100000

Laboratory tests at the time of obtaining Table 2-6 consent, according to the presence or absence of proton-pump inhibitors

Unique Subject Identifier=0078001000-0004

MEANS Procedure

Assay variable : PTINR_VAL1 PT-INR Value

N	Mean	Standard Deviation	Minimum	Maximum
4	1.8150000	0.1748333	1.5900000	2.0000000

Unique Subject Identifier=0078001000-0007

Assay variable : PTINR_VAL1 PT-INR Value

N	Mean	Standard Deviation	Minimum	Maximum
3	1.6066667	0.0321455	1.5700000	1.6300000

Unique Subject Identifier=0078001000-0008

Assay variable : PTINR_VAL1 PT-INR Value

N	Mean	Standard Deviation	Minimum	Maximum
5	2.1140000	0.3263893	1.7700000	2.5100000

Unique Subject Identifier=0078001000-0010

Assay variable : PTINR_VAL1 PT-INR Value

N	Mean	Standard Deviation	Minimum	Maximum
3	1.8933333	0.0251661	1.8700000	1.9200000

Laboratory tests at the time of obtaining Table 2-6 consent, according to the presence or absence of proton-pump inhibitors

Unique Subject Identifier=0078001000-0011

MEANS Procedure

Assay variable : PTINR_VAL1 PT-INR Value

N	Mean	Standard Deviation	Minimum	Maximum
3	2.1600000	0.3551056	1.7500000	2.3700000

Unique Subject Identifier=0078001000-0012

Assay variable : PTINR_VAL1 PT-INR Value

N	Mean	Standard Deviation	Minimum	Maximum
3	1.9400000	0.1212436	1.8700000	2.0800000

Unique Subject Identifier=0078001000-0013

Assay variable : PTINR_VAL1 PT-INR Value

N	Mean	Standard Deviation	Minimum	Maximum
3	1.9600000	0.0964365	1.8900000	2.0700000

Unique Subject Identifier=0079000000-0001

Assay variable : PTINR_VAL1 PT-INR Value

N	Mean	Standard Deviation	Minimum	Maximum
4	2.3750000	0.5081666	1.9000000	3.0600000

Laboratory tests at the time of obtaining Table 2-6 consent, according to the presence or absence of proton-pump inhibitors

Unique Subject Identifier=0079000000-0003

MEANS Procedure

Assay variable : PTINR_VAL1 PT-INR Value

N	Mean	Standard Deviation	Minimum	Maximum
4	1.7800000	0.1219289	1.6900000	1.9600000

Unique Subject Identifier=0079000000-0021

Assay variable : PTINR_VAL1 PT-INR Value

N	Mean	Standard Deviation	Minimum	Maximum
3	1.7900000	0.0300000	1.7600000	1.8200000

Unique Subject Identifier=0079000000-0022

Assay variable : PTINR_VAL1 PT-INR Value

N	Mean	Standard Deviation	Minimum	Maximum
2	2.1400000	0.5939697	1.7200000	2.5600000

Unique Subject Identifier=0079000000-0023

Assay variable : PTINR_VAL1 PT-INR Value

N	Mean	Standard Deviation	Minimum	Maximum
3	1.6000000	0.2007486	1.4600000	1.8300000

Laboratory tests at the time of obtaining Table 2-6 consent, according to the presence or absence of proton-pump inhibitors

Unique Subject Identifier=0079000000-0026

MEANS Procedure

Assay variable : PTINR_VAL1 PT-INR Value

N	Mean	Standard Deviation	Minimum	Maximum
1	1.4000000		1.4000000	1.4000000

Unique Subject Identifier=0079000000-0028

Assay variable : PTINR_VAL1 PT-INR Value

N	Mean	Standard Deviation	Minimum	Maximum
3	1.8666667	0.7486877	1.3200000	2.7200000

Unique Subject Identifier=0079000000-0030

Assay variable : PTINR_VAL1 PT-INR Value

N	Mean	Standard Deviation	Minimum	Maximum
3	2.1600000	1.1421033	1.0600000	3.3400000

Unique Subject Identifier=0085000000-0004

Assay variable : PTINR_VAL1 PT-INR Value

N	Mean	Standard Deviation	Minimum	Maximum
6	2.0100000	0.1941134	1.7200000	2.3100000

Laboratory tests at the time of obtaining Table 2-6 consent, according to the presence or absence of proton-pump inhibitors

Unique Subject Identifier=0086100000-0005

MEANS Procedure

Assay variable : PTINR_VAL1 PT-INR Value

N	Mean	Standard Deviation	Minimum	Maximum
1	1.4700000		1.4700000	1.4700000

Unique Subject Identifier=0086100000-0011

Assay variable : PTINR_VAL1 PT-INR Value

N	Mean	Standard Deviation	Minimum	Maximum
1	1.7600000		1.7600000	1.7600000

Unique Subject Identifier=0094001000-0012

Assay variable : PTINR_VAL1 PT-INR Value

N	Mean	Standard Deviation	Minimum	Maximum
4	2.2625000	0.3564992	1.8800000	2.7400000

Unique Subject Identifier=0094001000-0026

Assay variable : PTINR_VAL1 PT-INR Value

N	Mean	Standard Deviation	Minimum	Maximum
4	1.7525000	0.1004573	1.6400000	1.8600000

Laboratory tests at the time of obtaining Table 2-6 consent, according to the presence or absence of proton-pump inhibitors

Unique Subject Identifier=0094001000-0028

MEANS Procedure

Assay variable : PTINR_VAL1 PT-INR Value

N	Mean	Standard Deviation	Minimum	Maximum
3	1.9366667	0.1721434	1.8000000	2.1300000

Unique Subject Identifier=0094001000-0038

Assay variable : PTINR_VAL1 PT-INR Value

N	Mean	Standard Deviation	Minimum	Maximum
4	1.5600000	0.1336663	1.4200000	1.7400000

Unique Subject Identifier=0094001000-0047

Assay variable : PTINR_VAL1 PT-INR Value

N	Mean	Standard Deviation	Minimum	Maximum
4	2.0950000	0.1156143	1.9600000	2.2400000

Unique Subject Identifier=0094001000-0050

Assay variable : PTINR_VAL1 PT-INR Value

N	Mean	Standard Deviation	Minimum	Maximum
3	1.5833333	0.1069268	1.4900000	1.7000000

Laboratory tests at the time of obtaining Table 2-6 consent, according to the presence or absence of proton-pump inhibitors

Unique Subject Identifier=0098000000-0001

MEANS Procedure

Assay variable : PTINR_VAL1 PT-INR Value

N	Mean	Standard Deviation	Minimum	Maximum
5	1.9820000	0.1485598	1.7700000	2.0900000

Unique Subject Identifier=0098000000-0006

Assay variable : PTINR_VAL1 PT-INR Value

N	Mean	Standard Deviation	Minimum	Maximum
4	2.7275000	0.7020150	2.0900000	3.7300000

Unique Subject Identifier=0098000000-0008

Assay variable : PTINR_VAL1 PT-INR Value

N	Mean	Standard Deviation	Minimum	Maximum
6	2.4516667	1.3166232	0.9800000	4.0900000

Unique Subject Identifier=0098000000-0009

Assay variable : PTINR_VAL1 PT-INR Value

N	Mean	Standard Deviation	Minimum	Maximum
6	1.6316667	0.3060338	1.4500000	2.2300000

Laboratory tests at the time of obtaining Table 2-6 consent, according to the presence or absence of proton-pump inhibitors

Unique Subject Identifier=0098000000-0011

MEANS Procedure

Assay variable : PTINR_VAL1 PT-INR Value

N	Mean	Standard Deviation	Minimum	Maximum
3	2.3533333	0.2119748	2.1600000	2.5800000

Unique Subject Identifier=0098000000-0014

Assay variable : PTINR_VAL1 PT-INR Value

N	Mean	Standard Deviation	Minimum	Maximum
5	1.9940000	0.2743720	1.6700000	2.3200000

Unique Subject Identifier=0102000000-0003

Assay variable : PTINR_VAL1 PT-INR Value

N	Mean	Standard Deviation	Minimum	Maximum
6	1.6333333	0.3881580	1.1000000	2.2000000

Unique Subject Identifier=0102000000-0004

Assay variable : PTINR_VAL1 PT-INR Value

N	Mean	Standard Deviation	Minimum	Maximum
6	1.5000000	0.2097618	1.2000000	1.7000000

Laboratory tests at the time of obtaining Table 2-6 consent, according to the presence or absence of proton-pump inhibitors

Unique Subject Identifier=0102000000-0006

MEANS Procedure

Assay variable : PTINR_VAL1 PT-INR Value

N	Mean	Standard Deviation	Minimum	Maximum
5	1.8800000	0.2683282	1.6000000	2.3000000

Unique Subject Identifier=0102000000-0007

Assay variable : PTINR_VAL1 PT-INR Value

N	Mean	Standard Deviation	Minimum	Maximum
6	2.1500000	0.2073644	1.9000000	2.4000000

Unique Subject Identifier=0102000000-0010

Assay variable : PTINR_VAL1 PT-INR Value

N	Mean	Standard Deviation	Minimum	Maximum
5	1.9400000	0.4159327	1.4000000	2.3000000

Unique Subject Identifier=0103000000-0006

Assay variable : PTINR_VAL1 PT-INR Value

N	Mean	Standard Deviation	Minimum	Maximum
6	1.7466667	0.8857690	1.0800000	3.3600000

Laboratory tests at the time of obtaining Table 2-6 consent, according to the presence or absence of proton-pump inhibitors

Unique Subject Identifier=0103000000-0007

MEANS Procedure

Assay variable : PTINR_VAL1 PT-INR Value

N	Mean	Standard Deviation	Minimum	Maximum
6	2.1916667	0.9971443	1.3500000	4.0400000

Unique Subject Identifier=0103000000-0008

Assay variable : PTINR_VAL1 PT-INR Value

N	Mean	Standard Deviation	Minimum	Maximum
3	1.9300000	0.3304542	1.5900000	2.2500000

Unique Subject Identifier=0103000000-0010

Assay variable : PTINR_VAL1 PT-INR Value

N	Mean	Standard Deviation	Minimum	Maximum
4	1.8375000	0.3439356	1.4400000	2.2100000

Unique Subject Identifier=0103000000-0013

Assay variable : PTINR_VAL1 PT-INR Value

N	Mean	Standard Deviation	Minimum	Maximum
4	2.0525000	0.3345021	1.7700000	2.5100000

Laboratory tests at the time of obtaining Table 2-6 consent, according to the presence or absence of proton-pump inhibitors

Unique Subject Identifier=0103000000-0014

MEANS Procedure

Assay variable : PTINR_VAL1 PT-INR Value

N	Mean	Standard Deviation	Minimum	Maximum
5	1.8040000	0.2051341	1.6200000	2.1200000

Unique Subject Identifier=0103000000-0018

Assay variable : PTINR_VAL1 PT-INR Value

N	Mean	Standard Deviation	Minimum	Maximum
4	1.8650000	0.2754995	1.5000000	2.1600000

Unique Subject Identifier=0103000000-0019

Assay variable : PTINR_VAL1 PT-INR Value

N	Mean	Standard Deviation	Minimum	Maximum
4	1.7175000	0.1768945	1.5600000	1.9700000

Unique Subject Identifier=0103000000-0022

Assay variable : PTINR_VAL1 PT-INR Value

N	Mean	Standard Deviation	Minimum	Maximum
4	2.2825000	0.4733128	1.6600000	2.6800000

Laboratory tests at the time of obtaining Table 2-6 consent, according to the presence or absence of proton-pump inhibitors

Unique Subject Identifier=0116000000-0001

MEANS Procedure

Assay variable : PTINR_VAL1 PT-INR Value

N	Mean	Standard Deviation	Minimum	Maximum
3	2.3666667	0.5357549	1.9900000	2.9800000

Unique Subject Identifier=0120000000-0002

Assay variable : PTINR_VAL1 PT-INR Value

N	Mean	Standard Deviation	Minimum	Maximum
1	1.4800000		1.4800000	1.4800000

Unique Subject Identifier=0120000000-0007

Assay variable : PTINR_VAL1 PT-INR Value

N	Mean	Standard Deviation	Minimum	Maximum
1	1.7100000		1.7100000	1.7100000

Unique Subject Identifier=0121010000-0001

Assay variable : PTINR_VAL1 PT-INR Value

N	Mean	Standard Deviation	Minimum	Maximum
4	2.1250000	0.2753785	1.8000000	2.4000000

Laboratory tests at the time of obtaining Table 2-6 consent, according to the presence or absence of proton-pump inhibitors

Unique Subject Identifier=0121010000-0002

MEANS Procedure

Assay variable : PTINR_VAL1 PT-INR Value

N	Mean	Standard Deviation	Minimum	Maximum
4	1.6250000	0.0500000	1.6000000	1.7000000

Unique Subject Identifier=0121010000-0006

Assay variable : PTINR_VAL1 PT-INR Value

N	Mean	Standard Deviation	Minimum	Maximum
6	2.2166667	0.2562551	1.9000000	2.6000000

Unique Subject Identifier=0121010000-0016

Assay variable : PTINR_VAL1 PT-INR Value

N	Mean	Standard Deviation	Minimum	Maximum
5	1.6800000	0.2949576	1.3000000	2.1000000

Unique Subject Identifier=0121010000-0018

Assay variable : PTINR_VAL1 PT-INR Value

N	Mean	Standard Deviation	Minimum	Maximum
5	2.6400000	0.4722288	2.0000000	3.2000000

Laboratory tests at the time of obtaining Table 2-6 consent, according to the presence or absence of proton-pump inhibitors

Unique Subject Identifier=0121010000-0020

MEANS Procedure

Assay variable : PTINR_VAL1 PT-INR Value

N	Mean	Standard Deviation	Minimum	Maximum
4	2.2750000	0.0957427	2.2000000	2.4000000

Unique Subject Identifier=0121010000-0021

Assay variable : PTINR_VAL1 PT-INR Value

N	Mean	Standard Deviation	Minimum	Maximum
4	1.6500000	0.2645751	1.3000000	1.9000000

Unique Subject Identifier=0121010000-0028

Assay variable : PTINR_VAL1 PT-INR Value

N	Mean	Standard Deviation	Minimum	Maximum
6	2.3500000	0.6833740	1.9000000	3.7000000

Unique Subject Identifier=0122010000-0001

Assay variable : PTINR_VAL1 PT-INR Value

N	Mean	Standard Deviation	Minimum	Maximum
4	1.6000000	0.3741657	1.1000000	2.0000000

Laboratory tests at the time of obtaining Table 2-6 consent, according to the presence or absence of proton-pump inhibitors

Unique Subject Identifier=0122010000-0002

MEANS Procedure

Assay variable : PTINR_VAL1 PT-INR Value

N	Mean	Standard Deviation	Minimum	Maximum
6	1.8666667	0.5819507	1.1000000	2.9000000

Unique Subject Identifier=0124010000-0010

Assay variable : PTINR_VAL1 PT-INR Value

N	Mean	Standard Deviation	Minimum	Maximum
2	2.7000000	0.4242641	2.4000000	3.0000000

Unique Subject Identifier=0124010000-0012

Assay variable : PTINR_VAL1 PT-INR Value

N	Mean	Standard Deviation	Minimum	Maximum
3	1.6666667	0.0577350	1.6000000	1.7000000

Unique Subject Identifier=0124010000-0021

Assay variable : PTINR_VAL1 PT-INR Value

N	Mean	Standard Deviation	Minimum	Maximum
2	2.1000000	0.1414214	2.0000000	2.2000000

Laboratory tests at the time of obtaining Table 2-6 consent, according to the presence or absence of proton-pump inhibitors

Unique Subject Identifier=0132000000-0005

MEANS Procedure

Assay variable : PTINR_VAL1 PT-INR Value

N	Mean	Standard Deviation	Minimum	Maximum
3	1.5900000	0.3132092	1.2300000	1.8000000

Unique Subject Identifier=0133000000-0003

Assay variable : PTINR_VAL1 PT-INR Value

N	Mean	Standard Deviation	Minimum	Maximum
4	1.4125000	0.3732180	1.1300000	1.9600000

Unique Subject Identifier=0133000000-0020

Assay variable : PTINR_VAL1 PT-INR Value

N	Mean	Standard Deviation	Minimum	Maximum
2	1.9950000	0.1202082	1.9100000	2.0800000

Unique Subject Identifier=0133000000-0037

Assay variable : PTINR_VAL1 PT-INR Value

N	Mean	Standard Deviation	Minimum	Maximum
2	1.9100000	0.0141421	1.9000000	1.9200000

Laboratory tests at the time of obtaining Table 2-6 consent, according to the presence or absence of proton-pump inhibitors

Unique Subject Identifier=0133000000-0038

MEANS Procedure

Assay variable : PTINR_VAL1 PT-INR Value

N	Mean	Standard Deviation	Minimum	Maximum
2	1.9850000	0.0212132	1.9700000	2.0000000

Unique Subject Identifier=0134100000-0001

Assay variable : PTINR_VAL1 PT-INR Value

N	Mean	Standard Deviation	Minimum	Maximum
2	1.8950000	0.0353553	1.8700000	1.9200000

Unique Subject Identifier=0134100000-0002

Assay variable : PTINR_VAL1 PT-INR Value

N	Mean	Standard Deviation	Minimum	Maximum
3	2.0033333	0.5802011	1.5600000	2.6600000

Unique Subject Identifier=0134100000-0009

Assay variable : PTINR_VAL1 PT-INR Value

N	Mean	Standard Deviation	Minimum	Maximum
3	1.6433333	0.1159023	1.5100000	1.7200000

Laboratory tests at the time of obtaining Table 2-6 consent, according to the presence or absence of proton-pump inhibitors

Unique Subject Identifier=0134100000-0010

MEANS Procedure

Assay variable : PTINR_VAL1 PT-INR Value

N	Mean	Standard Deviation	Minimum	Maximum
3	2.5700000	0.2007486	2.3800000	2.7800000

Unique Subject Identifier=0134100000-0012

Assay variable : PTINR_VAL1 PT-INR Value

N	Mean	Standard Deviation	Minimum	Maximum
4	1.6850000	0.2500000	1.5000000	2.0400000

Unique Subject Identifier=0134100000-0014

Assay variable : PTINR_VAL1 PT-INR Value

N	Mean	Standard Deviation	Minimum	Maximum
6	2.4033333	1.2899716	1.0400000	4.2300000

Unique Subject Identifier=0134100000-0016

Assay variable : PTINR_VAL1 PT-INR Value

N	Mean	Standard Deviation	Minimum	Maximum
4	1.9800000	0.7144228	1.4800000	3.0400000

Laboratory tests at the time of obtaining Table 2-6 consent, according to the presence or absence of proton-pump inhibitors

Unique Subject Identifier=0134100000-0019

MEANS Procedure

Assay variable : PTINR_VAL1 PT-INR Value

N	Mean	Standard Deviation	Minimum	Maximum
3	1.8800000	0.3500000	1.5300000	2.2300000

Unique Subject Identifier=0134100000-0021

Assay variable : PTINR_VAL1 PT-INR Value

N	Mean	Standard Deviation	Minimum	Maximum
4	1.8950000	0.2353012	1.6400000	2.1000000

Unique Subject Identifier=0134100000-0023

Assay variable : PTINR_VAL1 PT-INR Value

N	Mean	Standard Deviation	Minimum	Maximum
4	2.0275000	0.2627261	1.7000000	2.2900000

Unique Subject Identifier=0135000000-0006

Assay variable : PTINR_VAL1 PT-INR Value

N	Mean	Standard Deviation	Minimum	Maximum
3	1.9733333	0.7523519	1.4200000	2.8300000

Laboratory tests at the time of obtaining Table 2-6 consent, according to the presence or absence of proton-pump inhibitors

Unique Subject Identifier=0135000000-0008

MEANS Procedure

Assay variable : PTINR_VAL1 PT-INR Value

N	Mean	Standard Deviation	Minimum	Maximum
4	1.7050000	0.3637307	1.2800000	2.1500000

Unique Subject Identifier=0135000000-0009

Assay variable : PTINR_VAL1 PT-INR Value

N	Mean	Standard Deviation	Minimum	Maximum
5	1.7160000	0.3756062	1.2000000	2.0600000

Unique Subject Identifier=0135000000-0013

Assay variable : PTINR_VAL1 PT-INR Value

N	Mean	Standard Deviation	Minimum	Maximum
3	1.5833333	0.0873689	1.5100000	1.6800000

Unique Subject Identifier=0135000000-0024

Assay variable : PTINR_VAL1 PT-INR Value

N	Mean	Standard Deviation	Minimum	Maximum
4	2.3400000	0.2906315	2.1100000	2.7600000

Laboratory tests at the time of obtaining Table 2-6 consent, according to the presence or absence of proton-pump inhibitors

Unique Subject Identifier=0136000010-0002

MEANS Procedure

Assay variable : PTINR_VAL1 PT-INR Value

N	Mean	Standard Deviation	Minimum	Maximum
6	2.0766667	0.2477633	1.7400000	2.5000000

Unique Subject Identifier=0138000010-0009

Assay variable : PTINR_VAL1 PT-INR Value

N	Mean	Standard Deviation	Minimum	Maximum
4	1.9200000	0.5023279	1.4800000	2.6000000

Unique Subject Identifier=0138000010-0024

Assay variable : PTINR_VAL1 PT-INR Value

N	Mean	Standard Deviation	Minimum	Maximum
6	1.7050000	0.2649340	1.3000000	2.0100000

Unique Subject Identifier=0138000010-0027

Assay variable : PTINR_VAL1 PT-INR Value

N	Mean	Standard Deviation	Minimum	Maximum
2	2.2700000	0.0424264	2.2400000	2.3000000

Laboratory tests at the time of obtaining Table 2-6 consent, according to the presence or absence of proton-pump inhibitors

Unique Subject Identifier=0138000010-0029

MEANS Procedure

Assay variable : PTINR_VAL1 PT-INR Value

N	Mean	Standard Deviation	Minimum	Maximum
2	1.7550000	0.0353553	1.7300000	1.7800000

Unique Subject Identifier=0138000010-0039

Assay variable : PTINR_VAL1 PT-INR Value

N	Mean	Standard Deviation	Minimum	Maximum
6	2.1250000	0.7332598	1.3000000	3.2100000

Unique Subject Identifier=0138000010-0042

Assay variable : PTINR_VAL1 PT-INR Value

N	Mean	Standard Deviation	Minimum	Maximum
4	1.7075000	0.3138338	1.3800000	2.1200000

Unique Subject Identifier=0138000010-0043

Assay variable : PTINR_VAL1 PT-INR Value

N	Mean	Standard Deviation	Minimum	Maximum
5	1.8960000	0.3864324	1.3100000	2.2800000

Laboratory tests at the time of obtaining Table 2-6 consent, according to the presence or absence of proton-pump inhibitors

Unique Subject Identifier=0138000010-0050

MEANS Procedure

Assay variable : PTINR_VAL1 PT-INR Value

N	Mean	Standard Deviation	Minimum	Maximum
1	1.0700000		1.0700000	1.0700000

Unique Subject Identifier=0139000010-0001

Assay variable : PTINR_VAL1 PT-INR Value

N	Mean	Standard Deviation	Minimum	Maximum
5	1.5100000	0.2919760	1.1000000	1.8800000

Unique Subject Identifier=0139000010-0003

Assay variable : PTINR_VAL1 PT-INR Value

N	Mean	Standard Deviation	Minimum	Maximum
6	1.4283333	0.2696232	1.1800000	1.9300000

Unique Subject Identifier=0139000010-0008

Assay variable : PTINR_VAL1 PT-INR Value

N	Mean	Standard Deviation	Minimum	Maximum
6	2.1450000	0.5838750	1.4600000	2.9500000

Laboratory tests at the time of obtaining Table 2-6 consent, according to the presence or absence of proton-pump inhibitors

Unique Subject Identifier=0139000010-0009

MEANS Procedure

Assay variable : PTINR_VAL1 PT-INR Value

N	Mean	Standard Deviation	Minimum	Maximum
6	1.5516667	0.3399068	1.2000000	2.1900000

Unique Subject Identifier=0139000010-0010

Assay variable : PTINR_VAL1 PT-INR Value

N	Mean	Standard Deviation	Minimum	Maximum
6	2.3816667	0.2837898	2.0400000	2.7900000

Unique Subject Identifier=0140000010-0003

Assay variable : PTINR_VAL1 PT-INR Value

N	Mean	Standard Deviation	Minimum	Maximum
4	2.2525000	0.9731863	1.5100000	3.6400000

Unique Subject Identifier=0140000010-0011

Assay variable : PTINR_VAL1 PT-INR Value

N	Mean	Standard Deviation	Minimum	Maximum
4	2.1425000	0.2451360	1.9700000	2.4900000

Laboratory tests at the time of obtaining Table 2-6 consent, according to the presence or absence of proton-pump inhibitors

Unique Subject Identifier=0140000010-0018

MEANS Procedure

Assay variable : PTINR_VAL1 PT-INR Value

N	Mean	Standard Deviation	Minimum	Maximum
3	2.1766667	0.1078579	2.1000000	2.3000000

Unique Subject Identifier=0140000010-0024

Assay variable : PTINR_VAL1 PT-INR Value

N	Mean	Standard Deviation	Minimum	Maximum
3	2.3200000	0.9263369	1.5100000	3.3300000

Unique Subject Identifier=0140000010-0026

Assay variable : PTINR_VAL1 PT-INR Value

N	Mean	Standard Deviation	Minimum	Maximum
4	2.6675000	0.5993538	2.0600000	3.4300000

Unique Subject Identifier=0141000000-0002

Assay variable : PTINR_VAL1 PT-INR Value

N	Mean	Standard Deviation	Minimum	Maximum
4	1.9500000	0.1732051	1.7200000	2.1400000

Laboratory tests at the time of obtaining Table 2-6 consent, according to the presence or absence of proton-pump inhibitors

Unique Subject Identifier=0142000000-0001

MEANS Procedure

Assay variable : PTINR_VAL1 PT-INR Value

N	Mean	Standard Deviation	Minimum	Maximum
6	2.0316667	0.2256029	1.7500000	2.3700000

Unique Subject Identifier=0143010000-0002

Assay variable : PTINR_VAL1 PT-INR Value

N	Mean	Standard Deviation	Minimum	Maximum
5	2.1980000	0.6462739	1.5900000	3.1700000

Unique Subject Identifier=0143010000-0004

Assay variable : PTINR_VAL1 PT-INR Value

N	Mean	Standard Deviation	Minimum	Maximum
3	1.1233333	0.0602771	1.0600000	1.1800000

Unique Subject Identifier=0143010000-0015

Assay variable : PTINR_VAL1 PT-INR Value

N	Mean	Standard Deviation	Minimum	Maximum
2	1.6050000	0.0353553	1.5800000	1.6300000

Laboratory tests at the time of obtaining Table 2-6 consent, according to the presence or absence of proton-pump inhibitors

Unique Subject Identifier=0143010000-0018

MEANS Procedure

Assay variable : PTINR_VAL1 PT-INR Value

N	Mean	Standard Deviation	Minimum	Maximum
6	2.2983333	0.1756607	2.1500000	2.5500000

Unique Subject Identifier=0143010000-0030

Assay variable : PTINR_VAL1 PT-INR Value

N	Mean	Standard Deviation	Minimum	Maximum
6	1.8116667	0.0953764	1.7000000	1.9600000

Unique Subject Identifier=0143010000-0032

Assay variable : PTINR_VAL1 PT-INR Value

N	Mean	Standard Deviation	Minimum	Maximum
3	1.9900000	0.1700000	1.8200000	2.1600000

Unique Subject Identifier=0143010000-0034

Assay variable : PTINR_VAL1 PT-INR Value

N	Mean	Standard Deviation	Minimum	Maximum
6	1.9183333	0.2634704	1.6700000	2.4300000

Laboratory tests at the time of obtaining Table 2-6 consent, according to the presence or absence of proton-pump inhibitors

Unique Subject Identifier=0143010000-0038

MEANS Procedure

Assay variable : PTINR_VAL1 PT-INR Value

N	Mean	Standard Deviation	Minimum	Maximum
6	2.1483333	0.2359167	1.8900000	2.5300000

Unique Subject Identifier=0143010000-0039

Assay variable : PTINR_VAL1 PT-INR Value

N	Mean	Standard Deviation	Minimum	Maximum
6	1.9333333	0.5831524	1.2100000	2.6100000

Unique Subject Identifier=0143010000-0069

Assay variable : PTINR_VAL1 PT-INR Value

N	Mean	Standard Deviation	Minimum	Maximum
6	2.2150000	0.4219360	1.5000000	2.6700000

Unique Subject Identifier=0143010000-0070

Assay variable : PTINR_VAL1 PT-INR Value

N	Mean	Standard Deviation	Minimum	Maximum
6	1.5666667	0.2264214	1.3200000	1.8800000

Laboratory tests at the time of obtaining Table 2-6 consent, according to the presence or absence of proton-pump inhibitors

Unique Subject Identifier=0143010000-0084

MEANS Procedure

Assay variable : PTINR_VAL1 PT-INR Value

N	Mean	Standard Deviation	Minimum	Maximum
3	1.6266667	0.2218859	1.3900000	1.8300000

Unique Subject Identifier=0143010000-0095

Assay variable : PTINR_VAL1 PT-INR Value

N	Mean	Standard Deviation	Minimum	Maximum
6	1.6300000	0.0651153	1.5700000	1.7200000

Unique Subject Identifier=0143010000-0106

Assay variable : PTINR_VAL1 PT-INR Value

N	Mean	Standard Deviation	Minimum	Maximum
6	2.1100000	0.3531572	1.5500000	2.5600000

Unique Subject Identifier=0143010000-0128

Assay variable : PTINR_VAL1 PT-INR Value

N	Mean	Standard Deviation	Minimum	Maximum
4	1.8725000	0.2088660	1.6900000	2.1700000

Laboratory tests at the time of obtaining Table 2-6 consent, according to the presence or absence of proton-pump inhibitors

Unique Subject Identifier=0143010000-0144

MEANS Procedure

Assay variable : PTINR_VAL1 PT-INR Value

N	Mean	Standard Deviation	Minimum	Maximum
3	2.1100000	0.0721110	2.0300000	2.1700000

Unique Subject Identifier=0143010000-0158

Assay variable : PTINR_VAL1 PT-INR Value

N	Mean	Standard Deviation	Minimum	Maximum
3	2.9500000	0.3026549	2.6100000	3.1900000

Unique Subject Identifier=0143010000-0166

Assay variable : PTINR_VAL1 PT-INR Value

N	Mean	Standard Deviation	Minimum	Maximum
6	1.9800000	0.2815670	1.5700000	2.3400000

Unique Subject Identifier=0143010000-0167

Assay variable : PTINR_VAL1 PT-INR Value

N	Mean	Standard Deviation	Minimum	Maximum
6	1.8383333	0.5036434	1.3600000	2.7100000

Laboratory tests at the time of obtaining Table 2-6 consent, according to the presence or absence of proton-pump inhibitors

Unique Subject Identifier=0143010000-0197

MEANS Procedure

Assay variable : PTINR_VAL1 PT-INR Value

N	Mean	Standard Deviation	Minimum	Maximum
3	2.2800000	0.3119295	1.9900000	2.6100000

Unique Subject Identifier=0143010000-0203

Assay variable : PTINR_VAL1 PT-INR Value

N	Mean	Standard Deviation	Minimum	Maximum
1	1.9600000		1.9600000	1.9600000

Unique Subject Identifier=0144000000-0008

Assay variable : PTINR_VAL1 PT-INR Value

N	Mean	Standard Deviation	Minimum	Maximum
1	1.4700000		1.4700000	1.4700000

Unique Subject Identifier=0144000000-0009

Assay variable : PTINR_VAL1 PT-INR Value

N	Mean	Standard Deviation	Minimum	Maximum
1	1.7900000		1.7900000	1.7900000

Laboratory tests at the time of obtaining Table 2-6 consent, according to the presence or absence of proton-pump inhibitors

Unique Subject Identifier=0145000000-0010

MEANS Procedure

Assay variable : PTINR_VAL1 PT-INR Value

N	Mean	Standard Deviation	Minimum	Maximum
3	2.2833333	0.6672581	1.5300000	2.8000000

Unique Subject Identifier=0145000000-0016

Assay variable : PTINR_VAL1 PT-INR Value

N	Mean	Standard Deviation	Minimum	Maximum
6	2.7600000	0.4118738	2.1800000	3.3000000

Unique Subject Identifier=0145000000-0022

Assay variable : PTINR_VAL1 PT-INR Value

N	Mean	Standard Deviation	Minimum	Maximum
6	1.5066667	0.2136040	1.2900000	1.7600000

Unique Subject Identifier=0146000000-0008

Assay variable : PTINR_VAL1 PT-INR Value

N	Mean	Standard Deviation	Minimum	Maximum
3	3.1033333	0.5450076	2.5000000	3.5600000

Laboratory tests at the time of obtaining Table 2-6 consent, according to the presence or absence of proton-pump inhibitors

Unique Subject Identifier=0146000000-0016

MEANS Procedure

Assay variable : PTINR_VAL1 PT-INR Value

N	Mean	Standard Deviation	Minimum	Maximum
4	2.1400000	0.5618422	1.4600000	2.8300000

Unique Subject Identifier=0147000000-0002

Assay variable : PTINR_VAL1 PT-INR Value

N	Mean	Standard Deviation	Minimum	Maximum
3	1.8766667	0.0642910	1.8300000	1.9500000

Unique Subject Identifier=0147000000-0003

Assay variable : PTINR_VAL1 PT-INR Value

N	Mean	Standard Deviation	Minimum	Maximum
4	2.1000000	0.3108054	1.7900000	2.5200000

Unique Subject Identifier=0147000000-0005

Assay variable : PTINR_VAL1 PT-INR Value

N	Mean	Standard Deviation	Minimum	Maximum
3	2.2733333	0.1803700	2.1000000	2.4600000

Laboratory tests at the time of obtaining Table 2-6 consent, according to the presence or absence of proton-pump inhibitors

Unique Subject Identifier=0147000000-0016

MEANS Procedure

Assay variable : PTINR_VAL1 PT-INR Value

N	Mean	Standard Deviation	Minimum	Maximum
3	1.7900000	0.4853864	1.2300000	2.0900000

Unique Subject Identifier=0154010000-0018

Assay variable : PTINR_VAL1 PT-INR Value

N	Mean	Standard Deviation	Minimum	Maximum
5	1.7200000	0.3033150	1.4000000	2.2000000

Unique Subject Identifier=0155010000-0002

Assay variable : PTINR_VAL1 PT-INR Value

N	Mean	Standard Deviation	Minimum	Maximum
3	2.5333333	0.3511885	2.2000000	2.9000000

Unique Subject Identifier=0161000000-0007

Assay variable : PTINR_VAL1 PT-INR Value

N	Mean	Standard Deviation	Minimum	Maximum
5	2.3540000	0.2361779	2.1000000	2.7000000

Laboratory tests at the time of obtaining Table 2-6 consent, according to the presence or absence of proton-pump inhibitors

Unique Subject Identifier=0161000000-0009

MEANS Procedure

Assay variable : PTINR_VAL1 PT-INR Value

N	Mean	Standard Deviation	Minimum	Maximum
6	2.1766667	0.1732820	2.0000000	2.4800000

Unique Subject Identifier=0161000000-0010

Assay variable : PTINR_VAL1 PT-INR Value

N	Mean	Standard Deviation	Minimum	Maximum
6	2.3066667	0.1711919	2.1300000	2.6000000

Unique Subject Identifier=0161000000-0014

Assay variable : PTINR_VAL1 PT-INR Value

N	Mean	Standard Deviation	Minimum	Maximum
6	2.1716667	0.2199470	1.8400000	2.3700000

Unique Subject Identifier=0162000000-0002

Assay variable : PTINR_VAL1 PT-INR Value

N	Mean	Standard Deviation	Minimum	Maximum
4	1.4850000	0.3445287	1.0400000	1.8800000

Laboratory tests at the time of obtaining Table 2-6 consent, according to the presence or absence of proton-pump inhibitors

Unique Subject Identifier=0165000000-0005

MEANS Procedure

Assay variable : PTINR_VAL1 PT-INR Value

N	Mean	Standard Deviation	Minimum	Maximum
4	1.9300000	0.1540563	1.7700000	2.0900000

Unique Subject Identifier=0165000000-0012

Assay variable : PTINR_VAL1 PT-INR Value

N	Mean	Standard Deviation	Minimum	Maximum
5	2.2620000	0.5316672	1.6800000	3.1300000

Unique Subject Identifier=0165000000-0015

Assay variable : PTINR_VAL1 PT-INR Value

N	Mean	Standard Deviation	Minimum	Maximum
3	2.6666667	0.8590887	1.9800000	3.6300000

Unique Subject Identifier=0166000000-0027

Assay variable : PTINR_VAL1 PT-INR Value

N	Mean	Standard Deviation	Minimum	Maximum
3	2.1666667	0.4495924	1.7800000	2.6600000

Laboratory tests at the time of obtaining Table 2-6 consent, according to the presence or absence of proton-pump inhibitors

Unique Subject Identifier=0166000000-0031

MEANS Procedure

Assay variable : PTINR_VAL1 PT-INR Value

N	Mean	Standard Deviation	Minimum	Maximum
3	2.0333333	0.3536005	1.6300000	2.2900000

Unique Subject Identifier=0166000000-0035

Assay variable : PTINR_VAL1 PT-INR Value

N	Mean	Standard Deviation	Minimum	Maximum
2	1.8450000	0.2474874	1.6700000	2.0200000

Unique Subject Identifier=0166000000-0036

Assay variable : PTINR_VAL1 PT-INR Value

N	Mean	Standard Deviation	Minimum	Maximum
2	1.8350000	0.5020458	1.4800000	2.1900000

Unique Subject Identifier=0166000000-0043

Assay variable : PTINR_VAL1 PT-INR Value

N	Mean	Standard Deviation	Minimum	Maximum
2	2.3600000	0.8485281	1.7600000	2.9600000

Laboratory tests at the time of obtaining Table 2-6 consent, according to the presence or absence of proton-pump inhibitors

Unique Subject Identifier=0166000000-0053

MEANS Procedure

Assay variable : PTINR_VAL1 PT-INR Value

N	Mean	Standard Deviation	Minimum	Maximum
6	2.4000000	0.3407052	2.1400000	3.0100000

Unique Subject Identifier=0166000000-0056

Assay variable : PTINR_VAL1 PT-INR Value

N	Mean	Standard Deviation	Minimum	Maximum
5	1.7760000	0.3803682	1.4300000	2.3900000

Unique Subject Identifier=0166000000-0059

Assay variable : PTINR_VAL1 PT-INR Value

N	Mean	Standard Deviation	Minimum	Maximum
2	1.5550000	0.0212132	1.5400000	1.5700000

Unique Subject Identifier=0166000000-0063

Assay variable : PTINR_VAL1 PT-INR Value

N	Mean	Standard Deviation	Minimum	Maximum
2	2.6200000	0.3959798	2.3400000	2.9000000

Laboratory tests at the time of obtaining Table 2-6 consent, according to the presence or absence of proton-pump inhibitors

Unique Subject Identifier=0168000000-0004

MEANS Procedure

Assay variable : PTINR_VAL1 PT-INR Value

N	Mean	Standard Deviation	Minimum	Maximum
1	1.6600000		1.6600000	1.6600000

Unique Subject Identifier=0170000000-0001

Assay variable : PTINR_VAL1 PT-INR Value

N	Mean	Standard Deviation	Minimum	Maximum
5	2.0320000	0.1811629	1.8300000	2.3100000

Unique Subject Identifier=0170000000-0003

Assay variable : PTINR_VAL1 PT-INR Value

N	Mean	Standard Deviation	Minimum	Maximum
3	1.7100000	0.0435890	1.6800000	1.7600000

Unique Subject Identifier=0170000000-0004

Assay variable : PTINR_VAL1 PT-INR Value

N	Mean	Standard Deviation	Minimum	Maximum
6	1.9316667	0.1543265	1.6500000	2.1000000

Laboratory tests at the time of obtaining Table 2-6 consent, according to the presence or absence of proton-pump inhibitors

Unique Subject Identifier=0170000000-0005

MEANS Procedure

Assay variable : PTINR_VAL1 PT-INR Value

N	Mean	Standard Deviation	Minimum	Maximum
5	1.6840000	0.2586117	1.2900000	1.9700000

Unique Subject Identifier=0170000000-0007

Assay variable : PTINR_VAL1 PT-INR Value

N	Mean	Standard Deviation	Minimum	Maximum
6	2.0150000	0.7322773	1.3900000	3.4300000

Unique Subject Identifier=0170000000-0011

Assay variable : PTINR_VAL1 PT-INR Value

N	Mean	Standard Deviation	Minimum	Maximum
6	1.8316667	0.2192183	1.5300000	2.1700000

Unique Subject Identifier=0170000000-0017

Assay variable : PTINR_VAL1 PT-INR Value

N	Mean	Standard Deviation	Minimum	Maximum
2	1.5650000	0.1626346	1.4500000	1.6800000

Laboratory tests at the time of obtaining Table 2-6 consent, according to the presence or absence of proton-pump inhibitors

Unique Subject Identifier=0170000000-0018

MEANS Procedure

Assay variable : PTINR_VAL1 PT-INR Value

N	Mean	Standard Deviation	Minimum	Maximum
3	1.5433333	0.2145538	1.4000000	1.7900000

Unique Subject Identifier=0170000000-0020

Assay variable : PTINR_VAL1 PT-INR Value

N	Mean	Standard Deviation	Minimum	Maximum
3	1.7366667	0.1457166	1.5700000	1.8400000

Unique Subject Identifier=0177100000-0004

Assay variable : PTINR_VAL1 PT-INR Value

N	Mean	Standard Deviation	Minimum	Maximum
4	1.6575000	0.3102015	1.2300000	1.9500000

Unique Subject Identifier=0177100000-0005

Assay variable : PTINR_VAL1 PT-INR Value

N	Mean	Standard Deviation	Minimum	Maximum
3	2.6233333	1.0400641	1.8700000	3.8100000

Laboratory tests at the time of obtaining Table 2-6 consent, according to the presence or absence of proton-pump inhibitors

Unique Subject Identifier=0177100000-0006

MEANS Procedure

Assay variable : PTINR_VAL1 PT-INR Value

N	Mean	Standard Deviation	Minimum	Maximum
2	2.0950000	0.0636396	2.0500000	2.1400000

Unique Subject Identifier=0177100000-0008

Assay variable : PTINR_VAL1 PT-INR Value

N	Mean	Standard Deviation	Minimum	Maximum
5	1.5720000	0.5142665	1.1600000	2.4600000

Unique Subject Identifier=0177100000-0021

Assay variable : PTINR_VAL1 PT-INR Value

N	Mean	Standard Deviation	Minimum	Maximum
6	1.7683333	0.1376106	1.5300000	1.8700000

Unique Subject Identifier=0177100000-0046

Assay variable : PTINR_VAL1 PT-INR Value

N	Mean	Standard Deviation	Minimum	Maximum
5	1.7960000	0.2356480	1.5000000	2.1600000

Laboratory tests at the time of obtaining Table 2-6 consent, according to the presence or absence of proton-pump inhibitors

Unique Subject Identifier=0177100000-0047

MEANS Procedure

Assay variable : PTINR_VAL1 PT-INR Value

N	Mean	Standard Deviation	Minimum	Maximum
4	2.0475000	0.7650000	1.2600000	3.0900000

Unique Subject Identifier=0177100000-0049

Assay variable : PTINR_VAL1 PT-INR Value

N	Mean	Standard Deviation	Minimum	Maximum
6	2.1883333	1.2622427	1.2800000	4.5700000

Unique Subject Identifier=0177100000-0059

Assay variable : PTINR_VAL1 PT-INR Value

N	Mean	Standard Deviation	Minimum	Maximum
4	2.1500000	0.5255473	1.6200000	2.7000000

Unique Subject Identifier=0178100000-0002

Assay variable : PTINR_VAL1 PT-INR Value

N	Mean	Standard Deviation	Minimum	Maximum
2	2.4250000	0.1767767	2.3000000	2.5500000

Laboratory tests at the time of obtaining Table 2-6 consent, according to the presence or absence of proton-pump inhibitors

Unique Subject Identifier=0178100000-0005

MEANS Procedure

Assay variable : PTINR_VAL1 PT-INR Value

N	Mean	Standard Deviation	Minimum	Maximum
4	1.8325000	0.1774589	1.6000000	1.9900000

Unique Subject Identifier=0178100000-0016

Assay variable : PTINR_VAL1 PT-INR Value

N	Mean	Standard Deviation	Minimum	Maximum
4	2.1400000	0.3703152	1.6800000	2.5700000

Unique Subject Identifier=0178100000-0018

Assay variable : PTINR_VAL1 PT-INR Value

N	Mean	Standard Deviation	Minimum	Maximum
5	1.9420000	0.3721156	1.2900000	2.2100000

Unique Subject Identifier=0178100000-0020

Assay variable : PTINR_VAL1 PT-INR Value

N	Mean	Standard Deviation	Minimum	Maximum
3	1.9466667	0.3900427	1.5000000	2.2200000

Laboratory tests at the time of obtaining Table 2-6 consent, according to the presence or absence of proton-pump inhibitors

Unique Subject Identifier=0178100000-0022

MEANS Procedure

Assay variable : PTINR_VAL1 PT-INR Value

N	Mean	Standard Deviation	Minimum	Maximum
4	2.1325000	0.2404683	1.9200000	2.4600000

Unique Subject Identifier=0178100000-0024

Assay variable : PTINR_VAL1 PT-INR Value

N	Mean	Standard Deviation	Minimum	Maximum
5	1.6500000	0.2331309	1.2500000	1.8600000

Unique Subject Identifier=0178100000-0025

Assay variable : PTINR_VAL1 PT-INR Value

N	Mean	Standard Deviation	Minimum	Maximum
4	2.5775000	0.5395291	2.0200000	3.1800000

Unique Subject Identifier=0178100000-0026

Assay variable : PTINR_VAL1 PT-INR Value

N	Mean	Standard Deviation	Minimum	Maximum
4	2.2275000	0.2595348	2.0500000	2.6000000

Laboratory tests at the time of obtaining Table 2-6 consent, according to the presence or absence of proton-pump inhibitors

Unique Subject Identifier=0178100000-0028

MEANS Procedure

Assay variable : PTINR_VAL1 PT-INR Value

N	Mean	Standard Deviation	Minimum	Maximum
4	2.4700000	0.6106281	1.9900000	3.3500000

Unique Subject Identifier=0178100000-0032

Assay variable : PTINR_VAL1 PT-INR Value

N	Mean	Standard Deviation	Minimum	Maximum
4	2.3750000	0.1332917	2.2300000	2.5200000

Unique Subject Identifier=0178100000-0034

Assay variable : PTINR_VAL1 PT-INR Value

N	Mean	Standard Deviation	Minimum	Maximum
4	2.1450000	0.3443351	1.8600000	2.5700000

Unique Subject Identifier=0178100000-0035

Assay variable : PTINR_VAL1 PT-INR Value

N	Mean	Standard Deviation	Minimum	Maximum
4	1.8975000	0.1925920	1.6400000	2.0500000

Laboratory tests at the time of obtaining Table 2-6 consent, according to the presence or absence of proton-pump inhibitors

Unique Subject Identifier=0178100000-0036

MEANS Procedure

Assay variable : PTINR_VAL1 PT-INR Value

N	Mean	Standard Deviation	Minimum	Maximum
4	2.1075000	0.1736615	1.9400000	2.3500000

Unique Subject Identifier=0178100000-0037

Assay variable : PTINR_VAL1 PT-INR Value

N	Mean	Standard Deviation	Minimum	Maximum
4	2.4725000	0.1510794	2.2600000	2.5900000

Unique Subject Identifier=0178100000-0052

Assay variable : PTINR_VAL1 PT-INR Value

N	Mean	Standard Deviation	Minimum	Maximum
4	1.8575000	0.1530523	1.7100000	2.0700000

Unique Subject Identifier=0178100000-0055

Assay variable : PTINR_VAL1 PT-INR Value

N	Mean	Standard Deviation	Minimum	Maximum
5	1.5840000	0.3295907	1.1900000	1.8600000

Laboratory tests at the time of obtaining Table 2-6 consent, according to the presence or absence of proton-pump inhibitors

Unique Subject Identifier=0178100000-0057

MEANS Procedure

Assay variable : PTINR_VAL1 PT-INR Value

N	Mean	Standard Deviation	Minimum	Maximum
2	1.8500000	0.1131371	1.7700000	1.9300000

Unique Subject Identifier=0178100000-0059

Assay variable : PTINR_VAL1 PT-INR Value

N	Mean	Standard Deviation	Minimum	Maximum
4	2.3575000	0.3051093	2.0200000	2.7500000

Unique Subject Identifier=0178100000-0063

Assay variable : PTINR_VAL1 PT-INR Value

N	Mean	Standard Deviation	Minimum	Maximum
5	2.3440000	0.5912106	1.7400000	3.3100000

Unique Subject Identifier=0178100000-0066

Assay variable : PTINR_VAL1 PT-INR Value

N	Mean	Standard Deviation	Minimum	Maximum
4	1.4700000	0.0983192	1.3800000	1.6000000

Laboratory tests at the time of obtaining Table 2-6 consent, according to the presence or absence of proton-pump inhibitors

Unique Subject Identifier=0178100000-0069

MEANS Procedure

Assay variable : PTINR_VAL1 PT-INR Value

N	Mean	Standard Deviation	Minimum	Maximum
3	2.0066667	0.1504438	1.9100000	2.1800000

Unique Subject Identifier=0180000000-0006

Assay variable : PTINR_VAL1 PT-INR Value

N	Mean	Standard Deviation	Minimum	Maximum
5	2.1520000	0.3898333	1.9200000	2.8400000

Unique Subject Identifier=0180000000-0007

Assay variable : PTINR_VAL1 PT-INR Value

N	Mean	Standard Deviation	Minimum	Maximum
3	1.8466667	0.7490216	1.3700000	2.7100000

Unique Subject Identifier=0180000000-0011

Assay variable : PTINR_VAL1 PT-INR Value

N	Mean	Standard Deviation	Minimum	Maximum
6	2.2966667	0.5308358	1.5600000	2.9700000

Laboratory tests at the time of obtaining Table 2-6 consent, according to the presence or absence of proton-pump inhibitors

Unique Subject Identifier=0180000000-0015

MEANS Procedure

Assay variable : PTINR_VAL1 PT-INR Value

N	Mean	Standard Deviation	Minimum	Maximum
4	1.9575000	0.2581182	1.6300000	2.2600000

Unique Subject Identifier=0181000000-0002

Assay variable : PTINR_VAL1 PT-INR Value

N	Mean	Standard Deviation	Minimum	Maximum
5	2.0420000	0.6430163	1.2100000	2.9700000

Unique Subject Identifier=0181000000-0003

Assay variable : PTINR_VAL1 PT-INR Value

N	Mean	Standard Deviation	Minimum	Maximum
2	1.8150000	0.2757716	1.6200000	2.0100000

Unique Subject Identifier=0181000000-0005

Assay variable : PTINR_VAL1 PT-INR Value

N	Mean	Standard Deviation	Minimum	Maximum
6	1.9800000	0.3001333	1.6100000	2.3100000

Laboratory tests at the time of obtaining Table 2-6 consent, according to the presence or absence of proton-pump inhibitors

Unique Subject Identifier=0189000000-0012

MEANS Procedure

Assay variable : PTINR_VAL1 PT-INR Value

N	Mean	Standard Deviation	Minimum	Maximum
3	1.8700000	0.1400000	1.7300000	2.0100000

Unique Subject Identifier=0189000000-0013

Assay variable : PTINR_VAL1 PT-INR Value

N	Mean	Standard Deviation	Minimum	Maximum
6	2.1350000	0.3800395	1.7000000	2.7400000

Unique Subject Identifier=0189000000-0026

Assay variable : PTINR_VAL1 PT-INR Value

N	Mean	Standard Deviation	Minimum	Maximum
2	2.0650000	0.2192031	1.9100000	2.2200000

Unique Subject Identifier=0189000000-0035

Assay variable : PTINR_VAL1 PT-INR Value

N	Mean	Standard Deviation	Minimum	Maximum
3	1.7900000	0.3850974	1.4100000	2.1800000

Laboratory tests at the time of obtaining Table 2-6 consent, according to the presence or absence of proton-pump inhibitors

Unique Subject Identifier=0189000000-0043

MEANS Procedure

Assay variable : PTINR_VAL1 PT-INR Value

N	Mean	Standard Deviation	Minimum	Maximum
4	1.6900000	0.1845716	1.5400000	1.9300000

Unique Subject Identifier=0189000000-0046

Assay variable : PTINR_VAL1 PT-INR Value

N	Mean	Standard Deviation	Minimum	Maximum
3	2.6200000	0.4150904	2.2100000	3.0400000

Unique Subject Identifier=0205100000-0001

Assay variable : PTINR_VAL1 PT-INR Value

N	Mean	Standard Deviation	Minimum	Maximum
3	1.8133333	0.1703917	1.7100000	2.0100000

Unique Subject Identifier=0205100000-0002

Assay variable : PTINR_VAL1 PT-INR Value

N	Mean	Standard Deviation	Minimum	Maximum
4	2.2575000	0.2478407	2.0300000	2.5700000

Laboratory tests at the time of obtaining Table 2-6 consent, according to the presence or absence of proton-pump inhibitors

Unique Subject Identifier=0205100000-0006

MEANS Procedure

Assay variable : PTINR_VAL1 PT-INR Value

N	Mean	Standard Deviation	Minimum	Maximum
3	1.8000000	0.3700000	1.4000000	2.1300000

Unique Subject Identifier=0205100000-0013

Assay variable : PTINR_VAL1 PT-INR Value

N	Mean	Standard Deviation	Minimum	Maximum
2	2.6600000	0.0989949	2.5900000	2.7300000

Unique Subject Identifier=0206100000-0004

Assay variable : PTINR_VAL1 PT-INR Value

N	Mean	Standard Deviation	Minimum	Maximum
6	2.1800000	0.3240988	1.8500000	2.5700000

Unique Subject Identifier=0206100000-0005

Assay variable : PTINR_VAL1 PT-INR Value

N	Mean	Standard Deviation	Minimum	Maximum
5	1.7040000	0.3691612	1.3300000	2.2300000

Laboratory tests at the time of obtaining Table 2-6 consent, according to the presence or absence of proton-pump inhibitors

Unique Subject Identifier=0206100000-0006

MEANS Procedure

Assay variable : PTINR_VAL1 PT-INR Value

N	Mean	Standard Deviation	Minimum	Maximum
6	1.8666667	0.3144307	1.3600000	2.2400000

Unique Subject Identifier=0207100000-0005

Assay variable : PTINR_VAL1 PT-INR Value

N	Mean	Standard Deviation	Minimum	Maximum
1	2.0600000		2.0600000	2.0600000

Unique Subject Identifier=0207100000-0011

Assay variable : PTINR_VAL1 PT-INR Value

N	Mean	Standard Deviation	Minimum	Maximum
1	1.9000000		1.9000000	1.9000000

Unique Subject Identifier=0210000000-0006

Assay variable : PTINR_VAL1 PT-INR Value

N	Mean	Standard Deviation	Minimum	Maximum
5	2.5000000	0.3316625	2.2000000	2.9000000

Laboratory tests at the time of obtaining Table 2-6 consent, according to the presence or absence of proton-pump inhibitors

Unique Subject Identifier=0210000000-0011

MEANS Procedure

Assay variable : PTINR_VAL1 PT-INR Value

N	Mean	Standard Deviation	Minimum	Maximum
5	2.0400000	0.2966479	1.7000000	2.4000000

Unique Subject Identifier=0211000000-0001

Assay variable : PTINR_VAL1 PT-INR Value

N	Mean	Standard Deviation	Minimum	Maximum
6	1.7983333	0.0679461	1.7100000	1.8800000

Unique Subject Identifier=0214000000-0003

Assay variable : PTINR_VAL1 PT-INR Value

N	Mean	Standard Deviation	Minimum	Maximum
4	1.9250000	0.1642153	1.7800000	2.1500000

Unique Subject Identifier=0214000000-0011

Assay variable : PTINR_VAL1 PT-INR Value

N	Mean	Standard Deviation	Minimum	Maximum
6	1.6166667	0.2517671	1.2900000	1.9000000

Laboratory tests at the time of obtaining Table 2-6 consent, according to the presence or absence of proton-pump inhibitors

Unique Subject Identifier=0218000000-0005

MEANS Procedure

Assay variable : PTINR_VAL1 PT-INR Value

N	Mean	Standard Deviation	Minimum	Maximum
5	1.7120000	0.0970567	1.6100000	1.8500000

Unique Subject Identifier=0218000000-0006

Assay variable : PTINR_VAL1 PT-INR Value

N	Mean	Standard Deviation	Minimum	Maximum
6	1.9866667	0.2605123	1.5900000	2.3100000

Unique Subject Identifier=0220000000-0001

Assay variable : PTINR_VAL1 PT-INR Value

N	Mean	Standard Deviation	Minimum	Maximum
3	1.4300000	0.2553429	1.2100000	1.7100000

Unique Subject Identifier=0220000000-0003

Assay variable : PTINR_VAL1 PT-INR Value

N	Mean	Standard Deviation	Minimum	Maximum
6	2.2166667	0.3910328	1.5100000	2.6300000

Laboratory tests at the time of obtaining Table 2-6 consent, according to the presence or absence of proton-pump inhibitors

Unique Subject Identifier=0220000000-0011

MEANS Procedure

Assay variable : PTINR_VAL1 PT-INR Value

N	Mean	Standard Deviation	Minimum	Maximum
4	2.2225000	0.2317146	1.9700000	2.5000000

Unique Subject Identifier=0220000000-0014

Assay variable : PTINR_VAL1 PT-INR Value

N	Mean	Standard Deviation	Minimum	Maximum
3	2.1600000	0.0556776	2.1000000	2.2100000

Unique Subject Identifier=0220000000-0018

Assay variable : PTINR_VAL1 PT-INR Value

N	Mean	Standard Deviation	Minimum	Maximum
4	2.2825000	0.3902456	2.0400000	2.8600000

Unique Subject Identifier=0220000000-0022

Assay variable : PTINR_VAL1 PT-INR Value

N	Mean	Standard Deviation	Minimum	Maximum
4	1.8825000	0.1030776	1.7300000	1.9500000

Laboratory tests at the time of obtaining Table 2-6 consent, according to the presence or absence of proton-pump inhibitors

Unique Subject Identifier=0220000000-0024

MEANS Procedure

Assay variable : PTINR_VAL1 PT-INR Value

N	Mean	Standard Deviation	Minimum	Maximum
3	2.1566667	0.2196209	2.0200000	2.4100000

Unique Subject Identifier=0220000000-0026

Assay variable : PTINR_VAL1 PT-INR Value

N	Mean	Standard Deviation	Minimum	Maximum
4	2.6825000	0.3057641	2.3900000	3.1000000

Unique Subject Identifier=0220000000-0028

Assay variable : PTINR_VAL1 PT-INR Value

N	Mean	Standard Deviation	Minimum	Maximum
3	1.9566667	0.2973774	1.6400000	2.2300000

Unique Subject Identifier=0220000000-0029

Assay variable : PTINR_VAL1 PT-INR Value

N	Mean	Standard Deviation	Minimum	Maximum
4	2.4250000	0.4878183	1.8000000	2.9800000

Laboratory tests at the time of obtaining Table 2-6 consent, according to the presence or absence of proton-pump inhibitors

Unique Subject Identifier=0220000000-0030

MEANS Procedure

Assay variable : PTINR_VAL1 PT-INR Value

N	Mean	Standard Deviation	Minimum	Maximum
5	2.3060000	0.2898793	2.0400000	2.7900000

Unique Subject Identifier=0220000000-0034

Assay variable : PTINR_VAL1 PT-INR Value

N	Mean	Standard Deviation	Minimum	Maximum
3	2.1133333	0.3843609	1.7000000	2.4600000

Unique Subject Identifier=0220000000-0035

Assay variable : PTINR_VAL1 PT-INR Value

N	Mean	Standard Deviation	Minimum	Maximum
5	2.1000000	0.6643041	1.4600000	2.9700000

Unique Subject Identifier=0222011000-0011

Assay variable : PTINR_VAL1 PT-INR Value

N	Mean	Standard Deviation	Minimum	Maximum
5	1.9480000	0.2641401	1.5600000	2.2100000

Laboratory tests at the time of obtaining Table 2-6 consent, according to the presence or absence of proton-pump inhibitors

Unique Subject Identifier=0222011000-0012

MEANS Procedure

Assay variable : PTINR_VAL1 PT-INR Value

N	Mean	Standard Deviation	Minimum	Maximum
6	2.0866667	0.3141125	1.5900000	2.4900000

Unique Subject Identifier=0222011000-0014

Assay variable : PTINR_VAL1 PT-INR Value

N	Mean	Standard Deviation	Minimum	Maximum
5	2.2420000	0.3429577	1.8000000	2.6400000

Unique Subject Identifier=0222011000-0015

Assay variable : PTINR_VAL1 PT-INR Value

N	Mean	Standard Deviation	Minimum	Maximum
6	2.5283333	0.7471658	1.5500000	3.7500000

Unique Subject Identifier=0222011000-0016

Assay variable : PTINR_VAL1 PT-INR Value

N	Mean	Standard Deviation	Minimum	Maximum
6	2.2533333	0.3077445	1.9000000	2.8200000

Laboratory tests at the time of obtaining Table 2-6 consent, according to the presence or absence of proton-pump inhibitors

Unique Subject Identifier=0225001000-0001

MEANS Procedure

Assay variable : PTINR_VAL1 PT-INR Value

N	Mean	Standard Deviation	Minimum	Maximum
3	1.8900000	0.0692820	1.8500000	1.9700000

Unique Subject Identifier=0226010000-0001

Assay variable : PTINR_VAL1 PT-INR Value

N	Mean	Standard Deviation	Minimum	Maximum
2	1.3250000	0.1060660	1.2500000	1.4000000

Unique Subject Identifier=0226010000-0014

Assay variable : PTINR_VAL1 PT-INR Value

N	Mean	Standard Deviation	Minimum	Maximum
3	2.1166667	0.6819335	1.5200000	2.8600000

Unique Subject Identifier=0228100000-0016

Assay variable : PTINR_VAL1 PT-INR Value

N	Mean	Standard Deviation	Minimum	Maximum
5	2.2440000	0.6254838	1.5400000	2.8200000

Laboratory tests at the time of obtaining Table 2-6 consent, according to the presence or absence of proton-pump inhibitors

Unique Subject Identifier=0228100000-0027

MEANS Procedure

Assay variable : PTINR_VAL1 PT-INR Value

N	Mean	Standard Deviation	Minimum	Maximum
1	1.9100000		1.9100000	1.9100000

Unique Subject Identifier=0228100000-0030

Assay variable : PTINR_VAL1 PT-INR Value

N	Mean	Standard Deviation	Minimum	Maximum
2	2.0750000	0.4596194	1.7500000	2.4000000

Unique Subject Identifier=0228100000-0042

Assay variable : PTINR_VAL1 PT-INR Value

N	Mean	Standard Deviation	Minimum	Maximum
3	1.8566667	0.1415392	1.7700000	2.0200000

Unique Subject Identifier=0229100000-0002

Assay variable : PTINR_VAL1 PT-INR Value

N	Mean	Standard Deviation	Minimum	Maximum
5	1.6040000	0.2085186	1.4300000	1.9500000

Laboratory tests at the time of obtaining Table 2-6 consent, according to the presence or absence of proton-pump inhibitors

Unique Subject Identifier=0229100000-0010

MEANS Procedure

Assay variable : PTINR_VAL1 PT-INR Value

N	Mean	Standard Deviation	Minimum	Maximum
6	2.0566667	0.2180520	1.6600000	2.3200000

Unique Subject Identifier=0229100000-0016

Assay variable : PTINR_VAL1 PT-INR Value

N	Mean	Standard Deviation	Minimum	Maximum
3	2.2333333	0.1553491	2.0600000	2.3600000

Unique Subject Identifier=0229100000-0021

Assay variable : PTINR_VAL1 PT-INR Value

N	Mean	Standard Deviation	Minimum	Maximum
3	2.0766667	0.2193931	1.8300000	2.2500000

Unique Subject Identifier=0230100000-0003

Assay variable : PTINR_VAL1 PT-INR Value

N	Mean	Standard Deviation	Minimum	Maximum
5	2.2280000	0.1807484	1.9500000	2.4000000

Laboratory tests at the time of obtaining Table 2-6 consent, according to the presence or absence of proton-pump inhibitors

Unique Subject Identifier=0231100000-0011

MEANS Procedure

Assay variable : PTINR_VAL1 PT-INR Value

N	Mean	Standard Deviation	Minimum	Maximum
4	2.4275000	0.2596632	2.1300000	2.7000000

Unique Subject Identifier=0231100000-0016

Assay variable : PTINR_VAL1 PT-INR Value

N	Mean	Standard Deviation	Minimum	Maximum
6	1.5616667	0.7512234	0.9600000	2.9800000

Unique Subject Identifier=0231100000-0018

Assay variable : PTINR_VAL1 PT-INR Value

N	Mean	Standard Deviation	Minimum	Maximum
3	2.4833333	0.5810622	1.8600000	3.0100000

Unique Subject Identifier=0231100000-0031

Assay variable : PTINR_VAL1 PT-INR Value

N	Mean	Standard Deviation	Minimum	Maximum
4	2.6050000	0.2838427	2.3500000	2.9400000

Laboratory tests at the time of obtaining Table 2-6 consent, according to the presence or absence of proton-pump inhibitors

Unique Subject Identifier=0231100000-0036

MEANS Procedure

Assay variable : PTINR_VAL1 PT-INR Value

N	Mean	Standard Deviation	Minimum	Maximum
3	2.3133333	0.3952636	1.9800000	2.7500000

Unique Subject Identifier=1003000000-0001

Assay variable : PTINR_VAL1 PT-INR Value

N	Mean	Standard Deviation	Minimum	Maximum
5	2.1320000	0.3756594	1.6700000	2.6500000

Unique Subject Identifier=1003000000-0005

Assay variable : PTINR_VAL1 PT-INR Value

N	Mean	Standard Deviation	Minimum	Maximum
5	1.9140000	0.3326860	1.6400000	2.3800000

Unique Subject Identifier=1003000000-0015

Assay variable : PTINR_VAL1 PT-INR Value

N	Mean	Standard Deviation	Minimum	Maximum
5	2.0080000	0.1275539	1.9200000	2.2100000

Laboratory tests at the time of obtaining Table 2-6 consent, according to the presence or absence of proton-pump inhibitors

Unique Subject Identifier=1004000000-0003

MEANS Procedure

Assay variable : PTINR_VAL1 PT-INR Value

N	Mean	Standard Deviation	Minimum	Maximum
6	1.7833333	0.5741661	1.2000000	2.6000000

Unique Subject Identifier=1005000000-0003

Assay variable : PTINR_VAL1 PT-INR Value

N	Mean	Standard Deviation	Minimum	Maximum
5	1.7580000	0.0813634	1.6800000	1.8800000

Unique Subject Identifier=1005000000-0007

Assay variable : PTINR_VAL1 PT-INR Value

N	Mean	Standard Deviation	Minimum	Maximum
5	2.0040000	0.1986957	1.6600000	2.1600000

Unique Subject Identifier=1005000000-0015

Assay variable : PTINR_VAL1 PT-INR Value

N	Mean	Standard Deviation	Minimum	Maximum
6	1.8366667	0.1015218	1.6900000	1.9400000

Laboratory tests at the time of obtaining Table 2-6 consent, according to the presence or absence of proton-pump inhibitors

Unique Subject Identifier=1006010010-0003

MEANS Procedure

Assay variable : PTINR_VAL1 PT-INR Value

N	Mean	Standard Deviation	Minimum	Maximum
6	2.0116667	0.2691778	1.7100000	2.4600000

Unique Subject Identifier=1006010010-0005

Assay variable : PTINR_VAL1 PT-INR Value

N	Mean	Standard Deviation	Minimum	Maximum
4	2.2850000	0.1613485	2.1400000	2.4700000

Unique Subject Identifier=1006010010-0013

Assay variable : PTINR_VAL1 PT-INR Value

N	Mean	Standard Deviation	Minimum	Maximum
4	2.3500000	0.1602082	2.2600000	2.5900000

Unique Subject Identifier=1009010010-0003

Assay variable : PTINR_VAL1 PT-INR Value

N	Mean	Standard Deviation	Minimum	Maximum
2	2.0650000	0.1767767	1.9400000	2.1900000

Laboratory tests at the time of obtaining Table 2-6 consent, according to the presence or absence of proton-pump inhibitors

Unique Subject Identifier=1009010010-0012

MEANS Procedure

Assay variable : PTINR_VAL1 PT-INR Value

N	Mean	Standard Deviation	Minimum	Maximum
6	2.2833333	0.3868678	1.9000000	2.9000000

Unique Subject Identifier=1009010010-0025

Assay variable : PTINR_VAL1 PT-INR Value

N	Mean	Standard Deviation	Minimum	Maximum
6	1.8500000	0.2880972	1.5000000	2.3000000

Unique Subject Identifier=1011000000-0004

Assay variable : PTINR_VAL1 PT-INR Value

N	Mean	Standard Deviation	Minimum	Maximum
4	2.1750000	0.3304038	1.7000000	2.4000000

Unique Subject Identifier=1013010110-0010

Assay variable : PTINR_VAL1 PT-INR Value

N	Mean	Standard Deviation	Minimum	Maximum
5	2.7000000	0.7035624	1.9000000	3.7000000

Laboratory tests at the time of obtaining Table 2-6 consent, according to the presence or absence of proton-pump inhibitors

Unique Subject Identifier=1018000000-0001

MEANS Procedure

Assay variable : PTINR_VAL1 PT-INR Value

N	Mean	Standard Deviation	Minimum	Maximum
3	2.2666667	0.1527525	2.1000000	2.4000000

Unique Subject Identifier=1019010100-0001

Assay variable : PTINR_VAL1 PT-INR Value

N	Mean	Standard Deviation	Minimum	Maximum
4	1.4700000	0.1834848	1.2600000	1.6900000

Unique Subject Identifier=1019010100-0005

Assay variable : PTINR_VAL1 PT-INR Value

N	Mean	Standard Deviation	Minimum	Maximum
5	1.4920000	0.2501400	1.1800000	1.8200000

Unique Subject Identifier=1024011100-0009

Assay variable : PTINR_VAL1 PT-INR Value

N	Mean	Standard Deviation	Minimum	Maximum
5	1.9780000	0.2764417	1.5000000	2.2000000

Laboratory tests at the time of obtaining Table 2-6 consent, according to the presence or absence of proton-pump inhibitors

Unique Subject Identifier=1026011000-0006

MEANS Procedure

Assay variable : PTINR_VAL1 PT-INR Value

N	Mean	Standard Deviation	Minimum	Maximum
4	1.9250000	0.3590265	1.5400000	2.2600000

Unique Subject Identifier=1026011000-0024

Assay variable : PTINR_VAL1 PT-INR Value

N	Mean	Standard Deviation	Minimum	Maximum
4	1.3275000	0.0505800	1.2700000	1.3900000

Unique Subject Identifier=1031010000-0037

Assay variable : PTINR_VAL1 PT-INR Value

N	Mean	Standard Deviation	Minimum	Maximum
6	2.5666667	0.1144844	2.4400000	2.7700000

Unique Subject Identifier=1031010000-0127

Assay variable : PTINR_VAL1 PT-INR Value

N	Mean	Standard Deviation	Minimum	Maximum
1	3.2000000		3.2000000	3.2000000

Laboratory tests at the time of obtaining Table 2-6 consent, according to the presence or absence of proton-pump inhibitors

Unique Subject Identifier=1031010000-0134

MEANS Procedure

Assay variable : PTINR_VAL1 PT-INR Value

N	Mean	Standard Deviation	Minimum	Maximum
3	2.4400000	0.3157531	2.0800000	2.6700000

Unique Subject Identifier=1031010000-0155

Assay variable : PTINR_VAL1 PT-INR Value

N	Mean	Standard Deviation	Minimum	Maximum
3	2.5033333	0.4366158	2.0100000	2.8400000

Unique Subject Identifier=1032000000-0002

Assay variable : PTINR_VAL1 PT-INR Value

N	Mean	Standard Deviation	Minimum	Maximum
6	1.6216667	0.1612968	1.3900000	1.8500000

Unique Subject Identifier=1032000000-0003

Assay variable : PTINR_VAL1 PT-INR Value

N	Mean	Standard Deviation	Minimum	Maximum
6	1.9133333	0.2606658	1.6200000	2.3700000

Laboratory tests at the time of obtaining Table 2-6 consent, according to the presence or absence of proton-pump inhibitors

Unique Subject Identifier=1032000000-0012

MEANS Procedure

Assay variable : PTINR_VAL1 PT-INR Value

N	Mean	Standard Deviation	Minimum	Maximum
6	1.5000000	0.1784377	1.3700000	1.8400000

Unique Subject Identifier=1032000000-0019

Assay variable : PTINR_VAL1 PT-INR Value

N	Mean	Standard Deviation	Minimum	Maximum
6	1.6533333	0.1123684	1.4600000	1.7600000

Unique Subject Identifier=1032000000-0020

Assay variable : PTINR_VAL1 PT-INR Value

N	Mean	Standard Deviation	Minimum	Maximum
6	2.0083333	0.3642206	1.4100000	2.5200000

Unique Subject Identifier=1032000000-0022

Assay variable : PTINR_VAL1 PT-INR Value

N	Mean	Standard Deviation	Minimum	Maximum
6	2.4200000	0.3159747	2.1000000	2.9200000

Laboratory tests at the time of obtaining Table 2-6 consent, according to the presence or absence of proton-pump inhibitors

Unique Subject Identifier=1032000000-0023

MEANS Procedure

Assay variable : PTINR_VAL1 PT-INR Value

N	Mean	Standard Deviation	Minimum	Maximum
6	1.5783333	0.3328914	1.1900000	2.0600000

Unique Subject Identifier=1032000000-0026

Assay variable : PTINR_VAL1 PT-INR Value

N	Mean	Standard Deviation	Minimum	Maximum
6	2.1383333	0.3119241	1.6300000	2.4500000

Unique Subject Identifier=1032000000-0029

Assay variable : PTINR_VAL1 PT-INR Value

N	Mean	Standard Deviation	Minimum	Maximum
5	2.3460000	0.5855169	1.4400000	3.0100000

Unique Subject Identifier=1034000000-0002

Assay variable : PTINR_VAL1 PT-INR Value

N	Mean	Standard Deviation	Minimum	Maximum
3	2.0666667	0.3511885	1.7000000	2.4000000

Laboratory tests at the time of obtaining Table 2-6 consent, according to the presence or absence of proton-pump inhibitors

Unique Subject Identifier=1034000000-0006

MEANS Procedure

Assay variable : PTINR_VAL1 PT-INR Value

N	Mean	Standard Deviation	Minimum	Maximum
4	2.5250000	0.4112988	2.0000000	2.9000000

Unique Subject Identifier=1035000000-0017

Assay variable : PTINR_VAL1 PT-INR Value

N	Mean	Standard Deviation	Minimum	Maximum
4	1.8325000	0.3057641	1.5400000	2.2500000

Unique Subject Identifier=1038000000-0002

Assay variable : PTINR_VAL1 PT-INR Value

N	Mean	Standard Deviation	Minimum	Maximum
6	2.7833333	0.3600926	2.3000000	3.3000000

Unique Subject Identifier=1038000000-0005

Assay variable : PTINR_VAL1 PT-INR Value

N	Mean	Standard Deviation	Minimum	Maximum
6	1.8000000	0.2966479	1.3000000	2.1000000

Laboratory tests at the time of obtaining Table 2-6 consent, according to the presence or absence of proton-pump inhibitors

Unique Subject Identifier=1038000000-0006

MEANS Procedure

Assay variable : PTINR_VAL1 PT-INR Value

N	Mean	Standard Deviation	Minimum	Maximum
4	1.8250000	0.1500000	1.7000000	2.0000000

Unique Subject Identifier=1038000000-0007

Assay variable : PTINR_VAL1 PT-INR Value

N	Mean	Standard Deviation	Minimum	Maximum
6	1.9000000	0.4939636	1.4000000	2.8000000

Unique Subject Identifier=1038000000-0008

Assay variable : PTINR_VAL1 PT-INR Value

N	Mean	Standard Deviation	Minimum	Maximum
6	2.1333333	0.5537749	1.5000000	3.0000000

Unique Subject Identifier=1041011110-0003

Assay variable : PTINR_VAL1 PT-INR Value

N	Mean	Standard Deviation	Minimum	Maximum
5	1.7000000	0.1581139	1.5000000	1.9000000

Laboratory tests at the time of obtaining Table 2-6 consent, according to the presence or absence of proton-pump inhibitors

Unique Subject Identifier=1041011110-0004

MEANS Procedure

Assay variable : PTINR_VAL1 PT-INR Value

N	Mean	Standard Deviation	Minimum	Maximum
6	1.9833333	0.1471960	1.8000000	2.2000000

Unique Subject Identifier=1041011110-0005

Assay variable : PTINR_VAL1 PT-INR Value

N	Mean	Standard Deviation	Minimum	Maximum
6	1.9333333	0.2338090	1.7000000	2.3000000

Unique Subject Identifier=1041011110-0011

Assay variable : PTINR_VAL1 PT-INR Value

N	Mean	Standard Deviation	Minimum	Maximum
6	2.2166667	0.3763863	1.8000000	2.9000000

Unique Subject Identifier=1041011110-0024

Assay variable : PTINR_VAL1 PT-INR Value

N	Mean	Standard Deviation	Minimum	Maximum
5	1.8600000	0.2701851	1.5000000	2.2000000

Laboratory tests at the time of obtaining Table 2-6 consent, according to the presence or absence of proton-pump inhibitors

Unique Subject Identifier=1041011110-0026

MEANS Procedure

Assay variable : PTINR_VAL1 PT-INR Value

N	Mean	Standard Deviation	Minimum	Maximum
6	1.8000000	0.2097618	1.6000000	2.1000000

Unique Subject Identifier=1041011110-0029

Assay variable : PTINR_VAL1 PT-INR Value

N	Mean	Standard Deviation	Minimum	Maximum
6	2.2666667	0.5202563	1.7000000	3.1000000

Unique Subject Identifier=1041011110-0034

Assay variable : PTINR_VAL1 PT-INR Value

N	Mean	Standard Deviation	Minimum	Maximum
3	1.6666667	0.3511885	1.3000000	2.0000000

Unique Subject Identifier=1041011110-0035

Assay variable : PTINR_VAL1 PT-INR Value

N	Mean	Standard Deviation	Minimum	Maximum
6	2.2500000	0.1870829	2.1000000	2.6000000

Laboratory tests at the time of obtaining Table 2-6 consent, according to the presence or absence of proton-pump inhibitors

Unique Subject Identifier=1049000000-0001

MEANS Procedure

Assay variable : PTINR_VAL1 PT-INR Value

N	Mean	Standard Deviation	Minimum	Maximum
4	1.6150000	0.0967815	1.5300000	1.7300000

Unique Subject Identifier=1049000000-0003

Assay variable : PTINR_VAL1 PT-INR Value

N	Mean	Standard Deviation	Minimum	Maximum
5	1.9400000	0.1164045	1.8400000	2.1400000

Unique Subject Identifier=1049000000-0004

Assay variable : PTINR_VAL1 PT-INR Value

N	Mean	Standard Deviation	Minimum	Maximum
3	1.9400000	0.4309292	1.6200000	2.4300000

Unique Subject Identifier=1055000000-0015

Assay variable : PTINR_VAL1 PT-INR Value

N	Mean	Standard Deviation	Minimum	Maximum
3	1.9600000	0.2749545	1.7200000	2.2600000

Laboratory tests at the time of obtaining Table 2-6 consent, according to the presence or absence of proton-pump inhibitors

Unique Subject Identifier=1055000000-0021

MEANS Procedure

Assay variable : PTINR_VAL1 PT-INR Value

N	Mean	Standard Deviation	Minimum	Maximum
2	2.5300000	0.1131371	2.4500000	2.6100000

Unique Subject Identifier=1055000000-0030

Assay variable : PTINR_VAL1 PT-INR Value

N	Mean	Standard Deviation	Minimum	Maximum
1	2.1600000		2.1600000	2.1600000

Unique Subject Identifier=1058011111-0002

Assay variable : PTINR_VAL1 PT-INR Value

N	Mean	Standard Deviation	Minimum	Maximum
6	1.4233333	0.1148332	1.3000000	1.6300000

Unique Subject Identifier=1058011111-0004

Assay variable : PTINR_VAL1 PT-INR Value

N	Mean	Standard Deviation	Minimum	Maximum
6	1.8550000	0.2703146	1.5800000	2.1900000

Laboratory tests at the time of obtaining Table 2-6 consent, according to the presence or absence of proton-pump inhibitors

Unique Subject Identifier=1060000000-0007

MEANS Procedure

Assay variable : PTINR_VAL1 PT-INR Value

N	Mean	Standard Deviation	Minimum	Maximum
2	1.5200000	0.3252691	1.2900000	1.7500000

Unique Subject Identifier=1060000000-0013

Assay variable : PTINR_VAL1 PT-INR Value

N	Mean	Standard Deviation	Minimum	Maximum
3	1.7833333	0.3274650	1.5200000	2.1500000

Unique Subject Identifier=1060000000-0027

Assay variable : PTINR_VAL1 PT-INR Value

N	Mean	Standard Deviation	Minimum	Maximum
2	1.7750000	0.1060660	1.7000000	1.8500000

Unique Subject Identifier=1061000000-0001

Assay variable : PTINR_VAL1 PT-INR Value

N	Mean	Standard Deviation	Minimum	Maximum
6	2.0333333	0.2338090	1.7000000	2.4000000

Laboratory tests at the time of obtaining Table 2-6 consent, according to the presence or absence of proton-pump inhibitors

Unique Subject Identifier=1061000000-0022

MEANS Procedure

Assay variable : PTINR_VAL1 PT-INR Value

N	Mean	Standard Deviation	Minimum	Maximum
6	1.9000000	0.1897367	1.7000000	2.2000000

Unique Subject Identifier=1065010011-0005

Assay variable : PTINR_VAL1 PT-INR Value

N	Mean	Standard Deviation	Minimum	Maximum
6	1.7666667	0.3011091	1.4000000	2.1000000

Unique Subject Identifier=1065010011-0006

Assay variable : PTINR_VAL1 PT-INR Value

N	Mean	Standard Deviation	Minimum	Maximum
6	2.0333333	0.3011091	1.6000000	2.4000000

Unique Subject Identifier=1065010011-0007

Assay variable : PTINR_VAL1 PT-INR Value

N	Mean	Standard Deviation	Minimum	Maximum
6	2.3000000	0.3898718	1.8000000	2.8000000

Laboratory tests at the time of obtaining Table 2-6 consent, according to the presence or absence of proton-pump inhibitors

Unique Subject Identifier=1065010011-0014

MEANS Procedure

Assay variable : PTINR_VAL1 PT-INR Value

N	Mean	Standard Deviation	Minimum	Maximum
6	2.4333333	0.2581989	2.1000000	2.8000000

Unique Subject Identifier=1065010011-0017

Assay variable : PTINR_VAL1 PT-INR Value

N	Mean	Standard Deviation	Minimum	Maximum
6	1.9666667	0.7229569	1.1000000	2.9000000

Unique Subject Identifier=1065010011-0019

Assay variable : PTINR_VAL1 PT-INR Value

N	Mean	Standard Deviation	Minimum	Maximum
4	1.6750000	0.0957427	1.6000000	1.8000000

Unique Subject Identifier=1065010011-0026

Assay variable : PTINR_VAL1 PT-INR Value

N	Mean	Standard Deviation	Minimum	Maximum
6	2.2666667	0.6713171	1.7000000	3.5000000

Laboratory tests at the time of obtaining Table 2-6 consent, according to the presence or absence of proton-pump inhibitors

Unique Subject Identifier=1065010011-0031

MEANS Procedure

Assay variable : PTINR_VAL1 PT-INR Value

N	Mean	Standard Deviation	Minimum	Maximum
6	1.9666667	0.4082483	1.6000000	2.7000000

Unique Subject Identifier=1065010011-0032

Assay variable : PTINR_VAL1 PT-INR Value

N	Mean	Standard Deviation	Minimum	Maximum
6	2.1833333	0.5706721	1.4000000	3.1000000

Unique Subject Identifier=1065010011-0037

Assay variable : PTINR_VAL1 PT-INR Value

N	Mean	Standard Deviation	Minimum	Maximum
3	1.6000000	0.3605551	1.2000000	1.9000000

Unique Subject Identifier=1065010011-0041

Assay variable : PTINR_VAL1 PT-INR Value

N	Mean	Standard Deviation	Minimum	Maximum
6	2.6500000	0.7395945	1.6000000	3.6000000

Laboratory tests at the time of obtaining Table 2-6 consent, according to the presence or absence of proton-pump inhibitors

Unique Subject Identifier=1065010011-0044

MEANS Procedure

Assay variable : PTINR_VAL1 PT-INR Value

N	Mean	Standard Deviation	Minimum	Maximum
6	2.0666667	0.7711463	1.3000000	3.4000000

Unique Subject Identifier=1065010011-0048

Assay variable : PTINR_VAL1 PT-INR Value

N	Mean	Standard Deviation	Minimum	Maximum
6	1.7500000	0.2345208	1.6000000	2.1000000

Unique Subject Identifier=1065010011-0054

Assay variable : PTINR_VAL1 PT-INR Value

N	Mean	Standard Deviation	Minimum	Maximum
6	1.9000000	0.4195235	1.5000000	2.6000000

Unique Subject Identifier=1065010011-0058

Assay variable : PTINR_VAL1 PT-INR Value

N	Mean	Standard Deviation	Minimum	Maximum
6	2.2500000	0.1643168	2.1000000	2.5000000

Laboratory tests at the time of obtaining Table 2-6 consent, according to the presence or absence of proton-pump inhibitors

Unique Subject Identifier=1065010011-0059

MEANS Procedure

Assay variable : PTINR_VAL1 PT-INR Value

N	Mean	Standard Deviation	Minimum	Maximum
6	1.8333333	0.4633213	1.4000000	2.6000000

Unique Subject Identifier=1065010011-0067

Assay variable : PTINR_VAL1 PT-INR Value

N	Mean	Standard Deviation	Minimum	Maximum
6	2.4333333	0.8286535	1.5000000	3.5000000

Unique Subject Identifier=1065010011-0072

Assay variable : PTINR_VAL1 PT-INR Value

N	Mean	Standard Deviation	Minimum	Maximum
6	1.6500000	0.2509980	1.2000000	1.9000000

Unique Subject Identifier=1068010111-0003

Assay variable : PTINR_VAL1 PT-INR Value

N	Mean	Standard Deviation	Minimum	Maximum
2	2.2000000	0.5232590	1.8300000	2.5700000

Laboratory tests at the time of obtaining Table 2-6 consent, according to the presence or absence of proton-pump inhibitors

Unique Subject Identifier=1068010111-0008

MEANS Procedure

Assay variable : PTINR_VAL1 PT-INR Value

N	Mean	Standard Deviation	Minimum	Maximum
3	1.8300000	0.3034798	1.6400000	2.1800000

Unique Subject Identifier=1068010111-0009

Assay variable : PTINR_VAL1 PT-INR Value

N	Mean	Standard Deviation	Minimum	Maximum
3	1.8366667	0.0513160	1.7800000	1.8800000

Unique Subject Identifier=1068010111-0013

Assay variable : PTINR_VAL1 PT-INR Value

N	Mean	Standard Deviation	Minimum	Maximum
6	1.3250000	0.1399643	1.2000000	1.5900000

Unique Subject Identifier=1068010111-0017

Assay variable : PTINR_VAL1 PT-INR Value

N	Mean	Standard Deviation	Minimum	Maximum
4	1.7875000	0.2692428	1.5400000	2.1200000

Laboratory tests at the time of obtaining Table 2-6 consent, according to the presence or absence of proton-pump inhibitors

Unique Subject Identifier=1068010111-0035

MEANS Procedure

Assay variable : PTINR_VAL1 PT-INR Value

N	Mean	Standard Deviation	Minimum	Maximum
4	1.9750000	0.3275668	1.6200000	2.4000000

Unique Subject Identifier=1068010111-0060

Assay variable : PTINR_VAL1 PT-INR Value

N	Mean	Standard Deviation	Minimum	Maximum
6	1.4650000	0.4579847	0.9700000	2.1600000

Unique Subject Identifier=1070000000-0002

Assay variable : PTINR_VAL1 PT-INR Value

N	Mean	Standard Deviation	Minimum	Maximum
6	2.0833333	0.4875107	1.6000000	2.9000000

Unique Subject Identifier=1070000000-0003

Assay variable : PTINR_VAL1 PT-INR Value

N	Mean	Standard Deviation	Minimum	Maximum
6	2.1500000	0.5319774	1.6000000	3.1000000

Laboratory tests at the time of obtaining Table 2-6 consent, according to the presence or absence of proton-pump inhibitors

Unique Subject Identifier=1071000000-0003

MEANS Procedure

Assay variable : PTINR_VAL1 PT-INR Value

N	Mean	Standard Deviation	Minimum	Maximum
1	1.0400000		1.0400000	1.0400000

Unique Subject Identifier=1071000000-0006

Assay variable : PTINR_VAL1 PT-INR Value

N	Mean	Standard Deviation	Minimum	Maximum
1	1.8400000		1.8400000	1.8400000

Unique Subject Identifier=1072000000-0026

Assay variable : PTINR_VAL1 PT-INR Value

N	Mean	Standard Deviation	Minimum	Maximum
6	1.7383333	0.1383353	1.6100000	1.8900000

Unique Subject Identifier=1072000000-0029

Assay variable : PTINR_VAL1 PT-INR Value

N	Mean	Standard Deviation	Minimum	Maximum
5	1.6960000	0.2785319	1.4300000	2.1700000

Laboratory tests at the time of obtaining Table 2-6 consent, according to the presence or absence of proton-pump inhibitors

Unique Subject Identifier=1072000000-0033

MEANS Procedure

Assay variable : PTINR_VAL1 PT-INR Value

N	Mean	Standard Deviation	Minimum	Maximum
6	1.8366667	0.1885382	1.5900000	2.1000000

Unique Subject Identifier=1074011010-0005

Assay variable : PTINR_VAL1 PT-INR Value

N	Mean	Standard Deviation	Minimum	Maximum
6	1.9383333	0.4533174	1.4500000	2.5200000

Unique Subject Identifier=1079000000-0003

Assay variable : PTINR_VAL1 PT-INR Value

N	Mean	Standard Deviation	Minimum	Maximum
3	1.8433333	0.2300725	1.6100000	2.0700000

Unique Subject Identifier=1079000000-0004

Assay variable : PTINR_VAL1 PT-INR Value

N	Mean	Standard Deviation	Minimum	Maximum
3	1.4633333	0.1738774	1.3300000	1.6600000

Laboratory tests at the time of obtaining Table 2-6 consent, according to the presence or absence of proton-pump inhibitors

Unique Subject Identifier=1080000000-0002

MEANS Procedure

Assay variable : PTINR_VAL1 PT-INR Value

N	Mean	Standard Deviation	Minimum	Maximum
6	2.3483333	0.4951532	1.5200000	2.9300000

Unique Subject Identifier=1080000000-0003

Assay variable : PTINR_VAL1 PT-INR Value

N	Mean	Standard Deviation	Minimum	Maximum
6	2.1516667	0.2041976	1.8200000	2.4400000

Unique Subject Identifier=1080000000-0005

Assay variable : PTINR_VAL1 PT-INR Value

N	Mean	Standard Deviation	Minimum	Maximum
6	2.1616667	0.4302286	1.6900000	2.8600000

Unique Subject Identifier=1085000000-0001

Assay variable : PTINR_VAL1 PT-INR Value

N	Mean	Standard Deviation	Minimum	Maximum
1	2.4700000		2.4700000	2.4700000

Laboratory tests at the time of obtaining Table 2-6 consent, according to the presence or absence of proton-pump inhibitors

Unique Subject Identifier=1085000000-0004

MEANS Procedure

Assay variable : PTINR_VAL1 PT-INR Value

N	Mean	Standard Deviation	Minimum	Maximum
2	1.5600000	0.3535534	1.3100000	1.8100000

Unique Subject Identifier=1085000000-0007

Assay variable : PTINR_VAL1 PT-INR Value

N	Mean	Standard Deviation	Minimum	Maximum
1	1.6200000		1.6200000	1.6200000

Unique Subject Identifier=1085000000-0009

Assay variable : PTINR_VAL1 PT-INR Value

N	Mean	Standard Deviation	Minimum	Maximum
1	2.4900000		2.4900000	2.4900000

Unique Subject Identifier=1085000000-0012

Assay variable : PTINR_VAL1 PT-INR Value

N	Mean	Standard Deviation	Minimum	Maximum
2	1.8250000	0.1060660	1.7500000	1.9000000

Laboratory tests at the time of obtaining Table 2-6 consent, according to the presence or absence of proton-pump inhibitors

Unique Subject Identifier=1087000000-0003

MEANS Procedure

Assay variable : PTINR_VAL1 PT-INR Value

N	Mean	Standard Deviation	Minimum	Maximum
3	1.9000000	0.1646208	1.7100000	2.0000000

Unique Subject Identifier=1087000000-0006

Assay variable : PTINR_VAL1 PT-INR Value

N	Mean	Standard Deviation	Minimum	Maximum
4	2.3725000	0.2224672	2.0500000	2.5500000

Unique Subject Identifier=1091010000-0008

Assay variable : PTINR_VAL1 PT-INR Value

N	Mean	Standard Deviation	Minimum	Maximum
6	1.6833333	0.2401388	1.3000000	1.9000000

Unique Subject Identifier=1091010000-0024

Assay variable : PTINR_VAL1 PT-INR Value

N	Mean	Standard Deviation	Minimum	Maximum
6	1.6500000	0.2880972	1.4000000	2.1000000

Laboratory tests at the time of obtaining Table 2-6 consent, according to the presence or absence of proton-pump inhibitors

Unique Subject Identifier=1091010000-0046

MEANS Procedure

Assay variable : PTINR_VAL1 PT-INR Value

N	Mean	Standard Deviation	Minimum	Maximum
6	1.7666667	0.3011091	1.3000000	2.0000000

Unique Subject Identifier=1091010000-0063

Assay variable : PTINR_VAL1 PT-INR Value

N	Mean	Standard Deviation	Minimum	Maximum
6	2.9333333	1.1639015	1.9000000	5.0000000

Unique Subject Identifier=1094000000-0004

Assay variable : PTINR_VAL1 PT-INR Value

N	Mean	Standard Deviation	Minimum	Maximum
5	1.8200000	0.1701470	1.5800000	1.9800000

Unique Subject Identifier=1094000000-0008

Assay variable : PTINR_VAL1 PT-INR Value

N	Mean	Standard Deviation	Minimum	Maximum
6	2.1550000	0.3011810	1.8100000	2.6300000

Laboratory tests at the time of obtaining Table 2-6 consent, according to the presence or absence of proton-pump inhibitors

Unique Subject Identifier=1095000000-0029

MEANS Procedure

Assay variable : PTINR_VAL1 PT-INR Value

N	Mean	Standard Deviation	Minimum	Maximum
6	2.3000000	0.2000000	2.2000000	2.7000000

Unique Subject Identifier=1095000000-0034

Assay variable : PTINR_VAL1 PT-INR Value

N	Mean	Standard Deviation	Minimum	Maximum
5	1.8600000	0.7469940	1.2000000	2.8000000

Unique Subject Identifier=1102000000-0005

Assay variable : PTINR_VAL1 PT-INR Value

N	Mean	Standard Deviation	Minimum	Maximum
2	1.7000000	0.1272792	1.6100000	1.7900000

Unique Subject Identifier=1102000000-0010

Assay variable : PTINR_VAL1 PT-INR Value

N	Mean	Standard Deviation	Minimum	Maximum
1	2.1700000		2.1700000	2.1700000

Laboratory tests at the time of obtaining Table 2-6 consent, according to the presence or absence of proton-pump inhibitors

Unique Subject Identifier=1102000000-0013

MEANS Procedure

Assay variable : PTINR_VAL1 PT-INR Value

N	Mean	Standard Deviation	Minimum	Maximum
2	1.6300000	0.0989949	1.5600000	1.7000000

Unique Subject Identifier=1102000000-0036

Assay variable : PTINR_VAL1 PT-INR Value

N	Mean	Standard Deviation	Minimum	Maximum
3	1.6500000	0.2475884	1.3700000	1.8400000

Unique Subject Identifier=1103001000-0011

Assay variable : PTINR_VAL1 PT-INR Value

N	Mean	Standard Deviation	Minimum	Maximum
2	1.5600000	0.2545584	1.3800000	1.7400000

Unique Subject Identifier=1107011010-0001

Assay variable : PTINR_VAL1 PT-INR Value

N	Mean	Standard Deviation	Minimum	Maximum
4	2.0250000	0.3545420	1.7700000	2.5500000

Laboratory tests at the time of obtaining Table 2-6 consent, according to the presence or absence of proton-pump inhibitors

Unique Subject Identifier=1107011010-0002

MEANS Procedure

Assay variable : PTINR_VAL1 PT-INR Value

N	Mean	Standard Deviation	Minimum	Maximum
6	1.8416667	0.1804901	1.6500000	2.1500000

Unique Subject Identifier=1107011010-0005

Assay variable : PTINR_VAL1 PT-INR Value

N	Mean	Standard Deviation	Minimum	Maximum
5	1.9100000	0.9907321	0.9700000	3.5100000

Unique Subject Identifier=1107011010-0006

Assay variable : PTINR_VAL1 PT-INR Value

N	Mean	Standard Deviation	Minimum	Maximum
4	1.8075000	0.3854327	1.2700000	2.1200000

Unique Subject Identifier=1107011010-0007

Assay variable : PTINR_VAL1 PT-INR Value

N	Mean	Standard Deviation	Minimum	Maximum
6	1.6666667	0.0886942	1.5600000	1.7700000

Laboratory tests at the time of obtaining Table 2-6 consent, according to the presence or absence of proton-pump inhibitors

Unique Subject Identifier=1107011010-0011

MEANS Procedure

Assay variable : PTINR_VAL1 PT-INR Value

N	Mean	Standard Deviation	Minimum	Maximum
6	1.7083333	0.2064380	1.4600000	1.9900000

Unique Subject Identifier=1110000000-0013

Assay variable : PTINR_VAL1 PT-INR Value

N	Mean	Standard Deviation	Minimum	Maximum
5	1.7800000	0.2588436	1.5000000	2.2000000

Unique Subject Identifier=1110000000-0016

Assay variable : PTINR_VAL1 PT-INR Value

N	Mean	Standard Deviation	Minimum	Maximum
4	2.2250000	0.1500000	2.1000000	2.4000000

Unique Subject Identifier=1110000000-0017

Assay variable : PTINR_VAL1 PT-INR Value

N	Mean	Standard Deviation	Minimum	Maximum
4	1.9250000	0.4573474	1.4000000	2.4000000

Laboratory tests at the time of obtaining Table 2-6 consent, according to the presence or absence of proton-pump inhibitors

Unique Subject Identifier=1110000000-0019

MEANS Procedure

Assay variable : PTINR_VAL1 PT-INR Value

N	Mean	Standard Deviation	Minimum	Maximum
1	1.7200000		1.7200000	1.7200000

Unique Subject Identifier=1110000000-0020

Assay variable : PTINR_VAL1 PT-INR Value

N	Mean	Standard Deviation	Minimum	Maximum
6	1.7000000	0.1788854	1.5000000	2.0000000

Unique Subject Identifier=1110000000-0022

Assay variable : PTINR_VAL1 PT-INR Value

N	Mean	Standard Deviation	Minimum	Maximum
5	1.7600000	0.1516575	1.6000000	2.0000000

Unique Subject Identifier=1111000000-0017

Assay variable : PTINR_VAL1 PT-INR Value

N	Mean	Standard Deviation	Minimum	Maximum
3	1.7166667	0.1050397	1.6100000	1.8200000

Laboratory tests at the time of obtaining Table 2-6 consent, according to the presence or absence of proton-pump inhibitors

Unique Subject Identifier=1111000000-0021

MEANS Procedure

Assay variable : PTINR_VAL1 PT-INR Value

N	Mean	Standard Deviation	Minimum	Maximum
6	1.8016667	0.2604931	1.4300000	2.1700000

Unique Subject Identifier=1112000000-0003

Assay variable : PTINR_VAL1 PT-INR Value

N	Mean	Standard Deviation	Minimum	Maximum
5	1.7200000	0.2181742	1.5100000	2.0700000

Unique Subject Identifier=1112000000-0009

Assay variable : PTINR_VAL1 PT-INR Value

N	Mean	Standard Deviation	Minimum	Maximum
6	2.5000000	1.1120611	1.4000000	4.2900000

Unique Subject Identifier=1112000000-0013

Assay variable : PTINR_VAL1 PT-INR Value

N	Mean	Standard Deviation	Minimum	Maximum
4	2.1475000	0.4944610	1.7800000	2.8600000

Laboratory tests at the time of obtaining Table 2-6 consent, according to the presence or absence of proton-pump inhibitors

Unique Subject Identifier=1118011000-0005

MEANS Procedure

Assay variable : PTINR_VAL1 PT-INR Value

N	Mean	Standard Deviation	Minimum	Maximum
5	2.4200000	0.3033150	2.0000000	2.8000000

Unique Subject Identifier=1118011000-0007

Assay variable : PTINR_VAL1 PT-INR Value

N	Mean	Standard Deviation	Minimum	Maximum
4	1.4750000	0.0957427	1.4000000	1.6000000

Unique Subject Identifier=1118011000-0008

Assay variable : PTINR_VAL1 PT-INR Value

N	Mean	Standard Deviation	Minimum	Maximum
6	2.1666667	0.5354126	1.8000000	3.2000000

Unique Subject Identifier=1118011000-0012

Assay variable : PTINR_VAL1 PT-INR Value

N	Mean	Standard Deviation	Minimum	Maximum
5	1.5000000	0.2549510	1.3000000	1.9000000

Laboratory tests at the time of obtaining Table 2-6 consent, according to the presence or absence of proton-pump inhibitors

Unique Subject Identifier=1118011000-0014

MEANS Procedure

Assay variable : PTINR_VAL1 PT-INR Value

N	Mean	Standard Deviation	Minimum	Maximum
5	2.0000000	0.0707107	1.9000000	2.1000000

Unique Subject Identifier=1118011000-0018

Assay variable : PTINR_VAL1 PT-INR Value

N	Mean	Standard Deviation	Minimum	Maximum
5	2.3000000	0.6324555	1.3000000	3.0000000

Unique Subject Identifier=1118011000-0019

Assay variable : PTINR_VAL1 PT-INR Value

N	Mean	Standard Deviation	Minimum	Maximum
6	2.0166667	0.2994439	1.7000000	2.5000000

Unique Subject Identifier=1118011000-0024

Assay variable : PTINR_VAL1 PT-INR Value

N	Mean	Standard Deviation	Minimum	Maximum
4	1.4750000	0.2629956	1.1000000	1.7000000

Laboratory tests at the time of obtaining Table 2-6 consent, according to the presence or absence of proton-pump inhibitors

Unique Subject Identifier=1118011000-0030

MEANS Procedure

Assay variable : PTINR_VAL1 PT-INR Value

N	Mean	Standard Deviation	Minimum	Maximum
5	1.9600000	0.2792848	1.5000000	2.2000000

Unique Subject Identifier=1118011000-0032

Assay variable : PTINR_VAL1 PT-INR Value

N	Mean	Standard Deviation	Minimum	Maximum
5	1.9800000	0.3114482	1.7000000	2.4000000

Unique Subject Identifier=1118011000-0035

Assay variable : PTINR_VAL1 PT-INR Value

N	Mean	Standard Deviation	Minimum	Maximum
5	2.1000000	0.4358899	1.6000000	2.8000000

Unique Subject Identifier=1118011000-0038

Assay variable : PTINR_VAL1 PT-INR Value

N	Mean	Standard Deviation	Minimum	Maximum
6	2.1166667	0.1722401	1.9000000	2.4000000

Laboratory tests at the time of obtaining Table 2-6 consent, according to the presence or absence of proton-pump inhibitors

Unique Subject Identifier=1118011000-0041

MEANS Procedure

Assay variable : PTINR_VAL1 PT-INR Value

N	Mean	Standard Deviation	Minimum	Maximum
6	2.6000000	0.3521363	2.2000000	3.2000000

Unique Subject Identifier=1118011000-0048

Assay variable : PTINR_VAL1 PT-INR Value

N	Mean	Standard Deviation	Minimum	Maximum
6	1.6000000	0.2000000	1.4000000	1.9000000

Unique Subject Identifier=1118011000-0049

Assay variable : PTINR_VAL1 PT-INR Value

N	Mean	Standard Deviation	Minimum	Maximum
6	2.2166667	0.2994439	1.8000000	2.6000000

Unique Subject Identifier=1118011000-0051

Assay variable : PTINR_VAL1 PT-INR Value

N	Mean	Standard Deviation	Minimum	Maximum
5	1.7400000	0.3714835	1.2000000	2.2000000

Laboratory tests at the time of obtaining Table 2-6 consent, according to the presence or absence of proton-pump inhibitors

Unique Subject Identifier=1118011000-0057

MEANS Procedure

Assay variable : PTINR_VAL1 PT-INR Value

N	Mean	Standard Deviation	Minimum	Maximum
6	1.6166667	0.1471960	1.5000000	1.8000000

Unique Subject Identifier=1118011000-0060

Assay variable : PTINR_VAL1 PT-INR Value

N	Mean	Standard Deviation	Minimum	Maximum
4	1.5000000	0.2828427	1.1000000	1.7000000

Unique Subject Identifier=1118011000-0062

Assay variable : PTINR_VAL1 PT-INR Value

N	Mean	Standard Deviation	Minimum	Maximum
6	2.1500000	0.4324350	1.3000000	2.5000000

Unique Subject Identifier=1118011000-0064

Assay variable : PTINR_VAL1 PT-INR Value

N	Mean	Standard Deviation	Minimum	Maximum
6	2.7500000	0.4415880	2.0000000	3.3000000

Laboratory tests at the time of obtaining Table 2-6 consent, according to the presence or absence of proton-pump inhibitors

Unique Subject Identifier=1118011000-0067

MEANS Procedure

Assay variable : PTINR_VAL1 PT-INR Value

N	Mean	Standard Deviation	Minimum	Maximum
4	1.8250000	0.1707825	1.6000000	2.0000000

Unique Subject Identifier=1118011000-0068

Assay variable : PTINR_VAL1 PT-INR Value

N	Mean	Standard Deviation	Minimum	Maximum
6	1.6833333	0.2041241	1.4000000	1.9000000

Unique Subject Identifier=1118011000-0069

Assay variable : PTINR_VAL1 PT-INR Value

N	Mean	Standard Deviation	Minimum	Maximum
6	1.8166667	0.2401388	1.6000000	2.2000000

Unique Subject Identifier=1118011000-0070

Assay variable : PTINR_VAL1 PT-INR Value

N	Mean	Standard Deviation	Minimum	Maximum
4	1.8000000	0.2160247	1.6000000	2.1000000

Laboratory tests at the time of obtaining Table 2-6 consent, according to the presence or absence of proton-pump inhibitors

Unique Subject Identifier=1118011000-0072

MEANS Procedure

Assay variable : PTINR_VAL1 PT-INR Value

N	Mean	Standard Deviation	Minimum	Maximum
6	2.0333333	0.1861899	1.8000000	2.3000000

Unique Subject Identifier=1118011000-0078

Assay variable : PTINR_VAL1 PT-INR Value

N	Mean	Standard Deviation	Minimum	Maximum
5	1.9600000	0.5504544	1.4000000	2.6000000

Unique Subject Identifier=1118011000-0079

Assay variable : PTINR_VAL1 PT-INR Value

N	Mean	Standard Deviation	Minimum	Maximum
5	2.2800000	0.6534524	1.7000000	3.4000000

Unique Subject Identifier=1118011000-0082

Assay variable : PTINR_VAL1 PT-INR Value

N	Mean	Standard Deviation	Minimum	Maximum
6	1.3833333	0.3250641	1.0000000	1.9000000

Laboratory tests at the time of obtaining Table 2-6 consent, according to the presence or absence of proton-pump inhibitors

Unique Subject Identifier=1118011000-0086

MEANS Procedure

Assay variable : PTINR_VAL1 PT-INR Value

N	Mean	Standard Deviation	Minimum	Maximum
5	2.0200000	0.5449771	1.3000000	2.8000000

Unique Subject Identifier=1118011000-0089

Assay variable : PTINR_VAL1 PT-INR Value

N	Mean	Standard Deviation	Minimum	Maximum
5	1.8600000	0.1949359	1.6000000	2.1000000

Unique Subject Identifier=1118011000-0091

Assay variable : PTINR_VAL1 PT-INR Value

N	Mean	Standard Deviation	Minimum	Maximum
6	2.0833333	0.5231316	1.5000000	2.9000000

Unique Subject Identifier=1118011000-0093

Assay variable : PTINR_VAL1 PT-INR Value

N	Mean	Standard Deviation	Minimum	Maximum
6	2.1000000	0.3794733	1.7000000	2.7000000

Laboratory tests at the time of obtaining Table 2-6 consent, according to the presence or absence of proton-pump inhibitors

Unique Subject Identifier=1118011000-0099

MEANS Procedure

Assay variable : PTINR_VAL1 PT-INR Value

N	Mean	Standard Deviation	Minimum	Maximum
6	1.4333333	0.1505545	1.3000000	1.7000000

Unique Subject Identifier=1118011000-0101

Assay variable : PTINR_VAL1 PT-INR Value

N	Mean	Standard Deviation	Minimum	Maximum
6	2.0333333	0.2658320	1.6000000	2.3000000

Unique Subject Identifier=1118011000-0105

Assay variable : PTINR_VAL1 PT-INR Value

N	Mean	Standard Deviation	Minimum	Maximum
4	1.7750000	0.2753785	1.5000000	2.1000000

Unique Subject Identifier=1118011000-0113

Assay variable : PTINR_VAL1 PT-INR Value

N	Mean	Standard Deviation	Minimum	Maximum
3	1.7666667	0.2516611	1.5000000	2.0000000

Laboratory tests at the time of obtaining Table 2-6 consent, according to the presence or absence of proton-pump inhibitors

Unique Subject Identifier=1118011000-0116

MEANS Procedure

Assay variable : PTINR_VAL1 PT-INR Value

N	Mean	Standard Deviation	Minimum	Maximum
3	1.6333333	0.3214550	1.4000000	2.0000000

Unique Subject Identifier=1118011000-0118

Assay variable : PTINR_VAL1 PT-INR Value

N	Mean	Standard Deviation	Minimum	Maximum
4	1.6000000	0.2828427	1.4000000	2.0000000

Unique Subject Identifier=1119000000-0001

Assay variable : PTINR_VAL1 PT-INR Value

N	Mean	Standard Deviation	Minimum	Maximum
6	1.6500000	0.1649242	1.4400000	1.9100000

Unique Subject Identifier=1119000000-0010

Assay variable : PTINR_VAL1 PT-INR Value

N	Mean	Standard Deviation	Minimum	Maximum
3	2.8000000	0.4358899	2.5000000	3.3000000

Laboratory tests at the time of obtaining Table 2-6 consent, according to the presence or absence of proton-pump inhibitors

Unique Subject Identifier=1119000000-0022

MEANS Procedure

Assay variable : PTINR_VAL1 PT-INR Value

N	Mean	Standard Deviation	Minimum	Maximum
6	2.1466667	0.3819249	1.7800000	2.8000000

Unique Subject Identifier=1119000000-0025

Assay variable : PTINR_VAL1 PT-INR Value

N	Mean	Standard Deviation	Minimum	Maximum
6	2.2500000	0.5244044	1.8000000	3.2000000

Unique Subject Identifier=1119000000-0030

Assay variable : PTINR_VAL1 PT-INR Value

N	Mean	Standard Deviation	Minimum	Maximum
4	2.4500000	0.2380476	2.3000000	2.8000000

Unique Subject Identifier=1119000000-0031

Assay variable : PTINR_VAL1 PT-INR Value

N	Mean	Standard Deviation	Minimum	Maximum
3	3.4333333	0.7505553	2.7000000	4.2000000

Laboratory tests at the time of obtaining Table 2-6 consent, according to the presence or absence of proton-pump inhibitors

Unique Subject Identifier=1119000000-0036

MEANS Procedure

Assay variable : PTINR_VAL1 PT-INR Value

N	Mean	Standard Deviation	Minimum	Maximum
5	2.7260000	0.2380756	2.4900000	3.0600000

Unique Subject Identifier=1119000000-0039

Assay variable : PTINR_VAL1 PT-INR Value

N	Mean	Standard Deviation	Minimum	Maximum
5	2.0000000	0.3605551	1.6000000	2.5000000

Unique Subject Identifier=1119000000-0041

Assay variable : PTINR_VAL1 PT-INR Value

N	Mean	Standard Deviation	Minimum	Maximum
6	2.1833333	0.5231316	1.5000000	2.9000000

Unique Subject Identifier=1119000000-0055

Assay variable : PTINR_VAL1 PT-INR Value

N	Mean	Standard Deviation	Minimum	Maximum
1	2.9000000		2.9000000	2.9000000

Laboratory tests at the time of obtaining Table 2-6 consent, according to the presence or absence of proton-pump inhibitors

Unique Subject Identifier=1119000000-0060

MEANS Procedure

Assay variable : PTINR_VAL1 PT-INR Value

N	Mean	Standard Deviation	Minimum	Maximum
2	3.0350000	0.0212132	3.0200000	3.0500000

Unique Subject Identifier=1122001010-0014

Assay variable : PTINR_VAL1 PT-INR Value

N	Mean	Standard Deviation	Minimum	Maximum
6	1.4283333	0.1697547	1.2700000	1.7000000

Unique Subject Identifier=1122001010-0032

Assay variable : PTINR_VAL1 PT-INR Value

N	Mean	Standard Deviation	Minimum	Maximum
6	1.8566667	0.2010638	1.6200000	2.1600000

Unique Subject Identifier=1122001010-0033

Assay variable : PTINR_VAL1 PT-INR Value

N	Mean	Standard Deviation	Minimum	Maximum
6	2.1500000	0.5616761	1.1100000	2.6100000

Laboratory tests at the time of obtaining Table 2-6 consent, according to the presence or absence of proton-pump inhibitors

Unique Subject Identifier=1122001010-0034

MEANS Procedure

Assay variable : PTINR_VAL1 PT-INR Value

N	Mean	Standard Deviation	Minimum	Maximum
6	2.6000000	1.0848594	1.1100000	4.3800000

Unique Subject Identifier=1122001010-0037

Assay variable : PTINR_VAL1 PT-INR Value

N	Mean	Standard Deviation	Minimum	Maximum
6	1.7116667	0.2398680	1.4300000	2.1300000

Unique Subject Identifier=1122001010-0039

Assay variable : PTINR_VAL1 PT-INR Value

N	Mean	Standard Deviation	Minimum	Maximum
1	2.0600000		2.0600000	2.0600000

Unique Subject Identifier=1122001010-0041

Assay variable : PTINR_VAL1 PT-INR Value

N	Mean	Standard Deviation	Minimum	Maximum
6	2.0583333	0.1683350	1.8300000	2.3300000

Laboratory tests at the time of obtaining Table 2-6 consent, according to the presence or absence of proton-pump inhibitors

Unique Subject Identifier=1122001010-0045

MEANS Procedure

Assay variable : PTINR_VAL1 PT-INR Value

N	Mean	Standard Deviation	Minimum	Maximum
6	1.5966667	0.2299275	1.3100000	1.9900000

Unique Subject Identifier=1122001010-0046

Assay variable : PTINR_VAL1 PT-INR Value

N	Mean	Standard Deviation	Minimum	Maximum
6	2.5183333	1.0548823	1.3100000	3.7600000

Unique Subject Identifier=1122001010-0047

Assay variable : PTINR_VAL1 PT-INR Value

N	Mean	Standard Deviation	Minimum	Maximum
6	2.1283333	0.3985181	1.5500000	2.5300000

Unique Subject Identifier=1122001010-0048

Assay variable : PTINR_VAL1 PT-INR Value

N	Mean	Standard Deviation	Minimum	Maximum
6	2.4100000	0.4795832	1.6800000	3.1200000

Laboratory tests at the time of obtaining Table 2-6 consent, according to the presence or absence of proton-pump inhibitors

Unique Subject Identifier=1122001010-0050

MEANS Procedure

Assay variable : PTINR_VAL1 PT-INR Value

N	Mean	Standard Deviation	Minimum	Maximum
6	1.7233333	0.3512074	1.4100000	2.3800000

Unique Subject Identifier=1122001010-0053

Assay variable : PTINR_VAL1 PT-INR Value

N	Mean	Standard Deviation	Minimum	Maximum
6	2.6066667	0.4033691	2.0400000	3.1200000

Unique Subject Identifier=1122001010-0054

Assay variable : PTINR_VAL1 PT-INR Value

N	Mean	Standard Deviation	Minimum	Maximum
6	2.3033333	1.1264221	1.1600000	4.3800000

Unique Subject Identifier=1122001010-0057

Assay variable : PTINR_VAL1 PT-INR Value

N	Mean	Standard Deviation	Minimum	Maximum
6	1.9350000	0.3984344	1.5500000	2.6400000

Laboratory tests at the time of obtaining Table 2-6 consent, according to the presence or absence of proton-pump inhibitors

Unique Subject Identifier=1122001010-0059

MEANS Procedure

Assay variable : PTINR_VAL1 PT-INR Value

N	Mean	Standard Deviation	Minimum	Maximum
6	2.1800000	0.4648010	1.8600000	3.0600000

Unique Subject Identifier=1122001010-0062

Assay variable : PTINR_VAL1 PT-INR Value

N	Mean	Standard Deviation	Minimum	Maximum
6	2.4733333	0.5971153	1.5500000	3.2000000

Unique Subject Identifier=1122001010-0064

Assay variable : PTINR_VAL1 PT-INR Value

N	Mean	Standard Deviation	Minimum	Maximum
6	2.0216667	0.4163372	1.4500000	2.5600000

Unique Subject Identifier=1122001010-0065

Assay variable : PTINR_VAL1 PT-INR Value

N	Mean	Standard Deviation	Minimum	Maximum
6	1.7000000	0.3573234	1.3100000	2.3300000

Laboratory tests at the time of obtaining Table 2-6 consent, according to the presence or absence of proton-pump inhibitors

Unique Subject Identifier=1122001010-0069

MEANS Procedure

Assay variable : PTINR_VAL1 PT-INR Value

N	Mean	Standard Deviation	Minimum	Maximum
6	1.7300000	0.1856879	1.5500000	2.0600000

Unique Subject Identifier=1122001010-0074

Assay variable : PTINR_VAL1 PT-INR Value

N	Mean	Standard Deviation	Minimum	Maximum
3	2.4233333	0.2294196	2.1600000	2.5800000

Unique Subject Identifier=1126011000-0002

Assay variable : PTINR_VAL1 PT-INR Value

N	Mean	Standard Deviation	Minimum	Maximum
6	1.5816667	0.3057722	1.2700000	2.0500000

Unique Subject Identifier=1126011000-0004

Assay variable : PTINR_VAL1 PT-INR Value

N	Mean	Standard Deviation	Minimum	Maximum
4	1.5700000	0.5963221	1.0600000	2.3400000

Laboratory tests at the time of obtaining Table 2-6 consent, according to the presence or absence of proton-pump inhibitors

Unique Subject Identifier=1126011000-0010

MEANS Procedure

Assay variable : PTINR_VAL1 PT-INR Value

N	Mean	Standard Deviation	Minimum	Maximum
6	1.9316667	0.3364174	1.5900000	2.5300000

Unique Subject Identifier=1126011000-0011

Assay variable : PTINR_VAL1 PT-INR Value

N	Mean	Standard Deviation	Minimum	Maximum
5	2.0320000	0.3856423	1.5500000	2.4900000

Unique Subject Identifier=1126011000-0013

Assay variable : PTINR_VAL1 PT-INR Value

N	Mean	Standard Deviation	Minimum	Maximum
6	1.7583333	0.2245366	1.6000000	2.2000000

Unique Subject Identifier=1126011000-0014

Assay variable : PTINR_VAL1 PT-INR Value

N	Mean	Standard Deviation	Minimum	Maximum
5	1.6700000	0.6279729	1.1000000	2.7300000

Laboratory tests at the time of obtaining Table 2-6 consent, according to the presence or absence of proton-pump inhibitors

Unique Subject Identifier=1126011000-0015

MEANS Procedure

Assay variable : PTINR_VAL1 PT-INR Value

N	Mean	Standard Deviation	Minimum	Maximum
6	1.9166667	0.4128276	1.4500000	2.4900000

Unique Subject Identifier=1127010000-0002

Assay variable : PTINR_VAL1 PT-INR Value

N	Mean	Standard Deviation	Minimum	Maximum
4	1.4500000	0.0577350	1.4000000	1.5000000

Unique Subject Identifier=1127010000-0003

Assay variable : PTINR_VAL1 PT-INR Value

N	Mean	Standard Deviation	Minimum	Maximum
5	2.1800000	0.1483240	2.0000000	2.4000000

Unique Subject Identifier=1127010000-0005

Assay variable : PTINR_VAL1 PT-INR Value

N	Mean	Standard Deviation	Minimum	Maximum
1	1.1000000		1.1000000	1.1000000

Laboratory tests at the time of obtaining Table 2-6 consent, according to the presence or absence of proton-pump inhibitors

Unique Subject Identifier=1128011000-0002

MEANS Procedure

Assay variable : PTINR_VAL1 PT-INR Value

N	Mean	Standard Deviation	Minimum	Maximum
6	1.9333333	0.3265986	1.6000000	2.5000000

Unique Subject Identifier=1128011000-0003

Assay variable : PTINR_VAL1 PT-INR Value

N	Mean	Standard Deviation	Minimum	Maximum
6	1.8666667	0.1032796	1.7000000	2.0000000

Unique Subject Identifier=1128011000-0004

Assay variable : PTINR_VAL1 PT-INR Value

N	Mean	Standard Deviation	Minimum	Maximum
5	1.8600000	0.1516575	1.7000000	2.1000000

Unique Subject Identifier=1128011000-0005

Assay variable : PTINR_VAL1 PT-INR Value

N	Mean	Standard Deviation	Minimum	Maximum
6	2.0666667	0.3983298	1.6000000	2.6000000

Laboratory tests at the time of obtaining Table 2-6 consent, according to the presence or absence of proton-pump inhibitors

Unique Subject Identifier=1128011000-0009

MEANS Procedure

Assay variable : PTINR_VAL1 PT-INR Value

N	Mean	Standard Deviation	Minimum	Maximum
4	2.2000000	0.2943920	1.9000000	2.6000000

Unique Subject Identifier=1128011000-0010

Assay variable : PTINR_VAL1 PT-INR Value

N	Mean	Standard Deviation	Minimum	Maximum
5	2.2400000	0.8988882	1.0000000	3.3000000

Unique Subject Identifier=1131000000-0001

Assay variable : PTINR_VAL1 PT-INR Value

N	Mean	Standard Deviation	Minimum	Maximum
6	1.5016667	0.3225161	1.1300000	1.8700000

Unique Subject Identifier=1131000000-0002

Assay variable : PTINR_VAL1 PT-INR Value

N	Mean	Standard Deviation	Minimum	Maximum
5	1.3300000	0.2933428	1.0500000	1.7900000

Laboratory tests at the time of obtaining Table 2-6 consent, according to the presence or absence of proton-pump inhibitors

Unique Subject Identifier=1131000000-0003

MEANS Procedure

Assay variable : PTINR_VAL1 PT-INR Value

N	Mean	Standard Deviation	Minimum	Maximum
6	2.0800000	0.2337520	1.7300000	2.3300000

Unique Subject Identifier=1131000000-0004

Assay variable : PTINR_VAL1 PT-INR Value

N	Mean	Standard Deviation	Minimum	Maximum
6	1.4550000	0.4079583	1.0800000	2.2300000

Unique Subject Identifier=1131000000-0005

Assay variable : PTINR_VAL1 PT-INR Value

N	Mean	Standard Deviation	Minimum	Maximum
6	2.2650000	0.4269309	1.8500000	2.8000000

Unique Subject Identifier=1131000000-0006

Assay variable : PTINR_VAL1 PT-INR Value

N	Mean	Standard Deviation	Minimum	Maximum
6	1.6383333	0.2778789	1.3600000	2.1700000

Laboratory tests at the time of obtaining Table 2-6 consent, according to the presence or absence of proton-pump inhibitors

Unique Subject Identifier=1131000000-0007

MEANS Procedure

Assay variable : PTINR_VAL1 PT-INR Value

N	Mean	Standard Deviation	Minimum	Maximum
6	2.1600000	0.6108355	1.6500000	3.1100000

Unique Subject Identifier=1131000000-0008

Assay variable : PTINR_VAL1 PT-INR Value

N	Mean	Standard Deviation	Minimum	Maximum
5	1.5220000	0.3168122	1.2400000	1.9700000

Unique Subject Identifier=1131000000-0009

Assay variable : PTINR_VAL1 PT-INR Value

N	Mean	Standard Deviation	Minimum	Maximum
3	1.9400000	0.2107131	1.7200000	2.1400000

Unique Subject Identifier=1137000000-0007

Assay variable : PTINR_VAL1 PT-INR Value

N	Mean	Standard Deviation	Minimum	Maximum
3	1.0366667	0.0208167	1.0200000	1.0600000

Laboratory tests at the time of obtaining Table 2-6 consent, according to the presence or absence of proton-pump inhibitors

Unique Subject Identifier=1137000000-0032

MEANS Procedure

Assay variable : PTINR_VAL1 PT-INR Value

N	Mean	Standard Deviation	Minimum	Maximum
2	1.0400000	0.0989949	0.9700000	1.1100000

Unique Subject Identifier=1141000000-0002

Assay variable : PTINR_VAL1 PT-INR Value

N	Mean	Standard Deviation	Minimum	Maximum
1	1.6800000		1.6800000	1.6800000

Unique Subject Identifier=1141000000-0005

Assay variable : PTINR_VAL1 PT-INR Value

N	Mean	Standard Deviation	Minimum	Maximum
1	1.2000000		1.2000000	1.2000000

Unique Subject Identifier=1146011000-0001

Assay variable : PTINR_VAL1 PT-INR Value

N	Mean	Standard Deviation	Minimum	Maximum
4	2.2750000	0.4607602	1.9800000	2.9500000

Laboratory tests at the time of obtaining Table 2-6 consent, according to the presence or absence of proton-pump inhibitors

Unique Subject Identifier=1146011000-0002

MEANS Procedure

Assay variable : PTINR_VAL1 PT-INR Value

N	Mean	Standard Deviation	Minimum	Maximum
3	2.0566667	0.6293118	1.5800000	2.7700000

Unique Subject Identifier=1146011000-0003

Assay variable : PTINR_VAL1 PT-INR Value

N	Mean	Standard Deviation	Minimum	Maximum
2	1.8400000	0.2969848	1.6300000	2.0500000

Unique Subject Identifier=1149000000-0001

Assay variable : PTINR_VAL1 PT-INR Value

N	Mean	Standard Deviation	Minimum	Maximum
6	1.7333333	0.2581989	1.4000000	2.1000000

Unique Subject Identifier=1149000000-0002

Assay variable : PTINR_VAL1 PT-INR Value

N	Mean	Standard Deviation	Minimum	Maximum
6	1.8500000	0.4135215	1.4000000	2.6000000

Laboratory tests at the time of obtaining Table 2-6 consent, according to the presence or absence of proton-pump inhibitors

Unique Subject Identifier=1149000000-0003

MEANS Procedure

Assay variable : PTINR_VAL1 PT-INR Value

N	Mean	Standard Deviation	Minimum	Maximum
6	2.2000000	0.0894427	2.1000000	2.3000000

Unique Subject Identifier=1149000000-0004

Assay variable : PTINR_VAL1 PT-INR Value

N	Mean	Standard Deviation	Minimum	Maximum
6	1.8666667	0.2804758	1.6000000	2.2000000

Unique Subject Identifier=1149000000-0005

Assay variable : PTINR_VAL1 PT-INR Value

N	Mean	Standard Deviation	Minimum	Maximum
6	2.1333333	0.6186006	1.4000000	3.2000000

Unique Subject Identifier=1149000000-0006

Assay variable : PTINR_VAL1 PT-INR Value

N	Mean	Standard Deviation	Minimum	Maximum
6	1.7166667	0.2041241	1.5000000	1.9000000

Laboratory tests at the time of obtaining Table 2-6 consent, according to the presence or absence of proton-pump inhibitors

Unique Subject Identifier=1149000000-0007

MEANS Procedure

Assay variable : PTINR_VAL1 PT-INR Value

N	Mean	Standard Deviation	Minimum	Maximum
6	1.8666667	0.4676181	1.0000000	2.4000000

Unique Subject Identifier=1156000000-0001

Assay variable : PTINR_VAL1 PT-INR Value

N	Mean	Standard Deviation	Minimum	Maximum
6	1.4766667	0.0945868	1.3600000	1.6100000

Unique Subject Identifier=1156000000-0002

Assay variable : PTINR_VAL1 PT-INR Value

N	Mean	Standard Deviation	Minimum	Maximum
5	1.7920000	0.4152349	1.3900000	2.2500000

Unique Subject Identifier=1156000000-0004

Assay variable : PTINR_VAL1 PT-INR Value

N	Mean	Standard Deviation	Minimum	Maximum
5	2.2100000	0.6031998	1.6600000	2.9700000

Laboratory tests at the time of obtaining Table 2-6 consent, according to the presence or absence of proton-pump inhibitors

Unique Subject Identifier=1156000000-0005

MEANS Procedure

Assay variable : PTINR_VAL1 PT-INR Value

N	Mean	Standard Deviation	Minimum	Maximum
1	1.4100000		1.4100000	1.4100000

Unique Subject Identifier=1156000000-0009

Assay variable : PTINR_VAL1 PT-INR Value

N	Mean	Standard Deviation	Minimum	Maximum
6	1.8250000	0.3733497	1.4200000	2.5000000

Unique Subject Identifier=1156000000-0014

Assay variable : PTINR_VAL1 PT-INR Value

N	Mean	Standard Deviation	Minimum	Maximum
6	1.3700000	0.1842824	1.2100000	1.6200000

Unique Subject Identifier=1156000000-0015

Assay variable : PTINR_VAL1 PT-INR Value

N	Mean	Standard Deviation	Minimum	Maximum
6	2.3333333	0.3844303	1.8600000	2.9400000

Laboratory tests at the time of obtaining Table 2-6 consent, according to the presence or absence of proton-pump inhibitors

Unique Subject Identifier=1156000000-0018

MEANS Procedure

Assay variable : PTINR_VAL1 PT-INR Value

N	Mean	Standard Deviation	Minimum	Maximum
6	1.3150000	0.1224337	1.1700000	1.4600000

Unique Subject Identifier=1156000000-0022

Assay variable : PTINR_VAL1 PT-INR Value

N	Mean	Standard Deviation	Minimum	Maximum
5	1.7720000	0.3772532	1.2000000	2.0900000

Unique Subject Identifier=1156000000-0023

Assay variable : PTINR_VAL1 PT-INR Value

N	Mean	Standard Deviation	Minimum	Maximum
5	1.8680000	0.4928184	0.9900000	2.1400000

Unique Subject Identifier=1156000000-0056

Assay variable : PTINR_VAL1 PT-INR Value

N	Mean	Standard Deviation	Minimum	Maximum
5	1.8560000	0.1073778	1.7100000	1.9700000

Laboratory tests at the time of obtaining Table 2-6 consent, according to the presence or absence of proton-pump inhibitors

Unique Subject Identifier=1156000000-0057

MEANS Procedure

Assay variable : PTINR_VAL1 PT-INR Value

N	Mean	Standard Deviation	Minimum	Maximum
2	1.2900000	0.0707107	1.2400000	1.3400000

Unique Subject Identifier=1156000000-0062

Assay variable : PTINR_VAL1 PT-INR Value

N	Mean	Standard Deviation	Minimum	Maximum
6	1.3416667	0.0584523	1.2500000	1.4000000

Unique Subject Identifier=1157000000-0002

Assay variable : PTINR_VAL1 PT-INR Value

N	Mean	Standard Deviation	Minimum	Maximum
6	2.5000000	0.5099020	1.7000000	3.1000000

Unique Subject Identifier=1157000000-0003

Assay variable : PTINR_VAL1 PT-INR Value

N	Mean	Standard Deviation	Minimum	Maximum
6	2.1333333	0.4760952	1.5000000	2.9000000

Laboratory tests at the time of obtaining Table 2-6 consent, according to the presence or absence of proton-pump inhibitors

Unique Subject Identifier=1157000000-0006

MEANS Procedure

Assay variable : PTINR_VAL1 PT-INR Value

N	Mean	Standard Deviation	Minimum	Maximum
6	2.0016667	0.4574021	1.5000000	2.7000000

Unique Subject Identifier=1157000000-0011

Assay variable : PTINR_VAL1 PT-INR Value

N	Mean	Standard Deviation	Minimum	Maximum
6	1.8666667	0.2732520	1.7000000	2.4000000

Unique Subject Identifier=1157000000-0013

Assay variable : PTINR_VAL1 PT-INR Value

N	Mean	Standard Deviation	Minimum	Maximum
4	2.3000000	0.4546061	1.8000000	2.9000000

Unique Subject Identifier=1157000000-0033

Assay variable : PTINR_VAL1 PT-INR Value

N	Mean	Standard Deviation	Minimum	Maximum
6	1.8666667	0.3076795	1.6000000	2.4000000

Laboratory tests at the time of obtaining Table 2-6 consent, according to the presence or absence of proton-pump inhibitors

Unique Subject Identifier=1157000000-0034

MEANS Procedure

Assay variable : PTINR_VAL1 PT-INR Value

N	Mean	Standard Deviation	Minimum	Maximum
6	2.1333333	0.1751190	1.9000000	2.4000000

Unique Subject Identifier=1158010000-0022

Assay variable : PTINR_VAL1 PT-INR Value

N	Mean	Standard Deviation	Minimum	Maximum
6	1.9133333	0.1423610	1.7700000	2.1300000

Unique Subject Identifier=1158010000-0023

Assay variable : PTINR_VAL1 PT-INR Value

N	Mean	Standard Deviation	Minimum	Maximum
6	1.8900000	0.4136665	1.6000000	2.7100000

Unique Subject Identifier=1158010000-0027

Assay variable : PTINR_VAL1 PT-INR Value

N	Mean	Standard Deviation	Minimum	Maximum
6	1.8600000	0.3269251	1.4300000	2.2500000

Laboratory tests at the time of obtaining Table 2-6 consent, according to the presence or absence of proton-pump inhibitors

Unique Subject Identifier=1158010000-0035

MEANS Procedure

Assay variable : PTINR_VAL1 PT-INR Value

N	Mean	Standard Deviation	Minimum	Maximum
6	2.3366667	0.5368302	1.6800000	3.2300000

Unique Subject Identifier=1158010000-0042

Assay variable : PTINR_VAL1 PT-INR Value

N	Mean	Standard Deviation	Minimum	Maximum
6	2.0683333	0.3535770	1.6400000	2.5000000

Unique Subject Identifier=1158010000-0045

Assay variable : PTINR_VAL1 PT-INR Value

N	Mean	Standard Deviation	Minimum	Maximum
6	1.9533333	0.5908525	1.4700000	3.1200000

Unique Subject Identifier=1158010000-0055

Assay variable : PTINR_VAL1 PT-INR Value

N	Mean	Standard Deviation	Minimum	Maximum
6	2.1183333	0.1340771	1.9900000	2.3500000

Laboratory tests at the time of obtaining Table 2-6 consent, according to the presence or absence of proton-pump inhibitors

Unique Subject Identifier=1158010000-0065

MEANS Procedure

Assay variable : PTINR_VAL1 PT-INR Value

N	Mean	Standard Deviation	Minimum	Maximum
6	2.0816667	0.3621832	1.4700000	2.4500000

Unique Subject Identifier=1158010000-0080

Assay variable : PTINR_VAL1 PT-INR Value

N	Mean	Standard Deviation	Minimum	Maximum
6	2.0250000	0.0875785	1.9000000	2.1600000

Unique Subject Identifier=1158010000-0082

Assay variable : PTINR_VAL1 PT-INR Value

N	Mean	Standard Deviation	Minimum	Maximum
6	2.4200000	0.3996498	1.8100000	2.9800000

Unique Subject Identifier=1158010000-0104

Assay variable : PTINR_VAL1 PT-INR Value

N	Mean	Standard Deviation	Minimum	Maximum
6	1.8666667	0.3042148	1.4700000	2.3800000

Laboratory tests at the time of obtaining Table 2-6 consent, according to the presence or absence of proton-pump inhibitors

Unique Subject Identifier=1158010000-0106

MEANS Procedure

Assay variable : PTINR_VAL1 PT-INR Value

N	Mean	Standard Deviation	Minimum	Maximum
6	2.2600000	0.4376300	1.7700000	3.0100000

Unique Subject Identifier=1158010000-0117

Assay variable : PTINR_VAL1 PT-INR Value

N	Mean	Standard Deviation	Minimum	Maximum
6	2.0533333	0.3923094	1.6400000	2.7100000

Unique Subject Identifier=1158010000-0126

Assay variable : PTINR_VAL1 PT-INR Value

N	Mean	Standard Deviation	Minimum	Maximum
6	2.1750000	0.4345457	1.4700000	2.6600000

Unique Subject Identifier=1159000000-0002

Assay variable : PTINR_VAL1 PT-INR Value

N	Mean	Standard Deviation	Minimum	Maximum
6	2.2666667	0.1966384	1.9000000	2.4000000

Laboratory tests at the time of obtaining Table 2-6 consent, according to the presence or absence of proton-pump inhibitors

Unique Subject Identifier=1159000000-0003

MEANS Procedure

Assay variable : PTINR_VAL1 PT-INR Value

N	Mean	Standard Deviation	Minimum	Maximum
6	2.0500000	0.6348228	1.1000000	2.8000000

Unique Subject Identifier=1159000000-0005

Assay variable : PTINR_VAL1 PT-INR Value

N	Mean	Standard Deviation	Minimum	Maximum
6	2.3666667	0.6121002	1.5000000	3.1000000

Unique Subject Identifier=1159000000-0010

Assay variable : PTINR_VAL1 PT-INR Value

N	Mean	Standard Deviation	Minimum	Maximum
5	2.1600000	0.4037326	1.8000000	2.8000000

Unique Subject Identifier=1159000000-0013

Assay variable : PTINR_VAL1 PT-INR Value

N	Mean	Standard Deviation	Minimum	Maximum
6	2.4833333	0.4956477	1.5000000	2.9000000

Laboratory tests at the time of obtaining Table 2-6 consent, according to the presence or absence of proton-pump inhibitors

Unique Subject Identifier=1159000000-0015

MEANS Procedure

Assay variable : PTINR_VAL1 PT-INR Value

N	Mean	Standard Deviation	Minimum	Maximum
6	2.3000000	0.2898275	1.9000000	2.7000000

Unique Subject Identifier=1159000000-0016

Assay variable : PTINR_VAL1 PT-INR Value

N	Mean	Standard Deviation	Minimum	Maximum
6	2.1500000	0.6220932	1.3000000	3.1000000

Unique Subject Identifier=1159000000-0017

Assay variable : PTINR_VAL1 PT-INR Value

N	Mean	Standard Deviation	Minimum	Maximum
6	2.2333333	0.4033196	1.8000000	2.9000000

Unique Subject Identifier=1159000000-0018

Assay variable : PTINR_VAL1 PT-INR Value

N	Mean	Standard Deviation	Minimum	Maximum
6	1.9333333	0.4718757	1.5000000	2.8000000

Laboratory tests at the time of obtaining Table 2-6 consent, according to the presence or absence of proton-pump inhibitors

Unique Subject Identifier=1159000000-0019

MEANS Procedure

Assay variable : PTINR_VAL1 PT-INR Value

N	Mean	Standard Deviation	Minimum	Maximum
6	2.0000000	0.2097618	1.8000000	2.3000000

Unique Subject Identifier=1160000000-0005

Assay variable : PTINR_VAL1 PT-INR Value

N	Mean	Standard Deviation	Minimum	Maximum
4	2.4125000	1.2308906	1.2400000	4.1300000

Unique Subject Identifier=1160000000-0035

Assay variable : PTINR_VAL1 PT-INR Value

N	Mean	Standard Deviation	Minimum	Maximum
5	1.9560000	0.2402707	1.6800000	2.2100000

Unique Subject Identifier=1160000000-0055

Assay variable : PTINR_VAL1 PT-INR Value

N	Mean	Standard Deviation	Minimum	Maximum
4	2.4800000	1.0526158	1.7000000	3.9400000

Laboratory tests at the time of obtaining Table 2-6 consent, according to the presence or absence of proton-pump inhibitors

Unique Subject Identifier=1160000000-0057

MEANS Procedure

Assay variable : PTINR_VAL1 PT-INR Value

N	Mean	Standard Deviation	Minimum	Maximum
5	1.8660000	0.2176695	1.6600000	2.2100000

Unique Subject Identifier=1160000000-0094

Assay variable : PTINR_VAL1 PT-INR Value

N	Mean	Standard Deviation	Minimum	Maximum
2	1.8650000	0.0777817	1.8100000	1.9200000

Unique Subject Identifier=1160000000-0104

Assay variable : PTINR_VAL1 PT-INR Value

N	Mean	Standard Deviation	Minimum	Maximum
4	2.1450000	0.2487971	1.8800000	2.4800000

Unique Subject Identifier=1160000000-0112

Assay variable : PTINR_VAL1 PT-INR Value

N	Mean	Standard Deviation	Minimum	Maximum
5	1.9240000	0.2338376	1.7200000	2.2500000

Laboratory tests at the time of obtaining Table 2-6 consent, according to the presence or absence of proton-pump inhibitors

Unique Subject Identifier=1160000000-0118

MEANS Procedure

Assay variable : PTINR_VAL1 PT-INR Value

N	Mean	Standard Deviation	Minimum	Maximum
5	1.8360000	0.7326527	1.2700000	3.0900000

Unique Subject Identifier=1160000000-0120

Assay variable : PTINR_VAL1 PT-INR Value

N	Mean	Standard Deviation	Minimum	Maximum
6	2.7600000	0.4822448	2.1300000	3.4900000

Unique Subject Identifier=1161010100-0001

Assay variable : PTINR_VAL1 PT-INR Value

N	Mean	Standard Deviation	Minimum	Maximum
1	2.4900000		2.4900000	2.4900000

Unique Subject Identifier=1161010100-0006

Assay variable : PTINR_VAL1 PT-INR Value

N	Mean	Standard Deviation	Minimum	Maximum
3	1.6566667	0.0321455	1.6200000	1.6800000

Laboratory tests at the time of obtaining Table 2-6 consent, according to the presence or absence of proton-pump inhibitors

Unique Subject Identifier=1161010100-0011

MEANS Procedure

Assay variable : PTINR_VAL1 PT-INR Value

N	Mean	Standard Deviation	Minimum	Maximum
2	1.8250000	0.0070711	1.8200000	1.8300000

Unique Subject Identifier=1161010100-0017

Assay variable : PTINR_VAL1 PT-INR Value

N	Mean	Standard Deviation	Minimum	Maximum
1	2.1900000		2.1900000	2.1900000

Unique Subject Identifier=1161010100-0022

Assay variable : PTINR_VAL1 PT-INR Value

N	Mean	Standard Deviation	Minimum	Maximum
1	2.0500000		2.0500000	2.0500000

Unique Subject Identifier=1162011100-0003

Assay variable : PTINR_VAL1 PT-INR Value

N	Mean	Standard Deviation	Minimum	Maximum
4	1.9900000	0.1618641	1.7700000	2.1600000

Laboratory tests at the time of obtaining Table 2-6 consent, according to the presence or absence of proton-pump inhibitors

Unique Subject Identifier=1167000000-0010

MEANS Procedure

Assay variable : PTINR_VAL1 PT-INR Value

N	Mean	Standard Deviation	Minimum	Maximum
1	2.2100000		2.2100000	2.2100000

Unique Subject Identifier=1168011000-0026

Assay variable : PTINR_VAL1 PT-INR Value

N	Mean	Standard Deviation	Minimum	Maximum
1	1.6400000		1.6400000	1.6400000

Unique Subject Identifier=1168011000-0028

Assay variable : PTINR_VAL1 PT-INR Value

N	Mean	Standard Deviation	Minimum	Maximum
1	1.6000000		1.6000000	1.6000000

Unique Subject Identifier=1168011000-0039

Assay variable : PTINR_VAL1 PT-INR Value

N	Mean	Standard Deviation	Minimum	Maximum
2	2.5300000	0.1272792	2.4400000	2.6200000

Laboratory tests at the time of obtaining Table 2-6 consent, according to the presence or absence of proton-pump inhibitors

Unique Subject Identifier=1168011000-0040

MEANS Procedure

Assay variable : PTINR_VAL1 PT-INR Value

N	Mean	Standard Deviation	Minimum	Maximum
2	2.6600000	0.3818377	2.3900000	2.9300000

Unique Subject Identifier=1168011000-0050

Assay variable : PTINR_VAL1 PT-INR Value

N	Mean	Standard Deviation	Minimum	Maximum
2	1.4200000	0.0424264	1.3900000	1.4500000

Unique Subject Identifier=1169010100-0013

Assay variable : PTINR_VAL1 PT-INR Value

N	Mean	Standard Deviation	Minimum	Maximum
5	2.4200000	0.2774887	2.1000000	2.8000000

Unique Subject Identifier=1171000000-0003

Assay variable : PTINR_VAL1 PT-INR Value

N	Mean	Standard Deviation	Minimum	Maximum
5	1.6760000	0.0391152	1.6400000	1.7300000

Laboratory tests at the time of obtaining Table 2-6 consent, according to the presence or absence of proton-pump inhibitors

Unique Subject Identifier=1172011000-0005

MEANS Procedure

Assay variable : PTINR_VAL1 PT-INR Value

N	Mean	Standard Deviation	Minimum	Maximum
6	2.1166667	0.1169045	2.0000000	2.3000000

Unique Subject Identifier=1173011100-0011

Assay variable : PTINR_VAL1 PT-INR Value

N	Mean	Standard Deviation	Minimum	Maximum
6	1.9000000	0.3033150	1.4000000	2.2000000

Unique Subject Identifier=1173011100-0015

Assay variable : PTINR_VAL1 PT-INR Value

N	Mean	Standard Deviation	Minimum	Maximum
6	1.6666667	0.3265986	1.3000000	2.2000000

Unique Subject Identifier=1173011100-0018

Assay variable : PTINR_VAL1 PT-INR Value

N	Mean	Standard Deviation	Minimum	Maximum
6	2.2833333	0.2316607	1.9000000	2.6000000

Laboratory tests at the time of obtaining Table 2-6 consent, according to the presence or absence of proton-pump inhibitors

Unique Subject Identifier=1173011100-0019

MEANS Procedure

Assay variable : PTINR_VAL1 PT-INR Value

N	Mean	Standard Deviation	Minimum	Maximum
6	2.3500000	1.0445095	1.1000000	3.6000000

Unique Subject Identifier=1173011100-0021

Assay variable : PTINR_VAL1 PT-INR Value

N	Mean	Standard Deviation	Minimum	Maximum
6	2.5000000	0.8221922	1.7000000	3.8000000

Unique Subject Identifier=1173011100-0026

Assay variable : PTINR_VAL1 PT-INR Value

N	Mean	Standard Deviation	Minimum	Maximum
6	2.2500000	1.0634848	1.5000000	4.3000000

Unique Subject Identifier=1173011100-0031

Assay variable : PTINR_VAL1 PT-INR Value

N	Mean	Standard Deviation	Minimum	Maximum
6	1.6833333	0.4445972	1.3000000	2.3000000

Laboratory tests at the time of obtaining Table 2-6 consent, according to the presence or absence of proton-pump inhibitors

Unique Subject Identifier=1175011000-0004

MEANS Procedure

Assay variable : PTINR_VAL1 PT-INR Value

N	Mean	Standard Deviation	Minimum	Maximum
6	1.7833333	0.2316607	1.5000000	2.1000000

Unique Subject Identifier=1175011000-0005

Assay variable : PTINR_VAL1 PT-INR Value

N	Mean	Standard Deviation	Minimum	Maximum
6	2.3666667	2.7645373	1.0000000	8.0000000

Unique Subject Identifier=1175011000-0009

Assay variable : PTINR_VAL1 PT-INR Value

N	Mean	Standard Deviation	Minimum	Maximum
6	2.0000000	0.2756810	1.6000000	2.4000000

Unique Subject Identifier=1175011000-0010

Assay variable : PTINR_VAL1 PT-INR Value

N	Mean	Standard Deviation	Minimum	Maximum
6	1.8500000	0.2428992	1.5000000	2.1000000

Laboratory tests at the time of obtaining Table 2-6 consent, according to the presence or absence of proton-pump inhibitors

Unique Subject Identifier=1175011000-0016

MEANS Procedure

Assay variable : PTINR_VAL1 PT-INR Value

N	Mean	Standard Deviation	Minimum	Maximum
6	1.7666667	0.1366260	1.6000000	2.0000000

Unique Subject Identifier=1175011000-0019

Assay variable : PTINR_VAL1 PT-INR Value

N	Mean	Standard Deviation	Minimum	Maximum
6	2.0500000	0.4764452	1.5000000	2.6000000

Unique Subject Identifier=1179000000-0002

Assay variable : PTINR_VAL1 PT-INR Value

N	Mean	Standard Deviation	Minimum	Maximum
3	1.8333333	0.3511885	1.5000000	2.2000000

Unique Subject Identifier=1179000000-0003

Assay variable : PTINR_VAL1 PT-INR Value

N	Mean	Standard Deviation	Minimum	Maximum
6	1.9500000	1.0310189	1.1000000	4.0000000

Laboratory tests at the time of obtaining Table 2-6 consent, according to the presence or absence of proton-pump inhibitors

Unique Subject Identifier=1179000000-0004

MEANS Procedure

Assay variable : PTINR_VAL1 PT-INR Value

N	Mean	Standard Deviation	Minimum	Maximum
3	2.0333333	0.2081666	1.8000000	2.2000000

Unique Subject Identifier=1179000000-0006

Assay variable : PTINR_VAL1 PT-INR Value

N	Mean	Standard Deviation	Minimum	Maximum
3	2.2333333	0.3214550	2.0000000	2.6000000

Unique Subject Identifier=1181000000-0003

Assay variable : PTINR_VAL1 PT-INR Value

N	Mean	Standard Deviation	Minimum	Maximum
1	2.0300000		2.0300000	2.0300000

Unique Subject Identifier=1181000000-0004

Assay variable : PTINR_VAL1 PT-INR Value

N	Mean	Standard Deviation	Minimum	Maximum
1	2.3900000		2.3900000	2.3900000

Laboratory tests at the time of obtaining Table 2-6 consent, according to the presence or absence of proton-pump inhibitors

Unique Subject Identifier=1181000000-0005

MEANS Procedure

Assay variable : PTINR_VAL1 PT-INR Value

N	Mean	Standard Deviation	Minimum	Maximum
2	2.7950000	0.3889087	2.5200000	3.0700000

Unique Subject Identifier=1181000000-0006

Assay variable : PTINR_VAL1 PT-INR Value

N	Mean	Standard Deviation	Minimum	Maximum
2	2.1000000	0.3676955	1.8400000	2.3600000

Unique Subject Identifier=1181000000-0007

Assay variable : PTINR_VAL1 PT-INR Value

N	Mean	Standard Deviation	Minimum	Maximum
3	1.6866667	0.3360556	1.3200000	1.9800000

Unique Subject Identifier=1181000000-0008

Assay variable : PTINR_VAL1 PT-INR Value

N	Mean	Standard Deviation	Minimum	Maximum
2	1.4650000	0.2050610	1.3200000	1.6100000

Laboratory tests at the time of obtaining Table 2-6 consent, according to the presence or absence of proton-pump inhibitors

Unique Subject Identifier=1181000000-0009

MEANS Procedure

Assay variable : PTINR_VAL1 PT-INR Value

N	Mean	Standard Deviation	Minimum	Maximum
1	3.5700000		3.5700000	3.5700000

Unique Subject Identifier=1181000000-0010

Assay variable : PTINR_VAL1 PT-INR Value

N	Mean	Standard Deviation	Minimum	Maximum
2	1.7950000	0.4030509	1.5100000	2.0800000

Unique Subject Identifier=1181000000-0012

Assay variable : PTINR_VAL1 PT-INR Value

N	Mean	Standard Deviation	Minimum	Maximum
1	1.6200000		1.6200000	1.6200000

Unique Subject Identifier=1181000000-0013

Assay variable : PTINR_VAL1 PT-INR Value

N	Mean	Standard Deviation	Minimum	Maximum
3	1.6600000	0.0754983	1.5900000	1.7400000

Laboratory tests at the time of obtaining Table 2-6 consent, according to the presence or absence of proton-pump inhibitors

Unique Subject Identifier=1181000000-0018

MEANS Procedure

Assay variable : PTINR_VAL1 PT-INR Value

N	Mean	Standard Deviation	Minimum	Maximum
2	2.5450000	0.2192031	2.3900000	2.7000000

Unique Subject Identifier=1181000000-0019

Assay variable : PTINR_VAL1 PT-INR Value

N	Mean	Standard Deviation	Minimum	Maximum
1	1.7200000		1.7200000	1.7200000

Unique Subject Identifier=1182000000-0001

Assay variable : PTINR_VAL1 PT-INR Value

N	Mean	Standard Deviation	Minimum	Maximum
4	1.4750000	0.3403430	1.2000000	1.9000000

Unique Subject Identifier=1182000000-0002

Assay variable : PTINR_VAL1 PT-INR Value

N	Mean	Standard Deviation	Minimum	Maximum
6	2.0000000	0.3741657	1.6000000	2.6000000

Laboratory tests at the time of obtaining Table 2-6 consent, according to the presence or absence of proton-pump inhibitors

Unique Subject Identifier=1186000000-0008

MEANS Procedure

Assay variable : PTINR_VAL1 PT-INR Value

N	Mean	Standard Deviation	Minimum	Maximum
4	2.2750000	0.9535023	1.7000000	3.7000000

Unique Subject Identifier=1188011110-0027

Assay variable : PTINR_VAL1 PT-INR Value

N	Mean	Standard Deviation	Minimum	Maximum
5	2.0420000	0.1953714	1.8100000	2.2700000

Unique Subject Identifier=1188011110-0068

Assay variable : PTINR_VAL1 PT-INR Value

N	Mean	Standard Deviation	Minimum	Maximum
3	2.1433333	0.0971253	2.0600000	2.2500000

Unique Subject Identifier=1188011110-0124

Assay variable : PTINR_VAL1 PT-INR Value

N	Mean	Standard Deviation	Minimum	Maximum
4	1.5925000	0.1569235	1.4200000	1.8000000

Laboratory tests at the time of obtaining Table 2-6 consent, according to the presence or absence of proton-pump inhibitors

Unique Subject Identifier=1189001100-0001

MEANS Procedure

Assay variable : PTINR_VAL1 PT-INR Value

N	Mean	Standard Deviation	Minimum	Maximum
2	1.3950000	0.0636396	1.3500000	1.4400000

Unique Subject Identifier=1189001100-0008

Assay variable : PTINR_VAL1 PT-INR Value

N	Mean	Standard Deviation	Minimum	Maximum
4	3.1475000	0.8403323	1.9500000	3.8500000

Unique Subject Identifier=1189001100-0017

Assay variable : PTINR_VAL1 PT-INR Value

N	Mean	Standard Deviation	Minimum	Maximum
1	1.8800000		1.8800000	1.8800000

Unique Subject Identifier=1191000000-0001

Assay variable : PTINR_VAL1 PT-INR Value

N	Mean	Standard Deviation	Minimum	Maximum
6	1.4550000	0.1266096	1.3100000	1.6200000

Laboratory tests at the time of obtaining Table 2-6 consent, according to the presence or absence of proton-pump inhibitors

Unique Subject Identifier=1194000000-0014

MEANS Procedure

Assay variable : PTINR_VAL1 PT-INR Value

N	Mean	Standard Deviation	Minimum	Maximum
2	1.8650000	0.1484924	1.7600000	1.9700000

Unique Subject Identifier=1195000000-0006

Assay variable : PTINR_VAL1 PT-INR Value

N	Mean	Standard Deviation	Minimum	Maximum
4	1.6700000	0.3436083	1.4300000	2.1700000

Unique Subject Identifier=1195000000-0007

Assay variable : PTINR_VAL1 PT-INR Value

N	Mean	Standard Deviation	Minimum	Maximum
5	1.4120000	0.1223520	1.2400000	1.5500000

Unique Subject Identifier=1195000000-0008

Assay variable : PTINR_VAL1 PT-INR Value

N	Mean	Standard Deviation	Minimum	Maximum
2	1.8800000	0.1414214	1.7800000	1.9800000

Laboratory tests at the time of obtaining Table 2-6 consent, according to the presence or absence of proton-pump inhibitors

Unique Subject Identifier=1195000000-0009

MEANS Procedure

Assay variable : PTINR_VAL1 PT-INR Value

N	Mean	Standard Deviation	Minimum	Maximum
4	2.1350000	0.4702836	1.8800000	2.8400000

Unique Subject Identifier=1195000000-0010

Assay variable : PTINR_VAL1 PT-INR Value

N	Mean	Standard Deviation	Minimum	Maximum
6	1.8816667	0.2770138	1.4300000	2.1500000

Unique Subject Identifier=1195000000-0011

Assay variable : PTINR_VAL1 PT-INR Value

N	Mean	Standard Deviation	Minimum	Maximum
5	1.9100000	0.2876630	1.5000000	2.2600000

Unique Subject Identifier=1195000000-0012

Assay variable : PTINR_VAL1 PT-INR Value

N	Mean	Standard Deviation	Minimum	Maximum
4	1.6900000	0.1166190	1.5900000	1.8100000

Laboratory tests at the time of obtaining Table 2-6 consent, according to the presence or absence of proton-pump inhibitors

Unique Subject Identifier=1195000000-0013

MEANS Procedure

Assay variable : PTINR_VAL1 PT-INR Value

N	Mean	Standard Deviation	Minimum	Maximum
4	1.3575000	0.1613227	1.1200000	1.4800000

Unique Subject Identifier=1195000000-0014

Assay variable : PTINR_VAL1 PT-INR Value

N	Mean	Standard Deviation	Minimum	Maximum
4	1.9225000	0.6399154	1.3400000	2.8300000

Unique Subject Identifier=1195000000-0015

Assay variable : PTINR_VAL1 PT-INR Value

N	Mean	Standard Deviation	Minimum	Maximum
4	1.2400000	0.0905539	1.1400000	1.3600000

Unique Subject Identifier=1195000000-0016

Assay variable : PTINR_VAL1 PT-INR Value

N	Mean	Standard Deviation	Minimum	Maximum
4	2.4325000	0.2061351	2.2300000	2.6200000

Laboratory tests at the time of obtaining Table 2-6 consent, according to the presence or absence of proton-pump inhibitors

Unique Subject Identifier=1195000000-0017

MEANS Procedure

Assay variable : PTINR_VAL1 PT-INR Value

N	Mean	Standard Deviation	Minimum	Maximum
4	1.8775000	0.1164403	1.7300000	2.0100000

Unique Subject Identifier=1195000000-0018

Assay variable : PTINR_VAL1 PT-INR Value

N	Mean	Standard Deviation	Minimum	Maximum
5	1.3260000	0.1223928	1.1600000	1.4200000

Unique Subject Identifier=1195000000-0019

Assay variable : PTINR_VAL1 PT-INR Value

N	Mean	Standard Deviation	Minimum	Maximum
5	1.4920000	0.0947101	1.3400000	1.6000000

Unique Subject Identifier=1195000000-0020

Assay variable : PTINR_VAL1 PT-INR Value

N	Mean	Standard Deviation	Minimum	Maximum
4	1.9200000	0.0621825	1.8700000	2.0100000

Laboratory tests at the time of obtaining Table 2-6 consent, according to the presence or absence of proton-pump inhibitors

Unique Subject Identifier=1198000000-0003

MEANS Procedure

Assay variable : PTINR_VAL1 PT-INR Value

N	Mean	Standard Deviation	Minimum	Maximum
6	2.0616667	0.3142876	1.6500000	2.6000000

Unique Subject Identifier=1198000000-0005

Assay variable : PTINR_VAL1 PT-INR Value

N	Mean	Standard Deviation	Minimum	Maximum
6	1.8083333	0.0970395	1.6500000	1.9000000

Unique Subject Identifier=1198000000-0008

Assay variable : PTINR_VAL1 PT-INR Value

N	Mean	Standard Deviation	Minimum	Maximum
6	1.9200000	0.1963670	1.7000000	2.1600000

Unique Subject Identifier=1198000000-0009

Assay variable : PTINR_VAL1 PT-INR Value

N	Mean	Standard Deviation	Minimum	Maximum
6	1.6250000	0.1635543	1.4000000	1.8500000

Laboratory tests at the time of obtaining Table 2-6 consent, according to the presence or absence of proton-pump inhibitors

Unique Subject Identifier=1198000000-0010

MEANS Procedure

Assay variable : PTINR_VAL1 PT-INR Value

N	Mean	Standard Deviation	Minimum	Maximum
5	1.6800000	0.3213254	1.1500000	2.0000000

Unique Subject Identifier=1198000000-0011

Assay variable : PTINR_VAL1 PT-INR Value

N	Mean	Standard Deviation	Minimum	Maximum
6	2.2183333	0.1158303	2.0100000	2.3100000

Unique Subject Identifier=1198000000-0014

Assay variable : PTINR_VAL1 PT-INR Value

N	Mean	Standard Deviation	Minimum	Maximum
5	2.3660000	0.0536656	2.3000000	2.4500000

Unique Subject Identifier=1198000000-0015

Assay variable : PTINR_VAL1 PT-INR Value

N	Mean	Standard Deviation	Minimum	Maximum
6	1.5350000	0.2105944	1.1500000	1.7500000

Laboratory tests at the time of obtaining Table 2-6 consent, according to the presence or absence of proton-pump inhibitors

Unique Subject Identifier=1198000000-0017

MEANS Procedure

Assay variable : PTINR_VAL1 PT-INR Value

N	Mean	Standard Deviation	Minimum	Maximum
5	2.3000000	0.0651920	2.2100000	2.3600000

Unique Subject Identifier=1198000000-0018

Assay variable : PTINR_VAL1 PT-INR Value

N	Mean	Standard Deviation	Minimum	Maximum
6	1.7033333	0.6047038	0.4900000	2.0600000

Unique Subject Identifier=1198000000-0021

Assay variable : PTINR_VAL1 PT-INR Value

N	Mean	Standard Deviation	Minimum	Maximum
2	1.9500000	0	1.9500000	1.9500000

Unique Subject Identifier=1198000000-0023

Assay variable : PTINR_VAL1 PT-INR Value

N	Mean	Standard Deviation	Minimum	Maximum
5	2.2400000	0.0908295	2.1100000	2.3600000

Laboratory tests at the time of obtaining Table 2-6 consent, according to the presence or absence of proton-pump inhibitors

Unique Subject Identifier=1198000000-0024

MEANS Procedure

Assay variable : PTINR_VAL1 PT-INR Value

N	Mean	Standard Deviation	Minimum	Maximum
2	2.2100000	0.0707107	2.1600000	2.2600000

Unique Subject Identifier=1198000000-0026

Assay variable : PTINR_VAL1 PT-INR Value

N	Mean	Standard Deviation	Minimum	Maximum
5	1.5820000	0.3864842	1.1500000	2.0600000

Unique Subject Identifier=1198000000-0029

Assay variable : PTINR_VAL1 PT-INR Value

N	Mean	Standard Deviation	Minimum	Maximum
5	1.7740000	0.3963332	1.3500000	2.3100000

Unique Subject Identifier=1198000000-0030

Assay variable : PTINR_VAL1 PT-INR Value

N	Mean	Standard Deviation	Minimum	Maximum
5	1.8860000	0.2642537	1.5500000	2.1100000

Laboratory tests at the time of obtaining Table 2-6 consent, according to the presence or absence of proton-pump inhibitors

Unique Subject Identifier=1198000000-0031

MEANS Procedure

Assay variable : PTINR_VAL1 PT-INR Value

N	Mean	Standard Deviation	Minimum	Maximum
5	1.9940000	0.4897755	1.4000000	2.6000000

Unique Subject Identifier=1198000000-0032

Assay variable : PTINR_VAL1 PT-INR Value

N	Mean	Standard Deviation	Minimum	Maximum
5	1.6720000	0.4745208	0.8500000	2.0600000

Unique Subject Identifier=1198000000-0033

Assay variable : PTINR_VAL1 PT-INR Value

N	Mean	Standard Deviation	Minimum	Maximum
2	0.4950000	0.5020458	0.1400000	0.8500000

Unique Subject Identifier=1198000000-0034

Assay variable : PTINR_VAL1 PT-INR Value

N	Mean	Standard Deviation	Minimum	Maximum
2	1.1450000	0.9263099	0.4900000	1.8000000

Laboratory tests at the time of obtaining Table 2-6 consent, according to the presence or absence of proton-pump inhibitors

Unique Subject Identifier=1198000000-0035

MEANS Procedure

Assay variable : PTINR_VAL1 PT-INR Value

N	Mean	Standard Deviation	Minimum	Maximum
2	2.3350000	0.0353553	2.3100000	2.3600000

Unique Subject Identifier=1198000000-0039

Assay variable : PTINR_VAL1 PT-INR Value

N	Mean	Standard Deviation	Minimum	Maximum
6	1.9050000	0.1755847	1.7000000	2.1100000

Unique Subject Identifier=1198000000-0041

Assay variable : PTINR_VAL1 PT-INR Value

N	Mean	Standard Deviation	Minimum	Maximum
6	2.3350000	0.0689202	2.2100000	2.4100000

Unique Subject Identifier=1200010111-0005

Assay variable : PTINR_VAL1 PT-INR Value

N	Mean	Standard Deviation	Minimum	Maximum
4	1.5250000	0.2500000	1.4000000	1.9000000

Laboratory tests at the time of obtaining Table 2-6 consent, according to the presence or absence of proton-pump inhibitors

Unique Subject Identifier=1200010111-0007

MEANS Procedure

Assay variable : PTINR_VAL1 PT-INR Value

N	Mean	Standard Deviation	Minimum	Maximum
3	1.9000000	0.1732051	1.7000000	2.0000000

Unique Subject Identifier=1200010111-0013

Assay variable : PTINR_VAL1 PT-INR Value

N	Mean	Standard Deviation	Minimum	Maximum
3	1.8000000	0.1000000	1.7000000	1.9000000

Unique Subject Identifier=1200010111-0019

Assay variable : PTINR_VAL1 PT-INR Value

N	Mean	Standard Deviation	Minimum	Maximum
3	1.6333333	0.2081666	1.4000000	1.8000000

Unique Subject Identifier=1200010111-0020

Assay variable : PTINR_VAL1 PT-INR Value

N	Mean	Standard Deviation	Minimum	Maximum
3	1.6000000	0.2000000	1.4000000	1.8000000

Laboratory tests at the time of obtaining Table 2-6 consent, according to the presence or absence of proton-pump inhibitors

Unique Subject Identifier=1200010111-0026

MEANS Procedure

Assay variable : PTINR_VAL1 PT-INR Value

N	Mean	Standard Deviation	Minimum	Maximum
1	1.6000000		1.6000000	1.6000000

Unique Subject Identifier=1201000000-0001

Assay variable : PTINR_VAL1 PT-INR Value

N	Mean	Standard Deviation	Minimum	Maximum
4	1.7175000	0.0997914	1.6100000	1.8400000

Unique Subject Identifier=1201000000-0003

Assay variable : PTINR_VAL1 PT-INR Value

N	Mean	Standard Deviation	Minimum	Maximum
4	1.5600000	0.1540563	1.4200000	1.7600000

Unique Subject Identifier=1201000000-0004

Assay variable : PTINR_VAL1 PT-INR Value

N	Mean	Standard Deviation	Minimum	Maximum
5	1.9460000	0.3261595	1.4400000	2.3000000

Laboratory tests at the time of obtaining Table 2-6 consent, according to the presence or absence of proton-pump inhibitors

Unique Subject Identifier=1201000000-0007

MEANS Procedure

Assay variable : PTINR_VAL1 PT-INR Value

N	Mean	Standard Deviation	Minimum	Maximum
5	1.9120000	0.3495283	1.4300000	2.2400000

Unique Subject Identifier=1201000000-0008

Assay variable : PTINR_VAL1 PT-INR Value

N	Mean	Standard Deviation	Minimum	Maximum
4	2.0050000	0.3845777	1.4800000	2.3700000

Unique Subject Identifier=1201000000-0010

Assay variable : PTINR_VAL1 PT-INR Value

N	Mean	Standard Deviation	Minimum	Maximum
4	1.8475000	0.1090489	1.6900000	1.9300000

Unique Subject Identifier=1201000000-0011

Assay variable : PTINR_VAL1 PT-INR Value

N	Mean	Standard Deviation	Minimum	Maximum
4	1.7400000	0.1476482	1.6000000	1.9300000

Laboratory tests at the time of obtaining Table 2-6 consent, according to the presence or absence of proton-pump inhibitors

Unique Subject Identifier=1203010000-0004

MEANS Procedure

Assay variable : PTINR_VAL1 PT-INR Value

N	Mean	Standard Deviation	Minimum	Maximum
6	2.3183333	0.3921947	1.8800000	3.0100000

Unique Subject Identifier=1203010000-0005

Assay variable : PTINR_VAL1 PT-INR Value

N	Mean	Standard Deviation	Minimum	Maximum
6	2.0366667	0.3356586	1.6800000	2.6100000

Unique Subject Identifier=1203010000-0006

Assay variable : PTINR_VAL1 PT-INR Value

N	Mean	Standard Deviation	Minimum	Maximum
6	2.7866667	0.2260678	2.4300000	3.0400000

Unique Subject Identifier=1203010000-0009

Assay variable : PTINR_VAL1 PT-INR Value

N	Mean	Standard Deviation	Minimum	Maximum
6	2.1983333	0.7104764	1.5100000	3.4000000

Laboratory tests at the time of obtaining Table 2-6 consent, according to the presence or absence of proton-pump inhibitors

Unique Subject Identifier=1203010000-0010

MEANS Procedure

Assay variable : PTINR_VAL1 PT-INR Value

N	Mean	Standard Deviation	Minimum	Maximum
6	2.8366667	0.6295289	2.1100000	3.8500000

Unique Subject Identifier=1203010000-0013

Assay variable : PTINR_VAL1 PT-INR Value

N	Mean	Standard Deviation	Minimum	Maximum
6	2.3300000	0.2451938	2.0200000	2.6900000

Unique Subject Identifier=1203010000-0017

Assay variable : PTINR_VAL1 PT-INR Value

N	Mean	Standard Deviation	Minimum	Maximum
6	2.4350000	1.0120623	1.1200000	3.8800000

Unique Subject Identifier=1203010000-0018

Assay variable : PTINR_VAL1 PT-INR Value

N	Mean	Standard Deviation	Minimum	Maximum
6	2.6266667	0.4180750	2.0200000	3.3200000

Laboratory tests at the time of obtaining Table 2-6 consent, according to the presence or absence of proton-pump inhibitors

Unique Subject Identifier=1203010000-0020

MEANS Procedure

Assay variable : PTINR_VAL1 PT-INR Value

N	Mean	Standard Deviation	Minimum	Maximum
6	2.3416667	0.3456829	1.9700000	2.7900000

Unique Subject Identifier=1203010000-0022

Assay variable : PTINR_VAL1 PT-INR Value

N	Mean	Standard Deviation	Minimum	Maximum
6	2.2500000	0.2279474	1.9900000	2.6300000

Unique Subject Identifier=1203010000-0026

Assay variable : PTINR_VAL1 PT-INR Value

N	Mean	Standard Deviation	Minimum	Maximum
6	2.0316667	0.1986370	1.7900000	2.3800000

Unique Subject Identifier=1203010000-0028

Assay variable : PTINR_VAL1 PT-INR Value

N	Mean	Standard Deviation	Minimum	Maximum
6	2.4366667	0.9070759	1.7500000	4.1600000

Laboratory tests at the time of obtaining Table 2-6 consent, according to the presence or absence of proton-pump inhibitors

Unique Subject Identifier=1203010000-0030

MEANS Procedure

Assay variable : PTINR_VAL1 PT-INR Value

N	Mean	Standard Deviation	Minimum	Maximum
6	2.0050000	0.3174114	1.6800000	2.5800000

Unique Subject Identifier=1203010000-0031

Assay variable : PTINR_VAL1 PT-INR Value

N	Mean	Standard Deviation	Minimum	Maximum
6	2.6183333	0.3869582	2.2100000	3.1200000

Unique Subject Identifier=1203010000-0038

Assay variable : PTINR_VAL1 PT-INR Value

N	Mean	Standard Deviation	Minimum	Maximum
6	2.6850000	0.5224844	2.0900000	3.4600000

Unique Subject Identifier=1203010000-0040

Assay variable : PTINR_VAL1 PT-INR Value

N	Mean	Standard Deviation	Minimum	Maximum
6	1.8400000	0.1788854	1.5500000	2.0600000

Laboratory tests at the time of obtaining Table 2-6 consent, according to the presence or absence of proton-pump inhibitors

Unique Subject Identifier=1206000000-0003

MEANS Procedure

Assay variable : PTINR_VAL1 PT-INR Value

N	Mean	Standard Deviation	Minimum	Maximum
3	2.3833333	0.2050203	2.1800000	2.5900000

Unique Subject Identifier=1212000000-0001

Assay variable : PTINR_VAL1 PT-INR Value

N	Mean	Standard Deviation	Minimum	Maximum
6	1.9650000	0.3414528	1.6200000	2.5300000

Unique Subject Identifier=1212000000-0002

Assay variable : PTINR_VAL1 PT-INR Value

N	Mean	Standard Deviation	Minimum	Maximum
6	2.0916667	0.7101385	1.4500000	3.4000000

Unique Subject Identifier=1212000000-0004

Assay variable : PTINR_VAL1 PT-INR Value

N	Mean	Standard Deviation	Minimum	Maximum
5	1.9220000	0.6746999	1.4900000	3.1200000

Laboratory tests at the time of obtaining Table 2-6 consent, according to the presence or absence of proton-pump inhibitors

Unique Subject Identifier=1212000000-0006

MEANS Procedure

Assay variable : PTINR_VAL1 PT-INR Value

N	Mean	Standard Deviation	Minimum	Maximum
6	2.3450000	0.3283748	1.8300000	2.7300000

Unique Subject Identifier=1212000000-0007

Assay variable : PTINR_VAL1 PT-INR Value

N	Mean	Standard Deviation	Minimum	Maximum
3	2.2766667	0.1101514	2.1500000	2.3500000

Unique Subject Identifier=1212000000-0012

Assay variable : PTINR_VAL1 PT-INR Value

N	Mean	Standard Deviation	Minimum	Maximum
4	1.7750000	0.4110556	1.4900000	2.3800000

Unique Subject Identifier=1212000000-0014

Assay variable : PTINR_VAL1 PT-INR Value

N	Mean	Standard Deviation	Minimum	Maximum
6	2.3200000	0.2384114	1.9800000	2.6300000

Laboratory tests at the time of obtaining Table 2-6 consent, according to the presence or absence of proton-pump inhibitors

Unique Subject Identifier=1212000000-0015

MEANS Procedure

Assay variable : PTINR_VAL1 PT-INR Value

N	Mean	Standard Deviation	Minimum	Maximum
5	2.0980000	0.4196070	1.7500000	2.5800000

Unique Subject Identifier=1216000000-0001

Assay variable : PTINR_VAL1 PT-INR Value

N	Mean	Standard Deviation	Minimum	Maximum
5	1.3360000	0.4370698	1.0600000	2.1100000

Unique Subject Identifier=1216000000-0002

Assay variable : PTINR_VAL1 PT-INR Value

N	Mean	Standard Deviation	Minimum	Maximum
5	1.5400000	0.2750455	1.0900000	1.7900000

Unique Subject Identifier=1216000000-0004

Assay variable : PTINR_VAL1 PT-INR Value

N	Mean	Standard Deviation	Minimum	Maximum
3	1.3433333	0.1619671	1.2400000	1.5300000

Laboratory tests at the time of obtaining Table 2-6 consent, according to the presence or absence of proton-pump inhibitors

Unique Subject Identifier=1219000000-0005

MEANS Procedure

Assay variable : PTINR_VAL1 PT-INR Value

N	Mean	Standard Deviation	Minimum	Maximum
2	1.5700000	0.1272792	1.4800000	1.6600000

Unique Subject Identifier=1219000000-0006

Assay variable : PTINR_VAL1 PT-INR Value

N	Mean	Standard Deviation	Minimum	Maximum
3	2.0300000	0.3315117	1.7600000	2.4000000

Unique Subject Identifier=1219000000-0009

Assay variable : PTINR_VAL1 PT-INR Value

N	Mean	Standard Deviation	Minimum	Maximum
4	1.9000000	0.0787401	1.8000000	1.9900000

Unique Subject Identifier=1219000000-0011

Assay variable : PTINR_VAL1 PT-INR Value

N	Mean	Standard Deviation	Minimum	Maximum
3	2.2500000	0.2007486	2.0600000	2.4600000

Laboratory tests at the time of obtaining Table 2-6 consent, according to the presence or absence of proton-pump inhibitors

Unique Subject Identifier=1219000000-0013

MEANS Procedure

Assay variable : PTINR_VAL1 PT-INR Value

N	Mean	Standard Deviation	Minimum	Maximum
3	2.2433333	0.2608320	2.0500000	2.5400000

Unique Subject Identifier=1219000000-0014

Assay variable : PTINR_VAL1 PT-INR Value

N	Mean	Standard Deviation	Minimum	Maximum
4	1.7850000	0.0723418	1.7200000	1.8700000

Unique Subject Identifier=1219000000-0016

Assay variable : PTINR_VAL1 PT-INR Value

N	Mean	Standard Deviation	Minimum	Maximum
3	2.2466667	0.4842864	1.9000000	2.8000000

Unique Subject Identifier=1219000000-0017

Assay variable : PTINR_VAL1 PT-INR Value

N	Mean	Standard Deviation	Minimum	Maximum
3	2.2833333	0.3089229	1.9500000	2.5600000

Laboratory tests at the time of obtaining Table 2-6 consent, according to the presence or absence of proton-pump inhibitors

Unique Subject Identifier=1219000000-0018

MEANS Procedure

Assay variable : PTINR_VAL1 PT-INR Value

N	Mean	Standard Deviation	Minimum	Maximum
4	2.0450000	1.3675647	1.0200000	4.0600000

Unique Subject Identifier=1219000000-0021

Assay variable : PTINR_VAL1 PT-INR Value

N	Mean	Standard Deviation	Minimum	Maximum
3	1.7666667	0.4128357	1.2900000	2.0100000

Unique Subject Identifier=1219000000-0023

Assay variable : PTINR_VAL1 PT-INR Value

N	Mean	Standard Deviation	Minimum	Maximum
3	2.2100000	0.1571623	2.0700000	2.3800000

Unique Subject Identifier=1219000000-0024

Assay variable : PTINR_VAL1 PT-INR Value

N	Mean	Standard Deviation	Minimum	Maximum
3	2.2800000	0.1571623	2.1400000	2.4500000

Laboratory tests at the time of obtaining Table 2-6 consent, according to the presence or absence of proton-pump inhibitors

Unique Subject Identifier=1219000000-0026

MEANS Procedure

Assay variable : PTINR_VAL1 PT-INR Value

N	Mean	Standard Deviation	Minimum	Maximum
2	1.7800000	0.1414214	1.6800000	1.8800000

Unique Subject Identifier=1219000000-0030

Assay variable : PTINR_VAL1 PT-INR Value

N	Mean	Standard Deviation	Minimum	Maximum
3	1.8000000	0.0100000	1.7900000	1.8100000

Unique Subject Identifier=1219000000-0033

Assay variable : PTINR_VAL1 PT-INR Value

N	Mean	Standard Deviation	Minimum	Maximum
3	1.8300000	0.8029321	1.1000000	2.6900000

Unique Subject Identifier=1219000000-0034

Assay variable : PTINR_VAL1 PT-INR Value

N	Mean	Standard Deviation	Minimum	Maximum
4	1.5650000	0.0704746	1.4700000	1.6400000

Laboratory tests at the time of obtaining Table 2-6 consent, according to the presence or absence of proton-pump inhibitors

Unique Subject Identifier=1219000000-0035

MEANS Procedure

Assay variable : PTINR_VAL1 PT-INR Value

N	Mean	Standard Deviation	Minimum	Maximum
1	1.9500000		1.9500000	1.9500000

Unique Subject Identifier=1219000000-0036

Assay variable : PTINR_VAL1 PT-INR Value

N	Mean	Standard Deviation	Minimum	Maximum
2	2.0200000	0.1697056	1.9000000	2.1400000

Unique Subject Identifier=1221000000-0001

Assay variable : PTINR_VAL1 PT-INR Value

N	Mean	Standard Deviation	Minimum	Maximum
6	2.1000000	0.5621388	1.5000000	3.1000000

Unique Subject Identifier=1222000000-0002

Assay variable : PTINR_VAL1 PT-INR Value

N	Mean	Standard Deviation	Minimum	Maximum
6	2.5166667	0.4020779	1.9000000	3.1000000

Laboratory tests at the time of obtaining Table 2-6 consent, according to the presence or absence of proton-pump inhibitors

Unique Subject Identifier=1222000000-0005

MEANS Procedure

Assay variable : PTINR_VAL1 PT-INR Value

N	Mean	Standard Deviation	Minimum	Maximum
6	1.9666667	0.3204164	1.7000000	2.5000000

Unique Subject Identifier=1222000000-0009

Assay variable : PTINR_VAL1 PT-INR Value

N	Mean	Standard Deviation	Minimum	Maximum
5	1.8600000	0.2880972	1.5000000	2.2000000

Unique Subject Identifier=1222000000-0012

Assay variable : PTINR_VAL1 PT-INR Value

N	Mean	Standard Deviation	Minimum	Maximum
6	1.8833333	0.3250641	1.6000000	2.5000000

Unique Subject Identifier=1223010000-0002

Assay variable : PTINR_VAL1 PT-INR Value

N	Mean	Standard Deviation	Minimum	Maximum
4	2.2250000	0.4645787	1.6000000	2.7000000

Laboratory tests at the time of obtaining Table 2-6 consent, according to the presence or absence of proton-pump inhibitors

Unique Subject Identifier=1223010000-0003

MEANS Procedure

Assay variable : PTINR_VAL1 PT-INR Value

N	Mean	Standard Deviation	Minimum	Maximum
4	2.4750000	0.8958236	1.6000000	3.7000000

Unique Subject Identifier=1223010000-0005

Assay variable : PTINR_VAL1 PT-INR Value

N	Mean	Standard Deviation	Minimum	Maximum
4	2.3250000	0.2986079	2.0000000	2.7000000

Unique Subject Identifier=1223010000-0006

Assay variable : PTINR_VAL1 PT-INR Value

N	Mean	Standard Deviation	Minimum	Maximum
4	1.6000000	0.2160247	1.3000000	1.8000000

Unique Subject Identifier=1223010000-0008

Assay variable : PTINR_VAL1 PT-INR Value

N	Mean	Standard Deviation	Minimum	Maximum
6	2.2000000	0.1788854	2.0000000	2.5000000

Laboratory tests at the time of obtaining Table 2-6 consent, according to the presence or absence of proton-pump inhibitors

Unique Subject Identifier=1223010000-0012

MEANS Procedure

Assay variable : PTINR_VAL1 PT-INR Value

N	Mean	Standard Deviation	Minimum	Maximum
5	1.8400000	0.2408319	1.6000000	2.1000000

Unique Subject Identifier=1223010000-0015

Assay variable : PTINR_VAL1 PT-INR Value

N	Mean	Standard Deviation	Minimum	Maximum
4	2.0500000	0.1290994	1.9000000	2.2000000

Unique Subject Identifier=1223010000-0023

Assay variable : PTINR_VAL1 PT-INR Value

N	Mean	Standard Deviation	Minimum	Maximum
4	1.8500000	0.2081666	1.6000000	2.1000000

Unique Subject Identifier=1223010000-0025

Assay variable : PTINR_VAL1 PT-INR Value

N	Mean	Standard Deviation	Minimum	Maximum
6	1.7500000	0.0836660	1.7000000	1.9000000

Laboratory tests at the time of obtaining Table 2-6 consent, according to the presence or absence of proton-pump inhibitors

Unique Subject Identifier=1223010000-0030

MEANS Procedure

Assay variable : PTINR_VAL1 PT-INR Value

N	Mean	Standard Deviation	Minimum	Maximum
4	1.9000000	0.1825742	1.7000000	2.1000000

Unique Subject Identifier=1223010000-0031

Assay variable : PTINR_VAL1 PT-INR Value

N	Mean	Standard Deviation	Minimum	Maximum
5	2.0000000	0.2236068	1.7000000	2.3000000

Unique Subject Identifier=1223010000-0033

Assay variable : PTINR_VAL1 PT-INR Value

N	Mean	Standard Deviation	Minimum	Maximum
4	2.3250000	0.3862210	1.8000000	2.7000000

Unique Subject Identifier=1223010000-0035

Assay variable : PTINR_VAL1 PT-INR Value

N	Mean	Standard Deviation	Minimum	Maximum
4	2.0500000	0.3872983	1.5000000	2.4000000

Laboratory tests at the time of obtaining Table 2-6 consent, according to the presence or absence of proton-pump inhibitors

Unique Subject Identifier=1223010000-0037

MEANS Procedure

Assay variable : PTINR_VAL1 PT-INR Value

N	Mean	Standard Deviation	Minimum	Maximum
4	1.6250000	0.1258306	1.5000000	1.8000000

Unique Subject Identifier=1223010000-0039

Assay variable : PTINR_VAL1 PT-INR Value

N	Mean	Standard Deviation	Minimum	Maximum
4	2.1250000	0.1500000	2.0000000	2.3000000

Unique Subject Identifier=1223010000-0041

Assay variable : PTINR_VAL1 PT-INR Value

N	Mean	Standard Deviation	Minimum	Maximum
6	2.1166667	0.3763863	1.7000000	2.8000000

Unique Subject Identifier=1223010000-0042

Assay variable : PTINR_VAL1 PT-INR Value

N	Mean	Standard Deviation	Minimum	Maximum
4	1.8500000	0.3696846	1.4000000	2.3000000

Laboratory tests at the time of obtaining Table 2-6 consent, according to the presence or absence of proton-pump inhibitors

Unique Subject Identifier=1223010000-0043

MEANS Procedure

Assay variable : PTINR_VAL1 PT-INR Value

N	Mean	Standard Deviation	Minimum	Maximum
5	2.3800000	0.4147288	2.0000000	3.0000000

Unique Subject Identifier=1226000000-0002

Assay variable : PTINR_VAL1 PT-INR Value

N	Mean	Standard Deviation	Minimum	Maximum
2	1.2300000	0.0707107	1.1800000	1.2800000

Unique Subject Identifier=1226000000-0003

Assay variable : PTINR_VAL1 PT-INR Value

N	Mean	Standard Deviation	Minimum	Maximum
5	1.9180000	0.2087343	1.6600000	2.2100000

Unique Subject Identifier=1226000000-0005

Assay variable : PTINR_VAL1 PT-INR Value

N	Mean	Standard Deviation	Minimum	Maximum
4	2.0700000	0.1626858	1.9200000	2.2300000

Laboratory tests at the time of obtaining Table 2-6 consent, according to the presence or absence of proton-pump inhibitors

Unique Subject Identifier=1226000000-0006

MEANS Procedure

Assay variable : PTINR_VAL1 PT-INR Value

N	Mean	Standard Deviation	Minimum	Maximum
5	1.9680000	0.1283355	1.8000000	2.1300000

Unique Subject Identifier=1226000000-0008

Assay variable : PTINR_VAL1 PT-INR Value

N	Mean	Standard Deviation	Minimum	Maximum
5	1.6360000	0.1327780	1.5000000	1.8400000

Unique Subject Identifier=1226000000-0010

Assay variable : PTINR_VAL1 PT-INR Value

N	Mean	Standard Deviation	Minimum	Maximum
5	1.8080000	0.0846759	1.7100000	1.9200000

Unique Subject Identifier=1226000000-0011

Assay variable : PTINR_VAL1 PT-INR Value

N	Mean	Standard Deviation	Minimum	Maximum
6	1.9116667	0.1921891	1.5600000	2.1400000

Laboratory tests at the time of obtaining Table 2-6 consent, according to the presence or absence of proton-pump inhibitors

Unique Subject Identifier=1226000000-0012

MEANS Procedure

Assay variable : PTINR_VAL1 PT-INR Value

N	Mean	Standard Deviation	Minimum	Maximum
3	2.0133333	0.4960175	1.5000000	2.4900000

Unique Subject Identifier=1226000000-0014

Assay variable : PTINR_VAL1 PT-INR Value

N	Mean	Standard Deviation	Minimum	Maximum
5	2.0760000	0.4936902	1.5800000	2.8900000

Unique Subject Identifier=1226000000-0031

Assay variable : PTINR_VAL1 PT-INR Value

N	Mean	Standard Deviation	Minimum	Maximum
5	1.7000000	0.2351595	1.4700000	2.0300000

Unique Subject Identifier=1226000000-0032

Assay variable : PTINR_VAL1 PT-INR Value

N	Mean	Standard Deviation	Minimum	Maximum
5	1.9840000	0.1597811	1.7900000	2.2100000

Laboratory tests at the time of obtaining Table 2-6 consent, according to the presence or absence of proton-pump inhibitors

Unique Subject Identifier=1226000000-0034

MEANS Procedure

Assay variable : PTINR_VAL1 PT-INR Value

N	Mean	Standard Deviation	Minimum	Maximum
4	2.1525000	0.3247948	1.8900000	2.5800000

Unique Subject Identifier=1227000000-0027

Assay variable : PTINR_VAL1 PT-INR Value

N	Mean	Standard Deviation	Minimum	Maximum
4	2.3250000	0.2500000	2.0000000	2.6000000

Unique Subject Identifier=1227000000-0029

Assay variable : PTINR_VAL1 PT-INR Value

N	Mean	Standard Deviation	Minimum	Maximum
4	2.3750000	0.5439056	1.6000000	2.8000000

Unique Subject Identifier=1227000000-0030

Assay variable : PTINR_VAL1 PT-INR Value

N	Mean	Standard Deviation	Minimum	Maximum
3	2.0666667	0.5507571	1.7000000	2.7000000

Laboratory tests at the time of obtaining Table 2-6 consent, according to the presence or absence of proton-pump inhibitors

Unique Subject Identifier=1227000000-0031

MEANS Procedure

Assay variable : PTINR_VAL1 PT-INR Value

N	Mean	Standard Deviation	Minimum	Maximum
5	2.1600000	0.2880972	1.9000000	2.6000000

Unique Subject Identifier=1227000000-0032

Assay variable : PTINR_VAL1 PT-INR Value

N	Mean	Standard Deviation	Minimum	Maximum
5	2.1000000	0.2000000	1.9000000	2.4000000

Unique Subject Identifier=1227000000-0035

Assay variable : PTINR_VAL1 PT-INR Value

N	Mean	Standard Deviation	Minimum	Maximum
4	2.0750000	0.3862210	1.7000000	2.6000000

Unique Subject Identifier=1227000000-0038

Assay variable : PTINR_VAL1 PT-INR Value

N	Mean	Standard Deviation	Minimum	Maximum
5	2.3000000	0.2915476	1.9000000	2.7000000

Laboratory tests at the time of obtaining Table 2-6 consent, according to the presence or absence of proton-pump inhibitors

Unique Subject Identifier=1227000000-0041

MEANS Procedure

Assay variable : PTINR_VAL1 PT-INR Value

N	Mean	Standard Deviation	Minimum	Maximum
4	2.3000000	0.4966555	1.7000000	2.8000000

Unique Subject Identifier=1227000000-0043

Assay variable : PTINR_VAL1 PT-INR Value

N	Mean	Standard Deviation	Minimum	Maximum
5	2.2000000	0.1414214	2.1000000	2.4000000

Unique Subject Identifier=1227000000-0047

Assay variable : PTINR_VAL1 PT-INR Value

N	Mean	Standard Deviation	Minimum	Maximum
5	2.0400000	0.3847077	1.6000000	2.6000000

Unique Subject Identifier=1227000000-0050

Assay variable : PTINR_VAL1 PT-INR Value

N	Mean	Standard Deviation	Minimum	Maximum
6	1.9000000	0.1673320	1.7000000	2.1000000

Laboratory tests at the time of obtaining Table 2-6 consent, according to the presence or absence of proton-pump inhibitors

Unique Subject Identifier=1227000000-0055

MEANS Procedure

Assay variable : PTINR_VAL1 PT-INR Value

N	Mean	Standard Deviation	Minimum	Maximum
6	2.4333333	0.7004760	1.4000000	3.3000000

Unique Subject Identifier=1227000000-0058

Assay variable : PTINR_VAL1 PT-INR Value

N	Mean	Standard Deviation	Minimum	Maximum
6	2.0500000	0.2664583	1.7000000	2.4000000

Unique Subject Identifier=1227000000-0064

Assay variable : PTINR_VAL1 PT-INR Value

N	Mean	Standard Deviation	Minimum	Maximum
4	2.9500000	0.5972158	2.4000000	3.8000000

Unique Subject Identifier=1227000000-0089

Assay variable : PTINR_VAL1 PT-INR Value

N	Mean	Standard Deviation	Minimum	Maximum
6	1.8166667	0.2994439	1.3000000	2.1000000

Laboratory tests at the time of obtaining Table 2-6 consent, according to the presence or absence of proton-pump inhibitors

Unique Subject Identifier=1227000000-0091

MEANS Procedure

Assay variable : PTINR_VAL1 PT-INR Value

N	Mean	Standard Deviation	Minimum	Maximum
6	2.3166667	0.3868678	1.9000000	3.0000000

Unique Subject Identifier=1228000000-0004

Assay variable : PTINR_VAL1 PT-INR Value

N	Mean	Standard Deviation	Minimum	Maximum
1	2.9800000		2.9800000	2.9800000

Unique Subject Identifier=1229000000-0006

Assay variable : PTINR_VAL1 PT-INR Value

N	Mean	Standard Deviation	Minimum	Maximum
6	2.0500000	0.1048809	1.9000000	2.2000000

Unique Subject Identifier=1229000000-0007

Assay variable : PTINR_VAL1 PT-INR Value

N	Mean	Standard Deviation	Minimum	Maximum
5	2.4200000	0.1643168	2.3000000	2.6000000

Laboratory tests at the time of obtaining Table 2-6 consent, according to the presence or absence of proton-pump inhibitors

Unique Subject Identifier=1229000000-0010

MEANS Procedure

Assay variable : PTINR_VAL1 PT-INR Value

N	Mean	Standard Deviation	Minimum	Maximum
6	2.2333333	0.1032796	2.1000000	2.4000000

Unique Subject Identifier=1229000000-0017

Assay variable : PTINR_VAL1 PT-INR Value

N	Mean	Standard Deviation	Minimum	Maximum
6	2.3666667	0.9114092	1.6000000	4.1000000

Unique Subject Identifier=1229000000-0020

Assay variable : PTINR_VAL1 PT-INR Value

N	Mean	Standard Deviation	Minimum	Maximum
6	2.1833333	0.4119061	1.6000000	2.6000000

Unique Subject Identifier=1229000000-0021

Assay variable : PTINR_VAL1 PT-INR Value

N	Mean	Standard Deviation	Minimum	Maximum
6	2.0500000	0.2073644	1.9000000	2.4000000

Laboratory tests at the time of obtaining Table 2-6 consent, according to the presence or absence of proton-pump inhibitors

Unique Subject Identifier=1232000000-0003

MEANS Procedure

Assay variable : PTINR_VAL1 PT-INR Value

N	Mean	Standard Deviation	Minimum	Maximum
6	1.8166667	0.5913262	1.1000000	2.6000000

Unique Subject Identifier=1232000000-0004

Assay variable : PTINR_VAL1 PT-INR Value

N	Mean	Standard Deviation	Minimum	Maximum
6	2.0166667	0.4708149	1.5000000	2.6000000

Unique Subject Identifier=1232000000-0014

Assay variable : PTINR_VAL1 PT-INR Value

N	Mean	Standard Deviation	Minimum	Maximum
6	2.0833333	0.5946988	1.5000000	3.2000000

Unique Subject Identifier=1232000000-0016

Assay variable : PTINR_VAL1 PT-INR Value

N	Mean	Standard Deviation	Minimum	Maximum
6	1.7500000	0.1048809	1.6000000	1.9000000

Laboratory tests at the time of obtaining Table 2-6 consent, according to the presence or absence of proton-pump inhibitors

Unique Subject Identifier=1232000000-0017

MEANS Procedure

Assay variable : PTINR_VAL1 PT-INR Value

N	Mean	Standard Deviation	Minimum	Maximum
5	1.8000000	0.1870829	1.6000000	2.1000000

Unique Subject Identifier=1232000000-0020

Assay variable : PTINR_VAL1 PT-INR Value

N	Mean	Standard Deviation	Minimum	Maximum
6	2.4000000	0.4604346	1.8000000	3.2000000

Unique Subject Identifier=1235000000-0001

Assay variable : PTINR_VAL1 PT-INR Value

N	Mean	Standard Deviation	Minimum	Maximum
5	1.6580000	0.1360882	1.4500000	1.7700000

Unique Subject Identifier=1235000000-0003

Assay variable : PTINR_VAL1 PT-INR Value

N	Mean	Standard Deviation	Minimum	Maximum
5	1.4020000	0.0952365	1.2500000	1.5000000

Laboratory tests at the time of obtaining Table 2-6 consent, according to the presence or absence of proton-pump inhibitors

Unique Subject Identifier=1235000000-0005

MEANS Procedure

Assay variable : PTINR_VAL1 PT-INR Value

N	Mean	Standard Deviation	Minimum	Maximum
6	1.4550000	0.0971082	1.3600000	1.6300000

Unique Subject Identifier=1235000000-0008

Assay variable : PTINR_VAL1 PT-INR Value

N	Mean	Standard Deviation	Minimum	Maximum
5	1.8520000	0.1255787	1.7100000	1.9800000

Unique Subject Identifier=1235000000-0011

Assay variable : PTINR_VAL1 PT-INR Value

N	Mean	Standard Deviation	Minimum	Maximum
6	1.5500000	0.2484351	1.3500000	2.0400000

Unique Subject Identifier=1235000000-0015

Assay variable : PTINR_VAL1 PT-INR Value

N	Mean	Standard Deviation	Minimum	Maximum
6	1.7150000	0.2714959	1.4700000	2.1800000

Laboratory tests at the time of obtaining Table 2-6 consent, according to the presence or absence of proton-pump inhibitors

Unique Subject Identifier=1235000000-0020

MEANS Procedure

Assay variable : PTINR_VAL1 PT-INR Value

N	Mean	Standard Deviation	Minimum	Maximum
6	1.6316667	0.1404872	1.4800000	1.8400000

Unique Subject Identifier=1235000000-0022

Assay variable : PTINR_VAL1 PT-INR Value

N	Mean	Standard Deviation	Minimum	Maximum
6	1.6166667	0.2487301	1.3500000	1.9500000

Unique Subject Identifier=1235000000-0023

Assay variable : PTINR_VAL1 PT-INR Value

N	Mean	Standard Deviation	Minimum	Maximum
6	1.7250000	0.1375136	1.5800000	1.9100000

Unique Subject Identifier=1235000000-0026

Assay variable : PTINR_VAL1 PT-INR Value

N	Mean	Standard Deviation	Minimum	Maximum
6	1.4750000	0.1710848	1.2200000	1.6600000

Laboratory tests at the time of obtaining Table 2-6 consent, according to the presence or absence of proton-pump inhibitors

Unique Subject Identifier=1235000000-0033

MEANS Procedure

Assay variable : PTINR_VAL1 PT-INR Value

N	Mean	Standard Deviation	Minimum	Maximum
6	1.9400000	0.4784976	1.3500000	2.5200000

Unique Subject Identifier=1235000000-0040

Assay variable : PTINR_VAL1 PT-INR Value

N	Mean	Standard Deviation	Minimum	Maximum
3	1.7733333	0.0404145	1.7300000	1.8100000

Unique Subject Identifier=1235000000-0043

Assay variable : PTINR_VAL1 PT-INR Value

N	Mean	Standard Deviation	Minimum	Maximum
5	2.0400000	0.3647602	1.6800000	2.5400000

Unique Subject Identifier=1235000000-0045

Assay variable : PTINR_VAL1 PT-INR Value

N	Mean	Standard Deviation	Minimum	Maximum
6	1.5666667	0.1751190	1.2900000	1.8000000

Laboratory tests at the time of obtaining Table 2-6 consent, according to the presence or absence of proton-pump inhibitors

Unique Subject Identifier=1235000000-0050

MEANS Procedure

Assay variable : PTINR_VAL1 PT-INR Value

N	Mean	Standard Deviation	Minimum	Maximum
6	1.9616667	0.5810479	1.0400000	2.5200000

Unique Subject Identifier=1237000000-0006

Assay variable : PTINR_VAL1 PT-INR Value

N	Mean	Standard Deviation	Minimum	Maximum
5	2.1000000	0.3162278	1.8000000	2.6000000

Unique Subject Identifier=1237000000-0007

Assay variable : PTINR_VAL1 PT-INR Value

N	Mean	Standard Deviation	Minimum	Maximum
5	1.9000000	0.3872983	1.5000000	2.4000000

Unique Subject Identifier=1237000000-0010

Assay variable : PTINR_VAL1 PT-INR Value

N	Mean	Standard Deviation	Minimum	Maximum
3	1.7333333	0.0577350	1.7000000	1.8000000

Laboratory tests at the time of obtaining Table 2-6 consent, according to the presence or absence of proton-pump inhibitors

Unique Subject Identifier=1237000000-0013

MEANS Procedure

Assay variable : PTINR_VAL1 PT-INR Value

N	Mean	Standard Deviation	Minimum	Maximum
4	1.5250000	0.1500000	1.3000000	1.6000000

Unique Subject Identifier=1237000000-0014

Assay variable : PTINR_VAL1 PT-INR Value

N	Mean	Standard Deviation	Minimum	Maximum
3	1.5666667	0.2516611	1.3000000	1.8000000

Unique Subject Identifier=1249000000-0002

Assay variable : PTINR_VAL1 PT-INR Value

N	Mean	Standard Deviation	Minimum	Maximum
6	2.2166667	0.2141650	1.9700000	2.5600000

Unique Subject Identifier=1249000000-0008

Assay variable : PTINR_VAL1 PT-INR Value

N	Mean	Standard Deviation	Minimum	Maximum
6	1.8083333	0.1435851	1.6400000	2.0400000

Laboratory tests at the time of obtaining Table 2-6 consent, according to the presence or absence of proton-pump inhibitors

Unique Subject Identifier=1254100000-0002

MEANS Procedure

Assay variable : PTINR_VAL1 PT-INR Value

N	Mean	Standard Deviation	Minimum	Maximum
5	1.9600000	0.4159327	1.5000000	2.6000000

Unique Subject Identifier=1254100000-0013

Assay variable : PTINR_VAL1 PT-INR Value

N	Mean	Standard Deviation	Minimum	Maximum
6	1.8000000	0.2190890	1.5000000	2.1000000

Unique Subject Identifier=1254100000-0018

Assay variable : PTINR_VAL1 PT-INR Value

N	Mean	Standard Deviation	Minimum	Maximum
6	2.1333333	0.1211060	2.0000000	2.3000000

Unique Subject Identifier=1254100000-0019

Assay variable : PTINR_VAL1 PT-INR Value

N	Mean	Standard Deviation	Minimum	Maximum
5	2.2200000	0.3563706	1.7000000	2.7000000

Laboratory tests at the time of obtaining Table 2-6 consent, according to the presence or absence of proton-pump inhibitors

Unique Subject Identifier=1254100000-0021

MEANS Procedure

Assay variable : PTINR_VAL1 PT-INR Value

N	Mean	Standard Deviation	Minimum	Maximum
6	2.2166667	0.3311596	1.9000000	2.8000000

Unique Subject Identifier=1254100000-0024

Assay variable : PTINR_VAL1 PT-INR Value

N	Mean	Standard Deviation	Minimum	Maximum
6	2.0666667	0.3204164	1.7000000	2.5000000

Unique Subject Identifier=1254100000-0025

Assay variable : PTINR_VAL1 PT-INR Value

N	Mean	Standard Deviation	Minimum	Maximum
6	2.1166667	0.4665476	1.3000000	2.6000000

Unique Subject Identifier=1262000000-0019

Assay variable : PTINR_VAL1 PT-INR Value

N	Mean	Standard Deviation	Minimum	Maximum
2	1.3500000	0.2121320	1.2000000	1.5000000

Laboratory tests at the time of obtaining Table 2-6 consent, according to the presence or absence of proton-pump inhibitors

Unique Subject Identifier=1262000000-0030

MEANS Procedure

Assay variable : PTINR_VAL1 PT-INR Value

N	Mean	Standard Deviation	Minimum	Maximum
3	1.7333333	0.2081666	1.5000000	1.9000000

Unique Subject Identifier=1262000000-0049

Assay variable : PTINR_VAL1 PT-INR Value

N	Mean	Standard Deviation	Minimum	Maximum
1	1.3000000		1.3000000	1.3000000

Unique Subject Identifier=1262000000-0071

Assay variable : PTINR_VAL1 PT-INR Value

N	Mean	Standard Deviation	Minimum	Maximum
5	1.8200000	0.1303840	1.6000000	1.9000000

Unique Subject Identifier=1263011000-0014

Assay variable : PTINR_VAL1 PT-INR Value

N	Mean	Standard Deviation	Minimum	Maximum
6	2.6133333	0.5203717	2.0400000	3.2300000

Laboratory tests at the time of obtaining Table 2-6 consent, according to the presence or absence of proton-pump inhibitors

Unique Subject Identifier=1263011000-0027

MEANS Procedure

Assay variable : PTINR_VAL1 PT-INR Value

N	Mean	Standard Deviation	Minimum	Maximum
6	2.0200000	0.1589969	1.8800000	2.2900000

Unique Subject Identifier=1263011000-0033

Assay variable : PTINR_VAL1 PT-INR Value

N	Mean	Standard Deviation	Minimum	Maximum
6	2.0183333	0.6587994	1.5100000	3.2600000

Unique Subject Identifier=1266000000-0005

Assay variable : PTINR_VAL1 PT-INR Value

N	Mean	Standard Deviation	Minimum	Maximum
6	2.2616667	0.2018333	2.1000000	2.6000000

Unique Subject Identifier=1266000000-0009

Assay variable : PTINR_VAL1 PT-INR Value

N	Mean	Standard Deviation	Minimum	Maximum
6	2.2583333	0.3009596	1.9000000	2.6000000

Laboratory tests at the time of obtaining Table 2-6 consent, according to the presence or absence of proton-pump inhibitors

Unique Subject Identifier=1266000000-0015

MEANS Procedure

Assay variable : PTINR_VAL1 PT-INR Value

N	Mean	Standard Deviation	Minimum	Maximum
6	2.0766667	0.5020624	1.4000000	2.6600000

Unique Subject Identifier=1266000000-0016

Assay variable : PTINR_VAL1 PT-INR Value

N	Mean	Standard Deviation	Minimum	Maximum
6	2.0350000	0.8365584	1.3000000	3.2000000

Unique Subject Identifier=1266000000-0022

Assay variable : PTINR_VAL1 PT-INR Value

N	Mean	Standard Deviation	Minimum	Maximum
4	2.1975000	0.2723203	2.0000000	2.6000000

Unique Subject Identifier=1266000000-0025

Assay variable : PTINR_VAL1 PT-INR Value

N	Mean	Standard Deviation	Minimum	Maximum
6	2.5566667	0.6370767	1.5000000	3.3100000

Laboratory tests at the time of obtaining Table 2-6 consent, according to the presence or absence of proton-pump inhibitors

Unique Subject Identifier=1266000000-0027

MEANS Procedure

Assay variable : PTINR_VAL1 PT-INR Value

N	Mean	Standard Deviation	Minimum	Maximum
6	2.2783333	0.1510519	2.1000000	2.5000000

Unique Subject Identifier=1266000000-0030

Assay variable : PTINR_VAL1 PT-INR Value

N	Mean	Standard Deviation	Minimum	Maximum
6	2.2450000	0.2847279	1.8000000	2.6000000

Unique Subject Identifier=1266000000-0031

Assay variable : PTINR_VAL1 PT-INR Value

N	Mean	Standard Deviation	Minimum	Maximum
6	1.7666667	0.1175868	1.7000000	2.0000000

Unique Subject Identifier=1266000000-0032

Assay variable : PTINR_VAL1 PT-INR Value

N	Mean	Standard Deviation	Minimum	Maximum
6	1.6983333	0.5077959	0.7200000	2.1000000

Laboratory tests at the time of obtaining Table 2-6 consent, according to the presence or absence of proton-pump inhibitors

Unique Subject Identifier=1266000000-0033

MEANS Procedure

Assay variable : PTINR_VAL1 PT-INR Value

N	Mean	Standard Deviation	Minimum	Maximum
6	1.8833333	0.1329160	1.7000000	2.0000000

Unique Subject Identifier=1266000000-0034

Assay variable : PTINR_VAL1 PT-INR Value

N	Mean	Standard Deviation	Minimum	Maximum
6	1.6466667	0.1782882	1.5000000	2.0000000

Unique Subject Identifier=1266000000-0036

Assay variable : PTINR_VAL1 PT-INR Value

N	Mean	Standard Deviation	Minimum	Maximum
6	1.8600000	0.1814387	1.7000000	2.1700000

Unique Subject Identifier=1266000000-0039

Assay variable : PTINR_VAL1 PT-INR Value

N	Mean	Standard Deviation	Minimum	Maximum
6	1.6566667	0.1787363	1.4000000	1.9000000

Laboratory tests at the time of obtaining Table 2-6 consent, according to the presence or absence of proton-pump inhibitors

Unique Subject Identifier=1266000000-0041

MEANS Procedure

Assay variable : PTINR_VAL1 PT-INR Value

N	Mean	Standard Deviation	Minimum	Maximum
6	1.9000000	0.2529822	1.6000000	2.2000000

Unique Subject Identifier=1266000000-0050

Assay variable : PTINR_VAL1 PT-INR Value

N	Mean	Standard Deviation	Minimum	Maximum
6	1.6283333	0.3717212	1.3000000	2.3600000

Unique Subject Identifier=1266000000-0052

Assay variable : PTINR_VAL1 PT-INR Value

N	Mean	Standard Deviation	Minimum	Maximum
6	1.9050000	0.1963415	1.6000000	2.1300000

Unique Subject Identifier=1266000000-0056

Assay variable : PTINR_VAL1 PT-INR Value

N	Mean	Standard Deviation	Minimum	Maximum
6	2.1200000	0.3891015	1.6000000	2.6000000

Laboratory tests at the time of obtaining Table 2-6 consent, according to the presence or absence of proton-pump inhibitors

Unique Subject Identifier=1266000000-0058

MEANS Procedure

Assay variable : PTINR_VAL1 PT-INR Value

N	Mean	Standard Deviation	Minimum	Maximum
5	1.6980000	0.1298846	1.4800000	1.8000000

Unique Subject Identifier=1266000000-0061

Assay variable : PTINR_VAL1 PT-INR Value

N	Mean	Standard Deviation	Minimum	Maximum
6	2.0400000	0.2387467	1.7000000	2.3900000

Unique Subject Identifier=1266000000-0062

Assay variable : PTINR_VAL1 PT-INR Value

N	Mean	Standard Deviation	Minimum	Maximum
6	2.2116667	0.2049797	1.9200000	2.4500000

Unique Subject Identifier=1266000000-0070

Assay variable : PTINR_VAL1 PT-INR Value

N	Mean	Standard Deviation	Minimum	Maximum
6	1.6433333	0.2367840	1.2000000	1.8000000

Laboratory tests at the time of obtaining Table 2-6 consent, according to the presence or absence of proton-pump inhibitors

Unique Subject Identifier=1266000000-0071

MEANS Procedure

Assay variable : PTINR_VAL1 PT-INR Value

N	Mean	Standard Deviation	Minimum	Maximum
6	2.0916667	0.3350473	1.8000000	2.7000000

Unique Subject Identifier=1266000000-0072

Assay variable : PTINR_VAL1 PT-INR Value

N	Mean	Standard Deviation	Minimum	Maximum
6	2.2333333	0.3023684	1.7000000	2.5000000

Unique Subject Identifier=1266000000-0077

Assay variable : PTINR_VAL1 PT-INR Value

N	Mean	Standard Deviation	Minimum	Maximum
6	1.5250000	0.1405347	1.4000000	1.7000000

Unique Subject Identifier=1266000000-0082

Assay variable : PTINR_VAL1 PT-INR Value

N	Mean	Standard Deviation	Minimum	Maximum
6	2.0666667	0.2726659	1.8500000	2.5900000

Laboratory tests at the time of obtaining Table 2-6 consent, according to the presence or absence of proton-pump inhibitors

Unique Subject Identifier=1268000000-0001

MEANS Procedure

Assay variable : PTINR_VAL1 PT-INR Value

N	Mean	Standard Deviation	Minimum	Maximum
3	2.0066667	0.3500476	1.6600000	2.3600000

Unique Subject Identifier=1268000000-0003

Assay variable : PTINR_VAL1 PT-INR Value

N	Mean	Standard Deviation	Minimum	Maximum
6	1.5916667	0.1704602	1.3800000	1.8900000

Unique Subject Identifier=1268000000-0004

Assay variable : PTINR_VAL1 PT-INR Value

N	Mean	Standard Deviation	Minimum	Maximum
4	1.6150000	0.1047219	1.5200000	1.7200000

Unique Subject Identifier=1268000000-0006

Assay variable : PTINR_VAL1 PT-INR Value

N	Mean	Standard Deviation	Minimum	Maximum
6	1.6616667	0.1433062	1.4300000	1.8200000

Laboratory tests at the time of obtaining Table 2-6 consent, according to the presence or absence of proton-pump inhibitors

Unique Subject Identifier=1268000000-0007

MEANS Procedure

Assay variable : PTINR_VAL1 PT-INR Value

N	Mean	Standard Deviation	Minimum	Maximum
5	1.5580000	0.1822635	1.3000000	1.7000000

Unique Subject Identifier=1269000000-0008

Assay variable : PTINR_VAL1 PT-INR Value

N	Mean	Standard Deviation	Minimum	Maximum
6	2.1200000	0.2794280	1.6200000	2.4400000

Unique Subject Identifier=1269000000-0018

Assay variable : PTINR_VAL1 PT-INR Value

N	Mean	Standard Deviation	Minimum	Maximum
6	1.8233333	0.4719181	1.2600000	2.5500000

Unique Subject Identifier=1269000000-0019

Assay variable : PTINR_VAL1 PT-INR Value

N	Mean	Standard Deviation	Minimum	Maximum
2	1.5500000	0.3252691	1.3200000	1.7800000

Laboratory tests at the time of obtaining Table 2-6 consent, according to the presence or absence of proton-pump inhibitors

Unique Subject Identifier=1270000000-0001

MEANS Procedure

Assay variable : PTINR_VAL1 PT-INR Value

N	Mean	Standard Deviation	Minimum	Maximum
2	1.9500000	0.2121320	1.8000000	2.1000000

Unique Subject Identifier=1270000000-0003

Assay variable : PTINR_VAL1 PT-INR Value

N	Mean	Standard Deviation	Minimum	Maximum
2	1.8750000	0.0353553	1.8500000	1.9000000

Unique Subject Identifier=1270000000-0005

Assay variable : PTINR_VAL1 PT-INR Value

N	Mean	Standard Deviation	Minimum	Maximum
4	1.8500000	0.3696846	1.4000000	2.2000000

Unique Subject Identifier=1270000000-0007

Assay variable : PTINR_VAL1 PT-INR Value

N	Mean	Standard Deviation	Minimum	Maximum
2	1.8500000	0.3535534	1.6000000	2.1000000

Laboratory tests at the time of obtaining Table 2-6 consent, according to the presence or absence of proton-pump inhibitors

Unique Subject Identifier=1270000000-0008

MEANS Procedure

Assay variable : PTINR_VAL1 PT-INR Value

N	Mean	Standard Deviation	Minimum	Maximum
2	1.8000000	0.2828427	1.6000000	2.0000000

Unique Subject Identifier=1270000000-0009

Assay variable : PTINR_VAL1 PT-INR Value

N	Mean	Standard Deviation	Minimum	Maximum
3	2.3333333	0.1154701	2.2000000	2.4000000

Unique Subject Identifier=1270000000-0010

Assay variable : PTINR_VAL1 PT-INR Value

N	Mean	Standard Deviation	Minimum	Maximum
3	1.9666667	0.2516611	1.7000000	2.2000000

Unique Subject Identifier=1270000000-0011

Assay variable : PTINR_VAL1 PT-INR Value

N	Mean	Standard Deviation	Minimum	Maximum
2	2.5000000	1.5556349	1.4000000	3.6000000

Laboratory tests at the time of obtaining Table 2-6 consent, according to the presence or absence of proton-pump inhibitors

Unique Subject Identifier=1270000000-0012

MEANS Procedure

Assay variable : PTINR_VAL1 PT-INR Value

N	Mean	Standard Deviation	Minimum	Maximum
2	2.5750000	0.0353553	2.5500000	2.6000000

Unique Subject Identifier=1270000000-0013

Assay variable : PTINR_VAL1 PT-INR Value

N	Mean	Standard Deviation	Minimum	Maximum
1	2.0000000		2.0000000	2.0000000

Unique Subject Identifier=1270000000-0015

Assay variable : PTINR_VAL1 PT-INR Value

N	Mean	Standard Deviation	Minimum	Maximum
4	1.5275000	0.1001249	1.4100000	1.6500000

Unique Subject Identifier=1270000000-0016

Assay variable : PTINR_VAL1 PT-INR Value

N	Mean	Standard Deviation	Minimum	Maximum
2	1.8500000	0.0707107	1.8000000	1.9000000

Laboratory tests at the time of obtaining Table 2-6 consent, according to the presence or absence of proton-pump inhibitors

Unique Subject Identifier=1270000000-0017

MEANS Procedure

Assay variable : PTINR_VAL1 PT-INR Value

N	Mean	Standard Deviation	Minimum	Maximum
3	2.8000000	1.1532563	1.9000000	4.1000000

Unique Subject Identifier=1270000000-0021

Assay variable : PTINR_VAL1 PT-INR Value

N	Mean	Standard Deviation	Minimum	Maximum
2	2.2300000	0.2404163	2.0600000	2.4000000

Unique Subject Identifier=1270000000-0023

Assay variable : PTINR_VAL1 PT-INR Value

N	Mean	Standard Deviation	Minimum	Maximum
3	1.7666667	0.6027714	1.2000000	2.4000000

Unique Subject Identifier=1274000000-0003

Assay variable : PTINR_VAL1 PT-INR Value

N	Mean	Standard Deviation	Minimum	Maximum
2	1.4550000	0.0494975	1.4200000	1.4900000

Laboratory tests at the time of obtaining Table 2-6 consent, according to the presence or absence of proton-pump inhibitors

Unique Subject Identifier=1276000000-0001

MEANS Procedure

Assay variable : PTINR_VAL1 PT-INR Value

N	Mean	Standard Deviation	Minimum	Maximum
6	2.5900000	0.7753193	1.8000000	3.9000000

Unique Subject Identifier=1276000000-0002

Assay variable : PTINR_VAL1 PT-INR Value

N	Mean	Standard Deviation	Minimum	Maximum
2	2.0500000	0.0565685	2.0100000	2.0900000

Unique Subject Identifier=1277010100-0001

Assay variable : PTINR_VAL1 PT-INR Value

N	Mean	Standard Deviation	Minimum	Maximum
6	2.1900000	0.0678233	2.1000000	2.3000000

Unique Subject Identifier=1277010100-0002

Assay variable : PTINR_VAL1 PT-INR Value

N	Mean	Standard Deviation	Minimum	Maximum
6	1.8416667	0.1357080	1.6000000	2.0000000

Laboratory tests at the time of obtaining Table 2-6 consent, according to the presence or absence of proton-pump inhibitors

Unique Subject Identifier=1277010100-0003

MEANS Procedure

Assay variable : PTINR_VAL1 PT-INR Value

N	Mean	Standard Deviation	Minimum	Maximum
6	2.3333333	0.1632993	2.2000000	2.6000000

Unique Subject Identifier=1277010100-0004

Assay variable : PTINR_VAL1 PT-INR Value

N	Mean	Standard Deviation	Minimum	Maximum
6	1.7750000	0.2444381	1.5000000	2.2000000

Unique Subject Identifier=1277010100-0005

Assay variable : PTINR_VAL1 PT-INR Value

N	Mean	Standard Deviation	Minimum	Maximum
6	2.1783333	0.1040032	2.0700000	2.3000000

Unique Subject Identifier=1277010100-0006

Assay variable : PTINR_VAL1 PT-INR Value

N	Mean	Standard Deviation	Minimum	Maximum
6	2.4333333	0.2732520	2.2000000	2.9000000

Laboratory tests at the time of obtaining Table 2-6 consent, according to the presence or absence of proton-pump inhibitors

Unique Subject Identifier=1277010100-0007

MEANS Procedure

Assay variable : PTINR_VAL1 PT-INR Value

N	Mean	Standard Deviation	Minimum	Maximum
6	1.9150000	0.2072438	1.5900000	2.2000000

Unique Subject Identifier=1277010100-0008

Assay variable : PTINR_VAL1 PT-INR Value

N	Mean	Standard Deviation	Minimum	Maximum
6	2.3000000	0.2529822	1.9000000	2.6000000

Unique Subject Identifier=1277010100-0010

Assay variable : PTINR_VAL1 PT-INR Value

N	Mean	Standard Deviation	Minimum	Maximum
6	1.9000000	0.0894427	1.8000000	2.0000000

Unique Subject Identifier=1277010100-0011

Assay variable : PTINR_VAL1 PT-INR Value

N	Mean	Standard Deviation	Minimum	Maximum
6	2.4100000	0.4259108	1.8000000	2.9000000

Laboratory tests at the time of obtaining Table 2-6 consent, according to the presence or absence of proton-pump inhibitors

Unique Subject Identifier=1277010100-0012

MEANS Procedure

Assay variable : PTINR_VAL1 PT-INR Value

N	Mean	Standard Deviation	Minimum	Maximum
6	1.8666667	0.2804758	1.5000000	2.2000000

Unique Subject Identifier=1277010100-0015

Assay variable : PTINR_VAL1 PT-INR Value

N	Mean	Standard Deviation	Minimum	Maximum
6	1.9000000	0.3577709	1.6000000	2.4000000

Unique Subject Identifier=1277010100-0016

Assay variable : PTINR_VAL1 PT-INR Value

N	Mean	Standard Deviation	Minimum	Maximum
6	2.8000000	0.8390471	1.5000000	3.8000000

Unique Subject Identifier=1277010100-0017

Assay variable : PTINR_VAL1 PT-INR Value

N	Mean	Standard Deviation	Minimum	Maximum
6	2.1950000	0.1554027	2.1000000	2.5000000

Laboratory tests at the time of obtaining Table 2-6 consent, according to the presence or absence of proton-pump inhibitors

Unique Subject Identifier=1278011100-0002

MEANS Procedure

Assay variable : PTINR_VAL1 PT-INR Value

N	Mean	Standard Deviation	Minimum	Maximum
4	2.1750000	0.3361051	1.8900000	2.6600000

Unique Subject Identifier=1278011100-0006

Assay variable : PTINR_VAL1 PT-INR Value

N	Mean	Standard Deviation	Minimum	Maximum
5	1.8340000	0.3822041	1.1900000	2.1200000

Unique Subject Identifier=1278011100-0009

Assay variable : PTINR_VAL1 PT-INR Value

N	Mean	Standard Deviation	Minimum	Maximum
2	2.1350000	0.1484924	2.0300000	2.2400000

Unique Subject Identifier=1278011100-0011

Assay variable : PTINR_VAL1 PT-INR Value

N	Mean	Standard Deviation	Minimum	Maximum
6	2.2816667	1.0698863	1.5800000	4.4200000

Laboratory tests at the time of obtaining Table 2-6 consent, according to the presence or absence of proton-pump inhibitors

Unique Subject Identifier=1278011100-0012

MEANS Procedure

Assay variable : PTINR_VAL1 PT-INR Value

N	Mean	Standard Deviation	Minimum	Maximum
2	1.9950000	0.0353553	1.9700000	2.0200000

Unique Subject Identifier=1279000000-0003

Assay variable : PTINR_VAL1 PT-INR Value

N	Mean	Standard Deviation	Minimum	Maximum
4	2.4500000	0.4358899	2.1000000	3.0000000

Unique Subject Identifier=1279000000-0007

Assay variable : PTINR_VAL1 PT-INR Value

N	Mean	Standard Deviation	Minimum	Maximum
1	1.6000000		1.6000000	1.6000000

Unique Subject Identifier=1279000000-0008

Assay variable : PTINR_VAL1 PT-INR Value

N	Mean	Standard Deviation	Minimum	Maximum
2	1.5750000	0.1202082	1.4900000	1.6600000

Laboratory tests at the time of obtaining Table 2-6 consent, according to the presence or absence of proton-pump inhibitors

Unique Subject Identifier=1280001000-0003

MEANS Procedure

Assay variable : PTINR_VAL1 PT-INR Value

N	Mean	Standard Deviation	Minimum	Maximum
4	1.4500000	0.2081666	1.2000000	1.7000000

Unique Subject Identifier=1280001000-0005

Assay variable : PTINR_VAL1 PT-INR Value

N	Mean	Standard Deviation	Minimum	Maximum
6	2.0333333	0.2250926	1.7000000	2.2000000

Unique Subject Identifier=1281000000-0001

Assay variable : PTINR_VAL1 PT-INR Value

N	Mean	Standard Deviation	Minimum	Maximum
6	2.1716667	0.1702253	2.0000000	2.4600000

Unique Subject Identifier=1281000000-0005

Assay variable : PTINR_VAL1 PT-INR Value

N	Mean	Standard Deviation	Minimum	Maximum
5	1.8200000	0.1673320	1.5600000	1.9700000

Laboratory tests at the time of obtaining Table 2-6 consent, according to the presence or absence of proton-pump inhibitors

Unique Subject Identifier=1282011111-0001

MEANS Procedure

Assay variable : PTINR_VAL1 PT-INR Value

N	Mean	Standard Deviation	Minimum	Maximum
6	2.3000000	0.2828427	2.0000000	2.8000000

Unique Subject Identifier=1282011111-0005

Assay variable : PTINR_VAL1 PT-INR Value

N	Mean	Standard Deviation	Minimum	Maximum
6	1.9166667	0.4445972	1.1000000	2.4000000

Unique Subject Identifier=1286000000-0004

Assay variable : PTINR_VAL1 PT-INR Value

N	Mean	Standard Deviation	Minimum	Maximum
6	1.9333333	0.3444803	1.6000000	2.5000000

Unique Subject Identifier=1286000000-0017

Assay variable : PTINR_VAL1 PT-INR Value

N	Mean	Standard Deviation	Minimum	Maximum
1	1.7000000		1.7000000	1.7000000

Laboratory tests at the time of obtaining Table 2-6 consent, according to the presence or absence of proton-pump inhibitors

Unique Subject Identifier=1287000000-0005

MEANS Procedure

Assay variable : PTINR_VAL1 PT-INR Value

N	Mean	Standard Deviation	Minimum	Maximum
6	1.8833333	0.1940790	1.6000000	2.1000000

Unique Subject Identifier=1287000000-0010

Assay variable : PTINR_VAL1 PT-INR Value

N	Mean	Standard Deviation	Minimum	Maximum
5	1.5200000	0.1095445	1.4000000	1.7000000

Unique Subject Identifier=1287000000-0014

Assay variable : PTINR_VAL1 PT-INR Value

N	Mean	Standard Deviation	Minimum	Maximum
6	1.9000000	0.4000000	1.4000000	2.5000000

Unique Subject Identifier=1287000000-0015

Assay variable : PTINR_VAL1 PT-INR Value

N	Mean	Standard Deviation	Minimum	Maximum
2	1.9000000	0.2828427	1.7000000	2.1000000

Laboratory tests at the time of obtaining Table 2-6 consent, according to the presence or absence of proton-pump inhibitors

Unique Subject Identifier=1289000000-0001

MEANS Procedure

Assay variable : PTINR_VAL1 PT-INR Value

N	Mean	Standard Deviation	Minimum	Maximum
3	1.3600000	0.1637071	1.1800000	1.5000000

Unique Subject Identifier=1289000000-0002

Assay variable : PTINR_VAL1 PT-INR Value

N	Mean	Standard Deviation	Minimum	Maximum
3	1.8566667	0.3137409	1.5000000	2.0900000

Unique Subject Identifier=1289000000-0003

Assay variable : PTINR_VAL1 PT-INR Value

N	Mean	Standard Deviation	Minimum	Maximum
2	1.4150000	0.0070711	1.4100000	1.4200000

Unique Subject Identifier=1289000000-0004

Assay variable : PTINR_VAL1 PT-INR Value

N	Mean	Standard Deviation	Minimum	Maximum
2	1.5700000	0.2404163	1.4000000	1.7400000

Laboratory tests at the time of obtaining Table 2-6 consent, according to the presence or absence of proton-pump inhibitors

Unique Subject Identifier=1289000000-0005

MEANS Procedure

Assay variable : PTINR_VAL1 PT-INR Value

N	Mean	Standard Deviation	Minimum	Maximum
2	1.6200000	0.0707107	1.5700000	1.6700000

Unique Subject Identifier=1289000000-0008

Assay variable : PTINR_VAL1 PT-INR Value

N	Mean	Standard Deviation	Minimum	Maximum
4	1.8825000	0.3341033	1.4000000	2.1600000

Unique Subject Identifier=1291000000-0002

Assay variable : PTINR_VAL1 PT-INR Value

N	Mean	Standard Deviation	Minimum	Maximum
2	1.3500000	0.2121320	1.2000000	1.5000000

Unique Subject Identifier=1291000000-0003

Assay variable : PTINR_VAL1 PT-INR Value

N	Mean	Standard Deviation	Minimum	Maximum
6	1.9833333	0.6112828	1.5000000	2.9000000

Laboratory tests at the time of obtaining Table 2-6 consent, according to the presence or absence of proton-pump inhibitors

Unique Subject Identifier=1291000000-0004

MEANS Procedure

Assay variable : PTINR_VAL1 PT-INR Value

N	Mean	Standard Deviation	Minimum	Maximum
6	1.6500000	0.1643168	1.4000000	1.9000000

Unique Subject Identifier=1291000000-0005

Assay variable : PTINR_VAL1 PT-INR Value

N	Mean	Standard Deviation	Minimum	Maximum
5	1.8000000	0.1870829	1.5000000	2.0000000

Unique Subject Identifier=1291000000-0009

Assay variable : PTINR_VAL1 PT-INR Value

N	Mean	Standard Deviation	Minimum	Maximum
5	1.6200000	0.1483240	1.4000000	1.8000000

Unique Subject Identifier=1291000000-0011

Assay variable : PTINR_VAL1 PT-INR Value

N	Mean	Standard Deviation	Minimum	Maximum
4	1.7750000	0.3095696	1.5000000	2.2000000

Laboratory tests at the time of obtaining Table 2-6 consent, according to the presence or absence of proton-pump inhibitors

Unique Subject Identifier=1291000000-0012

MEANS Procedure

Assay variable : PTINR_VAL1 PT-INR Value

N	Mean	Standard Deviation	Minimum	Maximum
5	1.6200000	0.4438468	1.2000000	2.3000000

Unique Subject Identifier=1291000000-0014

Assay variable : PTINR_VAL1 PT-INR Value

N	Mean	Standard Deviation	Minimum	Maximum
5	1.5200000	0.0836660	1.4000000	1.6000000

Unique Subject Identifier=1291000000-0015

Assay variable : PTINR_VAL1 PT-INR Value

N	Mean	Standard Deviation	Minimum	Maximum
2	2.0000000	0.8485281	1.4000000	2.6000000

Unique Subject Identifier=1291000000-0016

Assay variable : PTINR_VAL1 PT-INR Value

N	Mean	Standard Deviation	Minimum	Maximum
3	1.8333333	0.3055050	1.5000000	2.1000000

Laboratory tests at the time of obtaining Table 2-6 consent, according to the presence or absence of proton-pump inhibitors

Unique Subject Identifier=1291000000-0018

MEANS Procedure

Assay variable : PTINR_VAL1 PT-INR Value

N	Mean	Standard Deviation	Minimum	Maximum
1	1.9000000		1.9000000	1.9000000

Unique Subject Identifier=1291000000-0019

Assay variable : PTINR_VAL1 PT-INR Value

N	Mean	Standard Deviation	Minimum	Maximum
6	1.7166667	0.2786874	1.4000000	2.2000000

Unique Subject Identifier=1293000000-0001

Assay variable : PTINR_VAL1 PT-INR Value

N	Mean	Standard Deviation	Minimum	Maximum
6	1.3166667	0.1169045	1.2000000	1.5000000

Unique Subject Identifier=1293000000-0003

Assay variable : PTINR_VAL1 PT-INR Value

N	Mean	Standard Deviation	Minimum	Maximum
6	1.7833333	0.2041241	1.6000000	2.0000000

Laboratory tests at the time of obtaining Table 2-6 consent, according to the presence or absence of proton-pump inhibitors

Unique Subject Identifier=1293000000-0005

MEANS Procedure

Assay variable : PTINR_VAL1 PT-INR Value

N	Mean	Standard Deviation	Minimum	Maximum
6	1.2333333	0.1861899	1.1000000	1.6000000

Unique Subject Identifier=1293000000-0007

Assay variable : PTINR_VAL1 PT-INR Value

N	Mean	Standard Deviation	Minimum	Maximum
6	1.6166667	0.1329160	1.4000000	1.8000000

Unique Subject Identifier=1293000000-0008

Assay variable : PTINR_VAL1 PT-INR Value

N	Mean	Standard Deviation	Minimum	Maximum
6	1.7333333	0.1861899	1.5000000	2.0000000

Unique Subject Identifier=1293000000-0010

Assay variable : PTINR_VAL1 PT-INR Value

N	Mean	Standard Deviation	Minimum	Maximum
6	1.5333333	0.1632993	1.3000000	1.7000000

Laboratory tests at the time of obtaining Table 2-6 consent, according to the presence or absence of proton-pump inhibitors

Unique Subject Identifier=1293000000-0011

MEANS Procedure

Assay variable : PTINR_VAL1 PT-INR Value

N	Mean	Standard Deviation	Minimum	Maximum
5	1.3600000	0.0547723	1.3000000	1.4000000

Unique Subject Identifier=1293000000-0012

Assay variable : PTINR_VAL1 PT-INR Value

N	Mean	Standard Deviation	Minimum	Maximum
6	1.3666667	0.0816497	1.3000000	1.5000000

Unique Subject Identifier=1293000000-0013

Assay variable : PTINR_VAL1 PT-INR Value

N	Mean	Standard Deviation	Minimum	Maximum
1	1.6000000		1.6000000	1.6000000

Unique Subject Identifier=1293000000-0016

Assay variable : PTINR_VAL1 PT-INR Value

N	Mean	Standard Deviation	Minimum	Maximum
6	1.4333333	0.1632993	1.3000000	1.7000000

Laboratory tests at the time of obtaining Table 2-6 consent, according to the presence or absence of proton-pump inhibitors

Unique Subject Identifier=1293000000-0020

MEANS Procedure

Assay variable : PTINR_VAL1 PT-INR Value

N	Mean	Standard Deviation	Minimum	Maximum
5	1.4800000	0.2489980	1.3000000	1.9000000

Unique Subject Identifier=1293000000-0023

Assay variable : PTINR_VAL1 PT-INR Value

N	Mean	Standard Deviation	Minimum	Maximum
5	2.0200000	0.1923538	1.7000000	2.2000000

Unique Subject Identifier=1293000000-0024

Assay variable : PTINR_VAL1 PT-INR Value

N	Mean	Standard Deviation	Minimum	Maximum
5	1.4200000	0.1643168	1.3000000	1.7000000

Unique Subject Identifier=1293000000-0029

Assay variable : PTINR_VAL1 PT-INR Value

N	Mean	Standard Deviation	Minimum	Maximum
5	1.5000000	0.1581139	1.3000000	1.7000000

Laboratory tests at the time of obtaining Table 2-6 consent, according to the presence or absence of proton-pump inhibitors

Unique Subject Identifier=1296011000-0006

MEANS Procedure

Assay variable : PTINR_VAL1 PT-INR Value

N	Mean	Standard Deviation	Minimum	Maximum
1	2.0300000		2.0300000	2.0300000

Unique Subject Identifier=1296011000-0007

Assay variable : PTINR_VAL1 PT-INR Value

N	Mean	Standard Deviation	Minimum	Maximum
1	1.1200000		1.1200000	1.1200000

Unique Subject Identifier=1296011000-0013

Assay variable : PTINR_VAL1 PT-INR Value

N	Mean	Standard Deviation	Minimum	Maximum
1	2.2100000		2.2100000	2.2100000

Unique Subject Identifier=1296011000-0015

Assay variable : PTINR_VAL1 PT-INR Value

N	Mean	Standard Deviation	Minimum	Maximum
2	2.1050000	0.6576093	1.6400000	2.5700000

Laboratory tests at the time of obtaining Table 2-6 consent, according to the presence or absence of proton-pump inhibitors

Unique Subject Identifier=1296011000-0017

MEANS Procedure

Assay variable : PTINR_VAL1 PT-INR Value

N	Mean	Standard Deviation	Minimum	Maximum
5	1.9820000	0.5526482	1.0800000	2.5500000

Unique Subject Identifier=1296011000-0026

Assay variable : PTINR_VAL1 PT-INR Value

N	Mean	Standard Deviation	Minimum	Maximum
4	1.1550000	0.0793725	1.0400000	1.2200000

Unique Subject Identifier=1296011000-0028

Assay variable : PTINR_VAL1 PT-INR Value

N	Mean	Standard Deviation	Minimum	Maximum
1	1.5500000		1.5500000	1.5500000

Unique Subject Identifier=1297011000-0005

Assay variable : PTINR_VAL1 PT-INR Value

N	Mean	Standard Deviation	Minimum	Maximum
6	2.1833333	1.3166878	1.2000000	4.2000000

Laboratory tests at the time of obtaining Table 2-6 consent, according to the presence or absence of proton-pump inhibitors

Unique Subject Identifier=1297011000-0008

MEANS Procedure

Assay variable : PTINR_VAL1 PT-INR Value

N	Mean	Standard Deviation	Minimum	Maximum
4	1.8500000	0.2645751	1.6000000	2.2000000

Unique Subject Identifier=1297011000-0010

Assay variable : PTINR_VAL1 PT-INR Value

N	Mean	Standard Deviation	Minimum	Maximum
5	1.7600000	0.2408319	1.5000000	2.1000000

Unique Subject Identifier=1297011000-0017

Assay variable : PTINR_VAL1 PT-INR Value

N	Mean	Standard Deviation	Minimum	Maximum
6	1.8333333	0.5125102	1.2000000	2.6000000

Unique Subject Identifier=1298011000-0002

Assay variable : PTINR_VAL1 PT-INR Value

N	Mean	Standard Deviation	Minimum	Maximum
5	2.3200000	0.7049823	1.4000000	3.3000000

Laboratory tests at the time of obtaining Table 2-6 consent, according to the presence or absence of proton-pump inhibitors

Unique Subject Identifier=1298011000-0010

MEANS Procedure

Assay variable : PTINR_VAL1 PT-INR Value

N	Mean	Standard Deviation	Minimum	Maximum
6	1.6666667	0.1032796	1.5000000	1.8000000

Unique Subject Identifier=1298011000-0011

Assay variable : PTINR_VAL1 PT-INR Value

N	Mean	Standard Deviation	Minimum	Maximum
6	2.1166667	0.2483277	1.7000000	2.4000000

Unique Subject Identifier=1298011000-0014

Assay variable : PTINR_VAL1 PT-INR Value

N	Mean	Standard Deviation	Minimum	Maximum
5	1.8000000	0.3162278	1.5000000	2.3000000

Unique Subject Identifier=1298011000-0017

Assay variable : PTINR_VAL1 PT-INR Value

N	Mean	Standard Deviation	Minimum	Maximum
6	1.9500000	0.2073644	1.6000000	2.2000000

Laboratory tests at the time of obtaining Table 2-6 consent, according to the presence or absence of proton-pump inhibitors

Unique Subject Identifier=1298011000-0020

MEANS Procedure

Assay variable : PTINR_VAL1 PT-INR Value

N	Mean	Standard Deviation	Minimum	Maximum
6	1.7500000	0.4636809	1.1000000	2.5000000

Unique Subject Identifier=1298011000-0021

Assay variable : PTINR_VAL1 PT-INR Value

N	Mean	Standard Deviation	Minimum	Maximum
6	2.1000000	0.5138093	1.6000000	3.0000000

Unique Subject Identifier=1298011000-0022

Assay variable : PTINR_VAL1 PT-INR Value

N	Mean	Standard Deviation	Minimum	Maximum
6	1.9833333	0.2316607	1.6000000	2.2000000

Unique Subject Identifier=1299010000-0006

Assay variable : PTINR_VAL1 PT-INR Value

N	Mean	Standard Deviation	Minimum	Maximum
6	1.2783333	0.1197358	1.1800000	1.5000000

Laboratory tests at the time of obtaining Table 2-6 consent, according to the presence or absence of proton-pump inhibitors

Unique Subject Identifier=1299010000-0009

MEANS Procedure

Assay variable : PTINR_VAL1 PT-INR Value

N	Mean	Standard Deviation	Minimum	Maximum
1	1.9000000		1.9000000	1.9000000

Unique Subject Identifier=1299010000-0012

Assay variable : PTINR_VAL1 PT-INR Value

N	Mean	Standard Deviation	Minimum	Maximum
6	2.7700000	0.5978294	1.9000000	3.4000000

Unique Subject Identifier=1299010000-0013

Assay variable : PTINR_VAL1 PT-INR Value

N	Mean	Standard Deviation	Minimum	Maximum
6	1.5600000	0.1979899	1.2600000	1.8000000

Unique Subject Identifier=1299010000-0016

Assay variable : PTINR_VAL1 PT-INR Value

N	Mean	Standard Deviation	Minimum	Maximum
6	2.2666667	0.1211060	2.1000000	2.4000000

Laboratory tests at the time of obtaining Table 2-6 consent, according to the presence or absence of proton-pump inhibitors

Unique Subject Identifier=1299010000-0018

MEANS Procedure

Assay variable : PTINR_VAL1 PT-INR Value

N	Mean	Standard Deviation	Minimum	Maximum
6	1.9700000	0.3685105	1.6200000	2.6000000

Unique Subject Identifier=1299010000-0020

Assay variable : PTINR_VAL1 PT-INR Value

N	Mean	Standard Deviation	Minimum	Maximum
5	2.0580000	0.7316557	1.5000000	3.3000000

Unique Subject Identifier=1301000000-0001

Assay variable : PTINR_VAL1 PT-INR Value

N	Mean	Standard Deviation	Minimum	Maximum
6	2.2200000	0.5196152	1.7300000	3.0600000

Unique Subject Identifier=1301000000-0005

Assay variable : PTINR_VAL1 PT-INR Value

N	Mean	Standard Deviation	Minimum	Maximum
6	1.2366667	0.1616993	1.0700000	1.5100000

Laboratory tests at the time of obtaining Table 2-6 consent, according to the presence or absence of proton-pump inhibitors

Unique Subject Identifier=1301000000-0006

MEANS Procedure

Assay variable : PTINR_VAL1 PT-INR Value

N	Mean	Standard Deviation	Minimum	Maximum
4	1.3400000	0.1089342	1.2500000	1.4700000

Unique Subject Identifier=1303000000-0002

Assay variable : PTINR_VAL1 PT-INR Value

N	Mean	Standard Deviation	Minimum	Maximum
5	1.7800000	0.3271085	1.5000000	2.3000000

Unique Subject Identifier=1303000000-0003

Assay variable : PTINR_VAL1 PT-INR Value

N	Mean	Standard Deviation	Minimum	Maximum
3	2.5333333	0.3511885	2.2000000	2.9000000

Unique Subject Identifier=1303000000-0006

Assay variable : PTINR_VAL1 PT-INR Value

N	Mean	Standard Deviation	Minimum	Maximum
6	2.0000000	0.1264911	1.8000000	2.1000000

Laboratory tests at the time of obtaining Table 2-6 consent, according to the presence or absence of proton-pump inhibitors

Unique Subject Identifier=1303000000-0008

MEANS Procedure

Assay variable : PTINR_VAL1 PT-INR Value

N	Mean	Standard Deviation	Minimum	Maximum
4	2.0000000	0.3559026	1.7000000	2.5000000

Unique Subject Identifier=1303000000-0009

Assay variable : PTINR_VAL1 PT-INR Value

N	Mean	Standard Deviation	Minimum	Maximum
5	1.6800000	0.1095445	1.6000000	1.8000000

Unique Subject Identifier=1303000000-0015

Assay variable : PTINR_VAL1 PT-INR Value

N	Mean	Standard Deviation	Minimum	Maximum
6	1.7166667	0.1169045	1.6000000	1.9000000

Unique Subject Identifier=1303000000-0016

Assay variable : PTINR_VAL1 PT-INR Value

N	Mean	Standard Deviation	Minimum	Maximum
6	1.7500000	0.1048809	1.6000000	1.9000000

Laboratory tests at the time of obtaining Table 2-6 consent, according to the presence or absence of proton-pump inhibitors

Unique Subject Identifier=1303000000-0025

MEANS Procedure

Assay variable : PTINR_VAL1 PT-INR Value

N	Mean	Standard Deviation	Minimum	Maximum
6	2.0166667	0.4875107	1.3000000	2.7000000

Unique Subject Identifier=1303000000-0029

Assay variable : PTINR_VAL1 PT-INR Value

N	Mean	Standard Deviation	Minimum	Maximum
4	1.6000000	0.2449490	1.3000000	1.8000000

Unique Subject Identifier=1306000000-0007

Assay variable : PTINR_VAL1 PT-INR Value

N	Mean	Standard Deviation	Minimum	Maximum
1	1.5900000		1.5900000	1.5900000

Unique Subject Identifier=1308000000-0001

Assay variable : PTINR_VAL1 PT-INR Value

N	Mean	Standard Deviation	Minimum	Maximum
5	1.7320000	0.2258761	1.5100000	2.0600000

Laboratory tests at the time of obtaining Table 2-6 consent, according to the presence or absence of proton-pump inhibitors

Unique Subject Identifier=1308000000-0004

MEANS Procedure

Assay variable : PTINR_VAL1 PT-INR Value

N	Mean	Standard Deviation	Minimum	Maximum
2	1.1000000	0	1.1000000	1.1000000

Unique Subject Identifier=1312000000-0005

Assay variable : PTINR_VAL1 PT-INR Value

N	Mean	Standard Deviation	Minimum	Maximum
6	2.0433333	0.3311596	1.7000000	2.4000000

Unique Subject Identifier=1312000000-0014

Assay variable : PTINR_VAL1 PT-INR Value

N	Mean	Standard Deviation	Minimum	Maximum
6	2.3550000	0.3434967	1.7900000	2.6600000

Unique Subject Identifier=1313000000-0013

Assay variable : PTINR_VAL1 PT-INR Value

N	Mean	Standard Deviation	Minimum	Maximum
1	1.4200000		1.4200000	1.4200000

Laboratory tests at the time of obtaining Table 2-6 consent, according to the presence or absence of proton-pump inhibitors

Unique Subject Identifier=1313000000-0018

MEANS Procedure

Assay variable : PTINR_VAL1 PT-INR Value

N	Mean	Standard Deviation	Minimum	Maximum
1	1.3100000		1.3100000	1.3100000

Unique Subject Identifier=1319000000-0001

Assay variable : PTINR_VAL1 PT-INR Value

N	Mean	Standard Deviation	Minimum	Maximum
1	3.0000000		3.0000000	3.0000000

Unique Subject Identifier=1319000000-0005

Assay variable : PTINR_VAL1 PT-INR Value

N	Mean	Standard Deviation	Minimum	Maximum
6	1.9500000	0.2880972	1.6000000	2.3000000

Unique Subject Identifier=1319000000-0011

Assay variable : PTINR_VAL1 PT-INR Value

N	Mean	Standard Deviation	Minimum	Maximum
3	1.5366667	0.1096966	1.4100000	1.6000000

Laboratory tests at the time of obtaining Table 2-6 consent, according to the presence or absence of proton-pump inhibitors

Unique Subject Identifier=1319000000-0018

MEANS Procedure

Assay variable : PTINR_VAL1 PT-INR Value

N	Mean	Standard Deviation	Minimum	Maximum
6	2.4833333	0.4020779	1.8000000	3.0000000

Unique Subject Identifier=1319000000-0020

Assay variable : PTINR_VAL1 PT-INR Value

N	Mean	Standard Deviation	Minimum	Maximum
6	1.8500000	0.2073644	1.6000000	2.1000000

Unique Subject Identifier=1320000000-0002

Assay variable : PTINR_VAL1 PT-INR Value

N	Mean	Standard Deviation	Minimum	Maximum
6	2.0566667	0.2809033	1.7600000	2.4700000

Unique Subject Identifier=1320000000-0003

Assay variable : PTINR_VAL1 PT-INR Value

N	Mean	Standard Deviation	Minimum	Maximum
4	1.8650000	0.3162805	1.5900000	2.3200000

Laboratory tests at the time of obtaining Table 2-6 consent, according to the presence or absence of proton-pump inhibitors

Unique Subject Identifier=1320000000-0004

MEANS Procedure

Assay variable : PTINR_VAL1 PT-INR Value

N	Mean	Standard Deviation	Minimum	Maximum
6	2.3700000	0.3960303	1.9500000	3.0900000

Unique Subject Identifier=1320000000-0005

Assay variable : PTINR_VAL1 PT-INR Value

N	Mean	Standard Deviation	Minimum	Maximum
6	2.1666667	0.3539868	1.7100000	2.5700000

Unique Subject Identifier=1320000000-0007

Assay variable : PTINR_VAL1 PT-INR Value

N	Mean	Standard Deviation	Minimum	Maximum
4	1.8850000	0.5557877	1.3000000	2.6400000

Unique Subject Identifier=1320000000-0009

Assay variable : PTINR_VAL1 PT-INR Value

N	Mean	Standard Deviation	Minimum	Maximum
5	1.9780000	0.5148495	1.3300000	2.4600000

Laboratory tests at the time of obtaining Table 2-6 consent, according to the presence or absence of proton-pump inhibitors

Unique Subject Identifier=1320000000-0011

MEANS Procedure

Assay variable : PTINR_VAL1 PT-INR Value

N	Mean	Standard Deviation	Minimum	Maximum
5	1.7040000	0.2951779	1.3000000	2.0000000

Unique Subject Identifier=1320000000-0013

Assay variable : PTINR_VAL1 PT-INR Value

N	Mean	Standard Deviation	Minimum	Maximum
6	1.7350000	0.1534601	1.5200000	1.9100000

Unique Subject Identifier=1320000000-0014

Assay variable : PTINR_VAL1 PT-INR Value

N	Mean	Standard Deviation	Minimum	Maximum
5	1.7560000	0.3250846	1.4800000	2.2000000

Unique Subject Identifier=1320000000-0017

Assay variable : PTINR_VAL1 PT-INR Value

N	Mean	Standard Deviation	Minimum	Maximum
6	2.2066667	0.3567445	1.9000000	2.7000000

Laboratory tests at the time of obtaining Table 2-6 consent, according to the presence or absence of proton-pump inhibitors

Unique Subject Identifier=1320000000-0020

MEANS Procedure

Assay variable : PTINR_VAL1 PT-INR Value

N	Mean	Standard Deviation	Minimum	Maximum
6	2.1700000	0.6441739	1.4700000	3.1500000

Unique Subject Identifier=1320000000-0021

Assay variable : PTINR_VAL1 PT-INR Value

N	Mean	Standard Deviation	Minimum	Maximum
6	1.8733333	0.5328289	1.1700000	2.5000000

Unique Subject Identifier=1320000000-0022

Assay variable : PTINR_VAL1 PT-INR Value

N	Mean	Standard Deviation	Minimum	Maximum
5	2.2040000	0.2899655	1.8000000	2.6000000

Unique Subject Identifier=1320000000-0024

Assay variable : PTINR_VAL1 PT-INR Value

N	Mean	Standard Deviation	Minimum	Maximum
6	1.9266667	0.3090415	1.5900000	2.4600000

Laboratory tests at the time of obtaining Table 2-6 consent, according to the presence or absence of proton-pump inhibitors

Unique Subject Identifier=1320000000-0025

MEANS Procedure

Assay variable : PTINR_VAL1 PT-INR Value

N	Mean	Standard Deviation	Minimum	Maximum
6	2.2083333	0.7145185	1.5000000	3.5100000

Unique Subject Identifier=1320000000-0026

Assay variable : PTINR_VAL1 PT-INR Value

N	Mean	Standard Deviation	Minimum	Maximum
5	2.5820000	1.0081766	1.2000000	4.0000000

Unique Subject Identifier=1320000000-0029

Assay variable : PTINR_VAL1 PT-INR Value

N	Mean	Standard Deviation	Minimum	Maximum
6	1.7050000	0.4458139	1.0000000	2.1700000

Unique Subject Identifier=1321011110-0004

Assay variable : PTINR_VAL1 PT-INR Value

N	Mean	Standard Deviation	Minimum	Maximum
1	3.1600000		3.1600000	3.1600000

Laboratory tests at the time of obtaining Table 2-6 consent, according to the presence or absence of proton-pump inhibitors

Unique Subject Identifier=1321011110-0016

MEANS Procedure

Assay variable : PTINR_VAL1 PT-INR Value

N	Mean	Standard Deviation	Minimum	Maximum
1	2.2900000		2.2900000	2.2900000

Unique Subject Identifier=1321011110-0023

Assay variable : PTINR_VAL1 PT-INR Value

N	Mean	Standard Deviation	Minimum	Maximum
4	1.5975000	0.0660177	1.5100000	1.6700000

Unique Subject Identifier=1322000000-0001

Assay variable : PTINR_VAL1 PT-INR Value

N	Mean	Standard Deviation	Minimum	Maximum
3	1.6600000	0.4613025	1.1800000	2.1000000

Unique Subject Identifier=1322000000-0002

Assay variable : PTINR_VAL1 PT-INR Value

N	Mean	Standard Deviation	Minimum	Maximum
1	0.9800000		0.9800000	0.9800000

Laboratory tests at the time of obtaining Table 2-6 consent, according to the presence or absence of proton-pump inhibitors

Unique Subject Identifier=1322000000-0009

MEANS Procedure

Assay variable : PTINR_VAL1 PT-INR Value

N	Mean	Standard Deviation	Minimum	Maximum
2	1.1550000	0.0353553	1.1300000	1.1800000

Unique Subject Identifier=1324111000-0003

Assay variable : PTINR_VAL1 PT-INR Value

N	Mean	Standard Deviation	Minimum	Maximum
6	2.1666667	0.4179314	1.7000000	2.8000000

Unique Subject Identifier=1324111000-0009

Assay variable : PTINR_VAL1 PT-INR Value

N	Mean	Standard Deviation	Minimum	Maximum
6	2.5166667	0.3544949	2.0000000	3.1000000

Unique Subject Identifier=1324111000-0014

Assay variable : PTINR_VAL1 PT-INR Value

N	Mean	Standard Deviation	Minimum	Maximum
6	1.7166667	0.3060501	1.4000000	2.2000000

Laboratory tests at the time of obtaining Table 2-6 consent, according to the presence or absence of proton-pump inhibitors

Unique Subject Identifier=1326000000-0001

MEANS Procedure

Assay variable : PTINR_VAL1 PT-INR Value

N	Mean	Standard Deviation	Minimum	Maximum
5	1.5000000	0.1581139	1.3000000	1.7000000

Unique Subject Identifier=1328011111-0003

Assay variable : PTINR_VAL1 PT-INR Value

N	Mean	Standard Deviation	Minimum	Maximum
6	2.3183333	0.4281783	1.7900000	3.0400000

Unique Subject Identifier=1328011111-0005

Assay variable : PTINR_VAL1 PT-INR Value

N	Mean	Standard Deviation	Minimum	Maximum
6	2.4183333	0.3291454	1.8300000	2.7900000

Unique Subject Identifier=1328011111-0009

Assay variable : PTINR_VAL1 PT-INR Value

N	Mean	Standard Deviation	Minimum	Maximum
4	1.5900000	0.2597435	1.3900000	1.9500000

Laboratory tests at the time of obtaining Table 2-6 consent, according to the presence or absence of proton-pump inhibitors

Unique Subject Identifier=1328011111-0033

MEANS Procedure

Assay variable : PTINR_VAL1 PT-INR Value

N	Mean	Standard Deviation	Minimum	Maximum
2	1.2250000	0.1202082	1.1400000	1.3100000

Unique Subject Identifier=1328011111-0036

Assay variable : PTINR_VAL1 PT-INR Value

N	Mean	Standard Deviation	Minimum	Maximum
6	1.9633333	0.3518333	1.6200000	2.5600000

Unique Subject Identifier=1328011111-0044

Assay variable : PTINR_VAL1 PT-INR Value

N	Mean	Standard Deviation	Minimum	Maximum
2	1.7600000	0.0424264	1.7300000	1.7900000

Unique Subject Identifier=1328011111-0045

Assay variable : PTINR_VAL1 PT-INR Value

N	Mean	Standard Deviation	Minimum	Maximum
2	1.2450000	0.1767767	1.1200000	1.3700000

Laboratory tests at the time of obtaining Table 2-6 consent, according to the presence or absence of proton-pump inhibitors

Unique Subject Identifier=1328011111-0072

MEANS Procedure

Assay variable : PTINR_VAL1 PT-INR Value

N	Mean	Standard Deviation	Minimum	Maximum
5	2.2160000	0.2659511	1.8800000	2.5800000

Unique Subject Identifier=1328011111-0074

Assay variable : PTINR_VAL1 PT-INR Value

N	Mean	Standard Deviation	Minimum	Maximum
1	1.9900000		1.9900000	1.9900000

Unique Subject Identifier=1328011111-0079

Assay variable : PTINR_VAL1 PT-INR Value

N	Mean	Standard Deviation	Minimum	Maximum
4	2.3575000	0.1252664	2.1700000	2.4300000

Unique Subject Identifier=1328011111-0090

Assay variable : PTINR_VAL1 PT-INR Value

N	Mean	Standard Deviation	Minimum	Maximum
5	1.6440000	0.1130487	1.5100000	1.7700000

Laboratory tests at the time of obtaining Table 2-6 consent, according to the presence or absence of proton-pump inhibitors

Unique Subject Identifier=1336011110-0001

MEANS Procedure

Assay variable : PTINR_VAL1 PT-INR Value

N	Mean	Standard Deviation	Minimum	Maximum
4	1.4250000	0.2629956	1.2000000	1.8000000

Unique Subject Identifier=1341011111-0011

Assay variable : PTINR_VAL1 PT-INR Value

N	Mean	Standard Deviation	Minimum	Maximum
5	2.3500000	0.3421257	1.9100000	2.8700000

Unique Subject Identifier=1341011111-0014

Assay variable : PTINR_VAL1 PT-INR Value

N	Mean	Standard Deviation	Minimum	Maximum
1	2.4300000		2.4300000	2.4300000

Unique Subject Identifier=1341011111-0015

Assay variable : PTINR_VAL1 PT-INR Value

N	Mean	Standard Deviation	Minimum	Maximum
3	2.5100000	0.3950949	2.1100000	2.9000000

Laboratory tests at the time of obtaining Table 2-6 consent, according to the presence or absence of proton-pump inhibitors

Unique Subject Identifier=1342000000-0006

MEANS Procedure

Assay variable : PTINR_VAL1 PT-INR Value

N	Mean	Standard Deviation	Minimum	Maximum
6	2.3200000	0.3955250	1.7200000	2.8100000

Unique Subject Identifier=1351010111-0002

Assay variable : PTINR_VAL1 PT-INR Value

N	Mean	Standard Deviation	Minimum	Maximum
6	1.8166667	0.2714160	1.3000000	2.1000000

Unique Subject Identifier=1351010111-0009

Assay variable : PTINR_VAL1 PT-INR Value

N	Mean	Standard Deviation	Minimum	Maximum
6	1.8833333	0.3430258	1.4000000	2.3000000

Unique Subject Identifier=1351010111-0012

Assay variable : PTINR_VAL1 PT-INR Value

N	Mean	Standard Deviation	Minimum	Maximum
6	1.5666667	0.2581989	1.2000000	1.9000000

Laboratory tests at the time of obtaining Table 2-6 consent, according to the presence or absence of proton-pump inhibitors

Unique Subject Identifier=1351010111-0013

MEANS Procedure

Assay variable : PTINR_VAL1 PT-INR Value

N	Mean	Standard Deviation	Minimum	Maximum
6	1.7000000	0.2529822	1.5000000	2.2000000

Unique Subject Identifier=1351010111-0015

Assay variable : PTINR_VAL1 PT-INR Value

N	Mean	Standard Deviation	Minimum	Maximum
6	2.1500000	0.3146427	1.7000000	2.6000000

Unique Subject Identifier=1351010111-0022

Assay variable : PTINR_VAL1 PT-INR Value

N	Mean	Standard Deviation	Minimum	Maximum
6	1.9333333	0.7089899	1.0000000	3.1000000

Unique Subject Identifier=1352011010-0003

Assay variable : PTINR_VAL1 PT-INR Value

N	Mean	Standard Deviation	Minimum	Maximum
6	2.2833333	0.6493587	1.3000000	3.0000000

Laboratory tests at the time of obtaining Table 2-6 consent, according to the presence or absence of proton-pump inhibitors

Unique Subject Identifier=1352011010-0011

MEANS Procedure

Assay variable : PTINR_VAL1 PT-INR Value

N	Mean	Standard Deviation	Minimum	Maximum
6	2.0666667	0.3559026	1.6000000	2.7000000

Unique Subject Identifier=1352011010-0012

Assay variable : PTINR_VAL1 PT-INR Value

N	Mean	Standard Deviation	Minimum	Maximum
6	1.6333333	0.4760952	1.2000000	2.5000000

Unique Subject Identifier=1353000000-0001

Assay variable : PTINR_VAL1 PT-INR Value

N	Mean	Standard Deviation	Minimum	Maximum
2	1.8500000	0.0707107	1.8000000	1.9000000

Unique Subject Identifier=1353000000-0003

Assay variable : PTINR_VAL1 PT-INR Value

N	Mean	Standard Deviation	Minimum	Maximum
2	1.9000000	0.1414214	1.8000000	2.0000000

Laboratory tests at the time of obtaining Table 2-6 consent, according to the presence or absence of proton-pump inhibitors

Unique Subject Identifier=1353000000-0005

MEANS Procedure

Assay variable : PTINR_VAL1 PT-INR Value

N	Mean	Standard Deviation	Minimum	Maximum
1	2.2000000		2.2000000	2.2000000

Unique Subject Identifier=1353000000-0015

Assay variable : PTINR_VAL1 PT-INR Value

N	Mean	Standard Deviation	Minimum	Maximum
1	2.4000000		2.4000000	2.4000000

Unique Subject Identifier=1353000000-0017

Assay variable : PTINR_VAL1 PT-INR Value

N	Mean	Standard Deviation	Minimum	Maximum
1	1.9000000		1.9000000	1.9000000

Unique Subject Identifier=1353000000-0024

Assay variable : PTINR_VAL1 PT-INR Value

N	Mean	Standard Deviation	Minimum	Maximum
2	2.1500000	0.2121320	2.0000000	2.3000000

Laboratory tests at the time of obtaining Table 2-6 consent, according to the presence or absence of proton-pump inhibitors

Unique Subject Identifier=1353000000-0028

MEANS Procedure

Assay variable : PTINR_VAL1 PT-INR Value

N	Mean	Standard Deviation	Minimum	Maximum
2	1.8500000	0.2121320	1.7000000	2.0000000

Unique Subject Identifier=1353000000-0034

Assay variable : PTINR_VAL1 PT-INR Value

N	Mean	Standard Deviation	Minimum	Maximum
2	1.5500000	0.0707107	1.5000000	1.6000000

Unique Subject Identifier=1353000000-0039

Assay variable : PTINR_VAL1 PT-INR Value

N	Mean	Standard Deviation	Minimum	Maximum
1	2.2000000		2.2000000	2.2000000

Unique Subject Identifier=1353000000-0048

Assay variable : PTINR_VAL1 PT-INR Value

N	Mean	Standard Deviation	Minimum	Maximum
2	1.5000000	0.4242641	1.2000000	1.8000000

Laboratory tests at the time of obtaining Table 2-6 consent, according to the presence or absence of proton-pump inhibitors

Unique Subject Identifier=1353000000-0049

MEANS Procedure

Assay variable : PTINR_VAL1 PT-INR Value

N	Mean	Standard Deviation	Minimum	Maximum
2	1.6500000	0.0707107	1.6000000	1.7000000

Unique Subject Identifier=1353000000-0051

Assay variable : PTINR_VAL1 PT-INR Value

N	Mean	Standard Deviation	Minimum	Maximum
1	1.8000000		1.8000000	1.8000000

Unique Subject Identifier=1354010000-0001

Assay variable : PTINR_VAL1 PT-INR Value

N	Mean	Standard Deviation	Minimum	Maximum
6	1.6100000	0.1005982	1.5100000	1.7400000

Unique Subject Identifier=1354010000-0004

Assay variable : PTINR_VAL1 PT-INR Value

N	Mean	Standard Deviation	Minimum	Maximum
5	2.1960000	0.2434748	1.9300000	2.5500000

Laboratory tests at the time of obtaining Table 2-6 consent, according to the presence or absence of proton-pump inhibitors

Unique Subject Identifier=1354010000-0006

MEANS Procedure

Assay variable : PTINR_VAL1 PT-INR Value

N	Mean	Standard Deviation	Minimum	Maximum
5	1.8140000	0.1443260	1.5900000	1.9600000

Unique Subject Identifier=1354010000-0007

Assay variable : PTINR_VAL1 PT-INR Value

N	Mean	Standard Deviation	Minimum	Maximum
5	1.5680000	0.1219426	1.4800000	1.7800000

Unique Subject Identifier=1354010000-0011

Assay variable : PTINR_VAL1 PT-INR Value

N	Mean	Standard Deviation	Minimum	Maximum
3	1.4733333	0.0757188	1.4200000	1.5600000

Unique Subject Identifier=1354010000-0015

Assay variable : PTINR_VAL1 PT-INR Value

N	Mean	Standard Deviation	Minimum	Maximum
5	2.1240000	0.1504327	1.8600000	2.2100000

Laboratory tests at the time of obtaining Table 2-6 consent, according to the presence or absence of proton-pump inhibitors

Unique Subject Identifier=1354010000-0023

MEANS Procedure

Assay variable : PTINR_VAL1 PT-INR Value

N	Mean	Standard Deviation	Minimum	Maximum
5	1.5940000	0.1277889	1.4400000	1.7900000

Unique Subject Identifier=1354010000-0024

Assay variable : PTINR_VAL1 PT-INR Value

N	Mean	Standard Deviation	Minimum	Maximum
6	1.5616667	0.1185608	1.4100000	1.7500000

Unique Subject Identifier=1354010000-0027

Assay variable : PTINR_VAL1 PT-INR Value

N	Mean	Standard Deviation	Minimum	Maximum
6	1.7050000	0.3923137	1.3700000	2.4400000

Unique Subject Identifier=1354010000-0032

Assay variable : PTINR_VAL1 PT-INR Value

N	Mean	Standard Deviation	Minimum	Maximum
6	1.4733333	0.1116542	1.3000000	1.6100000

Laboratory tests at the time of obtaining Table 2-6 consent, according to the presence or absence of proton-pump inhibitors

Unique Subject Identifier=1354010000-0033

MEANS Procedure

Assay variable : PTINR_VAL1 PT-INR Value

N	Mean	Standard Deviation	Minimum	Maximum
6	1.6900000	0.3095158	1.2200000	2.0600000

Unique Subject Identifier=1354010000-0035

Assay variable : PTINR_VAL1 PT-INR Value

N	Mean	Standard Deviation	Minimum	Maximum
3	1.6100000	0.4330127	1.1100000	1.8600000

Unique Subject Identifier=1354010000-0046

Assay variable : PTINR_VAL1 PT-INR Value

N	Mean	Standard Deviation	Minimum	Maximum
5	2.0760000	0.2469413	1.6600000	2.2700000

Unique Subject Identifier=1354010000-0050

Assay variable : PTINR_VAL1 PT-INR Value

N	Mean	Standard Deviation	Minimum	Maximum
5	1.7380000	0.1728294	1.5400000	1.9800000

Laboratory tests at the time of obtaining Table 2-6 consent, according to the presence or absence of proton-pump inhibitors

Unique Subject Identifier=1355000000-0005

MEANS Procedure

Assay variable : PTINR_VAL1 PT-INR Value

N	Mean	Standard Deviation	Minimum	Maximum
6	2.1166667	0.3371449	1.6000000	2.6000000

Unique Subject Identifier=1355000000-0007

Assay variable : PTINR_VAL1 PT-INR Value

N	Mean	Standard Deviation	Minimum	Maximum
6	2.1166667	0.0752773	2.0000000	2.2000000

Unique Subject Identifier=1365010000-0004

Assay variable : PTINR_VAL1 PT-INR Value

N	Mean	Standard Deviation	Minimum	Maximum
4	1.9325000	0.0607591	1.8600000	2.0000000

Unique Subject Identifier=1365010000-0005

Assay variable : PTINR_VAL1 PT-INR Value

N	Mean	Standard Deviation	Minimum	Maximum
4	1.7650000	0.1040833	1.6400000	1.8900000

Laboratory tests at the time of obtaining Table 2-6 consent, according to the presence or absence of proton-pump inhibitors

Unique Subject Identifier=1365010000-0006

MEANS Procedure

Assay variable : PTINR_VAL1 PT-INR Value

N	Mean	Standard Deviation	Minimum	Maximum
6	1.7083333	0.1762290	1.4900000	1.8800000

Unique Subject Identifier=1365010000-0021

Assay variable : PTINR_VAL1 PT-INR Value

N	Mean	Standard Deviation	Minimum	Maximum
1	1.6500000		1.6500000	1.6500000

Unique Subject Identifier=1368011111-0001

Assay variable : PTINR_VAL1 PT-INR Value

N	Mean	Standard Deviation	Minimum	Maximum
2	1.3250000	0.1060660	1.2500000	1.4000000

Unique Subject Identifier=1368011111-0002

Assay variable : PTINR_VAL1 PT-INR Value

N	Mean	Standard Deviation	Minimum	Maximum
2	1.9600000	0.1131371	1.8800000	2.0400000

Laboratory tests at the time of obtaining Table 2-6 consent, according to the presence or absence of proton-pump inhibitors

Unique Subject Identifier=1369010010-0001

MEANS Procedure

Assay variable : PTINR_VAL1 PT-INR Value

N	Mean	Standard Deviation	Minimum	Maximum
5	1.9460000	0.2567684	1.7300000	2.3000000

Unique Subject Identifier=1371000000-0004

Assay variable : PTINR_VAL1 PT-INR Value

N	Mean	Standard Deviation	Minimum	Maximum
6	2.1233333	0.3201666	1.6800000	2.5800000

Unique Subject Identifier=1371000000-0005

Assay variable : PTINR_VAL1 PT-INR Value

N	Mean	Standard Deviation	Minimum	Maximum
6	1.6833333	0.2959504	1.4200000	2.1800000

Unique Subject Identifier=1371000000-0007

Assay variable : PTINR_VAL1 PT-INR Value

N	Mean	Standard Deviation	Minimum	Maximum
6	2.0300000	0.1908926	1.7400000	2.2900000

Laboratory tests at the time of obtaining Table 2-6 consent, according to the presence or absence of proton-pump inhibitors

Unique Subject Identifier=1371000000-0009

MEANS Procedure

Assay variable : PTINR_VAL1 PT-INR Value

N	Mean	Standard Deviation	Minimum	Maximum
6	1.7900000	0.1352036	1.6100000	1.9700000

Unique Subject Identifier=1371000000-0010

Assay variable : PTINR_VAL1 PT-INR Value

N	Mean	Standard Deviation	Minimum	Maximum
6	1.7700000	0.3878144	1.4200000	2.5300000

Unique Subject Identifier=1371000000-0015

Assay variable : PTINR_VAL1 PT-INR Value

N	Mean	Standard Deviation	Minimum	Maximum
5	1.8700000	0.2813361	1.5500000	2.1900000

Unique Subject Identifier=1371000000-0016

Assay variable : PTINR_VAL1 PT-INR Value

N	Mean	Standard Deviation	Minimum	Maximum
6	1.9400000	0.3752333	1.4000000	2.4300000

Laboratory tests at the time of obtaining Table 2-6 consent, according to the presence or absence of proton-pump inhibitors

Unique Subject Identifier=1371000000-0017

MEANS Procedure

Assay variable : PTINR_VAL1 PT-INR Value

N	Mean	Standard Deviation	Minimum	Maximum
6	1.6366667	0.1979562	1.4600000	1.9900000

Unique Subject Identifier=1371000000-0021

Assay variable : PTINR_VAL1 PT-INR Value

N	Mean	Standard Deviation	Minimum	Maximum
6	1.8866667	0.2130415	1.6000000	2.0900000

Unique Subject Identifier=1371000000-0023

Assay variable : PTINR_VAL1 PT-INR Value

N	Mean	Standard Deviation	Minimum	Maximum
6	1.7516667	0.6507662	1.0700000	2.9600000

Unique Subject Identifier=1371000000-0024

Assay variable : PTINR_VAL1 PT-INR Value

N	Mean	Standard Deviation	Minimum	Maximum
6	1.7283333	0.3864410	1.2100000	2.0400000

Laboratory tests at the time of obtaining Table 2-6 consent, according to the presence or absence of proton-pump inhibitors

Unique Subject Identifier=1372011010-0006

MEANS Procedure

Assay variable : PTINR_VAL1 PT-INR Value

N	Mean	Standard Deviation	Minimum	Maximum
4	1.8000000	0.7845593	1.2400000	2.9500000

Unique Subject Identifier=1377011000-0002

Assay variable : PTINR_VAL1 PT-INR Value

N	Mean	Standard Deviation	Minimum	Maximum
5	2.3140000	0.3150079	1.8800000	2.7600000

Unique Subject Identifier=1377011000-0004

Assay variable : PTINR_VAL1 PT-INR Value

N	Mean	Standard Deviation	Minimum	Maximum
4	2.1300000	1.0241419	1.1200000	3.3500000

Unique Subject Identifier=1377011000-0009

Assay variable : PTINR_VAL1 PT-INR Value

N	Mean	Standard Deviation	Minimum	Maximum
4	2.1400000	0.4913926	1.4500000	2.5300000

Laboratory tests at the time of obtaining Table 2-6 consent, according to the presence or absence of proton-pump inhibitors

Unique Subject Identifier=1377011000-0012

MEANS Procedure

Assay variable : PTINR_VAL1 PT-INR Value

N	Mean	Standard Deviation	Minimum	Maximum
5	1.4240000	0.2244549	1.0900000	1.6800000

Unique Subject Identifier=1377011000-0016

Assay variable : PTINR_VAL1 PT-INR Value

N	Mean	Standard Deviation	Minimum	Maximum
5	1.9560000	0.2370232	1.6200000	2.1300000

Unique Subject Identifier=1380000110-0016

Assay variable : PTINR_VAL1 PT-INR Value

N	Mean	Standard Deviation	Minimum	Maximum
6	2.1000000	0.7293833	1.3000000	3.3000000

Unique Subject Identifier=1380000110-0027

Assay variable : PTINR_VAL1 PT-INR Value

N	Mean	Standard Deviation	Minimum	Maximum
5	2.2800000	0.3033150	1.8000000	2.6000000

Laboratory tests at the time of obtaining Table 2-6 consent, according to the presence or absence of proton-pump inhibitors

Unique Subject Identifier=1381000000-0008

MEANS Procedure

Assay variable : PTINR_VAL1 PT-INR Value

N	Mean	Standard Deviation	Minimum	Maximum
2	2.1150000	0.4171930	1.8200000	2.4100000

Unique Subject Identifier=1385011000-0035

Assay variable : PTINR_VAL1 PT-INR Value

N	Mean	Standard Deviation	Minimum	Maximum
1	1.6800000		1.6800000	1.6800000

Unique Subject Identifier=1385011000-0057

Assay variable : PTINR_VAL1 PT-INR Value

N	Mean	Standard Deviation	Minimum	Maximum
1	1.8200000		1.8200000	1.8200000

Unique Subject Identifier=1385011000-0110

Assay variable : PTINR_VAL1 PT-INR Value

N	Mean	Standard Deviation	Minimum	Maximum
2	2.1000000	0.0282843	2.0800000	2.1200000

Laboratory tests at the time of obtaining Table 2-6 consent, according to the presence or absence of proton-pump inhibitors

Unique Subject Identifier=1386000000-0006

MEANS Procedure

Assay variable : PTINR_VAL1 PT-INR Value

N	Mean	Standard Deviation	Minimum	Maximum
6	2.6483333	0.8358808	1.4900000	3.7600000

Unique Subject Identifier=1386000000-0007

Assay variable : PTINR_VAL1 PT-INR Value

N	Mean	Standard Deviation	Minimum	Maximum
3	1.2500000	0.0500000	1.2000000	1.3000000

Unique Subject Identifier=1386000000-0021

Assay variable : PTINR_VAL1 PT-INR Value

N	Mean	Standard Deviation	Minimum	Maximum
1	3.3000000		3.3000000	3.3000000

Unique Subject Identifier=1386000000-0022

Assay variable : PTINR_VAL1 PT-INR Value

N	Mean	Standard Deviation	Minimum	Maximum
3	2.7933333	0.9222979	1.9700000	3.7900000

Laboratory tests at the time of obtaining Table 2-6 consent, according to the presence or absence of proton-pump inhibitors

Unique Subject Identifier=1392011110-0007

MEANS Procedure

Assay variable : PTINR_VAL1 PT-INR Value

N	Mean	Standard Deviation	Minimum	Maximum
6	1.8666667	0.3386247	1.6000000	2.5000000

Unique Subject Identifier=1392011110-0008

Assay variable : PTINR_VAL1 PT-INR Value

N	Mean	Standard Deviation	Minimum	Maximum
6	1.5666667	0.2065591	1.2000000	1.8000000

Unique Subject Identifier=1392011110-0012

Assay variable : PTINR_VAL1 PT-INR Value

N	Mean	Standard Deviation	Minimum	Maximum
6	1.9166667	0.3544949	1.7000000	2.6000000

Unique Subject Identifier=1392011110-0013

Assay variable : PTINR_VAL1 PT-INR Value

N	Mean	Standard Deviation	Minimum	Maximum
6	2.0666667	0.3011091	1.8000000	2.5000000

Laboratory tests at the time of obtaining Table 2-6 consent, according to the presence or absence of proton-pump inhibitors

Unique Subject Identifier=1392011110-0016

MEANS Procedure

Assay variable : PTINR_VAL1 PT-INR Value

N	Mean	Standard Deviation	Minimum	Maximum
6	2.2000000	0.3033150	1.8000000	2.6000000

Unique Subject Identifier=1392011110-0017

Assay variable : PTINR_VAL1 PT-INR Value

N	Mean	Standard Deviation	Minimum	Maximum
6	1.8500000	0.1048809	1.7000000	2.0000000

Unique Subject Identifier=1394011010-0001

Assay variable : PTINR_VAL1 PT-INR Value

N	Mean	Standard Deviation	Minimum	Maximum
2	3.4000000	1.1313708	2.6000000	4.2000000

Unique Subject Identifier=1396000000-0005

Assay variable : PTINR_VAL1 PT-INR Value

N	Mean	Standard Deviation	Minimum	Maximum
1	1.6000000		1.6000000	1.6000000

Laboratory tests at the time of obtaining Table 2-6 consent, according to the presence or absence of proton-pump inhibitors

Unique Subject Identifier=1396000000-0014

MEANS Procedure

Assay variable : PTINR_VAL1 PT-INR Value

N	Mean	Standard Deviation	Minimum	Maximum
6	2.0000000	0.2607681	1.5000000	2.2000000

Unique Subject Identifier=1398010000-0007

Assay variable : PTINR_VAL1 PT-INR Value

N	Mean	Standard Deviation	Minimum	Maximum
5	1.7400000	0.2302173	1.5000000	2.1000000

Unique Subject Identifier=1398010000-0016

Assay variable : PTINR_VAL1 PT-INR Value

N	Mean	Standard Deviation	Minimum	Maximum
4	1.7000000	0.3366502	1.3000000	2.1000000

Unique Subject Identifier=1398010000-0018

Assay variable : PTINR_VAL1 PT-INR Value

N	Mean	Standard Deviation	Minimum	Maximum
5	1.9800000	0.1923538	1.7000000	2.2000000

Laboratory tests at the time of obtaining Table 2-6 consent, according to the presence or absence of proton-pump inhibitors

Unique Subject Identifier=1398010000-0019

MEANS Procedure

Assay variable : PTINR_VAL1 PT-INR Value

N	Mean	Standard Deviation	Minimum	Maximum
3	1.9333333	0.2309401	1.8000000	2.2000000

Unique Subject Identifier=1398010000-0021

Assay variable : PTINR_VAL1 PT-INR Value

N	Mean	Standard Deviation	Minimum	Maximum
4	2.0500000	0.4509250	1.7000000	2.7000000

Unique Subject Identifier=1398010000-0026

Assay variable : PTINR_VAL1 PT-INR Value

N	Mean	Standard Deviation	Minimum	Maximum
5	2.1200000	0.5069517	1.6000000	2.9000000

Unique Subject Identifier=1398010000-0032

Assay variable : PTINR_VAL1 PT-INR Value

N	Mean	Standard Deviation	Minimum	Maximum
3	1.5333333	0.0577350	1.5000000	1.6000000

Laboratory tests at the time of obtaining Table 2-6 consent, according to the presence or absence of proton-pump inhibitors

Unique Subject Identifier=1398010000-0034

MEANS Procedure

Assay variable : PTINR_VAL1 PT-INR Value

N	Mean	Standard Deviation	Minimum	Maximum
4	1.8000000	0.1414214	1.6000000	1.9000000

Unique Subject Identifier=1398010000-0036

Assay variable : PTINR_VAL1 PT-INR Value

N	Mean	Standard Deviation	Minimum	Maximum
3	2.2333333	0.1527525	2.1000000	2.4000000

Unique Subject Identifier=1398010000-0038

Assay variable : PTINR_VAL1 PT-INR Value

N	Mean	Standard Deviation	Minimum	Maximum
4	1.2750000	0.1500000	1.2000000	1.5000000

Unique Subject Identifier=1398010000-0043

Assay variable : PTINR_VAL1 PT-INR Value

N	Mean	Standard Deviation	Minimum	Maximum
3	2.3000000	0.1000000	2.2000000	2.4000000

Laboratory tests at the time of obtaining Table 2-6 consent, according to the presence or absence of proton-pump inhibitors

Unique Subject Identifier=1400000000-0001

MEANS Procedure

Assay variable : PTINR_VAL1 PT-INR Value

N	Mean	Standard Deviation	Minimum	Maximum
5	2.3120000	0.5893810	1.6700000	3.0800000

Unique Subject Identifier=1400000000-0002

Assay variable : PTINR_VAL1 PT-INR Value

N	Mean	Standard Deviation	Minimum	Maximum
5	2.0620000	0.5821684	1.4600000	2.9300000

Unique Subject Identifier=1400000000-0005

Assay variable : PTINR_VAL1 PT-INR Value

N	Mean	Standard Deviation	Minimum	Maximum
1	2.7800000		2.7800000	2.7800000

Unique Subject Identifier=1400000000-0006

Assay variable : PTINR_VAL1 PT-INR Value

N	Mean	Standard Deviation	Minimum	Maximum
3	2.0666667	0.2514624	1.7800000	2.2500000

Laboratory tests at the time of obtaining Table 2-6 consent, according to the presence or absence of proton-pump inhibitors

Unique Subject Identifier=1400000000-0010

MEANS Procedure

Assay variable : PTINR_VAL1 PT-INR Value

N	Mean	Standard Deviation	Minimum	Maximum
3	2.3233333	0.6473278	1.7100000	3.0000000

Unique Subject Identifier=1401000000-0002

Assay variable : PTINR_VAL1 PT-INR Value

N	Mean	Standard Deviation	Minimum	Maximum
5	2.0780000	0.3787083	1.5800000	2.5100000

Unique Subject Identifier=1401000000-0003

Assay variable : PTINR_VAL1 PT-INR Value

N	Mean	Standard Deviation	Minimum	Maximum
6	2.3716667	0.4688888	1.5400000	2.9400000

Unique Subject Identifier=1401000000-0004

Assay variable : PTINR_VAL1 PT-INR Value

N	Mean	Standard Deviation	Minimum	Maximum
5	2.4740000	0.2583215	2.0800000	2.7500000

Laboratory tests at the time of obtaining Table 2-6 consent, according to the presence or absence of proton-pump inhibitors

Unique Subject Identifier=1401000000-0007

MEANS Procedure

Assay variable : PTINR_VAL1 PT-INR Value

N	Mean	Standard Deviation	Minimum	Maximum
5	2.8940000	0.4098536	2.3800000	3.3100000

Unique Subject Identifier=1401000000-0008

Assay variable : PTINR_VAL1 PT-INR Value

N	Mean	Standard Deviation	Minimum	Maximum
5	2.6740000	0.3907429	2.1900000	3.2600000

Unique Subject Identifier=1401000000-0009

Assay variable : PTINR_VAL1 PT-INR Value

N	Mean	Standard Deviation	Minimum	Maximum
4	1.9450000	0.4747280	1.3900000	2.5500000

Unique Subject Identifier=1401000000-0010

Assay variable : PTINR_VAL1 PT-INR Value

N	Mean	Standard Deviation	Minimum	Maximum
6	2.9566667	0.3767050	2.5200000	3.5300000

Laboratory tests at the time of obtaining Table 2-6 consent, according to the presence or absence of proton-pump inhibitors

Unique Subject Identifier=1405000000-0001

MEANS Procedure

Assay variable : PTINR_VAL1 PT-INR Value

N	Mean	Standard Deviation	Minimum	Maximum
5	2.1400000	0.2792848	1.9000000	2.6000000

Unique Subject Identifier=1405000000-0002

Assay variable : PTINR_VAL1 PT-INR Value

N	Mean	Standard Deviation	Minimum	Maximum
6	2.5333333	0.6218253	1.9000000	3.3000000

Unique Subject Identifier=1405000000-0004

Assay variable : PTINR_VAL1 PT-INR Value

N	Mean	Standard Deviation	Minimum	Maximum
6	2.0000000	0.1549193	1.8000000	2.2000000

Unique Subject Identifier=1405000000-0005

Assay variable : PTINR_VAL1 PT-INR Value

N	Mean	Standard Deviation	Minimum	Maximum
6	2.0833333	0.2401388	1.9000000	2.5000000

Laboratory tests at the time of obtaining Table 2-6 consent, according to the presence or absence of proton-pump inhibitors

Unique Subject Identifier=1405000000-0006

MEANS Procedure

Assay variable : PTINR_VAL1 PT-INR Value

N	Mean	Standard Deviation	Minimum	Maximum
6	2.1666667	0.4082483	1.6000000	2.7000000

Unique Subject Identifier=1405000000-0008

Assay variable : PTINR_VAL1 PT-INR Value

N	Mean	Standard Deviation	Minimum	Maximum
6	2.2666667	0.1861899	2.0000000	2.5000000

Unique Subject Identifier=1405000000-0010

Assay variable : PTINR_VAL1 PT-INR Value

N	Mean	Standard Deviation	Minimum	Maximum
6	2.4500000	0.1048809	2.3000000	2.6000000

Unique Subject Identifier=1405000000-0011

Assay variable : PTINR_VAL1 PT-INR Value

N	Mean	Standard Deviation	Minimum	Maximum
6	2.4666667	0.3502380	1.9000000	2.9000000

Laboratory tests at the time of obtaining Table 2-6 consent, according to the presence or absence of proton-pump inhibitors

Unique Subject Identifier=1405000000-0015

MEANS Procedure

Assay variable : PTINR_VAL1 PT-INR Value

N	Mean	Standard Deviation	Minimum	Maximum
6	2.6666667	0.6947422	1.6000000	3.6000000

Unique Subject Identifier=1405000000-0017

Assay variable : PTINR_VAL1 PT-INR Value

N	Mean	Standard Deviation	Minimum	Maximum
6	2.3666667	0.2658320	1.9000000	2.7000000

Unique Subject Identifier=1405000000-0018

Assay variable : PTINR_VAL1 PT-INR Value

N	Mean	Standard Deviation	Minimum	Maximum
6	1.8333333	0.2422120	1.4000000	2.0000000

Unique Subject Identifier=1405000000-0021

Assay variable : PTINR_VAL1 PT-INR Value

N	Mean	Standard Deviation	Minimum	Maximum
6	2.1666667	0.4501851	1.4000000	2.6000000

Laboratory tests at the time of obtaining Table 2-6 consent, according to the presence or absence of proton-pump inhibitors

Unique Subject Identifier=1405000000-0023

MEANS Procedure

Assay variable : PTINR_VAL1 PT-INR Value

N	Mean	Standard Deviation	Minimum	Maximum
6	2.0666667	0.1861899	1.9000000	2.4000000

Unique Subject Identifier=1405000000-0024

Assay variable : PTINR_VAL1 PT-INR Value

N	Mean	Standard Deviation	Minimum	Maximum
6	2.4666667	0.3723797	1.8000000	2.8000000

Unique Subject Identifier=1405000000-0025

Assay variable : PTINR_VAL1 PT-INR Value

N	Mean	Standard Deviation	Minimum	Maximum
6	2.3000000	0.1788854	2.1000000	2.6000000

Unique Subject Identifier=1405000000-0026

Assay variable : PTINR_VAL1 PT-INR Value

N	Mean	Standard Deviation	Minimum	Maximum
6	2.1833333	0.2136976	1.9000000	2.4000000

Laboratory tests at the time of obtaining Table 2-6 consent, according to the presence or absence of proton-pump inhibitors

Unique Subject Identifier=1405000000-0028

MEANS Procedure

Assay variable : PTINR_VAL1 PT-INR Value

N	Mean	Standard Deviation	Minimum	Maximum
6	2.3833333	0.5706721	1.7000000	3.4000000

Unique Subject Identifier=1405000000-0029

Assay variable : PTINR_VAL1 PT-INR Value

N	Mean	Standard Deviation	Minimum	Maximum
6	2.7666667	0.6470446	1.9000000	3.6000000

Unique Subject Identifier=1405000000-0030

Assay variable : PTINR_VAL1 PT-INR Value

N	Mean	Standard Deviation	Minimum	Maximum
6	2.5000000	0.2097618	2.3000000	2.9000000

Unique Subject Identifier=1406000000-0001

Assay variable : PTINR_VAL1 PT-INR Value

N	Mean	Standard Deviation	Minimum	Maximum
1	1.1400000		1.1400000	1.1400000

Laboratory tests at the time of obtaining Table 2-6 consent, according to the presence or absence of proton-pump inhibitors

Unique Subject Identifier=1406000000-0005

MEANS Procedure

Assay variable : PTINR_VAL1 PT-INR Value

N	Mean	Standard Deviation	Minimum	Maximum
1	1.2800000		1.2800000	1.2800000

Unique Subject Identifier=1406000000-0006

Assay variable : PTINR_VAL1 PT-INR Value

N	Mean	Standard Deviation	Minimum	Maximum
1	3.2600000		3.2600000	3.2600000

Unique Subject Identifier=1406000000-0007

Assay variable : PTINR_VAL1 PT-INR Value

N	Mean	Standard Deviation	Minimum	Maximum
1	2.4500000		2.4500000	2.4500000

Unique Subject Identifier=1406000000-0008

Assay variable : PTINR_VAL1 PT-INR Value

N	Mean	Standard Deviation	Minimum	Maximum
1	1.4300000		1.4300000	1.4300000

Laboratory tests at the time of obtaining Table 2-6 consent, according to the presence or absence of proton-pump inhibitors

Unique Subject Identifier=1406000000-0011

MEANS Procedure

Assay variable : PTINR_VAL1 PT-INR Value

N	Mean	Standard Deviation	Minimum	Maximum
1	2.5000000		2.5000000	2.5000000

Unique Subject Identifier=1406000000-0014

Assay variable : PTINR_VAL1 PT-INR Value

N	Mean	Standard Deviation	Minimum	Maximum
1	1.7700000		1.7700000	1.7700000

Unique Subject Identifier=1406000000-0018

Assay variable : PTINR_VAL1 PT-INR Value

N	Mean	Standard Deviation	Minimum	Maximum
1	1.5300000		1.5300000	1.5300000

Unique Subject Identifier=1406000000-0019

Assay variable : PTINR_VAL1 PT-INR Value

N	Mean	Standard Deviation	Minimum	Maximum
1	1.6000000		1.6000000	1.6000000

Laboratory tests at the time of obtaining Table 2-6 consent, according to the presence or absence of proton-pump inhibitors

Unique Subject Identifier=1406000000-0020

MEANS Procedure

Assay variable : PTINR_VAL1 PT-INR Value

N	Mean	Standard Deviation	Minimum	Maximum
1	1.2500000		1.2500000	1.2500000

Unique Subject Identifier=1407000000-0007

Assay variable : PTINR_VAL1 PT-INR Value

N	Mean	Standard Deviation	Minimum	Maximum
6	2.0166667	0.1189398	1.9200000	2.2400000

Unique Subject Identifier=1407000000-0010

Assay variable : PTINR_VAL1 PT-INR Value

N	Mean	Standard Deviation	Minimum	Maximum
6	1.6883333	0.2422739	1.5000000	2.1400000

Unique Subject Identifier=1407000000-0013

Assay variable : PTINR_VAL1 PT-INR Value

N	Mean	Standard Deviation	Minimum	Maximum
6	1.8750000	0.1488288	1.6800000	2.0600000

Laboratory tests at the time of obtaining Table 2-6 consent, according to the presence or absence of proton-pump inhibitors

Unique Subject Identifier=1408010000-0001

MEANS Procedure

Assay variable : PTINR_VAL1 PT-INR Value

N	Mean	Standard Deviation	Minimum	Maximum
5	2.1640000	0.2016928	1.8600000	2.3800000

Unique Subject Identifier=1408010000-0004

Assay variable : PTINR_VAL1 PT-INR Value

N	Mean	Standard Deviation	Minimum	Maximum
5	2.6160000	0.3574633	2.3700000	3.2300000

Unique Subject Identifier=1408010000-0006

Assay variable : PTINR_VAL1 PT-INR Value

N	Mean	Standard Deviation	Minimum	Maximum
6	2.6933333	0.4401666	1.9400000	3.1800000

Unique Subject Identifier=1408010000-0007

Assay variable : PTINR_VAL1 PT-INR Value

N	Mean	Standard Deviation	Minimum	Maximum
5	2.4700000	0.4941660	2.1700000	3.3500000

Laboratory tests at the time of obtaining Table 2-6 consent, according to the presence or absence of proton-pump inhibitors

Unique Subject Identifier=1408010000-0008

MEANS Procedure

Assay variable : PTINR_VAL1 PT-INR Value

N	Mean	Standard Deviation	Minimum	Maximum
4	1.7600000	0.0408248	1.7000000	1.7900000

Unique Subject Identifier=1408010000-0012

Assay variable : PTINR_VAL1 PT-INR Value

N	Mean	Standard Deviation	Minimum	Maximum
5	3.0720000	0.4856645	2.5200000	3.8500000

Unique Subject Identifier=1408010000-0013

Assay variable : PTINR_VAL1 PT-INR Value

N	Mean	Standard Deviation	Minimum	Maximum
4	2.1725000	0.2780138	1.7800000	2.4200000

Unique Subject Identifier=1408010000-0015

Assay variable : PTINR_VAL1 PT-INR Value

N	Mean	Standard Deviation	Minimum	Maximum
6	2.4200000	0.3701351	2.0200000	2.9100000

Laboratory tests at the time of obtaining Table 2-6 consent, according to the presence or absence of proton-pump inhibitors

Unique Subject Identifier=1408010000-0021

MEANS Procedure

Assay variable : PTINR_VAL1 PT-INR Value

N	Mean	Standard Deviation	Minimum	Maximum
4	1.8375000	0.1472809	1.6400000	1.9500000

Unique Subject Identifier=1408010000-0025

Assay variable : PTINR_VAL1 PT-INR Value

N	Mean	Standard Deviation	Minimum	Maximum
4	2.1075000	0.3824809	1.8900000	2.6800000

Unique Subject Identifier=1408010000-0033

Assay variable : PTINR_VAL1 PT-INR Value

N	Mean	Standard Deviation	Minimum	Maximum
5	1.9540000	0.1722788	1.7900000	2.2400000

Unique Subject Identifier=1412000000-0007

Assay variable : PTINR_VAL1 PT-INR Value

N	Mean	Standard Deviation	Minimum	Maximum
6	1.8333333	0.4273952	1.3000000	2.3000000

Laboratory tests at the time of obtaining Table 2-6 consent, according to the presence or absence of proton-pump inhibitors

Unique Subject Identifier=1412000000-0014

MEANS Procedure

Assay variable : PTINR_VAL1 PT-INR Value

N	Mean	Standard Deviation	Minimum	Maximum
6	1.8333333	0.2065591	1.6000000	2.1000000

Unique Subject Identifier=1412000000-0021

Assay variable : PTINR_VAL1 PT-INR Value

N	Mean	Standard Deviation	Minimum	Maximum
6	2.1666667	0.1632993	1.9000000	2.4000000

Unique Subject Identifier=1412000000-0025

Assay variable : PTINR_VAL1 PT-INR Value

N	Mean	Standard Deviation	Minimum	Maximum
6	1.6333333	0.1632993	1.4000000	1.9000000

Unique Subject Identifier=1412000000-0028

Assay variable : PTINR_VAL1 PT-INR Value

N	Mean	Standard Deviation	Minimum	Maximum
6	2.6000000	1.4926487	1.4000000	5.5000000

Laboratory tests at the time of obtaining Table 2-6 consent, according to the presence or absence of proton-pump inhibitors

Unique Subject Identifier=1412000000-0031

MEANS Procedure

Assay variable : PTINR_VAL1 PT-INR Value

N	Mean	Standard Deviation	Minimum	Maximum
6	2.2166667	0.3544949	2.0000000	2.9000000

Unique Subject Identifier=1420010000-0007

Assay variable : PTINR_VAL1 PT-INR Value

N	Mean	Standard Deviation	Minimum	Maximum
6	2.2666667	0.3777124	1.8000000	2.8000000

Unique Subject Identifier=1420010000-0008

Assay variable : PTINR_VAL1 PT-INR Value

N	Mean	Standard Deviation	Minimum	Maximum
5	1.7600000	0.1816590	1.5000000	2.0000000

Unique Subject Identifier=1420010000-0012

Assay variable : PTINR_VAL1 PT-INR Value

N	Mean	Standard Deviation	Minimum	Maximum
6	2.1166667	0.2714160	1.6000000	2.4000000

Laboratory tests at the time of obtaining Table 2-6 consent, according to the presence or absence of proton-pump inhibitors

Unique Subject Identifier=1420010000-0013

MEANS Procedure

Assay variable : PTINR_VAL1 PT-INR Value

N	Mean	Standard Deviation	Minimum	Maximum
5	1.6400000	0.3049590	1.1000000	1.8000000

Unique Subject Identifier=1420010000-0014

Assay variable : PTINR_VAL1 PT-INR Value

N	Mean	Standard Deviation	Minimum	Maximum
6	2.0833333	0.1169045	1.9000000	2.2000000

Unique Subject Identifier=1423000000-0001

Assay variable : PTINR_VAL1 PT-INR Value

N	Mean	Standard Deviation	Minimum	Maximum
2	1.9100000	0.4666905	1.5800000	2.2400000

Unique Subject Identifier=1423000000-0006

Assay variable : PTINR_VAL1 PT-INR Value

N	Mean	Standard Deviation	Minimum	Maximum
1	1.3900000		1.3900000	1.3900000

Laboratory tests at the time of obtaining Table 2-6 consent, according to the presence or absence of proton-pump inhibitors

Unique Subject Identifier=1423000000-0010

MEANS Procedure

Assay variable : PTINR_VAL1 PT-INR Value

N	Mean	Standard Deviation	Minimum	Maximum
3	1.4900000	0.2251666	1.3600000	1.7500000

Unique Subject Identifier=1423000000-0015

Assay variable : PTINR_VAL1 PT-INR Value

N	Mean	Standard Deviation	Minimum	Maximum
2	2.0550000	0.1626346	1.9400000	2.1700000

Unique Subject Identifier=1428011110-0001

Assay variable : PTINR_VAL1 PT-INR Value

N	Mean	Standard Deviation	Minimum	Maximum
2	1.7850000	0.2616295	1.6000000	1.9700000

Unique Subject Identifier=1428011110-0002

Assay variable : PTINR_VAL1 PT-INR Value

N	Mean	Standard Deviation	Minimum	Maximum
2	1.4650000	0.2474874	1.2900000	1.6400000

Laboratory tests at the time of obtaining Table 2-6 consent, according to the presence or absence of proton-pump inhibitors

Unique Subject Identifier=1428011110-0003

MEANS Procedure

Assay variable : PTINR_VAL1 PT-INR Value

N	Mean	Standard Deviation	Minimum	Maximum
1	1.6900000		1.6900000	1.6900000

Unique Subject Identifier=1428011110-0006

Assay variable : PTINR_VAL1 PT-INR Value

N	Mean	Standard Deviation	Minimum	Maximum
2	1.7300000	0.0424264	1.7000000	1.7600000

Unique Subject Identifier=1428011110-0008

Assay variable : PTINR_VAL1 PT-INR Value

N	Mean	Standard Deviation	Minimum	Maximum
2	2.4750000	0.7566043	1.9400000	3.0100000

Unique Subject Identifier=1428011110-0009

Assay variable : PTINR_VAL1 PT-INR Value

N	Mean	Standard Deviation	Minimum	Maximum
2	1.7150000	0.7990307	1.1500000	2.2800000

Laboratory tests at the time of obtaining Table 2-6 consent, according to the presence or absence of proton-pump inhibitors

Unique Subject Identifier=1428011110-0010

MEANS Procedure

Assay variable : PTINR_VAL1 PT-INR Value

N	Mean	Standard Deviation	Minimum	Maximum
2	2.2450000	0.1484924	2.1400000	2.3500000

Unique Subject Identifier=1428011110-0011

Assay variable : PTINR_VAL1 PT-INR Value

N	Mean	Standard Deviation	Minimum	Maximum
2	2.1850000	0.3040559	1.9700000	2.4000000

Unique Subject Identifier=1428011110-0013

Assay variable : PTINR_VAL1 PT-INR Value

N	Mean	Standard Deviation	Minimum	Maximum
1	1.6800000		1.6800000	1.6800000

Unique Subject Identifier=1428011110-0015

Assay variable : PTINR_VAL1 PT-INR Value

N	Mean	Standard Deviation	Minimum	Maximum
2	1.3700000	0.2687006	1.1800000	1.5600000

Laboratory tests at the time of obtaining Table 2-6 consent, according to the presence or absence of proton-pump inhibitors

Unique Subject Identifier=1428011110-0017

MEANS Procedure

Assay variable : PTINR_VAL1 PT-INR Value

N	Mean	Standard Deviation	Minimum	Maximum
1	1.4900000		1.4900000	1.4900000

Unique Subject Identifier=1428011110-0019

Assay variable : PTINR_VAL1 PT-INR Value

N	Mean	Standard Deviation	Minimum	Maximum
3	1.9833333	0.2309401	1.8500000	2.2500000

Unique Subject Identifier=1428011110-0021

Assay variable : PTINR_VAL1 PT-INR Value

N	Mean	Standard Deviation	Minimum	Maximum
1	2.8600000		2.8600000	2.8600000

Unique Subject Identifier=1428011110-0023

Assay variable : PTINR_VAL1 PT-INR Value

N	Mean	Standard Deviation	Minimum	Maximum
1	1.5800000		1.5800000	1.5800000

Laboratory tests at the time of obtaining Table 2-6 consent, according to the presence or absence of proton-pump inhibitors

Unique Subject Identifier=1428011110-0025

MEANS Procedure

Assay variable : PTINR_VAL1 PT-INR Value

N	Mean	Standard Deviation	Minimum	Maximum
2	1.8100000	0.4666905	1.4800000	2.1400000

Unique Subject Identifier=1428011110-0032

Assay variable : PTINR_VAL1 PT-INR Value

N	Mean	Standard Deviation	Minimum	Maximum
3	1.7866667	0.5345403	1.4200000	2.4000000

Unique Subject Identifier=1430011010-0004

Assay variable : PTINR_VAL1 PT-INR Value

N	Mean	Standard Deviation	Minimum	Maximum
6	1.8666667	0.3444803	1.6000000	2.4000000

Unique Subject Identifier=1432000000-0049

Assay variable : PTINR_VAL1 PT-INR Value

N	Mean	Standard Deviation	Minimum	Maximum
6	1.7833333	0.6112828	1.1000000	2.6000000

Laboratory tests at the time of obtaining Table 2-6 consent, according to the presence or absence of proton-pump inhibitors

Unique Subject Identifier=1432000000-0056

MEANS Procedure

Assay variable : PTINR_VAL1 PT-INR Value

N	Mean	Standard Deviation	Minimum	Maximum
6	2.0833333	0.5455884	1.2000000	2.6000000

Unique Subject Identifier=1432000000-0071

Assay variable : PTINR_VAL1 PT-INR Value

N	Mean	Standard Deviation	Minimum	Maximum
6	2.2833333	0.2483277	1.9000000	2.6000000

Unique Subject Identifier=1432000000-0073

Assay variable : PTINR_VAL1 PT-INR Value

N	Mean	Standard Deviation	Minimum	Maximum
6	2.1166667	0.1602082	1.9000000	2.3000000

Unique Subject Identifier=1435000000-0003

Assay variable : PTINR_VAL1 PT-INR Value

N	Mean	Standard Deviation	Minimum	Maximum
1	2.1400000		2.1400000	2.1400000

Laboratory tests at the time of obtaining Table 2-6 consent, according to the presence or absence of proton-pump inhibitors

Unique Subject Identifier=1435000000-0018

MEANS Procedure

Assay variable : PTINR_VAL1 PT-INR Value

N	Mean	Standard Deviation	Minimum	Maximum
1	1.6700000		1.6700000	1.6700000

Unique Subject Identifier=1435000000-0028

Assay variable : PTINR_VAL1 PT-INR Value

N	Mean	Standard Deviation	Minimum	Maximum
3	2.6600000	0.1081665	2.5700000	2.7800000

Unique Subject Identifier=1435000000-0039

Assay variable : PTINR_VAL1 PT-INR Value

N	Mean	Standard Deviation	Minimum	Maximum
3	1.7266667	0.2402776	1.4800000	1.9600000

Unique Subject Identifier=1435000000-0041

Assay variable : PTINR_VAL1 PT-INR Value

N	Mean	Standard Deviation	Minimum	Maximum
5	2.2780000	0.2723417	1.9700000	2.5800000

Laboratory tests at the time of obtaining Table 2-6 consent, according to the presence or absence of proton-pump inhibitors

Unique Subject Identifier=1435000000-0042

MEANS Procedure

Assay variable : PTINR_VAL1 PT-INR Value

N	Mean	Standard Deviation	Minimum	Maximum
5	2.1240000	0.1270039	2.0000000	2.2800000

Unique Subject Identifier=1435000000-0048

Assay variable : PTINR_VAL1 PT-INR Value

N	Mean	Standard Deviation	Minimum	Maximum
5	1.9720000	0.6191688	1.3900000	3.0200000

Unique Subject Identifier=1435000000-0051

Assay variable : PTINR_VAL1 PT-INR Value

N	Mean	Standard Deviation	Minimum	Maximum
1	2.0900000		2.0900000	2.0900000

Unique Subject Identifier=1435000000-0055

Assay variable : PTINR_VAL1 PT-INR Value

N	Mean	Standard Deviation	Minimum	Maximum
6	1.5500000	0.5843629	1.1600000	2.7100000

Laboratory tests at the time of obtaining Table 2-6 consent, according to the presence or absence of proton-pump inhibitors

Unique Subject Identifier=1435000000-0065

MEANS Procedure

Assay variable : PTINR_VAL1 PT-INR Value

N	Mean	Standard Deviation	Minimum	Maximum
1	2.1800000		2.1800000	2.1800000

Unique Subject Identifier=1435000000-0071

Assay variable : PTINR_VAL1 PT-INR Value

N	Mean	Standard Deviation	Minimum	Maximum
6	1.2450000	0.2508984	1.0600000	1.6400000

Unique Subject Identifier=1435000000-0075

Assay variable : PTINR_VAL1 PT-INR Value

N	Mean	Standard Deviation	Minimum	Maximum
3	1.1466667	0.0251661	1.1200000	1.1700000

Unique Subject Identifier=1435000000-0091

Assay variable : PTINR_VAL1 PT-INR Value

N	Mean	Standard Deviation	Minimum	Maximum
3	2.1500000	0.2505993	1.8900000	2.3900000

Laboratory tests at the time of obtaining Table 2-6 consent, according to the presence or absence of proton-pump inhibitors

Unique Subject Identifier=1435000000-0103

MEANS Procedure

Assay variable : PTINR_VAL1 PT-INR Value

N	Mean	Standard Deviation	Minimum	Maximum
2	2.7750000	0.0494975	2.7400000	2.8100000

Unique Subject Identifier=1435000000-0105

Assay variable : PTINR_VAL1 PT-INR Value

N	Mean	Standard Deviation	Minimum	Maximum
1	1.5300000		1.5300000	1.5300000

Unique Subject Identifier=1435000000-0107

Assay variable : PTINR_VAL1 PT-INR Value

N	Mean	Standard Deviation	Minimum	Maximum
3	1.5866667	0.2318045	1.3200000	1.7400000

Unique Subject Identifier=1435000000-0117

Assay variable : PTINR_VAL1 PT-INR Value

N	Mean	Standard Deviation	Minimum	Maximum
6	2.4450000	0.2246553	2.2400000	2.8400000

Laboratory tests at the time of obtaining Table 2-6 consent, according to the presence or absence of proton-pump inhibitors

Unique Subject Identifier=1435000000-0119

MEANS Procedure

Assay variable : PTINR_VAL1 PT-INR Value

N	Mean	Standard Deviation	Minimum	Maximum
1	2.4200000		2.4200000	2.4200000

Unique Subject Identifier=1435000000-0120

Assay variable : PTINR_VAL1 PT-INR Value

N	Mean	Standard Deviation	Minimum	Maximum
6	1.6683333	0.4972491	1.1800000	2.5300000

Unique Subject Identifier=1435000000-0132

Assay variable : PTINR_VAL1 PT-INR Value

N	Mean	Standard Deviation	Minimum	Maximum
2	2.4550000	0.4030509	2.1700000	2.7400000

Unique Subject Identifier=1435000000-0139

Assay variable : PTINR_VAL1 PT-INR Value

N	Mean	Standard Deviation	Minimum	Maximum
2	2.6450000	1.4919953	1.5900000	3.7000000

Laboratory tests at the time of obtaining Table 2-6 consent, according to the presence or absence of proton-pump inhibitors

Unique Subject Identifier=1435000000-0140

MEANS Procedure

Assay variable : PTINR_VAL1 PT-INR Value

N	Mean	Standard Deviation	Minimum	Maximum
6	1.4450000	0.1980656	1.2400000	1.8100000

Unique Subject Identifier=1435000000-0153

Assay variable : PTINR_VAL1 PT-INR Value

N	Mean	Standard Deviation	Minimum	Maximum
5	1.7040000	0.1050238	1.5800000	1.8500000

Unique Subject Identifier=1435000000-0188

Assay variable : PTINR_VAL1 PT-INR Value

N	Mean	Standard Deviation	Minimum	Maximum
4	1.4350000	0.3641886	1.2200000	1.9800000

Unique Subject Identifier=1435000000-0200

Assay variable : PTINR_VAL1 PT-INR Value

N	Mean	Standard Deviation	Minimum	Maximum
2	1.1650000	0.0636396	1.1200000	1.2100000

Laboratory tests at the time of obtaining Table 2-6 consent, according to the presence or absence of proton-pump inhibitors

Unique Subject Identifier=1435000000-0209

MEANS Procedure

Assay variable : PTINR_VAL1 PT-INR Value

N	Mean	Standard Deviation	Minimum	Maximum
6	1.4316667	0.3882482	1.2200000	2.2200000

Unique Subject Identifier=1435000000-0212

Assay variable : PTINR_VAL1 PT-INR Value

N	Mean	Standard Deviation	Minimum	Maximum
1	1.5800000		1.5800000	1.5800000

Unique Subject Identifier=1435000000-0215

Assay variable : PTINR_VAL1 PT-INR Value

N	Mean	Standard Deviation	Minimum	Maximum
3	1.8533333	0.2872862	1.6400000	2.1800000

Unique Subject Identifier=1436000000-0008

Assay variable : PTINR_VAL1 PT-INR Value

N	Mean	Standard Deviation	Minimum	Maximum
5	2.1300000	0.2152905	1.7700000	2.3400000

Laboratory tests at the time of obtaining Table 2-6 consent, according to the presence or absence of proton-pump inhibitors

Unique Subject Identifier=1436000000-0010

MEANS Procedure

Assay variable : PTINR_VAL1 PT-INR Value

N	Mean	Standard Deviation	Minimum	Maximum
1	3.3300000		3.3300000	3.3300000

Unique Subject Identifier=1436000000-0019

Assay variable : PTINR_VAL1 PT-INR Value

N	Mean	Standard Deviation	Minimum	Maximum
2	1.6350000	0.1484924	1.5300000	1.7400000

Unique Subject Identifier=1436000000-0023

Assay variable : PTINR_VAL1 PT-INR Value

N	Mean	Standard Deviation	Minimum	Maximum
1	1.7900000		1.7900000	1.7900000

Unique Subject Identifier=1436000000-0024

Assay variable : PTINR_VAL1 PT-INR Value

N	Mean	Standard Deviation	Minimum	Maximum
2	2.3150000	0.0212132	2.3000000	2.3300000

Laboratory tests at the time of obtaining Table 2-6 consent, according to the presence or absence of proton-pump inhibitors

Unique Subject Identifier=1436000000-0028

MEANS Procedure

Assay variable : PTINR_VAL1 PT-INR Value

N	Mean	Standard Deviation	Minimum	Maximum
2	2.0900000	0.2121320	1.9400000	2.2400000

Unique Subject Identifier=1436000000-0032

Assay variable : PTINR_VAL1 PT-INR Value

N	Mean	Standard Deviation	Minimum	Maximum
1	2.6300000		2.6300000	2.6300000

Unique Subject Identifier=1436000000-0038

Assay variable : PTINR_VAL1 PT-INR Value

N	Mean	Standard Deviation	Minimum	Maximum
3	2.5166667	0.6859543	1.7300000	2.9900000

Unique Subject Identifier=1436000000-0048

Assay variable : PTINR_VAL1 PT-INR Value

N	Mean	Standard Deviation	Minimum	Maximum
1	2.2200000		2.2200000	2.2200000

Laboratory tests at the time of obtaining Table 2-6 consent, according to the presence or absence of proton-pump inhibitors

Unique Subject Identifier=1437000000-0009

MEANS Procedure

Assay variable : PTINR_VAL1 PT-INR Value

N	Mean	Standard Deviation	Minimum	Maximum
6	1.8500000	0.2258318	1.6000000	2.2000000

Unique Subject Identifier=1437000000-0010

Assay variable : PTINR_VAL1 PT-INR Value

N	Mean	Standard Deviation	Minimum	Maximum
6	1.9166667	0.3710346	1.3000000	2.3000000

Unique Subject Identifier=1437000000-0011

Assay variable : PTINR_VAL1 PT-INR Value

N	Mean	Standard Deviation	Minimum	Maximum
6	1.7833333	0.3060501	1.4000000	2.3000000

Unique Subject Identifier=1438000000-0010

Assay variable : PTINR_VAL1 PT-INR Value

N	Mean	Standard Deviation	Minimum	Maximum
5	2.3000000	0.0764853	2.2100000	2.3900000

Laboratory tests at the time of obtaining Table 2-6 consent, according to the presence or absence of proton-pump inhibitors

Unique Subject Identifier=1438000000-0017

MEANS Procedure

Assay variable : PTINR_VAL1 PT-INR Value

N	Mean	Standard Deviation	Minimum	Maximum
2	2.8100000	0.2404163	2.6400000	2.9800000

Unique Subject Identifier=1438000000-0030

Assay variable : PTINR_VAL1 PT-INR Value

N	Mean	Standard Deviation	Minimum	Maximum
1	1.7200000		1.7200000	1.7200000

Unique Subject Identifier=1438000000-0036

Assay variable : PTINR_VAL1 PT-INR Value

N	Mean	Standard Deviation	Minimum	Maximum
1	1.1800000		1.1800000	1.1800000

Unique Subject Identifier=1438000000-0043

Assay variable : PTINR_VAL1 PT-INR Value

N	Mean	Standard Deviation	Minimum	Maximum
1	1.6200000		1.6200000	1.6200000

Laboratory tests at the time of obtaining Table 2-6 consent, according to the presence or absence of proton-pump inhibitors

Unique Subject Identifier=1438000000-0047

MEANS Procedure

Assay variable : PTINR_VAL1 PT-INR Value

N	Mean	Standard Deviation	Minimum	Maximum
5	1.3040000	0.0870632	1.1700000	1.4100000

Unique Subject Identifier=1438000000-0049

Assay variable : PTINR_VAL1 PT-INR Value

N	Mean	Standard Deviation	Minimum	Maximum
4	2.4200000	0.3988316	2.0100000	2.8700000

Unique Subject Identifier=1440000000-0013

Assay variable : PTINR_VAL1 PT-INR Value

N	Mean	Standard Deviation	Minimum	Maximum
3	1.9600000	0.1734935	1.8500000	2.1600000

Unique Subject Identifier=1440000000-0016

Assay variable : PTINR_VAL1 PT-INR Value

N	Mean	Standard Deviation	Minimum	Maximum
3	2.9566667	0.2858904	2.7100000	3.2700000

Laboratory tests at the time of obtaining Table 2-6 consent, according to the presence or absence of proton-pump inhibitors

Unique Subject Identifier=1440000000-0018

MEANS Procedure

Assay variable : PTINR_VAL1 PT-INR Value

N	Mean	Standard Deviation	Minimum	Maximum
5	1.3940000	0.1556599	1.2800000	1.6500000

Unique Subject Identifier=1440000000-0019

Assay variable : PTINR_VAL1 PT-INR Value

N	Mean	Standard Deviation	Minimum	Maximum
2	1.1050000	0.0212132	1.0900000	1.1200000

Unique Subject Identifier=1441000000-0001

Assay variable : PTINR_VAL1 PT-INR Value

N	Mean	Standard Deviation	Minimum	Maximum
5	1.7880000	0.2462113	1.5000000	2.1500000

Unique Subject Identifier=1444000000-0009

Assay variable : PTINR_VAL1 PT-INR Value

N	Mean	Standard Deviation	Minimum	Maximum
5	1.9260000	0.3471023	1.4400000	2.3000000

Laboratory tests at the time of obtaining Table 2-6 consent, according to the presence or absence of proton-pump inhibitors

Unique Subject Identifier=1444000000-0019

MEANS Procedure

Assay variable : PTINR_VAL1 PT-INR Value

N	Mean	Standard Deviation	Minimum	Maximum
6	1.9033333	0.1983599	1.7300000	2.2800000

Unique Subject Identifier=1444000000-0040

Assay variable : PTINR_VAL1 PT-INR Value

N	Mean	Standard Deviation	Minimum	Maximum
6	2.0050000	0.3040230	1.6500000	2.5200000

Unique Subject Identifier=1444000000-0045

Assay variable : PTINR_VAL1 PT-INR Value

N	Mean	Standard Deviation	Minimum	Maximum
6	1.9616667	0.5192462	1.3400000	2.8100000

Unique Subject Identifier=1444000000-0048

Assay variable : PTINR_VAL1 PT-INR Value

N	Mean	Standard Deviation	Minimum	Maximum
6	2.3050000	0.4026289	1.8500000	3.0000000

Laboratory tests at the time of obtaining Table 2-6 consent, according to the presence or absence of proton-pump inhibitors

Unique Subject Identifier=1451000000-0001

MEANS Procedure

Assay variable : PTINR_VAL1 PT-INR Value

N	Mean	Standard Deviation	Minimum	Maximum
6	1.9500000	0.1378405	1.8000000	2.1000000

Unique Subject Identifier=1451000000-0002

Assay variable : PTINR_VAL1 PT-INR Value

N	Mean	Standard Deviation	Minimum	Maximum
2	1.8000000	0.1414214	1.7000000	1.9000000

Unique Subject Identifier=1451000000-0003

Assay variable : PTINR_VAL1 PT-INR Value

N	Mean	Standard Deviation	Minimum	Maximum
4	1.6500000	0.3696846	1.4000000	2.2000000

Unique Subject Identifier=1451000000-0004

Assay variable : PTINR_VAL1 PT-INR Value

N	Mean	Standard Deviation	Minimum	Maximum
3	2.3000000	0.5567764	1.8000000	2.9000000

Laboratory tests at the time of obtaining Table 2-6 consent, according to the presence or absence of proton-pump inhibitors

Unique Subject Identifier=1451000000-0005

MEANS Procedure

Assay variable : PTINR_VAL1 PT-INR Value

N	Mean	Standard Deviation	Minimum	Maximum
5	2.2200000	0.2774887	1.9000000	2.5000000

Unique Subject Identifier=1451000000-0007

Assay variable : PTINR_VAL1 PT-INR Value

N	Mean	Standard Deviation	Minimum	Maximum
6	2.0000000	0.2190890	1.7000000	2.3000000

Unique Subject Identifier=1451000000-0012

Assay variable : PTINR_VAL1 PT-INR Value

N	Mean	Standard Deviation	Minimum	Maximum
6	1.8500000	0.1974842	1.6000000	2.2000000

Unique Subject Identifier=1451000000-0018

Assay variable : PTINR_VAL1 PT-INR Value

N	Mean	Standard Deviation	Minimum	Maximum
6	1.9666667	0.4082483	1.3000000	2.5000000

Laboratory tests at the time of obtaining Table 2-6 consent, according to the presence or absence of proton-pump inhibitors

Unique Subject Identifier=1451000000-0019

MEANS Procedure

Assay variable : PTINR_VAL1 PT-INR Value

N	Mean	Standard Deviation	Minimum	Maximum
2	1.9500000	0.6363961	1.5000000	2.4000000

Unique Subject Identifier=1451000000-0021

Assay variable : PTINR_VAL1 PT-INR Value

N	Mean	Standard Deviation	Minimum	Maximum
6	1.9500000	0.1760682	1.8000000	2.2000000

Unique Subject Identifier=1451000000-0024

Assay variable : PTINR_VAL1 PT-INR Value

N	Mean	Standard Deviation	Minimum	Maximum
6	2.5333333	0.6377042	2.0000000	3.8000000

Unique Subject Identifier=1451000000-0030

Assay variable : PTINR_VAL1 PT-INR Value

N	Mean	Standard Deviation	Minimum	Maximum
5	2.3600000	1.0334409	1.3000000	3.7000000

Laboratory tests at the time of obtaining Table 2-6 consent, according to the presence or absence of proton-pump inhibitors

Unique Subject Identifier=1451000000-0031

MEANS Procedure

Assay variable : PTINR_VAL1 PT-INR Value

N	Mean	Standard Deviation	Minimum	Maximum
6	1.7333333	0.1966384	1.4000000	2.0000000

Unique Subject Identifier=1451000000-0033

Assay variable : PTINR_VAL1 PT-INR Value

N	Mean	Standard Deviation	Minimum	Maximum
6	1.2450000	0.1819615	1.0600000	1.5000000

Unique Subject Identifier=1451000000-0034

Assay variable : PTINR_VAL1 PT-INR Value

N	Mean	Standard Deviation	Minimum	Maximum
6	1.8666667	0.1632993	1.6000000	2.1000000

Unique Subject Identifier=1451000000-0035

Assay variable : PTINR_VAL1 PT-INR Value

N	Mean	Standard Deviation	Minimum	Maximum
6	2.5333333	0.2338090	2.4000000	3.0000000

Laboratory tests at the time of obtaining Table 2-6 consent, according to the presence or absence of proton-pump inhibitors

Unique Subject Identifier=1451000000-0040

MEANS Procedure

Assay variable : PTINR_VAL1 PT-INR Value

N	Mean	Standard Deviation	Minimum	Maximum
6	2.2833333	0.8886319	1.5000000	4.0000000

Unique Subject Identifier=1451000000-0042

Assay variable : PTINR_VAL1 PT-INR Value

N	Mean	Standard Deviation	Minimum	Maximum
6	1.9333333	0.2160247	1.6000000	2.2000000

Unique Subject Identifier=1451000000-0044

Assay variable : PTINR_VAL1 PT-INR Value

N	Mean	Standard Deviation	Minimum	Maximum
6	2.2166667	0.5636193	1.6000000	2.9000000

Unique Subject Identifier=1451000000-0046

Assay variable : PTINR_VAL1 PT-INR Value

N	Mean	Standard Deviation	Minimum	Maximum
6	1.5000000	0.1673320	1.3000000	1.7000000

Laboratory tests at the time of obtaining Table 2-6 consent, according to the presence or absence of proton-pump inhibitors

Unique Subject Identifier=1451000000-0047

MEANS Procedure

Assay variable : PTINR_VAL1 PT-INR Value

N	Mean	Standard Deviation	Minimum	Maximum
6	2.4833333	0.8232051	1.4000000	3.5000000

Unique Subject Identifier=1451000000-0050

Assay variable : PTINR_VAL1 PT-INR Value

N	Mean	Standard Deviation	Minimum	Maximum
6	2.3166667	0.9537645	1.1000000	3.9000000

Unique Subject Identifier=1451000000-0051

Assay variable : PTINR_VAL1 PT-INR Value

N	Mean	Standard Deviation	Minimum	Maximum
6	1.9500000	0.3619392	1.5000000	2.4000000

Unique Subject Identifier=1451000000-0055

Assay variable : PTINR_VAL1 PT-INR Value

N	Mean	Standard Deviation	Minimum	Maximum
6	1.7666667	0.1861899	1.6000000	2.1000000

Laboratory tests at the time of obtaining Table 2-6 consent, according to the presence or absence of proton-pump inhibitors

Unique Subject Identifier=1451000000-0063

MEANS Procedure

Assay variable : PTINR_VAL1 PT-INR Value

N	Mean	Standard Deviation	Minimum	Maximum
6	1.4666667	0.2250926	1.1000000	1.7000000

Unique Subject Identifier=1452000000-0002

Assay variable : PTINR_VAL1 PT-INR Value

N	Mean	Standard Deviation	Minimum	Maximum
5	2.2240000	0.2228901	1.9100000	2.5400000

Unique Subject Identifier=1452000000-0003

Assay variable : PTINR_VAL1 PT-INR Value

N	Mean	Standard Deviation	Minimum	Maximum
6	2.4866667	0.4723840	1.9300000	3.2000000

Unique Subject Identifier=1452000000-0004

Assay variable : PTINR_VAL1 PT-INR Value

N	Mean	Standard Deviation	Minimum	Maximum
6	2.0100000	0.2126029	1.7400000	2.3200000

Laboratory tests at the time of obtaining Table 2-6 consent, according to the presence or absence of proton-pump inhibitors

Unique Subject Identifier=1452000000-0007

MEANS Procedure

Assay variable : PTINR_VAL1 PT-INR Value

N	Mean	Standard Deviation	Minimum	Maximum
5	1.8660000	0.1379493	1.6900000	2.0600000

Unique Subject Identifier=1452000000-0008

Assay variable : PTINR_VAL1 PT-INR Value

N	Mean	Standard Deviation	Minimum	Maximum
4	2.9475000	0.7501278	2.5000000	4.0700000

Unique Subject Identifier=1452000000-0010

Assay variable : PTINR_VAL1 PT-INR Value

N	Mean	Standard Deviation	Minimum	Maximum
4	1.8200000	0.4180112	1.3800000	2.3800000

Unique Subject Identifier=1452000000-0011

Assay variable : PTINR_VAL1 PT-INR Value

N	Mean	Standard Deviation	Minimum	Maximum
6	1.7016667	0.5537298	1.2000000	2.7800000

Laboratory tests at the time of obtaining Table 2-6 consent, according to the presence or absence of proton-pump inhibitors

Unique Subject Identifier=1452000000-0012

MEANS Procedure

Assay variable : PTINR_VAL1 PT-INR Value

N	Mean	Standard Deviation	Minimum	Maximum
5	1.9400000	0.2756810	1.5700000	2.2500000

Unique Subject Identifier=1452000000-0013

Assay variable : PTINR_VAL1 PT-INR Value

N	Mean	Standard Deviation	Minimum	Maximum
6	2.0800000	0.7291365	1.5500000	3.1700000

Unique Subject Identifier=1452000000-0014

Assay variable : PTINR_VAL1 PT-INR Value

N	Mean	Standard Deviation	Minimum	Maximum
6	2.6233333	0.5473451	1.9500000	3.2800000

Unique Subject Identifier=1452000000-0015

Assay variable : PTINR_VAL1 PT-INR Value

N	Mean	Standard Deviation	Minimum	Maximum
6	2.3266667	0.0886942	2.2300000	2.4500000

Laboratory tests at the time of obtaining Table 2-6 consent, according to the presence or absence of proton-pump inhibitors

Unique Subject Identifier=1452000000-0019

MEANS Procedure

Assay variable : PTINR_VAL1 PT-INR Value

N	Mean	Standard Deviation	Minimum	Maximum
5	2.5580000	0.2932917	2.1600000	2.8800000

Unique Subject Identifier=1452000000-0020

Assay variable : PTINR_VAL1 PT-INR Value

N	Mean	Standard Deviation	Minimum	Maximum
6	2.1000000	0.3241605	1.8300000	2.5700000

Unique Subject Identifier=1452000000-0021

Assay variable : PTINR_VAL1 PT-INR Value

N	Mean	Standard Deviation	Minimum	Maximum
6	2.1700000	0.2033716	1.8900000	2.4600000

Unique Subject Identifier=1452000000-0024

Assay variable : PTINR_VAL1 PT-INR Value

N	Mean	Standard Deviation	Minimum	Maximum
6	2.1966667	0.3460443	1.8500000	2.7800000

Laboratory tests at the time of obtaining Table 2-6 consent, according to the presence or absence of proton-pump inhibitors

Unique Subject Identifier=1452000000-0026

MEANS Procedure

Assay variable : PTINR_VAL1 PT-INR Value

N	Mean	Standard Deviation	Minimum	Maximum
4	1.6925000	0.4897193	1.1700000	2.1200000

Unique Subject Identifier=1452000000-0027

Assay variable : PTINR_VAL1 PT-INR Value

N	Mean	Standard Deviation	Minimum	Maximum
5	2.0800000	0.2931723	1.7400000	2.4400000

Unique Subject Identifier=1452000000-0029

Assay variable : PTINR_VAL1 PT-INR Value

N	Mean	Standard Deviation	Minimum	Maximum
6	2.4833333	0.2224110	2.2900000	2.8800000

Unique Subject Identifier=1452000000-0030

Assay variable : PTINR_VAL1 PT-INR Value

N	Mean	Standard Deviation	Minimum	Maximum
6	2.4333333	0.7724938	1.6900000	3.8500000

Laboratory tests at the time of obtaining Table 2-6 consent, according to the presence or absence of proton-pump inhibitors

Unique Subject Identifier=1452000000-0031

MEANS Procedure

Assay variable : PTINR_VAL1 PT-INR Value

N	Mean	Standard Deviation	Minimum	Maximum
6	1.8550000	0.1693222	1.6300000	2.1000000

Unique Subject Identifier=1452000000-0033

Assay variable : PTINR_VAL1 PT-INR Value

N	Mean	Standard Deviation	Minimum	Maximum
6	1.7133333	0.3641245	1.2900000	2.2700000

Unique Subject Identifier=1452000000-0035

Assay variable : PTINR_VAL1 PT-INR Value

N	Mean	Standard Deviation	Minimum	Maximum
6	2.4700000	0.5721888	1.6700000	3.3300000

Unique Subject Identifier=1452000000-0036

Assay variable : PTINR_VAL1 PT-INR Value

N	Mean	Standard Deviation	Minimum	Maximum
6	2.0600000	0.5149369	1.7000000	3.0700000

Laboratory tests at the time of obtaining Table 2-6 consent, according to the presence or absence of proton-pump inhibitors

Unique Subject Identifier=1452000000-0037

MEANS Procedure

Assay variable : PTINR_VAL1 PT-INR Value

N	Mean	Standard Deviation	Minimum	Maximum
6	1.9033333	0.4987852	1.2900000	2.5900000

Unique Subject Identifier=1452000000-0038

Assay variable : PTINR_VAL1 PT-INR Value

N	Mean	Standard Deviation	Minimum	Maximum
4	2.0875000	0.2972513	1.7600000	2.4600000

Unique Subject Identifier=1452000000-0041

Assay variable : PTINR_VAL1 PT-INR Value

N	Mean	Standard Deviation	Minimum	Maximum
6	2.1350000	0.7433102	1.2200000	3.0200000

Unique Subject Identifier=1452000000-0050

Assay variable : PTINR_VAL1 PT-INR Value

N	Mean	Standard Deviation	Minimum	Maximum
5	2.4340000	0.2597691	2.1000000	2.7800000

Laboratory tests at the time of obtaining Table 2-6 consent, according to the presence or absence of proton-pump inhibitors

Unique Subject Identifier=1452000000-0052

MEANS Procedure

Assay variable : PTINR_VAL1 PT-INR Value

N	Mean	Standard Deviation	Minimum	Maximum
4	2.4450000	0.3181719	2.0800000	2.8300000

Unique Subject Identifier=1452000000-0054

Assay variable : PTINR_VAL1 PT-INR Value

N	Mean	Standard Deviation	Minimum	Maximum
5	1.9160000	0.5048564	1.3600000	2.4200000

Unique Subject Identifier=1452000000-0055

Assay variable : PTINR_VAL1 PT-INR Value

N	Mean	Standard Deviation	Minimum	Maximum
5	2.1360000	0.8257300	1.2200000	3.2500000

Unique Subject Identifier=1452000000-0056

Assay variable : PTINR_VAL1 PT-INR Value

N	Mean	Standard Deviation	Minimum	Maximum
4	1.7975000	0.1203813	1.6300000	1.9100000

Laboratory tests at the time of obtaining Table 2-6 consent, according to the presence or absence of proton-pump inhibitors

Unique Subject Identifier=1452000000-0057

MEANS Procedure

Assay variable : PTINR_VAL1 PT-INR Value

N	Mean	Standard Deviation	Minimum	Maximum
3	1.3766667	0.3212994	1.1300000	1.7400000

Unique Subject Identifier=1452000000-0059

Assay variable : PTINR_VAL1 PT-INR Value

N	Mean	Standard Deviation	Minimum	Maximum
6	1.9800000	0.1879362	1.8400000	2.3400000

Unique Subject Identifier=1452000000-0060

Assay variable : PTINR_VAL1 PT-INR Value

N	Mean	Standard Deviation	Minimum	Maximum
5	1.6220000	0.2580116	1.2500000	1.9400000

Unique Subject Identifier=1452000000-0061

Assay variable : PTINR_VAL1 PT-INR Value

N	Mean	Standard Deviation	Minimum	Maximum
4	1.8200000	0.3842742	1.2500000	2.0800000

Laboratory tests at the time of obtaining Table 2-6 consent, according to the presence or absence of proton-pump inhibitors

Unique Subject Identifier=1452000000-0062

MEANS Procedure

Assay variable : PTINR_VAL1 PT-INR Value

N	Mean	Standard Deviation	Minimum	Maximum
5	2.0620000	0.4810094	1.3000000	2.5500000

Unique Subject Identifier=1452000000-0065

Assay variable : PTINR_VAL1 PT-INR Value

N	Mean	Standard Deviation	Minimum	Maximum
4	1.9900000	0.2694439	1.6400000	2.2700000

Unique Subject Identifier=1452000000-0066

Assay variable : PTINR_VAL1 PT-INR Value

N	Mean	Standard Deviation	Minimum	Maximum
5	2.2800000	0.5793099	1.7000000	3.1300000

Unique Subject Identifier=1452000000-0068

Assay variable : PTINR_VAL1 PT-INR Value

N	Mean	Standard Deviation	Minimum	Maximum
6	1.9450000	0.2251888	1.7600000	2.3800000

Laboratory tests at the time of obtaining Table 2-6 consent, according to the presence or absence of proton-pump inhibitors

Unique Subject Identifier=1452000000-0069

MEANS Procedure

Assay variable : PTINR_VAL1 PT-INR Value

N	Mean	Standard Deviation	Minimum	Maximum
6	2.1883333	0.1330288	2.0600000	2.4200000

Unique Subject Identifier=1452000000-0074

Assay variable : PTINR_VAL1 PT-INR Value

N	Mean	Standard Deviation	Minimum	Maximum
6	2.4333333	0.2188759	2.1300000	2.7300000

Unique Subject Identifier=1452000000-0080

Assay variable : PTINR_VAL1 PT-INR Value

N	Mean	Standard Deviation	Minimum	Maximum
6	2.2450000	0.3602638	1.8300000	2.8000000

Unique Subject Identifier=1452000000-0081

Assay variable : PTINR_VAL1 PT-INR Value

N	Mean	Standard Deviation	Minimum	Maximum
6	1.6683333	0.2830135	1.1400000	1.9400000

Laboratory tests at the time of obtaining Table 2-6 consent, according to the presence or absence of proton-pump inhibitors

Unique Subject Identifier=1452000000-0083

MEANS Procedure

Assay variable : PTINR_VAL1 PT-INR Value

N	Mean	Standard Deviation	Minimum	Maximum
6	2.3450000	0.1808591	2.0600000	2.5100000

Unique Subject Identifier=1452000000-0084

Assay variable : PTINR_VAL1 PT-INR Value

N	Mean	Standard Deviation	Minimum	Maximum
2	1.7850000	0.3747666	1.5200000	2.0500000

Unique Subject Identifier=1452000000-0086

Assay variable : PTINR_VAL1 PT-INR Value

N	Mean	Standard Deviation	Minimum	Maximum
2	1.7200000	0.2828427	1.5200000	1.9200000

Unique Subject Identifier=1452000000-0101

Assay variable : PTINR_VAL1 PT-INR Value

N	Mean	Standard Deviation	Minimum	Maximum
6	1.8083333	0.1702253	1.6600000	2.1100000

Laboratory tests at the time of obtaining Table 2-6 consent, according to the presence or absence of proton-pump inhibitors

Unique Subject Identifier=1452000000-0103

MEANS Procedure

Assay variable : PTINR_VAL1 PT-INR Value

N	Mean	Standard Deviation	Minimum	Maximum
6	2.3233333	0.2125716	2.0100000	2.6600000

Unique Subject Identifier=1452000000-0107

Assay variable : PTINR_VAL1 PT-INR Value

N	Mean	Standard Deviation	Minimum	Maximum
4	2.1375000	0.9040050	1.1400000	3.2200000

Unique Subject Identifier=1452000000-0109

Assay variable : PTINR_VAL1 PT-INR Value

N	Mean	Standard Deviation	Minimum	Maximum
6	1.8366667	0.2488909	1.4900000	2.2100000

Unique Subject Identifier=1454000000-0005

Assay variable : PTINR_VAL1 PT-INR Value

N	Mean	Standard Deviation	Minimum	Maximum
6	2.4533333	0.4206978	1.9000000	2.9500000

Laboratory tests at the time of obtaining Table 2-6 consent, according to the presence or absence of proton-pump inhibitors

Unique Subject Identifier=1454000000-0028

MEANS Procedure

Assay variable : PTINR_VAL1 PT-INR Value

N	Mean	Standard Deviation	Minimum	Maximum
6	2.1466667	0.3545514	1.8300000	2.8200000

Unique Subject Identifier=1454000000-0039

Assay variable : PTINR_VAL1 PT-INR Value

N	Mean	Standard Deviation	Minimum	Maximum
6	2.2066667	0.3184755	1.6400000	2.4800000

Unique Subject Identifier=1454000000-0064

Assay variable : PTINR_VAL1 PT-INR Value

N	Mean	Standard Deviation	Minimum	Maximum
6	1.9216667	0.1290607	1.7700000	2.1100000

Unique Subject Identifier=1454000000-0067

Assay variable : PTINR_VAL1 PT-INR Value

N	Mean	Standard Deviation	Minimum	Maximum
6	2.1033333	0.2608959	1.8100000	2.5600000

Laboratory tests at the time of obtaining Table 2-6 consent, according to the presence or absence of proton-pump inhibitors

Unique Subject Identifier=1457000000-0001

MEANS Procedure

Assay variable : PTINR_VAL1 PT-INR Value

N	Mean	Standard Deviation	Minimum	Maximum
5	1.8600000	0.4393177	1.3000000	2.5000000

Unique Subject Identifier=1463010000-0002

Assay variable : PTINR_VAL1 PT-INR Value

N	Mean	Standard Deviation	Minimum	Maximum
5	1.6840000	0.1745852	1.4400000	1.8400000

Unique Subject Identifier=1463010000-0009

Assay variable : PTINR_VAL1 PT-INR Value

N	Mean	Standard Deviation	Minimum	Maximum
5	2.0160000	0.6467070	1.3000000	2.8500000

Unique Subject Identifier=1463010000-0014

Assay variable : PTINR_VAL1 PT-INR Value

N	Mean	Standard Deviation	Minimum	Maximum
5	1.3880000	0.1267675	1.2800000	1.5900000

Laboratory tests at the time of obtaining Table 2-6 consent, according to the presence or absence of proton-pump inhibitors

Unique Subject Identifier=1463010000-0018

MEANS Procedure

Assay variable : PTINR_VAL1 PT-INR Value

N	Mean	Standard Deviation	Minimum	Maximum
6	2.0333333	0.3765988	1.4900000	2.6100000

Unique Subject Identifier=1463010000-0030

Assay variable : PTINR_VAL1 PT-INR Value

N	Mean	Standard Deviation	Minimum	Maximum
6	1.2266667	0.0121106	1.2100000	1.2400000

Unique Subject Identifier=1470000000-0001

Assay variable : PTINR_VAL1 PT-INR Value

N	Mean	Standard Deviation	Minimum	Maximum
4	1.0075000	0.0095743	1.0000000	1.0200000

Unique Subject Identifier=1470000000-0002

Assay variable : PTINR_VAL1 PT-INR Value

N	Mean	Standard Deviation	Minimum	Maximum
2	1.8550000	0.2474874	1.6800000	2.0300000

Laboratory tests at the time of obtaining Table 2-6 consent, according to the presence or absence of proton-pump inhibitors

Unique Subject Identifier=1470000000-0004

MEANS Procedure

Assay variable : PTINR_VAL1 PT-INR Value

N	Mean	Standard Deviation	Minimum	Maximum
2	1.6950000	0.1626346	1.5800000	1.8100000

Unique Subject Identifier=1470000000-0009

Assay variable : PTINR_VAL1 PT-INR Value

N	Mean	Standard Deviation	Minimum	Maximum
4	1.4350000	0.0675771	1.3600000	1.5100000

Unique Subject Identifier=1474000000-0002

Assay variable : PTINR_VAL1 PT-INR Value

N	Mean	Standard Deviation	Minimum	Maximum
5	1.9200000	0.4764452	1.5000000	2.7000000

Unique Subject Identifier=1475000000-0016

Assay variable : PTINR_VAL1 PT-INR Value

N	Mean	Standard Deviation	Minimum	Maximum
3	2.4333333	0.2516611	2.2000000	2.7000000

Laboratory tests at the time of obtaining Table 2-6 consent, according to the presence or absence of proton-pump inhibitors

Unique Subject Identifier=1477000000-0007

MEANS Procedure

Assay variable : PTINR_VAL1 PT-INR Value

N	Mean	Standard Deviation	Minimum	Maximum
5	2.1800000	0.2062765	1.9700000	2.5200000

Unique Subject Identifier=1477000000-0014

Assay variable : PTINR_VAL1 PT-INR Value

N	Mean	Standard Deviation	Minimum	Maximum
4	2.3300000	0.1902630	2.1400000	2.5900000

Unique Subject Identifier=1478011111-0011

Assay variable : PTINR_VAL1 PT-INR Value

N	Mean	Standard Deviation	Minimum	Maximum
4	2.2625000	1.0897209	1.1200000	3.5400000

Unique Subject Identifier=1478011111-0016

Assay variable : PTINR_VAL1 PT-INR Value

N	Mean	Standard Deviation	Minimum	Maximum
2	2.5600000	0.6646804	2.0900000	3.0300000

Laboratory tests at the time of obtaining Table 2-6 consent, according to the presence or absence of proton-pump inhibitors

Unique Subject Identifier=1478011111-0028

MEANS Procedure

Assay variable : PTINR_VAL1 PT-INR Value

N	Mean	Standard Deviation	Minimum	Maximum
4	2.5775000	1.1448835	1.4500000	4.1400000

Unique Subject Identifier=1478011111-0030

Assay variable : PTINR_VAL1 PT-INR Value

N	Mean	Standard Deviation	Minimum	Maximum
3	2.4766667	0.1193035	2.3800000	2.6100000

Unique Subject Identifier=1478011111-0031

Assay variable : PTINR_VAL1 PT-INR Value

N	Mean	Standard Deviation	Minimum	Maximum
5	2.7340000	1.1840524	1.9600000	4.7800000

Unique Subject Identifier=1478011111-0038

Assay variable : PTINR_VAL1 PT-INR Value

N	Mean	Standard Deviation	Minimum	Maximum
3	2.9666667	0.2538372	2.7600000	3.2500000

Laboratory tests at the time of obtaining Table 2-6 consent, according to the presence or absence of proton-pump inhibitors

Unique Subject Identifier=1478011111-0040

MEANS Procedure

Assay variable : PTINR_VAL1 PT-INR Value

N	Mean	Standard Deviation	Minimum	Maximum
3	2.1233333	0.0723418	2.0400000	2.1700000

Unique Subject Identifier=1478011111-0060

Assay variable : PTINR_VAL1 PT-INR Value

N	Mean	Standard Deviation	Minimum	Maximum
3	2.8366667	0.6034346	2.4300000	3.5300000

Unique Subject Identifier=1478011111-0066

Assay variable : PTINR_VAL1 PT-INR Value

N	Mean	Standard Deviation	Minimum	Maximum
3	1.8433333	0.3162805	1.4900000	2.1000000

Unique Subject Identifier=1478011111-0069

Assay variable : PTINR_VAL1 PT-INR Value

N	Mean	Standard Deviation	Minimum	Maximum
3	2.5466667	0.0305505	2.5200000	2.5800000

Laboratory tests at the time of obtaining Table 2-6 consent, according to the presence or absence of proton-pump inhibitors

Unique Subject Identifier=1478011111-0093

MEANS Procedure

Assay variable : PTINR_VAL1 PT-INR Value

N	Mean	Standard Deviation	Minimum	Maximum
4	1.6525000	0.2003954	1.4700000	1.9100000

Unique Subject Identifier=1478011111-0097

Assay variable : PTINR_VAL1 PT-INR Value

N	Mean	Standard Deviation	Minimum	Maximum
3	2.2300000	0.7454529	1.4700000	2.9600000

Unique Subject Identifier=1478011111-0108

Assay variable : PTINR_VAL1 PT-INR Value

N	Mean	Standard Deviation	Minimum	Maximum
4	2.1225000	0.2441823	1.8400000	2.4000000

Unique Subject Identifier=1478011111-0118

Assay variable : PTINR_VAL1 PT-INR Value

N	Mean	Standard Deviation	Minimum	Maximum
4	2.7350000	0.0911043	2.6400000	2.8500000

Laboratory tests at the time of obtaining Table 2-6 consent, according to the presence or absence of proton-pump inhibitors

Unique Subject Identifier=1478011111-0121

MEANS Procedure

Assay variable : PTINR_VAL1 PT-INR Value

N	Mean	Standard Deviation	Minimum	Maximum
3	2.0933333	0.1721434	1.9700000	2.2900000

Unique Subject Identifier=1478011111-0127

Assay variable : PTINR_VAL1 PT-INR Value

N	Mean	Standard Deviation	Minimum	Maximum
4	1.8225000	0.0350000	1.7700000	1.8400000

Unique Subject Identifier=1478011111-0147

Assay variable : PTINR_VAL1 PT-INR Value

N	Mean	Standard Deviation	Minimum	Maximum
3	2.1666667	0.2396525	1.8900000	2.3100000

Unique Subject Identifier=1480011111-0003

Assay variable : PTINR_VAL1 PT-INR Value

N	Mean	Standard Deviation	Minimum	Maximum
3	2.5600000	0.9497895	1.4700000	3.2100000

Laboratory tests at the time of obtaining Table 2-6 consent, according to the presence or absence of proton-pump inhibitors

Unique Subject Identifier=1480011111-0005

MEANS Procedure

Assay variable : PTINR_VAL1 PT-INR Value

N	Mean	Standard Deviation	Minimum	Maximum
3	2.2533333	0.8220300	1.7200000	3.2000000

Unique Subject Identifier=1487000000-0001

Assay variable : PTINR_VAL1 PT-INR Value

N	Mean	Standard Deviation	Minimum	Maximum
6	2.3333333	0.4320494	1.9000000	3.1000000

Unique Subject Identifier=1487000000-0010

Assay variable : PTINR_VAL1 PT-INR Value

N	Mean	Standard Deviation	Minimum	Maximum
6	2.2000000	0.5513620	1.5000000	3.1000000

Unique Subject Identifier=1487000000-0012

Assay variable : PTINR_VAL1 PT-INR Value

N	Mean	Standard Deviation	Minimum	Maximum
6	2.1333333	0.2581989	1.8000000	2.5000000

Laboratory tests at the time of obtaining Table 2-6 consent, according to the presence or absence of proton-pump inhibitors

Unique Subject Identifier=1487000000-0017

MEANS Procedure

Assay variable : PTINR_VAL1 PT-INR Value

N	Mean	Standard Deviation	Minimum	Maximum
6	2.0666667	0.3614784	1.6000000	2.7000000

Unique Subject Identifier=1487000000-0019

Assay variable : PTINR_VAL1 PT-INR Value

N	Mean	Standard Deviation	Minimum	Maximum
6	2.1500000	0.1516575	2.0000000	2.4000000

Unique Subject Identifier=1487000000-0022

Assay variable : PTINR_VAL1 PT-INR Value

N	Mean	Standard Deviation	Minimum	Maximum
5	2.1600000	0.4505552	1.7000000	2.8000000

Unique Subject Identifier=1487000000-0023

Assay variable : PTINR_VAL1 PT-INR Value

N	Mean	Standard Deviation	Minimum	Maximum
6	2.0333333	0.3141125	1.6000000	2.5000000

Laboratory tests at the time of obtaining Table 2-6 consent, according to the presence or absence of proton-pump inhibitors

Unique Subject Identifier=1487000000-0027

MEANS Procedure

Assay variable : PTINR_VAL1 PT-INR Value

N	Mean	Standard Deviation	Minimum	Maximum
6	2.1166667	0.3710346	1.6000000	2.6000000

Unique Subject Identifier=1487000000-0028

Assay variable : PTINR_VAL1 PT-INR Value

N	Mean	Standard Deviation	Minimum	Maximum
6	1.8666667	0.4273952	1.5000000	2.5000000

Unique Subject Identifier=1487000000-0029

Assay variable : PTINR_VAL1 PT-INR Value

N	Mean	Standard Deviation	Minimum	Maximum
6	2.3833333	0.9304121	1.5000000	4.0000000

Unique Subject Identifier=1487000000-0032

Assay variable : PTINR_VAL1 PT-INR Value

N	Mean	Standard Deviation	Minimum	Maximum
5	2.2800000	0.1643168	2.1000000	2.5000000

Laboratory tests at the time of obtaining Table 2-6 consent, according to the presence or absence of proton-pump inhibitors

Unique Subject Identifier=1487000000-0033

MEANS Procedure

Assay variable : PTINR_VAL1 PT-INR Value

N	Mean	Standard Deviation	Minimum	Maximum
6	2.4666667	0.4802777	2.0000000	3.2000000

Unique Subject Identifier=1491000000-0008

Assay variable : PTINR_VAL1 PT-INR Value

N	Mean	Standard Deviation	Minimum	Maximum
6	2.2966667	0.3324856	1.8900000	2.7700000

Unique Subject Identifier=1491000000-0009

Assay variable : PTINR_VAL1 PT-INR Value

N	Mean	Standard Deviation	Minimum	Maximum
5	2.1060000	0.5477043	1.5400000	2.9700000

Unique Subject Identifier=1491000000-0010

Assay variable : PTINR_VAL1 PT-INR Value

N	Mean	Standard Deviation	Minimum	Maximum
4	2.3600000	0.5832667	1.5100000	2.8300000

Laboratory tests at the time of obtaining Table 2-6 consent, according to the presence or absence of proton-pump inhibitors

Unique Subject Identifier=1491000000-0011

MEANS Procedure

Assay variable : PTINR_VAL1 PT-INR Value

N	Mean	Standard Deviation	Minimum	Maximum
4	2.4825000	0.8079759	1.5200000	3.3800000

Unique Subject Identifier=1491000000-0012

Assay variable : PTINR_VAL1 PT-INR Value

N	Mean	Standard Deviation	Minimum	Maximum
4	1.8975000	0.0427200	1.8700000	1.9600000

Unique Subject Identifier=1491000000-0019

Assay variable : PTINR_VAL1 PT-INR Value

N	Mean	Standard Deviation	Minimum	Maximum
4	2.2975000	0.3704389	1.9200000	2.7900000

Unique Subject Identifier=1491000000-0021

Assay variable : PTINR_VAL1 PT-INR Value

N	Mean	Standard Deviation	Minimum	Maximum
6	2.0583333	0.3400833	1.7500000	2.5600000

Laboratory tests at the time of obtaining Table 2-6 consent, according to the presence or absence of proton-pump inhibitors

Unique Subject Identifier=1491000000-0025

MEANS Procedure

Assay variable : PTINR_VAL1 PT-INR Value

N	Mean	Standard Deviation	Minimum	Maximum
3	2.3033333	0.0737111	2.2200000	2.3600000

Unique Subject Identifier=1491000000-0031

Assay variable : PTINR_VAL1 PT-INR Value

N	Mean	Standard Deviation	Minimum	Maximum
3	1.8466667	0.0513160	1.7900000	1.8900000

Unique Subject Identifier=1491000000-0032

Assay variable : PTINR_VAL1 PT-INR Value

N	Mean	Standard Deviation	Minimum	Maximum
3	1.8500000	0.2325941	1.6000000	2.0600000

Unique Subject Identifier=1491000000-0035

Assay variable : PTINR_VAL1 PT-INR Value

N	Mean	Standard Deviation	Minimum	Maximum
4	1.9475000	0.3264327	1.6300000	2.4000000

Laboratory tests at the time of obtaining Table 2-6 consent, according to the presence or absence of proton-pump inhibitors

Unique Subject Identifier=1491000000-0040

MEANS Procedure

Assay variable : PTINR_VAL1 PT-INR Value

N	Mean	Standard Deviation	Minimum	Maximum
6	2.6333333	0.5336915	1.8900000	3.5400000

Unique Subject Identifier=1491000000-0044

Assay variable : PTINR_VAL1 PT-INR Value

N	Mean	Standard Deviation	Minimum	Maximum
4	1.9550000	0.4466542	1.6600000	2.6200000

Unique Subject Identifier=1491000000-0049

Assay variable : PTINR_VAL1 PT-INR Value

N	Mean	Standard Deviation	Minimum	Maximum
6	1.8566667	0.4394390	1.3900000	2.6700000

Unique Subject Identifier=1491000000-0056

Assay variable : PTINR_VAL1 PT-INR Value

N	Mean	Standard Deviation	Minimum	Maximum
3	1.9766667	0.0378594	1.9500000	2.0200000

Laboratory tests at the time of obtaining Table 2-6 consent, according to the presence or absence of proton-pump inhibitors

Unique Subject Identifier=1491000000-0059

MEANS Procedure

Assay variable : PTINR_VAL1 PT-INR Value

N	Mean	Standard Deviation	Minimum	Maximum
4	2.3575000	0.2555223	2.1000000	2.6900000

Unique Subject Identifier=1491000000-0060

Assay variable : PTINR_VAL1 PT-INR Value

N	Mean	Standard Deviation	Minimum	Maximum
4	1.9075000	0.1946578	1.7500000	2.1500000

Unique Subject Identifier=1491000000-0062

Assay variable : PTINR_VAL1 PT-INR Value

N	Mean	Standard Deviation	Minimum	Maximum
5	1.8600000	0.2309762	1.6900000	2.2600000

Unique Subject Identifier=1491000000-0063

Assay variable : PTINR_VAL1 PT-INR Value

N	Mean	Standard Deviation	Minimum	Maximum
4	2.2475000	0.2644964	1.9000000	2.5300000

Laboratory tests at the time of obtaining Table 2-6 consent, according to the presence or absence of proton-pump inhibitors

Unique Subject Identifier=1491000000-0066

MEANS Procedure

Assay variable : PTINR_VAL1 PT-INR Value

N	Mean	Standard Deviation	Minimum	Maximum
4	2.0725000	0.2117192	1.8700000	2.3100000

Unique Subject Identifier=1491000000-0070

Assay variable : PTINR_VAL1 PT-INR Value

N	Mean	Standard Deviation	Minimum	Maximum
4	2.0075000	0.0865544	1.9300000	2.1300000

Unique Subject Identifier=1491000000-0073

Assay variable : PTINR_VAL1 PT-INR Value

N	Mean	Standard Deviation	Minimum	Maximum
3	1.9900000	0.1997498	1.7700000	2.1600000

Unique Subject Identifier=1491000000-0077

Assay variable : PTINR_VAL1 PT-INR Value

N	Mean	Standard Deviation	Minimum	Maximum
3	2.2766667	0.4441096	1.7900000	2.6600000

Laboratory tests at the time of obtaining Table 2-6 consent, according to the presence or absence of proton-pump inhibitors

Unique Subject Identifier=1491000000-0086

MEANS Procedure

Assay variable : PTINR_VAL1 PT-INR Value

N	Mean	Standard Deviation	Minimum	Maximum
4	2.1675000	0.2817061	1.9300000	2.5700000

Unique Subject Identifier=1491000000-0094

Assay variable : PTINR_VAL1 PT-INR Value

N	Mean	Standard Deviation	Minimum	Maximum
4	2.1475000	0.1078193	2.0500000	2.2900000

Unique Subject Identifier=1491000000-0097

Assay variable : PTINR_VAL1 PT-INR Value

N	Mean	Standard Deviation	Minimum	Maximum
3	2.6600000	0.6577994	2.0400000	3.3500000

Unique Subject Identifier=1491000000-0098

Assay variable : PTINR_VAL1 PT-INR Value

N	Mean	Standard Deviation	Minimum	Maximum
4	2.1300000	0.3281260	1.8600000	2.5700000

Laboratory tests at the time of obtaining Table 2-6 consent, according to the presence or absence of proton-pump inhibitors

Unique Subject Identifier=1491000000-0105

MEANS Procedure

Assay variable : PTINR_VAL1 PT-INR Value

N	Mean	Standard Deviation	Minimum	Maximum
5	1.8920000	0.3596804	1.5900000	2.5000000

Unique Subject Identifier=1494000000-0015

Assay variable : PTINR_VAL1 PT-INR Value

N	Mean	Standard Deviation	Minimum	Maximum
2	1.5600000	0.2121320	1.4100000	1.7100000

Unique Subject Identifier=1494000000-0021

Assay variable : PTINR_VAL1 PT-INR Value

N	Mean	Standard Deviation	Minimum	Maximum
4	1.9375000	0.6571847	1.4000000	2.8800000

Unique Subject Identifier=1494000000-0027

Assay variable : PTINR_VAL1 PT-INR Value

N	Mean	Standard Deviation	Minimum	Maximum
3	1.7400000	0.0200000	1.7200000	1.7600000

Laboratory tests at the time of obtaining Table 2-6 consent, according to the presence or absence of proton-pump inhibitors

Unique Subject Identifier=1495011010-0005

MEANS Procedure

Assay variable : PTINR_VAL1 PT-INR Value

N	Mean	Standard Deviation	Minimum	Maximum
2	2.6700000	0.5374012	2.2900000	3.0500000

Unique Subject Identifier=1495011010-0009

Assay variable : PTINR_VAL1 PT-INR Value

N	Mean	Standard Deviation	Minimum	Maximum
3	2.4166667	0.5589574	1.9600000	3.0400000

Unique Subject Identifier=1495011010-0013

Assay variable : PTINR_VAL1 PT-INR Value

N	Mean	Standard Deviation	Minimum	Maximum
2	2.7300000	0.9475231	2.0600000	3.4000000

Unique Subject Identifier=1495011010-0019

Assay variable : PTINR_VAL1 PT-INR Value

N	Mean	Standard Deviation	Minimum	Maximum
2	2.2950000	0.1767767	2.1700000	2.4200000

Laboratory tests at the time of obtaining Table 2-6 consent, according to the presence or absence of proton-pump inhibitors

Unique Subject Identifier=1495011010-0022

MEANS Procedure

Assay variable : PTINR_VAL1 PT-INR Value

N	Mean	Standard Deviation	Minimum	Maximum
3	2.1266667	0.7674199	1.5600000	3.0000000

Unique Subject Identifier=1495011010-0024

Assay variable : PTINR_VAL1 PT-INR Value

N	Mean	Standard Deviation	Minimum	Maximum
2	2.0500000	0.3535534	1.8000000	2.3000000

Unique Subject Identifier=1495011010-0026

Assay variable : PTINR_VAL1 PT-INR Value

N	Mean	Standard Deviation	Minimum	Maximum
3	2.2933333	0.3971566	1.8400000	2.5800000

Unique Subject Identifier=1495011010-0027

Assay variable : PTINR_VAL1 PT-INR Value

N	Mean	Standard Deviation	Minimum	Maximum
2	1.5200000	0.0565685	1.4800000	1.5600000

Laboratory tests at the time of obtaining Table 2-6 consent, according to the presence or absence of proton-pump inhibitors

Unique Subject Identifier=1496000000-0008

MEANS Procedure

Assay variable : PTINR_VAL1 PT-INR Value

N	Mean	Standard Deviation	Minimum	Maximum
1	2.3000000		2.3000000	2.3000000

Unique Subject Identifier=1496000000-0012

Assay variable : PTINR_VAL1 PT-INR Value

N	Mean	Standard Deviation	Minimum	Maximum
1	1.4000000		1.4000000	1.4000000

Unique Subject Identifier=1499000000-0008

Assay variable : PTINR_VAL1 PT-INR Value

N	Mean	Standard Deviation	Minimum	Maximum
3	1.7566667	0.3942503	1.4000000	2.1800000

Unique Subject Identifier=1501000000-0004

Assay variable : PTINR_VAL1 PT-INR Value

N	Mean	Standard Deviation	Minimum	Maximum
6	2.1033333	0.5933521	1.2000000	3.0000000

Laboratory tests at the time of obtaining Table 2-6 consent, according to the presence or absence of proton-pump inhibitors

Unique Subject Identifier=1501000000-0012

MEANS Procedure

Assay variable : PTINR_VAL1 PT-INR Value

N	Mean	Standard Deviation	Minimum	Maximum
6	2.2333333	0.3386247	1.7000000	2.6000000

Unique Subject Identifier=1501000000-0014

Assay variable : PTINR_VAL1 PT-INR Value

N	Mean	Standard Deviation	Minimum	Maximum
5	1.7620000	0.3475917	1.4000000	2.2900000

Unique Subject Identifier=1501000000-0022

Assay variable : PTINR_VAL1 PT-INR Value

N	Mean	Standard Deviation	Minimum	Maximum
6	1.8750000	0.3895510	1.5000000	2.5000000

Unique Subject Identifier=1501000000-0025

Assay variable : PTINR_VAL1 PT-INR Value

N	Mean	Standard Deviation	Minimum	Maximum
6	2.2166667	0.5115336	1.5000000	2.8000000

Laboratory tests at the time of obtaining Table 2-6 consent, according to the presence or absence of proton-pump inhibitors

Unique Subject Identifier=1501000000-0033

MEANS Procedure

Assay variable : PTINR_VAL1 PT-INR Value

N	Mean	Standard Deviation	Minimum	Maximum
3	1.6700000	0.0608276	1.6000000	1.7100000

Unique Subject Identifier=1501000000-0046

Assay variable : PTINR_VAL1 PT-INR Value

N	Mean	Standard Deviation	Minimum	Maximum
5	2.3360000	0.8537740	1.2000000	3.3900000

Unique Subject Identifier=1502000000-0005

Assay variable : PTINR_VAL1 PT-INR Value

N	Mean	Standard Deviation	Minimum	Maximum
1	2.1000000		2.1000000	2.1000000

Unique Subject Identifier=1502000000-0007

Assay variable : PTINR_VAL1 PT-INR Value

N	Mean	Standard Deviation	Minimum	Maximum
2	1.7200000	0.6646804	1.2500000	2.1900000

Laboratory tests at the time of obtaining Table 2-6 consent, according to the presence or absence of proton-pump inhibitors

Unique Subject Identifier=1503000000-0001

MEANS Procedure

Assay variable : PTINR_VAL1 PT-INR Value

N	Mean	Standard Deviation	Minimum	Maximum
1	1.7500000		1.7500000	1.7500000

Unique Subject Identifier=1503000000-0005

Assay variable : PTINR_VAL1 PT-INR Value

N	Mean	Standard Deviation	Minimum	Maximum
3	1.8066667	1.3367997	1.0100000	3.3500000

Unique Subject Identifier=1503000000-0007

Assay variable : PTINR_VAL1 PT-INR Value

N	Mean	Standard Deviation	Minimum	Maximum
4	2.1825000	0.8794458	1.4100000	3.1000000

Unique Subject Identifier=1503000000-0008

Assay variable : PTINR_VAL1 PT-INR Value

N	Mean	Standard Deviation	Minimum	Maximum
3	2.2033333	1.2630255	1.3200000	3.6500000

Laboratory tests at the time of obtaining Table 2-6 consent, according to the presence or absence of proton-pump inhibitors

Unique Subject Identifier=1504000000-0001

MEANS Procedure

Assay variable : PTINR_VAL1 PT-INR Value

N	Mean	Standard Deviation	Minimum	Maximum
3	2.1600000	0.3351119	1.8200000	2.4900000

Unique Subject Identifier=1504000000-0002

Assay variable : PTINR_VAL1 PT-INR Value

N	Mean	Standard Deviation	Minimum	Maximum
5	2.6700000	0.3653081	2.1400000	3.0900000

Unique Subject Identifier=1512000000-0004

Assay variable : PTINR_VAL1 PT-INR Value

N	Mean	Standard Deviation	Minimum	Maximum
5	1.8000000	0.3706751	1.2700000	2.3100000

Unique Subject Identifier=1512000000-0005

Assay variable : PTINR_VAL1 PT-INR Value

N	Mean	Standard Deviation	Minimum	Maximum
3	2.0000000	0.2364318	1.7500000	2.2200000

Laboratory tests at the time of obtaining Table 2-6 consent, according to the presence or absence of proton-pump inhibitors

Unique Subject Identifier=1512000000-0007

MEANS Procedure

Assay variable : PTINR_VAL1 PT-INR Value

N	Mean	Standard Deviation	Minimum	Maximum
6	2.2116667	0.2878483	1.8600000	2.6900000

Unique Subject Identifier=1513000000-0006

Assay variable : PTINR_VAL1 PT-INR Value

N	Mean	Standard Deviation	Minimum	Maximum
3	1.8700000	0.1664332	1.7500000	2.0600000

Unique Subject Identifier=1513000000-0010

Assay variable : PTINR_VAL1 PT-INR Value

N	Mean	Standard Deviation	Minimum	Maximum
3	2.1700000	0.0435890	2.1200000	2.2000000

Unique Subject Identifier=1514011100-0011

Assay variable : PTINR_VAL1 PT-INR Value

N	Mean	Standard Deviation	Minimum	Maximum
1	2.1800000		2.1800000	2.1800000

Laboratory tests at the time of obtaining Table 2-6 consent, according to the presence or absence of proton-pump inhibitors

Unique Subject Identifier=1514011100-0012

MEANS Procedure

Assay variable : PTINR_VAL1 PT-INR Value

N	Mean	Standard Deviation	Minimum	Maximum
2	1.8250000	0.2192031	1.6700000	1.9800000

Unique Subject Identifier=1516011000-0043

Assay variable : PTINR_VAL1 PT-INR Value

N	Mean	Standard Deviation	Minimum	Maximum
5	1.6140000	0.0876356	1.4900000	1.7000000

Unique Subject Identifier=1516011000-0045

Assay variable : PTINR_VAL1 PT-INR Value

N	Mean	Standard Deviation	Minimum	Maximum
6	1.5500000	0.1643168	1.4000000	1.8000000

Unique Subject Identifier=1516011000-0052

Assay variable : PTINR_VAL1 PT-INR Value

N	Mean	Standard Deviation	Minimum	Maximum
5	2.1600000	0.5683309	1.2000000	2.6000000

Laboratory tests at the time of obtaining Table 2-6 consent, according to the presence or absence of proton-pump inhibitors

Unique Subject Identifier=1517010100-0003

MEANS Procedure

Assay variable : PTINR_VAL1 PT-INR Value

N	Mean	Standard Deviation	Minimum	Maximum
2	1.6900000	0.1272792	1.6000000	1.7800000

Unique Subject Identifier=1517010100-0004

Assay variable : PTINR_VAL1 PT-INR Value

N	Mean	Standard Deviation	Minimum	Maximum
3	2.1733333	0.4986315	1.6300000	2.6100000

Unique Subject Identifier=1517010100-0006

Assay variable : PTINR_VAL1 PT-INR Value

N	Mean	Standard Deviation	Minimum	Maximum
3	2.2633333	0.6664333	1.5500000	2.8700000

Unique Subject Identifier=1517010100-0009

Assay variable : PTINR_VAL1 PT-INR Value

N	Mean	Standard Deviation	Minimum	Maximum
3	1.9500000	0.3502856	1.6400000	2.3300000

Laboratory tests at the time of obtaining Table 2-6 consent, according to the presence or absence of proton-pump inhibitors

Unique Subject Identifier=1517010100-0011

MEANS Procedure

Assay variable : PTINR_VAL1 PT-INR Value

N	Mean	Standard Deviation	Minimum	Maximum
3	1.6900000	0.1473092	1.5600000	1.8500000

Unique Subject Identifier=1521000000-0002

Assay variable : PTINR_VAL1 PT-INR Value

N	Mean	Standard Deviation	Minimum	Maximum
1	1.7300000		1.7300000	1.7300000

Unique Subject Identifier=1521000000-0003

Assay variable : PTINR_VAL1 PT-INR Value

N	Mean	Standard Deviation	Minimum	Maximum
1	2.6600000		2.6600000	2.6600000

Unique Subject Identifier=1521000000-0006

Assay variable : PTINR_VAL1 PT-INR Value

N	Mean	Standard Deviation	Minimum	Maximum
1	1.7100000		1.7100000	1.7100000

Laboratory tests at the time of obtaining Table 2-6 consent, according to the presence or absence of proton-pump inhibitors

Unique Subject Identifier=1522000000-0001

MEANS Procedure

Assay variable : PTINR_VAL1 PT-INR Value

N	Mean	Standard Deviation	Minimum	Maximum
4	2.1100000	0.5918896	1.6700000	2.9400000

Unique Subject Identifier=1522000000-0003

Assay variable : PTINR_VAL1 PT-INR Value

N	Mean	Standard Deviation	Minimum	Maximum
2	1.4150000	0.4596194	1.0900000	1.7400000

Unique Subject Identifier=1522000000-0005

Assay variable : PTINR_VAL1 PT-INR Value

N	Mean	Standard Deviation	Minimum	Maximum
5	1.9700000	0.3586781	1.6500000	2.4400000

Unique Subject Identifier=1522000000-0006

Assay variable : PTINR_VAL1 PT-INR Value

N	Mean	Standard Deviation	Minimum	Maximum
4	1.8775000	0.2466272	1.6400000	2.1500000

Laboratory tests at the time of obtaining Table 2-6 consent, according to the presence or absence of proton-pump inhibitors

Unique Subject Identifier=1522000000-0010

MEANS Procedure

Assay variable : PTINR_VAL1 PT-INR Value

N	Mean	Standard Deviation	Minimum	Maximum
6	2.4316667	0.7141545	1.5300000	3.4100000

Unique Subject Identifier=1522000000-0011

Assay variable : PTINR_VAL1 PT-INR Value

N	Mean	Standard Deviation	Minimum	Maximum
5	2.2160000	0.7098098	1.3800000	2.8600000

Unique Subject Identifier=1522000000-0016

Assay variable : PTINR_VAL1 PT-INR Value

N	Mean	Standard Deviation	Minimum	Maximum
4	1.7400000	0.1913113	1.5600000	1.9200000

Unique Subject Identifier=1522000000-0017

Assay variable : PTINR_VAL1 PT-INR Value

N	Mean	Standard Deviation	Minimum	Maximum
4	1.9975000	0.5490826	1.3600000	2.4900000

Laboratory tests at the time of obtaining Table 2-6 consent, according to the presence or absence of proton-pump inhibitors

Unique Subject Identifier=1522000000-0024

MEANS Procedure

Assay variable : PTINR_VAL1 PT-INR Value

N	Mean	Standard Deviation	Minimum	Maximum
4	1.9600000	0.5485131	1.2100000	2.5000000

Unique Subject Identifier=1522000000-0027

Assay variable : PTINR_VAL1 PT-INR Value

N	Mean	Standard Deviation	Minimum	Maximum
4	1.6675000	0.1985573	1.5300000	1.9600000

Unique Subject Identifier=1522000000-0031

Assay variable : PTINR_VAL1 PT-INR Value

N	Mean	Standard Deviation	Minimum	Maximum
3	1.4866667	0.0152753	1.4700000	1.5000000

Unique Subject Identifier=1522000000-0035

Assay variable : PTINR_VAL1 PT-INR Value

N	Mean	Standard Deviation	Minimum	Maximum
3	1.8600000	0.4430576	1.3500000	2.1500000

Laboratory tests at the time of obtaining Table 2-6 consent, according to the presence or absence of proton-pump inhibitors

Unique Subject Identifier=1523010111-0001

MEANS Procedure

Assay variable : PTINR_VAL1 PT-INR Value

N	Mean	Standard Deviation	Minimum	Maximum
6	1.6800000	0.3017284	1.4100000	2.2500000

Unique Subject Identifier=1523010111-0006

Assay variable : PTINR_VAL1 PT-INR Value

N	Mean	Standard Deviation	Minimum	Maximum
6	1.5683333	0.1646107	1.3300000	1.7300000

Unique Subject Identifier=1524010000-0002

Assay variable : PTINR_VAL1 PT-INR Value

N	Mean	Standard Deviation	Minimum	Maximum
2	1.8450000	0.2050610	1.7000000	1.9900000

Unique Subject Identifier=1524010000-0005

Assay variable : PTINR_VAL1 PT-INR Value

N	Mean	Standard Deviation	Minimum	Maximum
2	2.0050000	0.1484924	1.9000000	2.1100000

Laboratory tests at the time of obtaining Table 2-6 consent, according to the presence or absence of proton-pump inhibitors

Unique Subject Identifier=1524010000-0012

MEANS Procedure

Assay variable : PTINR_VAL1 PT-INR Value

N	Mean	Standard Deviation	Minimum	Maximum
4	2.2575000	0.2129750	1.9600000	2.4500000

Unique Subject Identifier=1524010000-0018

Assay variable : PTINR_VAL1 PT-INR Value

N	Mean	Standard Deviation	Minimum	Maximum
4	2.2350000	0.4086971	1.9100000	2.8300000

Unique Subject Identifier=1527010000-0001

Assay variable : PTINR_VAL1 PT-INR Value

N	Mean	Standard Deviation	Minimum	Maximum
6	1.7250000	0.2076295	1.4500000	1.9900000

Unique Subject Identifier=1527010000-0002

Assay variable : PTINR_VAL1 PT-INR Value

N	Mean	Standard Deviation	Minimum	Maximum
5	2.0180000	0.1130044	1.9300000	2.2000000

Laboratory tests at the time of obtaining Table 2-6 consent, according to the presence or absence of proton-pump inhibitors

Unique Subject Identifier=1527010000-0003

MEANS Procedure

Assay variable : PTINR_VAL1 PT-INR Value

N	Mean	Standard Deviation	Minimum	Maximum
6	1.8283333	0.3559448	1.5600000	2.5000000

Unique Subject Identifier=1527010000-0004

Assay variable : PTINR_VAL1 PT-INR Value

N	Mean	Standard Deviation	Minimum	Maximum
6	2.0766667	0.1527962	1.9300000	2.3000000

Unique Subject Identifier=1527010000-0006

Assay variable : PTINR_VAL1 PT-INR Value

N	Mean	Standard Deviation	Minimum	Maximum
6	2.0616667	0.2521441	1.7100000	2.4000000

Unique Subject Identifier=1527010000-0007

Assay variable : PTINR_VAL1 PT-INR Value

N	Mean	Standard Deviation	Minimum	Maximum
6	2.3883333	0.3098656	1.9300000	2.8000000

Laboratory tests at the time of obtaining Table 2-6 consent, according to the presence or absence of proton-pump inhibitors

Unique Subject Identifier=1527010000-0008

MEANS Procedure

Assay variable : PTINR_VAL1 PT-INR Value

N	Mean	Standard Deviation	Minimum	Maximum
6	2.0716667	0.3989695	1.7100000	2.8000000

Unique Subject Identifier=1527010000-0009

Assay variable : PTINR_VAL1 PT-INR Value

N	Mean	Standard Deviation	Minimum	Maximum
6	1.9333333	0.1298717	1.7100000	2.1100000

Unique Subject Identifier=1527010000-0010

Assay variable : PTINR_VAL1 PT-INR Value

N	Mean	Standard Deviation	Minimum	Maximum
6	1.8316667	0.1595515	1.5600000	1.9300000

Unique Subject Identifier=1527010000-0011

Assay variable : PTINR_VAL1 PT-INR Value

N	Mean	Standard Deviation	Minimum	Maximum
6	2.0683333	0.3099301	1.7100000	2.4000000

Laboratory tests at the time of obtaining Table 2-6 consent, according to the presence or absence of proton-pump inhibitors

Unique Subject Identifier=1527010000-0012

MEANS Procedure

Assay variable : PTINR_VAL1 PT-INR Value

N	Mean	Standard Deviation	Minimum	Maximum
4	1.7100000	0.2933712	1.4500000	2.1200000

Unique Subject Identifier=1527010000-0013

Assay variable : PTINR_VAL1 PT-INR Value

N	Mean	Standard Deviation	Minimum	Maximum
5	2.0380000	0.2314519	1.7100000	2.3000000

Unique Subject Identifier=1527010000-0016

Assay variable : PTINR_VAL1 PT-INR Value

N	Mean	Standard Deviation	Minimum	Maximum
6	1.8516667	0.1699902	1.7100000	2.1200000

Unique Subject Identifier=1527010000-0017

Assay variable : PTINR_VAL1 PT-INR Value

N	Mean	Standard Deviation	Minimum	Maximum
5	1.9500000	0.3967367	1.5600000	2.4000000

Laboratory tests at the time of obtaining Table 2-6 consent, according to the presence or absence of proton-pump inhibitors

Unique Subject Identifier=1527010000-0018

MEANS Procedure

Assay variable : PTINR_VAL1 PT-INR Value

N	Mean	Standard Deviation	Minimum	Maximum
6	2.2633333	0.2242023	1.9300000	2.5000000

Unique Subject Identifier=1527010000-0022

Assay variable : PTINR_VAL1 PT-INR Value

N	Mean	Standard Deviation	Minimum	Maximum
6	1.9150000	0.6305791	1.2500000	2.8000000

Unique Subject Identifier=1527010000-0024

Assay variable : PTINR_VAL1 PT-INR Value

N	Mean	Standard Deviation	Minimum	Maximum
6	1.9166667	0.4050021	1.4500000	2.6000000

Unique Subject Identifier=1527010000-0026

Assay variable : PTINR_VAL1 PT-INR Value

N	Mean	Standard Deviation	Minimum	Maximum
6	2.1050000	0.3042860	1.7100000	2.6000000

Laboratory tests at the time of obtaining Table 2-6 consent, according to the presence or absence of proton-pump inhibitors

Unique Subject Identifier=1528000000-0003

MEANS Procedure

Assay variable : PTINR_VAL1 PT-INR Value

N	Mean	Standard Deviation	Minimum	Maximum
6	2.2866667	0.5947324	1.3800000	3.1300000

Unique Subject Identifier=1530000000-0003

Assay variable : PTINR_VAL1 PT-INR Value

N	Mean	Standard Deviation	Minimum	Maximum
5	1.5800000	0.1788854	1.3000000	1.7000000

Unique Subject Identifier=1530000000-0026

Assay variable : PTINR_VAL1 PT-INR Value

N	Mean	Standard Deviation	Minimum	Maximum
2	4.0500000	2.3334524	2.4000000	5.7000000

Unique Subject Identifier=1530000000-0030

Assay variable : PTINR_VAL1 PT-INR Value

N	Mean	Standard Deviation	Minimum	Maximum
1	1.4000000		1.4000000	1.4000000

Laboratory tests at the time of obtaining Table 2-6 consent, according to the presence or absence of proton-pump inhibitors

Unique Subject Identifier=1531100000-0011

MEANS Procedure

Assay variable : PTINR_VAL1 PT-INR Value

N	Mean	Standard Deviation	Minimum	Maximum
6	2.2833333	0.2639444	1.9000000	2.6000000

Unique Subject Identifier=1600011000-0001

Assay variable : PTINR_VAL1 PT-INR Value

N	Mean	Standard Deviation	Minimum	Maximum
2	1.6600000	0.0848528	1.6000000	1.7200000

Unique Subject Identifier=1600011000-0006

Assay variable : PTINR_VAL1 PT-INR Value

N	Mean	Standard Deviation	Minimum	Maximum
1	2.5700000		2.5700000	2.5700000

Unique Subject Identifier=1602000000-0002

Assay variable : PTINR_VAL1 PT-INR Value

N	Mean	Standard Deviation	Minimum	Maximum
3	1.5000000	0.1000000	1.4000000	1.6000000

Laboratory tests at the time of obtaining Table 2-6 consent, according to the presence or absence of proton-pump inhibitors

Unique Subject Identifier=1602000000-0003

MEANS Procedure

Assay variable : PTINR_VAL1 PT-INR Value

N	Mean	Standard Deviation	Minimum	Maximum
3	1.8333333	0.4041452	1.4000000	2.2000000

Unique Subject Identifier=1603000000-0019

Assay variable : PTINR_VAL1 PT-INR Value

N	Mean	Standard Deviation	Minimum	Maximum
6	2.5000000	0.4939636	1.9000000	3.0000000

Unique Subject Identifier=1701010000-0003

Assay variable : PTINR_VAL1 PT-INR Value

N	Mean	Standard Deviation	Minimum	Maximum
6	1.5016667	0.0685322	1.4300000	1.5700000

Unique Subject Identifier=1705010000-0003

Assay variable : PTINR_VAL1 PT-INR Value

N	Mean	Standard Deviation	Minimum	Maximum
2	1.6600000	0.0989949	1.5900000	1.7300000

Laboratory tests at the time of obtaining Table 2-6 consent, according to the presence or absence of proton-pump inhibitors

Unique Subject Identifier=1708010000-0003

MEANS Procedure

Assay variable : PTINR_VAL1 PT-INR Value

N	Mean	Standard Deviation	Minimum	Maximum
5	2.3020000	0.3291960	2.0900000	2.8800000

Unique Subject Identifier=1710000000-0008

Assay variable : PTINR_VAL1 PT-INR Value

N	Mean	Standard Deviation	Minimum	Maximum
6	2.1583333	0.3340309	1.8100000	2.7200000

Unique Subject Identifier=1716010000-0004

Assay variable : PTINR_VAL1 PT-INR Value

N	Mean	Standard Deviation	Minimum	Maximum
3	1.6933333	0.4045162	1.3800000	2.1500000

Unique Subject Identifier=1718000000-0011

Assay variable : PTINR_VAL1 PT-INR Value

N	Mean	Standard Deviation	Minimum	Maximum
6	1.7000000	0.1264911	1.6000000	1.9000000

Laboratory tests at the time of obtaining Table 2-6 consent, according to the presence or absence of proton-pump inhibitors

Unique Subject Identifier=1718000000-0015

MEANS Procedure

Assay variable : PTINR_VAL1 PT-INR Value

N	Mean	Standard Deviation	Minimum	Maximum
5	1.9200000	0.2683282	1.5000000	2.2000000

Unique Subject Identifier=1718000000-0017

Assay variable : PTINR_VAL1 PT-INR Value

N	Mean	Standard Deviation	Minimum	Maximum
6	2.2333333	0.5819507	1.9000000	3.4000000

Unique Subject Identifier=1718000000-0018

Assay variable : PTINR_VAL1 PT-INR Value

N	Mean	Standard Deviation	Minimum	Maximum
6	1.7666667	0.3265986	1.4000000	2.2000000

Unique Subject Identifier=1718000000-0020

Assay variable : PTINR_VAL1 PT-INR Value

N	Mean	Standard Deviation	Minimum	Maximum
6	2.0333333	1.2274635	1.2000000	4.4000000

Laboratory tests at the time of obtaining Table 2-6 consent, according to the presence or absence of proton-pump inhibitors

Unique Subject Identifier=1750100000-0007

MEANS Procedure

Assay variable : PTINR_VAL1 PT-INR Value

N	Mean	Standard Deviation	Minimum	Maximum
3	3.4466667	0.3952636	3.0100000	3.7800000

Unique Subject Identifier=1750100000-0009

Assay variable : PTINR_VAL1 PT-INR Value

N	Mean	Standard Deviation	Minimum	Maximum
5	1.6380000	0.2701296	1.3600000	1.9500000

Unique Subject Identifier=1750100000-0012

Assay variable : PTINR_VAL1 PT-INR Value

N	Mean	Standard Deviation	Minimum	Maximum
4	1.6325000	0.1138347	1.5100000	1.7800000

Unique Subject Identifier=1750100000-0021

Assay variable : PTINR_VAL1 PT-INR Value

N	Mean	Standard Deviation	Minimum	Maximum
3	2.2633333	0.2419366	1.9900000	2.4500000

Laboratory tests at the time of obtaining Table 2-6 consent, according to the presence or absence of proton-pump inhibitors

Unique Subject Identifier=1750100000-0025

MEANS Procedure

Assay variable : PTINR_VAL1 PT-INR Value

N	Mean	Standard Deviation	Minimum	Maximum
3	1.8366667	0.6619919	1.4200000	2.6000000

Unique Subject Identifier=1750100000-0028

Assay variable : PTINR_VAL1 PT-INR Value

N	Mean	Standard Deviation	Minimum	Maximum
4	1.8700000	0.6558963	1.2100000	2.7700000

Unique Subject Identifier=1750100000-0035

Assay variable : PTINR_VAL1 PT-INR Value

N	Mean	Standard Deviation	Minimum	Maximum
4	2.0775000	0.4478374	1.7900000	2.7400000

Unique Subject Identifier=1750100000-0036

Assay variable : PTINR_VAL1 PT-INR Value

N	Mean	Standard Deviation	Minimum	Maximum
3	2.2033333	0.4614470	1.8700000	2.7300000

Laboratory tests at the time of obtaining Table 2-6 consent, according to the presence or absence of proton-pump inhibitors

Unique Subject Identifier=1750100000-0037

MEANS Procedure

Assay variable : PTINR_VAL1 PT-INR Value

N	Mean	Standard Deviation	Minimum	Maximum
2	1.4650000	0.0494975	1.4300000	1.5000000

Unique Subject Identifier=1750100000-0039

Assay variable : PTINR_VAL1 PT-INR Value

N	Mean	Standard Deviation	Minimum	Maximum
5	1.9720000	0.3858368	1.5900000	2.3900000

Unique Subject Identifier=1750100000-0045

Assay variable : PTINR_VAL1 PT-INR Value

N	Mean	Standard Deviation	Minimum	Maximum
6	1.8700000	0.4787901	1.4200000	2.6700000

Unique Subject Identifier=1750100000-0052

Assay variable : PTINR_VAL1 PT-INR Value

N	Mean	Standard Deviation	Minimum	Maximum
4	1.7825000	0.2523721	1.5700000	2.1200000

Laboratory tests at the time of obtaining Table 2-6 consent, according to the presence or absence of proton-pump inhibitors

Unique Subject Identifier=1800000000-0007

MEANS Procedure

Assay variable : PTINR_VAL1 PT-INR Value

N	Mean	Standard Deviation	Minimum	Maximum
4	2.1750000	0.4645787	1.7000000	2.8000000

Unique Subject Identifier=1800000000-0008

Assay variable : PTINR_VAL1 PT-INR Value

N	Mean	Standard Deviation	Minimum	Maximum
6	1.9166667	0.4622409	1.4000000	2.6000000

Unique Subject Identifier=1800000000-0012

Assay variable : PTINR_VAL1 PT-INR Value

N	Mean	Standard Deviation	Minimum	Maximum
6	2.0650000	0.3887287	1.5000000	2.5900000

Unique Subject Identifier=1800000000-0015

Assay variable : PTINR_VAL1 PT-INR Value

N	Mean	Standard Deviation	Minimum	Maximum
6	1.8300000	0.2909295	1.4000000	2.1800000

Laboratory tests at the time of obtaining Table 2-6 consent, according to the presence or absence of proton-pump inhibitors

Unique Subject Identifier=1800000000-0021

MEANS Procedure

Assay variable : PTINR_VAL1 PT-INR Value

N	Mean	Standard Deviation	Minimum	Maximum
2	2.4950000	0.1484924	2.3900000	2.6000000

Unique Subject Identifier=1800000000-0028

Assay variable : PTINR_VAL1 PT-INR Value

N	Mean	Standard Deviation	Minimum	Maximum
6	2.3250000	0.3151349	1.8500000	2.7300000

Unique Subject Identifier=1805000000-0006

Assay variable : PTINR_VAL1 PT-INR Value

N	Mean	Standard Deviation	Minimum	Maximum
1	2.3000000		2.3000000	2.3000000

Unique Subject Identifier=1810000000-0001

Assay variable : PTINR_VAL1 PT-INR Value

N	Mean	Standard Deviation	Minimum	Maximum
6	2.0850000	0.3365561	1.6200000	2.4400000

Laboratory tests at the time of obtaining Table 2-6 consent, according to the presence or absence of proton-pump inhibitors

Unique Subject Identifier=1810000000-0004

MEANS Procedure

Assay variable : PTINR_VAL1 PT-INR Value

N	Mean	Standard Deviation	Minimum	Maximum
5	1.8100000	0.4154516	1.3800000	2.4400000

Unique Subject Identifier=1810000000-0005

Assay variable : PTINR_VAL1 PT-INR Value

N	Mean	Standard Deviation	Minimum	Maximum
6	1.6716667	0.2863855	1.3200000	2.0700000

Unique Subject Identifier=1810000000-0006

Assay variable : PTINR_VAL1 PT-INR Value

N	Mean	Standard Deviation	Minimum	Maximum
6	2.0350000	0.1342758	1.9100000	2.2100000

Unique Subject Identifier=1810000000-0009

Assay variable : PTINR_VAL1 PT-INR Value

N	Mean	Standard Deviation	Minimum	Maximum
6	1.9783333	0.2976183	1.5600000	2.4600000

Laboratory tests at the time of obtaining Table 2-6 consent, according to the presence or absence of proton-pump inhibitors

Unique Subject Identifier=1810000000-0011

MEANS Procedure

Assay variable : PTINR_VAL1 PT-INR Value

N	Mean	Standard Deviation	Minimum	Maximum
6	1.8600000	0.4210463	1.4500000	2.6500000

Unique Subject Identifier=1810000000-0012

Assay variable : PTINR_VAL1 PT-INR Value

N	Mean	Standard Deviation	Minimum	Maximum
6	2.0400000	0.1073313	1.8900000	2.1700000

Unique Subject Identifier=1810000000-0017

Assay variable : PTINR_VAL1 PT-INR Value

N	Mean	Standard Deviation	Minimum	Maximum
6	2.0200000	0.2748818	1.7000000	2.3200000

Unique Subject Identifier=1810000000-0018

Assay variable : PTINR_VAL1 PT-INR Value

N	Mean	Standard Deviation	Minimum	Maximum
6	2.1750000	0.4289406	1.5900000	2.8300000

Laboratory tests at the time of obtaining Table 2-6 consent, according to the presence or absence of proton-pump inhibitors

Unique Subject Identifier=1811000000-0005

MEANS Procedure

Assay variable : PTINR_VAL1 PT-INR Value

N	Mean	Standard Deviation	Minimum	Maximum
6	1.5900000	0.0660303	1.5300000	1.7000000

Unique Subject Identifier=1811000000-0007

Assay variable : PTINR_VAL1 PT-INR Value

N	Mean	Standard Deviation	Minimum	Maximum
6	1.8766667	0.2086784	1.5400000	2.0800000

Unique Subject Identifier=1811000000-0023

Assay variable : PTINR_VAL1 PT-INR Value

N	Mean	Standard Deviation	Minimum	Maximum
5	1.7200000	0.1100000	1.6100000	1.8700000

Unique Subject Identifier=1811000000-0026

Assay variable : PTINR_VAL1 PT-INR Value

N	Mean	Standard Deviation	Minimum	Maximum
6	2.4616667	0.3459142	2.1300000	3.0900000

Laboratory tests at the time of obtaining Table 2-6 consent, according to the presence or absence of proton-pump inhibitors

Unique Subject Identifier=1812000000-0004

MEANS Procedure

Assay variable : PTINR_VAL1 PT-INR Value

N	Mean	Standard Deviation	Minimum	Maximum
6	2.2166667	0.4535049	1.8000000	2.8000000

Unique Subject Identifier=1812000000-0011

Assay variable : PTINR_VAL1 PT-INR Value

N	Mean	Standard Deviation	Minimum	Maximum
6	1.5000000	0.2190890	1.2000000	1.8000000

Unique Subject Identifier=1812000000-0013

Assay variable : PTINR_VAL1 PT-INR Value

N	Mean	Standard Deviation	Minimum	Maximum
6	2.1166667	1.4372427	1.0000000	4.9000000

Unique Subject Identifier=1812000000-0019

Assay variable : PTINR_VAL1 PT-INR Value

N	Mean	Standard Deviation	Minimum	Maximum
6	1.7333333	0.6742897	1.1000000	2.8000000

Laboratory tests at the time of obtaining Table 2-6 consent, according to the presence or absence of proton-pump inhibitors

Unique Subject Identifier=1813100000-0028

MEANS Procedure

Assay variable : PTINR_VAL1 PT-INR Value

N	Mean	Standard Deviation	Minimum	Maximum
4	1.6850000	0.2810101	1.2800000	1.9100000

Unique Subject Identifier=1814100000-0001

Assay variable : PTINR_VAL1 PT-INR Value

N	Mean	Standard Deviation	Minimum	Maximum
1	3.2100000		3.2100000	3.2100000

Unique Subject Identifier=1814100000-0002

Assay variable : PTINR_VAL1 PT-INR Value

N	Mean	Standard Deviation	Minimum	Maximum
1	2.5700000		2.5700000	2.5700000

Unique Subject Identifier=1814100000-0005

Assay variable : PTINR_VAL1 PT-INR Value

N	Mean	Standard Deviation	Minimum	Maximum
1	1.9500000		1.9500000	1.9500000

Laboratory tests at the time of obtaining Table 2-6 consent, according to the presence or absence of proton-pump inhibitors

Unique Subject Identifier=1814100000-0008

MEANS Procedure

Assay variable : PTINR_VAL1 PT-INR Value

N	Mean	Standard Deviation	Minimum	Maximum
2	1.5200000	0.0707107	1.4700000	1.5700000

Unique Subject Identifier=1814100000-0010

Assay variable : PTINR_VAL1 PT-INR Value

N	Mean	Standard Deviation	Minimum	Maximum
6	1.6450000	0.5125915	1.1400000	2.1900000

Unique Subject Identifier=1814100000-0038

Assay variable : PTINR_VAL1 PT-INR Value

N	Mean	Standard Deviation	Minimum	Maximum
6	2.2566667	0.2811168	1.8200000	2.5500000

Unique Subject Identifier=1814100000-0058

Assay variable : PTINR_VAL1 PT-INR Value

N	Mean	Standard Deviation	Minimum	Maximum
6	1.9416667	0.8153874	1.2700000	3.2300000

Laboratory tests at the time of obtaining Table 2-6 consent, according to the presence or absence of proton-pump inhibitors

Unique Subject Identifier=1814100000-0063

MEANS Procedure

Assay variable : PTINR_VAL1 PT-INR Value

N	Mean	Standard Deviation	Minimum	Maximum
6	1.1066667	0.0524087	1.0300000	1.1800000

Unique Subject Identifier=1814100000-0067

Assay variable : PTINR_VAL1 PT-INR Value

N	Mean	Standard Deviation	Minimum	Maximum
1	1.9900000		1.9900000	1.9900000

Unique Subject Identifier=1814100000-0069

Assay variable : PTINR_VAL1 PT-INR Value

N	Mean	Standard Deviation	Minimum	Maximum
6	2.3416667	1.3300739	1.3200000	4.9500000

Unique Subject Identifier=1815100000-0044

Assay variable : PTINR_VAL1 PT-INR Value

N	Mean	Standard Deviation	Minimum	Maximum
5	1.5640000	0.2307163	1.3000000	1.8800000

Laboratory tests at the time of obtaining Table 2-6 consent, according to the presence or absence of proton-pump inhibitors

Unique Subject Identifier=1815100000-0051

MEANS Procedure

Assay variable : PTINR_VAL1 PT-INR Value

N	Mean	Standard Deviation	Minimum	Maximum
3	1.8600000	0.7807689	1.1000000	2.6600000

Unique Subject Identifier=1815100000-0069

Assay variable : PTINR_VAL1 PT-INR Value

N	Mean	Standard Deviation	Minimum	Maximum
3	2.3200000	0.4063250	1.8700000	2.6600000

Unique Subject Identifier=1815100000-0079

Assay variable : PTINR_VAL1 PT-INR Value

N	Mean	Standard Deviation	Minimum	Maximum
1	1.0600000		1.0600000	1.0600000

Unique Subject Identifier=1815100000-0080

Assay variable : PTINR_VAL1 PT-INR Value

N	Mean	Standard Deviation	Minimum	Maximum
2	1.8400000	0.0848528	1.7800000	1.9000000

Laboratory tests at the time of obtaining Table 2-6 consent, according to the presence or absence of proton-pump inhibitors

Unique Subject Identifier=1815100000-0082

MEANS Procedure

Assay variable : PTINR_VAL1 PT-INR Value

N	Mean	Standard Deviation	Minimum	Maximum
1	1.3500000		1.3500000	1.3500000

Unique Subject Identifier=1815100000-0093

Assay variable : PTINR_VAL1 PT-INR Value

N	Mean	Standard Deviation	Minimum	Maximum
6	1.8450000	0.2425490	1.4200000	2.0700000

Unique Subject Identifier=1973000000-0003

Assay variable : PTINR_VAL1 PT-INR Value

N	Mean	Standard Deviation	Minimum	Maximum
6	1.6666667	0.2250926	1.4000000	1.9000000

Unique Subject Identifier=1973000000-0006

Assay variable : PTINR_VAL1 PT-INR Value

N	Mean	Standard Deviation	Minimum	Maximum
6	2.3000000	0.2828427	2.0000000	2.7000000

Laboratory tests at the time of obtaining Table 2-6 consent, according to the presence or absence of proton-pump inhibitors

Unique Subject Identifier=1973000000-0011

MEANS Procedure

Assay variable : PTINR_VAL1 PT-INR Value

N	Mean	Standard Deviation	Minimum	Maximum
4	1.7750000	0.2500000	1.5000000	2.1000000

Unique Subject Identifier=2008010000-0011

Assay variable : PTINR_VAL1 PT-INR Value

N	Mean	Standard Deviation	Minimum	Maximum
6	1.7000000	0.1673320	1.5000000	1.9000000

Unique Subject Identifier=2008010000-0016

Assay variable : PTINR_VAL1 PT-INR Value

N	Mean	Standard Deviation	Minimum	Maximum
6	1.6833333	0.2562551	1.3000000	2.0000000

Unique Subject Identifier=2008010000-0018

Assay variable : PTINR_VAL1 PT-INR Value

N	Mean	Standard Deviation	Minimum	Maximum
3	1.2000000	0.1000000	1.1000000	1.3000000

Laboratory tests at the time of obtaining Table 2-6 consent, according to the presence or absence of proton-pump inhibitors

Unique Subject Identifier=2008010000-0023

MEANS Procedure

Assay variable : PTINR_VAL1 PT-INR Value

N	Mean	Standard Deviation	Minimum	Maximum
2	1.2750000	0.0777817	1.2200000	1.3300000

Unique Subject Identifier=2008010000-0027

Assay variable : PTINR_VAL1 PT-INR Value

N	Mean	Standard Deviation	Minimum	Maximum
4	1.9500000	0.4123106	1.5000000	2.3000000

Unique Subject Identifier=2008010000-0037

Assay variable : PTINR_VAL1 PT-INR Value

N	Mean	Standard Deviation	Minimum	Maximum
6	1.9500000	0.2073644	1.8000000	2.3000000

Unique Subject Identifier=2008010000-0043

Assay variable : PTINR_VAL1 PT-INR Value

N	Mean	Standard Deviation	Minimum	Maximum
4	1.9250000	0.2753785	1.6000000	2.2000000

Laboratory tests at the time of obtaining Table 2-6 consent, according to the presence or absence of proton-pump inhibitors

Unique Subject Identifier=2008010000-0046

MEANS Procedure

Assay variable : PTINR_VAL1 PT-INR Value

N	Mean	Standard Deviation	Minimum	Maximum
4	1.6000000	0.0816497	1.5000000	1.7000000

Unique Subject Identifier=2008010000-0047

Assay variable : PTINR_VAL1 PT-INR Value

N	Mean	Standard Deviation	Minimum	Maximum
6	1.9500000	0.5991661	1.3000000	2.8000000

Unique Subject Identifier=2008010000-0052

Assay variable : PTINR_VAL1 PT-INR Value

N	Mean	Standard Deviation	Minimum	Maximum
3	1.7000000	0.2645751	1.4000000	1.9000000

Unique Subject Identifier=2008010000-0055

Assay variable : PTINR_VAL1 PT-INR Value

N	Mean	Standard Deviation	Minimum	Maximum
1	1.0200000		1.0200000	1.0200000

Laboratory tests at the time of obtaining Table 2-6 consent, according to the presence or absence of proton-pump inhibitors

Unique Subject Identifier=2016011111-0002

MEANS Procedure

Assay variable : PTINR_VAL1 PT-INR Value

N	Mean	Standard Deviation	Minimum	Maximum
6	1.9450000	0.2212465	1.7600000	2.3200000

Unique Subject Identifier=2016011111-0004

Assay variable : PTINR_VAL1 PT-INR Value

N	Mean	Standard Deviation	Minimum	Maximum
5	2.0080000	0.1116692	1.8500000	2.1300000

Unique Subject Identifier=2016011111-0006

Assay variable : PTINR_VAL1 PT-INR Value

N	Mean	Standard Deviation	Minimum	Maximum
5	2.4700000	0.5487714	1.7300000	3.0300000

Unique Subject Identifier=2016011111-0010

Assay variable : PTINR_VAL1 PT-INR Value

N	Mean	Standard Deviation	Minimum	Maximum
5	2.1640000	0.6723689	1.5600000	3.3200000

Laboratory tests at the time of obtaining Table 2-6 consent, according to the presence or absence of proton-pump inhibitors

Unique Subject Identifier=2016011111-0017

MEANS Procedure

Assay variable : PTINR_VAL1 PT-INR Value

N	Mean	Standard Deviation	Minimum	Maximum
6	1.8916667	0.2997610	1.5700000	2.1700000

Unique Subject Identifier=2016011111-0019

Assay variable : PTINR_VAL1 PT-INR Value

N	Mean	Standard Deviation	Minimum	Maximum
5	2.4900000	0.3794074	2.0800000	2.9900000

Unique Subject Identifier=2016011111-0021

Assay variable : PTINR_VAL1 PT-INR Value

N	Mean	Standard Deviation	Minimum	Maximum
4	2.5000000	0.5707889	2.0900000	3.3400000

Unique Subject Identifier=2016011111-0025

Assay variable : PTINR_VAL1 PT-INR Value

N	Mean	Standard Deviation	Minimum	Maximum
5	2.6040000	0.3591379	2.3300000	3.1100000

Laboratory tests at the time of obtaining Table 2-6 consent, according to the presence or absence of proton-pump inhibitors

Unique Subject Identifier=2016011111-0028

MEANS Procedure

Assay variable : PTINR_VAL1 PT-INR Value

N	Mean	Standard Deviation	Minimum	Maximum
3	1.5066667	0.2458319	1.3500000	1.7900000

Unique Subject Identifier=2016011111-0030

Assay variable : PTINR_VAL1 PT-INR Value

N	Mean	Standard Deviation	Minimum	Maximum
5	2.0160000	0.4263567	1.6100000	2.5500000

Unique Subject Identifier=2016011111-0031

Assay variable : PTINR_VAL1 PT-INR Value

N	Mean	Standard Deviation	Minimum	Maximum
6	2.1166667	0.2801190	1.7700000	2.4500000

Unique Subject Identifier=2016011111-0034

Assay variable : PTINR_VAL1 PT-INR Value

N	Mean	Standard Deviation	Minimum	Maximum
3	2.3833333	0.1159023	2.2500000	2.4600000

Laboratory tests at the time of obtaining Table 2-6 consent, according to the presence or absence of proton-pump inhibitors

Unique Subject Identifier=2016011111-0035

MEANS Procedure

Assay variable : PTINR_VAL1 PT-INR Value

N	Mean	Standard Deviation	Minimum	Maximum
4	2.4100000	0.8089911	1.7700000	3.5600000

Unique Subject Identifier=2016011111-0036

Assay variable : PTINR_VAL1 PT-INR Value

N	Mean	Standard Deviation	Minimum	Maximum
4	2.7700000	0.7043673	2.2200000	3.8000000

Unique Subject Identifier=2016011111-0037

Assay variable : PTINR_VAL1 PT-INR Value

N	Mean	Standard Deviation	Minimum	Maximum
6	2.0900000	0.4848092	1.6100000	2.8500000

Unique Subject Identifier=2016011111-0040

Assay variable : PTINR_VAL1 PT-INR Value

N	Mean	Standard Deviation	Minimum	Maximum
5	2.3620000	0.9487465	1.4300000	3.9100000

Laboratory tests at the time of obtaining Table 2-6 consent, according to the presence or absence of proton-pump inhibitors

Unique Subject Identifier=2016011111-0043

MEANS Procedure

Assay variable : PTINR_VAL1 PT-INR Value

N	Mean	Standard Deviation	Minimum	Maximum
5	2.7300000	0.4640582	2.1500000	3.1900000

Unique Subject Identifier=2016011111-0050

Assay variable : PTINR_VAL1 PT-INR Value

N	Mean	Standard Deviation	Minimum	Maximum
4	2.2000000	0.1363818	2.0800000	2.3700000

Unique Subject Identifier=2016011111-0057

Assay variable : PTINR_VAL1 PT-INR Value

N	Mean	Standard Deviation	Minimum	Maximum
6	1.8666667	0.5833924	1.0800000	2.6700000

Unique Subject Identifier=2016011111-0060

Assay variable : PTINR_VAL1 PT-INR Value

N	Mean	Standard Deviation	Minimum	Maximum
6	1.6800000	0.2228901	1.4800000	2.1200000

Laboratory tests at the time of obtaining Table 2-6 consent, according to the presence or absence of proton-pump inhibitors

Unique Subject Identifier=2016011111-0062

MEANS Procedure

Assay variable : PTINR_VAL1 PT-INR Value

N	Mean	Standard Deviation	Minimum	Maximum
4	1.8775000	0.1364734	1.7100000	2.0200000

Unique Subject Identifier=2016011111-0063

Assay variable : PTINR_VAL1 PT-INR Value

N	Mean	Standard Deviation	Minimum	Maximum
5	2.4540000	0.1879628	2.2500000	2.7600000

Unique Subject Identifier=2016011111-0070

Assay variable : PTINR_VAL1 PT-INR Value

N	Mean	Standard Deviation	Minimum	Maximum
3	2.2966667	0.3450121	1.9500000	2.6400000

Unique Subject Identifier=2016011111-0081

Assay variable : PTINR_VAL1 PT-INR Value

N	Mean	Standard Deviation	Minimum	Maximum
3	2.3100000	0.2651415	2.0800000	2.6000000

Laboratory tests at the time of obtaining Table 2-6 consent, according to the presence or absence of proton-pump inhibitors

Unique Subject Identifier=2016011111-0082

MEANS Procedure

Assay variable : PTINR_VAL1 PT-INR Value

N	Mean	Standard Deviation	Minimum	Maximum
3	1.8933333	0.1850225	1.7100000	2.0800000

Unique Subject Identifier=2016011111-0085

Assay variable : PTINR_VAL1 PT-INR Value

N	Mean	Standard Deviation	Minimum	Maximum
4	2.0225000	0.2652515	1.7700000	2.3700000

Unique Subject Identifier=2016011111-0091

Assay variable : PTINR_VAL1 PT-INR Value

N	Mean	Standard Deviation	Minimum	Maximum
4	2.3850000	0.2666458	2.1700000	2.7400000

Unique Subject Identifier=2016011111-0095

Assay variable : PTINR_VAL1 PT-INR Value

N	Mean	Standard Deviation	Minimum	Maximum
3	2.3833333	0.5108163	1.8100000	2.7900000

Laboratory tests at the time of obtaining Table 2-6 consent, according to the presence or absence of proton-pump inhibitors

Unique Subject Identifier=2016011111-0096

MEANS Procedure

Assay variable : PTINR_VAL1 PT-INR Value

N	Mean	Standard Deviation	Minimum	Maximum
3	1.8700000	0.2497999	1.6700000	2.1500000

Unique Subject Identifier=2017011100-0002

Assay variable : PTINR_VAL1 PT-INR Value

N	Mean	Standard Deviation	Minimum	Maximum
5	2.2540000	0.6454688	1.4000000	3.2000000

Unique Subject Identifier=2017011100-0004

Assay variable : PTINR_VAL1 PT-INR Value

N	Mean	Standard Deviation	Minimum	Maximum
6	2.0483333	0.4900782	1.4500000	2.8500000

Unique Subject Identifier=2017011100-0011

Assay variable : PTINR_VAL1 PT-INR Value

N	Mean	Standard Deviation	Minimum	Maximum
3	1.0866667	0.1975686	0.9100000	1.3000000

Laboratory tests at the time of obtaining Table 2-6 consent, according to the presence or absence of proton-pump inhibitors

Unique Subject Identifier=2017011100-0014

MEANS Procedure

Assay variable : PTINR_VAL1 PT-INR Value

N	Mean	Standard Deviation	Minimum	Maximum
6	2.1366667	0.4337588	1.6300000	2.7800000

Unique Subject Identifier=2017011100-0017

Assay variable : PTINR_VAL1 PT-INR Value

N	Mean	Standard Deviation	Minimum	Maximum
6	1.5383333	0.2977527	1.0900000	1.8400000

Unique Subject Identifier=2017011100-0019

Assay variable : PTINR_VAL1 PT-INR Value

N	Mean	Standard Deviation	Minimum	Maximum
6	1.8083333	0.1414803	1.6400000	1.9400000

Unique Subject Identifier=2017011100-0020

Assay variable : PTINR_VAL1 PT-INR Value

N	Mean	Standard Deviation	Minimum	Maximum
6	2.0466667	0.4457204	1.5000000	2.6400000

Laboratory tests at the time of obtaining Table 2-6 consent, according to the presence or absence of proton-pump inhibitors

Unique Subject Identifier=2017011100-0023

MEANS Procedure

Assay variable : PTINR_VAL1 PT-INR Value

N	Mean	Standard Deviation	Minimum	Maximum
6	2.0133333	0.1944908	1.8400000	2.3700000

Unique Subject Identifier=2017011100-0027

Assay variable : PTINR_VAL1 PT-INR Value

N	Mean	Standard Deviation	Minimum	Maximum
6	1.9050000	0.0850294	1.8300000	2.0100000

Unique Subject Identifier=2017011100-0033

Assay variable : PTINR_VAL1 PT-INR Value

N	Mean	Standard Deviation	Minimum	Maximum
5	1.8620000	0.1572260	1.7200000	2.0900000

Unique Subject Identifier=2017011100-0037

Assay variable : PTINR_VAL1 PT-INR Value

N	Mean	Standard Deviation	Minimum	Maximum
5	2.0360000	0.3995372	1.7300000	2.7200000

Laboratory tests at the time of obtaining Table 2-6 consent, according to the presence or absence of proton-pump inhibitors

Unique Subject Identifier=2017011100-0038

MEANS Procedure

Assay variable : PTINR_VAL1 PT-INR Value

N	Mean	Standard Deviation	Minimum	Maximum
6	2.0600000	0.2354570	1.6600000	2.2700000

Unique Subject Identifier=2017011100-0047

Assay variable : PTINR_VAL1 PT-INR Value

N	Mean	Standard Deviation	Minimum	Maximum
5	2.0060000	0.0250998	1.9800000	2.0300000

Unique Subject Identifier=2017011100-0048

Assay variable : PTINR_VAL1 PT-INR Value

N	Mean	Standard Deviation	Minimum	Maximum
6	2.0516667	0.4543310	1.4200000	2.6100000

Unique Subject Identifier=2017011100-0049

Assay variable : PTINR_VAL1 PT-INR Value

N	Mean	Standard Deviation	Minimum	Maximum
6	2.1283333	0.2574814	1.8400000	2.4300000

Laboratory tests at the time of obtaining Table 2-6 consent, according to the presence or absence of proton-pump inhibitors

Unique Subject Identifier=2017011100-0053

MEANS Procedure

Assay variable : PTINR_VAL1 PT-INR Value

N	Mean	Standard Deviation	Minimum	Maximum
6	2.0550000	0.5021454	1.0800000	2.4900000

Unique Subject Identifier=2017011100-0056

Assay variable : PTINR_VAL1 PT-INR Value

N	Mean	Standard Deviation	Minimum	Maximum
6	1.8083333	0.4073532	1.4000000	2.5900000

Unique Subject Identifier=2017011100-0058

Assay variable : PTINR_VAL1 PT-INR Value

N	Mean	Standard Deviation	Minimum	Maximum
5	2.0100000	0.2955503	1.6100000	2.3300000

Unique Subject Identifier=2017011100-0060

Assay variable : PTINR_VAL1 PT-INR Value

N	Mean	Standard Deviation	Minimum	Maximum
6	1.7566667	0.6880310	1.0900000	2.9800000

Laboratory tests at the time of obtaining Table 2-6 consent, according to the presence or absence of proton-pump inhibitors

Unique Subject Identifier=2017011100-0061

MEANS Procedure

Assay variable : PTINR_VAL1 PT-INR Value

N	Mean	Standard Deviation	Minimum	Maximum
6	2.3733333	0.5452950	1.7700000	3.3300000

Unique Subject Identifier=2017011100-0062

Assay variable : PTINR_VAL1 PT-INR Value

N	Mean	Standard Deviation	Minimum	Maximum
6	1.9933333	0.3309179	1.4500000	2.3300000

Unique Subject Identifier=2017011100-0064

Assay variable : PTINR_VAL1 PT-INR Value

N	Mean	Standard Deviation	Minimum	Maximum
6	1.9000000	0.3244072	1.5400000	2.3500000

Unique Subject Identifier=2017011100-0075

Assay variable : PTINR_VAL1 PT-INR Value

N	Mean	Standard Deviation	Minimum	Maximum
6	2.3216667	0.7310928	1.4500000	3.3300000

Laboratory tests at the time of obtaining Table 2-6 consent, according to the presence or absence of proton-pump inhibitors

Unique Subject Identifier=2017011100-0076

MEANS Procedure

Assay variable : PTINR_VAL1 PT-INR Value

N	Mean	Standard Deviation	Minimum	Maximum
5	1.9520000	0.4097804	1.5900000	2.5700000

Unique Subject Identifier=2017011100-0082

Assay variable : PTINR_VAL1 PT-INR Value

N	Mean	Standard Deviation	Minimum	Maximum
5	2.2040000	0.4834563	1.8400000	3.0200000

Unique Subject Identifier=2017011100-0085

Assay variable : PTINR_VAL1 PT-INR Value

N	Mean	Standard Deviation	Minimum	Maximum
6	1.8333333	0.5709524	1.1100000	2.4900000

Unique Subject Identifier=2017011100-0092

Assay variable : PTINR_VAL1 PT-INR Value

N	Mean	Standard Deviation	Minimum	Maximum
6	1.9066667	0.2593582	1.5600000	2.1900000

Laboratory tests at the time of obtaining Table 2-6 consent, according to the presence or absence of proton-pump inhibitors

Unique Subject Identifier=2017011100-0098

MEANS Procedure

Assay variable : PTINR_VAL1 PT-INR Value

N	Mean	Standard Deviation	Minimum	Maximum
5	2.0640000	0.4530232	1.5600000	2.5900000

Unique Subject Identifier=2017011100-0100

Assay variable : PTINR_VAL1 PT-INR Value

N	Mean	Standard Deviation	Minimum	Maximum
6	2.1566667	0.4348640	1.4200000	2.7600000

Unique Subject Identifier=2017011100-0109

Assay variable : PTINR_VAL1 PT-INR Value

N	Mean	Standard Deviation	Minimum	Maximum
5	1.6940000	0.1669731	1.4500000	1.8600000

Unique Subject Identifier=2017011100-0115

Assay variable : PTINR_VAL1 PT-INR Value

N	Mean	Standard Deviation	Minimum	Maximum
6	2.3416667	0.4252725	2.0000000	3.0400000

Laboratory tests at the time of obtaining Table 2-6 consent, according to the presence or absence of proton-pump inhibitors

Unique Subject Identifier=2017011100-0118

MEANS Procedure

Assay variable : PTINR_VAL1 PT-INR Value

N	Mean	Standard Deviation	Minimum	Maximum
6	2.1233333	0.2985074	1.8300000	2.6800000

Unique Subject Identifier=2017011100-0120

Assay variable : PTINR_VAL1 PT-INR Value

N	Mean	Standard Deviation	Minimum	Maximum
6	2.1383333	0.1697547	1.9200000	2.3700000

Unique Subject Identifier=2018000000-0007

Assay variable : PTINR_VAL1 PT-INR Value

N	Mean	Standard Deviation	Minimum	Maximum
6	1.2283333	0.0604704	1.1700000	1.3400000

Unique Subject Identifier=2018000000-0008

Assay variable : PTINR_VAL1 PT-INR Value

N	Mean	Standard Deviation	Minimum	Maximum
3	2.0666667	0.1289703	1.9600000	2.2100000

Laboratory tests at the time of obtaining Table 2-6 consent, according to the presence or absence of proton-pump inhibitors

Unique Subject Identifier=2018000000-0012

MEANS Procedure

Assay variable : PTINR_VAL1 PT-INR Value

N	Mean	Standard Deviation	Minimum	Maximum
2	1.4000000	0.0565685	1.3600000	1.4400000

Unique Subject Identifier=2018000000-0013

Assay variable : PTINR_VAL1 PT-INR Value

N	Mean	Standard Deviation	Minimum	Maximum
2	1.8000000	0.1414214	1.7000000	1.9000000

Unique Subject Identifier=2026011111-0008

Assay variable : PTINR_VAL1 PT-INR Value

N	Mean	Standard Deviation	Minimum	Maximum
6	2.2666667	0.2422120	1.9000000	2.5000000

Unique Subject Identifier=2038000000-0005

Assay variable : PTINR_VAL1 PT-INR Value

N	Mean	Standard Deviation	Minimum	Maximum
3	1.9933333	0.3225420	1.6500000	2.2900000

Laboratory tests at the time of obtaining Table 2-6 consent, according to the presence or absence of proton-pump inhibitors

Unique Subject Identifier=2038000000-0006

MEANS Procedure

Assay variable : PTINR_VAL1 PT-INR Value

N	Mean	Standard Deviation	Minimum	Maximum
3	2.0133333	0.6656075	1.2900000	2.6000000

Unique Subject Identifier=2038000000-0007

Assay variable : PTINR_VAL1 PT-INR Value

N	Mean	Standard Deviation	Minimum	Maximum
3	2.0933333	0.3108590	1.8800000	2.4500000

Unique Subject Identifier=2038000000-0009

Assay variable : PTINR_VAL1 PT-INR Value

N	Mean	Standard Deviation	Minimum	Maximum
4	2.1650000	0.5704092	1.3400000	2.6500000

Unique Subject Identifier=2047011111-0007

Assay variable : PTINR_VAL1 PT-INR Value

N	Mean	Standard Deviation	Minimum	Maximum
6	1.6216667	0.2102776	1.3700000	1.9300000

Laboratory tests at the time of obtaining Table 2-6 consent, according to the presence or absence of proton-pump inhibitors

Unique Subject Identifier=2048011011-0045

MEANS Procedure

Assay variable : PTINR_VAL1 PT-INR Value

N	Mean	Standard Deviation	Minimum	Maximum
3	2.0000000	0.2645751	1.8000000	2.3000000

Unique Subject Identifier=2048011011-0048

Assay variable : PTINR_VAL1 PT-INR Value

N	Mean	Standard Deviation	Minimum	Maximum
6	2.0166667	1.0068101	1.1000000	3.3000000

Unique Subject Identifier=2048011011-0049

Assay variable : PTINR_VAL1 PT-INR Value

N	Mean	Standard Deviation	Minimum	Maximum
3	2.1666667	0.2081666	2.0000000	2.4000000

Unique Subject Identifier=2048011011-0052

Assay variable : PTINR_VAL1 PT-INR Value

N	Mean	Standard Deviation	Minimum	Maximum
3	1.4000000	0.5000000	0.9000000	1.9000000

Laboratory tests at the time of obtaining Table 2-6 consent, according to the presence or absence of proton-pump inhibitors

Unique Subject Identifier=2048011011-0053

MEANS Procedure

Assay variable : PTINR_VAL1 PT-INR Value

N	Mean	Standard Deviation	Minimum	Maximum
2	2.7500000	0.0707107	2.7000000	2.8000000

Unique Subject Identifier=2048011011-0056

Assay variable : PTINR_VAL1 PT-INR Value

N	Mean	Standard Deviation	Minimum	Maximum
3	2.6666667	0.1527525	2.5000000	2.8000000

Unique Subject Identifier=2048011011-0057

Assay variable : PTINR_VAL1 PT-INR Value

N	Mean	Standard Deviation	Minimum	Maximum
1	2.0000000		2.0000000	2.0000000

Unique Subject Identifier=2048011011-0070

Assay variable : PTINR_VAL1 PT-INR Value

N	Mean	Standard Deviation	Minimum	Maximum
3	2.5000000	0.4582576	2.1000000	3.0000000

Laboratory tests at the time of obtaining Table 2-6 consent, according to the presence or absence of proton-pump inhibitors

Unique Subject Identifier=2058011111-0006

MEANS Procedure

Assay variable : PTINR_VAL1 PT-INR Value

N	Mean	Standard Deviation	Minimum	Maximum
1	1.7100000		1.7100000	1.7100000

Unique Subject Identifier=2077011111-0001

Assay variable : PTINR_VAL1 PT-INR Value

N	Mean	Standard Deviation	Minimum	Maximum
6	2.2450000	0.7159539	1.3900000	3.2200000

Unique Subject Identifier=2077011111-0010

Assay variable : PTINR_VAL1 PT-INR Value

N	Mean	Standard Deviation	Minimum	Maximum
6	1.6716667	0.1094380	1.5400000	1.8000000

Unique Subject Identifier=2077011111-0016

Assay variable : PTINR_VAL1 PT-INR Value

N	Mean	Standard Deviation	Minimum	Maximum
6	2.0883333	0.8065089	1.1900000	3.4000000

Laboratory tests at the time of obtaining Table 2-6 consent, according to the presence or absence of proton-pump inhibitors

Unique Subject Identifier=2092011111-0004

MEANS Procedure

Assay variable : PTINR_VAL1 PT-INR Value

N	Mean	Standard Deviation	Minimum	Maximum
1	3.0500000		3.0500000	3.0500000

Unique Subject Identifier=2096011111-0001

Assay variable : PTINR_VAL1 PT-INR Value

N	Mean	Standard Deviation	Minimum	Maximum
2	1.3400000	0.1131371	1.2600000	1.4200000

Unique Subject Identifier=2107011000-0036

Assay variable : PTINR_VAL1 PT-INR Value

N	Mean	Standard Deviation	Minimum	Maximum
3	2.0533333	0.6321656	1.3300000	2.5000000

Unique Subject Identifier=2108011000-0002

Assay variable : PTINR_VAL1 PT-INR Value

N	Mean	Standard Deviation	Minimum	Maximum
3	3.4333333	1.6041613	1.9000000	5.1000000

Laboratory tests at the time of obtaining Table 2-6 consent, according to the presence or absence of proton-pump inhibitors

Unique Subject Identifier=2112011011-0001

MEANS Procedure

Assay variable : PTINR_VAL1 PT-INR Value

N	Mean	Standard Deviation	Minimum	Maximum
3	2.2266667	0.0680686	2.1500000	2.2800000

Unique Subject Identifier=2112011011-0006

Assay variable : PTINR_VAL1 PT-INR Value

N	Mean	Standard Deviation	Minimum	Maximum
5	1.9400000	0.1846619	1.7500000	2.1700000

Unique Subject Identifier=2112011011-0008

Assay variable : PTINR_VAL1 PT-INR Value

N	Mean	Standard Deviation	Minimum	Maximum
5	2.4060000	0.3755396	2.0000000	2.8700000

Unique Subject Identifier=2113010111-0001

Assay variable : PTINR_VAL1 PT-INR Value

N	Mean	Standard Deviation	Minimum	Maximum
2	1.8700000	0.2121320	1.7200000	2.0200000

Laboratory tests at the time of obtaining Table 2-6 consent, according to the presence or absence of proton-pump inhibitors

Unique Subject Identifier=2113010111-0006

MEANS Procedure

Assay variable : PTINR_VAL1 PT-INR Value

N	Mean	Standard Deviation	Minimum	Maximum
3	1.8500000	0.2066398	1.6800000	2.0800000

Unique Subject Identifier=2113010111-0015

Assay variable : PTINR_VAL1 PT-INR Value

N	Mean	Standard Deviation	Minimum	Maximum
1	1.2100000		1.2100000	1.2100000

Unique Subject Identifier=2115011111-0025

Assay variable : PTINR_VAL1 PT-INR Value

N	Mean	Standard Deviation	Minimum	Maximum
3	1.6333333	0.1527525	1.5000000	1.8000000

Unique Subject Identifier=2118000000-0005

Assay variable : PTINR_VAL1 PT-INR Value

N	Mean	Standard Deviation	Minimum	Maximum
2	1.5550000	0.2192031	1.4000000	1.7100000

Laboratory tests at the time of obtaining Table 2-6 consent, according to the presence or absence of proton-pump inhibitors

Unique Subject Identifier=2119011111-0001

MEANS Procedure

Assay variable : PTINR_VAL1 PT-INR Value

N	Mean	Standard Deviation	Minimum	Maximum
5	1.8540000	0.1637987	1.6300000	2.0800000

Unique Subject Identifier=2123010000-0004

Assay variable : PTINR_VAL1 PT-INR Value

N	Mean	Standard Deviation	Minimum	Maximum
6	2.3166667	0.1722401	2.1000000	2.6000000

Unique Subject Identifier=2123010000-0007

Assay variable : PTINR_VAL1 PT-INR Value

N	Mean	Standard Deviation	Minimum	Maximum
6	1.8666667	0.2065591	1.7000000	2.2000000

Unique Subject Identifier=2123010000-0009

Assay variable : PTINR_VAL1 PT-INR Value

N	Mean	Standard Deviation	Minimum	Maximum
6	2.0500000	0.4183300	1.6000000	2.8000000

Laboratory tests at the time of obtaining Table 2-6 consent, according to the presence or absence of proton-pump inhibitors

Unique Subject Identifier=2123010000-0011

MEANS Procedure

Assay variable : PTINR_VAL1 PT-INR Value

N	Mean	Standard Deviation	Minimum	Maximum
6	1.9666667	0.2160247	1.7000000	2.3000000

Unique Subject Identifier=2123010000-0012

Assay variable : PTINR_VAL1 PT-INR Value

N	Mean	Standard Deviation	Minimum	Maximum
6	1.5833333	0.1722401	1.3000000	1.8000000

Unique Subject Identifier=2123010000-0013

Assay variable : PTINR_VAL1 PT-INR Value

N	Mean	Standard Deviation	Minimum	Maximum
5	1.9600000	0.2966479	1.5000000	2.3000000

Unique Subject Identifier=2123010000-0015

Assay variable : PTINR_VAL1 PT-INR Value

N	Mean	Standard Deviation	Minimum	Maximum
6	2.0833333	0.1834848	1.8000000	2.2000000

Laboratory tests at the time of obtaining Table 2-6 consent, according to the presence or absence of proton-pump inhibitors

Unique Subject Identifier=2123010000-0016

MEANS Procedure

Assay variable : PTINR_VAL1 PT-INR Value

N	Mean	Standard Deviation	Minimum	Maximum
6	1.9333333	0.2160247	1.7000000	2.2000000

Unique Subject Identifier=2123010000-0017

Assay variable : PTINR_VAL1 PT-INR Value

N	Mean	Standard Deviation	Minimum	Maximum
4	2.2250000	0.2986079	1.8000000	2.5000000

Unique Subject Identifier=2123010000-0020

Assay variable : PTINR_VAL1 PT-INR Value

N	Mean	Standard Deviation	Minimum	Maximum
4	2.0250000	0.4272002	1.5000000	2.5000000

Unique Subject Identifier=2123010000-0026

Assay variable : PTINR_VAL1 PT-INR Value

N	Mean	Standard Deviation	Minimum	Maximum
6	1.9666667	0.0816497	1.9000000	2.1000000

Laboratory tests at the time of obtaining Table 2-6 consent, according to the presence or absence of proton-pump inhibitors

Unique Subject Identifier=2123010000-0029

MEANS Procedure

Assay variable : PTINR_VAL1 PT-INR Value

N	Mean	Standard Deviation	Minimum	Maximum
6	1.6833333	0.1940790	1.5000000	2.0000000

Unique Subject Identifier=2125011111-0002

Assay variable : PTINR_VAL1 PT-INR Value

N	Mean	Standard Deviation	Minimum	Maximum
5	2.3140000	0.1633401	2.1500000	2.5400000

Unique Subject Identifier=2125011111-0003

Assay variable : PTINR_VAL1 PT-INR Value

N	Mean	Standard Deviation	Minimum	Maximum
5	2.2660000	0.2591910	1.9700000	2.6400000

Unique Subject Identifier=2125011111-0011

Assay variable : PTINR_VAL1 PT-INR Value

N	Mean	Standard Deviation	Minimum	Maximum
2	1.9150000	0.0353553	1.8900000	1.9400000

Laboratory tests at the time of obtaining Table 2-6 consent, according to the presence or absence of proton-pump inhibitors

Unique Subject Identifier=2125011111-0013

MEANS Procedure

Assay variable : PTINR_VAL1 PT-INR Value

N	Mean	Standard Deviation	Minimum	Maximum
5	2.3240000	0.7497866	1.4600000	3.4300000

Unique Subject Identifier=2125011111-0027

Assay variable : PTINR_VAL1 PT-INR Value

N	Mean	Standard Deviation	Minimum	Maximum
6	2.3683333	1.0923629	1.1900000	3.9600000

Unique Subject Identifier=2127011011-0004

Assay variable : PTINR_VAL1 PT-INR Value

N	Mean	Standard Deviation	Minimum	Maximum
4	2.0450000	0.3831014	1.7800000	2.6000000

Unique Subject Identifier=2127011011-0006

Assay variable : PTINR_VAL1 PT-INR Value

N	Mean	Standard Deviation	Minimum	Maximum
6	1.5500000	0.1516575	1.4000000	1.8000000

Laboratory tests at the time of obtaining Table 2-6 consent, according to the presence or absence of proton-pump inhibitors

Unique Subject Identifier=2127011011-0008

MEANS Procedure

Assay variable : PTINR_VAL1 PT-INR Value

N	Mean	Standard Deviation	Minimum	Maximum
5	1.5000000	0.1581139	1.3000000	1.7000000

Unique Subject Identifier=2127011011-0010

Assay variable : PTINR_VAL1 PT-INR Value

N	Mean	Standard Deviation	Minimum	Maximum
5	1.9200000	0.6180615	1.3000000	2.8000000

Unique Subject Identifier=2127011011-0012

Assay variable : PTINR_VAL1 PT-INR Value

N	Mean	Standard Deviation	Minimum	Maximum
6	1.5000000	0.1414214	1.3000000	1.7000000

Unique Subject Identifier=2127011011-0016

Assay variable : PTINR_VAL1 PT-INR Value

N	Mean	Standard Deviation	Minimum	Maximum
3	1.7000000	0.1000000	1.6000000	1.8000000

Laboratory tests at the time of obtaining Table 2-6 consent, according to the presence or absence of proton-pump inhibitors

Unique Subject Identifier=2127011011-0017

MEANS Procedure

Assay variable : PTINR_VAL1 PT-INR Value

N	Mean	Standard Deviation	Minimum	Maximum
3	1.1666667	0.1527525	1.0000000	1.3000000

Unique Subject Identifier=2127011011-0018

Assay variable : PTINR_VAL1 PT-INR Value

N	Mean	Standard Deviation	Minimum	Maximum
3	1.6333333	0.1527525	1.5000000	1.8000000

Unique Subject Identifier=2127011011-0019

Assay variable : PTINR_VAL1 PT-INR Value

N	Mean	Standard Deviation	Minimum	Maximum
5	1.5000000	0.2449490	1.3000000	1.9000000

Unique Subject Identifier=2127011011-0021

Assay variable : PTINR_VAL1 PT-INR Value

N	Mean	Standard Deviation	Minimum	Maximum
1	2.0000000		2.0000000	2.0000000

Laboratory tests at the time of obtaining Table 2-6 consent, according to the presence or absence of proton-pump inhibitors

Unique Subject Identifier=2127011011-0024

MEANS Procedure

Assay variable : PTINR_VAL1 PT-INR Value

N	Mean	Standard Deviation	Minimum	Maximum
6	1.8833333	0.1169045	1.7000000	2.0000000

Unique Subject Identifier=2127011011-0025

Assay variable : PTINR_VAL1 PT-INR Value

N	Mean	Standard Deviation	Minimum	Maximum
6	1.8833333	0.2401388	1.6000000	2.3000000

Unique Subject Identifier=2127011011-0026

Assay variable : PTINR_VAL1 PT-INR Value

N	Mean	Standard Deviation	Minimum	Maximum
5	1.9800000	0.4147288	1.4000000	2.4000000

Unique Subject Identifier=2127011011-0027

Assay variable : PTINR_VAL1 PT-INR Value

N	Mean	Standard Deviation	Minimum	Maximum
2	1.5500000	0.0707107	1.5000000	1.6000000

Laboratory tests at the time of obtaining Table 2-6 consent, according to the presence or absence of proton-pump inhibitors

Unique Subject Identifier=2127011011-0030

MEANS Procedure

Assay variable : PTINR_VAL1 PT-INR Value

N	Mean	Standard Deviation	Minimum	Maximum
3	1.3333333	0.1154701	1.2000000	1.4000000

Unique Subject Identifier=2127011011-0034

Assay variable : PTINR_VAL1 PT-INR Value

N	Mean	Standard Deviation	Minimum	Maximum
2	2.0000000	0.7071068	1.5000000	2.5000000

Unique Subject Identifier=2127011011-0036

Assay variable : PTINR_VAL1 PT-INR Value

N	Mean	Standard Deviation	Minimum	Maximum
6	1.6500000	0.2428992	1.3000000	1.9000000

Unique Subject Identifier=2127011011-0046

Assay variable : PTINR_VAL1 PT-INR Value

N	Mean	Standard Deviation	Minimum	Maximum
4	1.4600000	0.1720465	1.2800000	1.6600000

Laboratory tests at the time of obtaining Table 2-6 consent, according to the presence or absence of proton-pump inhibitors

Unique Subject Identifier=2128010111-0001

MEANS Procedure

Assay variable : PTINR_VAL1 PT-INR Value

N	Mean	Standard Deviation	Minimum	Maximum
1	1.7600000		1.7600000	1.7600000

Unique Subject Identifier=2134011111-0013

Assay variable : PTINR_VAL1 PT-INR Value

N	Mean	Standard Deviation	Minimum	Maximum
1	1.6000000		1.6000000	1.6000000

Unique Subject Identifier=2138011111-0004

Assay variable : PTINR_VAL1 PT-INR Value

N	Mean	Standard Deviation	Minimum	Maximum
3	1.9266667	0.0416333	1.8800000	1.9600000

Unique Subject Identifier=2138011111-0021

Assay variable : PTINR_VAL1 PT-INR Value

N	Mean	Standard Deviation	Minimum	Maximum
5	1.9760000	0.2956011	1.5700000	2.3700000

Laboratory tests at the time of obtaining Table 2-6 consent, according to the presence or absence of proton-pump inhibitors

Unique Subject Identifier=2138011111-0026

MEANS Procedure

Assay variable : PTINR_VAL1 PT-INR Value

N	Mean	Standard Deviation	Minimum	Maximum
6	1.8516667	0.2762185	1.4400000	2.1600000

Unique Subject Identifier=2138011111-0027

Assay variable : PTINR_VAL1 PT-INR Value

N	Mean	Standard Deviation	Minimum	Maximum
6	2.4216667	0.5288257	1.4700000	2.8100000

Unique Subject Identifier=2138011111-0030

Assay variable : PTINR_VAL1 PT-INR Value

N	Mean	Standard Deviation	Minimum	Maximum
3	2.0333333	0.1814754	1.8400000	2.2000000

Unique Subject Identifier=2138011111-0032

Assay variable : PTINR_VAL1 PT-INR Value

N	Mean	Standard Deviation	Minimum	Maximum
5	1.9460000	0.2081586	1.6900000	2.1600000

Laboratory tests at the time of obtaining Table 2-6 consent, according to the presence or absence of proton-pump inhibitors

Unique Subject Identifier=2138011111-0033

MEANS Procedure

Assay variable : PTINR_VAL1 PT-INR Value

N	Mean	Standard Deviation	Minimum	Maximum
1	1.2600000		1.2600000	1.2600000

Unique Subject Identifier=2138011111-0040

Assay variable : PTINR_VAL1 PT-INR Value

N	Mean	Standard Deviation	Minimum	Maximum
5	1.9100000	0.2065188	1.6000000	2.1700000

Unique Subject Identifier=2138011111-0044

Assay variable : PTINR_VAL1 PT-INR Value

N	Mean	Standard Deviation	Minimum	Maximum
3	1.6733333	0.0896289	1.5700000	1.7300000

Unique Subject Identifier=2138011111-0049

Assay variable : PTINR_VAL1 PT-INR Value

N	Mean	Standard Deviation	Minimum	Maximum
4	2.3825000	0.2698611	1.9900000	2.6000000

Laboratory tests at the time of obtaining Table 2-6 consent, according to the presence or absence of proton-pump inhibitors

Unique Subject Identifier=2138011111-0054

MEANS Procedure

Assay variable : PTINR_VAL1 PT-INR Value

N	Mean	Standard Deviation	Minimum	Maximum
3	2.2233333	0.5254839	1.9100000	2.8300000

Unique Subject Identifier=2138011111-0055

Assay variable : PTINR_VAL1 PT-INR Value

N	Mean	Standard Deviation	Minimum	Maximum
6	2.1650000	0.5103234	1.3100000	2.7600000

Unique Subject Identifier=2138011111-0056

Assay variable : PTINR_VAL1 PT-INR Value

N	Mean	Standard Deviation	Minimum	Maximum
6	1.2950000	0.2700926	1.0500000	1.6900000

Unique Subject Identifier=2138011111-0062

Assay variable : PTINR_VAL1 PT-INR Value

N	Mean	Standard Deviation	Minimum	Maximum
3	1.8233333	0.1154701	1.6900000	1.8900000

Laboratory tests at the time of obtaining Table 2-6 consent, according to the presence or absence of proton-pump inhibitors

Unique Subject Identifier=2138011111-0065

MEANS Procedure

Assay variable : PTINR_VAL1 PT-INR Value

N	Mean	Standard Deviation	Minimum	Maximum
6	2.1066667	0.2028464	1.8600000	2.3400000

Unique Subject Identifier=2140011100-0004

Assay variable : PTINR_VAL1 PT-INR Value

N	Mean	Standard Deviation	Minimum	Maximum
5	2.3000000	0.7905694	1.7000000	3.6000000

Unique Subject Identifier=2148011111-0003

Assay variable : PTINR_VAL1 PT-INR Value

N	Mean	Standard Deviation	Minimum	Maximum
1	1.2600000		1.2600000	1.2600000

Unique Subject Identifier=2148011111-0006

Assay variable : PTINR_VAL1 PT-INR Value

N	Mean	Standard Deviation	Minimum	Maximum
3	1.0333333	0.0577350	1.0000000	1.1000000

Laboratory tests at the time of obtaining Table 2-6 consent, according to the presence or absence of proton-pump inhibitors

Unique Subject Identifier=2155011111-0008

MEANS Procedure

Assay variable : PTINR_VAL1 PT-INR Value

N	Mean	Standard Deviation	Minimum	Maximum
4	2.6150000	0.4291464	2.1800000	3.2000000

Unique Subject Identifier=2155011111-0017

Assay variable : PTINR_VAL1 PT-INR Value

N	Mean	Standard Deviation	Minimum	Maximum
1	1.7000000		1.7000000	1.7000000

Unique Subject Identifier=2157011111-0004

Assay variable : PTINR_VAL1 PT-INR Value

N	Mean	Standard Deviation	Minimum	Maximum
6	2.2500000	0.2258318	2.0000000	2.6000000

Unique Subject Identifier=2163000000-0027

Assay variable : PTINR_VAL1 PT-INR Value

N	Mean	Standard Deviation	Minimum	Maximum
2	1.8500000	0.0707107	1.8000000	1.9000000

Laboratory tests at the time of obtaining Table 2-6 consent, according to the presence or absence of proton-pump inhibitors

Unique Subject Identifier=2163000000-0051

MEANS Procedure

Assay variable : PTINR_VAL1 PT-INR Value

N	Mean	Standard Deviation	Minimum	Maximum
1	2.3000000		2.3000000	2.3000000

Unique Subject Identifier=2163000000-0058

Assay variable : PTINR_VAL1 PT-INR Value

N	Mean	Standard Deviation	Minimum	Maximum
2	2.0000000	0	2.0000000	2.0000000

Unique Subject Identifier=2176011011-0028

Assay variable : PTINR_VAL1 PT-INR Value

N	Mean	Standard Deviation	Minimum	Maximum
1	1.7700000		1.7700000	1.7700000

Unique Subject Identifier=2183011011-0007

Assay variable : PTINR_VAL1 PT-INR Value

N	Mean	Standard Deviation	Minimum	Maximum
3	2.1333333	0.4509250	1.7000000	2.6000000

Laboratory tests at the time of obtaining Table 2-6 consent, according to the presence or absence of proton-pump inhibitors

Unique Subject Identifier=2183011011-0008

MEANS Procedure

Assay variable : PTINR_VAL1 PT-INR Value

N	Mean	Standard Deviation	Minimum	Maximum
3	1.4666667	0.0577350	1.4000000	1.5000000

Unique Subject Identifier=2183011011-0011

Assay variable : PTINR_VAL1 PT-INR Value

N	Mean	Standard Deviation	Minimum	Maximum
2	2.1000000	0	2.1000000	2.1000000

Unique Subject Identifier=2183011011-0012

Assay variable : PTINR_VAL1 PT-INR Value

N	Mean	Standard Deviation	Minimum	Maximum
3	1.8666667	0.2309401	1.6000000	2.0000000

Unique Subject Identifier=2183011011-0017

Assay variable : PTINR_VAL1 PT-INR Value

N	Mean	Standard Deviation	Minimum	Maximum
2	2.1000000	0.5656854	1.7000000	2.5000000

Laboratory tests at the time of obtaining Table 2-6 consent, according to the presence or absence of proton-pump inhibitors

Unique Subject Identifier=2185010010-0002

MEANS Procedure

Assay variable : PTINR_VAL1 PT-INR Value

N	Mean	Standard Deviation	Minimum	Maximum
1	1.7200000		1.7200000	1.7200000

Unique Subject Identifier=2185010010-0004

Assay variable : PTINR_VAL1 PT-INR Value

N	Mean	Standard Deviation	Minimum	Maximum
6	1.9833333	0.1471960	1.8000000	2.1000000

Unique Subject Identifier=2185010010-0007

Assay variable : PTINR_VAL1 PT-INR Value

N	Mean	Standard Deviation	Minimum	Maximum
6	2.3333333	0.4802777	1.6000000	2.8000000

Unique Subject Identifier=2185010010-0010

Assay variable : PTINR_VAL1 PT-INR Value

N	Mean	Standard Deviation	Minimum	Maximum
5	2.2200000	0.4658326	1.4000000	2.5000000

Laboratory tests at the time of obtaining Table 2-6 consent, according to the presence or absence of proton-pump inhibitors

Unique Subject Identifier=2191011011-0005

MEANS Procedure

Assay variable : PTINR_VAL1 PT-INR Value

N	Mean	Standard Deviation	Minimum	Maximum
2	1.5950000	0.0353553	1.5700000	1.6200000

Unique Subject Identifier=2191011011-0008

Assay variable : PTINR_VAL1 PT-INR Value

N	Mean	Standard Deviation	Minimum	Maximum
3	1.5233333	0.1422439	1.3600000	1.6200000

Unique Subject Identifier=2191011011-0012

Assay variable : PTINR_VAL1 PT-INR Value

N	Mean	Standard Deviation	Minimum	Maximum
1	1.5100000		1.5100000	1.5100000

Unique Subject Identifier=2191011011-0013

Assay variable : PTINR_VAL1 PT-INR Value

N	Mean	Standard Deviation	Minimum	Maximum
1	1.7300000		1.7300000	1.7300000

Laboratory tests at the time of obtaining Table 2-6 consent, according to the presence or absence of proton-pump inhibitors

Unique Subject Identifier=2192010011-0002

MEANS Procedure

Assay variable : PTINR_VAL1 PT-INR Value

N	Mean	Standard Deviation	Minimum	Maximum
6	2.4333333	0.2943920	2.1000000	2.7000000

Unique Subject Identifier=2200000000-0001

Assay variable : PTINR_VAL1 PT-INR Value

N	Mean	Standard Deviation	Minimum	Maximum
3	2.6166667	0.8975708	1.8900000	3.6200000

Unique Subject Identifier=2203011010-0002

Assay variable : PTINR_VAL1 PT-INR Value

N	Mean	Standard Deviation	Minimum	Maximum
5	1.7800000	0.8671793	1.0000000	3.0000000

Unique Subject Identifier=2205001111-0003

Assay variable : PTINR_VAL1 PT-INR Value

N	Mean	Standard Deviation	Minimum	Maximum
6	1.9000000	0.4690416	1.3000000	2.6000000

Laboratory tests at the time of obtaining Table 2-6 consent, according to the presence or absence of proton-pump inhibitors

Unique Subject Identifier=2205001111-0018

MEANS Procedure

Assay variable : PTINR_VAL1 PT-INR Value

N	Mean	Standard Deviation	Minimum	Maximum
6	2.4666667	0.3502380	1.9000000	2.9000000

Unique Subject Identifier=2205001111-0025

Assay variable : PTINR_VAL1 PT-INR Value

N	Mean	Standard Deviation	Minimum	Maximum
6	2.1333333	0.1032796	2.0000000	2.3000000

Unique Subject Identifier=2205001111-0041

Assay variable : PTINR_VAL1 PT-INR Value

N	Mean	Standard Deviation	Minimum	Maximum
6	1.8166667	0.1471960	1.7000000	2.0000000

Unique Subject Identifier=2205001111-0046

Assay variable : PTINR_VAL1 PT-INR Value

N	Mean	Standard Deviation	Minimum	Maximum
4	2.1325000	0.1699755	1.9000000	2.3000000

Laboratory tests at the time of obtaining Table 2-6 consent, according to the presence or absence of proton-pump inhibitors

Unique Subject Identifier=2211011111-0003

MEANS Procedure

Assay variable : PTINR_VAL1 PT-INR Value

N	Mean	Standard Deviation	Minimum	Maximum
1	1.1000000		1.1000000	1.1000000

Unique Subject Identifier=2211011111-0038

Assay variable : PTINR_VAL1 PT-INR Value

N	Mean	Standard Deviation	Minimum	Maximum
4	1.9250000	0.1892969	1.8000000	2.2000000

Unique Subject Identifier=2211011111-0054

Assay variable : PTINR_VAL1 PT-INR Value

N	Mean	Standard Deviation	Minimum	Maximum
1	1.3500000		1.3500000	1.3500000

Unique Subject Identifier=2211011111-0057

Assay variable : PTINR_VAL1 PT-INR Value

N	Mean	Standard Deviation	Minimum	Maximum
1	1.6000000		1.6000000	1.6000000

Laboratory tests at the time of obtaining Table 2-6 consent, according to the presence or absence of proton-pump inhibitors

Unique Subject Identifier=2214011111-0003

MEANS Procedure

Assay variable : PTINR_VAL1 PT-INR Value

N	Mean	Standard Deviation	Minimum	Maximum
6	2.1683333	0.2604163	1.8900000	2.5900000

Unique Subject Identifier=2227010011-0003

Assay variable : PTINR_VAL1 PT-INR Value

N	Mean	Standard Deviation	Minimum	Maximum
1	1.9000000		1.9000000	1.9000000

Unique Subject Identifier=2227010011-0011

Assay variable : PTINR_VAL1 PT-INR Value

N	Mean	Standard Deviation	Minimum	Maximum
1	2.8300000		2.8300000	2.8300000

Unique Subject Identifier=2233011111-0001

Assay variable : PTINR_VAL1 PT-INR Value

N	Mean	Standard Deviation	Minimum	Maximum
6	1.9966667	0.4012314	1.4500000	2.5700000

Laboratory tests at the time of obtaining Table 2-6 consent, according to the presence or absence of proton-pump inhibitors

Unique Subject Identifier=2233011111-0002

MEANS Procedure

Assay variable : PTINR_VAL1 PT-INR Value

N	Mean	Standard Deviation	Minimum	Maximum
6	1.9400000	0.1704113	1.7100000	2.1500000

Unique Subject Identifier=2233011111-0008

Assay variable : PTINR_VAL1 PT-INR Value

N	Mean	Standard Deviation	Minimum	Maximum
4	1.4000000	0.1157584	1.2900000	1.5600000

Unique Subject Identifier=2233011111-0009

Assay variable : PTINR_VAL1 PT-INR Value

N	Mean	Standard Deviation	Minimum	Maximum
4	1.7600000	0.2047763	1.5600000	2.0100000

Unique Subject Identifier=2233011111-0011

Assay variable : PTINR_VAL1 PT-INR Value

N	Mean	Standard Deviation	Minimum	Maximum
5	2.2540000	0.2861468	1.9000000	2.6000000

Laboratory tests at the time of obtaining Table 2-6 consent, according to the presence or absence of proton-pump inhibitors

Unique Subject Identifier=2233011111-0015

MEANS Procedure

Assay variable : PTINR_VAL1 PT-INR Value

N	Mean	Standard Deviation	Minimum	Maximum
6	1.6016667	0.1358553	1.4600000	1.8000000

Unique Subject Identifier=2233011111-0016

Assay variable : PTINR_VAL1 PT-INR Value

N	Mean	Standard Deviation	Minimum	Maximum
6	1.9616667	0.2359167	1.6500000	2.3600000

Unique Subject Identifier=2233011111-0018

Assay variable : PTINR_VAL1 PT-INR Value

N	Mean	Standard Deviation	Minimum	Maximum
4	1.5250000	0.1912241	1.3100000	1.7500000

Unique Subject Identifier=2233011111-0019

Assay variable : PTINR_VAL1 PT-INR Value

N	Mean	Standard Deviation	Minimum	Maximum
2	2.2500000	0.3535534	2.0000000	2.5000000

Laboratory tests at the time of obtaining Table 2-6 consent, according to the presence or absence of proton-pump inhibitors

Unique Subject Identifier=2233011111-0020

MEANS Procedure

Assay variable : PTINR_VAL1 PT-INR Value

N	Mean	Standard Deviation	Minimum	Maximum
4	1.9075000	0.3986122	1.3400000	2.2000000

Unique Subject Identifier=2233011111-0021

Assay variable : PTINR_VAL1 PT-INR Value

N	Mean	Standard Deviation	Minimum	Maximum
5	1.4320000	0.1439444	1.3100000	1.6300000

Unique Subject Identifier=2233011111-0022

Assay variable : PTINR_VAL1 PT-INR Value

N	Mean	Standard Deviation	Minimum	Maximum
5	1.5680000	0.1231666	1.4300000	1.7600000

Unique Subject Identifier=2233011111-0026

Assay variable : PTINR_VAL1 PT-INR Value

N	Mean	Standard Deviation	Minimum	Maximum
3	1.4666667	0.1159023	1.3600000	1.5900000

Laboratory tests at the time of obtaining Table 2-6 consent, according to the presence or absence of proton-pump inhibitors

Unique Subject Identifier=2233011111-0032

MEANS Procedure

Assay variable : PTINR_VAL1 PT-INR Value

N	Mean	Standard Deviation	Minimum	Maximum
3	2.0133333	0.2138535	1.7800000	2.2000000

Unique Subject Identifier=2233011111-0035

Assay variable : PTINR_VAL1 PT-INR Value

N	Mean	Standard Deviation	Minimum	Maximum
5	1.7220000	0.2888252	1.3300000	1.9800000

Unique Subject Identifier=2233011111-0036

Assay variable : PTINR_VAL1 PT-INR Value

N	Mean	Standard Deviation	Minimum	Maximum
4	1.6725000	0.0340343	1.6300000	1.7000000

Unique Subject Identifier=2233011111-0037

Assay variable : PTINR_VAL1 PT-INR Value

N	Mean	Standard Deviation	Minimum	Maximum
3	2.0200000	0.1200000	1.9000000	2.1400000

Laboratory tests at the time of obtaining Table 2-6 consent, according to the presence or absence of proton-pump inhibitors

Unique Subject Identifier=2236011111-0001

MEANS Procedure

Assay variable : PTINR_VAL1 PT-INR Value

N	Mean	Standard Deviation	Minimum	Maximum
6	2.3866667	0.2338090	2.0000000	2.6000000

Unique Subject Identifier=2236011111-0002

Assay variable : PTINR_VAL1 PT-INR Value

N	Mean	Standard Deviation	Minimum	Maximum
6	1.9500000	0.4324350	1.4000000	2.6000000

Unique Subject Identifier=2236011111-0005

Assay variable : PTINR_VAL1 PT-INR Value

N	Mean	Standard Deviation	Minimum	Maximum
4	1.7500000	0.0577350	1.7000000	1.8000000

Unique Subject Identifier=2236011111-0007

Assay variable : PTINR_VAL1 PT-INR Value

N	Mean	Standard Deviation	Minimum	Maximum
6	1.6783333	0.2898563	1.3000000	2.0000000

Laboratory tests at the time of obtaining Table 2-6 consent, according to the presence or absence of proton-pump inhibitors

Unique Subject Identifier=2236011111-0009

MEANS Procedure

Assay variable : PTINR_VAL1 PT-INR Value

N	Mean	Standard Deviation	Minimum	Maximum
5	1.9500000	0.2000000	1.6000000	2.1000000

Unique Subject Identifier=2236011111-0012

Assay variable : PTINR_VAL1 PT-INR Value

N	Mean	Standard Deviation	Minimum	Maximum
5	1.5800000	0.1788854	1.3000000	1.7000000

Unique Subject Identifier=2236011111-0013

Assay variable : PTINR_VAL1 PT-INR Value

N	Mean	Standard Deviation	Minimum	Maximum
4	2.3500000	0.5802298	1.8000000	3.1000000

Unique Subject Identifier=2236011111-0014

Assay variable : PTINR_VAL1 PT-INR Value

N	Mean	Standard Deviation	Minimum	Maximum
3	1.7366667	0.3611556	1.3600000	2.0800000

Laboratory tests at the time of obtaining Table 2-6 consent, according to the presence or absence of proton-pump inhibitors

Unique Subject Identifier=2236011111-0017

MEANS Procedure

Assay variable : PTINR_VAL1 PT-INR Value

N	Mean	Standard Deviation	Minimum	Maximum
6	1.7000000	0.3687818	1.4000000	2.3000000

Unique Subject Identifier=2236011111-0018

Assay variable : PTINR_VAL1 PT-INR Value

N	Mean	Standard Deviation	Minimum	Maximum
5	2.3400000	0.1341641	2.2000000	2.5000000

Unique Subject Identifier=2236011111-0021

Assay variable : PTINR_VAL1 PT-INR Value

N	Mean	Standard Deviation	Minimum	Maximum
4	1.7000000	0.1154701	1.6000000	1.8000000

Unique Subject Identifier=2236011111-0024

Assay variable : PTINR_VAL1 PT-INR Value

N	Mean	Standard Deviation	Minimum	Maximum
6	1.4000000	0.2000000	1.1000000	1.6000000

Laboratory tests at the time of obtaining Table 2-6 consent, according to the presence or absence of proton-pump inhibitors

Unique Subject Identifier=2236011111-0025

MEANS Procedure

Assay variable : PTINR_VAL1 PT-INR Value

N	Mean	Standard Deviation	Minimum	Maximum
1	1.5000000		1.5000000	1.5000000

Unique Subject Identifier=2236011111-0027

Assay variable : PTINR_VAL1 PT-INR Value

N	Mean	Standard Deviation	Minimum	Maximum
4	2.0500000	0.1732051	1.9000000	2.3000000

Unique Subject Identifier=2236011111-0028

Assay variable : PTINR_VAL1 PT-INR Value

N	Mean	Standard Deviation	Minimum	Maximum
3	2.2666667	0.3055050	2.0000000	2.6000000

Unique Subject Identifier=2236011111-0031

Assay variable : PTINR_VAL1 PT-INR Value

N	Mean	Standard Deviation	Minimum	Maximum
6	1.6000000	0.2449490	1.2000000	1.9000000

Laboratory tests at the time of obtaining Table 2-6 consent, according to the presence or absence of proton-pump inhibitors

Unique Subject Identifier=2236011111-0032

MEANS Procedure

Assay variable : PTINR_VAL1 PT-INR Value

N	Mean	Standard Deviation	Minimum	Maximum
6	1.9166667	0.3125167	1.5000000	2.3000000

Unique Subject Identifier=2236011111-0034

Assay variable : PTINR_VAL1 PT-INR Value

N	Mean	Standard Deviation	Minimum	Maximum
5	1.8000000	0.2121320	1.6000000	2.1000000

Unique Subject Identifier=2236011111-0039

Assay variable : PTINR_VAL1 PT-INR Value

N	Mean	Standard Deviation	Minimum	Maximum
4	1.8000000	0.1414214	1.7000000	2.0000000

Unique Subject Identifier=2236011111-0042

Assay variable : PTINR_VAL1 PT-INR Value

N	Mean	Standard Deviation	Minimum	Maximum
4	1.8250000	0.2629956	1.6000000	2.2000000

Laboratory tests at the time of obtaining Table 2-6 consent, according to the presence or absence of proton-pump inhibitors

Unique Subject Identifier=2236011111-0043

MEANS Procedure

Assay variable : PTINR_VAL1 PT-INR Value

N	Mean	Standard Deviation	Minimum	Maximum
6	1.7833333	0.2857738	1.4000000	2.2000000

Unique Subject Identifier=2236011111-0045

Assay variable : PTINR_VAL1 PT-INR Value

N	Mean	Standard Deviation	Minimum	Maximum
5	3.0000000	0.7681146	2.3000000	4.1000000

Unique Subject Identifier=2236011111-0052

Assay variable : PTINR_VAL1 PT-INR Value

N	Mean	Standard Deviation	Minimum	Maximum
6	1.7000000	0.5215362	1.2000000	2.3000000

Unique Subject Identifier=2236011111-0053

Assay variable : PTINR_VAL1 PT-INR Value

N	Mean	Standard Deviation	Minimum	Maximum
4	1.7750000	0.1707825	1.6000000	2.0000000

Laboratory tests at the time of obtaining Table 2-6 consent, according to the presence or absence of proton-pump inhibitors

Unique Subject Identifier=2236011111-0054

MEANS Procedure

Assay variable : PTINR_VAL1 PT-INR Value

N	Mean	Standard Deviation	Minimum	Maximum
5	1.7160000	0.1152389	1.5800000	1.8000000

Unique Subject Identifier=2236011111-0059

Assay variable : PTINR_VAL1 PT-INR Value

N	Mean	Standard Deviation	Minimum	Maximum
5	2.5720000	0.2567489	2.3600000	3.0000000

Unique Subject Identifier=2236011111-0062

Assay variable : PTINR_VAL1 PT-INR Value

N	Mean	Standard Deviation	Minimum	Maximum
4	2.9000000	1.7088007	1.8000000	5.4000000

Unique Subject Identifier=2236011111-0064

Assay variable : PTINR_VAL1 PT-INR Value

N	Mean	Standard Deviation	Minimum	Maximum
4	1.9000000	0.4966555	1.3000000	2.5000000

Laboratory tests at the time of obtaining Table 2-6 consent, according to the presence or absence of proton-pump inhibitors

Unique Subject Identifier=2236011111-0066

MEANS Procedure

Assay variable : PTINR_VAL1 PT-INR Value

N	Mean	Standard Deviation	Minimum	Maximum
5	2.3320000	0.4734131	1.6000000	2.9000000

Unique Subject Identifier=2236011111-0070

Assay variable : PTINR_VAL1 PT-INR Value

N	Mean	Standard Deviation	Minimum	Maximum
4	2.4500000	0.3415650	2.1000000	2.9000000

Unique Subject Identifier=2236011111-0073

Assay variable : PTINR_VAL1 PT-INR Value

N	Mean	Standard Deviation	Minimum	Maximum
6	2.1000000	0.2828427	1.7000000	2.4000000

Unique Subject Identifier=2236011111-0075

Assay variable : PTINR_VAL1 PT-INR Value

N	Mean	Standard Deviation	Minimum	Maximum
3	2.5000000	0.2000000	2.3000000	2.7000000

Laboratory tests at the time of obtaining Table 2-6 consent, according to the presence or absence of proton-pump inhibitors

Unique Subject Identifier=2236011111-0080

MEANS Procedure

Assay variable : PTINR_VAL1 PT-INR Value

N	Mean	Standard Deviation	Minimum	Maximum
6	2.2166667	0.5845226	1.6000000	2.9000000

Unique Subject Identifier=2241011111-0004

Assay variable : PTINR_VAL1 PT-INR Value

N	Mean	Standard Deviation	Minimum	Maximum
5	1.6100000	0.1189538	1.4700000	1.7800000

Unique Subject Identifier=2241011111-0009

Assay variable : PTINR_VAL1 PT-INR Value

N	Mean	Standard Deviation	Minimum	Maximum
6	2.2183333	0.1267149	2.0400000	2.4300000

Unique Subject Identifier=2243011111-0011

Assay variable : PTINR_VAL1 PT-INR Value

N	Mean	Standard Deviation	Minimum	Maximum
5	1.6260000	0.1171751	1.4500000	1.7600000

Laboratory tests at the time of obtaining Table 2-6 consent, according to the presence or absence of proton-pump inhibitors

Unique Subject Identifier=2243011111-0013

MEANS Procedure

Assay variable : PTINR_VAL1 PT-INR Value

N	Mean	Standard Deviation	Minimum	Maximum
6	1.7483333	0.1584193	1.5800000	2.0300000

Unique Subject Identifier=2243011111-0014

Assay variable : PTINR_VAL1 PT-INR Value

N	Mean	Standard Deviation	Minimum	Maximum
5	1.9360000	0.5542833	1.3400000	2.7400000

Unique Subject Identifier=2243011111-0015

Assay variable : PTINR_VAL1 PT-INR Value

N	Mean	Standard Deviation	Minimum	Maximum
5	1.7240000	0.1645600	1.5200000	1.9300000

Unique Subject Identifier=2247010011-0001

Assay variable : PTINR_VAL1 PT-INR Value

N	Mean	Standard Deviation	Minimum	Maximum
2	1.7100000	0.0424264	1.6800000	1.7400000

Laboratory tests at the time of obtaining Table 2-6 consent, according to the presence or absence of proton-pump inhibitors

Unique Subject Identifier=2247010011-0002

MEANS Procedure

Assay variable : PTINR_VAL1 PT-INR Value

N	Mean	Standard Deviation	Minimum	Maximum
3	1.7266667	0.6180885	1.3500000	2.4400000

Unique Subject Identifier=2247010011-0003

Assay variable : PTINR_VAL1 PT-INR Value

N	Mean	Standard Deviation	Minimum	Maximum
4	1.7700000	0.1732051	1.5600000	1.9400000

Unique Subject Identifier=2247010011-0004

Assay variable : PTINR_VAL1 PT-INR Value

N	Mean	Standard Deviation	Minimum	Maximum
3	1.7900000	0.4059557	1.4300000	2.2300000

Unique Subject Identifier=2247010011-0005

Assay variable : PTINR_VAL1 PT-INR Value

N	Mean	Standard Deviation	Minimum	Maximum
3	1.5466667	0.2837840	1.2400000	1.8000000

Laboratory tests at the time of obtaining Table 2-6 consent, according to the presence or absence of proton-pump inhibitors

Unique Subject Identifier=2247010011-0007

MEANS Procedure

Assay variable : PTINR_VAL1 PT-INR Value

N	Mean	Standard Deviation	Minimum	Maximum
4	2.0175000	0.4503610	1.4400000	2.4800000

Unique Subject Identifier=2247010011-0008

Assay variable : PTINR_VAL1 PT-INR Value

N	Mean	Standard Deviation	Minimum	Maximum
5	1.7020000	0.2327445	1.4800000	1.9700000

Unique Subject Identifier=2247010011-0009

Assay variable : PTINR_VAL1 PT-INR Value

N	Mean	Standard Deviation	Minimum	Maximum
4	2.4950000	0.5238638	1.7100000	2.7900000

Unique Subject Identifier=2247010011-0011

Assay variable : PTINR_VAL1 PT-INR Value

N	Mean	Standard Deviation	Minimum	Maximum
4	2.0500000	0.4388622	1.4100000	2.3800000

Laboratory tests at the time of obtaining Table 2-6 consent, according to the presence or absence of proton-pump inhibitors

Unique Subject Identifier=2247010011-0014

MEANS Procedure

Assay variable : PTINR_VAL1 PT-INR Value

N	Mean	Standard Deviation	Minimum	Maximum
4	1.5475000	0.2715849	1.3100000	1.8900000

Unique Subject Identifier=2247010011-0017

Assay variable : PTINR_VAL1 PT-INR Value

N	Mean	Standard Deviation	Minimum	Maximum
4	1.8850000	0.1502221	1.6600000	1.9700000

Unique Subject Identifier=2247010011-0019

Assay variable : PTINR_VAL1 PT-INR Value

N	Mean	Standard Deviation	Minimum	Maximum
6	2.0133333	0.3028971	1.4900000	2.3900000

Unique Subject Identifier=2247010011-0025

Assay variable : PTINR_VAL1 PT-INR Value

N	Mean	Standard Deviation	Minimum	Maximum
4	1.9700000	0.6274286	1.6300000	2.9100000

Laboratory tests at the time of obtaining Table 2-6 consent, according to the presence or absence of proton-pump inhibitors

Unique Subject Identifier=2247010011-0026

MEANS Procedure

Assay variable : PTINR_VAL1 PT-INR Value

N	Mean	Standard Deviation	Minimum	Maximum
4	2.2625000	0.1685972	2.0900000	2.4800000

Unique Subject Identifier=2247010011-0027

Assay variable : PTINR_VAL1 PT-INR Value

N	Mean	Standard Deviation	Minimum	Maximum
3	1.8466667	0.0513160	1.7900000	1.8900000

Unique Subject Identifier=2247010011-0031

Assay variable : PTINR_VAL1 PT-INR Value

N	Mean	Standard Deviation	Minimum	Maximum
4	1.5300000	0.0820569	1.4500000	1.6400000

Unique Subject Identifier=2247010011-0032

Assay variable : PTINR_VAL1 PT-INR Value

N	Mean	Standard Deviation	Minimum	Maximum
4	2.3200000	0.2585859	2.0100000	2.6200000

Laboratory tests at the time of obtaining Table 2-6 consent, according to the presence or absence of proton-pump inhibitors

Unique Subject Identifier=2247010011-0037

MEANS Procedure

Assay variable : PTINR_VAL1 PT-INR Value

N	Mean	Standard Deviation	Minimum	Maximum
5	1.8380000	0.1482228	1.6700000	2.0700000

Unique Subject Identifier=2247010011-0038

Assay variable : PTINR_VAL1 PT-INR Value

N	Mean	Standard Deviation	Minimum	Maximum
4	1.4225000	0.1906786	1.2600000	1.6500000

Unique Subject Identifier=2247010011-0049

Assay variable : PTINR_VAL1 PT-INR Value

N	Mean	Standard Deviation	Minimum	Maximum
4	1.8075000	0.1723127	1.6200000	2.0100000

Unique Subject Identifier=2247010011-0050

Assay variable : PTINR_VAL1 PT-INR Value

N	Mean	Standard Deviation	Minimum	Maximum
4	2.3525000	0.1746186	2.2000000	2.6000000

Laboratory tests at the time of obtaining Table 2-6 consent, according to the presence or absence of proton-pump inhibitors

Unique Subject Identifier=2247010011-0051

MEANS Procedure

Assay variable : PTINR_VAL1 PT-INR Value

N	Mean	Standard Deviation	Minimum	Maximum
5	1.6900000	0.1504992	1.5600000	1.9300000

Unique Subject Identifier=2247010011-0054

Assay variable : PTINR_VAL1 PT-INR Value

N	Mean	Standard Deviation	Minimum	Maximum
4	1.5150000	0.1167619	1.3700000	1.6400000

Unique Subject Identifier=2247010011-0055

Assay variable : PTINR_VAL1 PT-INR Value

N	Mean	Standard Deviation	Minimum	Maximum
4	1.6325000	0.2653771	1.3200000	1.9500000

Unique Subject Identifier=2247010011-0062

Assay variable : PTINR_VAL1 PT-INR Value

N	Mean	Standard Deviation	Minimum	Maximum
3	1.9200000	1.0142485	1.2900000	3.0900000

Laboratory tests at the time of obtaining Table 2-6 consent, according to the presence or absence of proton-pump inhibitors

Unique Subject Identifier=2247010011-0063

MEANS Procedure

Assay variable : PTINR_VAL1 PT-INR Value

N	Mean	Standard Deviation	Minimum	Maximum
3	1.5833333	0.2173323	1.3500000	1.7800000

Unique Subject Identifier=2247010011-0072

Assay variable : PTINR_VAL1 PT-INR Value

N	Mean	Standard Deviation	Minimum	Maximum
2	3.0400000	0.5939697	2.6200000	3.4600000

Unique Subject Identifier=2247010011-0075

Assay variable : PTINR_VAL1 PT-INR Value

N	Mean	Standard Deviation	Minimum	Maximum
3	1.2200000	0.1646208	1.1200000	1.4100000

Unique Subject Identifier=2247010011-0076

Assay variable : PTINR_VAL1 PT-INR Value

N	Mean	Standard Deviation	Minimum	Maximum
4	1.7900000	0.3667879	1.3600000	2.1600000

Laboratory tests at the time of obtaining Table 2-6 consent, according to the presence or absence of proton-pump inhibitors

Unique Subject Identifier=2247010011-0077

MEANS Procedure

Assay variable : PTINR_VAL1 PT-INR Value

N	Mean	Standard Deviation	Minimum	Maximum
2	1.9400000	0.5515433	1.5500000	2.3300000

Unique Subject Identifier=2249011111-0011

Assay variable : PTINR_VAL1 PT-INR Value

N	Mean	Standard Deviation	Minimum	Maximum
2	1.4500000	0.2121320	1.3000000	1.6000000

Unique Subject Identifier=2249011111-0017

Assay variable : PTINR_VAL1 PT-INR Value

N	Mean	Standard Deviation	Minimum	Maximum
3	2.0566667	0.2205297	1.8700000	2.3000000

Unique Subject Identifier=2258010111-0001

Assay variable : PTINR_VAL1 PT-INR Value

N	Mean	Standard Deviation	Minimum	Maximum
4	1.2500000	0.1290994	1.1000000	1.4000000

Laboratory tests at the time of obtaining Table 2-6 consent, according to the presence or absence of proton-pump inhibitors

Unique Subject Identifier=2259010111-0001

MEANS Procedure

Assay variable : PTINR_VAL1 PT-INR Value

N	Mean	Standard Deviation	Minimum	Maximum
3	1.5066667	0.1960442	1.3000000	1.6900000

Unique Subject Identifier=2259010111-0002

Assay variable : PTINR_VAL1 PT-INR Value

N	Mean	Standard Deviation	Minimum	Maximum
4	1.3350000	0.0881287	1.2400000	1.4400000

Unique Subject Identifier=2263011111-0001

Assay variable : PTINR_VAL1 PT-INR Value

N	Mean	Standard Deviation	Minimum	Maximum
2	2.0600000	0.8626703	1.4500000	2.6700000

Unique Subject Identifier=2264011111-0003

Assay variable : PTINR_VAL1 PT-INR Value

N	Mean	Standard Deviation	Minimum	Maximum
6	1.6116667	0.3074682	1.1800000	2.0100000

Laboratory tests at the time of obtaining Table 2-6 consent, according to the presence or absence of proton-pump inhibitors

Unique Subject Identifier=2264011111-0004

MEANS Procedure

Assay variable : PTINR_VAL1 PT-INR Value

N	Mean	Standard Deviation	Minimum	Maximum
6	1.7650000	0.2074367	1.4900000	2.0300000

Unique Subject Identifier=2264011111-0006

Assay variable : PTINR_VAL1 PT-INR Value

N	Mean	Standard Deviation	Minimum	Maximum
6	1.6650000	0.4120073	1.1500000	2.3200000

Unique Subject Identifier=2264011111-0008

Assay variable : PTINR_VAL1 PT-INR Value

N	Mean	Standard Deviation	Minimum	Maximum
6	1.6550000	0.1005485	1.5100000	1.7700000

Unique Subject Identifier=2264011111-0009

Assay variable : PTINR_VAL1 PT-INR Value

N	Mean	Standard Deviation	Minimum	Maximum
6	1.5383333	0.1626551	1.4100000	1.8200000

Laboratory tests at the time of obtaining Table 2-6 consent, according to the presence or absence of proton-pump inhibitors

Unique Subject Identifier=2264011111-0010

MEANS Procedure

Assay variable : PTINR_VAL1 PT-INR Value

N	Mean	Standard Deviation	Minimum	Maximum
6	1.0516667	0.0240139	1.0200000	1.0800000

Unique Subject Identifier=2264011111-0012

Assay variable : PTINR_VAL1 PT-INR Value

N	Mean	Standard Deviation	Minimum	Maximum
6	1.8066667	0.6258328	1.1600000	2.9600000

Unique Subject Identifier=2268011111-0021

Assay variable : PTINR_VAL1 PT-INR Value

N	Mean	Standard Deviation	Minimum	Maximum
5	2.1400000	0.3361547	1.6000000	2.5000000

Unique Subject Identifier=2268011111-0023

Assay variable : PTINR_VAL1 PT-INR Value

N	Mean	Standard Deviation	Minimum	Maximum
5	2.0000000	0.2121320	1.8000000	2.3000000

Laboratory tests at the time of obtaining Table 2-6 consent, according to the presence or absence of proton-pump inhibitors

Unique Subject Identifier=2268011111-0024

MEANS Procedure

Assay variable : PTINR_VAL1 PT-INR Value

N	Mean	Standard Deviation	Minimum	Maximum
5	1.6200000	0.1788854	1.5000000	1.9000000

Unique Subject Identifier=2268011111-0028

Assay variable : PTINR_VAL1 PT-INR Value

N	Mean	Standard Deviation	Minimum	Maximum
6	2.3166667	0.4708149	1.9000000	3.2000000

Unique Subject Identifier=2268011111-0031

Assay variable : PTINR_VAL1 PT-INR Value

N	Mean	Standard Deviation	Minimum	Maximum
3	2.3000000	0.2645751	2.1000000	2.6000000

Unique Subject Identifier=2268011111-0042

Assay variable : PTINR_VAL1 PT-INR Value

N	Mean	Standard Deviation	Minimum	Maximum
6	1.9666667	0.1505545	1.7000000	2.1000000

Laboratory tests at the time of obtaining Table 2-6 consent, according to the presence or absence of proton-pump inhibitors

Unique Subject Identifier=2282011001-0006

MEANS Procedure

Assay variable : PTINR_VAL1 PT-INR Value

N	Mean	Standard Deviation	Minimum	Maximum
1	1.9200000		1.9200000	1.9200000

Unique Subject Identifier=2283011000-0008

Assay variable : PTINR_VAL1 PT-INR Value

N	Mean	Standard Deviation	Minimum	Maximum
3	2.1833333	0.3501904	1.7800000	2.4100000

Unique Subject Identifier=2283011000-0020

Assay variable : PTINR_VAL1 PT-INR Value

N	Mean	Standard Deviation	Minimum	Maximum
4	1.7425000	0.2495830	1.5300000	2.0800000

Unique Subject Identifier=2284011111-0011

Assay variable : PTINR_VAL1 PT-INR Value

N	Mean	Standard Deviation	Minimum	Maximum
5	1.7440000	0.2388095	1.5200000	2.1100000

Laboratory tests at the time of obtaining Table 2-6 consent, according to the presence or absence of proton-pump inhibitors

Unique Subject Identifier=2289000000-0001

MEANS Procedure

Assay variable : PTINR_VAL1 PT-INR Value

N	Mean	Standard Deviation	Minimum	Maximum
1	1.7400000		1.7400000	1.7400000

Unique Subject Identifier=2292000000-0001

Assay variable : PTINR_VAL1 PT-INR Value

N	Mean	Standard Deviation	Minimum	Maximum
4	2.2250000	0.1500000	2.1000000	2.4000000

Unique Subject Identifier=2292000000-0005

Assay variable : PTINR_VAL1 PT-INR Value

N	Mean	Standard Deviation	Minimum	Maximum
6	1.6333333	0.1861899	1.3000000	1.8000000

Unique Subject Identifier=2292000000-0008

Assay variable : PTINR_VAL1 PT-INR Value

N	Mean	Standard Deviation	Minimum	Maximum
6	2.1833333	0.4020779	1.7000000	2.8000000

Laboratory tests at the time of obtaining Table 2-6 consent, according to the presence or absence of proton-pump inhibitors

Unique Subject Identifier=2292000000-0013

MEANS Procedure

Assay variable : PTINR_VAL1 PT-INR Value

N	Mean	Standard Deviation	Minimum	Maximum
6	1.9333333	0.3614784	1.5000000	2.5000000

Unique Subject Identifier=2296010111-0005

Assay variable : PTINR_VAL1 PT-INR Value

N	Mean	Standard Deviation	Minimum	Maximum
5	1.8600000	0.0547723	1.8000000	1.9000000

Unique Subject Identifier=2296010111-0006

Assay variable : PTINR_VAL1 PT-INR Value

N	Mean	Standard Deviation	Minimum	Maximum
5	1.9000000	0.2549510	1.6000000	2.2000000

Unique Subject Identifier=2296010111-0009

Assay variable : PTINR_VAL1 PT-INR Value

N	Mean	Standard Deviation	Minimum	Maximum
5	2.2000000	0.2000000	1.9000000	2.4000000

Laboratory tests at the time of obtaining Table 2-6 consent, according to the presence or absence of proton-pump inhibitors

Unique Subject Identifier=2296010111-0011

MEANS Procedure

Assay variable : PTINR_VAL1 PT-INR Value

N	Mean	Standard Deviation	Minimum	Maximum
6	2.1666667	0.1211060	2.0000000	2.3000000

Unique Subject Identifier=2296010111-0012

Assay variable : PTINR_VAL1 PT-INR Value

N	Mean	Standard Deviation	Minimum	Maximum
6	1.8500000	0.2509980	1.6000000	2.3000000

Unique Subject Identifier=2301011111-0002

Assay variable : PTINR_VAL1 PT-INR Value

N	Mean	Standard Deviation	Minimum	Maximum
1	2.3400000		2.3400000	2.3400000

Unique Subject Identifier=2301011111-0010

Assay variable : PTINR_VAL1 PT-INR Value

N	Mean	Standard Deviation	Minimum	Maximum
2	1.8950000	0.7707464	1.3500000	2.4400000

Laboratory tests at the time of obtaining Table 2-6 consent, according to the presence or absence of proton-pump inhibitors

Unique Subject Identifier=2301011111-0013

MEANS Procedure

Assay variable : PTINR_VAL1 PT-INR Value

N	Mean	Standard Deviation	Minimum	Maximum
3	1.7000000	0.1539480	1.5700000	1.8700000

Unique Subject Identifier=2308011000-0001

Assay variable : PTINR_VAL1 PT-INR Value

N	Mean	Standard Deviation	Minimum	Maximum
3	1.6333333	0.0577350	1.6000000	1.7000000

Unique Subject Identifier=2308011000-0002

Assay variable : PTINR_VAL1 PT-INR Value

N	Mean	Standard Deviation	Minimum	Maximum
5	1.7000000	0.3872983	1.2000000	2.1000000

Unique Subject Identifier=2308011000-0004

Assay variable : PTINR_VAL1 PT-INR Value

N	Mean	Standard Deviation	Minimum	Maximum
4	2.2250000	0.2629956	2.0000000	2.6000000

Laboratory tests at the time of obtaining Table 2-6 consent, according to the presence or absence of proton-pump inhibitors

Unique Subject Identifier=2308011000-0013

MEANS Procedure

Assay variable : PTINR_VAL1 PT-INR Value

N	Mean	Standard Deviation	Minimum	Maximum
4	1.6000000	0.1414214	1.4000000	1.7000000

Unique Subject Identifier=2308011000-0017

Assay variable : PTINR_VAL1 PT-INR Value

N	Mean	Standard Deviation	Minimum	Maximum
3	1.7666667	0.2886751	1.6000000	2.1000000

Unique Subject Identifier=2308011000-0018

Assay variable : PTINR_VAL1 PT-INR Value

N	Mean	Standard Deviation	Minimum	Maximum
2	1.8500000	0.7778175	1.3000000	2.4000000

Unique Subject Identifier=2309000000-0001

Assay variable : PTINR_VAL1 PT-INR Value

N	Mean	Standard Deviation	Minimum	Maximum
3	1.8766667	0.3707200	1.5200000	2.2600000

Laboratory tests at the time of obtaining Table 2-6 consent, according to the presence or absence of proton-pump inhibitors

Unique Subject Identifier=2309000000-0003

MEANS Procedure

Assay variable : PTINR_VAL1 PT-INR Value

N	Mean	Standard Deviation	Minimum	Maximum
1	2.2900000		2.2900000	2.2900000

Unique Subject Identifier=2310011111-0001

Assay variable : PTINR_VAL1 PT-INR Value

N	Mean	Standard Deviation	Minimum	Maximum
2	1.8100000	0.2404163	1.6400000	1.9800000

Unique Subject Identifier=2310011111-0008

Assay variable : PTINR_VAL1 PT-INR Value

N	Mean	Standard Deviation	Minimum	Maximum
4	1.9100000	0.8183316	1.0300000	2.6700000

Unique Subject Identifier=2310011111-0018

Assay variable : PTINR_VAL1 PT-INR Value

N	Mean	Standard Deviation	Minimum	Maximum
4	2.2775000	0.7902057	1.8100000	3.4600000

Laboratory tests at the time of obtaining Table 2-6 consent, according to the presence or absence of proton-pump inhibitors

Unique Subject Identifier=2312011111-0005

MEANS Procedure

Assay variable : PTINR_VAL1 PT-INR Value

N	Mean	Standard Deviation	Minimum	Maximum
3	2.3666667	0.4041452	1.9000000	2.6000000

Unique Subject Identifier=2312011111-0006

Assay variable : PTINR_VAL1 PT-INR Value

N	Mean	Standard Deviation	Minimum	Maximum
1	2.0000000		2.0000000	2.0000000

Unique Subject Identifier=2312011111-0008

Assay variable : PTINR_VAL1 PT-INR Value

N	Mean	Standard Deviation	Minimum	Maximum
2	1.8000000	0.2828427	1.6000000	2.0000000

Unique Subject Identifier=2321011011-0006

Assay variable : PTINR_VAL1 PT-INR Value

N	Mean	Standard Deviation	Minimum	Maximum
1	2.0100000		2.0100000	2.0100000

Laboratory tests at the time of obtaining Table 2-6 consent, according to the presence or absence of proton-pump inhibitors

Unique Subject Identifier=2327011011-0018

MEANS Procedure

Assay variable : PTINR_VAL1 PT-INR Value

N	Mean	Standard Deviation	Minimum	Maximum
3	2.4900000	0.5604463	1.8900000	3.0000000

Unique Subject Identifier=2327011011-0027

Assay variable : PTINR_VAL1 PT-INR Value

N	Mean	Standard Deviation	Minimum	Maximum
4	2.0950000	0.2829016	1.8500000	2.4900000

Unique Subject Identifier=2327011011-0032

Assay variable : PTINR_VAL1 PT-INR Value

N	Mean	Standard Deviation	Minimum	Maximum
4	1.8250000	0.1292285	1.6500000	1.9600000

Unique Subject Identifier=2327011011-0038

Assay variable : PTINR_VAL1 PT-INR Value

N	Mean	Standard Deviation	Minimum	Maximum
4	1.7275000	0.2396351	1.4900000	2.0200000

Laboratory tests at the time of obtaining Table 2-6 consent, according to the presence or absence of proton-pump inhibitors

Unique Subject Identifier=2327011011-0046

MEANS Procedure

Assay variable : PTINR_VAL1 PT-INR Value

N	Mean	Standard Deviation	Minimum	Maximum
4	1.8125000	0.2794489	1.4600000	2.0700000

Unique Subject Identifier=2341001000-0004

Assay variable : PTINR_VAL1 PT-INR Value

N	Mean	Standard Deviation	Minimum	Maximum
2	1.1450000	0.0070711	1.1400000	1.1500000

Unique Subject Identifier=2341001000-0008

Assay variable : PTINR_VAL1 PT-INR Value

N	Mean	Standard Deviation	Minimum	Maximum
1	1.9100000		1.9100000	1.9100000

Unique Subject Identifier=2341001000-0010

Assay variable : PTINR_VAL1 PT-INR Value

N	Mean	Standard Deviation	Minimum	Maximum
1	2.5900000		2.5900000	2.5900000

Laboratory tests at the time of obtaining Table 2-6 consent, according to the presence or absence of proton-pump inhibitors

Unique Subject Identifier=2347011111-0001

MEANS Procedure

Assay variable : PTINR_VAL1 PT-INR Value

N	Mean	Standard Deviation	Minimum	Maximum
6	1.8633333	0.2362767	1.5300000	2.2000000

Unique Subject Identifier=2347011111-0006

Assay variable : PTINR_VAL1 PT-INR Value

N	Mean	Standard Deviation	Minimum	Maximum
6	2.4033333	1.0029091	1.3200000	4.0000000

Unique Subject Identifier=2347011111-0007

Assay variable : PTINR_VAL1 PT-INR Value

N	Mean	Standard Deviation	Minimum	Maximum
5	1.9680000	0.2846401	1.5900000	2.2800000

Unique Subject Identifier=2347011111-0008

Assay variable : PTINR_VAL1 PT-INR Value

N	Mean	Standard Deviation	Minimum	Maximum
6	1.9616667	0.0646271	1.8600000	2.0500000

Laboratory tests at the time of obtaining Table 2-6 consent, according to the presence or absence of proton-pump inhibitors

Unique Subject Identifier=2347011111-0013

MEANS Procedure

Assay variable : PTINR_VAL1 PT-INR Value

N	Mean	Standard Deviation	Minimum	Maximum
6	1.5333333	0.2520053	1.2300000	1.9000000

Unique Subject Identifier=2347011111-0014

Assay variable : PTINR_VAL1 PT-INR Value

N	Mean	Standard Deviation	Minimum	Maximum
6	2.2316667	0.1570244	2.0500000	2.4700000

Unique Subject Identifier=2347011111-0020

Assay variable : PTINR_VAL1 PT-INR Value

N	Mean	Standard Deviation	Minimum	Maximum
6	1.8816667	0.1767956	1.5600000	2.0700000

Unique Subject Identifier=2347011111-0022

Assay variable : PTINR_VAL1 PT-INR Value

N	Mean	Standard Deviation	Minimum	Maximum
6	1.9066667	0.1297176	1.7200000	2.0700000

Laboratory tests at the time of obtaining Table 2-6 consent, according to the presence or absence of proton-pump inhibitors

Unique Subject Identifier=2347011111-0023

MEANS Procedure

Assay variable : PTINR_VAL1 PT-INR Value

N	Mean	Standard Deviation	Minimum	Maximum
6	2.0066667	0.3538738	1.5300000	2.3700000

Unique Subject Identifier=2347011111-0024

Assay variable : PTINR_VAL1 PT-INR Value

N	Mean	Standard Deviation	Minimum	Maximum
6	2.0033333	0.1116542	1.8400000	2.1500000

Unique Subject Identifier=2347011111-0029

Assay variable : PTINR_VAL1 PT-INR Value

N	Mean	Standard Deviation	Minimum	Maximum
6	1.6933333	0.2259794	1.3600000	2.0100000

Unique Subject Identifier=2347011111-0033

Assay variable : PTINR_VAL1 PT-INR Value

N	Mean	Standard Deviation	Minimum	Maximum
6	1.8200000	0.2174396	1.4900000	2.1100000

Laboratory tests at the time of obtaining Table 2-6 consent, according to the presence or absence of proton-pump inhibitors

Unique Subject Identifier=2347011111-0035

MEANS Procedure

Assay variable : PTINR_VAL1 PT-INR Value

N	Mean	Standard Deviation	Minimum	Maximum
6	1.9766667	0.2438579	1.7100000	2.4300000

Unique Subject Identifier=2347011111-0038

Assay variable : PTINR_VAL1 PT-INR Value

N	Mean	Standard Deviation	Minimum	Maximum
4	1.6950000	0.8847033	1.0400000	2.9700000

Unique Subject Identifier=2353011111-0002

Assay variable : PTINR_VAL1 PT-INR Value

N	Mean	Standard Deviation	Minimum	Maximum
6	1.8350000	0.2117310	1.6000000	2.2100000

Unique Subject Identifier=2353011111-0009

Assay variable : PTINR_VAL1 PT-INR Value

N	Mean	Standard Deviation	Minimum	Maximum
6	2.2916667	0.3473567	1.7900000	2.5800000

Laboratory tests at the time of obtaining Table 2-6 consent, according to the presence or absence of proton-pump inhibitors

Unique Subject Identifier=2353011111-0010

MEANS Procedure

Assay variable : PTINR_VAL1 PT-INR Value

N	Mean	Standard Deviation	Minimum	Maximum
6	1.8433333	0.1906480	1.6400000	2.1100000

Unique Subject Identifier=2353011111-0012

Assay variable : PTINR_VAL1 PT-INR Value

N	Mean	Standard Deviation	Minimum	Maximum
6	1.9350000	0.2247443	1.5300000	2.1300000

Unique Subject Identifier=2353011111-0015

Assay variable : PTINR_VAL1 PT-INR Value

N	Mean	Standard Deviation	Minimum	Maximum
4	1.8375000	0.7744407	1.0000000	2.7400000

Unique Subject Identifier=2353011111-0016

Assay variable : PTINR_VAL1 PT-INR Value

N	Mean	Standard Deviation	Minimum	Maximum
6	2.1850000	0.5272096	1.5100000	2.9000000

Laboratory tests at the time of obtaining Table 2-6 consent, according to the presence or absence of proton-pump inhibitors

Unique Subject Identifier=2354011111-0002

MEANS Procedure

Assay variable : PTINR_VAL1 PT-INR Value

N	Mean	Standard Deviation	Minimum	Maximum
4	1.6925000	0.0997914	1.6200000	1.8400000

Unique Subject Identifier=2354011111-0003

Assay variable : PTINR_VAL1 PT-INR Value

N	Mean	Standard Deviation	Minimum	Maximum
6	1.5100000	0.0848528	1.4300000	1.6700000

Unique Subject Identifier=2354011111-0004

Assay variable : PTINR_VAL1 PT-INR Value

N	Mean	Standard Deviation	Minimum	Maximum
6	1.7566667	0.2421294	1.4200000	2.0700000

Unique Subject Identifier=2354011111-0005

Assay variable : PTINR_VAL1 PT-INR Value

N	Mean	Standard Deviation	Minimum	Maximum
6	2.2216667	0.1820348	1.9700000	2.5100000

Laboratory tests at the time of obtaining Table 2-6 consent, according to the presence or absence of proton-pump inhibitors

Unique Subject Identifier=2354011111-0007

MEANS Procedure

Assay variable : PTINR_VAL1 PT-INR Value

N	Mean	Standard Deviation	Minimum	Maximum
5	2.0980000	0.9495894	1.1000000	3.1500000

Unique Subject Identifier=2354011111-0008

Assay variable : PTINR_VAL1 PT-INR Value

N	Mean	Standard Deviation	Minimum	Maximum
6	2.0533333	0.4229736	1.3300000	2.4900000

Unique Subject Identifier=2354011111-0009

Assay variable : PTINR_VAL1 PT-INR Value

N	Mean	Standard Deviation	Minimum	Maximum
5	2.2620000	0.2512369	2.0100000	2.5700000

Unique Subject Identifier=2354011111-0016

Assay variable : PTINR_VAL1 PT-INR Value

N	Mean	Standard Deviation	Minimum	Maximum
4	1.6175000	0.0892095	1.5100000	1.7200000

Laboratory tests at the time of obtaining Table 2-6 consent, according to the presence or absence of proton-pump inhibitors

Unique Subject Identifier=2354011111-0019

MEANS Procedure

Assay variable : PTINR_VAL1 PT-INR Value

N	Mean	Standard Deviation	Minimum	Maximum
3	1.4800000	0.1752142	1.3100000	1.6600000

Unique Subject Identifier=2355011111-0004

Assay variable : PTINR_VAL1 PT-INR Value

N	Mean	Standard Deviation	Minimum	Maximum
2	3.9300000	3.6203867	1.3700000	6.4900000

Unique Subject Identifier=2355011111-0005

Assay variable : PTINR_VAL1 PT-INR Value

N	Mean	Standard Deviation	Minimum	Maximum
2	2.6250000	0.3181981	2.4000000	2.8500000

Unique Subject Identifier=2355011111-0007

Assay variable : PTINR_VAL1 PT-INR Value

N	Mean	Standard Deviation	Minimum	Maximum
2	1.5600000	0.0141421	1.5500000	1.5700000

Laboratory tests at the time of obtaining Table 2-6 consent, according to the presence or absence of proton-pump inhibitors

Unique Subject Identifier=2356011111-0001

MEANS Procedure

Assay variable : PTINR_VAL1 PT-INR Value

N	Mean	Standard Deviation	Minimum	Maximum
2	1.6150000	0.1060660	1.5400000	1.6900000

Unique Subject Identifier=2356011111-0002

Assay variable : PTINR_VAL1 PT-INR Value

N	Mean	Standard Deviation	Minimum	Maximum
3	1.6333333	0.0416333	1.6000000	1.6800000

Unique Subject Identifier=2356011111-0004

Assay variable : PTINR_VAL1 PT-INR Value

N	Mean	Standard Deviation	Minimum	Maximum
3	1.8400000	0.3517101	1.4700000	2.1700000

Unique Subject Identifier=2356011111-0009

Assay variable : PTINR_VAL1 PT-INR Value

N	Mean	Standard Deviation	Minimum	Maximum
2	1.9750000	0.0636396	1.9300000	2.0200000

Laboratory tests at the time of obtaining Table 2-6 consent, according to the presence or absence of proton-pump inhibitors

Unique Subject Identifier=2356011111-0015

MEANS Procedure

Assay variable : PTINR_VAL1 PT-INR Value

N	Mean	Standard Deviation	Minimum	Maximum
2	1.8050000	0.6434672	1.3500000	2.2600000

Unique Subject Identifier=2356011111-0016

Assay variable : PTINR_VAL1 PT-INR Value

N	Mean	Standard Deviation	Minimum	Maximum
2	2.7200000	0.6081118	2.2900000	3.1500000

Unique Subject Identifier=2356011111-0021

Assay variable : PTINR_VAL1 PT-INR Value

N	Mean	Standard Deviation	Minimum	Maximum
1	1.6300000		1.6300000	1.6300000

Unique Subject Identifier=2357011010-0005

Assay variable : PTINR_VAL1 PT-INR Value

N	Mean	Standard Deviation	Minimum	Maximum
3	1.8500000	1.1435471	1.1600000	3.1700000

Laboratory tests at the time of obtaining Table 2-6 consent, according to the presence or absence of proton-pump inhibitors

Unique Subject Identifier=2357011010-0009

MEANS Procedure

Assay variable : PTINR_VAL1 PT-INR Value

N	Mean	Standard Deviation	Minimum	Maximum
4	1.5900000	0.4543860	1.2200000	2.2300000

Unique Subject Identifier=2357011010-0019

Assay variable : PTINR_VAL1 PT-INR Value

N	Mean	Standard Deviation	Minimum	Maximum
2	2.0500000	0	2.0500000	2.0500000

Unique Subject Identifier=2357011010-0020

Assay variable : PTINR_VAL1 PT-INR Value

N	Mean	Standard Deviation	Minimum	Maximum
2	1.6100000	0.5656854	1.2100000	2.0100000

Unique Subject Identifier=2358000000-0005

Assay variable : PTINR_VAL1 PT-INR Value

N	Mean	Standard Deviation	Minimum	Maximum
3	1.5666667	0.0321455	1.5300000	1.5900000

Laboratory tests at the time of obtaining Table 2-6 consent, according to the presence or absence of proton-pump inhibitors

Unique Subject Identifier=2359000100-0001

MEANS Procedure

Assay variable : PTINR_VAL1 PT-INR Value

N	Mean	Standard Deviation	Minimum	Maximum
1	1.7400000		1.7400000	1.7400000

Unique Subject Identifier=2361011111-0013

Assay variable : PTINR_VAL1 PT-INR Value

N	Mean	Standard Deviation	Minimum	Maximum
1	1.3500000		1.3500000	1.3500000

Unique Subject Identifier=2362011111-0001

Assay variable : PTINR_VAL1 PT-INR Value

N	Mean	Standard Deviation	Minimum	Maximum
3	1.6233333	0.2345918	1.3600000	1.8100000

Unique Subject Identifier=2362011111-0003

Assay variable : PTINR_VAL1 PT-INR Value

N	Mean	Standard Deviation	Minimum	Maximum
3	1.7966667	0.4718404	1.4900000	2.3400000

Laboratory tests at the time of obtaining Table 2-6 consent, according to the presence or absence of proton-pump inhibitors

Unique Subject Identifier=2362011111-0006

MEANS Procedure

Assay variable : PTINR_VAL1 PT-INR Value

N	Mean	Standard Deviation	Minimum	Maximum
3	1.3200000	0.1473092	1.1900000	1.4800000

Unique Subject Identifier=2362011111-0007

Assay variable : PTINR_VAL1 PT-INR Value

N	Mean	Standard Deviation	Minimum	Maximum
4	1.1975000	0.0736546	1.1100000	1.2800000

Unique Subject Identifier=2365011111-0003

Assay variable : PTINR_VAL1 PT-INR Value

N	Mean	Standard Deviation	Minimum	Maximum
6	2.0166667	0.8035339	1.2000000	3.5000000

Unique Subject Identifier=2370011111-0002

Assay variable : PTINR_VAL1 PT-INR Value

N	Mean	Standard Deviation	Minimum	Maximum
5	2.1660000	0.1866280	1.9800000	2.4100000

Laboratory tests at the time of obtaining Table 2-6 consent, according to the presence or absence of proton-pump inhibitors

Unique Subject Identifier=2370011111-0003

MEANS Procedure

Assay variable : PTINR_VAL1 PT-INR Value

N	Mean	Standard Deviation	Minimum	Maximum
5	2.1760000	0.4535747	1.7700000	2.7500000

Unique Subject Identifier=2378000010-0011

Assay variable : PTINR_VAL1 PT-INR Value

N	Mean	Standard Deviation	Minimum	Maximum
6	2.3833333	0.3763863	1.7000000	2.7000000

Unique Subject Identifier=2378000010-0012

Assay variable : PTINR_VAL1 PT-INR Value

N	Mean	Standard Deviation	Minimum	Maximum
6	1.9833333	0.4262237	1.4000000	2.7000000

Unique Subject Identifier=2378000010-0015

Assay variable : PTINR_VAL1 PT-INR Value

N	Mean	Standard Deviation	Minimum	Maximum
5	2.0400000	0.3286335	1.5000000	2.3000000

Laboratory tests at the time of obtaining Table 2-6 consent, according to the presence or absence of proton-pump inhibitors

Unique Subject Identifier=2378000010-0018

MEANS Procedure

Assay variable : PTINR_VAL1 PT-INR Value

N	Mean	Standard Deviation	Minimum	Maximum
6	1.3666667	0.1751190	1.1000000	1.6000000

Unique Subject Identifier=2380011111-0001

Assay variable : PTINR_VAL1 PT-INR Value

N	Mean	Standard Deviation	Minimum	Maximum
2	1.2650000	0.0636396	1.2200000	1.3100000

Unique Subject Identifier=2383011110-0031

Assay variable : PTINR_VAL1 PT-INR Value

N	Mean	Standard Deviation	Minimum	Maximum
3	2.2500000	0.5056679	1.7600000	2.7700000

Unique Subject Identifier=2383011110-0033

Assay variable : PTINR_VAL1 PT-INR Value

N	Mean	Standard Deviation	Minimum	Maximum
6	2.1300000	0.1433876	1.9800000	2.4000000

Laboratory tests at the time of obtaining Table 2-6 consent, according to the presence or absence of proton-pump inhibitors

Unique Subject Identifier=2386011111-0008

MEANS Procedure

Assay variable : PTINR_VAL1 PT-INR Value

N	Mean	Standard Deviation	Minimum	Maximum
6	2.3500000	0.7341662	1.5000000	3.2000000

Unique Subject Identifier=2389011111-0003

Assay variable : PTINR_VAL1 PT-INR Value

N	Mean	Standard Deviation	Minimum	Maximum
3	2.1133333	0.6242863	1.4000000	2.5600000

Unique Subject Identifier=2389011111-0005

Assay variable : PTINR_VAL1 PT-INR Value

N	Mean	Standard Deviation	Minimum	Maximum
4	1.7475000	0.3148942	1.4800000	2.0900000

Unique Subject Identifier=2389011111-0017

Assay variable : PTINR_VAL1 PT-INR Value

N	Mean	Standard Deviation	Minimum	Maximum
5	1.6260000	0.2085186	1.3600000	1.7900000

Laboratory tests at the time of obtaining Table 2-6 consent, according to the presence or absence of proton-pump inhibitors

Unique Subject Identifier=2392000000-0001

MEANS Procedure

Assay variable : PTINR_VAL1 PT-INR Value

N	Mean	Standard Deviation	Minimum	Maximum
4	2.1000000	0.1825742	1.9000000	2.3000000

Unique Subject Identifier=2392000000-0005

Assay variable : PTINR_VAL1 PT-INR Value

N	Mean	Standard Deviation	Minimum	Maximum
6	2.2333333	0.4676181	1.6000000	2.7000000

Unique Subject Identifier=2392000000-0006

Assay variable : PTINR_VAL1 PT-INR Value

N	Mean	Standard Deviation	Minimum	Maximum
5	1.9400000	0.5366563	1.4000000	2.8000000

Unique Subject Identifier=2392000000-0013

Assay variable : PTINR_VAL1 PT-INR Value

N	Mean	Standard Deviation	Minimum	Maximum
6	2.1666667	0.8453796	1.6000000	3.8000000

Laboratory tests at the time of obtaining Table 2-6 consent, according to the presence or absence of proton-pump inhibitors

Unique Subject Identifier=2392000000-0017

MEANS Procedure

Assay variable : PTINR_VAL1 PT-INR Value

N	Mean	Standard Deviation	Minimum	Maximum
5	2.0400000	0.1516575	1.8000000	2.2000000

Unique Subject Identifier=2392000000-0018

Assay variable : PTINR_VAL1 PT-INR Value

N	Mean	Standard Deviation	Minimum	Maximum
6	1.9166667	0.2786874	1.6000000	2.3000000

Unique Subject Identifier=2392000000-0020

Assay variable : PTINR_VAL1 PT-INR Value

N	Mean	Standard Deviation	Minimum	Maximum
4	2.0000000	0.1414214	1.9000000	2.2000000

Unique Subject Identifier=2393011111-0003

Assay variable : PTINR_VAL1 PT-INR Value

N	Mean	Standard Deviation	Minimum	Maximum
6	1.7283333	0.1621625	1.5200000	1.9900000

Laboratory tests at the time of obtaining Table 2-6 consent, according to the presence or absence of proton-pump inhibitors

Unique Subject Identifier=2393011111-0004

MEANS Procedure

Assay variable : PTINR_VAL1 PT-INR Value

N	Mean	Standard Deviation	Minimum	Maximum
3	1.2000000	0.1452584	1.0500000	1.3400000

Unique Subject Identifier=2393011111-0005

Assay variable : PTINR_VAL1 PT-INR Value

N	Mean	Standard Deviation	Minimum	Maximum
5	1.7680000	0.2751727	1.5000000	2.1300000

Unique Subject Identifier=2393011111-0010

Assay variable : PTINR_VAL1 PT-INR Value

N	Mean	Standard Deviation	Minimum	Maximum
3	2.0900000	0.5943063	1.6700000	2.7700000

Unique Subject Identifier=2393011111-0017

Assay variable : PTINR_VAL1 PT-INR Value

N	Mean	Standard Deviation	Minimum	Maximum
4	1.6000000	0.0616441	1.5100000	1.6400000

Laboratory tests at the time of obtaining Table 2-6 consent, according to the presence or absence of proton-pump inhibitors

Unique Subject Identifier=2393011111-0025

MEANS Procedure

Assay variable : PTINR_VAL1 PT-INR Value

N	Mean	Standard Deviation	Minimum	Maximum
1	3.4000000		3.4000000	3.4000000

Unique Subject Identifier=2399011111-0004

Assay variable : PTINR_VAL1 PT-INR Value

N	Mean	Standard Deviation	Minimum	Maximum
3	1.9300000	0.5273519	1.3300000	2.3200000

Unique Subject Identifier=2403010000-0004

Assay variable : PTINR_VAL1 PT-INR Value

N	Mean	Standard Deviation	Minimum	Maximum
6	2.4583333	0.8422213	1.8200000	4.1100000

Unique Subject Identifier=2403010000-0005

Assay variable : PTINR_VAL1 PT-INR Value

N	Mean	Standard Deviation	Minimum	Maximum
6	2.2833333	0.3762269	1.9600000	3.0300000

Laboratory tests at the time of obtaining Table 2-6 consent, according to the presence or absence of proton-pump inhibitors

Unique Subject Identifier=2403010000-0025

MEANS Procedure

Assay variable : PTINR_VAL1 PT-INR Value

N	Mean	Standard Deviation	Minimum	Maximum
3	1.6433333	0.2829016	1.3300000	1.8800000

Unique Subject Identifier=2403010000-0043

Assay variable : PTINR_VAL1 PT-INR Value

N	Mean	Standard Deviation	Minimum	Maximum
5	2.4040000	0.2707951	2.0300000	2.7400000

Unique Subject Identifier=2403010000-0051

Assay variable : PTINR_VAL1 PT-INR Value

N	Mean	Standard Deviation	Minimum	Maximum
3	1.5300000	0.1915724	1.3100000	1.6600000

Unique Subject Identifier=2403010000-0060

Assay variable : PTINR_VAL1 PT-INR Value

N	Mean	Standard Deviation	Minimum	Maximum
6	2.0750000	0.2072438	1.8800000	2.4600000

Laboratory tests at the time of obtaining Table 2-6 consent, according to the presence or absence of proton-pump inhibitors

Unique Subject Identifier=2403010000-0061

MEANS Procedure

Assay variable : PTINR_VAL1 PT-INR Value

N	Mean	Standard Deviation	Minimum	Maximum
4	1.9300000	0.4208721	1.5000000	2.3300000

Unique Subject Identifier=2403010000-0069

Assay variable : PTINR_VAL1 PT-INR Value

N	Mean	Standard Deviation	Minimum	Maximum
1	2.3100000		2.3100000	2.3100000

Unique Subject Identifier=2403110000-0008

Assay variable : PTINR_VAL1 PT-INR Value

N	Mean	Standard Deviation	Minimum	Maximum
2	2.3350000	0.1767767	2.2100000	2.4600000

Unique Subject Identifier=2403110000-0016

Assay variable : PTINR_VAL1 PT-INR Value

N	Mean	Standard Deviation	Minimum	Maximum
3	1.8866667	0.0850490	1.7900000	1.9500000

Laboratory tests at the time of obtaining Table 2-6 consent, according to the presence or absence of proton-pump inhibitors

Unique Subject Identifier=2403110000-0017

MEANS Procedure

Assay variable : PTINR_VAL1 PT-INR Value

N	Mean	Standard Deviation	Minimum	Maximum
1	1.8000000		1.8000000	1.8000000

Unique Subject Identifier=2404011111-0005

Assay variable : PTINR_VAL1 PT-INR Value

N	Mean	Standard Deviation	Minimum	Maximum
6	1.9500000	0.4415880	1.6000000	2.8000000

Unique Subject Identifier=2405011111-0002

Assay variable : PTINR_VAL1 PT-INR Value

N	Mean	Standard Deviation	Minimum	Maximum
6	1.8466667	0.3586456	1.4900000	2.4600000

Unique Subject Identifier=2405011111-0005

Assay variable : PTINR_VAL1 PT-INR Value

N	Mean	Standard Deviation	Minimum	Maximum
3	1.9866667	0.2369247	1.8400000	2.2600000

Laboratory tests at the time of obtaining Table 2-6 consent, according to the presence or absence of proton-pump inhibitors

Unique Subject Identifier=2405011111-0011

MEANS Procedure

Assay variable : PTINR_VAL1 PT-INR Value

N	Mean	Standard Deviation	Minimum	Maximum
5	2.5240000	0.4204521	1.8700000	2.9500000

Unique Subject Identifier=2405011111-0014

Assay variable : PTINR_VAL1 PT-INR Value

N	Mean	Standard Deviation	Minimum	Maximum
6	1.5200000	0.4461390	1.1300000	2.3900000

Unique Subject Identifier=2405011111-0015

Assay variable : PTINR_VAL1 PT-INR Value

N	Mean	Standard Deviation	Minimum	Maximum
6	1.8216667	0.0934701	1.6900000	1.9700000

Unique Subject Identifier=2410011111-0002

Assay variable : PTINR_VAL1 PT-INR Value

N	Mean	Standard Deviation	Minimum	Maximum
2	1.6800000	0.2545584	1.5000000	1.8600000

Laboratory tests at the time of obtaining Table 2-6 consent, according to the presence or absence of proton-pump inhibitors

Unique Subject Identifier=2410011111-0008

MEANS Procedure

Assay variable : PTINR_VAL1 PT-INR Value

N	Mean	Standard Deviation	Minimum	Maximum
3	1.6100000	0.3732292	1.2000000	1.9300000

Unique Subject Identifier=2410011111-0012

Assay variable : PTINR_VAL1 PT-INR Value

N	Mean	Standard Deviation	Minimum	Maximum
1	1.3800000		1.3800000	1.3800000

Unique Subject Identifier=2410011111-0022

Assay variable : PTINR_VAL1 PT-INR Value

N	Mean	Standard Deviation	Minimum	Maximum
2	2.6150000	0.3040559	2.4000000	2.8300000

Unique Subject Identifier=2410011111-0023

Assay variable : PTINR_VAL1 PT-INR Value

N	Mean	Standard Deviation	Minimum	Maximum
2	2.7650000	1.0818734	2.0000000	3.5300000

Laboratory tests at the time of obtaining Table 2-6 consent, according to the presence or absence of proton-pump inhibitors

Unique Subject Identifier=2410011111-0025

MEANS Procedure

Assay variable : PTINR_VAL1 PT-INR Value

N	Mean	Standard Deviation	Minimum	Maximum
2	2.2150000	0.0212132	2.2000000	2.2300000

Unique Subject Identifier=2410011111-0031

Assay variable : PTINR_VAL1 PT-INR Value

N	Mean	Standard Deviation	Minimum	Maximum
3	2.0833333	0.3547299	1.7000000	2.4000000

Unique Subject Identifier=2412011111-0002

Assay variable : PTINR_VAL1 PT-INR Value

N	Mean	Standard Deviation	Minimum	Maximum
1	2.3500000		2.3500000	2.3500000

Unique Subject Identifier=2412011111-0003

Assay variable : PTINR_VAL1 PT-INR Value

N	Mean	Standard Deviation	Minimum	Maximum
2	1.3600000	0.2121320	1.2100000	1.5100000

Laboratory tests at the time of obtaining Table 2-6 consent, according to the presence or absence of proton-pump inhibitors

Unique Subject Identifier=2412011111-0004

MEANS Procedure

Assay variable : PTINR_VAL1 PT-INR Value

N	Mean	Standard Deviation	Minimum	Maximum
2	2.5000000	0.0848528	2.4400000	2.5600000

Unique Subject Identifier=2412011111-0006

Assay variable : PTINR_VAL1 PT-INR Value

N	Mean	Standard Deviation	Minimum	Maximum
1	2.6300000		2.6300000	2.6300000

Unique Subject Identifier=2421010000-0007

Assay variable : PTINR_VAL1 PT-INR Value

N	Mean	Standard Deviation	Minimum	Maximum
1	1.6800000		1.6800000	1.6800000

Unique Subject Identifier=2421010000-0020

Assay variable : PTINR_VAL1 PT-INR Value

N	Mean	Standard Deviation	Minimum	Maximum
2	2.5000000	0.2545584	2.3200000	2.6800000

Laboratory tests at the time of obtaining Table 2-6 consent, according to the presence or absence of proton-pump inhibitors

Unique Subject Identifier=2421010000-0035

MEANS Procedure

Assay variable : PTINR_VAL1 PT-INR Value

N	Mean	Standard Deviation	Minimum	Maximum
1	1.4500000		1.4500000	1.4500000

Unique Subject Identifier=2421010000-0037

Assay variable : PTINR_VAL1 PT-INR Value

N	Mean	Standard Deviation	Minimum	Maximum
4	2.3900000	0.1471960	2.2700000	2.6000000

Unique Subject Identifier=2421010000-0047

Assay variable : PTINR_VAL1 PT-INR Value

N	Mean	Standard Deviation	Minimum	Maximum
1	2.0500000		2.0500000	2.0500000

Unique Subject Identifier=2421010000-0059

Assay variable : PTINR_VAL1 PT-INR Value

N	Mean	Standard Deviation	Minimum	Maximum
4	1.5750000	0.1367479	1.3800000	1.6700000

Laboratory tests at the time of obtaining Table 2-6 consent, according to the presence or absence of proton-pump inhibitors

Unique Subject Identifier=2421010000-0066

MEANS Procedure

Assay variable : PTINR_VAL1 PT-INR Value

N	Mean	Standard Deviation	Minimum	Maximum
5	1.2700000	0.2295648	1.1200000	1.6400000

Unique Subject Identifier=2421010000-0073

Assay variable : PTINR_VAL1 PT-INR Value

N	Mean	Standard Deviation	Minimum	Maximum
4	2.4000000	0.4198412	1.9800000	2.8000000

Unique Subject Identifier=2421010000-0077

Assay variable : PTINR_VAL1 PT-INR Value

N	Mean	Standard Deviation	Minimum	Maximum
1	1.1700000		1.1700000	1.1700000

Unique Subject Identifier=2421010000-0086

Assay variable : PTINR_VAL1 PT-INR Value

N	Mean	Standard Deviation	Minimum	Maximum
1	2.3000000		2.3000000	2.3000000

Laboratory tests at the time of obtaining Table 2-6 consent, according to the presence or absence of proton-pump inhibitors

Unique Subject Identifier=2421010000-0096

MEANS Procedure

Assay variable : PTINR_VAL1 PT-INR Value

N	Mean	Standard Deviation	Minimum	Maximum
1	1.9600000		1.9600000	1.9600000

Unique Subject Identifier=2421010000-0128

Assay variable : PTINR_VAL1 PT-INR Value

N	Mean	Standard Deviation	Minimum	Maximum
1	1.8800000		1.8800000	1.8800000

Unique Subject Identifier=2421010000-0135

Assay variable : PTINR_VAL1 PT-INR Value

N	Mean	Standard Deviation	Minimum	Maximum
1	2.0300000		2.0300000	2.0300000

Unique Subject Identifier=2421010000-0137

Assay variable : PTINR_VAL1 PT-INR Value

N	Mean	Standard Deviation	Minimum	Maximum
1	1.9600000		1.9600000	1.9600000

Laboratory tests at the time of obtaining Table 2-6 consent, according to the presence or absence of proton-pump inhibitors

Unique Subject Identifier=2421010000-0138

MEANS Procedure

Assay variable : PTINR_VAL1 PT-INR Value

N	Mean	Standard Deviation	Minimum	Maximum
3	1.5733333	0.0416333	1.5400000	1.6200000

Unique Subject Identifier=2421010000-0158

Assay variable : PTINR_VAL1 PT-INR Value

N	Mean	Standard Deviation	Minimum	Maximum
6	1.8050000	0.3218540	1.4600000	2.2900000

Unique Subject Identifier=2421010000-0168

Assay variable : PTINR_VAL1 PT-INR Value

N	Mean	Standard Deviation	Minimum	Maximum
4	1.6725000	0.7430736	1.2100000	2.7700000

Unique Subject Identifier=2421010000-0174

Assay variable : PTINR_VAL1 PT-INR Value

N	Mean	Standard Deviation	Minimum	Maximum
4	2.5150000	0.9033456	1.8800000	3.8400000

Laboratory tests at the time of obtaining Table 2-6 consent, according to the presence or absence of proton-pump inhibitors

Unique Subject Identifier=2421010000-0198

MEANS Procedure

Assay variable : PTINR_VAL1 PT-INR Value

N	Mean	Standard Deviation	Minimum	Maximum
1	2.7600000		2.7600000	2.7600000

Unique Subject Identifier=2421010000-0210

Assay variable : PTINR_VAL1 PT-INR Value

N	Mean	Standard Deviation	Minimum	Maximum
1	1.6700000		1.6700000	1.6700000

Unique Subject Identifier=2421010000-0216

Assay variable : PTINR_VAL1 PT-INR Value

N	Mean	Standard Deviation	Minimum	Maximum
2	2.0500000	0.2121320	1.9000000	2.2000000

Unique Subject Identifier=2423010010-0002

Assay variable : PTINR_VAL1 PT-INR Value

N	Mean	Standard Deviation	Minimum	Maximum
1	1.8000000		1.8000000	1.8000000

Laboratory tests at the time of obtaining Table 2-6 consent, according to the presence or absence of proton-pump inhibitors

Unique Subject Identifier=2423010010-0004

MEANS Procedure

Assay variable : PTINR_VAL1 PT-INR Value

N	Mean	Standard Deviation	Minimum	Maximum
2	2.3050000	0.0636396	2.2600000	2.3500000

Unique Subject Identifier=2425010000-0001

Assay variable : PTINR_VAL1 PT-INR Value

N	Mean	Standard Deviation	Minimum	Maximum
1	1.2200000		1.2200000	1.2200000

Unique Subject Identifier=2425010000-0002

Assay variable : PTINR_VAL1 PT-INR Value

N	Mean	Standard Deviation	Minimum	Maximum
1	1.1200000		1.1200000	1.1200000

Unique Subject Identifier=2425010000-0004

Assay variable : PTINR_VAL1 PT-INR Value

N	Mean	Standard Deviation	Minimum	Maximum
1	1.0600000		1.0600000	1.0600000

Laboratory tests at the time of obtaining Table 2-6 consent, according to the presence or absence of proton-pump inhibitors

Unique Subject Identifier=2425010000-0006

MEANS Procedure

Assay variable : PTINR_VAL1 PT-INR Value

N	Mean	Standard Deviation	Minimum	Maximum
1	1.1900000		1.1900000	1.1900000

Unique Subject Identifier=2425010000-0019

Assay variable : PTINR_VAL1 PT-INR Value

N	Mean	Standard Deviation	Minimum	Maximum
1	2.0700000		2.0700000	2.0700000

Unique Subject Identifier=2425010000-0025

Assay variable : PTINR_VAL1 PT-INR Value

N	Mean	Standard Deviation	Minimum	Maximum
1	1.4600000		1.4600000	1.4600000

Unique Subject Identifier=2427000000-0001

Assay variable : PTINR_VAL1 PT-INR Value

N	Mean	Standard Deviation	Minimum	Maximum
6	1.6200000	0.1907878	1.3700000	1.9400000

Laboratory tests at the time of obtaining Table 2-6 consent, according to the presence or absence of proton-pump inhibitors

Unique Subject Identifier=2428011111-0004

MEANS Procedure

Assay variable : PTINR_VAL1 PT-INR Value

N	Mean	Standard Deviation	Minimum	Maximum
6	1.7733333	0.1287892	1.6000000	1.9700000

Unique Subject Identifier=2434011000-0002

Assay variable : PTINR_VAL1 PT-INR Value

N	Mean	Standard Deviation	Minimum	Maximum
3	2.1333333	0.4932883	1.8000000	2.7000000

Unique Subject Identifier=2434011000-0004

Assay variable : PTINR_VAL1 PT-INR Value

N	Mean	Standard Deviation	Minimum	Maximum
4	2.2250000	0.8139410	1.5000000	3.3000000

Unique Subject Identifier=2434011000-0009

Assay variable : PTINR_VAL1 PT-INR Value

N	Mean	Standard Deviation	Minimum	Maximum
3	2.2666667	0.2516611	2.0000000	2.5000000

Laboratory tests at the time of obtaining Table 2-6 consent, according to the presence or absence of proton-pump inhibitors

Unique Subject Identifier=2434011000-0010

MEANS Procedure

Assay variable : PTINR_VAL1 PT-INR Value

N	Mean	Standard Deviation	Minimum	Maximum
4	2.2000000	0.3464102	1.7000000	2.5000000

Unique Subject Identifier=2434011000-0011

Assay variable : PTINR_VAL1 PT-INR Value

N	Mean	Standard Deviation	Minimum	Maximum
4	2.3750000	0.4991660	2.0000000	3.1000000

Unique Subject Identifier=2434011000-0016

Assay variable : PTINR_VAL1 PT-INR Value

N	Mean	Standard Deviation	Minimum	Maximum
4	2.4250000	0.2217356	2.2000000	2.7000000

Unique Subject Identifier=2434011000-0018

Assay variable : PTINR_VAL1 PT-INR Value

N	Mean	Standard Deviation	Minimum	Maximum
4	2.4750000	0.4031129	1.9000000	2.8000000

Laboratory tests at the time of obtaining Table 2-6 consent, according to the presence or absence of proton-pump inhibitors

Unique Subject Identifier=2437000000-0008

MEANS Procedure

Assay variable : PTINR_VAL1 PT-INR Value

N	Mean	Standard Deviation	Minimum	Maximum
2	1.8500000	0.3818377	1.5800000	2.1200000

Unique Subject Identifier=2437000000-0011

Assay variable : PTINR_VAL1 PT-INR Value

N	Mean	Standard Deviation	Minimum	Maximum
1	1.9500000		1.9500000	1.9500000

Unique Subject Identifier=2437000000-0013

Assay variable : PTINR_VAL1 PT-INR Value

N	Mean	Standard Deviation	Minimum	Maximum
3	1.2066667	0.0577350	1.1400000	1.2400000

Unique Subject Identifier=2438010011-0002

Assay variable : PTINR_VAL1 PT-INR Value

N	Mean	Standard Deviation	Minimum	Maximum
6	1.9016667	0.5045163	1.4400000	2.7400000

Laboratory tests at the time of obtaining Table 2-6 consent, according to the presence or absence of proton-pump inhibitors

Unique Subject Identifier=2438010011-0011

MEANS Procedure

Assay variable : PTINR_VAL1 PT-INR Value

N	Mean	Standard Deviation	Minimum	Maximum
6	1.6466667	0.2516082	1.4100000	1.9700000

Unique Subject Identifier=2438010011-0025

Assay variable : PTINR_VAL1 PT-INR Value

N	Mean	Standard Deviation	Minimum	Maximum
6	1.9216667	0.2240015	1.6500000	2.2500000

Unique Subject Identifier=2438010011-0027

Assay variable : PTINR_VAL1 PT-INR Value

N	Mean	Standard Deviation	Minimum	Maximum
6	1.8233333	0.2899425	1.3500000	2.1200000

Unique Subject Identifier=2438010011-0035

Assay variable : PTINR_VAL1 PT-INR Value

N	Mean	Standard Deviation	Minimum	Maximum
6	2.1566667	0.4078071	1.7900000	2.8100000

Laboratory tests at the time of obtaining Table 2-6 consent, according to the presence or absence of proton-pump inhibitors

Unique Subject Identifier=2438010011-0040

MEANS Procedure

Assay variable : PTINR_VAL1 PT-INR Value

N	Mean	Standard Deviation	Minimum	Maximum
5	2.0300000	0.1927434	1.8300000	2.2800000

Unique Subject Identifier=2438010011-0044

Assay variable : PTINR_VAL1 PT-INR Value

N	Mean	Standard Deviation	Minimum	Maximum
6	2.1583333	0.3638910	1.7700000	2.8100000

Unique Subject Identifier=2438010011-0053

Assay variable : PTINR_VAL1 PT-INR Value

N	Mean	Standard Deviation	Minimum	Maximum
3	1.5733333	0.5519360	1.0500000	2.1500000

Unique Subject Identifier=2438010011-0059

Assay variable : PTINR_VAL1 PT-INR Value

N	Mean	Standard Deviation	Minimum	Maximum
2	1.6000000	0.5656854	1.2000000	2.0000000

Laboratory tests at the time of obtaining Table 2-6 consent, according to the presence or absence of proton-pump inhibitors

Unique Subject Identifier=2438010011-0062

MEANS Procedure

Assay variable : PTINR_VAL1 PT-INR Value

N	Mean	Standard Deviation	Minimum	Maximum
6	2.3950000	0.4478727	1.9900000	3.1000000

Unique Subject Identifier=2438010011-0065

Assay variable : PTINR_VAL1 PT-INR Value

N	Mean	Standard Deviation	Minimum	Maximum
3	1.5933333	0.0873689	1.5200000	1.6900000

Unique Subject Identifier=2438010011-0066

Assay variable : PTINR_VAL1 PT-INR Value

N	Mean	Standard Deviation	Minimum	Maximum
3	2.0900000	0.6349803	1.6100000	2.8100000

Unique Subject Identifier=2438010011-0067

Assay variable : PTINR_VAL1 PT-INR Value

N	Mean	Standard Deviation	Minimum	Maximum
1	1.0000000		1.0000000	1.0000000

Laboratory tests at the time of obtaining Table 2-6 consent, according to the presence or absence of proton-pump inhibitors

Unique Subject Identifier=2448011111-0002

MEANS Procedure

Assay variable : PTINR_VAL1 PT-INR Value

N	Mean	Standard Deviation	Minimum	Maximum
2	1.4900000	0.4949747	1.1400000	1.8400000

Unique Subject Identifier=2449011111-0001

Assay variable : PTINR_VAL1 PT-INR Value

N	Mean	Standard Deviation	Minimum	Maximum
6	1.9333333	0.7633261	1.2000000	3.2000000

Unique Subject Identifier=2449011111-0003

Assay variable : PTINR_VAL1 PT-INR Value

N	Mean	Standard Deviation	Minimum	Maximum
6	2.3166667	0.4578937	1.6000000	2.7000000

Unique Subject Identifier=2449011111-0005

Assay variable : PTINR_VAL1 PT-INR Value

N	Mean	Standard Deviation	Minimum	Maximum
6	1.5000000	0.2756810	1.2000000	1.9000000

Laboratory tests at the time of obtaining Table 2-6 consent, according to the presence or absence of proton-pump inhibitors

Unique Subject Identifier=2449011111-0006

MEANS Procedure

Assay variable : PTINR_VAL1 PT-INR Value

N	Mean	Standard Deviation	Minimum	Maximum
6	2.1000000	0.1673320	1.9000000	2.3000000

Unique Subject Identifier=2449011111-0008

Assay variable : PTINR_VAL1 PT-INR Value

N	Mean	Standard Deviation	Minimum	Maximum
6	2.2166667	0.3868678	1.7000000	2.8000000

Unique Subject Identifier=2449011111-0010

Assay variable : PTINR_VAL1 PT-INR Value

N	Mean	Standard Deviation	Minimum	Maximum
6	2.3500000	0.1048809	2.2000000	2.5000000

Unique Subject Identifier=2449011111-0014

Assay variable : PTINR_VAL1 PT-INR Value

N	Mean	Standard Deviation	Minimum	Maximum
3	1.6333333	0.0577350	1.6000000	1.7000000

Laboratory tests at the time of obtaining Table 2-6 consent, according to the presence or absence of proton-pump inhibitors

Unique Subject Identifier=2449011111-0018

MEANS Procedure

Assay variable : PTINR_VAL1 PT-INR Value

N	Mean	Standard Deviation	Minimum	Maximum
5	2.2000000	0.2549510	1.9000000	2.6000000

Unique Subject Identifier=2449011111-0019

Assay variable : PTINR_VAL1 PT-INR Value

N	Mean	Standard Deviation	Minimum	Maximum
6	2.2833333	0.3600926	1.9000000	2.9000000

Unique Subject Identifier=2449011111-0020

Assay variable : PTINR_VAL1 PT-INR Value

N	Mean	Standard Deviation	Minimum	Maximum
4	1.5000000	0.1414214	1.3000000	1.6000000

Unique Subject Identifier=2449011111-0021

Assay variable : PTINR_VAL1 PT-INR Value

N	Mean	Standard Deviation	Minimum	Maximum
6	1.5500000	0.1378405	1.4000000	1.7000000

Laboratory tests at the time of obtaining Table 2-6 consent, according to the presence or absence of proton-pump inhibitors

Unique Subject Identifier=2449011111-0022

MEANS Procedure

Assay variable : PTINR_VAL1 PT-INR Value

N	Mean	Standard Deviation	Minimum	Maximum
6	1.9500000	0.6252999	1.2000000	3.1000000

Unique Subject Identifier=2449011111-0023

Assay variable : PTINR_VAL1 PT-INR Value

N	Mean	Standard Deviation	Minimum	Maximum
5	1.5200000	0.1095445	1.4000000	1.6000000

Unique Subject Identifier=2449011111-0026

Assay variable : PTINR_VAL1 PT-INR Value

N	Mean	Standard Deviation	Minimum	Maximum
6	1.8166667	0.2639444	1.6000000	2.3000000

Unique Subject Identifier=2449011111-0030

Assay variable : PTINR_VAL1 PT-INR Value

N	Mean	Standard Deviation	Minimum	Maximum
6	1.8500000	0.1870829	1.7000000	2.2000000

Laboratory tests at the time of obtaining Table 2-6 consent, according to the presence or absence of proton-pump inhibitors

Unique Subject Identifier=2449011111-0032

MEANS Procedure

Assay variable : PTINR_VAL1 PT-INR Value

N	Mean	Standard Deviation	Minimum	Maximum
5	1.0400000	0.0547723	1.0000000	1.1000000

Unique Subject Identifier=2449011111-0033

Assay variable : PTINR_VAL1 PT-INR Value

N	Mean	Standard Deviation	Minimum	Maximum
6	1.7833333	0.1834848	1.6000000	2.1000000

Unique Subject Identifier=2449011111-0034

Assay variable : PTINR_VAL1 PT-INR Value

N	Mean	Standard Deviation	Minimum	Maximum
5	1.9400000	0.5029911	1.2000000	2.5000000

Unique Subject Identifier=2449011111-0036

Assay variable : PTINR_VAL1 PT-INR Value

N	Mean	Standard Deviation	Minimum	Maximum
6	1.6833333	0.1169045	1.6000000	1.9000000

Laboratory tests at the time of obtaining Table 2-6 consent, according to the presence or absence of proton-pump inhibitors

Unique Subject Identifier=2449011111-0041

MEANS Procedure

Assay variable : PTINR_VAL1 PT-INR Value

N	Mean	Standard Deviation	Minimum	Maximum
6	2.2000000	0.2097618	1.9000000	2.5000000

Unique Subject Identifier=2449011111-0046

Assay variable : PTINR_VAL1 PT-INR Value

N	Mean	Standard Deviation	Minimum	Maximum
6	1.9500000	0.2880972	1.7000000	2.4000000

Unique Subject Identifier=2449011111-0047

Assay variable : PTINR_VAL1 PT-INR Value

N	Mean	Standard Deviation	Minimum	Maximum
5	1.9200000	0.3033150	1.5000000	2.3000000

Unique Subject Identifier=2449011111-0048

Assay variable : PTINR_VAL1 PT-INR Value

N	Mean	Standard Deviation	Minimum	Maximum
1	2.6000000		2.6000000	2.6000000

Laboratory tests at the time of obtaining Table 2-6 consent, according to the presence or absence of proton-pump inhibitors

Unique Subject Identifier=2456000000-0001

MEANS Procedure

Assay variable : PTINR_VAL1 PT-INR Value

N	Mean	Standard Deviation	Minimum	Maximum
3	1.6000000	0.0624500	1.5300000	1.6500000

Unique Subject Identifier=2456000000-0003

Assay variable : PTINR_VAL1 PT-INR Value

N	Mean	Standard Deviation	Minimum	Maximum
3	1.7033333	0.1908752	1.5600000	1.9200000

Unique Subject Identifier=2456000000-0006

Assay variable : PTINR_VAL1 PT-INR Value

N	Mean	Standard Deviation	Minimum	Maximum
2	1.8950000	0.0777817	1.8400000	1.9500000

Unique Subject Identifier=2456000000-0007

Assay variable : PTINR_VAL1 PT-INR Value

N	Mean	Standard Deviation	Minimum	Maximum
3	1.6466667	0.2177919	1.4700000	1.8900000

Laboratory tests at the time of obtaining Table 2-6 consent, according to the presence or absence of proton-pump inhibitors

Unique Subject Identifier=2471011111-0001

MEANS Procedure

Assay variable : PTINR_VAL1 PT-INR Value

N	Mean	Standard Deviation	Minimum	Maximum
6	1.6500000	0.1224745	1.5000000	1.8000000

Unique Subject Identifier=2471011111-0003

Assay variable : PTINR_VAL1 PT-INR Value

N	Mean	Standard Deviation	Minimum	Maximum
5	2.3800000	0.2774887	2.1000000	2.8000000

Unique Subject Identifier=2471011111-0004

Assay variable : PTINR_VAL1 PT-INR Value

N	Mean	Standard Deviation	Minimum	Maximum
6	2.3166667	0.3060501	1.9000000	2.6000000

Unique Subject Identifier=2471011111-0005

Assay variable : PTINR_VAL1 PT-INR Value

N	Mean	Standard Deviation	Minimum	Maximum
5	1.9800000	0.1483240	1.8000000	2.2000000

Laboratory tests at the time of obtaining Table 2-6 consent, according to the presence or absence of proton-pump inhibitors

Unique Subject Identifier=2480011111-0003

MEANS Procedure

Assay variable : PTINR_VAL1 PT-INR Value

N	Mean	Standard Deviation	Minimum	Maximum
6	2.3550000	0.2442744	2.0000000	2.7600000

Unique Subject Identifier=2480011111-0004

Assay variable : PTINR_VAL1 PT-INR Value

N	Mean	Standard Deviation	Minimum	Maximum
4	2.3025000	0.1479583	2.1100000	2.4600000

Unique Subject Identifier=2480011111-0011

Assay variable : PTINR_VAL1 PT-INR Value

N	Mean	Standard Deviation	Minimum	Maximum
2	1.3800000	0.0707107	1.3300000	1.4300000

Unique Subject Identifier=2487000000-0001

Assay variable : PTINR_VAL1 PT-INR Value

N	Mean	Standard Deviation	Minimum	Maximum
4	1.6000000	0.4242641	1.2000000	2.1000000

Laboratory tests at the time of obtaining Table 2-6 consent, according to the presence or absence of proton-pump inhibitors

Unique Subject Identifier=2487000000-0014

MEANS Procedure

Assay variable : PTINR_VAL1 PT-INR Value

N	Mean	Standard Deviation	Minimum	Maximum
5	1.7200000	0.2489980	1.4000000	2.1000000

Unique Subject Identifier=2487000000-0016

Assay variable : PTINR_VAL1 PT-INR Value

N	Mean	Standard Deviation	Minimum	Maximum
1	1.5000000		1.5000000	1.5000000

Unique Subject Identifier=2488011100-0001

Assay variable : PTINR_VAL1 PT-INR Value

N	Mean	Standard Deviation	Minimum	Maximum
1	1.6000000		1.6000000	1.6000000

Unique Subject Identifier=2488011100-0008

Assay variable : PTINR_VAL1 PT-INR Value

N	Mean	Standard Deviation	Minimum	Maximum
2	1.2300000	0.0424264	1.2000000	1.2600000

Laboratory tests at the time of obtaining Table 2-6 consent, according to the presence or absence of proton-pump inhibitors

Unique Subject Identifier=2488011100-0009

MEANS Procedure

Assay variable : PTINR_VAL1 PT-INR Value

N	Mean	Standard Deviation	Minimum	Maximum
2	1.3950000	0.0777817	1.3400000	1.4500000

Unique Subject Identifier=2490011000-0006

Assay variable : PTINR_VAL1 PT-INR Value

N	Mean	Standard Deviation	Minimum	Maximum
2	1.8350000	0.2050610	1.6900000	1.9800000

Unique Subject Identifier=2498010111-0001

Assay variable : PTINR_VAL1 PT-INR Value

N	Mean	Standard Deviation	Minimum	Maximum
5	1.6800000	0.1483240	1.5000000	1.9000000

Unique Subject Identifier=2498010111-0006

Assay variable : PTINR_VAL1 PT-INR Value

N	Mean	Standard Deviation	Minimum	Maximum
5	2.0800000	0.4438468	1.4000000	2.5000000

Laboratory tests at the time of obtaining Table 2-6 consent, according to the presence or absence of proton-pump inhibitors

Unique Subject Identifier=2498010111-0018

MEANS Procedure

Assay variable : PTINR_VAL1 PT-INR Value

N	Mean	Standard Deviation	Minimum	Maximum
4	1.8250000	0.3403430	1.4000000	2.1000000

Unique Subject Identifier=2507000000-0004

Assay variable : PTINR_VAL1 PT-INR Value

N	Mean	Standard Deviation	Minimum	Maximum
2	2.4150000	0.1202082	2.3300000	2.5000000

Unique Subject Identifier=2507000000-0008

Assay variable : PTINR_VAL1 PT-INR Value

N	Mean	Standard Deviation	Minimum	Maximum
3	2.1300000	0.4158125	1.8800000	2.6100000

Unique Subject Identifier=2507000000-0009

Assay variable : PTINR_VAL1 PT-INR Value

N	Mean	Standard Deviation	Minimum	Maximum
2	2.4100000	1.0606602	1.6600000	3.1600000

Laboratory tests at the time of obtaining Table 2-6 consent, according to the presence or absence of proton-pump inhibitors

Unique Subject Identifier=2507000000-0014

MEANS Procedure

Assay variable : PTINR_VAL1 PT-INR Value

N	Mean	Standard Deviation	Minimum	Maximum
5	2.1380000	0.1359044	1.9700000	2.3000000

Unique Subject Identifier=2507000000-0016

Assay variable : PTINR_VAL1 PT-INR Value

N	Mean	Standard Deviation	Minimum	Maximum
1	2.1100000		2.1100000	2.1100000

Unique Subject Identifier=2507000000-0019

Assay variable : PTINR_VAL1 PT-INR Value

N	Mean	Standard Deviation	Minimum	Maximum
5	1.7300000	0.2517936	1.5500000	2.1300000

Unique Subject Identifier=2511011000-0002

Assay variable : PTINR_VAL1 PT-INR Value

N	Mean	Standard Deviation	Minimum	Maximum
5	1.6800000	0.1303840	1.6000000	1.9000000

Laboratory tests at the time of obtaining Table 2-6 consent, according to the presence or absence of proton-pump inhibitors

Unique Subject Identifier=2511011000-0004

MEANS Procedure

Assay variable : PTINR_VAL1 PT-INR Value

N	Mean	Standard Deviation	Minimum	Maximum
5	2.2460000	0.3950696	1.9000000	2.9000000

Unique Subject Identifier=2511011000-0007

Assay variable : PTINR_VAL1 PT-INR Value

N	Mean	Standard Deviation	Minimum	Maximum
6	1.3333333	0.1632993	1.2000000	1.6000000

Unique Subject Identifier=2511011000-0008

Assay variable : PTINR_VAL1 PT-INR Value

N	Mean	Standard Deviation	Minimum	Maximum
5	2.2200000	0.2387467	2.0000000	2.6000000

Unique Subject Identifier=2511011000-0023

Assay variable : PTINR_VAL1 PT-INR Value

N	Mean	Standard Deviation	Minimum	Maximum
6	2.2333333	0.3747355	1.8800000	2.9000000

Laboratory tests at the time of obtaining Table 2-6 consent, according to the presence or absence of proton-pump inhibitors

Unique Subject Identifier=2511011000-0025

MEANS Procedure

Assay variable : PTINR_VAL1 PT-INR Value

N	Mean	Standard Deviation	Minimum	Maximum
6	1.5833333	0.1940790	1.3000000	1.8000000

Unique Subject Identifier=2511011000-0027

Assay variable : PTINR_VAL1 PT-INR Value

N	Mean	Standard Deviation	Minimum	Maximum
6	2.2033333	0.6986177	1.5000000	3.3000000

Unique Subject Identifier=2511011000-0033

Assay variable : PTINR_VAL1 PT-INR Value

N	Mean	Standard Deviation	Minimum	Maximum
6	1.3833333	0.2786874	1.1000000	1.9000000

Unique Subject Identifier=2511011000-0044

Assay variable : PTINR_VAL1 PT-INR Value

N	Mean	Standard Deviation	Minimum	Maximum
6	1.8000000	0.5549775	1.4000000	2.8000000

Laboratory tests at the time of obtaining Table 2-6 consent, according to the presence or absence of proton-pump inhibitors

Unique Subject Identifier=2511011000-0050

MEANS Procedure

Assay variable : PTINR_VAL1 PT-INR Value

N	Mean	Standard Deviation	Minimum	Maximum
5	2.1240000	0.4897755	1.9000000	3.0000000

Unique Subject Identifier=2511011000-0055

Assay variable : PTINR_VAL1 PT-INR Value

N	Mean	Standard Deviation	Minimum	Maximum
6	1.7000000	0.6164414	1.3000000	2.9000000

Unique Subject Identifier=2511011000-0056

Assay variable : PTINR_VAL1 PT-INR Value

N	Mean	Standard Deviation	Minimum	Maximum
6	1.7300000	0.1892089	1.5000000	2.0000000

Unique Subject Identifier=2511011000-0067

Assay variable : PTINR_VAL1 PT-INR Value

N	Mean	Standard Deviation	Minimum	Maximum
6	1.9500000	0.2073644	1.7000000	2.3000000

Laboratory tests at the time of obtaining Table 2-6 consent, according to the presence or absence of proton-pump inhibitors

Unique Subject Identifier=2511011000-0075

MEANS Procedure

Assay variable : PTINR_VAL1 PT-INR Value

N	Mean	Standard Deviation	Minimum	Maximum
5	2.1200000	0.2683282	1.9000000	2.5000000

Unique Subject Identifier=2524011001-0001

Assay variable : PTINR_VAL1 PT-INR Value

N	Mean	Standard Deviation	Minimum	Maximum
1	1.7700000		1.7700000	1.7700000

Unique Subject Identifier=2524011001-0002

Assay variable : PTINR_VAL1 PT-INR Value

N	Mean	Standard Deviation	Minimum	Maximum
2	1.8400000	0.2545584	1.6600000	2.0200000

Unique Subject Identifier=2527000000-0008

Assay variable : PTINR_VAL1 PT-INR Value

N	Mean	Standard Deviation	Minimum	Maximum
6	2.0666667	0.2658320	1.8000000	2.4000000

Laboratory tests at the time of obtaining Table 2-6 consent, according to the presence or absence of proton-pump inhibitors

Unique Subject Identifier=2527000000-0012

MEANS Procedure

Assay variable : PTINR_VAL1 PT-INR Value

N	Mean	Standard Deviation	Minimum	Maximum
5	1.6400000	0.1516575	1.5000000	1.8000000

Unique Subject Identifier=2527000000-0016

Assay variable : PTINR_VAL1 PT-INR Value

N	Mean	Standard Deviation	Minimum	Maximum
6	2.0666667	0.2658320	1.7000000	2.4000000

Unique Subject Identifier=2527000000-0023

Assay variable : PTINR_VAL1 PT-INR Value

N	Mean	Standard Deviation	Minimum	Maximum
6	2.3666667	0.3265986	2.1000000	3.0000000

Unique Subject Identifier=2527000000-0029

Assay variable : PTINR_VAL1 PT-INR Value

N	Mean	Standard Deviation	Minimum	Maximum
6	2.0166667	0.4445972	1.5000000	2.6000000

Laboratory tests at the time of obtaining Table 2-6 consent, according to the presence or absence of proton-pump inhibitors

Unique Subject Identifier=2527000000-0033

MEANS Procedure

Assay variable : PTINR_VAL1 PT-INR Value

N	Mean	Standard Deviation	Minimum	Maximum
5	1.7800000	0.2167948	1.5000000	2.1000000

Unique Subject Identifier=2527000000-0036

Assay variable : PTINR_VAL1 PT-INR Value

N	Mean	Standard Deviation	Minimum	Maximum
6	2.0333333	0.3265986	1.6000000	2.5000000

Unique Subject Identifier=2527000000-0058

Assay variable : PTINR_VAL1 PT-INR Value

N	Mean	Standard Deviation	Minimum	Maximum
6	1.5500000	0.2345208	1.3000000	2.0000000

Unique Subject Identifier=2527000000-0063

Assay variable : PTINR_VAL1 PT-INR Value

N	Mean	Standard Deviation	Minimum	Maximum
6	2.0666667	0.6055301	1.4000000	3.0000000

Laboratory tests at the time of obtaining Table 2-6 consent, according to the presence or absence of proton-pump inhibitors

Unique Subject Identifier=2527000000-0065

MEANS Procedure

Assay variable : PTINR_VAL1 PT-INR Value

N	Mean	Standard Deviation	Minimum	Maximum
6	2.3000000	0.5366563	1.7000000	3.2000000

Unique Subject Identifier=2527000000-0067

Assay variable : PTINR_VAL1 PT-INR Value

N	Mean	Standard Deviation	Minimum	Maximum
6	1.8833333	0.1169045	1.8000000	2.1000000

Unique Subject Identifier=2527000000-0068

Assay variable : PTINR_VAL1 PT-INR Value

N	Mean	Standard Deviation	Minimum	Maximum
5	1.8400000	0.3577709	1.3000000	2.2000000

Unique Subject Identifier=2527000000-0069

Assay variable : PTINR_VAL1 PT-INR Value

N	Mean	Standard Deviation	Minimum	Maximum
6	1.9000000	0.1897367	1.7000000	2.1000000

Laboratory tests at the time of obtaining Table 2-6 consent, according to the presence or absence of proton-pump inhibitors

Unique Subject Identifier=2527000000-0070

MEANS Procedure

Assay variable : PTINR_VAL1 PT-INR Value

N	Mean	Standard Deviation	Minimum	Maximum
6	1.7166667	0.1471960	1.5000000	1.9000000

Unique Subject Identifier=2527000000-0071

Assay variable : PTINR_VAL1 PT-INR Value

N	Mean	Standard Deviation	Minimum	Maximum
6	2.0166667	0.2639444	1.6000000	2.4000000

Unique Subject Identifier=2527000000-0073

Assay variable : PTINR_VAL1 PT-INR Value

N	Mean	Standard Deviation	Minimum	Maximum
6	2.1166667	0.3868678	1.6000000	2.6000000

Unique Subject Identifier=2527000000-0075

Assay variable : PTINR_VAL1 PT-INR Value

N	Mean	Standard Deviation	Minimum	Maximum
5	1.6600000	0.6107373	1.1000000	2.5000000

Laboratory tests at the time of obtaining Table 2-6 consent, according to the presence or absence of proton-pump inhibitors

Unique Subject Identifier=2535000000-0001

MEANS Procedure

Assay variable : PTINR_VAL1 PT-INR Value

N	Mean	Standard Deviation	Minimum	Maximum
6	2.3833333	0.4445972	1.6000000	2.7000000

Unique Subject Identifier=2560011100-0001

Assay variable : PTINR_VAL1 PT-INR Value

N	Mean	Standard Deviation	Minimum	Maximum
6	1.0983333	0.0495648	1.0300000	1.1400000

Unique Subject Identifier=2560011100-0003

Assay variable : PTINR_VAL1 PT-INR Value

N	Mean	Standard Deviation	Minimum	Maximum
2	1.5850000	0.1343503	1.4900000	1.6800000

Unique Subject Identifier=2560011100-0005

Assay variable : PTINR_VAL1 PT-INR Value

N	Mean	Standard Deviation	Minimum	Maximum
1	1.7000000		1.7000000	1.7000000

Laboratory tests at the time of obtaining Table 2-6 consent, according to the presence or absence of proton-pump inhibitors

Unique Subject Identifier=2560011100-0006

MEANS Procedure

Assay variable : PTINR_VAL1 PT-INR Value

N	Mean	Standard Deviation	Minimum	Maximum
1	1.6000000		1.6000000	1.6000000

Unique Subject Identifier=2560011100-0007

Assay variable : PTINR_VAL1 PT-INR Value

N	Mean	Standard Deviation	Minimum	Maximum
3	1.4000000	0.4000000	1.0000000	1.8000000

Unique Subject Identifier=2560011100-0008

Assay variable : PTINR_VAL1 PT-INR Value

N	Mean	Standard Deviation	Minimum	Maximum
1	1.5000000		1.5000000	1.5000000

Unique Subject Identifier=2582010000-0004

Assay variable : PTINR_VAL1 PT-INR Value

N	Mean	Standard Deviation	Minimum	Maximum
6	2.1350000	0.3440203	1.8000000	2.6400000

Laboratory tests at the time of obtaining Table 2-6 consent, according to the presence or absence of proton-pump inhibitors

Unique Subject Identifier=2582010000-0006

MEANS Procedure

Assay variable : PTINR_VAL1 PT-INR Value

N	Mean	Standard Deviation	Minimum	Maximum
6	1.9433333	0.2124774	1.6500000	2.2000000

Unique Subject Identifier=2582010000-0020

Assay variable : PTINR_VAL1 PT-INR Value

N	Mean	Standard Deviation	Minimum	Maximum
6	1.9683333	0.2100873	1.7300000	2.2100000

Unique Subject Identifier=2582010000-0029

Assay variable : PTINR_VAL1 PT-INR Value

N	Mean	Standard Deviation	Minimum	Maximum
5	1.5660000	0.2020643	1.3400000	1.7900000

Unique Subject Identifier=2600000000-0001

Assay variable : PTINR_VAL1 PT-INR Value

N	Mean	Standard Deviation	Minimum	Maximum
6	2.2500000	0.2738613	1.8000000	2.5000000

Laboratory tests at the time of obtaining Table 2-6 consent, according to the presence or absence of proton-pump inhibitors

Unique Subject Identifier=2603011011-0029

MEANS Procedure

Assay variable : PTINR_VAL1 PT-INR Value

N	Mean	Standard Deviation	Minimum	Maximum
6	2.1000000	0.5865151	1.5000000	2.8000000

Unique Subject Identifier=2603011011-0035

Assay variable : PTINR_VAL1 PT-INR Value

N	Mean	Standard Deviation	Minimum	Maximum
6	2.2166667	0.2136976	2.0000000	2.6000000

Unique Subject Identifier=2606011111-0002

Assay variable : PTINR_VAL1 PT-INR Value

N	Mean	Standard Deviation	Minimum	Maximum
2	1.4100000	0.1697056	1.2900000	1.5300000

Unique Subject Identifier=2606011111-0019

Assay variable : PTINR_VAL1 PT-INR Value

N	Mean	Standard Deviation	Minimum	Maximum
6	1.5516667	0.2847045	1.1100000	1.9400000

Laboratory tests at the time of obtaining Table 2-6 consent, according to the presence or absence of proton-pump inhibitors

Unique Subject Identifier=2606011111-0029

MEANS Procedure

Assay variable : PTINR_VAL1 PT-INR Value

N	Mean	Standard Deviation	Minimum	Maximum
6	2.1216667	0.5464583	1.1400000	2.6900000

Unique Subject Identifier=2606011111-0036

Assay variable : PTINR_VAL1 PT-INR Value

N	Mean	Standard Deviation	Minimum	Maximum
6	2.1183333	0.1847611	1.8600000	2.4200000

Unique Subject Identifier=2606011111-0038

Assay variable : PTINR_VAL1 PT-INR Value

N	Mean	Standard Deviation	Minimum	Maximum
3	1.8033333	0.0723418	1.7200000	1.8500000

Unique Subject Identifier=2606011111-0043

Assay variable : PTINR_VAL1 PT-INR Value

N	Mean	Standard Deviation	Minimum	Maximum
3	1.9833333	0.1844813	1.7800000	2.1400000

Laboratory tests at the time of obtaining Table 2-6 consent, according to the presence or absence of proton-pump inhibitors

Unique Subject Identifier=2606011111-0046

MEANS Procedure

Assay variable : PTINR_VAL1 PT-INR Value

N	Mean	Standard Deviation	Minimum	Maximum
3	2.3666667	0.0321455	2.3300000	2.3900000

Unique Subject Identifier=2606011111-0087

Assay variable : PTINR_VAL1 PT-INR Value

N	Mean	Standard Deviation	Minimum	Maximum
5	1.6600000	0.6224950	1.0100000	2.5700000

Unique Subject Identifier=2606011111-0090

Assay variable : PTINR_VAL1 PT-INR Value

N	Mean	Standard Deviation	Minimum	Maximum
5	1.8240000	0.2786216	1.3900000	2.1600000

Unique Subject Identifier=2606011111-0100

Assay variable : PTINR_VAL1 PT-INR Value

N	Mean	Standard Deviation	Minimum	Maximum
6	1.0500000	0.1219836	0.9500000	1.2900000

Laboratory tests at the time of obtaining Table 2-6 consent, according to the presence or absence of proton-pump inhibitors

Unique Subject Identifier=2606011111-0101

MEANS Procedure

Assay variable : PTINR_VAL1 PT-INR Value

N	Mean	Standard Deviation	Minimum	Maximum
6	1.4483333	0.3348084	0.9500000	1.8500000

Unique Subject Identifier=2606011111-0142

Assay variable : PTINR_VAL1 PT-INR Value

N	Mean	Standard Deviation	Minimum	Maximum
6	1.9100000	0.4762352	1.4500000	2.7600000

Unique Subject Identifier=2607010010-0020

Assay variable : PTINR_VAL1 PT-INR Value

N	Mean	Standard Deviation	Minimum	Maximum
3	2.2433333	0.6051722	1.6300000	2.8400000

Unique Subject Identifier=2607010010-0037

Assay variable : PTINR_VAL1 PT-INR Value

N	Mean	Standard Deviation	Minimum	Maximum
3	2.0500000	0.4887740	1.5300000	2.5000000

Laboratory tests at the time of obtaining Table 2-6 consent, according to the presence or absence of proton-pump inhibitors

Unique Subject Identifier=2607010010-0038

MEANS Procedure

Assay variable : PTINR_VAL1 PT-INR Value

N	Mean	Standard Deviation	Minimum	Maximum
4	2.1425000	0.0846069	2.0400000	2.2300000

Unique Subject Identifier=2607010010-0045

Assay variable : PTINR_VAL1 PT-INR Value

N	Mean	Standard Deviation	Minimum	Maximum
4	1.5425000	0.0505800	1.4800000	1.6000000

Unique Subject Identifier=2607010010-0047

Assay variable : PTINR_VAL1 PT-INR Value

N	Mean	Standard Deviation	Minimum	Maximum
3	1.8766667	0.4354691	1.4300000	2.3000000

Unique Subject Identifier=2607010010-0061

Assay variable : PTINR_VAL1 PT-INR Value

N	Mean	Standard Deviation	Minimum	Maximum
3	1.7800000	0.0556776	1.7200000	1.8300000

Laboratory tests at the time of obtaining Table 2-6 consent, according to the presence or absence of proton-pump inhibitors

Unique Subject Identifier=2607010010-0063

MEANS Procedure

Assay variable : PTINR_VAL1 PT-INR Value

N	Mean	Standard Deviation	Minimum	Maximum
4	2.0225000	0.3346018	1.6300000	2.4400000

Unique Subject Identifier=2608010010-0009

Assay variable : PTINR_VAL1 PT-INR Value

N	Mean	Standard Deviation	Minimum	Maximum
3	2.1333333	0.7247298	1.7000000	2.9700000

Unique Subject Identifier=2608010010-0020

Assay variable : PTINR_VAL1 PT-INR Value

N	Mean	Standard Deviation	Minimum	Maximum
4	1.9275000	0.1252664	1.8000000	2.0400000

Unique Subject Identifier=2608010010-0030

Assay variable : PTINR_VAL1 PT-INR Value

N	Mean	Standard Deviation	Minimum	Maximum
5	1.6700000	0.1734935	1.4800000	1.9500000

Laboratory tests at the time of obtaining Table 2-6 consent, according to the presence or absence of proton-pump inhibitors

Unique Subject Identifier=2608010010-0038

MEANS Procedure

Assay variable : PTINR_VAL1 PT-INR Value

N	Mean	Standard Deviation	Minimum	Maximum
2	1.4650000	0.1060660	1.3900000	1.5400000

Unique Subject Identifier=2608010010-0039

Assay variable : PTINR_VAL1 PT-INR Value

N	Mean	Standard Deviation	Minimum	Maximum
3	1.5266667	0.1305118	1.3900000	1.6500000

Unique Subject Identifier=2608010010-0042

Assay variable : PTINR_VAL1 PT-INR Value

N	Mean	Standard Deviation	Minimum	Maximum
4	1.6525000	0.0670199	1.5900000	1.7200000

Unique Subject Identifier=2608010010-0047

Assay variable : PTINR_VAL1 PT-INR Value

N	Mean	Standard Deviation	Minimum	Maximum
3	1.4900000	0.2206808	1.2800000	1.7200000

Laboratory tests at the time of obtaining Table 2-6 consent, according to the presence or absence of proton-pump inhibitors

Unique Subject Identifier=2608010010-0048

MEANS Procedure

Assay variable : PTINR_VAL1 PT-INR Value

N	Mean	Standard Deviation	Minimum	Maximum
6	1.9200000	0.2499600	1.6800000	2.3700000

Unique Subject Identifier=2608010010-0051

Assay variable : PTINR_VAL1 PT-INR Value

N	Mean	Standard Deviation	Minimum	Maximum
5	2.2680000	0.6436381	1.7400000	3.3600000

Unique Subject Identifier=2608010010-0052

Assay variable : PTINR_VAL1 PT-INR Value

N	Mean	Standard Deviation	Minimum	Maximum
5	2.0280000	0.4770430	1.6900000	2.8700000

Unique Subject Identifier=2608010010-0060

Assay variable : PTINR_VAL1 PT-INR Value

N	Mean	Standard Deviation	Minimum	Maximum
5	1.7520000	0.2411846	1.3900000	1.9600000

Laboratory tests at the time of obtaining Table 2-6 consent, according to the presence or absence of proton-pump inhibitors

Unique Subject Identifier=2608010010-0063

MEANS Procedure

Assay variable : PTINR_VAL1 PT-INR Value

N	Mean	Standard Deviation	Minimum	Maximum
4	2.0700000	1.0701090	1.0400000	3.4300000

Unique Subject Identifier=2608010010-0064

Assay variable : PTINR_VAL1 PT-INR Value

N	Mean	Standard Deviation	Minimum	Maximum
1	1.3400000		1.3400000	1.3400000

Unique Subject Identifier=2608010010-0066

Assay variable : PTINR_VAL1 PT-INR Value

N	Mean	Standard Deviation	Minimum	Maximum
5	1.9360000	0.1092245	1.7600000	2.0400000

Unique Subject Identifier=2608010010-0068

Assay variable : PTINR_VAL1 PT-INR Value

N	Mean	Standard Deviation	Minimum	Maximum
6	2.1433333	0.1938728	1.8400000	2.4500000

Laboratory tests at the time of obtaining Table 2-6 consent, according to the presence or absence of proton-pump inhibitors

Unique Subject Identifier=2608010010-0078

MEANS Procedure

Assay variable : PTINR_VAL1 PT-INR Value

N	Mean	Standard Deviation	Minimum	Maximum
4	1.7825000	0.2051625	1.4900000	1.9300000

Unique Subject Identifier=2608010010-0079

Assay variable : PTINR_VAL1 PT-INR Value

N	Mean	Standard Deviation	Minimum	Maximum
6	2.2350000	1.1811308	1.2600000	4.3300000

Unique Subject Identifier=2608010010-0082

Assay variable : PTINR_VAL1 PT-INR Value

N	Mean	Standard Deviation	Minimum	Maximum
2	2.2950000	0.0919239	2.2300000	2.3600000

Unique Subject Identifier=2608010010-0085

Assay variable : PTINR_VAL1 PT-INR Value

N	Mean	Standard Deviation	Minimum	Maximum
6	1.6133333	0.0752773	1.5400000	1.7100000

Laboratory tests at the time of obtaining Table 2-6 consent, according to the presence or absence of proton-pump inhibitors

Unique Subject Identifier=2608010010-0086

MEANS Procedure

Assay variable : PTINR_VAL1 PT-INR Value

N	Mean	Standard Deviation	Minimum	Maximum
6	2.0766667	0.4405754	1.5600000	2.6400000

Unique Subject Identifier=2612011111-0005

Assay variable : PTINR_VAL1 PT-INR Value

N	Mean	Standard Deviation	Minimum	Maximum
1	1.8000000		1.8000000	1.8000000

Unique Subject Identifier=2614000000-0007

Assay variable : PTINR_VAL1 PT-INR Value

N	Mean	Standard Deviation	Minimum	Maximum
5	1.4880000	0.2834078	1.2500000	1.9100000

Unique Subject Identifier=2614000000-0009

Assay variable : PTINR_VAL1 PT-INR Value

N	Mean	Standard Deviation	Minimum	Maximum
2	1.8650000	0.2474874	1.6900000	2.0400000

Laboratory tests at the time of obtaining Table 2-6 consent, according to the presence or absence of proton-pump inhibitors

Unique Subject Identifier=2614000000-0013

MEANS Procedure

Assay variable : PTINR_VAL1 PT-INR Value

N	Mean	Standard Deviation	Minimum	Maximum
6	2.1933333	0.5227874	1.5500000	3.0400000

Unique Subject Identifier=2614000000-0022

Assay variable : PTINR_VAL1 PT-INR Value

N	Mean	Standard Deviation	Minimum	Maximum
3	2.5300000	0.3726929	2.3000000	2.9600000

Unique Subject Identifier=2614000000-0028

Assay variable : PTINR_VAL1 PT-INR Value

N	Mean	Standard Deviation	Minimum	Maximum
3	2.2566667	0.7925486	1.7500000	3.1700000

Unique Subject Identifier=2614000000-0031

Assay variable : PTINR_VAL1 PT-INR Value

N	Mean	Standard Deviation	Minimum	Maximum
4	2.0800000	0.2438579	1.8400000	2.3000000

Laboratory tests at the time of obtaining Table 2-6 consent, according to the presence or absence of proton-pump inhibitors

Unique Subject Identifier=2614000000-0033

MEANS Procedure

Assay variable : PTINR_VAL1 PT-INR Value

N	Mean	Standard Deviation	Minimum	Maximum
2	2.2250000	0.1626346	2.1100000	2.3400000

Unique Subject Identifier=2614000000-0035

Assay variable : PTINR_VAL1 PT-INR Value

N	Mean	Standard Deviation	Minimum	Maximum
4	1.3325000	0.2056494	1.1600000	1.6300000

Unique Subject Identifier=2614000000-0036

Assay variable : PTINR_VAL1 PT-INR Value

N	Mean	Standard Deviation	Minimum	Maximum
1	1.7500000		1.7500000	1.7500000

Unique Subject Identifier=2614000000-0040

Assay variable : PTINR_VAL1 PT-INR Value

N	Mean	Standard Deviation	Minimum	Maximum
3	1.7600000	0.2920616	1.4500000	2.0300000

Laboratory tests at the time of obtaining Table 2-6 consent, according to the presence or absence of proton-pump inhibitors

Unique Subject Identifier=2614000000-0046

MEANS Procedure

Assay variable : PTINR_VAL1 PT-INR Value

N	Mean	Standard Deviation	Minimum	Maximum
4	1.7075000	0.2051625	1.5300000	1.9400000

Unique Subject Identifier=2614000000-0055

Assay variable : PTINR_VAL1 PT-INR Value

N	Mean	Standard Deviation	Minimum	Maximum
3	1.6266667	0.2150194	1.4100000	1.8400000

Unique Subject Identifier=2614000000-0069

Assay variable : PTINR_VAL1 PT-INR Value

N	Mean	Standard Deviation	Minimum	Maximum
3	1.4933333	0.1850225	1.2800000	1.6100000

Unique Subject Identifier=2614000000-0071

Assay variable : PTINR_VAL1 PT-INR Value

N	Mean	Standard Deviation	Minimum	Maximum
3	2.6266667	0.5005330	2.1400000	3.1400000

Laboratory tests at the time of obtaining Table 2-6 consent, according to the presence or absence of proton-pump inhibitors

Unique Subject Identifier=2616000000-0007

MEANS Procedure

Assay variable : PTINR_VAL1 PT-INR Value

N	Mean	Standard Deviation	Minimum	Maximum
6	2.5083333	1.2747614	1.5000000	4.9100000

Unique Subject Identifier=2616000000-0008

Assay variable : PTINR_VAL1 PT-INR Value

N	Mean	Standard Deviation	Minimum	Maximum
3	1.5800000	0.2000000	1.3800000	1.7800000

Unique Subject Identifier=2616000000-0014

Assay variable : PTINR_VAL1 PT-INR Value

N	Mean	Standard Deviation	Minimum	Maximum
3	1.8466667	0.6268439	1.2800000	2.5200000

Unique Subject Identifier=2616000000-0015

Assay variable : PTINR_VAL1 PT-INR Value

N	Mean	Standard Deviation	Minimum	Maximum
3	1.7533333	0.1700980	1.5600000	1.8800000

Laboratory tests at the time of obtaining Table 2-6 consent, according to the presence or absence of proton-pump inhibitors

Unique Subject Identifier=2616000000-0039

MEANS Procedure

Assay variable : PTINR_VAL1 PT-INR Value

N	Mean	Standard Deviation	Minimum	Maximum
1	1.7800000		1.7800000	1.7800000

Unique Subject Identifier=2616000000-0051

Assay variable : PTINR_VAL1 PT-INR Value

N	Mean	Standard Deviation	Minimum	Maximum
2	1.4600000	0.0848528	1.4000000	1.5200000

Unique Subject Identifier=2617000000-0006

Assay variable : PTINR_VAL1 PT-INR Value

N	Mean	Standard Deviation	Minimum	Maximum
3	1.5366667	0.2369247	1.3900000	1.8100000

Unique Subject Identifier=2617000000-0011

Assay variable : PTINR_VAL1 PT-INR Value

N	Mean	Standard Deviation	Minimum	Maximum
3	2.2266667	0.6164684	1.7800000	2.9300000

Laboratory tests at the time of obtaining Table 2-6 consent, according to the presence or absence of proton-pump inhibitors

Unique Subject Identifier=2617000000-0012

MEANS Procedure

Assay variable : PTINR_VAL1 PT-INR Value

N	Mean	Standard Deviation	Minimum	Maximum
4	2.0250000	0.2157931	1.7100000	2.1700000

Unique Subject Identifier=2617000000-0015

Assay variable : PTINR_VAL1 PT-INR Value

N	Mean	Standard Deviation	Minimum	Maximum
6	2.3516667	0.7882999	1.4300000	3.4000000

Unique Subject Identifier=2617000000-0016

Assay variable : PTINR_VAL1 PT-INR Value

N	Mean	Standard Deviation	Minimum	Maximum
2	1.0350000	0.0353553	1.0100000	1.0600000

Unique Subject Identifier=2617000000-0019

Assay variable : PTINR_VAL1 PT-INR Value

N	Mean	Standard Deviation	Minimum	Maximum
2	2.0050000	0.2616295	1.8200000	2.1900000

Laboratory tests at the time of obtaining Table 2-6 consent, according to the presence or absence of proton-pump inhibitors

Unique Subject Identifier=2617000000-0021

MEANS Procedure

Assay variable : PTINR_VAL1 PT-INR Value

N	Mean	Standard Deviation	Minimum	Maximum
6	2.0633333	1.2088452	1.2200000	4.4300000

Unique Subject Identifier=2617000000-0025

Assay variable : PTINR_VAL1 PT-INR Value

N	Mean	Standard Deviation	Minimum	Maximum
3	1.6633333	0.5430776	1.0900000	2.1700000

Unique Subject Identifier=2617000000-0029

Assay variable : PTINR_VAL1 PT-INR Value

N	Mean	Standard Deviation	Minimum	Maximum
3	2.1400000	0.0916515	2.0600000	2.2400000

Unique Subject Identifier=2617000000-0036

Assay variable : PTINR_VAL1 PT-INR Value

N	Mean	Standard Deviation	Minimum	Maximum
1	1.8400000		1.8400000	1.8400000

Laboratory tests at the time of obtaining Table 2-6 consent, according to the presence or absence of proton-pump inhibitors

Unique Subject Identifier=2617000000-0042

MEANS Procedure

Assay variable : PTINR_VAL1 PT-INR Value

N	Mean	Standard Deviation	Minimum	Maximum
3	1.4400000	0.1513275	1.2700000	1.5600000

Unique Subject Identifier=2617000000-0043

Assay variable : PTINR_VAL1 PT-INR Value

N	Mean	Standard Deviation	Minimum	Maximum
4	1.7350000	0.1040833	1.6200000	1.8500000

Unique Subject Identifier=2617000000-0045

Assay variable : PTINR_VAL1 PT-INR Value

N	Mean	Standard Deviation	Minimum	Maximum
2	1.8300000	0.0141421	1.8200000	1.8400000

Unique Subject Identifier=2617000000-0049

Assay variable : PTINR_VAL1 PT-INR Value

N	Mean	Standard Deviation	Minimum	Maximum
2	2.7650000	0.4313351	2.4600000	3.0700000

Laboratory tests at the time of obtaining Table 2-6 consent, according to the presence or absence of proton-pump inhibitors

Unique Subject Identifier=2617000000-0060

MEANS Procedure

Assay variable : PTINR_VAL1 PT-INR Value

N	Mean	Standard Deviation	Minimum	Maximum
5	2.1000000	0.3839271	1.6400000	2.6900000

Unique Subject Identifier=2617000000-0066

Assay variable : PTINR_VAL1 PT-INR Value

N	Mean	Standard Deviation	Minimum	Maximum
3	2.6033333	0.5877358	2.2200000	3.2800000

Unique Subject Identifier=2617000000-0072

Assay variable : PTINR_VAL1 PT-INR Value

N	Mean	Standard Deviation	Minimum	Maximum
3	2.8766667	0.3073001	2.5500000	3.1600000

Unique Subject Identifier=2617000000-0078

Assay variable : PTINR_VAL1 PT-INR Value

N	Mean	Standard Deviation	Minimum	Maximum
4	1.9100000	0.3522310	1.5700000	2.3600000

Laboratory tests at the time of obtaining Table 2-6 consent, according to the presence or absence of proton-pump inhibitors

Unique Subject Identifier=2617000000-0081

MEANS Procedure

Assay variable : PTINR_VAL1 PT-INR Value

N	Mean	Standard Deviation	Minimum	Maximum
4	2.4350000	0.4647939	1.9900000	3.0700000

Unique Subject Identifier=2617000000-0086

Assay variable : PTINR_VAL1 PT-INR Value

N	Mean	Standard Deviation	Minimum	Maximum
3	2.3933333	0.0763763	2.3100000	2.4600000

Unique Subject Identifier=2617000000-0089

Assay variable : PTINR_VAL1 PT-INR Value

N	Mean	Standard Deviation	Minimum	Maximum
2	1.9500000	0.0282843	1.9300000	1.9700000

Unique Subject Identifier=2617000000-0092

Assay variable : PTINR_VAL1 PT-INR Value

N	Mean	Standard Deviation	Minimum	Maximum
4	1.8475000	0.1652019	1.7000000	2.0800000

Laboratory tests at the time of obtaining Table 2-6 consent, according to the presence or absence of proton-pump inhibitors

Unique Subject Identifier=2617000000-0093

MEANS Procedure

Assay variable : PTINR_VAL1 PT-INR Value

N	Mean	Standard Deviation	Minimum	Maximum
1	1.6300000		1.6300000	1.6300000

Unique Subject Identifier=2617000000-0094

Assay variable : PTINR_VAL1 PT-INR Value

N	Mean	Standard Deviation	Minimum	Maximum
5	1.7620000	0.1003494	1.6500000	1.9000000

Unique Subject Identifier=2617000000-0096

Assay variable : PTINR_VAL1 PT-INR Value

N	Mean	Standard Deviation	Minimum	Maximum
3	1.8500000	0.3377869	1.6500000	2.2400000

Unique Subject Identifier=2617000000-0101

Assay variable : PTINR_VAL1 PT-INR Value

N	Mean	Standard Deviation	Minimum	Maximum
4	1.7575000	0.1405643	1.6400000	1.9200000

Laboratory tests at the time of obtaining Table 2-6 consent, according to the presence or absence of proton-pump inhibitors

Unique Subject Identifier=2617000000-0102

MEANS Procedure

Assay variable : PTINR_VAL1 PT-INR Value

N	Mean	Standard Deviation	Minimum	Maximum
4	1.7275000	0.2099802	1.5000000	1.9600000

Unique Subject Identifier=2617000000-0108

Assay variable : PTINR_VAL1 PT-INR Value

N	Mean	Standard Deviation	Minimum	Maximum
4	2.0475000	0.1701715	1.8100000	2.2100000

Unique Subject Identifier=2617000000-0114

Assay variable : PTINR_VAL1 PT-INR Value

N	Mean	Standard Deviation	Minimum	Maximum
3	1.6166667	0.1006645	1.5100000	1.7100000

Unique Subject Identifier=2617000000-0116

Assay variable : PTINR_VAL1 PT-INR Value

N	Mean	Standard Deviation	Minimum	Maximum
5	1.7680000	0.2922670	1.4900000	2.2500000

Laboratory tests at the time of obtaining Table 2-6 consent, according to the presence or absence of proton-pump inhibitors

Unique Subject Identifier=2617000000-0117

MEANS Procedure

Assay variable : PTINR_VAL1 PT-INR Value

N	Mean	Standard Deviation	Minimum	Maximum
6	2.0733333	0.5709174	1.3300000	2.9900000

Unique Subject Identifier=2617000000-0120

Assay variable : PTINR_VAL1 PT-INR Value

N	Mean	Standard Deviation	Minimum	Maximum
3	1.9566667	0.2400694	1.7800000	2.2300000

Unique Subject Identifier=2617000000-0121

Assay variable : PTINR_VAL1 PT-INR Value

N	Mean	Standard Deviation	Minimum	Maximum
3	1.9566667	0.2400694	1.7800000	2.2300000

Unique Subject Identifier=2617000000-0127

Assay variable : PTINR_VAL1 PT-INR Value

N	Mean	Standard Deviation	Minimum	Maximum
4	1.7925000	0.2643703	1.4100000	2.0000000

Laboratory tests at the time of obtaining Table 2-6 consent, according to the presence or absence of proton-pump inhibitors

Unique Subject Identifier=2617000000-0131

MEANS Procedure

Assay variable : PTINR_VAL1 PT-INR Value

N	Mean	Standard Deviation	Minimum	Maximum
2	1.9800000	0.3394113	1.7400000	2.2200000

Unique Subject Identifier=2617000000-0139

Assay variable : PTINR_VAL1 PT-INR Value

N	Mean	Standard Deviation	Minimum	Maximum
6	1.4883333	0.1599271	1.2100000	1.6900000

Unique Subject Identifier=2617000000-0143

Assay variable : PTINR_VAL1 PT-INR Value

N	Mean	Standard Deviation	Minimum	Maximum
4	1.9025000	0.1144188	1.8000000	2.0600000

Unique Subject Identifier=2617000000-0144

Assay variable : PTINR_VAL1 PT-INR Value

N	Mean	Standard Deviation	Minimum	Maximum
2	1.8250000	0.1060660	1.7500000	1.9000000

Laboratory tests at the time of obtaining Table 2-6 consent, according to the presence or absence of proton-pump inhibitors

Unique Subject Identifier=2617000000-0147

MEANS Procedure

Assay variable : PTINR_VAL1 PT-INR Value

N	Mean	Standard Deviation	Minimum	Maximum
4	2.5900000	0.3365511	2.1500000	2.9700000

Unique Subject Identifier=2617000000-0151

Assay variable : PTINR_VAL1 PT-INR Value

N	Mean	Standard Deviation	Minimum	Maximum
3	1.7833333	0.1778576	1.5800000	1.9100000

Unique Subject Identifier=2617000000-0155

Assay variable : PTINR_VAL1 PT-INR Value

N	Mean	Standard Deviation	Minimum	Maximum
2	2.5350000	0.3606245	2.2800000	2.7900000

Unique Subject Identifier=2617000000-0160

Assay variable : PTINR_VAL1 PT-INR Value

N	Mean	Standard Deviation	Minimum	Maximum
3	2.3133333	0.4781562	1.8700000	2.8200000

Laboratory tests at the time of obtaining Table 2-6 consent, according to the presence or absence of proton-pump inhibitors

Unique Subject Identifier=2617000000-0166

MEANS Procedure

Assay variable : PTINR_VAL1 PT-INR Value

N	Mean	Standard Deviation	Minimum	Maximum
4	2.2700000	0.3522310	1.9700000	2.7300000

Unique Subject Identifier=2617000000-0167

Assay variable : PTINR_VAL1 PT-INR Value

N	Mean	Standard Deviation	Minimum	Maximum
4	1.8075000	0.2192981	1.5700000	2.1000000

Unique Subject Identifier=2617000000-0180

Assay variable : PTINR_VAL1 PT-INR Value

N	Mean	Standard Deviation	Minimum	Maximum
2	1.5900000	0.4384062	1.2800000	1.9000000

Unique Subject Identifier=2617000000-0183

Assay variable : PTINR_VAL1 PT-INR Value

N	Mean	Standard Deviation	Minimum	Maximum
2	1.5750000	0.1767767	1.4500000	1.7000000

Laboratory tests at the time of obtaining Table 2-6 consent, according to the presence or absence of proton-pump inhibitors

Unique Subject Identifier=2617000000-0184

MEANS Procedure

Assay variable : PTINR_VAL1 PT-INR Value

N	Mean	Standard Deviation	Minimum	Maximum
3	2.3866667	0.3089229	2.0300000	2.5700000

Unique Subject Identifier=2617000000-0193

Assay variable : PTINR_VAL1 PT-INR Value

N	Mean	Standard Deviation	Minimum	Maximum
3	2.0300000	0.5040833	1.4800000	2.4700000

Unique Subject Identifier=2617000000-0195

Assay variable : PTINR_VAL1 PT-INR Value

N	Mean	Standard Deviation	Minimum	Maximum
4	2.3175000	0.1543535	2.1500000	2.5100000

Unique Subject Identifier=2617000000-0201

Assay variable : PTINR_VAL1 PT-INR Value

N	Mean	Standard Deviation	Minimum	Maximum
4	2.0100000	0.7092719	0.9600000	2.4600000

Laboratory tests at the time of obtaining Table 2-6 consent, according to the presence or absence of proton-pump inhibitors

Unique Subject Identifier=2617000000-0202

MEANS Procedure

Assay variable : PTINR_VAL1 PT-INR Value

N	Mean	Standard Deviation	Minimum	Maximum
2	2.3750000	0.1767767	2.2500000	2.5000000

Unique Subject Identifier=2617000000-0203

Assay variable : PTINR_VAL1 PT-INR Value

N	Mean	Standard Deviation	Minimum	Maximum
5	2.1980000	0.6521273	1.4300000	3.0900000

Unique Subject Identifier=2617000000-0205

Assay variable : PTINR_VAL1 PT-INR Value

N	Mean	Standard Deviation	Minimum	Maximum
2	2.1750000	0.5586144	1.7800000	2.5700000

Unique Subject Identifier=2617000000-0208

Assay variable : PTINR_VAL1 PT-INR Value

N	Mean	Standard Deviation	Minimum	Maximum
4	1.7650000	0.2292742	1.4800000	1.9700000

Laboratory tests at the time of obtaining Table 2-6 consent, according to the presence or absence of proton-pump inhibitors

Unique Subject Identifier=2617000000-0213

MEANS Procedure

Assay variable : PTINR_VAL1 PT-INR Value

N	Mean	Standard Deviation	Minimum	Maximum
4	1.8550000	0.1236932	1.7000000	2.0000000

Unique Subject Identifier=2617000000-0214

Assay variable : PTINR_VAL1 PT-INR Value

N	Mean	Standard Deviation	Minimum	Maximum
3	2.1000000	0.5456189	1.7800000	2.7300000

Unique Subject Identifier=2617000000-0215

Assay variable : PTINR_VAL1 PT-INR Value

N	Mean	Standard Deviation	Minimum	Maximum
2	2.1000000	0.3676955	1.8400000	2.3600000

Unique Subject Identifier=2617000000-0216

Assay variable : PTINR_VAL1 PT-INR Value

N	Mean	Standard Deviation	Minimum	Maximum
4	1.5450000	0.1310216	1.3500000	1.6300000

Laboratory tests at the time of obtaining Table 2-6 consent, according to the presence or absence of proton-pump inhibitors

Unique Subject Identifier=2617000000-0218

MEANS Procedure

Assay variable : PTINR_VAL1 PT-INR Value

N	Mean	Standard Deviation	Minimum	Maximum
6	2.1300000	0.1711140	2.0000000	2.4200000

Unique Subject Identifier=2617000000-0221

Assay variable : PTINR_VAL1 PT-INR Value

N	Mean	Standard Deviation	Minimum	Maximum
4	2.1575000	0.2085466	1.8800000	2.3500000

Unique Subject Identifier=2617000000-0222

Assay variable : PTINR_VAL1 PT-INR Value

N	Mean	Standard Deviation	Minimum	Maximum
3	1.9400000	0.9734988	1.1300000	3.0200000

Unique Subject Identifier=2617000000-0223

Assay variable : PTINR_VAL1 PT-INR Value

N	Mean	Standard Deviation	Minimum	Maximum
3	2.0233333	0.0757188	1.9700000	2.1100000

Laboratory tests at the time of obtaining Table 2-6 consent, according to the presence or absence of proton-pump inhibitors

Unique Subject Identifier=2617000000-0224

MEANS Procedure

Assay variable : PTINR_VAL1 PT-INR Value

N	Mean	Standard Deviation	Minimum	Maximum
3	1.6300000	0.1178983	1.5000000	1.7300000

Unique Subject Identifier=2617000000-0226

Assay variable : PTINR_VAL1 PT-INR Value

N	Mean	Standard Deviation	Minimum	Maximum
4	2.2600000	0.2978814	1.9500000	2.5200000

Unique Subject Identifier=2617000000-0230

Assay variable : PTINR_VAL1 PT-INR Value

N	Mean	Standard Deviation	Minimum	Maximum
3	2.4433333	0.8064945	1.5200000	3.0100000

Unique Subject Identifier=2617000000-0231

Assay variable : PTINR_VAL1 PT-INR Value

N	Mean	Standard Deviation	Minimum	Maximum
4	2.0550000	0.3812698	1.5300000	2.3700000

Laboratory tests at the time of obtaining Table 2-6 consent, according to the presence or absence of proton-pump inhibitors

Unique Subject Identifier=2617000000-0243

MEANS Procedure

Assay variable : PTINR_VAL1 PT-INR Value

N	Mean	Standard Deviation	Minimum	Maximum
3	1.8966667	0.2874601	1.6700000	2.2200000

Unique Subject Identifier=2617000000-0244

Assay variable : PTINR_VAL1 PT-INR Value

N	Mean	Standard Deviation	Minimum	Maximum
5	2.1760000	0.4657574	1.3900000	2.6100000

Unique Subject Identifier=2617000000-0255

Assay variable : PTINR_VAL1 PT-INR Value

N	Mean	Standard Deviation	Minimum	Maximum
2	2.3950000	0.1767767	2.2700000	2.5200000

Unique Subject Identifier=2617000000-0258

Assay variable : PTINR_VAL1 PT-INR Value

N	Mean	Standard Deviation	Minimum	Maximum
3	1.8700000	0.0458258	1.8300000	1.9200000

Laboratory tests at the time of obtaining Table 2-6 consent, according to the presence or absence of proton-pump inhibitors

Unique Subject Identifier=2617000000-0259

MEANS Procedure

Assay variable : PTINR_VAL1 PT-INR Value

N	Mean	Standard Deviation	Minimum	Maximum
6	1.9333333	0.2353437	1.6300000	2.2700000

Unique Subject Identifier=2617000000-0262

Assay variable : PTINR_VAL1 PT-INR Value

N	Mean	Standard Deviation	Minimum	Maximum
4	1.6925000	0.2657536	1.3800000	2.0300000

Unique Subject Identifier=2617000000-0264

Assay variable : PTINR_VAL1 PT-INR Value

N	Mean	Standard Deviation	Minimum	Maximum
5	2.0320000	1.1410828	1.1600000	3.7800000

Unique Subject Identifier=2617000000-0271

Assay variable : PTINR_VAL1 PT-INR Value

N	Mean	Standard Deviation	Minimum	Maximum
4	1.6975000	0.1609089	1.4800000	1.8500000

Laboratory tests at the time of obtaining Table 2-6 consent, according to the presence or absence of proton-pump inhibitors

Unique Subject Identifier=2617000000-0274

MEANS Procedure

Assay variable : PTINR_VAL1 PT-INR Value

N	Mean	Standard Deviation	Minimum	Maximum
2	1.8900000	0.1838478	1.7600000	2.0200000

Unique Subject Identifier=2617000000-0281

Assay variable : PTINR_VAL1 PT-INR Value

N	Mean	Standard Deviation	Minimum	Maximum
3	2.0833333	0.2821938	1.7600000	2.2800000

Unique Subject Identifier=2617000000-0284

Assay variable : PTINR_VAL1 PT-INR Value

N	Mean	Standard Deviation	Minimum	Maximum
6	1.7416667	0.2171098	1.5100000	2.0600000

Unique Subject Identifier=2617000000-0287

Assay variable : PTINR_VAL1 PT-INR Value

N	Mean	Standard Deviation	Minimum	Maximum
4	1.9300000	0.3068116	1.5900000	2.2700000

Laboratory tests at the time of obtaining Table 2-6 consent, according to the presence or absence of proton-pump inhibitors

Unique Subject Identifier=2617000000-0288

MEANS Procedure

Assay variable : PTINR_VAL1 PT-INR Value

N	Mean	Standard Deviation	Minimum	Maximum
3	1.9566667	0.3707200	1.6900000	2.3800000

Unique Subject Identifier=2617000000-0294

Assay variable : PTINR_VAL1 PT-INR Value

N	Mean	Standard Deviation	Minimum	Maximum
4	2.3175000	0.9911735	1.3900000	3.7000000

Unique Subject Identifier=2617000000-0298

Assay variable : PTINR_VAL1 PT-INR Value

N	Mean	Standard Deviation	Minimum	Maximum
4	1.0500000	0.0355903	1.0000000	1.0800000

Unique Subject Identifier=2617000000-0299

Assay variable : PTINR_VAL1 PT-INR Value

N	Mean	Standard Deviation	Minimum	Maximum
4	1.8250000	0.0310913	1.8000000	1.8700000

Laboratory tests at the time of obtaining Table 2-6 consent, according to the presence or absence of proton-pump inhibitors

Unique Subject Identifier=2617000000-0303

MEANS Procedure

Assay variable : PTINR_VAL1 PT-INR Value

N	Mean	Standard Deviation	Minimum	Maximum
5	1.9240000	0.1913897	1.7200000	2.1700000

Unique Subject Identifier=2617000000-0306

Assay variable : PTINR_VAL1 PT-INR Value

N	Mean	Standard Deviation	Minimum	Maximum
4	1.6600000	0.1957890	1.4000000	1.8300000

Unique Subject Identifier=2617000000-0308

Assay variable : PTINR_VAL1 PT-INR Value

N	Mean	Standard Deviation	Minimum	Maximum
3	1.7733333	0.1457166	1.6700000	1.9400000

Unique Subject Identifier=2617000000-0314

Assay variable : PTINR_VAL1 PT-INR Value

N	Mean	Standard Deviation	Minimum	Maximum
3	1.5533333	0.0450925	1.5100000	1.6000000

Laboratory tests at the time of obtaining Table 2-6 consent, according to the presence or absence of proton-pump inhibitors

Unique Subject Identifier=2617000000-0320

MEANS Procedure

Assay variable : PTINR_VAL1 PT-INR Value

N	Mean	Standard Deviation	Minimum	Maximum
4	2.1000000	0.7140028	1.5200000	3.0200000

Unique Subject Identifier=2617000000-0324

Assay variable : PTINR_VAL1 PT-INR Value

N	Mean	Standard Deviation	Minimum	Maximum
3	2.3500000	0.4386342	1.8700000	2.7300000

Unique Subject Identifier=2617000000-0331

Assay variable : PTINR_VAL1 PT-INR Value

N	Mean	Standard Deviation	Minimum	Maximum
2	2.1300000	0.0707107	2.0800000	2.1800000

Unique Subject Identifier=2617000000-0340

Assay variable : PTINR_VAL1 PT-INR Value

N	Mean	Standard Deviation	Minimum	Maximum
1	1.3300000		1.3300000	1.3300000

Laboratory tests at the time of obtaining Table 2-6 consent, according to the presence or absence of proton-pump inhibitors

Unique Subject Identifier=2617000000-0341

MEANS Procedure

Assay variable : PTINR_VAL1 PT-INR Value

N	Mean	Standard Deviation	Minimum	Maximum
3	2.4100000	0.7856208	1.7300000	3.2700000

Unique Subject Identifier=2617000000-0348

Assay variable : PTINR_VAL1 PT-INR Value

N	Mean	Standard Deviation	Minimum	Maximum
4	1.9100000	0.9333452	1.1400000	3.1900000

Unique Subject Identifier=2617000000-0350

Assay variable : PTINR_VAL1 PT-INR Value

N	Mean	Standard Deviation	Minimum	Maximum
6	1.8983333	0.4334243	1.1700000	2.3500000

Unique Subject Identifier=2617000000-0358

Assay variable : PTINR_VAL1 PT-INR Value

N	Mean	Standard Deviation	Minimum	Maximum
4	1.9600000	0.3086530	1.6400000	2.3700000

Laboratory tests at the time of obtaining Table 2-6 consent, according to the presence or absence of proton-pump inhibitors

Unique Subject Identifier=2619011010-0032

MEANS Procedure

Assay variable : PTINR_VAL1 PT-INR Value

N	Mean	Standard Deviation	Minimum	Maximum
5	2.1040000	0.2884961	1.7700000	2.4200000

Unique Subject Identifier=2619011010-0037

Assay variable : PTINR_VAL1 PT-INR Value

N	Mean	Standard Deviation	Minimum	Maximum
6	1.8666667	0.2574231	1.5100000	2.2800000

Unique Subject Identifier=2619011010-0039

Assay variable : PTINR_VAL1 PT-INR Value

N	Mean	Standard Deviation	Minimum	Maximum
3	2.4033333	0.2411086	2.1500000	2.6300000

Unique Subject Identifier=2619011010-0042

Assay variable : PTINR_VAL1 PT-INR Value

N	Mean	Standard Deviation	Minimum	Maximum
6	1.7050000	0.2647074	1.5500000	2.2400000

Laboratory tests at the time of obtaining Table 2-6 consent, according to the presence or absence of proton-pump inhibitors

Unique Subject Identifier=2619011010-0045

MEANS Procedure

Assay variable : PTINR_VAL1 PT-INR Value

N	Mean	Standard Deviation	Minimum	Maximum
3	2.1400000	0.8944831	1.3300000	3.1000000

Unique Subject Identifier=2619011010-0047

Assay variable : PTINR_VAL1 PT-INR Value

N	Mean	Standard Deviation	Minimum	Maximum
6	1.5916667	0.5448455	1.0900000	2.3200000

Unique Subject Identifier=2619011010-0048

Assay variable : PTINR_VAL1 PT-INR Value

N	Mean	Standard Deviation	Minimum	Maximum
4	1.9625000	0.5040751	1.3500000	2.5700000

Unique Subject Identifier=2621011111-0013

Assay variable : PTINR_VAL1 PT-INR Value

N	Mean	Standard Deviation	Minimum	Maximum
4	1.8100000	0.2930301	1.5100000	2.1500000

Laboratory tests at the time of obtaining Table 2-6 consent, according to the presence or absence of proton-pump inhibitors

Unique Subject Identifier=2621011111-0019

MEANS Procedure

Assay variable : PTINR_VAL1 PT-INR Value

N	Mean	Standard Deviation	Minimum	Maximum
5	2.0720000	0.7127903	1.3500000	3.2700000

Unique Subject Identifier=2623000000-0005

Assay variable : PTINR_VAL1 PT-INR Value

N	Mean	Standard Deviation	Minimum	Maximum
3	2.0366667	0.1159023	1.9300000	2.1600000

Unique Subject Identifier=2623000000-0006

Assay variable : PTINR_VAL1 PT-INR Value

N	Mean	Standard Deviation	Minimum	Maximum
4	1.5675000	0.1682013	1.3500000	1.7100000

Unique Subject Identifier=2623000000-0008

Assay variable : PTINR_VAL1 PT-INR Value

N	Mean	Standard Deviation	Minimum	Maximum
6	2.0266667	0.2779688	1.8200000	2.5800000

Laboratory tests at the time of obtaining Table 2-6 consent, according to the presence or absence of proton-pump inhibitors

Unique Subject Identifier=2623000000-0017

MEANS Procedure

Assay variable : PTINR_VAL1 PT-INR Value

N	Mean	Standard Deviation	Minimum	Maximum
3	1.6000000	0.0529150	1.5600000	1.6600000

Unique Subject Identifier=2623000000-0025

Assay variable : PTINR_VAL1 PT-INR Value

N	Mean	Standard Deviation	Minimum	Maximum
4	2.1900000	1.1400877	1.1300000	3.3200000

Unique Subject Identifier=2623000000-0030

Assay variable : PTINR_VAL1 PT-INR Value

N	Mean	Standard Deviation	Minimum	Maximum
4	2.0400000	0.2563851	1.7300000	2.2700000

Unique Subject Identifier=2623000000-0031

Assay variable : PTINR_VAL1 PT-INR Value

N	Mean	Standard Deviation	Minimum	Maximum
3	2.0033333	0.4675824	1.4800000	2.3800000

Laboratory tests at the time of obtaining Table 2-6 consent, according to the presence or absence of proton-pump inhibitors

Unique Subject Identifier=2623000000-0037

MEANS Procedure

Assay variable : PTINR_VAL1 PT-INR Value

N	Mean	Standard Deviation	Minimum	Maximum
3	2.0633333	0.1418920	1.9100000	2.1900000

Unique Subject Identifier=2623000000-0039

Assay variable : PTINR_VAL1 PT-INR Value

N	Mean	Standard Deviation	Minimum	Maximum
3	2.0533333	0.2324507	1.7900000	2.2300000

Unique Subject Identifier=2623000000-0040

Assay variable : PTINR_VAL1 PT-INR Value

N	Mean	Standard Deviation	Minimum	Maximum
4	1.6875000	0.4157223	1.2700000	2.0500000

Unique Subject Identifier=2623000000-0043

Assay variable : PTINR_VAL1 PT-INR Value

N	Mean	Standard Deviation	Minimum	Maximum
3	1.8200000	0.3051229	1.4800000	2.0700000

Laboratory tests at the time of obtaining Table 2-6 consent, according to the presence or absence of proton-pump inhibitors

Unique Subject Identifier=2623000000-0049

MEANS Procedure

Assay variable : PTINR_VAL1 PT-INR Value

N	Mean	Standard Deviation	Minimum	Maximum
3	2.2600000	0.1311488	2.1400000	2.4000000

Unique Subject Identifier=2623000000-0062

Assay variable : PTINR_VAL1 PT-INR Value

N	Mean	Standard Deviation	Minimum	Maximum
4	1.7575000	0.4713368	1.2600000	2.3400000

Unique Subject Identifier=2623000000-0080

Assay variable : PTINR_VAL1 PT-INR Value

N	Mean	Standard Deviation	Minimum	Maximum
3	1.8900000	0.1997498	1.7200000	2.1100000

Unique Subject Identifier=2623000000-0081

Assay variable : PTINR_VAL1 PT-INR Value

N	Mean	Standard Deviation	Minimum	Maximum
6	2.1366667	0.2517671	1.7700000	2.4600000

Laboratory tests at the time of obtaining Table 2-6 consent, according to the presence or absence of proton-pump inhibitors

Unique Subject Identifier=2623000000-0087

MEANS Procedure

Assay variable : PTINR_VAL1 PT-INR Value

N	Mean	Standard Deviation	Minimum	Maximum
2	2.5300000	0.2545584	2.3500000	2.7100000

Unique Subject Identifier=2626011111-0010

Assay variable : PTINR_VAL1 PT-INR Value

N	Mean	Standard Deviation	Minimum	Maximum
6	1.6633333	0.2174090	1.3400000	2.0200000

Unique Subject Identifier=2626011111-0017

Assay variable : PTINR_VAL1 PT-INR Value

N	Mean	Standard Deviation	Minimum	Maximum
3	1.8466667	0.4495924	1.4600000	2.3400000

Unique Subject Identifier=2626011111-0026

Assay variable : PTINR_VAL1 PT-INR Value

N	Mean	Standard Deviation	Minimum	Maximum
4	1.9150000	0.5519964	1.5100000	2.7200000

Laboratory tests at the time of obtaining Table 2-6 consent, according to the presence or absence of proton-pump inhibitors

Unique Subject Identifier=2626011111-0037

MEANS Procedure

Assay variable : PTINR_VAL1 PT-INR Value

N	Mean	Standard Deviation	Minimum	Maximum
4	2.1725000	0.6584008	1.2700000	2.8400000

Unique Subject Identifier=2626011111-0038

Assay variable : PTINR_VAL1 PT-INR Value

N	Mean	Standard Deviation	Minimum	Maximum
5	1.8500000	0.1440486	1.6900000	1.9800000

Unique Subject Identifier=2626011111-0044

Assay variable : PTINR_VAL1 PT-INR Value

N	Mean	Standard Deviation	Minimum	Maximum
6	1.5550000	0.1405347	1.2900000	1.7100000

Unique Subject Identifier=2626011111-0046

Assay variable : PTINR_VAL1 PT-INR Value

N	Mean	Standard Deviation	Minimum	Maximum
3	1.7933333	0.0907377	1.7100000	1.8900000

Laboratory tests at the time of obtaining Table 2-6 consent, according to the presence or absence of proton-pump inhibitors

Unique Subject Identifier=2626011111-0048

MEANS Procedure

Assay variable : PTINR_VAL1 PT-INR Value

N	Mean	Standard Deviation	Minimum	Maximum
4	2.0125000	0.1541374	1.8500000	2.2200000

Unique Subject Identifier=2627011111-0010

Assay variable : PTINR_VAL1 PT-INR Value

N	Mean	Standard Deviation	Minimum	Maximum
4	1.9200000	0.1009950	1.8500000	2.0700000

Unique Subject Identifier=2627011111-0012

Assay variable : PTINR_VAL1 PT-INR Value

N	Mean	Standard Deviation	Minimum	Maximum
4	2.0675000	0.3966001	1.7300000	2.5800000

Unique Subject Identifier=2628011111-0001

Assay variable : PTINR_VAL1 PT-INR Value

N	Mean	Standard Deviation	Minimum	Maximum
5	1.5540000	0.2530415	1.1900000	1.8400000

Laboratory tests at the time of obtaining Table 2-6 consent, according to the presence or absence of proton-pump inhibitors

Unique Subject Identifier=2628011111-0003

MEANS Procedure

Assay variable : PTINR_VAL1 PT-INR Value

N	Mean	Standard Deviation	Minimum	Maximum
4	1.7775000	0.2260347	1.4400000	1.9200000

Unique Subject Identifier=2628011111-0009

Assay variable : PTINR_VAL1 PT-INR Value

N	Mean	Standard Deviation	Minimum	Maximum
2	1.4950000	0.3040559	1.2800000	1.7100000

Unique Subject Identifier=2628011111-0012

Assay variable : PTINR_VAL1 PT-INR Value

N	Mean	Standard Deviation	Minimum	Maximum
4	2.0325000	0.3850000	1.6400000	2.4200000

Unique Subject Identifier=2628011111-0016

Assay variable : PTINR_VAL1 PT-INR Value

N	Mean	Standard Deviation	Minimum	Maximum
4	2.3575000	0.6473214	1.9100000	3.3100000

Laboratory tests at the time of obtaining Table 2-6 consent, according to the presence or absence of proton-pump inhibitors

Unique Subject Identifier=2628011111-0024

MEANS Procedure

Assay variable : PTINR_VAL1 PT-INR Value

N	Mean	Standard Deviation	Minimum	Maximum
2	2.3600000	0.0848528	2.3000000	2.4200000

Unique Subject Identifier=2628011111-0052

Assay variable : PTINR_VAL1 PT-INR Value

N	Mean	Standard Deviation	Minimum	Maximum
3	1.6333333	0.3000556	1.3300000	1.9300000

Unique Subject Identifier=2628011111-0065

Assay variable : PTINR_VAL1 PT-INR Value

N	Mean	Standard Deviation	Minimum	Maximum
3	2.6666667	0.7023769	2.0000000	3.4000000

Unique Subject Identifier=2628011111-0067

Assay variable : PTINR_VAL1 PT-INR Value

N	Mean	Standard Deviation	Minimum	Maximum
3	2.3866667	0.3300505	2.0600000	2.7200000

Laboratory tests at the time of obtaining Table 2-6 consent, according to the presence or absence of proton-pump inhibitors

Unique Subject Identifier=2630000000-0005

MEANS Procedure

Assay variable : PTINR_VAL1 PT-INR Value

N	Mean	Standard Deviation	Minimum	Maximum
2	1.5000000	0.1414214	1.4000000	1.6000000

Unique Subject Identifier=2630000000-0006

Assay variable : PTINR_VAL1 PT-INR Value

N	Mean	Standard Deviation	Minimum	Maximum
5	1.9600000	0.3286335	1.6000000	2.4000000

Unique Subject Identifier=2630000000-0011

Assay variable : PTINR_VAL1 PT-INR Value

N	Mean	Standard Deviation	Minimum	Maximum
1	2.4000000		2.4000000	2.4000000

Unique Subject Identifier=2630000000-0012

Assay variable : PTINR_VAL1 PT-INR Value

N	Mean	Standard Deviation	Minimum	Maximum
3	1.7333333	0.3214550	1.5000000	2.1000000

Laboratory tests at the time of obtaining Table 2-6 consent, according to the presence or absence of proton-pump inhibitors

Unique Subject Identifier=2630000000-0013

MEANS Procedure

Assay variable : PTINR_VAL1 PT-INR Value

N	Mean	Standard Deviation	Minimum	Maximum
2	2.0500000	0.0707107	2.0000000	2.1000000

Unique Subject Identifier=2630000000-0014

Assay variable : PTINR_VAL1 PT-INR Value

N	Mean	Standard Deviation	Minimum	Maximum
3	1.4666667	0.2516611	1.2000000	1.7000000

Unique Subject Identifier=2630000000-0019

Assay variable : PTINR_VAL1 PT-INR Value

N	Mean	Standard Deviation	Minimum	Maximum
2	2.2000000	0.2828427	2.0000000	2.4000000

Unique Subject Identifier=2630000000-0020

Assay variable : PTINR_VAL1 PT-INR Value

N	Mean	Standard Deviation	Minimum	Maximum
1	1.9000000		1.9000000	1.9000000

Laboratory tests at the time of obtaining Table 2-6 consent, according to the presence or absence of proton-pump inhibitors

Unique Subject Identifier=2630000000-0021

MEANS Procedure

Assay variable : PTINR_VAL1 PT-INR Value

N	Mean	Standard Deviation	Minimum	Maximum
5	1.9800000	0.6685806	1.5000000	3.1000000

Unique Subject Identifier=2630000000-0022

Assay variable : PTINR_VAL1 PT-INR Value

N	Mean	Standard Deviation	Minimum	Maximum
2	2.0500000	0.7778175	1.5000000	2.6000000

Unique Subject Identifier=2630000000-0027

Assay variable : PTINR_VAL1 PT-INR Value

N	Mean	Standard Deviation	Minimum	Maximum
1	1.7000000		1.7000000	1.7000000

Unique Subject Identifier=2630000000-0038

Assay variable : PTINR_VAL1 PT-INR Value

N	Mean	Standard Deviation	Minimum	Maximum
5	1.1200000	0.0836660	1.0000000	1.2000000

Laboratory tests at the time of obtaining Table 2-6 consent, according to the presence or absence of proton-pump inhibitors

Unique Subject Identifier=2630000000-0040

MEANS Procedure

Assay variable : PTINR_VAL1 PT-INR Value

N	Mean	Standard Deviation	Minimum	Maximum
4	1.8500000	0.2081666	1.6000000	2.1000000

Unique Subject Identifier=2634000000-0004

Assay variable : PTINR_VAL1 PT-INR Value

N	Mean	Standard Deviation	Minimum	Maximum
3	1.6633333	0.5024274	1.3200000	2.2400000

Unique Subject Identifier=2634000000-0008

Assay variable : PTINR_VAL1 PT-INR Value

N	Mean	Standard Deviation	Minimum	Maximum
3	1.8700000	0.2628688	1.6800000	2.1700000

Unique Subject Identifier=2634000000-0010

Assay variable : PTINR_VAL1 PT-INR Value

N	Mean	Standard Deviation	Minimum	Maximum
4	1.7850000	0.1744515	1.5400000	1.9400000

Laboratory tests at the time of obtaining Table 2-6 consent, according to the presence or absence of proton-pump inhibitors

Unique Subject Identifier=2634000000-0017

MEANS Procedure

Assay variable : PTINR_VAL1 PT-INR Value

N	Mean	Standard Deviation	Minimum	Maximum
3	2.0966667	0.1401190	1.9600000	2.2400000

Unique Subject Identifier=2634000000-0019

Assay variable : PTINR_VAL1 PT-INR Value

N	Mean	Standard Deviation	Minimum	Maximum
3	2.5266667	0.7311863	2.0700000	3.3700000

Unique Subject Identifier=2634000000-0020

Assay variable : PTINR_VAL1 PT-INR Value

N	Mean	Standard Deviation	Minimum	Maximum
4	2.2050000	0.5768015	1.6300000	3.0000000

Unique Subject Identifier=2634000000-0021

Assay variable : PTINR_VAL1 PT-INR Value

N	Mean	Standard Deviation	Minimum	Maximum
6	1.5566667	0.0674290	1.4900000	1.6400000

Laboratory tests at the time of obtaining Table 2-6 consent, according to the presence or absence of proton-pump inhibitors

Unique Subject Identifier=2636011000-0016

MEANS Procedure

Assay variable : PTINR_VAL1 PT-INR Value

N	Mean	Standard Deviation	Minimum	Maximum
4	1.9600000	0.2258318	1.7700000	2.2800000

Unique Subject Identifier=2636011000-0017

Assay variable : PTINR_VAL1 PT-INR Value

N	Mean	Standard Deviation	Minimum	Maximum
4	1.6250000	0.4017877	1.3200000	2.2000000

Unique Subject Identifier=2638000000-0029

Assay variable : PTINR_VAL1 PT-INR Value

N	Mean	Standard Deviation	Minimum	Maximum
1	2.1100000		2.1100000	2.1100000

Unique Subject Identifier=2640011000-0007

Assay variable : PTINR_VAL1 PT-INR Value

N	Mean	Standard Deviation	Minimum	Maximum
5	1.7860000	0.1795272	1.6100000	1.9800000

Laboratory tests at the time of obtaining Table 2-6 consent, according to the presence or absence of proton-pump inhibitors

Unique Subject Identifier=2640011000-0008

MEANS Procedure

Assay variable : PTINR_VAL1 PT-INR Value

N	Mean	Standard Deviation	Minimum	Maximum
6	2.1316667	0.5223568	1.6900000	2.9500000

Unique Subject Identifier=2640011000-0010

Assay variable : PTINR_VAL1 PT-INR Value

N	Mean	Standard Deviation	Minimum	Maximum
6	1.8850000	0.3490415	1.3900000	2.4500000

Unique Subject Identifier=2641010000-0003

Assay variable : PTINR_VAL1 PT-INR Value

N	Mean	Standard Deviation	Minimum	Maximum
3	1.9500000	0.3404409	1.6000000	2.2800000

Unique Subject Identifier=2641010000-0005

Assay variable : PTINR_VAL1 PT-INR Value

N	Mean	Standard Deviation	Minimum	Maximum
5	2.3960000	0.5628321	1.9300000	3.3700000

Laboratory tests at the time of obtaining Table 2-6 consent, according to the presence or absence of proton-pump inhibitors

Unique Subject Identifier=2641010000-0007

MEANS Procedure

Assay variable : PTINR_VAL1 PT-INR Value

N	Mean	Standard Deviation	Minimum	Maximum
3	2.2233333	0.2345918	2.0200000	2.4800000

Unique Subject Identifier=2641010000-0010

Assay variable : PTINR_VAL1 PT-INR Value

N	Mean	Standard Deviation	Minimum	Maximum
4	1.9875000	0.5950560	1.1300000	2.4000000

Unique Subject Identifier=2641010000-0012

Assay variable : PTINR_VAL1 PT-INR Value

N	Mean	Standard Deviation	Minimum	Maximum
3	1.8066667	0.2538372	1.6000000	2.0900000

Unique Subject Identifier=2655000000-0008

Assay variable : PTINR_VAL1 PT-INR Value

N	Mean	Standard Deviation	Minimum	Maximum
1	3.7100000		3.7100000	3.7100000

Laboratory tests at the time of obtaining Table 2-6 consent, according to the presence or absence of proton-pump inhibitors

Unique Subject Identifier=2656011000-0001

MEANS Procedure

Assay variable : PTINR_VAL1 PT-INR Value

N	Mean	Standard Deviation	Minimum	Maximum
3	1.9033333	0.2025669	1.7400000	2.1300000

Unique Subject Identifier=2656011000-0007

Assay variable : PTINR_VAL1 PT-INR Value

N	Mean	Standard Deviation	Minimum	Maximum
3	2.3766667	0.4518112	1.9500000	2.8500000

Unique Subject Identifier=2656011000-0008

Assay variable : PTINR_VAL1 PT-INR Value

N	Mean	Standard Deviation	Minimum	Maximum
3	1.9600000	0.3477068	1.7300000	2.3600000

Unique Subject Identifier=2660011111-0010

Assay variable : PTINR_VAL1 PT-INR Value

N	Mean	Standard Deviation	Minimum	Maximum
1	1.5000000		1.5000000	1.5000000

Laboratory tests at the time of obtaining Table 2-6 consent, according to the presence or absence of proton-pump inhibitors

Unique Subject Identifier=2662011111-0001

MEANS Procedure

Assay variable : PTINR_VAL1 PT-INR Value

N	Mean	Standard Deviation	Minimum	Maximum
6	1.7166667	0.3371449	1.3000000	2.1000000

Unique Subject Identifier=2662011111-0014

Assay variable : PTINR_VAL1 PT-INR Value

N	Mean	Standard Deviation	Minimum	Maximum
6	2.2333333	0.4273952	1.5000000	2.7000000

Unique Subject Identifier=2662011111-0018

Assay variable : PTINR_VAL1 PT-INR Value

N	Mean	Standard Deviation	Minimum	Maximum
6	2.1500000	0.5958188	1.5000000	3.2000000

Unique Subject Identifier=2662011111-0026

Assay variable : PTINR_VAL1 PT-INR Value

N	Mean	Standard Deviation	Minimum	Maximum
6	1.9500000	1.3187115	1.1000000	4.6000000

Laboratory tests at the time of obtaining Table 2-6 consent, according to the presence or absence of proton-pump inhibitors

Unique Subject Identifier=2662011111-0035

MEANS Procedure

Assay variable : PTINR_VAL1 PT-INR Value

N	Mean	Standard Deviation	Minimum	Maximum
5	2.0800000	0.2167948	1.9000000	2.4000000

Unique Subject Identifier=2662011111-0045

Assay variable : PTINR_VAL1 PT-INR Value

N	Mean	Standard Deviation	Minimum	Maximum
4	2.1750000	0.4991660	1.8000000	2.9000000

Unique Subject Identifier=2662011111-0052

Assay variable : PTINR_VAL1 PT-INR Value

N	Mean	Standard Deviation	Minimum	Maximum
3	2.0000000	0.1732051	1.9000000	2.2000000

Unique Subject Identifier=2668011000-0026

Assay variable : PTINR_VAL1 PT-INR Value

N	Mean	Standard Deviation	Minimum	Maximum
5	2.2740000	0.9598854	1.3400000	3.8600000

Laboratory tests at the time of obtaining Table 2-6 consent, according to the presence or absence of proton-pump inhibitors

Unique Subject Identifier=2668011000-0031

MEANS Procedure

Assay variable : PTINR_VAL1 PT-INR Value

N	Mean	Standard Deviation	Minimum	Maximum
3	1.8366667	0.1656301	1.6800000	2.0100000

Unique Subject Identifier=2668011000-0040

Assay variable : PTINR_VAL1 PT-INR Value

N	Mean	Standard Deviation	Minimum	Maximum
3	1.8866667	0.4429823	1.5000000	2.3700000

Unique Subject Identifier=2668011000-0073

Assay variable : PTINR_VAL1 PT-INR Value

N	Mean	Standard Deviation	Minimum	Maximum
3	1.8800000	0.2722132	1.6300000	2.1700000

Unique Subject Identifier=2669000000-0006

Assay variable : PTINR_VAL1 PT-INR Value

N	Mean	Standard Deviation	Minimum	Maximum
4	2.1150000	0.4051749	1.7100000	2.5800000

Laboratory tests at the time of obtaining Table 2-6 consent, according to the presence or absence of proton-pump inhibitors

Unique Subject Identifier=2669000000-0014

MEANS Procedure

Assay variable : PTINR_VAL1 PT-INR Value

N	Mean	Standard Deviation	Minimum	Maximum
3	2.0300000	0.1509967	1.8700000	2.1700000

Unique Subject Identifier=2671000000-0004

Assay variable : PTINR_VAL1 PT-INR Value

N	Mean	Standard Deviation	Minimum	Maximum
6	1.7166667	0.6198602	1.1800000	2.8400000

Unique Subject Identifier=2671000000-0006

Assay variable : PTINR_VAL1 PT-INR Value

N	Mean	Standard Deviation	Minimum	Maximum
2	1.9550000	0.4030509	1.6700000	2.2400000

Unique Subject Identifier=2671000000-0020

Assay variable : PTINR_VAL1 PT-INR Value

N	Mean	Standard Deviation	Minimum	Maximum
2	2.0550000	0.0353553	2.0300000	2.0800000

Laboratory tests at the time of obtaining Table 2-6 consent, according to the presence or absence of proton-pump inhibitors

Unique Subject Identifier=2671000000-0025

MEANS Procedure

Assay variable : PTINR_VAL1 PT-INR Value

N	Mean	Standard Deviation	Minimum	Maximum
3	2.3433333	0.2914332	2.0700000	2.6500000

Unique Subject Identifier=2671000000-0026

Assay variable : PTINR_VAL1 PT-INR Value

N	Mean	Standard Deviation	Minimum	Maximum
4	1.8500000	0.3157003	1.6400000	2.3200000

Unique Subject Identifier=2671000000-0027

Assay variable : PTINR_VAL1 PT-INR Value

N	Mean	Standard Deviation	Minimum	Maximum
3	2.1166667	0.3716629	1.8100000	2.5300000

Unique Subject Identifier=2671000000-0030

Assay variable : PTINR_VAL1 PT-INR Value

N	Mean	Standard Deviation	Minimum	Maximum
3	1.9900000	0.5574944	1.3500000	2.3700000

Laboratory tests at the time of obtaining Table 2-6 consent, according to the presence or absence of proton-pump inhibitors

Unique Subject Identifier=2671000000-0031

MEANS Procedure

Assay variable : PTINR_VAL1 PT-INR Value

N	Mean	Standard Deviation	Minimum	Maximum
3	1.8533333	0.2136196	1.7300000	2.1000000

Unique Subject Identifier=2671000000-0034

Assay variable : PTINR_VAL1 PT-INR Value

N	Mean	Standard Deviation	Minimum	Maximum
3	1.6900000	0.2325941	1.4800000	1.9400000

Unique Subject Identifier=2671000000-0051

Assay variable : PTINR_VAL1 PT-INR Value

N	Mean	Standard Deviation	Minimum	Maximum
4	2.2325000	0.2087063	2.0200000	2.4700000

Unique Subject Identifier=2671000000-0057

Assay variable : PTINR_VAL1 PT-INR Value

N	Mean	Standard Deviation	Minimum	Maximum
3	1.8533333	0.2919475	1.6700000	2.1900000

Laboratory tests at the time of obtaining Table 2-6 consent, according to the presence or absence of proton-pump inhibitors

Unique Subject Identifier=2671000000-0060

MEANS Procedure

Assay variable : PTINR_VAL1 PT-INR Value

N	Mean	Standard Deviation	Minimum	Maximum
3	2.4266667	0.2802380	2.2000000	2.7400000

Unique Subject Identifier=2671000000-0061

Assay variable : PTINR_VAL1 PT-INR Value

N	Mean	Standard Deviation	Minimum	Maximum
3	3.2533333	1.4408447	2.0500000	4.8500000

Unique Subject Identifier=2671000000-0062

Assay variable : PTINR_VAL1 PT-INR Value

N	Mean	Standard Deviation	Minimum	Maximum
1	2.3300000		2.3300000	2.3300000

Unique Subject Identifier=2671000000-0065

Assay variable : PTINR_VAL1 PT-INR Value

N	Mean	Standard Deviation	Minimum	Maximum
2	2.2000000	0.2969848	1.9900000	2.4100000

Laboratory tests at the time of obtaining Table 2-6 consent, according to the presence or absence of proton-pump inhibitors

Unique Subject Identifier=2671000000-0066

MEANS Procedure

Assay variable : PTINR_VAL1 PT-INR Value

N	Mean	Standard Deviation	Minimum	Maximum
3	2.0700000	0.6122091	1.4300000	2.6500000

Unique Subject Identifier=2671000000-0071

Assay variable : PTINR_VAL1 PT-INR Value

N	Mean	Standard Deviation	Minimum	Maximum
3	2.2866667	0.3165965	2.0700000	2.6500000

Unique Subject Identifier=2671000000-0072

Assay variable : PTINR_VAL1 PT-INR Value

N	Mean	Standard Deviation	Minimum	Maximum
3	1.8700000	0.3176476	1.6000000	2.2200000

Unique Subject Identifier=2671000000-0073

Assay variable : PTINR_VAL1 PT-INR Value

N	Mean	Standard Deviation	Minimum	Maximum
3	2.1100000	0.3559494	1.8800000	2.5200000

Laboratory tests at the time of obtaining Table 2-6 consent, according to the presence or absence of proton-pump inhibitors

Unique Subject Identifier=2672000000-0010

MEANS Procedure

Assay variable : PTINR_VAL1 PT-INR Value

N	Mean	Standard Deviation	Minimum	Maximum
3	2.1666667	0.1674316	2.0700000	2.3600000

Unique Subject Identifier=2672000000-0013

Assay variable : PTINR_VAL1 PT-INR Value

N	Mean	Standard Deviation	Minimum	Maximum
3	1.7433333	0.2956913	1.4200000	2.0000000

Unique Subject Identifier=2672000000-0022

Assay variable : PTINR_VAL1 PT-INR Value

N	Mean	Standard Deviation	Minimum	Maximum
2	2.1500000	0.2121320	2.0000000	2.3000000

Unique Subject Identifier=2673011101-0001

Assay variable : PTINR_VAL1 PT-INR Value

N	Mean	Standard Deviation	Minimum	Maximum
3	1.8266667	0.0568624	1.7800000	1.8900000

Laboratory tests at the time of obtaining Table 2-6 consent, according to the presence or absence of proton-pump inhibitors

Unique Subject Identifier=2673011101-0002

MEANS Procedure

Assay variable : PTINR_VAL1 PT-INR Value

N	Mean	Standard Deviation	Minimum	Maximum
2	1.5800000	0.0989949	1.5100000	1.6500000

Unique Subject Identifier=2675000000-0010

Assay variable : PTINR_VAL1 PT-INR Value

N	Mean	Standard Deviation	Minimum	Maximum
1	0.9700000		0.9700000	0.9700000

Unique Subject Identifier=2675000000-0016

Assay variable : PTINR_VAL1 PT-INR Value

N	Mean	Standard Deviation	Minimum	Maximum
2	1.1700000	0.0424264	1.1400000	1.2000000

Unique Subject Identifier=2675000000-0021

Assay variable : PTINR_VAL1 PT-INR Value

N	Mean	Standard Deviation	Minimum	Maximum
5	1.9820000	0.2265392	1.7300000	2.1800000

Laboratory tests at the time of obtaining Table 2-6 consent, according to the presence or absence of proton-pump inhibitors

Unique Subject Identifier=2675000000-0022

MEANS Procedure

Assay variable : PTINR_VAL1 PT-INR Value

N	Mean	Standard Deviation	Minimum	Maximum
3	1.7133333	0.2433790	1.4500000	1.9300000

Unique Subject Identifier=2678010000-0009

Assay variable : PTINR_VAL1 PT-INR Value

N	Mean	Standard Deviation	Minimum	Maximum
4	1.7750000	0.2776689	1.4800000	2.1500000

Unique Subject Identifier=2678010000-0012

Assay variable : PTINR_VAL1 PT-INR Value

N	Mean	Standard Deviation	Minimum	Maximum
4	1.9350000	0.1658312	1.7500000	2.0800000

Unique Subject Identifier=2678010000-0019

Assay variable : PTINR_VAL1 PT-INR Value

N	Mean	Standard Deviation	Minimum	Maximum
3	2.2633333	0.1331666	2.1500000	2.4100000

Laboratory tests at the time of obtaining Table 2-6 consent, according to the presence or absence of proton-pump inhibitors

Unique Subject Identifier=2678010000-0035

MEANS Procedure

Assay variable : PTINR_VAL1 PT-INR Value

N	Mean	Standard Deviation	Minimum	Maximum
3	2.0033333	0.3579572	1.6100000	2.3100000

Unique Subject Identifier=2678010000-0037

Assay variable : PTINR_VAL1 PT-INR Value

N	Mean	Standard Deviation	Minimum	Maximum
5	1.7880000	0.2058397	1.5700000	1.9900000

Unique Subject Identifier=2678010000-0039

Assay variable : PTINR_VAL1 PT-INR Value

N	Mean	Standard Deviation	Minimum	Maximum
5	2.1500000	0.4368066	1.6200000	2.5700000

Unique Subject Identifier=2678010000-0041

Assay variable : PTINR_VAL1 PT-INR Value

N	Mean	Standard Deviation	Minimum	Maximum
4	1.7000000	0.1230176	1.5400000	1.8300000

Laboratory tests at the time of obtaining Table 2-6 consent, according to the presence or absence of proton-pump inhibitors

Unique Subject Identifier=2687001000-0003

MEANS Procedure

Assay variable : PTINR_VAL1 PT-INR Value

N	Mean	Standard Deviation	Minimum	Maximum
6	2.1500000	0.4929503	1.5000000	3.0000000

Unique Subject Identifier=2687001000-0010

Assay variable : PTINR_VAL1 PT-INR Value

N	Mean	Standard Deviation	Minimum	Maximum
6	1.9500000	0.2258318	1.6000000	2.3000000

Unique Subject Identifier=2687001000-0013

Assay variable : PTINR_VAL1 PT-INR Value

N	Mean	Standard Deviation	Minimum	Maximum
3	2.0000000	0.4582576	1.5000000	2.4000000

Unique Subject Identifier=2687001000-0039

Assay variable : PTINR_VAL1 PT-INR Value

N	Mean	Standard Deviation	Minimum	Maximum
5	2.1180000	0.4267552	1.5000000	2.7000000

Laboratory tests at the time of obtaining Table 2-6 consent, according to the presence or absence of proton-pump inhibitors

Unique Subject Identifier=2687001000-0040

MEANS Procedure

Assay variable : PTINR_VAL1 PT-INR Value

N	Mean	Standard Deviation	Minimum	Maximum
5	2.3600000	0.4615192	1.6000000	2.8000000

Unique Subject Identifier=2687001000-0046

Assay variable : PTINR_VAL1 PT-INR Value

N	Mean	Standard Deviation	Minimum	Maximum
5	1.6800000	0.3346640	1.4000000	2.2000000

Unique Subject Identifier=2688011111-0002

Assay variable : PTINR_VAL1 PT-INR Value

N	Mean	Standard Deviation	Minimum	Maximum
6	1.7833333	0.2714160	1.5000000	2.2000000

Unique Subject Identifier=2688011111-0011

Assay variable : PTINR_VAL1 PT-INR Value

N	Mean	Standard Deviation	Minimum	Maximum
6	2.1833333	0.6493587	1.5000000	3.0000000

Laboratory tests at the time of obtaining Table 2-6 consent, according to the presence or absence of proton-pump inhibitors

Unique Subject Identifier=2688011111-0014

MEANS Procedure

Assay variable : PTINR_VAL1 PT-INR Value

N	Mean	Standard Deviation	Minimum	Maximum
6	1.7166667	0.2041241	1.4000000	2.0000000

Unique Subject Identifier=2688011111-0022

Assay variable : PTINR_VAL1 PT-INR Value

N	Mean	Standard Deviation	Minimum	Maximum
5	1.6800000	0.7563068	1.2000000	3.0000000

Unique Subject Identifier=2688011111-0024

Assay variable : PTINR_VAL1 PT-INR Value

N	Mean	Standard Deviation	Minimum	Maximum
5	2.2000000	0.3162278	1.9000000	2.7000000

Unique Subject Identifier=2688011111-0031

Assay variable : PTINR_VAL1 PT-INR Value

N	Mean	Standard Deviation	Minimum	Maximum
5	1.6400000	0.0894427	1.5000000	1.7000000

Laboratory tests at the time of obtaining Table 2-6 consent, according to the presence or absence of proton-pump inhibitors

Unique Subject Identifier=2688011111-0038

MEANS Procedure

Assay variable : PTINR_VAL1 PT-INR Value

N	Mean	Standard Deviation	Minimum	Maximum
5	2.3600000	0.1341641	2.2000000	2.5000000

Unique Subject Identifier=2688011111-0046

Assay variable : PTINR_VAL1 PT-INR Value

N	Mean	Standard Deviation	Minimum	Maximum
6	1.7000000	0.1897367	1.5000000	2.0000000

Unique Subject Identifier=2688011111-0067

Assay variable : PTINR_VAL1 PT-INR Value

N	Mean	Standard Deviation	Minimum	Maximum
5	1.7600000	0.0894427	1.7000000	1.9000000

Unique Subject Identifier=2688011111-0072

Assay variable : PTINR_VAL1 PT-INR Value

N	Mean	Standard Deviation	Minimum	Maximum
5	1.4600000	0.2509980	1.1000000	1.8000000

Laboratory tests at the time of obtaining Table 2-6 consent, according to the presence or absence of proton-pump inhibitors

Unique Subject Identifier=2688011111-0073

MEANS Procedure

Assay variable : PTINR_VAL1 PT-INR Value

N	Mean	Standard Deviation	Minimum	Maximum
5	1.7400000	0.2302173	1.5000000	2.0000000

Unique Subject Identifier=2688011111-0077

Assay variable : PTINR_VAL1 PT-INR Value

N	Mean	Standard Deviation	Minimum	Maximum
5	1.9000000	0.5000000	1.1000000	2.4000000

Unique Subject Identifier=2688011111-0079

Assay variable : PTINR_VAL1 PT-INR Value

N	Mean	Standard Deviation	Minimum	Maximum
5	1.9200000	0.2280351	1.7000000	2.3000000

Unique Subject Identifier=2688011111-0083

Assay variable : PTINR_VAL1 PT-INR Value

N	Mean	Standard Deviation	Minimum	Maximum
1	1.7000000		1.7000000	1.7000000

Laboratory tests at the time of obtaining Table 2-6 consent, according to the presence or absence of proton-pump inhibitors

Unique Subject Identifier=2690000000-0010

MEANS Procedure

Assay variable : PTINR_VAL1 PT-INR Value

N	Mean	Standard Deviation	Minimum	Maximum
6	1.7683333	0.0875024	1.6300000	1.8800000

Unique Subject Identifier=2692000000-0001

Assay variable : PTINR_VAL1 PT-INR Value

N	Mean	Standard Deviation	Minimum	Maximum
5	1.9120000	0.3250692	1.4700000	2.3600000

Unique Subject Identifier=2692000000-0015

Assay variable : PTINR_VAL1 PT-INR Value

N	Mean	Standard Deviation	Minimum	Maximum
6	1.7700000	0.2934621	1.4100000	2.1200000

Unique Subject Identifier=2692000000-0016

Assay variable : PTINR_VAL1 PT-INR Value

N	Mean	Standard Deviation	Minimum	Maximum
4	1.7650000	0.1771064	1.5800000	1.9900000

Laboratory tests at the time of obtaining Table 2-6 consent, according to the presence or absence of proton-pump inhibitors

Unique Subject Identifier=2692000000-0020

MEANS Procedure

Assay variable : PTINR_VAL1 PT-INR Value

N	Mean	Standard Deviation	Minimum	Maximum
6	2.0366667	0.1949017	1.7500000	2.3600000

Unique Subject Identifier=2692000000-0021

Assay variable : PTINR_VAL1 PT-INR Value

N	Mean	Standard Deviation	Minimum	Maximum
5	1.9980000	0.3875822	1.5600000	2.6200000

Unique Subject Identifier=2692000000-0029

Assay variable : PTINR_VAL1 PT-INR Value

N	Mean	Standard Deviation	Minimum	Maximum
4	1.9550000	0.1147461	1.8100000	2.0900000

Unique Subject Identifier=2692000000-0033

Assay variable : PTINR_VAL1 PT-INR Value

N	Mean	Standard Deviation	Minimum	Maximum
6	2.0916667	0.1158303	1.9000000	2.2100000

Laboratory tests at the time of obtaining Table 2-6 consent, according to the presence or absence of proton-pump inhibitors

Unique Subject Identifier=2699000000-0001

MEANS Procedure

Assay variable : PTINR_VAL1 PT-INR Value

N	Mean	Standard Deviation	Minimum	Maximum
3	2.1466667	0.4473626	1.6600000	2.5400000

Unique Subject Identifier=2699000000-0004

Assay variable : PTINR_VAL1 PT-INR Value

N	Mean	Standard Deviation	Minimum	Maximum
4	2.3075000	0.6827091	1.3600000	2.9600000

Unique Subject Identifier=2699000000-0005

Assay variable : PTINR_VAL1 PT-INR Value

N	Mean	Standard Deviation	Minimum	Maximum
3	1.9433333	0.0832666	1.8500000	2.0100000

Unique Subject Identifier=2699000000-0009

Assay variable : PTINR_VAL1 PT-INR Value

N	Mean	Standard Deviation	Minimum	Maximum
2	2.4450000	0.9828784	1.7500000	3.1400000

Laboratory tests at the time of obtaining Table 2-6 consent, according to the presence or absence of proton-pump inhibitors

Unique Subject Identifier=2699000000-0010

MEANS Procedure

Assay variable : PTINR_VAL1 PT-INR Value

N	Mean	Standard Deviation	Minimum	Maximum
3	1.7766667	0.2150194	1.5400000	1.9600000

Unique Subject Identifier=2701011011-0001

Assay variable : PTINR_VAL1 PT-INR Value

N	Mean	Standard Deviation	Minimum	Maximum
6	2.1500000	0.1516575	1.9000000	2.3000000

Unique Subject Identifier=2701011011-0002

Assay variable : PTINR_VAL1 PT-INR Value

N	Mean	Standard Deviation	Minimum	Maximum
6	1.5500000	0.0547723	1.5000000	1.6000000

Unique Subject Identifier=2701011011-0003

Assay variable : PTINR_VAL1 PT-INR Value

N	Mean	Standard Deviation	Minimum	Maximum
6	1.9333333	0.2250926	1.7000000	2.3000000

Laboratory tests at the time of obtaining Table 2-6 consent, according to the presence or absence of proton-pump inhibitors

Unique Subject Identifier=2703000000-0002

MEANS Procedure

Assay variable : PTINR_VAL1 PT-INR Value

N	Mean	Standard Deviation	Minimum	Maximum
2	1.9000000	0.1414214	1.8000000	2.0000000

Unique Subject Identifier=2703000000-0003

Assay variable : PTINR_VAL1 PT-INR Value

N	Mean	Standard Deviation	Minimum	Maximum
2	2.0500000	0.0707107	2.0000000	2.1000000

Unique Subject Identifier=2703000000-0005

Assay variable : PTINR_VAL1 PT-INR Value

N	Mean	Standard Deviation	Minimum	Maximum
1	1.2000000		1.2000000	1.2000000

Unique Subject Identifier=2703000000-0006

Assay variable : PTINR_VAL1 PT-INR Value

N	Mean	Standard Deviation	Minimum	Maximum
3	2.0333333	0.4725816	1.5000000	2.4000000

Laboratory tests at the time of obtaining Table 2-6 consent, according to the presence or absence of proton-pump inhibitors

Unique Subject Identifier=2703000000-0007

MEANS Procedure

Assay variable : PTINR_VAL1 PT-INR Value

N	Mean	Standard Deviation	Minimum	Maximum
1	2.1000000		2.1000000	2.1000000

Unique Subject Identifier=2703000000-0008

Assay variable : PTINR_VAL1 PT-INR Value

N	Mean	Standard Deviation	Minimum	Maximum
2	1.5500000	0.0707107	1.5000000	1.6000000

Unique Subject Identifier=2703000000-0009

Assay variable : PTINR_VAL1 PT-INR Value

N	Mean	Standard Deviation	Minimum	Maximum
6	1.6333333	0.1366260	1.4000000	1.8000000

Unique Subject Identifier=2703000000-0012

Assay variable : PTINR_VAL1 PT-INR Value

N	Mean	Standard Deviation	Minimum	Maximum
3	1.9333333	0.7505553	1.5000000	2.8000000

Laboratory tests at the time of obtaining Table 2-6 consent, according to the presence or absence of proton-pump inhibitors

Unique Subject Identifier=2703000000-0013

MEANS Procedure

Assay variable : PTINR_VAL1 PT-INR Value

N	Mean	Standard Deviation	Minimum	Maximum
2	1.6000000	0	1.6000000	1.6000000

Unique Subject Identifier=2703000000-0018

Assay variable : PTINR_VAL1 PT-INR Value

N	Mean	Standard Deviation	Minimum	Maximum
1	1.3000000		1.3000000	1.3000000

Unique Subject Identifier=2729000000-0001

Assay variable : PTINR_VAL1 PT-INR Value

N	Mean	Standard Deviation	Minimum	Maximum
6	1.7933333	0.2315743	1.3900000	2.0300000

Unique Subject Identifier=2729000000-0003

Assay variable : PTINR_VAL1 PT-INR Value

N	Mean	Standard Deviation	Minimum	Maximum
5	1.5800000	0.1763519	1.3700000	1.7800000

Laboratory tests at the time of obtaining Table 2-6 consent, according to the presence or absence of proton-pump inhibitors

Unique Subject Identifier=2729000000-0004

MEANS Procedure

Assay variable : PTINR_VAL1 PT-INR Value

N	Mean	Standard Deviation	Minimum	Maximum
6	2.4433333	0.2484888	2.1100000	2.8100000

Unique Subject Identifier=2729000000-0005

Assay variable : PTINR_VAL1 PT-INR Value

N	Mean	Standard Deviation	Minimum	Maximum
6	2.6083333	0.2908207	2.1800000	3.0100000

Unique Subject Identifier=2732010001-0004

Assay variable : PTINR_VAL1 PT-INR Value

N	Mean	Standard Deviation	Minimum	Maximum
6	2.5000000	0.3633180	2.0000000	3.0000000

Unique Subject Identifier=2732010001-0005

Assay variable : PTINR_VAL1 PT-INR Value

N	Mean	Standard Deviation	Minimum	Maximum
4	2.0750000	0.3403430	1.8000000	2.5000000

Laboratory tests at the time of obtaining Table 2-6 consent, according to the presence or absence of proton-pump inhibitors

Unique Subject Identifier=2733010000-0002

MEANS Procedure

Assay variable : PTINR_VAL1 PT-INR Value

N	Mean	Standard Deviation	Minimum	Maximum
6	1.9633333	0.2173170	1.7600000	2.3100000

Unique Subject Identifier=2733010000-0008

Assay variable : PTINR_VAL1 PT-INR Value

N	Mean	Standard Deviation	Minimum	Maximum
6	2.1783333	0.2780228	1.8100000	2.5600000

Unique Subject Identifier=2733010000-0009

Assay variable : PTINR_VAL1 PT-INR Value

N	Mean	Standard Deviation	Minimum	Maximum
6	1.9383333	0.2949859	1.5300000	2.3800000

Unique Subject Identifier=2733010000-0011

Assay variable : PTINR_VAL1 PT-INR Value

N	Mean	Standard Deviation	Minimum	Maximum
6	2.4550000	0.3565810	2.1400000	2.9500000

Laboratory tests at the time of obtaining Table 2-6 consent, according to the presence or absence of proton-pump inhibitors

Unique Subject Identifier=2733010000-0016

MEANS Procedure

Assay variable : PTINR_VAL1 PT-INR Value

N	Mean	Standard Deviation	Minimum	Maximum
5	2.4400000	0.7313686	1.6500000	3.4900000

Unique Subject Identifier=2733010000-0017

Assay variable : PTINR_VAL1 PT-INR Value

N	Mean	Standard Deviation	Minimum	Maximum
6	1.9500000	0.5913713	1.1400000	2.5900000

Unique Subject Identifier=2733010000-0018

Assay variable : PTINR_VAL1 PT-INR Value

N	Mean	Standard Deviation	Minimum	Maximum
6	2.1716667	0.3365957	1.8300000	2.7800000

Unique Subject Identifier=2737011111-0001

Assay variable : PTINR_VAL1 PT-INR Value

N	Mean	Standard Deviation	Minimum	Maximum
6	1.2666667	0.1032796	1.1000000	1.4000000

Laboratory tests at the time of obtaining Table 2-6 consent, according to the presence or absence of proton-pump inhibitors

Unique Subject Identifier=2737011111-0003

MEANS Procedure

Assay variable : PTINR_VAL1 PT-INR Value

N	Mean	Standard Deviation	Minimum	Maximum
6	1.4833333	0.0983192	1.4000000	1.6000000

Unique Subject Identifier=2737011111-0005

Assay variable : PTINR_VAL1 PT-INR Value

N	Mean	Standard Deviation	Minimum	Maximum
6	1.7500000	0.3987480	1.3000000	2.5000000

Unique Subject Identifier=2748011000-0004

Assay variable : PTINR_VAL1 PT-INR Value

N	Mean	Standard Deviation	Minimum	Maximum
5	1.7120000	0.2275302	1.5400000	1.9900000

Unique Subject Identifier=2748011000-0022

Assay variable : PTINR_VAL1 PT-INR Value

N	Mean	Standard Deviation	Minimum	Maximum
2	2.3750000	0.0353553	2.3500000	2.4000000

Laboratory tests at the time of obtaining Table 2-6 consent, according to the presence or absence of proton-pump inhibitors

Unique Subject Identifier=2748011000-0024

MEANS Procedure

Assay variable : PTINR_VAL1 PT-INR Value

N	Mean	Standard Deviation	Minimum	Maximum
4	1.5300000	0.2615339	1.1800000	1.7400000

Unique Subject Identifier=2748011000-0031

Assay variable : PTINR_VAL1 PT-INR Value

N	Mean	Standard Deviation	Minimum	Maximum
3	2.2200000	0.0435890	2.1900000	2.2700000

Unique Subject Identifier=2748011000-0045

Assay variable : PTINR_VAL1 PT-INR Value

N	Mean	Standard Deviation	Minimum	Maximum
6	2.5850000	0.4136061	2.0900000	3.2400000

Unique Subject Identifier=2748011000-0051

Assay variable : PTINR_VAL1 PT-INR Value

N	Mean	Standard Deviation	Minimum	Maximum
4	2.0875000	0.2372586	1.7900000	2.3200000

Laboratory tests at the time of obtaining Table 2-6 consent, according to the presence or absence of proton-pump inhibitors

Unique Subject Identifier=2748011000-0052

MEANS Procedure

Assay variable : PTINR_VAL1 PT-INR Value

N	Mean	Standard Deviation	Minimum	Maximum
6	1.8716667	0.4007202	1.3600000	2.2600000

Unique Subject Identifier=2748011000-0053

Assay variable : PTINR_VAL1 PT-INR Value

N	Mean	Standard Deviation	Minimum	Maximum
4	2.1175000	0.3013719	1.9000000	2.5600000

Unique Subject Identifier=2748011000-0054

Assay variable : PTINR_VAL1 PT-INR Value

N	Mean	Standard Deviation	Minimum	Maximum
4	2.2450000	0.2223361	1.9400000	2.4200000

Unique Subject Identifier=2748011000-0057

Assay variable : PTINR_VAL1 PT-INR Value

N	Mean	Standard Deviation	Minimum	Maximum
2	1.7600000	0.0424264	1.7300000	1.7900000

Laboratory tests at the time of obtaining Table 2-6 consent, according to the presence or absence of proton-pump inhibitors

Unique Subject Identifier=2748011000-0060

MEANS Procedure

Assay variable : PTINR_VAL1 PT-INR Value

N	Mean	Standard Deviation	Minimum	Maximum
4	2.0300000	0.3232130	1.7200000	2.4800000

Unique Subject Identifier=2763011100-0002

Assay variable : PTINR_VAL1 PT-INR Value

N	Mean	Standard Deviation	Minimum	Maximum
4	2.4250000	0.2753785	2.1000000	2.7000000

Unique Subject Identifier=2764000000-0009

Assay variable : PTINR_VAL1 PT-INR Value

N	Mean	Standard Deviation	Minimum	Maximum
1	2.0000000		2.0000000	2.0000000

Unique Subject Identifier=2765000000-0003

Assay variable : PTINR_VAL1 PT-INR Value

N	Mean	Standard Deviation	Minimum	Maximum
6	1.7600000	0.2596151	1.4400000	2.1800000

Laboratory tests at the time of obtaining Table 2-6 consent, according to the presence or absence of proton-pump inhibitors

Unique Subject Identifier=2766010111-0005

MEANS Procedure

Assay variable : PTINR_VAL1 PT-INR Value

N	Mean	Standard Deviation	Minimum	Maximum
6	2.1883333	0.4578828	1.7000000	2.9700000

Unique Subject Identifier=2766010111-0006

Assay variable : PTINR_VAL1 PT-INR Value

N	Mean	Standard Deviation	Minimum	Maximum
6	1.9250000	0.1385280	1.7800000	2.1000000

Unique Subject Identifier=2769000000-0001

Assay variable : PTINR_VAL1 PT-INR Value

N	Mean	Standard Deviation	Minimum	Maximum
1	1.7900000		1.7900000	1.7900000

Unique Subject Identifier=2777011010-0006

Assay variable : PTINR_VAL1 PT-INR Value

N	Mean	Standard Deviation	Minimum	Maximum
4	2.1275000	0.3636276	1.6400000	2.4200000

Laboratory tests at the time of obtaining Table 2-6 consent, according to the presence or absence of proton-pump inhibitors

Unique Subject Identifier=2777011010-0007

MEANS Procedure

Assay variable : PTINR_VAL1 PT-INR Value

N	Mean	Standard Deviation	Minimum	Maximum
4	1.4650000	0.2145538	1.2800000	1.7100000

Unique Subject Identifier=2777011010-0009

Assay variable : PTINR_VAL1 PT-INR Value

N	Mean	Standard Deviation	Minimum	Maximum
3	2.3400000	0.1732051	2.2400000	2.5400000

Unique Subject Identifier=2777011010-0011

Assay variable : PTINR_VAL1 PT-INR Value

N	Mean	Standard Deviation	Minimum	Maximum
5	2.3480000	0.1907092	2.0600000	2.5900000

Unique Subject Identifier=2777011010-0012

Assay variable : PTINR_VAL1 PT-INR Value

N	Mean	Standard Deviation	Minimum	Maximum
6	2.1333333	0.4344038	1.7700000	2.9800000

Laboratory tests at the time of obtaining Table 2-6 consent, according to the presence or absence of proton-pump inhibitors

Unique Subject Identifier=2777011010-0014

MEANS Procedure

Assay variable : PTINR_VAL1 PT-INR Value

N	Mean	Standard Deviation	Minimum	Maximum
3	2.1500000	0.1852026	1.9400000	2.2900000

Unique Subject Identifier=2777011010-0017

Assay variable : PTINR_VAL1 PT-INR Value

N	Mean	Standard Deviation	Minimum	Maximum
3	1.8500000	0.3559494	1.6200000	2.2600000

Unique Subject Identifier=2777011010-0020

Assay variable : PTINR_VAL1 PT-INR Value

N	Mean	Standard Deviation	Minimum	Maximum
3	2.2033333	0.2665208	1.9000000	2.4000000

Unique Subject Identifier=2777011010-0024

Assay variable : PTINR_VAL1 PT-INR Value

N	Mean	Standard Deviation	Minimum	Maximum
4	1.7400000	0.1739732	1.5700000	1.8900000

Laboratory tests at the time of obtaining Table 2-6 consent, according to the presence or absence of proton-pump inhibitors

Unique Subject Identifier=2777011010-0028

MEANS Procedure

Assay variable : PTINR_VAL1 PT-INR Value

N	Mean	Standard Deviation	Minimum	Maximum
3	1.8300000	0.2570992	1.6300000	2.1200000

Unique Subject Identifier=2777011010-0035

Assay variable : PTINR_VAL1 PT-INR Value

N	Mean	Standard Deviation	Minimum	Maximum
5	1.6860000	0.2124382	1.4000000	1.9900000

Unique Subject Identifier=2777011010-0036

Assay variable : PTINR_VAL1 PT-INR Value

N	Mean	Standard Deviation	Minimum	Maximum
3	2.1900000	0.3704052	1.8300000	2.5700000

Unique Subject Identifier=2777011010-0039

Assay variable : PTINR_VAL1 PT-INR Value

N	Mean	Standard Deviation	Minimum	Maximum
3	1.6566667	0.2020726	1.4400000	1.8400000

Laboratory tests at the time of obtaining Table 2-6 consent, according to the presence or absence of proton-pump inhibitors

Unique Subject Identifier=2777011010-0044

MEANS Procedure

Assay variable : PTINR_VAL1 PT-INR Value

N	Mean	Standard Deviation	Minimum	Maximum
3	2.2833333	0.4722641	1.8400000	2.7800000

Unique Subject Identifier=2777011010-0045

Assay variable : PTINR_VAL1 PT-INR Value

N	Mean	Standard Deviation	Minimum	Maximum
4	2.5600000	1.2616127	1.6600000	4.3600000

Unique Subject Identifier=2777011010-0049

Assay variable : PTINR_VAL1 PT-INR Value

N	Mean	Standard Deviation	Minimum	Maximum
3	2.0166667	0.4758501	1.6100000	2.5400000

Unique Subject Identifier=2777011010-0050

Assay variable : PTINR_VAL1 PT-INR Value

N	Mean	Standard Deviation	Minimum	Maximum
3	2.0166667	0.0568624	1.9700000	2.0800000

Laboratory tests at the time of obtaining Table 2-6 consent, according to the presence or absence of proton-pump inhibitors

Unique Subject Identifier=2780000000-0003

MEANS Procedure

Assay variable : PTINR_VAL1 PT-INR Value

N	Mean	Standard Deviation	Minimum	Maximum
6	1.9833333	0.1602082	1.9000000	2.3000000

Unique Subject Identifier=2780000000-0008

Assay variable : PTINR_VAL1 PT-INR Value

N	Mean	Standard Deviation	Minimum	Maximum
6	1.5166667	0.0752773	1.4000000	1.6000000

Unique Subject Identifier=2784011111-0002

Assay variable : PTINR_VAL1 PT-INR Value

N	Mean	Standard Deviation	Minimum	Maximum
5	2.2200000	0.3701351	1.8000000	2.7000000

Unique Subject Identifier=2784011111-0010

Assay variable : PTINR_VAL1 PT-INR Value

N	Mean	Standard Deviation	Minimum	Maximum
5	1.4400000	0.7635444	1.0000000	2.8000000

Laboratory tests at the time of obtaining Table 2-6 consent, according to the presence or absence of proton-pump inhibitors

Unique Subject Identifier=2800011010-0033

MEANS Procedure

Assay variable : PTINR_VAL1 PT-INR Value

N	Mean	Standard Deviation	Minimum	Maximum
2	1.5050000	0.0919239	1.4400000	1.5700000

Unique Subject Identifier=2800011010-0037

Assay variable : PTINR_VAL1 PT-INR Value

N	Mean	Standard Deviation	Minimum	Maximum
1	2.0600000		2.0600000	2.0600000

Unique Subject Identifier=2808000000-0002

Assay variable : PTINR_VAL1 PT-INR Value

N	Mean	Standard Deviation	Minimum	Maximum
5	1.6500000	0.3323402	1.1900000	1.9500000

Unique Subject Identifier=2809000000-0011

Assay variable : PTINR_VAL1 PT-INR Value

N	Mean	Standard Deviation	Minimum	Maximum
6	1.2133333	0.0618601	1.1400000	1.3000000

Laboratory tests at the time of obtaining Table 2-6 consent, according to the presence or absence of proton-pump inhibitors

Unique Subject Identifier=2828010110-0001

MEANS Procedure

Assay variable : PTINR_VAL1 PT-INR Value

N	Mean	Standard Deviation	Minimum	Maximum
2	1.6000000	0	1.6000000	1.6000000

Unique Subject Identifier=2861011011-0003

Assay variable : PTINR_VAL1 PT-INR Value

N	Mean	Standard Deviation	Minimum	Maximum
3	2.0500000	0.2861818	1.8700000	2.3800000

Unique Subject Identifier=2861011011-0006

Assay variable : PTINR_VAL1 PT-INR Value

N	Mean	Standard Deviation	Minimum	Maximum
5	1.3440000	0.2920274	1.1000000	1.8300000

Unique Subject Identifier=2861011011-0010

Assay variable : PTINR_VAL1 PT-INR Value

N	Mean	Standard Deviation	Minimum	Maximum
6	1.7433333	0.1201111	1.6200000	1.9200000

Laboratory tests at the time of obtaining Table 2-6 consent, according to the presence or absence of proton-pump inhibitors

Unique Subject Identifier=2862000000-0003

MEANS Procedure

Assay variable : PTINR_VAL1 PT-INR Value

N	Mean	Standard Deviation	Minimum	Maximum
3	2.2300000	0.0888819	2.1300000	2.3000000

Unique Subject Identifier=2862000000-0004

Assay variable : PTINR_VAL1 PT-INR Value

N	Mean	Standard Deviation	Minimum	Maximum
1	1.9000000		1.9000000	1.9000000

Unique Subject Identifier=2868000000-0002

Assay variable : PTINR_VAL1 PT-INR Value

N	Mean	Standard Deviation	Minimum	Maximum
4	1.9975000	0.3151058	1.5500000	2.2900000

Unique Subject Identifier=2868000000-0007

Assay variable : PTINR_VAL1 PT-INR Value

N	Mean	Standard Deviation	Minimum	Maximum
2	2.3150000	1.0677312	1.5600000	3.0700000

Laboratory tests at the time of obtaining Table 2-6 consent, according to the presence or absence of proton-pump inhibitors

Unique Subject Identifier=2868000000-0009

MEANS Procedure

Assay variable : PTINR_VAL1 PT-INR Value

N	Mean	Standard Deviation	Minimum	Maximum
5	2.1220000	0.5362555	1.5200000	2.8200000

Unique Subject Identifier=2868000000-0019

Assay variable : PTINR_VAL1 PT-INR Value

N	Mean	Standard Deviation	Minimum	Maximum
3	2.2800000	0.2389561	2.0200000	2.4900000

Unique Subject Identifier=2872000000-0004

Assay variable : PTINR_VAL1 PT-INR Value

N	Mean	Standard Deviation	Minimum	Maximum
3	1.6233333	0.2510644	1.4100000	1.9000000

Unique Subject Identifier=2872000000-0005

Assay variable : PTINR_VAL1 PT-INR Value

N	Mean	Standard Deviation	Minimum	Maximum
4	2.3275000	0.8367148	1.5600000	3.5000000

Laboratory tests at the time of obtaining Table 2-6 consent, according to the presence or absence of proton-pump inhibitors

Unique Subject Identifier=2875000000-0012

MEANS Procedure

Assay variable : PTINR_VAL1 PT-INR Value

N	Mean	Standard Deviation	Minimum	Maximum
6	1.5466667	0.2045157	1.3000000	1.8200000

Unique Subject Identifier=2875000000-0019

Assay variable : PTINR_VAL1 PT-INR Value

N	Mean	Standard Deviation	Minimum	Maximum
5	1.4840000	0.1283745	1.3100000	1.6000000

Unique Subject Identifier=2875000000-0023

Assay variable : PTINR_VAL1 PT-INR Value

N	Mean	Standard Deviation	Minimum	Maximum
2	2.1750000	0.2333452	2.0100000	2.3400000

Unique Subject Identifier=2875000000-0025

Assay variable : PTINR_VAL1 PT-INR Value

N	Mean	Standard Deviation	Minimum	Maximum
2	1.8700000	0.0848528	1.8100000	1.9300000

Laboratory tests at the time of obtaining Table 2-6 consent, according to the presence or absence of proton-pump inhibitors

Unique Subject Identifier=2875000000-0038

MEANS Procedure

Assay variable : PTINR_VAL1 PT-INR Value

N	Mean	Standard Deviation	Minimum	Maximum
3	2.0066667	0.2055075	1.8100000	2.2200000

Unique Subject Identifier=2875000000-0041

Assay variable : PTINR_VAL1 PT-INR Value

N	Mean	Standard Deviation	Minimum	Maximum
3	2.1566667	0.1069268	2.0400000	2.2500000

Unique Subject Identifier=2875000000-0049

Assay variable : PTINR_VAL1 PT-INR Value

N	Mean	Standard Deviation	Minimum	Maximum
4	1.9675000	0.3559377	1.6900000	2.4900000

Unique Subject Identifier=2889010000-0002

Assay variable : PTINR_VAL1 PT-INR Value

N	Mean	Standard Deviation	Minimum	Maximum
5	1.3520000	0.0164317	1.3300000	1.3700000

Laboratory tests at the time of obtaining Table 2-6 consent, according to the presence or absence of proton-pump inhibitors

Unique Subject Identifier=2890001000-0001

MEANS Procedure

Assay variable : PTINR_VAL1 PT-INR Value

N	Mean	Standard Deviation	Minimum	Maximum
4	3.1225000	0.4659310	2.5200000	3.6500000

Unique Subject Identifier=2898010000-0008

Assay variable : PTINR_VAL1 PT-INR Value

N	Mean	Standard Deviation	Minimum	Maximum
4	1.7500000	0.0594418	1.6800000	1.8200000

Unique Subject Identifier=2898010000-0017

Assay variable : PTINR_VAL1 PT-INR Value

N	Mean	Standard Deviation	Minimum	Maximum
6	2.1033333	0.2989091	1.6000000	2.4400000

Unique Subject Identifier=2910000000-0002

Assay variable : PTINR_VAL1 PT-INR Value

N	Mean	Standard Deviation	Minimum	Maximum
2	2.0550000	0.1484924	1.9500000	2.1600000

Laboratory tests at the time of obtaining Table 2-6 consent, according to the presence or absence of proton-pump inhibitors

Unique Subject Identifier=2910000000-0005

MEANS Procedure

Assay variable : PTINR_VAL1 PT-INR Value

N	Mean	Standard Deviation	Minimum	Maximum
3	2.0766667	0.2706166	1.8600000	2.3800000

Unique Subject Identifier=2910000000-0008

Assay variable : PTINR_VAL1 PT-INR Value

N	Mean	Standard Deviation	Minimum	Maximum
2	2.1650000	0.1484924	2.0600000	2.2700000

Unique Subject Identifier=2910000000-0014

Assay variable : PTINR_VAL1 PT-INR Value

N	Mean	Standard Deviation	Minimum	Maximum
4	2.2125000	0.6854864	1.5700000	2.8200000

Unique Subject Identifier=2910000000-0016

Assay variable : PTINR_VAL1 PT-INR Value

N	Mean	Standard Deviation	Minimum	Maximum
2	2.0950000	0.0919239	2.0300000	2.1600000

Laboratory tests at the time of obtaining Table 2-6 consent, according to the presence or absence of proton-pump inhibitors

Unique Subject Identifier=2910000000-0021

MEANS Procedure

Assay variable : PTINR_VAL1 PT-INR Value

N	Mean	Standard Deviation	Minimum	Maximum
2	1.4650000	0.0212132	1.4500000	1.4800000

Unique Subject Identifier=2910000000-0022

Assay variable : PTINR_VAL1 PT-INR Value

N	Mean	Standard Deviation	Minimum	Maximum
4	1.8650000	0.3049044	1.6100000	2.2600000

Unique Subject Identifier=2910000000-0024

Assay variable : PTINR_VAL1 PT-INR Value

N	Mean	Standard Deviation	Minimum	Maximum
3	1.8733333	0.3464583	1.5100000	2.2000000

Unique Subject Identifier=2910000000-0025

Assay variable : PTINR_VAL1 PT-INR Value

N	Mean	Standard Deviation	Minimum	Maximum
5	2.3640000	0.3633593	1.8000000	2.7600000

Laboratory tests at the time of obtaining Table 2-6 consent, according to the presence or absence of proton-pump inhibitors

Unique Subject Identifier=2910000000-0026

MEANS Procedure

Assay variable : PTINR_VAL1 PT-INR Value

N	Mean	Standard Deviation	Minimum	Maximum
2	2.2400000	0.0282843	2.2200000	2.2600000

Unique Subject Identifier=2913000000-0006

Assay variable : PTINR_VAL1 PT-INR Value

N	Mean	Standard Deviation	Minimum	Maximum
3	1.8966667	0.1504438	1.8000000	2.0700000

Unique Subject Identifier=2913000000-0014

Assay variable : PTINR_VAL1 PT-INR Value

N	Mean	Standard Deviation	Minimum	Maximum
3	1.8066667	0.4212284	1.3500000	2.1800000

Unique Subject Identifier=2914000000-0005

Assay variable : PTINR_VAL1 PT-INR Value

N	Mean	Standard Deviation	Minimum	Maximum
4	2.0550000	0.0785281	1.9400000	2.1100000

Laboratory tests at the time of obtaining Table 2-6 consent, according to the presence or absence of proton-pump inhibitors

Unique Subject Identifier=2914000000-0007

MEANS Procedure

Assay variable : PTINR_VAL1 PT-INR Value

N	Mean	Standard Deviation	Minimum	Maximum
4	2.1400000	0.1373560	1.9600000	2.2900000

Unique Subject Identifier=2937010011-0001

Assay variable : PTINR_VAL1 PT-INR Value

N	Mean	Standard Deviation	Minimum	Maximum
3	1.5133333	0.1960442	1.3300000	1.7200000

Unique Subject Identifier=2937010011-0009

Assay variable : PTINR_VAL1 PT-INR Value

N	Mean	Standard Deviation	Minimum	Maximum
4	1.7425000	0.3920353	1.3300000	2.1300000

Unique Subject Identifier=2937010011-0013

Assay variable : PTINR_VAL1 PT-INR Value

N	Mean	Standard Deviation	Minimum	Maximum
5	1.8080000	1.0148497	1.0200000	3.5500000

Laboratory tests at the time of obtaining Table 2-6 consent, according to the presence or absence of proton-pump inhibitors

Unique Subject Identifier=2937010011-0015

MEANS Procedure

Assay variable : PTINR_VAL1 PT-INR Value

N	Mean	Standard Deviation	Minimum	Maximum
6	1.7816667	0.1452469	1.6200000	1.9900000

Unique Subject Identifier=2937010011-0017

Assay variable : PTINR_VAL1 PT-INR Value

N	Mean	Standard Deviation	Minimum	Maximum
6	1.9016667	0.4646899	1.5100000	2.6300000

Unique Subject Identifier=2937010011-0018

Assay variable : PTINR_VAL1 PT-INR Value

N	Mean	Standard Deviation	Minimum	Maximum
6	2.0950000	0.2341581	1.7200000	2.4300000

Unique Subject Identifier=2937010011-0019

Assay variable : PTINR_VAL1 PT-INR Value

N	Mean	Standard Deviation	Minimum	Maximum
6	1.7683333	0.1248065	1.6000000	1.9500000

Laboratory tests at the time of obtaining Table 2-6 consent, according to the presence or absence of proton-pump inhibitors

Unique Subject Identifier=2937010011-0022

MEANS Procedure

Assay variable : PTINR_VAL1 PT-INR Value

N	Mean	Standard Deviation	Minimum	Maximum
4	1.9225000	0.3247948	1.6000000	2.2800000

Unique Subject Identifier=2941010110-0014

Assay variable : PTINR_VAL1 PT-INR Value

N	Mean	Standard Deviation	Minimum	Maximum
5	1.8600000	0.3209361	1.6000000	2.4000000

Unique Subject Identifier=2949000000-0001

Assay variable : PTINR_VAL1 PT-INR Value

N	Mean	Standard Deviation	Minimum	Maximum
3	1.6966667	0.3043572	1.4600000	2.0400000

Unique Subject Identifier=2949000000-0003

Assay variable : PTINR_VAL1 PT-INR Value

N	Mean	Standard Deviation	Minimum	Maximum
1	2.6500000		2.6500000	2.6500000

Laboratory tests at the time of obtaining Table 2-6 consent, according to the presence or absence of proton-pump inhibitors

Unique Subject Identifier=2949000000-0004

MEANS Procedure

Assay variable : PTINR_VAL1 PT-INR Value

N	Mean	Standard Deviation	Minimum	Maximum
3	1.8433333	0.3027100	1.5200000	2.1200000

Unique Subject Identifier=2949000000-0027

Assay variable : PTINR_VAL1 PT-INR Value

N	Mean	Standard Deviation	Minimum	Maximum
2	1.8300000	0.2404163	1.6600000	2.0000000

Unique Subject Identifier=2949000000-0033

Assay variable : PTINR_VAL1 PT-INR Value

N	Mean	Standard Deviation	Minimum	Maximum
2	2.1850000	0.4030509	1.9000000	2.4700000

Unique Subject Identifier=2949000000-0037

Assay variable : PTINR_VAL1 PT-INR Value

N	Mean	Standard Deviation	Minimum	Maximum
2	1.9250000	0.1060660	1.8500000	2.0000000

Laboratory tests at the time of obtaining Table 2-6 consent, according to the presence or absence of proton-pump inhibitors

Unique Subject Identifier=2953000000-0003

MEANS Procedure

Assay variable : PTINR_VAL1 PT-INR Value

N	Mean	Standard Deviation	Minimum	Maximum
5	2.5620000	0.1061603	2.4300000	2.7000000

Unique Subject Identifier=2953000000-0004

Assay variable : PTINR_VAL1 PT-INR Value

N	Mean	Standard Deviation	Minimum	Maximum
6	2.1066667	0.2170407	1.8400000	2.5000000

Unique Subject Identifier=2954000000-0009

Assay variable : PTINR_VAL1 PT-INR Value

N	Mean	Standard Deviation	Minimum	Maximum
5	2.2960000	0.7554337	1.2500000	3.3800000

Unique Subject Identifier=2954000000-0013

Assay variable : PTINR_VAL1 PT-INR Value

N	Mean	Standard Deviation	Minimum	Maximum
6	1.6766667	0.2069460	1.3800000	1.9800000

Laboratory tests at the time of obtaining Table 2-6 consent, according to the presence or absence of proton-pump inhibitors

Unique Subject Identifier=2957000000-0017

MEANS Procedure

Assay variable : PTINR_VAL1 PT-INR Value

N	Mean	Standard Deviation	Minimum	Maximum
4	2.1925000	0.1144188	2.0900000	2.3500000

Unique Subject Identifier=3001010001-0003

Assay variable : PTINR_VAL1 PT-INR Value

N	Mean	Standard Deviation	Minimum	Maximum
5	1.6920000	0.2604227	1.4900000	2.0200000

Unique Subject Identifier=3001010001-0004

Assay variable : PTINR_VAL1 PT-INR Value

N	Mean	Standard Deviation	Minimum	Maximum
6	1.8250000	0.1170897	1.6500000	1.9400000

Unique Subject Identifier=3001010001-0009

Assay variable : PTINR_VAL1 PT-INR Value

N	Mean	Standard Deviation	Minimum	Maximum
5	1.7360000	0.1699412	1.6100000	1.9800000

Laboratory tests at the time of obtaining Table 2-6 consent, according to the presence or absence of proton-pump inhibitors

Unique Subject Identifier=3001010001-0013

MEANS Procedure

Assay variable : PTINR_VAL1 PT-INR Value

N	Mean	Standard Deviation	Minimum	Maximum
4	2.3675000	0.4050823	1.8500000	2.8400000

Unique Subject Identifier=3001010001-0035

Assay variable : PTINR_VAL1 PT-INR Value

N	Mean	Standard Deviation	Minimum	Maximum
5	1.7440000	0.2411016	1.4800000	2.1300000

Unique Subject Identifier=3001010001-0044

Assay variable : PTINR_VAL1 PT-INR Value

N	Mean	Standard Deviation	Minimum	Maximum
4	2.1825000	0.1685972	2.0200000	2.4100000

Unique Subject Identifier=3001010001-0046

Assay variable : PTINR_VAL1 PT-INR Value

N	Mean	Standard Deviation	Minimum	Maximum
4	2.1900000	0.2171021	2.0300000	2.5000000

Laboratory tests at the time of obtaining Table 2-6 consent, according to the presence or absence of proton-pump inhibitors

Unique Subject Identifier=3001010001-0049

MEANS Procedure

Assay variable : PTINR_VAL1 PT-INR Value

N	Mean	Standard Deviation	Minimum	Maximum
5	2.1920000	0.2622403	1.8900000	2.4700000

Unique Subject Identifier=3001010001-0057

Assay variable : PTINR_VAL1 PT-INR Value

N	Mean	Standard Deviation	Minimum	Maximum
3	2.1066667	0.1803700	1.9200000	2.2800000

Unique Subject Identifier=3003011110-0001

Assay variable : PTINR_VAL1 PT-INR Value

N	Mean	Standard Deviation	Minimum	Maximum
3	1.5333333	0.4933896	1.1500000	2.0900000

Unique Subject Identifier=3009011111-0006

Assay variable : PTINR_VAL1 PT-INR Value

N	Mean	Standard Deviation	Minimum	Maximum
3	1.8800000	0.1473092	1.7500000	2.0400000

Laboratory tests at the time of obtaining Table 2-6 consent, according to the presence or absence of proton-pump inhibitors

Unique Subject Identifier=3010011111-0001

MEANS Procedure

Assay variable : PTINR_VAL1 PT-INR Value

N	Mean	Standard Deviation	Minimum	Maximum
5	1.9020000	0.2240982	1.6000000	2.2300000

Unique Subject Identifier=3011010000-0004

Assay variable : PTINR_VAL1 PT-INR Value

N	Mean	Standard Deviation	Minimum	Maximum
6	1.7550000	0.3245766	1.4300000	2.2000000

Unique Subject Identifier=3011010000-0006

Assay variable : PTINR_VAL1 PT-INR Value

N	Mean	Standard Deviation	Minimum	Maximum
6	1.8150000	0.3179151	1.3900000	2.2400000

Unique Subject Identifier=3011010000-0010

Assay variable : PTINR_VAL1 PT-INR Value

N	Mean	Standard Deviation	Minimum	Maximum
6	1.8383333	0.2523027	1.6300000	2.3100000

Laboratory tests at the time of obtaining Table 2-6 consent, according to the presence or absence of proton-pump inhibitors

Unique Subject Identifier=3016111111-0068

MEANS Procedure

Assay variable : PTINR_VAL1 PT-INR Value

N	Mean	Standard Deviation	Minimum	Maximum
2	2.5450000	0.1767767	2.4200000	2.6700000

Unique Subject Identifier=3019011110-0002

Assay variable : PTINR_VAL1 PT-INR Value

N	Mean	Standard Deviation	Minimum	Maximum
1	2.2700000		2.2700000	2.2700000

Unique Subject Identifier=3019011110-0005

Assay variable : PTINR_VAL1 PT-INR Value

N	Mean	Standard Deviation	Minimum	Maximum
3	1.9733333	0.6493330	1.2700000	2.5500000

Unique Subject Identifier=3019011110-0008

Assay variable : PTINR_VAL1 PT-INR Value

N	Mean	Standard Deviation	Minimum	Maximum
3	2.0133333	0.6242863	1.4800000	2.7000000

Laboratory tests at the time of obtaining Table 2-6 consent, according to the presence or absence of proton-pump inhibitors

Unique Subject Identifier=3019011110-0010

MEANS Procedure

Assay variable : PTINR_VAL1 PT-INR Value

N	Mean	Standard Deviation	Minimum	Maximum
6	2.0133333	0.1240430	1.8100000	2.1400000

Unique Subject Identifier=3019011110-0012

Assay variable : PTINR_VAL1 PT-INR Value

N	Mean	Standard Deviation	Minimum	Maximum
3	2.0366667	0.1709776	1.8400000	2.1500000

Unique Subject Identifier=3019011110-0014

Assay variable : PTINR_VAL1 PT-INR Value

N	Mean	Standard Deviation	Minimum	Maximum
2	1.8850000	0.0636396	1.8400000	1.9300000

Unique Subject Identifier=3019011110-0019

Assay variable : PTINR_VAL1 PT-INR Value

N	Mean	Standard Deviation	Minimum	Maximum
1	1.3000000		1.3000000	1.3000000

Laboratory tests at the time of obtaining Table 2-6 consent, according to the presence or absence of proton-pump inhibitors

Unique Subject Identifier=3019011110-0023

MEANS Procedure

Assay variable : PTINR_VAL1 PT-INR Value

N	Mean	Standard Deviation	Minimum	Maximum
3	2.1266667	0.2722744	1.8200000	2.3400000

Unique Subject Identifier=3019011110-0041

Assay variable : PTINR_VAL1 PT-INR Value

N	Mean	Standard Deviation	Minimum	Maximum
3	1.6933333	0.3043572	1.4700000	2.0400000

Unique Subject Identifier=3019011110-0043

Assay variable : PTINR_VAL1 PT-INR Value

N	Mean	Standard Deviation	Minimum	Maximum
3	1.6466667	0.3431229	1.2800000	1.9600000

Unique Subject Identifier=3019011110-0047

Assay variable : PTINR_VAL1 PT-INR Value

N	Mean	Standard Deviation	Minimum	Maximum
3	1.8766667	0.0513160	1.8200000	1.9200000

Laboratory tests at the time of obtaining Table 2-6 consent, according to the presence or absence of proton-pump inhibitors

Unique Subject Identifier=3019011110-0051

MEANS Procedure

Assay variable : PTINR_VAL1 PT-INR Value

N	Mean	Standard Deviation	Minimum	Maximum
3	1.6300000	0.1670329	1.4800000	1.8100000

Unique Subject Identifier=3026011100-0016

Assay variable : PTINR_VAL1 PT-INR Value

N	Mean	Standard Deviation	Minimum	Maximum
3	2.5066667	0.3962743	2.1300000	2.9200000

Unique Subject Identifier=3026011100-0025

Assay variable : PTINR_VAL1 PT-INR Value

N	Mean	Standard Deviation	Minimum	Maximum
3	1.9300000	0.3078961	1.5900000	2.1900000

Unique Subject Identifier=3026011100-0033

Assay variable : PTINR_VAL1 PT-INR Value

N	Mean	Standard Deviation	Minimum	Maximum
3	2.6233333	0.6903864	1.9200000	3.3000000

Laboratory tests at the time of obtaining Table 2-6 consent, according to the presence or absence of proton-pump inhibitors

Unique Subject Identifier=3026011100-0058

MEANS Procedure

Assay variable : PTINR_VAL1 PT-INR Value

N	Mean	Standard Deviation	Minimum	Maximum
4	1.7650000	0.1913984	1.5900000	1.9500000

Unique Subject Identifier=3056000000-0009

Assay variable : PTINR_VAL1 PT-INR Value

N	Mean	Standard Deviation	Minimum	Maximum
4	2.1175000	0.3069609	1.7600000	2.5100000

Unique Subject Identifier=3056000000-0010

Assay variable : PTINR_VAL1 PT-INR Value

N	Mean	Standard Deviation	Minimum	Maximum
6	1.9916667	0.6568232	1.2100000	2.8500000

Unique Subject Identifier=3056000000-0015

Assay variable : PTINR_VAL1 PT-INR Value

N	Mean	Standard Deviation	Minimum	Maximum
4	1.8200000	0.4496665	1.3500000	2.3700000

Laboratory tests at the time of obtaining Table 2-6 consent, according to the presence or absence of proton-pump inhibitors

Unique Subject Identifier=3056000000-0024

MEANS Procedure

Assay variable : PTINR_VAL1 PT-INR Value

N	Mean	Standard Deviation	Minimum	Maximum
3	2.3133333	0.7900211	1.5200000	3.1000000

Unique Subject Identifier=3056000000-0025

Assay variable : PTINR_VAL1 PT-INR Value

N	Mean	Standard Deviation	Minimum	Maximum
2	2.5600000	0.5374012	2.1800000	2.9400000

Unique Subject Identifier=3056000000-0028

Assay variable : PTINR_VAL1 PT-INR Value

N	Mean	Standard Deviation	Minimum	Maximum
2	1.6950000	0.0636396	1.6500000	1.7400000

Unique Subject Identifier=3060011100-0007

Assay variable : PTINR_VAL1 PT-INR Value

N	Mean	Standard Deviation	Minimum	Maximum
5	2.1800000	0.4086563	1.6000000	2.7000000

Laboratory tests at the time of obtaining Table 2-6 consent, according to the presence or absence of proton-pump inhibitors

Unique Subject Identifier=3060011100-0011

MEANS Procedure

Assay variable : PTINR_VAL1 PT-INR Value

N	Mean	Standard Deviation	Minimum	Maximum
6	1.5666667	0.1366260	1.4000000	1.7000000

Unique Subject Identifier=3060011100-0013

Assay variable : PTINR_VAL1 PT-INR Value

N	Mean	Standard Deviation	Minimum	Maximum
3	1.6000000	0.2645751	1.4000000	1.9000000

Unique Subject Identifier=3060011100-0017

Assay variable : PTINR_VAL1 PT-INR Value

N	Mean	Standard Deviation	Minimum	Maximum
6	1.7833333	0.4792355	1.4000000	2.7000000

Unique Subject Identifier=3060011100-0020

Assay variable : PTINR_VAL1 PT-INR Value

N	Mean	Standard Deviation	Minimum	Maximum
6	2.1000000	0.0632456	2.0000000	2.2000000

Laboratory tests at the time of obtaining Table 2-6 consent, according to the presence or absence of proton-pump inhibitors

Unique Subject Identifier=3076011000-0005

MEANS Procedure

Assay variable : PTINR_VAL1 PT-INR Value

N	Mean	Standard Deviation	Minimum	Maximum
5	1.9200000	0.3420526	1.5000000	2.4000000

Unique Subject Identifier=3077010011-0007

Assay variable : PTINR_VAL1 PT-INR Value

N	Mean	Standard Deviation	Minimum	Maximum
5	1.9840000	0.2069541	1.7200000	2.1700000

Unique Subject Identifier=3077010011-0011

Assay variable : PTINR_VAL1 PT-INR Value

N	Mean	Standard Deviation	Minimum	Maximum
5	1.7660000	0.0835464	1.7000000	1.9100000

Unique Subject Identifier=3084010000-0007

Assay variable : PTINR_VAL1 PT-INR Value

N	Mean	Standard Deviation	Minimum	Maximum
1	1.9500000		1.9500000	1.9500000

Laboratory tests at the time of obtaining Table 2-6 consent, according to the presence or absence of proton-pump inhibitors

Unique Subject Identifier=3084010000-0013

MEANS Procedure

Assay variable : PTINR_VAL1 PT-INR Value

N	Mean	Standard Deviation	Minimum	Maximum
1	1.5000000		1.5000000	1.5000000

Unique Subject Identifier=3084010000-0014

Assay variable : PTINR_VAL1 PT-INR Value

N	Mean	Standard Deviation	Minimum	Maximum
3	1.6133333	0.2289833	1.4300000	1.8700000

Unique Subject Identifier=3084010000-0015

Assay variable : PTINR_VAL1 PT-INR Value

N	Mean	Standard Deviation	Minimum	Maximum
3	1.6033333	0.0750555	1.5600000	1.6900000

Unique Subject Identifier=3084010000-0017

Assay variable : PTINR_VAL1 PT-INR Value

N	Mean	Standard Deviation	Minimum	Maximum
2	2.0550000	0.3889087	1.7800000	2.3300000

Laboratory tests at the time of obtaining Table 2-6 consent, according to the presence or absence of proton-pump inhibitors

Unique Subject Identifier=3084010000-0021

MEANS Procedure

Assay variable : PTINR_VAL1 PT-INR Value

N	Mean	Standard Deviation	Minimum	Maximum
3	1.1066667	0.0832666	1.0400000	1.2000000

Unique Subject Identifier=3085010000-0005

Assay variable : PTINR_VAL1 PT-INR Value

N	Mean	Standard Deviation	Minimum	Maximum
3	1.5466667	0.4792007	1.2700000	2.1000000

Unique Subject Identifier=3085010000-0009

Assay variable : PTINR_VAL1 PT-INR Value

N	Mean	Standard Deviation	Minimum	Maximum
3	2.1800000	0.4403408	1.7300000	2.6100000

Unique Subject Identifier=3085010000-0016

Assay variable : PTINR_VAL1 PT-INR Value

N	Mean	Standard Deviation	Minimum	Maximum
2	1.7850000	0.1626346	1.6700000	1.9000000

Laboratory tests at the time of obtaining Table 2-6 consent, according to the presence or absence of proton-pump inhibitors

Unique Subject Identifier=3085010000-0018

MEANS Procedure

Assay variable : PTINR_VAL1 PT-INR Value

N	Mean	Standard Deviation	Minimum	Maximum
5	1.8100000	0.2298913	1.5600000	2.0500000

Unique Subject Identifier=3085010000-0019

Assay variable : PTINR_VAL1 PT-INR Value

N	Mean	Standard Deviation	Minimum	Maximum
3	1.8066667	0.1266228	1.6700000	1.9200000

Unique Subject Identifier=3085010000-0028

Assay variable : PTINR_VAL1 PT-INR Value

N	Mean	Standard Deviation	Minimum	Maximum
3	1.7700000	0.0556776	1.7200000	1.8300000

Unique Subject Identifier=3092000000-0003

Assay variable : PTINR_VAL1 PT-INR Value

N	Mean	Standard Deviation	Minimum	Maximum
5	2.0920000	0.1235718	1.9200000	2.2600000

Laboratory tests at the time of obtaining Table 2-6 consent, according to the presence or absence of proton-pump inhibitors

Unique Subject Identifier=3092000000-0008

MEANS Procedure

Assay variable : PTINR_VAL1 PT-INR Value

N	Mean	Standard Deviation	Minimum	Maximum
1	1.6400000		1.6400000	1.6400000

Unique Subject Identifier=3096000000-0004

Assay variable : PTINR_VAL1 PT-INR Value

N	Mean	Standard Deviation	Minimum	Maximum
4	2.5750000	0.5678908	2.0000000	3.2000000

Unique Subject Identifier=3096000000-0007

Assay variable : PTINR_VAL1 PT-INR Value

N	Mean	Standard Deviation	Minimum	Maximum
4	2.2750000	0.5377422	1.6000000	2.8000000

Unique Subject Identifier=3096000000-0010

Assay variable : PTINR_VAL1 PT-INR Value

N	Mean	Standard Deviation	Minimum	Maximum
4	2.6250000	0.7365460	1.8000000	3.5000000

Laboratory tests at the time of obtaining Table 2-6 consent, according to the presence or absence of proton-pump inhibitors

Unique Subject Identifier=3096000000-0012

MEANS Procedure

Assay variable : PTINR_VAL1 PT-INR Value

N	Mean	Standard Deviation	Minimum	Maximum
4	1.9500000	0.1914854	1.7000000	2.1000000

Unique Subject Identifier=3098011000-0002

Assay variable : PTINR_VAL1 PT-INR Value

N	Mean	Standard Deviation	Minimum	Maximum
3	2.2566667	0.1429452	2.1000000	2.3800000

Unique Subject Identifier=3098011000-0004

Assay variable : PTINR_VAL1 PT-INR Value

N	Mean	Standard Deviation	Minimum	Maximum
3	1.4033333	0.0723418	1.3200000	1.4500000

Unique Subject Identifier=3098011000-0005

Assay variable : PTINR_VAL1 PT-INR Value

N	Mean	Standard Deviation	Minimum	Maximum
4	1.9300000	0.2954093	1.5100000	2.1600000

Laboratory tests at the time of obtaining Table 2-6 consent, according to the presence or absence of proton-pump inhibitors

Unique Subject Identifier=3098011000-0006

MEANS Procedure

Assay variable : PTINR_VAL1 PT-INR Value

N	Mean	Standard Deviation	Minimum	Maximum
4	2.3950000	0.7583535	1.3000000	2.9200000

Unique Subject Identifier=3102111011-0003

Assay variable : PTINR_VAL1 PT-INR Value

N	Mean	Standard Deviation	Minimum	Maximum
2	2.3450000	0.2474874	2.1700000	2.5200000

Unique Subject Identifier=3102111011-0009

Assay variable : PTINR_VAL1 PT-INR Value

N	Mean	Standard Deviation	Minimum	Maximum
2	1.0550000	0.0494975	1.0200000	1.0900000

Unique Subject Identifier=3102111011-0032

Assay variable : PTINR_VAL1 PT-INR Value

N	Mean	Standard Deviation	Minimum	Maximum
4	1.9475000	0.5564396	1.5400000	2.7700000

Laboratory tests at the time of obtaining Table 2-6 consent, according to the presence or absence of proton-pump inhibitors

Unique Subject Identifier=3102111011-0066

MEANS Procedure

Assay variable : PTINR_VAL1 PT-INR Value

N	Mean	Standard Deviation	Minimum	Maximum
1	1.9600000		1.9600000	1.9600000

Unique Subject Identifier=3102111011-0114

Assay variable : PTINR_VAL1 PT-INR Value

N	Mean	Standard Deviation	Minimum	Maximum
2	1.8450000	0.2192031	1.6900000	2.0000000

Unique Subject Identifier=3102111011-0138

Assay variable : PTINR_VAL1 PT-INR Value

N	Mean	Standard Deviation	Minimum	Maximum
4	2.2225000	0.1062623	2.0900000	2.3500000

Unique Subject Identifier=3105011000-0001

Assay variable : PTINR_VAL1 PT-INR Value

N	Mean	Standard Deviation	Minimum	Maximum
3	1.6533333	0.1361372	1.5000000	1.7600000

Laboratory tests at the time of obtaining Table 2-6 consent, according to the presence or absence of proton-pump inhibitors

Unique Subject Identifier=3105011000-0002

MEANS Procedure

Assay variable : PTINR_VAL1 PT-INR Value

N	Mean	Standard Deviation	Minimum	Maximum
3	1.9033333	0.0493288	1.8700000	1.9600000

Unique Subject Identifier=3105011000-0003

Assay variable : PTINR_VAL1 PT-INR Value

N	Mean	Standard Deviation	Minimum	Maximum
5	1.9400000	0.6307139	1.3600000	3.0100000

Unique Subject Identifier=3114011111-0027

Assay variable : PTINR_VAL1 PT-INR Value

N	Mean	Standard Deviation	Minimum	Maximum
5	2.6620000	0.5276078	1.7900000	3.1900000

Unique Subject Identifier=3117010110-0003

Assay variable : PTINR_VAL1 PT-INR Value

N	Mean	Standard Deviation	Minimum	Maximum
1	2.5000000		2.5000000	2.5000000

Laboratory tests at the time of obtaining Table 2-6 consent, according to the presence or absence of proton-pump inhibitors

Unique Subject Identifier=3117010110-0007

MEANS Procedure

Assay variable : PTINR_VAL1 PT-INR Value

N	Mean	Standard Deviation	Minimum	Maximum
2	1.9500000	0.0707107	1.9000000	2.0000000

Unique Subject Identifier=3121011111-0001

Assay variable : PTINR_VAL1 PT-INR Value

N	Mean	Standard Deviation	Minimum	Maximum
3	1.9733333	0.6285168	1.2700000	2.4800000

Unique Subject Identifier=3122010111-0002

Assay variable : PTINR_VAL1 PT-INR Value

N	Mean	Standard Deviation	Minimum	Maximum
2	2.3600000	0.8485281	1.7600000	2.9600000

Unique Subject Identifier=3122010111-0003

Assay variable : PTINR_VAL1 PT-INR Value

N	Mean	Standard Deviation	Minimum	Maximum
3	1.9433333	0.2663331	1.6500000	2.1700000

Laboratory tests at the time of obtaining Table 2-6 consent, according to the presence or absence of proton-pump inhibitors

Unique Subject Identifier=3122010111-0005

MEANS Procedure

Assay variable : PTINR_VAL1 PT-INR Value

N	Mean	Standard Deviation	Minimum	Maximum
1	2.8000000		2.8000000	2.8000000

Unique Subject Identifier=3122010111-0006

Assay variable : PTINR_VAL1 PT-INR Value

N	Mean	Standard Deviation	Minimum	Maximum
4	1.4600000	0.2253886	1.2500000	1.7100000

Unique Subject Identifier=3122010111-0007

Assay variable : PTINR_VAL1 PT-INR Value

N	Mean	Standard Deviation	Minimum	Maximum
1	2.5300000		2.5300000	2.5300000

Unique Subject Identifier=3122010111-0008

Assay variable : PTINR_VAL1 PT-INR Value

N	Mean	Standard Deviation	Minimum	Maximum
1	2.1300000		2.1300000	2.1300000

Laboratory tests at the time of obtaining Table 2-6 consent, according to the presence or absence of proton-pump inhibitors

Unique Subject Identifier=3122010111-0015

MEANS Procedure

Assay variable : PTINR_VAL1 PT-INR Value

N	Mean	Standard Deviation	Minimum	Maximum
1	2.0000000		2.0000000	2.0000000

Unique Subject Identifier=3122010111-0018

Assay variable : PTINR_VAL1 PT-INR Value

N	Mean	Standard Deviation	Minimum	Maximum
1	2.3600000		2.3600000	2.3600000

Unique Subject Identifier=3122010111-0019

Assay variable : PTINR_VAL1 PT-INR Value

N	Mean	Standard Deviation	Minimum	Maximum
3	2.0466667	0.6585843	1.5800000	2.8000000

Unique Subject Identifier=3122010111-0023

Assay variable : PTINR_VAL1 PT-INR Value

N	Mean	Standard Deviation	Minimum	Maximum
1	1.6500000		1.6500000	1.6500000

Laboratory tests at the time of obtaining Table 2-6 consent, according to the presence or absence of proton-pump inhibitors

Unique Subject Identifier=3122010111-0025

MEANS Procedure

Assay variable : PTINR_VAL1 PT-INR Value

N	Mean	Standard Deviation	Minimum	Maximum
1	2.2800000		2.2800000	2.2800000

Unique Subject Identifier=3122010111-0026

Assay variable : PTINR_VAL1 PT-INR Value

N	Mean	Standard Deviation	Minimum	Maximum
1	1.5000000		1.5000000	1.5000000

Unique Subject Identifier=3122010111-0028

Assay variable : PTINR_VAL1 PT-INR Value

N	Mean	Standard Deviation	Minimum	Maximum
2	2.2950000	0.1626346	2.1800000	2.4100000

Unique Subject Identifier=3122010111-0031

Assay variable : PTINR_VAL1 PT-INR Value

N	Mean	Standard Deviation	Minimum	Maximum
4	2.0875000	0.1719254	1.8800000	2.2800000

Laboratory tests at the time of obtaining Table 2-6 consent, according to the presence or absence of proton-pump inhibitors

Unique Subject Identifier=3122010111-0032

MEANS Procedure

Assay variable : PTINR_VAL1 PT-INR Value

N	Mean	Standard Deviation	Minimum	Maximum
5	1.9520000	0.2588822	1.6300000	2.2700000

Unique Subject Identifier=3122010111-0033

Assay variable : PTINR_VAL1 PT-INR Value

N	Mean	Standard Deviation	Minimum	Maximum
3	2.5533333	0.0493288	2.5200000	2.6100000

Unique Subject Identifier=3122010111-0034

Assay variable : PTINR_VAL1 PT-INR Value

N	Mean	Standard Deviation	Minimum	Maximum
3	2.4933333	0.0493288	2.4600000	2.5500000

Unique Subject Identifier=3122010111-0041

Assay variable : PTINR_VAL1 PT-INR Value

N	Mean	Standard Deviation	Minimum	Maximum
4	2.4675000	0.2072639	2.2100000	2.6500000

Laboratory tests at the time of obtaining Table 2-6 consent, according to the presence or absence of proton-pump inhibitors

Unique Subject Identifier=3122010111-0043

MEANS Procedure

Assay variable : PTINR_VAL1 PT-INR Value

N	Mean	Standard Deviation	Minimum	Maximum
1	1.8200000		1.8200000	1.8200000

Unique Subject Identifier=3122010111-0044

Assay variable : PTINR_VAL1 PT-INR Value

N	Mean	Standard Deviation	Minimum	Maximum
1	1.4900000		1.4900000	1.4900000

Unique Subject Identifier=3122010111-0047

Assay variable : PTINR_VAL1 PT-INR Value

N	Mean	Standard Deviation	Minimum	Maximum
1	2.0800000		2.0800000	2.0800000

Unique Subject Identifier=3122010111-0051

Assay variable : PTINR_VAL1 PT-INR Value

N	Mean	Standard Deviation	Minimum	Maximum
4	2.0825000	0.1195478	1.9400000	2.2300000

Laboratory tests at the time of obtaining Table 2-6 consent, according to the presence or absence of proton-pump inhibitors

Unique Subject Identifier=3122010111-0054

MEANS Procedure

Assay variable : PTINR_VAL1 PT-INR Value

N	Mean	Standard Deviation	Minimum	Maximum
5	2.0900000	0.4991493	1.2300000	2.4800000

Unique Subject Identifier=3122010111-0056

Assay variable : PTINR_VAL1 PT-INR Value

N	Mean	Standard Deviation	Minimum	Maximum
4	2.2175000	0.2944345	1.9200000	2.6200000

Unique Subject Identifier=3122010111-0059

Assay variable : PTINR_VAL1 PT-INR Value

N	Mean	Standard Deviation	Minimum	Maximum
1	1.8600000		1.8600000	1.8600000

Unique Subject Identifier=3122010111-0060

Assay variable : PTINR_VAL1 PT-INR Value

N	Mean	Standard Deviation	Minimum	Maximum
4	1.5825000	0.1676057	1.3900000	1.7900000

Laboratory tests at the time of obtaining Table 2-6 consent, according to the presence or absence of proton-pump inhibitors

Unique Subject Identifier=3122010111-0062

MEANS Procedure

Assay variable : PTINR_VAL1 PT-INR Value

N	Mean	Standard Deviation	Minimum	Maximum
1	2.4200000		2.4200000	2.4200000

Unique Subject Identifier=3122010111-0068

Assay variable : PTINR_VAL1 PT-INR Value

N	Mean	Standard Deviation	Minimum	Maximum
3	1.8633333	0.0907377	1.7600000	1.9300000

Unique Subject Identifier=3122010111-0069

Assay variable : PTINR_VAL1 PT-INR Value

N	Mean	Standard Deviation	Minimum	Maximum
1	2.0900000		2.0900000	2.0900000

Unique Subject Identifier=3122010111-0075

Assay variable : PTINR_VAL1 PT-INR Value

N	Mean	Standard Deviation	Minimum	Maximum
1	2.4000000		2.4000000	2.4000000

Laboratory tests at the time of obtaining Table 2-6 consent, according to the presence or absence of proton-pump inhibitors

Unique Subject Identifier=3122010111-0076

MEANS Procedure

Assay variable : PTINR_VAL1 PT-INR Value

N	Mean	Standard Deviation	Minimum	Maximum
1	1.4300000		1.4300000	1.4300000

Unique Subject Identifier=3122010111-0078

Assay variable : PTINR_VAL1 PT-INR Value

N	Mean	Standard Deviation	Minimum	Maximum
1	1.4700000		1.4700000	1.4700000

Unique Subject Identifier=3122010111-0081

Assay variable : PTINR_VAL1 PT-INR Value

N	Mean	Standard Deviation	Minimum	Maximum
3	2.1600000	0.2364318	1.9900000	2.4300000

Unique Subject Identifier=3122010111-0082

Assay variable : PTINR_VAL1 PT-INR Value

N	Mean	Standard Deviation	Minimum	Maximum
3	1.6000000	0.1228821	1.5100000	1.7400000

Laboratory tests at the time of obtaining Table 2-6 consent, according to the presence or absence of proton-pump inhibitors

Unique Subject Identifier=3122010111-0083

MEANS Procedure

Assay variable : PTINR_VAL1 PT-INR Value

N	Mean	Standard Deviation	Minimum	Maximum
3	1.7466667	0.3552933	1.4000000	2.1100000

Unique Subject Identifier=3122010111-0085

Assay variable : PTINR_VAL1 PT-INR Value

N	Mean	Standard Deviation	Minimum	Maximum
3	2.0600000	0.1513275	1.9400000	2.2300000

Unique Subject Identifier=3122010111-0088

Assay variable : PTINR_VAL1 PT-INR Value

N	Mean	Standard Deviation	Minimum	Maximum
4	2.3375000	1.0215144	1.6400000	3.8500000

Unique Subject Identifier=3122010111-0089

Assay variable : PTINR_VAL1 PT-INR Value

N	Mean	Standard Deviation	Minimum	Maximum
6	2.1166667	0.5361219	1.3000000	2.8700000

Laboratory tests at the time of obtaining Table 2-6 consent, according to the presence or absence of proton-pump inhibitors

Unique Subject Identifier=3122010111-0092

MEANS Procedure

Assay variable : PTINR_VAL1 PT-INR Value

N	Mean	Standard Deviation	Minimum	Maximum
3	2.7300000	0.8671217	2.0300000	3.7000000

Unique Subject Identifier=3122010111-0095

Assay variable : PTINR_VAL1 PT-INR Value

N	Mean	Standard Deviation	Minimum	Maximum
3	2.1266667	0.0808290	2.0400000	2.2000000

Unique Subject Identifier=3122010111-0098

Assay variable : PTINR_VAL1 PT-INR Value

N	Mean	Standard Deviation	Minimum	Maximum
3	1.6366667	0.1644182	1.4500000	1.7600000

Unique Subject Identifier=3122010111-0099

Assay variable : PTINR_VAL1 PT-INR Value

N	Mean	Standard Deviation	Minimum	Maximum
3	1.5500000	0.3764306	1.1400000	1.8800000

Laboratory tests at the time of obtaining Table 2-6 consent, according to the presence or absence of proton-pump inhibitors

Unique Subject Identifier=3122010111-0100

MEANS Procedure

Assay variable : PTINR_VAL1 PT-INR Value

N	Mean	Standard Deviation	Minimum	Maximum
3	1.8333333	0.4007909	1.3900000	2.1700000

Unique Subject Identifier=3122010111-0102

Assay variable : PTINR_VAL1 PT-INR Value

N	Mean	Standard Deviation	Minimum	Maximum
5	2.0740000	0.5116933	1.4500000	2.8000000

Unique Subject Identifier=3122010111-0103

Assay variable : PTINR_VAL1 PT-INR Value

N	Mean	Standard Deviation	Minimum	Maximum
1	4.4300000		4.4300000	4.4300000

Unique Subject Identifier=3122010111-0107

Assay variable : PTINR_VAL1 PT-INR Value

N	Mean	Standard Deviation	Minimum	Maximum
1	2.4400000		2.4400000	2.4400000

Laboratory tests at the time of obtaining Table 2-6 consent, according to the presence or absence of proton-pump inhibitors

Unique Subject Identifier=3122010111-0108

MEANS Procedure

Assay variable : PTINR_VAL1 PT-INR Value

N	Mean	Standard Deviation	Minimum	Maximum
1	2.1100000		2.1100000	2.1100000

Unique Subject Identifier=3122010111-0112

Assay variable : PTINR_VAL1 PT-INR Value

N	Mean	Standard Deviation	Minimum	Maximum
1	2.1400000		2.1400000	2.1400000

Unique Subject Identifier=3122010111-0117

Assay variable : PTINR_VAL1 PT-INR Value

N	Mean	Standard Deviation	Minimum	Maximum
4	1.9475000	0.2845318	1.6500000	2.3200000

Unique Subject Identifier=3122010111-0118

Assay variable : PTINR_VAL1 PT-INR Value

N	Mean	Standard Deviation	Minimum	Maximum
2	1.6900000	0.3111270	1.4700000	1.9100000

Laboratory tests at the time of obtaining Table 2-6 consent, according to the presence or absence of proton-pump inhibitors

Unique Subject Identifier=3122010111-0122

MEANS Procedure

Assay variable : PTINR_VAL1 PT-INR Value

N	Mean	Standard Deviation	Minimum	Maximum
1	2.0600000		2.0600000	2.0600000

Unique Subject Identifier=3122010111-0127

Assay variable : PTINR_VAL1 PT-INR Value

N	Mean	Standard Deviation	Minimum	Maximum
1	2.3200000		2.3200000	2.3200000

Unique Subject Identifier=3122010111-0128

Assay variable : PTINR_VAL1 PT-INR Value

N	Mean	Standard Deviation	Minimum	Maximum
3	1.6966667	0.3257811	1.4700000	2.0700000

Unique Subject Identifier=3122010111-0129

Assay variable : PTINR_VAL1 PT-INR Value

N	Mean	Standard Deviation	Minimum	Maximum
3	2.3266667	0.2685765	2.0200000	2.5200000

Laboratory tests at the time of obtaining Table 2-6 consent, according to the presence or absence of proton-pump inhibitors

Unique Subject Identifier=3122010111-0131

MEANS Procedure

Assay variable : PTINR_VAL1 PT-INR Value

N	Mean	Standard Deviation	Minimum	Maximum
6	3.0350000	2.4783442	1.2900000	7.8800000

Unique Subject Identifier=3122010111-0132

Assay variable : PTINR_VAL1 PT-INR Value

N	Mean	Standard Deviation	Minimum	Maximum
1	1.8000000		1.8000000	1.8000000

Unique Subject Identifier=3122010111-0136

Assay variable : PTINR_VAL1 PT-INR Value

N	Mean	Standard Deviation	Minimum	Maximum
1	2.1100000		2.1100000	2.1100000

Unique Subject Identifier=3122010111-0139

Assay variable : PTINR_VAL1 PT-INR Value

N	Mean	Standard Deviation	Minimum	Maximum
1	1.7100000		1.7100000	1.7100000

Laboratory tests at the time of obtaining Table 2-6 consent, according to the presence or absence of proton-pump inhibitors

Unique Subject Identifier=3122010111-0142

MEANS Procedure

Assay variable : PTINR_VAL1 PT-INR Value

N	Mean	Standard Deviation	Minimum	Maximum
6	2.2100000	0.7181643	1.3800000	3.3900000

Unique Subject Identifier=3122010111-0144

Assay variable : PTINR_VAL1 PT-INR Value

N	Mean	Standard Deviation	Minimum	Maximum
3	1.8666667	0.3074627	1.6600000	2.2200000

Unique Subject Identifier=3122010111-0149

Assay variable : PTINR_VAL1 PT-INR Value

N	Mean	Standard Deviation	Minimum	Maximum
1	3.2000000		3.2000000	3.2000000

Unique Subject Identifier=3122010111-0155

Assay variable : PTINR_VAL1 PT-INR Value

N	Mean	Standard Deviation	Minimum	Maximum
1	1.2900000		1.2900000	1.2900000

Laboratory tests at the time of obtaining Table 2-6 consent, according to the presence or absence of proton-pump inhibitors

Unique Subject Identifier=3122010111-0165

MEANS Procedure

Assay variable : PTINR_VAL1 PT-INR Value

N	Mean	Standard Deviation	Minimum	Maximum
1	1.6300000		1.6300000	1.6300000

Unique Subject Identifier=3122010111-0167

Assay variable : PTINR_VAL1 PT-INR Value

N	Mean	Standard Deviation	Minimum	Maximum
1	2.1000000		2.1000000	2.1000000

Unique Subject Identifier=3122010111-0174

Assay variable : PTINR_VAL1 PT-INR Value

N	Mean	Standard Deviation	Minimum	Maximum
1	2.3900000		2.3900000	2.3900000

Unique Subject Identifier=3122010111-0186

Assay variable : PTINR_VAL1 PT-INR Value

N	Mean	Standard Deviation	Minimum	Maximum
1	2.1300000		2.1300000	2.1300000

Laboratory tests at the time of obtaining Table 2-6 consent, according to the presence or absence of proton-pump inhibitors

Unique Subject Identifier=3122010111-0189

MEANS Procedure

Assay variable : PTINR_VAL1 PT-INR Value

N	Mean	Standard Deviation	Minimum	Maximum
1	2.0000000		2.0000000	2.0000000

Unique Subject Identifier=3122010111-0192

Assay variable : PTINR_VAL1 PT-INR Value

N	Mean	Standard Deviation	Minimum	Maximum
1	1.5200000		1.5200000	1.5200000

Unique Subject Identifier=3122010111-0194

Assay variable : PTINR_VAL1 PT-INR Value

N	Mean	Standard Deviation	Minimum	Maximum
5	1.8440000	0.0918150	1.7100000	1.9400000

Unique Subject Identifier=3122010111-0195

Assay variable : PTINR_VAL1 PT-INR Value

N	Mean	Standard Deviation	Minimum	Maximum
1	2.5200000		2.5200000	2.5200000

Laboratory tests at the time of obtaining Table 2-6 consent, according to the presence or absence of proton-pump inhibitors

Unique Subject Identifier=3122010111-0198

MEANS Procedure

Assay variable : PTINR_VAL1 PT-INR Value

N	Mean	Standard Deviation	Minimum	Maximum
1	1.7800000		1.7800000	1.7800000

Unique Subject Identifier=3122010111-0202

Assay variable : PTINR_VAL1 PT-INR Value

N	Mean	Standard Deviation	Minimum	Maximum
1	3.0900000		3.0900000	3.0900000

Unique Subject Identifier=3122010111-0206

Assay variable : PTINR_VAL1 PT-INR Value

N	Mean	Standard Deviation	Minimum	Maximum
1	1.8500000		1.8500000	1.8500000

Unique Subject Identifier=3122010111-0208

Assay variable : PTINR_VAL1 PT-INR Value

N	Mean	Standard Deviation	Minimum	Maximum
1	2.5500000		2.5500000	2.5500000

Laboratory tests at the time of obtaining Table 2-6 consent, according to the presence or absence of proton-pump inhibitors

Unique Subject Identifier=3122010111-0211

MEANS Procedure

Assay variable : PTINR_VAL1 PT-INR Value

N	Mean	Standard Deviation	Minimum	Maximum
1	1.8100000		1.8100000	1.8100000

Unique Subject Identifier=3122010111-0214

Assay variable : PTINR_VAL1 PT-INR Value

N	Mean	Standard Deviation	Minimum	Maximum
1	1.4900000		1.4900000	1.4900000

Unique Subject Identifier=3122010111-0217

Assay variable : PTINR_VAL1 PT-INR Value

N	Mean	Standard Deviation	Minimum	Maximum
1	2.5000000		2.5000000	2.5000000

Unique Subject Identifier=3122010111-0218

Assay variable : PTINR_VAL1 PT-INR Value

N	Mean	Standard Deviation	Minimum	Maximum
1	2.2200000		2.2200000	2.2200000

Laboratory tests at the time of obtaining Table 2-6 consent, according to the presence or absence of proton-pump inhibitors

Unique Subject Identifier=3122010111-0222

MEANS Procedure

Assay variable : PTINR_VAL1 PT-INR Value

N	Mean	Standard Deviation	Minimum	Maximum
1	1.8500000		1.8500000	1.8500000

Unique Subject Identifier=3122010111-0226

Assay variable : PTINR_VAL1 PT-INR Value

N	Mean	Standard Deviation	Minimum	Maximum
1	2.0700000		2.0700000	2.0700000

Unique Subject Identifier=3122010111-0230

Assay variable : PTINR_VAL1 PT-INR Value

N	Mean	Standard Deviation	Minimum	Maximum
1	1.6500000		1.6500000	1.6500000

Unique Subject Identifier=3122010111-0231

Assay variable : PTINR_VAL1 PT-INR Value

N	Mean	Standard Deviation	Minimum	Maximum
6	1.7316667	0.2162791	1.4500000	1.9400000

Laboratory tests at the time of obtaining Table 2-6 consent, according to the presence or absence of proton-pump inhibitors

Unique Subject Identifier=3122010111-0232

MEANS Procedure

Assay variable : PTINR_VAL1 PT-INR Value

N	Mean	Standard Deviation	Minimum	Maximum
1	1.4800000		1.4800000	1.4800000

Unique Subject Identifier=3122010111-0234

Assay variable : PTINR_VAL1 PT-INR Value

N	Mean	Standard Deviation	Minimum	Maximum
1	1.2000000		1.2000000	1.2000000

Unique Subject Identifier=3122010111-0237

Assay variable : PTINR_VAL1 PT-INR Value

N	Mean	Standard Deviation	Minimum	Maximum
1	2.2100000		2.2100000	2.2100000

Unique Subject Identifier=3122010111-0239

Assay variable : PTINR_VAL1 PT-INR Value

N	Mean	Standard Deviation	Minimum	Maximum
1	1.7800000		1.7800000	1.7800000

Laboratory tests at the time of obtaining Table 2-6 consent, according to the presence or absence of proton-pump inhibitors

Unique Subject Identifier=3122010111-0240

MEANS Procedure

Assay variable : PTINR_VAL1 PT-INR Value

N	Mean	Standard Deviation	Minimum	Maximum
1	2.0000000		2.0000000	2.0000000

Unique Subject Identifier=3122010111-0241

Assay variable : PTINR_VAL1 PT-INR Value

N	Mean	Standard Deviation	Minimum	Maximum
1	2.4100000		2.4100000	2.4100000

Unique Subject Identifier=3122010111-0242

Assay variable : PTINR_VAL1 PT-INR Value

N	Mean	Standard Deviation	Minimum	Maximum
1	1.7700000		1.7700000	1.7700000

Unique Subject Identifier=3122010111-0243

Assay variable : PTINR_VAL1 PT-INR Value

N	Mean	Standard Deviation	Minimum	Maximum
1	2.0100000		2.0100000	2.0100000

Laboratory tests at the time of obtaining Table 2-6 consent, according to the presence or absence of proton-pump inhibitors

Unique Subject Identifier=3122010111-0250

MEANS Procedure

Assay variable : PTINR_VAL1 PT-INR Value

N	Mean	Standard Deviation	Minimum	Maximum
1	1.8800000		1.8800000	1.8800000

Unique Subject Identifier=3122010111-0259

Assay variable : PTINR_VAL1 PT-INR Value

N	Mean	Standard Deviation	Minimum	Maximum
3	1.8866667	0.0321455	1.8500000	1.9100000

Unique Subject Identifier=3122010111-0260

Assay variable : PTINR_VAL1 PT-INR Value

N	Mean	Standard Deviation	Minimum	Maximum
2	1.9550000	0.1484924	1.8500000	2.0600000

Unique Subject Identifier=3122010111-0262

Assay variable : PTINR_VAL1 PT-INR Value

N	Mean	Standard Deviation	Minimum	Maximum
1	2.9600000		2.9600000	2.9600000

Laboratory tests at the time of obtaining Table 2-6 consent, according to the presence or absence of proton-pump inhibitors

Unique Subject Identifier=3126000000-0002

MEANS Procedure

Assay variable : PTINR_VAL1 PT-INR Value

N	Mean	Standard Deviation	Minimum	Maximum
1	2.1000000		2.1000000	2.1000000

Unique Subject Identifier=3129000000-0001

Assay variable : PTINR_VAL1 PT-INR Value

N	Mean	Standard Deviation	Minimum	Maximum
1	1.9000000		1.9000000	1.9000000

Unique Subject Identifier=3129000000-0002

Assay variable : PTINR_VAL1 PT-INR Value

N	Mean	Standard Deviation	Minimum	Maximum
6	2.4233333	0.2997777	1.8800000	2.7900000

Unique Subject Identifier=3129000000-0005

Assay variable : PTINR_VAL1 PT-INR Value

N	Mean	Standard Deviation	Minimum	Maximum
6	1.9966667	0.3787171	1.4700000	2.6300000

Laboratory tests at the time of obtaining Table 2-6 consent, according to the presence or absence of proton-pump inhibitors

Unique Subject Identifier=3129000000-0006

MEANS Procedure

Assay variable : PTINR_VAL1 PT-INR Value

N	Mean	Standard Deviation	Minimum	Maximum
6	2.3183333	0.6036031	1.6000000	3.3200000

Unique Subject Identifier=3129000000-0009

Assay variable : PTINR_VAL1 PT-INR Value

N	Mean	Standard Deviation	Minimum	Maximum
4	1.8650000	0.4133199	1.2700000	2.1600000

Unique Subject Identifier=3131010000-0017

Assay variable : PTINR_VAL1 PT-INR Value

N	Mean	Standard Deviation	Minimum	Maximum
2	2.7300000	0.3252691	2.5000000	2.9600000

Unique Subject Identifier=3131010000-0026

Assay variable : PTINR_VAL1 PT-INR Value

N	Mean	Standard Deviation	Minimum	Maximum
3	2.1433333	0.4606879	1.7300000	2.6400000

Laboratory tests at the time of obtaining Table 2-6 consent, according to the presence or absence of proton-pump inhibitors

Unique Subject Identifier=3131010000-0034

MEANS Procedure

Assay variable : PTINR_VAL1 PT-INR Value

N	Mean	Standard Deviation	Minimum	Maximum
2	1.6150000	0.1060660	1.5400000	1.6900000

Unique Subject Identifier=3131010000-0042

Assay variable : PTINR_VAL1 PT-INR Value

N	Mean	Standard Deviation	Minimum	Maximum
4	1.4400000	0.1439907	1.3000000	1.6400000

Unique Subject Identifier=3133000000-0005

Assay variable : PTINR_VAL1 PT-INR Value

N	Mean	Standard Deviation	Minimum	Maximum
3	2.3200000	0.2783882	2.0700000	2.6200000

Unique Subject Identifier=3133000000-0007

Assay variable : PTINR_VAL1 PT-INR Value

N	Mean	Standard Deviation	Minimum	Maximum
4	1.6875000	0.1793274	1.5100000	1.9300000

Laboratory tests at the time of obtaining Table 2-6 consent, according to the presence or absence of proton-pump inhibitors

Unique Subject Identifier=3133000000-0008

MEANS Procedure

Assay variable : PTINR_VAL1 PT-INR Value

N	Mean	Standard Deviation	Minimum	Maximum
4	1.2450000	0.1869938	1.0800000	1.4400000

Unique Subject Identifier=3133000000-0011

Assay variable : PTINR_VAL1 PT-INR Value

N	Mean	Standard Deviation	Minimum	Maximum
3	1.9033333	0.3464583	1.6600000	2.3000000

Unique Subject Identifier=3133000000-0014

Assay variable : PTINR_VAL1 PT-INR Value

N	Mean	Standard Deviation	Minimum	Maximum
4	1.7950000	0.1317826	1.7000000	1.9900000

Unique Subject Identifier=3133000000-0020

Assay variable : PTINR_VAL1 PT-INR Value

N	Mean	Standard Deviation	Minimum	Maximum
3	2.6300000	0.6584831	2.2300000	3.3900000

Laboratory tests at the time of obtaining Table 2-6 consent, according to the presence or absence of proton-pump inhibitors

Unique Subject Identifier=3133000000-0021

MEANS Procedure

Assay variable : PTINR_VAL1 PT-INR Value

N	Mean	Standard Deviation	Minimum	Maximum
4	1.8425000	0.1828251	1.6400000	2.0500000

Unique Subject Identifier=3133000000-0034

Assay variable : PTINR_VAL1 PT-INR Value

N	Mean	Standard Deviation	Minimum	Maximum
3	2.3266667	0.1814754	2.1600000	2.5200000

Unique Subject Identifier=3133000000-0040

Assay variable : PTINR_VAL1 PT-INR Value

N	Mean	Standard Deviation	Minimum	Maximum
5	2.0400000	0.2650472	1.7200000	2.3600000

Unique Subject Identifier=3133000000-0041

Assay variable : PTINR_VAL1 PT-INR Value

N	Mean	Standard Deviation	Minimum	Maximum
3	1.6133333	0.2804164	1.3700000	1.9200000

Laboratory tests at the time of obtaining Table 2-6 consent, according to the presence or absence of proton-pump inhibitors

Unique Subject Identifier=3133000000-0050

MEANS Procedure

Assay variable : PTINR_VAL1 PT-INR Value

N	Mean	Standard Deviation	Minimum	Maximum
3	2.0766667	0.2274496	1.8900000	2.3300000

Unique Subject Identifier=3133000000-0055

Assay variable : PTINR_VAL1 PT-INR Value

N	Mean	Standard Deviation	Minimum	Maximum
2	2.0550000	0.2333452	1.8900000	2.2200000

Unique Subject Identifier=3133000000-0056

Assay variable : PTINR_VAL1 PT-INR Value

N	Mean	Standard Deviation	Minimum	Maximum
3	1.4766667	0.3066486	1.2300000	1.8200000

Unique Subject Identifier=3133000000-0057

Assay variable : PTINR_VAL1 PT-INR Value

N	Mean	Standard Deviation	Minimum	Maximum
2	3.5100000	0.1414214	3.4100000	3.6100000

Laboratory tests at the time of obtaining Table 2-6 consent, according to the presence or absence of proton-pump inhibitors

Unique Subject Identifier=3133000000-0073

MEANS Procedure

Assay variable : PTINR_VAL1 PT-INR Value

N	Mean	Standard Deviation	Minimum	Maximum
4	2.2075000	0.2482438	2.0100000	2.5500000

Unique Subject Identifier=3133000000-0076

Assay variable : PTINR_VAL1 PT-INR Value

N	Mean	Standard Deviation	Minimum	Maximum
5	1.2160000	0.3338862	1.0100000	1.8100000

Unique Subject Identifier=3133000000-0081

Assay variable : PTINR_VAL1 PT-INR Value

N	Mean	Standard Deviation	Minimum	Maximum
3	1.4666667	0.1800926	1.2600000	1.5900000

Unique Subject Identifier=3133000000-0084

Assay variable : PTINR_VAL1 PT-INR Value

N	Mean	Standard Deviation	Minimum	Maximum
2	1.6200000	0.4949747	1.2700000	1.9700000

Laboratory tests at the time of obtaining Table 2-6 consent, according to the presence or absence of proton-pump inhibitors

Unique Subject Identifier=3134000000-0004

MEANS Procedure

Assay variable : PTINR_VAL1 PT-INR Value

N	Mean	Standard Deviation	Minimum	Maximum
1	1.9000000		1.9000000	1.9000000

Unique Subject Identifier=3135000000-0004

Assay variable : PTINR_VAL1 PT-INR Value

N	Mean	Standard Deviation	Minimum	Maximum
1	1.6400000		1.6400000	1.6400000

Unique Subject Identifier=3135000000-0005

Assay variable : PTINR_VAL1 PT-INR Value

N	Mean	Standard Deviation	Minimum	Maximum
2	1.5800000	0.4808326	1.2400000	1.9200000

Unique Subject Identifier=3139000000-0004

Assay variable : PTINR_VAL1 PT-INR Value

N	Mean	Standard Deviation	Minimum	Maximum
3	1.5166667	0.2514624	1.3200000	1.8000000

Laboratory tests at the time of obtaining Table 2-6 consent, according to the presence or absence of proton-pump inhibitors

Unique Subject Identifier=3139000000-0012

MEANS Procedure

Assay variable : PTINR_VAL1 PT-INR Value

N	Mean	Standard Deviation	Minimum	Maximum
1	2.1600000		2.1600000	2.1600000

Unique Subject Identifier=3143000000-0012

Assay variable : PTINR_VAL1 PT-INR Value

N	Mean	Standard Deviation	Minimum	Maximum
5	1.9100000	0.7227379	1.2800000	3.1100000

Unique Subject Identifier=3147011110-0004

Assay variable : PTINR_VAL1 PT-INR Value

N	Mean	Standard Deviation	Minimum	Maximum
3	2.2933333	0.1357694	2.1500000	2.4200000

Unique Subject Identifier=3147011110-0005

Assay variable : PTINR_VAL1 PT-INR Value

N	Mean	Standard Deviation	Minimum	Maximum
4	2.0050000	0.6614378	1.5300000	2.9800000

Laboratory tests at the time of obtaining Table 2-6 consent, according to the presence or absence of proton-pump inhibitors

Unique Subject Identifier=3147011110-0006

MEANS Procedure

Assay variable : PTINR_VAL1 PT-INR Value

N	Mean	Standard Deviation	Minimum	Maximum
2	1.8150000	0.2333452	1.6500000	1.9800000

Unique Subject Identifier=3147011110-0009

Assay variable : PTINR_VAL1 PT-INR Value

N	Mean	Standard Deviation	Minimum	Maximum
1	1.6800000		1.6800000	1.6800000

Unique Subject Identifier=3150000000-0001

Assay variable : PTINR_VAL1 PT-INR Value

N	Mean	Standard Deviation	Minimum	Maximum
6	2.2833333	0.5845226	1.8000000	3.4000000

Unique Subject Identifier=3150000000-0004

Assay variable : PTINR_VAL1 PT-INR Value

N	Mean	Standard Deviation	Minimum	Maximum
6	1.8666667	0.3204164	1.4000000	2.2000000

Laboratory tests at the time of obtaining Table 2-6 consent, according to the presence or absence of proton-pump inhibitors

Unique Subject Identifier=3150000000-0005

MEANS Procedure

Assay variable : PTINR_VAL1 PT-INR Value

N	Mean	Standard Deviation	Minimum	Maximum
6	2.1166667	0.5154286	1.3000000	2.7000000

Unique Subject Identifier=3150000000-0009

Assay variable : PTINR_VAL1 PT-INR Value

N	Mean	Standard Deviation	Minimum	Maximum
6	1.6833333	0.3250641	1.2000000	2.2000000

Unique Subject Identifier=3150000000-0010

Assay variable : PTINR_VAL1 PT-INR Value

N	Mean	Standard Deviation	Minimum	Maximum
6	1.6166667	0.2401388	1.5000000	2.1000000

Unique Subject Identifier=3151000000-0002

Assay variable : PTINR_VAL1 PT-INR Value

N	Mean	Standard Deviation	Minimum	Maximum
5	1.2800000	0.0845577	1.2200000	1.4200000

Laboratory tests at the time of obtaining Table 2-6 consent, according to the presence or absence of proton-pump inhibitors

Unique Subject Identifier=3151000000-0007

MEANS Procedure

Assay variable : PTINR_VAL1 PT-INR Value

N	Mean	Standard Deviation	Minimum	Maximum
6	1.5983333	0.1495883	1.3900000	1.7600000

Unique Subject Identifier=3151000000-0008

Assay variable : PTINR_VAL1 PT-INR Value

N	Mean	Standard Deviation	Minimum	Maximum
6	1.8533333	0.1834848	1.6000000	2.0800000

Unique Subject Identifier=3151000000-0010

Assay variable : PTINR_VAL1 PT-INR Value

N	Mean	Standard Deviation	Minimum	Maximum
1	1.4200000		1.4200000	1.4200000

Unique Subject Identifier=3152011000-0007

Assay variable : PTINR_VAL1 PT-INR Value

N	Mean	Standard Deviation	Minimum	Maximum
5	1.5000000	0.1224745	1.4000000	1.7000000

Laboratory tests at the time of obtaining Table 2-6 consent, according to the presence or absence of proton-pump inhibitors

Unique Subject Identifier=3152011000-0020

MEANS Procedure

Assay variable : PTINR_VAL1 PT-INR Value

N	Mean	Standard Deviation	Minimum	Maximum
4	1.9500000	0.2380476	1.8000000	2.3000000

Unique Subject Identifier=3158111111-0002

Assay variable : PTINR_VAL1 PT-INR Value

N	Mean	Standard Deviation	Minimum	Maximum
4	2.8050000	0.6251666	2.0900000	3.5500000

Unique Subject Identifier=3158111111-0005

Assay variable : PTINR_VAL1 PT-INR Value

N	Mean	Standard Deviation	Minimum	Maximum
5	2.1540000	0.5195479	1.7300000	3.0500000

Unique Subject Identifier=3158111111-0009

Assay variable : PTINR_VAL1 PT-INR Value

N	Mean	Standard Deviation	Minimum	Maximum
3	2.4000000	0.1473092	2.2300000	2.4900000

Laboratory tests at the time of obtaining Table 2-6 consent, according to the presence or absence of proton-pump inhibitors

Unique Subject Identifier=3158111111-0010

MEANS Procedure

Assay variable : PTINR_VAL1 PT-INR Value

N	Mean	Standard Deviation	Minimum	Maximum
5	2.2740000	0.2769115	1.9800000	2.6800000

Unique Subject Identifier=3158111111-0011

Assay variable : PTINR_VAL1 PT-INR Value

N	Mean	Standard Deviation	Minimum	Maximum
4	2.0000000	0.1329160	1.8200000	2.1100000

Unique Subject Identifier=3158111111-0014

Assay variable : PTINR_VAL1 PT-INR Value

N	Mean	Standard Deviation	Minimum	Maximum
5	1.9260000	0.0859069	1.8000000	2.0400000

Unique Subject Identifier=3158111111-0029

Assay variable : PTINR_VAL1 PT-INR Value

N	Mean	Standard Deviation	Minimum	Maximum
4	2.0500000	0.0697615	1.9900000	2.1500000

Laboratory tests at the time of obtaining Table 2-6 consent, according to the presence or absence of proton-pump inhibitors

Unique Subject Identifier=3158111111-0030

MEANS Procedure

Assay variable : PTINR_VAL1 PT-INR Value

N	Mean	Standard Deviation	Minimum	Maximum
5	2.9220000	0.3417163	2.5500000	3.2800000

Unique Subject Identifier=3158111111-0034

Assay variable : PTINR_VAL1 PT-INR Value

N	Mean	Standard Deviation	Minimum	Maximum
5	1.5860000	0.2169793	1.3200000	1.9200000

Unique Subject Identifier=3158111111-0035

Assay variable : PTINR_VAL1 PT-INR Value

N	Mean	Standard Deviation	Minimum	Maximum
4	2.3925000	0.6396027	1.8000000	3.2500000

Unique Subject Identifier=3158111111-0038

Assay variable : PTINR_VAL1 PT-INR Value

N	Mean	Standard Deviation	Minimum	Maximum
4	2.7600000	0.4667619	2.1000000	3.2000000

Laboratory tests at the time of obtaining Table 2-6 consent, according to the presence or absence of proton-pump inhibitors

Unique Subject Identifier=3158111111-0047

MEANS Procedure

Assay variable : PTINR_VAL1 PT-INR Value

N	Mean	Standard Deviation	Minimum	Maximum
5	2.3060000	0.2736421	1.9600000	2.5900000

Unique Subject Identifier=3158111111-0063

Assay variable : PTINR_VAL1 PT-INR Value

N	Mean	Standard Deviation	Minimum	Maximum
3	1.9700000	0.1322876	1.8200000	2.0700000

Unique Subject Identifier=3158111111-0069

Assay variable : PTINR_VAL1 PT-INR Value

N	Mean	Standard Deviation	Minimum	Maximum
1	1.4700000		1.4700000	1.4700000

Unique Subject Identifier=3158111111-0070

Assay variable : PTINR_VAL1 PT-INR Value

N	Mean	Standard Deviation	Minimum	Maximum
4	1.9250000	0.2880393	1.6500000	2.3300000

Laboratory tests at the time of obtaining Table 2-6 consent, according to the presence or absence of proton-pump inhibitors

Unique Subject Identifier=3161010111-0002

MEANS Procedure

Assay variable : PTINR_VAL1 PT-INR Value

N	Mean	Standard Deviation	Minimum	Maximum
3	2.0333333	0.0907377	1.9300000	2.1000000

Unique Subject Identifier=3161010111-0004

Assay variable : PTINR_VAL1 PT-INR Value

N	Mean	Standard Deviation	Minimum	Maximum
4	2.3975000	0.3927998	2.0200000	2.7800000

Unique Subject Identifier=3162011000-0002

Assay variable : PTINR_VAL1 PT-INR Value

N	Mean	Standard Deviation	Minimum	Maximum
2	2.4700000	0.6363961	2.0200000	2.9200000

Unique Subject Identifier=3162011000-0006

Assay variable : PTINR_VAL1 PT-INR Value

N	Mean	Standard Deviation	Minimum	Maximum
4	2.0025000	0.4342330	1.6400000	2.5400000

Laboratory tests at the time of obtaining Table 2-6 consent, according to the presence or absence of proton-pump inhibitors

Unique Subject Identifier=3162011000-0025

MEANS Procedure

Assay variable : PTINR_VAL1 PT-INR Value

N	Mean	Standard Deviation	Minimum	Maximum
2	1.8800000	0.1838478	1.7500000	2.0100000

Unique Subject Identifier=3163011011-0005

Assay variable : PTINR_VAL1 PT-INR Value

N	Mean	Standard Deviation	Minimum	Maximum
4	2.0500000	0.1732051	1.8000000	2.2000000

Unique Subject Identifier=3163011011-0006

Assay variable : PTINR_VAL1 PT-INR Value

N	Mean	Standard Deviation	Minimum	Maximum
5	1.9400000	0.3714835	1.4000000	2.4000000

Unique Subject Identifier=3163011011-0009

Assay variable : PTINR_VAL1 PT-INR Value

N	Mean	Standard Deviation	Minimum	Maximum
2	1.8500000	0.3535534	1.6000000	2.1000000

Laboratory tests at the time of obtaining Table 2-6 consent, according to the presence or absence of proton-pump inhibitors

Unique Subject Identifier=3166011110-0011

MEANS Procedure

Assay variable : PTINR_VAL1 PT-INR Value

N	Mean	Standard Deviation	Minimum	Maximum
1	1.6100000		1.6100000	1.6100000

Unique Subject Identifier=3166011110-0017

Assay variable : PTINR_VAL1 PT-INR Value

N	Mean	Standard Deviation	Minimum	Maximum
2	2.7150000	0.6151829	2.2800000	3.1500000

Unique Subject Identifier=3171010000-0005

Assay variable : PTINR_VAL1 PT-INR Value

N	Mean	Standard Deviation	Minimum	Maximum
4	1.7250000	0.1892969	1.6000000	2.0000000

Unique Subject Identifier=3175011000-0003

Assay variable : PTINR_VAL1 PT-INR Value

N	Mean	Standard Deviation	Minimum	Maximum
1	2.1300000		2.1300000	2.1300000

Laboratory tests at the time of obtaining Table 2-6 consent, according to the presence or absence of proton-pump inhibitors

Unique Subject Identifier=3180010000-0010

MEANS Procedure

Assay variable : PTINR_VAL1 PT-INR Value

N	Mean	Standard Deviation	Minimum	Maximum
5	2.4440000	1.1439755	1.2100000	4.1600000

Unique Subject Identifier=3185010010-0012

Assay variable : PTINR_VAL1 PT-INR Value

N	Mean	Standard Deviation	Minimum	Maximum
4	1.2725000	0.1826426	1.1100000	1.4500000

Unique Subject Identifier=3185010010-0023

Assay variable : PTINR_VAL1 PT-INR Value

N	Mean	Standard Deviation	Minimum	Maximum
6	1.3466667	0.1136075	1.1900000	1.5100000

Unique Subject Identifier=3185010010-0034

Assay variable : PTINR_VAL1 PT-INR Value

N	Mean	Standard Deviation	Minimum	Maximum
5	1.8280000	0.4853555	1.4000000	2.6100000

Laboratory tests at the time of obtaining Table 2-6 consent, according to the presence or absence of proton-pump inhibitors

Unique Subject Identifier=3185010010-0038

MEANS Procedure

Assay variable : PTINR_VAL1 PT-INR Value

N	Mean	Standard Deviation	Minimum	Maximum
4	1.9350000	0.5476313	1.4600000	2.7200000

Unique Subject Identifier=3187000000-0001

Assay variable : PTINR_VAL1 PT-INR Value

N	Mean	Standard Deviation	Minimum	Maximum
5	2.4800000	0.6445153	1.8100000	3.1800000

Unique Subject Identifier=3187000000-0003

Assay variable : PTINR_VAL1 PT-INR Value

N	Mean	Standard Deviation	Minimum	Maximum
3	2.1000000	0.5469004	1.6100000	2.6900000

Unique Subject Identifier=3187000000-0006

Assay variable : PTINR_VAL1 PT-INR Value

N	Mean	Standard Deviation	Minimum	Maximum
6	2.4600000	1.2437363	1.4400000	4.9300000

Laboratory tests at the time of obtaining Table 2-6 consent, according to the presence or absence of proton-pump inhibitors

Unique Subject Identifier=3187000000-0009

MEANS Procedure

Assay variable : PTINR_VAL1 PT-INR Value

N	Mean	Standard Deviation	Minimum	Maximum
3	2.4366667	0.1205543	2.3100000	2.5500000

Unique Subject Identifier=3190111111-0001

Assay variable : PTINR_VAL1 PT-INR Value

N	Mean	Standard Deviation	Minimum	Maximum
6	2.1833333	0.3253716	1.6600000	2.5800000

Unique Subject Identifier=3190111111-0005

Assay variable : PTINR_VAL1 PT-INR Value

N	Mean	Standard Deviation	Minimum	Maximum
5	1.8540000	0.1837934	1.5600000	2.0300000

Unique Subject Identifier=3190111111-0006

Assay variable : PTINR_VAL1 PT-INR Value

N	Mean	Standard Deviation	Minimum	Maximum
2	2.0050000	0.1060660	1.9300000	2.0800000

Laboratory tests at the time of obtaining Table 2-6 consent, according to the presence or absence of proton-pump inhibitors

Unique Subject Identifier=3197000000-0003

MEANS Procedure

Assay variable : PTINR_VAL1 PT-INR Value

N	Mean	Standard Deviation	Minimum	Maximum
2	1.3100000	0.2404163	1.1400000	1.4800000

Unique Subject Identifier=3197000000-0048

Assay variable : PTINR_VAL1 PT-INR Value

N	Mean	Standard Deviation	Minimum	Maximum
6	1.7950000	0.4539053	1.2700000	2.3300000

Unique Subject Identifier=3204010110-0002

Assay variable : PTINR_VAL1 PT-INR Value

N	Mean	Standard Deviation	Minimum	Maximum
3	2.1633333	0.0550757	2.1100000	2.2200000

Unique Subject Identifier=3205011100-0003

Assay variable : PTINR_VAL1 PT-INR Value

N	Mean	Standard Deviation	Minimum	Maximum
3	2.3000000	0.1732051	2.2000000	2.5000000

Laboratory tests at the time of obtaining Table 2-6 consent, according to the presence or absence of proton-pump inhibitors

Unique Subject Identifier=3205011100-0010

MEANS Procedure

Assay variable : PTINR_VAL1 PT-INR Value

N	Mean	Standard Deviation	Minimum	Maximum
3	1.7333333	0.0577350	1.7000000	1.8000000

Unique Subject Identifier=3205011100-0021

Assay variable : PTINR_VAL1 PT-INR Value

N	Mean	Standard Deviation	Minimum	Maximum
6	2.2166667	0.4833908	1.7000000	3.0000000

Unique Subject Identifier=3205011100-0027

Assay variable : PTINR_VAL1 PT-INR Value

N	Mean	Standard Deviation	Minimum	Maximum
6	2.1666667	0.3444803	1.6000000	2.5000000

Unique Subject Identifier=3206011010-0005

Assay variable : PTINR_VAL1 PT-INR Value

N	Mean	Standard Deviation	Minimum	Maximum
2	1.9650000	0.2333452	1.8000000	2.1300000

Laboratory tests at the time of obtaining Table 2-6 consent, according to the presence or absence of proton-pump inhibitors

Unique Subject Identifier=3208000000-0002

MEANS Procedure

Assay variable : PTINR_VAL1 PT-INR Value

N	Mean	Standard Deviation	Minimum	Maximum
6	1.4666667	0.2133229	1.2700000	1.8000000

Unique Subject Identifier=3208000000-0007

Assay variable : PTINR_VAL1 PT-INR Value

N	Mean	Standard Deviation	Minimum	Maximum
5	2.0140000	0.3176161	1.7200000	2.4600000

Unique Subject Identifier=3214111000-0005

Assay variable : PTINR_VAL1 PT-INR Value

N	Mean	Standard Deviation	Minimum	Maximum
1	1.0500000		1.0500000	1.0500000

Unique Subject Identifier=3301010000-0008

Assay variable : PTINR_VAL1 PT-INR Value

N	Mean	Standard Deviation	Minimum	Maximum
6	1.5500000	0.1870829	1.3000000	1.8000000

Laboratory tests at the time of obtaining Table 2-6 consent, according to the presence or absence of proton-pump inhibitors

Unique Subject Identifier=3301010000-0018

MEANS Procedure

Assay variable : PTINR_VAL1 PT-INR Value

N	Mean	Standard Deviation	Minimum	Maximum
5	2.1600000	0.2302173	1.8000000	2.4000000

Unique Subject Identifier=3301010000-0021

Assay variable : PTINR_VAL1 PT-INR Value

N	Mean	Standard Deviation	Minimum	Maximum
6	2.1833333	0.5419102	1.7000000	3.1000000

Unique Subject Identifier=3301010000-0026

Assay variable : PTINR_VAL1 PT-INR Value

N	Mean	Standard Deviation	Minimum	Maximum
6	1.3166667	0.1169045	1.2000000	1.5000000

Unique Subject Identifier=3301010000-0033

Assay variable : PTINR_VAL1 PT-INR Value

N	Mean	Standard Deviation	Minimum	Maximum
6	1.6500000	0.3146427	1.1000000	2.0000000

Laboratory tests at the time of obtaining Table 2-6 consent, according to the presence or absence of proton-pump inhibitors

Unique Subject Identifier=3302010000-0005

MEANS Procedure

Assay variable : PTINR_VAL1 PT-INR Value

N	Mean	Standard Deviation	Minimum	Maximum
4	1.3875000	0.1260622	1.2900000	1.5700000

Unique Subject Identifier=3302010000-0010

Assay variable : PTINR_VAL1 PT-INR Value

N	Mean	Standard Deviation	Minimum	Maximum
6	2.1733333	0.6104971	1.5800000	3.1000000

Unique Subject Identifier=3302010000-0021

Assay variable : PTINR_VAL1 PT-INR Value

N	Mean	Standard Deviation	Minimum	Maximum
6	1.8050000	0.2564176	1.3800000	2.1400000

Unique Subject Identifier=3302010000-0028

Assay variable : PTINR_VAL1 PT-INR Value

N	Mean	Standard Deviation	Minimum	Maximum
4	2.3175000	0.8429067	1.4100000	3.4500000

Laboratory tests at the time of obtaining Table 2-6 consent, according to the presence or absence of proton-pump inhibitors

Unique Subject Identifier=3304011000-0004

MEANS Procedure

Assay variable : PTINR_VAL1 PT-INR Value

N	Mean	Standard Deviation	Minimum	Maximum
5	2.4400000	0.9343447	1.7000000	4.0000000

Unique Subject Identifier=3347010000-0003

Assay variable : PTINR_VAL1 PT-INR Value

N	Mean	Standard Deviation	Minimum	Maximum
6	2.1900000	0.3221801	1.8400000	2.7000000

Unique Subject Identifier=3347010000-0004

Assay variable : PTINR_VAL1 PT-INR Value

N	Mean	Standard Deviation	Minimum	Maximum
6	2.1833333	0.1169045	2.0000000	2.3000000

Unique Subject Identifier=3347010000-0006

Assay variable : PTINR_VAL1 PT-INR Value

N	Mean	Standard Deviation	Minimum	Maximum
6	2.2333333	0.3881580	1.9000000	3.0000000

Laboratory tests at the time of obtaining Table 2-6 consent, according to the presence or absence of proton-pump inhibitors

Unique Subject Identifier=3347010000-0009

MEANS Procedure

Assay variable : PTINR_VAL1 PT-INR Value

N	Mean	Standard Deviation	Minimum	Maximum
6	1.7183333	0.1166905	1.5000000	1.8000000

Unique Subject Identifier=3347010000-0011

Assay variable : PTINR_VAL1 PT-INR Value

N	Mean	Standard Deviation	Minimum	Maximum
6	2.3800000	0.2332381	2.0000000	2.7000000

Unique Subject Identifier=3347010000-0012

Assay variable : PTINR_VAL1 PT-INR Value

N	Mean	Standard Deviation	Minimum	Maximum
6	2.3450000	0.3668106	1.9000000	2.7000000

Unique Subject Identifier=3347010000-0013

Assay variable : PTINR_VAL1 PT-INR Value

N	Mean	Standard Deviation	Minimum	Maximum
6	1.9150000	0.1433527	1.8000000	2.1900000

Laboratory tests at the time of obtaining Table 2-6 consent, according to the presence or absence of proton-pump inhibitors

Unique Subject Identifier=3347010000-0014

MEANS Procedure

Assay variable : PTINR_VAL1 PT-INR Value

N	Mean	Standard Deviation	Minimum	Maximum
6	2.5000000	0.3464102	2.0000000	3.0000000

Unique Subject Identifier=3347010000-0016

Assay variable : PTINR_VAL1 PT-INR Value

N	Mean	Standard Deviation	Minimum	Maximum
5	1.7920000	0.1879362	1.6000000	2.1000000

Unique Subject Identifier=3347010000-0021

Assay variable : PTINR_VAL1 PT-INR Value

N	Mean	Standard Deviation	Minimum	Maximum
6	1.8483333	0.2891655	1.6000000	2.3000000

Unique Subject Identifier=3347010000-0023

Assay variable : PTINR_VAL1 PT-INR Value

N	Mean	Standard Deviation	Minimum	Maximum
6	2.1666667	0.5125102	1.3000000	2.6000000

Laboratory tests at the time of obtaining Table 2-6 consent, according to the presence or absence of proton-pump inhibitors

Unique Subject Identifier=3347010000-0028

MEANS Procedure

Assay variable : PTINR_VAL1 PT-INR Value

N	Mean	Standard Deviation	Minimum	Maximum
4	1.5250000	0.2217356	1.3000000	1.8000000

Unique Subject Identifier=3347010000-0030

Assay variable : PTINR_VAL1 PT-INR Value

N	Mean	Standard Deviation	Minimum	Maximum
6	1.9666667	0.2250926	1.6000000	2.2000000

Unique Subject Identifier=3347010000-0037

Assay variable : PTINR_VAL1 PT-INR Value

N	Mean	Standard Deviation	Minimum	Maximum
4	1.7375000	0.1265899	1.5600000	1.8600000

Unique Subject Identifier=3347010000-0044

Assay variable : PTINR_VAL1 PT-INR Value

N	Mean	Standard Deviation	Minimum	Maximum
6	2.0783333	0.1371739	1.9000000	2.2000000

Laboratory tests at the time of obtaining Table 2-6 consent, according to the presence or absence of proton-pump inhibitors

Unique Subject Identifier=3347010000-0046

MEANS Procedure

Assay variable : PTINR_VAL1 PT-INR Value

N	Mean	Standard Deviation	Minimum	Maximum
6	2.3250000	0.6570769	1.5000000	3.1000000

Unique Subject Identifier=3349010000-0006

Assay variable : PTINR_VAL1 PT-INR Value

N	Mean	Standard Deviation	Minimum	Maximum
6	1.3333333	0.2065591	1.0000000	1.5000000

Unique Subject Identifier=3349010000-0008

Assay variable : PTINR_VAL1 PT-INR Value

N	Mean	Standard Deviation	Minimum	Maximum
6	1.7833333	0.2316607	1.6000000	2.2000000

Unique Subject Identifier=3349010000-0009

Assay variable : PTINR_VAL1 PT-INR Value

N	Mean	Standard Deviation	Minimum	Maximum
6	1.6666667	0.2338090	1.4000000	2.0000000

Laboratory tests at the time of obtaining Table 2-6 consent, according to the presence or absence of proton-pump inhibitors

Unique Subject Identifier=3350010000-0003

MEANS Procedure

Assay variable : PTINR_VAL1 PT-INR Value

N	Mean	Standard Deviation	Minimum	Maximum
5	2.1800000	0.5761944	1.6000000	2.8000000

Unique Subject Identifier=3350010000-0005

Assay variable : PTINR_VAL1 PT-INR Value

N	Mean	Standard Deviation	Minimum	Maximum
6	2.6333333	0.3265986	2.2000000	3.2000000

Unique Subject Identifier=3401000000-0004

Assay variable : PTINR_VAL1 PT-INR Value

N	Mean	Standard Deviation	Minimum	Maximum
4	2.1125000	0.3233548	1.7500000	2.4000000

Unique Subject Identifier=3401000000-0011

Assay variable : PTINR_VAL1 PT-INR Value

N	Mean	Standard Deviation	Minimum	Maximum
4	1.8850000	0.5774946	1.3900000	2.7200000

Laboratory tests at the time of obtaining Table 2-6 consent, according to the presence or absence of proton-pump inhibitors

Unique Subject Identifier=3401000000-0019

MEANS Procedure

Assay variable : PTINR_VAL1 PT-INR Value

N	Mean	Standard Deviation	Minimum	Maximum
4	1.4175000	0.0736546	1.3300000	1.5000000

Unique Subject Identifier=3401000000-0020

Assay variable : PTINR_VAL1 PT-INR Value

N	Mean	Standard Deviation	Minimum	Maximum
5	1.8460000	0.2968670	1.5100000	2.3200000

Unique Subject Identifier=3401000000-0021

Assay variable : PTINR_VAL1 PT-INR Value

N	Mean	Standard Deviation	Minimum	Maximum
5	2.1180000	0.3089013	1.7100000	2.5200000

Unique Subject Identifier=3401000000-0024

Assay variable : PTINR_VAL1 PT-INR Value

N	Mean	Standard Deviation	Minimum	Maximum
4	1.7325000	0.2478407	1.5700000	2.1000000

Laboratory tests at the time of obtaining Table 2-6 consent, according to the presence or absence of proton-pump inhibitors

Unique Subject Identifier=3401000000-0027

MEANS Procedure

Assay variable : PTINR_VAL1 PT-INR Value

N	Mean	Standard Deviation	Minimum	Maximum
6	2.0616667	0.5977095	1.4000000	3.0400000

Unique Subject Identifier=3401000000-0033

Assay variable : PTINR_VAL1 PT-INR Value

N	Mean	Standard Deviation	Minimum	Maximum
4	1.9625000	0.2766918	1.6200000	2.2900000

Unique Subject Identifier=3401000000-0034

Assay variable : PTINR_VAL1 PT-INR Value

N	Mean	Standard Deviation	Minimum	Maximum
4	1.7450000	0.5634122	1.2700000	2.4900000

Unique Subject Identifier=3401000000-0035

Assay variable : PTINR_VAL1 PT-INR Value

N	Mean	Standard Deviation	Minimum	Maximum
6	2.2150000	0.3503570	1.8400000	2.7200000

Laboratory tests at the time of obtaining Table 2-6 consent, according to the presence or absence of proton-pump inhibitors

Unique Subject Identifier=3401000000-0037

MEANS Procedure

Assay variable : PTINR_VAL1 PT-INR Value

N	Mean	Standard Deviation	Minimum	Maximum
6	1.8566667	0.4036170	1.5000000	2.6000000

Unique Subject Identifier=3401000000-0038

Assay variable : PTINR_VAL1 PT-INR Value

N	Mean	Standard Deviation	Minimum	Maximum
4	1.7975000	0.3088014	1.4800000	2.2200000

Unique Subject Identifier=3401000000-0039

Assay variable : PTINR_VAL1 PT-INR Value

N	Mean	Standard Deviation	Minimum	Maximum
6	1.8083333	0.1068488	1.6600000	1.9900000

Unique Subject Identifier=3401000000-0041

Assay variable : PTINR_VAL1 PT-INR Value

N	Mean	Standard Deviation	Minimum	Maximum
4	1.8075000	0.4439501	1.4300000	2.4400000

Laboratory tests at the time of obtaining Table 2-6 consent, according to the presence or absence of proton-pump inhibitors

Unique Subject Identifier=3401000000-0043

MEANS Procedure

Assay variable : PTINR_VAL1 PT-INR Value

N	Mean	Standard Deviation	Minimum	Maximum
5	1.5840000	0.1375863	1.4900000	1.8200000

Unique Subject Identifier=3401000000-0045

Assay variable : PTINR_VAL1 PT-INR Value

N	Mean	Standard Deviation	Minimum	Maximum
6	1.7283333	0.5647448	1.1400000	2.7900000

Unique Subject Identifier=3401000000-0046

Assay variable : PTINR_VAL1 PT-INR Value

N	Mean	Standard Deviation	Minimum	Maximum
5	1.7800000	0.4046604	1.1300000	2.2200000

Unique Subject Identifier=3402010011-0007

Assay variable : PTINR_VAL1 PT-INR Value

N	Mean	Standard Deviation	Minimum	Maximum
6	1.9833333	0.3060501	1.4000000	2.3000000

Laboratory tests at the time of obtaining Table 2-6 consent, according to the presence or absence of proton-pump inhibitors

Unique Subject Identifier=3402010011-0023

MEANS Procedure

Assay variable : PTINR_VAL1 PT-INR Value

N	Mean	Standard Deviation	Minimum	Maximum
5	1.8000000	0.2000000	1.6000000	2.1000000

Unique Subject Identifier=3402010011-0025

Assay variable : PTINR_VAL1 PT-INR Value

N	Mean	Standard Deviation	Minimum	Maximum
6	2.1666667	0.2503331	1.8000000	2.5000000

Unique Subject Identifier=3402010011-0027

Assay variable : PTINR_VAL1 PT-INR Value

N	Mean	Standard Deviation	Minimum	Maximum
6	2.0666667	0.4802777	1.7000000	2.9000000

Unique Subject Identifier=3403011000-0012

Assay variable : PTINR_VAL1 PT-INR Value

N	Mean	Standard Deviation	Minimum	Maximum
2	1.9000000	0.2828427	1.7000000	2.1000000

Laboratory tests at the time of obtaining Table 2-6 consent, according to the presence or absence of proton-pump inhibitors

Unique Subject Identifier=3406010000-0003

MEANS Procedure

Assay variable : PTINR_VAL1 PT-INR Value

N	Mean	Standard Deviation	Minimum	Maximum
4	2.1775000	0.1528343	2.0400000	2.3900000

Unique Subject Identifier=3407010011-0001

Assay variable : PTINR_VAL1 PT-INR Value

N	Mean	Standard Deviation	Minimum	Maximum
6	1.8250000	0.2720110	1.4700000	2.2700000

Unique Subject Identifier=3412000000-0002

Assay variable : PTINR_VAL1 PT-INR Value

N	Mean	Standard Deviation	Minimum	Maximum
4	1.7000000	0.1113553	1.5500000	1.8100000

Unique Subject Identifier=3412000000-0004

Assay variable : PTINR_VAL1 PT-INR Value

N	Mean	Standard Deviation	Minimum	Maximum
4	1.4925000	0.0471699	1.4600000	1.5600000

Laboratory tests at the time of obtaining Table 2-6 consent, according to the presence or absence of proton-pump inhibitors

Unique Subject Identifier=3417111111-0003

MEANS Procedure

Assay variable : PTINR_VAL1 PT-INR Value

N	Mean	Standard Deviation	Minimum	Maximum
4	1.4900000	0.1745470	1.2600000	1.6400000

Unique Subject Identifier=3417111111-0008

Assay variable : PTINR_VAL1 PT-INR Value

N	Mean	Standard Deviation	Minimum	Maximum
6	1.7100000	0.2366432	1.4000000	2.0100000

Unique Subject Identifier=3417111111-0009

Assay variable : PTINR_VAL1 PT-INR Value

N	Mean	Standard Deviation	Minimum	Maximum
6	1.9400000	0.2276840	1.6800000	2.2500000

Unique Subject Identifier=3417111111-0013

Assay variable : PTINR_VAL1 PT-INR Value

N	Mean	Standard Deviation	Minimum	Maximum
6	1.9016667	0.0511534	1.8500000	1.9900000

Laboratory tests at the time of obtaining Table 2-6 consent, according to the presence or absence of proton-pump inhibitors

Unique Subject Identifier=3421000000-0014

MEANS Procedure

Assay variable : PTINR_VAL1 PT-INR Value

N	Mean	Standard Deviation	Minimum	Maximum
6	2.0916667	0.3975634	1.4600000	2.6400000

Unique Subject Identifier=3421000000-0015

Assay variable : PTINR_VAL1 PT-INR Value

N	Mean	Standard Deviation	Minimum	Maximum
4	2.5025000	0.6128825	1.6400000	2.9400000

Unique Subject Identifier=3421000000-0022

Assay variable : PTINR_VAL1 PT-INR Value

N	Mean	Standard Deviation	Minimum	Maximum
4	1.9800000	0.1630951	1.7800000	2.1600000

Unique Subject Identifier=3421000000-0025

Assay variable : PTINR_VAL1 PT-INR Value

N	Mean	Standard Deviation	Minimum	Maximum
4	1.9800000	0.1858315	1.7900000	2.2300000

Laboratory tests at the time of obtaining Table 2-6 consent, according to the presence or absence of proton-pump inhibitors

Unique Subject Identifier=3421000000-0031

MEANS Procedure

Assay variable : PTINR_VAL1 PT-INR Value

N	Mean	Standard Deviation	Minimum	Maximum
3	2.1066667	0.9450044	1.0700000	2.9200000

Unique Subject Identifier=3422000000-0003

Assay variable : PTINR_VAL1 PT-INR Value

N	Mean	Standard Deviation	Minimum	Maximum
3	1.8666667	0.5507571	1.5000000	2.5000000

Unique Subject Identifier=3422000000-0004

Assay variable : PTINR_VAL1 PT-INR Value

N	Mean	Standard Deviation	Minimum	Maximum
5	1.4000000	0.1000000	1.3000000	1.5000000

Unique Subject Identifier=3422000000-0009

Assay variable : PTINR_VAL1 PT-INR Value

N	Mean	Standard Deviation	Minimum	Maximum
3	1.5666667	0.4041452	1.2000000	2.0000000

Laboratory tests at the time of obtaining Table 2-6 consent, according to the presence or absence of proton-pump inhibitors

Unique Subject Identifier=3422000000-0011

MEANS Procedure

Assay variable : PTINR_VAL1 PT-INR Value

N	Mean	Standard Deviation	Minimum	Maximum
3	2.1000000	0.4582576	1.7000000	2.6000000

Unique Subject Identifier=3422000000-0024

Assay variable : PTINR_VAL1 PT-INR Value

N	Mean	Standard Deviation	Minimum	Maximum
3	2.0666667	0.2081666	1.9000000	2.3000000

Unique Subject Identifier=3422000000-0025

Assay variable : PTINR_VAL1 PT-INR Value

N	Mean	Standard Deviation	Minimum	Maximum
5	1.8800000	0.8526429	1.4000000	3.4000000

Unique Subject Identifier=3422000000-0048

Assay variable : PTINR_VAL1 PT-INR Value

N	Mean	Standard Deviation	Minimum	Maximum
2	2.1000000	0.4242641	1.8000000	2.4000000

Laboratory tests at the time of obtaining Table 2-6 consent, according to the presence or absence of proton-pump inhibitors

Unique Subject Identifier=3422000000-0056

MEANS Procedure

Assay variable : PTINR_VAL1 PT-INR Value

N	Mean	Standard Deviation	Minimum	Maximum
3	2.2000000	0.7937254	1.6000000	3.1000000

Unique Subject Identifier=3422000000-0060

Assay variable : PTINR_VAL1 PT-INR Value

N	Mean	Standard Deviation	Minimum	Maximum
3	1.6333333	0.1527525	1.5000000	1.8000000

Unique Subject Identifier=3422000000-0069

Assay variable : PTINR_VAL1 PT-INR Value

N	Mean	Standard Deviation	Minimum	Maximum
3	1.2666667	0.1154701	1.2000000	1.4000000

Unique Subject Identifier=3422000000-0070

Assay variable : PTINR_VAL1 PT-INR Value

N	Mean	Standard Deviation	Minimum	Maximum
3	2.2000000	0.3605551	1.9000000	2.6000000

Laboratory tests at the time of obtaining Table 2-6 consent, according to the presence or absence of proton-pump inhibitors

Unique Subject Identifier=3422000000-0073

MEANS Procedure

Assay variable : PTINR_VAL1 PT-INR Value

N	Mean	Standard Deviation	Minimum	Maximum
3	2.1000000	0.4000000	1.7000000	2.5000000

Unique Subject Identifier=3422000000-0089

Assay variable : PTINR_VAL1 PT-INR Value

N	Mean	Standard Deviation	Minimum	Maximum
4	1.5250000	0.2217356	1.2000000	1.7000000

Unique Subject Identifier=3422000000-0090

Assay variable : PTINR_VAL1 PT-INR Value

N	Mean	Standard Deviation	Minimum	Maximum
3	1.9000000	0.3605551	1.5000000	2.2000000

Unique Subject Identifier=3423000000-0003

Assay variable : PTINR_VAL1 PT-INR Value

N	Mean	Standard Deviation	Minimum	Maximum
6	1.5466667	0.6122962	0.9900000	2.6700000

Laboratory tests at the time of obtaining Table 2-6 consent, according to the presence or absence of proton-pump inhibitors

Unique Subject Identifier=3423000000-0031

MEANS Procedure

Assay variable : PTINR_VAL1 PT-INR Value

N	Mean	Standard Deviation	Minimum	Maximum
5	1.8400000	0.6213292	1.4400000	2.9400000

Unique Subject Identifier=3424000000-0016

Assay variable : PTINR_VAL1 PT-INR Value

N	Mean	Standard Deviation	Minimum	Maximum
3	1.9466667	0.5093460	1.5900000	2.5300000

Unique Subject Identifier=3424000000-0021

Assay variable : PTINR_VAL1 PT-INR Value

N	Mean	Standard Deviation	Minimum	Maximum
3	1.5466667	0.4271222	1.2200000	2.0300000

Unique Subject Identifier=3424000000-0026

Assay variable : PTINR_VAL1 PT-INR Value

N	Mean	Standard Deviation	Minimum	Maximum
5	1.8960000	0.6223584	1.1400000	2.6900000

Laboratory tests at the time of obtaining Table 2-6 consent, according to the presence or absence of proton-pump inhibitors

Unique Subject Identifier=3424000000-0032

MEANS Procedure

Assay variable : PTINR_VAL1 PT-INR Value

N	Mean	Standard Deviation	Minimum	Maximum
3	2.2933333	0.1069268	2.2000000	2.4100000

Unique Subject Identifier=3424000000-0033

Assay variable : PTINR_VAL1 PT-INR Value

N	Mean	Standard Deviation	Minimum	Maximum
2	2.5600000	0.1979899	2.4200000	2.7000000

Unique Subject Identifier=3424000000-0041

Assay variable : PTINR_VAL1 PT-INR Value

N	Mean	Standard Deviation	Minimum	Maximum
3	2.0966667	0.5300314	1.6700000	2.6900000

Unique Subject Identifier=3424000000-0044

Assay variable : PTINR_VAL1 PT-INR Value

N	Mean	Standard Deviation	Minimum	Maximum
6	1.8483333	0.4343002	1.1400000	2.3600000

Laboratory tests at the time of obtaining Table 2-6 consent, according to the presence or absence of proton-pump inhibitors

Unique Subject Identifier=3424000000-0047

MEANS Procedure

Assay variable : PTINR_VAL1 PT-INR Value

N	Mean	Standard Deviation	Minimum	Maximum
2	2.2500000	0.1838478	2.1200000	2.3800000

Unique Subject Identifier=3424000000-0049

Assay variable : PTINR_VAL1 PT-INR Value

N	Mean	Standard Deviation	Minimum	Maximum
3	2.6100000	0.7644606	2.1100000	3.4900000

Unique Subject Identifier=3424000000-0055

Assay variable : PTINR_VAL1 PT-INR Value

N	Mean	Standard Deviation	Minimum	Maximum
3	1.8100000	0.3148015	1.5500000	2.1600000

Unique Subject Identifier=3425001000-0005

Assay variable : PTINR_VAL1 PT-INR Value

N	Mean	Standard Deviation	Minimum	Maximum
4	1.9175000	0.5340022	1.3300000	2.5900000

Laboratory tests at the time of obtaining Table 2-6 consent, according to the presence or absence of proton-pump inhibitors

Unique Subject Identifier=3425001000-0007

MEANS Procedure

Assay variable : PTINR_VAL1 PT-INR Value

N	Mean	Standard Deviation	Minimum	Maximum
4	2.2100000	0.2409703	1.9700000	2.5000000

Unique Subject Identifier=3425001000-0010

Assay variable : PTINR_VAL1 PT-INR Value

N	Mean	Standard Deviation	Minimum	Maximum
3	2.3200000	0.0818535	2.2300000	2.3900000

Unique Subject Identifier=3425001000-0015

Assay variable : PTINR_VAL1 PT-INR Value

N	Mean	Standard Deviation	Minimum	Maximum
3	2.1333333	0.2350177	1.9000000	2.3700000

Unique Subject Identifier=3425001000-0022

Assay variable : PTINR_VAL1 PT-INR Value

N	Mean	Standard Deviation	Minimum	Maximum
4	1.9200000	0.2708013	1.5200000	2.1200000

Laboratory tests at the time of obtaining Table 2-6 consent, according to the presence or absence of proton-pump inhibitors

Unique Subject Identifier=3425001000-0023

MEANS Procedure

Assay variable : PTINR_VAL1 PT-INR Value

N	Mean	Standard Deviation	Minimum	Maximum
4	2.0025000	0.1147098	1.8500000	2.1200000

Unique Subject Identifier=3425001000-0034

Assay variable : PTINR_VAL1 PT-INR Value

N	Mean	Standard Deviation	Minimum	Maximum
4	2.2450000	0.2392349	1.9100000	2.4600000

Unique Subject Identifier=3425001000-0037

Assay variable : PTINR_VAL1 PT-INR Value

N	Mean	Standard Deviation	Minimum	Maximum
4	1.7900000	0.0761577	1.6800000	1.8500000

Unique Subject Identifier=3425001000-0040

Assay variable : PTINR_VAL1 PT-INR Value

N	Mean	Standard Deviation	Minimum	Maximum
5	2.4440000	0.2576432	2.2200000	2.8700000

Laboratory tests at the time of obtaining Table 2-6 consent, according to the presence or absence of proton-pump inhibitors

Unique Subject Identifier=3427001000-0002

MEANS Procedure

Assay variable : PTINR_VAL1 PT-INR Value

N	Mean	Standard Deviation	Minimum	Maximum
5	1.9920000	0.2752635	1.6500000	2.4000000

Unique Subject Identifier=3427001000-0015

Assay variable : PTINR_VAL1 PT-INR Value

N	Mean	Standard Deviation	Minimum	Maximum
4	2.0675000	0.2777739	1.8000000	2.3500000

Unique Subject Identifier=3428000000-0001

Assay variable : PTINR_VAL1 PT-INR Value

N	Mean	Standard Deviation	Minimum	Maximum
6	1.9483333	0.3818595	1.4500000	2.4400000

Unique Subject Identifier=3428000000-0004

Assay variable : PTINR_VAL1 PT-INR Value

N	Mean	Standard Deviation	Minimum	Maximum
3	1.6466667	0.3980368	1.3200000	2.0900000

Laboratory tests at the time of obtaining Table 2-6 consent, according to the presence or absence of proton-pump inhibitors

Unique Subject Identifier=3428000000-0005

MEANS Procedure

Assay variable : PTINR_VAL1 PT-INR Value

N	Mean	Standard Deviation	Minimum	Maximum
4	2.1175000	0.1796988	1.8600000	2.2700000

Unique Subject Identifier=3442000011-0001

Assay variable : PTINR_VAL1 PT-INR Value

N	Mean	Standard Deviation	Minimum	Maximum
5	2.1200000	0.0796869	2.0400000	2.2400000

Unique Subject Identifier=3442000011-0003

Assay variable : PTINR_VAL1 PT-INR Value

N	Mean	Standard Deviation	Minimum	Maximum
4	1.5750000	0.1912241	1.2900000	1.6900000

Unique Subject Identifier=3442000011-0007

Assay variable : PTINR_VAL1 PT-INR Value

N	Mean	Standard Deviation	Minimum	Maximum
5	1.9040000	0.2250111	1.6300000	2.1600000

Laboratory tests at the time of obtaining Table 2-6 consent, according to the presence or absence of proton-pump inhibitors

Unique Subject Identifier=3442000011-0008

MEANS Procedure

Assay variable : PTINR_VAL1 PT-INR Value

N	Mean	Standard Deviation	Minimum	Maximum
3	2.0866667	0.1908752	1.8700000	2.2300000

Unique Subject Identifier=3443011001-0009

Assay variable : PTINR_VAL1 PT-INR Value

N	Mean	Standard Deviation	Minimum	Maximum
6	1.9483333	0.3301767	1.5000000	2.4200000

Unique Subject Identifier=3444000000-0002

Assay variable : PTINR_VAL1 PT-INR Value

N	Mean	Standard Deviation	Minimum	Maximum
4	2.0475000	0.1071992	1.9200000	2.1700000

Unique Subject Identifier=3444000000-0004

Assay variable : PTINR_VAL1 PT-INR Value

N	Mean	Standard Deviation	Minimum	Maximum
4	2.0975000	0.6357345	1.6000000	3.0000000

Laboratory tests at the time of obtaining Table 2-6 consent, according to the presence or absence of proton-pump inhibitors

Unique Subject Identifier=3444000000-0011

MEANS Procedure

Assay variable : PTINR_VAL1 PT-INR Value

N	Mean	Standard Deviation	Minimum	Maximum
3	2.4700000	0.4392038	2.0000000	2.8700000

Unique Subject Identifier=3444000000-0015

Assay variable : PTINR_VAL1 PT-INR Value

N	Mean	Standard Deviation	Minimum	Maximum
5	2.2020000	0.6975815	1.4500000	3.2200000

Unique Subject Identifier=3444000000-0017

Assay variable : PTINR_VAL1 PT-INR Value

N	Mean	Standard Deviation	Minimum	Maximum
4	1.9175000	0.4294473	1.4400000	2.4000000

Unique Subject Identifier=3444000000-0019

Assay variable : PTINR_VAL1 PT-INR Value

N	Mean	Standard Deviation	Minimum	Maximum
3	2.0633333	0.3189566	1.8500000	2.4300000

Laboratory tests at the time of obtaining Table 2-6 consent, according to the presence or absence of proton-pump inhibitors

Unique Subject Identifier=3444000000-0021

MEANS Procedure

Assay variable : PTINR_VAL1 PT-INR Value

N	Mean	Standard Deviation	Minimum	Maximum
3	2.3333333	0.3023795	1.9900000	2.5600000

Unique Subject Identifier=3444000000-0027

Assay variable : PTINR_VAL1 PT-INR Value

N	Mean	Standard Deviation	Minimum	Maximum
4	2.3850000	1.0434718	1.2700000	3.7900000

Unique Subject Identifier=3444000000-0029

Assay variable : PTINR_VAL1 PT-INR Value

N	Mean	Standard Deviation	Minimum	Maximum
3	2.1833333	0.4883987	1.7900000	2.7300000

Unique Subject Identifier=3444000000-0035

Assay variable : PTINR_VAL1 PT-INR Value

N	Mean	Standard Deviation	Minimum	Maximum
4	2.3025000	0.3510342	1.8800000	2.6600000

Laboratory tests at the time of obtaining Table 2-6 consent, according to the presence or absence of proton-pump inhibitors

Unique Subject Identifier=3444000000-0037

MEANS Procedure

Assay variable : PTINR_VAL1 PT-INR Value

N	Mean	Standard Deviation	Minimum	Maximum
4	2.3550000	0.9300358	1.8000000	3.7400000

Unique Subject Identifier=3444000000-0039

Assay variable : PTINR_VAL1 PT-INR Value

N	Mean	Standard Deviation	Minimum	Maximum
3	2.5333333	0.2967041	2.3100000	2.8700000

Unique Subject Identifier=3444000000-0048

Assay variable : PTINR_VAL1 PT-INR Value

N	Mean	Standard Deviation	Minimum	Maximum
3	1.9133333	0.1985783	1.6900000	2.0700000

Unique Subject Identifier=3444000000-0049

Assay variable : PTINR_VAL1 PT-INR Value

N	Mean	Standard Deviation	Minimum	Maximum
3	2.4100000	0.1819341	2.3000000	2.6200000

Laboratory tests at the time of obtaining Table 2-6 consent, according to the presence or absence of proton-pump inhibitors

Unique Subject Identifier=3444000000-0055

MEANS Procedure

Assay variable : PTINR_VAL1 PT-INR Value

N	Mean	Standard Deviation	Minimum	Maximum
4	1.5700000	0.1860108	1.3400000	1.7900000

Unique Subject Identifier=3444000000-0057

Assay variable : PTINR_VAL1 PT-INR Value

N	Mean	Standard Deviation	Minimum	Maximum
4	2.4625000	0.2615817	2.1400000	2.7800000

Unique Subject Identifier=3444000000-0060

Assay variable : PTINR_VAL1 PT-INR Value

N	Mean	Standard Deviation	Minimum	Maximum
3	1.8100000	0.3732292	1.5700000	2.2400000

Unique Subject Identifier=3444000000-0061

Assay variable : PTINR_VAL1 PT-INR Value

N	Mean	Standard Deviation	Minimum	Maximum
4	1.7900000	0.1378405	1.6300000	1.9600000

Laboratory tests at the time of obtaining Table 2-6 consent, according to the presence or absence of proton-pump inhibitors

Unique Subject Identifier=3444000000-0065

MEANS Procedure

Assay variable : PTINR_VAL1 PT-INR Value

N	Mean	Standard Deviation	Minimum	Maximum
3	1.5666667	0.0737111	1.5100000	1.6500000

Unique Subject Identifier=3444000000-0070

Assay variable : PTINR_VAL1 PT-INR Value

N	Mean	Standard Deviation	Minimum	Maximum
5	1.7880000	0.3751933	1.4100000	2.3200000

Unique Subject Identifier=3444000000-0074

Assay variable : PTINR_VAL1 PT-INR Value

N	Mean	Standard Deviation	Minimum	Maximum
5	1.6780000	0.4704997	1.1800000	2.3900000

Unique Subject Identifier=3444000000-0084

Assay variable : PTINR_VAL1 PT-INR Value

N	Mean	Standard Deviation	Minimum	Maximum
4	1.7800000	0.1435270	1.6000000	1.9500000

Laboratory tests at the time of obtaining Table 2-6 consent, according to the presence or absence of proton-pump inhibitors

Unique Subject Identifier=3444000000-0092

MEANS Procedure

Assay variable : PTINR_VAL1 PT-INR Value

N	Mean	Standard Deviation	Minimum	Maximum
6	2.4283333	1.0451682	1.1200000	3.8500000

Unique Subject Identifier=3448111011-0005

Assay variable : PTINR_VAL1 PT-INR Value

N	Mean	Standard Deviation	Minimum	Maximum
4	1.7225000	0.2860507	1.4100000	2.0900000

Unique Subject Identifier=3452000000-0002

Assay variable : PTINR_VAL1 PT-INR Value

N	Mean	Standard Deviation	Minimum	Maximum
3	1.7100000	0.1276715	1.5700000	1.8200000

Unique Subject Identifier=3452000000-0007

Assay variable : PTINR_VAL1 PT-INR Value

N	Mean	Standard Deviation	Minimum	Maximum
3	1.9633333	0.0750555	1.9200000	2.0500000

Laboratory tests at the time of obtaining Table 2-6 consent, according to the presence or absence of proton-pump inhibitors

Unique Subject Identifier=3452000000-0009

MEANS Procedure

Assay variable : PTINR_VAL1 PT-INR Value

N	Mean	Standard Deviation	Minimum	Maximum
3	2.3866667	0.5604760	1.8400000	2.9600000

Unique Subject Identifier=3453000000-0002

Assay variable : PTINR_VAL1 PT-INR Value

N	Mean	Standard Deviation	Minimum	Maximum
2	1.9450000	0.4313351	1.6400000	2.2500000

Unique Subject Identifier=3453000000-0005

Assay variable : PTINR_VAL1 PT-INR Value

N	Mean	Standard Deviation	Minimum	Maximum
2	2.0650000	0.3181981	1.8400000	2.2900000

Unique Subject Identifier=3453000000-0007

Assay variable : PTINR_VAL1 PT-INR Value

N	Mean	Standard Deviation	Minimum	Maximum
2	1.7100000	0.1697056	1.5900000	1.8300000

Laboratory tests at the time of obtaining Table 2-6 consent, according to the presence or absence of proton-pump inhibitors

Unique Subject Identifier=3453000000-0008

MEANS Procedure

Assay variable : PTINR_VAL1 PT-INR Value

N	Mean	Standard Deviation	Minimum	Maximum
2	2.0350000	0.0353553	2.0100000	2.0600000

Unique Subject Identifier=3472000000-0017

Assay variable : PTINR_VAL1 PT-INR Value

N	Mean	Standard Deviation	Minimum	Maximum
6	1.8333333	0.3983298	1.2000000	2.3000000

Unique Subject Identifier=3481010111-0002

Assay variable : PTINR_VAL1 PT-INR Value

N	Mean	Standard Deviation	Minimum	Maximum
5	1.4600000	0.0547723	1.4000000	1.5000000

Unique Subject Identifier=3481010111-0006

Assay variable : PTINR_VAL1 PT-INR Value

N	Mean	Standard Deviation	Minimum	Maximum
3	2.0666667	0.2516611	1.8000000	2.3000000

Laboratory tests at the time of obtaining Table 2-6 consent, according to the presence or absence of proton-pump inhibitors

Unique Subject Identifier=3481010111-0007

MEANS Procedure

Assay variable : PTINR_VAL1 PT-INR Value

N	Mean	Standard Deviation	Minimum	Maximum
3	2.1666667	0.0577350	2.1000000	2.2000000

Unique Subject Identifier=3481010111-0008

Assay variable : PTINR_VAL1 PT-INR Value

N	Mean	Standard Deviation	Minimum	Maximum
1	1.1000000		1.1000000	1.1000000

Unique Subject Identifier=3481010111-0009

Assay variable : PTINR_VAL1 PT-INR Value

N	Mean	Standard Deviation	Minimum	Maximum
5	1.3200000	0.0836660	1.2000000	1.4000000

Unique Subject Identifier=3481010111-0010

Assay variable : PTINR_VAL1 PT-INR Value

N	Mean	Standard Deviation	Minimum	Maximum
2	1.6000000	0.1414214	1.5000000	1.7000000

Laboratory tests at the time of obtaining Table 2-6 consent, according to the presence or absence of proton-pump inhibitors

Unique Subject Identifier=3481010111-0012

MEANS Procedure

Assay variable : PTINR_VAL1 PT-INR Value

N	Mean	Standard Deviation	Minimum	Maximum
4	1.6750000	0.0957427	1.6000000	1.8000000

Unique Subject Identifier=3481010111-0013

Assay variable : PTINR_VAL1 PT-INR Value

N	Mean	Standard Deviation	Minimum	Maximum
5	2.4400000	0.5319774	1.8000000	3.0000000

Unique Subject Identifier=3481010111-0015

Assay variable : PTINR_VAL1 PT-INR Value

N	Mean	Standard Deviation	Minimum	Maximum
3	2.4666667	0.1527525	2.3000000	2.6000000

Unique Subject Identifier=3481010111-0016

Assay variable : PTINR_VAL1 PT-INR Value

N	Mean	Standard Deviation	Minimum	Maximum
6	1.5000000	0.1264911	1.3000000	1.6000000

Laboratory tests at the time of obtaining Table 2-6 consent, according to the presence or absence of proton-pump inhibitors

Unique Subject Identifier=3481010111-0017

MEANS Procedure

Assay variable : PTINR_VAL1 PT-INR Value

N	Mean	Standard Deviation	Minimum	Maximum
4	1.9250000	0.8057088	1.4000000	3.1000000

Unique Subject Identifier=3481010111-0019

Assay variable : PTINR_VAL1 PT-INR Value

N	Mean	Standard Deviation	Minimum	Maximum
3	2.0000000	0.5000000	1.5000000	2.5000000

Unique Subject Identifier=3481010111-0022

Assay variable : PTINR_VAL1 PT-INR Value

N	Mean	Standard Deviation	Minimum	Maximum
3	1.5666667	0.3214550	1.2000000	1.8000000

Unique Subject Identifier=3481010111-0024

Assay variable : PTINR_VAL1 PT-INR Value

N	Mean	Standard Deviation	Minimum	Maximum
2	1.4000000	0.4242641	1.1000000	1.7000000

Laboratory tests at the time of obtaining Table 2-6 consent, according to the presence or absence of proton-pump inhibitors

Unique Subject Identifier=3481010111-0026

MEANS Procedure

Assay variable : PTINR_VAL1 PT-INR Value

N	Mean	Standard Deviation	Minimum	Maximum
3	2.5666667	0.6110101	1.9000000	3.1000000

Unique Subject Identifier=3481010111-0029

Assay variable : PTINR_VAL1 PT-INR Value

N	Mean	Standard Deviation	Minimum	Maximum
4	2.2000000	0.9933110	1.0000000	3.4000000

Unique Subject Identifier=3491001000-0031

Assay variable : PTINR_VAL1 PT-INR Value

N	Mean	Standard Deviation	Minimum	Maximum
1	1.7800000		1.7800000	1.7800000

Unique Subject Identifier=3501111111-0003

Assay variable : PTINR_VAL1 PT-INR Value

N	Mean	Standard Deviation	Minimum	Maximum
6	2.3950000	0.9346604	1.0600000	3.7800000

Laboratory tests at the time of obtaining Table 2-6 consent, according to the presence or absence of proton-pump inhibitors

Unique Subject Identifier=3501111111-0004

MEANS Procedure

Assay variable : PTINR_VAL1 PT-INR Value

N	Mean	Standard Deviation	Minimum	Maximum
6	1.4916667	0.3354649	1.0000000	2.0000000

Unique Subject Identifier=3501111111-0011

Assay variable : PTINR_VAL1 PT-INR Value

N	Mean	Standard Deviation	Minimum	Maximum
3	2.2133333	0.4027820	1.7500000	2.4800000

Unique Subject Identifier=3501111111-0019

Assay variable : PTINR_VAL1 PT-INR Value

N	Mean	Standard Deviation	Minimum	Maximum
5	1.9360000	0.2223286	1.6800000	2.1700000

Unique Subject Identifier=3501111111-0026

Assay variable : PTINR_VAL1 PT-INR Value

N	Mean	Standard Deviation	Minimum	Maximum
3	2.7700000	1.7963296	1.5300000	4.8300000

Laboratory tests at the time of obtaining Table 2-6 consent, according to the presence or absence of proton-pump inhibitors

Unique Subject Identifier=5001010000-0001

MEANS Procedure

Assay variable : PTINR_VAL1 PT-INR Value

N	Mean	Standard Deviation	Minimum	Maximum
1	2.2500000		2.2500000	2.2500000

Unique Subject Identifier=5001010000-0007

Assay variable : PTINR_VAL1 PT-INR Value

N	Mean	Standard Deviation	Minimum	Maximum
3	1.9333333	0.0723418	1.8500000	1.9800000

Unique Subject Identifier=5001010000-0012

Assay variable : PTINR_VAL1 PT-INR Value

N	Mean	Standard Deviation	Minimum	Maximum
3	1.5000000	0.1000000	1.4000000	1.6000000

Unique Subject Identifier=5001010000-0013

Assay variable : PTINR_VAL1 PT-INR Value

N	Mean	Standard Deviation	Minimum	Maximum
1	2.0300000		2.0300000	2.0300000

Laboratory tests at the time of obtaining Table 2-6 consent, according to the presence or absence of proton-pump inhibitors

Unique Subject Identifier=5001010000-0021

MEANS Procedure

Assay variable : PTINR_VAL1 PT-INR Value

N	Mean	Standard Deviation	Minimum	Maximum
4	2.2300000	0.2041241	1.9600000	2.4200000

Unique Subject Identifier=5001010000-0031

Assay variable : PTINR_VAL1 PT-INR Value

N	Mean	Standard Deviation	Minimum	Maximum
6	2.3466667	0.7982898	1.1800000	3.5400000

Unique Subject Identifier=5002010010-0006

Assay variable : PTINR_VAL1 PT-INR Value

N	Mean	Standard Deviation	Minimum	Maximum
2	1.6550000	0.3889087	1.3800000	1.9300000

Unique Subject Identifier=5002010010-0012

Assay variable : PTINR_VAL1 PT-INR Value

N	Mean	Standard Deviation	Minimum	Maximum
2	1.9050000	0.1484924	1.8000000	2.0100000

Laboratory tests at the time of obtaining Table 2-6 consent, according to the presence or absence of proton-pump inhibitors

Unique Subject Identifier=5002010010-0013

MEANS Procedure

Assay variable : PTINR_VAL1 PT-INR Value

N	Mean	Standard Deviation	Minimum	Maximum
2	1.6150000	0.1484924	1.5100000	1.7200000

Unique Subject Identifier=5002010010-0014

Assay variable : PTINR_VAL1 PT-INR Value

N	Mean	Standard Deviation	Minimum	Maximum
4	1.9625000	0.1658061	1.8000000	2.1200000

Unique Subject Identifier=5002010010-0022

Assay variable : PTINR_VAL1 PT-INR Value

N	Mean	Standard Deviation	Minimum	Maximum
4	1.7100000	0.6702736	1.2800000	2.7100000

Unique Subject Identifier=5002010010-0023

Assay variable : PTINR_VAL1 PT-INR Value

N	Mean	Standard Deviation	Minimum	Maximum
6	2.0566667	0.4529312	1.6400000	2.9000000

Laboratory tests at the time of obtaining Table 2-6 consent, according to the presence or absence of proton-pump inhibitors

Unique Subject Identifier=5002010010-0025

MEANS Procedure

Assay variable : PTINR_VAL1 PT-INR Value

N	Mean	Standard Deviation	Minimum	Maximum
3	2.1600000	0.7269113	1.5400000	2.9600000

Unique Subject Identifier=5002010010-0026

Assay variable : PTINR_VAL1 PT-INR Value

N	Mean	Standard Deviation	Minimum	Maximum
6	2.3400000	0.2816381	1.8900000	2.7300000

Unique Subject Identifier=5002010010-0031

Assay variable : PTINR_VAL1 PT-INR Value

N	Mean	Standard Deviation	Minimum	Maximum
6	1.8300000	0.2572159	1.5000000	2.2200000

Unique Subject Identifier=5002010010-0032

Assay variable : PTINR_VAL1 PT-INR Value

N	Mean	Standard Deviation	Minimum	Maximum
6	2.1933333	0.1517454	1.9500000	2.3400000

Laboratory tests at the time of obtaining Table 2-6 consent, according to the presence or absence of proton-pump inhibitors

Unique Subject Identifier=5002010010-0039

MEANS Procedure

Assay variable : PTINR_VAL1 PT-INR Value

N	Mean	Standard Deviation	Minimum	Maximum
3	1.7600000	0.3567913	1.5200000	2.1700000

Unique Subject Identifier=5002010010-0044

Assay variable : PTINR_VAL1 PT-INR Value

N	Mean	Standard Deviation	Minimum	Maximum
3	1.7700000	0.2233831	1.5900000	2.0200000

Unique Subject Identifier=5002010010-0053

Assay variable : PTINR_VAL1 PT-INR Value

N	Mean	Standard Deviation	Minimum	Maximum
2	2.2700000	0.1131371	2.1900000	2.3500000

Unique Subject Identifier=5002010010-0054

Assay variable : PTINR_VAL1 PT-INR Value

N	Mean	Standard Deviation	Minimum	Maximum
4	2.0025000	0.1297112	1.8800000	2.1800000

Laboratory tests at the time of obtaining Table 2-6 consent, according to the presence or absence of proton-pump inhibitors

Unique Subject Identifier=5002010010-0058

MEANS Procedure

Assay variable : PTINR_VAL1 PT-INR Value

N	Mean	Standard Deviation	Minimum	Maximum
5	1.5980000	0.5341535	1.0400000	2.2400000

Unique Subject Identifier=5002010010-0060

Assay variable : PTINR_VAL1 PT-INR Value

N	Mean	Standard Deviation	Minimum	Maximum
6	1.8800000	0.6824368	1.3900000	3.2500000

Unique Subject Identifier=5002010010-0063

Assay variable : PTINR_VAL1 PT-INR Value

N	Mean	Standard Deviation	Minimum	Maximum
2	2.2750000	0.1909188	2.1400000	2.4100000

Unique Subject Identifier=5002010010-0069

Assay variable : PTINR_VAL1 PT-INR Value

N	Mean	Standard Deviation	Minimum	Maximum
4	1.9350000	0.5406478	1.4700000	2.6000000

Laboratory tests at the time of obtaining Table 2-6 consent, according to the presence or absence of proton-pump inhibitors

Unique Subject Identifier=5002010010-0085

MEANS Procedure

Assay variable : PTINR_VAL1 PT-INR Value

N	Mean	Standard Deviation	Minimum	Maximum
3	1.6433333	0.2914332	1.3100000	1.8500000

Unique Subject Identifier=5002010010-0086

Assay variable : PTINR_VAL1 PT-INR Value

N	Mean	Standard Deviation	Minimum	Maximum
3	1.7300000	0.3304542	1.4100000	2.0700000

Unique Subject Identifier=5002010010-0092

Assay variable : PTINR_VAL1 PT-INR Value

N	Mean	Standard Deviation	Minimum	Maximum
3	1.6733333	0.2478575	1.3900000	1.8500000

Unique Subject Identifier=5002010010-0095

Assay variable : PTINR_VAL1 PT-INR Value

N	Mean	Standard Deviation	Minimum	Maximum
2	2.0000000	0.1979899	1.8600000	2.1400000

Laboratory tests at the time of obtaining Table 2-6 consent, according to the presence or absence of proton-pump inhibitors

Unique Subject Identifier=5004000111-0003

MEANS Procedure

Assay variable : PTINR_VAL1 PT-INR Value

N	Mean	Standard Deviation	Minimum	Maximum
6	2.0816667	0.3817023	1.3900000	2.5000000

Unique Subject Identifier=5004000111-0008

Assay variable : PTINR_VAL1 PT-INR Value

N	Mean	Standard Deviation	Minimum	Maximum
4	2.0650000	0.5967970	1.6500000	2.9200000

Unique Subject Identifier=5004000111-0012

Assay variable : PTINR_VAL1 PT-INR Value

N	Mean	Standard Deviation	Minimum	Maximum
3	2.1733333	0.2052641	2.0000000	2.4000000

Unique Subject Identifier=5004000111-0016

Assay variable : PTINR_VAL1 PT-INR Value

N	Mean	Standard Deviation	Minimum	Maximum
4	2.0350000	0.1181807	1.9500000	2.2100000

Laboratory tests at the time of obtaining Table 2-6 consent, according to the presence or absence of proton-pump inhibitors

Unique Subject Identifier=5004000111-0019

MEANS Procedure

Assay variable : PTINR_VAL1 PT-INR Value

N	Mean	Standard Deviation	Minimum	Maximum
6	2.2416667	0.7647069	1.7100000	3.7600000

Unique Subject Identifier=5004000111-0022

Assay variable : PTINR_VAL1 PT-INR Value

N	Mean	Standard Deviation	Minimum	Maximum
6	1.6416667	0.1238413	1.4400000	1.7900000

Unique Subject Identifier=5004000111-0026

Assay variable : PTINR_VAL1 PT-INR Value

N	Mean	Standard Deviation	Minimum	Maximum
5	1.9580000	0.9992847	1.0200000	3.2700000

Unique Subject Identifier=5004000111-0031

Assay variable : PTINR_VAL1 PT-INR Value

N	Mean	Standard Deviation	Minimum	Maximum
2	1.8100000	0.1697056	1.6900000	1.9300000

Laboratory tests at the time of obtaining Table 2-6 consent, according to the presence or absence of proton-pump inhibitors

Unique Subject Identifier=5004000111-0035

MEANS Procedure

Assay variable : PTINR_VAL1 PT-INR Value

N	Mean	Standard Deviation	Minimum	Maximum
6	1.9033333	0.3640696	1.4700000	2.3700000

Unique Subject Identifier=5004000111-0039

Assay variable : PTINR_VAL1 PT-INR Value

N	Mean	Standard Deviation	Minimum	Maximum
3	2.0966667	0.5300314	1.4900000	2.4700000

Unique Subject Identifier=5004000111-0041

Assay variable : PTINR_VAL1 PT-INR Value

N	Mean	Standard Deviation	Minimum	Maximum
4	1.7425000	0.0607591	1.6900000	1.8300000

Unique Subject Identifier=5004000111-0043

Assay variable : PTINR_VAL1 PT-INR Value

N	Mean	Standard Deviation	Minimum	Maximum
2	1.5650000	0.0636396	1.5200000	1.6100000

Laboratory tests at the time of obtaining Table 2-6 consent, according to the presence or absence of proton-pump inhibitors

Unique Subject Identifier=5004000111-0047

MEANS Procedure

Assay variable : PTINR_VAL1 PT-INR Value

N	Mean	Standard Deviation	Minimum	Maximum
4	1.9275000	0.1908533	1.7700000	2.1700000

Unique Subject Identifier=5004000111-0052

Assay variable : PTINR_VAL1 PT-INR Value

N	Mean	Standard Deviation	Minimum	Maximum
6	1.7916667	0.2430158	1.4900000	2.1800000

Unique Subject Identifier=5004000111-0057

Assay variable : PTINR_VAL1 PT-INR Value

N	Mean	Standard Deviation	Minimum	Maximum
4	2.5250000	0.8266599	1.6600000	3.6500000

Unique Subject Identifier=5004000111-0061

Assay variable : PTINR_VAL1 PT-INR Value

N	Mean	Standard Deviation	Minimum	Maximum
6	1.7600000	0.0834266	1.6600000	1.8700000

Laboratory tests at the time of obtaining Table 2-6 consent, according to the presence or absence of proton-pump inhibitors

Unique Subject Identifier=5004000111-0071

MEANS Procedure

Assay variable : PTINR_VAL1 PT-INR Value

N	Mean	Standard Deviation	Minimum	Maximum
4	2.7925000	0.6411643	1.9500000	3.4900000

Unique Subject Identifier=5004000111-0072

Assay variable : PTINR_VAL1 PT-INR Value

N	Mean	Standard Deviation	Minimum	Maximum
5	1.8100000	0.2359025	1.4800000	2.1100000

Unique Subject Identifier=5004000111-0078

Assay variable : PTINR_VAL1 PT-INR Value

N	Mean	Standard Deviation	Minimum	Maximum
4	1.9325000	0.3089094	1.5000000	2.2200000

Unique Subject Identifier=5004000111-0080

Assay variable : PTINR_VAL1 PT-INR Value

N	Mean	Standard Deviation	Minimum	Maximum
3	2.2766667	0.1553491	2.1500000	2.4500000

Laboratory tests at the time of obtaining Table 2-6 consent, according to the presence or absence of proton-pump inhibitors

Unique Subject Identifier=5007111000-0005

MEANS Procedure

Assay variable : PTINR_VAL1 PT-INR Value

N	Mean	Standard Deviation	Minimum	Maximum
3	1.8800000	0.2251666	1.6200000	2.0100000

Unique Subject Identifier=5007111000-0008

Assay variable : PTINR_VAL1 PT-INR Value

N	Mean	Standard Deviation	Minimum	Maximum
3	1.8800000	0.2251666	1.6200000	2.0100000

Unique Subject Identifier=5007111000-0010

Assay variable : PTINR_VAL1 PT-INR Value

N	Mean	Standard Deviation	Minimum	Maximum
3	1.9666667	0.0305505	1.9400000	2.0000000

Unique Subject Identifier=5007111000-0012

Assay variable : PTINR_VAL1 PT-INR Value

N	Mean	Standard Deviation	Minimum	Maximum
3	2.0566667	0.0680686	1.9800000	2.1100000

Laboratory tests at the time of obtaining Table 2-6 consent, according to the presence or absence of proton-pump inhibitors

Unique Subject Identifier=5007111000-0037

MEANS Procedure

Assay variable : PTINR_VAL1 PT-INR Value

N	Mean	Standard Deviation	Minimum	Maximum
3	1.9300000	0.0871780	1.8300000	1.9900000

Unique Subject Identifier=5007111000-0038

Assay variable : PTINR_VAL1 PT-INR Value

N	Mean	Standard Deviation	Minimum	Maximum
3	2.0533333	0.1569501	1.9300000	2.2300000

Unique Subject Identifier=5007111000-0044

Assay variable : PTINR_VAL1 PT-INR Value

N	Mean	Standard Deviation	Minimum	Maximum
4	2.0825000	0.5145467	1.3300000	2.4900000

Unique Subject Identifier=5007111000-0049

Assay variable : PTINR_VAL1 PT-INR Value

N	Mean	Standard Deviation	Minimum	Maximum
6	2.3400000	0.5876053	1.7700000	3.4400000

Laboratory tests at the time of obtaining Table 2-6 consent, according to the presence or absence of proton-pump inhibitors

Unique Subject Identifier=5007111000-0055

MEANS Procedure

Assay variable : PTINR_VAL1 PT-INR Value

N	Mean	Standard Deviation	Minimum	Maximum
5	1.9460000	0.2939898	1.6900000	2.4400000

Unique Subject Identifier=5007111000-0058

Assay variable : PTINR_VAL1 PT-INR Value

N	Mean	Standard Deviation	Minimum	Maximum
5	2.0260000	0.4407720	1.3300000	2.4500000

Unique Subject Identifier=5008011111-0007

Assay variable : PTINR_VAL1 PT-INR Value

N	Mean	Standard Deviation	Minimum	Maximum
5	2.3140000	0.5144706	1.8600000	3.2000000

Unique Subject Identifier=5008011111-0009

Assay variable : PTINR_VAL1 PT-INR Value

N	Mean	Standard Deviation	Minimum	Maximum
5	2.1320000	0.2678992	1.8300000	2.4600000

Laboratory tests at the time of obtaining Table 2-6 consent, according to the presence or absence of proton-pump inhibitors

Unique Subject Identifier=5008011111-0011

MEANS Procedure

Assay variable : PTINR_VAL1 PT-INR Value

N	Mean	Standard Deviation	Minimum	Maximum
4	2.3225000	0.3250000	2.0500000	2.7800000

Unique Subject Identifier=5009000010-0013

Assay variable : PTINR_VAL1 PT-INR Value

N	Mean	Standard Deviation	Minimum	Maximum
6	1.9050000	0.4008865	1.4700000	2.4000000

Unique Subject Identifier=5010000100-0018

Assay variable : PTINR_VAL1 PT-INR Value

N	Mean	Standard Deviation	Minimum	Maximum
1	2.1500000		2.1500000	2.1500000

Unique Subject Identifier=5010000100-0024

Assay variable : PTINR_VAL1 PT-INR Value

N	Mean	Standard Deviation	Minimum	Maximum
2	1.9200000	0.3535534	1.6700000	2.1700000

Laboratory tests at the time of obtaining Table 2-6 consent, according to the presence or absence of proton-pump inhibitors

Unique Subject Identifier=5011010000-0002

MEANS Procedure

Assay variable : PTINR_VAL1 PT-INR Value

N	Mean	Standard Deviation	Minimum	Maximum
5	1.9640000	0.5542833	1.5400000	2.9200000

Unique Subject Identifier=5011010000-0006

Assay variable : PTINR_VAL1 PT-INR Value

N	Mean	Standard Deviation	Minimum	Maximum
4	2.3650000	0.4891830	1.8600000	3.0000000

Unique Subject Identifier=5011010000-0014

Assay variable : PTINR_VAL1 PT-INR Value

N	Mean	Standard Deviation	Minimum	Maximum
5	1.7880000	0.2721580	1.3400000	2.0000000

Unique Subject Identifier=5011010000-0016

Assay variable : PTINR_VAL1 PT-INR Value

N	Mean	Standard Deviation	Minimum	Maximum
6	1.9633333	0.2535087	1.6300000	2.3000000

Laboratory tests at the time of obtaining Table 2-6 consent, according to the presence or absence of proton-pump inhibitors

Unique Subject Identifier=5011010000-0020

MEANS Procedure

Assay variable : PTINR_VAL1 PT-INR Value

N	Mean	Standard Deviation	Minimum	Maximum
6	1.6966667	0.3229654	1.4100000	2.1600000

Unique Subject Identifier=5011010000-0026

Assay variable : PTINR_VAL1 PT-INR Value

N	Mean	Standard Deviation	Minimum	Maximum
6	2.0316667	0.6728274	1.4900000	3.1100000

Unique Subject Identifier=5011010000-0045

Assay variable : PTINR_VAL1 PT-INR Value

N	Mean	Standard Deviation	Minimum	Maximum
5	1.8460000	0.4565413	1.2900000	2.3300000

Unique Subject Identifier=5011010000-0046

Assay variable : PTINR_VAL1 PT-INR Value

N	Mean	Standard Deviation	Minimum	Maximum
3	1.9400000	0.3204684	1.6300000	2.2700000

Laboratory tests at the time of obtaining Table 2-6 consent, according to the presence or absence of proton-pump inhibitors

Unique Subject Identifier=5012000000-0001

MEANS Procedure

Assay variable : PTINR_VAL1 PT-INR Value

N	Mean	Standard Deviation	Minimum	Maximum
6	2.5500000	0.4408174	2.1200000	3.2000000

Unique Subject Identifier=5012000000-0006

Assay variable : PTINR_VAL1 PT-INR Value

N	Mean	Standard Deviation	Minimum	Maximum
5	2.0240000	0.4243583	1.5900000	2.5900000

Unique Subject Identifier=5012000000-0007

Assay variable : PTINR_VAL1 PT-INR Value

N	Mean	Standard Deviation	Minimum	Maximum
6	2.1583333	0.5896920	1.6700000	3.1100000

Unique Subject Identifier=5012000000-0013

Assay variable : PTINR_VAL1 PT-INR Value

N	Mean	Standard Deviation	Minimum	Maximum
6	1.5616667	0.1090718	1.3900000	1.6900000

Laboratory tests at the time of obtaining Table 2-6 consent, according to the presence or absence of proton-pump inhibitors

Unique Subject Identifier=5012000000-0015

MEANS Procedure

Assay variable : PTINR_VAL1 PT-INR Value

N	Mean	Standard Deviation	Minimum	Maximum
4	2.2625000	0.3517930	1.8300000	2.6800000

Unique Subject Identifier=5012000000-0017

Assay variable : PTINR_VAL1 PT-INR Value

N	Mean	Standard Deviation	Minimum	Maximum
5	1.8700000	0.3196091	1.5100000	2.2000000

Unique Subject Identifier=5012000000-0018

Assay variable : PTINR_VAL1 PT-INR Value

N	Mean	Standard Deviation	Minimum	Maximum
6	1.5150000	0.2935132	1.1700000	1.8000000

Unique Subject Identifier=5012000000-0023

Assay variable : PTINR_VAL1 PT-INR Value

N	Mean	Standard Deviation	Minimum	Maximum
3	2.0266667	0.1250333	1.9000000	2.1500000

Laboratory tests at the time of obtaining Table 2-6 consent, according to the presence or absence of proton-pump inhibitors

Unique Subject Identifier=5012000000-0028

MEANS Procedure

Assay variable : PTINR_VAL1 PT-INR Value

N	Mean	Standard Deviation	Minimum	Maximum
4	1.9950000	0.0580230	1.9300000	2.0700000

Unique Subject Identifier=5013000000-0042

Assay variable : PTINR_VAL1 PT-INR Value

N	Mean	Standard Deviation	Minimum	Maximum
3	1.9866667	0.2657693	1.6800000	2.1500000

Unique Subject Identifier=5013000000-0059

Assay variable : PTINR_VAL1 PT-INR Value

N	Mean	Standard Deviation	Minimum	Maximum
3	2.2000000	0.0556776	2.1400000	2.2500000

Unique Subject Identifier=5013000000-0063

Assay variable : PTINR_VAL1 PT-INR Value

N	Mean	Standard Deviation	Minimum	Maximum
2	1.7350000	0.1060660	1.6600000	1.8100000

Laboratory tests at the time of obtaining Table 2-6 consent, according to the presence or absence of proton-pump inhibitors

Unique Subject Identifier=5013000000-0132

MEANS Procedure

Assay variable : PTINR_VAL1 PT-INR Value

N	Mean	Standard Deviation	Minimum	Maximum
6	1.8350000	0.2517737	1.4700000	2.1300000

Unique Subject Identifier=5013000000-0143

Assay variable : PTINR_VAL1 PT-INR Value

N	Mean	Standard Deviation	Minimum	Maximum
3	1.8100000	0.2685144	1.5000000	1.9700000

Unique Subject Identifier=5013000000-0156

Assay variable : PTINR_VAL1 PT-INR Value

N	Mean	Standard Deviation	Minimum	Maximum
5	1.9140000	0.1687602	1.7400000	2.1400000

Unique Subject Identifier=5013000000-0166

Assay variable : PTINR_VAL1 PT-INR Value

N	Mean	Standard Deviation	Minimum	Maximum
6	2.1516667	0.2797439	1.7400000	2.5400000

Laboratory tests at the time of obtaining Table 2-6 consent, according to the presence or absence of proton-pump inhibitors

Unique Subject Identifier=5013000000-0217

MEANS Procedure

Assay variable : PTINR_VAL1 PT-INR Value

N	Mean	Standard Deviation	Minimum	Maximum
3	2.3033333	0.3286842	1.9500000	2.6000000

Unique Subject Identifier=5013000000-0262

Assay variable : PTINR_VAL1 PT-INR Value

N	Mean	Standard Deviation	Minimum	Maximum
2	1.6900000	0.1555635	1.5800000	1.8000000

Unique Subject Identifier=5013000000-0279

Assay variable : PTINR_VAL1 PT-INR Value

N	Mean	Standard Deviation	Minimum	Maximum
6	1.7266667	0.2202423	1.5800000	2.1700000

Unique Subject Identifier=5013000000-0283

Assay variable : PTINR_VAL1 PT-INR Value

N	Mean	Standard Deviation	Minimum	Maximum
5	2.2880000	0.1943451	2.1600000	2.6300000

Laboratory tests at the time of obtaining Table 2-6 consent, according to the presence or absence of proton-pump inhibitors

Unique Subject Identifier=5013000000-0286

MEANS Procedure

Assay variable : PTINR_VAL1 PT-INR Value

N	Mean	Standard Deviation	Minimum	Maximum
2	2.4100000	0.2687006	2.2200000	2.6000000

Unique Subject Identifier=5013000000-0297

Assay variable : PTINR_VAL1 PT-INR Value

N	Mean	Standard Deviation	Minimum	Maximum
6	1.8650000	0.3950063	1.0800000	2.1300000

Unique Subject Identifier=5013000000-0304

Assay variable : PTINR_VAL1 PT-INR Value

N	Mean	Standard Deviation	Minimum	Maximum
6	1.7016667	0.1655798	1.4900000	1.9700000

Unique Subject Identifier=5013000000-0318

Assay variable : PTINR_VAL1 PT-INR Value

N	Mean	Standard Deviation	Minimum	Maximum
4	2.1500000	0.2152518	1.8400000	2.3100000

Laboratory tests at the time of obtaining Table 2-6 consent, according to the presence or absence of proton-pump inhibitors

Unique Subject Identifier=5013000000-0323

MEANS Procedure

Assay variable : PTINR_VAL1 PT-INR Value

N	Mean	Standard Deviation	Minimum	Maximum
6	1.4400000	0.2419917	1.1500000	1.7500000

Unique Subject Identifier=5013000000-0330

Assay variable : PTINR_VAL1 PT-INR Value

N	Mean	Standard Deviation	Minimum	Maximum
6	2.4500000	0.3728270	1.9000000	3.0000000

Unique Subject Identifier=5013000000-0347

Assay variable : PTINR_VAL1 PT-INR Value

N	Mean	Standard Deviation	Minimum	Maximum
5	1.3920000	0.2519325	1.0800000	1.7600000

Unique Subject Identifier=5013000000-0355

Assay variable : PTINR_VAL1 PT-INR Value

N	Mean	Standard Deviation	Minimum	Maximum
3	2.2200000	0.4744470	1.6800000	2.5700000

Laboratory tests at the time of obtaining Table 2-6 consent, according to the presence or absence of proton-pump inhibitors

Unique Subject Identifier=5013000000-0401

MEANS Procedure

Assay variable : PTINR_VAL1 PT-INR Value

N	Mean	Standard Deviation	Minimum	Maximum
1	2.0600000		2.0600000	2.0600000

Unique Subject Identifier=5013000000-0426

Assay variable : PTINR_VAL1 PT-INR Value

N	Mean	Standard Deviation	Minimum	Maximum
3	1.8133333	0.2013289	1.6000000	2.0000000

Unique Subject Identifier=5013000000-0435

Assay variable : PTINR_VAL1 PT-INR Value

N	Mean	Standard Deviation	Minimum	Maximum
2	1.9650000	0.1060660	1.8900000	2.0400000

Unique Subject Identifier=5013000000-0465

Assay variable : PTINR_VAL1 PT-INR Value

N	Mean	Standard Deviation	Minimum	Maximum
6	1.9433333	0.2307957	1.7000000	2.2800000

Laboratory tests at the time of obtaining Table 2-6 consent, according to the presence or absence of proton-pump inhibitors

Unique Subject Identifier=5013000000-0483

MEANS Procedure

Assay variable : PTINR_VAL1 PT-INR Value

N	Mean	Standard Deviation	Minimum	Maximum
4	2.4825000	0.1284199	2.3800000	2.6700000

Unique Subject Identifier=5013000000-0508

Assay variable : PTINR_VAL1 PT-INR Value

N	Mean	Standard Deviation	Minimum	Maximum
3	1.9533333	0.0709460	1.8900000	2.0300000

Unique Subject Identifier=5013000000-0518

Assay variable : PTINR_VAL1 PT-INR Value

N	Mean	Standard Deviation	Minimum	Maximum
4	2.4150000	0.3169122	2.0800000	2.7100000

Unique Subject Identifier=5013000000-0523

Assay variable : PTINR_VAL1 PT-INR Value

N	Mean	Standard Deviation	Minimum	Maximum
1	1.2200000		1.2200000	1.2200000

Laboratory tests at the time of obtaining Table 2-6 consent, according to the presence or absence of proton-pump inhibitors

Unique Subject Identifier=5013000000-0547

MEANS Procedure

Assay variable : PTINR_VAL1 PT-INR Value

N	Mean	Standard Deviation	Minimum	Maximum
2	1.3950000	0.1626346	1.2800000	1.5100000

Unique Subject Identifier=5013000000-0553

Assay variable : PTINR_VAL1 PT-INR Value

N	Mean	Standard Deviation	Minimum	Maximum
6	1.3106667	0.2588024	1.1100000	1.8000000

Unique Subject Identifier=5013000000-0556

Assay variable : PTINR_VAL1 PT-INR Value

N	Mean	Standard Deviation	Minimum	Maximum
1	1.5300000		1.5300000	1.5300000

Unique Subject Identifier=5013000000-0568

Assay variable : PTINR_VAL1 PT-INR Value

N	Mean	Standard Deviation	Minimum	Maximum
6	1.2783333	0.4282717	0.9300000	1.8400000

Laboratory tests at the time of obtaining Table 2-6 consent, according to the presence or absence of proton-pump inhibitors

Unique Subject Identifier=5013000000-0589

MEANS Procedure

Assay variable : PTINR_VAL1 PT-INR Value

N	Mean	Standard Deviation	Minimum	Maximum
6	1.2466667	0.2231293	0.9900000	1.6100000

Unique Subject Identifier=5013000000-0595

Assay variable : PTINR_VAL1 PT-INR Value

N	Mean	Standard Deviation	Minimum	Maximum
6	2.3666667	0.4015304	1.9400000	2.9900000

Unique Subject Identifier=5014010001-0009

Assay variable : PTINR_VAL1 PT-INR Value

N	Mean	Standard Deviation	Minimum	Maximum
3	1.7433333	0.2059935	1.5500000	1.9600000

Unique Subject Identifier=5014010001-0013

Assay variable : PTINR_VAL1 PT-INR Value

N	Mean	Standard Deviation	Minimum	Maximum
1	1.2500000		1.2500000	1.2500000

Laboratory tests at the time of obtaining Table 2-6 consent, according to the presence or absence of proton-pump inhibitors

Unique Subject Identifier=5014010001-0016

MEANS Procedure

Assay variable : PTINR_VAL1 PT-INR Value

N	Mean	Standard Deviation	Minimum	Maximum
5	1.9560000	0.3299697	1.6000000	2.5000000

Unique Subject Identifier=5014010001-0020

Assay variable : PTINR_VAL1 PT-INR Value

N	Mean	Standard Deviation	Minimum	Maximum
5	2.2520000	0.3851234	1.8500000	2.8700000

Unique Subject Identifier=5014010001-0022

Assay variable : PTINR_VAL1 PT-INR Value

N	Mean	Standard Deviation	Minimum	Maximum
5	2.4280000	0.2007984	2.1800000	2.6600000

Unique Subject Identifier=5014010001-0027

Assay variable : PTINR_VAL1 PT-INR Value

N	Mean	Standard Deviation	Minimum	Maximum
3	1.5366667	0.0702377	1.4700000	1.6100000

Laboratory tests at the time of obtaining Table 2-6 consent, according to the presence or absence of proton-pump inhibitors

Unique Subject Identifier=5015000000-0014

MEANS Procedure

Assay variable : PTINR_VAL1 PT-INR Value

N	Mean	Standard Deviation	Minimum	Maximum
3	2.3800000	0.3019934	2.1000000	2.7000000

Unique Subject Identifier=5015000000-0017

Assay variable : PTINR_VAL1 PT-INR Value

N	Mean	Standard Deviation	Minimum	Maximum
6	2.4000000	0.1836301	2.0600000	2.5800000

Unique Subject Identifier=5015000000-0021

Assay variable : PTINR_VAL1 PT-INR Value

N	Mean	Standard Deviation	Minimum	Maximum
5	1.8780000	0.2754451	1.4200000	2.1100000

Unique Subject Identifier=5016000000-0004

Assay variable : PTINR_VAL1 PT-INR Value

N	Mean	Standard Deviation	Minimum	Maximum
3	2.4700000	0.8685045	1.5600000	3.2900000

Laboratory tests at the time of obtaining Table 2-6 consent, according to the presence or absence of proton-pump inhibitors

Unique Subject Identifier=5016000000-0007

MEANS Procedure

Assay variable : PTINR_VAL1 PT-INR Value

N	Mean	Standard Deviation	Minimum	Maximum
2	2.0050000	0.3323402	1.7700000	2.2400000

Unique Subject Identifier=5016000000-0008

Assay variable : PTINR_VAL1 PT-INR Value

N	Mean	Standard Deviation	Minimum	Maximum
4	2.4450000	0.2111082	2.1300000	2.5800000

Unique Subject Identifier=5016000000-0013

Assay variable : PTINR_VAL1 PT-INR Value

N	Mean	Standard Deviation	Minimum	Maximum
4	1.2800000	0.1606238	1.0600000	1.4300000

Unique Subject Identifier=5016000000-0014

Assay variable : PTINR_VAL1 PT-INR Value

N	Mean	Standard Deviation	Minimum	Maximum
4	2.1325000	0.1918984	2.0300000	2.4200000

Laboratory tests at the time of obtaining Table 2-6 consent, according to the presence or absence of proton-pump inhibitors

Unique Subject Identifier=5016000000-0019

MEANS Procedure

Assay variable : PTINR_VAL1 PT-INR Value

N	Mean	Standard Deviation	Minimum	Maximum
3	2.8533333	0.0808290	2.7800000	2.9400000

Unique Subject Identifier=5016000000-0020

Assay variable : PTINR_VAL1 PT-INR Value

N	Mean	Standard Deviation	Minimum	Maximum
3	2.2800000	0.5765414	1.7600000	2.9000000

Unique Subject Identifier=5016000000-0023

Assay variable : PTINR_VAL1 PT-INR Value

N	Mean	Standard Deviation	Minimum	Maximum
1	1.6600000		1.6600000	1.6600000

Unique Subject Identifier=5016000000-0026

Assay variable : PTINR_VAL1 PT-INR Value

N	Mean	Standard Deviation	Minimum	Maximum
3	2.5066667	0.5472050	1.9900000	3.0800000

Laboratory tests at the time of obtaining Table 2-6 consent, according to the presence or absence of proton-pump inhibitors

Unique Subject Identifier=5016000000-0029

MEANS Procedure

Assay variable : PTINR_VAL1 PT-INR Value

N	Mean	Standard Deviation	Minimum	Maximum
1	1.6700000		1.6700000	1.6700000

Unique Subject Identifier=5016000000-0030

Assay variable : PTINR_VAL1 PT-INR Value

N	Mean	Standard Deviation	Minimum	Maximum
4	2.1525000	0.2244066	1.8500000	2.3800000

Unique Subject Identifier=5016000000-0039

Assay variable : PTINR_VAL1 PT-INR Value

N	Mean	Standard Deviation	Minimum	Maximum
2	2.2150000	0.2757716	2.0200000	2.4100000

Unique Subject Identifier=5016000000-0057

Assay variable : PTINR_VAL1 PT-INR Value

N	Mean	Standard Deviation	Minimum	Maximum
1	1.9300000		1.9300000	1.9300000

Laboratory tests at the time of obtaining Table 2-6 consent, according to the presence or absence of proton-pump inhibitors

Unique Subject Identifier=5016000000-0059

MEANS Procedure

Assay variable : PTINR_VAL1 PT-INR Value

N	Mean	Standard Deviation	Minimum	Maximum
5	2.5160000	0.4261807	2.2200000	3.2500000

Unique Subject Identifier=5016000000-0062

Assay variable : PTINR_VAL1 PT-INR Value

N	Mean	Standard Deviation	Minimum	Maximum
3	2.0366667	0.5862025	1.6400000	2.7100000

Unique Subject Identifier=5016000000-0073

Assay variable : PTINR_VAL1 PT-INR Value

N	Mean	Standard Deviation	Minimum	Maximum
2	2.2900000	0.1697056	2.1700000	2.4100000

Unique Subject Identifier=5016000000-0077

Assay variable : PTINR_VAL1 PT-INR Value

N	Mean	Standard Deviation	Minimum	Maximum
3	2.6766667	0.3442867	2.4300000	3.0700000

Laboratory tests at the time of obtaining Table 2-6 consent, according to the presence or absence of proton-pump inhibitors

Unique Subject Identifier=5016000000-0078

MEANS Procedure

Assay variable : PTINR_VAL1 PT-INR Value

N	Mean	Standard Deviation	Minimum	Maximum
4	1.7225000	0.3238698	1.2800000	2.0100000

Unique Subject Identifier=5016000000-0083

Assay variable : PTINR_VAL1 PT-INR Value

N	Mean	Standard Deviation	Minimum	Maximum
4	1.8875000	0.2458150	1.6900000	2.2300000

Unique Subject Identifier=5016000000-0093

Assay variable : PTINR_VAL1 PT-INR Value

N	Mean	Standard Deviation	Minimum	Maximum
5	1.9360000	0.2903963	1.6300000	2.2900000

Unique Subject Identifier=5016000000-0098

Assay variable : PTINR_VAL1 PT-INR Value

N	Mean	Standard Deviation	Minimum	Maximum
3	2.1166667	0.4239497	1.7300000	2.5700000

Laboratory tests at the time of obtaining Table 2-6 consent, according to the presence or absence of proton-pump inhibitors

Unique Subject Identifier=5016000000-0100

MEANS Procedure

Assay variable : PTINR_VAL1 PT-INR Value

N	Mean	Standard Deviation	Minimum	Maximum
3	1.8866667	0.0901850	1.8000000	1.9800000

Unique Subject Identifier=5016000000-0101

Assay variable : PTINR_VAL1 PT-INR Value

N	Mean	Standard Deviation	Minimum	Maximum
3	2.1433333	0.2400694	1.9000000	2.3800000

Unique Subject Identifier=5016000000-0102

Assay variable : PTINR_VAL1 PT-INR Value

N	Mean	Standard Deviation	Minimum	Maximum
3	2.3733333	0.0378594	2.3300000	2.4000000

Unique Subject Identifier=5016000000-0107

Assay variable : PTINR_VAL1 PT-INR Value

N	Mean	Standard Deviation	Minimum	Maximum
3	1.9866667	0.1950214	1.7900000	2.1800000

Laboratory tests at the time of obtaining Table 2-6 consent, according to the presence or absence of proton-pump inhibitors

Unique Subject Identifier=5016000000-0111

MEANS Procedure

Assay variable : PTINR_VAL1 PT-INR Value

N	Mean	Standard Deviation	Minimum	Maximum
4	1.6925000	0.2677530	1.4200000	2.0600000

Unique Subject Identifier=5016000000-0116

Assay variable : PTINR_VAL1 PT-INR Value

N	Mean	Standard Deviation	Minimum	Maximum
4	1.8425000	0.2160825	1.5200000	1.9800000

Unique Subject Identifier=5016000000-0117

Assay variable : PTINR_VAL1 PT-INR Value

N	Mean	Standard Deviation	Minimum	Maximum
3	1.7933333	0.1882374	1.6700000	2.0100000

Unique Subject Identifier=5016000000-0120

Assay variable : PTINR_VAL1 PT-INR Value

N	Mean	Standard Deviation	Minimum	Maximum
2	2.2850000	0.2899138	2.0800000	2.4900000

Laboratory tests at the time of obtaining Table 2-6 consent, according to the presence or absence of proton-pump inhibitors

Unique Subject Identifier=5016000000-0122

MEANS Procedure

Assay variable : PTINR_VAL1 PT-INR Value

N	Mean	Standard Deviation	Minimum	Maximum
3	1.6133333	0.2157159	1.4600000	1.8600000

Unique Subject Identifier=5016000000-0124

Assay variable : PTINR_VAL1 PT-INR Value

N	Mean	Standard Deviation	Minimum	Maximum
3	1.8066667	0.1305118	1.6600000	1.9100000

Unique Subject Identifier=5016000000-0125

Assay variable : PTINR_VAL1 PT-INR Value

N	Mean	Standard Deviation	Minimum	Maximum
1	2.1000000		2.1000000	2.1000000

Unique Subject Identifier=5016000000-0128

Assay variable : PTINR_VAL1 PT-INR Value

N	Mean	Standard Deviation	Minimum	Maximum
4	1.7575000	0.3929695	1.3000000	2.1000000

Laboratory tests at the time of obtaining Table 2-6 consent, according to the presence or absence of proton-pump inhibitors

Unique Subject Identifier=5016000000-0129

MEANS Procedure

Assay variable : PTINR_VAL1 PT-INR Value

N	Mean	Standard Deviation	Minimum	Maximum
4	1.9700000	0.5846366	1.4400000	2.7300000

Unique Subject Identifier=5016000000-0131

Assay variable : PTINR_VAL1 PT-INR Value

N	Mean	Standard Deviation	Minimum	Maximum
3	2.2300000	0.1442221	2.0700000	2.3500000

Unique Subject Identifier=5016000000-0140

Assay variable : PTINR_VAL1 PT-INR Value

N	Mean	Standard Deviation	Minimum	Maximum
3	2.2766667	0.7696969	1.7500000	3.1600000

Unique Subject Identifier=5016000000-0141

Assay variable : PTINR_VAL1 PT-INR Value

N	Mean	Standard Deviation	Minimum	Maximum
3	1.9566667	0.4554485	1.4900000	2.4000000

Laboratory tests at the time of obtaining Table 2-6 consent, according to the presence or absence of proton-pump inhibitors

Unique Subject Identifier=5016000000-0148

MEANS Procedure

Assay variable : PTINR_VAL1 PT-INR Value

N	Mean	Standard Deviation	Minimum	Maximum
3	2.4966667	0.8981277	1.7000000	3.4700000

Unique Subject Identifier=5016000000-0149

Assay variable : PTINR_VAL1 PT-INR Value

N	Mean	Standard Deviation	Minimum	Maximum
3	2.3366667	0.7788667	1.8000000	3.2300000

Unique Subject Identifier=5016000000-0155

Assay variable : PTINR_VAL1 PT-INR Value

N	Mean	Standard Deviation	Minimum	Maximum
5	1.8480000	0.1084896	1.6600000	1.9300000

Unique Subject Identifier=5016000000-0159

Assay variable : PTINR_VAL1 PT-INR Value

N	Mean	Standard Deviation	Minimum	Maximum
3	2.0733333	0.3056687	1.7800000	2.3900000

Laboratory tests at the time of obtaining Table 2-6 consent, according to the presence or absence of proton-pump inhibitors

Unique Subject Identifier=5016000000-0177

MEANS Procedure

Assay variable : PTINR_VAL1 PT-INR Value

N	Mean	Standard Deviation	Minimum	Maximum
6	1.8883333	0.3213980	1.4900000	2.3000000

Unique Subject Identifier=5016000000-0178

Assay variable : PTINR_VAL1 PT-INR Value

N	Mean	Standard Deviation	Minimum	Maximum
4	2.1100000	0.2019901	1.9100000	2.3900000

Unique Subject Identifier=5016000000-0179

Assay variable : PTINR_VAL1 PT-INR Value

N	Mean	Standard Deviation	Minimum	Maximum
4	2.0625000	0.1426826	1.8500000	2.1500000

Unique Subject Identifier=5016000000-0182

Assay variable : PTINR_VAL1 PT-INR Value

N	Mean	Standard Deviation	Minimum	Maximum
2	2.1800000	0.0565685	2.1400000	2.2200000

Laboratory tests at the time of obtaining Table 2-6 consent, according to the presence or absence of proton-pump inhibitors

Unique Subject Identifier=5016000000-0184

MEANS Procedure

Assay variable : PTINR_VAL1 PT-INR Value

N	Mean	Standard Deviation	Minimum	Maximum
1	1.4100000		1.4100000	1.4100000

Unique Subject Identifier=5016000000-0187

Assay variable : PTINR_VAL1 PT-INR Value

N	Mean	Standard Deviation	Minimum	Maximum
4	1.6200000	0.1089342	1.5300000	1.7500000

Unique Subject Identifier=5016000000-0191

Assay variable : PTINR_VAL1 PT-INR Value

N	Mean	Standard Deviation	Minimum	Maximum
3	2.0533333	0.1101514	1.9800000	2.1800000

Unique Subject Identifier=5016000000-0207

Assay variable : PTINR_VAL1 PT-INR Value

N	Mean	Standard Deviation	Minimum	Maximum
2	1.8150000	0.2757716	1.6200000	2.0100000

Laboratory tests at the time of obtaining Table 2-6 consent, according to the presence or absence of proton-pump inhibitors

Unique Subject Identifier=5016000000-0211

MEANS Procedure

Assay variable : PTINR_VAL1 PT-INR Value

N	Mean	Standard Deviation	Minimum	Maximum
6	2.3150000	0.7912206	1.6700000	3.8300000

Unique Subject Identifier=5016000000-0222

Assay variable : PTINR_VAL1 PT-INR Value

N	Mean	Standard Deviation	Minimum	Maximum
3	2.4900000	0.5367495	1.8800000	2.8900000

Unique Subject Identifier=5016000000-0235

Assay variable : PTINR_VAL1 PT-INR Value

N	Mean	Standard Deviation	Minimum	Maximum
6	2.0733333	0.6205052	1.5000000	3.2800000

Unique Subject Identifier=5016000000-0237

Assay variable : PTINR_VAL1 PT-INR Value

N	Mean	Standard Deviation	Minimum	Maximum
3	1.7733333	0.1607275	1.5900000	1.8900000

Laboratory tests at the time of obtaining Table 2-6 consent, according to the presence or absence of proton-pump inhibitors

Unique Subject Identifier=5016000000-0238

MEANS Procedure

Assay variable : PTINR_VAL1 PT-INR Value

N	Mean	Standard Deviation	Minimum	Maximum
4	1.7250000	0.1206924	1.6400000	1.9000000

Unique Subject Identifier=5016000000-0240

Assay variable : PTINR_VAL1 PT-INR Value

N	Mean	Standard Deviation	Minimum	Maximum
4	2.0850000	0.3399510	1.7500000	2.5500000

Unique Subject Identifier=5016000000-0241

Assay variable : PTINR_VAL1 PT-INR Value

N	Mean	Standard Deviation	Minimum	Maximum
3	2.6233333	0.9609544	2.0000000	3.7300000

Unique Subject Identifier=5016000000-0244

Assay variable : PTINR_VAL1 PT-INR Value

N	Mean	Standard Deviation	Minimum	Maximum
3	1.5933333	0.0680686	1.5400000	1.6700000

Laboratory tests at the time of obtaining Table 2-6 consent, according to the presence or absence of proton-pump inhibitors

Unique Subject Identifier=5016000000-0248

MEANS Procedure

Assay variable : PTINR_VAL1 PT-INR Value

N	Mean	Standard Deviation	Minimum	Maximum
3	1.9166667	0.2685765	1.6100000	2.1100000

Unique Subject Identifier=5016000000-0249

Assay variable : PTINR_VAL1 PT-INR Value

N	Mean	Standard Deviation	Minimum	Maximum
5	2.5460000	0.9822067	2.0500000	4.3000000

Unique Subject Identifier=5016000000-0252

Assay variable : PTINR_VAL1 PT-INR Value

N	Mean	Standard Deviation	Minimum	Maximum
3	2.3000000	0.1153256	2.2100000	2.4300000

Unique Subject Identifier=5016000000-0258

Assay variable : PTINR_VAL1 PT-INR Value

N	Mean	Standard Deviation	Minimum	Maximum
1	2.1800000		2.1800000	2.1800000

Laboratory tests at the time of obtaining Table 2-6 consent, according to the presence or absence of proton-pump inhibitors

Unique Subject Identifier=5016000000-0263

MEANS Procedure

Assay variable : PTINR_VAL1 PT-INR Value

N	Mean	Standard Deviation	Minimum	Maximum
1	1.4500000		1.4500000	1.4500000

Unique Subject Identifier=5016000000-0264

Assay variable : PTINR_VAL1 PT-INR Value

N	Mean	Standard Deviation	Minimum	Maximum
4	2.0650000	0.1506652	1.9300000	2.2100000

Unique Subject Identifier=5016000000-0267

Assay variable : PTINR_VAL1 PT-INR Value

N	Mean	Standard Deviation	Minimum	Maximum
5	1.8500000	0.4913247	1.4300000	2.6900000

Unique Subject Identifier=5016000000-0273

Assay variable : PTINR_VAL1 PT-INR Value

N	Mean	Standard Deviation	Minimum	Maximum
3	1.6266667	0.8285731	0.6800000	2.2200000

Laboratory tests at the time of obtaining Table 2-6 consent, according to the presence or absence of proton-pump inhibitors

Unique Subject Identifier=5016000000-0275

MEANS Procedure

Assay variable : PTINR_VAL1 PT-INR Value

N	Mean	Standard Deviation	Minimum	Maximum
3	2.0166667	0.0602771	1.9600000	2.0800000

Unique Subject Identifier=5016000000-0277

Assay variable : PTINR_VAL1 PT-INR Value

N	Mean	Standard Deviation	Minimum	Maximum
3	2.0033333	0.1903506	1.8200000	2.2000000

Unique Subject Identifier=5016000000-0279

Assay variable : PTINR_VAL1 PT-INR Value

N	Mean	Standard Deviation	Minimum	Maximum
2	2.1550000	0.8697413	1.5400000	2.7700000

Unique Subject Identifier=5016000000-0280

Assay variable : PTINR_VAL1 PT-INR Value

N	Mean	Standard Deviation	Minimum	Maximum
2	1.2850000	0.3606245	1.0300000	1.5400000

Laboratory tests at the time of obtaining Table 2-6 consent, according to the presence or absence of proton-pump inhibitors

Unique Subject Identifier=5016000000-0282

MEANS Procedure

Assay variable : PTINR_VAL1 PT-INR Value

N	Mean	Standard Deviation	Minimum	Maximum
2	2.1200000	0.0141421	2.1100000	2.1300000

Unique Subject Identifier=5016000000-0283

Assay variable : PTINR_VAL1 PT-INR Value

N	Mean	Standard Deviation	Minimum	Maximum
3	1.9733333	0.1401190	1.8300000	2.1100000

Unique Subject Identifier=5016000000-0290

Assay variable : PTINR_VAL1 PT-INR Value

N	Mean	Standard Deviation	Minimum	Maximum
4	2.8375000	0.6825626	2.0300000	3.7000000

Unique Subject Identifier=5016000000-0291

Assay variable : PTINR_VAL1 PT-INR Value

N	Mean	Standard Deviation	Minimum	Maximum
1	1.0200000		1.0200000	1.0200000

Laboratory tests at the time of obtaining Table 2-6 consent, according to the presence or absence of proton-pump inhibitors

Unique Subject Identifier=5016000000-0295

MEANS Procedure

Assay variable : PTINR_VAL1 PT-INR Value

N	Mean	Standard Deviation	Minimum	Maximum
3	1.5966667	0.0351188	1.5600000	1.6300000

Unique Subject Identifier=5016000000-0297

Assay variable : PTINR_VAL1 PT-INR Value

N	Mean	Standard Deviation	Minimum	Maximum
4	1.4025000	0.1357387	1.2200000	1.5200000

Unique Subject Identifier=5016000000-0306

Assay variable : PTINR_VAL1 PT-INR Value

N	Mean	Standard Deviation	Minimum	Maximum
2	1.5600000	0.0989949	1.4900000	1.6300000

Unique Subject Identifier=5016000000-0322

Assay variable : PTINR_VAL1 PT-INR Value

N	Mean	Standard Deviation	Minimum	Maximum
1	2.6100000		2.6100000	2.6100000

Laboratory tests at the time of obtaining Table 2-6 consent, according to the presence or absence of proton-pump inhibitors

Unique Subject Identifier=5016000000-0328

MEANS Procedure

Assay variable : PTINR_VAL1 PT-INR Value

N	Mean	Standard Deviation	Minimum	Maximum
6	2.3616667	0.1689280	2.1000000	2.5500000

Unique Subject Identifier=5016000000-0330

Assay variable : PTINR_VAL1 PT-INR Value

N	Mean	Standard Deviation	Minimum	Maximum
1	2.1900000		2.1900000	2.1900000

Unique Subject Identifier=5016000000-0333

Assay variable : PTINR_VAL1 PT-INR Value

N	Mean	Standard Deviation	Minimum	Maximum
3	2.8833333	0.3910669	2.5100000	3.2900000

Unique Subject Identifier=5016000000-0335

Assay variable : PTINR_VAL1 PT-INR Value

N	Mean	Standard Deviation	Minimum	Maximum
3	2.3833333	0.2631223	2.0800000	2.5500000

Laboratory tests at the time of obtaining Table 2-6 consent, according to the presence or absence of proton-pump inhibitors

Unique Subject Identifier=5016000000-0337

MEANS Procedure

Assay variable : PTINR_VAL1 PT-INR Value

N	Mean	Standard Deviation	Minimum	Maximum
2	1.9450000	0.2474874	1.7700000	2.1200000

Unique Subject Identifier=5017011110-0005

Assay variable : PTINR_VAL1 PT-INR Value

N	Mean	Standard Deviation	Minimum	Maximum
2	2.5000000	0.4242641	2.2000000	2.8000000

Unique Subject Identifier=5017011110-0012

Assay variable : PTINR_VAL1 PT-INR Value

N	Mean	Standard Deviation	Minimum	Maximum
4	2.1050000	0.2563201	1.7900000	2.3900000

Unique Subject Identifier=5017011110-0017

Assay variable : PTINR_VAL1 PT-INR Value

N	Mean	Standard Deviation	Minimum	Maximum
6	1.4250000	0.1671825	1.2400000	1.7000000

Laboratory tests at the time of obtaining Table 2-6 consent, according to the presence or absence of proton-pump inhibitors

Unique Subject Identifier=5017011110-0021

MEANS Procedure

Assay variable : PTINR_VAL1 PT-INR Value

N	Mean	Standard Deviation	Minimum	Maximum
5	1.9860000	0.2021880	1.8300000	2.3400000

Unique Subject Identifier=5020011000-0010

Assay variable : PTINR_VAL1 PT-INR Value

N	Mean	Standard Deviation	Minimum	Maximum
3	2.5300000	0.7458552	2.0600000	3.3900000

Unique Subject Identifier=5020011000-0012

Assay variable : PTINR_VAL1 PT-INR Value

N	Mean	Standard Deviation	Minimum	Maximum
3	2.1766667	0.2709859	1.9200000	2.4600000

Unique Subject Identifier=5020011000-0013

Assay variable : PTINR_VAL1 PT-INR Value

N	Mean	Standard Deviation	Minimum	Maximum
3	1.8500000	0.2705550	1.5900000	2.1300000

Laboratory tests at the time of obtaining Table 2-6 consent, according to the presence or absence of proton-pump inhibitors

Unique Subject Identifier=5020011000-0021

MEANS Procedure

Assay variable : PTINR_VAL1 PT-INR Value

N	Mean	Standard Deviation	Minimum	Maximum
3	2.0766667	0.2119748	1.8500000	2.2700000

Unique Subject Identifier=5020011000-0025

Assay variable : PTINR_VAL1 PT-INR Value

N	Mean	Standard Deviation	Minimum	Maximum
3	1.8966667	0.6645550	1.3600000	2.6400000

Unique Subject Identifier=5021011111-0028

Assay variable : PTINR_VAL1 PT-INR Value

N	Mean	Standard Deviation	Minimum	Maximum
6	2.1150000	0.2025586	1.9400000	2.5100000

Unique Subject Identifier=5021011111-0032

Assay variable : PTINR_VAL1 PT-INR Value

N	Mean	Standard Deviation	Minimum	Maximum
5	2.4120000	0.2942278	2.1000000	2.8800000

Laboratory tests at the time of obtaining Table 2-6 consent, according to the presence or absence of proton-pump inhibitors

Unique Subject Identifier=5021011111-0036

MEANS Procedure

Assay variable : PTINR_VAL1 PT-INR Value

N	Mean	Standard Deviation	Minimum	Maximum
4	2.4425000	0.2789713	2.1500000	2.7100000

Unique Subject Identifier=5021011111-0040

Assay variable : PTINR_VAL1 PT-INR Value

N	Mean	Standard Deviation	Minimum	Maximum
3	2.0500000	0.1513275	1.8800000	2.1700000

Unique Subject Identifier=5021011111-0045

Assay variable : PTINR_VAL1 PT-INR Value

N	Mean	Standard Deviation	Minimum	Maximum
4	1.4300000	0.4081666	0.9600000	1.8500000

Unique Subject Identifier=5021011111-0050

Assay variable : PTINR_VAL1 PT-INR Value

N	Mean	Standard Deviation	Minimum	Maximum
3	1.8200000	0.1053565	1.7200000	1.9300000

Laboratory tests at the time of obtaining Table 2-6 consent, according to the presence or absence of proton-pump inhibitors

Unique Subject Identifier=5024000000-0001

MEANS Procedure

Assay variable : PTINR_VAL1 PT-INR Value

N	Mean	Standard Deviation	Minimum	Maximum
2	1.7200000	0.1697056	1.6000000	1.8400000

Unique Subject Identifier=5025000100-0007

Assay variable : PTINR_VAL1 PT-INR Value

N	Mean	Standard Deviation	Minimum	Maximum
6	2.3650000	0.4174087	1.9800000	3.1100000

Unique Subject Identifier=5026101000-0001

Assay variable : PTINR_VAL1 PT-INR Value

N	Mean	Standard Deviation	Minimum	Maximum
4	1.8350000	0.1340398	1.7300000	2.0300000

Unique Subject Identifier=5026101000-0006

Assay variable : PTINR_VAL1 PT-INR Value

N	Mean	Standard Deviation	Minimum	Maximum
6	2.0266667	0.3228416	1.5800000	2.4300000

Laboratory tests at the time of obtaining Table 2-6 consent, according to the presence or absence of proton-pump inhibitors

Unique Subject Identifier=5026101000-0007

MEANS Procedure

Assay variable : PTINR_VAL1 PT-INR Value

N	Mean	Standard Deviation	Minimum	Maximum
3	1.7766667	0.2600641	1.5200000	2.0400000

Unique Subject Identifier=5026101000-0014

Assay variable : PTINR_VAL1 PT-INR Value

N	Mean	Standard Deviation	Minimum	Maximum
5	2.2200000	0.2930017	1.9000000	2.6200000

Unique Subject Identifier=5026101000-0019

Assay variable : PTINR_VAL1 PT-INR Value

N	Mean	Standard Deviation	Minimum	Maximum
5	2.3420000	0.3293478	1.9400000	2.8100000

Unique Subject Identifier=5026101000-0024

Assay variable : PTINR_VAL1 PT-INR Value

N	Mean	Standard Deviation	Minimum	Maximum
4	1.5175000	0.3023657	1.2100000	1.8200000

Laboratory tests at the time of obtaining Table 2-6 consent, according to the presence or absence of proton-pump inhibitors

Unique Subject Identifier=5026101000-0038

MEANS Procedure

Assay variable : PTINR_VAL1 PT-INR Value

N	Mean	Standard Deviation	Minimum	Maximum
4	2.0675000	0.1481834	1.8700000	2.2000000

Unique Subject Identifier=5026101000-0050

Assay variable : PTINR_VAL1 PT-INR Value

N	Mean	Standard Deviation	Minimum	Maximum
3	2.3033333	0.5601190	1.9700000	2.9500000

Unique Subject Identifier=5026101000-0063

Assay variable : PTINR_VAL1 PT-INR Value

N	Mean	Standard Deviation	Minimum	Maximum
3	1.7033333	0.1101514	1.6300000	1.8300000

Unique Subject Identifier=5026101000-0089

Assay variable : PTINR_VAL1 PT-INR Value

N	Mean	Standard Deviation	Minimum	Maximum
4	1.5200000	0.1516575	1.3600000	1.6900000

Laboratory tests at the time of obtaining Table 2-6 consent, according to the presence or absence of proton-pump inhibitors

Unique Subject Identifier=5026101000-0095

MEANS Procedure

Assay variable : PTINR_VAL1 PT-INR Value

N	Mean	Standard Deviation	Minimum	Maximum
2	1.8750000	0.1484924	1.7700000	1.9800000

Unique Subject Identifier=5026101000-0096

Assay variable : PTINR_VAL1 PT-INR Value

N	Mean	Standard Deviation	Minimum	Maximum
3	2.1033333	0.1193035	2.0200000	2.2400000

Unique Subject Identifier=5026101000-0105

Assay variable : PTINR_VAL1 PT-INR Value

N	Mean	Standard Deviation	Minimum	Maximum
4	2.4200000	0.1718527	2.2200000	2.6400000

Unique Subject Identifier=5026101000-0107

Assay variable : PTINR_VAL1 PT-INR Value

N	Mean	Standard Deviation	Minimum	Maximum
1	1.3000000		1.3000000	1.3000000

Laboratory tests at the time of obtaining Table 2-6 consent, according to the presence or absence of proton-pump inhibitors

Unique Subject Identifier=5026101000-0118

MEANS Procedure

Assay variable : PTINR_VAL1 PT-INR Value

N	Mean	Standard Deviation	Minimum	Maximum
5	2.5160000	0.5725644	1.7600000	3.3500000

Unique Subject Identifier=5027000100-0006

Assay variable : PTINR_VAL1 PT-INR Value

N	Mean	Standard Deviation	Minimum	Maximum
6	2.2366667	0.3651666	1.6000000	2.6400000

Unique Subject Identifier=5030010000-0007

Assay variable : PTINR_VAL1 PT-INR Value

N	Mean	Standard Deviation	Minimum	Maximum
5	1.7780000	0.3134007	1.2200000	1.9600000

Unique Subject Identifier=5030010000-0009

Assay variable : PTINR_VAL1 PT-INR Value

N	Mean	Standard Deviation	Minimum	Maximum
6	1.7266667	0.1522717	1.4700000	1.8600000

Laboratory tests at the time of obtaining Table 2-6 consent, according to the presence or absence of proton-pump inhibitors

Unique Subject Identifier=5030010000-0014

MEANS Procedure

Assay variable : PTINR_VAL1 PT-INR Value

N	Mean	Standard Deviation	Minimum	Maximum
6	2.0733333	0.7299224	1.4100000	3.2000000

Unique Subject Identifier=5030010000-0026

Assay variable : PTINR_VAL1 PT-INR Value

N	Mean	Standard Deviation	Minimum	Maximum
5	1.8320000	0.3149921	1.6500000	2.3900000

Unique Subject Identifier=5030010000-0036

Assay variable : PTINR_VAL1 PT-INR Value

N	Mean	Standard Deviation	Minimum	Maximum
6	1.7983333	0.4104347	1.2800000	2.4600000

Unique Subject Identifier=5030010000-0037

Assay variable : PTINR_VAL1 PT-INR Value

N	Mean	Standard Deviation	Minimum	Maximum
6	2.2283333	0.3453356	1.8900000	2.8400000

Laboratory tests at the time of obtaining Table 2-6 consent, according to the presence or absence of proton-pump inhibitors

Unique Subject Identifier=5030010000-0038

MEANS Procedure

Assay variable : PTINR_VAL1 PT-INR Value

N	Mean	Standard Deviation	Minimum	Maximum
1	2.8100000		2.8100000	2.8100000

Unique Subject Identifier=5030010000-0039

Assay variable : PTINR_VAL1 PT-INR Value

N	Mean	Standard Deviation	Minimum	Maximum
5	1.6400000	0.2021138	1.3600000	1.8500000

Unique Subject Identifier=5030010000-0040

Assay variable : PTINR_VAL1 PT-INR Value

N	Mean	Standard Deviation	Minimum	Maximum
6	1.7150000	0.2550882	1.5000000	2.1900000

Unique Subject Identifier=5030010000-0049

Assay variable : PTINR_VAL1 PT-INR Value

N	Mean	Standard Deviation	Minimum	Maximum
4	1.9925000	0.2573422	1.6800000	2.2800000

Laboratory tests at the time of obtaining Table 2-6 consent, according to the presence or absence of proton-pump inhibitors

Unique Subject Identifier=5030010000-0052

MEANS Procedure

Assay variable : PTINR_VAL1 PT-INR Value

N	Mean	Standard Deviation	Minimum	Maximum
6	1.9466667	0.2022540	1.7000000	2.2400000

Unique Subject Identifier=5030010000-0054

Assay variable : PTINR_VAL1 PT-INR Value

N	Mean	Standard Deviation	Minimum	Maximum
5	1.8960000	0.1393915	1.7500000	2.0600000

Unique Subject Identifier=5030010000-0055

Assay variable : PTINR_VAL1 PT-INR Value

N	Mean	Standard Deviation	Minimum	Maximum
1	1.6300000		1.6300000	1.6300000

Unique Subject Identifier=5030010000-0058

Assay variable : PTINR_VAL1 PT-INR Value

N	Mean	Standard Deviation	Minimum	Maximum
2	1.8000000	0.4242641	1.5000000	2.1000000

Laboratory tests at the time of obtaining Table 2-6 consent, according to the presence or absence of proton-pump inhibitors

Unique Subject Identifier=5031111000-0019

MEANS Procedure

Assay variable : PTINR_VAL1 PT-INR Value

N	Mean	Standard Deviation	Minimum	Maximum
3	1.8766667	0.1457166	1.7400000	2.0300000

Unique Subject Identifier=5033000000-0001

Assay variable : PTINR_VAL1 PT-INR Value

N	Mean	Standard Deviation	Minimum	Maximum
3	1.8100000	0.3732292	1.4900000	2.2200000

Unique Subject Identifier=5034000010-0018

Assay variable : PTINR_VAL1 PT-INR Value

N	Mean	Standard Deviation	Minimum	Maximum
4	1.4850000	0.2444722	1.2600000	1.7800000

Unique Subject Identifier=5035010010-0001

Assay variable : PTINR_VAL1 PT-INR Value

N	Mean	Standard Deviation	Minimum	Maximum
2	2.0600000	0.0282843	2.0400000	2.0800000

Laboratory tests at the time of obtaining Table 2-6 consent, according to the presence or absence of proton-pump inhibitors

Unique Subject Identifier=5035010010-0003

MEANS Procedure

Assay variable : PTINR_VAL1 PT-INR Value

N	Mean	Standard Deviation	Minimum	Maximum
3	1.9300000	0.3340659	1.5700000	2.2300000

Unique Subject Identifier=5035010010-0008

Assay variable : PTINR_VAL1 PT-INR Value

N	Mean	Standard Deviation	Minimum	Maximum
4	1.7075000	0.5095995	1.3300000	2.4600000

Unique Subject Identifier=5035010010-0017

Assay variable : PTINR_VAL1 PT-INR Value

N	Mean	Standard Deviation	Minimum	Maximum
1	2.2300000		2.2300000	2.2300000

Unique Subject Identifier=5035010010-0018

Assay variable : PTINR_VAL1 PT-INR Value

N	Mean	Standard Deviation	Minimum	Maximum
4	2.1350000	0.1554563	2.0000000	2.3300000

Laboratory tests at the time of obtaining Table 2-6 consent, according to the presence or absence of proton-pump inhibitors

Unique Subject Identifier=5035010010-0041

MEANS Procedure

Assay variable : PTINR_VAL1 PT-INR Value

N	Mean	Standard Deviation	Minimum	Maximum
5	2.1840000	0.5774340	1.2200000	2.6300000

Unique Subject Identifier=5035010010-0044

Assay variable : PTINR_VAL1 PT-INR Value

N	Mean	Standard Deviation	Minimum	Maximum
6	2.2566667	0.9109482	1.6000000	4.0800000

Unique Subject Identifier=5035010010-0056

Assay variable : PTINR_VAL1 PT-INR Value

N	Mean	Standard Deviation	Minimum	Maximum
3	2.3366667	0.2203028	2.1100000	2.5500000

Unique Subject Identifier=5036010000-0013

Assay variable : PTINR_VAL1 PT-INR Value

N	Mean	Standard Deviation	Minimum	Maximum
6	1.1466667	0.2329521	0.9300000	1.5400000

Laboratory tests at the time of obtaining Table 2-6 consent, according to the presence or absence of proton-pump inhibitors

Unique Subject Identifier=5036010000-0026

MEANS Procedure

Assay variable : PTINR_VAL1 PT-INR Value

N	Mean	Standard Deviation	Minimum	Maximum
4	1.8500000	0.0621825	1.8000000	1.9400000

Unique Subject Identifier=5036010000-0039

Assay variable : PTINR_VAL1 PT-INR Value

N	Mean	Standard Deviation	Minimum	Maximum
4	1.7600000	0.2146315	1.5800000	2.0700000

Unique Subject Identifier=5036010000-0054

Assay variable : PTINR_VAL1 PT-INR Value

N	Mean	Standard Deviation	Minimum	Maximum
6	2.1000000	0.1555635	1.8900000	2.3000000

Unique Subject Identifier=5036010000-0055

Assay variable : PTINR_VAL1 PT-INR Value

N	Mean	Standard Deviation	Minimum	Maximum
4	1.8000000	0.2293469	1.5000000	2.0500000

Laboratory tests at the time of obtaining Table 2-6 consent, according to the presence or absence of proton-pump inhibitors

Unique Subject Identifier=5037010000-0002

MEANS Procedure

Assay variable : PTINR_VAL1 PT-INR Value

N	Mean	Standard Deviation	Minimum	Maximum
1	1.4600000		1.4600000	1.4600000

Unique Subject Identifier=5037010000-0007

Assay variable : PTINR_VAL1 PT-INR Value

N	Mean	Standard Deviation	Minimum	Maximum
1	1.2000000		1.2000000	1.2000000

Unique Subject Identifier=5037010000-0026

Assay variable : PTINR_VAL1 PT-INR Value

N	Mean	Standard Deviation	Minimum	Maximum
2	1.5150000	0.1909188	1.3800000	1.6500000

Unique Subject Identifier=5037010000-0039

Assay variable : PTINR_VAL1 PT-INR Value

N	Mean	Standard Deviation	Minimum	Maximum
1	3.0300000		3.0300000	3.0300000

Laboratory tests at the time of obtaining Table 2-6 consent, according to the presence or absence of proton-pump inhibitors

Unique Subject Identifier=5040011000-0001

MEANS Procedure

Assay variable : PTINR_VAL1 PT-INR Value

N	Mean	Standard Deviation	Minimum	Maximum
4	2.4025000	0.1830073	2.1400000	2.5600000

Unique Subject Identifier=5040011000-0004

Assay variable : PTINR_VAL1 PT-INR Value

N	Mean	Standard Deviation	Minimum	Maximum
2	1.8000000	0.1272792	1.7100000	1.8900000

Unique Subject Identifier=5040011000-0007

Assay variable : PTINR_VAL1 PT-INR Value

N	Mean	Standard Deviation	Minimum	Maximum
4	1.3375000	0.1081280	1.2100000	1.4500000

Unique Subject Identifier=5040011000-0015

Assay variable : PTINR_VAL1 PT-INR Value

N	Mean	Standard Deviation	Minimum	Maximum
5	1.9920000	0.3459335	1.6000000	2.5200000

Laboratory tests at the time of obtaining Table 2-6 consent, according to the presence or absence of proton-pump inhibitors

Unique Subject Identifier=5041011000-0008

MEANS Procedure

Assay variable : PTINR_VAL1 PT-INR Value

N	Mean	Standard Deviation	Minimum	Maximum
6	1.8900000	0.5465528	1.3800000	2.5900000

Unique Subject Identifier=5044000000-0002

Assay variable : PTINR_VAL1 PT-INR Value

N	Mean	Standard Deviation	Minimum	Maximum
6	1.7016667	0.6760301	1.0900000	2.6400000

Unique Subject Identifier=5044000000-0016

Assay variable : PTINR_VAL1 PT-INR Value

N	Mean	Standard Deviation	Minimum	Maximum
5	2.0000000	0.2738613	1.7000000	2.4000000

Unique Subject Identifier=5044000000-0021

Assay variable : PTINR_VAL1 PT-INR Value

N	Mean	Standard Deviation	Minimum	Maximum
5	2.1140000	0.3083504	1.7900000	2.5600000

Laboratory tests at the time of obtaining Table 2-6 consent, according to the presence or absence of proton-pump inhibitors

Unique Subject Identifier=5044000000-0026

MEANS Procedure

Assay variable : PTINR_VAL1 PT-INR Value

N	Mean	Standard Deviation	Minimum	Maximum
3	2.3133333	0.3050137	2.0100000	2.6200000

Unique Subject Identifier=5044000000-0038

Assay variable : PTINR_VAL1 PT-INR Value

N	Mean	Standard Deviation	Minimum	Maximum
4	2.2325000	0.3142584	1.8600000	2.5800000

Unique Subject Identifier=5044000000-0044

Assay variable : PTINR_VAL1 PT-INR Value

N	Mean	Standard Deviation	Minimum	Maximum
5	2.2780000	0.5363488	1.5900000	2.9700000

Unique Subject Identifier=5044000000-0053

Assay variable : PTINR_VAL1 PT-INR Value

N	Mean	Standard Deviation	Minimum	Maximum
5	1.9900000	0.3931921	1.3500000	2.4300000

Laboratory tests at the time of obtaining Table 2-6 consent, according to the presence or absence of proton-pump inhibitors

Unique Subject Identifier=5045010110-0011

MEANS Procedure

Assay variable : PTINR_VAL1 PT-INR Value

N	Mean	Standard Deviation	Minimum	Maximum
4	1.8250000	0.1968925	1.5700000	1.9900000

Unique Subject Identifier=5046010000-0035

Assay variable : PTINR_VAL1 PT-INR Value

N	Mean	Standard Deviation	Minimum	Maximum
6	2.1500000	1.1623253	1.1000000	4.1000000

Unique Subject Identifier=5047000010-0003

Assay variable : PTINR_VAL1 PT-INR Value

N	Mean	Standard Deviation	Minimum	Maximum
4	1.6425000	0.1372042	1.5100000	1.7800000

Unique Subject Identifier=5047000010-0004

Assay variable : PTINR_VAL1 PT-INR Value

N	Mean	Standard Deviation	Minimum	Maximum
3	1.5266667	0.1342882	1.4300000	1.6800000

Laboratory tests at the time of obtaining Table 2-6 consent, according to the presence or absence of proton-pump inhibitors

Unique Subject Identifier=5047000010-0012

MEANS Procedure

Assay variable : PTINR_VAL1 PT-INR Value

N	Mean	Standard Deviation	Minimum	Maximum
4	2.2000000	0.1714643	2.0500000	2.4400000

Unique Subject Identifier=5048011000-0006

Assay variable : PTINR_VAL1 PT-INR Value

N	Mean	Standard Deviation	Minimum	Maximum
5	2.1380000	0.2873500	1.8900000	2.5500000

Unique Subject Identifier=5048011000-0008

Assay variable : PTINR_VAL1 PT-INR Value

N	Mean	Standard Deviation	Minimum	Maximum
3	1.9566667	0.5398457	1.3800000	2.4500000

Unique Subject Identifier=5048011000-0013

Assay variable : PTINR_VAL1 PT-INR Value

N	Mean	Standard Deviation	Minimum	Maximum
4	2.0225000	0.0485627	1.9600000	2.0700000

Laboratory tests at the time of obtaining Table 2-6 consent, according to the presence or absence of proton-pump inhibitors

Unique Subject Identifier=5048011000-0016

MEANS Procedure

Assay variable : PTINR_VAL1 PT-INR Value

N	Mean	Standard Deviation	Minimum	Maximum
4	1.2150000	0.0450925	1.1800000	1.2800000

Unique Subject Identifier=5048011000-0019

Assay variable : PTINR_VAL1 PT-INR Value

N	Mean	Standard Deviation	Minimum	Maximum
2	1.4800000	0.0848528	1.4200000	1.5400000

Unique Subject Identifier=5048011000-0022

Assay variable : PTINR_VAL1 PT-INR Value

N	Mean	Standard Deviation	Minimum	Maximum
3	1.6100000	0.6000833	1.0600000	2.2500000

Unique Subject Identifier=5048011000-0023

Assay variable : PTINR_VAL1 PT-INR Value

N	Mean	Standard Deviation	Minimum	Maximum
6	1.9800000	0.3051557	1.5600000	2.3100000

Laboratory tests at the time of obtaining Table 2-6 consent, according to the presence or absence of proton-pump inhibitors

Unique Subject Identifier=5048011000-0025

MEANS Procedure

Assay variable : PTINR_VAL1 PT-INR Value

N	Mean	Standard Deviation	Minimum	Maximum
3	1.2133333	0.1379613	1.1100000	1.3700000

Unique Subject Identifier=5048011000-0029

Assay variable : PTINR_VAL1 PT-INR Value

N	Mean	Standard Deviation	Minimum	Maximum
3	1.8800000	0.3223352	1.6600000	2.2500000

Unique Subject Identifier=5048011000-0031

Assay variable : PTINR_VAL1 PT-INR Value
[truncated: 276,119 more chars]
