# Supplementary material for: Current status of proton pump inhibitor use in Japanese elderly patients with non-valvular atrial fibrillation: A subanalysis of the ANAFIE Registry
Source: PLoS One. 2020 Nov 5;15(11):e0240859. doi: 10.1371/journal.pone.0240859 (PMC7644054; doi:10.1371/journal.pone.0240859)
Supplement: S5 File — (PDF) [file pone.0240859.s006.pdf]

Case composition diagram from the entire Figure 1-1 population to the enrolled population and the analysis population by the presence or absence of proton-pump inhibitors

FREQ Procedure

Cumulative

Percent of ALL power Percent of power

1	33278	100.00	33278	100.00

Case composition diagram from the entire Figure 1-1 population to the enrolled population and the analysis population by the presence or absence of proton-pump inhibitors

FREQ Procedure

Table : ALL * C345

ALL C345

Power |
Percent |
Percent of rows
Percentage of columns | 0| 1| Sum

	0	1	Sum
1	33115	163	33278
	99.51	0.49	100.00
	99.51	0.49	
	100.00	100.00	
Total	33115	163	33278
	99.51	0.49	100.00

Case composition diagram from the entire Figure 1-1 population to the enrolled population and the analysis population by the presence or absence of proton-pump inhibitors

FREQ Procedure

Table : ALL * C3

ALL C3(Other Criteria 3)

Power |
Percent |
Percent of rows
Percent of columns | None | With | Total
______________+________+________+
1 | 33202 | 76 | 33278
| 99.77 | 0.23 | 100.00
| 99.77 | 0.23 |
| 100.00 | 100.00 |
______________+________+________+
Total 33202 76 33278
99.77 0.23 100.00

Case composition diagram from the entire Figure 1-1 population to the enrolled population and the analysis population by the presence or absence of proton-pump inhibitors

FREQ Procedure

Table : ALL * C4

ALL C4(Other Criteria 4)

Power |
Percent |
Percent of rows
Percent of columns | None | With | Total
______________+________+________+
1 | 33193 | 85 | 33278
| 99.74 | 0.26 | 100.00
| 99.74 | 0.26 |
| 100.00 | 100.00 |
______________+________+________+
Total 33193 85 33278
99.74 0.26 100.00

Case composition diagram from the entire Figure 1-1 population to the enrolled population and the analysis population by the presence or absence of proton-pump inhibitors

FREQ Procedure

Table : ALL * C5

ALL C5(Other Criteria 5)

Power |
Percent |
Percent of rows
Percent of columns | None | With | Total

```
______________+________+________+
          1 |   33276 |       2 |   33278
            |   99.99 |    0.01 | 100.00
            |   99.99 |    0.01 |
            | 100.00 | 100.00 |
______________+________+________+
```

Total 33276 2 33278
99.99 0.01 100.00

Case composition diagram from the entire Figure 1-1 population to the enrolled population and the analysis population by the presence or absence of proton-pump inhibitors

FREQ Procedure

Table: ALL * _AD_FLAG

ALL _AD_FLAG

Power |
Percent |
Percent of rows
Percent of columns | 1| Total
______________+________+
1 | 389 | 389
| 100.00 | 100.00
| 100.00 |
| 100.00 |
______________+________+
Total 389 389
100.00 100.00

Frequency of missing values = 32726

Case composition diagram from the entire Figure 1-1 population to the enrolled population and the analysis population by the presence or absence of proton-pump inhibitors

FREQ Procedure

Table : ALL * A1

ALL A1(Inclusion Criteria 1)

Power |
Percent |
Percent of rows
Percent of columns | None | With | Total
______________+________+________+
1 | 33088 | 27 | 33115
| 99.92 | 0.08 | 100.00
| 99.92 | 0.08 |
| 100.00 | 100.00 |
______________+________+________+
Total 33088 27 33115
99.92 0.08 100.00

Case composition diagram from the entire Figure 1-1 population to the enrolled population and the analysis population by the presence or absence of proton-pump inhibitors

FREQ Procedure

Table : ALL * A2

ALL

A2(Inclusion Criteria 2)

Power |
Percent |
Percent of rows
Percent of columns | None | Total
______________+________+
1 | 33115 | 33115
| 100.00 | 100.00
| 100.00 |
| 100.00 |
______________+________+
Total 33115 33115
100.00 100.00

Case composition diagram from the entire Figure 1-1 population to the enrolled population and the analysis population by the presence or absence of proton-pump inhibitors

FREQ Procedure

Table : ALL * A3

ALL A3(Inclusion Criteria 3)

Power |
Percent |
Percent of rows
Percent of columns | None | With | Total

Power / Percent / Percent of rows / Percent of columns	None	With	Total
1	33105	10	33115
	99.97	0.03	100.00
	99.97	0.03	
	100.00	100.00	
Total	33105	10	33115
	99.97	0.03	100.00

Case composition diagram from the entire Figure 1-1 population to the enrolled population and the analysis population by the presence or absence of proton-pump inhibitors

FREQ Procedure

Table : ALL * A4

ALL A4(Inclusion Criteria 4)

Power |
Percent |
Percent of rows
Percent of columns | None | With | Total
______________+________+________+
1 | 33044 | 71 | 33115
| 99.79 | 0.21 | 100.00
| 99.79 | 0.21 |
| 100.00 | 100.00 |
______________+________+________+
Total 33044 71 33115
99.79 0.21 100.00

Case composition diagram from the entire Figure 1-1 population to the enrolled population and the analysis population by the presence or absence of proton-pump inhibitors

FREQ Procedure

Table : ALL * B1

ALL B1(Exclusion Criteria 1)

Power<br>Percent<br>Percent of rows<br>Percent of columns	None	With	Total
1	33104<br>99.97<br>99.97<br>100.00	11<br>0.03<br>0.03<br>100.00	33115<br>100.00
Total	33104<br>99.97	11<br>0.03	33115<br>100.00

Case composition diagram from the entire Figure 1-1 population to the enrolled population and the analysis population by the presence or absence of proton-pump inhibitors

FREQ Procedure

Table : ALL * B2

ALL B2(Exclusion Criteria 2)

```
Power |
Percent |
Percent of rows
Percent of columns | None | With | Total
______________+________+________+
             1 |  33111 |       4 |  33115
               |  99.99 |    0.01 | 100.00
               |  99.99 |    0.01 |
               | 100.00 | 100.00 |
______________+________+________+
Total 33111 4 33115
                 99.99      0.01    100.00
```

Case composition diagram from the entire Figure 1-1 population to the enrolled population and the analysis population by the presence or absence of proton-pump inhibitors

FREQ Procedure

Table : ALL * B3

ALL B3(Exclusion Criteria 3)

Power |
Percent |
Percent of rows
Percent of columns | None | With | Total

```
______________+________+________+
             1 |   33098 |       17 |   33115
               |   99.95 |     0.05 | 100.00
               |   99.95 |     0.05 |
               | 100.00 | 100.00 |
______________+________+________+
Total 33098 17 33115
                   99.95       0.05    100.00
```

Case composition diagram from the entire Figure 1-1 population to the enrolled population and the analysis population by the presence or absence of proton-pump inhibitors

FREQ Procedure

Table : ALL * B4

ALL B4(Exclusion Criteria 4)

Power / Percent / Percent of rows / Percent of columns	None	With	Total
1	33112	3	33115
	99.99	0.01	100.00
	99.99	0.01	
	100.00	100.00	
Total	33112	3	33115
	99.99	0.01	100.00

Case composition diagram from the entire Figure 1-1 population to the enrolled population and the analysis population by the presence or absence of proton-pump inhibitors

FREQ Procedure

Table : ALL * B5

ALL B5(Exclusion Criteria 5)

Power |
Percent |
Percent of rows
Percent of columns | None | With | Total
______________+________+________+
1 | 33114 | 1 | 33115
| 100.00 | 0.00 | 100.00
| 100.00 | 0.00 |
| 100.00 | 100.00 |
______________+________+________+
Total 33114 1 33115
100.00 0.00 100.00

Case composition diagram from the entire Figure 1-1 population to the enrolled population and the analysis population by the presence or absence of proton-pump inhibitors

FREQ Procedure

Table : ALL * B6

ALL B6(Exclusion Criteria 6)

```
Power |
Percent |
Percent of rows
Percent of columns | None | With | Total
______________+________+________+
             1 |   33102 |       13 |   33115
               |   99.96 |     0.04 | 100.00
               |   99.96 |     0.04 |
               | 100.00 | 100.00 |
______________+________+________+
Total 33102 13 33115
                   99.96       0.04     100.00
```

Case composition diagram from the entire Figure 1-1 population to the enrolled population and the analysis population by the presence or absence of proton-pump inhibitors

FREQ Procedure

Table : ALL * C1

ALL C1(Other Criteria 1)

```
Power |
Percent |
Percent of rows
Percent of columns | None | With | Total
______________+________+________+
            1 |  32883 |     232 |  33115
              |  99.30 |    0.70 | 100.00
              |  99.30 |    0.70 |
              | 100.00 | 100.00 |
______________+________+________+
Total 32883 232 33115
                 99.30     0.70   100.00
```

Case composition diagram from the entire Figure 1-1 population to the enrolled population and the analysis population by the presence or absence of proton-pump inhibitors

FREQ Procedure

Table : ALL * C2

ALL

C2(Other Criteria 2)

Power |
Percent |
Percent of rows
Percent of columns | None | Total
______________+________+
1 | 33115 | 33115
| 100.00 | 100.00
| 100.00 |
| 100.00 |
______________+________+
Total 33115 33115
100.00 100.00

Case composition diagram from the entire Figure 1-1 population to the enrolled population and the analysis population by the presence or absence of proton-pump inhibitors

FREQ Procedure

Table : ALL * f1

ALL f1

Power |
Percent |
Percent of rows
Percent of columns | 1| Total
______________+________+
1 | 32726 | 32726
| 100.00 | 100.00
| 100.00 |
| 100.00 |
______________+________+
Total 32726 32726
100.00 100.00

Frequency of missing values = 552

Case composition diagram from the entire Figure 1-1 population to the enrolled population and the analysis population by the presence or absence of proton-pump inhibitors

FREQ Procedure

Table : ALL * p1

ALL p1

Power |
Percent |
Percent of rows
Percentage of columns | 0| 1| Sum

1	20745	11981	32726
	63.39	36.61	100.00
	63.39	36.61	
	100.00	100.00	
Total	20745	11981	32726
	63.39	36.61	100.00

Case composition diagram from the entire Figure 1-1 population to the enrolled population and the analysis population by the presence or absence of proton-pump inhibitors

FREQ Procedure

Table : ALL * p2

ALL p2

```
Power |
Percent |
Percent of rows
Percentage of columns | 0| 1| Sum
______________+________+________+
            1 |   12217 |   20509 |   32726
              |   37.33 |   62.67 | 100.00
              |   37.33 |   62.67 |
              | 100.00 | 100.00 |
______________+________+________+
Total 12217 20509 32726
              37.33      62.67     100.00
```

Case composition diagram from the entire Figure 1-1 population to the enrolled population and the analysis population by the presence or absence of proton-pump inhibitors

FREQ Procedure

Table : ALL * p3

ALL p3

Power |
Percent |
Percent of rows
Percentage of columns | 0| 1| Sum

```
______________+________+________+
          1 |  32490 |     236 |  32726
            |  99.28 |    0.72 | 100.00
            |  99.28 |    0.72 |
            | 100.00 | 100.00 |
______________+________+________+
Total 32490 236 32726
               99.28     0.72   100.00
```

Distributions of patients with and without Table 1-2 proton-pump inhibitors

FREQ Procedure

			Cumulative	
Percent of ALL	power	Percent of power		
1	33278	100.00	33278	100.00

Distributions of patients with and without Table 1-2 proton-pump inhibitors

FREQ Procedure

Table : ALL * f1

ALL f1

Power |
Percent |
Percent of rows
Percent of columns | 1| Total
______________+________+
1 | 32726 | 32726
| 100.00 | 100.00
| 100.00 |
| 100.00 |
______________+________+
Total 32726 32726
100.00 100.00

Frequency of missing values = 552

Distributions of patients with and without Table 1-2 proton-pump inhibitors

FREQ Procedure

Table : ALL * p1

ALL p1

Power |
Percent |
Percent of rows
Percentage of columns | 0| 1| Sum

ALL	0	1	Sum
1	20745	11981	32726
	63.39	36.61	100.00
	63.39	36.61	
	100.00	100.00	
Total	20745	11981	32726
	63.39	36.61	100.00

Distributions of patients with and without Table 1-2 proton-pump inhibitors

FREQ Procedure

Table : ALL * p2

ALL p2

Power |
Percent |
Percent of rows
Percentage of columns | 0| 1| Sum

ALL	0	1	Sum
1	12217	20509	32726
	37.33	62.67	100.00
	37.33	62.67	
	100.00	100.00	
Total	12217	20509	32726
	37.33	62.67	100.00

Distributions of patients with and without Table 1-2 proton-pump inhibitors

FREQ Procedure

Table : ALL * p3

ALL p3

Power |
Percent |
Percent of rows
Percentage of columns | 0| 1| Sum
______________+________+________+
1 | 32490 | 236 | 32726
| 99.28 | 0.72 | 100.00
| 99.28 | 0.72 |
| 100.00 | 100.00 |
______________+________+________+
Total 32490 236 32726
99.28 0.72 100.00

Table 2-1 demographics according to the presence or absence of proton-pump inhibitors

FREQ Procedure

			Cumulative	
Percent of ALL	power	Percent of power		
1	32490	100.00	32490	100.00

Tables: ALL* SEXN

ALL SEXN(Sex (N))

Power |
Percent |

Percent of rows

Percentage of columns | 0| 1| Sum

_______________+________+________+

1 | 18601 | 13889 | 32490

| 57.25 | 42.75 | 100.00

| 57.25 | 42.75 |

| 100.00 | 100.00 |

_______________+________+________+

Total 18601 13889 32490

57.25 42.75 100.00

Table 2-1 demographics according to the presence or absence of proton-pump inhibitors

FREQ Procedure

			Cumulative	
Percent of ALL	power	Percent of	power	
1	32490	100.00	32490	100.00

Tables: ALL* AGEC

ALL AGEC(Age Category)

Power |
Percent |
Percent of rows
Percentage of column |75 years and older |80 years and older |85 years and older |90 years and older |95 years and older | 100 years and older |Total
|Ages under 80|Ages under 85|Ages under 90|Ages under 95|Ages under 100 Ages under |Above|
| | | | Full | | | |

1	12967	11022	6352	1864	274	11	32490
	39.91	33.92	19.55	5.74	0.84	0.03	100.00
	39.91	33.92	19.55	5.74	0.84	0.03	
	100.00	100.00	100.00	100.00	100.00	100.00	
Total	12967	11022	6352	1864	274	11	32490
	39.91	33.92	19.55	5.74	0.84	0.03	100.00

Table 2-1 demographics according to the presence or absence of proton-pump inhibitors

FREQ Procedure

Cumulative

Percent of ALL power Percent of power

1	32490	100.00	32490	100.00

Table: ALL * WGTC_BL

ALL

WGTC_BL(Weight Category at Informed Concent)

Power |

Percent |

Percent of rows

Percent of column |less than 60 kg|more than 60 kg|unknown|total

	less than 60 kg	more than 60 kg	unknown	total
1	17005	12638	2847	32490
	52.34	38.90	8.76	100.00
	52.34	38.90	8.76	
	100.00	100.00	100.00	
Total	17005	12638	2847	32490
	52.34	38.90	8.76	100.00

Table 2-1 demographics according to the presence or absence of proton-pump inhibitors

FREQ Procedure

Cumulative

Percent of ALL power Percent of power

1	32490	100.00	32490	100.00

Table : ALL * BMIC_BL

ALL BMIC_BL(BMI Category at Informed Consent)

Power |
Percent |
Percent of rows
Column percent|18.5kg/m|18.5kg/m|25.0kg/m|9| Total
<|2| □2> 25.|2 | |
| | 0 kg/m2 un| | |
| | Full| | | |

______________+________+________+________+________+

1	2077	17747	8120	4546	32490
	6.39	54.62	24.99	13.99	100.00
	6.39	54.62	24.99	13.99	
	100.00	100.00	100.00	100.00	

______________+________+________+________+________+

Total 2077 17747 8120 4546 32490

6.39 54.62 24.99 13.99 100.00

Table 2-1 demographics according to the presence or absence of proton-pump inhibitors

FREQ Procedure

			Cumulative	
Percent of ALL	power	Percent of	power	
1	32490	100.00	32490	100.00

Table : ALL * SMK1

ALL SMK1(Smoking Status Part 1)

Power |
Percent |
Percent of rows
Percent of column |Smoking|Stopped|Smoked|Unknown|Total
|No| | |

ALL	Smoking No	Stopped	Smoked	Unknown	Total
1	16252	9963	1244	5031	32490
	50.02	30.66	3.83	15.48	100.00
	50.02	30.66	3.83	15.48	
	100.00	100.00	100.00	100.00	
Total	16252	9963	1244	5031	32490
	50.02	30.66	3.83	15.48	100.00

Table 2-1 demographics according to the presence or absence of proton-pump inhibitors

FREQ Procedure

Cumulative
Percent of ALL power Percent of power

---

1 32490 100.00 32490 100.00

Table: ALL * DRK

ALL DRK(Drinking Habit)

Power |
Percent |
Percent of rows
Percentage of columns | routinely | occasionally | completely | unknown | total
|Drink|Drink|Do not drink| |
______________+________+________+________+________+
1 | 5804 | 5902 | 15369 | 5415 | 32490
| 17.86 | 18.17 | 47.30 | 16.67 | 100.00
| 17.86 | 18.17 | 47.30 | 16.67 |
| 100.00 | 100.00 | 100.00 | 100.00 |
______________+________+________+________+________+
Total 5804 5902 15369 5415 32490
17.86 18.17 47.30 16.67 100.00

Table 2-1 demographics according to the presence or absence of proton-pump inhibitors

FREQ Procedure

			Cumulative	
Percent of ALL	power	Percent of	power	
1	11981	100.00	11981	100.00

Tables: ALL* SEXN

ALL SEXN(Sex (N))

Power |
Percent |
Percent of rows
Percentage of columns | 0| 1| Sum

	0	1	Sum
1	6561	5420	11981
	54.76	45.24	100.00
	54.76	45.24	
	100.00	100.00	
Total	6561	5420	11981
	54.76	45.24	100.00

Table 2-1 demographics according to the presence or absence of proton-pump inhibitors

FREQ Procedure

			Cumulative	
Percent of ALL	power	Percent of power		
1	11981	100.00	11981	100.00

Tables: ALL* AGEC

ALL AGEC(Age Category)

Power |
Percent |
Percent of rows
Percentage of column |75 years and older |80 years and older |85 years and older |90 years and older |95 years and older | 100 years and older |Total
|Ages under 80|Ages under 85|Ages under 90|Ages under 95|Ages under 100 Ages under |Above|
| | | | Full | | | |

1	4559	4061	2481	767	110	3	11981
	38.05	33.90	20.71	6.40	0.92	0.03	100.00
	38.05	33.90	20.71	6.40	0.92	0.03	
	100.00	100.00	100.00	100.00	100.00	100.00	
Total	4559	4061	2481	767	110	3	11981
	38.05	33.90	20.71	6.40	0.92	0.03	100.00

Table 2-1 demographics according to the presence or absence of proton-pump inhibitors

FREQ Procedure

			Cumulative	
Percent of ALL	power	Percent of	power	
1	11981	100.00	11981	100.00

Table: ALL * WGTC_BL

ALL

WGTC_BL(Weight Category at Informed Concent)

Power<br>Percent<br>Percent of rows<br>Percent of column	less than 60 kg	more than 60 kg	unknown	total
1	6543<br>54.61<br>54.61<br>100.00	4503<br>37.58<br>37.58<br>100.00	935<br>7.80<br>7.80<br>100.00	11981<br>100.00
Total	6543<br>54.61	4503<br>37.58	935<br>7.80	11981<br>100.00

Table 2-1 demographics according to the presence or absence of proton-pump inhibitors

FREQ Procedure

			Cumulative	
	Percent of ALL power	Percent of power		
1	11981	100.00	11981	100.00

Table : ALL * BMIC_BL

ALL BMIC_BL(BMI Category at Informed Consent)

```
Power |
Percent |
Percent of rows
Column percent|18.5kg/m|18.5kg/m|25.0kg/m|9| Total
         <|2| □2> 25.|2 | |
         | | 0 kg/m2 un| | |
         | | Full| | | |
______________+________+________+________+________+
             1 |    805 |    6659 |    3046 |    1471 |   11981
               |   6.72 |   55.58 |   25.42 |   12.28 | 100.00
               |   6.72 |   55.58 |   25.42 |   12.28 |
               | 100.00 | 100.00 | 100.00 | 100.00 |
______________+________+________+________+________+
Total 805 6659 3046 1471 11981
                  6.72     55.58     25.42     12.28    100.00
```

Table 2-1 demographics according to the presence or absence of proton-pump inhibitors

FREQ Procedure

			Cumulative	
Percent of ALL	power	Percent of	power	
1	11981	100.00	11981	100.00

Table : ALL * SMK1

ALL SMK1(Smoking Status Part 1)

Power / Percent / Percent of rows / Percent of column	Smoking No	Stopped	Smoked	Unknown	Total
1	6084	3802	452	1643	11981
	50.78	31.73	3.77	13.71	100.00
	50.78	31.73	3.77	13.71	
	100.00	100.00	100.00	100.00	
Total	6084	3802	452	1643	11981
	50.78	31.73	3.77	13.71	100.00

Table 2-1 demographics according to the presence or absence of proton-pump inhibitors

FREQ Procedure

Cumulative

Percent of ALL power Percent of power

1	11981	100.00	11981	100.00

Table: ALL * DRK

ALL DRK(Drinking Habit)

Power |

Percent |

Percent of rows

Percentage of columns | routinely | occasionally | completely | unknown | total

|Drink|Drink|Do not drink| |

	routinely Drink	occasionally Drink	completely Do not drink	unknown	total
1	1979	2067	6131	1804	11981
	16.52	17.25	51.17	15.06	100.00
	16.52	17.25	51.17	15.06	
	100.00	100.00	100.00	100.00	
Total	1979	2067	6131	1804	11981
	16.52	17.25	51.17	15.06	100.00

Table 2-1 demographics according to the presence or absence of proton-pump inhibitors

FREQ Procedure

Percent of ALL	power	Percent	Cumulative power	Cumulative Percent of power
1	20509	100.00	20509	100.00

Tables: ALL* SEXN

ALL SEXN(Sex (N))

Power |
Percent |
Percent of rows
Percentage of columns | 0| 1| Sum

ALL	0	1	Sum
1	12040	8469	20509
	58.71	41.29	100.00
	58.71	41.29	
	100.00	100.00	
Total	12040	8469	20509
	58.71	41.29	100.00

Table 2-1 demographics according to the presence or absence of proton-pump inhibitors

FREQ Procedure

Cumulative

Percent of ALL power Percent of power

1	20509	100.00	20509	100.00

Tables: ALL* AGEC

ALL AGEC(Age Category)

Power |

Percent |

Percent of rows

Percentage of column |75 years and older |80 years and older |85 years and older |90 years and older |95 years and older | 100 years and older |Total

|Ages under 80|Ages under 85|Ages under 90|Ages under 95|Ages under 100 Ages under |Above|

| | | | Full | | | |

1	8408	6961	3871	1097	164	8	20509
	41.00	33.94	18.87	5.35	0.80	0.04	100.00
	41.00	33.94	18.87	5.35	0.80	0.04	
	100.00	100.00	100.00	100.00	100.00	100.00	
Total	8408	6961	3871	1097	164	8	20509
	41.00	33.94	18.87	5.35	0.80	0.04	100.00

Table 2-1 demographics according to the presence or absence of proton-pump inhibitors

FREQ Procedure

			Cumulative	
Percent of ALL power Percent of power				
1	20509	100.00	20509	100.00

Table: ALL * WGTC_BL

ALL

WGTC_BL(Weight Category at Informed Concent)

Power |

Percent |

Percent of rows

Percent of column	less than 60 kg	more than 60 kg	unknown	total
1	10462	8135	1912	20509
	51.01	39.67	9.32	100.00
	51.01	39.67	9.32	
	100.00	100.00	100.00	
Total	10462	8135	1912	20509
	51.01	39.67	9.32	100.00

Table 2-1 demographics according to the presence or absence of proton-pump inhibitors

FREQ Procedure

Cumulative

Percent of ALL power Percent of power

1	20509	100.00	20509	100.00

Table : ALL * BMIC_BL

ALL BMIC_BL(BMI Category at Informed Consent)

Power |

Percent |

Percent of rows

Column percent|18.5kg/m|18.5kg/m|25.0kg/m|9| Total

<|2| □2> 25.|2 | |

| | 0 kg/m2 un| | |

| | Full| | | |

______________+________+________+________+________+

1 | 1272 | 11088 | 5074 | 3075 | 20509

| 6.20 | 54.06 | 24.74 | 14.99 | 100.00

| 6.20 | 54.06 | 24.74 | 14.99 |

| 100.00 | 100.00 | 100.00 | 100.00 |

______________+________+________+________+________+

Total 1272 11088 5074 3075 20509

6.20 54.06 24.74 14.99 100.00

Table 2-1 demographics according to the presence or absence of proton-pump inhibitors

FREQ Procedure

Cumulative

Percent of ALL power Percent of power

1	20509	100.00	20509	100.00

Table : ALL * SMK1

ALL SMK1(Smoking Status Part 1)

Power |
Percent |
Percent of rows
Percent of column |Smoking|Stopped|Smoked|Unknown|Total
|No| | |

______________+________+________+________+________+
1 | 10168 | 6161 | 792 | 3388 | 20509
| 49.58 | 30.04 | 3.86 | 16.52 | 100.00
| 49.58 | 30.04 | 3.86 | 16.52 |
| 100.00 | 100.00 | 100.00 | 100.00 |
______________+________+________+________+________+

Total 10168 6161 792 3388 20509
49.58 30.04 3.86 16.52 100.00

Table 2-1 demographics according to the presence or absence of proton-pump inhibitors

FREQ Procedure

			Cumulative	
Percent of ALL	power	Percent of	power	
1	20509	100.00	20509	100.00

Table: ALL * DRK

ALL DRK(Drinking Habit)

Power |
Percent |
Percent of rows
Percentage of columns | routinely | occasionally | completely | unknown | total
|Drink|Drink|Do not drink| |

	routinely Drink	occasionally Drink	completely Do not drink	unknown	total
1	3825	3835	9238	3611	20509
	18.65	18.70	45.04	17.61	100.00
	18.65	18.70	45.04	17.61	
	100.00	100.00	100.00	100.00	
Total	3825	3835	9238	3611	20509
	18.65	18.70	45.04	17.61	100.00

Table 2-1 demographics according to the presence or absence of proton-pump inhibitors

UNIVARIATE Procedure
Variable: AGEs (Age at First Drug Intake)

Moment

Total of 32490 for N 32490 weight variable
Mean 81.472607 total 2647045
Standard deviation 4.82078117 variance 23.2399311
Skewness 0.64098604 kurtosis-0.1355405
Unmodified square sum 216416699 modified square sum 755042.12
Coefficient of variation 5.9170577 standard error of the mean 0.026745

Basic statistics

Positional variation

Mean 81.47261 standard deviation 4.82078
Median 81.00000 variance 23.23993
Mode 75.00000 Range 28.00000
Interquartile range 8.00000

Test for position H0: Mu0=0

Test-Statistics------p Value

Student's t-test t 3046.275 Pr > |t| <. 0001
Sign test M 16245 Pr >= |M| <. 0001
Signed rank test S 2. 6391E8 Pr >= |S| <. 0001

Quartile (Definition 5)

Level quantile

100% max. 103	
99%	94
95%	90
90%	88
75% Q3 85	
50% median 81	
25% Q1 77	
10%	76
5%	75
1%	75
0% minimum 75	

Table 2-1 demographics according to the presence or absence of proton-pump inhibitors

UNIVARIATE Procedure
Variable: AGEs (Age at First Drug Intake)

Extreme value

--- Minimum value----Maximum value----

Value	Obs	Value	Obs
75	32490	101	5225
75	32470	101	23819
75	32456	102	5655
75	32444	102	29497
75	32443	103	7812

Table 2-1 demographics according to the presence or absence of proton-pump inhibitors

UNIVARIATE Procedure
Variable: HGT_BL (Height at Informed Concent)

Moment

Total of 28210 for N 28210 weight variable
Mean 157.226389 total 4435356.43
Standard deviation 9.50007446 variance 90.2514148
Skewness-0.1810018 kurtosis-0.3935688
Unmodified square sum 699900977 modified square sum 2545902.16
Coefficient of variation 6.04229006 standard error of the mean 0.05656209

Basic statistics

Positional variation

Mean 157.2264 standard deviation 9.50007
Median 158.0000 variance 90.25141
Mode 160.0000 Range 99.90000
Interquartile range 14.60000

Test for position H0: Mu0=0

Test-Statistics------p Value

Student's t-test t 2779.713 Pr > |t| <. 0001
Sign test M 14105 Pr >= |M| <. 0001
Signed rank test S 1. 9896E8 Pr >= |S| <. 0001

Quartile (Definition 5)

Level quantile

100% max.	195.9
99%	176.3
95%	172.0
90%	169.0
75% Q3	164.6
50% median	158.0
25% Q1	150.0
10%	145.0
5%	141.5
1%	135.7
0% minimum	96.0

Table 2-1 demographics according to the presence or absence of proton-pump inhibitors

UNIVARIATE Procedure

Variable: HGT_BL (Height at Informed Concent)

Extreme value

---- Minimum value----Maximum value----

Value	Obs	Value	Obs
96	2035	184.6	702
120	29891	185.5	31487
120	23794	188.0	29308
120	23497	193.0	15473
120	12920	195.9	24973

Table 2-1 demographics according to the presence or absence of proton-pump inhibitors

UNIVARIATE Procedure
Variable: WGT_BL (Weight at Informed Concent)

Moment

Total of 29643 for N 29643 weight variable
Mean 57.8048885 total 1713510.31
Standard deviation 11.1616947 variance 124.583428
Skewness 0.30021343 kurtosis 0.2312245
Unmodified square sum 102742174 modified square sum 3692901.96
Coefficient of variation 19.3092573 standard error of the mean 0.06482896

Basic statistics

Positional variation

Mean 57.80489 standard deviation 11.16169
Median 57.60000 variance 124.58343
Most frequent value 60.00000 range 122.60000
Interquartile range 15.00000

Test for position H0: Mu0=0

Test-Statistics------p Value

Student's t-test t 891.6522 Pr > |t| <. 0001
Sign test M 14821.5 Pr >= |M| <. 0001
Signed rank test S 2. 1968E8 Pr >= |S| <. 0001

Quartile (Definition 5)

Level quantile

100% max.	149.0
99%	85.7
95%	76.3
90%	72.0
75% Q3	65.0
50% median	57.6
25% Q1	50.0
10%	43.5
5%	40.0
1%	35.0
0% minimum	26.4

Table 2-1 demographics according to the presence or absence of proton-pump inhibitors

UNIVARIATE Procedure
Variable: WGT_BL (Weight at Informed Concent)

Extreme value

---- Minimum value----Maximum value----

Value	Obs	Value	Obs
26.4	17111	110.0	21562
27.0	20372	113.0	27031
27.4	12654	116.0	4265
27.7	4530	118.6	702
28.1	22941	149.0	27363

Table 2-1 demographics according to the presence or absence of proton-pump inhibitors

UNIVARIATE Procedure
Variables: BMI_BL (BMI at Informed Consent)

Moment

Total of 27944 for N 27944 weight variable
Mean 23.3235796 total 651754.109
Standard deviation 3.55682834 variance 12.6510279
Skewness 0.54074056 kurtosis 1.30857061
Unmodified square sum 15554746.5 modified square sum 353507.672
Coefficient of variation 15.2499248 standard error of the mean 0.0212774

Basic statistics

Positional variation

Mean 23.32358 standard deviation 3.55683
Median 23.14050 variance 12.65103
Most frequent value 22.22222 range 36.95231
Interquartile range 4.52852

Note: There are two most frequent values (frequency: 49). The table shows the smallest of the most common values.

Test for position H0: Mu0=0

Test-Statistics------p Value

Student's t-test t 1096.167 Pr > |t| <. 0001
Sign test M 13972 Pr >= |M| <. 0001
Signed rank test S 1. 9522E8 Pr >= |S| <. 0001

Quartile (Definition 5)

Level quantile

100% max.	47.7828
99%	33.1646
95%	29.3748
90%	27.7671
75% Q3	25.4651
50% median	23.1405
25% Q1	20.9366
10%	18.9903
5%	17.8571

Table 2-1 demographics according to the presence or absence of proton-pump inhibitors

UNIVARIATE Procedure
Variables: BMI_BL (BMI at Informed Consent)

Quartile (Definition 5)

Level quantile

1% 15.9170
0% minimum 10.8304

Extreme value

------ Minimum value------Maximum value------

Value	Obs	Value	Obs
10.8304	24083	46.4669	4265
11.7539	29923	46.5212	19571
12.1123	14615	47.0615	12554
12.2474	7548	47.1137	3292
12.3555	31962	47.7828	19258

Table 2-1 demographics according to the presence or absence of proton-pump inhibitors

UNIVARIATE Procedure
Variable: SBP_BL (Systolic Blood Pressure at Informed Consent)

Moment

Total of 29915 for N 29915 weight variable
Mean 127.335684 total 3809247
Standard deviation 17.0193514 variance 289.658322
Skewness 0.25480656 kurtosis 0.65335071
Unmodified square sum 493717913 modified square sum 8664839.06
Coefficient of variation 13.365736 standard error of the mean 0.09840077

Basic statistics

Positional variation

Mean 127.3357 standard deviation 17.01935
Median 127.0000 variance 289.65832
Mode 130.0000 Range 158.00000
Interquartile range 22.00000

Test for position H0: Mu0=0

Test-Statistics------p Value

Student's t-test t 1294.052 Pr > |t| <. 0001
Sign test M 14957.5 Pr >= |M| <. 0001
Signed rank test S 2. 2373E8 Pr >= |S| <. 0001

Quartile (Definition 5)

Level quantile

100% max. 224

99% 172

95% 156

90% 148

75% Q3 138

50% median 127

25% Q1 116

10% 106

5% 100

1% 89

0% minimum 66

Table 2-1 demographics according to the presence or absence of proton-pump inhibitors

UNIVARIATE Procedure

Variable: SBP_BL (Systolic Blood Pressure at Informed Consent)

Extreme value

--- Minimum value----Maximum value----

Value	Obs	Value	Obs
66	22326	210	7806
66	19535	210	7813
67	17540	211	21426
68	26217	214	18497
70	22563	224	32115

Table 2-1 demographics according to the presence or absence of proton-pump inhibitors

UNIVARIATE Procedure
Variable: DBP_BL (Diastolic Blood Pressure at Informed Concent)

Moment

Total of 29915 for N 29915 weight variable
Mean 70.6440247 total 2113316
Standard deviation 11.6239152 variance 135.115405
Skewness 0.25007232 kurtosis 0.62914099
Unmodified square sum 153334990 modified square sum 4041842.22
Coefficient of variation 16.4542086 standard error of the mean 0.06720598

Basic statistics

Positional variation

Mean 70.64402 standard deviation 11.62392
Median 70.00000 variance 135.11540
Mode 70.00000 Range 126.00000
Interquartile range 16.00000

Test for position H0: Mu0=0

Test-Statistics------p Value

Student's t-test t 1051.157 Pr > |t| <. 0001
Sign test M 14957.5 Pr >= |M| <. 0001
Signed rank test S 2. 2373E8 Pr >= |S| <. 0001

Quartile (Definition 5)

Level quantile

100% max. 139	
99%	100
95%	90
90%	85
75% Q3 78	
50% median 70	
25% Q1 62	
10%	56
5%	52
1%	44
0% minimum 13	

Table 2-1 demographics according to the presence or absence of proton-pump inhibitors

UNIVARIATE Procedure

Variable: DBP_BL (Diastolic Blood Pressure at Informed Concent)

Extreme value

--- Minimum value----Maximum value----

Value	Obs	Value	Obs
13	25699	130	31090
20	26670	131	15323
22	10698	131	28505
24	18950	133	24388
24	7652	139	22464

Table 2-1 demographics according to the presence or absence of proton-pump inhibitors

UNIVARIATE Procedure
Variable: AGEs (Age at First Drug Intake)

Moment

Total of 11981 for N 11981 weight variable
Mean 81.7248143 total 979145
Standard deviation 4.86749047 variance 23.6924635
Skewness 0.58315188 kurtosis-0.2678585
Unmodified square sum 80304279 modified square sum 283835.713
Coefficient of variation 5.95595171 standard error of the mean 0.04446912

Basic statistics

Positional variation

Mean 81.72481 standard deviation 4.86749
Median 81.00000 variance 23.69246
Mode 75.00000 Range 25.00000
Interquartile range 7.00000

Test for position H0: Mu0=0

Test-Statistics------p Value

Student's t-test t 1837.788 Pr > |t| <. 0001
Sign test M 5990.5 Pr >= |M| <. 0001
Signed rank test S 35889086 Pr >= |S| <. 0001

Quartile (Definition 5)

Level quantile

100% max. 100	
99%	94
95%	91
90%	89
75% Q3 85	
50% median 81	
25% Q1 78	
10%	76
5%	75
1%	75
0% minimum 75	

Table 2-1 demographics according to the presence or absence of proton-pump inhibitors

UNIVARIATE Procedure
Variable: AGEs (Age at First Drug Intake)

Extreme value

--- Minimum value----Maximum value----

Value	Obs	Value	Obs
75	32490	99	12659
75	32443	99	23804
75	32378	100	4681
75	32310	100	10523
75	32256	100	12680

Table 2-1 demographics according to the presence or absence of proton-pump inhibitors

UNIVARIATE Procedure
Variable: HGT_BL (Height at Informed Concent)

Moment

Total of 10606 for N 10606 weight variable
Mean 156.564613 total 1660524.29
Standard deviation 9.60591131 variance 92.273532
Skewness-0.1527824 kurtosis-0.3446183
Unmodified square sum 260957904 modified square sum 978560.807
Coefficient of variation 6.13542939 standard error of the mean 0.09327446

Basic statistics

Positional variation

Mean 156.5646 standard deviation 9.60591
Median 157.0000 variance 92.27353
Mode 160.0000 Range 97.00000
Interquartile range 14.50000

Test for position H0: Mu0=0

Test-Statistics------p Value

Student's t-test t 1678.537 Pr > |t| <. 0001
Sign test M 5303 Pr >= |M| <. 0001
Signed rank test S 28124461 Pr >= |S| <. 0001

Quartile (Definition 5)

Level quantile

100% max.	193.0
99%	176.0
95%	171.3
90%	169.0
75% Q3	164.0
50% median	157.0
25% Q1	149.5
10%	144.0
5%	140.5
1%	135.0
0% minimum	96.0

Table 2-1 demographics according to the presence or absence of proton-pump inhibitors

UNIVARIATE Procedure

Variable: HGT_BL (Height at Informed Concent)

Extreme value

---- Minimum value----Maximum value----

Value	Obs	Value	Obs
96	2035	184.0	6024
120	29891	184.0	6921
120	12920	185.5	31487
123	14613	188.0	29308
125	17227	193.0	15473

Table 2-1 demographics according to the presence or absence of proton-pump inhibitors

UNIVARIATE Procedure
Variable: WGT_BL (Weight at Informed Concent)

Moment

Total of 11046 for N 11046 weight variable
Mean 57.2802671 total 632717.83
Standard deviation 11.1711529 variance 124.794657
Skewness 0.2778062 kurtosis-0.0154519
Unmodified square sum 37620603.3 modified square sum 1378356.99
Coefficient of variation 19.5026201 standard error of the mean 0.10629075

Basic statistics

Positional variation

Mean 57.28027 standard deviation 11.17115
Median 57.00000 variance 124.79466
Mode 60.00000 Range 82.60000
Interquartile range 16.00000

Test for position H0: Mu0=0

Test-Statistics------p Value

Student's t-test t 538.9017 Pr > |t| <. 0001
Sign test M 5523 Pr >= |M| <. 0001
Signed rank test S 30506291 Pr >= |S| <. 0001

Quartile (Definition 5)

Level quantile

100% max.	110.0
99%	85.0
95%	76.0
90%	72.0
75% Q3	65.0
50% median	57.0
25% Q1	49.0
10%	43.0
5%	40.0
1%	34.8
0% minimum	27.4

Table 2-1 demographics according to the presence or absence of proton-pump inhibitors

UNIVARIATE Procedure

Variable: WGT_BL (Weight at Informed Concent)

Extreme value

---- Minimum value----Maximum value----

Value	Obs	Value	Obs
27.4	12654	102.2	5693
28.1	20038	102.5	2266
28.5	25995	107.0	96
29.0	2035	110.0	11271
29.2	15206	110.0	21562

Table 2-1 demographics according to the presence or absence of proton-pump inhibitors

UNIVARIATE Procedure
Variables: BMI_BL (BMI at Informed Consent)

Moment

Total of 10510 for N 10510 weight variable
Mean 23.3148454 total 245039.025
Standard deviation 3.62336868 variance 13.1288006
Skewness 0.5704506 kurtosis 1.26153856
Unmodified square sum 5851017.55 modified square sum 137970.565
Coefficient of variation 15.5410367 standard error of the mean 0.03534363

Basic statistics

Positional variation

Mean 23.31485 standard deviation 3.62337
Median 23.10439 variance 13.12880
Most frequent value 21.48438 range 33.94461
Interquartile range 4.62234

Test for position H0: Mu0=0

Test-Statistics------p Value

Student's t-test t 659.6619 Pr > |t| <. 0001
Sign test M 5255 Pr >= |M| <. 0001
Signed rank test S 27617653 Pr >= |S| <. 0001

Quartile (Definition 5)

Level quantile

100% max.	46.5212
99%	33.4692
95%	29.5226
90%	27.8488
75% Q3	25.4933
50% median	23.1044
25% Q1	20.8709
10%	18.9189
5%	17.8329
1%	15.9131
0% minimum	12.5766

Table 2-1 demographics according to the presence or absence of proton-pump inhibitors

UNIVARIATE Procedure

Variables: BMI_BL (BMI at Informed Consent)

Extreme value

------ Minimum value------Maximum value------

Value	Obs	Value	Obs
12.5766	12924	42.6909	12079
13.0131	257	43.1349	23172
13.0143	28211	43.8892	14613
13.1618	22008	44.2585	19336
13.2511	30646	46.5212	19571

Table 2-1 demographics according to the presence or absence of proton-pump inhibitors

UNIVARIATE Procedure
Variable: SBP_BL (Systolic Blood Pressure at Informed Consent)

Moment

Total of 10942 for N 10942 weight variable
Mean 126.866295 total 1388171
Standard deviation 17.3752684 variance 301.899953
Skewness 0.29135138 kurtosis 0.72811297
Unmodified square sum 179415199 modified square sum 3303087.39
Coefficient of variation 13.6957325 standard error of the mean 0.16610518

Basic statistics

Positional variation

Mean 126.8663 standard deviation 17.37527
Median 126.0000 variance 301.89995
Mode 120.0000 Range 158.00000
Interquartile range 21.00000

Test for position H0: Mu0=0

Test-Statistics------p Value

Student's t-test t 763.7709 Pr > |t| <. 0001
Sign test M 5471 Pr >= |M| <. 0001
Signed rank test S 29934577 Pr >= |S| <. 0001

Quartile (Definition 5)

Level quantile

100% max. 224

99% 173

95% 157

90% 149

75% Q3 137

50% median 126

25% Q1 116

10% 105

5% 100

1% 88

0% minimum 66

Table 2-1 demographics according to the presence or absence of proton-pump inhibitors

UNIVARIATE Procedure
Variable: SBP_BL (Systolic Blood Pressure at Informed Consent)

Extreme value

--- Minimum value----Maximum value----

Value	Obs	Value	Obs
66	22326	204	13790
66	19535	208	10107
70	11019	211	21426
71	13585	214	18497
71	179	224	32115

Table 2-1 demographics according to the presence or absence of proton-pump inhibitors

UNIVARIATE Procedure
Variable: DBP_BL (Diastolic Blood Pressure at Informed Concent)

Moment

Total of 10942 for N 10942 weight variable
Mean 70.1461342 total 767539
Standard deviation 11.7787208 variance 138.738263
Skewness 0.31581935 kurtosis 0.7429483
Unmodified square sum 55357829 modified square sum 1517935.33
Coefficient of variation 16.7916891 standard error of the mean 0.11260295

Basic statistics

Positional variation

Mean 70.14613 standard deviation 11.77872
Median 70.00000 variance 138.73826
Mode 70.00000 Range 117.00000
Interquartile range 16.00000

Test for position H0: Mu0=0

Test-Statistics------p Value

Student's t-test t 622.9511 Pr > |t| <. 0001
Sign test M 5471 Pr >= |M| <. 0001
Signed rank test S 29934577 Pr >= |S| <. 0001

Quartile (Definition 5)

Level quantile

100% max.	139
99%	100
95%	90
90%	85
75% Q3	78
50% median	70
25% Q1	62
10%	56
5%	52
1%	44
0% minimum	22

Table 2-1 demographics according to the presence or absence of proton-pump inhibitors

UNIVARIATE Procedure
Variable: DBP_BL (Diastolic Blood Pressure at Informed Concent)

Extreme value

--- Minimum value----Maximum value----

Value	Obs	Value	Obs
22	10698	124	15335
24	7652	130	3039
31	23196	131	15323
34	29954	133	24388
34	29221	139	22464

Table 2-1 demographics according to the presence or absence of proton-pump inhibitors

UNIVARIATE Procedure
Variable: AGEs (Age at First Drug Intake)

Moment

Total of 20509 for N 20509 weight variable
Mean 81.3252718 total 1667900
Standard deviation 4.78725834 variance 22.9178424
Skewness 0.67575565 kurtosis-0.0469797
Unmodified square sum 136112420 modified square sum 469999.112
Coefficient of variation 5.88655682 standard error of the mean 0.03342833

Basic statistics

Positional variation

Mean 81.32527 standard deviation 4.78726
Median 81.00000 variance 22.91784
Mode 75.00000 Range 28.00000
Interquartile range 8.00000

Test for position H0: Mu0=0

Test-Statistics------p Value

Student's t-test t 2432.825 Pr > |t| <. 0001
Sign test M 10254.5 Pr >= |M| <. 0001
Signed rank test S 1. 0516E8 Pr >= |S| <. 0001

Quartile (Definition 5)

Level quantile

100% max. 103	
99%	94
95%	90
90%	88
75% Q3 85	
50% median 81	
25% Q1 77	
10%	76
5%	75
1%	75
0% minimum 75	

Table 2-1 demographics according to the presence or absence of proton-pump inhibitors

UNIVARIATE Procedure
Variable: AGEs (Age at First Drug Intake)

Extreme value

--- Minimum value----Maximum value----

Value	Obs	Value	Obs
75	32470	101	5225
75	32456	101	23819
75	32444	102	5655
75	32432	102	29497
75	32431	103	7812

Table 2-1 demographics according to the presence or absence of proton-pump inhibitors

UNIVARIATE Procedure
Variable: HGT_BL (Height at Informed Concent)

Moment

Total of 17604 for N 17604 weight variable
Mean 157.625093 total 2774832.14
Standard deviation 9.41357887 variance 88.6154671
Skewness-0.1949703 kurtosis-0.425521
Unmodified square sum 438943073 modified square sum 1559898.07
Coefficient of variation 5.97213215 standard error of the mean 0.07094946

Basic statistics

Positional variation

Mean 157.6251 standard deviation 9.41358
Median 158.0000 variance 88.61547
Most frequent value 160.0000 range 75.90000
Interquartile range 14.70000

Test for position H0: Mu0=0

Test-Statistics------p Value

Student's t-test t 2221.653 Pr > |t| <. 0001
Sign test M 8802 Pr >= |M| <. 0001
Signed rank test S 77479605 Pr >= |S| <. 0001

Quartile (Definition 5)

Level quantile

100% max. 195.9

99% 176.5

95% 172.0

90% 169.5

75% Q3 165.0

50% median 158.0

25% Q1 150.3

10% 145.0

5% 142.0

1% 136.4

0% minimum 120.0

Table 2-1 demographics according to the presence or absence of proton-pump inhibitors

UNIVARIATE Procedure

Variable: HGT_BL (Height at Informed Concent)

Extreme value

----- Minimum value----Maximum value----

Value	Obs	Value	Obs
120.0	23794	183.0	27283
120.0	23497	183.0	28042
120.0	11830	184.0	25870
121.5	27679	184.6	702
122.0	7115	195.9	24973

Table 2-1 demographics according to the presence or absence of proton-pump inhibitors

UNIVARIATE Procedure
Variable: WGT_BL (Weight at Informed Concent)

Moment

Total of 18597 for N 18597 weight variable
Mean 58.1164962 total 1080792.48
Standard deviation 11.1446884 variance 124.204079
Skewness 0.31502141 kurtosis 0.37647788
Unmodified square sum 65121571.1 modified square sum 2309699.05
Coefficient of variation 19.176463 standard error of the mean 0.08172341

Basic statistics

Positional variation

Mean 58.11650 standard deviation 11.14469
Median 58.00000 variance 124.20408
Most frequent value 60.00000 range 122.60000
Interquartile range 15.20000

Test for position H0: Mu0=0

Test-Statistics------p Value

Student's t-test t 711.1365 Pr > |t| <. 0001
Sign test M 9298.5 Pr >= |M| <. 0001
Signed rank test S 86466752 Pr >= |S| <. 0001

Quartile (Definition 5)

Level quantile

100% max.	149.0
99%	86.0
95%	76.6
90%	72.4
75% Q3	65.2
50% median	58.0
25% Q1	50.0
10%	44.0
5%	40.5
1%	35.1
0% minimum	26.4

Table 2-1 demographics according to the presence or absence of proton-pump inhibitors

UNIVARIATE Procedure
Variable: WGT_BL (Weight at Informed Concent)

Extreme value

---- Minimum value----Maximum value----

Value	Obs	Value	Obs
26.4	17111	108.0	22896
27.0	20372	113.0	27031
27.7	4530	116.0	4265
28.1	22941	118.6	702
28.5	4172	149.0	27363

Table 2-1 demographics according to the presence or absence of proton-pump inhibitors

UNIVARIATE Procedure
Variables: BMI_BL (BMI at Informed Consent)

Moment

Total of 17434 for N 17434 weight variable
Mean 23.328845 total 406715.084
Standard deviation 3.51620077 variance 12.3636678
Skewness 0.5211885 kurtosis 1.33574282
Unmodified square sum 9703728.98 modified square sum 215535.821
Coefficient of variation 15.0723311 standard error of the mean 0.02663024

Basic statistics

Positional variation

Mean 23.32885 standard deviation 3.51620
Median 23.15765 variance 12.36367
Most frequent value 22.22222 range 36.95231
Interquartile range 4.49371

Test for position H0: Mu0=0

Test-Statistics------p Value

Student's t-test t 876.0282 Pr > |t| <. 0001
Sign test M 8717 Pr >= |M| <. 0001
Signed rank test S 75990448 Pr >= |S| <. 0001

Quartile (Definition 5)

Level quantile

100% max.	47.7828
99%	32.8731
95%	29.3199
90%	27.7344
75% Q3	25.4581
50% median	23.1576
25% Q1	20.9644
10%	19.0274
5%	17.8980
1%	15.9194
0% minimum	10.8304

Table 2-1 demographics according to the presence or absence of proton-pump inhibitors

UNIVARIATE Procedure
Variables: BMI_BL (BMI at Informed Consent)

Extreme value

------ Minimum value------Maximum value------

Value	Obs	Value	Obs
10.8304	24083	42.3666	20570
11.7539	29923	46.4669	4265
12.1123	14615	47.0615	12554
12.2474	7548	47.1137	3292
12.3555	31962	47.7828	19258

Table 2-1 demographics according to the presence or absence of proton-pump inhibitors

UNIVARIATE Procedure
Variable: SBP_BL (Systolic Blood Pressure at Informed Consent)

Moment

Total of 18973 for N 18973 weight variable
Mean 127.606388 total 2421076
Standard deviation 16.8051654 variance 282.413583
Skewness 0.23498835 kurtosis 0.60474821
Unmodified square sum 314302714 modified square sum 5357950.51
Coefficient of variation 13.169533 standard error of the mean 0.1220042

Basic statistics

Positional variation

Mean 127.6064 standard deviation 16.80517
Median 128.0000 variance 282.41358
Mode 130.0000 Range 143.00000
Interquartile range 21.00000

Test for position H0: Mu0=0

Test-Statistics------p Value

Student's t-test t 1045.918 Pr > |t| <. 0001
Sign test M 9486.5 Pr >= |M| <. 0001
Signed rank test S 89998426 Pr >= |S| <. 0001

Quartile (Definition 5)

Level quantile

100% max. 210	
99%	172
95%	156
90%	148
75% Q3 138	
50% median 128	
25% Q1 117	
10%	106
5%	100
1%	90
0% minimum 67	

Table 2-1 demographics according to the presence or absence of proton-pump inhibitors

UNIVARIATE Procedure
Variable: SBP_BL (Systolic Blood Pressure at Informed Consent)

Extreme value

--- Minimum value----Maximum value----

Value	Obs	Value	Obs
67	17540	205	7600
68	26217	205	20799
70	22563	207	27937
70	18755	210	7806
74	27610	210	7813

Table 2-1 demographics according to the presence or absence of proton-pump inhibitors

UNIVARIATE Procedure
Variable: DBP_BL (Diastolic Blood Pressure at Informed Concent)

Moment

Total of 18973 for N 18973 weight variable
Mean 70.9311653 total 1345777
Standard deviation 11.5242278 variance 132.807827
Skewness 0.21355319 kurtosis 0.56977604
Unmodified square sum 97977161 modified square sum 2519630.1
Coefficient of variation 16.2470584 standard error of the mean 0.083665

Basic statistics

Positional variation

Mean 70.93117 standard deviation 11.52423
Median 70.00000 variance 132.80783
Mode 70.00000 Range 118.00000
Interquartile range 16.00000

Test for position H0: Mu0=0

Test-Statistics------p Value

Student's t-test t 847.7997 Pr > |t| <. 0001
Sign test M 9486.5 Pr >= |M| <. 0001
Signed rank test S 89998426 Pr >= |S| <. 0001

Quartile (Definition 5)

Level quantile

100% max. 131	
99%	100
95%	90
90%	85
75% Q3 79	
50% median 70	
25% Q1 63	
10%	57
5%	52
1%	45
0% minimum 13	

Table 2-1 demographics according to the presence or absence of proton-pump inhibitors

UNIVARIATE Procedure
Variable: DBP_BL (Diastolic Blood Pressure at Informed Concent)

Extreme value

--- Minimum value----Maximum value----

Value	Obs	Value	Obs
13	25699	123	3096
20	26670	123	19480
24	18950	124	17556
26	17540	130	31090
27	31091	131	28505

Table 2-1 demographics according to the presence or absence of proton-pump inhibitors

FREQ Procedure

Height at Informed Concent

HGT_BL	Frequency	Percentage	Cumulative	
999	4280	100.00	4280	100.00

Table 2-1 demographics according to the presence or absence of proton-pump inhibitors

FREQ Procedure

Weight at Informed Concent

WGT_BL	Frequency	Percentage	Cumulative Frequency	Cumulative Percentage
999	2847	100.00	2847	100.00

Table 2-1 demographics according to the presence or absence of proton-pump inhibitors

FREQ Procedure

BMI at Informed Consent

BMI_BL	Frequency	Percentage	Cumulative	
999	4546	100.00	4546	100.00

Table 2-1 demographics according to the presence or absence of proton-pump inhibitors

FREQ Procedure

Systolic Blood Pressure at Informed Consent

SBP_BL	Frequency	Percentage	Cumulative Frequency	Cumulative Percentage
999	2575	100.00	2575	100.00

Table 2-1 demographics according to the presence or absence of proton-pump inhibitors

FREQ Procedure

Diastolic Blood Pressure at Informed Concent

DBP_BL	Frequency	Percentage	Cumulative Frequency	Cumulative Percentage
999	2575	100.00	2575	100.00

Table 2-1 demographics according to the presence or absence of proton-pump inhibitors

FREQ Procedure

Height at Informed Concent

HGT_BL	Frequency	Percentage	Cumulative	
999	1375	100.00	1375	100.00

Table 2-1 demographics according to the presence or absence of proton-pump inhibitors

FREQ Procedure

Weight at Informed Concent

WGT_BL	Frequency	Percentage	Cumulative	
999	935	100.00	935	100.00

Table 2-1 demographics according to the presence or absence of proton-pump inhibitors

FREQ Procedure

BMI at Informed Consent

BMI_BL	Frequency	Percentage	Cumulative Frequency	Cumulative Percentage
999	1471	100.00	1471	100.00

Table 2-1 demographics according to the presence or absence of proton-pump inhibitors

FREQ Procedure

Systolic Blood Pressure at Informed Consent

SBP_BL	Frequency	Percentage	Cumulative	
999	1039	100.00	1039	100.00

Table 2-1 demographics according to the presence or absence of proton-pump inhibitors

FREQ Procedure

Diastolic Blood Pressure at Informed Concent

DBP_BL	Frequency	Percentage	Cumulative Frequency	Cumulative Percentage
999	1039	100.00	1039	100.00

Table 2-1 demographics according to the presence or absence of proton-pump inhibitors

FREQ Procedure

Height at Informed Concent

HGT_BL	Frequency	Percentage	Cumulative	
999	2905	100.00	2905	100.00

Table 2-1 demographics according to the presence or absence of proton-pump inhibitors

FREQ Procedure

Weight at Informed Concent

WGT_BL	Frequency	Percentage	Cumulative Frequency	Cumulative Percentage
999	1912	100.00	1912	100.00

Table 2-1 demographics according to the presence or absence of proton-pump inhibitors

FREQ Procedure

BMI at Informed Consent

BMI_BL	Frequency	Percentage	Cumulative Frequency	Cumulative Percentage
999	3075	100.00	3075	100.00

Table 2-1 demographics according to the presence or absence of proton-pump inhibitors

FREQ Procedure

Systolic Blood Pressure at Informed Consent

SBP_BL	Frequency	Percentage	Cumulative	
999	1536	100.00	1536	100.00

Table 2-1 demographics according to the presence or absence of proton-pump inhibitors

FREQ Procedure

Diastolic Blood Pressure at Informed Concent

DBP_BL	Frequency	Percentage	Cumulative	
999	1536	100.00	1536	100.00

Table 2-1 demographics according to the presence or absence of proton-pump inhibitors

FREQ Procedure

Number of levels of variables

Variables Label Level Number

GROUP1 2
SEXN Sex (N) 2

Table : GROUP1 * SEXN

GROUP1 SEXN(Sex (N))

Power / Percent / Percent of rows / Percentage of columns	0	1	Sum
1	6561	5420	11981
	20.19	16.68	36.88
	54.76	45.24	
	35.27	39.02	
2	12040	8469	20509
	37.06	26.07	63.12
	58.71	41.29	
	64.73	60.98	
Total	18601	13889	32490
	57.25	42.75	100.00

Table 2-1 demographics according to the presence or absence of proton-pump inhibitors

FREQ Procedure

Number of levels of variables

Variables Label Level Number

GROUP1 2
AGEC Age Category 6

Table : GROUP1 * AGEC

GROUP1 AGEC(Age Category)

Power |
Percent |
Percent of rows
Percentage of column |75 years and older |80 years and older |85 years and older |90 years and older |95 years and older | 100 years and older |Total
|Ages under 80|Ages under 85|Ages under 90|Ages under 95|Ages under 100 Ages under |Above|
| | | | Full | | | |

1	4559	4061	2481	767	110	3	11981
	14.03	12.50	7.64	2.36	0.34	0.01	36.88
	38.05	33.90	20.71	6.40	0.92	0.03	
	35.16	36.84	39.06	41.15	40.15	27.27	
2	8408	6961	3871	1097	164	8	20509
	25.88	21.43	11.91	3.38	0.50	0.02	63.12
	41.00	33.94	18.87	5.35	0.80	0.04	
	64.84	63.16	60.94	58.85	59.85	72.73	

Total 12967 11022 6352 1864 274 11 32490

39.91	33.92	19.55	5.74	0.84	0.03	100.00

Table 2-1 demographics according to the presence or absence of proton-pump inhibitors

FREQ Procedure

Number of levels of variables

Variables Label Level Number

GROUP1 2
WGTC_BL Weight Category at Informed Concent 2

Table : GROUP1 * WGTC_BL

GROUP1
WGTC_BL(Weight Category at Informed Concent)

Power / Percent / Percent of rows / Percent of column	less than 60 kg	more than 60 kg	Total
1	6543	4503	11046
	22.07	15.19	37.26
	59.23	40.77	
	38.48	35.63	
2	10462	8135	18597
	35.29	27.44	62.74
	56.26	43.74	
	61.52	64.37	
Total	17005	12638	29643
	57.37	42.63	100.00

Table 2-1 demographics according to the presence or absence of proton-pump inhibitors

FREQ Procedure

Number of levels of variables

Variables Label Level Number

GROUP1 2
BMIC_BL BMI Category at Informed Consent 3

Table : GROUP1 * BMIC_BL

GROUP1
BMIC_BL(BMI Category at Informed Consent)

Power |
Percent |
Percent of rows
Percent of column |18.5kg/m|18.5kg/m|25.0kg/m| Total
<2	>2	>25	>2	
	0 kg/m2 un	
	Full	

1	805	6659	3046	10510
	2.88	23.83	10.90	37.61
	7.66	63.36	28.98	
	38.76	37.52	37.51	
2	1272	11088	5074	17434
	4.55	39.68	18.16	62.39
	7.30	63.60	29.10	
	61.24	62.48	62.49	
Total	2077	17747	8120	27944

7.43	63.51	29.06	100.00

Table 2-1 demographics according to the presence or absence of proton-pump inhibitors

FREQ Procedure

Number of levels of variables

Variables Label Level Number

GROUP1 2
SMK1 Smoking Status Part 1 3

Table : GROUP1 * SMK1

GROUP1 SMK1(Smoking Status Part 1)

Power |
Percent |
Percent of rows
Percent of column |Smoking|Stopped|Smoked|Total
|no|||no||

	Smoking no	Stopped	Smoked no	Total
1	6084	3802	452	10338
	22.16	13.85	1.65	37.65
	58.85	36.78	4.37	
	37.44	38.16	36.33	
2	10168	6161	792	17121
	37.03	22.44	2.88	62.35
	59.39	35.99	4.63	
	62.56	61.84	63.67	
Total	16252	9963	1244	27459
	59.19	36.28	4.53	100.00

Table 2-1 demographics according to the presence or absence of proton-pump inhibitors

FREQ Procedure

Number of levels of variables

Variables Label Level Number

GROUP1 2
DRK Drinking Habit 3

Table : GROUP1 * DRK

GROUP1 DRK(Drinking Habit)

Power / Percent / Percent of rows / Percentage of columns	Drink routinely	Drink occasionally	Do not drink at all	total
1	1979	2067	6131	10177
	7.31	7.63	22.64	37.59
	19.45	20.31	60.24	
	34.10	35.02	39.89	
2	3825	3835	9238	16898
	14.13	14.16	34.12	62.41
	22.64	22.69	54.67	
	65.90	64.98	60.11	
Total	5804	5902	15369	27075
	21.44	21.80	56.76	100.00

Table 2-1 demographics according to the presence or absence of proton-pump inhibitors

TTEST Procedure

Variable: AGEs (Age at First Drug Intake)

GROUP1	N	Mean	Standard Deviation	Standard Error	Minimum Value	Maximum Value
1	11981	81.7248	4.8675	0.0445	75.0000	100.0
2	20509	81.3253	4.7873	0.0334	75.0000	103.0
Diff (1-2)		0.3995	4.8170	0.0554		

To the mean standard deviation

GROUP1	methods	Mean	95% confidence limits		standard deviation	95% confidence limits	
1		81.7248	81.6376	81.8120	4.8675	4.8066	4.9299
2		81.3253	81.2597	81.3908	4.7873	4.7414	4.8340
Diff (1-2)	Pooled	0.3995	0.2910	0.5081	4.8170	4.7802	4.8543
Diff (1-2)	Satterthwaite	0.3995	0.2905	0.5086			

Technique	Dispersion	degrees of freedom	t-value	Pr > |t|
Pooled	Equal	32488	7.21	<. 0001
Satterthwaite	Unequal	24732	7.18	<. 0001

Isodispersity

Technique	Degree of Freedom of Moleculars	Degree of Freedom of the denominator	F Value	Pr > F
Folded F	11980	20508	1.03	0.0405

Table 2-1 demographics according to the presence or absence of proton-pump inhibitors

TTEST Procedure

Variable: HGT_BL (Height at Informed Concent)

GROUP1	N	Mean	Standard Deviation	Standard Error	Minimum Value	Maximum Value
1	10606	156.6	9.6059	0.0933	96.0000	193.0
2	17604	157.6	9.4136	0.0709	120.0	195.9
Diff (1-2)		-1.0605	9.4863	0.1166		

			To the mean		standard deviation		
GROUP1	methods	Mean	95% confidence limits		standard deviation	95% confidence limits	
1		156.6	156.4	156.7	9.6059	9.4784	9.7370
2		157.6	157.5	157.8	9.4136	9.3163	9.5130
Diff (1-2)	Pooled	-1.0605	-1.2890	-0.8319	9.4863	9.4087	9.5653
Diff (1-2)	Satterthwaite	-1.0605	-1.2902	-0.8308			

Technique	Dispersion	degrees of freedom	t-value	Pr > |t|
Pooled	Equal	28208	-9.09	<. 0001
Satterthwaite	Unequal	21992	-9.05	<. 0001

Isodispersity

Technique	Degree of Freedom of Moleculars	Degree of Freedom of the denominator	F Value	Pr > F
Folded F	10605	17603	1.04	0.0197

Table 2-1 demographics according to the presence or absence of proton-pump inhibitors

TTEST Procedure

Variable: WGT_BL (Weight at Informed Concent)

GROUP1	N	Mean	Standard Deviation	Standard Error	Minimum Value	Maximum Value
1	11046	57.2803	11.1712	0.1063	27.4000	110.0
2	18597	58.1165	11.1447	0.0817	26.4000	149.0
Diff (1-2)		-0.8362	11.1546	0.1340		

GROUP1	methods	Mean	95% confidence limits		To the mean standard deviation<br>standard deviation	95% confidence limits	
1		57.2803	57.0719	57.4886	11.1712	11.0258	11.3204
2		58.1165	57.9563	58.2767	11.1447	11.0326	11.2591
Diff (1-2)	Pooled	-0.8362	-1.0989	-0.5736	11.1546	11.0655	11.2451
Diff (1-2)	Satterthwaite	-0.8362	-1.0990	-0.5734			

Technique	Dispersion	degrees of freedom	t-value	Pr > |t|
Pooled	Equal	29641	-6.24	<. 0001
Satterthwaite	Unequal	23157	-6.24	<. 0001

Isodispersity

Technique	Degree of Freedom of Moleculars	Degree of Freedom of the denominator	F Value	Pr > F
Folded F	11045	18596	1.00	0.7789

Table 2-1 demographics according to the presence or absence of proton-pump inhibitors

TTEST Procedure

Variables: BMI_BL (BMI at Informed Consent)

GROUP1	N	Mean	Standard Deviation	Standard Error	Minimum Value	Maximum Value
1	10510	23.3148	3.6234	0.0353	12.5766	46.5212
2	17434	23.3288	3.5162	0.0266	10.8304	47.7828

Diff (1-2) -0.0140 3.5569 0.0439

To the mean standard deviation

GROUP1 methods Mean 95% confidence limits standard deviation 95% confidence limits

1 23.3148 23.2456 23.3841 3.6234 3.5750 3.6730

2 23.3288 23.2766 23.3810 3.5162 3.4797 3.5535

Diff (1-2) Pooled -0.0140 -0.1001 0.0721 3.5569 3.5276 3.5866

Diff (1-2) Satterthwaite -0.0140 -0.1007 0.0727

Technique Dispersion degrees of freedom t-value Pr > |t|

Pooled Equal 27942 -0.32 0.7499

Satterthwaite Unequal 21626 -0.32 0.7517

Isodispersity

Technique Degree of Freedom of Moleculars Degree of Freedom of the denominator F Value Pr > F

Folded F 10509 17433 1.06 0.0006

Table 2-1 demographics according to the presence or absence of proton-pump inhibitors

TTEST Procedure

Variable: SBP_BL (Systolic Blood Pressure at Informed Consent)

GROUP1 N Mean Standard Deviation Standard Error Minimum Value Maximum Value

1 10942 126.9 17.3753 0.1661 66.0000 224.0
2 18973 127.6 16.8052 0.1220 67.0000 210.0
Diff (1-2) -0.7401 17.0159 0.2043

To the mean standard deviation
GROUP1 methods Mean 95% confidence limits standard deviation 95% confidence limits

1 126.9 126.5 127.2 17.3753 17.1481
17.6086
2 127.6 127.4 127.8 16.8052 16.6378
16.9760
Diff (1-2) Pooled -0.7401 -1.1405 -0.3397 17.0159 16.8806 17.1534
Diff (1-2) Satterthwaite -0.7401 -1.1441 -0.3361

Technique Dispersion degrees of freedom t-value Pr > |t|

Pooled Equal 29913 -3.62 0.0003
Satterthwaite Unequal 22204 -3.59 0.0003

Isodispersity

Technique Degree of Freedom of Moleculars Degree of Freedom of the denominator F Value Pr > F

Folded F 10941 18972 1.07 <. 0001

Table 2-1 demographics according to the presence or absence of proton-pump inhibitors

TTEST Procedure

Variable: DBP_BL (Diastolic Blood Pressure at Informed Concent)

GROUP1	N	Mean	Standard Deviation	Standard Error	Minimum Value	Maximum Value
1	10942	70.1461	11.7787	0.1126	22.0000	139.0
2	18973	70.9312	11.5242	0.0837	13.0000	131.0
Diff (1-2)		-0.7850	11.6180	0.1395		

GROUP1	methods	Mean	95% confidence limits		To the mean standard deviation<br>standard deviation	95% confidence limits	
1		70.1461	69.9254	70.3669	11.7787	11.6247	11.9369
2		70.9312	70.7672	71.0952	11.5242	11.4094	11.6414
Diff (1-2)	Pooled	-0.7850	-1.0584	-0.5117	11.6180	11.5256	11.7118
Diff (1-2)	Satterthwaite	-0.7850	-1.0600	-0.5101			

Technique	Dispersion	degrees of freedom	t-value	Pr > |t|
Pooled	Equal	29913	-5.63	<. 0001
Satterthwaite	Unequal	22416	-5.60	<. 0001

Isodispersity

Technique	Degree of Freedom of Moleculars	Degree of Freedom of the denominator	F Value	Pr > F
Folded F	10941	18972	1.04	0.0099

Treatment history at the time of obtaining Table 2-2 consent according to the presence or absence of proton-pump inhibitors

FREQ Procedure

Cumulative
Percent of ALL power Percent of power

1	32490	100.00	32490	100.00

Tables: ALL* HISAF

ALL
HISAF(History of Major Surgery for non-AF)

Power |
Percent |
Percent of rows
Percent of column |immediate 3 months |immediate 3 months |unknown |total
) within |Month within |Month| |
|Major surgery|Major surgery| |
Lip/lip | |

1	336	31690	464	32490
	1.03	97.54	1.43	100.00
	1.03	97.54	1.43	
	100.00	100.00	100.00	
Total	336	31690	464	32490
	1.03	97.54	1.43	100.00

Treatment history at the time of obtaining Table 2-2 consent according to the presence or absence of proton-pump inhibitors

FREQ Procedure

Cumulative

Percent of ALL power Percent of power

1	32490	100.00	32490	100.00

Tables: ALL* HISAF

ALL

HISAF(History of Major Surgery for non-AF)

Power |
Percent |
Percent of rows
Percent of column |immediate 3 months |immediate 3 months |unknown |total
) within |Month within |Month| |
|Major surgery|Major surgery| |
Lip/lip | |

1	336	31690	464	32490
	1.03	97.54	1.43	100.00
	1.03	97.54	1.43	
	100.00	100.00	100.00	
Total	336	31690	464	32490
	1.03	97.54	1.43	100.00

Treatment history at the time of obtaining Table 2-2 consent according to the presence or absence of proton-pump inhibitors

FREQ Procedure

Cumulative
Percent of ALL power Percent of power

1	32490	100.00	32490	100.00

Tables: ALL* HISAF

ALL
HISAF(History of Major Surgery for non-AF)

Power |
Percent |
Percent of rows
Percent of column |immediate 3 months |immediate 3 months |unknown |total
) within |Month within |Month| |
|Major surgery|Major surgery| |
Lip/lip | |

1	336	31690	464	32490
	1.03	97.54	1.43	100.00
	1.03	97.54	1.43	
	100.00	100.00	100.00	
Total	336	31690	464	32490
	1.03	97.54	1.43	100.00

Treatment history at the time of obtaining Table 2-2 consent according to the presence or absence of proton-pump inhibitors

FREQ Procedure

Cumulative

Percent of ALL power Percent of power

1	32490	100.00	32490	100.00

Tables: ALL* HISBDYN

ALL

HISBDYN(History of Major Beeding)

Power |
Percent |
Percent of rows
Percent of columns | None | With | Total

	None	With	Total
1	31229	1261	32490
	96.12	3.88	100.00
	96.12	3.88	
	100.00	100.00	
Total	31229	1261	32490
	96.12	3.88	100.00

Treatment history at the time of obtaining Table 2-2 consent according to the presence or absence of proton-pump inhibitors

FREQ Procedure

			Cumulative	
Percent of ALL	power	Percent of power		
1	32490	100.00	32490	100.00

Tables: ALL* HISBDYN

ALL

HISBDYN(History of Major Beeding)

Power |
Percent |
Percent of rows
Percent of columns | None | With | Total

	None	With	Total
1	31229	1261	32490
	96.12	3.88	100.00
	96.12	3.88	
	100.00	100.00	
Total	31229	1261	32490
	96.12	3.88	100.00

Treatment history at the time of obtaining Table 2-2 consent according to the presence or absence of proton-pump inhibitors

FREQ Procedure

			Cumulative	
Percent of ALL	power	Percent of	power	
1	32490	100.00	32490	100.00

Table : ALL * HISBD1

ALL

HISBD1(Intracranial Hemorrhage)

Power / Percent / Percent of rows / Percent of columns	None	With	Total
1	31841	649	32490
	98.00	2.00	100.00
	98.00	2.00	
	100.00	100.00	
Total	31841	649	32490
	98.00	2.00	100.00

Treatment history at the time of obtaining Table 2-2 consent according to the presence or absence of proton-pump inhibitors

FREQ Procedure

			Cumulative	
Percent of ALL	power	Percent of power		
1	32490	100.00	32490	100.00

Table : ALL * HISBD1

ALL

HISBD1(Intracranial Hemorrhage)

Power | Percent | Percent of rows Percent of columns	None	With	Total
1	31841	649	32490
	98.00	2.00	100.00
	98.00	2.00	
	100.00	100.00	
Total	31841	649	32490
	98.00	2.00	100.00

Treatment history at the time of obtaining Table 2-2 consent according to the presence or absence of proton-pump inhibitors

FREQ Procedure

			Cumulative	
	Percent of ALL power	Percent of power		
1	32490	100.00	32490	100.00

Table : ALL * HISBD2

ALL

HISBD2(Upper Gastrointestinal Bleeding)

Power |
Percent |
Percent of rows
Percent of columns | None | With | Total

	None	With	Total
1	32216	274	32490
	99.16	0.84	100.00
	99.16	0.84	
	100.00	100.00	
Total	32216	274	32490
	99.16	0.84	100.00

Treatment history at the time of obtaining Table 2-2 consent according to the presence or absence of proton-pump inhibitors

FREQ Procedure

			Cumulative	
	Percent of ALL	power	Percent of power	
1	32490	100.00	32490	100.00

Table : ALL * HISBD2

ALL

HISBD2(Upper Gastrointestinal Bleeding)

Power |
Percent |
Percent of rows
Percent of columns | None | With | Total

	None	With	Total
1	32216	274	32490
	99.16	0.84	100.00
	99.16	0.84	
	100.00	100.00	
Total	32216	274	32490
	99.16	0.84	100.00

Treatment history at the time of obtaining Table 2-2 consent according to the presence or absence of proton-pump inhibitors

FREQ Procedure

			Cumulative	
Percent of ALL	power	Percent of	power	
1	32490	100.00	32490	100.00

Table : ALL * HISBD3

ALL

HISBD3(Lower Gastrointestinal Bleeding)

Power<br>Percent<br>Percent of rows<br>Percent of columns	None	With	Total
1	32182<br>99.05<br>99.05<br>100.00	308<br>0.95<br>0.95<br>100.00	32490<br>100.00
Total	32182<br>99.05	308<br>0.95	32490<br>100.00

Treatment history at the time of obtaining Table 2-2 consent according to the presence or absence of proton-pump inhibitors

FREQ Procedure

			Cumulative	
Percent of ALL	power	Percent of power		
1	32490	100.00	32490	100.00

Table : ALL * HISBD3

ALL

HISBD3(Lower Gastrointestinal Bleeding)

Power / Percent / Percent of rows / Percent of columns	None	With	Total
1	32182	308	32490
	99.05	0.95	100.00
	99.05	0.95	
	100.00	100.00	
Total	32182	308	32490
	99.05	0.95	100.00

Treatment history at the time of obtaining Table 2-2 consent according to the presence or absence of proton-pump inhibitors

FREQ Procedure

			Cumulative	
Percent of ALL	power	Percent of	power	
1	32490	100.00	32490	100.00

Table : ALL * HISBD4

ALL HISBD4(Other Beeding)

Power |
Percent |
Percent of rows
Percent of columns | None | With | Total

	None	With	Total
1	32242	248	32490
	99.24	0.76	100.00
	99.24	0.76	
	100.00	100.00	
Total	32242	248	32490
	99.24	0.76	100.00

Treatment history at the time of obtaining Table 2-2 consent according to the presence or absence of proton-pump inhibitors

FREQ Procedure

			Cumulative	
Percent of ALL	power	Percent	of power	
1	32490	100.00	32490	100.00

Table : ALL * HISBD4

ALL HISBD4(Other Beeding)

Power<br>Percent<br>Percent of rows<br>Percent of columns	None	With	Total
1	32242<br>99.24<br>99.24<br>100.00	248<br>0.76<br>0.76<br>100.00	32490<br>100.00
Total	32242<br>99.24	248<br>0.76	32490<br>100.00

Treatment history at the time of obtaining Table 2-2 consent according to the presence or absence of proton-pump inhibitors

FREQ Procedure

			Cumulative	
Percent of ALL	power	Percent of power		
1	32490	100.00	32490	100.00

Tables: ALL* HISAL

ALL
HISAL(History of Drug Allergy)

Power |
Percent |
Percent of rows
Percent of columns | None | With | Total

	None	With	Total
1	30277	2213	32490
	93.19	6.81	100.00
	93.19	6.81	
	100.00	100.00	
Total	30277	2213	32490
	93.19	6.81	100.00

Treatment history at the time of obtaining Table 2-2 consent according to the presence or absence of proton-pump inhibitors

FREQ Procedure

Cumulative

Percent of ALL power Percent of power

1	32490	100.00	32490	100.00

Tables: ALL* HISAL

ALL

HISAL(History of Drug Allergy)

Power |
Percent |
Percent of rows
Percent of columns | None | With | Total

	None	With	Total
1	30277	2213	32490
	93.19	6.81	100.00
	93.19	6.81	
	100.00	100.00	
Total	30277	2213	32490
	93.19	6.81	100.00

Treatment history at the time of obtaining Table 2-2 consent according to the presence or absence of proton-pump inhibitors

FREQ Procedure

Cumulative
Percent of ALL power Percent of power

1	32490	100.00	32490	100.00

Tables: ALL* AFTYP

ALL AFTYP(Type of AF)

Power |
Percent |
Percent of rows
Percent of columns | Episodic | Persistent | Long lasting | Persistent | Total
| | sex| |

1	13639	5381	4399	9071	32490
	41.98	16.56	13.54	27.92	100.00
	41.98	16.56	13.54	27.92	
	100.00	100.00	100.00	100.00	
Total	13639	5381	4399	9071	32490
	41.98	16.56	13.54	27.92	100.00

Treatment history at the time of obtaining Table 2-2 consent according to the presence or absence of proton-pump inhibitors

FREQ Procedure

			Cumulative	
Percent of ALL	power	Percent of power		
1	32490	100.00	32490	100.00

Tables: ALL* AFTYP

ALL AFTYP(Type of AF)

Power |
Percent |
Percent of rows
Percent of columns | Episodic | Persistent | Long lasting | Persistent | Total
| | sex| |

1	13639	5381	4399	9071	32490
	41.98	16.56	13.54	27.92	100.00
	41.98	16.56	13.54	27.92	
	100.00	100.00	100.00	100.00	
Total	13639	5381	4399	9071	32490
	41.98	16.56	13.54	27.92	100.00

Treatment history at the time of obtaining Table 2-2 consent according to the presence or absence of proton-pump inhibitors

FREQ Procedure

			Cumulative	
Percent of ALL	power	Percent of	power	
1	32490	100.00	32490	100.00

Tables: ALL* AFTYP

ALL AFTYP(Type of AF)

Power |
Percent |
Percent of rows
Percent of columns | Episodic | Persistent | Long lasting | Persistent | Total
| | sex| |

	Episodic	Persistent	Long lasting sex	Persistent	Total
1	13639	5381	4399	9071	32490
	41.98	16.56	13.54	27.92	100.00
	41.98	16.56	13.54	27.92	
	100.00	100.00	100.00	100.00	
Total	13639	5381	4399	9071	32490
	41.98	16.56	13.54	27.92	100.00

Treatment history at the time of obtaining Table 2-2 consent according to the presence or absence of proton-pump inhibitors

FREQ Procedure

Cumulative

Percent of ALL power Percent of power

1	32490	100.00	32490	100.00

Tables: ALL* AFTYP

ALL AFTYP(Type of AF)

Power |
Percent |
Percent of rows
Percent of columns | Episodic | Persistent | Long lasting | Persistent | Total
| | sex| |

	Episodic	Persistent	Long lasting Persistent	sex	Total
1	13639	5381	4399	9071	32490
	41.98	16.56	13.54	27.92	100.00
	41.98	16.56	13.54	27.92	
	100.00	100.00	100.00	100.00	
Total	13639	5381	4399	9071	32490
	41.98	16.56	13.54	27.92	100.00

Treatment history at the time of obtaining Table 2-2 consent according to the presence or absence of proton-pump inhibitors

FREQ Procedure

Cumulative
Percent of ALL power Percent of power

1	32490	100.00	32490	100.00

Tables: ALL* AFTYP

ALL AFTYP(Type of AF)

Power |
Percent |
Percent of rows
Percent of columns | Episodic | Persistent | Long lasting | Persistent | Total
| | sex| |

1	13639	5381	4399	9071	32490
	41.98	16.56	13.54	27.92	100.00
	41.98	16.56	13.54	27.92	
	100.00	100.00	100.00	100.00	
Total	13639	5381	4399	9071	32490
	41.98	16.56	13.54	27.92	100.00

Treatment history at the time of obtaining Table 2-2 consent according to the presence or absence of proton-pump inhibitors

FREQ Procedure

Cumulative

Percent of ALL power Percent of power

1	32490	100.00	32490	100.00

Tables: ALL* AFTYPN

ALL AFTYPN

Power |
Percent |
Percent of rows
Percentage of columns | 5| Total

_______________+________+

1 | 9780 | 9780

| 100.00 | 100.00

| 100.00 |

| 100.00 |

_______________+________+

Total 9780 9780

100.00 100.00

Degree of missing values = 22710

Treatment history at the time of obtaining Table 2-2 consent according to the presence or absence of proton-pump inhibitors

FREQ Procedure

			Cumulative	
Percent of ALL power Percent of power				
1	32490	100.00	32490	100.00

Tables: ALL* AFTYP

ALL AFTYP(Type of AF)

Power |
Percent |
Percent of rows
Percent of columns | Episodic | Persistent | Long lasting | Persistent | Total
| | sex| |

	Episodic	Persistent	Long lasting sex	Persistent	Total
1	13639	5381	4399	9071	32490
	41.98	16.56	13.54	27.92	100.00
	41.98	16.56	13.54	27.92	
	100.00	100.00	100.00	100.00	
Total	13639	5381	4399	9071	32490
	41.98	16.56	13.54	27.92	100.00

Treatment history at the time of obtaining Table 2-2 consent according to the presence or absence of proton-pump inhibitors

FREQ Procedure

			Cumulative	
Percent of ALL	power	Percent of	power	
1	32490	100.00	32490	100.00

Tables: ALL* AFTHSPE

ALL

AFTHSPE(Non-drug Therapy for AF)

Power |
Percent |
Percent of rows
Percent of columns | None | With | Total

	None	With	Total
1	26863	5627	32490
	82.68	17.32	100.00
	82.68	17.32	
	100.00	100.00	
Total	26863	5627	32490
	82.68	17.32	100.00

Treatment history at the time of obtaining Table 2-2 consent according to the presence or absence of proton-pump inhibitors

FREQ Procedure

		Cumulative		
Percent of ALL power Percent of power				
1	32490	100.00	32490	100.00

Tables: ALL* AFTHSPE

ALL

AFTHSPE(Non-drug Therapy for AF)

Power | Percent | Percent of rows Percent of columns	None	With	Total
1	26863	5627	32490
	82.68	17.32	100.00
	82.68	17.32	
	100.00	100.00	
Total	26863	5627	32490
	82.68	17.32	100.00

Treatment history at the time of obtaining Table 2-2 consent according to the presence or absence of proton-pump inhibitors

FREQ Procedure

			Cumulative	
Percent of ALL	power	Percent of	power	
1	32490	100.00	32490	100.00

Table : ALL * AFTHSPE1

ALL

AFTHSPE1(Catheter Ablation)

Power |
Percent |
Percent of rows
Percent of columns | None | With | Total

	None	With	Total
1	29542	2948	32490
	90.93	9.07	100.00
	90.93	9.07	
	100.00	100.00	
Total	29542	2948	32490
	90.93	9.07	100.00

Treatment history at the time of obtaining Table 2-2 consent according to the presence or absence of proton-pump inhibitors

FREQ Procedure

			Cumulative	
Percent of ALL	power	Percent of power		
1	32490	100.00	32490	100.00

Table : ALL * AFTHSPE1

ALL

AFTHSPE1(Catheter Ablation)

Power |
Percent |
Percent of rows
Percent of columns | None | With | Total

	None	With	Total
1	29542	2948	32490
	90.93	9.07	100.00
	90.93	9.07	
	100.00	100.00	
Total	29542	2948	32490
	90.93	9.07	100.00

Treatment history at the time of obtaining Table 2-2 consent according to the presence or absence of proton-pump inhibitors

FREQ Procedure

			Cumulative	
Percent of ALL	power	Percent of	power	
1	32490	100.00	32490	100.00

Table : ALL * AFTHSPE2

ALL AFTHSPE2(Cardioversion)

Power<br>Percent<br>Percent of rows<br>Percent of columns	None	With	Total
1	31779<br>97.81<br>97.81<br>100.00	711<br>2.19<br>2.19<br>100.00	32490<br>100.00
Total	31779<br>97.81	711<br>2.19	32490<br>100.00

Treatment history at the time of obtaining Table 2-2 consent according to the presence or absence of proton-pump inhibitors

FREQ Procedure

			Cumulative	
Percent of ALL	power	Percent	of power	
1	32490	100.00	32490	100.00

Table : ALL * AFTHSPE2

ALL AFTHSPE2(Cardioversion)

Power |
Percent |
Percent of rows
Percent of columns | None | With | Total

	None	With	Total
1	31779	711	32490
	97.81	2.19	100.00
	97.81	2.19	
	100.00	100.00	
Total	31779	711	32490
	97.81	2.19	100.00

Treatment history at the time of obtaining Table 2-2 consent according to the presence or absence of proton-pump inhibitors

FREQ Procedure

Cumulative
Percent of ALL power Percent of power

1	32490	100.00	32490	100.00

Table : ALL * AFTHSPE3

ALL AFTHSPE3(ICD)

Power |
Percent |
Percent of rows
Percent of columns | None | With | Total

	None	With	Total
1	32337	153	32490
	99.53	0.47	100.00
	99.53	0.47	
	100.00	100.00	
Total	32337	153	32490
	99.53	0.47	100.00

Treatment history at the time of obtaining Table 2-2 consent according to the presence or absence of proton-pump inhibitors

FREQ Procedure

			Cumulative	
		Percent of ALL power	Percent of power	
1	32490	100.00	32490	100.00

Table : ALL * AFTHSPE3

ALL AFTHSPE3(ICD)

Power |
Percent |
Percent of rows
Percent of columns | None | With | Total

	None	With	Total
1	32337	153	32490
	99.53	0.47	100.00
	99.53	0.47	
	100.00	100.00	
Total	32337	153	32490
	99.53	0.47	100.00

Treatment history at the time of obtaining Table 2-2 consent according to the presence or absence of proton-pump inhibitors

FREQ Procedure

Cumulative
Percent of ALL power Percent of power

1	32490	100.00	32490	100.00

Table : ALL * AFTHSPE4

ALL
AFTHSPE4(Pacemaker Implantation)

Power |
Percent |
Percent of rows
Percent of columns | None | With | Total

1	30168	2322	32490
	92.85	7.15	100.00
	92.85	7.15	
	100.00	100.00	
Total	30168	2322	32490
	92.85	7.15	100.00

Treatment history at the time of obtaining Table 2-2 consent according to the presence or absence of proton-pump inhibitors

FREQ Procedure

			Cumulative	
Percent of ALL	power	Percent of	power	
1	32490	100.00	32490	100.00

Table : ALL * AFTHSPE4

ALL

AFTHSPE4(Pacemaker Implantation)

Power<br>Percent<br>Percent of rows<br>Percent of columns	None	With	Total
1	30168	2322	32490
	92.85	7.15	100.00
	92.85	7.15	
	100.00	100.00	
Total	30168	2322	32490
	92.85	7.15	100.00

Treatment history at the time of obtaining Table 2-2 consent according to the presence or absence of proton-pump inhibitors

FREQ Procedure

Cumulative
Percent of ALL power Percent of power

1	32490	100.00	32490	100.00

Table : ALL * AFTHSPE5

ALL
AFTHSPE5(Other Non-drug Therapy)

Power |
Percent |
Percent of rows
Percent of columns | None | With | Total

	None	With	Total
1	32377	113	32490
	99.65	0.35	100.00
	99.65	0.35	
	100.00	100.00	
Total	32377	113	32490
	99.65	0.35	100.00

Treatment history at the time of obtaining Table 2-2 consent according to the presence or absence of proton-pump inhibitors

FREQ Procedure

			Cumulative	
Percent of ALL	power	Percent	of power	
1	32490	100.00	32490	100.00

Table : ALL * AFTHSPE5

ALL

AFTHSPE5(Other Non-drug Therapy)

Power<br>Percent<br>Percent of rows<br>Percent of columns	None	With	Total
1	32377<br>99.65<br>99.65<br>100.00	113<br>0.35<br>0.35<br>100.00	32490<br>100.00
Total	32377<br>99.65	113<br>0.35	32490<br>100.00

Treatment history at the time of obtaining Table 2-2 consent according to the presence or absence of proton-pump inhibitors

FREQ Procedure

Cumulative
Percent of ALL power Percent of power

1	11981	100.00	11981	100.00

Tables: ALL* HISAF

ALL
HISAF(History of Major Surgery for non-AF)

Power |
Percent |
Percent of rows
Percent of column |immediate 3 months |immediate 3 months |unknown |total
) within |Month within |Month| |
|Major surgery|Major surgery| |
Lip/lip | |

1	149	11683	149	11981
	1.24	97.51	1.24	100.00
	1.24	97.51	1.24	
	100.00	100.00	100.00	
Total	149	11683	149	11981
	1.24	97.51	1.24	100.00

Treatment history at the time of obtaining Table 2-2 consent according to the presence or absence of proton-pump inhibitors

FREQ Procedure

Cumulative
Percent of ALL power Percent of power

1	11981	100.00	11981	100.00

Tables: ALL* HISAF

ALL
HISAF(History of Major Surgery for non-AF)

Power |
Percent |
Percent of rows
Percent of column |immediate 3 months |immediate 3 months |unknown |total
) within |Month within |Month| |
|Major surgery|Major surgery| |
Lip/lip | |

1	149	11683	149	11981
	1.24	97.51	1.24	100.00
	1.24	97.51	1.24	
	100.00	100.00	100.00	
Total	149	11683	149	11981
	1.24	97.51	1.24	100.00

Treatment history at the time of obtaining Table 2-2 consent according to the presence or absence of proton-pump inhibitors

FREQ Procedure

Cumulative
Percent of ALL power Percent of power

1	11981	100.00	11981	100.00

Tables: ALL* HISAF

ALL
HISAF(History of Major Surgery for non-AF)

Power |
Percent |
Percent of rows
Percent of column |immediate 3 months |immediate 3 months |unknown |total
) within |Month within |Month| |
|Major surgery|Major surgery| |
Lip/lip | |

1	149	11683	149	11981
	1.24	97.51	1.24	100.00
	1.24	97.51	1.24	
	100.00	100.00	100.00	
Total	149	11683	149	11981
	1.24	97.51	1.24	100.00

Treatment history at the time of obtaining Table 2-2 consent according to the presence or absence of proton-pump inhibitors

FREQ Procedure

			Cumulative	
Percent of ALL	power	Percent of	power	
1	11981	100.00	11981	100.00

Tables: ALL* HISBDYN

ALL

HISBDYN(History of Major Beeding)

Power |
Percent |
Percent of rows
Percent of columns | None | With | Total

	None	With	Total
1	11385	596	11981
	95.03	4.97	100.00
	95.03	4.97	
	100.00	100.00	
Total	11385	596	11981
	95.03	4.97	100.00

Treatment history at the time of obtaining Table 2-2 consent according to the presence or absence of proton-pump inhibitors

FREQ Procedure

			Cumulative	
Percent of ALL power Percent of power				
1	11981	100.00	11981	100.00

Tables: ALL* HISBDYN

ALL

HISBDYN(History of Major Beeding)

Power |
Percent |
Percent of rows
Percent of columns | None | With | Total

	None	With	Total
1	11385	596	11981
	95.03	4.97	100.00
	95.03	4.97	
	100.00	100.00	
Total	11385	596	11981
	95.03	4.97	100.00

Treatment history at the time of obtaining Table 2-2 consent according to the presence or absence of proton-pump inhibitors

FREQ Procedure

Cumulative

Percent of ALL power Percent of power

1	11981	100.00	11981	100.00

Table : ALL * HISBD1

ALL

HISBD1(Intracranial Hemorrhage)

Power |
Percent |
Percent of rows
Percent of columns | None | With | Total

1	11704	277	11981
	97.69	2.31	100.00
	97.69	2.31	
	100.00	100.00	
Total	11704	277	11981
	97.69	2.31	100.00

Treatment history at the time of obtaining Table 2-2 consent according to the presence or absence of proton-pump inhibitors

FREQ Procedure

			Cumulative	
Percent of ALL	power	Percent of	power	
1	11981	100.00	11981	100.00

Table : ALL * HISBD1

ALL

HISBD1(Intracranial Hemorrhage)

Power<br>Percent<br>Percent of rows<br>Percent of columns	None	With	Total
1	11704<br>97.69<br>97.69<br>100.00	277<br>2.31<br>2.31<br>100.00	11981<br>100.00
Total	11704<br>97.69	277<br>2.31	11981<br>100.00

Treatment history at the time of obtaining Table 2-2 consent according to the presence or absence of proton-pump inhibitors

FREQ Procedure

			Cumulative	
	Percent of ALL power	Percent of power		
1	11981	100.00	11981	100.00

Table : ALL * HISBD2

ALL

HISBD2(Upper Gastrointestinal Bleeding)

Power |
Percent |
Percent of rows
Percent of columns | None | With | Total

	None	With	Total
1	11813	168	11981
	98.60	1.40	100.00
	98.60	1.40	
	100.00	100.00	
Total	11813	168	11981
	98.60	1.40	100.00

Treatment history at the time of obtaining Table 2-2 consent according to the presence or absence of proton-pump inhibitors

FREQ Procedure

Cumulative
Percent of ALL power Percent of power

1	11981	100.00	11981	100.00

Table : ALL * HISBD2

ALL
HISBD2(Upper Gastrointestinal Bleeding)

Power |
Percent |
Percent of rows
Percent of columns | None | With | Total

	None	With	Total
1	11813	168	11981
	98.60	1.40	100.00
	98.60	1.40	
	100.00	100.00	
Total	11813	168	11981
	98.60	1.40	100.00

Treatment history at the time of obtaining Table 2-2 consent according to the presence or absence of proton-pump inhibitors

FREQ Procedure

			Cumulative	
Percent of ALL	power	Percent of	power	
1	11981	100.00	11981	100.00

Table : ALL * HISBD3

ALL

HISBD3(Lower Gastrointestinal Bleeding)

Power<br>Percent<br>Percent of rows<br>Percent of columns	None	With	Total
1	11831<br>98.75<br>98.75<br>100.00	150<br>1.25<br>1.25<br>100.00	11981<br>100.00
Total	11831<br>98.75	150<br>1.25	11981<br>100.00

Treatment history at the time of obtaining Table 2-2 consent according to the presence or absence of proton-pump inhibitors

FREQ Procedure

			Cumulative	
Percent of ALL	power	Percent of power		
1	11981	100.00	11981	100.00

Table : ALL * HISBD3

ALL

HISBD3(Lower Gastrointestinal Bleeding)

Power | Percent | Percent of rows Percent of columns	None	With	Total
1	11831	150	11981
	98.75	1.25	100.00
	98.75	1.25	
	100.00	100.00	
Total	11831	150	11981
	98.75	1.25	100.00

Treatment history at the time of obtaining Table 2-2 consent according to the presence or absence of proton-pump inhibitors

FREQ Procedure

Cumulative

Percent of ALL power Percent of power

1	11981	100.00	11981	100.00

Table : ALL * HISBD4

ALL HISBD4(Other Beeding)

```
Power |
Percent |
Percent of rows
Percent of columns | None | With | Total
______________+________+________+
             1 |   11874 |      107 |   11981
               |   99.11 |     0.89 | 100.00
               |   99.11 |     0.89 |
               | 100.00 | 100.00 |
______________+________+________+
Total 11874 107 11981
                  99.11        0.89     100.00
```

Treatment history at the time of obtaining Table 2-2 consent according to the presence or absence of proton-pump inhibitors

FREQ Procedure

			Cumulative	
Percent of ALL	power	Percent	of power	
1	11981	100.00	11981	100.00

Table : ALL * HISBD4

ALL HISBD4(Other Beeding)

Power |
Percent |
Percent of rows
Percent of columns | None | With | Total

	None	With	Total
1	11874	107	11981
	99.11	0.89	100.00
	99.11	0.89	
	100.00	100.00	
Total	11874	107	11981
	99.11	0.89	100.00

Treatment history at the time of obtaining Table 2-2 consent according to the presence or absence of proton-pump inhibitors

FREQ Procedure

			Cumulative	
	Percent of ALL power	Percent of power		
1	11981	100.00	11981	100.00

Tables: ALL* HISAL

ALL

HISAL(History of Drug Allergy)

Power | Percent | Percent of rows Percent of columns	None	With	Total
1	11073	908	11981
	92.42	7.58	100.00
	92.42	7.58	
	100.00	100.00	
Total	11073	908	11981
	92.42	7.58	100.00

Treatment history at the time of obtaining Table 2-2 consent according to the presence or absence of proton-pump inhibitors

FREQ Procedure

			Cumulative	
Percent of ALL	power	Percent of	power	
1	11981	100.00	11981	100.00

Tables: ALL* HISAL

ALL

HISAL(History of Drug Allergy)

Power |
Percent |
Percent of rows
Percent of columns | None | With | Total

	None	With	Total
1	11073	908	11981
	92.42	7.58	100.00
	92.42	7.58	
	100.00	100.00	
Total	11073	908	11981
	92.42	7.58	100.00

Treatment history at the time of obtaining Table 2-2 consent according to the presence or absence of proton-pump inhibitors

FREQ Procedure

Cumulative

Percent of ALL power Percent of power

1	11981	100.00	11981	100.00

Tables: ALL* AFTYP

ALL AFTYP(Type of AF)

Power |

Percent |

Percent of rows

Percent of columns | Episodic | Persistent | Long lasting | Persistent | Total

| | sex| |

	Episodic	Persistent	Long lasting sex	Persistent	Total
1	5351	1899	1486	3245	11981
	44.66	15.85	12.40	27.08	100.00
	44.66	15.85	12.40	27.08	
	100.00	100.00	100.00	100.00	
Total	5351	1899	1486	3245	11981
	44.66	15.85	12.40	27.08	100.00

Treatment history at the time of obtaining Table 2-2 consent according to the presence or absence of proton-pump inhibitors

FREQ Procedure

			Cumulative	
Percent of ALL	power	Percent of power		
1	11981	100.00	11981	100.00

Tables: ALL* AFTYP

ALL AFTYP(Type of AF)

Power |
Percent |
Percent of rows
Percent of columns | Episodic | Persistent | Long lasting | Persistent | Total
| | sex| |

1	5351	1899	1486	3245	11981
	44.66	15.85	12.40	27.08	100.00
	44.66	15.85	12.40	27.08	
	100.00	100.00	100.00	100.00	
Total	5351	1899	1486	3245	11981
	44.66	15.85	12.40	27.08	100.00

Treatment history at the time of obtaining Table 2-2 consent according to the presence or absence of proton-pump inhibitors

FREQ Procedure

Cumulative
Percent of ALL power Percent of power

1	11981	100.00	11981	100.00

Tables: ALL* AFTYP

ALL AFTYP(Type of AF)

Power |
Percent |
Percent of rows
Percent of columns | Episodic | Persistent | Long lasting | Persistent | Total
| | sex| |

1	5351	1899	1486	3245	11981
	44.66	15.85	12.40	27.08	100.00
	44.66	15.85	12.40	27.08	
	100.00	100.00	100.00	100.00	
Total	5351	1899	1486	3245	11981
	44.66	15.85	12.40	27.08	100.00

Treatment history at the time of obtaining Table 2-2 consent according to the presence or absence of proton-pump inhibitors

FREQ Procedure

			Cumulative	
Percent of ALL	power	Percent of	power	
1	11981	100.00	11981	100.00

Tables: ALL* AFTYP

ALL AFTYP(Type of AF)

Power |
Percent |
Percent of rows
Percent of columns | Episodic | Persistent | Long lasting | Persistent | Total
| | sex| |

1	5351	1899	1486	3245	11981
	44.66	15.85	12.40	27.08	100.00
	44.66	15.85	12.40	27.08	
	100.00	100.00	100.00	100.00	
Total	5351	1899	1486	3245	11981
	44.66	15.85	12.40	27.08	100.00

Treatment history at the time of obtaining Table 2-2 consent according to the presence or absence of proton-pump inhibitors

FREQ Procedure

Cumulative
Percent of ALL power Percent of power

1	11981	100.00	11981	100.00

Tables: ALL* AFTYP

ALL AFTYP(Type of AF)

Power |
Percent |
Percent of rows
Percent of columns | Episodic | Persistent | Long lasting | Persistent | Total
| | sex| |

	Episodic	Persistent	Long lasting Persistent sex	Persistent	Total
1	5351	1899	1486	3245	11981
	44.66	15.85	12.40	27.08	100.00
	44.66	15.85	12.40	27.08	
	100.00	100.00	100.00	100.00	
Total	5351	1899	1486	3245	11981
	44.66	15.85	12.40	27.08	100.00

Treatment history at the time of obtaining Table 2-2 consent according to the presence or absence of proton-pump inhibitors

FREQ Procedure

Cumulative

Percent of ALL power Percent of power

1	11981	100.00	11981	100.00

Tables: ALL* AFTYPN

ALL AFTYPN

Power |

Percent |

Percent of rows

Percentage of columns | 5| Total

_______________+________+

1 | 3385 | 3385

| 100.00 | 100.00

| 100.00 |

| 100.00 |

_______________+________+

Total 3385 3385

100.00 100.00

Degree of missing values = 8596

Treatment history at the time of obtaining Table 2-2 consent according to the presence or absence of proton-pump inhibitors

FREQ Procedure

			Cumulative	
Percent of ALL	power	Percent of power		
1	11981	100.00	11981	100.00

Tables: ALL* AFTYP

ALL AFTYP(Type of AF)

Power |
Percent |
Percent of rows
Percent of columns | Episodic | Persistent | Long lasting | Persistent | Total
| | sex| |

	Episodic	Persistent	Long lasting sex	Persistent	Total
1	5351	1899	1486	3245	11981
	44.66	15.85	12.40	27.08	100.00
	44.66	15.85	12.40	27.08	
	100.00	100.00	100.00	100.00	
Total	5351	1899	1486	3245	11981
	44.66	15.85	12.40	27.08	100.00

Treatment history at the time of obtaining Table 2-2 consent according to the presence or absence of proton-pump inhibitors

FREQ Procedure

Cumulative

Percent of ALL power Percent of power

1	11981	100.00	11981	100.00

Tables: ALL* AFTHSPE

ALL

AFTHSPE(Non-drug Therapy for AF)

Power |
Percent |
Percent of rows
Percent of columns | None | With | Total

	None	With	Total
1	9409	2572	11981
	78.53	21.47	100.00
	78.53	21.47	
	100.00	100.00	
Total	9409	2572	11981
	78.53	21.47	100.00

Treatment history at the time of obtaining Table 2-2 consent according to the presence or absence of proton-pump inhibitors

FREQ Procedure

			Cumulative	
Percent of ALL	power	Percent of power		
1	11981	100.00	11981	100.00

Tables: ALL* AFTHSPE

ALL

AFTHSPE(Non-drug Therapy for AF)

Power |
Percent |
Percent of rows
Percent of columns | None | With | Total

	None	With	Total
1	9409	2572	11981
	78.53	21.47	100.00
	78.53	21.47	
	100.00	100.00	
Total	9409	2572	11981
	78.53	21.47	100.00

Treatment history at the time of obtaining Table 2-2 consent according to the presence or absence of proton-pump inhibitors

FREQ Procedure

			Cumulative	
Percent of ALL	power	Percent of	power	
1	11981	100.00	11981	100.00

Table : ALL * AFTHSPE1

ALL

AFTHSPE1(Catheter Ablation)

Power |
Percent |
Percent of rows
Percent of columns | None | With | Total

	None	With	Total
1	10578	1403	11981
	88.29	11.71	100.00
	88.29	11.71	
	100.00	100.00	
Total	10578	1403	11981
	88.29	11.71	100.00

Treatment history at the time of obtaining Table 2-2 consent according to the presence or absence of proton-pump inhibitors

FREQ Procedure

Cumulative
Percent of ALL power Percent of power

1	11981	100.00	11981	100.00

Table : ALL * AFTHSPE1

ALL
AFTHSPE1(Catheter Ablation)

Power |
Percent |
Percent of rows
Percent of columns | None | With | Total

1	10578	1403	11981
	88.29	11.71	100.00
	88.29	11.71	
	100.00	100.00	
Total	10578	1403	11981
	88.29	11.71	100.00

Treatment history at the time of obtaining Table 2-2 consent according to the presence or absence of proton-pump inhibitors

FREQ Procedure

			Cumulative	
Percent of ALL	power	Percent of	power	
1	11981	100.00	11981	100.00

Table : ALL * AFTHSPE2

ALL AFTHSPE2(Cardioversion)

Power |
Percent |
Percent of rows
Percent of columns | None | With | Total

	None	With	Total
1	11683	298	11981
	97.51	2.49	100.00
	97.51	2.49	
	100.00	100.00	
Total	11683	298	11981
	97.51	2.49	100.00

Treatment history at the time of obtaining Table 2-2 consent according to the presence or absence of proton-pump inhibitors

FREQ Procedure

			Cumulative	
Percent of ALL	power	Percent of power		
1	11981	100.00	11981	100.00

Table : ALL * AFTHSPE2

ALL AFTHSPE2(Cardioversion)

Power |
Percent |
Percent of rows
Percent of columns | None | With | Total

	None	With	Total
1	11683	298	11981
	97.51	2.49	100.00
	97.51	2.49	
	100.00	100.00	
Total	11683	298	11981
	97.51	2.49	100.00

Treatment history at the time of obtaining Table 2-2 consent according to the presence or absence of proton-pump inhibitors

FREQ Procedure

			Cumulative	
Percent of ALL	power	Percent of power		
1	11981	100.00	11981	100.00

Table : ALL * AFTHSPE3

ALL AFTHSPE3(ICD)

Power |
Percent |
Percent of rows
Percent of columns | None | With | Total

	None	With	Total
1	11905	76	11981
	99.37	0.63	100.00
	99.37	0.63	
	100.00	100.00	
Total	11905	76	11981
	99.37	0.63	100.00

Treatment history at the time of obtaining Table 2-2 consent according to the presence or absence of proton-pump inhibitors

FREQ Procedure

			Cumulative	
	Percent of ALL power	Percent of power		
1	11981	100.00	11981	100.00

Table : ALL * AFTHSPE3

ALL AFTHSPE3(ICD)

Power |
Percent |
Percent of rows
Percent of columns | None | With | Total

	None	With	Total
1	11905	76	11981
	99.37	0.63	100.00
	99.37	0.63	
	100.00	100.00	
Total	11905	76	11981
	99.37	0.63	100.00

Treatment history at the time of obtaining Table 2-2 consent according to the presence or absence of proton-pump inhibitors

FREQ Procedure

			Cumulative	
Percent of ALL	power	Percent	of power	
1	11981	100.00	11981	100.00

Table : ALL * AFTHSPE4

ALL

AFTHSPE4(Pacemaker Implantation)

Power / Percent / Percent of rows / Percent of columns	None	With	Total
1	10926	1055	11981
	91.19	8.81	100.00
	91.19	8.81	
	100.00	100.00	
Total	10926	1055	11981
	91.19	8.81	100.00

Treatment history at the time of obtaining Table 2-2 consent according to the presence or absence of proton-pump inhibitors

FREQ Procedure

Cumulative

Percent of ALL power Percent of power

1	11981	100.00	11981	100.00

Table : ALL * AFTHSPE4

ALL

AFTHSPE4(Pacemaker Implantation)

Power |

Percent |

Percent of rows

Percent of columns | None | With | Total

	None	With	Total
1	10926	1055	11981
	91.19	8.81	100.00
	91.19	8.81	
	100.00	100.00	
Total	10926	1055	11981
	91.19	8.81	100.00

Treatment history at the time of obtaining Table 2-2 consent according to the presence or absence of proton-pump inhibitors

FREQ Procedure

Cumulative
Percent of ALL power Percent of power

1	11981	100.00	11981	100.00

Table : ALL * AFTHSPE5

ALL
AFTHSPE5(Other Non-drug Therapy)

Power |
Percent |
Percent of rows
Percent of columns | None | With | Total

	None	With	Total
1	11925	56	11981
	99.53	0.47	100.00
	99.53	0.47	
	100.00	100.00	
Total	11925	56	11981
	99.53	0.47	100.00

Treatment history at the time of obtaining Table 2-2 consent according to the presence or absence of proton-pump inhibitors

FREQ Procedure

		Cumulative		
Percent of ALL	power	Percent of power		
1	11981	100.00	11981	100.00

Table : ALL * AFTHSPE5

ALL

AFTHSPE5(Other Non-drug Therapy)

Power |
Percent |
Percent of rows
Percent of columns | None | With | Total

	None	With	Total
1	11925	56	11981
	99.53	0.47	100.00
	99.53	0.47	
	100.00	100.00	
Total	11925	56	11981
	99.53	0.47	100.00

Treatment history at the time of obtaining Table 2-2 consent according to the presence or absence of proton-pump inhibitors

FREQ Procedure

			Cumulative	
Percent of ALL power Percent of power				
1	20509	100.00	20509	100.00

Tables: ALL* HISAF

ALL

HISAF(History of Major Surgery for non-AF)

Power |
Percent |
Percent of rows
Percent of column |immediate 3 months |immediate 3 months |unknown |total
) within |Month within |Month| |
|Major surgery|Major surgery| |
Lip/lip | |

1	187	20007	315	20509
	0.91	97.55	1.54	100.00
	0.91	97.55	1.54	
	100.00	100.00	100.00	
Total 187 20007 315 20509				
	0.91	97.55	1.54	100.00

Treatment history at the time of obtaining Table 2-2 consent according to the presence or absence of proton-pump inhibitors

FREQ Procedure

Cumulative

Percent of ALL power Percent of power

1	20509	100.00	20509	100.00

Tables: ALL* HISAF

ALL

HISAF(History of Major Surgery for non-AF)

Power |
Percent |
Percent of rows
Percent of column |immediate 3 months |immediate 3 months |unknown |total
) within |Month within |Month| |
|Major surgery|Major surgery| |
Lip/lip | |

1	187	20007	315	20509
	0.91	97.55	1.54	100.00
	0.91	97.55	1.54	
	100.00	100.00	100.00	
Total	187	20007	315	20509
	0.91	97.55	1.54	100.00

Treatment history at the time of obtaining Table 2-2 consent according to the presence or absence of proton-pump inhibitors

FREQ Procedure

Cumulative
Percent of ALL power Percent of power

1	20509	100.00	20509	100.00

Tables: ALL* HISAF

ALL
HISAF(History of Major Surgery for non-AF)

Power |
Percent |
Percent of rows
Percent of column |immediate 3 months |immediate 3 months |unknown |total
) within |Month within |Month| |
|Major surgery|Major surgery| |
Lip/lip | |

1	187	20007	315	20509
	0.91	97.55	1.54	100.00
	0.91	97.55	1.54	
	100.00	100.00	100.00	
Total	187	20007	315	20509
	0.91	97.55	1.54	100.00

Treatment history at the time of obtaining Table 2-2 consent according to the presence or absence of proton-pump inhibitors

FREQ Procedure

			Cumulative	
Percent of ALL	power	Percent of	power	
1	20509	100.00	20509	100.00

Tables: ALL* HISBDYN

ALL

HISBDYN(History of Major Beeding)

Power |
Percent |
Percent of rows
Percent of columns | None | With | Total

	None	With	Total
1	19844	665	20509
	96.76	3.24	100.00
	96.76	3.24	
	100.00	100.00	
Total	19844	665	20509
	96.76	3.24	100.00

Treatment history at the time of obtaining Table 2-2 consent according to the presence or absence of proton-pump inhibitors

FREQ Procedure

			Cumulative	
Percent of ALL	power	Percent of	power	
1	20509	100.00	20509	100.00

Tables: ALL* HISBDYN

ALL
HISBDYN(History of Major Beeding)

Power |
Percent |
Percent of rows
Percent of columns | None | With | Total

	None	With	Total
1	19844	665	20509
	96.76	3.24	100.00
	96.76	3.24	
	100.00	100.00	
Total	19844	665	20509
	96.76	3.24	100.00

Treatment history at the time of obtaining Table 2-2 consent according to the presence or absence of proton-pump inhibitors

FREQ Procedure

			Cumulative	
Percent of ALL	power	Percent	of power	
1	20509	100.00	20509	100.00

Table : ALL * HISBD1

ALL

HISBD1(Intracranial Hemorrhage)

Power |
Percent |
Percent of rows
Percent of columns | None | With | Total

	None	With	Total
1	20137	372	20509
	98.19	1.81	100.00
	98.19	1.81	
	100.00	100.00	
Total	20137	372	20509
	98.19	1.81	100.00

Treatment history at the time of obtaining Table 2-2 consent according to the presence or absence of proton-pump inhibitors

FREQ Procedure

Cumulative
Percent of ALL power Percent of power

1	20509	100.00	20509	100.00

Table : ALL * HISBD1

ALL
HISBD1(Intracranial Hemorrhage)

Power |
Percent |
Percent of rows
Percent of columns | None | With | Total

	None	With	Total
1	20137	372	20509
	98.19	1.81	100.00
	98.19	1.81	
	100.00	100.00	
Total	20137	372	20509
	98.19	1.81	100.00

Treatment history at the time of obtaining Table 2-2 consent according to the presence or absence of proton-pump inhibitors

FREQ Procedure

Cumulative
Percent of ALL power Percent of power

1	20509	100.00	20509	100.00

Table : ALL * HISBD2

ALL
HISBD2(Upper Gastrointestinal Bleeding)

Power |
Percent |
Percent of rows
Percent of columns | None | With | Total

	None	With	Total
1	20403	106	20509
	99.48	0.52	100.00
	99.48	0.52	
	100.00	100.00	
Total	20403	106	20509
	99.48	0.52	100.00

Treatment history at the time of obtaining Table 2-2 consent according to the presence or absence of proton-pump inhibitors

FREQ Procedure

			Cumulative	
Percent of ALL	power	Percent of power		
1	20509	100.00	20509	100.00

Table : ALL * HISBD2

ALL

HISBD2(Upper Gastrointestinal Bleeding)

Power |
Percent |
Percent of rows
Percent of columns | None | With | Total

	None	With	Total
1	20403	106	20509
	99.48	0.52	100.00
	99.48	0.52	
	100.00	100.00	
Total	20403	106	20509
	99.48	0.52	100.00

Treatment history at the time of obtaining Table 2-2 consent according to the presence or absence of proton-pump inhibitors

FREQ Procedure

			Cumulative	
Percent of ALL power Percent of power				
1	20509	100.00	20509	100.00

Table : ALL * HISBD3

ALL

HISBD3(Lower Gastrointestinal Bleeding)

Power |
Percent |
Percent of rows
Percent of columns | None | With | Total

	None	With	Total
1	20351	158	20509
	99.23	0.77	100.00
	99.23	0.77	
	100.00	100.00	
Total	20351	158	20509
	99.23	0.77	100.00

Treatment history at the time of obtaining Table 2-2 consent according to the presence or absence of proton-pump inhibitors

FREQ Procedure

			Cumulative	
Percent of ALL	power	Percent of	power	
1	20509	100.00	20509	100.00

Table : ALL * HISBD3

ALL

HISBD3(Lower Gastrointestinal Bleeding)

Power |
Percent |
Percent of rows
Percent of columns | None | With | Total

	None	With	Total
1	20351	158	20509
	99.23	0.77	100.00
	99.23	0.77	
	100.00	100.00	
Total	20351	158	20509
	99.23	0.77	100.00

Treatment history at the time of obtaining Table 2-2 consent according to the presence or absence of proton-pump inhibitors

FREQ Procedure

Cumulative

Percent of ALL power Percent of power

1	20509	100.00	20509	100.00

Table : ALL * HISBD4

ALL HISBD4(Other Beeding)

Power |
Percent |
Percent of rows
Percent of columns | None | With | Total

1	20368	141	20509
	99.31	0.69	100.00
	99.31	0.69	
	100.00	100.00	
Total	20368	141	20509
	99.31	0.69	100.00

Treatment history at the time of obtaining Table 2-2 consent according to the presence or absence of proton-pump inhibitors

FREQ Procedure

			Cumulative	
Percent of ALL	power	Percent of power		
1	20509	100.00	20509	100.00

Table : ALL * HISBD4

ALL HISBD4(Other Beeding)

Power |
Percent |
Percent of rows
Percent of columns | None | With | Total

	None	With	Total
1	20368	141	20509
	99.31	0.69	100.00
	99.31	0.69	
	100.00	100.00	
Total	20368	141	20509
	99.31	0.69	100.00

Treatment history at the time of obtaining Table 2-2 consent according to the presence or absence of proton-pump inhibitors

FREQ Procedure

Cumulative
Percent of ALL power Percent of power

1	20509	100.00	20509	100.00

Tables: ALL* HISAL

ALL
HISAL(History of Drug Allergy)

Power |
Percent |
Percent of rows
Percent of columns | None | With | Total

1	19204	1305	20509
	93.64	6.36	100.00
	93.64	6.36	
	100.00	100.00	
Total	19204	1305	20509
	93.64	6.36	100.00

Treatment history at the time of obtaining Table 2-2 consent according to the presence or absence of proton-pump inhibitors

FREQ Procedure

Cumulative
Percent of ALL power Percent of power

1	20509	100.00	20509	100.00

Tables: ALL* HISAL

ALL
HISAL(History of Drug Allergy)

Power |
Percent |
Percent of rows
Percent of columns | None | With | Total

1	19204	1305	20509
	93.64	6.36	100.00
	93.64	6.36	
	100.00	100.00	
Total	19204	1305	20509
	93.64	6.36	100.00

Treatment history at the time of obtaining Table 2-2 consent according to the presence or absence of proton-pump inhibitors

FREQ Procedure

			Cumulative	
Percent of ALL	power	Percent	of power	
1	20509	100.00	20509	100.00

Tables: ALL* AFTYP

ALL AFTYP(Type of AF)

Power |
Percent |
Percent of rows
Percent of columns | Episodic | Persistent | Long lasting | Persistent | Total
| | sex| |

	Episodic	Persistent	Long lasting	Persistent	Total
1	8288	3482	2913	5826	20509
	40.41	16.98	14.20	28.41	100.00
	40.41	16.98	14.20	28.41	
	100.00	100.00	100.00	100.00	
Total	8288	3482	2913	5826	20509
	40.41	16.98	14.20	28.41	100.00

Treatment history at the time of obtaining Table 2-2 consent according to the presence or absence of proton-pump inhibitors

FREQ Procedure

Cumulative
Percent of ALL power Percent of power

1	20509	100.00	20509	100.00

Tables: ALL* AFTYP

ALL AFTYP(Type of AF)

Power |
Percent |
Percent of rows
Percent of columns | Episodic | Persistent | Long lasting | Persistent | Total
| | sex| |

	Episodic	Persistent	Long lasting Persistent	sex	Total
1	8288	3482	2913	5826	20509
	40.41	16.98	14.20	28.41	100.00
	40.41	16.98	14.20	28.41	
	100.00	100.00	100.00	100.00	
Total	8288	3482	2913	5826	20509
	40.41	16.98	14.20	28.41	100.00

Treatment history at the time of obtaining Table 2-2 consent according to the presence or absence of proton-pump inhibitors

FREQ Procedure

Cumulative
Percent of ALL power Percent of power

1	20509	100.00	20509	100.00

Tables: ALL* AFTYP

ALL AFTYP(Type of AF)

Power |
Percent |
Percent of rows
Percent of columns | Episodic | Persistent | Long lasting | Persistent | Total
| | sex| |
______________+________+________+________+________+
1 | 8288 | 3482 | 2913 | 5826 | 20509
| 40.41 | 16.98 | 14.20 | 28.41 | 100.00
| 40.41 | 16.98 | 14.20 | 28.41 |
| 100.00 | 100.00 | 100.00 | 100.00 |
______________+________+________+________+________+
Total 8288 3482 2913 5826 20509
40.41 16.98 14.20 28.41 100.00

Treatment history at the time of obtaining Table 2-2 consent according to the presence or absence of proton-pump inhibitors

FREQ Procedure

			Cumulative	
Percent of ALL	power	Percent of power		
1	20509	100.00	20509	100.00

Tables: ALL* AFTYP

ALL AFTYP(Type of AF)

Power |
Percent |
Percent of rows
Percent of columns | Episodic | Persistent | Long lasting | Persistent | Total
| | sex| |

	Episodic	Persistent	Long lasting	Persistent	Total
1	8288	3482	2913	5826	20509
	40.41	16.98	14.20	28.41	100.00
	40.41	16.98	14.20	28.41	
	100.00	100.00	100.00	100.00	
Total	8288	3482	2913	5826	20509
	40.41	16.98	14.20	28.41	100.00

Treatment history at the time of obtaining Table 2-2 consent according to the presence or absence of proton-pump inhibitors

FREQ Procedure

			Cumulative	
Percent of ALL	power	Percent of power		
1	20509	100.00	20509	100.00

Tables: ALL* AFTYP

ALL AFTYP(Type of AF)

Power |
Percent |
Percent of rows
Percent of columns | Episodic | Persistent | Long lasting | Persistent | Total
| | sex| |

1	8288	3482	2913	5826	20509
	40.41	16.98	14.20	28.41	100.00
	40.41	16.98	14.20	28.41	
	100.00	100.00	100.00	100.00	
Total	8288	3482	2913	5826	20509
	40.41	16.98	14.20	28.41	100.00

Treatment history at the time of obtaining Table 2-2 consent according to the presence or absence of proton-pump inhibitors

FREQ Procedure

Cumulative
Percent of ALL power Percent of power

1	20509	100.00	20509	100.00

Tables: ALL* AFTYPN

ALL AFTYPN

Power |
Percent |
Percent of rows
Percentage of columns | 5| Total
_______________+_________+
1 | 6395 | 6395
| 100.00 | 100.00
| 100.00 |
| 100.00 |
_______________+_________+
Total 6395 6395
100.00 100.00

Degree of missing values = 14114

Treatment history at the time of obtaining Table 2-2 consent according to the presence or absence of proton-pump inhibitors

FREQ Procedure

			Cumulative	
Percent of ALL	power	Percent of	power	
1	20509	100.00	20509	100.00

Tables: ALL* AFTYP

ALL AFTYP(Type of AF)

Power |
Percent |
Percent of rows
Percent of columns | Episodic | Persistent | Long lasting | Persistent | Total
| | sex| |

	Episodic	Persistent	Long lasting	Persistent	Total
1	8288	3482	2913	5826	20509
	40.41	16.98	14.20	28.41	100.00
	40.41	16.98	14.20	28.41	
	100.00	100.00	100.00	100.00	
Total	8288	3482	2913	5826	20509
	40.41	16.98	14.20	28.41	100.00

Treatment history at the time of obtaining Table 2-2 consent according to the presence or absence of proton-pump inhibitors

FREQ Procedure

			Cumulative	
Percent of ALL	power	Percent of	power	
1	20509	100.00	20509	100.00

Tables: ALL* AFTHSPE

ALL

AFTHSPE(Non-drug Therapy for AF)

Power |
Percent |
Percent of rows
Percent of columns | None | With | Total

ALL	None	With	Total
1	17454	3055	20509
	85.10	14.90	100.00
	85.10	14.90	
	100.00	100.00	
Total	17454	3055	20509
	85.10	14.90	100.00

Treatment history at the time of obtaining Table 2-2 consent according to the presence or absence of proton-pump inhibitors

FREQ Procedure

Cumulative
Percent of ALL power Percent of power

1	20509	100.00	20509	100.00

Tables: ALL* AFTHSPE

ALL
AFTHSPE(Non-drug Therapy for AF)

Power |
Percent |
Percent of rows
Percent of columns | None | With | Total

	None	With	Total
1	17454	3055	20509
	85.10	14.90	100.00
	85.10	14.90	
	100.00	100.00	
Total	17454	3055	20509
	85.10	14.90	100.00

Treatment history at the time of obtaining Table 2-2 consent according to the presence or absence of proton-pump inhibitors

FREQ Procedure

			Cumulative	
Percent of ALL	power	Percent of power		
1	20509	100.00	20509	100.00

Table : ALL * AFTHSPE1

ALL

AFTHSPE1(Catheter Ablation)

Power |
Percent |
Percent of rows
Percent of columns | None | With | Total

	None	With	Total
1	18964	1545	20509
	92.47	7.53	100.00
	92.47	7.53	
	100.00	100.00	
Total	18964	1545	20509
	92.47	7.53	100.00

Treatment history at the time of obtaining Table 2-2 consent according to the presence or absence of proton-pump inhibitors

FREQ Procedure

			Cumulative	
Percent of ALL	power	Percent of power		
1	20509	100.00	20509	100.00

Table : ALL * AFTHSPE1

ALL

AFTHSPE1(Catheter Ablation)

Power |
Percent |
Percent of rows
Percent of columns | None | With | Total

	None	With	Total
1	18964	1545	20509
	92.47	7.53	100.00
	92.47	7.53	
	100.00	100.00	
Total	18964	1545	20509
	92.47	7.53	100.00

Treatment history at the time of obtaining Table 2-2 consent according to the presence or absence of proton-pump inhibitors

FREQ Procedure

Cumulative

Percent of ALL power Percent of power

1	20509	100.00	20509	100.00

Table : ALL * AFTHSPE2

ALL AFTHSPE2(Cardioversion)

Power |

Percent |

Percent of rows

Percent of columns | None | With | Total

	None	With	Total
1	20096	413	20509
	97.99	2.01	100.00
	97.99	2.01	
	100.00	100.00	
Total	20096	413	20509
	97.99	2.01	100.00

Treatment history at the time of obtaining Table 2-2 consent according to the presence or absence of proton-pump inhibitors

FREQ Procedure

			Cumulative	
Percent of ALL	power	Percent	of power	
1	20509	100.00	20509	100.00

Table : ALL * AFTHSPE2

ALL AFTHSPE2(Cardioversion)

Power<br>Percent<br>Percent of rows<br>Percent of columns	None	With	Total
1	20096	413	20509
	97.99	2.01	100.00
	97.99	2.01	
	100.00	100.00	
Total	20096	413	20509
	97.99	2.01	100.00

Treatment history at the time of obtaining Table 2-2 consent according to the presence or absence of proton-pump inhibitors

FREQ Procedure

Cumulative
Percent of ALL power Percent of power

1	20509	100.00	20509	100.00

Table : ALL * AFTHSPE3

ALL AFTHSPE3(ICD)

Power |
Percent |
Percent of rows
Percent of columns | None | With | Total

	None	With	Total
1	20432	77	20509
	99.62	0.38	100.00
	99.62	0.38	
	100.00	100.00	
Total	20432	77	20509
	99.62	0.38	100.00

Treatment history at the time of obtaining Table 2-2 consent according to the presence or absence of proton-pump inhibitors

FREQ Procedure

			Cumulative	
	Percent of ALL power	Percent of power		
1	20509	100.00	20509	100.00

Table : ALL * AFTHSPE3

ALL AFTHSPE3(ICD)

Power |
Percent |
Percent of rows
Percent of columns | None | With | Total

	None	With	Total
1	20432	77	20509
	99.62	0.38	100.00
	99.62	0.38	
	100.00	100.00	
Total	20432	77	20509
	99.62	0.38	100.00

Treatment history at the time of obtaining Table 2-2 consent according to the presence or absence of proton-pump inhibitors

FREQ Procedure

			Cumulative	
Percent of ALL	power	Percent of power		
1	20509	100.00	20509	100.00

Table : ALL * AFTHSPE4

ALL

AFTHSPE4(Pacemaker Implantation)

Power | Percent | Percent of rows Percent of columns	None	With	Total
1	19242	1267	20509
	93.82	6.18	100.00
	93.82	6.18	
	100.00	100.00	
Total	19242	1267	20509
	93.82	6.18	100.00

Treatment history at the time of obtaining Table 2-2 consent according to the presence or absence of proton-pump inhibitors

FREQ Procedure

			Cumulative	
Percent of ALL	power	Percent of	power	
1	20509	100.00	20509	100.00

Table : ALL * AFTHSPE4

ALL

AFTHSPE4(Pacemaker Implantation)

Power |
Percent |
Percent of rows
Percent of columns | None | With | Total

	None	With	Total
1	19242	1267	20509
	93.82	6.18	100.00
	93.82	6.18	
	100.00	100.00	
Total	19242	1267	20509
	93.82	6.18	100.00

Treatment history at the time of obtaining Table 2-2 consent according to the presence or absence of proton-pump inhibitors

FREQ Procedure

			Cumulative	
		Percent of ALL power	Percent of power	
1	20509	100.00	20509	100.00

Table : ALL * AFTHSPE5

ALL

AFTHSPE5(Other Non-drug Therapy)

Power | Percent | Percent of rows Percent of columns	None	With	Total
1	20452	57	20509
	99.72	0.28	100.00
	99.72	0.28	
	100.00	100.00	
Total	20452	57	20509
	99.72	0.28	100.00

Treatment history at the time of obtaining Table 2-2 consent according to the presence or absence of proton-pump inhibitors

FREQ Procedure

Cumulative

Percent of ALL power Percent of power

1	20509	100.00	20509	100.00

Table : ALL * AFTHSPE5

ALL

AFTHSPE5(Other Non-drug Therapy)

Power |
Percent |
Percent of rows
Percent of columns | None | With | Total

	None	With	Total
1	20452	57	20509
	99.72	0.28	100.00
	99.72	0.28	
	100.00	100.00	
Total	20452	57	20509
	99.72	0.28	100.00

Treatment history at the time of obtaining Table 2-2 consent according to the presence or absence of proton-pump inhibitors

FREQ Procedure

Number of levels of variables

Variables Label Level Number

GROUP1 2
HISAF History of Major Surgery for non-AF 2

Table : GROUP1 * HISAF

GROUP1
HISAF(History of Major Surgery for non-AF)

```
Power |
Percent |
Percent of rows
Percent of column |immediate 3 months |immediate 3 months | total
                  |Within month|Within month|
                  |Major surgery|Major surgery|
                  Lines |
_______________+________+________+
              1 |     149 |  11683 |  11832
                |    0.47 |  36.48 |  36.94
                |    1.26 |  98.74 |
                |   44.35 |  36.87 |
_______________+________+________+
              2 |     187 |  20007 |  20194
                |    0.58 |  62.47 |  63.06
                |    0.93 |  99.07 |
                |   55.65 |  63.13 |
_______________+________+________+
```

Total	336	31690	32026
	1.05	98.95	100.00

Treatment history at the time of obtaining Table 2-2 consent according to the presence or absence of proton-pump inhibitors

FREQ Procedure

Number of levels of variables

Variables	Label	Level Number
GROUP1		2
HISBDYN	History of Major Beeding	2

Table : GROUP1 * HISBDYN

GROUP1

HISBDYN(History of Major Beeding)

Power<br>Percent<br>Percent of rows<br>Percent of columns	None	With	Total
1	11385<br>35.04<br>95.03<br>36.46	596<br>1.83<br>4.97<br>47.26	11981<br>36.88
2	19844<br>61.08<br>96.76<br>63.54	665<br>2.05<br>3.24<br>52.74	20509<br>63.12
Total	31229<br>96.12	1261<br>3.88	32490<br>100.00

Treatment history at the time of obtaining Table 2-2 consent according to the presence or absence of proton-pump inhibitors

FREQ Procedure

Number of levels of variables

Variables Label Level Number

GROUP1 2
HISBD1 Intracranial Hemorrhage 2

Table : GROUP1 * HISBD1

GROUP1
HISBD1(Intracranial Hemorrhage)

Power |
Percent |
Percent of rows
Percent of columns | None | With | Total

	None	With	Total
1	11704	277	11981
	36.02	0.85	36.88
	97.69	2.31	
	36.76	42.68	
2	20137	372	20509
	61.98	1.14	63.12
	98.19	1.81	
	63.24	57.32	
Total	31841	649	32490
	98.00	2.00	100.00

Treatment history at the time of obtaining Table 2-2 consent according to the presence or absence of proton-pump inhibitors

FREQ Procedure

Number of levels of variables

Variables Label Level Number

GROUP1 2
HISBD2 Upper Gastrointestinal Bleeding 2

Table : GROUP1 * HISBD2

GROUP1
HISBD2(Upper Gastrointestinal Bleeding)

Power | Percent | Percent of rows Percent of columns	None	With	Total
1	11813	168	11981
	36.36	0.52	36.88
	98.60	1.40	
	36.67	61.31	
2	20403	106	20509
	62.80	0.33	63.12
	99.48	0.52	
	63.33	38.69	
Total	32216	274	32490
	99.16	0.84	100.00

Treatment history at the time of obtaining Table 2-2 consent according to the presence or absence of proton-pump inhibitors

FREQ Procedure

Number of levels of variables

Variables	Label	Level Number
GROUP1		2
HISBD3	Lower Gastrointestinal Bleeding	2

Table : GROUP1 * HISBD3

GROUP1

HISBD3(Lower Gastrointestinal Bleeding)

Power<br>Percent<br>Percent of rows<br>Percent of columns	None	With	Total
1	11831<br>36.41<br>98.75<br>36.76	150<br>0.46<br>1.25<br>48.70	11981<br>36.88
2	20351<br>62.64<br>99.23<br>63.24	158<br>0.49<br>0.77<br>51.30	20509<br>63.12
Total	32182<br>99.05	308<br>0.95	32490<br>100.00

Treatment history at the time of obtaining Table 2-2 consent according to the presence or absence of proton-pump inhibitors

FREQ Procedure

Number of levels of variables

Variable	Label	Level Count
GROUP1		2
HISBD4	Other Beeding	2

Table : GROUP1 * HISBD4

GROUP1 HISBD4(Other Beeding)

Power<br>Percent<br>Percent of rows<br>Percent of columns	None	With	Total
1	11874<br>36.55<br>99.11<br>36.83	107<br>0.33<br>0.89<br>43.15	11981<br>36.88
2	20368<br>62.69<br>99.31<br>63.17	141<br>0.43<br>0.69<br>56.85	20509<br>63.12
Total	32242<br>99.24	248<br>0.76	32490<br>100.00

Treatment history at the time of obtaining Table 2-2 consent according to the presence or absence of proton-pump inhibitors

FREQ Procedure

Number of levels of variables

Variables Label Level Number

GROUP1 2
HISAL History of Drug Allergy 2

Table : GROUP1 * HISAL

GROUP1
HISAL(History of Drug Allergy)

Power |
Percent |
Percent of rows
Percent of columns | None | With | Total

1	11073	908	11981
	34.08	2.79	36.88
	92.42	7.58	
	36.57	41.03	
2	19204	1305	20509
	59.11	4.02	63.12
	93.64	6.36	
	63.43	58.97	
Total	30277	2213	32490
	93.19	6.81	100.00

Treatment history at the time of obtaining Table 2-2 consent according to the presence or absence of proton-pump inhibitors

FREQ Procedure

Number of levels of variables

Variables	Label	Level Number
GROUP1		2
AFTYP	Type of AF	4

Table : GROUP1 * AFTYP

GROUP1 AFTYP(Type of AF)

Power<br>Percent<br>Percent of rows<br>Percent of columns	Episodic	Persistent	Long lasting | | sex|	Persistent	Total
1	5351	1899	1486	3245	11981
	16.47	5.84	4.57	9.99	36.88
	44.66	15.85	12.40	27.08	
	39.23	35.29	33.78	35.77	
2	8288	3482	2913	5826	20509
	25.51	10.72	8.97	17.93	63.12
	40.41	16.98	14.20	28.41	
	60.77	64.71	66.22	64.23	
Total	13639	5381	4399	9071	32490
	41.98	16.56	13.54	27.92	100.00

Treatment history at the time of obtaining Table 2-2 consent according to the presence or absence of proton-pump inhibitors

FREQ Procedure

Number of levels of variables

Number of Variables Level

GROUP1	2
Af_typ	3

Table : GROUP1 * af_typ

GROUP1 af_typ

Power |
Percent |
Percent of rows
Percentage of columns | 1| 2| 3| Total

	1	2	3	Total
1	5351	3385	3245	11981
	16.47	10.42	9.99	36.88
	44.66	28.25	27.08	
	39.23	34.61	35.77	
2	8288	6395	5826	20509
	25.51	19.68	17.93	63.12
	40.41	31.18	28.41	
	60.77	65.39	64.23	
Total	13639	9780	9071	32490
	41.98	30.10	27.92	100.00

Treatment history at the time of obtaining Table 2-2 consent according to the presence or absence of proton-pump inhibitors

FREQ Procedure

Number of levels of variables

Variable Label Level Count

Variable	Label	Level Count
GROUP1		2
AFTHSPE	Non-drug Therapy for AF	2

Table : GROUP1 * AFTHSPE

GROUP1

AFTHSPE(Non-drug Therapy for AF)

Power<br>Percent<br>Percent of rows<br>Percent of columns	None	With	Total
1	9409<br>28.96<br>78.53<br>35.03	2572<br>7.92<br>21.47<br>45.71	11981<br>36.88
2	17454<br>53.72<br>85.10<br>64.97	3055<br>9.40<br>14.90<br>54.29	20509<br>63.12
Total	26863<br>82.68	5627<br>17.32	32490<br>100.00

Treatment history at the time of obtaining Table 2-2 consent according to the presence or absence of proton-pump inhibitors

FREQ Procedure

Number of levels of variables

Variables Label Level Number

Variables	Label	Level Number
GROUP1		2
AFTHSPE1	Catheter Ablation	2

Table : GROUP1 * AFTHSPE1

GROUP1

AFTHSPE1(Catheter Ablation)

Power / Percent / Percent of rows / Percent of columns	None	With	Total
1	10578	1403	11981
	32.56	4.32	36.88
	88.29	11.71	
	35.81	47.59	
2	18964	1545	20509
	58.37	4.76	63.12
	92.47	7.53	
	64.19	52.41	
Total	29542	2948	32490
	90.93	9.07	100.00

Treatment history at the time of obtaining Table 2-2 consent according to the presence or absence of proton-pump inhibitors

FREQ Procedure

Number of levels of variables

Variable Label Level Count

GROUP1 2
AFTHSPE2 Cardioversion 2

Table : GROUP1 * AFTHSPE2

GROUP1 AFTHSPE2(Cardioversion)

Power |
Percent |
Percent of rows
Percent of columns | None | With | Total

1	11683	298	11981
	35.96	0.92	36.88
	97.51	2.49	
	36.76	41.91	
2	20096	413	20509
	61.85	1.27	63.12
	97.99	2.01	
	63.24	58.09	
Total	31779	711	32490
	97.81	2.19	100.00

Treatment history at the time of obtaining Table 2-2 consent according to the presence or absence of proton-pump inhibitors

FREQ Procedure

Number of levels of variables

Variables	Label	Level Number
GROUP1		2
AFTHSPE3	ICD	2

Table : GROUP1 * AFTHSPE3

GROUP1 AFTHSPE3(ICD)

Power / Percent / Percent of rows / Percent of columns	None	With	Total
1	11905	76	11981
	36.64	0.23	36.88
	99.37	0.63	
	36.82	49.67	
2	20432	77	20509
	62.89	0.24	63.12
	99.62	0.38	
	63.18	50.33	
Total	32337	153	32490
	99.53	0.47	100.00

Treatment history at the time of obtaining Table 2-2 consent according to the presence or absence of proton-pump inhibitors

FREQ Procedure

Number of levels of variables

Variables Label Level Number

GROUP1 2
AFTHSPE4 Pacemaker Implantation 2

Table : GROUP1 * AFTHSPE4

GROUP1
AFTHSPE4(Pacemaker Implantation)

Power |
Percent |
Percent of rows
Percent of columns | None | With | Total

GROUP1	None	With	Total
1	10926	1055	11981
	33.63	3.25	36.88
	91.19	8.81	
	36.22	45.43	
2	19242	1267	20509
	59.22	3.90	63.12
	93.82	6.18	
	63.78	54.57	
Total	30168	2322	32490
	92.85	7.15	100.00

Treatment history at the time of obtaining Table 2-2 consent according to the presence or absence of proton-pump inhibitors

FREQ Procedure

Number of levels of variables

Variables Label Level Number

GROUP1 2
AFTHSPE5 Other Non-drug Therapy 2

Table : GROUP1 * AFTHSPE5

GROUP1
AFTHSPE5(Other Non-drug Therapy)

Power |
Percent |
Percent of rows
Percent of columns | None | With | Total

GROUP1	None	With	Total
1	11925	56	11981
	36.70	0.17	36.88
	99.53	0.47	
	36.83	49.56	
2	20452	57	20509
	62.95	0.18	63.12
	99.72	0.28	
	63.17	50.44	
Total	32377	113	32490
	99.65	0.35	100.00

Complications and medical history at the time of obtaining Table 2-3 informed consent by the

presence or absence of proton-pump inhibitors

FREQ Procedure

			Cumulative	
Percent of ALL	power	Percent of	power	
1	32490	100.00	32490	100.00

Tables: ALL * MHYN

ALL MHYN(Medical History)

Power |
Percent |
Percent of rows
Percent of columns | None | With | Total

	None	With	Total
1	884	31606	32490
	2.72	97.28	100.00
	2.72	97.28	
	100.00	100.00	
Total	884	31606	32490
	2.72	97.28	100.00

Complications and medical history at the time of obtaining Table 2-3 informed consent by the presence or absence of proton-pump inhibitors

FREQ Procedure

			Cumulative	
Percent of ALL	power	Percent of power		
1	32490	100.00	32490	100.00

Tables: ALL * MHYN

ALL MHYN(Medical History)

Power |
Percent |
Percent of rows
Percent of columns | None | With | Total

	None	With	Total
1	884	31606	32490
	2.72	97.28	100.00
	2.72	97.28	
	100.00	100.00	
Total	884	31606	32490
	2.72	97.28	100.00

Complications and medical history at the time of obtaining Table 2-3 informed consent by the presence or absence of proton-pump inhibitors

FREQ Procedure

Cumulative
Percent of ALL power Percent of power

1	32490	100.00	32490	100.00

Tables: ALL* HBPYN

ALL
HBPYN(High Blood Pressure)

Power |
Percent |
Percent of rows
Percent of columns | None | With | Total

	None	With	Total
1	8015	24475	32490
	24.67	75.33	100.00
	24.67	75.33	
	100.00	100.00	
Total	8015	24475	32490
	24.67	75.33	100.00

Complications and medical history at the time of obtaining Table 2-3 informed consent by the presence or absence of proton-pump inhibitors

FREQ Procedure

			Cumulative	
Percent of ALL	power	Percent of power		
1	32490	100.00	32490	100.00

Tables: ALL* HBPYN

ALL

HBPYN(High Blood Pressure)

Power |
Percent |
Percent of rows
Percent of columns | None | With | Total

	None	With	Total
1	8015	24475	32490
	24.67	75.33	100.00
	24.67	75.33	
	100.00	100.00	
Total	8015	24475	32490
	24.67	75.33	100.00

Complications and medical history at the time of obtaining Table 2-3 informed consent by the presence or absence of proton-pump inhibitors

FREQ Procedure

Cumulative

Percent of ALL power Percent of power

1	32490	100.00	32490	100.00

Tables: ALL * DBYN

ALL DBYN(Diabetes)

Power |
Percent |
Percent of rows
Percent of columns | None | With | Total

1	23740	8750	32490
	73.07	26.93	100.00
	73.07	26.93	
	100.00	100.00	
Total	23740	8750	32490
	73.07	26.93	100.00

Complications and medical history at the time of obtaining Table 2-3 informed consent by the presence or absence of proton-pump inhibitors

FREQ Procedure

Cumulative
Percent of ALL power Percent of power

1	32490	100.00	32490	100.00

Tables: ALL * DBYN

ALL DBYN(Diabetes)

Power |
Percent |
Percent of rows
Percent of columns | None | With | Total

1	23740	8750	32490
	73.07	26.93	100.00
	73.07	26.93	
	100.00	100.00	
Total	23740	8750	32490
	73.07	26.93	100.00

Complications and medical history at the time of obtaining Table 2-3 informed consent by the presence or absence of proton-pump inhibitors

FREQ Procedure

			Cumulative	
Percent of ALL	power	Percent of power		
1	32490	100.00	32490	100.00

Table: ALL * t_DB

ALL t_DB

Power |
Percent |
Percent of rows
Percentage of columns | 0| 1| Sum

	0	1	Sum
1	30830	1660	32490
	94.89	5.11	100.00
	94.89	5.11	
	100.00	100.00	
Total	30830	1660	32490
	94.89	5.11	100.00

Complications and medical history at the time of obtaining Table 2-3 informed consent by the presence or absence of proton-pump inhibitors

FREQ Procedure

Cumulative
Percent of ALL power Percent of power

1	32490	100.00	32490	100.00

Table: ALL * t_DB

ALL t_DB

Power |
Percent |
Percent of rows
Percentage of columns | 0| 1| Sum

```
______________+________+________+
         1 |   30830 |    1660 |   32490
           |   94.89 |    5.11 | 100.00
           |   94.89 |    5.11 |
           | 100.00 | 100.00 |
______________+________+________+
Total 30830 1660 32490
              94.89      5.11    100.00
```

Complications and medical history at the time of obtaining Table 2-3 informed consent by the presence or absence of proton-pump inhibitors

FREQ Procedure

Cumulative
Percent of ALL power Percent of power

1	32490	100.00	32490	100.00

Table : ALL * DB 1 YN

ALL
DB1YN(Diabetic Retinopathy)

Power |
Percent |
Percent of rows
Percent of columns | None | With | Total

	None	With	Total
1	31984	506	32490
	98.44	1.56	100.00
	98.44	1.56	
	100.00	100.00	
Total	31984	506	32490
	98.44	1.56	100.00

Complications and medical history at the time of obtaining Table 2-3 informed consent by the presence or absence of proton-pump inhibitors

FREQ Procedure

Cumulative
Percent of ALL power Percent of power

1	32490	100.00	32490	100.00

Table : ALL * DB 1 YN

ALL
DB1YN(Diabetic Retinopathy)

Power |
Percent |
Percent of rows
Percent of columns | None | With | Total

	None	With	Total
1	31984	506	32490
	98.44	1.56	100.00
	98.44	1.56	
	100.00	100.00	
Total	31984	506	32490
	98.44	1.56	100.00

Complications and medical history at the time of obtaining Table 2-3 informed consent by the presence or absence of proton-pump inhibitors

FREQ Procedure

			Cumulative	
Percent of ALL	power	Percent of power		
1	32490	100.00	32490	100.00

Table : ALL * DB 2 YN

ALL

DB2YN(Diabetic Nephropathy)

Power |
Percent |
Percent of rows
Percent of columns | None | With | Total

	None	With	Total
1	31287	1203	32490
	96.30	3.70	100.00
	96.30	3.70	
	100.00	100.00	
Total	31287	1203	32490
	96.30	3.70	100.00

Complications and medical history at the time of obtaining Table 2-3 informed consent by the presence or absence of proton-pump inhibitors

FREQ Procedure

			Cumulative	
Percent of ALL	power	Percent of power		
1	32490	100.00	32490	100.00

Table : ALL * DB 2 YN

ALL

DB2YN(Diabetic Nephropathy)

Power |
Percent |
Percent of rows
Percent of columns | None | With | Total

	None	With	Total
1	31287	1203	32490
	96.30	3.70	100.00
	96.30	3.70	
	100.00	100.00	
Total	31287	1203	32490
	96.30	3.70	100.00

Complications and medical history at the time of obtaining Table 2-3 informed consent by the presence or absence of proton-pump inhibitors

FREQ Procedure

Cumulative
Percent of ALL power Percent of power

1	32490	100.00	32490	100.00

Table : ALL * DB 3 YN

ALL
DB3YN(Diabetic Neuropathy)

Power |
Percent |
Percent of rows
Percent of columns | None | With | Total

	None	With	Total
1	32166	324	32490
	99.00	1.00	100.00
	99.00	1.00	
	100.00	100.00	
Total	32166	324	32490
	99.00	1.00	100.00

Complications and medical history at the time of obtaining Table 2-3 informed consent by the presence or absence of proton-pump inhibitors

FREQ Procedure

Cumulative
Percent of ALL power Percent of power

1	32490	100.00	32490	100.00

Table : ALL * DB 3 YN

ALL

DB3YN(Diabetic Neuropathy)

Power |
Percent |
Percent of rows
Percent of columns | None | With | Total

1	32166	324	32490
	99.00	1.00	100.00
	99.00	1.00	
	100.00	100.00	
Total	32166	324	32490
	99.00	1.00	100.00

Complications and medical history at the time of obtaining Table 2-3 informed consent by the presence or absence of proton-pump inhibitors

FREQ Procedure

Cumulative
Percent of ALL power Percent of power

1	32490	100.00	32490	100.00

Tables: ALL* LMDYN

ALL
LMDYN(Lipid metabolism disorders)

Power |
Percent |
Percent of rows
Percent of columns | None | With | Total

1	18675	13815	32490
	57.48	42.52	100.00
	57.48	42.52	
	100.00	100.00	
Total	18675	13815	32490
	57.48	42.52	100.00

Complications and medical history at the time of obtaining Table 2-3 informed consent by the presence or absence of proton-pump inhibitors

FREQ Procedure

			Cumulative	
Percent of ALL	power	Percent of power		
1	32490	100.00	32490	100.00

Tables: ALL* LMDYN

ALL

LMDYN(Lipid metabolism disorders)

Power |
Percent |
Percent of rows
Percent of columns | None | With | Total

	None	With	Total
1	18675	13815	32490
	57.48	42.52	100.00
	57.48	42.52	
	100.00	100.00	
Total	18675	13815	32490
	57.48	42.52	100.00

Complications and medical history at the time of obtaining Table 2-3 informed consent by the presence or absence of proton-pump inhibitors

FREQ Procedure

Cumulative
Percent of ALL power Percent of power

1	32490	100.00	32490	100.00

Tables: ALL * HYPURNYN

ALL HYPURNYN(Hyperuricemia)

Power |
Percent |
Percent of rows
Percent of columns | None | With | Total

	None	With	Total
1	25112	7378	32490
	77.29	22.71	100.00
	77.29	22.71	
	100.00	100.00	
Total	25112	7378	32490
	77.29	22.71	100.00

Complications and medical history at the time of obtaining Table 2-3 informed consent by the presence or absence of proton-pump inhibitors

FREQ Procedure

Cumulative
Percent of ALL power Percent of power

1	32490	100.00	32490	100.00

Tables: ALL * HYPURNYN

ALL HYPURNYN(Hyperuricemia)

Power |
Percent |
Percent of rows
Percent of columns | None | With | Total

	None	With	Total
1	25112	7378	32490
	77.29	22.71	100.00
	77.29	22.71	
	100.00	100.00	
Total	25112	7378	32490
	77.29	22.71	100.00

Complications and medical history at the time of obtaining Table 2-3 informed consent by the presence or absence of proton-pump inhibitors

FREQ Procedure

			Cumulative	
Percent of ALL	power	Percent of	power	
1	32490	100.00	32490	100.00

Tables: ALL * KDDSYN

ALL KDDSYN(Kidney Disease)

Power |
Percent |
Percent of rows
Percent of columns | None | With | Total

	None	With	Total
1	24883	7607	32490
	76.59	23.41	100.00
	76.59	23.41	
	100.00	100.00	
Total	24883	7607	32490
	76.59	23.41	100.00

Complications and medical history at the time of obtaining Table 2-3 informed consent by the presence or absence of proton-pump inhibitors

FREQ Procedure

Cumulative

Percent of ALL power Percent of power

1	32490	100.00	32490	100.00

Tables: ALL * KDDSYN

ALL KDDSYN(Kidney Disease)

Power |
Percent |
Percent of rows
Percent of columns | None | With | Total

1	24883	7607	32490
	76.59	23.41	100.00
	76.59	23.41	
	100.00	100.00	
Total	24883	7607	32490
	76.59	23.41	100.00

Complications and medical history at the time of obtaining Table 2-3 informed consent by the presence or absence of proton-pump inhibitors

FREQ Procedure

			Cumulative	
Percent of ALL	power	Percent of power		
1	32490	100.00	32490	100.00

Table : ALL * KDDS 1 YN

ALL

KDDS1YN(Chronic Kidney Disease)

Power | Percent | Percent of rows Percent of columns	None	With	Total
1	25732	6758	32490
	79.20	20.80	100.00
	79.20	20.80	
	100.00	100.00	
Total	25732	6758	32490
	79.20	20.80	100.00

Complications and medical history at the time of obtaining Table 2-3 informed consent by the presence or absence of proton-pump inhibitors

FREQ Procedure

			Cumulative	
Percent of ALL	power	Percent of	power	
1	32490	100.00	32490	100.00

Table : ALL * KDDS 1 YN

ALL

KDDS1YN(Chronic Kidney Disease)

Power |
Percent |
Percent of rows
Percent of columns | None | With | Total

1	25732	6758	32490
	79.20	20.80	100.00
	79.20	20.80	
	100.00	100.00	
Total	25732	6758	32490
	79.20	20.80	100.00

Complications and medical history at the time of obtaining Table 2-3 informed consent by the presence or absence of proton-pump inhibitors

FREQ Procedure

Cumulative

Percent of ALL power Percent of power

1	32490	100.00	32490	100.00

Table : ALL * KDDS 2 YN

ALL

KDDS2YN(Advanced Renal Dysfunction without Dyalysis)

Power | Percent | Percent of rows Percent of columns	None	With	Total
1	32018	472	32490
	98.55	1.45	100.00
	98.55	1.45	
	100.00	100.00	
Total	32018	472	32490
	98.55	1.45	100.00

Complications and medical history at the time of obtaining Table 2-3 informed consent by the presence or absence of proton-pump inhibitors

FREQ Procedure

			Cumulative	
ALL	power	Percent	power	Percent of power
1	32490	100.00	32490	100.00

Table : ALL * KDDS 2 YN

ALL

KDDS2YN(Advanced Renal Dysfunction without Dyalysis)

Power |
Percent |
Percent of rows
Percent of columns | None | With | Total

Percent of columns	None	With	Total
1	32018	472	32490
	98.55	1.45	100.00
	98.55	1.45	
	100.00	100.00	
Total	32018	472	32490
	98.55	1.45	100.00

Complications and medical history at the time of obtaining Table 2-3 informed consent by the presence or absence of proton-pump inhibitors

FREQ Procedure

			Cumulative	
Percent of ALL	power	Percent of power		
1	32490	100.00	32490	100.00

Table : ALL * KDDS 3 YN

ALL

KDDS3YN(Advanced Renal Dysfunction with Dyalysis)

Power | Percent | Percent of rows Percent of columns	None	With	Total
1	32372	118	32490
	99.64	0.36	100.00
	99.64	0.36	
	100.00	100.00	
Total	32372	118	32490
	99.64	0.36	100.00

Complications and medical history at the time of obtaining Table 2-3 informed consent by the presence or absence of proton-pump inhibitors

FREQ Procedure

			Cumulative	
Percent of ALL	power	Percent of	power	
1	32490	100.00	32490	100.00

Table : ALL * KDDS 3 YN

ALL

KDDS3YN(Advanced Renal Dysfunction with Dyalysis)

Power / Percent / Percent of rows / Percent of columns	None	With	Total
1	32372	118	32490
	99.64	0.36	100.00
	99.64	0.36	
	100.00	100.00	
Total	32372	118	32490
	99.64	0.36	100.00

Complications and medical history at the time of obtaining Table 2-3 informed consent by the presence or absence of proton-pump inhibitors

FREQ Procedure

			Cumulative	
Percent of ALL	power	Percent	of power	
1	32490	100.00	32490	100.00

Tables: ALL * SLVDYN

ALL

SLVDYN(Severe Liver Dysfunction)

Power |
Percent |
Percent of rows
Percent of columns | None | With | Total

	None	With	Total
1	32191	299	32490
	99.08	0.92	100.00
	99.08	0.92	
	100.00	100.00	
Total	32191	299	32490
	99.08	0.92	100.00

Complications and medical history at the time of obtaining Table 2-3 informed consent by the presence or absence of proton-pump inhibitors

FREQ Procedure

			Cumulative	
Percent of ALL	power	Percent of	power	
1	32490	100.00	32490	100.00

Tables: ALL * SLVDYN

ALL

SLVDYN(Severe Liver Dysfunction)

Power / Percent / Percent of rows / Percent of columns	None	With	Total
1	32191	299	32490
	99.08	0.92	100.00
	99.08	0.92	
	100.00	100.00	
Total	32191	299	32490
	99.08	0.92	100.00

Complications and medical history at the time of obtaining Table 2-3 informed consent by the presence or absence of proton-pump inhibitors

FREQ Procedure

			Cumulative	
Percent of ALL	power	Percent of power		
1	32490	100.00	32490	100.00

Tables: ALL* RESDYN

ALL

RESDYN(Respiratory Disease)

Power |
Percent |
Percent of rows
Percent of columns | None | With | Total

	None	With	Total
1	28326	4164	32490
	87.18	12.82	100.00
	87.18	12.82	
	100.00	100.00	
Total	28326	4164	32490
	87.18	12.82	100.00

Complications and medical history at the time of obtaining Table 2-3 informed consent by the presence or absence of proton-pump inhibitors

FREQ Procedure

			Cumulative	
Percent of ALL	power	Percent of power		
1	32490	100.00	32490	100.00

Tables: ALL* RESDYN

ALL

RESDYN(Respiratory Disease)

Power |
Percent |
Percent of rows
Percent of columns | None | With | Total

ALL	None	With	Total
1	28326	4164	32490
	87.18	12.82	100.00
	87.18	12.82	
	100.00	100.00	
Total	28326	4164	32490
	87.18	12.82	100.00

Complications and medical history at the time of obtaining Table 2-3 informed consent by the presence or absence of proton-pump inhibitors

FREQ Procedure

			Cumulative	
Percent of ALL	power	Percent of	power	
1	32490	100.00	32490	100.00

Tables: ALL* HTDYN

ALL HTDYN(Heart Disease)

Power |
Percent |
Percent of rows
Percent of columns | None | With | Total

	None	With	Total
1	13379	19111	32490
	41.18	58.82	100.00
	41.18	58.82	
	100.00	100.00	
Total	13379	19111	32490
	41.18	58.82	100.00

Complications and medical history at the time of obtaining Table 2-3 informed consent by the presence or absence of proton-pump inhibitors

FREQ Procedure

			Cumulative	
Percent of ALL	power	Percent of power		
1	32490	100.00	32490	100.00

Tables: ALL* HTDYN

ALL HTDYN(Heart Disease)

Power |
Percent |
Percent of rows
Percent of columns | None | With | Total

	None	With	Total
1	13379	19111	32490
	41.18	58.82	100.00
	41.18	58.82	
	100.00	100.00	
Total	13379	19111	32490
	41.18	58.82	100.00

Complications and medical history at the time of obtaining Table 2-3 informed consent by the presence or absence of proton-pump inhibitors

FREQ Procedure

Cumulative
Percent of ALL power Percent of power

1	32490	100.00	32490	100.00

Table : ALL * HTD 1 YN

ALL
HTD1YN(Myocardial Infarction)

Power |
Percent |
Percent of rows
Percent of columns | None | With | Total

	None	With	Total
1	30628	1862	32490
	94.27	5.73	100.00
	94.27	5.73	
	100.00	100.00	
Total	30628	1862	32490
	94.27	5.73	100.00

Complications and medical history at the time of obtaining Table 2-3 informed consent by the presence or absence of proton-pump inhibitors

FREQ Procedure

			Cumulative	
Percent of ALL	power	Percent of	power	
1	32490	100.00	32490	100.00

Table : ALL * HTD 1 YN

ALL

HTD1YN(Myocardial Infarction)

Power |
Percent |
Percent of rows
Percent of columns | None | With | Total

	None	With	Total
1	30628	1862	32490
	94.27	5.73	100.00
	94.27	5.73	
	100.00	100.00	
Total	30628	1862	32490
	94.27	5.73	100.00

Complications and medical history at the time of obtaining Table 2-3 informed consent by the presence or absence of proton-pump inhibitors

FREQ Procedure

			Cumulative	
Percent of ALL	power	Percent of power		
1	32490	100.00	32490	100.00

Table : ALL * HTD 2 YN

ALL HTD2YN(Angina Pectoris)

Power |
Percent |
Percent of rows
Percent of columns | None | With | Total

	None	With	Total
1	26938	5552	32490
	82.91	17.09	100.00
	82.91	17.09	
	100.00	100.00	
Total	26938	5552	32490
	82.91	17.09	100.00

Complications and medical history at the time of obtaining Table 2-3 informed consent by the presence or absence of proton-pump inhibitors

FREQ Procedure

Cumulative

Percent of ALL power Percent of power

1	32490	100.00	32490	100.00

Table : ALL * HTD 2 YN

ALL HTD2YN(Angina Pectoris)

Power |
Percent |
Percent of rows
Percent of columns | None | With | Total

1	26938	5552	32490
	82.91	17.09	100.00
	82.91	17.09	
	100.00	100.00	
Total	26938	5552	32490
	82.91	17.09	100.00

Complications and medical history at the time of obtaining Table 2-3 informed consent by the presence or absence of proton-pump inhibitors

FREQ Procedure

Cumulative
Percent of ALL power Percent of power

1	32490	100.00	32490	100.00

Table: ALL * t_HTD0

ALL t_HTD0

Power |
Percent |
Percent of rows
Percentage of columns | 0| 1| Sum

	0	1	Sum
1	25739	6751	32490
	79.22	20.78	100.00
	79.22	20.78	
	100.00	100.00	
Total	25739	6751	32490
	79.22	20.78	100.00

Complications and medical history at the time of obtaining Table 2-3 informed consent by the presence or absence of proton-pump inhibitors

FREQ Procedure

Cumulative

Percent of ALL power Percent of power

1	32490	100.00	32490	100.00

Table: ALL * t_HTD0

ALL t_HTD0

Power |
Percent |
Percent of rows
Percentage of columns | 0| 1| Sum

	0	1	Sum
1	25739	6751	32490
	79.22	20.78	100.00
	79.22	20.78	
	100.00	100.00	
Total	25739	6751	32490
	79.22	20.78	100.00

Complications and medical history at the time of obtaining Table 2-3 informed consent by the presence or absence of proton-pump inhibitors

FREQ Procedure

Cumulative
Percent of ALL power Percent of power

1	32490	100.00	32490	100.00

Table : ALL * HTD 3 YN

ALL HTD3YN(Heart Failure)

Power |
Percent |
Percent of rows
Percent of columns | None | With | Total

1	20302	12188	32490
	62.49	37.51	100.00
	62.49	37.51	
	100.00	100.00	
Total	20302	12188	32490
	62.49	37.51	100.00

Complications and medical history at the time of obtaining Table 2-3 informed consent by the presence or absence of proton-pump inhibitors

FREQ Procedure

			Cumulative	
Percent of ALL	power	Percent of power		
1	32490	100.00	32490	100.00

Table : ALL * HTD 3 YN

ALL HTD3YN(Heart Failure)

Power |
Percent |
Percent of rows
Percent of columns | None | With | Total

	None	With	Total
1	20302	12188	32490
	62.49	37.51	100.00
	62.49	37.51	
	100.00	100.00	
Total	20302	12188	32490
	62.49	37.51	100.00

Complications and medical history at the time of obtaining Table 2-3 informed consent by the presence or absence of proton-pump inhibitors

FREQ Procedure

			Cumulative	
Percent of ALL	power	Percent of power		
1	32490	100.00	32490	100.00

Table : ALL * HTD 4 YN

ALL

HTD4YN(Left Ventricular Systolic Dysfunction)

Power |
Percent |
Percent of rows
Percent of columns | None | With | Total

	None	With	Total
1	31801	689	32490
	97.88	2.12	100.00
	97.88	2.12	
	100.00	100.00	
Total	31801	689	32490
	97.88	2.12	100.00

Complications and medical history at the time of obtaining Table 2-3 informed consent by the presence or absence of proton-pump inhibitors

FREQ Procedure

			Cumulative	
	Percent of ALL power	Percent of power		
1	32490	100.00	32490	100.00

Table : ALL * HTD 4 YN

ALL

HTD4YN(Left Ventricular Systolic Dysfunction)

Power / Percent / Percent of rows / Percent of columns	None	With	Total
1	31801	689	32490
	97.88	2.12	100.00
	97.88	2.12	
	100.00	100.00	
Total	31801	689	32490
	97.88	2.12	100.00

Complications and medical history at the time of obtaining Table 2-3 informed consent by the presence or absence of proton-pump inhibitors

FREQ Procedure

Cumulative
Percent of ALL power Percent of power

1	32490	100.00	32490	100.00

Table: ALL * t_HTD

ALL t_HTD

Power |
Percent |
Percent of rows
Percentage of columns | 0| 1| Sum

	0	1	Sum
1	20140	12350	32490
	61.99	38.01	100.00
	61.99	38.01	
	100.00	100.00	
Total	20140	12350	32490
	61.99	38.01	100.00

Complications and medical history at the time of obtaining Table 2-3 informed consent by the presence or absence of proton-pump inhibitors

FREQ Procedure

Cumulative
Percent of ALL power Percent of power

1	32490	100.00	32490	100.00

Table: ALL * t_HTD

ALL t_HTD

Power |
Percent |
Percent of rows
Percentage of columns | 0| 1| Sum

	0	1	Sum
1	20140	12350	32490
	61.99	38.01	100.00
	61.99	38.01	
	100.00	100.00	
Total	20140	12350	32490
	61.99	38.01	100.00

Complications and medical history at the time of obtaining Table 2-3 informed consent by the presence or absence of proton-pump inhibitors

FREQ Procedure

Cumulative
Percent of ALL power Percent of power

1	32490	100.00	32490	100.00

Table : ALL * HTD 5 YN

ALL HTD5YN(Valvular Disease)

Power |
Percent |
Percent of rows
Percent of columns | None | With | Total

	None	With	Total
1	28515	3975	32490
	87.77	12.23	100.00
	87.77	12.23	
	100.00	100.00	
Total	28515	3975	32490
	87.77	12.23	100.00

Complications and medical history at the time of obtaining Table 2-3 informed consent by the presence or absence of proton-pump inhibitors

FREQ Procedure

Cumulative
Percent of ALL power Percent of power

1	32490	100.00	32490	100.00

Table : ALL * HTD 5 YN

ALL HTD5YN(Valvular Disease)

Power |
Percent |
Percent of rows
Percent of columns | None | With | Total

1	28515	3975	32490
	87.77	12.23	100.00
	87.77	12.23	
	100.00	100.00	
Total	28515	3975	32490
	87.77	12.23	100.00

Complications and medical history at the time of obtaining Table 2-3 informed consent by the presence or absence of proton-pump inhibitors

FREQ Procedure

			Cumulative	
Percent of ALL	power	Percent of power		
1	32490	100.00	32490	100.00

Table : ALL * HTD 6 YN

ALL HTD6YN(Cardiomyopathy)

Power |
Percent |
Percent of rows
Percent of columns | None | With | Total

	None	With	Total
1	31291	1199	32490
	96.31	3.69	100.00
	96.31	3.69	
	100.00	100.00	
Total	31291	1199	32490
	96.31	3.69	100.00

Complications and medical history at the time of obtaining Table 2-3 informed consent by the presence or absence of proton-pump inhibitors

FREQ Procedure

			Cumulative	
ALL	power	Percent	power	Percent of power
1	32490	100.00	32490	100.00

Table : ALL * HTD 6 YN

ALL HTD6YN(Cardiomyopathy)

Power |
Percent |
Percent of rows
Percent of columns | None | With | Total

ALL	None	With	Total
1	31291	1199	32490
	96.31	3.69	100.00
	96.31	3.69	
	100.00	100.00	
Total	31291	1199	32490
	96.31	3.69	100.00

Complications and medical history at the time of obtaining Table 2-3 informed consent by the presence or absence of proton-pump inhibitors

FREQ Procedure

Cumulative

Percent of ALL power Percent of power

1	32490	100.00	32490	100.00

Table : ALL * HTD 7 YN

ALL

HTD7YN(Hypertensive Heart Disease)

Power |
Percent |
Percent of rows
Percent of columns | None | With | Total

	None	With	Total
1	31427	1063	32490
	96.73	3.27	100.00
	96.73	3.27	
	100.00	100.00	
Total	31427	1063	32490
	96.73	3.27	100.00

Complications and medical history at the time of obtaining Table 2-3 informed consent by the presence or absence of proton-pump inhibitors

FREQ Procedure

Cumulative

Percent of ALL power Percent of power

1	32490	100.00	32490	100.00

Table : ALL * HTD 7 YN

ALL

HTD7YN(Hypertensive Heart Disease)

Power |

Percent |

Percent of rows

Percent of columns | None | With | Total

	None	With	Total
1	31427	1063	32490
	96.73	3.27	100.00
	96.73	3.27	
	100.00	100.00	
Total	31427	1063	32490
	96.73	3.27	100.00

Complications and medical history at the time of obtaining Table 2-3 informed consent by the presence or absence of proton-pump inhibitors

FREQ Procedure

Cumulative
Percent of ALL power Percent of power

1	32490	100.00	32490	100.00

Table : ALL * HTD 8 YN

ALL
HTD8YN(Cognenital Heart Disease)

Power |
Percent |
Percent of rows
Percent of columns | None | With | Total

Percent of columns	None	With	Total
1	32305	185	32490
	99.43	0.57	100.00
	99.43	0.57	
	100.00	100.00	
Total	32305	185	32490
	99.43	0.57	100.00

Complications and medical history at the time of obtaining Table 2-3 informed consent by the presence or absence of proton-pump inhibitors

FREQ Procedure

			Cumulative	
Percent of ALL	power	Percent of	power	
1	32490	100.00	32490	100.00

Table : ALL * HTD 8 YN

ALL

HTD8YN(Cognenital Heart Disease)

Power |
Percent |
Percent of rows
Percent of columns | None | With | Total

Power / Percent / Percent of rows / Percent of columns	None	With	Total
1	32305	185	32490
	99.43	0.57	100.00
	99.43	0.57	
	100.00	100.00	
Total	32305	185	32490
	99.43	0.57	100.00

Complications and medical history at the time of obtaining Table 2-3 informed consent by the presence or absence of proton-pump inhibitors

FREQ Procedure

Cumulative
Percent of ALL power Percent of power

1	32490	100.00	32490	100.00

Table : ALL * HTD 9 YN

ALL
HTD9YN(Other Heart Disease)

Power |
Percent |
Percent of rows
Percent of columns | None | With | Total

1	29797	2693	32490
	91.71	8.29	100.00
	91.71	8.29	
	100.00	100.00	
Total	29797	2693	32490
	91.71	8.29	100.00

Complications and medical history at the time of obtaining Table 2-3 informed consent by the presence or absence of proton-pump inhibitors

FREQ Procedure

			Cumulative	
Percent of ALL	power	Percent of power		
1	32490	100.00	32490	100.00

Table : ALL * HTD 9 YN

ALL

HTD9YN(Other Heart Disease)

Power |
Percent |
Percent of rows
Percent of columns | None | With | Total

	None	With	Total
1	29797	2693	32490
	91.71	8.29	100.00
	91.71	8.29	
	100.00	100.00	
Total	29797	2693	32490
	91.71	8.29	100.00

Complications and medical history at the time of obtaining Table 2-3 informed consent by the presence or absence of proton-pump inhibitors

FREQ Procedure

			Cumulative	
Percent of ALL	power	Percent of power		
1	32490	100.00	32490	100.00

Tables: ALL* CVACYN

ALL

CVACYN(Cerebrovascular Accident)

Power |
Percent |
Percent of rows
Percent of columns | None | With | Total

	None	With	Total
1	25133	7357	32490
	77.36	22.64	100.00
	77.36	22.64	
	100.00	100.00	
Total	25133	7357	32490
	77.36	22.64	100.00

Complications and medical history at the time of obtaining Table 2-3 informed consent by the presence or absence of proton-pump inhibitors

FREQ Procedure

Cumulative

Percent of ALL power Percent of power

1	32490	100.00	32490	100.00

Tables: ALL* CVACYN

ALL

CVACYN(Cerebrovascular Accident)

Power |

Percent |

Percent of rows

Percent of columns | None | With | Total

1	25133	7357	32490
	77.36	22.64	100.00
	77.36	22.64	
	100.00	100.00	
Total	25133	7357	32490
	77.36	22.64	100.00

Complications and medical history at the time of obtaining Table 2-3 informed consent by the presence or absence of proton-pump inhibitors

FREQ Procedure

			Cumulative	
Percent of ALL	power	Percent of	power	
1	32490	100.00	32490	100.00

Table : ALL * CVAC 1 YN

ALL

CVAC1YN(Atherosclerotic Cerebral Infarction)

Power |
Percent |
Percent of rows
Percent of columns | None | With | Total

	None	With	Total
1	31819	671	32490
	97.93	2.07	100.00
	97.93	2.07	
	100.00	100.00	
Total	31819	671	32490
	97.93	2.07	100.00

Complications and medical history at the time of obtaining Table 2-3 informed consent by the presence or absence of proton-pump inhibitors

FREQ Procedure

Percent of ALL	power	Percent	Cumulative power	Cumulative Percent of power
1	32490	100.00	32490	100.00

Table : ALL * CVAC 1 YN

ALL

CVAC1YN(Atherosclerotic Cerebral Infarction)

Power<br>Percent<br>Percent of rows<br>Percent of columns	None	With	Total
1	31819<br>97.93<br>97.93<br>100.00	671<br>2.07<br>2.07<br>100.00	32490<br>100.00
Total	31819<br>97.93	671<br>2.07	32490<br>100.00

Complications and medical history at the time of obtaining Table 2-3 informed consent by the presence or absence of proton-pump inhibitors

FREQ Procedure

Cumulative
Percent of ALL power Percent of power

1	32490	100.00	32490	100.00

Table : ALL * CVAC 2 YN

ALL
CVAC2YN(Cardiogenic Cerebral Infarction)

Power |
Percent |
Percent of rows
Percent of columns | None | With | Total

	None	With	Total
1	30096	2394	32490
	92.63	7.37	100.00
	92.63	7.37	
	100.00	100.00	
Total	30096	2394	32490
	92.63	7.37	100.00

Complications and medical history at the time of obtaining Table 2-3 informed consent by the presence or absence of proton-pump inhibitors

FREQ Procedure

			Cumulative	
Percent of ALL	power	Percent of	power	
1	32490	100.00	32490	100.00

Table : ALL * CVAC 2 YN

ALL

CVAC2YN(Cardiogenic Cerebral Infarction)

Power<br>Percent<br>Percent of rows<br>Percent of columns	None	With	Total
1	30096<br>92.63<br>92.63<br>100.00	2394<br>7.37<br>7.37<br>100.00	32490<br>100.00
Total	30096<br>92.63	2394<br>7.37	32490<br>100.00

Complications and medical history at the time of obtaining Table 2-3 informed consent by the presence or absence of proton-pump inhibitors

FREQ Procedure

Cumulative
Percent of ALL power Percent of power

1	32490	100.00	32490	100.00

Table : ALL * CVAC 3 YN

ALL
CVAC3YN(Lacunar Cerebral Infarction)

Power |
Percent |
Percent of rows
Percent of columns | None | With | Total

	None	With	Total
1	31043	1447	32490
	95.55	4.45	100.00
	95.55	4.45	
	100.00	100.00	
Total	31043	1447	32490
	95.55	4.45	100.00

Complications and medical history at the time of obtaining Table 2-3 informed consent by the presence or absence of proton-pump inhibitors

FREQ Procedure

Cumulative
Percent of ALL power Percent of power

1	32490	100.00	32490	100.00

Table : ALL * CVAC 3 YN

ALL
CVAC3YN(Lacunar Cerebral Infarction)

Power |
Percent |
Percent of rows
Percent of columns | None | With | Total

	None	With	Total
1	31043	1447	32490
	95.55	4.45	100.00
	95.55	4.45	
	100.00	100.00	
Total	31043	1447	32490
	95.55	4.45	100.00

Complications and medical history at the time of obtaining Table 2-3 informed consent by the presence or absence of proton-pump inhibitors

FREQ Procedure

			Cumulative	
Percent of ALL	power	Percent	of power	
1	32490	100.00	32490	100.00

Table : ALL * CVAC 4 YN

ALL

CVAC4YN(Hemorrhagic Stroke)

Power |
Percent |
Percent of rows
Percent of columns | None | With | Total

	None	With	Total
1	32120	370	32490
	98.86	1.14	100.00
	98.86	1.14	
	100.00	100.00	
Total	32120	370	32490
	98.86	1.14	100.00

Complications and medical history at the time of obtaining Table 2-3 informed consent by the presence or absence of proton-pump inhibitors

FREQ Procedure

Cumulative
Percent of ALL power Percent of power

1	32490	100.00	32490	100.00

Table : ALL * CVAC 4 YN

ALL
CVAC4YN(Hemorrhagic Stroke)

Power |
Percent |
Percent of rows
Percent of columns | None | With | Total

1	32120	370	32490
	98.86	1.14	100.00
	98.86	1.14	
	100.00	100.00	
Total	32120	370	32490
	98.86	1.14	100.00

Complications and medical history at the time of obtaining Table 2-3 informed consent by the presence or absence of proton-pump inhibitors

FREQ Procedure

			Cumulative	
Percent of ALL	power	Percent of power		
1	32490	100.00	32490	100.00

Table : ALL * CVAC 5 YN

ALL

CVAC5YN(Unclassified/Unknown Cerebral Infarction)

Power |
Percent |
Percent of rows
Percent of columns | None | With | Total

	None	With	Total
1	30728	1762	32490
	94.58	5.42	100.00
	94.58	5.42	
	100.00	100.00	
Total	30728	1762	32490
	94.58	5.42	100.00

Complications and medical history at the time of obtaining Table 2-3 informed consent by the presence or absence of proton-pump inhibitors

FREQ Procedure

			Cumulative	
Percent of ALL	power	Percent of power		
1	32490	100.00	32490	100.00

Table : ALL * CVAC 5 YN

ALL

CVAC5YN(Unclassified/Unknown Cerebral Infarction)

Power |
Percent |
Percent of rows
Percent of columns | None | With | Total

	None	With	Total
1	30728	1762	32490
	94.58	5.42	100.00
	94.58	5.42	
	100.00	100.00	
Total	30728	1762	32490
	94.58	5.42	100.00

Complications and medical history at the time of obtaining Table 2-3 informed consent by the presence or absence of proton-pump inhibitors

FREQ Procedure

ALL	power	Percent	Cumulative power	Cumulative Percent of power
1	32490	100.00	32490	100.00

Table : ALL * CVAC 6 YN

ALL

CVAC6YN(Transient Ischemic Attacks)

Power |
Percent |
Percent of rows
Percent of columns | None | With | Total

ALL	None	With	Total
1	31971	519	32490
	98.40	1.60	100.00
	98.40	1.60	
	100.00	100.00	
Total	31971	519	32490
	98.40	1.60	100.00

Complications and medical history at the time of obtaining Table 2-3 informed consent by the presence or absence of proton-pump inhibitors

FREQ Procedure

			Cumulative	
Percent of ALL	power	Percent of	power	
1	32490	100.00	32490	100.00

Table : ALL * CVAC 6 YN

ALL

CVAC6YN(Transient Ischemic Attacks)

Power |
Percent |
Percent of rows
Percent of columns | None | With | Total

	None	With	Total
1	31971	519	32490
	98.40	1.60	100.00
	98.40	1.60	
	100.00	100.00	
Total	31971	519	32490
	98.40	1.60	100.00

Complications and medical history at the time of obtaining Table 2-3 informed consent by the presence or absence of proton-pump inhibitors

FREQ Procedure

Cumulative
Percent of ALL power Percent of power

1	32490	100.00	32490	100.00

Table : ALL * CVAC 7 YN

ALL
CVAC7YN(Other Cerebrovascular Accident)

Power |
Percent |
Percent of rows
Percent of columns | None | With | Total

	None	With	Total
1	31681	809	32490
	97.51	2.49	100.00
	97.51	2.49	
	100.00	100.00	
Total	31681	809	32490
	97.51	2.49	100.00

Complications and medical history at the time of obtaining Table 2-3 informed consent by the presence or absence of proton-pump inhibitors

FREQ Procedure

			Cumulative	
Percent of ALL	power	Percent of power		
1	32490	100.00	32490	100.00

Table : ALL * CVAC 7 YN

ALL

CVAC7YN(Other Cerebrovascular Accident)

Power / Percent / Percent of rows / Percent of columns	None	With	Total
1	31681	809	32490
	97.51	2.49	100.00
	97.51	2.49	
	100.00	100.00	
Total	31681	809	32490
	97.51	2.49	100.00

Complications and medical history at the time of obtaining Table 2-3 informed consent by the presence or absence of proton-pump inhibitors

FREQ Procedure

Cumulative
Percent of ALL power Percent of power

1	32490	100.00	32490	100.00

Tables: ALL * OTVDYN

ALL
OTVDYN(Other Vascular Disease)

Power |
Percent |
Percent of rows
Percent of columns | None | With | Total

	None	With	Total
1	30760	1730	32490
	94.68	5.32	100.00
	94.68	5.32	
	100.00	100.00	
Total	30760	1730	32490
	94.68	5.32	100.00

Complications and medical history at the time of obtaining Table 2-3 informed consent by the presence or absence of proton-pump inhibitors

FREQ Procedure

			Cumulative	
Percent of ALL	power	Percent	of power	
1	32490	100.00	32490	100.00

Tables: ALL * OTVDYN

ALL

OTVDYN(Other Vascular Disease)

Power | Percent | Percent of rows Percent of columns	None	With	Total
1	30760	1730	32490
	94.68	5.32	100.00
	94.68	5.32	
	100.00	100.00	
Total	30760	1730	32490
	94.68	5.32	100.00

Complications and medical history at the time of obtaining Table 2-3 informed consent by the presence or absence of proton-pump inhibitors

FREQ Procedure

Cumulative
Percent of ALL power Percent of power

1	32490	100.00	32490	100.00

Table : ALL * OTVD 1 YN

ALL
OTVD1YN(Peripheral Vascular Disease)

Power |
Percent |
Percent of rows
Percent of columns | None | With | Total

	None	With	Total
1	31990	500	32490
	98.46	1.54	100.00
	98.46	1.54	
	100.00	100.00	
Total	31990	500	32490
	98.46	1.54	100.00

Complications and medical history at the time of obtaining Table 2-3 informed consent by the presence or absence of proton-pump inhibitors

FREQ Procedure

			Cumulative	
Percent of ALL	power	Percent of power		
1	32490	100.00	32490	100.00

Table : ALL * OTVD 1 YN

ALL

OTVD1YN(Peripheral Vascular Disease)

Power |
Percent |
Percent of rows
Percent of columns | None | With | Total

	None	With	Total
1	31990	500	32490
	98.46	1.54	100.00
	98.46	1.54	
	100.00	100.00	
Total	31990	500	32490
	98.46	1.54	100.00

Complications and medical history at the time of obtaining Table 2-3 informed consent by the presence or absence of proton-pump inhibitors

FREQ Procedure

			Cumulative	
Percent of ALL	power	Percent of power		
1	32490	100.00	32490	100.00

Table : ALL * OTVD 2 YN

ALL OTVD2YN(Aortic Plaque)

Power |
Percent |
Percent of rows
Percent of columns | None | With | Total

	None	With	Total
1	32249	241	32490
	99.26	0.74	100.00
	99.26	0.74	
	100.00	100.00	
Total	32249	241	32490
	99.26	0.74	100.00

Complications and medical history at the time of obtaining Table 2-3 informed consent by the presence or absence of proton-pump inhibitors

FREQ Procedure

Cumulative
Percent of ALL power Percent of power

1	32490	100.00	32490	100.00

Table : ALL * OTVD 2 YN

ALL OTVD2YN(Aortic Plaque)

Power |
Percent |
Percent of rows
Percent of columns | None | With | Total

1	32249	241	32490
	99.26	0.74	100.00
	99.26	0.74	
	100.00	100.00	
Total	32249	241	32490
	99.26	0.74	100.00

Complications and medical history at the time of obtaining Table 2-3 informed consent by the presence or absence of proton-pump inhibitors

FREQ Procedure

Cumulative
Percent of ALL power Percent of power

1	32490	100.00	32490	100.00

Table : ALL * TED 3 YN

ALL
TED3YN(Internal Carotid Artery Stenosis)

Power |
Percent |
Percent of rows
Percent of columns | None | With | Total

	None	With	Total
1	31733	757	32490
	97.67	2.33	100.00
	97.67	2.33	
	100.00	100.00	
Total	31733	757	32490
	97.67	2.33	100.00

Complications and medical history at the time of obtaining Table 2-3 informed consent by the presence or absence of proton-pump inhibitors

FREQ Procedure

			Cumulative	
Percent of ALL	power	Percent of power		
1	32490	100.00	32490	100.00

Table : ALL * TED 3 YN

ALL

TED3YN(Internal Carotid Artery Stenosis)

Power / Percent / Percent of rows / Percent of columns	None	With	Total
1	31733	757	32490
	97.67	2.33	100.00
	97.67	2.33	
	100.00	100.00	
Total	31733	757	32490
	97.67	2.33	100.00

Complications and medical history at the time of obtaining Table 2-3 informed consent by the presence or absence of proton-pump inhibitors

FREQ Procedure

			Cumulative	
Percent of ALL	power	Percent of	power	
1	32490	100.00	32490	100.00

Table : ALL * TED 4 YN

ALL

TED4YN(Arteriosclerosis Obliterans)

Power |
Percent |
Percent of rows
Percent of columns | None | With | Total

	None	With	Total
1	31372	1118	32490
	96.56	3.44	100.00
	96.56	3.44	
	100.00	100.00	
Total	31372	1118	32490
	96.56	3.44	100.00

Complications and medical history at the time of obtaining Table 2-3 informed consent by the presence or absence of proton-pump inhibitors

FREQ Procedure

			Cumulative	
Percent of ALL	power	Percent of	power	
1	32490	100.00	32490	100.00

Table : ALL * TED 4 YN

ALL

TED4YN(Arteriosclerosis Obliterans)

Power |
Percent |
Percent of rows
Percent of columns | None | With | Total

Percent of columns	None	With	Total
1	31372	1118	32490
	96.56	3.44	100.00
	96.56	3.44	
	100.00	100.00	
Total	31372	1118	32490
	96.56	3.44	100.00

Complications and medical history at the time of obtaining Table 2-3 informed consent by the presence or absence of proton-pump inhibitors

FREQ Procedure

			Cumulative	
Percent of ALL	power	Percent of power		
1	32490	100.00	32490	100.00

Table : ALL * OTVD 3 YN

ALL

OTVD3YN(Other Vascular Disease: Other)

Power | Percent | Percent of rows Percent of columns	None	With	Total
1	31451	1039	32490
	96.80	3.20	100.00
	96.80	3.20	
	100.00	100.00	
Total	31451	1039	32490
	96.80	3.20	100.00

Complications and medical history at the time of obtaining Table 2-3 informed consent by the presence or absence of proton-pump inhibitors

FREQ Procedure

			Cumulative	
Percent of ALL	power	Percent of power		
1	32490	100.00	32490	100.00

Table : ALL * OTVD 3 YN

ALL

OTVD3YN(Other Vascular Disease: Other)

Power |
Percent |
Percent of rows
Percent of columns | None | With | Total

	None	With	Total
1	31451	1039	32490
	96.80	3.20	100.00
	96.80	3.20	
	100.00	100.00	
Total	31451	1039	32490
	96.80	3.20	100.00

Complications and medical history at the time of obtaining Table 2-3 informed consent by the presence or absence of proton-pump inhibitors

FREQ Procedure

Cumulative

Percent of ALL power Percent of power

1	32490	100.00	32490	100.00

Tables: ALL * TEDYN

ALL

TEDYN(Thrombosis/Embolism-Related Disease)

Power |
Percent |
Percent of rows
Percent of columns | None | With | Total

	None	With	Total
1	29709	2781	32490
	91.44	8.56	100.00
	91.44	8.56	
	100.00	100.00	
Total	29709	2781	32490
	91.44	8.56	100.00

Complications and medical history at the time of obtaining Table 2-3 informed consent by the presence or absence of proton-pump inhibitors

FREQ Procedure

			Cumulative	
Percent of ALL power Percent of power				
1	32490	100.00	32490	100.00

Tables: ALL * TEDYN

ALL

TEDYN(Thrombosis/Embolism-Related Disease)

Power<br>Percent<br>Percent of rows<br>Percent of columns	None	With	Total
1	29709	2781	32490
	91.44	8.56	100.00
	91.44	8.56	
	100.00	100.00	
Total	29709	2781	32490
	91.44	8.56	100.00

Complications and medical history at the time of obtaining Table 2-3 informed consent by the presence or absence of proton-pump inhibitors

FREQ Procedure

			Cumulative	
Percent of ALL	power	Percent of power		
1	32490	100.00	32490	100.00

Table: ALL * t_TED

ALL t_TED

Power |
Percent |
Percent of rows
Percentage of columns | 0| 1| Sum

	0	1	Sum
1	32077	413	32490
	98.73	1.27	100.00
	98.73	1.27	
	100.00	100.00	
Total	32077	413	32490
	98.73	1.27	100.00

Complications and medical history at the time of obtaining Table 2-3 informed consent by the presence or absence of proton-pump inhibitors

FREQ Procedure

Cumulative
Percent of ALL power Percent of power

1	32490	100.00	32490	100.00

Table: ALL * t_TED

ALL t_TED

Power |
Percent |
Percent of rows
Percentage of columns | 0| 1| Sum

1	32077	413	32490
	98.73	1.27	100.00
	98.73	1.27	
	100.00	100.00	
Total	32077	413	32490
	98.73	1.27	100.00

Complications and medical history at the time of obtaining Table 2-3 informed consent by the presence or absence of proton-pump inhibitors

FREQ Procedure

Cumulative
Percent of ALL power Percent of power

1	32490	100.00	32490	100.00

Table : ALL * TED 1 YN

ALL
TED1YN(Pulmonary Embolism)

Power |
Percent |
Percent of rows
Percent of columns | None | With | Total

	None	With	Total
1	32375	115	32490
	99.65	0.35	100.00
	99.65	0.35	
	100.00	100.00	
Total	32375	115	32490
	99.65	0.35	100.00

Complications and medical history at the time of obtaining Table 2-3 informed consent by the presence or absence of proton-pump inhibitors

FREQ Procedure

			Cumulative	
	Percent of ALL power	Percent of power		
1	32490	100.00	32490	100.00

Table : ALL * TED 1 YN

ALL

TED1YN(Pulmonary Embolism)

Power |
Percent |
Percent of rows
Percent of columns | None | With | Total

	None	With	Total
1	32375	115	32490
	99.65	0.35	100.00
	99.65	0.35	
	100.00	100.00	
Total	32375	115	32490
	99.65	0.35	100.00

Complications and medical history at the time of obtaining Table 2-3 informed consent by the presence or absence of proton-pump inhibitors

FREQ Procedure

Cumulative
Percent of ALL power Percent of power

1	32490	100.00	32490	100.00

Table : ALL * TED 2 YN

ALL

TED2YN(Deep Vein Thrombosis)

Power |
Percent |
Percent of rows
Percent of columns | None | With | Total

1	32153	337	32490
	98.96	1.04	100.00
	98.96	1.04	
	100.00	100.00	
Total	32153	337	32490
	98.96	1.04	100.00

Complications and medical history at the time of obtaining Table 2-3 informed consent by the presence or absence of proton-pump inhibitors

FREQ Procedure

Cumulative
Percent of ALL power Percent of power

1	32490	100.00	32490	100.00

Table : ALL * TED 2 YN

ALL
TED2YN(Deep Vein Thrombosis)

Power |
Percent |
Percent of rows
Percent of columns | None | With | Total

	None	With	Total
1	32153	337	32490
	98.96	1.04	100.00
	98.96	1.04	
	100.00	100.00	
Total	32153	337	32490
	98.96	1.04	100.00

Complications and medical history at the time of obtaining Table 2-3 informed consent by the presence or absence of proton-pump inhibitors

FREQ Procedure

			Cumulative	
Percent of ALL	power	Percent of power		
1	32490	100.00	32490	100.00

Table : ALL * TED 5 YN

ALL

TED5YN(Other Thrombosis/Embolism-Related Disease)

Power |
Percent |
Percent of rows
Percent of columns | None | With | Total

	None	With	Total
1	31689	801	32490
	97.53	2.47	100.00
	97.53	2.47	
	100.00	100.00	
Total	31689	801	32490
	97.53	2.47	100.00

Complications and medical history at the time of obtaining Table 2-3 informed consent by the presence or absence of proton-pump inhibitors

FREQ Procedure

			Cumulative	
Percent of ALL	power	Percent of power		
1	32490	100.00	32490	100.00

Table : ALL * TED 5 YN

ALL

TED5YN(Other Thrombosis/Embolism-Related Disease)

Power |
Percent |
Percent of rows
Percent of columns | None | With | Total

	None	With	Total
1	31689	801	32490
	97.53	2.47	100.00
	97.53	2.47	
	100.00	100.00	
Total	31689	801	32490
	97.53	2.47	100.00

Complications and medical history at the time of obtaining Table 2-3 informed consent by the presence or absence of proton-pump inhibitors

FREQ Procedure

			Cumulative	
Percent of ALL	power	Percent	of power	
1	32490	100.00	32490	100.00

Tables: ALL * HTDMYN

ALL HTDMYN(Hyperthyroidism)

Power<br>Percent<br>Percent of rows<br>Percent of columns	None	With	Total
1	32009<br>98.52<br>98.52<br>100.00	481<br>1.48<br>1.48<br>100.00	32490<br>100.00
Total	32009<br>98.52	481<br>1.48	32490<br>100.00

Complications and medical history at the time of obtaining Table 2-3 informed consent by the presence or absence of proton-pump inhibitors

FREQ Procedure

Cumulative
Percent of ALL power Percent of power

1	32490	100.00	32490	100.00

Tables: ALL * HTDMYN

ALL HTDMYN(Hyperthyroidism)

Power |
Percent |
Percent of rows
Percent of columns | None | With | Total

	None	With	Total
1	32009	481	32490
	98.52	1.48	100.00
	98.52	1.48	
	100.00	100.00	
Total	32009	481	32490
	98.52	1.48	100.00

Complications and medical history at the time of obtaining Table 2-3 informed consent by the presence or absence of proton-pump inhibitors

FREQ Procedure

Cumulative
Percent of ALL power Percent of power

1	32490	100.00	32490	100.00

Tables: ALL * GASDYN

ALL
GASDYN(Gastrointestinal Disease)

Power |
Percent |
Percent of rows
Percent of columns | None | With | Total

	None	With	Total
1	22966	9524	32490
	70.69	29.31	100.00
	70.69	29.31	
	100.00	100.00	
Total	22966	9524	32490
	70.69	29.31	100.00

Complications and medical history at the time of obtaining Table 2-3 informed consent by the presence or absence of proton-pump inhibitors

FREQ Procedure

			Cumulative	
Percent of ALL	power	Percent of power		
1	32490	100.00	32490	100.00

Tables: ALL * GASDYN

ALL
GASDYN(Gastrointestinal Disease)

Power |
Percent |
Percent of rows
Percent of columns | None | With | Total

	None	With	Total
1	22966	9524	32490
	70.69	29.31	100.00
	70.69	29.31	
	100.00	100.00	
Total	22966	9524	32490
	70.69	29.31	100.00

Complications and medical history at the time of obtaining Table 2-3 informed consent by the presence or absence of proton-pump inhibitors

FREQ Procedure

Cumulative
Percent of ALL power Percent of power

1	32490	100.00	32490	100.00

Table : ALL * GASD 1 YN

ALL
GASD1YN(Upper Gastrointestinal Tract)

Power |
Percent |
Percent of rows
Percent of columns | None | With | Total

1	31774	716	32490
	97.80	2.20	100.00
	97.80	2.20	
	100.00	100.00	
Total	31774	716	32490
	97.80	2.20	100.00

Complications and medical history at the time of obtaining Table 2-3 informed consent by the presence or absence of proton-pump inhibitors

FREQ Procedure

Cumulative
Percent of ALL power Percent of power

1	32490	100.00	32490	100.00

Table : ALL * GASD 1 YN

ALL
GASD1YN(Upper Gastrointestinal Tract)

Power |
Percent |
Percent of rows
Percent of columns | None | With | Total

	None	With	Total
1	31774	716	32490
	97.80	2.20	100.00
	97.80	2.20	
	100.00	100.00	
Total	31774	716	32490
	97.80	2.20	100.00

Complications and medical history at the time of obtaining Table 2-3 informed consent by the presence or absence of proton-pump inhibitors

FREQ Procedure

			Cumulative	
Percent of ALL	power	Percent of	power	
1	32490	100.00	32490	100.00

Table : ALL * GASD 2 YN

ALL

GASD2YN(Lower Gastrointestinal Tract)

Power |
Percent |
Percent of rows
Percent of columns | None | With | Total

	None	With	Total
1	31764	726	32490
	97.77	2.23	100.00
	97.77	2.23	
	100.00	100.00	
Total	31764	726	32490
	97.77	2.23	100.00

Complications and medical history at the time of obtaining Table 2-3 informed consent by the presence or absence of proton-pump inhibitors

FREQ Procedure

			Cumulative	
Percent of ALL	power	Percent of power		
1	32490	100.00	32490	100.00

Table : ALL * GASD 2 YN

ALL

GASD2YN(Lower Gastrointestinal Tract)

Power |
Percent |
Percent of rows
Percent of columns | None | With | Total

	None	With	Total
1	31764	726	32490
	97.77	2.23	100.00
	97.77	2.23	
	100.00	100.00	
Total	31764	726	32490
	97.77	2.23	100.00

Complications and medical history at the time of obtaining Table 2-3 informed consent by the presence or absence of proton-pump inhibitors

FREQ Procedure

Cumulative
Percent of ALL power Percent of power

1	32490	100.00	32490	100.00

Table : ALL * GASD 3 YN

ALL
GASD3YN(Reflux esophagitis)

Power |
Percent |
Percent of rows
Percent of columns | None | With | Total

	None	With	Total
1	27371	5119	32490
	84.24	15.76	100.00
	84.24	15.76	
	100.00	100.00	
Total	27371	5119	32490
	84.24	15.76	100.00

Complications and medical history at the time of obtaining Table 2-3 informed consent by the presence or absence of proton-pump inhibitors

FREQ Procedure

			Cumulative	
Percent of ALL	power	Percent of power		
1	32490	100.00	32490	100.00

Table : ALL * GASD 3 YN

ALL

GASD3YN(Reflux esophagitis)

Power / Percent / Percent of rows / Percent of columns	None	With	Total
1	27371	5119	32490
	84.24	15.76	100.00
	84.24	15.76	
	100.00	100.00	
Total	27371	5119	32490
	84.24	15.76	100.00

Complications and medical history at the time of obtaining Table 2-3 informed consent by the presence or absence of proton-pump inhibitors

FREQ Procedure

Cumulative
Percent of ALL power Percent of power

1	32490	100.00	32490	100.00

Table : ALL * GASD 4 YN

ALL

GASD4YN(Other Gastrointestinal Disease)

Power |
Percent |
Percent of rows
Percent of columns | None | With | Total

	None	With	Total
1	28069	4421	32490
	86.39	13.61	100.00
	86.39	13.61	
	100.00	100.00	
Total	28069	4421	32490
	86.39	13.61	100.00

Complications and medical history at the time of obtaining Table 2-3 informed consent by the presence or absence of proton-pump inhibitors

FREQ Procedure

			Cumulative	
Percent of ALL	power	Percent of power		
1	32490	100.00	32490	100.00

Table : ALL * GASD 4 YN

ALL

GASD4YN(Other Gastrointestinal Disease)

Power |
Percent |
Percent of rows
Percent of columns | None | With | Total

	None	With	Total
1	28069	4421	32490
	86.39	13.61	100.00
	86.39	13.61	
	100.00	100.00	
Total	28069	4421	32490
	86.39	13.61	100.00

Complications and medical history at the time of obtaining Table 2-3 informed consent by the presence or absence of proton-pump inhibitors

FREQ Procedure

			Cumulative	
Percent of ALL	power	Percent of	power	
1	32490	100.00	32490	100.00

Tables: ALL* MGTYN

ALL MGTYN(Malignant Tumor)

Power |
Percent |
Percent of rows
Percent of columns | None | With | Total

	None	With	Total
1	28931	3559	32490
	89.05	10.95	100.00
	89.05	10.95	
	100.00	100.00	
Total	28931	3559	32490
	89.05	10.95	100.00

Complications and medical history at the time of obtaining Table 2-3 informed consent by the presence or absence of proton-pump inhibitors

FREQ Procedure

Cumulative
Percent of ALL power Percent of power

1	32490	100.00	32490	100.00

Tables: ALL* MGTYN

ALL MGTYN(Malignant Tumor)

Power |
Percent |
Percent of rows
Percent of columns | None | With | Total

	None	With	Total
1	28931	3559	32490
	89.05	10.95	100.00
	89.05	10.95	
	100.00	100.00	
Total	28931	3559	32490
	89.05	10.95	100.00

Complications and medical history at the time of obtaining Table 2-3 informed consent by the presence or absence of proton-pump inhibitors

FREQ Procedure

			Cumulative	
	Percent of ALL	power	Percent of power	
1	32490	100.00	32490	100.00

Table : ALL * MGT 1 YN

ALL MGT1YN(Gastric Cancer)

Power / Percent / Percent of rows / Percent of columns	None	With	Total
1	31755	735	32490
	97.74	2.26	100.00
	97.74	2.26	
	100.00	100.00	
Total	31755	735	32490
	97.74	2.26	100.00

Complications and medical history at the time of obtaining Table 2-3 informed consent by the presence or absence of proton-pump inhibitors

FREQ Procedure

Cumulative
Percent of ALL power Percent of power

1	32490	100.00	32490	100.00

Table : ALL * MGT 1 YN

ALL MGT1YN(Gastric Cancer)

Power |
Percent |
Percent of rows
Percent of columns | None | With | Total

	None	With	Total
1	31755	735	32490
	97.74	2.26	100.00
	97.74	2.26	
	100.00	100.00	
Total	31755	735	32490
	97.74	2.26	100.00

Complications and medical history at the time of obtaining Table 2-3 informed consent by the presence or absence of proton-pump inhibitors

FREQ Procedure

Cumulative

Percent of ALL power Percent of power

1	32490	100.00	32490	100.00

Table : ALL * MGT 2 YN

ALL MGT2YN(Colorectal Cancer)

Power |
Percent |
Percent of rows
Percent of columns | None | With | Total

	None	With	Total
1	31752	738	32490
	97.73	2.27	100.00
	97.73	2.27	
	100.00	100.00	
Total	31752	738	32490
	97.73	2.27	100.00

Complications and medical history at the time of obtaining Table 2-3 informed consent by the presence or absence of proton-pump inhibitors

FREQ Procedure

			Cumulative	
Percent of ALL	power	Percent of power		
1	32490	100.00	32490	100.00

Table : ALL * MGT 2 YN

ALL MGT2YN(Colorectal Cancer)

Power | Percent | Percent of rows Percent of columns	None	With	Total
1	31752	738	32490
	97.73	2.27	100.00
	97.73	2.27	
	100.00	100.00	
Total	31752	738	32490
	97.73	2.27	100.00

Complications and medical history at the time of obtaining Table 2-3 informed consent by the presence or absence of proton-pump inhibitors

FREQ Procedure

Cumulative

Percent of ALL power Percent of power

1	32490	100.00	32490	100.00

Table : ALL * MGT 3 YN

ALL MGT3YN(Lung Cancer)

Power |

Percent |

Percent of rows

Percent of columns | None | With | Total

	None	With	Total
1	32124	366	32490
	98.87	1.13	100.00
	98.87	1.13	
	100.00	100.00	
Total	32124	366	32490
	98.87	1.13	100.00

Complications and medical history at the time of obtaining Table 2-3 informed consent by the presence or absence of proton-pump inhibitors

FREQ Procedure

			Cumulative	
Percent of ALL	power	Percent of power		
1	32490	100.00	32490	100.00

Table : ALL * MGT 3 YN

ALL MGT3YN(Lung Cancer)

Power |
Percent |
Percent of rows
Percent of columns | None | With | Total

	None	With	Total
1	32124	366	32490
	98.87	1.13	100.00
	98.87	1.13	
	100.00	100.00	
Total	32124	366	32490
	98.87	1.13	100.00

Complications and medical history at the time of obtaining Table 2-3 informed consent by the presence or absence of proton-pump inhibitors

FREQ Procedure

			Cumulative	
Percent of ALL	power	Percent	of power	
1	32490	100.00	32490	100.00

Table : ALL * MGT 4 YN

ALL MGT4YN(Brest Cancer)

Power | Percent | Percent of rows Percent of columns	None	With	Total
1	32254	236	32490
	99.27	0.73	100.00
	99.27	0.73	
	100.00	100.00	
Total	32254	236	32490
	99.27	0.73	100.00

Complications and medical history at the time of obtaining Table 2-3 informed consent by the presence or absence of proton-pump inhibitors

FREQ Procedure

Cumulative

Percent of ALL power Percent of power

1	32490	100.00	32490	100.00

Table : ALL * MGT 4 YN

ALL MGT4YN(Brest Cancer)

Power |

Percent |

Percent of rows

Percent of columns | None | With | Total

1	32254	236	32490
	99.27	0.73	100.00
	99.27	0.73	
	100.00	100.00	
Total	32254	236	32490
	99.27	0.73	100.00

Complications and medical history at the time of obtaining Table 2-3 informed consent by the presence or absence of proton-pump inhibitors

FREQ Procedure

			Cumulative	
Percent of ALL	power	Percent of power		
1	32490	100.00	32490	100.00

Table : ALL * MGT 5 YN

ALL

MGT5YN(Uterus/Ovarian Cancer)

Power / Percent / Percent of rows / Percent of columns	None	With	Total
1	32397	93	32490
	99.71	0.29	100.00
	99.71	0.29	
	100.00	100.00	
Total	32397	93	32490
	99.71	0.29	100.00

Complications and medical history at the time of obtaining Table 2-3 informed consent by the presence or absence of proton-pump inhibitors

FREQ Procedure

			Cumulative	
Percent of ALL	power	Percent of	power	
1	32490	100.00	32490	100.00

Table : ALL * MGT 5 YN

ALL

MGT5YN(Uterus/Ovarian Cancer)

Power |
Percent |
Percent of rows
Percent of columns | None | With | Total

	None	With	Total
1	32397	93	32490
	99.71	0.29	100.00
	99.71	0.29	
	100.00	100.00	
Total	32397	93	32490
	99.71	0.29	100.00

Complications and medical history at the time of obtaining Table 2-3 informed consent by the presence or absence of proton-pump inhibitors

FREQ Procedure

			Cumulative	
Percent of ALL	power	Percent of power		
1	32490	100.00	32490	100.00

Table : ALL * MGT 6 YN

ALL MGT6YN(Pancreatic Cancer)

Power<br>Percent<br>Percent of rows<br>Percent of columns	None	With	Total
1	32456	34	32490
	99.90	0.10	100.00
	99.90	0.10	
	100.00	100.00	
Total	32456	34	32490
	99.90	0.10	100.00

Complications and medical history at the time of obtaining Table 2-3 informed consent by the presence or absence of proton-pump inhibitors

FREQ Procedure

Cumulative

Percent of ALL power Percent of power

1	32490	100.00	32490	100.00

Table : ALL * MGT 6 YN

ALL MGT6YN(Pancreatic Cancer)

Power |
Percent |
Percent of rows
Percent of columns | None | With | Total

	None	With	Total
1	32456	34	32490
	99.90	0.10	100.00
	99.90	0.10	
	100.00	100.00	
Total	32456	34	32490
	99.90	0.10	100.00

Complications and medical history at the time of obtaining Table 2-3 informed consent by the presence or absence of proton-pump inhibitors

FREQ Procedure

			Cumulative	
Percent of ALL	power	Percent of power		
1	32490	100.00	32490	100.00

Table : ALL * MGT 7 YN

ALL

MGT7YN(Other Malignant Tumor)

Power |
Percent |
Percent of rows
Percent of columns | None | With | Total

	None	With	Total
1	30756	1734	32490
	94.66	5.34	100.00
	94.66	5.34	
	100.00	100.00	
Total	30756	1734	32490
	94.66	5.34	100.00

Complications and medical history at the time of obtaining Table 2-3 informed consent by the presence or absence of proton-pump inhibitors

FREQ Procedure

			Cumulative	
Percent of ALL	power	Percent of power		
1	32490	100.00	32490	100.00

Table : ALL * MGT 7 YN

ALL

MGT7YN(Other Malignant Tumor)

Power |
Percent |
Percent of rows
Percent of columns | None | With | Total

	None	With	Total
1	30756	1734	32490
	94.66	5.34	100.00
	94.66	5.34	
	100.00	100.00	
Total	30756	1734	32490
	94.66	5.34	100.00

Complications and medical history at the time of obtaining Table 2-3 informed consent by the presence or absence of proton-pump inhibitors

FREQ Procedure

			Cumulative	
Percent of ALL	power	Percent of power		
1	32490	100.00	32490	100.00

Tables: ALL* ALZHYN

ALL ALZHYN(Alzheimer)

Power |
Percent |
Percent of rows
Percent of columns | None | With | Total

	None	With	Total
1	29937	2553	32490
	92.14	7.86	100.00
	92.14	7.86	
	100.00	100.00	
Total	29937	2553	32490
	92.14	7.86	100.00

Complications and medical history at the time of obtaining Table 2-3 informed consent by the presence or absence of proton-pump inhibitors

FREQ Procedure

Cumulative
Percent of ALL power Percent of power

1	32490	100.00	32490	100.00

Tables: ALL* ALZHYN

ALL ALZHYN(Alzheimer)

Power |
Percent |
Percent of rows
Percent of columns | None | With | Total

	None	With	Total
1	29937	2553	32490
	92.14	7.86	100.00
	92.14	7.86	
	100.00	100.00	
Total	29937	2553	32490
	92.14	7.86	100.00

Complications and medical history at the time of obtaining Table 2-3 informed consent by the presence or absence of proton-pump inhibitors

FREQ Procedure

Cumulative
Percent of ALL power Percent of power

1	32490	100.00	32490	100.00

Tables: ALL * OHCPYN

ALL
OHCPYN(Other Complications)

Power |
Percent |
Percent of rows
Percent of columns | None | With | Total

	None	With	Total
1	21955	10535	32490
	67.57	32.43	100.00
	67.57	32.43	
	100.00	100.00	
Total	21955	10535	32490
	67.57	32.43	100.00

Complications and medical history at the time of obtaining Table 2-3 informed consent by the presence or absence of proton-pump inhibitors

FREQ Procedure

			Cumulative	
Percent of ALL	power	Percent of	power	
1	32490	100.00	32490	100.00

Tables: ALL * OHCPYN

ALL

OHCPYN(Other Complications)

Power |
Percent |
Percent of rows
Percent of columns | None | With | Total

	None	With	Total
1	21955	10535	32490
	67.57	32.43	100.00
	67.57	32.43	
	100.00	100.00	
Total	21955	10535	32490
	67.57	32.43	100.00

Complications and medical history at the time of obtaining Table 2-3 informed consent by the presence or absence of proton-pump inhibitors

FREQ Procedure

Cumulative
Percent of ALL power Percent of power

1	32490	100.00	32490	100.00

Tables: ALL * IMGYN

ALL IMGYN(Brain Imaging Test)

Power |
Percent |
Percent of rows
Percentage of columns |None| | |Unknown| | Total

1	28921	2852	717	32490
	89.02	8.78	2.21	100.00
	89.02	8.78	2.21	
	100.00	100.00	100.00	
Total	28921	2852	717	32490
	89.02	8.78	2.21	100.00

Complications and medical history at the time of obtaining Table 2-3 informed consent by the presence or absence of proton-pump inhibitors

FREQ Procedure

			Cumulative	
Percent of ALL	power	Percent	of power	
1	32490	100.00	32490	100.00

Tables: ALL * IMGYN

ALL IMGYN(Brain Imaging Test)

Power |
Percent |
Percent of rows
Percentage of columns |None| | |Unknown| | Total

	None		Unknown	Total
1	28921	2852	717	32490
	89.02	8.78	2.21	100.00
	89.02	8.78	2.21	
	100.00	100.00	100.00	
Total	28921	2852	717	32490
	89.02	8.78	2.21	100.00

Complications and medical history at the time of obtaining Table 2-3 informed consent by the presence or absence of proton-pump inhibitors

FREQ Procedure

Cumulative
Percent of ALL power Percent of power

1	32490	100.00	32490	100.00

Tables: ALL * IMGYN

ALL IMGYN(Brain Imaging Test)

Power |
Percent |
Percent of rows
Percentage of columns |None| | |Unknown| | Total

1	28921	2852	717	32490
	89.02	8.78	2.21	100.00
	89.02	8.78	2.21	
	100.00	100.00	100.00	
Total	28921	2852	717	32490
	89.02	8.78	2.21	100.00

Complications and medical history at the time of obtaining Table 2-3 informed consent by the presence or absence of proton-pump inhibitors

FREQ Procedure

Cumulative

Percent of ALL power Percent of power

1	32490	100.00	32490	100.00

Tables: ALL* FALYN

ALL FALYN(Fall Down within one year)

Power |

Percent |

Percent of rows

Percentage of columns |None| | |Unknown| | Total

1	26525	2369	3596	32490
	81.64	7.29	11.07	100.00
	81.64	7.29	11.07	
	100.00	100.00	100.00	
Total	26525	2369	3596	32490
	81.64	7.29	11.07	100.00

Complications and medical history at the time of obtaining Table 2-3 informed consent by the presence or absence of proton-pump inhibitors

FREQ Procedure

Cumulative
Percent of ALL power Percent of power

1	32490	100.00	32490	100.00

Tables: ALL* FALYN

ALL FALYN(Fall Down within one year)

Power |
Percent |
Percent of rows
Percentage of columns |None| | |Unknown| | Total

1	26525	2369	3596	32490
	81.64	7.29	11.07	100.00
	81.64	7.29	11.07	
	100.00	100.00	100.00	
Total	26525	2369	3596	32490
	81.64	7.29	11.07	100.00

Complications and medical history at the time of obtaining Table 2-3 informed consent by the presence or absence of proton-pump inhibitors

FREQ Procedure

Cumulative

Percent of ALL power Percent of power

1	32490	100.00	32490	100.00

Tables: ALL* FALYN

ALL FALYN(Fall Down within one year)

Power |
Percent |
Percent of rows
Percentage of columns |None| | |Unknown| | Total

1	26525	2369	3596	32490
	81.64	7.29	11.07	100.00
	81.64	7.29	11.07	
	100.00	100.00	100.00	
Total	26525	2369	3596	32490
	81.64	7.29	11.07	100.00

Complications and medical history at the time of obtaining Table 2-3 informed consent by the presence or absence of proton-pump inhibitors

FREQ Procedure

Tables: ALL * FNDYN

ALL FNDYN(Brain Imaging Result)

Power |
Percent |
Percent of rows
Percentage of columns |None| | |Unknown| | Total
______________+________+________+________+
1 | 1329 | 1413 | 110 | 2852
| 46.60 | 49.54 | 3.86 | 100.00
| 46.60 | 49.54 | 3.86 |
| 100.00 | 100.00 | 100.00 |
______________+________+________+________+
Total 1329 1413 110 2852
46.60 49.54 3.86 100.00

Degree of missing values = 29638

Complications and medical history at the time of obtaining Table 2-3 informed consent by the presence or absence of proton-pump inhibitors

FREQ Procedure

Tables: ALL * FNDYN

ALL FNDYN(Brain Imaging Result)

Power |
Percent |
Percent of rows
Percentage of columns |None| | |Unknown| | Total

1	1329	1413	110	2852
	46.60	49.54	3.86	100.00
	46.60	49.54	3.86	
	100.00	100.00	100.00	
Total	1329	1413	110	2852
	46.60	49.54	3.86	100.00

Degree of missing values = 29638

Complications and medical history at the time of obtaining Table 2-3 informed consent by the presence or absence of proton-pump inhibitors

FREQ Procedure

Tables: ALL * FNDYN

ALL FNDYN(Brain Imaging Result)

Power |
Percent |
Percent of rows
Percentage of columns |None| | |Unknown| | Total

	None		Unknown	Total
1	1329	1413	110	2852
	46.60	49.54	3.86	100.00
	46.60	49.54	3.86	
	100.00	100.00	100.00	
Total	1329	1413	110	2852
	46.60	49.54	3.86	100.00

Degree of missing values = 29638

Complications and medical history at the time of obtaining Table 2-3 informed consent by the presence or absence of proton-pump inhibitors

FREQ Procedure

			Cumulative	
Percent of ALL	power	Percent of	power	
1	11981	100.00	11981	100.00

Tables: ALL * MHYN

ALL MHYN(Medical History)

Power |
Percent |
Percent of rows
Percent of columns | None | With | Total

	None	With	Total
1	179	11802	11981
	1.49	98.51	100.00
	1.49	98.51	
	100.00	100.00	
Total	179	11802	11981
	1.49	98.51	100.00

Complications and medical history at the time of obtaining Table 2-3 informed consent by the presence or absence of proton-pump inhibitors

FREQ Procedure

Cumulative
Percent of ALL power Percent of power

1	11981	100.00	11981	100.00

Tables: ALL * MHYN

ALL MHYN(Medical History)

Power |
Percent |
Percent of rows
Percent of columns | None | With | Total

	None	With	Total
1	179	11802	11981
	1.49	98.51	100.00
	1.49	98.51	
	100.00	100.00	
Total	179	11802	11981
	1.49	98.51	100.00

Complications and medical history at the time of obtaining Table 2-3 informed consent by the presence or absence of proton-pump inhibitors

FREQ Procedure

			Cumulative	
Percent of ALL power Percent of power				
1	11981	100.00	11981	100.00

Tables: ALL* HBPYN

ALL

HBPYN(High Blood Pressure)

Power | Percent | Percent of rows Percent of columns	None	With	Total
1	2641	9340	11981
	22.04	77.96	100.00
	22.04	77.96	
	100.00	100.00	
Total	2641	9340	11981
	22.04	77.96	100.00

Complications and medical history at the time of obtaining Table 2-3 informed consent by the presence or absence of proton-pump inhibitors

FREQ Procedure

			Cumulative	
ALL	power	Percent	power	Percent of power
1	11981	100.00	11981	100.00

Tables: ALL* HBPYN

ALL

HBPYN(High Blood Pressure)

Power / Percent / Percent of rows / Percent of columns	None	With	Total
1	2641	9340	11981
	22.04	77.96	100.00
	22.04	77.96	
	100.00	100.00	
Total	2641	9340	11981
	22.04	77.96	100.00

Complications and medical history at the time of obtaining Table 2-3 informed consent by the presence or absence of proton-pump inhibitors

FREQ Procedure

			Cumulative	
ALL	power	Percent of		Percent of power
1	11981	100.00	11981	100.00

Tables: ALL * DBYN

ALL DBYN(Diabetes)

Power |
Percent |
Percent of rows
Percent of columns | None | With | Total

	None	With	Total
1	8497	3484	11981
	70.92	29.08	100.00
	70.92	29.08	
	100.00	100.00	
Total	8497	3484	11981
	70.92	29.08	100.00

Complications and medical history at the time of obtaining Table 2-3 informed consent by the presence or absence of proton-pump inhibitors

FREQ Procedure

Cumulative
Percent of ALL power Percent of power

1	11981	100.00	11981	100.00

Tables: ALL * DBYN

ALL DBYN(Diabetes)

Power |
Percent |
Percent of rows
Percent of columns | None | With | Total

	None	With	Total
1	8497	3484	11981
	70.92	29.08	100.00
	70.92	29.08	
	100.00	100.00	
Total	8497	3484	11981
	70.92	29.08	100.00

Complications and medical history at the time of obtaining Table 2-3 informed consent by the presence or absence of proton-pump inhibitors

FREQ Procedure

			Cumulative	
Percent of ALL	power	Percent of	power	
1	11981	100.00	11981	100.00

Table: ALL * t_DB

ALL t_DB

Power |
Percent |
Percent of rows
Percentage of columns | 0| 1| Sum

	0	1	Sum
1	11220	761	11981
	93.65	6.35	100.00
	93.65	6.35	
	100.00	100.00	
Total	11220	761	11981
	93.65	6.35	100.00

Complications and medical history at the time of obtaining Table 2-3 informed consent by the presence or absence of proton-pump inhibitors

FREQ Procedure

Cumulative
Percent of ALL power Percent of power

1	11981	100.00	11981	100.00

Table: ALL * t_DB

ALL t_DB

```
Power |
Percent |
Percent of rows
Percentage of columns | 0| 1| Sum
_______________+_________+_________+
           1 |   11220 |      761 |   11981
             |   93.65 |     6.35 | 100.00
             |   93.65 |     6.35 |
             | 100.00 | 100.00 |
_______________+_________+_________+
Total 11220 761 11981
               93.65      6.35    100.00
```

Complications and medical history at the time of obtaining Table 2-3 informed consent by the presence or absence of proton-pump inhibitors

FREQ Procedure

Cumulative
Percent of ALL power Percent of power

1	11981	100.00	11981	100.00

Table : ALL * DB 1 YN

ALL
DB1YN(Diabetic Retinopathy)

Power |
Percent |
Percent of rows
Percent of columns | None | With | Total

	None	With	Total
1	11753	228	11981
	98.10	1.90	100.00
	98.10	1.90	
	100.00	100.00	
Total	11753	228	11981
	98.10	1.90	100.00

Complications and medical history at the time of obtaining Table 2-3 informed consent by the presence or absence of proton-pump inhibitors

FREQ Procedure

			Cumulative	
Percent of ALL	power	Percent of power		
1	11981	100.00	11981	100.00

Table : ALL * DB 1 YN

ALL

DB1YN(Diabetic Retinopathy)

Power |
Percent |
Percent of rows
Percent of columns | None | With | Total

	None	With	Total
1	11753	228	11981
	98.10	1.90	100.00
	98.10	1.90	
	100.00	100.00	
Total	11753	228	11981
	98.10	1.90	100.00

Complications and medical history at the time of obtaining Table 2-3 informed consent by the presence or absence of proton-pump inhibitors

FREQ Procedure

			Cumulative	
Percent of ALL	power	Percent of power		
1	11981	100.00	11981	100.00

Table : ALL * DB 2 YN

ALL

DB2YN(Diabetic Nephropathy)

Power |
Percent |
Percent of rows
Percent of columns | None | With | Total

	None	With	Total
1	11403	578	11981
	95.18	4.82	100.00
	95.18	4.82	
	100.00	100.00	
Total	11403	578	11981
	95.18	4.82	100.00

Complications and medical history at the time of obtaining Table 2-3 informed consent by the presence or absence of proton-pump inhibitors

FREQ Procedure

			Cumulative	
Percent of ALL	power	Percent of power		
1	11981	100.00	11981	100.00

Table : ALL * DB 2 YN

ALL

DB2YN(Diabetic Nephropathy)

Power |
Percent |
Percent of rows
Percent of columns | None | With | Total

	None	With	Total
1	11403	578	11981
	95.18	4.82	100.00
	95.18	4.82	
	100.00	100.00	
Total	11403	578	11981
	95.18	4.82	100.00

Complications and medical history at the time of obtaining Table 2-3 informed consent by the presence or absence of proton-pump inhibitors

FREQ Procedure

			Cumulative	
Percent of ALL	power	Percent of	power	
1	11981	100.00	11981	100.00

Table : ALL * DB 3 YN

ALL

DB3YN(Diabetic Neuropathy)

Power |
Percent |
Percent of rows
Percent of columns | None | With | Total

	None	With	Total
1	11839	142	11981
	98.81	1.19	100.00
	98.81	1.19	
	100.00	100.00	
Total	11839	142	11981
	98.81	1.19	100.00

Complications and medical history at the time of obtaining Table 2-3 informed consent by the presence or absence of proton-pump inhibitors

FREQ Procedure

			Cumulative	
Percent of ALL	power	Percent of power		
1	11981	100.00	11981	100.00

Table : ALL * DB 3 YN

ALL

DB3YN(Diabetic Neuropathy)

Power |
Percent |
Percent of rows
Percent of columns | None | With | Total

	None	With	Total
1	11839	142	11981
	98.81	1.19	100.00
	98.81	1.19	
	100.00	100.00	
Total	11839	142	11981
	98.81	1.19	100.00

Complications and medical history at the time of obtaining Table 2-3 informed consent by the presence or absence of proton-pump inhibitors

FREQ Procedure

			Cumulative	
Percent of ALL	power	Percent of power		
1	11981	100.00	11981	100.00

Tables: ALL* LMDYN

ALL

LMDYN(Lipid metabolism disorders)

Power / Percent / Percent of rows / Percent of columns	None	With	Total
1	6154	5827	11981
	51.36	48.64	100.00
	51.36	48.64	
	100.00	100.00	
Total	6154	5827	11981
	51.36	48.64	100.00

Complications and medical history at the time of obtaining Table 2-3 informed consent by the presence or absence of proton-pump inhibitors

FREQ Procedure

			Cumulative	
Percent of ALL	power	Percent of	power	
1	11981	100.00	11981	100.00

Tables: ALL* LMDYN

ALL

LMDYN(Lipid metabolism disorders)

Power / Percent / Percent of rows / Percent of columns	None	With	Total
1	6154	5827	11981
	51.36	48.64	100.00
	51.36	48.64	
	100.00	100.00	
Total	6154	5827	11981
	51.36	48.64	100.00

Complications and medical history at the time of obtaining Table 2-3 informed consent by the presence or absence of proton-pump inhibitors

FREQ Procedure

Cumulative
Percent of ALL power Percent of power

1	11981	100.00	11981	100.00

Tables: ALL * HYPURNYN

ALL HYPURNYN(Hyperuricemia)

Power |
Percent |
Percent of rows
Percent of columns | None | With | Total

	None	With	Total
1	8972	3009	11981
	74.89	25.11	100.00
	74.89	25.11	
	100.00	100.00	
Total	8972	3009	11981
	74.89	25.11	100.00

Complications and medical history at the time of obtaining Table 2-3 informed consent by the presence or absence of proton-pump inhibitors

FREQ Procedure

Cumulative
Percent of ALL power Percent of power

1	11981	100.00	11981	100.00

Tables: ALL * HYPURNYN

ALL HYPURNYN(Hyperuricemia)

Power |
Percent |
Percent of rows
Percent of columns | None | With | Total

	None	With	Total
1	8972	3009	11981
	74.89	25.11	100.00
	74.89	25.11	
	100.00	100.00	
Total	8972	3009	11981
	74.89	25.11	100.00

Complications and medical history at the time of obtaining Table 2-3 informed consent by the presence or absence of proton-pump inhibitors

FREQ Procedure

Cumulative

Percent of ALL power Percent of power

1	11981	100.00	11981	100.00

Tables: ALL * KDDSYN

ALL KDDSYN(Kidney Disease)

Power |

Percent |

Percent of rows

Percent of columns | None | With | Total

1	8596	3385	11981
	71.75	28.25	100.00
	71.75	28.25	
	100.00	100.00	
Total	8596	3385	11981
	71.75	28.25	100.00

Complications and medical history at the time of obtaining Table 2-3 informed consent by the presence or absence of proton-pump inhibitors

FREQ Procedure

Cumulative
Percent of ALL power Percent of power

1	11981	100.00	11981	100.00

Tables: ALL * KDDSYN

ALL KDDSYN(Kidney Disease)

Power |
Percent |
Percent of rows
Percent of columns | None | With | Total

	None	With	Total
1	8596	3385	11981
	71.75	28.25	100.00
	71.75	28.25	
	100.00	100.00	
Total	8596	3385	11981
	71.75	28.25	100.00

Complications and medical history at the time of obtaining Table 2-3 informed consent by the presence or absence of proton-pump inhibitors

FREQ Procedure

Cumulative
Percent of ALL power Percent of power

1	11981	100.00	11981	100.00

Table : ALL * KDDS 1 YN

ALL
KDDS1YN(Chronic Kidney Disease)

Power |
Percent |
Percent of rows
Percent of columns | None | With | Total

1	9001	2980	11981
	75.13	24.87	100.00
	75.13	24.87	
	100.00	100.00	
Total	9001	2980	11981
	75.13	24.87	100.00

Complications and medical history at the time of obtaining Table 2-3 informed consent by the presence or absence of proton-pump inhibitors

FREQ Procedure

			Cumulative	
Percent of ALL	power	Percent of	power	
1	11981	100.00	11981	100.00

Table : ALL * KDDS 1 YN

ALL

KDDS1YN(Chronic Kidney Disease)

Power |
Percent |
Percent of rows
Percent of columns | None | With | Total

	None	With	Total
1	9001	2980	11981
	75.13	24.87	100.00
	75.13	24.87	
	100.00	100.00	
Total	9001	2980	11981
	75.13	24.87	100.00

Complications and medical history at the time of obtaining Table 2-3 informed consent by the presence or absence of proton-pump inhibitors

FREQ Procedure

			Cumulative	
Percent of ALL	power	Percent of	power	
1	11981	100.00	11981	100.00

Table : ALL * KDDS 2 YN

ALL

KDDS2YN(Advanced Renal Dysfunction without Dyalysis)

Power |
Percent |
Percent of rows
Percent of columns | None | With | Total

	None	With	Total
1	11747	234	11981
	98.05	1.95	100.00
	98.05	1.95	
	100.00	100.00	
Total	11747	234	11981
	98.05	1.95	100.00

Complications and medical history at the time of obtaining Table 2-3 informed consent by the presence or absence of proton-pump inhibitors

FREQ Procedure

ALL	power	Percent	Cumulative power	Cumulative Percent of power
1	11981	100.00	11981	100.00

Table : ALL * KDDS 2 YN

ALL

KDDS2YN(Advanced Renal Dysfunction without Dyalysis)

Power |
Percent |
Percent of rows
Percent of columns | None | With | Total

ALL	None	With	Total
1	11747	234	11981
	98.05	1.95	100.00
	98.05	1.95	
	100.00	100.00	
Total	11747	234	11981
	98.05	1.95	100.00

Complications and medical history at the time of obtaining Table 2-3 informed consent by the presence or absence of proton-pump inhibitors

FREQ Procedure

			Cumulative	
Percent of ALL	power	Percent of	power	
1	11981	100.00	11981	100.00

Table : ALL * KDDS 3 YN

ALL

KDDS3YN(Advanced Renal Dysfunction with Dyalysis)

Power | Percent | Percent of rows Percent of columns	None	With	Total
1	11919	62	11981
	99.48	0.52	100.00
	99.48	0.52	
	100.00	100.00	
Total	11919	62	11981
	99.48	0.52	100.00

Complications and medical history at the time of obtaining Table 2-3 informed consent by the presence or absence of proton-pump inhibitors

FREQ Procedure

			Cumulative	
Percent of ALL	power	Percent of power		
1	11981	100.00	11981	100.00

Table : ALL * KDDS 3 YN

ALL

KDDS3YN(Advanced Renal Dysfunction with Dyalysis)

Power / Percent / Percent of rows / Percent of columns	None	With	Total
1	11919	62	11981
	99.48	0.52	100.00
	99.48	0.52	
	100.00	100.00	
Total	11919	62	11981
	99.48	0.52	100.00

Complications and medical history at the time of obtaining Table 2-3 informed consent by the presence or absence of proton-pump inhibitors

FREQ Procedure

Cumulative
Percent of ALL power Percent of power

1	11981	100.00	11981	100.00

Tables: ALL * SLVDYN

ALL
SLVDYN(Severe Liver Dysfunction)

Power |
Percent |
Percent of rows
Percent of columns | None | With | Total

	None	With	Total
1	11852	129	11981
	98.92	1.08	100.00
	98.92	1.08	
	100.00	100.00	
Total	11852	129	11981
	98.92	1.08	100.00

Complications and medical history at the time of obtaining Table 2-3 informed consent by the presence or absence of proton-pump inhibitors

FREQ Procedure

			Cumulative	
Percent of ALL	power	Percent	of power	
1	11981	100.00	11981	100.00

Tables: ALL * SLVDYN

ALL

SLVDYN(Severe Liver Dysfunction)

Power |
Percent |
Percent of rows
Percent of columns | None | With | Total

	None	With	Total
1	11852	129	11981
	98.92	1.08	100.00
	98.92	1.08	
	100.00	100.00	
Total	11852	129	11981
	98.92	1.08	100.00

Complications and medical history at the time of obtaining Table 2-3 informed consent by the presence or absence of proton-pump inhibitors

FREQ Procedure

			Cumulative	
Percent of ALL	power	Percent of power		
1	11981	100.00	11981	100.00

Tables: ALL* RESDYN

ALL

RESDYN(Respiratory Disease)

Power |
Percent |
Percent of rows
Percent of columns | None | With | Total

	None	With	Total
1	10183	1798	11981
	84.99	15.01	100.00
	84.99	15.01	
	100.00	100.00	
Total	10183	1798	11981
	84.99	15.01	100.00

Complications and medical history at the time of obtaining Table 2-3 informed consent by the presence or absence of proton-pump inhibitors

FREQ Procedure

Percent of ALL	power	Percent	Cumulative power	Cumulative Percent of power
1	11981	100.00	11981	100.00

Tables: ALL* RESDYN

ALL
RESDYN(Respiratory Disease)

Power |
Percent |
Percent of rows
Percent of columns | None | With | Total

	None	With	Total
1	10183	1798	11981
	84.99	15.01	100.00
	84.99	15.01	
	100.00	100.00	
Total	10183	1798	11981
	84.99	15.01	100.00

Complications and medical history at the time of obtaining Table 2-3 informed consent by the presence or absence of proton-pump inhibitors

FREQ Procedure

			Cumulative	
Percent of ALL	power	Percent of power		
1	11981	100.00	11981	100.00

Tables: ALL* HTDYN

ALL HTDYN(Heart Disease)

Power |
Percent |
Percent of rows
Percent of columns | None | With | Total

	None	With	Total
1	4172	7809	11981
	34.82	65.18	100.00
	34.82	65.18	
	100.00	100.00	
Total	4172	7809	11981
	34.82	65.18	100.00

Complications and medical history at the time of obtaining Table 2-3 informed consent by the presence or absence of proton-pump inhibitors

FREQ Procedure

Cumulative
Percent of ALL power Percent of power

1	11981	100.00	11981	100.00

Tables: ALL* HTDYN

ALL HTDYN(Heart Disease)

Power |
Percent |
Percent of rows
Percent of columns | None | With | Total

	None	With	Total
1	4172	7809	11981
	34.82	65.18	100.00
	34.82	65.18	
	100.00	100.00	
Total	4172	7809	11981
	34.82	65.18	100.00

Complications and medical history at the time of obtaining Table 2-3 informed consent by the presence or absence of proton-pump inhibitors

FREQ Procedure

			Cumulative	
Percent of ALL	power	Percent of power		
1	11981	100.00	11981	100.00

Table : ALL * HTD 1 YN

ALL

HTD1YN(Myocardial Infarction)

Power |
Percent |
Percent of rows
Percent of columns | None | With | Total

	None	With	Total
1	10924	1057	11981
	91.18	8.82	100.00
	91.18	8.82	
	100.00	100.00	
Total	10924	1057	11981
	91.18	8.82	100.00

Complications and medical history at the time of obtaining Table 2-3 informed consent by the presence or absence of proton-pump inhibitors

FREQ Procedure

			Cumulative	
Percent of ALL	power	Percent of power		
1	11981	100.00	11981	100.00

Table : ALL * HTD 1 YN

ALL

HTD1YN(Myocardial Infarction)

Power |
Percent |
Percent of rows
Percent of columns | None | With | Total

ALL	None	With	Total
1	10924	1057	11981
	91.18	8.82	100.00
	91.18	8.82	
	100.00	100.00	
Total	10924	1057	11981
	91.18	8.82	100.00

Complications and medical history at the time of obtaining Table 2-3 informed consent by the presence or absence of proton-pump inhibitors

FREQ Procedure

Cumulative
Percent of ALL power Percent of power

1	11981	100.00	11981	100.00

Table : ALL * HTD 2 YN

ALL HTD2YN(Angina Pectoris)

Power |
Percent |
Percent of rows
Percent of columns | None | With | Total

1	9362	2619	11981
	78.14	21.86	100.00
	78.14	21.86	
	100.00	100.00	
Total	9362	2619	11981
	78.14	21.86	100.00

Complications and medical history at the time of obtaining Table 2-3 informed consent by the presence or absence of proton-pump inhibitors

FREQ Procedure

			Cumulative	
	Percent of ALL power	Percent of power		
1	11981	100.00	11981	100.00

Table : ALL * HTD 2 YN

ALL HTD2YN(Angina Pectoris)

Power | Percent | Percent of rows Percent of columns	None	With	Total
1	9362	2619	11981
	78.14	21.86	100.00
	78.14	21.86	
	100.00	100.00	
Total	9362	2619	11981
	78.14	21.86	100.00

Complications and medical history at the time of obtaining Table 2-3 informed consent by the presence or absence of proton-pump inhibitors

FREQ Procedure

Cumulative
Percent of ALL power Percent of power

1	11981	100.00	11981	100.00

Table: ALL * t_HTD0

ALL t_HTD0

Power |
Percent |
Percent of rows
Percentage of columns | 0| 1| Sum

	0	1	Sum
1	8693	3288	11981
	72.56	27.44	100.00
	72.56	27.44	
	100.00	100.00	
Total	8693	3288	11981
	72.56	27.44	100.00

Complications and medical history at the time of obtaining Table 2-3 informed consent by the presence or absence of proton-pump inhibitors

FREQ Procedure

Cumulative
Percent of ALL power Percent of power

1	11981	100.00	11981	100.00

Table: ALL * t_HTD0

ALL t_HTD0

Power |
Percent |
Percent of rows
Percentage of columns | 0| 1| Sum

```
______________+________+________+
             1 |   8693 |   3288 |  11981
               |  72.56 |  27.44 | 100.00
               |  72.56 |  27.44 |
               | 100.00 | 100.00 |
______________+________+________+
Total 8693 3288 11981
                  72.56    27.44   100.00
```

Complications and medical history at the time of obtaining Table 2-3 informed consent by the presence or absence of proton-pump inhibitors

FREQ Procedure

			Cumulative	
Percent of ALL	power	Percent of	power	
1	11981	100.00	11981	100.00

Table : ALL * HTD 3 YN

ALL HTD3YN(Heart Failure)

Power |
Percent |
Percent of rows
Percent of columns | None | With | Total

	None	With	Total
1	7035	4946	11981
	58.72	41.28	100.00
	58.72	41.28	
	100.00	100.00	
Total	7035	4946	11981
	58.72	41.28	100.00

Complications and medical history at the time of obtaining Table 2-3 informed consent by the presence or absence of proton-pump inhibitors

FREQ Procedure

			Cumulative	
Percent of ALL	power	Percent of	power	
1	11981	100.00	11981	100.00

Table : ALL * HTD 3 YN

ALL HTD3YN(Heart Failure)

Power |
Percent |
Percent of rows
Percent of columns | None | With | Total

	None	With	Total
1	7035	4946	11981
	58.72	41.28	100.00
	58.72	41.28	
	100.00	100.00	
Total	7035	4946	11981
	58.72	41.28	100.00

Complications and medical history at the time of obtaining Table 2-3 informed consent by the presence or absence of proton-pump inhibitors

FREQ Procedure

			Cumulative	
Percent of ALL	power	Percent of	power	
1	11981	100.00	11981	100.00

Table : ALL * HTD 4 YN

ALL

HTD4YN(Left Ventricular Systolic Dysfunction)

Power / Percent / Percent of rows / Percent of columns	None	With	Total
1	11635	346	11981
	97.11	2.89	100.00
	97.11	2.89	
	100.00	100.00	
Total	11635	346	11981
	97.11	2.89	100.00

Complications and medical history at the time of obtaining Table 2-3 informed consent by the presence or absence of proton-pump inhibitors

FREQ Procedure

			Cumulative	
Percent of ALL	power	Percent of power		
1	11981	100.00	11981	100.00

Table : ALL * HTD 4 YN

ALL

HTD4YN(Left Ventricular Systolic Dysfunction)

Power / Percent / Percent of rows / Percent of columns	None	With	Total
1	11635	346	11981
	97.11	2.89	100.00
	97.11	2.89	
	100.00	100.00	
Total	11635	346	11981
	97.11	2.89	100.00

Complications and medical history at the time of obtaining Table 2-3 informed consent by the presence or absence of proton-pump inhibitors

FREQ Procedure

			Cumulative	
Percent of ALL	power	Percent of	power	
1	11981	100.00	11981	100.00

Table: ALL * t_HTD

ALL t_HTD

Power |
Percent |
Percent of rows
Percentage of columns | 0| 1| Sum

	0	1	Sum
1	6951	5030	11981
	58.02	41.98	100.00
	58.02	41.98	
	100.00	100.00	
Total	6951	5030	11981
	58.02	41.98	100.00

Complications and medical history at the time of obtaining Table 2-3 informed consent by the presence or absence of proton-pump inhibitors

FREQ Procedure

Cumulative

Percent of ALL power Percent of power

1	11981	100.00	11981	100.00

Table: ALL * t_HTD

ALL t_HTD

Power |
Percent |
Percent of rows
Percentage of columns | 0| 1| Sum

	0	1	Sum
1	6951	5030	11981
	58.02	41.98	100.00
	58.02	41.98	
	100.00	100.00	
Total	6951	5030	11981
	58.02	41.98	100.00

Complications and medical history at the time of obtaining Table 2-3 informed consent by the presence or absence of proton-pump inhibitors

FREQ Procedure

			Cumulative	
Percent of ALL	power	Percent of	power	
1	11981	100.00	11981	100.00

Table : ALL * HTD 5 YN

ALL HTD5YN(Valvular Disease)

Power |
Percent |
Percent of rows
Percent of columns | None | With | Total

	None	With	Total
1	10415	1566	11981
	86.93	13.07	100.00
	86.93	13.07	
	100.00	100.00	
Total	10415	1566	11981
	86.93	13.07	100.00

Complications and medical history at the time of obtaining Table 2-3 informed consent by the presence or absence of proton-pump inhibitors

FREQ Procedure

Cumulative

Percent of ALL power Percent of power

1	11981	100.00	11981	100.00

Table : ALL * HTD 5 YN

ALL HTD5YN(Valvular Disease)

Power |
Percent |
Percent of rows
Percent of columns | None | With | Total

	None	With	Total
1	10415	1566	11981
	86.93	13.07	100.00
	86.93	13.07	
	100.00	100.00	
Total	10415	1566	11981
	86.93	13.07	100.00

Complications and medical history at the time of obtaining Table 2-3 informed consent by the presence or absence of proton-pump inhibitors

FREQ Procedure

			Cumulative	
Percent of ALL	power	Percent of power		
1	11981	100.00	11981	100.00

Table : ALL * HTD 6 YN

ALL HTD6YN(Cardiomyopathy)

Power |
Percent |
Percent of rows
Percent of columns | None | With | Total

	None	With	Total
1	11510	471	11981
	96.07	3.93	100.00
	96.07	3.93	
	100.00	100.00	
Total	11510	471	11981
	96.07	3.93	100.00

Complications and medical history at the time of obtaining Table 2-3 informed consent by the presence or absence of proton-pump inhibitors

FREQ Procedure

			Cumulative	
Percent of ALL	power	Percent of	power	
1	11981	100.00	11981	100.00

Table : ALL * HTD 6 YN

ALL HTD6YN(Cardiomyopathy)

Power | Percent | Percent of rows Percent of columns	None	With	Total
1	11510	471	11981
	96.07	3.93	100.00
	96.07	3.93	
	100.00	100.00	
Total	11510	471	11981
	96.07	3.93	100.00

Complications and medical history at the time of obtaining Table 2-3 informed consent by the presence or absence of proton-pump inhibitors

FREQ Procedure

Cumulative
Percent of ALL power Percent of power

1	11981	100.00	11981	100.00

Table : ALL * HTD 7 YN

ALL
HTD7YN(Hypertensive Heart Disease)

Power |
Percent |
Percent of rows
Percent of columns | None | With | Total

	None	With	Total
1	11604	377	11981
	96.85	3.15	100.00
	96.85	3.15	
	100.00	100.00	
Total	11604	377	11981
	96.85	3.15	100.00

Complications and medical history at the time of obtaining Table 2-3 informed consent by the presence or absence of proton-pump inhibitors

FREQ Procedure

			Cumulative	
Percent of ALL	power	Percent of	power	
1	11981	100.00	11981	100.00

Table : ALL * HTD 7 YN

ALL

HTD7YN(Hypertensive Heart Disease)

Power / Percent / Percent of rows / Percent of columns	None	With	Total
1	11604	377	11981
	96.85	3.15	100.00
	96.85	3.15	
	100.00	100.00	
Total	11604	377	11981
	96.85	3.15	100.00

Complications and medical history at the time of obtaining Table 2-3 informed consent by the presence or absence of proton-pump inhibitors

FREQ Procedure

			Cumulative	
Percent of ALL	power	Percent of	power	
1	11981	100.00	11981	100.00

Table : ALL * HTD 8 YN

ALL

HTD8YN(Cognenital Heart Disease)

Power |
Percent |
Percent of rows
Percent of columns | None | With | Total

	None	With	Total
1	11902	79	11981
	99.34	0.66	100.00
	99.34	0.66	
	100.00	100.00	
Total	11902	79	11981
	99.34	0.66	100.00

Complications and medical history at the time of obtaining Table 2-3 informed consent by the presence or absence of proton-pump inhibitors

FREQ Procedure

			Cumulative	
Percent of ALL	power	Percent of power		
1	11981	100.00	11981	100.00

Table : ALL * HTD 8 YN

ALL

HTD8YN(Cognenital Heart Disease)

Power |
Percent |
Percent of rows
Percent of columns | None | With | Total

	None	With	Total
1	11902	79	11981
	99.34	0.66	100.00
	99.34	0.66	
	100.00	100.00	
Total	11902	79	11981
	99.34	0.66	100.00

Complications and medical history at the time of obtaining Table 2-3 informed consent by the presence or absence of proton-pump inhibitors

FREQ Procedure

Cumulative
Percent of ALL power Percent of power

1	11981	100.00	11981	100.00

Table : ALL * HTD 9 YN

ALL
HTD9YN(Other Heart Disease)

Power |
Percent |
Percent of rows
Percent of columns | None | With | Total

	None	With	Total
1	10900	1081	11981
	90.98	9.02	100.00
	90.98	9.02	
	100.00	100.00	
Total	10900	1081	11981
	90.98	9.02	100.00

Complications and medical history at the time of obtaining Table 2-3 informed consent by the presence or absence of proton-pump inhibitors

FREQ Procedure

			Cumulative	
Percent of ALL	power	Percent of	power	
1	11981	100.00	11981	100.00

Table : ALL * HTD 9 YN

ALL

HTD9YN(Other Heart Disease)

Power |
Percent |
Percent of rows
Percent of columns | None | With | Total

	None	With	Total
1	10900	1081	11981
	90.98	9.02	100.00
	90.98	9.02	
	100.00	100.00	
Total	10900	1081	11981
	90.98	9.02	100.00

Complications and medical history at the time of obtaining Table 2-3 informed consent by the presence or absence of proton-pump inhibitors

FREQ Procedure

Cumulative
Percent of ALL power Percent of power

1	11981	100.00	11981	100.00

Tables: ALL* CVACYN

ALL
CVACYN(Cerebrovascular Accident)

Power |
Percent |
Percent of rows
Percent of columns | None | With | Total

	None	With	Total
1	8856	3125	11981
	73.92	26.08	100.00
	73.92	26.08	
	100.00	100.00	
Total	8856	3125	11981
	73.92	26.08	100.00

Complications and medical history at the time of obtaining Table 2-3 informed consent by the presence or absence of proton-pump inhibitors

FREQ Procedure

			Cumulative	
Percent of ALL	power	Percent of power		
1	11981	100.00	11981	100.00

Tables: ALL* CVACYN

ALL

CVACYN(Cerebrovascular Accident)

Power |
Percent |
Percent of rows
Percent of columns | None | With | Total

	None	With	Total
1	8856	3125	11981
	73.92	26.08	100.00
	73.92	26.08	
	100.00	100.00	
Total	8856	3125	11981
	73.92	26.08	100.00

Complications and medical history at the time of obtaining Table 2-3 informed consent by the presence or absence of proton-pump inhibitors

FREQ Procedure

			Cumulative	
Percent of ALL	power	Percent of	power	
1	11981	100.00	11981	100.00

Table : ALL * CVAC 1 YN

ALL

CVAC1YN(Atherosclerotic Cerebral Infarction)

Power |
Percent |
Percent of rows
Percent of columns | None | With | Total

	None	With	Total
1	11679	302	11981
	97.48	2.52	100.00
	97.48	2.52	
	100.00	100.00	
Total	11679	302	11981
	97.48	2.52	100.00

Complications and medical history at the time of obtaining Table 2-3 informed consent by the presence or absence of proton-pump inhibitors

FREQ Procedure

			Cumulative	
Percent of ALL	power	Percent of	power	
1	11981	100.00	11981	100.00

Table : ALL * CVAC 1 YN

ALL

CVAC1YN(Atherosclerotic Cerebral Infarction)

Power |
Percent |
Percent of rows
Percent of columns | None | With | Total

	None	With	Total
1	11679	302	11981
	97.48	2.52	100.00
	97.48	2.52	
	100.00	100.00	
Total	11679	302	11981
	97.48	2.52	100.00

Complications and medical history at the time of obtaining Table 2-3 informed consent by the presence or absence of proton-pump inhibitors

FREQ Procedure

			Cumulative	
Percent of ALL	power	Percent of	power	
1	11981	100.00	11981	100.00

Table : ALL * CVAC 2 YN

ALL

CVAC2YN(Cardiogenic Cerebral Infarction)

Power |
Percent |
Percent of rows
Percent of columns | None | With | Total

	None	With	Total
1	10983	998	11981
	91.67	8.33	100.00
	91.67	8.33	
	100.00	100.00	
Total	10983	998	11981
	91.67	8.33	100.00

Complications and medical history at the time of obtaining Table 2-3 informed consent by the presence or absence of proton-pump inhibitors

FREQ Procedure

			Cumulative	
Percent of ALL	power	Percent of power		
1	11981	100.00	11981	100.00

Table : ALL * CVAC 2 YN

ALL

CVAC2YN(Cardiogenic Cerebral Infarction)

Power<br>Percent<br>Percent of rows<br>Percent of columns	None	With	Total
1	10983<br>91.67<br>91.67<br>100.00	998<br>8.33<br>8.33<br>100.00	11981<br>100.00
Total	10983<br>91.67	998<br>8.33	11981<br>100.00

Complications and medical history at the time of obtaining Table 2-3 informed consent by the presence or absence of proton-pump inhibitors

FREQ Procedure

			Cumulative	
Percent of ALL	power	Percent of power		
1	11981	100.00	11981	100.00

Table : ALL * CVAC 3 YN

ALL

CVAC3YN(Lacunar Cerebral Infarction)

Power<br>Percent<br>Percent of rows<br>Percent of columns	None	With	Total
1	11362<br>94.83<br>94.83<br>100.00	619<br>5.17<br>5.17<br>100.00	11981<br>100.00
Total	11362<br>94.83	619<br>5.17	11981<br>100.00

Complications and medical history at the time of obtaining Table 2-3 informed consent by the presence or absence of proton-pump inhibitors

FREQ Procedure

Cumulative

Percent of ALL power Percent of power

1	11981	100.00	11981	100.00

Table : ALL * CVAC 3 YN

ALL

CVAC3YN(Lacunar Cerebral Infarction)

Power |
Percent |
Percent of rows
Percent of columns | None | With | Total

	None	With	Total
1	11362	619	11981
	94.83	5.17	100.00
	94.83	5.17	
	100.00	100.00	
Total	11362	619	11981
	94.83	5.17	100.00

Complications and medical history at the time of obtaining Table 2-3 informed consent by the presence or absence of proton-pump inhibitors

FREQ Procedure

Percent of ALL	power	Percent	Cumulative power	Cumulative Percent of power
1	11981	100.00	11981	100.00

Table : ALL * CVAC 4 YN

ALL

CVAC4YN(Hemorrhagic Stroke)

Power |
Percent |
Percent of rows
Percent of columns | None | With | Total

	None	With	Total
1	11816	165	11981
	98.62	1.38	100.00
	98.62	1.38	
	100.00	100.00	
Total	11816	165	11981
	98.62	1.38	100.00

Complications and medical history at the time of obtaining Table 2-3 informed consent by the presence or absence of proton-pump inhibitors

FREQ Procedure

Cumulative

Percent of ALL power Percent of power

1	11981	100.00	11981	100.00

Table : ALL * CVAC 4 YN

ALL

CVAC4YN(Hemorrhagic Stroke)

Power |

Percent |

Percent of rows

Percent of columns | None | With | Total

	None	With	Total
1	11816	165	11981
	98.62	1.38	100.00
	98.62	1.38	
	100.00	100.00	
Total	11816	165	11981
	98.62	1.38	100.00

Complications and medical history at the time of obtaining Table 2-3 informed consent by the presence or absence of proton-pump inhibitors

FREQ Procedure

Cumulative
Percent of ALL power Percent of power

1	11981	100.00	11981	100.00

Table : ALL * CVAC 5 YN

ALL
CVAC5YN(Unclassified/Unknown Cerebral Infarction)

Power |
Percent |
Percent of rows
Percent of columns | None | With | Total

1	11249	732	11981
	93.89	6.11	100.00
	93.89	6.11	
	100.00	100.00	
Total	11249	732	11981
	93.89	6.11	100.00

Complications and medical history at the time of obtaining Table 2-3 informed consent by the presence or absence of proton-pump inhibitors

FREQ Procedure

			Cumulative	
Percent of ALL	power	Percent of	power	
1	11981	100.00	11981	100.00

Table : ALL * CVAC 5 YN

ALL

CVAC5YN(Unclassified/Unknown Cerebral Infarction)

Power |
Percent |
Percent of rows
Percent of columns | None | With | Total

ALL	None	With	Total
1	11249	732	11981
	93.89	6.11	100.00
	93.89	6.11	
	100.00	100.00	
Total	11249	732	11981
	93.89	6.11	100.00

Complications and medical history at the time of obtaining Table 2-3 informed consent by the presence or absence of proton-pump inhibitors

FREQ Procedure

			Cumulative	
Percent of ALL	power	Percent of	power	
1	11981	100.00	11981	100.00

Table : ALL * CVAC 6 YN

ALL

CVAC6YN(Transient Ischemic Attacks)

Power |
Percent |
Percent of rows
Percent of columns | None | With | Total

	None	With	Total
1	11753	228	11981
	98.10	1.90	100.00
	98.10	1.90	
	100.00	100.00	
Total	11753	228	11981
	98.10	1.90	100.00

Complications and medical history at the time of obtaining Table 2-3 informed consent by the presence or absence of proton-pump inhibitors

FREQ Procedure

			Cumulative	
Percent of ALL	power	Percent of power		
1	11981	100.00	11981	100.00

Table : ALL * CVAC 6 YN

ALL

CVAC6YN(Transient Ischemic Attacks)

Power |
Percent |
Percent of rows
Percent of columns | None | With | Total

ALL	None	With	Total
1	11753	228	11981
	98.10	1.90	100.00
	98.10	1.90	
	100.00	100.00	
Total	11753	228	11981
	98.10	1.90	100.00

Complications and medical history at the time of obtaining Table 2-3 informed consent by the presence or absence of proton-pump inhibitors

FREQ Procedure

			Cumulative	
	Percent of ALL power	Percent of power		
1	11981	100.00	11981	100.00

Table : ALL * CVAC 7 YN

ALL

CVAC7YN(Other Cerebrovascular Accident)

Power / Percent / Percent of rows / Percent of columns	None	With	Total
1	11631	350	11981
	97.08	2.92	100.00
	97.08	2.92	
	100.00	100.00	
Total	11631	350	11981
	97.08	2.92	100.00

Complications and medical history at the time of obtaining Table 2-3 informed consent by the presence or absence of proton-pump inhibitors

FREQ Procedure

Cumulative

Percent of ALL power Percent of power

1	11981	100.00	11981	100.00

Table : ALL * CVAC 7 YN

ALL

CVAC7YN(Other Cerebrovascular Accident)

Power |

Percent |

Percent of rows

Percent of columns | None | With | Total

	None	With	Total
1	11631	350	11981
	97.08	2.92	100.00
	97.08	2.92	
	100.00	100.00	
Total	11631	350	11981
	97.08	2.92	100.00

Complications and medical history at the time of obtaining Table 2-3 informed consent by the presence or absence of proton-pump inhibitors

FREQ Procedure

Cumulative
Percent of ALL power Percent of power

1	11981	100.00	11981	100.00

Tables: ALL * OTVDYN

ALL
OTVDYN(Other Vascular Disease)

Power |
Percent |
Percent of rows
Percent of columns | None | With | Total

	None	With	Total
1	11174	807	11981
	93.26	6.74	100.00
	93.26	6.74	
	100.00	100.00	
Total	11174	807	11981
	93.26	6.74	100.00

Complications and medical history at the time of obtaining Table 2-3 informed consent by the presence or absence of proton-pump inhibitors

FREQ Procedure

Cumulative
Percent of ALL power Percent of power

1	11981	100.00	11981	100.00

Tables: ALL * OTVDYN

ALL
OTVDYN(Other Vascular Disease)

Power |
Percent |
Percent of rows
Percent of columns | None | With | Total

	None	With	Total
1	11174	807	11981
	93.26	6.74	100.00
	93.26	6.74	
	100.00	100.00	
Total	11174	807	11981
	93.26	6.74	100.00

Complications and medical history at the time of obtaining Table 2-3 informed consent by the presence or absence of proton-pump inhibitors

FREQ Procedure

			Cumulative	
Percent of ALL	power	Percent of power		
1	11981	100.00	11981	100.00

Table : ALL * OTVD 1 YN

ALL

OTVD1YN(Peripheral Vascular Disease)

Power |
Percent |
Percent of rows
Percent of columns | None | With | Total

	None	With	Total
1	11740	241	11981
	97.99	2.01	100.00
	97.99	2.01	
	100.00	100.00	
Total	11740	241	11981
	97.99	2.01	100.00

Complications and medical history at the time of obtaining Table 2-3 informed consent by the presence or absence of proton-pump inhibitors

FREQ Procedure

			Cumulative	
Percent of ALL	power	Percent of power		
1	11981	100.00	11981	100.00

Table : ALL * OTVD 1 YN

ALL

OTVD1YN(Peripheral Vascular Disease)

Power / Percent / Percent of rows / Percent of columns	None	With	Total
1	11740	241	11981
	97.99	2.01	100.00
	97.99	2.01	
	100.00	100.00	
Total	11740	241	11981
	97.99	2.01	100.00

Complications and medical history at the time of obtaining Table 2-3 informed consent by the presence or absence of proton-pump inhibitors

FREQ Procedure

Cumulative
Percent of ALL power Percent of power

1	11981	100.00	11981	100.00

Table : ALL * OTVD 2 YN

ALL OTVD2YN(Aortic Plaque)

Power |
Percent |
Percent of rows
Percent of columns | None | With | Total

	None	With	Total
1	11893	88	11981
	99.27	0.73	100.00
	99.27	0.73	
	100.00	100.00	
Total	11893	88	11981
	99.27	0.73	100.00

Complications and medical history at the time of obtaining Table 2-3 informed consent by the presence or absence of proton-pump inhibitors

FREQ Procedure

			Cumulative	
	Percent of ALL power	Percent of power		
1	11981	100.00	11981	100.00

Table : ALL * OTVD 2 YN

ALL OTVD2YN(Aortic Plaque)

Power |
Percent |
Percent of rows
Percent of columns | None | With | Total

	None	With	Total
1	11893	88	11981
	99.27	0.73	100.00
	99.27	0.73	
	100.00	100.00	
Total	11893	88	11981
	99.27	0.73	100.00

Complications and medical history at the time of obtaining Table 2-3 informed consent by the presence or absence of proton-pump inhibitors

FREQ Procedure

			Cumulative	
Percent of ALL	power	Percent of	power	
1	11981	100.00	11981	100.00

Table : ALL * TED 3 YN

ALL

TED3YN(Internal Carotid Artery Stenosis)

Power |
Percent |
Percent of rows
Percent of columns | None | With | Total

	None	With	Total
1	11630	351	11981
	97.07	2.93	100.00
	97.07	2.93	
	100.00	100.00	
Total	11630	351	11981
	97.07	2.93	100.00

Complications and medical history at the time of obtaining Table 2-3 informed consent by the presence or absence of proton-pump inhibitors

FREQ Procedure

			Cumulative	
Percent of ALL	power	Percent of power		
1	11981	100.00	11981	100.00

Table : ALL * TED 3 YN

ALL

TED3YN(Internal Carotid Artery Stenosis)

Power<br>Percent<br>Percent of rows<br>Percent of columns	None	With	Total
1	11630<br>97.07<br>97.07<br>100.00	351<br>2.93<br>2.93<br>100.00	11981<br>100.00
Total	11630<br>97.07	351<br>2.93	11981<br>100.00

Complications and medical history at the time of obtaining Table 2-3 informed consent by the presence or absence of proton-pump inhibitors

FREQ Procedure

			Cumulative	
Percent of ALL	power	Percent of power		
1	11981	100.00	11981	100.00

Table : ALL * TED 4 YN

ALL

TED4YN(Arteriosclerosis Obliterans)

Power / Percent / Percent of rows / Percent of columns	None	With	Total
1	11472	509	11981
	95.75	4.25	100.00
	95.75	4.25	
	100.00	100.00	
Total	11472	509	11981
	95.75	4.25	100.00

Complications and medical history at the time of obtaining Table 2-3 informed consent by the presence or absence of proton-pump inhibitors

FREQ Procedure

			Cumulative	
Percent of ALL	power	Percent of power		
1	11981	100.00	11981	100.00

Table : ALL * TED 4 YN

ALL

TED4YN(Arteriosclerosis Obliterans)

Power |
Percent |
Percent of rows
Percent of columns | None | With | Total

	None	With	Total
1	11472	509	11981
	95.75	4.25	100.00
	95.75	4.25	
	100.00	100.00	
Total	11472	509	11981
	95.75	4.25	100.00

Complications and medical history at the time of obtaining Table 2-3 informed consent by the presence or absence of proton-pump inhibitors

FREQ Procedure

			Cumulative	
Percent of ALL	power	Percent of power		
1	11981	100.00	11981	100.00

Table : ALL * OTVD 3 YN

ALL

OTVD3YN(Other Vascular Disease: Other)

Power |
Percent |
Percent of rows
Percent of columns | None | With | Total

	None	With	Total
1	11483	498	11981
	95.84	4.16	100.00
	95.84	4.16	
	100.00	100.00	
Total	11483	498	11981
	95.84	4.16	100.00

Complications and medical history at the time of obtaining Table 2-3 informed consent by the presence or absence of proton-pump inhibitors

FREQ Procedure

			Cumulative	
ALL	power	Percent	of power	Percent of power
1	11981	100.00	11981	100.00

Table : ALL * OTVD 3 YN

ALL

OTVD3YN(Other Vascular Disease: Other)

Power |
Percent |
Percent of rows
Percent of columns | None | With | Total

	None	With	Total
1	11483	498	11981
	95.84	4.16	100.00
	95.84	4.16	
	100.00	100.00	
Total	11483	498	11981
	95.84	4.16	100.00

Complications and medical history at the time of obtaining Table 2-3 informed consent by the presence or absence of proton-pump inhibitors

FREQ Procedure

			Cumulative	
Percent of ALL power Percent of power				
1	11981	100.00	11981	100.00

Tables: ALL * TEDYN

ALL

TEDYN(Thrombosis/Embolism-Related Disease)

Power / Percent / Percent of rows / Percent of columns	None	With	Total
1	10756	1225	11981
	89.78	10.22	100.00
	89.78	10.22	
	100.00	100.00	
Total	10756	1225	11981
	89.78	10.22	100.00

Complications and medical history at the time of obtaining Table 2-3 informed consent by the presence or absence of proton-pump inhibitors

FREQ Procedure

			Cumulative	
Percent of ALL	power	Percent of	power	
1	11981	100.00	11981	100.00

Tables: ALL * TEDYN

ALL

TEDYN(Thrombosis/Embolism-Related Disease)

Power |
Percent |
Percent of rows
Percent of columns | None | With | Total

	None	With	Total
1	10756	1225	11981
	89.78	10.22	100.00
	89.78	10.22	
	100.00	100.00	
Total	10756	1225	11981
	89.78	10.22	100.00

Complications and medical history at the time of obtaining Table 2-3 informed consent by the presence or absence of proton-pump inhibitors

FREQ Procedure

Cumulative
Percent of ALL power Percent of power

1	11981	100.00	11981	100.00

Table: ALL * t_TED

ALL t_TED

Power |
Percent |
Percent of rows
Percentage of columns | 0| 1| Sum

```
______________+________+________+
         1 |  11793 |    188 |  11981
           |  98.43 |   1.57 | 100.00
           |  98.43 |   1.57 |
           | 100.00 | 100.00 |
______________+________+________+
Total 11793 188 11981
              98.43     1.57   100.00
```

Complications and medical history at the time of obtaining Table 2-3 informed consent by the presence or absence of proton-pump inhibitors

FREQ Procedure

			Cumulative	
Percent of ALL	power	Percent of power		
1	11981	100.00	11981	100.00

Table: ALL * t_TED

ALL t_TED

Power |
Percent |
Percent of rows
Percentage of columns | 0| 1| Sum

	0	1	Sum
1	11793	188	11981
	98.43	1.57	100.00
	98.43	1.57	
	100.00	100.00	
Total	11793	188	11981
	98.43	1.57	100.00

Complications and medical history at the time of obtaining Table 2-3 informed consent by the presence or absence of proton-pump inhibitors

FREQ Procedure

			Cumulative	
Percent of ALL	power	Percent of	power	
1	11981	100.00	11981	100.00

Table : ALL * TED 1 YN

ALL

TED1YN(Pulmonary Embolism)

Power |
Percent |
Percent of rows
Percent of columns | None | With | Total

	None	With	Total
1	11929	52	11981
	99.57	0.43	100.00
	99.57	0.43	
	100.00	100.00	
Total	11929	52	11981
	99.57	0.43	100.00

Complications and medical history at the time of obtaining Table 2-3 informed consent by the presence or absence of proton-pump inhibitors

FREQ Procedure

			Cumulative	
Percent of ALL	power	Percent of	power	
1	11981	100.00	11981	100.00

Table : ALL * TED 1 YN

ALL

TED1YN(Pulmonary Embolism)

Power | Percent | Percent of rows Percent of columns	None	With	Total
1	11929	52	11981
	99.57	0.43	100.00
	99.57	0.43	
	100.00	100.00	
Total	11929	52	11981
	99.57	0.43	100.00

Complications and medical history at the time of obtaining Table 2-3 informed consent by the presence or absence of proton-pump inhibitors

FREQ Procedure

			Cumulative	
Percent of ALL	power	Percent of	power	
1	11981	100.00	11981	100.00

Table : ALL * TED 2 YN

ALL

TED2YN(Deep Vein Thrombosis)

Power |
Percent |
Percent of rows
Percent of columns | None | With | Total

	None	With	Total
1	11829	152	11981
	98.73	1.27	100.00
	98.73	1.27	
	100.00	100.00	
Total	11829	152	11981
	98.73	1.27	100.00

Complications and medical history at the time of obtaining Table 2-3 informed consent by the presence or absence of proton-pump inhibitors

FREQ Procedure

			Cumulative	
Percent of ALL	power	Percent of	power	
1	11981	100.00	11981	100.00

Table : ALL * TED 2 YN

ALL

TED2YN(Deep Vein Thrombosis)

Power |
Percent |
Percent of rows
Percent of columns | None | With | Total

ALL	None	With	Total
1	11829	152	11981
	98.73	1.27	100.00
	98.73	1.27	
	100.00	100.00	
Total	11829	152	11981
	98.73	1.27	100.00

Complications and medical history at the time of obtaining Table 2-3 informed consent by the presence or absence of proton-pump inhibitors

FREQ Procedure

			Cumulative	
Percent of ALL	power	Percent of power		
1	11981	100.00	11981	100.00

Table : ALL * TED 5 YN

ALL

TED5YN(Other Thrombosis/Embolism-Related Disease)

Power | Percent | Percent of rows Percent of columns	None	With	Total
1	11646	335	11981
	97.20	2.80	100.00
	97.20	2.80	
	100.00	100.00	
Total	11646	335	11981
	97.20	2.80	100.00

Complications and medical history at the time of obtaining Table 2-3 informed consent by the presence or absence of proton-pump inhibitors

FREQ Procedure

ALL	power	Percent	Cumulative power	Cumulative Percent of power
1	11981	100.00	11981	100.00

Table : ALL * TED 5 YN

ALL

TED5YN(Other Thrombosis/Embolism-Related Disease)

Power<br>Percent<br>Percent of rows<br>Percent of columns	None	With	Total
1	11646<br>97.20<br>97.20<br>100.00	335<br>2.80<br>2.80<br>100.00	11981<br>100.00
Total	11646<br>97.20	335<br>2.80	11981<br>100.00

Complications and medical history at the time of obtaining Table 2-3 informed consent by the presence or absence of proton-pump inhibitors

FREQ Procedure

Cumulative
Percent of ALL power Percent of power

1	11981	100.00	11981	100.00

Tables: ALL * HTDMYN

ALL HTDMYN(Hyperthyroidism)

Power |
Percent |
Percent of rows
Percent of columns | None | With | Total

	None	With	Total
1	11800	181	11981
	98.49	1.51	100.00
	98.49	1.51	
	100.00	100.00	
Total	11800	181	11981
	98.49	1.51	100.00

Complications and medical history at the time of obtaining Table 2-3 informed consent by the presence or absence of proton-pump inhibitors

FREQ Procedure

			Cumulative	
Percent of ALL	power	Percent of	power	
1	11981	100.00	11981	100.00

Tables: ALL * HTDMYN

ALL HTDMYN(Hyperthyroidism)

Power |
Percent |
Percent of rows
Percent of columns | None | With | Total

	None	With	Total
1	11800	181	11981
	98.49	1.51	100.00
	98.49	1.51	
	100.00	100.00	
Total	11800	181	11981
	98.49	1.51	100.00

Complications and medical history at the time of obtaining Table 2-3 informed consent by the presence or absence of proton-pump inhibitors

FREQ Procedure

			Cumulative	
Percent of ALL	power	Percent of	power	
1	11981	100.00	11981	100.00

Tables: ALL * GASDYN

ALL

GASDYN(Gastrointestinal Disease)

Power |
Percent |
Percent of rows
Percent of columns | None | With | Total

	None	With	Total
1	6541	5440	11981
	54.59	45.41	100.00
	54.59	45.41	
	100.00	100.00	
Total	6541	5440	11981
	54.59	45.41	100.00

Complications and medical history at the time of obtaining Table 2-3 informed consent by the presence or absence of proton-pump inhibitors

FREQ Procedure

			Cumulative	
Percent of ALL	power	Percent of	power	
1	11981	100.00	11981	100.00

Tables: ALL * GASDYN

ALL

GASDYN(Gastrointestinal Disease)

Power / Percent / Percent of rows / Percent of columns	None	With	Total
1	6541	5440	11981
	54.59	45.41	100.00
	54.59	45.41	
	100.00	100.00	
Total	6541	5440	11981
	54.59	45.41	100.00

Complications and medical history at the time of obtaining Table 2-3 informed consent by the presence or absence of proton-pump inhibitors

FREQ Procedure

Cumulative
Percent of ALL power Percent of power

1	11981	100.00	11981	100.00

Table : ALL * GASD 1 YN

ALL
GASD1YN(Upper Gastrointestinal Tract)

Power |
Percent |
Percent of rows
Percent of columns | None | With | Total

	None	With	Total
1	11678	303	11981
	97.47	2.53	100.00
	97.47	2.53	
	100.00	100.00	
Total	11678	303	11981
	97.47	2.53	100.00

Complications and medical history at the time of obtaining Table 2-3 informed consent by the presence or absence of proton-pump inhibitors

FREQ Procedure

Cumulative
Percent of ALL power Percent of power

1	11981	100.00	11981	100.00

Table : ALL * GASD 1 YN

ALL
GASD1YN(Upper Gastrointestinal Tract)

Power |
Percent |
Percent of rows
Percent of columns | None | With | Total

	None	With	Total
1	11678	303	11981
	97.47	2.53	100.00
	97.47	2.53	
	100.00	100.00	
Total	11678	303	11981
	97.47	2.53	100.00

Complications and medical history at the time of obtaining Table 2-3 informed consent by the presence or absence of proton-pump inhibitors

FREQ Procedure

Cumulative

Percent of ALL power Percent of power

1	11981	100.00	11981	100.00

Table : ALL * GASD 2 YN

ALL

GASD2YN(Lower Gastrointestinal Tract)

Power |
Percent |
Percent of rows
Percent of columns | None | With | Total

	None	With	Total
1	11680	301	11981
	97.49	2.51	100.00
	97.49	2.51	
	100.00	100.00	
Total	11680	301	11981
	97.49	2.51	100.00

Complications and medical history at the time of obtaining Table 2-3 informed consent by the presence or absence of proton-pump inhibitors

FREQ Procedure

			Cumulative	
Percent of ALL power Percent of power				
1	11981	100.00	11981	100.00

Table : ALL * GASD 2 YN

ALL

GASD2YN(Lower Gastrointestinal Tract)

Power |
Percent |
Percent of rows
Percent of columns | None | With | Total

	None	With	Total
1	11680	301	11981
	97.49	2.51	100.00
	97.49	2.51	
	100.00	100.00	
Total	11680	301	11981
	97.49	2.51	100.00

Complications and medical history at the time of obtaining Table 2-3 informed consent by the presence or absence of proton-pump inhibitors

FREQ Procedure

			Cumulative	
Percent of ALL	power	Percent of power		
1	11981	100.00	11981	100.00

Table : ALL * GASD 3 YN

ALL

GASD3YN(Reflux esophagitis)

Power / Percent / Percent of rows / Percent of columns	None	With	Total
1	8140	3841	11981
	67.94	32.06	100.00
	67.94	32.06	
	100.00	100.00	
Total	8140	3841	11981
	67.94	32.06	100.00

Complications and medical history at the time of obtaining Table 2-3 informed consent by the presence or absence of proton-pump inhibitors

FREQ Procedure

			Cumulative	
Percent of ALL	power	Percent of	power	
1	11981	100.00	11981	100.00

Table : ALL * GASD 3 YN

ALL

GASD3YN(Reflux esophagitis)

Power |
Percent |
Percent of rows
Percent of columns | None | With | Total

	None	With	Total
1	8140	3841	11981
	67.94	32.06	100.00
	67.94	32.06	
	100.00	100.00	
Total	8140	3841	11981
	67.94	32.06	100.00

Complications and medical history at the time of obtaining Table 2-3 informed consent by the presence or absence of proton-pump inhibitors

FREQ Procedure

			Cumulative	
Percent of ALL	power	Percent of	power	
1	11981	100.00	11981	100.00

Table : ALL * GASD 4 YN

ALL

GASD4YN(Other Gastrointestinal Disease)

Power / Percent / Percent of rows / Percent of columns	None	With	Total
1	10043	1938	11981
	83.82	16.18	100.00
	83.82	16.18	
	100.00	100.00	
Total	10043	1938	11981
	83.82	16.18	100.00

Complications and medical history at the time of obtaining Table 2-3 informed consent by the presence or absence of proton-pump inhibitors

FREQ Procedure

			Cumulative	
Percent of ALL power Percent of power				
1	11981	100.00	11981	100.00

Table : ALL * GASD 4 YN

ALL

GASD4YN(Other Gastrointestinal Disease)

Power |
Percent |
Percent of rows
Percent of columns | None | With | Total

	None	With	Total
1	10043	1938	11981
	83.82	16.18	100.00
	83.82	16.18	
	100.00	100.00	
Total	10043	1938	11981
	83.82	16.18	100.00

Complications and medical history at the time of obtaining Table 2-3 informed consent by the presence or absence of proton-pump inhibitors

FREQ Procedure

			Cumulative	
Percent of ALL	power	Percent of power		
1	11981	100.00	11981	100.00

Tables: ALL* MGTYN

ALL MGTYN(Malignant Tumor)

Power<br>Percent<br>Percent of rows<br>Percent of columns	None	With	Total
1	10618<br>88.62<br>88.62<br>100.00	1363<br>11.38<br>11.38<br>100.00	11981<br>100.00
Total	10618<br>88.62	1363<br>11.38	11981<br>100.00

Complications and medical history at the time of obtaining Table 2-3 informed consent by the presence or absence of proton-pump inhibitors

FREQ Procedure

Cumulative

Percent of ALL power Percent of power

1	11981	100.00	11981	100.00

Tables: ALL* MGTYN

ALL MGTYN(Malignant Tumor)

Power |
Percent |
Percent of rows
Percent of columns | None | With | Total
______________+________+________+
1 | 10618 | 1363 | 11981
| 88.62 | 11.38 | 100.00
| 88.62 | 11.38 |
| 100.00 | 100.00 |
______________+________+________+
Total 10618 1363 11981
88.62 11.38 100.00

Complications and medical history at the time of obtaining Table 2-3 informed consent by the presence or absence of proton-pump inhibitors

FREQ Procedure

Cumulative

Percent of ALL power Percent of power

1	11981	100.00	11981	100.00

Table : ALL * MGT 1 YN

ALL MGT1YN(Gastric Cancer)

Power |

Percent |

Percent of rows

Percent of columns | None | With | Total

	None	With	Total
1	11688	293	11981
	97.55	2.45	100.00
	97.55	2.45	
	100.00	100.00	
Total	11688	293	11981
	97.55	2.45	100.00

Complications and medical history at the time of obtaining Table 2-3 informed consent by the presence or absence of proton-pump inhibitors

FREQ Procedure

Cumulative
Percent of ALL power Percent of power

1	11981	100.00	11981	100.00

Table : ALL * MGT 1 YN

ALL MGT1YN(Gastric Cancer)

Power |
Percent |
Percent of rows
Percent of columns | None | With | Total

	None	With	Total
1	11688	293	11981
	97.55	2.45	100.00
	97.55	2.45	
	100.00	100.00	
Total	11688	293	11981
	97.55	2.45	100.00

Complications and medical history at the time of obtaining Table 2-3 informed consent by the presence or absence of proton-pump inhibitors

FREQ Procedure

Cumulative

Percent of ALL power Percent of power

1	11981	100.00	11981	100.00

Table : ALL * MGT 2 YN

ALL MGT2YN(Colorectal Cancer)

Power |

Percent |

Percent of rows

Percent of columns | None | With | Total

	None	With	Total
1	11700	281	11981
	97.65	2.35	100.00
	97.65	2.35	
	100.00	100.00	
Total	11700	281	11981
	97.65	2.35	100.00

Complications and medical history at the time of obtaining Table 2-3 informed consent by the presence or absence of proton-pump inhibitors

FREQ Procedure

Cumulative

Percent of ALL power Percent of power

1	11981	100.00	11981	100.00

Table : ALL * MGT 2 YN

ALL MGT2YN(Colorectal Cancer)

Power |
Percent |
Percent of rows
Percent of columns | None | With | Total

	None	With	Total
1	11700	281	11981
	97.65	2.35	100.00
	97.65	2.35	
	100.00	100.00	
Total	11700	281	11981
	97.65	2.35	100.00

Complications and medical history at the time of obtaining Table 2-3 informed consent by the presence or absence of proton-pump inhibitors

FREQ Procedure

Cumulative

Percent of ALL power Percent of power

1	11981	100.00	11981	100.00

Table : ALL * MGT 3 YN

ALL MGT3YN(Lung Cancer)

Power |
Percent |
Percent of rows
Percent of columns | None | With | Total

	None	With	Total
1	11837	144	11981
	98.80	1.20	100.00
	98.80	1.20	
	100.00	100.00	
Total	11837	144	11981
	98.80	1.20	100.00

Complications and medical history at the time of obtaining Table 2-3 informed consent by the presence or absence of proton-pump inhibitors

FREQ Procedure

			Cumulative	
	Percent of ALL power	Percent of power		
1	11981	100.00	11981	100.00

Table : ALL * MGT 3 YN

ALL MGT3YN(Lung Cancer)

Power | Percent | Percent of rows Percent of columns	None	With	Total
1	11837	144	11981
	98.80	1.20	100.00
	98.80	1.20	
	100.00	100.00	
Total	11837	144	11981
	98.80	1.20	100.00

Complications and medical history at the time of obtaining Table 2-3 informed consent by the presence or absence of proton-pump inhibitors

FREQ Procedure

Cumulative
Percent of ALL power Percent of power

1	11981	100.00	11981	100.00

Table : ALL * MGT 4 YN

ALL MGT4YN(Brest Cancer)

Power |
Percent |
Percent of rows
Percent of columns | None | With | Total

	None	With	Total
1	11895	86	11981
	99.28	0.72	100.00
	99.28	0.72	
	100.00	100.00	
Total	11895	86	11981
	99.28	0.72	100.00

Complications and medical history at the time of obtaining Table 2-3 informed consent by the presence or absence of proton-pump inhibitors

FREQ Procedure

Cumulative
Percent of ALL power Percent of power

1	11981	100.00	11981	100.00

Table : ALL * MGT 4 YN

ALL MGT4YN(Brest Cancer)

Power |
Percent |
Percent of rows
Percent of columns | None | With | Total

1	11895	86	11981
	99.28	0.72	100.00
	99.28	0.72	
	100.00	100.00	
Total	11895	86	11981
	99.28	0.72	100.00

Complications and medical history at the time of obtaining Table 2-3 informed consent by the presence or absence of proton-pump inhibitors

FREQ Procedure

Cumulative

Percent of ALL power Percent of power

1	11981	100.00	11981	100.00

Table : ALL * MGT 5 YN

ALL

MGT5YN(Uterus/Ovarian Cancer)

Power |
Percent |
Percent of rows
Percent of columns | None | With | Total

	None	With	Total
1	11942	39	11981
	99.67	0.33	100.00
	99.67	0.33	
	100.00	100.00	
Total	11942	39	11981
	99.67	0.33	100.00

Complications and medical history at the time of obtaining Table 2-3 informed consent by the presence or absence of proton-pump inhibitors

FREQ Procedure

			Cumulative	
Percent of ALL	power	Percent of	power	
1	11981	100.00	11981	100.00

Table : ALL * MGT 5 YN

ALL

MGT5YN(Uterus/Ovarian Cancer)

Power |
Percent |
Percent of rows
Percent of columns | None | With | Total

	None	With	Total
1	11942	39	11981
	99.67	0.33	100.00
	99.67	0.33	
	100.00	100.00	
Total	11942	39	11981
	99.67	0.33	100.00

Complications and medical history at the time of obtaining Table 2-3 informed consent by the presence or absence of proton-pump inhibitors

FREQ Procedure

			Cumulative	
Percent of ALL	power	Percent	of power	
1	11981	100.00	11981	100.00

Table : ALL * MGT 6 YN

ALL MGT6YN(Pancreatic Cancer)

Power |
Percent |
Percent of rows
Percent of columns | None | With | Total

	None	With	Total
1	11968	13	11981
	99.89	0.11	100.00
	99.89	0.11	
	100.00	100.00	
Total	11968	13	11981
	99.89	0.11	100.00

Complications and medical history at the time of obtaining Table 2-3 informed consent by the presence or absence of proton-pump inhibitors

FREQ Procedure

			Cumulative	
Percent of ALL	power	Percent of	power	
1	11981	100.00	11981	100.00

Table : ALL * MGT 6 YN

ALL MGT6YN(Pancreatic Cancer)

Power |
Percent |
Percent of rows
Percent of columns | None | With | Total

	None	With	Total
1	11968	13	11981
	99.89	0.11	100.00
	99.89	0.11	
	100.00	100.00	
Total	11968	13	11981
	99.89	0.11	100.00

Complications and medical history at the time of obtaining Table 2-3 informed consent by the presence or absence of proton-pump inhibitors

FREQ Procedure

			Cumulative	
Percent of ALL power Percent of power				
1	11981	100.00	11981	100.00

Table : ALL * MGT 7 YN

ALL

MGT7YN(Other Malignant Tumor)

Power, Percent, Percent of rows, Percent of columns	None	With	Total
1	11331	650	11981
	94.57	5.43	100.00
	94.57	5.43	
	100.00	100.00	
Total	11331	650	11981
	94.57	5.43	100.00

Complications and medical history at the time of obtaining Table 2-3 informed consent by the presence or absence of proton-pump inhibitors

FREQ Procedure

			Cumulative	
Percent of ALL	power	Percent of	power	
1	11981	100.00	11981	100.00

Table : ALL * MGT 7 YN

ALL

MGT7YN(Other Malignant Tumor)

Power |
Percent |
Percent of rows
Percent of columns | None | With | Total

	None	With	Total
1	11331	650	11981
	94.57	5.43	100.00
	94.57	5.43	
	100.00	100.00	
Total	11331	650	11981
	94.57	5.43	100.00

Complications and medical history at the time of obtaining Table 2-3 informed consent by the presence or absence of proton-pump inhibitors

FREQ Procedure

Cumulative

Percent of ALL power Percent of power

1	11981	100.00	11981	100.00

Tables: ALL* ALZHYN

ALL ALZHYN(Alzheimer)

Power |
Percent |
Percent of rows
Percent of columns | None | With | Total

	None	With	Total
1	10995	986	11981
	91.77	8.23	100.00
	91.77	8.23	
	100.00	100.00	
Total	10995	986	11981
	91.77	8.23	100.00

Complications and medical history at the time of obtaining Table 2-3 informed consent by the presence or absence of proton-pump inhibitors

FREQ Procedure

Cumulative
Percent of ALL power Percent of power

1	11981	100.00	11981	100.00

Tables: ALL* ALZHYN

ALL ALZHYN(Alzheimer)

Power |
Percent |
Percent of rows
Percent of columns | None | With | Total

	None	With	Total
1	10995	986	11981
	91.77	8.23	100.00
	91.77	8.23	
	100.00	100.00	
Total	10995	986	11981
	91.77	8.23	100.00

Complications and medical history at the time of obtaining Table 2-3 informed consent by the presence or absence of proton-pump inhibitors

FREQ Procedure

Cumulative

Percent of ALL power Percent of power

1	11981	100.00	11981	100.00

Tables: ALL * OHCPYN

ALL

OHCPYN(Other Complications)

Power |
Percent |
Percent of rows
Percent of columns | None | With | Total

	None	With	Total
1	7822	4159	11981
	65.29	34.71	100.00
	65.29	34.71	
	100.00	100.00	
Total	7822	4159	11981
	65.29	34.71	100.00

Complications and medical history at the time of obtaining Table 2-3 informed consent by the presence or absence of proton-pump inhibitors

FREQ Procedure

Cumulative
Percent of ALL power Percent of power

1	11981	100.00	11981	100.00

Tables: ALL * OHCPYN

ALL
OHCPYN(Other Complications)

Power |
Percent |
Percent of rows
Percent of columns | None | With | Total

	None	With	Total
1	7822	4159	11981
	65.29	34.71	100.00
	65.29	34.71	
	100.00	100.00	
Total	7822	4159	11981
	65.29	34.71	100.00

Complications and medical history at the time of obtaining Table 2-3 informed consent by the presence or absence of proton-pump inhibitors

FREQ Procedure

			Cumulative	
	Percent of ALL power	Percent of power		
1	11981	100.00	11981	100.00

Tables: ALL * IMGYN

ALL IMGYN(Brain Imaging Test)

Power |
Percent |
Percent of rows
Percentage of columns |None| | |Unknown| | Total

	None		Unknown	Total
1	10487	1232	262	11981
	87.53	10.28	2.19	100.00
	87.53	10.28	2.19	
	100.00	100.00	100.00	
Total	10487	1232	262	11981
	87.53	10.28	2.19	100.00

Complications and medical history at the time of obtaining Table 2-3 informed consent by the presence or absence of proton-pump inhibitors

FREQ Procedure

			Cumulative	
Percent of ALL	power	Percent of power		
1	11981	100.00	11981	100.00

Tables: ALL * IMGYN

ALL IMGYN(Brain Imaging Test)

Power |
Percent |
Percent of rows
Percentage of columns |None| | |Unknown| | Total

1	10487	1232	262	11981
	87.53	10.28	2.19	100.00
	87.53	10.28	2.19	
	100.00	100.00	100.00	
Total	10487	1232	262	11981
	87.53	10.28	2.19	100.00

Complications and medical history at the time of obtaining Table 2-3 informed consent by the presence or absence of proton-pump inhibitors

FREQ Procedure

Cumulative
Percent of ALL power Percent of power

1	11981	100.00	11981	100.00

Tables: ALL * IMGYN

ALL IMGYN(Brain Imaging Test)

Power |
Percent |
Percent of rows
Percentage of columns |None| | |Unknown| | Total

1	10487	1232	262	11981
	87.53	10.28	2.19	100.00
	87.53	10.28	2.19	
	100.00	100.00	100.00	
Total	10487	1232	262	11981
	87.53	10.28	2.19	100.00

Complications and medical history at the time of obtaining Table 2-3 informed consent by the presence or absence of proton-pump inhibitors

FREQ Procedure

			Cumulative	
Percent of ALL	power	Percent of	power	
1	11981	100.00	11981	100.00

Tables: ALL* FALYN

ALL FALYN(Fall Down within one year)

Power |
Percent |
Percent of rows
Percentage of columns |None| | |Unknown| | Total

	None		Unknown	Total
1	9638	999	1344	11981
	80.44	8.34	11.22	100.00
	80.44	8.34	11.22	
	100.00	100.00	100.00	
Total	9638	999	1344	11981
	80.44	8.34	11.22	100.00

Complications and medical history at the time of obtaining Table 2-3 informed consent by the presence or absence of proton-pump inhibitors

FREQ Procedure

Cumulative
Percent of ALL power Percent of power

1	11981	100.00	11981	100.00

Tables: ALL* FALYN

ALL FALYN(Fall Down within one year)

Power |
Percent |
Percent of rows
Percentage of columns |None| | |Unknown| | Total

1	9638	999	1344	11981
	80.44	8.34	11.22	100.00
	80.44	8.34	11.22	
	100.00	100.00	100.00	
Total	9638	999	1344	11981
	80.44	8.34	11.22	100.00

Complications and medical history at the time of obtaining Table 2-3 informed consent by the presence or absence of proton-pump inhibitors

FREQ Procedure

			Cumulative	
Percent of ALL	power	Percent	of power	
1	11981	100.00	11981	100.00

Tables: ALL* FALYN

ALL FALYN(Fall Down within one year)

Power |
Percent |
Percent of rows
Percentage of columns |None| | |Unknown| | Total

1	9638	999	1344	11981
	80.44	8.34	11.22	100.00
	80.44	8.34	11.22	
	100.00	100.00	100.00	
Total	9638	999	1344	11981
	80.44	8.34	11.22	100.00

Complications and medical history at the time of obtaining Table 2-3 informed consent by the presence or absence of proton-pump inhibitors

FREQ Procedure

Tables: ALL * FNDYN

ALL FNDYN(Brain Imaging Result)

```
Power |
Percent |
Percent of rows
Percentage of columns |None| | |Unknown| | Total
______________+________+________+________+
            1 |    562 |    625 |     45 |   1232
              |  45.62 |  50.73 |   3.65 | 100.00
              |  45.62 |  50.73 |   3.65 |
              | 100.00 | 100.00 | 100.00 |
______________+________+________+________+
Total 562 625 45 1232
                 45.62    50.73     3.65   100.00
```

Degree of missing values = 10749

Complications and medical history at the time of obtaining Table 2-3 informed consent by the presence or absence of proton-pump inhibitors

FREQ Procedure

Tables: ALL * FNDYN

ALL FNDYN(Brain Imaging Result)

Power |
Percent |
Percent of rows
Percentage of columns |None| | |Unknown| | Total

1	562	625	45	1232
	45.62	50.73	3.65	100.00
	45.62	50.73	3.65	
	100.00	100.00	100.00	
Total	562	625	45	1232
	45.62	50.73	3.65	100.00

Degree of missing values = 10749

Complications and medical history at the time of obtaining Table 2-3 informed consent by the presence or absence of proton-pump inhibitors

FREQ Procedure

Tables: ALL * FNDYN

ALL FNDYN(Brain Imaging Result)

Power |
Percent |
Percent of rows
Percentage of columns |None| | |Unknown| | Total

1	562	625	45	1232
	45.62	50.73	3.65	100.00
	45.62	50.73	3.65	
	100.00	100.00	100.00	
Total	562	625	45	1232
	45.62	50.73	3.65	100.00

Degree of missing values = 10749

Complications and medical history at the time of obtaining Table 2-3 informed consent by the presence or absence of proton-pump inhibitors

FREQ Procedure

Cumulative
Percent of ALL power Percent of power

1	20509	100.00	20509	100.00

Tables: ALL * MHYN

ALL MHYN(Medical History)

Power |
Percent |
Percent of rows
Percent of columns | None | With | Total

______________+________+________+

1	705	19804	20509
	3.44	96.56	100.00
	3.44	96.56	
	100.00	100.00	

______________+________+________+

Total 705 19804 20509
3.44 96.56 100.00

Complications and medical history at the time of obtaining Table 2-3 informed consent by the presence or absence of proton-pump inhibitors

FREQ Procedure

Cumulative

Percent of ALL power Percent of power

1	20509	100.00	20509	100.00

Tables: ALL * MHYN

ALL MHYN(Medical History)

Power |

Percent |

Percent of rows

Percent of columns | None | With | Total

	None	With	Total
1	705	19804	20509
	3.44	96.56	100.00
	3.44	96.56	
	100.00	100.00	
Total	705	19804	20509
	3.44	96.56	100.00

Complications and medical history at the time of obtaining Table 2-3 informed consent by the presence or absence of proton-pump inhibitors

FREQ Procedure

			Cumulative	
Percent of ALL	power	Percent of	power	
1	20509	100.00	20509	100.00

Tables: ALL* HBPYN

ALL

HBPYN(High Blood Pressure)

Power |
Percent |
Percent of rows
Percent of columns | None | With | Total

	None	With	Total
1	5374	15135	20509
	26.20	73.80	100.00
	26.20	73.80	
	100.00	100.00	
Total	5374	15135	20509
	26.20	73.80	100.00

Complications and medical history at the time of obtaining Table 2-3 informed consent by the presence or absence of proton-pump inhibitors

FREQ Procedure

			Cumulative	
Percent of ALL	power	Percent of power		
1	20509	100.00	20509	100.00

Tables: ALL* HBPYN

ALL

HBPYN(High Blood Pressure)

Power |
Percent |
Percent of rows
Percent of columns | None | With | Total

	None	With	Total
1	5374	15135	20509
	26.20	73.80	100.00
	26.20	73.80	
	100.00	100.00	
Total	5374	15135	20509
	26.20	73.80	100.00

Complications and medical history at the time of obtaining Table 2-3 informed consent by the presence or absence of proton-pump inhibitors

FREQ Procedure

Cumulative
Percent of ALL power Percent of power

1	20509	100.00	20509	100.00

Tables: ALL * DBYN

ALL DBYN(Diabetes)

Power |
Percent |
Percent of rows
Percent of columns | None | With | Total

1	15243	5266	20509
	74.32	25.68	100.00
	74.32	25.68	
	100.00	100.00	
Total	15243	5266	20509
	74.32	25.68	100.00

Complications and medical history at the time of obtaining Table 2-3 informed consent by the presence or absence of proton-pump inhibitors

FREQ Procedure

Cumulative
Percent of ALL power Percent of power

1	20509	100.00	20509	100.00

Tables: ALL * DBYN

ALL DBYN(Diabetes)

Power |
Percent |
Percent of rows
Percent of columns | None | With | Total

	None	With	Total
1	15243	5266	20509
	74.32	25.68	100.00
	74.32	25.68	
	100.00	100.00	
Total	15243	5266	20509
	74.32	25.68	100.00

Complications and medical history at the time of obtaining Table 2-3 informed consent by the presence or absence of proton-pump inhibitors

FREQ Procedure

Cumulative
Percent of ALL power Percent of power

1	20509	100.00	20509	100.00

Table: ALL * t_DB

ALL t_DB

Power |
Percent |
Percent of rows
Percentage of columns | 0| 1| Sum

1	19610	899	20509
	95.62	4.38	100.00
	95.62	4.38	
	100.00	100.00	
Total	19610	899	20509
	95.62	4.38	100.00

Complications and medical history at the time of obtaining Table 2-3 informed consent by the presence or absence of proton-pump inhibitors

FREQ Procedure

Cumulative
Percent of ALL power Percent of power

1	20509	100.00	20509	100.00

Table: ALL * t_DB

ALL t_DB

Power |
Percent |
Percent of rows
Percentage of columns | 0| 1| Sum

	0	1	Sum
1	19610	899	20509
	95.62	4.38	100.00
	95.62	4.38	
	100.00	100.00	
Total	19610	899	20509
	95.62	4.38	100.00

Complications and medical history at the time of obtaining Table 2-3 informed consent by the presence or absence of proton-pump inhibitors

FREQ Procedure

Cumulative
Percent of ALL power Percent of power

1	20509	100.00	20509	100.00

Table : ALL * DB 1 YN

ALL
DB1YN(Diabetic Retinopathy)

Power |
Percent |
Percent of rows
Percent of columns | None | With | Total

1	20231	278	20509
	98.64	1.36	100.00
	98.64	1.36	
	100.00	100.00	
Total	20231	278	20509
	98.64	1.36	100.00

Complications and medical history at the time of obtaining Table 2-3 informed consent by the presence or absence of proton-pump inhibitors

FREQ Procedure

Cumulative
Percent of ALL power Percent of power

1	20509	100.00	20509	100.00

Table : ALL * DB 1 YN

ALL
DB1YN(Diabetic Retinopathy)

Power |
Percent |
Percent of rows
Percent of columns | None | With | Total

	None	With	Total
1	20231	278	20509
	98.64	1.36	100.00
	98.64	1.36	
	100.00	100.00	
Total	20231	278	20509
	98.64	1.36	100.00

Complications and medical history at the time of obtaining Table 2-3 informed consent by the presence or absence of proton-pump inhibitors

FREQ Procedure

			Cumulative	
Percent of ALL	power	Percent of	power	
1	20509	100.00	20509	100.00

Table : ALL * DB 2 YN

ALL

DB2YN(Diabetic Nephropathy)

Power |
Percent |
Percent of rows
Percent of columns | None | With | Total

	None	With	Total
1	19884	625	20509
	96.95	3.05	100.00
	96.95	3.05	
	100.00	100.00	
Total	19884	625	20509
	96.95	3.05	100.00

Complications and medical history at the time of obtaining Table 2-3 informed consent by the presence or absence of proton-pump inhibitors

FREQ Procedure

Cumulative

Percent of ALL power Percent of power

1	20509	100.00	20509	100.00

Table : ALL * DB 2 YN

ALL

DB2YN(Diabetic Nephropathy)

Power |
Percent |
Percent of rows
Percent of columns | None | With | Total

	None	With	Total
1	19884	625	20509
	96.95	3.05	100.00
	96.95	3.05	
	100.00	100.00	
Total	19884	625	20509
	96.95	3.05	100.00

Complications and medical history at the time of obtaining Table 2-3 informed consent by the presence or absence of proton-pump inhibitors

FREQ Procedure

			Cumulative	
Percent of ALL	power	Percent of	power	
1	20509	100.00	20509	100.00

Table : ALL * DB 3 YN

ALL

DB3YN(Diabetic Neuropathy)

Power |
Percent |
Percent of rows
Percent of columns | None | With | Total

	None	With	Total
1	20327	182	20509
	99.11	0.89	100.00
	99.11	0.89	
	100.00	100.00	
Total	20327	182	20509
	99.11	0.89	100.00

Complications and medical history at the time of obtaining Table 2-3 informed consent by the presence or absence of proton-pump inhibitors

FREQ Procedure

Cumulative
Percent of ALL power Percent of power

1	20509	100.00	20509	100.00

Table : ALL * DB 3 YN

ALL
DB3YN(Diabetic Neuropathy)

Power |
Percent |
Percent of rows
Percent of columns | None | With | Total

	None	With	Total
1	20327	182	20509
	99.11	0.89	100.00
	99.11	0.89	
	100.00	100.00	
Total	20327	182	20509
	99.11	0.89	100.00

Complications and medical history at the time of obtaining Table 2-3 informed consent by the presence or absence of proton-pump inhibitors

FREQ Procedure

			Cumulative	
Percent of ALL	power	Percent of power		
1	20509	100.00	20509	100.00

Tables: ALL* LMDYN

ALL

LMDYN(Lipid metabolism disorders)

Power |
Percent |
Percent of rows
Percent of columns | None | With | Total

	None	With	Total
1	12521	7988	20509
	61.05	38.95	100.00
	61.05	38.95	
	100.00	100.00	
Total	12521	7988	20509
	61.05	38.95	100.00

Complications and medical history at the time of obtaining Table 2-3 informed consent by the presence or absence of proton-pump inhibitors

FREQ Procedure

Cumulative
Percent of ALL power Percent of power

1	20509	100.00	20509	100.00

Tables: ALL* LMDYN

ALL
LMDYN(Lipid metabolism disorders)

Power |
Percent |
Percent of rows
Percent of columns | None | With | Total
______________+________+________+
1 | 12521 | 7988 | 20509
| 61.05 | 38.95 | 100.00
| 61.05 | 38.95 |
| 100.00 | 100.00 |
______________+________+________+
Total 12521 7988 20509
61.05 38.95 100.00

Complications and medical history at the time of obtaining Table 2-3 informed consent by the presence or absence of proton-pump inhibitors

FREQ Procedure

Cumulative
Percent of ALL power Percent of power

1	20509	100.00	20509	100.00

Tables: ALL * HYPURNYN

ALL HYPURNYN(Hyperuricemia)

Power |
Percent |
Percent of rows
Percent of columns | None | With | Total

	None	With	Total
1	16140	4369	20509
	78.70	21.30	100.00
	78.70	21.30	
	100.00	100.00	
Total	16140	4369	20509
	78.70	21.30	100.00

Complications and medical history at the time of obtaining Table 2-3 informed consent by the presence or absence of proton-pump inhibitors

FREQ Procedure

		Percent of ALL power	Cumulative	Percent of power
1	20509	100.00	20509	100.00

Tables: ALL * HYPURNYN

ALL HYPURNYN(Hyperuricemia)

Power / Percent / Percent of rows / Percent of columns	None	With	Total
1	16140	4369	20509
	78.70	21.30	100.00
	78.70	21.30	
	100.00	100.00	
Total	16140	4369	20509
	78.70	21.30	100.00

Complications and medical history at the time of obtaining Table 2-3 informed consent by the presence or absence of proton-pump inhibitors

FREQ Procedure

			Cumulative	
Percent of ALL	power	Percent of power		
1	20509	100.00	20509	100.00

Tables: ALL * KDDSYN

ALL KDDSYN(Kidney Disease)

Power |
Percent |
Percent of rows
Percent of columns | None | With | Total

	None	With	Total
1	16287	4222	20509
	79.41	20.59	100.00
	79.41	20.59	
	100.00	100.00	
Total	16287	4222	20509
	79.41	20.59	100.00

Complications and medical history at the time of obtaining Table 2-3 informed consent by the presence or absence of proton-pump inhibitors

FREQ Procedure

			Cumulative	
	Percent of ALL	power	Percent of power	
1	20509	100.00	20509	100.00

Tables: ALL * KDDSYN

ALL KDDSYN(Kidney Disease)

Power | Percent | Percent of rows Percent of columns	None	With	Total
1	16287	4222	20509
	79.41	20.59	100.00
	79.41	20.59	
	100.00	100.00	
Total	16287	4222	20509
	79.41	20.59	100.00

Complications and medical history at the time of obtaining Table 2-3 informed consent by the presence or absence of proton-pump inhibitors

FREQ Procedure

Cumulative
Percent of ALL power Percent of power

1	20509	100.00	20509	100.00

Table : ALL * KDDS 1 YN

ALL

KDDS1YN(Chronic Kidney Disease)

Power |
Percent |
Percent of rows
Percent of columns | None | With | Total

1	16731	3778	20509
	81.58	18.42	100.00
	81.58	18.42	
	100.00	100.00	
Total	16731	3778	20509
	81.58	18.42	100.00

Complications and medical history at the time of obtaining Table 2-3 informed consent by the presence or absence of proton-pump inhibitors

FREQ Procedure

Cumulative
Percent of ALL power Percent of power

1	20509	100.00	20509	100.00

Table : ALL * KDDS 1 YN

ALL
KDDS1YN(Chronic Kidney Disease)

Power |
Percent |
Percent of rows
Percent of columns | None | With | Total

1	16731	3778	20509
	81.58	18.42	100.00
	81.58	18.42	
	100.00	100.00	
Total	16731	3778	20509
	81.58	18.42	100.00

Complications and medical history at the time of obtaining Table 2-3 informed consent by the presence or absence of proton-pump inhibitors

FREQ Procedure

			Cumulative	
Percent of ALL	power	Percent	of power	
1	20509	100.00	20509	100.00

Table : ALL * KDDS 2 YN

ALL

KDDS2YN(Advanced Renal Dysfunction without Dyalysis)

Power |
Percent |
Percent of rows
Percent of columns | None | With | Total

	None	With	Total
1	20271	238	20509
	98.84	1.16	100.00
	98.84	1.16	
	100.00	100.00	
Total	20271	238	20509
	98.84	1.16	100.00

Complications and medical history at the time of obtaining Table 2-3 informed consent by the presence or absence of proton-pump inhibitors

FREQ Procedure

			Cumulative	
Percent of ALL	power	Percent	of power	
1	20509	100.00	20509	100.00

Table : ALL * KDDS 2 YN

ALL

KDDS2YN(Advanced Renal Dysfunction without Dyalysis)

Power |
Percent |
Percent of rows
Percent of columns | None | With | Total

	None	With	Total
1	20271	238	20509
	98.84	1.16	100.00
	98.84	1.16	
	100.00	100.00	
Total	20271	238	20509
	98.84	1.16	100.00

Complications and medical history at the time of obtaining Table 2-3 informed consent by the presence or absence of proton-pump inhibitors

FREQ Procedure

			Cumulative	
Percent of ALL	power	Percent of power		
1	20509	100.00	20509	100.00

Table : ALL * KDDS 3 YN

ALL

KDDS3YN(Advanced Renal Dysfunction with Dyalysis)

Power |
Percent |
Percent of rows
Percent of columns | None | With | Total

	None	With	Total
1	20453	56	20509
	99.73	0.27	100.00
	99.73	0.27	
	100.00	100.00	
Total	20453	56	20509
	99.73	0.27	100.00

Complications and medical history at the time of obtaining Table 2-3 informed consent by the presence or absence of proton-pump inhibitors

FREQ Procedure

			Cumulative	
Percent of ALL	power	Percent of power		
1	20509	100.00	20509	100.00

Table : ALL * KDDS 3 YN

ALL

KDDS3YN(Advanced Renal Dysfunction with Dyalysis)

Power |
Percent |
Percent of rows
Percent of columns | None | With | Total

	None	With	Total
1	20453	56	20509
	99.73	0.27	100.00
	99.73	0.27	
	100.00	100.00	
Total	20453	56	20509
	99.73	0.27	100.00

Complications and medical history at the time of obtaining Table 2-3 informed consent by the presence or absence of proton-pump inhibitors

FREQ Procedure

Cumulative
Percent of ALL power Percent of power

1	20509	100.00	20509	100.00

Tables: ALL * SLVDYN

ALL
SLVDYN(Severe Liver Dysfunction)

Power |
Percent |
Percent of rows
Percent of columns | None | With | Total

	None	With	Total
1	20339	170	20509
	99.17	0.83	100.00
	99.17	0.83	
	100.00	100.00	
Total	20339	170	20509
	99.17	0.83	100.00

Complications and medical history at the time of obtaining Table 2-3 informed consent by the presence or absence of proton-pump inhibitors

FREQ Procedure

			Cumulative	
Percent of ALL	power	Percent of	power	
1	20509	100.00	20509	100.00

Tables: ALL * SLVDYN

ALL

SLVDYN(Severe Liver Dysfunction)

Power |
Percent |
Percent of rows
Percent of columns | None | With | Total

	None	With	Total
1	20339	170	20509
	99.17	0.83	100.00
	99.17	0.83	
	100.00	100.00	
Total	20339	170	20509
	99.17	0.83	100.00

Complications and medical history at the time of obtaining Table 2-3 informed consent by the presence or absence of proton-pump inhibitors

FREQ Procedure

ALL	power	Percent	Cumulative power	Cumulative Percent of power
1	20509	100.00	20509	100.00

Tables: ALL* RESDYN

ALL

RESDYN(Respiratory Disease)

Power / Percent / Percent of rows / Percent of columns	None	With	Total
1	18143	2366	20509
	88.46	11.54	100.00
	88.46	11.54	
	100.00	100.00	
Total	18143	2366	20509
	88.46	11.54	100.00

Complications and medical history at the time of obtaining Table 2-3 informed consent by the presence or absence of proton-pump inhibitors

FREQ Procedure

			Cumulative	
Percent of ALL	power	Percent of	power	
1	20509	100.00	20509	100.00

Tables: ALL* RESDYN

ALL

RESDYN(Respiratory Disease)

Power |
Percent |
Percent of rows
Percent of columns | None | With | Total

	None	With	Total
1	18143	2366	20509
	88.46	11.54	100.00
	88.46	11.54	
	100.00	100.00	
Total	18143	2366	20509
	88.46	11.54	100.00

Complications and medical history at the time of obtaining Table 2-3 informed consent by the presence or absence of proton-pump inhibitors

FREQ Procedure

			Cumulative	
	Percent of ALL power	Percent of power		
1	20509	100.00	20509	100.00

Tables: ALL* HTDYN

ALL HTDYN(Heart Disease)

Power |
Percent |
Percent of rows
Percent of columns | None | With | Total

```
______________+________+________+
             1 |    9207 |   11302 |   20509
               |   44.89 |   55.11 | 100.00
               |   44.89 |   55.11 |
               | 100.00 | 100.00 |
______________+________+________+
Total 9207 11302 20509
                 44.89      55.11     100.00
```

Complications and medical history at the time of obtaining Table 2-3 informed consent by the presence or absence of proton-pump inhibitors

FREQ Procedure

			Cumulative	
	Percent of ALL power	Percent of power		
1	20509	100.00	20509	100.00

Tables: ALL* HTDYN

ALL HTDYN(Heart Disease)

Power |
Percent |
Percent of rows
Percent of columns | None | With | Total

	None	With	Total
1	9207	11302	20509
	44.89	55.11	100.00
	44.89	55.11	
	100.00	100.00	
Total	9207	11302	20509
	44.89	55.11	100.00

Complications and medical history at the time of obtaining Table 2-3 informed consent by the presence or absence of proton-pump inhibitors

FREQ Procedure

			Cumulative	
Percent of ALL	power	Percent of	power	
1	20509	100.00	20509	100.00

Table : ALL * HTD 1 YN

ALL

HTD1YN(Myocardial Infarction)

Power |
Percent |
Percent of rows
Percent of columns | None | With | Total

	None	With	Total
1	19704	805	20509
	96.07	3.93	100.00
	96.07	3.93	
	100.00	100.00	
Total	19704	805	20509
	96.07	3.93	100.00

Complications and medical history at the time of obtaining Table 2-3 informed consent by the presence or absence of proton-pump inhibitors

FREQ Procedure

			Cumulative	
Percent of ALL	power	Percent of	power	
1	20509	100.00	20509	100.00

Table : ALL * HTD 1 YN

ALL

HTD1YN(Myocardial Infarction)

Power |
Percent |
Percent of rows
Percent of columns | None | With | Total

	None	With	Total
1	19704	805	20509
	96.07	3.93	100.00
	96.07	3.93	
	100.00	100.00	
Total	19704	805	20509
	96.07	3.93	100.00

Complications and medical history at the time of obtaining Table 2-3 informed consent by the presence or absence of proton-pump inhibitors

FREQ Procedure

			Cumulative	
Percent of ALL	power	Percent	of power	
1	20509	100.00	20509	100.00

Table : ALL * HTD 2 YN

ALL HTD2YN(Angina Pectoris)

Power |
Percent |
Percent of rows
Percent of columns | None | With | Total

	None	With	Total
1	17576	2933	20509
	85.70	14.30	100.00
	85.70	14.30	
	100.00	100.00	
Total	17576	2933	20509
	85.70	14.30	100.00

Complications and medical history at the time of obtaining Table 2-3 informed consent by the presence or absence of proton-pump inhibitors

FREQ Procedure

			Cumulative	
Percent of ALL	power	Percent of	power	
1	20509	100.00	20509	100.00

Table : ALL * HTD 2 YN

ALL HTD2YN(Angina Pectoris)

Power |
Percent |
Percent of rows
Percent of columns | None | With | Total

	None	With	Total
1	17576	2933	20509
	85.70	14.30	100.00
	85.70	14.30	
	100.00	100.00	
Total	17576	2933	20509
	85.70	14.30	100.00

Complications and medical history at the time of obtaining Table 2-3 informed consent by the presence or absence of proton-pump inhibitors

FREQ Procedure

			Cumulative	
Percent of ALL	power	Percent	of power	
1	20509	100.00	20509	100.00

Table: ALL * t_HTD0

ALL t_HTD0

Power |
Percent |
Percent of rows
Percentage of columns | 0| 1| Sum

	0	1	Sum
1	17046	3463	20509
	83.11	16.89	100.00
	83.11	16.89	
	100.00	100.00	
Total	17046	3463	20509
	83.11	16.89	100.00

Complications and medical history at the time of obtaining Table 2-3 informed consent by the presence or absence of proton-pump inhibitors

FREQ Procedure

Cumulative

Percent of ALL power Percent of power

1	20509	100.00	20509	100.00

Table: ALL * t_HTD0

ALL t_HTD0

Power |
Percent |
Percent of rows
Percentage of columns | 0| 1| Sum

	0	1	Sum
1	17046	3463	20509
	83.11	16.89	100.00
	83.11	16.89	
	100.00	100.00	
Total	17046	3463	20509
	83.11	16.89	100.00

Complications and medical history at the time of obtaining Table 2-3 informed consent by the presence or absence of proton-pump inhibitors

FREQ Procedure

Cumulative
Percent of ALL power Percent of power

1	20509	100.00	20509	100.00

Table : ALL * HTD 3 YN

ALL HTD3YN(Heart Failure)

Power |
Percent |
Percent of rows
Percent of columns | None | With | Total

	None	With	Total
1	13267	7242	20509
	64.69	35.31	100.00
	64.69	35.31	
	100.00	100.00	
Total	13267	7242	20509
	64.69	35.31	100.00

Complications and medical history at the time of obtaining Table 2-3 informed consent by the presence or absence of proton-pump inhibitors

FREQ Procedure

Cumulative
Percent of ALL power Percent of power

1	20509	100.00	20509	100.00

Table : ALL * HTD 3 YN

ALL HTD3YN(Heart Failure)

Power |
Percent |
Percent of rows
Percent of columns | None | With | Total

	None	With	Total
1	13267	7242	20509
	64.69	35.31	100.00
	64.69	35.31	
	100.00	100.00	
Total	13267	7242	20509
	64.69	35.31	100.00

Complications and medical history at the time of obtaining Table 2-3 informed consent by the presence or absence of proton-pump inhibitors

FREQ Procedure

Cumulative
Percent of ALL power Percent of power

1	20509	100.00	20509	100.00

Table : ALL * HTD 4 YN

ALL
HTD4YN(Left Ventricular Systolic Dysfunction)

Power |
Percent |
Percent of rows
Percent of columns | None | With | Total

	None	With	Total
1	20166	343	20509
	98.33	1.67	100.00
	98.33	1.67	
	100.00	100.00	
Total	20166	343	20509
	98.33	1.67	100.00

Complications and medical history at the time of obtaining Table 2-3 informed consent by the presence or absence of proton-pump inhibitors

FREQ Procedure

Cumulative
Percent of ALL power Percent of power

1	20509	100.00	20509	100.00

Table : ALL * HTD 4 YN

ALL
HTD4YN(Left Ventricular Systolic Dysfunction)

Power |
Percent |
Percent of rows
Percent of columns | None | With | Total

	None	With	Total
1	20166	343	20509
	98.33	1.67	100.00
	98.33	1.67	
	100.00	100.00	
Total	20166	343	20509
	98.33	1.67	100.00

Complications and medical history at the time of obtaining Table 2-3 informed consent by the presence or absence of proton-pump inhibitors

FREQ Procedure

Cumulative
Percent of ALL power Percent of power

1	20509	100.00	20509	100.00

Table: ALL * t_HTD

ALL t_HTD

Power |
Percent |
Percent of rows
Percentage of columns | 0| 1| Sum

	0	1	Sum
1	13189	7320	20509
	64.31	35.69	100.00
	64.31	35.69	
	100.00	100.00	
Total	13189	7320	20509
	64.31	35.69	100.00

Complications and medical history at the time of obtaining Table 2-3 informed consent by the presence or absence of proton-pump inhibitors

FREQ Procedure

Cumulative
Percent of ALL power Percent of power

1	20509	100.00	20509	100.00

Table: ALL * t_HTD

ALL t_HTD

Power |
Percent |
Percent of rows
Percentage of columns | 0| 1| Sum

	0	1	Sum
1	13189	7320	20509
	64.31	35.69	100.00
	64.31	35.69	
	100.00	100.00	
Total	13189	7320	20509
	64.31	35.69	100.00

Complications and medical history at the time of obtaining Table 2-3 informed consent by the presence or absence of proton-pump inhibitors

FREQ Procedure

			Cumulative	
ALL	power	Percent	power	Percent of power
1	20509	100.00	20509	100.00

Table : ALL * HTD 5 YN

ALL HTD5YN(Valvular Disease)

Power / Percent / Percent of rows / Percent of columns	None	With	Total
1	18100	2409	20509
	88.25	11.75	100.00
	88.25	11.75	
	100.00	100.00	
Total	18100	2409	20509
	88.25	11.75	100.00

Complications and medical history at the time of obtaining Table 2-3 informed consent by the presence or absence of proton-pump inhibitors

FREQ Procedure

			Cumulative	
Percent of ALL	power	Percent of	power	
1	20509	100.00	20509	100.00

Table : ALL * HTD 5 YN

ALL HTD5YN(Valvular Disease)

Power |
Percent |
Percent of rows
Percent of columns | None | With | Total

	None	With	Total
1	18100	2409	20509
	88.25	11.75	100.00
	88.25	11.75	
	100.00	100.00	
Total	18100	2409	20509
	88.25	11.75	100.00

Complications and medical history at the time of obtaining Table 2-3 informed consent by the presence or absence of proton-pump inhibitors

FREQ Procedure

			Cumulative	
Percent of ALL	power	Percent of power		
1	20509	100.00	20509	100.00

Table : ALL * HTD 6 YN

ALL HTD6YN(Cardiomyopathy)

Power | Percent | Percent of rows Percent of columns	None	With	Total
1	19781	728	20509
	96.45	3.55	100.00
	96.45	3.55	
	100.00	100.00	
Total	19781	728	20509
	96.45	3.55	100.00

Complications and medical history at the time of obtaining Table 2-3 informed consent by the presence or absence of proton-pump inhibitors

FREQ Procedure

			Cumulative	
Percent of ALL	power	Percent of power		
1	20509	100.00	20509	100.00

Table : ALL * HTD 6 YN

ALL HTD6YN(Cardiomyopathy)

Power |
Percent |
Percent of rows
Percent of columns | None | With | Total

1	19781	728	20509
	96.45	3.55	100.00
	96.45	3.55	
	100.00	100.00	
Total	19781	728	20509
	96.45	3.55	100.00

Complications and medical history at the time of obtaining Table 2-3 informed consent by the presence or absence of proton-pump inhibitors

FREQ Procedure

			Cumulative	
Percent of ALL	power	Percent of	power	
1	20509	100.00	20509	100.00

Table : ALL * HTD 7 YN

ALL

HTD7YN(Hypertensive Heart Disease)

Power<br>Percent<br>Percent of rows<br>Percent of columns	None	With	Total
1	19823	686	20509
	96.66	3.34	100.00
	96.66	3.34	
	100.00	100.00	
Total	19823	686	20509
	96.66	3.34	100.00

Complications and medical history at the time of obtaining Table 2-3 informed consent by the presence or absence of proton-pump inhibitors

FREQ Procedure

			Cumulative	
Percent of ALL	power	Percent of power		
1	20509	100.00	20509	100.00

Table : ALL * HTD 7 YN

ALL

HTD7YN(Hypertensive Heart Disease)

Power |
Percent |
Percent of rows
Percent of columns | None | With | Total

	None	With	Total
1	19823	686	20509
	96.66	3.34	100.00
	96.66	3.34	
	100.00	100.00	
Total	19823	686	20509
	96.66	3.34	100.00

Complications and medical history at the time of obtaining Table 2-3 informed consent by the presence or absence of proton-pump inhibitors

FREQ Procedure

			Cumulative	
Percent of ALL	power	Percent	of power	
1	20509	100.00	20509	100.00

Table : ALL * HTD 7 YN

ALL

HTD7YN(Hypertensive Heart Disease)

Power |
Percent |
Percent of rows
Percent of columns | None | With | Total

	None	With	Total
1	19823	686	20509
	96.66	3.34	100.00
	96.66	3.34	
	100.00	100.00	
Total	19823	686	20509
	96.66	3.34	100.00

Complications and medical history at the time of obtaining Table 2-3 informed consent by the presence or absence of proton-pump inhibitors

FREQ Procedure

			Cumulative	
Percent of ALL	power	Percent of power		
1	20509	100.00	20509	100.00

Table : ALL * HTD 8 YN

ALL

HTD8YN(Cognenital Heart Disease)

Power |
Percent |
Percent of rows
Percent of columns | None | With | Total

	None	With	Total
1	20403	106	20509
	99.48	0.52	100.00
	99.48	0.52	
	100.00	100.00	
Total	20403	106	20509
	99.48	0.52	100.00

Complications and medical history at the time of obtaining Table 2-3 informed consent by the presence or absence of proton-pump inhibitors

FREQ Procedure

Cumulative
Percent of ALL power Percent of power

1	20509	100.00	20509	100.00

Table : ALL * HTD 8 YN

ALL
HTD8YN(Cognenital Heart Disease)

Power |
Percent |
Percent of rows
Percent of columns | None | With | Total

	None	With	Total
1	20403	106	20509
	99.48	0.52	100.00
	99.48	0.52	
	100.00	100.00	
Total	20403	106	20509
	99.48	0.52	100.00

Complications and medical history at the time of obtaining Table 2-3 informed consent by the presence or absence of proton-pump inhibitors

FREQ Procedure

			Cumulative	
Percent of ALL	power	Percent of power		
1	20509	100.00	20509	100.00

Table : ALL * HTD 9 YN

ALL

HTD9YN(Other Heart Disease)

Power |
Percent |
Percent of rows
Percent of columns | None | With | Total

	None	With	Total
1	18897	1612	20509
	92.14	7.86	100.00
	92.14	7.86	
	100.00	100.00	
Total	18897	1612	20509
	92.14	7.86	100.00

Complications and medical history at the time of obtaining Table 2-3 informed consent by the presence or absence of proton-pump inhibitors

FREQ Procedure

Cumulative
Percent of ALL power Percent of power

1	20509	100.00	20509	100.00

Table : ALL * HTD 9 YN

ALL
HTD9YN(Other Heart Disease)

Power |
Percent |
Percent of rows
Percent of columns | None | With | Total

1	18897	1612	20509
	92.14	7.86	100.00
	92.14	7.86	
	100.00	100.00	
Total	18897	1612	20509
	92.14	7.86	100.00

Complications and medical history at the time of obtaining Table 2-3 informed consent by the presence or absence of proton-pump inhibitors

FREQ Procedure

			Cumulative	
Percent of ALL	power	Percent of	power	
1	20509	100.00	20509	100.00

Tables: ALL* CVACYN

ALL

CVACYN(Cerebrovascular Accident)

Power |
Percent |
Percent of rows
Percent of columns | None | With | Total

	None	With	Total
1	16277	4232	20509
	79.37	20.63	100.00
	79.37	20.63	
	100.00	100.00	
Total	16277	4232	20509
	79.37	20.63	100.00

Complications and medical history at the time of obtaining Table 2-3 informed consent by the presence or absence of proton-pump inhibitors

FREQ Procedure

			Cumulative	
Percent of ALL	power	Percent of	power	
1	20509	100.00	20509	100.00

Tables: ALL* CVACYN

ALL

CVACYN(Cerebrovascular Accident)

Power | Percent | Percent of rows Percent of columns	None	With	Total
1	16277	4232	20509
	79.37	20.63	100.00
	79.37	20.63	
	100.00	100.00	
Total	16277	4232	20509
	79.37	20.63	100.00

Complications and medical history at the time of obtaining Table 2-3 informed consent by the presence or absence of proton-pump inhibitors

FREQ Procedure

Cumulative
Percent of ALL power Percent of power

1	20509	100.00	20509	100.00

Table : ALL * CVAC 1 YN

ALL
CVAC1YN(Atherosclerotic Cerebral Infarction)

Power |
Percent |
Percent of rows
Percent of columns | None | With | Total

1	20140	369	20509
	98.20	1.80	100.00
	98.20	1.80	
	100.00	100.00	
Total	20140	369	20509
	98.20	1.80	100.00

Complications and medical history at the time of obtaining Table 2-3 informed consent by the presence or absence of proton-pump inhibitors

FREQ Procedure

Cumulative
Percent of ALL power Percent of power

1	20509	100.00	20509	100.00

Table : ALL * CVAC 1 YN

ALL
CVAC1YN(Atherosclerotic Cerebral Infarction)

Power |
Percent |
Percent of rows
Percent of columns | None | With | Total

	None	With	Total
1	20140	369	20509
	98.20	1.80	100.00
	98.20	1.80	
	100.00	100.00	
Total	20140	369	20509
	98.20	1.80	100.00

Complications and medical history at the time of obtaining Table 2-3 informed consent by the presence or absence of proton-pump inhibitors

FREQ Procedure

			Cumulative	
Percent of ALL	power	Percent of power		
1	20509	100.00	20509	100.00

Table : ALL * CVAC 2 YN

ALL

CVAC2YN(Cardiogenic Cerebral Infarction)

Power |
Percent |
Percent of rows
Percent of columns | None | With | Total

	None	With	Total
1	19113	1396	20509
	93.19	6.81	100.00
	93.19	6.81	
	100.00	100.00	
Total	19113	1396	20509
	93.19	6.81	100.00

Complications and medical history at the time of obtaining Table 2-3 informed consent by the presence or absence of proton-pump inhibitors

FREQ Procedure

			Cumulative	
Percent of ALL	power	Percent of	power	
1	20509	100.00	20509	100.00

Table : ALL * CVAC 2 YN

ALL

CVAC2YN(Cardiogenic Cerebral Infarction)

Power |
Percent |
Percent of rows
Percent of columns | None | With | Total

	None	With	Total
1	19113	1396	20509
	93.19	6.81	100.00
	93.19	6.81	
	100.00	100.00	
Total	19113	1396	20509
	93.19	6.81	100.00

Complications and medical history at the time of obtaining Table 2-3 informed consent by the presence or absence of proton-pump inhibitors

FREQ Procedure

			Cumulative	
Percent of ALL	power	Percent of	power	
1	20509	100.00	20509	100.00

Table : ALL * CVAC 3 YN

ALL

CVAC3YN(Lacunar Cerebral Infarction)

Power<br>Percent<br>Percent of rows<br>Percent of columns	None	With	Total
1	19681<br>95.96<br>95.96<br>100.00	828<br>4.04<br>4.04<br>100.00	20509<br>100.00
Total	19681<br>95.96	828<br>4.04	20509<br>100.00

Complications and medical history at the time of obtaining Table 2-3 informed consent by the presence or absence of proton-pump inhibitors

FREQ Procedure

			Cumulative	
Percent of ALL	power	Percent of power		
1	20509	100.00	20509	100.00

Table : ALL * CVAC 3 YN

ALL

CVAC3YN(Lacunar Cerebral Infarction)

Power |
Percent |
Percent of rows
Percent of columns | None | With | Total

	None	With	Total
1	19681	828	20509
	95.96	4.04	100.00
	95.96	4.04	
	100.00	100.00	
Total	19681	828	20509
	95.96	4.04	100.00

Complications and medical history at the time of obtaining Table 2-3 informed consent by the presence or absence of proton-pump inhibitors

FREQ Procedure

			Cumulative	
Percent of ALL	power	Percent of power		
1	20509	100.00	20509	100.00

Table : ALL * CVAC 4 YN

ALL

CVAC4YN(Hemorrhagic Stroke)

Power<br>Percent<br>Percent of rows<br>Percent of columns	None	With	Total
1	20304	205	20509
	99.00	1.00	100.00
	99.00	1.00	
	100.00	100.00	
Total	20304	205	20509
	99.00	1.00	100.00

Complications and medical history at the time of obtaining Table 2-3 informed consent by the presence or absence of proton-pump inhibitors

FREQ Procedure

Cumulative
Percent of ALL power Percent of power

1	20509	100.00	20509	100.00

Table : ALL * CVAC 4 YN

ALL
CVAC4YN(Hemorrhagic Stroke)

Power |
Percent |
Percent of rows
Percent of columns | None | With | Total

	None	With	Total
1	20304	205	20509
	99.00	1.00	100.00
	99.00	1.00	
	100.00	100.00	
Total	20304	205	20509
	99.00	1.00	100.00

Complications and medical history at the time of obtaining Table 2-3 informed consent by the presence or absence of proton-pump inhibitors

FREQ Procedure

			Cumulative	
Percent of ALL	power	Percent of power		
1	20509	100.00	20509	100.00

Table : ALL * CVAC 5 YN

ALL

CVAC5YN(Unclassified/Unknown Cerebral Infarction)

Power<br>Percent<br>Percent of rows<br>Percent of columns	None	With	Total
1	19479<br>94.98<br>94.98<br>100.00	1030<br>5.02<br>5.02<br>100.00	20509<br>100.00
Total	19479<br>94.98	1030<br>5.02	20509<br>100.00

Complications and medical history at the time of obtaining Table 2-3 informed consent by the presence or absence of proton-pump inhibitors

FREQ Procedure

			Cumulative	
Percent of ALL	power	Percent of power		
1	20509	100.00	20509	100.00

Table : ALL * CVAC 5 YN

ALL

CVAC5YN(Unclassified/Unknown Cerebral Infarction)

Power<br>Percent<br>Percent of rows<br>Percent of columns	None	With	Total
1	19479<br>94.98<br>94.98<br>100.00	1030<br>5.02<br>5.02<br>100.00	20509<br>100.00
Total	19479<br>94.98	1030<br>5.02	20509<br>100.00

Complications and medical history at the time of obtaining Table 2-3 informed consent by the presence or absence of proton-pump inhibitors

FREQ Procedure

			Cumulative	
Percent of ALL	power	Percent of power		
1	20509	100.00	20509	100.00

Table : ALL * CVAC 6 YN

ALL

CVAC6YN(Transient Ischemic Attacks)

Power |
Percent |
Percent of rows
Percent of columns | None | With | Total

	None	With	Total
1	20218	291	20509
	98.58	1.42	100.00
	98.58	1.42	
	100.00	100.00	
Total	20218	291	20509
	98.58	1.42	100.00

Complications and medical history at the time of obtaining Table 2-3 informed consent by the presence or absence of proton-pump inhibitors

FREQ Procedure

			Cumulative	
Percent of ALL	power	Percent of	power	
1	20509	100.00	20509	100.00

Table : ALL * CVAC 6 YN

ALL

CVAC6YN(Transient Ischemic Attacks)

Power |
Percent |
Percent of rows
Percent of columns | None | With | Total

	None	With	Total
1	20218	291	20509
	98.58	1.42	100.00
	98.58	1.42	
	100.00	100.00	
Total	20218	291	20509
	98.58	1.42	100.00

Complications and medical history at the time of obtaining Table 2-3 informed consent by the presence or absence of proton-pump inhibitors

FREQ Procedure

Cumulative

Percent of ALL power Percent of power

1	20509	100.00	20509	100.00

Table : ALL * CVAC 7 YN

ALL

CVAC7YN(Other Cerebrovascular Accident)

Power |
Percent |
Percent of rows
Percent of columns | None | With | Total

	None	With	Total
1	20050	459	20509
	97.76	2.24	100.00
	97.76	2.24	
	100.00	100.00	
Total	20050	459	20509
	97.76	2.24	100.00

Complications and medical history at the time of obtaining Table 2-3 informed consent by the presence or absence of proton-pump inhibitors

FREQ Procedure

			Cumulative	
Percent of ALL	power	Percent of	power	
1	20509	100.00	20509	100.00

Table : ALL * CVAC 7 YN

ALL

CVAC7YN(Other Cerebrovascular Accident)

Power / Percent / Percent of rows / Percent of columns	None	With	Total
1	20050	459	20509
	97.76	2.24	100.00
	97.76	2.24	
	100.00	100.00	
Total	20050	459	20509
	97.76	2.24	100.00

Complications and medical history at the time of obtaining Table 2-3 informed consent by the presence or absence of proton-pump inhibitors

FREQ Procedure

			Cumulative	
Percent of ALL	power	Percent of	power	
1	20509	100.00	20509	100.00

Tables: ALL * OTVDYN

ALL

OTVDYN(Other Vascular Disease)

Power |
Percent |
Percent of rows
Percent of columns | None | With | Total

	None	With	Total
1	19586	923	20509
	95.50	4.50	100.00
	95.50	4.50	
	100.00	100.00	
Total	19586	923	20509
	95.50	4.50	100.00

Complications and medical history at the time of obtaining Table 2-3 informed consent by the presence or absence of proton-pump inhibitors

FREQ Procedure

Cumulative

Percent of ALL power Percent of power

1	20509	100.00	20509	100.00

Tables: ALL * OTVDYN

ALL

OTVDYN(Other Vascular Disease)

Power |
Percent |
Percent of rows
Percent of columns | None | With | Total

	None	With	Total
1	19586	923	20509
	95.50	4.50	100.00
	95.50	4.50	
	100.00	100.00	
Total	19586	923	20509
	95.50	4.50	100.00

Complications and medical history at the time of obtaining Table 2-3 informed consent by the presence or absence of proton-pump inhibitors

FREQ Procedure

			Cumulative	
Percent of ALL	power	Percent of power		
1	20509	100.00	20509	100.00

Table : ALL * OTVD 1 YN

ALL

OTVD1YN(Peripheral Vascular Disease)

Power<br>Percent<br>Percent of rows<br>Percent of columns	None	With	Total
1	20250<br>98.74<br>98.74<br>100.00	259<br>1.26<br>1.26<br>100.00	20509<br>100.00
Total	20250<br>98.74	259<br>1.26	20509<br>100.00

Complications and medical history at the time of obtaining Table 2-3 informed consent by the presence or absence of proton-pump inhibitors

FREQ Procedure

			Cumulative	
Percent of ALL	power	Percent of	power	
1	20509	100.00	20509	100.00

Table : ALL * OTVD 1 YN

ALL

OTVD1YN(Peripheral Vascular Disease)

Power |
Percent |
Percent of rows
Percent of columns | None | With | Total

	None	With	Total
1	20250	259	20509
	98.74	1.26	100.00
	98.74	1.26	
	100.00	100.00	
Total	20250	259	20509
	98.74	1.26	100.00

Complications and medical history at the time of obtaining Table 2-3 informed consent by the presence or absence of proton-pump inhibitors

FREQ Procedure

			Cumulative	
Percent of ALL	power	Percent of power		
1	20509	100.00	20509	100.00

Table : ALL * OTVD 2 YN

ALL OTVD2YN(Aortic Plaque)

Power |
Percent |
Percent of rows
Percent of columns | None | With | Total

	None	With	Total
1	20356	153	20509
	99.25	0.75	100.00
	99.25	0.75	
	100.00	100.00	
Total	20356	153	20509
	99.25	0.75	100.00

Complications and medical history at the time of obtaining Table 2-3 informed consent by the presence or absence of proton-pump inhibitors

FREQ Procedure

			Cumulative	
	Percent of ALL	power	Percent of power	
1	20509	100.00	20509	100.00

Table : ALL * OTVD 2 YN

ALL OTVD2YN(Aortic Plaque)

Power / Percent / Percent of rows / Percent of columns	None	With	Total
1	20356	153	20509
	99.25	0.75	100.00
	99.25	0.75	
	100.00	100.00	
Total	20356	153	20509
	99.25	0.75	100.00

Complications and medical history at the time of obtaining Table 2-3 informed consent by the presence or absence of proton-pump inhibitors

FREQ Procedure

Percent of ALL	power	Percent	Cumulative power	Cumulative Percent of power
1	20509	100.00	20509	100.00

Table : ALL * TED 3 YN

ALL

TED3YN(Internal Carotid Artery Stenosis)

Power / Percent / Percent of rows / Percent of columns	None	With	Total
1	20103	406	20509
	98.02	1.98	100.00
	98.02	1.98	
	100.00	100.00	
Total	20103	406	20509
	98.02	1.98	100.00

Complications and medical history at the time of obtaining Table 2-3 informed consent by the presence or absence of proton-pump inhibitors

FREQ Procedure

			Cumulative	
Percent of ALL	power	Percent	of power	
1	20509	100.00	20509	100.00

Table : ALL * TED 3 YN

ALL

TED3YN(Internal Carotid Artery Stenosis)

Power |
Percent |
Percent of rows
Percent of columns | None | With | Total

	None	With	Total
1	20103	406	20509
	98.02	1.98	100.00
	98.02	1.98	
	100.00	100.00	
Total	20103	406	20509
	98.02	1.98	100.00

Complications and medical history at the time of obtaining Table 2-3 informed consent by the presence or absence of proton-pump inhibitors

FREQ Procedure

Cumulative
Percent of ALL power Percent of power

1	20509	100.00	20509	100.00

Table : ALL * TED 4 YN

ALL
TED4YN(Arteriosclerosis Obliterans)

Power |
Percent |
Percent of rows
Percent of columns | None | With | Total

	None	With	Total
1	19900	609	20509
	97.03	2.97	100.00
	97.03	2.97	
	100.00	100.00	
Total	19900	609	20509
	97.03	2.97	100.00

Complications and medical history at the time of obtaining Table 2-3 informed consent by the presence or absence of proton-pump inhibitors

FREQ Procedure

			Cumulative	
Percent of ALL	power	Percent of power		
1	20509	100.00	20509	100.00

Table : ALL * TED 4 YN

ALL

TED4YN(Arteriosclerosis Obliterans)

Power |
Percent |
Percent of rows
Percent of columns | None | With | Total

	None	With	Total
1	19900	609	20509
	97.03	2.97	100.00
	97.03	2.97	
	100.00	100.00	
Total	19900	609	20509
	97.03	2.97	100.00

Complications and medical history at the time of obtaining Table 2-3 informed consent by the presence or absence of proton-pump inhibitors

FREQ Procedure

Cumulative
Percent of ALL power Percent of power

1	20509	100.00	20509	100.00

Table : ALL * OTVD 3 YN

ALL
OTVD3YN(Other Vascular Disease: Other)

Power |
Percent |
Percent of rows
Percent of columns | None | With | Total

	None	With	Total
1	19968	541	20509
	97.36	2.64	100.00
	97.36	2.64	
	100.00	100.00	
Total	19968	541	20509
	97.36	2.64	100.00

Complications and medical history at the time of obtaining Table 2-3 informed consent by the presence or absence of proton-pump inhibitors

FREQ Procedure

			Cumulative	
Percent of ALL	power	Percent of	power	
1	20509	100.00	20509	100.00

Table : ALL * OTVD 3 YN

ALL

OTVD3YN(Other Vascular Disease: Other)

```
Power |
Percent |
Percent of rows
Percent of columns | None | With | Total
______________+________+________+
             1 |  19968 |    541 |  20509
               |  97.36 |   2.64 | 100.00
               |  97.36 |   2.64 |
               | 100.00 | 100.00 |
______________+________+________+
Total 19968 541 20509
                 97.36     2.64   100.00
```

Complications and medical history at the time of obtaining Table 2-3 informed consent by the presence or absence of proton-pump inhibitors

FREQ Procedure

ALL	power	Percent	Cumulative power	Cumulative Percent of power
1	20509	100.00	20509	100.00

Tables: ALL * TEDYN

ALL

TEDYN(Thrombosis/Embolism-Related Disease)

Power / Percent / Percent of rows / Percent of columns	None	With	Total
1	18953	1556	20509
	92.41	7.59	100.00
	92.41	7.59	
	100.00	100.00	
Total	18953	1556	20509
	92.41	7.59	100.00

Complications and medical history at the time of obtaining Table 2-3 informed consent by the presence or absence of proton-pump inhibitors

FREQ Procedure

			Cumulative	
Percent of ALL	power	Percent of power		
1	20509	100.00	20509	100.00

Tables: ALL * TEDYN

ALL

TEDYN(Thrombosis/Embolism-Related Disease)

Power / Percent / Percent of rows / Percent of columns	None	With	Total
1	18953	1556	20509
	92.41	7.59	100.00
	92.41	7.59	
	100.00	100.00	
Total	18953	1556	20509
	92.41	7.59	100.00

Complications and medical history at the time of obtaining Table 2-3 informed consent by the presence or absence of proton-pump inhibitors

FREQ Procedure

Cumulative
Percent of ALL power Percent of power

1	20509	100.00	20509	100.00

Table: ALL * t_TED

ALL t_TED

Power |
Percent |
Percent of rows
Percentage of columns | 0| 1| Sum

	0	1	Sum
1	20284	225	20509
	98.90	1.10	100.00
	98.90	1.10	
	100.00	100.00	
Total	20284	225	20509
	98.90	1.10	100.00

Complications and medical history at the time of obtaining Table 2-3 informed consent by the presence or absence of proton-pump inhibitors

FREQ Procedure

Cumulative
Percent of ALL power Percent of power

1	20509	100.00	20509	100.00

Table: ALL * t_TED

ALL t_TED

Power |
Percent |
Percent of rows
Percentage of columns | 0| 1| Sum

	0	1	Sum
1	20284	225	20509
	98.90	1.10	100.00
	98.90	1.10	
	100.00	100.00	
Total	20284	225	20509
	98.90	1.10	100.00

Complications and medical history at the time of obtaining Table 2-3 informed consent by the presence or absence of proton-pump inhibitors

FREQ Procedure

			Cumulative	
Percent of ALL	power	Percent of power		
1	20509	100.00	20509	100.00

Table : ALL * TED 1 YN

ALL

TED1YN(Pulmonary Embolism)

Power |
Percent |
Percent of rows
Percent of columns | None | With | Total

	None	With	Total
1	20446	63	20509
	99.69	0.31	100.00
	99.69	0.31	
	100.00	100.00	
Total	20446	63	20509
	99.69	0.31	100.00

Complications and medical history at the time of obtaining Table 2-3 informed consent by the presence or absence of proton-pump inhibitors

FREQ Procedure

Cumulative
Percent of ALL power Percent of power

1	20509	100.00	20509	100.00

Table : ALL * TED 1 YN

ALL
TED1YN(Pulmonary Embolism)

Power |
Percent |
Percent of rows
Percent of columns | None | With | Total

	None	With	Total
1	20446	63	20509
	99.69	0.31	100.00
	99.69	0.31	
	100.00	100.00	
Total	20446	63	20509
	99.69	0.31	100.00

Complications and medical history at the time of obtaining Table 2-3 informed consent by the presence or absence of proton-pump inhibitors

FREQ Procedure

			Cumulative	
Percent of ALL	power	Percent of	power	
1	20509	100.00	20509	100.00

Table : ALL * TED 2 YN

ALL

TED2YN(Deep Vein Thrombosis)

Power |
Percent |
Percent of rows
Percent of columns | None | With | Total

	None	With	Total
1	20324	185	20509
	99.10	0.90	100.00
	99.10	0.90	
	100.00	100.00	
Total	20324	185	20509
	99.10	0.90	100.00

Complications and medical history at the time of obtaining Table 2-3 informed consent by the presence or absence of proton-pump inhibitors

FREQ Procedure

Cumulative
Percent of ALL power Percent of power

1	20509	100.00	20509	100.00

Table : ALL * TED 2 YN

ALL
TED2YN(Deep Vein Thrombosis)

Power |
Percent |
Percent of rows
Percent of columns | None | With | Total

	None	With	Total
1	20324	185	20509
	99.10	0.90	100.00
	99.10	0.90	
	100.00	100.00	
Total	20324	185	20509
	99.10	0.90	100.00

Complications and medical history at the time of obtaining Table 2-3 informed consent by the presence or absence of proton-pump inhibitors

FREQ Procedure

			Cumulative	
Percent of ALL	power	Percent of power		
1	20509	100.00	20509	100.00

Table : ALL * TED 5 YN

ALL

TED5YN(Other Thrombosis/Embolism-Related Disease)

Power |
Percent |
Percent of rows
Percent of columns | None | With | Total

	None	With	Total
1	20043	466	20509
	97.73	2.27	100.00
	97.73	2.27	
	100.00	100.00	
Total	20043	466	20509
	97.73	2.27	100.00

Complications and medical history at the time of obtaining Table 2-3 informed consent by the presence or absence of proton-pump inhibitors

FREQ Procedure

			Cumulative	
Percent of ALL	power	Percent of power		
1	20509	100.00	20509	100.00

Table : ALL * TED 5 YN

ALL

TED5YN(Other Thrombosis/Embolism-Related Disease)

Power |
Percent |
Percent of rows
Percent of columns | None | With | Total

	None	With	Total
1	20043	466	20509
	97.73	2.27	100.00
	97.73	2.27	
	100.00	100.00	
Total	20043	466	20509
	97.73	2.27	100.00

Complications and medical history at the time of obtaining Table 2-3 informed consent by the presence or absence of proton-pump inhibitors

FREQ Procedure

			Cumulative	
Percent of ALL	power	Percent of power		
1	20509	100.00	20509	100.00

Tables: ALL * HTDMYN

ALL HTDMYN(Hyperthyroidism)

Power |
Percent |
Percent of rows
Percent of columns | None | With | Total

	None	With	Total
1	20209	300	20509
	98.54	1.46	100.00
	98.54	1.46	
	100.00	100.00	
Total	20209	300	20509
	98.54	1.46	100.00

Complications and medical history at the time of obtaining Table 2-3 informed consent by the presence or absence of proton-pump inhibitors

FREQ Procedure

			Cumulative	
Percent of ALL	power	Percent of	power	
1	20509	100.00	20509	100.00

Tables: ALL * HTDMYN

ALL HTDMYN(Hyperthyroidism)

Power |
Percent |
Percent of rows
Percent of columns | None | With | Total

	None	With	Total
1	20209	300	20509
	98.54	1.46	100.00
	98.54	1.46	
	100.00	100.00	
Total	20209	300	20509
	98.54	1.46	100.00

Complications and medical history at the time of obtaining Table 2-3 informed consent by the presence or absence of proton-pump inhibitors

FREQ Procedure

Cumulative
Percent of ALL power Percent of power

1	20509	100.00	20509	100.00

Tables: ALL * GASDYN

ALL
GASDYN(Gastrointestinal Disease)

Power |
Percent |
Percent of rows
Percent of columns | None | With | Total

	None	With	Total
1	16425	4084	20509
	80.09	19.91	100.00
	80.09	19.91	
	100.00	100.00	
Total	16425	4084	20509
	80.09	19.91	100.00

Complications and medical history at the time of obtaining Table 2-3 informed consent by the presence or absence of proton-pump inhibitors

FREQ Procedure

			Cumulative	
Percent of ALL	power	Percent of power		
1	20509	100.00	20509	100.00

Tables: ALL * GASDYN

ALL

GASDYN(Gastrointestinal Disease)

```
Power |
Percent |
Percent of rows
Percent of columns | None | With | Total
______________+________+________+
             1 |   16425 |    4084 |   20509
               |   80.09 |   19.91 | 100.00
               |   80.09 |   19.91 |
               | 100.00 | 100.00 |
______________+________+________+
Total 16425 4084 20509
                80.09      19.91     100.00
```

Complications and medical history at the time of obtaining Table 2-3 informed consent by the presence or absence of proton-pump inhibitors

FREQ Procedure

			Cumulative	
Percent of ALL	power	Percent of	power	
1	20509	100.00	20509	100.00

Table : ALL * GASD 1 YN

ALL

GASD1YN(Upper Gastrointestinal Tract)

Power |
Percent |
Percent of rows
Percent of columns | None | With | Total

ALL	None	With	Total
1	20096	413	20509
	97.99	2.01	100.00
	97.99	2.01	
	100.00	100.00	
Total	20096	413	20509
	97.99	2.01	100.00

Complications and medical history at the time of obtaining Table 2-3 informed consent by the presence or absence of proton-pump inhibitors

FREQ Procedure

Cumulative
Percent of ALL power Percent of power

1	20509	100.00	20509	100.00

Table : ALL * GASD 1 YN

ALL

GASD1YN(Upper Gastrointestinal Tract)

Power |
Percent |
Percent of rows
Percent of columns | None | With | Total

	None	With	Total
1	20096	413	20509
	97.99	2.01	100.00
	97.99	2.01	
	100.00	100.00	
Total	20096	413	20509
	97.99	2.01	100.00

Complications and medical history at the time of obtaining Table 2-3 informed consent by the presence or absence of proton-pump inhibitors

FREQ Procedure

			Cumulative	
Percent of ALL	power	Percent of power		
1	20509	100.00	20509	100.00

Table : ALL * GASD 2 YN

ALL

GASD2YN(Lower Gastrointestinal Tract)

Power |
Percent |
Percent of rows
Percent of columns | None | With | Total

	None	With	Total
1	20084	425	20509
	97.93	2.07	100.00
	97.93	2.07	
	100.00	100.00	
Total	20084	425	20509
	97.93	2.07	100.00

Complications and medical history at the time of obtaining Table 2-3 informed consent by the presence or absence of proton-pump inhibitors

FREQ Procedure

			Cumulative	
Percent of ALL	power	Percent of	power	
1	20509	100.00	20509	100.00

Table : ALL * GASD 2 YN

ALL

GASD2YN(Lower Gastrointestinal Tract)

Power |
Percent |
Percent of rows
Percent of columns | None | With | Total

	None	With	Total
1	20084	425	20509
	97.93	2.07	100.00
	97.93	2.07	
	100.00	100.00	
Total	20084	425	20509
	97.93	2.07	100.00

Complications and medical history at the time of obtaining Table 2-3 informed consent by the presence or absence of proton-pump inhibitors

FREQ Procedure

			Cumulative	
Percent of ALL	power	Percent of	power	
1	20509	100.00	20509	100.00

Table : ALL * GASD 3 YN

ALL

GASD3YN(Reflux esophagitis)

Power | Percent | Percent of rows Percent of columns	None	With	Total
1	19231	1278	20509
	93.77	6.23	100.00
	93.77	6.23	
	100.00	100.00	
Total	19231	1278	20509
	93.77	6.23	100.00

Complications and medical history at the time of obtaining Table 2-3 informed consent by the presence or absence of proton-pump inhibitors

FREQ Procedure

			Cumulative	
Percent of ALL	power	Percent of	power	
1	20509	100.00	20509	100.00

Table : ALL * GASD 3 YN

ALL

GASD3YN(Reflux esophagitis)

Power |
Percent |
Percent of rows
Percent of columns | None | With | Total

	None	With	Total
1	19231	1278	20509
	93.77	6.23	100.00
	93.77	6.23	
	100.00	100.00	
Total	19231	1278	20509
	93.77	6.23	100.00

Complications and medical history at the time of obtaining Table 2-3 informed consent by the presence or absence of proton-pump inhibitors

FREQ Procedure

Cumulative
Percent of ALL power Percent of power

1	20509	100.00	20509	100.00

Table : ALL * GASD 4 YN

ALL
GASD4YN(Other Gastrointestinal Disease)

Power |
Percent |
Percent of rows
Percent of columns | None | With | Total

	None	With	Total
1	18026	2483	20509
	87.89	12.11	100.00
	87.89	12.11	
	100.00	100.00	
Total	18026	2483	20509
	87.89	12.11	100.00

Complications and medical history at the time of obtaining Table 2-3 informed consent by the presence or absence of proton-pump inhibitors

FREQ Procedure

			Cumulative	
Percent of ALL	power	Percent of power		
1	20509	100.00	20509	100.00

Table : ALL * GASD 4 YN

ALL

GASD4YN(Other Gastrointestinal Disease)

Power |
Percent |
Percent of rows
Percent of columns | None | With | Total

	None	With	Total
1	18026	2483	20509
	87.89	12.11	100.00
	87.89	12.11	
	100.00	100.00	
Total	18026	2483	20509
	87.89	12.11	100.00

Complications and medical history at the time of obtaining Table 2-3 informed consent by the presence or absence of proton-pump inhibitors

FREQ Procedure

			Cumulative	
Percent of ALL	power	Percent of power		
1	20509	100.00	20509	100.00

Tables: ALL* MGTYN

ALL MGTYN(Malignant Tumor)

Power / Percent / Percent of rows / Percent of columns	None	With	Total
1	18313	2196	20509
	89.29	10.71	100.00
	89.29	10.71	
	100.00	100.00	
Total	18313	2196	20509
	89.29	10.71	100.00

Complications and medical history at the time of obtaining Table 2-3 informed consent by the presence or absence of proton-pump inhibitors

FREQ Procedure

Cumulative
Percent of ALL power Percent of power

1	20509	100.00	20509	100.00

Tables: ALL* MGTYN

ALL MGTYN(Malignant Tumor)

Power |
Percent |
Percent of rows
Percent of columns | None | With | Total

```
______________+________+________+
          1 |   18313 |    2196 |   20509
            |   89.29 |   10.71 | 100.00
            |   89.29 |   10.71 |
            | 100.00 | 100.00 |
______________+________+________+
Total 18313 2196 20509
             89.29      10.71     100.00
```

Complications and medical history at the time of obtaining Table 2-3 informed consent by the presence or absence of proton-pump inhibitors

FREQ Procedure

		Cumulative		
Percent of ALL	power	Percent of power		
1	20509	100.00	20509	100.00

Table : ALL * MGT 1 YN

ALL MGT1YN(Gastric Cancer)

Power |
Percent |
Percent of rows
Percent of columns | None | With | Total

	None	With	Total
1	20067	442	20509
	97.84	2.16	100.00
	97.84	2.16	
	100.00	100.00	
Total	20067	442	20509
	97.84	2.16	100.00

Complications and medical history at the time of obtaining Table 2-3 informed consent by the presence or absence of proton-pump inhibitors

FREQ Procedure

			Cumulative	
Percent of ALL	power	Percent of	power	
1	20509	100.00	20509	100.00

Table : ALL * MGT 1 YN

ALL MGT1YN(Gastric Cancer)

Power |
Percent |
Percent of rows
Percent of columns | None | With | Total

	None	With	Total
1	20067	442	20509
	97.84	2.16	100.00
	97.84	2.16	
	100.00	100.00	
Total	20067	442	20509
	97.84	2.16	100.00

Complications and medical history at the time of obtaining Table 2-3 informed consent by the presence or absence of proton-pump inhibitors

FREQ Procedure

			Cumulative	
Percent of ALL	power	Percent of power		
1	20509	100.00	20509	100.00

Table : ALL * MGT 2 YN

ALL MGT2YN(Colorectal Cancer)

Power<br>Percent<br>Percent of rows<br>Percent of columns	None	With	Total
1	20052<br>97.77<br>97.77<br>100.00	457<br>2.23<br>2.23<br>100.00	20509<br>100.00
Total	20052<br>97.77	457<br>2.23	20509<br>100.00

Complications and medical history at the time of obtaining Table 2-3 informed consent by the presence or absence of proton-pump inhibitors

FREQ Procedure

			Cumulative	
Percent of ALL	power	Percent of power		
1	20509	100.00	20509	100.00

Table : ALL * MGT 2 YN

ALL MGT2YN(Colorectal Cancer)

Power |
Percent |
Percent of rows
Percent of columns | None | With | Total

	None	With	Total
1	20052	457	20509
	97.77	2.23	100.00
	97.77	2.23	
	100.00	100.00	
Total	20052	457	20509
	97.77	2.23	100.00

Complications and medical history at the time of obtaining Table 2-3 informed consent by the presence or absence of proton-pump inhibitors

FREQ Procedure

Cumulative
Percent of ALL power Percent of power

1	20509	100.00	20509	100.00

Table : ALL * MGT 3 YN

ALL MGT3YN(Lung Cancer)

Power |
Percent |
Percent of rows
Percent of columns | None | With | Total

	None	With	Total
1	20287	222	20509
	98.92	1.08	100.00
	98.92	1.08	
	100.00	100.00	
Total	20287	222	20509
	98.92	1.08	100.00

Complications and medical history at the time of obtaining Table 2-3 informed consent by the presence or absence of proton-pump inhibitors

FREQ Procedure

			Cumulative	
Percent of ALL	power	Percent of power		
1	20509	100.00	20509	100.00

Table : ALL * MGT 3 YN

ALL MGT3YN(Lung Cancer)

Power |
Percent |
Percent of rows
Percent of columns | None | With | Total

	None	With	Total
1	20287	222	20509
	98.92	1.08	100.00
	98.92	1.08	
	100.00	100.00	
Total	20287	222	20509
	98.92	1.08	100.00

Complications and medical history at the time of obtaining Table 2-3 informed consent by the presence or absence of proton-pump inhibitors

FREQ Procedure

Cumulative
Percent of ALL power Percent of power

1	20509	100.00	20509	100.00

Table : ALL * MGT 4 YN

ALL MGT4YN(Brest Cancer)

Power |
Percent |
Percent of rows
Percent of columns | None | With | Total

	None	With	Total
1	20359	150	20509
	99.27	0.73	100.00
	99.27	0.73	
	100.00	100.00	
Total	20359	150	20509
	99.27	0.73	100.00

Complications and medical history at the time of obtaining Table 2-3 informed consent by the presence or absence of proton-pump inhibitors

FREQ Procedure

			Cumulative	
Percent of ALL	power	Percent of power		
1	20509	100.00	20509	100.00

Table : ALL * MGT 4 YN

ALL MGT4YN(Brest Cancer)

Power / Percent / Percent of rows / Percent of columns	None	With	Total
1	20359	150	20509
	99.27	0.73	100.00
	99.27	0.73	
	100.00	100.00	
Total	20359	150	20509
	99.27	0.73	100.00

Complications and medical history at the time of obtaining Table 2-3 informed consent by the presence or absence of proton-pump inhibitors

FREQ Procedure

			Cumulative	
Percent of ALL power Percent of power				
1	20509	100.00	20509	100.00

Table : ALL * MGT 5 YN

ALL

MGT5YN(Uterus/Ovarian Cancer)

Power / Percent / Percent of rows / Percent of columns	None	With	Total
1	20455	54	20509
	99.74	0.26	100.00
	99.74	0.26	
	100.00	100.00	
Total	20455	54	20509
	99.74	0.26	100.00

Complications and medical history at the time of obtaining Table 2-3 informed consent by the presence or absence of proton-pump inhibitors

FREQ Procedure

			Cumulative	
Percent of ALL	power	Percent of power		
1	20509	100.00	20509	100.00

Table : ALL * MGT 5 YN

ALL

MGT5YN(Uterus/Ovarian Cancer)

Power |
Percent |
Percent of rows
Percent of columns | None | With | Total

	None	With	Total
1	20455	54	20509
	99.74	0.26	100.00
	99.74	0.26	
	100.00	100.00	
Total	20455	54	20509
	99.74	0.26	100.00

Complications and medical history at the time of obtaining Table 2-3 informed consent by the presence or absence of proton-pump inhibitors

FREQ Procedure

			Cumulative	
Percent of ALL	power	Percent of	power	
1	20509	100.00	20509	100.00

Table : ALL * MGT 6 YN

ALL MGT6YN(Pancreatic Cancer)

Power |
Percent |
Percent of rows
Percent of columns | None | With | Total

	None	With	Total
1	20488	21	20509
	99.90	0.10	100.00
	99.90	0.10	
	100.00	100.00	
Total	20488	21	20509
	99.90	0.10	100.00

Complications and medical history at the time of obtaining Table 2-3 informed consent by the presence or absence of proton-pump inhibitors

FREQ Procedure

			Cumulative	
Percent of ALL	power	Percent of power		
1	20509	100.00	20509	100.00

Table : ALL * MGT 6 YN

ALL MGT6YN(Pancreatic Cancer)

Power |
Percent |
Percent of rows
Percent of columns | None | With | Total

	None	With	Total
1	20488	21	20509
	99.90	0.10	100.00
	99.90	0.10	
	100.00	100.00	
Total	20488	21	20509
	99.90	0.10	100.00

Complications and medical history at the time of obtaining Table 2-3 informed consent by the presence or absence of proton-pump inhibitors

FREQ Procedure

			Cumulative	
Percent of ALL	power	Percent of power		
1	20509	100.00	20509	100.00

Table : ALL * MGT 7 YN

ALL

MGT7YN(Other Malignant Tumor)

Power |
Percent |
Percent of rows
Percent of columns | None | With | Total

	None	With	Total
1	19425	1084	20509
	94.71	5.29	100.00
	94.71	5.29	
	100.00	100.00	
Total	19425	1084	20509
	94.71	5.29	100.00

Complications and medical history at the time of obtaining Table 2-3 informed consent by the presence or absence of proton-pump inhibitors

FREQ Procedure

			Cumulative	
Percent of ALL	power	Percent of power		
1	20509	100.00	20509	100.00

Table : ALL * MGT 7 YN

ALL

MGT7YN(Other Malignant Tumor)

Power |
Percent |
Percent of rows
Percent of columns | None | With | Total

	None	With	Total
1	19425	1084	20509
	94.71	5.29	100.00
	94.71	5.29	
	100.00	100.00	
Total	19425	1084	20509
	94.71	5.29	100.00

Complications and medical history at the time of obtaining Table 2-3 informed consent by the presence or absence of proton-pump inhibitors

FREQ Procedure

Cumulative
Percent of ALL power Percent of power

1	20509	100.00	20509	100.00

Tables: ALL* ALZHYN

ALL ALZHYN(Alzheimer)

Power |
Percent |
Percent of rows
Percent of columns | None | With | Total

	None	With	Total
1	18942	1567	20509
	92.36	7.64	100.00
	92.36	7.64	
	100.00	100.00	
Total	18942	1567	20509
	92.36	7.64	100.00

Complications and medical history at the time of obtaining Table 2-3 informed consent by the presence or absence of proton-pump inhibitors

FREQ Procedure

Cumulative
Percent of ALL power Percent of power

1	20509	100.00	20509	100.00

Tables: ALL* ALZHYN

ALL ALZHYN(Alzheimer)

Power |
Percent |
Percent of rows
Percent of columns | None | With | Total

	None	With	Total
1	18942	1567	20509
	92.36	7.64	100.00
	92.36	7.64	
	100.00	100.00	
Total	18942	1567	20509
	92.36	7.64	100.00

Complications and medical history at the time of obtaining Table 2-3 informed consent by the presence or absence of proton-pump inhibitors

FREQ Procedure

Cumulative
Percent of ALL power Percent of power

1	20509	100.00	20509	100.00

Tables: ALL * OHCPYN

ALL
OHCPYN(Other Complications)

Power |
Percent |
Percent of rows
Percent of columns | None | With | Total

	None	With	Total
1	14133	6376	20509
	68.91	31.09	100.00
	68.91	31.09	
	100.00	100.00	
Total	14133	6376	20509
	68.91	31.09	100.00

Complications and medical history at the time of obtaining Table 2-3 informed consent by the presence or absence of proton-pump inhibitors

FREQ Procedure

			Cumulative	
Percent of ALL	power	Percent of power		
1	20509	100.00	20509	100.00

Tables: ALL * OHCPYN

ALL

OHCPYN(Other Complications)

Power |
Percent |
Percent of rows
Percent of columns | None | With | Total

	None	With	Total
1	14133	6376	20509
	68.91	31.09	100.00
	68.91	31.09	
	100.00	100.00	
Total	14133	6376	20509
	68.91	31.09	100.00

Complications and medical history at the time of obtaining Table 2-3 informed consent by the presence or absence of proton-pump inhibitors

FREQ Procedure

Cumulative

Percent of ALL power Percent of power

1	20509	100.00	20509	100.00

Tables: ALL * IMGYN

ALL IMGYN(Brain Imaging Test)

Power |

Percent |

Percent of rows

Percentage of columns |None| | |Unknown| | Total

1	18434	1620	455	20509
	89.88	7.90	2.22	100.00
	89.88	7.90	2.22	
	100.00	100.00	100.00	
Total	18434	1620	455	20509
	89.88	7.90	2.22	100.00

Complications and medical history at the time of obtaining Table 2-3 informed consent by the presence or absence of proton-pump inhibitors

FREQ Procedure

Cumulative
Percent of ALL power Percent of power

1	20509	100.00	20509	100.00

Tables: ALL * IMGYN

ALL IMGYN(Brain Imaging Test)

Power |
Percent |
Percent of rows
Percentage of columns |None| | |Unknown| | Total

1	18434	1620	455	20509
	89.88	7.90	2.22	100.00
	89.88	7.90	2.22	
	100.00	100.00	100.00	
Total	18434	1620	455	20509
	89.88	7.90	2.22	100.00

Complications and medical history at the time of obtaining Table 2-3 informed consent by the presence or absence of proton-pump inhibitors

FREQ Procedure

			Cumulative	
Percent of ALL	power	Percent of power		
1	20509	100.00	20509	100.00

Tables: ALL * IMGYN

ALL IMGYN(Brain Imaging Test)

Power |
Percent |
Percent of rows
Percentage of columns |None| | |Unknown| | Total

1	18434	1620	455	20509
	89.88	7.90	2.22	100.00
	89.88	7.90	2.22	
	100.00	100.00	100.00	
Total	18434	1620	455	20509
	89.88	7.90	2.22	100.00

Complications and medical history at the time of obtaining Table 2-3 informed consent by the presence or absence of proton-pump inhibitors

FREQ Procedure

			Cumulative	
Percent of ALL	power	Percent of power		
1	20509	100.00	20509	100.00

Tables: ALL* FALYN

ALL FALYN(Fall Down within one year)

Power |
Percent |
Percent of rows
Percentage of columns |None| | |Unknown| | Total

1	16887	1370	2252	20509
	82.34	6.68	10.98	100.00
	82.34	6.68	10.98	
	100.00	100.00	100.00	
Total	16887	1370	2252	20509
	82.34	6.68	10.98	100.00

Complications and medical history at the time of obtaining Table 2-3 informed consent by the presence or absence of proton-pump inhibitors

FREQ Procedure

Cumulative
Percent of ALL power Percent of power

1	20509	100.00	20509	100.00

Tables: ALL* FALYN

ALL FALYN(Fall Down within one year)

Power |
Percent |
Percent of rows
Percentage of columns |None| | |Unknown| | Total

1	16887	1370	2252	20509
	82.34	6.68	10.98	100.00
	82.34	6.68	10.98	
	100.00	100.00	100.00	
Total	16887	1370	2252	20509
	82.34	6.68	10.98	100.00

Complications and medical history at the time of obtaining Table 2-3 informed consent by the presence or absence of proton-pump inhibitors

FREQ Procedure

Cumulative

Percent of ALL power Percent of power

1	20509	100.00	20509	100.00

Tables: ALL* FALYN

ALL FALYN(Fall Down within one year)

Power |

Percent |

Percent of rows

Percentage of columns |None| | |Unknown| | Total

1	16887	1370	2252	20509
	82.34	6.68	10.98	100.00
	82.34	6.68	10.98	
	100.00	100.00	100.00	
Total	16887	1370	2252	20509
	82.34	6.68	10.98	100.00

Complications and medical history at the time of obtaining Table 2-3 informed consent by the presence or absence of proton-pump inhibitors

FREQ Procedure

Tables: ALL * FNDYN

ALL FNDYN(Brain Imaging Result)

Power |
Percent |
Percent of rows
Percentage of columns |None| | |Unknown| | Total

	None		Unknown	Total
1	767	788	65	1620
	47.35	48.64	4.01	100.00
	47.35	48.64	4.01	
	100.00	100.00	100.00	
Total	767	788	65	1620
	47.35	48.64	4.01	100.00

Degree of missing values = 18889

Complications and medical history at the time of obtaining Table 2-3 informed consent by the presence or absence of proton-pump inhibitors

FREQ Procedure

Tables: ALL * FNDYN

ALL FNDYN(Brain Imaging Result)

Power |
Percent |
Percent of rows
Percentage of columns |None| | |Unknown| | Total

1	767	788	65	1620
	47.35	48.64	4.01	100.00
	47.35	48.64	4.01	
	100.00	100.00	100.00	
Total	767	788	65	1620
	47.35	48.64	4.01	100.00

Degree of missing values = 18889

Complications and medical history at the time of obtaining Table 2-3 informed consent by the presence or absence of proton-pump inhibitors

FREQ Procedure

Tables: ALL * FNDYN

ALL FNDYN(Brain Imaging Result)

Power |
Percent |
Percent of rows
Percentage of columns |None| | |Unknown| | Total

1	767	788	65	1620
	47.35	48.64	4.01	100.00
	47.35	48.64	4.01	
	100.00	100.00	100.00	
Total	767	788	65	1620
	47.35	48.64	4.01	100.00

Degree of missing values = 18889

Complications and medical history at the time of obtaining Table 2-3 informed consent by the presence or absence of proton-pump inhibitors

FREQ Procedure

Number of levels of variables

Variable Label Level Count

GROUP1 2
MHYN Medical History 2

Table : GROUP1 * MHYN

GROUP1 MHYN(Medical History)

Power / Percent / Percent of rows / Percent of columns	None	With	Total
1	179	11802	11981
	0.55	36.33	36.88
	1.49	98.51	
	20.25	37.34	
2	705	19804	20509
	2.17	60.95	63.12
	3.44	96.56	
	79.75	62.66	
Total	884	31606	32490
	2.72	97.28	100.00

Complications and medical history at the time of obtaining Table 2-3 informed consent by the presence or absence of proton-pump inhibitors

FREQ Procedure

Number of levels of variables

Variables Label Level Number

GROUP1 2
HBPYN High Blood Pressure 2

Table : GROUP1 * HBPYN

GROUP1
HBPYN(High Blood Pressure)

Power |
Percent |
Percent of rows
Percent of columns | None | With | Total

Percent of columns	None	With	Total
1	2641	9340	11981
	8.13	28.75	36.88
	22.04	77.96	
	32.95	38.16	
2	5374	15135	20509
	16.54	46.58	63.12
	26.20	73.80	
	67.05	61.84	
Total	8015	24475	32490
	24.67	75.33	100.00

Complications and medical history at the time of obtaining Table 2-3 informed consent by the presence or absence of proton-pump inhibitors

FREQ Procedure

Number of levels of variables

Variables	Label	Level Number
GROUP1		2
DBYN	Diabetes	2

Table : GROUP1 * DBYN

GROUP1 DBYN(Diabetes)

Power<br>Percent<br>Percent of rows<br>Percent of columns	None	With	Total
1	8497<br>26.15<br>70.92<br>35.79	3484<br>10.72<br>29.08<br>39.82	11981<br>36.88
2	15243<br>46.92<br>74.32<br>64.21	5266<br>16.21<br>25.68<br>60.18	20509<br>63.12
Total	23740<br>73.07	8750<br>26.93	32490<br>100.00

Complications and medical history at the time of obtaining Table 2-3 informed consent by the presence or absence of proton-pump inhibitors

FREQ Procedure

Number of levels of variables

Number of Variables Level

GROUP1	2
t_DB	2

Table : GROUP1 * t_DB

GROUP1 t_DB

Power |
Percent |
Percent of rows
Percentage of columns | 0| 1| Sum

GROUP1	0	1	Sum
1	11220	761	11981
	34.53	2.34	36.88
	93.65	6.35	
	36.39	45.84	
2	19610	899	20509
	60.36	2.77	63.12
	95.62	4.38	
	63.61	54.16	
Total	30830	1660	32490
	94.89	5.11	100.00

Complications and medical history at the time of obtaining Table 2-3 informed consent by the presence or absence of proton-pump inhibitors

FREQ Procedure

Number of levels of variables

Variables	Label	Level Number
GROUP1		2
DB1YN	Diabetic Retinopathy	2

Table : GROUP 1 * DB 1 YN

GROUP1

DB1YN(Diabetic Retinopathy)

Power / Percent / Percent of rows / Percent of columns	None	With	Total
1	11753	228	11981
	36.17	0.70	36.88
	98.10	1.90	
	36.75	45.06	
2	20231	278	20509
	62.27	0.86	63.12
	98.64	1.36	
	63.25	54.94	
Total	31984	506	32490
	98.44	1.56	100.00

Complications and medical history at the time of obtaining Table 2-3 informed consent by the presence or absence of proton-pump inhibitors

FREQ Procedure

Number of levels of variables

Variables Label Level Number

GROUP1 2
DB2YN Diabetic Nephropathy 2

Table : GROUP 1 * DB 2 YN

GROUP1
DB2YN(Diabetic Nephropathy)

Power / Percent / Percent of rows / Percent of columns	None	With	Total
1	11403	578	11981
	35.10	1.78	36.88
	95.18	4.82	
	36.45	48.05	
2	19884	625	20509
	61.20	1.92	63.12
	96.95	3.05	
	63.55	51.95	
Total	31287	1203	32490
	96.30	3.70	100.00

Complications and medical history at the time of obtaining Table 2-3 informed consent by the presence or absence of proton-pump inhibitors

FREQ Procedure

Number of levels of variables

Variables Label Level Number

GROUP1 2
DB3YN Diabetic Neuropathy 2

Table : GROUP 1 * DB 3 YN

GROUP1
DB3YN(Diabetic Neuropathy)

Power | Percent | Percent of rows Percent of columns	None	With	Total
1	11839	142	11981
	36.44	0.44	36.88
	98.81	1.19	
	36.81	43.83	
2	20327	182	20509
	62.56	0.56	63.12
	99.11	0.89	
	63.19	56.17	
Total	32166	324	32490
	99.00	1.00	100.00

Complications and medical history at the time of obtaining Table 2-3 informed consent by the presence or absence of proton-pump inhibitors

FREQ Procedure

Number of levels of variables

Variables Label Level Number

GROUP1 2
LMDYN Lipid metabolism disorders 2

Table : GROUP1 * LMDYN

GROUP1
LMDYN(Lipid metabolism disorders)

Power / Percent / Percent of rows / Percent of columns	None	With	Total
1	6154	5827	11981
	18.94	17.93	36.88
	51.36	48.64	
	32.95	42.18	
2	12521	7988	20509
	38.54	24.59	63.12
	61.05	38.95	
	67.05	57.82	
Total	18675	13815	32490
	57.48	42.52	100.00

Complications and medical history at the time of obtaining Table 2-3 informed consent by the presence or absence of proton-pump inhibitors

FREQ Procedure

Number of levels of variables

Variable	Label	Level Count
GROUP1		2
HYPURNYN	Hyperuricemia	2

Table : GROUP1 * HYPURNYN

GROUP1 HYPURNYN(Hyperuricemia)

Power / Percent / Percent of rows / Percent of columns	None	With	Total
1	8972	3009	11981
	27.61	9.26	36.88
	74.89	25.11	
	35.73	40.78	
2	16140	4369	20509
	49.68	13.45	63.12
	78.70	21.30	
	64.27	59.22	
Total	25112	7378	32490
	77.29	22.71	100.00

Complications and medical history at the time of obtaining Table 2-3 informed consent by the presence or absence of proton-pump inhibitors

FREQ Procedure

Number of levels of variables

Variables	Label	Level Number
GROUP1		2
KDDSYN	Kidney Disease	2

Table : GROUP1 * KDDSYN

GROUP1 KDDSYN(Kidney Disease)

Power / Percent / Percent of rows / Percent of columns	None	With	Total
1	8596	3385	11981
	26.46	10.42	36.88
	71.75	28.25	
	34.55	44.50	
2	16287	4222	20509
	50.13	12.99	63.12
	79.41	20.59	
	65.45	55.50	
Total	24883	7607	32490
	76.59	23.41	100.00

Complications and medical history at the time of obtaining Table 2-3 informed consent by the presence or absence of proton-pump inhibitors

FREQ Procedure

Number of levels of variables

Variables Label Level Number

GROUP1 2
KDDS1YN Chronic Kidney Disease 2

Table : GROUP 1 * KDDS 1 YN

GROUP1
KDDS1YN(Chronic Kidney Disease)

Power |
Percent |
Percent of rows
Percent of columns | None | With | Total

	None	With	Total
1	9001	2980	11981
	27.70	9.17	36.88
	75.13	24.87	
	34.98	44.10	
2	16731	3778	20509
	51.50	11.63	63.12
	81.58	18.42	
	65.02	55.90	
Total	25732	6758	32490
	79.20	20.80	100.00

Complications and medical history at the time of obtaining Table 2-3 informed consent by the presence or absence of proton-pump inhibitors

FREQ Procedure

Number of levels of variables

Variables	Label	Level Number
GROUP1		2
KDDS2YN	Advanced Renal Dysfunction without Dyalysis	2

Table : GROUP 1 * KDDS 2 YN

GROUP1

KDDS2YN(Advanced Renal Dysfunction without Dyalysis)

Power / Percent / Percent of rows / Percent of columns	None	With	Total
1	11747	234	11981
	36.16	0.72	36.88
	98.05	1.95	
	36.69	49.58	
2	20271	238	20509
	62.39	0.73	63.12
	98.84	1.16	
	63.31	50.42	
Total	32018	472	32490
	98.55	1.45	100.00

Complications and medical history at the time of obtaining Table 2-3 informed consent by the presence or absence of proton-pump inhibitors

FREQ Procedure

Number of levels of variables

Variables	Label	Level Number
GROUP1		2
KDDS3YN	Advanced Renal Dysfunction with Dyalysis	2

Table : GROUP 1 * KDDS 3 YN

GROUP1

KDDS3YN(Advanced Renal Dysfunction with Dyalysis)

Power<br>Percent<br>Percent of rows<br>Percent of columns	None	With	Total
1	11919<br>36.69<br>99.48<br>36.82	62<br>0.19<br>0.52<br>52.54	11981<br>36.88
2	20453<br>62.95<br>99.73<br>63.18	56<br>0.17<br>0.27<br>47.46	20509<br>63.12
Total	32372<br>99.64	118<br>0.36	32490<br>100.00

Complications and medical history at the time of obtaining Table 2-3 informed consent by the presence or absence of proton-pump inhibitors

FREQ Procedure

Number of levels of variables

Variables Label Level Number

GROUP1 2
SLVDYN Severe Liver Dysfunction 2

Table : GROUP1 * SLVDYN

GROUP1
SLVDYN(Severe Liver Dysfunction)

Power / Percent / Percent of rows / Percent of columns	None	With	Total
1	11852	129	11981
	36.48	0.40	36.88
	98.92	1.08	
	36.82	43.14	
2	20339	170	20509
	62.60	0.52	63.12
	99.17	0.83	
	63.18	56.86	
Total	32191	299	32490
	99.08	0.92	100.00

Complications and medical history at the time of obtaining Table 2-3 informed consent by the presence or absence of proton-pump inhibitors

FREQ Procedure

Number of levels of variables

Variables Label Level Number

GROUP1 2
RESDYN Respiratory Disease 2

Table : GROUP1 * RESDYN

GROUP1
RESDYN(Respiratory Disease)

Power |
Percent |
Percent of rows
Percent of columns | None | With | Total

1	10183	1798	11981
	31.34	5.53	36.88
	84.99	15.01	
	35.95	43.18	
2	18143	2366	20509
	55.84	7.28	63.12
	88.46	11.54	
	64.05	56.82	
Total	28326	4164	32490
	87.18	12.82	100.00

Complications and medical history at the time of obtaining Table 2-3 informed consent by the presence or absence of proton-pump inhibitors

FREQ Procedure

Number of levels of variables

Variable	Label	Level Count
GROUP1		2
HTDYN	Heart Disease	2

Table : GROUP1 * HTDYN

GROUP1 HTDYN(Heart Disease)

Power / Percent / Percent of rows / Percent of columns	None	With	Total
1	4172	7809	11981
	12.84	24.04	36.88
	34.82	65.18	
	31.18	40.86	
2	9207	11302	20509
	28.34	34.79	63.12
	44.89	55.11	
	68.82	59.14	
Total	13379	19111	32490
	41.18	58.82	100.00

Complications and medical history at the time of obtaining Table 2-3 informed consent by the presence or absence of proton-pump inhibitors

FREQ Procedure

Number of levels of variables

Variables Label Level Number

GROUP1 2
HTD1YN Myocardial Infarction 2

Table : GROUP 1 * HTD 1 YN

GROUP1

HTD1YN(Myocardial Infarction)

Power |
Percent |
Percent of rows
Percent of columns | None | With | Total

	None	With	Total
1	10924	1057	11981
	33.62	3.25	36.88
	91.18	8.82	
	35.67	56.77	
2	19704	805	20509
	60.65	2.48	63.12
	96.07	3.93	
	64.33	43.23	
Total	30628	1862	32490
	94.27	5.73	100.00

Complications and medical history at the time of obtaining Table 2-3 informed consent by the presence or absence of proton-pump inhibitors

FREQ Procedure

Number of levels of variables

Variable Label Level Count

GROUP1 2
HTD2YN Angina Pectoris 2

Table : GROUP 1 * HTD 2 YN

GROUP1 HTD2YN(Angina Pectoris)

Power / Percent / Percent of rows / Percent of columns	None	With	Total
1	9362<br>28.82<br>78.14<br>34.75	2619<br>8.06<br>21.86<br>47.17	11981<br>36.88
2	17576<br>54.10<br>85.70<br>65.25	2933<br>9.03<br>14.30<br>52.83	20509<br>63.12
Total	26938<br>82.91	5552<br>17.09	32490<br>100.00

Complications and medical history at the time of obtaining Table 2-3 informed consent by the presence or absence of proton-pump inhibitors

FREQ Procedure

Number of levels of variables

Number of Variables Level

GROUP1	2
t_HTD0	2

Table : GROUP1 * t_HTD0

GROUP1 t_HTD0

Power |
Percent |
Percent of rows
Percentage of columns | 0| 1| Sum

	0	1	Sum
1	8693	3288	11981
	26.76	10.12	36.88
	72.56	27.44	
	33.77	48.70	
2	17046	3463	20509
	52.47	10.66	63.12
	83.11	16.89	
	66.23	51.30	
Total	25739	6751	32490
	79.22	20.78	100.00

Complications and medical history at the time of obtaining Table 2-3 informed consent by the presence or absence of proton-pump inhibitors

FREQ Procedure

Number of levels of variables

Variable	Label	Level Count
GROUP1		2
HTD3YN	Heart Failure	2

Table : GROUP 1 * HTD 3 YN

GROUP1 HTD3YN(Heart Failure)

Power / Percent / Percent of rows / Percent of columns	None	With	Total
1	7035	4946	11981
	21.65	15.22	36.88
	58.72	41.28	
	34.65	40.58	
2	13267	7242	20509
	40.83	22.29	63.12
	64.69	35.31	
	65.35	59.42	
Total	20302	12188	32490
	62.49	37.51	100.00

Complications and medical history at the time of obtaining Table 2-3 informed consent by the presence or absence of proton-pump inhibitors

FREQ Procedure

Number of levels of variables

Variables Label Level Number

GROUP1 2
HTD4YN Left Ventricular Systolic Dysfunction 2

Table : GROUP 1 * HTD 4 YN

GROUP1
HTD4YN(Left Ventricular Systolic Dysfunction)

Power |
Percent |
Percent of rows
Percent of columns | None | With | Total

	None	With	Total
1	11635	346	11981
	35.81	1.06	36.88
	97.11	2.89	
	36.59	50.22	
2	20166	343	20509
	62.07	1.06	63.12
	98.33	1.67	
	63.41	49.78	
Total	31801	689	32490
	97.88	2.12	100.00

Complications and medical history at the time of obtaining Table 2-3 informed consent by the presence or absence of proton-pump inhibitors

FREQ Procedure

Number of levels of variables

Number of Variables Level

Variable	Level
GROUP1	2
t_HTD	2

Table : GROUP1 * t_HTD

GROUP1 t_HTD

Power |
Percent |
Percent of rows
Percentage of columns | 0| 1| Sum

GROUP1	0	1	Sum
1	6951	5030	11981
	21.39	15.48	36.88
	58.02	41.98	
	34.51	40.73	
2	13189	7320	20509
	40.59	22.53	63.12
	64.31	35.69	
	65.49	59.27	
Total	20140	12350	32490
	61.99	38.01	100.00

Complications and medical history at the time of obtaining Table 2-3 informed consent by the presence or absence of proton-pump inhibitors

FREQ Procedure

Number of levels of variables

Variables Label Level Number

GROUP1 2
HTD5YN Valvular Disease 2

Table : GROUP 1 * HTD 5 YN

GROUP1 HTD5YN(Valvular Disease)

Power |
Percent |
Percent of rows
Percent of columns | None | With | Total

1	10415	1566	11981
	32.06	4.82	36.88
	86.93	13.07	
	36.52	39.40	
2	18100	2409	20509
	55.71	7.41	63.12
	88.25	11.75	
	63.48	60.60	
Total	28515	3975	32490
	87.77	12.23	100.00

Complications and medical history at the time of obtaining Table 2-3 informed consent by the presence or absence of proton-pump inhibitors

FREQ Procedure

Number of levels of variables

Variables	Label	Level Number
GROUP1		2
HTD6YN	Cardiomyopathy	2

Table : GROUP 1 * HTD 6 YN

GROUP1 HTD6YN(Cardiomyopathy)

Power / Percent / Percent of rows / Percent of columns	None	With	Total
1	11510	471	11981
	35.43	1.45	36.88
	96.07	3.93	
	36.78	39.28	
2	19781	728	20509
	60.88	2.24	63.12
	96.45	3.55	
	63.22	60.72	
Total	31291	1199	32490
	96.31	3.69	100.00

Complications and medical history at the time of obtaining Table 2-3 informed consent by the presence or absence of proton-pump inhibitors

FREQ Procedure

Number of levels of variables

Variables Label Level Number

GROUP1 2
HTD7YN Hypertensive Heart Disease 2

Table : GROUP 1 * HTD 7 YN

GROUP1
HTD7YN(Hypertensive Heart Disease)

Power | Percent | Percent of rows Percent of columns	None	With	Total
1	11604	377	11981
	35.72	1.16	36.88
	96.85	3.15	
	36.92	35.47	
2	19823	686	20509
	61.01	2.11	63.12
	96.66	3.34	
	63.08	64.53	
Total	31427	1063	32490
	96.73	3.27	100.00

Complications and medical history at the time of obtaining Table 2-3 informed consent by the presence or absence of proton-pump inhibitors

FREQ Procedure

Number of levels of variables

Variables	Label	Level Number
GROUP1		2
HTD8YN	Cognenital Heart Disease	2

Table : GROUP 1 * HTD 8 YN

GROUP1

HTD8YN(Cognenital Heart Disease)

Power / Percent / Percent of rows / Percent of columns	None	With	Total
1	11902	79	11981
	36.63	0.24	36.88
	99.34	0.66	
	36.84	42.70	
2	20403	106	20509
	62.80	0.33	63.12
	99.48	0.52	
	63.16	57.30	
Total	32305	185	32490
	99.43	0.57	100.00

Complications and medical history at the time of obtaining Table 2-3 informed consent by the presence or absence of proton-pump inhibitors

FREQ Procedure

Number of levels of variables

Variables Label Level Number

GROUP1 2
HTD9YN Other Heart Disease 2

Table : GROUP 1 * HTD 9 YN

GROUP1
HTD9YN(Other Heart Disease)

Power |
Percent |
Percent of rows
Percent of columns | None | With | Total

	None	With	Total
1	10900	1081	11981
	33.55	3.33	36.88
	90.98	9.02	
	36.58	40.14	
2	18897	1612	20509
	58.16	4.96	63.12
	92.14	7.86	
	63.42	59.86	
Total	29797	2693	32490
	91.71	8.29	100.00

Complications and medical history at the time of obtaining Table 2-3 informed consent by the presence or absence of proton-pump inhibitors

FREQ Procedure

Number of levels of variables

Variables	Label	Level Number
GROUP1		2
CVACYN	Cerebrovascular Accident	2

Table : GROUP1 * CVACYN

GROUP1

CVACYN(Cerebrovascular Accident)

Power<br>Percent<br>Percent of rows<br>Percent of columns	None	With	Total
1	8856<br>27.26<br>73.92<br>35.24	3125<br>9.62<br>26.08<br>42.48	11981<br>36.88
2	16277<br>50.10<br>79.37<br>64.76	4232<br>13.03<br>20.63<br>57.52	20509<br>63.12
Total	25133<br>77.36	7357<br>22.64	32490<br>100.00

Complications and medical history at the time of obtaining Table 2-3 informed consent by the presence or absence of proton-pump inhibitors

FREQ Procedure

Number of levels of variables

Variables Label Level Number

GROUP1 2
CVAC1YN Atherosclerotic Cerebral Infarction 2

Table : GROUP 1 * CVAC 1 YN

GROUP1

CVAC1YN(Atherosclerotic Cerebral Infarction)

Power | Percent | Percent of rows Percent of columns	None	With	Total
1	11679	302	11981
	35.95	0.93	36.88
	97.48	2.52	
	36.70	45.01	
2	20140	369	20509
	61.99	1.14	63.12
	98.20	1.80	
	63.30	54.99	
Total	31819	671	32490
	97.93	2.07	100.00

Complications and medical history at the time of obtaining Table 2-3 informed consent by the presence or absence of proton-pump inhibitors

FREQ Procedure

Number of levels of variables

Variables Label Level Number

GROUP1 2
CVAC2YN Cardiogenic Cerebral Infarction 2

Table : GROUP 1 * CVAC 2 YN

GROUP1
CVAC2YN(Cardiogenic Cerebral Infarction)

Power |
Percent |
Percent of rows
Percent of columns | None | With | Total

1	10983	998	11981
	33.80	3.07	36.88
	91.67	8.33	
	36.49	41.69	
2	19113	1396	20509
	58.83	4.30	63.12
	93.19	6.81	
	63.51	58.31	
Total	30096	2394	32490
	92.63	7.37	100.00

Complications and medical history at the time of obtaining Table 2-3 informed consent by the presence or absence of proton-pump inhibitors

FREQ Procedure

Number of levels of variables

Variables Label Level Number

GROUP1 2
CVAC3YN Lacunar Cerebral Infarction 2

Table : GROUP 1 * CVAC 3 YN

GROUP1

CVAC3YN(Lacunar Cerebral Infarction)

Power |
Percent |
Percent of rows
Percent of columns | None | With | Total

GROUP1	None	With	Total
1	11362	619	11981
	34.97	1.91	36.88
	94.83	5.17	
	36.60	42.78	
2	19681	828	20509
	60.58	2.55	63.12
	95.96	4.04	
	63.40	57.22	
Total	31043	1447	32490
	95.55	4.45	100.00

Complications and medical history at the time of obtaining Table 2-3 informed consent by the presence or absence of proton-pump inhibitors

FREQ Procedure

Number of levels of variables

Variables Label Level Number

GROUP1 2
CVAC4YN Hemorrhagic Stroke 2

Table : GROUP 1 * CVAC 4 YN

```
GROUP1
                CVAC4YN(Hemorrhagic Stroke)

Power |
Percent |
Percent of rows
Percent of columns | None | With | Total
______________+________+________+
           1 |  11816 |    165 |  11981
             |  36.37 |   0.51 |  36.88
             |  98.62 |   1.38 |
             |  36.79 |  44.59 |
______________+________+________+
           2 |  20304 |    205 |  20509
             |  62.49 |   0.63 |  63.12
             |  99.00 |   1.00 |
             |  63.21 |  55.41 |
______________+________+________+
Total 32120 370 32490
                98.86     1.14   100.00
```

Complications and medical history at the time of obtaining Table 2-3 informed consent by the presence or absence of proton-pump inhibitors

FREQ Procedure

Number of levels of variables

Variables Label Level Number

GROUP1 2
CVAC5YN Unclassified/Unknown Cerebral Infarction 2

Table : GROUP 1 * CVAC 5 YN

GROUP1
CVAC5YN(Unclassified/Unknown Cerebral Infarction)

Power |
Percent |
Percent of rows
Percent of columns | None | With | Total

1	11249	732	11981
	34.62	2.25	36.88
	93.89	6.11	
	36.61	41.54	
2	19479	1030	20509
	59.95	3.17	63.12
	94.98	5.02	
	63.39	58.46	
Total	30728	1762	32490
	94.58	5.42	100.00

Complications and medical history at the time of obtaining Table 2-3 informed consent by the presence or absence of proton-pump inhibitors

FREQ Procedure

Number of levels of variables

Variables Label Level Number

GROUP1 2
CVAC6YN Transient Ischemic Attacks 2

Table : GROUP 1 * CVAC 6 YN

GROUP1
CVAC6YN(Transient Ischemic Attacks)

Power | Percent | Percent of rows Percent of columns	None	With	Total
1	11753	228	11981
	36.17	0.70	36.88
	98.10	1.90	
	36.76	43.93	
2	20218	291	20509
	62.23	0.90	63.12
	98.58	1.42	
	63.24	56.07	
Total	31971	519	32490
	98.40	1.60	100.00

Complications and medical history at the time of obtaining Table 2-3 informed consent by the presence or absence of proton-pump inhibitors

FREQ Procedure

Number of levels of variables

Variables Label Level Number

GROUP1 2
CVAC7YN Other Cerebrovascular Accident 2

Table : GROUP 1 * CVAC 7 YN

GROUP1
CVAC7YN(Other Cerebrovascular Accident)

Power |
Percent |
Percent of rows
Percent of columns | None | With | Total

1	11631	350	11981
	35.80	1.08	36.88
	97.08	2.92	
	36.71	43.26	
2	20050	459	20509
	61.71	1.41	63.12
	97.76	2.24	
	63.29	56.74	
Total	31681	809	32490
	97.51	2.49	100.00

Complications and medical history at the time of obtaining Table 2-3 informed consent by the presence or absence of proton-pump inhibitors

FREQ Procedure

Number of levels of variables

Variables Label Level Number

GROUP1 2
OTVDYN Other Vascular Disease 2

Table : GROUP1 * OTVDYN

GROUP1
OTVDYN(Other Vascular Disease)

Power | Percent | Percent of rows Percent of columns	None	With	Total
1	11174	807	11981
	34.39	2.48	36.88
	93.26	6.74	
	36.33	46.65	
2	19586	923	20509
	60.28	2.84	63.12
	95.50	4.50	
	63.67	53.35	
Total	30760	1730	32490
	94.68	5.32	100.00

Complications and medical history at the time of obtaining Table 2-3 informed consent by the presence or absence of proton-pump inhibitors

FREQ Procedure

Number of levels of variables

Variables	Label	Level Number
GROUP1		2
OTVD1YN	Peripheral Vascular Disease	2

Table : GROUP 1 * OTVD 1 YN

GROUP1

OTVD1YN(Peripheral Vascular Disease)

Power / Percent / Percent of rows / Percent of columns	None	With	Total
1	11740	241	11981
	36.13	0.74	36.88
	97.99	2.01	
	36.70	48.20	
2	20250	259	20509
	62.33	0.80	63.12
	98.74	1.26	
	63.30	51.80	
Total	31990	500	32490
	98.46	1.54	100.00

Complications and medical history at the time of obtaining Table 2-3 informed consent by the presence or absence of proton-pump inhibitors

FREQ Procedure

Number of levels of variables

Variable	Label	Level Count
GROUP1		2
OTVD2YN	Aortic Plaque	2

Table : GROUP 1 * OTVD 2 YN

GROUP1 OTVD2YN(Aortic Plaque)

Power / Percent / Percent of rows / Percent of columns	None	With	Total
1	11893	88	11981
	36.61	0.27	36.88
	99.27	0.73	
	36.88	36.51	
2	20356	153	20509
	62.65	0.47	63.12
	99.25	0.75	
	63.12	63.49	
Total	32249	241	32490
	99.26	0.74	100.00

Complications and medical history at the time of obtaining Table 2-3 informed consent by the presence or absence of proton-pump inhibitors

FREQ Procedure

Number of levels of variables

Variables	Label	Level Number
GROUP1		2
TED3YN	Internal Carotid Artery Stenosis	2

Table : GROUP 1 * TED 3 YN

GROUP1

TED3YN(Internal Carotid Artery Stenosis)

Power<br>Percent<br>Percent of rows<br>Percent of columns	None	With	Total
1	11630<br>35.80<br>97.07<br>36.65	351<br>1.08<br>2.93<br>46.37	11981<br>36.88
2	20103<br>61.87<br>98.02<br>63.35	406<br>1.25<br>1.98<br>53.63	20509<br>63.12
Total	31733<br>97.67	757<br>2.33	32490<br>100.00

Complications and medical history at the time of obtaining Table 2-3 informed consent by the presence or absence of proton-pump inhibitors

FREQ Procedure

Number of levels of variables

Variables Label Level Number

GROUP1 2
TED4YN Arteriosclerosis Obliterans 2

Table : GROUP 1 * TED 4 YN

GROUP1
TED4YN(Arteriosclerosis Obliterans)

Power |
Percent |
Percent of rows
Percent of columns | None | With | Total

	None	With	Total
1	11472	509	11981
	35.31	1.57	36.88
	95.75	4.25	
	36.57	45.53	
2	19900	609	20509
	61.25	1.87	63.12
	97.03	2.97	
	63.43	54.47	
Total	31372	1118	32490
	96.56	3.44	100.00

Complications and medical history at the time of obtaining Table 2-3 informed consent by the presence or absence of proton-pump inhibitors

FREQ Procedure

Number of levels of variables

Variables	Label	Level Number
GROUP1		2
OTVD3YN	Other Vascular Disease: Other	2

Table : GROUP 1 * OTVD 3 YN

GROUP1

OTVD3YN(Other Vascular Disease: Other)

Power<br>Percent<br>Percent of rows<br>Percent of columns	None	With	Total
1	11483	498	11981
	35.34	1.53	36.88
	95.84	4.16	
	36.51	47.93	
2	19968	541	20509
	61.46	1.67	63.12
	97.36	2.64	
	63.49	52.07	
Total	31451	1039	32490
	96.80	3.20	100.00

Complications and medical history at the time of obtaining Table 2-3 informed consent by the presence or absence of proton-pump inhibitors

FREQ Procedure

Number of levels of variables

Variables	Label	Level Number
GROUP1		2
TEDYN	Thrombosis/Embolism-Related Disease	2

Table : GROUP1 * TEDYN

GROUP1

TEDYN(Thrombosis/Embolism-Related Disease)

Power / Percent / Percent of rows / Percent of columns	None	With	Total
1	10756	1225	11981
	33.11	3.77	36.88
	89.78	10.22	
	36.20	44.05	
2	18953	1556	20509
	58.33	4.79	63.12
	92.41	7.59	
	63.80	55.95	
Total	29709	2781	32490
	91.44	8.56	100.00

Complications and medical history at the time of obtaining Table 2-3 informed consent by the presence or absence of proton-pump inhibitors

FREQ Procedure

Number of levels of variables

Number of Variables Level

GROUP1	2
t_TED	2

Table : GROUP1 * t_TED

GROUP1 t_TED

Power |
Percent |
Percent of rows
Percentage of columns | 0| 1| Sum

GROUP1	0	1	Sum
1	11793	188	11981
	36.30	0.58	36.88
	98.43	1.57	
	36.76	45.52	
2	20284	225	20509
	62.43	0.69	63.12
	98.90	1.10	
	63.24	54.48	
Total	32077	413	32490
	98.73	1.27	100.00

Complications and medical history at the time of obtaining Table 2-3 informed consent by the presence or absence of proton-pump inhibitors

FREQ Procedure

Number of levels of variables

Variables	Label	Level Number
GROUP1		2
TED1YN	Pulmonary Embolism	2

Table : GROUP 1 * TED 1 YN

GROUP1

TED1YN(Pulmonary Embolism)

Power<br>Percent<br>Percent of rows<br>Percent of columns	None	With	Total
1	11929<br>36.72<br>99.57<br>36.85	52<br>0.16<br>0.43<br>45.22	11981<br>36.88
2	20446<br>62.93<br>99.69<br>63.15	63<br>0.19<br>0.31<br>54.78	20509<br>63.12
Total	32375<br>99.65	115<br>0.35	32490<br>100.00

Complications and medical history at the time of obtaining Table 2-3 informed consent by the presence or absence of proton-pump inhibitors

FREQ Procedure

Number of levels of variables

Variables Label Level Number

GROUP1 2
TED2YN Deep Vein Thrombosis 2

Table : GROUP 1 * TED 2 YN

GROUP1
TED2YN(Deep Vein Thrombosis)

Power / Percent / Percent of rows / Percent of columns	None	With	Total
1	11829	152	11981
	36.41	0.47	36.88
	98.73	1.27	
	36.79	45.10	
2	20324	185	20509
	62.55	0.57	63.12
	99.10	0.90	
	63.21	54.90	
Total	32153	337	32490
	98.96	1.04	100.00

Complications and medical history at the time of obtaining Table 2-3 informed consent by the presence or absence of proton-pump inhibitors

FREQ Procedure

Number of levels of variables

Variables Label Level Number

GROUP1 2
TED5YN Other Thrombosis/Embolism-Related Disease 2

Table : GROUP 1 * TED 5 YN

GROUP1
TED5YN(Other Thrombosis/Embolism-Related Disease)

Power / Percent / Percent of rows / Percent of columns	None	With	Total
1	11646	335	11981
	35.84	1.03	36.88
	97.20	2.80	
	36.75	41.82	
2	20043	466	20509
	61.69	1.43	63.12
	97.73	2.27	
	63.25	58.18	
Total	31689	801	32490
	97.53	2.47	100.00

Complications and medical history at the time of obtaining Table 2-3 informed consent by the presence or absence of proton-pump inhibitors

FREQ Procedure

Number of levels of variables

Variable Label Level Count

GROUP1 2
HTDMYN Hyperthyroidism 2

Table : GROUP1 * HTDMYN

GROUP1 HTDMYN(Hyperthyroidism)

Power / Percent / Percent of rows / Percent of columns	None	With	Total
1	11800	181	11981
	36.32	0.56	36.88
	98.49	1.51	
	36.86	37.63	
2	20209	300	20509
	62.20	0.92	63.12
	98.54	1.46	
	63.14	62.37	
Total	32009	481	32490
	98.52	1.48	100.00

Complications and medical history at the time of obtaining Table 2-3 informed consent by the presence or absence of proton-pump inhibitors

FREQ Procedure

Number of levels of variables

Variables Label Level Number

GROUP1 2
GASDYN Gastrointestinal Disease 2

Table : GROUP1 * GASDYN

GROUP1
GASDYN(Gastrointestinal Disease)

Power |
Percent |
Percent of rows
Percent of columns | None | With | Total

GROUP1	None	With	Total
1	6541	5440	11981
	20.13	16.74	36.88
	54.59	45.41	
	28.48	57.12	
2	16425	4084	20509
	50.55	12.57	63.12
	80.09	19.91	
	71.52	42.88	
Total	22966	9524	32490
	70.69	29.31	100.00

Complications and medical history at the time of obtaining Table 2-3 informed consent by the presence or absence of proton-pump inhibitors

FREQ Procedure

Number of levels of variables

Variables Label Level Number

GROUP1 2
GASD1YN Upper Gastrointestinal Tract 2

Table : GROUP 1 * GASD 1 YN

GROUP1

GASD1YN(Upper Gastrointestinal Tract)

Power<br>Percent<br>Percent of rows<br>Percent of columns	None	With	Total
1	11678<br>35.94<br>97.47<br>36.75	303<br>0.93<br>2.53<br>42.32	11981<br>36.88
2	20096<br>61.85<br>97.99<br>63.25	413<br>1.27<br>2.01<br>57.68	20509<br>63.12
Total	31774<br>97.80	716<br>2.20	32490<br>100.00

Complications and medical history at the time of obtaining Table 2-3 informed consent by the presence or absence of proton-pump inhibitors

FREQ Procedure

Number of levels of variables

Variables Label Level Number

Variables	Label	Level Number
GROUP1		2
GASD2YN	Lower Gastrointestinal Tract	2

Table : GROUP 1 * GASD 2 YN

GROUP1

GASD2YN(Lower Gastrointestinal Tract)

Power | Percent | Percent of rows Percent of columns	None	With	Total
1	11680	301	11981
	35.95	0.93	36.88
	97.49	2.51	
	36.77	41.46	
2	20084	425	20509
	61.82	1.31	63.12
	97.93	2.07	
	63.23	58.54	
Total	31764	726	32490
	97.77	2.23	100.00

Complications and medical history at the time of obtaining Table 2-3 informed consent by the presence or absence of proton-pump inhibitors

FREQ Procedure

Number of levels of variables

Variables Label Level Number

GROUP1 2
GASD3YN Reflux esophagitis 2

Table : GROUP 1 * GASD 3 YN

GROUP1
GASD3YN(Reflux esophagitis)

Power |
Percent |
Percent of rows
Percent of columns | None | With | Total

1	8140	3841	11981
	25.05	11.82	36.88
	67.94	32.06	
	29.74	75.03	
2	19231	1278	20509
	59.19	3.93	63.12
	93.77	6.23	
	70.26	24.97	
Total	27371	5119	32490
	84.24	15.76	100.00

Complications and medical history at the time of obtaining Table 2-3 informed consent by the presence or absence of proton-pump inhibitors

FREQ Procedure

Number of levels of variables

Variables Label Level Number

GROUP1 2
GASD4YN Other Gastrointestinal Disease 2

Table : GROUP 1 * GASD 4 YN

GROUP1

GASD4YN(Other Gastrointestinal Disease)

Power<br>Percent<br>Percent of rows<br>Percent of columns	None	With	Total
1	10043<br>30.91<br>83.82<br>35.78	1938<br>5.96<br>16.18<br>43.84	11981<br>36.88
2	18026<br>55.48<br>87.89<br>64.22	2483<br>7.64<br>12.11<br>56.16	20509<br>63.12
Total	28069<br>86.39	4421<br>13.61	32490<br>100.00

Complications and medical history at the time of obtaining Table 2-3 informed consent by the presence or absence of proton-pump inhibitors

FREQ Procedure

Number of levels of variables

Variable Label Level Count

GROUP1 2
MGTYN Malignant Tumor 2

Table : GROUP1 * MGTYN

GROUP1 MGTYN(Malignant Tumor)

Power |
Percent |
Percent of rows
Percent of columns | None | With | Total

Percent of columns	None	With	Total
1	10618	1363	11981
	32.68	4.20	36.88
	88.62	11.38	
	36.70	38.30	
2	18313	2196	20509
	56.37	6.76	63.12
	89.29	10.71	
	63.30	61.70	
Total	28931	3559	32490
	89.05	10.95	100.00

Complications and medical history at the time of obtaining Table 2-3 informed consent by the presence or absence of proton-pump inhibitors

FREQ Procedure

Number of levels of variables

Variables Label Level Number

GROUP1 2
MGT1YN Gastric Cancer 2

Table : GROUP 1 * MGT 1 YN

GROUP1 MGT1YN(Gastric Cancer)

Power / Percent / Percent of rows / Percent of columns	None	With	Total
1	11688	293	11981
	35.97	0.90	36.88
	97.55	2.45	
	36.81	39.86	
2	20067	442	20509
	61.76	1.36	63.12
	97.84	2.16	
	63.19	60.14	
Total	31755	735	32490
	97.74	2.26	100.00

Complications and medical history at the time of obtaining Table 2-3 informed consent by the presence or absence of proton-pump inhibitors

FREQ Procedure

Number of levels of variables

Variables Label Level Number

GROUP1 2
MGT2YN Colorectal Cancer 2

Table : GROUP 1 * MGT 2 YN

GROUP1 MGT2YN(Colorectal Cancer)

Power |
Percent |
Percent of rows
Percent of columns | None | With | Total

1	11700	281	11981
	36.01	0.86	36.88
	97.65	2.35	
	36.85	38.08	
2	20052	457	20509
	61.72	1.41	63.12
	97.77	2.23	
	63.15	61.92	
Total	31752	738	32490
	97.73	2.27	100.00

Complications and medical history at the time of obtaining Table 2-3 informed consent by the presence or absence of proton-pump inhibitors

FREQ Procedure

Number of levels of variables

Variable Label Level Count

GROUP1 2
MGT3YN Lung Cancer 2

Table : GROUP 1 * MGT 3 YN

GROUP1 MGT3YN(Lung Cancer)

Power<br>Percent<br>Percent of rows<br>Percent of columns	None	With	Total
1	11837	144	11981
	36.43	0.44	36.88
	98.80	1.20	
	36.85	39.34	
2	20287	222	20509
	62.44	0.68	63.12
	98.92	1.08	
	63.15	60.66	
Total	32124	366	32490
	98.87	1.13	100.00

Complications and medical history at the time of obtaining Table 2-3 informed consent by the presence or absence of proton-pump inhibitors

FREQ Procedure

Number of levels of variables

Variables Label Level Number

GROUP1 2
MGT4YN Brest Cancer 2

Table : GROUP 1 * MGT 4 YN

GROUP1 MGT4YN(Brest Cancer)

Power | Percent | Percent of rows Percent of columns	None	With	Total
1	11895	86	11981
	36.61	0.26	36.88
	99.28	0.72	
	36.88	36.44	
2	20359	150	20509
	62.66	0.46	63.12
	99.27	0.73	
	63.12	63.56	
Total	32254	236	32490
	99.27	0.73	100.00

Complications and medical history at the time of obtaining Table 2-3 informed consent by the presence or absence of proton-pump inhibitors

FREQ Procedure

Number of levels of variables

Variables Label Level Number

Variables	Label	Level Number
GROUP1		2
MGT5YN	Uterus/Ovarian Cancer	2

Table : GROUP 1 * MGT 5 YN

GROUP1

MGT5YN(Uterus/Ovarian Cancer)

Power / Percent / Percent of rows / Percent of columns	None	With	Total
1	11942	39	11981
	36.76	0.12	36.88
	99.67	0.33	
	36.86	41.94	
2	20455	54	20509
	62.96	0.17	63.12
	99.74	0.26	
	63.14	58.06	
Total	32397	93	32490
	99.71	0.29	100.00

Complications and medical history at the time of obtaining Table 2-3 informed consent by the presence or absence of proton-pump inhibitors

FREQ Procedure

Number of levels of variables

Variables Label Level Number

GROUP1 2
MGT6YN Pancreatic Cancer 2

Table : GROUP 1 * MGT 6 YN

GROUP1 MGT6YN(Pancreatic Cancer)

Power / Percent / Percent of rows / Percent of columns	None	With	Total
1	11968	13	11981
	36.84	0.04	36.88
	99.89	0.11	
	36.87	38.24	
2	20488	21	20509
	63.06	0.06	63.12
	99.90	0.10	
	63.13	61.76	
Total	32456	34	32490
	99.90	0.10	100.00

Complications and medical history at the time of obtaining Table 2-3 informed consent by the presence or absence of proton-pump inhibitors

FREQ Procedure

Number of levels of variables

Variables	Label	Level Number
GROUP1		2
MGT7YN	Other Malignant Tumor	2

Table : GROUP 1 * MGT 7 YN

GROUP1

MGT7YN(Other Malignant Tumor)

Power<br>Percent<br>Percent of rows<br>Percent of columns	None	With	Total
1	11331	650	11981
	34.88	2.00	36.88
	94.57	5.43	
	36.84	37.49	
2	19425	1084	20509
	59.79	3.34	63.12
	94.71	5.29	
	63.16	62.51	
Total	30756	1734	32490
	94.66	5.34	100.00

Complications and medical history at the time of obtaining Table 2-3 informed consent by the presence or absence of proton-pump inhibitors

FREQ Procedure

Number of levels of variables

Variable Label Level Count

Variable	Label	Level Count
GROUP1		2
ALZHYN	Alzheimer	2

Table : GROUP1 * ALZHYN

GROUP1 ALZHYN(Alzheimer)

Power / Percent / Percent of rows / Percent of columns	None	With	Total
1	10995	986	11981
	33.84	3.03	36.88
	91.77	8.23	
	36.73	38.62	
2	18942	1567	20509
	58.30	4.82	63.12
	92.36	7.64	
	63.27	61.38	
Total	29937	2553	32490
	92.14	7.86	100.00

Complications and medical history at the time of obtaining Table 2-3 informed consent by the presence or absence of proton-pump inhibitors

FREQ Procedure

Number of levels of variables

Variables Label Level Number

GROUP1 2

OHCPYN Other Complications 2

Table : GROUP1 * OHCPYN

GROUP1

OHCPYN(Other Complications)

Power |

Percent |

Percent of rows

Percent of columns	None	With	Total
1	7822	4159	11981
	24.08	12.80	36.88
	65.29	34.71	
	35.63	39.48	
2	14133	6376	20509
	43.50	19.62	63.12
	68.91	31.09	
	64.37	60.52	
Total	21955	10535	32490
	67.57	32.43	100.00

Complications and medical history at the time of obtaining Table 2-3 informed consent by the presence or absence of proton-pump inhibitors

FREQ Procedure

Number of levels of variables

Variables	Label	Level Number
GROUP1		2
IMGYN	Brain Imaging Test	2

Table : GROUP1 * IMGYN

GROUP1 IMGYN(Brain Imaging Test)

Power / Percent / Percent of rows / Percent of columns	None	With	Total
1	10487	1232	11719
	33.01	3.88	36.88
	89.49	10.51	
	36.26	43.20	
2	18434	1620	20054
	58.02	5.10	63.12
	91.92	8.08	
	63.74	56.80	
Total	28921	2852	31773
	91.02	8.98	100.00

Complications and medical history at the time of obtaining Table 2-3 informed consent by the presence or absence of proton-pump inhibitors

FREQ Procedure

Number of levels of variables

Variables	Label	Level Number
GROUP1		2
FNDYN	Brain Imaging Result	2

Table : GROUP1 * FNDYN

GROUP1

FNDYN(Brain Imaging Result)

Power | Percent | Percent of rows Percent of columns	None	With	Total
1	562	625	1187
	20.50	22.79	43.29
	47.35	52.65	
	42.29	44.23	
2	767	788	1555
	27.97	28.74	56.71
	49.32	50.68	
	57.71	55.77	
Total	1329	1413	2742
	48.47	51.53	100.00

Complications and medical history at the time of obtaining Table 2-3 informed consent by the presence or absence of proton-pump inhibitors

FREQ Procedure

Number of levels of variables

Variables Label Level Number

GROUP1 2
FALYN Fall Down within one year 2

Table : GROUP1 * FALYN

GROUP1

FALYN(Fall Down within one year)

Power |
Percent |
Percent of rows
Percent of columns | None | With | Total

GROUP1	None	With	Total
1	9638	999	10637
	33.36	3.46	36.81
	90.61	9.39	
	36.34	42.17	
2	16887	1370	18257
	58.44	4.74	63.19
	92.50	7.50	
	63.66	57.83	
Total	26525	2369	28894
	91.80	8.20	100.00

Administration of anticoagulants at the time of obtaining Table 2-4 consent, according to the

presence or absence of proton-pump inhibitors

FREQ Procedure

Cumulative

Percent of ALL power Percent of power

1	32490	100.00	32490	100.00

Tables: ALL * ADYN

ALL

ADYN(Administration of Anticoagulant)

Power |

Percent |

Percent of rows

Percentage of columns | with | total

______________+________+

1 | 29914 | 29914

| 100.00 | 100.00

| 100.00 |

| 100.00 |

______________+________+

Total 29914 29914

100.00 100.00

Degree of missing values = 2576

Administration of anticoagulants at the time of obtaining Table 2-4 consent, according to the presence or absence of proton-pump inhibitors

FREQ Procedure

Table 1 : ADYN * ACTYP
Stratification variable : ALL=1

ADYN(Administration of Anticoagulant) ACTYP(Type of AF)

Power |
Percent |
Percent of rows
Percent of column | Warfa| Dabigat| Riverro| Apixa| Edoxa| Parenteral Anti| Edoxa| Total
|phosphate|run|xaban|ban|ban|coagulant|ban (OD|)
| | | | | | |) |
______________+________+________+________+________+________+________+________+
Present | 8295 | 2339 | 6436 | 8039 | 4752 | 13 | 40 | 29914
| 27.73 | 7.82 | 21.52 | 26.87 | 15.89 | 0.04 | 0.13 | 100.00
| 27.73 | 7.82 | 21.52 | 26.87 | 15.89 | 0.04 | 0.13 |
| 100.00 | 100.00 | 100.00 | 100.00 | 100.00 | 100.00 | 100.00 |
______________+________+________+________+________+________+________+________+
Total 8295 2339 6436 8039 4752 13 40 29914
27.73 7.82 21.52 26.87 15.89 0.04 0.13 100.00

Degree of missing values = 2576

Table 1 : ADYN * ACTYP
Stratification variable : ALL=1

ADYN(Administration of Anticoagulant) ACTYP(Type of AF)

Power |

Percent |

Percent of rows

Percent of column | Warfa| Dabigat| Riverro| Apixa| Edoxa| Parenteral Anti| Edoxa| Total

|phosphate|run|xaban|ban|ban|coagulant|ban (OD|)

| | | | | | |) |

______________+________+________+________+________+________+________+________

_+

Present | 8295 | 2339 | 6436 | 8039 | 4752 | 13 | 40 | 29914

| 27.73 | 7.82 | 21.52 | 26.87 | 15.89 | 0.04 | 0.13 | 100.00

| 27.73 | 7.82 | 21.52 | 26.87 | 15.89 | 0.04 | 0.13 |

| 100.00 | 100.00 | 100.00 | 100.00 | 100.00 | 100.00 | 100.00 |

______________+________+________+________+________+________+________+________

_+

Total 8295 2339 6436 8039 4752 13 40 29914

27.73 7.82 21.52 26.87 15.89 0.04 0.13 100.00

Degree of missing values = 2576

Administration of anticoagulants at the time of obtaining Table 2-4 consent, according to the presence or absence of proton-pump inhibitors

FREQ Procedure

Table 1 : ADYN * ACTYP
Stratification variable : ALL=1

ADYN(Administration of Anticoagulant) ACTYP(Type of AF)

Power |
Percent |
Percent of rows
Percent of column | Warfa| Dabigat| Riverro| Apixa| Edoxa| Parenteral Anti| Edoxa| Total
|phosphate|run|xaban|ban|ban|coagulant|ban (OD|)
| | | | | | |) |
______________+________+________+________+________+________+________+________+
Present | 8295 | 2339 | 6436 | 8039 | 4752 | 13 | 40 | 29914
| 27.73 | 7.82 | 21.52 | 26.87 | 15.89 | 0.04 | 0.13 | 100.00
| 27.73 | 7.82 | 21.52 | 26.87 | 15.89 | 0.04 | 0.13 |
| 100.00 | 100.00 | 100.00 | 100.00 | 100.00 | 100.00 | 100.00 |
______________+________+________+________+________+________+________+________+
Total 8295 2339 6436 8039 4752 13 40 29914
27.73 7.82 21.52 26.87 15.89 0.04 0.13 100.00

Degree of missing values = 2576

Table 1 : ADYN * ACTYP
Stratification variable : ALL=1

ADYN(Administration of Anticoagulant) ACTYP(Type of AF)

Power |

Percent |

Percent of rows

Percent of column | Warfa| Dabigat| Riverro| Apixa| Edoxa| Parenteral Anti| Edoxa| Total

|phosphate|run|xaban|ban|ban|coagulant|ban (OD|)

| | | | | | |) |

______________+________+________+________+________+________+________+________
_+

Present | 8295 | 2339 | 6436 | 8039 | 4752 | 13 | 40 | 29914

| 27.73 | 7.82 | 21.52 | 26.87 | 15.89 | 0.04 | 0.13 | 100.00

| 27.73 | 7.82 | 21.52 | 26.87 | 15.89 | 0.04 | 0.13 |

| 100.00 | 100.00 | 100.00 | 100.00 | 100.00 | 100.00 | 100.00 |

______________+________+________+________+________+________+________+________
_+

Total 8295 2339 6436 8039 4752 13 40 29914

27.73 7.82 21.52 26.87 15.89 0.04 0.13 100.00

Degree of missing values = 2576

Administration of anticoagulants at the time of obtaining Table 2-4 consent, according to the presence or absence of proton-pump inhibitors

FREQ Procedure

Table 1 : ADYN * ACTYP
Stratification variable : ALL=1

ADYN(Administration of Anticoagulant) ACTYP(Type of AF)

Power |
Percent |
Percent of rows
Percent of column | Warfa| Dabigat| Riverro| Apixa| Edoxa| Parenteral Anti| Edoxa| Total
|phosphate|run|xaban|ban|ban|coagulant|ban (OD|)
| | | | | | |) |
______________+________+________+________+________+________+________+________
_+
Present | 8295 | 2339 | 6436 | 8039 | 4752 | 13 | 40 | 29914
| 27.73 | 7.82 | 21.52 | 26.87 | 15.89 | 0.04 | 0.13 | 100.00
| 27.73 | 7.82 | 21.52 | 26.87 | 15.89 | 0.04 | 0.13 |
| 100.00 | 100.00 | 100.00 | 100.00 | 100.00 | 100.00 | 100.00 |
______________+________+________+________+________+________+________+________
_+
Total 8295 2339 6436 8039 4752 13 40 29914
27.73 7.82 21.52 26.87 15.89 0.04 0.13 100.00

Degree of missing values = 2576

Table 1 : ADYN * ACTYP
Stratification variable : ALL=1

ADYN(Administration of Anticoagulant) ACTYP(Type of AF)

Power |

Percent |

Percent of rows

Percent of column | Warfa| Dabigat| Riverro| Apixa| Edoxa| Parenteral Anti| Edoxa| Total

|phosphate|run|xaban|ban|ban|coagulant|ban (OD|)

| | | | | | |) |

______________+________+________+________+________+________+________+________+

Present | 8295 | 2339 | 6436 | 8039 | 4752 | 13 | 40 | 29914

| 27.73 | 7.82 | 21.52 | 26.87 | 15.89 | 0.04 | 0.13 | 100.00

| 27.73 | 7.82 | 21.52 | 26.87 | 15.89 | 0.04 | 0.13 |

| 100.00 | 100.00 | 100.00 | 100.00 | 100.00 | 100.00 | 100.00 |

______________+________+________+________+________+________+________+________+

Total 8295 2339 6436 8039 4752 13 40 29914

27.73 7.82 21.52 26.87 15.89 0.04 0.13 100.00

Degree of missing values = 2576

Administration of anticoagulants at the time of obtaining Table 2-4 consent, according to the presence or absence of proton-pump inhibitors

FREQ Procedure

Table 1 : ADYN * ACTYP
Stratification variable : ALL=1

ADYN(Administration of Anticoagulant) ACTYP(Type of AF)

Power |
Percent |
Percent of rows
Percent of column | Warfa| Dabigat| Riverro| Apixa| Edoxa| Parenteral Anti| Edoxa| Total
|phosphate|run|xaban|ban|ban|coagulant|ban (OD|)
| | | | | | |) |
______________+________+________+________+________+________+________+________
_+
Present | 8295 | 2339 | 6436 | 8039 | 4752 | 13 | 40 | 29914
| 27.73 | 7.82 | 21.52 | 26.87 | 15.89 | 0.04 | 0.13 | 100.00
| 27.73 | 7.82 | 21.52 | 26.87 | 15.89 | 0.04 | 0.13 |
| 100.00 | 100.00 | 100.00 | 100.00 | 100.00 | 100.00 | 100.00 |
______________+________+________+________+________+________+________+________
_+
Total 8295 2339 6436 8039 4752 13 40 29914
27.73 7.82 21.52 26.87 15.89 0.04 0.13 100.00

Degree of missing values = 2576

Table 1 : ADYN * t_ACT0
Stratification variable : ALL=1

ADYN(Administration of Anticoagulant)
t_ACT0

Power |

Percent |

Percent of rows

Percentage of columns | 0| 1| Sum

______________+________+________+

Present | 25122 | 4792 | 29914

| 83.98 | 16.02 | 100.00

| 83.98 | 16.02 |

| 100.00 | 100.00 |

______________+________+________+

Total 25122 4792 29914

83.98 16.02 100.00

Degree of missing values = 2576

Administration of anticoagulants at the time of obtaining Table 2-4 consent, according to the presence or absence of proton-pump inhibitors

FREQ Procedure

Table: ALL * t_ADYN

ALL t_ADYN

Power |
Percent |
Percent of rows
Percentage of columns | 0| 1| Sum

```
______________+________+________+
          1 |    2576 |   29914 |   32490
            |    7.93 |   92.07 | 100.00
            |    7.93 |   92.07 |
            | 100.00 | 100.00 |
______________+________+________+
Total 2576 29914 32490
                 7.93      92.07     100.00
```

Administration of anticoagulants at the time of obtaining Table 2-4 consent, according to the presence or absence of proton-pump inhibitors

FREQ Procedure

Cumulative
Percent of ALL power Percent of power

1	32490	100.00	32490	100.00

Tables: ALL * ADYN

ALL
ADYN(Administration of Anticoagulant)

Power |
Percent |
Percent of rows
Percentage of columns | with | total

1	29914	29914
	100.00	100.00
	100.00	
	100.00	
Total	29914	29914
	100.00	100.00

Degree of missing values = 2576

Administration of anticoagulants at the time of obtaining Table 2-4 consent, according to the presence or absence of proton-pump inhibitors

FREQ Procedure

Table 1 : ADYN * ACTYP
Stratification variable : ALL=1

ADYN(Administration of Anticoagulant) ACTYP(Type of AF)

Power |
Percent |
Percent of rows
Percent of column | Warfa| Dabigat| Riverro| Apixa| Edoxa| Parenteral Anti| Edoxa| Total
|phosphate|run|xaban|ban|ban|coagulant|ban (OD|)
| | | | | | |) |
______________+________+________+________+________+________+________+________
_+
Present | 8295 | 2339 | 6436 | 8039 | 4752 | 13 | 40 | 29914
| 27.73 | 7.82 | 21.52 | 26.87 | 15.89 | 0.04 | 0.13 | 100.00
| 27.73 | 7.82 | 21.52 | 26.87 | 15.89 | 0.04 | 0.13 |
| 100.00 | 100.00 | 100.00 | 100.00 | 100.00 | 100.00 | 100.00 |
______________+________+________+________+________+________+________+________
_+
Total 8295 2339 6436 8039 4752 13 40 29914
27.73 7.82 21.52 26.87 15.89 0.04 0.13 100.00

Degree of missing values = 2576

Table 1 : ADYN * ACTYP
Stratification variable : ALL=1

ADYN(Administration of Anticoagulant) ACTYP(Type of AF)

Power |

Percent |

Percent of rows

Percent of column | Warfa| Dabigat| Riverro| Apixa| Edoxa| Parenteral Anti| Edoxa| Total

|phosphate|run|xaban|ban|ban|coagulant|ban (OD|)

| | | | | | |) |

______________+________+________+________+________+________+________+________

_+

Present | 8295 | 2339 | 6436 | 8039 | 4752 | 13 | 40 | 29914

| 27.73 | 7.82 | 21.52 | 26.87 | 15.89 | 0.04 | 0.13 | 100.00

| 27.73 | 7.82 | 21.52 | 26.87 | 15.89 | 0.04 | 0.13 |

| 100.00 | 100.00 | 100.00 | 100.00 | 100.00 | 100.00 | 100.00 |

______________+________+________+________+________+________+________+________

_+

Total 8295 2339 6436 8039 4752 13 40 29914

27.73 7.82 21.52 26.87 15.89 0.04 0.13 100.00

Degree of missing values = 2576

Administration of anticoagulants at the time of obtaining Table 2-4 consent, according to the presence or absence of proton-pump inhibitors

FREQ Procedure

Table 1 : ADYN * ACTYP
Stratification variable : ALL=1

ADYN(Administration of Anticoagulant) ACTYP(Type of AF)

Power |
Percent |
Percent of rows
Percent of column | Warfa| Dabigat| Riverro| Apixa| Edoxa| Parenteral Anti| Edoxa| Total
|phosphate|run|xaban|ban|ban|coagulant|ban (OD|)
| | | | | | |) |
______________+________+________+________+________+________+________+________
_+
Present | 8295 | 2339 | 6436 | 8039 | 4752 | 13 | 40 | 29914
| 27.73 | 7.82 | 21.52 | 26.87 | 15.89 | 0.04 | 0.13 | 100.00
| 27.73 | 7.82 | 21.52 | 26.87 | 15.89 | 0.04 | 0.13 |
| 100.00 | 100.00 | 100.00 | 100.00 | 100.00 | 100.00 | 100.00 |
______________+________+________+________+________+________+________+________
_+
Total 8295 2339 6436 8039 4752 13 40 29914
27.73 7.82 21.52 26.87 15.89 0.04 0.13 100.00

Degree of missing values = 2576

Table 1 : ADYN * ACTYP
Stratification variable : ALL=1

ADYN(Administration of Anticoagulant) ACTYP(Type of AF)

Power |

Percent |

Percent of rows

Percent of column | Warfa| Dabigat| Riverro| Apixa| Edoxa| Parenteral Anti| Edoxa| Total

|phosphate|run|xaban|ban|ban|coagulant|ban (OD|)

| | | | | | |) |

______________+________+________+________+________+________+________+________

_+

Present | 8295 | 2339 | 6436 | 8039 | 4752 | 13 | 40 | 29914

| 27.73 | 7.82 | 21.52 | 26.87 | 15.89 | 0.04 | 0.13 | 100.00

| 27.73 | 7.82 | 21.52 | 26.87 | 15.89 | 0.04 | 0.13 |

| 100.00 | 100.00 | 100.00 | 100.00 | 100.00 | 100.00 | 100.00 |

______________+________+________+________+________+________+________+________

_+

Total 8295 2339 6436 8039 4752 13 40 29914

27.73 7.82 21.52 26.87 15.89 0.04 0.13 100.00

Degree of missing values = 2576

Administration of anticoagulants at the time of obtaining Table 2-4 consent, according to the presence or absence of proton-pump inhibitors

FREQ Procedure

Table 1 : ADYN * ACTYP
Stratification variable : ALL=1

ADYN(Administration of Anticoagulant) ACTYP(Type of AF)

Power |
Percent |
Percent of rows
Percent of column | Warfa| Dabigat| Riverro| Apixa| Edoxa| Parenteral Anti| Edoxa| Total
|phosphate|run|xaban|ban|ban|coagulant|ban (OD|)
| | | | | | |) |
______________+________+________+________+________+________+________+________
_+
Present | 8295 | 2339 | 6436 | 8039 | 4752 | 13 | 40 | 29914
| 27.73 | 7.82 | 21.52 | 26.87 | 15.89 | 0.04 | 0.13 | 100.00
| 27.73 | 7.82 | 21.52 | 26.87 | 15.89 | 0.04 | 0.13 |
| 100.00 | 100.00 | 100.00 | 100.00 | 100.00 | 100.00 | 100.00 |
______________+________+________+________+________+________+________+________
_+
Total 8295 2339 6436 8039 4752 13 40 29914
27.73 7.82 21.52 26.87 15.89 0.04 0.13 100.00

Degree of missing values = 2576

Table 1 : ADYN * ACTYP
Stratification variable : ALL=1

ADYN(Administration of Anticoagulant) ACTYP(Type of AF)

Power |

Percent |

Percent of rows

Percent of column | Warfa| Dabigat| Riverro| Apixa| Edoxa| Parenteral Anti| Edoxa| Total

|phosphate|run|xaban|ban|ban|coagulant|ban (OD|)

| | | | | | |) |

______________+________+________+________+________+________+________+________
_+

Present | 8295 | 2339 | 6436 | 8039 | 4752 | 13 | 40 | 29914

| 27.73 | 7.82 | 21.52 | 26.87 | 15.89 | 0.04 | 0.13 | 100.00

| 27.73 | 7.82 | 21.52 | 26.87 | 15.89 | 0.04 | 0.13 |

| 100.00 | 100.00 | 100.00 | 100.00 | 100.00 | 100.00 | 100.00 |

______________+________+________+________+________+________+________+________
_+

Total 8295 2339 6436 8039 4752 13 40 29914

27.73 7.82 21.52 26.87 15.89 0.04 0.13 100.00

Degree of missing values = 2576

Administration of anticoagulants at the time of obtaining Table 2-4 consent, according to the presence or absence of proton-pump inhibitors

FREQ Procedure

Table 1 : ADYN * ACTYP
Stratification variable : ALL=1

ADYN(Administration of Anticoagulant) ACTYP(Type of AF)

Power |
Percent |
Percent of rows
Percent of column | Warfa| Dabigat| Riverro| Apixa| Edoxa| Parenteral Anti| Edoxa| Total
|phosphate|run|xaban|ban|ban|coagulant|ban (OD|)
| | | | | | |) |
______________+________+________+________+________+________+________+________
_+
Present | 8295 | 2339 | 6436 | 8039 | 4752 | 13 | 40 | 29914
| 27.73 | 7.82 | 21.52 | 26.87 | 15.89 | 0.04 | 0.13 | 100.00
| 27.73 | 7.82 | 21.52 | 26.87 | 15.89 | 0.04 | 0.13 |
| 100.00 | 100.00 | 100.00 | 100.00 | 100.00 | 100.00 | 100.00 |
______________+________+________+________+________+________+________+________
_+
Total 8295 2339 6436 8039 4752 13 40 29914
27.73 7.82 21.52 26.87 15.89 0.04 0.13 100.00

Degree of missing values = 2576

Table 1 : ADYN * t_ACT0
Stratification variable : ALL=1

ADYN(Administration of Anticoagulant)
t_ACT0

Power |

Percent |

Percent of rows

Percentage of columns | 0| 1| Sum

______________+________+________+

Present | 25122 | 4792 | 29914

| 83.98 | 16.02 | 100.00

| 83.98 | 16.02 |

| 100.00 | 100.00 |

______________+________+________+

Total 25122 4792 29914

83.98 16.02 100.00

Degree of missing values = 2576

Administration of anticoagulants at the time of obtaining Table 2-4 consent, according to the presence or absence of proton-pump inhibitors

FREQ Procedure

Table: ALL * t_ADYN

ALL t_ADYN

Power |
Percent |
Percent of rows
Percentage of columns | 0| 1| Sum

	0	1	Sum
1	2576	29914	32490
	7.93	92.07	100.00
	7.93	92.07	
	100.00	100.00	
Total	2576	29914	32490
	7.93	92.07	100.00

Administration of anticoagulants at the time of obtaining Table 2-4 consent, according to the presence or absence of proton-pump inhibitors

FREQ Procedure

Tables: ALL* ACTYP

ALL ACTYP(Type of AF)

Power |
Percent |
Percent of rows
Percent of column | Warfa| Dabigat| Riverro| Apixa| Edoxa| Parenteral Anti| Edoxa| Total
|phosphate|run|xaban|ban|ban|coagulant|ban (OD|)
| | | | | | |) |
______________+________+________+________+________+________+________+_______
_+
1 | 8295 | 2339 | 6436 | 8039 | 4752 | 13 | 40 | 29914
| 27.73 | 7.82 | 21.52 | 26.87 | 15.89 | 0.04 | 0.13 | 100.00
| 27.73 | 7.82 | 21.52 | 26.87 | 15.89 | 0.04 | 0.13 |
| 100.00 | 100.00 | 100.00 | 100.00 | 100.00 | 100.00 | 100.00 |
______________+________+________+________+________+________+________+_______
_+
Total 8295 2339 6436 8039 4752 13 40 29914
27.73 7.82 21.52 26.87 15.89 0.04 0.13 100.00

Administration of anticoagulants at the time of obtaining Table 2-4 consent, according to the presence or absence of proton-pump inhibitors

FREQ Procedure

Table : ALL * n_ACT1

ALL n_ACT1

Power |
Percent |
Percent of rows
Percent of columns | 1| Total

1	21619	21619
	100.00	100.00
	100.00	
	100.00	
Total	21619	21619
	100.00	100.00

Degree of missing values = 8295

Administration of anticoagulants at the time of obtaining Table 2-4 consent, according to the presence or absence of proton-pump inhibitors

FREQ Procedure

Tables: ALL* ACTYP

ALL ACTYP(Type of AF)

Power |
Percent |
Percent of rows
Percent of column | Warfa| Dabigat| Riverro| Apixa| Edoxa| Parenteral Anti| Edoxa| Total
|phosphate|run|xaban|ban|ban|coagulant|ban (OD|)
| | | | | | |) |
______________+________+________+________+________+________+________+________+
1 | 8295 | 2339 | 6436 | 8039 | 4752 | 13 | 40 | 29914
| 27.73 | 7.82 | 21.52 | 26.87 | 15.89 | 0.04 | 0.13 | 100.00
| 27.73 | 7.82 | 21.52 | 26.87 | 15.89 | 0.04 | 0.13 |
| 100.00 | 100.00 | 100.00 | 100.00 | 100.00 | 100.00 | 100.00 |
______________+________+________+________+________+________+________+________+
Total 8295 2339 6436 8039 4752 13 40 29914
27.73 7.82 21.52 26.87 15.89 0.04 0.13 100.00

Administration of anticoagulants at the time of obtaining Table 2-4 consent, according to the presence or absence of proton-pump inhibitors

FREQ Procedure

Table : ALL * n_ACT2

ALL n_ACT2

```
Power |
Percent |
Percent of rows
Percent of columns | 1| Total
______________+________+
             1 |   27575 |   27575
               | 100.00 | 100.00
               | 100.00 |
               | 100.00 |
______________+________+
Total 27575 27575
                 100.00     100.00
```

Frequency of missing values = 2339

Administration of anticoagulants at the time of obtaining Table 2-4 consent, according to the presence or absence of proton-pump inhibitors

FREQ Procedure

Tables: ALL* ACTYP

ALL ACTYP(Type of AF)

Power |
Percent |
Percent of rows
Percent of column | Warfa| Dabigat| Riverro| Apixa| Edoxa| Parenteral Anti| Edoxa| Total
|phosphate|run|xaban|ban|ban|coagulant|ban (OD|)
| | | | | | |) |
______________+________+________+________+________+________+________+_______
_+
1 | 8295 | 2339 | 6436 | 8039 | 4752 | 13 | 40 | 29914
| 27.73 | 7.82 | 21.52 | 26.87 | 15.89 | 0.04 | 0.13 | 100.00
| 27.73 | 7.82 | 21.52 | 26.87 | 15.89 | 0.04 | 0.13 |
| 100.00 | 100.00 | 100.00 | 100.00 | 100.00 | 100.00 | 100.00 |
______________+________+________+________+________+________+________+_______
_+
Total 8295 2339 6436 8039 4752 13 40 29914
27.73 7.82 21.52 26.87 15.89 0.04 0.13 100.00

Administration of anticoagulants at the time of obtaining Table 2-4 consent, according to the presence or absence of proton-pump inhibitors

FREQ Procedure

Table : ALL * n_ACT3

ALL n_ACT3

```
Power |
Percent |
Percent of rows
Percent of columns | 1| Total
______________+________+
             1 |   23478 |   23478
               | 100.00 | 100.00
               | 100.00 |
               | 100.00 |
______________+________+
Total 23478 23478
                 100.00     100.00
```

Frequency of missing values = 6436

Administration of anticoagulants at the time of obtaining Table 2-4 consent, according to the presence or absence of proton-pump inhibitors

FREQ Procedure

Tables: ALL* ACTYP

ALL ACTYP(Type of AF)

Power |
Percent |
Percent of rows
Percent of column | Warfa| Dabigat| Riverro| Apixa| Edoxa| Parenteral Anti| Edoxa| Total
|phosphate|run|xaban|ban|ban|coagulant|ban (OD|)
| | | | | | |) |
______________+________+________+________+________+________+________+________
_+
1 | 8295 | 2339 | 6436 | 8039 | 4752 | 13 | 40 | 29914
| 27.73 | 7.82 | 21.52 | 26.87 | 15.89 | 0.04 | 0.13 | 100.00
| 27.73 | 7.82 | 21.52 | 26.87 | 15.89 | 0.04 | 0.13 |
| 100.00 | 100.00 | 100.00 | 100.00 | 100.00 | 100.00 | 100.00 |
______________+________+________+________+________+________+________+________
_+
Total 8295 2339 6436 8039 4752 13 40 29914
27.73 7.82 21.52 26.87 15.89 0.04 0.13 100.00

Administration of anticoagulants at the time of obtaining Table 2-4 consent, according to the presence or absence of proton-pump inhibitors

FREQ Procedure

Table : ALL * n_ACT4

ALL n_ACT4

Power |
Percent |
Percent of rows
Percent of columns | 1| Total

1	21875	21875
	100.00	100.00
	100.00	
	100.00	
Total	21875	21875
	100.00	100.00

Frequency of missing values = 8039

Administration of anticoagulants at the time of obtaining Table 2-4 consent, according to the presence or absence of proton-pump inhibitors

FREQ Procedure

Table: ALL * t_ACT0

ALL t_ACT0

Power |
Percent |
Percent of rows
Percentage of columns | 0| 1| Sum

1	25122	4792	29914
	83.98	16.02	100.00
	83.98	16.02	
	100.00	100.00	
Total	25122	4792	29914
	83.98	16.02	100.00

Administration of anticoagulants at the time of obtaining Table 2-4 consent, according to the presence or absence of proton-pump inhibitors

FREQ Procedure

Table: ALL * t_ACT0

ALL t_ACT0

Power |
Percent |
Percent of rows
Percentage of columns | 0| 1| Sum

1	25122	4792	29914
	83.98	16.02	100.00
	83.98	16.02	
	100.00	100.00	
Total	25122	4792	29914
	83.98	16.02	100.00

Administration of anticoagulants at the time of obtaining Table 2-4 consent, according to the presence or absence of proton-pump inhibitors

FREQ Procedure

Tables: ALL* ACTYP

ALL ACTYP(Type of AF)

Power |
Percent |
Percent of rows
Percent of column | Warfa| Dabigat| Riverro| Apixa| Edoxa| Parenteral Anti| Edoxa| Total
|phosphate|run|xaban|ban|ban|coagulant|ban (OD|)
| | | | | | |) |

	Warfa phosphate	Dabigat run	Riverro xaban	Apixa ban	Edoxa ban	Parenteral Anti coagulant	Edoxa ban (OD)	Total
1	8295	2339	6436	8039	4752	13	40	29914
	27.73	7.82	21.52	26.87	15.89	0.04	0.13	100.00
	27.73	7.82	21.52	26.87	15.89	0.04	0.13	
	100.00	100.00	100.00	100.00	100.00	100.00	100.00	
Total	8295	2339	6436	8039	4752	13	40	29914
	27.73	7.82	21.52	26.87	15.89	0.04	0.13	100.00

Administration of anticoagulants at the time of obtaining Table 2-4 consent, according to the presence or absence of proton-pump inhibitors

FREQ Procedure

Table : ALL * n_ACT5

ALL n_ACT5

Power |
Percent |
Percent of rows
Percent of columns | 1| Total

1	25162	25162
	100.00	100.00
	100.00	
	100.00	
Total	25162	25162
	100.00	100.00

Degree of missing values = 4752

Administration of anticoagulants at the time of obtaining Table 2-4 consent, according to the presence or absence of proton-pump inhibitors

FREQ Procedure

Tables: ALL* ACTYP

ALL ACTYP(Type of AF)

Power |
Percent |
Percent of rows
Percent of column | Warfa| Dabigat| Riverro| Apixa| Edoxa| Parenteral Anti| Edoxa| Total
|phosphate|run|xaban|ban|ban|coagulant|ban (OD|)
| | | | | | |) |

	Warfa	Dabigat	Riverro	Apixa	Edoxa	Parenteral Anti	Edoxa	Total
1	8295	2339	6436	8039	4752	13	40	29914
	27.73	7.82	21.52	26.87	15.89	0.04	0.13	100.00
	27.73	7.82	21.52	26.87	15.89	0.04	0.13	
	100.00	100.00	100.00	100.00	100.00	100.00	100.00	
Total	8295	2339	6436	8039	4752	13	40	29914
	27.73	7.82	21.52	26.87	15.89	0.04	0.13	100.00

Administration of anticoagulants at the time of obtaining Table 2-4 consent, according to the presence or absence of proton-pump inhibitors

FREQ Procedure

Table : ALL * n_ACT7

ALL n_ACT7

```
Power |
Percent |
Percent of rows
Percent of columns | 1| Total
______________+________+
             1 |   29874 |   29874
               | 100.00 | 100.00
               | 100.00 |
               | 100.00 |
______________+________+
Total 29874 29874
               100.00     100.00
```

Degree of missing values = 40

Administration of anticoagulants at the time of obtaining Table 2-4 consent, according to the presence or absence of proton-pump inhibitors

FREQ Procedure

Tables: ALL* ACTYP

ALL ACTYP(Type of AF)

Power |
Percent |
Percent of rows
Percent of column | Warfa| Dabigat| Riverro| Apixa| Edoxa| Parenteral Anti| Edoxa| Total
|phosphate|run|xaban|ban|ban|coagulant|ban (OD|)
| | | | | | |) |
______________+________+________+________+________+________+________+________
_+

ALL	Warfa phosphate	Dabigatrun	Riverroxaban	Apixaban	Edoxaban	Parenteral Anticoagulant	Edoxaban (OD)	Total
1	8295	2339	6436	8039	4752	13	40	29914
	27.73	7.82	21.52	26.87	15.89	0.04	0.13	100.00
	27.73	7.82	21.52	26.87	15.89	0.04	0.13	
	100.00	100.00	100.00	100.00	100.00	100.00	100.00	

______________+________+________+________+________+________+________+________
_+

Total 8295 2339 6436 8039 4752 13 40 29914								
	27.73	7.82	21.52	26.87	15.89	0.04	0.13	100.00

Administration of anticoagulants at the time of obtaining Table 2-4 consent, according to the presence or absence of proton-pump inhibitors

FREQ Procedure

Table : ALL * n_ACT6

ALL n_ACT6

Power |
Percent |
Percent of rows
Percent of columns | 1| Total
______________+________+
1 | 29901 | 29901
| 100.00 | 100.00
| 100.00 |
| 100.00 |
______________+________+
Total 29901 29901
100.00 100.00

Degree of missing values = 13

Administration of anticoagulants at the time of obtaining Table 2-4 consent, according to the presence or absence of proton-pump inhibitors

FREQ Procedure

Table : ALL * t_ACT1

ALL t_ACT1

Power |
Percent |
Percent of rows
Percentage of columns | 0| 1| Sum

1	13	29901	29914
	0.04	99.96	100.00
	0.04	99.96	
	100.00	100.00	

Total 13 29901 29914
0.04 99.96 100.00

Administration of anticoagulants at the time of obtaining Table 2-4 consent, according to the presence or absence of proton-pump inhibitors

FREQ Procedure

Table : ALL * t_ACT1

ALL t_ACT1

Power |
Percent |
Percent of rows
Percentage of columns | 0| 1| Sum

1	13	29901	29914
	0.04	99.96	100.00
	0.04	99.96	
	100.00	100.00	
Total	13	29901	29914
	0.04	99.96	100.00

Administration of anticoagulants at the time of obtaining Table 2-4 consent, according to the presence or absence of proton-pump inhibitors

FREQ Procedure

Table : ALL * t_ACT2

ALL t_ACT2

Power |
Percent |
Percent of rows
Percentage of columns | 0| 1| Sum

1	8308	21606	29914
	27.77	72.23	100.00
	27.77	72.23	
	100.00	100.00	
Total	8308	21606	29914
	27.77	72.23	100.00

Administration of anticoagulants at the time of obtaining Table 2-4 consent, according to the presence or absence of proton-pump inhibitors

FREQ Procedure

Table : ALL * t_ACT2

ALL t_ACT2

Power |
Percent |
Percent of rows
Percentage of columns | 0| 1| Sum

1	8308	21606	29914
	27.77	72.23	100.00
	27.77	72.23	
	100.00	100.00	
Total	8308	21606	29914
	27.77	72.23	100.00

Administration of anticoagulants at the time of obtaining Table 2-4 consent, according to the presence or absence of proton-pump inhibitors

FREQ Procedure

Table : ALL * t_ACT3

ALL t_ACT3

Power |
Percent |
Percent of rows
Percentage of columns | 0| 1| Sum

Percentage of columns	0	1	Sum
1	10647	19267	29914
	35.59	64.41	100.00
	35.59	64.41	
	100.00	100.00	
Total	10647	19267	29914
	35.59	64.41	100.00

Administration of anticoagulants at the time of obtaining Table 2-4 consent, according to the presence or absence of proton-pump inhibitors

FREQ Procedure

Table : ALL * t_ACT3

ALL t_ACT3

Power |
Percent |
Percent of rows
Percentage of columns | 0| 1| Sum

1	10647	19267	29914
	35.59	64.41	100.00
	35.59	64.41	
	100.00	100.00	
Total	10647	19267	29914
	35.59	64.41	100.00

Administration of anticoagulants at the time of obtaining Table 2-4 consent, according to the presence or absence of proton-pump inhibitors

FREQ Procedure

Tables: ALL* VISITAD

ALL VISITAD(Visit and Drug Administration)

Power |
Percent |
Percent of rows
Percent of column |Month |Month |Month |First dose |Unknown | Total
Within | | |
______________+________+________+________+________+
1 | 8172 | 28 | 23 | 72 | 8295
| 98.52 | 0.34 | 0.28 | 0.87 | 100.00
| 98.52 | 0.34 | 0.28 | 0.87 |
| 100.00 | 100.00 | 100.00 | 100.00 |
______________+________+________+________+________+
Total 8172 28 23 72 8295
98.52 0.34 0.28 0.87 100.00

Administration of anticoagulants at the time of obtaining Table 2-4 consent, according to the presence or absence of proton-pump inhibitors

FREQ Procedure

Tables: ALL* VISITAD

ALL VISITAD(Visit and Drug Administration)

```
Power |
Percent |
Percent of rows
Percent of column |Month |Month |Month |First dose |Unknown | Total
                  Within | | |
______________+________+________+________+________+
             1 |   8172 |      28 |      23 |       72 |    8295
               |  98.52 |    0.34 |    0.28 |     0.87 | 100.00
               |  98.52 |    0.34 |    0.28 |     0.87 |
               | 100.00 | 100.00 | 100.00 | 100.00 |
______________+________+________+________+________+
Total 8172 28 23 72 8295
                  98.52      0.34      0.28      0.87    100.00
```

Administration of anticoagulants at the time of obtaining Table 2-4 consent, according to the presence or absence of proton-pump inhibitors

FREQ Procedure

Tables: ALL* VISITAD

ALL VISITAD(Visit and Drug Administration)

Power |
Percent |
Percent of rows
Percent of column |Month |Month |Month |First dose |Unknown | Total
Within | | |
______________+________+________+________+________+
1 | 8172 | 28 | 23 | 72 | 8295
| 98.52 | 0.34 | 0.28 | 0.87 | 100.00
| 98.52 | 0.34 | 0.28 | 0.87 |
| 100.00 | 100.00 | 100.00 | 100.00 |
______________+________+________+________+________+
Total 8172 28 23 72 8295
98.52 0.34 0.28 0.87 100.00

Administration of anticoagulants at the time of obtaining Table 2-4 consent, according to the presence or absence of proton-pump inhibitors

FREQ Procedure

Tables: ALL* VISITAD

ALL VISITAD(Visit and Drug Administration)

Power |
Percent |
Percent of rows
Percent of column |Month |Month |Month |First dose |Unknown | Total
Within | | |
______________+________+________+________+________+
1 | 8172 | 28 | 23 | 72 | 8295
| 98.52 | 0.34 | 0.28 | 0.87 | 100.00
| 98.52 | 0.34 | 0.28 | 0.87 |
| 100.00 | 100.00 | 100.00 | 100.00 |
______________+________+________+________+________+
Total 8172 28 23 72 8295
98.52 0.34 0.28 0.87 100.00

Administration of anticoagulants at the time of obtaining Table 2-4 consent, according to the presence or absence of proton-pump inhibitors

FREQ Procedure

Tables: ALL* VISITAD

ALL VISITAD(Visit and Drug Administration)

Power |
Percent |
Percent of rows
Percent of column |Month |Month |Month |First dose |Unknown | Total
Within | | |

1	2266	23	18	32	2339
	96.88	0.98	0.77	1.37	100.00
	96.88	0.98	0.77	1.37	
	100.00	100.00	100.00	100.00	
Total	2266	23	18	32	2339
	96.88	0.98	0.77	1.37	100.00

Administration of anticoagulants at the time of obtaining Table 2-4 consent, according to the presence or absence of proton-pump inhibitors

FREQ Procedure

Tables: ALL* VISITAD

ALL VISITAD(Visit and Drug Administration)

Power |
Percent |
Percent of rows
Percent of column |Month |Month |Month |First dose |Unknown | Total
Within | | |

______________+________+________+________+________+
1 | 2266 | 23 | 18 | 32 | 2339
| 96.88 | 0.98 | 0.77 | 1.37 | 100.00
| 96.88 | 0.98 | 0.77 | 1.37 |
| 100.00 | 100.00 | 100.00 | 100.00 |
______________+________+________+________+________+
Total 2266 23 18 32 2339
96.88 0.98 0.77 1.37 100.00

Administration of anticoagulants at the time of obtaining Table 2-4 consent, according to the presence or absence of proton-pump inhibitors

FREQ Procedure

Tables: ALL* VISITAD

ALL VISITAD(Visit and Drug Administration)

Power |
Percent |
Percent of rows
Percent of column |Month |Month |Month |First dose |Unknown | Total
Within | | |

```
______________+________+________+________+________+
          1 |    2266 |       23 |       18 |       32 |    2339
            |   96.88 |     0.98 |     0.77 |     1.37 | 100.00
            |   96.88 |     0.98 |     0.77 |     1.37 |
            | 100.00 | 100.00 | 100.00 | 100.00 |
______________+________+________+________+________+
Total 2266 23 18 32 2339
               96.88     0.98     0.77     1.37    100.00
```

Administration of anticoagulants at the time of obtaining Table 2-4 consent, according to the presence or absence of proton-pump inhibitors

FREQ Procedure

Tables: ALL* VISITAD

ALL VISITAD(Visit and Drug Administration)

Power |
Percent |
Percent of rows
Percent of column |Month |Month |Month |First dose |Unknown | Total
Within | | |

1	2266	23	18	32	2339
	96.88	0.98	0.77	1.37	100.00
	96.88	0.98	0.77	1.37	
	100.00	100.00	100.00	100.00	
Total	2266	23	18	32	2339
	96.88	0.98	0.77	1.37	100.00

Administration of anticoagulants at the time of obtaining Table 2-4 consent, according to the presence or absence of proton-pump inhibitors

FREQ Procedure

Tables: ALL* VISITAD

ALL VISITAD(Visit and Drug Administration)

```
Power |
Percent |
Percent of rows
Percent of column |Month |Month |Month |First dose |Unknown | Total
           Within | | |
_______________+________+________+________+________+
              1 |   6162 |    109 |    110 |     55 |   6436
                |  95.74 |   1.69 |   1.71 |   0.85 | 100.00
                |  95.74 |   1.69 |   1.71 |   0.85 |
                | 100.00 | 100.00 | 100.00 | 100.00 |
_______________+________+________+________+________+
Total 6162 109 110 55 6436
                   95.74     1.69     1.71     0.85   100.00
```

Administration of anticoagulants at the time of obtaining Table 2-4 consent, according to the presence or absence of proton-pump inhibitors

FREQ Procedure

Tables: ALL* VISITAD

ALL VISITAD(Visit and Drug Administration)

```
Power |
Percent |
Percent of rows
Percent of column |Month |Month |Month |First dose |Unknown | Total
           Within | | |
______________+________+________+________+________+
            1 |   6162 |    109 |    110 |     55 |   6436
              |  95.74 |   1.69 |   1.71 |   0.85 | 100.00
              |  95.74 |   1.69 |   1.71 |   0.85 |
              | 100.00 | 100.00 | 100.00 | 100.00 |
______________+________+________+________+________+
Total 6162 109 110 55 6436
                 95.74     1.69     1.71     0.85   100.00
```

Administration of anticoagulants at the time of obtaining Table 2-4 consent, according to the presence or absence of proton-pump inhibitors

FREQ Procedure

Tables: ALL* VISITAD

ALL VISITAD(Visit and Drug Administration)

```
Power |
Percent |
Percent of rows
Percent of column |Month |Month |Month |First dose |Unknown | Total
           Within | | |
______________+________+________+________+________+
          1 |   6162 |    109 |    110 |     55 |   6436
            |  95.74 |   1.69 |   1.71 |   0.85 | 100.00
            |  95.74 |   1.69 |   1.71 |   0.85 |
            | 100.00 | 100.00 | 100.00 | 100.00 |
______________+________+________+________+________+
Total 6162 109 110 55 6436
              95.74     1.69     1.71     0.85   100.00
```

Administration of anticoagulants at the time of obtaining Table 2-4 consent, according to the presence or absence of proton-pump inhibitors

FREQ Procedure

Tables: ALL* VISITAD

ALL VISITAD(Visit and Drug Administration)

Power |
Percent |
Percent of rows
Percent of column |Month |Month |Month |First dose |Unknown | Total
Within | | |
______________+________+________+________+________+
1 | 6162 | 109 | 110 | 55 | 6436
| 95.74 | 1.69 | 1.71 | 0.85 | 100.00
| 95.74 | 1.69 | 1.71 | 0.85 |
| 100.00 | 100.00 | 100.00 | 100.00 |
______________+________+________+________+________+
Total 6162 109 110 55 6436
95.74 1.69 1.71 0.85 100.00

Administration of anticoagulants at the time of obtaining Table 2-4 consent, according to the presence or absence of proton-pump inhibitors

FREQ Procedure

Tables: ALL* VISITAD

ALL VISITAD(Visit and Drug Administration)

```
Power |
Percent |
Percent of rows
Percent of column |Month |Month |Month |First dose |Unknown | Total
              Within | | |
______________+________+________+________+________+
            1 |   7747 |    128 |    130 |     34 |   8039
              |  96.37 |   1.59 |   1.62 |   0.42 | 100.00
              |  96.37 |   1.59 |   1.62 |   0.42 |
              | 100.00 | 100.00 | 100.00 | 100.00 |
______________+________+________+________+________+
Total 7747 128 130 34 8039
                 96.37     1.59     1.62     0.42   100.00
```

Administration of anticoagulants at the time of obtaining Table 2-4 consent, according to the presence or absence of proton-pump inhibitors

FREQ Procedure

Tables: ALL* VISITAD

ALL VISITAD(Visit and Drug Administration)

Power |
Percent |
Percent of rows
Percent of column |Month |Month |Month |First dose |Unknown | Total
Within | | |

1	7747	128	130	34	8039
	96.37	1.59	1.62	0.42	100.00
	96.37	1.59	1.62	0.42	
	100.00	100.00	100.00	100.00	
Total	7747	128	130	34	8039
	96.37	1.59	1.62	0.42	100.00

Administration of anticoagulants at the time of obtaining Table 2-4 consent, according to the presence or absence of proton-pump inhibitors

FREQ Procedure

Tables: ALL* VISITAD

ALL VISITAD(Visit and Drug Administration)

Power |
Percent |
Percent of rows
Percent of column |Month |Month |Month |First dose |Unknown | Total
Within | | |
______________+________+________+________+________+
1 | 7747 | 128 | 130 | 34 | 8039
| 96.37 | 1.59 | 1.62 | 0.42 | 100.00
| 96.37 | 1.59 | 1.62 | 0.42 |
| 100.00 | 100.00 | 100.00 | 100.00 |
______________+________+________+________+________+
Total 7747 128 130 34 8039
96.37 1.59 1.62 0.42 100.00

Administration of anticoagulants at the time of obtaining Table 2-4 consent, according to the presence or absence of proton-pump inhibitors

FREQ Procedure

Tables: ALL* VISITAD

ALL VISITAD(Visit and Drug Administration)

Power |
Percent |
Percent of rows
Percent of column |Month |Month |Month |First dose |Unknown | Total
Within | | |
______________+________+________+________+________+
1 | 7747 | 128 | 130 | 34 | 8039
| 96.37 | 1.59 | 1.62 | 0.42 | 100.00
| 96.37 | 1.59 | 1.62 | 0.42 |
| 100.00 | 100.00 | 100.00 | 100.00 |
______________+________+________+________+________+
Total 7747 128 130 34 8039
96.37 1.59 1.62 0.42 100.00

Administration of anticoagulants at the time of obtaining Table 2-4 consent, according to the presence or absence of proton-pump inhibitors

FREQ Procedure

Tables: ALL* VISITAD

ALL VISITAD(Visit and Drug Administration)

Power |
Percent |
Percent of rows
Percent of column |Month |Month |Month |First dose |Unknown | Total
Within | | |

1	4141	226	400	25	4792
	86.41	4.72	8.35	0.52	100.00
	86.41	4.72	8.35	0.52	
	100.00	100.00	100.00	100.00	
Total	4141	226	400	25	4792
	86.41	4.72	8.35	0.52	100.00

Administration of anticoagulants at the time of obtaining Table 2-4 consent, according to the presence or absence of proton-pump inhibitors

FREQ Procedure

Tables: ALL* VISITAD

ALL VISITAD(Visit and Drug Administration)

Power<br>Percent<br>Percent of rows<br>Percent of column Within	Month	Month	Month	First dose	Unknown	Total
1	4141<br>86.41<br>86.41<br>100.00	226<br>4.72<br>4.72<br>100.00	400<br>8.35<br>8.35<br>100.00	25<br>0.52<br>0.52<br>100.00		4792<br>100.00
Total	4141<br>86.41	226<br>4.72	400<br>8.35	25<br>0.52		4792<br>100.00

Administration of anticoagulants at the time of obtaining Table 2-4 consent, according to the presence or absence of proton-pump inhibitors

FREQ Procedure

Tables: ALL* VISITAD

ALL VISITAD(Visit and Drug Administration)

Power / Percent / Percent of rows / Percent of column	Month Within	Month	Month	First dose	Unknown	Total
1	4141	226	400	25	4792	
	86.41	4.72	8.35	0.52	100.00	
	86.41	4.72	8.35	0.52		
	100.00	100.00	100.00	100.00		
Total	4141	226	400	25	4792	
	86.41	4.72	8.35	0.52	100.00	

Administration of anticoagulants at the time of obtaining Table 2-4 consent, according to the presence or absence of proton-pump inhibitors

FREQ Procedure

Tables: ALL* VISITAD

ALL VISITAD(Visit and Drug Administration)

Power | Percent | Percent of rows Percent of column Within	Month	Month	Month	First dose	Unknown	Total
1	4141	226	400	25		4792
	86.41	4.72	8.35	0.52		100.00
	86.41	4.72	8.35	0.52		
	100.00	100.00	100.00	100.00		
Total	4141	226	400	25		4792
	86.41	4.72	8.35	0.52		100.00

Administration of anticoagulants at the time of obtaining Table 2-4 consent, according to the presence or absence of proton-pump inhibitors

FREQ Procedure

Tables: ALL* VISITAD

ALL VISITAD(Visit and Drug Administration)

```
Power |
Percent |
Percent of rows
Percent of column |Month |Month |Month |First dose |Unknown | Total
          Within | | |
______________+________+________+________+________+
        1 |   4105 |    225 |    397 |     25 |   4752
          |  86.38 |   4.73 |   8.35 |   0.53 | 100.00
          |  86.38 |   4.73 |   8.35 |   0.53 |
          | 100.00 | 100.00 | 100.00 | 100.00 |
______________+________+________+________+________+
Total 4105 225 397 25 4752
             86.38     4.73     8.35     0.53   100.00
```

Administration of anticoagulants at the time of obtaining Table 2-4 consent, according to the presence or absence of proton-pump inhibitors

FREQ Procedure

Tables: ALL* VISITAD

ALL VISITAD(Visit and Drug Administration)

Power |
Percent |
Percent of rows

Percent of column Within	Month	Month	Month	First dose	Unknown	Total
1	4105	225	397	25		4752
	86.38	4.73	8.35	0.53		100.00
	86.38	4.73	8.35	0.53		
	100.00	100.00	100.00	100.00		
Total	4105	225	397	25		4752
	86.38	4.73	8.35	0.53		100.00

Administration of anticoagulants at the time of obtaining Table 2-4 consent, according to the presence or absence of proton-pump inhibitors

FREQ Procedure

Tables: ALL* VISITAD

ALL VISITAD(Visit and Drug Administration)

Power |
Percent |
Percent of rows

Percent of column	Month Within	Month	Month	First dose	Unknown	Total
1	4105	225	397	25	4752	
	86.38	4.73	8.35	0.53	100.00	
	86.38	4.73	8.35	0.53		
	100.00	100.00	100.00	100.00		
Total	4105	225	397	25	4752	
	86.38	4.73	8.35	0.53	100.00	

Administration of anticoagulants at the time of obtaining Table 2-4 consent, according to the presence or absence of proton-pump inhibitors

FREQ Procedure

Tables: ALL* VISITAD

ALL VISITAD(Visit and Drug Administration)

Power |
Percent |
Percent of rows
Percent of column |Month |Month |Month |First dose |Unknown | Total
Within | | |

1	4105	225	397	25	4752
	86.38	4.73	8.35	0.53	100.00
	86.38	4.73	8.35	0.53	
	100.00	100.00	100.00	100.00	
Total	4105	225	397	25	4752
	86.38	4.73	8.35	0.53	100.00

Administration of anticoagulants at the time of obtaining Table 2-4 consent, according to the presence or absence of proton-pump inhibitors

FREQ Procedure

Tables: ALL* VISITAD

ALL

VISITAD(Visit and Drug Administration)

Power |
Percent |
Percent of rows
Percent of column |Month |Month |Month |First dose |Total
Within | |
_______________+_________+_________+_________+
1 | 36 | 1 | 3 | 40
| 90.00 | 2.50 | 7.50 | 100.00
| 90.00 | 2.50 | 7.50 |
| 100.00 | 100.00 | 100.00 |
_______________+_________+_________+_________+
Total 36 1 3 40
90.00 2.50 7.50 100.00

Administration of anticoagulants at the time of obtaining Table 2-4 consent, according to the presence or absence of proton-pump inhibitors

FREQ Procedure

Tables: ALL* VISITAD

ALL

VISITAD(Visit and Drug Administration)

Power |
Percent |
Percent of rows
Percent of column |Month |Month |Month |First dose |Total
Within | |

1	36	1	3	40
	90.00	2.50	7.50	100.00
	90.00	2.50	7.50	
	100.00	100.00	100.00	
Total	36	1	3	40
	90.00	2.50	7.50	100.00

Administration of anticoagulants at the time of obtaining Table 2-4 consent, according to the presence or absence of proton-pump inhibitors

FREQ Procedure

Tables: ALL* VISITAD

ALL

VISITAD(Visit and Drug Administration)

Power |
Percent |
Percent of rows
Percent of column |Month |Month |Month |First dose |Total
Within | |
______________+________+________+________+
1 | 36 | 1 | 3 | 40
| 90.00 | 2.50 | 7.50 | 100.00
| 90.00 | 2.50 | 7.50 |
| 100.00 | 100.00 | 100.00 |
______________+________+________+________+
Total 36 1 3 40
90.00 2.50 7.50 100.00

Administration of anticoagulants at the time of obtaining Table 2-4 consent, according to the presence or absence of proton-pump inhibitors

FREQ Procedure

Tables: ALL* VISITAD

ALL

VISITAD(Visit and Drug Administration)

Power |

Percent |

Percent of rows

Percent of column |Month |Month |Month |First dose |Total

Within | |

______________+________+________+________+

1 | 36 | 1 | 3 | 40

| 90.00 | 2.50 | 7.50 | 100.00

| 90.00 | 2.50 | 7.50 |

| 100.00 | 100.00 | 100.00 |

______________+________+________+________+

Total 36 1 3 40

90.00 2.50 7.50 100.00

Administration of anticoagulants at the time of obtaining Table 2-4 consent, according to the presence or absence of proton-pump inhibitors

FREQ Procedure

Tables: ALL* VISITAD

ALL

VISITAD(Visit and Drug Administration)

Power |
Percent |
Percent of rows
Percent of column |Month | Unknown | Total
) in front | |

______________+________+________+

1 | 11 | 2 | 13
| 84.62 | 15.38 | 100.00
| 84.62 | 15.38 |
| 100.00 | 100.00 |

______________+________+________+

Total 11 2 13
84.62 15.38 100.00

Administration of anticoagulants at the time of obtaining Table 2-4 consent, according to the presence or absence of proton-pump inhibitors

FREQ Procedure

Tables: ALL* VISITAD

ALL
VISITAD(Visit and Drug Administration)

Power |
Percent |
Percent of rows
Percent of column |Month | Unknown | Total
) in front | |

1	11	2	13
	84.62	15.38	100.00
	84.62	15.38	
	100.00	100.00	
Total	11	2	13
	84.62	15.38	100.00

Administration of anticoagulants at the time of obtaining Table 2-4 consent, according to the presence or absence of proton-pump inhibitors

FREQ Procedure

Tables: ALL* VISITAD

ALL

VISITAD(Visit and Drug Administration)

Power |
Percent |
Percent of rows
Percent of column |Month | Unknown | Total
) in front | |

1	11	2	13
	84.62	15.38	100.00
	84.62	15.38	
	100.00	100.00	
Total	11	2	13
	84.62	15.38	100.00

Administration of anticoagulants at the time of obtaining Table 2-4 consent, according to the presence or absence of proton-pump inhibitors

FREQ Procedure

Tables: ALL* VISITAD

ALL

VISITAD(Visit and Drug Administration)

Power |

Percent |

Percent of rows

Percent of column |Month | Unknown | Total

) in front | |

______________+________+________+

1 | 11 | 2 | 13

| 84.62 | 15.38 | 100.00

| 84.62 | 15.38 |

| 100.00 | 100.00 |

______________+________+________+

Total 11 2 13

84.62 15.38 100.00

Administration of anticoagulants at the time of obtaining Table 2-4 consent, according to the presence or absence of proton-pump inhibitors

FREQ Procedure

Tables: ALL * DOSE

ALL DOSE(Dose per day for DOAC only)

Power |
Percent |
Percent of rows

Column percent	0	50	75	110	150	185	220	300	Total
1	2	1	11	205	123	3	1846	148	2339
	0.09	0.04	0.47	8.76	5.26	0.13	78.92	6.33	100.00
	0.09	0.04	0.47	8.76	5.26	0.13	78.92	6.33	
	100.00	100.00	100.00	100.00	100.00	100.00	100.00	100.00	
Total	2	1	11	205	123	3	1846	148	2339
	0.09	0.04	0.47	8.76	5.26	0.13	78.92	6.33	100.00

Administration of anticoagulants at the time of obtaining Table 2-4 consent, according to the presence or absence of proton-pump inhibitors

FREQ Procedure

Tables: ALL * DOSE

ALL DOSE(Dose per day for DOAC only)

Power |
Percent |
Percent of rows

Column percent	0	50	75	110	150	185	220	300	Total
1	2	1	11	205	123	3	1846	148	2339
	0.09	0.04	0.47	8.76	5.26	0.13	78.92	6.33	100.00
	0.09	0.04	0.47	8.76	5.26	0.13	78.92	6.33	
	100.00	100.00	100.00	100.00	100.00	100.00	100.00	100.00	
Total	2	1	11	205	123	3	1846	148	2339
	0.09	0.04	0.47	8.76	5.26	0.13	78.92	6.33	100.00

Administration of anticoagulants at the time of obtaining Table 2-4 consent, according to the presence or absence of proton-pump inhibitors

FREQ Procedure

Tables: ALL * DOSE

ALL DOSE(Dose per day for DOAC only)

Power |
Percent |
Percent of rows
Column percent | 0| 50| 75| 110| 150| 185| 220| 300| Total

______________+________+________+________+________+________+________+________+________+

1	2	1	11	205	123	3	1846	148	2339
	0.09	0.04	0.47	8.76	5.26	0.13	78.92	6.33	100.00
	0.09	0.04	0.47	8.76	5.26	0.13	78.92	6.33	
	100.00	100.00	100.00	100.00	100.00	100.00	100.00	100.00	

______________+________+________+________+________+________+________+________+________+

Total 2 1 11 205 123 3 1846 148 2339
0.09 0.04 0.47 8.76 5.26 0.13 78.92 6.33
100.00

Administration of anticoagulants at the time of obtaining Table 2-4 consent, according to the presence or absence of proton-pump inhibitors

FREQ Procedure

Tables: ALL * DOSE

ALL DOSE(Dose per day for DOAC only)

Power |
Percent |
Percent of rows
Column percent | 0| 50| 75| 110| 150| 185| 220| 300| Total
______________+________+________+________+________+________+________+________+_______
_+________+
1 | 2 | 1 | 11 | 205 | 123 | 3 | 1846 | 148 | 2339
| 0.09 | 0.04 | 0.47 | 8.76 | 5.26 | 0.13 | 78.92 | 6.33 | 100.00
| 0.09 | 0.04 | 0.47 | 8.76 | 5.26 | 0.13 | 78.92 | 6.33 |
| 100.00 | 100.00 | 100.00 | 100.00 | 100.00 | 100.00 | 100.00 | 100.00 |
______________+________+________+________+________+________+________+________+_______
_+________+
Total 2 1 11 205 123 3 1846 148 2339
0.09 0.04 0.47 8.76 5.26 0.13 78.92 6.33
100.00

Administration of anticoagulants at the time of obtaining Table 2-4 consent, according to the presence or absence of proton-pump inhibitors

FREQ Procedure

Tables: ALL * DOSE

ALL DOSE(Dose per day for DOAC only)

Power |
Percent |
Percent of rows
Column percent | 0| 50| 75| 110| 150| 185| 220| 300| Total
______________+________+________+________+________+________+________+_______
_+________+
1 | 2 | 1 | 11 | 205 | 123 | 3 | 1846 | 148 | 2339
| 0.09 | 0.04 | 0.47 | 8.76 | 5.26 | 0.13 | 78.92 | 6.33 | 100.00
| 0.09 | 0.04 | 0.47 | 8.76 | 5.26 | 0.13 | 78.92 | 6.33 |
| 100.00 | 100.00 | 100.00 | 100.00 | 100.00 | 100.00 | 100.00 | 100.00 |
______________+________+________+________+________+________+________+_______
_+________+
Total 2 1 11 205 123 3 1846 148 2339
0.09 0.04 0.47 8.76 5.26 0.13 78.92 6.33
100.00

Administration of anticoagulants at the time of obtaining Table 2-4 consent, according to the presence or absence of proton-pump inhibitors

FREQ Procedure

Table : ALL * f_d2

ALL f_d2

Power |
Percent |
Percent of rows
Percent of columns | 1| Total

```
______________+________+
          1 |       6 |        6
            | 100.00 | 100.00
            | 100.00 |
            | 100.00 |
______________+________+
Total 6 6
              100.00    100.00
```

Degree of missing values = 2333

Administration of anticoagulants at the time of obtaining Table 2-4 consent, according to the presence or absence of proton-pump inhibitors

FREQ Procedure

Tables: ALL * DOSE

ALL DOSE(Dose per day for DOAC only)

Power |
Percent |
Percent of rows
Column percent | 0| 2.5| 5| 7.5| 10| 15| 30| 60| Total
______________+________+________+________+________+________+________+_______
_+________+
1 | 2 | 1 | 12 | 6 | 4298 | 2109 | 7 | 1 | 6436
| 0.03 | 0.02 | 0.19 | 0.09 | 66.78 | 32.77 | 0.11 | 0.02 | 100.00
| 0.03 | 0.02 | 0.19 | 0.09 | 66.78 | 32.77 | 0.11 | 0.02 |
| 100.00 | 100.00 | 100.00 | 100.00 | 100.00 | 100.00 | 100.00 | 100.00 |
______________+________+________+________+________+________+________+_______
_+________+
Total 2 1 12 6 4298 2109 7 1 6436
0.03 0.02 0.19 0.09 66.78 32.77 0.11 0.02
100.00

Administration of anticoagulants at the time of obtaining Table 2-4 consent, according to the presence or absence of proton-pump inhibitors

FREQ Procedure

Tables: ALL * DOSE

ALL DOSE(Dose per day for DOAC only)

Power |
Percent |
Percent of rows

Column percent	0	2.5	5	7.5	10	15	30	60	Total
1	2	1	12	6	4298	2109	7	1	6436
	0.03	0.02	0.19	0.09	66.78	32.77	0.11	0.02	100.00
	0.03	0.02	0.19	0.09	66.78	32.77	0.11	0.02	
	100.00	100.00	100.00	100.00	100.00	100.00	100.00	100.00	
Total	2	1	12	6	4298	2109	7	1	6436
	0.03	0.02	0.19	0.09	66.78	32.77	0.11	0.02	100.00

Administration of anticoagulants at the time of obtaining Table 2-4 consent, according to the presence or absence of proton-pump inhibitors

FREQ Procedure

Table : ALL * f_d3

ALL f_d3

Power |
Percent |
Percent of rows
Percent of columns | 1| Total

1	29	29
	100.00	100.00
	100.00	
	100.00	
Total	29	29
	100.00	100.00

Frequency of missing values = 6407

Administration of anticoagulants at the time of obtaining Table 2-4 consent, according to the presence or absence of proton-pump inhibitors

FREQ Procedure

Tables: ALL * DOSE

ALL DOSE(Dose per day for DOAC only)

Power |
Percent |
Percent of rows
Column percent | 0| 1.25| 2.5| 5| 7.5| 10| 15| 30| Total
______________+________+________+________+________+________+________+________
_+________+
1 | 2 | 1 | 518 | 5153 | 5 | 2358 | 1 | 1 | 8039
| 0.02 | 0.01 | 6.44 | 64.10 | 0.06 | 29.33 | 0.01 | 0.01 | 100.00
| 0.02 | 0.01 | 6.44 | 64.10 | 0.06 | 29.33 | 0.01 | 0.01 |
| 100.00 | 100.00 | 100.00 | 100.00 | 100.00 | 100.00 | 100.00 | 100.00 |
______________+________+________+________+________+________+________+________
_+________+
Total 2 1 518 5153 5 2358 1 1 8039
0.02 0.01 6.44 64.10 0.06 29.33 0.01 0.01
100.00

Administration of anticoagulants at the time of obtaining Table 2-4 consent, according to the presence or absence of proton-pump inhibitors

FREQ Procedure

Tables: ALL * DOSE

ALL DOSE(Dose per day for DOAC only)

Power |
Percent |
Percent of rows
Column percent

ALL	0	1.25	2.5	5	7.5	10	15	30	Total
1	2	1	518	5153	5	2358	1	1	8039
	0.02	0.01	6.44	64.10	0.06	29.33	0.01	0.01	100.00
	0.02	0.01	6.44	64.10	0.06	29.33	0.01	0.01	
	100.00	100.00	100.00	100.00	100.00	100.00	100.00	100.00	
Total	2	1	518	5153	5	2358	1	1	8039
	0.02	0.01	6.44	64.10	0.06	29.33	0.01	0.01	100.00

Administration of anticoagulants at the time of obtaining Table 2-4 consent, according to the presence or absence of proton-pump inhibitors

FREQ Procedure

Tables: ALL * DOSE

ALL DOSE(Dose per day for DOAC only)

Power |
Percent |
Percent of rows
Column percent | 0| 1.25| 2.5| 5| 7.5| 10| 15| 30| Total
______________+________+________+________+________+________+________+_______
_+________+
1 | 2 | 1 | 518 | 5153 | 5 | 2358 | 1 | 1 | 8039
| 0.02 | 0.01 | 6.44 | 64.10 | 0.06 | 29.33 | 0.01 | 0.01 | 100.00
| 0.02 | 0.01 | 6.44 | 64.10 | 0.06 | 29.33 | 0.01 | 0.01 |
| 100.00 | 100.00 | 100.00 | 100.00 | 100.00 | 100.00 | 100.00 | 100.00 |
______________+________+________+________+________+________+________+_______
_+________+
Total 2 1 518 5153 5 2358 1 1 8039
0.02 0.01 6.44 64.10 0.06 29.33 0.01 0.01
100.00

Administration of anticoagulants at the time of obtaining Table 2-4 consent, according to the presence or absence of proton-pump inhibitors

FREQ Procedure

Table : ALL * f_d4

ALL f_d4

Power |
Percent |
Percent of rows
Percent of columns | 1| Total

1	10	10
	100.00	100.00
	100.00	
	100.00	
Total	10	10
	100.00	100.00

Frequency of missing values = 8029

Administration of anticoagulants at the time of obtaining Table 2-4 consent, according to the presence or absence of proton-pump inhibitors

FREQ Procedure

Tables: ALL * DOSE

ALL DOSE(Dose per day for DOAC only)

Power |
Percent |
Percent of rows
Percentage of columns | 5| 7.5| 10| 15| 30| 60| Total

	5	7.5	10	15	30	60	Total
1	2	1	1	243	3939	606	4792
	0.04	0.02	0.02	5.07	82.20	12.65	100.00
	0.04	0.02	0.02	5.07	82.20	12.65	
	100.00	100.00	100.00	100.00	100.00	100.00	
Total	2	1	1	243	3939	606	4792
	0.04	0.02	0.02	5.07	82.20	12.65	100.00

Administration of anticoagulants at the time of obtaining Table 2-4 consent, according to the presence or absence of proton-pump inhibitors

FREQ Procedure

Tables: ALL * DOSE

ALL DOSE(Dose per day for DOAC only)

Power / Percent / Percent of rows / Percentage of columns	5	7.5	10	15	30	60	Total
1	2	1	1	243	3939	606	4792
	0.04	0.02	0.02	5.07	82.20	12.65	100.00
	0.04	0.02	0.02	5.07	82.20	12.65	
	100.00	100.00	100.00	100.00	100.00	100.00	
Total	2	1	1	243	3939	606	4792
	0.04	0.02	0.02	5.07	82.20	12.65	100.00

Administration of anticoagulants at the time of obtaining Table 2-4 consent, according to the presence or absence of proton-pump inhibitors

FREQ Procedure

Tables: ALL * DOSE

ALL DOSE(Dose per day for DOAC only)

Power |
Percent |
Percent of rows
Percentage of columns | 5| 7.5| 10| 15| 30| 60| Total

	5	7.5	10	15	30	60	Total
1	2	1	1	243	3939	606	4792
	0.04	0.02	0.02	5.07	82.20	12.65	100.00
	0.04	0.02	0.02	5.07	82.20	12.65	
	100.00	100.00	100.00	100.00	100.00	100.00	
Total	2	1	1	243	3939	606	4792
	0.04	0.02	0.02	5.07	82.20	12.65	100.00

Administration of anticoagulants at the time of obtaining Table 2-4 consent, according to the presence or absence of proton-pump inhibitors

FREQ Procedure

Table: ALL * f_d0

ALL f_d0

Power |
Percent |
Percent of rows
Percent of columns | 1| Total

1	4	4
	100.00	100.00
	100.00	
	100.00	
Total	4	4
	100.00	100.00

Degree of missing values = 4788

Administration of anticoagulants at the time of obtaining Table 2-4 consent, according to the presence or absence of proton-pump inhibitors

FREQ Procedure

Tables: ALL * DOSE

ALL DOSE(Dose per day for DOAC only)

Power |
Percent |
Percent of rows
Percentage of columns | 5| 7.5| 10| 15| 30| 60| Total

1	2	1	1	242	3911	595	4752
	0.04	0.02	0.02	5.09	82.30	12.52	100.00
	0.04	0.02	0.02	5.09	82.30	12.52	
	100.00	100.00	100.00	100.00	100.00	100.00	
Total	2	1	1	242	3911	595	4752
	0.04	0.02	0.02	5.09	82.30	12.52	100.00

Administration of anticoagulants at the time of obtaining Table 2-4 consent, according to the presence or absence of proton-pump inhibitors

FREQ Procedure

Tables: ALL * DOSE

ALL DOSE(Dose per day for DOAC only)

Power |
Percent |
Percent of rows
Percentage of columns | 5| 7.5| 10| 15| 30| 60| Total

1	2	1	1	242	3911	595	4752
	0.04	0.02	0.02	5.09	82.30	12.52	100.00
	0.04	0.02	0.02	5.09	82.30	12.52	
	100.00	100.00	100.00	100.00	100.00	100.00	
Total	2	1	1	242	3911	595	4752
	0.04	0.02	0.02	5.09	82.30	12.52	100.00

Administration of anticoagulants at the time of obtaining Table 2-4 consent, according to the presence or absence of proton-pump inhibitors

FREQ Procedure

Tables: ALL * DOSE

ALL DOSE(Dose per day for DOAC only)

Power |
Percent |
Percent of rows
Percentage of columns | 5| 7.5| 10| 15| 30| 60| Total

1	2	1	1	242	3911	595	4752
	0.04	0.02	0.02	5.09	82.30	12.52	100.00
	0.04	0.02	0.02	5.09	82.30	12.52	
	100.00	100.00	100.00	100.00	100.00	100.00	
Total	2	1	1	242	3911	595	4752
	0.04	0.02	0.02	5.09	82.30	12.52	100.00

Administration of anticoagulants at the time of obtaining Table 2-4 consent, according to the presence or absence of proton-pump inhibitors

FREQ Procedure

Table : ALL * f_d5

ALL f_d5

Power |
Percent |
Percent of rows
Percent of columns | 1| Total

1	4	4
	100.00	100.00
	100.00	
	100.00	
Total	4	4
	100.00	100.00

Degree of missing values = 4748

Administration of anticoagulants at the time of obtaining Table 2-4 consent, according to the presence or absence of proton-pump inhibitors

FREQ Procedure

Tables: ALL * DOSE

ALL DOSE(Dose per day for DOAC only)

Power |
Percent |
Percent of rows
Percentage of columns | 15| 30| 60| Total

1	1	28	11	40
	2.50	70.00	27.50	100.00
	2.50	70.00	27.50	
	100.00	100.00	100.00	
Total	1	28	11	40
	2.50	70.00	27.50	100.00

Administration of anticoagulants at the time of obtaining Table 2-4 consent, according to the presence or absence of proton-pump inhibitors

FREQ Procedure

Tables: ALL * DOSE

ALL DOSE(Dose per day for DOAC only)

Power |
Percent |
Percent of rows
Percentage of columns | 15| 30| 60| Total

1	1	28	11	40
	2.50	70.00	27.50	100.00
	2.50	70.00	27.50	
	100.00	100.00	100.00	
Total	1	28	11	40
	2.50	70.00	27.50	100.00

Administration of anticoagulants at the time of obtaining Table 2-4 consent, according to the presence or absence of proton-pump inhibitors

FREQ Procedure

Tables: ALL * DOSE

ALL DOSE(Dose per day for DOAC only)

Power |
Percent |
Percent of rows
Percentage of columns | 15| 30| 60| Total

ALL	15	30	60	Total
1	1	28	11	40
	2.50	70.00	27.50	100.00
	2.50	70.00	27.50	
	100.00	100.00	100.00	
Total	1	28	11	40
	2.50	70.00	27.50	100.00

Administration of anticoagulants at the time of obtaining Table 2-4 consent, according to the presence or absence of proton-pump inhibitors

FREQ Procedure

For ALL * f_d7,
Because all levels of the variable f_d7 are missing,
There is no data.

Administration of anticoagulants at the time of obtaining Table 2-4 consent, according to the presence or absence of proton-pump inhibitors

FREQ Procedure

Tables: ALL * DOSECNT

ALL

DOSECNT(Dose per 1time for DOAC only)

Power |

Percent |

Percent of rows

Percentage of columns | 0| 1| 2| Sum

______________+________+________+________+

1 | 2 | 15 | 2322 | 2339

| 0.09 | 0.64 | 99.27 | 100.00

| 0.09 | 0.64 | 99.27 |

| 100.00 | 100.00 | 100.00 |

______________+________+________+________+

Total 2 15 2322 2339

0.09 0.64 99.27 100.00

Administration of anticoagulants at the time of obtaining Table 2-4 consent, according to the presence or absence of proton-pump inhibitors

FREQ Procedure

Tables: ALL * DOSECNT

ALL

DOSECNT(Dose per 1time for DOAC only)

Power |
Percent |
Percent of rows
Percentage of columns | 0| 1| 2| Sum

```
______________+________+________+________+
          1 |      2 |      15 |   2322 |    2339
            |   0.09 |    0.64 |  99.27 | 100.00
            |   0.09 |    0.64 |  99.27 |
            | 100.00 | 100.00 | 100.00 |
______________+________+________+________+
```

Total 2 15 2322 2339

0.09 0.64 99.27 100.00

Administration of anticoagulants at the time of obtaining Table 2-4 consent, according to the presence or absence of proton-pump inhibitors

FREQ Procedure

Table : ALL * t_dc2

ALL t_dc2

Power |
Percent |
Percent of rows
Percent of columns | 1| Total
______________+________+
1 | 2 | 2
| 100.00 | 100.00
| 100.00 |
| 100.00 |
______________+________+
Total 2 2
100.00 100.00

Frequency of missing values = 2337

Administration of anticoagulants at the time of obtaining Table 2-4 consent, according to the presence or absence of proton-pump inhibitors

FREQ Procedure

Tables: ALL * DOSECNT

```
ALL
                DOSECNT(Dose per 1time for DOAC only)

Power |
Percent |
Percent of rows
Percentage of columns | 0| 1| 2| Sum
______________+________+________+________+
            1 |       2 |    6428 |       6 |    6436
              |    0.03 |   99.88 |    0.09 | 100.00
              |    0.03 |   99.88 |    0.09 |
              |  100.00 |  100.00 |  100.00 |
______________+________+________+________+
Total 2 6428 6 6436
                   0.03     99.88      0.09    100.00
```

Administration of anticoagulants at the time of obtaining Table 2-4 consent, according to the presence or absence of proton-pump inhibitors

FREQ Procedure

Tables: ALL * DOSECNT

ALL

DOSECNT(Dose per 1time for DOAC only)

Power |
Percent |
Percent of rows
Percentage of columns | 0| 1| 2| Sum

ALL	0	1	2	Sum
1	2	6428	6	6436
	0.03	99.88	0.09	100.00
	0.03	99.88	0.09	
	100.00	100.00	100.00	
Total	2	6428	6	6436
	0.03	99.88	0.09	100.00

Administration of anticoagulants at the time of obtaining Table 2-4 consent, according to the presence or absence of proton-pump inhibitors

FREQ Procedure

Table : ALL * t_dc3

ALL t_dc3

Power |
Percent |
Percent of rows
Percent of columns | 1| Total
______________+________+
1 | 2 | 2
| 100.00 | 100.00
| 100.00 |
| 100.00 |
______________+________+
Total 2 2
100.00 100.00

Frequency of missing values = 6434

Administration of anticoagulants at the time of obtaining Table 2-4 consent, according to the presence or absence of proton-pump inhibitors

FREQ Procedure

Tables: ALL * DOSECNT

ALL DOSECNT(Dose per 1time for DOAC only)

Power |
Percent |
Percent of rows
Column Percent | 0| 0.5| 1| 2| 3| Sum

1	2	1	64	7971	1	8039
	0.02	0.01	0.80	99.15	0.01	100.00
	0.02	0.01	0.80	99.15	0.01	
	100.00	100.00	100.00	100.00	100.00	
Total	2	1	64	7971	1	8039
	0.02	0.01	0.80	99.15	0.01	100.00

Administration of anticoagulants at the time of obtaining Table 2-4 consent, according to the presence or absence of proton-pump inhibitors

FREQ Procedure

Tables: ALL * DOSECNT

ALL DOSECNT(Dose per 1time for DOAC only)

Power |
Percent |
Percent of rows
Column Percent | 0| 0.5| 1| 2| 3| Sum

1	2	1	64	7971	1	8039
	0.02	0.01	0.80	99.15	0.01	100.00
	0.02	0.01	0.80	99.15	0.01	
	100.00	100.00	100.00	100.00	100.00	
Total	2	1	64	7971	1	8039
	0.02	0.01	0.80	99.15	0.01	100.00

Administration of anticoagulants at the time of obtaining Table 2-4 consent, according to the presence or absence of proton-pump inhibitors

FREQ Procedure

Table : ALL * t_dc4

ALL t_dc4

Power |
Percent |
Percent of rows
Percent of columns | 1| Total

1	4	4
	100.00	100.00
	100.00	
	100.00	
Total	4	4
	100.00	100.00

Frequency of missing values = 8035

Administration of anticoagulants at the time of obtaining Table 2-4 consent, according to the presence or absence of proton-pump inhibitors

FREQ Procedure

Tables: ALL * DOSECNT

ALL

DOSECNT(Dose per 1time for DOAC only)

Power / Percent / Percent of rows / Percentage of columns	0.5	1	2	Sum
1	1	4786	5	4792
	0.02	99.87	0.10	100.00
	0.02	99.87	0.10	
	100.00	100.00	100.00	
Total	1	4786	5	4792
	0.02	99.87	0.10	100.00

Administration of anticoagulants at the time of obtaining Table 2-4 consent, according to the presence or absence of proton-pump inhibitors

FREQ Procedure

Tables: ALL * DOSECNT

ALL

DOSECNT(Dose per 1time for DOAC only)

Power |
Percent |
Percent of rows
Percentage of columns | 0.5| 1| 2| Sum

ALL	0.5	1	2	Sum
1	1	4786	5	4792
	0.02	99.87	0.10	100.00
	0.02	99.87	0.10	
	100.00	100.00	100.00	
Total	1	4786	5	4792
	0.02	99.87	0.10	100.00

Administration of anticoagulants at the time of obtaining Table 2-4 consent, according to the presence or absence of proton-pump inhibitors

FREQ Procedure

Table: ALL * t_dc0

ALL t_dc0

Power |
Percent |
Percent of rows
Percent of columns | 1| Total

1	1	1
	100.00	100.00
	100.00	
	100.00	
Total	1	1
	100.00	100.00

Degree of missing values = 4791

Administration of anticoagulants at the time of obtaining Table 2-4 consent, according to the presence or absence of proton-pump inhibitors

FREQ Procedure

Tables: ALL * DOSECNT

ALL

DOSECNT(Dose per 1time for DOAC only)

Power<br>Percent<br>Percent of rows<br>Percentage of columns	0.5	1	2	Sum
1	1<br>0.02<br>0.02<br>100.00	4747<br>99.89<br>99.89<br>100.00	4<br>0.08<br>0.08<br>100.00	4752<br>100.00
Total	1<br>0.02	4747<br>99.89	4<br>0.08	4752<br>100.00

Administration of anticoagulants at the time of obtaining Table 2-4 consent, according to the presence or absence of proton-pump inhibitors

FREQ Procedure

Tables: ALL * DOSECNT

ALL

DOSECNT(Dose per 1time for DOAC only)

Power |

Percent |

Percent of rows

Percentage of columns | 0.5| 1| 2| Sum

	0.5	1	2	Sum
1	1	4747	4	4752
	0.02	99.89	0.08	100.00
	0.02	99.89	0.08	
	100.00	100.00	100.00	
Total	1	4747	4	4752
	0.02	99.89	0.08	100.00

Administration of anticoagulants at the time of obtaining Table 2-4 consent, according to the presence or absence of proton-pump inhibitors

FREQ Procedure

Table : ALL * t_dc5

ALL t_dc5

```
Power |
Percent |
Percent of rows
Percent of columns | 1| Total
______________+________+
             1 |        1 |        1
               | 100.00 | 100.00
               | 100.00 |
               | 100.00 |
______________+________+
Total 1 1
                 100.00    100.00
```

Degree of missing values = 4751

Administration of anticoagulants at the time of obtaining Table 2-4 consent, according to the presence or absence of proton-pump inhibitors

FREQ Procedure

Tables: ALL * DOSECNT

ALL

DOSECNT(Dose per 1time for DOAC only)

```
Power |
Percent |
Percent of rows
Percentage of columns | 1| 2| Total
______________+________+________+
            1 |     39 |      1 |     40
              |  97.50 |   2.50 | 100.00
              |  97.50 |   2.50 |
              | 100.00 | 100.00 |
______________+________+________+
Total 39 1 40
                 97.50     2.50   100.00
```

Administration of anticoagulants at the time of obtaining Table 2-4 consent, according to the presence or absence of proton-pump inhibitors

FREQ Procedure

Tables: ALL * DOSECNT

ALL

DOSECNT(Dose per 1time for DOAC only)

Power |
Percent |
Percent of rows
Percentage of columns | 1| 2| Total

1	39	1	40
	97.50	2.50	100.00
	97.50	2.50	
	100.00	100.00	
Total	39	1	40
	97.50	2.50	100.00

Administration of anticoagulants at the time of obtaining Table 2-4 consent, according to the presence or absence of proton-pump inhibitors

FREQ Procedure

For ALL * t_dc7,
Because all levels of variable t_dc7 are missing,
There is no data.

Administration of anticoagulants at the time of obtaining Table 2-4 consent, according to the presence or absence of proton-pump inhibitors

FREQ Procedure

Cumulative
Percent of ALL power Percent of power

1	11981	100.00	11981	100.00

Tables: ALL * ADYN

ALL
ADYN(Administration of Anticoagulant)

Power |
Percent |
Percent of rows
Percentage of columns | with | total
______________+________+
1 | 11163 | 11163
| 100.00 | 100.00
| 100.00 |
| 100.00 |
______________+________+
Total 11163 11163
100.00 100.00

Degree of missing values = 818

Administration of anticoagulants at the time of obtaining Table 2-4 consent, according to the presence or absence of proton-pump inhibitors

FREQ Procedure

Table 1 : ADYN * ACTYP
Stratification variable : ALL=1

ADYN(Administration of Anticoagulant) ACTYP(Type of AF)

Power |
Percent |
Percent of rows
Percent of column | Warfa| Dabigat| Riverro| Apixa| Edoxa| Parenteral Anti| Edoxa| Total
|phosphate|run|xaban|ban|ban|coagulant|ban (OD|)
| | | | | | |) |
______________+________+________+________+________+________+________+________+
Present | 2955 | 906 | 2388 | 3174 | 1718 | 2 | 20 | 11163
| 26.47 | 8.12 | 21.39 | 28.43 | 15.39 | 0.02 | 0.18 | 100.00
| 26.47 | 8.12 | 21.39 | 28.43 | 15.39 | 0.02 | 0.18 |
| 100.00 | 100.00 | 100.00 | 100.00 | 100.00 | 100.00 | 100.00 |
______________+________+________+________+________+________+________+________+
Total 2955 906 2388 3174 1718 2 20 11163
26.47 8.12 21.39 28.43 15.39 0.02 0.18 100.00

Degree of missing values = 818

Table 1 : ADYN * ACTYP
Stratification variable : ALL=1

ADYN(Administration of Anticoagulant) ACTYP(Type of AF)

Power |

Percent |

Percent of rows

Percent of column | Warfa| Dabigat| Riverro| Apixa| Edoxa| Parenteral Anti| Edoxa| Total

|phosphate|run|xaban|ban|ban|coagulant|ban (OD|)

| | | | | | |) |

______________+________+________+________+________+________+________+________+

Present | 2955 | 906 | 2388 | 3174 | 1718 | 2 | 20 | 11163

| 26.47 | 8.12 | 21.39 | 28.43 | 15.39 | 0.02 | 0.18 | 100.00

| 26.47 | 8.12 | 21.39 | 28.43 | 15.39 | 0.02 | 0.18 |

| 100.00 | 100.00 | 100.00 | 100.00 | 100.00 | 100.00 | 100.00 |

______________+________+________+________+________+________+________+________+

Total 2955 906 2388 3174 1718 2 20 11163

26.47 8.12 21.39 28.43 15.39 0.02 0.18 100.00

Degree of missing values = 818

Administration of anticoagulants at the time of obtaining Table 2-4 consent, according to the presence or absence of proton-pump inhibitors

FREQ Procedure

Table 1 : ADYN * ACTYP
Stratification variable : ALL=1

ADYN(Administration of Anticoagulant) ACTYP(Type of AF)

Power |
Percent |
Percent of rows
Percent of column | Warfa| Dabigat| Riverro| Apixa| Edoxa| Parenteral Anti| Edoxa| Total
|phosphate|run|xaban|ban|ban|coagulant|ban (OD|)
| | | | | | |) |
______________+________+________+________+________+________+________+________
_+
Present | 2955 | 906 | 2388 | 3174 | 1718 | 2 | 20 | 11163
| 26.47 | 8.12 | 21.39 | 28.43 | 15.39 | 0.02 | 0.18 | 100.00
| 26.47 | 8.12 | 21.39 | 28.43 | 15.39 | 0.02 | 0.18 |
| 100.00 | 100.00 | 100.00 | 100.00 | 100.00 | 100.00 | 100.00 |
______________+________+________+________+________+________+________+________
_+
Total 2955 906 2388 3174 1718 2 20 11163
26.47 8.12 21.39 28.43 15.39 0.02 0.18 100.00

Degree of missing values = 818

Table 1 : ADYN * ACTYP
Stratification variable : ALL=1

ADYN(Administration of Anticoagulant) ACTYP(Type of AF)

Power |

Percent |

Percent of rows

Percent of column | Warfa| Dabigat| Riverro| Apixa| Edoxa| Parenteral Anti| Edoxa| Total

|phosphate|run|xaban|ban|ban|coagulant|ban (OD|)

| | | | | | |) |

______________+________+________+________+________+________+________+________+

Present | 2955 | 906 | 2388 | 3174 | 1718 | 2 | 20 | 11163

| 26.47 | 8.12 | 21.39 | 28.43 | 15.39 | 0.02 | 0.18 | 100.00

| 26.47 | 8.12 | 21.39 | 28.43 | 15.39 | 0.02 | 0.18 |

| 100.00 | 100.00 | 100.00 | 100.00 | 100.00 | 100.00 | 100.00 |

______________+________+________+________+________+________+________+________+

Total 2955 906 2388 3174 1718 2 20 11163

26.47 8.12 21.39 28.43 15.39 0.02 0.18 100.00

Degree of missing values = 818

Administration of anticoagulants at the time of obtaining Table 2-4 consent, according to the presence or absence of proton-pump inhibitors

FREQ Procedure

Table 1 : ADYN * ACTYP
Stratification variable : ALL=1

ADYN(Administration of Anticoagulant) ACTYP(Type of AF)

Power |
Percent |
Percent of rows
Percent of column | Warfa| Dabigat| Riverro| Apixa| Edoxa| Parenteral Anti| Edoxa| Total
|phosphate|run|xaban|ban|ban|coagulant|ban (OD|)
| | | | | | |) |
_______________+_________+_________+_________+_________+_________+_________+________
_+
Present | 2955 | 906 | 2388 | 3174 | 1718 | 2 | 20 | 11163
| 26.47 | 8.12 | 21.39 | 28.43 | 15.39 | 0.02 | 0.18 | 100.00
| 26.47 | 8.12 | 21.39 | 28.43 | 15.39 | 0.02 | 0.18 |
| 100.00 | 100.00 | 100.00 | 100.00 | 100.00 | 100.00 | 100.00 |
_______________+_________+_________+_________+_________+_________+_________+________
_+
Total 2955 906 2388 3174 1718 2 20 11163
26.47 8.12 21.39 28.43 15.39 0.02 0.18 100.00

Degree of missing values = 818

Table 1 : ADYN * ACTYP
Stratification variable : ALL=1

ADYN(Administration of Anticoagulant) ACTYP(Type of AF)

Power |

Percent |

Percent of rows

Percent of column | Warfa| Dabigat| Riverro| Apixa| Edoxa| Parenteral Anti| Edoxa| Total

|phosphate|run|xaban|ban|ban|coagulant|ban (OD|)

| | | | | | |) |

______________+________+________+________+________+________+________+________

_+

Present | 2955 | 906 | 2388 | 3174 | 1718 | 2 | 20 | 11163

| 26.47 | 8.12 | 21.39 | 28.43 | 15.39 | 0.02 | 0.18 | 100.00

| 26.47 | 8.12 | 21.39 | 28.43 | 15.39 | 0.02 | 0.18 |

| 100.00 | 100.00 | 100.00 | 100.00 | 100.00 | 100.00 | 100.00 |

______________+________+________+________+________+________+________+________

_+

Total 2955 906 2388 3174 1718 2 20 11163

26.47 8.12 21.39 28.43 15.39 0.02 0.18 100.00

Degree of missing values = 818

Administration of anticoagulants at the time of obtaining Table 2-4 consent, according to the presence or absence of proton-pump inhibitors

FREQ Procedure

Table 1 : ADYN * ACTYP
Stratification variable : ALL=1

ADYN(Administration of Anticoagulant) ACTYP(Type of AF)

Power |
Percent |
Percent of rows
Percent of column | Warfa| Dabigat| Riverro| Apixa| Edoxa| Parenteral Anti| Edoxa| Total
|phosphate|run|xaban|ban|ban|coagulant|ban (OD|)
| | | | | | |) |
______________+________+________+________+________+________+________+________
_+
Present | 2955 | 906 | 2388 | 3174 | 1718 | 2 | 20 | 11163
| 26.47 | 8.12 | 21.39 | 28.43 | 15.39 | 0.02 | 0.18 | 100.00
| 26.47 | 8.12 | 21.39 | 28.43 | 15.39 | 0.02 | 0.18 |
| 100.00 | 100.00 | 100.00 | 100.00 | 100.00 | 100.00 | 100.00 |
______________+________+________+________+________+________+________+________
_+
Total 2955 906 2388 3174 1718 2 20 11163
26.47 8.12 21.39 28.43 15.39 0.02 0.18 100.00

Degree of missing values = 818

Table 1 : ADYN * t_ACT0
Stratification variable : ALL=1

ADYN(Administration of Anticoagulant)
t_ACT0

Power |

Percent |

Percent of rows

Percentage of columns | 0| 1| Sum

______________+________+________+

Present | 9425 | 1738 | 11163

| 84.43 | 15.57 | 100.00

| 84.43 | 15.57 |

| 100.00 | 100.00 |

______________+________+________+

Total 9425 1738 11163

84.43 15.57 100.00

Degree of missing values = 818

Administration of anticoagulants at the time of obtaining Table 2-4 consent, according to the presence or absence of proton-pump inhibitors

FREQ Procedure

Table: ALL * t_ADYN

ALL t_ADYN

Power |
Percent |
Percent of rows
Percentage of columns | 0| 1| Sum

```
______________+________+________+
             1 |     818 |   11163 |   11981
               |    6.83 |   93.17 | 100.00
               |    6.83 |   93.17 |
               | 100.00 | 100.00 |
______________+________+________+
Total 818 11163 11981
                  6.83      93.17     100.00
```

Administration of anticoagulants at the time of obtaining Table 2-4 consent, according to the presence or absence of proton-pump inhibitors

FREQ Procedure

Cumulative
Percent of ALL power Percent of power

1	11981	100.00	11981	100.00

Tables: ALL * ADYN

ALL
ADYN(Administration of Anticoagulant)

Power |
Percent |
Percent of rows
Percentage of columns | with | total
______________+________+
1 | 11163 | 11163
| 100.00 | 100.00
| 100.00 |
| 100.00 |
______________+________+
Total 11163 11163
100.00 100.00

Degree of missing values = 818

Administration of anticoagulants at the time of obtaining Table 2-4 consent, according to the presence or absence of proton-pump inhibitors

FREQ Procedure

Table 1 : ADYN * ACTYP
Stratification variable : ALL=1

ADYN(Administration of Anticoagulant) ACTYP(Type of AF)

Power |
Percent |
Percent of rows
Percent of column | Warfa| Dabigat| Riverro| Apixa| Edoxa| Parenteral Anti| Edoxa| Total
|phosphate|run|xaban|ban|ban|coagulant|ban (OD|)
| | | | | | |) |
______________+________+________+________+________+________+________+________
_+
Present | 2955 | 906 | 2388 | 3174 | 1718 | 2 | 20 | 11163
| 26.47 | 8.12 | 21.39 | 28.43 | 15.39 | 0.02 | 0.18 | 100.00
| 26.47 | 8.12 | 21.39 | 28.43 | 15.39 | 0.02 | 0.18 |
| 100.00 | 100.00 | 100.00 | 100.00 | 100.00 | 100.00 | 100.00 |
______________+________+________+________+________+________+________+________
_+
Total 2955 906 2388 3174 1718 2 20 11163
26.47 8.12 21.39 28.43 15.39 0.02 0.18 100.00

Degree of missing values = 818

Table 1 : ADYN * ACTYP
Stratification variable : ALL=1

ADYN(Administration of Anticoagulant) ACTYP(Type of AF)

Power |

Percent |

Percent of rows

Percent of column | Warfa| Dabigat| Riverro| Apixa| Edoxa| Parenteral Anti| Edoxa| Total

|phosphate|run|xaban|ban|ban|coagulant|ban (OD|)

| | | | | | |) |

______________+________+________+________+________+________+________+________

_+

Present | 2955 | 906 | 2388 | 3174 | 1718 | 2 | 20 | 11163

| 26.47 | 8.12 | 21.39 | 28.43 | 15.39 | 0.02 | 0.18 | 100.00

| 26.47 | 8.12 | 21.39 | 28.43 | 15.39 | 0.02 | 0.18 |

| 100.00 | 100.00 | 100.00 | 100.00 | 100.00 | 100.00 | 100.00 |

______________+________+________+________+________+________+________+________

_+

Total 2955 906 2388 3174 1718 2 20 11163

26.47 8.12 21.39 28.43 15.39 0.02 0.18 100.00

Degree of missing values = 818

Administration of anticoagulants at the time of obtaining Table 2-4 consent, according to the presence or absence of proton-pump inhibitors

FREQ Procedure

Table 1 : ADYN * ACTYP
Stratification variable : ALL=1

ADYN(Administration of Anticoagulant) ACTYP(Type of AF)

Power |
Percent |
Percent of rows
Percent of column | Warfa| Dabigat| Riverro| Apixa| Edoxa| Parenteral Anti| Edoxa| Total
|phosphate|run|xaban|ban|ban|coagulant|ban (OD|)
| | | | | | |) |
______________+________+________+________+________+________+________+________
_+
Present | 2955 | 906 | 2388 | 3174 | 1718 | 2 | 20 | 11163
| 26.47 | 8.12 | 21.39 | 28.43 | 15.39 | 0.02 | 0.18 | 100.00
| 26.47 | 8.12 | 21.39 | 28.43 | 15.39 | 0.02 | 0.18 |
| 100.00 | 100.00 | 100.00 | 100.00 | 100.00 | 100.00 | 100.00 |
______________+________+________+________+________+________+________+________
_+
Total 2955 906 2388 3174 1718 2 20 11163
26.47 8.12 21.39 28.43 15.39 0.02 0.18 100.00

Degree of missing values = 818

Table 1 : ADYN * ACTYP
Stratification variable : ALL=1

ADYN(Administration of Anticoagulant) ACTYP(Type of AF)

Power |

Percent |

Percent of rows

Percent of column | Warfa| Dabigat| Riverro| Apixa| Edoxa| Parenteral Anti| Edoxa| Total

|phosphate|run|xaban|ban|ban|coagulant|ban (OD|)

| | | | | | |) |

______________+________+________+________+________+________+________+_______

_+

Present | 2955 | 906 | 2388 | 3174 | 1718 | 2 | 20 | 11163

| 26.47 | 8.12 | 21.39 | 28.43 | 15.39 | 0.02 | 0.18 | 100.00

| 26.47 | 8.12 | 21.39 | 28.43 | 15.39 | 0.02 | 0.18 |

| 100.00 | 100.00 | 100.00 | 100.00 | 100.00 | 100.00 | 100.00 |

______________+________+________+________+________+________+________+_______

_+

Total 2955 906 2388 3174 1718 2 20 11163

26.47 8.12 21.39 28.43 15.39 0.02 0.18 100.00

Degree of missing values = 818

Administration of anticoagulants at the time of obtaining Table 2-4 consent, according to the presence or absence of proton-pump inhibitors

FREQ Procedure

Table 1 : ADYN * ACTYP
Stratification variable : ALL=1

ADYN(Administration of Anticoagulant) ACTYP(Type of AF)

Power |
Percent |
Percent of rows
Percent of column | Warfa| Dabigat| Riverro| Apixa| Edoxa| Parenteral Anti| Edoxa| Total
|phosphate|run|xaban|ban|ban|coagulant|ban (OD|)
| | | | | | |) |
______________+________+________+________+________+________+________+________
_+
Present | 2955 | 906 | 2388 | 3174 | 1718 | 2 | 20 | 11163
| 26.47 | 8.12 | 21.39 | 28.43 | 15.39 | 0.02 | 0.18 | 100.00
| 26.47 | 8.12 | 21.39 | 28.43 | 15.39 | 0.02 | 0.18 |
| 100.00 | 100.00 | 100.00 | 100.00 | 100.00 | 100.00 | 100.00 |
______________+________+________+________+________+________+________+________
_+
Total 2955 906 2388 3174 1718 2 20 11163
26.47 8.12 21.39 28.43 15.39 0.02 0.18 100.00

Degree of missing values = 818

Table 1 : ADYN * ACTYP
Stratification variable : ALL=1

ADYN(Administration of Anticoagulant) ACTYP(Type of AF)

Power |

Percent |

Percent of rows

Percent of column | Warfa| Dabigat| Riverro| Apixa| Edoxa| Parenteral Anti| Edoxa| Total

|phosphate|run|xaban|ban|ban|coagulant|ban (OD|)

| | | | | | |) |

______________+________+________+________+________+________+________+________+

Present | 2955 | 906 | 2388 | 3174 | 1718 | 2 | 20 | 11163

| 26.47 | 8.12 | 21.39 | 28.43 | 15.39 | 0.02 | 0.18 | 100.00

| 26.47 | 8.12 | 21.39 | 28.43 | 15.39 | 0.02 | 0.18 |

| 100.00 | 100.00 | 100.00 | 100.00 | 100.00 | 100.00 | 100.00 |

______________+________+________+________+________+________+________+________+

Total 2955 906 2388 3174 1718 2 20 11163

26.47 8.12 21.39 28.43 15.39 0.02 0.18 100.00

Degree of missing values = 818

Administration of anticoagulants at the time of obtaining Table 2-4 consent, according to the presence or absence of proton-pump inhibitors

FREQ Procedure

Table 1 : ADYN * ACTYP
Stratification variable : ALL=1

ADYN(Administration of Anticoagulant) ACTYP(Type of AF)

Power |
Percent |
Percent of rows
Percent of column | Warfa| Dabigat| Riverro| Apixa| Edoxa| Parenteral Anti| Edoxa| Total
|phosphate|run|xaban|ban|ban|coagulant|ban (OD|)
| | | | | | |) |
______________+________+________+________+________+________+________+________
_+
Present | 2955 | 906 | 2388 | 3174 | 1718 | 2 | 20 | 11163
| 26.47 | 8.12 | 21.39 | 28.43 | 15.39 | 0.02 | 0.18 | 100.00
| 26.47 | 8.12 | 21.39 | 28.43 | 15.39 | 0.02 | 0.18 |
| 100.00 | 100.00 | 100.00 | 100.00 | 100.00 | 100.00 | 100.00 |
______________+________+________+________+________+________+________+________
_+
Total 2955 906 2388 3174 1718 2 20 11163
26.47 8.12 21.39 28.43 15.39 0.02 0.18 100.00

Degree of missing values = 818

Table 1 : ADYN * t_ACT0
Stratification variable : ALL=1

ADYN(Administration of Anticoagulant)
t_ACT0

Power |

Percent |

Percent of rows

Percentage of columns | 0| 1| Sum

_______________+_________+_________+

Present | 9425 | 1738 | 11163

| 84.43 | 15.57 | 100.00

| 84.43 | 15.57 |

| 100.00 | 100.00 |

_______________+_________+_________+

Total 9425 1738 11163

84.43 15.57 100.00

Degree of missing values = 818

Administration of anticoagulants at the time of obtaining Table 2-4 consent, according to the presence or absence of proton-pump inhibitors

FREQ Procedure

Table: ALL * t_ADYN

ALL t_ADYN

Power |
Percent |
Percent of rows
Percentage of columns | 0| 1| Sum

```
______________+________+________+
             1 |     818 |   11163 |   11981
               |    6.83 |   93.17 | 100.00
               |    6.83 |   93.17 |
               | 100.00 | 100.00 |
______________+________+________+
Total 818 11163 11981
                  6.83      93.17     100.00
```

Administration of anticoagulants at the time of obtaining Table 2-4 consent, according to the presence or absence of proton-pump inhibitors

FREQ Procedure

Tables: ALL* ACTYP

ALL ACTYP(Type of AF)

```
Power |
Percent |
Percent of rows
Percent of column | Warfa| Dabigat| Riverro| Apixa| Edoxa| Parenteral Anti| Edoxa| Total
                 |phosphate|run|xaban|ban|ban|coagulant|ban (OD|)
                 | | | | | | |) |
______________+________+________+________+________+________+________+_______
_+
              1 |    2955 |     906 |    2388 |    3174 |    1718 |       2 |      20 |  11163
                |   26.47 |    8.12 |   21.39 |   28.43 |   15.39 |    0.02 |    0.18 | 100.00
                |   26.47 |    8.12 |   21.39 |   28.43 |   15.39 |    0.02 |    0.18 |
                | 100.00 | 100.00 | 100.00 | 100.00 | 100.00 | 100.00 | 100.00 |
______________+________+________+________+________+________+________+_______
_+
Total 2955 906 2388 3174 1718 2 20 11163
                   26.47      8.12      21.39      28.43      15.39      0.02      0.18    100.00
```

Administration of anticoagulants at the time of obtaining Table 2-4 consent, according to the presence or absence of proton-pump inhibitors

FREQ Procedure

Table : ALL * n_ACT1

ALL n_ACT1

Power |
Percent |
Percent of rows
Percent of columns | 1| Total

1	8208	8208
	100.00	100.00
	100.00	
	100.00	
Total	8208	8208
	100.00	100.00

Degree of missing values = 2955

Administration of anticoagulants at the time of obtaining Table 2-4 consent, according to the presence or absence of proton-pump inhibitors

FREQ Procedure

Tables: ALL* ACTYP

ALL ACTYP(Type of AF)

Power |
Percent |
Percent of rows
Percent of column | Warfa| Dabigat| Riverro| Apixa| Edoxa| Parenteral Anti| Edoxa| Total
|phosphate|run|xaban|ban|ban|coagulant|ban (OD|)
| | | | | | |) |
______________+________+________+________+________+________+________+________
_+
1 | 2955 | 906 | 2388 | 3174 | 1718 | 2 | 20 | 11163
| 26.47 | 8.12 | 21.39 | 28.43 | 15.39 | 0.02 | 0.18 | 100.00
| 26.47 | 8.12 | 21.39 | 28.43 | 15.39 | 0.02 | 0.18 |
| 100.00 | 100.00 | 100.00 | 100.00 | 100.00 | 100.00 | 100.00 |
______________+________+________+________+________+________+________+________
_+
Total 2955 906 2388 3174 1718 2 20 11163
26.47 8.12 21.39 28.43 15.39 0.02 0.18 100.00

Administration of anticoagulants at the time of obtaining Table 2-4 consent, according to the presence or absence of proton-pump inhibitors

FREQ Procedure

Table : ALL * n_ACT2

ALL n_ACT2

Power |
Percent |
Percent of rows
Percent of columns | 1| Total

1	10257	10257
	100.00	100.00
	100.00	
	100.00	
Total	10257	10257
	100.00	100.00

Frequency of missing values = 906

Administration of anticoagulants at the time of obtaining Table 2-4 consent, according to the presence or absence of proton-pump inhibitors

FREQ Procedure

Tables: ALL* ACTYP

ALL ACTYP(Type of AF)

Power |
Percent |
Percent of rows
Percent of column | Warfa| Dabigat| Riverro| Apixa| Edoxa| Parenteral Anti| Edoxa| Total
|phosphate|run|xaban|ban|ban|coagulant|ban (OD|)
| | | | | | |) |
______________+________+________+________+________+________+________+________
_+
1 | 2955 | 906 | 2388 | 3174 | 1718 | 2 | 20 | 11163
| 26.47 | 8.12 | 21.39 | 28.43 | 15.39 | 0.02 | 0.18 | 100.00
| 26.47 | 8.12 | 21.39 | 28.43 | 15.39 | 0.02 | 0.18 |
| 100.00 | 100.00 | 100.00 | 100.00 | 100.00 | 100.00 | 100.00 |
______________+________+________+________+________+________+________+________
_+
Total 2955 906 2388 3174 1718 2 20 11163
26.47 8.12 21.39 28.43 15.39 0.02 0.18 100.00

Administration of anticoagulants at the time of obtaining Table 2-4 consent, according to the presence or absence of proton-pump inhibitors

FREQ Procedure

Table : ALL * n_ACT3

ALL n_ACT3

```
Power |
Percent |
Percent of rows
Percent of columns | 1| Total
______________+________+
             1 |    8775 |    8775
               | 100.00 | 100.00
               | 100.00 |
               | 100.00 |
______________+________+
Total 8775 8775
                 100.00    100.00
```

Frequency of missing values = 2388

Administration of anticoagulants at the time of obtaining Table 2-4 consent, according to the presence or absence of proton-pump inhibitors

FREQ Procedure

Tables: ALL* ACTYP

ALL ACTYP(Type of AF)

Power | Percent | Percent of rows<br>Percent of column	Warfa<br>phosphate	Dabigat<br>run	Riverro<br>xaban	Apixa<br>ban	Edoxa<br>ban	Parenteral Anti<br>coagulant	Edoxa<br>ban (OD)	Total
1	2955<br>26.47<br>26.47<br>100.00	906<br>8.12<br>8.12<br>100.00	2388<br>21.39<br>21.39<br>100.00	3174<br>28.43<br>28.43<br>100.00	1718<br>15.39<br>15.39<br>100.00	2<br>0.02<br>0.02<br>100.00	20<br>0.18<br>0.18<br>100.00	11163<br>100.00
Total	2955<br>26.47	906<br>8.12	2388<br>21.39	3174<br>28.43	1718<br>15.39	2<br>0.02	20<br>0.18	11163<br>100.00

Administration of anticoagulants at the time of obtaining Table 2-4 consent, according to the presence or absence of proton-pump inhibitors

FREQ Procedure

Table : ALL * n_ACT4

ALL n_ACT4

Power |
Percent |
Percent of rows
Percent of columns | 1| Total

```
______________+________+
            1 |    7989 |    7989
              | 100.00 | 100.00
              | 100.00 |
              | 100.00 |
______________+________+
Total 7989 7989
                100.00    100.00
```

Degree of missing values = 3174

Administration of anticoagulants at the time of obtaining Table 2-4 consent, according to the presence or absence of proton-pump inhibitors

FREQ Procedure

Table: ALL * t_ACT0

ALL t_ACT0

Power |
Percent |
Percent of rows
Percentage of columns | 0| 1| Sum

1	9425	1738	11163
	84.43	15.57	100.00
	84.43	15.57	
	100.00	100.00	
Total	9425	1738	11163
	84.43	15.57	100.00

Administration of anticoagulants at the time of obtaining Table 2-4 consent, according to the presence or absence of proton-pump inhibitors

FREQ Procedure

Table: ALL * t_ACT0

ALL t_ACT0

Power |
Percent |
Percent of rows
Percentage of columns | 0| 1| Sum

	0	1	Sum
1	9425	1738	11163
	84.43	15.57	100.00
	84.43	15.57	
	100.00	100.00	
Total	9425	1738	11163
	84.43	15.57	100.00

Administration of anticoagulants at the time of obtaining Table 2-4 consent, according to the presence or absence of proton-pump inhibitors

FREQ Procedure

Tables: ALL* ACTYP

ALL ACTYP(Type of AF)

Power |
Percent |
Percent of rows
Percent of column | Warfa| Dabigat| Riverro| Apixa| Edoxa| Parenteral Anti| Edoxa| Total
|phosphate|run|xaban|ban|ban|coagulant|ban (OD|)
| | | | | | |) |
______________+________+________+________+________+________+________+________
_+
1 | 2955 | 906 | 2388 | 3174 | 1718 | 2 | 20 | 11163
| 26.47 | 8.12 | 21.39 | 28.43 | 15.39 | 0.02 | 0.18 | 100.00
| 26.47 | 8.12 | 21.39 | 28.43 | 15.39 | 0.02 | 0.18 |
| 100.00 | 100.00 | 100.00 | 100.00 | 100.00 | 100.00 | 100.00 |
______________+________+________+________+________+________+________+________
_+
Total 2955 906 2388 3174 1718 2 20 11163
26.47 8.12 21.39 28.43 15.39 0.02 0.18 100.00

Administration of anticoagulants at the time of obtaining Table 2-4 consent, according to the presence or absence of proton-pump inhibitors

FREQ Procedure

Table : ALL * n_ACT5

ALL n_ACT5

```
Power |
Percent |
Percent of rows
Percent of columns | 1| Total
______________+________+
            1 |    9445 |    9445
              | 100.00 | 100.00
              | 100.00 |
              | 100.00 |
______________+________+
Total 9445 9445
                100.00     100.00
```

Degree of missing values = 1718

Administration of anticoagulants at the time of obtaining Table 2-4 consent, according to the presence or absence of proton-pump inhibitors

FREQ Procedure

Tables: ALL* ACTYP

ALL ACTYP(Type of AF)

Power |
Percent |
Percent of rows
Percent of column | Warfa| Dabigat| Riverro| Apixa| Edoxa| Parenteral Anti| Edoxa| Total
|phosphate|run|xaban|ban|ban|coagulant|ban (OD|)
| | | | | | |) |
______________+________+________+________+________+________+________+________
_+
1 | 2955 | 906 | 2388 | 3174 | 1718 | 2 | 20 | 11163
| 26.47 | 8.12 | 21.39 | 28.43 | 15.39 | 0.02 | 0.18 | 100.00
| 26.47 | 8.12 | 21.39 | 28.43 | 15.39 | 0.02 | 0.18 |
| 100.00 | 100.00 | 100.00 | 100.00 | 100.00 | 100.00 | 100.00 |
______________+________+________+________+________+________+________+________
_+
Total 2955 906 2388 3174 1718 2 20 11163
26.47 8.12 21.39 28.43 15.39 0.02 0.18 100.00

Administration of anticoagulants at the time of obtaining Table 2-4 consent, according to the presence or absence of proton-pump inhibitors

FREQ Procedure

Table : ALL * n_ACT7

ALL n_ACT7

Power |
Percent |
Percent of rows
Percent of columns | 1| Total

1	11143	11143
	100.00	100.00
	100.00	
	100.00	
Total	11143	11143
	100.00	100.00

Degree of missing values = 20

Administration of anticoagulants at the time of obtaining Table 2-4 consent, according to the presence or absence of proton-pump inhibitors

FREQ Procedure

Tables: ALL* ACTYP

ALL ACTYP(Type of AF)

Power |
Percent |
Percent of rows
Percent of column | Warfa| Dabigat| Riverro| Apixa| Edoxa| Parenteral Anti| Edoxa| Total
|phosphate|run|xaban|ban|ban|coagulant|ban (OD|)
| | | | | | |) |
______________+________+________+________+________+________+________+________+
1 | 2955 | 906 | 2388 | 3174 | 1718 | 2 | 20 | 11163
| 26.47 | 8.12 | 21.39 | 28.43 | 15.39 | 0.02 | 0.18 | 100.00
| 26.47 | 8.12 | 21.39 | 28.43 | 15.39 | 0.02 | 0.18 |
| 100.00 | 100.00 | 100.00 | 100.00 | 100.00 | 100.00 | 100.00 |
______________+________+________+________+________+________+________+________+
Total 2955 906 2388 3174 1718 2 20 11163
26.47 8.12 21.39 28.43 15.39 0.02 0.18 100.00

Administration of anticoagulants at the time of obtaining Table 2-4 consent, according to the presence or absence of proton-pump inhibitors

FREQ Procedure

Table : ALL * n_ACT6

ALL n_ACT6

Power |
Percent |
Percent of rows
Percent of columns | 1| Total

1	11161	11161
	100.00	100.00
	100.00	
	100.00	
Total	11161	11161
	100.00	100.00

Degree of missing values = 2

Administration of anticoagulants at the time of obtaining Table 2-4 consent, according to the presence or absence of proton-pump inhibitors

FREQ Procedure

Table : ALL * t_ACT1

ALL t_ACT1

Power |
Percent |
Percent of rows
Percentage of columns | 0| 1| Sum

1	2	11161	11163
	0.02	99.98	100.00
	0.02	99.98	
	100.00	100.00	
Total	2	11161	11163
	0.02	99.98	100.00

Administration of anticoagulants at the time of obtaining Table 2-4 consent, according to the presence or absence of proton-pump inhibitors

FREQ Procedure

Table : ALL * t_ACT1

ALL t_ACT1

Power |
Percent |
Percent of rows
Percentage of columns | 0| 1| Sum

1	2	11161	11163
	0.02	99.98	100.00
	0.02	99.98	
	100.00	100.00	
Total	2	11161	11163
	0.02	99.98	100.00

Administration of anticoagulants at the time of obtaining Table 2-4 consent, according to the presence or absence of proton-pump inhibitors

FREQ Procedure

Table : ALL * t_ACT2

ALL t_ACT2

```
Power |
Percent |
Percent of rows
Percentage of columns | 0| 1| Sum
______________+________+________+
            1 |   2957 |   8206 |  11163
              |  26.49 |  73.51 | 100.00
              |  26.49 |  73.51 |
              | 100.00 | 100.00 |
______________+________+________+
Total 2957 8206 11163
                 26.49    73.51   100.00
```

Administration of anticoagulants at the time of obtaining Table 2-4 consent, according to the presence or absence of proton-pump inhibitors

FREQ Procedure

Table : ALL * t_ACT2

ALL t_ACT2

Power |
Percent |
Percent of rows
Percentage of columns | 0| 1| Sum

1	2957	8206	11163
	26.49	73.51	100.00
	26.49	73.51	
	100.00	100.00	
Total	2957	8206	11163
	26.49	73.51	100.00

Administration of anticoagulants at the time of obtaining Table 2-4 consent, according to the presence or absence of proton-pump inhibitors

FREQ Procedure

Table : ALL * t_ACT3

ALL t_ACT3

Power |
Percent |
Percent of rows
Percentage of columns | 0| 1| Sum

1	3863	7300	11163
	34.61	65.39	100.00
	34.61	65.39	
	100.00	100.00	
Total	3863	7300	11163
	34.61	65.39	100.00

Administration of anticoagulants at the time of obtaining Table 2-4 consent, according to the presence or absence of proton-pump inhibitors

FREQ Procedure

Table : ALL * t_ACT3

ALL t_ACT3

```
Power |
Percent |
Percent of rows
Percentage of columns | 0| 1| Sum
______________+________+________+
            1 |   3863 |   7300 |  11163
              |  34.61 |  65.39 | 100.00
              |  34.61 |  65.39 |
              | 100.00 | 100.00 |
______________+________+________+
Total 3863 7300 11163
                 34.61    65.39   100.00
```

Administration of anticoagulants at the time of obtaining Table 2-4 consent, according to the presence or absence of proton-pump inhibitors

FREQ Procedure

Tables: ALL* VISITAD

ALL VISITAD(Visit and Drug Administration)

Power |
Percent |
Percent of rows
Percent of column |Month |Month |Month |First dose |Unknown | Total
Within | | |
______________+________+________+________+________+
1 | 2914 | 8 | 8 | 25 | 2955
| 98.61 | 0.27 | 0.27 | 0.85 | 100.00
| 98.61 | 0.27 | 0.27 | 0.85 |
| 100.00 | 100.00 | 100.00 | 100.00 |
______________+________+________+________+________+
Total 2914 8 8 25 2955
98.61 0.27 0.27 0.85 100.00

Administration of anticoagulants at the time of obtaining Table 2-4 consent, according to the presence or absence of proton-pump inhibitors

FREQ Procedure

Tables: ALL* VISITAD

ALL VISITAD(Visit and Drug Administration)

```
Power |
Percent |
Percent of rows
Percent of column |Month |Month |Month |First dose |Unknown | Total
                  Within | | |
______________+________+________+________+________+
             1 |    2914 |         8 |         8 |        25 |    2955
               |   98.61 |     0.27 |     0.27 |     0.85 | 100.00
               |   98.61 |     0.27 |     0.27 |     0.85 |
               | 100.00 | 100.00 | 100.00 | 100.00 |
______________+________+________+________+________+
Total 2914 8 8 25 2955
                  98.61       0.27       0.27       0.85     100.00
```

Administration of anticoagulants at the time of obtaining Table 2-4 consent, according to the presence or absence of proton-pump inhibitors

FREQ Procedure

Tables: ALL* VISITAD

ALL VISITAD(Visit and Drug Administration)

Power |
Percent |
Percent of rows
Percent of column |Month |Month |Month |First dose |Unknown | Total
Within | | |

1	2914	8	8	25	2955
	98.61	0.27	0.27	0.85	100.00
	98.61	0.27	0.27	0.85	
	100.00	100.00	100.00	100.00	
Total	2914	8	8	25	2955
	98.61	0.27	0.27	0.85	100.00

Administration of anticoagulants at the time of obtaining Table 2-4 consent, according to the presence or absence of proton-pump inhibitors

FREQ Procedure

Tables: ALL* VISITAD

ALL VISITAD(Visit and Drug Administration)

Power |
Percent |
Percent of rows
Percent of column |Month |Month |Month |First dose |Unknown | Total
Within | | |

```
______________+________+________+________+________+
             1 |    2914 |        8 |        8 |       25 |    2955
               |   98.61 |     0.27 |     0.27 |     0.85 | 100.00
               |   98.61 |     0.27 |     0.27 |     0.85 |
               |  100.00 |   100.00 |   100.00 |   100.00 |
______________+________+________+________+________+
Total 2914 8 8 25 2955
                  98.61       0.27       0.27       0.85    100.00
```

Administration of anticoagulants at the time of obtaining Table 2-4 consent, according to the presence or absence of proton-pump inhibitors

FREQ Procedure

Tables: ALL* VISITAD

ALL VISITAD(Visit and Drug Administration)

Power |
Percent |
Percent of rows
Percent of column |Month |Month |Month |First dose |Unknown | Total
Within | | |

Percent of column	Month	Month	Month	First dose / Unknown	Total
1	875	9	8	14	906
	96.58	0.99	0.88	1.55	100.00
	96.58	0.99	0.88	1.55	
	100.00	100.00	100.00	100.00	
Total	875	9	8	14	906
	96.58	0.99	0.88	1.55	100.00

Administration of anticoagulants at the time of obtaining Table 2-4 consent, according to the presence or absence of proton-pump inhibitors

FREQ Procedure

Tables: ALL* VISITAD

ALL VISITAD(Visit and Drug Administration)

Power |
Percent |
Percent of rows
Percent of column |Month |Month |Month |First dose |Unknown | Total
Within | | |
______________+________+________+________+________+
1 | 875 | 9 | 8 | 14 | 906
| 96.58 | 0.99 | 0.88 | 1.55 | 100.00
| 96.58 | 0.99 | 0.88 | 1.55 |
| 100.00 | 100.00 | 100.00 | 100.00 |
______________+________+________+________+________+
Total 875 9 8 14 906
96.58 0.99 0.88 1.55 100.00

Administration of anticoagulants at the time of obtaining Table 2-4 consent, according to the presence or absence of proton-pump inhibitors

FREQ Procedure

Tables: ALL* VISITAD

ALL VISITAD(Visit and Drug Administration)

Power |
Percent |
Percent of rows
Percent of column |Month |Month |Month |First dose |Unknown | Total
Within | | |

1	875	9	8	14	906
	96.58	0.99	0.88	1.55	100.00
	96.58	0.99	0.88	1.55	
	100.00	100.00	100.00	100.00	
Total	875	9	8	14	906
	96.58	0.99	0.88	1.55	100.00

Administration of anticoagulants at the time of obtaining Table 2-4 consent, according to the presence or absence of proton-pump inhibitors

FREQ Procedure

Tables: ALL* VISITAD

ALL VISITAD(Visit and Drug Administration)

Power |
Percent |
Percent of rows
Percent of column |Month |Month |Month |First dose |Unknown | Total
Within | | |

1	875	9	8	14	906
	96.58	0.99	0.88	1.55	100.00
	96.58	0.99	0.88	1.55	
	100.00	100.00	100.00	100.00	
Total	875	9	8	14	906
	96.58	0.99	0.88	1.55	100.00

Administration of anticoagulants at the time of obtaining Table 2-4 consent, according to the presence or absence of proton-pump inhibitors

FREQ Procedure

Tables: ALL* VISITAD

ALL VISITAD(Visit and Drug Administration)

Power |
Percent |
Percent of rows
Percent of column |Month |Month |Month |First dose |Unknown | Total
Within | | |

1	2297	38	29	24	2388
	96.19	1.59	1.21	1.01	100.00
	96.19	1.59	1.21	1.01	
	100.00	100.00	100.00	100.00	
Total	2297	38	29	24	2388
	96.19	1.59	1.21	1.01	100.00

Administration of anticoagulants at the time of obtaining Table 2-4 consent, according to the presence or absence of proton-pump inhibitors

FREQ Procedure

Tables: ALL* VISITAD

ALL VISITAD(Visit and Drug Administration)

```
Power |
Percent |
Percent of rows
Percent of column |Month |Month |Month |First dose |Unknown | Total
           Within | | |
______________+________+________+________+________+
            1 |   2297 |     38 |     29 |     24 |  2388
              |  96.19 |   1.59 |   1.21 |   1.01 | 100.00
              |  96.19 |   1.59 |   1.21 |   1.01 |
              | 100.00 | 100.00 | 100.00 | 100.00 |
______________+________+________+________+________+
Total 2297 38 29 24 2388
                 96.19     1.59     1.21     1.01   100.00
```

Administration of anticoagulants at the time of obtaining Table 2-4 consent, according to the presence or absence of proton-pump inhibitors

FREQ Procedure

Tables: ALL* VISITAD

ALL VISITAD(Visit and Drug Administration)

Power |
Percent |
Percent of rows
Percent of column |Month |Month |Month |First dose |Unknown | Total
Within | | |

Power / Percent / Percent of rows / Percent of column	Month Within	Month	Month	First dose	Unknown	Total
1	2297	38	29	24	2388	
	96.19	1.59	1.21	1.01	100.00	
	96.19	1.59	1.21	1.01		
	100.00	100.00	100.00	100.00		
Total	2297	38	29	24	2388	
	96.19	1.59	1.21	1.01	100.00	

Administration of anticoagulants at the time of obtaining Table 2-4 consent, according to the presence or absence of proton-pump inhibitors

FREQ Procedure

Tables: ALL* VISITAD

ALL VISITAD(Visit and Drug Administration)

Power |
Percent |
Percent of rows
Percent of column |Month |Month |Month |First dose |Unknown | Total
Within | | |
______________+________+________+________+________+
1 | 2297 | 38 | 29 | 24 | 2388
| 96.19 | 1.59 | 1.21 | 1.01 | 100.00
| 96.19 | 1.59 | 1.21 | 1.01 |
| 100.00 | 100.00 | 100.00 | 100.00 |
______________+________+________+________+________+
Total 2297 38 29 24 2388
96.19 1.59 1.21 1.01 100.00

Administration of anticoagulants at the time of obtaining Table 2-4 consent, according to the presence or absence of proton-pump inhibitors

FREQ Procedure

Tables: ALL* VISITAD

ALL VISITAD(Visit and Drug Administration)

Power / Percent / Percent of rows / Percent of column	Month Within	Month	Month	First dose	Unknown	Total
1	3062	54	43	15	3174	
	96.47	1.70	1.35	0.47	100.00	
	96.47	1.70	1.35	0.47		
	100.00	100.00	100.00	100.00		
Total	3062	54	43	15	3174	
	96.47	1.70	1.35	0.47	100.00	

Administration of anticoagulants at the time of obtaining Table 2-4 consent, according to the presence or absence of proton-pump inhibitors

FREQ Procedure

Tables: ALL* VISITAD

ALL VISITAD(Visit and Drug Administration)

Power |
Percent |
Percent of rows
Percent of column |Month |Month |Month |First dose |Unknown | Total
Within | | |
______________+________+________+________+________+
1 | 3062 | 54 | 43 | 15 | 3174
| 96.47 | 1.70 | 1.35 | 0.47 | 100.00
| 96.47 | 1.70 | 1.35 | 0.47 |
| 100.00 | 100.00 | 100.00 | 100.00 |
______________+________+________+________+________+
Total 3062 54 43 15 3174
96.47 1.70 1.35 0.47 100.00

Administration of anticoagulants at the time of obtaining Table 2-4 consent, according to the presence or absence of proton-pump inhibitors

FREQ Procedure

Tables: ALL* VISITAD

ALL VISITAD(Visit and Drug Administration)

Power |
Percent |
Percent of rows
Percent of column |Month |Month |Month |First dose |Unknown | Total
Within | | |
______________+________+________+________+________+
1 | 3062 | 54 | 43 | 15 | 3174
| 96.47 | 1.70 | 1.35 | 0.47 | 100.00
| 96.47 | 1.70 | 1.35 | 0.47 |
| 100.00 | 100.00 | 100.00 | 100.00 |
______________+________+________+________+________+
Total 3062 54 43 15 3174
96.47 1.70 1.35 0.47 100.00

Administration of anticoagulants at the time of obtaining Table 2-4 consent, according to the presence or absence of proton-pump inhibitors

FREQ Procedure

Tables: ALL* VISITAD

ALL VISITAD(Visit and Drug Administration)

Power |
Percent |
Percent of rows
Percent of column |Month |Month |Month |First dose |Unknown | Total
Within | | |

1	3062	54	43	15	3174
	96.47	1.70	1.35	0.47	100.00
	96.47	1.70	1.35	0.47	
	100.00	100.00	100.00	100.00	
Total	3062	54	43	15	3174
	96.47	1.70	1.35	0.47	100.00

Administration of anticoagulants at the time of obtaining Table 2-4 consent, according to the presence or absence of proton-pump inhibitors

FREQ Procedure

Tables: ALL* VISITAD

ALL VISITAD(Visit and Drug Administration)

Power |
Percent |
Percent of rows
Percent of column |Month |Month |Month |First dose |Unknown | Total
Within | | |

1	1528	65	135	10	1738
	87.92	3.74	7.77	0.58	100.00
	87.92	3.74	7.77	0.58	
	100.00	100.00	100.00	100.00	
Total	1528	65	135	10	1738
	87.92	3.74	7.77	0.58	100.00

Administration of anticoagulants at the time of obtaining Table 2-4 consent, according to the presence or absence of proton-pump inhibitors

FREQ Procedure

Tables: ALL* VISITAD

ALL VISITAD(Visit and Drug Administration)

Power |
Percent |
Percent of rows
Percent of column |Month |Month |Month |First dose |Unknown | Total
Within | | |
______________+________+________+________+________+
1 | 1528 | 65 | 135 | 10 | 1738
| 87.92 | 3.74 | 7.77 | 0.58 | 100.00
| 87.92 | 3.74 | 7.77 | 0.58 |
| 100.00 | 100.00 | 100.00 | 100.00 |
______________+________+________+________+________+
Total 1528 65 135 10 1738
87.92 3.74 7.77 0.58 100.00

Administration of anticoagulants at the time of obtaining Table 2-4 consent, according to the presence or absence of proton-pump inhibitors

FREQ Procedure

Tables: ALL* VISITAD

ALL VISITAD(Visit and Drug Administration)

Power |
Percent |
Percent of rows
Percent of column |Month |Month |Month |First dose |Unknown | Total
Within | | |

1	1528	65	135	10	1738
	87.92	3.74	7.77	0.58	100.00
	87.92	3.74	7.77	0.58	
	100.00	100.00	100.00	100.00	
Total	1528	65	135	10	1738
	87.92	3.74	7.77	0.58	100.00

Administration of anticoagulants at the time of obtaining Table 2-4 consent, according to the presence or absence of proton-pump inhibitors

FREQ Procedure

Tables: ALL* VISITAD

ALL VISITAD(Visit and Drug Administration)

Power |
Percent |
Percent of rows
Percent of column |Month |Month |Month |First dose |Unknown | Total
Within | | |

	Month	Month	Month	First dose	Unknown	Total
1	1528	65	135	10	1738	
	87.92	3.74	7.77	0.58	100.00	
	87.92	3.74	7.77	0.58		
	100.00	100.00	100.00	100.00		
Total	1528	65	135	10	1738	
	87.92	3.74	7.77	0.58	100.00	

Administration of anticoagulants at the time of obtaining Table 2-4 consent, according to the presence or absence of proton-pump inhibitors

FREQ Procedure

Tables: ALL* VISITAD

ALL VISITAD(Visit and Drug Administration)

Power |
Percent |
Percent of rows
Percent of column |Month |Month |Month |First dose |Unknown | Total
Within | | |

1	1508	65	135	10	1718
	87.78	3.78	7.86	0.58	100.00
	87.78	3.78	7.86	0.58	
	100.00	100.00	100.00	100.00	
Total	1508	65	135	10	1718
	87.78	3.78	7.86	0.58	100.00

Administration of anticoagulants at the time of obtaining Table 2-4 consent, according to the presence or absence of proton-pump inhibitors

FREQ Procedure

Tables: ALL* VISITAD

ALL VISITAD(Visit and Drug Administration)

Power |
Percent |
Percent of rows
Percent of column |Month |Month |Month |First dose |Unknown | Total
Within | | |

1	1508	65	135	10	1718
	87.78	3.78	7.86	0.58	100.00
	87.78	3.78	7.86	0.58	
	100.00	100.00	100.00	100.00	
Total	1508	65	135	10	1718
	87.78	3.78	7.86	0.58	100.00

Administration of anticoagulants at the time of obtaining Table 2-4 consent, according to the presence or absence of proton-pump inhibitors

FREQ Procedure

Tables: ALL* VISITAD

ALL VISITAD(Visit and Drug Administration)

Power |
Percent |
Percent of rows
Percent of column |Month |Month |Month |First dose |Unknown | Total
Within | | |

1	1508	65	135	10	1718
	87.78	3.78	7.86	0.58	100.00
	87.78	3.78	7.86	0.58	
	100.00	100.00	100.00	100.00	
Total	1508	65	135	10	1718
	87.78	3.78	7.86	0.58	100.00

Administration of anticoagulants at the time of obtaining Table 2-4 consent, according to the presence or absence of proton-pump inhibitors

FREQ Procedure

Tables: ALL* VISITAD

ALL VISITAD(Visit and Drug Administration)

Power |
Percent |
Percent of rows
Percent of column |Month |Month |Month |First dose |Unknown | Total
Within | | |

1	1508	65	135	10	1718
	87.78	3.78	7.86	0.58	100.00
	87.78	3.78	7.86	0.58	
	100.00	100.00	100.00	100.00	
Total	1508	65	135	10	1718
	87.78	3.78	7.86	0.58	100.00

Administration of anticoagulants at the time of obtaining Table 2-4 consent, according to the presence or absence of proton-pump inhibitors

FREQ Procedure

Tables: ALL* VISITAD

ALL

VISITAD(Visit and Drug Administration)

```
Power |
Percent |
Percent of rows
Percent of column |Month | Total
          Breakforward |
______________+________+
        1 |      20 |      20
          | 100.00 | 100.00
          | 100.00 |
          | 100.00 |
______________+________+
Total 20 20
          100.00   100.00
```

Administration of anticoagulants at the time of obtaining Table 2-4 consent, according to the presence or absence of proton-pump inhibitors

FREQ Procedure

Tables: ALL* VISITAD

ALL

VISITAD(Visit and Drug Administration)

Power |
Percent |
Percent of rows
Percent of column |Month | Total
Breakforward |
______________+________+
1 | 20 | 20
| 100.00 | 100.00
| 100.00 |
| 100.00 |
______________+________+
Total 20 20
100.00 100.00

Administration of anticoagulants at the time of obtaining Table 2-4 consent, according to the presence or absence of proton-pump inhibitors

FREQ Procedure

Tables: ALL* VISITAD

ALL

VISITAD(Visit and Drug Administration)

Power |
Percent |
Percent of rows
Percent of column |Month | Total
Breakforward |
______________+________+
1 | 20 | 20
| 100.00 | 100.00
| 100.00 |
| 100.00 |
______________+________+
Total 20 20
100.00 100.00

Administration of anticoagulants at the time of obtaining Table 2-4 consent, according to the presence or absence of proton-pump inhibitors

FREQ Procedure

Tables: ALL* VISITAD

ALL
VISITAD(Visit and Drug Administration)

Power |
Percent |
Percent of rows
Percent of column |Month | Total
Breakforward |
______________+________+
1 | 20 | 20
| 100.00 | 100.00
| 100.00 |
| 100.00 |
______________+________+
Total 20 20
100.00 100.00

Administration of anticoagulants at the time of obtaining Table 2-4 consent, according to the presence or absence of proton-pump inhibitors

FREQ Procedure

Tables: ALL* VISITAD

ALL

VISITAD(Visit and Drug Administration)

Power |
Percent |
Percent of rows
Percent of column |Month | Unknown | Total
) in front | |
______________+________+________+
1 | 1 | 1 | 2
| 50.00 | 50.00 | 100.00
| 50.00 | 50.00 |
| 100.00 | 100.00 |
______________+________+________+
Total 1 1 2
50.00 50.00 100.00

Administration of anticoagulants at the time of obtaining Table 2-4 consent, according to the presence or absence of proton-pump inhibitors

FREQ Procedure

Tables: ALL* VISITAD

ALL

VISITAD(Visit and Drug Administration)

```
Power |
Percent |
Percent of rows
Percent of column |Month | Unknown | Total
           ) in front | |
______________+________+________+
            1 |      1 |      1 |     2
              |  50.00 |  50.00 | 100.00
              |  50.00 |  50.00 |
              | 100.00 | 100.00 |
______________+________+________+
Total 1 1 2
                 50.00    50.00   100.00
```

Administration of anticoagulants at the time of obtaining Table 2-4 consent, according to the presence or absence of proton-pump inhibitors

FREQ Procedure

Tables: ALL* VISITAD

ALL

VISITAD(Visit and Drug Administration)

Power |
Percent |
Percent of rows
Percent of column |Month | Unknown | Total
) in front | |
______________+________+________+
1 | 1 | 1 | 2
| 50.00 | 50.00 | 100.00
| 50.00 | 50.00 |
| 100.00 | 100.00 |
______________+________+________+
Total 1 1 2
50.00 50.00 100.00

Administration of anticoagulants at the time of obtaining Table 2-4 consent, according to the presence or absence of proton-pump inhibitors

FREQ Procedure

Tables: ALL* VISITAD

ALL

VISITAD(Visit and Drug Administration)

Power |
Percent |
Percent of rows
Percent of column |Month | Unknown | Total
) in front | |
______________+________+________+
1 | 1 | 1 | 2
| 50.00 | 50.00 | 100.00
| 50.00 | 50.00 |
| 100.00 | 100.00 |
______________+________+________+
Total 1 1 2
50.00 50.00 100.00

Administration of anticoagulants at the time of obtaining Table 2-4 consent, according to the presence or absence of proton-pump inhibitors

FREQ Procedure

Tables: ALL * DOSE

ALL DOSE(Dose per day for DOAC only)

Power |
Percent |
Percent of rows

Column percent	50	75	110	150	185	220	300	Total
1	1	5	74	45	1	725	55	906
	0.11	0.55	8.17	4.97	0.11	80.02	6.07	100.00
	0.11	0.55	8.17	4.97	0.11	80.02	6.07	
	100.00	100.00	100.00	100.00	100.00	100.00	100.00	
Total	1	5	74	45	1	725	55	906
	0.11	0.55	8.17	4.97	0.11	80.02	6.07	100.00

Administration of anticoagulants at the time of obtaining Table 2-4 consent, according to the presence or absence of proton-pump inhibitors

FREQ Procedure

Tables: ALL * DOSE

ALL DOSE(Dose per day for DOAC only)

Power |
Percent |
Percent of rows
Column percent | 50| 75| 110| 150| 185| 220| 300| Total
______________+________+________+________+________+________+________+_______
_+
1 | 1 | 5 | 74 | 45 | 1 | 725 | 55 | 906
| 0.11 | 0.55 | 8.17 | 4.97 | 0.11 | 80.02 | 6.07 | 100.00
| 0.11 | 0.55 | 8.17 | 4.97 | 0.11 | 80.02 | 6.07 |
| 100.00 | 100.00 | 100.00 | 100.00 | 100.00 | 100.00 | 100.00 |
______________+________+________+________+________+________+________+_______
_+
Total 1 5 74 45 1 725 55 906
0.11 0.55 8.17 4.97 0.11 80.02 6.07 100.00

Administration of anticoagulants at the time of obtaining Table 2-4 consent, according to the presence or absence of proton-pump inhibitors

FREQ Procedure

Tables: ALL * DOSE

ALL DOSE(Dose per day for DOAC only)

Power |
Percent |
Percent of rows
Column percent | 50| 75| 110| 150| 185| 220| 300| Total
______________+________+________+________+________+________+________+________
_+
1 | 1 | 5 | 74 | 45 | 1 | 725 | 55 | 906
| 0.11 | 0.55 | 8.17 | 4.97 | 0.11 | 80.02 | 6.07 | 100.00
| 0.11 | 0.55 | 8.17 | 4.97 | 0.11 | 80.02 | 6.07 |
| 100.00 | 100.00 | 100.00 | 100.00 | 100.00 | 100.00 | 100.00 |
______________+________+________+________+________+________+________+________
_+
Total 1 5 74 45 1 725 55 906
0.11 0.55 8.17 4.97 0.11 80.02 6.07 100.00

Administration of anticoagulants at the time of obtaining Table 2-4 consent, according to the presence or absence of proton-pump inhibitors

FREQ Procedure

Tables: ALL * DOSE

ALL DOSE(Dose per day for DOAC only)

Power |
Percent |
Percent of rows

Column percent	50	75	110	150	185	220	300	Total
1	1	5	74	45	1	725	55	906
	0.11	0.55	8.17	4.97	0.11	80.02	6.07	100.00
	0.11	0.55	8.17	4.97	0.11	80.02	6.07	
	100.00	100.00	100.00	100.00	100.00	100.00	100.00	
Total	1	5	74	45	1	725	55	906
	0.11	0.55	8.17	4.97	0.11	80.02	6.07	100.00

Administration of anticoagulants at the time of obtaining Table 2-4 consent, according to the presence or absence of proton-pump inhibitors

FREQ Procedure

Tables: ALL * DOSE

ALL DOSE(Dose per day for DOAC only)

Power |
Percent |
Percent of rows
Column percent | 50| 75| 110| 150| 185| 220| 300| Total
______________+________+________+________+________+________+________+________
_+
1 | 1 | 5 | 74 | 45 | 1 | 725 | 55 | 906
| 0.11 | 0.55 | 8.17 | 4.97 | 0.11 | 80.02 | 6.07 | 100.00
| 0.11 | 0.55 | 8.17 | 4.97 | 0.11 | 80.02 | 6.07 |
| 100.00 | 100.00 | 100.00 | 100.00 | 100.00 | 100.00 | 100.00 |
______________+________+________+________+________+________+________+________
_+
Total 1 5 74 45 1 725 55 906
0.11 0.55 8.17 4.97 0.11 80.02 6.07 100.00

Administration of anticoagulants at the time of obtaining Table 2-4 consent, according to the presence or absence of proton-pump inhibitors

FREQ Procedure

Table : ALL * f_d2

ALL f_d2

Power |
Percent |
Percent of rows
Percent of columns | 1| Total

______________+________+
1 | 2 | 2
| 100.00 | 100.00
| 100.00 |
| 100.00 |
______________+________+
Total 2 2
100.00 100.00

Frequency of missing values = 904

Administration of anticoagulants at the time of obtaining Table 2-4 consent, according to the presence or absence of proton-pump inhibitors

FREQ Procedure

Tables: ALL * DOSE

ALL DOSE(Dose per day for DOAC only)

Power |
Percent |
Percent of rows
Column percent | 0| 2.5| 5| 7.5| 10| 15| 30| Total
_______________+_________+_________+_________+_________+_________+_________+________
_+

1	1	1	8	3	1695	678	2	2388
	0.04	0.04	0.34	0.13	70.98	28.39	0.08	100.00
	0.04	0.04	0.34	0.13	70.98	28.39	0.08	
	100.00	100.00	100.00	100.00	100.00	100.00	100.00	

_______________+_________+_________+_________+_________+_________+_________+________
_+

Total 1 1 8 3 1695 678 2 2388

0.04 0.04 0.34 0.13 70.98 28.39 0.08 100.00

Administration of anticoagulants at the time of obtaining Table 2-4 consent, according to the presence or absence of proton-pump inhibitors

FREQ Procedure

Tables: ALL * DOSE

ALL DOSE(Dose per day for DOAC only)

Power |
Percent |
Percent of rows
Column percent | 0| 2.5| 5| 7.5| 10| 15| 30| Total

ALL	0	2.5	5	7.5	10	15	30	Total
1	1	1	8	3	1695	678	2	2388
	0.04	0.04	0.34	0.13	70.98	28.39	0.08	100.00
	0.04	0.04	0.34	0.13	70.98	28.39	0.08	
	100.00	100.00	100.00	100.00	100.00	100.00	100.00	
Total	1	1	8	3	1695	678	2	2388
	0.04	0.04	0.34	0.13	70.98	28.39	0.08	100.00

Administration of anticoagulants at the time of obtaining Table 2-4 consent, according to the presence or absence of proton-pump inhibitors

FREQ Procedure

Table : ALL * f_d3

ALL f_d3

Power |
Percent |
Percent of rows
Percent of columns | 1| Total
______________+________+
1 | 15 | 15
| 100.00 | 100.00
| 100.00 |
| 100.00 |
______________+________+
Total 15 15
100.00 100.00

Frequency of missing values = 2373

Administration of anticoagulants at the time of obtaining Table 2-4 consent, according to the presence or absence of proton-pump inhibitors

FREQ Procedure

Tables: ALL * DOSE

ALL DOSE(Dose per day for DOAC only)

Power |
Percent |
Percent of rows
Column percent | 0| 2.5| 5| 7.5| 10| 30| Total

1	1	198	2086	3	885	1	3174
	0.03	6.24	65.72	0.09	27.88	0.03	100.00
	0.03	6.24	65.72	0.09	27.88	0.03	
	100.00	100.00	100.00	100.00	100.00	100.00	
Total	1	198	2086	3	885	1	3174
	0.03	6.24	65.72	0.09	27.88	0.03	100.00

Administration of anticoagulants at the time of obtaining Table 2-4 consent, according to the presence or absence of proton-pump inhibitors

FREQ Procedure

Tables: ALL * DOSE

ALL DOSE(Dose per day for DOAC only)

Power |
Percent |
Percent of rows
Column percent | 0| 2.5| 5| 7.5| 10| 30| Total

1	1	198	2086	3	885	1	3174
	0.03	6.24	65.72	0.09	27.88	0.03	100.00
	0.03	6.24	65.72	0.09	27.88	0.03	
	100.00	100.00	100.00	100.00	100.00	100.00	
Total	1	198	2086	3	885	1	3174
	0.03	6.24	65.72	0.09	27.88	0.03	100.00

Administration of anticoagulants at the time of obtaining Table 2-4 consent, according to the presence or absence of proton-pump inhibitors

FREQ Procedure

Tables: ALL * DOSE

ALL DOSE(Dose per day for DOAC only)

Power |
Percent |
Percent of rows
Column percent | 0| 2.5| 5| 7.5| 10| 30| Total

1	1	198	2086	3	885	1	3174
	0.03	6.24	65.72	0.09	27.88	0.03	100.00
	0.03	6.24	65.72	0.09	27.88	0.03	
	100.00	100.00	100.00	100.00	100.00	100.00	
Total	1	198	2086	3	885	1	3174
	0.03	6.24	65.72	0.09	27.88	0.03	100.00

Administration of anticoagulants at the time of obtaining Table 2-4 consent, according to the presence or absence of proton-pump inhibitors

FREQ Procedure

Table : ALL * f_d4

ALL f_d4

Power |
Percent |
Percent of rows
Percent of columns | 1| Total
______________+________+
1 | 5 | 5
| 100.00 | 100.00
| 100.00 |
| 100.00 |
______________+________+
Total 5 5
100.00 100.00

Degree of missing values = 3169

Administration of anticoagulants at the time of obtaining Table 2-4 consent, according to the presence or absence of proton-pump inhibitors

FREQ Procedure

Tables: ALL * DOSE

ALL DOSE(Dose per day for DOAC only)

Power |
Percent |
Percent of rows
Percentage of columns | 15| 30| 60| Total

ALL	15	30	60	Total
1	106	1445	187	1738
	6.10	83.14	10.76	100.00
	6.10	83.14	10.76	
	100.00	100.00	100.00	
Total	106	1445	187	1738
	6.10	83.14	10.76	100.00

Administration of anticoagulants at the time of obtaining Table 2-4 consent, according to the presence or absence of proton-pump inhibitors

FREQ Procedure

Tables: ALL * DOSE

ALL DOSE(Dose per day for DOAC only)

Power |
Percent |
Percent of rows
Percentage of columns | 15| 30| 60| Total

1	106	1445	187	1738
	6.10	83.14	10.76	100.00
	6.10	83.14	10.76	
	100.00	100.00	100.00	
Total	106	1445	187	1738
	6.10	83.14	10.76	100.00

Administration of anticoagulants at the time of obtaining Table 2-4 consent, according to the presence or absence of proton-pump inhibitors

FREQ Procedure

Tables: ALL * DOSE

ALL DOSE(Dose per day for DOAC only)

Power |
Percent |
Percent of rows
Percentage of columns | 15| 30| 60| Total

1	106	1445	187	1738
	6.10	83.14	10.76	100.00
	6.10	83.14	10.76	
	100.00	100.00	100.00	
Total	106	1445	187	1738
	6.10	83.14	10.76	100.00

Administration of anticoagulants at the time of obtaining Table 2-4 consent, according to the presence or absence of proton-pump inhibitors

FREQ Procedure

For ALL * f_d0,
Because all levels of the variable f_d0 are missing,
There is no data.

Administration of anticoagulants at the time of obtaining Table 2-4 consent, according to the presence or absence of proton-pump inhibitors

FREQ Procedure

Tables: ALL * DOSE

ALL DOSE(Dose per day for DOAC only)

Power |
Percent |
Percent of rows
Percentage of columns | 15| 30| 60| Total

ALL	15	30	60	Total
1	105	1429	184	1718
	6.11	83.18	10.71	100.00
	6.11	83.18	10.71	
	100.00	100.00	100.00	
Total	105	1429	184	1718
	6.11	83.18	10.71	100.00

Administration of anticoagulants at the time of obtaining Table 2-4 consent, according to the presence or absence of proton-pump inhibitors

FREQ Procedure

Tables: ALL * DOSE

ALL DOSE(Dose per day for DOAC only)

Power |
Percent |
Percent of rows
Percentage of columns | 15| 30| 60| Total

	15	30	60	Total
1	105	1429	184	1718
	6.11	83.18	10.71	100.00
	6.11	83.18	10.71	
	100.00	100.00	100.00	
Total	105	1429	184	1718
	6.11	83.18	10.71	100.00

Administration of anticoagulants at the time of obtaining Table 2-4 consent, according to the presence or absence of proton-pump inhibitors

FREQ Procedure

Tables: ALL * DOSE

ALL DOSE(Dose per day for DOAC only)

Power |
Percent |
Percent of rows
Percentage of columns | 15| 30| 60| Total

ALL	15	30	60	Total
1	105	1429	184	1718
	6.11	83.18	10.71	100.00
	6.11	83.18	10.71	
	100.00	100.00	100.00	
Total	105	1429	184	1718
	6.11	83.18	10.71	100.00

Administration of anticoagulants at the time of obtaining Table 2-4 consent, according to the presence or absence of proton-pump inhibitors

FREQ Procedure

For ALL * f_d5,
Because all levels of the variable f_d5 are missing,
There is no data.

Administration of anticoagulants at the time of obtaining Table 2-4 consent, according to the presence or absence of proton-pump inhibitors

FREQ Procedure

Tables: ALL * DOSE

ALL DOSE(Dose per day for DOAC only)

Power |
Percent |
Percent of rows
Percentage of columns | 15| 30| 60| Total

1	1	16	3	20
	5.00	80.00	15.00	100.00
	5.00	80.00	15.00	
	100.00	100.00	100.00	
Total	1	16	3	20
	5.00	80.00	15.00	100.00

Administration of anticoagulants at the time of obtaining Table 2-4 consent, according to the presence or absence of proton-pump inhibitors

FREQ Procedure

Tables: ALL * DOSE

ALL DOSE(Dose per day for DOAC only)

Power |
Percent |
Percent of rows
Percentage of columns | 15| 30| 60| Total

1	1	16	3	20
	5.00	80.00	15.00	100.00
	5.00	80.00	15.00	
	100.00	100.00	100.00	
Total	1	16	3	20
	5.00	80.00	15.00	100.00

Administration of anticoagulants at the time of obtaining Table 2-4 consent, according to the presence or absence of proton-pump inhibitors

FREQ Procedure

Tables: ALL * DOSE

ALL DOSE(Dose per day for DOAC only)

Power |
Percent |
Percent of rows

Percentage of columns	15	30	60	Total
1	1	16	3	20
	5.00	80.00	15.00	100.00
	5.00	80.00	15.00	
	100.00	100.00	100.00	
Total	1	16	3	20
	5.00	80.00	15.00	100.00

Administration of anticoagulants at the time of obtaining Table 2-4 consent, according to the presence or absence of proton-pump inhibitors

FREQ Procedure

For ALL * f_d7,
Because all levels of the variable f_d7 are missing,
There is no data.

Administration of anticoagulants at the time of obtaining Table 2-4 consent, according to the presence or absence of proton-pump inhibitors

FREQ Procedure

Tables: ALL * DOSECNT

ALL

DOSECNT(Dose per 1time for DOAC only)

Power |
Percent |
Percent of rows
Percentage of columns | 1| 2| Total

1	5	901	906
	0.55	99.45	100.00
	0.55	99.45	
	100.00	100.00	
Total	5	901	906
	0.55	99.45	100.00

Administration of anticoagulants at the time of obtaining Table 2-4 consent, according to the presence or absence of proton-pump inhibitors

FREQ Procedure

Tables: ALL * DOSECNT

ALL

DOSECNT(Dose per 1time for DOAC only)

Power |
Percent |
Percent of rows
Percentage of columns | 1| 2| Total

1	5	901	906
	0.55	99.45	100.00
	0.55	99.45	
	100.00	100.00	
Total	5	901	906
	0.55	99.45	100.00

Administration of anticoagulants at the time of obtaining Table 2-4 consent, according to the presence or absence of proton-pump inhibitors

FREQ Procedure

For ALL * t_dc2,
Because all levels of variable t_dc2 are missing,
There is no data.

Administration of anticoagulants at the time of obtaining Table 2-4 consent, according to the presence or absence of proton-pump inhibitors

FREQ Procedure

Tables: ALL * DOSECNT

ALL

DOSECNT(Dose per 1time for DOAC only)

Power |
Percent |
Percent of rows
Percentage of columns | 0| 1| 2| Sum

	0	1	2	Sum
1	1	2384	3	2388
	0.04	99.83	0.13	100.00
	0.04	99.83	0.13	
	100.00	100.00	100.00	
Total	1	2384	3	2388
	0.04	99.83	0.13	100.00

Administration of anticoagulants at the time of obtaining Table 2-4 consent, according to the presence or absence of proton-pump inhibitors

FREQ Procedure

Tables: ALL * DOSECNT

ALL

DOSECNT(Dose per 1time for DOAC only)

Power |
Percent |
Percent of rows
Percentage of columns | 0| 1| 2| Sum

1	1	2384	3	2388
	0.04	99.83	0.13	100.00
	0.04	99.83	0.13	
	100.00	100.00	100.00	
Total	1	2384	3	2388
	0.04	99.83	0.13	100.00

Administration of anticoagulants at the time of obtaining Table 2-4 consent, according to the presence or absence of proton-pump inhibitors

FREQ Procedure

Table : ALL * t_dc3

ALL t_dc3

```
Power |
Percent |
Percent of rows
Percent of columns | 1| Total
______________+________+
          1 |       1 |       1
            | 100.00 | 100.00
            | 100.00 |
            | 100.00 |
______________+________+
Total 1 1
              100.00    100.00
```

Degree of missing values = 2387

Administration of anticoagulants at the time of obtaining Table 2-4 consent, according to the presence or absence of proton-pump inhibitors

FREQ Procedure

Tables: ALL * DOSECNT

ALL DOSECNT(Dose per 1time for DOAC only)

Power |
Percent |
Percent of rows
Percentage of columns | 0| 1| 2| 3| Total

1	1	26	3146	1	3174
	0.03	0.82	99.12	0.03	100.00
	0.03	0.82	99.12	0.03	
	100.00	100.00	100.00	100.00	
Total	1	26	3146	1	3174
	0.03	0.82	99.12	0.03	100.00

Administration of anticoagulants at the time of obtaining Table 2-4 consent, according to the presence or absence of proton-pump inhibitors

FREQ Procedure

Tables: ALL * DOSECNT

ALL DOSECNT(Dose per 1time for DOAC only)

Power |
Percent |
Percent of rows
Percentage of columns | 0| 1| 2| 3| Total

ALL	0	1	2	3	Total
1	1	26	3146	1	3174
	0.03	0.82	99.12	0.03	100.00
	0.03	0.82	99.12	0.03	
	100.00	100.00	100.00	100.00	
Total	1	26	3146	1	3174
	0.03	0.82	99.12	0.03	100.00

Administration of anticoagulants at the time of obtaining Table 2-4 consent, according to the presence or absence of proton-pump inhibitors

FREQ Procedure

Table : ALL * t_dc4

ALL t_dc4

Power |
Percent |
Percent of rows
Percent of columns | 1| Total
______________+________+
1 | 2 | 2
| 100.00 | 100.00
| 100.00 |
| 100.00 |
______________+________+
Total 2 2
100.00 100.00

Degree of missing values = 3172

Administration of anticoagulants at the time of obtaining Table 2-4 consent, according to the presence or absence of proton-pump inhibitors

FREQ Procedure

Tables: ALL * DOSECNT

ALL

DOSECNT(Dose per 1time for DOAC only)

Power |
Percent |
Percent of rows
Percentage of columns | 1| 2| Total

1	1736	2	1738
	99.88	0.12	100.00
	99.88	0.12	
	100.00	100.00	
Total	1736	2	1738
	99.88	0.12	100.00

Administration of anticoagulants at the time of obtaining Table 2-4 consent, according to the presence or absence of proton-pump inhibitors

FREQ Procedure

Tables: ALL * DOSECNT

ALL

DOSECNT(Dose per 1time for DOAC only)

Power |
Percent |
Percent of rows
Percentage of columns | 1| 2| Total

ALL	1	2	Total
1	1736	2	1738
	99.88	0.12	100.00
	99.88	0.12	
	100.00	100.00	
Total	1736	2	1738
	99.88	0.12	100.00

Administration of anticoagulants at the time of obtaining Table 2-4 consent, according to the presence or absence of proton-pump inhibitors

FREQ Procedure

For ALL * t_dc0,
Because all levels of variable t_dc0 are missing,
There is no data.

Administration of anticoagulants at the time of obtaining Table 2-4 consent, according to the presence or absence of proton-pump inhibitors

FREQ Procedure

Tables: ALL * DOSECNT

ALL

DOSECNT(Dose per 1time for DOAC only)

Power |

Percent |

Percent of rows

Percentage of columns | 1| 2| Total

______________+________+________+

1 | 1717 | 1 | 1718

| 99.94 | 0.06 | 100.00

| 99.94 | 0.06 |

| 100.00 | 100.00 |

______________+________+________+

Total 1717 1 1718

99.94 0.06 100.00

Administration of anticoagulants at the time of obtaining Table 2-4 consent, according to the presence or absence of proton-pump inhibitors

FREQ Procedure

Tables: ALL * DOSECNT

ALL

DOSECNT(Dose per 1time for DOAC only)

Power |
Percent |
Percent of rows
Percentage of columns | 1| 2| Total

1	1717	1	1718
	99.94	0.06	100.00
	99.94	0.06	
	100.00	100.00	
Total	1717	1	1718
	99.94	0.06	100.00

Administration of anticoagulants at the time of obtaining Table 2-4 consent, according to the presence or absence of proton-pump inhibitors

FREQ Procedure

For ALL * t_dc5,
Because all levels of variable t_dc5 are missing,
There is no data.

Administration of anticoagulants at the time of obtaining Table 2-4 consent, according to the presence or absence of proton-pump inhibitors

FREQ Procedure

Tables: ALL * DOSECNT

ALL

DOSECNT(Dose per 1time for DOAC only)

Power |
Percent |
Percent of rows
Percentage of columns | 1| 2| Total

ALL	1	2	Total
1	19	1	20
	95.00	5.00	100.00
	95.00	5.00	
	100.00	100.00	
Total	19	1	20
	95.00	5.00	100.00

Administration of anticoagulants at the time of obtaining Table 2-4 consent, according to the presence or absence of proton-pump inhibitors

FREQ Procedure

Tables: ALL * DOSECNT

ALL

DOSECNT(Dose per 1time for DOAC only)

Power |
Percent |
Percent of rows
Percentage of columns | 1| 2| Total

1	19	1	20
	95.00	5.00	100.00
	95.00	5.00	
	100.00	100.00	
Total	19	1	20
	95.00	5.00	100.00

Administration of anticoagulants at the time of obtaining Table 2-4 consent, according to the presence or absence of proton-pump inhibitors

FREQ Procedure

For ALL * t_dc7,
Because all levels of variable t_dc7 are missing,
There is no data.

Administration of anticoagulants at the time of obtaining Table 2-4 consent, according to the presence or absence of proton-pump inhibitors

FREQ Procedure

Cumulative
Percent of ALL power Percent of power

1	20509	100.00	20509	100.00

Tables: ALL * ADYN

ALL
ADYN(Administration of Anticoagulant)

Power |
Percent |
Percent of rows
Percentage of columns | with | total
______________+________+
1 | 18751 | 18751
| 100.00 | 100.00
| 100.00 |
| 100.00 |
______________+________+
Total 18751 18751
100.00 100.00

Degree of missing values = 1758

Administration of anticoagulants at the time of obtaining Table 2-4 consent, according to the presence or absence of proton-pump inhibitors

FREQ Procedure

Table 1 : ADYN * ACTYP
Stratification variable : ALL=1

ADYN(Administration of Anticoagulant) ACTYP(Type of AF)

Power |
Percent |
Percent of rows
Percent of column | Warfa| Dabigat| Riverro| Apixa| Edoxa| Parenteral Anti| Edoxa| Total
|phosphate|run|xaban|ban|ban|coagulant|ban (OD|)
| | | | | | |) |
______________+________+________+________+________+________+________+________+________+
Present | 5340 | 1433 | 4048 | 4865 | 3034 | 11 | 20 | 18751
| 28.48 | 7.64 | 21.59 | 25.95 | 16.18 | 0.06 | 0.11 | 100.00
| 28.48 | 7.64 | 21.59 | 25.95 | 16.18 | 0.06 | 0.11 |
| 100.00 | 100.00 | 100.00 | 100.00 | 100.00 | 100.00 | 100.00 |
______________+________+________+________+________+________+________+________+________+
Total 5340 1433 4048 4865 3034 11 20 18751
28.48 7.64 21.59 25.95 16.18 0.06 0.11 100.00

Degree of missing values = 1758

Table 1 : ADYN * ACTYP
Stratification variable : ALL=1

ADYN(Administration of Anticoagulant) ACTYP(Type of AF)

Power |

Percent |
Percent of rows
Percent of column | Warfa| Dabigat| Riverro| Apixa| Edoxa| Parenteral Anti| Edoxa| Total
|phosphate|run|xaban|ban|ban|coagulant|ban (OD|)
| | | | | | |) |
______________+________+________+________+________+________+________+________
_+
Present | 5340 | 1433 | 4048 | 4865 | 3034 | 11 | 20 | 18751
| 28.48 | 7.64 | 21.59 | 25.95 | 16.18 | 0.06 | 0.11 | 100.00
| 28.48 | 7.64 | 21.59 | 25.95 | 16.18 | 0.06 | 0.11 |
| 100.00 | 100.00 | 100.00 | 100.00 | 100.00 | 100.00 | 100.00 |
______________+________+________+________+________+________+________+________
_+
Total 5340 1433 4048 4865 3034 11 20 18751
28.48 7.64 21.59 25.95 16.18 0.06 0.11 100.00

Degree of missing values = 1758

Administration of anticoagulants at the time of obtaining Table 2-4 consent, according to the presence or absence of proton-pump inhibitors

FREQ Procedure

Table 1 : ADYN * ACTYP
Stratification variable : ALL=1

ADYN(Administration of Anticoagulant) ACTYP(Type of AF)

Power |
Percent |
Percent of rows
Percent of column | Warfa| Dabigat| Riverro| Apixa| Edoxa| Parenteral Anti| Edoxa| Total
|phosphate|run|xaban|ban|ban|coagulant|ban (OD|)
| | | | | | |) |
______________+________+________+________+________+________+________+________+________+
Present | 5340 | 1433 | 4048 | 4865 | 3034 | 11 | 20 | 18751
| 28.48 | 7.64 | 21.59 | 25.95 | 16.18 | 0.06 | 0.11 | 100.00
| 28.48 | 7.64 | 21.59 | 25.95 | 16.18 | 0.06 | 0.11 |
| 100.00 | 100.00 | 100.00 | 100.00 | 100.00 | 100.00 | 100.00 |
______________+________+________+________+________+________+________+________+________+
Total 5340 1433 4048 4865 3034 11 20 18751
28.48 7.64 21.59 25.95 16.18 0.06 0.11 100.00

Degree of missing values = 1758

Table 1 : ADYN * ACTYP
Stratification variable : ALL=1

ADYN(Administration of Anticoagulant) ACTYP(Type of AF)

Power |

Percent |

Percent of rows

Percent of column | Warfa| Dabigat| Riverro| Apixa| Edoxa| Parenteral Anti| Edoxa| Total

|phosphate|run|xaban|ban|ban|coagulant|ban (OD|)

| | | | | | |) |

______________+________+________+________+________+________+________+_______
_+

Present | 5340 | 1433 | 4048 | 4865 | 3034 | 11 | 20 | 18751

| 28.48 | 7.64 | 21.59 | 25.95 | 16.18 | 0.06 | 0.11 | 100.00

| 28.48 | 7.64 | 21.59 | 25.95 | 16.18 | 0.06 | 0.11 |

| 100.00 | 100.00 | 100.00 | 100.00 | 100.00 | 100.00 | 100.00 |

______________+________+________+________+________+________+________+_______
_+

Total 5340 1433 4048 4865 3034 11 20 18751

28.48 7.64 21.59 25.95 16.18 0.06 0.11 100.00

Degree of missing values = 1758

Administration of anticoagulants at the time of obtaining Table 2-4 consent, according to the presence or absence of proton-pump inhibitors

FREQ Procedure

Table 1 : ADYN * ACTYP
Stratification variable : ALL=1

ADYN(Administration of Anticoagulant) ACTYP(Type of AF)

Power |
Percent |
Percent of rows
Percent of column | Warfa| Dabigat| Riverro| Apixa| Edoxa| Parenteral Anti| Edoxa| Total
|phosphate|run|xaban|ban|ban|coagulant|ban (OD|)
| | | | | | |) |
______________+________+________+________+________+________+________+________+
Present | 5340 | 1433 | 4048 | 4865 | 3034 | 11 | 20 | 18751
| 28.48 | 7.64 | 21.59 | 25.95 | 16.18 | 0.06 | 0.11 | 100.00
| 28.48 | 7.64 | 21.59 | 25.95 | 16.18 | 0.06 | 0.11 |
| 100.00 | 100.00 | 100.00 | 100.00 | 100.00 | 100.00 | 100.00 |
______________+________+________+________+________+________+________+________+
Total 5340 1433 4048 4865 3034 11 20 18751
28.48 7.64 21.59 25.95 16.18 0.06 0.11 100.00

Degree of missing values = 1758

Table 1 : ADYN * ACTYP
Stratification variable : ALL=1

ADYN(Administration of Anticoagulant) ACTYP(Type of AF)

Power |

Percent |

Percent of rows

Percent of column | Warfa| Dabigat| Riverro| Apixa| Edoxa| Parenteral Anti| Edoxa| Total

|phosphate|run|xaban|ban|ban|coagulant|ban (OD|)

| | | | | | |) |

______________+________+________+________+________+________+________+________+

Present | 5340 | 1433 | 4048 | 4865 | 3034 | 11 | 20 | 18751

| 28.48 | 7.64 | 21.59 | 25.95 | 16.18 | 0.06 | 0.11 | 100.00

| 28.48 | 7.64 | 21.59 | 25.95 | 16.18 | 0.06 | 0.11 |

| 100.00 | 100.00 | 100.00 | 100.00 | 100.00 | 100.00 | 100.00 |

______________+________+________+________+________+________+________+________+

Total 5340 1433 4048 4865 3034 11 20 18751

28.48 7.64 21.59 25.95 16.18 0.06 0.11 100.00

Degree of missing values = 1758

Administration of anticoagulants at the time of obtaining Table 2-4 consent, according to the presence or absence of proton-pump inhibitors

FREQ Procedure

Table 1 : ADYN * ACTYP
Stratification variable : ALL=1

ADYN(Administration of Anticoagulant) ACTYP(Type of AF)

Power |
Percent |
Percent of rows
Percent of column | Warfa| Dabigat| Riverro| Apixa| Edoxa| Parenteral Anti| Edoxa| Total
|phosphate|run|xaban|ban|ban|coagulant|ban (OD|)
| | | | | | |) |
______________+________+________+________+________+________+________+________+
Present | 5340 | 1433 | 4048 | 4865 | 3034 | 11 | 20 | 18751
| 28.48 | 7.64 | 21.59 | 25.95 | 16.18 | 0.06 | 0.11 | 100.00
| 28.48 | 7.64 | 21.59 | 25.95 | 16.18 | 0.06 | 0.11 |
| 100.00 | 100.00 | 100.00 | 100.00 | 100.00 | 100.00 | 100.00 |
______________+________+________+________+________+________+________+________+
Total 5340 1433 4048 4865 3034 11 20 18751
28.48 7.64 21.59 25.95 16.18 0.06 0.11 100.00

Degree of missing values = 1758

Table 1 : ADYN * t_ACT0
Stratification variable : ALL=1

ADYN(Administration of Anticoagulant)
t_ACT0

Power |

Percent |

Percent of rows

Percentage of columns | 0| 1| Sum

______________+________+________+

Present | 15697 | 3054 | 18751

| 83.71 | 16.29 | 100.00

| 83.71 | 16.29 |

| 100.00 | 100.00 |

______________+________+________+

Total 15697 3054 18751

83.71 16.29 100.00

Degree of missing values = 1758

Administration of anticoagulants at the time of obtaining Table 2-4 consent, according to the presence or absence of proton-pump inhibitors

FREQ Procedure

Table: ALL * t_ADYN

ALL t_ADYN

Power |
Percent |
Percent of rows
Percentage of columns | 0| 1| Sum

1	1758	18751	20509
	8.57	91.43	100.00
	8.57	91.43	
	100.00	100.00	
Total	1758	18751	20509
	8.57	91.43	100.00

Administration of anticoagulants at the time of obtaining Table 2-4 consent, according to the presence or absence of proton-pump inhibitors

FREQ Procedure

Cumulative
Percent of ALL power Percent of power

1	20509	100.00	20509	100.00

Tables: ALL * ADYN

ALL
ADYN(Administration of Anticoagulant)

Power |
Percent |
Percent of rows
Percentage of columns | with | total
______________+________+
1 | 18751 | 18751
| 100.00 | 100.00
| 100.00 |
| 100.00 |
______________+________+
Total 18751 18751
100.00 100.00

Degree of missing values = 1758

Administration of anticoagulants at the time of obtaining Table 2-4 consent, according to the presence or absence of proton-pump inhibitors

FREQ Procedure

Table 1 : ADYN * ACTYP
Stratification variable : ALL=1

ADYN(Administration of Anticoagulant) ACTYP(Type of AF)

Power |
Percent |
Percent of rows
Percent of column | Warfa| Dabigat| Riverro| Apixa| Edoxa| Parenteral Anti| Edoxa| Total
|phosphate|run|xaban|ban|ban|coagulant|ban (OD|)
| | | | | | |) |
______________+________+________+________+________+________+________+________+
Present | 5340 | 1433 | 4048 | 4865 | 3034 | 11 | 20 | 18751
| 28.48 | 7.64 | 21.59 | 25.95 | 16.18 | 0.06 | 0.11 | 100.00
| 28.48 | 7.64 | 21.59 | 25.95 | 16.18 | 0.06 | 0.11 |
| 100.00 | 100.00 | 100.00 | 100.00 | 100.00 | 100.00 | 100.00 |
______________+________+________+________+________+________+________+________+
Total 5340 1433 4048 4865 3034 11 20 18751
28.48 7.64 21.59 25.95 16.18 0.06 0.11 100.00

Degree of missing values = 1758

Table 1 : ADYN * ACTYP
Stratification variable : ALL=1

ADYN(Administration of Anticoagulant) ACTYP(Type of AF)

Power |

Percent |

Percent of rows

Percent of column | Warfa| Dabigat| Riverro| Apixa| Edoxa| Parenteral Anti| Edoxa| Total

|phosphate|run|xaban|ban|ban|coagulant|ban (OD|)

| | | | | | |) |

______________+________+________+________+________+________+________+________+

Present | 5340 | 1433 | 4048 | 4865 | 3034 | 11 | 20 | 18751

| 28.48 | 7.64 | 21.59 | 25.95 | 16.18 | 0.06 | 0.11 | 100.00

| 28.48 | 7.64 | 21.59 | 25.95 | 16.18 | 0.06 | 0.11 |

| 100.00 | 100.00 | 100.00 | 100.00 | 100.00 | 100.00 | 100.00 |

______________+________+________+________+________+________+________+________+

Total 5340 1433 4048 4865 3034 11 20 18751

28.48 7.64 21.59 25.95 16.18 0.06 0.11 100.00

Degree of missing values = 1758

Administration of anticoagulants at the time of obtaining Table 2-4 consent, according to the presence or absence of proton-pump inhibitors

FREQ Procedure

Table 1 : ADYN * ACTYP
Stratification variable : ALL=1

ADYN(Administration of Anticoagulant) ACTYP(Type of AF)

Power |
Percent |
Percent of rows
Percent of column | Warfa| Dabigat| Riverro| Apixa| Edoxa| Parenteral Anti| Edoxa| Total
|phosphate|run|xaban|ban|ban|coagulant|ban (OD|)
| | | | | | |) |
______________+________+________+________+________+________+________+________
_+
Present | 5340 | 1433 | 4048 | 4865 | 3034 | 11 | 20 | 18751
| 28.48 | 7.64 | 21.59 | 25.95 | 16.18 | 0.06 | 0.11 | 100.00
| 28.48 | 7.64 | 21.59 | 25.95 | 16.18 | 0.06 | 0.11 |
| 100.00 | 100.00 | 100.00 | 100.00 | 100.00 | 100.00 | 100.00 |
______________+________+________+________+________+________+________+________
_+
Total 5340 1433 4048 4865 3034 11 20 18751
28.48 7.64 21.59 25.95 16.18 0.06 0.11 100.00

Degree of missing values = 1758

Table 1 : ADYN * ACTYP
Stratification variable : ALL=1

ADYN(Administration of Anticoagulant) ACTYP(Type of AF)

Power |

Percent |
Percent of rows
Percent of column | Warfa| Dabigat| Riverro| Apixa| Edoxa| Parenteral Anti| Edoxa| Total
|phosphate|run|xaban|ban|ban|coagulant|ban (OD|)
| | | | | | |) |

______________+________+________+________+________+________+________+________
_+

Present | 5340 | 1433 | 4048 | 4865 | 3034 | 11 | 20 | 18751
| 28.48 | 7.64 | 21.59 | 25.95 | 16.18 | 0.06 | 0.11 | 100.00
| 28.48 | 7.64 | 21.59 | 25.95 | 16.18 | 0.06 | 0.11 |
| 100.00 | 100.00 | 100.00 | 100.00 | 100.00 | 100.00 | 100.00 |

______________+________+________+________+________+________+________+________
_+

Total 5340 1433 4048 4865 3034 11 20 18751
28.48 7.64 21.59 25.95 16.18 0.06 0.11 100.00

Degree of missing values = 1758

Administration of anticoagulants at the time of obtaining Table 2-4 consent, according to the presence or absence of proton-pump inhibitors

FREQ Procedure

Table 1 : ADYN * ACTYP
Stratification variable : ALL=1

ADYN(Administration of Anticoagulant) ACTYP(Type of AF)

Power |
Percent |
Percent of rows
Percent of column | Warfa| Dabigat| Riverro| Apixa| Edoxa| Parenteral Anti| Edoxa| Total
|phosphate|run|xaban|ban|ban|coagulant|ban (OD|)
| | | | | | |) |
______________+________+________+________+________+________+________+________+
Present | 5340 | 1433 | 4048 | 4865 | 3034 | 11 | 20 | 18751
| 28.48 | 7.64 | 21.59 | 25.95 | 16.18 | 0.06 | 0.11 | 100.00
| 28.48 | 7.64 | 21.59 | 25.95 | 16.18 | 0.06 | 0.11 |
| 100.00 | 100.00 | 100.00 | 100.00 | 100.00 | 100.00 | 100.00 |
______________+________+________+________+________+________+________+________+
Total 5340 1433 4048 4865 3034 11 20 18751
28.48 7.64 21.59 25.95 16.18 0.06 0.11 100.00

Degree of missing values = 1758

Table 1 : ADYN * ACTYP
Stratification variable : ALL=1

ADYN(Administration of Anticoagulant) ACTYP(Type of AF)

Power |

Percent |

Percent of rows

Percent of column | Warfa| Dabigat| Riverro| Apixa| Edoxa| Parenteral Anti| Edoxa| Total

|phosphate|run|xaban|ban|ban|coagulant|ban (OD|)

| | | | | | |) |

______________+________+________+________+________+________+________+_______

_+

Present | 5340 | 1433 | 4048 | 4865 | 3034 | 11 | 20 | 18751

| 28.48 | 7.64 | 21.59 | 25.95 | 16.18 | 0.06 | 0.11 | 100.00

| 28.48 | 7.64 | 21.59 | 25.95 | 16.18 | 0.06 | 0.11 |

| 100.00 | 100.00 | 100.00 | 100.00 | 100.00 | 100.00 | 100.00 |

______________+________+________+________+________+________+________+_______

_+

Total 5340 1433 4048 4865 3034 11 20 18751

28.48 7.64 21.59 25.95 16.18 0.06 0.11 100.00

Degree of missing values = 1758

Administration of anticoagulants at the time of obtaining Table 2-4 consent, according to the presence or absence of proton-pump inhibitors

FREQ Procedure

Table 1 : ADYN * ACTYP
Stratification variable : ALL=1

ADYN(Administration of Anticoagulant) ACTYP(Type of AF)

Power |
Percent |
Percent of rows
Percent of column | Warfa| Dabigat| Riverro| Apixa| Edoxa| Parenteral Anti| Edoxa| Total
|phosphate|run|xaban|ban|ban|coagulant|ban (OD|)
| | | | | | |) |
______________+________+________+________+________+________+________+________+
Present | 5340 | 1433 | 4048 | 4865 | 3034 | 11 | 20 | 18751
| 28.48 | 7.64 | 21.59 | 25.95 | 16.18 | 0.06 | 0.11 | 100.00
| 28.48 | 7.64 | 21.59 | 25.95 | 16.18 | 0.06 | 0.11 |
| 100.00 | 100.00 | 100.00 | 100.00 | 100.00 | 100.00 | 100.00 |
______________+________+________+________+________+________+________+________+
Total 5340 1433 4048 4865 3034 11 20 18751
28.48 7.64 21.59 25.95 16.18 0.06 0.11 100.00

Degree of missing values = 1758

Table 1 : ADYN * t_ACT0
Stratification variable : ALL=1

ADYN(Administration of Anticoagulant)
t_ACT0

Power |

Percent |

Percent of rows

Percentage of columns | 0| 1| Sum

______________+________+________+

Present | 15697 | 3054 | 18751

| 83.71 | 16.29 | 100.00

| 83.71 | 16.29 |

| 100.00 | 100.00 |

______________+________+________+

Total 15697 3054 18751

83.71 16.29 100.00

Degree of missing values = 1758

Administration of anticoagulants at the time of obtaining Table 2-4 consent, according to the presence or absence of proton-pump inhibitors

FREQ Procedure

Table: ALL * t_ADYN

ALL t_ADYN

Power |
Percent |
Percent of rows
Percentage of columns | 0| 1| Sum

1	1758	18751	20509
	8.57	91.43	100.00
	8.57	91.43	
	100.00	100.00	
Total	1758	18751	20509
	8.57	91.43	100.00

Administration of anticoagulants at the time of obtaining Table 2-4 consent, according to the presence or absence of proton-pump inhibitors

FREQ Procedure

Tables: ALL* ACTYP

ALL ACTYP(Type of AF)

Power |
Percent |
Percent of rows
Percent of column | Warfa| Dabigat| Riverro| Apixa| Edoxa| Parenteral Anti| Edoxa| Total
|phosphate|run|xaban|ban|ban|coagulant|ban (OD|)
| | | | | | |) |

ALL	Warfa phosphate	Dabigat run	Riverro xaban	Apixa ban	Edoxa ban	Parenteral Anti coagulant	Edoxa ban (OD)	Total
1	5340	1433	4048	4865	3034	11	20	18751
	28.48	7.64	21.59	25.95	16.18	0.06	0.11	100.00
	28.48	7.64	21.59	25.95	16.18	0.06	0.11	
	100.00	100.00	100.00	100.00	100.00	100.00	100.00	
Total	5340	1433	4048	4865	3034	11	20	18751
	28.48	7.64	21.59	25.95	16.18	0.06	0.11	100.00

Administration of anticoagulants at the time of obtaining Table 2-4 consent, according to the presence or absence of proton-pump inhibitors

FREQ Procedure

Table : ALL * n_ACT1

ALL n_ACT1

Power |
Percent |
Percent of rows
Percent of columns | 1| Total

1	13411	13411
	100.00	100.00
	100.00	
	100.00	
Total	13411	13411
	100.00	100.00

Frequency of missing values = 5340

Administration of anticoagulants at the time of obtaining Table 2-4 consent, according to the presence or absence of proton-pump inhibitors

FREQ Procedure

Tables: ALL* ACTYP

ALL ACTYP(Type of AF)

```
Power |
Percent |
Percent of rows
Percent of column | Warfa| Dabigat| Riverro| Apixa| Edoxa| Parenteral Anti| Edoxa| Total
                  |phosphate|run|xaban|ban|ban|coagulant|ban (OD|)
                  | | | | | | |) |
______________+________+________+________+________+________+________+________
_+
               1 |    5340 |    1433 |    4048 |    4865 |    3034 |       11 |       20 |   18751
                 |   28.48 |    7.64 |   21.59 |   25.95 |   16.18 |    0.06 |    0.11 | 100.00
                 |   28.48 |    7.64 |   21.59 |   25.95 |   16.18 |    0.06 |    0.11 |
                 | 100.00 | 100.00 | 100.00 | 100.00 | 100.00 | 100.00 | 100.00 |
______________+________+________+________+________+________+________+________
_+
Total 5340 1433 4048 4865 3034 11 20 18751
                   28.48      7.64     21.59     25.95     16.18      0.06      0.11    100.00
```

Administration of anticoagulants at the time of obtaining Table 2-4 consent, according to the presence or absence of proton-pump inhibitors

FREQ Procedure

Table : ALL * n_ACT2

ALL n_ACT2

```
Power |
Percent |
Percent of rows
Percent of columns | 1| Total
______________+________+
             1 |   17318 |   17318
               | 100.00 | 100.00
               | 100.00 |
               | 100.00 |
______________+________+
Total 17318 17318
                 100.00     100.00
```

Degree of missing values = 1433

Administration of anticoagulants at the time of obtaining Table 2-4 consent, according to the presence or absence of proton-pump inhibitors

FREQ Procedure

Tables: ALL* ACTYP

ALL ACTYP(Type of AF)

Power |
Percent |
Percent of rows
Percent of column | Warfa| Dabigat| Riverro| Apixa| Edoxa| Parenteral Anti| Edoxa| Total
|phosphate|run|xaban|ban|ban|coagulant|ban (OD|)
| | | | | | |) |
______________+________+________+________+________+________+________+________
_+
1 | 5340 | 1433 | 4048 | 4865 | 3034 | 11 | 20 | 18751
| 28.48 | 7.64 | 21.59 | 25.95 | 16.18 | 0.06 | 0.11 | 100.00
| 28.48 | 7.64 | 21.59 | 25.95 | 16.18 | 0.06 | 0.11 |
| 100.00 | 100.00 | 100.00 | 100.00 | 100.00 | 100.00 | 100.00 |
______________+________+________+________+________+________+________+________
_+
Total 5340 1433 4048 4865 3034 11 20 18751
28.48 7.64 21.59 25.95 16.18 0.06 0.11 100.00

Administration of anticoagulants at the time of obtaining Table 2-4 consent, according to the presence or absence of proton-pump inhibitors

FREQ Procedure

Table : ALL * n_ACT3

ALL n_ACT3

```
Power |
Percent |
Percent of rows
Percent of columns | 1| Total
______________+________+
             1 |  14703 |  14703
               | 100.00 | 100.00
               | 100.00 |
               | 100.00 |
______________+________+
Total 14703 14703
                 100.00    100.00
```

Degree of missing values = 4048

Administration of anticoagulants at the time of obtaining Table 2-4 consent, according to the presence or absence of proton-pump inhibitors

FREQ Procedure

Tables: ALL* ACTYP

ALL ACTYP(Type of AF)

Power |
Percent |
Percent of rows
Percent of column | Warfa| Dabigat| Riverro| Apixa| Edoxa| Parenteral Anti| Edoxa| Total
|phosphate|run|xaban|ban|ban|coagulant|ban (OD|)
| | | | | | |) |
______________+________+________+________+________+________+________+________
_+
1 | 5340 | 1433 | 4048 | 4865 | 3034 | 11 | 20 | 18751
| 28.48 | 7.64 | 21.59 | 25.95 | 16.18 | 0.06 | 0.11 | 100.00
| 28.48 | 7.64 | 21.59 | 25.95 | 16.18 | 0.06 | 0.11 |
| 100.00 | 100.00 | 100.00 | 100.00 | 100.00 | 100.00 | 100.00 |
______________+________+________+________+________+________+________+________
_+
Total 5340 1433 4048 4865 3034 11 20 18751
28.48 7.64 21.59 25.95 16.18 0.06 0.11 100.00

Administration of anticoagulants at the time of obtaining Table 2-4 consent, according to the presence or absence of proton-pump inhibitors

FREQ Procedure

Table : ALL * n_ACT4

ALL n_ACT4

```
Power |
Percent |
Percent of rows
Percent of columns | 1| Total
______________+________+
             1 |   13886 |   13886
               | 100.00 | 100.00
               | 100.00 |
               | 100.00 |
______________+________+
Total 13886 13886
                  100.00    100.00
```

Degree of missing values = 4865

Administration of anticoagulants at the time of obtaining Table 2-4 consent, according to the presence or absence of proton-pump inhibitors

FREQ Procedure

Table: ALL * t_ACT0

ALL t_ACT0

Power |
Percent |
Percent of rows
Percentage of columns | 0| 1| Sum

1	15697	3054	18751
	83.71	16.29	100.00
	83.71	16.29	
	100.00	100.00	
Total	15697	3054	18751
	83.71	16.29	100.00

Administration of anticoagulants at the time of obtaining Table 2-4 consent, according to the presence or absence of proton-pump inhibitors

FREQ Procedure

Table: ALL * t_ACT0

ALL t_ACT0

Power |
Percent |
Percent of rows
Percentage of columns | 0| 1| Sum

1	15697	3054	18751
	83.71	16.29	100.00
	83.71	16.29	
	100.00	100.00	
Total	15697	3054	18751
	83.71	16.29	100.00

Administration of anticoagulants at the time of obtaining Table 2-4 consent, according to the presence or absence of proton-pump inhibitors

FREQ Procedure

Tables: ALL* ACTYP

ALL ACTYP(Type of AF)

```
Power |
Percent |
Percent of rows
Percent of column | Warfa| Dabigat| Riverro| Apixa| Edoxa| Parenteral Anti| Edoxa| Total
              |phosphate|run|xaban|ban|ban|coagulant|ban (OD|)
              | | | | | | |) |
______________+________+________+________+________+________+________+_______
_+
            1 |    5340 |    1433 |    4048 |    4865 |    3034 |       11 |       20 |   18751
              |   28.48 |    7.64 |   21.59 |   25.95 |   16.18 |     0.06 |     0.11 | 100.00
              |   28.48 |    7.64 |   21.59 |   25.95 |   16.18 |     0.06 |     0.11 |
              | 100.00 | 100.00 | 100.00 | 100.00 | 100.00 | 100.00 | 100.00 |
______________+________+________+________+________+________+________+_______
_+
Total 5340 1433 4048 4865 3034 11 20 18751
                 28.48      7.64     21.59     25.95     16.18      0.06      0.11    100.00
```

Administration of anticoagulants at the time of obtaining Table 2-4 consent, according to the presence or absence of proton-pump inhibitors

FREQ Procedure

Table : ALL * n_ACT5

ALL n_ACT5

```
Power |
Percent |
Percent of rows
Percent of columns | 1| Total
______________+________+
            1 |  15717 |  15717
              | 100.00 | 100.00
              | 100.00 |
              | 100.00 |
______________+________+
Total 15717 15717
                100.00   100.00
```

Degree of missing values = 3034

Administration of anticoagulants at the time of obtaining Table 2-4 consent, according to the presence or absence of proton-pump inhibitors

FREQ Procedure

Tables: ALL* ACTYP

ALL ACTYP(Type of AF)

Power |
Percent |
Percent of rows
Percent of column | Warfa| Dabigat| Riverro| Apixa| Edoxa| Parenteral Anti| Edoxa| Total
|phosphate|run|xaban|ban|ban|coagulant|ban (OD|)
| | | | | | |) |
______________+________+________+________+________+________+________+_______
_+
1 | 5340 | 1433 | 4048 | 4865 | 3034 | 11 | 20 | 18751
| 28.48 | 7.64 | 21.59 | 25.95 | 16.18 | 0.06 | 0.11 | 100.00
| 28.48 | 7.64 | 21.59 | 25.95 | 16.18 | 0.06 | 0.11 |
| 100.00 | 100.00 | 100.00 | 100.00 | 100.00 | 100.00 | 100.00 |
______________+________+________+________+________+________+________+_______
_+
Total 5340 1433 4048 4865 3034 11 20 18751
28.48 7.64 21.59 25.95 16.18 0.06 0.11 100.00

Administration of anticoagulants at the time of obtaining Table 2-4 consent, according to the presence or absence of proton-pump inhibitors

FREQ Procedure

Table : ALL * n_ACT7

ALL n_ACT7

Power |
Percent |
Percent of rows
Percent of columns | 1| Total

	1	Total
1	18731	18731
	100.00	100.00
	100.00	
	100.00	
Total	18731	18731
	100.00	100.00

Degree of missing values = 20

Administration of anticoagulants at the time of obtaining Table 2-4 consent, according to the presence or absence of proton-pump inhibitors

FREQ Procedure

Tables: ALL* ACTYP

ALL ACTYP(Type of AF)

Power |
Percent |
Percent of rows
Percent of column | Warfa| Dabigat| Riverro| Apixa| Edoxa| Parenteral Anti| Edoxa| Total
|phosphate|run|xaban|ban|ban|coagulant|ban (OD|)
| | | | | | |) |
______________+________+________+________+________+________+________+_______
_+

1	5340	1433	4048	4865	3034	11	20	18751
	28.48	7.64	21.59	25.95	16.18	0.06	0.11	100.00
	28.48	7.64	21.59	25.95	16.18	0.06	0.11	
	100.00	100.00	100.00	100.00	100.00	100.00	100.00	

______________+________+________+________+________+________+________+_______
_+

Total	5340	1433	4048	4865	3034	11	20	18751
	28.48	7.64	21.59	25.95	16.18	0.06	0.11	100.00

Administration of anticoagulants at the time of obtaining Table 2-4 consent, according to the presence or absence of proton-pump inhibitors

FREQ Procedure

Table : ALL * n_ACT6

ALL n_ACT6

```
Power |
Percent |
Percent of rows
Percent of columns | 1| Total
______________+________+
             1 |   18740 |   18740
               | 100.00 | 100.00
               | 100.00 |
               | 100.00 |
______________+________+
Total 18740 18740
                 100.00     100.00
```

Degree of missing values = 11

Administration of anticoagulants at the time of obtaining Table 2-4 consent, according to the presence or absence of proton-pump inhibitors

FREQ Procedure

Table : ALL * t_ACT1

ALL t_ACT1

Power |
Percent |
Percent of rows
Percentage of columns | 0| 1| Sum

1	11	18740	18751
	0.06	99.94	100.00
	0.06	99.94	
	100.00	100.00	
Total	11	18740	18751
	0.06	99.94	100.00

Administration of anticoagulants at the time of obtaining Table 2-4 consent, according to the presence or absence of proton-pump inhibitors

FREQ Procedure

Table : ALL * t_ACT1

ALL t_ACT1

Power / Percent / Percent of rows / Percentage of columns	0	1	Sum
1	11	18740	18751
	0.06	99.94	100.00
	0.06	99.94	
	100.00	100.00	
Total	11	18740	18751
	0.06	99.94	100.00

Administration of anticoagulants at the time of obtaining Table 2-4 consent, according to the presence or absence of proton-pump inhibitors

FREQ Procedure

Table : ALL * t_ACT2

ALL t_ACT2

Power |
Percent |
Percent of rows
Percentage of columns | 0| 1| Sum

	0	1	Sum
1	5351	13400	18751
	28.54	71.46	100.00
	28.54	71.46	
	100.00	100.00	
Total	5351	13400	18751
	28.54	71.46	100.00

Administration of anticoagulants at the time of obtaining Table 2-4 consent, according to the presence or absence of proton-pump inhibitors

FREQ Procedure

Table : ALL * t_ACT2

ALL t_ACT2

Power |
Percent |
Percent of rows
Percentage of columns | 0| 1| Sum
______________+________+________+
1 | 5351 | 13400 | 18751
| 28.54 | 71.46 | 100.00
| 28.54 | 71.46 |
| 100.00 | 100.00 |
______________+________+________+
Total 5351 13400 18751
28.54 71.46 100.00

Administration of anticoagulants at the time of obtaining Table 2-4 consent, according to the presence or absence of proton-pump inhibitors

FREQ Procedure

Table : ALL * t_ACT3

ALL t_ACT3

Power |
Percent |
Percent of rows
Percentage of columns | 0| 1| Sum

1	6784	11967	18751
	36.18	63.82	100.00
	36.18	63.82	
	100.00	100.00	
Total	6784	11967	18751
	36.18	63.82	100.00

Administration of anticoagulants at the time of obtaining Table 2-4 consent, according to the presence or absence of proton-pump inhibitors

FREQ Procedure

Table : ALL * t_ACT3

ALL t_ACT3

Power |
Percent |
Percent of rows
Percentage of columns | 0| 1| Sum

1	6784	11967	18751
	36.18	63.82	100.00
	36.18	63.82	
	100.00	100.00	
Total	6784	11967	18751
	36.18	63.82	100.00

Administration of anticoagulants at the time of obtaining Table 2-4 consent, according to the presence or absence of proton-pump inhibitors

FREQ Procedure

Tables: ALL* VISITAD

ALL VISITAD(Visit and Drug Administration)

Power |
Percent |
Percent of rows
Percent of column |Month |Month |Month |First dose |Unknown | Total
Within | | |

1	5258	20	15	47	5340
	98.46	0.37	0.28	0.88	100.00
	98.46	0.37	0.28	0.88	
	100.00	100.00	100.00	100.00	
Total	5258	20	15	47	5340
	98.46	0.37	0.28	0.88	100.00

Administration of anticoagulants at the time of obtaining Table 2-4 consent, according to the presence or absence of proton-pump inhibitors

FREQ Procedure

Tables: ALL* VISITAD

ALL VISITAD(Visit and Drug Administration)

Power |
Percent |
Percent of rows
Percent of column |Month |Month |Month |First dose |Unknown | Total
Within | | |

ALL	Month	Month	Month	First dose	Unknown	Total
1	5258	20	15	47	5340	
	98.46	0.37	0.28	0.88	100.00	
	98.46	0.37	0.28	0.88		
	100.00	100.00	100.00	100.00		
Total	5258	20	15	47	5340	
	98.46	0.37	0.28	0.88	100.00	

Administration of anticoagulants at the time of obtaining Table 2-4 consent, according to the presence or absence of proton-pump inhibitors

FREQ Procedure

Tables: ALL* VISITAD

ALL VISITAD(Visit and Drug Administration)

Power / Percent / Percent of rows / Percent of column	Month Within	Month	Month	First dose	Unknown	Total
1	5258	20	15	47		5340
	98.46	0.37	0.28	0.88		100.00
	98.46	0.37	0.28	0.88		
	100.00	100.00	100.00	100.00		
Total	5258	20	15	47		5340
	98.46	0.37	0.28	0.88		100.00

Administration of anticoagulants at the time of obtaining Table 2-4 consent, according to the presence or absence of proton-pump inhibitors

FREQ Procedure

Tables: ALL* VISITAD

ALL VISITAD(Visit and Drug Administration)

Power |
Percent |
Percent of rows
Percent of column |Month |Month |Month |First dose |Unknown | Total
Within | | |

1	5258	20	15	47	5340
	98.46	0.37	0.28	0.88	100.00
	98.46	0.37	0.28	0.88	
	100.00	100.00	100.00	100.00	
Total	5258	20	15	47	5340
	98.46	0.37	0.28	0.88	100.00

Administration of anticoagulants at the time of obtaining Table 2-4 consent, according to the presence or absence of proton-pump inhibitors

FREQ Procedure

Tables: ALL* VISITAD

ALL VISITAD(Visit and Drug Administration)

Power |
Percent |
Percent of rows
Percent of column |Month |Month |Month |First dose |Unknown | Total
Within | | |
______________+________+________+________+________+
1 | 1391 | 14 | 10 | 18 | 1433
| 97.07 | 0.98 | 0.70 | 1.26 | 100.00
| 97.07 | 0.98 | 0.70 | 1.26 |
| 100.00 | 100.00 | 100.00 | 100.00 |
______________+________+________+________+________+
Total 1391 14 10 18 1433
97.07 0.98 0.70 1.26 100.00

Administration of anticoagulants at the time of obtaining Table 2-4 consent, according to the presence or absence of proton-pump inhibitors

FREQ Procedure

Tables: ALL* VISITAD

ALL VISITAD(Visit and Drug Administration)

Power |
Percent |
Percent of rows
Percent of column |Month |Month |Month |First dose |Unknown | Total
Within | | |

1	1391	14	10	18	1433
	97.07	0.98	0.70	1.26	100.00
	97.07	0.98	0.70	1.26	
	100.00	100.00	100.00	100.00	
Total	1391	14	10	18	1433
	97.07	0.98	0.70	1.26	100.00

Administration of anticoagulants at the time of obtaining Table 2-4 consent, according to the presence or absence of proton-pump inhibitors

FREQ Procedure

Tables: ALL* VISITAD

ALL VISITAD(Visit and Drug Administration)

Power |
Percent |
Percent of rows
Percent of column |Month |Month |Month |First dose |Unknown | Total
Within | | |
______________+________+________+________+________+
1 | 1391 | 14 | 10 | 18 | 1433
| 97.07 | 0.98 | 0.70 | 1.26 | 100.00
| 97.07 | 0.98 | 0.70 | 1.26 |
| 100.00 | 100.00 | 100.00 | 100.00 |
______________+________+________+________+________+
Total 1391 14 10 18 1433
97.07 0.98 0.70 1.26 100.00

Administration of anticoagulants at the time of obtaining Table 2-4 consent, according to the presence or absence of proton-pump inhibitors

FREQ Procedure

Tables: ALL* VISITAD

ALL VISITAD(Visit and Drug Administration)

Power |
Percent |
Percent of rows
Percent of column |Month |Month |Month |First dose |Unknown | Total
Within | | |

ALL	Month	Month	Month	First dose	Unknown	Total
1	1391	14	10	18		1433
	97.07	0.98	0.70	1.26		100.00
	97.07	0.98	0.70	1.26		
	100.00	100.00	100.00	100.00		
Total	1391	14	10	18		1433
	97.07	0.98	0.70	1.26		100.00

Administration of anticoagulants at the time of obtaining Table 2-4 consent, according to the presence or absence of proton-pump inhibitors

FREQ Procedure

Tables: ALL* VISITAD

ALL VISITAD(Visit and Drug Administration)

Power |
Percent |
Percent of rows

Percent of column Within	Month	Month	Month	First dose	Unknown	Total
1	3865	71	81	31	4048	
	95.48	1.75	2.00	0.77	100.00	
	95.48	1.75	2.00	0.77		
	100.00	100.00	100.00	100.00		
Total	3865	71	81	31	4048	
	95.48	1.75	2.00	0.77	100.00	

Administration of anticoagulants at the time of obtaining Table 2-4 consent, according to the presence or absence of proton-pump inhibitors

FREQ Procedure

Tables: ALL* VISITAD

ALL VISITAD(Visit and Drug Administration)

Power<br>Percent<br>Percent of rows<br>Percent of column Within	Month	Month	Month	First dose	Unknown	Total
1	3865<br>95.48<br>95.48<br>100.00	71<br>1.75<br>1.75<br>100.00	81<br>2.00<br>2.00<br>100.00	31<br>0.77<br>0.77<br>100.00		4048<br>100.00
Total	3865<br>95.48	71<br>1.75	81<br>2.00	31<br>0.77		4048<br>100.00

Administration of anticoagulants at the time of obtaining Table 2-4 consent, according to the presence or absence of proton-pump inhibitors

FREQ Procedure

Tables: ALL* VISITAD

ALL VISITAD(Visit and Drug Administration)

Power / Percent / Percent of rows / Percent of column Within	Month	Month	Month	First dose	Unknown	Total
1	3865	71	81	31	4048	
	95.48	1.75	2.00	0.77	100.00	
	95.48	1.75	2.00	0.77		
	100.00	100.00	100.00	100.00		
Total	3865	71	81	31	4048	
	95.48	1.75	2.00	0.77	100.00	

Administration of anticoagulants at the time of obtaining Table 2-4 consent, according to the presence or absence of proton-pump inhibitors

FREQ Procedure

Tables: ALL* VISITAD

ALL VISITAD(Visit and Drug Administration)

Power |
Percent |
Percent of rows
Percent of column |Month |Month |Month |First dose |Unknown | Total
Within | | |

1	3865	71	81	31	4048
	95.48	1.75	2.00	0.77	100.00
	95.48	1.75	2.00	0.77	
	100.00	100.00	100.00	100.00	
Total	3865	71	81	31	4048
	95.48	1.75	2.00	0.77	100.00

Administration of anticoagulants at the time of obtaining Table 2-4 consent, according to the presence or absence of proton-pump inhibitors

FREQ Procedure

Tables: ALL* VISITAD

ALL VISITAD(Visit and Drug Administration)

Power |
Percent |
Percent of rows
Percent of column |Month |Month |Month |First dose |Unknown | Total
Within | | |
______________+________+________+________+________+
1 | 4685 | 74 | 87 | 19 | 4865
| 96.30 | 1.52 | 1.79 | 0.39 | 100.00
| 96.30 | 1.52 | 1.79 | 0.39 |
| 100.00 | 100.00 | 100.00 | 100.00 |
______________+________+________+________+________+
Total 4685 74 87 19 4865
96.30 1.52 1.79 0.39 100.00

Administration of anticoagulants at the time of obtaining Table 2-4 consent, according to the presence or absence of proton-pump inhibitors

FREQ Procedure

Tables: ALL* VISITAD

ALL VISITAD(Visit and Drug Administration)

Power |
Percent |
Percent of rows
Percent of column |Month |Month |Month |First dose |Unknown | Total
Within | | |

1	4685	74	87	19	4865
	96.30	1.52	1.79	0.39	100.00
	96.30	1.52	1.79	0.39	
	100.00	100.00	100.00	100.00	
Total	4685	74	87	19	4865
	96.30	1.52	1.79	0.39	100.00

Administration of anticoagulants at the time of obtaining Table 2-4 consent, according to the presence or absence of proton-pump inhibitors

FREQ Procedure

Tables: ALL* VISITAD

ALL VISITAD(Visit and Drug Administration)

Power |
Percent |
Percent of rows
Percent of column |Month |Month |Month |First dose |Unknown | Total
Within | | |

1	4685	74	87	19	4865
	96.30	1.52	1.79	0.39	100.00
	96.30	1.52	1.79	0.39	
	100.00	100.00	100.00	100.00	
Total	4685	74	87	19	4865
	96.30	1.52	1.79	0.39	100.00

Administration of anticoagulants at the time of obtaining Table 2-4 consent, according to the presence or absence of proton-pump inhibitors

FREQ Procedure

Tables: ALL* VISITAD

ALL VISITAD(Visit and Drug Administration)

Power |
Percent |
Percent of rows
Percent of column |Month |Month |Month |First dose |Unknown | Total
Within | | |

1	4685	74	87	19	4865
	96.30	1.52	1.79	0.39	100.00
	96.30	1.52	1.79	0.39	
	100.00	100.00	100.00	100.00	
Total	4685	74	87	19	4865
	96.30	1.52	1.79	0.39	100.00

Administration of anticoagulants at the time of obtaining Table 2-4 consent, according to the presence or absence of proton-pump inhibitors

FREQ Procedure

Tables: ALL* VISITAD

ALL VISITAD(Visit and Drug Administration)

```
Power |
Percent |
Percent of rows
Percent of column |Month |Month |Month |First dose |Unknown | Total
                 Within | | |
______________+________+________+________+________+
            1 |   2613 |    161 |    265 |     15 |   3054
              |  85.56 |   5.27 |   8.68 |   0.49 | 100.00
              |  85.56 |   5.27 |   8.68 |   0.49 |
              | 100.00 | 100.00 | 100.00 | 100.00 |
______________+________+________+________+________+
Total 2613 161 265 15 3054
                 85.56     5.27     8.68     0.49   100.00
```

Administration of anticoagulants at the time of obtaining Table 2-4 consent, according to the presence or absence of proton-pump inhibitors

FREQ Procedure

Tables: ALL* VISITAD

ALL VISITAD(Visit and Drug Administration)

Power |
Percent |
Percent of rows
Percent of column |Month |Month |Month |First dose |Unknown | Total
Within | | |

1	2613	161	265	15	3054
	85.56	5.27	8.68	0.49	100.00
	85.56	5.27	8.68	0.49	
	100.00	100.00	100.00	100.00	
Total	2613	161	265	15	3054
	85.56	5.27	8.68	0.49	100.00

Administration of anticoagulants at the time of obtaining Table 2-4 consent, according to the presence or absence of proton-pump inhibitors

FREQ Procedure

Tables: ALL* VISITAD

ALL VISITAD(Visit and Drug Administration)

Power |
Percent |
Percent of rows
Percent of column |Month |Month |Month |First dose |Unknown | Total
Within | | |

```
______________+________+________+________+________+
            1 |   2613 |    161 |    265 |     15 |   3054
              |  85.56 |   5.27 |   8.68 |   0.49 | 100.00
              |  85.56 |   5.27 |   8.68 |   0.49 |
              | 100.00 | 100.00 | 100.00 | 100.00 |
______________+________+________+________+________+
Total 2613 161 265 15 3054
                 85.56     5.27     8.68     0.49   100.00
```

Administration of anticoagulants at the time of obtaining Table 2-4 consent, according to the presence or absence of proton-pump inhibitors

FREQ Procedure

Tables: ALL* VISITAD

ALL VISITAD(Visit and Drug Administration)

```
Power |
Percent |
Percent of rows
Percent of column |Month |Month |Month |First dose |Unknown | Total
              Within | | |
______________+________+________+________+________+
            1 |   2613 |    161 |    265 |     15 |   3054
              |  85.56 |   5.27 |   8.68 |   0.49 | 100.00
              |  85.56 |   5.27 |   8.68 |   0.49 |
              | 100.00 | 100.00 | 100.00 | 100.00 |
______________+________+________+________+________+
Total 2613 161 265 15 3054
                 85.56     5.27     8.68     0.49   100.00
```

Administration of anticoagulants at the time of obtaining Table 2-4 consent, according to the presence or absence of proton-pump inhibitors

FREQ Procedure

Tables: ALL* VISITAD

ALL VISITAD(Visit and Drug Administration)

Power |
Percent |
Percent of rows
Percent of column |Month |Month |Month |First dose |Unknown | Total
Within | | |
______________+________+________+________+________+
1 | 2597 | 160 | 262 | 15 | 3034
| 85.60 | 5.27 | 8.64 | 0.49 | 100.00
| 85.60 | 5.27 | 8.64 | 0.49 |
| 100.00 | 100.00 | 100.00 | 100.00 |
______________+________+________+________+________+
Total 2597 160 262 15 3034
85.60 5.27 8.64 0.49 100.00

Administration of anticoagulants at the time of obtaining Table 2-4 consent, according to the presence or absence of proton-pump inhibitors

FREQ Procedure

Tables: ALL* VISITAD

ALL VISITAD(Visit and Drug Administration)

Power |
Percent |
Percent of rows
Percent of column |Month |Month |Month |First dose |Unknown | Total
Within | | |

1	2597	160	262	15	3034
	85.60	5.27	8.64	0.49	100.00
	85.60	5.27	8.64	0.49	
	100.00	100.00	100.00	100.00	
Total	2597	160	262	15	3034
	85.60	5.27	8.64	0.49	100.00

Administration of anticoagulants at the time of obtaining Table 2-4 consent, according to the presence or absence of proton-pump inhibitors

FREQ Procedure

Tables: ALL* VISITAD

ALL VISITAD(Visit and Drug Administration)

Power |
Percent |
Percent of rows
Percent of column |Month |Month |Month |First dose |Unknown | Total
Within | | |

______________+________+________+________+________+
1 | 2597 | 160 | 262 | 15 | 3034
| 85.60 | 5.27 | 8.64 | 0.49 | 100.00
| 85.60 | 5.27 | 8.64 | 0.49 |
| 100.00 | 100.00 | 100.00 | 100.00 |
______________+________+________+________+________+
Total 2597 160 262 15 3034
85.60 5.27 8.64 0.49 100.00

Administration of anticoagulants at the time of obtaining Table 2-4 consent, according to the presence or absence of proton-pump inhibitors

FREQ Procedure

Tables: ALL* VISITAD

ALL VISITAD(Visit and Drug Administration)

Power | Percent | Percent of rows Percent of column	Month Within	Month	Month	First dose	Unknown	Total
1	2597	160	262	15	3034	
	85.60	5.27	8.64	0.49	100.00	
	85.60	5.27	8.64	0.49		
	100.00	100.00	100.00	100.00		
Total	2597	160	262	15	3034	
	85.60	5.27	8.64	0.49	100.00	

Administration of anticoagulants at the time of obtaining Table 2-4 consent, according to the presence or absence of proton-pump inhibitors

FREQ Procedure

Tables: ALL* VISITAD

ALL

VISITAD(Visit and Drug Administration)

Power |
Percent |
Percent of rows
Percent of column |Month |Month |Month |First dose |Total
Within | |
______________+________+________+________+
1 | 16 | 1 | 3 | 20
| 80.00 | 5.00 | 15.00 | 100.00
| 80.00 | 5.00 | 15.00 |
| 100.00 | 100.00 | 100.00 |
______________+________+________+________+
Total 16 1 3 20
80.00 5.00 15.00 100.00

Administration of anticoagulants at the time of obtaining Table 2-4 consent, according to the presence or absence of proton-pump inhibitors

FREQ Procedure

Tables: ALL* VISITAD

ALL

VISITAD(Visit and Drug Administration)

Power |

Percent |

Percent of rows

Percent of column |Month |Month |Month |First dose |Total

Within | |

______________+________+________+________+

1 | 16 | 1 | 3 | 20

| 80.00 | 5.00 | 15.00 | 100.00

| 80.00 | 5.00 | 15.00 |

| 100.00 | 100.00 | 100.00 |

______________+________+________+________+

Total 16 1 3 20

80.00 5.00 15.00 100.00

Administration of anticoagulants at the time of obtaining Table 2-4 consent, according to the presence or absence of proton-pump inhibitors

FREQ Procedure

Tables: ALL* VISITAD

ALL

VISITAD(Visit and Drug Administration)

Power / Percent / Percent of rows / Percent of column Within	Month	Month	Month	First dose	Total
1		16	1	3	20
		80.00	5.00	15.00	100.00
		80.00	5.00	15.00	
		100.00	100.00	100.00	
Total		16	1	3	20
		80.00	5.00	15.00	100.00

Administration of anticoagulants at the time of obtaining Table 2-4 consent, according to the presence or absence of proton-pump inhibitors

FREQ Procedure

Tables: ALL* VISITAD

ALL

VISITAD(Visit and Drug Administration)

Power |
Percent |
Percent of rows
Percent of column |Month |Month |Month |First dose |Total
Within | |

______________+________+________+________+

1	16	1	3	20
	80.00	5.00	15.00	100.00
	80.00	5.00	15.00	
	100.00	100.00	100.00	

______________+________+________+________+

Total 16 1 3 20

80.00 5.00 15.00 100.00

Administration of anticoagulants at the time of obtaining Table 2-4 consent, according to the presence or absence of proton-pump inhibitors

FREQ Procedure

Tables: ALL* VISITAD

ALL

VISITAD(Visit and Drug Administration)

```
Power |
Percent |
Percent of rows
Percent of column |Month | Unknown | Total
          ) in front | |
______________+________+________+
         1 |     10 |      1 |     11
           |  90.91 |   9.09 | 100.00
           |  90.91 |   9.09 |
           | 100.00 | 100.00 |
______________+________+________+
Total 10 1 11
              90.91     9.09   100.00
```

Administration of anticoagulants at the time of obtaining Table 2-4 consent, according to the presence or absence of proton-pump inhibitors

FREQ Procedure

Tables: ALL* VISITAD

ALL

VISITAD(Visit and Drug Administration)

Power |
Percent |
Percent of rows
Percent of column |Month | Unknown | Total
) in front | |
______________+________+________+
1 | 10 | 1 | 11
| 90.91 | 9.09 | 100.00
| 90.91 | 9.09 |
| 100.00 | 100.00 |
______________+________+________+
Total 10 1 11
90.91 9.09 100.00

Administration of anticoagulants at the time of obtaining Table 2-4 consent, according to the presence or absence of proton-pump inhibitors

FREQ Procedure

Tables: ALL* VISITAD

ALL

VISITAD(Visit and Drug Administration)

Power |

Percent |

Percent of rows

Percent of column |Month | Unknown | Total

) in front | |

______________+________+________+

1 | 10 | 1 | 11

| 90.91 | 9.09 | 100.00

| 90.91 | 9.09 |

| 100.00 | 100.00 |

______________+________+________+

Total 10 1 11

90.91 9.09 100.00

Administration of anticoagulants at the time of obtaining Table 2-4 consent, according to the presence or absence of proton-pump inhibitors

FREQ Procedure

Tables: ALL* VISITAD

ALL
VISITAD(Visit and Drug Administration)

Power<br>Percent<br>Percent of rows<br>Percent of column<br>) in front	Month	Unknown	Total
1	10<br>90.91<br>90.91<br>100.00	1<br>9.09<br>9.09<br>100.00	11<br>100.00
Total	10<br>90.91	1<br>9.09	11<br>100.00

Administration of anticoagulants at the time of obtaining Table 2-4 consent, according to the presence or absence of proton-pump inhibitors

FREQ Procedure

Tables: ALL * DOSE

ALL DOSE(Dose per day for DOAC only)

Power |
Percent |
Percent of rows
Column percent | 0| 75| 110| 150| 185| 220| 300| Total

ALL	0	75	110	150	185	220	300	Total
1	2	6	131	78	2	1121	93	1433
	0.14	0.42	9.14	5.44	0.14	78.23	6.49	100.00
	0.14	0.42	9.14	5.44	0.14	78.23	6.49	
	100.00	100.00	100.00	100.00	100.00	100.00	100.00	
Total	2	6	131	78	2	1121	93	1433
	0.14	0.42	9.14	5.44	0.14	78.23	6.49	100.00

Administration of anticoagulants at the time of obtaining Table 2-4 consent, according to the presence or absence of proton-pump inhibitors

FREQ Procedure

Tables: ALL * DOSE

ALL DOSE(Dose per day for DOAC only)

Power / Percent / Percent of rows / Column percent	0	75	110	150	185	220	300	Total
1	2	6	131	78	2	1121	93	1433
	0.14	0.42	9.14	5.44	0.14	78.23	6.49	100.00
	0.14	0.42	9.14	5.44	0.14	78.23	6.49	
	100.00	100.00	100.00	100.00	100.00	100.00	100.00	
Total	2	6	131	78	2	1121	93	1433
	0.14	0.42	9.14	5.44	0.14	78.23	6.49	100.00

Administration of anticoagulants at the time of obtaining Table 2-4 consent, according to the presence or absence of proton-pump inhibitors

FREQ Procedure

Tables: ALL * DOSE

ALL DOSE(Dose per day for DOAC only)

Power / Percent / Percent of rows / Column percent	0	75	110	150	185	220	300	Total
1	2	6	131	78	2	1121	93	1433
	0.14	0.42	9.14	5.44	0.14	78.23	6.49	100.00
	0.14	0.42	9.14	5.44	0.14	78.23	6.49	
	100.00	100.00	100.00	100.00	100.00	100.00	100.00	
Total	2	6	131	78	2	1121	93	1433
	0.14	0.42	9.14	5.44	0.14	78.23	6.49	100.00

Administration of anticoagulants at the time of obtaining Table 2-4 consent, according to the presence or absence of proton-pump inhibitors

FREQ Procedure

Tables: ALL * DOSE

ALL DOSE(Dose per day for DOAC only)

Power / Percent / Percent of rows / Column percent	0	75	110	150	185	220	300	Total
1	2	6	131	78	2	1121	93	1433
	0.14	0.42	9.14	5.44	0.14	78.23	6.49	100.00
	0.14	0.42	9.14	5.44	0.14	78.23	6.49	
	100.00	100.00	100.00	100.00	100.00	100.00	100.00	
Total	2	6	131	78	2	1121	93	1433
	0.14	0.42	9.14	5.44	0.14	78.23	6.49	100.00

Administration of anticoagulants at the time of obtaining Table 2-4 consent, according to the presence or absence of proton-pump inhibitors

FREQ Procedure

Tables: ALL * DOSE

ALL DOSE(Dose per day for DOAC only)

Power<br>Percent<br>Percent of rows<br>Column percent	0	75	110	150	185	220	300	Total
1	2<br>0.14<br>0.14<br>100.00	6<br>0.42<br>0.42<br>100.00	131<br>9.14<br>9.14<br>100.00	78<br>5.44<br>5.44<br>100.00	2<br>0.14<br>0.14<br>100.00	1121<br>78.23<br>78.23<br>100.00	93<br>6.49<br>6.49<br>100.00	1433<br>100.00
Total	2<br>0.14	6<br>0.42	131<br>9.14	78<br>5.44	2<br>0.14	1121<br>78.23	93<br>6.49	1433<br>100.00

Administration of anticoagulants at the time of obtaining Table 2-4 consent, according to the presence or absence of proton-pump inhibitors

FREQ Procedure

Table : ALL * f_d2

ALL f_d2

Power |
Percent |
Percent of rows
Percent of columns | 1| Total

1	4	4
	100.00	100.00
	100.00	
	100.00	
Total	4	4
	100.00	100.00

Frequency of missing values = 1429

Administration of anticoagulants at the time of obtaining Table 2-4 consent, according to the presence or absence of proton-pump inhibitors

FREQ Procedure

Tables: ALL * DOSE

ALL DOSE(Dose per day for DOAC only)

Power |
Percent |
Percent of rows
Column percent | 0| 5| 7.5| 10| 15| 30| 60| Total

	0	5	7.5	10	15	30	60	Total
1	1	4	3	2603	1431	5	1	4048
	0.02	0.10	0.07	64.30	35.35	0.12	0.02	100.00
	0.02	0.10	0.07	64.30	35.35	0.12	0.02	
	100.00	100.00	100.00	100.00	100.00	100.00	100.00	
Total	1	4	3	2603	1431	5	1	4048
	0.02	0.10	0.07	64.30	35.35	0.12	0.02	100.00

Administration of anticoagulants at the time of obtaining Table 2-4 consent, according to the presence or absence of proton-pump inhibitors

FREQ Procedure

Tables: ALL * DOSE

ALL DOSE(Dose per day for DOAC only)

Power |
Percent |
Percent of rows
Column percent

ALL	0	5	7.5	10	15	30	60	Total
1	1	4	3	2603	1431	5	1	4048
	0.02	0.10	0.07	64.30	35.35	0.12	0.02	100.00
	0.02	0.10	0.07	64.30	35.35	0.12	0.02	
	100.00	100.00	100.00	100.00	100.00	100.00	100.00	
Total	1	4	3	2603	1431	5	1	4048
	0.02	0.10	0.07	64.30	35.35	0.12	0.02	100.00

Administration of anticoagulants at the time of obtaining Table 2-4 consent, according to the presence or absence of proton-pump inhibitors

FREQ Procedure

Table : ALL * f_d3

ALL f_d3

Power |
Percent |
Percent of rows
Percent of columns | 1| Total

1	14	14
	100.00	100.00
	100.00	
	100.00	
Total	14	14
	100.00	100.00

Degree of missing values = 4034

Administration of anticoagulants at the time of obtaining Table 2-4 consent, according to the presence or absence of proton-pump inhibitors

FREQ Procedure

Tables: ALL * DOSE

ALL DOSE(Dose per day for DOAC only)

Power |
Percent |
Percent of rows
Column percent | 0| 1.25| 2.5| 5| 7.5| 10| 15| Total

ALL	0	1.25	2.5	5	7.5	10	15	Total
1	1	1	320	3067	2	1473	1	4865
	0.02	0.02	6.58	63.04	0.04	30.28	0.02	100.00
	0.02	0.02	6.58	63.04	0.04	30.28	0.02	
	100.00	100.00	100.00	100.00	100.00	100.00	100.00	
Total	1	1	320	3067	2	1473	1	4865
	0.02	0.02	6.58	63.04	0.04	30.28	0.02	100.00

Administration of anticoagulants at the time of obtaining Table 2-4 consent, according to the presence or absence of proton-pump inhibitors

FREQ Procedure

Tables: ALL * DOSE

ALL DOSE(Dose per day for DOAC only)

Power |
Percent |
Percent of rows
Column percent | 0| 1.25| 2.5| 5| 7.5| 10| 15| Total

1	1	1	320	3067	2	1473	1	4865
	0.02	0.02	6.58	63.04	0.04	30.28	0.02	100.00
	0.02	0.02	6.58	63.04	0.04	30.28	0.02	
	100.00	100.00	100.00	100.00	100.00	100.00	100.00	
Total	1	1	320	3067	2	1473	1	4865
	0.02	0.02	6.58	63.04	0.04	30.28	0.02	100.00

Administration of anticoagulants at the time of obtaining Table 2-4 consent, according to the presence or absence of proton-pump inhibitors

FREQ Procedure

Tables: ALL * DOSE

ALL DOSE(Dose per day for DOAC only)

Power |
Percent |
Percent of rows
Column percent | 0| 1.25| 2.5| 5| 7.5| 10| 15| Total

ALL	0	1.25	2.5	5	7.5	10	15	Total
1	1	1	320	3067	2	1473	1	4865
	0.02	0.02	6.58	63.04	0.04	30.28	0.02	100.00
	0.02	0.02	6.58	63.04	0.04	30.28	0.02	
	100.00	100.00	100.00	100.00	100.00	100.00	100.00	
Total	1	1	320	3067	2	1473	1	4865
	0.02	0.02	6.58	63.04	0.04	30.28	0.02	100.00

Administration of anticoagulants at the time of obtaining Table 2-4 consent, according to the presence or absence of proton-pump inhibitors

FREQ Procedure

Table : ALL * f_d4

ALL f_d4

Power |
Percent |
Percent of rows
Percent of columns | 1| Total

1	5	5
	100.00	100.00
	100.00	
	100.00	
Total	5	5
	100.00	100.00

Degree of missing values = 4860

Administration of anticoagulants at the time of obtaining Table 2-4 consent, according to the presence or absence of proton-pump inhibitors

FREQ Procedure

Tables: ALL * DOSE

ALL DOSE(Dose per day for DOAC only)

Power |
Percent |
Percent of rows
Percentage of columns | 5| 7.5| 10| 15| 30| 60| Total

1	2	1	1	137	2494	419	3054
	0.07	0.03	0.03	4.49	81.66	13.72	100.00
	0.07	0.03	0.03	4.49	81.66	13.72	
	100.00	100.00	100.00	100.00	100.00	100.00	
Total	2	1	1	137	2494	419	3054
	0.07	0.03	0.03	4.49	81.66	13.72	100.00

Administration of anticoagulants at the time of obtaining Table 2-4 consent, according to the presence or absence of proton-pump inhibitors

FREQ Procedure

Tables: ALL * DOSE

ALL DOSE(Dose per day for DOAC only)

Power |
Percent |
Percent of rows
Percentage of columns | 5| 7.5| 10| 15| 30| 60| Total

1	2	1	1	137	2494	419	3054
	0.07	0.03	0.03	4.49	81.66	13.72	100.00
	0.07	0.03	0.03	4.49	81.66	13.72	
	100.00	100.00	100.00	100.00	100.00	100.00	
Total	2	1	1	137	2494	419	3054
	0.07	0.03	0.03	4.49	81.66	13.72	100.00

Administration of anticoagulants at the time of obtaining Table 2-4 consent, according to the presence or absence of proton-pump inhibitors

FREQ Procedure

Tables: ALL * DOSE

ALL DOSE(Dose per day for DOAC only)

Power |
Percent |
Percent of rows
Percentage of columns | 5| 7.5| 10| 15| 30| 60| Total

ALL	5	7.5	10	15	30	60	Total
1	2	1	1	137	2494	419	3054
	0.07	0.03	0.03	4.49	81.66	13.72	100.00
	0.07	0.03	0.03	4.49	81.66	13.72	
	100.00	100.00	100.00	100.00	100.00	100.00	
Total	2	1	1	137	2494	419	3054
	0.07	0.03	0.03	4.49	81.66	13.72	100.00

Administration of anticoagulants at the time of obtaining Table 2-4 consent, according to the presence or absence of proton-pump inhibitors

FREQ Procedure

Table: ALL * f_d0

ALL f_d0

Power |
Percent |
Percent of rows
Percent of columns | 1| Total

______________+________+

1	4	4
	100.00	100.00
	100.00	
	100.00	

______________+________+

Total 4 4
100.00 100.00

Degree of missing values = 3050

Administration of anticoagulants at the time of obtaining Table 2-4 consent, according to the presence or absence of proton-pump inhibitors

FREQ Procedure

Tables: ALL * DOSE

ALL DOSE(Dose per day for DOAC only)

Power |
Percent |
Percent of rows
Percentage of columns | 5| 7.5| 10| 15| 30| 60| Total
______________+________+________+________+________+________+________+
1 | 2 | 1 | 1 | 137 | 2482 | 411 | 3034
| 0.07 | 0.03 | 0.03 | 4.52 | 81.81 | 13.55 | 100.00
| 0.07 | 0.03 | 0.03 | 4.52 | 81.81 | 13.55 |
| 100.00 | 100.00 | 100.00 | 100.00 | 100.00 | 100.00 |
______________+________+________+________+________+________+________+
Total 2 1 1 137 2482 411 3034
0.07 0.03 0.03 4.52 81.81 13.55 100.00

Administration of anticoagulants at the time of obtaining Table 2-4 consent, according to the presence or absence of proton-pump inhibitors

FREQ Procedure

Tables: ALL * DOSE

ALL DOSE(Dose per day for DOAC only)

Power / Percent / Percent of rows / Percentage of columns	5	7.5	10	15	30	60	Total
1	2	1	1	137	2482	411	3034
	0.07	0.03	0.03	4.52	81.81	13.55	100.00
	0.07	0.03	0.03	4.52	81.81	13.55	
	100.00	100.00	100.00	100.00	100.00	100.00	
Total	2	1	1	137	2482	411	3034
	0.07	0.03	0.03	4.52	81.81	13.55	100.00

Administration of anticoagulants at the time of obtaining Table 2-4 consent, according to the presence or absence of proton-pump inhibitors

FREQ Procedure

Tables: ALL * DOSE

ALL DOSE(Dose per day for DOAC only)

Power |
Percent |
Percent of rows
Percentage of columns | 5| 7.5| 10| 15| 30| 60| Total

ALL	5	7.5	10	15	30	60	Total
1	2	1	1	137	2482	411	3034
	0.07	0.03	0.03	4.52	81.81	13.55	100.00
	0.07	0.03	0.03	4.52	81.81	13.55	
	100.00	100.00	100.00	100.00	100.00	100.00	
Total	2	1	1	137	2482	411	3034
	0.07	0.03	0.03	4.52	81.81	13.55	100.00

Administration of anticoagulants at the time of obtaining Table 2-4 consent, according to the presence or absence of proton-pump inhibitors

FREQ Procedure

Table : ALL * f_d5

ALL f_d5

Power |
Percent |
Percent of rows
Percent of columns | 1| Total

1	4	4
	100.00	100.00
	100.00	
	100.00	
Total	4	4
	100.00	100.00

Degree of missing values = 3030

Administration of anticoagulants at the time of obtaining Table 2-4 consent, according to the presence or absence of proton-pump inhibitors

FREQ Procedure

Tables: ALL * DOSE

ALL

DOSE(Dose per day for DOAC only)

Power |
Percent |
Percent of rows
Percentage of columns | 30| 60| Total

1	12	8	20
	60.00	40.00	100.00
	60.00	40.00	
	100.00	100.00	
Total	12	8	20
	60.00	40.00	100.00

Administration of anticoagulants at the time of obtaining Table 2-4 consent, according to the presence or absence of proton-pump inhibitors

FREQ Procedure

Tables: ALL * DOSE

ALL

DOSE(Dose per day for DOAC only)

Power |
Percent |
Percent of rows
Percentage of columns | 30| 60| Total

1	12	8	20
	60.00	40.00	100.00
	60.00	40.00	
	100.00	100.00	
Total	12	8	20
	60.00	40.00	100.00

Administration of anticoagulants at the time of obtaining Table 2-4 consent, according to the presence or absence of proton-pump inhibitors

FREQ Procedure

Tables: ALL * DOSE

ALL

DOSE(Dose per day for DOAC only)

Power | Percent | Percent of rows Percentage of columns	30	60	Total
1	12	8	20
	60.00	40.00	100.00
	60.00	40.00	
	100.00	100.00	
Total	12	8	20
	60.00	40.00	100.00

Administration of anticoagulants at the time of obtaining Table 2-4 consent, according to the presence or absence of proton-pump inhibitors

FREQ Procedure

For ALL * f_d7,
Because all levels of the variable f_d7 are missing,
There is no data.

Administration of anticoagulants at the time of obtaining Table 2-4 consent, according to the presence or absence of proton-pump inhibitors

FREQ Procedure

Tables: ALL * DOSECNT

ALL

DOSECNT(Dose per 1time for DOAC only)

Power |
Percent |
Percent of rows
Percentage of columns | 0| 1| 2| Sum

1	2	10	1421	1433
	0.14	0.70	99.16	100.00
	0.14	0.70	99.16	
	100.00	100.00	100.00	
Total	2	10	1421	1433
	0.14	0.70	99.16	100.00

Administration of anticoagulants at the time of obtaining Table 2-4 consent, according to the presence or absence of proton-pump inhibitors

FREQ Procedure

Tables: ALL * DOSECNT

ALL

DOSECNT(Dose per 1time for DOAC only)

Power |
Percent |
Percent of rows
Percentage of columns | 0| 1| 2| Sum

1	2	10	1421	1433
	0.14	0.70	99.16	100.00
	0.14	0.70	99.16	
	100.00	100.00	100.00	
Total	2	10	1421	1433
	0.14	0.70	99.16	100.00

Administration of anticoagulants at the time of obtaining Table 2-4 consent, according to the presence or absence of proton-pump inhibitors

FREQ Procedure

Table : ALL * t_dc2

ALL t_dc2

Power |
Percent |
Percent of rows
Percent of columns | 1| Total

```
______________+________+
          1 |        2 |        2
            | 100.00 | 100.00
            | 100.00 |
            | 100.00 |
______________+________+
Total 2 2
              100.00    100.00
```

Frequency of missing values = 1431

Administration of anticoagulants at the time of obtaining Table 2-4 consent, according to the presence or absence of proton-pump inhibitors

FREQ Procedure

Tables: ALL * DOSECNT

ALL

DOSECNT(Dose per 1time for DOAC only)

Power / Percent / Percent of rows / Percentage of columns	0	1	2	Sum
1	1	4044	3	4048
	0.02	99.90	0.07	100.00
	0.02	99.90	0.07	
	100.00	100.00	100.00	
Total	1	4044	3	4048
	0.02	99.90	0.07	100.00

Administration of anticoagulants at the time of obtaining Table 2-4 consent, according to the presence or absence of proton-pump inhibitors

FREQ Procedure

Tables: ALL * DOSECNT

ALL

DOSECNT(Dose per 1time for DOAC only)

Power |
Percent |
Percent of rows
Percentage of columns | 0| 1| 2| Sum

	0	1	2	Sum
1	1	4044	3	4048
	0.02	99.90	0.07	100.00
	0.02	99.90	0.07	
	100.00	100.00	100.00	
Total	1	4044	3	4048
	0.02	99.90	0.07	100.00

Administration of anticoagulants at the time of obtaining Table 2-4 consent, according to the presence or absence of proton-pump inhibitors

FREQ Procedure

Table : ALL * t_dc3

ALL t_dc3

Power |
Percent |
Percent of rows
Percent of columns | 1| Total
______________+________+
1 | 1 | 1
| 100.00 | 100.00
| 100.00 |
| 100.00 |
______________+________+
Total 1 1
100.00 100.00

Degree of missing values = 4047

Administration of anticoagulants at the time of obtaining Table 2-4 consent, according to the presence or absence of proton-pump inhibitors

FREQ Procedure

Tables: ALL * DOSECNT

ALL DOSECNT(Dose per 1time for DOAC only)

Power |
Percent |
Percent of rows
Percentage of columns | 0| 0.5| 1| 2| Sum

	0	0.5	1	2	Sum
1	1	1	38	4825	4865
	0.02	0.02	0.78	99.18	100.00
	0.02	0.02	0.78	99.18	
	100.00	100.00	100.00	100.00	
Total	1	1	38	4825	4865
	0.02	0.02	0.78	99.18	100.00

Administration of anticoagulants at the time of obtaining Table 2-4 consent, according to the presence or absence of proton-pump inhibitors

FREQ Procedure

Tables: ALL * DOSECNT

ALL DOSECNT(Dose per 1time for DOAC only)

Power |
Percent |
Percent of rows
Percentage of columns | 0| 0.5| 1| 2| Sum

1	1	1	38	4825	4865
	0.02	0.02	0.78	99.18	100.00
	0.02	0.02	0.78	99.18	
	100.00	100.00	100.00	100.00	
Total	1	1	38	4825	4865
	0.02	0.02	0.78	99.18	100.00

Administration of anticoagulants at the time of obtaining Table 2-4 consent, according to the presence or absence of proton-pump inhibitors

FREQ Procedure

Table : ALL * t_dc4

ALL t_dc4

Power |
Percent |
Percent of rows
Percent of columns | 1| Total
______________+________+
1 | 2 | 2
| 100.00 | 100.00
| 100.00 |
| 100.00 |
______________+________+
Total 2 2
100.00 100.00

Degree of missing values = 4863

Administration of anticoagulants at the time of obtaining Table 2-4 consent, according to the presence or absence of proton-pump inhibitors

FREQ Procedure

Tables: ALL * DOSECNT

ALL

DOSECNT(Dose per 1time for DOAC only)

Power |
Percent |
Percent of rows
Percentage of columns | 0.5| 1| 2| Sum

1	1	3050	3	3054
	0.03	99.87	0.10	100.00
	0.03	99.87	0.10	
	100.00	100.00	100.00	
Total	1	3050	3	3054
	0.03	99.87	0.10	100.00

Administration of anticoagulants at the time of obtaining Table 2-4 consent, according to the presence or absence of proton-pump inhibitors

FREQ Procedure

Tables: ALL * DOSECNT

ALL

DOSECNT(Dose per 1time for DOAC only)

Power |
Percent |
Percent of rows
Percentage of columns | 0.5| 1| 2| Sum

ALL	0.5	1	2	Sum
1	1	3050	3	3054
	0.03	99.87	0.10	100.00
	0.03	99.87	0.10	
	100.00	100.00	100.00	
Total	1	3050	3	3054
	0.03	99.87	0.10	100.00

Administration of anticoagulants at the time of obtaining Table 2-4 consent, according to the presence or absence of proton-pump inhibitors

FREQ Procedure

Table: ALL * t_dc0

ALL t_dc0

Power |
Percent |
Percent of rows
Percent of columns | 1| Total

1	1	1
	100.00	100.00
	100.00	
	100.00	
Total	1	1
	100.00	100.00

Degree of missing values = 3053

Administration of anticoagulants at the time of obtaining Table 2-4 consent, according to the presence or absence of proton-pump inhibitors

FREQ Procedure

Tables: ALL * DOSECNT

ALL

DOSECNT(Dose per 1time for DOAC only)

Power / Percent / Percent of rows / Percentage of columns	0.5	1	2	Sum
1	1	3030	3	3034
	0.03	99.87	0.10	100.00
	0.03	99.87	0.10	
	100.00	100.00	100.00	
Total	1	3030	3	3034
	0.03	99.87	0.10	100.00

Administration of anticoagulants at the time of obtaining Table 2-4 consent, according to the presence or absence of proton-pump inhibitors

FREQ Procedure

Tables: ALL * DOSECNT

ALL

DOSECNT(Dose per 1time for DOAC only)

Power / Percent / Percent of rows / Percentage of columns	0.5	1	2	Sum
1	1	3030	3	3034
	0.03	99.87	0.10	100.00
	0.03	99.87	0.10	
	100.00	100.00	100.00	
Total	1	3030	3	3034
	0.03	99.87	0.10	100.00

Administration of anticoagulants at the time of obtaining Table 2-4 consent, according to the presence or absence of proton-pump inhibitors

FREQ Procedure

Table : ALL * t_dc5

ALL t_dc5

Power |
Percent |
Percent of rows
Percent of columns | 1| Total

```
______________+________+
         1 |      1 |      1
           | 100.00 | 100.00
           | 100.00 |
           | 100.00 |
______________+________+
Total 1 1
             100.00   100.00
```

Degree of missing values = 3033

Administration of anticoagulants at the time of obtaining Table 2-4 consent, according to the presence or absence of proton-pump inhibitors

FREQ Procedure

Tables: ALL * DOSECNT

ALL
DOSECNT(Dose per 1time for DOAC only)

Power |
Percent |
Percent of rows
Percent of columns | 1| Total

1	20	20
	100.00	100.00
	100.00	
	100.00	
Total	20	20
	100.00	100.00

Administration of anticoagulants at the time of obtaining Table 2-4 consent, according to the presence or absence of proton-pump inhibitors

FREQ Procedure

Tables: ALL * DOSECNT

ALL
DOSECNT(Dose per 1time for DOAC only)

Power |
Percent |
Percent of rows
Percent of columns | 1| Total

1	20	20
	100.00	100.00
	100.00	
	100.00	
Total	20	20
	100.00	100.00

Administration of anticoagulants at the time of obtaining Table 2-4 consent, according to the presence or absence of proton-pump inhibitors

FREQ Procedure

For ALL * t_dc7,
Because all levels of variable t_dc7 are missing,
There is no data.

Administration of anticoagulants at the time of obtaining Table 2-4 consent, according to the presence or absence of proton-pump inhibitors

UNIVARIATE Procedure
Variable: DOSE (Dose per day for DOAC only)

Moment

Total of 2339 for N 2339 weight variable
Mean 210.752458 total 492950
Standard deviation 42.3387378 variance 1792.56872
Skewness-1.0210372 kurtosis 2.40797329
Unmodified square sum 108081450 modified square sum 4191025.67
Coefficient of variation 20.089321 standard error of the mean 0.87543276

Basic statistics

Positional variation

Mean 210.7525 standard deviation 42.33874
Median 220.0000 variance 1793
Mode 220.0000 Range 300.00000
Interquartile range 0

Test for position H0: Mu0=0

Test-Statistics------p Value

Student's t-test t 240.7409 Pr > |t| <. 0001
Sign test M 1168.5 Pr >= |M| <. 0001
Signed rank test S 1365977 Pr >= |S| <. 0001

Quartile (Definition 5)

Level quantile

100% max. 300	
99%	300
95%	300
90%	220
75% Q3 220	
50% median 220	
25% Q1 220	
10%	150
5%	110
1%	110
0% minimum 0	

Administration of anticoagulants at the time of obtaining Table 2-4 consent, according to the presence or absence of proton-pump inhibitors

UNIVARIATE Procedure
Variable: DOSE (Dose per day for DOAC only)

Extreme value

--- Minimum value----Maximum value----

Value	Obs	Value	Obs
0	16400	300	29307
0	16399	300	29710
50	15254	300	30753
75	31507	300	31236
75	30747	300	31382

Administration of anticoagulants at the time of obtaining Table 2-4 consent, according to the presence or absence of proton-pump inhibitors

UNIVARIATE Procedure
Variable: DOSE (Dose per day for DOAC only)

Moment

Total of 6436 for N 6436 weight variable
Mean 11.6520354 total 74992.5
Standard deviation 2.5258103 variance 6.37971769
Skewness 2.00774957 kurtosis 22.3114367
Unmodified square sum 914868.75 modified square sum 41053.4833
Coefficient of variation 21.6769879 standard error of the mean 0.0314842

Basic statistics

Positional variation

Mean 11.65204 standard deviation 2.52581
Median 10.00000 variance 6.37972
Mode 10.00000 Range 60.00000
Interquartile range 5.00000

Test for position H0: Mu0=0

Test-Statistics------p Value

Student's t-test t 370.0915 Pr > |t| <. 0001
Sign test M 3217 Pr >= |M| <. 0001
Signed rank test S 10350698 Pr >= |S| <. 0001

Quartile (Definition 5)

Level quantile

100% max 60	
99%	15
95%	15
90%	15
75% Q3 15	
50% median 10	
25% Q1 10	
10%	10
5%	10
1%	10
0% minimum 0	

Administration of anticoagulants at the time of obtaining Table 2-4 consent, according to the presence or absence of proton-pump inhibitors

UNIVARIATE Procedure
Variable: DOSE (Dose per day for DOAC only)

Extreme value

---- Minimum value--Maximum value----

Value	Obs	Value	Obs
0.0	21132	30	7339
0.0	21127	30	16123
2.5	24892	30	20502
5.0	25751	30	30152
5.0	25736	60	8437

Administration of anticoagulants at the time of obtaining Table 2-4 consent, according to the presence or absence of proton-pump inhibitors

UNIVARIATE Procedure
Variable: DOSE (Dose per day for DOAC only)

Moment

Total of 8039 for N 8039 weight variable
Mean 6.30970892 total 50723.75
Standard deviation 2.47450837 variance 6.12319168
Skewness 0.7523919 kurtosis-0.0586932
Unmodified square sum 369270.313 modified square sum 49218.2147
Coefficient of variation 39.2174727 standard error of the mean 0.02759865

Basic statistics

Positional variation

Mean 6.309709 standard deviation 2.47451
Median 5.000000 variance 6.12319
Mode 5.000000 Range 30.00000
Interquartile range 5.00000

Test for position H0: Mu0=0

Test-Statistics------p Value

Student's t-test t 228.6238 Pr > |t| <. 0001
Sign test M 4018.5 Pr >= |M| <. 0001
Signed rank test S 16150352 Pr >= |S| <. 0001

Quartile (Definition 5)

Level quantile

100% max.	30.0
99%	10.0
95%	10.0
90%	10.0
75% Q3	10.0
50% median	5.0
25% Q1	5.0
10%	5.0
5%	2.5
1%	2.5
0% minimum	0.0

Administration of anticoagulants at the time of obtaining Table 2-4 consent, according to the presence or absence of proton-pump inhibitors

UNIVARIATE Procedure

Variable: DOSE (Dose per day for DOAC only)

Extreme value

---- Minimum value----Maximum value----

Value	Obs	Value	Obs
0.00	21138	10	32436
0.00	21126	10	32445
1.25	20710	10	32478
2.50	32242	15	4367
2.50	32240	30	21384

Administration of anticoagulants at the time of obtaining Table 2-4 consent, according to the presence or absence of proton-pump inhibitors

UNIVARIATE Procedure
Variable: DOSE (Dose per day for DOAC only)

Moment

Total of 4792 for N 4792 weight variable
Mean 33.0138773 total 158202.5
Standard deviation 10.7988509 variance 116.61518
Skewness 1.70929156 kurtosis 2.36512876
Unmodified square sum 5781581.25 modified square sum 558703.327
Coefficient of variation 32.7100351 standard error of the mean 0.15599804

Basic statistics

Positional variation

Mean 33.01388 standard deviation 10.79885
Median 30.00000 variance 116.61518
Mode 30.00000 Range 55.00000
Interquartile range 0

Test for position H0: Mu0=0

Test-Statistics------p Value

Student's t-test t 211.6301 Pr > |t| <. 0001
Sign test M 2396 Pr >= |M| <. 0001
Signed rank test S 5742014 Pr >= |S| <. 0001

Quartile (Definition 5)

Level quantile

100% max 60	
99%	60
95%	60
90%	60
75% Q3 30	
50% median 30	
25% Q1 30	
10%	30
5%	15
1%	15
0% minimum 5	

Administration of anticoagulants at the time of obtaining Table 2-4 consent, according to the presence or absence of proton-pump inhibitors

UNIVARIATE Procedure
Variable: DOSE (Dose per day for DOAC only)

Extreme value

---- Minimum value----Maximum value----

Value	Obs	Value	Obs
5.0	20709	60	32152
5.0	14343	60	32202
7.5	1331	60	32253
10.0	14342	60	32416
15.0	32442	60	32489

Administration of anticoagulants at the time of obtaining Table 2-4 consent, according to the presence or absence of proton-pump inhibitors

UNIVARIATE Procedure
Variable: DOSE (Dose per day for DOAC only)

Moment

Total of 4752 for N 4752 weight variable
Mean 32.9729588 total 156687.5
Standard deviation 10.7604538 variance 115.787366
Skewness 1.71820116 kurtosis 2.42069509
Unmodified square sum 5716556.25 modified square sum 550105.775
Coefficient of variation 32.6341772 standard error of the mean 0.15609621

Basic statistics

Positional variation

Mean 32.97296 standard deviation 10.76045
Median 30.00000 variance 115.78737
Mode 30.00000 Range 55.00000
Interquartile range 0

Test for position H0: Mu0=0

Test-Statistics------p Value

Student's t-test t 211.2348 Pr > |t| <. 0001
Sign test M 2376 Pr >= |M| <. 0001
Signed rank test S 5646564 Pr >= |S| <. 0001

Quartile (Definition 5)

Level quantile

100% max 60	
99%	60
95%	60
90%	60
75% Q3 30	
50% median 30	
25% Q1 30	
10%	30
5%	15
1%	15
0% minimum 5	

Administration of anticoagulants at the time of obtaining Table 2-4 consent, according to the presence or absence of proton-pump inhibitors

UNIVARIATE Procedure
Variable: DOSE (Dose per day for DOAC only)

Extreme value

---- Minimum value----Maximum value----

Value	Obs	Value	Obs
5.0	20709	60	32149
5.0	14343	60	32152
7.5	1331	60	32202
10.0	14342	60	32416
15.0	32442	60	32489

Administration of anticoagulants at the time of obtaining Table 2-4 consent, according to the presence or absence of proton-pump inhibitors

UNIVARIATE Procedure
Variable: DOSE (Dose per day for DOAC only)

Moment

Sum of 40 N 40 weight variables
Mean 37.875 total 1515
Standard deviation 14.0003434 variance 196.009615
Skewness 0.91953628 kurtosis-0.8964563
Unmodified square sum 65025 modified square sum 7644.375
Coefficient of variation 36.964603 standard error of the mean 2.21364866

Basic statistics

Positional variation

Mean 37.87500 standard deviation 14.00034
Median 30.00000 variance 196.00962
Mode 30.00000 Range 45.00000
Interquartile range 30.00000

Test for position H0: Mu0=0

Test-Statistics------p Value

Student's t-test t 17.10976 Pr > |t| <. 0001
Sign test M 20 Pr >= |M| <. 0001
Signed rank test S 410 Pr >= |S| <. 0001

Quartile (Definition 5)

Level quantile

100% max	60
99%	60
95%	60
90%	60
75% Q3	60
50% median	30
25% Q1	30
10%	30
5%	30
1%	15
0% minimum	15

Administration of anticoagulants at the time of obtaining Table 2-4 consent, according to the presence or absence of proton-pump inhibitors

UNIVARIATE Procedure

Variable: DOSE (Dose per day for DOAC only)

Extreme value

--- Minimum value--Maximum value----

Value	Obs	Value	Obs
15	18632	60	23574
30	31156	60	26685
30	30986	60	30154
30	30251	60	30474
30	28756	60	32253

Administration of anticoagulants at the time of obtaining Table 2-4 consent, according to the presence or absence of proton-pump inhibitors

UNIVARIATE Procedure
Variable: DOSE (Dose per day for DOAC only)

Moment

Total of 906 for N 906 weight variable
Mean 211.368653 total 191500
Standard deviation 41.1928877 variance 1696.854
Skewness-1.0396528 kurtosis 2.49791488
Unmodified square sum 42012750 modified square sum 1535652.87
Coefficient of variation 19.4886456 standard error of the mean 1.36854203

Basic statistics

Positional variation

Mean 211.3687 standard deviation 41.19289
Median 220.0000 variance 1697
Mode 220.0000 Range 250.00000
Interquartile range 0

Test for position H0: Mu0=0

Test-Statistics------p Value

Student's t-test t 154.4481 Pr > |t| <. 0001
Sign test M 453 Pr >= |M| <. 0001
Signed rank test S 205435.5 Pr >= |S| <. 0001

Quartile (Definition 5)

Level quantile

100% max.	300
99%	300
95%	300
90%	220
75% Q3	220
50% median	220
25% Q1	220
10%	150
5%	110
1%	110
0% minimum	50

Administration of anticoagulants at the time of obtaining Table 2-4 consent, according to the presence or absence of proton-pump inhibitors

UNIVARIATE Procedure
Variable: DOSE (Dose per day for DOAC only)

Extreme value

--- Minimum value----Maximum value----

Value	Obs	Value	Obs
50	15254	300	28039
75	31507	300	29256
75	30747	300	29710
75	26708	300	31236
75	26699	300	31382

Administration of anticoagulants at the time of obtaining Table 2-4 consent, according to the presence or absence of proton-pump inhibitors

UNIVARIATE Procedure
Variable: DOSE (Dose per day for DOAC only)

Moment

Total of 2388 for N 2388 weight variable
Mean 11.409129 total 27245
Standard deviation 2.36785492 variance 5.6067369
Skewness 1.10612099 kurtosis 2.28373271
Unmodified square sum 324225 modified square sum 13383.281
Coefficient of variation 20.7540376 standard error of the mean 0.04845493

Basic statistics

Positional variation

Mean 11.40913 standard deviation 2.36785
Median 10.00000 variance 5.60674
Mode 10.00000 Range 30.00000
Interquartile range 5.00000

Test for position H0: Mu0=0

Test-Statistics------p Value

Student's t-test t 235.4586 Pr > |t| <. 0001
Sign test M 1193.5 Pr >= |M| <. 0001
Signed rank test S 1425039 Pr >= |S| <. 0001

Quartile (Definition 5)

Level quantile

100% max. 30	
99%	15
95%	15
90%	15
75% Q3 15	
50% median 10	
25% Q1 10	
10%	10
5%	10
1%	10
0% minimum 0	

Administration of anticoagulants at the time of obtaining Table 2-4 consent, according to the presence or absence of proton-pump inhibitors

UNIVARIATE Procedure
Variable: DOSE (Dose per day for DOAC only)

Extreme value

---- Minimum value--Maximum value----

Value	Obs	Value	Obs
0.0	21132	15	32268
2.5	24892	15	32303
5.0	25751	15	32409
5.0	24889	30	2441
5.0	24299	30	2443

Administration of anticoagulants at the time of obtaining Table 2-4 consent, according to the presence or absence of proton-pump inhibitors

UNIVARIATE Procedure
Variable: DOSE (Dose per day for DOAC only)

Moment

Total of 3174 for N 3174 weight variable
Mean 6.2468494 total 19827.5
Standard deviation 2.45327232 variance 6.01854507
Skewness 0.97165249 kurtosis 1.70027487
Unmodified square sum 142956.25 modified square sum 19096.8435
Coefficient of variation 39.272154 standard error of the mean 0.0435454

Basic statistics

Positional variation

Mean 6.246849 standard deviation 2.45327
Median 5.000000 variance 6.01855
Mode 5.000000 Range 30.00000
Interquartile range 5.00000

Test for position H0: Mu0=0

Test-Statistics------p Value

Student's t-test t 143.456 Pr > |t| <. 0001
Sign test M 1586.5 Pr >= |M| <. 0001
Signed rank test S 2517776 Pr >= |S| <. 0001

Quartile (Definition 5)

Level quantile

100% max. 30.0	
99%	10.0
95%	10.0
90%	10.0
75% Q3 10.0	
50% median 5.0	
25% Q1 5.0	
10%	5.0
5%	2.5
1%	2.5
0% minimum 0.0	

Administration of anticoagulants at the time of obtaining Table 2-4 consent, according to the presence or absence of proton-pump inhibitors

UNIVARIATE Procedure
Variable: DOSE (Dose per day for DOAC only)

Extreme value

---- Minimum value--Maximum value----

Value	Obs	Value	Obs
0.0	21126	10	32297
2.5	32242	10	32390
2.5	32240	10	32436
2.5	31611	10	32445
2.5	31419	30	21384

Administration of anticoagulants at the time of obtaining Table 2-4 consent, according to the presence or absence of proton-pump inhibitors

UNIVARIATE Procedure
Variable: DOSE (Dose per day for DOAC only)

Moment

Total of 1738 for N 1738 weight variable
Mean 32.3130035 total 56160
Standard deviation 10.2600534 variance 105.268697
Skewness 1.8148864 kurtosis 3.22202087
Unmodified square sum 1997550 modified square sum 182851.726
Coefficient of variation 31.7520885 standard error of the mean 0.24610744

Basic statistics

Positional variation

Mean 32.31300 standard deviation 10.26005
Median 30.00000 variance 105.26870
Mode 30.00000 Range 45.00000
Interquartile range 0

Test for position H0: Mu0=0

Test-Statistics------p Value

Student's t-test t 131.2963 Pr > |t| <. 0001
Sign test M 869 Pr >= |M| <. 0001
Signed rank test S 755595.5 Pr >= |S| <. 0001

Quartile (Definition 5)

Level quantile

100% max 60	
99%	60
95%	60
90%	60
75% Q3 30	
50% median 30	
25% Q1 30	
10%	30
5%	15
1%	15
0% minimum 15	

Administration of anticoagulants at the time of obtaining Table 2-4 consent, according to the presence or absence of proton-pump inhibitors

UNIVARIATE Procedure
Variable: DOSE (Dose per day for DOAC only)

Extreme value

--- Minimum value--Maximum value----

Value	Obs	Value	Obs
15	32442	60	30944
15	32382	60	31501
15	32372	60	32130
15	32278	60	32149
15	31783	60	32152

Administration of anticoagulants at the time of obtaining Table 2-4 consent, according to the presence or absence of proton-pump inhibitors

UNIVARIATE Procedure
Variable: DOSE (Dose per day for DOAC only)

Moment

Total of 1718 for N 1718 weight variable
Mean 32.2962747 total 55485
Standard deviation 10.243574 variance 104.930807
Skewness 1.81821374 kurtosis 3.24890777
Unmodified square sum 1972125 modified square sum 180166.196
Coefficient of variation 31.7175093 standard error of the mean 0.24713823

Basic statistics

Positional variation

Mean 32.29627 standard deviation 10.24357
Median 30.00000 variance 104.93081
Mode 30.00000 Range 45.00000
Interquartile range 0

Test for position H0: Mu0=0

Test-Statistics------p Value

Student's t-test t 130.681 Pr > |t| <. 0001
Sign test M 859 Pr >= |M| <. 0001
Signed rank test S 738310.5 Pr >= |S| <. 0001

Quartile (Definition 5)

Level quantile

100% max 60	
99%	60
95%	60
90%	60
75% Q3 30	
50% median 30	
25% Q1 30	
10%	30
5%	15
1%	15
0% minimum 15	

Administration of anticoagulants at the time of obtaining Table 2-4 consent, according to the presence or absence of proton-pump inhibitors

UNIVARIATE Procedure
Variable: DOSE (Dose per day for DOAC only)

Extreme value

--- Minimum value--Maximum value----

Value	Obs	Value	Obs
15	32442	60	30944
15	32382	60	31501
15	32372	60	32130
15	32278	60	32149
15	31783	60	32152

Administration of anticoagulants at the time of obtaining Table 2-4 consent, according to the presence or absence of proton-pump inhibitors

UNIVARIATE Procedure
Variable: DOSE (Dose per day for DOAC only)

Moment

Sum of 20 N 20 weight variables
Mean 33.75 total 675
Standard deviation 11.7959627 variance 139.144737
Skewness 1.66843958 kurtosis 2.24844886
Unmodified square sum 25425 modified square sum 2643.75
Coefficient of variation 34.9510007 standard error of the mean 2.63765745

Basic statistics

Positional variation

Mean 33.75000 standard deviation 11.79596
Median 30.00000 variance 139.14474
Mode 30.00000 Range 45.00000
Interquartile range 0

Test for position H0: Mu0=0

Test-Statistics------p Value

Student's t-test t 12.79544 Pr > |t| <. 0001
Sign test M 10 Pr >= |M| <. 0001
Signed rank test S 105 Pr >= |S| <. 0001

Quartile (Definition 5)

Level quantile

100% max.	60.0
99%	60.0
95%	60.0
90%	60.0
75% Q3	30.0
50% median	30.0
25% Q1	30.0
10%	30.0
5%	22.5
1%	15.0
0% minimum	15.0

Administration of anticoagulants at the time of obtaining Table 2-4 consent, according to the presence or absence of proton-pump inhibitors

UNIVARIATE Procedure
Variable: DOSE (Dose per day for DOAC only)

Extreme value

--- Minimum value--Maximum value----

Value	Obs	Value	Obs
15	18632	30	30251
30	31156	30	31156
30	30251	60	23222
30	28756	60	23564
30	26684	60	23574

Administration of anticoagulants at the time of obtaining Table 2-4 consent, according to the presence or absence of proton-pump inhibitors

UNIVARIATE Procedure
Variable: DOSE (Dose per day for DOAC only)

Moment

Total of 1433 for N 1433 weight variable
Mean 210.362875 total 301450
Standard deviation 43.057154 variance 1853.91851
Skewness-1.0086429 kurtosis 2.35402754
Unmodified square sum 66068700 modified square sum 2654811.3
Coefficient of variation 20.4680384 standard error of the mean 1.13742357

Basic statistics

Positional variation

Mean 210.3629 standard deviation 43.05715
Median 220.0000 variance 1854
Mode 220.0000 Range 300.00000
Interquartile range 0

Test for position H0: Mu0=0

Test-Statistics------p Value

Student's t-test t 184.9468 Pr > |t| <. 0001
Sign test M 715.5 Pr >= |M| <. 0001
Signed rank test S 512298 Pr >= |S| <. 0001

Quartile (Definition 5)

Level quantile

100% max. 300	
99%	300
95%	300
90%	220
75% Q3 220	
50% median 220	
25% Q1 220	
10%	150
5%	110
1%	110
0% minimum 0	

Administration of anticoagulants at the time of obtaining Table 2-4 consent, according to the presence or absence of proton-pump inhibitors

UNIVARIATE Procedure
Variable: DOSE (Dose per day for DOAC only)

Extreme value

--- Minimum value----Maximum value----

Value	Obs	Value	Obs
0	16400	300	28293
0	16399	300	28505
75	26696	300	29009
75	25847	300	29307
75	19451	300	30753

Administration of anticoagulants at the time of obtaining Table 2-4 consent, according to the presence or absence of proton-pump inhibitors

UNIVARIATE Procedure
Variable: DOSE (Dose per day for DOAC only)

Moment

Total of 4048 for N 4048 weight variable
Mean 11.795331 total 47747.5
Standard deviation 2.60420018 variance 6.7818586
Skewness 2.39471424 kurtosis 30.1171418
Unmodified square sum 590643.75 modified square sum 27446.1818
Coefficient of variation 22.0782289 standard error of the mean 0.04093117

Basic statistics

Positional variation

Mean 11.79533 standard deviation 2.60420
Median 10.00000 variance 6.78186
Mode 10.00000 Range 60.00000
Interquartile range 5.00000

Test for position H0: Mu0=0

Test-Statistics------p Value

Student's t-test t 288.1748 Pr > |t| <. 0001
Sign test M 2023.5 Pr >= |M| <. 0001
Signed rank test S 4095564 Pr >= |S| <. 0001

Quartile (Definition 5)

Level quantile

100% max 60	
99%	15
95%	15
90%	15
75% Q3 15	
50% median 10	
25% Q1 10	
10%	10
5%	10
1%	10
0% minimum 0	

Administration of anticoagulants at the time of obtaining Table 2-4 consent, according to the presence or absence of proton-pump inhibitors

UNIVARIATE Procedure
Variable: DOSE (Dose per day for DOAC only)

Extreme value

--- Minimum value--Maximum value----

Value Obs Value Obs

0	21127	30	7339
5	25736	30	16123
5	24894	30	20502
5	19094	30	30152
5	7666	60	8437

Administration of anticoagulants at the time of obtaining Table 2-4 consent, according to the presence or absence of proton-pump inhibitors

UNIVARIATE Procedure
Variable: DOSE (Dose per day for DOAC only)

Moment

Total of 4865 for N 4865 weight variable
Mean 6.35071942 total 30896.25
Standard deviation 2.48766065 variance 6.18845549
Skewness 0.61479516 kurtosis-1.1221838
Unmodified square sum 226314.063 modified square sum 30100.6475
Coefficient of variation 39.1713203 standard error of the mean 0.03566561

Basic statistics

Positional variation

Mean 6.350719 standard deviation 2.48766
Median 5.000000 variance 6.18846
Mode 5.000000 Range 15.00000
Interquartile range 5.00000

Test for position H0: Mu0=0

Test-Statistics------p Value

Student's t-test t 178.0628 Pr > |t| <. 0001
Sign test M 2432 Pr >= |M| <. 0001
Signed rank test S 5915840 Pr >= |S| <. 0001

Quartile (Definition 5)

Level quantile

100% max.	15.0
99%	10.0
95%	10.0
90%	10.0
75% Q3	10.0
50% median	5.0
25% Q1	5.0
10%	5.0
5%	2.5
1%	2.5
0% minimum	0.0

Administration of anticoagulants at the time of obtaining Table 2-4 consent, according to the presence or absence of proton-pump inhibitors

UNIVARIATE Procedure
Variable: DOSE (Dose per day for DOAC only)

Extreme value

---- Minimum value----Maximum value----

Value	Obs	Value	Obs
0.00	21138	10	32377
1.25	20710	10	32380
2.50	31908	10	32405
2.50	31907	10	32478
2.50	31787	15	4367

Administration of anticoagulants at the time of obtaining Table 2-4 consent, according to the presence or absence of proton-pump inhibitors

UNIVARIATE Procedure
Variable: DOSE (Dose per day for DOAC only)

Moment

Total of 3054 for N 3054 weight variable
Mean 33.4127374 total 102042.5
Standard deviation 11.0756561 variance 122.670159
Skewness 1.65110986 kurtosis 1.95890114
Unmodified square sum 3784031.25 modified square sum 374511.995
Coefficient of variation 33.1480058 standard error of the mean 0.20041718

Basic statistics

Positional variation

Mean 33.41274 standard deviation 11.07566
Median 30.00000 variance 122.67016
Mode 30.00000 Range 55.00000
Interquartile range 0

Test for position H0: Mu0=0

Test-Statistics------p Value

Student's t-test t 166.7159 Pr > |t| <. 0001
Sign test M 1527 Pr >= |M| <. 0001
Signed rank test S 2332493 Pr >= |S| <. 0001

Quartile (Definition 5)

Level quantile

100% max	60
99%	60
95%	60
90%	60
75% Q3	30
50% median	30
25% Q1	30
10%	30
5%	30
1%	15
0% minimum	5

Administration of anticoagulants at the time of obtaining Table 2-4 consent, according to the presence or absence of proton-pump inhibitors

UNIVARIATE Procedure
Variable: DOSE (Dose per day for DOAC only)

Extreme value

---- Minimum value----Maximum value----

Value	Obs	Value	Obs
5.0	20709	60	32123
5.0	14343	60	32202
7.5	1331	60	32253
10.0	14342	60	32416
15.0	32374	60	32489

Administration of anticoagulants at the time of obtaining Table 2-4 consent, according to the presence or absence of proton-pump inhibitors

UNIVARIATE Procedure
Variable: DOSE (Dose per day for DOAC only)

Moment

Total of 3034 for N 3034 weight variable
Mean 33.3561305 total 101202.5
Standard deviation 11.025664 variance 121.565266
Skewness 1.66323547 kurtosis 2.02751468
Unmodified square sum 3744431.25 modified square sum 368707.451
Coefficient of variation 33.0543855 standard error of the mean 0.20016907

Basic statistics

Positional variation

Mean 33.35613 standard deviation 11.02566
Median 30.00000 variance 121.56527
Mode 30.00000 Range 55.00000
Interquartile range 0

Test for position H0: Mu0=0

Test-Statistics------p Value

Student's t-test t 166.6398 Pr > |t| <. 0001
Sign test M 1517 Pr >= |M| <. 0001
Signed rank test S 2302048 Pr >= |S| <. 0001

Quartile (Definition 5)

Level quantile

100% max	60
99%	60
95%	60
90%	60
75% Q3	30
50% median	30
25% Q1	30
10%	30
5%	30
1%	15
0% minimum	5

Administration of anticoagulants at the time of obtaining Table 2-4 consent, according to the presence or absence of proton-pump inhibitors

UNIVARIATE Procedure
Variable: DOSE (Dose per day for DOAC only)

Extreme value

---- Minimum value----Maximum value----

Value	Obs	Value	Obs
5.0	20709	60	32082
5.0	14343	60	32123
7.5	1331	60	32202
10.0	14342	60	32416
15.0	32374	60	32489

Administration of anticoagulants at the time of obtaining Table 2-4 consent, according to the presence or absence of proton-pump inhibitors

UNIVARIATE Procedure
Variable: DOSE (Dose per day for DOAC only)

Moment

Sum of 20 N 20 weight variables
Mean 42 840 total
Standard deviation 15.0787407 variance 227.368421
Skewness 0.44212357 kurtosis-2.0179739
Modified Square 39600 Modified Square Sum 4320
Coefficient of variation 35.9017636 standard error of the mean 3.37170892

Basic statistics

Positional variation

Mean 42.00000 standard deviation 15.07874
Median 30.00000 variance 227.36842
Mode 30.00000 Range 30.00000
Interquartile range 30.00000

Test for position H0: Mu0=0

Test-Statistics------p Value

Student's t-test t 12.45659 Pr > |t| <. 0001
Sign test M 10 Pr >= |M| <. 0001
Signed rank test S 105 Pr >= |S| <. 0001

Quartile (Definition 5)

Level quantile

100% max	60
99%	60
95%	60
90%	60
75% Q3	60
50% median	30
25% Q1	30
10%	30
5%	30
1%	30
0% minimum	30

Administration of anticoagulants at the time of obtaining Table 2-4 consent, according to the presence or absence of proton-pump inhibitors

UNIVARIATE Procedure
Variable: DOSE (Dose per day for DOAC only)

Extreme value

--- Minimum value--Maximum value----

Value	Obs	Value	Obs
30	30986	60	23562
30	27075	60	26685
30	23579	60	30154
30	17268	60	30474
30	16290	60	32253

Administration of anticoagulants at the time of obtaining Table 2-4 consent, according to the presence or absence of proton-pump inhibitors

FREQ Procedure

		Cumulative
t_d2	Frequency	Percentage

Frequency of missing values = 32490

Administration of anticoagulants at the time of obtaining Table 2-4 consent, according to the presence or absence of proton-pump inhibitors

FREQ Procedure

t_d3	Frequency	Cumulative Percentage

Frequency of missing values = 32490

Administration of anticoagulants at the time of obtaining Table 2-4 consent, according to the presence or absence of proton-pump inhibitors

FREQ Procedure

t_d4	Frequency	Cumulative Percentage

Frequency of missing values = 32490

Administration of anticoagulants at the time of obtaining Table 2-4 consent, according to the presence or absence of proton-pump inhibitors

FREQ Procedure

t_d0	Frequency	Percentage	Cumulative

Frequency of missing values = 32490

Administration of anticoagulants at the time of obtaining Table 2-4 consent, according to the presence or absence of proton-pump inhibitors

FREQ Procedure

t_d5	Frequency	Cumulative Percentage

Frequency of missing values = 32490

Administration of anticoagulants at the time of obtaining Table 2-4 consent, according to the presence or absence of proton-pump inhibitors

FREQ Procedure

t_d7	Frequency	Percentage	Cumulative

Frequency of missing values = 32490

Administration of anticoagulants at the time of obtaining Table 2-4 consent, according to the presence or absence of proton-pump inhibitors

FREQ Procedure

t_d2	Frequency	Cumulative Percentage

Frequency of missing values = 11981

Administration of anticoagulants at the time of obtaining Table 2-4 consent, according to the presence or absence of proton-pump inhibitors

FREQ Procedure

t_d3	Frequency	Percentage	Cumulative

Frequency of missing values = 11981

Administration of anticoagulants at the time of obtaining Table 2-4 consent, according to the presence or absence of proton-pump inhibitors

FREQ Procedure

t_d4	Frequency	Percentage	Cumulative

Frequency of missing values = 11981

Administration of anticoagulants at the time of obtaining Table 2-4 consent, according to the presence or absence of proton-pump inhibitors

FREQ Procedure

t_d0	Frequency	Cumulative Percentage

Frequency of missing values = 11981

Administration of anticoagulants at the time of obtaining Table 2-4 consent, according to the presence or absence of proton-pump inhibitors

FREQ Procedure

t_d5	Frequency	Cumulative Percentage

Frequency of missing values = 11981

Administration of anticoagulants at the time of obtaining Table 2-4 consent, according to the presence or absence of proton-pump inhibitors

FREQ Procedure

t_d7	Frequency	Percentage	Cumulative

Frequency of missing values = 11981

Administration of anticoagulants at the time of obtaining Table 2-4 consent, according to the presence or absence of proton-pump inhibitors

FREQ Procedure

t_d2	Frequency	Percentage	Cumulative

Degree of missing values = 20509

Administration of anticoagulants at the time of obtaining Table 2-4 consent, according to the presence or absence of proton-pump inhibitors

FREQ Procedure

		Cumulative
t_d3	Frequency	Percentage

Degree of missing values = 20509

Administration of anticoagulants at the time of obtaining Table 2-4 consent, according to the presence or absence of proton-pump inhibitors

FREQ Procedure

t_d4	Frequency	Percentage	Cumulative

Degree of missing values = 20509

Administration of anticoagulants at the time of obtaining Table 2-4 consent, according to the presence or absence of proton-pump inhibitors

FREQ Procedure

t_d0	Frequency	Percentage	Cumulative

Degree of missing values = 20509

Administration of anticoagulants at the time of obtaining Table 2-4 consent, according to the presence or absence of proton-pump inhibitors

FREQ Procedure

t_d5	Frequency	Percentage	Cumulative

Degree of missing values = 20509

Administration of anticoagulants at the time of obtaining Table 2-4 consent, according to the presence or absence of proton-pump inhibitors

FREQ Procedure

t_d7	Frequency	Percentage	Cumulative

Degree of missing values = 20509

Administration of anticoagulants at the time of obtaining Table 2-4 consent, according to the presence or absence of proton-pump inhibitors

FREQ Procedure

Number of levels of variables

Number of Variables Level

GROUP1	2
t_ADYN	2

Table : GROUP1 * t_ADYN

GROUP1 t_ADYN

Power |
Percent |
Percent of rows
Percentage of columns | 0| 1| Sum

	0	1	Sum
1	818	11163	11981
	2.52	34.36	36.88
	6.83	93.17	
	31.75	37.32	
2	1758	18751	20509
	5.41	57.71	63.12
	8.57	91.43	
	68.25	62.68	
Total	2576	29914	32490
	7.93	92.07	100.00

Administration of anticoagulants at the time of obtaining Table 2-4 consent, according to the presence or absence of proton-pump inhibitors

FREQ Procedure

Number of levels of variables

Number of Variables Level

GROUP1 2
Tf_ACT1 2

Table : GROUP1 * tf_ACT1

GROUP1 tf_ACT1

Power |
Percent |
Percent of rows
Percentage of columns | 0| 1| Sum

	0	1	Sum
1	8208	2955	11163
	27.44	9.88	37.32
	73.53	26.47	
	37.97	35.62	
2	13411	5340	18751
	44.83	17.85	62.68
	71.52	28.48	
	62.03	64.38	
Total	21619	8295	29914
	72.27	27.73	100.00

Administration of anticoagulants at the time of obtaining Table 2-4 consent, according to the presence or absence of proton-pump inhibitors

FREQ Procedure

Number of levels of variables

Number of Variables Level

GROUP1	2
Tf_ACT2	2

Table : GROUP1 * tf_ACT2

GROUP1 tf_ACT2

Power |
Percent |
Percent of rows
Percentage of columns | 0| 1| Sum

	0	1	Sum
1	10257	906	11163
	34.29	3.03	37.32
	91.88	8.12	
	37.20	38.73	
2	17318	1433	18751
	57.89	4.79	62.68
	92.36	7.64	
	62.80	61.27	
Total	27575	2339	29914
	92.18	7.82	100.00

Administration of anticoagulants at the time of obtaining Table 2-4 consent, according to the presence or absence of proton-pump inhibitors

FREQ Procedure

Number of levels of variables

Number of Variables Level

GROUP1	2
Tf_ACT3	2

Table : GROUP1 * tf_ACT3

GROUP1 tf_ACT3

Power |
Percent |
Percent of rows
Percentage of columns | 0| 1| Sum

	0	1	Sum
1	8775	2388	11163
	29.33	7.98	37.32
	78.61	21.39	
	37.38	37.10	
2	14703	4048	18751
	49.15	13.53	62.68
	78.41	21.59	
	62.62	62.90	
Total	23478	6436	29914
	78.48	21.52	100.00

Administration of anticoagulants at the time of obtaining Table 2-4 consent, according to the presence or absence of proton-pump inhibitors

FREQ Procedure

Number of levels of variables

Number of Variables Level

GROUP1 2
Tf_ACT4 2

Table : GROUP1 * tf_ACT4

GROUP1 tf_ACT4

Power |
Percent |
Percent of rows
Percentage of columns | 0| 1| Sum

GROUP1	0	1	Sum
1	7989	3174	11163
	26.71	10.61	37.32
	71.57	28.43	
	36.52	39.48	
2	13886	4865	18751
	46.42	16.26	62.68
	74.05	25.95	
	63.48	60.52	
Total	21875	8039	29914
	73.13	26.87	100.00

Administration of anticoagulants at the time of obtaining Table 2-4 consent, according to the presence or absence of proton-pump inhibitors

FREQ Procedure

Number of levels of variables

Number of Variables Level

GROUP1 2
t_ACT0 2

Table : GROUP1 * t_ACT0

GROUP1 t_ACT0

Power |
Percent |
Percent of rows
Percentage of columns | 0| 1| Sum

GROUP1	0	1	Sum
1	9425	1738	11163
	31.51	5.81	37.32
	84.43	15.57	
	37.52	36.27	
2	15697	3054	18751
	52.47	10.21	62.68
	83.71	16.29	
	62.48	63.73	
Total	25122	4792	29914
	83.98	16.02	100.00

Administration of anticoagulants at the time of obtaining Table 2-4 consent, according to the presence or absence of proton-pump inhibitors

FREQ Procedure

Number of levels of variables

Number of Variables Level

GROUP1	2
Tf_ACT5	2

Table : GROUP1 * tf_ACT5

GROUP1 tf_ACT5

Power |
Percent |
Percent of rows
Percentage of columns | 0| 1| Sum

GROUP1	0	1	Sum
1	9445	1718	11163
	31.57	5.74	37.32
	84.61	15.39	
	37.54	36.15	
2	15717	3034	18751
	52.54	10.14	62.68
	83.82	16.18	
	62.46	63.85	
Total	25162	4752	29914
	84.11	15.89	100.00

Administration of anticoagulants at the time of obtaining Table 2-4 consent, according to the presence or absence of proton-pump inhibitors

FREQ Procedure

Number of levels of variables

Number of Variables Level

GROUP1	2
Tf_ACT7	2

Table : GROUP1 * tf_ACT7

GROUP1 tf_ACT7

Power / Percent / Percent of rows / Percentage of columns	0	1	Sum
1	11143	20	11163
	37.25	0.07	37.32
	99.82	0.18	
	37.30	50.00	
2	18731	20	18751
	62.62	0.07	62.68
	99.89	0.11	
	62.70	50.00	
Total	29874	40	29914
	99.87	0.13	100.00

Administration of anticoagulants at the time of obtaining Table 2-4 consent, according to the presence or absence of proton-pump inhibitors

FREQ Procedure

Number of levels of variables

Number of Variables Level

GROUP1	2
Tf_ACT6	2

Table : GROUP1 * tf_ACT6

GROUP1 tf_ACT6

Power |
Percent |
Percent of rows
Percentage of columns | 0| 1| Sum

	0	1	Sum
1	11161	2	11163
	37.31	0.01	37.32
	99.98	0.02	
	37.33	15.38	
2	18740	11	18751
	62.65	0.04	62.68
	99.94	0.06	
	62.67	84.62	
Total	29901	13	29914
	99.96	0.04	100.00

Administration of anticoagulants at the time of obtaining Table 2-4 consent, according to the presence or absence of proton-pump inhibitors

FREQ Procedure

Number of levels of variables

Number of Variables Level

GROUP1	2
t_ACT1	2

Table : GROUP1 * t_ACT1

GROUP1 t_ACT1

Power |
Percent |
Percent of rows
Percentage of columns | 0| 1| Sum

	0	1	Sum
1	2	11161	11163
	0.01	37.31	37.32
	0.02	99.98	
	15.38	37.33	
2	11	18740	18751
	0.04	62.65	62.68
	0.06	99.94	
	84.62	62.67	
Total	13	29901	29914
	0.04	99.96	100.00

Administration of anticoagulants at the time of obtaining Table 2-4 consent, according to the presence or absence of proton-pump inhibitors

FREQ Procedure

Number of levels of variables

Number of Variables Level

GROUP1	2
t_ACT2	2

Table : GROUP1 * t_ACT2

GROUP1 t_ACT2

Power |
Percent |
Percent of rows
Percentage of columns | 0| 1| Sum

	0	1	Sum
1	2957	8206	11163
	9.89	27.43	37.32
	26.49	73.51	
	35.59	37.98	
2	5351	13400	18751
	17.89	44.80	62.68
	28.54	71.46	
	64.41	62.02	
Total	8308	21606	29914
	27.77	72.23	100.00

Administration of anticoagulants at the time of obtaining Table 2-4 consent, according to the presence or absence of proton-pump inhibitors

FREQ Procedure

Number of levels of variables

Number of Variables Level

GROUP1	2
t_ACT3	2

Table : GROUP1 * t_ACT3

GROUP1 t_ACT3

Power |
Percent |
Percent of rows
Percentage of columns | 0| 1| Sum

	0	1	Sum
1	3863	7300	11163
	12.91	24.40	37.32
	34.61	65.39	
	36.28	37.89	
2	6784	11967	18751
	22.68	40.00	62.68
	36.18	63.82	
	63.72	62.11	
Total	10647	19267	29914
	35.59	64.41	100.00

Administration of anticoagulants at the time of obtaining Table 2-4 consent, according to the presence or absence of proton-pump inhibitors

FREQ Procedure

Number of levels of variables

Variables Label Level Number

GROUP1 2
VISITAD Visit and Drug Administration 3

Table : GROUP1 * VISITAD

GROUP1
VISITAD(Visit and Drug Administration)

Power |
Percent |
Percent of rows
Percent of column |Month |Month |Month |First dose |Total
Within | |

1	2914	8	8	2930
	35.44	0.10	0.10	35.63
	99.45	0.27	0.27	
	35.66	28.57	34.78	
2	5258	20	15	5293
	63.94	0.24	0.18	64.37
	99.34	0.38	0.28	
	64.34	71.43	65.22	
Total	8172	28	23	8223
	99.38	0.34	0.28	100.00

Administration of anticoagulants at the time of obtaining Table 2-4 consent, according to the presence or absence of proton-pump inhibitors

FREQ Procedure

Number of levels of variables

Variables Label Level Number

GROUP1 2
VISITAD Visit and Drug Administration 3

Table : GROUP1 * VISITAD

GROUP1
VISITAD(Visit and Drug Administration)

Power | Percent | Percent of rows Percent of column Within	Month	Month	Month | First dose	Total
1	875	9	8	892
	37.93	0.39	0.35	38.66
	98.09	1.01	0.90	
	38.61	39.13	44.44	
2	1391	14	10	1415
	60.29	0.61	0.43	61.34
	98.30	0.99	0.71	
	61.39	60.87	55.56	
Total	2266	23	18	2307
	98.22	1.00	0.78	100.00

Administration of anticoagulants at the time of obtaining Table 2-4 consent, according to the presence or absence of proton-pump inhibitors

FREQ Procedure

Number of levels of variables

Variables Label Level Number

GROUP1 2
VISITAD Visit and Drug Administration 3

Table : GROUP1 * VISITAD

GROUP1
VISITAD(Visit and Drug Administration)

Power |
Percent |
Percent of rows
Percent of column |Month |Month |Month |First dose |Total
Within | |

1	2297	38	29	2364
	36.00	0.60	0.45	37.05
	97.17	1.61	1.23	
	37.28	34.86	26.36	
2	3865	71	81	4017
	60.57	1.11	1.27	62.95
	96.22	1.77	2.02	
	62.72	65.14	73.64	
Total	6162	109	110	6381
	96.57	1.71	1.72	100.00

Administration of anticoagulants at the time of obtaining Table 2-4 consent, according to the presence or absence of proton-pump inhibitors

FREQ Procedure

Number of levels of variables

Variables	Label	Level Number
GROUP1		2
VISITAD	Visit and Drug Administration	3

Table : GROUP1 * VISITAD

GROUP1

VISITAD(Visit and Drug Administration)

Power<br>Percent<br>Percent of rows<br>Percent of column Within	Month	Month	Month First dose	Total
1	3062<br>38.25<br>96.93<br>39.52	54<br>0.67<br>1.71<br>42.19	43<br>0.54<br>1.36<br>33.08	3159<br>39.46
2	4685<br>58.53<br>96.68<br>60.48	74<br>0.92<br>1.53<br>57.81	87<br>1.09<br>1.80<br>66.92	4846<br>60.54
Total	7747<br>96.78	128<br>1.60	130<br>1.62	8005<br>100.00

Administration of anticoagulants at the time of obtaining Table 2-4 consent, according to the presence or absence of proton-pump inhibitors

FREQ Procedure

Number of levels of variables

Variables Label Level Number

GROUP1	2
VISITAD Visit and Drug Administration	3

Table : GROUP1 * VISITAD

GROUP1

VISITAD(Visit and Drug Administration)

Power |
Percent |
Percent of rows
Percent of column |Month |Month |Month |First dose |Total
Within | |

1	1528	65	135	1728
	32.05	1.36	2.83	36.25
	88.43	3.76	7.81	
	36.90	28.76	33.75	
2	2613	161	265	3039
	54.81	3.38	5.56	63.75
	85.98	5.30	8.72	
	63.10	71.24	66.25	
Total	4141	226	400	4767
	86.87	4.74	8.39	100.00

Administration of anticoagulants at the time of obtaining Table 2-4 consent, according to the presence or absence of proton-pump inhibitors

FREQ Procedure

Number of levels of variables

Variables Label Level Number

GROUP1 2
VISITAD Visit and Drug Administration 3

Table : GROUP1 * VISITAD

GROUP1
VISITAD(Visit and Drug Administration)

Power |
Percent |
Percent of rows
Percent of column |Month |Month |Month |First dose |Total
Within | |

Percent of column	Month Within	Month	Month First dose	Total
1	1508	65	135	1708
	31.90	1.38	2.86	36.13
	88.29	3.81	7.90	
	36.74	28.89	34.01	
2	2597	160	262	3019
	54.94	3.38	5.54	63.87
	86.02	5.30	8.68	
	63.26	71.11	65.99	
Total	4105	225	397	4727
	86.84	4.76	8.40	100.00

Administration of anticoagulants at the time of obtaining Table 2-4 consent, according to the presence or absence of proton-pump inhibitors

FREQ Procedure

Number of levels of variables

Variables Label Level Number

GROUP1 2
VISITAD Visit and Drug Administration 3

Table : GROUP1 * VISITAD

GROUP1
VISITAD(Visit and Drug Administration)

Power |
Percent |
Percent of rows
Percent of column |Month |Month |Month |First dose |Total
Within | |

GROUP1	Month	Month	Month / First dose Within	Total
1	20	0	0	20
	50.00	0.00	0.00	50.00
	100.00	0.00	0.00	
	55.56	0.00	0.00	
2	16	1	3	20
	40.00	2.50	7.50	50.00
	80.00	5.00	15.00	
	44.44	100.00	100.00	
Total	36	1	3	40
	90.00	2.50	7.50	100.00

Administration of anticoagulants at the time of obtaining Table 2-4 consent, according to the presence or absence of proton-pump inhibitors

FREQ Procedure

Number of levels of variables

Variables Label Level Number

GROUP1 2
VISITAD Visit and Drug Administration 1

Table : GROUP1 * VISITAD

GROUP1
VISITAD(Visit and Drug Administration)

Power |
Percent |
Percent of rows
Percent of column |Month | Total
Breakforward |

1	1	1
	9.09	9.09
	100.00	
	9.09	
2	10	10
	90.91	90.91
	100.00	
	90.91	
Total	11	11
	100.00	100.00

Administration of anticoagulants at the time of obtaining Table 2-4 consent, according to the presence or absence of proton-pump inhibitors

FREQ Procedure

Number of levels of variables

Variables Label Level Number

Variables	Label	Level Number
GROUP1		2
DOSE	Dose per day for DOAC only	5

Table : GROUP1 * DOSE

GROUP1 DOSE(Dose per day for DOAC only)

Power |
Percent |
Percent of rows
Column percent| 75| 110| 150| 220| 300| Total

GROUP1	75	110	150	220	300	Total
1	5	74	45	725	55	904
	0.21	3.17	1.93	31.08	2.36	38.75
	0.55	8.19	4.98	80.20	6.08	
	45.45	36.10	36.59	39.27	37.16	
2	6	131	78	1121	93	1429
	0.26	5.62	3.34	48.05	3.99	61.25
	0.42	9.17	5.46	78.45	6.51	
	54.55	63.90	63.41	60.73	62.84	
Total	11	205	123	1846	148	2333
	0.47	8.79	5.27	79.13	6.34	100.00

Administration of anticoagulants at the time of obtaining Table 2-4 consent, according to the presence or absence of proton-pump inhibitors

FREQ Procedure

Number of levels of variables

Variables Label Level Number

GROUP1 2
DOSE Dose per day for DOAC only 2

Table : GROUP1 * DOSE

GROUP1
DOSE(Dose per day for DOAC only)

Power<br>Percent<br>Percent of rows<br>Percentage of columns	10	15	Total
1	1695<br>26.46<br>71.43<br>39.44	678<br>10.58<br>28.57<br>32.15	2373<br>37.04
2	2603<br>40.63<br>64.53<br>60.56	1431<br>22.33<br>35.47<br>67.85	4034<br>62.96
Total	4298<br>67.08	2109<br>32.92	6407<br>100.00

Administration of anticoagulants at the time of obtaining Table 2-4 consent, according to the presence or absence of proton-pump inhibitors

FREQ Procedure

Number of levels of variables

Variables	Label	Level Number
GROUP1		2
DOSE	Dose per day for DOAC only	3

Table : GROUP1 * DOSE

GROUP1 DOSE(Dose per day for DOAC only)

Power / Percent / Percent of rows / Percent of columns	2.5	5	10	Total
1	198	2086	885	3169
	2.47	25.98	11.02	39.47
	6.25	65.83	27.93	
	38.22	40.48	37.53	
2	320	3067	1473	4860
	3.99	38.20	18.35	60.53
	6.58	63.11	30.31	
	61.78	59.52	62.47	
Total	518	5153	2358	8029
	6.45	64.18	29.37	100.00

Administration of anticoagulants at the time of obtaining Table 2-4 consent, according to the presence or absence of proton-pump inhibitors

FREQ Procedure

Number of levels of variables

Variables Label Level Number

GROUP1 2
DOSE Dose per day for DOAC only 3

Table : GROUP1 * DOSE

GROUP1 DOSE(Dose per day for DOAC only)

Power |
Percent |
Percent of rows
Percentage of columns | 15| 30| 60| Total

1	106	1445	187	1738
	2.21	30.18	3.91	36.30
	6.10	83.14	10.76	
	43.62	36.68	30.86	
2	137	2494	419	3050
	2.86	52.09	8.75	63.70
	4.49	81.77	13.74	
	56.38	63.32	69.14	
Total	243	3939	606	4788
	5.08	82.27	12.66	100.00

Administration of anticoagulants at the time of obtaining Table 2-4 consent, according to the presence or absence of proton-pump inhibitors

FREQ Procedure

Number of levels of variables

Variables Label Level Number

GROUP1 2
DOSE Dose per day for DOAC only 3

Table : GROUP1 * DOSE

GROUP1 DOSE(Dose per day for DOAC only)

Power |
Percent |
Percent of rows
Percentage of columns | 15| 30| 60| Total

1	105	1429	184	1718
	2.21	30.10	3.88	36.18
	6.11	83.18	10.71	
	43.39	36.54	30.92	
2	137	2482	411	3030
	2.89	52.27	8.66	63.82
	4.52	81.91	13.56	
	56.61	63.46	69.08	
Total	242	3911	595	4748
	5.10	82.37	12.53	100.00

Administration of anticoagulants at the time of obtaining Table 2-4 consent, according to the presence or absence of proton-pump inhibitors

FREQ Procedure

Number of levels of variables

Variables Label Level Number

Variables	Label	Level Number
GROUP1		2
DOSE	Dose per day for DOAC only	3

Table : GROUP1 * DOSE

GROUP1 DOSE(Dose per day for DOAC only)

Power |
Percent |
Percent of rows
Percentage of columns | 15| 30| 60| Total

GROUP1	15	30	60	Total
1	1	16	3	20
	2.50	40.00	7.50	50.00
	5.00	80.00	15.00	
	100.00	57.14	27.27	
2	0	12	8	20
	0.00	30.00	20.00	50.00
	0.00	60.00	40.00	
	0.00	42.86	72.73	
Total	1	28	11	40
	2.50	70.00	27.50	100.00

Administration of anticoagulants at the time of obtaining Table 2-4 consent, according to the presence or absence of proton-pump inhibitors

FREQ Procedure

Number of levels of variables

Variables Label Level Number

Variables	Label	Level Number
GROUP1		2
DOSECNT	Dose per 1time for DOAC only	2

Table : GROUP1 * DOSECNT

GROUP1

DOSECNT(Dose per 1time for DOAC only)

Power |
Percent |
Percent of rows
Percentage of columns | 1| 2| Total

GROUP1	1	2	Total
1	5	901	906
	0.21	38.55	38.77
	0.55	99.45	
	33.33	38.80	
2	10	1421	1431
	0.43	60.80	61.23
	0.70	99.30	
	66.67	61.20	
Total	15	2322	2337
	0.64	99.36	100.00

Administration of anticoagulants at the time of obtaining Table 2-4 consent, according to the presence or absence of proton-pump inhibitors

FREQ Procedure

Number of levels of variables

Variables	Label	Level Number
GROUP1		2
DOSECNT	Dose per 1time for DOAC only	2

Table : GROUP1 * DOSECNT

GROUP1

DOSECNT(Dose per 1time for DOAC only)

Power |
Percent |
Percent of rows
Percentage of columns | 1| 2| Total

GROUP1	1	2	Total
1	2384	3	2387
	37.05	0.05	37.10
	99.87	0.13	
	37.09	50.00	
2	4044	3	4047
	62.85	0.05	62.90
	99.93	0.07	
	62.91	50.00	
Total	6428	6	6434
	99.91	0.09	100.00

Administration of anticoagulants at the time of obtaining Table 2-4 consent, according to the presence or absence of proton-pump inhibitors

FREQ Procedure

Number of levels of variables

Variables Label Level Number

GROUP1 2

DOSECNT Dose per 1time for DOAC only 2

Table : GROUP1 * DOSECNT

GROUP1

DOSECNT(Dose per 1time for DOAC only)

Power / Percent / Percent of rows / Percentage of columns	1	2	Total
1	26	3146	3172
	0.32	39.15	39.48
	0.82	99.18	
	40.63	39.47	
2	38	4825	4863
	0.47	60.05	60.52
	0.78	99.22	
	59.38	60.53	
Total	64	7971	8035
	0.80	99.20	100.00

Administration of anticoagulants at the time of obtaining Table 2-4 consent, according to the presence or absence of proton-pump inhibitors

FREQ Procedure

Number of levels of variables

Variables Label Level Number

GROUP1 2
DOSECNT Dose per 1time for DOAC only 2

Table : GROUP1 * DOSECNT

GROUP1
DOSECNT(Dose per 1time for DOAC only)

Power |
Percent |
Percent of rows
Percentage of columns | 1| 2| Total

GROUP1	1	2	Total
1	1736	2	1738
	36.23	0.04	36.28
	99.88	0.12	
	36.27	40.00	
2	3050	3	3053
	63.66	0.06	63.72
	99.90	0.10	
	63.73	60.00	
Total	4786	5	4791
	99.90	0.10	100.00

Administration of anticoagulants at the time of obtaining Table 2-4 consent, according to the presence or absence of proton-pump inhibitors

FREQ Procedure

Number of levels of variables

Variables Label Level Number

GROUP1 2
DOSECNT Dose per 1time for DOAC only 2

Table : GROUP1 * DOSECNT

GROUP1
DOSECNT(Dose per 1time for DOAC only)

Power |
Percent |
Percent of rows
Percentage of columns | 1| 2| Total

GROUP1	1	2	Total
1	1717	1	1718
	36.14	0.02	36.16
	99.94	0.06	
	36.17	25.00	
2	3030	3	3033
	63.78	0.06	63.84
	99.90	0.10	
	63.83	75.00	
Total	4747	4	4751
	99.92	0.08	100.00

Administration of anticoagulants at the time of obtaining Table 2-4 consent, according to the presence or absence of proton-pump inhibitors

FREQ Procedure

Number of levels of variables

Variables	Label	Level Number
GROUP1		2
DOSECNT	Dose per 1time for DOAC only	2

Table : GROUP1 * DOSECNT

GROUP1

DOSECNT(Dose per 1time for DOAC only)

Power |
Percent |
Percent of rows
Percentage of columns | 1| 2| Total

GROUP1	1	2	Total
1	19	1	20
	47.50	2.50	50.00
	95.00	5.00	
	48.72	100.00	
2	20	0	20
	50.00	0.00	50.00
	100.00	0.00	
	51.28	0.00	
Total	39	1	40
	97.50	2.50	100.00

Administration of anticoagulants at the time of obtaining Table 2-4 consent, according to the presence or absence of proton-pump inhibitors

TTEST Procedure

Variable: DOSE (Dose per day for DOAC only)

GROUP1 N Mean Standard Deviation Standard Error Minimum Value Maximum Value

1	906	211.4	41.1929	1.3685	50.0000	300.0
2	1433	210.4	43.0572	1.1374	0	300.0

Diff (1-2) 1.0058 42.3450 1.7973

To the mean standard deviation

GROUP1 methods Mean 95% confidence limits standard deviation 95% confidence limits

1		211.4	208.7	214.1	41.1929	39.3796	43.1825
2		210.4	208.1	212.6	43.0572	41.5365	44.6942

Diff (1-2) Pooled 1.0058 -2.5188 4.5303 42.3450 41.1651 43.5949
Diff (1-2) Satterthwaite 1.0058 -2.4841 4.4957

Technique Dispersion degrees of freedom t-value Pr > |t|

Pooled Equal 2337 0.56 0.5758
Satterthwaite Unequal 1987.7 0.57 0.5720

Isodispersity

Technique Degree of Freedom of Moleculars Degree of Freedom of the denominator F Value Pr > F

Folded F 1432 905 1.09 0.1432

Administration of anticoagulants at the time of obtaining Table 2-4 consent, according to the presence or absence of proton-pump inhibitors

TTEST Procedure

Variable: DOSE (Dose per day for DOAC only)

GROUP1 N Mean Standard Deviation Standard Error Minimum Value Maximum Value

1	2388	11.4091	2.3679	0.0485	0	30.0000
2	4048	11.7953	2.6042	0.0409	0	60.0000

Diff (1-2) -0.3862 2.5191 0.0650

To the mean standard deviation

GROUP1 methods Mean 95% confidence limits standard deviation 95% confidence limits

1	11.4091	11.3141	11.5041	2.3679	2.3026	2.4370
2	11.7953	11.7151	11.8756	2.6042	2.5487	2.6622

Diff (1-2) Pooled -0.3862 -0.5136 -0.2588 2.5191 2.4763 2.5634

Diff (1-2) Satterthwaite -0.3862 -0.5105 -0.2619

Technique Dispersion degrees of freedom t-value Pr > |t|

Pooled Equal 6434 -5.94 <. 0001

Satterthwaite Unequal 5390.2 -6.09 <. 0001

Isodispersity

Technique Degree of Freedom of Moleculars Degree of Freedom of the denominator F Value Pr > F

Folded F 4047 2387 1.21 <. 0001

Administration of anticoagulants at the time of obtaining Table 2-4 consent, according to the presence or absence of proton-pump inhibitors

TTEST Procedure

Variable: DOSE (Dose per day for DOAC only)

GROUP1 N Mean Standard Deviation Standard Error Minimum Value Maximum Value

1	3174	6.2468	2.4533	0.0435	0	30.0000
2	4865	6.3507	2.4877	0.0357	0	15.0000

Diff (1-2) -0.1039 2.4741 0.0565

To the mean standard deviation

GROUP1 methods Mean 95% confidence limits standard deviation 95% confidence limits

1	6.2468	6.1615	6.3322	2.4533	2.3944	2.5152
2	6.3507	6.2808	6.4206	2.4877	2.4392	2.5381

Diff (1-2) Pooled -0.1039 -0.2145 0.00679 2.4741 2.4365 2.5130

Diff (1-2) Satterthwaite -0.1039 -0.2142 0.00647

Technique Dispersion degrees of freedom t-value Pr > |t|

Pooled Equal 8037 -1.84 0.0658

Satterthwaite Unequal 6847.7 -1.85 0.0650

Isodispersity

Technique Degree of Freedom of Moleculars Degree of Freedom of the denominator F Value Pr > F

Folded F 4864 3173 1.03 0.3901

Administration of anticoagulants at the time of obtaining Table 2-4 consent, according to the presence or absence of proton-pump inhibitors

TTEST Procedure

Variable: DOSE (Dose per day for DOAC only)

GROUP1 N Mean Standard Deviation Standard Error Minimum Value Maximum Value

1	1738	32.3130	10.2601	0.2461	15.0000	60.0000
2	3054	33.4127	11.0757	0.2004	5.0000	60.0000

Diff (1-2) -1.0997 10.7870 0.3241

To the mean standard deviation

GROUP1 methods Mean 95% confidence limits standard deviation 95% confidence limits

1		32.3130	31.8303	32.7957	10.2601	9.9300	10.6130
2		33.4127	33.0198	33.8057	11.0757	10.8047	11.3606

Diff (1-2) Pooled -1.0997 -1.7352 -0.4643 10.7870 10.5753 11.0075

Diff (1-2) Satterthwaite -1.0997 -1.7220 -0.4775

Technique Dispersion degrees of freedom t-value Pr > |t|

Pooled Equal 4790 -3.39 0.0007

Satterthwaite Unequal 3843.1 -3.46 0.0005

Isodispersity

Technique Degree of Freedom of Moleculars Degree of Freedom of the denominator F Value Pr > F

Folded F 3053 1737 1.17 0.0004

Administration of anticoagulants at the time of obtaining Table 2-4 consent, according to the presence or absence of proton-pump inhibitors

TTEST Procedure

Variable: DOSE (Dose per day for DOAC only)

GROUP1	N	Mean	Standard Deviation	Standard Error	Minimum Value	Maximum Value
1	1718	32.2963	10.2436	0.2471	15.0000	60.0000
2	3034	33.3561	11.0257	0.2002	5.0000	60.0000
Diff (1-2)		-1.0599	10.7495	0.3246		

To the mean standard deviation

GROUP1	methods	Mean	95% confidence limits		standard deviation	95% confidence limits	
1		32.2963	31.8116	32.7810	10.2436	9.9122	10.5981
2		33.3561	32.9636	33.7486	11.0257	10.7551	11.3103
Diff (1-2)	Pooled	-1.0599	-1.6962	-0.4235	10.7495	10.5377	10.9701
Diff (1-2)	Satterthwaite	-1.0599	-1.6834	-0.4363			

Technique	Dispersion	degrees of freedom	t-value	Pr > |t|
Pooled	Equal	4750	-3.27	0.0011
Satterthwaite	Unequal	3786.2	-3.33	0.0009

Isodispersity

Technique	Degree of Freedom of Moleculars	Degree of Freedom of the denominator	F Value	Pr > F
Folded F	3033	1717	1.16	0.0006

Administration of anticoagulants at the time of obtaining Table 2-4 consent, according to the presence or absence of proton-pump inhibitors

TTEST Procedure

Variable: DOSE (Dose per day for DOAC only)

GROUP1	N	Mean	Standard Deviation	Standard Error	Minimum Value	Maximum Value
1	20	33.7500	11.7960	2.6377	15.0000	60.0000
2	20	42.0000	15.0787	3.3717	30.0000	60.0000
Diff (1-2)		-8.2500	13.5372	4.2808		

To the mean standard deviation

GROUP1	methods	Mean	95% confidence limits		standard deviation	95% confidence limits	
1		33.7500	28.2293	39.2707	11.7960	8.9707	17.2288
2		42.0000	34.9429	49.0571	15.0787	11.4672	22.0236
Diff (1-2)	Pooled	-8.2500	-16.9161	0.4161	13.5372	11.0632	17.4465
Diff (1-2)	Satterthwaite	-8.2500	-16.9326	0.4326			

Technique	Dispersion	degrees of freedom	t-value	Pr > |t|
Pooled	Equal	38	-1.93	0.0615
Satterthwaite	Unequal	35.919	-1.93	0.0619

Isodispersity

Technique	Degree of Freedom of Moleculars	Degree of Freedom of the denominator	F Value	Pr > F
Folded F	19	19	1.63	0.2932

Concomitant medications other than anticoagulants at the time of obtaining Table 2-5 consent, according to the presence or absence of proton-pump inhibitors

FREQ Procedure

			Cumulative	
Percent of ALL	power	Percent of	power	
1	32490	100.00	32490	100.00

Tables: ALL * CMYN

ALL

CMYN(Administration of Concomitant Drug)

Power<br>Percent<br>Percent of rows<br>Percent of columns	None	With	Total
1	1445<br>4.45<br>4.45<br>100.00	31045<br>95.55<br>95.55<br>100.00	32490<br>100.00
Total	1445<br>4.45	31045<br>95.55	32490<br>100.00

Concomitant medications other than anticoagulants at the time of obtaining Table 2-5 consent, according to the presence or absence of proton-pump inhibitors

FREQ Procedure

Tables: ALL * CMYN

ALL
CMYN(Administration of Concomitant Drug)

Power<br>Percent<br>Percent of rows<br>Percent of columns	None	With	Total
1	1445<br>4.45<br>4.45<br>100.00	31045<br>95.55<br>95.55<br>100.00	32490<br>100.00
Total	1445<br>4.45	31045<br>95.55	32490<br>100.00

Concomitant medications other than anticoagulants at the time of obtaining Table 2-5 consent, according to the presence or absence of proton-pump inhibitors

FREQ Procedure

			Cumulative	
Percent of ALL	power	Percent of power		
1	32490	100.00	32490	100.00

Tables: ALL * CMYN

ALL

CMYN(Administration of Concomitant Drug)

Power | Percent | Percent of rows Percent of columns	None	With	Total
1	1445	31045	32490
	4.45	95.55	100.00
	4.45	95.55	
	100.00	100.00	
Total	1445	31045	32490
	4.45	95.55	100.00

Concomitant medications other than anticoagulants at the time of obtaining Table 2-5 consent, according to the presence or absence of proton-pump inhibitors

FREQ Procedure

Tables: ALL * CMYN

ALL

CMYN(Administration of Concomitant Drug)

Power / Percent / Percent of rows / Percent of columns	None	With	Total
1	1445	31045	32490
	4.45	95.55	100.00
	4.45	95.55	
	100.00	100.00	
Total	1445	31045	32490
	4.45	95.55	100.00

Concomitant medications other than anticoagulants at the time of obtaining Table 2-5 consent, according to the presence or absence of proton-pump inhibitors

FREQ Procedure

			Cumulative	
Percent of ALL	power	Percent of	power	
1	32490	100.00	32490	100.00

Tables: ALL * CMYN

ALL

CMYN(Administration of Concomitant Drug)

Power | Percent | Percent of rows Percent of columns	None	With	Total
1	1445	31045	32490
	4.45	95.55	100.00
	4.45	95.55	
	100.00	100.00	
Total	1445	31045	32490
	4.45	95.55	100.00

Concomitant medications other than anticoagulants at the time of obtaining Table 2-5 consent, according to the presence or absence of proton-pump inhibitors

FREQ Procedure

Tables: ALL * CMYN

ALL

CMYN(Administration of Concomitant Drug)

Power |
Percent |
Percent of rows
Percent of columns | None | With | Total

______________+________+________+

1 | 1445 | 31045 | 32490
| 4.45 | 95.55 | 100.00
| 4.45 | 95.55 |
| 100.00 | 100.00 |

______________+________+________+

Total 1445 31045 32490
4.45 95.55 100.00

Concomitant medications other than anticoagulants at the time of obtaining Table 2-5 consent, according to the presence or absence of proton-pump inhibitors

FREQ Procedure

			Cumulative	
Percent of ALL	power	Percent of	power	
1	32490	100.00	32490	100.00

Tables: ALL * CMYN

ALL

CMYN(Administration of Concomitant Drug)

Power | Percent | Percent of rows Percent of columns	None	With	Total
1	1445	31045	32490
	4.45	95.55	100.00
	4.45	95.55	
	100.00	100.00	
Total	1445	31045	32490
	4.45	95.55	100.00

Concomitant medications other than anticoagulants at the time of obtaining Table 2-5 consent, according to the presence or absence of proton-pump inhibitors

FREQ Procedure

Tables: ALL* ARTD

ALL ARTD(Arrhythmic Drugs)

Power |
Percent |
Percent of rows
Percent of columns | None | With | Total

1	12671	18374	31045
	40.81	59.19	100.00
	40.81	59.19	
	100.00	100.00	
Total	12671	18374	31045
	40.81	59.19	100.00

Concomitant medications other than anticoagulants at the time of obtaining Table 2-5 consent, according to the presence or absence of proton-pump inhibitors

FREQ Procedure

Percent of ALL	power	Percent	Cumulative power	Cumulative Percent of power
1	32490	100.00	32490	100.00

Tables: ALL * CMYN

ALL

CMYN(Administration of Concomitant Drug)

Power |
Percent |
Percent of rows
Percent of columns | None | With | Total

ALL	None	With	Total
1	1445	31045	32490
	4.45	95.55	100.00
	4.45	95.55	
	100.00	100.00	
Total	1445	31045	32490
	4.45	95.55	100.00

Concomitant medications other than anticoagulants at the time of obtaining Table 2-5 consent, according to the presence or absence of proton-pump inhibitors

FREQ Procedure

Tables: ALL* ARTD

ALL ARTD(Arrhythmic Drugs)

Power |
Percent |
Percent of rows
Percent of columns | None | With | Total

1	12671	18374	31045
	40.81	59.19	100.00
	40.81	59.19	
	100.00	100.00	
Total	12671	18374	31045
	40.81	59.19	100.00

Concomitant medications other than anticoagulants at the time of obtaining Table 2-5 consent, according to the presence or absence of proton-pump inhibitors

FREQ Procedure

			Cumulative	
Percent of ALL	power	Percent of power		
1	32490	100.00	32490	100.00

Tables: ALL * CMYN

ALL

CMYN(Administration of Concomitant Drug)

Power<br>Percent<br>Percent of rows<br>Percent of columns	None	With	Total
1	1445<br>4.45<br>4.45<br>100.00	31045<br>95.55<br>95.55<br>100.00	32490<br>100.00
Total	1445<br>4.45	31045<br>95.55	32490<br>100.00

Concomitant medications other than anticoagulants at the time of obtaining Table 2-5 consent, according to the presence or absence of proton-pump inhibitors

FREQ Procedure

Table : ALL * ARTD1

ALL ARTD1(Rhythm Control)

Power |
Percent |
Percent of rows
Percent of columns | None | With | Total

1	24676	6369	31045
	79.48	20.52	100.00
	79.48	20.52	
	100.00	100.00	
Total	24676	6369	31045
	79.48	20.52	100.00

Concomitant medications other than anticoagulants at the time of obtaining Table 2-5 consent, according to the presence or absence of proton-pump inhibitors

FREQ Procedure

			Cumulative	
Percent of ALL power Percent of power				
1	32490	100.00	32490	100.00

Tables: ALL * CMYN

ALL

CMYN(Administration of Concomitant Drug)

Power<br>Percent<br>Percent of rows<br>Percent of columns	None	With	Total
1	1445<br>4.45<br>4.45<br>100.00	31045<br>95.55<br>95.55<br>100.00	32490<br>100.00
Total	1445<br>4.45	31045<br>95.55	32490<br>100.00

Concomitant medications other than anticoagulants at the time of obtaining Table 2-5 consent, according to the presence or absence of proton-pump inhibitors

FREQ Procedure

Table : ALL * ARTD1

ALL ARTD1(Rhythm Control)

Power<br>Percent<br>Percent of rows<br>Percent of columns	None	With	Total
1	24676<br>79.48<br>79.48<br>100.00	6369<br>20.52<br>20.52<br>100.00	31045<br>100.00
Total	24676<br>79.48	6369<br>20.52	31045<br>100.00

Concomitant medications other than anticoagulants at the time of obtaining Table 2-5 consent, according to the presence or absence of proton-pump inhibitors

FREQ Procedure

			Cumulative	
Percent of ALL	power	Percent of power		
1	32490	100.00	32490	100.00

Tables: ALL * CMYN

ALL

CMYN(Administration of Concomitant Drug)

Power |
Percent |
Percent of rows
Percent of columns | None | With | Total

	None	With	Total
1	1445	31045	32490
	4.45	95.55	100.00
	4.45	95.55	
	100.00	100.00	
Total	1445	31045	32490
	4.45	95.55	100.00

Concomitant medications other than anticoagulants at the time of obtaining Table 2-5 consent, according to the presence or absence of proton-pump inhibitors

FREQ Procedure

Table : ALL * ARTD2

ALL ARTD2(Rate Control)

Power<br>Percent<br>Percent of rows<br>Percent of columns	None	With	Total
1	17579<br>56.62<br>56.62<br>100.00	13466<br>43.38<br>43.38<br>100.00	31045<br>100.00
Total	17579<br>56.62	13466<br>43.38	31045<br>100.00

Concomitant medications other than anticoagulants at the time of obtaining Table 2-5 consent, according to the presence or absence of proton-pump inhibitors

FREQ Procedure

			Cumulative	
Percent of ALL	power	Percent	of power	
1	32490	100.00	32490	100.00

Tables: ALL * CMYN

ALL

CMYN(Administration of Concomitant Drug)

Power |
Percent |
Percent of rows
Percent of columns | None | With | Total

	None	With	Total
1	1445	31045	32490
	4.45	95.55	100.00
	4.45	95.55	
	100.00	100.00	
Total	1445	31045	32490
	4.45	95.55	100.00

Concomitant medications other than anticoagulants at the time of obtaining Table 2-5 consent, according to the presence or absence of proton-pump inhibitors

FREQ Procedure

Table : ALL * ARTD2

ALL ARTD2(Rate Control)

Power<br>Percent<br>Percent of rows<br>Percent of columns	None	With	Total
1	17579<br>56.62<br>56.62<br>100.00	13466<br>43.38<br>43.38<br>100.00	31045<br>100.00
Total	17579<br>56.62	13466<br>43.38	31045<br>100.00

Concomitant medications other than anticoagulants at the time of obtaining Table 2-5 consent, according to the presence or absence of proton-pump inhibitors

FREQ Procedure

			Cumulative	
Percent of ALL	power	Percent of power		
1	32490	100.00	32490	100.00

Tables: ALL * CMYN

ALL

CMYN(Administration of Concomitant Drug)

Power |
Percent |
Percent of rows
Percent of columns | None | With | Total

	None	With	Total
1	1445	31045	32490
	4.45	95.55	100.00
	4.45	95.55	
	100.00	100.00	
Total	1445	31045	32490
	4.45	95.55	100.00

Concomitant medications other than anticoagulants at the time of obtaining Table 2-5 consent, according to the presence or absence of proton-pump inhibitors

FREQ Procedure

Tables: ALL* APLD

ALL APLD(Anti-Platelet Drugs)

Power | Percent | Percent of rows Percent of columns	None	With	Total
1	25252	5793	31045
	81.34	18.66	100.00
	81.34	18.66	
	100.00	100.00	
Total	25252	5793	31045
	81.34	18.66	100.00

Concomitant medications other than anticoagulants at the time of obtaining Table 2-5 consent, according to the presence or absence of proton-pump inhibitors

FREQ Procedure

			Cumulative	
Percent of ALL	power	Percent of	power	
1	32490	100.00	32490	100.00

Tables: ALL * CMYN

ALL

CMYN(Administration of Concomitant Drug)

Power |
Percent |
Percent of rows
Percent of columns | None | With | Total

	None	With	Total
1	1445	31045	32490
	4.45	95.55	100.00
	4.45	95.55	
	100.00	100.00	
Total	1445	31045	32490
	4.45	95.55	100.00

Concomitant medications other than anticoagulants at the time of obtaining Table 2-5 consent, according to the presence or absence of proton-pump inhibitors

FREQ Procedure

Tables: ALL* APLD

ALL APLD(Anti-Platelet Drugs)

Power |
Percent |
Percent of rows
Percent of columns | None | With | Total

1	25252	5793	31045
	81.34	18.66	100.00
	81.34	18.66	
	100.00	100.00	
Total	25252	5793	31045
	81.34	18.66	100.00

Concomitant medications other than anticoagulants at the time of obtaining Table 2-5 consent, according to the presence or absence of proton-pump inhibitors

FREQ Procedure

			Cumulative	
Percent of ALL power Percent of power				
1	32490	100.00	32490	100.00

Tables: ALL * CMYN

ALL

CMYN(Administration of Concomitant Drug)

Power | Percent | Percent of rows Percent of columns	None	With	Total
1	1445	31045	32490
	4.45	95.55	100.00
	4.45	95.55	
	100.00	100.00	
Total	1445	31045	32490
	4.45	95.55	100.00

Concomitant medications other than anticoagulants at the time of obtaining Table 2-5 consent, according to the presence or absence of proton-pump inhibitors

FREQ Procedure

Table: ALL * t_AP

ALL t_AP

Power |
Percent |
Percent of rows
Percentage of columns | 0| 1| Sum

	0	1	Sum
1	30802	243	31045
	99.22	0.78	100.00
	99.22	0.78	
	100.00	100.00	
Total	30802	243	31045
	99.22	0.78	100.00

Concomitant medications other than anticoagulants at the time of obtaining Table 2-5 consent, according to the presence or absence of proton-pump inhibitors

FREQ Procedure

Percent of ALL	power	Percent of power	Cumulative	
1	32490	100.00	32490	100.00

Tables: ALL * CMYN

ALL

CMYN(Administration of Concomitant Drug)

Power |
Percent |
Percent of rows
Percent of columns | None | With | Total

	None	With	Total
1	1445	31045	32490
	4.45	95.55	100.00
	4.45	95.55	
	100.00	100.00	
Total	1445	31045	32490
	4.45	95.55	100.00

Concomitant medications other than anticoagulants at the time of obtaining Table 2-5 consent, according to the presence or absence of proton-pump inhibitors

FREQ Procedure

Table: ALL * t_AP

ALL t_AP

Power |
Percent |
Percent of rows
Percentage of columns | 0| 1| Sum

	0	1	Sum
1	30802	243	31045
	99.22	0.78	100.00
	99.22	0.78	
	100.00	100.00	
Total	30802	243	31045
	99.22	0.78	100.00

Concomitant medications other than anticoagulants at the time of obtaining Table 2-5 consent, according to the presence or absence of proton-pump inhibitors

FREQ Procedure

			Cumulative	
Percent of ALL	power	Percent of power		
1	32490	100.00	32490	100.00

Tables: ALL * CMYN

ALL

CMYN(Administration of Concomitant Drug)

Power | Percent | Percent of rows Percent of columns	None	With	Total
1	1445	31045	32490
	4.45	95.55	100.00
	4.45	95.55	
	100.00	100.00	
Total	1445	31045	32490
	4.45	95.55	100.00

Concomitant medications other than anticoagulants at the time of obtaining Table 2-5 consent, according to the presence or absence of proton-pump inhibitors

FREQ Procedure

Table : ALL * APLD1

ALL APLD1(ASA)

Power<br>Percent<br>Percent of rows<br>Percent of columns	None	With	Total
1	27508<br>88.61<br>88.61<br>100.00	3537<br>11.39<br>11.39<br>100.00	31045<br>100.00
Total	27508<br>88.61	3537<br>11.39	31045<br>100.00

Concomitant medications other than anticoagulants at the time of obtaining Table 2-5 consent, according to the presence or absence of proton-pump inhibitors

FREQ Procedure

			Cumulative	
Percent of ALL	power	Percent of	power	
1	32490	100.00	32490	100.00

Tables: ALL * CMYN

ALL

CMYN(Administration of Concomitant Drug)

Power | Percent | Percent of rows Percent of columns	None	With	Total
1	1445	31045	32490
	4.45	95.55	100.00
	4.45	95.55	
	100.00	100.00	
Total	1445	31045	32490
	4.45	95.55	100.00

Concomitant medications other than anticoagulants at the time of obtaining Table 2-5 consent, according to the presence or absence of proton-pump inhibitors

FREQ Procedure

Table : ALL * APLD1

ALL APLD1(ASA)

Power / Percent / Percent of rows / Percent of columns	None	With	Total
1	27508	3537	31045
	88.61	11.39	100.00
	88.61	11.39	
	100.00	100.00	
Total	27508	3537	31045
	88.61	11.39	100.00

Concomitant medications other than anticoagulants at the time of obtaining Table 2-5 consent, according to the presence or absence of proton-pump inhibitors

FREQ Procedure

			Cumulative	
Percent of ALL	power	Percent of	power	
1	32490	100.00	32490	100.00

Tables: ALL * CMYN

ALL

CMYN(Administration of Concomitant Drug)

Power |
Percent |
Percent of rows
Percent of columns | None | With | Total

	None	With	Total
1	1445	31045	32490
	4.45	95.55	100.00
	4.45	95.55	
	100.00	100.00	
Total	1445	31045	32490
	4.45	95.55	100.00

Concomitant medications other than anticoagulants at the time of obtaining Table 2-5 consent, according to the presence or absence of proton-pump inhibitors

FREQ Procedure

Table : ALL * APLD2

ALL APLD2(P2Y12-I)

Power |
Percent |
Percent of rows
Percent of columns | None | With | Total

```
______________+________+________+
            1 |  29650 |   1395 |  31045
              |  95.51 |   4.49 | 100.00
              |  95.51 |   4.49 |
              | 100.00 | 100.00 |
______________+________+________+
Total 29650 1395 31045
                 95.51     4.49   100.00
```

Concomitant medications other than anticoagulants at the time of obtaining Table 2-5 consent, according to the presence or absence of proton-pump inhibitors

FREQ Procedure

			Cumulative	
Percent of ALL	power	Percent of power		
1	32490	100.00	32490	100.00

Tables: ALL * CMYN

ALL

CMYN(Administration of Concomitant Drug)

Power |
Percent |
Percent of rows
Percent of columns | None | With | Total

	None	With	Total
1	1445	31045	32490
	4.45	95.55	100.00
	4.45	95.55	
	100.00	100.00	
Total	1445	31045	32490
	4.45	95.55	100.00

Concomitant medications other than anticoagulants at the time of obtaining Table 2-5 consent, according to the presence or absence of proton-pump inhibitors

FREQ Procedure

Table : ALL * APLD2

ALL APLD2(P2Y12-I)

Power |
Percent |
Percent of rows
Percent of columns | None | With | Total

ALL	None	With	Total
1	29650	1395	31045
	95.51	4.49	100.00
	95.51	4.49	
	100.00	100.00	
Total	29650	1395	31045
	95.51	4.49	100.00

Concomitant medications other than anticoagulants at the time of obtaining Table 2-5 consent, according to the presence or absence of proton-pump inhibitors

FREQ Procedure

			Cumulative	
Percent of ALL	power	Percent of	power	
1	32490	100.00	32490	100.00

Tables: ALL * CMYN

ALL

CMYN(Administration of Concomitant Drug)

Power | Percent | Percent of rows Percent of columns	None	With	Total
1	1445	31045	32490
	4.45	95.55	100.00
	4.45	95.55	
	100.00	100.00	
Total	1445	31045	32490
	4.45	95.55	100.00

Concomitant medications other than anticoagulants at the time of obtaining Table 2-5 consent, according to the presence or absence of proton-pump inhibitors

FREQ Procedure

Table : ALL * APLD3

ALL

APLD3(Other Anti-Platelet Drugs)

Power |
Percent |
Percent of rows
Percent of columns | None | With | Total

1	29743	1302	31045
	95.81	4.19	100.00
	95.81	4.19	
	100.00	100.00	
Total	29743	1302	31045
	95.81	4.19	100.00

Concomitant medications other than anticoagulants at the time of obtaining Table 2-5 consent, according to the presence or absence of proton-pump inhibitors

FREQ Procedure

			Cumulative	
Percent of ALL	power	Percent of	power	
1	32490	100.00	32490	100.00

Tables: ALL * CMYN

ALL

CMYN(Administration of Concomitant Drug)

Power | Percent | Percent of rows Percent of columns	None	With	Total
1	1445	31045	32490
	4.45	95.55	100.00
	4.45	95.55	
	100.00	100.00	
Total	1445	31045	32490
	4.45	95.55	100.00

Concomitant medications other than anticoagulants at the time of obtaining Table 2-5 consent, according to the presence or absence of proton-pump inhibitors

FREQ Procedure

Table : ALL * APLD3

ALL

APLD3(Other Anti-Platelet Drugs)

Power |
Percent |
Percent of rows
Percent of columns | None | With | Total

	None	With	Total
1	29743	1302	31045
	95.81	4.19	100.00
	95.81	4.19	
	100.00	100.00	
Total	29743	1302	31045
	95.81	4.19	100.00

Concomitant medications other than anticoagulants at the time of obtaining Table 2-5 consent, according to the presence or absence of proton-pump inhibitors

FREQ Procedure

			Cumulative	
Percent of ALL	power	Percent of	power	
1	32490	100.00	32490	100.00

Tables: ALL * CMYN

ALL

CMYN(Administration of Concomitant Drug)

Power |
Percent |
Percent of rows
Percent of columns | None | With | Total

1	1445	31045	32490
	4.45	95.55	100.00
	4.45	95.55	
	100.00	100.00	
Total	1445	31045	32490
	4.45	95.55	100.00

Concomitant medications other than anticoagulants at the time of obtaining Table 2-5 consent, according to the presence or absence of proton-pump inhibitors

FREQ Procedure

Tables: ALL * AHPD

ALL

AHPD(Antihypertensive Drugs)

Power |
Percent |
Percent of rows
Percent of columns | None | With | Total

Power / Percent / Percent of rows / Percent of columns	None	With	Total
1	7865	23180	31045
	25.33	74.67	100.00
	25.33	74.67	
	100.00	100.00	
Total	7865	23180	31045
	25.33	74.67	100.00

Concomitant medications other than anticoagulants at the time of obtaining Table 2-5 consent, according to the presence or absence of proton-pump inhibitors

FREQ Procedure

			Cumulative	
Percent of ALL	power	Percent of	power	
1	32490	100.00	32490	100.00

Tables: ALL * CMYN

ALL

CMYN(Administration of Concomitant Drug)

Power | Percent | Percent of rows Percent of columns	None	With	Total
1	1445	31045	32490
	4.45	95.55	100.00
	4.45	95.55	
	100.00	100.00	
Total	1445	31045	32490
	4.45	95.55	100.00

Concomitant medications other than anticoagulants at the time of obtaining Table 2-5 consent, according to the presence or absence of proton-pump inhibitors

FREQ Procedure

Tables: ALL * AHPD

ALL

AHPD(Antihypertensive Drugs)

Power |
Percent |
Percent of rows
Percent of columns | None | With | Total

Power / Percent / Percent of rows / Percent of columns	None	With	Total
1	7865	23180	31045
	25.33	74.67	100.00
	25.33	74.67	
	100.00	100.00	
Total	7865	23180	31045
	25.33	74.67	100.00

Concomitant medications other than anticoagulants at the time of obtaining Table 2-5 consent, according to the presence or absence of proton-pump inhibitors

FREQ Procedure

			Cumulative	
Percent of ALL	power	Percent of	power	
1	32490	100.00	32490	100.00

Tables: ALL * CMYN

ALL

CMYN(Administration of Concomitant Drug)

Power<br>Percent<br>Percent of rows<br>Percent of columns	None	With	Total
1	1445<br>4.45<br>4.45<br>100.00	31045<br>95.55<br>95.55<br>100.00	32490<br>100.00
Total	1445<br>4.45	31045<br>95.55	32490<br>100.00

Concomitant medications other than anticoagulants at the time of obtaining Table 2-5 consent, according to the presence or absence of proton-pump inhibitors

FREQ Procedure

Table: ALL * LTD

ALL LTD(Lipid Therapy)

Power |
Percent |
Percent of rows
Percent of columns | None | With | Total

	None	With	Total
1	18916	12129	31045
	60.93	39.07	100.00
	60.93	39.07	
	100.00	100.00	
Total	18916	12129	31045
	60.93	39.07	100.00

Concomitant medications other than anticoagulants at the time of obtaining Table 2-5 consent, according to the presence or absence of proton-pump inhibitors

FREQ Procedure

			Cumulative	
Percent of ALL	power	Percent of	power	
1	32490	100.00	32490	100.00

Tables: ALL * CMYN

ALL

CMYN(Administration of Concomitant Drug)

Power / Percent / Percent of rows / Percent of columns	None	With	Total
1	1445	31045	32490
	4.45	95.55	100.00
	4.45	95.55	
	100.00	100.00	
Total	1445	31045	32490
	4.45	95.55	100.00

Concomitant medications other than anticoagulants at the time of obtaining Table 2-5 consent, according to the presence or absence of proton-pump inhibitors

FREQ Procedure

Table: ALL * LTD

ALL LTD(Lipid Therapy)

Power |
Percent |
Percent of rows
Percent of columns | None | With | Total

	None	With	Total
1	18916	12129	31045
	60.93	39.07	100.00
	60.93	39.07	
	100.00	100.00	
Total	18916	12129	31045
	60.93	39.07	100.00

Concomitant medications other than anticoagulants at the time of obtaining Table 2-5 consent, according to the presence or absence of proton-pump inhibitors

FREQ Procedure

			Cumulative	
Percent of ALL	power	Percent of power		
1	32490	100.00	32490	100.00

Tables: ALL * CMYN

ALL

CMYN(Administration of Concomitant Drug)

Power / Percent / Percent of rows / Percent of columns	None	With	Total
1	1445	31045	32490
	4.45	95.55	100.00
	4.45	95.55	
	100.00	100.00	
Total	1445	31045	32490
	4.45	95.55	100.00

Concomitant medications other than anticoagulants at the time of obtaining Table 2-5 consent, according to the presence or absence of proton-pump inhibitors

FREQ Procedure

Table : ALL * LTD1

ALL LTD1(Statins)

Power<br>Percent<br>Percent of rows<br>Percent of columns	None	With	Total
1	20182<br>65.01<br>65.01<br>100.00	10863<br>34.99<br>34.99<br>100.00	31045<br>100.00
Total	20182<br>65.01	10863<br>34.99	31045<br>100.00

Concomitant medications other than anticoagulants at the time of obtaining Table 2-5 consent, according to the presence or absence of proton-pump inhibitors

FREQ Procedure

			Cumulative	
Percent of ALL	power	Percent of	power	
1	32490	100.00	32490	100.00

Tables: ALL * CMYN

ALL

CMYN(Administration of Concomitant Drug)

Power |

Percent |

Percent of rows

Percent of columns | None | With | Total

	None	With	Total
1	1445	31045	32490
	4.45	95.55	100.00
	4.45	95.55	
	100.00	100.00	
Total	1445	31045	32490
	4.45	95.55	100.00

Concomitant medications other than anticoagulants at the time of obtaining Table 2-5 consent, according to the presence or absence of proton-pump inhibitors

FREQ Procedure

Table : ALL * LTD1

ALL LTD1(Statins)

Power<br>Percent<br>Percent of rows<br>Percent of columns	None	With	Total
1	20182<br>65.01<br>65.01<br>100.00	10863<br>34.99<br>34.99<br>100.00	31045<br>100.00
Total	20182<br>65.01	10863<br>34.99	31045<br>100.00

Concomitant medications other than anticoagulants at the time of obtaining Table 2-5 consent, according to the presence or absence of proton-pump inhibitors

FREQ Procedure

			Cumulative	
Percent of ALL	power	Percent of	power	
1	32490	100.00	32490	100.00

Tables: ALL * CMYN

ALL

CMYN(Administration of Concomitant Drug)

Power | Percent | Percent of rows Percent of columns	None	With	Total
1	1445	31045	32490
	4.45	95.55	100.00
	4.45	95.55	
	100.00	100.00	
Total	1445	31045	32490
	4.45	95.55	100.00

Concomitant medications other than anticoagulants at the time of obtaining Table 2-5 consent, according to the presence or absence of proton-pump inhibitors

FREQ Procedure

Table : ALL * LTD2

ALL LTD2(Other Than Statins)

Power |
Percent |
Percent of rows
Percent of columns | None | With | Total

	None	With	Total
1	29323	1722	31045
	94.45	5.55	100.00
	94.45	5.55	
	100.00	100.00	
Total	29323	1722	31045
	94.45	5.55	100.00

Concomitant medications other than anticoagulants at the time of obtaining Table 2-5 consent, according to the presence or absence of proton-pump inhibitors

FREQ Procedure

			Cumulative	
Percent of ALL	power	Percent of	power	
1	32490	100.00	32490	100.00

Tables: ALL * CMYN

ALL
CMYN(Administration of Concomitant Drug)

Power / Percent / Percent of rows / Percent of columns	None	With	Total
1	1445	31045	32490
	4.45	95.55	100.00
	4.45	95.55	
	100.00	100.00	
Total	1445	31045	32490
	4.45	95.55	100.00

Concomitant medications other than anticoagulants at the time of obtaining Table 2-5 consent, according to the presence or absence of proton-pump inhibitors

FREQ Procedure

Table : ALL * LTD2

ALL LTD2(Other Than Statins)

Power<br>Percent<br>Percent of rows<br>Percent of columns	None	With	Total
1	29323<br>94.45<br>94.45<br>100.00	1722<br>5.55<br>5.55<br>100.00	31045<br>100.00
Total	29323<br>94.45	1722<br>5.55	31045<br>100.00

Concomitant medications other than anticoagulants at the time of obtaining Table 2-5 consent, according to the presence or absence of proton-pump inhibitors

FREQ Procedure

			Cumulative	
Percent of ALL	power	Percent of	power	
1	32490	100.00	32490	100.00

Tables: ALL * CMYN

ALL

CMYN(Administration of Concomitant Drug)

Power | Percent | Percent of rows Percent of columns	None	With	Total
1	1445	31045	32490
	4.45	95.55	100.00
	4.45	95.55	
	100.00	100.00	
Total	1445	31045	32490
	4.45	95.55	100.00

Concomitant medications other than anticoagulants at the time of obtaining Table 2-5 consent, according to the presence or absence of proton-pump inhibitors

FREQ Procedure

Tables: ALL * DBTD

ALL DBTD(Diabetes Drugs)

Power |
Percent |
Percent of rows
Percent of columns | None | With | Total

	None	With	Total
1	25814	5231	31045
	83.15	16.85	100.00
	83.15	16.85	
	100.00	100.00	
Total	25814	5231	31045
	83.15	16.85	100.00

Concomitant medications other than anticoagulants at the time of obtaining Table 2-5 consent, according to the presence or absence of proton-pump inhibitors

FREQ Procedure

			Cumulative	
Percent of ALL	power	Percent of	power	
1	32490	100.00	32490	100.00

Tables: ALL * CMYN

ALL

CMYN(Administration of Concomitant Drug)

Power<br>Percent<br>Percent of rows<br>Percent of columns	None	With	Total
1	1445<br>4.45<br>4.45<br>100.00	31045<br>95.55<br>95.55<br>100.00	32490<br>100.00
Total	1445<br>4.45	31045<br>95.55	32490<br>100.00

Concomitant medications other than anticoagulants at the time of obtaining Table 2-5 consent, according to the presence or absence of proton-pump inhibitors

FREQ Procedure

Tables: ALL * DBTD

ALL DBTD(Diabetes Drugs)

Power |
Percent |
Percent of rows
Percent of columns | None | With | Total

	None	With	Total
1	25814	5231	31045
	83.15	16.85	100.00
	83.15	16.85	
	100.00	100.00	
Total	25814	5231	31045
	83.15	16.85	100.00

Concomitant medications other than anticoagulants at the time of obtaining Table 2-5 consent, according to the presence or absence of proton-pump inhibitors

FREQ Procedure

			Cumulative	
Percent of ALL	power	Percent of power		
1	32490	100.00	32490	100.00

Tables: ALL * CMYN

ALL

CMYN(Administration of Concomitant Drug)

Power | Percent | Percent of rows Percent of columns	None	With	Total
1	1445	31045	32490
	4.45	95.55	100.00
	4.45	95.55	
	100.00	100.00	
Total	1445	31045	32490
	4.45	95.55	100.00

Concomitant medications other than anticoagulants at the time of obtaining Table 2-5 consent, according to the presence or absence of proton-pump inhibitors

FREQ Procedure

Table: ALL * t_DBT

ALL t_DBT

Power |
Percent |
Percent of rows
Percentage of columns | 0| 1| Sum

1	26081	4964	31045
	84.01	15.99	100.00
	84.01	15.99	
	100.00	100.00	
Total	26081	4964	31045
	84.01	15.99	100.00

Concomitant medications other than anticoagulants at the time of obtaining Table 2-5 consent, according to the presence or absence of proton-pump inhibitors

FREQ Procedure

			Cumulative	
Percent of ALL	power	Percent of	power	
1	32490	100.00	32490	100.00

Tables: ALL * CMYN

ALL

CMYN(Administration of Concomitant Drug)

Power | Percent | Percent of rows Percent of columns	None	With	Total
1	1445	31045	32490
	4.45	95.55	100.00
	4.45	95.55	
	100.00	100.00	
Total	1445	31045	32490
	4.45	95.55	100.00

Concomitant medications other than anticoagulants at the time of obtaining Table 2-5 consent, according to the presence or absence of proton-pump inhibitors

FREQ Procedure

Table: ALL * t_DBT

ALL t_DBT

Power |
Percent |
Percent of rows
Percentage of columns | 0| 1| Sum

1	26081	4964	31045
	84.01	15.99	100.00
	84.01	15.99	
	100.00	100.00	
Total	26081	4964	31045
	84.01	15.99	100.00

Concomitant medications other than anticoagulants at the time of obtaining Table 2-5 consent, according to the presence or absence of proton-pump inhibitors

FREQ Procedure

			Cumulative	
Percent of ALL	power	Percent of	power	
1	32490	100.00	32490	100.00

Tables: ALL * CMYN

ALL

CMYN(Administration of Concomitant Drug)

Power<br>Percent<br>Percent of rows<br>Percent of columns	None	With	Total
1	1445<br>4.45<br>4.45<br>100.00	31045<br>95.55<br>95.55<br>100.00	32490<br>100.00
Total	1445<br>4.45	31045<br>95.55	32490<br>100.00

Concomitant medications other than anticoagulants at the time of obtaining Table 2-5 consent, according to the presence or absence of proton-pump inhibitors

FREQ Procedure

Table : ALL * DBTD1

ALL DBTD1(Sulfonylurea Drugs)

Power |
Percent |
Percent of rows
Percent of columns | None | With | Total

1	29824	1221	31045
	96.07	3.93	100.00
	96.07	3.93	
	100.00	100.00	
Total	29824	1221	31045
	96.07	3.93	100.00

Concomitant medications other than anticoagulants at the time of obtaining Table 2-5 consent, according to the presence or absence of proton-pump inhibitors

FREQ Procedure

			Cumulative	
Percent of ALL	power	Percent of power		
1	32490	100.00	32490	100.00

Tables: ALL * CMYN

ALL

CMYN(Administration of Concomitant Drug)

Power |
Percent |
Percent of rows
Percent of columns | None | With | Total

	None	With	Total
1	1445	31045	32490
	4.45	95.55	100.00
	4.45	95.55	
	100.00	100.00	
Total	1445	31045	32490
	4.45	95.55	100.00

Concomitant medications other than anticoagulants at the time of obtaining Table 2-5 consent, according to the presence or absence of proton-pump inhibitors

FREQ Procedure

Table : ALL * DBTD1

ALL DBTD1(Sulfonylurea Drugs)

Power |
Percent |
Percent of rows
Percent of columns | None | With | Total

1	29824	1221	31045
	96.07	3.93	100.00
	96.07	3.93	
	100.00	100.00	
Total	29824	1221	31045
	96.07	3.93	100.00

Concomitant medications other than anticoagulants at the time of obtaining Table 2-5 consent, according to the presence or absence of proton-pump inhibitors

FREQ Procedure

			Cumulative	
Percent of ALL	power	Percent of	power	
1	32490	100.00	32490	100.00

Tables: ALL * CMYN

ALL

CMYN(Administration of Concomitant Drug)

Power | Percent | Percent of rows Percent of columns	None	With	Total
1	1445	31045	32490
	4.45	95.55	100.00
	4.45	95.55	
	100.00	100.00	
Total	1445	31045	32490
	4.45	95.55	100.00

Concomitant medications other than anticoagulants at the time of obtaining Table 2-5 consent, according to the presence or absence of proton-pump inhibitors

FREQ Procedure

Table : ALL * DBTD2

ALL

DBTD2(α-Glucosidase Inhibitor)

Power / Percent / Percent of rows / Percent of columns	None	With	Total
1	30061	984	31045
	96.83	3.17	100.00
	96.83	3.17	
	100.00	100.00	
Total	30061	984	31045
	96.83	3.17	100.00

Concomitant medications other than anticoagulants at the time of obtaining Table 2-5 consent, according to the presence or absence of proton-pump inhibitors

FREQ Procedure

			Cumulative	
Percent of ALL	power	Percent of	power	
1	32490	100.00	32490	100.00

Tables: ALL * CMYN

ALL

CMYN(Administration of Concomitant Drug)

Power | Percent | Percent of rows Percent of columns	None	With	Total
1	1445	31045	32490
	4.45	95.55	100.00
	4.45	95.55	
	100.00	100.00	
Total	1445	31045	32490
	4.45	95.55	100.00

Concomitant medications other than anticoagulants at the time of obtaining Table 2-5 consent, according to the presence or absence of proton-pump inhibitors

FREQ Procedure

Table : ALL * DBTD2

ALL

DBTD2(α-Glucosidase Inhibitor)

Power |
Percent |
Percent of rows
Percent of columns | None | With | Total

1	30061	984	31045
	96.83	3.17	100.00
	96.83	3.17	
	100.00	100.00	
Total	30061	984	31045
	96.83	3.17	100.00

Concomitant medications other than anticoagulants at the time of obtaining Table 2-5 consent, according to the presence or absence of proton-pump inhibitors

FREQ Procedure

			Cumulative	
Percent of ALL	power	Percent of power		
1	32490	100.00	32490	100.00

Tables: ALL * CMYN

ALL

CMYN(Administration of Concomitant Drug)

Power |
Percent |
Percent of rows
Percent of columns | None | With | Total

	None	With	Total
1	1445	31045	32490
	4.45	95.55	100.00
	4.45	95.55	
	100.00	100.00	
Total	1445	31045	32490
	4.45	95.55	100.00

Concomitant medications other than anticoagulants at the time of obtaining Table 2-5 consent, according to the presence or absence of proton-pump inhibitors

FREQ Procedure

Table : ALL * DBTD3

ALL DBTD3(Thiazolidine)

Power<br>Percent<br>Percent of rows<br>Percent of columns	None	With	Total
1	30674<br>98.80<br>98.80<br>100.00	371<br>1.20<br>1.20<br>100.00	31045<br>100.00
Total	30674<br>98.80	371<br>1.20	31045<br>100.00

Concomitant medications other than anticoagulants at the time of obtaining Table 2-5 consent, according to the presence or absence of proton-pump inhibitors

FREQ Procedure

Percent of ALL	power	Percent	Cumulative of power	
1	32490	100.00	32490	100.00

Tables: ALL * CMYN

ALL

CMYN(Administration of Concomitant Drug)

Power<br>Percent<br>Percent of rows<br>Percent of columns	None	With	Total
1	1445<br>4.45<br>4.45<br>100.00	31045<br>95.55<br>95.55<br>100.00	32490<br>100.00
Total	1445<br>4.45	31045<br>95.55	32490<br>100.00

Concomitant medications other than anticoagulants at the time of obtaining Table 2-5 consent, according to the presence or absence of proton-pump inhibitors

FREQ Procedure

Table : ALL * DBTD3

ALL DBTD3(Thiazolidine)

Power<br>Percent<br>Percent of rows<br>Percent of columns	None	With	Total
1	30674<br>98.80<br>98.80<br>100.00	371<br>1.20<br>1.20<br>100.00	31045<br>100.00
Total	30674<br>98.80	371<br>1.20	31045<br>100.00

Concomitant medications other than anticoagulants at the time of obtaining Table 2-5 consent, according to the presence or absence of proton-pump inhibitors

FREQ Procedure

			Cumulative	
Percent of ALL	power	Percent of power		
1	32490	100.00	32490	100.00

Tables: ALL * CMYN

ALL

CMYN(Administration of Concomitant Drug)

Power | Percent | Percent of rows Percent of columns	None	With	Total
1	1445	31045	32490
	4.45	95.55	100.00
	4.45	95.55	
	100.00	100.00	
Total	1445	31045	32490
	4.45	95.55	100.00

Concomitant medications other than anticoagulants at the time of obtaining Table 2-5 consent, according to the presence or absence of proton-pump inhibitors

FREQ Procedure

Table : ALL * DBTD4

ALL DBTD4(DPP-4 Inhabitors)

Power / Percent / Percent of rows / Percent of columns	None	With	Total
1	27220	3825	31045
	87.68	12.32	100.00
	87.68	12.32	
	100.00	100.00	
Total	27220	3825	31045
	87.68	12.32	100.00

Concomitant medications other than anticoagulants at the time of obtaining Table 2-5 consent, according to the presence or absence of proton-pump inhibitors

FREQ Procedure

			Cumulative	
Percent of ALL	power	Percent of	power	
1	32490	100.00	32490	100.00

Tables: ALL * CMYN

ALL

CMYN(Administration of Concomitant Drug)

Power |
Percent |
Percent of rows
Percent of columns | None | With | Total

	None	With	Total
1	1445	31045	32490
	4.45	95.55	100.00
	4.45	95.55	
	100.00	100.00	
Total	1445	31045	32490
	4.45	95.55	100.00

Concomitant medications other than anticoagulants at the time of obtaining Table 2-5 consent, according to the presence or absence of proton-pump inhibitors

FREQ Procedure

Table : ALL * DBTD4

ALL DBTD4(DPP-4 Inhabitors)

Power<br>Percent<br>Percent of rows<br>Percent of columns	None	With	Total
1	27220<br>87.68<br>87.68<br>100.00	3825<br>12.32<br>12.32<br>100.00	31045<br>100.00
Total	27220<br>87.68	3825<br>12.32	31045<br>100.00

Concomitant medications other than anticoagulants at the time of obtaining Table 2-5 consent, according to the presence or absence of proton-pump inhibitors

FREQ Procedure

			Cumulative	
Percent of ALL	power	Percent	of power	
1	32490	100.00	32490	100.00

Tables: ALL * CMYN

ALL

CMYN(Administration of Concomitant Drug)

Power |
Percent |
Percent of rows
Percent of columns | None | With | Total

	None	With	Total
1	1445	31045	32490
	4.45	95.55	100.00
	4.45	95.55	
	100.00	100.00	
Total	1445	31045	32490
	4.45	95.55	100.00

Concomitant medications other than anticoagulants at the time of obtaining Table 2-5 consent, according to the presence or absence of proton-pump inhibitors

FREQ Procedure

Table : ALL * DBTD5

ALL DBTD5(GLP-1 Inhabitors)

Power |
Percent |
Percent of rows
Percent of columns | None | With | Total

Power / Percent / Percent of rows / Percent of columns	None	With	Total
1	30968	77	31045
	99.75	0.25	100.00
	99.75	0.25	
	100.00	100.00	
Total	30968	77	31045
	99.75	0.25	100.00

Concomitant medications other than anticoagulants at the time of obtaining Table 2-5 consent, according to the presence or absence of proton-pump inhibitors

FREQ Procedure

			Cumulative	
Percent of ALL	power	Percent of	power	
1	32490	100.00	32490	100.00

Tables: ALL * CMYN

ALL

CMYN(Administration of Concomitant Drug)

Power | Percent | Percent of rows Percent of columns	None	With	Total
1	1445	31045	32490
	4.45	95.55	100.00
	4.45	95.55	
	100.00	100.00	
Total	1445	31045	32490
	4.45	95.55	100.00

Concomitant medications other than anticoagulants at the time of obtaining Table 2-5 consent, according to the presence or absence of proton-pump inhibitors

FREQ Procedure

Table : ALL * DBTD5

ALL DBTD5(GLP-1 Inhabitors)

Power<br>Percent<br>Percent of rows<br>Percent of columns	None	With	Total
1	30968<br>99.75<br>99.75<br>100.00	77<br>0.25<br>0.25<br>100.00	31045<br>100.00
Total	30968<br>99.75	77<br>0.25	31045<br>100.00

Concomitant medications other than anticoagulants at the time of obtaining Table 2-5 consent, according to the presence or absence of proton-pump inhibitors

FREQ Procedure

			Cumulative	
Percent of ALL	power	Percent of	power	
1	32490	100.00	32490	100.00

Tables: ALL * CMYN

ALL

CMYN(Administration of Concomitant Drug)

Power |
Percent |
Percent of rows
Percent of columns | None | With | Total

	None	With	Total
1	1445	31045	32490
	4.45	95.55	100.00
	4.45	95.55	
	100.00	100.00	
Total	1445	31045	32490
	4.45	95.55	100.00

Concomitant medications other than anticoagulants at the time of obtaining Table 2-5 consent, according to the presence or absence of proton-pump inhibitors

FREQ Procedure

Table : ALL * DBTD6

ALL DBTD6(SGLT2 Inhabitors)

Power<br>Percent<br>Percent of rows<br>Percent of columns	None	With	Total
1	30638<br>98.69<br>98.69<br>100.00	407<br>1.31<br>1.31<br>100.00	31045<br>100.00
Total	30638<br>98.69	407<br>1.31	31045<br>100.00

Concomitant medications other than anticoagulants at the time of obtaining Table 2-5 consent, according to the presence or absence of proton-pump inhibitors

FREQ Procedure

			Cumulative	
Percent of ALL	power	Percent of	power	
1	32490	100.00	32490	100.00

Tables: ALL * CMYN

ALL

CMYN(Administration of Concomitant Drug)

Power | Percent | Percent of rows Percent of columns	None	With	Total
1	1445	31045	32490
	4.45	95.55	100.00
	4.45	95.55	
	100.00	100.00	
Total	1445	31045	32490
	4.45	95.55	100.00

Concomitant medications other than anticoagulants at the time of obtaining Table 2-5 consent, according to the presence or absence of proton-pump inhibitors

FREQ Procedure

Table : ALL * DBTD6

ALL DBTD6(SGLT2 Inhabitors)

Power<br>Percent<br>Percent of rows<br>Percent of columns	None	With	Total
1	30638<br>98.69<br>98.69<br>100.00	407<br>1.31<br>1.31<br>100.00	31045<br>100.00
Total	30638<br>98.69	407<br>1.31	31045<br>100.00

Concomitant medications other than anticoagulants at the time of obtaining Table 2-5 consent, according to the presence or absence of proton-pump inhibitors

FREQ Procedure

ALL	power	Percent	Cumulative power	Cumulative Percent of power
1	32490	100.00	32490	100.00

Tables: ALL * CMYN

ALL

CMYN(Administration of Concomitant Drug)

Power |
Percent |
Percent of rows
Percent of columns | None | With | Total

ALL	None	With	Total
1	1445	31045	32490
	4.45	95.55	100.00
	4.45	95.55	
	100.00	100.00	
Total	1445	31045	32490
	4.45	95.55	100.00

Concomitant medications other than anticoagulants at the time of obtaining Table 2-5 consent, according to the presence or absence of proton-pump inhibitors

FREQ Procedure

Table : ALL * DBTD7

ALL DBTD7(Insuline Related)

Power |
Percent |
Percent of rows
Percent of columns | None | With | Total

	None	With	Total
1	30406	639	31045
	97.94	2.06	100.00
	97.94	2.06	
	100.00	100.00	
Total	30406	639	31045
	97.94	2.06	100.00

Concomitant medications other than anticoagulants at the time of obtaining Table 2-5 consent, according to the presence or absence of proton-pump inhibitors

FREQ Procedure

			Cumulative	
Percent of ALL	power	Percent of	power	
1	32490	100.00	32490	100.00

Tables: ALL * CMYN

ALL

CMYN(Administration of Concomitant Drug)

Power |
Percent |
Percent of rows
Percent of columns | None | With | Total

ALL	None	With	Total
1	1445	31045	32490
	4.45	95.55	100.00
	4.45	95.55	
	100.00	100.00	
Total	1445	31045	32490
	4.45	95.55	100.00

Concomitant medications other than anticoagulants at the time of obtaining Table 2-5 consent, according to the presence or absence of proton-pump inhibitors

FREQ Procedure

Table : ALL * DBTD7

ALL DBTD7(Insuline Related)

Power |
Percent |
Percent of rows
Percent of columns | None | With | Total

	None	With	Total
1	30406	639	31045
	97.94	2.06	100.00
	97.94	2.06	
	100.00	100.00	
Total	30406	639	31045
	97.94	2.06	100.00

Concomitant medications other than anticoagulants at the time of obtaining Table 2-5 consent, according to the presence or absence of proton-pump inhibitors

FREQ Procedure

			Cumulative	
Percent of ALL	power	Percent of power		
1	32490	100.00	32490	100.00

Tables: ALL * CMYN

ALL

CMYN(Administration of Concomitant Drug)

Power | Percent | Percent of rows Percent of columns	None	With	Total
1	1445	31045	32490
	4.45	95.55	100.00
	4.45	95.55	
	100.00	100.00	
Total	1445	31045	32490
	4.45	95.55	100.00

Concomitant medications other than anticoagulants at the time of obtaining Table 2-5 consent, according to the presence or absence of proton-pump inhibitors

FREQ Procedure

Table : ALL * DBTD8

ALL

DBTD8(Other Diabetes Drugs)

Power |
Percent |
Percent of rows
Percent of columns | None | With | Total

ALL	None	With	Total
1	30059	986	31045
	96.82	3.18	100.00
	96.82	3.18	
	100.00	100.00	
Total	30059	986	31045
	96.82	3.18	100.00

Concomitant medications other than anticoagulants at the time of obtaining Table 2-5 consent, according to the presence or absence of proton-pump inhibitors

FREQ Procedure

			Cumulative	
Percent of ALL	power	Percent of	power	
1	32490	100.00	32490	100.00

Tables: ALL * CMYN

ALL

CMYN(Administration of Concomitant Drug)

Power | Percent | Percent of rows Percent of columns	None	With	Total
1	1445	31045	32490
	4.45	95.55	100.00
	4.45	95.55	
	100.00	100.00	
Total	1445	31045	32490
	4.45	95.55	100.00

Concomitant medications other than anticoagulants at the time of obtaining Table 2-5 consent, according to the presence or absence of proton-pump inhibitors

FREQ Procedure

Table : ALL * DBTD8

ALL

DBTD8(Other Diabetes Drugs)

Power | Percent | Percent of rows Percent of columns	None	With	Total
1	30059	986	31045
	96.82	3.18	100.00
	96.82	3.18	
	100.00	100.00	
Total	30059	986	31045
	96.82	3.18	100.00

Concomitant medications other than anticoagulants at the time of obtaining Table 2-5 consent, according to the presence or absence of proton-pump inhibitors

FREQ Procedure

			Cumulative	
Percent of ALL	power	Percent	of power	
1	32490	100.00	32490	100.00

Tables: ALL * CMYN

ALL

CMYN(Administration of Concomitant Drug)

Power |
Percent |
Percent of rows
Percent of columns | None | With | Total

	None	With	Total
1	1445	31045	32490
	4.45	95.55	100.00
	4.45	95.55	
	100.00	100.00	
Total	1445	31045	32490
	4.45	95.55	100.00

Concomitant medications other than anticoagulants at the time of obtaining Table 2-5 consent, according to the presence or absence of proton-pump inhibitors

FREQ Procedure

Table: ALL * DMM

ALL DMM(Dementia Medications)

Power / Percent / Percent of rows / Percent of columns	None	With	Total
1	29842	1203	31045
	96.12	3.88	100.00
	96.12	3.88	
	100.00	100.00	
Total	29842	1203	31045
	96.12	3.88	100.00

Concomitant medications other than anticoagulants at the time of obtaining Table 2-5 consent, according to the presence or absence of proton-pump inhibitors

FREQ Procedure

			Cumulative	
Percent of ALL	power	Percent of	power	
1	32490	100.00	32490	100.00

Tables: ALL * CMYN

ALL

CMYN(Administration of Concomitant Drug)

Power / Percent / Percent of rows / Percent of columns	None	With	Total
1	1445	31045	32490
	4.45	95.55	100.00
	4.45	95.55	
	100.00	100.00	
Total	1445	31045	32490
	4.45	95.55	100.00

Concomitant medications other than anticoagulants at the time of obtaining Table 2-5 consent, according to the presence or absence of proton-pump inhibitors

FREQ Procedure

Table: ALL * DMM

ALL DMM(Dementia Medications)

Power / Percent / Percent of rows / Percent of columns	None	With	Total
1	29842	1203	31045
	96.12	3.88	100.00
	96.12	3.88	
	100.00	100.00	
Total	29842	1203	31045
	96.12	3.88	100.00

Concomitant medications other than anticoagulants at the time of obtaining Table 2-5 consent, according to the presence or absence of proton-pump inhibitors

FREQ Procedure

			Cumulative	
Percent of ALL	power	Percent of	power	
1	32490	100.00	32490	100.00

Tables: ALL * CMYN

ALL
CMYN(Administration of Concomitant Drug)

Power |
Percent |
Percent of rows
Percent of columns | None | With | Total

	None	With	Total
1	1445	31045	32490
	4.45	95.55	100.00
	4.45	95.55	
	100.00	100.00	
Total	1445	31045	32490
	4.45	95.55	100.00

Concomitant medications other than anticoagulants at the time of obtaining Table 2-5 consent, according to the presence or absence of proton-pump inhibitors

FREQ Procedure

Tables: ALL* ACNA

ALL ACNA(Anti-Cancer Agent)

Power |
Percent |
Percent of rows
Percent of columns | None | With | Total

1	30756	289	31045
	99.07	0.93	100.00
	99.07	0.93	
	100.00	100.00	
Total	30756	289	31045
	99.07	0.93	100.00

Concomitant medications other than anticoagulants at the time of obtaining Table 2-5 consent, according to the presence or absence of proton-pump inhibitors

FREQ Procedure

ALL	power	Percent	Cumulative power	Cumulative Percent of power
1	32490	100.00	32490	100.00

Tables: ALL * CMYN

ALL
CMYN(Administration of Concomitant Drug)

Power |
Percent |
Percent of rows
Percent of columns | None | With | Total

	None	With	Total
1	1445	31045	32490
	4.45	95.55	100.00
	4.45	95.55	
	100.00	100.00	
Total	1445	31045	32490
	4.45	95.55	100.00

Concomitant medications other than anticoagulants at the time of obtaining Table 2-5 consent, according to the presence or absence of proton-pump inhibitors

FREQ Procedure

Tables: ALL* ACNA

ALL ACNA(Anti-Cancer Agent)

Power |
Percent |
Percent of rows
Percent of columns | None | With | Total

1	30756	289	31045
	99.07	0.93	100.00
	99.07	0.93	
	100.00	100.00	
Total	30756	289	31045
	99.07	0.93	100.00

Concomitant medications other than anticoagulants at the time of obtaining Table 2-5 consent, according to the presence or absence of proton-pump inhibitors

FREQ Procedure

			Cumulative	
Percent of ALL	power	Percent of	power	
1	32490	100.00	32490	100.00

Tables: ALL * CMYN

ALL

CMYN(Administration of Concomitant Drug)

Power |
Percent |
Percent of rows
Percent of columns | None | With | Total

	None	With	Total
1	1445	31045	32490
	4.45	95.55	100.00
	4.45	95.55	
	100.00	100.00	
Total	1445	31045	32490
	4.45	95.55	100.00

Concomitant medications other than anticoagulants at the time of obtaining Table 2-5 consent, according to the presence or absence of proton-pump inhibitors

FREQ Procedure

Tables: ALL* COPD

ALL COPD(COPD)

Power |
Percent |
Percent of rows
Percent of columns | None | With | Total

1	30206	839	31045
	97.30	2.70	100.00
	97.30	2.70	
	100.00	100.00	
Total	30206	839	31045
	97.30	2.70	100.00

Concomitant medications other than anticoagulants at the time of obtaining Table 2-5 consent, according to the presence or absence of proton-pump inhibitors

FREQ Procedure

			Cumulative	
Percent of ALL	power	Percent of	power	
1	32490	100.00	32490	100.00

Tables: ALL * CMYN

ALL

CMYN(Administration of Concomitant Drug)

Power | Percent | Percent of rows Percent of columns	None	With	Total
1	1445	31045	32490
	4.45	95.55	100.00
	4.45	95.55	
	100.00	100.00	
Total	1445	31045	32490
	4.45	95.55	100.00

Concomitant medications other than anticoagulants at the time of obtaining Table 2-5 consent, according to the presence or absence of proton-pump inhibitors

FREQ Procedure

Tables: ALL* COPD

ALL COPD(COPD)

Power<br>Percent<br>Percent of rows<br>Percent of columns	None	With	Total
1	30206<br>97.30<br>97.30<br>100.00	839<br>2.70<br>2.70<br>100.00	31045<br>100.00
Total	30206<br>97.30	839<br>2.70	31045<br>100.00

Concomitant medications other than anticoagulants at the time of obtaining Table 2-5 consent, according to the presence or absence of proton-pump inhibitors

FREQ Procedure

			Cumulative	
Percent of ALL power Percent of power				
1	32490	100.00	32490	100.00

Tables: ALL * CMYN

ALL

CMYN(Administration of Concomitant Drug)

Power | Percent | Percent of rows Percent of columns	None	With	Total
1	1445	31045	32490
	4.45	95.55	100.00
	4.45	95.55	
	100.00	100.00	
Total	1445	31045	32490
	4.45	95.55	100.00

Concomitant medications other than anticoagulants at the time of obtaining Table 2-5 consent, according to the presence or absence of proton-pump inhibitors

FREQ Procedure

Tables: ALL * PSYD

ALL PSYD(Psychotropic Drugs)

Power<br>Percent<br>Percent of rows<br>Percent of columns	None	With	Total
1	28522<br>91.87<br>91.87<br>100.00	2523<br>8.13<br>8.13<br>100.00	31045<br>100.00
Total	28522<br>91.87	2523<br>8.13	31045<br>100.00

Concomitant medications other than anticoagulants at the time of obtaining Table 2-5 consent, according to the presence or absence of proton-pump inhibitors

FREQ Procedure

			Cumulative	
Percent of ALL	power	Percent of power		
1	32490	100.00	32490	100.00

Tables: ALL * CMYN

ALL

CMYN(Administration of Concomitant Drug)

Power / Percent / Percent of rows / Percent of columns	None	With	Total
1	1445	31045	32490
	4.45	95.55	100.00
	4.45	95.55	
	100.00	100.00	
Total	1445	31045	32490
	4.45	95.55	100.00

Concomitant medications other than anticoagulants at the time of obtaining Table 2-5 consent, according to the presence or absence of proton-pump inhibitors

FREQ Procedure

Tables: ALL * PSYD

ALL PSYD(Psychotropic Drugs)

Power / Percent / Percent of rows / Percent of columns	None	With	Total
1	28522	2523	31045
	91.87	8.13	100.00
	91.87	8.13	
	100.00	100.00	
Total	28522	2523	31045
	91.87	8.13	100.00

Concomitant medications other than anticoagulants at the time of obtaining Table 2-5 consent, according to the presence or absence of proton-pump inhibitors

FREQ Procedure

			Cumulative	
Percent of ALL	power	Percent of	power	
1	32490	100.00	32490	100.00

Tables: ALL * CMYN

ALL

CMYN(Administration of Concomitant Drug)

Power |
Percent |
Percent of rows
Percent of columns | None | With | Total

	None	With	Total
1	1445	31045	32490
	4.45	95.55	100.00
	4.45	95.55	
	100.00	100.00	
Total	1445	31045	32490
	4.45	95.55	100.00

Concomitant medications other than anticoagulants at the time of obtaining Table 2-5 consent, according to the presence or absence of proton-pump inhibitors

FREQ Procedure

Tables: ALL* PRPI

ALL

PRPI(Proton Pump Inhabitors)

Power |
Percent |
Percent of rows
Percent of columns | None | With | Total

1	19064	11981	31045
	61.41	38.59	100.00
	61.41	38.59	
	100.00	100.00	
Total	19064	11981	31045
	61.41	38.59	100.00

Concomitant medications other than anticoagulants at the time of obtaining Table 2-5 consent, according to the presence or absence of proton-pump inhibitors

FREQ Procedure

			Cumulative	
Percent of ALL	power	Percent of power		
1	32490	100.00	32490	100.00

Tables: ALL * CMYN

ALL

CMYN(Administration of Concomitant Drug)

Power |
Percent |
Percent of rows
Percent of columns | None | With | Total

	None	With	Total
1	1445	31045	32490
	4.45	95.55	100.00
	4.45	95.55	
	100.00	100.00	
Total	1445	31045	32490
	4.45	95.55	100.00

Concomitant medications other than anticoagulants at the time of obtaining Table 2-5 consent, according to the presence or absence of proton-pump inhibitors

FREQ Procedure

Tables: ALL* PRPI

ALL

PRPI(Proton Pump Inhabitors)

Power / Percent / Percent of rows / Percent of columns	None	With	Total
1	19064	11981	31045
	61.41	38.59	100.00
	61.41	38.59	
	100.00	100.00	
Total	19064	11981	31045
	61.41	38.59	100.00

Concomitant medications other than anticoagulants at the time of obtaining Table 2-5 consent, according to the presence or absence of proton-pump inhibitors

FREQ Procedure

			Cumulative	
Percent of ALL	power	Percent of	power	
1	32490	100.00	32490	100.00

Tables: ALL * CMYN

ALL

CMYN(Administration of Concomitant Drug)

Power |
Percent |
Percent of rows
Percent of columns | None | With | Total

	None	With	Total
1	1445	31045	32490
	4.45	95.55	100.00
	4.45	95.55	
	100.00	100.00	
Total	1445	31045	32490
	4.45	95.55	100.00

Concomitant medications other than anticoagulants at the time of obtaining Table 2-5 consent, according to the presence or absence of proton-pump inhibitors

FREQ Procedure

Tables: ALL* PGPI

ALL PGPI(P-gp Inhabitors)

Power |
Percent |
Percent of rows
Percent of columns | None | With | Total

	None	With	Total
1	30503	542	31045
	98.25	1.75	100.00
	98.25	1.75	
	100.00	100.00	
Total	30503	542	31045
	98.25	1.75	100.00

Concomitant medications other than anticoagulants at the time of obtaining Table 2-5 consent, according to the presence or absence of proton-pump inhibitors

FREQ Procedure

			Cumulative	
Percent of ALL	power	Percent of power		
1	32490	100.00	32490	100.00

Tables: ALL * CMYN

ALL

CMYN(Administration of Concomitant Drug)

Power |
Percent |
Percent of rows
Percent of columns | None | With | Total

	None	With	Total
1	1445	31045	32490
	4.45	95.55	100.00
	4.45	95.55	
	100.00	100.00	
Total	1445	31045	32490
	4.45	95.55	100.00

Concomitant medications other than anticoagulants at the time of obtaining Table 2-5 consent, according to the presence or absence of proton-pump inhibitors

FREQ Procedure

Tables: ALL* PGPI

ALL PGPI(P-gp Inhabitors)

Power |
Percent |
Percent of rows
Percent of columns | None | With | Total

1	30503	542	31045
	98.25	1.75	100.00
	98.25	1.75	
	100.00	100.00	
Total	30503	542	31045
	98.25	1.75	100.00

Concomitant medications other than anticoagulants at the time of obtaining Table 2-5 consent, according to the presence or absence of proton-pump inhibitors

FREQ Procedure

			Cumulative	
Percent of ALL	power	Percent of	power	
1	32490	100.00	32490	100.00

Tables: ALL * CMYN

ALL

CMYN(Administration of Concomitant Drug)

Power |
Percent |
Percent of rows
Percent of columns | None | With | Total

	None	With	Total
1	1445	31045	32490
	4.45	95.55	100.00
	4.45	95.55	
	100.00	100.00	
Total	1445	31045	32490
	4.45	95.55	100.00

Concomitant medications other than anticoagulants at the time of obtaining Table 2-5 consent, according to the presence or absence of proton-pump inhibitors

FREQ Procedure

Tables: ALL * TCMCNT

ALL TCMCNT(Total Number of Drugs Other than Anticoagulant)

Power |
Percent |
Percent of rows
Percent|0|1|2|3|4|5|6|7|8|9| Total
______________+________+________+________+________+________+________+________
_+________+________+________+
1 | 52 | 688 | 1602 | 2622 | 3597 | 4105 | 4171 | 3747 | 3187 | 2516 | 31831
| 0.16 | 2.16 | 5.03 | 8.24 | 11.30 | 12.90 | 13.10 | 11.77 | 10.01 | 7.90 | 100.00
| 0.16 | 2.16 | 5.03 | 8.24 | 11.30 | 12.90 | 13.10 | 11.77 | 10.01 | 7.90 |
| 100.00 | 100.00 | 100.00 | 100.00 | 100.00 | 100.00 | 100.00 | 100.00 | 100.00 | 100.00 |
______________+________+________+________+________+________+________+________
_+________+________+________+
Total 52 688 1602 2622 3597 4105 4171 3747 3187 2516 31831
0.16 2.16 5.03 8.24 11.30 12.90 13.10 11.77 10.01 7.90 100.00
(continued)

Tables: ALL * TCMCNT

ALL TCMCNT(Total Number of Drugs Other than Anticoagulant)

Power |
Percent |

Percent of rows

Percent |10|11|12|13|14|15|16|17|18|19|Total

______________+________+________+________+________+________+________+________
_+________+________+________+

1 | 1825 | 1360 | 881 | 598 | 366 | 206 | 121 | 79 | 47 |
24 | 31831

| 5.73 | 4.27 | 2.77 | 1.88 | 1.15 | 0.65 | 0.38 | 0.25 | 0.15 |
0.08 | 100.00

| 5.73 | 4.27 | 2.77 | 1.88 | 1.15 | 0.65 | 0.38 | 0.25 | 0.15 |
0.08 |

| 100.00 | 100.00 | 100.00 | 100.00 | 100.00 | 100.00 | 100.00 | 100.00 | 100.00 |
100.00 |

______________+________+________+________+________+________+________+________
_+________+________+________+

Total 1825 1360 881 598 366 206 121 79 47 24 31831

5.73 4.27 2.77 1.88 1.15 0.65 0.38 0.25
0.15 0.08 100.00

(continued)

Concomitant medications other than anticoagulants at the time of obtaining Table 2-5 consent, according to the presence or absence of proton-pump inhibitors

FREQ Procedure

Tables: ALL * TCMCNT

ALL TCMCNT(Total Number of Drugs Other than Anticoagulant)

Power |
Percent |
Percent of rows
Percent | 20| 21| 22| 23| 24| 25| 26| 27| Total

1	19	6	2	5	1	1	2	1	31831
	0.06	0.02	0.01	0.02	0.00	0.00	0.01	0.00	100.00
	0.06	0.02	0.01	0.02	0.00	0.00	0.01	0.00	
	100.00	100.00	100.00	100.00	100.00	100.00	100.00	100.00	
Total	19	6	2	5	1	1	2	1	31831
	0.06	0.02	0.01	0.02	0.00	0.00	0.01	0.00	100.00

Degree of missing values = 659

Concomitant medications other than anticoagulants at the time of obtaining Table 2-5 consent, according to the presence or absence of proton-pump inhibitors

FREQ Procedure

			Cumulative	
Percent of ALL	power	Percent of	power	
1	32490	100.00	32490	100.00

Tables: ALL * CMYN

ALL

CMYN(Administration of Concomitant Drug)

Power |
Percent |
Percent of rows
Percent of columns | None | With | Total

	None	With	Total
1	1445	31045	32490
	4.45	95.55	100.00
	4.45	95.55	
	100.00	100.00	
Total	1445	31045	32490
	4.45	95.55	100.00

Concomitant medications other than anticoagulants at the time of obtaining Table 2-5 consent, according to the presence or absence of proton-pump inhibitors

FREQ Procedure

Tables: ALL * TCMCNT

ALL TCMCNT(Total Number of Drugs Other than Anticoagulant)

Power |
Percent |
Percent of rows
Percent|0|1|2|3|4|5|6|7|8|9| Total
______________+________+________+________+________+________+________+________
_+________+________+________+
1 | 52 | 688 | 1602 | 2622 | 3597 | 4105 | 4171 | 3747 | 3187 | 2516 | 31831
| 0.16 | 2.16 | 5.03 | 8.24 | 11.30 | 12.90 | 13.10 | 11.77 | 10.01 | 7.90 | 100.00
| 0.16 | 2.16 | 5.03 | 8.24 | 11.30 | 12.90 | 13.10 | 11.77 | 10.01 | 7.90 |
| 100.00 | 100.00 | 100.00 | 100.00 | 100.00 | 100.00 | 100.00 | 100.00 | 100.00 | 100.00 |
______________+________+________+________+________+________+________+________
_+________+________+________+
Total 52 688 1602 2622 3597 4105 4171 3747 3187 2516 31831
0.16 2.16 5.03 8.24 11.30 12.90 13.10 11.77 10.01 7.90 100.00
(continued)

Tables: ALL * TCMCNT

ALL TCMCNT(Total Number of Drugs Other than Anticoagulant)

Power |
Percent |

Percent of rows

Percent |10|11|12|13|14|15|16|17|18|19|Total

______________+________+________+________+________+________+________+_______
_+________+________+________+

1 | 1825 | 1360 | 881 | 598 | 366 | 206 | 121 | 79 | 47 |
24 | 31831

| 5.73 | 4.27 | 2.77 | 1.88 | 1.15 | 0.65 | 0.38 | 0.25 | 0.15 |
0.08 | 100.00

| 5.73 | 4.27 | 2.77 | 1.88 | 1.15 | 0.65 | 0.38 | 0.25 | 0.15 |
0.08 |

| 100.00 | 100.00 | 100.00 | 100.00 | 100.00 | 100.00 | 100.00 | 100.00 | 100.00 |
100.00 |

______________+________+________+________+________+________+________+_______
_+________+________+________+

Total 1825 1360 881 598 366 206 121 79 47 24 31831

5.73 4.27 2.77 1.88 1.15 0.65 0.38 0.25
0.15 0.08 100.00

(continued)

Concomitant medications other than anticoagulants at the time of obtaining Table 2-5 consent, according to the presence or absence of proton-pump inhibitors

FREQ Procedure

Tables: ALL * TCMCNT

ALL TCMCNT(Total Number of Drugs Other than Anticoagulant)

Power<br>Percent<br>Percent of rows<br>Percent	20	21	22	23	24	25	26	27	Total
1	19<br>0.06<br>0.06<br>100.00	6<br>0.02<br>0.02<br>100.00	2<br>0.01<br>0.01<br>100.00	5<br>0.02<br>0.02<br>100.00	1<br>0.00<br>0.00<br>100.00	1<br>0.00<br>0.00<br>100.00	2<br>0.01<br>0.01<br>100.00	1<br>0.00<br>0.00<br>100.00	31831<br>100.00
Total	19<br>0.06	6<br>0.02	2<br>0.01	5<br>0.02	1<br>0.00	1<br>0.00	2<br>0.01	1<br>0.00	31831<br>100.00

Degree of missing values = 659

Concomitant medications other than anticoagulants at the time of obtaining Table 2-5 consent, according to the presence or absence of proton-pump inhibitors

FREQ Procedure

			Cumulative	
Percent of ALL	power	Percent of	power	
1	32490	100.00	32490	100.00

Tables: ALL * CMYN

ALL

CMYN(Administration of Concomitant Drug)

Power | Percent | Percent of rows Percent of columns	None	With	Total
1	1445	31045	32490
	4.45	95.55	100.00
	4.45	95.55	
	100.00	100.00	
Total	1445	31045	32490
	4.45	95.55	100.00

Concomitant medications other than anticoagulants at the time of obtaining Table 2-5 consent, according to the presence or absence of proton-pump inhibitors

FREQ Procedure

Tables: ALL * TCMCNT

ALL TCMCNT(Total Number of Drugs Other than Anticoagulant)

Power |
Percent |
Percent of rows
Percent|0|1|2|3|4|5|6|7|8|9| Total
______________+________+________+________+________+________+________+________
_+________+________+________+
1 | 52 | 688 | 1602 | 2622 | 3597 | 4105 | 4171 | 3747 | 3187 | 2516 | 31831
| 0.16 | 2.16 | 5.03 | 8.24 | 11.30 | 12.90 | 13.10 | 11.77 | 10.01 | 7.90 | 100.00
| 0.16 | 2.16 | 5.03 | 8.24 | 11.30 | 12.90 | 13.10 | 11.77 | 10.01 | 7.90 |
| 100.00 | 100.00 | 100.00 | 100.00 | 100.00 | 100.00 | 100.00 | 100.00 | 100.00 | 100.00 |
______________+________+________+________+________+________+________+________
_+________+________+________+
Total 52 688 1602 2622 3597 4105 4171 3747 3187 2516 31831
0.16 2.16 5.03 8.24 11.30 12.90 13.10 11.77 10.01 7.90 100.00
(continued)

Tables: ALL * TCMCNT

ALL TCMCNT(Total Number of Drugs Other than Anticoagulant)

Power |
Percent |

Percent of rows

Percent |10|11|12|13|14|15|16|17|18|19|Total

______________+________+________+________+________+________+________+_______
_+________+________+________+

1 | 1825 | 1360 | 881 | 598 | 366 | 206 | 121 | 79 | 47 |
24 | 31831

| 5.73 | 4.27 | 2.77 | 1.88 | 1.15 | 0.65 | 0.38 | 0.25 | 0.15 |
0.08 | 100.00

| 5.73 | 4.27 | 2.77 | 1.88 | 1.15 | 0.65 | 0.38 | 0.25 | 0.15 |
0.08 |

| 100.00 | 100.00 | 100.00 | 100.00 | 100.00 | 100.00 | 100.00 | 100.00 | 100.00 |
100.00 |

______________+________+________+________+________+________+________+_______
_+________+________+________+

Total 1825 1360 881 598 366 206 121 79 47 24 31831

5.73 4.27 2.77 1.88 1.15 0.65 0.38 0.25
0.15 0.08 100.00

(continued)

Concomitant medications other than anticoagulants at the time of obtaining Table 2-5 consent, according to the presence or absence of proton-pump inhibitors

FREQ Procedure

Tables: ALL * TCMCNT

ALL TCMCNT(Total Number of Drugs Other than Anticoagulant)

Power / Percent / Percent of rows / Percent	20	21	22	23	24	25	26	27	Total
1	19	6	2	5	1	1	2	1	31831
	0.06	0.02	0.01	0.02	0.00	0.00	0.01	0.00	100.00
	0.06	0.02	0.01	0.02	0.00	0.00	0.01	0.00	
	100.00	100.00	100.00	100.00	100.00	100.00	100.00	100.00	
Total	19	6	2	5	1	1	2	1	31831
	0.06	0.02	0.01	0.02	0.00	0.00	0.01	0.00	100.00

Degree of missing values = 659

Concomitant medications other than anticoagulants at the time of obtaining Table 2-5 consent, according to the presence or absence of proton-pump inhibitors

FREQ Procedure

			Cumulative	
Percent of ALL	power	Percent of power		
1	32490	100.00	32490	100.00

Tables: ALL * CMYN

ALL

CMYN(Administration of Concomitant Drug)

Power<br>Percent<br>Percent of rows<br>Percent of columns	None	With	Total
1	1445<br>4.45<br>4.45<br>100.00	31045<br>95.55<br>95.55<br>100.00	32490<br>100.00
Total	1445<br>4.45	31045<br>95.55	32490<br>100.00

Concomitant medications other than anticoagulants at the time of obtaining Table 2-5 consent, according to the presence or absence of proton-pump inhibitors

FREQ Procedure

Tables: ALL * TCMCNT

ALL TCMCNT(Total Number of Drugs Other than Anticoagulant)

Power |
Percent |
Percent of rows
Percent|0|1|2|3|4|5|6|7|8|9| Total
______________+________+________+________+________+________+________+________
_+________+________+________+
1 | 52 | 688 | 1602 | 2622 | 3597 | 4105 | 4171 | 3747 | 3187 | 2516 | 31831
| 0.16 | 2.16 | 5.03 | 8.24 | 11.30 | 12.90 | 13.10 | 11.77 | 10.01 | 7.90 | 100.00
| 0.16 | 2.16 | 5.03 | 8.24 | 11.30 | 12.90 | 13.10 | 11.77 | 10.01 | 7.90 |
| 100.00 | 100.00 | 100.00 | 100.00 | 100.00 | 100.00 | 100.00 | 100.00 | 100.00 | 100.00 |
______________+________+________+________+________+________+________+________
_+________+________+________+
Total 52 688 1602 2622 3597 4105 4171 3747 3187 2516 31831
0.16 2.16 5.03 8.24 11.30 12.90 13.10 11.77 10.01 7.90 100.00
(continued)

Tables: ALL * TCMCNT

ALL TCMCNT(Total Number of Drugs Other than Anticoagulant)

Power |
Percent |

Percent of rows

Percent |10|11|12|13|14|15|16|17|18|19|Total

______________+________+________+________+________+________+________+_______
_+________+________+________+

1 | 1825 | 1360 | 881 | 598 | 366 | 206 | 121 | 79 | 47 |
24 | 31831

| 5.73 | 4.27 | 2.77 | 1.88 | 1.15 | 0.65 | 0.38 | 0.25 | 0.15 |
0.08 | 100.00

| 5.73 | 4.27 | 2.77 | 1.88 | 1.15 | 0.65 | 0.38 | 0.25 | 0.15 |
0.08 |

| 100.00 | 100.00 | 100.00 | 100.00 | 100.00 | 100.00 | 100.00 | 100.00 | 100.00 |
100.00 |

______________+________+________+________+________+________+________+_______
_+________+________+________+

Total 1825 1360 881 598 366 206 121 79 47 24 31831

5.73 4.27 2.77 1.88 1.15 0.65 0.38 0.25
0.15 0.08 100.00

(continued)

Concomitant medications other than anticoagulants at the time of obtaining Table 2-5 consent, according to the presence or absence of proton-pump inhibitors

FREQ Procedure

Tables: ALL * TCMCNT

ALL TCMCNT(Total Number of Drugs Other than Anticoagulant)

Power |
Percent |
Percent of rows
Percent | 20| 21| 22| 23| 24| 25| 26| 27| Total
______________+________+________+________+________+________+________+________+________+

1	19	6	2	5	1	1	2	1	31831
	0.06	0.02	0.01	0.02	0.00	0.00	0.01	0.00	100.00
	0.06	0.02	0.01	0.02	0.00	0.00	0.01	0.00	
	100.00	100.00	100.00	100.00	100.00	100.00	100.00	100.00	

______________+________+________+________+________+________+________+________+________+

Total 19 6 2 5 1 1 2 1 31831
0.06 0.02 0.01 0.02 0.00 0.00 0.01 0.00 100.00

Degree of missing values = 659

Concomitant medications other than anticoagulants at the time of obtaining Table 2-5 consent, according to the presence or absence of proton-pump inhibitors

FREQ Procedure

ALL	power	Percent	Cumulative power	Cumulative Percent
1	32490	100.00	32490	100.00

Tables: ALL * CMYN

ALL

CMYN(Administration of Concomitant Drug)

Power |
Percent |
Percent of rows
Percent of columns | None | With | Total

ALL	None	With	Total
1	1445	31045	32490
	4.45	95.55	100.00
	4.45	95.55	
	100.00	100.00	
Total	1445	31045	32490
	4.45	95.55	100.00

Concomitant medications other than anticoagulants at the time of obtaining Table 2-5 consent, according to the presence or absence of proton-pump inhibitors

FREQ Procedure

Tables: ALL * TCMCNT

ALL TCMCNT(Total Number of Drugs Other than Anticoagulant)

Power |
Percent |
Percent of rows
Percent|0|1|2|3|4|5|6|7|8|9| Total
______________+________+________+________+________+________+________+________
_+________+________+________+
1 | 52 | 688 | 1602 | 2622 | 3597 | 4105 | 4171 | 3747 | 3187 | 2516 | 31831
| 0.16 | 2.16 | 5.03 | 8.24 | 11.30 | 12.90 | 13.10 | 11.77 | 10.01 | 7.90 | 100.00
| 0.16 | 2.16 | 5.03 | 8.24 | 11.30 | 12.90 | 13.10 | 11.77 | 10.01 | 7.90 |
| 100.00 | 100.00 | 100.00 | 100.00 | 100.00 | 100.00 | 100.00 | 100.00 | 100.00 | 100.00 |
______________+________+________+________+________+________+________+________
_+________+________+________+
Total 52 688 1602 2622 3597 4105 4171 3747 3187 2516 31831
0.16 2.16 5.03 8.24 11.30 12.90 13.10 11.77 10.01 7.90 100.00
(continued)

Tables: ALL * TCMCNT

ALL TCMCNT(Total Number of Drugs Other than Anticoagulant)

Power |
Percent |

Percent of rows

Percent |10|11|12|13|14|15|16|17|18|19|Total

______________+________+________+________+________+________+________+________+________+________+________+

1	1825	1360	881	598	366	206	121	79	47	24	31831
	5.73	4.27	2.77	1.88	1.15	0.65	0.38	0.25	0.15	0.08	100.00
	5.73	4.27	2.77	1.88	1.15	0.65	0.38	0.25	0.15	0.08	
	100.00	100.00	100.00	100.00	100.00	100.00	100.00	100.00	100.00	100.00	

______________+________+________+________+________+________+________+________+________+________+________+

Total	1825	1360	881	598	366	206	121	79	47	24	31831
	5.73	4.27	2.77	1.88	1.15	0.65	0.38	0.25	0.15	0.08	100.00

(continued)

Concomitant medications other than anticoagulants at the time of obtaining Table 2-5 consent, according to the presence or absence of proton-pump inhibitors

FREQ Procedure

Tables: ALL * TCMCNT

ALL TCMCNT(Total Number of Drugs Other than Anticoagulant)

Power |
Percent |
Percent of rows
Percent | 20| 21| 22| 23| 24| 25| 26| 27| Total

	20	21	22	23	24	25	26	27	Total
1	19	6	2	5	1	1	2	1	31831
	0.06	0.02	0.01	0.02	0.00	0.00	0.01	0.00	100.00
	0.06	0.02	0.01	0.02	0.00	0.00	0.01	0.00	
	100.00	100.00	100.00	100.00	100.00	100.00	100.00	100.00	
Total	19	6	2	5	1	1	2	1	31831
	0.06	0.02	0.01	0.02	0.00	0.00	0.01	0.00	100.00

Degree of missing values = 659

Concomitant medications other than anticoagulants at the time of obtaining Table 2-5 consent, according to the presence or absence of proton-pump inhibitors

FREQ Procedure

Percent of ALL	power	Percent	Cumulative power	Cumulative Percent of power
1	32490	100.00	32490	100.00

Tables: ALL * CMYN

ALL

CMYN(Administration of Concomitant Drug)

Power |
Percent |
Percent of rows
Percent of columns | None | With | Total

	None	With	Total
1	1445	31045	32490
	4.45	95.55	100.00
	4.45	95.55	
	100.00	100.00	
Total	1445	31045	32490
	4.45	95.55	100.00

Concomitant medications other than anticoagulants at the time of obtaining Table 2-5 consent, according to the presence or absence of proton-pump inhibitors

FREQ Procedure

Tables: ALL * TCMCNT

ALL TCMCNT(Total Number of Drugs Other than Anticoagulant)

Power |
Percent |
Percent of rows
Percent|0|1|2|3|4|5|6|7|8|9| Total
______________+________+________+________+________+________+________+________
_+________+________+________+
1 | 52 | 688 | 1602 | 2622 | 3597 | 4105 | 4171 | 3747 | 3187 | 2516 | 31831
| 0.16 | 2.16 | 5.03 | 8.24 | 11.30 | 12.90 | 13.10 | 11.77 | 10.01 | 7.90 | 100.00
| 0.16 | 2.16 | 5.03 | 8.24 | 11.30 | 12.90 | 13.10 | 11.77 | 10.01 | 7.90 |
| 100.00 | 100.00 | 100.00 | 100.00 | 100.00 | 100.00 | 100.00 | 100.00 | 100.00 | 100.00 |
______________+________+________+________+________+________+________+________
_+________+________+________+
Total 52 688 1602 2622 3597 4105 4171 3747 3187 2516 31831
0.16 2.16 5.03 8.24 11.30 12.90 13.10 11.77 10.01 7.90 100.00
(continued)

Tables: ALL * TCMCNT

ALL TCMCNT(Total Number of Drugs Other than Anticoagulant)

Power |
Percent |

Percent of rows

Percent |10|11|12|13|14|15|16|17|18|19|Total

______________+________+________+________+________+________+________+________

_+________+________+________+

1 | 1825 | 1360 | 881 | 598 | 366 | 206 | 121 | 79 | 47 |

24 | 31831

| 5.73 | 4.27 | 2.77 | 1.88 | 1.15 | 0.65 | 0.38 | 0.25 | 0.15 |

0.08 | 100.00

| 5.73 | 4.27 | 2.77 | 1.88 | 1.15 | 0.65 | 0.38 | 0.25 | 0.15 |

0.08 |

| 100.00 | 100.00 | 100.00 | 100.00 | 100.00 | 100.00 | 100.00 | 100.00 | 100.00 |

100.00 |

______________+________+________+________+________+________+________+________

_+________+________+________+

Total 1825 1360 881 598 366 206 121 79 47 24 31831

5.73 4.27 2.77 1.88 1.15 0.65 0.38 0.25

0.15 0.08 100.00

(continued)

Concomitant medications other than anticoagulants at the time of obtaining Table 2-5 consent, according to the presence or absence of proton-pump inhibitors

FREQ Procedure

Tables: ALL * TCMCNT

ALL TCMCNT(Total Number of Drugs Other than Anticoagulant)

Power |
Percent |
Percent of rows
Percent | 20| 21| 22| 23| 24| 25| 26| 27| Total

ALL	20	21	22	23	24	25	26	27	Total
1	19	6	2	5	1	1	2	1	31831
	0.06	0.02	0.01	0.02	0.00	0.00	0.01	0.00	100.00
	0.06	0.02	0.01	0.02	0.00	0.00	0.01	0.00	
	100.00	100.00	100.00	100.00	100.00	100.00	100.00	100.00	
Total	19	6	2	5	1	1	2	1	31831
	0.06	0.02	0.01	0.02	0.00	0.00	0.01	0.00	100.00

Degree of missing values = 659

Concomitant medications other than anticoagulants at the time of obtaining Table 2-5 consent, according to the presence or absence of proton-pump inhibitors

FREQ Procedure

			Cumulative	
Percent of ALL	power	Percent of	power	
1	32490	100.00	32490	100.00

Tables: ALL * CMYN

ALL

CMYN(Administration of Concomitant Drug)

Power / Percent / Percent of rows / Percent of columns	None	With	Total
1	1445	31045	32490
	4.45	95.55	100.00
	4.45	95.55	
	100.00	100.00	
Total	1445	31045	32490
	4.45	95.55	100.00

Concomitant medications other than anticoagulants at the time of obtaining Table 2-5 consent, according to the presence or absence of proton-pump inhibitors

FREQ Procedure

Tables: ALL * TCMCNT

ALL TCMCNT(Total Number of Drugs Other than Anticoagulant)

Power |
Percent |
Percent of rows
Percent|0|1|2|3|4|5|6|7|8|9| Total
______________+________+________+________+________+________+________+________
_+________+________+________+
1 | 52 | 688 | 1602 | 2622 | 3597 | 4105 | 4171 | 3747 | 3187 |
2516 | 31831
| 0.16 | 2.16 | 5.03 | 8.24 | 11.30 | 12.90 | 13.10 | 11.77 | 10.01 |
7.90 | 100.00
| 0.16 | 2.16 | 5.03 | 8.24 | 11.30 | 12.90 | 13.10 | 11.77 | 10.01 |
7.90 |
| 100.00 | 100.00 | 100.00 | 100.00 | 100.00 | 100.00 | 100.00 | 100.00 | 100.00 |
100.00 |
______________+________+________+________+________+________+________+________
_+________+________+________+
Total 52 688 1602 2622 3597 4105 4171 3747 3187 2516 31831
0.16 2.16 5.03 8.24 11.30 12.90 13.10 11.77
10.01 7.90 100.00
(continued)

Tables: ALL * TCMCNT

ALL TCMCNT(Total Number of Drugs Other than Anticoagulant)

Power |
Percent |

Percent of rows

Percent |10|11|12|13|14|15|16|17|18|19|Total

______________+________+________+________+________+________+________+________
_+________+________+________+

1 | 1825 | 1360 | 881 | 598 | 366 | 206 | 121 | 79 | 47 |
24 | 31831

| 5.73 | 4.27 | 2.77 | 1.88 | 1.15 | 0.65 | 0.38 | 0.25 | 0.15 |
0.08 | 100.00

| 5.73 | 4.27 | 2.77 | 1.88 | 1.15 | 0.65 | 0.38 | 0.25 | 0.15 |
0.08 |

| 100.00 | 100.00 | 100.00 | 100.00 | 100.00 | 100.00 | 100.00 | 100.00 | 100.00 |
100.00 |

______________+________+________+________+________+________+________+________
_+________+________+________+

Total 1825 1360 881 598 366 206 121 79 47 24 31831

5.73 4.27 2.77 1.88 1.15 0.65 0.38 0.25
0.15 0.08 100.00

(continued)

Concomitant medications other than anticoagulants at the time of obtaining Table 2-5 consent, according to the presence or absence of proton-pump inhibitors

FREQ Procedure

Tables: ALL * TCMCNT

ALL TCMCNT(Total Number of Drugs Other than Anticoagulant)

Power |
Percent |
Percent of rows
Percent | 20| 21| 22| 23| 24| 25| 26| 27| Total

1	19	6	2	5	1	1	2	1	31831
	0.06	0.02	0.01	0.02	0.00	0.00	0.01	0.00	100.00
	0.06	0.02	0.01	0.02	0.00	0.00	0.01	0.00	
	100.00	100.00	100.00	100.00	100.00	100.00	100.00	100.00	
Total	19	6	2	5	1	1	2	1	31831
	0.06	0.02	0.01	0.02	0.00	0.00	0.01	0.00	100.00

Degree of missing values = 659

Concomitant medications other than anticoagulants at the time of obtaining Table 2-5 consent, according to the presence or absence of proton-pump inhibitors

FREQ Procedure

Cumulative
Percent of ALL power Percent of power

1	32490	100.00	32490	100.00

Tables: ALL * CMYN

ALL
CMYN(Administration of Concomitant Drug)

Power |
Percent |
Percent of rows
Percent of columns | None | With | Total

1	1445	31045	32490
	4.45	95.55	100.00
	4.45	95.55	
	100.00	100.00	
Total	1445	31045	32490
	4.45	95.55	100.00

Concomitant medications other than anticoagulants at the time of obtaining Table 2-5 consent, according to the presence or absence of proton-pump inhibitors

FREQ Procedure

Tables: ALL * TCMCNT

ALL TCMCNT(Total Number of Drugs Other than Anticoagulant)

Power |
Percent |
Percent of rows
Percent|0|1|2|3|4|5|6|7|8|9| Total
______________+________+________+________+________+________+________+________
_+________+________+________+
1 | 52 | 688 | 1602 | 2622 | 3597 | 4105 | 4171 | 3747 | 3187 |
2516 | 31831
| 0.16 | 2.16 | 5.03 | 8.24 | 11.30 | 12.90 | 13.10 | 11.77 | 10.01 |
7.90 | 100.00
| 0.16 | 2.16 | 5.03 | 8.24 | 11.30 | 12.90 | 13.10 | 11.77 | 10.01 |
7.90 |
| 100.00 | 100.00 | 100.00 | 100.00 | 100.00 | 100.00 | 100.00 | 100.00 | 100.00 |
100.00 |
______________+________+________+________+________+________+________+________
_+________+________+________+
Total 52 688 1602 2622 3597 4105 4171 3747 3187 2516 31831
0.16 2.16 5.03 8.24 11.30 12.90 13.10 11.77
10.01 7.90 100.00
(continued)

Tables: ALL * TCMCNT

ALL TCMCNT(Total Number of Drugs Other than Anticoagulant)

Power |
Percent |

Percent of rows

Percent |10|11|12|13|14|15|16|17|18|19|Total

______________+________+________+________+________+________+________+________
_+________+________+________+

1 | 1825 | 1360 | 881 | 598 | 366 | 206 | 121 | 79 | 47 |
24 | 31831

| 5.73 | 4.27 | 2.77 | 1.88 | 1.15 | 0.65 | 0.38 | 0.25 | 0.15 |
0.08 | 100.00

| 5.73 | 4.27 | 2.77 | 1.88 | 1.15 | 0.65 | 0.38 | 0.25 | 0.15 |
0.08 |

| 100.00 | 100.00 | 100.00 | 100.00 | 100.00 | 100.00 | 100.00 | 100.00 | 100.00 |
100.00 |

______________+________+________+________+________+________+________+________
_+________+________+________+

Total 1825 1360 881 598 366 206 121 79 47 24 31831

5.73 4.27 2.77 1.88 1.15 0.65 0.38 0.25
0.15 0.08 100.00

(continued)

Concomitant medications other than anticoagulants at the time of obtaining Table 2-5 consent, according to the presence or absence of proton-pump inhibitors

FREQ Procedure

Tables: ALL * TCMCNT

ALL TCMCNT(Total Number of Drugs Other than Anticoagulant)

Power |
Percent |
Percent of rows
Percent | 20| 21| 22| 23| 24| 25| 26| 27| Total

ALL	20	21	22	23	24	25	26	27	Total
1	19	6	2	5	1	1	2	1	31831
	0.06	0.02	0.01	0.02	0.00	0.00	0.01	0.00	100.00
	0.06	0.02	0.01	0.02	0.00	0.00	0.01	0.00	
	100.00	100.00	100.00	100.00	100.00	100.00	100.00	100.00	
Total	19	6	2	5	1	1	2	1	31831
	0.06	0.02	0.01	0.02	0.00	0.00	0.01	0.00	100.00

Degree of missing values = 659

Concomitant medications other than anticoagulants at the time of obtaining Table 2-5 consent, according to the presence or absence of proton-pump inhibitors

FREQ Procedure

			Cumulative	
Percent of ALL	power	Percent of power		
1	32490	100.00	32490	100.00

Tables: ALL * CMYN

ALL

CMYN(Administration of Concomitant Drug)

Power / Percent / Percent of rows / Percent of columns	None	With	Total
1	1445	31045	32490
	4.45	95.55	100.00
	4.45	95.55	
	100.00	100.00	
Total	1445	31045	32490
	4.45	95.55	100.00

Concomitant medications other than anticoagulants at the time of obtaining Table 2-5 consent, according to the presence or absence of proton-pump inhibitors

FREQ Procedure

Tables: ALL * TCMCNT

ALL TCMCNT(Total Number of Drugs Other than Anticoagulant)

Power |
Percent |
Percent of rows
Percent|0|1|2|3|4|5|6|7|8|9| Total
______________+________+________+________+________+________+________+________+________+________+________+
1 | 52 | 688 | 1602 | 2622 | 3597 | 4105 | 4171 | 3747 | 3187 | 2516 | 31831
| 0.16 | 2.16 | 5.03 | 8.24 | 11.30 | 12.90 | 13.10 | 11.77 | 10.01 | 7.90 | 100.00
| 0.16 | 2.16 | 5.03 | 8.24 | 11.30 | 12.90 | 13.10 | 11.77 | 10.01 | 7.90 |
| 100.00 | 100.00 | 100.00 | 100.00 | 100.00 | 100.00 | 100.00 | 100.00 | 100.00 | 100.00 |
______________+________+________+________+________+________+________+________+________+________+________+
Total 52 688 1602 2622 3597 4105 4171 3747 3187 2516 31831
0.16 2.16 5.03 8.24 11.30 12.90 13.10 11.77 10.01 7.90 100.00
(continued)

Tables: ALL * TCMCNT

ALL TCMCNT(Total Number of Drugs Other than Anticoagulant)

Power |
Percent |

Percent of rows

Percent |10|11|12|13|14|15|16|17|18|19|Total

______________+________+________+________+________+________+________+_______
_+________+________+________+

1 | 1825 | 1360 | 881 | 598 | 366 | 206 | 121 | 79 | 47 |
24 | 31831

| 5.73 | 4.27 | 2.77 | 1.88 | 1.15 | 0.65 | 0.38 | 0.25 | 0.15 |
0.08 | 100.00

| 5.73 | 4.27 | 2.77 | 1.88 | 1.15 | 0.65 | 0.38 | 0.25 | 0.15 |
0.08 |

| 100.00 | 100.00 | 100.00 | 100.00 | 100.00 | 100.00 | 100.00 | 100.00 | 100.00 |
100.00 |

______________+________+________+________+________+________+________+_______
_+________+________+________+

Total 1825 1360 881 598 366 206 121 79 47 24 31831

5.73 4.27 2.77 1.88 1.15 0.65 0.38 0.25
0.15 0.08 100.00

(continued)

Concomitant medications other than anticoagulants at the time of obtaining Table 2-5 consent, according to the presence or absence of proton-pump inhibitors

FREQ Procedure

Tables: ALL * TCMCNT

ALL TCMCNT(Total Number of Drugs Other than Anticoagulant)

Power / Percent / Percent of rows / Percent	20	21	22	23	24	25	26	27	Total
1	19	6	2	5	1	1	2	1	31831
	0.06	0.02	0.01	0.02	0.00	0.00	0.01	0.00	100.00
	0.06	0.02	0.01	0.02	0.00	0.00	0.01	0.00	
	100.00	100.00	100.00	100.00	100.00	100.00	100.00	100.00	
Total	19	6	2	5	1	1	2	1	31831
	0.06	0.02	0.01	0.02	0.00	0.00	0.01	0.00	100.00

Degree of missing values = 659

Concomitant medications other than anticoagulants at the time of obtaining Table 2-5 consent, according to the presence or absence of proton-pump inhibitors

FREQ Procedure

			Cumulative	
Percent of ALL	power	Percent of	power	
1	32490	100.00	32490	100.00

Tables: ALL * CMYN

ALL

CMYN(Administration of Concomitant Drug)

Power |
Percent |
Percent of rows
Percent of columns | None | With | Total

	None	With	Total
1	1445	31045	32490
	4.45	95.55	100.00
	4.45	95.55	
	100.00	100.00	
Total	1445	31045	32490
	4.45	95.55	100.00

Concomitant medications other than anticoagulants at the time of obtaining Table 2-5 consent, according to the presence or absence of proton-pump inhibitors

FREQ Procedure

Tables: ALL * TCMCNT

ALL TCMCNT(Total Number of Drugs Other than Anticoagulant)

Power |
Percent |
Percent of rows
Percent|0|1|2|3|4|5|6|7|8|9| Total
______________+________+________+________+________+________+________+________
_+________+________+________+
1 | 52 | 688 | 1602 | 2622 | 3597 | 4105 | 4171 | 3747 | 3187 | 2516 | 31831
| 0.16 | 2.16 | 5.03 | 8.24 | 11.30 | 12.90 | 13.10 | 11.77 | 10.01 | 7.90 | 100.00
| 0.16 | 2.16 | 5.03 | 8.24 | 11.30 | 12.90 | 13.10 | 11.77 | 10.01 | 7.90 |
| 100.00 | 100.00 | 100.00 | 100.00 | 100.00 | 100.00 | 100.00 | 100.00 | 100.00 | 100.00 |
______________+________+________+________+________+________+________+________
_+________+________+________+
Total 52 688 1602 2622 3597 4105 4171 3747 3187 2516 31831
0.16 2.16 5.03 8.24 11.30 12.90 13.10 11.77 10.01 7.90 100.00
(continued)

Tables: ALL * TCMCNT

ALL TCMCNT(Total Number of Drugs Other than Anticoagulant)

Power |
Percent |

Percent of rows

```
Percent |10|11|12|13|14|15|16|17|18|19|Total
______________+________+________+________+________+________+________+________
_+________+________+________+
              1 |    1825 |    1360 |     881 |     598 |     366 |     206 |     121 |      79 |      47 |
24 |   31831
                |    5.73 |    4.27 |    2.77 |    1.88 |    1.15 |    0.65 |    0.38 |    0.25 |    0.15 |
0.08 | 100.00
                |    5.73 |    4.27 |    2.77 |    1.88 |    1.15 |    0.65 |    0.38 |    0.25 |    0.15 |
0.08 |
                | 100.00 | 100.00 | 100.00 | 100.00 | 100.00 | 100.00 | 100.00 | 100.00 | 100.00 |
100.00 |
______________+________+________+________+________+________+________+________
_+________+________+________+
Total 1825 1360 881 598 366 206 121 79 47 24 31831
                  5.73        4.27        2.77        1.88        1.15        0.65        0.38        0.25
0.15       0.08     100.00
```

(continued)

Concomitant medications other than anticoagulants at the time of obtaining Table 2-5 consent, according to the presence or absence of proton-pump inhibitors

FREQ Procedure

Tables: ALL * TCMCNT

ALL TCMCNT(Total Number of Drugs Other than Anticoagulant)

Power |
Percent |
Percent of rows
Percent | 20| 21| 22| 23| 24| 25| 26| 27| Total

1	19	6	2	5	1	1	2	1	31831
	0.06	0.02	0.01	0.02	0.00	0.00	0.01	0.00	100.00
	0.06	0.02	0.01	0.02	0.00	0.00	0.01	0.00	
	100.00	100.00	100.00	100.00	100.00	100.00	100.00	100.00	
Total	19	6	2	5	1	1	2	1	31831
	0.06	0.02	0.01	0.02	0.00	0.00	0.01	0.00	100.00

Degree of missing values = 659

Concomitant medications other than anticoagulants at the time of obtaining Table 2-5 consent, according to the presence or absence of proton-pump inhibitors

FREQ Procedure

			Cumulative	
Percent of ALL	power	Percent of	power	
1	32490	100.00	32490	100.00

Tables: ALL * CMYN

ALL

CMYN(Administration of Concomitant Drug)

Power / Percent / Percent of rows / Percent of columns	None	With	Total
1	1445	31045	32490
	4.45	95.55	100.00
	4.45	95.55	
	100.00	100.00	
Total	1445	31045	32490
	4.45	95.55	100.00

Concomitant medications other than anticoagulants at the time of obtaining Table 2-5 consent, according to the presence or absence of proton-pump inhibitors

FREQ Procedure

Tables: ALL * TCMCNT

ALL TCMCNT(Total Number of Drugs Other than Anticoagulant)

Power |
Percent |
Percent of rows
Percent|0|1|2|3|4|5|6|7|8|9| Total
______________+________+________+________+________+________+________+________+________+________+________+
1 | 52 | 688 | 1602 | 2622 | 3597 | 4105 | 4171 | 3747 | 3187 | 2516 | 31831
| 0.16 | 2.16 | 5.03 | 8.24 | 11.30 | 12.90 | 13.10 | 11.77 | 10.01 | 7.90 | 100.00
| 0.16 | 2.16 | 5.03 | 8.24 | 11.30 | 12.90 | 13.10 | 11.77 | 10.01 | 7.90 |
| 100.00 | 100.00 | 100.00 | 100.00 | 100.00 | 100.00 | 100.00 | 100.00 | 100.00 | 100.00 |
______________+________+________+________+________+________+________+________+________+________+________+
Total 52 688 1602 2622 3597 4105 4171 3747 3187 2516 31831
0.16 2.16 5.03 8.24 11.30 12.90 13.10 11.77 10.01 7.90 100.00
(continued)

Tables: ALL * TCMCNT

ALL TCMCNT(Total Number of Drugs Other than Anticoagulant)

Power |
Percent |

Percent of rows

Percent |10|11|12|13|14|15|16|17|18|19|Total

______________+________+________+________+________+________+________+________
_+________+________+________+

1 | 1825 | 1360 | 881 | 598 | 366 | 206 | 121 | 79 | 47 |
24 | 31831

| 5.73 | 4.27 | 2.77 | 1.88 | 1.15 | 0.65 | 0.38 | 0.25 | 0.15 |
0.08 | 100.00

| 5.73 | 4.27 | 2.77 | 1.88 | 1.15 | 0.65 | 0.38 | 0.25 | 0.15 |
0.08 |

| 100.00 | 100.00 | 100.00 | 100.00 | 100.00 | 100.00 | 100.00 | 100.00 | 100.00 |
100.00 |

______________+________+________+________+________+________+________+________
_+________+________+________+

Total 1825 1360 881 598 366 206 121 79 47 24 31831

5.73 4.27 2.77 1.88 1.15 0.65 0.38 0.25
0.15 0.08 100.00

(continued)

Concomitant medications other than anticoagulants at the time of obtaining Table 2-5 consent, according to the presence or absence of proton-pump inhibitors

FREQ Procedure

Tables: ALL * TCMCNT

ALL TCMCNT(Total Number of Drugs Other than Anticoagulant)

Power |
Percent |
Percent of rows
Percent | 20| 21| 22| 23| 24| 25| 26| 27| Total

1	19	6	2	5	1	1	2	1	31831
	0.06	0.02	0.01	0.02	0.00	0.00	0.01	0.00	100.00
	0.06	0.02	0.01	0.02	0.00	0.00	0.01	0.00	
	100.00	100.00	100.00	100.00	100.00	100.00	100.00	100.00	
Total	19	6	2	5	1	1	2	1	31831
	0.06	0.02	0.01	0.02	0.00	0.00	0.01	0.00	100.00

Degree of missing values = 659

Concomitant medications other than anticoagulants at the time of obtaining Table 2-5 consent, according to the presence or absence of proton-pump inhibitors

FREQ Procedure

			Cumulative	
Percent of ALL	power	Percent of power		
1	32490	100.00	32490	100.00

Tables: ALL * CMYN

ALL

CMYN(Administration of Concomitant Drug)

Power | Percent | Percent of rows Percent of columns	None	With	Total
1	1445	31045	32490
	4.45	95.55	100.00
	4.45	95.55	
	100.00	100.00	
Total	1445	31045	32490
	4.45	95.55	100.00

Concomitant medications other than anticoagulants at the time of obtaining Table 2-5 consent, according to the presence or absence of proton-pump inhibitors

FREQ Procedure

Tables: ALL * TCMCNT

ALL TCMCNT(Total Number of Drugs Other than Anticoagulant)

Power |
Percent |
Percent of rows
Percent|0|1|2|3|4|5|6|7|8|9| Total
______________+________+________+________+________+________+________+________
_+________+________+________+
1 | 52 | 688 | 1602 | 2622 | 3597 | 4105 | 4171 | 3747 | 3187 |
2516 | 31831
| 0.16 | 2.16 | 5.03 | 8.24 | 11.30 | 12.90 | 13.10 | 11.77 | 10.01 |
7.90 | 100.00
| 0.16 | 2.16 | 5.03 | 8.24 | 11.30 | 12.90 | 13.10 | 11.77 | 10.01 |
7.90 |
| 100.00 | 100.00 | 100.00 | 100.00 | 100.00 | 100.00 | 100.00 | 100.00 | 100.00 |
100.00 |
______________+________+________+________+________+________+________+________
_+________+________+________+
Total 52 688 1602 2622 3597 4105 4171 3747 3187 2516 31831
0.16 2.16 5.03 8.24 11.30 12.90 13.10 11.77
10.01 7.90 100.00
(continued)

Tables: ALL * TCMCNT

ALL TCMCNT(Total Number of Drugs Other than Anticoagulant)

Power |
Percent |

Percent of rows

Percent |10|11|12|13|14|15|16|17|18|19|Total

______________+________+________+________+________+________+________+________+________+________+________+

1	1825	1360	881	598	366	206	121	79	47	24	31831
	5.73	4.27	2.77	1.88	1.15	0.65	0.38	0.25	0.15	0.08	100.00
	5.73	4.27	2.77	1.88	1.15	0.65	0.38	0.25	0.15	0.08	
	100.00	100.00	100.00	100.00	100.00	100.00	100.00	100.00	100.00	100.00	

______________+________+________+________+________+________+________+________+________+________+________+

Total	1825	1360	881	598	366	206	121	79	47	24	31831
	5.73	4.27	2.77	1.88	1.15	0.65	0.38	0.25	0.15	0.08	100.00

(continued)

Concomitant medications other than anticoagulants at the time of obtaining Table 2-5 consent, according to the presence or absence of proton-pump inhibitors

FREQ Procedure

Tables: ALL * TCMCNT

ALL TCMCNT(Total Number of Drugs Other than Anticoagulant)

Power |
Percent |
Percent of rows
Percent | 20| 21| 22| 23| 24| 25| 26| 27| Total

1	19	6	2	5	1	1	2	1	31831
	0.06	0.02	0.01	0.02	0.00	0.00	0.01	0.00	100.00
	0.06	0.02	0.01	0.02	0.00	0.00	0.01	0.00	
	100.00	100.00	100.00	100.00	100.00	100.00	100.00	100.00	
Total	19	6	2	5	1	1	2	1	31831
	0.06	0.02	0.01	0.02	0.00	0.00	0.01	0.00	100.00

Degree of missing values = 659

Concomitant medications other than anticoagulants at the time of obtaining Table 2-5 consent, according to the presence or absence of proton-pump inhibitors

FREQ Procedure

ALL	power	Percent	Cumulative power	Cumulative Percent of power
1	32490	100.00	32490	100.00

Tables: ALL * CMYN

ALL

CMYN(Administration of Concomitant Drug)

Power / Percent / Percent of rows / Percent of columns	None	With	Total
1	1445	31045	32490
	4.45	95.55	100.00
	4.45	95.55	
	100.00	100.00	
Total	1445	31045	32490
	4.45	95.55	100.00

Concomitant medications other than anticoagulants at the time of obtaining Table 2-5 consent, according to the presence or absence of proton-pump inhibitors

FREQ Procedure

Tables: ALL * TCMCNT

ALL TCMCNT(Total Number of Drugs Other than Anticoagulant)

Power |
Percent |
Percent of rows
Percent|0|1|2|3|4|5|6|7|8|9| Total
______________+________+________+________+________+________+________+________+________+________+________+
1 | 52 | 688 | 1602 | 2622 | 3597 | 4105 | 4171 | 3747 | 3187 | 2516 | 31831
| 0.16 | 2.16 | 5.03 | 8.24 | 11.30 | 12.90 | 13.10 | 11.77 | 10.01 | 7.90 | 100.00
| 0.16 | 2.16 | 5.03 | 8.24 | 11.30 | 12.90 | 13.10 | 11.77 | 10.01 | 7.90 |
| 100.00 | 100.00 | 100.00 | 100.00 | 100.00 | 100.00 | 100.00 | 100.00 | 100.00 | 100.00 |
______________+________+________+________+________+________+________+________+________+________+________+
Total 52 688 1602 2622 3597 4105 4171 3747 3187 2516 31831
0.16 2.16 5.03 8.24 11.30 12.90 13.10 11.77 10.01 7.90 100.00
(continued)

Tables: ALL * TCMCNT

ALL TCMCNT(Total Number of Drugs Other than Anticoagulant)

Power |
Percent |

Percent of rows

Percent |10|11|12|13|14|15|16|17|18|19|Total

______________+________+________+________+________+________+________+_______
_+________+________+________+

1 | 1825 | 1360 | 881 | 598 | 366 | 206 | 121 | 79 | 47 |
24 | 31831

| 5.73 | 4.27 | 2.77 | 1.88 | 1.15 | 0.65 | 0.38 | 0.25 | 0.15 |
0.08 | 100.00

| 5.73 | 4.27 | 2.77 | 1.88 | 1.15 | 0.65 | 0.38 | 0.25 | 0.15 |
0.08 |

| 100.00 | 100.00 | 100.00 | 100.00 | 100.00 | 100.00 | 100.00 | 100.00 | 100.00 |
100.00 |

______________+________+________+________+________+________+________+_______
_+________+________+________+

Total 1825 1360 881 598 366 206 121 79 47 24 31831

5.73 4.27 2.77 1.88 1.15 0.65 0.38 0.25
0.15 0.08 100.00

(continued)

Concomitant medications other than anticoagulants at the time of obtaining Table 2-5 consent, according to the presence or absence of proton-pump inhibitors

FREQ Procedure

Tables: ALL * TCMCNT

ALL TCMCNT(Total Number of Drugs Other than Anticoagulant)

Power |
Percent |
Percent of rows
Percent | 20| 21| 22| 23| 24| 25| 26| 27| Total

ALL	20	21	22	23	24	25	26	27	Total
1	19	6	2	5	1	1	2	1	31831
	0.06	0.02	0.01	0.02	0.00	0.00	0.01	0.00	100.00
	0.06	0.02	0.01	0.02	0.00	0.00	0.01	0.00	
	100.00	100.00	100.00	100.00	100.00	100.00	100.00	100.00	
Total	19	6	2	5	1	1	2	1	31831
	0.06	0.02	0.01	0.02	0.00	0.00	0.01	0.00	100.00

Degree of missing values = 659

Concomitant medications other than anticoagulants at the time of obtaining Table 2-5 consent, according to the presence or absence of proton-pump inhibitors

FREQ Procedure

			Cumulative	
Percent of ALL	power	Percent of power		
1	32490	100.00	32490	100.00

Tables: ALL * CMYN

ALL

CMYN(Administration of Concomitant Drug)

Power |
Percent |
Percent of rows
Percent of columns | None | With | Total

	None	With	Total
1	1445	31045	32490
	4.45	95.55	100.00
	4.45	95.55	
	100.00	100.00	
Total	1445	31045	32490
	4.45	95.55	100.00

Concomitant medications other than anticoagulants at the time of obtaining Table 2-5 consent, according to the presence or absence of proton-pump inhibitors

FREQ Procedure

Tables: ALL * TCMCNT

ALL TCMCNT(Total Number of Drugs Other than Anticoagulant)

Power |
Percent |
Percent of rows
Percent|0|1|2|3|4|5|6|7|8|9| Total
______________+________+________+________+________+________+________+________
_+________+________+________+
1 | 52 | 688 | 1602 | 2622 | 3597 | 4105 | 4171 | 3747 | 3187 |
2516 | 31831
| 0.16 | 2.16 | 5.03 | 8.24 | 11.30 | 12.90 | 13.10 | 11.77 | 10.01 |
7.90 | 100.00
| 0.16 | 2.16 | 5.03 | 8.24 | 11.30 | 12.90 | 13.10 | 11.77 | 10.01 |
7.90 |
| 100.00 | 100.00 | 100.00 | 100.00 | 100.00 | 100.00 | 100.00 | 100.00 | 100.00 |
100.00 |
______________+________+________+________+________+________+________+________
_+________+________+________+
Total 52 688 1602 2622 3597 4105 4171 3747 3187 2516 31831
0.16 2.16 5.03 8.24 11.30 12.90 13.10 11.77
10.01 7.90 100.00
(continued)

Tables: ALL * TCMCNT

ALL TCMCNT(Total Number of Drugs Other than Anticoagulant)

Power |
Percent |

Percent of rows

Percent |10|11|12|13|14|15|16|17|18|19|Total

______________+________+________+________+________+________+________+_______
_+________+________+________+

1 | 1825 | 1360 | 881 | 598 | 366 | 206 | 121 | 79 | 47 |
24 | 31831

| 5.73 | 4.27 | 2.77 | 1.88 | 1.15 | 0.65 | 0.38 | 0.25 | 0.15 |
0.08 | 100.00

| 5.73 | 4.27 | 2.77 | 1.88 | 1.15 | 0.65 | 0.38 | 0.25 | 0.15 |
0.08 |

| 100.00 | 100.00 | 100.00 | 100.00 | 100.00 | 100.00 | 100.00 | 100.00 | 100.00 |
100.00 |

______________+________+________+________+________+________+________+_______
_+________+________+________+

Total 1825 1360 881 598 366 206 121 79 47 24 31831

5.73 4.27 2.77 1.88 1.15 0.65 0.38 0.25
0.15 0.08 100.00

(continued)

Concomitant medications other than anticoagulants at the time of obtaining Table 2-5 consent, according to the presence or absence of proton-pump inhibitors

FREQ Procedure

Tables: ALL * TCMCNT

ALL TCMCNT(Total Number of Drugs Other than Anticoagulant)

Power / Percent / Percent of rows / Percent	20	21	22	23	24	25	26	27	Total
1	19	6	2	5	1	1	2	1	31831
	0.06	0.02	0.01	0.02	0.00	0.00	0.01	0.00	100.00
	0.06	0.02	0.01	0.02	0.00	0.00	0.01	0.00	
	100.00	100.00	100.00	100.00	100.00	100.00	100.00	100.00	
Total	19	6	2	5	1	1	2	1	31831
	0.06	0.02	0.01	0.02	0.00	0.00	0.01	0.00	100.00

Degree of missing values = 659

Concomitant medications other than anticoagulants at the time of obtaining Table 2-5 consent, according to the presence or absence of proton-pump inhibitors

FREQ Procedure

ALL	power	Percent	Cumulative power	Cumulative Percent of power
1	32490	100.00	32490	100.00

Tables: ALL * CMYN

ALL

CMYN(Administration of Concomitant Drug)

Power |
Percent |
Percent of rows
Percent of columns | None | With | Total

ALL	None	With	Total
1	1445	31045	32490
	4.45	95.55	100.00
	4.45	95.55	
	100.00	100.00	
Total	1445	31045	32490
	4.45	95.55	100.00

Concomitant medications other than anticoagulants at the time of obtaining Table 2-5 consent, according to the presence or absence of proton-pump inhibitors

FREQ Procedure

Tables: ALL * TCMCNT

ALL TCMCNT(Total Number of Drugs Other than Anticoagulant)

Power |
Percent |
Percent of rows
Percent|0|1|2|3|4|5|6|7|8|9| Total
______________+________+________+________+________+________+________+________
_+________+________+________+
1 | 52 | 688 | 1602 | 2622 | 3597 | 4105 | 4171 | 3747 | 3187 | 2516 | 31831
| 0.16 | 2.16 | 5.03 | 8.24 | 11.30 | 12.90 | 13.10 | 11.77 | 10.01 | 7.90 | 100.00
| 0.16 | 2.16 | 5.03 | 8.24 | 11.30 | 12.90 | 13.10 | 11.77 | 10.01 | 7.90 |
| 100.00 | 100.00 | 100.00 | 100.00 | 100.00 | 100.00 | 100.00 | 100.00 | 100.00 | 100.00 |
______________+________+________+________+________+________+________+________
_+________+________+________+
Total 52 688 1602 2622 3597 4105 4171 3747 3187 2516 31831
0.16 2.16 5.03 8.24 11.30 12.90 13.10 11.77 10.01 7.90 100.00
(continued)

Tables: ALL * TCMCNT

ALL TCMCNT(Total Number of Drugs Other than Anticoagulant)

Power |
Percent |

Percent of rows

Percent |10|11|12|13|14|15|16|17|18|19|Total

______________+________+________+________+________+________+________+_______

_+________+________+________+

1 | 1825 | 1360 | 881 | 598 | 366 | 206 | 121 | 79 | 47 |

24 | 31831

| 5.73 | 4.27 | 2.77 | 1.88 | 1.15 | 0.65 | 0.38 | 0.25 | 0.15 |

0.08 | 100.00

| 5.73 | 4.27 | 2.77 | 1.88 | 1.15 | 0.65 | 0.38 | 0.25 | 0.15 |

0.08 |

| 100.00 | 100.00 | 100.00 | 100.00 | 100.00 | 100.00 | 100.00 | 100.00 | 100.00 |

100.00 |

______________+________+________+________+________+________+________+_______

_+________+________+________+

Total 1825 1360 881 598 366 206 121 79 47 24 31831

5.73 4.27 2.77 1.88 1.15 0.65 0.38 0.25

0.15 0.08 100.00

(continued)

Concomitant medications other than anticoagulants at the time of obtaining Table 2-5 consent, according to the presence or absence of proton-pump inhibitors

FREQ Procedure

Tables: ALL * TCMCNT

ALL TCMCNT(Total Number of Drugs Other than Anticoagulant)

Power / Percent / Percent of rows / Percent	20	21	22	23	24	25	26	27	Total
1	19	6	2	5	1	1	2	1	31831
	0.06	0.02	0.01	0.02	0.00	0.00	0.01	0.00	100.00
	0.06	0.02	0.01	0.02	0.00	0.00	0.01	0.00	
	100.00	100.00	100.00	100.00	100.00	100.00	100.00	100.00	
Total	19	6	2	5	1	1	2	1	31831
	0.06	0.02	0.01	0.02	0.00	0.00	0.01	0.00	100.00

Degree of missing values = 659

Concomitant medications other than anticoagulants at the time of obtaining Table 2-5 consent, according to the presence or absence of proton-pump inhibitors

FREQ Procedure

			Cumulative	
Percent of ALL	power	Percent of	power	
1	32490	100.00	32490	100.00

Tables: ALL * CMYN

ALL

CMYN(Administration of Concomitant Drug)

Power | Percent | Percent of rows Percent of columns	None	With	Total
1	1445	31045	32490
	4.45	95.55	100.00
	4.45	95.55	
	100.00	100.00	
Total	1445	31045	32490
	4.45	95.55	100.00

Concomitant medications other than anticoagulants at the time of obtaining Table 2-5 consent, according to the presence or absence of proton-pump inhibitors

FREQ Procedure

Tables: ALL * TCMCNT

ALL TCMCNT(Total Number of Drugs Other than Anticoagulant)

Power |
Percent |
Percent of rows
Percent|0|1|2|3|4|5|6|7|8|9| Total
______________+________+________+________+________+________+________+________
_+________+________+________+
1 | 52 | 688 | 1602 | 2622 | 3597 | 4105 | 4171 | 3747 | 3187 | 2516 | 31831
| 0.16 | 2.16 | 5.03 | 8.24 | 11.30 | 12.90 | 13.10 | 11.77 | 10.01 | 7.90 | 100.00
| 0.16 | 2.16 | 5.03 | 8.24 | 11.30 | 12.90 | 13.10 | 11.77 | 10.01 | 7.90 |
| 100.00 | 100.00 | 100.00 | 100.00 | 100.00 | 100.00 | 100.00 | 100.00 | 100.00 | 100.00 |
______________+________+________+________+________+________+________+________
_+________+________+________+
Total 52 688 1602 2622 3597 4105 4171 3747 3187 2516 31831
0.16 2.16 5.03 8.24 11.30 12.90 13.10 11.77 10.01 7.90 100.00
(continued)

Tables: ALL * TCMCNT

ALL TCMCNT(Total Number of Drugs Other than Anticoagulant)

Power |
Percent |

Percent of rows

Percent |10|11|12|13|14|15|16|17|18|19|Total

______________+________+________+________+________+________+________+_______

_+________+________+________+

1 | 1825 | 1360 | 881 | 598 | 366 | 206 | 121 | 79 | 47 |

24 | 31831

| 5.73 | 4.27 | 2.77 | 1.88 | 1.15 | 0.65 | 0.38 | 0.25 | 0.15 |

0.08 | 100.00

| 5.73 | 4.27 | 2.77 | 1.88 | 1.15 | 0.65 | 0.38 | 0.25 | 0.15 |

0.08 |

| 100.00 | 100.00 | 100.00 | 100.00 | 100.00 | 100.00 | 100.00 | 100.00 | 100.00 |

100.00 |

______________+________+________+________+________+________+________+_______

_+________+________+________+

Total 1825 1360 881 598 366 206 121 79 47 24 31831

5.73 4.27 2.77 1.88 1.15 0.65 0.38 0.25

0.15 0.08 100.00

(continued)

Concomitant medications other than anticoagulants at the time of obtaining Table 2-5 consent, according to the presence or absence of proton-pump inhibitors

FREQ Procedure

Tables: ALL * TCMCNT

ALL TCMCNT(Total Number of Drugs Other than Anticoagulant)

Power |
Percent |
Percent of rows
Percent | 20| 21| 22| 23| 24| 25| 26| 27| Total

ALL	20	21	22	23	24	25	26	27	Total
1	19	6	2	5	1	1	2	1	31831
	0.06	0.02	0.01	0.02	0.00	0.00	0.01	0.00	100.00
	0.06	0.02	0.01	0.02	0.00	0.00	0.01	0.00	
	100.00	100.00	100.00	100.00	100.00	100.00	100.00	100.00	
Total	19	6	2	5	1	1	2	1	31831
	0.06	0.02	0.01	0.02	0.00	0.00	0.01	0.00	100.00

Degree of missing values = 659

Concomitant medications other than anticoagulants at the time of obtaining Table 2-5 consent, according to the presence or absence of proton-pump inhibitors

FREQ Procedure

			Cumulative	
Percent of ALL	power	Percent of	power	
1	32490	100.00	32490	100.00

Tables: ALL * CMYN

ALL

CMYN(Administration of Concomitant Drug)

Power | Percent | Percent of rows Percent of columns	None	With	Total
1	1445	31045	32490
	4.45	95.55	100.00
	4.45	95.55	
	100.00	100.00	
Total	1445	31045	32490
	4.45	95.55	100.00

Concomitant medications other than anticoagulants at the time of obtaining Table 2-5 consent, according to the presence or absence of proton-pump inhibitors

FREQ Procedure

Tables: ALL * TCMCNT

ALL TCMCNT(Total Number of Drugs Other than Anticoagulant)

Power |
Percent |
Percent of rows
Percent|0|1|2|3|4|5|6|7|8|9| Total
______________+________+________+________+________+________+________+________
_+________+________+________+
1 | 52 | 688 | 1602 | 2622 | 3597 | 4105 | 4171 | 3747 | 3187 |
2516 | 31831
| 0.16 | 2.16 | 5.03 | 8.24 | 11.30 | 12.90 | 13.10 | 11.77 | 10.01 |
7.90 | 100.00
| 0.16 | 2.16 | 5.03 | 8.24 | 11.30 | 12.90 | 13.10 | 11.77 | 10.01 |
7.90 |
| 100.00 | 100.00 | 100.00 | 100.00 | 100.00 | 100.00 | 100.00 | 100.00 | 100.00 |
100.00 |
______________+________+________+________+________+________+________+________
_+________+________+________+
Total 52 688 1602 2622 3597 4105 4171 3747 3187 2516 31831
0.16 2.16 5.03 8.24 11.30 12.90 13.10 11.77
10.01 7.90 100.00
(continued)

Tables: ALL * TCMCNT

ALL TCMCNT(Total Number of Drugs Other than Anticoagulant)

Power |
Percent |

Percent of rows

Percent |10|11|12|13|14|15|16|17|18|19|Total

______________+________+________+________+________+________+________+________

_+________+________+________+

1 | 1825 | 1360 | 881 | 598 | 366 | 206 | 121 | 79 | 47 |

24 | 31831

| 5.73 | 4.27 | 2.77 | 1.88 | 1.15 | 0.65 | 0.38 | 0.25 | 0.15 |

0.08 | 100.00

| 5.73 | 4.27 | 2.77 | 1.88 | 1.15 | 0.65 | 0.38 | 0.25 | 0.15 |

0.08 |

| 100.00 | 100.00 | 100.00 | 100.00 | 100.00 | 100.00 | 100.00 | 100.00 | 100.00 |

100.00 |

______________+________+________+________+________+________+________+________

_+________+________+________+

Total 1825 1360 881 598 366 206 121 79 47 24 31831

5.73 4.27 2.77 1.88 1.15 0.65 0.38 0.25

0.15 0.08 100.00

(continued)

Concomitant medications other than anticoagulants at the time of obtaining Table 2-5 consent, according to the presence or absence of proton-pump inhibitors

FREQ Procedure

Tables: ALL * TCMCNT

ALL TCMCNT(Total Number of Drugs Other than Anticoagulant)

Power |
Percent |
Percent of rows
Percent | 20| 21| 22| 23| 24| 25| 26| 27| Total

	20	21	22	23	24	25	26	27	Total
1	19	6	2	5	1	1	2	1	31831
	0.06	0.02	0.01	0.02	0.00	0.00	0.01	0.00	100.00
	0.06	0.02	0.01	0.02	0.00	0.00	0.01	0.00	
	100.00	100.00	100.00	100.00	100.00	100.00	100.00	100.00	
Total	19	6	2	5	1	1	2	1	31831
	0.06	0.02	0.01	0.02	0.00	0.00	0.01	0.00	100.00

Degree of missing values = 659

Concomitant medications other than anticoagulants at the time of obtaining Table 2-5 consent, according to the presence or absence of proton-pump inhibitors

FREQ Procedure

			Cumulative	
Percent of ALL	power	Percent of	power	
1	32490	100.00	32490	100.00

Tables: ALL * CMYN

ALL

CMYN(Administration of Concomitant Drug)

Power | Percent | Percent of rows Percent of columns	None	With	Total
1	1445	31045	32490
	4.45	95.55	100.00
	4.45	95.55	
	100.00	100.00	
Total	1445	31045	32490
	4.45	95.55	100.00

Concomitant medications other than anticoagulants at the time of obtaining Table 2-5 consent, according to the presence or absence of proton-pump inhibitors

FREQ Procedure

Tables: ALL * TCMCNT

ALL TCMCNT(Total Number of Drugs Other than Anticoagulant)

Power |
Percent |
Percent of rows
Percent|0|1|2|3|4|5|6|7|8|9| Total
______________+________+________+________+________+________+________+________
_+________+________+________+
1 | 52 | 688 | 1602 | 2622 | 3597 | 4105 | 4171 | 3747 | 3187 |
2516 | 31831
| 0.16 | 2.16 | 5.03 | 8.24 | 11.30 | 12.90 | 13.10 | 11.77 | 10.01 |
7.90 | 100.00
| 0.16 | 2.16 | 5.03 | 8.24 | 11.30 | 12.90 | 13.10 | 11.77 | 10.01 |
7.90 |
| 100.00 | 100.00 | 100.00 | 100.00 | 100.00 | 100.00 | 100.00 | 100.00 | 100.00 |
100.00 |
______________+________+________+________+________+________+________+________
_+________+________+________+
Total 52 688 1602 2622 3597 4105 4171 3747 3187 2516 31831
0.16 2.16 5.03 8.24 11.30 12.90 13.10 11.77
10.01 7.90 100.00
(continued)

Tables: ALL * TCMCNT

ALL TCMCNT(Total Number of Drugs Other than Anticoagulant)

Power |
Percent |

Percent of rows

Percent |10|11|12|13|14|15|16|17|18|19|Total

______________+________+________+________+________+________+________+________+________+________+________+

1	1825	1360	881	598	366	206	121	79	47	24	31831
	5.73	4.27	2.77	1.88	1.15	0.65	0.38	0.25	0.15	0.08	100.00
	5.73	4.27	2.77	1.88	1.15	0.65	0.38	0.25	0.15	0.08	
	100.00	100.00	100.00	100.00	100.00	100.00	100.00	100.00	100.00	100.00	

______________+________+________+________+________+________+________+________+________+________+________+

Total	1825	1360	881	598	366	206	121	79	47	24	31831
	5.73	4.27	2.77	1.88	1.15	0.65	0.38	0.25	0.15	0.08	100.00

(continued)

Concomitant medications other than anticoagulants at the time of obtaining Table 2-5 consent, according to the presence or absence of proton-pump inhibitors

FREQ Procedure

Tables: ALL * TCMCNT

ALL TCMCNT(Total Number of Drugs Other than Anticoagulant)

Power |
Percent |
Percent of rows
Percent | 20| 21| 22| 23| 24| 25| 26| 27| Total

ALL	20	21	22	23	24	25	26	27	Total
1	19	6	2	5	1	1	2	1	31831
	0.06	0.02	0.01	0.02	0.00	0.00	0.01	0.00	100.00
	0.06	0.02	0.01	0.02	0.00	0.00	0.01	0.00	
	100.00	100.00	100.00	100.00	100.00	100.00	100.00	100.00	
Total	19	6	2	5	1	1	2	1	31831
	0.06	0.02	0.01	0.02	0.00	0.00	0.01	0.00	100.00

Degree of missing values = 659

Concomitant medications other than anticoagulants at the time of obtaining Table 2-5 consent, according to the presence or absence of proton-pump inhibitors

FREQ Procedure

			Cumulative	
Percent of ALL	power	Percent of	power	
1	32490	100.00	32490	100.00

Tables: ALL * CMYN

ALL

CMYN(Administration of Concomitant Drug)

Power / Percent / Percent of rows / Percent of columns	None	With	Total
1	1445	31045	32490
	4.45	95.55	100.00
	4.45	95.55	
	100.00	100.00	
Total	1445	31045	32490
	4.45	95.55	100.00

Concomitant medications other than anticoagulants at the time of obtaining Table 2-5 consent, according to the presence or absence of proton-pump inhibitors

FREQ Procedure

Tables: ALL * TCMCNT

ALL TCMCNT(Total Number of Drugs Other than Anticoagulant)

Power |
Percent |
Percent of rows
Percent|0|1|2|3|4|5|6|7|8|9| Total
______________+________+________+________+________+________+________+________
_+________+________+________+
1 | 52 | 688 | 1602 | 2622 | 3597 | 4105 | 4171 | 3747 | 3187 | 2516 | 31831
| 0.16 | 2.16 | 5.03 | 8.24 | 11.30 | 12.90 | 13.10 | 11.77 | 10.01 | 7.90 | 100.00
| 0.16 | 2.16 | 5.03 | 8.24 | 11.30 | 12.90 | 13.10 | 11.77 | 10.01 | 7.90 |
| 100.00 | 100.00 | 100.00 | 100.00 | 100.00 | 100.00 | 100.00 | 100.00 | 100.00 | 100.00 |
______________+________+________+________+________+________+________+________
_+________+________+________+
Total 52 688 1602 2622 3597 4105 4171 3747 3187 2516 31831
0.16 2.16 5.03 8.24 11.30 12.90 13.10 11.77 10.01 7.90 100.00
(continued)

Tables: ALL * TCMCNT

ALL TCMCNT(Total Number of Drugs Other than Anticoagulant)

Power |
Percent |

Percent of rows

Percent |10|11|12|13|14|15|16|17|18|19|Total

______________+________+________+________+________+________+________+_______

_+________+________+________+

1 | 1825 | 1360 | 881 | 598 | 366 | 206 | 121 | 79 | 47 |

24 | 31831

| 5.73 | 4.27 | 2.77 | 1.88 | 1.15 | 0.65 | 0.38 | 0.25 | 0.15 |

0.08 | 100.00

| 5.73 | 4.27 | 2.77 | 1.88 | 1.15 | 0.65 | 0.38 | 0.25 | 0.15 |

0.08 |

| 100.00 | 100.00 | 100.00 | 100.00 | 100.00 | 100.00 | 100.00 | 100.00 | 100.00 |

100.00 |

______________+________+________+________+________+________+________+_______

_+________+________+________+

Total 1825 1360 881 598 366 206 121 79 47 24 31831

5.73 4.27 2.77 1.88 1.15 0.65 0.38 0.25

0.15 0.08 100.00

(continued)

Concomitant medications other than anticoagulants at the time of obtaining Table 2-5 consent, according to the presence or absence of proton-pump inhibitors

FREQ Procedure

Tables: ALL * TCMCNT

ALL TCMCNT(Total Number of Drugs Other than Anticoagulant)

Power |
Percent |
Percent of rows
Percent | 20| 21| 22| 23| 24| 25| 26| 27| Total

	20	21	22	23	24	25	26	27	Total
1	19	6	2	5	1	1	2	1	31831
	0.06	0.02	0.01	0.02	0.00	0.00	0.01	0.00	100.00
	0.06	0.02	0.01	0.02	0.00	0.00	0.01	0.00	
	100.00	100.00	100.00	100.00	100.00	100.00	100.00	100.00	
Total	19	6	2	5	1	1	2	1	31831
	0.06	0.02	0.01	0.02	0.00	0.00	0.01	0.00	100.00

Degree of missing values = 659

Concomitant medications other than anticoagulants at the time of obtaining Table 2-5 consent, according to the presence or absence of proton-pump inhibitors

FREQ Procedure

			Cumulative	
Percent of ALL	power	Percent of power		
1	32490	100.00	32490	100.00

Tables: ALL * CMYN

ALL

CMYN(Administration of Concomitant Drug)

Power |
Percent |
Percent of rows
Percent of columns | None | With | Total

	None	With	Total
1	1445	31045	32490
	4.45	95.55	100.00
	4.45	95.55	
	100.00	100.00	
Total	1445	31045	32490
	4.45	95.55	100.00

Concomitant medications other than anticoagulants at the time of obtaining Table 2-5 consent, according to the presence or absence of proton-pump inhibitors

FREQ Procedure

Tables: ALL * TCMCNT

ALL TCMCNT(Total Number of Drugs Other than Anticoagulant)

Power |
Percent |
Percent of rows
Percent|0|1|2|3|4|5|6|7|8|9| Total
______________+________+________+________+________+________+________+________
_+________+________+________+
1 | 52 | 688 | 1602 | 2622 | 3597 | 4105 | 4171 | 3747 | 3187 | 2516 | 31831
| 0.16 | 2.16 | 5.03 | 8.24 | 11.30 | 12.90 | 13.10 | 11.77 | 10.01 | 7.90 | 100.00
| 0.16 | 2.16 | 5.03 | 8.24 | 11.30 | 12.90 | 13.10 | 11.77 | 10.01 | 7.90 |
| 100.00 | 100.00 | 100.00 | 100.00 | 100.00 | 100.00 | 100.00 | 100.00 | 100.00 | 100.00 |
______________+________+________+________+________+________+________+________
_+________+________+________+
Total 52 688 1602 2622 3597 4105 4171 3747 3187 2516 31831
0.16 2.16 5.03 8.24 11.30 12.90 13.10 11.77 10.01 7.90 100.00
(continued)

Tables: ALL * TCMCNT

ALL TCMCNT(Total Number of Drugs Other than Anticoagulant)

Power |
Percent |

Percent of rows

Percent |10|11|12|13|14|15|16|17|18|19|Total

______________+________+________+________+________+________+________+_______

_+________+________+________+

1 | 1825 | 1360 | 881 | 598 | 366 | 206 | 121 | 79 | 47 |

24 | 31831

| 5.73 | 4.27 | 2.77 | 1.88 | 1.15 | 0.65 | 0.38 | 0.25 | 0.15 |

0.08 | 100.00

| 5.73 | 4.27 | 2.77 | 1.88 | 1.15 | 0.65 | 0.38 | 0.25 | 0.15 |

0.08 |

| 100.00 | 100.00 | 100.00 | 100.00 | 100.00 | 100.00 | 100.00 | 100.00 | 100.00 |

100.00 |

______________+________+________+________+________+________+________+_______

_+________+________+________+

Total 1825 1360 881 598 366 206 121 79 47 24 31831

5.73 4.27 2.77 1.88 1.15 0.65 0.38 0.25

0.15 0.08 100.00

(continued)

Concomitant medications other than anticoagulants at the time of obtaining Table 2-5 consent, according to the presence or absence of proton-pump inhibitors

FREQ Procedure

Tables: ALL * TCMCNT

ALL TCMCNT(Total Number of Drugs Other than Anticoagulant)

Power |
Percent |
Percent of rows
Percent | 20| 21| 22| 23| 24| 25| 26| 27| Total
______________+________+________+________+________+________+________+________+________+
1 | 19 | 6 | 2 | 5 | 1 | 1 | 2 | 1 | 31831
| 0.06 | 0.02 | 0.01 | 0.02 | 0.00 | 0.00 | 0.01 | 0.00 | 100.00
| 0.06 | 0.02 | 0.01 | 0.02 | 0.00 | 0.00 | 0.01 | 0.00 |
| 100.00 | 100.00 | 100.00 | 100.00 | 100.00 | 100.00 | 100.00 | 100.00 |
______________+________+________+________+________+________+________+________+________+
Total 19 6 2 5 1 1 2 1 31831
0.06 0.02 0.01 0.02 0.00 0.00 0.01 0.00 100.00

Degree of missing values = 659

Concomitant medications other than anticoagulants at the time of obtaining Table 2-5 consent, according to the presence or absence of proton-pump inhibitors

FREQ Procedure

			Cumulative	
Percent of ALL	power	Percent of	power	
1	32490	100.00	32490	100.00

Tables: ALL * CMYN

ALL

CMYN(Administration of Concomitant Drug)

Power |
Percent |
Percent of rows
Percent of columns | None | With | Total

	None	With	Total
1	1445	31045	32490
	4.45	95.55	100.00
	4.45	95.55	
	100.00	100.00	
Total	1445	31045	32490
	4.45	95.55	100.00

Concomitant medications other than anticoagulants at the time of obtaining Table 2-5 consent, according to the presence or absence of proton-pump inhibitors

FREQ Procedure

Table: ALL * t_cm

ALL t_cm

Power |
Percent |
Percent of rows
Percent of columns | 1| Total
______________+________+
1 | 37 | 37
| 100.00 | 100.00
| 100.00 |
| 100.00 |
______________+________+
Total 37 37
100.00 100.00

Frequency of missing values = 32453

Concomitant medications other than anticoagulants at the time of obtaining Table 2-5 consent, according to the presence or absence of proton-pump inhibitors

FREQ Procedure

			Cumulative	
Percent of ALL	power	Percent of	power	
1	32490	100.00	32490	100.00

Tables: ALL * CMYN

ALL
CMYN(Administration of Concomitant Drug)

Power |
Percent |
Percent of rows
Percent of columns | None | With | Total

	None	With	Total
1	1445	31045	32490
	4.45	95.55	100.00
	4.45	95.55	
	100.00	100.00	
Total	1445	31045	32490
	4.45	95.55	100.00

Concomitant medications other than anticoagulants at the time of obtaining Table 2-5 consent, according to the presence or absence of proton-pump inhibitors

FREQ Procedure

Table: ALL * t_TCMCNT

ALL t_TCMCNT

Power |
Percent |
Percent of rows
Percent of columns | 9| Total

1	659	659
	100.00	100.00
	100.00	
	100.00	
Total	659	659
	100.00	100.00

Degree of missing values = 31831

Concomitant medications other than anticoagulants at the time of obtaining Table 2-5 consent, according to the presence or absence of proton-pump inhibitors

FREQ Procedure

			Cumulative	
Percent of ALL power Percent of power				
1	32490	100.00	32490	100.00

Tables: ALL * CMYN

ALL

CMYN(Administration of Concomitant Drug)

Power / Percent / Percent of rows / Percent of columns	None	With	Total
1	1445	31045	32490
	4.45	95.55	100.00
	4.45	95.55	
	100.00	100.00	
Total	1445	31045	32490
	4.45	95.55	100.00

Concomitant medications other than anticoagulants at the time of obtaining Table 2-5 consent, according to the presence or absence of proton-pump inhibitors

FREQ Procedure

Tables: ALL* TCMCNTCT

ALL

TCMCNTCT(Category for Total Number of Drugs Other than Anticoagulant)

Power |

Percent |

Percent of rows

Percentage of column	0 to 4 or more Bottom	5 to 9 or more Bottom	10 or more	unknown	total
1	8561	17726	5544	659	32490
	26.35	54.56	17.06	2.03	100.00
	26.35	54.56	17.06	2.03	
	100.00	100.00	100.00	100.00	
Total	8561	17726	5544	659	32490
	26.35	54.56	17.06	2.03	100.00

Concomitant medications other than anticoagulants at the time of obtaining Table 2-5 consent, according to the presence or absence of proton-pump inhibitors

FREQ Procedure

			Cumulative	
Percent of ALL	power	Percent of	power	
1	32490	100.00	32490	100.00

Tables: ALL * CMYN

ALL

CMYN(Administration of Concomitant Drug)

Power | Percent | Percent of rows Percent of columns	None	With	Total
1	1445	31045	32490
	4.45	95.55	100.00
	4.45	95.55	
	100.00	100.00	
Total	1445	31045	32490
	4.45	95.55	100.00

Concomitant medications other than anticoagulants at the time of obtaining Table 2-5 consent, according to the presence or absence of proton-pump inhibitors

FREQ Procedure

Tables: ALL* TCMCNTCT

ALL

TCMCNTCT(Category for Total Number of Drugs Other than Anticoagulant)

Power / Percent / Percent of rows / Percentage of column	0 to 4 or more Bottom	5 to 9 or more Bottom	10 or more	unknown	total
1	8561	17726	5544	659	32490
	26.35	54.56	17.06	2.03	100.00
	26.35	54.56	17.06	2.03	
	100.00	100.00	100.00	100.00	
Total	8561	17726	5544	659	32490
	26.35	54.56	17.06	2.03	100.00

Concomitant medications other than anticoagulants at the time of obtaining Table 2-5 consent, according to the presence or absence of proton-pump inhibitors

FREQ Procedure

			Cumulative	
Percent of ALL	power	Percent of	power	
1	32490	100.00	32490	100.00

Tables: ALL * CMYN

ALL

CMYN(Administration of Concomitant Drug)

Power |
Percent |
Percent of rows
Percent of columns | None | With | Total

	None	With	Total
1	1445	31045	32490
	4.45	95.55	100.00
	4.45	95.55	
	100.00	100.00	
Total	1445	31045	32490
	4.45	95.55	100.00

Concomitant medications other than anticoagulants at the time of obtaining Table 2-5 consent, according to the presence or absence of proton-pump inhibitors

FREQ Procedure

Tables: ALL* TCMCNTCT

ALL

TCMCNTCT(Category for Total Number of Drugs Other than Anticoagulant)

Power |
Percent |
Percent of rows
Percentage of column |0 to 4 or more|5 to 9 or more|10 or more|unknown|total
|Bottom|Bottom | | |

1	8561	17726	5544	659	32490
	26.35	54.56	17.06	2.03	100.00
	26.35	54.56	17.06	2.03	
	100.00	100.00	100.00	100.00	
Total	8561	17726	5544	659	32490
	26.35	54.56	17.06	2.03	100.00

Concomitant medications other than anticoagulants at the time of obtaining Table 2-5 consent, according to the presence or absence of proton-pump inhibitors

FREQ Procedure

			Cumulative	
Percent of ALL	power	Percent of power		
1	32490	100.00	32490	100.00

Tables: ALL * CMYN

ALL

CMYN(Administration of Concomitant Drug)

Power | Percent | Percent of rows Percent of columns	None	With	Total
1	1445	31045	32490
	4.45	95.55	100.00
	4.45	95.55	
	100.00	100.00	
Total	1445	31045	32490
	4.45	95.55	100.00

Concomitant medications other than anticoagulants at the time of obtaining Table 2-5 consent, according to the presence or absence of proton-pump inhibitors

FREQ Procedure

Tables: ALL* TCMCNTCT

ALL

TCMCNTCT(Category for Total Number of Drugs Other than Anticoagulant)

Power |
Percent |
Percent of rows
Percentage of column |0 to 4 or more|5 to 9 or more|10 or more|unknown|total
|Bottom|Bottom | | |

1	8561	17726	5544	659	32490
	26.35	54.56	17.06	2.03	100.00
	26.35	54.56	17.06	2.03	
	100.00	100.00	100.00	100.00	
Total	8561	17726	5544	659	32490
	26.35	54.56	17.06	2.03	100.00

Concomitant medications other than anticoagulants at the time of obtaining Table 2-5 consent, according to the presence or absence of proton-pump inhibitors

FREQ Procedure

			Cumulative	
Percent of ALL power Percent of power				
1	11981	100.00	11981	100.00

Tables: ALL * CMYN

ALL

CMYN(Administration of Concomitant Drug)

Power |
Percent |
Percent of rows
Percentage of columns | with | total

```
______________+________+
         1 |   11981 |   11981
           | 100.00 | 100.00
           | 100.00 |
           | 100.00 |
______________+________+
Total 11981 11981
            100.00    100.00
```

Concomitant medications other than anticoagulants at the time of obtaining Table 2-5 consent, according to the presence or absence of proton-pump inhibitors

FREQ Procedure

Tables: ALL * CMYN

ALL
CMYN(Administration of Concomitant Drug)

Power |
Percent |
Percent of rows
Percentage of columns | with | total

1	11981	11981
	100.00	100.00
	100.00	
	100.00	
Total	11981	11981
	100.00	100.00

Concomitant medications other than anticoagulants at the time of obtaining Table 2-5 consent, according to the presence or absence of proton-pump inhibitors

FREQ Procedure

			Cumulative	
Percent of ALL	power	Percent of power		
1	11981	100.00	11981	100.00

Tables: ALL * CMYN

ALL

CMYN(Administration of Concomitant Drug)

Power |
Percent |
Percent of rows
Percentage of columns | with | total

1	11981	11981
	100.00	100.00
	100.00	
	100.00	
Total	11981	11981
	100.00	100.00

Concomitant medications other than anticoagulants at the time of obtaining Table 2-5 consent, according to the presence or absence of proton-pump inhibitors

FREQ Procedure

Tables: ALL * CMYN

ALL
CMYN(Administration of Concomitant Drug)

Power |
Percent |
Percent of rows
Percentage of columns | with | total

______________+________+
1 | 11981 | 11981
| 100.00 | 100.00
| 100.00 |
| 100.00 |
______________+________+
Total 11981 11981
100.00 100.00

Concomitant medications other than anticoagulants at the time of obtaining Table 2-5 consent, according to the presence or absence of proton-pump inhibitors

FREQ Procedure

			Cumulative	
Percent of ALL	power	Percent of power		
1	11981	100.00	11981	100.00

Tables: ALL * CMYN

ALL

CMYN(Administration of Concomitant Drug)

Power |
Percent |
Percent of rows
Percentage of columns | with | total

1	11981	11981
	100.00	100.00
	100.00	
	100.00	
Total	11981	11981
	100.00	100.00

Concomitant medications other than anticoagulants at the time of obtaining Table 2-5 consent, according to the presence or absence of proton-pump inhibitors

FREQ Procedure

Tables: ALL * CMYN

ALL

CMYN(Administration of Concomitant Drug)

Power |
Percent |
Percent of rows
Percentage of columns | with | total

______________+________+

1 | 11981 | 11981
| 100.00 | 100.00
| 100.00 |
| 100.00 |

______________+________+

Total 11981 11981
100.00 100.00

Concomitant medications other than anticoagulants at the time of obtaining Table 2-5 consent, according to the presence or absence of proton-pump inhibitors

FREQ Procedure

			Cumulative	
Percent of ALL	power	Percent of	power	
1	11981	100.00	11981	100.00

Tables: ALL * CMYN

ALL

CMYN(Administration of Concomitant Drug)

Power |
Percent |
Percent of rows
Percentage of columns | with | total

1	11981	11981
	100.00	100.00
	100.00	
	100.00	
Total	11981	11981
	100.00	100.00

Concomitant medications other than anticoagulants at the time of obtaining Table 2-5 consent, according to the presence or absence of proton-pump inhibitors

FREQ Procedure

Tables: ALL* ARTD

ALL ARTD(Arrhythmic Drugs)

Power |
Percent |
Percent of rows
Percent of columns | None | With | Total

1	4835	7146	11981
	40.36	59.64	100.00
	40.36	59.64	
	100.00	100.00	
Total	4835	7146	11981
	40.36	59.64	100.00

Concomitant medications other than anticoagulants at the time of obtaining Table 2-5 consent, according to the presence or absence of proton-pump inhibitors

FREQ Procedure

Cumulative

Percent of ALL power Percent of power

1	11981	100.00	11981	100.00

Tables: ALL * CMYN

ALL

CMYN(Administration of Concomitant Drug)

Power |

Percent |

Percent of rows

Percentage of columns | with | total

______________+________+

1 | 11981 | 11981

| 100.00 | 100.00

| 100.00 |

| 100.00 |

______________+________+

Total 11981 11981

100.00 100.00

Concomitant medications other than anticoagulants at the time of obtaining Table 2-5 consent, according to the presence or absence of proton-pump inhibitors

FREQ Procedure

Tables: ALL* ARTD

ALL ARTD(Arrhythmic Drugs)

Power / Percent / Percent of rows / Percent of columns	None	With	Total
1	4835	7146	11981
	40.36	59.64	100.00
	40.36	59.64	
	100.00	100.00	
Total	4835	7146	11981
	40.36	59.64	100.00

Concomitant medications other than anticoagulants at the time of obtaining Table 2-5 consent, according to the presence or absence of proton-pump inhibitors

FREQ Procedure

			Cumulative	
Percent of ALL	power	Percent	of power	
1	11981	100.00	11981	100.00

Tables: ALL * CMYN

ALL

CMYN(Administration of Concomitant Drug)

Power |
Percent |
Percent of rows
Percentage of columns | with | total

1	11981	11981
	100.00	100.00
	100.00	
	100.00	
Total	11981	11981
	100.00	100.00

Concomitant medications other than anticoagulants at the time of obtaining Table 2-5 consent, according to the presence or absence of proton-pump inhibitors

FREQ Procedure

Table : ALL * ARTD1

ALL ARTD1(Rhythm Control)

Power |
Percent |
Percent of rows
Percent of columns | None | With | Total

1	9496	2485	11981
	79.26	20.74	100.00
	79.26	20.74	
	100.00	100.00	
Total	9496	2485	11981
	79.26	20.74	100.00

Concomitant medications other than anticoagulants at the time of obtaining Table 2-5 consent, according to the presence or absence of proton-pump inhibitors

FREQ Procedure

			Cumulative	
Percent of ALL	power	Percent of	power	
1	11981	100.00	11981	100.00

Tables: ALL * CMYN

ALL

CMYN(Administration of Concomitant Drug)

Power |
Percent |
Percent of rows
Percentage of columns | with | total

1	11981	11981
	100.00	100.00
	100.00	
	100.00	
Total	11981	11981
	100.00	100.00

Concomitant medications other than anticoagulants at the time of obtaining Table 2-5 consent, according to the presence or absence of proton-pump inhibitors

FREQ Procedure

Table : ALL * ARTD1

ALL ARTD1(Rhythm Control)

Power<br>Percent<br>Percent of rows<br>Percent of columns	None	With	Total
1	9496<br>79.26<br>79.26<br>100.00	2485<br>20.74<br>20.74<br>100.00	11981<br>100.00
Total	9496<br>79.26	2485<br>20.74	11981<br>100.00

Concomitant medications other than anticoagulants at the time of obtaining Table 2-5 consent, according to the presence or absence of proton-pump inhibitors

FREQ Procedure

			Cumulative	
Percent of ALL	power	Percent of	power	
1	11981	100.00	11981	100.00

Tables: ALL * CMYN

ALL

CMYN(Administration of Concomitant Drug)

Power |
Percent |
Percent of rows
Percentage of columns | with | total
______________+________+
1 | 11981 | 11981
| 100.00 | 100.00
| 100.00 |
| 100.00 |
______________+________+
Total 11981 11981
100.00 100.00

Concomitant medications other than anticoagulants at the time of obtaining Table 2-5 consent, according to the presence or absence of proton-pump inhibitors

FREQ Procedure

Table : ALL * ARTD2

ALL ARTD2(Rate Control)

Power |
Percent |
Percent of rows
Percent of columns | None | With | Total

1	6708	5273	11981
	55.99	44.01	100.00
	55.99	44.01	
	100.00	100.00	
Total	6708	5273	11981
	55.99	44.01	100.00

Concomitant medications other than anticoagulants at the time of obtaining Table 2-5 consent, according to the presence or absence of proton-pump inhibitors

FREQ Procedure

			Cumulative	
Percent of ALL	power	Percent of power		
1	11981	100.00	11981	100.00

Tables: ALL * CMYN

ALL

CMYN(Administration of Concomitant Drug)

Power |
Percent |
Percent of rows
Percentage of columns | with | total

1	11981	11981
	100.00	100.00
	100.00	
	100.00	
Total	11981	11981
	100.00	100.00

Concomitant medications other than anticoagulants at the time of obtaining Table 2-5 consent, according to the presence or absence of proton-pump inhibitors

FREQ Procedure

Table : ALL * ARTD2

ALL ARTD2(Rate Control)

Power<br>Percent<br>Percent of rows<br>Percent of columns	None	With	Total
1	6708<br>55.99<br>55.99<br>100.00	5273<br>44.01<br>44.01<br>100.00	11981<br>100.00
Total	6708<br>55.99	5273<br>44.01	11981<br>100.00

Concomitant medications other than anticoagulants at the time of obtaining Table 2-5 consent, according to the presence or absence of proton-pump inhibitors

FREQ Procedure

Cumulative
Percent of ALL power Percent of power

1	11981	100.00	11981	100.00

Tables: ALL * CMYN

ALL
CMYN(Administration of Concomitant Drug)

Power |
Percent |
Percent of rows
Percentage of columns | with | total

1	11981<br>100.00<br>100.00<br>100.00	11981<br>100.00
Total	11981<br>100.00	11981<br>100.00

Concomitant medications other than anticoagulants at the time of obtaining Table 2-5 consent, according to the presence or absence of proton-pump inhibitors

FREQ Procedure

Tables: ALL* APLD

ALL APLD(Anti-Platelet Drugs)

Power / Percent / Percent of rows / Percent of columns	None	With	Total
1	8965	3016	11981
	74.83	25.17	100.00
	74.83	25.17	
	100.00	100.00	
Total	8965	3016	11981
	74.83	25.17	100.00

Concomitant medications other than anticoagulants at the time of obtaining Table 2-5 consent, according to the presence or absence of proton-pump inhibitors

FREQ Procedure

			Cumulative	
Percent of ALL	power	Percent of power		
1	11981	100.00	11981	100.00

Tables: ALL * CMYN

ALL

CMYN(Administration of Concomitant Drug)

Power |
Percent |
Percent of rows
Percentage of columns | with | total

______________+________+
1 | 11981 | 11981
| 100.00 | 100.00
| 100.00 |
| 100.00 |
______________+________+
Total 11981 11981
100.00 100.00

Concomitant medications other than anticoagulants at the time of obtaining Table 2-5 consent, according to the presence or absence of proton-pump inhibitors

FREQ Procedure

Tables: ALL* APLD

ALL APLD(Anti-Platelet Drugs)

Power |
Percent |
Percent of rows
Percent of columns | None | With | Total

1	8965	3016	11981
	74.83	25.17	100.00
	74.83	25.17	
	100.00	100.00	
Total	8965	3016	11981
	74.83	25.17	100.00

Concomitant medications other than anticoagulants at the time of obtaining Table 2-5 consent, according to the presence or absence of proton-pump inhibitors

FREQ Procedure

			Cumulative	
Percent of ALL	power	Percent of	power	
1	11981	100.00	11981	100.00

Tables: ALL * CMYN

ALL

CMYN(Administration of Concomitant Drug)

Power |
Percent |
Percent of rows
Percentage of columns | with | total

1	11981	11981
	100.00	100.00
	100.00	
	100.00	
Total	11981	11981
	100.00	100.00

Concomitant medications other than anticoagulants at the time of obtaining Table 2-5 consent, according to the presence or absence of proton-pump inhibitors

FREQ Procedure

Table: ALL * t_AP

ALL t_AP

Power |
Percent |
Percent of rows
Percentage of columns | 0| 1| Sum

1	11824	157	11981
	98.69	1.31	100.00
	98.69	1.31	
	100.00	100.00	
Total	11824	157	11981
	98.69	1.31	100.00

Concomitant medications other than anticoagulants at the time of obtaining Table 2-5 consent, according to the presence or absence of proton-pump inhibitors

FREQ Procedure

			Cumulative	
Percent of ALL	power	Percent of power		
1	11981	100.00	11981	100.00

Tables: ALL * CMYN

ALL

CMYN(Administration of Concomitant Drug)

Power |
Percent |
Percent of rows
Percentage of columns | with | total

1	11981	11981
	100.00	100.00
	100.00	
	100.00	
Total	11981	11981
	100.00	100.00

Concomitant medications other than anticoagulants at the time of obtaining Table 2-5 consent, according to the presence or absence of proton-pump inhibitors

FREQ Procedure

Table: ALL * t_AP

ALL t_AP

Power / Percent / Percent of rows / Percentage of columns	0	1	Sum
1	11824	157	11981
	98.69	1.31	100.00
	98.69	1.31	
	100.00	100.00	
Total	11824	157	11981
	98.69	1.31	100.00

Concomitant medications other than anticoagulants at the time of obtaining Table 2-5 consent, according to the presence or absence of proton-pump inhibitors

FREQ Procedure

			Cumulative	
Percent of ALL	power	Percent of	power	
1	11981	100.00	11981	100.00

Tables: ALL * CMYN

ALL

CMYN(Administration of Concomitant Drug)

Power |
Percent |
Percent of rows
Percentage of columns | with | total

1	11981	11981
	100.00	100.00
	100.00	
	100.00	
Total	11981	11981
	100.00	100.00

Concomitant medications other than anticoagulants at the time of obtaining Table 2-5 consent, according to the presence or absence of proton-pump inhibitors

FREQ Procedure

Table : ALL * APLD1

ALL APLD1(ASA)

Power<br>Percent<br>Percent of rows<br>Percent of columns	None	With	Total
1	10026<br>83.68<br>83.68<br>100.00	1955<br>16.32<br>16.32<br>100.00	11981<br>100.00
Total	10026<br>83.68	1955<br>16.32	11981<br>100.00

Concomitant medications other than anticoagulants at the time of obtaining Table 2-5 consent, according to the presence or absence of proton-pump inhibitors

FREQ Procedure

			Cumulative	
Percent of ALL	power	Percent of	power	
1	11981	100.00	11981	100.00

Tables: ALL * CMYN

ALL

CMYN(Administration of Concomitant Drug)

Power |
Percent |
Percent of rows
Percentage of columns | with | total

1	11981	11981
	100.00	100.00
	100.00	
	100.00	
Total	11981	11981
	100.00	100.00

Concomitant medications other than anticoagulants at the time of obtaining Table 2-5 consent, according to the presence or absence of proton-pump inhibitors

FREQ Procedure

Table : ALL * APLD1

ALL APLD1(ASA)

Power / Percent / Percent of rows / Percent of columns	None	With	Total
1	10026	1955	11981
	83.68	16.32	100.00
	83.68	16.32	
	100.00	100.00	
Total	10026	1955	11981
	83.68	16.32	100.00

Concomitant medications other than anticoagulants at the time of obtaining Table 2-5 consent, according to the presence or absence of proton-pump inhibitors

FREQ Procedure

			Cumulative	
Percent of ALL	power	Percent of power		
1	11981	100.00	11981	100.00

Tables: ALL * CMYN

ALL

CMYN(Administration of Concomitant Drug)

Power |
Percent |
Percent of rows
Percentage of columns | with | total

1	11981	11981
	100.00	100.00
	100.00	
	100.00	
Total	11981	11981
	100.00	100.00

Concomitant medications other than anticoagulants at the time of obtaining Table 2-5 consent, according to the presence or absence of proton-pump inhibitors

FREQ Procedure

Table : ALL * APLD2

ALL APLD2(P2Y12-I)

Power |
Percent |
Percent of rows
Percent of columns | None | With | Total

1	11206	775	11981
	93.53	6.47	100.00
	93.53	6.47	
	100.00	100.00	
Total	11206	775	11981
	93.53	6.47	100.00

Concomitant medications other than anticoagulants at the time of obtaining Table 2-5 consent, according to the presence or absence of proton-pump inhibitors

FREQ Procedure

			Cumulative	
Percent of ALL power Percent of power				
1	11981	100.00	11981	100.00

Tables: ALL * CMYN

ALL

CMYN(Administration of Concomitant Drug)

Power |
Percent |
Percent of rows
Percentage of columns | with | total

1	11981	11981
	100.00	100.00
	100.00	
	100.00	
Total	11981	11981
	100.00	100.00

Concomitant medications other than anticoagulants at the time of obtaining Table 2-5 consent, according to the presence or absence of proton-pump inhibitors

FREQ Procedure

Table : ALL * APLD2

ALL APLD2(P2Y12-I)

Power |
Percent |
Percent of rows
Percent of columns | None | With | Total

1	11206	775	11981
	93.53	6.47	100.00
	93.53	6.47	
	100.00	100.00	
Total	11206	775	11981
	93.53	6.47	100.00

Concomitant medications other than anticoagulants at the time of obtaining Table 2-5 consent, according to the presence or absence of proton-pump inhibitors

FREQ Procedure

			Cumulative	
Percent of ALL	power	Percent of power		
1	11981	100.00	11981	100.00

Tables: ALL * CMYN

ALL

CMYN(Administration of Concomitant Drug)

Power |

Percent |

Percent of rows

Percentage of columns | with | total

______________+________+

1 | 11981 | 11981

| 100.00 | 100.00

| 100.00 |

| 100.00 |

______________+________+

Total 11981 11981

100.00 100.00

Concomitant medications other than anticoagulants at the time of obtaining Table 2-5 consent, according to the presence or absence of proton-pump inhibitors

FREQ Procedure

Table : ALL * APLD3

ALL

APLD3(Other Anti-Platelet Drugs)

Power<br>Percent<br>Percent of rows<br>Percent of columns	None	With	Total
1	11421<br>95.33<br>95.33<br>100.00	560<br>4.67<br>4.67<br>100.00	11981<br>100.00
Total	11421<br>95.33	560<br>4.67	11981<br>100.00

Concomitant medications other than anticoagulants at the time of obtaining Table 2-5 consent, according to the presence or absence of proton-pump inhibitors

FREQ Procedure

			Cumulative	
Percent of ALL	power	Percent of power		
1	11981	100.00	11981	100.00

Tables: ALL * CMYN

ALL

CMYN(Administration of Concomitant Drug)

Power |
Percent |
Percent of rows
Percentage of columns | with | total

______________+________+
1 | 11981 | 11981
| 100.00 | 100.00
| 100.00 |
| 100.00 |
______________+________+
Total 11981 11981
100.00 100.00

Concomitant medications other than anticoagulants at the time of obtaining Table 2-5 consent, according to the presence or absence of proton-pump inhibitors

FREQ Procedure

Table : ALL * APLD3

ALL

APLD3(Other Anti-Platelet Drugs)

Power |
Percent |
Percent of rows
Percent of columns | None | With | Total

1	11421	560	11981
	95.33	4.67	100.00
	95.33	4.67	
	100.00	100.00	
Total	11421	560	11981
	95.33	4.67	100.00

Concomitant medications other than anticoagulants at the time of obtaining Table 2-5 consent, according to the presence or absence of proton-pump inhibitors

FREQ Procedure

Percent of ALL	power	Percent of	Cumulative power	Cumulative Percent of power
1	11981	100.00	11981	100.00

Tables: ALL * CMYN

ALL

CMYN(Administration of Concomitant Drug)

Power |
Percent |
Percent of rows
Percentage of columns | with | total

1	11981<br>100.00<br>100.00<br>100.00	11981<br>100.00
Total	11981<br>100.00	11981<br>100.00

Concomitant medications other than anticoagulants at the time of obtaining Table 2-5 consent, according to the presence or absence of proton-pump inhibitors

FREQ Procedure

Tables: ALL * AHPD

ALL

AHPD(Antihypertensive Drugs)

Power |
Percent |
Percent of rows
Percent of columns | None | With | Total

1	3096	8885	11981
	25.84	74.16	100.00
	25.84	74.16	
	100.00	100.00	
Total	3096	8885	11981
	25.84	74.16	100.00

Concomitant medications other than anticoagulants at the time of obtaining Table 2-5 consent, according to the presence or absence of proton-pump inhibitors

FREQ Procedure

			Cumulative	
Percent of ALL	power	Percent	of power	
1	11981	100.00	11981	100.00

Tables: ALL * CMYN

ALL

CMYN(Administration of Concomitant Drug)

Power |
Percent |
Percent of rows
Percentage of columns | with | total

1	11981	11981
	100.00	100.00
	100.00	
	100.00	
Total	11981	11981
	100.00	100.00

Concomitant medications other than anticoagulants at the time of obtaining Table 2-5 consent, according to the presence or absence of proton-pump inhibitors

FREQ Procedure

Tables: ALL * AHPD

ALL

AHPD(Antihypertensive Drugs)

Power / Percent / Percent of rows / Percent of columns	None	With	Total
1	3096	8885	11981
	25.84	74.16	100.00
	25.84	74.16	
	100.00	100.00	
Total	3096	8885	11981
	25.84	74.16	100.00

Concomitant medications other than anticoagulants at the time of obtaining Table 2-5 consent, according to the presence or absence of proton-pump inhibitors

FREQ Procedure

			Cumulative	
Percent of ALL	power	Percent of	power	
1	11981	100.00	11981	100.00

Tables: ALL * CMYN

ALL

CMYN(Administration of Concomitant Drug)

Power |
Percent |
Percent of rows
Percentage of columns | with | total

1	11981	11981
	100.00	100.00
	100.00	
	100.00	
Total	11981	11981
	100.00	100.00

Concomitant medications other than anticoagulants at the time of obtaining Table 2-5 consent, according to the presence or absence of proton-pump inhibitors

FREQ Procedure

Table: ALL * LTD

ALL LTD(Lipid Therapy)

Power |
Percent |
Percent of rows
Percent of columns | None | With | Total

	None	With	Total
1	6650	5331	11981
	55.50	44.50	100.00
	55.50	44.50	
	100.00	100.00	
Total	6650	5331	11981
	55.50	44.50	100.00

Concomitant medications other than anticoagulants at the time of obtaining Table 2-5 consent, according to the presence or absence of proton-pump inhibitors

FREQ Procedure

			Cumulative	
Percent of ALL	power	Percent of power		
1	11981	100.00	11981	100.00

Tables: ALL * CMYN

ALL

CMYN(Administration of Concomitant Drug)

Power |
Percent |
Percent of rows
Percentage of columns | with | total

1	11981	11981
	100.00	100.00
	100.00	
	100.00	
Total	11981	11981
	100.00	100.00

Concomitant medications other than anticoagulants at the time of obtaining Table 2-5 consent, according to the presence or absence of proton-pump inhibitors

FREQ Procedure

Table: ALL * LTD

ALL LTD(Lipid Therapy)

Power |
Percent |
Percent of rows
Percent of columns | None | With | Total

	None	With	Total
1	6650	5331	11981
	55.50	44.50	100.00
	55.50	44.50	
	100.00	100.00	
Total	6650	5331	11981
	55.50	44.50	100.00

Concomitant medications other than anticoagulants at the time of obtaining Table 2-5 consent, according to the presence or absence of proton-pump inhibitors

FREQ Procedure

			Cumulative	
Percent of ALL	power	Percent of power		
1	11981	100.00	11981	100.00

Tables: ALL * CMYN

ALL
CMYN(Administration of Concomitant Drug)

Power |
Percent |
Percent of rows
Percentage of columns | with | total

1	11981	11981
	100.00	100.00
	100.00	
	100.00	
Total	11981	11981
	100.00	100.00

Concomitant medications other than anticoagulants at the time of obtaining Table 2-5 consent, according to the presence or absence of proton-pump inhibitors

FREQ Procedure

Table : ALL * LTD1

ALL LTD1(Statins)

Power |
Percent |
Percent of rows
Percent of columns | None | With | Total

1	7179	4802	11981
	59.92	40.08	100.00
	59.92	40.08	
	100.00	100.00	
Total	7179	4802	11981
	59.92	40.08	100.00

Concomitant medications other than anticoagulants at the time of obtaining Table 2-5 consent, according to the presence or absence of proton-pump inhibitors

FREQ Procedure

			Cumulative	
Percent of ALL	power	Percent of power		
1	11981	100.00	11981	100.00

Tables: ALL * CMYN

ALL

CMYN(Administration of Concomitant Drug)

Power |
Percent |
Percent of rows
Percentage of columns | with | total

1	11981	11981
	100.00	100.00
	100.00	
	100.00	
Total	11981	11981
	100.00	100.00

Concomitant medications other than anticoagulants at the time of obtaining Table 2-5 consent, according to the presence or absence of proton-pump inhibitors

FREQ Procedure

Table : ALL * LTD1

ALL LTD1(Statins)

Power / Percent / Percent of rows / Percent of columns	None	With	Total
1	7179	4802	11981
	59.92	40.08	100.00
	59.92	40.08	
	100.00	100.00	
Total	7179	4802	11981
	59.92	40.08	100.00

Concomitant medications other than anticoagulants at the time of obtaining Table 2-5 consent, according to the presence or absence of proton-pump inhibitors

FREQ Procedure

			Cumulative	
Percent of ALL	power	Percent of power		
1	11981	100.00	11981	100.00

Tables: ALL * CMYN

ALL

CMYN(Administration of Concomitant Drug)

Power |
Percent |
Percent of rows
Percentage of columns | with | total

```
______________+________+
          1 |  11981 |  11981
            | 100.00 | 100.00
            | 100.00 |
            | 100.00 |
______________+________+
Total 11981 11981
             100.00   100.00
```

Concomitant medications other than anticoagulants at the time of obtaining Table 2-5 consent, according to the presence or absence of proton-pump inhibitors

FREQ Procedure

Table : ALL * LTD2

ALL LTD2(Other Than Statins)

Power / Percent / Percent of rows / Percent of columns	None	With	Total
1	11227	754	11981
	93.71	6.29	100.00
	93.71	6.29	
	100.00	100.00	
Total	11227	754	11981
	93.71	6.29	100.00

Concomitant medications other than anticoagulants at the time of obtaining Table 2-5 consent, according to the presence or absence of proton-pump inhibitors

FREQ Procedure

			Cumulative	
Percent of ALL	power	Percent of power		
1	11981	100.00	11981	100.00

Tables: ALL * CMYN

ALL

CMYN(Administration of Concomitant Drug)

Power |
Percent |
Percent of rows
Percentage of columns | with | total

1	11981	11981
	100.00	100.00
	100.00	
	100.00	
Total	11981	11981
	100.00	100.00

Concomitant medications other than anticoagulants at the time of obtaining Table 2-5 consent, according to the presence or absence of proton-pump inhibitors

FREQ Procedure

Table : ALL * LTD2

ALL LTD2(Other Than Statins)

Power<br>Percent<br>Percent of rows<br>Percent of columns	None	With	Total
1	11227<br>93.71<br>93.71<br>100.00	754<br>6.29<br>6.29<br>100.00	11981<br>100.00
Total	11227<br>93.71	754<br>6.29	11981<br>100.00

Concomitant medications other than anticoagulants at the time of obtaining Table 2-5 consent, according to the presence or absence of proton-pump inhibitors

FREQ Procedure

			Cumulative	
Percent of ALL	power	Percent of	power	
1	11981	100.00	11981	100.00

Tables: ALL * CMYN

ALL

CMYN(Administration of Concomitant Drug)

Power |
Percent |
Percent of rows
Percentage of columns | with | total

1	11981	11981
	100.00	100.00
	100.00	
	100.00	
Total	11981	11981
	100.00	100.00

Concomitant medications other than anticoagulants at the time of obtaining Table 2-5 consent, according to the presence or absence of proton-pump inhibitors

FREQ Procedure

Tables: ALL * DBTD

ALL DBTD(Diabetes Drugs)

Power |
Percent |
Percent of rows
Percent of columns | None | With | Total

1	9802	2179	11981
	81.81	18.19	100.00
	81.81	18.19	
	100.00	100.00	
Total	9802	2179	11981
	81.81	18.19	100.00

Concomitant medications other than anticoagulants at the time of obtaining Table 2-5 consent, according to the presence or absence of proton-pump inhibitors

FREQ Procedure

			Cumulative	
Percent of ALL	power	Percent of	power	
1	11981	100.00	11981	100.00

Tables: ALL * CMYN

ALL

CMYN(Administration of Concomitant Drug)

Power |
Percent |
Percent of rows
Percentage of columns | with | total

1	11981	11981
	100.00	100.00
	100.00	
	100.00	
Total	11981	11981
	100.00	100.00

Concomitant medications other than anticoagulants at the time of obtaining Table 2-5 consent, according to the presence or absence of proton-pump inhibitors

FREQ Procedure

Tables: ALL * DBTD

ALL DBTD(Diabetes Drugs)

Power |
Percent |
Percent of rows
Percent of columns | None | With | Total

Power / Percent / Percent of rows / Percent of columns	None	With	Total
1	9802	2179	11981
	81.81	18.19	100.00
	81.81	18.19	
	100.00	100.00	
Total	9802	2179	11981
	81.81	18.19	100.00

Concomitant medications other than anticoagulants at the time of obtaining Table 2-5 consent, according to the presence or absence of proton-pump inhibitors

FREQ Procedure

Cumulative
Percent of ALL power Percent of power

1	11981	100.00	11981	100.00

Tables: ALL * CMYN

ALL
CMYN(Administration of Concomitant Drug)

Power |
Percent |
Percent of rows
Percentage of columns | with | total

1	11981	11981
	100.00	100.00
	100.00	
	100.00	
Total	11981	11981
	100.00	100.00

Concomitant medications other than anticoagulants at the time of obtaining Table 2-5 consent, according to the presence or absence of proton-pump inhibitors

FREQ Procedure

Table: ALL * t_DBT

ALL t_DBT

Power |
Percent |
Percent of rows
Percentage of columns | 0| 1| Sum

1	9919	2062	11981
	82.79	17.21	100.00
	82.79	17.21	
	100.00	100.00	
Total	9919	2062	11981
	82.79	17.21	100.00

Concomitant medications other than anticoagulants at the time of obtaining Table 2-5 consent, according to the presence or absence of proton-pump inhibitors

FREQ Procedure

			Cumulative	
	Percent of ALL power	Percent of power		
1	11981	100.00	11981	100.00

Tables: ALL * CMYN

ALL

CMYN(Administration of Concomitant Drug)

Power |

Percent |

Percent of rows

Percentage of columns | with | total

______________+________+

1 | 11981 | 11981

| 100.00 | 100.00

| 100.00 |

| 100.00 |

______________+________+

Total 11981 11981

100.00 100.00

Concomitant medications other than anticoagulants at the time of obtaining Table 2-5 consent, according to the presence or absence of proton-pump inhibitors

FREQ Procedure

Table: ALL * t_DBT

ALL t_DBT

Power |
Percent |
Percent of rows
Percentage of columns | 0| 1| Sum

	0	1	Sum
1	9919	2062	11981
	82.79	17.21	100.00
	82.79	17.21	
	100.00	100.00	
Total	9919	2062	11981
	82.79	17.21	100.00

Concomitant medications other than anticoagulants at the time of obtaining Table 2-5 consent, according to the presence or absence of proton-pump inhibitors

FREQ Procedure

			Cumulative	
Percent of ALL	power	Percent of power		
1	11981	100.00	11981	100.00

Tables: ALL * CMYN

ALL

CMYN(Administration of Concomitant Drug)

Power |
Percent |
Percent of rows
Percentage of columns | with | total
______________+________+
1 | 11981 | 11981
| 100.00 | 100.00
| 100.00 |
| 100.00 |
______________+________+
Total 11981 11981
100.00 100.00

Concomitant medications other than anticoagulants at the time of obtaining Table 2-5 consent, according to the presence or absence of proton-pump inhibitors

FREQ Procedure

Table : ALL * DBTD1

ALL DBTD1(Sulfonylurea Drugs)

Power<br>Percent<br>Percent of rows<br>Percent of columns	None	With	Total
1	11508<br>96.05<br>96.05<br>100.00	473<br>3.95<br>3.95<br>100.00	11981<br>100.00
Total	11508<br>96.05	473<br>3.95	11981<br>100.00

Concomitant medications other than anticoagulants at the time of obtaining Table 2-5 consent, according to the presence or absence of proton-pump inhibitors

FREQ Procedure

			Cumulative	
Percent of ALL	power	Percent of	power	
1	11981	100.00	11981	100.00

Tables: ALL * CMYN

ALL

CMYN(Administration of Concomitant Drug)

Power |
Percent |
Percent of rows
Percentage of columns | with | total

1	11981	11981
	100.00	100.00
	100.00	
	100.00	
Total	11981	11981
	100.00	100.00

Concomitant medications other than anticoagulants at the time of obtaining Table 2-5 consent, according to the presence or absence of proton-pump inhibitors

FREQ Procedure

Table : ALL * DBTD1

ALL DBTD1(Sulfonylurea Drugs)

Power |
Percent |
Percent of rows
Percent of columns | None | With | Total

	None	With	Total
1	11508	473	11981
	96.05	3.95	100.00
	96.05	3.95	
	100.00	100.00	
Total	11508	473	11981
	96.05	3.95	100.00

Concomitant medications other than anticoagulants at the time of obtaining Table 2-5 consent, according to the presence or absence of proton-pump inhibitors

FREQ Procedure

			Cumulative	
Percent of ALL power Percent of power				
1	11981	100.00	11981	100.00

Tables: ALL * CMYN

ALL

CMYN(Administration of Concomitant Drug)

Power |
Percent |
Percent of rows
Percentage of columns | with | total

1	11981	11981
	100.00	100.00
	100.00	
	100.00	
Total	11981	11981
	100.00	100.00

Concomitant medications other than anticoagulants at the time of obtaining Table 2-5 consent, according to the presence or absence of proton-pump inhibitors

FREQ Procedure

Table : ALL * DBTD2

ALL

DBTD2(α-Glucosidase Inhibitor)

Power / Percent / Percent of rows / Percent of columns	None	With	Total
1	11592	389	11981
	96.75	3.25	100.00
	96.75	3.25	
	100.00	100.00	
Total	11592	389	11981
	96.75	3.25	100.00

Concomitant medications other than anticoagulants at the time of obtaining Table 2-5 consent, according to the presence or absence of proton-pump inhibitors

FREQ Procedure

			Cumulative	
Percent of ALL	power	Percent of power		
1	11981	100.00	11981	100.00

Tables: ALL * CMYN

ALL

CMYN(Administration of Concomitant Drug)

Power |
Percent |
Percent of rows
Percentage of columns | with | total
______________+________+
1 | 11981 | 11981
| 100.00 | 100.00
| 100.00 |
| 100.00 |
______________+________+
Total 11981 11981
100.00 100.00

Concomitant medications other than anticoagulants at the time of obtaining Table 2-5 consent, according to the presence or absence of proton-pump inhibitors

FREQ Procedure

Table : ALL * DBTD2

ALL

DBTD2(α-Glucosidase Inhibitor)

Power / Percent / Percent of rows / Percent of columns	None	With	Total
1	11592	389	11981
	96.75	3.25	100.00
	96.75	3.25	
	100.00	100.00	
Total	11592	389	11981
	96.75	3.25	100.00

Concomitant medications other than anticoagulants at the time of obtaining Table 2-5 consent, according to the presence or absence of proton-pump inhibitors

FREQ Procedure

			Cumulative	
Percent of ALL	power	Percent of	power	
1	11981	100.00	11981	100.00

Tables: ALL * CMYN

ALL

CMYN(Administration of Concomitant Drug)

Power |
Percent |
Percent of rows
Percentage of columns | with | total

______________+________+

1 | 11981 | 11981
| 100.00 | 100.00
| 100.00 |
| 100.00 |

______________+________+

Total 11981 11981
100.00 100.00

Concomitant medications other than anticoagulants at the time of obtaining Table 2-5 consent, according to the presence or absence of proton-pump inhibitors

FREQ Procedure

Table : ALL * DBTD3

ALL DBTD3(Thiazolidine)

Power |
Percent |
Percent of rows
Percent of columns | None | With | Total

	None	With	Total
1	11856	125	11981
	98.96	1.04	100.00
	98.96	1.04	
	100.00	100.00	
Total	11856	125	11981
	98.96	1.04	100.00

Concomitant medications other than anticoagulants at the time of obtaining Table 2-5 consent, according to the presence or absence of proton-pump inhibitors

FREQ Procedure

			Cumulative	
Percent of ALL	power	Percent of	power	
1	11981	100.00	11981	100.00

Tables: ALL * CMYN

ALL

CMYN(Administration of Concomitant Drug)

Power |
Percent |
Percent of rows
Percentage of columns | with | total

______________+________+
1 | 11981 | 11981
| 100.00 | 100.00
| 100.00 |
| 100.00 |
______________+________+
Total 11981 11981
100.00 100.00

Concomitant medications other than anticoagulants at the time of obtaining Table 2-5 consent, according to the presence or absence of proton-pump inhibitors

FREQ Procedure

Table : ALL * DBTD3

ALL DBTD3(Thiazolidine)

Power / Percent / Percent of rows / Percent of columns	None	With	Total
1	11856	125	11981
	98.96	1.04	100.00
	98.96	1.04	
	100.00	100.00	
Total	11856	125	11981
	98.96	1.04	100.00

Concomitant medications other than anticoagulants at the time of obtaining Table 2-5 consent, according to the presence or absence of proton-pump inhibitors

FREQ Procedure

			Cumulative	
Percent of ALL	power	Percent of power		
1	11981	100.00	11981	100.00

Tables: ALL * CMYN

ALL

CMYN(Administration of Concomitant Drug)

Power |
Percent |
Percent of rows
Percentage of columns | with | total
______________+________+
1 | 11981 | 11981
| 100.00 | 100.00
| 100.00 |
| 100.00 |
______________+________+
Total 11981 11981
100.00 100.00

Concomitant medications other than anticoagulants at the time of obtaining Table 2-5 consent, according to the presence or absence of proton-pump inhibitors

FREQ Procedure

Table : ALL * DBTD4

ALL DBTD4(DPP-4 Inhabitors)

Power |
Percent |
Percent of rows
Percent of columns | None | With | Total

1	10310	1671	11981
	86.05	13.95	100.00
	86.05	13.95	
	100.00	100.00	
Total	10310	1671	11981
	86.05	13.95	100.00

Concomitant medications other than anticoagulants at the time of obtaining Table 2-5 consent, according to the presence or absence of proton-pump inhibitors

FREQ Procedure

		Cumulative		
Percent of ALL power Percent of power				
1	11981	100.00	11981	100.00

Tables: ALL * CMYN

ALL

CMYN(Administration of Concomitant Drug)

Power |
Percent |
Percent of rows
Percentage of columns | with | total

1	11981	11981
	100.00	100.00
	100.00	
	100.00	
Total	11981	11981
	100.00	100.00

Concomitant medications other than anticoagulants at the time of obtaining Table 2-5 consent, according to the presence or absence of proton-pump inhibitors

FREQ Procedure

Table : ALL * DBTD4

ALL DBTD4(DPP-4 Inhabitors)

Power |
Percent |
Percent of rows
Percent of columns | None | With | Total

	None	With	Total
1	10310	1671	11981
	86.05	13.95	100.00
	86.05	13.95	
	100.00	100.00	
Total	10310	1671	11981
	86.05	13.95	100.00

Concomitant medications other than anticoagulants at the time of obtaining Table 2-5 consent, according to the presence or absence of proton-pump inhibitors

FREQ Procedure

			Cumulative	
Percent of ALL power Percent of power				
1	11981	100.00	11981	100.00

Tables: ALL * CMYN

ALL

CMYN(Administration of Concomitant Drug)

Power |
Percent |
Percent of rows
Percentage of columns | with | total

1	11981	11981
	100.00	100.00
	100.00	
	100.00	
Total	11981	11981
	100.00	100.00

Concomitant medications other than anticoagulants at the time of obtaining Table 2-5 consent, according to the presence or absence of proton-pump inhibitors

FREQ Procedure

Table : ALL * DBTD5

ALL DBTD5(GLP-1 Inhabitors)

Power |
Percent |
Percent of rows
Percent of columns | None | With | Total

1	11943	38	11981
	99.68	0.32	100.00
	99.68	0.32	
	100.00	100.00	
Total	11943	38	11981
	99.68	0.32	100.00

Concomitant medications other than anticoagulants at the time of obtaining Table 2-5 consent, according to the presence or absence of proton-pump inhibitors

FREQ Procedure

			Cumulative	
Percent of ALL	power	Percent of power		
1	11981	100.00	11981	100.00

Tables: ALL * CMYN

ALL

CMYN(Administration of Concomitant Drug)

Power |
Percent |
Percent of rows
Percentage of columns | with | total

1	11981	11981
	100.00	100.00
	100.00	
	100.00	
Total	11981	11981
	100.00	100.00

Concomitant medications other than anticoagulants at the time of obtaining Table 2-5 consent, according to the presence or absence of proton-pump inhibitors

FREQ Procedure

Table : ALL * DBTD5

ALL DBTD5(GLP-1 Inhabitors)

Power |
Percent |
Percent of rows
Percent of columns | None | With | Total

1	11943	38	11981
	99.68	0.32	100.00
	99.68	0.32	
	100.00	100.00	
Total	11943	38	11981
	99.68	0.32	100.00

Concomitant medications other than anticoagulants at the time of obtaining Table 2-5 consent, according to the presence or absence of proton-pump inhibitors

FREQ Procedure

			Cumulative	
Percent of ALL	power	Percent of power		
1	11981	100.00	11981	100.00

Tables: ALL * CMYN

ALL

CMYN(Administration of Concomitant Drug)

Power |
Percent |
Percent of rows
Percentage of columns | with | total

1	11981	11981
	100.00	100.00
	100.00	
	100.00	
Total	11981	11981
	100.00	100.00

Concomitant medications other than anticoagulants at the time of obtaining Table 2-5 consent, according to the presence or absence of proton-pump inhibitors

FREQ Procedure

Table : ALL * DBTD6

ALL DBTD6(SGLT2 Inhabitors)

Power / Percent / Percent of rows / Percent of columns	None	With	Total
1	11799	182	11981
	98.48	1.52	100.00
	98.48	1.52	
	100.00	100.00	
Total	11799	182	11981
	98.48	1.52	100.00

Concomitant medications other than anticoagulants at the time of obtaining Table 2-5 consent, according to the presence or absence of proton-pump inhibitors

FREQ Procedure

			Cumulative	
Percent of ALL	power	Percent of power		
1	11981	100.00	11981	100.00

Tables: ALL * CMYN

ALL

CMYN(Administration of Concomitant Drug)

Power |
Percent |
Percent of rows
Percentage of columns | with | total

1	11981	11981
	100.00	100.00
	100.00	
	100.00	
Total	11981	11981
	100.00	100.00

Concomitant medications other than anticoagulants at the time of obtaining Table 2-5 consent, according to the presence or absence of proton-pump inhibitors

FREQ Procedure

Table : ALL * DBTD6

ALL DBTD6(SGLT2 Inhabitors)

Power / Percent / Percent of rows / Percent of columns	None	With	Total
1	11799	182	11981
	98.48	1.52	100.00
	98.48	1.52	
	100.00	100.00	
Total	11799	182	11981
	98.48	1.52	100.00

Concomitant medications other than anticoagulants at the time of obtaining Table 2-5 consent, according to the presence or absence of proton-pump inhibitors

FREQ Procedure

			Cumulative	
Percent of ALL	power	Percent of	power	
1	11981	100.00	11981	100.00

Tables: ALL * CMYN

ALL

CMYN(Administration of Concomitant Drug)

Power |
Percent |
Percent of rows
Percentage of columns | with | total

1	11981	11981
	100.00	100.00
	100.00	
	100.00	
Total	11981	11981
	100.00	100.00

Concomitant medications other than anticoagulants at the time of obtaining Table 2-5 consent, according to the presence or absence of proton-pump inhibitors

FREQ Procedure

Table : ALL * DBTD7

ALL DBTD7(Insuline Related)

Power<br>Percent<br>Percent of rows<br>Percent of columns	None	With	Total
1	11706<br>97.70<br>97.70<br>100.00	275<br>2.30<br>2.30<br>100.00	11981<br>100.00
Total	11706<br>97.70	275<br>2.30	11981<br>100.00

Concomitant medications other than anticoagulants at the time of obtaining Table 2-5 consent, according to the presence or absence of proton-pump inhibitors

FREQ Procedure

Cumulative
Percent of ALL power Percent of power

1	11981	100.00	11981	100.00

Tables: ALL * CMYN

ALL
CMYN(Administration of Concomitant Drug)

Power |
Percent |
Percent of rows
Percentage of columns | with | total

1	11981	11981
	100.00	100.00
	100.00	
	100.00	
Total	11981	11981
	100.00	100.00

Concomitant medications other than anticoagulants at the time of obtaining Table 2-5 consent, according to the presence or absence of proton-pump inhibitors

FREQ Procedure

Table : ALL * DBTD7

ALL DBTD7(Insuline Related)

Power<br>Percent<br>Percent of rows<br>Percent of columns	None	With	Total
1	11706<br>97.70<br>97.70<br>100.00	275<br>2.30<br>2.30<br>100.00	11981<br>100.00
Total	11706<br>97.70	275<br>2.30	11981<br>100.00

Concomitant medications other than anticoagulants at the time of obtaining Table 2-5 consent, according to the presence or absence of proton-pump inhibitors

FREQ Procedure

			Cumulative	
Percent of ALL	power	Percent of	power	
1	11981	100.00	11981	100.00

Tables: ALL * CMYN

ALL

CMYN(Administration of Concomitant Drug)

Power |
Percent |
Percent of rows
Percentage of columns | with | total

______________+________+
1 | 11981 | 11981
| 100.00 | 100.00
| 100.00 |
| 100.00 |
______________+________+
Total 11981 11981
100.00 100.00

Concomitant medications other than anticoagulants at the time of obtaining Table 2-5 consent, according to the presence or absence of proton-pump inhibitors

FREQ Procedure

Table : ALL * DBTD8

ALL

DBTD8(Other Diabetes Drugs)

Power / Percent / Percent of rows / Percent of columns	None	With	Total
1	11613	368	11981
	96.93	3.07	100.00
	96.93	3.07	
	100.00	100.00	
Total	11613	368	11981
	96.93	3.07	100.00

Concomitant medications other than anticoagulants at the time of obtaining Table 2-5 consent, according to the presence or absence of proton-pump inhibitors

FREQ Procedure

Cumulative
Percent of ALL power Percent of power

1	11981	100.00	11981	100.00

Tables: ALL * CMYN

ALL
CMYN(Administration of Concomitant Drug)

Power |
Percent |
Percent of rows
Percentage of columns | with | total

1	11981	11981
	100.00	100.00
	100.00	
	100.00	
Total	11981	11981
	100.00	100.00

Concomitant medications other than anticoagulants at the time of obtaining Table 2-5 consent, according to the presence or absence of proton-pump inhibitors

FREQ Procedure

Table : ALL * DBTD8

ALL

DBTD8(Other Diabetes Drugs)

Power |
Percent |
Percent of rows
Percent of columns | None | With | Total

1	11613	368	11981
	96.93	3.07	100.00
	96.93	3.07	
	100.00	100.00	
Total	11613	368	11981
	96.93	3.07	100.00

Concomitant medications other than anticoagulants at the time of obtaining Table 2-5 consent, according to the presence or absence of proton-pump inhibitors

FREQ Procedure

			Cumulative	
Percent of ALL	power	Percent	of power	
1	11981	100.00	11981	100.00

Tables: ALL * CMYN

ALL
CMYN(Administration of Concomitant Drug)

Power |
Percent |
Percent of rows
Percentage of columns | with | total

1	11981	11981
	100.00	100.00
	100.00	
	100.00	
Total	11981	11981
	100.00	100.00

Concomitant medications other than anticoagulants at the time of obtaining Table 2-5 consent, according to the presence or absence of proton-pump inhibitors

FREQ Procedure

Table: ALL * DMM

ALL DMM(Dementia Medications)

Power / Percent / Percent of rows / Percent of columns	None	With	Total
1	11538	443	11981
	96.30	3.70	100.00
	96.30	3.70	
	100.00	100.00	
Total	11538	443	11981
	96.30	3.70	100.00

Concomitant medications other than anticoagulants at the time of obtaining Table 2-5 consent, according to the presence or absence of proton-pump inhibitors

FREQ Procedure

			Cumulative	
Percent of ALL	power	Percent of power		
1	11981	100.00	11981	100.00

Tables: ALL * CMYN

ALL

CMYN(Administration of Concomitant Drug)

Power |
Percent |
Percent of rows
Percentage of columns | with | total

1	11981	11981
	100.00	100.00
	100.00	
	100.00	
Total	11981	11981
	100.00	100.00

Concomitant medications other than anticoagulants at the time of obtaining Table 2-5 consent, according to the presence or absence of proton-pump inhibitors

FREQ Procedure

Table: ALL * DMM

ALL DMM(Dementia Medications)

Power |
Percent |
Percent of rows
Percent of columns | None | With | Total

	None	With	Total
1	11538	443	11981
	96.30	3.70	100.00
	96.30	3.70	
	100.00	100.00	
Total	11538	443	11981
	96.30	3.70	100.00

Concomitant medications other than anticoagulants at the time of obtaining Table 2-5 consent, according to the presence or absence of proton-pump inhibitors

FREQ Procedure

			Cumulative	
Percent of ALL	power	Percent of power		
1	11981	100.00	11981	100.00

Tables: ALL * CMYN

ALL

CMYN(Administration of Concomitant Drug)

Power |
Percent |
Percent of rows
Percentage of columns | with | total

1	11981	11981
	100.00	100.00
	100.00	
	100.00	
Total	11981	11981
	100.00	100.00

Concomitant medications other than anticoagulants at the time of obtaining Table 2-5 consent, according to the presence or absence of proton-pump inhibitors

FREQ Procedure

Tables: ALL* ACNA

ALL ACNA(Anti-Cancer Agent)

Power<br>Percent<br>Percent of rows<br>Percent of columns	None	With	Total
1	11871<br>99.08<br>99.08<br>100.00	110<br>0.92<br>0.92<br>100.00	11981<br>100.00
Total	11871<br>99.08	110<br>0.92	11981<br>100.00

Concomitant medications other than anticoagulants at the time of obtaining Table 2-5 consent, according to the presence or absence of proton-pump inhibitors

FREQ Procedure

			Cumulative	
Percent of ALL	power	Percent of	power	
1	11981	100.00	11981	100.00

Tables: ALL * CMYN

ALL
CMYN(Administration of Concomitant Drug)

Power |
Percent |
Percent of rows
Percentage of columns | with | total

1	11981	11981
	100.00	100.00
	100.00	
	100.00	
Total	11981	11981
	100.00	100.00

Concomitant medications other than anticoagulants at the time of obtaining Table 2-5 consent, according to the presence or absence of proton-pump inhibitors

FREQ Procedure

Tables: ALL* ACNA

ALL ACNA(Anti-Cancer Agent)

Power |
Percent |
Percent of rows
Percent of columns | None | With | Total

1	11871	110	11981
	99.08	0.92	100.00
	99.08	0.92	
	100.00	100.00	
Total	11871	110	11981
	99.08	0.92	100.00

Concomitant medications other than anticoagulants at the time of obtaining Table 2-5 consent, according to the presence or absence of proton-pump inhibitors

FREQ Procedure

Cumulative
Percent of ALL power Percent of power

1	11981	100.00	11981	100.00

Tables: ALL * CMYN

ALL
CMYN(Administration of Concomitant Drug)

Power |
Percent |
Percent of rows
Percentage of columns | with | total

1	11981	11981
	100.00	100.00
	100.00	
	100.00	
Total	11981	11981
	100.00	100.00

Concomitant medications other than anticoagulants at the time of obtaining Table 2-5 consent, according to the presence or absence of proton-pump inhibitors

FREQ Procedure

Tables: ALL* COPD

ALL COPD(COPD)

Power |
Percent |
Percent of rows
Percent of columns | None | With | Total

1	11601	380	11981
	96.83	3.17	100.00
	96.83	3.17	
	100.00	100.00	
Total	11601	380	11981
	96.83	3.17	100.00

Concomitant medications other than anticoagulants at the time of obtaining Table 2-5 consent, according to the presence or absence of proton-pump inhibitors

FREQ Procedure

			Cumulative	
Percent of ALL	power	Percent of	power	
1	11981	100.00	11981	100.00

Tables: ALL * CMYN

ALL
CMYN(Administration of Concomitant Drug)

Power |
Percent |
Percent of rows
Percentage of columns | with | total

1	11981	11981
	100.00	100.00
	100.00	
	100.00	
Total	11981	11981
	100.00	100.00

Concomitant medications other than anticoagulants at the time of obtaining Table 2-5 consent, according to the presence or absence of proton-pump inhibitors

FREQ Procedure

Tables: ALL* COPD

ALL COPD(COPD)

Power |
Percent |
Percent of rows
Percent of columns | None | With | Total

1	11601	380	11981
	96.83	3.17	100.00
	96.83	3.17	
	100.00	100.00	
Total	11601	380	11981
	96.83	3.17	100.00

Concomitant medications other than anticoagulants at the time of obtaining Table 2-5 consent, according to the presence or absence of proton-pump inhibitors

FREQ Procedure

			Cumulative	
Percent of ALL	power	Percent of	power	
1	11981	100.00	11981	100.00

Tables: ALL * CMYN

ALL

CMYN(Administration of Concomitant Drug)

Power |
Percent |
Percent of rows
Percentage of columns | with | total

1	11981	11981
	100.00	100.00
	100.00	
	100.00	
Total	11981	11981
	100.00	100.00

Concomitant medications other than anticoagulants at the time of obtaining Table 2-5 consent, according to the presence or absence of proton-pump inhibitors

FREQ Procedure

Tables: ALL * PSYD

ALL PSYD(Psychotropic Drugs)

Power | Percent | Percent of rows Percent of columns	None	With	Total
1	10834	1147	11981
	90.43	9.57	100.00
	90.43	9.57	
	100.00	100.00	
Total	10834	1147	11981
	90.43	9.57	100.00

Concomitant medications other than anticoagulants at the time of obtaining Table 2-5 consent, according to the presence or absence of proton-pump inhibitors

FREQ Procedure

			Cumulative	
Percent of ALL	power	Percent of	power	
1	11981	100.00	11981	100.00

Tables: ALL * CMYN

ALL

CMYN(Administration of Concomitant Drug)

Power |

Percent |

Percent of rows

Percentage of columns | with | total

______________+________+

1 | 11981 | 11981

| 100.00 | 100.00

| 100.00 |

| 100.00 |

______________+________+

Total 11981 11981

100.00 100.00

Concomitant medications other than anticoagulants at the time of obtaining Table 2-5 consent, according to the presence or absence of proton-pump inhibitors

FREQ Procedure

Tables: ALL * PSYD

ALL PSYD(Psychotropic Drugs)

Power<br>Percent<br>Percent of rows<br>Percent of columns	None	With	Total
1	10834<br>90.43<br>90.43<br>100.00	1147<br>9.57<br>9.57<br>100.00	11981<br>100.00
Total	10834<br>90.43	1147<br>9.57	11981<br>100.00

Concomitant medications other than anticoagulants at the time of obtaining Table 2-5 consent, according to the presence or absence of proton-pump inhibitors

FREQ Procedure

			Cumulative	
Percent of ALL	power	Percent of power		
1	11981	100.00	11981	100.00

Tables: ALL * CMYN

ALL

CMYN(Administration of Concomitant Drug)

Power |
Percent |
Percent of rows
Percentage of columns | with | total

1	11981	11981
	100.00	100.00
	100.00	
	100.00	
Total	11981	11981
	100.00	100.00

Concomitant medications other than anticoagulants at the time of obtaining Table 2-5 consent, according to the presence or absence of proton-pump inhibitors

FREQ Procedure

Tables: ALL* PRPI

ALL

PRPI(Proton Pump Inhabitors)

Power |
Percent |
Percent of rows
Percentage of columns | with | total

```
______________+________+
         1 |   11981 |   11981
           | 100.00 | 100.00
           | 100.00 |
           | 100.00 |
______________+________+
Total 11981 11981
            100.00    100.00
```

Concomitant medications other than anticoagulants at the time of obtaining Table 2-5 consent, according to the presence or absence of proton-pump inhibitors

FREQ Procedure

			Cumulative	
Percent of ALL	power	Percent of power		
1	11981	100.00	11981	100.00

Tables: ALL * CMYN

ALL

CMYN(Administration of Concomitant Drug)

Power |
Percent |
Percent of rows
Percentage of columns | with | total

1	11981	11981
	100.00	100.00
	100.00	
	100.00	
Total	11981	11981
	100.00	100.00

Concomitant medications other than anticoagulants at the time of obtaining Table 2-5 consent, according to the presence or absence of proton-pump inhibitors

FREQ Procedure

Tables: ALL* PRPI

ALL
PRPI(Proton Pump Inhabitors)

Power |
Percent |
Percent of rows
Percentage of columns | with | total

______________+________+
1 | 11981 | 11981
| 100.00 | 100.00
| 100.00 |
| 100.00 |
______________+________+
Total 11981 11981
100.00   100.00

Concomitant medications other than anticoagulants at the time of obtaining Table 2-5 consent, according to the presence or absence of proton-pump inhibitors

FREQ Procedure

			Cumulative	
Percent of ALL	power	Percent	of power	
1	11981	100.00	11981	100.00

Tables: ALL * CMYN

ALL

CMYN(Administration of Concomitant Drug)

Power |
Percent |
Percent of rows
Percentage of columns | with | total
_______________+_________+
1 | 11981 | 11981
| 100.00 | 100.00
| 100.00 |
| 100.00 |
_______________+_________+
Total 11981 11981
100.00 100.00

Concomitant medications other than anticoagulants at the time of obtaining Table 2-5 consent, according to the presence or absence of proton-pump inhibitors

FREQ Procedure

Tables: ALL* PGPI

ALL PGPI(P-gp Inhabitors)

Power |
Percent |
Percent of rows
Percent of columns | None | With | Total

1	11741	240	11981
	98.00	2.00	100.00
	98.00	2.00	
	100.00	100.00	
Total	11741	240	11981
	98.00	2.00	100.00

Concomitant medications other than anticoagulants at the time of obtaining Table 2-5 consent, according to the presence or absence of proton-pump inhibitors

FREQ Procedure

			Cumulative	
Percent of ALL	power	Percent	of power	
1	11981	100.00	11981	100.00

Tables: ALL * CMYN

ALL

CMYN(Administration of Concomitant Drug)

Power |
Percent |
Percent of rows
Percentage of columns | with | total
______________+________+
1 | 11981 | 11981
| 100.00 | 100.00
| 100.00 |
| 100.00 |
______________+________+
Total 11981 11981
100.00 100.00

Concomitant medications other than anticoagulants at the time of obtaining Table 2-5 consent, according to the presence or absence of proton-pump inhibitors

FREQ Procedure

Tables: ALL* PGPI

ALL PGPI(P-gp Inhabitors)

Power |
Percent |
Percent of rows
Percent of columns | None | With | Total
_______________+_________+_________+
1 | 11741 | 240 | 11981
| 98.00 | 2.00 | 100.00
| 98.00 | 2.00 |
| 100.00 | 100.00 |
_______________+_________+_________+
Total 11741 240 11981
98.00 2.00 100.00

Concomitant medications other than anticoagulants at the time of obtaining Table 2-5 consent, according to the presence or absence of proton-pump inhibitors

FREQ Procedure

			Cumulative	
Percent of ALL	power	Percent of power		
1	11981	100.00	11981	100.00

Tables: ALL * CMYN

ALL

CMYN(Administration of Concomitant Drug)

Power |
Percent |
Percent of rows
Percentage of columns | with | total

1	11981	11981
	100.00	100.00
	100.00	
	100.00	
Total	11981	11981
	100.00	100.00

Concomitant medications other than anticoagulants at the time of obtaining Table 2-5 consent, according to the presence or absence of proton-pump inhibitors

FREQ Procedure

Tables: ALL * TCMCNT

ALL TCMCNT(Total Number of Drugs Other than Anticoagulant)

Power |
Percent |
Percent of rows
Percent | 1| 2| 3| 4| 5| 6| 7| 8| 9| 10| 11| 12| Total
______________+________+________+________+________+________+________+________+________+________+________+________+
1 | 28 | 165 | 420 | 794 | 1257 | 1528 | 1604 | 1495 | 1265 | 996 | 777 | 516 | 11795
| 0.24 | 1.40 | 3.56 | 6.73 | 10.66 | 12.95 | 13.60 | 12.67 | 10.72 | 8.44 | 6.59 | 4.37 | 100.00
| 0.24 | 1.40 | 3.56 | 6.73 | 10.66 | 12.95 | 13.60 | 12.67 | 10.72 | 8.44 | 6.59 | 4.37 |
| 100.00 | 100.00 | 100.00 | 100.00 | 100.00 | 100.00 | 100.00 | 100.00 | 100.00 | 100.00 | 100.00 | 100.00 |
______________+________+________+________+________+________+________+________+________+________+________+________+
Total 28 165 420 794 1257 1528 1604 1495 1265 996 777 516 11795
0.24 1.40 3.56 6.73 10.66 12.95 13.60 12.67 10.72 8.44 6.59 4.37 100.00
(continued)

Tables: ALL * TCMCNT

ALL TCMCNT(Total Number of Drugs Other than Anticoagulant)

Power |
Percent |

Percent of rows

Percent | 13| 14| 15| 16| 17| 18| 19| 20| 21| 22| 24| 26| Total

______________+________+________+________+________+________+________+________
_+________+________+________+________+________+

1 | 373 | 248 | 128 | 77 | 54 | 29 | 20 | 12 | 5 |
2 | 1 | 1 | 11795

| 3.16 | 2.10 | 1.09 | 0.65 | 0.46 | 0.25 | 0.17 | 0.10 | 0.04 |
0.02 | 0.01 | 0.01 | 100.00

| 3.16 | 2.10 | 1.09 | 0.65 | 0.46 | 0.25 | 0.17 | 0.10 | 0.04 |
0.02 | 0.01 | 0.01 |

| 100.00 | 100.00 | 100.00 | 100.00 | 100.00 | 100.00 | 100.00 | 100.00 | 100.00 |
100.00 | 100.00 | 100.00 |

______________+________+________+________+________+________+________+________
_+________+________+________+________+________+

Total 373 248 128 77 54 29 20 12 5 2 1 1 11795

3.16 2.10 1.09 0.65 0.46 0.25 0.17 0.10
0.04 0.02 0.01 0.01 100.00

Degree of missing values = 186

Concomitant medications other than anticoagulants at the time of obtaining Table 2-5 consent, according to the presence or absence of proton-pump inhibitors

FREQ Procedure

			Cumulative	
Percent of ALL power Percent of power				
1	11981	100.00	11981	100.00

Tables: ALL * CMYN

ALL

CMYN(Administration of Concomitant Drug)

Power |
Percent |
Percent of rows
Percentage of columns | with | total

1	11981	11981
	100.00	100.00
	100.00	
	100.00	
Total	11981	11981
	100.00	100.00

Concomitant medications other than anticoagulants at the time of obtaining Table 2-5 consent, according to the presence or absence of proton-pump inhibitors

FREQ Procedure

Tables: ALL * TCMCNT

ALL TCMCNT(Total Number of Drugs Other than Anticoagulant)

Power |
Percent |
Percent of rows
Percent | 1| 2| 3| 4| 5| 6| 7| 8| 9| 10| 11| 12| Total
______________+________+________+________+________+________+________+_______
_+________+________+________+________+________+
1 | 28 | 165 | 420 | 794 | 1257 | 1528 | 1604 | 1495 | 1265 |
996 | 777 | 516 | 11795
| 0.24 | 1.40 | 3.56 | 6.73 | 10.66 | 12.95 | 13.60 | 12.67 | 10.72 |
8.44 | 6.59 | 4.37 | 100.00
| 0.24 | 1.40 | 3.56 | 6.73 | 10.66 | 12.95 | 13.60 | 12.67 | 10.72 |
8.44 | 6.59 | 4.37 |
| 100.00 | 100.00 | 100.00 | 100.00 | 100.00 | 100.00 | 100.00 | 100.00 | 100.00 |
100.00 | 100.00 | 100.00 |
______________+________+________+________+________+________+________+_______
_+________+________+________+________+________+
Total 28 165 420 794 1257 1528 1604 1495 1265 996 777 516 11795
0.24 1.40 3.56 6.73 10.66 12.95 13.60 12.67
10.72 8.44 6.59 4.37 100.00
(continued)

Tables: ALL * TCMCNT

ALL TCMCNT(Total Number of Drugs Other than Anticoagulant)

Power |
Percent |

Percent of rows

Percent | 13| 14| 15| 16| 17| 18| 19| 20| 21| 22| 24| 26| Total

______________+________+________+________+________+________+________+_______
_+________+________+________+________+________+

1 | 373 | 248 | 128 | 77 | 54 | 29 | 20 | 12 | 5 |
2 | 1 | 1 | 11795

| 3.16 | 2.10 | 1.09 | 0.65 | 0.46 | 0.25 | 0.17 | 0.10 | 0.04 |
0.02 | 0.01 | 0.01 | 100.00

| 3.16 | 2.10 | 1.09 | 0.65 | 0.46 | 0.25 | 0.17 | 0.10 | 0.04 |
0.02 | 0.01 | 0.01 |

| 100.00 | 100.00 | 100.00 | 100.00 | 100.00 | 100.00 | 100.00 | 100.00 | 100.00 |
100.00 | 100.00 | 100.00 |

______________+________+________+________+________+________+________+_______
_+________+________+________+________+________+

Total 373 248 128 77 54 29 20 12 5 2 1 1 11795

3.16 2.10 1.09 0.65 0.46 0.25 0.17 0.10
0.04 0.02 0.01 0.01 100.00

Degree of missing values = 186

Concomitant medications other than anticoagulants at the time of obtaining Table 2-5 consent, according to the presence or absence of proton-pump inhibitors

FREQ Procedure

			Cumulative	
Percent of ALL power Percent of power				
1	11981	100.00	11981	100.00

Tables: ALL * CMYN

ALL

CMYN(Administration of Concomitant Drug)

Power |
Percent |
Percent of rows
Percentage of columns | with | total

1	11981	11981
	100.00	100.00
	100.00	
	100.00	
Total	11981	11981
	100.00	100.00

Concomitant medications other than anticoagulants at the time of obtaining Table 2-5 consent, according to the presence or absence of proton-pump inhibitors

FREQ Procedure

Tables: ALL * TCMCNT

ALL TCMCNT(Total Number of Drugs Other than Anticoagulant)

Power |
Percent |
Percent of rows
Percent | 1| 2| 3| 4| 5| 6| 7| 8| 9| 10| 11| 12| Total
______________+________+________+________+________+________+________+________
_+________+________+________+________+________+
1 | 28 | 165 | 420 | 794 | 1257 | 1528 | 1604 | 1495 | 1265 |
996 | 777 | 516 | 11795
| 0.24 | 1.40 | 3.56 | 6.73 | 10.66 | 12.95 | 13.60 | 12.67 | 10.72 |
8.44 | 6.59 | 4.37 | 100.00
| 0.24 | 1.40 | 3.56 | 6.73 | 10.66 | 12.95 | 13.60 | 12.67 | 10.72 |
8.44 | 6.59 | 4.37 |
| 100.00 | 100.00 | 100.00 | 100.00 | 100.00 | 100.00 | 100.00 | 100.00 | 100.00 |
100.00 | 100.00 | 100.00 |
______________+________+________+________+________+________+________+________
_+________+________+________+________+________+
Total 28 165 420 794 1257 1528 1604 1495 1265 996 777 516 11795
0.24 1.40 3.56 6.73 10.66 12.95 13.60 12.67
10.72 8.44 6.59 4.37 100.00
(continued)

Tables: ALL * TCMCNT

ALL TCMCNT(Total Number of Drugs Other than Anticoagulant)

Power |
Percent |

Percent of rows

Percent | 13| 14| 15| 16| 17| 18| 19| 20| 21| 22| 24| 26| Total

______________+________+________+________+________+________+________+_______

_+________+________+________+________+________+

1 | 373 | 248 | 128 | 77 | 54 | 29 | 20 | 12 | 5 |

2 | 1 | 1 | 11795

| 3.16 | 2.10 | 1.09 | 0.65 | 0.46 | 0.25 | 0.17 | 0.10 | 0.04 |

0.02 | 0.01 | 0.01 | 100.00

| 3.16 | 2.10 | 1.09 | 0.65 | 0.46 | 0.25 | 0.17 | 0.10 | 0.04 |

0.02 | 0.01 | 0.01 |

| 100.00 | 100.00 | 100.00 | 100.00 | 100.00 | 100.00 | 100.00 | 100.00 | 100.00 |

100.00 | 100.00 | 100.00 |

______________+________+________+________+________+________+________+_______

_+________+________+________+________+________+

Total 373 248 128 77 54 29 20 12 5 2 1 1 11795

3.16 2.10 1.09 0.65 0.46 0.25 0.17 0.10

0.04 0.02 0.01 0.01 100.00

Degree of missing values = 186

Concomitant medications other than anticoagulants at the time of obtaining Table 2-5 consent, according to the presence or absence of proton-pump inhibitors

FREQ Procedure

			Cumulative	
Percent of ALL	power	Percent of power		
1	11981	100.00	11981	100.00

Tables: ALL * CMYN

ALL

CMYN(Administration of Concomitant Drug)

Power |
Percent |
Percent of rows
Percentage of columns | with | total

______________+________+
1 | 11981 | 11981
| 100.00 | 100.00
| 100.00 |
| 100.00 |
______________+________+
Total 11981 11981
100.00 100.00

Concomitant medications other than anticoagulants at the time of obtaining Table 2-5 consent, according to the presence or absence of proton-pump inhibitors

FREQ Procedure

Tables: ALL * TCMCNT

ALL TCMCNT(Total Number of Drugs Other than Anticoagulant)

Power |
Percent |
Percent of rows
Percent | 1| 2| 3| 4| 5| 6| 7| 8| 9| 10| 11| 12| Total
______________+________+________+________+________+________+________+________+________+________+________+________+________+
1 | 28 | 165 | 420 | 794 | 1257 | 1528 | 1604 | 1495 | 1265 | 996 | 777 | 516 | 11795
| 0.24 | 1.40 | 3.56 | 6.73 | 10.66 | 12.95 | 13.60 | 12.67 | 10.72 | 8.44 | 6.59 | 4.37 | 100.00
| 0.24 | 1.40 | 3.56 | 6.73 | 10.66 | 12.95 | 13.60 | 12.67 | 10.72 | 8.44 | 6.59 | 4.37 |
| 100.00 | 100.00 | 100.00 | 100.00 | 100.00 | 100.00 | 100.00 | 100.00 | 100.00 | 100.00 | 100.00 | 100.00 |
______________+________+________+________+________+________+________+________+________+________+________+________+________+
Total 28 165 420 794 1257 1528 1604 1495 1265 996 777 516 11795
0.24 1.40 3.56 6.73 10.66 12.95 13.60 12.67 10.72 8.44 6.59 4.37 100.00
(continued)

Tables: ALL * TCMCNT

ALL TCMCNT(Total Number of Drugs Other than Anticoagulant)

Power |
Percent |

Percent of rows

Percent | 13| 14| 15| 16| 17| 18| 19| 20| 21| 22| 24| 26| Total

______________+________+________+________+________+________+________+_______
_+________+________+________+________+________+

1 | 373 | 248 | 128 | 77 | 54 | 29 | 20 | 12 | 5 |
2 | 1 | 1 | 11795

| 3.16 | 2.10 | 1.09 | 0.65 | 0.46 | 0.25 | 0.17 | 0.10 | 0.04 |
0.02 | 0.01 | 0.01 | 100.00

| 3.16 | 2.10 | 1.09 | 0.65 | 0.46 | 0.25 | 0.17 | 0.10 | 0.04 |
0.02 | 0.01 | 0.01 |

| 100.00 | 100.00 | 100.00 | 100.00 | 100.00 | 100.00 | 100.00 | 100.00 | 100.00 |
100.00 | 100.00 | 100.00 |

______________+________+________+________+________+________+________+_______
_+________+________+________+________+________+

Total 373 248 128 77 54 29 20 12 5 2 1 1 11795

3.16 2.10 1.09 0.65 0.46 0.25 0.17 0.10
0.04 0.02 0.01 0.01 100.00

Degree of missing values = 186

Concomitant medications other than anticoagulants at the time of obtaining Table 2-5 consent, according to the presence or absence of proton-pump inhibitors

FREQ Procedure

			Cumulative	
Percent of ALL	power	Percent of power		
1	11981	100.00	11981	100.00

Tables: ALL * CMYN

ALL

CMYN(Administration of Concomitant Drug)

Power |
Percent |
Percent of rows
Percentage of columns | with | total
______________+________+
1 | 11981 | 11981
| 100.00 | 100.00
| 100.00 |
| 100.00 |
______________+________+
Total 11981 11981
100.00 100.00

Concomitant medications other than anticoagulants at the time of obtaining Table 2-5 consent, according to the presence or absence of proton-pump inhibitors

FREQ Procedure

Tables: ALL * TCMCNT

ALL TCMCNT(Total Number of Drugs Other than Anticoagulant)

Power |
Percent |
Percent of rows
Percent | 1| 2| 3| 4| 5| 6| 7| 8| 9| 10| 11| 12| Total
______________+________+________+________+________+________+________+_______
_+________+________+________+________+________+
1 | 28 | 165 | 420 | 794 | 1257 | 1528 | 1604 | 1495 | 1265 |
996 | 777 | 516 | 11795
| 0.24 | 1.40 | 3.56 | 6.73 | 10.66 | 12.95 | 13.60 | 12.67 | 10.72 |
8.44 | 6.59 | 4.37 | 100.00
| 0.24 | 1.40 | 3.56 | 6.73 | 10.66 | 12.95 | 13.60 | 12.67 | 10.72 |
8.44 | 6.59 | 4.37 |
| 100.00 | 100.00 | 100.00 | 100.00 | 100.00 | 100.00 | 100.00 | 100.00 | 100.00 |
100.00 | 100.00 | 100.00 |
______________+________+________+________+________+________+________+_______
_+________+________+________+________+________+
Total 28 165 420 794 1257 1528 1604 1495 1265 996 777 516 11795
0.24 1.40 3.56 6.73 10.66 12.95 13.60 12.67
10.72 8.44 6.59 4.37 100.00
(continued)

Tables: ALL * TCMCNT

ALL TCMCNT(Total Number of Drugs Other than Anticoagulant)

Power |
Percent |

Percent of rows

Percent | 13| 14| 15| 16| 17| 18| 19| 20| 21| 22| 24| 26| Total

______________+________+________+________+________+________+________+________
_+________+________+________+________+________+

1 | 373 | 248 | 128 | 77 | 54 | 29 | 20 | 12 | 5 |
2 | 1 | 1 | 11795

| 3.16 | 2.10 | 1.09 | 0.65 | 0.46 | 0.25 | 0.17 | 0.10 | 0.04 |
0.02 | 0.01 | 0.01 | 100.00

| 3.16 | 2.10 | 1.09 | 0.65 | 0.46 | 0.25 | 0.17 | 0.10 | 0.04 |
0.02 | 0.01 | 0.01 |

| 100.00 | 100.00 | 100.00 | 100.00 | 100.00 | 100.00 | 100.00 | 100.00 | 100.00 |
100.00 | 100.00 | 100.00 |

______________+________+________+________+________+________+________+________
_+________+________+________+________+________+

Total 373 248 128 77 54 29 20 12 5 2 1 1 11795

3.16 2.10 1.09 0.65 0.46 0.25 0.17 0.10
0.04 0.02 0.01 0.01 100.00

Degree of missing values = 186

Concomitant medications other than anticoagulants at the time of obtaining Table 2-5 consent, according to the presence or absence of proton-pump inhibitors

FREQ Procedure

			Cumulative	
Percent of ALL	power	Percent of power		
1	11981	100.00	11981	100.00

Tables: ALL * CMYN

ALL

CMYN(Administration of Concomitant Drug)

Power |
Percent |
Percent of rows
Percentage of columns | with | total

1	11981	11981
	100.00	100.00
	100.00	
	100.00	
Total	11981	11981
	100.00	100.00

Concomitant medications other than anticoagulants at the time of obtaining Table 2-5 consent, according to the presence or absence of proton-pump inhibitors

FREQ Procedure

Tables: ALL * TCMCNT

ALL TCMCNT(Total Number of Drugs Other than Anticoagulant)

Power |
Percent |
Percent of rows
Percent | 1| 2| 3| 4| 5| 6| 7| 8| 9| 10| 11| 12| Total
______________+________+________+________+________+________+________+________
_+________+________+________+________+________+
1 | 28 | 165 | 420 | 794 | 1257 | 1528 | 1604 | 1495 | 1265 |
996 | 777 | 516 | 11795
| 0.24 | 1.40 | 3.56 | 6.73 | 10.66 | 12.95 | 13.60 | 12.67 | 10.72 |
8.44 | 6.59 | 4.37 | 100.00
| 0.24 | 1.40 | 3.56 | 6.73 | 10.66 | 12.95 | 13.60 | 12.67 | 10.72 |
8.44 | 6.59 | 4.37 |
| 100.00 | 100.00 | 100.00 | 100.00 | 100.00 | 100.00 | 100.00 | 100.00 | 100.00 |
100.00 | 100.00 | 100.00 |
______________+________+________+________+________+________+________+________
_+________+________+________+________+________+
Total 28 165 420 794 1257 1528 1604 1495 1265 996 777 516 11795
0.24 1.40 3.56 6.73 10.66 12.95 13.60 12.67
10.72 8.44 6.59 4.37 100.00
(continued)

Tables: ALL * TCMCNT

ALL TCMCNT(Total Number of Drugs Other than Anticoagulant)

Power |
Percent |

Percent of rows

Percent | 13| 14| 15| 16| 17| 18| 19| 20| 21| 22| 24| 26| Total

______________+________+________+________+________+________+________+________
_+________+________+________+________+________+

1 | 373 | 248 | 128 | 77 | 54 | 29 | 20 | 12 | 5 |
2 | 1 | 1 | 11795

| 3.16 | 2.10 | 1.09 | 0.65 | 0.46 | 0.25 | 0.17 | 0.10 | 0.04 |
0.02 | 0.01 | 0.01 | 100.00

| 3.16 | 2.10 | 1.09 | 0.65 | 0.46 | 0.25 | 0.17 | 0.10 | 0.04 |
0.02 | 0.01 | 0.01 |

| 100.00 | 100.00 | 100.00 | 100.00 | 100.00 | 100.00 | 100.00 | 100.00 | 100.00 |
100.00 | 100.00 | 100.00 |

______________+________+________+________+________+________+________+________
_+________+________+________+________+________+

Total 373 248 128 77 54 29 20 12 5 2 1 1 11795

3.16 2.10 1.09 0.65 0.46 0.25 0.17 0.10
0.04 0.02 0.01 0.01 100.00

Degree of missing values = 186

Concomitant medications other than anticoagulants at the time of obtaining Table 2-5 consent, according to the presence or absence of proton-pump inhibitors

FREQ Procedure

			Cumulative	
Percent of ALL	power	Percent	of power	
1	11981	100.00	11981	100.00

Tables: ALL * CMYN

ALL

CMYN(Administration of Concomitant Drug)

Power |
Percent |
Percent of rows
Percentage of columns | with | total

1	11981	11981
	100.00	100.00
	100.00	
	100.00	
Total	11981	11981
	100.00	100.00

Concomitant medications other than anticoagulants at the time of obtaining Table 2-5 consent, according to the presence or absence of proton-pump inhibitors

FREQ Procedure

Tables: ALL * TCMCNT

ALL TCMCNT(Total Number of Drugs Other than Anticoagulant)

Power |
Percent |
Percent of rows
Percent | 1| 2| 3| 4| 5| 6| 7| 8| 9| 10| 11| 12| Total
______________+________+________+________+________+________+________+_______
_+________+________+________+________+________+
1 | 28 | 165 | 420 | 794 | 1257 | 1528 | 1604 | 1495 | 1265 |
996 | 777 | 516 | 11795
| 0.24 | 1.40 | 3.56 | 6.73 | 10.66 | 12.95 | 13.60 | 12.67 | 10.72 |
8.44 | 6.59 | 4.37 | 100.00
| 0.24 | 1.40 | 3.56 | 6.73 | 10.66 | 12.95 | 13.60 | 12.67 | 10.72 |
8.44 | 6.59 | 4.37 |
| 100.00 | 100.00 | 100.00 | 100.00 | 100.00 | 100.00 | 100.00 | 100.00 | 100.00 |
100.00 | 100.00 | 100.00 |
______________+________+________+________+________+________+________+_______
_+________+________+________+________+________+
Total 28 165 420 794 1257 1528 1604 1495 1265 996 777 516 11795
0.24 1.40 3.56 6.73 10.66 12.95 13.60 12.67
10.72 8.44 6.59 4.37 100.00
(continued)

Tables: ALL * TCMCNT

ALL TCMCNT(Total Number of Drugs Other than Anticoagulant)

Power |
Percent |

Percent of rows

Percent | 13| 14| 15| 16| 17| 18| 19| 20| 21| 22| 24| 26| Total

_______________+_________+_________+_________+_________+_________+_________+________

_+_________+_________+_________+_________+_________+

1 | 373 | 248 | 128 | 77 | 54 | 29 | 20 | 12 | 5 |

2 | 1 | 1 | 11795

| 3.16 | 2.10 | 1.09 | 0.65 | 0.46 | 0.25 | 0.17 | 0.10 | 0.04 |

0.02 | 0.01 | 0.01 | 100.00

| 3.16 | 2.10 | 1.09 | 0.65 | 0.46 | 0.25 | 0.17 | 0.10 | 0.04 |

0.02 | 0.01 | 0.01 |

| 100.00 | 100.00 | 100.00 | 100.00 | 100.00 | 100.00 | 100.00 | 100.00 | 100.00 |

100.00 | 100.00 | 100.00 |

_______________+_________+_________+_________+_________+_________+_________+________

_+_________+_________+_________+_________+_________+

Total 373 248 128 77 54 29 20 12 5 2 1 1 11795

3.16 2.10 1.09 0.65 0.46 0.25 0.17 0.10

0.04 0.02 0.01 0.01 100.00

Degree of missing values = 186

Concomitant medications other than anticoagulants at the time of obtaining Table 2-5 consent, according to the presence or absence of proton-pump inhibitors

FREQ Procedure

			Cumulative	
Percent of ALL	power	Percent of	power	
1	11981	100.00	11981	100.00

Tables: ALL * CMYN

ALL

CMYN(Administration of Concomitant Drug)

Power |
Percent |
Percent of rows
Percentage of columns | with | total

```
______________+________+
          1 |  11981 |  11981
            | 100.00 | 100.00
            | 100.00 |
            | 100.00 |
______________+________+
Total 11981 11981
              100.00   100.00
```

Concomitant medications other than anticoagulants at the time of obtaining Table 2-5 consent, according to the presence or absence of proton-pump inhibitors

FREQ Procedure

Tables: ALL * TCMCNT

ALL TCMCNT(Total Number of Drugs Other than Anticoagulant)

Power |
Percent |
Percent of rows
Percent | 1| 2| 3| 4| 5| 6| 7| 8| 9| 10| 11| 12| Total
______________+________+________+________+________+________+________+________+________+________+________+________+________+
1 | 28 | 165 | 420 | 794 | 1257 | 1528 | 1604 | 1495 | 1265 | 996 | 777 | 516 | 11795
| 0.24 | 1.40 | 3.56 | 6.73 | 10.66 | 12.95 | 13.60 | 12.67 | 10.72 | 8.44 | 6.59 | 4.37 | 100.00
| 0.24 | 1.40 | 3.56 | 6.73 | 10.66 | 12.95 | 13.60 | 12.67 | 10.72 | 8.44 | 6.59 | 4.37 |
| 100.00 | 100.00 | 100.00 | 100.00 | 100.00 | 100.00 | 100.00 | 100.00 | 100.00 | 100.00 | 100.00 | 100.00 |
______________+________+________+________+________+________+________+________+________+________+________+________+________+
Total 28 165 420 794 1257 1528 1604 1495 1265 996 777 516 11795
0.24 1.40 3.56 6.73 10.66 12.95 13.60 12.67 10.72 8.44 6.59 4.37 100.00
(continued)

Tables: ALL * TCMCNT

ALL TCMCNT(Total Number of Drugs Other than Anticoagulant)

Power |
Percent |

Percent of rows

Percent | 13| 14| 15| 16| 17| 18| 19| 20| 21| 22| 24| 26| Total

______________+________+________+________+________+________+________+_______
_+________+________+________+________+________+

1 | 373 | 248 | 128 | 77 | 54 | 29 | 20 | 12 | 5 |
2 | 1 | 1 | 11795

| 3.16 | 2.10 | 1.09 | 0.65 | 0.46 | 0.25 | 0.17 | 0.10 | 0.04 |
0.02 | 0.01 | 0.01 | 100.00

| 3.16 | 2.10 | 1.09 | 0.65 | 0.46 | 0.25 | 0.17 | 0.10 | 0.04 |
0.02 | 0.01 | 0.01 |

| 100.00 | 100.00 | 100.00 | 100.00 | 100.00 | 100.00 | 100.00 | 100.00 | 100.00 |
100.00 | 100.00 | 100.00 |

______________+________+________+________+________+________+________+_______
_+________+________+________+________+________+

Total 373 248 128 77 54 29 20 12 5 2 1 1 11795

3.16 2.10 1.09 0.65 0.46 0.25 0.17 0.10
0.04 0.02 0.01 0.01 100.00

Degree of missing values = 186

Concomitant medications other than anticoagulants at the time of obtaining Table 2-5 consent, according to the presence or absence of proton-pump inhibitors

FREQ Procedure

			Cumulative	
Percent of ALL power Percent of power				
1	11981	100.00	11981	100.00

Tables: ALL * CMYN

ALL

CMYN(Administration of Concomitant Drug)

Power |
Percent |
Percent of rows
Percentage of columns | with | total

1	11981	11981
	100.00	100.00
	100.00	
	100.00	
Total	11981	11981
	100.00	100.00

Concomitant medications other than anticoagulants at the time of obtaining Table 2-5 consent, according to the presence or absence of proton-pump inhibitors

FREQ Procedure

Tables: ALL * TCMCNT

ALL TCMCNT(Total Number of Drugs Other than Anticoagulant)

Power |
Percent |
Percent of rows
Percent | 1| 2| 3| 4| 5| 6| 7| 8| 9| 10| 11| 12| Total
______________+________+________+________+________+________+________+_______
_+________+________+________+________+________+
1 | 28 | 165 | 420 | 794 | 1257 | 1528 | 1604 | 1495 | 1265 |
996 | 777 | 516 | 11795
| 0.24 | 1.40 | 3.56 | 6.73 | 10.66 | 12.95 | 13.60 | 12.67 | 10.72 |
8.44 | 6.59 | 4.37 | 100.00
| 0.24 | 1.40 | 3.56 | 6.73 | 10.66 | 12.95 | 13.60 | 12.67 | 10.72 |
8.44 | 6.59 | 4.37 |
| 100.00 | 100.00 | 100.00 | 100.00 | 100.00 | 100.00 | 100.00 | 100.00 | 100.00 |
100.00 | 100.00 | 100.00 |
______________+________+________+________+________+________+________+_______
_+________+________+________+________+________+
Total 28 165 420 794 1257 1528 1604 1495 1265 996 777 516 11795
0.24 1.40 3.56 6.73 10.66 12.95 13.60 12.67
10.72 8.44 6.59 4.37 100.00
(continued)

Tables: ALL * TCMCNT

ALL TCMCNT(Total Number of Drugs Other than Anticoagulant)

Power |
Percent |

Percent of rows

Percent | 13| 14| 15| 16| 17| 18| 19| 20| 21| 22| 24| 26| Total

______________+________+________+________+________+________+________+________
_+________+________+________+________+________+

1 | 373 | 248 | 128 | 77 | 54 | 29 | 20 | 12 | 5 |
2 | 1 | 1 | 11795

| 3.16 | 2.10 | 1.09 | 0.65 | 0.46 | 0.25 | 0.17 | 0.10 | 0.04 |
0.02 | 0.01 | 0.01 | 100.00

| 3.16 | 2.10 | 1.09 | 0.65 | 0.46 | 0.25 | 0.17 | 0.10 | 0.04 |
0.02 | 0.01 | 0.01 |

| 100.00 | 100.00 | 100.00 | 100.00 | 100.00 | 100.00 | 100.00 | 100.00 | 100.00 |
100.00 | 100.00 | 100.00 |

______________+________+________+________+________+________+________+________
_+________+________+________+________+________+

Total 373 248 128 77 54 29 20 12 5 2 1 1 11795

3.16 2.10 1.09 0.65 0.46 0.25 0.17 0.10
0.04 0.02 0.01 0.01 100.00

Degree of missing values = 186

Concomitant medications other than anticoagulants at the time of obtaining Table 2-5 consent, according to the presence or absence of proton-pump inhibitors

FREQ Procedure

Cumulative
Percent of ALL power Percent of power

1	11981	100.00	11981	100.00

Tables: ALL * CMYN

ALL
CMYN(Administration of Concomitant Drug)

Power |
Percent |
Percent of rows
Percentage of columns | with | total
______________+________+
1 | 11981 | 11981
| 100.00 | 100.00
| 100.00 |
| 100.00 |
______________+________+
Total 11981 11981
100.00 100.00

Concomitant medications other than anticoagulants at the time of obtaining Table 2-5 consent, according to the presence or absence of proton-pump inhibitors

FREQ Procedure

Tables: ALL * TCMCNT

ALL TCMCNT(Total Number of Drugs Other than Anticoagulant)

Power |
Percent |
Percent of rows
Percent | 1| 2| 3| 4| 5| 6| 7| 8| 9| 10| 11| 12| Total
______________+________+________+________+________+________+________+________
_+________+________+________+________+________+
1 | 28 | 165 | 420 | 794 | 1257 | 1528 | 1604 | 1495 | 1265 |
996 | 777 | 516 | 11795
| 0.24 | 1.40 | 3.56 | 6.73 | 10.66 | 12.95 | 13.60 | 12.67 | 10.72 |
8.44 | 6.59 | 4.37 | 100.00
| 0.24 | 1.40 | 3.56 | 6.73 | 10.66 | 12.95 | 13.60 | 12.67 | 10.72 |
8.44 | 6.59 | 4.37 |
| 100.00 | 100.00 | 100.00 | 100.00 | 100.00 | 100.00 | 100.00 | 100.00 | 100.00 |
100.00 | 100.00 | 100.00 |
______________+________+________+________+________+________+________+________
_+________+________+________+________+________+
Total 28 165 420 794 1257 1528 1604 1495 1265 996 777 516 11795
0.24 1.40 3.56 6.73 10.66 12.95 13.60 12.67
10.72 8.44 6.59 4.37 100.00
(continued)

Tables: ALL * TCMCNT

ALL TCMCNT(Total Number of Drugs Other than Anticoagulant)

Power |
Percent |

Percent of rows

Percent	13	14	15	16	17	18	19	20	21	22	24	26	Total
1	373	248	128	77	54	29	20	12	5	2	1	1	11795
	3.16	2.10	1.09	0.65	0.46	0.25	0.17	0.10	0.04	0.02	0.01	0.01	100.00
	3.16	2.10	1.09	0.65	0.46	0.25	0.17	0.10	0.04	0.02	0.01	0.01	
	100.00	100.00	100.00	100.00	100.00	100.00	100.00	100.00	100.00	100.00	100.00	100.00	
Total	373	248	128	77	54	29	20	12	5	2	1	1	11795
	3.16	2.10	1.09	0.65	0.46	0.25	0.17	0.10	0.04	0.02	0.01	0.01	100.00

Degree of missing values = 186

Concomitant medications other than anticoagulants at the time of obtaining Table 2-5 consent, according to the presence or absence of proton-pump inhibitors

FREQ Procedure

			Cumulative	
Percent of ALL	power	Percent of power		
1	11981	100.00	11981	100.00

Tables: ALL * CMYN

ALL

CMYN(Administration of Concomitant Drug)

Power |
Percent |
Percent of rows
Percentage of columns | with | total

1	11981	11981
	100.00	100.00
	100.00	
	100.00	
Total	11981	11981
	100.00	100.00

Concomitant medications other than anticoagulants at the time of obtaining Table 2-5 consent, according to the presence or absence of proton-pump inhibitors

FREQ Procedure

Tables: ALL * TCMCNT

ALL TCMCNT(Total Number of Drugs Other than Anticoagulant)

Power |
Percent |
Percent of rows
Percent | 1| 2| 3| 4| 5| 6| 7| 8| 9| 10| 11| 12| Total
______________+________+________+________+________+________+________+________
_+________+________+________+________+________+
1 | 28 | 165 | 420 | 794 | 1257 | 1528 | 1604 | 1495 | 1265 |
996 | 777 | 516 | 11795
| 0.24 | 1.40 | 3.56 | 6.73 | 10.66 | 12.95 | 13.60 | 12.67 | 10.72 |
8.44 | 6.59 | 4.37 | 100.00
| 0.24 | 1.40 | 3.56 | 6.73 | 10.66 | 12.95 | 13.60 | 12.67 | 10.72 |
8.44 | 6.59 | 4.37 |
| 100.00 | 100.00 | 100.00 | 100.00 | 100.00 | 100.00 | 100.00 | 100.00 | 100.00 |
100.00 | 100.00 | 100.00 |
______________+________+________+________+________+________+________+________
_+________+________+________+________+________+
Total 28 165 420 794 1257 1528 1604 1495 1265 996 777 516 11795
0.24 1.40 3.56 6.73 10.66 12.95 13.60 12.67
10.72 8.44 6.59 4.37 100.00
(continued)

Tables: ALL * TCMCNT

ALL TCMCNT(Total Number of Drugs Other than Anticoagulant)

Power |
Percent |

Percent of rows

Percent | 13| 14| 15| 16| 17| 18| 19| 20| 21| 22| 24| 26| Total

______________+________+________+________+________+________+________+________+________+________+________+________+

1 | 373 | 248 | 128 | 77 | 54 | 29 | 20 | 12 | 5 | 2 | 1 | 1 | 11795

| 3.16 | 2.10 | 1.09 | 0.65 | 0.46 | 0.25 | 0.17 | 0.10 | 0.04 | 0.02 | 0.01 | 0.01 | 100.00

| 3.16 | 2.10 | 1.09 | 0.65 | 0.46 | 0.25 | 0.17 | 0.10 | 0.04 | 0.02 | 0.01 | 0.01 |

| 100.00 | 100.00 | 100.00 | 100.00 | 100.00 | 100.00 | 100.00 | 100.00 | 100.00 | 100.00 | 100.00 | 100.00 |

______________+________+________+________+________+________+________+________+________+________+________+________+

Total 373 248 128 77 54 29 20 12 5 2 1 1 11795

3.16 2.10 1.09 0.65 0.46 0.25 0.17 0.10 0.04 0.02 0.01 0.01 100.00

Degree of missing values = 186

Concomitant medications other than anticoagulants at the time of obtaining Table 2-5 consent, according to the presence or absence of proton-pump inhibitors

FREQ Procedure

			Cumulative	
Percent of ALL	power	Percent of power		
1	11981	100.00	11981	100.00

Tables: ALL * CMYN

ALL

CMYN(Administration of Concomitant Drug)

Power |
Percent |
Percent of rows
Percentage of columns | with | total

1	11981	11981
	100.00	100.00
	100.00	
	100.00	
Total	11981	11981
	100.00	100.00

Concomitant medications other than anticoagulants at the time of obtaining Table 2-5 consent, according to the presence or absence of proton-pump inhibitors

FREQ Procedure

Tables: ALL * TCMCNT

ALL TCMCNT(Total Number of Drugs Other than Anticoagulant)

Power |
Percent |
Percent of rows
Percent | 1| 2| 3| 4| 5| 6| 7| 8| 9| 10| 11| 12| Total
______________+________+________+________+________+________+________+________+________+________+________+________+
1 | 28 | 165 | 420 | 794 | 1257 | 1528 | 1604 | 1495 | 1265 | 996 | 777 | 516 | 11795
| 0.24 | 1.40 | 3.56 | 6.73 | 10.66 | 12.95 | 13.60 | 12.67 | 10.72 | 8.44 | 6.59 | 4.37 | 100.00
| 0.24 | 1.40 | 3.56 | 6.73 | 10.66 | 12.95 | 13.60 | 12.67 | 10.72 | 8.44 | 6.59 | 4.37 |
| 100.00 | 100.00 | 100.00 | 100.00 | 100.00 | 100.00 | 100.00 | 100.00 | 100.00 | 100.00 | 100.00 | 100.00 |
______________+________+________+________+________+________+________+________+________+________+________+________+
Total 28 165 420 794 1257 1528 1604 1495 1265 996 777 516 11795
0.24 1.40 3.56 6.73 10.66 12.95 13.60 12.67 10.72 8.44 6.59 4.37 100.00
(continued)

Tables: ALL * TCMCNT

ALL TCMCNT(Total Number of Drugs Other than Anticoagulant)

Power |
Percent |

Percent of rows

Percent | 13| 14| 15| 16| 17| 18| 19| 20| 21| 22| 24| 26| Total

______________+________+________+________+________+________+________+________+________+________+________+________+________+

1 | 373 | 248 | 128 | 77 | 54 | 29 | 20 | 12 | 5 | 2 | 1 | 1 | 11795

| 3.16 | 2.10 | 1.09 | 0.65 | 0.46 | 0.25 | 0.17 | 0.10 | 0.04 | 0.02 | 0.01 | 0.01 | 100.00

| 3.16 | 2.10 | 1.09 | 0.65 | 0.46 | 0.25 | 0.17 | 0.10 | 0.04 | 0.02 | 0.01 | 0.01 |

| 100.00 | 100.00 | 100.00 | 100.00 | 100.00 | 100.00 | 100.00 | 100.00 | 100.00 | 100.00 | 100.00 | 100.00 |

______________+________+________+________+________+________+________+________+________+________+________+________+________+

Total 373 248 128 77 54 29 20 12 5 2 1 1 11795

3.16 2.10 1.09 0.65 0.46 0.25 0.17 0.10 0.04 0.02 0.01 0.01 100.00

Degree of missing values = 186

Concomitant medications other than anticoagulants at the time of obtaining Table 2-5 consent, according to the presence or absence of proton-pump inhibitors

FREQ Procedure

			Cumulative	
Percent of ALL power Percent of power				
1	11981	100.00	11981	100.00

Tables: ALL * CMYN

ALL

CMYN(Administration of Concomitant Drug)

Power |
Percent |
Percent of rows
Percentage of columns | with | total
______________+________+
1 | 11981 | 11981
| 100.00 | 100.00
| 100.00 |
| 100.00 |
______________+________+
Total 11981 11981
100.00 100.00

Concomitant medications other than anticoagulants at the time of obtaining Table 2-5 consent, according to the presence or absence of proton-pump inhibitors

FREQ Procedure

Tables: ALL * TCMCNT

ALL TCMCNT(Total Number of Drugs Other than Anticoagulant)

Power |
Percent |
Percent of rows
Percent | 1| 2| 3| 4| 5| 6| 7| 8| 9| 10| 11| 12| Total
______________+________+________+________+________+________+________+________
_+________+________+________+________+________+
1 | 28 | 165 | 420 | 794 | 1257 | 1528 | 1604 | 1495 | 1265 |
996 | 777 | 516 | 11795
| 0.24 | 1.40 | 3.56 | 6.73 | 10.66 | 12.95 | 13.60 | 12.67 | 10.72 |
8.44 | 6.59 | 4.37 | 100.00
| 0.24 | 1.40 | 3.56 | 6.73 | 10.66 | 12.95 | 13.60 | 12.67 | 10.72 |
8.44 | 6.59 | 4.37 |
| 100.00 | 100.00 | 100.00 | 100.00 | 100.00 | 100.00 | 100.00 | 100.00 | 100.00 |
100.00 | 100.00 | 100.00 |
______________+________+________+________+________+________+________+________
_+________+________+________+________+________+
Total 28 165 420 794 1257 1528 1604 1495 1265 996 777 516 11795
0.24 1.40 3.56 6.73 10.66 12.95 13.60 12.67
10.72 8.44 6.59 4.37 100.00
(continued)

Tables: ALL * TCMCNT

ALL TCMCNT(Total Number of Drugs Other than Anticoagulant)

Power |
Percent |

Percent of rows

Percent | 13| 14| 15| 16| 17| 18| 19| 20| 21| 22| 24| 26| Total

______________+________+________+________+________+________+________+_______
_+________+________+________+________+________+

1 | 373 | 248 | 128 | 77 | 54 | 29 | 20 | 12 | 5 |
2 | 1 | 1 | 11795

| 3.16 | 2.10 | 1.09 | 0.65 | 0.46 | 0.25 | 0.17 | 0.10 | 0.04 |
0.02 | 0.01 | 0.01 | 100.00

| 3.16 | 2.10 | 1.09 | 0.65 | 0.46 | 0.25 | 0.17 | 0.10 | 0.04 |
0.02 | 0.01 | 0.01 |

| 100.00 | 100.00 | 100.00 | 100.00 | 100.00 | 100.00 | 100.00 | 100.00 | 100.00 |
100.00 | 100.00 | 100.00 |

______________+________+________+________+________+________+________+_______
_+________+________+________+________+________+

Total 373 248 128 77 54 29 20 12 5 2 1 1 11795

3.16 2.10 1.09 0.65 0.46 0.25 0.17 0.10
0.04 0.02 0.01 0.01 100.00

Degree of missing values = 186

Concomitant medications other than anticoagulants at the time of obtaining Table 2-5 consent, according to the presence or absence of proton-pump inhibitors

FREQ Procedure

			Cumulative	
ALL	power	Percent	power	Percent of power
1	11981	100.00	11981	100.00

Tables: ALL * CMYN

ALL
CMYN(Administration of Concomitant Drug)

Power |
Percent |
Percent of rows
Percentage of columns | with | total
______________+________+
1 | 11981 | 11981
| 100.00 | 100.00
| 100.00 |
| 100.00 |
______________+________+
Total 11981 11981
100.00 100.00

Concomitant medications other than anticoagulants at the time of obtaining Table 2-5 consent, according to the presence or absence of proton-pump inhibitors

FREQ Procedure

Tables: ALL * TCMCNT

ALL TCMCNT(Total Number of Drugs Other than Anticoagulant)

Power |
Percent |
Percent of rows
Percent | 1| 2| 3| 4| 5| 6| 7| 8| 9| 10| 11| 12| Total
______________+________+________+________+________+________+________+________+________+________+________+________+

1 | 28 | 165 | 420 | 794 | 1257 | 1528 | 1604 | 1495 | 1265 | 996 | 777 | 516 | 11795
| 0.24 | 1.40 | 3.56 | 6.73 | 10.66 | 12.95 | 13.60 | 12.67 | 10.72 | 8.44 | 6.59 | 4.37 | 100.00
| 0.24 | 1.40 | 3.56 | 6.73 | 10.66 | 12.95 | 13.60 | 12.67 | 10.72 | 8.44 | 6.59 | 4.37 |
| 100.00 | 100.00 | 100.00 | 100.00 | 100.00 | 100.00 | 100.00 | 100.00 | 100.00 | 100.00 | 100.00 | 100.00 |
______________+________+________+________+________+________+________+________+________+________+________+________+

Total 28 165 420 794 1257 1528 1604 1495 1265 996 777 516 11795
0.24 1.40 3.56 6.73 10.66 12.95 13.60 12.67 10.72 8.44 6.59 4.37 100.00
(continued)

Tables: ALL * TCMCNT

ALL TCMCNT(Total Number of Drugs Other than Anticoagulant)

Power |
Percent |

Percent of rows

Percent | 13| 14| 15| 16| 17| 18| 19| 20| 21| 22| 24| 26| Total

______________+________+________+________+________+________+________+_______
_+________+________+________+________+________+

1 | 373 | 248 | 128 | 77 | 54 | 29 | 20 | 12 | 5 |
2 | 1 | 1 | 11795

| 3.16 | 2.10 | 1.09 | 0.65 | 0.46 | 0.25 | 0.17 | 0.10 | 0.04 |
0.02 | 0.01 | 0.01 | 100.00

| 3.16 | 2.10 | 1.09 | 0.65 | 0.46 | 0.25 | 0.17 | 0.10 | 0.04 |
0.02 | 0.01 | 0.01 |

| 100.00 | 100.00 | 100.00 | 100.00 | 100.00 | 100.00 | 100.00 | 100.00 | 100.00 |
100.00 | 100.00 | 100.00 |

______________+________+________+________+________+________+________+_______
_+________+________+________+________+________+

Total 373 248 128 77 54 29 20 12 5 2 1 1 11795

3.16 2.10 1.09 0.65 0.46 0.25 0.17 0.10
0.04 0.02 0.01 0.01 100.00

Degree of missing values = 186

Concomitant medications other than anticoagulants at the time of obtaining Table 2-5 consent, according to the presence or absence of proton-pump inhibitors

FREQ Procedure

			Cumulative	
Percent of ALL	power	Percent of power		
1	11981	100.00	11981	100.00

Tables: ALL * CMYN

ALL
CMYN(Administration of Concomitant Drug)

Power |
Percent |
Percent of rows
Percentage of columns | with | total

1	11981	11981
	100.00	100.00
	100.00	
	100.00	
Total	11981	11981
	100.00	100.00

Concomitant medications other than anticoagulants at the time of obtaining Table 2-5 consent, according to the presence or absence of proton-pump inhibitors

FREQ Procedure

Tables: ALL * TCMCNT

ALL TCMCNT(Total Number of Drugs Other than Anticoagulant)

Power |
Percent |
Percent of rows
Percent | 1| 2| 3| 4| 5| 6| 7| 8| 9| 10| 11| 12| Total
______________+________+________+________+________+________+________+________
_+________+________+________+________+________+
1 | 28 | 165 | 420 | 794 | 1257 | 1528 | 1604 | 1495 | 1265 |
996 | 777 | 516 | 11795
| 0.24 | 1.40 | 3.56 | 6.73 | 10.66 | 12.95 | 13.60 | 12.67 | 10.72 |
8.44 | 6.59 | 4.37 | 100.00
| 0.24 | 1.40 | 3.56 | 6.73 | 10.66 | 12.95 | 13.60 | 12.67 | 10.72 |
8.44 | 6.59 | 4.37 |
| 100.00 | 100.00 | 100.00 | 100.00 | 100.00 | 100.00 | 100.00 | 100.00 | 100.00 |
100.00 | 100.00 | 100.00 |
______________+________+________+________+________+________+________+________
_+________+________+________+________+________+
Total 28 165 420 794 1257 1528 1604 1495 1265 996 777 516 11795
0.24 1.40 3.56 6.73 10.66 12.95 13.60 12.67
10.72 8.44 6.59 4.37 100.00
(continued)

Tables: ALL * TCMCNT

ALL TCMCNT(Total Number of Drugs Other than Anticoagulant)

Power |
Percent |

Percent of rows

Percent | 13| 14| 15| 16| 17| 18| 19| 20| 21| 22| 24| 26| Total

_______________+________+________+________+________+________+________+________
_+________+________+________+________+________+

1 | 373 | 248 | 128 | 77 | 54 | 29 | 20 | 12 | 5 |
2 | 1 | 1 | 11795

| 3.16 | 2.10 | 1.09 | 0.65 | 0.46 | 0.25 | 0.17 | 0.10 | 0.04 |
0.02 | 0.01 | 0.01 | 100.00

| 3.16 | 2.10 | 1.09 | 0.65 | 0.46 | 0.25 | 0.17 | 0.10 | 0.04 |
0.02 | 0.01 | 0.01 |

| 100.00 | 100.00 | 100.00 | 100.00 | 100.00 | 100.00 | 100.00 | 100.00 | 100.00 |
100.00 | 100.00 | 100.00 |

_______________+________+________+________+________+________+________+________
_+________+________+________+________+________+

Total 373 248 128 77 54 29 20 12 5 2 1 1 11795

3.16 2.10 1.09 0.65 0.46 0.25 0.17 0.10
0.04 0.02 0.01 0.01 100.00

Degree of missing values = 186

Concomitant medications other than anticoagulants at the time of obtaining Table 2-5 consent, according to the presence or absence of proton-pump inhibitors

FREQ Procedure

			Cumulative	
Percent of ALL	power	Percent of power		
1	11981	100.00	11981	100.00

Tables: ALL * CMYN

ALL

CMYN(Administration of Concomitant Drug)

Power |
Percent |
Percent of rows
Percentage of columns | with | total

1	11981	11981
	100.00	100.00
	100.00	
	100.00	
Total	11981	11981
	100.00	100.00

Concomitant medications other than anticoagulants at the time of obtaining Table 2-5 consent, according to the presence or absence of proton-pump inhibitors

FREQ Procedure

Tables: ALL * TCMCNT

ALL TCMCNT(Total Number of Drugs Other than Anticoagulant)

Power |
Percent |
Percent of rows
Percent | 1| 2| 3| 4| 5| 6| 7| 8| 9| 10| 11| 12| Total
______________+________+________+________+________+________+________+________
_+________+________+________+________+________+
1 | 28 | 165 | 420 | 794 | 1257 | 1528 | 1604 | 1495 | 1265 | 996 | 777 | 516 | 11795
| 0.24 | 1.40 | 3.56 | 6.73 | 10.66 | 12.95 | 13.60 | 12.67 | 10.72 | 8.44 | 6.59 | 4.37 | 100.00
| 0.24 | 1.40 | 3.56 | 6.73 | 10.66 | 12.95 | 13.60 | 12.67 | 10.72 | 8.44 | 6.59 | 4.37 |
| 100.00 | 100.00 | 100.00 | 100.00 | 100.00 | 100.00 | 100.00 | 100.00 | 100.00 | 100.00 | 100.00 | 100.00 |
______________+________+________+________+________+________+________+________
_+________+________+________+________+________+
Total 28 165 420 794 1257 1528 1604 1495 1265 996 777 516 11795
0.24 1.40 3.56 6.73 10.66 12.95 13.60 12.67 10.72 8.44 6.59 4.37 100.00
(continued)

Tables: ALL * TCMCNT

ALL TCMCNT(Total Number of Drugs Other than Anticoagulant)

Power |
Percent |

Percent of rows

Percent	13	14	15	16	17	18	19	20	21	22	24	26	Total
1	373	248	128	77	54	29	20	12	5	2	1	1	11795
	3.16	2.10	1.09	0.65	0.46	0.25	0.17	0.10	0.04	0.02	0.01	0.01	100.00
	3.16	2.10	1.09	0.65	0.46	0.25	0.17	0.10	0.04	0.02	0.01	0.01	
	100.00	100.00	100.00	100.00	100.00	100.00	100.00	100.00	100.00	100.00	100.00	100.00	
Total	373	248	128	77	54	29	20	12	5	2	1	1	11795
	3.16	2.10	1.09	0.65	0.46	0.25	0.17	0.10	0.04	0.02	0.01	0.01	100.00

Degree of missing values = 186

Concomitant medications other than anticoagulants at the time of obtaining Table 2-5 consent, according to the presence or absence of proton-pump inhibitors

FREQ Procedure

			Cumulative	
Percent of ALL	power	Percent of	power	
1	11981	100.00	11981	100.00

Tables: ALL * CMYN

ALL

CMYN(Administration of Concomitant Drug)

Power |
Percent |
Percent of rows
Percentage of columns | with | total

1	11981	11981
	100.00	100.00
	100.00	
	100.00	
Total	11981	11981
	100.00	100.00

Concomitant medications other than anticoagulants at the time of obtaining Table 2-5 consent, according to the presence or absence of proton-pump inhibitors

FREQ Procedure

Tables: ALL * TCMCNT

ALL TCMCNT(Total Number of Drugs Other than Anticoagulant)

Power |
Percent |
Percent of rows
Percent | 1| 2| 3| 4| 5| 6| 7| 8| 9| 10| 11| 12| Total
______________+________+________+________+________+________+________+________
_+________+________+________+________+________+
1 | 28 | 165 | 420 | 794 | 1257 | 1528 | 1604 | 1495 | 1265 |
996 | 777 | 516 | 11795
| 0.24 | 1.40 | 3.56 | 6.73 | 10.66 | 12.95 | 13.60 | 12.67 | 10.72 |
8.44 | 6.59 | 4.37 | 100.00
| 0.24 | 1.40 | 3.56 | 6.73 | 10.66 | 12.95 | 13.60 | 12.67 | 10.72 |
8.44 | 6.59 | 4.37 |
| 100.00 | 100.00 | 100.00 | 100.00 | 100.00 | 100.00 | 100.00 | 100.00 | 100.00 |
100.00 | 100.00 | 100.00 |
______________+________+________+________+________+________+________+________
_+________+________+________+________+________+
Total 28 165 420 794 1257 1528 1604 1495 1265 996 777 516 11795
0.24 1.40 3.56 6.73 10.66 12.95 13.60 12.67
10.72 8.44 6.59 4.37 100.00
(continued)

Tables: ALL * TCMCNT

ALL TCMCNT(Total Number of Drugs Other than Anticoagulant)

Power |
Percent |

Percent of rows

Percent | 13| 14| 15| 16| 17| 18| 19| 20| 21| 22| 24| 26| Total

______________+________+________+________+________+________+________+________+________+________+________+________+________+

1 | 373 | 248 | 128 | 77 | 54 | 29 | 20 | 12 | 5 | 2 | 1 | 1 | 11795

| 3.16 | 2.10 | 1.09 | 0.65 | 0.46 | 0.25 | 0.17 | 0.10 | 0.04 | 0.02 | 0.01 | 0.01 | 100.00

| 3.16 | 2.10 | 1.09 | 0.65 | 0.46 | 0.25 | 0.17 | 0.10 | 0.04 | 0.02 | 0.01 | 0.01 |

| 100.00 | 100.00 | 100.00 | 100.00 | 100.00 | 100.00 | 100.00 | 100.00 | 100.00 | 100.00 | 100.00 | 100.00 |

______________+________+________+________+________+________+________+________+________+________+________+________+________+

Total 373 248 128 77 54 29 20 12 5 2 1 1 11795

3.16 2.10 1.09 0.65 0.46 0.25 0.17 0.10 0.04 0.02 0.01 0.01 100.00

Degree of missing values = 186

Concomitant medications other than anticoagulants at the time of obtaining Table 2-5 consent, according to the presence or absence of proton-pump inhibitors

FREQ Procedure

			Cumulative	
Percent of ALL	power	Percent of power		
1	11981	100.00	11981	100.00

Tables: ALL * CMYN

ALL

CMYN(Administration of Concomitant Drug)

Power |
Percent |
Percent of rows
Percentage of columns | with | total

1	11981	11981
	100.00	100.00
	100.00	
	100.00	
Total	11981	11981
	100.00	100.00

Concomitant medications other than anticoagulants at the time of obtaining Table 2-5 consent, according to the presence or absence of proton-pump inhibitors

FREQ Procedure

Tables: ALL * TCMCNT

ALL TCMCNT(Total Number of Drugs Other than Anticoagulant)

Power |
Percent |
Percent of rows
Percent | 1| 2| 3| 4| 5| 6| 7| 8| 9| 10| 11| 12| Total
______________+________+________+________+________+________+________+________+________+________+________+________+
1 | 28 | 165 | 420 | 794 | 1257 | 1528 | 1604 | 1495 | 1265 | 996 | 777 | 516 | 11795
| 0.24 | 1.40 | 3.56 | 6.73 | 10.66 | 12.95 | 13.60 | 12.67 | 10.72 | 8.44 | 6.59 | 4.37 | 100.00
| 0.24 | 1.40 | 3.56 | 6.73 | 10.66 | 12.95 | 13.60 | 12.67 | 10.72 | 8.44 | 6.59 | 4.37 |
| 100.00 | 100.00 | 100.00 | 100.00 | 100.00 | 100.00 | 100.00 | 100.00 | 100.00 | 100.00 | 100.00 | 100.00 |
______________+________+________+________+________+________+________+________+________+________+________+________+
Total 28 165 420 794 1257 1528 1604 1495 1265 996 777 516 11795
0.24 1.40 3.56 6.73 10.66 12.95 13.60 12.67 10.72 8.44 6.59 4.37 100.00
(continued)

Tables: ALL * TCMCNT

ALL TCMCNT(Total Number of Drugs Other than Anticoagulant)

Power |
Percent |

Percent of rows

Percent | 13| 14| 15| 16| 17| 18| 19| 20| 21| 22| 24| 26| Total

______________+________+________+________+________+________+________+_______
_+________+________+________+________+________+

1 | 373 | 248 | 128 | 77 | 54 | 29 | 20 | 12 | 5 |
2 | 1 | 1 | 11795

| 3.16 | 2.10 | 1.09 | 0.65 | 0.46 | 0.25 | 0.17 | 0.10 | 0.04 |
0.02 | 0.01 | 0.01 | 100.00

| 3.16 | 2.10 | 1.09 | 0.65 | 0.46 | 0.25 | 0.17 | 0.10 | 0.04 |
0.02 | 0.01 | 0.01 |

| 100.00 | 100.00 | 100.00 | 100.00 | 100.00 | 100.00 | 100.00 | 100.00 | 100.00 |
100.00 | 100.00 | 100.00 |

______________+________+________+________+________+________+________+_______
_+________+________+________+________+________+

Total 373 248 128 77 54 29 20 12 5 2 1 1 11795

3.16 2.10 1.09 0.65 0.46 0.25 0.17 0.10
0.04 0.02 0.01 0.01 100.00

Degree of missing values = 186

Concomitant medications other than anticoagulants at the time of obtaining Table 2-5 consent, according to the presence or absence of proton-pump inhibitors

FREQ Procedure

			Cumulative	
Percent of ALL power Percent of power				
1	11981	100.00	11981	100.00

Tables: ALL * CMYN

ALL

CMYN(Administration of Concomitant Drug)

Power |
Percent |
Percent of rows
Percentage of columns | with | total
_______________+_________+
1 | 11981 | 11981
| 100.00 | 100.00
| 100.00 |
| 100.00 |
_______________+_________+
Total 11981 11981
100.00 100.00

Concomitant medications other than anticoagulants at the time of obtaining Table 2-5 consent, according to the presence or absence of proton-pump inhibitors

FREQ Procedure

Tables: ALL * TCMCNT

ALL TCMCNT(Total Number of Drugs Other than Anticoagulant)

Power |
Percent |
Percent of rows
Percent | 1| 2| 3| 4| 5| 6| 7| 8| 9| 10| 11| 12| Total
______________+________+________+________+________+________+________+________
_+________+________+________+________+________+
1 | 28 | 165 | 420 | 794 | 1257 | 1528 | 1604 | 1495 | 1265 |
996 | 777 | 516 | 11795
| 0.24 | 1.40 | 3.56 | 6.73 | 10.66 | 12.95 | 13.60 | 12.67 | 10.72 |
8.44 | 6.59 | 4.37 | 100.00
| 0.24 | 1.40 | 3.56 | 6.73 | 10.66 | 12.95 | 13.60 | 12.67 | 10.72 |
8.44 | 6.59 | 4.37 |
| 100.00 | 100.00 | 100.00 | 100.00 | 100.00 | 100.00 | 100.00 | 100.00 | 100.00 |
100.00 | 100.00 | 100.00 |
______________+________+________+________+________+________+________+________
_+________+________+________+________+________+
Total 28 165 420 794 1257 1528 1604 1495 1265 996 777 516 11795
0.24 1.40 3.56 6.73 10.66 12.95 13.60 12.67
10.72 8.44 6.59 4.37 100.00
(continued)

Tables: ALL * TCMCNT

ALL TCMCNT(Total Number of Drugs Other than Anticoagulant)

Power |
Percent |

Percent of rows
Percent | 13| 14| 15| 16| 17| 18| 19| 20| 21| 22| 24| 26| Total
______________+________+________+________+________+________+________+_______
_+________+________+________+________+________+
1 | 373 | 248 | 128 | 77 | 54 | 29 | 20 | 12 | 5 |
2 | 1 | 1 | 11795
| 3.16 | 2.10 | 1.09 | 0.65 | 0.46 | 0.25 | 0.17 | 0.10 | 0.04 |
0.02 | 0.01 | 0.01 | 100.00
| 3.16 | 2.10 | 1.09 | 0.65 | 0.46 | 0.25 | 0.17 | 0.10 | 0.04 |
0.02 | 0.01 | 0.01 |
| 100.00 | 100.00 | 100.00 | 100.00 | 100.00 | 100.00 | 100.00 | 100.00 | 100.00 |
100.00 | 100.00 | 100.00 |
______________+________+________+________+________+________+________+_______
_+________+________+________+________+________+
Total 373 248 128 77 54 29 20 12 5 2 1 1 11795
3.16 2.10 1.09 0.65 0.46 0.25 0.17 0.10
0.04 0.02 0.01 0.01 100.00

Degree of missing values = 186

Concomitant medications other than anticoagulants at the time of obtaining Table 2-5 consent, according to the presence or absence of proton-pump inhibitors

FREQ Procedure

			Cumulative	
Percent of ALL	power	Percent	of power	
1	11981	100.00	11981	100.00

Tables: ALL * CMYN

ALL

CMYN(Administration of Concomitant Drug)

Power |
Percent |
Percent of rows
Percentage of columns | with | total
______________+________+
1 | 11981 | 11981
| 100.00 | 100.00
| 100.00 |
| 100.00 |
______________+________+
Total 11981 11981
100.00 100.00

Concomitant medications other than anticoagulants at the time of obtaining Table 2-5 consent, according to the presence or absence of proton-pump inhibitors

FREQ Procedure

Tables: ALL * TCMCNT

ALL TCMCNT(Total Number of Drugs Other than Anticoagulant)

Power |
Percent |
Percent of rows
Percent | 1| 2| 3| 4| 5| 6| 7| 8| 9| 10| 11| 12| Total
______________+________+________+________+________+________+________+________+________+________+________+________+
1 | 28 | 165 | 420 | 794 | 1257 | 1528 | 1604 | 1495 | 1265 | 996 | 777 | 516 | 11795
| 0.24 | 1.40 | 3.56 | 6.73 | 10.66 | 12.95 | 13.60 | 12.67 | 10.72 | 8.44 | 6.59 | 4.37 | 100.00
| 0.24 | 1.40 | 3.56 | 6.73 | 10.66 | 12.95 | 13.60 | 12.67 | 10.72 | 8.44 | 6.59 | 4.37 |
| 100.00 | 100.00 | 100.00 | 100.00 | 100.00 | 100.00 | 100.00 | 100.00 | 100.00 | 100.00 | 100.00 | 100.00 |
______________+________+________+________+________+________+________+________+________+________+________+________+
Total 28 165 420 794 1257 1528 1604 1495 1265 996 777 516 11795
0.24 1.40 3.56 6.73 10.66 12.95 13.60 12.67 10.72 8.44 6.59 4.37 100.00
(continued)

Tables: ALL * TCMCNT

ALL TCMCNT(Total Number of Drugs Other than Anticoagulant)

Power |
Percent |

Percent of rows

Percent | 13| 14| 15| 16| 17| 18| 19| 20| 21| 22| 24| 26| Total

______________+________+________+________+________+________+________+_______
_+________+________+________+________+________+

1 | 373 | 248 | 128 | 77 | 54 | 29 | 20 | 12 | 5 |
2 | 1 | 1 | 11795

| 3.16 | 2.10 | 1.09 | 0.65 | 0.46 | 0.25 | 0.17 | 0.10 | 0.04 |
0.02 | 0.01 | 0.01 | 100.00

| 3.16 | 2.10 | 1.09 | 0.65 | 0.46 | 0.25 | 0.17 | 0.10 | 0.04 |
0.02 | 0.01 | 0.01 |

| 100.00 | 100.00 | 100.00 | 100.00 | 100.00 | 100.00 | 100.00 | 100.00 | 100.00 |
100.00 | 100.00 | 100.00 |

______________+________+________+________+________+________+________+_______
_+________+________+________+________+________+

Total 373 248 128 77 54 29 20 12 5 2 1 1 11795

3.16 2.10 1.09 0.65 0.46 0.25 0.17 0.10
0.04 0.02 0.01 0.01 100.00

Degree of missing values = 186

Concomitant medications other than anticoagulants at the time of obtaining Table 2-5 consent, according to the presence or absence of proton-pump inhibitors

FREQ Procedure

			Cumulative	
Percent of ALL	power	Percent of power		
1	11981	100.00	11981	100.00

Tables: ALL * CMYN

ALL

CMYN(Administration of Concomitant Drug)

Power |
Percent |
Percent of rows
Percentage of columns | with | total

1	11981	11981
	100.00	100.00
	100.00	
	100.00	
Total	11981	11981
	100.00	100.00

Concomitant medications other than anticoagulants at the time of obtaining Table 2-5 consent, according to the presence or absence of proton-pump inhibitors

FREQ Procedure

Table: ALL * t_cm

ALL t_cm

Power |
Percent |
Percent of rows
Percent of columns | 1| Total
______________+________+
1 | 21 | 21
| 100.00 | 100.00
| 100.00 |
| 100.00 |
______________+________+
Total 21 21
100.00 100.00

Frequency of missing values = 11960

Concomitant medications other than anticoagulants at the time of obtaining Table 2-5 consent, according to the presence or absence of proton-pump inhibitors

FREQ Procedure

			Cumulative	
Percent of ALL	power	Percent of power		
1	11981	100.00	11981	100.00

Tables: ALL * CMYN

ALL

CMYN(Administration of Concomitant Drug)

Power |
Percent |
Percent of rows
Percentage of columns | with | total

1	11981	11981
	100.00	100.00
	100.00	
	100.00	
Total	11981	11981
	100.00	100.00

Concomitant medications other than anticoagulants at the time of obtaining Table 2-5 consent, according to the presence or absence of proton-pump inhibitors

FREQ Procedure

Table: ALL * t_TCMCNT

ALL t_TCMCNT

Power |
Percent |
Percent of rows
Percent of columns | 9| Total
______________+________+
1 | 186 | 186
| 100.00 | 100.00
| 100.00 |
| 100.00 |
______________+________+
Total 186 186
100.00 100.00

Frequency of missing values = 11795

Concomitant medications other than anticoagulants at the time of obtaining Table 2-5 consent, according to the presence or absence of proton-pump inhibitors

FREQ Procedure

			Cumulative	
Percent of ALL	power	Percent of power		
1	11981	100.00	11981	100.00

Tables: ALL * CMYN

ALL

CMYN(Administration of Concomitant Drug)

Power |
Percent |
Percent of rows
Percentage of columns | with | total

1	11981	11981
	100.00	100.00
	100.00	
	100.00	
Total	11981	11981
	100.00	100.00

Concomitant medications other than anticoagulants at the time of obtaining Table 2-5 consent, according to the presence or absence of proton-pump inhibitors

FREQ Procedure

Tables: ALL* TCMCNTCT

ALL

TCMCNTCT(Category for Total Number of Drugs Other than Anticoagulant)

Power |
Percent |
Percent of rows
Percentage of column |0 to 4 or more|5 to 9 or more|10 or more|unknown|total
|Bottom|Bottom | | |

	0 to 4 or more Bottom	5 to 9 or more Bottom	10 or more	unknown	total
1	1407	7149	3239	186	11981
	11.74	59.67	27.03	1.55	100.00
	11.74	59.67	27.03	1.55	
	100.00	100.00	100.00	100.00	
Total	1407	7149	3239	186	11981
	11.74	59.67	27.03	1.55	100.00

Concomitant medications other than anticoagulants at the time of obtaining Table 2-5 consent, according to the presence or absence of proton-pump inhibitors

FREQ Procedure

			Cumulative	
Percent of ALL	power	Percent of power		
1	11981	100.00	11981	100.00

Tables: ALL * CMYN

ALL

CMYN(Administration of Concomitant Drug)

Power |
Percent |
Percent of rows
Percentage of columns | with | total

1	11981	11981
	100.00	100.00
	100.00	
	100.00	
Total	11981	11981
	100.00	100.00

Concomitant medications other than anticoagulants at the time of obtaining Table 2-5 consent, according to the presence or absence of proton-pump inhibitors

FREQ Procedure

Tables: ALL* TCMCNTCT

ALL

TCMCNTCT(Category for Total Number of Drugs Other than Anticoagulant)

ALL	0 to 4 or more Bottom	5 to 9 or more Bottom	10 or more	unknown	total
Power / Percent / Percent of rows / Percentage of column					
1	1407	7149	3239	186	11981
	11.74	59.67	27.03	1.55	100.00
	11.74	59.67	27.03	1.55	
	100.00	100.00	100.00	100.00	
Total	1407	7149	3239	186	11981
	11.74	59.67	27.03	1.55	100.00

Concomitant medications other than anticoagulants at the time of obtaining Table 2-5 consent, according to the presence or absence of proton-pump inhibitors

FREQ Procedure

			Cumulative	
Percent of ALL	power	Percent of power		
1	11981	100.00	11981	100.00

Tables: ALL * CMYN

ALL

CMYN(Administration of Concomitant Drug)

Power |
Percent |
Percent of rows
Percentage of columns | with | total

1	11981	11981
	100.00	100.00
	100.00	
	100.00	
Total	11981	11981
	100.00	100.00

Concomitant medications other than anticoagulants at the time of obtaining Table 2-5 consent, according to the presence or absence of proton-pump inhibitors

FREQ Procedure

Tables: ALL* TCMCNTCT

ALL

TCMCNTCT(Category for Total Number of Drugs Other than Anticoagulant)

Power |
Percent |
Percent of rows
Percentage of column |0 to 4 or more|5 to 9 or more|10 or more|unknown|total
|Bottom|Bottom | | |

1	1407	7149	3239	186	11981
	11.74	59.67	27.03	1.55	100.00
	11.74	59.67	27.03	1.55	
	100.00	100.00	100.00	100.00	
Total	1407	7149	3239	186	11981
	11.74	59.67	27.03	1.55	100.00

Concomitant medications other than anticoagulants at the time of obtaining Table 2-5 consent, according to the presence or absence of proton-pump inhibitors

FREQ Procedure

			Cumulative	
Percent of ALL	power	Percent of power		
1	11981	100.00	11981	100.00

Tables: ALL * CMYN

ALL

CMYN(Administration of Concomitant Drug)

Power |
Percent |
Percent of rows
Percentage of columns | with | total

______________+________+

1 | 11981 | 11981
| 100.00 | 100.00
| 100.00 |
| 100.00 |

______________+________+

Total 11981 11981
100.00 100.00

Concomitant medications other than anticoagulants at the time of obtaining Table 2-5 consent, according to the presence or absence of proton-pump inhibitors

FREQ Procedure

Tables: ALL* TCMCNTCT

ALL

TCMCNTCT(Category for Total Number of Drugs Other than Anticoagulant)

Power / Percent / Percent of rows / Percentage of column	0 to 4 or more Bottom	5 to 9 or more Bottom	10 or more	unknown	total
1	1407	7149	3239	186	11981
	11.74	59.67	27.03	1.55	100.00
	11.74	59.67	27.03	1.55	
	100.00	100.00	100.00	100.00	
Total	1407	7149	3239	186	11981
	11.74	59.67	27.03	1.55	100.00

Concomitant medications other than anticoagulants at the time of obtaining Table 2-5 consent, according to the presence or absence of proton-pump inhibitors

FREQ Procedure

			Cumulative	
Percent of ALL	power	Percent of	power	
1	20509	100.00	20509	100.00

Tables: ALL * CMYN

ALL

CMYN(Administration of Concomitant Drug)

Power |
Percent |
Percent of rows
Percent of columns | None | With | Total

	None	With	Total
1	1445	19064	20509
	7.05	92.95	100.00
	7.05	92.95	
	100.00	100.00	
Total	1445	19064	20509
	7.05	92.95	100.00

Concomitant medications other than anticoagulants at the time of obtaining Table 2-5 consent, according to the presence or absence of proton-pump inhibitors

FREQ Procedure

Tables: ALL * CMYN

ALL

CMYN(Administration of Concomitant Drug)

Power / Percent / Percent of rows / Percent of columns	None	With	Total
1	1445	19064	20509
	7.05	92.95	100.00
	7.05	92.95	
	100.00	100.00	
Total	1445	19064	20509
	7.05	92.95	100.00

Concomitant medications other than anticoagulants at the time of obtaining Table 2-5 consent, according to the presence or absence of proton-pump inhibitors

FREQ Procedure

			Cumulative	
Percent of ALL	power	Percent of	power	
1	20509	100.00	20509	100.00

Tables: ALL * CMYN

ALL

CMYN(Administration of Concomitant Drug)

Power | Percent | Percent of rows Percent of columns	None	With	Total
1	1445	19064	20509
	7.05	92.95	100.00
	7.05	92.95	
	100.00	100.00	
Total	1445	19064	20509
	7.05	92.95	100.00

Concomitant medications other than anticoagulants at the time of obtaining Table 2-5 consent, according to the presence or absence of proton-pump inhibitors

FREQ Procedure

Tables: ALL * CMYN

ALL
CMYN(Administration of Concomitant Drug)

Power / Percent / Percent of rows / Percent of columns	None	With	Total
1	1445	19064	20509
	7.05	92.95	100.00
	7.05	92.95	
	100.00	100.00	
Total	1445	19064	20509
	7.05	92.95	100.00

Concomitant medications other than anticoagulants at the time of obtaining Table 2-5 consent, according to the presence or absence of proton-pump inhibitors

FREQ Procedure

ALL	power	Percent	Cumulative power	Cumulative Percent of power
1	20509	100.00	20509	100.00

Tables: ALL * CMYN

ALL
CMYN(Administration of Concomitant Drug)

Power |
Percent |
Percent of rows
Percent of columns | None | With | Total

	None	With	Total
1	1445	19064	20509
	7.05	92.95	100.00
	7.05	92.95	
	100.00	100.00	
Total	1445	19064	20509
	7.05	92.95	100.00

Concomitant medications other than anticoagulants at the time of obtaining Table 2-5 consent, according to the presence or absence of proton-pump inhibitors

FREQ Procedure

Tables: ALL * CMYN

ALL

CMYN(Administration of Concomitant Drug)

Power |
Percent |
Percent of rows
Percent of columns | None | With | Total

1	1445	19064	20509
	7.05	92.95	100.00
	7.05	92.95	
	100.00	100.00	
Total	1445	19064	20509
	7.05	92.95	100.00

Concomitant medications other than anticoagulants at the time of obtaining Table 2-5 consent, according to the presence or absence of proton-pump inhibitors

FREQ Procedure

ALL	power	Percent	Cumulative power	Cumulative Percent of power
1	20509	100.00	20509	100.00

Tables: ALL * CMYN

ALL

CMYN(Administration of Concomitant Drug)

Power |
Percent |
Percent of rows
Percent of columns | None | With | Total

ALL	None	With	Total
1	1445	19064	20509
	7.05	92.95	100.00
	7.05	92.95	
	100.00	100.00	
Total	1445	19064	20509
	7.05	92.95	100.00

Concomitant medications other than anticoagulants at the time of obtaining Table 2-5 consent, according to the presence or absence of proton-pump inhibitors

FREQ Procedure

Tables: ALL* ARTD

ALL ARTD(Arrhythmic Drugs)

Power |
Percent |
Percent of rows
Percent of columns | None | With | Total

```
______________+________+________+
             1 |    7836 |   11228 |   19064
               |   41.10 |   58.90 | 100.00
               |   41.10 |   58.90 |
               | 100.00 | 100.00 |
______________+________+________+
Total 7836 11228 19064
                  41.10      58.90     100.00
```

Concomitant medications other than anticoagulants at the time of obtaining Table 2-5 consent, according to the presence or absence of proton-pump inhibitors

FREQ Procedure

			Cumulative	
Percent of ALL	power	Percent of power		
1	20509	100.00	20509	100.00

Tables: ALL * CMYN

ALL

CMYN(Administration of Concomitant Drug)

Power / Percent / Percent of rows / Percent of columns	None	With	Total
1	1445	19064	20509
	7.05	92.95	100.00
	7.05	92.95	
	100.00	100.00	
Total	1445	19064	20509
	7.05	92.95	100.00

Concomitant medications other than anticoagulants at the time of obtaining Table 2-5 consent, according to the presence or absence of proton-pump inhibitors

FREQ Procedure

Tables: ALL* ARTD

ALL ARTD(Arrhythmic Drugs)

Power |
Percent |
Percent of rows
Percent of columns | None | With | Total

1	7836	11228	19064
	41.10	58.90	100.00
	41.10	58.90	
	100.00	100.00	
Total	7836	11228	19064
	41.10	58.90	100.00

Concomitant medications other than anticoagulants at the time of obtaining Table 2-5 consent, according to the presence or absence of proton-pump inhibitors

FREQ Procedure

			Cumulative	
Percent of ALL	power	Percent of power		
1	20509	100.00	20509	100.00

Tables: ALL * CMYN

ALL

CMYN(Administration of Concomitant Drug)

Power / Percent / Percent of rows / Percent of columns	None	With	Total
1	1445	19064	20509
	7.05	92.95	100.00
	7.05	92.95	
	100.00	100.00	
Total	1445	19064	20509
	7.05	92.95	100.00

Concomitant medications other than anticoagulants at the time of obtaining Table 2-5 consent, according to the presence or absence of proton-pump inhibitors

FREQ Procedure

Table : ALL * ARTD1

ALL ARTD1(Rhythm Control)

Power<br>Percent<br>Percent of rows<br>Percent of columns	None	With	Total
1	15180<br>79.63<br>79.63<br>100.00	3884<br>20.37<br>20.37<br>100.00	19064<br>100.00
Total	15180<br>79.63	3884<br>20.37	19064<br>100.00

Concomitant medications other than anticoagulants at the time of obtaining Table 2-5 consent, according to the presence or absence of proton-pump inhibitors

FREQ Procedure

			Cumulative	
Percent of ALL	power	Percent of	power	
1	20509	100.00	20509	100.00

Tables: ALL * CMYN

ALL

CMYN(Administration of Concomitant Drug)

Power / Percent / Percent of rows / Percent of columns	None	With	Total
1	1445	19064	20509
	7.05	92.95	100.00
	7.05	92.95	
	100.00	100.00	
Total	1445	19064	20509
	7.05	92.95	100.00

Concomitant medications other than anticoagulants at the time of obtaining Table 2-5 consent, according to the presence or absence of proton-pump inhibitors

FREQ Procedure

Table : ALL * ARTD1

ALL ARTD1(Rhythm Control)

Power / Percent / Percent of rows / Percent of columns	None	With	Total
1	15180	3884	19064
	79.63	20.37	100.00
	79.63	20.37	
	100.00	100.00	
Total	15180	3884	19064
	79.63	20.37	100.00

Concomitant medications other than anticoagulants at the time of obtaining Table 2-5 consent, according to the presence or absence of proton-pump inhibitors

FREQ Procedure

ALL	power	Percent	Cumulative power	Cumulative Percent of power
1	20509	100.00	20509	100.00

Tables: ALL * CMYN

ALL / Power / Percent / Percent of rows / Percent of columns	CMYN(Administration of Concomitant Drug) None	With	Total
1	1445	19064	20509
	7.05	92.95	100.00
	7.05	92.95	
	100.00	100.00	
Total	1445	19064	20509
	7.05	92.95	100.00

Concomitant medications other than anticoagulants at the time of obtaining Table 2-5 consent, according to the presence or absence of proton-pump inhibitors

FREQ Procedure

Table : ALL * ARTD2

ALL ARTD2(Rate Control)

Power<br>Percent<br>Percent of rows<br>Percent of columns	None	With	Total
1	10871<br>57.02<br>57.02<br>100.00	8193<br>42.98<br>42.98<br>100.00	19064<br>100.00
Total	10871<br>57.02	8193<br>42.98	19064<br>100.00

Concomitant medications other than anticoagulants at the time of obtaining Table 2-5 consent, according to the presence or absence of proton-pump inhibitors

FREQ Procedure

			Cumulative	
Percent of ALL	power	Percent of	power	
1	20509	100.00	20509	100.00

Tables: ALL * CMYN

ALL
CMYN(Administration of Concomitant Drug)

Power<br>Percent<br>Percent of rows<br>Percent of columns	None	With	Total
1	1445<br>7.05<br>7.05<br>100.00	19064<br>92.95<br>92.95<br>100.00	20509<br>100.00
Total	1445<br>7.05	19064<br>92.95	20509<br>100.00

Concomitant medications other than anticoagulants at the time of obtaining Table 2-5 consent, according to the presence or absence of proton-pump inhibitors

FREQ Procedure

Table : ALL * ARTD2

ALL ARTD2(Rate Control)

Power |
Percent |
Percent of rows
Percent of columns | None | With | Total

1	10871	8193	19064
	57.02	42.98	100.00
	57.02	42.98	
	100.00	100.00	
Total	10871	8193	19064
	57.02	42.98	100.00

Concomitant medications other than anticoagulants at the time of obtaining Table 2-5 consent, according to the presence or absence of proton-pump inhibitors

FREQ Procedure

ALL	power	Percent	Cumulative power	Cumulative Percent of power
1	20509	100.00	20509	100.00

Tables: ALL * CMYN

ALL
CMYN(Administration of Concomitant Drug)

Power |
Percent |
Percent of rows
Percent of columns | None | With | Total

ALL	None	With	Total
1	1445	19064	20509
	7.05	92.95	100.00
	7.05	92.95	
	100.00	100.00	
Total	1445	19064	20509
	7.05	92.95	100.00

Concomitant medications other than anticoagulants at the time of obtaining Table 2-5 consent, according to the presence or absence of proton-pump inhibitors

FREQ Procedure

Tables: ALL* APLD

ALL APLD(Anti-Platelet Drugs)

Power / Percent / Percent of rows / Percent of columns	None	With	Total
1	16287	2777	19064
	85.43	14.57	100.00
	85.43	14.57	
	100.00	100.00	
Total	16287	2777	19064
	85.43	14.57	100.00

Concomitant medications other than anticoagulants at the time of obtaining Table 2-5 consent, according to the presence or absence of proton-pump inhibitors

FREQ Procedure

Percent of ALL	power	Percent	Cumulative power	Cumulative Percent of power
1	20509	100.00	20509	100.00

Tables: ALL * CMYN

ALL

CMYN(Administration of Concomitant Drug)

Power / Percent / Percent of rows / Percent of columns	None	With	Total
1	1445	19064	20509
	7.05	92.95	100.00
	7.05	92.95	
	100.00	100.00	
Total	1445	19064	20509
	7.05	92.95	100.00

Concomitant medications other than anticoagulants at the time of obtaining Table 2-5 consent, according to the presence or absence of proton-pump inhibitors

FREQ Procedure

Tables: ALL* APLD

ALL APLD(Anti-Platelet Drugs)

Power |
Percent |
Percent of rows
Percent of columns | None | With | Total
______________+________+________+
1 | 16287 | 2777 | 19064
| 85.43 | 14.57 | 100.00
| 85.43 | 14.57 |
| 100.00 | 100.00 |
______________+________+________+
Total 16287 2777 19064
85.43 14.57 100.00

Concomitant medications other than anticoagulants at the time of obtaining Table 2-5 consent, according to the presence or absence of proton-pump inhibitors

FREQ Procedure

			Cumulative	
Percent of ALL	power	Percent of	power	
1	20509	100.00	20509	100.00

Tables: ALL * CMYN

ALL

CMYN(Administration of Concomitant Drug)

Power |
Percent |
Percent of rows
Percent of columns | None | With | Total

	None	With	Total
1	1445	19064	20509
	7.05	92.95	100.00
	7.05	92.95	
	100.00	100.00	
Total	1445	19064	20509
	7.05	92.95	100.00

Concomitant medications other than anticoagulants at the time of obtaining Table 2-5 consent, according to the presence or absence of proton-pump inhibitors

FREQ Procedure

Table: ALL * t_AP

ALL t_AP

Power |
Percent |
Percent of rows
Percentage of columns | 0| 1| Sum

	0	1	Sum
1	18978	86	19064
	99.55	0.45	100.00
	99.55	0.45	
	100.00	100.00	
Total	18978	86	19064
	99.55	0.45	100.00

Concomitant medications other than anticoagulants at the time of obtaining Table 2-5 consent, according to the presence or absence of proton-pump inhibitors

FREQ Procedure

			Cumulative	
Percent of ALL	power	Percent of	power	
1	20509	100.00	20509	100.00

Tables: ALL * CMYN

ALL

CMYN(Administration of Concomitant Drug)

Power<br>Percent<br>Percent of rows<br>Percent of columns	None	With	Total
1	1445<br>7.05<br>7.05<br>100.00	19064<br>92.95<br>92.95<br>100.00	20509<br>100.00
Total	1445<br>7.05	19064<br>92.95	20509<br>100.00

Concomitant medications other than anticoagulants at the time of obtaining Table 2-5 consent, according to the presence or absence of proton-pump inhibitors

FREQ Procedure

Table: ALL * t_AP

ALL t_AP

Power |
Percent |
Percent of rows
Percentage of columns | 0| 1| Sum

1	18978	86	19064
	99.55	0.45	100.00
	99.55	0.45	
	100.00	100.00	
Total	18978	86	19064
	99.55	0.45	100.00

Concomitant medications other than anticoagulants at the time of obtaining Table 2-5 consent, according to the presence or absence of proton-pump inhibitors

FREQ Procedure

			Cumulative	
Percent of ALL	power	Percent of	power	
1	20509	100.00	20509	100.00

Tables: ALL * CMYN

ALL

CMYN(Administration of Concomitant Drug)

Power | Percent | Percent of rows Percent of columns	None	With	Total
1	1445	19064	20509
	7.05	92.95	100.00
	7.05	92.95	
	100.00	100.00	
Total	1445	19064	20509
	7.05	92.95	100.00

Concomitant medications other than anticoagulants at the time of obtaining Table 2-5 consent, according to the presence or absence of proton-pump inhibitors

FREQ Procedure

Table : ALL * APLD1

ALL APLD1(ASA)

Power |
Percent |
Percent of rows
Percent of columns | None | With | Total

1	17482	1582	19064
	91.70	8.30	100.00
	91.70	8.30	
	100.00	100.00	
Total	17482	1582	19064
	91.70	8.30	100.00

Concomitant medications other than anticoagulants at the time of obtaining Table 2-5 consent, according to the presence or absence of proton-pump inhibitors

FREQ Procedure

			Cumulative	
Percent of ALL	power	Percent of power		
1	20509	100.00	20509	100.00

Tables: ALL * CMYN

ALL

CMYN(Administration of Concomitant Drug)

Power |
Percent |
Percent of rows
Percent of columns | None | With | Total

	None	With	Total
1	1445	19064	20509
	7.05	92.95	100.00
	7.05	92.95	
	100.00	100.00	
Total	1445	19064	20509
	7.05	92.95	100.00

Concomitant medications other than anticoagulants at the time of obtaining Table 2-5 consent, according to the presence or absence of proton-pump inhibitors

FREQ Procedure

Table : ALL * APLD1

ALL APLD1(ASA)

Power<br>Percent<br>Percent of rows<br>Percent of columns	None	With	Total
1	17482<br>91.70<br>91.70<br>100.00	1582<br>8.30<br>8.30<br>100.00	19064<br>100.00
Total	17482<br>91.70	1582<br>8.30	19064<br>100.00

Concomitant medications other than anticoagulants at the time of obtaining Table 2-5 consent, according to the presence or absence of proton-pump inhibitors

FREQ Procedure

			Cumulative	
Percent of ALL	power	Percent of	power	
1	20509	100.00	20509	100.00

Tables: ALL * CMYN

ALL

CMYN(Administration of Concomitant Drug)

Power / Percent / Percent of rows / Percent of columns	None	With	Total
1	1445	19064	20509
	7.05	92.95	100.00
	7.05	92.95	
	100.00	100.00	
Total	1445	19064	20509
	7.05	92.95	100.00

Concomitant medications other than anticoagulants at the time of obtaining Table 2-5 consent, according to the presence or absence of proton-pump inhibitors

FREQ Procedure

Table : ALL * APLD2

ALL APLD2(P2Y12-I)

Power<br>Percent<br>Percent of rows<br>Percent of columns	None	With	Total
1	18444<br>96.75<br>96.75<br>100.00	620<br>3.25<br>3.25<br>100.00	19064<br>100.00
Total	18444<br>96.75	620<br>3.25	19064<br>100.00

Concomitant medications other than anticoagulants at the time of obtaining Table 2-5 consent, according to the presence or absence of proton-pump inhibitors

FREQ Procedure

			Cumulative	
Percent of ALL	power	Percent of power		
1	20509	100.00	20509	100.00

Tables: ALL * CMYN

ALL

CMYN(Administration of Concomitant Drug)

Power / Percent / Percent of rows / Percent of columns	None	With	Total
1	1445	19064	20509
	7.05	92.95	100.00
	7.05	92.95	
	100.00	100.00	
Total	1445	19064	20509
	7.05	92.95	100.00

Concomitant medications other than anticoagulants at the time of obtaining Table 2-5 consent, according to the presence or absence of proton-pump inhibitors

FREQ Procedure

Table : ALL * APLD2

ALL APLD2(P2Y12-I)

Power |
Percent |
Percent of rows
Percent of columns | None | With | Total

1	18444	620	19064
	96.75	3.25	100.00
	96.75	3.25	
	100.00	100.00	
Total	18444	620	19064
	96.75	3.25	100.00

Concomitant medications other than anticoagulants at the time of obtaining Table 2-5 consent, according to the presence or absence of proton-pump inhibitors

FREQ Procedure

			Cumulative	
Percent of ALL	power	Percent of	power	
1	20509	100.00	20509	100.00

Tables: ALL * CMYN

ALL

CMYN(Administration of Concomitant Drug)

Power |
Percent |
Percent of rows
Percent of columns | None | With | Total

	None	With	Total
1	1445	19064	20509
	7.05	92.95	100.00
	7.05	92.95	
	100.00	100.00	
Total	1445	19064	20509
	7.05	92.95	100.00

Concomitant medications other than anticoagulants at the time of obtaining Table 2-5 consent, according to the presence or absence of proton-pump inhibitors

FREQ Procedure

Table : ALL * APLD3

ALL

APLD3(Other Anti-Platelet Drugs)

Power / Percent / Percent of rows / Percent of columns	None	With	Total
1	18322	742	19064
	96.11	3.89	100.00
	96.11	3.89	
	100.00	100.00	
Total	18322	742	19064
	96.11	3.89	100.00

Concomitant medications other than anticoagulants at the time of obtaining Table 2-5 consent, according to the presence or absence of proton-pump inhibitors

FREQ Procedure

			Cumulative	
Percent of ALL	power	Percent	of power	
1	20509	100.00	20509	100.00

Tables: ALL * CMYN

ALL

CMYN(Administration of Concomitant Drug)

Power / Percent / Percent of rows / Percent of columns	None	With	Total
1	1445	19064	20509
	7.05	92.95	100.00
	7.05	92.95	
	100.00	100.00	
Total	1445	19064	20509
	7.05	92.95	100.00

Concomitant medications other than anticoagulants at the time of obtaining Table 2-5 consent, according to the presence or absence of proton-pump inhibitors

FREQ Procedure

Table : ALL * APLD3

ALL

APLD3(Other Anti-Platelet Drugs)

Power | Percent | Percent of rows Percent of columns	None	With	Total
1	18322	742	19064
	96.11	3.89	100.00
	96.11	3.89	
	100.00	100.00	
Total	18322	742	19064
	96.11	3.89	100.00

Concomitant medications other than anticoagulants at the time of obtaining Table 2-5 consent, according to the presence or absence of proton-pump inhibitors

FREQ Procedure

			Cumulative	
Percent of ALL	power	Percent of	power	
1	20509	100.00	20509	100.00

Tables: ALL * CMYN

ALL

CMYN(Administration of Concomitant Drug)

Power |
Percent |
Percent of rows
Percent of columns | None | With | Total

	None	With	Total
1	1445	19064	20509
	7.05	92.95	100.00
	7.05	92.95	
	100.00	100.00	
Total	1445	19064	20509
	7.05	92.95	100.00

Concomitant medications other than anticoagulants at the time of obtaining Table 2-5 consent, according to the presence or absence of proton-pump inhibitors

FREQ Procedure

Tables: ALL * AHPD

ALL

AHPD(Antihypertensive Drugs)

Power | Percent | Percent of rows Percent of columns	None	With	Total
1	4769	14295	19064
	25.02	74.98	100.00
	25.02	74.98	
	100.00	100.00	
Total	4769	14295	19064
	25.02	74.98	100.00

Concomitant medications other than anticoagulants at the time of obtaining Table 2-5 consent, according to the presence or absence of proton-pump inhibitors

FREQ Procedure

			Cumulative	
Percent of ALL	power	Percent of	power	
1	20509	100.00	20509	100.00

Tables: ALL * CMYN

ALL
CMYN(Administration of Concomitant Drug)

Power / Percent / Percent of rows / Percent of columns	None	With	Total
1	1445	19064	20509
	7.05	92.95	100.00
	7.05	92.95	
	100.00	100.00	
Total	1445	19064	20509
	7.05	92.95	100.00

Concomitant medications other than anticoagulants at the time of obtaining Table 2-5 consent, according to the presence or absence of proton-pump inhibitors

FREQ Procedure

Tables: ALL * AHPD

ALL

AHPD(Antihypertensive Drugs)

Power<br>Percent<br>Percent of rows<br>Percent of columns	None	With	Total
1	4769<br>25.02<br>25.02<br>100.00	14295<br>74.98<br>74.98<br>100.00	19064<br>100.00
Total	4769<br>25.02	14295<br>74.98	19064<br>100.00

Concomitant medications other than anticoagulants at the time of obtaining Table 2-5 consent, according to the presence or absence of proton-pump inhibitors

FREQ Procedure

			Cumulative	
Percent of ALL	power	Percent	of power	
1	20509	100.00	20509	100.00

Tables: ALL * CMYN

ALL

CMYN(Administration of Concomitant Drug)

Power |
Percent |
Percent of rows
Percent of columns | None | With | Total

	None	With	Total
1	1445	19064	20509
	7.05	92.95	100.00
	7.05	92.95	
	100.00	100.00	
Total	1445	19064	20509
	7.05	92.95	100.00

Concomitant medications other than anticoagulants at the time of obtaining Table 2-5 consent, according to the presence or absence of proton-pump inhibitors

FREQ Procedure

Table: ALL * LTD

ALL LTD(Lipid Therapy)

Power |
Percent |
Percent of rows
Percent of columns | None | With | Total

ALL	None	With	Total
1	12266	6798	19064
	64.34	35.66	100.00
	64.34	35.66	
	100.00	100.00	
Total	12266	6798	19064
	64.34	35.66	100.00

Concomitant medications other than anticoagulants at the time of obtaining Table 2-5 consent, according to the presence or absence of proton-pump inhibitors

FREQ Procedure

			Cumulative	
Percent of ALL	power	Percent of	power	
1	20509	100.00	20509	100.00

Tables: ALL * CMYN

ALL

CMYN(Administration of Concomitant Drug)

Power |
Percent |
Percent of rows
Percent of columns | None | With | Total

	None	With	Total
1	1445	19064	20509
	7.05	92.95	100.00
	7.05	92.95	
	100.00	100.00	
Total	1445	19064	20509
	7.05	92.95	100.00

Concomitant medications other than anticoagulants at the time of obtaining Table 2-5 consent, according to the presence or absence of proton-pump inhibitors

FREQ Procedure

Table: ALL * LTD

ALL LTD(Lipid Therapy)

Power / Percent / Percent of rows / Percent of columns	None	With	Total
1	12266	6798	19064
	64.34	35.66	100.00
	64.34	35.66	
	100.00	100.00	
Total	12266	6798	19064
	64.34	35.66	100.00

Concomitant medications other than anticoagulants at the time of obtaining Table 2-5 consent, according to the presence or absence of proton-pump inhibitors

FREQ Procedure

ALL	power	Percent	Cumulative power	Cumulative Percent
1	20509	100.00	20509	100.00

Tables: ALL * CMYN

ALL
CMYN(Administration of Concomitant Drug)

Power |
Percent |
Percent of rows
Percent of columns | None | With | Total

ALL	None	With	Total
1	1445	19064	20509
	7.05	92.95	100.00
	7.05	92.95	
	100.00	100.00	
Total	1445	19064	20509
	7.05	92.95	100.00

Concomitant medications other than anticoagulants at the time of obtaining Table 2-5 consent, according to the presence or absence of proton-pump inhibitors

FREQ Procedure

Table : ALL * LTD1

ALL LTD1(Statins)

Power |
Percent |
Percent of rows
Percent of columns | None | With | Total
______________+________+________+
1 | 13003 | 6061 | 19064
| 68.21 | 31.79 | 100.00
| 68.21 | 31.79 |
| 100.00 | 100.00 |
______________+________+________+
Total 13003 6061 19064
68.21 31.79 100.00

Concomitant medications other than anticoagulants at the time of obtaining Table 2-5 consent, according to the presence or absence of proton-pump inhibitors

FREQ Procedure

ALL	power	Percent of power	Cumulative power	Cumulative Percent of power
1	20509	100.00	20509	100.00

Tables: ALL * CMYN

ALL
CMYN(Administration of Concomitant Drug)

Power |
Percent |
Percent of rows
Percent of columns | None | With | Total

ALL	None	With	Total
1	1445	19064	20509
	7.05	92.95	100.00
	7.05	92.95	
	100.00	100.00	
Total	1445	19064	20509
	7.05	92.95	100.00

Concomitant medications other than anticoagulants at the time of obtaining Table 2-5 consent, according to the presence or absence of proton-pump inhibitors

FREQ Procedure

Table : ALL * LTD1

ALL LTD1(Statins)

Power |
Percent |
Percent of rows
Percent of columns | None | With | Total

	None	With	Total
1	13003	6061	19064
	68.21	31.79	100.00
	68.21	31.79	
	100.00	100.00	
Total	13003	6061	19064
	68.21	31.79	100.00

Concomitant medications other than anticoagulants at the time of obtaining Table 2-5 consent, according to the presence or absence of proton-pump inhibitors

FREQ Procedure

Percent of ALL	power	Percent of power	Cumulative	
1	20509	100.00	20509	100.00

Tables: ALL * CMYN

ALL

CMYN(Administration of Concomitant Drug)

Power | Percent | Percent of rows Percent of columns	None	With	Total
1	1445	19064	20509
	7.05	92.95	100.00
	7.05	92.95	
	100.00	100.00	
Total	1445	19064	20509
	7.05	92.95	100.00

Concomitant medications other than anticoagulants at the time of obtaining Table 2-5 consent, according to the presence or absence of proton-pump inhibitors

FREQ Procedure

Table : ALL * LTD2

ALL LTD2(Other Than Statins)

Power<br>Percent<br>Percent of rows<br>Percent of columns	None	With	Total
1	18096<br>94.92<br>94.92<br>100.00	968<br>5.08<br>5.08<br>100.00	19064<br>100.00
Total	18096<br>94.92	968<br>5.08	19064<br>100.00

Concomitant medications other than anticoagulants at the time of obtaining Table 2-5 consent, according to the presence or absence of proton-pump inhibitors

FREQ Procedure

			Cumulative	
Percent of ALL	power	Percent of	power	
1	20509	100.00	20509	100.00

Tables: ALL * CMYN

ALL

CMYN(Administration of Concomitant Drug)

Power / Percent / Percent of rows / Percent of columns	None	With	Total
1	1445	19064	20509
	7.05	92.95	100.00
	7.05	92.95	
	100.00	100.00	
Total	1445	19064	20509
	7.05	92.95	100.00

Concomitant medications other than anticoagulants at the time of obtaining Table 2-5 consent, according to the presence or absence of proton-pump inhibitors

FREQ Procedure

Table : ALL * LTD2

ALL LTD2(Other Than Statins)

Power<br>Percent<br>Percent of rows<br>Percent of columns	None	With	Total
1	18096<br>94.92<br>94.92<br>100.00	968<br>5.08<br>5.08<br>100.00	19064<br>100.00
Total	18096<br>94.92	968<br>5.08	19064<br>100.00

Concomitant medications other than anticoagulants at the time of obtaining Table 2-5 consent, according to the presence or absence of proton-pump inhibitors

FREQ Procedure

			Cumulative	
Percent of ALL	power	Percent of	power	
1	20509	100.00	20509	100.00

Tables: ALL * CMYN

ALL

CMYN(Administration of Concomitant Drug)

Power / Percent / Percent of rows / Percent of columns	None	With	Total
1	1445	19064	20509
	7.05	92.95	100.00
	7.05	92.95	
	100.00	100.00	
Total	1445	19064	20509
	7.05	92.95	100.00

Concomitant medications other than anticoagulants at the time of obtaining Table 2-5 consent, according to the presence or absence of proton-pump inhibitors

FREQ Procedure

Tables: ALL * DBTD

ALL DBTD(Diabetes Drugs)

Power |
Percent |
Percent of rows
Percent of columns | None | With | Total

	None	With	Total
1	16012	3052	19064
	83.99	16.01	100.00
	83.99	16.01	
	100.00	100.00	
Total	16012	3052	19064
	83.99	16.01	100.00

Concomitant medications other than anticoagulants at the time of obtaining Table 2-5 consent, according to the presence or absence of proton-pump inhibitors

FREQ Procedure

			Cumulative	
Percent of ALL	power	Percent of power		
1	20509	100.00	20509	100.00

Tables: ALL * CMYN

ALL

CMYN(Administration of Concomitant Drug)

Power |
Percent |
Percent of rows
Percent of columns | None | With | Total

	None	With	Total
1	1445	19064	20509
	7.05	92.95	100.00
	7.05	92.95	
	100.00	100.00	
Total	1445	19064	20509
	7.05	92.95	100.00

Concomitant medications other than anticoagulants at the time of obtaining Table 2-5 consent, according to the presence or absence of proton-pump inhibitors

FREQ Procedure

Tables: ALL * DBTD

ALL DBTD(Diabetes Drugs)

Power / Percent / Percent of rows / Percent of columns	None	With	Total
1	16012	3052	19064
	83.99	16.01	100.00
	83.99	16.01	
	100.00	100.00	
Total	16012	3052	19064
	83.99	16.01	100.00

Concomitant medications other than anticoagulants at the time of obtaining Table 2-5 consent, according to the presence or absence of proton-pump inhibitors

FREQ Procedure

ALL	power	Percent of power	Cumulative power	Cumulative Percent of power
1	20509	100.00	20509	100.00

Tables: ALL * CMYN

ALL

CMYN(Administration of Concomitant Drug)

Power |
Percent |
Percent of rows
Percent of columns | None | With | Total

ALL	None	With	Total
1	1445	19064	20509
	7.05	92.95	100.00
	7.05	92.95	
	100.00	100.00	
Total	1445	19064	20509
	7.05	92.95	100.00

Concomitant medications other than anticoagulants at the time of obtaining Table 2-5 consent, according to the presence or absence of proton-pump inhibitors

FREQ Procedure

Table: ALL * t_DBT

ALL t_DBT

Power |
Percent |
Percent of rows
Percentage of columns | 0| 1| Sum

	0	1	Sum
1	16162	2902	19064
	84.78	15.22	100.00
	84.78	15.22	
	100.00	100.00	
Total	16162	2902	19064
	84.78	15.22	100.00

Concomitant medications other than anticoagulants at the time of obtaining Table 2-5 consent, according to the presence or absence of proton-pump inhibitors

FREQ Procedure

			Cumulative	
Percent of ALL	power	Percent	of power	
1	20509	100.00	20509	100.00

Tables: ALL * CMYN

ALL
CMYN(Administration of Concomitant Drug)

Power / Percent / Percent of rows / Percent of columns	None	With	Total
1	1445	19064	20509
	7.05	92.95	100.00
	7.05	92.95	
	100.00	100.00	
Total	1445	19064	20509
	7.05	92.95	100.00

Concomitant medications other than anticoagulants at the time of obtaining Table 2-5 consent, according to the presence or absence of proton-pump inhibitors

FREQ Procedure

Table: ALL * t_DBT

ALL t_DBT

Power |
Percent |
Percent of rows
Percentage of columns | 0| 1| Sum

	0	1	Sum
1	16162	2902	19064
	84.78	15.22	100.00
	84.78	15.22	
	100.00	100.00	
Total	16162	2902	19064
	84.78	15.22	100.00

Concomitant medications other than anticoagulants at the time of obtaining Table 2-5 consent, according to the presence or absence of proton-pump inhibitors

FREQ Procedure

			Cumulative	
Percent of ALL	power	Percent of power		
1	20509	100.00	20509	100.00

Tables: ALL * CMYN

ALL

CMYN(Administration of Concomitant Drug)

Power<br>Percent<br>Percent of rows<br>Percent of columns	None	With	Total
1	1445<br>7.05<br>7.05<br>100.00	19064<br>92.95<br>92.95<br>100.00	20509<br>100.00
Total	1445<br>7.05	19064<br>92.95	20509<br>100.00

Concomitant medications other than anticoagulants at the time of obtaining Table 2-5 consent, according to the presence or absence of proton-pump inhibitors

FREQ Procedure

Table : ALL * DBTD1

ALL DBTD1(Sulfonylurea Drugs)

Power |
Percent |
Percent of rows
Percent of columns | None | With | Total

1	18316	748	19064
	96.08	3.92	100.00
	96.08	3.92	
	100.00	100.00	
Total	18316	748	19064
	96.08	3.92	100.00

Concomitant medications other than anticoagulants at the time of obtaining Table 2-5 consent, according to the presence or absence of proton-pump inhibitors

FREQ Procedure

ALL	power	Percent	Cumulative power	Cumulative Percent
1	20509	100.00	20509	100.00

Tables: ALL * CMYN

ALL

CMYN(Administration of Concomitant Drug)

Power / Percent / Percent of rows / Percent of columns	None	With	Total
1	1445	19064	20509
	7.05	92.95	100.00
	7.05	92.95	
	100.00	100.00	
Total	1445	19064	20509
	7.05	92.95	100.00

Concomitant medications other than anticoagulants at the time of obtaining Table 2-5 consent, according to the presence or absence of proton-pump inhibitors

FREQ Procedure

Table : ALL * DBTD1

ALL DBTD1(Sulfonylurea Drugs)

Power |
Percent |
Percent of rows
Percent of columns | None | With | Total

	None	With	Total
1	18316	748	19064
	96.08	3.92	100.00
	96.08	3.92	
	100.00	100.00	
Total	18316	748	19064
	96.08	3.92	100.00

Concomitant medications other than anticoagulants at the time of obtaining Table 2-5 consent, according to the presence or absence of proton-pump inhibitors

FREQ Procedure

ALL	power	Percent	Cumulative power	Cumulative Percent of power
1	20509	100.00	20509	100.00

Tables: ALL * CMYN

ALL
CMYN(Administration of Concomitant Drug)

Power |
Percent |
Percent of rows
Percent of columns | None | With | Total

ALL	None	With	Total
1	1445	19064	20509
	7.05	92.95	100.00
	7.05	92.95	
	100.00	100.00	
Total	1445	19064	20509
	7.05	92.95	100.00

Concomitant medications other than anticoagulants at the time of obtaining Table 2-5 consent, according to the presence or absence of proton-pump inhibitors

FREQ Procedure

Table : ALL * DBTD2

ALL

DBTD2(α-Glucosidase Inhibitor)

Power / Percent / Percent of rows / Percent of columns	None	With	Total
1	18469	595	19064
	96.88	3.12	100.00
	96.88	3.12	
	100.00	100.00	
Total	18469	595	19064
	96.88	3.12	100.00

Concomitant medications other than anticoagulants at the time of obtaining Table 2-5 consent, according to the presence or absence of proton-pump inhibitors

FREQ Procedure

ALL	power	Percent	Cumulative power	Cumulative Percent
1	20509	100.00	20509	100.00

Tables: ALL * CMYN

ALL
CMYN(Administration of Concomitant Drug)

Power |
Percent |
Percent of rows
Percent of columns | None | With | Total

	None	With	Total
1	1445	19064	20509
	7.05	92.95	100.00
	7.05	92.95	
	100.00	100.00	
Total	1445	19064	20509
	7.05	92.95	100.00

Concomitant medications other than anticoagulants at the time of obtaining Table 2-5 consent, according to the presence or absence of proton-pump inhibitors

FREQ Procedure

Table : ALL * DBTD2

ALL

DBTD2(α-Glucosidase Inhibitor)

Power / Percent / Percent of rows / Percent of columns	None	With	Total
1	18469	595	19064
	96.88	3.12	100.00
	96.88	3.12	
	100.00	100.00	
Total	18469	595	19064
	96.88	3.12	100.00

Concomitant medications other than anticoagulants at the time of obtaining Table 2-5 consent, according to the presence or absence of proton-pump inhibitors

FREQ Procedure

			Cumulative	
Percent of ALL	power	Percent of power		
1	20509	100.00	20509	100.00

Tables: ALL * CMYN

ALL

CMYN(Administration of Concomitant Drug)

Power<br>Percent<br>Percent of rows<br>Percent of columns	None	With	Total
1	1445<br>7.05<br>7.05<br>100.00	19064<br>92.95<br>92.95<br>100.00	20509<br>100.00
Total	1445<br>7.05	19064<br>92.95	20509<br>100.00

Concomitant medications other than anticoagulants at the time of obtaining Table 2-5 consent, according to the presence or absence of proton-pump inhibitors

FREQ Procedure

Table : ALL * DBTD3

ALL DBTD3(Thiazolidine)

Power |
Percent |
Percent of rows
Percent of columns | None | With | Total

1	18818	246	19064
	98.71	1.29	100.00
	98.71	1.29	
	100.00	100.00	
Total	18818	246	19064
	98.71	1.29	100.00

Concomitant medications other than anticoagulants at the time of obtaining Table 2-5 consent, according to the presence or absence of proton-pump inhibitors

FREQ Procedure

			Cumulative	
Percent of ALL	power	Percent of	power	
1	20509	100.00	20509	100.00

Tables: ALL * CMYN

ALL

CMYN(Administration of Concomitant Drug)

Power<br>Percent<br>Percent of rows<br>Percent of columns	None	With	Total
1	1445<br>7.05<br>7.05<br>100.00	19064<br>92.95<br>92.95<br>100.00	20509<br>100.00
Total	1445<br>7.05	19064<br>92.95	20509<br>100.00

Concomitant medications other than anticoagulants at the time of obtaining Table 2-5 consent, according to the presence or absence of proton-pump inhibitors

FREQ Procedure

Table : ALL * DBTD3

ALL DBTD3(Thiazolidine)

Power | Percent | Percent of rows Percent of columns	None	With	Total
1	18818	246	19064
	98.71	1.29	100.00
	98.71	1.29	
	100.00	100.00	
Total	18818	246	19064
	98.71	1.29	100.00

Concomitant medications other than anticoagulants at the time of obtaining Table 2-5 consent, according to the presence or absence of proton-pump inhibitors

FREQ Procedure

Percent of ALL	power	Percent	Cumulative power	Cumulative Percent of power
1	20509	100.00	20509	100.00

Tables: ALL * CMYN

ALL

CMYN(Administration of Concomitant Drug)

Power / Percent / Percent of rows / Percent of columns	None	With	Total
1	1445	19064	20509
	7.05	92.95	100.00
	7.05	92.95	
	100.00	100.00	
Total	1445	19064	20509
	7.05	92.95	100.00

Concomitant medications other than anticoagulants at the time of obtaining Table 2-5 consent, according to the presence or absence of proton-pump inhibitors

FREQ Procedure

Table : ALL * DBTD4

ALL DBTD4(DPP-4 Inhabitors)

Power |
Percent |
Percent of rows
Percent of columns | None | With | Total

1	16910	2154	19064
	88.70	11.30	100.00
	88.70	11.30	
	100.00	100.00	
Total	16910	2154	19064
	88.70	11.30	100.00

Concomitant medications other than anticoagulants at the time of obtaining Table 2-5 consent, according to the presence or absence of proton-pump inhibitors

FREQ Procedure

			Cumulative	
Percent of ALL	power	Percent of power		
1	20509	100.00	20509	100.00

Tables: ALL * CMYN

ALL

CMYN(Administration of Concomitant Drug)

Power |
Percent |
Percent of rows
Percent of columns | None | With | Total

	None	With	Total
1	1445	19064	20509
	7.05	92.95	100.00
	7.05	92.95	
	100.00	100.00	
Total	1445	19064	20509
	7.05	92.95	100.00

Concomitant medications other than anticoagulants at the time of obtaining Table 2-5 consent, according to the presence or absence of proton-pump inhibitors

FREQ Procedure

Table : ALL * DBTD4

ALL DBTD4(DPP-4 Inhabitors)

Power |
Percent |
Percent of rows
Percent of columns | None | With | Total

1	16910	2154	19064
	88.70	11.30	100.00
	88.70	11.30	
	100.00	100.00	
Total	16910	2154	19064
	88.70	11.30	100.00

Concomitant medications other than anticoagulants at the time of obtaining Table 2-5 consent, according to the presence or absence of proton-pump inhibitors

FREQ Procedure

			Cumulative	
Percent of ALL	power	Percent of	power	
1	20509	100.00	20509	100.00

Tables: ALL * CMYN

ALL

CMYN(Administration of Concomitant Drug)

Power |
Percent |
Percent of rows
Percent of columns | None | With | Total

	None	With	Total
1	1445	19064	20509
	7.05	92.95	100.00
	7.05	92.95	
	100.00	100.00	
Total	1445	19064	20509
	7.05	92.95	100.00

Concomitant medications other than anticoagulants at the time of obtaining Table 2-5 consent, according to the presence or absence of proton-pump inhibitors

FREQ Procedure

Table : ALL * DBTD5

ALL DBTD5(GLP-1 Inhabitors)

Power / Percent / Percent of rows / Percent of columns	None	With	Total
1	19025	39	19064
	99.80	0.20	100.00
	99.80	0.20	
	100.00	100.00	
Total	19025	39	19064
	99.80	0.20	100.00

Concomitant medications other than anticoagulants at the time of obtaining Table 2-5 consent, according to the presence or absence of proton-pump inhibitors

FREQ Procedure

ALL	power	Percent	Cumulative power	Cumulative Percent of power
1	20509	100.00	20509	100.00

Tables: ALL * CMYN

ALL

CMYN(Administration of Concomitant Drug)

Power |
Percent |
Percent of rows
Percent of columns | None | With | Total

	None	With	Total
1	1445	19064	20509
	7.05	92.95	100.00
	7.05	92.95	
	100.00	100.00	
Total	1445	19064	20509
	7.05	92.95	100.00

Concomitant medications other than anticoagulants at the time of obtaining Table 2-5 consent, according to the presence or absence of proton-pump inhibitors

FREQ Procedure

Table : ALL * DBTD5

ALL DBTD5(GLP-1 Inhabitors)

Power |
Percent |
Percent of rows
Percent of columns | None | With | Total
______________+________+________+
1 | 19025 | 39 | 19064
| 99.80 | 0.20 | 100.00
| 99.80 | 0.20 |
| 100.00 | 100.00 |
______________+________+________+
Total 19025 39 19064
99.80 0.20 100.00

Concomitant medications other than anticoagulants at the time of obtaining Table 2-5 consent, according to the presence or absence of proton-pump inhibitors

FREQ Procedure

ALL	power	Percent	Cumulative power	Cumulative Percent
1	20509	100.00	20509	100.00

Tables: ALL * CMYN

ALL

CMYN(Administration of Concomitant Drug)

Power |
Percent |
Percent of rows
Percent of columns | None | With | Total

	None	With	Total
1	1445	19064	20509
	7.05	92.95	100.00
	7.05	92.95	
	100.00	100.00	
Total	1445	19064	20509
	7.05	92.95	100.00

Concomitant medications other than anticoagulants at the time of obtaining Table 2-5 consent, according to the presence or absence of proton-pump inhibitors

FREQ Procedure

Table : ALL * DBTD6

ALL DBTD6(SGLT2 Inhabitors)

Power<br>Percent<br>Percent of rows<br>Percent of columns	None	With	Total
1	18839<br>98.82<br>98.82<br>100.00	225<br>1.18<br>1.18<br>100.00	19064<br>100.00
Total	18839<br>98.82	225<br>1.18	19064<br>100.00

Concomitant medications other than anticoagulants at the time of obtaining Table 2-5 consent, according to the presence or absence of proton-pump inhibitors

FREQ Procedure

			Cumulative	
Percent of ALL	power	Percent of power		
1	20509	100.00	20509	100.00

Tables: ALL * CMYN

ALL

CMYN(Administration of Concomitant Drug)

Power | Percent | Percent of rows Percent of columns	None	With	Total
1	1445	19064	20509
	7.05	92.95	100.00
	7.05	92.95	
	100.00	100.00	
Total	1445	19064	20509
	7.05	92.95	100.00

Concomitant medications other than anticoagulants at the time of obtaining Table 2-5 consent, according to the presence or absence of proton-pump inhibitors

FREQ Procedure

Table : ALL * DBTD6

ALL DBTD6(SGLT2 Inhabitors)

Power |
Percent |
Percent of rows
Percent of columns | None | With | Total

	None	With	Total
1	18839	225	19064
	98.82	1.18	100.00
	98.82	1.18	
	100.00	100.00	
Total	18839	225	19064
	98.82	1.18	100.00

Concomitant medications other than anticoagulants at the time of obtaining Table 2-5 consent, according to the presence or absence of proton-pump inhibitors

FREQ Procedure

ALL	power	Percent	Cumulative power	Cumulative Percent
1	20509	100.00	20509	100.00

Tables: ALL * CMYN

ALL
CMYN(Administration of Concomitant Drug)

Power |
Percent |
Percent of rows
Percent of columns | None | With | Total

ALL	None	With	Total
1	1445	19064	20509
	7.05	92.95	100.00
	7.05	92.95	
	100.00	100.00	
Total	1445	19064	20509
	7.05	92.95	100.00

Concomitant medications other than anticoagulants at the time of obtaining Table 2-5 consent, according to the presence or absence of proton-pump inhibitors

FREQ Procedure

Table : ALL * DBTD7

ALL DBTD7(Insuline Related)

Power<br>Percent<br>Percent of rows<br>Percent of columns	None	With	Total
1	18700<br>98.09<br>98.09<br>100.00	364<br>1.91<br>1.91<br>100.00	19064<br>100.00
Total	18700<br>98.09	364<br>1.91	19064<br>100.00

Concomitant medications other than anticoagulants at the time of obtaining Table 2-5 consent, according to the presence or absence of proton-pump inhibitors

FREQ Procedure

			Cumulative	
Percent of ALL	power	Percent of	power	
1	20509	100.00	20509	100.00

Tables: ALL * CMYN

ALL

CMYN(Administration of Concomitant Drug)

Power |
Percent |
Percent of rows
Percent of columns | None | With | Total

1	1445	19064	20509
	7.05	92.95	100.00
	7.05	92.95	
	100.00	100.00	
Total	1445	19064	20509
	7.05	92.95	100.00

Concomitant medications other than anticoagulants at the time of obtaining Table 2-5 consent, according to the presence or absence of proton-pump inhibitors

FREQ Procedure

Table : ALL * DBTD7

ALL DBTD7(Insuline Related)

Power<br>Percent<br>Percent of rows<br>Percent of columns	None	With	Total
1	18700<br>98.09<br>98.09<br>100.00	364<br>1.91<br>1.91<br>100.00	19064<br>100.00
Total	18700<br>98.09	364<br>1.91	19064<br>100.00

Concomitant medications other than anticoagulants at the time of obtaining Table 2-5 consent, according to the presence or absence of proton-pump inhibitors

FREQ Procedure

ALL	power	Percent	Cumulative power	Cumulative Percent
1	20509	100.00	20509	100.00

Tables: ALL * CMYN

ALL
CMYN(Administration of Concomitant Drug)

Power |
Percent |
Percent of rows
Percent of columns | None | With | Total

ALL	None	With	Total
1	1445	19064	20509
	7.05	92.95	100.00
	7.05	92.95	
	100.00	100.00	
Total	1445	19064	20509
	7.05	92.95	100.00

Concomitant medications other than anticoagulants at the time of obtaining Table 2-5 consent, according to the presence or absence of proton-pump inhibitors

FREQ Procedure

Table : ALL * DBTD8

ALL

DBTD8(Other Diabetes Drugs)

Power / Percent / Percent of rows / Percent of columns	None	With	Total
1	18446	618	19064
	96.76	3.24	100.00
	96.76	3.24	
	100.00	100.00	
Total	18446	618	19064
	96.76	3.24	100.00

Concomitant medications other than anticoagulants at the time of obtaining Table 2-5 consent, according to the presence or absence of proton-pump inhibitors

FREQ Procedure

			Cumulative	
Percent of ALL	power	Percent of	power	
1	20509	100.00	20509	100.00

Tables: ALL * CMYN

ALL

CMYN(Administration of Concomitant Drug)

Power | Percent | Percent of rows Percent of columns	None	With	Total
1	1445	19064	20509
	7.05	92.95	100.00
	7.05	92.95	
	100.00	100.00	
Total	1445	19064	20509
	7.05	92.95	100.00

Concomitant medications other than anticoagulants at the time of obtaining Table 2-5 consent, according to the presence or absence of proton-pump inhibitors

FREQ Procedure

Table : ALL * DBTD8

ALL

DBTD8(Other Diabetes Drugs)

Power |
Percent |
Percent of rows
Percent of columns | None | With | Total

1	18446	618	19064
	96.76	3.24	100.00
	96.76	3.24	
	100.00	100.00	
Total	18446	618	19064
	96.76	3.24	100.00

Concomitant medications other than anticoagulants at the time of obtaining Table 2-5 consent, according to the presence or absence of proton-pump inhibitors

FREQ Procedure

			Cumulative	
Percent of ALL	power	Percent of power		
1	20509	100.00	20509	100.00

Tables: ALL * CMYN

ALL

CMYN(Administration of Concomitant Drug)

Power |
Percent |
Percent of rows
Percent of columns | None | With | Total

	None	With	Total
1	1445	19064	20509
	7.05	92.95	100.00
	7.05	92.95	
	100.00	100.00	
Total	1445	19064	20509
	7.05	92.95	100.00

Concomitant medications other than anticoagulants at the time of obtaining Table 2-5 consent, according to the presence or absence of proton-pump inhibitors

FREQ Procedure

Table: ALL * DMM

ALL DMM(Dementia Medications)

Power / Percent / Percent of rows / Percent of columns	None	With	Total
1	18304	760	19064
	96.01	3.99	100.00
	96.01	3.99	
	100.00	100.00	
Total	18304	760	19064
	96.01	3.99	100.00

Concomitant medications other than anticoagulants at the time of obtaining Table 2-5 consent, according to the presence or absence of proton-pump inhibitors

FREQ Procedure

			Cumulative	
Percent of ALL	power	Percent of power		
1	20509	100.00	20509	100.00

Tables: ALL * CMYN

ALL

CMYN(Administration of Concomitant Drug)

Power |
Percent |
Percent of rows
Percent of columns | None | With | Total

	None	With	Total
1	1445	19064	20509
	7.05	92.95	100.00
	7.05	92.95	
	100.00	100.00	
Total	1445	19064	20509
	7.05	92.95	100.00

Concomitant medications other than anticoagulants at the time of obtaining Table 2-5 consent, according to the presence or absence of proton-pump inhibitors

FREQ Procedure

Table: ALL * DMM

ALL DMM(Dementia Medications)

Power |
Percent |
Percent of rows
Percent of columns | None | With | Total

1	18304	760	19064
	96.01	3.99	100.00
	96.01	3.99	
	100.00	100.00	
Total	18304	760	19064
	96.01	3.99	100.00

Concomitant medications other than anticoagulants at the time of obtaining Table 2-5 consent, according to the presence or absence of proton-pump inhibitors

FREQ Procedure

			Cumulative	
Percent of ALL	power	Percent of power		
1	20509	100.00	20509	100.00

Tables: ALL * CMYN

ALL

CMYN(Administration of Concomitant Drug)

Power / Percent / Percent of rows / Percent of columns	None	With	Total
1	1445	19064	20509
	7.05	92.95	100.00
	7.05	92.95	
	100.00	100.00	
Total	1445	19064	20509
	7.05	92.95	100.00

Concomitant medications other than anticoagulants at the time of obtaining Table 2-5 consent, according to the presence or absence of proton-pump inhibitors

FREQ Procedure

Tables: ALL* ACNA

ALL ACNA(Anti-Cancer Agent)

Power / Percent / Percent of rows / Percent of columns	None	With	Total
1	18885	179	19064
	99.06	0.94	100.00
	99.06	0.94	
	100.00	100.00	
Total	18885	179	19064
	99.06	0.94	100.00

Concomitant medications other than anticoagulants at the time of obtaining Table 2-5 consent, according to the presence or absence of proton-pump inhibitors

FREQ Procedure

			Cumulative	
Percent of ALL	power	Percent of power		
1	20509	100.00	20509	100.00

Tables: ALL * CMYN

ALL

CMYN(Administration of Concomitant Drug)

Power |
Percent |
Percent of rows
Percent of columns | None | With | Total

	None	With	Total
1	1445	19064	20509
	7.05	92.95	100.00
	7.05	92.95	
	100.00	100.00	
Total	1445	19064	20509
	7.05	92.95	100.00

Concomitant medications other than anticoagulants at the time of obtaining Table 2-5 consent, according to the presence or absence of proton-pump inhibitors

FREQ Procedure

Tables: ALL* ACNA

ALL ACNA(Anti-Cancer Agent)

Power<br>Percent<br>Percent of rows<br>Percent of columns	None	With	Total
1	18885<br>99.06<br>99.06<br>100.00	179<br>0.94<br>0.94<br>100.00	19064<br>100.00
Total	18885<br>99.06	179<br>0.94	19064<br>100.00

Concomitant medications other than anticoagulants at the time of obtaining Table 2-5 consent, according to the presence or absence of proton-pump inhibitors

FREQ Procedure

ALL	power	Percent	Cumulative power	Cumulative Percent of power
1	20509	100.00	20509	100.00

Cumulative
Percent of ALL power Percent of power

Tables: ALL * CMYN

ALL
CMYN(Administration of Concomitant Drug)

Power |
Percent |
Percent of rows
Percent of columns | None | With | Total

ALL	None	With	Total
1	1445	19064	20509
	7.05	92.95	100.00
	7.05	92.95	
	100.00	100.00	
Total	1445	19064	20509
	7.05	92.95	100.00

Concomitant medications other than anticoagulants at the time of obtaining Table 2-5 consent, according to the presence or absence of proton-pump inhibitors

FREQ Procedure

Tables: ALL* COPD

ALL COPD(COPD)

Power<br>Percent<br>Percent of rows<br>Percent of columns	None	With	Total
1	18605<br>97.59<br>97.59<br>100.00	459<br>2.41<br>2.41<br>100.00	19064<br>100.00
Total	18605<br>97.59	459<br>2.41	19064<br>100.00

Concomitant medications other than anticoagulants at the time of obtaining Table 2-5 consent, according to the presence or absence of proton-pump inhibitors

FREQ Procedure

			Cumulative	
Percent of ALL	power	Percent of power		
1	20509	100.00	20509	100.00

Tables: ALL * CMYN

ALL

CMYN(Administration of Concomitant Drug)

Power | Percent | Percent of rows Percent of columns	None	With	Total
1	1445	19064	20509
	7.05	92.95	100.00
	7.05	92.95	
	100.00	100.00	
Total	1445	19064	20509
	7.05	92.95	100.00

Concomitant medications other than anticoagulants at the time of obtaining Table 2-5 consent, according to the presence or absence of proton-pump inhibitors

FREQ Procedure

Tables: ALL* COPD

ALL COPD(COPD)

Power |
Percent |
Percent of rows
Percent of columns | None | With | Total

	None	With	Total
1	18605	459	19064
	97.59	2.41	100.00
	97.59	2.41	
	100.00	100.00	
Total	18605	459	19064
	97.59	2.41	100.00

Concomitant medications other than anticoagulants at the time of obtaining Table 2-5 consent, according to the presence or absence of proton-pump inhibitors

FREQ Procedure

			Cumulative	
Percent of ALL	power	Percent of	power	
1	20509	100.00	20509	100.00

Tables: ALL * CMYN

ALL

CMYN(Administration of Concomitant Drug)

Power | Percent | Percent of rows Percent of columns	None	With	Total
1	1445	19064	20509
	7.05	92.95	100.00
	7.05	92.95	
	100.00	100.00	
Total	1445	19064	20509
	7.05	92.95	100.00

Concomitant medications other than anticoagulants at the time of obtaining Table 2-5 consent, according to the presence or absence of proton-pump inhibitors

FREQ Procedure

Tables: ALL * PSYD

ALL PSYD(Psychotropic Drugs)

Power |
Percent |
Percent of rows
Percent of columns | None | With | Total

	None	With	Total
1	17688	1376	19064
	92.78	7.22	100.00
	92.78	7.22	
	100.00	100.00	
Total	17688	1376	19064
	92.78	7.22	100.00

Concomitant medications other than anticoagulants at the time of obtaining Table 2-5 consent, according to the presence or absence of proton-pump inhibitors

FREQ Procedure

			Cumulative	
Percent of ALL	power	Percent of power		
1	20509	100.00	20509	100.00

Tables: ALL * CMYN

ALL

CMYN(Administration of Concomitant Drug)

Power<br>Percent<br>Percent of rows<br>Percent of columns	None	With	Total
1	1445<br>7.05<br>7.05<br>100.00	19064<br>92.95<br>92.95<br>100.00	20509<br>100.00
Total	1445<br>7.05	19064<br>92.95	20509<br>100.00

Concomitant medications other than anticoagulants at the time of obtaining Table 2-5 consent, according to the presence or absence of proton-pump inhibitors

FREQ Procedure

Tables: ALL * PSYD

ALL PSYD(Psychotropic Drugs)

```
Power |
Percent |
Percent of rows
Percent of columns | None | With | Total
______________+________+________+
            1 |  17688 |   1376 |  19064
              |  92.78 |   7.22 | 100.00
              |  92.78 |   7.22 |
              | 100.00 | 100.00 |
______________+________+________+
Total 17688 1376 19064
                 92.78     7.22   100.00
```

Concomitant medications other than anticoagulants at the time of obtaining Table 2-5 consent, according to the presence or absence of proton-pump inhibitors

FREQ Procedure

			Cumulative	
Percent of ALL	power	Percent of	power	
1	20509	100.00	20509	100.00

Tables: ALL * CMYN

ALL

CMYN(Administration of Concomitant Drug)

Power / Percent / Percent of rows / Percent of columns	None	With	Total
1	1445	19064	20509
	7.05	92.95	100.00
	7.05	92.95	
	100.00	100.00	
Total	1445	19064	20509
	7.05	92.95	100.00

Concomitant medications other than anticoagulants at the time of obtaining Table 2-5 consent, according to the presence or absence of proton-pump inhibitors

FREQ Procedure

Tables: ALL* PRPI

ALL
PRPI(Proton Pump Inhabitors)

Power |
Percent |
Percent of rows
Percent of columns | None | Total
______________+________+
1 | 19064 | 19064
| 100.00 | 100.00
| 100.00 |
| 100.00 |
______________+________+
Total 19064 19064
100.00 100.00

Concomitant medications other than anticoagulants at the time of obtaining Table 2-5 consent, according to the presence or absence of proton-pump inhibitors

FREQ Procedure

			Cumulative	
Percent of ALL	power	Percent	of power	
1	20509	100.00	20509	100.00

Tables: ALL * CMYN

ALL

CMYN(Administration of Concomitant Drug)

Power / Percent / Percent of rows / Percent of columns	None	With	Total
1	1445	19064	20509
	7.05	92.95	100.00
	7.05	92.95	
	100.00	100.00	
Total	1445	19064	20509
	7.05	92.95	100.00

Concomitant medications other than anticoagulants at the time of obtaining Table 2-5 consent, according to the presence or absence of proton-pump inhibitors

FREQ Procedure

Tables: ALL* PRPI

ALL

PRPI(Proton Pump Inhabitors)

Power |
Percent |
Percent of rows
Percent of columns | None | Total
______________+________+
1 | 19064 | 19064
| 100.00 | 100.00
| 100.00 |
| 100.00 |
______________+________+
Total 19064 19064
100.00 100.00

Concomitant medications other than anticoagulants at the time of obtaining Table 2-5 consent, according to the presence or absence of proton-pump inhibitors

FREQ Procedure

			Cumulative	
Percent of ALL	power	Percent of	power	
1	20509	100.00	20509	100.00

Tables: ALL * CMYN

ALL

CMYN(Administration of Concomitant Drug)

Power |
Percent |
Percent of rows
Percent of columns | None | With | Total

	None	With	Total
1	1445	19064	20509
	7.05	92.95	100.00
	7.05	92.95	
	100.00	100.00	
Total	1445	19064	20509
	7.05	92.95	100.00

Concomitant medications other than anticoagulants at the time of obtaining Table 2-5 consent, according to the presence or absence of proton-pump inhibitors

FREQ Procedure

Tables: ALL* PGPI

ALL PGPI(P-gp Inhabitors)

Power<br>Percent<br>Percent of rows<br>Percent of columns	None	With	Total
1	18762<br>98.42<br>98.42<br>100.00	302<br>1.58<br>1.58<br>100.00	19064<br>100.00
Total	18762<br>98.42	302<br>1.58	19064<br>100.00

Concomitant medications other than anticoagulants at the time of obtaining Table 2-5 consent, according to the presence or absence of proton-pump inhibitors

FREQ Procedure

			Cumulative	
Percent of ALL	power	Percent of	power	
1	20509	100.00	20509	100.00

Tables: ALL * CMYN

ALL

CMYN(Administration of Concomitant Drug)

Power |
Percent |
Percent of rows
Percent of columns | None | With | Total

	None	With	Total
1	1445	19064	20509
	7.05	92.95	100.00
	7.05	92.95	
	100.00	100.00	
Total	1445	19064	20509
	7.05	92.95	100.00

Concomitant medications other than anticoagulants at the time of obtaining Table 2-5 consent, according to the presence or absence of proton-pump inhibitors

FREQ Procedure

Tables: ALL* PGPI

ALL PGPI(P-gp Inhabitors)

Power<br>Percent<br>Percent of rows<br>Percent of columns	None	With	Total
1	18762<br>98.42<br>98.42<br>100.00	302<br>1.58<br>1.58<br>100.00	19064<br>100.00
Total	18762<br>98.42	302<br>1.58	19064<br>100.00

Concomitant medications other than anticoagulants at the time of obtaining Table 2-5 consent, according to the presence or absence of proton-pump inhibitors

FREQ Procedure

Percent of ALL	power	Percent	Cumulative power	Cumulative Percent of power
1	20509	100.00	20509	100.00

Tables: ALL * CMYN

ALL
CMYN(Administration of Concomitant Drug)

Power |
Percent |
Percent of rows
Percent of columns | None | With | Total

	None	With	Total
1	1445	19064	20509
	7.05	92.95	100.00
	7.05	92.95	
	100.00	100.00	
Total	1445	19064	20509
	7.05	92.95	100.00

Concomitant medications other than anticoagulants at the time of obtaining Table 2-5 consent, according to the presence or absence of proton-pump inhibitors

FREQ Procedure

Tables: ALL * TCMCNT

ALL TCMCNT(Total Number of Drugs Other than Anticoagulant)

Power |
Percent |
Percent of rows
Percent|0|1|2|3|4|5|6|7|8|9|10|11|12| Total
______________+________+________+________+________+________+________+________+________+________+________+________+________+
1 | 52 | 660 | 1437 | 2202 | 2803 | 2848 | 2643 | 2143 | 1692 | 1251 | 829 | 583 | 365 | 20036
| 0.26 | 3.29 | 7.17 | 10.99 | 13.99 | 14.21 | 13.19 | 10.70 | 8.44 | 6.24 | 4.14 | 2.91 | 1.82 | 100.00
| 0.26 | 3.29 | 7.17 | 10.99 | 13.99 | 14.21 | 13.19 | 10.70 | 8.44 | 6.24 | 4.14 | 2.91 | 1.82 |
| 100.00 | 100.00 | 100.00 | 100.00 | 100.00 | 100.00 | 100.00 | 100.00 | 100.00 | 100.00 | 100.00 | 100.00 | 100.00 |
______________+________+________+________+________+________+________+________+________+________+________+________+________+
Total 52 660 1437 2202 2803 2848 2643 2143 1692 1251 829 583 365 20036
0.26 3.29 7.17 10.99 13.99 14.21 13.19 10.70 8.44 6.24 4.14 2.91 1.82 100.00
(continued)

Tables: ALL * TCMCNT

ALL TCMCNT(Total Number of Drugs Other than Anticoagulant)

Power |
Percent |

Percent of rows

|13|14|15|16|17|18|19|20|21|23|25|26|27|Total

______________+________+________+________+________+________+________+_______

_+________+________+________+________+________+________+

1 | 225 | 118 | 78 | 44 | 25 | 18 | 4 | 7 | 1 |

5 | 1 | 1 | 1 | 20036

| 1.12 | 0.59 | 0.39 | 0.22 | 0.12 | 0.09 | 0.02 | 0.03 | 0.00 |

0.02 | 0.00 | 0.00 | 0.00 | 100.00

| 1.12 | 0.59 | 0.39 | 0.22 | 0.12 | 0.09 | 0.02 | 0.03 | 0.00 |

0.02 | 0.00 | 0.00 | 0.00 |

| 100.00 | 100.00 | 100.00 | 100.00 | 100.00 | 100.00 | 100.00 | 100.00 | 100.00 |

100.00 | 100.00 | 100.00 | 100.00 |

______________+________+________+________+________+________+________+_______

_+________+________+________+________+________+________+

Total 225 118 78 44 25 18 4 7 1 5 1 1 1 20036

1.12 0.59 0.39 0.22 0.12 0.09 0.02 0.03

0.00 0.02 0.00 0.00 0.00 100.00

Degree of missing values = 473

Concomitant medications other than anticoagulants at the time of obtaining Table 2-5 consent, according to the presence or absence of proton-pump inhibitors

FREQ Procedure

			Cumulative	
Percent of ALL	power	Percent of	power	
1	20509	100.00	20509	100.00

Tables: ALL * CMYN

ALL

CMYN(Administration of Concomitant Drug)

Power |
Percent |
Percent of rows
Percent of columns | None | With | Total

	None	With	Total
1	1445	19064	20509
	7.05	92.95	100.00
	7.05	92.95	
	100.00	100.00	
Total	1445	19064	20509
	7.05	92.95	100.00

Concomitant medications other than anticoagulants at the time of obtaining Table 2-5 consent, according to the presence or absence of proton-pump inhibitors

FREQ Procedure

Tables: ALL * TCMCNT

ALL TCMCNT(Total Number of Drugs Other than Anticoagulant)

Power |
Percent |
Percent of rows
Percent|0|1|2|3|4|5|6|7|8|9|10|11|12| Total
______________+________+________+________+________+________+________+________
_+________+________+________+________+________+________+
1 | 52 | 660 | 1437 | 2202 | 2803 | 2848 | 2643 | 2143 | 1692 |
1251 | 829 | 583 | 365 | 20036
| 0.26 | 3.29 | 7.17 | 10.99 | 13.99 | 14.21 | 13.19 | 10.70 | 8.44 |
6.24 | 4.14 | 2.91 | 1.82 | 100.00
| 0.26 | 3.29 | 7.17 | 10.99 | 13.99 | 14.21 | 13.19 | 10.70 | 8.44 |
6.24 | 4.14 | 2.91 | 1.82 |
| 100.00 | 100.00 | 100.00 | 100.00 | 100.00 | 100.00 | 100.00 | 100.00 | 100.00 |
100.00 | 100.00 | 100.00 | 100.00 |
______________+________+________+________+________+________+________+________
_+________+________+________+________+________+________+
Total 52 660 1437 2202 2803 2848 2643 2143 1692 1251 829 583 365 20036
0.26 3.29 7.17 10.99 13.99 14.21 13.19 10.70
8.44 6.24 4.14 2.91 1.82 100.00
(continued)

Tables: ALL * TCMCNT

ALL TCMCNT(Total Number of Drugs Other than Anticoagulant)

Power |
Percent |

Percent of rows

|13|14|15|16|17|18|19|20|21|23|25|26|27|Total

______________+________+________+________+________+________+________+_______
_+________+________+________+________+________+________+

1 | 225 | 118 | 78 | 44 | 25 | 18 | 4 | 7 | 1 |
5 | 1 | 1 | 1 | 20036

| 1.12 | 0.59 | 0.39 | 0.22 | 0.12 | 0.09 | 0.02 | 0.03 | 0.00 |
0.02 | 0.00 | 0.00 | 0.00 | 100.00

| 1.12 | 0.59 | 0.39 | 0.22 | 0.12 | 0.09 | 0.02 | 0.03 | 0.00 |
0.02 | 0.00 | 0.00 | 0.00 |

| 100.00 | 100.00 | 100.00 | 100.00 | 100.00 | 100.00 | 100.00 | 100.00 | 100.00 |
100.00 | 100.00 | 100.00 | 100.00 |

______________+________+________+________+________+________+________+_______
_+________+________+________+________+________+________+

Total 225 118 78 44 25 18 4 7 1 5 1 1 1 20036

1.12 0.59 0.39 0.22 0.12 0.09 0.02 0.03
0.00 0.02 0.00 0.00 0.00 100.00

Degree of missing values = 473

Concomitant medications other than anticoagulants at the time of obtaining Table 2-5 consent, according to the presence or absence of proton-pump inhibitors

FREQ Procedure

			Cumulative	
Percent of ALL	power	Percent	of power	
1	20509	100.00	20509	100.00

Tables: ALL * CMYN

ALL
CMYN(Administration of Concomitant Drug)

Power / Percent / Percent of rows / Percent of columns	None	With	Total
1	1445	19064	20509
	7.05	92.95	100.00
	7.05	92.95	
	100.00	100.00	
Total	1445	19064	20509
	7.05	92.95	100.00

Concomitant medications other than anticoagulants at the time of obtaining Table 2-5 consent, according to the presence or absence of proton-pump inhibitors

FREQ Procedure

Tables: ALL * TCMCNT

ALL TCMCNT(Total Number of Drugs Other than Anticoagulant)

Power |
Percent |
Percent of rows
Percent|0|1|2|3|4|5|6|7|8|9|10|11|12| Total
______________+________+________+________+________+________+________+________+________+________+________+________+________+________+
1 | 52 | 660 | 1437 | 2202 | 2803 | 2848 | 2643 | 2143 | 1692 | 1251 | 829 | 583 | 365 | 20036
| 0.26 | 3.29 | 7.17 | 10.99 | 13.99 | 14.21 | 13.19 | 10.70 | 8.44 | 6.24 | 4.14 | 2.91 | 1.82 | 100.00
| 0.26 | 3.29 | 7.17 | 10.99 | 13.99 | 14.21 | 13.19 | 10.70 | 8.44 | 6.24 | 4.14 | 2.91 | 1.82 |
| 100.00 | 100.00 | 100.00 | 100.00 | 100.00 | 100.00 | 100.00 | 100.00 | 100.00 | 100.00 | 100.00 | 100.00 | 100.00 |
______________+________+________+________+________+________+________+________+________+________+________+________+________+________+
Total 52 660 1437 2202 2803 2848 2643 2143 1692 1251 829 583 365 20036
0.26 3.29 7.17 10.99 13.99 14.21 13.19 10.70 8.44 6.24 4.14 2.91 1.82 100.00
(continued)

Tables: ALL * TCMCNT

ALL TCMCNT(Total Number of Drugs Other than Anticoagulant)

Power |
Percent |

Percent of rows

|13|14|15|16|17|18|19|20|21|23|25|26|27|Total

______________+________+________+________+________+________+________+_______

_+________+________+________+________+________+________+

1 | 225 | 118 | 78 | 44 | 25 | 18 | 4 | 7 | 1 |

5 | 1 | 1 | 1 | 20036

| 1.12 | 0.59 | 0.39 | 0.22 | 0.12 | 0.09 | 0.02 | 0.03 | 0.00 |

0.02 | 0.00 | 0.00 | 0.00 | 100.00

| 1.12 | 0.59 | 0.39 | 0.22 | 0.12 | 0.09 | 0.02 | 0.03 | 0.00 |

0.02 | 0.00 | 0.00 | 0.00 |

| 100.00 | 100.00 | 100.00 | 100.00 | 100.00 | 100.00 | 100.00 | 100.00 | 100.00 |

100.00 | 100.00 | 100.00 | 100.00 |

______________+________+________+________+________+________+________+_______

_+________+________+________+________+________+________+

Total 225 118 78 44 25 18 4 7 1 5 1 1 1 20036

1.12 0.59 0.39 0.22 0.12 0.09 0.02 0.03

0.00 0.02 0.00 0.00 0.00 100.00

Degree of missing values = 473

Concomitant medications other than anticoagulants at the time of obtaining Table 2-5 consent, according to the presence or absence of proton-pump inhibitors

FREQ Procedure

ALL	power	Percent	Cumulative power	Cumulative Percent of power
1	20509	100.00	20509	100.00

Tables: ALL * CMYN

ALL

CMYN(Administration of Concomitant Drug)

Power / Percent / Percent of rows / Percent of columns	None	With	Total
1	1445	19064	20509
	7.05	92.95	100.00
	7.05	92.95	
	100.00	100.00	
Total	1445	19064	20509
	7.05	92.95	100.00

Concomitant medications other than anticoagulants at the time of obtaining Table 2-5 consent, according to the presence or absence of proton-pump inhibitors

FREQ Procedure

Tables: ALL * TCMCNT

ALL TCMCNT(Total Number of Drugs Other than Anticoagulant)

Power |
Percent |
Percent of rows
Percent|0|1|2|3|4|5|6|7|8|9|10|11|12| Total
______________+________+________+________+________+________+________+________
_+________+________+________+________+________+________+
1 | 52 | 660 | 1437 | 2202 | 2803 | 2848 | 2643 | 2143 | 1692 | 1251 | 829 | 583 | 365 | 20036
| 0.26 | 3.29 | 7.17 | 10.99 | 13.99 | 14.21 | 13.19 | 10.70 | 8.44 | 6.24 | 4.14 | 2.91 | 1.82 | 100.00
| 0.26 | 3.29 | 7.17 | 10.99 | 13.99 | 14.21 | 13.19 | 10.70 | 8.44 | 6.24 | 4.14 | 2.91 | 1.82 |
| 100.00 | 100.00 | 100.00 | 100.00 | 100.00 | 100.00 | 100.00 | 100.00 | 100.00 | 100.00 | 100.00 | 100.00 | 100.00 |
______________+________+________+________+________+________+________+________
_+________+________+________+________+________+________+
Total 52 660 1437 2202 2803 2848 2643 2143 1692 1251 829 583 365 20036
0.26 3.29 7.17 10.99 13.99 14.21 13.19 10.70 8.44 6.24 4.14 2.91 1.82 100.00
(continued)

Tables: ALL * TCMCNT

ALL TCMCNT(Total Number of Drugs Other than Anticoagulant)

Power |
Percent |

Percent of rows

|13|14|15|16|17|18|19|20|21|23|25|26|27|Total

______________+________+________+________+________+________+________+_______

_+________+________+________+________+________+________+

1 | 225 | 118 | 78 | 44 | 25 | 18 | 4 | 7 | 1 |

5 | 1 | 1 | 1 | 20036

| 1.12 | 0.59 | 0.39 | 0.22 | 0.12 | 0.09 | 0.02 | 0.03 | 0.00 |

0.02 | 0.00 | 0.00 | 0.00 | 100.00

| 1.12 | 0.59 | 0.39 | 0.22 | 0.12 | 0.09 | 0.02 | 0.03 | 0.00 |

0.02 | 0.00 | 0.00 | 0.00 |

| 100.00 | 100.00 | 100.00 | 100.00 | 100.00 | 100.00 | 100.00 | 100.00 | 100.00 |

100.00 | 100.00 | 100.00 | 100.00 |

______________+________+________+________+________+________+________+_______

_+________+________+________+________+________+________+

Total 225 118 78 44 25 18 4 7 1 5 1 1 1 20036

1.12 0.59 0.39 0.22 0.12 0.09 0.02 0.03

0.00 0.02 0.00 0.00 0.00 100.00

Degree of missing values = 473

Concomitant medications other than anticoagulants at the time of obtaining Table 2-5 consent, according to the presence or absence of proton-pump inhibitors

FREQ Procedure

			Cumulative	
Percent of ALL	power	Percent of	power	
1	20509	100.00	20509	100.00

Tables: ALL * CMYN

ALL

CMYN(Administration of Concomitant Drug)

Power | Percent | Percent of rows Percent of columns	None	With	Total
1	1445	19064	20509
	7.05	92.95	100.00
	7.05	92.95	
	100.00	100.00	
Total	1445	19064	20509
	7.05	92.95	100.00

Concomitant medications other than anticoagulants at the time of obtaining Table 2-5 consent, according to the presence or absence of proton-pump inhibitors

FREQ Procedure

Tables: ALL * TCMCNT

ALL TCMCNT(Total Number of Drugs Other than Anticoagulant)

Power |
Percent |
Percent of rows
Percent|0|1|2|3|4|5|6|7|8|9|10|11|12| Total
______________+________+________+________+________+________+________+________
_+________+________+________+________+________+________+
1 | 52 | 660 | 1437 | 2202 | 2803 | 2848 | 2643 | 2143 | 1692 |
1251 | 829 | 583 | 365 | 20036
| 0.26 | 3.29 | 7.17 | 10.99 | 13.99 | 14.21 | 13.19 | 10.70 | 8.44 |
6.24 | 4.14 | 2.91 | 1.82 | 100.00
| 0.26 | 3.29 | 7.17 | 10.99 | 13.99 | 14.21 | 13.19 | 10.70 | 8.44 |
6.24 | 4.14 | 2.91 | 1.82 |
| 100.00 | 100.00 | 100.00 | 100.00 | 100.00 | 100.00 | 100.00 | 100.00 | 100.00 |
100.00 | 100.00 | 100.00 | 100.00 |
______________+________+________+________+________+________+________+________
_+________+________+________+________+________+________+
Total 52 660 1437 2202 2803 2848 2643 2143 1692 1251 829 583 365 20036
0.26 3.29 7.17 10.99 13.99 14.21 13.19 10.70
8.44 6.24 4.14 2.91 1.82 100.00
(continued)

Tables: ALL * TCMCNT

ALL TCMCNT(Total Number of Drugs Other than Anticoagulant)

Power |
Percent |

Percent of rows

|13|14|15|16|17|18|19|20|21|23|25|26|27|Total

______________+________+________+________+________+________+________+_______

_+________+________+________+________+________+________+

1 | 225 | 118 | 78 | 44 | 25 | 18 | 4 | 7 | 1 |

5 | 1 | 1 | 1 | 20036

| 1.12 | 0.59 | 0.39 | 0.22 | 0.12 | 0.09 | 0.02 | 0.03 | 0.00 |

0.02 | 0.00 | 0.00 | 0.00 | 100.00

| 1.12 | 0.59 | 0.39 | 0.22 | 0.12 | 0.09 | 0.02 | 0.03 | 0.00 |

0.02 | 0.00 | 0.00 | 0.00 |

| 100.00 | 100.00 | 100.00 | 100.00 | 100.00 | 100.00 | 100.00 | 100.00 | 100.00 |

100.00 | 100.00 | 100.00 | 100.00 |

______________+________+________+________+________+________+________+_______

_+________+________+________+________+________+________+

Total 225 118 78 44 25 18 4 7 1 5 1 1 1 20036

1.12 0.59 0.39 0.22 0.12 0.09 0.02 0.03

0.00 0.02 0.00 0.00 0.00 100.00

Degree of missing values = 473

Concomitant medications other than anticoagulants at the time of obtaining Table 2-5 consent, according to the presence or absence of proton-pump inhibitors

FREQ Procedure

			Cumulative	
Percent of ALL	power	Percent of	power	
1	20509	100.00	20509	100.00

Tables: ALL * CMYN

ALL

CMYN(Administration of Concomitant Drug)

Power<br>Percent<br>Percent of rows<br>Percent of columns	None	With	Total
1	1445<br>7.05<br>7.05<br>100.00	19064<br>92.95<br>92.95<br>100.00	20509<br>100.00
Total	1445<br>7.05	19064<br>92.95	20509<br>100.00

Concomitant medications other than anticoagulants at the time of obtaining Table 2-5 consent, according to the presence or absence of proton-pump inhibitors

FREQ Procedure

Tables: ALL * TCMCNT

ALL TCMCNT(Total Number of Drugs Other than Anticoagulant)

Power |
Percent |
Percent of rows
Percent|0|1|2|3|4|5|6|7|8|9|10|11|12| Total
______________+________+________+________+________+________+________+________
_+________+________+________+________+________+________+
1 | 52 | 660 | 1437 | 2202 | 2803 | 2848 | 2643 | 2143 | 1692 |
1251 | 829 | 583 | 365 | 20036
| 0.26 | 3.29 | 7.17 | 10.99 | 13.99 | 14.21 | 13.19 | 10.70 | 8.44 |
6.24 | 4.14 | 2.91 | 1.82 | 100.00
| 0.26 | 3.29 | 7.17 | 10.99 | 13.99 | 14.21 | 13.19 | 10.70 | 8.44 |
6.24 | 4.14 | 2.91 | 1.82 |
| 100.00 | 100.00 | 100.00 | 100.00 | 100.00 | 100.00 | 100.00 | 100.00 | 100.00 |
100.00 | 100.00 | 100.00 | 100.00 |
______________+________+________+________+________+________+________+________
_+________+________+________+________+________+________+
Total 52 660 1437 2202 2803 2848 2643 2143 1692 1251 829 583 365 20036
0.26 3.29 7.17 10.99 13.99 14.21 13.19 10.70
8.44 6.24 4.14 2.91 1.82 100.00
(continued)

Tables: ALL * TCMCNT

ALL TCMCNT(Total Number of Drugs Other than Anticoagulant)

Power |
Percent |

Percent of rows

|13|14|15|16|17|18|19|20|21|23|25|26|27|Total

______________+________+________+________+________+________+________+________

_+________+________+________+________+________+________+

1 | 225 | 118 | 78 | 44 | 25 | 18 | 4 | 7 | 1 |

5 | 1 | 1 | 1 | 20036

| 1.12 | 0.59 | 0.39 | 0.22 | 0.12 | 0.09 | 0.02 | 0.03 | 0.00 |

0.02 | 0.00 | 0.00 | 0.00 | 100.00

| 1.12 | 0.59 | 0.39 | 0.22 | 0.12 | 0.09 | 0.02 | 0.03 | 0.00 |

0.02 | 0.00 | 0.00 | 0.00 |

| 100.00 | 100.00 | 100.00 | 100.00 | 100.00 | 100.00 | 100.00 | 100.00 | 100.00 |

100.00 | 100.00 | 100.00 | 100.00 |

______________+________+________+________+________+________+________+________

_+________+________+________+________+________+________+

Total 225 118 78 44 25 18 4 7 1 5 1 1 1 20036

1.12 0.59 0.39 0.22 0.12 0.09 0.02 0.03

0.00 0.02 0.00 0.00 0.00 100.00

Degree of missing values = 473

Concomitant medications other than anticoagulants at the time of obtaining Table 2-5 consent, according to the presence or absence of proton-pump inhibitors

FREQ Procedure

ALL	power	Percent	Cumulative power	Cumulative Percent
1	20509	100.00	20509	100.00

Tables: ALL * CMYN

ALL
CMYN(Administration of Concomitant Drug)

Power |
Percent |
Percent of rows
Percent of columns | None | With | Total

ALL	None	With	Total
1	1445	19064	20509
	7.05	92.95	100.00
	7.05	92.95	
	100.00	100.00	
Total	1445	19064	20509
	7.05	92.95	100.00

Concomitant medications other than anticoagulants at the time of obtaining Table 2-5 consent, according to the presence or absence of proton-pump inhibitors

FREQ Procedure

Tables: ALL * TCMCNT

ALL TCMCNT(Total Number of Drugs Other than Anticoagulant)

Power |
Percent |
Percent of rows
Percent|0|1|2|3|4|5|6|7|8|9|10|11|12| Total
______________+________+________+________+________+________+________+________+________+________+________+________+________+
1 | 52 | 660 | 1437 | 2202 | 2803 | 2848 | 2643 | 2143 | 1692 | 1251 | 829 | 583 | 365 | 20036
| 0.26 | 3.29 | 7.17 | 10.99 | 13.99 | 14.21 | 13.19 | 10.70 | 8.44 | 6.24 | 4.14 | 2.91 | 1.82 | 100.00
| 0.26 | 3.29 | 7.17 | 10.99 | 13.99 | 14.21 | 13.19 | 10.70 | 8.44 | 6.24 | 4.14 | 2.91 | 1.82 |
| 100.00 | 100.00 | 100.00 | 100.00 | 100.00 | 100.00 | 100.00 | 100.00 | 100.00 | 100.00 | 100.00 | 100.00 | 100.00 |
______________+________+________+________+________+________+________+________+________+________+________+________+________+
Total 52 660 1437 2202 2803 2848 2643 2143 1692 1251 829 583 365 20036
0.26 3.29 7.17 10.99 13.99 14.21 13.19 10.70 8.44 6.24 4.14 2.91 1.82 100.00
(continued)

Tables: ALL * TCMCNT

ALL TCMCNT(Total Number of Drugs Other than Anticoagulant)

Power |
Percent |

Percent of rows

|13|14|15|16|17|18|19|20|21|23|25|26|27|Total

______________+________+________+________+________+________+________+________

_+________+________+________+________+________+________+

1 | 225 | 118 | 78 | 44 | 25 | 18 | 4 | 7 | 1 |

5 | 1 | 1 | 1 | 20036

| 1.12 | 0.59 | 0.39 | 0.22 | 0.12 | 0.09 | 0.02 | 0.03 | 0.00 |

0.02 | 0.00 | 0.00 | 0.00 | 100.00

| 1.12 | 0.59 | 0.39 | 0.22 | 0.12 | 0.09 | 0.02 | 0.03 | 0.00 |

0.02 | 0.00 | 0.00 | 0.00 |

| 100.00 | 100.00 | 100.00 | 100.00 | 100.00 | 100.00 | 100.00 | 100.00 | 100.00 |

100.00 | 100.00 | 100.00 | 100.00 |

______________+________+________+________+________+________+________+________

_+________+________+________+________+________+________+

Total 225 118 78 44 25 18 4 7 1 5 1 1 1 20036

1.12 0.59 0.39 0.22 0.12 0.09 0.02 0.03

0.00 0.02 0.00 0.00 0.00 100.00

Degree of missing values = 473

Concomitant medications other than anticoagulants at the time of obtaining Table 2-5 consent, according to the presence or absence of proton-pump inhibitors

FREQ Procedure

			Cumulative	
Percent of ALL	power	Percent of	power	
1	20509	100.00	20509	100.00

Tables: ALL * CMYN

ALL

CMYN(Administration of Concomitant Drug)

Power<br>Percent<br>Percent of rows<br>Percent of columns	None	With	Total
1	1445<br>7.05<br>7.05<br>100.00	19064<br>92.95<br>92.95<br>100.00	20509<br>100.00
Total	1445<br>7.05	19064<br>92.95	20509<br>100.00

Concomitant medications other than anticoagulants at the time of obtaining Table 2-5 consent, according to the presence or absence of proton-pump inhibitors

FREQ Procedure

Tables: ALL * TCMCNT

ALL TCMCNT(Total Number of Drugs Other than Anticoagulant)

Power |
Percent |
Percent of rows
Percent|0|1|2|3|4|5|6|7|8|9|10|11|12| Total
______________+________+________+________+________+________+________+________
_+________+________+________+________+________+________+
1 | 52 | 660 | 1437 | 2202 | 2803 | 2848 | 2643 | 2143 | 1692 |
1251 | 829 | 583 | 365 | 20036
| 0.26 | 3.29 | 7.17 | 10.99 | 13.99 | 14.21 | 13.19 | 10.70 | 8.44 |
6.24 | 4.14 | 2.91 | 1.82 | 100.00
| 0.26 | 3.29 | 7.17 | 10.99 | 13.99 | 14.21 | 13.19 | 10.70 | 8.44 |
6.24 | 4.14 | 2.91 | 1.82 |
| 100.00 | 100.00 | 100.00 | 100.00 | 100.00 | 100.00 | 100.00 | 100.00 | 100.00 |
100.00 | 100.00 | 100.00 | 100.00 |
______________+________+________+________+________+________+________+________
_+________+________+________+________+________+________+
Total 52 660 1437 2202 2803 2848 2643 2143 1692 1251 829 583 365 20036
0.26 3.29 7.17 10.99 13.99 14.21 13.19 10.70
8.44 6.24 4.14 2.91 1.82 100.00
(continued)

Tables: ALL * TCMCNT

ALL TCMCNT(Total Number of Drugs Other than Anticoagulant)

Power |
Percent |

Percent of rows

|13|14|15|16|17|18|19|20|21|23|25|26|27|Total

______________+________+________+________+________+________+________+________+________+________+________+________+________+

	1	225	118	78	44	25	18	4	7	1	5	1	1	1	20036
		1.12	0.59	0.39	0.22	0.12	0.09	0.02	0.03	0.00	0.02	0.00	0.00	0.00	100.00
		1.12	0.59	0.39	0.22	0.12	0.09	0.02	0.03	0.00	0.02	0.00	0.00	0.00	
		100.00	100.00	100.00	100.00	100.00	100.00	100.00	100.00	100.00	100.00	100.00	100.00	100.00	

______________+________+________+________+________+________+________+________+________+________+________+________+________+

Total 225 118 78 44 25 18 4 7 1 5 1 1 1 20036

1.12 0.59 0.39 0.22 0.12 0.09 0.02 0.03 0.00 0.02 0.00 0.00 0.00 100.00

Degree of missing values = 473

Concomitant medications other than anticoagulants at the time of obtaining Table 2-5 consent, according to the presence or absence of proton-pump inhibitors

FREQ Procedure

			Cumulative	
Percent of ALL	power	Percent	of power	
1	20509	100.00	20509	100.00

Tables: ALL * CMYN

ALL

CMYN(Administration of Concomitant Drug)

Power / Percent / Percent of rows / Percent of columns	None	With	Total
1	1445	19064	20509
	7.05	92.95	100.00
	7.05	92.95	
	100.00	100.00	
Total	1445	19064	20509
	7.05	92.95	100.00

Concomitant medications other than anticoagulants at the time of obtaining Table 2-5 consent, according to the presence or absence of proton-pump inhibitors

FREQ Procedure

Tables: ALL * TCMCNT

ALL TCMCNT(Total Number of Drugs Other than Anticoagulant)

Power |
Percent |
Percent of rows
Percent|0|1|2|3|4|5|6|7|8|9|10|11|12| Total
______________+________+________+________+________+________+________+________+________+________+________+________+________+
1 | 52 | 660 | 1437 | 2202 | 2803 | 2848 | 2643 | 2143 | 1692 | 1251 | 829 | 583 | 365 | 20036
| 0.26 | 3.29 | 7.17 | 10.99 | 13.99 | 14.21 | 13.19 | 10.70 | 8.44 | 6.24 | 4.14 | 2.91 | 1.82 | 100.00
| 0.26 | 3.29 | 7.17 | 10.99 | 13.99 | 14.21 | 13.19 | 10.70 | 8.44 | 6.24 | 4.14 | 2.91 | 1.82 |
| 100.00 | 100.00 | 100.00 | 100.00 | 100.00 | 100.00 | 100.00 | 100.00 | 100.00 | 100.00 | 100.00 | 100.00 | 100.00 |
______________+________+________+________+________+________+________+________+________+________+________+________+________+
Total 52 660 1437 2202 2803 2848 2643 2143 1692 1251 829 583 365 20036
0.26 3.29 7.17 10.99 13.99 14.21 13.19 10.70 8.44 6.24 4.14 2.91 1.82 100.00
(continued)

Tables: ALL * TCMCNT

ALL TCMCNT(Total Number of Drugs Other than Anticoagulant)

Power |
Percent |

Percent of rows

|13|14|15|16|17|18|19|20|21|23|25|26|27|Total

______________+________+________+________+________+________+________+_______

_+________+________+________+________+________+________+

1 | 225 | 118 | 78 | 44 | 25 | 18 | 4 | 7 | 1 |

5 | 1 | 1 | 1 | 20036

| 1.12 | 0.59 | 0.39 | 0.22 | 0.12 | 0.09 | 0.02 | 0.03 | 0.00 |

0.02 | 0.00 | 0.00 | 0.00 | 100.00

| 1.12 | 0.59 | 0.39 | 0.22 | 0.12 | 0.09 | 0.02 | 0.03 | 0.00 |

0.02 | 0.00 | 0.00 | 0.00 |

| 100.00 | 100.00 | 100.00 | 100.00 | 100.00 | 100.00 | 100.00 | 100.00 | 100.00 |

100.00 | 100.00 | 100.00 | 100.00 |

______________+________+________+________+________+________+________+_______

_+________+________+________+________+________+________+

Total 225 118 78 44 25 18 4 7 1 5 1 1 1 20036

1.12 0.59 0.39 0.22 0.12 0.09 0.02 0.03

0.00 0.02 0.00 0.00 0.00 100.00

Degree of missing values = 473

Concomitant medications other than anticoagulants at the time of obtaining Table 2-5 consent, according to the presence or absence of proton-pump inhibitors

FREQ Procedure

			Cumulative	
Percent of ALL	power	Percent of	power	
1	20509	100.00	20509	100.00

Tables: ALL * CMYN

ALL

CMYN(Administration of Concomitant Drug)

Power |
Percent |
Percent of rows
Percent of columns | None | With | Total

	None	With	Total
1	1445	19064	20509
	7.05	92.95	100.00
	7.05	92.95	
	100.00	100.00	
Total	1445	19064	20509
	7.05	92.95	100.00

Concomitant medications other than anticoagulants at the time of obtaining Table 2-5 consent, according to the presence or absence of proton-pump inhibitors

FREQ Procedure

Tables: ALL * TCMCNT

ALL TCMCNT(Total Number of Drugs Other than Anticoagulant)

Power |
Percent |
Percent of rows
Percent|0|1|2|3|4|5|6|7|8|9|10|11|12| Total

ALL	0	1	2	3	4	5	6	7	8	9	10	11	12	Total
1	52	660	1437	2202	2803	2848	2643	2143	1692	1251	829	583	365	20036
	0.26	3.29	7.17	10.99	13.99	14.21	13.19	10.70	8.44	6.24	4.14	2.91	1.82	100.00
	0.26	3.29	7.17	10.99	13.99	14.21	13.19	10.70	8.44	6.24	4.14	2.91	1.82	
	100.00	100.00	100.00	100.00	100.00	100.00	100.00	100.00	100.00	100.00	100.00	100.00	100.00	
Total	52	660	1437	2202	2803	2848	2643	2143	1692	1251	829	583	365	20036
	0.26	3.29	7.17	10.99	13.99	14.21	13.19	10.70	8.44	6.24	4.14	2.91	1.82	100.00

(continued)

Tables: ALL * TCMCNT

ALL TCMCNT(Total Number of Drugs Other than Anticoagulant)

Power |
Percent |

Percent of rows

|13|14|15|16|17|18|19|20|21|23|25|26|27|Total

______________+________+________+________+________+________+________+________
_+________+________+________+________+________+________+

1 | 225 | 118 | 78 | 44 | 25 | 18 | 4 | 7 | 1 |
5 | 1 | 1 | 1 | 20036

| 1.12 | 0.59 | 0.39 | 0.22 | 0.12 | 0.09 | 0.02 | 0.03 | 0.00 |
0.02 | 0.00 | 0.00 | 0.00 | 100.00

| 1.12 | 0.59 | 0.39 | 0.22 | 0.12 | 0.09 | 0.02 | 0.03 | 0.00 |
0.02 | 0.00 | 0.00 | 0.00 |

| 100.00 | 100.00 | 100.00 | 100.00 | 100.00 | 100.00 | 100.00 | 100.00 | 100.00 |
100.00 | 100.00 | 100.00 | 100.00 |

______________+________+________+________+________+________+________+________
_+________+________+________+________+________+________+

Total 225 118 78 44 25 18 4 7 1 5 1 1 1 20036

1.12 0.59 0.39 0.22 0.12 0.09 0.02 0.03
0.00 0.02 0.00 0.00 0.00 100.00

Degree of missing values = 473

Concomitant medications other than anticoagulants at the time of obtaining Table 2-5 consent, according to the presence or absence of proton-pump inhibitors

FREQ Procedure

			Cumulative	
Percent of ALL	power	Percent	of power	
1	20509	100.00	20509	100.00

Tables: ALL * CMYN

ALL

CMYN(Administration of Concomitant Drug)

Power | Percent | Percent of rows Percent of columns	None	With	Total
1	1445	19064	20509
	7.05	92.95	100.00
	7.05	92.95	
	100.00	100.00	
Total	1445	19064	20509
	7.05	92.95	100.00

Concomitant medications other than anticoagulants at the time of obtaining Table 2-5 consent, according to the presence or absence of proton-pump inhibitors

FREQ Procedure

Tables: ALL * TCMCNT

ALL TCMCNT(Total Number of Drugs Other than Anticoagulant)

Power |
Percent |
Percent of rows
Percent|0|1|2|3|4|5|6|7|8|9|10|11|12| Total
______________+________+________+________+________+________+________+________
_+________+________+________+________+________+________+

ALL	0	1	2	3	4	5	6	7	8	9	10	11	12	Total
1	52	660	1437	2202	2803	2848	2643	2143	1692	1251	829	583	365	20036
	0.26	3.29	7.17	10.99	13.99	14.21	13.19	10.70	8.44	6.24	4.14	2.91	1.82	100.00
	0.26	3.29	7.17	10.99	13.99	14.21	13.19	10.70	8.44	6.24	4.14	2.91	1.82	
	100.00	100.00	100.00	100.00	100.00	100.00	100.00	100.00	100.00	100.00	100.00	100.00	100.00	

______________+________+________+________+________+________+________+________
_+________+________+________+________+________+________+

Total	52	660	1437	2202	2803	2848	2643	2143	1692	1251	829	583	365	20036
	0.26	3.29	7.17	10.99	13.99	14.21	13.19	10.70	8.44	6.24	4.14	2.91	1.82	100.00

(continued)

Tables: ALL * TCMCNT

ALL TCMCNT(Total Number of Drugs Other than Anticoagulant)

Power |
Percent |

Percent of rows

|13|14|15|16|17|18|19|20|21|23|25|26|27|Total

_______________+_________+_________+_________+_________+_________+_________+________

_+_________+_________+_________+_________+_________+_________+

1 | 225 | 118 | 78 | 44 | 25 | 18 | 4 | 7 | 1 |

5 | 1 | 1 | 1 | 20036

| 1.12 | 0.59 | 0.39 | 0.22 | 0.12 | 0.09 | 0.02 | 0.03 | 0.00 |

0.02 | 0.00 | 0.00 | 0.00 | 100.00

| 1.12 | 0.59 | 0.39 | 0.22 | 0.12 | 0.09 | 0.02 | 0.03 | 0.00 |

0.02 | 0.00 | 0.00 | 0.00 |

| 100.00 | 100.00 | 100.00 | 100.00 | 100.00 | 100.00 | 100.00 | 100.00 | 100.00 |

100.00 | 100.00 | 100.00 | 100.00 |

_______________+_________+_________+_________+_________+_________+_________+________

_+_________+_________+_________+_________+_________+_________+

Total 225 118 78 44 25 18 4 7 1 5 1 1 1 20036

1.12 0.59 0.39 0.22 0.12 0.09 0.02 0.03

0.00 0.02 0.00 0.00 0.00 100.00

Degree of missing values = 473

Concomitant medications other than anticoagulants at the time of obtaining Table 2-5 consent, according to the presence or absence of proton-pump inhibitors

FREQ Procedure

Percent of ALL	power	Percent	Cumulative power	Cumulative Percent of power
1	20509	100.00	20509	100.00

Tables: ALL * CMYN

ALL

CMYN(Administration of Concomitant Drug)

Power / Percent / Percent of rows / Percent of columns	None	With	Total
1	1445	19064	20509
	7.05	92.95	100.00
	7.05	92.95	
	100.00	100.00	
Total	1445	19064	20509
	7.05	92.95	100.00

Concomitant medications other than anticoagulants at the time of obtaining Table 2-5 consent, according to the presence or absence of proton-pump inhibitors

FREQ Procedure

Tables: ALL * TCMCNT

ALL TCMCNT(Total Number of Drugs Other than Anticoagulant)

Power |
Percent |
Percent of rows
Percent|0|1|2|3|4|5|6|7|8|9|10|11|12| Total
______________+________+________+________+________+________+________+________
_+________+________+________+________+________+________+
1 | 52 | 660 | 1437 | 2202 | 2803 | 2848 | 2643 | 2143 | 1692 |
1251 | 829 | 583 | 365 | 20036
| 0.26 | 3.29 | 7.17 | 10.99 | 13.99 | 14.21 | 13.19 | 10.70 | 8.44 |
6.24 | 4.14 | 2.91 | 1.82 | 100.00
| 0.26 | 3.29 | 7.17 | 10.99 | 13.99 | 14.21 | 13.19 | 10.70 | 8.44 |
6.24 | 4.14 | 2.91 | 1.82 |
| 100.00 | 100.00 | 100.00 | 100.00 | 100.00 | 100.00 | 100.00 | 100.00 | 100.00 |
100.00 | 100.00 | 100.00 | 100.00 |
______________+________+________+________+________+________+________+________
_+________+________+________+________+________+________+
Total 52 660 1437 2202 2803 2848 2643 2143 1692 1251 829 583 365 20036
0.26 3.29 7.17 10.99 13.99 14.21 13.19 10.70
8.44 6.24 4.14 2.91 1.82 100.00
(continued)

Tables: ALL * TCMCNT

ALL TCMCNT(Total Number of Drugs Other than Anticoagulant)

Power |
Percent |

Percent of rows

|13|14|15|16|17|18|19|20|21|23|25|26|27|Total

______________+________+________+________+________+________+________+_______

_+________+________+________+________+________+________+

1 | 225 | 118 | 78 | 44 | 25 | 18 | 4 | 7 | 1 |

5 | 1 | 1 | 1 | 20036

| 1.12 | 0.59 | 0.39 | 0.22 | 0.12 | 0.09 | 0.02 | 0.03 | 0.00 |

0.02 | 0.00 | 0.00 | 0.00 | 100.00

| 1.12 | 0.59 | 0.39 | 0.22 | 0.12 | 0.09 | 0.02 | 0.03 | 0.00 |

0.02 | 0.00 | 0.00 | 0.00 |

| 100.00 | 100.00 | 100.00 | 100.00 | 100.00 | 100.00 | 100.00 | 100.00 | 100.00 |

100.00 | 100.00 | 100.00 | 100.00 |

______________+________+________+________+________+________+________+_______

_+________+________+________+________+________+________+

Total 225 118 78 44 25 18 4 7 1 5 1 1 1 20036

1.12 0.59 0.39 0.22 0.12 0.09 0.02 0.03

0.00 0.02 0.00 0.00 0.00 100.00

Degree of missing values = 473

Concomitant medications other than anticoagulants at the time of obtaining Table 2-5 consent, according to the presence or absence of proton-pump inhibitors

FREQ Procedure

			Cumulative	
Percent of ALL	power	Percent of	power	
1	20509	100.00	20509	100.00

Tables: ALL * CMYN

ALL

CMYN(Administration of Concomitant Drug)

Power |
Percent |
Percent of rows
Percent of columns | None | With | Total

	None	With	Total
1	1445	19064	20509
	7.05	92.95	100.00
	7.05	92.95	
	100.00	100.00	
Total	1445	19064	20509
	7.05	92.95	100.00

Concomitant medications other than anticoagulants at the time of obtaining Table 2-5 consent, according to the presence or absence of proton-pump inhibitors

FREQ Procedure

Tables: ALL * TCMCNT

ALL TCMCNT(Total Number of Drugs Other than Anticoagulant)

Power |
Percent |
Percent of rows
Percent|0|1|2|3|4|5|6|7|8|9|10|11|12| Total
______________+________+________+________+________+________+________+________
_+________+________+________+________+________+________+
1 | 52 | 660 | 1437 | 2202 | 2803 | 2848 | 2643 | 2143 | 1692 | 1251 | 829 | 583 | 365 | 20036
| 0.26 | 3.29 | 7.17 | 10.99 | 13.99 | 14.21 | 13.19 | 10.70 | 8.44 | 6.24 | 4.14 | 2.91 | 1.82 | 100.00
| 0.26 | 3.29 | 7.17 | 10.99 | 13.99 | 14.21 | 13.19 | 10.70 | 8.44 | 6.24 | 4.14 | 2.91 | 1.82 |
| 100.00 | 100.00 | 100.00 | 100.00 | 100.00 | 100.00 | 100.00 | 100.00 | 100.00 | 100.00 | 100.00 | 100.00 | 100.00 |
______________+________+________+________+________+________+________+________
_+________+________+________+________+________+________+
Total 52 660 1437 2202 2803 2848 2643 2143 1692 1251 829 583 365 20036
0.26 3.29 7.17 10.99 13.99 14.21 13.19 10.70 8.44 6.24 4.14 2.91 1.82 100.00
(continued)

Tables: ALL * TCMCNT

ALL TCMCNT(Total Number of Drugs Other than Anticoagulant)

Power |
Percent |

Percent of rows

|13|14|15|16|17|18|19|20|21|23|25|26|27|Total

______________+________+________+________+________+________+________+_______

_+________+________+________+________+________+________+

1 | 225 | 118 | 78 | 44 | 25 | 18 | 4 | 7 | 1 |

5 | 1 | 1 | 1 | 20036

| 1.12 | 0.59 | 0.39 | 0.22 | 0.12 | 0.09 | 0.02 | 0.03 | 0.00 |

0.02 | 0.00 | 0.00 | 0.00 | 100.00

| 1.12 | 0.59 | 0.39 | 0.22 | 0.12 | 0.09 | 0.02 | 0.03 | 0.00 |

0.02 | 0.00 | 0.00 | 0.00 |

| 100.00 | 100.00 | 100.00 | 100.00 | 100.00 | 100.00 | 100.00 | 100.00 | 100.00 |

100.00 | 100.00 | 100.00 | 100.00 |

______________+________+________+________+________+________+________+_______

_+________+________+________+________+________+________+

Total 225 118 78 44 25 18 4 7 1 5 1 1 1 20036

1.12 0.59 0.39 0.22 0.12 0.09 0.02 0.03

0.00 0.02 0.00 0.00 0.00 100.00

Degree of missing values = 473

Concomitant medications other than anticoagulants at the time of obtaining Table 2-5 consent, according to the presence or absence of proton-pump inhibitors

FREQ Procedure

			Cumulative	
Percent of ALL	power	Percent of	power	
1	20509	100.00	20509	100.00

Tables: ALL * CMYN

ALL

CMYN(Administration of Concomitant Drug)

Power / Percent / Percent of rows / Percent of columns	None	With	Total
1	1445	19064	20509
	7.05	92.95	100.00
	7.05	92.95	
	100.00	100.00	
Total	1445	19064	20509
	7.05	92.95	100.00

Concomitant medications other than anticoagulants at the time of obtaining Table 2-5 consent, according to the presence or absence of proton-pump inhibitors

FREQ Procedure

Tables: ALL * TCMCNT

ALL TCMCNT(Total Number of Drugs Other than Anticoagulant)

Power |
Percent |
Percent of rows
Percent|0|1|2|3|4|5|6|7|8|9|10|11|12| Total
______________+________+________+________+________+________+________+________
_+________+________+________+________+________+________+
1 | 52 | 660 | 1437 | 2202 | 2803 | 2848 | 2643 | 2143 | 1692 |
1251 | 829 | 583 | 365 | 20036
| 0.26 | 3.29 | 7.17 | 10.99 | 13.99 | 14.21 | 13.19 | 10.70 | 8.44 |
6.24 | 4.14 | 2.91 | 1.82 | 100.00
| 0.26 | 3.29 | 7.17 | 10.99 | 13.99 | 14.21 | 13.19 | 10.70 | 8.44 |
6.24 | 4.14 | 2.91 | 1.82 |
| 100.00 | 100.00 | 100.00 | 100.00 | 100.00 | 100.00 | 100.00 | 100.00 | 100.00 |
100.00 | 100.00 | 100.00 | 100.00 |
______________+________+________+________+________+________+________+________
_+________+________+________+________+________+________+
Total 52 660 1437 2202 2803 2848 2643 2143 1692 1251 829 583 365 20036
0.26 3.29 7.17 10.99 13.99 14.21 13.19 10.70
8.44 6.24 4.14 2.91 1.82 100.00
(continued)

Tables: ALL * TCMCNT

ALL TCMCNT(Total Number of Drugs Other than Anticoagulant)

Power |
Percent |

Percent of rows

|13|14|15|16|17|18|19|20|21|23|25|26|27|Total

______________+________+________+________+________+________+________+_______

_+________+________+________+________+________+________+

1 | 225 | 118 | 78 | 44 | 25 | 18 | 4 | 7 | 1 |

5 | 1 | 1 | 1 | 20036

| 1.12 | 0.59 | 0.39 | 0.22 | 0.12 | 0.09 | 0.02 | 0.03 | 0.00 |

0.02 | 0.00 | 0.00 | 0.00 | 100.00

| 1.12 | 0.59 | 0.39 | 0.22 | 0.12 | 0.09 | 0.02 | 0.03 | 0.00 |

0.02 | 0.00 | 0.00 | 0.00 |

| 100.00 | 100.00 | 100.00 | 100.00 | 100.00 | 100.00 | 100.00 | 100.00 | 100.00 |

100.00 | 100.00 | 100.00 | 100.00 |

______________+________+________+________+________+________+________+_______

_+________+________+________+________+________+________+

Total 225 118 78 44 25 18 4 7 1 5 1 1 1 20036

1.12 0.59 0.39 0.22 0.12 0.09 0.02 0.03

0.00 0.02 0.00 0.00 0.00 100.00

Degree of missing values = 473

Concomitant medications other than anticoagulants at the time of obtaining Table 2-5 consent, according to the presence or absence of proton-pump inhibitors

FREQ Procedure

			Cumulative	
Percent of ALL	power	Percent	of power	
1	20509	100.00	20509	100.00

Tables: ALL * CMYN

ALL

CMYN(Administration of Concomitant Drug)

Power | Percent | Percent of rows Percent of columns	None	With	Total
1	1445	19064	20509
	7.05	92.95	100.00
	7.05	92.95	
	100.00	100.00	
Total	1445	19064	20509
	7.05	92.95	100.00

Concomitant medications other than anticoagulants at the time of obtaining Table 2-5 consent, according to the presence or absence of proton-pump inhibitors

FREQ Procedure

Tables: ALL * TCMCNT

ALL TCMCNT(Total Number of Drugs Other than Anticoagulant)

Power |
Percent |
Percent of rows
Percent|0|1|2|3|4|5|6|7|8|9|10|11|12| Total
______________+________+________+________+________+________+________+________+________+________+________+________+________+
1 | 52 | 660 | 1437 | 2202 | 2803 | 2848 | 2643 | 2143 | 1692 | 1251 | 829 | 583 | 365 | 20036
| 0.26 | 3.29 | 7.17 | 10.99 | 13.99 | 14.21 | 13.19 | 10.70 | 8.44 | 6.24 | 4.14 | 2.91 | 1.82 | 100.00
| 0.26 | 3.29 | 7.17 | 10.99 | 13.99 | 14.21 | 13.19 | 10.70 | 8.44 | 6.24 | 4.14 | 2.91 | 1.82 |
| 100.00 | 100.00 | 100.00 | 100.00 | 100.00 | 100.00 | 100.00 | 100.00 | 100.00 | 100.00 | 100.00 | 100.00 | 100.00 |
______________+________+________+________+________+________+________+________+________+________+________+________+________+
Total 52 660 1437 2202 2803 2848 2643 2143 1692 1251 829 583 365 20036
0.26 3.29 7.17 10.99 13.99 14.21 13.19 10.70 8.44 6.24 4.14 2.91 1.82 100.00
(continued)

Tables: ALL * TCMCNT

ALL TCMCNT(Total Number of Drugs Other than Anticoagulant)

Power |
Percent |

Percent of rows

|13|14|15|16|17|18|19|20|21|23|25|26|27|Total

______________+________+________+________+________+________+________+_______

_+________+________+________+________+________+________+

1 | 225 | 118 | 78 | 44 | 25 | 18 | 4 | 7 | 1 |

5 | 1 | 1 | 1 | 20036

| 1.12 | 0.59 | 0.39 | 0.22 | 0.12 | 0.09 | 0.02 | 0.03 | 0.00 |

0.02 | 0.00 | 0.00 | 0.00 | 100.00

| 1.12 | 0.59 | 0.39 | 0.22 | 0.12 | 0.09 | 0.02 | 0.03 | 0.00 |

0.02 | 0.00 | 0.00 | 0.00 |

| 100.00 | 100.00 | 100.00 | 100.00 | 100.00 | 100.00 | 100.00 | 100.00 | 100.00 |

100.00 | 100.00 | 100.00 | 100.00 |

______________+________+________+________+________+________+________+_______

_+________+________+________+________+________+________+

Total 225 118 78 44 25 18 4 7 1 5 1 1 1 20036

1.12 0.59 0.39 0.22 0.12 0.09 0.02 0.03

0.00 0.02 0.00 0.00 0.00 100.00

Degree of missing values = 473

Concomitant medications other than anticoagulants at the time of obtaining Table 2-5 consent, according to the presence or absence of proton-pump inhibitors

FREQ Procedure

			Cumulative	
Percent of ALL	power	Percent of power		
1	20509	100.00	20509	100.00

Tables: ALL * CMYN

ALL

CMYN(Administration of Concomitant Drug)

Power / Percent / Percent of rows / Percent of columns	None	With	Total
1	1445	19064	20509
	7.05	92.95	100.00
	7.05	92.95	
	100.00	100.00	
Total	1445	19064	20509
	7.05	92.95	100.00

Concomitant medications other than anticoagulants at the time of obtaining Table 2-5 consent, according to the presence or absence of proton-pump inhibitors

FREQ Procedure

Tables: ALL * TCMCNT

ALL TCMCNT(Total Number of Drugs Other than Anticoagulant)

Power |
Percent |
Percent of rows
Percent|0|1|2|3|4|5|6|7|8|9|10|11|12| Total
______________+________+________+________+________+________+________+________
_+________+________+________+________+________+________+
1 | 52 | 660 | 1437 | 2202 | 2803 | 2848 | 2643 | 2143 | 1692 |
1251 | 829 | 583 | 365 | 20036
| 0.26 | 3.29 | 7.17 | 10.99 | 13.99 | 14.21 | 13.19 | 10.70 | 8.44 |
6.24 | 4.14 | 2.91 | 1.82 | 100.00
| 0.26 | 3.29 | 7.17 | 10.99 | 13.99 | 14.21 | 13.19 | 10.70 | 8.44 |
6.24 | 4.14 | 2.91 | 1.82 |
| 100.00 | 100.00 | 100.00 | 100.00 | 100.00 | 100.00 | 100.00 | 100.00 | 100.00 |
100.00 | 100.00 | 100.00 | 100.00 |
______________+________+________+________+________+________+________+________
_+________+________+________+________+________+________+
Total 52 660 1437 2202 2803 2848 2643 2143 1692 1251 829 583 365 20036
0.26 3.29 7.17 10.99 13.99 14.21 13.19 10.70
8.44 6.24 4.14 2.91 1.82 100.00
(continued)

Tables: ALL * TCMCNT

ALL TCMCNT(Total Number of Drugs Other than Anticoagulant)

Power |
Percent |

Percent of rows

|13|14|15|16|17|18|19|20|21|23|25|26|27|Total

______________+________+________+________+________+________+________+_______

_+________+________+________+________+________+________+

1 | 225 | 118 | 78 | 44 | 25 | 18 | 4 | 7 | 1 |

5 | 1 | 1 | 1 | 20036

| 1.12 | 0.59 | 0.39 | 0.22 | 0.12 | 0.09 | 0.02 | 0.03 | 0.00 |

0.02 | 0.00 | 0.00 | 0.00 | 100.00

| 1.12 | 0.59 | 0.39 | 0.22 | 0.12 | 0.09 | 0.02 | 0.03 | 0.00 |

0.02 | 0.00 | 0.00 | 0.00 |

| 100.00 | 100.00 | 100.00 | 100.00 | 100.00 | 100.00 | 100.00 | 100.00 | 100.00 |

100.00 | 100.00 | 100.00 | 100.00 |

______________+________+________+________+________+________+________+_______

_+________+________+________+________+________+________+

Total 225 118 78 44 25 18 4 7 1 5 1 1 1 20036

1.12 0.59 0.39 0.22 0.12 0.09 0.02 0.03

0.00 0.02 0.00 0.00 0.00 100.00

Degree of missing values = 473

Concomitant medications other than anticoagulants at the time of obtaining Table 2-5 consent, according to the presence or absence of proton-pump inhibitors

FREQ Procedure

Percent of ALL	power	Percent	Cumulative power	Cumulative Percent of power
1	20509	100.00	20509	100.00

Tables: ALL * CMYN

ALL

CMYN(Administration of Concomitant Drug)

Power |
Percent |
Percent of rows
Percent of columns | None | With | Total

ALL	None	With	Total
1	1445	19064	20509
	7.05	92.95	100.00
	7.05	92.95	
	100.00	100.00	
Total	1445	19064	20509
	7.05	92.95	100.00

Concomitant medications other than anticoagulants at the time of obtaining Table 2-5 consent, according to the presence or absence of proton-pump inhibitors

FREQ Procedure

Tables: ALL * TCMCNT

ALL TCMCNT(Total Number of Drugs Other than Anticoagulant)

Power |
Percent |
Percent of rows
Percent|0|1|2|3|4|5|6|7|8|9|10|11|12| Total
______________+________+________+________+________+________+________+________
_+________+________+________+________+________+________+
1 | 52 | 660 | 1437 | 2202 | 2803 | 2848 | 2643 | 2143 | 1692 | 1251 | 829 | 583 | 365 | 20036
| 0.26 | 3.29 | 7.17 | 10.99 | 13.99 | 14.21 | 13.19 | 10.70 | 8.44 | 6.24 | 4.14 | 2.91 | 1.82 | 100.00
| 0.26 | 3.29 | 7.17 | 10.99 | 13.99 | 14.21 | 13.19 | 10.70 | 8.44 | 6.24 | 4.14 | 2.91 | 1.82 |
| 100.00 | 100.00 | 100.00 | 100.00 | 100.00 | 100.00 | 100.00 | 100.00 | 100.00 | 100.00 | 100.00 | 100.00 | 100.00 |
______________+________+________+________+________+________+________+________
_+________+________+________+________+________+________+
Total 52 660 1437 2202 2803 2848 2643 2143 1692 1251 829 583 365 20036
0.26 3.29 7.17 10.99 13.99 14.21 13.19 10.70 8.44 6.24 4.14 2.91 1.82 100.00
(continued)

Tables: ALL * TCMCNT

ALL TCMCNT(Total Number of Drugs Other than Anticoagulant)

Power |
Percent |

Percent of rows

|13|14|15|16|17|18|19|20|21|23|25|26|27|Total

______________+________+________+________+________+________+________+_______

_+________+________+________+________+________+________+

1 | 225 | 118 | 78 | 44 | 25 | 18 | 4 | 7 | 1 |

5 | 1 | 1 | 1 | 20036

| 1.12 | 0.59 | 0.39 | 0.22 | 0.12 | 0.09 | 0.02 | 0.03 | 0.00 |

0.02 | 0.00 | 0.00 | 0.00 | 100.00

| 1.12 | 0.59 | 0.39 | 0.22 | 0.12 | 0.09 | 0.02 | 0.03 | 0.00 |

0.02 | 0.00 | 0.00 | 0.00 |

| 100.00 | 100.00 | 100.00 | 100.00 | 100.00 | 100.00 | 100.00 | 100.00 | 100.00 |

100.00 | 100.00 | 100.00 | 100.00 |

______________+________+________+________+________+________+________+_______

_+________+________+________+________+________+________+

Total 225 118 78 44 25 18 4 7 1 5 1 1 1 20036

1.12 0.59 0.39 0.22 0.12 0.09 0.02 0.03

0.00 0.02 0.00 0.00 0.00 100.00

Degree of missing values = 473

Concomitant medications other than anticoagulants at the time of obtaining Table 2-5 consent, according to the presence or absence of proton-pump inhibitors

FREQ Procedure

			Cumulative	
Percent of ALL	power	Percent of	power	
1	20509	100.00	20509	100.00

Tables: ALL * CMYN

ALL

CMYN(Administration of Concomitant Drug)

Power | Percent | Percent of rows Percent of columns	None	With	Total
1	1445	19064	20509
	7.05	92.95	100.00
	7.05	92.95	
	100.00	100.00	
Total	1445	19064	20509
	7.05	92.95	100.00

Concomitant medications other than anticoagulants at the time of obtaining Table 2-5 consent, according to the presence or absence of proton-pump inhibitors

FREQ Procedure

Tables: ALL * TCMCNT

ALL TCMCNT(Total Number of Drugs Other than Anticoagulant)

Power |
Percent |
Percent of rows
Percent|0|1|2|3|4|5|6|7|8|9|10|11|12| Total
______________+________+________+________+________+________+________+_______
_+________+________+________+________+________+________+
1 | 52 | 660 | 1437 | 2202 | 2803 | 2848 | 2643 | 2143 | 1692 |
1251 | 829 | 583 | 365 | 20036
| 0.26 | 3.29 | 7.17 | 10.99 | 13.99 | 14.21 | 13.19 | 10.70 | 8.44 |
6.24 | 4.14 | 2.91 | 1.82 | 100.00
| 0.26 | 3.29 | 7.17 | 10.99 | 13.99 | 14.21 | 13.19 | 10.70 | 8.44 |
6.24 | 4.14 | 2.91 | 1.82 |
| 100.00 | 100.00 | 100.00 | 100.00 | 100.00 | 100.00 | 100.00 | 100.00 | 100.00 |
100.00 | 100.00 | 100.00 | 100.00 |
______________+________+________+________+________+________+________+_______
_+________+________+________+________+________+________+
Total 52 660 1437 2202 2803 2848 2643 2143 1692 1251 829 583 365 20036
0.26 3.29 7.17 10.99 13.99 14.21 13.19 10.70
8.44 6.24 4.14 2.91 1.82 100.00
(continued)

Tables: ALL * TCMCNT

ALL TCMCNT(Total Number of Drugs Other than Anticoagulant)

Power |
Percent |

Percent of rows

|13|14|15|16|17|18|19|20|21|23|25|26|27|Total

______________+________+________+________+________+________+________+________

_+________+________+________+________+________+________+________+

1 | 225 | 118 | 78 | 44 | 25 | 18 | 4 | 7 | 1 |

5 | 1 | 1 | 1 | 20036

| 1.12 | 0.59 | 0.39 | 0.22 | 0.12 | 0.09 | 0.02 | 0.03 | 0.00 |

0.02 | 0.00 | 0.00 | 0.00 | 100.00

| 1.12 | 0.59 | 0.39 | 0.22 | 0.12 | 0.09 | 0.02 | 0.03 | 0.00 |

0.02 | 0.00 | 0.00 | 0.00 |

| 100.00 | 100.00 | 100.00 | 100.00 | 100.00 | 100.00 | 100.00 | 100.00 | 100.00 |

100.00 | 100.00 | 100.00 | 100.00 |

______________+________+________+________+________+________+________+________

_+________+________+________+________+________+________+________+

Total 225 118 78 44 25 18 4 7 1 5 1 1 1 20036

1.12 0.59 0.39 0.22 0.12 0.09 0.02 0.03

0.00 0.02 0.00 0.00 0.00 100.00

Degree of missing values = 473

Concomitant medications other than anticoagulants at the time of obtaining Table 2-5 consent, according to the presence or absence of proton-pump inhibitors

FREQ Procedure

			Cumulative	
Percent of ALL	power	Percent of	power	
1	20509	100.00	20509	100.00

Tables: ALL * CMYN

ALL

CMYN(Administration of Concomitant Drug)

Power | Percent | Percent of rows Percent of columns	None	With	Total
1	1445	19064	20509
	7.05	92.95	100.00
	7.05	92.95	
	100.00	100.00	
Total	1445	19064	20509
	7.05	92.95	100.00

Concomitant medications other than anticoagulants at the time of obtaining Table 2-5 consent, according to the presence or absence of proton-pump inhibitors

FREQ Procedure

Tables: ALL * TCMCNT

ALL TCMCNT(Total Number of Drugs Other than Anticoagulant)

Power |
Percent |
Percent of rows
Percent|0|1|2|3|4|5|6|7|8|9|10|11|12| Total
______________+________+________+________+________+________+________+________
_+________+________+________+________+________+________+
1 | 52 | 660 | 1437 | 2202 | 2803 | 2848 | 2643 | 2143 | 1692 | 1251 | 829 | 583 | 365 | 20036
| 0.26 | 3.29 | 7.17 | 10.99 | 13.99 | 14.21 | 13.19 | 10.70 | 8.44 | 6.24 | 4.14 | 2.91 | 1.82 | 100.00
| 0.26 | 3.29 | 7.17 | 10.99 | 13.99 | 14.21 | 13.19 | 10.70 | 8.44 | 6.24 | 4.14 | 2.91 | 1.82 |
| 100.00 | 100.00 | 100.00 | 100.00 | 100.00 | 100.00 | 100.00 | 100.00 | 100.00 | 100.00 | 100.00 | 100.00 | 100.00 |
______________+________+________+________+________+________+________+________
_+________+________+________+________+________+________+
Total 52 660 1437 2202 2803 2848 2643 2143 1692 1251 829 583 365 20036
0.26 3.29 7.17 10.99 13.99 14.21 13.19 10.70 8.44 6.24 4.14 2.91 1.82 100.00
(continued)

Tables: ALL * TCMCNT

ALL TCMCNT(Total Number of Drugs Other than Anticoagulant)

Power |
Percent |

Percent of rows

|13|14|15|16|17|18|19|20|21|23|25|26|27|Total

______________+________+________+________+________+________+________+________

_+________+________+________+________+________+________+

1 | 225 | 118 | 78 | 44 | 25 | 18 | 4 | 7 | 1 |

5 | 1 | 1 | 1 | 20036

| 1.12 | 0.59 | 0.39 | 0.22 | 0.12 | 0.09 | 0.02 | 0.03 | 0.00 |

0.02 | 0.00 | 0.00 | 0.00 | 100.00

| 1.12 | 0.59 | 0.39 | 0.22 | 0.12 | 0.09 | 0.02 | 0.03 | 0.00 |

0.02 | 0.00 | 0.00 | 0.00 |

| 100.00 | 100.00 | 100.00 | 100.00 | 100.00 | 100.00 | 100.00 | 100.00 | 100.00 |

100.00 | 100.00 | 100.00 | 100.00 |

______________+________+________+________+________+________+________+________

_+________+________+________+________+________+________+

Total 225 118 78 44 25 18 4 7 1 5 1 1 1 20036

1.12 0.59 0.39 0.22 0.12 0.09 0.02 0.03

0.00 0.02 0.00 0.00 0.00 100.00

Degree of missing values = 473

Concomitant medications other than anticoagulants at the time of obtaining Table 2-5 consent, according to the presence or absence of proton-pump inhibitors

FREQ Procedure

			Cumulative	
Percent of ALL	power	Percent of	power	
1	20509	100.00	20509	100.00

Tables: ALL * CMYN

ALL

CMYN(Administration of Concomitant Drug)

Power |
Percent |
Percent of rows
Percent of columns | None | With | Total

	None	With	Total
1	1445	19064	20509
	7.05	92.95	100.00
	7.05	92.95	
	100.00	100.00	
Total	1445	19064	20509
	7.05	92.95	100.00

Concomitant medications other than anticoagulants at the time of obtaining Table 2-5 consent, according to the presence or absence of proton-pump inhibitors

FREQ Procedure

Tables: ALL * TCMCNT

ALL TCMCNT(Total Number of Drugs Other than Anticoagulant)

Power |
Percent |
Percent of rows
Percent|0|1|2|3|4|5|6|7|8|9|10|11|12| Total
______________+________+________+________+________+________+________+________
_+________+________+________+________+________+________+
1 | 52 | 660 | 1437 | 2202 | 2803 | 2848 | 2643 | 2143 | 1692 |
1251 | 829 | 583 | 365 | 20036
| 0.26 | 3.29 | 7.17 | 10.99 | 13.99 | 14.21 | 13.19 | 10.70 | 8.44 |
6.24 | 4.14 | 2.91 | 1.82 | 100.00
| 0.26 | 3.29 | 7.17 | 10.99 | 13.99 | 14.21 | 13.19 | 10.70 | 8.44 |
6.24 | 4.14 | 2.91 | 1.82 |
| 100.00 | 100.00 | 100.00 | 100.00 | 100.00 | 100.00 | 100.00 | 100.00 | 100.00 |
100.00 | 100.00 | 100.00 | 100.00 |
______________+________+________+________+________+________+________+________
_+________+________+________+________+________+________+
Total 52 660 1437 2202 2803 2848 2643 2143 1692 1251 829 583 365 20036
0.26 3.29 7.17 10.99 13.99 14.21 13.19 10.70
8.44 6.24 4.14 2.91 1.82 100.00
(continued)

Tables: ALL * TCMCNT

ALL TCMCNT(Total Number of Drugs Other than Anticoagulant)

Power |
Percent |

Percent of rows

|13|14|15|16|17|18|19|20|21|23|25|26|27|Total

______________+________+________+________+________+________+________+________+________+________+________+________+________+________+

1 | 225 | 118 | 78 | 44 | 25 | 18 | 4 | 7 | 1 | 5 | 1 | 1 | 1 | 20036

| 1.12 | 0.59 | 0.39 | 0.22 | 0.12 | 0.09 | 0.02 | 0.03 | 0.00 | 0.02 | 0.00 | 0.00 | 0.00 | 100.00

| 1.12 | 0.59 | 0.39 | 0.22 | 0.12 | 0.09 | 0.02 | 0.03 | 0.00 | 0.02 | 0.00 | 0.00 | 0.00 |

| 100.00 | 100.00 | 100.00 | 100.00 | 100.00 | 100.00 | 100.00 | 100.00 | 100.00 | 100.00 | 100.00 | 100.00 | 100.00 |

______________+________+________+________+________+________+________+________+________+________+________+________+________+________+

Total 225 118 78 44 25 18 4 7 1 5 1 1 1 20036

1.12 0.59 0.39 0.22 0.12 0.09 0.02 0.03 0.00 0.02 0.00 0.00 0.00 100.00

Degree of missing values = 473

Concomitant medications other than anticoagulants at the time of obtaining Table 2-5 consent, according to the presence or absence of proton-pump inhibitors

FREQ Procedure

			Cumulative	
Percent of ALL	power	Percent of power		
1	20509	100.00	20509	100.00

Tables: ALL * CMYN

ALL

CMYN(Administration of Concomitant Drug)

Power | Percent | Percent of rows Percent of columns	None	With	Total
1	1445	19064	20509
	7.05	92.95	100.00
	7.05	92.95	
	100.00	100.00	
Total	1445	19064	20509
	7.05	92.95	100.00

Concomitant medications other than anticoagulants at the time of obtaining Table 2-5 consent, according to the presence or absence of proton-pump inhibitors

FREQ Procedure

Table: ALL * t_cm

ALL t_cm

Power |
Percent |
Percent of rows
Percent of columns | 1| Total

1	16	16
	100.00	100.00
	100.00	
	100.00	
Total	16	16
	100.00	100.00

Degree of missing values = 20493

Concomitant medications other than anticoagulants at the time of obtaining Table 2-5 consent, according to the presence or absence of proton-pump inhibitors

FREQ Procedure

			Cumulative	
Percent of ALL	power	Percent of	power	
1	20509	100.00	20509	100.00

Tables: ALL * CMYN

ALL

CMYN(Administration of Concomitant Drug)

Power | Percent | Percent of rows Percent of columns	None	With	Total
1	1445	19064	20509
	7.05	92.95	100.00
	7.05	92.95	
	100.00	100.00	
Total	1445	19064	20509
	7.05	92.95	100.00

Concomitant medications other than anticoagulants at the time of obtaining Table 2-5 consent, according to the presence or absence of proton-pump inhibitors

FREQ Procedure

Table: ALL * t_TCMCNT

ALL t_TCMCNT

Power |
Percent |
Percent of rows
Percent of columns | 9| Total

1	473	473
	100.00	100.00
	100.00	
	100.00	
Total	473	473
	100.00	100.00

Degree of missing values = 20036

Concomitant medications other than anticoagulants at the time of obtaining Table 2-5 consent, according to the presence or absence of proton-pump inhibitors

FREQ Procedure

			Cumulative	
Percent of ALL	power	Percent of	power	
1	20509	100.00	20509	100.00

Tables: ALL * CMYN

ALL

CMYN(Administration of Concomitant Drug)

Power / Percent / Percent of rows / Percent of columns	None	With	Total
1	1445	19064	20509
	7.05	92.95	100.00
	7.05	92.95	
	100.00	100.00	
Total	1445	19064	20509
	7.05	92.95	100.00

Concomitant medications other than anticoagulants at the time of obtaining Table 2-5 consent, according to the presence or absence of proton-pump inhibitors

FREQ Procedure

Tables: ALL* TCMCNTCT

ALL

TCMCNTCT(Category for Total Number of Drugs Other than Anticoagulant)

Power |
Percent |
Percent of rows
Percentage of column |0 to 4 or more|5 to 9 or more|10 or more|unknown|total
|Bottom|Bottom | | |

	0 to 4 or more Bottom	5 to 9 or more Bottom	10 or more	unknown	total
1	7154	10577	2305	473	20509
	34.88	51.57	11.24	2.31	100.00
	34.88	51.57	11.24	2.31	
	100.00	100.00	100.00	100.00	
Total	7154	10577	2305	473	20509
	34.88	51.57	11.24	2.31	100.00

Concomitant medications other than anticoagulants at the time of obtaining Table 2-5 consent, according to the presence or absence of proton-pump inhibitors

FREQ Procedure

			Cumulative	
Percent of ALL	power	Percent of	power	
1	20509	100.00	20509	100.00

Tables: ALL * CMYN

ALL
CMYN(Administration of Concomitant Drug)

Power |
Percent |
Percent of rows
Percent of columns | None | With | Total

	None	With	Total
1	1445	19064	20509
	7.05	92.95	100.00
	7.05	92.95	
	100.00	100.00	
Total	1445	19064	20509
	7.05	92.95	100.00

Concomitant medications other than anticoagulants at the time of obtaining Table 2-5 consent, according to the presence or absence of proton-pump inhibitors

FREQ Procedure

Tables: ALL* TCMCNTCT

ALL

TCMCNTCT(Category for Total Number of Drugs Other than Anticoagulant)

Power / Percent / Percent of rows / Percentage of column	0 to 4 or more Bottom	5 to 9 or more Bottom	10 or more	unknown	total
1	7154	10577	2305	473	20509
	34.88	51.57	11.24	2.31	100.00
	34.88	51.57	11.24	2.31	
	100.00	100.00	100.00	100.00	
Total	7154	10577	2305	473	20509
	34.88	51.57	11.24	2.31	100.00

Concomitant medications other than anticoagulants at the time of obtaining Table 2-5 consent, according to the presence or absence of proton-pump inhibitors

FREQ Procedure

ALL	power	Percent	Cumulative power	Cumulative Percent
1	20509	100.00	20509	100.00

Tables: ALL * CMYN

ALL
CMYN(Administration of Concomitant Drug)

Power / Percent / Percent of rows / Percent of columns	None	With	Total
1	1445	19064	20509
	7.05	92.95	100.00
	7.05	92.95	
	100.00	100.00	
Total	1445	19064	20509
	7.05	92.95	100.00

Concomitant medications other than anticoagulants at the time of obtaining Table 2-5 consent, according to the presence or absence of proton-pump inhibitors

FREQ Procedure

Tables: ALL* TCMCNTCT

ALL

TCMCNTCT(Category for Total Number of Drugs Other than Anticoagulant)

Power |
Percent |
Percent of rows
Percentage of column |0 to 4 or more|5 to 9 or more|10 or more|unknown|total
|Bottom|Bottom | | |

1	7154	10577	2305	473	20509
	34.88	51.57	11.24	2.31	100.00
	34.88	51.57	11.24	2.31	
	100.00	100.00	100.00	100.00	
Total	7154	10577	2305	473	20509
	34.88	51.57	11.24	2.31	100.00

Concomitant medications other than anticoagulants at the time of obtaining Table 2-5 consent, according to the presence or absence of proton-pump inhibitors

FREQ Procedure

			Cumulative	
Percent of ALL	power	Percent of	power	
1	20509	100.00	20509	100.00

Tables: ALL * CMYN

ALL
CMYN(Administration of Concomitant Drug)

Power |
Percent |
Percent of rows
Percent of columns | None | With | Total

	None	With	Total
1	1445	19064	20509
	7.05	92.95	100.00
	7.05	92.95	
	100.00	100.00	
Total	1445	19064	20509
	7.05	92.95	100.00

Concomitant medications other than anticoagulants at the time of obtaining Table 2-5 consent, according to the presence or absence of proton-pump inhibitors

FREQ Procedure

Tables: ALL* TCMCNTCT

ALL

TCMCNTCT(Category for Total Number of Drugs Other than Anticoagulant)

Power |
Percent |
Percent of rows
Percentage of column |0 to 4 or more|5 to 9 or more|10 or more|unknown|total
|Bottom|Bottom | | |

	0 to 4 or more Bottom	5 to 9 or more Bottom	10 or more	unknown	total
1	7154	10577	2305	473	20509
	34.88	51.57	11.24	2.31	100.00
	34.88	51.57	11.24	2.31	
	100.00	100.00	100.00	100.00	
Total	7154	10577	2305	473	20509
	34.88	51.57	11.24	2.31	100.00

Concomitant medications other than anticoagulants at the time of obtaining Table 2-5 consent, according to the presence or absence of proton-pump inhibitors

UNIVARIATE Procedure
Variables: TCMCNT (Total Number of Drugs Other than Anticoagulant)

Moment

Total of 31831 for N 31831 weight variable
Mean 6.63535547 total 211210
Standard deviation 3.16502134 variance 10.0173601
Skewness 0.69445812 kurtosis 0.75298246
Unmodified square sum 1720306 modified square sum 318852.571
Coefficient of variation 47.6993486 standard error of the mean 0.01773991

Basic statistics

Positional variation

Mean 6.635355 standard deviation 3.16502
Median 6.000000 variance 10.01736
Mode 6.000000 Range 27.00000
Interquartile range 5.00000

Test for position H0: Mu0=0

Test-Statistics------p Value

Student's t-test t 374.0354 Pr > |t| <. 0001
Sign test M 15889.5 Pr >= |M| <. 0001
Signed rank test S 2. 5248E8 Pr >= |S| <. 0001

Quartile (Definition 5)

Level quantile

100% max 27	
99%	15
95%	12
90%	11
75% Q3 9	
Median 50% 6	
25% Q1 4	
10%	3
5%	2
1%	1
0% minimum 0	

Concomitant medications other than anticoagulants at the time of obtaining Table 2-5 consent, according to the presence or absence of proton-pump inhibitors

UNIVARIATE Procedure
Variables: TCMCNT (Total Number of Drugs Other than Anticoagulant)

Extreme value

--- Minimum value--Maximum value----

Value Obs Value Obs

0	31987	24	11360
0	31978	25	11392
0	31891	26	23911
0	31640	26	28331
0	31625	27	23233

Missing Values

----- Percent----
Missing Values Overall Missing Values

659	2.03	100.00

Concomitant medications other than anticoagulants at the time of obtaining Table 2-5 consent, according to the presence or absence of proton-pump inhibitors

UNIVARIATE Procedure
Variables: TCMCNT (Total Number of Drugs Other than Anticoagulant)

Moment

Total of 11795 for N 11795 weight variable
Mean 7.90597711 total 93251
Standard deviation 3.09028038 variance 9.54983285
Skewness 0.64463054 kurtosis 0.64432289
Unmodified square sum 849871 modified square sum 112630.729
Coefficient of variation 39.0878995 standard error of the mean 0.02845437

Basic statistics

Positional variation

Mean 7.905977 standard deviation 3.09028
Median 8.000000 variance 9.54983
Mode 7.000000 Range 25.00000
Interquartile range 4.00000

Test for position H0: Mu0=0

Test-Statistics------p Value

Student's t-test t 277.8476 Pr > |t| <. 0001
Sign test M 5897.5 Pr >= |M| <. 0001
Signed rank test S 34783455 Pr >= |S| <. 0001

Quartile (Definition 5)

Level quantile

100% max.	26
99%	17
95%	13
90%	12
75% Q3	10
Median 50%	8
25% Q1	6
10%	4
5%	3
1%	2
0% minimum	1

Concomitant medications other than anticoagulants at the time of obtaining Table 2-5 consent, according to the presence or absence of proton-pump inhibitors

UNIVARIATE Procedure

Variables: TCMCNT (Total Number of Drugs Other than Anticoagulant)

Extreme value

--- Minimum value--Maximum value----

Value	Obs	Value	Obs
1	11837	21	10673
1	11210	22	5310
1	10063	22	6199
1	9459	24	3864
1	9284	26	8576

Missing Values

Missing Values	----- Percent---- Overall	Missing Values
186	1.55	100.00

Concomitant medications other than anticoagulants at the time of obtaining Table 2-5 consent, according to the presence or absence of proton-pump inhibitors

UNIVARIATE Procedure
Variables: TCMCNT (Total Number of Drugs Other than Anticoagulant)

Moment

Total of 20036 for N 20036 weight variable
Mean 5.88735277 total 117959
Standard deviation 2.96362403 variance 8.78306739
Skewness 0.81730219 kurtosis 1.19570963
Unmodified square sum 870435 modified square sum 175968.755
Coefficient of variation 50.338822 standard error of the mean 0.02093715

Basic statistics

Positional variation

Mean 5.887353 standard deviation 2.96362
Median 6.000000 variance 8.78307
Mode 5.000000 Range 27.00000
Interquartile range 4.00000

Test for position H0: Mu0=0

Test-Statistics------p Value

Student's t-test t 281. 1917 Pr > |t| <. 0001
Sign test M 9992 Pr >= |M| <. 0001
Signed rank test S 99845060 Pr >= |S| <. 0001

Quartile (Definition 5)

Level quantile

100% max 27	
99%	14
95%	11
90%	10
75% Q3 8	
Median 50% 6	
25% Q1 4	
10%	2
5%	2
1%	1
0% minimum 0	

Concomitant medications other than anticoagulants at the time of obtaining Table 2-5 consent, according to the presence or absence of proton-pump inhibitors

UNIVARIATE Procedure

Variables: TCMCNT (Total Number of Drugs Other than Anticoagulant)

Extreme value

--- Minimum value--Maximum value----

Value	Obs	Value	Obs
0	20199	23	14042
0	20193	23	15129
0	20129	25	7515
0	19954	26	18014
0	19946	27	14915

Missing Values

Missing Values	----- Percent---- Overall	Missing Values
473	2.31	100.00

Concomitant medications other than anticoagulants at the time of obtaining Table 2-5 consent, according to the presence or absence of proton-pump inhibitors

FREQ Procedure

Number of levels of variables

Variables	Label	Level Number
GROUP1		2
CMYN	Administration of Concomitant Drug	2

Table : GROUP1 * CMYN

GROUP1

CMYN(Administration of Concomitant Drug)

Power / Percent / Percent of rows / Percent of columns	None	With	Total
1	0	11981	11981
	0.00	36.88	36.88
	0.00	100.00	
	0.00	38.59	
2	1445	19064	20509
	4.45	58.68	63.12
	7.05	92.95	
	100.00	61.41	
Total	1445	31045	32490
	4.45	95.55	100.00

Concomitant medications other than anticoagulants at the time of obtaining Table 2-5 consent, according to the presence or absence of proton-pump inhibitors

FREQ Procedure

Number of levels of variables

Variables Label Level Number

GROUP1 2
ARTD Arrhythmic Drugs 2

Table : GROUP1 * ARTD

GROUP1 ARTD(Arrhythmic Drugs)

Power / Percent / Percent of rows / Percent of columns	None	With	Total
1	4835	7146	11981
	15.57	23.02	38.59
	40.36	59.64	
	38.16	38.89	
2	7836	11228	19064
	25.24	36.17	61.41
	41.10	58.90	
	61.84	61.11	
Total	12671	18374	31045
	40.81	59.19	100.00

Concomitant medications other than anticoagulants at the time of obtaining Table 2-5 consent, according to the presence or absence of proton-pump inhibitors

FREQ Procedure

Number of levels of variables

Variables Label Level Number

GROUP1 2
ARTD1 Rhythm Control 2

Table : GROUP1 * ARTD1

GROUP1 ARTD1(Rhythm Control)

Power |
Percent |
Percent of rows
Percent of columns | None | With | Total

	None	With	Total
1	9496	2485	11981
	30.59	8.00	38.59
	79.26	20.74	
	38.48	39.02	
2	15180	3884	19064
	48.90	12.51	61.41
	79.63	20.37	
	61.52	60.98	
Total	24676	6369	31045
	79.48	20.52	100.00

Concomitant medications other than anticoagulants at the time of obtaining Table 2-5 consent, according to the presence or absence of proton-pump inhibitors

FREQ Procedure

Number of levels of variables

Variables	Label	Level Number
GROUP1		2
ARTD2	Rate Control	2

Table : GROUP1 * ARTD2

GROUP1 ARTD2(Rate Control)

Power<br>Percent<br>Percent of rows<br>Percent of columns	None	With	Total
1	6708	5273	11981
	21.61	16.99	38.59
	55.99	44.01	
	38.16	39.16	
2	10871	8193	19064
	35.02	26.39	61.41
	57.02	42.98	
	61.84	60.84	
Total	17579	13466	31045
	56.62	43.38	100.00

Concomitant medications other than anticoagulants at the time of obtaining Table 2-5 consent, according to the presence or absence of proton-pump inhibitors

FREQ Procedure

Number of levels of variables

Variables Label Level Number

GROUP1 2
APLD Anti-Platelet Drugs 2

Table : GROUP1 * APLD

GROUP1 APLD(Anti-Platelet Drugs)

Power | Percent | Percent of rows Percent of columns	None	With	Total
1	8965	3016	11981
	28.88	9.71	38.59
	74.83	25.17	
	35.50	52.06	
2	16287	2777	19064
	52.46	8.95	61.41
	85.43	14.57	
	64.50	47.94	
Total	25252	5793	31045
	81.34	18.66	100.00

Concomitant medications other than anticoagulants at the time of obtaining Table 2-5 consent, according to the presence or absence of proton-pump inhibitors

FREQ Procedure

Number of levels of variables

Number of Variables Level

GROUP1	2
t_AP	2

Table : GROUP1 * t_AP

GROUP1 t_AP

Power |
Percent |
Percent of rows
Percentage of columns | 0| 1| Sum

	0	1	Sum
1	11824	157	11981
	38.09	0.51	38.59
	98.69	1.31	
	38.39	64.61	
2	18978	86	19064
	61.13	0.28	61.41
	99.55	0.45	
	61.61	35.39	
Total	30802	243	31045
	99.22	0.78	100.00

Concomitant medications other than anticoagulants at the time of obtaining Table 2-5 consent, according to the presence or absence of proton-pump inhibitors

FREQ Procedure

Number of levels of variables

Variables	Label	Level Number
GROUP1		2
APLD1	ASA	2

Table : GROUP1 * APLD1

GROUP1 APLD1(ASA)

Power<br>Percent<br>Percent of rows<br>Percent of columns	None	With	Total
1	10026<br>32.30<br>83.68<br>36.45	1955<br>6.30<br>16.32<br>55.27	11981<br>38.59
2	17482<br>56.31<br>91.70<br>63.55	1582<br>5.10<br>8.30<br>44.73	19064<br>61.41
Total	27508<br>88.61	3537<br>11.39	31045<br>100.00

Concomitant medications other than anticoagulants at the time of obtaining Table 2-5 consent, according to the presence or absence of proton-pump inhibitors

FREQ Procedure

Number of levels of variables

Variables Label Level Number

GROUP1 2
APLD2 P2Y12-I 2

Table : GROUP1 * APLD2

GROUP1 APLD2(P2Y12-I)

Power |
Percent |
Percent of rows
Percent of columns | None | With | Total

1	11206	775	11981
	36.10	2.50	38.59
	93.53	6.47	
	37.79	55.56	
2	18444	620	19064
	59.41	2.00	61.41
	96.75	3.25	
	62.21	44.44	
Total	29650	1395	31045
	95.51	4.49	100.00

Concomitant medications other than anticoagulants at the time of obtaining Table 2-5 consent, according to the presence or absence of proton-pump inhibitors

FREQ Procedure

Number of levels of variables

Variables Label Level Number

GROUP1 2
APLD3 Other Anti-Platelet Drugs 2

Table : GROUP1 * APLD3

GROUP1
APLD3(Other Anti-Platelet Drugs)

Power / Percent / Percent of rows / Percent of columns	None	With	Total
1	11421	560	11981
	36.79	1.80	38.59
	95.33	4.67	
	38.40	43.01	
2	18322	742	19064
	59.02	2.39	61.41
	96.11	3.89	
	61.60	56.99	
Total	29743	1302	31045
	95.81	4.19	100.00

Concomitant medications other than anticoagulants at the time of obtaining Table 2-5 consent, according to the presence or absence of proton-pump inhibitors

FREQ Procedure

Number of levels of variables

Variables	Label	Level Number
GROUP1		2
AHPD	Antihypertensive Drugs	2

Table : GROUP1 * AHPD

GROUP1

AHPD(Antihypertensive Drugs)

Power<br>Percent<br>Percent of rows<br>Percent of columns	None	With	Total
1	3096<br>9.97<br>25.84<br>39.36	8885<br>28.62<br>74.16<br>38.33	11981<br>38.59
2	4769<br>15.36<br>25.02<br>60.64	14295<br>46.05<br>74.98<br>61.67	19064<br>61.41
Total	7865<br>25.33	23180<br>74.67	31045<br>100.00

Concomitant medications other than anticoagulants at the time of obtaining Table 2-5 consent, according to the presence or absence of proton-pump inhibitors

FREQ Procedure

Number of levels of variables

Variable	Label	Level Count
GROUP1		2
LTD	Lipid Therapy	2

Table : GROUP1 * LTD

GROUP1 LTD(Lipid Therapy)

Power / Percent / Percent of rows / Percent of columns	None	With	Total
1	6650	5331	11981
	21.42	17.17	38.59
	55.50	44.50	
	35.16	43.95	
2	12266	6798	19064
	39.51	21.90	61.41
	64.34	35.66	
	64.84	56.05	
Total	18916	12129	31045
	60.93	39.07	100.00

Concomitant medications other than anticoagulants at the time of obtaining Table 2-5 consent, according to the presence or absence of proton-pump inhibitors

FREQ Procedure

Number of levels of variables

Variables Label Level Number

GROUP1 2
LTD1 Statins 2

Table : GROUP1 * LTD1

GROUP1 LTD1(Statins)

```
Power |
Percent |
Percent of rows
Percent of columns | None | With | Total
______________+________+________+
            1 |   7179 |   4802 |  11981
              |  23.12 |  15.47 |  38.59
              |  59.92 |  40.08 |
              |  35.57 |  44.21 |
______________+________+________+
            2 |  13003 |   6061 |  19064
              |  41.88 |  19.52 |  61.41
              |  68.21 |  31.79 |
              |  64.43 |  55.79 |
______________+________+________+
Total 20182 10863 31045
         65.01    34.99   100.00
```

Concomitant medications other than anticoagulants at the time of obtaining Table 2-5 consent, according to the presence or absence of proton-pump inhibitors

FREQ Procedure

Number of levels of variables

Variables Label Level Number

GROUP1 2
LTD2 Other Than Statins 2

Table : GROUP1 * LTD2

GROUP1 LTD2(Other Than Statins)

Power<br>Percent<br>Percent of rows<br>Percent of columns	None	With	Total
1	11227<br>36.16<br>93.71<br>38.29	754<br>2.43<br>6.29<br>43.79	11981<br>38.59
2	18096<br>58.29<br>94.92<br>61.71	968<br>3.12<br>5.08<br>56.21	19064<br>61.41
Total	29323<br>94.45	1722<br>5.55	31045<br>100.00

Concomitant medications other than anticoagulants at the time of obtaining Table 2-5 consent, according to the presence or absence of proton-pump inhibitors

FREQ Procedure

Number of levels of variables

Variables	Label	Level Number
GROUP1		2
DBTD	Diabetes Drugs	2

Table : GROUP1 * DBTD

GROUP1 DBTD(Diabetes Drugs)

Power<br>Percent<br>Percent of rows<br>Percent of columns	None	With	Total
1	9802<br>31.57<br>81.81<br>37.97	2179<br>7.02<br>18.19<br>41.66	11981<br>38.59
2	16012<br>51.58<br>83.99<br>62.03	3052<br>9.83<br>16.01<br>58.34	19064<br>61.41
Total	25814<br>83.15	5231<br>16.85	31045<br>100.00

Concomitant medications other than anticoagulants at the time of obtaining Table 2-5 consent, according to the presence or absence of proton-pump inhibitors

FREQ Procedure

Number of levels of variables

Number of Variables Level

GROUP1	2
t_DBT	2

Table : GROUP1 * t_DBT

GROUP1 t_DBT

Power |
Percent |
Percent of rows
Percentage of columns | 0| 1| Sum

	0	1	Sum
1	9919	2062	11981
	31.95	6.64	38.59
	82.79	17.21	
	38.03	41.54	
2	16162	2902	19064
	52.06	9.35	61.41
	84.78	15.22	
	61.97	58.46	
Total	26081	4964	31045
	84.01	15.99	100.00

Concomitant medications other than anticoagulants at the time of obtaining Table 2-5 consent, according to the presence or absence of proton-pump inhibitors

FREQ Procedure

Number of levels of variables

Variables	Label	Level Number
GROUP1		2
DBTD1	Sulfonylurea Drugs	2

Table : GROUP1 * DBTD1

GROUP1 DBTD1(Sulfonylurea Drugs)

Power / Percent / Percent of rows / Percent of columns	None	With	Total
1	11508	473	11981
	37.07	1.52	38.59
	96.05	3.95	
	38.59	38.74	
2	18316	748	19064
	59.00	2.41	61.41
	96.08	3.92	
	61.41	61.26	
Total	29824	1221	31045
	96.07	3.93	100.00

Concomitant medications other than anticoagulants at the time of obtaining Table 2-5 consent, according to the presence or absence of proton-pump inhibitors

FREQ Procedure

Number of levels of variables

Variables	Label	Level Number
GROUP1		2
DBTD2	α-Glucosidase Inhibitor	2

Table : GROUP1 * DBTD2

GROUP1

DBTD2(α-Glucosidase Inhibitor)

Power<br>Percent<br>Percent of rows<br>Percent of columns	None	With	Total
1	11592<br>37.34<br>96.75<br>38.56	389<br>1.25<br>3.25<br>39.53	11981<br>38.59
2	18469<br>59.49<br>96.88<br>61.44	595<br>1.92<br>3.12<br>60.47	19064<br>61.41
Total	30061<br>96.83	984<br>3.17	31045<br>100.00

Concomitant medications other than anticoagulants at the time of obtaining Table 2-5 consent, according to the presence or absence of proton-pump inhibitors

FREQ Procedure

Number of levels of variables

Variables Label Level Number

GROUP1 2
DBTD3 Thiazolidine 2

Table : GROUP1 * DBTD3

GROUP1 DBTD3(Thiazolidine)

Power<br>Percent<br>Percent of rows<br>Percent of columns	None	With	Total
1	11856<br>38.19<br>98.96<br>38.65	125<br>0.40<br>1.04<br>33.69	11981<br>38.59
2	18818<br>60.62<br>98.71<br>61.35	246<br>0.79<br>1.29<br>66.31	19064<br>61.41
Total	30674<br>98.80	371<br>1.20	31045<br>100.00

Concomitant medications other than anticoagulants at the time of obtaining Table 2-5 consent, according to the presence or absence of proton-pump inhibitors

FREQ Procedure

Number of levels of variables

Variables Label Level Number
GROUP1 2
DBTD4 DPP-4 Inhabitors 2

Table : GROUP1 * DBTD4

GROUP1 DBTD4(DPP-4 Inhabitors)

Power | Percent | Percent of rows<br>Percent of columns	None	With	Total
1	10310<br>33.21<br>86.05<br>37.88	1671<br>5.38<br>13.95<br>43.69	11981<br>38.59
2	16910<br>54.47<br>88.70<br>62.12	2154<br>6.94<br>11.30<br>56.31	19064<br>61.41
Total	27220<br>87.68	3825<br>12.32	31045<br>100.00

Concomitant medications other than anticoagulants at the time of obtaining Table 2-5 consent, according to the presence or absence of proton-pump inhibitors

FREQ Procedure

Number of levels of variables

Variables Label Level Number

GROUP1 2
DBTD5 GLP-1 Inhabitors 2

Table : GROUP1 * DBTD5

GROUP1 DBTD5(GLP-1 Inhabitors)

Power / Percent / Percent of rows / Percent of columns	None	With	Total
1	11943	38	11981
	38.47	0.12	38.59
	99.68	0.32	
	38.57	49.35	
2	19025	39	19064
	61.28	0.13	61.41
	99.80	0.20	
	61.43	50.65	
Total	30968	77	31045
	99.75	0.25	100.00

Concomitant medications other than anticoagulants at the time of obtaining Table 2-5 consent, according to the presence or absence of proton-pump inhibitors

FREQ Procedure

Number of levels of variables

Variables Label Level Number

GROUP1 2
DBTD6 SGLT2 Inhabitors 2

Table : GROUP1 * DBTD6

GROUP1 DBTD6(SGLT2 Inhabitors)

Power |
Percent |
Percent of rows
Percent of columns | None | With | Total

GROUP1	None	With	Total
1	11799	182	11981
	38.01	0.59	38.59
	98.48	1.52	
	38.51	44.72	
2	18839	225	19064
	60.68	0.72	61.41
	98.82	1.18	
	61.49	55.28	
Total	30638	407	31045
	98.69	1.31	100.00

Concomitant medications other than anticoagulants at the time of obtaining Table 2-5 consent, according to the presence or absence of proton-pump inhibitors

FREQ Procedure

Number of levels of variables

Variables	Label	Level Number
GROUP1		2
DBTD7	Insuline Related	2

Table : GROUP1 * DBTD7

GROUP1 DBTD7(Insuline Related)

Power<br>Percent<br>Percent of rows<br>Percent of columns	None	With	Total
1	11706<br>37.71<br>97.70<br>38.50	275<br>0.89<br>2.30<br>43.04	11981<br>38.59
2	18700<br>60.24<br>98.09<br>61.50	364<br>1.17<br>1.91<br>56.96	19064<br>61.41
Total	30406<br>97.94	639<br>2.06	31045<br>100.00

Concomitant medications other than anticoagulants at the time of obtaining Table 2-5 consent, according to the presence or absence of proton-pump inhibitors

FREQ Procedure

Number of levels of variables

Variables Label Level Number

GROUP1 2
DBTD8 Other Diabetes Drugs 2

Table : GROUP1 * DBTD8

GROUP1
DBTD8(Other Diabetes Drugs)

Power / Percent / Percent of rows / Percent of columns	None	With	Total
1	11613	368	11981
	37.41	1.19	38.59
	96.93	3.07	
	38.63	37.32	
2	18446	618	19064
	59.42	1.99	61.41
	96.76	3.24	
	61.37	62.68	
Total	30059	986	31045
	96.82	3.18	100.00

Concomitant medications other than anticoagulants at the time of obtaining Table 2-5 consent, according to the presence or absence of proton-pump inhibitors

FREQ Procedure

Number of levels of variables

Variables Label Level Number

GROUP1 2
DMM Dementia Medications 2

Table : GROUP1 * DMM

GROUP1 DMM(Dementia Medications)

Power / Percent / Percent of rows / Percent of columns	None	With	Total
1	11538	443	11981
	37.17	1.43	38.59
	96.30	3.70	
	38.66	36.82	
2	18304	760	19064
	58.96	2.45	61.41
	96.01	3.99	
	61.34	63.18	
Total	29842	1203	31045
	96.12	3.88	100.00

Concomitant medications other than anticoagulants at the time of obtaining Table 2-5 consent, according to the presence or absence of proton-pump inhibitors

FREQ Procedure

Number of levels of variables

Variables Label Level Number

GROUP1 2
ACNA Anti-Cancer Agent 2

Table : GROUP1 * ACNA

GROUP1 ACNA(Anti-Cancer Agent)

Power |
Percent |
Percent of rows
Percent of columns | None | With | Total

GROUP1	None	With	Total
1	11871	110	11981
	38.24	0.35	38.59
	99.08	0.92	
	38.60	38.06	
2	18885	179	19064
	60.83	0.58	61.41
	99.06	0.94	
	61.40	61.94	
Total	30756	289	31045
	99.07	0.93	100.00

Concomitant medications other than anticoagulants at the time of obtaining Table 2-5 consent, according to the presence or absence of proton-pump inhibitors

FREQ Procedure

Number of levels of variables

Variables	Label	Level Number
GROUP1		2
COPD	COPD	2

Table : GROUP1 * COPD

GROUP1 COPD(COPD)

Power<br>Percent<br>Percent of rows<br>Percent of columns	None	With	Total
1	11601	380	11981
	37.37	1.22	38.59
	96.83	3.17	
	38.41	45.29	
2	18605	459	19064
	59.93	1.48	61.41
	97.59	2.41	
	61.59	54.71	
Total	30206	839	31045
	97.30	2.70	100.00

Concomitant medications other than anticoagulants at the time of obtaining Table 2-5 consent, according to the presence or absence of proton-pump inhibitors

FREQ Procedure

Number of levels of variables

Variables Label Level Number

GROUP1 2
PSYD Psychotropic Drugs 2

Table : GROUP1 * PSYD

GROUP1 PSYD(Psychotropic Drugs)

Power<br>Percent<br>Percent of rows<br>Percent of columns	None	With	Total
1	10834<br>34.90<br>90.43<br>37.98	1147<br>3.69<br>9.57<br>45.46	11981<br>38.59
2	17688<br>56.98<br>92.78<br>62.02	1376<br>4.43<br>7.22<br>54.54	19064<br>61.41
Total	28522<br>91.87	2523<br>8.13	31045<br>100.00

Concomitant medications other than anticoagulants at the time of obtaining Table 2-5 consent, according to the presence or absence of proton-pump inhibitors

FREQ Procedure

Number of levels of variables

Variables	Label	Level Number
GROUP1		2
PRPI	Proton Pump Inhabitors	2

Table : GROUP1 * PRPI

GROUP1

PRPI(Proton Pump Inhabitors)

Power<br>Percent<br>Percent of rows<br>Percent of columns	None	With	Total
1	0<br>0.00<br>0.00<br>0.00	11981<br>38.59<br>100.00<br>100.00	11981<br>38.59
2	19064<br>61.41<br>100.00<br>100.00	0<br>0.00<br>0.00<br>0.00	19064<br>61.41
Total	19064<br>61.41	11981<br>38.59	31045<br>100.00

Concomitant medications other than anticoagulants at the time of obtaining Table 2-5 consent, according to the presence or absence of proton-pump inhibitors

FREQ Procedure

Number of levels of variables

Variable	Label	Level Count
GROUP1		2
PGPI	P-gp Inhabitors	2

Table : GROUP1 * PGPI

GROUP1 PGPI(P-gp Inhabitors)

Power / Percent / Percent of rows / Percent of columns	None	With	Total
1	11741	240	11981
	37.82	0.77	38.59
	98.00	2.00	
	38.49	44.28	
2	18762	302	19064
	60.43	0.97	61.41
	98.42	1.58	
	61.51	55.72	
Total	30503	542	31045
	98.25	1.75	100.00

Concomitant medications other than anticoagulants at the time of obtaining Table 2-5 consent, according to the presence or absence of proton-pump inhibitors

FREQ Procedure

Number of levels of variables

Number of Variables Level

____

GROUP1 2
f_cmcnt1 21

Table : GROUP1 * f_cmcnt1

GROUP1 f_cmcnt1

Power |
Percent |
Percent of rows
Percent|0|1|2|3|4|5|6|7|8|9|10| Total
______________+________+________+________+________+________+________+________
_+________+________+________+________+
1 | 0 | 28 | 165 | 420 | 794 | 1257 | 1528 | 1604 | 1495 |
1265 | 996 | 11795
| 0.00 | 0.09 | 0.52 | 1.32 | 2.49 | 3.95 | 4.80 | 5.04 | 4.70 |
3.97 | 3.13 | 37.06
| 0.00 | 0.24 | 1.40 | 3.56 | 6.73 | 10.66 | 12.95 | 13.60 | 12.67 |
10.72 | 8.44 |
| 0.00 | 4.07 | 10.30 | 16.02 | 22.07 | 30.62 | 36.63 | 42.81 | 46.91 |
50.28 | 54.58 |
______________+________+________+________+________+________+________+________
_+________+________+________+________+
2 | 52 | 660 | 1437 | 2202 | 2803 | 2848 | 2643 | 2143 | 1692 |
1251 | 829 | 20036
| 0.16 | 2.07 | 4.51 | 6.92 | 8.81 | 8.95 | 8.30 | 6.73 | 5.32 |

3.93 | 2.60 | 62.94

| 0.26 | 3.29 | 7.17 | 10.99 | 13.99 | 14.21 | 13.19 | 10.70 | 8.44 |

6.24 | 4.14 |

| 100.00 | 95.93 | 89.70 | 83.98 | 77.93 | 69.38 | 63.37 | 57.19 | 53.09 |

49.72 | 45.42 |

_______________+_________+_________+_________+_________+_________+_________+________

_+_________+_________+_________+_________+

Total 52 688 1602 2622 3597 4105 4171 3747 3187 2516 1825 31831

0.16 2.16 5.03 8.24 11.30 12.90 13.10 11.77

10.01 7.90 5.73 100.00

(continued)

Concomitant medications other than anticoagulants at the time of obtaining Table 2-5 consent, according to the presence or absence of proton-pump inhibitors

FREQ Procedure

Table : GROUP1 * f_cmcnt1

GROUP1 f_cmcnt1

Power |
Percent |
Percent of rows
Percent

GROUP1	11	12	13	14	15	16	17	18	19	20	Total
1	777	516	373	248	128	77	54	29	20	21	11795
	2.44	1.62	1.17	0.78	0.40	0.24	0.17	0.09	0.06	0.07	37.06
	6.59	4.37	3.16	2.10	1.09	0.65	0.46	0.25	0.17	0.18	
	57.13	58.57	62.37	67.76	62.14	63.64	68.35	61.70	83.33	56.76	
2	583	365	225	118	78	44	25	18	4	16	20036
	1.83	1.15	0.71	0.37	0.25	0.14	0.08	0.06	0.01	0.05	62.94
	2.91	1.82	1.12	0.59	0.39	0.22	0.12	0.09	0.02	0.08	
	42.87	41.43	37.63	32.24	37.86	36.36	31.65	38.30	16.67	43.24	
Total	1360	881	598	366	206	121	79	47	24	37	31831

4.27 2.77 1.88 1.15 0.65 0.38 0.25 0.15
0.08 0.12 100.00

Concomitant medications other than anticoagulants at the time of obtaining Table 2-5 consent, according to the presence or absence of proton-pump inhibitors

FREQ Procedure

Number of levels of variables

Variables Label Level Number

GROUP1 2

TCMCNTCT Category for Total Number of Drugs Other than Anticoagulant 3

Table : GROUP1 * TCMCNTCT

GROUP1

TCMCNTCT(Category for Total Number of Drugs Other than Anticoagulant)

Power |
Percent |
Percent of rows
Percentage of column |0 to 4 or more|5 to 9 or more|10 or more|Total
|Bottom|Bottom | |

GROUP1	0 to 4 or more Bottom	5 to 9 or more Bottom	10 or more	Total
1	1407	7149	3239	11795
	4.42	22.46	10.18	37.06
	11.93	60.61	27.46	
	16.43	40.33	58.42	
2	7154	10577	2305	20036
	22.47	33.23	7.24	62.94
	35.71	52.79	11.50	
	83.57	59.67	41.58	
Total	8561	17726	5544	31831

26.90	55.69	17.42	100.00

Concomitant medications other than anticoagulants at the time of obtaining Table 2-5 consent, according to the presence or absence of proton-pump inhibitors

TTEST Procedure

Variables: TCMCNT (Total Number of Drugs Other than Anticoagulant)

GROUP1 N Mean Standard Deviation Standard Error Minimum Value Maximum Value

1	11795	7.9060	3.0903	0.0285	1.0000	26.0000
2	20036	5.8874	2.9636	0.0209	0	27.0000

Diff (1-2) 2.0186 3.0112 0.0349

To the mean standard deviation

GROUP1 methods Mean 95% confidence limits standard deviation 95% confidence limits

1	7.9060	7.8502	7.9618	3.0903	3.0513	3.1302
2	5.8874	5.8463	5.9284	2.9636	2.9349	2.9929

Diff (1-2) Pooled 2.0186 1.9501 2.0871 3.0112 2.9880 3.0348
Diff (1-2) Satterthwaite 2.0186 1.9494 2.0879

Technique Dispersion degrees of freedom t-value Pr > |t|

Pooled Equal 31829 57.76 <. 0001
Satterthwaite Unequal 23898 57.14 <. 0001

Isodispersity

Technique Degree of Freedom of Moleculars Degree of Freedom of the denominator F Value Pr > F

Folded F 11794 20035 1.09 <. 0001

Laboratory tests at the time of obtaining Table 2-6 consent, according to the presence or absence of proton-pump inhibitors

FREQ Procedure

Percent of ALL power		Percent of power	Cumulative	
1	32490	100.00	32490	100.00

Laboratory tests at the time of obtaining Table 2-6 consent, according to the presence or absence of proton-pump inhibitors

FREQ Procedure

Tables: ALL* ACTYP

ALL
ACTYP(Type of AF)

Power |
Percent |
Percent of rows
Percent of column|Warfa| Total
|Phosphate|
| |
______________+________+
1 | 8295 | 8295
| 100.00 | 100.00
| 100.00 |
| 100.00 |
______________+________+
Total 8295 8295
100.00 100.00

Laboratory tests at the time of obtaining Table 2-6 consent, according to the presence or absence of proton-pump inhibitors

Unique Subject Identifier=0001110010-0003

MEANS Procedure

Assay variable : PTINR_VAL1 PT-INR Value

N	Mean	Standard Deviation	Minimum	Maximum
3	2.0366667	0.2542309	1.7600000	2.2600000

Unique Subject Identifier=0001110010-0004

Assay variable : PTINR_VAL1 PT-INR Value

N	Mean	Standard Deviation	Minimum	Maximum
4	2.7500000	0.3389199	2.3000000	3.0700000

Unique Subject Identifier=0001110010-0005

Assay variable : PTINR_VAL1 PT-INR Value

N	Mean	Standard Deviation	Minimum	Maximum
5	2.1760000	0.2298478	2.0100000	2.5600000

Unique Subject Identifier=0001110010-0015

Assay variable : PTINR_VAL1 PT-INR Value

N	Mean	Standard Deviation	Minimum	Maximum
3	2.0866667	0.1006645	1.9800000	2.1800000

Laboratory tests at the time of obtaining Table 2-6 consent, according to the presence or absence of proton-pump inhibitors

Unique Subject Identifier=0001110010-0018

MEANS Procedure

Assay variable : PTINR_VAL1 PT-INR Value

N	Mean	Standard Deviation	Minimum	Maximum
4	2.7475000	0.4163632	2.4500000	3.3500000

Unique Subject Identifier=0001110010-0027

Assay variable : PTINR_VAL1 PT-INR Value

N	Mean	Standard Deviation	Minimum	Maximum
4	2.2725000	0.0665207	2.1900000	2.3400000

Unique Subject Identifier=0001110010-0032

Assay variable : PTINR_VAL1 PT-INR Value

N	Mean	Standard Deviation	Minimum	Maximum
4	2.3300000	0.4302712	1.8600000	2.7700000

Unique Subject Identifier=0004110010-0007

Assay variable : PTINR_VAL1 PT-INR Value

N	Mean	Standard Deviation	Minimum	Maximum
5	1.7880000	0.1658915	1.5600000	1.9300000

Laboratory tests at the time of obtaining Table 2-6 consent, according to the presence or absence of proton-pump inhibitors

Unique Subject Identifier=0004110010-0008

MEANS Procedure

Assay variable : PTINR_VAL1 PT-INR Value

N	Mean	Standard Deviation	Minimum	Maximum
2	1.6800000	0.3535534	1.4300000	1.9300000

Unique Subject Identifier=0004110010-0009

Assay variable : PTINR_VAL1 PT-INR Value

N	Mean	Standard Deviation	Minimum	Maximum
6	2.1000000	0.4030385	1.7400000	2.8000000

Unique Subject Identifier=0004110010-0010

Assay variable : PTINR_VAL1 PT-INR Value

N	Mean	Standard Deviation	Minimum	Maximum
6	1.8166667	0.2108712	1.5500000	2.0100000

Unique Subject Identifier=0004110010-0011

Assay variable : PTINR_VAL1 PT-INR Value

N	Mean	Standard Deviation	Minimum	Maximum
4	2.0425000	0.1662077	1.8600000	2.2200000

Laboratory tests at the time of obtaining Table 2-6 consent, according to the presence or absence of proton-pump inhibitors

Unique Subject Identifier=0004110010-0017

MEANS Procedure

Assay variable : PTINR_VAL1 PT-INR Value

N	Mean	Standard Deviation	Minimum	Maximum
5	1.9240000	0.8984320	1.3900000	3.5100000

Unique Subject Identifier=0004110010-0021

Assay variable : PTINR_VAL1 PT-INR Value

N	Mean	Standard Deviation	Minimum	Maximum
3	1.8900000	0.2233831	1.7100000	2.1400000

Unique Subject Identifier=0004110010-0022

Assay variable : PTINR_VAL1 PT-INR Value

N	Mean	Standard Deviation	Minimum	Maximum
4	1.6350000	0.3158586	1.2900000	2.0100000

Unique Subject Identifier=0004110010-0026

Assay variable : PTINR_VAL1 PT-INR Value

N	Mean	Standard Deviation	Minimum	Maximum
6	1.8650000	0.2438647	1.4900000	2.1800000

Laboratory tests at the time of obtaining Table 2-6 consent, according to the presence or absence of proton-pump inhibitors

Unique Subject Identifier=0004110010-0029

MEANS Procedure

Assay variable : PTINR_VAL1 PT-INR Value

N	Mean	Standard Deviation	Minimum	Maximum
4	2.0075000	0.2956772	1.5900000	2.2800000

Unique Subject Identifier=0004110010-0031

Assay variable : PTINR_VAL1 PT-INR Value

N	Mean	Standard Deviation	Minimum	Maximum
4	2.1100000	0.1602082	1.9300000	2.3000000

Unique Subject Identifier=0004110010-0032

Assay variable : PTINR_VAL1 PT-INR Value

N	Mean	Standard Deviation	Minimum	Maximum
3	2.2100000	0.1900000	2.0200000	2.4000000

Unique Subject Identifier=0004110010-0033

Assay variable : PTINR_VAL1 PT-INR Value

N	Mean	Standard Deviation	Minimum	Maximum
4	2.1400000	0.1019804	2.0000000	2.2400000

Laboratory tests at the time of obtaining Table 2-6 consent, according to the presence or absence of proton-pump inhibitors

Unique Subject Identifier=0004110010-0034

MEANS Procedure

Assay variable : PTINR_VAL1 PT-INR Value

N	Mean	Standard Deviation	Minimum	Maximum
4	1.9650000	0.1391642	1.8000000	2.1400000

Unique Subject Identifier=0004110010-0037

Assay variable : PTINR_VAL1 PT-INR Value

N	Mean	Standard Deviation	Minimum	Maximum
3	2.4966667	0.5108163	2.0900000	3.0700000

Unique Subject Identifier=0004110010-0040

Assay variable : PTINR_VAL1 PT-INR Value

N	Mean	Standard Deviation	Minimum	Maximum
4	2.3200000	0.2684524	1.9700000	2.6200000

Unique Subject Identifier=0004110010-0042

Assay variable : PTINR_VAL1 PT-INR Value

N	Mean	Standard Deviation	Minimum	Maximum
4	1.7100000	0.1927001	1.4800000	1.9100000

Laboratory tests at the time of obtaining Table 2-6 consent, according to the presence or absence of proton-pump inhibitors

Unique Subject Identifier=0004110010-0043

MEANS Procedure

Assay variable : PTINR_VAL1 PT-INR Value

N	Mean	Standard Deviation	Minimum	Maximum
3	2.5533333	0.0776745	2.4900000	2.6400000

Unique Subject Identifier=0004110010-0044

Assay variable : PTINR_VAL1 PT-INR Value

N	Mean	Standard Deviation	Minimum	Maximum
3	2.5333333	0.1386843	2.3800000	2.6500000

Unique Subject Identifier=0004110010-0046

Assay variable : PTINR_VAL1 PT-INR Value

N	Mean	Standard Deviation	Minimum	Maximum
3	2.4433333	0.5312564	1.8300000	2.7600000

Unique Subject Identifier=0004110010-0047

Assay variable : PTINR_VAL1 PT-INR Value

N	Mean	Standard Deviation	Minimum	Maximum
3	1.8500000	0.3874274	1.4900000	2.2600000

Laboratory tests at the time of obtaining Table 2-6 consent, according to the presence or absence of proton-pump inhibitors

Unique Subject Identifier=0004110010-0048

MEANS Procedure

Assay variable : PTINR_VAL1 PT-INR Value

N	Mean	Standard Deviation	Minimum	Maximum
4	1.7050000	0.2266422	1.5400000	2.0400000

Unique Subject Identifier=0004110010-0049

Assay variable : PTINR_VAL1 PT-INR Value

N	Mean	Standard Deviation	Minimum	Maximum
4	2.3000000	0.2121320	2.0500000	2.5000000

Unique Subject Identifier=0004110010-0051

Assay variable : PTINR_VAL1 PT-INR Value

N	Mean	Standard Deviation	Minimum	Maximum
6	2.4366667	0.3922074	1.8300000	2.9100000

Unique Subject Identifier=0004110010-0052

Assay variable : PTINR_VAL1 PT-INR Value

N	Mean	Standard Deviation	Minimum	Maximum
6	2.1983333	0.2008399	1.9600000	2.4600000

Laboratory tests at the time of obtaining Table 2-6 consent, according to the presence or absence of proton-pump inhibitors

Unique Subject Identifier=0004110010-0053

MEANS Procedure

Assay variable : PTINR_VAL1 PT-INR Value

N	Mean	Standard Deviation	Minimum	Maximum
6	2.4883333	0.3756816	2.2400000	3.1700000

Unique Subject Identifier=0005010000-0003

Assay variable : PTINR_VAL1 PT-INR Value

N	Mean	Standard Deviation	Minimum	Maximum
6	1.9066667	0.4583739	1.3500000	2.6800000

Unique Subject Identifier=0005010000-0007

Assay variable : PTINR_VAL1 PT-INR Value

N	Mean	Standard Deviation	Minimum	Maximum
3	1.9800000	0.4900000	1.4900000	2.4700000

Unique Subject Identifier=0005010000-0012

Assay variable : PTINR_VAL1 PT-INR Value

N	Mean	Standard Deviation	Minimum	Maximum
6	2.6266667	0.3273327	2.1800000	3.1700000

Laboratory tests at the time of obtaining Table 2-6 consent, according to the presence or absence of proton-pump inhibitors

Unique Subject Identifier=0005010000-0020

MEANS Procedure

Assay variable : PTINR_VAL1 PT-INR Value

N	Mean	Standard Deviation	Minimum	Maximum
3	1.8400000	0.1216553	1.7000000	1.9200000

Unique Subject Identifier=0005010000-0022

Assay variable : PTINR_VAL1 PT-INR Value

N	Mean	Standard Deviation	Minimum	Maximum
6	2.2233333	0.7559012	1.6600000	3.6100000

Unique Subject Identifier=0005010000-0028

Assay variable : PTINR_VAL1 PT-INR Value

N	Mean	Standard Deviation	Minimum	Maximum
3	1.9700000	0.6065476	1.6000000	2.6700000

Unique Subject Identifier=0005010000-0034

Assay variable : PTINR_VAL1 PT-INR Value

N	Mean	Standard Deviation	Minimum	Maximum
6	1.2433333	0.0997330	1.1400000	1.4000000

Laboratory tests at the time of obtaining Table 2-6 consent, according to the presence or absence of proton-pump inhibitors

Unique Subject Identifier=0005010000-0036

MEANS Procedure

Assay variable : PTINR_VAL1 PT-INR Value

N	Mean	Standard Deviation	Minimum	Maximum
3	2.1866667	0.1721434	1.9900000	2.3100000

Unique Subject Identifier=0005010000-0045

Assay variable : PTINR_VAL1 PT-INR Value

N	Mean	Standard Deviation	Minimum	Maximum
4	1.8525000	0.2305609	1.5800000	2.1400000

Unique Subject Identifier=0005010000-0050

Assay variable : PTINR_VAL1 PT-INR Value

N	Mean	Standard Deviation	Minimum	Maximum
6	1.7866667	0.2182353	1.5600000	2.1200000

Unique Subject Identifier=0005010000-0052

Assay variable : PTINR_VAL1 PT-INR Value

N	Mean	Standard Deviation	Minimum	Maximum
2	1.0900000	0.0707107	1.0400000	1.1400000

Laboratory tests at the time of obtaining Table 2-6 consent, according to the presence or absence of proton-pump inhibitors

Unique Subject Identifier=0005010000-0053

MEANS Procedure

Assay variable : PTINR_VAL1 PT-INR Value

N	Mean	Standard Deviation	Minimum	Maximum
6	1.9983333	0.3748822	1.4700000	2.5400000

Unique Subject Identifier=0005010000-0058

Assay variable : PTINR_VAL1 PT-INR Value

N	Mean	Standard Deviation	Minimum	Maximum
2	1.6950000	0.1484924	1.5900000	1.8000000

Unique Subject Identifier=0006100000-0006

Assay variable : PTINR_VAL1 PT-INR Value

N	Mean	Standard Deviation	Minimum	Maximum
3	2.3633333	0.2098412	2.2000000	2.6000000

Unique Subject Identifier=0006100000-0012

Assay variable : PTINR_VAL1 PT-INR Value

N	Mean	Standard Deviation	Minimum	Maximum
2	1.9450000	0.3181981	1.7200000	2.1700000

Laboratory tests at the time of obtaining Table 2-6 consent, according to the presence or absence of proton-pump inhibitors

Unique Subject Identifier=0006100000-0014

MEANS Procedure

Assay variable : PTINR_VAL1 PT-INR Value

N	Mean	Standard Deviation	Minimum	Maximum
4	2.1925000	0.0713559	2.1300000	2.2900000

Unique Subject Identifier=0006100000-0016

Assay variable : PTINR_VAL1 PT-INR Value

N	Mean	Standard Deviation	Minimum	Maximum
3	1.9400000	1.0544667	1.0900000	3.1200000

Unique Subject Identifier=0006100000-0021

Assay variable : PTINR_VAL1 PT-INR Value

N	Mean	Standard Deviation	Minimum	Maximum
1	1.5100000		1.5100000	1.5100000

Unique Subject Identifier=0006100000-0032

Assay variable : PTINR_VAL1 PT-INR Value

N	Mean	Standard Deviation	Minimum	Maximum
6	2.3083333	0.3635611	1.8600000	2.7700000

Laboratory tests at the time of obtaining Table 2-6 consent, according to the presence or absence of proton-pump inhibitors

Unique Subject Identifier=0006100000-0034

MEANS Procedure

Assay variable : PTINR_VAL1 PT-INR Value

N	Mean	Standard Deviation	Minimum	Maximum
1	2.8900000		2.8900000	2.8900000

Unique Subject Identifier=0006100000-0036

Assay variable : PTINR_VAL1 PT-INR Value

N	Mean	Standard Deviation	Minimum	Maximum
3	1.7233333	0.6992377	1.2900000	2.5300000

Unique Subject Identifier=0006100000-0050

Assay variable : PTINR_VAL1 PT-INR Value

N	Mean	Standard Deviation	Minimum	Maximum
4	2.6000000	0.2682039	2.3800000	2.9500000

Unique Subject Identifier=0006100000-0054

Assay variable : PTINR_VAL1 PT-INR Value

N	Mean	Standard Deviation	Minimum	Maximum
5	1.7740000	0.1986957	1.5800000	2.0200000

Laboratory tests at the time of obtaining Table 2-6 consent, according to the presence or absence of proton-pump inhibitors

Unique Subject Identifier=0006100000-0055

MEANS Procedure

Assay variable : PTINR_VAL1 PT-INR Value

N	Mean	Standard Deviation	Minimum	Maximum
4	1.8725000	0.1350000	1.6800000	1.9800000

Unique Subject Identifier=0006100000-0056

Assay variable : PTINR_VAL1 PT-INR Value

N	Mean	Standard Deviation	Minimum	Maximum
3	1.7166667	0.0986577	1.6500000	1.8300000

Unique Subject Identifier=0006100000-0057

Assay variable : PTINR_VAL1 PT-INR Value

N	Mean	Standard Deviation	Minimum	Maximum
3	2.4100000	0.2206808	2.2000000	2.6400000

Unique Subject Identifier=0006100000-0062

Assay variable : PTINR_VAL1 PT-INR Value

N	Mean	Standard Deviation	Minimum	Maximum
6	1.9066667	0.3507516	1.7100000	2.6200000

Laboratory tests at the time of obtaining Table 2-6 consent, according to the presence or absence of proton-pump inhibitors

Unique Subject Identifier=0006100000-0064

MEANS Procedure

Assay variable : PTINR_VAL1 PT-INR Value

N	Mean	Standard Deviation	Minimum	Maximum
4	1.7800000	0.1888562	1.5600000	1.9700000

Unique Subject Identifier=0006100000-0065

Assay variable : PTINR_VAL1 PT-INR Value

N	Mean	Standard Deviation	Minimum	Maximum
6	1.8983333	1.0817470	1.2700000	4.0900000

Unique Subject Identifier=0006100000-0068

Assay variable : PTINR_VAL1 PT-INR Value

N	Mean	Standard Deviation	Minimum	Maximum
1	1.7700000		1.7700000	1.7700000

Unique Subject Identifier=0006100000-0069

Assay variable : PTINR_VAL1 PT-INR Value

N	Mean	Standard Deviation	Minimum	Maximum
4	2.0075000	0.4908751	1.7100000	2.7400000

Laboratory tests at the time of obtaining Table 2-6 consent, according to the presence or absence of proton-pump inhibitors

Unique Subject Identifier=0007011000-0001

MEANS Procedure

Assay variable : PTINR_VAL1 PT-INR Value

N	Mean	Standard Deviation	Minimum	Maximum
3	1.8966667	0.2950141	1.6000000	2.1900000

Unique Subject Identifier=0007011000-0002

Assay variable : PTINR_VAL1 PT-INR Value

N	Mean	Standard Deviation	Minimum	Maximum
3	1.9133333	0.8891194	1.4000000	2.9400000

Unique Subject Identifier=0007011000-0004

Assay variable : PTINR_VAL1 PT-INR Value

N	Mean	Standard Deviation	Minimum	Maximum
4	1.8250000	0.2629956	1.6000000	2.1000000

Unique Subject Identifier=0007011000-0005

Assay variable : PTINR_VAL1 PT-INR Value

N	Mean	Standard Deviation	Minimum	Maximum
5	2.0900000	0.3537655	1.7300000	2.4700000

Laboratory tests at the time of obtaining Table 2-6 consent, according to the presence or absence of proton-pump inhibitors

Unique Subject Identifier=0007011000-0011

MEANS Procedure

Assay variable : PTINR_VAL1 PT-INR Value

N	Mean	Standard Deviation	Minimum	Maximum
3	2.5433333	0.1193035	2.4600000	2.6800000

Unique Subject Identifier=0007011000-0012

Assay variable : PTINR_VAL1 PT-INR Value

N	Mean	Standard Deviation	Minimum	Maximum
5	1.7060000	0.0861974	1.6100000	1.8200000

Unique Subject Identifier=0007011000-0014

Assay variable : PTINR_VAL1 PT-INR Value

N	Mean	Standard Deviation	Minimum	Maximum
3	1.8666667	0.0404145	1.8200000	1.8900000

Unique Subject Identifier=0007011000-0022

Assay variable : PTINR_VAL1 PT-INR Value

N	Mean	Standard Deviation	Minimum	Maximum
6	2.0483333	0.5314665	1.3000000	2.8300000

Laboratory tests at the time of obtaining Table 2-6 consent, according to the presence or absence of proton-pump inhibitors

Unique Subject Identifier=0007011000-0028

MEANS Procedure

Assay variable : PTINR_VAL1 PT-INR Value

N	Mean	Standard Deviation	Minimum	Maximum
3	2.0833333	0.5838093	1.4500000	2.6000000

Unique Subject Identifier=0007011000-0031

Assay variable : PTINR_VAL1 PT-INR Value

N	Mean	Standard Deviation	Minimum	Maximum
3	1.9800000	0.4073082	1.5500000	2.3600000

Unique Subject Identifier=0007011000-0034

Assay variable : PTINR_VAL1 PT-INR Value

N	Mean	Standard Deviation	Minimum	Maximum
4	1.7350000	0.2042058	1.5700000	2.0100000

Unique Subject Identifier=0007011000-0037

Assay variable : PTINR_VAL1 PT-INR Value

N	Mean	Standard Deviation	Minimum	Maximum
3	2.2366667	0.6185736	1.5300000	2.6800000

Laboratory tests at the time of obtaining Table 2-6 consent, according to the presence or absence of proton-pump inhibitors

Unique Subject Identifier=0007011000-0042

MEANS Procedure

Assay variable : PTINR_VAL1 PT-INR Value

N	Mean	Standard Deviation	Minimum	Maximum
1	1.7900000		1.7900000	1.7900000

Unique Subject Identifier=0007011000-0043

Assay variable : PTINR_VAL1 PT-INR Value

N	Mean	Standard Deviation	Minimum	Maximum
3	1.6100000	0.1014889	1.5000000	1.7000000

Unique Subject Identifier=0007011000-0048

Assay variable : PTINR_VAL1 PT-INR Value

N	Mean	Standard Deviation	Minimum	Maximum
3	1.7733333	0.0945163	1.7000000	1.8800000

Unique Subject Identifier=0007011000-0053

Assay variable : PTINR_VAL1 PT-INR Value

N	Mean	Standard Deviation	Minimum	Maximum
3	1.8866667	0.0230940	1.8600000	1.9000000

Laboratory tests at the time of obtaining Table 2-6 consent, according to the presence or absence of proton-pump inhibitors

Unique Subject Identifier=0007011000-0057

MEANS Procedure

Assay variable : PTINR_VAL1 PT-INR Value

N	Mean	Standard Deviation	Minimum	Maximum
4	2.0075000	0.1024288	1.9400000	2.1600000

Unique Subject Identifier=0007011000-0058

Assay variable : PTINR_VAL1 PT-INR Value

N	Mean	Standard Deviation	Minimum	Maximum
4	2.1775000	0.2439091	1.8400000	2.4000000

Unique Subject Identifier=0007011000-0067

Assay variable : PTINR_VAL1 PT-INR Value

N	Mean	Standard Deviation	Minimum	Maximum
1	1.2900000		1.2900000	1.2900000

Unique Subject Identifier=0007011000-0070

Assay variable : PTINR_VAL1 PT-INR Value

N	Mean	Standard Deviation	Minimum	Maximum
2	3.1950000	1.1101576	2.4100000	3.9800000

Laboratory tests at the time of obtaining Table 2-6 consent, according to the presence or absence of proton-pump inhibitors

Unique Subject Identifier=0007011000-0072

MEANS Procedure

Assay variable : PTINR_VAL1 PT-INR Value

N	Mean	Standard Deviation	Minimum	Maximum
2	2.2050000	0.4454773	1.8900000	2.5200000

Unique Subject Identifier=0007011000-0075

Assay variable : PTINR_VAL1 PT-INR Value

N	Mean	Standard Deviation	Minimum	Maximum
6	1.9633333	0.6006219	1.4100000	2.8300000

Unique Subject Identifier=0007011000-0078

Assay variable : PTINR_VAL1 PT-INR Value

N	Mean	Standard Deviation	Minimum	Maximum
4	1.7925000	0.1004573	1.7000000	1.9300000

Unique Subject Identifier=0007011000-0079

Assay variable : PTINR_VAL1 PT-INR Value

N	Mean	Standard Deviation	Minimum	Maximum
2	2.2000000	0.4242641	1.9000000	2.5000000

Laboratory tests at the time of obtaining Table 2-6 consent, according to the presence or absence of proton-pump inhibitors

Unique Subject Identifier=0007011000-0094

MEANS Procedure

Assay variable : PTINR_VAL1 PT-INR Value

N	Mean	Standard Deviation	Minimum	Maximum
4	1.6375000	0.4458980	1.0400000	2.1200000

Unique Subject Identifier=0007011000-0095

Assay variable : PTINR_VAL1 PT-INR Value

N	Mean	Standard Deviation	Minimum	Maximum
5	1.1160000	0.0545894	1.0500000	1.1800000

Unique Subject Identifier=0007011000-0097

Assay variable : PTINR_VAL1 PT-INR Value

N	Mean	Standard Deviation	Minimum	Maximum
6	1.6150000	0.1943965	1.3700000	1.9600000

Unique Subject Identifier=0007011000-0100

Assay variable : PTINR_VAL1 PT-INR Value

N	Mean	Standard Deviation	Minimum	Maximum
4	2.0300000	0.6737457	1.3000000	2.7700000

Laboratory tests at the time of obtaining Table 2-6 consent, according to the presence or absence of proton-pump inhibitors

Unique Subject Identifier=0007011000-0102

MEANS Procedure

Assay variable : PTINR_VAL1 PT-INR Value

N	Mean	Standard Deviation	Minimum	Maximum
1	1.6600000		1.6600000	1.6600000

Unique Subject Identifier=0007011000-0112

Assay variable : PTINR_VAL1 PT-INR Value

N	Mean	Standard Deviation	Minimum	Maximum
3	2.1133333	0.5084617	1.8000000	2.7000000

Unique Subject Identifier=0007011000-0116

Assay variable : PTINR_VAL1 PT-INR Value

N	Mean	Standard Deviation	Minimum	Maximum
3	2.0733333	0.5900282	1.4000000	2.5000000

Unique Subject Identifier=0007011000-0118

Assay variable : PTINR_VAL1 PT-INR Value

N	Mean	Standard Deviation	Minimum	Maximum
3	1.5900000	0.3000000	1.2900000	1.8900000

Laboratory tests at the time of obtaining Table 2-6 consent, according to the presence or absence of proton-pump inhibitors

Unique Subject Identifier=0007011000-0120

MEANS Procedure

Assay variable : PTINR_VAL1 PT-INR Value

N	Mean	Standard Deviation	Minimum	Maximum
3	1.9666667	0.1527525	1.8000000	2.1000000

Unique Subject Identifier=0007011000-0121

Assay variable : PTINR_VAL1 PT-INR Value

N	Mean	Standard Deviation	Minimum	Maximum
3	2.1766667	0.5600298	1.5300000	2.5000000

Unique Subject Identifier=0007011000-0122

Assay variable : PTINR_VAL1 PT-INR Value

N	Mean	Standard Deviation	Minimum	Maximum
6	1.7816667	0.3644402	1.2000000	2.2000000

Unique Subject Identifier=0007011000-0130

Assay variable : PTINR_VAL1 PT-INR Value

N	Mean	Standard Deviation	Minimum	Maximum
1	1.7600000		1.7600000	1.7600000

Laboratory tests at the time of obtaining Table 2-6 consent, according to the presence or absence of proton-pump inhibitors

Unique Subject Identifier=0007011000-0135

MEANS Procedure

Assay variable : PTINR_VAL1 PT-INR Value

N	Mean	Standard Deviation	Minimum	Maximum
5	2.1000000	0.4484417	1.5700000	2.8100000

Unique Subject Identifier=0007011000-0137

Assay variable : PTINR_VAL1 PT-INR Value

N	Mean	Standard Deviation	Minimum	Maximum
3	1.3900000	0.0781025	1.3400000	1.4800000

Unique Subject Identifier=0007011000-0139

Assay variable : PTINR_VAL1 PT-INR Value

N	Mean	Standard Deviation	Minimum	Maximum
3	2.9266667	0.2052641	2.7000000	3.1000000

Unique Subject Identifier=0008000110-0001

Assay variable : PTINR_VAL1 PT-INR Value

N	Mean	Standard Deviation	Minimum	Maximum
1	2.2800000		2.2800000	2.2800000

Laboratory tests at the time of obtaining Table 2-6 consent, according to the presence or absence of proton-pump inhibitors

Unique Subject Identifier=0008000110-0007

MEANS Procedure

Assay variable : PTINR_VAL1 PT-INR Value

N	Mean	Standard Deviation	Minimum	Maximum
6	1.9166667	0.3415650	1.3700000	2.3000000

Unique Subject Identifier=0008000110-0016

Assay variable : PTINR_VAL1 PT-INR Value

N	Mean	Standard Deviation	Minimum	Maximum
4	1.8175000	0.2196019	1.5600000	2.0100000

Unique Subject Identifier=0009010000-0002

Assay variable : PTINR_VAL1 PT-INR Value

N	Mean	Standard Deviation	Minimum	Maximum
4	2.0575000	0.1802544	1.8400000	2.2700000

Unique Subject Identifier=0009010000-0004

Assay variable : PTINR_VAL1 PT-INR Value

N	Mean	Standard Deviation	Minimum	Maximum
3	2.0366667	0.1985783	1.8800000	2.2600000

Laboratory tests at the time of obtaining Table 2-6 consent, according to the presence or absence of proton-pump inhibitors

Unique Subject Identifier=0009010000-0006

MEANS Procedure

Assay variable : PTINR_VAL1 PT-INR Value

N	Mean	Standard Deviation	Minimum	Maximum
6	1.6366667	0.0915787	1.5500000	1.7500000

Unique Subject Identifier=0009010000-0008

Assay variable : PTINR_VAL1 PT-INR Value

N	Mean	Standard Deviation	Minimum	Maximum
2	2.4600000	0.1131371	2.3800000	2.5400000

Unique Subject Identifier=0009010000-0010

Assay variable : PTINR_VAL1 PT-INR Value

N	Mean	Standard Deviation	Minimum	Maximum
2	2.1800000	0.0282843	2.1600000	2.2000000

Unique Subject Identifier=0009010000-0013

Assay variable : PTINR_VAL1 PT-INR Value

N	Mean	Standard Deviation	Minimum	Maximum
3	2.4933333	0.3326159	2.2400000	2.8700000

Laboratory tests at the time of obtaining Table 2-6 consent, according to the presence or absence of proton-pump inhibitors

Unique Subject Identifier=0009010000-0018

MEANS Procedure

Assay variable : PTINR_VAL1 PT-INR Value

N	Mean	Standard Deviation	Minimum	Maximum
6	2.0583333	0.2135806	1.7500000	2.3100000

Unique Subject Identifier=0009010000-0019

Assay variable : PTINR_VAL1 PT-INR Value

N	Mean	Standard Deviation	Minimum	Maximum
5	2.4300000	0.5772781	1.9300000	3.4200000

Unique Subject Identifier=0009010000-0021

Assay variable : PTINR_VAL1 PT-INR Value

N	Mean	Standard Deviation	Minimum	Maximum
6	1.8750000	0.5326443	1.1600000	2.7900000

Unique Subject Identifier=0009010000-0024

Assay variable : PTINR_VAL1 PT-INR Value

N	Mean	Standard Deviation	Minimum	Maximum
6	2.0600000	0.1930803	1.7100000	2.2700000

Laboratory tests at the time of obtaining Table 2-6 consent, according to the presence or absence of proton-pump inhibitors

Unique Subject Identifier=0009010000-0029

MEANS Procedure

Assay variable : PTINR_VAL1 PT-INR Value

N	Mean	Standard Deviation	Minimum	Maximum
5	2.8740000	0.5623433	2.3800000	3.5500000

Unique Subject Identifier=0009010000-0032

Assay variable : PTINR_VAL1 PT-INR Value

N	Mean	Standard Deviation	Minimum	Maximum
4	1.7675000	0.1362290	1.6300000	1.9500000

Unique Subject Identifier=0009010000-0033

Assay variable : PTINR_VAL1 PT-INR Value

N	Mean	Standard Deviation	Minimum	Maximum
3	2.3200000	0.0264575	2.2900000	2.3400000

Unique Subject Identifier=0009010000-0036

Assay variable : PTINR_VAL1 PT-INR Value

N	Mean	Standard Deviation	Minimum	Maximum
3	2.3766667	0.4796179	1.9400000	2.8900000

Laboratory tests at the time of obtaining Table 2-6 consent, according to the presence or absence of proton-pump inhibitors

Unique Subject Identifier=0009010000-0037

MEANS Procedure

Assay variable : PTINR_VAL1 PT-INR Value

N	Mean	Standard Deviation	Minimum	Maximum
3	1.6200000	0.1664332	1.4300000	1.7400000

Unique Subject Identifier=0009010000-0039

Assay variable : PTINR_VAL1 PT-INR Value

N	Mean	Standard Deviation	Minimum	Maximum
3	1.7366667	0.1357694	1.5800000	1.8200000

Unique Subject Identifier=0009010000-0041

Assay variable : PTINR_VAL1 PT-INR Value

N	Mean	Standard Deviation	Minimum	Maximum
3	1.5933333	0.0723418	1.5100000	1.6400000

Unique Subject Identifier=0009010000-0043

Assay variable : PTINR_VAL1 PT-INR Value

N	Mean	Standard Deviation	Minimum	Maximum
2	2.5200000	0.4525483	2.2000000	2.8400000

Laboratory tests at the time of obtaining Table 2-6 consent, according to the presence or absence of proton-pump inhibitors

Unique Subject Identifier=0009010000-0044

MEANS Procedure

Assay variable : PTINR_VAL1 PT-INR Value

N	Mean	Standard Deviation	Minimum	Maximum
2	2.7550000	0.4737615	2.4200000	3.0900000

Unique Subject Identifier=0009010000-0046

Assay variable : PTINR_VAL1 PT-INR Value

N	Mean	Standard Deviation	Minimum	Maximum
3	2.1500000	0.4313931	1.7000000	2.5600000

Unique Subject Identifier=0009010000-0047

Assay variable : PTINR_VAL1 PT-INR Value

N	Mean	Standard Deviation	Minimum	Maximum
3	2.0633333	0.7159143	1.6500000	2.8900000

Unique Subject Identifier=0009010000-0048

Assay variable : PTINR_VAL1 PT-INR Value

N	Mean	Standard Deviation	Minimum	Maximum
3	1.9066667	0.3288363	1.6600000	2.2800000

Laboratory tests at the time of obtaining Table 2-6 consent, according to the presence or absence of proton-pump inhibitors

Unique Subject Identifier=0009010000-0050

MEANS Procedure

Assay variable : PTINR_VAL1 PT-INR Value

N	Mean	Standard Deviation	Minimum	Maximum
3	2.1800000	0.1300000	2.1000000	2.3300000

Unique Subject Identifier=0009010000-0061

Assay variable : PTINR_VAL1 PT-INR Value

N	Mean	Standard Deviation	Minimum	Maximum
2	2.2950000	0.5161880	1.9300000	2.6600000

Unique Subject Identifier=0009010000-0062

Assay variable : PTINR_VAL1 PT-INR Value

N	Mean	Standard Deviation	Minimum	Maximum
3	2.0800000	0.2690725	1.7800000	2.3000000

Unique Subject Identifier=0009010000-0072

Assay variable : PTINR_VAL1 PT-INR Value

N	Mean	Standard Deviation	Minimum	Maximum
6	2.0783333	0.4169612	1.6500000	2.8700000

Laboratory tests at the time of obtaining Table 2-6 consent, according to the presence or absence of proton-pump inhibitors

Unique Subject Identifier=0009010000-0078

MEANS Procedure

Assay variable : PTINR_VAL1 PT-INR Value

N	Mean	Standard Deviation	Minimum	Maximum
3	1.9533333	0.1517674	1.7900000	2.0900000

Unique Subject Identifier=0009010000-0079

Assay variable : PTINR_VAL1 PT-INR Value

N	Mean	Standard Deviation	Minimum	Maximum
4	2.4000000	0.3892728	1.9700000	2.8400000

Unique Subject Identifier=0009010000-0084

Assay variable : PTINR_VAL1 PT-INR Value

N	Mean	Standard Deviation	Minimum	Maximum
5	1.9940000	0.1761533	1.8200000	2.2600000

Unique Subject Identifier=0009010000-0085

Assay variable : PTINR_VAL1 PT-INR Value

N	Mean	Standard Deviation	Minimum	Maximum
3	2.0700000	0.2946184	1.8100000	2.3900000

Laboratory tests at the time of obtaining Table 2-6 consent, according to the presence or absence of proton-pump inhibitors

Unique Subject Identifier=0009010000-0087

MEANS Procedure

Assay variable : PTINR_VAL1 PT-INR Value

N	Mean	Standard Deviation	Minimum	Maximum
5	1.4800000	0.0930054	1.3800000	1.6100000

Unique Subject Identifier=0009010000-0089

Assay variable : PTINR_VAL1 PT-INR Value

N	Mean	Standard Deviation	Minimum	Maximum
3	2.4633333	0.5451911	1.8800000	2.9600000

Unique Subject Identifier=0009010000-0090

Assay variable : PTINR_VAL1 PT-INR Value

N	Mean	Standard Deviation	Minimum	Maximum
5	1.8860000	0.4982269	1.2900000	2.5700000

Unique Subject Identifier=0009010000-0093

Assay variable : PTINR_VAL1 PT-INR Value

N	Mean	Standard Deviation	Minimum	Maximum
6	2.1100000	0.2913417	1.7500000	2.4600000

Laboratory tests at the time of obtaining Table 2-6 consent, according to the presence or absence of proton-pump inhibitors

Unique Subject Identifier=0009010000-0097

MEANS Procedure

Assay variable : PTINR_VAL1 PT-INR Value

N	Mean	Standard Deviation	Minimum	Maximum
3	2.3400000	0.2594224	2.1300000	2.6300000

Unique Subject Identifier=0009010000-0099

Assay variable : PTINR_VAL1 PT-INR Value

N	Mean	Standard Deviation	Minimum	Maximum
3	2.7266667	0.6957250	2.3200000	3.5300000

Unique Subject Identifier=0009010000-0102

Assay variable : PTINR_VAL1 PT-INR Value

N	Mean	Standard Deviation	Minimum	Maximum
3	1.6266667	0.3937427	1.3700000	2.0800000

Unique Subject Identifier=0009010000-0104

Assay variable : PTINR_VAL1 PT-INR Value

N	Mean	Standard Deviation	Minimum	Maximum
6	2.1083333	0.6375395	1.3300000	3.0000000

Laboratory tests at the time of obtaining Table 2-6 consent, according to the presence or absence of proton-pump inhibitors

Unique Subject Identifier=0009010000-0106

MEANS Procedure

Assay variable : PTINR_VAL1 PT-INR Value

N	Mean	Standard Deviation	Minimum	Maximum
3	1.9833333	0.3879863	1.7300000	2.4300000

Unique Subject Identifier=0009010000-0107

Assay variable : PTINR_VAL1 PT-INR Value

N	Mean	Standard Deviation	Minimum	Maximum
3	2.2800000	0.3174902	2.0400000	2.6400000

Unique Subject Identifier=0009010000-0108

Assay variable : PTINR_VAL1 PT-INR Value

N	Mean	Standard Deviation	Minimum	Maximum
3	1.9233333	0.0568624	1.8600000	1.9700000

Unique Subject Identifier=0009010000-0109

Assay variable : PTINR_VAL1 PT-INR Value

N	Mean	Standard Deviation	Minimum	Maximum
4	2.4000000	0.2871701	2.0300000	2.7000000

Laboratory tests at the time of obtaining Table 2-6 consent, according to the presence or absence of proton-pump inhibitors

Unique Subject Identifier=0009010000-0112

MEANS Procedure

Assay variable : PTINR_VAL1 PT-INR Value

N	Mean	Standard Deviation	Minimum	Maximum
3	1.8366667	0.5005330	1.3500000	2.3500000

Unique Subject Identifier=0009010000-0114

Assay variable : PTINR_VAL1 PT-INR Value

N	Mean	Standard Deviation	Minimum	Maximum
3	2.2966667	0.6668083	1.6600000	2.9900000

Unique Subject Identifier=0009010000-0117

Assay variable : PTINR_VAL1 PT-INR Value

N	Mean	Standard Deviation	Minimum	Maximum
3	2.1800000	0.0435890	2.1500000	2.2300000

Unique Subject Identifier=0009010000-0130

Assay variable : PTINR_VAL1 PT-INR Value

N	Mean	Standard Deviation	Minimum	Maximum
4	1.2725000	0.2150000	1.0400000	1.5600000

Laboratory tests at the time of obtaining Table 2-6 consent, according to the presence or absence of proton-pump inhibitors

Unique Subject Identifier=0009010000-0137

MEANS Procedure

Assay variable : PTINR_VAL1 PT-INR Value

N	Mean	Standard Deviation	Minimum	Maximum
5	1.6860000	0.3005495	1.2200000	1.9700000

Unique Subject Identifier=0009010000-0139

Assay variable : PTINR_VAL1 PT-INR Value

N	Mean	Standard Deviation	Minimum	Maximum
3	2.1466667	0.6335877	1.6900000	2.8700000

Unique Subject Identifier=0009010000-0141

Assay variable : PTINR_VAL1 PT-INR Value

N	Mean	Standard Deviation	Minimum	Maximum
4	2.4450000	0.3221283	2.0300000	2.7000000

Unique Subject Identifier=0009010000-0142

Assay variable : PTINR_VAL1 PT-INR Value

N	Mean	Standard Deviation	Minimum	Maximum
4	2.0500000	0.2815434	1.7200000	2.3800000

Laboratory tests at the time of obtaining Table 2-6 consent, according to the presence or absence of proton-pump inhibitors

Unique Subject Identifier=0009010000-0143

MEANS Procedure

Assay variable : PTINR_VAL1 PT-INR Value

N	Mean	Standard Deviation	Minimum	Maximum
2	1.8850000	0.2474874	1.7100000	2.0600000

Unique Subject Identifier=0009010000-0147

Assay variable : PTINR_VAL1 PT-INR Value

N	Mean	Standard Deviation	Minimum	Maximum
4	1.7300000	0.1726268	1.5900000	1.9700000

Unique Subject Identifier=0009010000-0149

Assay variable : PTINR_VAL1 PT-INR Value

N	Mean	Standard Deviation	Minimum	Maximum
4	2.3825000	0.2696139	2.0200000	2.6600000

Unique Subject Identifier=0009010000-0150

Assay variable : PTINR_VAL1 PT-INR Value

N	Mean	Standard Deviation	Minimum	Maximum
2	1.8450000	0.2050610	1.7000000	1.9900000

Laboratory tests at the time of obtaining Table 2-6 consent, according to the presence or absence of proton-pump inhibitors

Unique Subject Identifier=0009010000-0151

MEANS Procedure

Assay variable : PTINR_VAL1 PT-INR Value

N	Mean	Standard Deviation	Minimum	Maximum
3	1.5366667	0.1504438	1.4400000	1.7100000

Unique Subject Identifier=0009010000-0153

Assay variable : PTINR_VAL1 PT-INR Value

N	Mean	Standard Deviation	Minimum	Maximum
4	2.2850000	0.2056696	2.0300000	2.5300000

Unique Subject Identifier=0009010000-0154

Assay variable : PTINR_VAL1 PT-INR Value

N	Mean	Standard Deviation	Minimum	Maximum
5	2.0180000	0.3054832	1.7100000	2.3700000

Unique Subject Identifier=0009010000-0156

Assay variable : PTINR_VAL1 PT-INR Value

N	Mean	Standard Deviation	Minimum	Maximum
3	2.3700000	0.1345362	2.2200000	2.4800000

Laboratory tests at the time of obtaining Table 2-6 consent, according to the presence or absence of proton-pump inhibitors

Unique Subject Identifier=0009010000-0162

MEANS Procedure

Assay variable : PTINR_VAL1 PT-INR Value

N	Mean	Standard Deviation	Minimum	Maximum
3	1.6933333	0.1205543	1.5800000	1.8200000

Unique Subject Identifier=0009010000-0163

Assay variable : PTINR_VAL1 PT-INR Value

N	Mean	Standard Deviation	Minimum	Maximum
3	2.2966667	0.1677299	2.1900000	2.4900000

Unique Subject Identifier=0009010000-0166

Assay variable : PTINR_VAL1 PT-INR Value

N	Mean	Standard Deviation	Minimum	Maximum
3	1.8800000	0.1479865	1.7100000	1.9800000

Unique Subject Identifier=0009010000-0167

Assay variable : PTINR_VAL1 PT-INR Value

N	Mean	Standard Deviation	Minimum	Maximum
3	2.0033333	0.3251666	1.7500000	2.3700000

Laboratory tests at the time of obtaining Table 2-6 consent, according to the presence or absence of proton-pump inhibitors

Unique Subject Identifier=0009010000-0168

MEANS Procedure

Assay variable : PTINR_VAL1 PT-INR Value

N	Mean	Standard Deviation	Minimum	Maximum
3	2.2400000	0.0400000	2.2000000	2.2800000

Unique Subject Identifier=0009010000-0178

Assay variable : PTINR_VAL1 PT-INR Value

N	Mean	Standard Deviation	Minimum	Maximum
2	1.9800000	0.3959798	1.7000000	2.2600000

Unique Subject Identifier=0009010000-0180

Assay variable : PTINR_VAL1 PT-INR Value

N	Mean	Standard Deviation	Minimum	Maximum
3	2.2466667	0.0585947	2.1800000	2.2900000

Unique Subject Identifier=0009010000-0197

Assay variable : PTINR_VAL1 PT-INR Value

N	Mean	Standard Deviation	Minimum	Maximum
3	1.8233333	0.0351188	1.7900000	1.8600000

Laboratory tests at the time of obtaining Table 2-6 consent, according to the presence or absence of proton-pump inhibitors

Unique Subject Identifier=0009010000-0198

MEANS Procedure

Assay variable : PTINR_VAL1 PT-INR Value

N	Mean	Standard Deviation	Minimum	Maximum
6	1.5600000	0.1628496	1.3500000	1.8200000

Unique Subject Identifier=0009010000-0200

Assay variable : PTINR_VAL1 PT-INR Value

N	Mean	Standard Deviation	Minimum	Maximum
2	2.3700000	0.1131371	2.2900000	2.4500000

Unique Subject Identifier=0009010000-0204

Assay variable : PTINR_VAL1 PT-INR Value

N	Mean	Standard Deviation	Minimum	Maximum
5	2.2680000	0.1293058	2.1400000	2.4400000

Unique Subject Identifier=0009010000-0217

Assay variable : PTINR_VAL1 PT-INR Value

N	Mean	Standard Deviation	Minimum	Maximum
6	1.3750000	0.0768765	1.2500000	1.4700000

Laboratory tests at the time of obtaining Table 2-6 consent, according to the presence or absence of proton-pump inhibitors

Unique Subject Identifier=0009010000-0220

MEANS Procedure

Assay variable : PTINR_VAL1 PT-INR Value

N	Mean	Standard Deviation	Minimum	Maximum
6	2.0316667	0.0970395	1.8700000	2.1300000

Unique Subject Identifier=0009010000-0222

Assay variable : PTINR_VAL1 PT-INR Value

N	Mean	Standard Deviation	Minimum	Maximum
6	1.7883333	0.1853016	1.5200000	2.0600000

Unique Subject Identifier=0009010000-0223

Assay variable : PTINR_VAL1 PT-INR Value

N	Mean	Standard Deviation	Minimum	Maximum
6	2.1433333	0.2331237	1.8100000	2.4600000

Unique Subject Identifier=0009010000-0224

Assay variable : PTINR_VAL1 PT-INR Value

N	Mean	Standard Deviation	Minimum	Maximum
5	2.2180000	0.0772658	2.1000000	2.3100000

Laboratory tests at the time of obtaining Table 2-6 consent, according to the presence or absence of proton-pump inhibitors

Unique Subject Identifier=0009010000-0229

MEANS Procedure

Assay variable : PTINR_VAL1 PT-INR Value

N	Mean	Standard Deviation	Minimum	Maximum
3	1.8700000	0.1307670	1.7800000	2.0200000

Unique Subject Identifier=0009010000-0234

Assay variable : PTINR_VAL1 PT-INR Value

N	Mean	Standard Deviation	Minimum	Maximum
1	2.7500000		2.7500000	2.7500000

Unique Subject Identifier=0009010000-0235

Assay variable : PTINR_VAL1 PT-INR Value

N	Mean	Standard Deviation	Minimum	Maximum
6	1.5416667	0.1703428	1.3300000	1.7400000

Unique Subject Identifier=0009010000-0238

Assay variable : PTINR_VAL1 PT-INR Value

N	Mean	Standard Deviation	Minimum	Maximum
3	2.2100000	0.5456189	1.8900000	2.8400000

Laboratory tests at the time of obtaining Table 2-6 consent, according to the presence or absence of proton-pump inhibitors

Unique Subject Identifier=0009010000-0239

MEANS Procedure

Assay variable : PTINR_VAL1 PT-INR Value

N	Mean	Standard Deviation	Minimum	Maximum
3	1.6366667	0.0493288	1.5800000	1.6700000

Unique Subject Identifier=0009010000-0244

Assay variable : PTINR_VAL1 PT-INR Value

N	Mean	Standard Deviation	Minimum	Maximum
4	2.1200000	0.6906519	1.5500000	3.0400000

Unique Subject Identifier=0009010000-0246

Assay variable : PTINR_VAL1 PT-INR Value

N	Mean	Standard Deviation	Minimum	Maximum
4	2.5500000	0.3987480	1.9700000	2.8800000

Unique Subject Identifier=0009010000-0247

Assay variable : PTINR_VAL1 PT-INR Value

N	Mean	Standard Deviation	Minimum	Maximum
3	1.6566667	0.3817504	1.3700000	2.0900000

Laboratory tests at the time of obtaining Table 2-6 consent, according to the presence or absence of proton-pump inhibitors

Unique Subject Identifier=0009010000-0254

MEANS Procedure

Assay variable : PTINR_VAL1 PT-INR Value

N	Mean	Standard Deviation	Minimum	Maximum
2	1.3400000	0.1131371	1.2600000	1.4200000

Unique Subject Identifier=0013011100-0002

Assay variable : PTINR_VAL1 PT-INR Value

N	Mean	Standard Deviation	Minimum	Maximum
5	2.1560000	0.0944987	2.0300000	2.2800000

Unique Subject Identifier=0013011100-0008

Assay variable : PTINR_VAL1 PT-INR Value

N	Mean	Standard Deviation	Minimum	Maximum
6	1.8916667	0.1126795	1.7700000	2.0700000

Unique Subject Identifier=0013011100-0016

Assay variable : PTINR_VAL1 PT-INR Value

N	Mean	Standard Deviation	Minimum	Maximum
4	1.9625000	0.1768945	1.7600000	2.1700000

Laboratory tests at the time of obtaining Table 2-6 consent, according to the presence or absence of proton-pump inhibitors

Unique Subject Identifier=0013011100-0022

MEANS Procedure

Assay variable : PTINR_VAL1 PT-INR Value

N	Mean	Standard Deviation	Minimum	Maximum
3	1.4700000	0.2351595	1.2000000	1.6300000

Unique Subject Identifier=0013011100-0027

Assay variable : PTINR_VAL1 PT-INR Value

N	Mean	Standard Deviation	Minimum	Maximum
3	2.4366667	0.3259346	2.1700000	2.8000000

Unique Subject Identifier=0014010000-0001

Assay variable : PTINR_VAL1 PT-INR Value

N	Mean	Standard Deviation	Minimum	Maximum
4	2.2700000	0.2674572	2.1100000	2.6700000

Unique Subject Identifier=0014010000-0004

Assay variable : PTINR_VAL1 PT-INR Value

N	Mean	Standard Deviation	Minimum	Maximum
6	1.5366667	0.3691432	1.2200000	2.2400000

Laboratory tests at the time of obtaining Table 2-6 consent, according to the presence or absence of proton-pump inhibitors

Unique Subject Identifier=0016011100-0001

MEANS Procedure

Assay variable : PTINR_VAL1 PT-INR Value

N	Mean	Standard Deviation	Minimum	Maximum
2	2.2550000	0.0070711	2.2500000	2.2600000

Unique Subject Identifier=0017010000-0001

Assay variable : PTINR_VAL1 PT-INR Value

N	Mean	Standard Deviation	Minimum	Maximum
3	1.7100000	0.1969772	1.5500000	1.9300000

Unique Subject Identifier=0017010000-0003

Assay variable : PTINR_VAL1 PT-INR Value

N	Mean	Standard Deviation	Minimum	Maximum
4	2.1775000	0.1350000	1.9900000	2.2900000

Unique Subject Identifier=0017010000-0007

Assay variable : PTINR_VAL1 PT-INR Value

N	Mean	Standard Deviation	Minimum	Maximum
2	2.1550000	0.2192031	2.0000000	2.3100000

Laboratory tests at the time of obtaining Table 2-6 consent, according to the presence or absence of proton-pump inhibitors

Unique Subject Identifier=0017010000-0014

MEANS Procedure

Assay variable : PTINR_VAL1 PT-INR Value

N	Mean	Standard Deviation	Minimum	Maximum
6	2.1916667	0.2579470	1.9200000	2.5500000

Unique Subject Identifier=0017010000-0015

Assay variable : PTINR_VAL1 PT-INR Value

N	Mean	Standard Deviation	Minimum	Maximum
4	2.0900000	0.2738613	1.8500000	2.4600000

Unique Subject Identifier=0017010000-0017

Assay variable : PTINR_VAL1 PT-INR Value

N	Mean	Standard Deviation	Minimum	Maximum
3	2.1833333	0.2482606	2.0400000	2.4700000

Unique Subject Identifier=0017010000-0020

Assay variable : PTINR_VAL1 PT-INR Value

N	Mean	Standard Deviation	Minimum	Maximum
3	1.9500000	0.1228821	1.8100000	2.0400000

Laboratory tests at the time of obtaining Table 2-6 consent, according to the presence or absence of proton-pump inhibitors

Unique Subject Identifier=0017010000-0024

MEANS Procedure

Assay variable : PTINR_VAL1 PT-INR Value

N	Mean	Standard Deviation	Minimum	Maximum
3	2.0533333	0.0208167	2.0300000	2.0700000

Unique Subject Identifier=0017010000-0028

Assay variable : PTINR_VAL1 PT-INR Value

N	Mean	Standard Deviation	Minimum	Maximum
4	2.5500000	0.5575542	1.7700000	3.0400000

Unique Subject Identifier=0017010000-0029

Assay variable : PTINR_VAL1 PT-INR Value

N	Mean	Standard Deviation	Minimum	Maximum
4	2.6250000	0.7736709	1.8500000	3.6000000

Unique Subject Identifier=0017010000-0031

Assay variable : PTINR_VAL1 PT-INR Value

N	Mean	Standard Deviation	Minimum	Maximum
2	2.3350000	0.0070711	2.3300000	2.3400000

Laboratory tests at the time of obtaining Table 2-6 consent, according to the presence or absence of proton-pump inhibitors

Unique Subject Identifier=0017010000-0032

MEANS Procedure

Assay variable : PTINR_VAL1 PT-INR Value

N	Mean	Standard Deviation	Minimum	Maximum
2	2.0100000	0.1131371	1.9300000	2.0900000

Unique Subject Identifier=0017010000-0035

Assay variable : PTINR_VAL1 PT-INR Value

N	Mean	Standard Deviation	Minimum	Maximum
2	1.8100000	0.5232590	1.4400000	2.1800000

Unique Subject Identifier=0017010000-0038

Assay variable : PTINR_VAL1 PT-INR Value

N	Mean	Standard Deviation	Minimum	Maximum
3	2.1200000	0.1228821	1.9800000	2.2100000

Unique Subject Identifier=0017010000-0039

Assay variable : PTINR_VAL1 PT-INR Value

N	Mean	Standard Deviation	Minimum	Maximum
4	1.7600000	0.1032796	1.6400000	1.8800000

Laboratory tests at the time of obtaining Table 2-6 consent, according to the presence or absence of proton-pump inhibitors

Unique Subject Identifier=0017010000-0040

MEANS Procedure

Assay variable : PTINR_VAL1 PT-INR Value

N	Mean	Standard Deviation	Minimum	Maximum
3	1.6533333	0.2050203	1.4500000	1.8600000

Unique Subject Identifier=0017010000-0042

Assay variable : PTINR_VAL1 PT-INR Value

N	Mean	Standard Deviation	Minimum	Maximum
3	2.2000000	0.0888819	2.1300000	2.3000000

Unique Subject Identifier=0017010000-0044

Assay variable : PTINR_VAL1 PT-INR Value

N	Mean	Standard Deviation	Minimum	Maximum
5	1.9300000	0.1246996	1.7900000	2.1300000

Unique Subject Identifier=0017010000-0056

Assay variable : PTINR_VAL1 PT-INR Value

N	Mean	Standard Deviation	Minimum	Maximum
3	1.9366667	0.5896892	1.2700000	2.3900000

Laboratory tests at the time of obtaining Table 2-6 consent, according to the presence or absence of proton-pump inhibitors

Unique Subject Identifier=0017010000-0060

MEANS Procedure

Assay variable : PTINR_VAL1 PT-INR Value

N	Mean	Standard Deviation	Minimum	Maximum
3	2.2133333	0.6601767	1.6300000	2.9300000

Unique Subject Identifier=0017010000-0069

Assay variable : PTINR_VAL1 PT-INR Value

N	Mean	Standard Deviation	Minimum	Maximum
3	1.7033333	0.2052641	1.5300000	1.9300000

Unique Subject Identifier=0017010000-0071

Assay variable : PTINR_VAL1 PT-INR Value

N	Mean	Standard Deviation	Minimum	Maximum
3	1.9066667	0.0472582	1.8700000	1.9600000

Unique Subject Identifier=0017010000-0076

Assay variable : PTINR_VAL1 PT-INR Value

N	Mean	Standard Deviation	Minimum	Maximum
3	2.5266667	0.2640707	2.2400000	2.7600000

Laboratory tests at the time of obtaining Table 2-6 consent, according to the presence or absence of proton-pump inhibitors

Unique Subject Identifier=0017010000-0082

MEANS Procedure

Assay variable : PTINR_VAL1 PT-INR Value

N	Mean	Standard Deviation	Minimum	Maximum
3	2.3800000	0.7171471	1.7700000	3.1700000

Unique Subject Identifier=0017010000-0084

Assay variable : PTINR_VAL1 PT-INR Value

N	Mean	Standard Deviation	Minimum	Maximum
3	1.8366667	0.1965536	1.6100000	1.9600000

Unique Subject Identifier=0019010000-0003

Assay variable : PTINR_VAL1 PT-INR Value

N	Mean	Standard Deviation	Minimum	Maximum
3	1.8966667	0.4006661	1.5100000	2.3100000

Unique Subject Identifier=0019010000-0010

Assay variable : PTINR_VAL1 PT-INR Value

N	Mean	Standard Deviation	Minimum	Maximum
2	1.8950000	0.0777817	1.8400000	1.9500000

Laboratory tests at the time of obtaining Table 2-6 consent, according to the presence or absence of proton-pump inhibitors

Unique Subject Identifier=0019010000-0012

MEANS Procedure

Assay variable : PTINR_VAL1 PT-INR Value

N	Mean	Standard Deviation	Minimum	Maximum
6	1.8150000	0.3632492	1.3100000	2.3500000

Unique Subject Identifier=0019010000-0017

Assay variable : PTINR_VAL1 PT-INR Value

N	Mean	Standard Deviation	Minimum	Maximum
6	1.9300000	0.4606083	1.0600000	2.3900000

Unique Subject Identifier=0019010000-0026

Assay variable : PTINR_VAL1 PT-INR Value

N	Mean	Standard Deviation	Minimum	Maximum
4	1.8475000	0.2385197	1.5600000	2.1400000

Unique Subject Identifier=0019010000-0031

Assay variable : PTINR_VAL1 PT-INR Value

N	Mean	Standard Deviation	Minimum	Maximum
2	1.6300000	0.0989949	1.5600000	1.7000000

Laboratory tests at the time of obtaining Table 2-6 consent, according to the presence or absence of proton-pump inhibitors

Unique Subject Identifier=0019010000-0035

MEANS Procedure

Assay variable : PTINR_VAL1 PT-INR Value

N	Mean	Standard Deviation	Minimum	Maximum
3	1.9733333	0.2138535	1.7400000	2.1600000

Unique Subject Identifier=0019010000-0038

Assay variable : PTINR_VAL1 PT-INR Value

N	Mean	Standard Deviation	Minimum	Maximum
3	1.3966667	0.2657693	1.1200000	1.6500000

Unique Subject Identifier=0019010000-0039

Assay variable : PTINR_VAL1 PT-INR Value

N	Mean	Standard Deviation	Minimum	Maximum
2	2.0800000	0.2121320	1.9300000	2.2300000

Unique Subject Identifier=0019010000-0041

Assay variable : PTINR_VAL1 PT-INR Value

N	Mean	Standard Deviation	Minimum	Maximum
3	2.2533333	0.2742870	1.9400000	2.4500000

Laboratory tests at the time of obtaining Table 2-6 consent, according to the presence or absence of proton-pump inhibitors

Unique Subject Identifier=0019010000-0043

MEANS Procedure

Assay variable : PTINR_VAL1 PT-INR Value

N	Mean	Standard Deviation	Minimum	Maximum
2	2.2850000	0.0636396	2.2400000	2.3300000

Unique Subject Identifier=0019010000-0045

Assay variable : PTINR_VAL1 PT-INR Value

N	Mean	Standard Deviation	Minimum	Maximum
4	1.8050000	0.3639139	1.6000000	2.3500000

Unique Subject Identifier=0019010000-0056

Assay variable : PTINR_VAL1 PT-INR Value

N	Mean	Standard Deviation	Minimum	Maximum
4	2.4125000	0.4027716	1.8800000	2.7800000

Unique Subject Identifier=0019010000-0057

Assay variable : PTINR_VAL1 PT-INR Value

N	Mean	Standard Deviation	Minimum	Maximum
3	1.8700000	0.0173205	1.8600000	1.8900000

Laboratory tests at the time of obtaining Table 2-6 consent, according to the presence or absence of proton-pump inhibitors

Unique Subject Identifier=0019010000-0065

MEANS Procedure

Assay variable : PTINR_VAL1 PT-INR Value

N	Mean	Standard Deviation	Minimum	Maximum
4	1.4900000	0.2083267	1.2400000	1.6900000

Unique Subject Identifier=0019010000-0070

Assay variable : PTINR_VAL1 PT-INR Value

N	Mean	Standard Deviation	Minimum	Maximum
2	2.1300000	0.2545584	1.9500000	2.3100000

Unique Subject Identifier=0019010000-0086

Assay variable : PTINR_VAL1 PT-INR Value

N	Mean	Standard Deviation	Minimum	Maximum
3	2.5833333	0.4772141	2.0500000	2.9700000

Unique Subject Identifier=0019010000-0092

Assay variable : PTINR_VAL1 PT-INR Value

N	Mean	Standard Deviation	Minimum	Maximum
2	2.0850000	0.2192031	1.9300000	2.2400000

Laboratory tests at the time of obtaining Table 2-6 consent, according to the presence or absence of proton-pump inhibitors

Unique Subject Identifier=0019010000-0094

MEANS Procedure

Assay variable : PTINR_VAL1 PT-INR Value

N	Mean	Standard Deviation	Minimum	Maximum
3	1.4366667	0.1680278	1.2900000	1.6200000

Unique Subject Identifier=0019010000-0096

Assay variable : PTINR_VAL1 PT-INR Value

N	Mean	Standard Deviation	Minimum	Maximum
2	1.8300000	0.4101219	1.5400000	2.1200000

Unique Subject Identifier=0019010000-0097

Assay variable : PTINR_VAL1 PT-INR Value

N	Mean	Standard Deviation	Minimum	Maximum
3	2.3900000	0.0888819	2.3200000	2.4900000

Unique Subject Identifier=0019010000-0098

Assay variable : PTINR_VAL1 PT-INR Value

N	Mean	Standard Deviation	Minimum	Maximum
2	2.0450000	0.0070711	2.0400000	2.0500000

Laboratory tests at the time of obtaining Table 2-6 consent, according to the presence or absence of proton-pump inhibitors

Unique Subject Identifier=0019010000-0112

MEANS Procedure

Assay variable : PTINR_VAL1 PT-INR Value

N	Mean	Standard Deviation	Minimum	Maximum
3	2.9333333	1.3891124	1.4100000	4.1300000

Unique Subject Identifier=0019010000-0114

Assay variable : PTINR_VAL1 PT-INR Value

N	Mean	Standard Deviation	Minimum	Maximum
5	2.1180000	0.2438647	1.9400000	2.5400000

Unique Subject Identifier=0019010000-0118

Assay variable : PTINR_VAL1 PT-INR Value

N	Mean	Standard Deviation	Minimum	Maximum
3	1.8733333	0.3617089	1.6400000	2.2900000

Unique Subject Identifier=0021000000-0008

Assay variable : PTINR_VAL1 PT-INR Value

N	Mean	Standard Deviation	Minimum	Maximum
6	1.9783333	0.5227013	1.6500000	3.0300000

Laboratory tests at the time of obtaining Table 2-6 consent, according to the presence or absence of proton-pump inhibitors

Unique Subject Identifier=0021000000-0009

MEANS Procedure

Assay variable : PTINR_VAL1 PT-INR Value

N	Mean	Standard Deviation	Minimum	Maximum
6	2.1216667	0.1859480	1.9100000	2.3800000

Unique Subject Identifier=0021000000-0012

Assay variable : PTINR_VAL1 PT-INR Value

N	Mean	Standard Deviation	Minimum	Maximum
3	1.7833333	0.1365040	1.6900000	1.9400000

Unique Subject Identifier=0021000000-0017

Assay variable : PTINR_VAL1 PT-INR Value

N	Mean	Standard Deviation	Minimum	Maximum
6	1.6866667	0.1303329	1.6000000	1.9500000

Unique Subject Identifier=0021000000-0027

Assay variable : PTINR_VAL1 PT-INR Value

N	Mean	Standard Deviation	Minimum	Maximum
5	2.2300000	0.2109502	1.9200000	2.4600000

Laboratory tests at the time of obtaining Table 2-6 consent, according to the presence or absence of proton-pump inhibitors

Unique Subject Identifier=0024010000-0001

MEANS Procedure

Assay variable : PTINR_VAL1 PT-INR Value

N	Mean	Standard Deviation	Minimum	Maximum
6	2.1833333	0.1940790	1.9000000	2.4000000

Unique Subject Identifier=0027000000-0001

Assay variable : PTINR_VAL1 PT-INR Value

N	Mean	Standard Deviation	Minimum	Maximum
4	4.1550000	1.3986779	2.1300000	5.1600000

Unique Subject Identifier=0027000000-0012

Assay variable : PTINR_VAL1 PT-INR Value

N	Mean	Standard Deviation	Minimum	Maximum
6	1.9250000	0.4174087	1.2100000	2.4200000

Unique Subject Identifier=0027000000-0018

Assay variable : PTINR_VAL1 PT-INR Value

N	Mean	Standard Deviation	Minimum	Maximum
6	2.2250000	0.6775470	1.0600000	3.0800000

Laboratory tests at the time of obtaining Table 2-6 consent, according to the presence or absence of proton-pump inhibitors

Unique Subject Identifier=0027000000-0022

MEANS Procedure

Assay variable : PTINR_VAL1 PT-INR Value

N	Mean	Standard Deviation	Minimum	Maximum
3	1.9033333	0.1656301	1.7300000	2.0600000

Unique Subject Identifier=0027000000-0027

Assay variable : PTINR_VAL1 PT-INR Value

N	Mean	Standard Deviation	Minimum	Maximum
2	2.9300000	0.2262742	2.7700000	3.0900000

Unique Subject Identifier=0027000000-0028

Assay variable : PTINR_VAL1 PT-INR Value

N	Mean	Standard Deviation	Minimum	Maximum
6	2.0700000	0.1446375	1.9100000	2.2700000

Unique Subject Identifier=0027000000-0029

Assay variable : PTINR_VAL1 PT-INR Value

N	Mean	Standard Deviation	Minimum	Maximum
2	2.5500000	0.2545584	2.3700000	2.7300000

Laboratory tests at the time of obtaining Table 2-6 consent, according to the presence or absence of proton-pump inhibitors

Unique Subject Identifier=0030010010-0001

MEANS Procedure

Assay variable : PTINR_VAL1 PT-INR Value

N	Mean	Standard Deviation	Minimum	Maximum
3	1.7333333	0.3326159	1.4800000	2.1100000

Unique Subject Identifier=0030010010-0005

Assay variable : PTINR_VAL1 PT-INR Value

N	Mean	Standard Deviation	Minimum	Maximum
3	2.1000000	0.1442221	1.9400000	2.2200000

Unique Subject Identifier=0030010010-0007

Assay variable : PTINR_VAL1 PT-INR Value

N	Mean	Standard Deviation	Minimum	Maximum
6	1.8966667	0.4332974	1.5100000	2.4700000

Unique Subject Identifier=0030010010-0011

Assay variable : PTINR_VAL1 PT-INR Value

N	Mean	Standard Deviation	Minimum	Maximum
3	2.4633333	0.5852635	1.7900000	2.8500000

Laboratory tests at the time of obtaining Table 2-6 consent, according to the presence or absence of proton-pump inhibitors

Unique Subject Identifier=0030010010-0012

MEANS Procedure

Assay variable : PTINR_VAL1 PT-INR Value

N	Mean	Standard Deviation	Minimum	Maximum
6	1.7750000	0.1925357	1.4900000	2.0500000

Unique Subject Identifier=0031110001-0005

Assay variable : PTINR_VAL1 PT-INR Value

N	Mean	Standard Deviation	Minimum	Maximum
5	2.1180000	0.4717203	1.7100000	2.8900000

Unique Subject Identifier=0031110001-0007

Assay variable : PTINR_VAL1 PT-INR Value

N	Mean	Standard Deviation	Minimum	Maximum
3	2.6366667	0.3496188	2.4200000	3.0400000

Unique Subject Identifier=0031110001-0008

Assay variable : PTINR_VAL1 PT-INR Value

N	Mean	Standard Deviation	Minimum	Maximum
2	2.3400000	0.3535534	2.0900000	2.5900000

Laboratory tests at the time of obtaining Table 2-6 consent, according to the presence or absence of proton-pump inhibitors

Unique Subject Identifier=0031110001-0016

MEANS Procedure

Assay variable : PTINR_VAL1 PT-INR Value

N	Mean	Standard Deviation	Minimum	Maximum
3	2.0766667	0.2354428	1.8500000	2.3200000

Unique Subject Identifier=0031110001-0018

Assay variable : PTINR_VAL1 PT-INR Value

N	Mean	Standard Deviation	Minimum	Maximum
5	2.0640000	0.4643598	1.3600000	2.5100000

Unique Subject Identifier=0031110001-0020

Assay variable : PTINR_VAL1 PT-INR Value

N	Mean	Standard Deviation	Minimum	Maximum
3	2.9833333	0.1939931	2.7600000	3.1100000

Unique Subject Identifier=0031110001-0023

Assay variable : PTINR_VAL1 PT-INR Value

N	Mean	Standard Deviation	Minimum	Maximum
3	2.1600000	0.0781025	2.0700000	2.2100000

Laboratory tests at the time of obtaining Table 2-6 consent, according to the presence or absence of proton-pump inhibitors

Unique Subject Identifier=0031110001-0025

MEANS Procedure

Assay variable : PTINR_VAL1 PT-INR Value

N	Mean	Standard Deviation	Minimum	Maximum
3	2.2066667	0.2615977	1.9300000	2.4500000

Unique Subject Identifier=0031110001-0026

Assay variable : PTINR_VAL1 PT-INR Value

N	Mean	Standard Deviation	Minimum	Maximum
4	1.6300000	0.3492850	1.1600000	2.0000000

Unique Subject Identifier=0032110000-0004

Assay variable : PTINR_VAL1 PT-INR Value

N	Mean	Standard Deviation	Minimum	Maximum
4	1.6025000	0.1512999	1.4900000	1.8200000

Unique Subject Identifier=0032110000-0007

Assay variable : PTINR_VAL1 PT-INR Value

N	Mean	Standard Deviation	Minimum	Maximum
4	2.0075000	0.2425387	1.7700000	2.3100000

Laboratory tests at the time of obtaining Table 2-6 consent, according to the presence or absence of proton-pump inhibitors

Unique Subject Identifier=0032110000-0008

MEANS Procedure

Assay variable : PTINR_VAL1 PT-INR Value

N	Mean	Standard Deviation	Minimum	Maximum
6	1.3133333	0.1148332	1.1300000	1.4200000

Unique Subject Identifier=0032110000-0009

Assay variable : PTINR_VAL1 PT-INR Value

N	Mean	Standard Deviation	Minimum	Maximum
6	1.5466667	0.1057670	1.4200000	1.6900000

Unique Subject Identifier=0038111111-0002

Assay variable : PTINR_VAL1 PT-INR Value

N	Mean	Standard Deviation	Minimum	Maximum
5	1.7800000	0.1973575	1.5500000	1.9800000

Unique Subject Identifier=0038111111-0008

Assay variable : PTINR_VAL1 PT-INR Value

N	Mean	Standard Deviation	Minimum	Maximum
5	2.3140000	0.2132604	1.9600000	2.4800000

Laboratory tests at the time of obtaining Table 2-6 consent, according to the presence or absence of proton-pump inhibitors

Unique Subject Identifier=0038111111-0011

MEANS Procedure

Assay variable : PTINR_VAL1 PT-INR Value

N	Mean	Standard Deviation	Minimum	Maximum
6	1.5683333	0.1190658	1.4300000	1.7500000

Unique Subject Identifier=0038111111-0019

Assay variable : PTINR_VAL1 PT-INR Value

N	Mean	Standard Deviation	Minimum	Maximum
5	2.0840000	0.3230015	1.7300000	2.4800000

Unique Subject Identifier=0038111111-0022

Assay variable : PTINR_VAL1 PT-INR Value

N	Mean	Standard Deviation	Minimum	Maximum
6	2.3133333	0.6361027	1.2200000	3.1000000

Unique Subject Identifier=0038111111-0023

Assay variable : PTINR_VAL1 PT-INR Value

N	Mean	Standard Deviation	Minimum	Maximum
5	2.2580000	0.6187649	1.3000000	2.8500000

Laboratory tests at the time of obtaining Table 2-6 consent, according to the presence or absence of proton-pump inhibitors

Unique Subject Identifier=0041110000-0002

MEANS Procedure

Assay variable : PTINR_VAL1 PT-INR Value

N	Mean	Standard Deviation	Minimum	Maximum
6	1.9350000	0.4455895	1.5400000	2.7500000

Unique Subject Identifier=0041110000-0003

Assay variable : PTINR_VAL1 PT-INR Value

N	Mean	Standard Deviation	Minimum	Maximum
3	2.1166667	0.0611010	2.0500000	2.1700000

Unique Subject Identifier=0041110000-0011

Assay variable : PTINR_VAL1 PT-INR Value

N	Mean	Standard Deviation	Minimum	Maximum
2	1.9750000	0.3889087	1.7000000	2.2500000

Unique Subject Identifier=0041110000-0017

Assay variable : PTINR_VAL1 PT-INR Value

N	Mean	Standard Deviation	Minimum	Maximum
2	2.2000000	0.1838478	2.0700000	2.3300000

Laboratory tests at the time of obtaining Table 2-6 consent, according to the presence or absence of proton-pump inhibitors

Unique Subject Identifier=0041110000-0018

MEANS Procedure

Assay variable : PTINR_VAL1 PT-INR Value

N	Mean	Standard Deviation	Minimum	Maximum
4	1.6275000	0.5288588	1.1700000	2.1800000

Unique Subject Identifier=0041110000-0020

Assay variable : PTINR_VAL1 PT-INR Value

N	Mean	Standard Deviation	Minimum	Maximum
3	2.0300000	0.5715768	1.3700000	2.3600000

Unique Subject Identifier=0041110000-0022

Assay variable : PTINR_VAL1 PT-INR Value

N	Mean	Standard Deviation	Minimum	Maximum
4	1.6175000	0.2218671	1.3100000	1.7800000

Unique Subject Identifier=0042100010-0001

Assay variable : PTINR_VAL1 PT-INR Value

N	Mean	Standard Deviation	Minimum	Maximum
4	2.0150000	0.7527948	1.3900000	3.1000000

Laboratory tests at the time of obtaining Table 2-6 consent, according to the presence or absence of proton-pump inhibitors

Unique Subject Identifier=0042100010-0002

MEANS Procedure

Assay variable : PTINR_VAL1 PT-INR Value

N	Mean	Standard Deviation	Minimum	Maximum
3	1.7866667	0.3384277	1.5000000	2.1600000

Unique Subject Identifier=0042100010-0003

Assay variable : PTINR_VAL1 PT-INR Value

N	Mean	Standard Deviation	Minimum	Maximum
5	1.8320000	0.2375289	1.5900000	2.1300000

Unique Subject Identifier=0042100010-0004

Assay variable : PTINR_VAL1 PT-INR Value

N	Mean	Standard Deviation	Minimum	Maximum
5	1.9240000	0.0756968	1.8200000	2.0200000

Unique Subject Identifier=0042100010-0006

Assay variable : PTINR_VAL1 PT-INR Value

N	Mean	Standard Deviation	Minimum	Maximum
6	2.2916667	0.3726884	1.9300000	2.8300000

Laboratory tests at the time of obtaining Table 2-6 consent, according to the presence or absence of proton-pump inhibitors

Unique Subject Identifier=0043010010-0001

MEANS Procedure

Assay variable : PTINR_VAL1 PT-INR Value

N	Mean	Standard Deviation	Minimum	Maximum
4	1.8025000	0.2833578	1.4100000	2.0200000

Unique Subject Identifier=0043010010-0003

Assay variable : PTINR_VAL1 PT-INR Value

N	Mean	Standard Deviation	Minimum	Maximum
2	1.4700000	0.0565685	1.4300000	1.5100000

Unique Subject Identifier=0043010010-0005

Assay variable : PTINR_VAL1 PT-INR Value

N	Mean	Standard Deviation	Minimum	Maximum
3	1.7700000	0.1587451	1.6500000	1.9500000

Unique Subject Identifier=0043010010-0007

Assay variable : PTINR_VAL1 PT-INR Value

N	Mean	Standard Deviation	Minimum	Maximum
5	2.0780000	0.7081102	1.4600000	3.2300000

Laboratory tests at the time of obtaining Table 2-6 consent, according to the presence or absence of proton-pump inhibitors

Unique Subject Identifier=0043010010-0009

MEANS Procedure

Assay variable : PTINR_VAL1 PT-INR Value

N	Mean	Standard Deviation	Minimum	Maximum
3	2.5600000	0.1824829	2.3500000	2.6800000

Unique Subject Identifier=0044110000-0014

Assay variable : PTINR_VAL1 PT-INR Value

N	Mean	Standard Deviation	Minimum	Maximum
3	1.6800000	0.2605763	1.3800000	1.8500000

Unique Subject Identifier=0046100001-0002

Assay variable : PTINR_VAL1 PT-INR Value

N	Mean	Standard Deviation	Minimum	Maximum
6	2.2733333	0.2268627	2.0200000	2.6100000

Unique Subject Identifier=0046100001-0003

Assay variable : PTINR_VAL1 PT-INR Value

N	Mean	Standard Deviation	Minimum	Maximum
4	2.0625000	0.3096638	1.7700000	2.3400000

Laboratory tests at the time of obtaining Table 2-6 consent, according to the presence or absence of proton-pump inhibitors

Unique Subject Identifier=0046100001-0011

MEANS Procedure

Assay variable : PTINR_VAL1 PT-INR Value

N	Mean	Standard Deviation	Minimum	Maximum
6	1.7933333	0.5809877	1.1400000	2.7400000

Unique Subject Identifier=0046100001-0015

Assay variable : PTINR_VAL1 PT-INR Value

N	Mean	Standard Deviation	Minimum	Maximum
2	1.5500000	0.0707107	1.5000000	1.6000000

Unique Subject Identifier=0046100001-0018

Assay variable : PTINR_VAL1 PT-INR Value

N	Mean	Standard Deviation	Minimum	Maximum
3	2.1766667	0.5727419	1.5300000	2.6200000

Unique Subject Identifier=0046100001-0022

Assay variable : PTINR_VAL1 PT-INR Value

N	Mean	Standard Deviation	Minimum	Maximum
6	3.1633333	1.9392335	1.1800000	6.4300000

Laboratory tests at the time of obtaining Table 2-6 consent, according to the presence or absence of proton-pump inhibitors

Unique Subject Identifier=0046100001-0027

MEANS Procedure

Assay variable : PTINR_VAL1 PT-INR Value

N	Mean	Standard Deviation	Minimum	Maximum
6	1.8350000	0.4404884	1.3100000	2.4700000

Unique Subject Identifier=0046100001-0047

Assay variable : PTINR_VAL1 PT-INR Value

N	Mean	Standard Deviation	Minimum	Maximum
3	1.6033333	0.1365040	1.4800000	1.7500000

Unique Subject Identifier=0046100001-0049

Assay variable : PTINR_VAL1 PT-INR Value

N	Mean	Standard Deviation	Minimum	Maximum
3	1.7633333	0.3695042	1.5500000	2.1900000

Unique Subject Identifier=0046100001-0051

Assay variable : PTINR_VAL1 PT-INR Value

N	Mean	Standard Deviation	Minimum	Maximum
2	1.6000000	0.3111270	1.3800000	1.8200000

Laboratory tests at the time of obtaining Table 2-6 consent, according to the presence or absence of proton-pump inhibitors

Unique Subject Identifier=0046100001-0065

MEANS Procedure

Assay variable : PTINR_VAL1 PT-INR Value

N	Mean	Standard Deviation	Minimum	Maximum
1	1.9200000		1.9200000	1.9200000

Unique Subject Identifier=0046100001-0066

Assay variable : PTINR_VAL1 PT-INR Value

N	Mean	Standard Deviation	Minimum	Maximum
2	2.2950000	0.4879037	1.9500000	2.6400000

Unique Subject Identifier=0046100001-0073

Assay variable : PTINR_VAL1 PT-INR Value

N	Mean	Standard Deviation	Minimum	Maximum
4	1.2925000	0.0750000	1.2300000	1.3800000

Unique Subject Identifier=0046100001-0079

Assay variable : PTINR_VAL1 PT-INR Value

N	Mean	Standard Deviation	Minimum	Maximum
5	2.0460000	0.8770006	1.3100000	3.4600000

Laboratory tests at the time of obtaining Table 2-6 consent, according to the presence or absence of proton-pump inhibitors

Unique Subject Identifier=0046100001-0082

MEANS Procedure

Assay variable : PTINR_VAL1 PT-INR Value

N	Mean	Standard Deviation	Minimum	Maximum
1	2.3100000		2.3100000	2.3100000

Unique Subject Identifier=0048000010-0002

Assay variable : PTINR_VAL1 PT-INR Value

N	Mean	Standard Deviation	Minimum	Maximum
3	2.1433333	0.7721615	1.2600000	2.6900000

Unique Subject Identifier=0048000010-0003

Assay variable : PTINR_VAL1 PT-INR Value

N	Mean	Standard Deviation	Minimum	Maximum
3	1.6566667	0.2050203	1.4500000	1.8600000

Unique Subject Identifier=0048000010-0004

Assay variable : PTINR_VAL1 PT-INR Value

N	Mean	Standard Deviation	Minimum	Maximum
5	2.2000000	0.0984886	2.0300000	2.2700000

Laboratory tests at the time of obtaining Table 2-6 consent, according to the presence or absence of proton-pump inhibitors

Unique Subject Identifier=0048000010-0010

MEANS Procedure

Assay variable : PTINR_VAL1 PT-INR Value

N	Mean	Standard Deviation	Minimum	Maximum
1	1.7300000		1.7300000	1.7300000

Unique Subject Identifier=0049000010-0002

Assay variable : PTINR_VAL1 PT-INR Value

N	Mean	Standard Deviation	Minimum	Maximum
6	1.8866667	0.2731056	1.5600000	2.3300000

Unique Subject Identifier=0049000010-0004

Assay variable : PTINR_VAL1 PT-INR Value

N	Mean	Standard Deviation	Minimum	Maximum
6	1.9850000	0.1707337	1.7600000	2.1700000

Unique Subject Identifier=0049000010-0005

Assay variable : PTINR_VAL1 PT-INR Value

N	Mean	Standard Deviation	Minimum	Maximum
6	2.2850000	0.2167718	2.0400000	2.5900000

Laboratory tests at the time of obtaining Table 2-6 consent, according to the presence or absence of proton-pump inhibitors

Unique Subject Identifier=0049000010-0006

MEANS Procedure

Assay variable : PTINR_VAL1 PT-INR Value

N	Mean	Standard Deviation	Minimum	Maximum
5	1.9750000	0.6737025	1.3400000	3.1000000

Unique Subject Identifier=0049000010-0008

Assay variable : PTINR_VAL1 PT-INR Value

N	Mean	Standard Deviation	Minimum	Maximum
5	1.9020000	0.0957601	1.7700000	2.0200000

Unique Subject Identifier=0049000010-0011

Assay variable : PTINR_VAL1 PT-INR Value

N	Mean	Standard Deviation	Minimum	Maximum
4	1.8350000	0.2335951	1.6200000	2.1200000

Unique Subject Identifier=0049000010-0015

Assay variable : PTINR_VAL1 PT-INR Value

N	Mean	Standard Deviation	Minimum	Maximum
6	1.8416667	0.4710166	1.4100000	2.7300000

Laboratory tests at the time of obtaining Table 2-6 consent, according to the presence or absence of proton-pump inhibitors

Unique Subject Identifier=0049000010-0029

MEANS Procedure

Assay variable : PTINR_VAL1 PT-INR Value

N	Mean	Standard Deviation	Minimum	Maximum
6	1.9883333	0.2657380	1.6800000	2.2900000

Unique Subject Identifier=0049000010-0031

Assay variable : PTINR_VAL1 PT-INR Value

N	Mean	Standard Deviation	Minimum	Maximum
6	1.9050000	0.2059854	1.5900000	2.1400000

Unique Subject Identifier=0049000010-0036

Assay variable : PTINR_VAL1 PT-INR Value

N	Mean	Standard Deviation	Minimum	Maximum
6	2.2200000	0.4799167	1.7700000	3.0900000

Unique Subject Identifier=0049000010-0037

Assay variable : PTINR_VAL1 PT-INR Value

N	Mean	Standard Deviation	Minimum	Maximum
6	2.5400000	0.3212476	2.1400000	2.9400000

Laboratory tests at the time of obtaining Table 2-6 consent, according to the presence or absence of proton-pump inhibitors

Unique Subject Identifier=0052000000-0001

MEANS Procedure

Assay variable : PTINR_VAL1 PT-INR Value

N	Mean	Standard Deviation	Minimum	Maximum
5	2.0080000	0.6680344	1.0900000	2.6800000

Unique Subject Identifier=0052000000-0003

Assay variable : PTINR_VAL1 PT-INR Value

N	Mean	Standard Deviation	Minimum	Maximum
6	1.7916667	0.1185608	1.6900000	2.0000000

Unique Subject Identifier=0052000000-0004

Assay variable : PTINR_VAL1 PT-INR Value

N	Mean	Standard Deviation	Minimum	Maximum
6	1.9250000	0.1330789	1.6900000	2.0600000

Unique Subject Identifier=0054010000-0004

Assay variable : PTINR_VAL1 PT-INR Value

N	Mean	Standard Deviation	Minimum	Maximum
3	2.4300000	0.6701492	1.8900000	3.1800000

Laboratory tests at the time of obtaining Table 2-6 consent, according to the presence or absence of proton-pump inhibitors

Unique Subject Identifier=0054010000-0005

MEANS Procedure

Assay variable : PTINR_VAL1 PT-INR Value

N	Mean	Standard Deviation	Minimum	Maximum
5	2.2800000	0.1093161	2.1800000	2.4300000

Unique Subject Identifier=0054010000-0007

Assay variable : PTINR_VAL1 PT-INR Value

N	Mean	Standard Deviation	Minimum	Maximum
3	2.1000000	0.1931321	1.9300000	2.3100000

Unique Subject Identifier=0054010000-0017

Assay variable : PTINR_VAL1 PT-INR Value

N	Mean	Standard Deviation	Minimum	Maximum
4	1.8950000	0.3164912	1.6100000	2.3400000

Unique Subject Identifier=0054010000-0020

Assay variable : PTINR_VAL1 PT-INR Value

N	Mean	Standard Deviation	Minimum	Maximum
1	2.2300000		2.2300000	2.2300000

Laboratory tests at the time of obtaining Table 2-6 consent, according to the presence or absence of proton-pump inhibitors

Unique Subject Identifier=0054010000-0022

MEANS Procedure

Assay variable : PTINR_VAL1 PT-INR Value

N	Mean	Standard Deviation	Minimum	Maximum
6	1.3066667	0.1929421	1.1300000	1.6700000

Unique Subject Identifier=0054010000-0026

Assay variable : PTINR_VAL1 PT-INR Value

N	Mean	Standard Deviation	Minimum	Maximum
3	1.6966667	0.3189566	1.4300000	2.0500000

Unique Subject Identifier=0054010000-0027

Assay variable : PTINR_VAL1 PT-INR Value

N	Mean	Standard Deviation	Minimum	Maximum
3	1.7700000	0.1646208	1.6700000	1.9600000

Unique Subject Identifier=0054010000-0028

Assay variable : PTINR_VAL1 PT-INR Value

N	Mean	Standard Deviation	Minimum	Maximum
5	2.3900000	0.2317326	2.0800000	2.7100000

Laboratory tests at the time of obtaining Table 2-6 consent, according to the presence or absence of proton-pump inhibitors

Unique Subject Identifier=0054010000-0029

MEANS Procedure

Assay variable : PTINR_VAL1 PT-INR Value

N	Mean	Standard Deviation	Minimum	Maximum
3	2.4200000	0.2351595	2.2600000	2.6900000

Unique Subject Identifier=0054010000-0033

Assay variable : PTINR_VAL1 PT-INR Value

N	Mean	Standard Deviation	Minimum	Maximum
3	2.5566667	0.1266228	2.4600000	2.7000000

Unique Subject Identifier=0055100000-0001

Assay variable : PTINR_VAL1 PT-INR Value

N	Mean	Standard Deviation	Minimum	Maximum
4	1.8175000	0.0754431	1.7700000	1.9300000

Unique Subject Identifier=0058000100-0007

Assay variable : PTINR_VAL1 PT-INR Value

N	Mean	Standard Deviation	Minimum	Maximum
2	1.6900000	0.8343860	1.1000000	2.2800000

Laboratory tests at the time of obtaining Table 2-6 consent, according to the presence or absence of proton-pump inhibitors

Unique Subject Identifier=0059100000-0001

MEANS Procedure

Assay variable : PTINR_VAL1 PT-INR Value

N	Mean	Standard Deviation	Minimum	Maximum
3	1.6000000	0.0953939	1.5000000	1.6900000

Unique Subject Identifier=0059100000-0002

Assay variable : PTINR_VAL1 PT-INR Value

N	Mean	Standard Deviation	Minimum	Maximum
5	1.5640000	0.1342758	1.3600000	1.7300000

Unique Subject Identifier=0059100000-0003

Assay variable : PTINR_VAL1 PT-INR Value

N	Mean	Standard Deviation	Minimum	Maximum
6	1.7716667	0.1251266	1.6400000	1.9700000

Unique Subject Identifier=0059100000-0005

Assay variable : PTINR_VAL1 PT-INR Value

N	Mean	Standard Deviation	Minimum	Maximum
5	1.5380000	0.2161481	1.3400000	1.8400000

Laboratory tests at the time of obtaining Table 2-6 consent, according to the presence or absence of proton-pump inhibitors

Unique Subject Identifier=0061000000-0001

MEANS Procedure

Assay variable : PTINR_VAL1 PT-INR Value

N	Mean	Standard Deviation	Minimum	Maximum
3	1.9800000	0.2170253	1.8400000	2.2300000

Unique Subject Identifier=0061000000-0005

Assay variable : PTINR_VAL1 PT-INR Value

N	Mean	Standard Deviation	Minimum	Maximum
3	2.1266667	0.4099187	1.8900000	2.6000000

Unique Subject Identifier=0061000000-0006

Assay variable : PTINR_VAL1 PT-INR Value

N	Mean	Standard Deviation	Minimum	Maximum
2	2.2100000	0.2828427	2.0100000	2.4100000

Unique Subject Identifier=0061000000-0010

Assay variable : PTINR_VAL1 PT-INR Value

N	Mean	Standard Deviation	Minimum	Maximum
4	2.0625000	0.4855495	1.6000000	2.7400000

Laboratory tests at the time of obtaining Table 2-6 consent, according to the presence or absence of proton-pump inhibitors

Unique Subject Identifier=0061000000-0018

MEANS Procedure

Assay variable : PTINR_VAL1 PT-INR Value

N	Mean	Standard Deviation	Minimum	Maximum
1	1.0800000		1.0800000	1.0800000

Unique Subject Identifier=0061000000-0019

Assay variable : PTINR_VAL1 PT-INR Value

N	Mean	Standard Deviation	Minimum	Maximum
2	2.4500000	0.9616652	1.7700000	3.1300000

Unique Subject Identifier=0061000000-0021

Assay variable : PTINR_VAL1 PT-INR Value

N	Mean	Standard Deviation	Minimum	Maximum
6	1.9633333	0.6763333	1.2700000	3.1700000

Unique Subject Identifier=0063010000-0012

Assay variable : PTINR_VAL1 PT-INR Value

N	Mean	Standard Deviation	Minimum	Maximum
3	1.6033333	0.0757188	1.5500000	1.6900000

Laboratory tests at the time of obtaining Table 2-6 consent, according to the presence or absence of proton-pump inhibitors

Unique Subject Identifier=0063010000-0013

MEANS Procedure

Assay variable : PTINR_VAL1 PT-INR Value

N	Mean	Standard Deviation	Minimum	Maximum
1	2.7000000		2.7000000	2.7000000

Unique Subject Identifier=0063010000-0015

Assay variable : PTINR_VAL1 PT-INR Value

N	Mean	Standard Deviation	Minimum	Maximum
2	1.5100000	0.0707107	1.4600000	1.5600000

Unique Subject Identifier=0063010000-0016

Assay variable : PTINR_VAL1 PT-INR Value

N	Mean	Standard Deviation	Minimum	Maximum
3	1.5333333	0.1761628	1.3700000	1.7200000

Unique Subject Identifier=0063010000-0018

Assay variable : PTINR_VAL1 PT-INR Value

N	Mean	Standard Deviation	Minimum	Maximum
4	1.9150000	0.4522905	1.5300000	2.5700000

Laboratory tests at the time of obtaining Table 2-6 consent, according to the presence or absence of proton-pump inhibitors

Unique Subject Identifier=0063010000-0024

MEANS Procedure

Assay variable : PTINR_VAL1 PT-INR Value

N	Mean	Standard Deviation	Minimum	Maximum
6	2.0466667	0.4078071	1.6100000	2.6000000

Unique Subject Identifier=0063010000-0029

Assay variable : PTINR_VAL1 PT-INR Value

N	Mean	Standard Deviation	Minimum	Maximum
3	1.9566667	0.2571640	1.7700000	2.2500000

Unique Subject Identifier=0063010000-0030

Assay variable : PTINR_VAL1 PT-INR Value

N	Mean	Standard Deviation	Minimum	Maximum
3	1.9133333	0.2902298	1.6300000	2.2100000

Unique Subject Identifier=0063010000-0033

Assay variable : PTINR_VAL1 PT-INR Value

N	Mean	Standard Deviation	Minimum	Maximum
3	1.7900000	0.3328663	1.5500000	2.1700000

Laboratory tests at the time of obtaining Table 2-6 consent, according to the presence or absence of proton-pump inhibitors

Unique Subject Identifier=0063010000-0036

MEANS Procedure

Assay variable : PTINR_VAL1 PT-INR Value

N	Mean	Standard Deviation	Minimum	Maximum
3	2.0900000	0.1509967	1.9300000	2.2300000

Unique Subject Identifier=0063010000-0041

Assay variable : PTINR_VAL1 PT-INR Value

N	Mean	Standard Deviation	Minimum	Maximum
2	1.7050000	0.5586144	1.3100000	2.1000000

Unique Subject Identifier=0063010000-0044

Assay variable : PTINR_VAL1 PT-INR Value

N	Mean	Standard Deviation	Minimum	Maximum
3	2.4366667	0.3066486	2.1900000	2.7800000

Unique Subject Identifier=0063110000-0004

Assay variable : PTINR_VAL1 PT-INR Value

N	Mean	Standard Deviation	Minimum	Maximum
6	1.8750000	0.0531977	1.8300000	1.9800000

Laboratory tests at the time of obtaining Table 2-6 consent, according to the presence or absence of proton-pump inhibitors

Unique Subject Identifier=0063110000-0005

MEANS Procedure

Assay variable : PTINR_VAL1 PT-INR Value

N	Mean	Standard Deviation	Minimum	Maximum
5	2.7820000	0.5848248	1.9100000	3.3800000

Unique Subject Identifier=0063110000-0007

Assay variable : PTINR_VAL1 PT-INR Value

N	Mean	Standard Deviation	Minimum	Maximum
2	1.8050000	0.0919239	1.7400000	1.8700000

Unique Subject Identifier=0063110000-0012

Assay variable : PTINR_VAL1 PT-INR Value

N	Mean	Standard Deviation	Minimum	Maximum
5	1.9900000	0.9109336	1.2200000	3.5600000

Unique Subject Identifier=0063110000-0015

Assay variable : PTINR_VAL1 PT-INR Value

N	Mean	Standard Deviation	Minimum	Maximum
4	2.0775000	1.0203717	1.3600000	3.5900000

Laboratory tests at the time of obtaining Table 2-6 consent, according to the presence or absence of proton-pump inhibitors

Unique Subject Identifier=0064010110-0003

MEANS Procedure

Assay variable : PTINR_VAL1 PT-INR Value

N	Mean	Standard Deviation	Minimum	Maximum
6	2.4583333	0.1922932	2.2600000	2.7800000

Unique Subject Identifier=0064010110-0011

Assay variable : PTINR_VAL1 PT-INR Value

N	Mean	Standard Deviation	Minimum	Maximum
6	1.7750000	0.1013410	1.6100000	1.9100000

Unique Subject Identifier=0064010110-0012

Assay variable : PTINR_VAL1 PT-INR Value

N	Mean	Standard Deviation	Minimum	Maximum
3	1.5666667	0.3768731	1.1700000	1.9200000

Unique Subject Identifier=0064010110-0014

Assay variable : PTINR_VAL1 PT-INR Value

N	Mean	Standard Deviation	Minimum	Maximum
6	1.8866667	0.1789600	1.7200000	2.1100000

Laboratory tests at the time of obtaining Table 2-6 consent, according to the presence or absence of proton-pump inhibitors

Unique Subject Identifier=0064010110-0015

MEANS Procedure

Assay variable : PTINR_VAL1 PT-INR Value

N	Mean	Standard Deviation	Minimum	Maximum
5	1.6900000	0.3061862	1.4100000	2.1700000

Unique Subject Identifier=0069000000-0003

Assay variable : PTINR_VAL1 PT-INR Value

N	Mean	Standard Deviation	Minimum	Maximum
6	1.8816667	0.5136698	1.5700000	2.9000000

Unique Subject Identifier=0069000000-0014

Assay variable : PTINR_VAL1 PT-INR Value

N	Mean	Standard Deviation	Minimum	Maximum
6	3.1800000	2.1056116	1.4200000	6.8400000

Unique Subject Identifier=0070000000-0003

Assay variable : PTINR_VAL1 PT-INR Value

N	Mean	Standard Deviation	Minimum	Maximum
6	2.0000000	0.3207491	1.7300000	2.4700000

Laboratory tests at the time of obtaining Table 2-6 consent, according to the presence or absence of proton-pump inhibitors

Unique Subject Identifier=0070000000-0005

MEANS Procedure

Assay variable : PTINR_VAL1 PT-INR Value

N	Mean	Standard Deviation	Minimum	Maximum
3	1.8533333	0.4343194	1.4800000	2.3300000

Unique Subject Identifier=0070000000-0008

Assay variable : PTINR_VAL1 PT-INR Value

N	Mean	Standard Deviation	Minimum	Maximum
4	2.2175000	0.1260622	2.0600000	2.3600000

Unique Subject Identifier=0070000000-0009

Assay variable : PTINR_VAL1 PT-INR Value

N	Mean	Standard Deviation	Minimum	Maximum
5	2.0500000	0.6826419	1.4600000	3.0000000

Unique Subject Identifier=0070000000-0013

Assay variable : PTINR_VAL1 PT-INR Value

N	Mean	Standard Deviation	Minimum	Maximum
3	2.1933333	0.2554082	1.9900000	2.4800000

Laboratory tests at the time of obtaining Table 2-6 consent, according to the presence or absence of proton-pump inhibitors

Unique Subject Identifier=0070000000-0016

MEANS Procedure

Assay variable : PTINR_VAL1 PT-INR Value

N	Mean	Standard Deviation	Minimum	Maximum
5	2.7660000	0.8777984	1.9700000	4.2300000

Unique Subject Identifier=0070000000-0018

Assay variable : PTINR_VAL1 PT-INR Value

N	Mean	Standard Deviation	Minimum	Maximum
5	1.5540000	0.3332867	1.0600000	1.8600000

Unique Subject Identifier=0070000000-0022

Assay variable : PTINR_VAL1 PT-INR Value

N	Mean	Standard Deviation	Minimum	Maximum
4	2.1075000	0.3629853	1.7900000	2.5300000

Unique Subject Identifier=0070000000-0026

Assay variable : PTINR_VAL1 PT-INR Value

N	Mean	Standard Deviation	Minimum	Maximum
6	2.6550000	0.1619568	2.4700000	2.8500000

Laboratory tests at the time of obtaining Table 2-6 consent, according to the presence or absence of proton-pump inhibitors

Unique Subject Identifier=0070000000-0028

MEANS Procedure

Assay variable : PTINR_VAL1 PT-INR Value

N	Mean	Standard Deviation	Minimum	Maximum
3	1.8166667	0.2800595	1.6500000	2.1400000

Unique Subject Identifier=0070000000-0029

Assay variable : PTINR_VAL1 PT-INR Value

N	Mean	Standard Deviation	Minimum	Maximum
4	2.5775000	0.5146763	2.1100000	3.2100000

Unique Subject Identifier=0070000000-0030

Assay variable : PTINR_VAL1 PT-INR Value

N	Mean	Standard Deviation	Minimum	Maximum
3	1.9400000	0.3465545	1.7300000	2.3400000

Unique Subject Identifier=0070000000-0031

Assay variable : PTINR_VAL1 PT-INR Value

N	Mean	Standard Deviation	Minimum	Maximum
3	1.7400000	0.1967232	1.5300000	1.9200000

Laboratory tests at the time of obtaining Table 2-6 consent, according to the presence or absence of proton-pump inhibitors

Unique Subject Identifier=0070000000-0032

MEANS Procedure

Assay variable : PTINR_VAL1 PT-INR Value

N	Mean	Standard Deviation	Minimum	Maximum
3	2.1766667	0.1250333	2.0900000	2.3200000

Unique Subject Identifier=0070000000-0033

Assay variable : PTINR_VAL1 PT-INR Value

N	Mean	Standard Deviation	Minimum	Maximum
3	1.8100000	0.0655744	1.7500000	1.8800000

Unique Subject Identifier=0070000000-0035

Assay variable : PTINR_VAL1 PT-INR Value

N	Mean	Standard Deviation	Minimum	Maximum
3	1.6000000	0.1000000	1.5000000	1.7000000

Unique Subject Identifier=0070000000-0039

Assay variable : PTINR_VAL1 PT-INR Value

N	Mean	Standard Deviation	Minimum	Maximum
4	1.5500000	0.4002499	1.1300000	2.0700000

Laboratory tests at the time of obtaining Table 2-6 consent, according to the presence or absence of proton-pump inhibitors

Unique Subject Identifier=0070000000-0041

MEANS Procedure

Assay variable : PTINR_VAL1 PT-INR Value

N	Mean	Standard Deviation	Minimum	Maximum
4	2.1800000	0.2409703	1.8200000	2.3300000

Unique Subject Identifier=0070000000-0043

Assay variable : PTINR_VAL1 PT-INR Value

N	Mean	Standard Deviation	Minimum	Maximum
4	1.7825000	0.1664081	1.5700000	1.9700000

Unique Subject Identifier=0070000000-0046

Assay variable : PTINR_VAL1 PT-INR Value

N	Mean	Standard Deviation	Minimum	Maximum
3	2.0866667	0.1504438	1.9300000	2.2300000

Unique Subject Identifier=0070000000-0048

Assay variable : PTINR_VAL1 PT-INR Value

N	Mean	Standard Deviation	Minimum	Maximum
4	1.8150000	0.1554563	1.6800000	2.0200000

Laboratory tests at the time of obtaining Table 2-6 consent, according to the presence or absence of proton-pump inhibitors

Unique Subject Identifier=0070000000-0049

MEANS Procedure

Assay variable : PTINR_VAL1 PT-INR Value

N	Mean	Standard Deviation	Minimum	Maximum
5	2.0280000	0.4447134	1.5700000	2.6600000

Unique Subject Identifier=0070000000-0050

Assay variable : PTINR_VAL1 PT-INR Value

N	Mean	Standard Deviation	Minimum	Maximum
6	2.2550000	1.2352935	1.4300000	4.7300000

Unique Subject Identifier=0070000000-0053

Assay variable : PTINR_VAL1 PT-INR Value

N	Mean	Standard Deviation	Minimum	Maximum
3	1.7166667	0.1001665	1.6400000	1.8300000

Unique Subject Identifier=0070000000-0054

Assay variable : PTINR_VAL1 PT-INR Value

N	Mean	Standard Deviation	Minimum	Maximum
5	2.2600000	0.3611094	1.8900000	2.6900000

Laboratory tests at the time of obtaining Table 2-6 consent, according to the presence or absence of proton-pump inhibitors

Unique Subject Identifier=0070000000-0056

MEANS Procedure

Assay variable : PTINR_VAL1 PT-INR Value

N	Mean	Standard Deviation	Minimum	Maximum
3	2.4266667	0.7861510	1.5900000	3.1500000

Unique Subject Identifier=0070000000-0058

Assay variable : PTINR_VAL1 PT-INR Value

N	Mean	Standard Deviation	Minimum	Maximum
3	2.2500000	0.3811824	2.0200000	2.6900000

Unique Subject Identifier=0070000000-0061

Assay variable : PTINR_VAL1 PT-INR Value

N	Mean	Standard Deviation	Minimum	Maximum
5	1.8480000	0.3226763	1.3500000	2.2100000

Unique Subject Identifier=0070000000-0063

Assay variable : PTINR_VAL1 PT-INR Value

N	Mean	Standard Deviation	Minimum	Maximum
3	1.5533333	0.0838650	1.5000000	1.6500000

Laboratory tests at the time of obtaining Table 2-6 consent, according to the presence or absence of proton-pump inhibitors

Unique Subject Identifier=0070000000-0064

MEANS Procedure

Assay variable : PTINR_VAL1 PT-INR Value

N	Mean	Standard Deviation	Minimum	Maximum
3	2.1933333	0.3875994	1.8900000	2.6300000

Unique Subject Identifier=0070000000-0066

Assay variable : PTINR_VAL1 PT-INR Value

N	Mean	Standard Deviation	Minimum	Maximum
3	1.8166667	0.1484363	1.6900000	1.9800000

Unique Subject Identifier=0070000000-0067

Assay variable : PTINR_VAL1 PT-INR Value

N	Mean	Standard Deviation	Minimum	Maximum
4	2.3400000	0.0496655	2.2900000	2.4000000

Unique Subject Identifier=0070000000-0070

Assay variable : PTINR_VAL1 PT-INR Value

N	Mean	Standard Deviation	Minimum	Maximum
4	2.2600000	0.6172520	1.6200000	3.0400000

Laboratory tests at the time of obtaining Table 2-6 consent, according to the presence or absence of proton-pump inhibitors

Unique Subject Identifier=0070000000-0071

MEANS Procedure

Assay variable : PTINR_VAL1 PT-INR Value

N	Mean	Standard Deviation	Minimum	Maximum
3	1.9133333	0.2454248	1.6600000	2.1500000

Unique Subject Identifier=0070000000-0073

Assay variable : PTINR_VAL1 PT-INR Value

N	Mean	Standard Deviation	Minimum	Maximum
5	1.8960000	0.4698191	1.4000000	2.4600000

Unique Subject Identifier=0070000000-0074

Assay variable : PTINR_VAL1 PT-INR Value

N	Mean	Standard Deviation	Minimum	Maximum
4	1.7525000	0.0991211	1.6100000	1.8200000

Unique Subject Identifier=0071000000-0001

Assay variable : PTINR_VAL1 PT-INR Value

N	Mean	Standard Deviation	Minimum	Maximum
4	1.6275000	0.1065755	1.4900000	1.7300000

Laboratory tests at the time of obtaining Table 2-6 consent, according to the presence or absence of proton-pump inhibitors

Unique Subject Identifier=0071000000-0002

MEANS Procedure

Assay variable : PTINR_VAL1 PT-INR Value

N	Mean	Standard Deviation	Minimum	Maximum
1	2.1300000		2.1300000	2.1300000

Unique Subject Identifier=0071000000-0003

Assay variable : PTINR_VAL1 PT-INR Value

N	Mean	Standard Deviation	Minimum	Maximum
4	2.5250000	0.7388505	1.9500000	3.5600000

Unique Subject Identifier=0071000000-0004

Assay variable : PTINR_VAL1 PT-INR Value

N	Mean	Standard Deviation	Minimum	Maximum
4	2.1550000	0.2315887	1.9500000	2.4800000

Unique Subject Identifier=0071000000-0005

Assay variable : PTINR_VAL1 PT-INR Value

N	Mean	Standard Deviation	Minimum	Maximum
6	1.9083333	0.1915637	1.7200000	2.2000000

Laboratory tests at the time of obtaining Table 2-6 consent, according to the presence or absence of proton-pump inhibitors

Unique Subject Identifier=0071000000-0006

MEANS Procedure

Assay variable : PTINR_VAL1 PT-INR Value

N	Mean	Standard Deviation	Minimum	Maximum
3	1.8133333	0.4452340	1.3600000	2.2500000

Unique Subject Identifier=0071000000-0007

Assay variable : PTINR_VAL1 PT-INR Value

N	Mean	Standard Deviation	Minimum	Maximum
6	1.8166667	0.3808762	1.4000000	2.3400000

Unique Subject Identifier=0071000000-0008

Assay variable : PTINR_VAL1 PT-INR Value

N	Mean	Standard Deviation	Minimum	Maximum
3	1.7433333	0.1556706	1.5800000	1.8900000

Unique Subject Identifier=0071000000-0009

Assay variable : PTINR_VAL1 PT-INR Value

N	Mean	Standard Deviation	Minimum	Maximum
4	1.7650000	0.1239624	1.6100000	1.8700000

Laboratory tests at the time of obtaining Table 2-6 consent, according to the presence or absence of proton-pump inhibitors

Unique Subject Identifier=0071000000-0010

MEANS Procedure

Assay variable : PTINR_VAL1 PT-INR Value

N	Mean	Standard Deviation	Minimum	Maximum
4	1.8125000	0.2233644	1.5500000	2.0700000

Unique Subject Identifier=0071000000-0013

Assay variable : PTINR_VAL1 PT-INR Value

N	Mean	Standard Deviation	Minimum	Maximum
4	2.0150000	0.1584298	1.9200000	2.2500000

Unique Subject Identifier=0071000000-0015

Assay variable : PTINR_VAL1 PT-INR Value

N	Mean	Standard Deviation	Minimum	Maximum
4	1.8825000	0.1547848	1.7500000	2.0900000

Unique Subject Identifier=0071000000-0016

Assay variable : PTINR_VAL1 PT-INR Value

N	Mean	Standard Deviation	Minimum	Maximum
1	2.0500000		2.0500000	2.0500000

Laboratory tests at the time of obtaining Table 2-6 consent, according to the presence or absence of proton-pump inhibitors

Unique Subject Identifier=0071000000-0018

MEANS Procedure

Assay variable : PTINR_VAL1 PT-INR Value

N	Mean	Standard Deviation	Minimum	Maximum
3	1.8100000	0.2343075	1.6600000	2.0800000

Unique Subject Identifier=0071000000-0019

Assay variable : PTINR_VAL1 PT-INR Value

N	Mean	Standard Deviation	Minimum	Maximum
5	1.9220000	0.0683374	1.8700000	2.0400000

Unique Subject Identifier=0071000000-0020

Assay variable : PTINR_VAL1 PT-INR Value

N	Mean	Standard Deviation	Minimum	Maximum
4	2.4150000	0.4956813	2.0700000	3.1500000

Unique Subject Identifier=0071000000-0021

Assay variable : PTINR_VAL1 PT-INR Value

N	Mean	Standard Deviation	Minimum	Maximum
5	1.9080000	0.2037646	1.7200000	2.1900000

Laboratory tests at the time of obtaining Table 2-6 consent, according to the presence or absence of proton-pump inhibitors

Unique Subject Identifier=0071000000-0022

MEANS Procedure

Assay variable : PTINR_VAL1 PT-INR Value

N	Mean	Standard Deviation	Minimum	Maximum
5	1.9380000	0.1781011	1.7000000	2.2000000

Unique Subject Identifier=0071000000-0025

Assay variable : PTINR_VAL1 PT-INR Value

N	Mean	Standard Deviation	Minimum	Maximum
3	1.4066667	0.2466441	1.2400000	1.6900000

Unique Subject Identifier=0071000000-0027

Assay variable : PTINR_VAL1 PT-INR Value

N	Mean	Standard Deviation	Minimum	Maximum
4	1.5950000	0.3139533	1.2600000	1.9100000

Unique Subject Identifier=0071000000-0028

Assay variable : PTINR_VAL1 PT-INR Value

N	Mean	Standard Deviation	Minimum	Maximum
3	2.1533333	0.6351640	1.4200000	2.5300000

Laboratory tests at the time of obtaining Table 2-6 consent, according to the presence or absence of proton-pump inhibitors

Unique Subject Identifier=0071000000-0030

MEANS Procedure

Assay variable : PTINR_VAL1 PT-INR Value

N	Mean	Standard Deviation	Minimum	Maximum
3	2.5500000	0.3465545	2.3400000	2.9500000

Unique Subject Identifier=0072000000-0004

Assay variable : PTINR_VAL1 PT-INR Value

N	Mean	Standard Deviation	Minimum	Maximum
6	1.8583333	0.3816499	1.4300000	2.2900000

Unique Subject Identifier=0072000000-0006

Assay variable : PTINR_VAL1 PT-INR Value

N	Mean	Standard Deviation	Minimum	Maximum
5	2.2060000	0.1829754	2.0400000	2.5200000

Unique Subject Identifier=0072000000-0010

Assay variable : PTINR_VAL1 PT-INR Value

N	Mean	Standard Deviation	Minimum	Maximum
6	2.3000000	0.4325737	1.6100000	2.7300000

Laboratory tests at the time of obtaining Table 2-6 consent, according to the presence or absence of proton-pump inhibitors

Unique Subject Identifier=0072000000-0011

MEANS Procedure

Assay variable : PTINR_VAL1 PT-INR Value

N	Mean	Standard Deviation	Minimum	Maximum
6	1.6583333	0.2003414	1.4000000	2.0200000

Unique Subject Identifier=0073000000-0003

Assay variable : PTINR_VAL1 PT-INR Value

N	Mean	Standard Deviation	Minimum	Maximum
5	1.8840000	0.3686190	1.3200000	2.3000000

Unique Subject Identifier=0073000000-0022

Assay variable : PTINR_VAL1 PT-INR Value

N	Mean	Standard Deviation	Minimum	Maximum
6	1.4116667	0.5947577	1.0300000	2.5800000

Unique Subject Identifier=0073000000-0035

Assay variable : PTINR_VAL1 PT-INR Value

N	Mean	Standard Deviation	Minimum	Maximum
3	2.1766667	0.1150362	2.0600000	2.2900000

Laboratory tests at the time of obtaining Table 2-6 consent, according to the presence or absence of proton-pump inhibitors

Unique Subject Identifier=0076011000-0003

MEANS Procedure

Assay variable : PTINR_VAL1 PT-INR Value

N	Mean	Standard Deviation	Minimum	Maximum
1	1.5300000		1.5300000	1.5300000

Unique Subject Identifier=0076011000-0004

Assay variable : PTINR_VAL1 PT-INR Value

N	Mean	Standard Deviation	Minimum	Maximum
1	1.6900000		1.6900000	1.6900000

Unique Subject Identifier=0076011000-0005

Assay variable : PTINR_VAL1 PT-INR Value

N	Mean	Standard Deviation	Minimum	Maximum
5	1.8240000	1.0294319	1.0100000	3.5100000

Unique Subject Identifier=0076011000-0008

Assay variable : PTINR_VAL1 PT-INR Value

N	Mean	Standard Deviation	Minimum	Maximum
6	1.4433333	0.3104620	1.2000000	1.9800000

Laboratory tests at the time of obtaining Table 2-6 consent, according to the presence or absence of proton-pump inhibitors

Unique Subject Identifier=0076011000-0009

MEANS Procedure

Assay variable : PTINR_VAL1 PT-INR Value

N	Mean	Standard Deviation	Minimum	Maximum
1	1.2100000		1.2100000	1.2100000

Unique Subject Identifier=0076011000-0015

Assay variable : PTINR_VAL1 PT-INR Value

N	Mean	Standard Deviation	Minimum	Maximum
2	1.8200000	0.1131371	1.7400000	1.9000000

Unique Subject Identifier=0076011000-0017

Assay variable : PTINR_VAL1 PT-INR Value

N	Mean	Standard Deviation	Minimum	Maximum
4	1.2225000	0.0330404	1.2000000	1.2700000

Unique Subject Identifier=0076011000-0020

Assay variable : PTINR_VAL1 PT-INR Value

N	Mean	Standard Deviation	Minimum	Maximum
2	2.1250000	0.2757716	1.9300000	2.3200000

Laboratory tests at the time of obtaining Table 2-6 consent, according to the presence or absence of proton-pump inhibitors

Unique Subject Identifier=0076011000-0024

MEANS Procedure

Assay variable : PTINR_VAL1 PT-INR Value

N	Mean	Standard Deviation	Minimum	Maximum
1	2.0300000		2.0300000	2.0300000

Unique Subject Identifier=0076011000-0025

Assay variable : PTINR_VAL1 PT-INR Value

N	Mean	Standard Deviation	Minimum	Maximum
2	2.0250000	0.6858936	1.5400000	2.5100000

Unique Subject Identifier=0077000000-0004

Assay variable : PTINR_VAL1 PT-INR Value

N	Mean	Standard Deviation	Minimum	Maximum
4	2.1100000	1.0274564	1.0300000	3.4900000

Unique Subject Identifier=0077000000-0005

Assay variable : PTINR_VAL1 PT-INR Value

N	Mean	Standard Deviation	Minimum	Maximum
1	1.6400000		1.6400000	1.6400000

Laboratory tests at the time of obtaining Table 2-6 consent, according to the presence or absence of proton-pump inhibitors

Unique Subject Identifier=0077000000-0010

MEANS Procedure

Assay variable : PTINR_VAL1 PT-INR Value

N	Mean	Standard Deviation	Minimum	Maximum
3	2.1966667	0.7328256	1.4100000	2.8600000

Unique Subject Identifier=0078001000-0001

Assay variable : PTINR_VAL1 PT-INR Value

N	Mean	Standard Deviation	Minimum	Maximum
6	2.1783333	0.1894642	1.8300000	2.3400000

Unique Subject Identifier=0078001000-0003

Assay variable : PTINR_VAL1 PT-INR Value

N	Mean	Standard Deviation	Minimum	Maximum
3	2.0466667	0.5746593	1.7000000	2.7100000

Unique Subject Identifier=0078001000-0004

Assay variable : PTINR_VAL1 PT-INR Value

N	Mean	Standard Deviation	Minimum	Maximum
4	1.8150000	0.1748333	1.5900000	2.0000000

Laboratory tests at the time of obtaining Table 2-6 consent, according to the presence or absence of proton-pump inhibitors

Unique Subject Identifier=0078001000-0005

MEANS Procedure

Assay variable : PTINR_VAL1 PT-INR Value

N	Mean	Standard Deviation	Minimum	Maximum
3	2.1133333	0.2804164	1.8700000	2.4200000

Unique Subject Identifier=0078001000-0007

Assay variable : PTINR_VAL1 PT-INR Value

N	Mean	Standard Deviation	Minimum	Maximum
3	1.6066667	0.0321455	1.5700000	1.6300000

Unique Subject Identifier=0078001000-0008

Assay variable : PTINR_VAL1 PT-INR Value

N	Mean	Standard Deviation	Minimum	Maximum
5	2.1140000	0.3263893	1.7700000	2.5100000

Unique Subject Identifier=0078001000-0010

Assay variable : PTINR_VAL1 PT-INR Value

N	Mean	Standard Deviation	Minimum	Maximum
3	1.8933333	0.0251661	1.8700000	1.9200000

Laboratory tests at the time of obtaining Table 2-6 consent, according to the presence or absence of proton-pump inhibitors

Unique Subject Identifier=0078001000-0011

MEANS Procedure

Assay variable : PTINR_VAL1 PT-INR Value

N	Mean	Standard Deviation	Minimum	Maximum
3	2.1600000	0.3551056	1.7500000	2.3700000

Unique Subject Identifier=0078001000-0012

Assay variable : PTINR_VAL1 PT-INR Value

N	Mean	Standard Deviation	Minimum	Maximum
3	1.9400000	0.1212436	1.8700000	2.0800000

Unique Subject Identifier=0078001000-0013

Assay variable : PTINR_VAL1 PT-INR Value

N	Mean	Standard Deviation	Minimum	Maximum
3	1.9600000	0.0964365	1.8900000	2.0700000

Unique Subject Identifier=0078001000-0016

Assay variable : PTINR_VAL1 PT-INR Value

N	Mean	Standard Deviation	Minimum	Maximum
4	2.2425000	1.1359101	1.5000000	3.9200000

Laboratory tests at the time of obtaining Table 2-6 consent, according to the presence or absence of proton-pump inhibitors

Unique Subject Identifier=0079000000-0001

MEANS Procedure

Assay variable : PTINR_VAL1 PT-INR Value

N	Mean	Standard Deviation	Minimum	Maximum
4	2.3750000	0.5081666	1.9000000	3.0600000

Unique Subject Identifier=0079000000-0002

Assay variable : PTINR_VAL1 PT-INR Value

N	Mean	Standard Deviation	Minimum	Maximum
3	1.9466667	0.2103172	1.7300000	2.1500000

Unique Subject Identifier=0079000000-0003

Assay variable : PTINR_VAL1 PT-INR Value

N	Mean	Standard Deviation	Minimum	Maximum
4	1.7800000	0.1219289	1.6900000	1.9600000

Unique Subject Identifier=0079000000-0009

Assay variable : PTINR_VAL1 PT-INR Value

N	Mean	Standard Deviation	Minimum	Maximum
3	1.7866667	0.0850490	1.6900000	1.8500000

Laboratory tests at the time of obtaining Table 2-6 consent, according to the presence or absence of proton-pump inhibitors

Unique Subject Identifier=0079000000-0020

MEANS Procedure

Assay variable : PTINR_VAL1 PT-INR Value

N	Mean	Standard Deviation	Minimum	Maximum
4	1.8125000	0.2628529	1.4700000	2.0600000

Unique Subject Identifier=0079000000-0021

Assay variable : PTINR_VAL1 PT-INR Value

N	Mean	Standard Deviation	Minimum	Maximum
3	1.7900000	0.0300000	1.7600000	1.8200000

Unique Subject Identifier=0079000000-0022

Assay variable : PTINR_VAL1 PT-INR Value

N	Mean	Standard Deviation	Minimum	Maximum
2	2.1400000	0.5939697	1.7200000	2.5600000

Unique Subject Identifier=0079000000-0023

Assay variable : PTINR_VAL1 PT-INR Value

N	Mean	Standard Deviation	Minimum	Maximum
3	1.6000000	0.2007486	1.4600000	1.8300000

Laboratory tests at the time of obtaining Table 2-6 consent, according to the presence or absence of proton-pump inhibitors

Unique Subject Identifier=0079000000-0026

MEANS Procedure

Assay variable : PTINR_VAL1 PT-INR Value

N	Mean	Standard Deviation	Minimum	Maximum
1	1.4000000		1.4000000	1.4000000

Unique Subject Identifier=0079000000-0028

Assay variable : PTINR_VAL1 PT-INR Value

N	Mean	Standard Deviation	Minimum	Maximum
3	1.8666667	0.7486877	1.3200000	2.7200000

Unique Subject Identifier=0079000000-0029

Assay variable : PTINR_VAL1 PT-INR Value

N	Mean	Standard Deviation	Minimum	Maximum
3	1.9366667	0.5179125	1.3400000	2.2700000

Unique Subject Identifier=0079000000-0030

Assay variable : PTINR_VAL1 PT-INR Value

N	Mean	Standard Deviation	Minimum	Maximum
3	2.1600000	1.1421033	1.0600000	3.3400000

Laboratory tests at the time of obtaining Table 2-6 consent, according to the presence or absence of proton-pump inhibitors

Unique Subject Identifier=0081000000-0005

MEANS Procedure

Assay variable : PTINR_VAL1 PT-INR Value

N	Mean	Standard Deviation	Minimum	Maximum
4	1.6250000	0.2651415	1.3800000	2.0000000

Unique Subject Identifier=0081000000-0006

Assay variable : PTINR_VAL1 PT-INR Value

N	Mean	Standard Deviation	Minimum	Maximum
3	1.6066667	0.2400694	1.4300000	1.8800000

Unique Subject Identifier=0081000000-0010

Assay variable : PTINR_VAL1 PT-INR Value

N	Mean	Standard Deviation	Minimum	Maximum
4	2.0850000	0.1552417	1.8900000	2.2700000

Unique Subject Identifier=0081000000-0011

Assay variable : PTINR_VAL1 PT-INR Value

N	Mean	Standard Deviation	Minimum	Maximum
6	2.3900000	0.7361522	1.3800000	3.6200000

Laboratory tests at the time of obtaining Table 2-6 consent, according to the presence or absence of proton-pump inhibitors

Unique Subject Identifier=0085000000-0004

MEANS Procedure

Assay variable : PTINR_VAL1 PT-INR Value

N	Mean	Standard Deviation	Minimum	Maximum
6	2.0100000	0.1941134	1.7200000	2.3100000

Unique Subject Identifier=0085000000-0007

Assay variable : PTINR_VAL1 PT-INR Value

N	Mean	Standard Deviation	Minimum	Maximum
6	1.8800000	0.5933633	1.2700000	2.9200000

Unique Subject Identifier=0086100000-0005

Assay variable : PTINR_VAL1 PT-INR Value

N	Mean	Standard Deviation	Minimum	Maximum
1	1.4700000		1.4700000	1.4700000

Unique Subject Identifier=0086100000-0010

Assay variable : PTINR_VAL1 PT-INR Value

N	Mean	Standard Deviation	Minimum	Maximum
1	2.5800000		2.5800000	2.5800000

Laboratory tests at the time of obtaining Table 2-6 consent, according to the presence or absence of proton-pump inhibitors

Unique Subject Identifier=0086100000-0011

MEANS Procedure

Assay variable : PTINR_VAL1 PT-INR Value

N	Mean	Standard Deviation	Minimum	Maximum
1	1.7600000		1.7600000	1.7600000

Unique Subject Identifier=0086100000-0019

Assay variable : PTINR_VAL1 PT-INR Value

N	Mean	Standard Deviation	Minimum	Maximum
4	1.6750000	0.2030599	1.4400000	1.8900000

Unique Subject Identifier=0094001000-0012

Assay variable : PTINR_VAL1 PT-INR Value

N	Mean	Standard Deviation	Minimum	Maximum
4	2.2625000	0.3564992	1.8800000	2.7400000

Unique Subject Identifier=0094001000-0016

Assay variable : PTINR_VAL1 PT-INR Value

N	Mean	Standard Deviation	Minimum	Maximum
4	1.5175000	0.2064582	1.2500000	1.6900000

Laboratory tests at the time of obtaining Table 2-6 consent, according to the presence or absence of proton-pump inhibitors

Unique Subject Identifier=0094001000-0018

MEANS Procedure

Assay variable : PTINR_VAL1 PT-INR Value

N	Mean	Standard Deviation	Minimum	Maximum
3	2.0966667	0.1850225	1.9800000	2.3100000

Unique Subject Identifier=0094001000-0023

Assay variable : PTINR_VAL1 PT-INR Value

N	Mean	Standard Deviation	Minimum	Maximum
3	1.9300000	0.0818535	1.8600000	2.0200000

Unique Subject Identifier=0094001000-0026

Assay variable : PTINR_VAL1 PT-INR Value

N	Mean	Standard Deviation	Minimum	Maximum
4	1.7525000	0.1004573	1.6400000	1.8600000

Unique Subject Identifier=0094001000-0028

Assay variable : PTINR_VAL1 PT-INR Value

N	Mean	Standard Deviation	Minimum	Maximum
3	1.9366667	0.1721434	1.8000000	2.1300000

Laboratory tests at the time of obtaining Table 2-6 consent, according to the presence or absence of proton-pump inhibitors

Unique Subject Identifier=0094001000-0038

MEANS Procedure

Assay variable : PTINR_VAL1 PT-INR Value

N	Mean	Standard Deviation	Minimum	Maximum
4	1.5600000	0.1336663	1.4200000	1.7400000

Unique Subject Identifier=0094001000-0047

Assay variable : PTINR_VAL1 PT-INR Value

N	Mean	Standard Deviation	Minimum	Maximum
4	2.0950000	0.1156143	1.9600000	2.2400000

Unique Subject Identifier=0094001000-0049

Assay variable : PTINR_VAL1 PT-INR Value

N	Mean	Standard Deviation	Minimum	Maximum
4	1.9250000	0.4018706	1.4100000	2.3400000

Unique Subject Identifier=0094001000-0050

Assay variable : PTINR_VAL1 PT-INR Value

N	Mean	Standard Deviation	Minimum	Maximum
3	1.5833333	0.1069268	1.4900000	1.7000000

Laboratory tests at the time of obtaining Table 2-6 consent, according to the presence or absence of proton-pump inhibitors

Unique Subject Identifier=0098000000-0001

MEANS Procedure

Assay variable : PTINR_VAL1 PT-INR Value

N	Mean	Standard Deviation	Minimum	Maximum
5	1.9820000	0.1485598	1.7700000	2.0900000

Unique Subject Identifier=0098000000-0002

Assay variable : PTINR_VAL1 PT-INR Value

N	Mean	Standard Deviation	Minimum	Maximum
6	1.9750000	0.3007158	1.5600000	2.4900000

Unique Subject Identifier=0098000000-0006

Assay variable : PTINR_VAL1 PT-INR Value

N	Mean	Standard Deviation	Minimum	Maximum
4	2.7275000	0.7020150	2.0900000	3.7300000

Unique Subject Identifier=0098000000-0007

Assay variable : PTINR_VAL1 PT-INR Value

N	Mean	Standard Deviation	Minimum	Maximum
5	1.9360000	0.3231563	1.5900000	2.4600000

Laboratory tests at the time of obtaining Table 2-6 consent, according to the presence or absence of proton-pump inhibitors

Unique Subject Identifier=0098000000-0008

MEANS Procedure

Assay variable : PTINR_VAL1 PT-INR Value

N	Mean	Standard Deviation	Minimum	Maximum
6	2.4516667	1.3166232	0.9800000	4.0900000

Unique Subject Identifier=0098000000-0009

Assay variable : PTINR_VAL1 PT-INR Value

N	Mean	Standard Deviation	Minimum	Maximum
6	1.6316667	0.3060338	1.4500000	2.2300000

Unique Subject Identifier=0098000000-0011

Assay variable : PTINR_VAL1 PT-INR Value

N	Mean	Standard Deviation	Minimum	Maximum
3	2.3533333	0.2119748	2.1600000	2.5800000

Unique Subject Identifier=0098000000-0012

Assay variable : PTINR_VAL1 PT-INR Value

N	Mean	Standard Deviation	Minimum	Maximum
6	2.0800000	0.2728369	1.8000000	2.4900000

Laboratory tests at the time of obtaining Table 2-6 consent, according to the presence or absence of proton-pump inhibitors

Unique Subject Identifier=0098000000-0013

MEANS Procedure

Assay variable : PTINR_VAL1 PT-INR Value

N	Mean	Standard Deviation	Minimum	Maximum
6	1.4966667	0.3375599	1.2000000	1.9700000

Unique Subject Identifier=0098000000-0014

Assay variable : PTINR_VAL1 PT-INR Value

N	Mean	Standard Deviation	Minimum	Maximum
5	1.9940000	0.2743720	1.6700000	2.3200000

Unique Subject Identifier=0098000000-0016

Assay variable : PTINR_VAL1 PT-INR Value

N	Mean	Standard Deviation	Minimum	Maximum
6	2.1116667	0.4949108	1.5200000	2.9500000

Unique Subject Identifier=0098000000-0017

Assay variable : PTINR_VAL1 PT-INR Value

N	Mean	Standard Deviation	Minimum	Maximum
6	1.5916667	0.2320704	1.1500000	1.8100000

Laboratory tests at the time of obtaining Table 2-6 consent, according to the presence or absence of proton-pump inhibitors

Unique Subject Identifier=0102000000-0002

MEANS Procedure

Assay variable : PTINR_VAL1 PT-INR Value

N	Mean	Standard Deviation	Minimum	Maximum
6	1.5333333	0.1966384	1.2000000	1.7000000

Unique Subject Identifier=0102000000-0003

Assay variable : PTINR_VAL1 PT-INR Value

N	Mean	Standard Deviation	Minimum	Maximum
6	1.6333333	0.3881580	1.1000000	2.2000000

Unique Subject Identifier=0102000000-0004

Assay variable : PTINR_VAL1 PT-INR Value

N	Mean	Standard Deviation	Minimum	Maximum
6	1.5000000	0.2097618	1.2000000	1.7000000

Unique Subject Identifier=0102000000-0006

Assay variable : PTINR_VAL1 PT-INR Value

N	Mean	Standard Deviation	Minimum	Maximum
5	1.8800000	0.2683282	1.6000000	2.3000000

Laboratory tests at the time of obtaining Table 2-6 consent, according to the presence or absence of proton-pump inhibitors

Unique Subject Identifier=0102000000-0007

MEANS Procedure

Assay variable : PTINR_VAL1 PT-INR Value

N	Mean	Standard Deviation	Minimum	Maximum
6	2.1500000	0.2073644	1.9000000	2.4000000

Unique Subject Identifier=0102000000-0008

Assay variable : PTINR_VAL1 PT-INR Value

N	Mean	Standard Deviation	Minimum	Maximum
6	1.8833333	0.8035339	1.2000000	3.2000000

Unique Subject Identifier=0102000000-0009

Assay variable : PTINR_VAL1 PT-INR Value

N	Mean	Standard Deviation	Minimum	Maximum
6	1.7166667	0.3188521	1.4000000	2.3000000

Unique Subject Identifier=0102000000-0010

Assay variable : PTINR_VAL1 PT-INR Value

N	Mean	Standard Deviation	Minimum	Maximum
5	1.9400000	0.4159327	1.4000000	2.3000000

Laboratory tests at the time of obtaining Table 2-6 consent, according to the presence or absence of proton-pump inhibitors

Unique Subject Identifier=0103000000-0006

MEANS Procedure

Assay variable : PTINR_VAL1 PT-INR Value

N	Mean	Standard Deviation	Minimum	Maximum
6	1.7466667	0.8857690	1.0800000	3.3600000

Unique Subject Identifier=0103000000-0007

Assay variable : PTINR_VAL1 PT-INR Value

N	Mean	Standard Deviation	Minimum	Maximum
6	2.1916667	0.9971443	1.3500000	4.0400000

Unique Subject Identifier=0103000000-0008

Assay variable : PTINR_VAL1 PT-INR Value

N	Mean	Standard Deviation	Minimum	Maximum
3	1.9300000	0.3304542	1.5900000	2.2500000

Unique Subject Identifier=0103000000-0009

Assay variable : PTINR_VAL1 PT-INR Value

N	Mean	Standard Deviation	Minimum	Maximum
6	1.8450000	0.2712748	1.5500000	2.3300000

Laboratory tests at the time of obtaining Table 2-6 consent, according to the presence or absence of proton-pump inhibitors

Unique Subject Identifier=0103000000-0010

MEANS Procedure

Assay variable : PTINR_VAL1 PT-INR Value

N	Mean	Standard Deviation	Minimum	Maximum
4	1.8375000	0.3439356	1.4400000	2.2100000

Unique Subject Identifier=0103000000-0013

Assay variable : PTINR_VAL1 PT-INR Value

N	Mean	Standard Deviation	Minimum	Maximum
4	2.0525000	0.3345021	1.7700000	2.5100000

Unique Subject Identifier=0103000000-0014

Assay variable : PTINR_VAL1 PT-INR Value

N	Mean	Standard Deviation	Minimum	Maximum
5	1.8040000	0.2051341	1.6200000	2.1200000

Unique Subject Identifier=0103000000-0015

Assay variable : PTINR_VAL1 PT-INR Value

N	Mean	Standard Deviation	Minimum	Maximum
6	1.8083333	0.1024532	1.6900000	1.9800000

Laboratory tests at the time of obtaining Table 2-6 consent, according to the presence or absence of proton-pump inhibitors

Unique Subject Identifier=0103000000-0017

MEANS Procedure

Assay variable : PTINR_VAL1 PT-INR Value

N	Mean	Standard Deviation	Minimum	Maximum
4	2.3025000	0.2242580	1.9800000	2.5000000

Unique Subject Identifier=0103000000-0018

Assay variable : PTINR_VAL1 PT-INR Value

N	Mean	Standard Deviation	Minimum	Maximum
4	1.8650000	0.2754995	1.5000000	2.1600000

Unique Subject Identifier=0103000000-0019

Assay variable : PTINR_VAL1 PT-INR Value

N	Mean	Standard Deviation	Minimum	Maximum
4	1.7175000	0.1768945	1.5600000	1.9700000

Unique Subject Identifier=0103000000-0022

Assay variable : PTINR_VAL1 PT-INR Value

N	Mean	Standard Deviation	Minimum	Maximum
4	2.2825000	0.4733128	1.6600000	2.6800000

Laboratory tests at the time of obtaining Table 2-6 consent, according to the presence or absence of proton-pump inhibitors

Unique Subject Identifier=0116000000-0001

MEANS Procedure

Assay variable : PTINR_VAL1 PT-INR Value

N	Mean	Standard Deviation	Minimum	Maximum
3	2.3666667	0.5357549	1.9900000	2.9800000

Unique Subject Identifier=0120000000-0002

Assay variable : PTINR_VAL1 PT-INR Value

N	Mean	Standard Deviation	Minimum	Maximum
1	1.4800000		1.4800000	1.4800000

Unique Subject Identifier=0120000000-0003

Assay variable : PTINR_VAL1 PT-INR Value

N	Mean	Standard Deviation	Minimum	Maximum
2	2.0250000	0.3323402	1.7900000	2.2600000

Unique Subject Identifier=0120000000-0004

Assay variable : PTINR_VAL1 PT-INR Value

N	Mean	Standard Deviation	Minimum	Maximum
3	1.7433333	0.2494661	1.4600000	1.9300000

Laboratory tests at the time of obtaining Table 2-6 consent, according to the presence or absence of proton-pump inhibitors

Unique Subject Identifier=0120000000-0007

MEANS Procedure

Assay variable : PTINR_VAL1 PT-INR Value

N	Mean	Standard Deviation	Minimum	Maximum
1	1.7100000		1.7100000	1.7100000

Unique Subject Identifier=0121010000-0001

Assay variable : PTINR_VAL1 PT-INR Value

N	Mean	Standard Deviation	Minimum	Maximum
4	2.1250000	0.2753785	1.8000000	2.4000000

Unique Subject Identifier=0121010000-0002

Assay variable : PTINR_VAL1 PT-INR Value

N	Mean	Standard Deviation	Minimum	Maximum
4	1.6250000	0.0500000	1.6000000	1.7000000

Unique Subject Identifier=0121010000-0005

Assay variable : PTINR_VAL1 PT-INR Value

N	Mean	Standard Deviation	Minimum	Maximum
6	1.9666667	0.2250926	1.6000000	2.3000000

Laboratory tests at the time of obtaining Table 2-6 consent, according to the presence or absence of proton-pump inhibitors

Unique Subject Identifier=0121010000-0006

MEANS Procedure

Assay variable : PTINR_VAL1 PT-INR Value

N	Mean	Standard Deviation	Minimum	Maximum
6	2.2166667	0.2562551	1.9000000	2.6000000

Unique Subject Identifier=0121010000-0012

Assay variable : PTINR_VAL1 PT-INR Value

N	Mean	Standard Deviation	Minimum	Maximum
6	2.2166667	0.2857738	1.8000000	2.6000000

Unique Subject Identifier=0121010000-0014

Assay variable : PTINR_VAL1 PT-INR Value

N	Mean	Standard Deviation	Minimum	Maximum
6	2.1500000	0.1048809	2.0000000	2.3000000

Unique Subject Identifier=0121010000-0016

Assay variable : PTINR_VAL1 PT-INR Value

N	Mean	Standard Deviation	Minimum	Maximum
5	1.6800000	0.2949576	1.3000000	2.1000000

Laboratory tests at the time of obtaining Table 2-6 consent, according to the presence or absence of proton-pump inhibitors

Unique Subject Identifier=0121010000-0018

MEANS Procedure

Assay variable : PTINR_VAL1 PT-INR Value

N	Mean	Standard Deviation	Minimum	Maximum
5	2.6400000	0.4722288	2.0000000	3.2000000

Unique Subject Identifier=0121010000-0020

Assay variable : PTINR_VAL1 PT-INR Value

N	Mean	Standard Deviation	Minimum	Maximum
4	2.2750000	0.0957427	2.2000000	2.4000000

Unique Subject Identifier=0121010000-0021

Assay variable : PTINR_VAL1 PT-INR Value

N	Mean	Standard Deviation	Minimum	Maximum
4	1.6500000	0.2645751	1.3000000	1.9000000

Unique Subject Identifier=0121010000-0023

Assay variable : PTINR_VAL1 PT-INR Value

N	Mean	Standard Deviation	Minimum	Maximum
6	2.1500000	0.6379655	1.6000000	3.4000000

Laboratory tests at the time of obtaining Table 2-6 consent, according to the presence or absence of proton-pump inhibitors

Unique Subject Identifier=0121010000-0028

MEANS Procedure

Assay variable : PTINR_VAL1 PT-INR Value

N	Mean	Standard Deviation	Minimum	Maximum
6	2.3500000	0.6833740	1.9000000	3.7000000

Unique Subject Identifier=0122010000-0001

Assay variable : PTINR_VAL1 PT-INR Value

N	Mean	Standard Deviation	Minimum	Maximum
4	1.6000000	0.3741657	1.1000000	2.0000000

Unique Subject Identifier=0122010000-0002

Assay variable : PTINR_VAL1 PT-INR Value

N	Mean	Standard Deviation	Minimum	Maximum
6	1.8666667	0.5819507	1.1000000	2.9000000

Unique Subject Identifier=0124010000-0010

Assay variable : PTINR_VAL1 PT-INR Value

N	Mean	Standard Deviation	Minimum	Maximum
2	2.7000000	0.4242641	2.4000000	3.0000000

Laboratory tests at the time of obtaining Table 2-6 consent, according to the presence or absence of proton-pump inhibitors

Unique Subject Identifier=0124010000-0012

MEANS Procedure

Assay variable : PTINR_VAL1 PT-INR Value

N	Mean	Standard Deviation	Minimum	Maximum
3	1.6666667	0.0577350	1.6000000	1.7000000

Unique Subject Identifier=0124010000-0021

Assay variable : PTINR_VAL1 PT-INR Value

N	Mean	Standard Deviation	Minimum	Maximum
2	2.1000000	0.1414214	2.0000000	2.2000000

Unique Subject Identifier=0132000000-0003

Assay variable : PTINR_VAL1 PT-INR Value

N	Mean	Standard Deviation	Minimum	Maximum
2	2.4950000	0.1909188	2.3600000	2.6300000

Unique Subject Identifier=0132000000-0005

Assay variable : PTINR_VAL1 PT-INR Value

N	Mean	Standard Deviation	Minimum	Maximum
3	1.5900000	0.3132092	1.2300000	1.8000000

Laboratory tests at the time of obtaining Table 2-6 consent, according to the presence or absence of proton-pump inhibitors

Unique Subject Identifier=0132000000-0009

MEANS Procedure

Assay variable : PTINR_VAL1 PT-INR Value

N	Mean	Standard Deviation	Minimum	Maximum
4	1.6600000	0.3465064	1.1700000	1.9100000

Unique Subject Identifier=0132000000-0012

Assay variable : PTINR_VAL1 PT-INR Value

N	Mean	Standard Deviation	Minimum	Maximum
3	1.5266667	0.3661056	1.1200000	1.8300000

Unique Subject Identifier=0133000000-0001

Assay variable : PTINR_VAL1 PT-INR Value

N	Mean	Standard Deviation	Minimum	Maximum
4	1.3675000	0.2090255	1.0800000	1.5800000

Unique Subject Identifier=0133000000-0003

Assay variable : PTINR_VAL1 PT-INR Value

N	Mean	Standard Deviation	Minimum	Maximum
4	1.4125000	0.3732180	1.1300000	1.9600000

Laboratory tests at the time of obtaining Table 2-6 consent, according to the presence or absence of proton-pump inhibitors

Unique Subject Identifier=0133000000-0005

MEANS Procedure

Assay variable : PTINR_VAL1 PT-INR Value

N	Mean	Standard Deviation	Minimum	Maximum
3	1.8500000	0.0556776	1.7900000	1.9000000

Unique Subject Identifier=0133000000-0010

Assay variable : PTINR_VAL1 PT-INR Value

N	Mean	Standard Deviation	Minimum	Maximum
3	2.2966667	0.6230837	1.7600000	2.9800000

Unique Subject Identifier=0133000000-0011

Assay variable : PTINR_VAL1 PT-INR Value

N	Mean	Standard Deviation	Minimum	Maximum
5	1.7120000	0.1485598	1.5300000	1.9300000

Unique Subject Identifier=0133000000-0012

Assay variable : PTINR_VAL1 PT-INR Value

N	Mean	Standard Deviation	Minimum	Maximum
3	1.5366667	0.0288675	1.5200000	1.5700000

Laboratory tests at the time of obtaining Table 2-6 consent, according to the presence or absence of proton-pump inhibitors

Unique Subject Identifier=0133000000-0013

MEANS Procedure

Assay variable : PTINR_VAL1 PT-INR Value

N	Mean	Standard Deviation	Minimum	Maximum
2	1.4750000	0.0494975	1.4400000	1.5100000

Unique Subject Identifier=0133000000-0015

Assay variable : PTINR_VAL1 PT-INR Value

N	Mean	Standard Deviation	Minimum	Maximum
1	2.0400000		2.0400000	2.0400000

Unique Subject Identifier=0133000000-0020

Assay variable : PTINR_VAL1 PT-INR Value

N	Mean	Standard Deviation	Minimum	Maximum
2	1.9950000	0.1202082	1.9100000	2.0800000

Unique Subject Identifier=0133000000-0037

Assay variable : PTINR_VAL1 PT-INR Value

N	Mean	Standard Deviation	Minimum	Maximum
2	1.9100000	0.0141421	1.9000000	1.9200000

Laboratory tests at the time of obtaining Table 2-6 consent, according to the presence or absence of proton-pump inhibitors

Unique Subject Identifier=0133000000-0038

MEANS Procedure

Assay variable : PTINR_VAL1 PT-INR Value

N	Mean	Standard Deviation	Minimum	Maximum
2	1.9850000	0.0212132	1.9700000	2.0000000

Unique Subject Identifier=0133000000-0045

Assay variable : PTINR_VAL1 PT-INR Value

N	Mean	Standard Deviation	Minimum	Maximum
3	2.2766667	0.7092484	1.5000000	2.8900000

Unique Subject Identifier=0133000000-0052

Assay variable : PTINR_VAL1 PT-INR Value

N	Mean	Standard Deviation	Minimum	Maximum
4	1.6150000	0.0974679	1.5200000	1.7500000

Unique Subject Identifier=0133000000-0061

Assay variable : PTINR_VAL1 PT-INR Value

N	Mean	Standard Deviation	Minimum	Maximum
3	1.8633333	0.3716629	1.4500000	2.1700000

Laboratory tests at the time of obtaining Table 2-6 consent, according to the presence or absence of proton-pump inhibitors

Unique Subject Identifier=0133000000-0068

MEANS Procedure

Assay variable : PTINR_VAL1 PT-INR Value

N	Mean	Standard Deviation	Minimum	Maximum
3	1.7433333	0.3837100	1.4600000	2.1800000

Unique Subject Identifier=0134100000-0001

Assay variable : PTINR_VAL1 PT-INR Value

N	Mean	Standard Deviation	Minimum	Maximum
2	1.8950000	0.0353553	1.8700000	1.9200000

Unique Subject Identifier=0134100000-0002

Assay variable : PTINR_VAL1 PT-INR Value

N	Mean	Standard Deviation	Minimum	Maximum
3	2.0033333	0.5802011	1.5600000	2.6600000

Unique Subject Identifier=0134100000-0009

Assay variable : PTINR_VAL1 PT-INR Value

N	Mean	Standard Deviation	Minimum	Maximum
3	1.6433333	0.1159023	1.5100000	1.7200000

Laboratory tests at the time of obtaining Table 2-6 consent, according to the presence or absence of proton-pump inhibitors

Unique Subject Identifier=0134100000-0010

MEANS Procedure

Assay variable : PTINR_VAL1 PT-INR Value

N	Mean	Standard Deviation	Minimum	Maximum
3	2.5700000	0.2007486	2.3800000	2.7800000

Unique Subject Identifier=0134100000-0012

Assay variable : PTINR_VAL1 PT-INR Value

N	Mean	Standard Deviation	Minimum	Maximum
4	1.6850000	0.2500000	1.5000000	2.0400000

Unique Subject Identifier=0134100000-0014

Assay variable : PTINR_VAL1 PT-INR Value

N	Mean	Standard Deviation	Minimum	Maximum
6	2.4033333	1.2899716	1.0400000	4.2300000

Unique Subject Identifier=0134100000-0016

Assay variable : PTINR_VAL1 PT-INR Value

N	Mean	Standard Deviation	Minimum	Maximum
4	1.9800000	0.7144228	1.4800000	3.0400000

Laboratory tests at the time of obtaining Table 2-6 consent, according to the presence or absence of proton-pump inhibitors

Unique Subject Identifier=0134100000-0019

MEANS Procedure

Assay variable : PTINR_VAL1 PT-INR Value

N	Mean	Standard Deviation	Minimum	Maximum
3	1.8800000	0.3500000	1.5300000	2.2300000

Unique Subject Identifier=0134100000-0021

Assay variable : PTINR_VAL1 PT-INR Value

N	Mean	Standard Deviation	Minimum	Maximum
4	1.8950000	0.2353012	1.6400000	2.1000000

Unique Subject Identifier=0134100000-0023

Assay variable : PTINR_VAL1 PT-INR Value

N	Mean	Standard Deviation	Minimum	Maximum
4	2.0275000	0.2627261	1.7000000	2.2900000

Unique Subject Identifier=0135000000-0002

Assay variable : PTINR_VAL1 PT-INR Value

N	Mean	Standard Deviation	Minimum	Maximum
3	1.7400000	0.1571623	1.6000000	1.9100000

Laboratory tests at the time of obtaining Table 2-6 consent, according to the presence or absence of proton-pump inhibitors

Unique Subject Identifier=0135000000-0006

MEANS Procedure

Assay variable : PTINR_VAL1 PT-INR Value

N	Mean	Standard Deviation	Minimum	Maximum
3	1.9733333	0.7523519	1.4200000	2.8300000

Unique Subject Identifier=0135000000-0008

Assay variable : PTINR_VAL1 PT-INR Value

N	Mean	Standard Deviation	Minimum	Maximum
4	1.7050000	0.3637307	1.2800000	2.1500000

Unique Subject Identifier=0135000000-0009

Assay variable : PTINR_VAL1 PT-INR Value

N	Mean	Standard Deviation	Minimum	Maximum
5	1.7160000	0.3756062	1.2000000	2.0600000

Unique Subject Identifier=0135000000-0010

Assay variable : PTINR_VAL1 PT-INR Value

N	Mean	Standard Deviation	Minimum	Maximum
3	1.6866667	0.0378594	1.6600000	1.7300000

Laboratory tests at the time of obtaining Table 2-6 consent, according to the presence or absence of proton-pump inhibitors

Unique Subject Identifier=0135000000-0013

MEANS Procedure

Assay variable : PTINR_VAL1 PT-INR Value

N	Mean	Standard Deviation	Minimum	Maximum
3	1.5833333	0.0873689	1.5100000	1.6800000

Unique Subject Identifier=0135000000-0020

Assay variable : PTINR_VAL1 PT-INR Value

N	Mean	Standard Deviation	Minimum	Maximum
3	1.7466667	0.1101514	1.6400000	1.8600000

Unique Subject Identifier=0135000000-0024

Assay variable : PTINR_VAL1 PT-INR Value

N	Mean	Standard Deviation	Minimum	Maximum
4	2.3400000	0.2906315	2.1100000	2.7600000

Unique Subject Identifier=0135000000-0026

Assay variable : PTINR_VAL1 PT-INR Value

N	Mean	Standard Deviation	Minimum	Maximum
3	1.6366667	0.1436431	1.5300000	1.8000000

Laboratory tests at the time of obtaining Table 2-6 consent, according to the presence or absence of proton-pump inhibitors

Unique Subject Identifier=0135000000-0028

MEANS Procedure

Assay variable : PTINR_VAL1 PT-INR Value

N	Mean	Standard Deviation	Minimum	Maximum
4	2.0575000	0.2653771	1.7000000	2.3000000

Unique Subject Identifier=0136000010-0002

Assay variable : PTINR_VAL1 PT-INR Value

N	Mean	Standard Deviation	Minimum	Maximum
6	2.0766667	0.2477633	1.7400000	2.5000000

Unique Subject Identifier=0136000010-0003

Assay variable : PTINR_VAL1 PT-INR Value

N	Mean	Standard Deviation	Minimum	Maximum
6	2.1200000	0.4215211	1.3000000	2.4500000

Unique Subject Identifier=0138000010-0001

Assay variable : PTINR_VAL1 PT-INR Value

N	Mean	Standard Deviation	Minimum	Maximum
6	1.6950000	0.2682350	1.4500000	2.0600000

Laboratory tests at the time of obtaining Table 2-6 consent, according to the presence or absence of proton-pump inhibitors

Unique Subject Identifier=0138000010-0002

MEANS Procedure

Assay variable : PTINR_VAL1 PT-INR Value

N	Mean	Standard Deviation	Minimum	Maximum
6	1.7716667	0.3066214	1.3300000	2.1600000

Unique Subject Identifier=0138000010-0003

Assay variable : PTINR_VAL1 PT-INR Value

N	Mean	Standard Deviation	Minimum	Maximum
3	1.9933333	0.4215843	1.6400000	2.4600000

Unique Subject Identifier=0138000010-0004

Assay variable : PTINR_VAL1 PT-INR Value

N	Mean	Standard Deviation	Minimum	Maximum
3	1.8566667	0.1096966	1.7300000	1.9200000

Unique Subject Identifier=0138000010-0009

Assay variable : PTINR_VAL1 PT-INR Value

N	Mean	Standard Deviation	Minimum	Maximum
4	1.9200000	0.5023279	1.4800000	2.6000000

Laboratory tests at the time of obtaining Table 2-6 consent, according to the presence or absence of proton-pump inhibitors

Unique Subject Identifier=0138000010-0014

MEANS Procedure

Assay variable : PTINR_VAL1 PT-INR Value

N	Mean	Standard Deviation	Minimum	Maximum
4	1.8300000	0.4009988	1.3500000	2.1900000

Unique Subject Identifier=0138000010-0015

Assay variable : PTINR_VAL1 PT-INR Value

N	Mean	Standard Deviation	Minimum	Maximum
3	2.1300000	0.0953939	2.0400000	2.2300000

Unique Subject Identifier=0138000010-0016

Assay variable : PTINR_VAL1 PT-INR Value

N	Mean	Standard Deviation	Minimum	Maximum
3	1.2733333	0.0288675	1.2400000	1.2900000

Unique Subject Identifier=0138000010-0022

Assay variable : PTINR_VAL1 PT-INR Value

N	Mean	Standard Deviation	Minimum	Maximum
2	1.7550000	0.1202082	1.6700000	1.8400000

Laboratory tests at the time of obtaining Table 2-6 consent, according to the presence or absence of proton-pump inhibitors

Unique Subject Identifier=0138000010-0024

MEANS Procedure

Assay variable : PTINR_VAL1 PT-INR Value

N	Mean	Standard Deviation	Minimum	Maximum
6	1.7050000	0.2649340	1.3000000	2.0100000

Unique Subject Identifier=0138000010-0027

Assay variable : PTINR_VAL1 PT-INR Value

N	Mean	Standard Deviation	Minimum	Maximum
2	2.2700000	0.0424264	2.2400000	2.3000000

Unique Subject Identifier=0138000010-0029

Assay variable : PTINR_VAL1 PT-INR Value

N	Mean	Standard Deviation	Minimum	Maximum
2	1.7550000	0.0353553	1.7300000	1.7800000

Unique Subject Identifier=0138000010-0031

Assay variable : PTINR_VAL1 PT-INR Value

N	Mean	Standard Deviation	Minimum	Maximum
3	1.5700000	0.1500000	1.4200000	1.7200000

Laboratory tests at the time of obtaining Table 2-6 consent, according to the presence or absence of proton-pump inhibitors

Unique Subject Identifier=0138000010-0033

MEANS Procedure

Assay variable : PTINR_VAL1 PT-INR Value

N	Mean	Standard Deviation	Minimum	Maximum
3	1.9066667	0.2657693	1.6300000	2.1600000

Unique Subject Identifier=0138000010-0035

Assay variable : PTINR_VAL1 PT-INR Value

N	Mean	Standard Deviation	Minimum	Maximum
2	1.5750000	0.2192031	1.4200000	1.7300000

Unique Subject Identifier=0138000010-0036

Assay variable : PTINR_VAL1 PT-INR Value

N	Mean	Standard Deviation	Minimum	Maximum
3	1.8566667	0.1501111	1.7700000	2.0300000

Unique Subject Identifier=0138000010-0037

Assay variable : PTINR_VAL1 PT-INR Value

N	Mean	Standard Deviation	Minimum	Maximum
3	1.8733333	0.1285820	1.7800000	2.0200000

Laboratory tests at the time of obtaining Table 2-6 consent, according to the presence or absence of proton-pump inhibitors

Unique Subject Identifier=0138000010-0039

MEANS Procedure

Assay variable : PTINR_VAL1 PT-INR Value

N	Mean	Standard Deviation	Minimum	Maximum
6	2.1250000	0.7332598	1.3000000	3.2100000

Unique Subject Identifier=0138000010-0042

Assay variable : PTINR_VAL1 PT-INR Value

N	Mean	Standard Deviation	Minimum	Maximum
4	1.7075000	0.3138338	1.3800000	2.1200000

Unique Subject Identifier=0138000010-0043

Assay variable : PTINR_VAL1 PT-INR Value

N	Mean	Standard Deviation	Minimum	Maximum
5	1.8960000	0.3864324	1.3100000	2.2800000

Unique Subject Identifier=0138000010-0050

Assay variable : PTINR_VAL1 PT-INR Value

N	Mean	Standard Deviation	Minimum	Maximum
1	1.0700000		1.0700000	1.0700000

Laboratory tests at the time of obtaining Table 2-6 consent, according to the presence or absence of proton-pump inhibitors

Unique Subject Identifier=0139000010-0001

MEANS Procedure

Assay variable : PTINR_VAL1 PT-INR Value

N	Mean	Standard Deviation	Minimum	Maximum
5	1.5100000	0.2919760	1.1000000	1.8800000

Unique Subject Identifier=0139000010-0003

Assay variable : PTINR_VAL1 PT-INR Value

N	Mean	Standard Deviation	Minimum	Maximum
6	1.4283333	0.2696232	1.1800000	1.9300000

Unique Subject Identifier=0139000010-0008

Assay variable : PTINR_VAL1 PT-INR Value

N	Mean	Standard Deviation	Minimum	Maximum
6	2.1450000	0.5838750	1.4600000	2.9500000

Unique Subject Identifier=0139000010-0009

Assay variable : PTINR_VAL1 PT-INR Value

N	Mean	Standard Deviation	Minimum	Maximum
6	1.5516667	0.3399068	1.2000000	2.1900000

Laboratory tests at the time of obtaining Table 2-6 consent, according to the presence or absence of proton-pump inhibitors

Unique Subject Identifier=0139000010-0010

MEANS Procedure

Assay variable : PTINR_VAL1 PT-INR Value

N	Mean	Standard Deviation	Minimum	Maximum
6	2.3816667	0.2837898	2.0400000	2.7900000

Unique Subject Identifier=0139000010-0017

Assay variable : PTINR_VAL1 PT-INR Value

N	Mean	Standard Deviation	Minimum	Maximum
2	1.4250000	0.5020458	1.0700000	1.7800000

Unique Subject Identifier=0140000010-0002

Assay variable : PTINR_VAL1 PT-INR Value

N	Mean	Standard Deviation	Minimum	Maximum
6	2.2400000	0.5415903	1.3400000	2.8500000

Unique Subject Identifier=0140000010-0003

Assay variable : PTINR_VAL1 PT-INR Value

N	Mean	Standard Deviation	Minimum	Maximum
4	2.2525000	0.9731863	1.5100000	3.6400000

Laboratory tests at the time of obtaining Table 2-6 consent, according to the presence or absence of proton-pump inhibitors

Unique Subject Identifier=0140000010-0008

MEANS Procedure

Assay variable : PTINR_VAL1 PT-INR Value

N	Mean	Standard Deviation	Minimum	Maximum
4	2.3675000	0.2835930	2.0100000	2.6100000

Unique Subject Identifier=0140000010-0011

Assay variable : PTINR_VAL1 PT-INR Value

N	Mean	Standard Deviation	Minimum	Maximum
4	2.1425000	0.2451360	1.9700000	2.4900000

Unique Subject Identifier=0140000010-0016

Assay variable : PTINR_VAL1 PT-INR Value

N	Mean	Standard Deviation	Minimum	Maximum
4	1.7725000	0.6115758	1.3100000	2.6000000

Unique Subject Identifier=0140000010-0017

Assay variable : PTINR_VAL1 PT-INR Value

N	Mean	Standard Deviation	Minimum	Maximum
6	1.8600000	0.7626008	1.4200000	3.4000000

Laboratory tests at the time of obtaining Table 2-6 consent, according to the presence or absence of proton-pump inhibitors

Unique Subject Identifier=0140000010-0018

MEANS Procedure

Assay variable : PTINR_VAL1 PT-INR Value

N	Mean	Standard Deviation	Minimum	Maximum
3	2.1766667	0.1078579	2.1000000	2.3000000

Unique Subject Identifier=0140000010-0024

Assay variable : PTINR_VAL1 PT-INR Value

N	Mean	Standard Deviation	Minimum	Maximum
3	2.3200000	0.9263369	1.5100000	3.3300000

Unique Subject Identifier=0140000010-0025

Assay variable : PTINR_VAL1 PT-INR Value

N	Mean	Standard Deviation	Minimum	Maximum
3	1.4900000	0.0624500	1.4200000	1.5400000

Unique Subject Identifier=0140000010-0026

Assay variable : PTINR_VAL1 PT-INR Value

N	Mean	Standard Deviation	Minimum	Maximum
4	2.6675000	0.5993538	2.0600000	3.4300000

Laboratory tests at the time of obtaining Table 2-6 consent, according to the presence or absence of proton-pump inhibitors

Unique Subject Identifier=0141000000-0002

MEANS Procedure

Assay variable : PTINR_VAL1 PT-INR Value

N	Mean	Standard Deviation	Minimum	Maximum
4	1.9500000	0.1732051	1.7200000	2.1400000

Unique Subject Identifier=0142000000-0001

Assay variable : PTINR_VAL1 PT-INR Value

N	Mean	Standard Deviation	Minimum	Maximum
6	2.0316667	0.2256029	1.7500000	2.3700000

Unique Subject Identifier=0143010000-0002

Assay variable : PTINR_VAL1 PT-INR Value

N	Mean	Standard Deviation	Minimum	Maximum
5	2.1980000	0.6462739	1.5900000	3.1700000

Unique Subject Identifier=0143010000-0003

Assay variable : PTINR_VAL1 PT-INR Value

N	Mean	Standard Deviation	Minimum	Maximum
6	2.1150000	0.3043518	1.8500000	2.5100000

Laboratory tests at the time of obtaining Table 2-6 consent, according to the presence or absence of proton-pump inhibitors

Unique Subject Identifier=0143010000-0004

MEANS Procedure

Assay variable : PTINR_VAL1 PT-INR Value

N	Mean	Standard Deviation	Minimum	Maximum
3	1.1233333	0.0602771	1.0600000	1.1800000

Unique Subject Identifier=0143010000-0011

Assay variable : PTINR_VAL1 PT-INR Value

N	Mean	Standard Deviation	Minimum	Maximum
5	2.3660000	0.6961537	1.5700000	3.4500000

Unique Subject Identifier=0143010000-0012

Assay variable : PTINR_VAL1 PT-INR Value

N	Mean	Standard Deviation	Minimum	Maximum
6	1.6550000	0.1595932	1.5100000	1.9200000

Unique Subject Identifier=0143010000-0015

Assay variable : PTINR_VAL1 PT-INR Value

N	Mean	Standard Deviation	Minimum	Maximum
2	1.6050000	0.0353553	1.5800000	1.6300000

Laboratory tests at the time of obtaining Table 2-6 consent, according to the presence or absence of proton-pump inhibitors

Unique Subject Identifier=0143010000-0018

MEANS Procedure

Assay variable : PTINR_VAL1 PT-INR Value

N	Mean	Standard Deviation	Minimum	Maximum
6	2.2983333	0.1756607	2.1500000	2.5500000

Unique Subject Identifier=0143010000-0030

Assay variable : PTINR_VAL1 PT-INR Value

N	Mean	Standard Deviation	Minimum	Maximum
6	1.8116667	0.0953764	1.7000000	1.9600000

Unique Subject Identifier=0143010000-0031

Assay variable : PTINR_VAL1 PT-INR Value

N	Mean	Standard Deviation	Minimum	Maximum
6	2.4433333	0.3251256	1.8200000	2.7800000

Unique Subject Identifier=0143010000-0032

Assay variable : PTINR_VAL1 PT-INR Value

N	Mean	Standard Deviation	Minimum	Maximum
3	1.9900000	0.1700000	1.8200000	2.1600000

Laboratory tests at the time of obtaining Table 2-6 consent, according to the presence or absence of proton-pump inhibitors

Unique Subject Identifier=0143010000-0034

MEANS Procedure

Assay variable : PTINR_VAL1 PT-INR Value

N	Mean	Standard Deviation	Minimum	Maximum
6	1.9183333	0.2634704	1.6700000	2.4300000

Unique Subject Identifier=0143010000-0036

Assay variable : PTINR_VAL1 PT-INR Value

N	Mean	Standard Deviation	Minimum	Maximum
6	1.8383333	0.1904118	1.5600000	2.0500000

Unique Subject Identifier=0143010000-0038

Assay variable : PTINR_VAL1 PT-INR Value

N	Mean	Standard Deviation	Minimum	Maximum
6	2.1483333	0.2359167	1.8900000	2.5300000

Unique Subject Identifier=0143010000-0039

Assay variable : PTINR_VAL1 PT-INR Value

N	Mean	Standard Deviation	Minimum	Maximum
6	1.9333333	0.5831524	1.2100000	2.6100000

Laboratory tests at the time of obtaining Table 2-6 consent, according to the presence or absence of proton-pump inhibitors

Unique Subject Identifier=0143010000-0043

MEANS Procedure

Assay variable : PTINR_VAL1 PT-INR Value

N	Mean	Standard Deviation	Minimum	Maximum
6	1.7733333	0.3512074	1.4200000	2.2500000

Unique Subject Identifier=0143010000-0044

Assay variable : PTINR_VAL1 PT-INR Value

N	Mean	Standard Deviation	Minimum	Maximum
6	1.7583333	0.2982225	1.3700000	1.9800000

Unique Subject Identifier=0143010000-0056

Assay variable : PTINR_VAL1 PT-INR Value

N	Mean	Standard Deviation	Minimum	Maximum
4	2.0625000	0.3895617	1.7200000	2.6200000

Unique Subject Identifier=0143010000-0064

Assay variable : PTINR_VAL1 PT-INR Value

N	Mean	Standard Deviation	Minimum	Maximum
6	2.2883333	0.3831144	1.9200000	2.8400000

Laboratory tests at the time of obtaining Table 2-6 consent, according to the presence or absence of proton-pump inhibitors

Unique Subject Identifier=0143010000-0069

MEANS Procedure

Assay variable : PTINR_VAL1 PT-INR Value

N	Mean	Standard Deviation	Minimum	Maximum
6	2.2150000	0.4219360	1.5000000	2.6700000

Unique Subject Identifier=0143010000-0070

Assay variable : PTINR_VAL1 PT-INR Value

N	Mean	Standard Deviation	Minimum	Maximum
6	1.5666667	0.2264214	1.3200000	1.8800000

Unique Subject Identifier=0143010000-0072

Assay variable : PTINR_VAL1 PT-INR Value

N	Mean	Standard Deviation	Minimum	Maximum
6	2.1366667	0.6937915	1.4600000	3.3900000

Unique Subject Identifier=0143010000-0073

Assay variable : PTINR_VAL1 PT-INR Value

N	Mean	Standard Deviation	Minimum	Maximum
6	2.0133333	0.2307957	1.7300000	2.2800000

Laboratory tests at the time of obtaining Table 2-6 consent, according to the presence or absence of proton-pump inhibitors

Unique Subject Identifier=0143010000-0075

MEANS Procedure

Assay variable : PTINR_VAL1 PT-INR Value

N	Mean	Standard Deviation	Minimum	Maximum
6	2.1666667	0.2559427	1.8800000	2.6100000

Unique Subject Identifier=0143010000-0076

Assay variable : PTINR_VAL1 PT-INR Value

N	Mean	Standard Deviation	Minimum	Maximum
6	1.9283333	0.3119241	1.5600000	2.4000000

Unique Subject Identifier=0143010000-0079

Assay variable : PTINR_VAL1 PT-INR Value

N	Mean	Standard Deviation	Minimum	Maximum
3	2.4166667	0.2302897	2.1800000	2.6400000

Unique Subject Identifier=0143010000-0084

Assay variable : PTINR_VAL1 PT-INR Value

N	Mean	Standard Deviation	Minimum	Maximum
3	1.6266667	0.2218859	1.3900000	1.8300000

Laboratory tests at the time of obtaining Table 2-6 consent, according to the presence or absence of proton-pump inhibitors

Unique Subject Identifier=0143010000-0093

MEANS Procedure

Assay variable : PTINR_VAL1 PT-INR Value

N	Mean	Standard Deviation	Minimum	Maximum
5	2.0500000	0.4650269	1.6700000	2.6900000

Unique Subject Identifier=0143010000-0095

Assay variable : PTINR_VAL1 PT-INR Value

N	Mean	Standard Deviation	Minimum	Maximum
6	1.6300000	0.0651153	1.5700000	1.7200000

Unique Subject Identifier=0143010000-0099

Assay variable : PTINR_VAL1 PT-INR Value

N	Mean	Standard Deviation	Minimum	Maximum
6	2.2566667	0.2694934	1.8800000	2.6700000

Unique Subject Identifier=0143010000-0100

Assay variable : PTINR_VAL1 PT-INR Value

N	Mean	Standard Deviation	Minimum	Maximum
6	2.0233333	0.5109468	1.2100000	2.6900000

Laboratory tests at the time of obtaining Table 2-6 consent, according to the presence or absence of proton-pump inhibitors

Unique Subject Identifier=0143010000-0106

MEANS Procedure

Assay variable : PTINR_VAL1 PT-INR Value

N	Mean	Standard Deviation	Minimum	Maximum
6	2.1100000	0.3531572	1.5500000	2.5600000

Unique Subject Identifier=0143010000-0108

Assay variable : PTINR_VAL1 PT-INR Value

N	Mean	Standard Deviation	Minimum	Maximum
6	2.1850000	0.6755664	1.8200000	3.5600000

Unique Subject Identifier=0143010000-0111

Assay variable : PTINR_VAL1 PT-INR Value

N	Mean	Standard Deviation	Minimum	Maximum
5	1.7480000	0.1564289	1.5800000	1.9800000

Unique Subject Identifier=0143010000-0112

Assay variable : PTINR_VAL1 PT-INR Value

N	Mean	Standard Deviation	Minimum	Maximum
6	1.8850000	0.1078425	1.7700000	2.0600000

Laboratory tests at the time of obtaining Table 2-6 consent, according to the presence or absence of proton-pump inhibitors

Unique Subject Identifier=0143010000-0118

MEANS Procedure

Assay variable : PTINR_VAL1 PT-INR Value

N	Mean	Standard Deviation	Minimum	Maximum
6	1.7116667	0.3252332	1.4400000	2.3400000

Unique Subject Identifier=0143010000-0128

Assay variable : PTINR_VAL1 PT-INR Value

N	Mean	Standard Deviation	Minimum	Maximum
4	1.8725000	0.2088660	1.6900000	2.1700000

Unique Subject Identifier=0143010000-0129

Assay variable : PTINR_VAL1 PT-INR Value

N	Mean	Standard Deviation	Minimum	Maximum
4	2.1300000	0.1134313	1.9800000	2.2500000

Unique Subject Identifier=0143010000-0132

Assay variable : PTINR_VAL1 PT-INR Value

N	Mean	Standard Deviation	Minimum	Maximum
4	1.3600000	0.5643285	1.0000000	2.2000000

Laboratory tests at the time of obtaining Table 2-6 consent, according to the presence or absence of proton-pump inhibitors

Unique Subject Identifier=0143010000-0141

MEANS Procedure

Assay variable : PTINR_VAL1 PT-INR Value

N	Mean	Standard Deviation	Minimum	Maximum
4	2.1150000	0.1844813	2.0000000	2.3900000

Unique Subject Identifier=0143010000-0144

Assay variable : PTINR_VAL1 PT-INR Value

N	Mean	Standard Deviation	Minimum	Maximum
3	2.1100000	0.0721110	2.0300000	2.1700000

Unique Subject Identifier=0143010000-0153

Assay variable : PTINR_VAL1 PT-INR Value

N	Mean	Standard Deviation	Minimum	Maximum
6	1.9733333	0.4101544	1.4300000	2.5900000

Unique Subject Identifier=0143010000-0154

Assay variable : PTINR_VAL1 PT-INR Value

N	Mean	Standard Deviation	Minimum	Maximum
6	2.0316667	0.6977798	1.1400000	2.8200000

Laboratory tests at the time of obtaining Table 2-6 consent, according to the presence or absence of proton-pump inhibitors

Unique Subject Identifier=0143010000-0155

MEANS Procedure

Assay variable : PTINR_VAL1 PT-INR Value

N	Mean	Standard Deviation	Minimum	Maximum
6	2.3250000	0.2429609	1.9500000	2.6100000

Unique Subject Identifier=0143010000-0158

Assay variable : PTINR_VAL1 PT-INR Value

N	Mean	Standard Deviation	Minimum	Maximum
3	2.9500000	0.3026549	2.6100000	3.1900000

Unique Subject Identifier=0143010000-0163

Assay variable : PTINR_VAL1 PT-INR Value

N	Mean	Standard Deviation	Minimum	Maximum
5	1.9640000	0.1141490	1.7900000	2.0800000

Unique Subject Identifier=0143010000-0164

Assay variable : PTINR_VAL1 PT-INR Value

N	Mean	Standard Deviation	Minimum	Maximum
6	2.4100000	0.2503597	2.0900000	2.7800000

Laboratory tests at the time of obtaining Table 2-6 consent, according to the presence or absence of proton-pump inhibitors

Unique Subject Identifier=0143010000-0166

MEANS Procedure

Assay variable : PTINR_VAL1 PT-INR Value

N	Mean	Standard Deviation	Minimum	Maximum
6	1.9800000	0.2815670	1.5700000	2.3400000

Unique Subject Identifier=0143010000-0167

Assay variable : PTINR_VAL1 PT-INR Value

N	Mean	Standard Deviation	Minimum	Maximum
6	1.8383333	0.5036434	1.3600000	2.7100000

Unique Subject Identifier=0143010000-0173

Assay variable : PTINR_VAL1 PT-INR Value

N	Mean	Standard Deviation	Minimum	Maximum
6	2.3616667	0.3055105	1.8700000	2.8200000

Unique Subject Identifier=0143010000-0175

Assay variable : PTINR_VAL1 PT-INR Value

N	Mean	Standard Deviation	Minimum	Maximum
2	1.8800000	0.2828427	1.6800000	2.0800000

Laboratory tests at the time of obtaining Table 2-6 consent, according to the presence or absence of proton-pump inhibitors

Unique Subject Identifier=0143010000-0182

MEANS Procedure

Assay variable : PTINR_VAL1 PT-INR Value

N	Mean	Standard Deviation	Minimum	Maximum
6	2.0133333	0.7044336	1.6300000	3.4200000

Unique Subject Identifier=0143010000-0188

Assay variable : PTINR_VAL1 PT-INR Value

N	Mean	Standard Deviation	Minimum	Maximum
1	1.6400000		1.6400000	1.6400000

Unique Subject Identifier=0143010000-0196

Assay variable : PTINR_VAL1 PT-INR Value

N	Mean	Standard Deviation	Minimum	Maximum
2	1.8900000	0.0141421	1.8800000	1.9000000

Unique Subject Identifier=0143010000-0197

Assay variable : PTINR_VAL1 PT-INR Value

N	Mean	Standard Deviation	Minimum	Maximum
3	2.2800000	0.3119295	1.9900000	2.6100000

Laboratory tests at the time of obtaining Table 2-6 consent, according to the presence or absence of proton-pump inhibitors

Unique Subject Identifier=0143010000-0203

MEANS Procedure

Assay variable : PTINR_VAL1 PT-INR Value

N	Mean	Standard Deviation	Minimum	Maximum
1	1.9600000		1.9600000	1.9600000

Unique Subject Identifier=0144000000-0006

Assay variable : PTINR_VAL1 PT-INR Value

N	Mean	Standard Deviation	Minimum	Maximum
6	1.9683333	0.2527779	1.6300000	2.4100000

Unique Subject Identifier=0144000000-0008

Assay variable : PTINR_VAL1 PT-INR Value

N	Mean	Standard Deviation	Minimum	Maximum
1	1.4700000		1.4700000	1.4700000

Unique Subject Identifier=0144000000-0009

Assay variable : PTINR_VAL1 PT-INR Value

N	Mean	Standard Deviation	Minimum	Maximum
1	1.7900000		1.7900000	1.7900000

Laboratory tests at the time of obtaining Table 2-6 consent, according to the presence or absence of proton-pump inhibitors

Unique Subject Identifier=0144000000-0014

MEANS Procedure

Assay variable : PTINR_VAL1 PT-INR Value

N	Mean	Standard Deviation	Minimum	Maximum
2	1.2000000	0.1555635	1.0900000	1.3100000

Unique Subject Identifier=0145000000-0005

Assay variable : PTINR_VAL1 PT-INR Value

N	Mean	Standard Deviation	Minimum	Maximum
4	2.6175000	0.3463500	2.2700000	3.0100000

Unique Subject Identifier=0145000000-0008

Assay variable : PTINR_VAL1 PT-INR Value

N	Mean	Standard Deviation	Minimum	Maximum
2	2.4200000	0.5232590	2.0500000	2.7900000

Unique Subject Identifier=0145000000-0010

Assay variable : PTINR_VAL1 PT-INR Value

N	Mean	Standard Deviation	Minimum	Maximum
3	2.2833333	0.6672581	1.5300000	2.8000000

Laboratory tests at the time of obtaining Table 2-6 consent, according to the presence or absence of proton-pump inhibitors

Unique Subject Identifier=0145000000-0014

MEANS Procedure

Assay variable : PTINR_VAL1 PT-INR Value

N	Mean	Standard Deviation	Minimum	Maximum
3	2.2100000	0.2861818	2.0300000	2.5400000

Unique Subject Identifier=0145000000-0015

Assay variable : PTINR_VAL1 PT-INR Value

N	Mean	Standard Deviation	Minimum	Maximum
4	1.7450000	0.0974679	1.6100000	1.8300000

Unique Subject Identifier=0145000000-0016

Assay variable : PTINR_VAL1 PT-INR Value

N	Mean	Standard Deviation	Minimum	Maximum
6	2.7600000	0.4118738	2.1800000	3.3000000

Unique Subject Identifier=0145000000-0022

Assay variable : PTINR_VAL1 PT-INR Value

N	Mean	Standard Deviation	Minimum	Maximum
6	1.5066667	0.2136040	1.2900000	1.7600000

Laboratory tests at the time of obtaining Table 2-6 consent, according to the presence or absence of proton-pump inhibitors

Unique Subject Identifier=0146000000-0007

MEANS Procedure

Assay variable : PTINR_VAL1 PT-INR Value

N	Mean	Standard Deviation	Minimum	Maximum
3	2.2566667	0.2608320	1.9600000	2.4500000

Unique Subject Identifier=0146000000-0008

Assay variable : PTINR_VAL1 PT-INR Value

N	Mean	Standard Deviation	Minimum	Maximum
3	3.1033333	0.5450076	2.5000000	3.5600000

Unique Subject Identifier=0146000000-0009

Assay variable : PTINR_VAL1 PT-INR Value

N	Mean	Standard Deviation	Minimum	Maximum
3	1.8233333	0.1700980	1.6300000	1.9500000

Unique Subject Identifier=0146000000-0015

Assay variable : PTINR_VAL1 PT-INR Value

N	Mean	Standard Deviation	Minimum	Maximum
4	2.3250000	0.3706301	1.8300000	2.7200000

Laboratory tests at the time of obtaining Table 2-6 consent, according to the presence or absence of proton-pump inhibitors

Unique Subject Identifier=0146000000-0016

MEANS Procedure

Assay variable : PTINR_VAL1 PT-INR Value

N	Mean	Standard Deviation	Minimum	Maximum
4	2.1400000	0.5618422	1.4600000	2.8300000

Unique Subject Identifier=0146000000-0021

Assay variable : PTINR_VAL1 PT-INR Value

N	Mean	Standard Deviation	Minimum	Maximum
2	2.1050000	0.0212132	2.0900000	2.1200000

Unique Subject Identifier=0147000000-0002

Assay variable : PTINR_VAL1 PT-INR Value

N	Mean	Standard Deviation	Minimum	Maximum
3	1.8766667	0.0642910	1.8300000	1.9500000

Unique Subject Identifier=0147000000-0003

Assay variable : PTINR_VAL1 PT-INR Value

N	Mean	Standard Deviation	Minimum	Maximum
4	2.1000000	0.3108054	1.7900000	2.5200000

Laboratory tests at the time of obtaining Table 2-6 consent, according to the presence or absence of proton-pump inhibitors

Unique Subject Identifier=0147000000-0005

MEANS Procedure

Assay variable : PTINR_VAL1 PT-INR Value

N	Mean	Standard Deviation	Minimum	Maximum
3	2.2733333	0.1803700	2.1000000	2.4600000

Unique Subject Identifier=0147000000-0009

Assay variable : PTINR_VAL1 PT-INR Value

N	Mean	Standard Deviation	Minimum	Maximum
3	1.8133333	0.0152753	1.8000000	1.8300000

Unique Subject Identifier=0147000000-0013

Assay variable : PTINR_VAL1 PT-INR Value

N	Mean	Standard Deviation	Minimum	Maximum
4	1.9850000	0.5911853	1.6500000	2.8700000

Unique Subject Identifier=0147000000-0016

Assay variable : PTINR_VAL1 PT-INR Value

N	Mean	Standard Deviation	Minimum	Maximum
3	1.7900000	0.4853864	1.2300000	2.0900000

Laboratory tests at the time of obtaining Table 2-6 consent, according to the presence or absence of proton-pump inhibitors

Unique Subject Identifier=0154010000-0018

MEANS Procedure

Assay variable : PTINR_VAL1 PT-INR Value

N	Mean	Standard Deviation	Minimum	Maximum
5	1.7200000	0.3033150	1.4000000	2.2000000

Unique Subject Identifier=0155010000-0002

Assay variable : PTINR_VAL1 PT-INR Value

N	Mean	Standard Deviation	Minimum	Maximum
3	2.5333333	0.3511885	2.2000000	2.9000000

Unique Subject Identifier=0155010000-0004

Assay variable : PTINR_VAL1 PT-INR Value

N	Mean	Standard Deviation	Minimum	Maximum
3	2.1666667	0.1154701	2.1000000	2.3000000

Unique Subject Identifier=0155010000-0006

Assay variable : PTINR_VAL1 PT-INR Value

N	Mean	Standard Deviation	Minimum	Maximum
4	2.1250000	0.2872281	1.7000000	2.3000000

Laboratory tests at the time of obtaining Table 2-6 consent, according to the presence or absence of proton-pump inhibitors

Unique Subject Identifier=0155010000-0007

MEANS Procedure

Assay variable : PTINR_VAL1 PT-INR Value

N	Mean	Standard Deviation	Minimum	Maximum
3	1.9333333	0.1154701	1.8000000	2.0000000

Unique Subject Identifier=0155010000-0008

Assay variable : PTINR_VAL1 PT-INR Value

N	Mean	Standard Deviation	Minimum	Maximum
4	2.7000000	0.4760952	2.2000000	3.2000000

Unique Subject Identifier=0155010000-0009

Assay variable : PTINR_VAL1 PT-INR Value

N	Mean	Standard Deviation	Minimum	Maximum
4	1.7750000	0.1500000	1.6000000	1.9000000

Unique Subject Identifier=0155010000-0010

Assay variable : PTINR_VAL1 PT-INR Value

N	Mean	Standard Deviation	Minimum	Maximum
4	2.2000000	0.2943920	1.9000000	2.5000000

Laboratory tests at the time of obtaining Table 2-6 consent, according to the presence or absence of proton-pump inhibitors

Unique Subject Identifier=0161000000-0007

MEANS Procedure

Assay variable : PTINR_VAL1 PT-INR Value

N	Mean	Standard Deviation	Minimum	Maximum
5	2.3540000	0.2361779	2.1000000	2.7000000

Unique Subject Identifier=0161000000-0009

Assay variable : PTINR_VAL1 PT-INR Value

N	Mean	Standard Deviation	Minimum	Maximum
6	2.1766667	0.1732820	2.0000000	2.4800000

Unique Subject Identifier=0161000000-0010

Assay variable : PTINR_VAL1 PT-INR Value

N	Mean	Standard Deviation	Minimum	Maximum
6	2.3066667	0.1711919	2.1300000	2.6000000

Unique Subject Identifier=0161000000-0011

Assay variable : PTINR_VAL1 PT-INR Value

N	Mean	Standard Deviation	Minimum	Maximum
6	2.0733333	0.3310992	1.5700000	2.5900000

Laboratory tests at the time of obtaining Table 2-6 consent, according to the presence or absence of proton-pump inhibitors

Unique Subject Identifier=0161000000-0014

MEANS Procedure

Assay variable : PTINR_VAL1 PT-INR Value

N	Mean	Standard Deviation	Minimum	Maximum
6	2.1716667	0.2199470	1.8400000	2.3700000

Unique Subject Identifier=0161000000-0017

Assay variable : PTINR_VAL1 PT-INR Value

N	Mean	Standard Deviation	Minimum	Maximum
6	1.8400000	0.3245921	1.3300000	2.2500000

Unique Subject Identifier=0162000000-0002

Assay variable : PTINR_VAL1 PT-INR Value

N	Mean	Standard Deviation	Minimum	Maximum
4	1.4850000	0.3445287	1.0400000	1.8800000

Unique Subject Identifier=0165000000-0005

Assay variable : PTINR_VAL1 PT-INR Value

N	Mean	Standard Deviation	Minimum	Maximum
4	1.9300000	0.1540563	1.7700000	2.0900000

Laboratory tests at the time of obtaining Table 2-6 consent, according to the presence or absence of proton-pump inhibitors

Unique Subject Identifier=0165000000-0012

MEANS Procedure

Assay variable : PTINR_VAL1 PT-INR Value

N	Mean	Standard Deviation	Minimum	Maximum
5	2.2620000	0.5316672	1.6800000	3.1300000

Unique Subject Identifier=0165000000-0013

Assay variable : PTINR_VAL1 PT-INR Value

N	Mean	Standard Deviation	Minimum	Maximum
5	2.2620000	0.5316672	1.6800000	3.1300000

Unique Subject Identifier=0165000000-0015

Assay variable : PTINR_VAL1 PT-INR Value

N	Mean	Standard Deviation	Minimum	Maximum
3	2.6666667	0.8590887	1.9800000	3.6300000

Unique Subject Identifier=0165000000-0017

Assay variable : PTINR_VAL1 PT-INR Value

N	Mean	Standard Deviation	Minimum	Maximum
6	1.7750000	0.1590912	1.5600000	2.0400000

Laboratory tests at the time of obtaining Table 2-6 consent, according to the presence or absence of proton-pump inhibitors

Unique Subject Identifier=0166000000-0009

MEANS Procedure

Assay variable : PTINR_VAL1 PT-INR Value

N	Mean	Standard Deviation	Minimum	Maximum
2	2.1050000	0.1060660	2.0300000	2.1800000

Unique Subject Identifier=0166000000-0017

Assay variable : PTINR_VAL1 PT-INR Value

N	Mean	Standard Deviation	Minimum	Maximum
6	1.1783333	0.1292156	1.0700000	1.4300000

Unique Subject Identifier=0166000000-0021

Assay variable : PTINR_VAL1 PT-INR Value

N	Mean	Standard Deviation	Minimum	Maximum
2	2.6400000	0.3394113	2.4000000	2.8800000

Unique Subject Identifier=0166000000-0023

Assay variable : PTINR_VAL1 PT-INR Value

N	Mean	Standard Deviation	Minimum	Maximum
1	1.6900000		1.6900000	1.6900000

Laboratory tests at the time of obtaining Table 2-6 consent, according to the presence or absence of proton-pump inhibitors

Unique Subject Identifier=0166000000-0027

MEANS Procedure

Assay variable : PTINR_VAL1 PT-INR Value

N	Mean	Standard Deviation	Minimum	Maximum
3	2.1666667	0.4495924	1.7800000	2.6600000

Unique Subject Identifier=0166000000-0031

Assay variable : PTINR_VAL1 PT-INR Value

N	Mean	Standard Deviation	Minimum	Maximum
3	2.0333333	0.3536005	1.6300000	2.2900000

Unique Subject Identifier=0166000000-0035

Assay variable : PTINR_VAL1 PT-INR Value

N	Mean	Standard Deviation	Minimum	Maximum
2	1.8450000	0.2474874	1.6700000	2.0200000

Unique Subject Identifier=0166000000-0036

Assay variable : PTINR_VAL1 PT-INR Value

N	Mean	Standard Deviation	Minimum	Maximum
2	1.8350000	0.5020458	1.4800000	2.1900000

Laboratory tests at the time of obtaining Table 2-6 consent, according to the presence or absence of proton-pump inhibitors

Unique Subject Identifier=0166000000-0039

MEANS Procedure

Assay variable : PTINR_VAL1 PT-INR Value

N	Mean	Standard Deviation	Minimum	Maximum
3	2.1466667	0.1357694	1.9900000	2.2300000

Unique Subject Identifier=0166000000-0040

Assay variable : PTINR_VAL1 PT-INR Value

N	Mean	Standard Deviation	Minimum	Maximum
3	2.2566667	0.2030599	2.1200000	2.4900000

Unique Subject Identifier=0166000000-0043

Assay variable : PTINR_VAL1 PT-INR Value

N	Mean	Standard Deviation	Minimum	Maximum
2	2.3600000	0.8485281	1.7600000	2.9600000

Unique Subject Identifier=0166000000-0047

Assay variable : PTINR_VAL1 PT-INR Value

N	Mean	Standard Deviation	Minimum	Maximum
2	2.1550000	0.0212132	2.1400000	2.1700000

Laboratory tests at the time of obtaining Table 2-6 consent, according to the presence or absence of proton-pump inhibitors

Unique Subject Identifier=0166000000-0053

MEANS Procedure

Assay variable : PTINR_VAL1 PT-INR Value

N	Mean	Standard Deviation	Minimum	Maximum
6	2.4000000	0.3407052	2.1400000	3.0100000

Unique Subject Identifier=0166000000-0054

Assay variable : PTINR_VAL1 PT-INR Value

N	Mean	Standard Deviation	Minimum	Maximum
2	1.8250000	0.0353553	1.8000000	1.8500000

Unique Subject Identifier=0166000000-0056

Assay variable : PTINR_VAL1 PT-INR Value

N	Mean	Standard Deviation	Minimum	Maximum
5	1.7760000	0.3803682	1.4300000	2.3900000

Unique Subject Identifier=0166000000-0059

Assay variable : PTINR_VAL1 PT-INR Value

N	Mean	Standard Deviation	Minimum	Maximum
2	1.5550000	0.0212132	1.5400000	1.5700000

Laboratory tests at the time of obtaining Table 2-6 consent, according to the presence or absence of proton-pump inhibitors

Unique Subject Identifier=0166000000-0062

MEANS Procedure

Assay variable : PTINR_VAL1 PT-INR Value

N	Mean	Standard Deviation	Minimum	Maximum
2	2.0200000	0.2969848	1.8100000	2.2300000

Unique Subject Identifier=0166000000-0063

Assay variable : PTINR_VAL1 PT-INR Value

N	Mean	Standard Deviation	Minimum	Maximum
2	2.6200000	0.3959798	2.3400000	2.9000000

Unique Subject Identifier=0168000000-0004

Assay variable : PTINR_VAL1 PT-INR Value

N	Mean	Standard Deviation	Minimum	Maximum
1	1.6600000		1.6600000	1.6600000

Unique Subject Identifier=0170000000-0001

Assay variable : PTINR_VAL1 PT-INR Value

N	Mean	Standard Deviation	Minimum	Maximum
5	2.0320000	0.1811629	1.8300000	2.3100000

Laboratory tests at the time of obtaining Table 2-6 consent, according to the presence or absence of proton-pump inhibitors

Unique Subject Identifier=0170000000-0003

MEANS Procedure

Assay variable : PTINR_VAL1 PT-INR Value

N	Mean	Standard Deviation	Minimum	Maximum
3	1.7100000	0.0435890	1.6800000	1.7600000

Unique Subject Identifier=0170000000-0004

Assay variable : PTINR_VAL1 PT-INR Value

N	Mean	Standard Deviation	Minimum	Maximum
6	1.9316667	0.1543265	1.6500000	2.1000000

Unique Subject Identifier=0170000000-0005

Assay variable : PTINR_VAL1 PT-INR Value

N	Mean	Standard Deviation	Minimum	Maximum
5	1.6840000	0.2586117	1.2900000	1.9700000

Unique Subject Identifier=0170000000-0007

Assay variable : PTINR_VAL1 PT-INR Value

N	Mean	Standard Deviation	Minimum	Maximum
6	2.0150000	0.7322773	1.3900000	3.4300000

Laboratory tests at the time of obtaining Table 2-6 consent, according to the presence or absence of proton-pump inhibitors

Unique Subject Identifier=0170000000-0008

MEANS Procedure

Assay variable : PTINR_VAL1 PT-INR Value

N	Mean	Standard Deviation	Minimum	Maximum
4	1.6825000	0.2304163	1.4600000	1.9600000

Unique Subject Identifier=0170000000-0011

Assay variable : PTINR_VAL1 PT-INR Value

N	Mean	Standard Deviation	Minimum	Maximum
6	1.8316667	0.2192183	1.5300000	2.1700000

Unique Subject Identifier=0170000000-0013

Assay variable : PTINR_VAL1 PT-INR Value

N	Mean	Standard Deviation	Minimum	Maximum
3	1.7366667	0.0986577	1.6700000	1.8500000

Unique Subject Identifier=0170000000-0017

Assay variable : PTINR_VAL1 PT-INR Value

N	Mean	Standard Deviation	Minimum	Maximum
2	1.5650000	0.1626346	1.4500000	1.6800000

Laboratory tests at the time of obtaining Table 2-6 consent, according to the presence or absence of proton-pump inhibitors

Unique Subject Identifier=0170000000-0018

MEANS Procedure

Assay variable : PTINR_VAL1 PT-INR Value

N	Mean	Standard Deviation	Minimum	Maximum
3	1.5433333	0.2145538	1.4000000	1.7900000

Unique Subject Identifier=0170000000-0020

Assay variable : PTINR_VAL1 PT-INR Value

N	Mean	Standard Deviation	Minimum	Maximum
3	1.7366667	0.1457166	1.5700000	1.8400000

Unique Subject Identifier=0173000000-0001

Assay variable : PTINR_VAL1 PT-INR Value

N	Mean	Standard Deviation	Minimum	Maximum
4	1.6525000	0.1883923	1.4300000	1.8900000

Unique Subject Identifier=0177100000-0004

Assay variable : PTINR_VAL1 PT-INR Value

N	Mean	Standard Deviation	Minimum	Maximum
4	1.6575000	0.3102015	1.2300000	1.9500000

Laboratory tests at the time of obtaining Table 2-6 consent, according to the presence or absence of proton-pump inhibitors

Unique Subject Identifier=0177100000-0005

MEANS Procedure

Assay variable : PTINR_VAL1 PT-INR Value

N	Mean	Standard Deviation	Minimum	Maximum
3	2.6233333	1.0400641	1.8700000	3.8100000

Unique Subject Identifier=0177100000-0006

Assay variable : PTINR_VAL1 PT-INR Value

N	Mean	Standard Deviation	Minimum	Maximum
2	2.0950000	0.0636396	2.0500000	2.1400000

Unique Subject Identifier=0177100000-0007

Assay variable : PTINR_VAL1 PT-INR Value

N	Mean	Standard Deviation	Minimum	Maximum
5	1.8680000	0.4621904	1.5500000	2.6700000

Unique Subject Identifier=0177100000-0008

Assay variable : PTINR_VAL1 PT-INR Value

N	Mean	Standard Deviation	Minimum	Maximum
5	1.5720000	0.5142665	1.1600000	2.4600000

Laboratory tests at the time of obtaining Table 2-6 consent, according to the presence or absence of proton-pump inhibitors

Unique Subject Identifier=0177100000-0021

MEANS Procedure

Assay variable : PTINR_VAL1 PT-INR Value

N	Mean	Standard Deviation	Minimum	Maximum
6	1.7683333	0.1376106	1.5300000	1.8700000

Unique Subject Identifier=0177100000-0046

Assay variable : PTINR_VAL1 PT-INR Value

N	Mean	Standard Deviation	Minimum	Maximum
5	1.7960000	0.2356480	1.5000000	2.1600000

Unique Subject Identifier=0177100000-0047

Assay variable : PTINR_VAL1 PT-INR Value

N	Mean	Standard Deviation	Minimum	Maximum
4	2.0475000	0.7650000	1.2600000	3.0900000

Unique Subject Identifier=0177100000-0049

Assay variable : PTINR_VAL1 PT-INR Value

N	Mean	Standard Deviation	Minimum	Maximum
6	2.1883333	1.2622427	1.2800000	4.5700000

Laboratory tests at the time of obtaining Table 2-6 consent, according to the presence or absence of proton-pump inhibitors

Unique Subject Identifier=0177100000-0059

MEANS Procedure

Assay variable : PTINR_VAL1 PT-INR Value

N	Mean	Standard Deviation	Minimum	Maximum
4	2.1500000	0.5255473	1.6200000	2.7000000

Unique Subject Identifier=0177100000-0065

Assay variable : PTINR_VAL1 PT-INR Value

N	Mean	Standard Deviation	Minimum	Maximum
4	2.3825000	0.4011130	1.9700000	2.8500000

Unique Subject Identifier=0178100000-0002

Assay variable : PTINR_VAL1 PT-INR Value

N	Mean	Standard Deviation	Minimum	Maximum
2	2.4250000	0.1767767	2.3000000	2.5500000

Unique Subject Identifier=0178100000-0004

Assay variable : PTINR_VAL1 PT-INR Value

N	Mean	Standard Deviation	Minimum	Maximum
4	2.2875000	0.1978846	2.1200000	2.5400000

Laboratory tests at the time of obtaining Table 2-6 consent, according to the presence or absence of proton-pump inhibitors

Unique Subject Identifier=0178100000-0005

MEANS Procedure

Assay variable : PTINR_VAL1 PT-INR Value

N	Mean	Standard Deviation	Minimum	Maximum
4	1.8325000	0.1774589	1.6000000	1.9900000

Unique Subject Identifier=0178100000-0007

Assay variable : PTINR_VAL1 PT-INR Value

N	Mean	Standard Deviation	Minimum	Maximum
5	2.1760000	0.5141790	1.7100000	2.9900000

Unique Subject Identifier=0178100000-0011

Assay variable : PTINR_VAL1 PT-INR Value

N	Mean	Standard Deviation	Minimum	Maximum
5	1.6000000	0.1048809	1.4400000	1.7200000

Unique Subject Identifier=0178100000-0013

Assay variable : PTINR_VAL1 PT-INR Value

N	Mean	Standard Deviation	Minimum	Maximum
4	2.2000000	0.0898146	2.1100000	2.3200000

Laboratory tests at the time of obtaining Table 2-6 consent, according to the presence or absence of proton-pump inhibitors

Unique Subject Identifier=0178100000-0016

MEANS Procedure

Assay variable : PTINR_VAL1 PT-INR Value

N	Mean	Standard Deviation	Minimum	Maximum
4	2.1400000	0.3703152	1.6800000	2.5700000

Unique Subject Identifier=0178100000-0017

Assay variable : PTINR_VAL1 PT-INR Value

N	Mean	Standard Deviation	Minimum	Maximum
6	2.0333333	0.2592811	1.5800000	2.2600000

Unique Subject Identifier=0178100000-0018

Assay variable : PTINR_VAL1 PT-INR Value

N	Mean	Standard Deviation	Minimum	Maximum
5	1.9420000	0.3721156	1.2900000	2.2100000

Unique Subject Identifier=0178100000-0020

Assay variable : PTINR_VAL1 PT-INR Value

N	Mean	Standard Deviation	Minimum	Maximum
3	1.9466667	0.3900427	1.5000000	2.2200000

Laboratory tests at the time of obtaining Table 2-6 consent, according to the presence or absence of proton-pump inhibitors

Unique Subject Identifier=0178100000-0022

MEANS Procedure

Assay variable : PTINR_VAL1 PT-INR Value

N	Mean	Standard Deviation	Minimum	Maximum
4	2.1325000	0.2404683	1.9200000	2.4600000

Unique Subject Identifier=0178100000-0024

Assay variable : PTINR_VAL1 PT-INR Value

N	Mean	Standard Deviation	Minimum	Maximum
5	1.6500000	0.2331309	1.2500000	1.8600000

Unique Subject Identifier=0178100000-0025

Assay variable : PTINR_VAL1 PT-INR Value

N	Mean	Standard Deviation	Minimum	Maximum
4	2.5775000	0.5395291	2.0200000	3.1800000

Unique Subject Identifier=0178100000-0026

Assay variable : PTINR_VAL1 PT-INR Value

N	Mean	Standard Deviation	Minimum	Maximum
4	2.2275000	0.2595348	2.0500000	2.6000000

Laboratory tests at the time of obtaining Table 2-6 consent, according to the presence or absence of proton-pump inhibitors

Unique Subject Identifier=0178100000-0028

MEANS Procedure

Assay variable : PTINR_VAL1 PT-INR Value

N	Mean	Standard Deviation	Minimum	Maximum
4	2.4700000	0.6106281	1.9900000	3.3500000

Unique Subject Identifier=0178100000-0032

Assay variable : PTINR_VAL1 PT-INR Value

N	Mean	Standard Deviation	Minimum	Maximum
4	2.3750000	0.1332917	2.2300000	2.5200000

Unique Subject Identifier=0178100000-0034

Assay variable : PTINR_VAL1 PT-INR Value

N	Mean	Standard Deviation	Minimum	Maximum
4	2.1450000	0.3443351	1.8600000	2.5700000

Unique Subject Identifier=0178100000-0035

Assay variable : PTINR_VAL1 PT-INR Value

N	Mean	Standard Deviation	Minimum	Maximum
4	1.8975000	0.1925920	1.6400000	2.0500000

Laboratory tests at the time of obtaining Table 2-6 consent, according to the presence or absence of proton-pump inhibitors

Unique Subject Identifier=0178100000-0036

MEANS Procedure

Assay variable : PTINR_VAL1 PT-INR Value

N	Mean	Standard Deviation	Minimum	Maximum
4	2.1075000	0.1736615	1.9400000	2.3500000

Unique Subject Identifier=0178100000-0037

Assay variable : PTINR_VAL1 PT-INR Value

N	Mean	Standard Deviation	Minimum	Maximum
4	2.4725000	0.1510794	2.2600000	2.5900000

Unique Subject Identifier=0178100000-0041

Assay variable : PTINR_VAL1 PT-INR Value

N	Mean	Standard Deviation	Minimum	Maximum
3	1.9866667	0.2354428	1.7600000	2.2300000

Unique Subject Identifier=0178100000-0042

Assay variable : PTINR_VAL1 PT-INR Value

N	Mean	Standard Deviation	Minimum	Maximum
4	1.9625000	0.1602862	1.8500000	2.1900000

Laboratory tests at the time of obtaining Table 2-6 consent, according to the presence or absence of proton-pump inhibitors

Unique Subject Identifier=0178100000-0052

MEANS Procedure

Assay variable : PTINR_VAL1 PT-INR Value

N	Mean	Standard Deviation	Minimum	Maximum
4	1.8575000	0.1530523	1.7100000	2.0700000

Unique Subject Identifier=0178100000-0053

Assay variable : PTINR_VAL1 PT-INR Value

N	Mean	Standard Deviation	Minimum	Maximum
6	1.8166667	0.3120684	1.5200000	2.4000000

Unique Subject Identifier=0178100000-0055

Assay variable : PTINR_VAL1 PT-INR Value

N	Mean	Standard Deviation	Minimum	Maximum
5	1.5840000	0.3295907	1.1900000	1.8600000

Unique Subject Identifier=0178100000-0057

Assay variable : PTINR_VAL1 PT-INR Value

N	Mean	Standard Deviation	Minimum	Maximum
2	1.8500000	0.1131371	1.7700000	1.9300000

Laboratory tests at the time of obtaining Table 2-6 consent, according to the presence or absence of proton-pump inhibitors

Unique Subject Identifier=0178100000-0058

MEANS Procedure

Assay variable : PTINR_VAL1 PT-INR Value

N	Mean	Standard Deviation	Minimum	Maximum
4	2.0675000	0.3244868	1.7600000	2.5200000

Unique Subject Identifier=0178100000-0059

Assay variable : PTINR_VAL1 PT-INR Value

N	Mean	Standard Deviation	Minimum	Maximum
4	2.3575000	0.3051093	2.0200000	2.7500000

Unique Subject Identifier=0178100000-0063

Assay variable : PTINR_VAL1 PT-INR Value

N	Mean	Standard Deviation	Minimum	Maximum
5	2.3440000	0.5912106	1.7400000	3.3100000

Unique Subject Identifier=0178100000-0065

Assay variable : PTINR_VAL1 PT-INR Value

N	Mean	Standard Deviation	Minimum	Maximum
4	2.3025000	0.7097124	1.5900000	3.2800000

Laboratory tests at the time of obtaining Table 2-6 consent, according to the presence or absence of proton-pump inhibitors

Unique Subject Identifier=0178100000-0066

MEANS Procedure

Assay variable : PTINR_VAL1 PT-INR Value

N	Mean	Standard Deviation	Minimum	Maximum
4	1.4700000	0.0983192	1.3800000	1.6000000

Unique Subject Identifier=0178100000-0069

Assay variable : PTINR_VAL1 PT-INR Value

N	Mean	Standard Deviation	Minimum	Maximum
3	2.0066667	0.1504438	1.9100000	2.1800000

Unique Subject Identifier=0180000000-0001

Assay variable : PTINR_VAL1 PT-INR Value

N	Mean	Standard Deviation	Minimum	Maximum
5	1.7280000	0.2413918	1.4800000	1.9900000

Unique Subject Identifier=0180000000-0004

Assay variable : PTINR_VAL1 PT-INR Value

N	Mean	Standard Deviation	Minimum	Maximum
6	2.2966667	0.1778389	2.1000000	2.5200000

Laboratory tests at the time of obtaining Table 2-6 consent, according to the presence or absence of proton-pump inhibitors

Unique Subject Identifier=0180000000-0006

MEANS Procedure

Assay variable : PTINR_VAL1 PT-INR Value

N	Mean	Standard Deviation	Minimum	Maximum
5	2.1520000	0.3898333	1.9200000	2.8400000

Unique Subject Identifier=0180000000-0007

Assay variable : PTINR_VAL1 PT-INR Value

N	Mean	Standard Deviation	Minimum	Maximum
3	1.8466667	0.7490216	1.3700000	2.7100000

Unique Subject Identifier=0180000000-0011

Assay variable : PTINR_VAL1 PT-INR Value

N	Mean	Standard Deviation	Minimum	Maximum
6	2.2966667	0.5308358	1.5600000	2.9700000

Unique Subject Identifier=0180000000-0015

Assay variable : PTINR_VAL1 PT-INR Value

N	Mean	Standard Deviation	Minimum	Maximum
4	1.9575000	0.2581182	1.6300000	2.2600000

Laboratory tests at the time of obtaining Table 2-6 consent, according to the presence or absence of proton-pump inhibitors

Unique Subject Identifier=0180000000-0024

MEANS Procedure

Assay variable : PTINR_VAL1 PT-INR Value

N	Mean	Standard Deviation	Minimum	Maximum
2	1.8900000	0.0282843	1.8700000	1.9100000

Unique Subject Identifier=0180000000-0026

Assay variable : PTINR_VAL1 PT-INR Value

N	Mean	Standard Deviation	Minimum	Maximum
6	2.2150000	0.4043637	1.7900000	2.9400000

Unique Subject Identifier=0181000000-0002

Assay variable : PTINR_VAL1 PT-INR Value

N	Mean	Standard Deviation	Minimum	Maximum
5	2.0420000	0.6430163	1.2100000	2.9700000

Unique Subject Identifier=0181000000-0003

Assay variable : PTINR_VAL1 PT-INR Value

N	Mean	Standard Deviation	Minimum	Maximum
2	1.8150000	0.2757716	1.6200000	2.0100000

Laboratory tests at the time of obtaining Table 2-6 consent, according to the presence or absence of proton-pump inhibitors

Unique Subject Identifier=0181000000-0005

MEANS Procedure

Assay variable : PTINR_VAL1 PT-INR Value

N	Mean	Standard Deviation	Minimum	Maximum
6	1.9800000	0.3001333	1.6100000	2.3100000

Unique Subject Identifier=0189000000-0012

Assay variable : PTINR_VAL1 PT-INR Value

N	Mean	Standard Deviation	Minimum	Maximum
3	1.8700000	0.1400000	1.7300000	2.0100000

Unique Subject Identifier=0189000000-0013

Assay variable : PTINR_VAL1 PT-INR Value

N	Mean	Standard Deviation	Minimum	Maximum
6	2.1350000	0.3800395	1.7000000	2.7400000

Unique Subject Identifier=0189000000-0020

Assay variable : PTINR_VAL1 PT-INR Value

N	Mean	Standard Deviation	Minimum	Maximum
3	2.4733333	0.6214767	1.9100000	3.1400000

Laboratory tests at the time of obtaining Table 2-6 consent, according to the presence or absence of proton-pump inhibitors

Unique Subject Identifier=0189000000-0026

MEANS Procedure

Assay variable : PTINR_VAL1 PT-INR Value

N	Mean	Standard Deviation	Minimum	Maximum
2	2.0650000	0.2192031	1.9100000	2.2200000

Unique Subject Identifier=0189000000-0030

Assay variable : PTINR_VAL1 PT-INR Value

N	Mean	Standard Deviation	Minimum	Maximum
4	1.8200000	0.0743864	1.7100000	1.8700000

Unique Subject Identifier=0189000000-0033

Assay variable : PTINR_VAL1 PT-INR Value

N	Mean	Standard Deviation	Minimum	Maximum
3	1.9033333	0.2003331	1.7100000	2.1100000

Unique Subject Identifier=0189000000-0035

Assay variable : PTINR_VAL1 PT-INR Value

N	Mean	Standard Deviation	Minimum	Maximum
3	1.7900000	0.3850974	1.4100000	2.1800000

Laboratory tests at the time of obtaining Table 2-6 consent, according to the presence or absence of proton-pump inhibitors

Unique Subject Identifier=0189000000-0043

MEANS Procedure

Assay variable : PTINR_VAL1 PT-INR Value

N	Mean	Standard Deviation	Minimum	Maximum
4	1.6900000	0.1845716	1.5400000	1.9300000

Unique Subject Identifier=0189000000-0046

Assay variable : PTINR_VAL1 PT-INR Value

N	Mean	Standard Deviation	Minimum	Maximum
3	2.6200000	0.4150904	2.2100000	3.0400000

Unique Subject Identifier=0195011000-0016

Assay variable : PTINR_VAL1 PT-INR Value

N	Mean	Standard Deviation	Minimum	Maximum
3	1.7366667	0.1305118	1.6000000	1.8600000

Unique Subject Identifier=0201000000-0006

Assay variable : PTINR_VAL1 PT-INR Value

N	Mean	Standard Deviation	Minimum	Maximum
3	2.2900000	0.5406478	1.8700000	2.9000000

Laboratory tests at the time of obtaining Table 2-6 consent, according to the presence or absence of proton-pump inhibitors

Unique Subject Identifier=0205100000-0001

MEANS Procedure

Assay variable : PTINR_VAL1 PT-INR Value

N	Mean	Standard Deviation	Minimum	Maximum
3	1.8133333	0.1703917	1.7100000	2.0100000

Unique Subject Identifier=0205100000-0002

Assay variable : PTINR_VAL1 PT-INR Value

N	Mean	Standard Deviation	Minimum	Maximum
4	2.2575000	0.2478407	2.0300000	2.5700000

Unique Subject Identifier=0205100000-0006

Assay variable : PTINR_VAL1 PT-INR Value

N	Mean	Standard Deviation	Minimum	Maximum
3	1.8000000	0.3700000	1.4000000	2.1300000

Unique Subject Identifier=0205100000-0013

Assay variable : PTINR_VAL1 PT-INR Value

N	Mean	Standard Deviation	Minimum	Maximum
2	2.6600000	0.0989949	2.5900000	2.7300000

Laboratory tests at the time of obtaining Table 2-6 consent, according to the presence or absence of proton-pump inhibitors

Unique Subject Identifier=0206100000-0004

MEANS Procedure

Assay variable : PTINR_VAL1 PT-INR Value

N	Mean	Standard Deviation	Minimum	Maximum
6	2.1800000	0.3240988	1.8500000	2.5700000

Unique Subject Identifier=0206100000-0005

Assay variable : PTINR_VAL1 PT-INR Value

N	Mean	Standard Deviation	Minimum	Maximum
5	1.7040000	0.3691612	1.3300000	2.2300000

Unique Subject Identifier=0206100000-0006

Assay variable : PTINR_VAL1 PT-INR Value

N	Mean	Standard Deviation	Minimum	Maximum
6	1.8666667	0.3144307	1.3600000	2.2400000

Unique Subject Identifier=0207100000-0002

Assay variable : PTINR_VAL1 PT-INR Value

N	Mean	Standard Deviation	Minimum	Maximum
6	1.9150000	0.3571414	1.6400000	2.6100000

Laboratory tests at the time of obtaining Table 2-6 consent, according to the presence or absence of proton-pump inhibitors

Unique Subject Identifier=0207100000-0005

MEANS Procedure

Assay variable : PTINR_VAL1 PT-INR Value

N	Mean	Standard Deviation	Minimum	Maximum
1	2.0600000		2.0600000	2.0600000

Unique Subject Identifier=0207100000-0008

Assay variable : PTINR_VAL1 PT-INR Value

N	Mean	Standard Deviation	Minimum	Maximum
1	1.6500000		1.6500000	1.6500000

Unique Subject Identifier=0207100000-0011

Assay variable : PTINR_VAL1 PT-INR Value

N	Mean	Standard Deviation	Minimum	Maximum
1	1.9000000		1.9000000	1.9000000

Unique Subject Identifier=0207100000-0013

Assay variable : PTINR_VAL1 PT-INR Value

N	Mean	Standard Deviation	Minimum	Maximum
3	1.7533333	0.1193035	1.6700000	1.8900000

Laboratory tests at the time of obtaining Table 2-6 consent, according to the presence or absence of proton-pump inhibitors

Unique Subject Identifier=0207100000-0015

MEANS Procedure

Assay variable : PTINR_VAL1 PT-INR Value

N	Mean	Standard Deviation	Minimum	Maximum
2	3.0850000	0.1060660	3.0100000	3.1600000

Unique Subject Identifier=0207100000-0017

Assay variable : PTINR_VAL1 PT-INR Value

N	Mean	Standard Deviation	Minimum	Maximum
3	2.2733333	0.4908496	1.9800000	2.8400000

Unique Subject Identifier=0210000000-0003

Assay variable : PTINR_VAL1 PT-INR Value

N	Mean	Standard Deviation	Minimum	Maximum
6	2.4666667	0.5715476	1.7000000	3.3000000

Unique Subject Identifier=0210000000-0006

Assay variable : PTINR_VAL1 PT-INR Value

N	Mean	Standard Deviation	Minimum	Maximum
5	2.5000000	0.3316625	2.2000000	2.9000000

Laboratory tests at the time of obtaining Table 2-6 consent, according to the presence or absence of proton-pump inhibitors

Unique Subject Identifier=0210000000-0011

MEANS Procedure

Assay variable : PTINR_VAL1 PT-INR Value

N	Mean	Standard Deviation	Minimum	Maximum
5	2.0400000	0.2966479	1.7000000	2.4000000

Unique Subject Identifier=0210000000-0017

Assay variable : PTINR_VAL1 PT-INR Value

N	Mean	Standard Deviation	Minimum	Maximum
5	1.7600000	0.1140175	1.6000000	1.9000000

Unique Subject Identifier=0211000000-0001

Assay variable : PTINR_VAL1 PT-INR Value

N	Mean	Standard Deviation	Minimum	Maximum
6	1.7983333	0.0679461	1.7100000	1.8800000

Unique Subject Identifier=0213000000-0001

Assay variable : PTINR_VAL1 PT-INR Value

N	Mean	Standard Deviation	Minimum	Maximum
6	2.1350000	0.1533297	1.9100000	2.3700000

Laboratory tests at the time of obtaining Table 2-6 consent, according to the presence or absence of proton-pump inhibitors

Unique Subject Identifier=0214000000-0003

MEANS Procedure

Assay variable : PTINR_VAL1 PT-INR Value

N	Mean	Standard Deviation	Minimum	Maximum
4	1.9250000	0.1642153	1.7800000	2.1500000

Unique Subject Identifier=0214000000-0011

Assay variable : PTINR_VAL1 PT-INR Value

N	Mean	Standard Deviation	Minimum	Maximum
6	1.6166667	0.2517671	1.2900000	1.9000000

Unique Subject Identifier=0217000000-0004

Assay variable : PTINR_VAL1 PT-INR Value

N	Mean	Standard Deviation	Minimum	Maximum
6	1.7750000	0.2757354	1.5000000	2.2200000

Unique Subject Identifier=0217000000-0006

Assay variable : PTINR_VAL1 PT-INR Value

N	Mean	Standard Deviation	Minimum	Maximum
1	1.6500000		1.6500000	1.6500000

Laboratory tests at the time of obtaining Table 2-6 consent, according to the presence or absence of proton-pump inhibitors

Unique Subject Identifier=0218000000-0005

MEANS Procedure

Assay variable : PTINR_VAL1 PT-INR Value

N	Mean	Standard Deviation	Minimum	Maximum
5	1.7120000	0.0970567	1.6100000	1.8500000

Unique Subject Identifier=0218000000-0006

Assay variable : PTINR_VAL1 PT-INR Value

N	Mean	Standard Deviation	Minimum	Maximum
6	1.9866667	0.2605123	1.5900000	2.3100000

Unique Subject Identifier=0220000000-0001

Assay variable : PTINR_VAL1 PT-INR Value

N	Mean	Standard Deviation	Minimum	Maximum
3	1.4300000	0.2553429	1.2100000	1.7100000

Unique Subject Identifier=0220000000-0003

Assay variable : PTINR_VAL1 PT-INR Value

N	Mean	Standard Deviation	Minimum	Maximum
6	2.2166667	0.3910328	1.5100000	2.6300000

Laboratory tests at the time of obtaining Table 2-6 consent, according to the presence or absence of proton-pump inhibitors

Unique Subject Identifier=0220000000-0004

MEANS Procedure

Assay variable : PTINR_VAL1 PT-INR Value

N	Mean	Standard Deviation	Minimum	Maximum
5	2.3160000	0.4197976	1.9700000	2.9400000

Unique Subject Identifier=0220000000-0005

Assay variable : PTINR_VAL1 PT-INR Value

N	Mean	Standard Deviation	Minimum	Maximum
5	2.5600000	0.7336212	1.8100000	3.7900000

Unique Subject Identifier=0220000000-0007

Assay variable : PTINR_VAL1 PT-INR Value

N	Mean	Standard Deviation	Minimum	Maximum
6	1.9116667	0.0919601	1.8000000	2.0800000

Unique Subject Identifier=0220000000-0008

Assay variable : PTINR_VAL1 PT-INR Value

N	Mean	Standard Deviation	Minimum	Maximum
4	2.2100000	0.4927474	1.7800000	2.9200000

Laboratory tests at the time of obtaining Table 2-6 consent, according to the presence or absence of proton-pump inhibitors

Unique Subject Identifier=0220000000-0010

MEANS Procedure

Assay variable : PTINR_VAL1 PT-INR Value

N	Mean	Standard Deviation	Minimum	Maximum
4	2.0350000	0.1047219	1.9300000	2.1700000

Unique Subject Identifier=0220000000-0011

Assay variable : PTINR_VAL1 PT-INR Value

N	Mean	Standard Deviation	Minimum	Maximum
4	2.2225000	0.2317146	1.9700000	2.5000000

Unique Subject Identifier=0220000000-0013

Assay variable : PTINR_VAL1 PT-INR Value

N	Mean	Standard Deviation	Minimum	Maximum
6	2.1783333	0.2587212	1.9100000	2.4800000

Unique Subject Identifier=0220000000-0014

Assay variable : PTINR_VAL1 PT-INR Value

N	Mean	Standard Deviation	Minimum	Maximum
3	2.1600000	0.0556776	2.1000000	2.2100000

Laboratory tests at the time of obtaining Table 2-6 consent, according to the presence or absence of proton-pump inhibitors

Unique Subject Identifier=0220000000-0015

MEANS Procedure

Assay variable : PTINR_VAL1 PT-INR Value

N	Mean	Standard Deviation	Minimum	Maximum
4	1.6975000	0.4239792	1.1300000	2.0600000

Unique Subject Identifier=0220000000-0016

Assay variable : PTINR_VAL1 PT-INR Value

N	Mean	Standard Deviation	Minimum	Maximum
5	2.1600000	0.4509989	1.6400000	2.6600000

Unique Subject Identifier=0220000000-0018

Assay variable : PTINR_VAL1 PT-INR Value

N	Mean	Standard Deviation	Minimum	Maximum
4	2.2825000	0.3902456	2.0400000	2.8600000

Unique Subject Identifier=0220000000-0020

Assay variable : PTINR_VAL1 PT-INR Value

N	Mean	Standard Deviation	Minimum	Maximum
4	1.9100000	0.2201515	1.7300000	2.2300000

Laboratory tests at the time of obtaining Table 2-6 consent, according to the presence or absence of proton-pump inhibitors

Unique Subject Identifier=0220000000-0022

MEANS Procedure

Assay variable : PTINR_VAL1 PT-INR Value

N	Mean	Standard Deviation	Minimum	Maximum
4	1.8825000	0.1030776	1.7300000	1.9500000

Unique Subject Identifier=0220000000-0024

Assay variable : PTINR_VAL1 PT-INR Value

N	Mean	Standard Deviation	Minimum	Maximum
3	2.1566667	0.2196209	2.0200000	2.4100000

Unique Subject Identifier=0220000000-0025

Assay variable : PTINR_VAL1 PT-INR Value

N	Mean	Standard Deviation	Minimum	Maximum
4	2.1150000	0.4969574	1.6500000	2.6600000

Unique Subject Identifier=0220000000-0026

Assay variable : PTINR_VAL1 PT-INR Value

N	Mean	Standard Deviation	Minimum	Maximum
4	2.6825000	0.3057641	2.3900000	3.1000000

Laboratory tests at the time of obtaining Table 2-6 consent, according to the presence or absence of proton-pump inhibitors

Unique Subject Identifier=0220000000-0028

MEANS Procedure

Assay variable : PTINR_VAL1 PT-INR Value

N	Mean	Standard Deviation	Minimum	Maximum
3	1.9566667	0.2973774	1.6400000	2.2300000

Unique Subject Identifier=0220000000-0029

Assay variable : PTINR_VAL1 PT-INR Value

N	Mean	Standard Deviation	Minimum	Maximum
4	2.4250000	0.4878183	1.8000000	2.9800000

Unique Subject Identifier=0220000000-0030

Assay variable : PTINR_VAL1 PT-INR Value

N	Mean	Standard Deviation	Minimum	Maximum
5	2.3060000	0.2898793	2.0400000	2.7900000

Unique Subject Identifier=0220000000-0032

Assay variable : PTINR_VAL1 PT-INR Value

N	Mean	Standard Deviation	Minimum	Maximum
3	2.6033333	0.2802380	2.3300000	2.8900000

Laboratory tests at the time of obtaining Table 2-6 consent, according to the presence or absence of proton-pump inhibitors

Unique Subject Identifier=0220000000-0034

MEANS Procedure

Assay variable : PTINR_VAL1 PT-INR Value

N	Mean	Standard Deviation	Minimum	Maximum
3	2.1133333	0.3843609	1.7000000	2.4600000

Unique Subject Identifier=0220000000-0035

Assay variable : PTINR_VAL1 PT-INR Value

N	Mean	Standard Deviation	Minimum	Maximum
5	2.1000000	0.6643041	1.4600000	2.9700000

Unique Subject Identifier=0222011000-0001

Assay variable : PTINR_VAL1 PT-INR Value

N	Mean	Standard Deviation	Minimum	Maximum
6	1.9800000	0.3700811	1.5700000	2.3900000

Unique Subject Identifier=0222011000-0005

Assay variable : PTINR_VAL1 PT-INR Value

N	Mean	Standard Deviation	Minimum	Maximum
5	2.0880000	0.2135884	1.8900000	2.4000000

Laboratory tests at the time of obtaining Table 2-6 consent, according to the presence or absence of proton-pump inhibitors

Unique Subject Identifier=0222011000-0009

MEANS Procedure

Assay variable : PTINR_VAL1 PT-INR Value

N	Mean	Standard Deviation	Minimum	Maximum
4	2.0425000	0.2069420	1.8600000	2.3400000

Unique Subject Identifier=0222011000-0010

Assay variable : PTINR_VAL1 PT-INR Value

N	Mean	Standard Deviation	Minimum	Maximum
5	2.4220000	0.7858562	1.8800000	3.8100000

Unique Subject Identifier=0222011000-0011

Assay variable : PTINR_VAL1 PT-INR Value

N	Mean	Standard Deviation	Minimum	Maximum
5	1.9480000	0.2641401	1.5600000	2.2100000

Unique Subject Identifier=0222011000-0012

Assay variable : PTINR_VAL1 PT-INR Value

N	Mean	Standard Deviation	Minimum	Maximum
6	2.0866667	0.3141125	1.5900000	2.4900000

Laboratory tests at the time of obtaining Table 2-6 consent, according to the presence or absence of proton-pump inhibitors

Unique Subject Identifier=0222011000-0013

MEANS Procedure

Assay variable : PTINR_VAL1 PT-INR Value

N	Mean	Standard Deviation	Minimum	Maximum
5	2.3140000	0.1659217	2.0500000	2.4600000

Unique Subject Identifier=0222011000-0014

Assay variable : PTINR_VAL1 PT-INR Value

N	Mean	Standard Deviation	Minimum	Maximum
5	2.2420000	0.3429577	1.8000000	2.6400000

Unique Subject Identifier=0222011000-0015

Assay variable : PTINR_VAL1 PT-INR Value

N	Mean	Standard Deviation	Minimum	Maximum
6	2.5283333	0.7471658	1.5500000	3.7500000

Unique Subject Identifier=0222011000-0016

Assay variable : PTINR_VAL1 PT-INR Value

N	Mean	Standard Deviation	Minimum	Maximum
6	2.2533333	0.3077445	1.9000000	2.8200000

Laboratory tests at the time of obtaining Table 2-6 consent, according to the presence or absence of proton-pump inhibitors

Unique Subject Identifier=0225001000-0001

MEANS Procedure

Assay variable : PTINR_VAL1 PT-INR Value

N	Mean	Standard Deviation	Minimum	Maximum
3	1.8900000	0.0692820	1.8500000	1.9700000

Unique Subject Identifier=0226010000-0001

Assay variable : PTINR_VAL1 PT-INR Value

N	Mean	Standard Deviation	Minimum	Maximum
2	1.3250000	0.1060660	1.2500000	1.4000000

Unique Subject Identifier=0226010000-0014

Assay variable : PTINR_VAL1 PT-INR Value

N	Mean	Standard Deviation	Minimum	Maximum
3	2.1166667	0.6819335	1.5200000	2.8600000

Unique Subject Identifier=0228100000-0012

Assay variable : PTINR_VAL1 PT-INR Value

N	Mean	Standard Deviation	Minimum	Maximum
6	1.3550000	0.1380942	1.1200000	1.5100000

Laboratory tests at the time of obtaining Table 2-6 consent, according to the presence or absence of proton-pump inhibitors

Unique Subject Identifier=0228100000-0014

MEANS Procedure

Assay variable : PTINR_VAL1 PT-INR Value

N	Mean	Standard Deviation	Minimum	Maximum
6	2.0833333	0.2852134	1.8300000	2.6200000

Unique Subject Identifier=0228100000-0016

Assay variable : PTINR_VAL1 PT-INR Value

N	Mean	Standard Deviation	Minimum	Maximum
5	2.2440000	0.6254838	1.5400000	2.8200000

Unique Subject Identifier=0228100000-0027

Assay variable : PTINR_VAL1 PT-INR Value

N	Mean	Standard Deviation	Minimum	Maximum
1	1.9100000		1.9100000	1.9100000

Unique Subject Identifier=0228100000-0030

Assay variable : PTINR_VAL1 PT-INR Value

N	Mean	Standard Deviation	Minimum	Maximum
2	2.0750000	0.4596194	1.7500000	2.4000000

Laboratory tests at the time of obtaining Table 2-6 consent, according to the presence or absence of proton-pump inhibitors

Unique Subject Identifier=0228100000-0042

MEANS Procedure

Assay variable : PTINR_VAL1 PT-INR Value

N	Mean	Standard Deviation	Minimum	Maximum
3	1.8566667	0.1415392	1.7700000	2.0200000

Unique Subject Identifier=0228100000-0052

Assay variable : PTINR_VAL1 PT-INR Value

N	Mean	Standard Deviation	Minimum	Maximum
1	1.8000000		1.8000000	1.8000000

Unique Subject Identifier=0229100000-0001

Assay variable : PTINR_VAL1 PT-INR Value

N	Mean	Standard Deviation	Minimum	Maximum
4	1.7550000	0.1178983	1.6400000	1.8800000

Unique Subject Identifier=0229100000-0002

Assay variable : PTINR_VAL1 PT-INR Value

N	Mean	Standard Deviation	Minimum	Maximum
5	1.6040000	0.2085186	1.4300000	1.9500000

Laboratory tests at the time of obtaining Table 2-6 consent, according to the presence or absence of proton-pump inhibitors

Unique Subject Identifier=0229100000-0004

MEANS Procedure

Assay variable : PTINR_VAL1 PT-INR Value

N	Mean	Standard Deviation	Minimum	Maximum
6	1.5983333	0.4860213	1.1700000	2.4600000

Unique Subject Identifier=0229100000-0010

Assay variable : PTINR_VAL1 PT-INR Value

N	Mean	Standard Deviation	Minimum	Maximum
6	2.0566667	0.2180520	1.6600000	2.3200000

Unique Subject Identifier=0229100000-0016

Assay variable : PTINR_VAL1 PT-INR Value

N	Mean	Standard Deviation	Minimum	Maximum
3	2.2333333	0.1553491	2.0600000	2.3600000

Unique Subject Identifier=0229100000-0018

Assay variable : PTINR_VAL1 PT-INR Value

N	Mean	Standard Deviation	Minimum	Maximum
6	1.9466667	0.1880071	1.7800000	2.2800000

Laboratory tests at the time of obtaining Table 2-6 consent, according to the presence or absence of proton-pump inhibitors

Unique Subject Identifier=0229100000-0021

MEANS Procedure

Assay variable : PTINR_VAL1 PT-INR Value

N	Mean	Standard Deviation	Minimum	Maximum
3	2.0766667	0.2193931	1.8300000	2.2500000

Unique Subject Identifier=0229100000-0029

Assay variable : PTINR_VAL1 PT-INR Value

N	Mean	Standard Deviation	Minimum	Maximum
6	2.1550000	0.2954827	1.7900000	2.5900000

Unique Subject Identifier=0229100000-0030

Assay variable : PTINR_VAL1 PT-INR Value

N	Mean	Standard Deviation	Minimum	Maximum
4	2.0775000	0.1252664	1.9500000	2.1900000

Unique Subject Identifier=0230100000-0003

Assay variable : PTINR_VAL1 PT-INR Value

N	Mean	Standard Deviation	Minimum	Maximum
5	2.2280000	0.1807484	1.9500000	2.4000000

Laboratory tests at the time of obtaining Table 2-6 consent, according to the presence or absence of proton-pump inhibitors

Unique Subject Identifier=0230100000-0004

MEANS Procedure

Assay variable : PTINR_VAL1 PT-INR Value

N	Mean	Standard Deviation	Minimum	Maximum
5	2.4640000	0.3543021	2.0000000	2.8100000

Unique Subject Identifier=0231100000-0008

Assay variable : PTINR_VAL1 PT-INR Value

N	Mean	Standard Deviation	Minimum	Maximum
5	1.7260000	0.2921986	1.2700000	2.0600000

Unique Subject Identifier=0231100000-0011

Assay variable : PTINR_VAL1 PT-INR Value

N	Mean	Standard Deviation	Minimum	Maximum
4	2.4275000	0.2596632	2.1300000	2.7000000

Unique Subject Identifier=0231100000-0013

Assay variable : PTINR_VAL1 PT-INR Value

N	Mean	Standard Deviation	Minimum	Maximum
4	1.8125000	0.2224672	1.4900000	1.9700000

Laboratory tests at the time of obtaining Table 2-6 consent, according to the presence or absence of proton-pump inhibitors

Unique Subject Identifier=0231100000-0016

MEANS Procedure

Assay variable : PTINR_VAL1 PT-INR Value

N	Mean	Standard Deviation	Minimum	Maximum
6	1.5616667	0.7512234	0.9600000	2.9800000

Unique Subject Identifier=0231100000-0017

Assay variable : PTINR_VAL1 PT-INR Value

N	Mean	Standard Deviation	Minimum	Maximum
2	1.3900000	0.0282843	1.3700000	1.4100000

Unique Subject Identifier=0231100000-0018

Assay variable : PTINR_VAL1 PT-INR Value

N	Mean	Standard Deviation	Minimum	Maximum
3	2.4833333	0.5810622	1.8600000	3.0100000

Unique Subject Identifier=0231100000-0019

Assay variable : PTINR_VAL1 PT-INR Value

N	Mean	Standard Deviation	Minimum	Maximum
5	1.7680000	0.3922627	1.4200000	2.3500000

Laboratory tests at the time of obtaining Table 2-6 consent, according to the presence or absence of proton-pump inhibitors

Unique Subject Identifier=0231100000-0031

MEANS Procedure

Assay variable : PTINR_VAL1 PT-INR Value

N	Mean	Standard Deviation	Minimum	Maximum
4	2.6050000	0.2838427	2.3500000	2.9400000

Unique Subject Identifier=0231100000-0036

Assay variable : PTINR_VAL1 PT-INR Value

N	Mean	Standard Deviation	Minimum	Maximum
3	2.3133333	0.3952636	1.9800000	2.7500000

Unique Subject Identifier=0231100000-0038

Assay variable : PTINR_VAL1 PT-INR Value

N	Mean	Standard Deviation	Minimum	Maximum
4	2.0575000	0.2807579	1.7800000	2.3800000

Unique Subject Identifier=0231100000-0046

Assay variable : PTINR_VAL1 PT-INR Value

N	Mean	Standard Deviation	Minimum	Maximum
6	2.1883333	0.1718623	1.8800000	2.4000000

Laboratory tests at the time of obtaining Table 2-6 consent, according to the presence or absence of proton-pump inhibitors

Unique Subject Identifier=1003000000-0001

MEANS Procedure

Assay variable : PTINR_VAL1 PT-INR Value

N	Mean	Standard Deviation	Minimum	Maximum
5	2.1320000	0.3756594	1.6700000	2.6500000

Unique Subject Identifier=1003000000-0005

Assay variable : PTINR_VAL1 PT-INR Value

N	Mean	Standard Deviation	Minimum	Maximum
5	1.9140000	0.3326860	1.6400000	2.3800000

Unique Subject Identifier=1003000000-0010

Assay variable : PTINR_VAL1 PT-INR Value

N	Mean	Standard Deviation	Minimum	Maximum
5	2.2020000	0.2882187	1.8400000	2.5900000

Unique Subject Identifier=1003000000-0015

Assay variable : PTINR_VAL1 PT-INR Value

N	Mean	Standard Deviation	Minimum	Maximum
5	2.0080000	0.1275539	1.9200000	2.2100000

Laboratory tests at the time of obtaining Table 2-6 consent, according to the presence or absence of proton-pump inhibitors

Unique Subject Identifier=1004000000-0003

MEANS Procedure

Assay variable : PTINR_VAL1 PT-INR Value

N	Mean	Standard Deviation	Minimum	Maximum
6	1.7833333	0.5741661	1.2000000	2.6000000

Unique Subject Identifier=1005000000-0003

Assay variable : PTINR_VAL1 PT-INR Value

N	Mean	Standard Deviation	Minimum	Maximum
5	1.7580000	0.0813634	1.6800000	1.8800000

Unique Subject Identifier=1005000000-0007

Assay variable : PTINR_VAL1 PT-INR Value

N	Mean	Standard Deviation	Minimum	Maximum
5	2.0040000	0.1986957	1.6600000	2.1600000

Unique Subject Identifier=1005000000-0010

Assay variable : PTINR_VAL1 PT-INR Value

N	Mean	Standard Deviation	Minimum	Maximum
6	1.5200000	0.0850882	1.4200000	1.6700000

Laboratory tests at the time of obtaining Table 2-6 consent, according to the presence or absence of proton-pump inhibitors

Unique Subject Identifier=1005000000-0015

MEANS Procedure

Assay variable : PTINR_VAL1 PT-INR Value

N	Mean	Standard Deviation	Minimum	Maximum
6	1.8366667	0.1015218	1.6900000	1.9400000

Unique Subject Identifier=1006010010-0003

Assay variable : PTINR_VAL1 PT-INR Value

N	Mean	Standard Deviation	Minimum	Maximum
6	2.0116667	0.2691778	1.7100000	2.4600000

Unique Subject Identifier=1006010010-0005

Assay variable : PTINR_VAL1 PT-INR Value

N	Mean	Standard Deviation	Minimum	Maximum
4	2.2850000	0.1613485	2.1400000	2.4700000

Unique Subject Identifier=1006010010-0013

Assay variable : PTINR_VAL1 PT-INR Value

N	Mean	Standard Deviation	Minimum	Maximum
4	2.3500000	0.1602082	2.2600000	2.5900000

Laboratory tests at the time of obtaining Table 2-6 consent, according to the presence or absence of proton-pump inhibitors

Unique Subject Identifier=1009010010-0003

MEANS Procedure

Assay variable : PTINR_VAL1 PT-INR Value

N	Mean	Standard Deviation	Minimum	Maximum
2	2.0650000	0.1767767	1.9400000	2.1900000

Unique Subject Identifier=1009010010-0007

Assay variable : PTINR_VAL1 PT-INR Value

N	Mean	Standard Deviation	Minimum	Maximum
3	2.3333333	0.2309401	2.2000000	2.6000000

Unique Subject Identifier=1009010010-0012

Assay variable : PTINR_VAL1 PT-INR Value

N	Mean	Standard Deviation	Minimum	Maximum
6	2.2833333	0.3868678	1.9000000	2.9000000

Unique Subject Identifier=1009010010-0013

Assay variable : PTINR_VAL1 PT-INR Value

N	Mean	Standard Deviation	Minimum	Maximum
6	2.5333333	0.1966384	2.3000000	2.8000000

Laboratory tests at the time of obtaining Table 2-6 consent, according to the presence or absence of proton-pump inhibitors

Unique Subject Identifier=1009010010-0014

MEANS Procedure

Assay variable : PTINR_VAL1 PT-INR Value

N	Mean	Standard Deviation	Minimum	Maximum
6	2.3833333	0.6080022	1.9000000	3.5000000

Unique Subject Identifier=1009010010-0025

Assay variable : PTINR_VAL1 PT-INR Value

N	Mean	Standard Deviation	Minimum	Maximum
6	1.8500000	0.2880972	1.5000000	2.3000000

Unique Subject Identifier=1009010010-0027

Assay variable : PTINR_VAL1 PT-INR Value

N	Mean	Standard Deviation	Minimum	Maximum
6	2.1166667	0.7250287	1.6000000	3.5000000

Unique Subject Identifier=1010010000-0003

Assay variable : PTINR_VAL1 PT-INR Value

N	Mean	Standard Deviation	Minimum	Maximum
6	1.7833333	0.1329160	1.6000000	2.0000000

Laboratory tests at the time of obtaining Table 2-6 consent, according to the presence or absence of proton-pump inhibitors

Unique Subject Identifier=1010010000-0005

MEANS Procedure

Assay variable : PTINR_VAL1 PT-INR Value

N	Mean	Standard Deviation	Minimum	Maximum
1	2.0000000		2.0000000	2.0000000

Unique Subject Identifier=1011000000-0004

Assay variable : PTINR_VAL1 PT-INR Value

N	Mean	Standard Deviation	Minimum	Maximum
4	2.1750000	0.3304038	1.7000000	2.4000000

Unique Subject Identifier=1013010110-0006

Assay variable : PTINR_VAL1 PT-INR Value

N	Mean	Standard Deviation	Minimum	Maximum
6	1.9666667	0.2160247	1.6000000	2.2000000

Unique Subject Identifier=1013010110-0010

Assay variable : PTINR_VAL1 PT-INR Value

N	Mean	Standard Deviation	Minimum	Maximum
5	2.7000000	0.7035624	1.9000000	3.7000000

Laboratory tests at the time of obtaining Table 2-6 consent, according to the presence or absence of proton-pump inhibitors

Unique Subject Identifier=1018000000-0001

MEANS Procedure

Assay variable : PTINR_VAL1 PT-INR Value

N	Mean	Standard Deviation	Minimum	Maximum
3	2.2666667	0.1527525	2.1000000	2.4000000

Unique Subject Identifier=1019010100-0001

Assay variable : PTINR_VAL1 PT-INR Value

N	Mean	Standard Deviation	Minimum	Maximum
4	1.4700000	0.1834848	1.2600000	1.6900000

Unique Subject Identifier=1019010100-0005

Assay variable : PTINR_VAL1 PT-INR Value

N	Mean	Standard Deviation	Minimum	Maximum
5	1.4920000	0.2501400	1.1800000	1.8200000

Unique Subject Identifier=1019010100-0009

Assay variable : PTINR_VAL1 PT-INR Value

N	Mean	Standard Deviation	Minimum	Maximum
6	3.2066667	1.1550180	1.7200000	4.9100000

Laboratory tests at the time of obtaining Table 2-6 consent, according to the presence or absence of proton-pump inhibitors

Unique Subject Identifier=1019010100-0013

MEANS Procedure

Assay variable : PTINR_VAL1 PT-INR Value

N	Mean	Standard Deviation	Minimum	Maximum
5	1.6940000	0.3864324	1.2700000	2.1700000

Unique Subject Identifier=1024011100-0009

Assay variable : PTINR_VAL1 PT-INR Value

N	Mean	Standard Deviation	Minimum	Maximum
5	1.9780000	0.2764417	1.5000000	2.2000000

Unique Subject Identifier=1026011000-0006

Assay variable : PTINR_VAL1 PT-INR Value

N	Mean	Standard Deviation	Minimum	Maximum
4	1.9250000	0.3590265	1.5400000	2.2600000

Unique Subject Identifier=1026011000-0024

Assay variable : PTINR_VAL1 PT-INR Value

N	Mean	Standard Deviation	Minimum	Maximum
4	1.3275000	0.0505800	1.2700000	1.3900000

Laboratory tests at the time of obtaining Table 2-6 consent, according to the presence or absence of proton-pump inhibitors

Unique Subject Identifier=1028000000-0019

MEANS Procedure

Assay variable : PTINR_VAL1 PT-INR Value

N	Mean	Standard Deviation	Minimum	Maximum
2	1.9950000	0.0212132	1.9800000	2.0100000

Unique Subject Identifier=1031010000-0037

Assay variable : PTINR_VAL1 PT-INR Value

N	Mean	Standard Deviation	Minimum	Maximum
6	2.5666667	0.1144844	2.4400000	2.7700000

Unique Subject Identifier=1031010000-0111

Assay variable : PTINR_VAL1 PT-INR Value

N	Mean	Standard Deviation	Minimum	Maximum
6	1.7900000	0.2429815	1.5900000	2.2300000

Unique Subject Identifier=1031010000-0120

Assay variable : PTINR_VAL1 PT-INR Value

N	Mean	Standard Deviation	Minimum	Maximum
2	1.7400000	0.3535534	1.4900000	1.9900000

Laboratory tests at the time of obtaining Table 2-6 consent, according to the presence or absence of proton-pump inhibitors

Unique Subject Identifier=1031010000-0127

MEANS Procedure

Assay variable : PTINR_VAL1 PT-INR Value

N	Mean	Standard Deviation	Minimum	Maximum
1	3.2000000		3.2000000	3.2000000

Unique Subject Identifier=1031010000-0134

Assay variable : PTINR_VAL1 PT-INR Value

N	Mean	Standard Deviation	Minimum	Maximum
3	2.4400000	0.3157531	2.0800000	2.6700000

Unique Subject Identifier=1031010000-0155

Assay variable : PTINR_VAL1 PT-INR Value

N	Mean	Standard Deviation	Minimum	Maximum
3	2.5033333	0.4366158	2.0100000	2.8400000

Unique Subject Identifier=1032000000-0001

Assay variable : PTINR_VAL1 PT-INR Value

N	Mean	Standard Deviation	Minimum	Maximum
3	1.6900000	0.0300000	1.6600000	1.7200000

Laboratory tests at the time of obtaining Table 2-6 consent, according to the presence or absence of proton-pump inhibitors

Unique Subject Identifier=1032000000-0002

MEANS Procedure

Assay variable : PTINR_VAL1 PT-INR Value

N	Mean	Standard Deviation	Minimum	Maximum
6	1.6216667	0.1612968	1.3900000	1.8500000

Unique Subject Identifier=1032000000-0003

Assay variable : PTINR_VAL1 PT-INR Value

N	Mean	Standard Deviation	Minimum	Maximum
6	1.9133333	0.2606658	1.6200000	2.3700000

Unique Subject Identifier=1032000000-0004

Assay variable : PTINR_VAL1 PT-INR Value

N	Mean	Standard Deviation	Minimum	Maximum
3	2.0666667	0.5291818	1.4900000	2.5300000

Unique Subject Identifier=1032000000-0006

Assay variable : PTINR_VAL1 PT-INR Value

N	Mean	Standard Deviation	Minimum	Maximum
6	1.7933333	0.4071200	1.4600000	2.5000000

Laboratory tests at the time of obtaining Table 2-6 consent, according to the presence or absence of proton-pump inhibitors

Unique Subject Identifier=1032000000-0007

MEANS Procedure

Assay variable : PTINR_VAL1 PT-INR Value

N	Mean	Standard Deviation	Minimum	Maximum
6	2.4366667	0.6166252	1.6900000	3.1000000

Unique Subject Identifier=1032000000-0008

Assay variable : PTINR_VAL1 PT-INR Value

N	Mean	Standard Deviation	Minimum	Maximum
6	1.6883333	0.5840348	1.2900000	2.5600000

Unique Subject Identifier=1032000000-0010

Assay variable : PTINR_VAL1 PT-INR Value

N	Mean	Standard Deviation	Minimum	Maximum
6	2.2250000	0.2714959	1.8800000	2.6200000

Unique Subject Identifier=1032000000-0011

Assay variable : PTINR_VAL1 PT-INR Value

N	Mean	Standard Deviation	Minimum	Maximum
6	2.1600000	0.1288410	1.9200000	2.2900000

Laboratory tests at the time of obtaining Table 2-6 consent, according to the presence or absence of proton-pump inhibitors

Unique Subject Identifier=1032000000-0012

MEANS Procedure

Assay variable : PTINR_VAL1 PT-INR Value

N	Mean	Standard Deviation	Minimum	Maximum
6	1.5000000	0.1784377	1.3700000	1.8400000

Unique Subject Identifier=1032000000-0013

Assay variable : PTINR_VAL1 PT-INR Value

N	Mean	Standard Deviation	Minimum	Maximum
6	1.9833333	0.4921653	1.6500000	2.9300000

Unique Subject Identifier=1032000000-0017

Assay variable : PTINR_VAL1 PT-INR Value

N	Mean	Standard Deviation	Minimum	Maximum
6	2.1833333	0.3101398	1.7600000	2.7000000

Unique Subject Identifier=1032000000-0018

Assay variable : PTINR_VAL1 PT-INR Value

N	Mean	Standard Deviation	Minimum	Maximum
6	1.9116667	0.3806529	1.4900000	2.5500000

Laboratory tests at the time of obtaining Table 2-6 consent, according to the presence or absence of proton-pump inhibitors

Unique Subject Identifier=1032000000-0019

MEANS Procedure

Assay variable : PTINR_VAL1 PT-INR Value

N	Mean	Standard Deviation	Minimum	Maximum
6	1.6533333	0.1123684	1.4600000	1.7600000

Unique Subject Identifier=1032000000-0020

Assay variable : PTINR_VAL1 PT-INR Value

N	Mean	Standard Deviation	Minimum	Maximum
6	2.0083333	0.3642206	1.4100000	2.5200000

Unique Subject Identifier=1032000000-0021

Assay variable : PTINR_VAL1 PT-INR Value

N	Mean	Standard Deviation	Minimum	Maximum
6	1.8950000	0.1712016	1.6500000	2.0500000

Unique Subject Identifier=1032000000-0022

Assay variable : PTINR_VAL1 PT-INR Value

N	Mean	Standard Deviation	Minimum	Maximum
6	2.4200000	0.3159747	2.1000000	2.9200000

Laboratory tests at the time of obtaining Table 2-6 consent, according to the presence or absence of proton-pump inhibitors

Unique Subject Identifier=1032000000-0023

MEANS Procedure

Assay variable : PTINR_VAL1 PT-INR Value

N	Mean	Standard Deviation	Minimum	Maximum
6	1.5783333	0.3328914	1.1900000	2.0600000

Unique Subject Identifier=1032000000-0025

Assay variable : PTINR_VAL1 PT-INR Value

N	Mean	Standard Deviation	Minimum	Maximum
6	1.5883333	0.3950907	1.2800000	2.3500000

Unique Subject Identifier=1032000000-0026

Assay variable : PTINR_VAL1 PT-INR Value

N	Mean	Standard Deviation	Minimum	Maximum
6	2.1383333	0.3119241	1.6300000	2.4500000

Unique Subject Identifier=1032000000-0028

Assay variable : PTINR_VAL1 PT-INR Value

N	Mean	Standard Deviation	Minimum	Maximum
6	2.3250000	0.2562616	1.9800000	2.7200000

Laboratory tests at the time of obtaining Table 2-6 consent, according to the presence or absence of proton-pump inhibitors

Unique Subject Identifier=1032000000-0029

MEANS Procedure

Assay variable : PTINR_VAL1 PT-INR Value

N	Mean	Standard Deviation	Minimum	Maximum
5	2.3460000	0.5855169	1.4400000	3.0100000

Unique Subject Identifier=1032000000-0030

Assay variable : PTINR_VAL1 PT-INR Value

N	Mean	Standard Deviation	Minimum	Maximum
6	1.7050000	0.0939681	1.5900000	1.8300000

Unique Subject Identifier=1034000000-0002

Assay variable : PTINR_VAL1 PT-INR Value

N	Mean	Standard Deviation	Minimum	Maximum
3	2.0666667	0.3511885	1.7000000	2.4000000

Unique Subject Identifier=1034000000-0006

Assay variable : PTINR_VAL1 PT-INR Value

N	Mean	Standard Deviation	Minimum	Maximum
4	2.5250000	0.4112988	2.0000000	2.9000000

Laboratory tests at the time of obtaining Table 2-6 consent, according to the presence or absence of proton-pump inhibitors

Unique Subject Identifier=1034000000-0009

MEANS Procedure

Assay variable : PTINR_VAL1 PT-INR Value

N	Mean	Standard Deviation	Minimum	Maximum
1	1.2000000		1.2000000	1.2000000

Unique Subject Identifier=1035000000-0013

Assay variable : PTINR_VAL1 PT-INR Value

N	Mean	Standard Deviation	Minimum	Maximum
5	2.0740000	0.1984439	1.8000000	2.3300000

Unique Subject Identifier=1035000000-0017

Assay variable : PTINR_VAL1 PT-INR Value

N	Mean	Standard Deviation	Minimum	Maximum
4	1.8325000	0.3057641	1.5400000	2.2500000

Unique Subject Identifier=1038000000-0002

Assay variable : PTINR_VAL1 PT-INR Value

N	Mean	Standard Deviation	Minimum	Maximum
6	2.7833333	0.3600926	2.3000000	3.3000000

Laboratory tests at the time of obtaining Table 2-6 consent, according to the presence or absence of proton-pump inhibitors

Unique Subject Identifier=1038000000-0004

MEANS Procedure

Assay variable : PTINR_VAL1 PT-INR Value

N	Mean	Standard Deviation	Minimum	Maximum
6	2.0833333	0.1722401	1.9000000	2.3000000

Unique Subject Identifier=1038000000-0005

Assay variable : PTINR_VAL1 PT-INR Value

N	Mean	Standard Deviation	Minimum	Maximum
6	1.8000000	0.2966479	1.3000000	2.1000000

Unique Subject Identifier=1038000000-0006

Assay variable : PTINR_VAL1 PT-INR Value

N	Mean	Standard Deviation	Minimum	Maximum
4	1.8250000	0.1500000	1.7000000	2.0000000

Unique Subject Identifier=1038000000-0007

Assay variable : PTINR_VAL1 PT-INR Value

N	Mean	Standard Deviation	Minimum	Maximum
6	1.9000000	0.4939636	1.4000000	2.8000000

Laboratory tests at the time of obtaining Table 2-6 consent, according to the presence or absence of proton-pump inhibitors

Unique Subject Identifier=1038000000-0008

MEANS Procedure

Assay variable : PTINR_VAL1 PT-INR Value

N	Mean	Standard Deviation	Minimum	Maximum
6	2.1333333	0.5537749	1.5000000	3.0000000

Unique Subject Identifier=1041011110-0003

Assay variable : PTINR_VAL1 PT-INR Value

N	Mean	Standard Deviation	Minimum	Maximum
5	1.7000000	0.1581139	1.5000000	1.9000000

Unique Subject Identifier=1041011110-0004

Assay variable : PTINR_VAL1 PT-INR Value

N	Mean	Standard Deviation	Minimum	Maximum
6	1.9833333	0.1471960	1.8000000	2.2000000

Unique Subject Identifier=1041011110-0005

Assay variable : PTINR_VAL1 PT-INR Value

N	Mean	Standard Deviation	Minimum	Maximum
6	1.9333333	0.2338090	1.7000000	2.3000000

Laboratory tests at the time of obtaining Table 2-6 consent, according to the presence or absence of proton-pump inhibitors

Unique Subject Identifier=1041011110-0011

MEANS Procedure

Assay variable : PTINR_VAL1 PT-INR Value

N	Mean	Standard Deviation	Minimum	Maximum
6	2.2166667	0.3763863	1.8000000	2.9000000

Unique Subject Identifier=1041011110-0024

Assay variable : PTINR_VAL1 PT-INR Value

N	Mean	Standard Deviation	Minimum	Maximum
5	1.8600000	0.2701851	1.5000000	2.2000000

Unique Subject Identifier=1041011110-0026

Assay variable : PTINR_VAL1 PT-INR Value

N	Mean	Standard Deviation	Minimum	Maximum
6	1.8000000	0.2097618	1.6000000	2.1000000

Unique Subject Identifier=1041011110-0029

Assay variable : PTINR_VAL1 PT-INR Value

N	Mean	Standard Deviation	Minimum	Maximum
6	2.2666667	0.5202563	1.7000000	3.1000000

Laboratory tests at the time of obtaining Table 2-6 consent, according to the presence or absence of proton-pump inhibitors

Unique Subject Identifier=1041011110-0033

MEANS Procedure

Assay variable : PTINR_VAL1 PT-INR Value

N	Mean	Standard Deviation	Minimum	Maximum
6	1.9833333	0.1471960	1.8000000	2.2000000

Unique Subject Identifier=1041011110-0034

Assay variable : PTINR_VAL1 PT-INR Value

N	Mean	Standard Deviation	Minimum	Maximum
3	1.6666667	0.3511885	1.3000000	2.0000000

Unique Subject Identifier=1041011110-0035

Assay variable : PTINR_VAL1 PT-INR Value

N	Mean	Standard Deviation	Minimum	Maximum
6	2.2500000	0.1870829	2.1000000	2.6000000

Unique Subject Identifier=1041011110-0036

Assay variable : PTINR_VAL1 PT-INR Value

N	Mean	Standard Deviation	Minimum	Maximum
6	2.2666667	0.6186006	1.2000000	2.9000000

Laboratory tests at the time of obtaining Table 2-6 consent, according to the presence or absence of proton-pump inhibitors

Unique Subject Identifier=1041011110-0043

MEANS Procedure

Assay variable : PTINR_VAL1 PT-INR Value

N	Mean	Standard Deviation	Minimum	Maximum
5	1.7200000	0.1303840	1.6000000	1.9000000

Unique Subject Identifier=1049000000-0001

Assay variable : PTINR_VAL1 PT-INR Value

N	Mean	Standard Deviation	Minimum	Maximum
4	1.6150000	0.0967815	1.5300000	1.7300000

Unique Subject Identifier=1049000000-0003

Assay variable : PTINR_VAL1 PT-INR Value

N	Mean	Standard Deviation	Minimum	Maximum
5	1.9400000	0.1164045	1.8400000	2.1400000

Unique Subject Identifier=1049000000-0004

Assay variable : PTINR_VAL1 PT-INR Value

N	Mean	Standard Deviation	Minimum	Maximum
3	1.9400000	0.4309292	1.6200000	2.4300000

Laboratory tests at the time of obtaining Table 2-6 consent, according to the presence or absence of proton-pump inhibitors

Unique Subject Identifier=1052000000-0003

MEANS Procedure

Assay variable : PTINR_VAL1 PT-INR Value

N	Mean	Standard Deviation	Minimum	Maximum
5	1.7960000	0.1668233	1.6300000	2.0000000

Unique Subject Identifier=1055000000-0015

Assay variable : PTINR_VAL1 PT-INR Value

N	Mean	Standard Deviation	Minimum	Maximum
3	1.9600000	0.2749545	1.7200000	2.2600000

Unique Subject Identifier=1055000000-0021

Assay variable : PTINR_VAL1 PT-INR Value

N	Mean	Standard Deviation	Minimum	Maximum
2	2.5300000	0.1131371	2.4500000	2.6100000

Unique Subject Identifier=1055000000-0030

Assay variable : PTINR_VAL1 PT-INR Value

N	Mean	Standard Deviation	Minimum	Maximum
1	2.1600000		2.1600000	2.1600000

Laboratory tests at the time of obtaining Table 2-6 consent, according to the presence or absence of proton-pump inhibitors

Unique Subject Identifier=1058011111-0002

MEANS Procedure

Assay variable : PTINR_VAL1 PT-INR Value

N	Mean	Standard Deviation	Minimum	Maximum
6	1.4233333	0.1148332	1.3000000	1.6300000

Unique Subject Identifier=1058011111-0004

Assay variable : PTINR_VAL1 PT-INR Value

N	Mean	Standard Deviation	Minimum	Maximum
6	1.8550000	0.2703146	1.5800000	2.1900000

Unique Subject Identifier=1060000000-0007

Assay variable : PTINR_VAL1 PT-INR Value

N	Mean	Standard Deviation	Minimum	Maximum
2	1.5200000	0.3252691	1.2900000	1.7500000

Unique Subject Identifier=1060000000-0010

Assay variable : PTINR_VAL1 PT-INR Value

N	Mean	Standard Deviation	Minimum	Maximum
6	1.9666667	0.2771041	1.6800000	2.3600000

Laboratory tests at the time of obtaining Table 2-6 consent, according to the presence or absence of proton-pump inhibitors

Unique Subject Identifier=1060000000-0013

MEANS Procedure

Assay variable : PTINR_VAL1 PT-INR Value

N	Mean	Standard Deviation	Minimum	Maximum
3	1.7833333	0.3274650	1.5200000	2.1500000

Unique Subject Identifier=1060000000-0022

Assay variable : PTINR_VAL1 PT-INR Value

N	Mean	Standard Deviation	Minimum	Maximum
4	1.8100000	0.1570563	1.6200000	2.0000000

Unique Subject Identifier=1060000000-0027

Assay variable : PTINR_VAL1 PT-INR Value

N	Mean	Standard Deviation	Minimum	Maximum
2	1.7750000	0.1060660	1.7000000	1.8500000

Unique Subject Identifier=1060000000-0043

Assay variable : PTINR_VAL1 PT-INR Value

N	Mean	Standard Deviation	Minimum	Maximum
2	1.8950000	0.2899138	1.6900000	2.1000000

Laboratory tests at the time of obtaining Table 2-6 consent, according to the presence or absence of proton-pump inhibitors

Unique Subject Identifier=1061000000-0001

MEANS Procedure

Assay variable : PTINR_VAL1 PT-INR Value

N	Mean	Standard Deviation	Minimum	Maximum
6	2.0333333	0.2338090	1.7000000	2.4000000

Unique Subject Identifier=1061000000-0022

Assay variable : PTINR_VAL1 PT-INR Value

N	Mean	Standard Deviation	Minimum	Maximum
6	1.9000000	0.1897367	1.7000000	2.2000000

Unique Subject Identifier=1065010011-0005

Assay variable : PTINR_VAL1 PT-INR Value

N	Mean	Standard Deviation	Minimum	Maximum
6	1.7666667	0.3011091	1.4000000	2.1000000

Unique Subject Identifier=1065010011-0006

Assay variable : PTINR_VAL1 PT-INR Value

N	Mean	Standard Deviation	Minimum	Maximum
6	2.0333333	0.3011091	1.6000000	2.4000000

Laboratory tests at the time of obtaining Table 2-6 consent, according to the presence or absence of proton-pump inhibitors

Unique Subject Identifier=1065010011-0007

MEANS Procedure

Assay variable : PTINR_VAL1 PT-INR Value

N	Mean	Standard Deviation	Minimum	Maximum
6	2.3000000	0.3898718	1.8000000	2.8000000

Unique Subject Identifier=1065010011-0008

Assay variable : PTINR_VAL1 PT-INR Value

N	Mean	Standard Deviation	Minimum	Maximum
6	2.4666667	0.5163978	1.6000000	3.0000000

Unique Subject Identifier=1065010011-0012

Assay variable : PTINR_VAL1 PT-INR Value

N	Mean	Standard Deviation	Minimum	Maximum
6	2.2333333	0.7580677	1.5000000	3.4000000

Unique Subject Identifier=1065010011-0014

Assay variable : PTINR_VAL1 PT-INR Value

N	Mean	Standard Deviation	Minimum	Maximum
6	2.4333333	0.2581989	2.1000000	2.8000000

Laboratory tests at the time of obtaining Table 2-6 consent, according to the presence or absence of proton-pump inhibitors

Unique Subject Identifier=1065010011-0017

MEANS Procedure

Assay variable : PTINR_VAL1 PT-INR Value

N	Mean	Standard Deviation	Minimum	Maximum
6	1.9666667	0.7229569	1.1000000	2.9000000

Unique Subject Identifier=1065010011-0019

Assay variable : PTINR_VAL1 PT-INR Value

N	Mean	Standard Deviation	Minimum	Maximum
4	1.6750000	0.0957427	1.6000000	1.8000000

Unique Subject Identifier=1065010011-0026

Assay variable : PTINR_VAL1 PT-INR Value

N	Mean	Standard Deviation	Minimum	Maximum
6	2.2666667	0.6713171	1.7000000	3.5000000

Unique Subject Identifier=1065010011-0031

Assay variable : PTINR_VAL1 PT-INR Value

N	Mean	Standard Deviation	Minimum	Maximum
6	1.9666667	0.4082483	1.6000000	2.7000000

Laboratory tests at the time of obtaining Table 2-6 consent, according to the presence or absence of proton-pump inhibitors

Unique Subject Identifier=1065010011-0032

MEANS Procedure

Assay variable : PTINR_VAL1 PT-INR Value

N	Mean	Standard Deviation	Minimum	Maximum
6	2.1833333	0.5706721	1.4000000	3.1000000

Unique Subject Identifier=1065010011-0037

Assay variable : PTINR_VAL1 PT-INR Value

N	Mean	Standard Deviation	Minimum	Maximum
3	1.6000000	0.3605551	1.2000000	1.9000000

Unique Subject Identifier=1065010011-0041

Assay variable : PTINR_VAL1 PT-INR Value

N	Mean	Standard Deviation	Minimum	Maximum
6	2.6500000	0.7395945	1.6000000	3.6000000

Unique Subject Identifier=1065010011-0044

Assay variable : PTINR_VAL1 PT-INR Value

N	Mean	Standard Deviation	Minimum	Maximum
6	2.0666667	0.7711463	1.3000000	3.4000000

Laboratory tests at the time of obtaining Table 2-6 consent, according to the presence or absence of proton-pump inhibitors

Unique Subject Identifier=1065010011-0048

MEANS Procedure

Assay variable : PTINR_VAL1 PT-INR Value

N	Mean	Standard Deviation	Minimum	Maximum
6	1.7500000	0.2345208	1.6000000	2.1000000

Unique Subject Identifier=1065010011-0054

Assay variable : PTINR_VAL1 PT-INR Value

N	Mean	Standard Deviation	Minimum	Maximum
6	1.9000000	0.4195235	1.5000000	2.6000000

Unique Subject Identifier=1065010011-0058

Assay variable : PTINR_VAL1 PT-INR Value

N	Mean	Standard Deviation	Minimum	Maximum
6	2.2500000	0.1643168	2.1000000	2.5000000

Unique Subject Identifier=1065010011-0059

Assay variable : PTINR_VAL1 PT-INR Value

N	Mean	Standard Deviation	Minimum	Maximum
6	1.8333333	0.4633213	1.4000000	2.6000000

Laboratory tests at the time of obtaining Table 2-6 consent, according to the presence or absence of proton-pump inhibitors

Unique Subject Identifier=1065010011-0067

MEANS Procedure

Assay variable : PTINR_VAL1 PT-INR Value

N	Mean	Standard Deviation	Minimum	Maximum
6	2.4333333	0.8286535	1.5000000	3.5000000

Unique Subject Identifier=1065010011-0072

Assay variable : PTINR_VAL1 PT-INR Value

N	Mean	Standard Deviation	Minimum	Maximum
6	1.6500000	0.2509980	1.2000000	1.9000000

Unique Subject Identifier=1068010111-0003

Assay variable : PTINR_VAL1 PT-INR Value

N	Mean	Standard Deviation	Minimum	Maximum
2	2.2000000	0.5232590	1.8300000	2.5700000

Unique Subject Identifier=1068010111-0006

Assay variable : PTINR_VAL1 PT-INR Value

N	Mean	Standard Deviation	Minimum	Maximum
3	1.9266667	0.1331666	1.7800000	2.0400000

Laboratory tests at the time of obtaining Table 2-6 consent, according to the presence or absence of proton-pump inhibitors

Unique Subject Identifier=1068010111-0008

MEANS Procedure

Assay variable : PTINR_VAL1 PT-INR Value

N	Mean	Standard Deviation	Minimum	Maximum
3	1.8300000	0.3034798	1.6400000	2.1800000

Unique Subject Identifier=1068010111-0009

Assay variable : PTINR_VAL1 PT-INR Value

N	Mean	Standard Deviation	Minimum	Maximum
3	1.8366667	0.0513160	1.7800000	1.8800000

Unique Subject Identifier=1068010111-0013

Assay variable : PTINR_VAL1 PT-INR Value

N	Mean	Standard Deviation	Minimum	Maximum
6	1.3250000	0.1399643	1.2000000	1.5900000

Unique Subject Identifier=1068010111-0017

Assay variable : PTINR_VAL1 PT-INR Value

N	Mean	Standard Deviation	Minimum	Maximum
4	1.7875000	0.2692428	1.5400000	2.1200000

Laboratory tests at the time of obtaining Table 2-6 consent, according to the presence or absence of proton-pump inhibitors

Unique Subject Identifier=1068010111-0026

MEANS Procedure

Assay variable : PTINR_VAL1 PT-INR Value

N	Mean	Standard Deviation	Minimum	Maximum
5	2.1220000	0.7623451	1.4100000	3.2600000

Unique Subject Identifier=1068010111-0035

Assay variable : PTINR_VAL1 PT-INR Value

N	Mean	Standard Deviation	Minimum	Maximum
4	1.9750000	0.3275668	1.6200000	2.4000000

Unique Subject Identifier=1068010111-0060

Assay variable : PTINR_VAL1 PT-INR Value

N	Mean	Standard Deviation	Minimum	Maximum
6	1.4650000	0.4579847	0.9700000	2.1600000

Unique Subject Identifier=1070000000-0002

Assay variable : PTINR_VAL1 PT-INR Value

N	Mean	Standard Deviation	Minimum	Maximum
6	2.0833333	0.4875107	1.6000000	2.9000000

Laboratory tests at the time of obtaining Table 2-6 consent, according to the presence or absence of proton-pump inhibitors

Unique Subject Identifier=1070000000-0003

MEANS Procedure

Assay variable : PTINR_VAL1 PT-INR Value

N	Mean	Standard Deviation	Minimum	Maximum
6	2.1500000	0.5319774	1.6000000	3.1000000

Unique Subject Identifier=1071000000-0003

Assay variable : PTINR_VAL1 PT-INR Value

N	Mean	Standard Deviation	Minimum	Maximum
1	1.0400000		1.0400000	1.0400000

Unique Subject Identifier=1071000000-0006

Assay variable : PTINR_VAL1 PT-INR Value

N	Mean	Standard Deviation	Minimum	Maximum
1	1.8400000		1.8400000	1.8400000

Unique Subject Identifier=1072000000-0022

Assay variable : PTINR_VAL1 PT-INR Value

N	Mean	Standard Deviation	Minimum	Maximum
6	1.7950000	0.1415274	1.6500000	2.0600000

Laboratory tests at the time of obtaining Table 2-6 consent, according to the presence or absence of proton-pump inhibitors

Unique Subject Identifier=1072000000-0026

MEANS Procedure

Assay variable : PTINR_VAL1 PT-INR Value

N	Mean	Standard Deviation	Minimum	Maximum
6	1.7383333	0.1383353	1.6100000	1.8900000

Unique Subject Identifier=1072000000-0029

Assay variable : PTINR_VAL1 PT-INR Value

N	Mean	Standard Deviation	Minimum	Maximum
5	1.6960000	0.2785319	1.4300000	2.1700000

Unique Subject Identifier=1072000000-0033

Assay variable : PTINR_VAL1 PT-INR Value

N	Mean	Standard Deviation	Minimum	Maximum
6	1.8366667	0.1885382	1.5900000	2.1000000

Unique Subject Identifier=1072000000-0034

Assay variable : PTINR_VAL1 PT-INR Value

N	Mean	Standard Deviation	Minimum	Maximum
6	1.9483333	0.1212298	1.7300000	2.0400000

Laboratory tests at the time of obtaining Table 2-6 consent, according to the presence or absence of proton-pump inhibitors

Unique Subject Identifier=1074011010-0002

MEANS Procedure

Assay variable : PTINR_VAL1 PT-INR Value

N	Mean	Standard Deviation	Minimum	Maximum
5	2.3260000	0.6975529	1.7600000	3.5000000

Unique Subject Identifier=1074011010-0005

Assay variable : PTINR_VAL1 PT-INR Value

N	Mean	Standard Deviation	Minimum	Maximum
6	1.9383333	0.4533174	1.4500000	2.5200000

Unique Subject Identifier=1074011010-0010

Assay variable : PTINR_VAL1 PT-INR Value

N	Mean	Standard Deviation	Minimum	Maximum
5	1.7600000	0.2302173	1.5100000	2.1000000

Unique Subject Identifier=1079000000-0003

Assay variable : PTINR_VAL1 PT-INR Value

N	Mean	Standard Deviation	Minimum	Maximum
3	1.8433333	0.2300725	1.6100000	2.0700000

Laboratory tests at the time of obtaining Table 2-6 consent, according to the presence or absence of proton-pump inhibitors

Unique Subject Identifier=1079000000-0004

MEANS Procedure

Assay variable : PTINR_VAL1 PT-INR Value

N	Mean	Standard Deviation	Minimum	Maximum
3	1.4633333	0.1738774	1.3300000	1.6600000

Unique Subject Identifier=1080000000-0001

Assay variable : PTINR_VAL1 PT-INR Value

N	Mean	Standard Deviation	Minimum	Maximum
6	2.1783333	0.2145150	1.9600000	2.4400000

Unique Subject Identifier=1080000000-0002

Assay variable : PTINR_VAL1 PT-INR Value

N	Mean	Standard Deviation	Minimum	Maximum
6	2.3483333	0.4951532	1.5200000	2.9300000

Unique Subject Identifier=1080000000-0003

Assay variable : PTINR_VAL1 PT-INR Value

N	Mean	Standard Deviation	Minimum	Maximum
6	2.1516667	0.2041976	1.8200000	2.4400000

Laboratory tests at the time of obtaining Table 2-6 consent, according to the presence or absence of proton-pump inhibitors

Unique Subject Identifier=1080000000-0005

MEANS Procedure

Assay variable : PTINR_VAL1 PT-INR Value

N	Mean	Standard Deviation	Minimum	Maximum
6	2.1616667	0.4302286	1.6900000	2.8600000

Unique Subject Identifier=1080000000-0007

Assay variable : PTINR_VAL1 PT-INR Value

N	Mean	Standard Deviation	Minimum	Maximum
6	2.5350000	0.1775106	2.2700000	2.7300000

Unique Subject Identifier=1085000000-0001

Assay variable : PTINR_VAL1 PT-INR Value

N	Mean	Standard Deviation	Minimum	Maximum
1	2.4700000		2.4700000	2.4700000

Unique Subject Identifier=1085000000-0004

Assay variable : PTINR_VAL1 PT-INR Value

N	Mean	Standard Deviation	Minimum	Maximum
2	1.5600000	0.3535534	1.3100000	1.8100000

Laboratory tests at the time of obtaining Table 2-6 consent, according to the presence or absence of proton-pump inhibitors

Unique Subject Identifier=1085000000-0007

MEANS Procedure

Assay variable : PTINR_VAL1 PT-INR Value

N	Mean	Standard Deviation	Minimum	Maximum
1	1.6200000		1.6200000	1.6200000

Unique Subject Identifier=1085000000-0009

Assay variable : PTINR_VAL1 PT-INR Value

N	Mean	Standard Deviation	Minimum	Maximum
1	2.4900000		2.4900000	2.4900000

Unique Subject Identifier=1085000000-0012

Assay variable : PTINR_VAL1 PT-INR Value

N	Mean	Standard Deviation	Minimum	Maximum
2	1.8250000	0.1060660	1.7500000	1.9000000

Unique Subject Identifier=1087000000-0003

Assay variable : PTINR_VAL1 PT-INR Value

N	Mean	Standard Deviation	Minimum	Maximum
3	1.9000000	0.1646208	1.7100000	2.0000000

Laboratory tests at the time of obtaining Table 2-6 consent, according to the presence or absence of proton-pump inhibitors

Unique Subject Identifier=1087000000-0004

MEANS Procedure

Assay variable : PTINR_VAL1 PT-INR Value

N	Mean	Standard Deviation	Minimum	Maximum
6	1.9983333	0.4471875	1.5400000	2.5400000

Unique Subject Identifier=1087000000-0006

Assay variable : PTINR_VAL1 PT-INR Value

N	Mean	Standard Deviation	Minimum	Maximum
4	2.3725000	0.2224672	2.0500000	2.5500000

Unique Subject Identifier=1090001110-0011

Assay variable : PTINR_VAL1 PT-INR Value

N	Mean	Standard Deviation	Minimum	Maximum
6	2.4000000	0.3098387	1.9000000	2.8000000

Unique Subject Identifier=1090001110-0012

Assay variable : PTINR_VAL1 PT-INR Value

N	Mean	Standard Deviation	Minimum	Maximum
6	1.8666667	0.2732520	1.5000000	2.3000000

Laboratory tests at the time of obtaining Table 2-6 consent, according to the presence or absence of proton-pump inhibitors

Unique Subject Identifier=1091010000-0007

MEANS Procedure

Assay variable : PTINR_VAL1 PT-INR Value

N	Mean	Standard Deviation	Minimum	Maximum
6	2.3666667	0.5046451	1.6000000	3.1000000

Unique Subject Identifier=1091010000-0008

Assay variable : PTINR_VAL1 PT-INR Value

N	Mean	Standard Deviation	Minimum	Maximum
6	1.6833333	0.2401388	1.3000000	1.9000000

Unique Subject Identifier=1091010000-0010

Assay variable : PTINR_VAL1 PT-INR Value

N	Mean	Standard Deviation	Minimum	Maximum
6	2.0500000	0.2073644	1.8000000	2.4000000

Unique Subject Identifier=1091010000-0024

Assay variable : PTINR_VAL1 PT-INR Value

N	Mean	Standard Deviation	Minimum	Maximum
6	1.6500000	0.2880972	1.4000000	2.1000000

Laboratory tests at the time of obtaining Table 2-6 consent, according to the presence or absence of proton-pump inhibitors

Unique Subject Identifier=1091010000-0028

MEANS Procedure

Assay variable : PTINR_VAL1 PT-INR Value

N	Mean	Standard Deviation	Minimum	Maximum
5	2.1800000	0.8043631	1.4000000	3.3000000

Unique Subject Identifier=1091010000-0030

Assay variable : PTINR_VAL1 PT-INR Value

N	Mean	Standard Deviation	Minimum	Maximum
6	1.7000000	0.2000000	1.5000000	2.0000000

Unique Subject Identifier=1091010000-0038

Assay variable : PTINR_VAL1 PT-INR Value

N	Mean	Standard Deviation	Minimum	Maximum
6	1.8666667	0.2065591	1.7000000	2.2000000

Unique Subject Identifier=1091010000-0042

Assay variable : PTINR_VAL1 PT-INR Value

N	Mean	Standard Deviation	Minimum	Maximum
6	1.8833333	0.2401388	1.6000000	2.3000000

Laboratory tests at the time of obtaining Table 2-6 consent, according to the presence or absence of proton-pump inhibitors

Unique Subject Identifier=1091010000-0046

MEANS Procedure

Assay variable : PTINR_VAL1 PT-INR Value

N	Mean	Standard Deviation	Minimum	Maximum
6	1.7666667	0.3011091	1.3000000	2.0000000

Unique Subject Identifier=1091010000-0063

Assay variable : PTINR_VAL1 PT-INR Value

N	Mean	Standard Deviation	Minimum	Maximum
6	2.9333333	1.1639015	1.9000000	5.0000000

Unique Subject Identifier=1094000000-0003

Assay variable : PTINR_VAL1 PT-INR Value

N	Mean	Standard Deviation	Minimum	Maximum
6	1.5350000	0.0736885	1.4400000	1.6300000

Unique Subject Identifier=1094000000-0004

Assay variable : PTINR_VAL1 PT-INR Value

N	Mean	Standard Deviation	Minimum	Maximum
5	1.8200000	0.1701470	1.5800000	1.9800000

Laboratory tests at the time of obtaining Table 2-6 consent, according to the presence or absence of proton-pump inhibitors

Unique Subject Identifier=1094000000-0005

MEANS Procedure

Assay variable : PTINR_VAL1 PT-INR Value

N	Mean	Standard Deviation	Minimum	Maximum
5	2.5000000	0.4803644	2.1200000	3.3000000

Unique Subject Identifier=1094000000-0008

Assay variable : PTINR_VAL1 PT-INR Value

N	Mean	Standard Deviation	Minimum	Maximum
6	2.1550000	0.3011810	1.8100000	2.6300000

Unique Subject Identifier=1095000000-0025

Assay variable : PTINR_VAL1 PT-INR Value

N	Mean	Standard Deviation	Minimum	Maximum
6	2.0666667	0.3204164	1.8000000	2.6000000

Unique Subject Identifier=1095000000-0029

Assay variable : PTINR_VAL1 PT-INR Value

N	Mean	Standard Deviation	Minimum	Maximum
6	2.3000000	0.2000000	2.2000000	2.7000000

Laboratory tests at the time of obtaining Table 2-6 consent, according to the presence or absence of proton-pump inhibitors

Unique Subject Identifier=1095000000-0034

MEANS Procedure

Assay variable : PTINR_VAL1 PT-INR Value

N	Mean	Standard Deviation	Minimum	Maximum
5	1.8600000	0.7469940	1.2000000	2.8000000

Unique Subject Identifier=1096000000-0001

Assay variable : PTINR_VAL1 PT-INR Value

N	Mean	Standard Deviation	Minimum	Maximum
1	2.0600000		2.0600000	2.0600000

Unique Subject Identifier=1096000000-0018

Assay variable : PTINR_VAL1 PT-INR Value

N	Mean	Standard Deviation	Minimum	Maximum
3	1.5666667	0.2516611	1.3000000	1.8000000

Unique Subject Identifier=1099000000-0002

Assay variable : PTINR_VAL1 PT-INR Value

N	Mean	Standard Deviation	Minimum	Maximum
3	1.4300000	0.1300000	1.3500000	1.5800000

Laboratory tests at the time of obtaining Table 2-6 consent, according to the presence or absence of proton-pump inhibitors

Unique Subject Identifier=1099000000-0004

MEANS Procedure

Assay variable : PTINR_VAL1 PT-INR Value

N	Mean	Standard Deviation	Minimum	Maximum
1	1.6400000		1.6400000	1.6400000

Unique Subject Identifier=1102000000-0005

Assay variable : PTINR_VAL1 PT-INR Value

N	Mean	Standard Deviation	Minimum	Maximum
2	1.7000000	0.1272792	1.6100000	1.7900000

Unique Subject Identifier=1102000000-0008

Assay variable : PTINR_VAL1 PT-INR Value

N	Mean	Standard Deviation	Minimum	Maximum
2	1.5700000	0.1131371	1.4900000	1.6500000

Unique Subject Identifier=1102000000-0010

Assay variable : PTINR_VAL1 PT-INR Value

N	Mean	Standard Deviation	Minimum	Maximum
1	2.1700000		2.1700000	2.1700000

Laboratory tests at the time of obtaining Table 2-6 consent, according to the presence or absence of proton-pump inhibitors

Unique Subject Identifier=1102000000-0013

MEANS Procedure

Assay variable : PTINR_VAL1 PT-INR Value

N	Mean	Standard Deviation	Minimum	Maximum
2	1.6300000	0.0989949	1.5600000	1.7000000

Unique Subject Identifier=1102000000-0021

Assay variable : PTINR_VAL1 PT-INR Value

N	Mean	Standard Deviation	Minimum	Maximum
2	1.7950000	0.0353553	1.7700000	1.8200000

Unique Subject Identifier=1102000000-0022

Assay variable : PTINR_VAL1 PT-INR Value

N	Mean	Standard Deviation	Minimum	Maximum
3	2.0666667	0.4441096	1.5800000	2.4500000

Unique Subject Identifier=1102000000-0036

Assay variable : PTINR_VAL1 PT-INR Value

N	Mean	Standard Deviation	Minimum	Maximum
3	1.6500000	0.2475884	1.3700000	1.8400000

Laboratory tests at the time of obtaining Table 2-6 consent, according to the presence or absence of proton-pump inhibitors

Unique Subject Identifier=1103001000-0011

MEANS Procedure

Assay variable : PTINR_VAL1 PT-INR Value

N	Mean	Standard Deviation	Minimum	Maximum
2	1.5600000	0.2545584	1.3800000	1.7400000

Unique Subject Identifier=1107011010-0001

Assay variable : PTINR_VAL1 PT-INR Value

N	Mean	Standard Deviation	Minimum	Maximum
4	2.0250000	0.3545420	1.7700000	2.5500000

Unique Subject Identifier=1107011010-0002

Assay variable : PTINR_VAL1 PT-INR Value

N	Mean	Standard Deviation	Minimum	Maximum
6	1.8416667	0.1804901	1.6500000	2.1500000

Unique Subject Identifier=1107011010-0005

Assay variable : PTINR_VAL1 PT-INR Value

N	Mean	Standard Deviation	Minimum	Maximum
5	1.9100000	0.9907321	0.9700000	3.5100000

Laboratory tests at the time of obtaining Table 2-6 consent, according to the presence or absence of proton-pump inhibitors

Unique Subject Identifier=1107011010-0006

MEANS Procedure

Assay variable : PTINR_VAL1 PT-INR Value

N	Mean	Standard Deviation	Minimum	Maximum
4	1.8075000	0.3854327	1.2700000	2.1200000

Unique Subject Identifier=1107011010-0007

Assay variable : PTINR_VAL1 PT-INR Value

N	Mean	Standard Deviation	Minimum	Maximum
6	1.6666667	0.0886942	1.5600000	1.7700000

Unique Subject Identifier=1107011010-0011

Assay variable : PTINR_VAL1 PT-INR Value

N	Mean	Standard Deviation	Minimum	Maximum
6	1.7083333	0.2064380	1.4600000	1.9900000

Unique Subject Identifier=1110000000-0002

Assay variable : PTINR_VAL1 PT-INR Value

N	Mean	Standard Deviation	Minimum	Maximum
5	2.1000000	0.1581139	1.9000000	2.3000000

Laboratory tests at the time of obtaining Table 2-6 consent, according to the presence or absence of proton-pump inhibitors

Unique Subject Identifier=1110000000-0013

MEANS Procedure

Assay variable : PTINR_VAL1 PT-INR Value

N	Mean	Standard Deviation	Minimum	Maximum
5	1.7800000	0.2588436	1.5000000	2.2000000

Unique Subject Identifier=1110000000-0016

Assay variable : PTINR_VAL1 PT-INR Value

N	Mean	Standard Deviation	Minimum	Maximum
4	2.2250000	0.1500000	2.1000000	2.4000000

Unique Subject Identifier=1110000000-0017

Assay variable : PTINR_VAL1 PT-INR Value

N	Mean	Standard Deviation	Minimum	Maximum
4	1.9250000	0.4573474	1.4000000	2.4000000

Unique Subject Identifier=1110000000-0019

Assay variable : PTINR_VAL1 PT-INR Value

N	Mean	Standard Deviation	Minimum	Maximum
1	1.7200000		1.7200000	1.7200000

Laboratory tests at the time of obtaining Table 2-6 consent, according to the presence or absence of proton-pump inhibitors

Unique Subject Identifier=1110000000-0020

MEANS Procedure

Assay variable : PTINR_VAL1 PT-INR Value

N	Mean	Standard Deviation	Minimum	Maximum
6	1.7000000	0.1788854	1.5000000	2.0000000

Unique Subject Identifier=1110000000-0022

Assay variable : PTINR_VAL1 PT-INR Value

N	Mean	Standard Deviation	Minimum	Maximum
5	1.7600000	0.1516575	1.6000000	2.0000000

Unique Subject Identifier=1111000000-0005

Assay variable : PTINR_VAL1 PT-INR Value

N	Mean	Standard Deviation	Minimum	Maximum
6	1.5600000	0.1181524	1.4800000	1.7800000

Unique Subject Identifier=1111000000-0012

Assay variable : PTINR_VAL1 PT-INR Value

N	Mean	Standard Deviation	Minimum	Maximum
2	2.1050000	0.1484924	2.0000000	2.2100000

Laboratory tests at the time of obtaining Table 2-6 consent, according to the presence or absence of proton-pump inhibitors

Unique Subject Identifier=1111000000-0017

MEANS Procedure

Assay variable : PTINR_VAL1 PT-INR Value

N	Mean	Standard Deviation	Minimum	Maximum
3	1.7166667	0.1050397	1.6100000	1.8200000

Unique Subject Identifier=1111000000-0021

Assay variable : PTINR_VAL1 PT-INR Value

N	Mean	Standard Deviation	Minimum	Maximum
6	1.8016667	0.2604931	1.4300000	2.1700000

Unique Subject Identifier=1112000000-0002

Assay variable : PTINR_VAL1 PT-INR Value

N	Mean	Standard Deviation	Minimum	Maximum
6	1.8966667	0.5702865	1.3100000	2.7600000

Unique Subject Identifier=1112000000-0003

Assay variable : PTINR_VAL1 PT-INR Value

N	Mean	Standard Deviation	Minimum	Maximum
5	1.7200000	0.2181742	1.5100000	2.0700000

Laboratory tests at the time of obtaining Table 2-6 consent, according to the presence or absence of proton-pump inhibitors

Unique Subject Identifier=1112000000-0009

MEANS Procedure

Assay variable : PTINR_VAL1 PT-INR Value

N	Mean	Standard Deviation	Minimum	Maximum
6	2.5000000	1.1120611	1.4000000	4.2900000

Unique Subject Identifier=1112000000-0013

Assay variable : PTINR_VAL1 PT-INR Value

N	Mean	Standard Deviation	Minimum	Maximum
4	2.1475000	0.4944610	1.7800000	2.8600000

Unique Subject Identifier=1112000000-0015

Assay variable : PTINR_VAL1 PT-INR Value

N	Mean	Standard Deviation	Minimum	Maximum
6	2.1833333	0.6064212	1.5500000	3.1900000

Unique Subject Identifier=1118011000-0002

Assay variable : PTINR_VAL1 PT-INR Value

N	Mean	Standard Deviation	Minimum	Maximum
5	2.1000000	0.2549510	1.8000000	2.4000000

Laboratory tests at the time of obtaining Table 2-6 consent, according to the presence or absence of proton-pump inhibitors

Unique Subject Identifier=1118011000-0005

MEANS Procedure

Assay variable : PTINR_VAL1 PT-INR Value

N	Mean	Standard Deviation	Minimum	Maximum
5	2.4200000	0.3033150	2.0000000	2.8000000

Unique Subject Identifier=1118011000-0006

Assay variable : PTINR_VAL1 PT-INR Value

N	Mean	Standard Deviation	Minimum	Maximum
6	2.1833333	0.5307228	1.6000000	3.0000000

Unique Subject Identifier=1118011000-0007

Assay variable : PTINR_VAL1 PT-INR Value

N	Mean	Standard Deviation	Minimum	Maximum
4	1.4750000	0.0957427	1.4000000	1.6000000

Unique Subject Identifier=1118011000-0008

Assay variable : PTINR_VAL1 PT-INR Value

N	Mean	Standard Deviation	Minimum	Maximum
6	2.1666667	0.5354126	1.8000000	3.2000000

Laboratory tests at the time of obtaining Table 2-6 consent, according to the presence or absence of proton-pump inhibitors

Unique Subject Identifier=1118011000-0009

MEANS Procedure

Assay variable : PTINR_VAL1 PT-INR Value

N	Mean	Standard Deviation	Minimum	Maximum
6	1.9666667	0.2503331	1.7000000	2.3000000

Unique Subject Identifier=1118011000-0012

Assay variable : PTINR_VAL1 PT-INR Value

N	Mean	Standard Deviation	Minimum	Maximum
5	1.5000000	0.2549510	1.3000000	1.9000000

Unique Subject Identifier=1118011000-0014

Assay variable : PTINR_VAL1 PT-INR Value

N	Mean	Standard Deviation	Minimum	Maximum
5	2.0000000	0.0707107	1.9000000	2.1000000

Unique Subject Identifier=1118011000-0018

Assay variable : PTINR_VAL1 PT-INR Value

N	Mean	Standard Deviation	Minimum	Maximum
5	2.3000000	0.6324555	1.3000000	3.0000000

Laboratory tests at the time of obtaining Table 2-6 consent, according to the presence or absence of proton-pump inhibitors

Unique Subject Identifier=1118011000-0019

MEANS Procedure

Assay variable : PTINR_VAL1 PT-INR Value

N	Mean	Standard Deviation	Minimum	Maximum
6	2.0166667	0.2994439	1.7000000	2.5000000

Unique Subject Identifier=1118011000-0021

Assay variable : PTINR_VAL1 PT-INR Value

N	Mean	Standard Deviation	Minimum	Maximum
5	1.5600000	0.1140175	1.4000000	1.7000000

Unique Subject Identifier=1118011000-0023

Assay variable : PTINR_VAL1 PT-INR Value

N	Mean	Standard Deviation	Minimum	Maximum
6	2.0000000	0.5513620	1.5000000	2.8000000

Unique Subject Identifier=1118011000-0024

Assay variable : PTINR_VAL1 PT-INR Value

N	Mean	Standard Deviation	Minimum	Maximum
4	1.4750000	0.2629956	1.1000000	1.7000000

Laboratory tests at the time of obtaining Table 2-6 consent, according to the presence or absence of proton-pump inhibitors

Unique Subject Identifier=1118011000-0027

MEANS Procedure

Assay variable : PTINR_VAL1 PT-INR Value

N	Mean	Standard Deviation	Minimum	Maximum
5	2.0800000	0.4549725	1.6000000	2.8000000

Unique Subject Identifier=1118011000-0030

Assay variable : PTINR_VAL1 PT-INR Value

N	Mean	Standard Deviation	Minimum	Maximum
5	1.9600000	0.2792848	1.5000000	2.2000000

Unique Subject Identifier=1118011000-0032

Assay variable : PTINR_VAL1 PT-INR Value

N	Mean	Standard Deviation	Minimum	Maximum
5	1.9800000	0.3114482	1.7000000	2.4000000

Unique Subject Identifier=1118011000-0035

Assay variable : PTINR_VAL1 PT-INR Value

N	Mean	Standard Deviation	Minimum	Maximum
5	2.1000000	0.4358899	1.6000000	2.8000000

Laboratory tests at the time of obtaining Table 2-6 consent, according to the presence or absence of proton-pump inhibitors

Unique Subject Identifier=1118011000-0038

MEANS Procedure

Assay variable : PTINR_VAL1 PT-INR Value

N	Mean	Standard Deviation	Minimum	Maximum
6	2.1166667	0.1722401	1.9000000	2.4000000

Unique Subject Identifier=1118011000-0041

Assay variable : PTINR_VAL1 PT-INR Value

N	Mean	Standard Deviation	Minimum	Maximum
6	2.6000000	0.3521363	2.2000000	3.2000000

Unique Subject Identifier=1118011000-0045

Assay variable : PTINR_VAL1 PT-INR Value

N	Mean	Standard Deviation	Minimum	Maximum
6	1.8833333	0.1169045	1.7000000	2.0000000

Unique Subject Identifier=1118011000-0048

Assay variable : PTINR_VAL1 PT-INR Value

N	Mean	Standard Deviation	Minimum	Maximum
6	1.6000000	0.2000000	1.4000000	1.9000000

Laboratory tests at the time of obtaining Table 2-6 consent, according to the presence or absence of proton-pump inhibitors

Unique Subject Identifier=1118011000-0049

MEANS Procedure

Assay variable : PTINR_VAL1 PT-INR Value

N	Mean	Standard Deviation	Minimum	Maximum
6	2.2166667	0.2994439	1.8000000	2.6000000

Unique Subject Identifier=1118011000-0051

Assay variable : PTINR_VAL1 PT-INR Value

N	Mean	Standard Deviation	Minimum	Maximum
5	1.7400000	0.3714835	1.2000000	2.2000000

Unique Subject Identifier=1118011000-0057

Assay variable : PTINR_VAL1 PT-INR Value

N	Mean	Standard Deviation	Minimum	Maximum
6	1.6166667	0.1471960	1.5000000	1.8000000

Unique Subject Identifier=1118011000-0060

Assay variable : PTINR_VAL1 PT-INR Value

N	Mean	Standard Deviation	Minimum	Maximum
4	1.5000000	0.2828427	1.1000000	1.7000000

Laboratory tests at the time of obtaining Table 2-6 consent, according to the presence or absence of proton-pump inhibitors

Unique Subject Identifier=1118011000-0062

MEANS Procedure

Assay variable : PTINR_VAL1 PT-INR Value

N	Mean	Standard Deviation	Minimum	Maximum
6	2.1500000	0.4324350	1.3000000	2.5000000

Unique Subject Identifier=1118011000-0064

Assay variable : PTINR_VAL1 PT-INR Value

N	Mean	Standard Deviation	Minimum	Maximum
6	2.7500000	0.4415880	2.0000000	3.3000000

Unique Subject Identifier=1118011000-0067

Assay variable : PTINR_VAL1 PT-INR Value

N	Mean	Standard Deviation	Minimum	Maximum
4	1.8250000	0.1707825	1.6000000	2.0000000

Unique Subject Identifier=1118011000-0068

Assay variable : PTINR_VAL1 PT-INR Value

N	Mean	Standard Deviation	Minimum	Maximum
6	1.6833333	0.2041241	1.4000000	1.9000000

Laboratory tests at the time of obtaining Table 2-6 consent, according to the presence or absence of proton-pump inhibitors

Unique Subject Identifier=1118011000-0069

MEANS Procedure

Assay variable : PTINR_VAL1 PT-INR Value

N	Mean	Standard Deviation	Minimum	Maximum
6	1.8166667	0.2401388	1.6000000	2.2000000

Unique Subject Identifier=1118011000-0070

Assay variable : PTINR_VAL1 PT-INR Value

N	Mean	Standard Deviation	Minimum	Maximum
4	1.8000000	0.2160247	1.6000000	2.1000000

Unique Subject Identifier=1118011000-0072

Assay variable : PTINR_VAL1 PT-INR Value

N	Mean	Standard Deviation	Minimum	Maximum
6	2.0333333	0.1861899	1.8000000	2.3000000

Unique Subject Identifier=1118011000-0078

Assay variable : PTINR_VAL1 PT-INR Value

N	Mean	Standard Deviation	Minimum	Maximum
5	1.9600000	0.5504544	1.4000000	2.6000000

Laboratory tests at the time of obtaining Table 2-6 consent, according to the presence or absence of proton-pump inhibitors

Unique Subject Identifier=1118011000-0079

MEANS Procedure

Assay variable : PTINR_VAL1 PT-INR Value

N	Mean	Standard Deviation	Minimum	Maximum
5	2.2800000	0.6534524	1.7000000	3.4000000

Unique Subject Identifier=1118011000-0082

Assay variable : PTINR_VAL1 PT-INR Value

N	Mean	Standard Deviation	Minimum	Maximum
6	1.3833333	0.3250641	1.0000000	1.9000000

Unique Subject Identifier=1118011000-0083

Assay variable : PTINR_VAL1 PT-INR Value

N	Mean	Standard Deviation	Minimum	Maximum
6	2.2833333	0.4792355	1.9000000	2.9000000

Unique Subject Identifier=1118011000-0086

Assay variable : PTINR_VAL1 PT-INR Value

N	Mean	Standard Deviation	Minimum	Maximum
5	2.0200000	0.5449771	1.3000000	2.8000000

Laboratory tests at the time of obtaining Table 2-6 consent, according to the presence or absence of proton-pump inhibitors

Unique Subject Identifier=1118011000-0087

MEANS Procedure

Assay variable : PTINR_VAL1 PT-INR Value

N	Mean	Standard Deviation	Minimum	Maximum
5	1.8000000	0.3000000	1.5000000	2.1000000

Unique Subject Identifier=1118011000-0089

Assay variable : PTINR_VAL1 PT-INR Value

N	Mean	Standard Deviation	Minimum	Maximum
5	1.8600000	0.1949359	1.6000000	2.1000000

Unique Subject Identifier=1118011000-0090

Assay variable : PTINR_VAL1 PT-INR Value

N	Mean	Standard Deviation	Minimum	Maximum
6	2.5833333	0.2483277	2.3000000	2.9000000

Unique Subject Identifier=1118011000-0091

Assay variable : PTINR_VAL1 PT-INR Value

N	Mean	Standard Deviation	Minimum	Maximum
6	2.0833333	0.5231316	1.5000000	2.9000000

Laboratory tests at the time of obtaining Table 2-6 consent, according to the presence or absence of proton-pump inhibitors

Unique Subject Identifier=1118011000-0093

MEANS Procedure

Assay variable : PTINR_VAL1 PT-INR Value

N	Mean	Standard Deviation	Minimum	Maximum
6	2.1000000	0.3794733	1.7000000	2.7000000

Unique Subject Identifier=1118011000-0099

Assay variable : PTINR_VAL1 PT-INR Value

N	Mean	Standard Deviation	Minimum	Maximum
6	1.4333333	0.1505545	1.3000000	1.7000000

Unique Subject Identifier=1118011000-0101

Assay variable : PTINR_VAL1 PT-INR Value

N	Mean	Standard Deviation	Minimum	Maximum
6	2.0333333	0.2658320	1.6000000	2.3000000

Unique Subject Identifier=1118011000-0102

Assay variable : PTINR_VAL1 PT-INR Value

N	Mean	Standard Deviation	Minimum	Maximum
6	1.9000000	0.3521363	1.7000000	2.6000000

Laboratory tests at the time of obtaining Table 2-6 consent, according to the presence or absence of proton-pump inhibitors

Unique Subject Identifier=1118011000-0105

MEANS Procedure

Assay variable : PTINR_VAL1 PT-INR Value

N	Mean	Standard Deviation	Minimum	Maximum
4	1.7750000	0.2753785	1.5000000	2.1000000

Unique Subject Identifier=1118011000-0109

Assay variable : PTINR_VAL1 PT-INR Value

N	Mean	Standard Deviation	Minimum	Maximum
5	2.2200000	0.2774887	1.8000000	2.5000000

Unique Subject Identifier=1118011000-0113

Assay variable : PTINR_VAL1 PT-INR Value

N	Mean	Standard Deviation	Minimum	Maximum
3	1.7666667	0.2516611	1.5000000	2.0000000

Unique Subject Identifier=1118011000-0116

Assay variable : PTINR_VAL1 PT-INR Value

N	Mean	Standard Deviation	Minimum	Maximum
3	1.6333333	0.3214550	1.4000000	2.0000000

Laboratory tests at the time of obtaining Table 2-6 consent, according to the presence or absence of proton-pump inhibitors

Unique Subject Identifier=1118011000-0118

MEANS Procedure

Assay variable : PTINR_VAL1 PT-INR Value

N	Mean	Standard Deviation	Minimum	Maximum
4	1.6000000	0.2828427	1.4000000	2.0000000

Unique Subject Identifier=1118011000-0120

Assay variable : PTINR_VAL1 PT-INR Value

N	Mean	Standard Deviation	Minimum	Maximum
6	1.8166667	0.2136976	1.6000000	2.2000000

Unique Subject Identifier=1119000000-0001

Assay variable : PTINR_VAL1 PT-INR Value

N	Mean	Standard Deviation	Minimum	Maximum
6	1.6500000	0.1649242	1.4400000	1.9100000

Unique Subject Identifier=1119000000-0005

Assay variable : PTINR_VAL1 PT-INR Value

N	Mean	Standard Deviation	Minimum	Maximum
1	1.6300000		1.6300000	1.6300000

Laboratory tests at the time of obtaining Table 2-6 consent, according to the presence or absence of proton-pump inhibitors

Unique Subject Identifier=1119000000-0010

MEANS Procedure

Assay variable : PTINR_VAL1 PT-INR Value

N	Mean	Standard Deviation	Minimum	Maximum
3	2.8000000	0.4358899	2.5000000	3.3000000

Unique Subject Identifier=1119000000-0016

Assay variable : PTINR_VAL1 PT-INR Value

N	Mean	Standard Deviation	Minimum	Maximum
2	1.9800000	0.0282843	1.9600000	2.0000000

Unique Subject Identifier=1119000000-0019

Assay variable : PTINR_VAL1 PT-INR Value

N	Mean	Standard Deviation	Minimum	Maximum
6	2.1466667	0.3225937	1.9000000	2.7400000

Unique Subject Identifier=1119000000-0022

Assay variable : PTINR_VAL1 PT-INR Value

N	Mean	Standard Deviation	Minimum	Maximum
6	2.1466667	0.3819249	1.7800000	2.8000000

Laboratory tests at the time of obtaining Table 2-6 consent, according to the presence or absence of proton-pump inhibitors

Unique Subject Identifier=1119000000-0025

MEANS Procedure

Assay variable : PTINR_VAL1 PT-INR Value

N	Mean	Standard Deviation	Minimum	Maximum
6	2.2500000	0.5244044	1.8000000	3.2000000

Unique Subject Identifier=1119000000-0030

Assay variable : PTINR_VAL1 PT-INR Value

N	Mean	Standard Deviation	Minimum	Maximum
4	2.4500000	0.2380476	2.3000000	2.8000000

Unique Subject Identifier=1119000000-0031

Assay variable : PTINR_VAL1 PT-INR Value

N	Mean	Standard Deviation	Minimum	Maximum
3	3.4333333	0.7505553	2.7000000	4.2000000

Unique Subject Identifier=1119000000-0036

Assay variable : PTINR_VAL1 PT-INR Value

N	Mean	Standard Deviation	Minimum	Maximum
5	2.7260000	0.2380756	2.4900000	3.0600000

Laboratory tests at the time of obtaining Table 2-6 consent, according to the presence or absence of proton-pump inhibitors

Unique Subject Identifier=1119000000-0039

MEANS Procedure

Assay variable : PTINR_VAL1 PT-INR Value

N	Mean	Standard Deviation	Minimum	Maximum
5	2.0000000	0.3605551	1.6000000	2.5000000

Unique Subject Identifier=1119000000-0041

Assay variable : PTINR_VAL1 PT-INR Value

N	Mean	Standard Deviation	Minimum	Maximum
6	2.1833333	0.5231316	1.5000000	2.9000000

Unique Subject Identifier=1119000000-0044

Assay variable : PTINR_VAL1 PT-INR Value

N	Mean	Standard Deviation	Minimum	Maximum
5	1.8400000	0.2966479	1.5000000	2.3000000

Unique Subject Identifier=1119000000-0055

Assay variable : PTINR_VAL1 PT-INR Value

N	Mean	Standard Deviation	Minimum	Maximum
1	2.9000000		2.9000000	2.9000000

Laboratory tests at the time of obtaining Table 2-6 consent, according to the presence or absence of proton-pump inhibitors

Unique Subject Identifier=1119000000-0060

MEANS Procedure

Assay variable : PTINR_VAL1 PT-INR Value

N	Mean	Standard Deviation	Minimum	Maximum
2	3.0350000	0.0212132	3.0200000	3.0500000

Unique Subject Identifier=1122001010-0014

Assay variable : PTINR_VAL1 PT-INR Value

N	Mean	Standard Deviation	Minimum	Maximum
6	1.4283333	0.1697547	1.2700000	1.7000000

Unique Subject Identifier=1122001010-0018

Assay variable : PTINR_VAL1 PT-INR Value

N	Mean	Standard Deviation	Minimum	Maximum
1	1.6400000		1.6400000	1.6400000

Unique Subject Identifier=1122001010-0031

Assay variable : PTINR_VAL1 PT-INR Value

N	Mean	Standard Deviation	Minimum	Maximum
6	2.4833333	0.4684727	1.7500000	2.9500000

Laboratory tests at the time of obtaining Table 2-6 consent, according to the presence or absence of proton-pump inhibitors

Unique Subject Identifier=1122001010-0032

MEANS Procedure

Assay variable : PTINR_VAL1 PT-INR Value

N	Mean	Standard Deviation	Minimum	Maximum
6	1.8566667	0.2010638	1.6200000	2.1600000

Unique Subject Identifier=1122001010-0033

Assay variable : PTINR_VAL1 PT-INR Value

N	Mean	Standard Deviation	Minimum	Maximum
6	2.1500000	0.5616761	1.1100000	2.6100000

Unique Subject Identifier=1122001010-0034

Assay variable : PTINR_VAL1 PT-INR Value

N	Mean	Standard Deviation	Minimum	Maximum
6	2.6000000	1.0848594	1.1100000	4.3800000

Unique Subject Identifier=1122001010-0037

Assay variable : PTINR_VAL1 PT-INR Value

N	Mean	Standard Deviation	Minimum	Maximum
6	1.7116667	0.2398680	1.4300000	2.1300000

Laboratory tests at the time of obtaining Table 2-6 consent, according to the presence or absence of proton-pump inhibitors

Unique Subject Identifier=1122001010-0038

MEANS Procedure

Assay variable : PTINR_VAL1 PT-INR Value

N	Mean	Standard Deviation	Minimum	Maximum
6	2.0683333	0.1979310	1.8600000	2.3300000

Unique Subject Identifier=1122001010-0039

Assay variable : PTINR_VAL1 PT-INR Value

N	Mean	Standard Deviation	Minimum	Maximum
1	2.0600000		2.0600000	2.0600000

Unique Subject Identifier=1122001010-0041

Assay variable : PTINR_VAL1 PT-INR Value

N	Mean	Standard Deviation	Minimum	Maximum
6	2.0583333	0.1683350	1.8300000	2.3300000

Unique Subject Identifier=1122001010-0045

Assay variable : PTINR_VAL1 PT-INR Value

N	Mean	Standard Deviation	Minimum	Maximum
6	1.5966667	0.2299275	1.3100000	1.9900000

Laboratory tests at the time of obtaining Table 2-6 consent, according to the presence or absence of proton-pump inhibitors

Unique Subject Identifier=1122001010-0046

MEANS Procedure

Assay variable : PTINR_VAL1 PT-INR Value

N	Mean	Standard Deviation	Minimum	Maximum
6	2.5183333	1.0548823	1.3100000	3.7600000

Unique Subject Identifier=1122001010-0047

Assay variable : PTINR_VAL1 PT-INR Value

N	Mean	Standard Deviation	Minimum	Maximum
6	2.1283333	0.3985181	1.5500000	2.5300000

Unique Subject Identifier=1122001010-0048

Assay variable : PTINR_VAL1 PT-INR Value

N	Mean	Standard Deviation	Minimum	Maximum
6	2.4100000	0.4795832	1.6800000	3.1200000

Unique Subject Identifier=1122001010-0050

Assay variable : PTINR_VAL1 PT-INR Value

N	Mean	Standard Deviation	Minimum	Maximum
6	1.7233333	0.3512074	1.4100000	2.3800000

Laboratory tests at the time of obtaining Table 2-6 consent, according to the presence or absence of proton-pump inhibitors

Unique Subject Identifier=1122001010-0052

MEANS Procedure

Assay variable : PTINR_VAL1 PT-INR Value

N	Mean	Standard Deviation	Minimum	Maximum
6	1.9266667	0.1954141	1.6200000	2.1300000

Unique Subject Identifier=1122001010-0053

Assay variable : PTINR_VAL1 PT-INR Value

N	Mean	Standard Deviation	Minimum	Maximum
6	2.6066667	0.4033691	2.0400000	3.1200000

Unique Subject Identifier=1122001010-0054

Assay variable : PTINR_VAL1 PT-INR Value

N	Mean	Standard Deviation	Minimum	Maximum
6	2.3033333	1.1264221	1.1600000	4.3800000

Unique Subject Identifier=1122001010-0055

Assay variable : PTINR_VAL1 PT-INR Value

N	Mean	Standard Deviation	Minimum	Maximum
6	1.7900000	0.1943193	1.6000000	2.1300000

Laboratory tests at the time of obtaining Table 2-6 consent, according to the presence or absence of proton-pump inhibitors

Unique Subject Identifier=1122001010-0056

MEANS Procedure

Assay variable : PTINR_VAL1 PT-INR Value

N	Mean	Standard Deviation	Minimum	Maximum
5	2.4940000	0.3922754	2.1100000	2.9300000

Unique Subject Identifier=1122001010-0057

Assay variable : PTINR_VAL1 PT-INR Value

N	Mean	Standard Deviation	Minimum	Maximum
6	1.9350000	0.3984344	1.5500000	2.6400000

Unique Subject Identifier=1122001010-0059

Assay variable : PTINR_VAL1 PT-INR Value

N	Mean	Standard Deviation	Minimum	Maximum
6	2.1800000	0.4648010	1.8600000	3.0600000

Unique Subject Identifier=1122001010-0060

Assay variable : PTINR_VAL1 PT-INR Value

N	Mean	Standard Deviation	Minimum	Maximum
6	1.7200000	0.7457882	1.1800000	3.1800000

Laboratory tests at the time of obtaining Table 2-6 consent, according to the presence or absence of proton-pump inhibitors

Unique Subject Identifier=1122001010-0061

MEANS Procedure

Assay variable : PTINR_VAL1 PT-INR Value

N	Mean	Standard Deviation	Minimum	Maximum
6	2.4133333	0.2000667	2.1300000	2.7100000

Unique Subject Identifier=1122001010-0062

Assay variable : PTINR_VAL1 PT-INR Value

N	Mean	Standard Deviation	Minimum	Maximum
6	2.4733333	0.5971153	1.5500000	3.2000000

Unique Subject Identifier=1122001010-0063

Assay variable : PTINR_VAL1 PT-INR Value

N	Mean	Standard Deviation	Minimum	Maximum
6	2.2633333	0.2497732	1.8100000	2.5600000

Unique Subject Identifier=1122001010-0064

Assay variable : PTINR_VAL1 PT-INR Value

N	Mean	Standard Deviation	Minimum	Maximum
6	2.0216667	0.4163372	1.4500000	2.5600000

Laboratory tests at the time of obtaining Table 2-6 consent, according to the presence or absence of proton-pump inhibitors

Unique Subject Identifier=1122001010-0065

MEANS Procedure

Assay variable : PTINR_VAL1 PT-INR Value

N	Mean	Standard Deviation	Minimum	Maximum
6	1.7000000	0.3573234	1.3100000	2.3300000

Unique Subject Identifier=1122001010-0066

Assay variable : PTINR_VAL1 PT-INR Value

N	Mean	Standard Deviation	Minimum	Maximum
6	2.0233333	0.2823945	1.6200000	2.3800000

Unique Subject Identifier=1122001010-0069

Assay variable : PTINR_VAL1 PT-INR Value

N	Mean	Standard Deviation	Minimum	Maximum
6	1.7300000	0.1856879	1.5500000	2.0600000

Unique Subject Identifier=1122001010-0072

Assay variable : PTINR_VAL1 PT-INR Value

N	Mean	Standard Deviation	Minimum	Maximum
3	1.6566667	0.4454586	1.2000000	2.0900000

Laboratory tests at the time of obtaining Table 2-6 consent, according to the presence or absence of proton-pump inhibitors

Unique Subject Identifier=1122001010-0073

MEANS Procedure

Assay variable : PTINR_VAL1 PT-INR Value

N	Mean	Standard Deviation	Minimum	Maximum
6	1.7566667	0.2808321	1.4700000	2.2500000

Unique Subject Identifier=1122001010-0074

Assay variable : PTINR_VAL1 PT-INR Value

N	Mean	Standard Deviation	Minimum	Maximum
3	2.4233333	0.2294196	2.1600000	2.5800000

Unique Subject Identifier=1126011000-0002

Assay variable : PTINR_VAL1 PT-INR Value

N	Mean	Standard Deviation	Minimum	Maximum
6	1.5816667	0.3057722	1.2700000	2.0500000

Unique Subject Identifier=1126011000-0004

Assay variable : PTINR_VAL1 PT-INR Value

N	Mean	Standard Deviation	Minimum	Maximum
4	1.5700000	0.5963221	1.0600000	2.3400000

Laboratory tests at the time of obtaining Table 2-6 consent, according to the presence or absence of proton-pump inhibitors

Unique Subject Identifier=1126011000-0010

MEANS Procedure

Assay variable : PTINR_VAL1 PT-INR Value

N	Mean	Standard Deviation	Minimum	Maximum
6	1.9316667	0.3364174	1.5900000	2.5300000

Unique Subject Identifier=1126011000-0011

Assay variable : PTINR_VAL1 PT-INR Value

N	Mean	Standard Deviation	Minimum	Maximum
5	2.0320000	0.3856423	1.5500000	2.4900000

Unique Subject Identifier=1126011000-0013

Assay variable : PTINR_VAL1 PT-INR Value

N	Mean	Standard Deviation	Minimum	Maximum
6	1.7583333	0.2245366	1.6000000	2.2000000

Unique Subject Identifier=1126011000-0014

Assay variable : PTINR_VAL1 PT-INR Value

N	Mean	Standard Deviation	Minimum	Maximum
5	1.6700000	0.6279729	1.1000000	2.7300000

Laboratory tests at the time of obtaining Table 2-6 consent, according to the presence or absence of proton-pump inhibitors

Unique Subject Identifier=1126011000-0015

MEANS Procedure

Assay variable : PTINR_VAL1 PT-INR Value

N	Mean	Standard Deviation	Minimum	Maximum
6	1.9166667	0.4128276	1.4500000	2.4900000

Unique Subject Identifier=1127010000-0001

Assay variable : PTINR_VAL1 PT-INR Value

N	Mean	Standard Deviation	Minimum	Maximum
6	1.6833333	0.1834848	1.4000000	1.9000000

Unique Subject Identifier=1127010000-0002

Assay variable : PTINR_VAL1 PT-INR Value

N	Mean	Standard Deviation	Minimum	Maximum
4	1.4500000	0.0577350	1.4000000	1.5000000

Unique Subject Identifier=1127010000-0003

Assay variable : PTINR_VAL1 PT-INR Value

N	Mean	Standard Deviation	Minimum	Maximum
5	2.1800000	0.1483240	2.0000000	2.4000000

Laboratory tests at the time of obtaining Table 2-6 consent, according to the presence or absence of proton-pump inhibitors

Unique Subject Identifier=1127010000-0005

MEANS Procedure

Assay variable : PTINR_VAL1 PT-INR Value

N	Mean	Standard Deviation	Minimum	Maximum
1	1.1000000		1.1000000	1.1000000

Unique Subject Identifier=1128011000-0002

Assay variable : PTINR_VAL1 PT-INR Value

N	Mean	Standard Deviation	Minimum	Maximum
6	1.9333333	0.3265986	1.6000000	2.5000000

Unique Subject Identifier=1128011000-0003

Assay variable : PTINR_VAL1 PT-INR Value

N	Mean	Standard Deviation	Minimum	Maximum
6	1.8666667	0.1032796	1.7000000	2.0000000

Unique Subject Identifier=1128011000-0004

Assay variable : PTINR_VAL1 PT-INR Value

N	Mean	Standard Deviation	Minimum	Maximum
5	1.8600000	0.1516575	1.7000000	2.1000000

Laboratory tests at the time of obtaining Table 2-6 consent, according to the presence or absence of proton-pump inhibitors

Unique Subject Identifier=1128011000-0005

MEANS Procedure

Assay variable : PTINR_VAL1 PT-INR Value

N	Mean	Standard Deviation	Minimum	Maximum
6	2.0666667	0.3983298	1.6000000	2.6000000

Unique Subject Identifier=1128011000-0009

Assay variable : PTINR_VAL1 PT-INR Value

N	Mean	Standard Deviation	Minimum	Maximum
4	2.2000000	0.2943920	1.9000000	2.6000000

Unique Subject Identifier=1128011000-0010

Assay variable : PTINR_VAL1 PT-INR Value

N	Mean	Standard Deviation	Minimum	Maximum
5	2.2400000	0.8988882	1.0000000	3.3000000

Unique Subject Identifier=1131000000-0001

Assay variable : PTINR_VAL1 PT-INR Value

N	Mean	Standard Deviation	Minimum	Maximum
6	1.5016667	0.3225161	1.1300000	1.8700000

Laboratory tests at the time of obtaining Table 2-6 consent, according to the presence or absence of proton-pump inhibitors

Unique Subject Identifier=1131000000-0002

MEANS Procedure

Assay variable : PTINR_VAL1 PT-INR Value

N	Mean	Standard Deviation	Minimum	Maximum
5	1.3300000	0.2933428	1.0500000	1.7900000

Unique Subject Identifier=1131000000-0003

Assay variable : PTINR_VAL1 PT-INR Value

N	Mean	Standard Deviation	Minimum	Maximum
6	2.0800000	0.2337520	1.7300000	2.3300000

Unique Subject Identifier=1131000000-0004

Assay variable : PTINR_VAL1 PT-INR Value

N	Mean	Standard Deviation	Minimum	Maximum
6	1.4550000	0.4079583	1.0800000	2.2300000

Unique Subject Identifier=1131000000-0005

Assay variable : PTINR_VAL1 PT-INR Value

N	Mean	Standard Deviation	Minimum	Maximum
6	2.2650000	0.4269309	1.8500000	2.8000000

Laboratory tests at the time of obtaining Table 2-6 consent, according to the presence or absence of proton-pump inhibitors

Unique Subject Identifier=1131000000-0006

MEANS Procedure

Assay variable : PTINR_VAL1 PT-INR Value

N	Mean	Standard Deviation	Minimum	Maximum
6	1.6383333	0.2778789	1.3600000	2.1700000

Unique Subject Identifier=1131000000-0007

Assay variable : PTINR_VAL1 PT-INR Value

N	Mean	Standard Deviation	Minimum	Maximum
6	2.1600000	0.6108355	1.6500000	3.1100000

Unique Subject Identifier=1131000000-0008

Assay variable : PTINR_VAL1 PT-INR Value

N	Mean	Standard Deviation	Minimum	Maximum
5	1.5220000	0.3168122	1.2400000	1.9700000

Unique Subject Identifier=1131000000-0009

Assay variable : PTINR_VAL1 PT-INR Value

N	Mean	Standard Deviation	Minimum	Maximum
3	1.9400000	0.2107131	1.7200000	2.1400000

Laboratory tests at the time of obtaining Table 2-6 consent, according to the presence or absence of proton-pump inhibitors

Unique Subject Identifier=1137000000-0007

MEANS Procedure

Assay variable : PTINR_VAL1 PT-INR Value

N	Mean	Standard Deviation	Minimum	Maximum
3	1.0366667	0.0208167	1.0200000	1.0600000

Unique Subject Identifier=1137000000-0032

Assay variable : PTINR_VAL1 PT-INR Value

N	Mean	Standard Deviation	Minimum	Maximum
2	1.0400000	0.0989949	0.9700000	1.1100000

Unique Subject Identifier=1141000000-0002

Assay variable : PTINR_VAL1 PT-INR Value

N	Mean	Standard Deviation	Minimum	Maximum
1	1.6800000		1.6800000	1.6800000

Unique Subject Identifier=1141000000-0005

Assay variable : PTINR_VAL1 PT-INR Value

N	Mean	Standard Deviation	Minimum	Maximum
1	1.2000000		1.2000000	1.2000000

Laboratory tests at the time of obtaining Table 2-6 consent, according to the presence or absence of proton-pump inhibitors

Unique Subject Identifier=1146011000-0001

MEANS Procedure

Assay variable : PTINR_VAL1 PT-INR Value

N	Mean	Standard Deviation	Minimum	Maximum
4	2.2750000	0.4607602	1.9800000	2.9500000

Unique Subject Identifier=1146011000-0002

Assay variable : PTINR_VAL1 PT-INR Value

N	Mean	Standard Deviation	Minimum	Maximum
3	2.0566667	0.6293118	1.5800000	2.7700000

Unique Subject Identifier=1146011000-0003

Assay variable : PTINR_VAL1 PT-INR Value

N	Mean	Standard Deviation	Minimum	Maximum
2	1.8400000	0.2969848	1.6300000	2.0500000

Unique Subject Identifier=1149000000-0001

Assay variable : PTINR_VAL1 PT-INR Value

N	Mean	Standard Deviation	Minimum	Maximum
6	1.7333333	0.2581989	1.4000000	2.1000000

Laboratory tests at the time of obtaining Table 2-6 consent, according to the presence or absence of proton-pump inhibitors

Unique Subject Identifier=1149000000-0002

MEANS Procedure

Assay variable : PTINR_VAL1 PT-INR Value

N	Mean	Standard Deviation	Minimum	Maximum
6	1.8500000	0.4135215	1.4000000	2.6000000

Unique Subject Identifier=1149000000-0003

Assay variable : PTINR_VAL1 PT-INR Value

N	Mean	Standard Deviation	Minimum	Maximum
6	2.2000000	0.0894427	2.1000000	2.3000000

Unique Subject Identifier=1149000000-0004

Assay variable : PTINR_VAL1 PT-INR Value

N	Mean	Standard Deviation	Minimum	Maximum
6	1.8666667	0.2804758	1.6000000	2.2000000

Unique Subject Identifier=1149000000-0005

Assay variable : PTINR_VAL1 PT-INR Value

N	Mean	Standard Deviation	Minimum	Maximum
6	2.1333333	0.6186006	1.4000000	3.2000000

Laboratory tests at the time of obtaining Table 2-6 consent, according to the presence or absence of proton-pump inhibitors

Unique Subject Identifier=1149000000-0006

MEANS Procedure

Assay variable : PTINR_VAL1 PT-INR Value

N	Mean	Standard Deviation	Minimum	Maximum
6	1.7166667	0.2041241	1.5000000	1.9000000

Unique Subject Identifier=1149000000-0007

Assay variable : PTINR_VAL1 PT-INR Value

N	Mean	Standard Deviation	Minimum	Maximum
6	1.8666667	0.4676181	1.0000000	2.4000000

Unique Subject Identifier=1154000000-0006

Assay variable : PTINR_VAL1 PT-INR Value

N	Mean	Standard Deviation	Minimum	Maximum
5	1.5900000	0.1168332	1.4500000	1.7200000

Unique Subject Identifier=1156000000-0001

Assay variable : PTINR_VAL1 PT-INR Value

N	Mean	Standard Deviation	Minimum	Maximum
6	1.4766667	0.0945868	1.3600000	1.6100000

Laboratory tests at the time of obtaining Table 2-6 consent, according to the presence or absence of proton-pump inhibitors

Unique Subject Identifier=1156000000-0002

MEANS Procedure

Assay variable : PTINR_VAL1 PT-INR Value

N	Mean	Standard Deviation	Minimum	Maximum
5	1.7920000	0.4152349	1.3900000	2.2500000

Unique Subject Identifier=1156000000-0003

Assay variable : PTINR_VAL1 PT-INR Value

N	Mean	Standard Deviation	Minimum	Maximum
6	1.1816667	0.2081746	0.9600000	1.4500000

Unique Subject Identifier=1156000000-0004

Assay variable : PTINR_VAL1 PT-INR Value

N	Mean	Standard Deviation	Minimum	Maximum
5	2.2100000	0.6031998	1.6600000	2.9700000

Unique Subject Identifier=1156000000-0005

Assay variable : PTINR_VAL1 PT-INR Value

N	Mean	Standard Deviation	Minimum	Maximum
1	1.4100000		1.4100000	1.4100000

Laboratory tests at the time of obtaining Table 2-6 consent, according to the presence or absence of proton-pump inhibitors

Unique Subject Identifier=1156000000-0006

MEANS Procedure

Assay variable : PTINR_VAL1 PT-INR Value

N	Mean	Standard Deviation	Minimum	Maximum
5	2.6020000	0.5514708	2.0500000	3.4700000

Unique Subject Identifier=1156000000-0007

Assay variable : PTINR_VAL1 PT-INR Value

N	Mean	Standard Deviation	Minimum	Maximum
5	3.6280000	2.4042400	1.6800000	7.8100000

Unique Subject Identifier=1156000000-0008

Assay variable : PTINR_VAL1 PT-INR Value

N	Mean	Standard Deviation	Minimum	Maximum
6	2.1600000	0.5060040	1.7400000	3.1300000

Unique Subject Identifier=1156000000-0009

Assay variable : PTINR_VAL1 PT-INR Value

N	Mean	Standard Deviation	Minimum	Maximum
6	1.8250000	0.3733497	1.4200000	2.5000000

Laboratory tests at the time of obtaining Table 2-6 consent, according to the presence or absence of proton-pump inhibitors

Unique Subject Identifier=1156000000-0010

MEANS Procedure

Assay variable : PTINR_VAL1 PT-INR Value

N	Mean	Standard Deviation	Minimum	Maximum
6	1.6116667	0.2249815	1.3200000	1.8600000

Unique Subject Identifier=1156000000-0011

Assay variable : PTINR_VAL1 PT-INR Value

N	Mean	Standard Deviation	Minimum	Maximum
4	2.6725000	0.2568235	2.4200000	3.0300000

Unique Subject Identifier=1156000000-0012

Assay variable : PTINR_VAL1 PT-INR Value

N	Mean	Standard Deviation	Minimum	Maximum
5	1.4780000	0.2141728	1.3100000	1.8400000

Unique Subject Identifier=1156000000-0014

Assay variable : PTINR_VAL1 PT-INR Value

N	Mean	Standard Deviation	Minimum	Maximum
6	1.3700000	0.1842824	1.2100000	1.6200000

Laboratory tests at the time of obtaining Table 2-6 consent, according to the presence or absence of proton-pump inhibitors

Unique Subject Identifier=1156000000-0015

MEANS Procedure

Assay variable : PTINR_VAL1 PT-INR Value

N	Mean	Standard Deviation	Minimum	Maximum
6	2.3333333	0.3844303	1.8600000	2.9400000

Unique Subject Identifier=1156000000-0018

Assay variable : PTINR_VAL1 PT-INR Value

N	Mean	Standard Deviation	Minimum	Maximum
6	1.3150000	0.1224337	1.1700000	1.4600000

Unique Subject Identifier=1156000000-0021

Assay variable : PTINR_VAL1 PT-INR Value

N	Mean	Standard Deviation	Minimum	Maximum
2	1.2700000	0.3394113	1.0300000	1.5100000

Unique Subject Identifier=1156000000-0022

Assay variable : PTINR_VAL1 PT-INR Value

N	Mean	Standard Deviation	Minimum	Maximum
5	1.7720000	0.3772532	1.2000000	2.0900000

Laboratory tests at the time of obtaining Table 2-6 consent, according to the presence or absence of proton-pump inhibitors

Unique Subject Identifier=1156000000-0023

MEANS Procedure

Assay variable : PTINR_VAL1 PT-INR Value

N	Mean	Standard Deviation	Minimum	Maximum
5	1.8680000	0.4928184	0.9900000	2.1400000

Unique Subject Identifier=1156000000-0056

Assay variable : PTINR_VAL1 PT-INR Value

N	Mean	Standard Deviation	Minimum	Maximum
5	1.8560000	0.1073778	1.7100000	1.9700000

Unique Subject Identifier=1156000000-0057

Assay variable : PTINR_VAL1 PT-INR Value

N	Mean	Standard Deviation	Minimum	Maximum
2	1.2900000	0.0707107	1.2400000	1.3400000

Unique Subject Identifier=1156000000-0059

Assay variable : PTINR_VAL1 PT-INR Value

N	Mean	Standard Deviation	Minimum	Maximum
6	1.6850000	0.2930358	1.2700000	1.9800000

Laboratory tests at the time of obtaining Table 2-6 consent, according to the presence or absence of proton-pump inhibitors

Unique Subject Identifier=1156000000-0062

MEANS Procedure

Assay variable : PTINR_VAL1 PT-INR Value

N	Mean	Standard Deviation	Minimum	Maximum
6	1.3416667	0.0584523	1.2500000	1.4000000

Unique Subject Identifier=1157000000-0002

Assay variable : PTINR_VAL1 PT-INR Value

N	Mean	Standard Deviation	Minimum	Maximum
6	2.5000000	0.5099020	1.7000000	3.1000000

Unique Subject Identifier=1157000000-0003

Assay variable : PTINR_VAL1 PT-INR Value

N	Mean	Standard Deviation	Minimum	Maximum
6	2.1333333	0.4760952	1.5000000	2.9000000

Unique Subject Identifier=1157000000-0006

Assay variable : PTINR_VAL1 PT-INR Value

N	Mean	Standard Deviation	Minimum	Maximum
6	2.0016667	0.4574021	1.5000000	2.7000000

Laboratory tests at the time of obtaining Table 2-6 consent, according to the presence or absence of proton-pump inhibitors

Unique Subject Identifier=1157000000-0011

MEANS Procedure

Assay variable : PTINR_VAL1 PT-INR Value

N	Mean	Standard Deviation	Minimum	Maximum
6	1.8666667	0.2732520	1.7000000	2.4000000

Unique Subject Identifier=1157000000-0013

Assay variable : PTINR_VAL1 PT-INR Value

N	Mean	Standard Deviation	Minimum	Maximum
4	2.3000000	0.4546061	1.8000000	2.9000000

Unique Subject Identifier=1157000000-0014

Assay variable : PTINR_VAL1 PT-INR Value

N	Mean	Standard Deviation	Minimum	Maximum
6	2.2783333	0.4660651	1.8000000	3.0000000

Unique Subject Identifier=1157000000-0028

Assay variable : PTINR_VAL1 PT-INR Value

N	Mean	Standard Deviation	Minimum	Maximum
6	1.6500000	0.2509980	1.5000000	2.1000000

Laboratory tests at the time of obtaining Table 2-6 consent, according to the presence or absence of proton-pump inhibitors

Unique Subject Identifier=1157000000-0029

MEANS Procedure

Assay variable : PTINR_VAL1 PT-INR Value

N	Mean	Standard Deviation	Minimum	Maximum
6	2.1000000	0.4560702	1.5000000	2.8000000

Unique Subject Identifier=1157000000-0033

Assay variable : PTINR_VAL1 PT-INR Value

N	Mean	Standard Deviation	Minimum	Maximum
6	1.8666667	0.3076795	1.6000000	2.4000000

Unique Subject Identifier=1157000000-0034

Assay variable : PTINR_VAL1 PT-INR Value

N	Mean	Standard Deviation	Minimum	Maximum
6	2.1333333	0.1751190	1.9000000	2.4000000

Unique Subject Identifier=1158010000-0010

Assay variable : PTINR_VAL1 PT-INR Value

N	Mean	Standard Deviation	Minimum	Maximum
6	2.1350000	0.4554887	1.6400000	2.9800000

Laboratory tests at the time of obtaining Table 2-6 consent, according to the presence or absence of proton-pump inhibitors

Unique Subject Identifier=1158010000-0015

MEANS Procedure

Assay variable : PTINR_VAL1 PT-INR Value

N	Mean	Standard Deviation	Minimum	Maximum
6	2.4800000	0.4456905	1.8800000	2.8700000

Unique Subject Identifier=1158010000-0022

Assay variable : PTINR_VAL1 PT-INR Value

N	Mean	Standard Deviation	Minimum	Maximum
6	1.9133333	0.1423610	1.7700000	2.1300000

Unique Subject Identifier=1158010000-0023

Assay variable : PTINR_VAL1 PT-INR Value

N	Mean	Standard Deviation	Minimum	Maximum
6	1.8900000	0.4136665	1.6000000	2.7100000

Unique Subject Identifier=1158010000-0026

Assay variable : PTINR_VAL1 PT-INR Value

N	Mean	Standard Deviation	Minimum	Maximum
6	2.0000000	0.3034469	1.4700000	2.3300000

Laboratory tests at the time of obtaining Table 2-6 consent, according to the presence or absence of proton-pump inhibitors

Unique Subject Identifier=1158010000-0027

MEANS Procedure

Assay variable : PTINR_VAL1 PT-INR Value

N	Mean	Standard Deviation	Minimum	Maximum
6	1.8600000	0.3269251	1.4300000	2.2500000

Unique Subject Identifier=1158010000-0031

Assay variable : PTINR_VAL1 PT-INR Value

N	Mean	Standard Deviation	Minimum	Maximum
6	2.3666667	0.3695763	1.9000000	3.0100000

Unique Subject Identifier=1158010000-0035

Assay variable : PTINR_VAL1 PT-INR Value

N	Mean	Standard Deviation	Minimum	Maximum
6	2.3366667	0.5368302	1.6800000	3.2300000

Unique Subject Identifier=1158010000-0042

Assay variable : PTINR_VAL1 PT-INR Value

N	Mean	Standard Deviation	Minimum	Maximum
6	2.0683333	0.3535770	1.6400000	2.5000000

Laboratory tests at the time of obtaining Table 2-6 consent, according to the presence or absence of proton-pump inhibitors

Unique Subject Identifier=1158010000-0043

MEANS Procedure

Assay variable : PTINR_VAL1 PT-INR Value

N	Mean	Standard Deviation	Minimum	Maximum
6	2.3216667	0.2634704	1.8300000	2.6300000

Unique Subject Identifier=1158010000-0045

Assay variable : PTINR_VAL1 PT-INR Value

N	Mean	Standard Deviation	Minimum	Maximum
6	1.9533333	0.5908525	1.4700000	3.1200000

Unique Subject Identifier=1158010000-0055

Assay variable : PTINR_VAL1 PT-INR Value

N	Mean	Standard Deviation	Minimum	Maximum
6	2.1183333	0.1340771	1.9900000	2.3500000

Unique Subject Identifier=1158010000-0058

Assay variable : PTINR_VAL1 PT-INR Value

N	Mean	Standard Deviation	Minimum	Maximum
6	1.9850000	0.1488288	1.8800000	2.2800000

Laboratory tests at the time of obtaining Table 2-6 consent, according to the presence or absence of proton-pump inhibitors

Unique Subject Identifier=1158010000-0065

MEANS Procedure

Assay variable : PTINR_VAL1 PT-INR Value

N	Mean	Standard Deviation	Minimum	Maximum
6	2.0816667	0.3621832	1.4700000	2.4500000

Unique Subject Identifier=1158010000-0067

Assay variable : PTINR_VAL1 PT-INR Value

N	Mean	Standard Deviation	Minimum	Maximum
6	2.2433333	0.6172412	1.4900000	3.2600000

Unique Subject Identifier=1158010000-0071

Assay variable : PTINR_VAL1 PT-INR Value

N	Mean	Standard Deviation	Minimum	Maximum
6	2.4450000	0.6404295	1.4800000	3.2000000

Unique Subject Identifier=1158010000-0076

Assay variable : PTINR_VAL1 PT-INR Value

N	Mean	Standard Deviation	Minimum	Maximum
6	2.0983333	0.4266810	1.6000000	2.6300000

Laboratory tests at the time of obtaining Table 2-6 consent, according to the presence or absence of proton-pump inhibitors

Unique Subject Identifier=1158010000-0080

MEANS Procedure

Assay variable : PTINR_VAL1 PT-INR Value

N	Mean	Standard Deviation	Minimum	Maximum
6	2.0250000	0.0875785	1.9000000	2.1600000

Unique Subject Identifier=1158010000-0082

Assay variable : PTINR_VAL1 PT-INR Value

N	Mean	Standard Deviation	Minimum	Maximum
6	2.4200000	0.3996498	1.8100000	2.9800000

Unique Subject Identifier=1158010000-0093

Assay variable : PTINR_VAL1 PT-INR Value

N	Mean	Standard Deviation	Minimum	Maximum
6	2.0983333	0.4264935	1.5700000	2.8400000

Unique Subject Identifier=1158010000-0095

Assay variable : PTINR_VAL1 PT-INR Value

N	Mean	Standard Deviation	Minimum	Maximum
6	2.2450000	0.2022622	1.9700000	2.4800000

Laboratory tests at the time of obtaining Table 2-6 consent, according to the presence or absence of proton-pump inhibitors

Unique Subject Identifier=1158010000-0104

MEANS Procedure

Assay variable : PTINR_VAL1 PT-INR Value

N	Mean	Standard Deviation	Minimum	Maximum
6	1.8666667	0.3042148	1.4700000	2.3800000

Unique Subject Identifier=1158010000-0105

Assay variable : PTINR_VAL1 PT-INR Value

N	Mean	Standard Deviation	Minimum	Maximum
6	2.0800000	0.3533836	1.5500000	2.5300000

Unique Subject Identifier=1158010000-0106

Assay variable : PTINR_VAL1 PT-INR Value

N	Mean	Standard Deviation	Minimum	Maximum
6	2.2600000	0.4376300	1.7700000	3.0100000

Unique Subject Identifier=1158010000-0114

Assay variable : PTINR_VAL1 PT-INR Value

N	Mean	Standard Deviation	Minimum	Maximum
6	2.3900000	0.3985975	1.7700000	2.9800000

Laboratory tests at the time of obtaining Table 2-6 consent, according to the presence or absence of proton-pump inhibitors

Unique Subject Identifier=1158010000-0117

MEANS Procedure

Assay variable : PTINR_VAL1 PT-INR Value

N	Mean	Standard Deviation	Minimum	Maximum
6	2.0533333	0.3923094	1.6400000	2.7100000

Unique Subject Identifier=1158010000-0124

Assay variable : PTINR_VAL1 PT-INR Value

N	Mean	Standard Deviation	Minimum	Maximum
6	1.9900000	0.7699610	1.1200000	3.2900000

Unique Subject Identifier=1158010000-0125

Assay variable : PTINR_VAL1 PT-INR Value

N	Mean	Standard Deviation	Minimum	Maximum
6	2.2600000	0.9979780	1.6400000	4.2600000

Unique Subject Identifier=1158010000-0126

Assay variable : PTINR_VAL1 PT-INR Value

N	Mean	Standard Deviation	Minimum	Maximum
6	2.1750000	0.4345457	1.4700000	2.6600000

Laboratory tests at the time of obtaining Table 2-6 consent, according to the presence or absence of proton-pump inhibitors

Unique Subject Identifier=1159000000-0001

MEANS Procedure

Assay variable : PTINR_VAL1 PT-INR Value

N	Mean	Standard Deviation	Minimum	Maximum
6	2.1000000	0.1897367	1.8000000	2.3000000

Unique Subject Identifier=1159000000-0002

Assay variable : PTINR_VAL1 PT-INR Value

N	Mean	Standard Deviation	Minimum	Maximum
6	2.2666667	0.1966384	1.9000000	2.4000000

Unique Subject Identifier=1159000000-0003

Assay variable : PTINR_VAL1 PT-INR Value

N	Mean	Standard Deviation	Minimum	Maximum
6	2.0500000	0.6348228	1.1000000	2.8000000

Unique Subject Identifier=1159000000-0005

Assay variable : PTINR_VAL1 PT-INR Value

N	Mean	Standard Deviation	Minimum	Maximum
6	2.3666667	0.6121002	1.5000000	3.1000000

Laboratory tests at the time of obtaining Table 2-6 consent, according to the presence or absence of proton-pump inhibitors

Unique Subject Identifier=1159000000-0007

MEANS Procedure

Assay variable : PTINR_VAL1 PT-INR Value

N	Mean	Standard Deviation	Minimum	Maximum
6	2.3000000	0.5549775	1.8000000	3.1000000

Unique Subject Identifier=1159000000-0010

Assay variable : PTINR_VAL1 PT-INR Value

N	Mean	Standard Deviation	Minimum	Maximum
5	2.1600000	0.4037326	1.8000000	2.8000000

Unique Subject Identifier=1159000000-0013

Assay variable : PTINR_VAL1 PT-INR Value

N	Mean	Standard Deviation	Minimum	Maximum
6	2.4833333	0.4956477	1.5000000	2.9000000

Unique Subject Identifier=1159000000-0015

Assay variable : PTINR_VAL1 PT-INR Value

N	Mean	Standard Deviation	Minimum	Maximum
6	2.3000000	0.2898275	1.9000000	2.7000000

Laboratory tests at the time of obtaining Table 2-6 consent, according to the presence or absence of proton-pump inhibitors

Unique Subject Identifier=1159000000-0016

MEANS Procedure

Assay variable : PTINR_VAL1 PT-INR Value

N	Mean	Standard Deviation	Minimum	Maximum
6	2.1500000	0.6220932	1.3000000	3.1000000

Unique Subject Identifier=1159000000-0017

Assay variable : PTINR_VAL1 PT-INR Value

N	Mean	Standard Deviation	Minimum	Maximum
6	2.2333333	0.4033196	1.8000000	2.9000000

Unique Subject Identifier=1159000000-0018

Assay variable : PTINR_VAL1 PT-INR Value

N	Mean	Standard Deviation	Minimum	Maximum
6	1.9333333	0.4718757	1.5000000	2.8000000

Unique Subject Identifier=1159000000-0019

Assay variable : PTINR_VAL1 PT-INR Value

N	Mean	Standard Deviation	Minimum	Maximum
6	2.0000000	0.2097618	1.8000000	2.3000000

Laboratory tests at the time of obtaining Table 2-6 consent, according to the presence or absence of proton-pump inhibitors

Unique Subject Identifier=1160000000-0005

MEANS Procedure

Assay variable : PTINR_VAL1 PT-INR Value

N	Mean	Standard Deviation	Minimum	Maximum
4	2.4125000	1.2308906	1.2400000	4.1300000

Unique Subject Identifier=1160000000-0035

Assay variable : PTINR_VAL1 PT-INR Value

N	Mean	Standard Deviation	Minimum	Maximum
5	1.9560000	0.2402707	1.6800000	2.2100000

Unique Subject Identifier=1160000000-0055

Assay variable : PTINR_VAL1 PT-INR Value

N	Mean	Standard Deviation	Minimum	Maximum
4	2.4800000	1.0526158	1.7000000	3.9400000

Unique Subject Identifier=1160000000-0057

Assay variable : PTINR_VAL1 PT-INR Value

N	Mean	Standard Deviation	Minimum	Maximum
5	1.8660000	0.2176695	1.6600000	2.2100000

Laboratory tests at the time of obtaining Table 2-6 consent, according to the presence or absence of proton-pump inhibitors

Unique Subject Identifier=1160000000-0094

MEANS Procedure

Assay variable : PTINR_VAL1 PT-INR Value

N	Mean	Standard Deviation	Minimum	Maximum
2	1.8650000	0.0777817	1.8100000	1.9200000

Unique Subject Identifier=1160000000-0104

Assay variable : PTINR_VAL1 PT-INR Value

N	Mean	Standard Deviation	Minimum	Maximum
4	2.1450000	0.2487971	1.8800000	2.4800000

Unique Subject Identifier=1160000000-0112

Assay variable : PTINR_VAL1 PT-INR Value

N	Mean	Standard Deviation	Minimum	Maximum
5	1.9240000	0.2338376	1.7200000	2.2500000

Unique Subject Identifier=1160000000-0118

Assay variable : PTINR_VAL1 PT-INR Value

N	Mean	Standard Deviation	Minimum	Maximum
5	1.8360000	0.7326527	1.2700000	3.0900000

Laboratory tests at the time of obtaining Table 2-6 consent, according to the presence or absence of proton-pump inhibitors

Unique Subject Identifier=1160000000-0120

MEANS Procedure

Assay variable : PTINR_VAL1 PT-INR Value

N	Mean	Standard Deviation	Minimum	Maximum
6	2.7600000	0.4822448	2.1300000	3.4900000

Unique Subject Identifier=1161010100-0001

Assay variable : PTINR_VAL1 PT-INR Value

N	Mean	Standard Deviation	Minimum	Maximum
1	2.4900000		2.4900000	2.4900000

Unique Subject Identifier=1161010100-0006

Assay variable : PTINR_VAL1 PT-INR Value

N	Mean	Standard Deviation	Minimum	Maximum
3	1.6566667	0.0321455	1.6200000	1.6800000

Unique Subject Identifier=1161010100-0011

Assay variable : PTINR_VAL1 PT-INR Value

N	Mean	Standard Deviation	Minimum	Maximum
2	1.8250000	0.0070711	1.8200000	1.8300000

Laboratory tests at the time of obtaining Table 2-6 consent, according to the presence or absence of proton-pump inhibitors

Unique Subject Identifier=1161010100-0017

MEANS Procedure

Assay variable : PTINR_VAL1 PT-INR Value

N	Mean	Standard Deviation	Minimum	Maximum
1	2.1900000		2.1900000	2.1900000

Unique Subject Identifier=1161010100-0022

Assay variable : PTINR_VAL1 PT-INR Value

N	Mean	Standard Deviation	Minimum	Maximum
1	2.0500000		2.0500000	2.0500000

Unique Subject Identifier=1161010100-0025

Assay variable : PTINR_VAL1 PT-INR Value

N	Mean	Standard Deviation	Minimum	Maximum
2	2.3350000	0.8980256	1.7000000	2.9700000

Unique Subject Identifier=1162011100-0003

Assay variable : PTINR_VAL1 PT-INR Value

N	Mean	Standard Deviation	Minimum	Maximum
4	1.9900000	0.1618641	1.7700000	2.1600000

Laboratory tests at the time of obtaining Table 2-6 consent, according to the presence or absence of proton-pump inhibitors

Unique Subject Identifier=1167000000-0010

MEANS Procedure

Assay variable : PTINR_VAL1 PT-INR Value

N	Mean	Standard Deviation	Minimum	Maximum
1	2.2100000		2.2100000	2.2100000

Unique Subject Identifier=1168011000-0006

Assay variable : PTINR_VAL1 PT-INR Value

N	Mean	Standard Deviation	Minimum	Maximum
3	2.3966667	0.1674316	2.3000000	2.5900000

Unique Subject Identifier=1168011000-0011

Assay variable : PTINR_VAL1 PT-INR Value

N	Mean	Standard Deviation	Minimum	Maximum
3	2.1066667	0.1934770	1.9900000	2.3300000

Unique Subject Identifier=1168011000-0021

Assay variable : PTINR_VAL1 PT-INR Value

N	Mean	Standard Deviation	Minimum	Maximum
2	2.2450000	0.0777817	2.1900000	2.3000000

Laboratory tests at the time of obtaining Table 2-6 consent, according to the presence or absence of proton-pump inhibitors

Unique Subject Identifier=1168011000-0026

MEANS Procedure

Assay variable : PTINR_VAL1 PT-INR Value

N	Mean	Standard Deviation	Minimum	Maximum
1	1.6400000		1.6400000	1.6400000

Unique Subject Identifier=1168011000-0028

Assay variable : PTINR_VAL1 PT-INR Value

N	Mean	Standard Deviation	Minimum	Maximum
1	1.6000000		1.6000000	1.6000000

Unique Subject Identifier=1168011000-0039

Assay variable : PTINR_VAL1 PT-INR Value

N	Mean	Standard Deviation	Minimum	Maximum
2	2.5300000	0.1272792	2.4400000	2.6200000

Unique Subject Identifier=1168011000-0040

Assay variable : PTINR_VAL1 PT-INR Value

N	Mean	Standard Deviation	Minimum	Maximum
2	2.6600000	0.3818377	2.3900000	2.9300000

Laboratory tests at the time of obtaining Table 2-6 consent, according to the presence or absence of proton-pump inhibitors

Unique Subject Identifier=1168011000-0050

MEANS Procedure

Assay variable : PTINR_VAL1 PT-INR Value

N	Mean	Standard Deviation	Minimum	Maximum
2	1.4200000	0.0424264	1.3900000	1.4500000

Unique Subject Identifier=1169010100-0013

Assay variable : PTINR_VAL1 PT-INR Value

N	Mean	Standard Deviation	Minimum	Maximum
5	2.4200000	0.2774887	2.1000000	2.8000000

Unique Subject Identifier=1171000000-0003

Assay variable : PTINR_VAL1 PT-INR Value

N	Mean	Standard Deviation	Minimum	Maximum
5	1.6760000	0.0391152	1.6400000	1.7300000

Unique Subject Identifier=1171000000-0004

Assay variable : PTINR_VAL1 PT-INR Value

N	Mean	Standard Deviation	Minimum	Maximum
6	1.3383333	0.0617792	1.2900000	1.4400000

Laboratory tests at the time of obtaining Table 2-6 consent, according to the presence or absence of proton-pump inhibitors

Unique Subject Identifier=1172011000-0005

MEANS Procedure

Assay variable : PTINR_VAL1 PT-INR Value

N	Mean	Standard Deviation	Minimum	Maximum
6	2.1166667	0.1169045	2.0000000	2.3000000

Unique Subject Identifier=1172011000-0007

Assay variable : PTINR_VAL1 PT-INR Value

N	Mean	Standard Deviation	Minimum	Maximum
6	1.6833333	0.1329160	1.5000000	1.9000000

Unique Subject Identifier=1173011100-0002

Assay variable : PTINR_VAL1 PT-INR Value

N	Mean	Standard Deviation	Minimum	Maximum
6	1.7833333	0.1329160	1.6000000	2.0000000

Unique Subject Identifier=1173011100-0011

Assay variable : PTINR_VAL1 PT-INR Value

N	Mean	Standard Deviation	Minimum	Maximum
6	1.9000000	0.3033150	1.4000000	2.2000000

Laboratory tests at the time of obtaining Table 2-6 consent, according to the presence or absence of proton-pump inhibitors

Unique Subject Identifier=1173011100-0015

MEANS Procedure

Assay variable : PTINR_VAL1 PT-INR Value

N	Mean	Standard Deviation	Minimum	Maximum
6	1.6666667	0.3265986	1.3000000	2.2000000

Unique Subject Identifier=1173011100-0018

Assay variable : PTINR_VAL1 PT-INR Value

N	Mean	Standard Deviation	Minimum	Maximum
6	2.2833333	0.2316607	1.9000000	2.6000000

Unique Subject Identifier=1173011100-0019

Assay variable : PTINR_VAL1 PT-INR Value

N	Mean	Standard Deviation	Minimum	Maximum
6	2.3500000	1.0445095	1.1000000	3.6000000

Unique Subject Identifier=1173011100-0021

Assay variable : PTINR_VAL1 PT-INR Value

N	Mean	Standard Deviation	Minimum	Maximum
6	2.5000000	0.8221922	1.7000000	3.8000000

Laboratory tests at the time of obtaining Table 2-6 consent, according to the presence or absence of proton-pump inhibitors

Unique Subject Identifier=1173011100-0026

MEANS Procedure

Assay variable : PTINR_VAL1 PT-INR Value

N	Mean	Standard Deviation	Minimum	Maximum
6	2.2500000	1.0634848	1.5000000	4.3000000

Unique Subject Identifier=1173011100-0027

Assay variable : PTINR_VAL1 PT-INR Value

N	Mean	Standard Deviation	Minimum	Maximum
6	2.1666667	0.7174027	1.6000000	3.6000000

Unique Subject Identifier=1173011100-0031

Assay variable : PTINR_VAL1 PT-INR Value

N	Mean	Standard Deviation	Minimum	Maximum
6	1.6833333	0.4445972	1.3000000	2.3000000

Unique Subject Identifier=1173011100-0041

Assay variable : PTINR_VAL1 PT-INR Value

N	Mean	Standard Deviation	Minimum	Maximum
5	1.6800000	0.3346640	1.4000000	2.2000000

Laboratory tests at the time of obtaining Table 2-6 consent, according to the presence or absence of proton-pump inhibitors

Unique Subject Identifier=1175011000-0002

MEANS Procedure

Assay variable : PTINR_VAL1 PT-INR Value

N	Mean	Standard Deviation	Minimum	Maximum
6	1.7333333	0.0516398	1.7000000	1.8000000

Unique Subject Identifier=1175011000-0004

Assay variable : PTINR_VAL1 PT-INR Value

N	Mean	Standard Deviation	Minimum	Maximum
6	1.7833333	0.2316607	1.5000000	2.1000000

Unique Subject Identifier=1175011000-0005

Assay variable : PTINR_VAL1 PT-INR Value

N	Mean	Standard Deviation	Minimum	Maximum
6	2.3666667	2.7645373	1.0000000	8.0000000

Unique Subject Identifier=1175011000-0008

Assay variable : PTINR_VAL1 PT-INR Value

N	Mean	Standard Deviation	Minimum	Maximum
6	2.0166667	0.2041241	1.7000000	2.3000000

Laboratory tests at the time of obtaining Table 2-6 consent, according to the presence or absence of proton-pump inhibitors

Unique Subject Identifier=1175011000-0009

MEANS Procedure

Assay variable : PTINR_VAL1 PT-INR Value

N	Mean	Standard Deviation	Minimum	Maximum
6	2.0000000	0.2756810	1.6000000	2.4000000

Unique Subject Identifier=1175011000-0010

Assay variable : PTINR_VAL1 PT-INR Value

N	Mean	Standard Deviation	Minimum	Maximum
6	1.8500000	0.2428992	1.5000000	2.1000000

Unique Subject Identifier=1175011000-0011

Assay variable : PTINR_VAL1 PT-INR Value

N	Mean	Standard Deviation	Minimum	Maximum
6	2.0666667	0.4885352	1.6000000	3.0000000

Unique Subject Identifier=1175011000-0016

Assay variable : PTINR_VAL1 PT-INR Value

N	Mean	Standard Deviation	Minimum	Maximum
6	1.7666667	0.1366260	1.6000000	2.0000000

Laboratory tests at the time of obtaining Table 2-6 consent, according to the presence or absence of proton-pump inhibitors

Unique Subject Identifier=1175011000-0018

MEANS Procedure

Assay variable : PTINR_VAL1 PT-INR Value

N	Mean	Standard Deviation	Minimum	Maximum
6	2.3500000	0.6090977	1.5000000	3.3000000

Unique Subject Identifier=1175011000-0019

Assay variable : PTINR_VAL1 PT-INR Value

N	Mean	Standard Deviation	Minimum	Maximum
6	2.0500000	0.4764452	1.5000000	2.6000000

Unique Subject Identifier=1179000000-0001

Assay variable : PTINR_VAL1 PT-INR Value

N	Mean	Standard Deviation	Minimum	Maximum
6	1.7000000	0.2280351	1.3000000	1.9000000

Unique Subject Identifier=1179000000-0002

Assay variable : PTINR_VAL1 PT-INR Value

N	Mean	Standard Deviation	Minimum	Maximum
3	1.8333333	0.3511885	1.5000000	2.2000000

Laboratory tests at the time of obtaining Table 2-6 consent, according to the presence or absence of proton-pump inhibitors

Unique Subject Identifier=1179000000-0003

MEANS Procedure

Assay variable : PTINR_VAL1 PT-INR Value

N	Mean	Standard Deviation	Minimum	Maximum
6	1.9500000	1.0310189	1.1000000	4.0000000

Unique Subject Identifier=1179000000-0004

Assay variable : PTINR_VAL1 PT-INR Value

N	Mean	Standard Deviation	Minimum	Maximum
3	2.0333333	0.2081666	1.8000000	2.2000000

Unique Subject Identifier=1179000000-0006

Assay variable : PTINR_VAL1 PT-INR Value

N	Mean	Standard Deviation	Minimum	Maximum
3	2.2333333	0.3214550	2.0000000	2.6000000

Unique Subject Identifier=1181000000-0003

Assay variable : PTINR_VAL1 PT-INR Value

N	Mean	Standard Deviation	Minimum	Maximum
1	2.0300000		2.0300000	2.0300000

Laboratory tests at the time of obtaining Table 2-6 consent, according to the presence or absence of proton-pump inhibitors

Unique Subject Identifier=1181000000-0004

MEANS Procedure

Assay variable : PTINR_VAL1 PT-INR Value

N	Mean	Standard Deviation	Minimum	Maximum
1	2.3900000		2.3900000	2.3900000

Unique Subject Identifier=1181000000-0005

Assay variable : PTINR_VAL1 PT-INR Value

N	Mean	Standard Deviation	Minimum	Maximum
2	2.7950000	0.3889087	2.5200000	3.0700000

Unique Subject Identifier=1181000000-0006

Assay variable : PTINR_VAL1 PT-INR Value

N	Mean	Standard Deviation	Minimum	Maximum
2	2.1000000	0.3676955	1.8400000	2.3600000

Unique Subject Identifier=1181000000-0007

Assay variable : PTINR_VAL1 PT-INR Value

N	Mean	Standard Deviation	Minimum	Maximum
3	1.6866667	0.3360556	1.3200000	1.9800000

Laboratory tests at the time of obtaining Table 2-6 consent, according to the presence or absence of proton-pump inhibitors

Unique Subject Identifier=1181000000-0008

MEANS Procedure

Assay variable : PTINR_VAL1 PT-INR Value

N	Mean	Standard Deviation	Minimum	Maximum
2	1.4650000	0.2050610	1.3200000	1.6100000

Unique Subject Identifier=1181000000-0009

Assay variable : PTINR_VAL1 PT-INR Value

N	Mean	Standard Deviation	Minimum	Maximum
1	3.5700000		3.5700000	3.5700000

Unique Subject Identifier=1181000000-0010

Assay variable : PTINR_VAL1 PT-INR Value

N	Mean	Standard Deviation	Minimum	Maximum
2	1.7950000	0.4030509	1.5100000	2.0800000

Unique Subject Identifier=1181000000-0011

Assay variable : PTINR_VAL1 PT-INR Value

N	Mean	Standard Deviation	Minimum	Maximum
2	1.3500000	0.0282843	1.3300000	1.3700000

Laboratory tests at the time of obtaining Table 2-6 consent, according to the presence or absence of proton-pump inhibitors

Unique Subject Identifier=1181000000-0012

MEANS Procedure

Assay variable : PTINR_VAL1 PT-INR Value

N	Mean	Standard Deviation	Minimum	Maximum
1	1.6200000		1.6200000	1.6200000

Unique Subject Identifier=1181000000-0013

Assay variable : PTINR_VAL1 PT-INR Value

N	Mean	Standard Deviation	Minimum	Maximum
3	1.6600000	0.0754983	1.5900000	1.7400000

Unique Subject Identifier=1181000000-0014

Assay variable : PTINR_VAL1 PT-INR Value

N	Mean	Standard Deviation	Minimum	Maximum
3	2.1600000	0.4396590	1.8000000	2.6500000

Unique Subject Identifier=1181000000-0018

Assay variable : PTINR_VAL1 PT-INR Value

N	Mean	Standard Deviation	Minimum	Maximum
2	2.5450000	0.2192031	2.3900000	2.7000000

Laboratory tests at the time of obtaining Table 2-6 consent, according to the presence or absence of proton-pump inhibitors

Unique Subject Identifier=1181000000-0019

MEANS Procedure

Assay variable : PTINR_VAL1 PT-INR Value

N	Mean	Standard Deviation	Minimum	Maximum
1	1.7200000		1.7200000	1.7200000

Unique Subject Identifier=1182000000-0001

Assay variable : PTINR_VAL1 PT-INR Value

N	Mean	Standard Deviation	Minimum	Maximum
4	1.4750000	0.3403430	1.2000000	1.9000000

Unique Subject Identifier=1182000000-0002

Assay variable : PTINR_VAL1 PT-INR Value

N	Mean	Standard Deviation	Minimum	Maximum
6	2.0000000	0.3741657	1.6000000	2.6000000

Unique Subject Identifier=1186000000-0008

Assay variable : PTINR_VAL1 PT-INR Value

N	Mean	Standard Deviation	Minimum	Maximum
4	2.2750000	0.9535023	1.7000000	3.7000000

Laboratory tests at the time of obtaining Table 2-6 consent, according to the presence or absence of proton-pump inhibitors

Unique Subject Identifier=1188011110-0027

MEANS Procedure

Assay variable : PTINR_VAL1 PT-INR Value

N	Mean	Standard Deviation	Minimum	Maximum
5	2.0420000	0.1953714	1.8100000	2.2700000

Unique Subject Identifier=1188011110-0068

Assay variable : PTINR_VAL1 PT-INR Value

N	Mean	Standard Deviation	Minimum	Maximum
3	2.1433333	0.0971253	2.0600000	2.2500000

Unique Subject Identifier=1188011110-0124

Assay variable : PTINR_VAL1 PT-INR Value

N	Mean	Standard Deviation	Minimum	Maximum
4	1.5925000	0.1569235	1.4200000	1.8000000

Unique Subject Identifier=1189001100-0001

Assay variable : PTINR_VAL1 PT-INR Value

N	Mean	Standard Deviation	Minimum	Maximum
2	1.3950000	0.0636396	1.3500000	1.4400000

Laboratory tests at the time of obtaining Table 2-6 consent, according to the presence or absence of proton-pump inhibitors

Unique Subject Identifier=1189001100-0008

MEANS Procedure

Assay variable : PTINR_VAL1 PT-INR Value

N	Mean	Standard Deviation	Minimum	Maximum
4	3.1475000	0.8403323	1.9500000	3.8500000

Unique Subject Identifier=1189001100-0017

Assay variable : PTINR_VAL1 PT-INR Value

N	Mean	Standard Deviation	Minimum	Maximum
1	1.8800000		1.8800000	1.8800000

Unique Subject Identifier=1191000000-0001

Assay variable : PTINR_VAL1 PT-INR Value

N	Mean	Standard Deviation	Minimum	Maximum
6	1.4550000	0.1266096	1.3100000	1.6200000

Unique Subject Identifier=1194000000-0008

Assay variable : PTINR_VAL1 PT-INR Value

N	Mean	Standard Deviation	Minimum	Maximum
3	2.3300000	0.4942671	1.9000000	2.8700000

Laboratory tests at the time of obtaining Table 2-6 consent, according to the presence or absence of proton-pump inhibitors

Unique Subject Identifier=1194000000-0014

MEANS Procedure

Assay variable : PTINR_VAL1 PT-INR Value

N	Mean	Standard Deviation	Minimum	Maximum
2	1.8650000	0.1484924	1.7600000	1.9700000

Unique Subject Identifier=1195000000-0006

Assay variable : PTINR_VAL1 PT-INR Value

N	Mean	Standard Deviation	Minimum	Maximum
4	1.6700000	0.3436083	1.4300000	2.1700000

Unique Subject Identifier=1195000000-0007

Assay variable : PTINR_VAL1 PT-INR Value

N	Mean	Standard Deviation	Minimum	Maximum
5	1.4120000	0.1223520	1.2400000	1.5500000

Unique Subject Identifier=1195000000-0008

Assay variable : PTINR_VAL1 PT-INR Value

N	Mean	Standard Deviation	Minimum	Maximum
2	1.8800000	0.1414214	1.7800000	1.9800000

Laboratory tests at the time of obtaining Table 2-6 consent, according to the presence or absence of proton-pump inhibitors

Unique Subject Identifier=1195000000-0009

MEANS Procedure

Assay variable : PTINR_VAL1 PT-INR Value

N	Mean	Standard Deviation	Minimum	Maximum
4	2.1350000	0.4702836	1.8800000	2.8400000

Unique Subject Identifier=1195000000-0010

Assay variable : PTINR_VAL1 PT-INR Value

N	Mean	Standard Deviation	Minimum	Maximum
6	1.8816667	0.2770138	1.4300000	2.1500000

Unique Subject Identifier=1195000000-0011

Assay variable : PTINR_VAL1 PT-INR Value

N	Mean	Standard Deviation	Minimum	Maximum
5	1.9100000	0.2876630	1.5000000	2.2600000

Unique Subject Identifier=1195000000-0012

Assay variable : PTINR_VAL1 PT-INR Value

N	Mean	Standard Deviation	Minimum	Maximum
4	1.6900000	0.1166190	1.5900000	1.8100000

Laboratory tests at the time of obtaining Table 2-6 consent, according to the presence or absence of proton-pump inhibitors

Unique Subject Identifier=1195000000-0013

MEANS Procedure

Assay variable : PTINR_VAL1 PT-INR Value

N	Mean	Standard Deviation	Minimum	Maximum
4	1.3575000	0.1613227	1.1200000	1.4800000

Unique Subject Identifier=1195000000-0014

Assay variable : PTINR_VAL1 PT-INR Value

N	Mean	Standard Deviation	Minimum	Maximum
4	1.9225000	0.6399154	1.3400000	2.8300000

Unique Subject Identifier=1195000000-0015

Assay variable : PTINR_VAL1 PT-INR Value

N	Mean	Standard Deviation	Minimum	Maximum
4	1.2400000	0.0905539	1.1400000	1.3600000

Unique Subject Identifier=1195000000-0016

Assay variable : PTINR_VAL1 PT-INR Value

N	Mean	Standard Deviation	Minimum	Maximum
4	2.4325000	0.2061351	2.2300000	2.6200000

Laboratory tests at the time of obtaining Table 2-6 consent, according to the presence or absence of proton-pump inhibitors

Unique Subject Identifier=1195000000-0017

MEANS Procedure

Assay variable : PTINR_VAL1 PT-INR Value

N	Mean	Standard Deviation	Minimum	Maximum
4	1.8775000	0.1164403	1.7300000	2.0100000

Unique Subject Identifier=1195000000-0018

Assay variable : PTINR_VAL1 PT-INR Value

N	Mean	Standard Deviation	Minimum	Maximum
5	1.3260000	0.1223928	1.1600000	1.4200000

Unique Subject Identifier=1195000000-0019

Assay variable : PTINR_VAL1 PT-INR Value

N	Mean	Standard Deviation	Minimum	Maximum
5	1.4920000	0.0947101	1.3400000	1.6000000

Unique Subject Identifier=1195000000-0020

Assay variable : PTINR_VAL1 PT-INR Value

N	Mean	Standard Deviation	Minimum	Maximum
4	1.9200000	0.0621825	1.8700000	2.0100000

Laboratory tests at the time of obtaining Table 2-6 consent, according to the presence or absence of proton-pump inhibitors

Unique Subject Identifier=1198000000-0003

MEANS Procedure

Assay variable : PTINR_VAL1 PT-INR Value

N	Mean	Standard Deviation	Minimum	Maximum
6	2.0616667	0.3142876	1.6500000	2.6000000

Unique Subject Identifier=1198000000-0005

Assay variable : PTINR_VAL1 PT-INR Value

N	Mean	Standard Deviation	Minimum	Maximum
6	1.8083333	0.0970395	1.6500000	1.9000000

Unique Subject Identifier=1198000000-0008

Assay variable : PTINR_VAL1 PT-INR Value

N	Mean	Standard Deviation	Minimum	Maximum
6	1.9200000	0.1963670	1.7000000	2.1600000

Unique Subject Identifier=1198000000-0009

Assay variable : PTINR_VAL1 PT-INR Value

N	Mean	Standard Deviation	Minimum	Maximum
6	1.6250000	0.1635543	1.4000000	1.8500000

Laboratory tests at the time of obtaining Table 2-6 consent, according to the presence or absence of proton-pump inhibitors

Unique Subject Identifier=1198000000-0010

MEANS Procedure

Assay variable : PTINR_VAL1 PT-INR Value

N	Mean	Standard Deviation	Minimum	Maximum
5	1.6800000	0.3213254	1.1500000	2.0000000

Unique Subject Identifier=1198000000-0011

Assay variable : PTINR_VAL1 PT-INR Value

N	Mean	Standard Deviation	Minimum	Maximum
6	2.2183333	0.1158303	2.0100000	2.3100000

Unique Subject Identifier=1198000000-0014

Assay variable : PTINR_VAL1 PT-INR Value

N	Mean	Standard Deviation	Minimum	Maximum
5	2.3660000	0.0536656	2.3000000	2.4500000

Unique Subject Identifier=1198000000-0015

Assay variable : PTINR_VAL1 PT-INR Value

N	Mean	Standard Deviation	Minimum	Maximum
6	1.5350000	0.2105944	1.1500000	1.7500000

Laboratory tests at the time of obtaining Table 2-6 consent, according to the presence or absence of proton-pump inhibitors

Unique Subject Identifier=1198000000-0017

MEANS Procedure

Assay variable : PTINR_VAL1 PT-INR Value

N	Mean	Standard Deviation	Minimum	Maximum
5	2.3000000	0.0651920	2.2100000	2.3600000

Unique Subject Identifier=1198000000-0018

Assay variable : PTINR_VAL1 PT-INR Value

N	Mean	Standard Deviation	Minimum	Maximum
6	1.7033333	0.6047038	0.4900000	2.0600000

Unique Subject Identifier=1198000000-0021

Assay variable : PTINR_VAL1 PT-INR Value

N	Mean	Standard Deviation	Minimum	Maximum
2	1.9500000	0	1.9500000	1.9500000

Unique Subject Identifier=1198000000-0023

Assay variable : PTINR_VAL1 PT-INR Value

N	Mean	Standard Deviation	Minimum	Maximum
5	2.2400000	0.0908295	2.1100000	2.3600000

Laboratory tests at the time of obtaining Table 2-6 consent, according to the presence or absence of proton-pump inhibitors

Unique Subject Identifier=1198000000-0024

MEANS Procedure

Assay variable : PTINR_VAL1 PT-INR Value

N	Mean	Standard Deviation	Minimum	Maximum
2	2.2100000	0.0707107	2.1600000	2.2600000

Unique Subject Identifier=1198000000-0026

Assay variable : PTINR_VAL1 PT-INR Value

N	Mean	Standard Deviation	Minimum	Maximum
5	1.5820000	0.3864842	1.1500000	2.0600000

Unique Subject Identifier=1198000000-0029

Assay variable : PTINR_VAL1 PT-INR Value

N	Mean	Standard Deviation	Minimum	Maximum
5	1.7740000	0.3963332	1.3500000	2.3100000

Unique Subject Identifier=1198000000-0030

Assay variable : PTINR_VAL1 PT-INR Value

N	Mean	Standard Deviation	Minimum	Maximum
5	1.8860000	0.2642537	1.5500000	2.1100000

Laboratory tests at the time of obtaining Table 2-6 consent, according to the presence or absence of proton-pump inhibitors

Unique Subject Identifier=1198000000-0031

MEANS Procedure

Assay variable : PTINR_VAL1 PT-INR Value

N	Mean	Standard Deviation	Minimum	Maximum
5	1.9940000	0.4897755	1.4000000	2.6000000

Unique Subject Identifier=1198000000-0032

Assay variable : PTINR_VAL1 PT-INR Value

N	Mean	Standard Deviation	Minimum	Maximum
5	1.6720000	0.4745208	0.8500000	2.0600000

Unique Subject Identifier=1198000000-0033

Assay variable : PTINR_VAL1 PT-INR Value

N	Mean	Standard Deviation	Minimum	Maximum
2	0.4950000	0.5020458	0.1400000	0.8500000

Unique Subject Identifier=1198000000-0034

Assay variable : PTINR_VAL1 PT-INR Value

N	Mean	Standard Deviation	Minimum	Maximum
2	1.1450000	0.9263099	0.4900000	1.8000000

Laboratory tests at the time of obtaining Table 2-6 consent, according to the presence or absence of proton-pump inhibitors

Unique Subject Identifier=1198000000-0035

MEANS Procedure

Assay variable : PTINR_VAL1 PT-INR Value

N	Mean	Standard Deviation	Minimum	Maximum
2	2.3350000	0.0353553	2.3100000	2.3600000

Unique Subject Identifier=1198000000-0039

Assay variable : PTINR_VAL1 PT-INR Value

N	Mean	Standard Deviation	Minimum	Maximum
6	1.9050000	0.1755847	1.7000000	2.1100000

Unique Subject Identifier=1198000000-0041

Assay variable : PTINR_VAL1 PT-INR Value

N	Mean	Standard Deviation	Minimum	Maximum
6	2.3350000	0.0689202	2.2100000	2.4100000

Unique Subject Identifier=1200010111-0005

Assay variable : PTINR_VAL1 PT-INR Value

N	Mean	Standard Deviation	Minimum	Maximum
4	1.5250000	0.2500000	1.4000000	1.9000000

Laboratory tests at the time of obtaining Table 2-6 consent, according to the presence or absence of proton-pump inhibitors

Unique Subject Identifier=1200010111-0007

MEANS Procedure

Assay variable : PTINR_VAL1 PT-INR Value

N	Mean	Standard Deviation	Minimum	Maximum
3	1.9000000	0.1732051	1.7000000	2.0000000

Unique Subject Identifier=1200010111-0013

Assay variable : PTINR_VAL1 PT-INR Value

N	Mean	Standard Deviation	Minimum	Maximum
3	1.8000000	0.1000000	1.7000000	1.9000000

Unique Subject Identifier=1200010111-0019

Assay variable : PTINR_VAL1 PT-INR Value

N	Mean	Standard Deviation	Minimum	Maximum
3	1.6333333	0.2081666	1.4000000	1.8000000

Unique Subject Identifier=1200010111-0020

Assay variable : PTINR_VAL1 PT-INR Value

N	Mean	Standard Deviation	Minimum	Maximum
3	1.6000000	0.2000000	1.4000000	1.8000000

Laboratory tests at the time of obtaining Table 2-6 consent, according to the presence or absence of proton-pump inhibitors

Unique Subject Identifier=1200010111-0026

MEANS Procedure

Assay variable : PTINR_VAL1 PT-INR Value

N	Mean	Standard Deviation	Minimum	Maximum
1	1.6000000		1.6000000	1.6000000

Unique Subject Identifier=1201000000-0001

Assay variable : PTINR_VAL1 PT-INR Value

N	Mean	Standard Deviation	Minimum	Maximum
4	1.7175000	0.0997914	1.6100000	1.8400000

Unique Subject Identifier=1201000000-0003

Assay variable : PTINR_VAL1 PT-INR Value

N	Mean	Standard Deviation	Minimum	Maximum
4	1.5600000	0.1540563	1.4200000	1.7600000

Unique Subject Identifier=1201000000-0004

Assay variable : PTINR_VAL1 PT-INR Value

N	Mean	Standard Deviation	Minimum	Maximum
5	1.9460000	0.3261595	1.4400000	2.3000000

Laboratory tests at the time of obtaining Table 2-6 consent, according to the presence or absence of proton-pump inhibitors

Unique Subject Identifier=1201000000-0007

MEANS Procedure

Assay variable : PTINR_VAL1 PT-INR Value

N	Mean	Standard Deviation	Minimum	Maximum
5	1.9120000	0.3495283	1.4300000	2.2400000

Unique Subject Identifier=1201000000-0008

Assay variable : PTINR_VAL1 PT-INR Value

N	Mean	Standard Deviation	Minimum	Maximum
4	2.0050000	0.3845777	1.4800000	2.3700000

Unique Subject Identifier=1201000000-0010

Assay variable : PTINR_VAL1 PT-INR Value

N	Mean	Standard Deviation	Minimum	Maximum
4	1.8475000	0.1090489	1.6900000	1.9300000

Unique Subject Identifier=1201000000-0011

Assay variable : PTINR_VAL1 PT-INR Value

N	Mean	Standard Deviation	Minimum	Maximum
4	1.7400000	0.1476482	1.6000000	1.9300000

Laboratory tests at the time of obtaining Table 2-6 consent, according to the presence or absence of proton-pump inhibitors

Unique Subject Identifier=1203010000-0001

MEANS Procedure

Assay variable : PTINR_VAL1 PT-INR Value

N	Mean	Standard Deviation	Minimum	Maximum
5	2.3280000	0.3598194	2.0300000	2.9400000

Unique Subject Identifier=1203010000-0004

Assay variable : PTINR_VAL1 PT-INR Value

N	Mean	Standard Deviation	Minimum	Maximum
6	2.3183333	0.3921947	1.8800000	3.0100000

Unique Subject Identifier=1203010000-0005

Assay variable : PTINR_VAL1 PT-INR Value

N	Mean	Standard Deviation	Minimum	Maximum
6	2.0366667	0.3356586	1.6800000	2.6100000

Unique Subject Identifier=1203010000-0006

Assay variable : PTINR_VAL1 PT-INR Value

N	Mean	Standard Deviation	Minimum	Maximum
6	2.7866667	0.2260678	2.4300000	3.0400000

Laboratory tests at the time of obtaining Table 2-6 consent, according to the presence or absence of proton-pump inhibitors

Unique Subject Identifier=1203010000-0007

MEANS Procedure

Assay variable : PTINR_VAL1 PT-INR Value

N	Mean	Standard Deviation	Minimum	Maximum
6	2.6633333	0.5982530	2.0900000	3.7300000

Unique Subject Identifier=1203010000-0009

Assay variable : PTINR_VAL1 PT-INR Value

N	Mean	Standard Deviation	Minimum	Maximum
6	2.1983333	0.7104764	1.5100000	3.4000000

Unique Subject Identifier=1203010000-0010

Assay variable : PTINR_VAL1 PT-INR Value

N	Mean	Standard Deviation	Minimum	Maximum
6	2.8366667	0.6295289	2.1100000	3.8500000

Unique Subject Identifier=1203010000-0012

Assay variable : PTINR_VAL1 PT-INR Value

N	Mean	Standard Deviation	Minimum	Maximum
6	2.4400000	1.1452161	1.0100000	4.1600000

Laboratory tests at the time of obtaining Table 2-6 consent, according to the presence or absence of proton-pump inhibitors

Unique Subject Identifier=1203010000-0013

MEANS Procedure

Assay variable : PTINR_VAL1 PT-INR Value

N	Mean	Standard Deviation	Minimum	Maximum
6	2.3300000	0.2451938	2.0200000	2.6900000

Unique Subject Identifier=1203010000-0016

Assay variable : PTINR_VAL1 PT-INR Value

N	Mean	Standard Deviation	Minimum	Maximum
6	2.3400000	0.4595215	1.6400000	3.0600000

Unique Subject Identifier=1203010000-0017

Assay variable : PTINR_VAL1 PT-INR Value

N	Mean	Standard Deviation	Minimum	Maximum
6	2.4350000	1.0120623	1.1200000	3.8800000

Unique Subject Identifier=1203010000-0018

Assay variable : PTINR_VAL1 PT-INR Value

N	Mean	Standard Deviation	Minimum	Maximum
6	2.6266667	0.4180750	2.0200000	3.3200000

Laboratory tests at the time of obtaining Table 2-6 consent, according to the presence or absence of proton-pump inhibitors

Unique Subject Identifier=1203010000-0020

MEANS Procedure

Assay variable : PTINR_VAL1 PT-INR Value

N	Mean	Standard Deviation	Minimum	Maximum
6	2.3416667	0.3456829	1.9700000	2.7900000

Unique Subject Identifier=1203010000-0022

Assay variable : PTINR_VAL1 PT-INR Value

N	Mean	Standard Deviation	Minimum	Maximum
6	2.2500000	0.2279474	1.9900000	2.6300000

Unique Subject Identifier=1203010000-0024

Assay variable : PTINR_VAL1 PT-INR Value

N	Mean	Standard Deviation	Minimum	Maximum
6	2.2000000	0.0822192	2.0600000	2.3000000

Unique Subject Identifier=1203010000-0026

Assay variable : PTINR_VAL1 PT-INR Value

N	Mean	Standard Deviation	Minimum	Maximum
6	2.0316667	0.1986370	1.7900000	2.3800000

Laboratory tests at the time of obtaining Table 2-6 consent, according to the presence or absence of proton-pump inhibitors

Unique Subject Identifier=1203010000-0028

MEANS Procedure

Assay variable : PTINR_VAL1 PT-INR Value

N	Mean	Standard Deviation	Minimum	Maximum
6	2.4366667	0.9070759	1.7500000	4.1600000

Unique Subject Identifier=1203010000-0030

Assay variable : PTINR_VAL1 PT-INR Value

N	Mean	Standard Deviation	Minimum	Maximum
6	2.0050000	0.3174114	1.6800000	2.5800000

Unique Subject Identifier=1203010000-0031

Assay variable : PTINR_VAL1 PT-INR Value

N	Mean	Standard Deviation	Minimum	Maximum
6	2.6183333	0.3869582	2.2100000	3.1200000

Unique Subject Identifier=1203010000-0038

Assay variable : PTINR_VAL1 PT-INR Value

N	Mean	Standard Deviation	Minimum	Maximum
6	2.6850000	0.5224844	2.0900000	3.4600000

Laboratory tests at the time of obtaining Table 2-6 consent, according to the presence or absence of proton-pump inhibitors

Unique Subject Identifier=1203010000-0040

MEANS Procedure

Assay variable : PTINR_VAL1 PT-INR Value

N	Mean	Standard Deviation	Minimum	Maximum
6	1.8400000	0.1788854	1.5500000	2.0600000

Unique Subject Identifier=1206000000-0003

Assay variable : PTINR_VAL1 PT-INR Value

N	Mean	Standard Deviation	Minimum	Maximum
3	2.3833333	0.2050203	2.1800000	2.5900000

Unique Subject Identifier=1211000000-0028

Assay variable : PTINR_VAL1 PT-INR Value

N	Mean	Standard Deviation	Minimum	Maximum
5	2.1600000	0.3847077	1.7000000	2.5000000

Unique Subject Identifier=1211000000-0036

Assay variable : PTINR_VAL1 PT-INR Value

N	Mean	Standard Deviation	Minimum	Maximum
5	1.5200000	0.1303840	1.4000000	1.7000000

Laboratory tests at the time of obtaining Table 2-6 consent, according to the presence or absence of proton-pump inhibitors

Unique Subject Identifier=1212000000-0001

MEANS Procedure

Assay variable : PTINR_VAL1 PT-INR Value

N	Mean	Standard Deviation	Minimum	Maximum
6	1.9650000	0.3414528	1.6200000	2.5300000

Unique Subject Identifier=1212000000-0002

Assay variable : PTINR_VAL1 PT-INR Value

N	Mean	Standard Deviation	Minimum	Maximum
6	2.0916667	0.7101385	1.4500000	3.4000000

Unique Subject Identifier=1212000000-0003

Assay variable : PTINR_VAL1 PT-INR Value

N	Mean	Standard Deviation	Minimum	Maximum
6	2.2216667	0.3413747	1.9000000	2.6500000

Unique Subject Identifier=1212000000-0004

Assay variable : PTINR_VAL1 PT-INR Value

N	Mean	Standard Deviation	Minimum	Maximum
5	1.9220000	0.6746999	1.4900000	3.1200000

Laboratory tests at the time of obtaining Table 2-6 consent, according to the presence or absence of proton-pump inhibitors

Unique Subject Identifier=1212000000-0005

MEANS Procedure

Assay variable : PTINR_VAL1 PT-INR Value

N	Mean	Standard Deviation	Minimum	Maximum
6	1.9483333	0.2242692	1.7200000	2.3500000

Unique Subject Identifier=1212000000-0006

Assay variable : PTINR_VAL1 PT-INR Value

N	Mean	Standard Deviation	Minimum	Maximum
6	2.3450000	0.3283748	1.8300000	2.7300000

Unique Subject Identifier=1212000000-0007

Assay variable : PTINR_VAL1 PT-INR Value

N	Mean	Standard Deviation	Minimum	Maximum
3	2.2766667	0.1101514	2.1500000	2.3500000

Unique Subject Identifier=1212000000-0011

Assay variable : PTINR_VAL1 PT-INR Value

N	Mean	Standard Deviation	Minimum	Maximum
6	1.7366667	0.2789743	1.3100000	2.1300000

Laboratory tests at the time of obtaining Table 2-6 consent, according to the presence or absence of proton-pump inhibitors

Unique Subject Identifier=1212000000-0012

MEANS Procedure

Assay variable : PTINR_VAL1 PT-INR Value

N	Mean	Standard Deviation	Minimum	Maximum
4	1.7750000	0.4110556	1.4900000	2.3800000

Unique Subject Identifier=1212000000-0013

Assay variable : PTINR_VAL1 PT-INR Value

N	Mean	Standard Deviation	Minimum	Maximum
6	1.8283333	0.2626341	1.4700000	2.0900000

Unique Subject Identifier=1212000000-0014

Assay variable : PTINR_VAL1 PT-INR Value

N	Mean	Standard Deviation	Minimum	Maximum
6	2.3200000	0.2384114	1.9800000	2.6300000

Unique Subject Identifier=1212000000-0015

Assay variable : PTINR_VAL1 PT-INR Value

N	Mean	Standard Deviation	Minimum	Maximum
5	2.0980000	0.4196070	1.7500000	2.5800000

Laboratory tests at the time of obtaining Table 2-6 consent, according to the presence or absence of proton-pump inhibitors

Unique Subject Identifier=1212000000-0017

MEANS Procedure

Assay variable : PTINR_VAL1 PT-INR Value

N	Mean	Standard Deviation	Minimum	Maximum
5	2.4480000	0.3210452	2.1600000	2.9800000

Unique Subject Identifier=1216000000-0001

Assay variable : PTINR_VAL1 PT-INR Value

N	Mean	Standard Deviation	Minimum	Maximum
5	1.3360000	0.4370698	1.0600000	2.1100000

Unique Subject Identifier=1216000000-0002

Assay variable : PTINR_VAL1 PT-INR Value

N	Mean	Standard Deviation	Minimum	Maximum
5	1.5400000	0.2750455	1.0900000	1.7900000

Unique Subject Identifier=1216000000-0003

Assay variable : PTINR_VAL1 PT-INR Value

N	Mean	Standard Deviation	Minimum	Maximum
5	1.7820000	0.6529701	1.2300000	2.6000000

Laboratory tests at the time of obtaining Table 2-6 consent, according to the presence or absence of proton-pump inhibitors

Unique Subject Identifier=1216000000-0004

MEANS Procedure

Assay variable : PTINR_VAL1 PT-INR Value

N	Mean	Standard Deviation	Minimum	Maximum
3	1.3433333	0.1619671	1.2400000	1.5300000

Unique Subject Identifier=1219000000-0002

Assay variable : PTINR_VAL1 PT-INR Value

N	Mean	Standard Deviation	Minimum	Maximum
4	2.3425000	0.6785954	1.7700000	3.2500000

Unique Subject Identifier=1219000000-0004

Assay variable : PTINR_VAL1 PT-INR Value

N	Mean	Standard Deviation	Minimum	Maximum
3	2.2533333	0.1289703	2.1100000	2.3600000

Unique Subject Identifier=1219000000-0005

Assay variable : PTINR_VAL1 PT-INR Value

N	Mean	Standard Deviation	Minimum	Maximum
2	1.5700000	0.1272792	1.4800000	1.6600000

Laboratory tests at the time of obtaining Table 2-6 consent, according to the presence or absence of proton-pump inhibitors

Unique Subject Identifier=1219000000-0006

MEANS Procedure

Assay variable : PTINR_VAL1 PT-INR Value

N	Mean	Standard Deviation	Minimum	Maximum
3	2.0300000	0.3315117	1.7600000	2.4000000

Unique Subject Identifier=1219000000-0007

Assay variable : PTINR_VAL1 PT-INR Value

N	Mean	Standard Deviation	Minimum	Maximum
2	2.1700000	0	2.1700000	2.1700000

Unique Subject Identifier=1219000000-0009

Assay variable : PTINR_VAL1 PT-INR Value

N	Mean	Standard Deviation	Minimum	Maximum
4	1.9000000	0.0787401	1.8000000	1.9900000

Unique Subject Identifier=1219000000-0010

Assay variable : PTINR_VAL1 PT-INR Value

N	Mean	Standard Deviation	Minimum	Maximum
3	1.2733333	0.1700980	1.0800000	1.4000000

Laboratory tests at the time of obtaining Table 2-6 consent, according to the presence or absence of proton-pump inhibitors

Unique Subject Identifier=1219000000-0011

MEANS Procedure

Assay variable : PTINR_VAL1 PT-INR Value

N	Mean	Standard Deviation	Minimum	Maximum
3	2.2500000	0.2007486	2.0600000	2.4600000

Unique Subject Identifier=1219000000-0013

Assay variable : PTINR_VAL1 PT-INR Value

N	Mean	Standard Deviation	Minimum	Maximum
3	2.2433333	0.2608320	2.0500000	2.5400000

Unique Subject Identifier=1219000000-0014

Assay variable : PTINR_VAL1 PT-INR Value

N	Mean	Standard Deviation	Minimum	Maximum
4	1.7850000	0.0723418	1.7200000	1.8700000

Unique Subject Identifier=1219000000-0016

Assay variable : PTINR_VAL1 PT-INR Value

N	Mean	Standard Deviation	Minimum	Maximum
3	2.2466667	0.4842864	1.9000000	2.8000000

Laboratory tests at the time of obtaining Table 2-6 consent, according to the presence or absence of proton-pump inhibitors

Unique Subject Identifier=1219000000-0017

MEANS Procedure

Assay variable : PTINR_VAL1 PT-INR Value

N	Mean	Standard Deviation	Minimum	Maximum
3	2.2833333	0.3089229	1.9500000	2.5600000

Unique Subject Identifier=1219000000-0018

Assay variable : PTINR_VAL1 PT-INR Value

N	Mean	Standard Deviation	Minimum	Maximum
4	2.0450000	1.3675647	1.0200000	4.0600000

Unique Subject Identifier=1219000000-0021

Assay variable : PTINR_VAL1 PT-INR Value

N	Mean	Standard Deviation	Minimum	Maximum
3	1.7666667	0.4128357	1.2900000	2.0100000

Unique Subject Identifier=1219000000-0023

Assay variable : PTINR_VAL1 PT-INR Value

N	Mean	Standard Deviation	Minimum	Maximum
3	2.2100000	0.1571623	2.0700000	2.3800000

Laboratory tests at the time of obtaining Table 2-6 consent, according to the presence or absence of proton-pump inhibitors

Unique Subject Identifier=1219000000-0024

MEANS Procedure

Assay variable : PTINR_VAL1 PT-INR Value

N	Mean	Standard Deviation	Minimum	Maximum
3	2.2800000	0.1571623	2.1400000	2.4500000

Unique Subject Identifier=1219000000-0026

Assay variable : PTINR_VAL1 PT-INR Value

N	Mean	Standard Deviation	Minimum	Maximum
2	1.7800000	0.1414214	1.6800000	1.8800000

Unique Subject Identifier=1219000000-0028

Assay variable : PTINR_VAL1 PT-INR Value

N	Mean	Standard Deviation	Minimum	Maximum
4	2.2550000	0.4258717	1.6700000	2.6300000

Unique Subject Identifier=1219000000-0030

Assay variable : PTINR_VAL1 PT-INR Value

N	Mean	Standard Deviation	Minimum	Maximum
3	1.8000000	0.0100000	1.7900000	1.8100000

Laboratory tests at the time of obtaining Table 2-6 consent, according to the presence or absence of proton-pump inhibitors

Unique Subject Identifier=1219000000-0033

MEANS Procedure

Assay variable : PTINR_VAL1 PT-INR Value

N	Mean	Standard Deviation	Minimum	Maximum
3	1.8300000	0.8029321	1.1000000	2.6900000

Unique Subject Identifier=1219000000-0034

Assay variable : PTINR_VAL1 PT-INR Value

N	Mean	Standard Deviation	Minimum	Maximum
4	1.5650000	0.0704746	1.4700000	1.6400000

Unique Subject Identifier=1219000000-0035

Assay variable : PTINR_VAL1 PT-INR Value

N	Mean	Standard Deviation	Minimum	Maximum
1	1.9500000		1.9500000	1.9500000

Unique Subject Identifier=1219000000-0036

Assay variable : PTINR_VAL1 PT-INR Value

N	Mean	Standard Deviation	Minimum	Maximum
2	2.0200000	0.1697056	1.9000000	2.1400000

Laboratory tests at the time of obtaining Table 2-6 consent, according to the presence or absence of proton-pump inhibitors

Unique Subject Identifier=1219000000-0037

MEANS Procedure

Assay variable : PTINR_VAL1 PT-INR Value

N	Mean	Standard Deviation	Minimum	Maximum
4	2.3400000	0.2230097	2.0900000	2.6300000

Unique Subject Identifier=1221000000-0001

Assay variable : PTINR_VAL1 PT-INR Value

N	Mean	Standard Deviation	Minimum	Maximum
6	2.1000000	0.5621388	1.5000000	3.1000000

Unique Subject Identifier=1221000000-0003

Assay variable : PTINR_VAL1 PT-INR Value

N	Mean	Standard Deviation	Minimum	Maximum
5	1.9200000	0.3271085	1.7000000	2.5000000

Unique Subject Identifier=1222000000-0002

Assay variable : PTINR_VAL1 PT-INR Value

N	Mean	Standard Deviation	Minimum	Maximum
6	2.5166667	0.4020779	1.9000000	3.1000000

Laboratory tests at the time of obtaining Table 2-6 consent, according to the presence or absence of proton-pump inhibitors

Unique Subject Identifier=1222000000-0003

MEANS Procedure

Assay variable : PTINR_VAL1 PT-INR Value

N	Mean	Standard Deviation	Minimum	Maximum
5	2.3000000	0.1224745	2.1000000	2.4000000

Unique Subject Identifier=1222000000-0005

Assay variable : PTINR_VAL1 PT-INR Value

N	Mean	Standard Deviation	Minimum	Maximum
6	1.9666667	0.3204164	1.7000000	2.5000000

Unique Subject Identifier=1222000000-0007

Assay variable : PTINR_VAL1 PT-INR Value

N	Mean	Standard Deviation	Minimum	Maximum
4	2.0750000	0.0957427	2.0000000	2.2000000

Unique Subject Identifier=1222000000-0008

Assay variable : PTINR_VAL1 PT-INR Value

N	Mean	Standard Deviation	Minimum	Maximum
5	1.8200000	0.0836660	1.7000000	1.9000000

Laboratory tests at the time of obtaining Table 2-6 consent, according to the presence or absence of proton-pump inhibitors

Unique Subject Identifier=1222000000-0009

MEANS Procedure

Assay variable : PTINR_VAL1 PT-INR Value

N	Mean	Standard Deviation	Minimum	Maximum
5	1.8600000	0.2880972	1.5000000	2.2000000

Unique Subject Identifier=1222000000-0011

Assay variable : PTINR_VAL1 PT-INR Value

N	Mean	Standard Deviation	Minimum	Maximum
5	1.6800000	0.1788854	1.5000000	1.9000000

Unique Subject Identifier=1222000000-0012

Assay variable : PTINR_VAL1 PT-INR Value

N	Mean	Standard Deviation	Minimum	Maximum
6	1.8833333	0.3250641	1.6000000	2.5000000

Unique Subject Identifier=1223010000-0002

Assay variable : PTINR_VAL1 PT-INR Value

N	Mean	Standard Deviation	Minimum	Maximum
4	2.2250000	0.4645787	1.6000000	2.7000000

Laboratory tests at the time of obtaining Table 2-6 consent, according to the presence or absence of proton-pump inhibitors

Unique Subject Identifier=1223010000-0003

MEANS Procedure

Assay variable : PTINR_VAL1 PT-INR Value

N	Mean	Standard Deviation	Minimum	Maximum
4	2.4750000	0.8958236	1.6000000	3.7000000

Unique Subject Identifier=1223010000-0005

Assay variable : PTINR_VAL1 PT-INR Value

N	Mean	Standard Deviation	Minimum	Maximum
4	2.3250000	0.2986079	2.0000000	2.7000000

Unique Subject Identifier=1223010000-0006

Assay variable : PTINR_VAL1 PT-INR Value

N	Mean	Standard Deviation	Minimum	Maximum
4	1.6000000	0.2160247	1.3000000	1.8000000

Unique Subject Identifier=1223010000-0007

Assay variable : PTINR_VAL1 PT-INR Value

N	Mean	Standard Deviation	Minimum	Maximum
6	1.8666667	0.3932768	1.1000000	2.2000000

Laboratory tests at the time of obtaining Table 2-6 consent, according to the presence or absence of proton-pump inhibitors

Unique Subject Identifier=1223010000-0008

MEANS Procedure

Assay variable : PTINR_VAL1 PT-INR Value

N	Mean	Standard Deviation	Minimum	Maximum
6	2.2000000	0.1788854	2.0000000	2.5000000

Unique Subject Identifier=1223010000-0012

Assay variable : PTINR_VAL1 PT-INR Value

N	Mean	Standard Deviation	Minimum	Maximum
5	1.8400000	0.2408319	1.6000000	2.1000000

Unique Subject Identifier=1223010000-0013

Assay variable : PTINR_VAL1 PT-INR Value

N	Mean	Standard Deviation	Minimum	Maximum
5	1.7800000	0.1303840	1.6000000	1.9000000

Unique Subject Identifier=1223010000-0015

Assay variable : PTINR_VAL1 PT-INR Value

N	Mean	Standard Deviation	Minimum	Maximum
4	2.0500000	0.1290994	1.9000000	2.2000000

Laboratory tests at the time of obtaining Table 2-6 consent, according to the presence or absence of proton-pump inhibitors

Unique Subject Identifier=1223010000-0018

MEANS Procedure

Assay variable : PTINR_VAL1 PT-INR Value

N	Mean	Standard Deviation	Minimum	Maximum
5	2.0200000	0.5761944	1.5000000	2.9000000

Unique Subject Identifier=1223010000-0021

Assay variable : PTINR_VAL1 PT-INR Value

N	Mean	Standard Deviation	Minimum	Maximum
6	1.8666667	0.1632993	1.7000000	2.1000000

Unique Subject Identifier=1223010000-0023

Assay variable : PTINR_VAL1 PT-INR Value

N	Mean	Standard Deviation	Minimum	Maximum
4	1.8500000	0.2081666	1.6000000	2.1000000

Unique Subject Identifier=1223010000-0025

Assay variable : PTINR_VAL1 PT-INR Value

N	Mean	Standard Deviation	Minimum	Maximum
6	1.7500000	0.0836660	1.7000000	1.9000000

Laboratory tests at the time of obtaining Table 2-6 consent, according to the presence or absence of proton-pump inhibitors

Unique Subject Identifier=1223010000-0030

MEANS Procedure

Assay variable : PTINR_VAL1 PT-INR Value

N	Mean	Standard Deviation	Minimum	Maximum
4	1.9000000	0.1825742	1.7000000	2.1000000

Unique Subject Identifier=1223010000-0031

Assay variable : PTINR_VAL1 PT-INR Value

N	Mean	Standard Deviation	Minimum	Maximum
5	2.0000000	0.2236068	1.7000000	2.3000000

Unique Subject Identifier=1223010000-0033

Assay variable : PTINR_VAL1 PT-INR Value

N	Mean	Standard Deviation	Minimum	Maximum
4	2.3250000	0.3862210	1.8000000	2.7000000

Unique Subject Identifier=1223010000-0035

Assay variable : PTINR_VAL1 PT-INR Value

N	Mean	Standard Deviation	Minimum	Maximum
4	2.0500000	0.3872983	1.5000000	2.4000000

Laboratory tests at the time of obtaining Table 2-6 consent, according to the presence or absence of proton-pump inhibitors

Unique Subject Identifier=1223010000-0037

MEANS Procedure

Assay variable : PTINR_VAL1 PT-INR Value

N	Mean	Standard Deviation	Minimum	Maximum
4	1.6250000	0.1258306	1.5000000	1.8000000

Unique Subject Identifier=1223010000-0038

Assay variable : PTINR_VAL1 PT-INR Value

N	Mean	Standard Deviation	Minimum	Maximum
4	2.1500000	0.7937254	1.0000000	2.8000000

Unique Subject Identifier=1223010000-0039

Assay variable : PTINR_VAL1 PT-INR Value

N	Mean	Standard Deviation	Minimum	Maximum
4	2.1250000	0.1500000	2.0000000	2.3000000

Unique Subject Identifier=1223010000-0041

Assay variable : PTINR_VAL1 PT-INR Value

N	Mean	Standard Deviation	Minimum	Maximum
6	2.1166667	0.3763863	1.7000000	2.8000000

Laboratory tests at the time of obtaining Table 2-6 consent, according to the presence or absence of proton-pump inhibitors

Unique Subject Identifier=1223010000-0042

MEANS Procedure

Assay variable : PTINR_VAL1 PT-INR Value

N	Mean	Standard Deviation	Minimum	Maximum
4	1.8500000	0.3696846	1.4000000	2.3000000

Unique Subject Identifier=1223010000-0043

Assay variable : PTINR_VAL1 PT-INR Value

N	Mean	Standard Deviation	Minimum	Maximum
5	2.3800000	0.4147288	2.0000000	3.0000000

Unique Subject Identifier=1226000000-0002

Assay variable : PTINR_VAL1 PT-INR Value

N	Mean	Standard Deviation	Minimum	Maximum
2	1.2300000	0.0707107	1.1800000	1.2800000

Unique Subject Identifier=1226000000-0003

Assay variable : PTINR_VAL1 PT-INR Value

N	Mean	Standard Deviation	Minimum	Maximum
5	1.9180000	0.2087343	1.6600000	2.2100000

Laboratory tests at the time of obtaining Table 2-6 consent, according to the presence or absence of proton-pump inhibitors

Unique Subject Identifier=1226000000-0005

MEANS Procedure

Assay variable : PTINR_VAL1 PT-INR Value

N	Mean	Standard Deviation	Minimum	Maximum
4	2.0700000	0.1626858	1.9200000	2.2300000

Unique Subject Identifier=1226000000-0006

Assay variable : PTINR_VAL1 PT-INR Value

N	Mean	Standard Deviation	Minimum	Maximum
5	1.9680000	0.1283355	1.8000000	2.1300000

Unique Subject Identifier=1226000000-0007

Assay variable : PTINR_VAL1 PT-INR Value

N	Mean	Standard Deviation	Minimum	Maximum
3	1.7700000	0.2137756	1.6000000	2.0100000

Unique Subject Identifier=1226000000-0008

Assay variable : PTINR_VAL1 PT-INR Value

N	Mean	Standard Deviation	Minimum	Maximum
5	1.6360000	0.1327780	1.5000000	1.8400000

Laboratory tests at the time of obtaining Table 2-6 consent, according to the presence or absence of proton-pump inhibitors

Unique Subject Identifier=1226000000-0010

MEANS Procedure

Assay variable : PTINR_VAL1 PT-INR Value

N	Mean	Standard Deviation	Minimum	Maximum
5	1.8080000	0.0846759	1.7100000	1.9200000

Unique Subject Identifier=1226000000-0011

Assay variable : PTINR_VAL1 PT-INR Value

N	Mean	Standard Deviation	Minimum	Maximum
6	1.9116667	0.1921891	1.5600000	2.1400000

Unique Subject Identifier=1226000000-0012

Assay variable : PTINR_VAL1 PT-INR Value

N	Mean	Standard Deviation	Minimum	Maximum
3	2.0133333	0.4960175	1.5000000	2.4900000

Unique Subject Identifier=1226000000-0013

Assay variable : PTINR_VAL1 PT-INR Value

N	Mean	Standard Deviation	Minimum	Maximum
4	1.7850000	0.1103026	1.6400000	1.9000000

Laboratory tests at the time of obtaining Table 2-6 consent, according to the presence or absence of proton-pump inhibitors

Unique Subject Identifier=1226000000-0014

MEANS Procedure

Assay variable : PTINR_VAL1 PT-INR Value

N	Mean	Standard Deviation	Minimum	Maximum
5	2.0760000	0.4936902	1.5800000	2.8900000

Unique Subject Identifier=1226000000-0030

Assay variable : PTINR_VAL1 PT-INR Value

N	Mean	Standard Deviation	Minimum	Maximum
4	2.1225000	0.4256270	1.6100000	2.6500000

Unique Subject Identifier=1226000000-0031

Assay variable : PTINR_VAL1 PT-INR Value

N	Mean	Standard Deviation	Minimum	Maximum
5	1.7000000	0.2351595	1.4700000	2.0300000

Unique Subject Identifier=1226000000-0032

Assay variable : PTINR_VAL1 PT-INR Value

N	Mean	Standard Deviation	Minimum	Maximum
5	1.9840000	0.1597811	1.7900000	2.2100000

Laboratory tests at the time of obtaining Table 2-6 consent, according to the presence or absence of proton-pump inhibitors

Unique Subject Identifier=1226000000-0034

MEANS Procedure

Assay variable : PTINR_VAL1 PT-INR Value

N	Mean	Standard Deviation	Minimum	Maximum
4	2.1525000	0.3247948	1.8900000	2.5800000

Unique Subject Identifier=1227000000-0027

Assay variable : PTINR_VAL1 PT-INR Value

N	Mean	Standard Deviation	Minimum	Maximum
4	2.3250000	0.2500000	2.0000000	2.6000000

Unique Subject Identifier=1227000000-0028

Assay variable : PTINR_VAL1 PT-INR Value

N	Mean	Standard Deviation	Minimum	Maximum
6	2.3833333	0.2228602	2.1000000	2.7000000

Unique Subject Identifier=1227000000-0029

Assay variable : PTINR_VAL1 PT-INR Value

N	Mean	Standard Deviation	Minimum	Maximum
4	2.3750000	0.5439056	1.6000000	2.8000000

Laboratory tests at the time of obtaining Table 2-6 consent, according to the presence or absence of proton-pump inhibitors

Unique Subject Identifier=1227000000-0030

MEANS Procedure

Assay variable : PTINR_VAL1 PT-INR Value

N	Mean	Standard Deviation	Minimum	Maximum
3	2.0666667	0.5507571	1.7000000	2.7000000

Unique Subject Identifier=1227000000-0031

Assay variable : PTINR_VAL1 PT-INR Value

N	Mean	Standard Deviation	Minimum	Maximum
5	2.1600000	0.2880972	1.9000000	2.6000000

Unique Subject Identifier=1227000000-0032

Assay variable : PTINR_VAL1 PT-INR Value

N	Mean	Standard Deviation	Minimum	Maximum
5	2.1000000	0.2000000	1.9000000	2.4000000

Unique Subject Identifier=1227000000-0034

Assay variable : PTINR_VAL1 PT-INR Value

N	Mean	Standard Deviation	Minimum	Maximum
5	2.2200000	0.5263079	1.4000000	2.8000000

Laboratory tests at the time of obtaining Table 2-6 consent, according to the presence or absence of proton-pump inhibitors

Unique Subject Identifier=1227000000-0035

MEANS Procedure

Assay variable : PTINR_VAL1 PT-INR Value

N	Mean	Standard Deviation	Minimum	Maximum
4	2.0750000	0.3862210	1.7000000	2.6000000

Unique Subject Identifier=1227000000-0038

Assay variable : PTINR_VAL1 PT-INR Value

N	Mean	Standard Deviation	Minimum	Maximum
5	2.3000000	0.2915476	1.9000000	2.7000000

Unique Subject Identifier=1227000000-0041

Assay variable : PTINR_VAL1 PT-INR Value

N	Mean	Standard Deviation	Minimum	Maximum
4	2.3000000	0.4966555	1.7000000	2.8000000

Unique Subject Identifier=1227000000-0043

Assay variable : PTINR_VAL1 PT-INR Value

N	Mean	Standard Deviation	Minimum	Maximum
5	2.2000000	0.1414214	2.1000000	2.4000000

Laboratory tests at the time of obtaining Table 2-6 consent, according to the presence or absence of proton-pump inhibitors

Unique Subject Identifier=1227000000-0047

MEANS Procedure

Assay variable : PTINR_VAL1 PT-INR Value

N	Mean	Standard Deviation	Minimum	Maximum
5	2.0400000	0.3847077	1.6000000	2.6000000

Unique Subject Identifier=1227000000-0050

Assay variable : PTINR_VAL1 PT-INR Value

N	Mean	Standard Deviation	Minimum	Maximum
6	1.9000000	0.1673320	1.7000000	2.1000000

Unique Subject Identifier=1227000000-0055

Assay variable : PTINR_VAL1 PT-INR Value

N	Mean	Standard Deviation	Minimum	Maximum
6	2.4333333	0.7004760	1.4000000	3.3000000

Unique Subject Identifier=1227000000-0058

Assay variable : PTINR_VAL1 PT-INR Value

N	Mean	Standard Deviation	Minimum	Maximum
6	2.0500000	0.2664583	1.7000000	2.4000000

Laboratory tests at the time of obtaining Table 2-6 consent, according to the presence or absence of proton-pump inhibitors

Unique Subject Identifier=1227000000-0064

MEANS Procedure

Assay variable : PTINR_VAL1 PT-INR Value

N	Mean	Standard Deviation	Minimum	Maximum
4	2.9500000	0.5972158	2.4000000	3.8000000

Unique Subject Identifier=1227000000-0078

Assay variable : PTINR_VAL1 PT-INR Value

N	Mean	Standard Deviation	Minimum	Maximum
5	1.8600000	0.0894427	1.8000000	2.0000000

Unique Subject Identifier=1227000000-0089

Assay variable : PTINR_VAL1 PT-INR Value

N	Mean	Standard Deviation	Minimum	Maximum
6	1.8166667	0.2994439	1.3000000	2.1000000

Unique Subject Identifier=1227000000-0091

Assay variable : PTINR_VAL1 PT-INR Value

N	Mean	Standard Deviation	Minimum	Maximum
6	2.3166667	0.3868678	1.9000000	3.0000000

Laboratory tests at the time of obtaining Table 2-6 consent, according to the presence or absence of proton-pump inhibitors

Unique Subject Identifier=1228000000-0004

MEANS Procedure

Assay variable : PTINR_VAL1 PT-INR Value

N	Mean	Standard Deviation	Minimum	Maximum
1	2.9800000		2.9800000	2.9800000

Unique Subject Identifier=1229000000-0006

Assay variable : PTINR_VAL1 PT-INR Value

N	Mean	Standard Deviation	Minimum	Maximum
6	2.0500000	0.1048809	1.9000000	2.2000000

Unique Subject Identifier=1229000000-0007

Assay variable : PTINR_VAL1 PT-INR Value

N	Mean	Standard Deviation	Minimum	Maximum
5	2.4200000	0.1643168	2.3000000	2.6000000

Unique Subject Identifier=1229000000-0010

Assay variable : PTINR_VAL1 PT-INR Value

N	Mean	Standard Deviation	Minimum	Maximum
6	2.2333333	0.1032796	2.1000000	2.4000000

Laboratory tests at the time of obtaining Table 2-6 consent, according to the presence or absence of proton-pump inhibitors

Unique Subject Identifier=1229000000-0016

MEANS Procedure

Assay variable : PTINR_VAL1 PT-INR Value

N	Mean	Standard Deviation	Minimum	Maximum
6	2.1666667	0.2732520	1.8000000	2.5000000

Unique Subject Identifier=1229000000-0017

Assay variable : PTINR_VAL1 PT-INR Value

N	Mean	Standard Deviation	Minimum	Maximum
6	2.3666667	0.9114092	1.6000000	4.1000000

Unique Subject Identifier=1229000000-0020

Assay variable : PTINR_VAL1 PT-INR Value

N	Mean	Standard Deviation	Minimum	Maximum
6	2.1833333	0.4119061	1.6000000	2.6000000

Unique Subject Identifier=1229000000-0021

Assay variable : PTINR_VAL1 PT-INR Value

N	Mean	Standard Deviation	Minimum	Maximum
6	2.0500000	0.2073644	1.9000000	2.4000000

Laboratory tests at the time of obtaining Table 2-6 consent, according to the presence or absence of proton-pump inhibitors

Unique Subject Identifier=1232000000-0003

MEANS Procedure

Assay variable : PTINR_VAL1 PT-INR Value

N	Mean	Standard Deviation	Minimum	Maximum
6	1.8166667	0.5913262	1.1000000	2.6000000

Unique Subject Identifier=1232000000-0004

Assay variable : PTINR_VAL1 PT-INR Value

N	Mean	Standard Deviation	Minimum	Maximum
6	2.0166667	0.4708149	1.5000000	2.6000000

Unique Subject Identifier=1232000000-0005

Assay variable : PTINR_VAL1 PT-INR Value

N	Mean	Standard Deviation	Minimum	Maximum
6	2.0833333	0.3920034	1.6000000	2.6000000

Unique Subject Identifier=1232000000-0008

Assay variable : PTINR_VAL1 PT-INR Value

N	Mean	Standard Deviation	Minimum	Maximum
5	2.0400000	0.2073644	1.7000000	2.2000000

Laboratory tests at the time of obtaining Table 2-6 consent, according to the presence or absence of proton-pump inhibitors

Unique Subject Identifier=1232000000-0014

MEANS Procedure

Assay variable : PTINR_VAL1 PT-INR Value

N	Mean	Standard Deviation	Minimum	Maximum
6	2.0833333	0.5946988	1.5000000	3.2000000

Unique Subject Identifier=1232000000-0016

Assay variable : PTINR_VAL1 PT-INR Value

N	Mean	Standard Deviation	Minimum	Maximum
6	1.7500000	0.1048809	1.6000000	1.9000000

Unique Subject Identifier=1232000000-0017

Assay variable : PTINR_VAL1 PT-INR Value

N	Mean	Standard Deviation	Minimum	Maximum
5	1.8000000	0.1870829	1.6000000	2.1000000

Unique Subject Identifier=1232000000-0020

Assay variable : PTINR_VAL1 PT-INR Value

N	Mean	Standard Deviation	Minimum	Maximum
6	2.4000000	0.4604346	1.8000000	3.2000000

Laboratory tests at the time of obtaining Table 2-6 consent, according to the presence or absence of proton-pump inhibitors

Unique Subject Identifier=1234001000-0006

MEANS Procedure

Assay variable : PTINR_VAL1 PT-INR Value

N	Mean	Standard Deviation	Minimum	Maximum
1	1.0300000		1.0300000	1.0300000

Unique Subject Identifier=1235000000-0001

Assay variable : PTINR_VAL1 PT-INR Value

N	Mean	Standard Deviation	Minimum	Maximum
5	1.6580000	0.1360882	1.4500000	1.7700000

Unique Subject Identifier=1235000000-0003

Assay variable : PTINR_VAL1 PT-INR Value

N	Mean	Standard Deviation	Minimum	Maximum
5	1.4020000	0.0952365	1.2500000	1.5000000

Unique Subject Identifier=1235000000-0005

Assay variable : PTINR_VAL1 PT-INR Value

N	Mean	Standard Deviation	Minimum	Maximum
6	1.4550000	0.0971082	1.3600000	1.6300000

Laboratory tests at the time of obtaining Table 2-6 consent, according to the presence or absence of proton-pump inhibitors

Unique Subject Identifier=1235000000-0006

MEANS Procedure

Assay variable : PTINR_VAL1 PT-INR Value

N	Mean	Standard Deviation	Minimum	Maximum
3	1.4233333	0.1234234	1.3200000	1.5600000

Unique Subject Identifier=1235000000-0008

Assay variable : PTINR_VAL1 PT-INR Value

N	Mean	Standard Deviation	Minimum	Maximum
5	1.8520000	0.1255787	1.7100000	1.9800000

Unique Subject Identifier=1235000000-0009

Assay variable : PTINR_VAL1 PT-INR Value

N	Mean	Standard Deviation	Minimum	Maximum
3	1.6466667	0.1616581	1.5000000	1.8200000

Unique Subject Identifier=1235000000-0010

Assay variable : PTINR_VAL1 PT-INR Value

N	Mean	Standard Deviation	Minimum	Maximum
6	1.6300000	0.2368122	1.2000000	1.8800000

Laboratory tests at the time of obtaining Table 2-6 consent, according to the presence or absence of proton-pump inhibitors

Unique Subject Identifier=1235000000-0011

MEANS Procedure

Assay variable : PTINR_VAL1 PT-INR Value

N	Mean	Standard Deviation	Minimum	Maximum
6	1.5500000	0.2484351	1.3500000	2.0400000

Unique Subject Identifier=1235000000-0013

Assay variable : PTINR_VAL1 PT-INR Value

N	Mean	Standard Deviation	Minimum	Maximum
6	1.4433333	0.0871015	1.3600000	1.5500000

Unique Subject Identifier=1235000000-0015

Assay variable : PTINR_VAL1 PT-INR Value

N	Mean	Standard Deviation	Minimum	Maximum
6	1.7150000	0.2714959	1.4700000	2.1800000

Unique Subject Identifier=1235000000-0017

Assay variable : PTINR_VAL1 PT-INR Value

N	Mean	Standard Deviation	Minimum	Maximum
5	1.5700000	0.1234909	1.4200000	1.7300000

Laboratory tests at the time of obtaining Table 2-6 consent, according to the presence or absence of proton-pump inhibitors

Unique Subject Identifier=1235000000-0020

MEANS Procedure

Assay variable : PTINR_VAL1 PT-INR Value

N	Mean	Standard Deviation	Minimum	Maximum
6	1.6316667	0.1404872	1.4800000	1.8400000

Unique Subject Identifier=1235000000-0021

Assay variable : PTINR_VAL1 PT-INR Value

N	Mean	Standard Deviation	Minimum	Maximum
6	1.2966667	0.0920145	1.2100000	1.4100000

Unique Subject Identifier=1235000000-0022

Assay variable : PTINR_VAL1 PT-INR Value

N	Mean	Standard Deviation	Minimum	Maximum
6	1.6166667	0.2487301	1.3500000	1.9500000

Unique Subject Identifier=1235000000-0023

Assay variable : PTINR_VAL1 PT-INR Value

N	Mean	Standard Deviation	Minimum	Maximum
6	1.7250000	0.1375136	1.5800000	1.9100000

Laboratory tests at the time of obtaining Table 2-6 consent, according to the presence or absence of proton-pump inhibitors

Unique Subject Identifier=1235000000-0024

MEANS Procedure

Assay variable : PTINR_VAL1 PT-INR Value

N	Mean	Standard Deviation	Minimum	Maximum
6	1.3716667	0.0793515	1.2700000	1.5000000

Unique Subject Identifier=1235000000-0026

Assay variable : PTINR_VAL1 PT-INR Value

N	Mean	Standard Deviation	Minimum	Maximum
6	1.4750000	0.1710848	1.2200000	1.6600000

Unique Subject Identifier=1235000000-0027

Assay variable : PTINR_VAL1 PT-INR Value

N	Mean	Standard Deviation	Minimum	Maximum
2	2.0100000	0.1131371	1.9300000	2.0900000

Unique Subject Identifier=1235000000-0033

Assay variable : PTINR_VAL1 PT-INR Value

N	Mean	Standard Deviation	Minimum	Maximum
6	1.9400000	0.4784976	1.3500000	2.5200000

Laboratory tests at the time of obtaining Table 2-6 consent, according to the presence or absence of proton-pump inhibitors

Unique Subject Identifier=1235000000-0034

MEANS Procedure

Assay variable : PTINR_VAL1 PT-INR Value

N	Mean	Standard Deviation	Minimum	Maximum
6	1.6650000	0.3907301	1.3300000	2.3500000

Unique Subject Identifier=1235000000-0040

Assay variable : PTINR_VAL1 PT-INR Value

N	Mean	Standard Deviation	Minimum	Maximum
3	1.7733333	0.0404145	1.7300000	1.8100000

Unique Subject Identifier=1235000000-0042

Assay variable : PTINR_VAL1 PT-INR Value

N	Mean	Standard Deviation	Minimum	Maximum
6	1.7666667	0.6168198	1.2600000	2.9900000

Unique Subject Identifier=1235000000-0043

Assay variable : PTINR_VAL1 PT-INR Value

N	Mean	Standard Deviation	Minimum	Maximum
5	2.0400000	0.3647602	1.6800000	2.5400000

Laboratory tests at the time of obtaining Table 2-6 consent, according to the presence or absence of proton-pump inhibitors

Unique Subject Identifier=1235000000-0045

MEANS Procedure

Assay variable : PTINR_VAL1 PT-INR Value

N	Mean	Standard Deviation	Minimum	Maximum
6	1.5666667	0.1751190	1.2900000	1.8000000

Unique Subject Identifier=1235000000-0050

Assay variable : PTINR_VAL1 PT-INR Value

N	Mean	Standard Deviation	Minimum	Maximum
6	1.9616667	0.5810479	1.0400000	2.5200000

Unique Subject Identifier=1237000000-0006

Assay variable : PTINR_VAL1 PT-INR Value

N	Mean	Standard Deviation	Minimum	Maximum
5	2.1000000	0.3162278	1.8000000	2.6000000

Unique Subject Identifier=1237000000-0007

Assay variable : PTINR_VAL1 PT-INR Value

N	Mean	Standard Deviation	Minimum	Maximum
5	1.9000000	0.3872983	1.5000000	2.4000000

Laboratory tests at the time of obtaining Table 2-6 consent, according to the presence or absence of proton-pump inhibitors

Unique Subject Identifier=1237000000-0010

MEANS Procedure

Assay variable : PTINR_VAL1 PT-INR Value

N	Mean	Standard Deviation	Minimum	Maximum
3	1.7333333	0.0577350	1.7000000	1.8000000

Unique Subject Identifier=1237000000-0011

Assay variable : PTINR_VAL1 PT-INR Value

N	Mean	Standard Deviation	Minimum	Maximum
3	2.0000000	0.1732051	1.9000000	2.2000000

Unique Subject Identifier=1237000000-0012

Assay variable : PTINR_VAL1 PT-INR Value

N	Mean	Standard Deviation	Minimum	Maximum
3	1.5333333	0.1527525	1.4000000	1.7000000

Unique Subject Identifier=1237000000-0013

Assay variable : PTINR_VAL1 PT-INR Value

N	Mean	Standard Deviation	Minimum	Maximum
4	1.5250000	0.1500000	1.3000000	1.6000000

Laboratory tests at the time of obtaining Table 2-6 consent, according to the presence or absence of proton-pump inhibitors

Unique Subject Identifier=1237000000-0014

MEANS Procedure

Assay variable : PTINR_VAL1 PT-INR Value

N	Mean	Standard Deviation	Minimum	Maximum
3	1.5666667	0.2516611	1.3000000	1.8000000

Unique Subject Identifier=1241000000-0005

Assay variable : PTINR_VAL1 PT-INR Value

N	Mean	Standard Deviation	Minimum	Maximum
1	2.7900000		2.7900000	2.7900000

Unique Subject Identifier=1241000000-0006

Assay variable : PTINR_VAL1 PT-INR Value

N	Mean	Standard Deviation	Minimum	Maximum
5	2.8220000	0.7436868	1.5500000	3.4700000

Unique Subject Identifier=1248000000-0001

Assay variable : PTINR_VAL1 PT-INR Value

N	Mean	Standard Deviation	Minimum	Maximum
2	2.4450000	0.3323402	2.2100000	2.6800000

Laboratory tests at the time of obtaining Table 2-6 consent, according to the presence or absence of proton-pump inhibitors

Unique Subject Identifier=1249000000-0002

MEANS Procedure

Assay variable : PTINR_VAL1 PT-INR Value

N	Mean	Standard Deviation	Minimum	Maximum
6	2.2166667	0.2141650	1.9700000	2.5600000

Unique Subject Identifier=1249000000-0008

Assay variable : PTINR_VAL1 PT-INR Value

N	Mean	Standard Deviation	Minimum	Maximum
6	1.8083333	0.1435851	1.6400000	2.0400000

Unique Subject Identifier=1254100000-0002

Assay variable : PTINR_VAL1 PT-INR Value

N	Mean	Standard Deviation	Minimum	Maximum
5	1.9600000	0.4159327	1.5000000	2.6000000

Unique Subject Identifier=1254100000-0013

Assay variable : PTINR_VAL1 PT-INR Value

N	Mean	Standard Deviation	Minimum	Maximum
6	1.8000000	0.2190890	1.5000000	2.1000000

Laboratory tests at the time of obtaining Table 2-6 consent, according to the presence or absence of proton-pump inhibitors

Unique Subject Identifier=1254100000-0018

MEANS Procedure

Assay variable : PTINR_VAL1 PT-INR Value

N	Mean	Standard Deviation	Minimum	Maximum
6	2.1333333	0.1211060	2.0000000	2.3000000

Unique Subject Identifier=1254100000-0019

Assay variable : PTINR_VAL1 PT-INR Value

N	Mean	Standard Deviation	Minimum	Maximum
5	2.2200000	0.3563706	1.7000000	2.7000000

Unique Subject Identifier=1254100000-0021

Assay variable : PTINR_VAL1 PT-INR Value

N	Mean	Standard Deviation	Minimum	Maximum
6	2.2166667	0.3311596	1.9000000	2.8000000

Unique Subject Identifier=1254100000-0024

Assay variable : PTINR_VAL1 PT-INR Value

N	Mean	Standard Deviation	Minimum	Maximum
6	2.0666667	0.3204164	1.7000000	2.5000000

Laboratory tests at the time of obtaining Table 2-6 consent, according to the presence or absence of proton-pump inhibitors

Unique Subject Identifier=1254100000-0025

MEANS Procedure

Assay variable : PTINR_VAL1 PT-INR Value

N	Mean	Standard Deviation	Minimum	Maximum
6	2.1166667	0.4665476	1.3000000	2.6000000

Unique Subject Identifier=1255011110-0002

Assay variable : PTINR_VAL1 PT-INR Value

N	Mean	Standard Deviation	Minimum	Maximum
2	2.4200000	0.3535534	2.1700000	2.6700000

Unique Subject Identifier=1255011110-0004

Assay variable : PTINR_VAL1 PT-INR Value

N	Mean	Standard Deviation	Minimum	Maximum
1	2.3000000		2.3000000	2.3000000

Unique Subject Identifier=1258000000-0005

Assay variable : PTINR_VAL1 PT-INR Value

N	Mean	Standard Deviation	Minimum	Maximum
6	2.0050000	0.3219161	1.8500000	2.6600000

Laboratory tests at the time of obtaining Table 2-6 consent, according to the presence or absence of proton-pump inhibitors

Unique Subject Identifier=1262000000-0016

MEANS Procedure

Assay variable : PTINR_VAL1 PT-INR Value

N	Mean	Standard Deviation	Minimum	Maximum
2	1.5000000	0	1.5000000	1.5000000

Unique Subject Identifier=1262000000-0019

Assay variable : PTINR_VAL1 PT-INR Value

N	Mean	Standard Deviation	Minimum	Maximum
2	1.3500000	0.2121320	1.2000000	1.5000000

Unique Subject Identifier=1262000000-0020

Assay variable : PTINR_VAL1 PT-INR Value

N	Mean	Standard Deviation	Minimum	Maximum
2	1.6000000	0.1414214	1.5000000	1.7000000

Unique Subject Identifier=1262000000-0023

Assay variable : PTINR_VAL1 PT-INR Value

N	Mean	Standard Deviation	Minimum	Maximum
1	1.7000000		1.7000000	1.7000000

Laboratory tests at the time of obtaining Table 2-6 consent, according to the presence or absence of proton-pump inhibitors

Unique Subject Identifier=1262000000-0027

MEANS Procedure

Assay variable : PTINR_VAL1 PT-INR Value

N	Mean	Standard Deviation	Minimum	Maximum
2	1.9500000	0.6363961	1.5000000	2.4000000

Unique Subject Identifier=1262000000-0030

Assay variable : PTINR_VAL1 PT-INR Value

N	Mean	Standard Deviation	Minimum	Maximum
3	1.7333333	0.2081666	1.5000000	1.9000000

Unique Subject Identifier=1262000000-0049

Assay variable : PTINR_VAL1 PT-INR Value

N	Mean	Standard Deviation	Minimum	Maximum
1	1.3000000		1.3000000	1.3000000

Unique Subject Identifier=1262000000-0058

Assay variable : PTINR_VAL1 PT-INR Value

N	Mean	Standard Deviation	Minimum	Maximum
1	1.3000000		1.3000000	1.3000000

Laboratory tests at the time of obtaining Table 2-6 consent, according to the presence or absence of proton-pump inhibitors

Unique Subject Identifier=1262000000-0064

MEANS Procedure

Assay variable : PTINR_VAL1 PT-INR Value

N	Mean	Standard Deviation	Minimum	Maximum
2	1.9000000	0.2828427	1.7000000	2.1000000

Unique Subject Identifier=1262000000-0071

Assay variable : PTINR_VAL1 PT-INR Value

N	Mean	Standard Deviation	Minimum	Maximum
5	1.8200000	0.1303840	1.6000000	1.9000000

Unique Subject Identifier=1262000000-0075

Assay variable : PTINR_VAL1 PT-INR Value

N	Mean	Standard Deviation	Minimum	Maximum
2	1.4500000	0.2121320	1.3000000	1.6000000

Unique Subject Identifier=1262000000-0079

Assay variable : PTINR_VAL1 PT-INR Value

N	Mean	Standard Deviation	Minimum	Maximum
2	2.2500000	0.3535534	2.0000000	2.5000000

Laboratory tests at the time of obtaining Table 2-6 consent, according to the presence or absence of proton-pump inhibitors

Unique Subject Identifier=1263011000-0004

MEANS Procedure

Assay variable : PTINR_VAL1 PT-INR Value

N	Mean	Standard Deviation	Minimum	Maximum
6	2.3133333	0.2189673	2.0900000	2.5800000

Unique Subject Identifier=1263011000-0007

Assay variable : PTINR_VAL1 PT-INR Value

N	Mean	Standard Deviation	Minimum	Maximum
4	2.5700000	0.9076343	1.6000000	3.6700000

Unique Subject Identifier=1263011000-0014

Assay variable : PTINR_VAL1 PT-INR Value

N	Mean	Standard Deviation	Minimum	Maximum
6	2.6133333	0.5203717	2.0400000	3.2300000

Unique Subject Identifier=1263011000-0018

Assay variable : PTINR_VAL1 PT-INR Value

N	Mean	Standard Deviation	Minimum	Maximum
6	2.3416667	0.2632426	1.9700000	2.6100000

Laboratory tests at the time of obtaining Table 2-6 consent, according to the presence or absence of proton-pump inhibitors

Unique Subject Identifier=1263011000-0027

MEANS Procedure

Assay variable : PTINR_VAL1 PT-INR Value

N	Mean	Standard Deviation	Minimum	Maximum
6	2.0200000	0.1589969	1.8800000	2.2900000

Unique Subject Identifier=1263011000-0033

Assay variable : PTINR_VAL1 PT-INR Value

N	Mean	Standard Deviation	Minimum	Maximum
6	2.0183333	0.6587994	1.5100000	3.2600000

Unique Subject Identifier=1264001000-0007

Assay variable : PTINR_VAL1 PT-INR Value

N	Mean	Standard Deviation	Minimum	Maximum
6	2.1166667	0.6080022	1.4000000	2.8000000

Unique Subject Identifier=1266000000-0001

Assay variable : PTINR_VAL1 PT-INR Value

N	Mean	Standard Deviation	Minimum	Maximum
6	2.0283333	0.3773283	1.4000000	2.4500000

Laboratory tests at the time of obtaining Table 2-6 consent, according to the presence or absence of proton-pump inhibitors

Unique Subject Identifier=1266000000-0005

MEANS Procedure

Assay variable : PTINR_VAL1 PT-INR Value

N	Mean	Standard Deviation	Minimum	Maximum
6	2.2616667	0.2018333	2.1000000	2.6000000

Unique Subject Identifier=1266000000-0009

Assay variable : PTINR_VAL1 PT-INR Value

N	Mean	Standard Deviation	Minimum	Maximum
6	2.2583333	0.3009596	1.9000000	2.6000000

Unique Subject Identifier=1266000000-0015

Assay variable : PTINR_VAL1 PT-INR Value

N	Mean	Standard Deviation	Minimum	Maximum
6	2.0766667	0.5020624	1.4000000	2.6600000

Unique Subject Identifier=1266000000-0016

Assay variable : PTINR_VAL1 PT-INR Value

N	Mean	Standard Deviation	Minimum	Maximum
6	2.0350000	0.8365584	1.3000000	3.2000000

Laboratory tests at the time of obtaining Table 2-6 consent, according to the presence or absence of proton-pump inhibitors

Unique Subject Identifier=1266000000-0020

MEANS Procedure

Assay variable : PTINR_VAL1 PT-INR Value

N	Mean	Standard Deviation	Minimum	Maximum
6	1.4316667	0.1837843	1.2000000	1.7000000

Unique Subject Identifier=1266000000-0021

Assay variable : PTINR_VAL1 PT-INR Value

N	Mean	Standard Deviation	Minimum	Maximum
6	2.1716667	0.3237540	1.8000000	2.7000000

Unique Subject Identifier=1266000000-0022

Assay variable : PTINR_VAL1 PT-INR Value

N	Mean	Standard Deviation	Minimum	Maximum
4	2.1975000	0.2723203	2.0000000	2.6000000

Unique Subject Identifier=1266000000-0025

Assay variable : PTINR_VAL1 PT-INR Value

N	Mean	Standard Deviation	Minimum	Maximum
6	2.5566667	0.6370767	1.5000000	3.3100000

Laboratory tests at the time of obtaining Table 2-6 consent, according to the presence or absence of proton-pump inhibitors

Unique Subject Identifier=1266000000-0027

MEANS Procedure

Assay variable : PTINR_VAL1 PT-INR Value

N	Mean	Standard Deviation	Minimum	Maximum
6	2.2783333	0.1510519	2.1000000	2.5000000

Unique Subject Identifier=1266000000-0029

Assay variable : PTINR_VAL1 PT-INR Value

N	Mean	Standard Deviation	Minimum	Maximum
6	1.9283333	0.2204011	1.6400000	2.2800000

Unique Subject Identifier=1266000000-0030

Assay variable : PTINR_VAL1 PT-INR Value

N	Mean	Standard Deviation	Minimum	Maximum
6	2.2450000	0.2847279	1.8000000	2.6000000

Unique Subject Identifier=1266000000-0031

Assay variable : PTINR_VAL1 PT-INR Value

N	Mean	Standard Deviation	Minimum	Maximum
6	1.7666667	0.1175868	1.7000000	2.0000000

Laboratory tests at the time of obtaining Table 2-6 consent, according to the presence or absence of proton-pump inhibitors

Unique Subject Identifier=1266000000-0032

MEANS Procedure

Assay variable : PTINR_VAL1 PT-INR Value

N	Mean	Standard Deviation	Minimum	Maximum
6	1.6983333	0.5077959	0.7200000	2.1000000

Unique Subject Identifier=1266000000-0033

Assay variable : PTINR_VAL1 PT-INR Value

N	Mean	Standard Deviation	Minimum	Maximum
6	1.8833333	0.1329160	1.7000000	2.0000000

Unique Subject Identifier=1266000000-0034

Assay variable : PTINR_VAL1 PT-INR Value

N	Mean	Standard Deviation	Minimum	Maximum
6	1.6466667	0.1782882	1.5000000	2.0000000

Unique Subject Identifier=1266000000-0036

Assay variable : PTINR_VAL1 PT-INR Value

N	Mean	Standard Deviation	Minimum	Maximum
6	1.8600000	0.1814387	1.7000000	2.1700000

Laboratory tests at the time of obtaining Table 2-6 consent, according to the presence or absence of proton-pump inhibitors

Unique Subject Identifier=1266000000-0038

MEANS Procedure

Assay variable : PTINR_VAL1 PT-INR Value

N	Mean	Standard Deviation	Minimum	Maximum
6	2.1250000	0.1740977	1.9000000	2.4000000

Unique Subject Identifier=1266000000-0039

Assay variable : PTINR_VAL1 PT-INR Value

N	Mean	Standard Deviation	Minimum	Maximum
6	1.6566667	0.1787363	1.4000000	1.9000000

Unique Subject Identifier=1266000000-0041

Assay variable : PTINR_VAL1 PT-INR Value

N	Mean	Standard Deviation	Minimum	Maximum
6	1.9000000	0.2529822	1.6000000	2.2000000

Unique Subject Identifier=1266000000-0047

Assay variable : PTINR_VAL1 PT-INR Value

N	Mean	Standard Deviation	Minimum	Maximum
6	1.4850000	0.2843062	1.1900000	1.8700000

Laboratory tests at the time of obtaining Table 2-6 consent, according to the presence or absence of proton-pump inhibitors

Unique Subject Identifier=1266000000-0049

MEANS Procedure

Assay variable : PTINR_VAL1 PT-INR Value

N	Mean	Standard Deviation	Minimum	Maximum
6	2.5066667	1.1082719	1.5000000	4.6000000

Unique Subject Identifier=1266000000-0050

Assay variable : PTINR_VAL1 PT-INR Value

N	Mean	Standard Deviation	Minimum	Maximum
6	1.6283333	0.3717212	1.3000000	2.3600000

Unique Subject Identifier=1266000000-0051

Assay variable : PTINR_VAL1 PT-INR Value

N	Mean	Standard Deviation	Minimum	Maximum
5	2.0340000	0.3240062	1.6100000	2.5000000

Unique Subject Identifier=1266000000-0052

Assay variable : PTINR_VAL1 PT-INR Value

N	Mean	Standard Deviation	Minimum	Maximum
6	1.9050000	0.1963415	1.6000000	2.1300000

Laboratory tests at the time of obtaining Table 2-6 consent, according to the presence or absence of proton-pump inhibitors

Unique Subject Identifier=1266000000-0053

MEANS Procedure

Assay variable : PTINR_VAL1 PT-INR Value

N	Mean	Standard Deviation	Minimum	Maximum
6	2.4983333	0.1980320	2.2000000	2.8000000

Unique Subject Identifier=1266000000-0055

Assay variable : PTINR_VAL1 PT-INR Value

N	Mean	Standard Deviation	Minimum	Maximum
6	2.1800000	0.2497999	1.8000000	2.5800000

Unique Subject Identifier=1266000000-0056

Assay variable : PTINR_VAL1 PT-INR Value

N	Mean	Standard Deviation	Minimum	Maximum
6	2.1200000	0.3891015	1.6000000	2.6000000

Unique Subject Identifier=1266000000-0058

Assay variable : PTINR_VAL1 PT-INR Value

N	Mean	Standard Deviation	Minimum	Maximum
5	1.6980000	0.1298846	1.4800000	1.8000000

Laboratory tests at the time of obtaining Table 2-6 consent, according to the presence or absence of proton-pump inhibitors

Unique Subject Identifier=1266000000-0059

MEANS Procedure

Assay variable : PTINR_VAL1 PT-INR Value

N	Mean	Standard Deviation	Minimum	Maximum
6	2.2900000	0.4923007	1.9000000	3.2000000

Unique Subject Identifier=1266000000-0061

Assay variable : PTINR_VAL1 PT-INR Value

N	Mean	Standard Deviation	Minimum	Maximum
6	2.0400000	0.2387467	1.7000000	2.3900000

Unique Subject Identifier=1266000000-0062

Assay variable : PTINR_VAL1 PT-INR Value

N	Mean	Standard Deviation	Minimum	Maximum
6	2.2116667	0.2049797	1.9200000	2.4500000

Unique Subject Identifier=1266000000-0063

Assay variable : PTINR_VAL1 PT-INR Value

N	Mean	Standard Deviation	Minimum	Maximum
6	2.3433333	0.5746883	1.6000000	2.9000000

Laboratory tests at the time of obtaining Table 2-6 consent, according to the presence or absence of proton-pump inhibitors

Unique Subject Identifier=1266000000-0068

MEANS Procedure

Assay variable : PTINR_VAL1 PT-INR Value

N	Mean	Standard Deviation	Minimum	Maximum
6	1.8616667	0.1397736	1.7000000	2.1000000

Unique Subject Identifier=1266000000-0070

Assay variable : PTINR_VAL1 PT-INR Value

N	Mean	Standard Deviation	Minimum	Maximum
6	1.6433333	0.2367840	1.2000000	1.8000000

Unique Subject Identifier=1266000000-0071

Assay variable : PTINR_VAL1 PT-INR Value

N	Mean	Standard Deviation	Minimum	Maximum
6	2.0916667	0.3350473	1.8000000	2.7000000

Unique Subject Identifier=1266000000-0072

Assay variable : PTINR_VAL1 PT-INR Value

N	Mean	Standard Deviation	Minimum	Maximum
6	2.2333333	0.3023684	1.7000000	2.5000000

Laboratory tests at the time of obtaining Table 2-6 consent, according to the presence or absence of proton-pump inhibitors

Unique Subject Identifier=1266000000-0077

MEANS Procedure

Assay variable : PTINR_VAL1 PT-INR Value

N	Mean	Standard Deviation	Minimum	Maximum
6	1.5250000	0.1405347	1.4000000	1.7000000

Unique Subject Identifier=1266000000-0082

Assay variable : PTINR_VAL1 PT-INR Value

N	Mean	Standard Deviation	Minimum	Maximum
6	2.0666667	0.2726659	1.8500000	2.5900000

Unique Subject Identifier=1268000000-0001

Assay variable : PTINR_VAL1 PT-INR Value

N	Mean	Standard Deviation	Minimum	Maximum
3	2.0066667	0.3500476	1.6600000	2.3600000

Unique Subject Identifier=1268000000-0003

Assay variable : PTINR_VAL1 PT-INR Value

N	Mean	Standard Deviation	Minimum	Maximum
6	1.5916667	0.1704602	1.3800000	1.8900000

Laboratory tests at the time of obtaining Table 2-6 consent, according to the presence or absence of proton-pump inhibitors

Unique Subject Identifier=1268000000-0004

MEANS Procedure

Assay variable : PTINR_VAL1 PT-INR Value

N	Mean	Standard Deviation	Minimum	Maximum
4	1.6150000	0.1047219	1.5200000	1.7200000

Unique Subject Identifier=1268000000-0006

Assay variable : PTINR_VAL1 PT-INR Value

N	Mean	Standard Deviation	Minimum	Maximum
6	1.6616667	0.1433062	1.4300000	1.8200000

Unique Subject Identifier=1268000000-0007

Assay variable : PTINR_VAL1 PT-INR Value

N	Mean	Standard Deviation	Minimum	Maximum
5	1.5580000	0.1822635	1.3000000	1.7000000

Unique Subject Identifier=1269000000-0002

Assay variable : PTINR_VAL1 PT-INR Value

N	Mean	Standard Deviation	Minimum	Maximum
6	1.6083333	0.4269153	1.0500000	2.2500000

Laboratory tests at the time of obtaining Table 2-6 consent, according to the presence or absence of proton-pump inhibitors

Unique Subject Identifier=1269000000-0007

MEANS Procedure

Assay variable : PTINR_VAL1 PT-INR Value

N	Mean	Standard Deviation	Minimum	Maximum
4	1.8625000	0.1990603	1.6400000	2.1200000

Unique Subject Identifier=1269000000-0008

Assay variable : PTINR_VAL1 PT-INR Value

N	Mean	Standard Deviation	Minimum	Maximum
6	2.1200000	0.2794280	1.6200000	2.4400000

Unique Subject Identifier=1269000000-0009

Assay variable : PTINR_VAL1 PT-INR Value

N	Mean	Standard Deviation	Minimum	Maximum
6	1.8533333	0.3991324	1.1900000	2.1600000

Unique Subject Identifier=1269000000-0010

Assay variable : PTINR_VAL1 PT-INR Value

N	Mean	Standard Deviation	Minimum	Maximum
5	1.8400000	0.2164486	1.5600000	2.0500000

Laboratory tests at the time of obtaining Table 2-6 consent, according to the presence or absence of proton-pump inhibitors

Unique Subject Identifier=1269000000-0012

MEANS Procedure

Assay variable : PTINR_VAL1 PT-INR Value

N	Mean	Standard Deviation	Minimum	Maximum
6	1.8933333	0.1742029	1.7200000	2.1600000

Unique Subject Identifier=1269000000-0018

Assay variable : PTINR_VAL1 PT-INR Value

N	Mean	Standard Deviation	Minimum	Maximum
6	1.8233333	0.4719181	1.2600000	2.5500000

Unique Subject Identifier=1269000000-0019

Assay variable : PTINR_VAL1 PT-INR Value

N	Mean	Standard Deviation	Minimum	Maximum
2	1.5500000	0.3252691	1.3200000	1.7800000

Unique Subject Identifier=1269000000-0020

Assay variable : PTINR_VAL1 PT-INR Value

N	Mean	Standard Deviation	Minimum	Maximum
6	1.5016667	0.1826928	1.2100000	1.7000000

Laboratory tests at the time of obtaining Table 2-6 consent, according to the presence or absence of proton-pump inhibitors

Unique Subject Identifier=1270000000-0001

MEANS Procedure

Assay variable : PTINR_VAL1 PT-INR Value

N	Mean	Standard Deviation	Minimum	Maximum
2	1.9500000	0.2121320	1.8000000	2.1000000

Unique Subject Identifier=1270000000-0002

Assay variable : PTINR_VAL1 PT-INR Value

N	Mean	Standard Deviation	Minimum	Maximum
3	2.0933333	0.0404145	2.0500000	2.1300000

Unique Subject Identifier=1270000000-0003

Assay variable : PTINR_VAL1 PT-INR Value

N	Mean	Standard Deviation	Minimum	Maximum
2	1.8750000	0.0353553	1.8500000	1.9000000

Unique Subject Identifier=1270000000-0004

Assay variable : PTINR_VAL1 PT-INR Value

N	Mean	Standard Deviation	Minimum	Maximum
2	1.9000000	0.1414214	1.8000000	2.0000000

Laboratory tests at the time of obtaining Table 2-6 consent, according to the presence or absence of proton-pump inhibitors

Unique Subject Identifier=1270000000-0005

MEANS Procedure

Assay variable : PTINR_VAL1 PT-INR Value

N	Mean	Standard Deviation	Minimum	Maximum
4	1.8500000	0.3696846	1.4000000	2.2000000

Unique Subject Identifier=1270000000-0007

Assay variable : PTINR_VAL1 PT-INR Value

N	Mean	Standard Deviation	Minimum	Maximum
2	1.8500000	0.3535534	1.6000000	2.1000000

Unique Subject Identifier=1270000000-0008

Assay variable : PTINR_VAL1 PT-INR Value

N	Mean	Standard Deviation	Minimum	Maximum
2	1.8000000	0.2828427	1.6000000	2.0000000

Unique Subject Identifier=1270000000-0009

Assay variable : PTINR_VAL1 PT-INR Value

N	Mean	Standard Deviation	Minimum	Maximum
3	2.3333333	0.1154701	2.2000000	2.4000000

Laboratory tests at the time of obtaining Table 2-6 consent, according to the presence or absence of proton-pump inhibitors

Unique Subject Identifier=1270000000-0010

MEANS Procedure

Assay variable : PTINR_VAL1 PT-INR Value

N	Mean	Standard Deviation	Minimum	Maximum
3	1.9666667	0.2516611	1.7000000	2.2000000

Unique Subject Identifier=1270000000-0011

Assay variable : PTINR_VAL1 PT-INR Value

N	Mean	Standard Deviation	Minimum	Maximum
2	2.5000000	1.5556349	1.4000000	3.6000000

Unique Subject Identifier=1270000000-0012

Assay variable : PTINR_VAL1 PT-INR Value

N	Mean	Standard Deviation	Minimum	Maximum
2	2.5750000	0.0353553	2.5500000	2.6000000

Unique Subject Identifier=1270000000-0013

Assay variable : PTINR_VAL1 PT-INR Value

N	Mean	Standard Deviation	Minimum	Maximum
1	2.0000000		2.0000000	2.0000000

Laboratory tests at the time of obtaining Table 2-6 consent, according to the presence or absence of proton-pump inhibitors

Unique Subject Identifier=1270000000-0015

MEANS Procedure

Assay variable : PTINR_VAL1 PT-INR Value

N	Mean	Standard Deviation	Minimum	Maximum
4	1.5275000	0.1001249	1.4100000	1.6500000

Unique Subject Identifier=1270000000-0016

Assay variable : PTINR_VAL1 PT-INR Value

N	Mean	Standard Deviation	Minimum	Maximum
2	1.8500000	0.0707107	1.8000000	1.9000000

Unique Subject Identifier=1270000000-0017

Assay variable : PTINR_VAL1 PT-INR Value

N	Mean	Standard Deviation	Minimum	Maximum
3	2.8000000	1.1532563	1.9000000	4.1000000

Unique Subject Identifier=1270000000-0021

Assay variable : PTINR_VAL1 PT-INR Value

N	Mean	Standard Deviation	Minimum	Maximum
2	2.2300000	0.2404163	2.0600000	2.4000000

Laboratory tests at the time of obtaining Table 2-6 consent, according to the presence or absence of proton-pump inhibitors

Unique Subject Identifier=1270000000-0023

MEANS Procedure

Assay variable : PTINR_VAL1 PT-INR Value

N	Mean	Standard Deviation	Minimum	Maximum
3	1.7666667	0.6027714	1.2000000	2.4000000

Unique Subject Identifier=1274000000-0003

Assay variable : PTINR_VAL1 PT-INR Value

N	Mean	Standard Deviation	Minimum	Maximum
2	1.4550000	0.0494975	1.4200000	1.4900000

Unique Subject Identifier=1276000000-0001

Assay variable : PTINR_VAL1 PT-INR Value

N	Mean	Standard Deviation	Minimum	Maximum
6	2.5900000	0.7753193	1.8000000	3.9000000

Unique Subject Identifier=1276000000-0002

Assay variable : PTINR_VAL1 PT-INR Value

N	Mean	Standard Deviation	Minimum	Maximum
2	2.0500000	0.0565685	2.0100000	2.0900000

Laboratory tests at the time of obtaining Table 2-6 consent, according to the presence or absence of proton-pump inhibitors

Unique Subject Identifier=1276000000-0004

MEANS Procedure

Assay variable : PTINR_VAL1 PT-INR Value

N	Mean	Standard Deviation	Minimum	Maximum
4	1.7250000	0.2747726	1.4300000	2.0600000

Unique Subject Identifier=1276000000-0005

Assay variable : PTINR_VAL1 PT-INR Value

N	Mean	Standard Deviation	Minimum	Maximum
3	2.1000000	0.2951271	1.8000000	2.3900000

Unique Subject Identifier=1276000000-0008

Assay variable : PTINR_VAL1 PT-INR Value

N	Mean	Standard Deviation	Minimum	Maximum
3	1.8633333	0.0665833	1.8200000	1.9400000

Unique Subject Identifier=1277010100-0001

Assay variable : PTINR_VAL1 PT-INR Value

N	Mean	Standard Deviation	Minimum	Maximum
6	2.1900000	0.0678233	2.1000000	2.3000000

Laboratory tests at the time of obtaining Table 2-6 consent, according to the presence or absence of proton-pump inhibitors

Unique Subject Identifier=1277010100-0002

MEANS Procedure

Assay variable : PTINR_VAL1 PT-INR Value

N	Mean	Standard Deviation	Minimum	Maximum
6	1.8416667	0.1357080	1.6000000	2.0000000

Unique Subject Identifier=1277010100-0003

Assay variable : PTINR_VAL1 PT-INR Value

N	Mean	Standard Deviation	Minimum	Maximum
6	2.3333333	0.1632993	2.2000000	2.6000000

Unique Subject Identifier=1277010100-0004

Assay variable : PTINR_VAL1 PT-INR Value

N	Mean	Standard Deviation	Minimum	Maximum
6	1.7750000	0.2444381	1.5000000	2.2000000

Unique Subject Identifier=1277010100-0005

Assay variable : PTINR_VAL1 PT-INR Value

N	Mean	Standard Deviation	Minimum	Maximum
6	2.1783333	0.1040032	2.0700000	2.3000000

Laboratory tests at the time of obtaining Table 2-6 consent, according to the presence or absence of proton-pump inhibitors

Unique Subject Identifier=1277010100-0006

MEANS Procedure

Assay variable : PTINR_VAL1 PT-INR Value

N	Mean	Standard Deviation	Minimum	Maximum
6	2.4333333	0.2732520	2.2000000	2.9000000

Unique Subject Identifier=1277010100-0007

Assay variable : PTINR_VAL1 PT-INR Value

N	Mean	Standard Deviation	Minimum	Maximum
6	1.9150000	0.2072438	1.5900000	2.2000000

Unique Subject Identifier=1277010100-0008

Assay variable : PTINR_VAL1 PT-INR Value

N	Mean	Standard Deviation	Minimum	Maximum
6	2.3000000	0.2529822	1.9000000	2.6000000

Unique Subject Identifier=1277010100-0010

Assay variable : PTINR_VAL1 PT-INR Value

N	Mean	Standard Deviation	Minimum	Maximum
6	1.9000000	0.0894427	1.8000000	2.0000000

Laboratory tests at the time of obtaining Table 2-6 consent, according to the presence or absence of proton-pump inhibitors

Unique Subject Identifier=1277010100-0011

MEANS Procedure

Assay variable : PTINR_VAL1 PT-INR Value

N	Mean	Standard Deviation	Minimum	Maximum
6	2.4100000	0.4259108	1.8000000	2.9000000

Unique Subject Identifier=1277010100-0012

Assay variable : PTINR_VAL1 PT-INR Value

N	Mean	Standard Deviation	Minimum	Maximum
6	1.8666667	0.2804758	1.5000000	2.2000000

Unique Subject Identifier=1277010100-0015

Assay variable : PTINR_VAL1 PT-INR Value

N	Mean	Standard Deviation	Minimum	Maximum
6	1.9000000	0.3577709	1.6000000	2.4000000

Unique Subject Identifier=1277010100-0016

Assay variable : PTINR_VAL1 PT-INR Value

N	Mean	Standard Deviation	Minimum	Maximum
6	2.8000000	0.8390471	1.5000000	3.8000000

Laboratory tests at the time of obtaining Table 2-6 consent, according to the presence or absence of proton-pump inhibitors

Unique Subject Identifier=1277010100-0017

MEANS Procedure

Assay variable : PTINR_VAL1 PT-INR Value

N	Mean	Standard Deviation	Minimum	Maximum
6	2.1950000	0.1554027	2.1000000	2.5000000

Unique Subject Identifier=1278011100-0002

Assay variable : PTINR_VAL1 PT-INR Value

N	Mean	Standard Deviation	Minimum	Maximum
4	2.1750000	0.3361051	1.8900000	2.6600000

Unique Subject Identifier=1278011100-0006

Assay variable : PTINR_VAL1 PT-INR Value

N	Mean	Standard Deviation	Minimum	Maximum
5	1.8340000	0.3822041	1.1900000	2.1200000

Unique Subject Identifier=1278011100-0009

Assay variable : PTINR_VAL1 PT-INR Value

N	Mean	Standard Deviation	Minimum	Maximum
2	2.1350000	0.1484924	2.0300000	2.2400000

Laboratory tests at the time of obtaining Table 2-6 consent, according to the presence or absence of proton-pump inhibitors

Unique Subject Identifier=1278011100-0011

MEANS Procedure

Assay variable : PTINR_VAL1 PT-INR Value

N	Mean	Standard Deviation	Minimum	Maximum
6	2.2816667	1.0698863	1.5800000	4.4200000

Unique Subject Identifier=1278011100-0012

Assay variable : PTINR_VAL1 PT-INR Value

N	Mean	Standard Deviation	Minimum	Maximum
2	1.9950000	0.0353553	1.9700000	2.0200000

Unique Subject Identifier=1278011100-0013

Assay variable : PTINR_VAL1 PT-INR Value

N	Mean	Standard Deviation	Minimum	Maximum
2	1.6900000	0.0141421	1.6800000	1.7000000

Unique Subject Identifier=1279000000-0003

Assay variable : PTINR_VAL1 PT-INR Value

N	Mean	Standard Deviation	Minimum	Maximum
4	2.4500000	0.4358899	2.1000000	3.0000000

Laboratory tests at the time of obtaining Table 2-6 consent, according to the presence or absence of proton-pump inhibitors

Unique Subject Identifier=1279000000-0007

MEANS Procedure

Assay variable : PTINR_VAL1 PT-INR Value

N	Mean	Standard Deviation	Minimum	Maximum
1	1.6000000		1.6000000	1.6000000

Unique Subject Identifier=1279000000-0008

Assay variable : PTINR_VAL1 PT-INR Value

N	Mean	Standard Deviation	Minimum	Maximum
2	1.5750000	0.1202082	1.4900000	1.6600000

Unique Subject Identifier=1280001000-0003

Assay variable : PTINR_VAL1 PT-INR Value

N	Mean	Standard Deviation	Minimum	Maximum
4	1.4500000	0.2081666	1.2000000	1.7000000

Unique Subject Identifier=1280001000-0005

Assay variable : PTINR_VAL1 PT-INR Value

N	Mean	Standard Deviation	Minimum	Maximum
6	2.0333333	0.2250926	1.7000000	2.2000000

Laboratory tests at the time of obtaining Table 2-6 consent, according to the presence or absence of proton-pump inhibitors

Unique Subject Identifier=1281000000-0001

MEANS Procedure

Assay variable : PTINR_VAL1 PT-INR Value

N	Mean	Standard Deviation	Minimum	Maximum
6	2.1716667	0.1702253	2.0000000	2.4600000

Unique Subject Identifier=1281000000-0005

Assay variable : PTINR_VAL1 PT-INR Value

N	Mean	Standard Deviation	Minimum	Maximum
5	1.8200000	0.1673320	1.5600000	1.9700000

Unique Subject Identifier=1282011111-0001

Assay variable : PTINR_VAL1 PT-INR Value

N	Mean	Standard Deviation	Minimum	Maximum
6	2.3000000	0.2828427	2.0000000	2.8000000

Unique Subject Identifier=1282011111-0005

Assay variable : PTINR_VAL1 PT-INR Value

N	Mean	Standard Deviation	Minimum	Maximum
6	1.9166667	0.4445972	1.1000000	2.4000000

Laboratory tests at the time of obtaining Table 2-6 consent, according to the presence or absence of proton-pump inhibitors

Unique Subject Identifier=1286000000-0004

MEANS Procedure

Assay variable : PTINR_VAL1 PT-INR Value

N	Mean	Standard Deviation	Minimum	Maximum
6	1.9333333	0.3444803	1.6000000	2.5000000

Unique Subject Identifier=1286000000-0017

Assay variable : PTINR_VAL1 PT-INR Value

N	Mean	Standard Deviation	Minimum	Maximum
1	1.7000000		1.7000000	1.7000000

Unique Subject Identifier=1286000000-0026

Assay variable : PTINR_VAL1 PT-INR Value

N	Mean	Standard Deviation	Minimum	Maximum
6	2.2833333	0.9453394	1.4000000	4.0000000

Unique Subject Identifier=1286000000-0027

Assay variable : PTINR_VAL1 PT-INR Value

N	Mean	Standard Deviation	Minimum	Maximum
6	2.1166667	0.1602082	2.0000000	2.4000000

Laboratory tests at the time of obtaining Table 2-6 consent, according to the presence or absence of proton-pump inhibitors

Unique Subject Identifier=1286000000-0028

MEANS Procedure

Assay variable : PTINR_VAL1 PT-INR Value

N	Mean	Standard Deviation	Minimum	Maximum
6	1.9333333	0.7501111	1.3000000	3.4000000

Unique Subject Identifier=1287000000-0005

Assay variable : PTINR_VAL1 PT-INR Value

N	Mean	Standard Deviation	Minimum	Maximum
6	1.8833333	0.1940790	1.6000000	2.1000000

Unique Subject Identifier=1287000000-0010

Assay variable : PTINR_VAL1 PT-INR Value

N	Mean	Standard Deviation	Minimum	Maximum
5	1.5200000	0.1095445	1.4000000	1.7000000

Unique Subject Identifier=1287000000-0014

Assay variable : PTINR_VAL1 PT-INR Value

N	Mean	Standard Deviation	Minimum	Maximum
6	1.9000000	0.4000000	1.4000000	2.5000000

Laboratory tests at the time of obtaining Table 2-6 consent, according to the presence or absence of proton-pump inhibitors

Unique Subject Identifier=1287000000-0015

MEANS Procedure

Assay variable : PTINR_VAL1 PT-INR Value

N	Mean	Standard Deviation	Minimum	Maximum
2	1.9000000	0.2828427	1.7000000	2.1000000

Unique Subject Identifier=1289000000-0001

Assay variable : PTINR_VAL1 PT-INR Value

N	Mean	Standard Deviation	Minimum	Maximum
3	1.3600000	0.1637071	1.1800000	1.5000000

Unique Subject Identifier=1289000000-0002

Assay variable : PTINR_VAL1 PT-INR Value

N	Mean	Standard Deviation	Minimum	Maximum
3	1.8566667	0.3137409	1.5000000	2.0900000

Unique Subject Identifier=1289000000-0003

Assay variable : PTINR_VAL1 PT-INR Value

N	Mean	Standard Deviation	Minimum	Maximum
2	1.4150000	0.0070711	1.4100000	1.4200000

Laboratory tests at the time of obtaining Table 2-6 consent, according to the presence or absence of proton-pump inhibitors

Unique Subject Identifier=1289000000-0004

MEANS Procedure

Assay variable : PTINR_VAL1 PT-INR Value

N	Mean	Standard Deviation	Minimum	Maximum
2	1.5700000	0.2404163	1.4000000	1.7400000

Unique Subject Identifier=1289000000-0005

Assay variable : PTINR_VAL1 PT-INR Value

N	Mean	Standard Deviation	Minimum	Maximum
2	1.6200000	0.0707107	1.5700000	1.6700000

Unique Subject Identifier=1289000000-0006

Assay variable : PTINR_VAL1 PT-INR Value

N	Mean	Standard Deviation	Minimum	Maximum
3	1.6366667	0.1833939	1.4300000	1.7800000

Unique Subject Identifier=1289000000-0008

Assay variable : PTINR_VAL1 PT-INR Value

N	Mean	Standard Deviation	Minimum	Maximum
4	1.8825000	0.3341033	1.4000000	2.1600000

Laboratory tests at the time of obtaining Table 2-6 consent, according to the presence or absence of proton-pump inhibitors

Unique Subject Identifier=1289000000-0009

MEANS Procedure

Assay variable : PTINR_VAL1 PT-INR Value

N	Mean	Standard Deviation	Minimum	Maximum
3	2.1033333	0.4000417	1.7000000	2.5000000

Unique Subject Identifier=1290011000-0004

Assay variable : PTINR_VAL1 PT-INR Value

N	Mean	Standard Deviation	Minimum	Maximum
3	1.5233333	0.0680686	1.4700000	1.6000000

Unique Subject Identifier=1290011000-0005

Assay variable : PTINR_VAL1 PT-INR Value

N	Mean	Standard Deviation	Minimum	Maximum
2	1.7150000	0.2333452	1.5500000	1.8800000

Unique Subject Identifier=1290011000-0011

Assay variable : PTINR_VAL1 PT-INR Value

N	Mean	Standard Deviation	Minimum	Maximum
2	1.7600000	0.6929646	1.2700000	2.2500000

Laboratory tests at the time of obtaining Table 2-6 consent, according to the presence or absence of proton-pump inhibitors

Unique Subject Identifier=1291000000-0001

MEANS Procedure

Assay variable : PTINR_VAL1 PT-INR Value

N	Mean	Standard Deviation	Minimum	Maximum
4	2.0500000	0.4509250	1.7000000	2.7000000

Unique Subject Identifier=1291000000-0002

Assay variable : PTINR_VAL1 PT-INR Value

N	Mean	Standard Deviation	Minimum	Maximum
2	1.3500000	0.2121320	1.2000000	1.5000000

Unique Subject Identifier=1291000000-0003

Assay variable : PTINR_VAL1 PT-INR Value

N	Mean	Standard Deviation	Minimum	Maximum
6	1.9833333	0.6112828	1.5000000	2.9000000

Unique Subject Identifier=1291000000-0004

Assay variable : PTINR_VAL1 PT-INR Value

N	Mean	Standard Deviation	Minimum	Maximum
6	1.6500000	0.1643168	1.4000000	1.9000000

Laboratory tests at the time of obtaining Table 2-6 consent, according to the presence or absence of proton-pump inhibitors

Unique Subject Identifier=1291000000-0005

MEANS Procedure

Assay variable : PTINR_VAL1 PT-INR Value

N	Mean	Standard Deviation	Minimum	Maximum
5	1.8000000	0.1870829	1.5000000	2.0000000

Unique Subject Identifier=1291000000-0006

Assay variable : PTINR_VAL1 PT-INR Value

N	Mean	Standard Deviation	Minimum	Maximum
5	2.1400000	0.5727128	1.4000000	2.8000000

Unique Subject Identifier=1291000000-0007

Assay variable : PTINR_VAL1 PT-INR Value

N	Mean	Standard Deviation	Minimum	Maximum
4	2.5500000	0.6557439	2.0000000	3.5000000

Unique Subject Identifier=1291000000-0008

Assay variable : PTINR_VAL1 PT-INR Value

N	Mean	Standard Deviation	Minimum	Maximum
3	2.1333333	0.3511885	1.8000000	2.5000000

Laboratory tests at the time of obtaining Table 2-6 consent, according to the presence or absence of proton-pump inhibitors

Unique Subject Identifier=1291000000-0009

MEANS Procedure

Assay variable : PTINR_VAL1 PT-INR Value

N	Mean	Standard Deviation	Minimum	Maximum
5	1.6200000	0.1483240	1.4000000	1.8000000

Unique Subject Identifier=1291000000-0010

Assay variable : PTINR_VAL1 PT-INR Value

N	Mean	Standard Deviation	Minimum	Maximum
4	1.8500000	0.1732051	1.6000000	2.0000000

Unique Subject Identifier=1291000000-0011

Assay variable : PTINR_VAL1 PT-INR Value

N	Mean	Standard Deviation	Minimum	Maximum
4	1.7750000	0.3095696	1.5000000	2.2000000

Unique Subject Identifier=1291000000-0012

Assay variable : PTINR_VAL1 PT-INR Value

N	Mean	Standard Deviation	Minimum	Maximum
5	1.6200000	0.4438468	1.2000000	2.3000000

Laboratory tests at the time of obtaining Table 2-6 consent, according to the presence or absence of proton-pump inhibitors

Unique Subject Identifier=1291000000-0013

MEANS Procedure

Assay variable : PTINR_VAL1 PT-INR Value

N	Mean	Standard Deviation	Minimum	Maximum
6	1.9166667	0.6013873	1.2000000	3.0000000

Unique Subject Identifier=1291000000-0014

Assay variable : PTINR_VAL1 PT-INR Value

N	Mean	Standard Deviation	Minimum	Maximum
5	1.5200000	0.0836660	1.4000000	1.6000000

Unique Subject Identifier=1291000000-0015

Assay variable : PTINR_VAL1 PT-INR Value

N	Mean	Standard Deviation	Minimum	Maximum
2	2.0000000	0.8485281	1.4000000	2.6000000

Unique Subject Identifier=1291000000-0016

Assay variable : PTINR_VAL1 PT-INR Value

N	Mean	Standard Deviation	Minimum	Maximum
3	1.8333333	0.3055050	1.5000000	2.1000000

Laboratory tests at the time of obtaining Table 2-6 consent, according to the presence or absence of proton-pump inhibitors

Unique Subject Identifier=1291000000-0017

MEANS Procedure

Assay variable : PTINR_VAL1 PT-INR Value

N	Mean	Standard Deviation	Minimum	Maximum
6	1.5333333	0.2732520	1.1000000	1.9000000

Unique Subject Identifier=1291000000-0018

Assay variable : PTINR_VAL1 PT-INR Value

N	Mean	Standard Deviation	Minimum	Maximum
1	1.9000000		1.9000000	1.9000000

Unique Subject Identifier=1291000000-0019

Assay variable : PTINR_VAL1 PT-INR Value

N	Mean	Standard Deviation	Minimum	Maximum
6	1.7166667	0.2786874	1.4000000	2.2000000

Unique Subject Identifier=1293000000-0001

Assay variable : PTINR_VAL1 PT-INR Value

N	Mean	Standard Deviation	Minimum	Maximum
6	1.3166667	0.1169045	1.2000000	1.5000000

Laboratory tests at the time of obtaining Table 2-6 consent, according to the presence or absence of proton-pump inhibitors

Unique Subject Identifier=1293000000-0003

MEANS Procedure

Assay variable : PTINR_VAL1 PT-INR Value

N	Mean	Standard Deviation	Minimum	Maximum
6	1.7833333	0.2041241	1.6000000	2.0000000

Unique Subject Identifier=1293000000-0005

Assay variable : PTINR_VAL1 PT-INR Value

N	Mean	Standard Deviation	Minimum	Maximum
6	1.2333333	0.1861899	1.1000000	1.6000000

Unique Subject Identifier=1293000000-0006

Assay variable : PTINR_VAL1 PT-INR Value

N	Mean	Standard Deviation	Minimum	Maximum
3	1.3333333	0.4163332	1.0000000	1.8000000

Unique Subject Identifier=1293000000-0007

Assay variable : PTINR_VAL1 PT-INR Value

N	Mean	Standard Deviation	Minimum	Maximum
6	1.6166667	0.1329160	1.4000000	1.8000000

Laboratory tests at the time of obtaining Table 2-6 consent, according to the presence or absence of proton-pump inhibitors

Unique Subject Identifier=1293000000-0008

MEANS Procedure

Assay variable : PTINR_VAL1 PT-INR Value

N	Mean	Standard Deviation	Minimum	Maximum
6	1.7333333	0.1861899	1.5000000	2.0000000

Unique Subject Identifier=1293000000-0010

Assay variable : PTINR_VAL1 PT-INR Value

N	Mean	Standard Deviation	Minimum	Maximum
6	1.5333333	0.1632993	1.3000000	1.7000000

Unique Subject Identifier=1293000000-0011

Assay variable : PTINR_VAL1 PT-INR Value

N	Mean	Standard Deviation	Minimum	Maximum
5	1.3600000	0.0547723	1.3000000	1.4000000

Unique Subject Identifier=1293000000-0012

Assay variable : PTINR_VAL1 PT-INR Value

N	Mean	Standard Deviation	Minimum	Maximum
6	1.3666667	0.0816497	1.3000000	1.5000000

Laboratory tests at the time of obtaining Table 2-6 consent, according to the presence or absence of proton-pump inhibitors

Unique Subject Identifier=1293000000-0013

MEANS Procedure

Assay variable : PTINR_VAL1 PT-INR Value

N	Mean	Standard Deviation	Minimum	Maximum
1	1.6000000		1.6000000	1.6000000

Unique Subject Identifier=1293000000-0016

Assay variable : PTINR_VAL1 PT-INR Value

N	Mean	Standard Deviation	Minimum	Maximum
6	1.4333333	0.1632993	1.3000000	1.7000000

Unique Subject Identifier=1293000000-0020

Assay variable : PTINR_VAL1 PT-INR Value

N	Mean	Standard Deviation	Minimum	Maximum
5	1.4800000	0.2489980	1.3000000	1.9000000

Unique Subject Identifier=1293000000-0022

Assay variable : PTINR_VAL1 PT-INR Value

N	Mean	Standard Deviation	Minimum	Maximum
5	1.3000000	0.1000000	1.2000000	1.4000000

Laboratory tests at the time of obtaining Table 2-6 consent, according to the presence or absence of proton-pump inhibitors

Unique Subject Identifier=1293000000-0023

MEANS Procedure

Assay variable : PTINR_VAL1 PT-INR Value

N	Mean	Standard Deviation	Minimum	Maximum
5	2.0200000	0.1923538	1.7000000	2.2000000

Unique Subject Identifier=1293000000-0024

Assay variable : PTINR_VAL1 PT-INR Value

N	Mean	Standard Deviation	Minimum	Maximum
5	1.4200000	0.1643168	1.3000000	1.7000000

Unique Subject Identifier=1293000000-0029

Assay variable : PTINR_VAL1 PT-INR Value

N	Mean	Standard Deviation	Minimum	Maximum
5	1.5000000	0.1581139	1.3000000	1.7000000

Unique Subject Identifier=1293000000-0030

Assay variable : PTINR_VAL1 PT-INR Value

N	Mean	Standard Deviation	Minimum	Maximum
6	1.3333333	0.1751190	1.0000000	1.5000000

Laboratory tests at the time of obtaining Table 2-6 consent, according to the presence or absence of proton-pump inhibitors

Unique Subject Identifier=1294011001-0009

MEANS Procedure

Assay variable : PTINR_VAL1 PT-INR Value

N	Mean	Standard Deviation	Minimum	Maximum
6	2.4700000	0.3459480	1.9100000	2.9000000

Unique Subject Identifier=1296011000-0006

Assay variable : PTINR_VAL1 PT-INR Value

N	Mean	Standard Deviation	Minimum	Maximum
1	2.0300000		2.0300000	2.0300000

Unique Subject Identifier=1296011000-0007

Assay variable : PTINR_VAL1 PT-INR Value

N	Mean	Standard Deviation	Minimum	Maximum
1	1.1200000		1.1200000	1.1200000

Unique Subject Identifier=1296011000-0013

Assay variable : PTINR_VAL1 PT-INR Value

N	Mean	Standard Deviation	Minimum	Maximum
1	2.2100000		2.2100000	2.2100000

Laboratory tests at the time of obtaining Table 2-6 consent, according to the presence or absence of proton-pump inhibitors

Unique Subject Identifier=1296011000-0015

MEANS Procedure

Assay variable : PTINR_VAL1 PT-INR Value

N	Mean	Standard Deviation	Minimum	Maximum
2	2.1050000	0.6576093	1.6400000	2.5700000

Unique Subject Identifier=1296011000-0017

Assay variable : PTINR_VAL1 PT-INR Value

N	Mean	Standard Deviation	Minimum	Maximum
5	1.9820000	0.5526482	1.0800000	2.5500000

Unique Subject Identifier=1296011000-0026

Assay variable : PTINR_VAL1 PT-INR Value

N	Mean	Standard Deviation	Minimum	Maximum
4	1.1550000	0.0793725	1.0400000	1.2200000

Unique Subject Identifier=1296011000-0028

Assay variable : PTINR_VAL1 PT-INR Value

N	Mean	Standard Deviation	Minimum	Maximum
1	1.5500000		1.5500000	1.5500000

Laboratory tests at the time of obtaining Table 2-6 consent, according to the presence or absence of proton-pump inhibitors

Unique Subject Identifier=1297011000-0005

MEANS Procedure

Assay variable : PTINR_VAL1 PT-INR Value

N	Mean	Standard Deviation	Minimum	Maximum
6	2.1833333	1.3166878	1.2000000	4.2000000

Unique Subject Identifier=1297011000-0008

Assay variable : PTINR_VAL1 PT-INR Value

N	Mean	Standard Deviation	Minimum	Maximum
4	1.8500000	0.2645751	1.6000000	2.2000000

Unique Subject Identifier=1297011000-0010

Assay variable : PTINR_VAL1 PT-INR Value

N	Mean	Standard Deviation	Minimum	Maximum
5	1.7600000	0.2408319	1.5000000	2.1000000

Unique Subject Identifier=1297011000-0017

Assay variable : PTINR_VAL1 PT-INR Value

N	Mean	Standard Deviation	Minimum	Maximum
6	1.8333333	0.5125102	1.2000000	2.6000000

Laboratory tests at the time of obtaining Table 2-6 consent, according to the presence or absence of proton-pump inhibitors

Unique Subject Identifier=1298011000-0002

MEANS Procedure

Assay variable : PTINR_VAL1 PT-INR Value

N	Mean	Standard Deviation	Minimum	Maximum
5	2.3200000	0.7049823	1.4000000	3.3000000

Unique Subject Identifier=1298011000-0006

Assay variable : PTINR_VAL1 PT-INR Value

N	Mean	Standard Deviation	Minimum	Maximum
6	2.2666667	0.3723797	1.9000000	2.9000000

Unique Subject Identifier=1298011000-0010

Assay variable : PTINR_VAL1 PT-INR Value

N	Mean	Standard Deviation	Minimum	Maximum
6	1.6666667	0.1032796	1.5000000	1.8000000

Unique Subject Identifier=1298011000-0011

Assay variable : PTINR_VAL1 PT-INR Value

N	Mean	Standard Deviation	Minimum	Maximum
6	2.1166667	0.2483277	1.7000000	2.4000000

Laboratory tests at the time of obtaining Table 2-6 consent, according to the presence or absence of proton-pump inhibitors

Unique Subject Identifier=1298011000-0014

MEANS Procedure

Assay variable : PTINR_VAL1 PT-INR Value

N	Mean	Standard Deviation	Minimum	Maximum
5	1.8000000	0.3162278	1.5000000	2.3000000

Unique Subject Identifier=1298011000-0017

Assay variable : PTINR_VAL1 PT-INR Value

N	Mean	Standard Deviation	Minimum	Maximum
6	1.9500000	0.2073644	1.6000000	2.2000000

Unique Subject Identifier=1298011000-0020

Assay variable : PTINR_VAL1 PT-INR Value

N	Mean	Standard Deviation	Minimum	Maximum
6	1.7500000	0.4636809	1.1000000	2.5000000

Unique Subject Identifier=1298011000-0021

Assay variable : PTINR_VAL1 PT-INR Value

N	Mean	Standard Deviation	Minimum	Maximum
6	2.1000000	0.5138093	1.6000000	3.0000000

Laboratory tests at the time of obtaining Table 2-6 consent, according to the presence or absence of proton-pump inhibitors

Unique Subject Identifier=1298011000-0022

MEANS Procedure

Assay variable : PTINR_VAL1 PT-INR Value

N	Mean	Standard Deviation	Minimum	Maximum
6	1.9833333	0.2316607	1.6000000	2.2000000

Unique Subject Identifier=1298011000-0025

Assay variable : PTINR_VAL1 PT-INR Value

N	Mean	Standard Deviation	Minimum	Maximum
5	1.5800000	0.2774887	1.2000000	1.9000000

Unique Subject Identifier=1298011000-0027

Assay variable : PTINR_VAL1 PT-INR Value

N	Mean	Standard Deviation	Minimum	Maximum
6	2.4666667	0.2943920	2.1000000	2.8000000

Unique Subject Identifier=1299010000-0006

Assay variable : PTINR_VAL1 PT-INR Value

N	Mean	Standard Deviation	Minimum	Maximum
6	1.2783333	0.1197358	1.1800000	1.5000000

Laboratory tests at the time of obtaining Table 2-6 consent, according to the presence or absence of proton-pump inhibitors

Unique Subject Identifier=1299010000-0009

MEANS Procedure

Assay variable : PTINR_VAL1 PT-INR Value

N	Mean	Standard Deviation	Minimum	Maximum
1	1.9000000		1.9000000	1.9000000

Unique Subject Identifier=1299010000-0012

Assay variable : PTINR_VAL1 PT-INR Value

N	Mean	Standard Deviation	Minimum	Maximum
6	2.7700000	0.5978294	1.9000000	3.4000000

Unique Subject Identifier=1299010000-0013

Assay variable : PTINR_VAL1 PT-INR Value

N	Mean	Standard Deviation	Minimum	Maximum
6	1.5600000	0.1979899	1.2600000	1.8000000

Unique Subject Identifier=1299010000-0016

Assay variable : PTINR_VAL1 PT-INR Value

N	Mean	Standard Deviation	Minimum	Maximum
6	2.2666667	0.1211060	2.1000000	2.4000000

Laboratory tests at the time of obtaining Table 2-6 consent, according to the presence or absence of proton-pump inhibitors

Unique Subject Identifier=1299010000-0018

MEANS Procedure

Assay variable : PTINR_VAL1 PT-INR Value

N	Mean	Standard Deviation	Minimum	Maximum
6	1.9700000	0.3685105	1.6200000	2.6000000

Unique Subject Identifier=1299010000-0020

Assay variable : PTINR_VAL1 PT-INR Value

N	Mean	Standard Deviation	Minimum	Maximum
5	2.0580000	0.7316557	1.5000000	3.3000000

Unique Subject Identifier=1301000000-0001

Assay variable : PTINR_VAL1 PT-INR Value

N	Mean	Standard Deviation	Minimum	Maximum
6	2.2200000	0.5196152	1.7300000	3.0600000

Unique Subject Identifier=1301000000-0005

Assay variable : PTINR_VAL1 PT-INR Value

N	Mean	Standard Deviation	Minimum	Maximum
6	1.2366667	0.1616993	1.0700000	1.5100000

Laboratory tests at the time of obtaining Table 2-6 consent, according to the presence or absence of proton-pump inhibitors

Unique Subject Identifier=1301000000-0006

MEANS Procedure

Assay variable : PTINR_VAL1 PT-INR Value

N	Mean	Standard Deviation	Minimum	Maximum
4	1.3400000	0.1089342	1.2500000	1.4700000

Unique Subject Identifier=1303000000-0001

Assay variable : PTINR_VAL1 PT-INR Value

N	Mean	Standard Deviation	Minimum	Maximum
5	1.8600000	0.4159327	1.4000000	2.5000000

Unique Subject Identifier=1303000000-0002

Assay variable : PTINR_VAL1 PT-INR Value

N	Mean	Standard Deviation	Minimum	Maximum
5	1.7800000	0.3271085	1.5000000	2.3000000

Unique Subject Identifier=1303000000-0003

Assay variable : PTINR_VAL1 PT-INR Value

N	Mean	Standard Deviation	Minimum	Maximum
3	2.5333333	0.3511885	2.2000000	2.9000000

Laboratory tests at the time of obtaining Table 2-6 consent, according to the presence or absence of proton-pump inhibitors

Unique Subject Identifier=1303000000-0004

MEANS Procedure

Assay variable : PTINR_VAL1 PT-INR Value

N	Mean	Standard Deviation	Minimum	Maximum
5	2.1800000	0.2774887	1.8000000	2.5000000

Unique Subject Identifier=1303000000-0006

Assay variable : PTINR_VAL1 PT-INR Value

N	Mean	Standard Deviation	Minimum	Maximum
6	2.0000000	0.1264911	1.8000000	2.1000000

Unique Subject Identifier=1303000000-0008

Assay variable : PTINR_VAL1 PT-INR Value

N	Mean	Standard Deviation	Minimum	Maximum
4	2.0000000	0.3559026	1.7000000	2.5000000

Unique Subject Identifier=1303000000-0009

Assay variable : PTINR_VAL1 PT-INR Value

N	Mean	Standard Deviation	Minimum	Maximum
5	1.6800000	0.1095445	1.6000000	1.8000000

Laboratory tests at the time of obtaining Table 2-6 consent, according to the presence or absence of proton-pump inhibitors

Unique Subject Identifier=1303000000-0012

MEANS Procedure

Assay variable : PTINR_VAL1 PT-INR Value

N	Mean	Standard Deviation	Minimum	Maximum
5	1.9800000	0.5848077	1.6000000	3.0000000

Unique Subject Identifier=1303000000-0013

Assay variable : PTINR_VAL1 PT-INR Value

N	Mean	Standard Deviation	Minimum	Maximum
6	1.8500000	0.2073644	1.5000000	2.1000000

Unique Subject Identifier=1303000000-0015

Assay variable : PTINR_VAL1 PT-INR Value

N	Mean	Standard Deviation	Minimum	Maximum
6	1.7166667	0.1169045	1.6000000	1.9000000

Unique Subject Identifier=1303000000-0016

Assay variable : PTINR_VAL1 PT-INR Value

N	Mean	Standard Deviation	Minimum	Maximum
6	1.7500000	0.1048809	1.6000000	1.9000000

Laboratory tests at the time of obtaining Table 2-6 consent, according to the presence or absence of proton-pump inhibitors

Unique Subject Identifier=1303000000-0025

MEANS Procedure

Assay variable : PTINR_VAL1 PT-INR Value

N	Mean	Standard Deviation	Minimum	Maximum
6	2.0166667	0.4875107	1.3000000	2.7000000

Unique Subject Identifier=1303000000-0026

Assay variable : PTINR_VAL1 PT-INR Value

N	Mean	Standard Deviation	Minimum	Maximum
5	1.9200000	0.3271085	1.5000000	2.3000000

Unique Subject Identifier=1303000000-0027

Assay variable : PTINR_VAL1 PT-INR Value

N	Mean	Standard Deviation	Minimum	Maximum
5	1.7400000	0.3577709	1.4000000	2.2000000

Unique Subject Identifier=1303000000-0029

Assay variable : PTINR_VAL1 PT-INR Value

N	Mean	Standard Deviation	Minimum	Maximum
4	1.6000000	0.2449490	1.3000000	1.8000000

Laboratory tests at the time of obtaining Table 2-6 consent, according to the presence or absence of proton-pump inhibitors

Unique Subject Identifier=1306000000-0007

MEANS Procedure

Assay variable : PTINR_VAL1 PT-INR Value

N	Mean	Standard Deviation	Minimum	Maximum
1	1.5900000		1.5900000	1.5900000

Unique Subject Identifier=1308000000-0001

Assay variable : PTINR_VAL1 PT-INR Value

N	Mean	Standard Deviation	Minimum	Maximum
5	1.7320000	0.2258761	1.5100000	2.0600000

Unique Subject Identifier=1308000000-0003

Assay variable : PTINR_VAL1 PT-INR Value

N	Mean	Standard Deviation	Minimum	Maximum
6	1.9550000	0.3385705	1.4200000	2.4200000

Unique Subject Identifier=1308000000-0004

Assay variable : PTINR_VAL1 PT-INR Value

N	Mean	Standard Deviation	Minimum	Maximum
2	1.1000000	0	1.1000000	1.1000000

Laboratory tests at the time of obtaining Table 2-6 consent, according to the presence or absence of proton-pump inhibitors

Unique Subject Identifier=1312000000-0005

MEANS Procedure

Assay variable : PTINR_VAL1 PT-INR Value

N	Mean	Standard Deviation	Minimum	Maximum
6	2.0433333	0.3311596	1.7000000	2.4000000

Unique Subject Identifier=1312000000-0014

Assay variable : PTINR_VAL1 PT-INR Value

N	Mean	Standard Deviation	Minimum	Maximum
6	2.3550000	0.3434967	1.7900000	2.6600000

Unique Subject Identifier=1313000000-0013

Assay variable : PTINR_VAL1 PT-INR Value

N	Mean	Standard Deviation	Minimum	Maximum
1	1.4200000		1.4200000	1.4200000

Unique Subject Identifier=1313000000-0014

Assay variable : PTINR_VAL1 PT-INR Value

N	Mean	Standard Deviation	Minimum	Maximum
1	1.5100000		1.5100000	1.5100000

Laboratory tests at the time of obtaining Table 2-6 consent, according to the presence or absence of proton-pump inhibitors

Unique Subject Identifier=1313000000-0018

MEANS Procedure

Assay variable : PTINR_VAL1 PT-INR Value

N	Mean	Standard Deviation	Minimum	Maximum
1	1.3100000		1.3100000	1.3100000

Unique Subject Identifier=1314010000-0007

Assay variable : PTINR_VAL1 PT-INR Value

N	Mean	Standard Deviation	Minimum	Maximum
1	1.5000000		1.5000000	1.5000000

Unique Subject Identifier=1319000000-0001

Assay variable : PTINR_VAL1 PT-INR Value

N	Mean	Standard Deviation	Minimum	Maximum
1	3.0000000		3.0000000	3.0000000

Unique Subject Identifier=1319000000-0005

Assay variable : PTINR_VAL1 PT-INR Value

N	Mean	Standard Deviation	Minimum	Maximum
6	1.9500000	0.2880972	1.6000000	2.3000000

Laboratory tests at the time of obtaining Table 2-6 consent, according to the presence or absence of proton-pump inhibitors

Unique Subject Identifier=1319000000-0009

MEANS Procedure

Assay variable : PTINR_VAL1 PT-INR Value

N	Mean	Standard Deviation	Minimum	Maximum
5	1.9000000	0.3464102	1.4000000	2.2000000

Unique Subject Identifier=1319000000-0011

Assay variable : PTINR_VAL1 PT-INR Value

N	Mean	Standard Deviation	Minimum	Maximum
3	1.5366667	0.1096966	1.4100000	1.6000000

Unique Subject Identifier=1319000000-0018

Assay variable : PTINR_VAL1 PT-INR Value

N	Mean	Standard Deviation	Minimum	Maximum
6	2.4833333	0.4020779	1.8000000	3.0000000

Unique Subject Identifier=1319000000-0019

Assay variable : PTINR_VAL1 PT-INR Value

N	Mean	Standard Deviation	Minimum	Maximum
6	2.5166667	1.0068101	1.7000000	4.4000000

Laboratory tests at the time of obtaining Table 2-6 consent, according to the presence or absence of proton-pump inhibitors

Unique Subject Identifier=1319000000-0020

MEANS Procedure

Assay variable : PTINR_VAL1 PT-INR Value

N	Mean	Standard Deviation	Minimum	Maximum
6	1.8500000	0.2073644	1.6000000	2.1000000

Unique Subject Identifier=1320000000-0002

Assay variable : PTINR_VAL1 PT-INR Value

N	Mean	Standard Deviation	Minimum	Maximum
6	2.0566667	0.2809033	1.7600000	2.4700000

Unique Subject Identifier=1320000000-0003

Assay variable : PTINR_VAL1 PT-INR Value

N	Mean	Standard Deviation	Minimum	Maximum
4	1.8650000	0.3162805	1.5900000	2.3200000

Unique Subject Identifier=1320000000-0004

Assay variable : PTINR_VAL1 PT-INR Value

N	Mean	Standard Deviation	Minimum	Maximum
6	2.3700000	0.3960303	1.9500000	3.0900000

Laboratory tests at the time of obtaining Table 2-6 consent, according to the presence or absence of proton-pump inhibitors

Unique Subject Identifier=1320000000-0005

MEANS Procedure

Assay variable : PTINR_VAL1 PT-INR Value

N	Mean	Standard Deviation	Minimum	Maximum
6	2.1666667	0.3539868	1.7100000	2.5700000

Unique Subject Identifier=1320000000-0006

Assay variable : PTINR_VAL1 PT-INR Value

N	Mean	Standard Deviation	Minimum	Maximum
4	1.9150000	0.3267517	1.4400000	2.1400000

Unique Subject Identifier=1320000000-0007

Assay variable : PTINR_VAL1 PT-INR Value

N	Mean	Standard Deviation	Minimum	Maximum
4	1.8850000	0.5557877	1.3000000	2.6400000

Unique Subject Identifier=1320000000-0008

Assay variable : PTINR_VAL1 PT-INR Value

N	Mean	Standard Deviation	Minimum	Maximum
5	2.0800000	0.2589401	1.7800000	2.3200000

Laboratory tests at the time of obtaining Table 2-6 consent, according to the presence or absence of proton-pump inhibitors

Unique Subject Identifier=1320000000-0009

MEANS Procedure

Assay variable : PTINR_VAL1 PT-INR Value

N	Mean	Standard Deviation	Minimum	Maximum
5	1.9780000	0.5148495	1.3300000	2.4600000

Unique Subject Identifier=1320000000-0011

Assay variable : PTINR_VAL1 PT-INR Value

N	Mean	Standard Deviation	Minimum	Maximum
5	1.7040000	0.2951779	1.3000000	2.0000000

Unique Subject Identifier=1320000000-0012

Assay variable : PTINR_VAL1 PT-INR Value

N	Mean	Standard Deviation	Minimum	Maximum
6	1.7816667	0.0879583	1.6700000	1.9000000

Unique Subject Identifier=1320000000-0013

Assay variable : PTINR_VAL1 PT-INR Value

N	Mean	Standard Deviation	Minimum	Maximum
6	1.7350000	0.1534601	1.5200000	1.9100000

Laboratory tests at the time of obtaining Table 2-6 consent, according to the presence or absence of proton-pump inhibitors

Unique Subject Identifier=1320000000-0014

MEANS Procedure

Assay variable : PTINR_VAL1 PT-INR Value

N	Mean	Standard Deviation	Minimum	Maximum
5	1.7560000	0.3250846	1.4800000	2.2000000

Unique Subject Identifier=1320000000-0016

Assay variable : PTINR_VAL1 PT-INR Value

N	Mean	Standard Deviation	Minimum	Maximum
6	2.5616667	0.6723218	1.6800000	3.2600000

Unique Subject Identifier=1320000000-0017

Assay variable : PTINR_VAL1 PT-INR Value

N	Mean	Standard Deviation	Minimum	Maximum
6	2.2066667	0.3567445	1.9000000	2.7000000

Unique Subject Identifier=1320000000-0018

Assay variable : PTINR_VAL1 PT-INR Value

N	Mean	Standard Deviation	Minimum	Maximum
5	1.9200000	0.3896794	1.3300000	2.2700000

Laboratory tests at the time of obtaining Table 2-6 consent, according to the presence or absence of proton-pump inhibitors

Unique Subject Identifier=1320000000-0020

MEANS Procedure

Assay variable : PTINR_VAL1 PT-INR Value

N	Mean	Standard Deviation	Minimum	Maximum
6	2.1700000	0.6441739	1.4700000	3.1500000

Unique Subject Identifier=1320000000-0021

Assay variable : PTINR_VAL1 PT-INR Value

N	Mean	Standard Deviation	Minimum	Maximum
6	1.8733333	0.5328289	1.1700000	2.5000000

Unique Subject Identifier=1320000000-0022

Assay variable : PTINR_VAL1 PT-INR Value

N	Mean	Standard Deviation	Minimum	Maximum
5	2.2040000	0.2899655	1.8000000	2.6000000

Unique Subject Identifier=1320000000-0024

Assay variable : PTINR_VAL1 PT-INR Value

N	Mean	Standard Deviation	Minimum	Maximum
6	1.9266667	0.3090415	1.5900000	2.4600000

Laboratory tests at the time of obtaining Table 2-6 consent, according to the presence or absence of proton-pump inhibitors

Unique Subject Identifier=1320000000-0025

MEANS Procedure

Assay variable : PTINR_VAL1 PT-INR Value

N	Mean	Standard Deviation	Minimum	Maximum
6	2.2083333	0.7145185	1.5000000	3.5100000

Unique Subject Identifier=1320000000-0026

Assay variable : PTINR_VAL1 PT-INR Value

N	Mean	Standard Deviation	Minimum	Maximum
5	2.5820000	1.0081766	1.2000000	4.0000000

Unique Subject Identifier=1320000000-0028

Assay variable : PTINR_VAL1 PT-INR Value

N	Mean	Standard Deviation	Minimum	Maximum
6	1.9716667	0.1146153	1.8000000	2.1000000

Unique Subject Identifier=1320000000-0029

Assay variable : PTINR_VAL1 PT-INR Value

N	Mean	Standard Deviation	Minimum	Maximum
6	1.7050000	0.4458139	1.0000000	2.1700000

Laboratory tests at the time of obtaining Table 2-6 consent, according to the presence or absence of proton-pump inhibitors

Unique Subject Identifier=1320000000-0030

MEANS Procedure

Assay variable : PTINR_VAL1 PT-INR Value

N	Mean	Standard Deviation	Minimum	Maximum
6	1.9950000	0.1315675	1.7700000	2.1500000

Unique Subject Identifier=1321011110-0004

Assay variable : PTINR_VAL1 PT-INR Value

N	Mean	Standard Deviation	Minimum	Maximum
1	3.1600000		3.1600000	3.1600000

Unique Subject Identifier=1321011110-0016

Assay variable : PTINR_VAL1 PT-INR Value

N	Mean	Standard Deviation	Minimum	Maximum
1	2.2900000		2.2900000	2.2900000

Unique Subject Identifier=1321011110-0023

Assay variable : PTINR_VAL1 PT-INR Value

N	Mean	Standard Deviation	Minimum	Maximum
4	1.5975000	0.0660177	1.5100000	1.6700000

Laboratory tests at the time of obtaining Table 2-6 consent, according to the presence or absence of proton-pump inhibitors

Unique Subject Identifier=1321011110-0025

MEANS Procedure

Assay variable : PTINR_VAL1 PT-INR Value

N	Mean	Standard Deviation	Minimum	Maximum
6	2.9550000	0.5216416	2.4000000	3.7400000

Unique Subject Identifier=1322000000-0001

Assay variable : PTINR_VAL1 PT-INR Value

N	Mean	Standard Deviation	Minimum	Maximum
3	1.6600000	0.4613025	1.1800000	2.1000000

Unique Subject Identifier=1322000000-0002

Assay variable : PTINR_VAL1 PT-INR Value

N	Mean	Standard Deviation	Minimum	Maximum
1	0.9800000		0.9800000	0.9800000

Unique Subject Identifier=1322000000-0009

Assay variable : PTINR_VAL1 PT-INR Value

N	Mean	Standard Deviation	Minimum	Maximum
2	1.1550000	0.0353553	1.1300000	1.1800000

Laboratory tests at the time of obtaining Table 2-6 consent, according to the presence or absence of proton-pump inhibitors

Unique Subject Identifier=1324111000-0003

MEANS Procedure

Assay variable : PTINR_VAL1 PT-INR Value

N	Mean	Standard Deviation	Minimum	Maximum
6	2.1666667	0.4179314	1.7000000	2.8000000

Unique Subject Identifier=1324111000-0004

Assay variable : PTINR_VAL1 PT-INR Value

N	Mean	Standard Deviation	Minimum	Maximum
6	2.0333333	0.6501282	1.2000000	3.1000000

Unique Subject Identifier=1324111000-0009

Assay variable : PTINR_VAL1 PT-INR Value

N	Mean	Standard Deviation	Minimum	Maximum
6	2.5166667	0.3544949	2.0000000	3.1000000

Unique Subject Identifier=1324111000-0013

Assay variable : PTINR_VAL1 PT-INR Value

N	Mean	Standard Deviation	Minimum	Maximum
6	1.6500000	0.2428992	1.4000000	2.0000000

Laboratory tests at the time of obtaining Table 2-6 consent, according to the presence or absence of proton-pump inhibitors

Unique Subject Identifier=1324111000-0014

MEANS Procedure

Assay variable : PTINR_VAL1 PT-INR Value

N	Mean	Standard Deviation	Minimum	Maximum
6	1.7166667	0.3060501	1.4000000	2.2000000

Unique Subject Identifier=1324111000-0015

Assay variable : PTINR_VAL1 PT-INR Value

N	Mean	Standard Deviation	Minimum	Maximum
5	2.0800000	0.3962323	1.6000000	2.6000000

Unique Subject Identifier=1324111000-0019

Assay variable : PTINR_VAL1 PT-INR Value

N	Mean	Standard Deviation	Minimum	Maximum
6	1.2333333	0.0516398	1.2000000	1.3000000

Unique Subject Identifier=1326000000-0001

Assay variable : PTINR_VAL1 PT-INR Value

N	Mean	Standard Deviation	Minimum	Maximum
5	1.5000000	0.1581139	1.3000000	1.7000000

Laboratory tests at the time of obtaining Table 2-6 consent, according to the presence or absence of proton-pump inhibitors

Unique Subject Identifier=1326000000-0005

MEANS Procedure

Assay variable : PTINR_VAL1 PT-INR Value

N	Mean	Standard Deviation	Minimum	Maximum
5	0.9000000	0	0.9000000	0.9000000

Unique Subject Identifier=1326000000-0008

Assay variable : PTINR_VAL1 PT-INR Value

N	Mean	Standard Deviation	Minimum	Maximum
6	1.3500000	0.0836660	1.3000000	1.5000000

Unique Subject Identifier=1326000000-0009

Assay variable : PTINR_VAL1 PT-INR Value

N	Mean	Standard Deviation	Minimum	Maximum
6	1.0000000	0	1.0000000	1.0000000

Unique Subject Identifier=1326000000-0010

Assay variable : PTINR_VAL1 PT-INR Value

N	Mean	Standard Deviation	Minimum	Maximum
6	1.1000000	0.0632456	1.0000000	1.2000000

Laboratory tests at the time of obtaining Table 2-6 consent, according to the presence or absence of proton-pump inhibitors

Unique Subject Identifier=1328011111-0003

MEANS Procedure

Assay variable : PTINR_VAL1 PT-INR Value

N	Mean	Standard Deviation	Minimum	Maximum
6	2.3183333	0.4281783	1.7900000	3.0400000

Unique Subject Identifier=1328011111-0005

Assay variable : PTINR_VAL1 PT-INR Value

N	Mean	Standard Deviation	Minimum	Maximum
6	2.4183333	0.3291454	1.8300000	2.7900000

Unique Subject Identifier=1328011111-0009

Assay variable : PTINR_VAL1 PT-INR Value

N	Mean	Standard Deviation	Minimum	Maximum
4	1.5900000	0.2597435	1.3900000	1.9500000

Unique Subject Identifier=1328011111-0033

Assay variable : PTINR_VAL1 PT-INR Value

N	Mean	Standard Deviation	Minimum	Maximum
2	1.2250000	0.1202082	1.1400000	1.3100000

Laboratory tests at the time of obtaining Table 2-6 consent, according to the presence or absence of proton-pump inhibitors

Unique Subject Identifier=1328011111-0036

MEANS Procedure

Assay variable : PTINR_VAL1 PT-INR Value

N	Mean	Standard Deviation	Minimum	Maximum
6	1.9633333	0.3518333	1.6200000	2.5600000

Unique Subject Identifier=1328011111-0043

Assay variable : PTINR_VAL1 PT-INR Value

N	Mean	Standard Deviation	Minimum	Maximum
6	1.8166667	0.2701604	1.4700000	2.2800000

Unique Subject Identifier=1328011111-0044

Assay variable : PTINR_VAL1 PT-INR Value

N	Mean	Standard Deviation	Minimum	Maximum
2	1.7600000	0.0424264	1.7300000	1.7900000

Unique Subject Identifier=1328011111-0045

Assay variable : PTINR_VAL1 PT-INR Value

N	Mean	Standard Deviation	Minimum	Maximum
2	1.2450000	0.1767767	1.1200000	1.3700000

Laboratory tests at the time of obtaining Table 2-6 consent, according to the presence or absence of proton-pump inhibitors

Unique Subject Identifier=1328011111-0050

MEANS Procedure

Assay variable : PTINR_VAL1 PT-INR Value

N	Mean	Standard Deviation	Minimum	Maximum
6	1.7950000	0.0926823	1.6300000	1.9000000

Unique Subject Identifier=1328011111-0060

Assay variable : PTINR_VAL1 PT-INR Value

N	Mean	Standard Deviation	Minimum	Maximum
2	1.2050000	0.2333452	1.0400000	1.3700000

Unique Subject Identifier=1328011111-0072

Assay variable : PTINR_VAL1 PT-INR Value

N	Mean	Standard Deviation	Minimum	Maximum
5	2.2160000	0.2659511	1.8800000	2.5800000

Unique Subject Identifier=1328011111-0074

Assay variable : PTINR_VAL1 PT-INR Value

N	Mean	Standard Deviation	Minimum	Maximum
1	1.9900000		1.9900000	1.9900000

Laboratory tests at the time of obtaining Table 2-6 consent, according to the presence or absence of proton-pump inhibitors

Unique Subject Identifier=1328011111-0075

MEANS Procedure

Assay variable : PTINR_VAL1 PT-INR Value

N	Mean	Standard Deviation	Minimum	Maximum
1	1.9500000		1.9500000	1.9500000

Unique Subject Identifier=1328011111-0079

Assay variable : PTINR_VAL1 PT-INR Value

N	Mean	Standard Deviation	Minimum	Maximum
4	2.3575000	0.1252664	2.1700000	2.4300000

Unique Subject Identifier=1328011111-0086

Assay variable : PTINR_VAL1 PT-INR Value

N	Mean	Standard Deviation	Minimum	Maximum
6	1.6766667	0.2522433	1.3700000	2.0900000

Unique Subject Identifier=1328011111-0090

Assay variable : PTINR_VAL1 PT-INR Value

N	Mean	Standard Deviation	Minimum	Maximum
5	1.6440000	0.1130487	1.5100000	1.7700000

Laboratory tests at the time of obtaining Table 2-6 consent, according to the presence or absence of proton-pump inhibitors

Unique Subject Identifier=1336011110-0001

MEANS Procedure

Assay variable : PTINR_VAL1 PT-INR Value

N	Mean	Standard Deviation	Minimum	Maximum
4	1.4250000	0.2629956	1.2000000	1.8000000

Unique Subject Identifier=1341011111-0008

Assay variable : PTINR_VAL1 PT-INR Value

N	Mean	Standard Deviation	Minimum	Maximum
5	1.9160000	0.1283745	1.7000000	2.0400000

Unique Subject Identifier=1341011111-0011

Assay variable : PTINR_VAL1 PT-INR Value

N	Mean	Standard Deviation	Minimum	Maximum
5	2.3500000	0.3421257	1.9100000	2.8700000

Unique Subject Identifier=1341011111-0014

Assay variable : PTINR_VAL1 PT-INR Value

N	Mean	Standard Deviation	Minimum	Maximum
1	2.4300000		2.4300000	2.4300000

Laboratory tests at the time of obtaining Table 2-6 consent, according to the presence or absence of proton-pump inhibitors

Unique Subject Identifier=1341011111-0015

MEANS Procedure

Assay variable : PTINR_VAL1 PT-INR Value

N	Mean	Standard Deviation	Minimum	Maximum
3	2.5100000	0.3950949	2.1100000	2.9000000

Unique Subject Identifier=1342000000-0006

Assay variable : PTINR_VAL1 PT-INR Value

N	Mean	Standard Deviation	Minimum	Maximum
6	2.3200000	0.3955250	1.7200000	2.8100000

Unique Subject Identifier=1351010111-0002

Assay variable : PTINR_VAL1 PT-INR Value

N	Mean	Standard Deviation	Minimum	Maximum
6	1.8166667	0.2714160	1.3000000	2.1000000

Unique Subject Identifier=1351010111-0003

Assay variable : PTINR_VAL1 PT-INR Value

N	Mean	Standard Deviation	Minimum	Maximum
6	2.1666667	0.3386247	1.9000000	2.8000000

Laboratory tests at the time of obtaining Table 2-6 consent, according to the presence or absence of proton-pump inhibitors

Unique Subject Identifier=1351010111-0009

MEANS Procedure

Assay variable : PTINR_VAL1 PT-INR Value

N	Mean	Standard Deviation	Minimum	Maximum
6	1.8833333	0.3430258	1.4000000	2.3000000

Unique Subject Identifier=1351010111-0012

Assay variable : PTINR_VAL1 PT-INR Value

N	Mean	Standard Deviation	Minimum	Maximum
6	1.5666667	0.2581989	1.2000000	1.9000000

Unique Subject Identifier=1351010111-0013

Assay variable : PTINR_VAL1 PT-INR Value

N	Mean	Standard Deviation	Minimum	Maximum
6	1.7000000	0.2529822	1.5000000	2.2000000

Unique Subject Identifier=1351010111-0015

Assay variable : PTINR_VAL1 PT-INR Value

N	Mean	Standard Deviation	Minimum	Maximum
6	2.1500000	0.3146427	1.7000000	2.6000000

Laboratory tests at the time of obtaining Table 2-6 consent, according to the presence or absence of proton-pump inhibitors

Unique Subject Identifier=1351010111-0018

MEANS Procedure

Assay variable : PTINR_VAL1 PT-INR Value

N	Mean	Standard Deviation	Minimum	Maximum
6	2.1166667	0.2994439	1.7000000	2.5000000

Unique Subject Identifier=1351010111-0022

Assay variable : PTINR_VAL1 PT-INR Value

N	Mean	Standard Deviation	Minimum	Maximum
6	1.9333333	0.7089899	1.0000000	3.1000000

Unique Subject Identifier=1352011010-0003

Assay variable : PTINR_VAL1 PT-INR Value

N	Mean	Standard Deviation	Minimum	Maximum
6	2.2833333	0.6493587	1.3000000	3.0000000

Unique Subject Identifier=1352011010-0011

Assay variable : PTINR_VAL1 PT-INR Value

N	Mean	Standard Deviation	Minimum	Maximum
6	2.0666667	0.3559026	1.6000000	2.7000000

Laboratory tests at the time of obtaining Table 2-6 consent, according to the presence or absence of proton-pump inhibitors

Unique Subject Identifier=1352011010-0012

MEANS Procedure

Assay variable : PTINR_VAL1 PT-INR Value

N	Mean	Standard Deviation	Minimum	Maximum
6	1.6333333	0.4760952	1.2000000	2.5000000

Unique Subject Identifier=1353000000-0001

Assay variable : PTINR_VAL1 PT-INR Value

N	Mean	Standard Deviation	Minimum	Maximum
2	1.8500000	0.0707107	1.8000000	1.9000000

Unique Subject Identifier=1353000000-0003

Assay variable : PTINR_VAL1 PT-INR Value

N	Mean	Standard Deviation	Minimum	Maximum
2	1.9000000	0.1414214	1.8000000	2.0000000

Unique Subject Identifier=1353000000-0005

Assay variable : PTINR_VAL1 PT-INR Value

N	Mean	Standard Deviation	Minimum	Maximum
1	2.2000000		2.2000000	2.2000000

Laboratory tests at the time of obtaining Table 2-6 consent, according to the presence or absence of proton-pump inhibitors

Unique Subject Identifier=1353000000-0015

MEANS Procedure

Assay variable : PTINR_VAL1 PT-INR Value

N	Mean	Standard Deviation	Minimum	Maximum
1	2.4000000		2.4000000	2.4000000

Unique Subject Identifier=1353000000-0017

Assay variable : PTINR_VAL1 PT-INR Value

N	Mean	Standard Deviation	Minimum	Maximum
1	1.9000000		1.9000000	1.9000000

Unique Subject Identifier=1353000000-0024

Assay variable : PTINR_VAL1 PT-INR Value

N	Mean	Standard Deviation	Minimum	Maximum
2	2.1500000	0.2121320	2.0000000	2.3000000

Unique Subject Identifier=1353000000-0028

Assay variable : PTINR_VAL1 PT-INR Value

N	Mean	Standard Deviation	Minimum	Maximum
2	1.8500000	0.2121320	1.7000000	2.0000000

Laboratory tests at the time of obtaining Table 2-6 consent, according to the presence or absence of proton-pump inhibitors

Unique Subject Identifier=1353000000-0034

MEANS Procedure

Assay variable : PTINR_VAL1 PT-INR Value

N	Mean	Standard Deviation	Minimum	Maximum
2	1.5500000	0.0707107	1.5000000	1.6000000

Unique Subject Identifier=1353000000-0039

Assay variable : PTINR_VAL1 PT-INR Value

N	Mean	Standard Deviation	Minimum	Maximum
1	2.2000000		2.2000000	2.2000000

Unique Subject Identifier=1353000000-0048

Assay variable : PTINR_VAL1 PT-INR Value

N	Mean	Standard Deviation	Minimum	Maximum
2	1.5000000	0.4242641	1.2000000	1.8000000

Unique Subject Identifier=1353000000-0049

Assay variable : PTINR_VAL1 PT-INR Value

N	Mean	Standard Deviation	Minimum	Maximum
2	1.6500000	0.0707107	1.6000000	1.7000000

Laboratory tests at the time of obtaining Table 2-6 consent, according to the presence or absence of proton-pump inhibitors

Unique Subject Identifier=1353000000-0050

MEANS Procedure

Assay variable : PTINR_VAL1 PT-INR Value

N	Mean	Standard Deviation	Minimum	Maximum
1	1.2000000		1.2000000	1.2000000

Unique Subject Identifier=1353000000-0051

Assay variable : PTINR_VAL1 PT-INR Value

N	Mean	Standard Deviation	Minimum	Maximum
1	1.8000000		1.8000000	1.8000000

Unique Subject Identifier=1354010000-0001

Assay variable : PTINR_VAL1 PT-INR Value

N	Mean	Standard Deviation	Minimum	Maximum
6	1.6100000	0.1005982	1.5100000	1.7400000

Unique Subject Identifier=1354010000-0004

Assay variable : PTINR_VAL1 PT-INR Value

N	Mean	Standard Deviation	Minimum	Maximum
5	2.1960000	0.2434748	1.9300000	2.5500000

Laboratory tests at the time of obtaining Table 2-6 consent, according to the presence or absence of proton-pump inhibitors

Unique Subject Identifier=1354010000-0006

MEANS Procedure

Assay variable : PTINR_VAL1 PT-INR Value

N	Mean	Standard Deviation	Minimum	Maximum
5	1.8140000	0.1443260	1.5900000	1.9600000

Unique Subject Identifier=1354010000-0007

Assay variable : PTINR_VAL1 PT-INR Value

N	Mean	Standard Deviation	Minimum	Maximum
5	1.5680000	0.1219426	1.4800000	1.7800000

Unique Subject Identifier=1354010000-0011

Assay variable : PTINR_VAL1 PT-INR Value

N	Mean	Standard Deviation	Minimum	Maximum
3	1.4733333	0.0757188	1.4200000	1.5600000

Unique Subject Identifier=1354010000-0012

Assay variable : PTINR_VAL1 PT-INR Value

N	Mean	Standard Deviation	Minimum	Maximum
6	1.8100000	0.1186592	1.6400000	1.9700000

Laboratory tests at the time of obtaining Table 2-6 consent, according to the presence or absence of proton-pump inhibitors

Unique Subject Identifier=1354010000-0013

MEANS Procedure

Assay variable : PTINR_VAL1 PT-INR Value

N	Mean	Standard Deviation	Minimum	Maximum
5	1.9380000	0.4510211	1.6000000	2.7000000

Unique Subject Identifier=1354010000-0015

Assay variable : PTINR_VAL1 PT-INR Value

N	Mean	Standard Deviation	Minimum	Maximum
5	2.1240000	0.1504327	1.8600000	2.2100000

Unique Subject Identifier=1354010000-0016

Assay variable : PTINR_VAL1 PT-INR Value

N	Mean	Standard Deviation	Minimum	Maximum
3	1.9233333	0.2315887	1.7300000	2.1800000

Unique Subject Identifier=1354010000-0023

Assay variable : PTINR_VAL1 PT-INR Value

N	Mean	Standard Deviation	Minimum	Maximum
5	1.5940000	0.1277889	1.4400000	1.7900000

Laboratory tests at the time of obtaining Table 2-6 consent, according to the presence or absence of proton-pump inhibitors

Unique Subject Identifier=1354010000-0024

MEANS Procedure

Assay variable : PTINR_VAL1 PT-INR Value

N	Mean	Standard Deviation	Minimum	Maximum
6	1.5616667	0.1185608	1.4100000	1.7500000

Unique Subject Identifier=1354010000-0027

Assay variable : PTINR_VAL1 PT-INR Value

N	Mean	Standard Deviation	Minimum	Maximum
6	1.7050000	0.3923137	1.3700000	2.4400000

Unique Subject Identifier=1354010000-0032

Assay variable : PTINR_VAL1 PT-INR Value

N	Mean	Standard Deviation	Minimum	Maximum
6	1.4733333	0.1116542	1.3000000	1.6100000

Unique Subject Identifier=1354010000-0033

Assay variable : PTINR_VAL1 PT-INR Value

N	Mean	Standard Deviation	Minimum	Maximum
6	1.6900000	0.3095158	1.2200000	2.0600000

Laboratory tests at the time of obtaining Table 2-6 consent, according to the presence or absence of proton-pump inhibitors

Unique Subject Identifier=1354010000-0034

MEANS Procedure

Assay variable : PTINR_VAL1 PT-INR Value

N	Mean	Standard Deviation	Minimum	Maximum
6	1.8083333	0.1801573	1.5800000	2.0800000

Unique Subject Identifier=1354010000-0035

Assay variable : PTINR_VAL1 PT-INR Value

N	Mean	Standard Deviation	Minimum	Maximum
3	1.6100000	0.4330127	1.1100000	1.8600000

Unique Subject Identifier=1354010000-0037

Assay variable : PTINR_VAL1 PT-INR Value

N	Mean	Standard Deviation	Minimum	Maximum
6	1.4383333	0.1360025	1.3000000	1.6500000

Unique Subject Identifier=1354010000-0045

Assay variable : PTINR_VAL1 PT-INR Value

N	Mean	Standard Deviation	Minimum	Maximum
6	1.8366667	0.3957609	1.3400000	2.5100000

Laboratory tests at the time of obtaining Table 2-6 consent, according to the presence or absence of proton-pump inhibitors

Unique Subject Identifier=1354010000-0046

MEANS Procedure

Assay variable : PTINR_VAL1 PT-INR Value

N	Mean	Standard Deviation	Minimum	Maximum
5	2.0760000	0.2469413	1.6600000	2.2700000

Unique Subject Identifier=1354010000-0050

Assay variable : PTINR_VAL1 PT-INR Value

N	Mean	Standard Deviation	Minimum	Maximum
5	1.7380000	0.1728294	1.5400000	1.9800000

Unique Subject Identifier=1354010000-0052

Assay variable : PTINR_VAL1 PT-INR Value

N	Mean	Standard Deviation	Minimum	Maximum
6	2.2083333	1.4976838	1.1600000	5.1000000

Unique Subject Identifier=1354010000-0056

Assay variable : PTINR_VAL1 PT-INR Value

N	Mean	Standard Deviation	Minimum	Maximum
6	1.7100000	0.1572260	1.5300000	1.9800000

Laboratory tests at the time of obtaining Table 2-6 consent, according to the presence or absence of proton-pump inhibitors

Unique Subject Identifier=1355000000-0005

MEANS Procedure

Assay variable : PTINR_VAL1 PT-INR Value

N	Mean	Standard Deviation	Minimum	Maximum
6	2.1166667	0.3371449	1.6000000	2.6000000

Unique Subject Identifier=1355000000-0007

Assay variable : PTINR_VAL1 PT-INR Value

N	Mean	Standard Deviation	Minimum	Maximum
6	2.1166667	0.0752773	2.0000000	2.2000000

Unique Subject Identifier=1365010000-0002

Assay variable : PTINR_VAL1 PT-INR Value

N	Mean	Standard Deviation	Minimum	Maximum
4	2.0400000	0.2174090	1.7600000	2.2900000

Unique Subject Identifier=1365010000-0004

Assay variable : PTINR_VAL1 PT-INR Value

N	Mean	Standard Deviation	Minimum	Maximum
4	1.9325000	0.0607591	1.8600000	2.0000000

Laboratory tests at the time of obtaining Table 2-6 consent, according to the presence or absence of proton-pump inhibitors

Unique Subject Identifier=1365010000-0005

MEANS Procedure

Assay variable : PTINR_VAL1 PT-INR Value

N	Mean	Standard Deviation	Minimum	Maximum
4	1.7650000	0.1040833	1.6400000	1.8900000

Unique Subject Identifier=1365010000-0006

Assay variable : PTINR_VAL1 PT-INR Value

N	Mean	Standard Deviation	Minimum	Maximum
6	1.7083333	0.1762290	1.4900000	1.8800000

Unique Subject Identifier=1365010000-0007

Assay variable : PTINR_VAL1 PT-INR Value

N	Mean	Standard Deviation	Minimum	Maximum
6	2.0833333	0.4950421	1.5000000	2.7000000

Unique Subject Identifier=1365010000-0008

Assay variable : PTINR_VAL1 PT-INR Value

N	Mean	Standard Deviation	Minimum	Maximum
5	1.8940000	0.3383489	1.6100000	2.3200000

Laboratory tests at the time of obtaining Table 2-6 consent, according to the presence or absence of proton-pump inhibitors

Unique Subject Identifier=1365010000-0019

MEANS Procedure

Assay variable : PTINR_VAL1 PT-INR Value

N	Mean	Standard Deviation	Minimum	Maximum
3	1.7700000	0.2000000	1.5700000	1.9700000

Unique Subject Identifier=1365010000-0021

Assay variable : PTINR_VAL1 PT-INR Value

N	Mean	Standard Deviation	Minimum	Maximum
1	1.6500000		1.6500000	1.6500000

Unique Subject Identifier=1368011111-0001

Assay variable : PTINR_VAL1 PT-INR Value

N	Mean	Standard Deviation	Minimum	Maximum
2	1.3250000	0.1060660	1.2500000	1.4000000

Unique Subject Identifier=1368011111-0002

Assay variable : PTINR_VAL1 PT-INR Value

N	Mean	Standard Deviation	Minimum	Maximum
2	1.9600000	0.1131371	1.8800000	2.0400000

Laboratory tests at the time of obtaining Table 2-6 consent, according to the presence or absence of proton-pump inhibitors

Unique Subject Identifier=1369010010-0001

MEANS Procedure

Assay variable : PTINR_VAL1 PT-INR Value

N	Mean	Standard Deviation	Minimum	Maximum
5	1.9460000	0.2567684	1.7300000	2.3000000

Unique Subject Identifier=1370010000-0012

Assay variable : PTINR_VAL1 PT-INR Value

N	Mean	Standard Deviation	Minimum	Maximum
1	2.1800000		2.1800000	2.1800000

Unique Subject Identifier=1371000000-0004

Assay variable : PTINR_VAL1 PT-INR Value

N	Mean	Standard Deviation	Minimum	Maximum
6	2.1233333	0.3201666	1.6800000	2.5800000

Unique Subject Identifier=1371000000-0005

Assay variable : PTINR_VAL1 PT-INR Value

N	Mean	Standard Deviation	Minimum	Maximum
6	1.6833333	0.2959504	1.4200000	2.1800000

Laboratory tests at the time of obtaining Table 2-6 consent, according to the presence or absence of proton-pump inhibitors

Unique Subject Identifier=1371000000-0007

MEANS Procedure

Assay variable : PTINR_VAL1 PT-INR Value

N	Mean	Standard Deviation	Minimum	Maximum
6	2.0300000	0.1908926	1.7400000	2.2900000

Unique Subject Identifier=1371000000-0009

Assay variable : PTINR_VAL1 PT-INR Value

N	Mean	Standard Deviation	Minimum	Maximum
6	1.7900000	0.1352036	1.6100000	1.9700000

Unique Subject Identifier=1371000000-0010

Assay variable : PTINR_VAL1 PT-INR Value

N	Mean	Standard Deviation	Minimum	Maximum
6	1.7700000	0.3878144	1.4200000	2.5300000

Unique Subject Identifier=1371000000-0011

Assay variable : PTINR_VAL1 PT-INR Value

N	Mean	Standard Deviation	Minimum	Maximum
5	1.8440000	0.2234502	1.4800000	2.0200000

Laboratory tests at the time of obtaining Table 2-6 consent, according to the presence or absence of proton-pump inhibitors

Unique Subject Identifier=1371000000-0015

MEANS Procedure

Assay variable : PTINR_VAL1 PT-INR Value

N	Mean	Standard Deviation	Minimum	Maximum
5	1.8700000	0.2813361	1.5500000	2.1900000

Unique Subject Identifier=1371000000-0016

Assay variable : PTINR_VAL1 PT-INR Value

N	Mean	Standard Deviation	Minimum	Maximum
6	1.9400000	0.3752333	1.4000000	2.4300000

Unique Subject Identifier=1371000000-0017

Assay variable : PTINR_VAL1 PT-INR Value

N	Mean	Standard Deviation	Minimum	Maximum
6	1.6366667	0.1979562	1.4600000	1.9900000

Unique Subject Identifier=1371000000-0021

Assay variable : PTINR_VAL1 PT-INR Value

N	Mean	Standard Deviation	Minimum	Maximum
6	1.8866667	0.2130415	1.6000000	2.0900000

Laboratory tests at the time of obtaining Table 2-6 consent, according to the presence or absence of proton-pump inhibitors

Unique Subject Identifier=1371000000-0023

MEANS Procedure

Assay variable : PTINR_VAL1 PT-INR Value

N	Mean	Standard Deviation	Minimum	Maximum
6	1.7516667	0.6507662	1.0700000	2.9600000

Unique Subject Identifier=1371000000-0024

Assay variable : PTINR_VAL1 PT-INR Value

N	Mean	Standard Deviation	Minimum	Maximum
6	1.7283333	0.3864410	1.2100000	2.0400000

Unique Subject Identifier=1372011010-0006

Assay variable : PTINR_VAL1 PT-INR Value

N	Mean	Standard Deviation	Minimum	Maximum
4	1.8000000	0.7845593	1.2400000	2.9500000

Unique Subject Identifier=1373000000-0002

Assay variable : PTINR_VAL1 PT-INR Value

N	Mean	Standard Deviation	Minimum	Maximum
5	1.4800000	0.2774887	1.2000000	1.9000000

Laboratory tests at the time of obtaining Table 2-6 consent, according to the presence or absence of proton-pump inhibitors

Unique Subject Identifier=1377011000-0002

MEANS Procedure

Assay variable : PTINR_VAL1 PT-INR Value

N	Mean	Standard Deviation	Minimum	Maximum
5	2.3140000	0.3150079	1.8800000	2.7600000

Unique Subject Identifier=1377011000-0003

Assay variable : PTINR_VAL1 PT-INR Value

N	Mean	Standard Deviation	Minimum	Maximum
4	1.5925000	0.0780491	1.4900000	1.6800000

Unique Subject Identifier=1377011000-0004

Assay variable : PTINR_VAL1 PT-INR Value

N	Mean	Standard Deviation	Minimum	Maximum
4	2.1300000	1.0241419	1.1200000	3.3500000

Unique Subject Identifier=1377011000-0009

Assay variable : PTINR_VAL1 PT-INR Value

N	Mean	Standard Deviation	Minimum	Maximum
4	2.1400000	0.4913926	1.4500000	2.5300000

Laboratory tests at the time of obtaining Table 2-6 consent, according to the presence or absence of proton-pump inhibitors

Unique Subject Identifier=1377011000-0011

MEANS Procedure

Assay variable : PTINR_VAL1 PT-INR Value

N	Mean	Standard Deviation	Minimum	Maximum
6	2.2766667	0.4296821	1.7900000	2.8700000

Unique Subject Identifier=1377011000-0012

Assay variable : PTINR_VAL1 PT-INR Value

N	Mean	Standard Deviation	Minimum	Maximum
5	1.4240000	0.2244549	1.0900000	1.6800000

Unique Subject Identifier=1377011000-0016

Assay variable : PTINR_VAL1 PT-INR Value

N	Mean	Standard Deviation	Minimum	Maximum
5	1.9560000	0.2370232	1.6200000	2.1300000

Unique Subject Identifier=1380000110-0008

Assay variable : PTINR_VAL1 PT-INR Value

N	Mean	Standard Deviation	Minimum	Maximum
6	1.8500000	0.5576737	1.4000000	2.8000000

Laboratory tests at the time of obtaining Table 2-6 consent, according to the presence or absence of proton-pump inhibitors

Unique Subject Identifier=1380000110-0011

MEANS Procedure

Assay variable : PTINR_VAL1 PT-INR Value

N	Mean	Standard Deviation	Minimum	Maximum
6	2.0000000	0.2366432	1.6000000	2.3000000

Unique Subject Identifier=1380000110-0016

Assay variable : PTINR_VAL1 PT-INR Value

N	Mean	Standard Deviation	Minimum	Maximum
6	2.1000000	0.7293833	1.3000000	3.3000000

Unique Subject Identifier=1380000110-0022

Assay variable : PTINR_VAL1 PT-INR Value

N	Mean	Standard Deviation	Minimum	Maximum
6	2.0666667	0.3444803	1.6000000	2.5000000

Unique Subject Identifier=1380000110-0024

Assay variable : PTINR_VAL1 PT-INR Value

N	Mean	Standard Deviation	Minimum	Maximum
6	1.8666667	0.2875181	1.4000000	2.2000000

Laboratory tests at the time of obtaining Table 2-6 consent, according to the presence or absence of proton-pump inhibitors

Unique Subject Identifier=1380000110-0027

MEANS Procedure

Assay variable : PTINR_VAL1 PT-INR Value

N	Mean	Standard Deviation	Minimum	Maximum
5	2.2800000	0.3033150	1.8000000	2.6000000

Unique Subject Identifier=1381000000-0008

Assay variable : PTINR_VAL1 PT-INR Value

N	Mean	Standard Deviation	Minimum	Maximum
2	2.1150000	0.4171930	1.8200000	2.4100000

Unique Subject Identifier=1385011000-0011

Assay variable : PTINR_VAL1 PT-INR Value

N	Mean	Standard Deviation	Minimum	Maximum
5	2.0820000	0.2811050	1.7200000	2.4100000

Unique Subject Identifier=1385011000-0014

Assay variable : PTINR_VAL1 PT-INR Value

N	Mean	Standard Deviation	Minimum	Maximum
3	2.4733333	0.7379928	1.7000000	3.1700000

Laboratory tests at the time of obtaining Table 2-6 consent, according to the presence or absence of proton-pump inhibitors

Unique Subject Identifier=1385011000-0016

MEANS Procedure

Assay variable : PTINR_VAL1 PT-INR Value

N	Mean	Standard Deviation	Minimum	Maximum
3	2.1500000	0.0888819	2.0800000	2.2500000

Unique Subject Identifier=1385011000-0027

Assay variable : PTINR_VAL1 PT-INR Value

N	Mean	Standard Deviation	Minimum	Maximum
2	2.1200000	0.0282843	2.1000000	2.1400000

Unique Subject Identifier=1385011000-0028

Assay variable : PTINR_VAL1 PT-INR Value

N	Mean	Standard Deviation	Minimum	Maximum
3	2.2933333	0.0450925	2.2500000	2.3400000

Unique Subject Identifier=1385011000-0035

Assay variable : PTINR_VAL1 PT-INR Value

N	Mean	Standard Deviation	Minimum	Maximum
1	1.6800000		1.6800000	1.6800000

Laboratory tests at the time of obtaining Table 2-6 consent, according to the presence or absence of proton-pump inhibitors

Unique Subject Identifier=1385011000-0044

MEANS Procedure

Assay variable : PTINR_VAL1 PT-INR Value

N	Mean	Standard Deviation	Minimum	Maximum
3	2.1200000	0.6100000	1.5100000	2.7300000

Unique Subject Identifier=1385011000-0057

Assay variable : PTINR_VAL1 PT-INR Value

N	Mean	Standard Deviation	Minimum	Maximum
1	1.8200000		1.8200000	1.8200000

Unique Subject Identifier=1385011000-0059

Assay variable : PTINR_VAL1 PT-INR Value

N	Mean	Standard Deviation	Minimum	Maximum
4	1.6200000	0.1346601	1.4200000	1.7000000

Unique Subject Identifier=1385011000-0061

Assay variable : PTINR_VAL1 PT-INR Value

N	Mean	Standard Deviation	Minimum	Maximum
3	2.2866667	0.0152753	2.2700000	2.3000000

Laboratory tests at the time of obtaining Table 2-6 consent, according to the presence or absence of proton-pump inhibitors

Unique Subject Identifier=1385011000-0065

MEANS Procedure

Assay variable : PTINR_VAL1 PT-INR Value

N	Mean	Standard Deviation	Minimum	Maximum
3	2.2500000	0.0600000	2.1900000	2.3100000

Unique Subject Identifier=1385011000-0110

Assay variable : PTINR_VAL1 PT-INR Value

N	Mean	Standard Deviation	Minimum	Maximum
2	2.1000000	0.0282843	2.0800000	2.1200000

Unique Subject Identifier=1386000000-0006

Assay variable : PTINR_VAL1 PT-INR Value

N	Mean	Standard Deviation	Minimum	Maximum
6	2.6483333	0.8358808	1.4900000	3.7600000

Unique Subject Identifier=1386000000-0007

Assay variable : PTINR_VAL1 PT-INR Value

N	Mean	Standard Deviation	Minimum	Maximum
3	1.2500000	0.0500000	1.2000000	1.3000000

Laboratory tests at the time of obtaining Table 2-6 consent, according to the presence or absence of proton-pump inhibitors

Unique Subject Identifier=1386000000-0017

MEANS Procedure

Assay variable : PTINR_VAL1 PT-INR Value

N	Mean	Standard Deviation	Minimum	Maximum
2	1.8150000	0.5444722	1.4300000	2.2000000

Unique Subject Identifier=1386000000-0018

Assay variable : PTINR_VAL1 PT-INR Value

N	Mean	Standard Deviation	Minimum	Maximum
4	1.9650000	0.3481858	1.6000000	2.3900000

Unique Subject Identifier=1386000000-0021

Assay variable : PTINR_VAL1 PT-INR Value

N	Mean	Standard Deviation	Minimum	Maximum
1	3.3000000		3.3000000	3.3000000

Unique Subject Identifier=1386000000-0022

Assay variable : PTINR_VAL1 PT-INR Value

N	Mean	Standard Deviation	Minimum	Maximum
3	2.7933333	0.9222979	1.9700000	3.7900000

Laboratory tests at the time of obtaining Table 2-6 consent, according to the presence or absence of proton-pump inhibitors

Unique Subject Identifier=1392011110-0003

MEANS Procedure

Assay variable : PTINR_VAL1 PT-INR Value

N	Mean	Standard Deviation	Minimum	Maximum
6	1.6166667	0.2483277	1.3000000	2.0000000

Unique Subject Identifier=1392011110-0006

Assay variable : PTINR_VAL1 PT-INR Value

N	Mean	Standard Deviation	Minimum	Maximum
6	2.0833333	0.4020779	1.6000000	2.6000000

Unique Subject Identifier=1392011110-0007

Assay variable : PTINR_VAL1 PT-INR Value

N	Mean	Standard Deviation	Minimum	Maximum
6	1.8666667	0.3386247	1.6000000	2.5000000

Unique Subject Identifier=1392011110-0008

Assay variable : PTINR_VAL1 PT-INR Value

N	Mean	Standard Deviation	Minimum	Maximum
6	1.5666667	0.2065591	1.2000000	1.8000000

Laboratory tests at the time of obtaining Table 2-6 consent, according to the presence or absence of proton-pump inhibitors

Unique Subject Identifier=1392011110-0011

MEANS Procedure

Assay variable : PTINR_VAL1 PT-INR Value

N	Mean	Standard Deviation	Minimum	Maximum
6	1.8166667	0.1602082	1.6000000	2.0000000

Unique Subject Identifier=1392011110-0012

Assay variable : PTINR_VAL1 PT-INR Value

N	Mean	Standard Deviation	Minimum	Maximum
6	1.9166667	0.3544949	1.7000000	2.6000000

Unique Subject Identifier=1392011110-0013

Assay variable : PTINR_VAL1 PT-INR Value

N	Mean	Standard Deviation	Minimum	Maximum
6	2.0666667	0.3011091	1.8000000	2.5000000

Unique Subject Identifier=1392011110-0016

Assay variable : PTINR_VAL1 PT-INR Value

N	Mean	Standard Deviation	Minimum	Maximum
6	2.2000000	0.3033150	1.8000000	2.6000000

Laboratory tests at the time of obtaining Table 2-6 consent, according to the presence or absence of proton-pump inhibitors

Unique Subject Identifier=1392011110-0017

MEANS Procedure

Assay variable : PTINR_VAL1 PT-INR Value

N	Mean	Standard Deviation	Minimum	Maximum
6	1.8500000	0.1048809	1.7000000	2.0000000

Unique Subject Identifier=1394011010-0001

Assay variable : PTINR_VAL1 PT-INR Value

N	Mean	Standard Deviation	Minimum	Maximum
2	3.4000000	1.1313708	2.6000000	4.2000000

Unique Subject Identifier=1394011010-0004

Assay variable : PTINR_VAL1 PT-INR Value

N	Mean	Standard Deviation	Minimum	Maximum
3	1.9666667	1.0016653	1.2000000	3.1000000

Unique Subject Identifier=1395000000-0007

Assay variable : PTINR_VAL1 PT-INR Value

N	Mean	Standard Deviation	Minimum	Maximum
5	2.3880000	0.4996699	1.9000000	3.2300000

Laboratory tests at the time of obtaining Table 2-6 consent, according to the presence or absence of proton-pump inhibitors

Unique Subject Identifier=1396000000-0005

MEANS Procedure

Assay variable : PTINR_VAL1 PT-INR Value

N	Mean	Standard Deviation	Minimum	Maximum
1	1.6000000		1.6000000	1.6000000

Unique Subject Identifier=1396000000-0006

Assay variable : PTINR_VAL1 PT-INR Value

N	Mean	Standard Deviation	Minimum	Maximum
1	1.5000000		1.5000000	1.5000000

Unique Subject Identifier=1396000000-0007

Assay variable : PTINR_VAL1 PT-INR Value

N	Mean	Standard Deviation	Minimum	Maximum
1	2.0000000		2.0000000	2.0000000

Unique Subject Identifier=1396000000-0014

Assay variable : PTINR_VAL1 PT-INR Value

N	Mean	Standard Deviation	Minimum	Maximum
6	2.0000000	0.2607681	1.5000000	2.2000000

Laboratory tests at the time of obtaining Table 2-6 consent, according to the presence or absence of proton-pump inhibitors

Unique Subject Identifier=1398010000-0007

MEANS Procedure

Assay variable : PTINR_VAL1 PT-INR Value

N	Mean	Standard Deviation	Minimum	Maximum
5	1.7400000	0.2302173	1.5000000	2.1000000

Unique Subject Identifier=1398010000-0016

Assay variable : PTINR_VAL1 PT-INR Value

N	Mean	Standard Deviation	Minimum	Maximum
4	1.7000000	0.3366502	1.3000000	2.1000000

Unique Subject Identifier=1398010000-0018

Assay variable : PTINR_VAL1 PT-INR Value

N	Mean	Standard Deviation	Minimum	Maximum
5	1.9800000	0.1923538	1.7000000	2.2000000

Unique Subject Identifier=1398010000-0019

Assay variable : PTINR_VAL1 PT-INR Value

N	Mean	Standard Deviation	Minimum	Maximum
3	1.9333333	0.2309401	1.8000000	2.2000000

Laboratory tests at the time of obtaining Table 2-6 consent, according to the presence or absence of proton-pump inhibitors

Unique Subject Identifier=1398010000-0021

MEANS Procedure

Assay variable : PTINR_VAL1 PT-INR Value

N	Mean	Standard Deviation	Minimum	Maximum
4	2.0500000	0.4509250	1.7000000	2.7000000

Unique Subject Identifier=1398010000-0022

Assay variable : PTINR_VAL1 PT-INR Value

N	Mean	Standard Deviation	Minimum	Maximum
5	2.2000000	0.3082207	1.9000000	2.6000000

Unique Subject Identifier=1398010000-0026

Assay variable : PTINR_VAL1 PT-INR Value

N	Mean	Standard Deviation	Minimum	Maximum
5	2.1200000	0.5069517	1.6000000	2.9000000

Unique Subject Identifier=1398010000-0032

Assay variable : PTINR_VAL1 PT-INR Value

N	Mean	Standard Deviation	Minimum	Maximum
3	1.5333333	0.0577350	1.5000000	1.6000000

Laboratory tests at the time of obtaining Table 2-6 consent, according to the presence or absence of proton-pump inhibitors

Unique Subject Identifier=1398010000-0034

MEANS Procedure

Assay variable : PTINR_VAL1 PT-INR Value

N	Mean	Standard Deviation	Minimum	Maximum
4	1.8000000	0.1414214	1.6000000	1.9000000

Unique Subject Identifier=1398010000-0036

Assay variable : PTINR_VAL1 PT-INR Value

N	Mean	Standard Deviation	Minimum	Maximum
3	2.2333333	0.1527525	2.1000000	2.4000000

Unique Subject Identifier=1398010000-0038

Assay variable : PTINR_VAL1 PT-INR Value

N	Mean	Standard Deviation	Minimum	Maximum
4	1.2750000	0.1500000	1.2000000	1.5000000

Unique Subject Identifier=1398010000-0043

Assay variable : PTINR_VAL1 PT-INR Value

N	Mean	Standard Deviation	Minimum	Maximum
3	2.3000000	0.1000000	2.2000000	2.4000000

Laboratory tests at the time of obtaining Table 2-6 consent, according to the presence or absence of proton-pump inhibitors

Unique Subject Identifier=1400000000-0001

MEANS Procedure

Assay variable : PTINR_VAL1 PT-INR Value

N	Mean	Standard Deviation	Minimum	Maximum
5	2.3120000	0.5893810	1.6700000	3.0800000

Unique Subject Identifier=1400000000-0002

Assay variable : PTINR_VAL1 PT-INR Value

N	Mean	Standard Deviation	Minimum	Maximum
5	2.0620000	0.5821684	1.4600000	2.9300000

Unique Subject Identifier=1400000000-0005

Assay variable : PTINR_VAL1 PT-INR Value

N	Mean	Standard Deviation	Minimum	Maximum
1	2.7800000		2.7800000	2.7800000

Unique Subject Identifier=1400000000-0006

Assay variable : PTINR_VAL1 PT-INR Value

N	Mean	Standard Deviation	Minimum	Maximum
3	2.0666667	0.2514624	1.7800000	2.2500000

Laboratory tests at the time of obtaining Table 2-6 consent, according to the presence or absence of proton-pump inhibitors

Unique Subject Identifier=1400000000-0007

MEANS Procedure

Assay variable : PTINR_VAL1 PT-INR Value

N	Mean	Standard Deviation	Minimum	Maximum
3	1.7200000	0.0781025	1.6300000	1.7700000

Unique Subject Identifier=1400000000-0010

Assay variable : PTINR_VAL1 PT-INR Value

N	Mean	Standard Deviation	Minimum	Maximum
3	2.3233333	0.6473278	1.7100000	3.0000000

Unique Subject Identifier=1401000000-0002

Assay variable : PTINR_VAL1 PT-INR Value

N	Mean	Standard Deviation	Minimum	Maximum
5	2.0780000	0.3787083	1.5800000	2.5100000

Unique Subject Identifier=1401000000-0003

Assay variable : PTINR_VAL1 PT-INR Value

N	Mean	Standard Deviation	Minimum	Maximum
6	2.3716667	0.4688888	1.5400000	2.9400000

Laboratory tests at the time of obtaining Table 2-6 consent, according to the presence or absence of proton-pump inhibitors

Unique Subject Identifier=1401000000-0004

MEANS Procedure

Assay variable : PTINR_VAL1 PT-INR Value

N	Mean	Standard Deviation	Minimum	Maximum
5	2.4740000	0.2583215	2.0800000	2.7500000

Unique Subject Identifier=1401000000-0007

Assay variable : PTINR_VAL1 PT-INR Value

N	Mean	Standard Deviation	Minimum	Maximum
5	2.8940000	0.4098536	2.3800000	3.3100000

Unique Subject Identifier=1401000000-0008

Assay variable : PTINR_VAL1 PT-INR Value

N	Mean	Standard Deviation	Minimum	Maximum
5	2.6740000	0.3907429	2.1900000	3.2600000

Unique Subject Identifier=1401000000-0009

Assay variable : PTINR_VAL1 PT-INR Value

N	Mean	Standard Deviation	Minimum	Maximum
4	1.9450000	0.4747280	1.3900000	2.5500000

Laboratory tests at the time of obtaining Table 2-6 consent, according to the presence or absence of proton-pump inhibitors

Unique Subject Identifier=1401000000-0010

MEANS Procedure

Assay variable : PTINR_VAL1 PT-INR Value

N	Mean	Standard Deviation	Minimum	Maximum
6	2.9566667	0.3767050	2.5200000	3.5300000

Unique Subject Identifier=1405000000-0001

Assay variable : PTINR_VAL1 PT-INR Value

N	Mean	Standard Deviation	Minimum	Maximum
5	2.1400000	0.2792848	1.9000000	2.6000000

Unique Subject Identifier=1405000000-0002

Assay variable : PTINR_VAL1 PT-INR Value

N	Mean	Standard Deviation	Minimum	Maximum
6	2.5333333	0.6218253	1.9000000	3.3000000

Unique Subject Identifier=1405000000-0003

Assay variable : PTINR_VAL1 PT-INR Value

N	Mean	Standard Deviation	Minimum	Maximum
6	2.2500000	0.3674235	1.9000000	2.8000000

Laboratory tests at the time of obtaining Table 2-6 consent, according to the presence or absence of proton-pump inhibitors

Unique Subject Identifier=1405000000-0004

MEANS Procedure

Assay variable : PTINR_VAL1 PT-INR Value

N	Mean	Standard Deviation	Minimum	Maximum
6	2.0000000	0.1549193	1.8000000	2.2000000

Unique Subject Identifier=1405000000-0005

Assay variable : PTINR_VAL1 PT-INR Value

N	Mean	Standard Deviation	Minimum	Maximum
6	2.0833333	0.2401388	1.9000000	2.5000000

Unique Subject Identifier=1405000000-0006

Assay variable : PTINR_VAL1 PT-INR Value

N	Mean	Standard Deviation	Minimum	Maximum
6	2.1666667	0.4082483	1.6000000	2.7000000

Unique Subject Identifier=1405000000-0008

Assay variable : PTINR_VAL1 PT-INR Value

N	Mean	Standard Deviation	Minimum	Maximum
6	2.2666667	0.1861899	2.0000000	2.5000000

Laboratory tests at the time of obtaining Table 2-6 consent, according to the presence or absence of proton-pump inhibitors

Unique Subject Identifier=1405000000-0009

MEANS Procedure

Assay variable : PTINR_VAL1 PT-INR Value

N	Mean	Standard Deviation	Minimum	Maximum
6	2.2166667	0.6306082	1.7000000	3.4000000

Unique Subject Identifier=1405000000-0010

Assay variable : PTINR_VAL1 PT-INR Value

N	Mean	Standard Deviation	Minimum	Maximum
6	2.4500000	0.1048809	2.3000000	2.6000000

Unique Subject Identifier=1405000000-0011

Assay variable : PTINR_VAL1 PT-INR Value

N	Mean	Standard Deviation	Minimum	Maximum
6	2.4666667	0.3502380	1.9000000	2.9000000

Unique Subject Identifier=1405000000-0013

Assay variable : PTINR_VAL1 PT-INR Value

N	Mean	Standard Deviation	Minimum	Maximum
6	2.0333333	0.1366260	1.9000000	2.3000000

Laboratory tests at the time of obtaining Table 2-6 consent, according to the presence or absence of proton-pump inhibitors

Unique Subject Identifier=1405000000-0015

MEANS Procedure

Assay variable : PTINR_VAL1 PT-INR Value

N	Mean	Standard Deviation	Minimum	Maximum
6	2.6666667	0.6947422	1.6000000	3.6000000

Unique Subject Identifier=1405000000-0016

Assay variable : PTINR_VAL1 PT-INR Value

N	Mean	Standard Deviation	Minimum	Maximum
6	2.4233333	0.4233989	1.8000000	2.8000000

Unique Subject Identifier=1405000000-0017

Assay variable : PTINR_VAL1 PT-INR Value

N	Mean	Standard Deviation	Minimum	Maximum
6	2.3666667	0.2658320	1.9000000	2.7000000

Unique Subject Identifier=1405000000-0018

Assay variable : PTINR_VAL1 PT-INR Value

N	Mean	Standard Deviation	Minimum	Maximum
6	1.8333333	0.2422120	1.4000000	2.0000000

Laboratory tests at the time of obtaining Table 2-6 consent, according to the presence or absence of proton-pump inhibitors

Unique Subject Identifier=1405000000-0019

MEANS Procedure

Assay variable : PTINR_VAL1 PT-INR Value

N	Mean	Standard Deviation	Minimum	Maximum
6	2.4666667	0.6742897	1.7000000	3.6000000

Unique Subject Identifier=1405000000-0020

Assay variable : PTINR_VAL1 PT-INR Value

N	Mean	Standard Deviation	Minimum	Maximum
6	2.0500000	0.3271085	1.7000000	2.5000000

Unique Subject Identifier=1405000000-0021

Assay variable : PTINR_VAL1 PT-INR Value

N	Mean	Standard Deviation	Minimum	Maximum
6	2.1666667	0.4501851	1.4000000	2.6000000

Unique Subject Identifier=1405000000-0022

Assay variable : PTINR_VAL1 PT-INR Value

N	Mean	Standard Deviation	Minimum	Maximum
6	2.0166667	0.4996666	1.6000000	3.0000000

Laboratory tests at the time of obtaining Table 2-6 consent, according to the presence or absence of proton-pump inhibitors

Unique Subject Identifier=1405000000-0023

MEANS Procedure

Assay variable : PTINR_VAL1 PT-INR Value

N	Mean	Standard Deviation	Minimum	Maximum
6	2.0666667	0.1861899	1.9000000	2.4000000

Unique Subject Identifier=1405000000-0024

Assay variable : PTINR_VAL1 PT-INR Value

N	Mean	Standard Deviation	Minimum	Maximum
6	2.4666667	0.3723797	1.8000000	2.8000000

Unique Subject Identifier=1405000000-0025

Assay variable : PTINR_VAL1 PT-INR Value

N	Mean	Standard Deviation	Minimum	Maximum
6	2.3000000	0.1788854	2.1000000	2.6000000

Unique Subject Identifier=1405000000-0026

Assay variable : PTINR_VAL1 PT-INR Value

N	Mean	Standard Deviation	Minimum	Maximum
6	2.1833333	0.2136976	1.9000000	2.4000000

Laboratory tests at the time of obtaining Table 2-6 consent, according to the presence or absence of proton-pump inhibitors

Unique Subject Identifier=1405000000-0027

MEANS Procedure

Assay variable : PTINR_VAL1 PT-INR Value

N	Mean	Standard Deviation	Minimum	Maximum
6	2.6833333	1.0534072	1.3000000	4.4000000

Unique Subject Identifier=1405000000-0028

Assay variable : PTINR_VAL1 PT-INR Value

N	Mean	Standard Deviation	Minimum	Maximum
6	2.3833333	0.5706721	1.7000000	3.4000000

Unique Subject Identifier=1405000000-0029

Assay variable : PTINR_VAL1 PT-INR Value

N	Mean	Standard Deviation	Minimum	Maximum
6	2.7666667	0.6470446	1.9000000	3.6000000

Unique Subject Identifier=1405000000-0030

Assay variable : PTINR_VAL1 PT-INR Value

N	Mean	Standard Deviation	Minimum	Maximum
6	2.5000000	0.2097618	2.3000000	2.9000000

Laboratory tests at the time of obtaining Table 2-6 consent, according to the presence or absence of proton-pump inhibitors

Unique Subject Identifier=1405000000-0033

MEANS Procedure

Assay variable : PTINR_VAL1 PT-INR Value

N	Mean	Standard Deviation	Minimum	Maximum
6	1.9833333	0.4622409	1.4000000	2.6000000

Unique Subject Identifier=1406000000-0001

Assay variable : PTINR_VAL1 PT-INR Value

N	Mean	Standard Deviation	Minimum	Maximum
1	1.1400000		1.1400000	1.1400000

Unique Subject Identifier=1406000000-0005

Assay variable : PTINR_VAL1 PT-INR Value

N	Mean	Standard Deviation	Minimum	Maximum
1	1.2800000		1.2800000	1.2800000

Unique Subject Identifier=1406000000-0006

Assay variable : PTINR_VAL1 PT-INR Value

N	Mean	Standard Deviation	Minimum	Maximum
1	3.2600000		3.2600000	3.2600000

Laboratory tests at the time of obtaining Table 2-6 consent, according to the presence or absence of proton-pump inhibitors

Unique Subject Identifier=1406000000-0007

MEANS Procedure

Assay variable : PTINR_VAL1 PT-INR Value

N	Mean	Standard Deviation	Minimum	Maximum
1	2.4500000		2.4500000	2.4500000

Unique Subject Identifier=1406000000-0008

Assay variable : PTINR_VAL1 PT-INR Value

N	Mean	Standard Deviation	Minimum	Maximum
1	1.4300000		1.4300000	1.4300000

Unique Subject Identifier=1406000000-0009

Assay variable : PTINR_VAL1 PT-INR Value

N	Mean	Standard Deviation	Minimum	Maximum
1	1.2900000		1.2900000	1.2900000

Unique Subject Identifier=1406000000-0011

Assay variable : PTINR_VAL1 PT-INR Value

N	Mean	Standard Deviation	Minimum	Maximum
1	2.5000000		2.5000000	2.5000000

Laboratory tests at the time of obtaining Table 2-6 consent, according to the presence or absence of proton-pump inhibitors

Unique Subject Identifier=1406000000-0014

MEANS Procedure

Assay variable : PTINR_VAL1 PT-INR Value

N	Mean	Standard Deviation	Minimum	Maximum
1	1.7700000		1.7700000	1.7700000

Unique Subject Identifier=1406000000-0018

Assay variable : PTINR_VAL1 PT-INR Value

N	Mean	Standard Deviation	Minimum	Maximum
1	1.5300000		1.5300000	1.5300000

Unique Subject Identifier=1406000000-0019

Assay variable : PTINR_VAL1 PT-INR Value

N	Mean	Standard Deviation	Minimum	Maximum
1	1.6000000		1.6000000	1.6000000

Unique Subject Identifier=1406000000-0020

Assay variable : PTINR_VAL1 PT-INR Value

N	Mean	Standard Deviation	Minimum	Maximum
1	1.2500000		1.2500000	1.2500000

Laboratory tests at the time of obtaining Table 2-6 consent, according to the presence or absence of proton-pump inhibitors

Unique Subject Identifier=1406000000-0021

MEANS Procedure

Assay variable : PTINR_VAL1 PT-INR Value

N	Mean	Standard Deviation	Minimum	Maximum
1	1.0500000		1.0500000	1.0500000

Unique Subject Identifier=1407000000-0004

Assay variable : PTINR_VAL1 PT-INR Value

N	Mean	Standard Deviation	Minimum	Maximum
6	1.9650000	0.1980656	1.7300000	2.3300000

Unique Subject Identifier=1407000000-0007

Assay variable : PTINR_VAL1 PT-INR Value

N	Mean	Standard Deviation	Minimum	Maximum
6	2.0166667	0.1189398	1.9200000	2.2400000

Unique Subject Identifier=1407000000-0010

Assay variable : PTINR_VAL1 PT-INR Value

N	Mean	Standard Deviation	Minimum	Maximum
6	1.6883333	0.2422739	1.5000000	2.1400000

Laboratory tests at the time of obtaining Table 2-6 consent, according to the presence or absence of proton-pump inhibitors

Unique Subject Identifier=1407000000-0013

MEANS Procedure

Assay variable : PTINR_VAL1 PT-INR Value

N	Mean	Standard Deviation	Minimum	Maximum
6	1.8750000	0.1488288	1.6800000	2.0600000

Unique Subject Identifier=1407000000-0016

Assay variable : PTINR_VAL1 PT-INR Value

N	Mean	Standard Deviation	Minimum	Maximum
6	2.3616667	0.2377744	2.0800000	2.7100000

Unique Subject Identifier=1407000000-0027

Assay variable : PTINR_VAL1 PT-INR Value

N	Mean	Standard Deviation	Minimum	Maximum
5	2.1520000	0.2169562	1.8900000	2.4900000

Unique Subject Identifier=1407000000-0030

Assay variable : PTINR_VAL1 PT-INR Value

N	Mean	Standard Deviation	Minimum	Maximum
6	1.8800000	0.3470447	1.3400000	2.2600000

Laboratory tests at the time of obtaining Table 2-6 consent, according to the presence or absence of proton-pump inhibitors

Unique Subject Identifier=1408010000-0001

MEANS Procedure

Assay variable : PTINR_VAL1 PT-INR Value

N	Mean	Standard Deviation	Minimum	Maximum
5	2.1640000	0.2016928	1.8600000	2.3800000

Unique Subject Identifier=1408010000-0004

Assay variable : PTINR_VAL1 PT-INR Value

N	Mean	Standard Deviation	Minimum	Maximum
5	2.6160000	0.3574633	2.3700000	3.2300000

Unique Subject Identifier=1408010000-0006

Assay variable : PTINR_VAL1 PT-INR Value

N	Mean	Standard Deviation	Minimum	Maximum
6	2.6933333	0.4401666	1.9400000	3.1800000

Unique Subject Identifier=1408010000-0007

Assay variable : PTINR_VAL1 PT-INR Value

N	Mean	Standard Deviation	Minimum	Maximum
5	2.4700000	0.4941660	2.1700000	3.3500000

Laboratory tests at the time of obtaining Table 2-6 consent, according to the presence or absence of proton-pump inhibitors

Unique Subject Identifier=1408010000-0008

MEANS Procedure

Assay variable : PTINR_VAL1 PT-INR Value

N	Mean	Standard Deviation	Minimum	Maximum
4	1.7600000	0.0408248	1.7000000	1.7900000

Unique Subject Identifier=1408010000-0012

Assay variable : PTINR_VAL1 PT-INR Value

N	Mean	Standard Deviation	Minimum	Maximum
5	3.0720000	0.4856645	2.5200000	3.8500000

Unique Subject Identifier=1408010000-0013

Assay variable : PTINR_VAL1 PT-INR Value

N	Mean	Standard Deviation	Minimum	Maximum
4	2.1725000	0.2780138	1.7800000	2.4200000

Unique Subject Identifier=1408010000-0015

Assay variable : PTINR_VAL1 PT-INR Value

N	Mean	Standard Deviation	Minimum	Maximum
6	2.4200000	0.3701351	2.0200000	2.9100000

Laboratory tests at the time of obtaining Table 2-6 consent, according to the presence or absence of proton-pump inhibitors

Unique Subject Identifier=1408010000-0021

MEANS Procedure

Assay variable : PTINR_VAL1 PT-INR Value

N	Mean	Standard Deviation	Minimum	Maximum
4	1.8375000	0.1472809	1.6400000	1.9500000

Unique Subject Identifier=1408010000-0025

Assay variable : PTINR_VAL1 PT-INR Value

N	Mean	Standard Deviation	Minimum	Maximum
4	2.1075000	0.3824809	1.8900000	2.6800000

Unique Subject Identifier=1408010000-0029

Assay variable : PTINR_VAL1 PT-INR Value

N	Mean	Standard Deviation	Minimum	Maximum
4	2.5700000	0.1476482	2.4800000	2.7900000

Unique Subject Identifier=1408010000-0033

Assay variable : PTINR_VAL1 PT-INR Value

N	Mean	Standard Deviation	Minimum	Maximum
5	1.9540000	0.1722788	1.7900000	2.2400000

Laboratory tests at the time of obtaining Table 2-6 consent, according to the presence or absence of proton-pump inhibitors

Unique Subject Identifier=1412000000-0002

MEANS Procedure

Assay variable : PTINR_VAL1 PT-INR Value

N	Mean	Standard Deviation	Minimum	Maximum
6	2.0500000	0.0836660	1.9000000	2.1000000

Unique Subject Identifier=1412000000-0007

Assay variable : PTINR_VAL1 PT-INR Value

N	Mean	Standard Deviation	Minimum	Maximum
6	1.8333333	0.4273952	1.3000000	2.3000000

Unique Subject Identifier=1412000000-0014

Assay variable : PTINR_VAL1 PT-INR Value

N	Mean	Standard Deviation	Minimum	Maximum
6	1.8333333	0.2065591	1.6000000	2.1000000

Unique Subject Identifier=1412000000-0021

Assay variable : PTINR_VAL1 PT-INR Value

N	Mean	Standard Deviation	Minimum	Maximum
6	2.1666667	0.1632993	1.9000000	2.4000000

Laboratory tests at the time of obtaining Table 2-6 consent, according to the presence or absence of proton-pump inhibitors

Unique Subject Identifier=1412000000-0023

MEANS Procedure

Assay variable : PTINR_VAL1 PT-INR Value

N	Mean	Standard Deviation	Minimum	Maximum
6	1.7833333	0.1329160	1.7000000	2.0000000

Unique Subject Identifier=1412000000-0025

Assay variable : PTINR_VAL1 PT-INR Value

N	Mean	Standard Deviation	Minimum	Maximum
6	1.6333333	0.1632993	1.4000000	1.9000000

Unique Subject Identifier=1412000000-0028

Assay variable : PTINR_VAL1 PT-INR Value

N	Mean	Standard Deviation	Minimum	Maximum
6	2.6000000	1.4926487	1.4000000	5.5000000

Unique Subject Identifier=1412000000-0029

Assay variable : PTINR_VAL1 PT-INR Value

N	Mean	Standard Deviation	Minimum	Maximum
5	1.5400000	0.1140175	1.4000000	1.7000000

Laboratory tests at the time of obtaining Table 2-6 consent, according to the presence or absence of proton-pump inhibitors

Unique Subject Identifier=1412000000-0031

MEANS Procedure

Assay variable : PTINR_VAL1 PT-INR Value

N	Mean	Standard Deviation	Minimum	Maximum
6	2.2166667	0.3544949	2.0000000	2.9000000

Unique Subject Identifier=1420010000-0004

Assay variable : PTINR_VAL1 PT-INR Value

N	Mean	Standard Deviation	Minimum	Maximum
6	1.8333333	0.3881580	1.4000000	2.4000000

Unique Subject Identifier=1420010000-0007

Assay variable : PTINR_VAL1 PT-INR Value

N	Mean	Standard Deviation	Minimum	Maximum
6	2.2666667	0.3777124	1.8000000	2.8000000

Unique Subject Identifier=1420010000-0008

Assay variable : PTINR_VAL1 PT-INR Value

N	Mean	Standard Deviation	Minimum	Maximum
5	1.7600000	0.1816590	1.5000000	2.0000000

Laboratory tests at the time of obtaining Table 2-6 consent, according to the presence or absence of proton-pump inhibitors

Unique Subject Identifier=1420010000-0010

MEANS Procedure

Assay variable : PTINR_VAL1 PT-INR Value

N	Mean	Standard Deviation	Minimum	Maximum
6	1.8333333	0.1751190	1.5000000	2.0000000

Unique Subject Identifier=1420010000-0012

Assay variable : PTINR_VAL1 PT-INR Value

N	Mean	Standard Deviation	Minimum	Maximum
6	2.1166667	0.2714160	1.6000000	2.4000000

Unique Subject Identifier=1420010000-0013

Assay variable : PTINR_VAL1 PT-INR Value

N	Mean	Standard Deviation	Minimum	Maximum
5	1.6400000	0.3049590	1.1000000	1.8000000

Unique Subject Identifier=1420010000-0014

Assay variable : PTINR_VAL1 PT-INR Value

N	Mean	Standard Deviation	Minimum	Maximum
6	2.0833333	0.1169045	1.9000000	2.2000000

Laboratory tests at the time of obtaining Table 2-6 consent, according to the presence or absence of proton-pump inhibitors

Unique Subject Identifier=1420010000-0017

MEANS Procedure

Assay variable : PTINR_VAL1 PT-INR Value

N	Mean	Standard Deviation	Minimum	Maximum
3	1.6333333	0.4041452	1.2000000	2.0000000

Unique Subject Identifier=1423000000-0001

Assay variable : PTINR_VAL1 PT-INR Value

N	Mean	Standard Deviation	Minimum	Maximum
2	1.9100000	0.4666905	1.5800000	2.2400000

Unique Subject Identifier=1423000000-0006

Assay variable : PTINR_VAL1 PT-INR Value

N	Mean	Standard Deviation	Minimum	Maximum
1	1.3900000		1.3900000	1.3900000

Unique Subject Identifier=1423000000-0010

Assay variable : PTINR_VAL1 PT-INR Value

N	Mean	Standard Deviation	Minimum	Maximum
3	1.4900000	0.2251666	1.3600000	1.7500000

Laboratory tests at the time of obtaining Table 2-6 consent, according to the presence or absence of proton-pump inhibitors

Unique Subject Identifier=1423000000-0015

MEANS Procedure

Assay variable : PTINR_VAL1 PT-INR Value

N	Mean	Standard Deviation	Minimum	Maximum
2	2.0550000	0.1626346	1.9400000	2.1700000

Unique Subject Identifier=1423000000-0021

Assay variable : PTINR_VAL1 PT-INR Value

N	Mean	Standard Deviation	Minimum	Maximum
1	2.0200000		2.0200000	2.0200000

Unique Subject Identifier=1423000000-0022

Assay variable : PTINR_VAL1 PT-INR Value

N	Mean	Standard Deviation	Minimum	Maximum
2	1.2700000	0.0282843	1.2500000	1.2900000

Unique Subject Identifier=1427000000-0003

Assay variable : PTINR_VAL1 PT-INR Value

N	Mean	Standard Deviation	Minimum	Maximum
6	1.8000000	0.3521363	1.4000000	2.3000000

Laboratory tests at the time of obtaining Table 2-6 consent, according to the presence or absence of proton-pump inhibitors

Unique Subject Identifier=1428011110-0001

MEANS Procedure

Assay variable : PTINR_VAL1 PT-INR Value

N	Mean	Standard Deviation	Minimum	Maximum
2	1.7850000	0.2616295	1.6000000	1.9700000

Unique Subject Identifier=1428011110-0002

Assay variable : PTINR_VAL1 PT-INR Value

N	Mean	Standard Deviation	Minimum	Maximum
2	1.4650000	0.2474874	1.2900000	1.6400000

Unique Subject Identifier=1428011110-0003

Assay variable : PTINR_VAL1 PT-INR Value

N	Mean	Standard Deviation	Minimum	Maximum
1	1.6900000		1.6900000	1.6900000

Unique Subject Identifier=1428011110-0006

Assay variable : PTINR_VAL1 PT-INR Value

N	Mean	Standard Deviation	Minimum	Maximum
2	1.7300000	0.0424264	1.7000000	1.7600000

Laboratory tests at the time of obtaining Table 2-6 consent, according to the presence or absence of proton-pump inhibitors

Unique Subject Identifier=1428011110-0008

MEANS Procedure

Assay variable : PTINR_VAL1 PT-INR Value

N	Mean	Standard Deviation	Minimum	Maximum
2	2.4750000	0.7566043	1.9400000	3.0100000

Unique Subject Identifier=1428011110-0009

Assay variable : PTINR_VAL1 PT-INR Value

N	Mean	Standard Deviation	Minimum	Maximum
2	1.7150000	0.7990307	1.1500000	2.2800000

Unique Subject Identifier=1428011110-0010

Assay variable : PTINR_VAL1 PT-INR Value

N	Mean	Standard Deviation	Minimum	Maximum
2	2.2450000	0.1484924	2.1400000	2.3500000

Unique Subject Identifier=1428011110-0011

Assay variable : PTINR_VAL1 PT-INR Value

N	Mean	Standard Deviation	Minimum	Maximum
2	2.1850000	0.3040559	1.9700000	2.4000000

Laboratory tests at the time of obtaining Table 2-6 consent, according to the presence or absence of proton-pump inhibitors

Unique Subject Identifier=1428011110-0013

MEANS Procedure

Assay variable : PTINR_VAL1 PT-INR Value

N	Mean	Standard Deviation	Minimum	Maximum
1	1.6800000		1.6800000	1.6800000

Unique Subject Identifier=1428011110-0015

Assay variable : PTINR_VAL1 PT-INR Value

N	Mean	Standard Deviation	Minimum	Maximum
2	1.3700000	0.2687006	1.1800000	1.5600000

Unique Subject Identifier=1428011110-0017

Assay variable : PTINR_VAL1 PT-INR Value

N	Mean	Standard Deviation	Minimum	Maximum
1	1.4900000		1.4900000	1.4900000

Unique Subject Identifier=1428011110-0019

Assay variable : PTINR_VAL1 PT-INR Value

N	Mean	Standard Deviation	Minimum	Maximum
3	1.9833333	0.2309401	1.8500000	2.2500000

Laboratory tests at the time of obtaining Table 2-6 consent, according to the presence or absence of proton-pump inhibitors

Unique Subject Identifier=1428011110-0021

MEANS Procedure

Assay variable : PTINR_VAL1 PT-INR Value

N	Mean	Standard Deviation	Minimum	Maximum
1	2.8600000		2.8600000	2.8600000

Unique Subject Identifier=1428011110-0023

Assay variable : PTINR_VAL1 PT-INR Value

N	Mean	Standard Deviation	Minimum	Maximum
1	1.5800000		1.5800000	1.5800000

Unique Subject Identifier=1428011110-0025

Assay variable : PTINR_VAL1 PT-INR Value

N	Mean	Standard Deviation	Minimum	Maximum
2	1.8100000	0.4666905	1.4800000	2.1400000

Unique Subject Identifier=1428011110-0032

Assay variable : PTINR_VAL1 PT-INR Value

N	Mean	Standard Deviation	Minimum	Maximum
3	1.7866667	0.5345403	1.4200000	2.4000000

Laboratory tests at the time of obtaining Table 2-6 consent, according to the presence or absence of proton-pump inhibitors

Unique Subject Identifier=1430011010-0003

MEANS Procedure

Assay variable : PTINR_VAL1 PT-INR Value

N	Mean	Standard Deviation	Minimum	Maximum
6	1.8166667	0.2786874	1.5000000	2.1000000

Unique Subject Identifier=1430011010-0004

Assay variable : PTINR_VAL1 PT-INR Value

N	Mean	Standard Deviation	Minimum	Maximum
6	1.8666667	0.3444803	1.6000000	2.4000000

Unique Subject Identifier=1432000000-0011

Assay variable : PTINR_VAL1 PT-INR Value

N	Mean	Standard Deviation	Minimum	Maximum
6	1.1833333	0.0752773	1.1000000	1.3000000

Unique Subject Identifier=1432000000-0015

Assay variable : PTINR_VAL1 PT-INR Value

N	Mean	Standard Deviation	Minimum	Maximum
6	1.8333333	0.7865537	1.1000000	3.3000000

Laboratory tests at the time of obtaining Table 2-6 consent, according to the presence or absence of proton-pump inhibitors

Unique Subject Identifier=1432000000-0017

MEANS Procedure

Assay variable : PTINR_VAL1 PT-INR Value

N	Mean	Standard Deviation	Minimum	Maximum
6	2.3500000	0.4549725	1.8000000	3.0000000

Unique Subject Identifier=1432000000-0020

Assay variable : PTINR_VAL1 PT-INR Value

N	Mean	Standard Deviation	Minimum	Maximum
5	1.9400000	0.4449719	1.6000000	2.6000000

Unique Subject Identifier=1432000000-0023

Assay variable : PTINR_VAL1 PT-INR Value

N	Mean	Standard Deviation	Minimum	Maximum
6	1.8500000	0.5648008	1.2000000	2.7000000

Unique Subject Identifier=1432000000-0028

Assay variable : PTINR_VAL1 PT-INR Value

N	Mean	Standard Deviation	Minimum	Maximum
6	2.3333333	0.5465040	1.7000000	3.2000000

Laboratory tests at the time of obtaining Table 2-6 consent, according to the presence or absence of proton-pump inhibitors

Unique Subject Identifier=1432000000-0034

MEANS Procedure

Assay variable : PTINR_VAL1 PT-INR Value

N	Mean	Standard Deviation	Minimum	Maximum
6	2.1000000	0.3162278	1.7000000	2.6000000

Unique Subject Identifier=1432000000-0037

Assay variable : PTINR_VAL1 PT-INR Value

N	Mean	Standard Deviation	Minimum	Maximum
6	1.4333333	0.0516398	1.4000000	1.5000000

Unique Subject Identifier=1432000000-0042

Assay variable : PTINR_VAL1 PT-INR Value

N	Mean	Standard Deviation	Minimum	Maximum
5	1.7600000	0.5176872	1.3000000	2.6000000

Unique Subject Identifier=1432000000-0049

Assay variable : PTINR_VAL1 PT-INR Value

N	Mean	Standard Deviation	Minimum	Maximum
6	1.7833333	0.6112828	1.1000000	2.6000000

Laboratory tests at the time of obtaining Table 2-6 consent, according to the presence or absence of proton-pump inhibitors

Unique Subject Identifier=1432000000-0056

MEANS Procedure

Assay variable : PTINR_VAL1 PT-INR Value

N	Mean	Standard Deviation	Minimum	Maximum
6	2.0833333	0.5455884	1.2000000	2.6000000

Unique Subject Identifier=1432000000-0071

Assay variable : PTINR_VAL1 PT-INR Value

N	Mean	Standard Deviation	Minimum	Maximum
6	2.2833333	0.2483277	1.9000000	2.6000000

Unique Subject Identifier=1432000000-0072

Assay variable : PTINR_VAL1 PT-INR Value

N	Mean	Standard Deviation	Minimum	Maximum
6	1.7000000	0.3033150	1.3000000	2.2000000

Unique Subject Identifier=1432000000-0073

Assay variable : PTINR_VAL1 PT-INR Value

N	Mean	Standard Deviation	Minimum	Maximum
6	2.1166667	0.1602082	1.9000000	2.3000000

Laboratory tests at the time of obtaining Table 2-6 consent, according to the presence or absence of proton-pump inhibitors

Unique Subject Identifier=1435000000-0003

MEANS Procedure

Assay variable : PTINR_VAL1 PT-INR Value

N	Mean	Standard Deviation	Minimum	Maximum
1	2.1400000		2.1400000	2.1400000

Unique Subject Identifier=1435000000-0018

Assay variable : PTINR_VAL1 PT-INR Value

N	Mean	Standard Deviation	Minimum	Maximum
1	1.6700000		1.6700000	1.6700000

Unique Subject Identifier=1435000000-0021

Assay variable : PTINR_VAL1 PT-INR Value

N	Mean	Standard Deviation	Minimum	Maximum
1	1.1600000		1.1600000	1.1600000

Unique Subject Identifier=1435000000-0028

Assay variable : PTINR_VAL1 PT-INR Value

N	Mean	Standard Deviation	Minimum	Maximum
3	2.6600000	0.1081665	2.5700000	2.7800000

Laboratory tests at the time of obtaining Table 2-6 consent, according to the presence or absence of proton-pump inhibitors

Unique Subject Identifier=1435000000-0039

MEANS Procedure

Assay variable : PTINR_VAL1 PT-INR Value

N	Mean	Standard Deviation	Minimum	Maximum
3	1.7266667	0.2402776	1.4800000	1.9600000

Unique Subject Identifier=1435000000-0041

Assay variable : PTINR_VAL1 PT-INR Value

N	Mean	Standard Deviation	Minimum	Maximum
5	2.2780000	0.2723417	1.9700000	2.5800000

Unique Subject Identifier=1435000000-0042

Assay variable : PTINR_VAL1 PT-INR Value

N	Mean	Standard Deviation	Minimum	Maximum
5	2.1240000	0.1270039	2.0000000	2.2800000

Unique Subject Identifier=1435000000-0048

Assay variable : PTINR_VAL1 PT-INR Value

N	Mean	Standard Deviation	Minimum	Maximum
5	1.9720000	0.6191688	1.3900000	3.0200000

Laboratory tests at the time of obtaining Table 2-6 consent, according to the presence or absence of proton-pump inhibitors

Unique Subject Identifier=1435000000-0051

MEANS Procedure

Assay variable : PTINR_VAL1 PT-INR Value

N	Mean	Standard Deviation	Minimum	Maximum
1	2.0900000		2.0900000	2.0900000

Unique Subject Identifier=1435000000-0055

Assay variable : PTINR_VAL1 PT-INR Value

N	Mean	Standard Deviation	Minimum	Maximum
6	1.5500000	0.5843629	1.1600000	2.7100000

Unique Subject Identifier=1435000000-0065

Assay variable : PTINR_VAL1 PT-INR Value

N	Mean	Standard Deviation	Minimum	Maximum
1	2.1800000		2.1800000	2.1800000

Unique Subject Identifier=1435000000-0071

Assay variable : PTINR_VAL1 PT-INR Value

N	Mean	Standard Deviation	Minimum	Maximum
6	1.2450000	0.2508984	1.0600000	1.6400000

Laboratory tests at the time of obtaining Table 2-6 consent, according to the presence or absence of proton-pump inhibitors

Unique Subject Identifier=1435000000-0072

MEANS Procedure

Assay variable : PTINR_VAL1 PT-INR Value

N	Mean	Standard Deviation	Minimum	Maximum
4	1.8275000	0.2848830	1.5900000	2.2000000

Unique Subject Identifier=1435000000-0075

Assay variable : PTINR_VAL1 PT-INR Value

N	Mean	Standard Deviation	Minimum	Maximum
3	1.1466667	0.0251661	1.1200000	1.1700000

Unique Subject Identifier=1435000000-0091

Assay variable : PTINR_VAL1 PT-INR Value

N	Mean	Standard Deviation	Minimum	Maximum
3	2.1500000	0.2505993	1.8900000	2.3900000

Unique Subject Identifier=1435000000-0103

Assay variable : PTINR_VAL1 PT-INR Value

N	Mean	Standard Deviation	Minimum	Maximum
2	2.7750000	0.0494975	2.7400000	2.8100000

Laboratory tests at the time of obtaining Table 2-6 consent, according to the presence or absence of proton-pump inhibitors

Unique Subject Identifier=1435000000-0105

MEANS Procedure

Assay variable : PTINR_VAL1 PT-INR Value

N	Mean	Standard Deviation	Minimum	Maximum
1	1.5300000		1.5300000	1.5300000

Unique Subject Identifier=1435000000-0107

Assay variable : PTINR_VAL1 PT-INR Value

N	Mean	Standard Deviation	Minimum	Maximum
3	1.5866667	0.2318045	1.3200000	1.7400000

Unique Subject Identifier=1435000000-0117

Assay variable : PTINR_VAL1 PT-INR Value

N	Mean	Standard Deviation	Minimum	Maximum
6	2.4450000	0.2246553	2.2400000	2.8400000

Unique Subject Identifier=1435000000-0119

Assay variable : PTINR_VAL1 PT-INR Value

N	Mean	Standard Deviation	Minimum	Maximum
1	2.4200000		2.4200000	2.4200000

Laboratory tests at the time of obtaining Table 2-6 consent, according to the presence or absence of proton-pump inhibitors

Unique Subject Identifier=1435000000-0120

MEANS Procedure

Assay variable : PTINR_VAL1 PT-INR Value

N	Mean	Standard Deviation	Minimum	Maximum
6	1.6683333	0.4972491	1.1800000	2.5300000

Unique Subject Identifier=1435000000-0132

Assay variable : PTINR_VAL1 PT-INR Value

N	Mean	Standard Deviation	Minimum	Maximum
2	2.4550000	0.4030509	2.1700000	2.7400000

Unique Subject Identifier=1435000000-0139

Assay variable : PTINR_VAL1 PT-INR Value

N	Mean	Standard Deviation	Minimum	Maximum
2	2.6450000	1.4919953	1.5900000	3.7000000

Unique Subject Identifier=1435000000-0140

Assay variable : PTINR_VAL1 PT-INR Value

N	Mean	Standard Deviation	Minimum	Maximum
6	1.4450000	0.1980656	1.2400000	1.8100000

Laboratory tests at the time of obtaining Table 2-6 consent, according to the presence or absence of proton-pump inhibitors

Unique Subject Identifier=1435000000-0145

MEANS Procedure

Assay variable : PTINR_VAL1 PT-INR Value

N	Mean	Standard Deviation	Minimum	Maximum
6	1.5983333	0.3660829	1.1900000	2.1900000

Unique Subject Identifier=1435000000-0153

Assay variable : PTINR_VAL1 PT-INR Value

N	Mean	Standard Deviation	Minimum	Maximum
5	1.7040000	0.1050238	1.5800000	1.8500000

Unique Subject Identifier=1435000000-0177

Assay variable : PTINR_VAL1 PT-INR Value

N	Mean	Standard Deviation	Minimum	Maximum
5	1.9480000	0.3900897	1.5500000	2.4600000

Unique Subject Identifier=1435000000-0188

Assay variable : PTINR_VAL1 PT-INR Value

N	Mean	Standard Deviation	Minimum	Maximum
4	1.4350000	0.3641886	1.2200000	1.9800000

Laboratory tests at the time of obtaining Table 2-6 consent, according to the presence or absence of proton-pump inhibitors

Unique Subject Identifier=1435000000-0200

MEANS Procedure

Assay variable : PTINR_VAL1 PT-INR Value

N	Mean	Standard Deviation	Minimum	Maximum
2	1.1650000	0.0636396	1.1200000	1.2100000

Unique Subject Identifier=1435000000-0209

Assay variable : PTINR_VAL1 PT-INR Value

N	Mean	Standard Deviation	Minimum	Maximum
6	1.4316667	0.3882482	1.2200000	2.2200000

Unique Subject Identifier=1435000000-0212

Assay variable : PTINR_VAL1 PT-INR Value

N	Mean	Standard Deviation	Minimum	Maximum
1	1.5800000		1.5800000	1.5800000

Unique Subject Identifier=1435000000-0215

Assay variable : PTINR_VAL1 PT-INR Value

N	Mean	Standard Deviation	Minimum	Maximum
3	1.8533333	0.2872862	1.6400000	2.1800000

Laboratory tests at the time of obtaining Table 2-6 consent, according to the presence or absence of proton-pump inhibitors

Unique Subject Identifier=1436000000-0003

MEANS Procedure

Assay variable : PTINR_VAL1 PT-INR Value

N	Mean	Standard Deviation	Minimum	Maximum
3	1.7666667	0.1709776	1.5700000	1.8800000

Unique Subject Identifier=1436000000-0008

Assay variable : PTINR_VAL1 PT-INR Value

N	Mean	Standard Deviation	Minimum	Maximum
5	2.1300000	0.2152905	1.7700000	2.3400000

Unique Subject Identifier=1436000000-0010

Assay variable : PTINR_VAL1 PT-INR Value

N	Mean	Standard Deviation	Minimum	Maximum
1	3.3300000		3.3300000	3.3300000

Unique Subject Identifier=1436000000-0013

Assay variable : PTINR_VAL1 PT-INR Value

N	Mean	Standard Deviation	Minimum	Maximum
1	2.2400000		2.2400000	2.2400000

Laboratory tests at the time of obtaining Table 2-6 consent, according to the presence or absence of proton-pump inhibitors

Unique Subject Identifier=1436000000-0018

MEANS Procedure

Assay variable : PTINR_VAL1 PT-INR Value

N	Mean	Standard Deviation	Minimum	Maximum
1	1.7200000		1.7200000	1.7200000

Unique Subject Identifier=1436000000-0019

Assay variable : PTINR_VAL1 PT-INR Value

N	Mean	Standard Deviation	Minimum	Maximum
2	1.6350000	0.1484924	1.5300000	1.7400000

Unique Subject Identifier=1436000000-0020

Assay variable : PTINR_VAL1 PT-INR Value

N	Mean	Standard Deviation	Minimum	Maximum
2	2.4400000	0.3252691	2.2100000	2.6700000

Unique Subject Identifier=1436000000-0021

Assay variable : PTINR_VAL1 PT-INR Value

N	Mean	Standard Deviation	Minimum	Maximum
1	1.3700000		1.3700000	1.3700000

Laboratory tests at the time of obtaining Table 2-6 consent, according to the presence or absence of proton-pump inhibitors

Unique Subject Identifier=1436000000-0022

MEANS Procedure

Assay variable : PTINR_VAL1 PT-INR Value

N	Mean	Standard Deviation	Minimum	Maximum
1	2.2200000		2.2200000	2.2200000

Unique Subject Identifier=1436000000-0023

Assay variable : PTINR_VAL1 PT-INR Value

N	Mean	Standard Deviation	Minimum	Maximum
1	1.7900000		1.7900000	1.7900000

Unique Subject Identifier=1436000000-0024

Assay variable : PTINR_VAL1 PT-INR Value

N	Mean	Standard Deviation	Minimum	Maximum
2	2.3150000	0.0212132	2.3000000	2.3300000

Unique Subject Identifier=1436000000-0028

Assay variable : PTINR_VAL1 PT-INR Value

N	Mean	Standard Deviation	Minimum	Maximum
2	2.0900000	0.2121320	1.9400000	2.2400000

Laboratory tests at the time of obtaining Table 2-6 consent, according to the presence or absence of proton-pump inhibitors

Unique Subject Identifier=1436000000-0032

MEANS Procedure

Assay variable : PTINR_VAL1 PT-INR Value

N	Mean	Standard Deviation	Minimum	Maximum
1	2.6300000		2.6300000	2.6300000

Unique Subject Identifier=1436000000-0034

Assay variable : PTINR_VAL1 PT-INR Value

N	Mean	Standard Deviation	Minimum	Maximum
1	1.3800000		1.3800000	1.3800000

Unique Subject Identifier=1436000000-0038

Assay variable : PTINR_VAL1 PT-INR Value

N	Mean	Standard Deviation	Minimum	Maximum
3	2.5166667	0.6859543	1.7300000	2.9900000

Unique Subject Identifier=1436000000-0047

Assay variable : PTINR_VAL1 PT-INR Value

N	Mean	Standard Deviation	Minimum	Maximum
1	2.0700000		2.0700000	2.0700000

Laboratory tests at the time of obtaining Table 2-6 consent, according to the presence or absence of proton-pump inhibitors

Unique Subject Identifier=1436000000-0048

MEANS Procedure

Assay variable : PTINR_VAL1 PT-INR Value

N	Mean	Standard Deviation	Minimum	Maximum
1	2.2200000		2.2200000	2.2200000

Unique Subject Identifier=1436000000-0063

Assay variable : PTINR_VAL1 PT-INR Value

N	Mean	Standard Deviation	Minimum	Maximum
1	1.8600000		1.8600000	1.8600000

Unique Subject Identifier=1437000000-0009

Assay variable : PTINR_VAL1 PT-INR Value

N	Mean	Standard Deviation	Minimum	Maximum
6	1.8500000	0.2258318	1.6000000	2.2000000

Unique Subject Identifier=1437000000-0010

Assay variable : PTINR_VAL1 PT-INR Value

N	Mean	Standard Deviation	Minimum	Maximum
6	1.9166667	0.3710346	1.3000000	2.3000000

Laboratory tests at the time of obtaining Table 2-6 consent, according to the presence or absence of proton-pump inhibitors

Unique Subject Identifier=1437000000-0011

MEANS Procedure

Assay variable : PTINR_VAL1 PT-INR Value

N	Mean	Standard Deviation	Minimum	Maximum
6	1.7833333	0.3060501	1.4000000	2.3000000

Unique Subject Identifier=1438000000-0001

Assay variable : PTINR_VAL1 PT-INR Value

N	Mean	Standard Deviation	Minimum	Maximum
2	1.6550000	0.1343503	1.5600000	1.7500000

Unique Subject Identifier=1438000000-0010

Assay variable : PTINR_VAL1 PT-INR Value

N	Mean	Standard Deviation	Minimum	Maximum
5	2.3000000	0.0764853	2.2100000	2.3900000

Unique Subject Identifier=1438000000-0017

Assay variable : PTINR_VAL1 PT-INR Value

N	Mean	Standard Deviation	Minimum	Maximum
2	2.8100000	0.2404163	2.6400000	2.9800000

Laboratory tests at the time of obtaining Table 2-6 consent, according to the presence or absence of proton-pump inhibitors

Unique Subject Identifier=1438000000-0030

MEANS Procedure

Assay variable : PTINR_VAL1 PT-INR Value

N	Mean	Standard Deviation	Minimum	Maximum
1	1.7200000		1.7200000	1.7200000

Unique Subject Identifier=1438000000-0036

Assay variable : PTINR_VAL1 PT-INR Value

N	Mean	Standard Deviation	Minimum	Maximum
1	1.1800000		1.1800000	1.1800000

Unique Subject Identifier=1438000000-0043

Assay variable : PTINR_VAL1 PT-INR Value

N	Mean	Standard Deviation	Minimum	Maximum
1	1.6200000		1.6200000	1.6200000

Unique Subject Identifier=1438000000-0047

Assay variable : PTINR_VAL1 PT-INR Value

N	Mean	Standard Deviation	Minimum	Maximum
5	1.3040000	0.0870632	1.1700000	1.4100000

Laboratory tests at the time of obtaining Table 2-6 consent, according to the presence or absence of proton-pump inhibitors

Unique Subject Identifier=1438000000-0049

MEANS Procedure

Assay variable : PTINR_VAL1 PT-INR Value

N	Mean	Standard Deviation	Minimum	Maximum
4	2.4200000	0.3988316	2.0100000	2.8700000

Unique Subject Identifier=1440000000-0001

Assay variable : PTINR_VAL1 PT-INR Value

N	Mean	Standard Deviation	Minimum	Maximum
4	1.3000000	0.1920069	1.1200000	1.4900000

Unique Subject Identifier=1440000000-0011

Assay variable : PTINR_VAL1 PT-INR Value

N	Mean	Standard Deviation	Minimum	Maximum
3	1.9133333	0.5744853	1.2500000	2.2500000

Unique Subject Identifier=1440000000-0013

Assay variable : PTINR_VAL1 PT-INR Value

N	Mean	Standard Deviation	Minimum	Maximum
3	1.9600000	0.1734935	1.8500000	2.1600000

Laboratory tests at the time of obtaining Table 2-6 consent, according to the presence or absence of proton-pump inhibitors

Unique Subject Identifier=1440000000-0014

MEANS Procedure

Assay variable : PTINR_VAL1 PT-INR Value

N	Mean	Standard Deviation	Minimum	Maximum
5	2.0280000	0.2251000	1.7000000	2.3000000

Unique Subject Identifier=1440000000-0016

Assay variable : PTINR_VAL1 PT-INR Value

N	Mean	Standard Deviation	Minimum	Maximum
3	2.9566667	0.2858904	2.7100000	3.2700000

Unique Subject Identifier=1440000000-0018

Assay variable : PTINR_VAL1 PT-INR Value

N	Mean	Standard Deviation	Minimum	Maximum
5	1.3940000	0.1556599	1.2800000	1.6500000

Unique Subject Identifier=1440000000-0019

Assay variable : PTINR_VAL1 PT-INR Value

N	Mean	Standard Deviation	Minimum	Maximum
2	1.1050000	0.0212132	1.0900000	1.1200000

Laboratory tests at the time of obtaining Table 2-6 consent, according to the presence or absence of proton-pump inhibitors

Unique Subject Identifier=1441000000-0001

MEANS Procedure

Assay variable : PTINR_VAL1 PT-INR Value

N	Mean	Standard Deviation	Minimum	Maximum
5	1.7880000	0.2462113	1.5000000	2.1500000

Unique Subject Identifier=1444000000-0009

Assay variable : PTINR_VAL1 PT-INR Value

N	Mean	Standard Deviation	Minimum	Maximum
5	1.9260000	0.3471023	1.4400000	2.3000000

Unique Subject Identifier=1444000000-0019

Assay variable : PTINR_VAL1 PT-INR Value

N	Mean	Standard Deviation	Minimum	Maximum
6	1.9033333	0.1983599	1.7300000	2.2800000

Unique Subject Identifier=1444000000-0040

Assay variable : PTINR_VAL1 PT-INR Value

N	Mean	Standard Deviation	Minimum	Maximum
6	2.0050000	0.3040230	1.6500000	2.5200000

Laboratory tests at the time of obtaining Table 2-6 consent, according to the presence or absence of proton-pump inhibitors

Unique Subject Identifier=1444000000-0045

MEANS Procedure

Assay variable : PTINR_VAL1 PT-INR Value

N	Mean	Standard Deviation	Minimum	Maximum
6	1.9616667	0.5192462	1.3400000	2.8100000

Unique Subject Identifier=1444000000-0048

Assay variable : PTINR_VAL1 PT-INR Value

N	Mean	Standard Deviation	Minimum	Maximum
6	2.3050000	0.4026289	1.8500000	3.0000000

Unique Subject Identifier=1446000000-0004

Assay variable : PTINR_VAL1 PT-INR Value

N	Mean	Standard Deviation	Minimum	Maximum
5	1.6200000	0.1095445	1.5000000	1.8000000

Unique Subject Identifier=1446000000-0010

Assay variable : PTINR_VAL1 PT-INR Value

N	Mean	Standard Deviation	Minimum	Maximum
3	1.7666667	0.2081666	1.6000000	2.0000000

Laboratory tests at the time of obtaining Table 2-6 consent, according to the presence or absence of proton-pump inhibitors

Unique Subject Identifier=1446000000-0012

MEANS Procedure

Assay variable : PTINR_VAL1 PT-INR Value

N	Mean	Standard Deviation	Minimum	Maximum
3	1.8666667	0.0577350	1.8000000	1.9000000

Unique Subject Identifier=1446000000-0014

Assay variable : PTINR_VAL1 PT-INR Value

N	Mean	Standard Deviation	Minimum	Maximum
6	1.9666667	0.2160247	1.6000000	2.2000000

Unique Subject Identifier=1448000000-0002

Assay variable : PTINR_VAL1 PT-INR Value

N	Mean	Standard Deviation	Minimum	Maximum
1	1.4300000		1.4300000	1.4300000

Unique Subject Identifier=1448000000-0004

Assay variable : PTINR_VAL1 PT-INR Value

N	Mean	Standard Deviation	Minimum	Maximum
2	1.6800000	0.1697056	1.5600000	1.8000000

Laboratory tests at the time of obtaining Table 2-6 consent, according to the presence or absence of proton-pump inhibitors

Unique Subject Identifier=1448000000-0009

MEANS Procedure

Assay variable : PTINR_VAL1 PT-INR Value

N	Mean	Standard Deviation	Minimum	Maximum
2	1.6750000	0.0070711	1.6700000	1.6800000

Unique Subject Identifier=1448000000-0016

Assay variable : PTINR_VAL1 PT-INR Value

N	Mean	Standard Deviation	Minimum	Maximum
2	2.1150000	0.6434672	1.6600000	2.5700000

Unique Subject Identifier=1449000000-0003

Assay variable : PTINR_VAL1 PT-INR Value

N	Mean	Standard Deviation	Minimum	Maximum
5	2.2800000	0.2863564	1.9000000	2.7000000

Unique Subject Identifier=1451000000-0001

Assay variable : PTINR_VAL1 PT-INR Value

N	Mean	Standard Deviation	Minimum	Maximum
6	1.9500000	0.1378405	1.8000000	2.1000000

Laboratory tests at the time of obtaining Table 2-6 consent, according to the presence or absence of proton-pump inhibitors

Unique Subject Identifier=1451000000-0002

MEANS Procedure

Assay variable : PTINR_VAL1 PT-INR Value

N	Mean	Standard Deviation	Minimum	Maximum
2	1.8000000	0.1414214	1.7000000	1.9000000

Unique Subject Identifier=1451000000-0003

Assay variable : PTINR_VAL1 PT-INR Value

N	Mean	Standard Deviation	Minimum	Maximum
4	1.6500000	0.3696846	1.4000000	2.2000000

Unique Subject Identifier=1451000000-0004

Assay variable : PTINR_VAL1 PT-INR Value

N	Mean	Standard Deviation	Minimum	Maximum
3	2.3000000	0.5567764	1.8000000	2.9000000

Unique Subject Identifier=1451000000-0005

Assay variable : PTINR_VAL1 PT-INR Value

N	Mean	Standard Deviation	Minimum	Maximum
5	2.2200000	0.2774887	1.9000000	2.5000000

Laboratory tests at the time of obtaining Table 2-6 consent, according to the presence or absence of proton-pump inhibitors

Unique Subject Identifier=1451000000-0007

MEANS Procedure

Assay variable : PTINR_VAL1 PT-INR Value

N	Mean	Standard Deviation	Minimum	Maximum
6	2.0000000	0.2190890	1.7000000	2.3000000

Unique Subject Identifier=1451000000-0012

Assay variable : PTINR_VAL1 PT-INR Value

N	Mean	Standard Deviation	Minimum	Maximum
6	1.8500000	0.1974842	1.6000000	2.2000000

Unique Subject Identifier=1451000000-0013

Assay variable : PTINR_VAL1 PT-INR Value

N	Mean	Standard Deviation	Minimum	Maximum
6	1.8166667	0.2714160	1.5000000	2.3000000

Unique Subject Identifier=1451000000-0018

Assay variable : PTINR_VAL1 PT-INR Value

N	Mean	Standard Deviation	Minimum	Maximum
6	1.9666667	0.4082483	1.3000000	2.5000000

Laboratory tests at the time of obtaining Table 2-6 consent, according to the presence or absence of proton-pump inhibitors

Unique Subject Identifier=1451000000-0019

MEANS Procedure

Assay variable : PTINR_VAL1 PT-INR Value

N	Mean	Standard Deviation	Minimum	Maximum
2	1.9500000	0.6363961	1.5000000	2.4000000

Unique Subject Identifier=1451000000-0021

Assay variable : PTINR_VAL1 PT-INR Value

N	Mean	Standard Deviation	Minimum	Maximum
6	1.9500000	0.1760682	1.8000000	2.2000000

Unique Subject Identifier=1451000000-0023

Assay variable : PTINR_VAL1 PT-INR Value

N	Mean	Standard Deviation	Minimum	Maximum
6	1.7833333	0.1329160	1.7000000	2.0000000

Unique Subject Identifier=1451000000-0024

Assay variable : PTINR_VAL1 PT-INR Value

N	Mean	Standard Deviation	Minimum	Maximum
6	2.5333333	0.6377042	2.0000000	3.8000000

Laboratory tests at the time of obtaining Table 2-6 consent, according to the presence or absence of proton-pump inhibitors

Unique Subject Identifier=1451000000-0025

MEANS Procedure

Assay variable : PTINR_VAL1 PT-INR Value

N	Mean	Standard Deviation	Minimum	Maximum
1	2.9000000		2.9000000	2.9000000

Unique Subject Identifier=1451000000-0026

Assay variable : PTINR_VAL1 PT-INR Value

N	Mean	Standard Deviation	Minimum	Maximum
6	1.9000000	0.4098780	1.4000000	2.5000000

Unique Subject Identifier=1451000000-0027

Assay variable : PTINR_VAL1 PT-INR Value

N	Mean	Standard Deviation	Minimum	Maximum
5	2.0800000	0.1788854	1.9000000	2.3000000

Unique Subject Identifier=1451000000-0028

Assay variable : PTINR_VAL1 PT-INR Value

N	Mean	Standard Deviation	Minimum	Maximum
6	2.2500000	0.4183300	1.6000000	2.8000000

Laboratory tests at the time of obtaining Table 2-6 consent, according to the presence or absence of proton-pump inhibitors

Unique Subject Identifier=1451000000-0029

MEANS Procedure

Assay variable : PTINR_VAL1 PT-INR Value

N	Mean	Standard Deviation	Minimum	Maximum
6	1.8333333	0.2250926	1.6000000	2.2000000

Unique Subject Identifier=1451000000-0030

Assay variable : PTINR_VAL1 PT-INR Value

N	Mean	Standard Deviation	Minimum	Maximum
5	2.3600000	1.0334409	1.3000000	3.7000000

Unique Subject Identifier=1451000000-0031

Assay variable : PTINR_VAL1 PT-INR Value

N	Mean	Standard Deviation	Minimum	Maximum
6	1.7333333	0.1966384	1.4000000	2.0000000

Unique Subject Identifier=1451000000-0032

Assay variable : PTINR_VAL1 PT-INR Value

N	Mean	Standard Deviation	Minimum	Maximum
6	1.8500000	0.3728270	1.4000000	2.3000000

Laboratory tests at the time of obtaining Table 2-6 consent, according to the presence or absence of proton-pump inhibitors

Unique Subject Identifier=1451000000-0033

MEANS Procedure

Assay variable : PTINR_VAL1 PT-INR Value

N	Mean	Standard Deviation	Minimum	Maximum
6	1.2450000	0.1819615	1.0600000	1.5000000

Unique Subject Identifier=1451000000-0034

Assay variable : PTINR_VAL1 PT-INR Value

N	Mean	Standard Deviation	Minimum	Maximum
6	1.8666667	0.1632993	1.6000000	2.1000000

Unique Subject Identifier=1451000000-0035

Assay variable : PTINR_VAL1 PT-INR Value

N	Mean	Standard Deviation	Minimum	Maximum
6	2.5333333	0.2338090	2.4000000	3.0000000

Unique Subject Identifier=1451000000-0040

Assay variable : PTINR_VAL1 PT-INR Value

N	Mean	Standard Deviation	Minimum	Maximum
6	2.2833333	0.8886319	1.5000000	4.0000000

Laboratory tests at the time of obtaining Table 2-6 consent, according to the presence or absence of proton-pump inhibitors

Unique Subject Identifier=1451000000-0042

MEANS Procedure

Assay variable : PTINR_VAL1 PT-INR Value

N	Mean	Standard Deviation	Minimum	Maximum
6	1.9333333	0.2160247	1.6000000	2.2000000

Unique Subject Identifier=1451000000-0043

Assay variable : PTINR_VAL1 PT-INR Value

N	Mean	Standard Deviation	Minimum	Maximum
6	1.8833333	0.2786874	1.6000000	2.3000000

Unique Subject Identifier=1451000000-0044

Assay variable : PTINR_VAL1 PT-INR Value

N	Mean	Standard Deviation	Minimum	Maximum
6	2.2166667	0.5636193	1.6000000	2.9000000

Unique Subject Identifier=1451000000-0046

Assay variable : PTINR_VAL1 PT-INR Value

N	Mean	Standard Deviation	Minimum	Maximum
6	1.5000000	0.1673320	1.3000000	1.7000000

Laboratory tests at the time of obtaining Table 2-6 consent, according to the presence or absence of proton-pump inhibitors

Unique Subject Identifier=1451000000-0047

MEANS Procedure

Assay variable : PTINR_VAL1 PT-INR Value

N	Mean	Standard Deviation	Minimum	Maximum
6	2.4833333	0.8232051	1.4000000	3.5000000

Unique Subject Identifier=1451000000-0048

Assay variable : PTINR_VAL1 PT-INR Value

N	Mean	Standard Deviation	Minimum	Maximum
6	1.8000000	0.6066300	1.1000000	2.9000000

Unique Subject Identifier=1451000000-0049

Assay variable : PTINR_VAL1 PT-INR Value

N	Mean	Standard Deviation	Minimum	Maximum
6	2.0833333	0.3544949	1.7000000	2.6000000

Unique Subject Identifier=1451000000-0050

Assay variable : PTINR_VAL1 PT-INR Value

N	Mean	Standard Deviation	Minimum	Maximum
6	2.3166667	0.9537645	1.1000000	3.9000000

Laboratory tests at the time of obtaining Table 2-6 consent, according to the presence or absence of proton-pump inhibitors

Unique Subject Identifier=1451000000-0051

MEANS Procedure

Assay variable : PTINR_VAL1 PT-INR Value

N	Mean	Standard Deviation	Minimum	Maximum
6	1.9500000	0.3619392	1.5000000	2.4000000

Unique Subject Identifier=1451000000-0052

Assay variable : PTINR_VAL1 PT-INR Value

N	Mean	Standard Deviation	Minimum	Maximum
6	2.0666667	0.6314006	1.1000000	2.8000000

Unique Subject Identifier=1451000000-0053

Assay variable : PTINR_VAL1 PT-INR Value

N	Mean	Standard Deviation	Minimum	Maximum
6	1.8666667	0.2338090	1.5000000	2.2000000

Unique Subject Identifier=1451000000-0054

Assay variable : PTINR_VAL1 PT-INR Value

N	Mean	Standard Deviation	Minimum	Maximum
6	2.2000000	0.4472136	1.4000000	2.7000000

Laboratory tests at the time of obtaining Table 2-6 consent, according to the presence or absence of proton-pump inhibitors

Unique Subject Identifier=1451000000-0055

MEANS Procedure

Assay variable : PTINR_VAL1 PT-INR Value

N	Mean	Standard Deviation	Minimum	Maximum
6	1.7666667	0.1861899	1.6000000	2.1000000

Unique Subject Identifier=1451000000-0063

Assay variable : PTINR_VAL1 PT-INR Value

N	Mean	Standard Deviation	Minimum	Maximum
6	1.4666667	0.2250926	1.1000000	1.7000000

Unique Subject Identifier=1452000000-0002

Assay variable : PTINR_VAL1 PT-INR Value

N	Mean	Standard Deviation	Minimum	Maximum
5	2.2240000	0.2228901	1.9100000	2.5400000

Unique Subject Identifier=1452000000-0003

Assay variable : PTINR_VAL1 PT-INR Value

N	Mean	Standard Deviation	Minimum	Maximum
6	2.4866667	0.4723840	1.9300000	3.2000000

Laboratory tests at the time of obtaining Table 2-6 consent, according to the presence or absence of proton-pump inhibitors

Unique Subject Identifier=1452000000-0004

MEANS Procedure

Assay variable : PTINR_VAL1 PT-INR Value

N	Mean	Standard Deviation	Minimum	Maximum
6	2.0100000	0.2126029	1.7400000	2.3200000

Unique Subject Identifier=1452000000-0006

Assay variable : PTINR_VAL1 PT-INR Value

N	Mean	Standard Deviation	Minimum	Maximum
6	2.5516667	0.2155381	2.3600000	2.9500000

Unique Subject Identifier=1452000000-0007

Assay variable : PTINR_VAL1 PT-INR Value

N	Mean	Standard Deviation	Minimum	Maximum
5	1.8660000	0.1379493	1.6900000	2.0600000

Unique Subject Identifier=1452000000-0008

Assay variable : PTINR_VAL1 PT-INR Value

N	Mean	Standard Deviation	Minimum	Maximum
4	2.9475000	0.7501278	2.5000000	4.0700000

Laboratory tests at the time of obtaining Table 2-6 consent, according to the presence or absence of proton-pump inhibitors

Unique Subject Identifier=1452000000-0010

MEANS Procedure

Assay variable : PTINR_VAL1 PT-INR Value

N	Mean	Standard Deviation	Minimum	Maximum
4	1.8200000	0.4180112	1.3800000	2.3800000

Unique Subject Identifier=1452000000-0011

Assay variable : PTINR_VAL1 PT-INR Value

N	Mean	Standard Deviation	Minimum	Maximum
6	1.7016667	0.5537298	1.2000000	2.7800000

Unique Subject Identifier=1452000000-0012

Assay variable : PTINR_VAL1 PT-INR Value

N	Mean	Standard Deviation	Minimum	Maximum
5	1.9400000	0.2756810	1.5700000	2.2500000

Unique Subject Identifier=1452000000-0013

Assay variable : PTINR_VAL1 PT-INR Value

N	Mean	Standard Deviation	Minimum	Maximum
6	2.0800000	0.7291365	1.5500000	3.1700000

Laboratory tests at the time of obtaining Table 2-6 consent, according to the presence or absence of proton-pump inhibitors

Unique Subject Identifier=1452000000-0014

MEANS Procedure

Assay variable : PTINR_VAL1 PT-INR Value

N	Mean	Standard Deviation	Minimum	Maximum
6	2.6233333	0.5473451	1.9500000	3.2800000

Unique Subject Identifier=1452000000-0015

Assay variable : PTINR_VAL1 PT-INR Value

N	Mean	Standard Deviation	Minimum	Maximum
6	2.3266667	0.0886942	2.2300000	2.4500000

Unique Subject Identifier=1452000000-0018

Assay variable : PTINR_VAL1 PT-INR Value

N	Mean	Standard Deviation	Minimum	Maximum
5	2.4340000	0.4462398	1.6900000	2.8000000

Unique Subject Identifier=1452000000-0019

Assay variable : PTINR_VAL1 PT-INR Value

N	Mean	Standard Deviation	Minimum	Maximum
5	2.5580000	0.2932917	2.1600000	2.8800000

Laboratory tests at the time of obtaining Table 2-6 consent, according to the presence or absence of proton-pump inhibitors

Unique Subject Identifier=1452000000-0020

MEANS Procedure

Assay variable : PTINR_VAL1 PT-INR Value

N	Mean	Standard Deviation	Minimum	Maximum
6	2.1000000	0.3241605	1.8300000	2.5700000

Unique Subject Identifier=1452000000-0021

Assay variable : PTINR_VAL1 PT-INR Value

N	Mean	Standard Deviation	Minimum	Maximum
6	2.1700000	0.2033716	1.8900000	2.4600000

Unique Subject Identifier=1452000000-0024

Assay variable : PTINR_VAL1 PT-INR Value

N	Mean	Standard Deviation	Minimum	Maximum
6	2.1966667	0.3460443	1.8500000	2.7800000

Unique Subject Identifier=1452000000-0025

Assay variable : PTINR_VAL1 PT-INR Value

N	Mean	Standard Deviation	Minimum	Maximum
6	1.7983333	0.2129241	1.5100000	2.1600000

Laboratory tests at the time of obtaining Table 2-6 consent, according to the presence or absence of proton-pump inhibitors

Unique Subject Identifier=1452000000-0026

MEANS Procedure

Assay variable : PTINR_VAL1 PT-INR Value

N	Mean	Standard Deviation	Minimum	Maximum
4	1.6925000	0.4897193	1.1700000	2.1200000

Unique Subject Identifier=1452000000-0027

Assay variable : PTINR_VAL1 PT-INR Value

N	Mean	Standard Deviation	Minimum	Maximum
5	2.0800000	0.2931723	1.7400000	2.4400000

Unique Subject Identifier=1452000000-0028

Assay variable : PTINR_VAL1 PT-INR Value

N	Mean	Standard Deviation	Minimum	Maximum
6	2.2400000	0.5422914	1.6100000	3.2200000

Unique Subject Identifier=1452000000-0029

Assay variable : PTINR_VAL1 PT-INR Value

N	Mean	Standard Deviation	Minimum	Maximum
6	2.4833333	0.2224110	2.2900000	2.8800000

Laboratory tests at the time of obtaining Table 2-6 consent, according to the presence or absence of proton-pump inhibitors

Unique Subject Identifier=1452000000-0030

MEANS Procedure

Assay variable : PTINR_VAL1 PT-INR Value

N	Mean	Standard Deviation	Minimum	Maximum
6	2.4333333	0.7724938	1.6900000	3.8500000

Unique Subject Identifier=1452000000-0031

Assay variable : PTINR_VAL1 PT-INR Value

N	Mean	Standard Deviation	Minimum	Maximum
6	1.8550000	0.1693222	1.6300000	2.1000000

Unique Subject Identifier=1452000000-0033

Assay variable : PTINR_VAL1 PT-INR Value

N	Mean	Standard Deviation	Minimum	Maximum
6	1.7133333	0.3641245	1.2900000	2.2700000

Unique Subject Identifier=1452000000-0034

Assay variable : PTINR_VAL1 PT-INR Value

N	Mean	Standard Deviation	Minimum	Maximum
6	2.0716667	0.2058559	1.8700000	2.3000000

Laboratory tests at the time of obtaining Table 2-6 consent, according to the presence or absence of proton-pump inhibitors

Unique Subject Identifier=1452000000-0035

MEANS Procedure

Assay variable : PTINR_VAL1 PT-INR Value

N	Mean	Standard Deviation	Minimum	Maximum
6	2.4700000	0.5721888	1.6700000	3.3300000

Unique Subject Identifier=1452000000-0036

Assay variable : PTINR_VAL1 PT-INR Value

N	Mean	Standard Deviation	Minimum	Maximum
6	2.0600000	0.5149369	1.7000000	3.0700000

Unique Subject Identifier=1452000000-0037

Assay variable : PTINR_VAL1 PT-INR Value

N	Mean	Standard Deviation	Minimum	Maximum
6	1.9033333	0.4987852	1.2900000	2.5900000

Unique Subject Identifier=1452000000-0038

Assay variable : PTINR_VAL1 PT-INR Value

N	Mean	Standard Deviation	Minimum	Maximum
4	2.0875000	0.2972513	1.7600000	2.4600000

Laboratory tests at the time of obtaining Table 2-6 consent, according to the presence or absence of proton-pump inhibitors

Unique Subject Identifier=1452000000-0040

MEANS Procedure

Assay variable : PTINR_VAL1 PT-INR Value

N	Mean	Standard Deviation	Minimum	Maximum
3	1.8100000	0.3704052	1.5300000	2.2300000

Unique Subject Identifier=1452000000-0041

Assay variable : PTINR_VAL1 PT-INR Value

N	Mean	Standard Deviation	Minimum	Maximum
6	2.1350000	0.7433102	1.2200000	3.0200000

Unique Subject Identifier=1452000000-0042

Assay variable : PTINR_VAL1 PT-INR Value

N	Mean	Standard Deviation	Minimum	Maximum
6	1.8716667	0.3365363	1.5500000	2.3800000

Unique Subject Identifier=1452000000-0048

Assay variable : PTINR_VAL1 PT-INR Value

N	Mean	Standard Deviation	Minimum	Maximum
5	2.0620000	0.6736245	1.3600000	3.0500000

Laboratory tests at the time of obtaining Table 2-6 consent, according to the presence or absence of proton-pump inhibitors

Unique Subject Identifier=1452000000-0049

MEANS Procedure

Assay variable : PTINR_VAL1 PT-INR Value

N	Mean	Standard Deviation	Minimum	Maximum
5	2.5280000	0.4078235	2.0800000	3.1000000

Unique Subject Identifier=1452000000-0050

Assay variable : PTINR_VAL1 PT-INR Value

N	Mean	Standard Deviation	Minimum	Maximum
5	2.4340000	0.2597691	2.1000000	2.7800000

Unique Subject Identifier=1452000000-0052

Assay variable : PTINR_VAL1 PT-INR Value

N	Mean	Standard Deviation	Minimum	Maximum
4	2.4450000	0.3181719	2.0800000	2.8300000

Unique Subject Identifier=1452000000-0054

Assay variable : PTINR_VAL1 PT-INR Value

N	Mean	Standard Deviation	Minimum	Maximum
5	1.9160000	0.5048564	1.3600000	2.4200000

Laboratory tests at the time of obtaining Table 2-6 consent, according to the presence or absence of proton-pump inhibitors

Unique Subject Identifier=1452000000-0055

MEANS Procedure

Assay variable : PTINR_VAL1 PT-INR Value

N	Mean	Standard Deviation	Minimum	Maximum
5	2.1360000	0.8257300	1.2200000	3.2500000

Unique Subject Identifier=1452000000-0056

Assay variable : PTINR_VAL1 PT-INR Value

N	Mean	Standard Deviation	Minimum	Maximum
4	1.7975000	0.1203813	1.6300000	1.9100000

Unique Subject Identifier=1452000000-0057

Assay variable : PTINR_VAL1 PT-INR Value

N	Mean	Standard Deviation	Minimum	Maximum
3	1.3766667	0.3212994	1.1300000	1.7400000

Unique Subject Identifier=1452000000-0059

Assay variable : PTINR_VAL1 PT-INR Value

N	Mean	Standard Deviation	Minimum	Maximum
6	1.9800000	0.1879362	1.8400000	2.3400000

Laboratory tests at the time of obtaining Table 2-6 consent, according to the presence or absence of proton-pump inhibitors

Unique Subject Identifier=1452000000-0060

MEANS Procedure

Assay variable : PTINR_VAL1 PT-INR Value

N	Mean	Standard Deviation	Minimum	Maximum
5	1.6220000	0.2580116	1.2500000	1.9400000

Unique Subject Identifier=1452000000-0061

Assay variable : PTINR_VAL1 PT-INR Value

N	Mean	Standard Deviation	Minimum	Maximum
4	1.8200000	0.3842742	1.2500000	2.0800000

Unique Subject Identifier=1452000000-0062

Assay variable : PTINR_VAL1 PT-INR Value

N	Mean	Standard Deviation	Minimum	Maximum
5	2.0620000	0.4810094	1.3000000	2.5500000

Unique Subject Identifier=1452000000-0065

Assay variable : PTINR_VAL1 PT-INR Value

N	Mean	Standard Deviation	Minimum	Maximum
4	1.9900000	0.2694439	1.6400000	2.2700000

Laboratory tests at the time of obtaining Table 2-6 consent, according to the presence or absence of proton-pump inhibitors

Unique Subject Identifier=1452000000-0066

MEANS Procedure

Assay variable : PTINR_VAL1 PT-INR Value

N	Mean	Standard Deviation	Minimum	Maximum
5	2.2800000	0.5793099	1.7000000	3.1300000

Unique Subject Identifier=1452000000-0067

Assay variable : PTINR_VAL1 PT-INR Value

N	Mean	Standard Deviation	Minimum	Maximum
6	2.0500000	0.1951410	1.8000000	2.3000000

Unique Subject Identifier=1452000000-0068

Assay variable : PTINR_VAL1 PT-INR Value

N	Mean	Standard Deviation	Minimum	Maximum
6	1.9450000	0.2251888	1.7600000	2.3800000

Unique Subject Identifier=1452000000-0069

Assay variable : PTINR_VAL1 PT-INR Value

N	Mean	Standard Deviation	Minimum	Maximum
6	2.1883333	0.1330288	2.0600000	2.4200000

Laboratory tests at the time of obtaining Table 2-6 consent, according to the presence or absence of proton-pump inhibitors

Unique Subject Identifier=1452000000-0074

MEANS Procedure

Assay variable : PTINR_VAL1 PT-INR Value

N	Mean	Standard Deviation	Minimum	Maximum
6	2.4333333	0.2188759	2.1300000	2.7300000

Unique Subject Identifier=1452000000-0080

Assay variable : PTINR_VAL1 PT-INR Value

N	Mean	Standard Deviation	Minimum	Maximum
6	2.2450000	0.3602638	1.8300000	2.8000000

Unique Subject Identifier=1452000000-0081

Assay variable : PTINR_VAL1 PT-INR Value

N	Mean	Standard Deviation	Minimum	Maximum
6	1.6683333	0.2830135	1.1400000	1.9400000

Unique Subject Identifier=1452000000-0083

Assay variable : PTINR_VAL1 PT-INR Value

N	Mean	Standard Deviation	Minimum	Maximum
6	2.3450000	0.1808591	2.0600000	2.5100000

Laboratory tests at the time of obtaining Table 2-6 consent, according to the presence or absence of proton-pump inhibitors

Unique Subject Identifier=1452000000-0084

MEANS Procedure

Assay variable : PTINR_VAL1 PT-INR Value

N	Mean	Standard Deviation	Minimum	Maximum
2	1.7850000	0.3747666	1.5200000	2.0500000

Unique Subject Identifier=1452000000-0086

Assay variable : PTINR_VAL1 PT-INR Value

N	Mean	Standard Deviation	Minimum	Maximum
2	1.7200000	0.2828427	1.5200000	1.9200000

Unique Subject Identifier=1452000000-0090

Assay variable : PTINR_VAL1 PT-INR Value

N	Mean	Standard Deviation	Minimum	Maximum
6	1.9233333	0.3040833	1.6300000	2.3100000

Unique Subject Identifier=1452000000-0095

Assay variable : PTINR_VAL1 PT-INR Value

N	Mean	Standard Deviation	Minimum	Maximum
3	1.9766667	0.5643876	1.3300000	2.3700000

Laboratory tests at the time of obtaining Table 2-6 consent, according to the presence or absence of proton-pump inhibitors

Unique Subject Identifier=1452000000-0101

MEANS Procedure

Assay variable : PTINR_VAL1 PT-INR Value

N	Mean	Standard Deviation	Minimum	Maximum
6	1.8083333	0.1702253	1.6600000	2.1100000

Unique Subject Identifier=1452000000-0103

Assay variable : PTINR_VAL1 PT-INR Value

N	Mean	Standard Deviation	Minimum	Maximum
6	2.3233333	0.2125716	2.0100000	2.6600000

Unique Subject Identifier=1452000000-0107

Assay variable : PTINR_VAL1 PT-INR Value

N	Mean	Standard Deviation	Minimum	Maximum
4	2.1375000	0.9040050	1.1400000	3.2200000

Unique Subject Identifier=1452000000-0109

Assay variable : PTINR_VAL1 PT-INR Value

N	Mean	Standard Deviation	Minimum	Maximum
6	1.8366667	0.2488909	1.4900000	2.2100000

Laboratory tests at the time of obtaining Table 2-6 consent, according to the presence or absence of proton-pump inhibitors

Unique Subject Identifier=1454000000-0005

MEANS Procedure

Assay variable : PTINR_VAL1 PT-INR Value

N	Mean	Standard Deviation	Minimum	Maximum
6	2.4533333	0.4206978	1.9000000	2.9500000

Unique Subject Identifier=1454000000-0022

Assay variable : PTINR_VAL1 PT-INR Value

N	Mean	Standard Deviation	Minimum	Maximum
6	1.9100000	0.6382789	1.1400000	2.5600000

Unique Subject Identifier=1454000000-0028

Assay variable : PTINR_VAL1 PT-INR Value

N	Mean	Standard Deviation	Minimum	Maximum
6	2.1466667	0.3545514	1.8300000	2.8200000

Unique Subject Identifier=1454000000-0039

Assay variable : PTINR_VAL1 PT-INR Value

N	Mean	Standard Deviation	Minimum	Maximum
6	2.2066667	0.3184755	1.6400000	2.4800000

Laboratory tests at the time of obtaining Table 2-6 consent, according to the presence or absence of proton-pump inhibitors

Unique Subject Identifier=1454000000-0061

MEANS Procedure

Assay variable : PTINR_VAL1 PT-INR Value

N	Mean	Standard Deviation	Minimum	Maximum
5	2.0200000	0.2285826	1.7200000	2.3300000

Unique Subject Identifier=1454000000-0064

Assay variable : PTINR_VAL1 PT-INR Value

N	Mean	Standard Deviation	Minimum	Maximum
6	1.9216667	0.1290607	1.7700000	2.1100000

Unique Subject Identifier=1454000000-0066

Assay variable : PTINR_VAL1 PT-INR Value

N	Mean	Standard Deviation	Minimum	Maximum
1	2.1100000		2.1100000	2.1100000

Unique Subject Identifier=1454000000-0067

Assay variable : PTINR_VAL1 PT-INR Value

N	Mean	Standard Deviation	Minimum	Maximum
6	2.1033333	0.2608959	1.8100000	2.5600000

Laboratory tests at the time of obtaining Table 2-6 consent, according to the presence or absence of proton-pump inhibitors

Unique Subject Identifier=1457000000-0001

MEANS Procedure

Assay variable : PTINR_VAL1 PT-INR Value

N	Mean	Standard Deviation	Minimum	Maximum
5	1.8600000	0.4393177	1.3000000	2.5000000

Unique Subject Identifier=1463010000-0002

Assay variable : PTINR_VAL1 PT-INR Value

N	Mean	Standard Deviation	Minimum	Maximum
5	1.6840000	0.1745852	1.4400000	1.8400000

Unique Subject Identifier=1463010000-0009

Assay variable : PTINR_VAL1 PT-INR Value

N	Mean	Standard Deviation	Minimum	Maximum
5	2.0160000	0.6467070	1.3000000	2.8500000

Unique Subject Identifier=1463010000-0014

Assay variable : PTINR_VAL1 PT-INR Value

N	Mean	Standard Deviation	Minimum	Maximum
5	1.3880000	0.1267675	1.2800000	1.5900000

Laboratory tests at the time of obtaining Table 2-6 consent, according to the presence or absence of proton-pump inhibitors

Unique Subject Identifier=1463010000-0017

MEANS Procedure

Assay variable : PTINR_VAL1 PT-INR Value

N	Mean	Standard Deviation	Minimum	Maximum
6	1.7950000	0.1617096	1.6400000	2.0700000

Unique Subject Identifier=1463010000-0018

Assay variable : PTINR_VAL1 PT-INR Value

N	Mean	Standard Deviation	Minimum	Maximum
6	2.0333333	0.3765988	1.4900000	2.6100000

Unique Subject Identifier=1463010000-0025

Assay variable : PTINR_VAL1 PT-INR Value

N	Mean	Standard Deviation	Minimum	Maximum
6	2.2816667	0.2688804	1.8900000	2.6000000

Unique Subject Identifier=1463010000-0030

Assay variable : PTINR_VAL1 PT-INR Value

N	Mean	Standard Deviation	Minimum	Maximum
6	1.2266667	0.0121106	1.2100000	1.2400000

Laboratory tests at the time of obtaining Table 2-6 consent, according to the presence or absence of proton-pump inhibitors

Unique Subject Identifier=1463010000-0035

MEANS Procedure

Assay variable : PTINR_VAL1 PT-INR Value

N	Mean	Standard Deviation	Minimum	Maximum
5	2.4900000	0.4252646	1.8200000	2.8700000

Unique Subject Identifier=1469010110-0011

Assay variable : PTINR_VAL1 PT-INR Value

N	Mean	Standard Deviation	Minimum	Maximum
3	1.5800000	0.1252996	1.4500000	1.7000000

Unique Subject Identifier=1470000000-0001

Assay variable : PTINR_VAL1 PT-INR Value

N	Mean	Standard Deviation	Minimum	Maximum
4	1.0075000	0.0095743	1.0000000	1.0200000

Unique Subject Identifier=1470000000-0002

Assay variable : PTINR_VAL1 PT-INR Value

N	Mean	Standard Deviation	Minimum	Maximum
2	1.8550000	0.2474874	1.6800000	2.0300000

Laboratory tests at the time of obtaining Table 2-6 consent, according to the presence or absence of proton-pump inhibitors

Unique Subject Identifier=1470000000-0003

MEANS Procedure

Assay variable : PTINR_VAL1 PT-INR Value

N	Mean	Standard Deviation	Minimum	Maximum
4	1.6775000	0.1960230	1.4700000	1.9400000

Unique Subject Identifier=1470000000-0004

Assay variable : PTINR_VAL1 PT-INR Value

N	Mean	Standard Deviation	Minimum	Maximum
2	1.6950000	0.1626346	1.5800000	1.8100000

Unique Subject Identifier=1470000000-0009

Assay variable : PTINR_VAL1 PT-INR Value

N	Mean	Standard Deviation	Minimum	Maximum
4	1.4350000	0.0675771	1.3600000	1.5100000

Unique Subject Identifier=1474000000-0002

Assay variable : PTINR_VAL1 PT-INR Value

N	Mean	Standard Deviation	Minimum	Maximum
5	1.9200000	0.4764452	1.5000000	2.7000000

Laboratory tests at the time of obtaining Table 2-6 consent, according to the presence or absence of proton-pump inhibitors

Unique Subject Identifier=1475000000-0005

MEANS Procedure

Assay variable : PTINR_VAL1 PT-INR Value

N	Mean	Standard Deviation	Minimum	Maximum
3	1.9000000	0.1000000	1.8000000	2.0000000

Unique Subject Identifier=1475000000-0006

Assay variable : PTINR_VAL1 PT-INR Value

N	Mean	Standard Deviation	Minimum	Maximum
3	2.6333333	0.8020806	1.8000000	3.4000000

Unique Subject Identifier=1475000000-0012

Assay variable : PTINR_VAL1 PT-INR Value

N	Mean	Standard Deviation	Minimum	Maximum
2	1.7000000	0	1.7000000	1.7000000

Unique Subject Identifier=1475000000-0016

Assay variable : PTINR_VAL1 PT-INR Value

N	Mean	Standard Deviation	Minimum	Maximum
3	2.4333333	0.2516611	2.2000000	2.7000000

Laboratory tests at the time of obtaining Table 2-6 consent, according to the presence or absence of proton-pump inhibitors

Unique Subject Identifier=1477000000-0005

MEANS Procedure

Assay variable : PTINR_VAL1 PT-INR Value

N	Mean	Standard Deviation	Minimum	Maximum
6	2.0833333	0.1717750	1.9900000	2.4300000

Unique Subject Identifier=1477000000-0007

Assay variable : PTINR_VAL1 PT-INR Value

N	Mean	Standard Deviation	Minimum	Maximum
5	2.1800000	0.2062765	1.9700000	2.5200000

Unique Subject Identifier=1477000000-0014

Assay variable : PTINR_VAL1 PT-INR Value

N	Mean	Standard Deviation	Minimum	Maximum
4	2.3300000	0.1902630	2.1400000	2.5900000

Unique Subject Identifier=1478011111-0011

Assay variable : PTINR_VAL1 PT-INR Value

N	Mean	Standard Deviation	Minimum	Maximum
4	2.2625000	1.0897209	1.1200000	3.5400000

Laboratory tests at the time of obtaining Table 2-6 consent, according to the presence or absence of proton-pump inhibitors

Unique Subject Identifier=1478011111-0016

MEANS Procedure

Assay variable : PTINR_VAL1 PT-INR Value

N	Mean	Standard Deviation	Minimum	Maximum
2	2.5600000	0.6646804	2.0900000	3.0300000

Unique Subject Identifier=1478011111-0028

Assay variable : PTINR_VAL1 PT-INR Value

N	Mean	Standard Deviation	Minimum	Maximum
4	2.5775000	1.1448835	1.4500000	4.1400000

Unique Subject Identifier=1478011111-0030

Assay variable : PTINR_VAL1 PT-INR Value

N	Mean	Standard Deviation	Minimum	Maximum
3	2.4766667	0.1193035	2.3800000	2.6100000

Unique Subject Identifier=1478011111-0031

Assay variable : PTINR_VAL1 PT-INR Value

N	Mean	Standard Deviation	Minimum	Maximum
5	2.7340000	1.1840524	1.9600000	4.7800000

Laboratory tests at the time of obtaining Table 2-6 consent, according to the presence or absence of proton-pump inhibitors

Unique Subject Identifier=1478011111-0034

MEANS Procedure

Assay variable : PTINR_VAL1 PT-INR Value

N	Mean	Standard Deviation	Minimum	Maximum
3	1.6966667	0.2200757	1.4800000	1.9200000

Unique Subject Identifier=1478011111-0037

Assay variable : PTINR_VAL1 PT-INR Value

N	Mean	Standard Deviation	Minimum	Maximum
3	2.6400000	0.3451087	2.3000000	2.9900000

Unique Subject Identifier=1478011111-0038

Assay variable : PTINR_VAL1 PT-INR Value

N	Mean	Standard Deviation	Minimum	Maximum
3	2.9666667	0.2538372	2.7600000	3.2500000

Unique Subject Identifier=1478011111-0040

Assay variable : PTINR_VAL1 PT-INR Value

N	Mean	Standard Deviation	Minimum	Maximum
3	2.1233333	0.0723418	2.0400000	2.1700000

Laboratory tests at the time of obtaining Table 2-6 consent, according to the presence or absence of proton-pump inhibitors

Unique Subject Identifier=1478011111-0041

MEANS Procedure

Assay variable : PTINR_VAL1 PT-INR Value

N	Mean	Standard Deviation	Minimum	Maximum
3	2.3966667	0.0602771	2.3400000	2.4600000

Unique Subject Identifier=1478011111-0051

Assay variable : PTINR_VAL1 PT-INR Value

N	Mean	Standard Deviation	Minimum	Maximum
2	1.1900000	0.0565685	1.1500000	1.2300000

Unique Subject Identifier=1478011111-0060

Assay variable : PTINR_VAL1 PT-INR Value

N	Mean	Standard Deviation	Minimum	Maximum
3	2.8366667	0.6034346	2.4300000	3.5300000

Unique Subject Identifier=1478011111-0066

Assay variable : PTINR_VAL1 PT-INR Value

N	Mean	Standard Deviation	Minimum	Maximum
3	1.8433333	0.3162805	1.4900000	2.1000000

Laboratory tests at the time of obtaining Table 2-6 consent, according to the presence or absence of proton-pump inhibitors

Unique Subject Identifier=1478011111-0069

MEANS Procedure

Assay variable : PTINR_VAL1 PT-INR Value

N	Mean	Standard Deviation	Minimum	Maximum
3	2.5466667	0.0305505	2.5200000	2.5800000

Unique Subject Identifier=1478011111-0070

Assay variable : PTINR_VAL1 PT-INR Value

N	Mean	Standard Deviation	Minimum	Maximum
3	1.9900000	0.4450843	1.4800000	2.3000000

Unique Subject Identifier=1478011111-0079

Assay variable : PTINR_VAL1 PT-INR Value

N	Mean	Standard Deviation	Minimum	Maximum
4	2.7100000	0.9296236	1.9100000	3.9700000

Unique Subject Identifier=1478011111-0093

Assay variable : PTINR_VAL1 PT-INR Value

N	Mean	Standard Deviation	Minimum	Maximum
4	1.6525000	0.2003954	1.4700000	1.9100000

Laboratory tests at the time of obtaining Table 2-6 consent, according to the presence or absence of proton-pump inhibitors

Unique Subject Identifier=1478011111-0096

MEANS Procedure

Assay variable : PTINR_VAL1 PT-INR Value

N	Mean	Standard Deviation	Minimum	Maximum
2	2.5350000	0.1626346	2.4200000	2.6500000

Unique Subject Identifier=1478011111-0097

Assay variable : PTINR_VAL1 PT-INR Value

N	Mean	Standard Deviation	Minimum	Maximum
3	2.2300000	0.7454529	1.4700000	2.9600000

Unique Subject Identifier=1478011111-0108

Assay variable : PTINR_VAL1 PT-INR Value

N	Mean	Standard Deviation	Minimum	Maximum
4	2.1225000	0.2441823	1.8400000	2.4000000

Unique Subject Identifier=1478011111-0118

Assay variable : PTINR_VAL1 PT-INR Value

N	Mean	Standard Deviation	Minimum	Maximum
4	2.7350000	0.0911043	2.6400000	2.8500000

Laboratory tests at the time of obtaining Table 2-6 consent, according to the presence or absence of proton-pump inhibitors

Unique Subject Identifier=1478011111-0120

MEANS Procedure

Assay variable : PTINR_VAL1 PT-INR Value

N	Mean	Standard Deviation	Minimum	Maximum
4	2.0225000	0.5209846	1.4000000	2.5100000

Unique Subject Identifier=1478011111-0121

Assay variable : PTINR_VAL1 PT-INR Value

N	Mean	Standard Deviation	Minimum	Maximum
3	2.0933333	0.1721434	1.9700000	2.2900000

Unique Subject Identifier=1478011111-0127

Assay variable : PTINR_VAL1 PT-INR Value

N	Mean	Standard Deviation	Minimum	Maximum
4	1.8225000	0.0350000	1.7700000	1.8400000

Unique Subject Identifier=1478011111-0144

Assay variable : PTINR_VAL1 PT-INR Value

N	Mean	Standard Deviation	Minimum	Maximum
2	2.2250000	0.1909188	2.0900000	2.3600000

Laboratory tests at the time of obtaining Table 2-6 consent, according to the presence or absence of proton-pump inhibitors

Unique Subject Identifier=1478011111-0147

MEANS Procedure

Assay variable : PTINR_VAL1 PT-INR Value

N	Mean	Standard Deviation	Minimum	Maximum
3	2.1666667	0.2396525	1.8900000	2.3100000

Unique Subject Identifier=1480011111-0002

Assay variable : PTINR_VAL1 PT-INR Value

N	Mean	Standard Deviation	Minimum	Maximum
1	1.0800000		1.0800000	1.0800000

Unique Subject Identifier=1480011111-0003

Assay variable : PTINR_VAL1 PT-INR Value

N	Mean	Standard Deviation	Minimum	Maximum
3	2.5600000	0.9497895	1.4700000	3.2100000

Unique Subject Identifier=1480011111-0005

Assay variable : PTINR_VAL1 PT-INR Value

N	Mean	Standard Deviation	Minimum	Maximum
3	2.2533333	0.8220300	1.7200000	3.2000000

Laboratory tests at the time of obtaining Table 2-6 consent, according to the presence or absence of proton-pump inhibitors

Unique Subject Identifier=1487000000-0001

MEANS Procedure

Assay variable : PTINR_VAL1 PT-INR Value

N	Mean	Standard Deviation	Minimum	Maximum
6	2.3333333	0.4320494	1.9000000	3.1000000

Unique Subject Identifier=1487000000-0002

Assay variable : PTINR_VAL1 PT-INR Value

N	Mean	Standard Deviation	Minimum	Maximum
6	2.1166667	0.3710346	1.7000000	2.7000000

Unique Subject Identifier=1487000000-0007

Assay variable : PTINR_VAL1 PT-INR Value

N	Mean	Standard Deviation	Minimum	Maximum
6	1.9166667	0.1834848	1.6000000	2.1000000

Unique Subject Identifier=1487000000-0010

Assay variable : PTINR_VAL1 PT-INR Value

N	Mean	Standard Deviation	Minimum	Maximum
6	2.2000000	0.5513620	1.5000000	3.1000000

Laboratory tests at the time of obtaining Table 2-6 consent, according to the presence or absence of proton-pump inhibitors

Unique Subject Identifier=1487000000-0012

MEANS Procedure

Assay variable : PTINR_VAL1 PT-INR Value

N	Mean	Standard Deviation	Minimum	Maximum
6	2.1333333	0.2581989	1.8000000	2.5000000

Unique Subject Identifier=1487000000-0017

Assay variable : PTINR_VAL1 PT-INR Value

N	Mean	Standard Deviation	Minimum	Maximum
6	2.0666667	0.3614784	1.6000000	2.7000000

Unique Subject Identifier=1487000000-0019

Assay variable : PTINR_VAL1 PT-INR Value

N	Mean	Standard Deviation	Minimum	Maximum
6	2.1500000	0.1516575	2.0000000	2.4000000

Unique Subject Identifier=1487000000-0022

Assay variable : PTINR_VAL1 PT-INR Value

N	Mean	Standard Deviation	Minimum	Maximum
5	2.1600000	0.4505552	1.7000000	2.8000000

Laboratory tests at the time of obtaining Table 2-6 consent, according to the presence or absence of proton-pump inhibitors

Unique Subject Identifier=1487000000-0023

MEANS Procedure

Assay variable : PTINR_VAL1 PT-INR Value

N	Mean	Standard Deviation	Minimum	Maximum
6	2.0333333	0.3141125	1.6000000	2.5000000

Unique Subject Identifier=1487000000-0027

Assay variable : PTINR_VAL1 PT-INR Value

N	Mean	Standard Deviation	Minimum	Maximum
6	2.1166667	0.3710346	1.6000000	2.6000000

Unique Subject Identifier=1487000000-0028

Assay variable : PTINR_VAL1 PT-INR Value

N	Mean	Standard Deviation	Minimum	Maximum
6	1.8666667	0.4273952	1.5000000	2.5000000

Unique Subject Identifier=1487000000-0029

Assay variable : PTINR_VAL1 PT-INR Value

N	Mean	Standard Deviation	Minimum	Maximum
6	2.3833333	0.9304121	1.5000000	4.0000000

Laboratory tests at the time of obtaining Table 2-6 consent, according to the presence or absence of proton-pump inhibitors

Unique Subject Identifier=1487000000-0031

MEANS Procedure

Assay variable : PTINR_VAL1 PT-INR Value

N	Mean	Standard Deviation	Minimum	Maximum
6	2.0833333	0.4622409	1.5000000	2.8000000

Unique Subject Identifier=1487000000-0032

Assay variable : PTINR_VAL1 PT-INR Value

N	Mean	Standard Deviation	Minimum	Maximum
5	2.2800000	0.1643168	2.1000000	2.5000000

Unique Subject Identifier=1487000000-0033

Assay variable : PTINR_VAL1 PT-INR Value

N	Mean	Standard Deviation	Minimum	Maximum
6	2.4666667	0.4802777	2.0000000	3.2000000

Unique Subject Identifier=1491000000-0002

Assay variable : PTINR_VAL1 PT-INR Value

N	Mean	Standard Deviation	Minimum	Maximum
4	2.3225000	0.9042262	1.7200000	3.6500000

Laboratory tests at the time of obtaining Table 2-6 consent, according to the presence or absence of proton-pump inhibitors

Unique Subject Identifier=1491000000-0006

MEANS Procedure

Assay variable : PTINR_VAL1 PT-INR Value

N	Mean	Standard Deviation	Minimum	Maximum
4	2.1075000	0.2436357	1.7600000	2.3300000

Unique Subject Identifier=1491000000-0007

Assay variable : PTINR_VAL1 PT-INR Value

N	Mean	Standard Deviation	Minimum	Maximum
6	1.6833333	0.2338090	1.4200000	2.0600000

Unique Subject Identifier=1491000000-0008

Assay variable : PTINR_VAL1 PT-INR Value

N	Mean	Standard Deviation	Minimum	Maximum
6	2.2966667	0.3324856	1.8900000	2.7700000

Unique Subject Identifier=1491000000-0009

Assay variable : PTINR_VAL1 PT-INR Value

N	Mean	Standard Deviation	Minimum	Maximum
5	2.1060000	0.5477043	1.5400000	2.9700000

Laboratory tests at the time of obtaining Table 2-6 consent, according to the presence or absence of proton-pump inhibitors

Unique Subject Identifier=1491000000-0010

MEANS Procedure

Assay variable : PTINR_VAL1 PT-INR Value

N	Mean	Standard Deviation	Minimum	Maximum
4	2.3600000	0.5832667	1.5100000	2.8300000

Unique Subject Identifier=1491000000-0011

Assay variable : PTINR_VAL1 PT-INR Value

N	Mean	Standard Deviation	Minimum	Maximum
4	2.4825000	0.8079759	1.5200000	3.3800000

Unique Subject Identifier=1491000000-0012

Assay variable : PTINR_VAL1 PT-INR Value

N	Mean	Standard Deviation	Minimum	Maximum
4	1.8975000	0.0427200	1.8700000	1.9600000

Unique Subject Identifier=1491000000-0019

Assay variable : PTINR_VAL1 PT-INR Value

N	Mean	Standard Deviation	Minimum	Maximum
4	2.2975000	0.3704389	1.9200000	2.7900000

Laboratory tests at the time of obtaining Table 2-6 consent, according to the presence or absence of proton-pump inhibitors

Unique Subject Identifier=1491000000-0020

MEANS Procedure

Assay variable : PTINR_VAL1 PT-INR Value

N	Mean	Standard Deviation	Minimum	Maximum
3	1.9133333	0.1601041	1.7500000	2.0700000

Unique Subject Identifier=1491000000-0021

Assay variable : PTINR_VAL1 PT-INR Value

N	Mean	Standard Deviation	Minimum	Maximum
6	2.0583333	0.3400833	1.7500000	2.5600000

Unique Subject Identifier=1491000000-0025

Assay variable : PTINR_VAL1 PT-INR Value

N	Mean	Standard Deviation	Minimum	Maximum
3	2.3033333	0.0737111	2.2200000	2.3600000

Unique Subject Identifier=1491000000-0031

Assay variable : PTINR_VAL1 PT-INR Value

N	Mean	Standard Deviation	Minimum	Maximum
3	1.8466667	0.0513160	1.7900000	1.8900000

Laboratory tests at the time of obtaining Table 2-6 consent, according to the presence or absence of proton-pump inhibitors

Unique Subject Identifier=1491000000-0032

MEANS Procedure

Assay variable : PTINR_VAL1 PT-INR Value

N	Mean	Standard Deviation	Minimum	Maximum
3	1.8500000	0.2325941	1.6000000	2.0600000

Unique Subject Identifier=1491000000-0035

Assay variable : PTINR_VAL1 PT-INR Value

N	Mean	Standard Deviation	Minimum	Maximum
4	1.9475000	0.3264327	1.6300000	2.4000000

Unique Subject Identifier=1491000000-0040

Assay variable : PTINR_VAL1 PT-INR Value

N	Mean	Standard Deviation	Minimum	Maximum
6	2.6333333	0.5336915	1.8900000	3.5400000

Unique Subject Identifier=1491000000-0041

Assay variable : PTINR_VAL1 PT-INR Value

N	Mean	Standard Deviation	Minimum	Maximum
4	2.0400000	0.5090514	1.6000000	2.6300000

Laboratory tests at the time of obtaining Table 2-6 consent, according to the presence or absence of proton-pump inhibitors

Unique Subject Identifier=1491000000-0044

MEANS Procedure

Assay variable : PTINR_VAL1 PT-INR Value

N	Mean	Standard Deviation	Minimum	Maximum
4	1.9550000	0.4466542	1.6600000	2.6200000

Unique Subject Identifier=1491000000-0045

Assay variable : PTINR_VAL1 PT-INR Value

N	Mean	Standard Deviation	Minimum	Maximum
4	1.9100000	0.2947315	1.6100000	2.3000000

Unique Subject Identifier=1491000000-0047

Assay variable : PTINR_VAL1 PT-INR Value

N	Mean	Standard Deviation	Minimum	Maximum
6	1.8016667	0.1619156	1.6200000	2.0900000

Unique Subject Identifier=1491000000-0049

Assay variable : PTINR_VAL1 PT-INR Value

N	Mean	Standard Deviation	Minimum	Maximum
6	1.8566667	0.4394390	1.3900000	2.6700000

Laboratory tests at the time of obtaining Table 2-6 consent, according to the presence or absence of proton-pump inhibitors

Unique Subject Identifier=1491000000-0050

MEANS Procedure

Assay variable : PTINR_VAL1 PT-INR Value

N	Mean	Standard Deviation	Minimum	Maximum
3	1.8200000	0.0818535	1.7300000	1.8900000

Unique Subject Identifier=1491000000-0056

Assay variable : PTINR_VAL1 PT-INR Value

N	Mean	Standard Deviation	Minimum	Maximum
3	1.9766667	0.0378594	1.9500000	2.0200000

Unique Subject Identifier=1491000000-0057

Assay variable : PTINR_VAL1 PT-INR Value

N	Mean	Standard Deviation	Minimum	Maximum
3	2.0800000	0.2718455	1.7800000	2.3100000

Unique Subject Identifier=1491000000-0059

Assay variable : PTINR_VAL1 PT-INR Value

N	Mean	Standard Deviation	Minimum	Maximum
4	2.3575000	0.2555223	2.1000000	2.6900000

Laboratory tests at the time of obtaining Table 2-6 consent, according to the presence or absence of proton-pump inhibitors

Unique Subject Identifier=1491000000-0060

MEANS Procedure

Assay variable : PTINR_VAL1 PT-INR Value

N	Mean	Standard Deviation	Minimum	Maximum
4	1.9075000	0.1946578	1.7500000	2.1500000

Unique Subject Identifier=1491000000-0062

Assay variable : PTINR_VAL1 PT-INR Value

N	Mean	Standard Deviation	Minimum	Maximum
5	1.8600000	0.2309762	1.6900000	2.2600000

Unique Subject Identifier=1491000000-0063

Assay variable : PTINR_VAL1 PT-INR Value

N	Mean	Standard Deviation	Minimum	Maximum
4	2.2475000	0.2644964	1.9000000	2.5300000

Unique Subject Identifier=1491000000-0066

Assay variable : PTINR_VAL1 PT-INR Value

N	Mean	Standard Deviation	Minimum	Maximum
4	2.0725000	0.2117192	1.8700000	2.3100000

Laboratory tests at the time of obtaining Table 2-6 consent, according to the presence or absence of proton-pump inhibitors

Unique Subject Identifier=1491000000-0070

MEANS Procedure

Assay variable : PTINR_VAL1 PT-INR Value

N	Mean	Standard Deviation	Minimum	Maximum
4	2.0075000	0.0865544	1.9300000	2.1300000

Unique Subject Identifier=1491000000-0073

Assay variable : PTINR_VAL1 PT-INR Value

N	Mean	Standard Deviation	Minimum	Maximum
3	1.9900000	0.1997498	1.7700000	2.1600000

Unique Subject Identifier=1491000000-0074

Assay variable : PTINR_VAL1 PT-INR Value

N	Mean	Standard Deviation	Minimum	Maximum
4	2.0675000	0.1901534	1.8100000	2.2300000

Unique Subject Identifier=1491000000-0077

Assay variable : PTINR_VAL1 PT-INR Value

N	Mean	Standard Deviation	Minimum	Maximum
3	2.2766667	0.4441096	1.7900000	2.6600000

Laboratory tests at the time of obtaining Table 2-6 consent, according to the presence or absence of proton-pump inhibitors

Unique Subject Identifier=1491000000-0082

MEANS Procedure

Assay variable : PTINR_VAL1 PT-INR Value

N	Mean	Standard Deviation	Minimum	Maximum
4	2.3575000	0.3834384	2.1200000	2.9300000

Unique Subject Identifier=1491000000-0084

Assay variable : PTINR_VAL1 PT-INR Value

N	Mean	Standard Deviation	Minimum	Maximum
3	2.0066667	0.2441994	1.8100000	2.2800000

Unique Subject Identifier=1491000000-0086

Assay variable : PTINR_VAL1 PT-INR Value

N	Mean	Standard Deviation	Minimum	Maximum
4	2.1675000	0.2817061	1.9300000	2.5700000

Unique Subject Identifier=1491000000-0088

Assay variable : PTINR_VAL1 PT-INR Value

N	Mean	Standard Deviation	Minimum	Maximum
4	2.7900000	0.8003749	2.3600000	3.9900000

Laboratory tests at the time of obtaining Table 2-6 consent, according to the presence or absence of proton-pump inhibitors

Unique Subject Identifier=1491000000-0089

MEANS Procedure

Assay variable : PTINR_VAL1 PT-INR Value

N	Mean	Standard Deviation	Minimum	Maximum
4	1.5925000	0.1152895	1.4200000	1.6600000

Unique Subject Identifier=1491000000-0094

Assay variable : PTINR_VAL1 PT-INR Value

N	Mean	Standard Deviation	Minimum	Maximum
4	2.1475000	0.1078193	2.0500000	2.2900000

Unique Subject Identifier=1491000000-0097

Assay variable : PTINR_VAL1 PT-INR Value

N	Mean	Standard Deviation	Minimum	Maximum
3	2.6600000	0.6577994	2.0400000	3.3500000

Unique Subject Identifier=1491000000-0098

Assay variable : PTINR_VAL1 PT-INR Value

N	Mean	Standard Deviation	Minimum	Maximum
4	2.1300000	0.3281260	1.8600000	2.5700000

Laboratory tests at the time of obtaining Table 2-6 consent, according to the presence or absence of proton-pump inhibitors

Unique Subject Identifier=1491000000-0101

MEANS Procedure

Assay variable : PTINR_VAL1 PT-INR Value

N	Mean	Standard Deviation	Minimum	Maximum
4	1.7700000	0.2044505	1.4700000	1.9300000

Unique Subject Identifier=1491000000-0103

Assay variable : PTINR_VAL1 PT-INR Value

N	Mean	Standard Deviation	Minimum	Maximum
4	2.5075000	0.2325762	2.2900000	2.8200000

Unique Subject Identifier=1491000000-0105

Assay variable : PTINR_VAL1 PT-INR Value

N	Mean	Standard Deviation	Minimum	Maximum
5	1.8920000	0.3596804	1.5900000	2.5000000

Unique Subject Identifier=1492011100-0027

Assay variable : PTINR_VAL1 PT-INR Value

N	Mean	Standard Deviation	Minimum	Maximum
6	2.1500000	0.2509980	1.8000000	2.4000000

Laboratory tests at the time of obtaining Table 2-6 consent, according to the presence or absence of proton-pump inhibitors

Unique Subject Identifier=1494000000-0005

MEANS Procedure

Assay variable : PTINR_VAL1 PT-INR Value

N	Mean	Standard Deviation	Minimum	Maximum
2	0.9500000	0.0424264	0.9200000	0.9800000

Unique Subject Identifier=1494000000-0007

Assay variable : PTINR_VAL1 PT-INR Value

N	Mean	Standard Deviation	Minimum	Maximum
4	1.5500000	0.2585859	1.2000000	1.7900000

Unique Subject Identifier=1494000000-0008

Assay variable : PTINR_VAL1 PT-INR Value

N	Mean	Standard Deviation	Minimum	Maximum
3	1.9500000	0.1058301	1.8300000	2.0300000

Unique Subject Identifier=1494000000-0015

Assay variable : PTINR_VAL1 PT-INR Value

N	Mean	Standard Deviation	Minimum	Maximum
2	1.5600000	0.2121320	1.4100000	1.7100000

Laboratory tests at the time of obtaining Table 2-6 consent, according to the presence or absence of proton-pump inhibitors

Unique Subject Identifier=1494000000-0017

MEANS Procedure

Assay variable : PTINR_VAL1 PT-INR Value

N	Mean	Standard Deviation	Minimum	Maximum
4	1.6825000	0.2443188	1.3900000	1.9300000

Unique Subject Identifier=1494000000-0021

Assay variable : PTINR_VAL1 PT-INR Value

N	Mean	Standard Deviation	Minimum	Maximum
4	1.9375000	0.6571847	1.4000000	2.8800000

Unique Subject Identifier=1494000000-0027

Assay variable : PTINR_VAL1 PT-INR Value

N	Mean	Standard Deviation	Minimum	Maximum
3	1.7400000	0.0200000	1.7200000	1.7600000

Unique Subject Identifier=1494000000-0033

Assay variable : PTINR_VAL1 PT-INR Value

N	Mean	Standard Deviation	Minimum	Maximum
2	1.8150000	0.1484924	1.7100000	1.9200000

Laboratory tests at the time of obtaining Table 2-6 consent, according to the presence or absence of proton-pump inhibitors

Unique Subject Identifier=1495011010-0003

MEANS Procedure

Assay variable : PTINR_VAL1 PT-INR Value

N	Mean	Standard Deviation	Minimum	Maximum
3	2.3600000	0.2193171	2.1700000	2.6000000

Unique Subject Identifier=1495011010-0005

Assay variable : PTINR_VAL1 PT-INR Value

N	Mean	Standard Deviation	Minimum	Maximum
2	2.6700000	0.5374012	2.2900000	3.0500000

Unique Subject Identifier=1495011010-0009

Assay variable : PTINR_VAL1 PT-INR Value

N	Mean	Standard Deviation	Minimum	Maximum
3	2.4166667	0.5589574	1.9600000	3.0400000

Unique Subject Identifier=1495011010-0013

Assay variable : PTINR_VAL1 PT-INR Value

N	Mean	Standard Deviation	Minimum	Maximum
2	2.7300000	0.9475231	2.0600000	3.4000000

Laboratory tests at the time of obtaining Table 2-6 consent, according to the presence or absence of proton-pump inhibitors

Unique Subject Identifier=1495011010-0019

MEANS Procedure

Assay variable : PTINR_VAL1 PT-INR Value

N	Mean	Standard Deviation	Minimum	Maximum
2	2.2950000	0.1767767	2.1700000	2.4200000

Unique Subject Identifier=1495011010-0022

Assay variable : PTINR_VAL1 PT-INR Value

N	Mean	Standard Deviation	Minimum	Maximum
3	2.1266667	0.7674199	1.5600000	3.0000000

Unique Subject Identifier=1495011010-0024

Assay variable : PTINR_VAL1 PT-INR Value

N	Mean	Standard Deviation	Minimum	Maximum
2	2.0500000	0.3535534	1.8000000	2.3000000

Unique Subject Identifier=1495011010-0026

Assay variable : PTINR_VAL1 PT-INR Value

N	Mean	Standard Deviation	Minimum	Maximum
3	2.2933333	0.3971566	1.8400000	2.5800000

Laboratory tests at the time of obtaining Table 2-6 consent, according to the presence or absence of proton-pump inhibitors

Unique Subject Identifier=1495011010-0027

MEANS Procedure

Assay variable : PTINR_VAL1 PT-INR Value

N	Mean	Standard Deviation	Minimum	Maximum
2	1.5200000	0.0565685	1.4800000	1.5600000

Unique Subject Identifier=1496000000-0008

Assay variable : PTINR_VAL1 PT-INR Value

N	Mean	Standard Deviation	Minimum	Maximum
1	2.3000000		2.3000000	2.3000000

Unique Subject Identifier=1496000000-0012

Assay variable : PTINR_VAL1 PT-INR Value

N	Mean	Standard Deviation	Minimum	Maximum
1	1.4000000		1.4000000	1.4000000

Unique Subject Identifier=1499000000-0001

Assay variable : PTINR_VAL1 PT-INR Value

N	Mean	Standard Deviation	Minimum	Maximum
2	2.0850000	0.1343503	1.9900000	2.1800000

Laboratory tests at the time of obtaining Table 2-6 consent, according to the presence or absence of proton-pump inhibitors

Unique Subject Identifier=1499000000-0008

MEANS Procedure

Assay variable : PTINR_VAL1 PT-INR Value

N	Mean	Standard Deviation	Minimum	Maximum
3	1.7566667	0.3942503	1.4000000	2.1800000

Unique Subject Identifier=1501000000-0004

Assay variable : PTINR_VAL1 PT-INR Value

N	Mean	Standard Deviation	Minimum	Maximum
6	2.1033333	0.5933521	1.2000000	3.0000000

Unique Subject Identifier=1501000000-0009

Assay variable : PTINR_VAL1 PT-INR Value

N	Mean	Standard Deviation	Minimum	Maximum
5	1.5920000	0.0729383	1.5000000	1.7000000

Unique Subject Identifier=1501000000-0010

Assay variable : PTINR_VAL1 PT-INR Value

N	Mean	Standard Deviation	Minimum	Maximum
4	1.9700000	0.5339788	1.5100000	2.6700000

Laboratory tests at the time of obtaining Table 2-6 consent, according to the presence or absence of proton-pump inhibitors

Unique Subject Identifier=1501000000-0012

MEANS Procedure

Assay variable : PTINR_VAL1 PT-INR Value

N	Mean	Standard Deviation	Minimum	Maximum
6	2.2333333	0.3386247	1.7000000	2.6000000

Unique Subject Identifier=1501000000-0014

Assay variable : PTINR_VAL1 PT-INR Value

N	Mean	Standard Deviation	Minimum	Maximum
5	1.7620000	0.3475917	1.4000000	2.2900000

Unique Subject Identifier=1501000000-0022

Assay variable : PTINR_VAL1 PT-INR Value

N	Mean	Standard Deviation	Minimum	Maximum
6	1.8750000	0.3895510	1.5000000	2.5000000

Unique Subject Identifier=1501000000-0025

Assay variable : PTINR_VAL1 PT-INR Value

N	Mean	Standard Deviation	Minimum	Maximum
6	2.2166667	0.5115336	1.5000000	2.8000000

Laboratory tests at the time of obtaining Table 2-6 consent, according to the presence or absence of proton-pump inhibitors

Unique Subject Identifier=1501000000-0033

MEANS Procedure

Assay variable : PTINR_VAL1 PT-INR Value

N	Mean	Standard Deviation	Minimum	Maximum
3	1.6700000	0.0608276	1.6000000	1.7100000

Unique Subject Identifier=1501000000-0035

Assay variable : PTINR_VAL1 PT-INR Value

N	Mean	Standard Deviation	Minimum	Maximum
2	1.6400000	0.2262742	1.4800000	1.8000000

Unique Subject Identifier=1501000000-0046

Assay variable : PTINR_VAL1 PT-INR Value

N	Mean	Standard Deviation	Minimum	Maximum
5	2.3360000	0.8537740	1.2000000	3.3900000

Unique Subject Identifier=1502000000-0001

Assay variable : PTINR_VAL1 PT-INR Value

N	Mean	Standard Deviation	Minimum	Maximum
4	1.6900000	0.2336664	1.4600000	1.9700000

Laboratory tests at the time of obtaining Table 2-6 consent, according to the presence or absence of proton-pump inhibitors

Unique Subject Identifier=1502000000-0003

MEANS Procedure

Assay variable : PTINR_VAL1 PT-INR Value

N	Mean	Standard Deviation	Minimum	Maximum
1	1.5600000		1.5600000	1.5600000

Unique Subject Identifier=1502000000-0004

Assay variable : PTINR_VAL1 PT-INR Value

N	Mean	Standard Deviation	Minimum	Maximum
4	2.1600000	0.4730750	1.8000000	2.8500000

Unique Subject Identifier=1502000000-0005

Assay variable : PTINR_VAL1 PT-INR Value

N	Mean	Standard Deviation	Minimum	Maximum
1	2.1000000		2.1000000	2.1000000

Unique Subject Identifier=1502000000-0007

Assay variable : PTINR_VAL1 PT-INR Value

N	Mean	Standard Deviation	Minimum	Maximum
2	1.7200000	0.6646804	1.2500000	2.1900000

Laboratory tests at the time of obtaining Table 2-6 consent, according to the presence or absence of proton-pump inhibitors

Unique Subject Identifier=1502000000-0008

MEANS Procedure

Assay variable : PTINR_VAL1 PT-INR Value

N	Mean	Standard Deviation	Minimum	Maximum
2	1.9900000	0.0141421	1.9800000	2.0000000

Unique Subject Identifier=1502000000-0010

Assay variable : PTINR_VAL1 PT-INR Value

N	Mean	Standard Deviation	Minimum	Maximum
1	1.3700000		1.3700000	1.3700000

Unique Subject Identifier=1503000000-0001

Assay variable : PTINR_VAL1 PT-INR Value

N	Mean	Standard Deviation	Minimum	Maximum
1	1.7500000		1.7500000	1.7500000

Unique Subject Identifier=1503000000-0005

Assay variable : PTINR_VAL1 PT-INR Value

N	Mean	Standard Deviation	Minimum	Maximum
3	1.8066667	1.3367997	1.0100000	3.3500000

Laboratory tests at the time of obtaining Table 2-6 consent, according to the presence or absence of proton-pump inhibitors

Unique Subject Identifier=1503000000-0007

MEANS Procedure

Assay variable : PTINR_VAL1 PT-INR Value

N	Mean	Standard Deviation	Minimum	Maximum
4	2.1825000	0.8794458	1.4100000	3.1000000

Unique Subject Identifier=1503000000-0008

Assay variable : PTINR_VAL1 PT-INR Value

N	Mean	Standard Deviation	Minimum	Maximum
3	2.2033333	1.2630255	1.3200000	3.6500000

Unique Subject Identifier=1503000000-0010

Assay variable : PTINR_VAL1 PT-INR Value

N	Mean	Standard Deviation	Minimum	Maximum
2	1.4200000	0.1838478	1.2900000	1.5500000

Unique Subject Identifier=1504000000-0001

Assay variable : PTINR_VAL1 PT-INR Value

N	Mean	Standard Deviation	Minimum	Maximum
3	2.1600000	0.3351119	1.8200000	2.4900000

Laboratory tests at the time of obtaining Table 2-6 consent, according to the presence or absence of proton-pump inhibitors

Unique Subject Identifier=1504000000-0002

MEANS Procedure

Assay variable : PTINR_VAL1 PT-INR Value

N	Mean	Standard Deviation	Minimum	Maximum
5	2.6700000	0.3653081	2.1400000	3.0900000

Unique Subject Identifier=1504000000-0009

Assay variable : PTINR_VAL1 PT-INR Value

N	Mean	Standard Deviation	Minimum	Maximum
1	2.3800000		2.3800000	2.3800000

Unique Subject Identifier=1512000000-0004

Assay variable : PTINR_VAL1 PT-INR Value

N	Mean	Standard Deviation	Minimum	Maximum
5	1.8000000	0.3706751	1.2700000	2.3100000

Unique Subject Identifier=1512000000-0005

Assay variable : PTINR_VAL1 PT-INR Value

N	Mean	Standard Deviation	Minimum	Maximum
3	2.0000000	0.2364318	1.7500000	2.2200000

Laboratory tests at the time of obtaining Table 2-6 consent, according to the presence or absence of proton-pump inhibitors

Unique Subject Identifier=1512000000-0007

MEANS Procedure

Assay variable : PTINR_VAL1 PT-INR Value

N	Mean	Standard Deviation	Minimum	Maximum
6	2.2116667	0.2878483	1.8600000	2.6900000

Unique Subject Identifier=1513000000-0006

Assay variable : PTINR_VAL1 PT-INR Value

N	Mean	Standard Deviation	Minimum	Maximum
3	1.8700000	0.1664332	1.7500000	2.0600000

Unique Subject Identifier=1513000000-0010

Assay variable : PTINR_VAL1 PT-INR Value

N	Mean	Standard Deviation	Minimum	Maximum
3	2.1700000	0.0435890	2.1200000	2.2000000

Unique Subject Identifier=1513000000-0011

Assay variable : PTINR_VAL1 PT-INR Value

N	Mean	Standard Deviation	Minimum	Maximum
3	1.5200000	0.2306513	1.2600000	1.7000000

Laboratory tests at the time of obtaining Table 2-6 consent, according to the presence or absence of proton-pump inhibitors

Unique Subject Identifier=1514011100-0011

MEANS Procedure

Assay variable : PTINR_VAL1 PT-INR Value

N	Mean	Standard Deviation	Minimum	Maximum
1	2.1800000		2.1800000	2.1800000

Unique Subject Identifier=1514011100-0012

Assay variable : PTINR_VAL1 PT-INR Value

N	Mean	Standard Deviation	Minimum	Maximum
2	1.8250000	0.2192031	1.6700000	1.9800000

Unique Subject Identifier=1516011000-0013

Assay variable : PTINR_VAL1 PT-INR Value

N	Mean	Standard Deviation	Minimum	Maximum
6	2.0500000	0.1516575	1.9000000	2.3000000

Unique Subject Identifier=1516011000-0020

Assay variable : PTINR_VAL1 PT-INR Value

N	Mean	Standard Deviation	Minimum	Maximum
5	2.1400000	0.5549775	1.3000000	2.7000000

Laboratory tests at the time of obtaining Table 2-6 consent, according to the presence or absence of proton-pump inhibitors

Unique Subject Identifier=1516011000-0041

MEANS Procedure

Assay variable : PTINR_VAL1 PT-INR Value

N	Mean	Standard Deviation	Minimum	Maximum
6	2.1233333	0.4757590	1.4500000	2.5600000

Unique Subject Identifier=1516011000-0043

Assay variable : PTINR_VAL1 PT-INR Value

N	Mean	Standard Deviation	Minimum	Maximum
5	1.6140000	0.0876356	1.4900000	1.7000000

Unique Subject Identifier=1516011000-0045

Assay variable : PTINR_VAL1 PT-INR Value

N	Mean	Standard Deviation	Minimum	Maximum
6	1.5500000	0.1643168	1.4000000	1.8000000

Unique Subject Identifier=1516011000-0051

Assay variable : PTINR_VAL1 PT-INR Value

N	Mean	Standard Deviation	Minimum	Maximum
2	2.0000000	0.9899495	1.3000000	2.7000000

Laboratory tests at the time of obtaining Table 2-6 consent, according to the presence or absence of proton-pump inhibitors

Unique Subject Identifier=1516011000-0052

MEANS Procedure

Assay variable : PTINR_VAL1 PT-INR Value

N	Mean	Standard Deviation	Minimum	Maximum
5	2.1600000	0.5683309	1.2000000	2.6000000

Unique Subject Identifier=1516011000-0079

Assay variable : PTINR_VAL1 PT-INR Value

N	Mean	Standard Deviation	Minimum	Maximum
6	1.8500000	0.4086563	1.5000000	2.6000000

Unique Subject Identifier=1517010100-0003

Assay variable : PTINR_VAL1 PT-INR Value

N	Mean	Standard Deviation	Minimum	Maximum
2	1.6900000	0.1272792	1.6000000	1.7800000

Unique Subject Identifier=1517010100-0004

Assay variable : PTINR_VAL1 PT-INR Value

N	Mean	Standard Deviation	Minimum	Maximum
3	2.1733333	0.4986315	1.6300000	2.6100000

Laboratory tests at the time of obtaining Table 2-6 consent, according to the presence or absence of proton-pump inhibitors

Unique Subject Identifier=1517010100-0005

MEANS Procedure

Assay variable : PTINR_VAL1 PT-INR Value

N	Mean	Standard Deviation	Minimum	Maximum
3	1.8766667	0.2650157	1.6100000	2.1400000

Unique Subject Identifier=1517010100-0006

Assay variable : PTINR_VAL1 PT-INR Value

N	Mean	Standard Deviation	Minimum	Maximum
3	2.2633333	0.6664333	1.5500000	2.8700000

Unique Subject Identifier=1517010100-0008

Assay variable : PTINR_VAL1 PT-INR Value

N	Mean	Standard Deviation	Minimum	Maximum
4	1.2925000	0.0330404	1.2600000	1.3300000

Unique Subject Identifier=1517010100-0009

Assay variable : PTINR_VAL1 PT-INR Value

N	Mean	Standard Deviation	Minimum	Maximum
3	1.9500000	0.3502856	1.6400000	2.3300000

Laboratory tests at the time of obtaining Table 2-6 consent, according to the presence or absence of proton-pump inhibitors

Unique Subject Identifier=1517010100-0011

MEANS Procedure

Assay variable : PTINR_VAL1 PT-INR Value

N	Mean	Standard Deviation	Minimum	Maximum
3	1.6900000	0.1473092	1.5600000	1.8500000

Unique Subject Identifier=1521000000-0002

Assay variable : PTINR_VAL1 PT-INR Value

N	Mean	Standard Deviation	Minimum	Maximum
1	1.7300000		1.7300000	1.7300000

Unique Subject Identifier=1521000000-0003

Assay variable : PTINR_VAL1 PT-INR Value

N	Mean	Standard Deviation	Minimum	Maximum
1	2.6600000		2.6600000	2.6600000

Unique Subject Identifier=1521000000-0006

Assay variable : PTINR_VAL1 PT-INR Value

N	Mean	Standard Deviation	Minimum	Maximum
1	1.7100000		1.7100000	1.7100000

Laboratory tests at the time of obtaining Table 2-6 consent, according to the presence or absence of proton-pump inhibitors

Unique Subject Identifier=1522000000-0001

MEANS Procedure

Assay variable : PTINR_VAL1 PT-INR Value

N	Mean	Standard Deviation	Minimum	Maximum
4	2.1100000	0.5918896	1.6700000	2.9400000

Unique Subject Identifier=1522000000-0003

Assay variable : PTINR_VAL1 PT-INR Value

N	Mean	Standard Deviation	Minimum	Maximum
2	1.4150000	0.4596194	1.0900000	1.7400000

Unique Subject Identifier=1522000000-0005

Assay variable : PTINR_VAL1 PT-INR Value

N	Mean	Standard Deviation	Minimum	Maximum
5	1.9700000	0.3586781	1.6500000	2.4400000

Unique Subject Identifier=1522000000-0006

Assay variable : PTINR_VAL1 PT-INR Value

N	Mean	Standard Deviation	Minimum	Maximum
4	1.8775000	0.2466272	1.6400000	2.1500000

Laboratory tests at the time of obtaining Table 2-6 consent, according to the presence or absence of proton-pump inhibitors

Unique Subject Identifier=1522000000-0009

MEANS Procedure

Assay variable : PTINR_VAL1 PT-INR Value

N	Mean	Standard Deviation	Minimum	Maximum
6	2.0883333	0.5870747	1.5500000	3.1100000

Unique Subject Identifier=1522000000-0010

Assay variable : PTINR_VAL1 PT-INR Value

N	Mean	Standard Deviation	Minimum	Maximum
6	2.4316667	0.7141545	1.5300000	3.4100000

Unique Subject Identifier=1522000000-0011

Assay variable : PTINR_VAL1 PT-INR Value

N	Mean	Standard Deviation	Minimum	Maximum
5	2.2160000	0.7098098	1.3800000	2.8600000

Unique Subject Identifier=1522000000-0016

Assay variable : PTINR_VAL1 PT-INR Value

N	Mean	Standard Deviation	Minimum	Maximum
4	1.7400000	0.1913113	1.5600000	1.9200000

Laboratory tests at the time of obtaining Table 2-6 consent, according to the presence or absence of proton-pump inhibitors

Unique Subject Identifier=1522000000-0017

MEANS Procedure

Assay variable : PTINR_VAL1 PT-INR Value

N	Mean	Standard Deviation	Minimum	Maximum
4	1.9975000	0.5490826	1.3600000	2.4900000

Unique Subject Identifier=1522000000-0022

Assay variable : PTINR_VAL1 PT-INR Value

N	Mean	Standard Deviation	Minimum	Maximum
4	2.5100000	0.3154890	2.1600000	2.8900000

Unique Subject Identifier=1522000000-0023

Assay variable : PTINR_VAL1 PT-INR Value

N	Mean	Standard Deviation	Minimum	Maximum
5	2.0600000	0.1140175	1.8900000	2.2100000

Unique Subject Identifier=1522000000-0024

Assay variable : PTINR_VAL1 PT-INR Value

N	Mean	Standard Deviation	Minimum	Maximum
4	1.9600000	0.5485131	1.2100000	2.5000000

Laboratory tests at the time of obtaining Table 2-6 consent, according to the presence or absence of proton-pump inhibitors

Unique Subject Identifier=1522000000-0027

MEANS Procedure

Assay variable : PTINR_VAL1 PT-INR Value

N	Mean	Standard Deviation	Minimum	Maximum
4	1.6675000	0.1985573	1.5300000	1.9600000

Unique Subject Identifier=1522000000-0031

Assay variable : PTINR_VAL1 PT-INR Value

N	Mean	Standard Deviation	Minimum	Maximum
3	1.4866667	0.0152753	1.4700000	1.5000000

Unique Subject Identifier=1522000000-0033

Assay variable : PTINR_VAL1 PT-INR Value

N	Mean	Standard Deviation	Minimum	Maximum
3	1.2466667	0.3384277	0.9600000	1.6200000

Unique Subject Identifier=1522000000-0035

Assay variable : PTINR_VAL1 PT-INR Value

N	Mean	Standard Deviation	Minimum	Maximum
3	1.8600000	0.4430576	1.3500000	2.1500000

Laboratory tests at the time of obtaining Table 2-6 consent, according to the presence or absence of proton-pump inhibitors

Unique Subject Identifier=1523010111-0001

MEANS Procedure

Assay variable : PTINR_VAL1 PT-INR Value

N	Mean	Standard Deviation	Minimum	Maximum
6	1.6800000	0.3017284	1.4100000	2.2500000

Unique Subject Identifier=1523010111-0006

Assay variable : PTINR_VAL1 PT-INR Value

N	Mean	Standard Deviation	Minimum	Maximum
6	1.5683333	0.1646107	1.3300000	1.7300000

Unique Subject Identifier=1523010111-0011

Assay variable : PTINR_VAL1 PT-INR Value

N	Mean	Standard Deviation	Minimum	Maximum
6	1.8616667	0.3711828	1.5900000	2.5800000

Unique Subject Identifier=1524010000-0002

Assay variable : PTINR_VAL1 PT-INR Value

N	Mean	Standard Deviation	Minimum	Maximum
2	1.8450000	0.2050610	1.7000000	1.9900000

Laboratory tests at the time of obtaining Table 2-6 consent, according to the presence or absence of proton-pump inhibitors

Unique Subject Identifier=1524010000-0003

MEANS Procedure

Assay variable : PTINR_VAL1 PT-INR Value

N	Mean	Standard Deviation	Minimum	Maximum
1	2.3200000		2.3200000	2.3200000

Unique Subject Identifier=1524010000-0005

Assay variable : PTINR_VAL1 PT-INR Value

N	Mean	Standard Deviation	Minimum	Maximum
2	2.0050000	0.1484924	1.9000000	2.1100000

Unique Subject Identifier=1524010000-0006

Assay variable : PTINR_VAL1 PT-INR Value

N	Mean	Standard Deviation	Minimum	Maximum
1	2.4200000		2.4200000	2.4200000

Unique Subject Identifier=1524010000-0007

Assay variable : PTINR_VAL1 PT-INR Value

N	Mean	Standard Deviation	Minimum	Maximum
3	2.2233333	0.1154701	2.0900000	2.2900000

Laboratory tests at the time of obtaining Table 2-6 consent, according to the presence or absence of proton-pump inhibitors

Unique Subject Identifier=1524010000-0008

MEANS Procedure

Assay variable : PTINR_VAL1 PT-INR Value

N	Mean	Standard Deviation	Minimum	Maximum
4	1.8575000	0.3262284	1.5700000	2.3000000

Unique Subject Identifier=1524010000-0009

Assay variable : PTINR_VAL1 PT-INR Value

N	Mean	Standard Deviation	Minimum	Maximum
4	1.8200000	0.4307358	1.2300000	2.2000000

Unique Subject Identifier=1524010000-0012

Assay variable : PTINR_VAL1 PT-INR Value

N	Mean	Standard Deviation	Minimum	Maximum
4	2.2575000	0.2129750	1.9600000	2.4500000

Unique Subject Identifier=1524010000-0018

Assay variable : PTINR_VAL1 PT-INR Value

N	Mean	Standard Deviation	Minimum	Maximum
4	2.2350000	0.4086971	1.9100000	2.8300000

Laboratory tests at the time of obtaining Table 2-6 consent, according to the presence or absence of proton-pump inhibitors

Unique Subject Identifier=1527010000-0001

MEANS Procedure

Assay variable : PTINR_VAL1 PT-INR Value

N	Mean	Standard Deviation	Minimum	Maximum
6	1.7250000	0.2076295	1.4500000	1.9900000

Unique Subject Identifier=1527010000-0002

Assay variable : PTINR_VAL1 PT-INR Value

N	Mean	Standard Deviation	Minimum	Maximum
5	2.0180000	0.1130044	1.9300000	2.2000000

Unique Subject Identifier=1527010000-0003

Assay variable : PTINR_VAL1 PT-INR Value

N	Mean	Standard Deviation	Minimum	Maximum
6	1.8283333	0.3559448	1.5600000	2.5000000

Unique Subject Identifier=1527010000-0004

Assay variable : PTINR_VAL1 PT-INR Value

N	Mean	Standard Deviation	Minimum	Maximum
6	2.0766667	0.1527962	1.9300000	2.3000000

Laboratory tests at the time of obtaining Table 2-6 consent, according to the presence or absence of proton-pump inhibitors

Unique Subject Identifier=1527010000-0005

MEANS Procedure

Assay variable : PTINR_VAL1 PT-INR Value

N	Mean	Standard Deviation	Minimum	Maximum
5	2.4140000	0.5662861	2.0300000	3.3900000

Unique Subject Identifier=1527010000-0006

Assay variable : PTINR_VAL1 PT-INR Value

N	Mean	Standard Deviation	Minimum	Maximum
6	2.0616667	0.2521441	1.7100000	2.4000000

Unique Subject Identifier=1527010000-0007

Assay variable : PTINR_VAL1 PT-INR Value

N	Mean	Standard Deviation	Minimum	Maximum
6	2.3883333	0.3098656	1.9300000	2.8000000

Unique Subject Identifier=1527010000-0008

Assay variable : PTINR_VAL1 PT-INR Value

N	Mean	Standard Deviation	Minimum	Maximum
6	2.0716667	0.3989695	1.7100000	2.8000000

Laboratory tests at the time of obtaining Table 2-6 consent, according to the presence or absence of proton-pump inhibitors

Unique Subject Identifier=1527010000-0009

MEANS Procedure

Assay variable : PTINR_VAL1 PT-INR Value

N	Mean	Standard Deviation	Minimum	Maximum
6	1.9333333	0.1298717	1.7100000	2.1100000

Unique Subject Identifier=1527010000-0010

Assay variable : PTINR_VAL1 PT-INR Value

N	Mean	Standard Deviation	Minimum	Maximum
6	1.8316667	0.1595515	1.5600000	1.9300000

Unique Subject Identifier=1527010000-0011

Assay variable : PTINR_VAL1 PT-INR Value

N	Mean	Standard Deviation	Minimum	Maximum
6	2.0683333	0.3099301	1.7100000	2.4000000

Unique Subject Identifier=1527010000-0012

Assay variable : PTINR_VAL1 PT-INR Value

N	Mean	Standard Deviation	Minimum	Maximum
4	1.7100000	0.2933712	1.4500000	2.1200000

Laboratory tests at the time of obtaining Table 2-6 consent, according to the presence or absence of proton-pump inhibitors

Unique Subject Identifier=1527010000-0013

MEANS Procedure

Assay variable : PTINR_VAL1 PT-INR Value

N	Mean	Standard Deviation	Minimum	Maximum
5	2.0380000	0.2314519	1.7100000	2.3000000

Unique Subject Identifier=1527010000-0016

Assay variable : PTINR_VAL1 PT-INR Value

N	Mean	Standard Deviation	Minimum	Maximum
6	1.8516667	0.1699902	1.7100000	2.1200000

Unique Subject Identifier=1527010000-0017

Assay variable : PTINR_VAL1 PT-INR Value

N	Mean	Standard Deviation	Minimum	Maximum
5	1.9500000	0.3967367	1.5600000	2.4000000

Unique Subject Identifier=1527010000-0018

Assay variable : PTINR_VAL1 PT-INR Value

N	Mean	Standard Deviation	Minimum	Maximum
6	2.2633333	0.2242023	1.9300000	2.5000000

Laboratory tests at the time of obtaining Table 2-6 consent, according to the presence or absence of proton-pump inhibitors

Unique Subject Identifier=1527010000-0022

MEANS Procedure

Assay variable : PTINR_VAL1 PT-INR Value

N	Mean	Standard Deviation	Minimum	Maximum
6	1.9150000	0.6305791	1.2500000	2.8000000

Unique Subject Identifier=1527010000-0024

Assay variable : PTINR_VAL1 PT-INR Value

N	Mean	Standard Deviation	Minimum	Maximum
6	1.9166667	0.4050021	1.4500000	2.6000000

Unique Subject Identifier=1527010000-0026

Assay variable : PTINR_VAL1 PT-INR Value

N	Mean	Standard Deviation	Minimum	Maximum
6	2.1050000	0.3042860	1.7100000	2.6000000

Unique Subject Identifier=1528000000-0001

Assay variable : PTINR_VAL1 PT-INR Value

N	Mean	Standard Deviation	Minimum	Maximum
6	1.7533333	0.1539697	1.5100000	1.9700000

Laboratory tests at the time of obtaining Table 2-6 consent, according to the presence or absence of proton-pump inhibitors

Unique Subject Identifier=1528000000-0003

MEANS Procedure

Assay variable : PTINR_VAL1 PT-INR Value

N	Mean	Standard Deviation	Minimum	Maximum
6	2.2866667	0.5947324	1.3800000	3.1300000

Unique Subject Identifier=1528000000-0004

Assay variable : PTINR_VAL1 PT-INR Value

N	Mean	Standard Deviation	Minimum	Maximum
4	1.8200000	0.2580698	1.4900000	2.1000000

Unique Subject Identifier=1530000000-0002

Assay variable : PTINR_VAL1 PT-INR Value

N	Mean	Standard Deviation	Minimum	Maximum
5	1.5800000	0.1923538	1.4000000	1.9000000

Unique Subject Identifier=1530000000-0003

Assay variable : PTINR_VAL1 PT-INR Value

N	Mean	Standard Deviation	Minimum	Maximum
5	1.5800000	0.1788854	1.3000000	1.7000000

Laboratory tests at the time of obtaining Table 2-6 consent, according to the presence or absence of proton-pump inhibitors

Unique Subject Identifier=1530000000-0026

MEANS Procedure

Assay variable : PTINR_VAL1 PT-INR Value

N	Mean	Standard Deviation	Minimum	Maximum
2	4.0500000	2.3334524	2.4000000	5.7000000

Unique Subject Identifier=1530000000-0030

Assay variable : PTINR_VAL1 PT-INR Value

N	Mean	Standard Deviation	Minimum	Maximum
1	1.4000000		1.4000000	1.4000000

Unique Subject Identifier=1531100000-0011

Assay variable : PTINR_VAL1 PT-INR Value

N	Mean	Standard Deviation	Minimum	Maximum
6	2.2833333	0.2639444	1.9000000	2.6000000

Unique Subject Identifier=1600011000-0001

Assay variable : PTINR_VAL1 PT-INR Value

N	Mean	Standard Deviation	Minimum	Maximum
2	1.6600000	0.0848528	1.6000000	1.7200000

Laboratory tests at the time of obtaining Table 2-6 consent, according to the presence or absence of proton-pump inhibitors

Unique Subject Identifier=1600011000-0006

MEANS Procedure

Assay variable : PTINR_VAL1 PT-INR Value

N	Mean	Standard Deviation	Minimum	Maximum
1	2.5700000		2.5700000	2.5700000

Unique Subject Identifier=1600011000-0016

Assay variable : PTINR_VAL1 PT-INR Value

N	Mean	Standard Deviation	Minimum	Maximum
3	1.3533333	0.0680686	1.3000000	1.4300000

Unique Subject Identifier=1600011000-0018

Assay variable : PTINR_VAL1 PT-INR Value

N	Mean	Standard Deviation	Minimum	Maximum
6	2.0333333	0.5442671	1.3900000	2.7200000

Unique Subject Identifier=1600011000-0023

Assay variable : PTINR_VAL1 PT-INR Value

N	Mean	Standard Deviation	Minimum	Maximum
3	2.0266667	0.2706166	1.8100000	2.3300000

Laboratory tests at the time of obtaining Table 2-6 consent, according to the presence or absence of proton-pump inhibitors

Unique Subject Identifier=1600011000-0024

MEANS Procedure

Assay variable : PTINR_VAL1 PT-INR Value

N	Mean	Standard Deviation	Minimum	Maximum
1	1.8300000		1.8300000	1.8300000

Unique Subject Identifier=1600011000-0027

Assay variable : PTINR_VAL1 PT-INR Value

N	Mean	Standard Deviation	Minimum	Maximum
6	1.7750000	0.3326710	1.3100000	2.1700000

Unique Subject Identifier=1602000000-0002

Assay variable : PTINR_VAL1 PT-INR Value

N	Mean	Standard Deviation	Minimum	Maximum
3	1.5000000	0.1000000	1.4000000	1.6000000

Unique Subject Identifier=1602000000-0003

Assay variable : PTINR_VAL1 PT-INR Value

N	Mean	Standard Deviation	Minimum	Maximum
3	1.8333333	0.4041452	1.4000000	2.2000000

Laboratory tests at the time of obtaining Table 2-6 consent, according to the presence or absence of proton-pump inhibitors

Unique Subject Identifier=1603000000-0015

MEANS Procedure

Assay variable : PTINR_VAL1 PT-INR Value

N	Mean	Standard Deviation	Minimum	Maximum
5	1.7600000	0.4669047	1.3000000	2.4000000

Unique Subject Identifier=1603000000-0019

Assay variable : PTINR_VAL1 PT-INR Value

N	Mean	Standard Deviation	Minimum	Maximum
6	2.5000000	0.4939636	1.9000000	3.0000000

Unique Subject Identifier=1701010000-0003

Assay variable : PTINR_VAL1 PT-INR Value

N	Mean	Standard Deviation	Minimum	Maximum
6	1.5016667	0.0685322	1.4300000	1.5700000

Unique Subject Identifier=1701010000-0011

Assay variable : PTINR_VAL1 PT-INR Value

N	Mean	Standard Deviation	Minimum	Maximum
4	1.4125000	0.2327194	1.1000000	1.5900000

Laboratory tests at the time of obtaining Table 2-6 consent, according to the presence or absence of proton-pump inhibitors

Unique Subject Identifier=1702010000-0009

MEANS Procedure

Assay variable : PTINR_VAL1 PT-INR Value

N	Mean	Standard Deviation	Minimum	Maximum
2	2.1850000	0.1343503	2.0900000	2.2800000

Unique Subject Identifier=1705010000-0002

Assay variable : PTINR_VAL1 PT-INR Value

N	Mean	Standard Deviation	Minimum	Maximum
6	1.8116667	0.1802683	1.5000000	1.9700000

Unique Subject Identifier=1705010000-0003

Assay variable : PTINR_VAL1 PT-INR Value

N	Mean	Standard Deviation	Minimum	Maximum
2	1.6600000	0.0989949	1.5900000	1.7300000

Unique Subject Identifier=1708010000-0003

Assay variable : PTINR_VAL1 PT-INR Value

N	Mean	Standard Deviation	Minimum	Maximum
5	2.3020000	0.3291960	2.0900000	2.8800000

Laboratory tests at the time of obtaining Table 2-6 consent, according to the presence or absence of proton-pump inhibitors

Unique Subject Identifier=1710000000-0001

MEANS Procedure

Assay variable : PTINR_VAL1 PT-INR Value

N	Mean	Standard Deviation	Minimum	Maximum
6	2.2450000	0.2115420	1.9800000	2.4700000

Unique Subject Identifier=1710000000-0008

Assay variable : PTINR_VAL1 PT-INR Value

N	Mean	Standard Deviation	Minimum	Maximum
6	2.1583333	0.3340309	1.8100000	2.7200000

Unique Subject Identifier=1710000000-0009

Assay variable : PTINR_VAL1 PT-INR Value

N	Mean	Standard Deviation	Minimum	Maximum
5	1.9600000	0.4259108	1.5300000	2.5900000

Unique Subject Identifier=1716010000-0004

Assay variable : PTINR_VAL1 PT-INR Value

N	Mean	Standard Deviation	Minimum	Maximum
3	1.6933333	0.4045162	1.3800000	2.1500000

Laboratory tests at the time of obtaining Table 2-6 consent, according to the presence or absence of proton-pump inhibitors

Unique Subject Identifier=1716010000-0006

MEANS Procedure

Assay variable : PTINR_VAL1 PT-INR Value

N	Mean	Standard Deviation	Minimum	Maximum
2	2.0500000	0.4808326	1.7100000	2.3900000

Unique Subject Identifier=1718000000-0008

Assay variable : PTINR_VAL1 PT-INR Value

N	Mean	Standard Deviation	Minimum	Maximum
6	1.7333333	0.3559026	1.2000000	2.1000000

Unique Subject Identifier=1718000000-0011

Assay variable : PTINR_VAL1 PT-INR Value

N	Mean	Standard Deviation	Minimum	Maximum
6	1.7000000	0.1264911	1.6000000	1.9000000

Unique Subject Identifier=1718000000-0015

Assay variable : PTINR_VAL1 PT-INR Value

N	Mean	Standard Deviation	Minimum	Maximum
5	1.9200000	0.2683282	1.5000000	2.2000000

Laboratory tests at the time of obtaining Table 2-6 consent, according to the presence or absence of proton-pump inhibitors

Unique Subject Identifier=1718000000-0017

MEANS Procedure

Assay variable : PTINR_VAL1 PT-INR Value

N	Mean	Standard Deviation	Minimum	Maximum
6	2.2333333	0.5819507	1.9000000	3.4000000

Unique Subject Identifier=1718000000-0018

Assay variable : PTINR_VAL1 PT-INR Value

N	Mean	Standard Deviation	Minimum	Maximum
6	1.7666667	0.3265986	1.4000000	2.2000000

Unique Subject Identifier=1718000000-0020

Assay variable : PTINR_VAL1 PT-INR Value

N	Mean	Standard Deviation	Minimum	Maximum
6	2.0333333	1.2274635	1.2000000	4.4000000

Unique Subject Identifier=1750100000-0005

Assay variable : PTINR_VAL1 PT-INR Value

N	Mean	Standard Deviation	Minimum	Maximum
3	1.8033333	0.1550269	1.6500000	1.9600000

Laboratory tests at the time of obtaining Table 2-6 consent, according to the presence or absence of proton-pump inhibitors

Unique Subject Identifier=1750100000-0006

MEANS Procedure

Assay variable : PTINR_VAL1 PT-INR Value

N	Mean	Standard Deviation	Minimum	Maximum
6	1.7966667	0.1076414	1.6300000	1.9000000

Unique Subject Identifier=1750100000-0007

Assay variable : PTINR_VAL1 PT-INR Value

N	Mean	Standard Deviation	Minimum	Maximum
3	3.4466667	0.3952636	3.0100000	3.7800000

Unique Subject Identifier=1750100000-0009

Assay variable : PTINR_VAL1 PT-INR Value

N	Mean	Standard Deviation	Minimum	Maximum
5	1.6380000	0.2701296	1.3600000	1.9500000

Unique Subject Identifier=1750100000-0012

Assay variable : PTINR_VAL1 PT-INR Value

N	Mean	Standard Deviation	Minimum	Maximum
4	1.6325000	0.1138347	1.5100000	1.7800000

Laboratory tests at the time of obtaining Table 2-6 consent, according to the presence or absence of proton-pump inhibitors

Unique Subject Identifier=1750100000-0018

MEANS Procedure

Assay variable : PTINR_VAL1 PT-INR Value

N	Mean	Standard Deviation	Minimum	Maximum
3	2.0766667	0.2402776	1.8300000	2.3100000

Unique Subject Identifier=1750100000-0021

Assay variable : PTINR_VAL1 PT-INR Value

N	Mean	Standard Deviation	Minimum	Maximum
3	2.2633333	0.2419366	1.9900000	2.4500000

Unique Subject Identifier=1750100000-0025

Assay variable : PTINR_VAL1 PT-INR Value

N	Mean	Standard Deviation	Minimum	Maximum
3	1.8366667	0.6619919	1.4200000	2.6000000

Unique Subject Identifier=1750100000-0028

Assay variable : PTINR_VAL1 PT-INR Value

N	Mean	Standard Deviation	Minimum	Maximum
4	1.8700000	0.6558963	1.2100000	2.7700000

Laboratory tests at the time of obtaining Table 2-6 consent, according to the presence or absence of proton-pump inhibitors

Unique Subject Identifier=1750100000-0035

MEANS Procedure

Assay variable : PTINR_VAL1 PT-INR Value

N	Mean	Standard Deviation	Minimum	Maximum
4	2.0775000	0.4478374	1.7900000	2.7400000

Unique Subject Identifier=1750100000-0036

Assay variable : PTINR_VAL1 PT-INR Value

N	Mean	Standard Deviation	Minimum	Maximum
3	2.2033333	0.4614470	1.8700000	2.7300000

Unique Subject Identifier=1750100000-0037

Assay variable : PTINR_VAL1 PT-INR Value

N	Mean	Standard Deviation	Minimum	Maximum
2	1.4650000	0.0494975	1.4300000	1.5000000

Unique Subject Identifier=1750100000-0039

Assay variable : PTINR_VAL1 PT-INR Value

N	Mean	Standard Deviation	Minimum	Maximum
5	1.9720000	0.3858368	1.5900000	2.3900000

Laboratory tests at the time of obtaining Table 2-6 consent, according to the presence or absence of proton-pump inhibitors

Unique Subject Identifier=1750100000-0040

MEANS Procedure

Assay variable : PTINR_VAL1 PT-INR Value

N	Mean	Standard Deviation	Minimum	Maximum
6	1.8750000	0.1408190	1.6800000	2.0400000

Unique Subject Identifier=1750100000-0043

Assay variable : PTINR_VAL1 PT-INR Value

N	Mean	Standard Deviation	Minimum	Maximum
5	1.8100000	0.2511971	1.6400000	2.2500000

Unique Subject Identifier=1750100000-0045

Assay variable : PTINR_VAL1 PT-INR Value

N	Mean	Standard Deviation	Minimum	Maximum
6	1.8700000	0.4787901	1.4200000	2.6700000

Unique Subject Identifier=1750100000-0047

Assay variable : PTINR_VAL1 PT-INR Value

N	Mean	Standard Deviation	Minimum	Maximum
4	1.8800000	0.7425631	1.4300000	2.9900000

Laboratory tests at the time of obtaining Table 2-6 consent, according to the presence or absence of proton-pump inhibitors

Unique Subject Identifier=1750100000-0052

MEANS Procedure

Assay variable : PTINR_VAL1 PT-INR Value

N	Mean	Standard Deviation	Minimum	Maximum
4	1.7825000	0.2523721	1.5700000	2.1200000

Unique Subject Identifier=1800000000-0007

Assay variable : PTINR_VAL1 PT-INR Value

N	Mean	Standard Deviation	Minimum	Maximum
4	2.1750000	0.4645787	1.7000000	2.8000000

Unique Subject Identifier=1800000000-0008

Assay variable : PTINR_VAL1 PT-INR Value

N	Mean	Standard Deviation	Minimum	Maximum
6	1.9166667	0.4622409	1.4000000	2.6000000

Unique Subject Identifier=1800000000-0012

Assay variable : PTINR_VAL1 PT-INR Value

N	Mean	Standard Deviation	Minimum	Maximum
6	2.0650000	0.3887287	1.5000000	2.5900000

Laboratory tests at the time of obtaining Table 2-6 consent, according to the presence or absence of proton-pump inhibitors

Unique Subject Identifier=1800000000-0015

MEANS Procedure

Assay variable : PTINR_VAL1 PT-INR Value

N	Mean	Standard Deviation	Minimum	Maximum
6	1.8300000	0.2909295	1.4000000	2.1800000

Unique Subject Identifier=1800000000-0021

Assay variable : PTINR_VAL1 PT-INR Value

N	Mean	Standard Deviation	Minimum	Maximum
2	2.4950000	0.1484924	2.3900000	2.6000000

Unique Subject Identifier=1800000000-0028

Assay variable : PTINR_VAL1 PT-INR Value

N	Mean	Standard Deviation	Minimum	Maximum
6	2.3250000	0.3151349	1.8500000	2.7300000

Unique Subject Identifier=1805000000-0006

Assay variable : PTINR_VAL1 PT-INR Value

N	Mean	Standard Deviation	Minimum	Maximum
1	2.3000000		2.3000000	2.3000000

Laboratory tests at the time of obtaining Table 2-6 consent, according to the presence or absence of proton-pump inhibitors

Unique Subject Identifier=1810000000-0001

MEANS Procedure

Assay variable : PTINR_VAL1 PT-INR Value

N	Mean	Standard Deviation	Minimum	Maximum
6	2.0850000	0.3365561	1.6200000	2.4400000

Unique Subject Identifier=1810000000-0004

Assay variable : PTINR_VAL1 PT-INR Value

N	Mean	Standard Deviation	Minimum	Maximum
5	1.8100000	0.4154516	1.3800000	2.4400000

Unique Subject Identifier=1810000000-0005

Assay variable : PTINR_VAL1 PT-INR Value

N	Mean	Standard Deviation	Minimum	Maximum
6	1.6716667	0.2863855	1.3200000	2.0700000

Unique Subject Identifier=1810000000-0006

Assay variable : PTINR_VAL1 PT-INR Value

N	Mean	Standard Deviation	Minimum	Maximum
6	2.0350000	0.1342758	1.9100000	2.2100000

Laboratory tests at the time of obtaining Table 2-6 consent, according to the presence or absence of proton-pump inhibitors

Unique Subject Identifier=1810000000-0009

MEANS Procedure

Assay variable : PTINR_VAL1 PT-INR Value

N	Mean	Standard Deviation	Minimum	Maximum
6	1.9783333	0.2976183	1.5600000	2.4600000

Unique Subject Identifier=1810000000-0011

Assay variable : PTINR_VAL1 PT-INR Value

N	Mean	Standard Deviation	Minimum	Maximum
6	1.8600000	0.4210463	1.4500000	2.6500000

Unique Subject Identifier=1810000000-0012

Assay variable : PTINR_VAL1 PT-INR Value

N	Mean	Standard Deviation	Minimum	Maximum
6	2.0400000	0.1073313	1.8900000	2.1700000

Unique Subject Identifier=1810000000-0014

Assay variable : PTINR_VAL1 PT-INR Value

N	Mean	Standard Deviation	Minimum	Maximum
6	1.9666667	0.2732520	1.5900000	2.2300000

Laboratory tests at the time of obtaining Table 2-6 consent, according to the presence or absence of proton-pump inhibitors

Unique Subject Identifier=1810000000-0017

MEANS Procedure

Assay variable : PTINR_VAL1 PT-INR Value

N	Mean	Standard Deviation	Minimum	Maximum
6	2.0200000	0.2748818	1.7000000	2.3200000

Unique Subject Identifier=1810000000-0018

Assay variable : PTINR_VAL1 PT-INR Value

N	Mean	Standard Deviation	Minimum	Maximum
6	2.1750000	0.4289406	1.5900000	2.8300000

Unique Subject Identifier=1811000000-0005

Assay variable : PTINR_VAL1 PT-INR Value

N	Mean	Standard Deviation	Minimum	Maximum
6	1.5900000	0.0660303	1.5300000	1.7000000

Unique Subject Identifier=1811000000-0006

Assay variable : PTINR_VAL1 PT-INR Value

N	Mean	Standard Deviation	Minimum	Maximum
6	2.0950000	0.7683684	1.2700000	3.2500000

Laboratory tests at the time of obtaining Table 2-6 consent, according to the presence or absence of proton-pump inhibitors

Unique Subject Identifier=1811000000-0007

MEANS Procedure

Assay variable : PTINR_VAL1 PT-INR Value

N	Mean	Standard Deviation	Minimum	Maximum
6	1.8766667	0.2086784	1.5400000	2.0800000

Unique Subject Identifier=1811000000-0015

Assay variable : PTINR_VAL1 PT-INR Value

N	Mean	Standard Deviation	Minimum	Maximum
4	2.5000000	0.3900427	2.1300000	3.0500000

Unique Subject Identifier=1811000000-0021

Assay variable : PTINR_VAL1 PT-INR Value

N	Mean	Standard Deviation	Minimum	Maximum
5	2.3140000	0.4056230	1.8800000	2.9500000

Unique Subject Identifier=1811000000-0023

Assay variable : PTINR_VAL1 PT-INR Value

N	Mean	Standard Deviation	Minimum	Maximum
5	1.7200000	0.1100000	1.6100000	1.8700000

Laboratory tests at the time of obtaining Table 2-6 consent, according to the presence or absence of proton-pump inhibitors

Unique Subject Identifier=1811000000-0026

MEANS Procedure

Assay variable : PTINR_VAL1 PT-INR Value

N	Mean	Standard Deviation	Minimum	Maximum
6	2.4616667	0.3459142	2.1300000	3.0900000

Unique Subject Identifier=1811000000-0028

Assay variable : PTINR_VAL1 PT-INR Value

N	Mean	Standard Deviation	Minimum	Maximum
3	2.5866667	0.4980295	2.0300000	2.9900000

Unique Subject Identifier=1812000000-0004

Assay variable : PTINR_VAL1 PT-INR Value

N	Mean	Standard Deviation	Minimum	Maximum
6	2.2166667	0.4535049	1.8000000	2.8000000

Unique Subject Identifier=1812000000-0011

Assay variable : PTINR_VAL1 PT-INR Value

N	Mean	Standard Deviation	Minimum	Maximum
6	1.5000000	0.2190890	1.2000000	1.8000000

Laboratory tests at the time of obtaining Table 2-6 consent, according to the presence or absence of proton-pump inhibitors

Unique Subject Identifier=1812000000-0013

MEANS Procedure

Assay variable : PTINR_VAL1 PT-INR Value

N	Mean	Standard Deviation	Minimum	Maximum
6	2.1166667	1.4372427	1.0000000	4.9000000

Unique Subject Identifier=1812000000-0018

Assay variable : PTINR_VAL1 PT-INR Value

N	Mean	Standard Deviation	Minimum	Maximum
6	1.5166667	0.0983192	1.4000000	1.6000000

Unique Subject Identifier=1812000000-0019

Assay variable : PTINR_VAL1 PT-INR Value

N	Mean	Standard Deviation	Minimum	Maximum
6	1.7333333	0.6742897	1.1000000	2.8000000

Unique Subject Identifier=1813100000-0009

Assay variable : PTINR_VAL1 PT-INR Value

N	Mean	Standard Deviation	Minimum	Maximum
6	2.0850000	0.3976305	1.7300000	2.6200000

Laboratory tests at the time of obtaining Table 2-6 consent, according to the presence or absence of proton-pump inhibitors

Unique Subject Identifier=1813100000-0015

MEANS Procedure

Assay variable : PTINR_VAL1 PT-INR Value

N	Mean	Standard Deviation	Minimum	Maximum
4	1.9550000	0.1250333	1.8100000	2.1100000

Unique Subject Identifier=1813100000-0024

Assay variable : PTINR_VAL1 PT-INR Value

N	Mean	Standard Deviation	Minimum	Maximum
3	2.1166667	0.1201388	2.0000000	2.2400000

Unique Subject Identifier=1813100000-0025

Assay variable : PTINR_VAL1 PT-INR Value

N	Mean	Standard Deviation	Minimum	Maximum
1	1.6400000		1.6400000	1.6400000

Unique Subject Identifier=1813100000-0028

Assay variable : PTINR_VAL1 PT-INR Value

N	Mean	Standard Deviation	Minimum	Maximum
4	1.6850000	0.2810101	1.2800000	1.9100000

Laboratory tests at the time of obtaining Table 2-6 consent, according to the presence or absence of proton-pump inhibitors

Unique Subject Identifier=1813100000-0046

MEANS Procedure

Assay variable : PTINR_VAL1 PT-INR Value

N	Mean	Standard Deviation	Minimum	Maximum
2	2.1900000	0.1697056	2.0700000	2.3100000

Unique Subject Identifier=1813100000-0048

Assay variable : PTINR_VAL1 PT-INR Value

N	Mean	Standard Deviation	Minimum	Maximum
4	1.7750000	0.2340228	1.4300000	1.9300000

Unique Subject Identifier=1814100000-0001

Assay variable : PTINR_VAL1 PT-INR Value

N	Mean	Standard Deviation	Minimum	Maximum
1	3.2100000		3.2100000	3.2100000

Unique Subject Identifier=1814100000-0002

Assay variable : PTINR_VAL1 PT-INR Value

N	Mean	Standard Deviation	Minimum	Maximum
1	2.5700000		2.5700000	2.5700000

Laboratory tests at the time of obtaining Table 2-6 consent, according to the presence or absence of proton-pump inhibitors

Unique Subject Identifier=1814100000-0005

MEANS Procedure

Assay variable : PTINR_VAL1 PT-INR Value

N	Mean	Standard Deviation	Minimum	Maximum
1	1.9500000		1.9500000	1.9500000

Unique Subject Identifier=1814100000-0008

Assay variable : PTINR_VAL1 PT-INR Value

N	Mean	Standard Deviation	Minimum	Maximum
2	1.5200000	0.0707107	1.4700000	1.5700000

Unique Subject Identifier=1814100000-0010

Assay variable : PTINR_VAL1 PT-INR Value

N	Mean	Standard Deviation	Minimum	Maximum
6	1.6450000	0.5125915	1.1400000	2.1900000

Unique Subject Identifier=1814100000-0017

Assay variable : PTINR_VAL1 PT-INR Value

N	Mean	Standard Deviation	Minimum	Maximum
6	2.0050000	0.6320680	1.2700000	2.9600000

Laboratory tests at the time of obtaining Table 2-6 consent, according to the presence or absence of proton-pump inhibitors

Unique Subject Identifier=1814100000-0022

MEANS Procedure

Assay variable : PTINR_VAL1 PT-INR Value

N	Mean	Standard Deviation	Minimum	Maximum
6	1.8650000	0.2738430	1.4500000	2.1600000

Unique Subject Identifier=1814100000-0026

Assay variable : PTINR_VAL1 PT-INR Value

N	Mean	Standard Deviation	Minimum	Maximum
6	2.6150000	0.3059902	2.2300000	2.9600000

Unique Subject Identifier=1814100000-0038

Assay variable : PTINR_VAL1 PT-INR Value

N	Mean	Standard Deviation	Minimum	Maximum
6	2.2566667	0.2811168	1.8200000	2.5500000

Unique Subject Identifier=1814100000-0050

Assay variable : PTINR_VAL1 PT-INR Value

N	Mean	Standard Deviation	Minimum	Maximum
4	1.7250000	0.9980815	1.1600000	3.2200000

Laboratory tests at the time of obtaining Table 2-6 consent, according to the presence or absence of proton-pump inhibitors

Unique Subject Identifier=1814100000-0053

MEANS Procedure

Assay variable : PTINR_VAL1 PT-INR Value

N	Mean	Standard Deviation	Minimum	Maximum
5	1.7200000	0.2049390	1.5000000	1.9000000

Unique Subject Identifier=1814100000-0055

Assay variable : PTINR_VAL1 PT-INR Value

N	Mean	Standard Deviation	Minimum	Maximum
3	2.1200000	0.4233202	1.6400000	2.4400000

Unique Subject Identifier=1814100000-0058

Assay variable : PTINR_VAL1 PT-INR Value

N	Mean	Standard Deviation	Minimum	Maximum
6	1.9416667	0.8153874	1.2700000	3.2300000

Unique Subject Identifier=1814100000-0063

Assay variable : PTINR_VAL1 PT-INR Value

N	Mean	Standard Deviation	Minimum	Maximum
6	1.1066667	0.0524087	1.0300000	1.1800000

Laboratory tests at the time of obtaining Table 2-6 consent, according to the presence or absence of proton-pump inhibitors

Unique Subject Identifier=1814100000-0067

MEANS Procedure

Assay variable : PTINR_VAL1 PT-INR Value

N	Mean	Standard Deviation	Minimum	Maximum
1	1.9900000		1.9900000	1.9900000

Unique Subject Identifier=1814100000-0069

Assay variable : PTINR_VAL1 PT-INR Value

N	Mean	Standard Deviation	Minimum	Maximum
6	2.3416667	1.3300739	1.3200000	4.9500000

Unique Subject Identifier=1815100000-0004

Assay variable : PTINR_VAL1 PT-INR Value

N	Mean	Standard Deviation	Minimum	Maximum
1	1.0500000		1.0500000	1.0500000

Unique Subject Identifier=1815100000-0044

Assay variable : PTINR_VAL1 PT-INR Value

N	Mean	Standard Deviation	Minimum	Maximum
5	1.5640000	0.2307163	1.3000000	1.8800000

Laboratory tests at the time of obtaining Table 2-6 consent, according to the presence or absence of proton-pump inhibitors

Unique Subject Identifier=1815100000-0051

MEANS Procedure

Assay variable : PTINR_VAL1 PT-INR Value

N	Mean	Standard Deviation	Minimum	Maximum
3	1.8600000	0.7807689	1.1000000	2.6600000

Unique Subject Identifier=1815100000-0069

Assay variable : PTINR_VAL1 PT-INR Value

N	Mean	Standard Deviation	Minimum	Maximum
3	2.3200000	0.4063250	1.8700000	2.6600000

Unique Subject Identifier=1815100000-0079

Assay variable : PTINR_VAL1 PT-INR Value

N	Mean	Standard Deviation	Minimum	Maximum
1	1.0600000		1.0600000	1.0600000

Unique Subject Identifier=1815100000-0080

Assay variable : PTINR_VAL1 PT-INR Value

N	Mean	Standard Deviation	Minimum	Maximum
2	1.8400000	0.0848528	1.7800000	1.9000000

Laboratory tests at the time of obtaining Table 2-6 consent, according to the presence or absence of proton-pump inhibitors

Unique Subject Identifier=1815100000-0082

MEANS Procedure

Assay variable : PTINR_VAL1 PT-INR Value

N	Mean	Standard Deviation	Minimum	Maximum
1	1.3500000		1.3500000	1.3500000

Unique Subject Identifier=1815100000-0093

Assay variable : PTINR_VAL1 PT-INR Value

N	Mean	Standard Deviation	Minimum	Maximum
6	1.8450000	0.2425490	1.4200000	2.0700000

Unique Subject Identifier=1973000000-0002

Assay variable : PTINR_VAL1 PT-INR Value

N	Mean	Standard Deviation	Minimum	Maximum
5	1.8800000	0.1923538	1.6000000	2.1000000

Unique Subject Identifier=1973000000-0003

Assay variable : PTINR_VAL1 PT-INR Value

N	Mean	Standard Deviation	Minimum	Maximum
6	1.6666667	0.2250926	1.4000000	1.9000000

Laboratory tests at the time of obtaining Table 2-6 consent, according to the presence or absence of proton-pump inhibitors

Unique Subject Identifier=1973000000-0004

MEANS Procedure

Assay variable : PTINR_VAL1 PT-INR Value

N	Mean	Standard Deviation	Minimum	Maximum
6	2.3000000	0.8602325	1.7000000	4.0000000

Unique Subject Identifier=1973000000-0006

Assay variable : PTINR_VAL1 PT-INR Value

N	Mean	Standard Deviation	Minimum	Maximum
6	2.3000000	0.2828427	2.0000000	2.7000000

Unique Subject Identifier=1973000000-0011

Assay variable : PTINR_VAL1 PT-INR Value

N	Mean	Standard Deviation	Minimum	Maximum
4	1.7750000	0.2500000	1.5000000	2.1000000

Unique Subject Identifier=1973000000-0016

Assay variable : PTINR_VAL1 PT-INR Value

N	Mean	Standard Deviation	Minimum	Maximum
6	1.7166667	0.2926887	1.5000000	2.3000000

Laboratory tests at the time of obtaining Table 2-6 consent, according to the presence or absence of proton-pump inhibitors

Unique Subject Identifier=1973000000-0017

MEANS Procedure

Assay variable : PTINR_VAL1 PT-INR Value

N	Mean	Standard Deviation	Minimum	Maximum
5	1.6000000	0.5787918	1.2000000	2.6000000

Unique Subject Identifier=2008010000-0011

Assay variable : PTINR_VAL1 PT-INR Value

N	Mean	Standard Deviation	Minimum	Maximum
6	1.7000000	0.1673320	1.5000000	1.9000000

Unique Subject Identifier=2008010000-0016

Assay variable : PTINR_VAL1 PT-INR Value

N	Mean	Standard Deviation	Minimum	Maximum
6	1.6833333	0.2562551	1.3000000	2.0000000

Unique Subject Identifier=2008010000-0018

Assay variable : PTINR_VAL1 PT-INR Value

N	Mean	Standard Deviation	Minimum	Maximum
3	1.2000000	0.1000000	1.1000000	1.3000000

Laboratory tests at the time of obtaining Table 2-6 consent, according to the presence or absence of proton-pump inhibitors

Unique Subject Identifier=2008010000-0023

MEANS Procedure

Assay variable : PTINR_VAL1 PT-INR Value

N	Mean	Standard Deviation	Minimum	Maximum
2	1.2750000	0.0777817	1.2200000	1.3300000

Unique Subject Identifier=2008010000-0027

Assay variable : PTINR_VAL1 PT-INR Value

N	Mean	Standard Deviation	Minimum	Maximum
4	1.9500000	0.4123106	1.5000000	2.3000000

Unique Subject Identifier=2008010000-0031

Assay variable : PTINR_VAL1 PT-INR Value

N	Mean	Standard Deviation	Minimum	Maximum
6	1.8166667	0.3656045	1.4000000	2.3000000

Unique Subject Identifier=2008010000-0037

Assay variable : PTINR_VAL1 PT-INR Value

N	Mean	Standard Deviation	Minimum	Maximum
6	1.9500000	0.2073644	1.8000000	2.3000000

Laboratory tests at the time of obtaining Table 2-6 consent, according to the presence or absence of proton-pump inhibitors

Unique Subject Identifier=2008010000-0038

MEANS Procedure

Assay variable : PTINR_VAL1 PT-INR Value

N	Mean	Standard Deviation	Minimum	Maximum
6	1.9500000	0.1643168	1.8000000	2.2000000

Unique Subject Identifier=2008010000-0041

Assay variable : PTINR_VAL1 PT-INR Value

N	Mean	Standard Deviation	Minimum	Maximum
6	1.8166667	0.5913262	1.5000000	3.0000000

Unique Subject Identifier=2008010000-0043

Assay variable : PTINR_VAL1 PT-INR Value

N	Mean	Standard Deviation	Minimum	Maximum
4	1.9250000	0.2753785	1.6000000	2.2000000

Unique Subject Identifier=2008010000-0046

Assay variable : PTINR_VAL1 PT-INR Value

N	Mean	Standard Deviation	Minimum	Maximum
4	1.6000000	0.0816497	1.5000000	1.7000000

Laboratory tests at the time of obtaining Table 2-6 consent, according to the presence or absence of proton-pump inhibitors

Unique Subject Identifier=2008010000-0047

MEANS Procedure

Assay variable : PTINR_VAL1 PT-INR Value

N	Mean	Standard Deviation	Minimum	Maximum
6	1.9500000	0.5991661	1.3000000	2.8000000

Unique Subject Identifier=2008010000-0052

Assay variable : PTINR_VAL1 PT-INR Value

N	Mean	Standard Deviation	Minimum	Maximum
3	1.7000000	0.2645751	1.4000000	1.9000000

Unique Subject Identifier=2008010000-0055

Assay variable : PTINR_VAL1 PT-INR Value

N	Mean	Standard Deviation	Minimum	Maximum
1	1.0200000		1.0200000	1.0200000

Unique Subject Identifier=2016011111-0002

Assay variable : PTINR_VAL1 PT-INR Value

N	Mean	Standard Deviation	Minimum	Maximum
6	1.9450000	0.2212465	1.7600000	2.3200000

Laboratory tests at the time of obtaining Table 2-6 consent, according to the presence or absence of proton-pump inhibitors

Unique Subject Identifier=2016011111-0004

MEANS Procedure

Assay variable : PTINR_VAL1 PT-INR Value

N	Mean	Standard Deviation	Minimum	Maximum
5	2.0080000	0.1116692	1.8500000	2.1300000

Unique Subject Identifier=2016011111-0006

Assay variable : PTINR_VAL1 PT-INR Value

N	Mean	Standard Deviation	Minimum	Maximum
5	2.4700000	0.5487714	1.7300000	3.0300000

Unique Subject Identifier=2016011111-0010

Assay variable : PTINR_VAL1 PT-INR Value

N	Mean	Standard Deviation	Minimum	Maximum
5	2.1640000	0.6723689	1.5600000	3.3200000

Unique Subject Identifier=2016011111-0017

Assay variable : PTINR_VAL1 PT-INR Value

N	Mean	Standard Deviation	Minimum	Maximum
6	1.8916667	0.2997610	1.5700000	2.1700000

Laboratory tests at the time of obtaining Table 2-6 consent, according to the presence or absence of proton-pump inhibitors

Unique Subject Identifier=2016011111-0018

MEANS Procedure

Assay variable : PTINR_VAL1 PT-INR Value

N	Mean	Standard Deviation	Minimum	Maximum
4	1.7375000	0.3456757	1.5200000	2.2500000

Unique Subject Identifier=2016011111-0019

Assay variable : PTINR_VAL1 PT-INR Value

N	Mean	Standard Deviation	Minimum	Maximum
5	2.4900000	0.3794074	2.0800000	2.9900000

Unique Subject Identifier=2016011111-0021

Assay variable : PTINR_VAL1 PT-INR Value

N	Mean	Standard Deviation	Minimum	Maximum
4	2.5000000	0.5707889	2.0900000	3.3400000

Unique Subject Identifier=2016011111-0023

Assay variable : PTINR_VAL1 PT-INR Value

N	Mean	Standard Deviation	Minimum	Maximum
6	2.3000000	0.6515213	1.6300000	3.1600000

Laboratory tests at the time of obtaining Table 2-6 consent, according to the presence or absence of proton-pump inhibitors

Unique Subject Identifier=2016011111-0025

MEANS Procedure

Assay variable : PTINR_VAL1 PT-INR Value

N	Mean	Standard Deviation	Minimum	Maximum
5	2.6040000	0.3591379	2.3300000	3.1100000

Unique Subject Identifier=2016011111-0028

Assay variable : PTINR_VAL1 PT-INR Value

N	Mean	Standard Deviation	Minimum	Maximum
3	1.5066667	0.2458319	1.3500000	1.7900000

Unique Subject Identifier=2016011111-0030

Assay variable : PTINR_VAL1 PT-INR Value

N	Mean	Standard Deviation	Minimum	Maximum
5	2.0160000	0.4263567	1.6100000	2.5500000

Unique Subject Identifier=2016011111-0031

Assay variable : PTINR_VAL1 PT-INR Value

N	Mean	Standard Deviation	Minimum	Maximum
6	2.1166667	0.2801190	1.7700000	2.4500000

Laboratory tests at the time of obtaining Table 2-6 consent, according to the presence or absence of proton-pump inhibitors

Unique Subject Identifier=2016011111-0034

MEANS Procedure

Assay variable : PTINR_VAL1 PT-INR Value

N	Mean	Standard Deviation	Minimum	Maximum
3	2.3833333	0.1159023	2.2500000	2.4600000

Unique Subject Identifier=2016011111-0035

Assay variable : PTINR_VAL1 PT-INR Value

N	Mean	Standard Deviation	Minimum	Maximum
4	2.4100000	0.8089911	1.7700000	3.5600000

Unique Subject Identifier=2016011111-0036

Assay variable : PTINR_VAL1 PT-INR Value

N	Mean	Standard Deviation	Minimum	Maximum
4	2.7700000	0.7043673	2.2200000	3.8000000

Unique Subject Identifier=2016011111-0037

Assay variable : PTINR_VAL1 PT-INR Value

N	Mean	Standard Deviation	Minimum	Maximum
6	2.0900000	0.4848092	1.6100000	2.8500000

Laboratory tests at the time of obtaining Table 2-6 consent, according to the presence or absence of proton-pump inhibitors

Unique Subject Identifier=2016011111-0040

MEANS Procedure

Assay variable : PTINR_VAL1 PT-INR Value

N	Mean	Standard Deviation	Minimum	Maximum
5	2.3620000	0.9487465	1.4300000	3.9100000

Unique Subject Identifier=2016011111-0043

Assay variable : PTINR_VAL1 PT-INR Value

N	Mean	Standard Deviation	Minimum	Maximum
5	2.7300000	0.4640582	2.1500000	3.1900000

Unique Subject Identifier=2016011111-0050

Assay variable : PTINR_VAL1 PT-INR Value

N	Mean	Standard Deviation	Minimum	Maximum
4	2.2000000	0.1363818	2.0800000	2.3700000

Unique Subject Identifier=2016011111-0057

Assay variable : PTINR_VAL1 PT-INR Value

N	Mean	Standard Deviation	Minimum	Maximum
6	1.8666667	0.5833924	1.0800000	2.6700000

Laboratory tests at the time of obtaining Table 2-6 consent, according to the presence or absence of proton-pump inhibitors

Unique Subject Identifier=2016011111-0060

MEANS Procedure

Assay variable : PTINR_VAL1 PT-INR Value

N	Mean	Standard Deviation	Minimum	Maximum
6	1.6800000	0.2228901	1.4800000	2.1200000

Unique Subject Identifier=2016011111-0062

Assay variable : PTINR_VAL1 PT-INR Value

N	Mean	Standard Deviation	Minimum	Maximum
4	1.8775000	0.1364734	1.7100000	2.0200000

Unique Subject Identifier=2016011111-0063

Assay variable : PTINR_VAL1 PT-INR Value

N	Mean	Standard Deviation	Minimum	Maximum
5	2.4540000	0.1879628	2.2500000	2.7600000

Unique Subject Identifier=2016011111-0070

Assay variable : PTINR_VAL1 PT-INR Value

N	Mean	Standard Deviation	Minimum	Maximum
3	2.2966667	0.3450121	1.9500000	2.6400000

Laboratory tests at the time of obtaining Table 2-6 consent, according to the presence or absence of proton-pump inhibitors

Unique Subject Identifier=2016011111-0081

MEANS Procedure

Assay variable : PTINR_VAL1 PT-INR Value

N	Mean	Standard Deviation	Minimum	Maximum
3	2.3100000	0.2651415	2.0800000	2.6000000

Unique Subject Identifier=2016011111-0082

Assay variable : PTINR_VAL1 PT-INR Value

N	Mean	Standard Deviation	Minimum	Maximum
3	1.8933333	0.1850225	1.7100000	2.0800000

Unique Subject Identifier=2016011111-0085

Assay variable : PTINR_VAL1 PT-INR Value

N	Mean	Standard Deviation	Minimum	Maximum
4	2.0225000	0.2652515	1.7700000	2.3700000

Unique Subject Identifier=2016011111-0091

Assay variable : PTINR_VAL1 PT-INR Value

N	Mean	Standard Deviation	Minimum	Maximum
4	2.3850000	0.2666458	2.1700000	2.7400000

Laboratory tests at the time of obtaining Table 2-6 consent, according to the presence or absence of proton-pump inhibitors

Unique Subject Identifier=2016011111-0095

MEANS Procedure

Assay variable : PTINR_VAL1 PT-INR Value

N	Mean	Standard Deviation	Minimum	Maximum
3	2.3833333	0.5108163	1.8100000	2.7900000

Unique Subject Identifier=2016011111-0096

Assay variable : PTINR_VAL1 PT-INR Value

N	Mean	Standard Deviation	Minimum	Maximum
3	1.8700000	0.2497999	1.6700000	2.1500000

Unique Subject Identifier=2017011100-0002

Assay variable : PTINR_VAL1 PT-INR Value

N	Mean	Standard Deviation	Minimum	Maximum
5	2.2540000	0.6454688	1.4000000	3.2000000

Unique Subject Identifier=2017011100-0004

Assay variable : PTINR_VAL1 PT-INR Value

N	Mean	Standard Deviation	Minimum	Maximum
6	2.0483333	0.4900782	1.4500000	2.8500000

Laboratory tests at the time of obtaining Table 2-6 consent, according to the presence or absence of proton-pump inhibitors

Unique Subject Identifier=2017011100-0006

MEANS Procedure

Assay variable : PTINR_VAL1 PT-INR Value

N	Mean	Standard Deviation	Minimum	Maximum
5	1.9760000	0.1540454	1.8400000	2.2100000

Unique Subject Identifier=2017011100-0011

Assay variable : PTINR_VAL1 PT-INR Value

N	Mean	Standard Deviation	Minimum	Maximum
3	1.0866667	0.1975686	0.9100000	1.3000000

Unique Subject Identifier=2017011100-0012

Assay variable : PTINR_VAL1 PT-INR Value

N	Mean	Standard Deviation	Minimum	Maximum
6	2.0150000	0.3148174	1.6300000	2.4300000

Unique Subject Identifier=2017011100-0014

Assay variable : PTINR_VAL1 PT-INR Value

N	Mean	Standard Deviation	Minimum	Maximum
6	2.1366667	0.4337588	1.6300000	2.7800000

Laboratory tests at the time of obtaining Table 2-6 consent, according to the presence or absence of proton-pump inhibitors

Unique Subject Identifier=2017011100-0017

MEANS Procedure

Assay variable : PTINR_VAL1 PT-INR Value

N	Mean	Standard Deviation	Minimum	Maximum
6	1.5383333	0.2977527	1.0900000	1.8400000

Unique Subject Identifier=2017011100-0019

Assay variable : PTINR_VAL1 PT-INR Value

N	Mean	Standard Deviation	Minimum	Maximum
6	1.8083333	0.1414803	1.6400000	1.9400000

Unique Subject Identifier=2017011100-0020

Assay variable : PTINR_VAL1 PT-INR Value

N	Mean	Standard Deviation	Minimum	Maximum
6	2.0466667	0.4457204	1.5000000	2.6400000

Unique Subject Identifier=2017011100-0023

Assay variable : PTINR_VAL1 PT-INR Value

N	Mean	Standard Deviation	Minimum	Maximum
6	2.0133333	0.1944908	1.8400000	2.3700000

Laboratory tests at the time of obtaining Table 2-6 consent, according to the presence or absence of proton-pump inhibitors

Unique Subject Identifier=2017011100-0025

MEANS Procedure

Assay variable : PTINR_VAL1 PT-INR Value

N	Mean	Standard Deviation	Minimum	Maximum
4	1.7850000	0.1317826	1.6400000	1.9600000

Unique Subject Identifier=2017011100-0027

Assay variable : PTINR_VAL1 PT-INR Value

N	Mean	Standard Deviation	Minimum	Maximum
6	1.9050000	0.0850294	1.8300000	2.0100000

Unique Subject Identifier=2017011100-0033

Assay variable : PTINR_VAL1 PT-INR Value

N	Mean	Standard Deviation	Minimum	Maximum
5	1.8620000	0.1572260	1.7200000	2.0900000

Unique Subject Identifier=2017011100-0035

Assay variable : PTINR_VAL1 PT-INR Value

N	Mean	Standard Deviation	Minimum	Maximum
1	2.2700000		2.2700000	2.2700000

Laboratory tests at the time of obtaining Table 2-6 consent, according to the presence or absence of proton-pump inhibitors

Unique Subject Identifier=2017011100-0037

MEANS Procedure

Assay variable : PTINR_VAL1 PT-INR Value

N	Mean	Standard Deviation	Minimum	Maximum
5	2.0360000	0.3995372	1.7300000	2.7200000

Unique Subject Identifier=2017011100-0038

Assay variable : PTINR_VAL1 PT-INR Value

N	Mean	Standard Deviation	Minimum	Maximum
6	2.0600000	0.2354570	1.6600000	2.2700000

Unique Subject Identifier=2017011100-0047

Assay variable : PTINR_VAL1 PT-INR Value

N	Mean	Standard Deviation	Minimum	Maximum
5	2.0060000	0.0250998	1.9800000	2.0300000

Unique Subject Identifier=2017011100-0048

Assay variable : PTINR_VAL1 PT-INR Value

N	Mean	Standard Deviation	Minimum	Maximum
6	2.0516667	0.4543310	1.4200000	2.6100000

Laboratory tests at the time of obtaining Table 2-6 consent, according to the presence or absence of proton-pump inhibitors

Unique Subject Identifier=2017011100-0049

MEANS Procedure

Assay variable : PTINR_VAL1 PT-INR Value

N	Mean	Standard Deviation	Minimum	Maximum
6	2.1283333	0.2574814	1.8400000	2.4300000

Unique Subject Identifier=2017011100-0053

Assay variable : PTINR_VAL1 PT-INR Value

N	Mean	Standard Deviation	Minimum	Maximum
6	2.0550000	0.5021454	1.0800000	2.4900000

Unique Subject Identifier=2017011100-0056

Assay variable : PTINR_VAL1 PT-INR Value

N	Mean	Standard Deviation	Minimum	Maximum
6	1.8083333	0.4073532	1.4000000	2.5900000

Unique Subject Identifier=2017011100-0058

Assay variable : PTINR_VAL1 PT-INR Value

N	Mean	Standard Deviation	Minimum	Maximum
5	2.0100000	0.2955503	1.6100000	2.3300000

Laboratory tests at the time of obtaining Table 2-6 consent, according to the presence or absence of proton-pump inhibitors

Unique Subject Identifier=2017011100-0059

MEANS Procedure

Assay variable : PTINR_VAL1 PT-INR Value

N	Mean	Standard Deviation	Minimum	Maximum
5	2.0640000	0.5264314	1.4000000	2.8500000

Unique Subject Identifier=2017011100-0060

Assay variable : PTINR_VAL1 PT-INR Value

N	Mean	Standard Deviation	Minimum	Maximum
6	1.7566667	0.6880310	1.0900000	2.9800000

Unique Subject Identifier=2017011100-0061

Assay variable : PTINR_VAL1 PT-INR Value

N	Mean	Standard Deviation	Minimum	Maximum
6	2.3733333	0.5452950	1.7700000	3.3300000

Unique Subject Identifier=2017011100-0062

Assay variable : PTINR_VAL1 PT-INR Value

N	Mean	Standard Deviation	Minimum	Maximum
6	1.9933333	0.3309179	1.4500000	2.3300000

Laboratory tests at the time of obtaining Table 2-6 consent, according to the presence or absence of proton-pump inhibitors

Unique Subject Identifier=2017011100-0064

MEANS Procedure

Assay variable : PTINR_VAL1 PT-INR Value

N	Mean	Standard Deviation	Minimum	Maximum
6	1.9000000	0.3244072	1.5400000	2.3500000

Unique Subject Identifier=2017011100-0075

Assay variable : PTINR_VAL1 PT-INR Value

N	Mean	Standard Deviation	Minimum	Maximum
6	2.3216667	0.7310928	1.4500000	3.3300000

Unique Subject Identifier=2017011100-0076

Assay variable : PTINR_VAL1 PT-INR Value

N	Mean	Standard Deviation	Minimum	Maximum
5	1.9520000	0.4097804	1.5900000	2.5700000

Unique Subject Identifier=2017011100-0082

Assay variable : PTINR_VAL1 PT-INR Value

N	Mean	Standard Deviation	Minimum	Maximum
5	2.2040000	0.4834563	1.8400000	3.0200000

Laboratory tests at the time of obtaining Table 2-6 consent, according to the presence or absence of proton-pump inhibitors

Unique Subject Identifier=2017011100-0085

MEANS Procedure

Assay variable : PTINR_VAL1 PT-INR Value

N	Mean	Standard Deviation	Minimum	Maximum
6	1.8333333	0.5709524	1.1100000	2.4900000

Unique Subject Identifier=2017011100-0088

Assay variable : PTINR_VAL1 PT-INR Value

N	Mean	Standard Deviation	Minimum	Maximum
6	2.1233333	0.2399722	1.8400000	2.5100000

Unique Subject Identifier=2017011100-0092

Assay variable : PTINR_VAL1 PT-INR Value

N	Mean	Standard Deviation	Minimum	Maximum
6	1.9066667	0.2593582	1.5600000	2.1900000

Unique Subject Identifier=2017011100-0093

Assay variable : PTINR_VAL1 PT-INR Value

N	Mean	Standard Deviation	Minimum	Maximum
6	1.8216667	0.1090718	1.6400000	1.9600000

Laboratory tests at the time of obtaining Table 2-6 consent, according to the presence or absence of proton-pump inhibitors

Unique Subject Identifier=2017011100-0098

MEANS Procedure

Assay variable : PTINR_VAL1 PT-INR Value

N	Mean	Standard Deviation	Minimum	Maximum
5	2.0640000	0.4530232	1.5600000	2.5900000

Unique Subject Identifier=2017011100-0100

Assay variable : PTINR_VAL1 PT-INR Value

N	Mean	Standard Deviation	Minimum	Maximum
6	2.1566667	0.4348640	1.4200000	2.7600000

Unique Subject Identifier=2017011100-0109

Assay variable : PTINR_VAL1 PT-INR Value

N	Mean	Standard Deviation	Minimum	Maximum
5	1.6940000	0.1669731	1.4500000	1.8600000

Unique Subject Identifier=2017011100-0113

Assay variable : PTINR_VAL1 PT-INR Value

N	Mean	Standard Deviation	Minimum	Maximum
4	1.3725000	0.1711481	1.2000000	1.5900000

Laboratory tests at the time of obtaining Table 2-6 consent, according to the presence or absence of proton-pump inhibitors

Unique Subject Identifier=2017011100-0115

MEANS Procedure

Assay variable : PTINR_VAL1 PT-INR Value

N	Mean	Standard Deviation	Minimum	Maximum
6	2.3416667	0.4252725	2.0000000	3.0400000

Unique Subject Identifier=2017011100-0116

Assay variable : PTINR_VAL1 PT-INR Value

N	Mean	Standard Deviation	Minimum	Maximum
6	1.9816667	0.2195829	1.7300000	2.3700000

Unique Subject Identifier=2017011100-0118

Assay variable : PTINR_VAL1 PT-INR Value

N	Mean	Standard Deviation	Minimum	Maximum
6	2.1233333	0.2985074	1.8300000	2.6800000

Unique Subject Identifier=2017011100-0120

Assay variable : PTINR_VAL1 PT-INR Value

N	Mean	Standard Deviation	Minimum	Maximum
6	2.1383333	0.1697547	1.9200000	2.3700000

Laboratory tests at the time of obtaining Table 2-6 consent, according to the presence or absence of proton-pump inhibitors

Unique Subject Identifier=2018000000-0005

MEANS Procedure

Assay variable : PTINR_VAL1 PT-INR Value

N	Mean	Standard Deviation	Minimum	Maximum
5	1.8820000	0.1771158	1.6800000	2.0500000

Unique Subject Identifier=2018000000-0006

Assay variable : PTINR_VAL1 PT-INR Value

N	Mean	Standard Deviation	Minimum	Maximum
2	2.5900000	0.2828427	2.3900000	2.7900000

Unique Subject Identifier=2018000000-0007

Assay variable : PTINR_VAL1 PT-INR Value

N	Mean	Standard Deviation	Minimum	Maximum
6	1.2283333	0.0604704	1.1700000	1.3400000

Unique Subject Identifier=2018000000-0008

Assay variable : PTINR_VAL1 PT-INR Value

N	Mean	Standard Deviation	Minimum	Maximum
3	2.0666667	0.1289703	1.9600000	2.2100000

Laboratory tests at the time of obtaining Table 2-6 consent, according to the presence or absence of proton-pump inhibitors

Unique Subject Identifier=2018000000-0010

MEANS Procedure

Assay variable : PTINR_VAL1 PT-INR Value

N	Mean	Standard Deviation	Minimum	Maximum
5	1.8100000	0.3000000	1.3900000	2.1500000

Unique Subject Identifier=2018000000-0012

Assay variable : PTINR_VAL1 PT-INR Value

N	Mean	Standard Deviation	Minimum	Maximum
2	1.4000000	0.0565685	1.3600000	1.4400000

Unique Subject Identifier=2018000000-0013

Assay variable : PTINR_VAL1 PT-INR Value

N	Mean	Standard Deviation	Minimum	Maximum
2	1.8000000	0.1414214	1.7000000	1.9000000

Unique Subject Identifier=2026011111-0004

Assay variable : PTINR_VAL1 PT-INR Value

N	Mean	Standard Deviation	Minimum	Maximum
6	1.6000000	0.2000000	1.3000000	1.8000000

Laboratory tests at the time of obtaining Table 2-6 consent, according to the presence or absence of proton-pump inhibitors

Unique Subject Identifier=2026011111-0006

MEANS Procedure

Assay variable : PTINR_VAL1 PT-INR Value

N	Mean	Standard Deviation	Minimum	Maximum
6	1.9500000	0.1870829	1.7000000	2.2000000

Unique Subject Identifier=2026011111-0008

Assay variable : PTINR_VAL1 PT-INR Value

N	Mean	Standard Deviation	Minimum	Maximum
6	2.2666667	0.2422120	1.9000000	2.5000000

Unique Subject Identifier=2027010010-0004

Assay variable : PTINR_VAL1 PT-INR Value

N	Mean	Standard Deviation	Minimum	Maximum
6	2.0166667	0.1966384	1.6600000	2.1700000

Unique Subject Identifier=2027010010-0007

Assay variable : PTINR_VAL1 PT-INR Value

N	Mean	Standard Deviation	Minimum	Maximum
3	1.6766667	0.0665833	1.6000000	1.7200000

Laboratory tests at the time of obtaining Table 2-6 consent, according to the presence or absence of proton-pump inhibitors

Unique Subject Identifier=2038000000-0005

MEANS Procedure

Assay variable : PTINR_VAL1 PT-INR Value

N	Mean	Standard Deviation	Minimum	Maximum
3	1.9933333	0.3225420	1.6500000	2.2900000

Unique Subject Identifier=2038000000-0006

Assay variable : PTINR_VAL1 PT-INR Value

N	Mean	Standard Deviation	Minimum	Maximum
3	2.0133333	0.6656075	1.2900000	2.6000000

Unique Subject Identifier=2038000000-0007

Assay variable : PTINR_VAL1 PT-INR Value

N	Mean	Standard Deviation	Minimum	Maximum
3	2.0933333	0.3108590	1.8800000	2.4500000

Unique Subject Identifier=2038000000-0009

Assay variable : PTINR_VAL1 PT-INR Value

N	Mean	Standard Deviation	Minimum	Maximum
4	2.1650000	0.5704092	1.3400000	2.6500000

Laboratory tests at the time of obtaining Table 2-6 consent, according to the presence or absence of proton-pump inhibitors

Unique Subject Identifier=2047011111-0007

MEANS Procedure

Assay variable : PTINR_VAL1 PT-INR Value

N	Mean	Standard Deviation	Minimum	Maximum
6	1.6216667	0.2102776	1.3700000	1.9300000

Unique Subject Identifier=2047011111-0012

Assay variable : PTINR_VAL1 PT-INR Value

N	Mean	Standard Deviation	Minimum	Maximum
2	2.4900000	0.2687006	2.3000000	2.6800000

Unique Subject Identifier=2048011011-0035

Assay variable : PTINR_VAL1 PT-INR Value

N	Mean	Standard Deviation	Minimum	Maximum
3	1.9000000	0.1000000	1.8000000	2.0000000

Unique Subject Identifier=2048011011-0045

Assay variable : PTINR_VAL1 PT-INR Value

N	Mean	Standard Deviation	Minimum	Maximum
3	2.0000000	0.2645751	1.8000000	2.3000000

Laboratory tests at the time of obtaining Table 2-6 consent, according to the presence or absence of proton-pump inhibitors

Unique Subject Identifier=2048011011-0048

MEANS Procedure

Assay variable : PTINR_VAL1 PT-INR Value

N	Mean	Standard Deviation	Minimum	Maximum
6	2.0166667	1.0068101	1.1000000	3.3000000

Unique Subject Identifier=2048011011-0049

Assay variable : PTINR_VAL1 PT-INR Value

N	Mean	Standard Deviation	Minimum	Maximum
3	2.1666667	0.2081666	2.0000000	2.4000000

Unique Subject Identifier=2048011011-0052

Assay variable : PTINR_VAL1 PT-INR Value

N	Mean	Standard Deviation	Minimum	Maximum
3	1.4000000	0.5000000	0.9000000	1.9000000

Unique Subject Identifier=2048011011-0053

Assay variable : PTINR_VAL1 PT-INR Value

N	Mean	Standard Deviation	Minimum	Maximum
2	2.7500000	0.0707107	2.7000000	2.8000000

Laboratory tests at the time of obtaining Table 2-6 consent, according to the presence or absence of proton-pump inhibitors

Unique Subject Identifier=2048011011-0056

MEANS Procedure

Assay variable : PTINR_VAL1 PT-INR Value

N	Mean	Standard Deviation	Minimum	Maximum
3	2.6666667	0.1527525	2.5000000	2.8000000

Unique Subject Identifier=2048011011-0057

Assay variable : PTINR_VAL1 PT-INR Value

N	Mean	Standard Deviation	Minimum	Maximum
1	2.0000000		2.0000000	2.0000000

Unique Subject Identifier=2048011011-0067

Assay variable : PTINR_VAL1 PT-INR Value

N	Mean	Standard Deviation	Minimum	Maximum
4	2.2000000	0.1825742	2.0000000	2.4000000

Unique Subject Identifier=2048011011-0070

Assay variable : PTINR_VAL1 PT-INR Value

N	Mean	Standard Deviation	Minimum	Maximum
3	2.5000000	0.4582576	2.1000000	3.0000000

Laboratory tests at the time of obtaining Table 2-6 consent, according to the presence or absence of proton-pump inhibitors

Unique Subject Identifier=2058011111-0006

MEANS Procedure

Assay variable : PTINR_VAL1 PT-INR Value

N	Mean	Standard Deviation	Minimum	Maximum
1	1.7100000		1.7100000	1.7100000

Unique Subject Identifier=2064011111-0008

Assay variable : PTINR_VAL1 PT-INR Value

N	Mean	Standard Deviation	Minimum	Maximum
1	1.9700000		1.9700000	1.9700000

Unique Subject Identifier=2064011111-0011

Assay variable : PTINR_VAL1 PT-INR Value

N	Mean	Standard Deviation	Minimum	Maximum
3	2.6533333	0.8775154	1.7400000	3.4900000

Unique Subject Identifier=2069000000-0002

Assay variable : PTINR_VAL1 PT-INR Value

N	Mean	Standard Deviation	Minimum	Maximum
4	2.2425000	0.2984823	1.8600000	2.5600000

Laboratory tests at the time of obtaining Table 2-6 consent, according to the presence or absence of proton-pump inhibitors

Unique Subject Identifier=2077011111-0001

MEANS Procedure

Assay variable : PTINR_VAL1 PT-INR Value

N	Mean	Standard Deviation	Minimum	Maximum
6	2.2450000	0.7159539	1.3900000	3.2200000

Unique Subject Identifier=2077011111-0003

Assay variable : PTINR_VAL1 PT-INR Value

N	Mean	Standard Deviation	Minimum	Maximum
5	2.2580000	0.3998375	1.8400000	2.8100000

Unique Subject Identifier=2077011111-0004

Assay variable : PTINR_VAL1 PT-INR Value

N	Mean	Standard Deviation	Minimum	Maximum
6	1.9366667	0.6416749	1.3200000	2.6800000

Unique Subject Identifier=2077011111-0010

Assay variable : PTINR_VAL1 PT-INR Value

N	Mean	Standard Deviation	Minimum	Maximum
6	1.6716667	0.1094380	1.5400000	1.8000000

Laboratory tests at the time of obtaining Table 2-6 consent, according to the presence or absence of proton-pump inhibitors

Unique Subject Identifier=2077011111-0016

MEANS Procedure

Assay variable : PTINR_VAL1 PT-INR Value

N	Mean	Standard Deviation	Minimum	Maximum
6	2.0883333	0.8065089	1.1900000	3.4000000

Unique Subject Identifier=2077011111-0022

Assay variable : PTINR_VAL1 PT-INR Value

N	Mean	Standard Deviation	Minimum	Maximum
5	1.9780000	0.1329286	1.8300000	2.1600000

Unique Subject Identifier=2092011111-0004

Assay variable : PTINR_VAL1 PT-INR Value

N	Mean	Standard Deviation	Minimum	Maximum
1	3.0500000		3.0500000	3.0500000

Unique Subject Identifier=2096011111-0001

Assay variable : PTINR_VAL1 PT-INR Value

N	Mean	Standard Deviation	Minimum	Maximum
2	1.3400000	0.1131371	1.2600000	1.4200000

Laboratory tests at the time of obtaining Table 2-6 consent, according to the presence or absence of proton-pump inhibitors

Unique Subject Identifier=2107011000-0024

MEANS Procedure

Assay variable : PTINR_VAL1 PT-INR Value

N	Mean	Standard Deviation	Minimum	Maximum
6	2.1416667	0.4149418	1.5100000	2.6100000

Unique Subject Identifier=2107011000-0036

Assay variable : PTINR_VAL1 PT-INR Value

N	Mean	Standard Deviation	Minimum	Maximum
3	2.0533333	0.6321656	1.3300000	2.5000000

Unique Subject Identifier=2107011000-0037

Assay variable : PTINR_VAL1 PT-INR Value

N	Mean	Standard Deviation	Minimum	Maximum
3	2.4533333	0.6792888	1.9700000	3.2300000

Unique Subject Identifier=2108011000-0002

Assay variable : PTINR_VAL1 PT-INR Value

N	Mean	Standard Deviation	Minimum	Maximum
3	3.4333333	1.6041613	1.9000000	5.1000000

Laboratory tests at the time of obtaining Table 2-6 consent, according to the presence or absence of proton-pump inhibitors

Unique Subject Identifier=2112011011-0001

MEANS Procedure

Assay variable : PTINR_VAL1 PT-INR Value

N	Mean	Standard Deviation	Minimum	Maximum
3	2.2266667	0.0680686	2.1500000	2.2800000

Unique Subject Identifier=2112011011-0006

Assay variable : PTINR_VAL1 PT-INR Value

N	Mean	Standard Deviation	Minimum	Maximum
5	1.9400000	0.1846619	1.7500000	2.1700000

Unique Subject Identifier=2112011011-0008

Assay variable : PTINR_VAL1 PT-INR Value

N	Mean	Standard Deviation	Minimum	Maximum
5	2.4060000	0.3755396	2.0000000	2.8700000

Unique Subject Identifier=2113010111-0001

Assay variable : PTINR_VAL1 PT-INR Value

N	Mean	Standard Deviation	Minimum	Maximum
2	1.8700000	0.2121320	1.7200000	2.0200000

Laboratory tests at the time of obtaining Table 2-6 consent, according to the presence or absence of proton-pump inhibitors

Unique Subject Identifier=2113010111-0006

MEANS Procedure

Assay variable : PTINR_VAL1 PT-INR Value

N	Mean	Standard Deviation	Minimum	Maximum
3	1.8500000	0.2066398	1.6800000	2.0800000

Unique Subject Identifier=2113010111-0015

Assay variable : PTINR_VAL1 PT-INR Value

N	Mean	Standard Deviation	Minimum	Maximum
1	1.2100000		1.2100000	1.2100000

Unique Subject Identifier=2115011111-0025

Assay variable : PTINR_VAL1 PT-INR Value

N	Mean	Standard Deviation	Minimum	Maximum
3	1.6333333	0.1527525	1.5000000	1.8000000

Unique Subject Identifier=2117011111-0001

Assay variable : PTINR_VAL1 PT-INR Value

N	Mean	Standard Deviation	Minimum	Maximum
6	1.7750000	0.4429334	1.1200000	2.3800000

Laboratory tests at the time of obtaining Table 2-6 consent, according to the presence or absence of proton-pump inhibitors

Unique Subject Identifier=2118000000-0005

MEANS Procedure

Assay variable : PTINR_VAL1 PT-INR Value

N	Mean	Standard Deviation	Minimum	Maximum
2	1.5550000	0.2192031	1.4000000	1.7100000

Unique Subject Identifier=2119011111-0001

Assay variable : PTINR_VAL1 PT-INR Value

N	Mean	Standard Deviation	Minimum	Maximum
5	1.8540000	0.1637987	1.6300000	2.0800000

Unique Subject Identifier=2123010000-0004

Assay variable : PTINR_VAL1 PT-INR Value

N	Mean	Standard Deviation	Minimum	Maximum
6	2.3166667	0.1722401	2.1000000	2.6000000

Unique Subject Identifier=2123010000-0007

Assay variable : PTINR_VAL1 PT-INR Value

N	Mean	Standard Deviation	Minimum	Maximum
6	1.8666667	0.2065591	1.7000000	2.2000000

Laboratory tests at the time of obtaining Table 2-6 consent, according to the presence or absence of proton-pump inhibitors

Unique Subject Identifier=2123010000-0009

MEANS Procedure

Assay variable : PTINR_VAL1 PT-INR Value

N	Mean	Standard Deviation	Minimum	Maximum
6	2.0500000	0.4183300	1.6000000	2.8000000

Unique Subject Identifier=2123010000-0011

Assay variable : PTINR_VAL1 PT-INR Value

N	Mean	Standard Deviation	Minimum	Maximum
6	1.9666667	0.2160247	1.7000000	2.3000000

Unique Subject Identifier=2123010000-0012

Assay variable : PTINR_VAL1 PT-INR Value

N	Mean	Standard Deviation	Minimum	Maximum
6	1.5833333	0.1722401	1.3000000	1.8000000

Unique Subject Identifier=2123010000-0013

Assay variable : PTINR_VAL1 PT-INR Value

N	Mean	Standard Deviation	Minimum	Maximum
5	1.9600000	0.2966479	1.5000000	2.3000000

Laboratory tests at the time of obtaining Table 2-6 consent, according to the presence or absence of proton-pump inhibitors

Unique Subject Identifier=2123010000-0015

MEANS Procedure

Assay variable : PTINR_VAL1 PT-INR Value

N	Mean	Standard Deviation	Minimum	Maximum
6	2.0833333	0.1834848	1.8000000	2.2000000

Unique Subject Identifier=2123010000-0016

Assay variable : PTINR_VAL1 PT-INR Value

N	Mean	Standard Deviation	Minimum	Maximum
6	1.9333333	0.2160247	1.7000000	2.2000000

Unique Subject Identifier=2123010000-0017

Assay variable : PTINR_VAL1 PT-INR Value

N	Mean	Standard Deviation	Minimum	Maximum
4	2.2250000	0.2986079	1.8000000	2.5000000

Unique Subject Identifier=2123010000-0020

Assay variable : PTINR_VAL1 PT-INR Value

N	Mean	Standard Deviation	Minimum	Maximum
4	2.0250000	0.4272002	1.5000000	2.5000000

Laboratory tests at the time of obtaining Table 2-6 consent, according to the presence or absence of proton-pump inhibitors

Unique Subject Identifier=2123010000-0026

MEANS Procedure

Assay variable : PTINR_VAL1 PT-INR Value

N	Mean	Standard Deviation	Minimum	Maximum
6	1.9666667	0.0816497	1.9000000	2.1000000

Unique Subject Identifier=2123010000-0029

Assay variable : PTINR_VAL1 PT-INR Value

N	Mean	Standard Deviation	Minimum	Maximum
6	1.6833333	0.1940790	1.5000000	2.0000000

Unique Subject Identifier=2125011111-0002

Assay variable : PTINR_VAL1 PT-INR Value

N	Mean	Standard Deviation	Minimum	Maximum
5	2.3140000	0.1633401	2.1500000	2.5400000

Unique Subject Identifier=2125011111-0003

Assay variable : PTINR_VAL1 PT-INR Value

N	Mean	Standard Deviation	Minimum	Maximum
5	2.2660000	0.2591910	1.9700000	2.6400000

Laboratory tests at the time of obtaining Table 2-6 consent, according to the presence or absence of proton-pump inhibitors

Unique Subject Identifier=2125011111-0011

MEANS Procedure

Assay variable : PTINR_VAL1 PT-INR Value

N	Mean	Standard Deviation	Minimum	Maximum
2	1.9150000	0.0353553	1.8900000	1.9400000

Unique Subject Identifier=2125011111-0013

Assay variable : PTINR_VAL1 PT-INR Value

N	Mean	Standard Deviation	Minimum	Maximum
5	2.3240000	0.7497866	1.4600000	3.4300000

Unique Subject Identifier=2125011111-0014

Assay variable : PTINR_VAL1 PT-INR Value

N	Mean	Standard Deviation	Minimum	Maximum
5	1.8920000	0.3536524	1.4200000	2.2900000

Unique Subject Identifier=2125011111-0027

Assay variable : PTINR_VAL1 PT-INR Value

N	Mean	Standard Deviation	Minimum	Maximum
6	2.3683333	1.0923629	1.1900000	3.9600000

Laboratory tests at the time of obtaining Table 2-6 consent, according to the presence or absence of proton-pump inhibitors

Unique Subject Identifier=2127011011-0003

MEANS Procedure

Assay variable : PTINR_VAL1 PT-INR Value

N	Mean	Standard Deviation	Minimum	Maximum
2	1.4000000	0	1.4000000	1.4000000

Unique Subject Identifier=2127011011-0004

Assay variable : PTINR_VAL1 PT-INR Value

N	Mean	Standard Deviation	Minimum	Maximum
4	2.0450000	0.3831014	1.7800000	2.6000000

Unique Subject Identifier=2127011011-0006

Assay variable : PTINR_VAL1 PT-INR Value

N	Mean	Standard Deviation	Minimum	Maximum
6	1.5500000	0.1516575	1.4000000	1.8000000

Unique Subject Identifier=2127011011-0008

Assay variable : PTINR_VAL1 PT-INR Value

N	Mean	Standard Deviation	Minimum	Maximum
5	1.5000000	0.1581139	1.3000000	1.7000000

Laboratory tests at the time of obtaining Table 2-6 consent, according to the presence or absence of proton-pump inhibitors

Unique Subject Identifier=2127011011-0009

MEANS Procedure

Assay variable : PTINR_VAL1 PT-INR Value

N	Mean	Standard Deviation	Minimum	Maximum
3	1.5000000	0.4358899	1.2000000	2.0000000

Unique Subject Identifier=2127011011-0010

Assay variable : PTINR_VAL1 PT-INR Value

N	Mean	Standard Deviation	Minimum	Maximum
5	1.9200000	0.6180615	1.3000000	2.8000000

Unique Subject Identifier=2127011011-0011

Assay variable : PTINR_VAL1 PT-INR Value

N	Mean	Standard Deviation	Minimum	Maximum
6	1.5333333	0.2943920	1.1000000	1.9000000

Unique Subject Identifier=2127011011-0012

Assay variable : PTINR_VAL1 PT-INR Value

N	Mean	Standard Deviation	Minimum	Maximum
6	1.5000000	0.1414214	1.3000000	1.7000000

Laboratory tests at the time of obtaining Table 2-6 consent, according to the presence or absence of proton-pump inhibitors

Unique Subject Identifier=2127011011-0016

MEANS Procedure

Assay variable : PTINR_VAL1 PT-INR Value

N	Mean	Standard Deviation	Minimum	Maximum
3	1.7000000	0.1000000	1.6000000	1.8000000

Unique Subject Identifier=2127011011-0017

Assay variable : PTINR_VAL1 PT-INR Value

N	Mean	Standard Deviation	Minimum	Maximum
3	1.1666667	0.1527525	1.0000000	1.3000000

Unique Subject Identifier=2127011011-0018

Assay variable : PTINR_VAL1 PT-INR Value

N	Mean	Standard Deviation	Minimum	Maximum
3	1.6333333	0.1527525	1.5000000	1.8000000

Unique Subject Identifier=2127011011-0019

Assay variable : PTINR_VAL1 PT-INR Value

N	Mean	Standard Deviation	Minimum	Maximum
5	1.5000000	0.2449490	1.3000000	1.9000000

Laboratory tests at the time of obtaining Table 2-6 consent, according to the presence or absence of proton-pump inhibitors

Unique Subject Identifier=2127011011-0021

MEANS Procedure

Assay variable : PTINR_VAL1 PT-INR Value

N	Mean	Standard Deviation	Minimum	Maximum
1	2.0000000		2.0000000	2.0000000

Unique Subject Identifier=2127011011-0023

Assay variable : PTINR_VAL1 PT-INR Value

N	Mean	Standard Deviation	Minimum	Maximum
5	1.6400000	0.1341641	1.5000000	1.8000000

Unique Subject Identifier=2127011011-0024

Assay variable : PTINR_VAL1 PT-INR Value

N	Mean	Standard Deviation	Minimum	Maximum
6	1.8833333	0.1169045	1.7000000	2.0000000

Unique Subject Identifier=2127011011-0025

Assay variable : PTINR_VAL1 PT-INR Value

N	Mean	Standard Deviation	Minimum	Maximum
6	1.8833333	0.2401388	1.6000000	2.3000000

Laboratory tests at the time of obtaining Table 2-6 consent, according to the presence or absence of proton-pump inhibitors

Unique Subject Identifier=2127011011-0026

MEANS Procedure

Assay variable : PTINR_VAL1 PT-INR Value

N	Mean	Standard Deviation	Minimum	Maximum
5	1.9800000	0.4147288	1.4000000	2.4000000

Unique Subject Identifier=2127011011-0027

Assay variable : PTINR_VAL1 PT-INR Value

N	Mean	Standard Deviation	Minimum	Maximum
2	1.5500000	0.0707107	1.5000000	1.6000000

Unique Subject Identifier=2127011011-0030

Assay variable : PTINR_VAL1 PT-INR Value

N	Mean	Standard Deviation	Minimum	Maximum
3	1.3333333	0.1154701	1.2000000	1.4000000

Unique Subject Identifier=2127011011-0034

Assay variable : PTINR_VAL1 PT-INR Value

N	Mean	Standard Deviation	Minimum	Maximum
2	2.0000000	0.7071068	1.5000000	2.5000000

Laboratory tests at the time of obtaining Table 2-6 consent, according to the presence or absence of proton-pump inhibitors

Unique Subject Identifier=2127011011-0036

MEANS Procedure

Assay variable : PTINR_VAL1 PT-INR Value

N	Mean	Standard Deviation	Minimum	Maximum
6	1.6500000	0.2428992	1.3000000	1.9000000

Unique Subject Identifier=2127011011-0039

Assay variable : PTINR_VAL1 PT-INR Value

N	Mean	Standard Deviation	Minimum	Maximum
5	1.2400000	0.1140175	1.1000000	1.4000000

Unique Subject Identifier=2127011011-0041

Assay variable : PTINR_VAL1 PT-INR Value

N	Mean	Standard Deviation	Minimum	Maximum
6	2.0166667	0.4578937	1.6000000	2.6000000

Unique Subject Identifier=2127011011-0046

Assay variable : PTINR_VAL1 PT-INR Value

N	Mean	Standard Deviation	Minimum	Maximum
4	1.4600000	0.1720465	1.2800000	1.6600000

Laboratory tests at the time of obtaining Table 2-6 consent, according to the presence or absence of proton-pump inhibitors

Unique Subject Identifier=2128010111-0001

MEANS Procedure

Assay variable : PTINR_VAL1 PT-INR Value

N	Mean	Standard Deviation	Minimum	Maximum
1	1.7600000		1.7600000	1.7600000

Unique Subject Identifier=2134011111-0013

Assay variable : PTINR_VAL1 PT-INR Value

N	Mean	Standard Deviation	Minimum	Maximum
1	1.6000000		1.6000000	1.6000000

Unique Subject Identifier=2138011111-0002

Assay variable : PTINR_VAL1 PT-INR Value

N	Mean	Standard Deviation	Minimum	Maximum
4	1.4375000	0.1021029	1.3100000	1.5600000

Unique Subject Identifier=2138011111-0004

Assay variable : PTINR_VAL1 PT-INR Value

N	Mean	Standard Deviation	Minimum	Maximum
3	1.9266667	0.0416333	1.8800000	1.9600000

Laboratory tests at the time of obtaining Table 2-6 consent, according to the presence or absence of proton-pump inhibitors

Unique Subject Identifier=2138011111-0009

MEANS Procedure

Assay variable : PTINR_VAL1 PT-INR Value

N	Mean	Standard Deviation	Minimum	Maximum
5	2.0520000	0.1943451	1.8100000	2.3400000

Unique Subject Identifier=2138011111-0021

Assay variable : PTINR_VAL1 PT-INR Value

N	Mean	Standard Deviation	Minimum	Maximum
5	1.9760000	0.2956011	1.5700000	2.3700000

Unique Subject Identifier=2138011111-0026

Assay variable : PTINR_VAL1 PT-INR Value

N	Mean	Standard Deviation	Minimum	Maximum
6	1.8516667	0.2762185	1.4400000	2.1600000

Unique Subject Identifier=2138011111-0027

Assay variable : PTINR_VAL1 PT-INR Value

N	Mean	Standard Deviation	Minimum	Maximum
6	2.4216667	0.5288257	1.4700000	2.8100000

Laboratory tests at the time of obtaining Table 2-6 consent, according to the presence or absence of proton-pump inhibitors

Unique Subject Identifier=2138011111-0028

MEANS Procedure

Assay variable : PTINR_VAL1 PT-INR Value

N	Mean	Standard Deviation	Minimum	Maximum
5	1.5300000	0.2331309	1.1900000	1.7800000

Unique Subject Identifier=2138011111-0030

Assay variable : PTINR_VAL1 PT-INR Value

N	Mean	Standard Deviation	Minimum	Maximum
3	2.0333333	0.1814754	1.8400000	2.2000000

Unique Subject Identifier=2138011111-0031

Assay variable : PTINR_VAL1 PT-INR Value

N	Mean	Standard Deviation	Minimum	Maximum
4	1.7425000	0.1851801	1.6000000	2.0100000

Unique Subject Identifier=2138011111-0032

Assay variable : PTINR_VAL1 PT-INR Value

N	Mean	Standard Deviation	Minimum	Maximum
5	1.9460000	0.2081586	1.6900000	2.1600000

Laboratory tests at the time of obtaining Table 2-6 consent, according to the presence or absence of proton-pump inhibitors

Unique Subject Identifier=2138011111-0033

MEANS Procedure

Assay variable : PTINR_VAL1 PT-INR Value

N	Mean	Standard Deviation	Minimum	Maximum
1	1.2600000		1.2600000	1.2600000

Unique Subject Identifier=2138011111-0040

Assay variable : PTINR_VAL1 PT-INR Value

N	Mean	Standard Deviation	Minimum	Maximum
5	1.9100000	0.2065188	1.6000000	2.1700000

Unique Subject Identifier=2138011111-0044

Assay variable : PTINR_VAL1 PT-INR Value

N	Mean	Standard Deviation	Minimum	Maximum
3	1.6733333	0.0896289	1.5700000	1.7300000

Unique Subject Identifier=2138011111-0046

Assay variable : PTINR_VAL1 PT-INR Value

N	Mean	Standard Deviation	Minimum	Maximum
3	1.6033333	0.3744774	1.1900000	1.9200000

Laboratory tests at the time of obtaining Table 2-6 consent, according to the presence or absence of proton-pump inhibitors

Unique Subject Identifier=2138011111-0049

MEANS Procedure

Assay variable : PTINR_VAL1 PT-INR Value

N	Mean	Standard Deviation	Minimum	Maximum
4	2.3825000	0.2698611	1.9900000	2.6000000

Unique Subject Identifier=2138011111-0054

Assay variable : PTINR_VAL1 PT-INR Value

N	Mean	Standard Deviation	Minimum	Maximum
3	2.2233333	0.5254839	1.9100000	2.8300000

Unique Subject Identifier=2138011111-0055

Assay variable : PTINR_VAL1 PT-INR Value

N	Mean	Standard Deviation	Minimum	Maximum
6	2.1650000	0.5103234	1.3100000	2.7600000

Unique Subject Identifier=2138011111-0056

Assay variable : PTINR_VAL1 PT-INR Value

N	Mean	Standard Deviation	Minimum	Maximum
6	1.2950000	0.2700926	1.0500000	1.6900000

Laboratory tests at the time of obtaining Table 2-6 consent, according to the presence or absence of proton-pump inhibitors

Unique Subject Identifier=2138011111-0059

MEANS Procedure

Assay variable : PTINR_VAL1 PT-INR Value

N	Mean	Standard Deviation	Minimum	Maximum
6	1.8566667	0.4131182	1.2200000	2.3000000

Unique Subject Identifier=2138011111-0062

Assay variable : PTINR_VAL1 PT-INR Value

N	Mean	Standard Deviation	Minimum	Maximum
3	1.8233333	0.1154701	1.6900000	1.8900000

Unique Subject Identifier=2138011111-0065

Assay variable : PTINR_VAL1 PT-INR Value

N	Mean	Standard Deviation	Minimum	Maximum
6	2.1066667	0.2028464	1.8600000	2.3400000

Unique Subject Identifier=2140011100-0001

Assay variable : PTINR_VAL1 PT-INR Value

N	Mean	Standard Deviation	Minimum	Maximum
3	2.2000000	0.9165151	1.4000000	3.2000000

Laboratory tests at the time of obtaining Table 2-6 consent, according to the presence or absence of proton-pump inhibitors

Unique Subject Identifier=2140011100-0004

MEANS Procedure

Assay variable : PTINR_VAL1 PT-INR Value

N	Mean	Standard Deviation	Minimum	Maximum
5	2.3000000	0.7905694	1.7000000	3.6000000

Unique Subject Identifier=2148011111-0003

Assay variable : PTINR_VAL1 PT-INR Value

N	Mean	Standard Deviation	Minimum	Maximum
1	1.2600000		1.2600000	1.2600000

Unique Subject Identifier=2148011111-0006

Assay variable : PTINR_VAL1 PT-INR Value

N	Mean	Standard Deviation	Minimum	Maximum
3	1.0333333	0.0577350	1.0000000	1.1000000

Unique Subject Identifier=2155011111-0008

Assay variable : PTINR_VAL1 PT-INR Value

N	Mean	Standard Deviation	Minimum	Maximum
4	2.6150000	0.4291464	2.1800000	3.2000000

Laboratory tests at the time of obtaining Table 2-6 consent, according to the presence or absence of proton-pump inhibitors

Unique Subject Identifier=2155011111-0017

MEANS Procedure

Assay variable : PTINR_VAL1 PT-INR Value

N	Mean	Standard Deviation	Minimum	Maximum
1	1.7000000		1.7000000	1.7000000

Unique Subject Identifier=2157011111-0004

Assay variable : PTINR_VAL1 PT-INR Value

N	Mean	Standard Deviation	Minimum	Maximum
6	2.2500000	0.2258318	2.0000000	2.6000000

Unique Subject Identifier=2163000000-0027

Assay variable : PTINR_VAL1 PT-INR Value

N	Mean	Standard Deviation	Minimum	Maximum
2	1.8500000	0.0707107	1.8000000	1.9000000

Unique Subject Identifier=2163000000-0035

Assay variable : PTINR_VAL1 PT-INR Value

N	Mean	Standard Deviation	Minimum	Maximum
1	1.1000000		1.1000000	1.1000000

Laboratory tests at the time of obtaining Table 2-6 consent, according to the presence or absence of proton-pump inhibitors

Unique Subject Identifier=2163000000-0047

MEANS Procedure

Assay variable : PTINR_VAL1 PT-INR Value

N	Mean	Standard Deviation	Minimum	Maximum
2	3.4000000	1.2727922	2.5000000	4.3000000

Unique Subject Identifier=2163000000-0051

Assay variable : PTINR_VAL1 PT-INR Value

N	Mean	Standard Deviation	Minimum	Maximum
1	2.3000000		2.3000000	2.3000000

Unique Subject Identifier=2163000000-0058

Assay variable : PTINR_VAL1 PT-INR Value

N	Mean	Standard Deviation	Minimum	Maximum
2	2.0000000	0	2.0000000	2.0000000

Unique Subject Identifier=2163000000-0060

Assay variable : PTINR_VAL1 PT-INR Value

N	Mean	Standard Deviation	Minimum	Maximum
2	1.6500000	0.0707107	1.6000000	1.7000000

Laboratory tests at the time of obtaining Table 2-6 consent, according to the presence or absence of proton-pump inhibitors

Unique Subject Identifier=2174011011-0021

MEANS Procedure

Assay variable : PTINR_VAL1 PT-INR Value

N	Mean	Standard Deviation	Minimum	Maximum
6	2.6333333	0.2732520	2.2000000	3.0000000

Unique Subject Identifier=2176011011-0013

Assay variable : PTINR_VAL1 PT-INR Value

N	Mean	Standard Deviation	Minimum	Maximum
2	2.2250000	0.8697413	1.6100000	2.8400000

Unique Subject Identifier=2176011011-0015

Assay variable : PTINR_VAL1 PT-INR Value

N	Mean	Standard Deviation	Minimum	Maximum
4	1.2325000	0.0419325	1.1900000	1.2900000

Unique Subject Identifier=2176011011-0022

Assay variable : PTINR_VAL1 PT-INR Value

N	Mean	Standard Deviation	Minimum	Maximum
4	1.8175000	0.2969147	1.4000000	2.1000000

Laboratory tests at the time of obtaining Table 2-6 consent, according to the presence or absence of proton-pump inhibitors

Unique Subject Identifier=2176011011-0026

MEANS Procedure

Assay variable : PTINR_VAL1 PT-INR Value

N	Mean	Standard Deviation	Minimum	Maximum
2	2.1450000	0.0636396	2.1000000	2.1900000

Unique Subject Identifier=2176011011-0028

Assay variable : PTINR_VAL1 PT-INR Value

N	Mean	Standard Deviation	Minimum	Maximum
1	1.7700000		1.7700000	1.7700000

Unique Subject Identifier=2176011011-0034

Assay variable : PTINR_VAL1 PT-INR Value

N	Mean	Standard Deviation	Minimum	Maximum
1	2.5200000		2.5200000	2.5200000

Unique Subject Identifier=2176011011-0044

Assay variable : PTINR_VAL1 PT-INR Value

N	Mean	Standard Deviation	Minimum	Maximum
1	2.2900000		2.2900000	2.2900000

Laboratory tests at the time of obtaining Table 2-6 consent, according to the presence or absence of proton-pump inhibitors

Unique Subject Identifier=2183011011-0007

MEANS Procedure

Assay variable : PTINR_VAL1 PT-INR Value

N	Mean	Standard Deviation	Minimum	Maximum
3	2.1333333	0.4509250	1.7000000	2.6000000

Unique Subject Identifier=2183011011-0008

Assay variable : PTINR_VAL1 PT-INR Value

N	Mean	Standard Deviation	Minimum	Maximum
3	1.4666667	0.0577350	1.4000000	1.5000000

Unique Subject Identifier=2183011011-0009

Assay variable : PTINR_VAL1 PT-INR Value

N	Mean	Standard Deviation	Minimum	Maximum
3	1.5333333	0.2081666	1.3000000	1.7000000

Unique Subject Identifier=2183011011-0011

Assay variable : PTINR_VAL1 PT-INR Value

N	Mean	Standard Deviation	Minimum	Maximum
2	2.1000000	0	2.1000000	2.1000000

Laboratory tests at the time of obtaining Table 2-6 consent, according to the presence or absence of proton-pump inhibitors

Unique Subject Identifier=2183011011-0012

MEANS Procedure

Assay variable : PTINR_VAL1 PT-INR Value

N	Mean	Standard Deviation	Minimum	Maximum
3	1.8666667	0.2309401	1.6000000	2.0000000

Unique Subject Identifier=2183011011-0014

Assay variable : PTINR_VAL1 PT-INR Value

N	Mean	Standard Deviation	Minimum	Maximum
2	1.6000000	0.5656854	1.2000000	2.0000000

Unique Subject Identifier=2183011011-0017

Assay variable : PTINR_VAL1 PT-INR Value

N	Mean	Standard Deviation	Minimum	Maximum
2	2.1000000	0.5656854	1.7000000	2.5000000

Unique Subject Identifier=2185010010-0002

Assay variable : PTINR_VAL1 PT-INR Value

N	Mean	Standard Deviation	Minimum	Maximum
1	1.7200000		1.7200000	1.7200000

Laboratory tests at the time of obtaining Table 2-6 consent, according to the presence or absence of proton-pump inhibitors

Unique Subject Identifier=2185010010-0004

MEANS Procedure

Assay variable : PTINR_VAL1 PT-INR Value

N	Mean	Standard Deviation	Minimum	Maximum
6	1.9833333	0.1471960	1.8000000	2.1000000

Unique Subject Identifier=2185010010-0005

Assay variable : PTINR_VAL1 PT-INR Value

N	Mean	Standard Deviation	Minimum	Maximum
6	1.9500000	0.3391165	1.6000000	2.5000000

Unique Subject Identifier=2185010010-0007

Assay variable : PTINR_VAL1 PT-INR Value

N	Mean	Standard Deviation	Minimum	Maximum
6	2.3333333	0.4802777	1.6000000	2.8000000

Unique Subject Identifier=2185010010-0008

Assay variable : PTINR_VAL1 PT-INR Value

N	Mean	Standard Deviation	Minimum	Maximum
6	1.9666667	0.5645057	1.4000000	2.9000000

Laboratory tests at the time of obtaining Table 2-6 consent, according to the presence or absence of proton-pump inhibitors

Unique Subject Identifier=2185010010-0010

MEANS Procedure

Assay variable : PTINR_VAL1 PT-INR Value

N	Mean	Standard Deviation	Minimum	Maximum
5	2.2200000	0.4658326	1.4000000	2.5000000

Unique Subject Identifier=2191011011-0005

Assay variable : PTINR_VAL1 PT-INR Value

N	Mean	Standard Deviation	Minimum	Maximum
2	1.5950000	0.0353553	1.5700000	1.6200000

Unique Subject Identifier=2191011011-0008

Assay variable : PTINR_VAL1 PT-INR Value

N	Mean	Standard Deviation	Minimum	Maximum
3	1.5233333	0.1422439	1.3600000	1.6200000

Unique Subject Identifier=2191011011-0012

Assay variable : PTINR_VAL1 PT-INR Value

N	Mean	Standard Deviation	Minimum	Maximum
1	1.5100000		1.5100000	1.5100000

Laboratory tests at the time of obtaining Table 2-6 consent, according to the presence or absence of proton-pump inhibitors

Unique Subject Identifier=2191011011-0013

MEANS Procedure

Assay variable : PTINR_VAL1 PT-INR Value

N	Mean	Standard Deviation	Minimum	Maximum
1	1.7300000		1.7300000	1.7300000

Unique Subject Identifier=2192010011-0002

Assay variable : PTINR_VAL1 PT-INR Value

N	Mean	Standard Deviation	Minimum	Maximum
6	2.4333333	0.2943920	2.1000000	2.7000000

Unique Subject Identifier=2200000000-0001

Assay variable : PTINR_VAL1 PT-INR Value

N	Mean	Standard Deviation	Minimum	Maximum
3	2.6166667	0.8975708	1.8900000	3.6200000

Unique Subject Identifier=2203011010-0002

Assay variable : PTINR_VAL1 PT-INR Value

N	Mean	Standard Deviation	Minimum	Maximum
5	1.7800000	0.8671793	1.0000000	3.0000000

Laboratory tests at the time of obtaining Table 2-6 consent, according to the presence or absence of proton-pump inhibitors

Unique Subject Identifier=2205001111-0003

MEANS Procedure

Assay variable : PTINR_VAL1 PT-INR Value

N	Mean	Standard Deviation	Minimum	Maximum
6	1.9000000	0.4690416	1.3000000	2.6000000

Unique Subject Identifier=2205001111-0018

Assay variable : PTINR_VAL1 PT-INR Value

N	Mean	Standard Deviation	Minimum	Maximum
6	2.4666667	0.3502380	1.9000000	2.9000000

Unique Subject Identifier=2205001111-0025

Assay variable : PTINR_VAL1 PT-INR Value

N	Mean	Standard Deviation	Minimum	Maximum
6	2.1333333	0.1032796	2.0000000	2.3000000

Unique Subject Identifier=2205001111-0027

Assay variable : PTINR_VAL1 PT-INR Value

N	Mean	Standard Deviation	Minimum	Maximum
6	1.7333333	0.1632993	1.6000000	2.0000000

Laboratory tests at the time of obtaining Table 2-6 consent, according to the presence or absence of proton-pump inhibitors

Unique Subject Identifier=2205001111-0041

MEANS Procedure

Assay variable : PTINR_VAL1 PT-INR Value

N	Mean	Standard Deviation	Minimum	Maximum
6	1.8166667	0.1471960	1.7000000	2.0000000

Unique Subject Identifier=2205001111-0046

Assay variable : PTINR_VAL1 PT-INR Value

N	Mean	Standard Deviation	Minimum	Maximum
4	2.1325000	0.1699755	1.9000000	2.3000000

Unique Subject Identifier=2205001111-0049

Assay variable : PTINR_VAL1 PT-INR Value

N	Mean	Standard Deviation	Minimum	Maximum
6	1.9450000	0.7634854	1.1700000	3.2300000

Unique Subject Identifier=2211011111-0001

Assay variable : PTINR_VAL1 PT-INR Value

N	Mean	Standard Deviation	Minimum	Maximum
1	2.6000000		2.6000000	2.6000000

Laboratory tests at the time of obtaining Table 2-6 consent, according to the presence or absence of proton-pump inhibitors

Unique Subject Identifier=2211011111-0003

MEANS Procedure

Assay variable : PTINR_VAL1 PT-INR Value

N	Mean	Standard Deviation	Minimum	Maximum
1	1.1000000		1.1000000	1.1000000

Unique Subject Identifier=2211011111-0037

Assay variable : PTINR_VAL1 PT-INR Value

N	Mean	Standard Deviation	Minimum	Maximum
6	1.7000000	0.6131884	1.0000000	2.6000000

Unique Subject Identifier=2211011111-0038

Assay variable : PTINR_VAL1 PT-INR Value

N	Mean	Standard Deviation	Minimum	Maximum
4	1.9250000	0.1892969	1.8000000	2.2000000

Unique Subject Identifier=2211011111-0039

Assay variable : PTINR_VAL1 PT-INR Value

N	Mean	Standard Deviation	Minimum	Maximum
5	1.4000000	0.1870829	1.2000000	1.7000000

Laboratory tests at the time of obtaining Table 2-6 consent, according to the presence or absence of proton-pump inhibitors

Unique Subject Identifier=2211011111-0041

MEANS Procedure

Assay variable : PTINR_VAL1 PT-INR Value

N	Mean	Standard Deviation	Minimum	Maximum
1	2.5000000		2.5000000	2.5000000

Unique Subject Identifier=2211011111-0044

Assay variable : PTINR_VAL1 PT-INR Value

N	Mean	Standard Deviation	Minimum	Maximum
5	1.0940000	0.0531977	1.0500000	1.1800000

Unique Subject Identifier=2211011111-0054

Assay variable : PTINR_VAL1 PT-INR Value

N	Mean	Standard Deviation	Minimum	Maximum
1	1.3500000		1.3500000	1.3500000

Unique Subject Identifier=2211011111-0057

Assay variable : PTINR_VAL1 PT-INR Value

N	Mean	Standard Deviation	Minimum	Maximum
1	1.6000000		1.6000000	1.6000000

Laboratory tests at the time of obtaining Table 2-6 consent, according to the presence or absence of proton-pump inhibitors

Unique Subject Identifier=2211011111-0058

MEANS Procedure

Assay variable : PTINR_VAL1 PT-INR Value

N	Mean	Standard Deviation	Minimum	Maximum
1	1.1900000		1.1900000	1.1900000

Unique Subject Identifier=2211011111-0060

Assay variable : PTINR_VAL1 PT-INR Value

N	Mean	Standard Deviation	Minimum	Maximum
1	1.9100000		1.9100000	1.9100000

Unique Subject Identifier=2214011111-0003

Assay variable : PTINR_VAL1 PT-INR Value

N	Mean	Standard Deviation	Minimum	Maximum
6	2.1683333	0.2604163	1.8900000	2.5900000

Unique Subject Identifier=2227010011-0003

Assay variable : PTINR_VAL1 PT-INR Value

N	Mean	Standard Deviation	Minimum	Maximum
1	1.9000000		1.9000000	1.9000000

Laboratory tests at the time of obtaining Table 2-6 consent, according to the presence or absence of proton-pump inhibitors

Unique Subject Identifier=2227010011-0011

MEANS Procedure

Assay variable : PTINR_VAL1 PT-INR Value

N	Mean	Standard Deviation	Minimum	Maximum
1	2.8300000		2.8300000	2.8300000

Unique Subject Identifier=2233011111-0001

Assay variable : PTINR_VAL1 PT-INR Value

N	Mean	Standard Deviation	Minimum	Maximum
6	1.9966667	0.4012314	1.4500000	2.5700000

Unique Subject Identifier=2233011111-0002

Assay variable : PTINR_VAL1 PT-INR Value

N	Mean	Standard Deviation	Minimum	Maximum
6	1.9400000	0.1704113	1.7100000	2.1500000

Unique Subject Identifier=2233011111-0008

Assay variable : PTINR_VAL1 PT-INR Value

N	Mean	Standard Deviation	Minimum	Maximum
4	1.4000000	0.1157584	1.2900000	1.5600000

Laboratory tests at the time of obtaining Table 2-6 consent, according to the presence or absence of proton-pump inhibitors

Unique Subject Identifier=2233011111-0009

MEANS Procedure

Assay variable : PTINR_VAL1 PT-INR Value

N	Mean	Standard Deviation	Minimum	Maximum
4	1.7600000	0.2047763	1.5600000	2.0100000

Unique Subject Identifier=2233011111-0011

Assay variable : PTINR_VAL1 PT-INR Value

N	Mean	Standard Deviation	Minimum	Maximum
5	2.2540000	0.2861468	1.9000000	2.6000000

Unique Subject Identifier=2233011111-0014

Assay variable : PTINR_VAL1 PT-INR Value

N	Mean	Standard Deviation	Minimum	Maximum
4	1.4800000	0.2044505	1.1800000	1.6100000

Unique Subject Identifier=2233011111-0015

Assay variable : PTINR_VAL1 PT-INR Value

N	Mean	Standard Deviation	Minimum	Maximum
6	1.6016667	0.1358553	1.4600000	1.8000000

Laboratory tests at the time of obtaining Table 2-6 consent, according to the presence or absence of proton-pump inhibitors

Unique Subject Identifier=2233011111-0016

MEANS Procedure

Assay variable : PTINR_VAL1 PT-INR Value

N	Mean	Standard Deviation	Minimum	Maximum
6	1.9616667	0.2359167	1.6500000	2.3600000

Unique Subject Identifier=2233011111-0018

Assay variable : PTINR_VAL1 PT-INR Value

N	Mean	Standard Deviation	Minimum	Maximum
4	1.5250000	0.1912241	1.3100000	1.7500000

Unique Subject Identifier=2233011111-0019

Assay variable : PTINR_VAL1 PT-INR Value

N	Mean	Standard Deviation	Minimum	Maximum
2	2.2500000	0.3535534	2.0000000	2.5000000

Unique Subject Identifier=2233011111-0020

Assay variable : PTINR_VAL1 PT-INR Value

N	Mean	Standard Deviation	Minimum	Maximum
4	1.9075000	0.3986122	1.3400000	2.2000000

Laboratory tests at the time of obtaining Table 2-6 consent, according to the presence or absence of proton-pump inhibitors

Unique Subject Identifier=2233011111-0021

MEANS Procedure

Assay variable : PTINR_VAL1 PT-INR Value

N	Mean	Standard Deviation	Minimum	Maximum
5	1.4320000	0.1439444	1.3100000	1.6300000

Unique Subject Identifier=2233011111-0022

Assay variable : PTINR_VAL1 PT-INR Value

N	Mean	Standard Deviation	Minimum	Maximum
5	1.5680000	0.1231666	1.4300000	1.7600000

Unique Subject Identifier=2233011111-0023

Assay variable : PTINR_VAL1 PT-INR Value

N	Mean	Standard Deviation	Minimum	Maximum
5	1.7000000	0.2354782	1.3500000	1.9700000

Unique Subject Identifier=2233011111-0024

Assay variable : PTINR_VAL1 PT-INR Value

N	Mean	Standard Deviation	Minimum	Maximum
6	1.5616667	0.2039036	1.3400000	1.8800000

Laboratory tests at the time of obtaining Table 2-6 consent, according to the presence or absence of proton-pump inhibitors

Unique Subject Identifier=2233011111-0025

MEANS Procedure

Assay variable : PTINR_VAL1 PT-INR Value

N	Mean	Standard Deviation	Minimum	Maximum
3	1.5566667	0.1556706	1.4100000	1.7200000

Unique Subject Identifier=2233011111-0026

Assay variable : PTINR_VAL1 PT-INR Value

N	Mean	Standard Deviation	Minimum	Maximum
3	1.4666667	0.1159023	1.3600000	1.5900000

Unique Subject Identifier=2233011111-0032

Assay variable : PTINR_VAL1 PT-INR Value

N	Mean	Standard Deviation	Minimum	Maximum
3	2.0133333	0.2138535	1.7800000	2.2000000

Unique Subject Identifier=2233011111-0034

Assay variable : PTINR_VAL1 PT-INR Value

N	Mean	Standard Deviation	Minimum	Maximum
4	1.3150000	0.2056696	1.1200000	1.5800000

Laboratory tests at the time of obtaining Table 2-6 consent, according to the presence or absence of proton-pump inhibitors

Unique Subject Identifier=2233011111-0035

MEANS Procedure

Assay variable : PTINR_VAL1 PT-INR Value

N	Mean	Standard Deviation	Minimum	Maximum
5	1.7220000	0.2888252	1.3300000	1.9800000

Unique Subject Identifier=2233011111-0036

Assay variable : PTINR_VAL1 PT-INR Value

N	Mean	Standard Deviation	Minimum	Maximum
4	1.6725000	0.0340343	1.6300000	1.7000000

Unique Subject Identifier=2233011111-0037

Assay variable : PTINR_VAL1 PT-INR Value

N	Mean	Standard Deviation	Minimum	Maximum
3	2.0200000	0.1200000	1.9000000	2.1400000

Unique Subject Identifier=2236011111-0001

Assay variable : PTINR_VAL1 PT-INR Value

N	Mean	Standard Deviation	Minimum	Maximum
6	2.3866667	0.2338090	2.0000000	2.6000000

Laboratory tests at the time of obtaining Table 2-6 consent, according to the presence or absence of proton-pump inhibitors

Unique Subject Identifier=2236011111-0002

MEANS Procedure

Assay variable : PTINR_VAL1 PT-INR Value

N	Mean	Standard Deviation	Minimum	Maximum
6	1.9500000	0.4324350	1.4000000	2.6000000

Unique Subject Identifier=2236011111-0005

Assay variable : PTINR_VAL1 PT-INR Value

N	Mean	Standard Deviation	Minimum	Maximum
4	1.7500000	0.0577350	1.7000000	1.8000000

Unique Subject Identifier=2236011111-0007

Assay variable : PTINR_VAL1 PT-INR Value

N	Mean	Standard Deviation	Minimum	Maximum
6	1.6783333	0.2898563	1.3000000	2.0000000

Unique Subject Identifier=2236011111-0009

Assay variable : PTINR_VAL1 PT-INR Value

N	Mean	Standard Deviation	Minimum	Maximum
5	1.9500000	0.2000000	1.6000000	2.1000000

Laboratory tests at the time of obtaining Table 2-6 consent, according to the presence or absence of proton-pump inhibitors

Unique Subject Identifier=2236011111-0012

MEANS Procedure

Assay variable : PTINR_VAL1 PT-INR Value

N	Mean	Standard Deviation	Minimum	Maximum
5	1.5800000	0.1788854	1.3000000	1.7000000

Unique Subject Identifier=2236011111-0013

Assay variable : PTINR_VAL1 PT-INR Value

N	Mean	Standard Deviation	Minimum	Maximum
4	2.3500000	0.5802298	1.8000000	3.1000000

Unique Subject Identifier=2236011111-0014

Assay variable : PTINR_VAL1 PT-INR Value

N	Mean	Standard Deviation	Minimum	Maximum
3	1.7366667	0.3611556	1.3600000	2.0800000

Unique Subject Identifier=2236011111-0017

Assay variable : PTINR_VAL1 PT-INR Value

N	Mean	Standard Deviation	Minimum	Maximum
6	1.7000000	0.3687818	1.4000000	2.3000000

Laboratory tests at the time of obtaining Table 2-6 consent, according to the presence or absence of proton-pump inhibitors

Unique Subject Identifier=2236011111-0018

MEANS Procedure

Assay variable : PTINR_VAL1 PT-INR Value

N	Mean	Standard Deviation	Minimum	Maximum
5	2.3400000	0.1341641	2.2000000	2.5000000

Unique Subject Identifier=2236011111-0021

Assay variable : PTINR_VAL1 PT-INR Value

N	Mean	Standard Deviation	Minimum	Maximum
4	1.7000000	0.1154701	1.6000000	1.8000000

Unique Subject Identifier=2236011111-0024

Assay variable : PTINR_VAL1 PT-INR Value

N	Mean	Standard Deviation	Minimum	Maximum
6	1.4000000	0.2000000	1.1000000	1.6000000

Unique Subject Identifier=2236011111-0025

Assay variable : PTINR_VAL1 PT-INR Value

N	Mean	Standard Deviation	Minimum	Maximum
1	1.5000000		1.5000000	1.5000000

Laboratory tests at the time of obtaining Table 2-6 consent, according to the presence or absence of proton-pump inhibitors

Unique Subject Identifier=2236011111-0027

MEANS Procedure

Assay variable : PTINR_VAL1 PT-INR Value

N	Mean	Standard Deviation	Minimum	Maximum
4	2.0500000	0.1732051	1.9000000	2.3000000

Unique Subject Identifier=2236011111-0028

Assay variable : PTINR_VAL1 PT-INR Value

N	Mean	Standard Deviation	Minimum	Maximum
3	2.2666667	0.3055050	2.0000000	2.6000000

Unique Subject Identifier=2236011111-0031

Assay variable : PTINR_VAL1 PT-INR Value

N	Mean	Standard Deviation	Minimum	Maximum
6	1.6000000	0.2449490	1.2000000	1.9000000

Unique Subject Identifier=2236011111-0032

Assay variable : PTINR_VAL1 PT-INR Value

N	Mean	Standard Deviation	Minimum	Maximum
6	1.9166667	0.3125167	1.5000000	2.3000000

Laboratory tests at the time of obtaining Table 2-6 consent, according to the presence or absence of proton-pump inhibitors

Unique Subject Identifier=2236011111-0034

MEANS Procedure

Assay variable : PTINR_VAL1 PT-INR Value

N	Mean	Standard Deviation	Minimum	Maximum
5	1.8000000	0.2121320	1.6000000	2.1000000

Unique Subject Identifier=2236011111-0039

Assay variable : PTINR_VAL1 PT-INR Value

N	Mean	Standard Deviation	Minimum	Maximum
4	1.8000000	0.1414214	1.7000000	2.0000000

Unique Subject Identifier=2236011111-0042

Assay variable : PTINR_VAL1 PT-INR Value

N	Mean	Standard Deviation	Minimum	Maximum
4	1.8250000	0.2629956	1.6000000	2.2000000

Unique Subject Identifier=2236011111-0043

Assay variable : PTINR_VAL1 PT-INR Value

N	Mean	Standard Deviation	Minimum	Maximum
6	1.7833333	0.2857738	1.4000000	2.2000000

Laboratory tests at the time of obtaining Table 2-6 consent, according to the presence or absence of proton-pump inhibitors

Unique Subject Identifier=2236011111-0045

MEANS Procedure

Assay variable : PTINR_VAL1 PT-INR Value

N	Mean	Standard Deviation	Minimum	Maximum
5	3.0000000	0.7681146	2.3000000	4.1000000

Unique Subject Identifier=2236011111-0052

Assay variable : PTINR_VAL1 PT-INR Value

N	Mean	Standard Deviation	Minimum	Maximum
6	1.7000000	0.5215362	1.2000000	2.3000000

Unique Subject Identifier=2236011111-0053

Assay variable : PTINR_VAL1 PT-INR Value

N	Mean	Standard Deviation	Minimum	Maximum
4	1.7750000	0.1707825	1.6000000	2.0000000

Unique Subject Identifier=2236011111-0054

Assay variable : PTINR_VAL1 PT-INR Value

N	Mean	Standard Deviation	Minimum	Maximum
5	1.7160000	0.1152389	1.5800000	1.8000000

Laboratory tests at the time of obtaining Table 2-6 consent, according to the presence or absence of proton-pump inhibitors

Unique Subject Identifier=2236011111-0059

MEANS Procedure

Assay variable : PTINR_VAL1 PT-INR Value

N	Mean	Standard Deviation	Minimum	Maximum
5	2.5720000	0.2567489	2.3600000	3.0000000

Unique Subject Identifier=2236011111-0062

Assay variable : PTINR_VAL1 PT-INR Value

N	Mean	Standard Deviation	Minimum	Maximum
4	2.9000000	1.7088007	1.8000000	5.4000000

Unique Subject Identifier=2236011111-0064

Assay variable : PTINR_VAL1 PT-INR Value

N	Mean	Standard Deviation	Minimum	Maximum
4	1.9000000	0.4966555	1.3000000	2.5000000

Unique Subject Identifier=2236011111-0066

Assay variable : PTINR_VAL1 PT-INR Value

N	Mean	Standard Deviation	Minimum	Maximum
5	2.3320000	0.4734131	1.6000000	2.9000000

Laboratory tests at the time of obtaining Table 2-6 consent, according to the presence or absence of proton-pump inhibitors

Unique Subject Identifier=2236011111-0070

MEANS Procedure

Assay variable : PTINR_VAL1 PT-INR Value

N	Mean	Standard Deviation	Minimum	Maximum
4	2.4500000	0.3415650	2.1000000	2.9000000

Unique Subject Identifier=2236011111-0073

Assay variable : PTINR_VAL1 PT-INR Value

N	Mean	Standard Deviation	Minimum	Maximum
6	2.1000000	0.2828427	1.7000000	2.4000000

Unique Subject Identifier=2236011111-0075

Assay variable : PTINR_VAL1 PT-INR Value

N	Mean	Standard Deviation	Minimum	Maximum
3	2.5000000	0.2000000	2.3000000	2.7000000

Unique Subject Identifier=2236011111-0080

Assay variable : PTINR_VAL1 PT-INR Value

N	Mean	Standard Deviation	Minimum	Maximum
6	2.2166667	0.5845226	1.6000000	2.9000000

Laboratory tests at the time of obtaining Table 2-6 consent, according to the presence or absence of proton-pump inhibitors

Unique Subject Identifier=2241011111-0004

MEANS Procedure

Assay variable : PTINR_VAL1 PT-INR Value

N	Mean	Standard Deviation	Minimum	Maximum
5	1.6100000	0.1189538	1.4700000	1.7800000

Unique Subject Identifier=2241011111-0009

Assay variable : PTINR_VAL1 PT-INR Value

N	Mean	Standard Deviation	Minimum	Maximum
6	2.2183333	0.1267149	2.0400000	2.4300000

Unique Subject Identifier=2241011111-0011

Assay variable : PTINR_VAL1 PT-INR Value

N	Mean	Standard Deviation	Minimum	Maximum
5	1.9700000	0.1996246	1.8000000	2.2600000

Unique Subject Identifier=2243011111-0011

Assay variable : PTINR_VAL1 PT-INR Value

N	Mean	Standard Deviation	Minimum	Maximum
5	1.6260000	0.1171751	1.4500000	1.7600000

Laboratory tests at the time of obtaining Table 2-6 consent, according to the presence or absence of proton-pump inhibitors

Unique Subject Identifier=2243011111-0013

MEANS Procedure

Assay variable : PTINR_VAL1 PT-INR Value

N	Mean	Standard Deviation	Minimum	Maximum
6	1.7483333	0.1584193	1.5800000	2.0300000

Unique Subject Identifier=2243011111-0014

Assay variable : PTINR_VAL1 PT-INR Value

N	Mean	Standard Deviation	Minimum	Maximum
5	1.9360000	0.5542833	1.3400000	2.7400000

Unique Subject Identifier=2243011111-0015

Assay variable : PTINR_VAL1 PT-INR Value

N	Mean	Standard Deviation	Minimum	Maximum
5	1.7240000	0.1645600	1.5200000	1.9300000

Unique Subject Identifier=2247010011-0001

Assay variable : PTINR_VAL1 PT-INR Value

N	Mean	Standard Deviation	Minimum	Maximum
2	1.7100000	0.0424264	1.6800000	1.7400000

Laboratory tests at the time of obtaining Table 2-6 consent, according to the presence or absence of proton-pump inhibitors

Unique Subject Identifier=2247010011-0002

MEANS Procedure

Assay variable : PTINR_VAL1 PT-INR Value

N	Mean	Standard Deviation	Minimum	Maximum
3	1.7266667	0.6180885	1.3500000	2.4400000

Unique Subject Identifier=2247010011-0003

Assay variable : PTINR_VAL1 PT-INR Value

N	Mean	Standard Deviation	Minimum	Maximum
4	1.7700000	0.1732051	1.5600000	1.9400000

Unique Subject Identifier=2247010011-0004

Assay variable : PTINR_VAL1 PT-INR Value

N	Mean	Standard Deviation	Minimum	Maximum
3	1.7900000	0.4059557	1.4300000	2.2300000

Unique Subject Identifier=2247010011-0005

Assay variable : PTINR_VAL1 PT-INR Value

N	Mean	Standard Deviation	Minimum	Maximum
3	1.5466667	0.2837840	1.2400000	1.8000000

Laboratory tests at the time of obtaining Table 2-6 consent, according to the presence or absence of proton-pump inhibitors

Unique Subject Identifier=2247010011-0006

MEANS Procedure

Assay variable : PTINR_VAL1 PT-INR Value

N	Mean	Standard Deviation	Minimum	Maximum
4	1.8550000	0.1123981	1.7100000	1.9700000

Unique Subject Identifier=2247010011-0007

Assay variable : PTINR_VAL1 PT-INR Value

N	Mean	Standard Deviation	Minimum	Maximum
4	2.0175000	0.4503610	1.4400000	2.4800000

Unique Subject Identifier=2247010011-0008

Assay variable : PTINR_VAL1 PT-INR Value

N	Mean	Standard Deviation	Minimum	Maximum
5	1.7020000	0.2327445	1.4800000	1.9700000

Unique Subject Identifier=2247010011-0009

Assay variable : PTINR_VAL1 PT-INR Value

N	Mean	Standard Deviation	Minimum	Maximum
4	2.4950000	0.5238638	1.7100000	2.7900000

Laboratory tests at the time of obtaining Table 2-6 consent, according to the presence or absence of proton-pump inhibitors

Unique Subject Identifier=2247010011-0010

MEANS Procedure

Assay variable : PTINR_VAL1 PT-INR Value

N	Mean	Standard Deviation	Minimum	Maximum
4	2.5575000	0.3375772	2.1200000	2.8700000

Unique Subject Identifier=2247010011-0011

Assay variable : PTINR_VAL1 PT-INR Value

N	Mean	Standard Deviation	Minimum	Maximum
4	2.0500000	0.4388622	1.4100000	2.3800000

Unique Subject Identifier=2247010011-0012

Assay variable : PTINR_VAL1 PT-INR Value

N	Mean	Standard Deviation	Minimum	Maximum
4	1.5250000	0.2338803	1.1900000	1.7200000

Unique Subject Identifier=2247010011-0013

Assay variable : PTINR_VAL1 PT-INR Value

N	Mean	Standard Deviation	Minimum	Maximum
4	2.3450000	0.2517274	2.0700000	2.6600000

Laboratory tests at the time of obtaining Table 2-6 consent, according to the presence or absence of proton-pump inhibitors

Unique Subject Identifier=2247010011-0014

MEANS Procedure

Assay variable : PTINR_VAL1 PT-INR Value

N	Mean	Standard Deviation	Minimum	Maximum
4	1.5475000	0.2715849	1.3100000	1.8900000

Unique Subject Identifier=2247010011-0015

Assay variable : PTINR_VAL1 PT-INR Value

N	Mean	Standard Deviation	Minimum	Maximum
5	1.8440000	0.1425833	1.6400000	2.0300000

Unique Subject Identifier=2247010011-0016

Assay variable : PTINR_VAL1 PT-INR Value

N	Mean	Standard Deviation	Minimum	Maximum
4	1.5300000	0.1219289	1.4300000	1.6900000

Unique Subject Identifier=2247010011-0017

Assay variable : PTINR_VAL1 PT-INR Value

N	Mean	Standard Deviation	Minimum	Maximum
4	1.8850000	0.1502221	1.6600000	1.9700000

Laboratory tests at the time of obtaining Table 2-6 consent, according to the presence or absence of proton-pump inhibitors

Unique Subject Identifier=2247010011-0018

MEANS Procedure

Assay variable : PTINR_VAL1 PT-INR Value

N	Mean	Standard Deviation	Minimum	Maximum
4	1.7825000	0.3397425	1.2800000	2.0300000

Unique Subject Identifier=2247010011-0019

Assay variable : PTINR_VAL1 PT-INR Value

N	Mean	Standard Deviation	Minimum	Maximum
6	2.0133333	0.3028971	1.4900000	2.3900000

Unique Subject Identifier=2247010011-0020

Assay variable : PTINR_VAL1 PT-INR Value

N	Mean	Standard Deviation	Minimum	Maximum
3	1.9966667	0.1965536	1.7700000	2.1200000

Unique Subject Identifier=2247010011-0021

Assay variable : PTINR_VAL1 PT-INR Value

N	Mean	Standard Deviation	Minimum	Maximum
6	1.4100000	0.3959798	1.0800000	2.0700000

Laboratory tests at the time of obtaining Table 2-6 consent, according to the presence or absence of proton-pump inhibitors

Unique Subject Identifier=2247010011-0022

MEANS Procedure

Assay variable : PTINR_VAL1 PT-INR Value

N	Mean	Standard Deviation	Minimum	Maximum
4	1.5900000	0.1227464	1.4400000	1.7400000

Unique Subject Identifier=2247010011-0023

Assay variable : PTINR_VAL1 PT-INR Value

N	Mean	Standard Deviation	Minimum	Maximum
4	2.3800000	0.3067029	2.1700000	2.8200000

Unique Subject Identifier=2247010011-0024

Assay variable : PTINR_VAL1 PT-INR Value

N	Mean	Standard Deviation	Minimum	Maximum
4	2.0825000	0.1840969	1.8500000	2.3000000

Unique Subject Identifier=2247010011-0025

Assay variable : PTINR_VAL1 PT-INR Value

N	Mean	Standard Deviation	Minimum	Maximum
4	1.9700000	0.6274286	1.6300000	2.9100000

Laboratory tests at the time of obtaining Table 2-6 consent, according to the presence or absence of proton-pump inhibitors

Unique Subject Identifier=2247010011-0026

MEANS Procedure

Assay variable : PTINR_VAL1 PT-INR Value

N	Mean	Standard Deviation	Minimum	Maximum
4	2.2625000	0.1685972	2.0900000	2.4800000

Unique Subject Identifier=2247010011-0027

Assay variable : PTINR_VAL1 PT-INR Value

N	Mean	Standard Deviation	Minimum	Maximum
3	1.8466667	0.0513160	1.7900000	1.8900000

Unique Subject Identifier=2247010011-0028

Assay variable : PTINR_VAL1 PT-INR Value

N	Mean	Standard Deviation	Minimum	Maximum
4	2.2100000	0.1311488	2.0200000	2.3200000

Unique Subject Identifier=2247010011-0029

Assay variable : PTINR_VAL1 PT-INR Value

N	Mean	Standard Deviation	Minimum	Maximum
4	2.2475000	0.3852597	1.8100000	2.7400000

Laboratory tests at the time of obtaining Table 2-6 consent, according to the presence or absence of proton-pump inhibitors

Unique Subject Identifier=2247010011-0031

MEANS Procedure

Assay variable : PTINR_VAL1 PT-INR Value

N	Mean	Standard Deviation	Minimum	Maximum
4	1.5300000	0.0820569	1.4500000	1.6400000

Unique Subject Identifier=2247010011-0032

Assay variable : PTINR_VAL1 PT-INR Value

N	Mean	Standard Deviation	Minimum	Maximum
4	2.3200000	0.2585859	2.0100000	2.6200000

Unique Subject Identifier=2247010011-0033

Assay variable : PTINR_VAL1 PT-INR Value

N	Mean	Standard Deviation	Minimum	Maximum
4	1.6950000	0.3319136	1.2900000	2.0000000

Unique Subject Identifier=2247010011-0035

Assay variable : PTINR_VAL1 PT-INR Value

N	Mean	Standard Deviation	Minimum	Maximum
5	2.4340000	0.4240637	2.0900000	3.1500000

Laboratory tests at the time of obtaining Table 2-6 consent, according to the presence or absence of proton-pump inhibitors

Unique Subject Identifier=2247010011-0036

MEANS Procedure

Assay variable : PTINR_VAL1 PT-INR Value

N	Mean	Standard Deviation	Minimum	Maximum
6	1.6916667	0.1110705	1.5600000	1.8800000

Unique Subject Identifier=2247010011-0037

Assay variable : PTINR_VAL1 PT-INR Value

N	Mean	Standard Deviation	Minimum	Maximum
5	1.8380000	0.1482228	1.6700000	2.0700000

Unique Subject Identifier=2247010011-0038

Assay variable : PTINR_VAL1 PT-INR Value

N	Mean	Standard Deviation	Minimum	Maximum
4	1.4225000	0.1906786	1.2600000	1.6500000

Unique Subject Identifier=2247010011-0039

Assay variable : PTINR_VAL1 PT-INR Value

N	Mean	Standard Deviation	Minimum	Maximum
4	1.9850000	0.2522565	1.7200000	2.2600000

Laboratory tests at the time of obtaining Table 2-6 consent, according to the presence or absence of proton-pump inhibitors

Unique Subject Identifier=2247010011-0040

MEANS Procedure

Assay variable : PTINR_VAL1 PT-INR Value

N	Mean	Standard Deviation	Minimum	Maximum
4	1.8800000	0.1446836	1.7100000	2.0100000

Unique Subject Identifier=2247010011-0041

Assay variable : PTINR_VAL1 PT-INR Value

N	Mean	Standard Deviation	Minimum	Maximum
4	2.4325000	0.6373578	1.5500000	3.0200000

Unique Subject Identifier=2247010011-0042

Assay variable : PTINR_VAL1 PT-INR Value

N	Mean	Standard Deviation	Minimum	Maximum
5	1.7160000	0.4074678	1.3000000	2.2000000

Unique Subject Identifier=2247010011-0043

Assay variable : PTINR_VAL1 PT-INR Value

N	Mean	Standard Deviation	Minimum	Maximum
4	1.8125000	0.2784930	1.6000000	2.2000000

Laboratory tests at the time of obtaining Table 2-6 consent, according to the presence or absence of proton-pump inhibitors

Unique Subject Identifier=2247010011-0044

MEANS Procedure

Assay variable : PTINR_VAL1 PT-INR Value

N	Mean	Standard Deviation	Minimum	Maximum
4	2.3600000	0.2565151	1.9800000	2.5200000

Unique Subject Identifier=2247010011-0045

Assay variable : PTINR_VAL1 PT-INR Value

N	Mean	Standard Deviation	Minimum	Maximum
4	1.9025000	0.1508587	1.7500000	2.1000000

Unique Subject Identifier=2247010011-0046

Assay variable : PTINR_VAL1 PT-INR Value

N	Mean	Standard Deviation	Minimum	Maximum
4	2.0975000	0.2850000	1.7900000	2.4600000

Unique Subject Identifier=2247010011-0047

Assay variable : PTINR_VAL1 PT-INR Value

N	Mean	Standard Deviation	Minimum	Maximum
4	2.1025000	0.0942956	1.9900000	2.1900000

Laboratory tests at the time of obtaining Table 2-6 consent, according to the presence or absence of proton-pump inhibitors

Unique Subject Identifier=2247010011-0048

MEANS Procedure

Assay variable : PTINR_VAL1 PT-INR Value

N	Mean	Standard Deviation	Minimum	Maximum
4	3.2375000	0.8573360	2.4900000	4.4400000

Unique Subject Identifier=2247010011-0049

Assay variable : PTINR_VAL1 PT-INR Value

N	Mean	Standard Deviation	Minimum	Maximum
4	1.8075000	0.1723127	1.6200000	2.0100000

Unique Subject Identifier=2247010011-0050

Assay variable : PTINR_VAL1 PT-INR Value

N	Mean	Standard Deviation	Minimum	Maximum
4	2.3525000	0.1746186	2.2000000	2.6000000

Unique Subject Identifier=2247010011-0051

Assay variable : PTINR_VAL1 PT-INR Value

N	Mean	Standard Deviation	Minimum	Maximum
5	1.6900000	0.1504992	1.5600000	1.9300000

Laboratory tests at the time of obtaining Table 2-6 consent, according to the presence or absence of proton-pump inhibitors

Unique Subject Identifier=2247010011-0052

MEANS Procedure

Assay variable : PTINR_VAL1 PT-INR Value

N	Mean	Standard Deviation	Minimum	Maximum
1	1.4300000		1.4300000	1.4300000

Unique Subject Identifier=2247010011-0053

Assay variable : PTINR_VAL1 PT-INR Value

N	Mean	Standard Deviation	Minimum	Maximum
6	2.3283333	0.4026620	1.5600000	2.6900000

Unique Subject Identifier=2247010011-0054

Assay variable : PTINR_VAL1 PT-INR Value

N	Mean	Standard Deviation	Minimum	Maximum
4	1.5150000	0.1167619	1.3700000	1.6400000

Unique Subject Identifier=2247010011-0055

Assay variable : PTINR_VAL1 PT-INR Value

N	Mean	Standard Deviation	Minimum	Maximum
4	1.6325000	0.2653771	1.3200000	1.9500000

Laboratory tests at the time of obtaining Table 2-6 consent, according to the presence or absence of proton-pump inhibitors

Unique Subject Identifier=2247010011-0056

MEANS Procedure

Assay variable : PTINR_VAL1 PT-INR Value

N	Mean	Standard Deviation	Minimum	Maximum
6	2.1866667	0.2207865	1.8800000	2.5200000

Unique Subject Identifier=2247010011-0057

Assay variable : PTINR_VAL1 PT-INR Value

N	Mean	Standard Deviation	Minimum	Maximum
4	2.1075000	0.6416840	1.6100000	3.0500000

Unique Subject Identifier=2247010011-0058

Assay variable : PTINR_VAL1 PT-INR Value

N	Mean	Standard Deviation	Minimum	Maximum
3	1.5500000	0.0700000	1.4700000	1.6000000

Unique Subject Identifier=2247010011-0060

Assay variable : PTINR_VAL1 PT-INR Value

N	Mean	Standard Deviation	Minimum	Maximum
6	2.1866667	0.6503743	1.4200000	2.9700000

Laboratory tests at the time of obtaining Table 2-6 consent, according to the presence or absence of proton-pump inhibitors

Unique Subject Identifier=2247010011-0061

MEANS Procedure

Assay variable : PTINR_VAL1 PT-INR Value

N	Mean	Standard Deviation	Minimum	Maximum
6	2.1000000	0.1716974	1.8400000	2.2900000

Unique Subject Identifier=2247010011-0062

Assay variable : PTINR_VAL1 PT-INR Value

N	Mean	Standard Deviation	Minimum	Maximum
3	1.9200000	1.0142485	1.2900000	3.0900000

Unique Subject Identifier=2247010011-0063

Assay variable : PTINR_VAL1 PT-INR Value

N	Mean	Standard Deviation	Minimum	Maximum
3	1.5833333	0.2173323	1.3500000	1.7800000

Unique Subject Identifier=2247010011-0064

Assay variable : PTINR_VAL1 PT-INR Value

N	Mean	Standard Deviation	Minimum	Maximum
4	1.6325000	0.2839454	1.2800000	1.9400000

Laboratory tests at the time of obtaining Table 2-6 consent, according to the presence or absence of proton-pump inhibitors

Unique Subject Identifier=2247010011-0066

MEANS Procedure

Assay variable : PTINR_VAL1 PT-INR Value

N	Mean	Standard Deviation	Minimum	Maximum
5	1.9060000	0.2160555	1.7300000	2.1700000

Unique Subject Identifier=2247010011-0067

Assay variable : PTINR_VAL1 PT-INR Value

N	Mean	Standard Deviation	Minimum	Maximum
4	1.4775000	0.1908533	1.2100000	1.6600000

Unique Subject Identifier=2247010011-0068

Assay variable : PTINR_VAL1 PT-INR Value

N	Mean	Standard Deviation	Minimum	Maximum
5	2.0100000	0.3930649	1.6100000	2.5200000

Unique Subject Identifier=2247010011-0069

Assay variable : PTINR_VAL1 PT-INR Value

N	Mean	Standard Deviation	Minimum	Maximum
5	1.5540000	0.2886694	1.2900000	1.9200000

Laboratory tests at the time of obtaining Table 2-6 consent, according to the presence or absence of proton-pump inhibitors

Unique Subject Identifier=2247010011-0070

MEANS Procedure

Assay variable : PTINR_VAL1 PT-INR Value

N	Mean	Standard Deviation	Minimum	Maximum
4	2.2375000	0.3467348	2.0200000	2.7500000

Unique Subject Identifier=2247010011-0071

Assay variable : PTINR_VAL1 PT-INR Value

N	Mean	Standard Deviation	Minimum	Maximum
4	2.2725000	0.6687987	1.7000000	3.0300000

Unique Subject Identifier=2247010011-0072

Assay variable : PTINR_VAL1 PT-INR Value

N	Mean	Standard Deviation	Minimum	Maximum
2	3.0400000	0.5939697	2.6200000	3.4600000

Unique Subject Identifier=2247010011-0073

Assay variable : PTINR_VAL1 PT-INR Value

N	Mean	Standard Deviation	Minimum	Maximum
3	1.2500000	0.2981610	1.0000000	1.5800000

Laboratory tests at the time of obtaining Table 2-6 consent, according to the presence or absence of proton-pump inhibitors

Unique Subject Identifier=2247010011-0074

MEANS Procedure

Assay variable : PTINR_VAL1 PT-INR Value

N	Mean	Standard Deviation	Minimum	Maximum
3	1.6533333	0.2177919	1.4100000	1.8300000

Unique Subject Identifier=2247010011-0075

Assay variable : PTINR_VAL1 PT-INR Value

N	Mean	Standard Deviation	Minimum	Maximum
3	1.2200000	0.1646208	1.1200000	1.4100000

Unique Subject Identifier=2247010011-0076

Assay variable : PTINR_VAL1 PT-INR Value

N	Mean	Standard Deviation	Minimum	Maximum
4	1.7900000	0.3667879	1.3600000	2.1600000

Unique Subject Identifier=2247010011-0077

Assay variable : PTINR_VAL1 PT-INR Value

N	Mean	Standard Deviation	Minimum	Maximum
2	1.9400000	0.5515433	1.5500000	2.3300000

Laboratory tests at the time of obtaining Table 2-6 consent, according to the presence or absence of proton-pump inhibitors

Unique Subject Identifier=2247010011-0078

MEANS Procedure

Assay variable : PTINR_VAL1 PT-INR Value

N	Mean	Standard Deviation	Minimum	Maximum
3	1.7633333	0.0750555	1.6900000	1.8400000

Unique Subject Identifier=2249011111-0011

Assay variable : PTINR_VAL1 PT-INR Value

N	Mean	Standard Deviation	Minimum	Maximum
2	1.4500000	0.2121320	1.3000000	1.6000000

Unique Subject Identifier=2249011111-0017

Assay variable : PTINR_VAL1 PT-INR Value

N	Mean	Standard Deviation	Minimum	Maximum
3	2.0566667	0.2205297	1.8700000	2.3000000

Unique Subject Identifier=2258010111-0001

Assay variable : PTINR_VAL1 PT-INR Value

N	Mean	Standard Deviation	Minimum	Maximum
4	1.2500000	0.1290994	1.1000000	1.4000000

Laboratory tests at the time of obtaining Table 2-6 consent, according to the presence or absence of proton-pump inhibitors

Unique Subject Identifier=2258010111-0014

MEANS Procedure

Assay variable : PTINR_VAL1 PT-INR Value

N	Mean	Standard Deviation	Minimum	Maximum
1	1.1000000		1.1000000	1.1000000

Unique Subject Identifier=2259010111-0001

Assay variable : PTINR_VAL1 PT-INR Value

N	Mean	Standard Deviation	Minimum	Maximum
3	1.5066667	0.1960442	1.3000000	1.6900000

Unique Subject Identifier=2259010111-0002

Assay variable : PTINR_VAL1 PT-INR Value

N	Mean	Standard Deviation	Minimum	Maximum
4	1.3350000	0.0881287	1.2400000	1.4400000

Unique Subject Identifier=2263011111-0001

Assay variable : PTINR_VAL1 PT-INR Value

N	Mean	Standard Deviation	Minimum	Maximum
2	2.0600000	0.8626703	1.4500000	2.6700000

Laboratory tests at the time of obtaining Table 2-6 consent, according to the presence or absence of proton-pump inhibitors

Unique Subject Identifier=2264011111-0003

MEANS Procedure

Assay variable : PTINR_VAL1 PT-INR Value

N	Mean	Standard Deviation	Minimum	Maximum
6	1.6116667	0.3074682	1.1800000	2.0100000

Unique Subject Identifier=2264011111-0004

Assay variable : PTINR_VAL1 PT-INR Value

N	Mean	Standard Deviation	Minimum	Maximum
6	1.7650000	0.2074367	1.4900000	2.0300000

Unique Subject Identifier=2264011111-0006

Assay variable : PTINR_VAL1 PT-INR Value

N	Mean	Standard Deviation	Minimum	Maximum
6	1.6650000	0.4120073	1.1500000	2.3200000

Unique Subject Identifier=2264011111-0008

Assay variable : PTINR_VAL1 PT-INR Value

N	Mean	Standard Deviation	Minimum	Maximum
6	1.6550000	0.1005485	1.5100000	1.7700000

Laboratory tests at the time of obtaining Table 2-6 consent, according to the presence or absence of proton-pump inhibitors

Unique Subject Identifier=2264011111-0009

MEANS Procedure

Assay variable : PTINR_VAL1 PT-INR Value

N	Mean	Standard Deviation	Minimum	Maximum
6	1.5383333	0.1626551	1.4100000	1.8200000

Unique Subject Identifier=2264011111-0010

Assay variable : PTINR_VAL1 PT-INR Value

N	Mean	Standard Deviation	Minimum	Maximum
6	1.0516667	0.0240139	1.0200000	1.0800000

Unique Subject Identifier=2264011111-0012

Assay variable : PTINR_VAL1 PT-INR Value

N	Mean	Standard Deviation	Minimum	Maximum
6	1.8066667	0.6258328	1.1600000	2.9600000

Unique Subject Identifier=2268011111-0013

Assay variable : PTINR_VAL1 PT-INR Value

N	Mean	Standard Deviation	Minimum	Maximum
4	1.8500000	0.1290994	1.7000000	2.0000000

Laboratory tests at the time of obtaining Table 2-6 consent, according to the presence or absence of proton-pump inhibitors

Unique Subject Identifier=2268011111-0021

MEANS Procedure

Assay variable : PTINR_VAL1 PT-INR Value

N	Mean	Standard Deviation	Minimum	Maximum
5	2.1400000	0.3361547	1.6000000	2.5000000

Unique Subject Identifier=2268011111-0023

Assay variable : PTINR_VAL1 PT-INR Value

N	Mean	Standard Deviation	Minimum	Maximum
5	2.0000000	0.2121320	1.8000000	2.3000000

Unique Subject Identifier=2268011111-0024

Assay variable : PTINR_VAL1 PT-INR Value

N	Mean	Standard Deviation	Minimum	Maximum
5	1.6200000	0.1788854	1.5000000	1.9000000

Unique Subject Identifier=2268011111-0028

Assay variable : PTINR_VAL1 PT-INR Value

N	Mean	Standard Deviation	Minimum	Maximum
6	2.3166667	0.4708149	1.9000000	3.2000000

Laboratory tests at the time of obtaining Table 2-6 consent, according to the presence or absence of proton-pump inhibitors

Unique Subject Identifier=2268011111-0029

MEANS Procedure

Assay variable : PTINR_VAL1 PT-INR Value

N	Mean	Standard Deviation	Minimum	Maximum
6	1.8833333	0.5776389	1.5000000	3.0000000

Unique Subject Identifier=2268011111-0031

Assay variable : PTINR_VAL1 PT-INR Value

N	Mean	Standard Deviation	Minimum	Maximum
3	2.3000000	0.2645751	2.1000000	2.6000000

Unique Subject Identifier=2268011111-0042

Assay variable : PTINR_VAL1 PT-INR Value

N	Mean	Standard Deviation	Minimum	Maximum
6	1.9666667	0.1505545	1.7000000	2.1000000

Unique Subject Identifier=2282011001-0006

Assay variable : PTINR_VAL1 PT-INR Value

N	Mean	Standard Deviation	Minimum	Maximum
1	1.9200000		1.9200000	1.9200000

Laboratory tests at the time of obtaining Table 2-6 consent, according to the presence or absence of proton-pump inhibitors

Unique Subject Identifier=2283011000-0008

MEANS Procedure

Assay variable : PTINR_VAL1 PT-INR Value

N	Mean	Standard Deviation	Minimum	Maximum
3	2.1833333	0.3501904	1.7800000	2.4100000

Unique Subject Identifier=2283011000-0020

Assay variable : PTINR_VAL1 PT-INR Value

N	Mean	Standard Deviation	Minimum	Maximum
4	1.7425000	0.2495830	1.5300000	2.0800000

Unique Subject Identifier=2284011111-0011

Assay variable : PTINR_VAL1 PT-INR Value

N	Mean	Standard Deviation	Minimum	Maximum
5	1.7440000	0.2388095	1.5200000	2.1100000

Unique Subject Identifier=2289000000-0001

Assay variable : PTINR_VAL1 PT-INR Value

N	Mean	Standard Deviation	Minimum	Maximum
1	1.7400000		1.7400000	1.7400000

Laboratory tests at the time of obtaining Table 2-6 consent, according to the presence or absence of proton-pump inhibitors

Unique Subject Identifier=2292000000-0001

MEANS Procedure

Assay variable : PTINR_VAL1 PT-INR Value

N	Mean	Standard Deviation	Minimum	Maximum
4	2.2250000	0.1500000	2.1000000	2.4000000

Unique Subject Identifier=2292000000-0005

Assay variable : PTINR_VAL1 PT-INR Value

N	Mean	Standard Deviation	Minimum	Maximum
6	1.6333333	0.1861899	1.3000000	1.8000000

Unique Subject Identifier=2292000000-0008

Assay variable : PTINR_VAL1 PT-INR Value

N	Mean	Standard Deviation	Minimum	Maximum
6	2.1833333	0.4020779	1.7000000	2.8000000

Unique Subject Identifier=2292000000-0013

Assay variable : PTINR_VAL1 PT-INR Value

N	Mean	Standard Deviation	Minimum	Maximum
6	1.9333333	0.3614784	1.5000000	2.5000000

Laboratory tests at the time of obtaining Table 2-6 consent, according to the presence or absence of proton-pump inhibitors

Unique Subject Identifier=2292000000-0014

MEANS Procedure

Assay variable : PTINR_VAL1 PT-INR Value

N	Mean	Standard Deviation	Minimum	Maximum
6	2.2500000	0.3619392	1.8000000	2.9000000

Unique Subject Identifier=2296010111-0002

Assay variable : PTINR_VAL1 PT-INR Value

N	Mean	Standard Deviation	Minimum	Maximum
5	1.7000000	0.0707107	1.6000000	1.8000000

Unique Subject Identifier=2296010111-0005

Assay variable : PTINR_VAL1 PT-INR Value

N	Mean	Standard Deviation	Minimum	Maximum
5	1.8600000	0.0547723	1.8000000	1.9000000

Unique Subject Identifier=2296010111-0006

Assay variable : PTINR_VAL1 PT-INR Value

N	Mean	Standard Deviation	Minimum	Maximum
5	1.9000000	0.2549510	1.6000000	2.2000000

Laboratory tests at the time of obtaining Table 2-6 consent, according to the presence or absence of proton-pump inhibitors

Unique Subject Identifier=2296010111-0009

MEANS Procedure

Assay variable : PTINR_VAL1 PT-INR Value

N	Mean	Standard Deviation	Minimum	Maximum
5	2.2000000	0.2000000	1.9000000	2.4000000

Unique Subject Identifier=2296010111-0011

Assay variable : PTINR_VAL1 PT-INR Value

N	Mean	Standard Deviation	Minimum	Maximum
6	2.1666667	0.1211060	2.0000000	2.3000000

Unique Subject Identifier=2296010111-0012

Assay variable : PTINR_VAL1 PT-INR Value

N	Mean	Standard Deviation	Minimum	Maximum
6	1.8500000	0.2509980	1.6000000	2.3000000

Unique Subject Identifier=2301011111-0002

Assay variable : PTINR_VAL1 PT-INR Value

N	Mean	Standard Deviation	Minimum	Maximum
1	2.3400000		2.3400000	2.3400000

Laboratory tests at the time of obtaining Table 2-6 consent, according to the presence or absence of proton-pump inhibitors

Unique Subject Identifier=2301011111-0010

MEANS Procedure

Assay variable : PTINR_VAL1 PT-INR Value

N	Mean	Standard Deviation	Minimum	Maximum
2	1.8950000	0.7707464	1.3500000	2.4400000

Unique Subject Identifier=2301011111-0013

Assay variable : PTINR_VAL1 PT-INR Value

N	Mean	Standard Deviation	Minimum	Maximum
3	1.7000000	0.1539480	1.5700000	1.8700000

Unique Subject Identifier=2301011111-0022

Assay variable : PTINR_VAL1 PT-INR Value

N	Mean	Standard Deviation	Minimum	Maximum
2	1.4950000	0.0919239	1.4300000	1.5600000

Unique Subject Identifier=2301011111-0027

Assay variable : PTINR_VAL1 PT-INR Value

N	Mean	Standard Deviation	Minimum	Maximum
4	1.7500000	0.2745906	1.3600000	2.0000000

Laboratory tests at the time of obtaining Table 2-6 consent, according to the presence or absence of proton-pump inhibitors

Unique Subject Identifier=2301011111-0031

MEANS Procedure

Assay variable : PTINR_VAL1 PT-INR Value

N	Mean	Standard Deviation	Minimum	Maximum
1	1.9500000		1.9500000	1.9500000

Unique Subject Identifier=2308011000-0001

Assay variable : PTINR_VAL1 PT-INR Value

N	Mean	Standard Deviation	Minimum	Maximum
3	1.6333333	0.0577350	1.6000000	1.7000000

Unique Subject Identifier=2308011000-0002

Assay variable : PTINR_VAL1 PT-INR Value

N	Mean	Standard Deviation	Minimum	Maximum
5	1.7000000	0.3872983	1.2000000	2.1000000

Unique Subject Identifier=2308011000-0004

Assay variable : PTINR_VAL1 PT-INR Value

N	Mean	Standard Deviation	Minimum	Maximum
4	2.2250000	0.2629956	2.0000000	2.6000000

Laboratory tests at the time of obtaining Table 2-6 consent, according to the presence or absence of proton-pump inhibitors

Unique Subject Identifier=2308011000-0013

MEANS Procedure

Assay variable : PTINR_VAL1 PT-INR Value

N	Mean	Standard Deviation	Minimum	Maximum
4	1.6000000	0.1414214	1.4000000	1.7000000

Unique Subject Identifier=2308011000-0017

Assay variable : PTINR_VAL1 PT-INR Value

N	Mean	Standard Deviation	Minimum	Maximum
3	1.7666667	0.2886751	1.6000000	2.1000000

Unique Subject Identifier=2308011000-0018

Assay variable : PTINR_VAL1 PT-INR Value

N	Mean	Standard Deviation	Minimum	Maximum
2	1.8500000	0.7778175	1.3000000	2.4000000

Unique Subject Identifier=2309000000-0001

Assay variable : PTINR_VAL1 PT-INR Value

N	Mean	Standard Deviation	Minimum	Maximum
3	1.8766667	0.3707200	1.5200000	2.2600000

Laboratory tests at the time of obtaining Table 2-6 consent, according to the presence or absence of proton-pump inhibitors

Unique Subject Identifier=2309000000-0003

MEANS Procedure

Assay variable : PTINR_VAL1 PT-INR Value

N	Mean	Standard Deviation	Minimum	Maximum
1	2.2900000		2.2900000	2.2900000

Unique Subject Identifier=2310011111-0001

Assay variable : PTINR_VAL1 PT-INR Value

N	Mean	Standard Deviation	Minimum	Maximum
2	1.8100000	0.2404163	1.6400000	1.9800000

Unique Subject Identifier=2310011111-0008

Assay variable : PTINR_VAL1 PT-INR Value

N	Mean	Standard Deviation	Minimum	Maximum
4	1.9100000	0.8183316	1.0300000	2.6700000

Unique Subject Identifier=2310011111-0011

Assay variable : PTINR_VAL1 PT-INR Value

N	Mean	Standard Deviation	Minimum	Maximum
3	1.7933333	0.1800926	1.6100000	1.9700000

Laboratory tests at the time of obtaining Table 2-6 consent, according to the presence or absence of proton-pump inhibitors

Unique Subject Identifier=2310011111-0018

MEANS Procedure

Assay variable : PTINR_VAL1 PT-INR Value

N	Mean	Standard Deviation	Minimum	Maximum
4	2.2775000	0.7902057	1.8100000	3.4600000

Unique Subject Identifier=2312011111-0005

Assay variable : PTINR_VAL1 PT-INR Value

N	Mean	Standard Deviation	Minimum	Maximum
3	2.3666667	0.4041452	1.9000000	2.6000000

Unique Subject Identifier=2312011111-0006

Assay variable : PTINR_VAL1 PT-INR Value

N	Mean	Standard Deviation	Minimum	Maximum
1	2.0000000		2.0000000	2.0000000

Unique Subject Identifier=2312011111-0008

Assay variable : PTINR_VAL1 PT-INR Value

N	Mean	Standard Deviation	Minimum	Maximum
2	1.8000000	0.2828427	1.6000000	2.0000000

Laboratory tests at the time of obtaining Table 2-6 consent, according to the presence or absence of proton-pump inhibitors

Unique Subject Identifier=2312011111-0010

MEANS Procedure

Assay variable : PTINR_VAL1 PT-INR Value

N	Mean	Standard Deviation	Minimum	Maximum
4	1.6750000	0.0957427	1.6000000	1.8000000

Unique Subject Identifier=2321011011-0006

Assay variable : PTINR_VAL1 PT-INR Value

N	Mean	Standard Deviation	Minimum	Maximum
1	2.0100000		2.0100000	2.0100000

Unique Subject Identifier=2327011011-0005

Assay variable : PTINR_VAL1 PT-INR Value

N	Mean	Standard Deviation	Minimum	Maximum
4	1.7100000	0.2096028	1.4600000	1.9400000

Unique Subject Identifier=2327011011-0007

Assay variable : PTINR_VAL1 PT-INR Value

N	Mean	Standard Deviation	Minimum	Maximum
3	2.0733333	0.9042308	1.3300000	3.0800000

Laboratory tests at the time of obtaining Table 2-6 consent, according to the presence or absence of proton-pump inhibitors

Unique Subject Identifier=2327011011-0018

MEANS Procedure

Assay variable : PTINR_VAL1 PT-INR Value

N	Mean	Standard Deviation	Minimum	Maximum
3	2.4900000	0.5604463	1.8900000	3.0000000

Unique Subject Identifier=2327011011-0020

Assay variable : PTINR_VAL1 PT-INR Value

N	Mean	Standard Deviation	Minimum	Maximum
4	2.2000000	0.1512173	2.0000000	2.3400000

Unique Subject Identifier=2327011011-0022

Assay variable : PTINR_VAL1 PT-INR Value

N	Mean	Standard Deviation	Minimum	Maximum
4	1.9525000	0.1477329	1.7700000	2.1000000

Unique Subject Identifier=2327011011-0027

Assay variable : PTINR_VAL1 PT-INR Value

N	Mean	Standard Deviation	Minimum	Maximum
4	2.0950000	0.2829016	1.8500000	2.4900000

Laboratory tests at the time of obtaining Table 2-6 consent, according to the presence or absence of proton-pump inhibitors

Unique Subject Identifier=2327011011-0028

MEANS Procedure

Assay variable : PTINR_VAL1 PT-INR Value

N	Mean	Standard Deviation	Minimum	Maximum
3	1.9733333	0.1137248	1.8800000	2.1000000

Unique Subject Identifier=2327011011-0031

Assay variable : PTINR_VAL1 PT-INR Value

N	Mean	Standard Deviation	Minimum	Maximum
4	1.8800000	0.2592296	1.5600000	2.1600000

Unique Subject Identifier=2327011011-0032

Assay variable : PTINR_VAL1 PT-INR Value

N	Mean	Standard Deviation	Minimum	Maximum
4	1.8250000	0.1292285	1.6500000	1.9600000

Unique Subject Identifier=2327011011-0034

Assay variable : PTINR_VAL1 PT-INR Value

N	Mean	Standard Deviation	Minimum	Maximum
4	1.8725000	0.0531507	1.8300000	1.9500000

Laboratory tests at the time of obtaining Table 2-6 consent, according to the presence or absence of proton-pump inhibitors

Unique Subject Identifier=2327011011-0038

MEANS Procedure

Assay variable : PTINR_VAL1 PT-INR Value

N	Mean	Standard Deviation	Minimum	Maximum
4	1.7275000	0.2396351	1.4900000	2.0200000

Unique Subject Identifier=2327011011-0039

Assay variable : PTINR_VAL1 PT-INR Value

N	Mean	Standard Deviation	Minimum	Maximum
4	2.1100000	0.2201515	1.9000000	2.4200000

Unique Subject Identifier=2327011011-0041

Assay variable : PTINR_VAL1 PT-INR Value

N	Mean	Standard Deviation	Minimum	Maximum
4	1.9600000	0.5112729	1.5600000	2.7100000

Unique Subject Identifier=2327011011-0046

Assay variable : PTINR_VAL1 PT-INR Value

N	Mean	Standard Deviation	Minimum	Maximum
4	1.8125000	0.2794489	1.4600000	2.0700000

Laboratory tests at the time of obtaining Table 2-6 consent, according to the presence or absence of proton-pump inhibitors

Unique Subject Identifier=2327011011-0047

MEANS Procedure

Assay variable : PTINR_VAL1 PT-INR Value

N	Mean	Standard Deviation	Minimum	Maximum
6	2.3916667	0.1847611	2.0900000	2.6100000

Unique Subject Identifier=2341001000-0003

Assay variable : PTINR_VAL1 PT-INR Value

N	Mean	Standard Deviation	Minimum	Maximum
1	1.4200000		1.4200000	1.4200000

Unique Subject Identifier=2341001000-0004

Assay variable : PTINR_VAL1 PT-INR Value

N	Mean	Standard Deviation	Minimum	Maximum
2	1.1450000	0.0070711	1.1400000	1.1500000

Unique Subject Identifier=2341001000-0008

Assay variable : PTINR_VAL1 PT-INR Value

N	Mean	Standard Deviation	Minimum	Maximum
1	1.9100000		1.9100000	1.9100000

Laboratory tests at the time of obtaining Table 2-6 consent, according to the presence or absence of proton-pump inhibitors

Unique Subject Identifier=2341001000-0010

MEANS Procedure

Assay variable : PTINR_VAL1 PT-INR Value

N	Mean	Standard Deviation	Minimum	Maximum
1	2.5900000		2.5900000	2.5900000

Unique Subject Identifier=2341001000-0013

Assay variable : PTINR_VAL1 PT-INR Value

N	Mean	Standard Deviation	Minimum	Maximum
1	1.6000000		1.6000000	1.6000000

Unique Subject Identifier=2341001000-0015

Assay variable : PTINR_VAL1 PT-INR Value

N	Mean	Standard Deviation	Minimum	Maximum
1	1.4600000		1.4600000	1.4600000

Unique Subject Identifier=2347011111-0001

Assay variable : PTINR_VAL1 PT-INR Value

N	Mean	Standard Deviation	Minimum	Maximum
6	1.8633333	0.2362767	1.5300000	2.2000000

Laboratory tests at the time of obtaining Table 2-6 consent, according to the presence or absence of proton-pump inhibitors

Unique Subject Identifier=2347011111-0002

MEANS Procedure

Assay variable : PTINR_VAL1 PT-INR Value

N	Mean	Standard Deviation	Minimum	Maximum
6	2.6183333	0.4615373	2.1500000	3.4700000

Unique Subject Identifier=2347011111-0005

Assay variable : PTINR_VAL1 PT-INR Value

N	Mean	Standard Deviation	Minimum	Maximum
5	1.4620000	0.3902179	0.9700000	2.0500000

Unique Subject Identifier=2347011111-0006

Assay variable : PTINR_VAL1 PT-INR Value

N	Mean	Standard Deviation	Minimum	Maximum
6	2.4033333	1.0029091	1.3200000	4.0000000

Unique Subject Identifier=2347011111-0007

Assay variable : PTINR_VAL1 PT-INR Value

N	Mean	Standard Deviation	Minimum	Maximum
5	1.9680000	0.2846401	1.5900000	2.2800000

Laboratory tests at the time of obtaining Table 2-6 consent, according to the presence or absence of proton-pump inhibitors

Unique Subject Identifier=2347011111-0008

MEANS Procedure

Assay variable : PTINR_VAL1 PT-INR Value

N	Mean	Standard Deviation	Minimum	Maximum
6	1.9616667	0.0646271	1.8600000	2.0500000

Unique Subject Identifier=2347011111-0009

Assay variable : PTINR_VAL1 PT-INR Value

N	Mean	Standard Deviation	Minimum	Maximum
6	2.5550000	0.4134126	2.0100000	3.1600000

Unique Subject Identifier=2347011111-0011

Assay variable : PTINR_VAL1 PT-INR Value

N	Mean	Standard Deviation	Minimum	Maximum
6	2.3883333	0.4908937	1.8900000	3.0600000

Unique Subject Identifier=2347011111-0012

Assay variable : PTINR_VAL1 PT-INR Value

N	Mean	Standard Deviation	Minimum	Maximum
5	2.3960000	0.3449348	1.9700000	2.7800000

Laboratory tests at the time of obtaining Table 2-6 consent, according to the presence or absence of proton-pump inhibitors

Unique Subject Identifier=2347011111-0013

MEANS Procedure

Assay variable : PTINR_VAL1 PT-INR Value

N	Mean	Standard Deviation	Minimum	Maximum
6	1.5333333	0.2520053	1.2300000	1.9000000

Unique Subject Identifier=2347011111-0014

Assay variable : PTINR_VAL1 PT-INR Value

N	Mean	Standard Deviation	Minimum	Maximum
6	2.2316667	0.1570244	2.0500000	2.4700000

Unique Subject Identifier=2347011111-0016

Assay variable : PTINR_VAL1 PT-INR Value

N	Mean	Standard Deviation	Minimum	Maximum
6	1.9300000	0.1825377	1.6200000	2.1500000

Unique Subject Identifier=2347011111-0018

Assay variable : PTINR_VAL1 PT-INR Value

N	Mean	Standard Deviation	Minimum	Maximum
6	1.4783333	0.1919809	1.2800000	1.8100000

Laboratory tests at the time of obtaining Table 2-6 consent, according to the presence or absence of proton-pump inhibitors

Unique Subject Identifier=2347011111-0019

MEANS Procedure

Assay variable : PTINR_VAL1 PT-INR Value

N	Mean	Standard Deviation	Minimum	Maximum
6	1.7483333	0.2143284	1.4900000	1.9500000

Unique Subject Identifier=2347011111-0020

Assay variable : PTINR_VAL1 PT-INR Value

N	Mean	Standard Deviation	Minimum	Maximum
6	1.8816667	0.1767956	1.5600000	2.0700000

Unique Subject Identifier=2347011111-0021

Assay variable : PTINR_VAL1 PT-INR Value

N	Mean	Standard Deviation	Minimum	Maximum
6	1.8633333	0.1841376	1.5400000	2.0700000

Unique Subject Identifier=2347011111-0022

Assay variable : PTINR_VAL1 PT-INR Value

N	Mean	Standard Deviation	Minimum	Maximum
6	1.9066667	0.1297176	1.7200000	2.0700000

Laboratory tests at the time of obtaining Table 2-6 consent, according to the presence or absence of proton-pump inhibitors

Unique Subject Identifier=2347011111-0023

MEANS Procedure

Assay variable : PTINR_VAL1 PT-INR Value

N	Mean	Standard Deviation	Minimum	Maximum
6	2.0066667	0.3538738	1.5300000	2.3700000

Unique Subject Identifier=2347011111-0024

Assay variable : PTINR_VAL1 PT-INR Value

N	Mean	Standard Deviation	Minimum	Maximum
6	2.0033333	0.1116542	1.8400000	2.1500000

Unique Subject Identifier=2347011111-0026

Assay variable : PTINR_VAL1 PT-INR Value

N	Mean	Standard Deviation	Minimum	Maximum
6	1.8900000	0.3367492	1.3300000	2.2000000

Unique Subject Identifier=2347011111-0029

Assay variable : PTINR_VAL1 PT-INR Value

N	Mean	Standard Deviation	Minimum	Maximum
6	1.6933333	0.2259794	1.3600000	2.0100000

Laboratory tests at the time of obtaining Table 2-6 consent, according to the presence or absence of proton-pump inhibitors

Unique Subject Identifier=2347011111-0030

MEANS Procedure

Assay variable : PTINR_VAL1 PT-INR Value

N	Mean	Standard Deviation	Minimum	Maximum
6	1.3700000	0.1685230	1.1600000	1.6000000

Unique Subject Identifier=2347011111-0032

Assay variable : PTINR_VAL1 PT-INR Value

N	Mean	Standard Deviation	Minimum	Maximum
6	1.9483333	0.3411402	1.3800000	2.3000000

Unique Subject Identifier=2347011111-0033

Assay variable : PTINR_VAL1 PT-INR Value

N	Mean	Standard Deviation	Minimum	Maximum
6	1.8200000	0.2174396	1.4900000	2.1100000

Unique Subject Identifier=2347011111-0034

Assay variable : PTINR_VAL1 PT-INR Value

N	Mean	Standard Deviation	Minimum	Maximum
6	2.0850000	0.3237746	1.6800000	2.4400000

Laboratory tests at the time of obtaining Table 2-6 consent, according to the presence or absence of proton-pump inhibitors

Unique Subject Identifier=2347011111-0035

MEANS Procedure

Assay variable : PTINR_VAL1 PT-INR Value

N	Mean	Standard Deviation	Minimum	Maximum
6	1.9766667	0.2438579	1.7100000	2.4300000

Unique Subject Identifier=2347011111-0038

Assay variable : PTINR_VAL1 PT-INR Value

N	Mean	Standard Deviation	Minimum	Maximum
4	1.6950000	0.8847033	1.0400000	2.9700000

Unique Subject Identifier=2347011111-0043

Assay variable : PTINR_VAL1 PT-INR Value

N	Mean	Standard Deviation	Minimum	Maximum
6	1.9233333	0.2250926	1.6900000	2.3300000

Unique Subject Identifier=2348000000-0006

Assay variable : PTINR_VAL1 PT-INR Value

N	Mean	Standard Deviation	Minimum	Maximum
3	1.7866667	0.1680278	1.6400000	1.9700000

Laboratory tests at the time of obtaining Table 2-6 consent, according to the presence or absence of proton-pump inhibitors

Unique Subject Identifier=2353011111-0002

MEANS Procedure

Assay variable : PTINR_VAL1 PT-INR Value

N	Mean	Standard Deviation	Minimum	Maximum
6	1.8350000	0.2117310	1.6000000	2.2100000

Unique Subject Identifier=2353011111-0009

Assay variable : PTINR_VAL1 PT-INR Value

N	Mean	Standard Deviation	Minimum	Maximum
6	2.2916667	0.3473567	1.7900000	2.5800000

Unique Subject Identifier=2353011111-0010

Assay variable : PTINR_VAL1 PT-INR Value

N	Mean	Standard Deviation	Minimum	Maximum
6	1.8433333	0.1906480	1.6400000	2.1100000

Unique Subject Identifier=2353011111-0012

Assay variable : PTINR_VAL1 PT-INR Value

N	Mean	Standard Deviation	Minimum	Maximum
6	1.9350000	0.2247443	1.5300000	2.1300000

Laboratory tests at the time of obtaining Table 2-6 consent, according to the presence or absence of proton-pump inhibitors

Unique Subject Identifier=2353011111-0015

MEANS Procedure

Assay variable : PTINR_VAL1 PT-INR Value

N	Mean	Standard Deviation	Minimum	Maximum
4	1.8375000	0.7744407	1.0000000	2.7400000

Unique Subject Identifier=2353011111-0016

Assay variable : PTINR_VAL1 PT-INR Value

N	Mean	Standard Deviation	Minimum	Maximum
6	2.1850000	0.5272096	1.5100000	2.9000000

Unique Subject Identifier=2354011111-0001

Assay variable : PTINR_VAL1 PT-INR Value

N	Mean	Standard Deviation	Minimum	Maximum
5	1.7280000	0.2139392	1.4100000	1.9500000

Unique Subject Identifier=2354011111-0002

Assay variable : PTINR_VAL1 PT-INR Value

N	Mean	Standard Deviation	Minimum	Maximum
4	1.6925000	0.0997914	1.6200000	1.8400000

Laboratory tests at the time of obtaining Table 2-6 consent, according to the presence or absence of proton-pump inhibitors

Unique Subject Identifier=2354011111-0003

MEANS Procedure

Assay variable : PTINR_VAL1 PT-INR Value

N	Mean	Standard Deviation	Minimum	Maximum
6	1.5100000	0.0848528	1.4300000	1.6700000

Unique Subject Identifier=2354011111-0004

Assay variable : PTINR_VAL1 PT-INR Value

N	Mean	Standard Deviation	Minimum	Maximum
6	1.7566667	0.2421294	1.4200000	2.0700000

Unique Subject Identifier=2354011111-0005

Assay variable : PTINR_VAL1 PT-INR Value

N	Mean	Standard Deviation	Minimum	Maximum
6	2.2216667	0.1820348	1.9700000	2.5100000

Unique Subject Identifier=2354011111-0007

Assay variable : PTINR_VAL1 PT-INR Value

N	Mean	Standard Deviation	Minimum	Maximum
5	2.0980000	0.9495894	1.1000000	3.1500000

Laboratory tests at the time of obtaining Table 2-6 consent, according to the presence or absence of proton-pump inhibitors

Unique Subject Identifier=2354011111-0008

MEANS Procedure

Assay variable : PTINR_VAL1 PT-INR Value

N	Mean	Standard Deviation	Minimum	Maximum
6	2.0533333	0.4229736	1.3300000	2.4900000

Unique Subject Identifier=2354011111-0009

Assay variable : PTINR_VAL1 PT-INR Value

N	Mean	Standard Deviation	Minimum	Maximum
5	2.2620000	0.2512369	2.0100000	2.5700000

Unique Subject Identifier=2354011111-0012

Assay variable : PTINR_VAL1 PT-INR Value

N	Mean	Standard Deviation	Minimum	Maximum
4	2.3900000	0.0824621	2.2800000	2.4800000

Unique Subject Identifier=2354011111-0014

Assay variable : PTINR_VAL1 PT-INR Value

N	Mean	Standard Deviation	Minimum	Maximum
6	1.5150000	0.2960912	1.1900000	1.9300000

Laboratory tests at the time of obtaining Table 2-6 consent, according to the presence or absence of proton-pump inhibitors

Unique Subject Identifier=2354011111-0016

MEANS Procedure

Assay variable : PTINR_VAL1 PT-INR Value

N	Mean	Standard Deviation	Minimum	Maximum
4	1.6175000	0.0892095	1.5100000	1.7200000

Unique Subject Identifier=2354011111-0019

Assay variable : PTINR_VAL1 PT-INR Value

N	Mean	Standard Deviation	Minimum	Maximum
3	1.4800000	0.1752142	1.3100000	1.6600000

Unique Subject Identifier=2355011111-0004

Assay variable : PTINR_VAL1 PT-INR Value

N	Mean	Standard Deviation	Minimum	Maximum
2	3.9300000	3.6203867	1.3700000	6.4900000

Unique Subject Identifier=2355011111-0005

Assay variable : PTINR_VAL1 PT-INR Value

N	Mean	Standard Deviation	Minimum	Maximum
2	2.6250000	0.3181981	2.4000000	2.8500000

Laboratory tests at the time of obtaining Table 2-6 consent, according to the presence or absence of proton-pump inhibitors

Unique Subject Identifier=2355011111-0007

MEANS Procedure

Assay variable : PTINR_VAL1 PT-INR Value

N	Mean	Standard Deviation	Minimum	Maximum
2	1.5600000	0.0141421	1.5500000	1.5700000

Unique Subject Identifier=2356011111-0001

Assay variable : PTINR_VAL1 PT-INR Value

N	Mean	Standard Deviation	Minimum	Maximum
2	1.6150000	0.1060660	1.5400000	1.6900000

Unique Subject Identifier=2356011111-0002

Assay variable : PTINR_VAL1 PT-INR Value

N	Mean	Standard Deviation	Minimum	Maximum
3	1.6333333	0.0416333	1.6000000	1.6800000

Unique Subject Identifier=2356011111-0004

Assay variable : PTINR_VAL1 PT-INR Value

N	Mean	Standard Deviation	Minimum	Maximum
3	1.8400000	0.3517101	1.4700000	2.1700000

Laboratory tests at the time of obtaining Table 2-6 consent, according to the presence or absence of proton-pump inhibitors

Unique Subject Identifier=2356011111-0007

MEANS Procedure

Assay variable : PTINR_VAL1 PT-INR Value

N	Mean	Standard Deviation	Minimum	Maximum
2	2.0700000	0.5232590	1.7000000	2.4400000

Unique Subject Identifier=2356011111-0009

Assay variable : PTINR_VAL1 PT-INR Value

N	Mean	Standard Deviation	Minimum	Maximum
2	1.9750000	0.0636396	1.9300000	2.0200000

Unique Subject Identifier=2356011111-0015

Assay variable : PTINR_VAL1 PT-INR Value

N	Mean	Standard Deviation	Minimum	Maximum
2	1.8050000	0.6434672	1.3500000	2.2600000

Unique Subject Identifier=2356011111-0016

Assay variable : PTINR_VAL1 PT-INR Value

N	Mean	Standard Deviation	Minimum	Maximum
2	2.7200000	0.6081118	2.2900000	3.1500000

Laboratory tests at the time of obtaining Table 2-6 consent, according to the presence or absence of proton-pump inhibitors

Unique Subject Identifier=2356011111-0019

MEANS Procedure

Assay variable : PTINR_VAL1 PT-INR Value

N	Mean	Standard Deviation	Minimum	Maximum
2	1.9900000	0.1555635	1.8800000	2.1000000

Unique Subject Identifier=2356011111-0021

Assay variable : PTINR_VAL1 PT-INR Value

N	Mean	Standard Deviation	Minimum	Maximum
1	1.6300000		1.6300000	1.6300000

Unique Subject Identifier=2357011010-0005

Assay variable : PTINR_VAL1 PT-INR Value

N	Mean	Standard Deviation	Minimum	Maximum
3	1.8500000	1.1435471	1.1600000	3.1700000

Unique Subject Identifier=2357011010-0009

Assay variable : PTINR_VAL1 PT-INR Value

N	Mean	Standard Deviation	Minimum	Maximum
4	1.5900000	0.4543860	1.2200000	2.2300000

Laboratory tests at the time of obtaining Table 2-6 consent, according to the presence or absence of proton-pump inhibitors

Unique Subject Identifier=2357011010-0018

MEANS Procedure

Assay variable : PTINR_VAL1 PT-INR Value

N	Mean	Standard Deviation	Minimum	Maximum
3	2.3866667	1.3743847	1.0800000	3.8200000

Unique Subject Identifier=2357011010-0019

Assay variable : PTINR_VAL1 PT-INR Value

N	Mean	Standard Deviation	Minimum	Maximum
2	2.0500000	0	2.0500000	2.0500000

Unique Subject Identifier=2357011010-0020

Assay variable : PTINR_VAL1 PT-INR Value

N	Mean	Standard Deviation	Minimum	Maximum
2	1.6100000	0.5656854	1.2100000	2.0100000

Unique Subject Identifier=2357011010-0021

Assay variable : PTINR_VAL1 PT-INR Value

N	Mean	Standard Deviation	Minimum	Maximum
4	1.8350000	0.8066598	1.2700000	3.0200000

Laboratory tests at the time of obtaining Table 2-6 consent, according to the presence or absence of proton-pump inhibitors

Unique Subject Identifier=2357011010-0027

MEANS Procedure

Assay variable : PTINR_VAL1 PT-INR Value

N	Mean	Standard Deviation	Minimum	Maximum
3	2.5733333	0.2154840	2.3300000	2.7400000

Unique Subject Identifier=2358000000-0001

Assay variable : PTINR_VAL1 PT-INR Value

N	Mean	Standard Deviation	Minimum	Maximum
6	1.6133333	0.1127239	1.4400000	1.7500000

Unique Subject Identifier=2358000000-0005

Assay variable : PTINR_VAL1 PT-INR Value

N	Mean	Standard Deviation	Minimum	Maximum
3	1.5666667	0.0321455	1.5300000	1.5900000

Unique Subject Identifier=2359000100-0001

Assay variable : PTINR_VAL1 PT-INR Value

N	Mean	Standard Deviation	Minimum	Maximum
1	1.7400000		1.7400000	1.7400000

Laboratory tests at the time of obtaining Table 2-6 consent, according to the presence or absence of proton-pump inhibitors

Unique Subject Identifier=2361011111-0012

MEANS Procedure

Assay variable : PTINR_VAL1 PT-INR Value

N	Mean	Standard Deviation	Minimum	Maximum
1	1.4900000		1.4900000	1.4900000

Unique Subject Identifier=2361011111-0013

Assay variable : PTINR_VAL1 PT-INR Value

N	Mean	Standard Deviation	Minimum	Maximum
1	1.3500000		1.3500000	1.3500000

Unique Subject Identifier=2362011111-0001

Assay variable : PTINR_VAL1 PT-INR Value

N	Mean	Standard Deviation	Minimum	Maximum
3	1.6233333	0.2345918	1.3600000	1.8100000

Unique Subject Identifier=2362011111-0003

Assay variable : PTINR_VAL1 PT-INR Value

N	Mean	Standard Deviation	Minimum	Maximum
3	1.7966667	0.4718404	1.4900000	2.3400000

Laboratory tests at the time of obtaining Table 2-6 consent, according to the presence or absence of proton-pump inhibitors

Unique Subject Identifier=2362011111-0006

MEANS Procedure

Assay variable : PTINR_VAL1 PT-INR Value

N	Mean	Standard Deviation	Minimum	Maximum
3	1.3200000	0.1473092	1.1900000	1.4800000

Unique Subject Identifier=2362011111-0007

Assay variable : PTINR_VAL1 PT-INR Value

N	Mean	Standard Deviation	Minimum	Maximum
4	1.1975000	0.0736546	1.1100000	1.2800000

Unique Subject Identifier=2365011111-0003

Assay variable : PTINR_VAL1 PT-INR Value

N	Mean	Standard Deviation	Minimum	Maximum
6	2.0166667	0.8035339	1.2000000	3.5000000

Unique Subject Identifier=2366011111-0017

Assay variable : PTINR_VAL1 PT-INR Value

N	Mean	Standard Deviation	Minimum	Maximum
1	2.1000000		2.1000000	2.1000000

Laboratory tests at the time of obtaining Table 2-6 consent, according to the presence or absence of proton-pump inhibitors

Unique Subject Identifier=2370011111-0002

MEANS Procedure

Assay variable : PTINR_VAL1 PT-INR Value

N	Mean	Standard Deviation	Minimum	Maximum
5	2.1660000	0.1866280	1.9800000	2.4100000

Unique Subject Identifier=2370011111-0003

Assay variable : PTINR_VAL1 PT-INR Value

N	Mean	Standard Deviation	Minimum	Maximum
5	2.1760000	0.4535747	1.7700000	2.7500000

Unique Subject Identifier=2370011111-0006

Assay variable : PTINR_VAL1 PT-INR Value

N	Mean	Standard Deviation	Minimum	Maximum
6	2.0500000	0.2866357	1.6200000	2.3800000

Unique Subject Identifier=2378000010-0011

Assay variable : PTINR_VAL1 PT-INR Value

N	Mean	Standard Deviation	Minimum	Maximum
6	2.3833333	0.3763863	1.7000000	2.7000000

Laboratory tests at the time of obtaining Table 2-6 consent, according to the presence or absence of proton-pump inhibitors

Unique Subject Identifier=2378000010-0012

MEANS Procedure

Assay variable : PTINR_VAL1 PT-INR Value

N	Mean	Standard Deviation	Minimum	Maximum
6	1.9833333	0.4262237	1.4000000	2.7000000

Unique Subject Identifier=2378000010-0013

Assay variable : PTINR_VAL1 PT-INR Value

N	Mean	Standard Deviation	Minimum	Maximum
6	1.5733333	0.1177568	1.4000000	1.7000000

Unique Subject Identifier=2378000010-0015

Assay variable : PTINR_VAL1 PT-INR Value

N	Mean	Standard Deviation	Minimum	Maximum
5	2.0400000	0.3286335	1.5000000	2.3000000

Unique Subject Identifier=2378000010-0018

Assay variable : PTINR_VAL1 PT-INR Value

N	Mean	Standard Deviation	Minimum	Maximum
6	1.3666667	0.1751190	1.1000000	1.6000000

Laboratory tests at the time of obtaining Table 2-6 consent, according to the presence or absence of proton-pump inhibitors

Unique Subject Identifier=2378000010-0034

MEANS Procedure

Assay variable : PTINR_VAL1 PT-INR Value

N	Mean	Standard Deviation	Minimum	Maximum
6	1.3833333	0.1169045	1.2000000	1.5000000

Unique Subject Identifier=2378000010-0043

Assay variable : PTINR_VAL1 PT-INR Value

N	Mean	Standard Deviation	Minimum	Maximum
5	1.3000000	0.0707107	1.2000000	1.4000000

Unique Subject Identifier=2380011111-0001

Assay variable : PTINR_VAL1 PT-INR Value

N	Mean	Standard Deviation	Minimum	Maximum
2	1.2650000	0.0636396	1.2200000	1.3100000

Unique Subject Identifier=2383011110-0013

Assay variable : PTINR_VAL1 PT-INR Value

N	Mean	Standard Deviation	Minimum	Maximum
4	2.0225000	0.2067809	1.8300000	2.2300000

Laboratory tests at the time of obtaining Table 2-6 consent, according to the presence or absence of proton-pump inhibitors

Unique Subject Identifier=2383011110-0030

MEANS Procedure
[truncated: 324,272 more chars]
