# Supplementary material for: Current status of proton pump inhibitor use in Japanese elderly patients with non-valvular atrial fibrillation: A subanalysis of the ANAFIE Registry
Source: PLoS One. 2020 Nov 5;15(11):e0240859. doi: 10.1371/journal.pone.0240859 (PMC7644054; doi:10.1371/journal.pone.0240859)
Supplement: S2 File — (PDF) [file pone.0240859.s003.pdf]

臨床研究実施計画書番号: LIX-DS-15022

非弁膜症性心房細動を有する後期高齢患者を対象とした前向き観察研究

All Nippon AF In Elderly Registry
－ANAFIE Registry－

（企業主導臨床研究）

臨床研究実施計画書

代 表 世 話 人 : 井上 博
富山県済生会富山病院

研究代表責任医師 : 山下 武志
心臓血管研究所

研 究 依 頼 者 : 福知　良和
第一三共株式会社

2017 年 4 月 10 日作成（Ver 1.21）

目　次

目　次 1
表目次 4
図目次 4
付録目次 4
研究の概要 5
略号及び定義の一覧 8
1　研究の背景 10
2　研究の目的 12
3　研究デザイン 13
4　対象患者 13
5　選択基準 13
6　除外基準 13
7　インフォームド・コンセント 14
8　研究参加の手続き及び研究対象者の登録 15
8.1　研究参加の手続き 15
8.2　契約手続き 15
8.3　研究対象者の登録 15
9　研究方法 16
10　有害事象 17
10.1　有害事象の定義 17
10.1.1　有害事象 17
10.1.2　重篤な有害事象 17
10.2　有害事象の調査項目及び収集手順 17
10.2.1　有害事象の調査項目 17
10.2.2　有害事象発生時の対応手順 19
10.3　重篤な有害事象への対応 19
10.3.1　研究協力者等の対応 19
10.3.2　研究責任医師等の対応 19
10.3.3　研究実施医療機関の長の対応 20
11　観察項目 20
11.1　登録時調査 20
11.2　ベースライン調査 20
11.3　12 ヵ月及び 24 ヵ月調査 22
12　調査スケジュール 26
13　研究対象者の目標登録数 27
14　研究実施期間 27

15　評価項目 27
15.1　主要評価項目 27
15.2　副次評価項目 28
15.3　その他の評価項目 28
16　サブコホート研究の実施（サブ研究 1） 28
16.1　サブコホート研究 A：アセスメントマーカー 28
16.2　サブコホート研究 B：心エコー 29
16.3　サブコホート研究 C：心拍数 30
16.4　サブコホート研究 D：高血圧 30
16.5　サブコホート研究 E：認知機能 30
16.6　サブコホート研究 F：フレイル 30
16.7　サブコホート研究 G：アドヒアランス 31
17　統計解析 31
17.1　解析対象集団 31
17.2　ベースライン調査項目の解析 31
17.3　追跡データの解析 32
17.4　サブグループ解析（サブ研究 2） 32
17.5　サブコホート研究の解析 33
17.5.1　サブコホート研究 A：アセスメントマーカー 33
17.5.2　サブコホート研究 B：心エコー 33
17.5.3　サブコホート研究 C：心拍数 33
17.5.4　サブコホート研究 D：高血圧 33
17.5.5　サブコホート研究 E：認知機能 34
17.5.6　サブコホート研究 F：フレイル 34
17.5.7　サブコホート研究 G：アドヒアランス 34
18　症例報告書の作成と提出 35
18.1　症例報告書の様式と提出 35
18.2　症例報告書の作成方法 35
19　モニタリング及び監査 35
19.1　モニタリング及び監査 35
19.2　モニタリング 35
19.3　監査 36
19.4　原資料の直接閲覧 36
20　倫理 36
20.1　倫理審査委員会 36
20.2　研究対象者に予想される利益 36
20.3　研究対象者に予想される危険や不利益 37

20.4　健康被害に対する補償 37
20.5　個人情報等の保護 37
21　研究の費用に関する事項 38
21.1　研究資金及び利益相反 38
21.2　研究対象者の費用負担 38
22　研究実施計画書の変更・改訂 38
22.1　研究実施計画書の変更 38
22.2　研究実施計画書の改訂 38
23　研究の終了と中断・中止 39
23.1　研究の終了 39
23.2　研究の中断・中止 39
24　研究に係る資料等の保管 39
25　研究対象者等及びその関係者からの相談等への対応 40
26　研究の公表と成果の帰属 40
26.1　臨床研究登録 40
26.2　研究の公表 40
26.3　成果の帰属 40
27　研究組織 41
27.1　代表世話人 41
27.2　研究顧問 41
27.3　研究代表責任医師 41
27.4　研究中央委員会 41
27.5　研究推進・パブリケーション委員会 41
27.6　イベント判定委員 42
27.7　サブ研究責任医師 42
27.8　地区推進担当医師 42
27.9　統計解析責任者 42
27.10 研究責任機関 42
27.11 研究事務局 42
27.12 研究依頼者 43
27.13 データセンター 43
27.14 モニタリング担当機関 43
27.15 監査担当機関 44
28　参考文献 45
付　録 47

表目次

表 1　観察・検査スケジュール 7
表 2　AF の病型 21
表 3　診察室血圧測定法 22
表 4　出血分類の定義 25
表 5　観察・検査スケジュール 26

図目次

図 1　本研究の流れ 13

付録目次

付録 1　$CHADS_2$スコア 47
付録 2　CHA_2DS_2-VASc スコア 47
付録 3　HAS-BLED スコア 48
付録 4　各 DOAC との相互作用が認められた P 糖蛋白阻害剤 49

研究の概要

<table>
<tr><td colspan="2">研究の目的</td><td>本研究は、非弁膜症性心房細動（NVAF）を有する後期高齢患者（75 歳以上）における抗凝固療法の実態及びその予後を明らかにするとともに、脳卒中／全身性塞栓症及び頭蓋内出血のリスク因子を特定し、直接経口抗凝固薬（DOAC）に最適な治療対象集団及びその使用法を明確にすることを主目的とする。また、副次的に NVAF に関連する種々のクリニカルクエスチョンについて検討する。</td></tr>
<tr><td colspan="2">研究デザイン</td><td>多施設共同、前向き登録、観察研究であり、本研究による治療への介入はない。
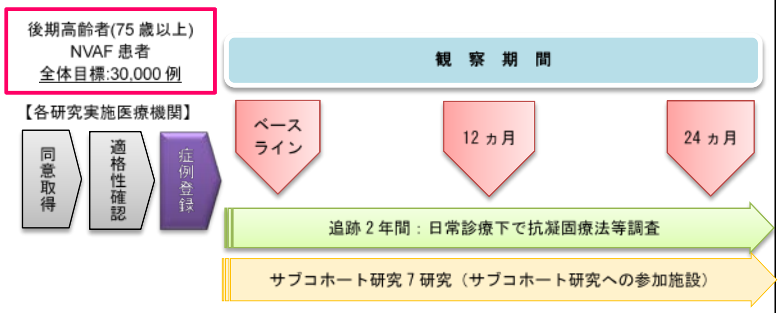
</td></tr>
<tr><td colspan="2">対象患者</td><td>NVAF を有する後期高齢患者（75 歳以上）
（抗凝固薬の投与の有無、抗凝固薬の種類は問わない）</td></tr>
<tr><td rowspan="2">適格基準</td><td>選択基準</td><td>(1) NVAF と確定診断され、通院が可能な患者
(2) 同意取得時の年齢が 75 歳以上の患者（性別不問）
(3) 文書による同意が得られた患者</td></tr>
<tr><td>除外基準</td><td>(1) 現在介入試験に参加している又は参加を予定している患者
(2) 僧帽弁狭窄症と確定診断されている患者
(3) 人工弁（機械弁、生体弁）留置患者
(4) 登録前 1 ヵ月以内に心血管イベント（脳卒中、心筋梗塞、心筋梗塞以外の心インターベンション、入院を要する心不全）又は入院を伴う出血を発現した患者
(5) 何らかの疾患により余命 1 年以内と診断された患者
(6) その他、担当医師により研究参加に不適当と判断された患者</td></tr>
<tr><td colspan="2">研究方法</td><td>前向き観察研究</td></tr>
<tr><td colspan="2">登録方法</td><td>連続登録方式</td></tr>
<tr><td colspan="2">追跡データ収集方法</td><td>主研究のデータは、Electric data capturing system（EDC）にて収集する。サブコホート研究のデータは、電子カルテ等から</td></tr>
</table>

		の出力データ、EDC 又は、紙アンケート調査票等より収集する。
調査・観察及び検査項目		患者背景、薬剤の投与状況、健在確認、抗凝固薬以外の併用薬及び併用療法の状況、NVAF に対する非薬物療法の実施状況、NVAF 治療以外の侵襲的手技（小手術※を含む）の実施状況、血液凝固検査、臨床経過及び臨床検査値、心血管イベントの発症、その他の出血性有害事象の発症、並びに転倒及び骨折の発生、その他の有害事象<br><br>※小手術には生検・穿刺を伴う処置を含む
研究対象者ごとの追跡期間		2 年間
評価項目	主要評価項目	観察期間中における脳卒中／全身性塞栓症の発現状況
	副次評価項目	観察期間中における以下のイベントの発現状況<br>(1) 出血性有害事象（Major Bleeding）<br>(2) 脳卒中<br>(3) 全身性塞栓症<br>(4) 虚血性脳卒中<br>(5) 出血性脳卒中<br>(6) 頭蓋内出血<br>(7) 心血管系イベント（脳卒中、心筋梗塞、心筋梗塞以外の心インターベンション、入院を要する心不全）<br>(8) 心血管系疾患による死亡（心血管系の要因による死亡が否定できない死亡）<br>(9) すべての死亡
	他の評価項目	転倒、骨折
主な解析方法		生存時間解析（COX 比例ハザード回帰分析等）
研究実施予定期間（観察期間・患者登録期間）		研究実施医療機関の倫理審査委員会による承認日～2020 年 9 月<br>（登録期間：研究実施医療機関の倫理審査委員会による承認日～2018 年 9 月、追跡期間 2 年）<br>ただし、研究代表責任医師と研究依頼者の合意により、登録期間、及び予定実施期間の短縮又は延長がなされる場合がある。
参加予定医療機関		約 1,000 施設（研究対象者の登録状況に応じて、適宜追加する）

表 1　観察・検査スケジュール

調査項目は以下のとおりとし、日常診療の範囲内にて実施する。

調査時点 / 調査項目	適格性確認	ベースライン調査（-180～0 日）	12 ヵ月調査（±60 日）	24 ヵ月調査（±60 日）
同意、選択・除外基準の確認	○			
同意撤回・来院状況・健在確認			○	○
患者背景・AF の罹患及び過去の治療歴、手術歴、出血の既往、既往歴（出血以外）・合併症等		○		
抗凝固薬の投与状況、服薬状況		○	○	○
抗凝固薬以外の薬剤の投与状況、服薬状況		○	○	○
AF に対する非薬物療法の実施状況		○	○	○
AF 治療以外の侵襲的手技（小手術[1]を含む）の実施状況			○	○
血液凝固検査（PT-INR）		○	○	○
臨床経過・臨床検査値		○	○	○
心血管系イベント、消化管出血、出血性有害事象、転倒、骨折、死亡、その他有害事象の有無			○	○
**サブコホート研究**				
A. アセスメントマーカー		○（中央測定）		
B. 心エコー		○	○	○
C. 心拍数		○	○	○
D. 高血圧		○		
E. 認知機能		○		○
F. フレイル		○		
G. アドヒアランス		○		○

AF：心房細動、PT-INR：プロトロンビン-国際標準比

1 小手術には生検・穿刺を伴う処置を含む

略号及び定義の一覧

略号の一覧

略号	省略していない表現	
	英語	日本語
A		左心室流入血流心房収縮期最大流速
AF	Atrial fibrillation	心房細動
AHA	American Heart Association	アメリカ心臓協会
APTT	Activated partial thromboplastin time	活性化部分トロンボプラスチン時間
Ccr	Creatinine clearance	クレアチニンクリアランス
$CHADS_2$	-	$CHADS_2$スコア
CHA_2DS_2-VASc	-	CHA_2DS_2-VASc スコア
COPD	Chronic obstructive pulmonary disease	慢性閉塞性肺疾患
CYP2C9	Cytochrome P450 2C9	シトクロム P450 2C9
DOAC	Direct oral anticoagulant	直接経口抗凝固薬
E		左心室流入血流拡張早期最大流速
eCRF	Electronic case report form	電子症例報告書
EDC	Electronic data capturing	電子的データ収集
F1+2	Prothrombin fragment 1+2	プロトロンビンフラグメント 1+2
HbA1c	Hemoglobin A1c	ヘモグロビン A1c
IVCD	Inferior vena cava diameter	下大静脈径
IVSTD	Interventricular septum thickness	心室中隔壁厚
LA	Left atrium	左房
LAD	Left atrial dimension	左房径
LV	Left ventricle	左心室
LVDd	Left ventricular end-diastolic dimension	左室拡張末期径
LVDs	Left ventricular end-systolic dimension	左室収縮末期径
LVEDV	Left ventricular end-diastolic volume	左室拡張末期容積
LVEF	Left ventricular ejection fraction	左室駆出率
LVESV	Left ventricular end-systolic volume	左室収縮末期容積
MMSE	Mini-Mental State Examination	ミニメンタルステート検査
MMAS-8	Morisky Medication Adherence Scale	モリスキー服薬アドヒアランス尺度
NT-proBNP	N-terminal pro B-type natriuretic peptide	脳性ナトリウム利尿ペプチド前駆体 N 端フラグメント
NVAF	Non-valvular atrial fibrillation	非弁膜症性心房細動
PASP	Pulmonary artery systolic pressure	肺動脈収縮期圧
PDC	Proportion of days covered	処方日数の割合
P-gp	P-glycoprotein	P-糖タンパク
PT	Prothrombin time	プロトロンビン時間
PT-INR	Prothrombin time-international normalized ratio	プロトロンビン時間 - 国際標準比
RV	Right ventricle	右心室
SFMC	Soluble fibrinmonomer complex	可溶性フィブリンモノマー複合体
SRI	Severe renal impairment	高度腎機能障害
SV	Stroke volume	一回拍出量
TAPSE	Tricuspid annual plane systolic excursion	三尖弁輪部収縮期移動距離
TAT	Thrombin-antithrombin complex	トロンビン・アンチトロンビン複合体
TDI	Tissue doppler imaging	組織ドプラ法
TDIa’	-	僧帽弁輪心房収縮期速度
TDIe’	-	僧帽弁輪拡張早期速度
TIA	Transient ischemic attacks	一過性脳虚血発作

略号	省略していない表現	
	英語	日本語
TRPG	Tricuspid regurgitation pressure gradient	三尖弁収縮期圧較差
TTR	Time in therapeutic range	国際標準化比至適範囲内時間
TVS	Tricuspid valve systolic speed	三尖弁輪収縮期運動速度
$t_{1/2}$	-	消失半減期
UMIN	University hospital medical information network	大学病院医療情報ネットワーク
VKORC1	Vitamin K epoxide reductase complex, subunit 1	ビタミン K エポキシド還元酵素

用語の定義の一覧

用語	定義
予定実施期間	研究対象者の登録が開始され、すべての研究対象者が 2 年間の観察を完了するまでの期間
症例登録期間	研究対象者の登録後、ベースライン調査が完了するまで
研究代表責任医師	「人を対象とする医学系研究に関する倫理指針ガイダンス（平成 27 年 3 月 31 日一部改訂）」における、共同研究機関における研究を統括する研究代表者にあたる者
研究責任医師	本研究に参加する研究実施医療機関の責任医師
研究責任医師等	本研究に参加する研究実施医療機関の責任医師及び担当医師
研究協力者	研究責任医師から指名された臨床研究コーディネーター（看護師、薬剤師、等）
患者登録識別番号	研究実施医療機関の患者カルテ番号から置き換えられた本研究特有の研究対象者を識別する番号
研究業務受託機関	当該研究の準備又は管理に係る業務の全部又は一部を受託する企業

※ 本研究計画書に定めのない用語については、「人を対象とする医学系研究に関する倫理指針」に準ずるものとする。

1　研究の背景

心房細動（AF）の有病率は加齢とともに増加することが知られている。米国の ATRIA study[1]における年齢別 AF 有病率は 75 歳未満で男性 5.0%以下、女性 3.4%以下であったのに対し、75～80 歳で男性 7.3%、女性 5.0%、80 歳以上で男性 10.6%、女性 8.0%と特に 75 歳以上で高かった。また、本邦においては、70 歳代で男性 3.4%、女性 1.1%、80 歳以上で男性 4.4%、女性 2.2%と推定されている[2]。高齢化と共に NVAF 患者数も増加しており、NVAF 患者の脳卒中発症率が年間約 6%と洞調律を示した患者と比べて約 5 倍も高いことが報告されている[3]。

NVAF が主要な危険因子である心原性脳塞栓症は、脳卒中の中でも重症化しやすく予後の悪い病型である。そのため、AF 患者の日常診療では、抗凝固療法により塞栓症を予防することが重要となる。一方で、AF 患者が高年齢であること自体が塞栓症リスクであり、75 歳以上では心原性脳塞栓症の発現リスクが高いことが報告されているが[4]、同時に出血性有害事象の発現リスクも高いとの懸念もあり、抗凝固療法を受けている患者は半数程度である[5]。

1962 年の国内販売開始以降、ワルファリンは抗凝固療法として長年使用されている[6]。AF 患者を対象とした国内大規模試験である J-RHYTHM Registry では、血栓塞栓症のリスクが低い被験者の多くがワルファリンを使用しており、脳梗塞の発症率も低かった[7]。一方で、ワルファリン服用下では頭蓋内出血や消化管出血等の出血性合併症が最も懸念されている。また、ワルファリンには薬剤相互作用による併用注意薬が非常に多いことや[8]、ビタミン K 依存性凝固因子の生合成を阻害する作用機序によりビタミン K 含有食品を摂取するとワルファリンの抗凝固作用が減弱されるため、ビタミン K 含有食品の食事制限が求められる、といった患者の QOL 低下につながる要因も多い[9]。

ワルファリンの標的分子はビタミン K 依存性凝固因子の生成に関与するビタミン K エポキシド還元酵素（VKORC1）であり、薬理作用を示す S 体ワルファリンの主な代謝酵素はチトクローム P450 2C9（CYP2C9）であるが、これらの酵素には遺伝子多型がある[10]。つまり、ワルファリンの薬効発現及び代謝能には個体差が大きく、また、薬物や食物との相互作用により個人内でも変動する。そのため、安全性に配慮した上で十分な有効性を得るには、プロトロンビン時間－国際標準比（PT-INR）を定期的にモニタリングして、投与量の決定や調整を行う必要がある[8,11]。

こうしたワルファリン投与における問題点を解消すべく、2010 年代に、直接経口抗凝固薬（DOAC）として、直接トロンビン阻害剤のダビガトラン[12]、第 Xa 因子阻害剤のリバーロキサバン[13]、アピキサバン[14]及びエドキサバン[15]が臨床の現場で使用可能となった。ワルファリンと比較したこれらの DOAC のメリットは、効果判定のための定期的な検査や患者ごとの投与量の調整が不要であり、かつ一定の抗凝固効果が得られること、直接凝固因子に作用すること、頭蓋内出血の発現率が低いこと、薬剤がビタミン K の代

謝に直接関係しないため食事の影響がほとんどないこと、他の薬剤との相互作用が少ないこと、半減期が短いこと等である[16]。特に、これらの薬剤を単回投与した際の半減期は、ワルファリンの 55～133 時間[8]に対し、第 Xa 因子阻害剤では 10～14 時間[16,17]と短いため、薬物動態のコントロールが容易になった。しかし、これらの DOAC は、使用経験が浅く、また、高度腎機能低下患者［クレアチニンクリアランス（Ccr）15 mL/分未満］では投与できないこと、半減期が短いため服用忘れによる効果低下が早いこと、重大な出血性有害事象が発現した際の対策が十分確立していないこと、患者の費用負担増加の可能性があることといったデメリットもあり[18]、より適切に使いこなすには使用経験に基づくデータをより多く蓄積する必要がある。最新の「心房細動治療（薬物）ガイドライン（2013 年改訂版）」[18]においても、限られた使用経験により推奨度を決めることが難しく、臨床開発治験として行われた大規模試験の結果や、2013 年 12 月の時点までに得られた情報に基づいた推奨度として定められているのが実情であり、各薬剤の使用調査情報の早急な収集が望まれている。

NVAF では脳梗塞発症のリスクが集積すると脳梗塞の発症率が上昇するため、そのリスク評価として $CHADS_2$ スコア及び CHA_2DS_2-VASc スコアが提唱されており、いずれも NVAF に対する抗凝固療法の方針を決定する際に用いられる。$CHADS_2$ スコアは、Congestive heart failure（心不全）、Hypertension（高血圧）、Age ≥ 75（年齢 75 歳以上）、Diabetics（糖尿病）、Stroke/TIA［脳梗塞や一過性脳虚血発作（TIA）の既往］の頭文字をとって命名されたスコアであり、前 4 項目を各 1 点、脳梗塞や TIA の既往を 2 点とし、各項目の合計スコア（0～6 点）によりリスクを評価する。CHA_2DS_2-VASc スコアは、Congestive heart failure（心不全）、Hypertension（高血圧）、Age ≥ 75（年齢 75 歳以上）、Diabetics（糖尿病）、Stroke/TIA（脳梗塞、TIA、血栓塞栓症の既往）、Vascular Disease（血管疾患）、Age 65 – 74（年齢 65～74 歳）、Sex category［性別（女性）］の頭文字をとって命名されたスコアであり，年齢 75 歳以上、脳梗塞、TIA 及び血栓塞栓症の既往を各 2 点、その他の項目を各 1 点とし、各項目の合計スコア（0～9 点）によりリスクを評価する。これらのスコア評価で 1 点以上、又は 0 点であってもその他のリスク（心筋症、年齢 65 歳以上 74 歳以下、血管疾患）がある場合は、DOAC が推奨又は考慮可とされている[18]。

しかし、抗凝固療法を進める上では、出血性リスクも併せて評価し、出血性イベントを予防することが重要となる。各種出血危険因子から出血を予測するスコアとして、比較的簡便かつ臨床的に重大な出血事象を評価できる HAS-BLED スコアが提唱された[19]。HAS-BLED スコアは 2010 年の欧州心臓病学会でガイドラインに採用され、本邦でも取り入れられた。HAS-BLED のリスク因子は、高血圧、腎機能障害、肝機能障害、脳卒中、出血の既往／出血傾向、不安定な INR、高齢者（65 歳以上）、薬剤（抗血小板薬や NSAIDs）又はアルコール依存の項目に該当する場合は各 1 点とし、合計スコアにより出血の低リスク（0 点）、中等度リスク（1～2 点）、高リスク（3 点以上）として評価する

[18]。ただし、HAS-BLED スコアは、PT-INR のコントロール不良等ワルファリン療法を想定しており、DOAC に応用した場合の検証は充分に行われていないのが現状である。一方で、抗凝固療法中の重大な出血関連因子として、75 歳以上の高齢、50 kg 以下の低体重、腎機能障害及び抗血小板薬の併用が、頭蓋内出血因子として、年齢、脳梗塞及び TIA の既往、アスピリンの服用、ワルファリン投与ならびに非白人であることが指摘されている [20]。NVAF の抗凝固療法では、これらのリスク因子も同様に注意深く観察しなくてはならない。

特に高齢者においては、疾患の現れ方や治療に対する反応も若年者とは異なること、加齢による複数の疾患の合併、それに伴う多剤併用、生活機能の変化、生活環境の多様性等考慮すべき点が多い [21]。「高齢者に使用される医薬品の臨床評価法に関するガイドライン」[22]（薬新薬第 104 号 平成 5 年 12 月 2 日）では、高齢者の老化に伴う腎機能等の生理機能の低下や多臓器疾患を伴う場合が多いこと、薬物併用に基づく相互作用発現の可能性が増すこと等から、高齢者の薬物療法に対してより適切な情報を提供する必要があると提言されている。また、2015 年に刊行された日本老年医学会「高齢者の安全な薬物療法ガイドライン 2015」も、高齢者では薬物有害事象の発現頻度が高く、重症例が多いことを背景として、高齢者に対する薬物療法の安全性を高める目的で作成されている [23]。これらのガイドライン制定からも、高齢者、特に 75 歳以上の後期高齢者が増加している現代の日本社会において、安全で有効な高齢者医療の需要が高まっていることは明らかである。

このような背景から、本研究では NVAF を有する後期高齢患者をターゲットとし、治療における問題点や最適な抗凝固療法の明確化に繋がる情報が得られるように計画した。また、実際に NVAF の治療を行っている臨床医からのクリニカルクエスチョンに着目し、アセスメントマーカー、心拍コントロール、心不全、認知機能、血圧管理、フレイル、アドヒアランス等を多面的に評価することとした。これらの検討及び評価によって、NVAF 患者のトータルケアに資する情報の創出に繋がることが期待される。

2　研究の目的

本研究は、非弁膜症性心房細動（NVAF）を有する後期高齢患者（75 歳以上）における抗凝固療法の実態及びその予後を明らかにするとともに、脳卒中／全身性塞栓症及び頭蓋内出血のリスク因子を特定し、DOAC に最適な治療対象集団及びその使用法を明確にすることを主目的とする。また、副次的に NVAF に関連する種々のクリニカルクエスチョンについて検討する。

3　研究デザイン

多施設共同、前向き登録、観察研究であり、本研究による治療への介入はない。

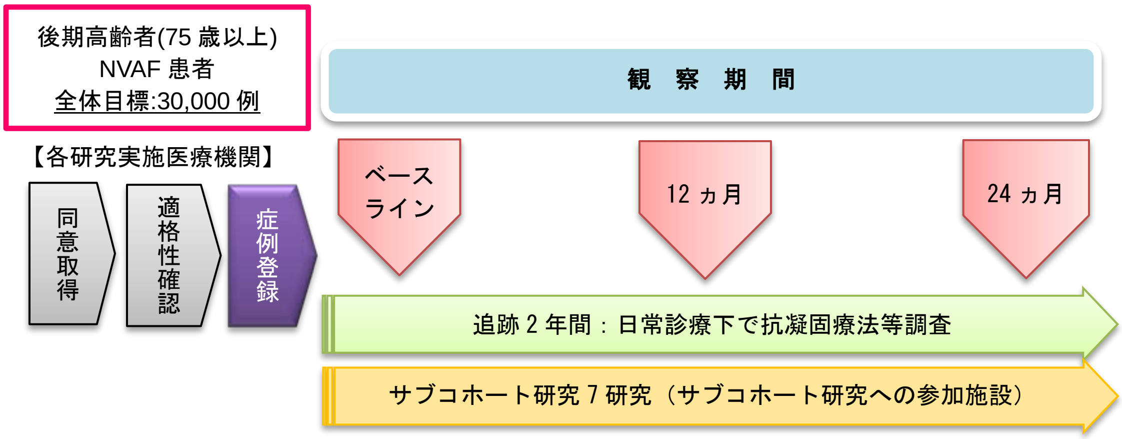

図 1　　本研究の流れ

4　対象患者

NVAF を有する後期高齢患者（75 歳以上）
（抗凝固薬の投与の有無、抗凝固薬の種類は問わない）

5　選択基準

(1) NVAF と確定診断され、通院が可能な患者。なお、新たに NVAF と診断する場合は、心電図、ホルター心電図、携帯型心電図、心臓超音波等を実施し、確定診断する。
(2) 同意取得時の年齢が 75 歳以上の患者（性別不問）
(3) 文書による同意が得られた患者

《設定根拠》

(1) 2 年間という観察期間を考慮し、登録時点で通院が可能である患者とした。
(2) 「2. 研究の背景」に記載のとおり、本研究は NVAF を有する後期高齢患者に対する適切な治療法の検討を目的として実施することとしているため、登録時点で 75 歳以上である患者を研究対象者とした。
(3) 「人を対象とする医学系研究に関する倫理指針」[24] に遵守して設定した。

6　除外基準

(1) 現在介入試験に参加している、又は参加を予定している患者
(2) 僧帽弁狭窄症と確定診断されている患者
(3) 人工弁（機械弁、生体弁）留置患者
(4) 登録前 1 ヵ月以内に心血管系イベント（脳卒中、心筋梗塞、心筋梗塞以外の心イン

ターベンション、入院を要する心不全)、又は入院を伴う出血を発現した患者

(5) 何らかの疾患により余命 1 年以内と診断された患者

(6) その他、担当医師により研究参加に不適当と判断された患者

《設定根拠》

(1) 一般診療下で行われる治療に関する観察研究のため設定した。

(2), (3) 観察対象疾患を NVAF としたため設定した。

(4), (5) 主要評価に与える影響を考慮し、観察期間を完了できる研究対象者を確保するため設定した。

7 インフォームド・コンセント

研究責任医師は、本研究開始前に本研究への参加に関する同意説明文書及び同意書について、倫理審査委員会の承認及び研究機関の長の許可を得る。また、同意説明文書及び同意書の改訂を行う場合、改訂した同意説明文書及び同意書について、同様に倫理審査委員会の承認及び研究機関の長の許可を得る。なお、本研究の対象者は後期高齢者であり、認知機能の低下などの理由により、研究対象者本人より同意を得ることが困難な場合は、代諾者からの同意取得も可能とする。ただし、代諾者は、被験者の親権を行う者、配偶者、後見人その他これに準じる者に限る。

《説明事項》

インフォームド・コンセントを受ける際に研究対象者または代諾者等に対し説明すべき事項は、以下のとおりとする。

(1) 本臨床研究の名称及び当該研究の実施について研究実施医療機関の長の許可を受けている旨

(2) 研究実施医療機関の名称及び研究責任医師の氏名（本臨床研究に共同参加している研究実施医療機関の名称及び研究責任医師の氏名を含む）

(3) 本臨床研究の目的及び意義

(4) 本臨床研究の方法（研究対象者から取得された試料・情報の利用目的を含む）及び期間

(5) 研究対象者として選定された理由

(6) 研究対象者に生じる負担並びに予測されるリスク及び利益

(7) 本臨床研究が実施又は継続されることに同意した場合であっても、随時これを撤回できる旨（研究対象者等からの撤回の内容に従った措置を講じることが困難となる場合があるときは、その旨及びその理由）

(8) 本臨床研究が実施又は継続されることに同意しないこと又は同意を撤回することによって、研究対象者等が不利益な取扱いを受けない旨

(9) 本臨床研究に関する情報公開の方法

(10) 研究対象者等の求めに応じて、他の研究対象者等の個人情報等の保護及び当該研究の

独創性の確保に支障がない範囲内で本臨床研究実施計画書及び研究の方法に関する資料を入手又は閲覧できる旨、並びにその入手又は閲覧の方法

(11) 個人情報等の取扱い（匿名化する場合にはその方法も含む）

(12) 試料・情報の保管及び廃棄の方法

(13) 本臨床研究の資金源等、研究機関の研究に係る利益相反及び個人の収益等、研究者等の研究に係る利益相反に関する状況

(14) 研究対象者等及びその関係者からの相談等への対応

(15) 研究対象者等に経済的負担又は謝礼がある場合には、その旨及びその内容

(16) 研究対象者から取得された試料・情報について、研究対象者等から同意を受ける時点では特定されない将来の研究のために用いられる可能性又は他の研究機関に提供する可能性がある場合には、その旨と同意を受ける時点において想定される内容

8 研究参加の手続き及び研究対象者の登録

8.1 研究参加の手続き

各研究実施医療機関の研究責任医師は、本研究への参加に際し、「臨床研究実施計画書の合意書」に所属、実施医療機関名等の必要事項を記載し、研究事務局へ提出する。その際、各サブコホート研究（16. サブコホート研究の実施）への参加の有無についても併せて明記する（サブコホート研究への参加は任意とする)。

本研究への参加決定後、臨床研究実施計画書、参加するサブコホート研究の臨床研究実施計画書（Appendix 版）及びサブコホート研究の内容を含めた同意説明文書及び同意書について、倫理審査委員会へ審査を依頼し承認を得る。

8.2 契約手続き

各研究実施医療機関にて倫理審査委員会による承認後、研究責任機関または研究実施業務を委託する CRO と各研究実施医療機関との間で本研究の実施に関する契約を締結する。

8.3 研究対象者の登録

研究対象者の登録は、本研究の EDC システムにて行う。研究責任医師等は本研究の対象となりうる NVAF 患者から文書による同意を取得し、速やかに登録システムへアクセスし、以下の情報をすべて入力する。

・性別
・生年月日または同意取得時年齢
・同意取得日

登録された情報は、本研究の適格基準に適合された場合に限り、患者登録識別番号が同システムから即時に発行される（詳細は「EDC システム手順書」に従う)。

9　研究方法

(1) EDC システムへのアカウント申請

研究責任医師は、研究担当医師及び研究協力者（臨床研究コーディネーター等）を指名し、契約締結後に本研究の EDC システムのアカウントを申請する。アカウント及びパスワードの発行後、研究担当医師及び研究協力者は、EDC システムにログインの上、ユーザー名を設定する。研究責任医師等及び研究協力者は、可能な限り、研究開始前に予め操作方法等のトレーニングを受ける。

(2) 患者等からの文書による同意取得

研究責任医師等は、本研究の実施に際し、適格基準に合致する患者に対して、本研究及び研究実施医療機関が参加する本研究のサブコホート研究に応じた同意説明文書を用いて患者に説明し、文書による同意を取得する。患者本人に判断能力がない場合には、代諾者による同意も可とする（詳細は、「7. インフォームド・コンセント」参照）。

(3) 研究対象者の登録

本研究の対象となる患者から文書による同意を取得後、症例登録システムにより速やかに症例登録を行う。同時に、研究責任医師等は、患者データを連結可能匿名化のため、患者カルテ番号と患者登録識別番号の対応表「患者登録識別番号管理表」を作成し、個人を特定できない状態とする。また、研究実施医療機関内で施錠可能な場所へ厳重に保管する。

(4) 観察項目の調査

本研究では、EDC システム上に、以下の観察項目の調査情報を入力するフォームを作成する。

- ベースライン調査 : 登録時（以下、ベースライン時）の研究対象者の背景に関するデータ（同意取得日から 180 日以内の直近のデータ）
- 12 ヵ月調査 : 同意取得日から 12 ヵ月後の直近のデータ（ベースライン時から 12 ヵ月経過後の直近のデータが望ましい（許容範囲 : 同意取得日の 1 年後の同日±60 日））
- 24 ヵ月調査 : 同意取得日から 24 ヵ月後の直近のデータ（ベースライン時から 24 ヵ月経過後の直近のデータが望ましい（許容範囲 : 同意取得日の 2 年後の同日±60 日））

研究責任医師等及び研究協力者は、登録したすべての研究対象者について、上記の各時点における診療情報を毎回 EDC システムへデータを入力し、研究責任医師等が電子署名を行った上で、eCRF を提出（送信）する。サブコホート研究における電子カルテ等からの出力データ、患者アンケート等、書面にて入手したデータについては研究事務局がデータをデータベースに登録する。

(5) 健在確認（イベント・有害事象発現の有無の確認を含む）

研究責任医師等は、来院しない研究対象者に対し、可能な限り登録後 12 ヵ月及び 24

ヵ月を経過した時点で、健在及びイベント・有害事象発現等を確認する。来院のない研究対象者については電話･手紙等の連絡手段を用い可能な限りイベント及び有害事象の情報を調査する。また、他科・他医療機関への転院の場合も、可能な限り転院先の治療を担当した医師よりイベント及び有害事象、その他必要情報を聴取し、EDC システムへ入力する。

(6) クエリー対応

研究責任医師等及び研究協力者は、データセンターから EDC システム入力内容の確認、追加、修正を要するクエリーが発行された場合、EDC システムのデータを確認後、適宜追加、修正し、研究責任医師等が電子署名した上で eCRF を提出（送信）する。サブコホート研究のデータについては、各サブコホート研究 Appendix に記載の方法にてデータを取り扱う。

10 有害事象

10.1 有害事象の定義

10.1.1 有害事象

有害事象とは、同意取得日から観察期間中に発生する、あらゆる好ましくないあるいは意図しない徴候（臨床検査値の異常を含む）、症状又は病気のことであり、薬剤等との因果関係は問わない。有害事象には手術侵襲に関連するものも含まれる。

10.1.2 重篤な有害事象

重篤な有害事象とは、有害事象のうち以下のものをいう。

(1) 死亡
(2) 死亡につながるおそれのあるもの
(3) 治療のために病院又は診療所への入院又は入院期間の延長が必要となるもの
(4) 障害
(5) 障害につながるおそれのあるもの
(6) その他、1～5 に準じて重篤であるもの
(7) 後世代における先天性の疾病又は異常

10.2 有害事象の調査項目及び収集手順

10.2.1 有害事象の調査項目

研究責任医師等は有害事象の発生を確認した場合、以下の項目を調査する。

(1) 有害事象名
(2) 発現日
(3) 重篤性
- 重篤又は非重篤

・　重篤区分

重篤な有害事象は「10.1.2. 重篤な有害事象」に定義した。

(4) 転帰、転帰確認日

各有害事象の転帰及び転帰確認日を収集する。

・　転帰

転帰は、「回復」、「軽快」、「回復したが後遺症あり（及び後遺症名）」、「未回復」、「当該事象による死亡」、又は「不明（及びその理由）」のいずれかとする

・　転帰確認日

転帰が「回復」の場合は消失日、「軽快」、「回復したが後遺症あり」、「未回復」又は「不明」の場合は転帰確認日、「当該事象により死亡」の場合は死亡日とする

(5) 因果関係のある薬剤の有無

・　因果関係のある薬剤名

薬剤との因果関係の判断は、以下の基準を参考とする。

【因果関係あり】

有害事象発現と当該薬剤の投与に合理的な時間相関があり、研究対象者の状態や当該薬剤以外の要因（原疾患、合併症、併用薬等）によると判断することが妥当ではなく、当該薬剤との関連性を否定できない場合

【因果関係なし】

有害事象 発現と当該薬剤投与に合理的な時間相関がない場合、または有害事象発現が研究対象者の状態や当該薬剤以外の要因（原疾患、合併症、併用薬等）によると 判断することが妥当であり、当該薬剤の関連性を否定できる場合

当該有害事象発生前 1 週間以内にリクシアナの投与がある場合、又は因果関係のある薬剤が第一三共株式会社が製造販売承認を有する製品又は他社提携販売品の場合、以下の項目についても調査する。

(6) 処置

・　抗凝固薬の投与（「継続（減量なし）」、「減量」、「中止・休薬」、又は「投与終了後に発現」）

・　その他の処置の有無

　　ありの場合は、薬物治療等の詳細を記載する

(7) その他考えられる要因

・　その他考えられる要因の有無

　　ありの場合、その詳細を記載する

(8) 因果関係がある薬剤の投与状況の詳細（因果関係がある薬剤が「あり」の場合）

・　薬剤名

・　1 日投与量

・　投与期間

また、有害事象発生前の 1 週間以内にリクシアナの投与がある場合又は因果関係のある薬剤が第一三共株式会社の製品の場合で、かつ重篤な有害事象の場合、上記に加えて以下の項目について調査を行う。

- 有害事象発現時に使用されていた併用薬の詳細：薬剤名、1 日投与量、投与期間
- 有害事象発現時の関連する臨床検査値：検査項目名、検査日、検査値
- 臨床経過

10.2.2 有害事象発生時の対応手順

研究責任医師等は有害事象が発生した場合、研究対象者等への説明及び必要な措置を講じる。また、研究責任医師を含む研究協力者等は、有害事象に対して必要な項目（「10.2.1. 有害事象の調査項目」参照）を調査した上で、当該調査結果及び判定結果を速やかに EDC へ入力する。なお、薬剤との因果関係は研究責任医師等が判定する。

10.3 重篤な有害事象への対応

本研究の中で、軽微な侵襲を伴う検査を実施するのはサブコホート研究 A のみであるため、本項で記載する重篤な有害事象への対応はサブコホート研究 A に対してのみ適用する。

10.3.1 研究協力者等の対応

研究協力者等は、本臨床研究期間中にサブコホート研究 A で行う採血に由来する重篤な有害事象の発生を知った場合、研究実施医療機関の長が作成する手順書に従い、研究対象者等への説明及び必要な措置を講じる。研究協力者等は、その事象について速やかに研究責任医師に報告する。

10.3.2 研究責任医師等の対応

研究責任医師等は、本臨床研究期間中にサブコホート研究 A で行う採血に由来する重篤な有害事象の発生を知った場合は、速やかにその旨を研究実施医療機関の長に報告すると共に、研究実施医療機関の長が作成する手順書に従い、適切な措置を講じる。

研究責任医師等は、速やかに当該有害事象を研究事務局に報告（又は EDC システムへ入力）する。その後、研究責任医師等から報告を受けた研究事務局は、速やかに本サブコホート研究に携わる研究協力者等及び共同参加しているその他の研究実施医療機関の研究責任医師等に、当該有害事象の発生に係る情報を共有する。

（研究依頼者への協力事項）
研究責任医師等は、本臨床研究期間中に医薬品に関連する副作用を認めたときは、当該医薬品を製造販売する製薬企業にその事象を報告し、市販後安全対策に協力する。第一

三共株式会社が販売する製品の「医薬品、医療機器等の品質、有効性及び安全性の確保等に関する法律」で報告が義務づけられている副作用については、第一三共株式会社より独立行政法人医薬品医療機器総合機構に報告を行なう。報告した研究対象者は、独立行政法人医薬品医療機器総合機構のホームページ（http://www.pmda.go.jp/）内に「副作用が疑われる症例報告に関する情報」として掲載される。なお、その場合には報告書の出所や研究対象者のプライバシーに関連する部分は非公開の扱いとされる。

10.3.3　研究実施医療機関の長の対応

サブコホート研究 A に参加する研究実施医療機関の長は、予め、サブコホート研究 A で行う採血に由来する重篤な有害事象が発生した際に研究責任医師等が実施すべき事項に関する手順書を作成し、当該手順書に従って適正かつ円滑に対応が行われるよう必要な措置を講じる。研究責任医師等からサブコホート研究 A で行う採血に由来する重篤な有害事象の発生について報告がなされた場合は、手順書に従って速やかに必要な対応を行うとともに、当該有害事象について倫理審査委員会の意見を聴き、必要な措置を講じる。

11 観察項目

研究責任医師等及び研究協力者は、登録したすべての研究対象者について、「12. 調査スケジュール」に従って、以下の項目を調査し、EDC システムの症例調査フォームへ入力し、研究責任医師等が電子署名を行った上で eCRF を提出（送信）する。

11.1 登録時調査

(1) 患者識別情報

- 患者登録識別番号（患者カルテ番号を除く）
- 性別
- 生年月日、もしくは同意取得時点の年齢
- 同意取得日

11.2 ベースライン調査（同意取得日から 180 日以内の直近のデータ）

(1) 患者背景

- 身体所見
  身長、体重
- AF の治療歴
  AF の病型（表 2)、AF に対する非薬物療法の治療歴

表 2　AF の病型

病型 [1]	基準
発作性 AF	発症後 7 日以内に洞調律に復したもの
持続性 AF	発症後 7 日を超えて AF が持続しているもの
長期持続性 AF	持続性 AF のうち発症後 1 年以上、AF が持続しているもの
永続性 AF	電気的あるいは薬理学的に除細動不能のもの

1 この分類は、薬物療法、非薬物療法の有無にかかわらず適応し、持続時間が広範にわたる場合には、その患者が示す代表的な AF 持続時間で代用する。
参照元：心房細動治療（薬物）ガイドライン、p.14[16]

- AF 治療以外の治療歴・既往歴
  心房細動以外の大手術歴、大出血の既往、薬物アレルギー歴
- 合併症・既往歴
  高血圧症、糖尿病（糖尿病性合併症含む）、脂質代謝異常症、高尿酸血症、腎臓病、高度肝機能障害、呼吸器疾患、心疾患、脳血管障害、その他血管疾患、血栓・塞栓関連疾患、甲状腺機能亢進症、消化器疾患、悪性腫瘍（担がん状態）、認知症、脳画像検査実施の有無、1 年以内の転倒の有無
- 生活習慣
  喫煙習慣、飲酒習慣

(2) 登録時の抗凝固薬の投与状況
- 抗凝固薬の投与の有無
- 抗凝固薬の種類
- 投与開始時期
- 登録時点の 1 日投与量及び 1 日投与回数（DOAC のみ）

(3) 登録時の抗凝固薬以外の併用薬
- 以下の併用薬使用の有無を調査する。
  不整脈薬、抗血小板薬、降圧薬、脂質治療薬、糖尿病薬、認知症治療薬、抗がん剤、慢性閉塞性肺疾患（COPD）治療薬、向精神薬、プロトンポンプ阻害薬（PPI）、P-糖タンパク（p-gp）阻害薬（付録 4）
- 登録時点の内服薬剤種類数（抗凝固薬、抗血小板薬を含むすべての薬剤。ただし、外用薬、市販薬は除く）

(4) 血液凝固検査（ワルファリン服用患者のみ）
日常診療において以下の検査項目が測定された場合、eCRF に入力する。
- PT-INR 値、および測定日
  （登録前半年間に測定された値を最大 6 点記載する [1 ヶ月間隔を目安]）

(5) 臨床経過・臨床検査値
日常診療において以下の検査項目が測定された場合、eCRF に入力する。
- 診察室血圧
  収縮期及び拡張期血圧を測定する（測定方法は表 3 を参照）
- ヘモグロビン

・　血清クレアチニン

・　HbA1c

表 3　　診察室血圧測定法

**1. 装置**	a. 精度検定された水銀血圧計、アネロイド血圧計による聴診法が用いられる。精度検定された電子血圧計も使用可 [1]<br>b. カフ内ゴム嚢の幅 13 cm、長さ 22〜24 cm のカフを用いる<br>［小児上腕周 27 cm 未満では小児用カフ、太い腕（腕周 34 cm 以上）で成人用大型カフを使用］
**2. 測定時の条件**	a. 静かで適当な室温の環境<br>b. 背もたれつきの椅子に足を組まずに座って数分の安静後<br>c. 会話をかわさない<br>d. 測定前に喫煙、飲酒、カフェインの摂取を行わない
**3. 測定法**	a. カフ位置は、心臓の高さに維持<br>b. 急速にカフを加圧する<br>c. カフ排気速度は、2〜3 mmHg/拍あるいは秒<br>d. 聴診法ではコロトコフ第 I 相の開始を収縮期血圧、第 V 相を拡張期血圧とする
**4. 測定回数**	1〜2 分の間隔をあけて少なくとも 2 回測定。この 2 回の測定値が大きく異なっている場合には、追加測定を行う
**5. 判定**	a. 安定した値 [2] を示した 2 回の平均値を血圧値とする<br>b. 高血圧の診断は少なくとも 2 回以上の異なる機会における血圧値に基づいて行う
**6. その他の注意**	a. 初診時には、上腕の血圧左右差を確認<br>b. 厚手のシャツ、上着の上からカフを巻いてはいけない。厚地のシャツをたくしあげて上腕を圧迫してはいけない<br>c. 糖尿病、高齢者等起立性低血圧の認められる病態では、立位 1 分及び 3 分の血圧測定を行い、起立性低血圧の有無を確認<br>d. 聴診者は十分な聴力を有する者で、かつ測定のための十分な指導を受けた者でなくてはならない<br>e. 脈拍数も必ず測定し記録

1. 最近では水銀の環境への影響、水銀柱の精度管理、アネロイド血圧計の精度の問題等から、電子血圧計の使用が勧められている。水銀計の代わりに電子式のアナログ柱を用いたハイブリッド血圧計も入手可能である。自動巻き付け式血圧計を待合室等で使用する場合、十分な指導と管理のもとで測定されなければ大きな誤差が生じる
2. 安定した値とは、目安として測定値の差がおよそ 5 mmHg 未満の近似した値をいう

参照元：高血圧治療ガイドライン 2014、p.16

11.3 12 ヵ月及び 24 ヵ月調査（許容範囲：同意取得日から 1 年後、2 年後の同日±60 日以内）

(1) 患者識別情報

・　患者登録識別番号（患者カルテ番号を除く）

(2) 同意継続状況確認

・　同意撤回の有無、同意撤回日

(3) 来院状況・健在確認

・　健在確認（確認日、確認方法、確認結果）

(4) 抗凝固薬の投与状況

- 当該観察期間開始時の抗凝固薬投与の有無
- 当該観察期間開始時の抗凝固薬の種類
- 当該観察期間開始時の抗凝固薬 1 日投与量及び 1 日投与回数（DOAC のみ）
- 抗凝固薬投与開始日
- 抗凝固薬投与終了日
- 投与変更・中止理由

(5) 抗凝固薬以外の併用薬・併用療法の状況調査

- 各観察期間終了時の、以下の薬剤の使用の有無を調査する
  抗血小板薬、p-gp 阻害薬
- 観察期間中の最大内服薬剤種類数（抗凝固薬、抗血小板薬を含むすべての薬剤。ただし、外用薬、市販薬は除く）

(6) AF に対する非薬物療法の実施状況

- 調査期間中の非薬物療法の有無
- 有の場合は、実施日、手術前後の抗血小板薬の投与状況、ヘパリンブリッジの有無

(7) AF 治療以外の侵襲的手技（小手術※を含む）の実施状況

- 調査期間中の NVAF 治療以外の侵襲的手技（小手術※を含む）の有無
  有の場合は、実施日、侵襲的手術（大手術、小手術※）の種類、手術前後の抗血小板薬の投与状況、ヘパリンブリッジの有無

(8) 血液凝固検査（ワルファリン服用患者のみ）

日常診療において以下の検査項目が測定された場合、eCRF に入力する。

- PT-INR 値、測定日
  （観察期間中に測定された値を最大 6 点記載する［2 ヶ月間隔を目安］）

(9) 臨床経過・臨床検査値

日常診療において以下の検査項目が測定された場合、eCRF に入力する。

- 体重
- 診察室血圧（収縮期及び拡張期血圧を測定。測定方法は表 3 を参照）
- 脈拍数
- ヘモグロビン
- 血清クレアチニン
- HbA1c

(10) 心血管系イベント

以下の①～⑧について、事象名、発現日、イベントの詳細（以下①～⑧の各イベント別の詳細）、イベント直前の PT-INR 値、イベント直前の抗血小板薬投与の有無、種類、イベント直前の内服薬剤種類数、重篤性、処置、その他の処置、転帰、転帰日（転

※ 小手術には生検・穿刺を伴う処置を含む

帰が「死亡」の場合は、死亡日を記載）、因果関係がある薬剤、薬剤以外に考えられる要因、有害事象に関するコメントを調査する。

① 脳卒中（一過性虚血発作（TIA）含む）
イベントの詳細：脳卒中種類（アテローム性、心原性、ラクナ、出血性、分類不能、その他脳卒中、TIA）、神経症状有無、画像診断有無、日本版 modified Rankin Scale（mRS）、National Institutes of Health Stroke Scale　（NIHSS）、急性期治療プロセス、急性期転帰

② 全身性塞栓症
イベントの詳細：画像診断有無

③ 出血性脳卒中以外の頭蓋内出血（くも膜下出血、硬膜下/硬膜外出血を含む）
イベントの詳細：頭蓋内出血種類（くも膜下出血、硬膜下/硬膜外出血、その他）、神経症状有無、画像診断有無、急性期治療プロセス、急性期転帰

④ 心臓／突然死

⑤ 心筋梗塞
イベントの詳細：臨床症状、診断根拠、心インターベンションの有無、種類

⑥ 心筋梗塞以外の心インターベンション施行
イベントの詳細：原疾患名、心インターベンションの種類

⑦ 入院を要する心不全
イベントの詳細：臨床症状

⑧ ①～⑦以外の死亡
イベントの詳細：死因種別（心血管死、非心血管死、不明）、原死因名

(11) 消化管出血
部位（有害事象名）、潰瘍・逆流性食道炎の有無、出血分類（表 4）、ショック指数、処置、ピロリ菌の有無、発現日、処置、出血前後のヘモグロビン値、イベント直前の PT-INR 値、イベント直前の抗血小板薬投与の有無、種類、イベント直前の PPI 投与の有無、イベント直前の内服薬剤種類数、重篤性、処置、その他の処置、転帰、転帰日、因果関係がある薬剤、薬剤以外に考えられる要因、有害事象に関するコメント

(12) その他（出血性脳卒中、頭蓋内出血、消化管出血以外）の出血性有害事象
有害事象名、出血分類（表 4）、発現日、イベント直前の PT-INR 値、イベント直前の抗血小板薬投与の有無、種類、イベント直前の内服薬剤種類数、重篤性、処置、その他の処置、転帰、転帰日、因果関係がある薬剤、薬剤以外に考えられる要因、有害事象に関するコメント

表 4　出血分類の定義

出血分類[1]	定義
大出血<br>(Major bleeding)	以下の少なくとも 1 つを満たす、臨床的に明らかな出血（検査又は放射線画像診断によって視覚的に確認される出血）<br>・致死的な出血<br>・後腹膜、頭蓋内、眼内、髄腔内、関節内、心膜、コンパートメント症候群を伴う筋肉内での症候性出血<br>・ヘモグロビンが 2.0 g/dL 以上低下し、輸血を必要とした臨床的に明らかな出血※<br><br>※濃縮赤血球又は全血 2 単位（400 cc）を輸血した場合は、1.0 g/dL のヘモグロビン減少と換算する。外科的処置に関連する出血は、通常の手術・処置で認められる出血量を上回る出血とする。ヘモグロビンのデータがない場合は、ヘマトクリット値が 6.0%以上低下し、輸血を必要とした出血とする。
臨床的に重要な出血<br>(Clinically relevant non-major bleeding)	治療を必要とする臨床的に明らかな出血とする。例として、以下に示した診断のための検査や治療に至ったものとするが、この限りではない。なお、以下又はそれに類する医学的処置（診断のための検査や治療）を伴わない外来受診は「治療を要する」に該当しない。<br><br>・入院又は入院期間の延長<br>・臨床検査<br>・画像検査<br>・内視鏡検査、結腸鏡検査、膀胱鏡検査、気管支鏡検査<br>・鼻腔パッキング<br>・圧迫止血<br>・超音波ガイド下での動脈瘤閉鎖<br>・コイル塞栓術<br>・強心療法<br>・手術<br>・医師の指示による抗凝固薬投与の中断又は中止<br>・医師の指示による抗凝固薬以外の併用治療の変更（アスピリンの減量や中止等）
小出血<br>［Minor (not clinically relevant) bleeding］	他の明らかな出血で、大出血又は臨床的に重要な出血の基準に該当しないもの（治療を必要としない鼻出血等）。

1 第 III 相、国際共同 AF 試験の定義を一部改変[25]

(13) その他のイベント

転倒の有無、骨折（外因性、内因性）の有無

有の場合、

有害事象名、発現日、イベント直前の PT-INR 値、イベント直前の抗血小板薬投与の有無、種類、重篤性、処置、その他の処置、転帰、転帰日、因果関係がある薬剤、薬剤以外に考えられる要因、有害事象に関するコメント

(14) その他の有害事象

重篤な有害事象が発生した場合、又は有害事象発生前の 1 週間以内にリクシアナの投与がある場合は、以下の項目を調査する。

有害事象名、発現日、重篤性、処置、その他の処置、転帰、転帰日、因果関係がある薬剤、薬剤以外に考えられる要因、有害事象に関するコメント

(10)～(14)については、来院がない場合でも、電話や手紙等で研究対象者に連絡を取り、12 ヵ月及び 24 ヵ月時点でのイベント発現状況を確認する。

12 調査スケジュール

表 5　観察・検査スケジュール

調査項目は以下のとおりとし、日常診療の範囲内にて実施する。

調査時点 / 調査項目	適格性確認	ベースライン調査（-180～0 日）	12 ヵ月調査（±60 日）	24 ヵ月調査（±60 日）
同意、選択・除外基準の確認	○			
同意撤回・来院状況・健在確認			○	○
患者背景・AF の罹患及び過去の治療歴、手術歴、出血の既往、既往歴（出血以外）・合併症等		○		
抗凝固薬の投与状況、服薬状況		○	○	○
抗凝固薬以外の薬剤の投与状況、服薬状況		○	○	○
AF に対する非薬物療法の実施状況		○	○	○
AF 治療以外の侵襲的手技（小手術[1]を含む）の実施状況			○	○
血液凝固検査（PT-INR）		○	○	○
臨床経過・臨床検査値		○	○	○
心血管系イベント、消化管出血、出血性有害事象、転倒、骨折、死亡、その他有害事象の有無			○	○
サブコホート研究				
A. アセスメントマーカー		○（中央測定）		
B. 心エコー		○	○	○
C. 心拍数		○	○	○
D. 高血圧		○		
E. 認知機能		○		○
F. フレイル		○		
G. アドヒアランス		○		○

AF：心房細動、PT-INR：プロトロンビン-国際標準比

1 小手術には生検・穿刺を伴う処置を含む

13 研究対象者の目標登録数

本研究の全体で 30,000 例（連続登録）

《目標登録数の設定根拠》

Fushimi AF Registry[5] における脳卒中／全身性塞栓症の発症率は、85 歳以上の患者で 5.1/100 人年、84 歳以下の患者で 2.0/100 人年、75 歳以上 84 歳以下の患者に対する 85 歳以上の患者のハザード比は 1.77 と報告されている。

これを元に、75 歳以上 84 歳以下の患者と 85 歳以上の患者における発症率を、それぞれ 2.9/100 人年、5.1/100 人年とし、本研究の患者構成比を 3:1 と仮定した場合、全体での発症率は 3.5/100 人年程度と推定される。

本研究のリスク因子なしの患者における脳卒中／全身性塞栓症の発症率を 3.4/100 人年と推定する。発症までの時間に指数分布（ハザード一定）を仮定して、リスク因子ありの患者の割合を 5%とした場合でも、30,000 例を登録することで、有意水準両側 5%、検出力 80%以上で、追跡期間 2 年間におけるリスク因子なしに対するリスク因子ありの脳卒中／全身性塞栓症の発症のハザード比 1.3 を検出することができる。

また、30,000 例であれば、検討を予定しているイベントの中でも、発症率は低いが特に注目すべき頭蓋内出血に関しても、ハザード比 1.5 程度のリスク因子が検出力 80%で検出可能となる。その際、頭蓋内出血の推定発症率 1.2%/年、リスク因子ありの場合を 5%と仮定した［例として、高度腎機能障害（SRI）を有する患者において、SRI がない患者の 1.5 倍の頭蓋内出血リスクが潜在的にあるとした場合、SRI が患者全体の 5%程度であっても統計学的に有意なリスク上昇を 80%の検出力で検出できる]。

14 研究実施期間

研究責任実施医療機関の倫理審査委員会による承認日～2020 年 9 月

（登録期間：研究責任機関の倫理審査委員会による承認日～2018 年 9 月、追跡期間 2 年）

ただし、研究中央委員会、研究代表責任医師と研究依頼者の合意により、登録期間の短縮、及び予定実施期間の短縮又は延長がなされる場合がある。

15 評価項目

15.1 主要評価項目

観察期間中における脳卒中／全身性塞栓症の発現状況

15.2副次評価項目

観察期間中における以下のイベントの発現状況

(1) 出血性有害事象（Major Bleeding）
(2) 脳卒中
(3) 全身性塞栓症
(4) 虚血性脳卒中
(5) 出血性脳卒中
(6) 頭蓋内出血
(7) 心血管系イベント（脳卒中、心筋梗塞、心筋梗塞以外の心インターベンション、入院を要する心不全）
(8) 心血管系疾患による死亡（心血管系の要因による死亡が否定できない死亡）
(9) すべての死亡

15.3その他の評価項目

転倒、骨折の発現状況

16 サブコホート研究の実施（サブ研究 1）

本研究の実施と共に、以下のサブコホート研究を実施する。本臨床研究への参加時にそれぞれのサブコホート研究への協力を表明した研究実施医療機関の研究責任医師等は、可能な限り、当該研究実施医療機関に登録されたすべての研究対象者について、以下のサブコホート研究ごとに設定された調査を実施する。

各サブコホート研究は、「表 1　観察・検査スケジュール」の調査時点で行うこととし、詳細は別途作成される「サブコホート研究実施計画書（Appendix 版）」に従うこととする。

［許容範囲］

ベースライン（特に指定がない場合は、同意取得日から 180 日以内の直近の情報）
12 ヵ月調査及び 24 ヵ月調査：いずれも±60 日

16.1サブコホート研究 A：アセスメントマーカー

凝固線溶分子マーカー、抗血栓作用活性と脳卒中／全身性塞栓症及び出血性有害事象との関連を、抗凝固薬非投与、ワルファリン、DOAC 別に比較検討する。

評価項目：

① プロトロンビン時間（PT）、PT-INR、活性化部分トロンボプラスチン時間（APTT）、TTR、D-dimer、可溶性フィブリンモノマー複合体（SFMC）、プロトロンビンフラグメント 1+2（F1+2）、トロンビン-アンチトロンビン複合体（TAT）、高感度

トロポニン I、脳性ナトリウム利尿ポリペプチド(BNP) 前駆体 N 端フラグメント（NT-pro BNP）

② 脳卒中／全身性塞栓症

③ 出血性有害事象

16.2 サブコホート研究 B：心エコー

心エコーによる基礎心疾患、左室（LV）及び左房（LA）の構造と機能、並びにこれらと脳卒中／全身性塞栓症との関連を検討する。

評価項目：

① LV の構造及び機能：
- 左室拡張末期径（LVDd）/左室収縮末期径（LVDs）/心室中隔壁厚（IVSTD）/左室後壁厚（PWTD）
- 左室拡張末期容積（LVEDV）/左室収縮末期容積（LVESV）/左室駆出率（LVEF）
- 一回拍出量（SV）
- 左室心筋重量係数（LV mass index）
- LV sphericity index（①左室拡張末期容積（LVEDV） /π*LV length^3/6, ② LVDd/LV length）
- LV 壁異常運動

② LA の構造及び機能：
- 左房径（LAD）
- 左房容積*（LA volume）（最大 LA volume [LA_{max}]、最小 LA volume [LA_{min}] 及び心房収縮前 LA volume [$LA_{pre\text{-}A}$]）による LA expansion index、total/passive/active LA emptying fraction の算出値

③ 右室（RV）の構造及び機能：
- 三尖弁収縮期圧較差 （TRPG）、下大静脈径（IVCD）、呼吸性変動からの推定右房圧(RAP)、推定肺動脈収縮期圧（PASP）
- RV function [三尖弁輪収縮期移動距離（TAPSE）及び三尖弁輪収縮期運動速度（TVS）]

④ ドプラ法：
- LV 流入血流拡張早期波（E）、LV 流入血流心房収縮期波（A）、僧帽弁輪拡張早期速度（TDI e’）、僧帽弁輪心房収縮期速度（TDI a’）

⑤ 心臓弁膜病変（僧帽弁、大動脈弁、三尖弁、肺動脈弁、人工弁、弁修復）

⑥ 脳卒中／全身性塞栓症

16.3サブコホート研究 C：心拍数

心電図で記録された AF 発現中の心拍数と脳卒中／全身性塞栓症の発現リスクとの間の関連性を評価する。

評価項目：

① 心拍数
② 脳卒中／全身性塞栓症

16.4サブコホート研究 D：高血圧

血圧（診察室血圧及び家庭血圧）と脳卒中／全身性塞栓症及び出血性有害事象との関連性を検討する。

評価項目：

① 診察室血圧値及び家庭血圧値
② 脳卒中／全身性塞栓症
③ 出血性有害事象

16.5サブコホート研究 E：認知機能

NVAF を有する後期高齢患者における認知機能の実態を明らかにするとともに、認知機能に影響を与える因子を検討する。認知機能を測定するミニメンタルステート検査（Mini Mental State Examination，MMSE）（サブコホート研究 E 実施計画書，付録 1 参照）及び社会活動度に関する質問票（サブコホート研究 E 実施計画書，付録 2 参照）を用いて、認知機能及び社会活動度に関する情報を収集する。

評価項目：

① Mini-Mental State Examination（MMSE）スコア
② 社会活動度

16.6サブコホート研究 F：フレイル

NVAF を有する後期高齢患者におけるフレイル患者の割合を把握するとともに、フレイル患者の抗凝固療法の使用状況及び実態を確認する。また、フレイルと脳卒中／全身性塞栓症及び出血性有害事象との関連性を探索的に検討する。フレイルに関する情報の収集には、フレイルに関連する質問票［簡易フレイルスケール（国立長寿医療研究センター）（サブコホート研究 F 実施計画書，付録 1 参照)、高齢者基本チェックリスト（厚生労働省）（サブコホート研究 F 実施計画書，付録 2 参照)、生活状況に関するアンケート（要介護認定の有無及びグレード、同居人の有無、同居家族の構成)］を用いる。

評価項目：

① フレイルティスコア
② 高齢者基本チェックリスト評価値
③ 生活状況に関するアンケート
④ 脳卒中／全身性塞栓症
⑤ 出血性有害事象

16.7 サブコホート研究 G：アドヒアランス

NVAF を有する後期高齢患者における服薬アドヒアランスと NVAF の理解度を調査し、服薬アドヒアランスと NVAF の理解度、脳卒中／全身性塞栓症及び出血性有害事象との関連性を検討する。アドヒアランスに関する情報の収集には、アドヒアランス質問票［Morisky Medication Adherence Scale（MMAS）[26] スコア及び NVAF の理解度に関する質問票］により、アドヒアランスに関する情報を収集する。なお、MMAS には、4 項目の質問から構成される MMAS-4 及び 8 項目の質問から構成される MMAS-8（サブコホート研究 G 実施計画書，付録 1 参照）の 2 種類がある。当該サブコホート研究では、患者の服薬アドヒアランスをより正確に把握できる MMAS-8 を採用する。また、登録時までの処方日数の割合（Proportion of days covered , PDC）を調査する。

評価項目：

① MMAS-8 スコア
② NVAF の理解度
③ 処方日数の割合（Proportion of days covered , PDC）
④ 脳卒中／全身性塞栓症
⑤ 出血性有害事象

17 統計解析

以下の規定に基づいて、調査情報を集計し統計解析を実施する。詳細な解析方法及び以下に記載のない解析項目ついては、別途作成される「統計解析計画書」に従う。

17.1 解析対象集団

本研究に登録されたすべての研究対象者のうち、以下の研究実施計画書からの重大な逸脱を除いた研究対象者を含めた集団とする。

- 選択基準の不適合
- 除外基準の抵触

17.2 ベースライン調査項目の解析

以下について、カテゴリ変数については頻度表を作成し、連続変数については要約統計量（例数、平均値、標準偏差、最小値、中央値、最大値）を求める。

(1) 背景因子の分布
(2) 登録時に使用していた抗凝固療法の種類、用量、1 日投与回数
(3) 登録時に使用していた抗血小板薬の種類
(4) 登録時に使用していたその他併用薬の種類
(5) 登録時の患者情報等より算出した $CHADS_2$スコア（付録 1）又は CHA_2DS_2-VASc スコア（付録 2）

17.3追跡データの解析

以下について、項目に応じ頻度やイベント発生率とその 95%信頼区間を算出する。また、リスク因子の探索では、Cox 比例ハザードモデルやロジスティック回帰分析等を用いる。

(1) 抗凝固薬及び抗血小板薬の種類、用量並びに 1 日投与回数、変更頻度［12 ヵ月、24 ヵ月調査時］
(2) 脳卒中／全身性塞栓症の発現率［24 ヵ月調査時］
- 脳卒中（アテローム性、心原性、ラクナ、出血性、分類不能、その他脳卒中、TIA）
- 全身性塞栓症

(3) 出血性有害事象の発現率［24 ヵ月調査時］（出血分類及び定義は、表 4 を参照）
- 大出血
- 臨床的に重要な出血
- 小出血
- 頭蓋内出血
- 消化管出血（上部・下部）

(4) 転倒回数
(5) 骨折発生率
(6) 死亡率（すべての死亡、心血管死、非心血管死）
(7) 心筋梗塞、心インターベンションの実施、入院を要する心不全の発生率

(1)～(7)に関して、$CHADS_2$スコア、CHA_2DS_2-VASc スコア、HAS-BLED スコア及びその構成因子や、ガイドライン等でリスク因子として特定されている因子、本研究でリスク因子として特定された因子、抗凝固療法の有無及び種類（DOAC、ワルファリン）、用量、PT-INR 等のサブグループ別に検討する。

17.4サブグループ解析（サブ研究 2）

サブグループ解析により検討する項目を以下に示す。

(1) 腎機能（Ccr 値）別
(2) HbA1c 別

(3) 最大投与薬剤数別
(4) 虚血性心疾患発症例
(5) 消化管出血発症例
(6) 脳卒中発症例

17.5サブコホート研究の解析

集計項目に応じて、17.2 項及び 17.3 項に準じた解析を実施する。詳細な解析方法及び以下に記載のない解析項目については、別途作成される「統計解析計画書」に従う。

17.5.1 サブコホート研究 A：アセスメントマーカー

(1) PT、PT-INR、APTT、TTR、D-dimer、SFMC、F1+2、TAT、高感度トロポニンⅠ 、NT-pro BNP の分布（ベースライン時）
(2) TTR 別の大出血、臨床的に重要な出血、及び脳卒中／全身性塞栓症の発現率（ワルファリン投与例）（24 ヵ月調査時）
(3) PT 及び APTT 値別に要約した、大出血、臨床的に重要な出血、脳卒中／全身性塞栓症の発現率（24 ヵ月調査時）
(4) D-dimer、SFMC、F1+2、TAT、高感度トロポニン I、NT-pro BNP の値別に要約した、大出血、臨床的に重要な出血、脳卒中／全身性塞栓症の発現率（24 ヵ月調査時）

17.5.2 サブコホート研究 B：心エコー

(1) 基礎心疾患の内訳
(2) NVAF の種類（発作性、持続性、永続性）別、心エコーパラメータ値（ベースライン時）
(3) $CHADS_2$スコア別、心エコーパラメータ値（ベースライン時）
(4) 心エコーパラメータの経時的変化（ベースライン時、12 ヵ月調査時、24 ヵ月調査時）
(5) $CHADS_2$高スコアにおける心エコー規定因子（ベースライン時）
(6) 脳卒中を含むイベントの心エコー規定因子（予測因子）（24 ヵ月調査時）

17.5.3 サブコホート研究 C：心拍数

(1) 安静時 12 誘導心電図で記録された AF 発現中の心拍数の分布（ベースライン時）
(2) 安静時 12 誘導心電図で記録された AF の頻度の推移（ベースライン時、12 ヵ月調査時、24 ヵ月調査時）
(3) 安静時 12 誘導心電図で記録された AF 発現中の心拍数の推移（ベースライン時、12 ヵ月調査時、24 ヵ月調査時）
(4) 安静時 12 誘導心電図で記録された AF 発現中の脳卒中及び全身性塞栓症の心拍数別発現状況（24 ヵ月調査時）

17.5.4 サブコホート研究 D：高血圧

(1) 登録時の診察室血圧値及び家庭血圧値の分布（ベースライン時）

(2) 登録時の診察室血圧値及び家庭血圧値の分布に基づく、脳卒中及び全身性塞栓症の発現率（24 ヵ月調査時）

(3) 登録時の診察室血圧値及び家庭血圧値の分布に基づく、出血性有害事象の発現率（24 ヵ月調査時）

17.5.5 サブコホート研究 E：認知機能

(1) MMSE スコアの各時点での分布（ベースライン時、24 ヵ月調査時）

(2) 登録時の社会活動度の分布（ベースライン時）

(3) MMSE スコアのベースラインからの変化（24 ヵ月調査時）

17.5.6 サブコホート研究 F：フレイル

(1) 登録時のフレイル患者及び非フレイル患者の割合（ベースライン時）

(2) 登録時のフレイル患者及び非フレイル患者の患者背景（ベースライン時）

(3) 登録時のフレイル患者及び非フレイル患者別、抗凝固薬の種類、用量（DOAC のみ）、投与期間（ベースライン時）

(4) 高齢者基本チェックリスト評価値の分布（ベースライン時）

(5) 生活状況に関するアンケートの分布（ベースライン時）

(6) 登録時のフレイル患者及び非フレイル患者別、脳卒中／全身性塞栓症の発現率（24 ヵ月調査時）

(7) 登録時のフレイル患者及び非フレイル患者別、出血性有害事象の発現率（24 ヵ月調査時）

17.5.7 サブコホート研究 G：アドヒアランス

(1) MMAS-8 スコアの各時点での分布（ベースライン時、24 ヵ月調査時）

(2) PDC の分布（ベースライン時）

(3) MMAS-8 スコアが改善した研究対象者の背景因子（ベースライン時、24 ヵ月調査時）

(4) MMAS-8 スコア、PDC と NVAF の理解度、及びそれらの関連性（ベースライン時、24 ヵ月調査時）

(5) MMAS-8 スコアと脳卒中／全身性塞栓症及び出血性有害事象との関連性（24 ヵ月調査時）

(6) NVAF の理解度と脳卒中／全身性塞栓症及び出血性有害事象との関連性（24 ヵ月調査時）

(7) PDC と脳卒中／全身性塞栓症及び出血性有害事象との関連性（24 ヵ月調査時）

18 症例報告書の作成と提出

18.1 症例報告書の様式と提出

本研究実施計画書に規定される調査項目については、本研究専用の症例報告書（eCRF）へ記録する。eCRF の様式は以下のとおりであり、ベースライン調査時、12 ヵ月調査時及び 24 ヵ月調査時に入力し、速やかに提出（送信）する。

- 研究対象者の登録情報
- ベースライン調査時
- 12 ヵ月及び 24 ヵ月調査時
- 同意撤回時及び有害事象発現時 : 随時（ただし、緊急を要する重篤な有害事象が発現した場合を除く）

18.2 症例報告書の作成方法

研究責任医師等及び研究協力者は、発行された ID 及びパスワードを用いて本研究専用の EDC システムへアクセスし、調査情報を入力する（EDC への入力手順については、別途作成される「EDC 入力マニュアル」に従うものとする）。

19 モニタリング及び監査

19.1 モニタリング及び監査

研究依頼者は研究代表責任医師と協議し、研究の実施及びその記録の信頼性を確保し、これを保証するためにモニタリング及び監査を研究業務受託機関等に委託する。研究責任医師等及び研究実施医療機関は、モニタリング及び監査に協力する。また、研究責任医師等は、研究対象者に原資料がモニタリング担当者及び監査担当者に閲覧される旨を、同意説明文書にて説明し同意を取得する。

19.2 モニタリング

モニタリング担当者は、別途作成される「モニタリング手順書」に従って、本研究が「人を対象とする医学系研究に関する倫理指針」及び本研究実施計画書を遵守して実施されていることを確認する。直接閲覧では、本研究で定められている各種手順書を確認の上、原資料と EDC に入力されている内容とを照合し、収集データの完全性、正確性及び一貫性を確認する。モニタリング業務完了後、モニタリング報告書を作成し、研究代表責任医師へ報告する。また、モニタリング担当者は、その業務に従事しなくなった後であっても、業務上知り得た情報を正当な理由なく漏らしてはならない。

研究依頼者（第一三共株式会社）は、本研究に対する中立性を保持するため、モニタリング業務には直接関係しないこととする。

19.3 監査

監査担当者は、モニタリング業務とは独立した立場を保持し、別途作成される「監査業務手順書」に従って、書面及び実地調査を行う。モニタリング報告書を検証し、研究の実施及びその記録の信頼性を確保し、これを保証する。各研究責任医師等は、監査担当者の求めに応じて研究対象者のすべての研究関連記録を直接閲覧に供しなければならない。監査担当者は、研究代表責任医師及び研究実施医療機関の長へ監査報告書及び監査証明書を提出する。また、監査担当者は、その業務に従事しなくなった後であっても、業務上知り得た情報を正当な理由なく漏らしてはならない。

19.4 原資料の直接閲覧

実施医療機関の長及び研究責任医師は、モニタリング、監査並びに規制当局及び倫理審査委員会による調査の際に原資料等、臨床研究関連記録を直接閲覧に供する。研究依頼者（第一三共株式会社）は、直接閲覧を実施しない。

20 倫理

本研究は、最新の「世界医師会ヘルシンキ宣言」の精神に基づき、研修対象者の人権及び福祉を守り、「人を対象とする医学系研究に関する倫理指針」（平成 26 年 文部科学省、厚生労働省告示第 415 号）[24] 及び関連通知を遵守して実施する。

20.1 倫理審査委員会

研究依頼者は、研究代表責任医師及び研究中央委員会と協議の上、研究実施計画書及び同意説明文書（見本）を作成し、研究依頼者が指定する外部倫理審査委員会、および研究代表責任施設を管轄する心臓血管研究所倫理審査委員会の承認を得る。研究計画を改訂する場合には、研究依頼者と研究代表責任医師が必要に応じ研究中央委員会に意見を求めた上で研究実施計画書等（必要に応じて、同意説明文書）の改訂版を作成し、それぞれの倫理審査委員会へ再審査を依頼し承認を得た後、改訂版を施行する。なお、本研究開始後、少なくとも 1 年に 1 回は、それぞれの倫理審査委員会へ研究継続の報告を行う。

各参加施設の研究責任医師は、研究実施計画書、同意説明文書（見本）等の研究資料について、所属する各参加施設の倫理審査委員会の承認を得た後に本研究を開始する。なお、倫理審査委員会の設置がない施設の場合には、当該施設が指定する外部倫理審査委員会か、研究依頼者が指定する外部倫理審査委員会または心臓血管研究所倫理審査委員会のいずれかで代行審議を受けることとする。

20.2 研究対象者に予想される利益

本研究は、既承認薬を適応範囲内で使用する登録研究であるため、研究対象者が本研究へ参加することで治療方針の変更はなく、本研究に参加することによる特別な利益はな

い。本研究から得られた成果により、研究対象者を含めた将来の AF 患者に利益が生じる可能性が期待される。

20.3研究対象者に予想される危険や不利益

本研究は、NVAF を有する後期高齢患者の現在の治療状況を前向きに観察するものであり、治療に伴う有害事象は一般診療下で通常行われる治療で起こり得るもの以外にはない。また、研究対象者に対して行われる採血、各検査及び調査アンケートは、一般診療下で通常行われる治療の範囲を逸脱するものではない。

20.4健康被害に対する補償

本研究は一般診療下における前向き観察研究であり、本研究参加後に健康被害が発生した場合には、原則として研究対象者が加入する健康保険を使用して治療することとし、研究実施医療機関から研究対象者への治療に係る費用の支払いは行わないこととする。

健康被害が発生した場合には、研究実施医療機関の研究責任医師等は、速やかに治療及びその他の必要な処置を講じる。研究責任医師等の指示に従った用法・用量で服薬し、発生した副作用に起因する健康被害は、医薬品副作用被害救済制度による救済給付申請の対象となる。

なお、研究依頼者は、本研究に起因する健康被害について、本研究中に健康被害による補償または賠償責任が生じた場合の履行措置として、また本研究計画に起因する健康被害（死亡、後遺障害 1 級又は 2 級）による賠償への備えとして、臨床研究保険に加入する。

20.5個人情報等の保護

本研究に関わる関係者は、研究対象者の個人情報等の保護について適用される「個人情報の保護に関する法律」（平成 15 年法律第 57 号）及び関連通知を遵守する。

関係者は、研究対象者の個人情報及びプライバシーの保護に充分配慮し、患者情報を識別する患者カルテ番号は、本研究の登録時に付与される患者登録識別番号で匿名化する（連結可能匿名化）。EDC には、患者カルテ番号、氏名、連絡先等の個人を特定できる情報は一切記載しない。患者カルテ番号と患者登録識別番号との対応表は、研究実施医療機関内の施錠可能な場所で厳重に保管する。

本研究で得られた研究対象者のデータは、本研究の目的以外には使用せず、結果を公表する際は、研究対象者を特定する情報は含めない。

本研究を行う上で知り得た個人情報を正当な理由なく漏らしてはならない。関係者がその職を退いた後も同様とする。

21 研究の費用に関する事項

21.1 研究資金及び利益相反

本研究は、第一三共株式会社の資金により実施する企業主導臨床研究である。

資金提供者は、研究実施計画書の作成、研究業務受託機関の選定及び当該機関への助言、統計解析計画の立案、研究結果の解釈には関与するが、データマネジメント、直接閲覧及び統計解析には直接関与しない。

資金提供者は、本研究の実施及び結果の公表に際しては、利益相反関係を適切に管理し、中立性と公明性を維持して適正に研究を遂行する。

研究代表医師及び研究責任医師等は、本研究の計画、結果の解釈に影響を及ぼすような利益相反の状況について、施設の規定に従い倫理審査委員会又は利益相反委員会の審査を受ける。その内容については、説明文書に記載し、研究対象者への説明後に、本研究への参加の同意を取得する。また、研究結果の公表に際しては、結果を発表する学術団体及び機関紙の指針等を遵守し、自己申告によって正確な状況を開示するものとする。

21.2 研究対象者の費用負担

薬剤の投薬、血液学的検査、その他の治療や検査等、通常の保険診療の範囲内で施行される診療費については、研究対象者の自己負担とする。
この研究による交通費の支給は行わないが、各施設の規定に従い、研究協力への謝礼を試験終了時に提供する場合もある。研究協力謝礼を提供する施設については、同意説明文書に内容を記載するとともに、各施設の倫理審査委員会の承認を得ることとする。

22 研究実施計画書の変更・改訂

22.1 研究実施計画書の変更

研究責任医師等は、研究実施計画書の変更が必要と判断した場合は、研究代表責任医師の了承後、倫理審査委員会の承認を得る。研究実施計画書の変更内容により同意説明文書の変更が必要と判断された場合は、変更した同意説明文書を研究実施計画書と併せて倫理審査委員会の承認を受け、研究対象者に当該同意説明文書を用いて文書による再同意を得る。

22.2 研究実施計画書の改訂

研究代表責任医師は、研究実施計画書の改訂を行う場合、改訂した研究実施計画書を研究責任実施医療機関の長に提出し、倫理審査委員会の承認を得なければならない。

改訂の承認を得た後、研究代表責任医師は、改訂内容を研究事務局経由で研究実施医療機関の各研究責任医師、データセンター等、研究関係者へ速やかに伝達する。

23 研究の終了と中断・中止

23.1研究の終了

研究終了後、各研究実施医療機関の研究責任医師は速やかに研究終了報告書を研究実施医療機関の長に提出する。

研究実施医療機関の長は、各研究実施医療機関の研究責任医師から上記のとおり報告を受けたときは、本研究に関する審査を行った倫理審査委員会に、研究終了報告書を提出する。

23.2研究の中断・中止

研究代表責任医師は、以下の事項に該当する場合は中央委員会の意見を得た上で研究実施継続の可否を検討する。研究代表責任医師は、本研究を中断又は中止を決定した場合は、各々の理由及び研究対象者への対応方法について文書を作成し、速やかに研究実施医療機関へ通知する。

なお、研究実施医療機関（研究責任医師等を含む）が「人に対する医学系研究に関する倫理指針」、本研究実施計画書又は契約書に重大な違反を犯したことが判明した場合、又は適正な研究の実施ができなくなった場合、当該研究実施医療機関は研究代表責任医師から研究の中止を求められることがある。

また、研究実施医療機関で生じた以下の事由等により研究中断又は中止した場合は、当該研究責任医師は中断又は中止した情報を速やかに研究代表責任医師へ報告する。

(1) 本研究に関連する医薬品の品質、安全性、有効性に関する重大な情報が得られたとき
(2) 研究対象者のリクルートが困難で、予定していた研究対象者数の登録を達成することが到底困難であると判断されたとき
(3) 倫理審査委員会により、中止の勧告あるいは指示があったとき
(4) 倫理審査委員会により、研究実施計画等の変更の指示があり、これを受け入れることが困難と判断されたとき

24 研究に係る資料等の保管

研究者等は、研究等の実施に係る文書（申請書類の控え、研究実施医療機関の長からの通知文書、各種申請書及び報告書の控え、連結可能匿名化した研究対象者の患者登録識別番号リスト、同意書、その他データの信頼性を保証するのに必要な書類又は記録等）を正確なものにしなければならない。

研究責任医師は、研究対象者から取得された試料や研究の実施に係る文書等を保管するときは、研究実施医療機関の長が作成した保管に関する手順書に基づき、研究者等が研究等の実施に係る文書を正確なものにするよう指導・管理し、これらの漏洩、混交、盗

難、紛失等が起こらないように管理する。また、手順書に従って、管理状況について研究実施医療機関の長へ報告することとする。

研究実施医療機関の長は、研究対象者から取得された試料や研究の実施に係る文書等の保管に関する手順書を作成し、その手順書に従って試料や文書等を適切に保管する。また、研究実施医療機関の長は、研究の実施に係る文書等について、少なくとも、本研究の終了について報告された日から 5 年を経過した日又は当該研究の結果の最終の公表について報告された日から 3 年を経過した日のいずれか遅い日までの期間、適切に保管する。連結可能匿名化された情報の対応表についても、同様に対応する。保存期間が経過した後は、研究対象者の個人情報が特定されない適切な方法にて速やかに廃棄する。

各研究実施医療機関では個人情報管理者を設置し、個人情報管理を行う。

なお、本研究で得られた研究対象者のデータ及び検体は、本実施計画書で定めた目的以外に使用しない。

25 研究対象者等及びその関係者からの相談等への対応

研究代表責任医師は、研究対象者及びその関係者からの本研究に係る相談窓口を設置し、連絡方法を説明文書に記載する。研究者等は、研究対象者から受けた全ての質問に対し、速やかに回答し詳細を説明する。但し、研究対象者及びその関係者の人権又は研究者等及びその関係者の権利利益の保護のため非公開とすることが必要な内容として、倫理審査委員会の意見を受けて研究機関の長が許可したものについては、この限りでない。

26 研究の公表と成果の帰属

26.1 臨床研究登録

研究代表責任医師は、研究開始前（最初の研究対象者の登録開始前）に、研究計画内容を公開登録システム［大学病院医療情報ネットワーク（UMIN）］に登録し、公開する。

26.2 研究の公表

研究代表責任医師は、本研究終了後、遅滞なくその結果を公表する。研究成果を発表する者は、事前に研究推進・パブリケーション委員会の承認を得る。論文・学会発表等の著者は、International Committee of Medical Journal Editors の著者要件（authorship）に従い、研究推進・パブリケーション委員会が適切に決定する。

26.3 成果の帰属

研究で得られた結果は、第一三共株式会社が排他的に利用する権利を有する。研究代表責任医師等が研究成果を対外的に公表する場合、契約書の規定に基づき利益相反に関する記載が論文等に適切になされるよう、その公表前に研究代表責任医師等に依頼する。

なお、学会発表や論文として報告する際には、研究対象者の名前や傷病名等の個人情報に留意する。

27 研究組織

27.1代表世話人

研究組織の代表者として多施設間の調整を行う。

井上 博　　富山県済生会富山病院　院長

〒931-8533 富山県富山市楠木 33−1

電話：076-437-1111（代）

27.2研究顧問

研究顧問として多施設間の調整を行う。

山口 武典　 国立循環器病研究センター　名誉総長

〒565-8565 大阪府吹田市藤白台 5 丁目 7 番 1 号

電話：06-6833-5012（代）

27.3研究代表責任医師

「人を対象とする医学系研究に関する倫理指針ガイダンス（平成 27 年 3 月 31 日一部改訂）」における、共同研究機関における研究を統括する研究代表者にあたり、本研究の実施上の責任を担う。

山下 武志　 心臓血管研究所　所長

〒106-0031 東京都港区西麻布 3-2-19

電話：03-3408-2151（代） Fax：03-3408-2159

27.4研究中央委員会

研究計画に対し医学的な判断を行うとともに、本研究が円滑に推進されるよう研究組織の運営方針を策定する。

※委員のリストは別冊参照。

27.5研究推進・パブリケーション委員会

患者登録やフォローアップ等の研究の推進を行う。また、研究結果等の公表方針、および公表内容を決定する。

※委員のリストは別冊参照。

27.6イベント判定委員

脳卒中イベント、心疾患イベント、出血性有害事象を本研究における評価イベントに該当するかどうかの判定を行う。

※委員のリストは別冊参照。

27.7サブ研究責任医師

16 項の各サブコホート研究（サブ研究 1）および 17,4 サブグループ解析（サブ研究 2）の計画立案、およびサブ研究実施の推進の責任を担う。 また、サブ研究結果の解釈、および結果公表内容の提案を行う。

※サブ研究責任医師のリストは別冊参照。

27.8地区推進担当医師

地域における関連施設の取りまとめ役として、患者登録および患者フォローアップの推進を担当する。

※地区推進担当医師のリストは別冊参照。

27.9統計解析責任者

統計解析計画の立案、実施、統計解析結果への責任を担う。また、研究結果の解釈への助言を行う。

手良向 聡　　京都府立医科大学大学院医学研究科生物統計学　教授

〒602-8566 京都府京都市上京区河原町通広小路上ル梶井町 465

電話：075-251-5966、075-251-5944 Fax：075-251-6588

27.10　研究責任機関

本研究の実施上の責任機関として「人を対象とする医学系研究に関する倫理指針」上の「研究機関」の役割を担う。本研究責任機関の倫理審査委員会を、本研究の中央倫理委員会とする。

心臓血管研究所付属病院

〒106-0031 東京都港区西麻布 3-2-19　公益財団法人心臓血管研究所内

電話：03-3408-2151（代） Fax：03-3408-2159

27.11　研究事務局

研究代表責任医師の業務を補佐し、試験が円滑に遂行されるために必要な対応を行う。

心臓血管研究所　CVI-ARO

〒106-0031 東京都港区西麻布 3-2-19　公益財団法人心臓血管研究所内

電話：03-3408-2151（代）　　　Fax：03-3408-2159

なお、以下の機関にて研究運営に関する事務的業務の実務を行う。

クインタイルズ・トランスナショナル・ジャパン株式会社

〒532-0003 大阪府大阪市淀川区宮原 3-4-30 ニッセイ新大阪ビル

電話：06-7668-9053　 Fax：06-4807-9801

E-mail: ANAFIE_office@quintiles.com

27.12　研究依頼者

研究依頼者として、本研究に関する全体責任を負う。

福知　良和　　第一三共株式会社　メディカルサイエンス部

〒103-8426 東京都中央区日本橋本町三丁目 5 番 1 号

電話：03-6225-1053　 Fax：03-6225-1959

27.13　データセンター

各参加施設より収集されたデータおよび資料を適切に管理する。

クインタイルズ・トランスナショナル・ジャパン株式会社

〒532-0003 大阪府大阪市淀川区宮原 3-4-30 ニッセイ新大阪ビル

電話：06-7668-9053　 Fax：06-4807-9801

27.14　モニタリング担当機関

研究委託者からの委託により、モニタリング業務を行う機関。

① クインタイルズ・トランスナショナル・ジャパン株式会社

〒532-0003 大阪府大阪市淀川区宮原 3-4-30 ニッセイ新大阪ビル

電話：06-7668-9053　 Fax：06-4807-9801

②EP クルーズ株式会社

〒162-0821 東京都新宿区新小川町 6 番 29 号アクロポリス 東京

電話：03-5804-5045　Fax：03-5684-8052

27.15　監査担当機関

研究委託者からの委託により、監査業務を行う機関。

株式会社リニカル

〒532-0003 大阪市淀川区宮原一丁目 6 番 1 号 新大阪ブリックビル 10 階

電話：06-6150-2478　Fax：06-6150-2675

28 参考文献

1. Go AS, Hylek EM, Phillips KA, et al.: Prevalence of diagnosed atrial fibrillation in adults. National implications for rhythm management and stroke prevention: the AnTicoagulation and Risk Factors in Atrial Fibrillation (ATRIA) study. JAMA 2001; 285(18):2370-5.
2. Inoue H, Fujiki A, Origasa H, et al. Prevalence of atrial fibrillation in the general population of Japan: an analysis based on periodic health examination. Int J Cardiol 2009;137:102-7.
3. Wolf PA, Abbott RD, Kannel WB: Atrial fibrillation as an independent risk factor for stroke: the Framingham Study. Stroke. 1991;22(8):983-8.
4. Hart RG, Pearce LA, Rothbart RM, et al.: Stroke with intermittent atrial fibrillation: incidence and predictors during aspirin therapy. Stroke Prevention in Atrial Fibrillation Investigators. J Am Coll Cardiol. 2000;35(1):183-7.
5. Yamashita Y, Hamatani Y, Esato M, Chun YH, Tsuji H, Wada H, Hasegawa K, Abe M, Lip GY, Akao M. Clinical Characteristics and Outcomes in Extreme Elderly (Age ≥ 85 Years) Japanese Patients With Atrial Fibrillation: The Fushimi AF Registry. Chest. 2016 ;149:401-12.
6. 添付文書「ワーファリン錠」第 23 版
7. Atarashi H, Inoue H, Okumura K, et al. Present status of anticoagulation treatment in Japanese patients with atrial fibrillation. Circ J. 2011;75:1328-33.
8. Ogawa S, Yamashita T, Yamazaki T, et al. Optimal treatment strategy for patients with paroxysmal atrial fibrillation: J-RHYTHM Study. Circ J. 2009 Feb;73(2):242-8.
9. 佐藤陽子，村田美由貴，千葉剛，梅垣敬三．ワルファリン服用者におけるビタミン K 摂取量の許容範囲に関する系統的レビュー．食品衛生学雑誌．2015;56(4):157-65.
10. 厚生労働省．医薬品・医療機器等安全性情報 No.235，参考資料 2「ファーマコゲノミクスの展望（ワルファリンの治療に関する遺伝子多型．2007;235:22-3.
11. 厚生労働省．重篤副作用疾患別対策マニュアルー出血傾向（平成 19 年 6 月）．2007:11.
12. 添付文書「プラザキサ®」第 8 版
13. 添付文書「イグザレルト®錠」第 6 版
14. 添付文書「エリキュース®錠」第 6 版
15. 添付文書「リクシアナ®錠」第 5 版
16. Mendell J, Zahir H, Matsushima N, et al. Drug-drug interaction studies of cardiovascular drugs involving P-glycoprotein, an efflux transporter, on the pharmacokinetics of edoxaban, an oral factor Xa inhibitor.Am J Cardiovasc Drugs. 2013 Oct;13(5):331-2.
17. Abstracts of the 60th Annual Meeting of the Scientific and Standardization Committee of the International Society on Thrombosis and Haemostasis, June 23-26, 2014, Milwaukee, WI. J Thromb Haemost. 2014;12(Suppl 1):17.
18. 循環器病の診断と治療に関するガイドライン（2012 年度合同研究班報告）. 非弁膜症性心房細動治療（薬物）ガイドライン（2013 年改訂版）.2013
19. Apostolakis S, Lane DA, Buller H, et al. Comparison of the $CHADS_2$, CHA_2DS_2-VASc and HAS-

BLED scores for the prediction of clinically relevant bleeding in anticoagulated patients with atrial fibrillation: the AMADEUS trial. Thromb Haemost. 2013 Nov;110(5):1074-9.

20. Connolly SJ, Ezekowitz MD, Yusuf S, et al. RE-LY Steering Committee and Investigators. Dabigatran versus warfarin in patients with atrial fibrillation. N Engl J Med. 2009;361(12):1139-51.
21. 日本老年医学会.「高齢者に対する適切な医療提供の指針」2011.
22. 厚生労働省.「高齢者に使用される医薬品の臨床評価法に関するガイドラインについて」(平成 5 年 12 月 2 日薬新薬第 104 号) 1997.
23. 日本老年医学会．高齢者の安全な薬物療法ガイドライン 2015． 2015
24. 文部科学省，厚生労働省．人を対象とする医学系研究に関する倫理指針（平成 26 年 12 月 22 日）．2014
25. Ruff CT, Giugliano RP, Antman EM, et al. Evaluation of the novel factor Xa inhibitor edoxaban compared with warfarin in patients with atrial fibrillation: design and rationale for the Effective aNticoaGulation with factor xA next GEneration in Atrial Fibrillation-Thrombolysis In Myocardial Infarction study 48 (ENGAGE AF-TIMI 48). Am Heart J. 2010 Oct;160(4):635-41.
26. Moriski DE, Green LW, Levine DM. Concurrent and predictive validity of a self-reported measure of medicatio adherence. Med Care. 1986;24:67-74

付　録

付録 1　CHADS$_2$スコア

	危険因子		スコア
**C**	Congestive heart failure/LV dysfunction	心不全、左室機能不全	1
**H**	Hypertension	高血圧	1
**A**	Age ≥ 75 years	75 歳以上	1
**D**	Diabetes mellitus	糖尿病	1
**S$_2$**	Stroke/TIA	脳梗塞、TIA の既往	2
	合計		0～6

TIA：一過性脳虚血発作
参照元：心房細動治療（薬物）ガイドライン（2013 年改訂版）、p.22

付録 2　CHA$_2$DS$_2$-VASc スコア

	危険因子		スコア
**C**	Congestive heart failure/LV dysfunction	心不全、左室機能不全	1
**H**	Hypertension	高血圧	1
**A$_2$**	Age ≥ 75 years	75 歳以上	2
**D**	Diabetes mellitus	糖尿病	1
**S$_2$**	Stroke/TIA/TE	脳梗塞、TIA、血栓塞栓症の既往	2
**V**	Vascular disease (prior myocardial infarction, peripheral artery desease, or aortic plaque)	血管疾患（心筋梗塞の既往、末梢動脈疾患、大動脈プラーク）	1
**A**	Age 65 – 74 years		1
**Sc**	Sex category (i.e., female gender)	性別（女性）	1
	合計		0～9*

TIA：一過性脳虚血発作
* 年齢によって 0、1、2 点が配点されるので合計は最高で 9 点にとどまる。
参照元：心房細動治療（薬物）ガイドライン（2013 年改訂版）、p.24

付録 3　HAS-BLED スコア

	臨床像	ポイント
**H**	高血圧 [1]	1
**A**	腎機能障害、肝機能障害（各 1 点）[2]	2
**S**	脳卒中	1
**B**	出血 [3]	1
**L**	不安定な国際標準比（INR）[4]	1
**E**	高齢者（> 65 歳）	1
**D**	薬剤、アルコール（各 1 点）[5]	2
	合計	9

1.　収縮期血圧> 160 mmHg
2.　腎機能障害：慢性透析や腎移植、血清クレアチニン 200 μmol/L
　　（2.26 mg/dL）以上
　　肝機能障害：慢性肝障害（肝硬変等）又は検査値異常
　　（ビリルビン値 ＞ 正常上限の 2 倍、AST、ALT、ALP
　　＞ 正常上限の 3 倍）
3.　出血歴、出血傾向（出血素因、貧血等）
4.　INR 不安定、高値又は TTR（time in therapeutic range）　< 60%
5.　抗血小板薬や NSAIDs 併用、アルコール依存症

参照元：心房細動治療（薬物）ガイドライン（2013 年改訂版）、p.25

付録 4　各 DOAC との相互作用が認められた P 糖蛋白阻害剤

DOAC 名	p-gp 阻害剤	
イグザレルト	併用禁忌	リトナビル<br>ロピナビル・リトナビル<br>アタザナビル<br>インジナビル<br>サキナビル<br>ダルナビル<br>ホスアンプレナビル<br>ネルフィナビル<br>イトラコナゾール<br>ボリコナゾール<br>ミコナゾール<br>ケトコナゾール（国内未発売）
	併用注意	クラリスロマイシン<br>エリスロマイシン
エリキュース	併用注意	イトラコナゾール<br>ボリコナゾール<br>HIV プロテアーゼ阻害剤（リトナビル等）<br>マクロライド系抗菌薬（クラリスロマイシン、エリスロマイシン等）<br>フルコナゾール<br>ナプロキセン<br>ジルチアゼム
プラザキサ	併用禁忌	イトラコナゾール
	併用注意	ベラパミル塩酸塩<br>アミオダロン塩酸塩<br>キニジン硫酸塩水和物<br>タクロリムス<br>シクロスポリン<br>クラリスロマイシン<br>リトナビル<br>ネルフィナビル<br>サキナビル等
リクシアナ	併用注意	キニジン硫酸塩水和物<br>ベラパミル塩酸塩<br>エリスロマイシン<br>シクロスポリン<br>アジスロマイシン<br>クラリスロマイリン<br>イトラコナゾール<br>ジルチアゼム<br>アミオダロン塩酸塩<br>HIV プロテアーゼ阻害剤（リトナビル等）等

参照元：添付文書「プラザキサ®」第 8 版 [12]，添付文書「イグザレルト®錠」第 6 版 [13]，添付文書「エリキュース®錠」第 6 版 [14]，添付文書「リクシアナ®錠」第 5 版 [15]
