# Supplementary material for: Current status of proton pump inhibitor use in Japanese elderly patients with non-valvular atrial fibrillation: A subanalysis of the ANAFIE Registry
Source: PLoS One. 2020 Nov 5;15(11):e0240859. doi: 10.1371/journal.pone.0240859 (PMC7644054; doi:10.1371/journal.pone.0240859)
Supplement: S3 File — (PDF) [file pone.0240859.s004.pdf]

ADCL Subject Criteria Subcohort Dataset

Variable Name	Variable Label	Variable Label (Japanese)	Type	Codelist	Details
STUDYID	Study Identifier	Study Identifier	C	$200	
USUBJID	Unique Subject Identifier	Subject number	C	$200	
SITE_NO	Study Site Number	Facility No.	C	$4	
SITE	Study Site	Name of facility	C	$200	
PID_SHORT	Study Identifier for SRL	Clintrial_Case number_SRL_Subject identification code	C	$10	
SUBA_F	Participate Subcohort A Flag	SubA_Join Flag	N		Sub Cohort A join flag<br>"1" = subject cases, "0" = not subject
SUBB_F	Participate Subcohort B Flag	SubB_Join Flag	N		Sub Cohort B join flag<br>"1" = subject cases, "0" = not subject
SUBC_F	Participate Subcohort C Flag	SubC_Join Flag	N		Sub Cohort C join flag<br>"1" = subject cases, "0" = not subject
SUBD_F	Participate Subcohort D Flag	SubD_Join Flag	N		Sub Cohort D join flag<br>"1" = subject cases, "0" = not subject
SUBE_F	Participate Subcohort E Flag	SubE_join flag	N		Sub Cohort E join flag<br>"1" = subject cases, "0" = not subject
SUBF_F	Participate Subcohort F Flag	SubF_Join Flag	N		Sub Cohort F join flag<br>"1" = subject cases, "0" = not subject
SUBG_F	Participate Subcohort G Flag	SubG_Join Flag	N		Sub Cohort G join flag<br>"1" = subject cases, "0" = not subject
STATUS_B	Status at Baseline	Baseline Status	C	$20	
PT_FLAG	Main Baseline Qualified	Target case flag	N		Main-Baseline case flag<br>"1" = subject cases, "0" = not subject

Sort by USUBJID

ADAD Subject Inclusion/Exclusion Criteria Dataset

Variable Name	Variable Label	Variable Label (Japanese)	Type	Codelist	Details
STUDYID	Study Identifier	Study Identifier	C	$200	
USUBJID	Unique Subject Identifier	Subject number	C	$200	
SITE_NO	Study Site Number	Facility No.	C	4	
A1	Inclusion Criteria 1	Failure of selection criteria_A1	N	YN [0="None", 1="Yes"]	Patients who were found to have not been definitively diagnosed with NVAF after obtaining informed consent [Inclusion criteria nonconformity = 1, inclusion criteria conformity = 0}
A2	Inclusion Criteria 2	Failure of selection criteria_A2	N	YN [0="None", 1="Yes"]	Patients who are unable to attend the hospital [Inclusion criteria nonconformity = 1, inclusion criteria conformity = 0].
A3	Inclusion Criteria 3	Failure of selection criteria_A3	N	YN [0="None", 1="Yes"]	Patients younger than 75 years at the time of informed consent [Inclusion criteria nonconformity = 1, inclusion criteria conformity = 0].
A4	Inclusion Criteria 4	Failure of selection criteria_A4	N	YN [0="None", 1="Yes"]	Patients for whom written informed consent has not been obtained [Inclusion criteria nonconformity = 1, inclusion criteria conformity = 0].
B1	Exclusion Criteria 1	To meet the exclusion criterion _B1	N	YN [0="None", 1="Yes"]	Patients who were or were known to have participated in an intervention trial after obtaining informed consent [Conflict with exclusion criteria = 1, non-conflict with exclusion criteria = 0].
B2	Exclusion Criteria 2	To meet the exclusion criterion _B2	N	YN [0="None", 1="Yes"]	Patients who were found to have a confirmed diagnosis of mitral stenosis after obtaining informed consent [Conflict with exclusion criteria = 1, non-conflict with exclusion criteria = 0].
B3	Exclusion Criteria 3	To meet the exclusion criterion _B3	N	YN [0="None", 1="Yes"]	Patients who were confirmed to have a prosthetic valve (mechanical valve, bioprosthesis) in place before obtaining informed consent after obtaining informed consent [Conflict with exclusion criteria = 1, non-conflict with exclusion criteria = 0].
B4	Exclusion Criteria 4	To meet the exclusion criterion _B4	N	YN [0="None", 1="Yes"]	Patients who have experienced a cardiovascular event (stroke, myocardial infarction, cardiac intervention other than myocardial infarction, heart failure requiring hospitalization) or bleeding with hospitalization within 1 month before enrollment after obtaining informed consent. [Conflict with exclusion criteria = 1, non-conflict with exclusion criteria = 0].
B5	Exclusion Criteria 5	To meet the exclusion criterion _B5	N	YN [0="None", 1="Yes"]	Patients who were confirmed to have been diagnosed within 1 year of life expectancy due to any disease before obtaining informed consent after obtaining informed consent [Conflict with exclusion criteria = 1, non-conflict with exclusion criteria = 0].
B6	Exclusion Criteria 6	To meet the exclusion criterion _B6	N	YN [0="None", 1="Yes"]	Other patients who are deemed unsuitable to participate in the study by their physician [Conflict with exclusion criteria = 1, non-conflict with exclusion criteria = 0].
C1	Other Criteria 1	Others _C1	N	YN [0="None", 1="Yes"]	Cases in Facilities with Significant Contract Violations [Conflict = 1, non-conflict = 0]
C2	Other Criteria 2	Others _C2	N	YN [0="None", 1="Yes"]	Patients who are known to be ineligible after registration [Conflict = 1, non-conflict = 0]
C3	Other Criteria 3	Other _C3	N	YN [0="None", 1="Yes"]	Data not available in cases where consent has been withdrawn [Conflict = 1, non-conflict = 0]
C4	Other Criteria 4	Others _C4	N	YN [0="None", 1="Yes"]	Cases that were incorrectly registered in the system, such as duplicate, etc. [Conflict = 1, non-conflict = 0]
C5	Other Criteria 5	Others _C5	N	YN [0="None", 1="Yes"]	Case of baseline data not recalled (including during requesting review) [Conflict = 1, non-conflict = 0]
AD_FLAG	Criteria Flag	Case withdrawal flags	N	YN [0="None", 1="Yes"]	=1 when either of the inclusion criteria is 1. All 0 = 0

Sort by USUBJID

Data set specifications for analysis

ADSL Subject-Level Analysis Dataset

Variable Name	Variable Label	Variable Label (Japanese)	Type	Codelist
STUDYID	Study Identifier		C	$200
USUBJID	Unique Subject Identifier	Subject number	C	$200
SITE	Study Site	Name of facility	C	$200
AGE	Age at First Drug Intake	Age	N	
AGEU	Unit of Age	Units of age	C	$200
AGEC	Age Category	Age category	N	AGECT [1 = "75 years of age or older and younger than 80 years of age", 2 = "80 years old or older and younger than 85 years old", 3 = "85 years old or older and younger than 90 years old", 4 = "90 years and older and younger than 95 years," 5 = "aged ≧95 years and <100 years," 6 = "100 years or older"]
SEX	Sex	Sex	N	SEXN [0 = male, 1 = female].
SEXN	Sex (N)	Gender (number)	N	
ICDTC	Date of Informed Consent	Date of consent	YYMMDD10.	
ENRLFL	Enrolled Population Flag	Registration confirmed case flag: Flag of cases with date of informed consent	N	1
INCFL	Inclusion Flag	Selection criteria flag	N	1
EXCFL	Exclusion Flag	Exclusion criteria flag	N	1
UQLFYD	Unqualified after Enrollment	Disqualification after Registration	N	1
FASFL	Full Analysis Set Population Flag	Flags for Efficacy Analysis	N	1
NDGADENL	Non-Drug Administration after Enrollment	Anticoagulant-naive patients after registration	N	1
TRSDT	Date of First Exposure	Starting date of drug [illegible]		
TREDT	Date of Last Exposure	Last day of drug administration		
TRSDUR	Duration of Drug Exposure	Duration of drug use (days)	N	
HGT_BL	Height at Informed Concent	Height (cm) at baseline	N	
WGT_BL	Weight at Informed Concent	Baseline body weight (kg)	N	
WGTC_BL	Weight Category at Informed Concent	Baseline weight (kg) categories	N	WGTC [1 = "less than 60 kg" and 2 = "more than 60 kg]]
BMI_BL	BMI at Informed Consent	BMI (kg per m^2) at baseline.	N	
BMIC_BL	BMI Category at Informed Consent	BMI(kg/m^2) categories at baseline.	N	BMIC [1 = "<18.5kg/m2", 2 = "18.5kg/m2 or more and <25.0kg/m2", 3 = "25.0kg/m2 or more"]
AFTYP	Type of AF	Type of atrial fibrillation	N	AFTYP [1 = "paroxysmal", 2 = "persistent", 3 = "long-lasting", 4 = "Persistence"]
AFTYP1	Type of AF Alternate	Atrial fibrillation type 1	N	AFTYPA: [1 = "paroxysmal", 2 = "persistent" and "long-lasting", 4 = "Persistence"]
HISAF	History of Major Surgery for non-AF	History of major surgery other than AF	N	HISAF [1 = "Major surgery within the last 3 months", 2="No major surgery within the
HISBDYN	History of Major Beeding	History of major bleeding	N	YN [0 = none, 1 = yes]
HISBD1	Intracranial Hemorrhage	Major hemorrhage: Intracranial hemorrhage	N	YN [0 = none, 1 = yes]
HISBD2	Upper Gastrointestinal Bleeding	Major bleeding: Upper gastrointestinal bleeding	N	YN [0 = none, 1 = yes]
HISBD3	Lower Gastrointestinal Bleeding	Major bleeding: Lower gastrointestinal bleeding	N	YN [0 = none, 1 = yes]

Data set specifications for analysis

ADSL Subject-Level Analysis Dataset

Variable Name	Variable Label	Variable Label (Japanese)	Type	Codelist
HISBD4	Other Beeding	Major bleeding: Other	N	YN<br>[0 = none, 1 = yes]
HISAL	History of Drug Allergy	History of drug allergy	N	YN<br>[0 = none, 1 = yes]
SMK	Smoking Status	Smoking habit	N	SMK<br>[1 = "never smoked",<br>2 = "1 year or more after stopping,"<br>3="Stopped within 1 year",<br>4="Smoking"; 9="Unknown"]
SMK1	Smoking Status Part 1	Smoking habit 1	N	SMKA<br>[1 = "never smoked",<br>2="stopped",<br>4="Smoking,"<br>9 = "unknown"]
DRK	Drinking Habit	Drinking habit	N	DRK [1 = "drink on a daily basis,"<br>2="Sometimes I drink."<br>3 = "never drink",<br>9 = "unknown"]
AFTHSPE	Non-drug Therapy for AF	Prior nonpharmacologic therapy for AF	N	YN<br>[0 = none, 1 = yes]
AFTHSPE1	Catheter Ablation	Catheter ablation	N	YN<br>[0 = none, 1 = yes]
AFTHSPE2	Cardioversion	Electric defibrillation	N	YN<br>[0 = none, 1 = yes]
AFTHSPE3	ICD	ICD	N	YN<br>[0 = none, 1 = yes]
AFTHSPE4	Pacemaker Implantation	Pacemaker implantation	N	YN<br>[0 = none, 1 = yes]
AFTHSPE5	Other Non-drug Therapy	Non-Pharmacotherapy: Other	N	YN<br>[0 = none, 1 = yes]
EXBLDTC	Date of Examinations at Baseline	Baseline test date	YYMMDD10.	
EXBLDUR	Days from Examination and Informed Consent	Period from the date of examination to the time of obtaining informed consent	N	
EXBLFL	Examination Range Flag	Acceptable range adoption flag for test date	N	RNG<br>[1 = within acceptable limits]
SBP_BL	Systolic Blood Pressure at Informed Consent	Baseline office BP:systolic (mmHg)	N	
DBP_BL	Diastolic Blood Pressure at Informed Concent	Baseline office BP:diastolic (mmHg)	N	
HGB_BL	Hemoglobin at Baseline	Haemoglobin (g/dL) at baseline.	N	
CRE_BL	Serum Creatinine at Baseline	Serum creatinine (g/dL) at baseline.	N	
CRC_BL	Creatinine Clearance at Baseline	Baseline creatinine clearance (mL/min	N	
CRCC_BL	Category of Creatinine Clearance at Baseline	Creatinine clearance (mL/min) categories.	N	CRCCT<br>[1 = "less than 15mL/min",<br>2 = "15mL/min or more and less than 30mL/min",<br>3 = "30mL/min or more and less than 50mL/min",<br>4 = "50mL/min or more and less
EGFR_BL	Estimated Glomerular Filtration Rate at Baseline	eGFR (mL/min/1.73m²) at baseline.	N	

Data set specifications for analysis

ADSL Subject-Level Analysis Dataset

Variable Name	Variable Label	Variable Label (Japanese)	Type	Codelist
EGFRC_BL	Category of Estimated Glomerular Filtration Rate at Baseline	eGFR (mL/min/1.73m^2) categories at baseline.	N	EGFRCT<br>[1="less than 15,"<br>2 = "15 or more and less than 30",<br>3="30 to less than 45",<br>4 = "45 to less than 60",<br>5 = "60 to less than 90",<br>6="more than 90",<br>9 = "missing"]
HBA_BL	Hemoglobin A1c at Baseline	Baseline HbA 1 c(%)	N	
HBAC_BL	Category of Hemoglobin A1c at Baseline	HbA1c (%) categories at baseline.		HBACT<br>[1="less than 6.0%,"<br>2 = "6.0% or more but less than 7.0%,"<br>3="7.0% to 8.0%,"<br>4 = "8.0% or more",<br>9 = "missing"]
CHFDFL	Congestive Heart Failure/LV Dysfunction Flag	C: Heart failure or left ventricular dysfunction flag	N	YN<br>[0="None", 1="Yes"]
HYPTFL	Hypertension Flag	H: Hypertension flag	N	YN<br>[0="None", 1="Yes"]
AGE75FL	Age 75 Years or Older Flag	A:75 Flag for age or older	N	YN<br>[0="None", 1="Yes"]
DIAMFL	Diabetes Mellitus Flag	D: Diabetes flag	N	YN<br>[0="None", 1="Yes"]
CERIFL	Cerebral Infarction Flag	S: Previous flag for cerebral infarction	N	Y2N<br>[0="None", 2="Yes"]
CHADS2	CHADS2 Score	CHADS2 Score	N	
AGE75VFL	Age 75 Years or Older Flag	A2: Flag over 75	N	Y2N<br>[0="None", 2="Yes"]
TIATEFL	Stroke/TIA/TE	S2: Patients with a history of atherosclerotic, cardiogenic, lacunar, unclassified/unspecified cerebral infarction, TIA, pulmonary embolism, deep vein thrombosis, and other thromboembolic-related disorders	N	Y2N<br>[0="None", 2="Yes"]
VSDSFL	Vascular disease	V: Patients with a history of vascular disease (myocardial infarction, peripheral vascular disorder, internal carotid artery stenosis, arteriosclerosis obliterans or aortic plaque)	N	YN<br>[0="None", 1="Yes"]
AGE65FL	Age 65 Years to Less Than 75 Years Old Flag	A: 65 years and older and younger than 75 years	N	YN<br>[0="None", 1="Yes"]
SEXFL	Sex Flag	Sc: Female	N	YN<br>[0="None", 1="Yes"]
CHA2DS2V	CHA2DS2-VASc Score	CHA 2 DS 2-VASc Score	N	
SBP160FL	SBP at Baseline greater than 160 Flag	H: Hypertension (systolic blood pressure >=160mmHg)	N	YN<br>[0="None", 1="Yes"]
RNDFL	Renal Dysfunction Flag	A: Chronic kidney disease, severe renal impairment (chronic dialysis/kidney transplantation, serum creatinine 2.26 mg/dL)	N	YN<br>[0="None", 1="Yes"]
STRFL	Stroke Flag	S: Stroke	N	YN<br>[0="None", 1="Yes"]
BLDFL	Bleed Flag	B: Bleeding (bleeding history, bleeding tendency (bleeding diathesis, anaemia, etc.))	N	YN<br>[0="None", 1="Yes"]
PTINRFL	PT-INR Flag	L: Unstable International Normalized Ratio (INR) (INR unstable, high or TTR < 60%)	N	YN<br>[0="None", 1="Yes"]
AGE65EFL	Age 65 Years or Older Flag	E: Elderly (above age 65)	N	YN<br>[0="None", 1="Yes"]
DGALFL	Drug or Alcohol	D: Drugs, alcohol (antiplatelet drugs, combination NSAIDs, alcoholism)	N	YN<br>[0="None", 1="Yes"]
HASBLED	HAS-BLED Score	HAS-BLED scoring	N	

Sort by USUBJID

Data set specifications for analysis

ADMH Medical History Analysis Dataset

Variable Name	Variable Label	Variable Label (Japanese)	Type	Codelist
STUDYID	Study Identifier		C	$200
USUBJID	Unique Subject Identifier	Subject number	C	$200
SITE	Study Site	Name of facility	C	$200
AGE	Age at First Drug Intake	Age	N	
AGEU	Unit of Age	Units of age	C	$200
SEX	Sex	Sex	N	SEXN [0 = male, 1 = female].
SEXN	Sex (N)	Gender (number)	N	
ICDTC	Date of Informed Consent	Date of consent	YYMMDD10.	
FASFL	Full Analysis Set Population Flag	Flags for Efficacy Analysis	N	1
TRSDT	Date of First Exposure	Starting date of drug		
TREDT	Date of Last Exposure	Last day of drug administration		
TRSDUR	Duration of Drug Exposure	Duration of drug use (days)	N	
MHYN	Medical History	Presence or absence of complications or medical history	N	YN [0="None", 1="Yes"]
HBPYN	High Blood Pressure	Hypertension	N	YN [0="None", 1="Yes"]
DBYN	Diabetes	Diabetes mellitus	N	YN [0="None", 1="Yes"]
DB1YN	Diabetic Retinopathy	Diabetes: Diabetic retinopathy	N	YN [0="None", 1="Yes"]
DB2YN	Diabetic Nephropathy	Diabetes: Diabetic nephropathy	N	YN [0="None", 1="Yes"]
DB3YN	Diabetic Neuropathy	Diabetes Mellitus: Diabetic Neuropathy	N	YN [0="None", 1="Yes"]
LMDYN	Lipid metabolism disorders	Abnormality of lipid metabolism	N	YN [0="None", 1="Yes"]
HYPURNYN	Hyperuricemia	Hyperuricemia	N	YN [0="None", 1="Yes"]
KDDSYN	Kidney Disease	Kidney disease	N	YN [0="None", 1="Yes"]
KDDS1YN	Chronic Kidney Disease	Kidney Disease: Chronic Kidney Disease	N	YN [0="None", 1="Yes"]
KDDS2YN	Advanced Renal Dysfunction without Dyalysis	Kidney disease: Severe renal impairment (no dialysis)	N	YN [0="None", 1="Yes"]
KDDS3YN	Advanced Renal Dysfunction with Dyalysis	Kidney disease: Severe renal impairment (with dialysis)	N	YN [0="None", 1="Yes"]
SLVDYN	Severe Liver Dysfunction	Severe liver dysfunction	N	YN [0="None", 1="Yes"]
RESDYN	Respiratory Disease	Respiratory disease	N	YN [0="None", 1="Yes"]
HTDYN	Heart Disease	Cardiac disorders	N	YN [0="None", 1="Yes"]
HTD1YN	Myocardial Infarction	Cardiac Disease: Myocardial Infarction	N	YN [0="None", 1="Yes"]
HTD2YN	Angina Pectoris	Heart Disease: Angina	N	YN [0="None", 1="Yes"]
HTD3YN	Heart Failure	Heart disease: Heart failure (including those without or with controlled congestion)	N	YN [0="None", 1="Yes"]
HTD4YN	Left Ventricular Systolic Dysfunction	Heart Disease: Left Ventricular Systolic Dysfunction	N	YN [0="None", 1="Yes"]
HTD5YN	Valvular Disease	Cardiac disease: Valvular disease (including postoperative)	N	YN [0="None", 1="Yes"]
HTD6YN	Cardiomyopathy	Cardiac Disease: Cardiomyopathy	N	YN [0="None", 1="Yes"]
HTD7YN	Hypertensive Heart Disease	Heart Disease: Hypertensive Heart Disease	N	YN [0="None", 1="Yes"]
HTD8YN	Cognenital Heart Disease	Heart Disease: Congenital Heart Disease	N	YN [0="None", 1="Yes"]
HTD9YN	Other Heart Disease	Heart Diseases: Other	N	YN [0="None", 1="Yes"]
CVACYN	Cerebrovascular Accident	Cerebrovascular disorders	N	YN [0="None", 1="Yes"]
CVAC1YN	Atherosclerotic Cerebral Infarction	Cerebrovascular disorders: atherosclerotics cerebral infarction	N	YN [0="None", 1="Yes"]
CVAC2YN	Cardiogenic Cerebral Infarction	Cerebrovascular disorders: Cardiogenic cerebral infarction	N	YN [0="None", 1="Yes"]
CVAC3YN	Lacunar Cerebral Infarction	Cerebrovascular disorders: Lacunar cerebral infarction	N	YN [0="None", 1="Yes"]
CVAC4YN	Hemorrhagic Stroke	Cerebrovascular disorders: Hemorrhagic stroke	N	YN [0="None", 1="Yes"]
CVAC5YN	Unclassified/Unknown Cerebral Infarction	Cerebrovascular disorders: Unclassified/unspecified cerebral infarction	N	YN [0="None", 1="Yes"]

ADMH Medical History Analysis Dataset

Variable Name	Variable Label	Variable Label (Japanese)	Type	Codelist
CVAC6YN	Transient Ischemic Attacks	Cerebrovascular disorders; TIA	N	YN [0="None", 1="Yes"]
CVAC7YN	Other Cerebrovascular Accident	Cerebrovascular disorders: Other	N	YN [0="None", 1="Yes"]
OTVDYN	Other Vascular Disease	Other vascular diseases	N	YN [0="None", 1="Yes"]
OTVD1YN	Peripheral Vascular Disease	Other vascular diseases: Peripheral vascular disorders	N	YN [0="None", 1="Yes"]
OTVD2YN	Aortic Plaque	Other Vascular Diseases: Aortic Plaque	N	YN [0="None", 1="Yes"]
OTVD3YN	Other Vascular Disease: Other	Other vascular diseases: Other	N	YN [0="None", 1="Yes"]
TEDYN	Thrombosis/Embolism-Related Disease	Thrombotic and embolic disease	N	YN [0="None", 1="Yes"]
TED1YN	Pulmonary Embolism	Thromboembolic disease: Pulmonary embolism	N	YN [0="None", 1="Yes"]
TED2YN	Deep Vein Thrombosis	Thrombosis/embolism-related disorders: Deep vein thrombosis	N	YN [0="None", 1="Yes"]
TED3YN	Internal Carotid Artery Stenosis	Thromboembolic Disorders: Internal Carotid Artery Stenosis	N	YN [0="None", 1="Yes"]
TED4YN	Arteriosclerosis Obliterans	Thromboembolism-related disease: arteriosclerosis obliterans	N	YN [0="None", 1="Yes"]
TED5YN	Other Thrombosis/Embolism-Related Disease	Thrombus and embolism-related diseases: Other	N	YN [0="None", 1="Yes"]
HTDMYN	Hyperthyroidism	Hyperthyroidism	N	YN [0="None", 1="Yes"]
GASDYN	Gastrointestinal Disease	Digestive disorder	N	YN [0="None", 1="Yes"]
GASD1YN	Upper Gastrointestinal Tract	Gastrointestinal disease: Upper gastrointestinal tract resected	N	YN [0="None", 1="Yes"]
GASD2YN	Lower Gastrointestinal Tract	Gastrointestinal disease: Lower gastrointestinal tract resected	N	YN [0="None", 1="Yes"]
GASD3YN	Reflux esophagitis	Gastrointestinal Diseases: Reflux Esophagitis	N	YN [0="None", 1="Yes"]
GASD4YN	Other Gastrointestinal Disease	Gastrointestinal Diseases: Other	N	YN [0="None", 1="Yes"]
MGTYN	Malignant Tumor	Malignant tumor (tumor-bearing condition) Primary cancer only	N	YN [0="None", 1="Yes"]
MGT1YN	Gastric Cancer	Malignancy: Gastric cancer	N	YN [0="None", 1="Yes"]
MGT2YN	Colorectal Cancer	Malignancy: Colorectal Cancer	N	YN [0="None", 1="Yes"]
MGT3YN	Lung Cancer	Malignancy: Lung Cancer	N	YN [0="None", 1="Yes"]
MGT4YN	Brest Cancer	Malignancy: Breast Cancer	N	YN [0="None", 1="Yes"]
MGT5YN	Uterus/Ovarian Cancer	Malignancy: Uterine and ovarian cancer	N	YN [0="None", 1="Yes"]
MGT6YN	Pancreatic Cancer	Malignancy: Pancreatic Cancer	N	YN [0="None", 1="Yes"]
MGT7YN	Other Malignant Tumor	Malignancy: Other	N	YN [0="None", 1="Yes"]
ALZHYN	Alzheimer	Dementia	N	YN [0="None", 1="Yes"]
OHCPYN	Other Complications	Other complications other than those listed above	N	YN [0="None", 1="Yes"]
IMGYN	Brain Imaging Test	Implementation of brain imaging	N	YNU [0 = "none", "1 = "present", "9 = "unknown]]
FNDYN	Brain Imaging Result	Abnormal brain imaging findings	N	YNU [0="None" and 1="With", 9 = "unknown"]
FALYN	Fall Down within one year	Falls within 1 year	N	YNU [0="None" and 1="With", 9 = "unknown"]

Sort by USUBJID

Data set specifications for analysis

ADCM1 Concomitant Drug Analysis Dataset Concomitant Dirug Analysis Dataset

Variable Name	Variable Label	Variable Label (Japanese)	Type	Codelist
STUDYID	Study Identifier		C	$200
USUBJID	Unique Subject Identifier	Subject number	C	$200
SITE	Study Site	Name of facility	C	$200
AGE	Age at First Drug Intake	Age	N	
AGEU	Unit of Age	Units of age	C	$200
SEX	Sex	Sex	N	SEXN<br>[0 = male, 1 = female].
SEXN	Sex (N)	Gender (number)	N	
ICDTC	Date of Informed Consent	Date of consent	YYMMDD10.	
FASFL	Full Analysis Set Population Flag	Flags for Efficacy Analysis	N	1
TRSDT	Date of First Exposure	Starting date of drug [illegible]		
TREDT	Date of Last Exposure	Last day of drug administration		
TRSDUR	Duration of Drug Exposure	Duration of drug use (days)	N	
VISITCT	Visit	Survey period	N	VISITN<br>[1="Baseline"<br>2="12 months"<br>3 = "after 24 months"]
VISITCTN	Visit (N)	Survey Timing Categories	N	
CMCNT1	Number of Types of Internal Medicines	Number of oral medications	N	
CMCNT1NK	Unknown Number of Types of Internal Medicines	Oral medication type unknown	N	NK<br>[1 = "unknown"]]
CMCNT1CT	Category for Number of Types of Internal Medicines	Several categories of oral medications	N	CMCT<br>[1 = "0 or more and 4 or less",<br>2="more than 5 and less than 9",<br>3="10 or more",<br>9 = "unknown"]
CMYN	Administration of Concomitant Drug	Dose of concomitant drugs other than anticoagulants	N	YNU<br>[0="None" and 1="With",<br>9 = "unknown"]
ARTD	Arrhythmic Drugs	Arrhythmic drug	N	YN<br>[0 = none, 1 = yes]
ARTD1	Rhythm Control	Arrhythmia Drugs: Rhythm Control	N	YN<br>[0 = none, 1 = yes]
ARTD2	Rate Control	Arrhythmia Drugs: Rate Control	N	YN<br>[0 = none, 1 = yes]
APLD	Anti-Platelet Drugs	Antiplatelet drugs	N	YN<br>[0 = none, 1 = yes]
APLD1	ASA	Antiplatelet agents; ASA:	N	YN<br>[0 = none, 1 = yes]
APLD2	P2Y12-I	Antiplatelet drugs :P 2 Y 12-I	N	YN<br>[0 = none, 1 = yes]
APLD3	Other Anti-Platelet Drugs	Antiplatelet agents: Other	N	YN<br>[0 = none, 1 = yes]
AHPD	Antihypertensive Drugs	Antihypertensives	N	YN<br>[0 = none, 1 = yes]
LTD	Lipid Therapy	Lipid therapeutics	N	YN<br>[0 = none, 1 = yes]
LTD1	Statins	Lipid treatments: Statins	N	YN<br>[0 = none, 1 = yes]
LTD2	Other Than Statins	Drugs for lipid treatment: Beyond statins	N	YN<br>[0 = none, 1 = yes]
DBTD	Diabetes Drugs	Diabetes pills	N	YN<br>[0 = none, 1 = yes]
DBTD1	Sulfonylurea Drugs	Diabetes Drugs: SU Agents	N	YN<br>[0 = none, 1 = yes]
DBTD2	α-Glucosidase Inhibitor	Diabetes pills : α GI	N	YN<br>[0 = none, 1 = yes]

Data set specifications for analysis

ADCM1	Concomitant Drug Analysis Dataset	Concomitant Dirug Analysis Dataset		
Variable Name	Variable Label	Variable Label (Japanese)	Type	Codelist
DBTD3	Thiazolidine	Diabetes: Thiazolidines	N	YN [0 = none, 1 = yes]
DBTD4	DPP-4 Inhabitors	Diabetes pills :DPP-4	N	YN [0 = none, 1 = yes]
DBTD5	GLP-1 Inhabitors	Diabetes pills :GLP-1	N	YN [0 = none, 1 = yes]
DBTD6	SGLT2 Inhabitors	Diabetes pills :SGLT2	N	YN [0 = none, 1 = yes]
DBTD7	Insuline Related	Diabetes Drugs: Insulin	N	YN [0 = none, 1 = yes]
DBTD8	Other Diabetes Drugs	Diabetes Drugs: Other	N	YN [0 = none, 1 = yes]
DMM	Dementia Medications	Drugs for dementia	N	YN [0 = none, 1 = yes]
ACNA	Anti-Cancer Agent	Anti-cancer drugs	N	YN [0 = none, 1 = yes]
COPD	COPD	Drugs for chronic obstructive pulmonary disease (COPD)	N	YN [0 = none, 1 = yes]
PSYD	Psychotropic Drugs	Psychoactive drugs	N	YN [0 = none, 1 = yes]
PRPI	Proton Pump Inhabitors	Proton pump inhibitors	N	YN [0 = none, 1 = yes]
PGPI	P-gp Inhabitors	P-glycoprotein (P-gp) inhibitors	N	YN [0 = none, 1 = yes]
TCMCNT	Total Number of Drugs Including Anticoagulant	Total number of drugs for use in combination with anticoagulants	N	
TCMCNTCT	Category for Total Number of Drugs Other than Anticoagulant	Total number of concomitant medications other than anticoagulants category	N	TCMCT [1 = "0 or more and 4 or less", 2="more than 5 and less than 9", 3="10 or more" 999 = "unknown"]

Sort by USUBJID VISITCTN

Data set specifications for analysis

ADCM2 Administration Status of Anticoagulant Analysis Dataset

Variable Name	Variable Label	Variable Label (Japanese)	Type	Codelist
STUDYID	Study Identifier		C	$200
USUBJID	Unique Subject Identifier	Subject number	C	$200
SITE	Study Site	Name of facility	C	$200
AGE	Age at First Drug Intake	Age	N	
AGEU	Unit of Age	Units of age	C	$200
SEX	Sex	Sex	N	SEXN [0 = male, 1 = female].
SEXN	Sex (N)	Gender (number)	N	
ICDTC	Date of Informed Consent	Date of consent	YYMMDD10.	
FASFL	Full Analysis Set Population Flag	Flags for Efficacy Analysis	N	1
TRSDT	Date of First Exposure	Starting date of drug		
TREDT	Date of Last Exposure	Last day of drug administration		
TRSDUR	Duration of Drug Exposure	Duration of drug use (days)	N	
CM2NUM	Administration Number	Dose No.	N	
VISITCT	Visit	Survey period	N	VISITN [1="Baseline" 2="12 months" 3 = "after 24 months"]
VISITCTN	Visit (N)	Survey Timing Categories	N	
ADYN	Administration of Anticoagulant	Presence of anticoagulants	N	YN [0="None", 1="Yes"]
VISITAD	Visit and Drug Administration	Time of the start of administration	N	VISITAD [1 = "before 1 month", 2 = "within 1 month", 3 = "first dose", 9 = "unknown"]
ACTYP	Type of Anticoagulant	Type of anticoagulant	N	ACTYP [1 = "warfarin", 2="Dabigatran," 3 = "rivaroxaban", 4 = "apixaban", 5 = "edoxaban", 6 = "parenteral anticoagulants", 7 = "edoxaban (OD)"]
DOSE	Dose per day for DOAC only	Daily dose (DOAC only)/(mg)	N	
DOSECNT	Dose per 1time for DOAC only	Number of daily doses (DOAC only)/(times)	N	
STDTC	Date of Start Administration	Day of start of administration	YYMMDD10.	
STADTC	Around Date of Start Administration	Approximately 1	N	AROUND [1="Around"]
STCNT	Continuation at start	Continue from previous Visit	N	STCONT [1 = "continued from the previous Visit]]
ENDTC	Date of End Administration	Completion of administration	YYMMDD10.	
ENADTC	Around Date of End Administration	Approximately 2	N	AROUND [1="Around"]
ENCNT	Continuation	Continuation	N	ENCONT [1 = "continued]]
DSRSN	Reason for Discontinuation	Change/discontinuation of administration	N	YN [0="None",1="Yes"]
DSRSN1	Reason for Adverse Events	Event/AE	N	YN [0="None",1="Yes"]
DSRSN2	Reason for AF	Nonpharmacologic therapy for AF	N	YN [0="None",1="Yes"]
DSRSN3	Reason for Invasive Procedures	Performing invasive procedures	N	YN [0="None",1="Yes"]
DSRSN4	Reason for Decreased Renal Function	Poor kidney function	N	YN [0="None",1="Yes"]
DSRSN5	Reason for Decreased Weight	Weight loss	N	YN [0="None",1="Yes"]
DSRSN6	Reason for P-glycoprotein Inhibitors	Combination of P-glycoprotein inhil	N	YN [0="None",1="Yes"]
DSRSN7	Reason for Fear of Bleeding	Fear of bleeding without a definite cause of bleeding	N	YN [0="None",1="Yes"]

ADCM2 Administration Status of Anticoagulant Analysis Dataset

Variable Name	Variable Label	Variable Label (Japanese)	Type	Codelist
DSRSN8	Reason for Patient	Patient preferences	N	YN [0="None",1="Yes"]
DSRSN9	Reason for Patient Improved Convenience	Improving patient convenience	N	YN [0="None",1="Yes"]
DSRSN10	Other Reason	Other	N	YN [0="None",1="Yes"]

Sort by USUBJID VISITCTN CM2NUM

Data set specifications for analysis

ADLB1 PI-INR Analysis Dataset

Variable Name	Variable Label	Variable Label (Japanese)	Type	Codelist
STUDYID	Study Identifier		C	$200
USUBJID	Unique Subject Identifier	Subject number	C	$200
SITE	Study Site	Name of facility	C	$200
AGE	Age at First Drug Intake	Age	N	
AGEU	Unit of Age	Units of age	C	$200
SEX	Sex	Sex	N	SEXN<br>[0 = male, 1 = female].
SEXN	Sex (N)	Gender (number)	N	
ICDTC	Date of Informed Consent	Date of consent	YYMMDD10.	
FASFL	Full Analysis Set Population Flag	Flags for Efficacy Analysis	N	1
TRSDT	Date of First Exposure	Starting date of drug [illegible]		
TREDT	Date of Last Exposure	Last day of drug administration		
TRSDUR	Duration of Drug Exposure	Duration of drug use (days)	N	
VISITCT	Visit	Survey period	N	VISITN<br>[1="Baseline"<br>2="12 months"<br>3 = "after 24 months"]
VISITCTN	Visit (N)	Survey Timing Categories	N	
LB1NO	LB1 Number	Order of CRFs for PT-INR level	N	
LBYN1		Presence or absence of PT-INR readings	N	YNU<br>[0="None" and 1="With",<br>9 = "unknown"]
POINT	PT-INR Point	Sum of PTINR measured points	N	
WFFG	Warfarin Flag	Warfarin dosing flag	N	
PTINRFR	PT-INR Flag Range	PT-INR range	N	
PTINRFGB	PT-INR Flag of Baseline	PT-INR use flag at baseline	N	
PTINR_DTC1	Date/Time of Specimen Collection	Date of PT-INR determination	YYMMDD10.	
PTINR_DTC0	Date/Time of Previous Specimen Collection	Date of last PT-INR determination	YYMMDD10.	
PTINR_VAL1	PT-INR Value	PT-INR level	N	

ADLB1 PI-INR Analysis Dataset

Variable Name	Variable Label	Variable Label (Japanese)	Type	Codelist
PTINR_VAL0	PT-INR Previous Value	Previous PT-INR	N	
PTINR_NKND	PT-INR Unknown	PT-INR not measured/unknown	N	NK<br>[1 = "unknown]]
DSLDTC	Days Since Last Date/Time of Specimen Collection	Number of days	N	
PTINRDIFF	PT-INR Diff	PT-INR difference	N	
PINRWR	Previous PT-INR Within Range	Flags within and outside the previous PT-INR level	N	RGCT<br>[0="less than 1.6",<br>1 = "1.6 to 2.6" or less,<br>2="greater than 2.6"]
CINRWR	Current PT-INR Within Range	PT-INR Out-of-Range Flag	N	RGCT<br>[0="less than 1.6",<br>1 = "1.6 to 2.6" or less,<br>2="greater than 2.6"]
SCENARIO	Scenario	TTR Calculation Flag	N	
FIRFG1	FIR Flag1	FIR calculation flag 1	N	
FIRFG2	FIR Flag2	FIR calculation flag 2	N	
PINRDAR	PT-INR Diff Above Range	Difference greater than the range	N	
PINRDBR	PT-INR Diff Below Range	Difference less than the range	N	
PINRDWR	PT-INR Diff Within Range	Differences within the range	N	

ADLB1 PI-INR Analysis Dataset

Variable Name	Variable Label	Variable Label (Japanese)	Type	Codelist
DWRSLPER	Days within Range since Last Date/Time of Specimen Collection	Percentage of days within the range	N	
DWRSL	Days within Range since Last Date/Time of Specimen Collection	Days within the range	N	

Sort by USUBJID VISITCTN PTINR_DTC1 LB1NO

Methods for calculating TTRs using Rosendaal method

Data set specifications for analysis

Format

FMTNAME	DESCRIPTION	TYPE	CODE	LABEL
AGECT	Age	N	1	75 years and older and younger than 80 years
AGECT	Age	N	2	80 years and older and younger than 85 years
AGECT	Age	N	3	85 years old or older and younger than 90 years old
AGECT	Age	N	4	90 years and older and younger than 95 years
AGECT	Age	N	5	Age ≧ 95 and < 100 years
AGECT	Age	N	6	100 years or older
SEXN	Sex	N	0	Man
SEXN	Sex	N	1	Woman
WGTC	Weight Category	N	1	Less than 60 kg
WGTC	Weight Category	N	2	60 kg or more
BMIC	BMI Category	N	1	Less than 18.5kg/m2
BMIC	BMI Category	N	2	18.5kg/m2 and less than 25.0kg/m2
BMIC	BMI Category	N	3	25.0kg/m2 or more
AFTYP	Type of AF	N	1	Paroxysmal
AFTYP	Type of AF	N	2	Persistence
AFTYP	Type of AF	N	3	Long-term persistence
AFTYP	Type of AF	N	4	Persistence
HISAF	History of Sugery for non-AF	N	1	Major surgery within the last 3 months
HISAF	History of Sugery for non-AF	N	2	No major surgery within the last 3 months
HISAF	History of Sugery for non-AF	N	9	Unknown
SMK	Smoking Status	N	1	Have never smoked
SMK	Smoking Status	N	2	More than a year after quitting
SMK	Smoking Status	N	3	Quit within a year
SMK	Smoking Status	N	4	Be breathing
SMK	Smoking Status	N	9	Unknown
SMKA	Smoking Status Part1	N	1	Have never smoked
SMKA	Smoking Status Part1	N	2	To discontinue
SMKA	Smoking Status Part1	N	4	Be breathing
SMKA	Smoking Habit	N	9	Unknown
DRK	Drinking Habit	N	1	Drink routinely
DRK	Drinking Habit	N	2	Occasional drinking
DRK	Drinking Habit	N	3	Never drink
DRK	Drinking Habit	N	9	Unknown
RNG	Range	N	1	Within acceptable limits
CRCCT	Category of Creatinine Clearance	N	1	<15 mL/min
CRCCT	Category of Creatinine Clearance	N	2	15 mL/min or more and less than 30mL/min
CRCCT	Category of Creatinine Clearance	N	3	30 mL/min or more and less than 50mL/min
CRCCT	Category of Creatinine Clearance	N	4	50 mL/min to less than 80mL/min
CRCCT	Category of Creatinine Clearance	N	5	80 mL/min or more
CRCCT	Category of Creatinine Clearance	N	9	Missing data
EGFRCT	Category of eGFR	N	1	Less than 15
EGFRCT	Category of eGFR	N	2	15 to less than 30
EGFRCT	Category of eGFR	N	3	30 to less than 45
EGFRCT	Category of eGFR	N	4	45 to less than 60
EGFRCT	Category of eGFR	N	5	60 to less than 90
EGFRCT	Category of eGFR	N	6	90 or more
EGFRCT	Category of eGFR	N	9	Missing data
HBACT	Category of Hemoglobin A1c	N	1	<6.0%
HBACT	Category of Hemoglobin A1c	N	2	6.0% to less than 7.0%
HBACT	Category of Hemoglobin A1c	N	3	Not less than 7.0% and not more than 8.0%.
HBACT	Category of Hemoglobin A1c	N	4	8.0% or more
HBACT	Category of Hemoglobin A1c	N	9	Missing data
YN	Yes or No	N	0	None
YN	Yes or No	N	1	Present
Y2N	Yes or No	N	0	None
Y2N	Yes or No	N	2	Present
YNU	Yes or No	N	0	None
YNU	Yes or No	N	1	Present
YNU	Yes or No	N	9	Unknown
VISITN	Category of VISIT	N	1	Baseline
VISITN	Category of VISIT	N	2	After 12 months
VISITN	Category of VISIT	N	3	After 24 months
VISITAD	Category for Timing of Administration	N	1	Before 1 month
VISITAD	Category for Timing of Administration	N	2	Within 1 month
VISITAD	Category for Timing of Administration	N	3	First dose
VISITAD	Category for Timing of Administration	N	9	Unknown
CMCT	Category for Type of Concomitant Drug	N	1	0 to 4 or less
CMCT	Category for Type of Concomitant Drug	N	2	5 or more and 9 or less

Data set specifications for analysis

Format

FMTNAME	DESCRIPTION	TYPE	CODE	LABEL
CMCT	Category for Type of Concomitant Drug	N	3	10 or more
CMCT	Category for Type of Concomitant Drug	N	9	Unknown
NK	Unknown	N	1	Unknown
TCMCT	Total Category for Type of Concomitant Drug	N	1	0 to 4 or less
TCMCT	Total Category for Type of Concomitant Drug	N	2	5 or more and 9 or less
TCMCT	Total Category for Type of Concomitant Drug	N	3	10 or more
TCMCT	Total Category for Type of Concomitant Drug	N	999	Unknown
ACTYP	Type of AC	N	1	Warfarin
ACTYP	Type of AC	N	2	Dabigatran
ACTYP	Type of AC	N	3	Rivaroxaban
ACTYP	Type of AC	N	4	Apixaban
ACTYP	Type of AC	N	5	Edoxaban
ACTYP	Type of AC	N	6	Parenteral anticoagulants
ACTYP	Type of AC	N	7	Edoxaban (OD
STCONT	Continuation at start	N	1	Continue from previous Visit
ENCONT	Continuation	N	1	Continuation
RGCT	Category of Range	N	0	Less than 1.6
RGCT	Category of Range	N	1	1.6 ≧2.6 or less
RGCT	Category of Range	N	2	Greater than 2.6
AROUND	Around Date	N	1	Around
